# Inferring the hosts of coronavirus using dual statistical models based on nucleotide composition

**DOI:** 10.1038/srep17155

**Published:** 2015-11-26

**Authors:** Qin Tang, Yulong Song, Mijuan Shi, Yingyin Cheng, Wanting Zhang, Xiao-Qin Xia

**Affiliations:** 1 Center for Molecular and Cellular Biology of Aquatic Organisms, Institute of Hydrobiology, the Chinese Academy of Sciences, Wuhan 430072, China; 2 University of Chinese Academy of Sciences, Beijing, China

## Abstract

Many coronaviruses are capable of interspecies transmission. Some of them have caused worldwide panic as emerging human pathogens in recent years, e.g., severe acute respiratory syndrome coronavirus (SARS-CoV) and Middle East respiratory syndrome coronavirus (MERS-CoV). In order to assess their threat to humans, we explored to infer the potential hosts of coronaviruses using a dual-model approach based on nineteen parameters computed from spike genes of coronaviruses. Both the support vector machine (SVM) model and the Mahalanobis distance (MD) discriminant model achieved high accuracies in leave-one-out cross-validation of training data consisting of 730 representative coronaviruses (99.86% and 98.08% respectively). Predictions on 47 additional coronaviruses precisely conformed to conclusions or speculations by other researchers. Our approach is implemented as a web server that can be accessed at http://bioinfo.ihb.ac.cn/seq2hosts.

Emerging infectious diseases (EIDs) and their determinants have recently attracted substantial scientific and popular attention. Over 75% of EIDs consist of zoonosis^1^. Among these pathogens are a group of viruses that belong to *Coronaviridae*. *Coronaviridae* is a family of enveloped, positive-sense, single-stranded RNA viruses that are usually characterized by an enveloped, spherical particle with a diameter in the range of 120–160 nm and a crown-like appearance^2^. Coronaviruses usually cause respiratory tract infections, pneumonia, gastroenteritis, epidemic diarrhoea, enteric infections, hepatitis, encephalomyelitis, and kidney failure. Their hosts include humans, porcines, bovines, murines, avians, and other animals. In the past 12 years, two emerging infectious diseases—severe acute respiratory syndrome (SARS) and Middle East respiratory syndrome (MERS)—attacked humans and animals worldwide and caused approximately 774 human deaths and 315 human deaths, respectively (http://www.who.int/csr/sars/country/table2004_04_21/en/, http://www.who.int/csr/don/2014_07_23_mers/en/). Especially MERS is still persistently bringing human infections and deaths in the outbreak in Korea recently (http://www.who.int/csr/don/19-june-2015-mers-korea/en/). These diseases, which are spread by respiratory means, caused significant panic around the world.

Coronaviruses are currently classified into four major genera or groups: the alpha-coronavirus, the beta-coronavirus, the gamma-coronavirus, and the delta-coronavirus^1^,^2^,^3^,^4^. Alpha-coronavirus and beta-coronavirus usually infect mammalians, whereas gamma-coronavirus and delta-coronavirus usually infect birds^5^. Among all proteins encoded by the coronavirus, the spike protein on the virion surface is the most critical protein, as it mediates both cell attachment and membrane fusion; a few nucleotide changes on the spike gene can cause interspecies transmission^6^. The spike protein primarily consists of three segments, i.e., an ectodomain, a transmembrane anchor, and a short intracellular tail. The ectodomain has two subunits for invading hosts: S1 is responsible for binding receptors and S2 is responsible for membrane fusion^7^. A receptor-binding domain (RBD) near the C-terminal of S1 is primarily responsible for receptor recognition. Coronaviruses recognize a variety of molecules as receptors, including proteins, sugars and heparan sulfates on surfaces of host cells^8^. As the spike gene mediates host recognition and invasion, its sequence must encode the information related to specific hosts; therefore, it is especially useful in identifying hosts of given coronaviruses.

As the result of natural selection and evolution, different genomes are characterized with different preferences for nucleotides. According to probability principles, a shorter nucleotide fragment has a lower chance of variation due to evolution, and the copies of this fragment in a genome tend not to change significantly. This phenomenon is helpful for evolutionary analysis. Dinucleotides are the most stable of these fragments because they are the shortest, and their bias values are usually diverse among species and they are highly invariant for a given individual genome^9^. Dinucleotide abundance has been proven to be reliable in the identification and classification of sequences from viral genomes^10^,^11^,^12^,^13^,^14^,^15^,^16^,^17^.

Support vector machines (SVMs) are a group of supervised machine learning methods that were originally introduced by Vapnik as a linear classifier^18^. Their current standard incarnation (soft margin) comprises associated learning algorithms for classification and regression analysis^19^. The basic principle of class separation for a SVM is mapping vectors into a high-dimensional feature space and finding an optimal separating hyperplane between the two classes in this space by maximizing the margin between the classes’ closest points. The points on the boundaries are referred to as support vectors, and the middle of the margin is the optimal separating hyperplane, which forms the largest gap between two sets of data^20^. Based on this gap, the points of different attributes fall into different classes. Several types of algorithms exist for a SVM to address classification problems for multiple classes and high-dimension data. SVMs perform well in multiple areas of biological analysis, including the evaluation of microarray expression data, the detection of remote protein homologies, and the recognition of translation initiation sites^21^. Instances in which the established classification is questionable or wrong can be identified if an SVM is used for prediction of training samples.

Mahalanobis distance (MD) discrimination is a classical and accurate method that is extensively applied in cluster analysis and classification techniques^22^. MD measures the distance between a point and a population and considers the variance of the population distribution; the points are sorted to the closest population in distance. Another method—Fisher’s linear discriminant analysis—has been applied to infer hosts for three novel Picorna-like viruses^23^. As it requires data that have a normal distribution, which is not the case for our data, MD is adopted in this study.

Previous studies of coronaviruses were primarily focused on the evolution of genomes or specific genes, serum-neutralization assays for identification of receptors, and crystal structure analysis of spike protein and receptor binding domains. In this study, we analysed the compositions of mononucleotides and dinucleotides in coronavirus spike genes. Based on the data matrix of nucleotide composition, the MD and SVM were applied to predict hosts of coronaviruses. The results of this technique may provide hints regarding natural hosts or potential hosts of the virus and can be used to guide the selection of the cells for virus isolation or to explore the probability of interspecies transmission of coronaviruses.

## Results

### Nucleotide composition analysis

Nineteen parameters, including three mononucleotide frequencies (G, C and T) and 16 dinucleotide biases, were computed from 777 spike gene sequences (see Supplementary Table S1). All parameters show significant differences across the host groups (Kruskal-Wallis tests, *p* < 2.2e–16); therefore, they were subsequently employed as factors in statistical models for discriminant analyses. Empirically, a dinucleotide relative abundance or dinucleotide bias (e.g., 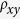) is significantly high if 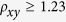 or extremely low if 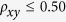
24. Among the 16 dinucleotides in this study, the CpA and TpG show an average abundance that is significantly higher than the expected values (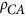 = 1.29, 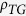 = 1.28), whereas the average bias of CpG is extremely low (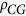 = 0.44). This result indicates that the observed abundances of CpA and TpG are significantly higher than their expected values, and the observed abundance of CpG is significantly lower than the expected value. The G+C content is minimal (31–47%). This finding indicates that coronaviruses exhibit a low density of nucleotide sequences and may be sensitive to heat or alkali. The low G+C content also indicates a preference for codons ending with A or T and a higher mutability.

### Training and validation of statistical models

The data matrix with 19 factors as columns and 730 samples as rows was fitted to SVM and MD models, all predictions in leave-one-out cross-validations were listed in Supplementary Table S2 and summarized in Table 1 according to host species. The validations indicate that both models achieved high accuracies on the training data set: 99.86% for the SVM and 98.08% for the MD. All incorrect cases in unsupervised predictions are listed in Table 2. The only incorrect prediction by the SVM is sample NC_016996.1, which is isolated from an avian species but was predicted to infect humans. Among all 14 incorrect predictions by MD, bats are the common predicted hosts. No sample was incorrectly predicted by both models.

### Predictions for viruses capable of interspecies transmission

The trained models were applied to 47 additional samples, and the predictions unveiled clues regarding potential interspecies transmission (See Table 3). Sequences 1–31 comprise spike genes of coronaviruses that were primarily isolated from palm civets from restaurants, animal markets, or farms in southern China when SARS wreaked havoc in 2003. The sequences of these coronaviruses (civet-CoVs) are similar not only to each other but also to SARS-CoV. Cross-host evolution research of SARS-CoV in palm civet and humans indicated that the variations in spike genes seemed to be essential for the transition of coronavirus from animal-to-human transmission to human-to-human transmission^25^. In addition to cross-neutralization with SARS-CoV, these SARS-like civet-CoVs can use human ACE2 as an entry receptor^26^. Bats are the reservoir hosts of a number of coronaviruses, and a recent study also suggests that bats are natural reservoirs of these SARS-like coronaviruses, whereas palm civets and humans are intermediate hosts^1^. All hosts predicted by the SVM are humans, which supports the previously mentioned research. The MD identified both bats and humans as hosts of these samples, but bats are the preferable hosts for samples 1–26 and the second choice for samples 27–31. This finding is also expected as bats are considered to be natural hosts of these viruses.

Sequences 32–40 comprise spike genes of MERS-CoVs from dromedaries after the outbreak in the Middle East in 2012. MERS-CoVs are similar to the bat coronaviruses HKU5 and HKU4 in their amino acid sequences^27^, and they can use human DPP4 as an entry receptor^28^. MERS-CoVs was assumed to originate from HKU5 in pipistrelle, which is a type of Japanese bat^3^. In our study, these MERS-CoVs isolated from camels were predicted to be capable of infecting humans; and bats are also likely hosts next to humans in predictions by MD. This result is obviously consistent with above speculations, and also supports the WHO advices about avoiding close contact with camels (http://www.who.int/csr/don/2014_07_23_mers/en/).

The 41st sample was a SARS-associated coronavirus that was transmitted from human to pig^29^, and both SVM and MD detected its threat to humans. Bat and avian might be potential hosts since both models suggest that they are more vulnerable than porcine. Samples 42–44 (RsSHC014, Rs3367 and SL-CoV-WIV1) consist of three SARS-like coronaviruses from bats^30^. Analyses based on the sequence similarities and cultures in the cell lines suggest that Rs3367 and SL-CoV-WIV1 are capable of using a SARS-CoV receptor for cell entry and pose a threat to humans, whereas RsSHC014 cannot^30^. Our study provides a precise support to these conclusions. The MD correctly predicts bats as the natural hosts of the three viruses, and the SVM indicates that Rs3367 and SL-CoV-WIV1 are harmful to humans.

The 45th sample was isolated from an alpaca by Jin *et al.* in 2007 with a serotype of bovine; the phylogenetic analysis suggests that it shares the same ancestor with bovine-coronaviruses^31^. Our analysis supports the finding that this coronavirus is capable of infecting bovine. These analyses imply that this strain is capable of interspecies transmission between bovines and alpacas. Samples 46 and 47 are enteric coronaviruses from bovines and humans; they have been identified as the same strain named “Human enteric coronavirus 4408” in the NCBI database due to the similarity between their spike protein sequences of 99.9%. Although they are similar to the human coronavirus OC43 and the bovine coronavirus, evidences from morphological, immunological, and genomic studies indicate that they are closer to bovine coronavirus than to human coronavirus (unpublished research, from personal communication). This finding is consistent with our analysis. In addition, avian and bat are worthy of attentions as potential hosts due to the small MD values.

### Tendencies of MD and SVM in predictions

**Figure 1:**
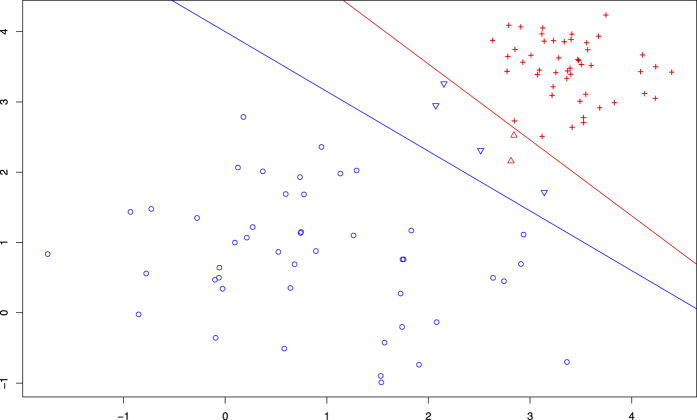
Tendencies of MD and SVM models.

Two groups of two-dimensional data are plotted in Fig. 1. The blue points represent a “loose” population with a larger standard deviation (SD) of *N*(1, 1), and the red points represent a “tight” population with a smaller SD of *N*(3.5, 0.5). The red line separates the two groups classified by the MD, and the groups predicted by the SVM are delimited by the blue line. In this figure, two individuals (the red triangles between the two lines) from the “tight” population were classified into the “loose” group by the MD, whereas the SVM accidentally excluded four points (the blue reversed triangles between the two lines) from the “loose” population. This example shows that MD and SVM have inverse tendencies in some cases, i.e., when a “loose” population is close to a “tight” population, MD intends to classify outliers of the “tight” population into the former. The opposite situation is valid for the SVM.

## Discussion

Nucleotide composition analysis revealed the overrepresentation of CpA and TpG dinucleotides and the suppression of CpG dinucleotides (see Supplementary Table S1), which indicates that coronaviruses generally prefer motifs that contain CpAs and TpGs and avoid CpGs in sequences. These dinucleotide biases are common characteristics of RNA viruses in vertebrates^11^,^12^,^15^,^16^. As most vertebrates exhibit a very low CpG representation in genomes, RNA viruses may gradually adapt to the accumulation of host mutations and mimic the host gene’s dinucleotide patterns for survival^11^. For DNA viruses, the most-accepted mechanisms for the suppression of CpG dinucleotides are the methylation of CpG nucleotides and the subsequent deamination of 5-methylcytosine, which renders CpG a mutational hotspot^24^. For RNA viruses, a different hypothesis is that the RNA viruses encounter different selection pressures when they switch to a new host, and viral RNA genes mimic host mRNAs to avoid immune detection^11^. Similar to other human ssRNA viruses, coronaviruses show a strong correlation between CpG pressure and C+G content (Pearson’s correlation coefficient, r = 0.5443, *p* < 2.2e-16, our data). A lower C+G content usually indicates that the nucleotide sequence of the virus is unstable or is highly variable under evolutionary selection pressure. Considering that the mutation rates for RNA viruses are significantly higher than the mutation rates for DNA viruses^32^, mutational pressure may be the most important determinant of the bias in codon usage in human RNA viruses, such as coronaviruses^14^.

The capabilities to bind with receptors and to replicate in host cells are essential for any virus to infect hosts. Different genes contribute to these biological processes. Variations on these genes may enable a virus to transmit cross-species. One famous example would be the polymerase 2 (PB2) of influenza A virus, in which amino acid change from E to K at its 627th position would render the virus to replicate in mammalian cells^33^,^34^,^35^. In coronaviruses, the spike protein is functionally associated with recognition of hosts and the RNA-dependent RNA polymerase (RdRp) is related to proliferation of virus. However, there are two obstacles limiting the use of RdRp gene: (1) The similarities among nucleotide sequences is too high to train MD model, i.e., the variation rate of RdRp sequence is slower and cannot provide enough resolution to discriminate different coronaviruses; (2) Even worse, available full-length CDSs in public databases are very limited — only 23 or so. On the contrary, the spike gene perfectly satisfied the requirements for variation rate and availability, therefore was adopted as markers in this study.

MD and SVM show opposite tendencies in judging outliers (See Fig. 1), which reflects the different principles of the two classification approaches. Unlike the Euclidian distance (ED), which measures the absolute distance between points or mass centres in space, the Mahalanobis distance considers the variances within a population and the covariance between variables. In some cases, especially when a population with individuals who are scattered across a wide range is located close to a “tight” population with smaller internal variations, the MD may classify marginal individuals from the latter into the “loose” population even if they are “close” to a “tight” population according to the ED. The MD enables “loose” populations to have a greater number of points. The SVM has a different philosophy. SVM separates populations by finding a hyperplane that maximizes the distances between populations. When a “loose” population is close to the boundary of a “tight” population, SVM is more likely to find this hyperplane within the former. This finding explains SVM’s tendency to exclude outliers from a “loose” population.

Bats are the reservoir hosts of a number of coronaviruses that can survive in bats and accumulate variations in the long evolutionary process^1^,^36^,^37^. Thus, coronaviruses in bats constitute a “loose” population with larger internal gaps. We assume that some strains of viruses in bats gain sufficient variation to enable them to infect other organisms; these viruses form a new “tight” population at the edge of the original group. In this case, the MD emphasizes the connection of a virus with the original source, whereas the SVM may be more sensitive to the possibility of infecting new hosts. Therefore, the incorporation of analyses using the MD and SVM can be especially helpful for revealing the profile of interspecies transmission.

According to the predictions by MD, bats are not only the hosts in all 14 incorrect cases from training data set (See Table 2), but also in the host list of each coronaviruses for testing (See Table 3). Furthermore, bats were predicted to host of 64.02% training samples isolated from other hosts (See Table 1). These facts convincingly support the notion that these viruses originated from bats and shifted to other hosts.

Next to bats, avians could be infected by 36.64% samples from other hosts. If bats are the only reservoir hosts and coronaviruses spread from bats to avians and other animals, according to the stochastic event model, the probability of co-infectivity to both bat and avian can be the product of the infectivity probabilities to each of them, i.e., 0.3494 (0.5164 × 0.6767, see Table 1), then 255 (0.3494 × 730) samples are expected to be of co-infectivity. However, only 173 samples were predicted to be of co-infectivity to bats and avians. So avians might be the second independent source of coronavirus in parallel to bats. If this speculation is true, people will have to maintain vigilance to avian coronaviruses apart from avian influenza viruses. Especially, due to the high accuracy of the SVM in cross-validation, we should seriously consider its only “wrong” prediction: perhaps it is sensible to investigate whether the NC_016996.1 virus from avian is capable of infecting humans.

For the viruses that are capable of spreading across a host species barrier, the combination of the MD and the SVM is valuable for assessing their potential threat. The origin and interspecies transmission of coronaviruses have been extensively discussed in the past ten years, and the coronaviruses of most mammals are believed to originate from their ancestors in bats^1^,^36^,^37^. Our analysis with dual statistical models support the finding that SARS-CoVs and MERS-CoVs spread from bats to humans and other animals. In most cases, our approach provided convincing predictions. The dual-model approach can be expected to become a useful tool in future studies. Typically, when a novel coronavirus is isolated, the combination of the MD and the SVM may provide meaningful hints regarding its origin and potential threat to humans or other animals. As soon as more virus genomes are sequenced, this approach can be applied to investigate the interspecies transmission route of other threatening viruses, including the recent Ebola outbreak in West Africa.

## Methods

### Data preparation

All genome sequences and complete coding sequences (CDSs) of spike genes were downloaded from the National Centre for Biotechnology Information (NCBI) database (http://www.ncbi.nlm.nih.gov/) on July 17, 2014. Sequences of spike genes were extracted from the 1044 coronavirus genomes and pooled with 1380 downloaded CDSs. Then, we removed replicate sequences and sequences that contained non-standard bases or were incapable of coding complete products. The length of each sequence is longer than 3,000 bases. Among all 777 valid nucleotide sequences that are listed in Supplementary Data S1, 730 sequences fall into six categories according to different hosts: 196 for humans, 182 for porcines, 77 for bovines, 74 for bats, 28 for murines and 173 for avians. The majority of the remaining 47 viruses were isolated from the two epidemic diseases caused by the coronavirus in the past 12 years. Although we only listed the hosts from which they were isolated, these viruses have been verified or suspected to have the ability to infect different hosts; thus, all 47 sequences were employed to explore interspecies transmission of coronaviruses. Viruses from other mammals, including canines, felines, rabbits, equines, alpacas and whales, were excluded from the data set as the number of spike sequences for each host is insufficient for establishing a separate group.

### Nucleotide composition analysis

The mononucleotide frequencies and dinucleotide biases of the spike sequences were computed using our original Python scripts. Dinucleotide bias is the ratio of the observed value to the expected frequency of each of the 16 dinucleotides: 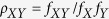, where 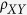 is the dinucleotide bias, *f*_*XY*_ is the frequency of dinucleotide XY, *f*_*X*_ and *f*_*Y*_ are the frequencies of nucleotide X and nucleotide Y^38^, respectively.

In this study, we considered 19 factors, including three mononucleotide frequencies (G, C and T) and 16 dinucleotide biases. As none of the frequencies has a normal distribution, the nonparametric “Kruskal-Wallis Test” was employed to investigate the difference in each factor among six categories. As a result, significant differences across categories were detected for each factor; thus, all 19 factors were employed for modelling.

### Modelling, validation and prediction

As a classifier, the SVM can efficiently perform a nonlinear classification using a kernel technique that is rooted in structural risk minimization. In this study, the R package e1071 (Version: 1.6–3)^20^ was employed for the SVM analysis. “C-classification” was adopted as the model type and “Radial” was adopted as the SVM kernel in our analysis. The MD is a measure of the distance from a point to the centre of a distribution; the principle of this discriminant is that individuals belong to the closest group in the distance. The MD is defined as 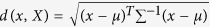, where *X* denotes the population, *x* denotes the individual, *μ* is the mean value of the population, *T* denotes the matrix transpose, and 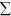 denotes the covariance matrix of population^39^. The R program “distinguish.distance.R”^40^ was employed in the MD analysis. Leave-one-out cross-validation was employed for both SVM and MD analyses.

When the trained models are applied to a sequence for testing, each of the six categories of hosts will obtain a *p* value from SVM and a MD value. Based on *p* values and MD values, three steps will be taken to determine candidate hosts. First, the host of minimal *p* value or MD value is reasonably regarded as the preferable host. Then, two adjustable empirical thresholds can be used for each model to pick out other potential hosts. In this study, we adopted 0.05 and 0.01 for *p* value, 200 and 100 for MD value; i.e., likely hosts were determined if *p* <= 0.05 or MD <= 200, and very likely hosts were defined by *p* <= 0.01 or MD <= 100. The two steps are unsupervised prediction. In case that the isolate source is among the six host groups for modelling, a supervised prediction can be applied as the third step, i.e., all host species with *p* values or MD values no more than those of the observed host will be listed as potential hosts, which can be practical references for researchers to evaluate a virus’s threats to human or other animals.

### Compare the tendencies of MD and SVM in predictions

Two groups of two-dimensional vectors were generated *in silico* as two populations. The number of vectors in the first population are randomly generated from the normal distribution *N*(1, 1), and the number of vectors in the second population are randomly generated from *N*(3.5, 0.5). As the first population has a larger standard deviation (SD), we refer to it as the “loose” population and refer to the second population as the “tight” population. The two groups of data are employed for the leave-one-out cross-validations of MD and SVM.

All Python and R scripts employed in this study are available from the authors upon request. The prediction can be performed using the spike gene sequences of the coronaviruses on our web server, which is available to the public at no cost at http://bioinfo.ihb.ac.cn/seq2hosts.

## Additional Information

**How to cite this article**: Tang, Q. *et al.* Inferring the hosts of coronavirus using dual statistical models based on nucleotide composition. *Sci. Rep.* **5**, 17155; doi: 10.1038/srep17155 (2015).

## Supplementary Material

Supplementary Table S1

Supplementary Table S2

Supplementary Data S1

## Figures and Tables

**Table 1:** Summary of the hosts predicted for the 730 samples by MD in leave-one-out cross-validation.

Host species	Viruses isolated (A)	Other viruses (B = 730 − A)	Total predictions (C)	Infectivity probability (P = C/730)	Predictions in others (D = C – A)	Percentage in others (E = D/B × 100%)
avian	173	557	377	0.5164	204	36.62%
bat	74	656	494	0.6767	420	64.02%
bovine	77	653	77	0.1055	0	0
human	196	534	202	0.2767	6	1.12%
murine	28	702	28	0.0384	0	0
porcine	182	548	185	0.2534	3	0.55%

**Table 2:** The incorrect predictions of MD and SVM in leave-one-out cross-validation.

NCBI Access No.	Virus sources	Wrong predictions by MD	Wrong predictions by SVM
AB008940.1; AB551247.1; AF190406.1;AF201929.1; AF208066.1; FJ647223.1;FJ647224.1;FJ938068.1;JF792616.1	murine	9 (bat)		
NC_011549.1; NC_011550.1; NC_016993.1;NC_016994.1; NC_016995.1	avian	5 (bat)		
NC_016996.1	avian		1 (human)	
In total		14	1	
Accuracy rate		(730–14)/730 = 98.08%	(730–1)/730 = 99.86%	

**Table 3:** The isolate sources and predicted hosts of 47 coronaviruses.

Serial number	Test sample AccNum	SVM prediction	MD prediction	Isolate source
1	AY572034.1	human^*^	bat^**^, human^*^	palm civet
2	AY572036.1	human^*^	bat^**^, human^*^	palm civet
3	AY572037.1	human^*^	bat^**^, human^*^	palm civet
4	AY687355.1	human^*^	bat^**^, human^*^	palm civet
5	AY687356.1	human^*^	bat^**^, human^**^	palm civet
6	AY687358.1	human^*^	bat^**^, human^*^	raccoon dog
7	AY687359.1	human^*^	bat^**^, human^**^	palm civet
8	AY687360.1	human^*^	bat^**^, human^*^	palm civet
9	AY687361.1	human^*^	bat^**^, human^*^	palm civet
10	AY687362.1	human^*^	bat^**^, human^*^	palm civet
11	AY687363.1	human^*^	bat^**^, human^*^	palm civet
12	AY687365.1	human^*^	bat^**^, human^*^	palm civet
13	AY687367.1	human^*^	bat^**^, human^*^	palm civet
14	AY687368.1	human^*^	bat^**^, human^*^	palm civet
15	AY687369.1	human^*^	bat^**^, human^*^	palm civet
16	AY687370.1	human^*^	bat^**^, human^*^	palm civet
17	AY687371.1	human^*^	bat^**^, human^*^	palm civet
18	AY687372.1	human^*^	bat^**^, human^*^	palm civet
19	AY627044.1	human^*^	bat^**^, human^**^	palm civet
20	AY627045.1	human^*^	bat^**^, human^**^	palm civet
21	AY627046.1	human^*^	bat^**^, human^**^	palm civet
22	AY627047.1	human^*^	bat^**^, human^**^	palm civet
23	AY627048.1	human^*^	bat^**^, human^**^	palm civet
24	AY613952.1	human^*^	bat^**^, human^*^	palm civet
25	AY613951.1	human^*^	bat^**^, human^*^	palm civet
26	AY525636.1	human^*^	bat^**^, human^*^	palm civet
27	DQ514528.1	human^*^	human^**^, bat^**^	palm civet
28	DQ514529.1	human^*^	human^**^, bat^**^	palm civet
29	DQ514530.1	human^*^	human^**^, bat^**^	palm civet
30	DQ514531.1	human^*^	human^**^, bat^**^	palm civet
31	DQ514532.1	human^*^	human^**^, bat^**^	palm civet
32	KJ477102.1	human^*^	human^**^, bat^*^	dromedary
33	KJ650098.1	human^*^	human^**^, bat^**^	dromedary
34	KJ650295.1	human^*^	human^**^, bat^**^	dromedary
35	KJ713295.1	human^*^	human^**^, bat^**^	dromedary
36	KJ713296.1	human^*^	human^**^, bat^**^	dromedary
37	KJ713297.1	human^*^	human^**^, bat^**^	dromedary
38	KJ713298.1	human^*^	human^**^, bat^**^	dromedary
39	KJ713299.1	human^*^	human^**^, bat^**^	dromedary
40	KF917527.1	human^*^	human^**^, bat^**^	dromedary
41	AY654624.1	human^*^, bat, avian, porcine	human^**^, bat^*^, avian, porcine	porcine
42	KC881005.1	bat	bat^**^, avian^*^	bat
43	KC881006.1	human, bat	bat^**^	bat
44	KC881007.1	human, bat	bat^**^	bat
45	DQ915164.2	bovine^*^	bovine^**^, avian^*^, bat^*^	alpaca
46	FJ415324.1	bovine^*^, human	bovine^**^, avian^*^, bat, human	human
47	FJ938067.1	bovine^*^, human	bovine^**^, avian^*^, bat, human	human, bovine

Predictions consist of hosts with minimal MD or *p* values, those with MD <= 200 or *p* <= 0.05 for SVM, and those with MD or *p* values no greater than corresponding values of isolate sources if the isolate sources are among the six categories of hosts. All predictions are listed in ascending of MD or *p* values. ^*^p <= 0.05 or MD <= 200. ^**^p <= 0.01 or MD <= 100.
