# Supplementary material for: Inferring the hosts of coronavirus using dual statistical models based on nucleotide composition
Source: Sci Rep. 2015 Nov 26;5:17155. doi: 10.1038/srep17155 (PMC4660426; doi:10.1038/srep17155)
Supplement: Supplementary Data S1 [file srep17155-s3.pdf]

Inferring the hosts of coronavirus using dual statistical models based on nucleotide composition

Qin Tang[1,2], Yulong Song[1,2], Mijuan Shi[1], Yingyin Cheng[1], Wanting Zhang[1], Xiao-Qin Xia[1]*

1 *Center for Molecular and Cellular Biology of Aquatic Organisms, Institute of Hydrobiology, the Chinese Academy of Sciences, Wuhan 430072, China*, 2 *University of Chinese Academy of Sciences, Beijing, China*

*Corresponding author. Mailing address: No. 7 Donghu South Road, Wuchang District, Wuhan, Hubei Province, 430072, China. Phone and Fax: 86-027-68780915. E-mail: xqxia@ihb.ac.cn

>gi|371940428|dbj|AB691763.1|_Human_coronavirus_229E_S_gene_for_spike_protein_complete_cds_strain:_ATCC_VR740

ATGTTTGTTTTGCTTGTTGCATATGCCTTGTTGCATATTGCTGGTTGTCAAACTACAAATGGGCTGAACACTAGT
TACTCTGTTTGCAACGGCTGTGTTGGTTATTCAGAAAATGTATTTGCTGTTGAGAGTGGTGGTTATATACCCTCCGACT
TTGCATTCAATAATTGGTTCCTTCTAACTAATACCTCATCTGTTGTAGATGGTGTTGTGAGGAGTTTTCAGCCTTTGTT
GCTTAATTGCTTATGGTCTGTTTCTGGCTTGCGGTTTACTACTGGTTTTGTCTATTTTAATGGTACTGGGAGAGGTGAT
TGTAAAGGTTTTTCCTCAGATGTTTTGTCTGATGTCATACGTTACAACCTCAATTTTGAAGAAAACCTTAGACGTGGAA
CCATTTTGTTTAAAACATCTTATGGTGTTGTTGTGTTTTATTGTACCAACAACACTTTAGTTTCAGGTGATGCTCACAT
ACCATTTGGTACAGTTTTGGGCAATTTTTATTGCTTTGTAAATACTACTATTGGCAATGAAACTACGTCTGCTTTTGTG
GGTGCACTACCTAAGACAGTTCGTGAGTTTGTTATTTCACGCACAGGACATTTTTATATTAATGGCTATCGCTATTTCA
CTTTAGGTAATGTAGAAGCCGTTAATTTCAATGTCACTACTGCAGAAACCACTGATTTTTTTACTGTTGCGTTAGCTTC
TTATGCTGACGTTTTGGTTAATGTGTCACAAACCTCTATTGCTAATATAATTTATTGCAACTCTGTTATTAACAGACTG
AGATGTGACCAGTTGTCCTTTGATGTACCAGATGGTTTTTATTCTACAAGCCCTATTCAATCCGTTGAGCTACCTGTGT
CTATTGTGTCGCTACCTGTTTATCATAAACATACGTTTATTGTGTTGTACGTTGACTTCAAACCTCAGAGTGGCGGTGG
CAAGTGCTTTAACTGTTATCCTGCTGGTGTTAATATTACACTGGCCAATTTTAATGAAACTAAAGGGCCTTTGTGTGTT
GACACATCACACTTCACTACCAAATACGTTGCTGTTTATGCCAATGTTGGTAGGTGGAGTGCTAGTATTAACACGGGAA
ATTGCCCTTTTTCTTTTGGCAAAGTTAATAACTTTGTTAAATTTGGCAGTGTATGTTTTTCGCTAAAGGATATACCCGG
TGGTTGCGCAATGCCTATAGTGGCTAATTGGGCTTATAGTAAGTACTATACTATAGGCTCATTGTATGTTTCTTGGAGT
GATGGTGATGGAATTACTGGCGTCCCACAACCTGTTGAGGGTGTTAGTTCCTTTATGAATGTTACATTGGACAAATGTA
CTAAATATAATATTTATGATGTATCTGGTGTGGGTGTTATTCGCGTTAGCAATGACACCTTTCTTAATGGAATTACGTA
CACATCAACTTCAGGTAACCTTCTGGGTTTTAAAGATGTTACTAAGGGCACCATCTACTCTATCACTCCTTGTAACCCA
CCAGATCAGCTTGTTGTTTATCAGCAAGCTGTTGTTGGTGCTATGTTGTCTGAAAATTTTACTAGTTACGGCTTTTCTA
ATGTTGTAGAACTGCCGAAATTTTTCTATGCGTCCAATGGCACTTATAATTGCACAGACGCTGTTTTAACTTATTCTAG
TTTTGGCGTTTGTGCAGATGGTTCTATAATTGCTGTTCAACCACGTAATGTTTCATATGATAGTGTTTCAGCTATCGTC
ACAGCTAATTTGTCTATACCTTCCAATTGGACCACTTCGGTCCAGGTTGAGTATTTACAAATTACAAGTACACCTATCG
TAGTTGATTGCTCCACTTATGTTTGCAATGGTAATGTGCGCTGTGTTGAATTGCTTAAGCAGTATACTTCTGCTTGTAA
AACTATTGAAGACGCCTTAAGAAATAGCGCCAGGCTGGAGTCTGCAGATGTTAGTGAGATGCTCACTTTTGACAAGAAA
GCGTTTACACTTGCTAATGTTAGTAGTTTTGGTGACTACAACCTTAGCAGCGTCATACCTAGCTTGCCCACAAGTGGTA
GTAGAGTGGCTGGTCGCAGTGCCATAGAAGACATACTTTTTAGCAAAATTGTTACTTCTGGACTTGGCACTGTGGACGC
AGACTACAAAAATTGCACTAAGGGTCTTTCCATTGCTGACTTGGCTTGTGCTCAATATTATAATGGCATTATGGTTTTG
CCTGGCGTCGCTGATGCTGAACGAATGGCCATGTATACAGGTTCTTTAATTGGTGGAATTGCTTTAGGAGGTCTAACAT
CAGCCGTTTCAATACCATTTTCATTAGCAATTCAGGCACGTTTAAATTATGTTGCATTGCAGACTGATGTTTTACAAGA
AAATCAGAAAATTCTTGCTGCATCTTTTAACAAAGCAATGACCAACATAGTAGATGCCTTTACTGGTGTTAATGATGCT
ATTACACAAACTTCACAAGCCCTACAAACAGTTGCTACTGCACTTAACAAGATCCAGGATGTTGTTAATCAACAAGGCA
ACTTATTGAACCATTTAAATTCTCAGTTGAGGCAGAATTTTCAAGCTATCTCTAGCTCTATTCAGGCTATCTATGACAG
ACTTGACACTATTCAGGCTGATCAACAAGTAGATAGGCTGATTACTGGTAGATTGGCTGCTTTGAATGTATTCGTTTCT
CATACATTGACTAAGTACACTGAAGTTCGTGCTTCCAGACAGCTTGCACAACAAAAAGTGAATGAGTGTGTCAAATCCC
AGTCTAAGCGTTATGGCTTCTGTGGAAATGGCACTCACATTTTCTCAATTGTTAATGCTGCTCCTGAGGGGCTTGTTTT
TCTCCACACTGTCTTGTTGCCGACACAATATAAGGATGTTGAAGCGTGGTCTGGGTTGTGCGTTGATGGTACAAACGGT
TATGTGTTGCGACAACCTAATCTTGCTCTTTACAAAGAAGGCAATTATTATAGAATCACATCTCGCATAATGTTTGAAC
CACGTATTCCTACCATGGCAGATTTTGTTCAAATTGAAAATTGCAATGTCACATTTGTTAACATTTCTCGCTCTGAGTT
GCAAACCATTGTGCCAGAGTATATTGATGTTAATAAGACGCTGCAAGAATTAAGTTACAAATTGCCAAATTACACTGTT
CCAGACCTAGTTGTCGAACAGTACAACCAGACTATTTTGAATTTGACCAGTGAAATTAGCACCCTTGAAAATAAATCTG

CGGAGCTTAATTACACTGTTCAAAAATTGCAAACTCTGATTGACAACATAAATAGCACATTAGTCGACTTAAAGTGGCT
CAACCGGGTTGAGACTTACATCAAGTGGCCGTGGTGGGTGTGGTTGTGCATTTCAGTCGTGCTCATCTTTGTGGTGAGT
ATGTTGCTATTATGTTGTTGTTCTACTGGTTGCTGTGGCTTCTTTAGTTGTTTTGCATCTTCTATTAGAGGTTGTTGTG
AATCAACTAAACTTCCTTATTACGACGTTGAAAAGATCCACATACAGTAA

>gi|371940430|dbj|AB691764.1|_Human_coronavirus_229E_S_gene_for_spike_protein_complete_cds_isolate:_SendaiH/1121/04

ATGTTTGTTTTACTTGTTGCATATGCCTTGTTGCATATTGCTGGTTGTCAAACTACAAATGGGATGAACACTAGT
CACTCTGTTTGCAACGGCTGTGTTGGTCATTCGGAAAATGTATTTGCTGTTGAGAGTGGTGGTTATATACCATCCAACT
TTGCATTTAATAATTGGTTCCTCTTAACTAATACCTCATCTGTTGTAGATGGTGTTGTGAGGAGTTTTCAGCCTTTGTT
GCTTAATTGCTTATGGTCTGTTTCTGGCTCGCGGCTTACTACTGGTTTTGTCTATTTTAATGGTACTGGGAGAGGTGAT
TGTAAAGGTTTTTATTCAAATGCTTCGTCTGATGTCATACGTTACAACATCAATTTTGAAGAAAACCTTAGACGTGGAA
CCATTTTGTTTAAAACATCTTATGGTGCTGTTGTGTTTTATTGTACCAACAACACTTTGGTTTCAGGTGATGCTCACAT
ACCATCTGGTACAGTTTTGGGCAATTTTTATTGCTTTGTAAATACTACTATTGGCAATGAAACTACATCTGCTTTTGTG
GGTGCACTACCTAAGACAGTTCGTGAGTTTGTTATTTCACGCACAGGACATTTCTATATTAATGGCTATCGCTATTTCT
CTTTAGGTAATGTAGAAGCCGTTAATTTCAATGTCACTAATGCTGCAACCACTGTCTGTACTGTTGCGTTAGCTTCTTA
TGCTGACGTTTTGGTTAATGTGTCACAAACTGCTATTGCTAATATAATTTATTGCAATTCTGTTATTAACAGACTGAGA
TGTGACCAGTTGTCCTTTGATGTACCAGATGGTTTTTATTCCACAAGCCCTATTCAATCCGTTGAGCTACCTGTGTCTA
TTGTGTCGCTACCTGTTTATCACAAACACACGTTTATTGTGTTGTATGTTAACTTCGAACTTCGGCGTGGCCCTGGCAC
GTGCTATAACTGTCGTCCTGCTGTTGTTAATATTACACTGGCCAATTTTAATGAAACTAAAGGGCCCTTGTGTGTTGAC
ACATCACACTTCACTACCCAATTCGTTGGTGTCAAGTTTGATAGGTGGAGTGCTAGTATTAACACGGGAAATTGCCCTT
TTTCTTTTGGCAAAGTTAATAACTTTGTTAAATTTGGCAGTGTATGTTTTTCGCTAAAGGATATACCCGGTGGTTGCGC
AATGCCTATAATGGCTAATTTGGCTAATCTTAATTCTCATAATATAGGCTCATTGTATGTTTCTTGGAGTGATGGTGAT
GTAATTACTGGCGTCCCGAAACCTGTTGAGGGTGTTAGTTCCTTTATGAATGTTACATTGAATAAATGTACTAAATATA
ACATTTATGATGTAGTTGGTGTGGGTGTTATTCGCATTAGCAATGAAACCTTTCTTAATGGAATTACGTACACATCAAC
TTCAGGTAACCTTCTGGGTTTTAAAGATGTTACTAATGGCACCATCTACTCTATCACTCCTTGTAACCCACCAGATCAG
CTTGTTGTTTATCAGCAAGCTGTTGTTGGTGCTATGTTGTCTGAAAACTTTACTAGTTACGGCTTTTCTAATGTTGTAG
AAATGCCGAAATTTTTCTATGCGTCCAATGGCACTTATAATTGCACAGACGCTGTTTTAACTTATTCTAGTTTTGGCGT
TTGTGCAGATGGTTCTATAATTGCTGTTCAACCACGTAATGTTTCATATGATAGTGTTTCAGCTATTGTCACAGCTAAT
TTGTCTATACCCTCCAATTGGACCACTTCGGTCCAGGTTGAGTATTTACAAATTACAAGTACACCTATCGTAGTTGATT
GCTCCACTTATGTTTGCAATGGTAATGTGCGCTGTGTTGAATTGCTTAAGCAGTATACTTCTGCTTGTAAAACTATTGA
AGACGCCTTAAGAAATAGCGCCATGCTGGAGTCTGCAGATGTTAGTGAGATGCTCACTTTTGACAAGAAAGCGTTTACA
CTTGCTAATGTTAGTAGTTTTGGTGACTACAACCTTAGCAGTGTTATACCTAGCTTGCCCAGAAGTGGTAGTAGAGTGG
CTGGTCGCAGTGCCATAGAAGACATACTTTTTAGCAAACTTGTTACGTCTGGACTTGGCACTGTGGACGCAGACTACAA
AAAGTGTACTAAGGGTCTTTCCATTGCTGACTTGGCTTGTGCCCAATATTATAATGGCATTATGGTTTTGCCTGGCGTC
GCTGATGCTGAACGTATGGCCATGTATACAGGTTCTTTAATTGGTGGAATTGCTTTAGGAGGTCTTACATCAGCCGCTT
CAATACCATTTTCATTAGCAATTCAGTCACGTTTAAATTATGTTGCATTGCAGACTGATGTTTTACAAGAAAATCAGAA
AATTCTTGCTGCATCTTTTAATAAAGCAATGACCAACATAGTAGATGCCTTTACTGGTGTTAATGATGCTATTACACAA
ACTTCACAAGCTCTACAAACAGTTGCTACTGCACTTAATAAGATCCAGGATGTTGTTAATCAACAAGGCAACTCATTGA
ACCATTTAACTTCTCAGTTGAGGCAGAATTTTCAAGCCATTTCTAGCTCTATTCAGGCTATCTATGACAGACTTGATAT
TATTCAGGCTGATCAACAAGTAGATAGGCTGATTACTGGTAGATTGGCTGCTCTGAATGTATTCGTTTCTCATACATTG
ACTAAGTACACTGAAGTTCGTGCTTCCAGACAGCTTGCACAACAAAAAGTGAATGAGTGTGTCAAATCCCAGTCTAAGC

GGTATGGCTTCTGTGGAAATGGCACGCACATTTTCTCACTTGTTAATGCTGCTCCTGAGGGGCTTGTTTTTCTCCATAC
TGTCTTGTTGCCGACACAATATAAGGATGTTGAAGCGTGGTCTGGGTTGTGCGTTGATGGTAGAAACGGTTATGTGTTG
AGACAACCTAATCTTGCTCTTTACAAAGAAGGCAATTATTATAGAATTACATCTCGCATAATGTTTGAACCACGTATTC
CTACCATAGCAGATTTTGTCCAAATTGAAAATTGCAATGTCACATTTGTTAACATTTCTCGCTCTGAGTTGCAAACTAT
TGTGCCAGAGTATATTGATGTTAATAAGACGCTGCAAGAATTAAGTTACAAATTGCCAAATTACACTGTTCCAGACCTA
GTTGTCGAACAGTACAACCAGACTATTTTGAATTTGACCAGTGAAATTAGCACCCTTGAAAATAAATCTGCGGAGCTTA
ATTACACTGTTCAAAAACTGCAAACTTTGATCGACAACATAAATAGCACATTAGTTGACTTAAAGTGGCTCAACCGTGT
TGAGACTTATATCAAGTGGCCGTGGTGGGTGTGGTTGTGCATTTCAGTCGTGCTCATCTTTGTGGTGAGTATGTTGCTA
TTATGTTGTTGTTCTACTGGTTGCTGCGGCTTCTTTAGTTGTTTTGCATCTTCTATTAGAGGTTGTTGTGAATCAACTA
AACTTCCTTATTACGACGTTGAAAAGATCCACATACAGTAA

>gi|371940432|dbj|AB691765.1|_Human_coronavirus_229E_S_gene_for_spike_protein_complete_cds_isolate:_SendaiH/826/04

ATGTTTGTTTTACTTGTTGCATATGCCTTGTTGCATATTGCTGGTTGTCAAACTACAAATGGGATGAACACTAGT
CACTCTGTTTGCAACGGCTGTGTTGGTCATTCGGAAAATGTATTTGCTGTTGAGAGTGGTGGTTATATACCATCCAACT
TTGCATTTAATAATTGGTTCCTCTTAACTAATACCTCATCTGTTGTAGATGGTGTTGTGAGGAGTTTTCAGCCTTTGTT
GCTTAATTGCTTATGGTCTGTTTCTGGCTCACGGCTTACTACTGGTTTTGTCTATTTTAATGGTACTGGGAGAGGTGAT
TGTCAAGGTTTTTATTCAAATGCTTCGTCTGATGTCATACATTACAACATCAATTTTGAAGAAAACCTTAGACGTGGAA
CCATTTTGTTTAAAACATCTTATGGTGCTGTTGTGTTTTATTGTACCAACAACACTTTGGTTTCAGGTGATGCTCACAT
ACCATCTGGTACAGTTTTGGGCAATTTTTATTGCTTTGTAAATACTACTATTGGCAATGAAACTACGTCTGCTTTTGTG
GGTGCACTACCTAAGACAGTTCGTGAGTTTGTTATTTCACGCACAGGACATTTCTATATTAATGGCTATCGCTATTTCT
CTTTAGGTAATGTAGAAGCCGTTAATTTCAATGTCACTAATGCTGCAACCACTGTTTGTACTGTTGCGTTAGCTTCTTA
TGCTGACGTTTTGGTTAATGTGTCACAAACTGCTATTGCTAATATAATTTATTGCAATTCTGTTATTAACAGACTGAGA
TGTGACCAGTTGTCCTTTGATGTACCAGATGGTTTTTATTCCACAAGCCCTATTCAATCCGTTGAGCTACCTGTGTCTA
TTGTGTCGCTACCTGTTTATCACAAACACACGTTTATTGTGTTGTATGTTAACTTCGAACTTCGGCGTGGCCCTGGCAG
GTGCTATAACTGTCGTCCTGCTGTTGTTAATATTACACTGGCCAATTTTAATGAAACTAAAGGGCCCTTGTGTGTTGAC
ACATCACACTTCACTACCCAATTCGTTGGTGTCAAGTTTGATAGGTGGAGTGCTAGTATTAACACGGGAAATTGCCCTT
TTTCTTTTGGCAAAGTTAATAACTTTGTTAAATTTGGCAGTGTATGTTTTTCGCTAAAGGATATACCCGGTGGTTGCGC
AATGCCTATAATGGCTAATTTGGCTAATCTTAATTCTCATAATATAGGCTCATTGTATGTTTCTTGGAGTGATGGTGAT
GTAATTACTGGCGTCCCGAAACCTGTTGAGGGTGTTAGTTCCTTTATGAATGTTACATTGAATAAATGTACTAAATATA
ACATTTATGATGGATCTGGTGTGGGTGTTATTCGCATTAGCAATGAAACCTTTCTTAATGGAATTACGTACACATCAAC
TTCAGGTAACCTTCTGGGTTTTAAAGATGTTACTAATGGCACCATCTACTCTATCACTCCTTGTAACCCACCAGATCAG
CTTGTTGTTTATCAGCAAGCTGTTGTTGGTGCTATGTTGTCTGAAAACTTTACTAGTTACGGCTTTTCTAATGTTGTAG
AAATGCCGAAATTTTTCTATGCGTCCAATGGCACTTATAATTGCACAGACGCTGTTTTAACTTATTCTAGTTTTGGCGT
TTGTGCAGATGGTTCTATAATTGCTGTTCAACCACGTAATGTTTCATATGATAGTGTTTCAGCTATTGTCACAGCTAAT
TTGTCTATACCCTCCAATTGGACCACTTCGGTCCAGGTTGAGTATTTACAAATTACAAGTACACCTATCGTAGTTGATT
GCTCCACTTATGTTTGCAATGGTAATGTGCGCTGTGTTGAATTGCTTAAGCAGTATACTTCTGCTTGTAAAACTATTGA
AGACGCCTTAAGAAATAGCGCCATGCTGGAGTCTGCAGATGTTAGTGAGATGCTCACTTTTGACAAGAAAGCGTTTACA
CTTGCTAATGTTAGTAGTTTTGGTGACTACAACCTTAGCAGTGTTATACCTAGCTTGCCCAGAAGTGGTAGTAGAGTGG
CTGGTCGCAGTGCCATAGAAGACATACTTTTTAGCAAACTTGTTACGTCTGGACTTGGCACTGTGGACGCAGACTACAA
AAAGTGTACTAAGGGTCTTTCCATTGCTGACTTGGCTTGTGCCCAATATTATAATGGCATTATGGTTGTGCCTGGGGTC
GCTGATGCTGAACGTATGGCCATGTATACAGGTTCTTTAATTGGTGGAATTGCTTTAGGAGGTCTTACATCAGCCGCTT

CAATACCATTTTCATTAGCAATTCAGTCACGTTTAAATTATGTTGCATTGCAGACTGATGTTTTACAAGAAGATCAGAA
AATTCTTGCTGCATCTTTTAATAAAGCAATGACCAACATAGTAGATGCCTTTACTGGTGTTAATGATGCTATTACACAA
ACTTCACAAGCTCTACAAACAGTTGCTACTGCACTTAATAAGATCCAGGATGTTGTTAATCAACAAGGCAACTCATTGA
ACCATTTAACTTCTCAGTTGAGGCAGAATTTTCAAGCCATTTCTAGCTCTATTCAGGCTATCTATGACAGACTTGATAT
TATTCAGGCTGATCAACAAGTAGATAGGCTGATTACTGGTAGATTGGCTGCTCTGAATGTATTCGTTTCTCATACATTG
ACTAAGTACACTGAAGTTCGTGCTTCCAGACAGCTTGCACAACAAAAAGTGAATGAGTGTGTCAAATCCCAGTCTAAGC
GGTATGGCTTCTGTGGAAATGGCACGCACATTTTCTCACTTGTTAATGCTGCTCCTGAGGGGCTTGTTTTTCTCCATAC
TGTCTTGTTGCCGACACAATATAAGGATGTTGAAGCGTGGTCTGGGTTGTGCGTTGATGGTAGAAACGGTTATGTGTTG
AGACAACCTAATCTTGCTCTTTACAAAGAAGGCAATTATTATAGAATTACATCTCGCATAATGTTTGAACCACGTATTC
CTACCATAGCAGATTTTGTCCAAATTGAAAATTGCAATGTCACATTTGTTAACATTTCTCGCTCTGAGTTGCAAACTAT
TGTGCCAGAGTATATTGATGTTAATAAGACGCTGCAAGAATTAAGTTACAAATTGCCAAATTACACTGTTCCAGACCTA
GTTGTCGAACAGTACAACCAGACTATTTTGAATTTGACCAGTGAAATTAGCACCCTTGAAAATAAATCTGCGGAGCTTA
ATTACACTGTTCAAAAACTGCAAACTTTGATTGACAACATAAATAGCACATTAGTTGACTTAAAGTGGCTCAACCGTGT
TGAGACTTATATCAAGTGGCCGTGGTGGGTGTGGTTGTGCATTTCAGTCGTGCTCATCTTTGTGGTGAGTATGGTGCTA
TTATGTTGTTGTTCTACTGGTTGCTGCGGCTTCTTTAGTTGTTTTGCATCTTCTATTAGAGGTTGTTGTGAATCAACTA
AACTTCCTTATTACGACGTTGAAAAGATCCACATACAGTAA

>gi|371940434|dbj|AB691766.1|_Human_coronavirus_229E_S_gene_for_spike_protein_complete_cds_isolate:_SendaiH/1948/04

ATGTTTGTTTTACTTGTTGCATATGCCTTGTTGCATATTGCTGGTTGTCAAACTACAAATGGGATGAACACTAGT
CACTCTGTTTGCAACGGCTGTGTTGGTCATTCGGAAAATGTATTTGCTGTTGAGAGTGGTGGTTATATACCATCCAACT
TTGCATTTAATAATTGGTTCCTCTTAACTAATACCTCATCTGTTGTAGATGGTGTTGTGAGGAGTTTTCAGCCTTTGTT
GCTTAATTGCTTATGGTCTGTTTCTGGCTCACGGCTTACTACTGGTTTTGTCTATTTTAATGGTACTGGGAGAGGTGAT
TGTAAAGGTTTTTATTCAAATGCTTCGTCTGATGTCATACGTTACAACATCAATTTTGAAGAAAACCTTAGACGTGGAA
CCATTTTGTTTAAAACATCTTATGGTGCTGTTGTGTTTTATTGTACCAACACCACTTTGGTTTCAGGTGATGCTCACAT
ACCATCTGGTACAGTTTTGGGCAATTTTTATTGCTTTGTAAATACTACTATTGGCAATGAAACTACGTCTGCTTTTGTG
GGTGCACTACCTAAGACAGTTCGTGAGTTTGTTATTTCACGCACAGGACATTTCTATATTAATGGCTATCGCTATTTCT
CTTTAGGTAATGTAGAAGCCGTTAATTTCAATGTCACTAATGCTGCAACCACTGTTTGTACTGTTGCGTTAGCTTCTTA
TGCTGACGTTTTGGTTAATGTGTCACAAACTGCTATTGCTAATATAATTTATTGCAATTCTGTTATTAACAGACTGAGA
TGTGACCAGTTGTCCTTTGATGTACCAGATGGTTTTTATTCCACAAGCCCTATTCAATCCGTTGAGCTACCTGTGTCTA
TTGTGTCGCTACCTGTTTATCACAAACACACGTTTATTGTGTTGTATGTTAACTTCGAACTTCGGCGTGGCCCTGGCAG
GTGCTATAACTGTCGTCCTGCTGTTGTTAATATTACACTGGCCAATTTTAATGAAACTAAAGGGCCCTTGTGTGTTGAC
ACATCACACTTCACTACCCAATTCGTTGGTGTCAAGTTTGATAGGTGGAGTGCTAGTATTAACACGGGAAATTGCCCTT
TTTCTTTTGGCACAGTTAATAACTTTGTTAAATTTGGCAGTGTATGTTTTTCGCTAAAGGATATACCCGGTGGTTGCGC
AATGCCTATAATGGCTAATTTGGCTAATCTTAATTCTCATAATATAGGCTCATTGTATGTTTCTTGGAGTGATGGTGAT
GTAATTACTGGCGTCCCGAAACCTGTTGAGGGTGTTAGTTCCTTTATGAATGTTACATTGAATAAATGTACTAAATATA
ACATTTATGATGTATCTGGTGTGGGTGTTATTCGCATTAGCAATGAAACCTTTCTTAATGGAATTACGTACACATCAAC
TTCAGGTAACCTTCTGGGTTTTAAAGATGTTACTAATGGCACCATCTACTCTATCACTCCTTGTAACCCACCAGATCAG
CTTGTTGTTTATCAGCAAGCTGTTGTTGGTGCTATGTTGTCTGAAAACTTTACTAGTTACGGCTTTTCTAATGTTGTAG
AAATGCCGAAATTTTTCTATGCGTCCAATGGCACTTATAATTGCACAGACGCTGTTTTAACTTATTCTAGTTTTGGCGT
TTGTGCAGATGGTTCTATAATTGCTGTTCAACCACGTAATGTTTTATATGATAGTGTTTCAGCTATTGTCACAGCTAAT
TTGTCTATACCCTCCAATTGGACCACTTCGGTCCAGGTTGAGTATTTACAAATTACAAGTACACCTATCGTAGTTGATT

GCTCCACTTATGTTTGCAATGGTAATGTGCGCTGTGTTGAATTGCTTAAGCAGTATACTTCTGCTTGTAAAACTATTGA
AGACGCCTTAAGAAATAGCGCCATGCTGGAGTCTGCAGATGTTAGTGAGATGCTCACTTTTGACAAGAAAGCGTTTACA
CTTGCTAATGTTAGTAGTTTTGGTGACTACAACCTTAGCAGTGTTATACCTAGCTTGCCCAGAAGTGGTAGTAGAGTGG
CTGGTCGCAGTGCCATAGAAGACATACTTTTTAGCAAACTTGTTACGTCTGGACTTGGCACTGTGGACGCAGACTACAA
AAAGTGTACTAAGGGTCTTTCCATTGCTGACTTGGCTTGTGCCCAATATTATAATGGCATTATGGTTTTGCCTGGCGTC
GCTGATGCTGAACGTATGGCCATGTATACAGGTTCTTTAATTGGTGGAATTGCTTTAGGAGGTCTTACATCAGCCGCTT
CAATACCATTTTCATTAGCAATTCAGTCACGTTTAAATTATGTTGCATTGCAGACTGATGTTTTACAAGAAAATCAGAA
AATTCTTGCTGCATCTTTTAATAAAGCAATGACCAACATAGTAGATGCCTTTACTGGTGTTAATGATGCTATTACACAA
ACTTCACAAGCTCTACAAACAGTTGCTACTGCACTTAATAAGATCCAGGATGTTGTTAATCAACAAGGCAACTCATTGA
ACCATTTAACTTCTCAGTTGAGGCAGAATTTTCAAGCCATTTCTAGCTCTATTCAGGCTATCTATGACAGACTTGATAT
TATTCAGGCTGATCAACAAGTAGATAGGCTGATTACTGGTAGATTGGCTGCTCTGAATGTATTCGTTTCTCATACATTG
ACTAAGTACACTGAAGTTCGTGCTTCCAGACAGCTTGCACAACAAAAAGTGAATGAGTGTGTCAAATCCCAGTCTAAGC
GGTATGGCTTCTGTGGAAATGGCACGCACATTTTCTCACTTGTTAATGCTGCTCCTGAGGGGCTTGTTTTTCTCCATAC
TGTCTTGTTGCCGACACAATATAAGGATGTTGAAGCGTGGTCTGGGTTGTGCGTTGATGGTAGAAACGGTTATGTGTTG
AGACAACCTAATCTTGCTCTTTACAAAGAAGGCAATTATTATAGAATTACATCTCGCGTAATGTTTGAACCACGTATTC
CTACCATAGCAGATTTTGTCCAAATTGAAAATTGCGATGTCACATTTGTTAACATTTCTCGCTCTGAGTTGCAAACTAT
TGTGCCAGAGTATATTGATGTTAATAAGACGCTGCAAGAATTAAGTTACAAATTGCCAAATTACACTGTTCCAGACCTA
GTTGTCGAACAGTACAACCAGACTATTTTGAATTTGACCAGTGAAATTAGCACCCGTGAGAATAAATCTGCGGAGCTTA
ATTACACTGTTCAAAAACTGCAAAGTTTGATTGACAACATAAATAGCACATTAGTTGACTTAAAGTGGCTCAACCGTGT
TGAGACTTATATCAAGTGGCCGTGGTGGGTGTGGTTGTGCATTTCAGTCGTGCTCATCTTTGTGGTGAGTATGTTGCTA
TTATGTTGTTGTTCTACTGGTTGCTGCGGCTTCTTTAGTTGTTTTGCATCTTCTATTAGAGGTTGTTGTGAATCAACTA
AACTTCCTTATTACGACGTTAAAAAGATCCACATACAGTAA

>gi|371940436|dbj|AB691767.1|_Human_coronavirus_229E_S_gene_for_spike_protein_complete_cds_isolate:_Niigata/01/08

ATGTTTGTTTTACTTGTTGCATATGCCTTGTTGCATATTGCTGGTTGTCAAACTACAAATGGGACGAACACTAGT
CACTCTGTTTGCAACGGCTGTGTTGGTCATTCGGAAAATGTATTTGCTGTTGAGAGTGGTGGTTATATACCCTCCAACT
TTGCATTTAATAATTGGTTCCTCTTAACTAATACCTCATCTGTTGTAGATGGTGTTGTGAGGAGTTTTCAGCCTTTGTT
GCTTAATTGCTTATGGTCTGTTTCTGGCTCGCAGTTTACTACTGGTTTTGTCTATTTTAATGGTACTGGGAGAGGTGAT
TGTAAAGGTTTTTATTCAAATGCTTCGTCTGATGTCATACGTTACAACATCAATTTTGAAGAAAACCTTAGACGTGGAA
CCATTTTGTTTAAAACATCTTATGGTGCTGTTGTGTTTTATTGTACCAACAACACTTTGGTTTCAGGTGATGCTCACAT
ACCATCTGGTACAGTTTTGGGCAATTTTTATTGCTTTGTAAATACTACTATTGGCAATGAAACTACGTCCGCTTTTGTG
GGTGCACTACCTAAGACAGTTCGTGAGTTTGTTATTTCACGCACAGGACATTTTTATATTAATGGCTATCGCTATTTCT
CTTTAGGTAATGTAGAAGCCGTTAATTTCAATGTCACTAATGCTGCAACCACTGTTTGTACTGTTGCGTTAGCTTCTTA
TGCTGACGTTTTGGTTAATGTGTCACAAACTGCTATTGCTAATATAATTTATTGCAATTCTGTTATTAACAGACTGAGA
TGTGACCAGTTGTCCTTTGATGTACCAGATGGTTTTTATTCCACAAGCCCTATTCAACCCGTTGAGCTACCTGTGTCTA
TTGTGTCGCTACCTGTTTATCACAAACACACGTTTATTGTGTTGTATGTTAACTTCGAACTTCGGCGTGGCCCTGGCAG
GTGCTATAACTGTCGTCCTGCTGTTATTAATATTACACTGGCCAATTTTAATGAAACTAAAGGGCCCTTGTGTGTTGAC
ACATCACACTTCACTACCCAATTCGTTGGTGTCAAGTTTGATAGGTGGAGTGCTAGTATTAACACGGGAAATTGCCCTT
TTTCTTTTGGCAAAGTTAATAACTTTGTTAAATTTGGCAGTGTATGTTTTTCGCTAAAGGATATACCCGGTGGTTGCGC
AATGCCTATAATGGCTAATTTGGTTAATCATAAATCTCATAATATAGGCTCATTGTATGTTTCTTGGAGTGATGGTGAT
GTAATTACTGGCGTCCCGAAACCTGTTGAGGGTGTTAGTTCCTTTATGAATGTTACATTGAATAAATGTACTAAATATA

ACATTTATGATGTATCTGGTGTGGGTGTTATTCGCATTAGCAATGACACCTTTCTTAATGGAATTACGTACACATCAAC
TTCAGGTAACCTTCTGGGTTTTAAAGATGTTACTAATGGCACCATCTACTCTATCACTCCTTGTAACCCACCAGATCAG
CTTGTTGTTTATCAGCAAGCTGTTGTTGGTGCTATGTTGTCTGAAAACTTTACTAGTTACGGCTTTTCTAATGTTGTAG
AAATGCCGAAATTTTTCTATGCGTCCAATGGCACTTATAATTGCACAGACGCTGTTTTAACTTATTCTAGTTTTGGCGT
TTGTGCAGATGGTTCTATAATTGCTGTTCAACCACGTAATGTTTCATATGATAGTGTTTCAGCTATTGTCACAGCTAAT
TTATCTATACCCTCCAATTGGACCACTTCGGTCCAGGTTGAGTATTTACAAATTACAAGTACACCTATCGTAGTTGATT
GCTCCACTTATGTTTGCAATGGTAATGTGCGCTGTGTTGAATTGCTTAAGCAGTATACTTCTGCTTGTAAAACTATTGA
AGACGCCTTAAGAAATAGCGCCATGCTGGAGTCTGCAGATGTTAGTGAGATGCTCACTTTTGACAAGAAAGCGTTTACA
CTTGCTAATGTTAGTAGTTTTGGTGACTACAACCTTAGCAGTGTTATACCTAGCTTGCCCAGAAGTGGTAGTAGAGTGG
CTGGTCGCAGTGCCATAGAAGACATACTTTTTAGCAAACTTGTTACGTCTGGACTTGGCACTGTGGACGCAGACTACAA
AAAGTGTACTAAGGGTCTTTCCATTGCTGACTTGGCTTGTGCCCAATATTATAATGGCATTATGGTTTTGCCTGGCGTC
GCTGATGCTGAACGTATGGCCATGTATACAGGTTCTTTAATTGGTGGAATTGCTTTAGGAGGTCTTACATCAGCCGCTT
CAATACCATTTTCATTAGCAATTCAGTCACGTTTAAATTATGTTGCATTGCAGACTGATGTTTTACAAGAAAATCAGAA
AATTCTTGCTGCATCTTTTAATAAAGCAATGACCAACATAGTAGATGCCTTTACTGGTGTTAATGATGCTATTACACAA
ACTTCACAAGCTCTACAAACAGTTGCTACTGCACTTAATAAGATCCAGGATGTTGTTAATCAACAAGGCAACTCATTGA
ACCATTTAACTTCTCAGTTGAGGCAGAATTTTCAAGCCATTTCTAGCTCTATTCAGGCTATCTATGACAGACTTGATAT
TATTCAGGCTGATCAACAAGTAGATAGGCTGATTACTGGTAGATTGGCTGCTCTGAATGTATTCGTTTCTCATACATTG
ACTAAGTACACTGAAGTTCGTGCTTCCAGACAGCTTGCACAACAAAAAGTGAATGAGTGTGTCAAATCCCAGTCTAAGC
GGTATGGCTTCTGTGGAAATGGCACGCACATTTTCTCACTTGTTAATGCTGCTCCTGAGGGGCTTGTTTTTCTCCATAC
TGTCTTGTTGCCGACACAATATAAGGATGTTGAAGCGTGGTCTGGGTTGTGCGTTGATGGTATAAACGGTTATGTGTTG
AGACAACCTAATCTTGCTCTTTACAAAGAAGGCAATTATTATAGAATTACATCTCGCATAATGTTTGAACCACGTATTC
CTACCATAGCAGATTTTGTCCAAATTGAAAATTGCAATGTCACATTTGTTAACATTTCTCGCTCTGAGTTGCAAACTAT
TGTGCCAGAGTATATTGATGTTAATAAGACGCTGCAAGAATTAAGTTACAAATTGCCAAATTACACTGTTCCAGACCTA
GTTGTCGAACAGTACAACCAGACTATTTTGAATTTGACCAGTGAAATTAGCACCCTTGAAAATAAATCTGCGGAGCTTA
ATTACACTGTTCAAAAACTGCAAACTTTGATCGACAACATAAATAGCACATTAGTTGACTTAAAGTGGCTCAACCGTGT
TGAGACTTATATTAAGTGGCCGTGGTGGGTGTGGTTGTGCATTTCAGTCGTGCTCATCTTTGTGGTGAGTATGTTGCTA
TTATGTTGTTGTTCTACTGGTTGCTGCGGCTTCTTTAGTTGTTTTGCATCTTCTATTAGAGGTTGTTGTGAATCAACTA
AACTTCCTTATTACGACGTTGAAAAGATCCACATACAGTAA

>gi|13604331|gb|AF344186.1| Human coronavirus 229E RW Stock spike glycoprotein (S) gene, complete cds

ATGTTTGTTTTGCTTGTTGCATATGCCTTGTTGCATATTGCTGGTTGTCAAACTACAAATGGGCTGAACACTAGT
TACTCTGTTTGCAACGGCTGTGTTGGTTATTCAGAAAATGTATTTGCTGTTGAGAGTGGTGGTTATATACCCTCCGACT
TTGCATTCAATAATTGGTTCCTTCTAACTAATACCTCATCTGTTGTAGATGGTGTTGTGAGGAGTTTTCAGCCTTTGTT
GCTTAATTGCTTATGGTCTGTTTCTGGCTTGCGGTTTACTACTGGTTTTGTCTATTTTAATGGTACTGGGAGAGGTGAT
TGTAAAGGTTTTTCCTCAGATGTTTTGTCTGATGTCATACGTTACAACCTCAATTTTGAAGAAAACCTTAGACGTGGAA
CCATTTTGTTTAAAACATCTTATGGTGTTGTTGTGTTTTATTGTACCAACAACACTTTAGTTTCAGGTGATGCTCACAT
ACCATTTGGTACAGTTTTGGGCAATTTTTATTGCTTTGTAAATACTACTATTGGCAATGAAACTACGTCTGCTTTTGTG
GGTGCACTACCTAAGACAGTTCGTGAGTTTGTTATTTCACGCACAGGACATTTTTATATTAATGGCTATCGCTATTTCA
CTTTAGGTAATGTAGAAGCCGTTAATTTCAATGTCACTACTGCAGAAACCACTGATTTTTTTACTGTTGCGTTAGCTTC
TTATGCTGACGTTTTGGTTAATGTGTCACAAACCTCTATTGCTAATATAATTTATTGCAACTCTGTTATTAACAGACTG
AGATGTGACCAGTTGTCCTTTGATGTACCAGATGGTTTTTATTCTACAAGCCCTATTCAATCCGTTGAGCTACCTGTGT

CTATTGTGTCGCTACCTGTTTATCATAAACATACGTTTATTGTGTTGTACGTTGACTTCAAACCTCAGAGTGGCGGTGG
CAAGTGCTTTAACTGTTATCCTGCTGGTGTTAATATTACACTGGCCAATTTTAATGAAACTAAAGGGCCTTTGTGTGTT
GACACATCACACTTCACTACCAAATACGTTGCTGTTTATGCCAATGTTGGTAGGTGGAGTGCTAGTATTAACACGGGAA
ATTGCCCTTTTTCTTTTGGCAAAGTTAATAACTTTGTTAAATTTGGCAGTGTATGTTTTTCGCTAAAGGATATACCCGG
TGGTTGCGCAATGCCTATAGTGGCTAATTGGGCTTATAGTAAGTACTATACTATAGGCTCATTGTATGTTTCTTGGAGT
GATGGTGATGGAATTACTGGCGTCCCACAACCTGTTGAGGGTGTTAGTTCCTTTATGAATGTTACATTGGACAAATGTA
CTAAATATAATATTTATGATGTATCTGGTGTGGGTGTTATTCGCGTTAGCAATGACACCTTTCTTAATGGAATTACGTA
CACATCAACTTCAGGTAACCTTCTGGGTTTTAAAGATGTTACTAAGGGCACCATCTACTCTATCACTCCTTGTAACCCA
CCAGATCAGCTTGTTGTTTATCAGCAAGCTGTTGTTGGTGCTATGTTGTCTGAAAATTTTACTAGTTACGGCTTTTCTA
ATGTTGTAGAACTGCCGAAATTTTTCTATGCGTCCAATGGCACTTATAATTGCACAGACGCTGTTTTAACTTATTCTAG
TTTTGGCGTTTGTGCAGATGGTTCTATAATTGCTGTTCAACCACGTAATGTTTCATATGATAGTGTTTCAGCTATCGTC
ACAGCTAATTTGTCTATACCTTCCAATTGGACCACTTCGGTCCAGGTTGAGTATTTACAAATTACAAGTACACCTATCG
TAGTTGATTGCTCCACTTATGTTTGCAATGGTAATGTGCGCTGTGTTGAATTGCTTAAGCAGTATACTTCTGCTTGTAA
AACTATTGAAGACGCCTTAAGAAATAGCGCCAGGCTGGAGTCTGCAGATGTTAGTGAGATGCTCACTTTTGACAAGAAA
GCGTTTACACTTGCTAATGTTAGTAGTTTTGGTGACTACAACCTTAGCAGCGTCATACCTAGCTTGCCCACAAGTGGTA
GTAGAGTGGCTGGTCGCAGTGCCATAGAAGACATACTTTTTAGCAAAATTGTTACTTCTGGACTTGGCACTGTGGACGC
AGACTACAAAAACTGCACTAAGGGTCTTTCCATTGCTGACTTGGCTTGTGCTCAATATTATAATGGCATTATGGTTTTG
CCTGGCGTCGCTGATGCTGAACGAATGGCCATGTATACAGGTTCTTTAATTGGTGGAATTGCTTTAGGAGGTCTAACAT
CAGCCGTTTCAATACCATTTTCATTAGCAATTCAGGCACGTTTAAATTATGTTGCATTGCAGACTGATGTTTTACAAGA
AAATCAGAAAATTCTTGCTGCATCTTTTAACAAAGCAATGACCAACATAGTAGATGCCTTTACTGGTGTTAATGATGCT
ATTACACAAACTTCACAAGCCCTACAAACAGTTGCTACTGCACTTAACAAGATCCAGGATGTTGTTAATCAACAAGGCA
ACTCATTGAACCATTTAACTTCTCAGTTGAGGCAGAATTTTCAAGCTATCTCTAGCTCTATTCAGGCTATCTATGACAG
ACTTGACACTATTCAGGCTGATCAACAAGTAGATAGGCTGATTACTGGTAGATTGGCTGCTTTGAATGTATTCGTTTCT
CATACATTGACTAAGTACACTGAAGTTCGTGCTTCCAGACAGCTTGCACAACAAAAAGTGAATGAGTGTGTCAAATCCC
AGTCTAAGCGTTATGGCTTCTGTGGAAATGGCACTCACATTTTCTCAATTGTTAATGCTGCTCCTGAGGGGCTTGTTTT
TCTCCACACTGTCTTGTTGCCGACACAATATAAGGATGTTGAAGCGTGGTCTGGGTTGTGCGTTGATGGTACAAACGGT
TATGTGTTGCGACAACCTAATCTTGCTCTTTACAAAGAAGGCAATTATTATAGAATCACATCTCGCATAATGTTTGAAC
CACGTATTCCTACCATGGCAGATTTTGTTCAAATTGAAAATTGCAATGTCACATTTGTTAACATTTCTCGCTCTGAGTT
GCAAACCATTGTGCCAGAGTATATTGATGTTAATAAGACGCTGCAAGAATTAAGTTACAAATTGCCAAATTACACTGTT
CCAGACCTAGTTGTCGAACAGTACAACCAGACTATTTTGAATTTGACCAGTGAAATTAGCACCCTTGAAAATAAATCTG
CGGAGCTTAATTACACTGTTCAAAAATTGCAAACTCTGATTGACAACATAAATAGCACATTAGTCGACTTAAAGTGGCT
CAACCGGGTTGAGACTTACATCAAGTGGCCGTGGTGGGTGTGGTTGTGCATTTCAGTCGTGCTCATCTTTGTGGTGAGT
ATGTTGCTATTATGTTGTTGTTCTACTGGTTGCTGTGGCTTCTTTAGTTGTTTTGCATCTTCTATTAGAGGTTGTTGTG
AATCAACTAAACTTCCTTATTACGACGTTGAAAAGATCCACATACAGTAA

>gi|13604333|gb|AF344187.1| Human coronavirus 229E P11A spike glycoprotein (S) gene, complete cds

ATGTTTGTTTTGCTTGTTGCATATGCCTTGTTGCATATTGCTGGTTGTCAAACTACAAATGGGCTGAACACTAGT
TACTCTGTTTGCAACGGCTGTGTTGGTTATTCAGAAAATGTATTTGCTGTTGAGAGTGGTGGTTATATACCCTCCGACT
TTGCATTCAATAATTGGTTCCTTCTAACTAATACCTCATCTGTTGTAGATGGTGTTGTGAGGAGTTTTCAGCCTTTGTT
GCTTAATTGCTTATGGTCTGTTTCTGGCTTGCGGTTTACTACTGGTTTTGTCTATTTTAATGGTACTGGGAGAGGTGAT
TGTAAAGGTTTTTCCTCAGATGTTTTGTCTGATGTCATACGTTACAACCTCAATTTTGAAGAAAACCTTAGACGTGGAA

CCATTTTGTTTAAAACATCTTATGGTGTTGTTGTGTTTTATTGTACCAACAACACTTTAGTTTCAGGTGATGCTCACAT
ACCATTTGGTACAGTTTTGGGCAATTTTTATTGCTTTGTAAATACTACTATTGGCAATGAAACTACGTCTGCTTTTGTG
GGTGCACTACCTAAGACAGTTCGTGAGTTTGTTATTTCACGCACAGGACATTTTTATATTAATGGCTATCGCTATTTCA
CTTTAGGTAATGTAGAAGCCGTTAATTTCAATGTCACTACTGCAGAAACCACTGATTTTTTTACTGTTGCGTTAGCTTC
TTATGCTGACGTTTTGGTTAATGTGTCACAAACCTCTATTGCTAATATAATTTATTGCAACTCTGTTATTAACAGACTG
AGATGTGACCAGTTGTCCTTTGATGTACCAGATGGTTTTTATTCTACAAGCCCTATTCAATCCGTTGAGCTACCTGTGT
CTATTGTGTCGCTACCTGTTTATCATAAACATACGTTTATTGTGTTGTACGTTGACTTCAAACCTCAGAGTGGCGGTGG
CAAGTGCTTTAACTGTTATCCTGCTGGTGTTAATATTACACTGGCCAATTTTAATGAAACTAAAGGGCCTTTGTGTGTT
GACACATCACACTTCACTACCAAATACGTTGCTGTTTATGCCAATGTTGGTAGGTGGAGTGCTAGTATTAACACGGGAA
ATTGCCCTTTTTCTTTTGGCAAAGTTAATAACTTTGTTAAATTTGGCAGTGTATGTTTTTCGCTAAAGGATATACCCGG
TGGTTGCGCAATGCCTATAGTGGCTAATTGGGCTTATAGTAAGTACTATACTATAGGCTCATTGTATGTTTCTTGGAGT
GATGGTGATGGAATTACTGGCGTCCCACAACCTGTTGAGGGTGTTAGTTCCTTTATGAATGTTACATTGGACAAATGTA
CTAAATATAATATTTATGATGTATCTGGTGTGGGTGTTATTCGCGTTAGCAATGACACCTTTCTTAATGGAATTACGTA
CACATCAACTTCAGGTAACCTTCTGGGTTTTAAAGATGTTACTAAGGGCACCATCTACTCTATCACTCCTTGTAACCCA
CCAGATCAGCTTGTTGTTTATCAGCAAGCTGTTGTTGGTGCTATGTTGTCTGAAAATTTTACTAGTTACGGCTTTTCTA
ATGTTGTAGAACTGCCGAAATTTTTCTATGCGTCCAATGGCACTTATAATTGCACAGACGCTGTTTTAACTTATTCTAG
TTTTGGCGTTTGTGCAGATGGTTCTATAATTGCTGTTCAACCACGTAATGTTTCATATGATAGTGTTTCAGCTACCGTC
ACAGCTAATTTGTCTATACCTTCCAATTGGACCACTTCGGTCCAGGTTGAGTATTTACAAATTACAAGTACACCTATCG
TAGTTGATTGCTCCACTTATGTTTGCAATGGTAATGTGCGCTGTGTTGAATTGCTTAAGCAGTATACTTCTGCTTGTAA
AACTATTGAAGACGCCTTAAGAAATAGCGCCAGGCTGGAGTCTGCAGATGTTAGTGAGATGCTCACTTTTGACAAGAAA
GCGTTTACACTTGCTAATGTTAGTAGTTTTGGTGACTACAACCTTAGCAGCGTCATACCTAGCTTGCCCACAAGTGGTA
GTAGAGTGGCTGGTCGCAGTGCCATAGAAGACATACTTTTTAGCAAAATTGTTACTTCTGGACTTGGCACTGTGGACGC
AGACTACAAAAACTGCACTAAGGGTCTTTCCATTGCTGACTTGGCTTGTGCTCAATATTATAATGGCATTATGGTTTTG
CCTGGCGTCGCTGATGCTGAACGAATGGCCATGTATACAGGTTCTTTAATTGGTGGAATTGCTTTAGGAGGTCTAACAT
CAGCCGTTTCAATACCATTTTCATTAGCAATTCAGGCACGTTTAAATTATGTTGCATTGCAGACTGATGTTTTACAAGA
AAATCAGAAAATTCTTGCTGCATCTTTTAACAAAGCAATGACCAACATAGTAGATGCCTTTACTGGTGTTAATGATGCT
ATTACACAAACTTCACAAGCCCTACAAACAGTTGCTACTGCACTTAACAAGATCCAGGATGTTGTTAATCAACAAGGCA
ACTCATTGAACTATTTAACTTCTCAGTTGAGGCAGAATTTTCAAGCTATCTCTAGCTCTATTCAGGCTATCTATGACAG
ACTTGACACTATTCAGGCTGATCAACAAGTAGATAGGCTGATTACTGGTAGATTGGCTGCTTTGAATGTATTCGTTTCT
CATACATTGACTAAGTACACTGAAGTTCGTGCTTCCAGACAGCTTGCACAACAAAAAGTGAATGAGTGTGTCAAATCCC
AGTCTAAGCGTTATGGCTTCTGTGGAAATGGCACTCACATTTTCTCAATTGTTAATGCTGCTCCTGAGGGGCTTGTTTT
TCTCCACACTGTCTTGTTGCCGACACAATATAAGGATGTTGAAGCGTGGTCTGGGTTGTGCGTTGATGGTACAAACGGT
TATGTGTTGCGACAACCTAATCTTGCTCTTTACAAAGAAGGCAATTATTATAGAATCACATCTCGCATAATGTTTGAAC
CACGTATTCCTACCATGGCAGATTTTGTTCAAATTGAAAATTGCAATGTCACATTTGTTAACATTTCTCGCTCTGAGTT
GCAAACCATTGTGCCAGAGTATATTGATGTTAATAAGACGCTGCAAGAATTAAGTTACAAATTGCCAAATTACACTGTT
CCAGACCTAGTTGTCGAACAGTACAACCAGACTATTTTGAATTTGACCAGTGAAATTAGCACCCTTGAAAATAAATCTG
CGGAGCTTAATTACACTGTTCAAAAATTGCAAACTCTGATTGACAACATAAATAGCACATTAGTCGACTTAAAGTGGCT
CAACCGGGTTGAGACTTACATCAAGTGGCCGTGGTGGGTGTGGTTGTGCATTTCAGTCGTGCTCATCTTTGTGGTGAGT
ATGTTGCTATTATGTTGTTGTTCTACTGGTTGCTGTGGCTTCTTTAGTTGTTTTGCATCTTCTATTAGAGGTTGTTGTG
AATCAACTAAACTTCCTTATTACGACGTTGAAAAGATCCACATACAGTAA

>gi|13604335|gb|AF344188.1| Human coronavirus 229E P11B spike glycoprotein (S) gene， complete cds

ATGTTTGTTTTGCTTGTTGCATATGCCTTGTTGCATATTGCTGGTTGTCAAACTACAAATGGGCTGAACACTAGT
TACTCTGTTTGCAACGGCTGTGTTGGTTATTCAGAAAATGTATTTGCTGTTGAGAGTGGTGGTTATATACCCTCCGACT
TTGCATTCAATAATTGGTTCCTTCTAACTAATACCTCATCTGTTGTAGATGGTGTTGTGAGGAGTTTTCAGCCTTTGTT
GCTTAATTGCTTATGGTCTGTTTCTGGCTTGCGGTTTACTACTGGTTTTGTCTATTTTAATGGTACTGGGAGAGGTGAT
TGTAAAGGTTTTTCCTCAGATGTTTTGTCTGATGTCATACGTTACAACCTCAATTTTGAAGAAAACCTTAGACGTGGAA
CCATTTTGTTTAAAACATCTTATGGTGTTGTTGTGTTTTATTGTACCAACAACACTTTAGTTTCAGGTGATGCTCACAT
ACCATTTGGTACAGTTTTGGGCAATTTTTATTGCTTTGTAAATACTACTATTGGCAATGAAACTACGTCTGCTTTTGTG
GGTGCACTACCTAAGACAGTTCGTGAGTTTGTTATTTCACGCACAGGACATTTTTATATTAATGGCTATCGCTATTTCA
CTTTAGGTAATGTAGAAGCCGTTAATTTCAATGTCACTACTGCAGAAACCACTGATTTTTTTACTGTTGCGTTAGCTTC
TTATGCTGACGTTTTGGTTAATGTGTCACAAACCTCTATTGCTAATATAATTTATTGCAACTCTGTTATTAACAGACTG
AGATGTGACCAGTTGTCCTTTGATGTACCAGATGGTTTTTATTCTACAAGCCCTATTCAATCCGTTGAGCTACCTGTGT
CTATTGTGTCGCTACCTGTTTATCATAAACATACGTTTATTGTGTTGTACGTTGACTTCAAACCTCAGAGTGGCGGTGG
CAAGTGCTTTAACTGTTATCCTGCTGGTGTTAATATTACACTGGCCAATTTTAATGAAACTAAAGGGCCTTTGTGTGTT
GACACATCACACTTCACTACCAAATACGTTGCTGTTTATGCCAATGTTGGTAGGTGGAGTGCTAGTATTAACACGGGAA
ATTGCCCTTTTTCTTTTGGCAAAGTTAATAACTTTGTTAAATTTGGCAGTGTATGTTTTTCGCTAAAGGATATACCCGG
TGGTTGCGCAATGCCTATAGTGGCTAATTGGGCTTATAGTAAGTACTATACTATAGGCTCATTGTATGTTTCTTGGAGT
GATGGTGATGGAATTACTGGCGTCCCACAACCTGTTGAGGGTGTTAGTTCCTTTATGAATGTTACATTGGACAAATGTA
CTAAATATAATATTTATGATGTATCTGGTGTGGGTGTTATTCGCGTTAGCAATGACACCTTTCTTAATGGAATTACGTA
CACATCAACTTCAGGTAACCTTCTGGGTTTTAAAGATGTTACTAAGGGCACCATCTACTCTATCACTCCTTGTAACCCA
CCAGATCAGCTTGTTGTTTATCAGCAAGCTGTTGTTGGTGCTATGTTGTCTGAAAATTTTACTAGTTACGGCTTTTCTA
ATGTTGTAGAACTGCCGAAATTTTTCTATGCGTCCAATGGCACTTATAATTGCACAGACGCTGTTTTAACTTATTCTAG
TTTTGGCGTTTGTGCAGATGGTTCTATAATTGCTGTTCAACCACGTAATGTTTCATATGATAGTGTTTCAGCTATCGGC
ACAGCTAATTTGTCTATACCTTCCAATTGGACCACTTCGGTCCAGGTTGAGTATTTACAAATTACAAGTACACCTATCG
TAGTTGATTGCTCCACTTATGTTTGCAATGGTAATGTGCGCTGTGTTGAATTGCTTAAGCAGTATACTTCTGCTTGTAA
AACTATTGAAGACGCCTTAAGAAATAGCGCCAGGCTGGAGTCTGCAGATGTTAGTGAGATGCTCACTTTTGACAAGAAA
GCGTTTACACTTGCTAATGTTAGTAGTTTTGGTGACTACAACCTTAGCAGCGTCATACCTAGCTTGCCCACAAGTGGTA
GTAGAGTGGCTGGTCGCAGTGCCATAGAAGACATACTTTTTAGCAAAATTGTTACTTCTGGACTTGGCACTGTGGACGC
AGACTACAAAAACTGCACTAAGGGTCTTTCCATTGCTGACTTGGCTTGTGCTCAATATTATAATGGCATTATGGTTTTG
CCTGGCGTCGCTGATGCTGAACGAATGGCCATGTATACAGGTTCTTTAATTGGTGGAATTGCTTTAGGAGGTCTAACAT
CAGCCGTTTCAATACCATTTTCATTAGCAATTCAGGCACGTTTAAATTATGTTGCATTGCAGACTGATGTTTTACAAGA
AAATCAGAAAATTCTTGCTGCATCTTTTAACAAAGCAATGACCAACATAGTAGATGCCTTTACTGGTGTTAATGATGCT
ATTACACAAACTTCACAAGCCCTACAAACAGTTGCTACTGCACTTAACAAGATCCAGGATGTTGTTAATCAACAAGGCA
ACTCATTGAACTATTTAACTTCTCAGTTGAGGCAGAATTTTCAAGCTATCTCTAGCTCTATTCAGGCTATCTATGACAG
ACTTGACACTATTCAGGCTGATCAACAAGTAGATAGGCTGATTACTGGTAGATTGGCTGCTTTGAATGTATTCGTTTCT
CATACATTGACTAAGTACACTGAAGTTCGTGCTTCCAGACAGCTTGCACAACAAAAAGTGAATGAGTGTGTCAAATCCC
AGTCTAAGCGTTATGGCTTCTGTGGAAATGGCACTCACATTTTCTCAATTGTTAATGCTGCTCCTGAGGGGCTTGTTTT
TCTCCACACTGTCTTGTTGCCGACACAATATAAGGATGTTGAAGCGTGGTCTGGGTTGTGCGTTGATGGTACAAACGGT
TATGTGTTGCGACAACCTAATCTTGCTCTTTACAAAGAAGGCAATTATTATAGAATCACATCTCGCATAATGTTTGAAC
CACGTATTCCTACCATGGCAGATTTTGTTCAAATTGAAAATTGCAATGTCACATTTGTTAACATTTCTCGCTCTGAGTT
GCAAACCATTGTGCCAGAGTATATTGATGTTAATAAGACGCTGCAAGAATTAAGTTACAAATTGCCAAATTACACTGTT
CCAGACCTAGTTGTCGAACAGTACAACCAGACTATTTTGAATTTGACCAGTGAAATTAGCACCCTTGAAAATAAATCTG
CGGAGCTTAATTACACTGTTCAAAAATTGCAAACTCTGATTGACAACATAAATAGCACATTAGTCGACTTAAAGTGGCT
CAACCGGGTTGAGACTTACATCAAGTGGCCGTGGTGGGTGTGGTTGTGCATTTCAGTCGTGCTCATCTTTGTGGTGAGT
ATGTTGCTATTATGTTGTTGTTCTACTGGTTGCTGTGGCTTCTTTAGTTGTTTTGCATCTTCTATTAGAGGTTGTTGTG

AATCAACTAAACTTCCTTATTACGACGTTGAAAAGATCCACATACAGTAA

>gi|13604337|gb|AF344189.1| Human coronavirus 229E P100E spike glycoprotein (S) gene, complete cds

ATGTTTGTTTTGCTTGTTGCATATGCCTTGTTGCATATTGCTGGTTGTCAAACTACAAATGGGCTGAACACTAGT
TACTCTGTTTGCAACGGCTGTGTTGGTTATTCAGAAAATGTATTTGCTGTTGAGAGTGGTGGTTATATACCCTCCGACT
TTGCATTCAATAATTGGTTCCTTCTAACTAATACCTCATCTGTTGTAGATGGTGTTGTGAGGAGTTTTCAGCCTTTGTT
GCTTAATTGCTTATGGTCTGTTTCTGGCTTGCGGTTTACTACTGGTTTTGTCTATTTTAATGGTACTGGGAGAGGTGAT
TGTAAAGGTTTTTCCTCAGATGTTTTGTCTGATGTCATACGTTACAACCTCAATTTTGAAGAAAACCTTAGACGTGGAA
CCATTTTGTTTAAAACATCTTATGGTGTTGTTGTGTTTTATTGTACCAACAACACTTTAGTTTCAGGTGATGCTCACAT
ACCATTTGGTACAGTTTTGGGCAATTTTTATTGCTTTGTAAATACTACTATTGGCACTGAAACTACGTCTGCTTTTGTG
GGTGCACTACCTAAGACAGTTCGTGAGTTTGTTATTTCACGCACAGGACATTTTTATATTAATGGCTATCGCTATTTCA
CTTTAGGTAATGTAGAAGCCGTTAATTTCAATGTCACTACTGCAGAAACCACTGATTTTTTTACTGTTGCGTTAGCTTC
TTATGCTGACGTTTTGGTTAATGTGTCACAAACCTCTATTGCTAATATAATTTATTGCAACTCTGTTATTAACAGACTG
AGATGTGACCAGTTGTCCTTTTATGTACCAGATGGTTTTTATTCTACAAGCCCTATTCAATCCGTTGAGCTACCTGTGT
CTATTGTGTCGCTACCTGTTTATCATAAACATATGTTTATTGTGTTGTACGTTGACTTCAAACCTCAGAGTGGCGGTGG
CAAGTGCTTTAACTGTTATCCTGCTGGTGTTAATATTACACTGGCCAATTTTAATGAAACTAAAGGGCCTTTGTGTGTT
GACACATCACACTTCACTACCAAATACGTTGCTGTTTATGCCAATGTTGGTAGGTGGAGTGCTAGTATTAACACGGGAA
ATTGCCCTTTTTCTTTTGGCAAAGTTAATAACTTTGTTAAATTTGGCAGTGTATGTTTTTCGCTAAAGGATATACCCGG
TGGTTGCGCAATGCCTATAGTGGCTAATTGGGCTTATAGTAAGTACTATACTATAGGCACATTGTATGTTTCTTGGAGT
GATGGTGATGGAATTACTGGCGTCCCACAACCTGTTGAGGGTGTTAGTTCCTTTATGAATGTTACATTGGACAAATGTA
CTAAATATAATATTTATGATGTATCTGGTGTGGGTGTTATTCGCGTTAGCAATGACACCTTTCTTAATGGAATTACGTA
CACATCAACTTCAGGTAACCTTCTGGGTTTTAAAGATGTTACTAAGGGCACCATCTACTCTATCACTCCTTGTAACCCA
CCAGATCAGCTTGTTGTTTATCAGCAAGCTGTTGTTGGTGCTATGTTGTCTGAAAATTTTACTAGTTACGGCTTTTCTA
ATGTTGTAGAACTGCCGAAATTTTTCTATGCGTCCAATGGCACTTATAATTGCACAGACGCTGTTTTAACTTATTCTAG
TTTTGGCGTTTGTGCAGATGGTTCTATAATTGCTGTTCAACCACGTAATGTTTCATATGATAGTGTTTCAGCTATCGTC
ACAGCTAATTTGTCTATACCTTCCAATTGGACCATTTCGGTCCAGGTTGAGTATTTACAAATTACAAGTACACCTATCG
TAGTTGATTGCTCCACTTATGTTTGCAATGGTAATGTGCGCTGTGTTGAATTGCTTAAGCAGTATACTTCTGCTTGTAA
AACTATTGAAGACGCCTTAAGAAATAGCGCCAGGCTGGAGTCTGCAGATGTTAGTGAGATGCTCACTTTTGACAAGAAA
GCGTTTACACTTGCTAATGTTAGTAGTTTTGGTGACTACAACCTTAGCAGCGTCATACCTAGCTTGCCCACAAGTGGTA
GTAGAGTGGCTGGTCGCAGTGCCATAGAAGACATACTTTTTAGCAAAATTGTTACTTCTGGACTTGGCACTGTGGACGC
AGACTACAAAAACTGCACTAAGGGTCTTTCCATTGCTGACTTGGCTTGTGCTCAATATTATAATGGCATTATGGTTTTG
CCTGGCGTCGCTGATGCTGAACGAATGGCCATGTATACAGGTTCTTTAATTGGTGGAATTGCTTTAGGAGGTCTAACAT
CAGCCGTTTCAATACCATTTTCATTAGCAATTCAGGCACGTTTAAATTATGTTGCATTGCAGACTGATGTTTTACAAGA
AAATCAGAAAATTCTTGCTGCATCTTTTAACAAAGCAATGACCAACATAGTAGATGCCTTTACTGGTGTTAATGATGCT
ATTACACAAACTTCACAAGCCCTACAAACAGTTGCTACTGCACTTAACAAGATCCAGGATGTTGTTAATCAACAAGGCA
ACTCATTGAACCATTTAACTTCTCAGTTGAGGCAGAATTTTCAAGCTATCTCTAGCTCTATTCAGGCTATCTATGACAG
ACTTGACACTATTCAGGCTGATCAACAAGTAGATAGGCTGATTACTGGTAGATTGGCTGCTTTGAATGTATTCGTTTCT
CATACATTGACTAAGTACACTGAAGTTCGTGCTTCCAGACAGCTTGCACAACAAAAAGTGAATGAGTGTGTCAAATCCC
AGTCTAAGCGTTATGGCTTCTGTGGAAATGGCACTCACATTTTCTCAATTGTTAATGCTGCTCCTGAGGGGCTTGTTTT
TCTCCACACTGTCTTGTTGCCGACACAATATAAGGATGTTGAAGCGTGGTCTGGGTTGTGCGTTGATGGTACAAACGGT
TATGTGTTGCGACAACCTAATCTTGCTCTTTACAAAGAAGGCAATTATTATAGAATCACATCTCGCATAATGTTTGAAC

CACGTATTCCTACCATGGCAGATTTTGTTCAAATTGAAAATTGCAATGTCACATTTGTTAACATTTCTCGCTCTGAGTT
GCAAACCATTGTGCCAGAGTATATTGATGTTAATAAGACGCTGCAAGAATTAAGTTACAAATTGCCAAATTACACTGTT
CCAGACCTAGTTGTCGAACAGTACAACCAGACTATTTTGAATTTGACCAGTGAAATTAGCACCCTTGAAAATAAATCTG
CGGAGCTTAATTACACTGTTCAAAAATTGCAAACTCTGATTGACAACATAAATAGCACATTAGTCGACTTAAAGTGGCT
CAACCGGGTTGAGACTTACATCAAGTGGCCGTGGTGGGTGTGGTTGTGCATTTCAGTCGTGCTCATCTTTGTGGTGAGT
ATGTTGCTATTATGTTGTTGTTCTACTGGTTGCTGTGGCTTCTTTAGTTGTTTTGCATCTTCTATTAGAGGTTGTTGTG
AATCAACTAAACTTCCTTATTACGACGTTGAAAAGATCCACATACAGTAA

>gi|404553537|gb|JX503061.1| Human coronavirus 229E isolate J0304, complete genome

ATGTTTGTTTTACTTGTTGCATATGCCTTGTTGCATATTGCTGGTTGTCAAACTACAAATGGGATGAACACTAGT
CACTCTGTTTGCAACGGCTGTGTTGGTCATTCGGAAAATGTATTTGCTGTTGAGAGTGGTGGTTATATACCCTCCAACT
TTGCATTTAATAATTGGTTCCTCTTAACTAATACCTCATCTGTTGTAGATGGTTTTGTGAGGAGTTTTCAGCCTTTGTT
GCTTAATTGCTTATGGTCTGTTTCTGGCTCGCGGCTTACTACTGGTTTTGTCTATTTTAATGGTACTGGGAGAGGTGAT
TGTAAAGGTTTTTATTCAAATGCTTCGTCTGATGTCATACGTTACAACATCAATTTTGAAGAAAACCTTAGACGTGGAA
CCATTTTGTTTAAAACATCTTATGGTGCTGTTGTGTTTTATTGTACCAACAACACTTTGGTTTCAGGTGATGCTCACAT
ACCATCTGGTACAGTTTTGGGCAATTTTTATTGCTTTGTAAATACTACTATTGGCAATGAAACTACGTCTGCTTTTGTG
GGTGCACTACCTAAGACAGTTCGTGAGTTTGTTATTTCACGCACAGGACATTTCTATATTAATGGCTATCGCTATTTCT
CTTTAGGTAATGTAGAAGCCGTTAATTTCAATGTCACTAATGCTGCAACCACTGTTTGTACTGTTGCGTTAGCTTCTTA
TGCTGACGTTTTGGTTAATGTGTCACAAACTGCTATTGCTAATATAATTTATTGCAATTCTGTTATTAACAGACTGAGA
TGTGACCAGTTGGCCTTTGATGTACCAGATGGTTTTTATTCCACAAGCCCTATTCAATCCGTTGAGCTACCTGTGTCTA
TTGTGTCGCTACCTGTTTATCACAAACACACGTTTATTGTGTTGTATGTTAACTTCGAACTTCGGCGTGGCCCTGGCAG
GTGCTATAACTGTCGTCCTGCTGTTGTTAATATTACACTGGCCAATTTTAATGAAACTAAAGGGCCCTTGTGTGTTGAC
ACATCACACTTCACTACCCAATTCGTTGGTGTCAAGTTTGATAGGTGGAGTGCTAGTATTAACACGGGAAATTGCCCTT
TTTCTTTTGGCAAAGTTAATAACTTTGTTAAATTTGGCAGTGTATGTTTTTCGCTAAAGGATATACCCGGTGGTTGCGC
AATGCCTATAATGGCTAATTTGGCTAATCTTAATTCTCATAATATAGGCTCATTGTATGTTTCTTGGAGTGATGGTGAT
GTAATTACTGGCGTCCCGAAACCTGTTGAGGGTGTTAGTTCCTTTATGAATGTTACATTGAATAAATGTACTAAATATA
ACATTTATGATGTATCTGGTGTGGGTGTTATTCGCATTAGCAATGACACCTTTCTTAACGGAATTACGTACACATCAAC
TTCAGGTAACCTTCTGGGTTTTAAAGATGTTACTAATGGCACCATCTACTCTATCACTCCTTGTAACCCACCAGATCAG
CTTGTTGTTTATCAGCAAGCTGTTGTTGGTGCTATGTTGTCTGAAAACTTTACTAGTTACGGCTTTTCTAATGTTGTAG
AAATGCCGAAATTTTTCTATGCGTCCAATGGCACTTATAATTGCACAGACGCTGTTTTAACTTATTCTAGTTTTGGCGT
TTGTGCAGATGGTTCTATAATTGCTGTTCAACCACGTAATGTTTCATATGATAGTGTTTCAGCTATTGTCACAGCTAAT
TTGTCTATACCCTCCAATTGGACCACTTCGGTCCAGGTTGAGTATTTACAAATTACAAGTACACCTATCGTAGTTGATT
GCTCCACTTATGTTTGCAATGGTAATGTGCGTTGTGTTGAATTGCTTAAGCAGTATACTTCTGCTTGTAAAACTATTGA
AGATGCCTTAAGAAATAGCGCCATGCTGGAGTCTGCAGATGTTAGTGAGATGCTCACTTTTGACAAGAAAGCGTTTACA
CTTGCTAATGTTAGTAGTTTTGGTGACTACAACCTTAGCAGTGTTATACCTAGCTTGCCCAGAAGTGGTAGTAGAGTGG
CTGGTCGCAGTGCCATAGAAGACATACTTTTTAGTAAACTTGTTACGTCTGGACTTGGCACTGTGGACGCAGACTACAA
AAAGTGTACTAAGGGTCTTTCTATTGCTGACTTGGCTTGTGCCCAATATTATAATGGCATTATGGTTTTGCCTGGCGTC
GCTGATGCTGAACGTATGGCCATGTATACAGGTTCTTTAATTGGTGGAATTGCTTTAGGAGGTCTTACATCAGCCGCTT
CAATACCATTTTCATTAGCAATCCAGTCACGTTTAAATTATGTTGCATTGCAGACTGATGTTTTACAAGAAAATCAGAA
AATTCTTGCTGCATCTTTTAATAAAGCAATGACCAACATAGTAGATGCCTTTACTGGTGTTAATGATGCTATTACACAA
ACTTCACAAGCTCTACAAACAGTTGCTACTGCACTTAATAAGATCCAGGATGTTGTTAATCAACAAGGCAACTCATTGA

ACCATTTAACTTCTCAGTTGAGGCAGAATTTTCAAGCCATTTCTAGCTCTATTCAGGCTATCTATGACAGACTTGATAT
TATTCAGGCTGATCAACAAGTAGATAGGCTGATTACTGGTAGATTGGCTGCTTTGAATGTATTCGTTTCTCATACATTG
ACTAAGTACACTGAAGTTCGTGCTTCCAGACAGCTTGCACAACAAAAAGTGAATGAGTGTGTCAAATCCCAGTCTAAGC
GGTATGGCTTCTGTGGAAATGGCACGCACATTTTCTCACTTGTTAATGCTGCTCCTGAGGGGCTTGTTTTTCTCCATAC
TGTCTTGTTGCCGACACAATATAAGGATGTTGAAGCGTGGTCTGGGTTGTGCGTTGATGGTAGAAACGGTTATGTGTTG
AGACAACCTAATCTTGCTCTTTACAAAGAAGGCAATTATTATAGAATTACATCTCGCATAATGTTTGAACCACGTATTC
CTACCATAGCAGATTTTGTCCAAATTGAAAATTGCAATGTCACATTTGTTAACATTTCTCGCTCTGAGTTGCAAACTAT
TGTGCCAGAGTATATTGATGTTAATAAGACGCTGCAAGAATTAAGTTACAAATTGCCAAATTACACTGTTCCAGACCTA
GTTGTCGAACAGTACAACCAGACTATTTTGAATTTGACCAGTGAAATTAGCACCCTTGAAAATAAATCTGCGGAGCTTA
ATTACACTGTTCAAAAACTGCAAACTTTGATCGACAACATAAATAGCACATTAGTTGACTTAAAGTGGCTCAACCGTGT
TGAGACTTACATCAAGTGGCCGTGGTGGGTGTGGTTGTGCATTTCAGTCGTGCTCATCTTTGTGGTGAGTATGTTGCTA
TTATGTTGTTGTTCTACTGGTTGCTGCGGTTTCTTTAGTTGTTTTGCATCTTCTATTAGAGGTTGTTGTGAATCAACTA
AACTTCCTTATTACGACGTTGAAAAGATCCACATACAGTAA

>gi|530341159|gb|KF514430.1| Human coronavirus 229E strain 229E/human/USA/933-50/1993, complete genome

ATGTTTGTTTTACTTGTTGCATATGCCTTGTTGCATATTGCTGGTTGTCAAACTACAAATGGGATGAACACTAGT
CACTCTGTTTGCAACGGCTGTGTTGGTCATTCAGAAAATGTATTTGCTGTTGAGAGTGGTGGTTATATACCCTCCGACT
TTGCATTTAATAATTGGTTCCTCTTAACTAATACCTCATCTGTTGTAGATGGTGTTGTGAGGAGTTTTCAGCCTTTGTT
GCTTAATTGCTTATGGTCTGTTTCTGGCTCGCAGTTTACTACTGGTTTTGTCTATTTTAATGGTACTGGGAGAGGTGAT
TGTAAAGGTTTTTATTCAAATGCTTCGTCTGATGTCATACGTTACAACATCAATTTTGAAGAAAACCTTAGACGTGGAA
CCATTTTGTTTAAAACATCTTATGGTGCTGTTGTGTTTTATTGTACCAACAACACTTTGGTTTCAGGTGATGCTCACAT
ACCATCTGGTACAGTTTTGGGCAATTTTTATTGCTTTGTAAATACTACTATTGGCAATGAAACTACGTCTGCTTTTGTG
GGTGCACTACCTAAGACAGTTCGTGAGTTTGTTATTTCACGCACAGGACATTTCTATATTAATGGCTATCGCTATTTTA
CTTTAGGTAATGTAGAAGCCGTTAATTTCAATGTCACTAATGCAGAAACCACTGTTTGTACTGTTGCGTTAGCTTCTTA
TGCTGACGTTTTGGTTAATGTGTCACAAACTGCTATTGCTAATATAATTTATTGCAACTCTGTTATTAACAGACTGAGA
TGTGACCAGTTGTCCTTTGATGTACCAGATGGTTTTTATTCCACAAGCCCTATTCAATCCGTTGAGCTACCTGTGTCTA
TTGTGTCGCTACCTGTTTATCACAAACACACGTTTATTGTGTTGTATGTTAACTTCAAACTTCGGAGTGGCGTTGGCAG
GTGCTATAACTGTCGTCCTGCTGTTGTTAATATTACACTGGCCAATTTTAATGAAACTAAAGGGCCTTTGTGTGTTGAC
ACATCACACTTCACTACCCAATTCGTTGGTGCCAAGTTTGATAGGTGGAGTGCTAGTATTAACACGGGAAATTGCCCTT
TTTCTTTTGGCAAAGTTAATAACTTTGTTAAATTTGGCAGTGTATGTTTTTCGCTAAAGGATATACCCGGTGGTTGCGC
AATGCCTATAATGGCTAATTTGGCTAATCTTAATTCTCATAATATAGGCTCATTGTATGTTTCTTGGAGTGATGGTGAT
GTAATTACTGGCGTCCCAAAACCTGTTGAGGGTGTTAGTTCCTTTATGAATGTTACATTGAATAAATGTACTAAATATA
ACATTTATGATGTATCTGGTGTGGGTGTTATTCGCATTAGCAATGACACCTTTCTTAATGGAATTACGTACACATCAAC
TTCAGGTAACCTTCTGGGTTTTAAAGATGTTACTAATGGCACCATCTACTCTATCACTCCTTGTAACCCACCAGATCAG
CTTGTTGTTTATCAGCAAGCTGTTGTTGGTGCTATGTTGTCTGAAAACTTTACTAGTTACGGCTTTTCTAATGTTGTAG
AAATGCCGAAATTTTTCTATGCGTCCAATGGCACTTATAATTGCACAGACGCTGTTTTAACTTATTCTAGTTTTGGCGT
TTGTGCAGATGGTTCTATAATTGCTGTTCAACCACGTAATGTTTCATATGATAGTGTTTCAGCTATCGTCACAGCTAAT
TTGTCTATACCCTCCAATTGGACCACTTCGGTCCAGGTTGAGTATTTACAAATTACAAGTACACCTATCGTAGTTGATT
GCTCCACTTATGTTTGCAATGGTAATGTGCGCTGTGTTGAATTGCTTAAGCAGTATACTTCTGCTTGTAAAACTATTGA
AGACGCCTTAAGAAATAGCGCCATGCTGGAGTCTGCAGATGTTAGTGAGATGCTCACTTTTGACAAGAAAGCGTTTACA
CTTGCTAATGTTAGTAGTTTTGGTGACTACAACCTTAGCAGTGTTATACCTAGCTTGCCCAGAAGTGGTAGTAGAGTGG

CTGGTCGCAGTGCCATAGAAGACATACTTTTTAGCAAACTTGTTACGTCTGGACTTGGCACTGTGGACGCAGACTACAA
AAAGTGCACTAAGGGTCTTTCCATTGCTGACTTGGCTTGTGCCCAATATTATAATGGCATTATGGTTTTGCCTGGCGTC
GCTGATGCTGAACGAATGGCCATGTATACAGGTTCTTTAATTGGTGGAATTGCGTTAGGAGGTCTTACATCAGCCGCTT
CAATACCATTCTCATTAGCAATTCAGTCACGTTTAAATTATGTTGCATTGCAGACTGATGTTTTACAAGAAAATCAGAA
AATTCTTGCTGCATCTTTTAATAAAGCAATGACCAACATAGTAGATGCCTTTACTGGTGTTAATGATGCTATTACACAA
ACTTCACAAGCTCTACAAACAGTTGCTACTGCACTTAATAAGATCCAGGATGTTGTTAATCAACAAGGCAACTCATTGA
ACCATTTAACTTCTCAGTTGAGGCAGAATTTTCAAGCCATCTCTAGCTCTATTCAGGCTATCTATGACAGACTTGATAT
TATTCAGGCTGATCAACAAGTAGATAGGCTGATTACTGGTAGATTGGCTGCTCTGAATGTATTCGTTTCTCATACATTG
ACTAAGTACACTGAAGTTCGTGCTTCCAGACAGCTTGCACAACAAAAAGTGAATGAGTGTGTCAAATCCCAGTCTAAGC
GGTATGGCTTCTGTGGAAATGGCACGCACATTTTCTCACTTGTTAATGCTGCTCCTGAGGGGCTTGTTTTTCTCCATAC
TGTCTTGTTGCCGACACAATATAAGGATGTTGAAGCGTGGTCTGGGTTGTGCGTTGATGGTAGAAACGGTTATGTGTTG
AGACAACCTAATCTTGCTCTTTACAAAGAAGGCAATTATTATAGAATTACATCTCGCATAATGTTTGAACCACGTATTC
CTACCATAGCAGATTTTGTCCAAATTGAAAATTGCAATGTCACATTTGTTAACATTTCTCGCTCTGAGTTGCAAACTAT
TGTGCCAGAGTATATTGATGTTAATAAGACGCTGCAAGAATTAAGTTACAAATTGCCAAATTACACTGTTCCAGACCTA
GTTGTCGAACAGTACAACCAGACTATTTTGAATTTGACCAGTGAAATTAGCACCCTTGAAAATAAATCTGCGGAGCTTA
ATTACACTGTTCAAAAACTGCAAACTTTGATTGACAACATAAATAGCACATTAGTTGACTTAAAGTGGCTCAACCGTGT
TGAGACTTATATCAAGTGGCCGTGGTGGGTGTGGTTGTGCATTTCAGTCGTGCTCATCTTTGTGGTGAGTATGTTGCTA
TTATGTTGTTGTTCTACTGGTTGCTGTGGCTTCTTTAGTTGTTTTGCATCTTCTATTAGAGGTTGTTGTGAATCAACTA
AACTTCCTTATTACGACGTTGAAAAGATCCACATACAGTAA

>gi|530341176|gb|KF514432.1| Human coronavirus 229E strain 229E/human/USA/932-72/1993, complete genome

ATGTTTGTTTTACTTGTTGCATATGCCTTGTTGCATATTGCTGGTTGTCAAACTACAAATGGGATGAACACTAGT
CACTCTGTTTGCAACGGCTGTGTTGGTCATTCAGAAAATGTATTTGCTGTTGAGAGTGGTGGTTATATACCCTCCGACT
TTGCATTTAATAATTGGTTCCTCTTAACTAATACCTCATCTGTTGTAGATGGTGTTGTGAGGAGTTTTCAGCCTTTGTT
GCTTAATTGCTTATGGTCTGTTTCTGGCTCGCGGTTTACTACTGGTTTTGTCTATTTTAATGGTACTGGGAGAGGTGAT
TGTAAAGGTTTTTATTCAAATGCTTCGTCTGATGTCATACGTTACAACATCAATTTTGAAGAAAACCTTAGACGTGGAA
CCATTTTGTTTAAAACATCTTATGGTGCTGTTGTGTTTTATTGTACCAACAACACTTTGGTTTCAGGTGATGCTCACAT
ACCATCTGGTACAGTTTTGGGCAATTTTTATTGCTTTGTAAATACTACTATTGGCAATGAAACTACGTCTGCTTTTGTG
GGTGCACTACCTAAGACAGTTCGTGAGTTTGTTATTTCACGCACAGGACATTTCTATATTAATGGCTATCGCTATTTCA
CTTTAGGTAATGTAGAAGCCGTTAATTTCAATGTCACTAATGCAGAAACCACTGTTTGTACTGTTGCGTTAGCTTCTTA
TGCTGACGTTTTGGTTAATGTGTCACAAACTGCTATTGCTAATATAATTTATTGCAACTCTGTTATTAACAGACTGAGA
TGTGACCAGTTGTCCTTTGATGTACCAGATGGTTTTTATTCCACAAGCCCTATTCAATCCGTTGAGCTACCTGTGTCTA
TTGTGTCACTACCTGTTTATCACAAACACACGTTTATTGTGTTGTATGTTAACTTCAAACTTCGGAGTGGCGTTGGCAG
GTGCTATAACTGTCGTCCTGCTGTTGTTAATATTACACTGGCCAATTTTAATGAAACTAAAGGGCCTTTGTGTGTTGAC
ACATCACACTTCACTACCCAATTCGTTGGTGCCAAGTTTGATAGGTGGAGTGCTAGTATTAACACGGGAAATTGCCCTT
TTTCTTTTGGCAAAGTTAATAACTTTGTTAAATTTGGCAGTGTATGTTTTTCGCTAAAGGATATACCCGGTGGTTGCGC
AATGCCTATAATGGCTAATTTGGCTAATCTTAATTCTCATACTATAGGCTCATTGTATGTTTCTTGGAGTGATGGTGAT
GTAATTACTGGCGTCCCAAAACCTGTTGAGGGTGTTAGTTCCTTTATGAATGTTACATTGAATAAATGTACTAAATATA
ACATTTATGATGTATCTGGTGTGGGTGTTATTCGCATTAGCAATGACACCTTTCTTAATGGAATTACGTACACATCAAC
TTCAGGTAACCTTCTGGGTTTTAAAGATGTTACTAATGGCACCATCTACTCTATCACTCCTTGTAACCCACCAGATCAG
CTTGTTGTTTATCAGCAAGCTGTTGTTGGTGCTATGTTGTCTGAAAACTTTACTAGTTACGGCTTTTCTAATGTTGTAG

AAATGCCGAAATTTTTCTATGCGTCCAATGGCACTTATAATTGCACAGACGCTGTTTTAACTTATTCTAGTTTTGGCGT
TTGTGCAGATGGTTCTATAATTGCTGTTCAACCACGTAATGTTTCATATGATAGTGTTTCAGCTATCGTCACAGCTAAT
TTGTCTATACCCTCCAATTGGACCACTTCGGTCCAGGTTGAGTATTTACAAATTACAAGTACACCTATCGTAGTTGATT
GCTCCACTTATGTTTGCAATGGTAATGTGCGCTGTGTTGAATTGCTTAAGCAGTATACTTCTGCTTGTAAAACTATTGA
AGATGCCTTAAGAAATAGCGCCATGCTGGAGTCTGCAGATGTTAGTGAGATGCTCACTTTTGACAAGAAAGCGTTTACA
CTTGCTAATGTTAGTAGTTTTGGTGACTACAACCTTAGCAGTGTTATACCTAGCTTGCCCAGAAGTGGTAGTAGAGTGG
CTGGTCGCAGTGCCATAGAAGACATACTTTTTAGCAAACTTGTTACGTCTGGACTTGGCACTGTGGACGCAGACTACAA
AAAGTGCACTAAGGGTCTTTCCATTGCTGACTTGGCTTGTGCCCAATATTATAATGGCATTATGGTTTTGCCTGGCGTC
GCTGATGCTGAACGAATGGCCATGTATACAGGTTCTTTAATTGGTGGAATTGCTTTAGGAGGTCTTACATCAGCCGCTT
CAATACCATTTTCATTAGCAATTCAGTCACGTTTAAATTATGTTGCATTGCAGACTGATGTTTTACAAGAAAATCAGAA
AATTCTTGCTGCATCTTTTAATAAAGCAATGACCAACATAGTAGATGCCTTTACTGGTGTTAATGATGCTATTACACAA
ACTTCACAAGCTCTACAAACAGTTGCTACTGCACTTAATAAGATCCAGGATGTTGTTAATCAACAAGGCAACTCATTGA
ACCATTTAACTTCTCAGTTGAGGCAGAATTTTCAAGCCATTTCTAGCTCTATTCAGGCTATCTATGACAGACTTGATAT
TATTCAGGCTGATCAACAAGTAGATAGGCTGATTACTGGTAGATTGGCTGCTCTGAATGTATTCGTTTCTCATACATTG
ACTAAGTACACTGAAGTTCGTGCTTCCAGACAGCTTGCACAACAAAAAGTGAATGAGTGTGTCAAATCCCAGTCTAAGC
GGTATGGCTTCTGTGGAAATGGCACGCACATTTTCTCACTTGTTAATGCTGCTCCTGAGGGGCTTGTTTTTCTCCATAC
TGTCTTGTTGCCGACACAATATAAGGATGTTGAAGCGTGGTCTGGGTTGTGCGTTGATGGTAGAAACGGTTATGTGTTG
AGACAACCTAATCTTGCTCTTTATAAAGAAGGCAATTATTATAGAATTACATCTCGCATAATGTTTGAACCACGTATTC
CTACCATAGCAGATTTTGTCCAAATTGAAAATTGCAATGTCACATTTGTTAACATTTCTCGCTCTGAGTTGCAAACTAT
TGTGCCAGAGTATATTGATGTTAATAAGACGCTGCAAGAATTAAGTTACAAATTGCCAAATTACACTGTTCCAGACCTA
GTTGTCGAACAGTACAACCAGACTATTTTGAATTTGACCAGTGAAATTAGCACCCTTGAAAATAAATCTGCGGAGCTTA
ATTACACTGTTCAAAAACTGCAAACTTTGATTGACAACATAAATAGCACATTAGTTGACTTAAAGTGGCTCAACCGGGT
TGAGACTTATATCAAGTGGCCGTGGTGGGTGTGGTTGTGCATTTCAGTCGTGCTCATCTTTGTGGTGAGTATGTTGCTA
TTATGTTGTTGTTCTACTGGTTGCTGTGGCTTCTTTAGTTGTTTTGCATCTTCTATTAGAGGTTGTTGTGAATCAACTA
AACTTCCTTATTACGACGTTGAAAAGATCCACATACAGTAA

>gi|530341184|gb|KF514433.1| Human coronavirus 229E strain 229E/human/USA/933-40/1993, complete genome

ATGTTTGTTTTACTTGTTGCATATGCCTTGTTGCATATTGCTGGTTGTCAAACTACAAATGGGATGAACACTAGT
CACTCTGTTTGCAACGGCTGTGTTGGTCATTCGGAAAATGTATTTGCTGTTGAGAGTGGTGGTTATATACCCTCCGACT
TTGCATTTAATAATTGGTTCCTCTTAACTAATACCTCATCTGTTGTAGATGGTGTTGTGAGGAGTTTTCAGCCTTTGTT
GCTTAATTGCTTATGGTCTGTTTCTGGCTCGCGGTTTACTACTGGTTTTGTCTATTTTAATGGTACTGGGAGAGGTGAT
TGTAAAGGTTTTTATTCAAATGCTTCGTCTGATGTCATACGTTACAACATCAATTTTGAAGAAAACCTTAGACGTGGAA
CCATTTTGTTTAAAACATCTTATGGTGCTGTTGTGTTTTATTGTACCAACAACACTTTGGTTTCAGGTGATGCTCACAT
ACCATCTGGTACAGTTTTGGGCAATTTTTATTGCTTTGTAAATACTACTATTGGCAATGAAACTACGTCTGCTTTTGTG
GGTGCACTACCTAAGACAGTTCGTGAGTTTGTTATTTCACGCACAGGACATTTCTATATTAATGGCTATCGCTATTTCT
CTTTAGGTAATGTAGAAGCCGTTAATTTCAATGTCACTAATGCAGAAACCACTGTTTGTACTGTTGCGTTAGCTTCTTA
TGCTGACGTTTTGGTTAATGTGTCACAAACTGCTATTGCTAATATAATTTATTGCAACTCTGTTATTAACAGACTGAGA
TGTGACCAGTTGTCCTTTGATGTACCAGATGGTTTTTATTCCACAAGCCCTATTCAATCCGTTGAGCTACCTGTGTCTA
TTGTGTCGCTACCTGTTTATCACAAACACACGTTTATTGTGTTGTATGTTAACTTCAAACTTCGGAGTGGCGTTGGCAG
GTGCTATAACTGTCGTCCTGCTGTTGTTAATATTACACTGGCCAATTTTAATGAAACTAAAGGGCCTTTGTGTGTTGAC
ACATCACACTTCACTACCCAATTCGTTGGTGCCAAGTTTGATAGGTGGAGTGCTAGTATTAACACGGGAAATTGCCCTT

TTTCTTTTGGCAAAGTTAATAACTTTGTTAAATTTGGCAGTGTATGTTTTTCGCTAAAGGATATACCCGGTGGTTGCGC
AATGCCTATAATGGCTAATTTGGCTAATCTTAATTCTCATAATATAGGCTCATTGTATGTTTCTTGGAGTGATGGTGAT
GTAATTACTGGCGTCCCAAAACCTGTTGAGGGTGTTAGTTCCTTTATGAATGTTACATTGAATAAATGTACTAAATATA
ACATTTATGATGTATCTGGTGTGGGTGTTATTCGCATTAGCAATGACACCTTTCTTAATGGAATTACGTACACATCAAC
CTCAGGTAACCTTCTGGGTTTTAAAGATGTTACTAATGGCACCATCTACTCTATCACTCCTTGTAACCCACCAGATCAG
CTTGTTGTTTATCAGCAAGCTGTTGTTGGTGCTATGTTGTCTGAAAACTTTACTAGTTACGGCTTTTCTAATGTTGTAG
AAATGCCGAAATTTTTCTATGCGTCCAATGGCACTTATAATTGCACAGACGCTGTTTTAACTTATTCTAGTTTTGGCGT
TTGTGCAGATGGTTCTATAATTGCTGTTCAACCACGTAATGTTTCATATGATAGTGTTTCAGCTATCGTCACAGCTAAT
TTGTCTATACCCTCCAATTGGACCACTTCGGTCCAGGTTGAGTATTTACAAATTACAAGTACACCTATCGTAGTTGATT
GCTCCACTTATGTTTGCAATGGTAATGTGCGCTGTGTTGAATTGCTTAAGCAGTATACTTCTGCTTGTAAAACTATTGA
AGACGCCTTAAGAAATAGCGCCATGCTGGAGTCTGCAGATGTTAGTGAGATGCTCACTTTTGACAAGAAAGCGTTTACA
CTTGCTAATGTTAGTAGTTTTGGTGACTACAACCTTAGCAGTGTTATACCTAGCTTGCCCAGAAGTGGTAGTAGAGTGG
CTGGTCGCAGTGCCATAGAAGACATACTTTTTAGCAAACTTGTTACGTCTGGACTTGGCACTGTGGACGCAGACTACAA
AAAGTGCACTAAGGGTCTTTCCATTGCTGACTTGGCTTGTGCCCAATATTATAATGGCATTATGGTTTTGCCTGGCGTC
GCTGATGCTGAACGAATGGCCATGTATACAGGTTCTTTAATTGGTGGAATTGCTTTAGGAGGTCTTACATCAGCCGCTT
CAATACCATTTTCATTAGCAATTCAGTCACGTTTAAATTATGTTGCATTGCAGACTGATGTTTTACAAGAAAATCAGAA
AATTCTTGCTGCATCTTTTAATAAAGCAATGACCAACATAGTAGATGCCTTTACTGGTGTTAATGATGCTATTACACAA
ACTTCACAAGCTCTACAAACAGTTGCTACTGCACTTAATAAGATCCAGGATGTTGTTAATCAACAAGGCAACTCATTGA
ACCATTTAACTTCTCAGTTGAGGCAGAATTTTCAAGCCATTTCTAGCTCTATTCAGGCTATCTATGACAGACTTGATAT
TATTCAGGCTGATCAACAAGTAGATAGGCTGATTACTGGTAGATTGGCTGCTCTGAATGTATTCGTTTCTCATACATTG
ACTAAGTACACTGAAGTTCGTGCTTCCAGACAGCTTGCACAACAAAAAGTGAATGAGTGTGTCAAATCCCAGTCTAAGC
GGTATGGCTTCTGTGGAAATGGCACGCACATTTTCTCACTTGTTAATGCTGCTCCTGAGGGGCTTGTTTTTCTCCATAC
TGTCTTGTTGCCGACACAATATAAGGATGTTGAAGCGTGGTCTGGGTTGTGCGTTGATGGTAGAAACGGTTATGTGTTG
AGACAACCTAATCTTGCTCTTTACAAAGAAGGCAATTATTATAGAATTACATCTCGCATAATGTTTGAACCACGTATTC
CTACCATAGCAGATTTTGTCCAAATTGAAAATTGCAATGTCACATTTGTTAACATTTCTCGCTCTGAGTTGCAAACTAT
TGTGCCAGAGTATATTGATGTTAATAAGACGCTGCAAGAATTAAGTTACAAATTGCCAAATTACACTGTTCCAGACCTA
GTTGTCGAACAGTACAACCAGACTATTTTGAATTTGACCAGTGAAATTAGCACCCTTGAAAATAAATCTGCGGAGCTTA
ATTACACTGTTCAAAAACTGCAAACTTTGATCGACAACATAAATAGCACATTAGTTGACTTAAAGTGGCTCAACCGGGT
TGAGACTTATATCAAGTGGCCGTGGTGGGTGTGGTTGTGCATTTCAGTCGTGCTCATCTTTGTGGTGAGTATGTTGCTA
TTATGTTGTTGTTCTACTGGTTGCTGTGGTTTCTTTAGTTGTTTTGCATCTTCTATTAGAGGTTGTTGTGAATCAACTA
AACTTCCTTATTACGACGTTGAAAAGATCCACATACAGTAA

>gi|12175745|ref|NC_002645.1| Human coronavirus 229E, complete genome

ATGTTTGTTTTGCTTGTTGCATATGCCTTGTTGCATATTGCTGGTTGTCAAACTACAAATGGGCTGAACACTAGT
TACTCTGTTTGCAACGGCTGTGTTGGTTATTCAGAAAATGTATTTGCTGTTGAGAGTGGTGGTTATATACCCTCCGACT
TTGCATTCAATAATTGGTTCCTTCTAACTAATACCTCATCTGTTGTAGATGGTGTTGTGAGGAGTTTTCAGCCTTTGTT
GCTTAATTGCTTATGGTCTGTTTCTGGCTTGCGGTTTACTACTGGTTTTGTCTATTTTAATGGTACTGGGAGAGGTGAT
TGTAAAGGTTTTTCCTCAGATGTTTTGTCTGATGTCATACGTTACAACCTCAATTTTGAAGAAAACCTTAGACGTGGAA
CCATTTTGTTTAAAACATCTTATGGTGTTGTTGTGTTTTATTGTACCAACAACACTTTAGTTTCAGGTGATGCTCACAT
ACCATTTGGTACAGTTTTGGGCAATTTTTATTGCTTTGTAAATACTACTATTGGCAATGAAACTACGTCTGCTTTTGTG
GGTGCACTACCTAAGACAGTTCGTGAGTTTGTTATTTCACGCACAGGACATTTTTATATTAATGGCTATCGCTATTTCA
CTTTAGGTAATGTAGAAGCCGTTAATTTCAATGTCACTACTGCAGAAACCACTGATTTTTGTACTGTTGCGTTAGCTTC

TTATGCTGACGTTTTGGTTAATGTGTCACAAACCTCTATTGCTAATATAATTTATTGCAACTCTGTTATTAACAGACTG
AGATGTGACCAGTTGTCCTTTGATGTACCAGATGGTTTTTATTCTACAAGCCCTATTCAATCCGTTGAGCTACCTGTGT
CTATTGTGTCGCTACCTGTTTATCATAAACATACGTTTATTGTGTTGTACGTTGACTTCAAACCTCAGAGTGGCGGTGG
CAAGTGCTTTAACTGTTATCCTGCTGGTGTTAATATTACACTGGCCAATTTTAATGAAACTAAAGGGCCTTTGTGTGTT
GACACATCACACTTCACTACCAAATACGTTGCTGTTTATGCCAATGTTGGTAGGTGGAGTGCTAGTATTAACACGGGAA
ATTGCCCTTTTTCTTTTGGCAAAGTTAATAACTTTGTTAAATTTGGCAGTGTATGTTTTTCGCTAAAGGATATACCCGG
TGGTTGCGCAATGCCTATAGTGGCTAATTGGGCTTATAGTAAGTACTATACTATAGGCTCATTGTATGTTTCTTGGAGT
GATGGTGATGGAATTACTGGCGTCCCACAACCTGTTGAGGGTGTTAGTTCCTTTATGAATGTTACATTGGACAAATGTA
CTAAATATAATATTTATGATGTATCTGGTGTGGGTGTTATTCGCGTTAGCAATGACACCTTTCTTAATGGAATTACGTA
CACATCAACTTCAGGTAACCTTCTGGGTTTTAAAGATGTTACTAAGGGCACCATCTACTCTATCACTCCTTGTAACCCA
CCAGATCAGCTTGTTGTTTATCAGCAAGCTGTTGTTGGTGCTATGTTGTCTGAAAATTTTACTAGTTACGGCTTTTCTA
ATGTTGTAGAACTGCCGAAATTTTTCTATGCGTCCAATGGCACTTATAATTGCACAGACGCTGTTTTAACTTATTCTAG
TTTTGGCGTTTGTGCAGATGGTTCTATAATTGCTGTTCAACCACGTAATGTTTCATATGATAGTGTTTCAGCTATCGTC
ACAGCTAATTTGTCTATACCTTCCAATTGGACCACTTCGGTCCAGGTTGAGTATTTACAAATTACAAGTACACCTATCG
TAGTTGATTGCTCCACTTATGTTTGCAATGGTAATGTGCGCTGTGTTGAATTGCTTAAGCAGTATACTTCTGCTTGTAA
AACTATTGAAGACGCCTTAAGAAATAGCGCCAGGCTGGAGTCTGCAGATGTTAGTGAGATGCTCACTTTTGACAAGAAA
GCGTTTACACTTGCTAATGTTAGTAGTTTTGGTGACTACAACCTTAGCAGCGTCATACCTAGCTTGCCCACAAGTGGTA
GTAGAGTGGCTGGTCGCAGTGCCATAGAAGACATACTTTTTAGCAAACTTGTTACTTCTGGACTTGGCACTGTGGACGC
AGACTACAAAAAGTGCACTAAGGGTCTTTCCATTGCTGACTTGGCTTGTGCTCAATATTATAATGGCATTATGGTTTTG
CCTGGCGTCGCTGATGCTGAACGAATGGCCATGTATACAGGTTCTTTAATTGGTGGAATTGCTTTAGGAGGTCTAACAT
CAGCCGTTTCAATACCATTTTCATTAGCAATTCAGGCACGTTTAAATTATGTTGCATTGCAGACTGATGTTTTACAAGA
AAATCAGAAAATTCTTGCTGCATCTTTTAACAAAGCAATGACCAACATAGTAGATGCCTTTACTGGTGTTAATGATGCT
ATTACACAAACTTCACAAGCCCTACAAACAGTTGCTACTGCACTTAACAAGATCCAGGATGTTGTTAATCAACAAGGCA
ACTCATTGAACCATTTAACTTCTCAGTTGAGGCAGAATTTTCAAGCTATCTCTAGCTCTATTCAGGCTATCTATGACAG
ACTTGACACTATTCAGGCTGATCAACAAGTAGATAGGCTGATTACTGGTAGATTGGCTGCTTTGAATGTATTCGTTTCT
CATACATTGACTAAGTACACTGAAGTTCGTGCTTCCAGACAGCTTGCACAACAAAAAGTGAATGAGTGTGTCAAATCCC
AGTCTAAGCGTTATGGCTTCTGTGGAAATGGCACTCACATTTTCTCAATTGTTAATGCTGCTCCTGAGGGGCTTGTTTT
TCTCCACACTGTCTTGTTGCCGACACAATATAAGGATGTTGAAGCGTGGTCTGGGTTGTGCGTTGATGGTACAAACGGT
TATGTGTTGCGACAACCTAATCTTGCTCTTTACAAAGAAGGCAATTATTATAGAATCACATCTCGCATAATGTTTGAAC
CACGTATTCCTACCATGGCAGATTTTGTTCAAATTGAAAATTGCAATGTCACATTTGTTAACATTTCTCGCTCTGAGTT
GCAAACCATTGTGCCAGAGTATATTGATGTTAATAAGACGCTGCAAGAATTAAGTTACAAATTGCCAAATTACACTGTT
CCAGACCTAGTTGTCGAACAGTACAACCAGACTATTTTGAATTTGACCAGTGAAATTAGCACCCTTGAAAATAAATCTG
CGGAGCTTAATTACACTGTTCAAAAATTGCAAACTCTGATTGACAACATAAATAGCACATTAGTCGACTTAAAGTGGCT
CAACCGGGTTGAGACTTACATCAAGTGGCCGTGGTGGGTGTGGTTGTGCATTTCAGTCGTGCTCATCTTTGTGGTGAGT
ATGTTGCTATTATGTTGTTGTTCTACTGGTTGCTGTGGCTTCTTTAGTTGTTTTGCATCTTCTATTAGAGGTTGTTGTG
AATCAACTAAACTTCCTTATTACGACGTTGAAAAGATCCACATACAGTAA

>gi|82780452|gb|DQ243963.1|_Human_coronavirus_229E_strain_ATCC_VR740_spike_glycoprotein_(S)_gene_complete_cds

ATGTTTGTTTTGCTTGTTGCATATGCCTTGTTGCATATTGCTGGTTGTCAAACTACAAATGGGCTGAACACTAGT
TACTCTGTTTGCAACGGCTGTGTTGGTTATTCAGAAAATGTATTTGCTGTTGAGAGTGGTGGTTATATACCCTCCGACT
TTGCATTCAATAATTGGTTCCTTCTAACTAATACCTCATCTGTTGTAGATGGTGTTGTGAGGAGTTTTCAGCCTTTGTT

GCTTAATTGCTTATGGTCTGTTTCTGGCTTGCGGTTTACTACTGGTTTTGTCTATTTTAATGGTACTGGGAGAGGTGAT
TGTAAAGGTTTTTCCTCAGATGTTTTGTCTGATGTCATACGTTACAACCTCAATTTTGAAGAAAACCTTAGACGTGGAA
CCATTTTGTTTAAAACATCTTATGGTGTTGTTGTGTTTTATTGTACCAACAACACTTTAGTTTCAGGTGATGCTCACAT
ACCATTTGGTACAGTTTTGGGCAATTTTTATTGCTTTGTAAATACTACTATTGGCAATGAAACTACGTCTGCTTTTGTG
GGTGCACTACCTAAGACAGTTCGTGAGTTTGTTATTTCACGCACAGGACATTTTTATATTAATGGCTATCGCTATTTCA
CTTTAGGTAATGTAGAAGCCGTTAATTTCAATGTCACTACTGCAGAAACCACTGATTTTTGTACTGTTGCGTTAGCTTC
TTATGCTGACGTTTTGGTTAATGTGTCACAAACCTCTATTGCTAATATAATTTATTGCAACTCTGTTATTAACAGACTG
AGATGTGACCAGTTGTCCTTTGATGTACCAGATGGTTTTTATTCTACAAGCCCTATTCAATCCGTTGAGCTACCTGTGT
CTATTGTGTCGCTACCTGTTTATCATAAACATACGTTTATTGTGTTGTACGTTGACTTCAAACCTCAGAGTGGCGGTGG
CAAGTGCTTTAACTGTTATCCTGCTGGTGTTAATATTACACTGGCCAATTTTAATGAAACTAAAGGGCCTTTGTGTGTT
GACACATCACACTTCACTACCAAATACGTTGCTGTTTATGCCAATGTTGGTAGGTGGAGTGCTAGTATTAACACGGGAA
ATTGCCCTTTTTCTTTTGGCAAAGTTAATAACTTTGTTAAATTTGGCAGTGTATGTTTTTCGCTAAAGGATATACCCGG
TGGTTGCGCAATGCCTATAGTGGCTAATTGGGCTTATAGTAAGTACTATACTATAGGCTCATTGTATGTTTCTTGGAGT
GATGGTGATGGAATTACTGGCGTCCCACAACCTGTTGAGGGTGTTAGTTCCTTTATGAATGTTACATTGGACAAATGTA
CTAAATATAATATTTATGATGTATCTGGTGTGGGTGTTATTCGCGTTAGCAATGACACCTTTCTTAATGGAATTACGTA
CACATCAACTTCAGGTAACCTTCTGGGTTTTAAAGATGTTACTAAGGGCACCATCTACTCTATCACTCCTTGTAACCCA
CCAGATCAGCTTGTTGTTTATCAGCAAGCTGTTGTTGGTGCTATGTTGTCTGAAAATTTTACTAGTTACGGCTTTTCTA
ATGTTGTAGAACTGCCGAAATTTTTCTATGCGTCCAATGGCACTTATAATTGCACAGACGCTGTTTTAACTTATTCTAG
TTTTGGCGTTTGTGCAGATGGTTCTATAATTGCTGTTCAACCACGTAATGTTTCATATGATAGTGTTTCAGCTATCGTC
ACAGCTAATTTGTCTATACCTTCCAATTGGACCACTTCGGTCCAGGTTGAGTATTTACAAATTACAAGTACACCTATCG
TAGTTGATTGCTCCACTTATGTTTGCAATGGTAATGTGCGCTGTGTTGAATTGCTTAAGCAGTATACTTCTGCTTGTAA
AACTATTGAAGACGCCTTAAGAAATAGCGCCAGGCTGGAGTCTGCAGATGTTAGTGAGATGCTCACTTTTGACAAGAAA
GCGTTTACACTTGCTAATGTTAGTAGTTTTGGTGACTACAACCTTAGCAGCGTCATACCTAGCTTGCCCACAAGTGGTA
GTAGAGTGGCTGGTCGCAGTGCCATAGAAGACATACTTTTTAGCAAACTTGTTACTTCTGGACTTGGCACTGTGGACGC
AGACTACAAAAACTGCACTAAGGGTCTTTCCATTGCTGACTTGGCTTGTGCTCAATATTATAATGGCATTATGGTTTTG
CCTGGCGTCGCTGATGCTGAACGAATGGCCATGTATACAGGTTCTTTAATTGGTGGAATTGCTTTAGGAGGTCTAACAT
CAGCCGTTTCAATACCATTTTCATTAGCAATTCAGGCACGTTTAAATTATGTTGCATTGCAGACTGATGTTTTACAAGA
AAATCAGAAAATTCTTGCTGCATCTTTTAACAAAGCAATGACCAACATAGTAGATGCCTTTACTGGTGTTAATGATGCT
ATTACACAAACTTCACAAGCCCTACAAACAGTTGCTACTGCACTTAACAAGATCCAGGATGTTGTTAATCAACAAGGCA
ACTCATTGAACCATTTAACTTCTCAGTTGAGGCAGAATTTTCAAGCTATCTCTAGCTCTATTCAGGCTATCTATGACAG
ACTTGACACTATTCAGGCTGATCAACAAGTAGATAGGCTGATTACTGGTAGATTGGCTGCTTTGAATGTATTCGTTTCT
CATACATTGACTAAGTACACTGAAGTTCGTGCTTCCAGACAGCTTGCACAACAAAAAGTGAATGAGTGTGTCAAATCCC
AGTCTAAGCGTTATGGCTTCTGTGGAAATGGCACTCACATTTTCTCAATTGTTAATGCTGCTCCTGAGGGGCTTGTTTT
TCTCCACACTGTCTTGTTGCCGACACAATATAAGGATGTTGAAGCGTGGTCTGGGTTGTGCGTTGATGGTACAAACGGT
TATGTGTTGCGACAACCTAATCTTGCTCTTTACAAAGAAGGCAATTATTATAGAATCACATCTCGCATAATGTTTGAAC
CACGTATTCCTACCATGGCAGATTTTGTTCAAATTGAAAATTGCAATGTCACATTTGTTAACATTTCTCGCTCTGAGTT
GCAAACCATTGTGCCAGAGTATATTGATGTTAATAAGACGCTGCAAGAATTAAGTTACAAATTGCCAAATTACACTGTT
CCAGACCTAGTTGTCGAACAGTACAACCAGACTATTTTGAATTTGACCAGTGAAATTAGCACCCTTGAAAATAAATCTG
CGGAGCTTAATTACACTGTTCAAAAATTGCAAACTCTGATTGACAACATAAATAGCACATTAGTCGACTTAAAGTGGCT
CAACCGGGTTGAGACTTACATCAAGTGGCCGTGGTGGGTGTGGTTGTGCATTTCAGTCGTGCTCATCTTTGTGGTGAGT
ATGTTGCTATTATGTTGTTGTTCTACTGGTTGCTGTGGCTTCTTTAGTTGTTTTGCATCTTCTATTAGAGGTTGTTGTG
AATCAACTAAACTTCCTTATTACGACGTTGAAAAGATCCACATACAGTAA

>gi|82780454|gb|DQ243964.1|_Human_coronavirus_229E_isolate_HCoV229E11/6/79_spike_glycoprotein_(S)_gene_complete_cds

ATGTTTGTTTTACTTGTTGCATATGCCTTGTTGCATATTGCTGGTTGTCAAACTACAAATGGGCTGAACACTAGT
CACTCTGTTTGCAACGGCTGTGTTGGTTATTCAGAAAATGTATTTGCTGTTGAGAGTGGTGGTTATATACCCTCCGACT
TTGCATTTAATAATTGGTTCCTTCTAACTAATACCTCATCTGTTGTAGACGGTGTTGTGAGGAGTTTTCAGCCTTTGTT
GCTTAATTGCTTATGGTCTGTTTCTGGCTCGCGGTTTACTACTGGTTTTGTCTATTTTAATGGTACTGGGAGAGGTGAT
TGTAAAGGTTTTTCTTCAAACGTTTCGTCTGATGTCATACGTTACAACATCAATTTTGAAGAAAACCTTAGACGTGGAA
CCATTTTGTTTAAAACATCTTATGGTGTTGTTGTGTTTTATTGTACCAACAACACTTTAGTTTCAGGTGATGCTCACAT
ACCATTTGGTACAGTTTTGGGCAATTTTTATTGCTTTGTAAATACTACTATTGGCAATGAAACTACGTCTTCTTTTGTG
GGTGCACTACCTAAGACAGTTCGTGAGTTTGTTATTTCACGCACAGGACATTTCTATATTAATGGCTATCGCTATTTCA
CTTTAGGTAATGTAGAAGCCGTTAATTTCAATGTCACTAATGCAGAAACCACTGTTTGTACTGTTGCGTTAGCTTCTTA
TGCTGACGTTTTGGTTAATGTGTCACAAACCGCTATTGCTAATATAATTTATTGCAACTCTGTTATTAACAGACTGAGA
TGTGACCAGTTGTCCTTTGATGTACCAGATGGTTTTTATTCCACAAGCCCTATTCAATCCGTTGAGCTACCTGTGTCTA
TTGTGTCGCTACCTGTTTATCACAAACATACGTTTATTGTGTTGTACGTTGACTTCAAACCTCAGAGTGGCGGTGGCAA
GTGCTATAACTGTCGTCCTGCTGTTGTTAATATTACACTTGCCAATTTTAATGAAACTAAAGGGCCTTTGTGTGTTGAA
ACATCACACTTCACTACCAAATACGTTGCTGCCAATGTTGGTAGGTGGAGTGCTAGTATTAACACGGGAAATTGCCCTT
TTTCTTTTGGCAAAGTTAATAACTTTGTTAAATTTGGCAGTGTATGTTTTTCGCTAAAGGATATACCCGGTGGTTGCGC
AATGCCTATAGTGGCTAATTTGGCTTATATTAATTCTTATACTATAGGCTCATTGTATGTTTCTTGGAGTGATGGTGAT
GGAATTACTGGCGTCCCAAAACCTGTTGAGGGTGTTAGTTCCTTTATGAATGTTACATTGAACAAATGTACTAAATATA
ACATTTATGATGTATCTGGTGTGGGTGTTATTCGCATTAGCAATGACACCTTTCTTAATGGAATTACGTACACATCAAC
TTCAGGTAACCTTCTGGGTTTTAAAGATGTTACTAATGGCACCATCTACTCTATCACTCCTTGTAACCCACCAGATCAG
CTTGTTGTTTATCAGCAAGCTGTTGTTGGTGCTATGTTGTCTGAAAATTTTACTAGTTACGGCTTTTCTAATGTTGTAG
AACTGCCGAAATTTTTCTATGCGTCCAATGGCACTTATAATTGCACAGACGCTGTTTTAACTTATTCTAGTTTTGGCGT
TTGTGCAGATGGTTCTATAATTGCTGTTCAACCACGTAATGTTTCATATGATAGTGTTTCAGCTATCGTCACAGCTAAT
TTGTCTATACCTTCCAATTGGACCACTTCGGTCCAGGTTGAGTATTTACAAATTACAAGTACACCTATCGTTGTTGATT
GCTCCACTTATGTTTGCAATGGTAATGTGCGCTGTGTTGAATTGCTTAAGCAGTATACTTCTGCTTGTAAAACTATTGA
AGACGCCTTAAGAAATAGCGCCATGCTGGAGTCTGCAGATGTTAGTGAGATGCTCACTTTTGACAAGAAAGCGTTTACA
CTTGCTAATGTTAGTAGTTTTGGTGACTACAACCTTAGCAGCGTTATACCTAGCTTGCCCACAAGTGGTAGTAGAGTGG
CTGGTCGCAGTGCCATAGAAGACATACTTTTTAGCAAACTTGTTACGTCTGGACTTGGCACTGTGGACGCAGACTACAA
AAAGTGCACTAAGGGTCTTTCCATTGCTGACTTGGCTTGTGCCCAATATTATAATGGCATTATGGTTTTGCCTGGCGTC
GCTGATGCTGAACGAATGGCCATGTATACAGGTTCTTTAATTGGTGGAATTGCTTTAGGAGGTCTTACATCAGCCGCTT
CAATACCATTTTCATTAGCAATTCAGGCACGTTTAAATTATGTTGCATTGCAGACTGATGTTTTACAAGAAAATCAGAA
AATTCTTGCTGCATCTTTTAATAAAGCAATGACCAACATAGTAGATGCCTTTACTGGTGTTAATGATGCTATTACACAA
ACTTCACAAGCTCTACAAACAGTTGCTACTGCACTTAACAAGATCCAGGATGTTGTTAATCAACAAGGCAACTCATTGA
ACCATTTAACTTCTCAGTTGAGGCAGAATTTTCAAGCCATCTCTAGCTCTATTCAGGCTATCTATGACAGACTTGATAT
TATTCAGGCTGATCAACAAGTAGATAGGCTGATTACTGGTAGATTGGCTGCTTTGAATGTATTCGTTTCTCATACATTG
ACTAAGTACACTGAAGTTCGTGCTTCCAGACAGCTTGCACAACAAAAAGTGAATGAGTGTGTCAAATCCCAGTCTAAGC
GCTATGGCTTCTGTGGAAATGGCACGCACATTTTCTCACTTGTTAATGCTGCTCCTGAGGGGCTTGTTTTTCTCCATAC
TGTCTTGTTGCCGACACAATATAAGGATGTTGAAGCGTGGTCTGGGTTGTGCGTTGATGGTAGAAACGGTTATGTGTTG
CGACAACCTAATCTTGCTCTTTACAAAGAAGGCAATTATTATAGAATTACATCTCGCATAATGTTTGAACCACGTATTC
CTACTATGGCAGATTTTGTCCAAATTGAAAATTGCAATGTCACATTTGTTAACATTTCTCGCTCTGAGTTGCAAACCAT
TGTGCCAGAGTATATTGATGTTAATAAGACGCTGCAAGAATTAAGTTACAAATTGCCAAATTACACTGTTCCAGACCTA
GTTGTCGAACAGTACAACCAGACTATTTTGAATTTGACCAGTGAAATTAGCACCCTTGAAAATAAATCTGCGGAGCTTA

ATTACACTGTTCAAAAACTGCAAACTCTGATTGACAACATAAATAGCACATTAGTTGACTTAAAGTGGCTCAACCGGGT
TGAGACTTATATCAAGTGGCCGTGGTGGGTGTGGTTGTGCATTTCAGTCGTGCTCATCTTTGTGGTGAGTATGTTGCTA
TTATGTTGTTGTTCTACTGGTTGCTGTGGCTTCTTTAGTTGTTTTGCATCTTCTATTAGAGGTTGTTGTGAATCAACTA
AACTTCCTTATTACGACGTTGAAAAGATCCACATACAGTAA

>gi|82780456|gb|DQ243965.1|_Human_coronavirus_229E_isolate_HCoV229E16/6/82_spike_glycoprotein_(S)_gene_complete_cds

ATGTTTGTTTTACTTGTTGCATATGCCTTGTTGCATATTGCTGGTTGTCAAACTACAAATGGGCTGAACACTAGT
CACTCTGTTTGCAACGGCTGTGTTGGTCATTCAGAAAATGTATTTGCTGTTGAGAGTGGTGGTTATATACCCTCCGACT
TTGCATTTAATAATTGGTTCCTTCTAACTAATACCTCATCTGTTGTAGACGGTGTTGTGAGGAGTTTTCAGCCTTTGTT
GCTTAATTGCTTATGGTCTGTTTCTGGCTCGCGGTTTACTACTGGTTTTGTCTATTTTAATGGTACTGGGAGAGGTGAT
TGTAAAGGTTTTTCTTCAAACGTTTCGTCTGATGTCATACGTTACAACATCAATTTTGAAGAAAACCTTAGACGTGGAA
CCATTTTGTTTAAAACATCTTATGGTGTTGTTGTGTTTTATTGTACCAACAACACTTTAGTTTCAGGTGATGCTCACAT
ACCATTTGGTACAGTTTTGGGCAATTTTTATTGCTTTGTAAATACTACTATTGGCAATGAAACTACGTCTTCTTTTGTG
GGTGCACTACCTAAGACAGTTCGTGAGTTTGTTATTGCACGCACAGGACATTTCTATATTAATGGCTATCGCTATTTCA
CTTTAGGTAATGTAGAAGCCGTTAATTTCAATGTCACTAATGCAGAAACCACTGTTTGTACTGTTGCGTTAGCTTCTTA
TGCTGACGTTTTGGTTAATGTGTCACAAACTGCTATTGCTAATATAATTTATTGCAACTCTGTTATTAACAGACTGAGA
TGTGACCAGTTGTCCTTTGATGTACCAGATGGTTTTTATTCCACAAGCCCTATTCAATCCGTTGAGCTACCTGTGTCTA
TTGTGTCGCTACCTGTTTATCATAAACATACGTTTATTGTGTTGTACGTTGACTTCAAACCTCAGAGTGGCGGTGGCAG
GTGCTATAACTGTCGTCCTGCTGTTGTTAATATTACACTGGCCAATTTTAATGAAACTAAAGGGCCTTTGTGTGTTGAA
ACATCACACTTCACTACCAAATACGTTGCTGCCAATGTTGGTAGGTGGAGTGCTAGTATTAACACTGGAAATTGCCCTT
TTTCTTTTGGCAAAGTTAATAACTTTGTTAAATTTGGCAGTGTATGTTTTTCGCTAAAGGATATACCCGGTGGTTGCGC
AATGCCTATAGTGGCTAATTTGGCTTATATTAATTCTTATACTATAGGCTCATTGTATGTTTCTTGGAGTGATGGTGAT
GGAATTACTGGCGTCCCAAAACCTGTTGAGGGTGTTAGTTCCTTTATGAATGTTACATTGAACAAATGTACTAAATATA
ACATTTATGATGTATCTGGTGTGGGTGTTATTCGCATTAGCAATGACACCTTTCTTAATGGAATTACGTACACATCAAC
TTCAGGTAACCTTCTGGGTTTTAAAGATGTTACTAATGGCACCATCTACTCTATCACTCCTTGTAACCCACCAGATCAG
CTTGTTGTTTATCAGCAAGCTGTTGTTGGTGCTATGTTGTCTGAAAATTTTACTAGTTACGGCTTTTCTAATGTTGTAG
AACTGCCGAAATTTTTCTATGCGTCCAATGGCACTTATAATTGCACAGACGCTGTTTTAACTTATTCTAGTTTTGGCGT
TTGTGCAGATGGTTCTATAATTGCTGTTCAACCACGTAATGTTTCATATGATAGTGTTTCAGCTATCGTCACAGCTAAT
TTGTCTATACCTTCCAATTGGACCACTTCGGTCCAGGTTGAGTATTTACAAATTACAAGTACACCTATCGTAGTTGATT
GCTCCACTTATGTTTGCAATGGTAATGTGCGCTGTGTTGAATTGCTTAAGCAGTATACTTCTGCTTGTAAAACTATTGA
AGACGCCTTAAGAAATAGCGCCATGCTGGAGTCTGCAGATGTTAGTGAGATGCTCACTTTTGACAAGAAAGCGTTTACA
CTTGCTAATGTTAGTAGTTTTGGTGAATACAACCTCAGCAGCGTTGTACCTAGCTTGCCCACAAGTGGTAGTAGAGTGG
CTGGTCGCAGTGCCATAGAAGACATACTTTTTAGCAAACTTGTTACGTCTGGACTTGGCACTGTGGATGCAGACTACAA
AAAGTGCACTAAGGGTCTTTCCATTGCTGACTTGGCTTGTGCTCAATATTATAATGGCATTATGGTTTTGCCTGGCGTC
GCTGATGCTGAACGAATGGCCATGTATACAGGTTCTTTAATTGGTGGAATTGCTTTAGGAGGTCTTACATCAGCCGCTT
CAATACCATTTTCATTAGCAATTCAGGCACGTTTAAATTATGTTGCATTGCAGACTGATGTTTTACAAGAAAATCAGAA
AATTCTTGCTGCATCTTTTAATAAAGCAATGACCAACATAGTAGATGCCTTTACTGGTGTTAATGATGCTATTACACAA
ACTTCACAAGCTCTACAAACAGTTGCTACTGCACTTAACAAGATCCAGGATGTTGTTAATCAACAAGGCAACTCATTGA
ACCATTTAACTTCTCAGTTGAGGCAGAATTTTCAAGCCATCTCTAGCTCTATTCAGGCTATCTATGACAGACTTGATAT
TATTCAGGCTGATCAACAAGTAGATAGGCTGATTACTGGTAGATTGGCTGCTTTGAATGTATTCGTTTCTCATACATTG
ACTAAGTACACTGAAGTTCGTGCTTCCAGACAGCTTGCACAACAAAAAGTGAATGAGTGTGTCAAATCCCAGTCTAAGC

GCTATGGCTTCTGTGGAAATGGCACGCACATTTTCTCACTTGTTAATGCTGCTCCTGAGGGGCTTGTTTTCCTCCATAC
TGTCTTGTTGCCGACACAATATAAGGATGTTGAAGCGTGGTCTGGGTTGTGCGTTGATGGTAGAAACGGTTATGTGTTG
CGACAACCTAATCTTGCTCTTTACAAAGAAGGCAATTATTATAGAATTACATCTCGCATAATGTTTGAACCACGTGTTC
CTACCATGGCAGATTTTGTCCAAATTGAAAATTGCAATGTCACATTTGTTAACATTTCTCGCTCTGAGTTGCAAACCAT
TGTGCCAGAGTATATTGATGTTAATAAGACGCTGCAAGAATTAAGTTACAAATTGCCAAATTACACTGTTCCAGACCTA
GTTGTCGAACAGTACAATCAGACTATTTTGAATTTGACCAGTGAAATTAGCACCCTTGAAAATAAATCTGCGGAGCTTA
ATTACACTGTTCAAAAACTGCAAACTCTGATTGACAACATAAATAGCACATTAGTTGACTTAAAGTGGCTCAACCGGGT
TGAGACTTATATCAAGTGGCCGTGGTGGGTGTGGTTGTGCATTTCAGTCGTGCTCATCTTTGTGGTGAGTATGTTGCTA
TTATGTTGTTGTTCTACTGGTTGCTGTGGCTTCTTTAGTTGTTTTGCATCTTCTATTAGAGGTTGTTGTGAATCAACTA
AACTTCCTTATTACGACGTTGAAAAGATCCACATACAGTAA

>gi|82780458|gb|DQ243966.1|_Human_coronavirus_229E_isolate_HCoV229E22/9/82_spike_glycoprotein_(S)_gene_complete_cds

ATGTTTGTTTTACTTGTTGCATATGCCTTGTTGCATATTGCTGATTGTCAAACTACAAATGGGCTGAACACTAGT
CACTCTGTTTGCAACGGCTGTGTTGGTCATTCAGAAAATGTATTTGCTGTTGAGAGTGGTGGTTATATACCCTCCGACT
TTGCATTTAATAATTGGTTCCTTTTAACTAATACCTCATCTGTTGTAGACGGTGTTGTGAGGAGTTTTCAGCCTTTGTT
GCTTAATTGCTTATGGTCTGTTTCTGGCTCGCGGTTTACTACTGGTTTTGTCTATTTTAATGGTACTGGGAGAGGTGAT
TGTAAAGGTTTTTCTTCAAACGTTTCGTCTGATGTCATACGTTACAACATCAATTTTGAAGAAAACCTTAGACGTGGAA
CCATTTTGTTTAAAACATCTTATGGTGTTGTTGTGTTTTATTGTACCAACAACACTTTAGTTTCAGGTGATGCTCACAT
ACCATTTGGTACAGTTTTGGGCAATTTTTATTGCTTTGTAAATACTACTATTGGCAATGAAACTACGTCTTCTTTTGTT
GGTGCACTACCTAAGACAGTTCGTGAGTTTGTTATTTCACGCACAGGACATTTTTATATTAATGGCTATCGCTATTTCA
CTTTAGGTAATGTAGAAGCCGTTAATTTCAATGTCACTAATGCAGAAACCACTGTTTGTACTGTTGCGTTAGCTTCTTA
TGCTGACGTTTTGGTTAATGTGTCACAAACCGCTATTGCTAATATAATTTATTGCAACTCTGTTATTAACAGACTGAGA
TGTGACCAGTTGTCCTTTGATGTACCAGATGGTTTTTATTCCACAAGCCCTATTCAATCCGTTGAGCTACCTGTGTCTA
TTGTGTCGCTACCTGTTTATCACAAACATACGTTTATTGTGTTGTACGTTGACTTCAAACCTCAGAGTGGCGGTGGCAA
GTGCTATAACTGTCGTCCTGCTGTTGTTAATATTACACTGGCCAATTTTAATGAAACTAAAGGGCCTTTGTGTGTTGAA
ACATCACACTTCACTACCAAATACGTTGCTGCCAATGTTGGTAGGTGGAGTGCTAGTATTAACACGGGAAATTGCCCTT
TTTCTTTTGGCAAAGTTAATAACTTTGTTAAATTTGGCAGTGTATGTTTTTCGCTAAAGGATATACCCGGTGGTTGCGC
AATGCCTATAGTGGCTAATTTGGCTTATATTAATTCTTATACTATAGGCTCATTGTATGTTTCTTGGAGTGATGGTGAT
GGAATTACTGGCGTCCCAAAACCTGTTGAGGGTGTTAGTTCCTTTATGAATGTTACATTGAACAAATGTACTAAATATA
ACATTTATGATGTATCTGGTGTGGGTGTTATTCGCATTAGCAATGACACCTTTCTTAATGGAATTACGTACACATCAAC
TTCAGGTAACCTTCTGGGTTTTAAAGATGTTACTAATGGCACCATCTACTCTATCACTCCTTGTAACCCACCAGATCAG
CTTGTTGTTTATCAGCAAGCTATTGTTGGTGCTATGTTGTCTGAAAATTTTACTAGTTACGGCTTTTCTAATGTTGTAG
AACTGCCGAAATTTTTCTATGCGTCCAATGGCACTTATAATTGCACAGACGCTGTTTTAACTTATTCTAGTTTTGGCGT
TTGTGCAGATGGTTCTATAATTGCTGTTCAACCACGTAATGTTTCATATGATAGTGTTTCAGCTATCGTCACAGCTAAT
TTGTCTATACCTTCCAATTGGACCACTTCGGTCCAGGTTGAGTATTTACAAATTACAAGTACACCTATCGTAGTTGATT
GCTCCACTTATGTTTGCAATGGTAATGTGCGCTGTGTTGAATTGCTTAAGCAGTATACTTCTGCTTGTAAAACTATTGA
AGACGCCTTAAGAAATAGCGCCATGCTGGAGTCTGCAGATGTTAGTGAGATGCTCACTTTTGACAAGAAAGCGTTTACA
CTTGCTAATGTTAGTAGTTTTGGTGACTACAACCTTAGCAGCGTTATACCTAGCTTGCCCACAAGTGGTAGTAGAGTGG
CTGGTCGCAGTGCCATAGAAGACATACTTTTTAGCAAACTTGTTACGTCTGGACTTGGCACTGTGGACGCAGACTACAA
AAAGTGCACTAAGGGTCTTTCCATTGCTGACTTGGCTTGTGCCCAATATTATAATGGCATTATGGTTTTGCCTGGCGTC
GCTGATGCTGAACGAATGGCCATGTATACAGGTTCTTTAATTGGTGGAATTGCTTTAGGAGGTCTTACATCAGCCGCTT

CAATACCATTTTCATTAGCAATTCAGGCACGTTTAAATTATGTTGCATTGCAGACTGATGTTTTACAAGAAAATCAGAA
AATTCTTGCTGCATCTTTTAATAAAGCAATGACCAACATAGTAGATGCCTTTACTGGTGTTAATGATGCTATTACACAA
ACTTCACAAGCTCTACAAACAGTTGCTACTGCACTTAACAAGATCCAGGATGTTGTTAATCAACAAGGCAACTCATTGA
ACCATTTAACTTCTCAGTTGAGGCAGAATTTTCAAGCCATCTCTAGCTCTATTCAGGCTATTTATGACAGACTTGATAT
TATTCAGGCTGATCAACAAGTAGATAGGCTGATTACTGGTAGATTGGCTGCTTTGAATGTATTCGTTTCTCATACATTG
ACTAAGTACACTGAAGTTCGTGCTTCCAGACAGCTTGCACAACAAAAAGTGAATGAGTGTGTCAAATCCCAGTCTAAGC
GCTATGGCTTCTGTGGAAATGGCACGCACATTTTCTCACTTGTTAATGCTGCTCCTGAGGGGCTTGTTTTTCTCCATAC
TGTCTTGTTGCCGACACAATATAAGGATGTTGAAGCGTGGTCTGGGTTGTGCGTTGATGGTAGAAACGGTTATGTGTTG
CGACAACCTAATCTTGCTCTTTACAAAGAAGGCAATTATTATAGAATTACATCTCGCATAATGTTTGAACCACGTATTC
CTACCATGGCAGATTTTGTCCAAATTGAAAATTGCAATGTCACATTTGTTAACATTTCTCGCTCTGAGTTGCAAACCAT
TGTGCCAGAGTATATTGATGTTAATAAGACGCTGCAAGAATTAAGTTACAAATTGCCAAATTACACTGTTCCAGACCTA
GTTGTCGAACAGTACAACCAGACTATTTTGAATTTGACCAGTGAAATTAGCACCCTTGAAAATAAATCTGCGGAGCTTA
ATTACACTGTTCAAAAACTGCAAACTCTGATTGACAACATAAATAGCACATTAGTTGACTTAAAGTGGCTCAACCGGGT
TGAGACTTATATCAAGTGGCCGTGGTGGGTGTGGTTGTGCATTTCAGTCGTGCTCATCTTTGTGGTGAGTATGTTGCTA
TTATGTTGTTGTTCTACTGGTTGCTGTGGCTTCTTTAGTTGTTTTGCATCTTCTATTAGAGGTTGTTGTGAATCAACTA
AACTTCCTTATTACGACGTTGAAAAGATCCACATACAGTAA

>gi|82780462|gb|DQ243968.1|_Human_coronavirus_229E_isolate_HCoV229E21/10/82_spike_glycoprotein_(S)_gene_complete_cds

ATGTTTGTTTTACTTGTTGCATATGCCTTGTTGCATATTGCTGGTTGTCAAACTACAAATGGGCTGAACACTAGT
CACTCTGTTTGCAACGGCTGTGTTGGTCATTCAGAAAATGTATTTGCTGTTGAGAGTGGTGGTTATATACCCTCCGACT
TTGCATTTAATAATTGGTTCCTCTTAACTAATACCTCATCTGTTGTAGATGGTGTTGTGAGGAGTTTTCAGCCTTTGTT
GCTTAATTGCTTATGGTCTGTTTCTGGCTCGCGGTTTACTACTGGTTTTGTCTATTTTAATGGTACTGGGAGAGGTGAT
TGTAAAGGTTTTTATTCAAATGCTTCGTCTGATGTCATACGTTACAACATCAATTTTGAAGAAAACCTTAGACGTGGAA
CCATTTTGTTTAAAACATCTTATGGTGTTGTTGTGTTTTATTGTACCAACAACACTTTAGTTTCAGGTGATGCTCACAT
ACCATTTGGTACAGTTTTGGGCAATTTTTATTGCTTTGTAAATACTACTATTGGCAATGAAACTACGTCTGCTTTTGTG
GGTGCACTACCTAAGACAGTTCGTGAGTTTGTTATTTCACGCACAGGACATTTCTATATTAATGGCTATCGCTATTTCA
CTTTAGGTAATGTAGAAGCCGTTAATTTCAATGTCACTAATGCAGAAACCACTGTTTGTACTGTTGCGTTAGCTTCTTA
TGCTGACGTTTTGGTTAATGTGTCACAAACCGCTATTGCTAATATAATTTATTGCAACTCTGTTATTAACAGACTGAGA
TGTGACCAGTTGTCCTTTGATGTACCAGATGGTTTTTATTCCACAAGCCCTATTCAATCCGTTGAGCTACCTGTGTCTA
TTGTGTCGCTACCTGTTTATCACAAACACACGTTTATTGTGTTGTATGTTGACTTCAAACTTCAGAGTGGCGTTGGCAG
GTGCTTTAACTGTCGTCCTGCTGTTGTTAATATTACACTGGCCAATTTTAATGAAACTAAAGGGCCTTTGTGTGTTGAC
ACATCACACTTCACTACCAAATACGTTGGTGCCAATTTTGGTAGGTGGAGTGCTAGTATTAACACGGGAAATTGCCCTT
TTTCTTTTGGCAAAGTTAATAACTTTGTTAAATTTGGCAGTGTATGTTTTTCGCTAAAGGATATACCCGGTGGTTGCGC
AATGCCTATAGTGGCTAATTTGGCTTATCTTAATTCTTATACTATAGGCTCATTGTATGTTTCTTGGAGTGATGGTGAT
GTAATTACTGGCGTCCCAAAACCTGTTGAGGGTGTTAGTTCCTTTATGAATGTTACATTGAACAAATGTACTAAATATA
ACATTTATGATGTATCTGGTGTGGGTGTTATTCGCATTAGCAATGACACCTTTCTTAATGGAATTACGTACACATCAAC
TTCAGGTAACCTTCTGGGTTTTAAAGATGTTACTAATGGCACCATCTACTCTATCACTCCTTGTAACCCACCAGATCAG
CTTGTTGTTTATCAGCAAGCTGTTGTTGGTGCTATGTTGTCTGAAAACTTTACTAGTTACGGCTTTTCTAATGTTGTAG
AAATGCCGAAATTTTTCTATGCGTCCAATGGCACTTATAATTGCACAGACGCTGTTTTAACTTATTCTAGTTTTGGCGT
TTGTGCAGATGGTTCTATAATTGCTGTTCAACCACGTAATGTTTCATATGATAGTGTTTCAGCTATCGTCACAGCTAAT
TTGTCTATACCTTCCAATTGGACCACTTCGGTCCAGGTTGAGTATTTACAAATTACAAGTACACCTATCGTAGTTGATT

GCTCCACTTATGTTTGCAATGGTAATGTGCGCTGTGTTGAATTGCTTAAGCAGTATACTTCTGCTTGTAAAACTATTGA
AGACGCCTTAAGAAATAGCGCCATGCTGGAGTCTGCAGATGTTAGTGAGATGCTCACTTTTGACAAGAAAGCGTTTACA
CTTGCTAATGTTAGTAGTTTTGGTGACTACAACCTTAGCAGTGTTATACCTAGCTTGCCCACAAGTGGTAGTAGAGTGG
CTGGTCGCAGTGCCATAGAAGACATACTTTTTAGCAAACTTGTTACGTCTGGACTTGGCACTGTGGACGCAGACTACAA
AAAGTGCACTAAGGGTCTTTCCATTGCTGACTTGGCTTGTGCCCAATATTATAATGGCATTATGGTTTTGCCTGGCGTC
GCTGATGCTGAACGAATGGCCATGTATACAGGTTCTTTAATTGGTGGAATTGCTTTAGGAGGTCTTACATCAGCCGCTT
CAATACCATTTTCATTAGCAATTCAGTCACGTTTAAATTATGTTGCATTGCAGACTGATGTTTTACAAGAAAATCAGAA
AATTCTTGCTGCATCTTTTAATAAAGCAATGACCAACATAGTAGATGCCTTTACTGGTGTTAATGATGCTATTACACAA
ACTTCACAAGCTCTACAAACAGTTGCTACTGCACTTAATAAGATCCAGGATGTTGTTAATCAACAAGGCAACTCATTGA
ACCATTTAACTTCTCAGTTGAGGCAGAATTTTCAAGCCATCTCTAGCTCTATTCAGGCTATCTATGACAGACTTGATAT
TATTCAGGCTGATCAACAAGTAGATAGGCTGATTACTGGTAGATTGGCTGCTTTGAATGTATTCGTTTCTCATACATTG
ACTAAGTACACTGAAGTTCGTGCTTCCAGACAGCTTGCACAACAAAAAGTGAATGAGTGTGTCAAATCCCAGTCTAAGC
GCTATGGCTTCTGTGGAAATGGCACGCACATTTTCTCAATTGTTAATGCTGCTCCTGAGGGGCTTGTTTTTCTCCATAC
TGTCTTGTTGCCGACACAATATAAGGATGTTGAAGCGTGGTCTGGGTTGTGCGTTGATGGTAGAAACGGTTATGTGTTG
CGACAACCTAATCTTGCTCTTTACAAAGAAGGCAATTATTATAGAATTACATCTCGCATAATGTTTGAACCACGTATTC
CTACCATGGCAGATTTTGTCCAAATTGAAAATTGCAATGTCACGTTTGTTAACATTTCTCGCTCTGAGTTGCAAACCAT
TGTGCCAGAGTATATTGATGTTAATAAGACGCTGCAAGAATTAAGTTACAAATTGCCAAATTACACTGTTCCAGACCTA
GTTGTCGAACAGTACAACCAGACTATTTTGAATTTGACCAGTGAAATTAGCACCCTTGAAAATAAATCTGCGGAGCTTA
ATTACACTGTTCAAAAACTGCAAACTCTGATTGACAACATAAATAGCACATTAGTTGACTTAAAGTGGCTCAACCGGGT
TGAGACTTATATCAAGTGGCCGTGGTGGGTGTGGTTGTGCATTTCAGTCGTGCTCATCTTTGTGGTGAGTATGTTGCTA
TTATGTTGTTGTTCTACTGGTTGCTGTGGCTTCTTTAGTTGTTTTGCATCTTCTATTAGAGGTTGTTGTGAATCAACTA
AACTTCCTTATTACGACGTTGAAAAGATCCACATACAGTAA

>gi|82780464|gb|DQ243969.1|_Human_coronavirus_229E_isolate_HCoV229E8/11/82a_spike_glycoprotein_(S)_gene_complete_cds

ATGTTTGTTTTACTTGTTGCATATGCCTTGTTGCATATTGCTGGTTGTCAAACTACAAATGGGCTGAACACTAGT
CACTCTGTTTGCAACGGCTGTGTTGGTCATTCAGAAAATGTATTTGCTGTTGAGAGTGGTGGTTATATACCCTCCGACT
TTGCATTTAATAATTGGTTCCTTCTAACTAATACCTCATCTGTTGTAGACGGTGTTGTGAGGAGTTTTCAGCCTTTGTT
GCTTAATTGCTTATGGTCTGTTTCTGGCTCGCGGTTTACTACTGGTTTTGTCTATTTTAATGGTACTGGGAGAGGTGAT
TGTAAAGGTTTTTCTTCAAACGTTTCGTCTGATGTCATACGTTACAACATCAATTTTGAAGAAAACCTTAGACGTGGAA
CCATTTTGTTTAAAACATCTTATGGTGTTGTTGTGTTTTATTGTACCAACAACACTTTAGTTTCAGGTGATGCTCACAT
ACCATTTGGTACAGTTTTGGGCAATTTTTATTGCTTTGTAAATACTACTATTGGCAATGAAACTACGTCTTCTTTTGTG
GGTGCACTACCTAAGACAGTTCGTGAGTTTGTTATTGCACGCACAGGACATTTCTATATTAATGGCTATCGTTATTTCA
CTTTAGGTAATGTAGAAGCCGTTAATTTCAATGTCACTAATGCAGAAACCACTGTTTGTACTGTTGCGTTAGCTTCTTA
TGCTGACGTTTTGGTTAATGTGTCACAAACTGCTATTGCTAATATAATTTATTGCAACTCTGTTATTAACAGACTGAGA
TGTGACCAGTTGTCCTTTGATGTACCAGATGGTTTTTATTCCACAAGCCCTATTCAATCCGTTGAGCTACCTGTGTCTA
TTGTGTCGCTACCTGTTTATCATAAACATACGTTTATTGTGTTGTACGTTGACTTCAAACCTCAGAGTGGCGGTGGCAG
GTGCTATAACTGTCGTCCTGCTGTTGTTAATATTACACTGGCCAATTTTAATGAAACTAAAGGGCCTTTGTGTGTTGAA
ACATCACACTTCACTACCAAATACGTTGCTGCCAATGTTGGTAGGTGGAGTGCTAGTATTAACACTGGAAATTGCCCTT
TTTCTTTTGGCAAAGTTAATAACTTTGTTAAATTTGGCAGTGTATGTTTTTCTCTAAAGGATATACCCGGTGGTTGCGC
AATGCCTATAGTGGCTAATTTGGCTTATATTAATTCTTATACTATAGGCTCATTGTATGTTTCTTGGAGTGATGGTGAT
GGAATTACTGGCGTCCCAAAACCTGTTGAGGGTGTTAGTTCCTTTATGAATGTTACATTGAACAAATGTACTAAATATA

ACATTTATGATGTATCTGGTGTGGGTGTTATTCGCATTAGCAATGACACCTTTCTTAATGGAATTACGTACACATCAAC
TTCAGGTAACCTTCTGGGTTTTAAAGATGTTACTAATGGCACCATCTACTCTATCACTCCTTGTAACCCACCAGATCAG
CTTGTTGTTTATCAGCAAGCTGTTGTTGGTGCTATGTTGTCTGAAAATTTTACTAGTTACGGCTTTTCTAATGTTGTAG
AACTGCCGAAATTTTTCTATGCGTCCAATGGCACTTATAATTGCACAGACGCTGTTTTAACTTATTCTAGTTTTGGCGT
TTGTGCAGATGGTTCTATAATTGCTGTTCAACCACGTAATGTTTCATATGATAGTGTTTCAGCTATCGTCACAGCTAAT
TTGTCTATACCTTCCAATTGGACCACTTCGGTCCAGGTTGAGTATTTACAAATTACAAGTACACCTATCGTAGTTGATT
GCTCCACTTATGTTTGCAATGGTAATGTGCGCTGTGTTGAATTGCTTAAGCAGTATACTTCTGCTTGTAAAACTATTGA
AGACGCCTTAAGAAATAGCGCCATGCTGGAGTCTGCAGATGTTAGTGAGATGCTCACTTTTGACAAGAAAGCGTTTACA
CTTGCTAATGTTAGTAGTTTTGGTGAATACAACCTTAGCAGTGTTGTACCTAGCTTGCCCACAAGTGGTAGTAGAGTGG
CTGGTCGCAGTGCCATAGAAGACATACTTTTTAGCAAACTTGTTACGTCTGGACTTGGCACTGTGGATGCAGACTACAA
AAAGTGCACTAAGGGTCTTTCCATTGCTGACTTGGCTTGTGCTCAATATTATAATGGCATTATGGTTTTGCCTGGCGTC
GCTGATGCTGAACGAATGGCCATGTATACAGGTTCTTTAATTGGTGGAATTGCTTTAGGAGGTCTTACATCAGCCGCTT
CAATACCATTTTCATTAGCAATTCAGGCACGTTTAAATTATGTTGCATTGCAGACTGATGTTTTACAAGAAAATCAGAA
AATTCTTGCTGCATCTTTTAATAAAGCAATGACCAACATAGTAGATGCCTTTACTGGTGTTAATGATGCTATTACACAA
ACTTCACAAGCTCTACAAACAGTTGCTACTGCACTTAACAAGATCCAGGATGTTGTTAATCAACAAGGCAACTCATTGA
ACCATTTAACTTCTCAGTTGAGGCAGAATTTTCAAGCCATCTCTAGCTCTATTCAGGCTATCTATGACAGACTTGATAT
TATTCAGGCTGATCAACAAGTAGATAGGCTGATTACTGGTAGATTGGCTGCTTTGAATGTATTCGTTTCTCATACATTG
ACTAAGTACACTGAAGTTCGTGCTTCCAGACAGCTTGCACAACAAAAAGTGAATGAGTGTGTCAAATCCCAGTCTAAGC
GCTATGGCTTCTGTGGAAATGGCACGCACATTTTCTCACTTGTTAATGCTGCTCCTGAGGGGCTTGTTTTCCTCCATAC
TGTCTTGTTGCCGACACAATATAAGGATGTTGAAGCGTGGTCTGGGTTGTGCGTTGATGGTAGAAACGGTTATGTGTTG
CGACAACCTAATCTTGCTCTTTACAAAGAAGGCAATTATTATAGAATTACATCTCGCATAATGTTTGAACCACGTGTTC
CTACCATGGCAGATTTTGTCCAAATTGAAAATTGCAATGTCACATTTGTTAACATTTCTCGCTCTGAGTTGCAAACCAT
TGTGCCAGAGTATATTGATGTTAATAAGACGCTGCAAGAATTAAGTTACAAATTGCCAAATTACACTGTTCCAGACCTA
GTTGTCGAACAGTACAATCAGACTATTTTGAATTTGACCAGTGAAATTAGCACCCTTGAAAATAAATCTGCGGAGCTTA
ATTACACTGTTCAAAAACTGCAAACTCTGATTGACAACATAAATAGCACATTAGTTGACTTAAAGTGGCTCAACCGGGT
TGAGACTTATATCAAGTGGCCGTGGTGGGTGTGGTTGTGCATTTCAGTCGTGCTCATCTTTGTGGTGAGTATGTTGCTA
TTATGTTGTTGTTCTACTGGTTGCTGTGGCTTCTTTAGTTGTTTTGCATCTTCTATTAGAGGTTGTTGTGAATCAACTA
AACTTCCTTATTACGACGTTGAAAAGATCCACATACAGTAA

>gi|82780466|gb|DQ243970.1|_Human_coronavirus_229E_isolate_HCoV229E8/11/82b_spike_glycoprotein_(S)_gene_complete_cds

ATGTTTGTTTTACTTGTTGCATATGCCTTGTTGCATATTGCTGGTTGTCAAACTACAAATGGGCTGAACACTAGT
CACTCTGTTTGCAACGGCTGTGTTGGTCATTCAGAAAATGTATTTGCTGTTGAGAGTGGTGGTTATATACCCTCCGACT
TTGCATTTAATAATTGGTTCCTTCTAACTAATACCTCATCTGTTGTAGACGGTGTTGTGAGGAGTTTTCAGCCTTTGTT
GCTTAATTGCTTATGGTCTGTTTCTGGCTCGCGGTTTACTACTGGTTTTGTCTATTTTAATGGTACTGGGAGAGGTGAT
TGTAAAGGTTTTTCTTCAAACGTTTCGTCTGATGTCATACGTTACAACATCAATTTTGAAGAAAACCTTAGACGTGGAA
CCATTTTGTTTAAAACATCTTATGGTGTTGTTGTGTTTTATTGTACCAACAACACTTTAGTTTCAGGTGATGCTCACAT
ACCATTTGGTACAGTTTTGGGCAATTTTTATTGCTTTGTAAATACTACTATTGGCAATGAAACTACGTCTTCTTTTGTG
GGTGCACTACCTAAGACAGTTCGTGAGTTTGTTATTGCACGCACAGGACATTTCTATATTAATGGCTATCGTTATTTCA
CTTTAGGTAATGTAGAAGCCGTTAATTTCAATGTCACTAATGCAGAAACCACTGTTTGTACTGTTGCGTTAGCTTCTTA
TGCTGACGTTTTGGTTAATGTGTCACAAACTGCTATTGCTAATATAATTTATTGCAACTCTGTTATTAACAGACTGAGA
TGTGACCAGTTGTCCTTTGATGTACCAGATGGTTTTTATTCCACAAGCCCTATTCAATCCGTTGAGCTACCTGTGTCTA

TTGTGTCGCTACCTGTTTATCATAAACATACGTTTATTGTGTTGTACGTTGACTTCAAACCTCAGAGTGGCGGTGGCAA
GTGCTATAACTGTCGTCCTGCTGTTGTTAATATTACACTGGCCAATTTTAATGAAACTAAAGGGCCTTTGTGTGTTGAA
ACATCACACTTCACTACCAAATACGTTGCTGCCAATGTTGGTAGGTGGAGTGCTAGTATTAACACTGGAAATTGCCCTT
TTTCTTTTGGCAAAGTTAATAACTTTGTTAAATTTGGCAGTGTATGTTTTTCTCTAAAGGATATACCCGGTGGTTGCGC
AATGCCTATAGTGGCTAATTTGGCTTATATTAATTCTTATACTATAGGCTCATTGTATGTTTCTTGGAGTGATGGTGAT
GGAATTACTGGCGTCCCAAAACCTGTTGAGGGTGTTAGTTCCTTTATGAATGTTACATTGAACAAATGTACTAAATATA
ACATTTATGATGTATCTGGTGTGGGTGTTATTCGCATTAGCAATGACACCTTTCTTAATGGAATTACGTACACATCAAC
TTCAGGTAACCTTCTGGGTTTTAAAGATGTTACTAATGGCACCATCTACTCTATCACTCCTTGTAACCCACCAGATCAG
CTTGTTGTTTATCAGCAAGCTGTTGTTGGTGCTATGTTGTCTGAAAATTTTACTAGTTACGGCTTTTCTAATGTTGTAG
AACTGCCGAAATTTTTCTATGCGTCCAATGGCACTTATAATTGCACAGACGCTGTTTTAACTTATTCTAGTTTTGGCGT
TTGTGCAGATGGTTCTATAATTGCTGTTCAACCACGTAATGTTTCATATGATAGTGTTTCAGCTATCGTCACAGCTAAT
TTGTCTATACCTTCCAATTGGACCACTTCGGTCCAGGTTGAGTATTTACAAATTACAAGTACACCTATCGTAGTTGATT
GCTCCACTTATGTTTGCAATGGTAATGTGCGCTGTGTTGAATTGCTTAAGCAGTATACTTCTGCTTGTAAAACTATTGA
AGACGCCTTAAGAAATAGCGCCATGCTGGAGTCTGCAGATGTTAGTGAGATGCTCACTTTTGACAAGAAAGCGTTTACA
CTTGCTAATGTTAGTAGTTTTGGTGAATACAACCTTAGCAGTGTTGTACCTAGCTTGCCCACAAGTGGTAGTAGAGTGG
CTGGTCGCAGTGCCATAGAAGACATACTTTTTAGCAAACTTGTTACGTCTGGACTTGGCACTGTGGATGCAGACTACAA
AAAGTGCACTAAGGGTCTTTCCATTGCTGACTTGGCTTGTGCTCAATATTATAATGGCATTATGGTTTTGCCTGGCGTC
GCTGATGCTGAACGAATGGCCATGTATACAGGTTCTTTAATTGGTGGAATTGCTTTAGGAGGTCTTACATCAGCCGCTT
CAATACCATTTTCATTAGCAATTCAGGCACGTTTAAATTATGTTGCATTGCAGACTGATGTTTTACAAGAAAATCAGAA
AATTCTTGCTGCATCTTTTAATAAAGCAATGACCAACATAGTAGATGCCTTTACTGGTGTTAATGATGCTATTACACAA
ACTTCACAAGCTCTACAAACAGTTGCTACTGCACTTAACAAGATCCAGGATGTTGTTAATCAACAAGGCAACTCATTGA
ACCATTTAACTTCTCAGTTGAGGCAGAATTTTCAAGCCATCTCTAGCTCTATTCAGGCTATCTATGACAGACTTGATAT
TATTCAGGCTGATCAACAAGTAGATAGGCTGATTACTGGTAGATTGGCTGCTTTGAATGTATTCGTTTCTCATACATTG
ACTAAGTACACTGAAGTTCGTGCTTCCAGACAGCTTGCACAACAAAAAGTGAATGAGTGTGTCAAATCCCAGTCTAAGC
GCTATGGCTTCTGTGGAAATGGCACGCACATTTTCTCACTTGTTAATGCTGCTCCTGAGGGGCTTGTTTTCCTCCATAC
TGTCTTGTTGCCGACACAATATAAGGATGTTGAAGCGTGGTCTGGGTTGTGCGTTGATGGTAGAAACGGTTATGTGTTG
CGACAACCTAATCTTGCTCTTTACAAAGAAGGCAATTATTATAGAATTACATCTCGCATAATGTTTGAACCACGTGTTC
CTACCATGGCAGATTTTGTCCAAATTGAAAATTGCAATGTCACATTTGTTAACATTTCTCGCTCTGAGTTGCAAACCAT
TGTGCCAGAGTATATTGATGTTAATAAGACGCTGCAAGAATTAAGTTACAAATTGCCAAATTACACTGTTCCAGACCTA
GTTGTCGAACAGTACAATCAGACTATTTTGAATTTGACCAGTGAAATTAGCACCCTTGAAAATAAATCTGCGGAGCTTA
ATTACACTGTTCAAAAACTGCAAACTCTGATTGACAACATAAATAGCACATTAGTTGACTTAAAGTGGCTCAACCGGGT
TGAGACTTATATCAAGTGGCCGTGGTGGGTGTGGTTGTGCATTTCAGTCGTGCTCATCTTTGTGGTGAGTATGTTGCTA
TTATGTTGTTGTTCTACTGGTTGCTGTGGCTTCTTTAGTTGTTTTGCATCTTCTATTAGAGGTTGTTGTGAATCAACTA
AACTTCCTTATTACGACGTTGAAAAGATCCACATACAGTAA

>gi|82780468|gb|DQ243971.1|_Human_coronavirus_229E_isolate_HCoV229E29/7/84_spike_glycoprotein_(S)_gene_complete_cds

ATGTTTGTTTTACTTGTTGCATATGCCTTGTTGCATATTGCTGGTTGTCAAACTACAAATGGGATGAACACTAGT
CACTCTGTTTGCAACGGCTGTGTTGGTCATTCAGAAAATGTATTTGCTGTTGAGAGTGGTGGTTATATACCCTCCGACT
TTGCATTTAATAATTGGTTCCTCTTAACTAATACCTCATCTGTTGTAGATGGTGTTGTGAGGAGTTTTCAGCCTTTGTT
GCTTAATTGCTTATGGTCTGTTTCTGGCTCGCGGTTTACTACTGGTTTTGTCTATTTTAATGGTACTGGGAGAGGTGAT
TGTAAAGGTTTTTATTCAAATGCTTCGTCTGATGTCATACGTTACAACATCAATTTTGAAGAAAACCTTAGACGTGGAA

CCATTTTGTTTAAAACATCTTATGGTGTTGTTGTGTTTTATTGTACCAACAACACTTTAGTTTCAGGTGATGCTCACAT
ACCATTTGGTACAGTTTTGGGCAATTTTTATTGCTTTGTAAATACTACTATTGGCAATGAAACTACGTTTGCTTTTGTG
GGTGCACTACCTAAGACAGTTCGTGAGTTTGTTATTTCACGCACAGGACATTTCTATATTAATGGCTATCGCTATTTCA
CTTTAGGTAATGTAGAAGCCGTTAATTTCAATGTCACTAATGCAGAAACCACTGTTTGTACTGTTGCGTTAGCTTCTTA
TGCTGACGTTTTGGTTAATGTGTCACAAACCGCTATTGCTAATATAATTTATTGCAACTCTGTTATTAACAGACTGAGA
TGTGACCAGTTGTCCTTTGATGTACCAGATGGTTTTTATTCCACAAGCCCTATTCAATCCGTTGAGCTACCTGTGTCTA
TTGTGTCGCTACCTGTTTATCACAAACACACGTTTATTGTGTTGTATGTTGACTTCAAACTTCAGAGTGGCGTTGGCAG
GTGCTTTAACTGTCGTCCTGCTGTTGTTAATATTACACTGGCCAATTTTAATGAAACTAAAGGGCCTTTGTGTGTTGAC
ACATCACACTTCACTACCAAATTCGTTGGTGCCAATTTTGGTAGGTGGAGTGCTAGTATTAACACGGGAAATTGCCCTT
TTTCTTTTGGCAAAGTTAATAACTTTGTTAAATTTGGCAGTGTATGTTTTTCGCTAAAGGATATACCCGGTGGTTGCGC
AATGCCTATAGTGGCTAATTTGGCTTATCTTAATTCTTATACTATAGGCTCATTGTATGTTTCTTGGAGTGATGGTGAT
GTAATTACTGGCGTCCCAAAACCTGTTGAGGGTGTTAGTTCCTTTATGAATGTTACATTGAACAAATGTACTAAATATA
ACATTTATGATGTATCTGGTGTGGGTGTTATTCGCATTAGCAATGACACCTTTCTTAATGGAATTACGTACACATCAAC
TTCAGGTAACCTTCTGGGTTTTAAAGATGTTACTAATGGCACCATCTACTCTATCACTCCTTGTAACCCACCAGACCAG
CTTGTTGTTTATCAGCAAGCTGTTGTTGGTGCTATGTTGTCTGAAAACTTTACTAGTTACGGCTTTTCTAATGTTGTAG
AAATGCCGAAATTTTTCTATGCGTCCAATGGCACTTATAATTGCACAGACGCTGTTTTAACTTATTCTAGTTTTGGCGT
TTGTGCAGATGGTTCTATAATTGCTGTTCAACCACGTAATGTTTCATATGATAGTGTTTCAGCTATCGTCACAGCTAAT
TTGTCTATACCTTCCAATTGGACCACTTCGGTCCAGGTTGAGTATTTACAAATTACAAGTACACCTATCGTAGTTGATT
GCTCCACTTATGTTTGCAATGGTAATGTGCGCTGTGTTGAATTGCTTAAGCAGTATACTTCTGCTTGTAAAACTATTGA
AGACGCCTTAAGAAATAGCGCCATGCTGGAGTCTGCAGATGTTAGTGAGATGCTCACTTTTGACAAGAAAGCGTTTACA
CTTGCTAATGTTAGTAGTTTTGGTGACTACAACCTTAGCAGTGTTATACCTAGCTTGCCCACAAGTGGTAGTAGAGTGG
CTGGTCGCAGTGCCATAGAAGACATACTTTTTAGCAAACTTGTTACGTCTGGACTTGGCACTGTGGACGCAGACTACAA
AAAGTGCACTAAGGGTCTTTCCATTGCTGACTTGGCTTGTGCCCAATATTATAATGGCATTATGGTTTTGCCTGGCGTC
GCTGATGCTGAACGAATGGCCATGTATACAGGTTCTTTAATTGGTGGAATTGCTTTAGGAGGTCTTACATCAGCCGCTT
CAATACCATTTTCATTAGCAATTCAGTCACGTTTAAATTATGTTGCATTGCAGACTGATGTTTTACAAGAAAATCAGAA
AATTCTTGCTGCATCTTTTAATAAAGCAATGACCAACATAGTAGATGCCTTTACTGGTGTTAATGATGCTATTACACAA
ACTTCACAAGCTCTACAAACAGTTGCTACTGCACTTAATAAGATCCAGGATGTTGTTAATCAACAAGGCAACTCATTGA
ACCATTTAACTTCTCAGTTGAGGCAGAATTTTCAAGCCATCTCTAGCTCTATTCAGGCTATCTATGACAGACTTGATAT
TATTCAGGCTGATCAACAAGTAGATAGGCTGATTACTGGTAGATTGGCTGCTTTGAATGTATTCGTTTCTCATACATTG
ACTAAGTACACTGAAGTTCGTGCTTCCAGACAGCTTGCACAACAAAAAGTGAATGAGTGTGTCAAATCCCAGTCTAAGC
GCTATGGCTTCTGTGGAAATGGCACGCACATTTTCTCACTTGTTAATGCTGCTCCTGAGGGGCTTGTTTTTCTCCATAC
TGTCTTGTTGCCGACACAATATAAGGATGTTGAAGCGTGGTCTGGGTTGTGCGTTGATGGTAGAAACGGTTATGTGTTG
CGACAACCTAATCTTGCTCTTTACAAAGAAGGCAATTATTATAGAATTACATCTCGCATAATGTTTGAACCACGTATTC
CTACCATGGCAGATTTTGTCCAAATTGAAAATTGCAATGTCACATTTGTTAACATTTCTCGCTCTGAGTTGCAAACCAT
TGTGCCAGAGTATATTGATGTTAATAAGACGCTGCAAGAATTAAGTTACAAATTGCCAAATTACACTGTTCCAGACCTA
GTTGTCGAACAGTACAACCAGATTATTTTGAATTTGACCAGTGAAATTAGTACCCTTGAAAATAAATCTGCGGAGCTTA
ATTACACTGTTCAAAAACTGCAAACTCTGATTGACAACATAAATAGCACATTAGTTGACTTAAAGTGGCTCAACCGGGT
TGAAACTTATATCAAGTGGCCGTGGTGGGTGTGGTTGTGCATTTCAGTCGTGCTCATCTTTGTGGTGAGTATGTTGCTA
TTATGTTGTTGTTCTACTGGTTGCTGTGGCTTCTTTAGTTGTTTTGCATCTTCTATTAGAGGTTGTTGTGAATCAACTA
AACTTCCTTATTACGACGTTGAAAAGATCCACATACAGTAA

>gi|82780470|gb|DQ243972.1|_Human_coronavirus_229E_isolate_HCoV229E5/9/84_spike_glycoprotein_(S)_gene_complete_cds

ATGTTTGTTTTACTTGTTGCATATGCCTTGTTGCATATTGCTGGTTGTCAAACTACAAATGGGCTGAACACTAGT
CACTCTGTTTGCAACGGCTGTGTTGGTCATTCAGAAAATGTATTTGCTGTTGAGAGTGGTGGTTATATACCCTCCGACT
TTGCATTTAATAATTGGTTCCTCTTAACTAATACCTCATCTGTTGTAGATGGTGTTGTGAGGAGTTTTCAGCCTTTGTT
GCTTAATTGCTTATGGTCTGTTTCTGGCTCGCGGTTTACTACTGGTTTTGTCTATTTTAATGGTACTGGGAGAGGTGAT
TGTAAAGGTTTTTATTCAAATGCTTCGTCTGATGTCATACGTTACAACATCAATTTTGAACGAAACCTTAGACGTGGAA
CCATTTTGTTCAAAACATCTTATGGTGTTGTTGTGTTTTATTGTACCAACAACACTTTAGTTTCAGGTGATGCTCACAT
ACCATTTGGTACAGTTTTGGGCAATTTTTATTGCTTTGTAAATACTACTATTGGCAATGAAACTACGTCTGCTTTTGTG
GGTGCACTACCTAAGACAGTTCGTGAGTTTGTTATTTCACGCACAGGACATTTCTATATTAATGGCTATCGCTATTTCA
CTTTAGGTAATGTAGAAGCCGTTAATTTCAATGTCACTAATGCAGAAACCACTGTTTGTACTGTTGCGTTAGCTTCTTA
TGCTGACGTTTTGGTTAATGTGTCACAAACCGCTATTGCTAATATAATTTATTGCAACTCTGTTATTAACAGACTGAGA
TGTGACCAGTTGTCCTTTGATGTACCAGATGGTTTTTATTCCACAAGCCCTATTCAATCCGTTGAGCTACCTGTGTCTA
TTGTGTCGCTACCTGTTTATCACAAACACACGTTTATTGTGTTGTATGTTGACTTCAAACTTCAGAGTGGCGTTGGCAG
GTGCTTTAACTGTCGTCCTGCTGTTGTTAATATTACACTGGCCAATTTTAATGAAACTAAAGGGCCTTTGTGTGTTGAC
ACATCACACTTCACTACCAAATTCGTTGGTGCCAATTTTGGTAGGTGGAGTGCTAGTATTAACACGGGAAATTGCCCTT
TTTCTTTTGGCAAAGTTAATAACTTTGTTAAATTTGGCAGTGTATGTTTTTCGCTAAAGGATATACCCGGTGGTTGCGC
AATGCCTATAGTGGCTAATTTGGCTTATCTTAATTCTTATACTATAGGCTCATTGTATGTTTCTTGGAGTGATGGTGAT
GTAATTACTGGCGTCCCAAAACCTGTTGAGGGTGTTAGTTCCTTTATGAATGTTACATTGAACAAATGTACTAAATATA
ACATTTATGATGTATCTGGTGTGGGTGTTATTCGCATTAGCAATGACACCTTTCTTAATGGAATTACGTACACATCAAC
TTCAGGTAACCTTCTGGGTTTTAAAGATGTTACTAATGGCACCATCTACTCTATCACTCCTTGTAACCCACCAGATCAG
CTTGTTGTTTATCAGCAAGCTGTTGTTGGTGCTATGTTGTCTGAAAACTTTACTAGTTACGGCTTTTCTAATGTTGTAG
AAATGCCGAAATTTTTCTATGCGTCCAATGGCACTTATAATTGCACAGACGCTGTTTTAACTTATTCTAGTTTTGGCGT
TTGTGCAGATGGTTCTATAATTGCTGTTCAACCACGTAATGTTTCATATGATAGTGTTTCAGCTATCGTCACAGCTAAT
TTGTCTATACCTTCCAATTGGACCACTTCGGTCCAGGTTGAGTATTTACAAATTACAAGTACACCTATCGTAGTTGATT
GCTCCACTTATGTTTGCAATGGTAATGTGCGCTGTGTTGAATTGCTTAAGCAGTATACTTCTGCTTGTAAAACTATTGA
AGACGCCTTAAGAAATAGCGCCATGCTGGAGTCTGCAGATGTTAGTGAGATGCTCACTTTTGACAAGAAAGCGTTTACA
CTTGCTAATGTTAGTAGTTTTGGTGACTACAACCTTAGCAGTGTTATACCTAGCTTGCCCACAAGTGGTAGTAGAGTGG
CTGGTCGCAGTGCCATAGAAGACATACTTTTTAGCAAACTTGTTACGTCTGGACTTGGCACTGTGGACGCAGACTACAA
AAAGTGCACTAAGGGTCTTTCCATTGCTGACTTGGCTTGTGCCCAATATTATAATGGCATTATGGTTTTGCCTGGCGTC
GCTGATGCTGAACGAATGGCCATGTATACAGGTTCTTTAATTGGTGGAATTGCTTTAGGAGGTCTTACATCAGCCGCTT
CAATACCATTTTCATTAGCAATTCAGTCACGTTTAAATTATGTTGCATTGCAGACTGATGTTTTACAAGAAAATCAGAA
AATTCTTGCTGCATCTTTTAATAAAGCAATGACCAACATAGTAGATGCCTTTACTGGTGTTAATGATGCTATTACACAA
ACTTCACAAGCTCTACAAACAGTTGCTACTGCACTTAATAAGATCCAGGATGTTGTTAATCAACAAGGCAACTCATTGA
ACCATTTAACTTCTCAGTTGAGGCAGAATTTTCAAGCCATCTCTAGCTCTATTCAGGCTATCTATGACAGACTTGATAT
TATTCAGGCTGATCAACAAGTAGATAGGCTGATTACTGGTAGATTGGCTGCTTTGAATGTATTCGTTTCTCATACATTG
ACTAAGTACACTGAAGTTCGTGCTTCCAGACAGCTTGCACAACAAAAAGTGAATGAGTGTGTCAAATCCCAGTCTAAGC
GCTATGGCTTCTGTGGAAATGGCACGCACATTTTCTCAATTGTTAATGCTGCTCCTGAGGGGCTTGTTTTTCTCCATAC
TGTCTTGTTGCCGACACAATATAAGGATGTTGAAGCGTGGTCTGGGTTGTGCGTTGATGGTAGAAACGGTTATGTGTTG
CGACAACCTAATCTTGCTCTTTACAAAGAAGGCAATTATTATAGAATTACATCTCGCATAATGTTTGAACCACGTATTC
CTACCATGGCAGATTTTGTCCAAATTGAAAATTGCAATGTCACGTTTGTTAACATTTCTCGCTCTGAGTTGCAAACCAT
TGTGCCAGAGTATATTGATGTTAATAAGACGCTGCAAGAATTAAGTTACAAATTGCCAAATTACACTGTTCCAGACCTA
GTTGTCGAACAGTACAACCAGACTATTTTGAATTTGACCAGTGAAATTAGCACCCTTGAAAATAAATCTGCGGAGCTTA
ATTACACTGTTCAAAAACTGCAAACTCTGATTGACAACATAAATAGCACATTAGTTGACTTAAAGTGGCTCAACCGTGT
TGAGACTTATATCAAGTGGCCGTGGTGGGTGTGGTTGTGCATTTCAGTCGTGCTCATCTTTGTGGTGAGTATGTTGCTA
TTATGTTGTTGTTCTACTGGTTGCTGTGGCTTCTTTAGTTGTTTTGCATCTTCTATTAGAGGTTGTTGTGAATCAACTA

AACTTCCTTATTACGACGTTGAAAAGATCCACATACAGTAA

>gi|82780472|gb|DQ243973.1|_Human_coronavirus_229E_isolate_HCoV229E24/6/90_spike_glycoprotein_(S)_gene_complete_cds

ATGTTTGTTTTACTTGTTGCATATGCCTTGTTGCATATTGCTGGTTGTCAAACTACAAATGGGATGAACACTAGTCACTCTGTTTGCAACGGCTGTGTTGGTCATTCAGAAAATGTATTTGCTGTTGAGAGTGGTGGTTATATACCCTCCGACTTTGCATTTAATAATTGGTTCCTCTTAACTAATACCTCATCTGTTGTAGATGGTGTTGTGAGGAGTTTTCAGCCTTTGTTGCTTAATTGCTTATGGTCTGTTTCTGGCTCGCGGTTTACTACTGGTTTTGTCTATTTTAATGGTACTGGGAGAGGTGATTGTAAAGGTTTTTATTCAAATGCTTCGTCTGATGTCATACGTTACAACATCAATTTTGAAGAAAACCTTAGACGTGGAACCATTTTGTTTAAAACATCTTATGGTGCTGTTGTGTTTTATTGTACCAACAACACTTTGGTTTCAGGTGATGCTCACATACCATCTGGTACAGTTTTGGGCAATTTTTATTGCTTTGTAAATACTACTATTGGCAATGAAACTACGTCTGCTTTTGTGGGTGCACTACCTAAGACAGTTCGTGAGTTTGTTATTTCACGCACAGGACATTTCTATATTAATGGCTATCGCTATTTCACTTTAGGTAATGTAGAAGCCGTTAATTTCAATGTCACTAATGCAGAAACCACTGTTTGTACTGTTGCGTTAGCTTCTTATGCTGACGTTTTGGTTAATGTGTCACAAACTGCTATTGCTAATATAATTTATTGCAACTCTGTTATTAACAGACTGAGATGTGACCAGTTGTCCTTTGATGTACCAGATGGTTTTTATTCCACAAGCCCTATTCAATCCGTTGAGCTACCTGTGTCTATTGTGTCGCTACCTGTTTATCACAAACACACGTTTATTGTGTTGTATGTTAACTTCAAACTTCGGAGTGGCGTTGGCAGGTGCTATAACTGTCGTCCTGCTGTTGTTAATATTACACTGGCCAATTTTAATGAAACTAAAGGGCCTTTGTGTGTTGACACATCACACTTCACTACCCAATTCGTTGGTGCCAAGTTTGATAGGTGGAGTGCTAGTATTAACACGGGAAATTGCCCTTTTTCTTTTGGCAAAGTTAATAACTTTGTTAAATTTGGCAGTGTATGTTTTTCGCTAAAGGATATACCCGGTGGTTGCGCAATGCCTATAATGGCTAATTTGGCTAATCTTAATTCTCATACTATAGGCTCATTGTATGTTTCTTGGAGTGATGGTGATGTAATTACTGGCGTCCCAAAACCTGTTGAGGGTGTTAGTTCCTTTATGAATGTTACATTGAATAAATGTACTAAATATAACATTTATGATGTATCTGGTGTGGGTGTTATTCGCATTAGCAATGACACCTTTCTTAATGGAATTACGTACACATCAACTTCAGGTAACCTTCTGGGTTTTAAAGATGTTACTAATGGCACCATCTACTCTATCACTCCTTGTAACCCACCAGATCAGCTTGTTGTTTATCAGCAAGCTGTTGTTGGTGCTATGTTGTCTGAAAACTTTACTAGTTACGGCTTTTCTAATGTTGTAGAAATGCCGAAATTTTTCTATGCGTCCAATGGCACTTATAATTGCACAGACGCTGTTTTAACTTATTCTAGTTTTGGCGTTTGTGCAGATGGTTCTATAATTGCTGTTCAACCACGTAATGTTTCATATGATAGTGTTTCAGCTATCGTCACAGCTAATTTGTCTATACCCTCCAATTGGACCACTTCGGTCCAGGTTGAGTATTTACAAATTACAAGTACACCTATCGTAGTTGATTGCTCCACTTATGTTTGCAATGGTAATGTGCGCTGTGTTGAATTGCTTAAGCAGTATACTTCTGCTTGTAAAACTATTGAAGACGCCTTAAGAAATAGCGCCATGCTGGAGTCTGCAGATGTTAGTGAGATGCTCACTTTTGACAAGAAAGCGTTTACACTTGCTAATGTTAGTAGTTTTGGTGACTACAACCTTAGCAGTGTTATACCTAGCTTGCCCAGAAGTGGTAGTAGAGTGGCTGGTCGCAGTGCCATAGAAGACATACTTTTTAGCAAACTTGTTACGTCTGGACTTGGCACTGTGGACGCAGACTACAAAAAGTGCACTAAGGGTCTTTCCATTGCTGACTTGGCTTGTGCCCAATATTATAATGGCATTATGGTTTTGCCTGGCGTCGCTGATGCTGAACGAATGGCCATGTATACAGGTTCTTTAATTGGTGGAATTGCTTTAGGAGGTCTTACATCAGCCGCTTCAATACCATTTTCATTAGCAATTCAGTCACGTTTAAATTATGTTGCATTGCAGACTGATGTTTTACAAGAAAATCAGAAAATTCTTGCTGCATCTTTTAATAAAGCAATGACCAACATAGTAGATGCCTTTACTGGTGTTAATGATGCTATTACACAAACTTCACAAGCTCTACAAACAGTTGCTACTGCACTTAATAAGATCCAGGATGTTGTTAATCAACAAGGCAACTCATTGAACCATTTAACTTCTCAGTTGAGGCAGAATTTTCAAGCCATCTCTAGCTCTATTCAGGCTATCTATGACAGACTTGATATTATTCAGGCTGATCAACAAGTAGATAGGCTGATTACTGGTAGATTGGCTGCTCTGAATGTATTCGTTTCTCATACATTGACTAAGTACACTGAAGTTCGTGCTTCCAGACAGCTTGCACAACAAAAAGTGAATGAGTGTGTCAAATCCCAGTCTAAGCGGTATGGCTTCTGTGGAAATGGCACGCACATTTTCTCACTTGTTAATGCTGCTCCTGAGGGGCTTGTTTTTCTCCATACTGTCTTGTTGCCGACACAATATAAGGATGTTGAAGCGTGGTCTGGGTTGTGCGTTGATGGTAGAAACGGTTATGTGTTGAGACAACCTAATCTTGCTCTTTACAAAGAAGGCAATTATTATAGAATTACATCTCGCATAATGTTTGAACCACGTATTC

CTACCATAGCAGATTTTGTCCAAATTGAAAATTGCAATGTCACATTTGTTAACATTTCTCGCTCTGAGTTGCAAACTAT
TGTGCCAGAGTATATTGATGTTAATAAGACGCTGCAAGAATTAAGTTACAAATTGCCAAATTACACTGTTCCAGACCTA
GTTGTCGAACAGTACAACCAGACTATTTTGAATTTGACCAGTGAAATTAGCACCCTTGAAAATAAATCTGCGGAGCTTA
ATTACACTGTTCAAAAACTGCAAACTTTGATTGACAACATAAATAGCACATTAGTTGACTTAAAGTGGCTCAACCGGGT
TGAGACTTATATCAAGTGGCCGTGGTGGGTGTGGTTGTGCATTTCAGTCGTGCTCATCTTTGTGGTGAGTATGTTGCTA
TTATGTTGTTGTTCTACTGGTTGCTGTGGCTTCTTTAGTTGTTTTGCATCTTCTATTAGAGGTTGTTGTGAATCAACTA
AACTTCCTTATTACGACGTTGAAAAGATCCACATACAGTAA

>gi|82780474|gb|DQ243974.1|_Human_coronavirus_229E_isolate_HCoV22925/6/92_spike_glycoprotein_(S)_gene_complete_cds

ATGTTTGTTTTACTTGTTGCATATGCCTTGTTGCATATTGCTGGTTGTCAAACTACAAATGGGATGAACACTAGT
CACTCTGTTTGCAACGGCTGTGTTGGTCATTCAGAAAATGTATTTGCTGTTGAGAGTGGTGGTTATATACCCTCCGACT
TTGCATTTAATAATTGGTTCCTCTTAACTAATACCTCATCTGTTGTAGATGGTGTTGTGAGGAGTTTTCAGCCTTTGTT
GCTTAATTGCTTATGGTCTGTTTCTGGCTCGCGGTTTACTACTGGTTTTGTCTATTTTAATGGTACTGGGAGAGGTGAT
TGTAAAGGTTTTTATTCAAATGCTTCGTCTGATGTCATACGTTACAACATCAATTTTGAAGAAAACCTTAGACGTGGAA
CCATTTTGTTTAAAACATCTTATGGTGCTGTTGTGTTTTATTGTACCAACAACACTTTGGTTTCAGGTGATGCTCACAT
ACCATCTGGTACAGTTTTGGGCAATTTTTATTGCTTTGTAAATACTACTATTGGCAATGAAACTACATCTGCTTTTGTG
GGTGCACTACCTAAGACAGTTCGTGAGTTTGTTATTTCACGCACAGGACATTTCTATATTAATGGCTATCGCTATTTCA
CTTTAGGTAATGTAGAAGCCGTTAATTTCAATGTCACTAATGCAGAAACCACTGTTTGTACTGTTGCGTTAGCTTCTTA
TGCTGACGTTTTGGTTAATGTGTCACAAACTGCTATTGCTAATATAATTTATTGCAACTCTGTTATTAACAGACTGAGA
TGTGACCAGTTGTCCTTTGATGTACCAGATGGTTTTTATTCCACAAGCCCTATTCAATCCGTTGAGCTACCTGTGTCTA
TTGTGTCGCTACCTGTTTATCACAAACACACGTTTATTGTGTTGTATGTTAACTTCAAACTTCGGAGTGGCGTTGGCAG
GTGCTATAACTGTCGTCCTGCTGTTGTTAATATTACACTGGCCAATTTTAATGAAACTAAAGGGCCTTTGTGTGTTGAC
ACATCACACTTCACTACCCAATTCGTTGGTGCCAAGTTTGATAGGTGGAGTGCTAGTATTAACACGGGAAATTGCCCTT
TTTCTTTTGGCAAAGTTAATAACTTTGTTAAATTTGGCAGTGTATGTTTTTCGCTAAAGGATATACCCGGTGGTTGCGC
AATGCCTATAATGGCTAATTTGGCTAATCTTAATTCTCATACTATAGGCTCATTGTATGTTTCTTGGAGTGATGGTGAT
GTAATTACTGGCGTCCCAAAACCTGTTGAGGGTGTTAGTTCCTTTATGAATGTTACATTGAATAAATGTACTAAATATA
ACATTTATGATGTATCTGGTGTGGGTGTTATTCGCATTAGCAATGACACCTTTCTTAATGGAATTACGTACACATCAAC
TTCAGGTAACCTTCTGGGTTTTAAAGATGTTACTAATGGCACCATCTACTCTATCACTCCTTGTAACCCACCAGATCAG
CTTGTTGTTTATCAGCAAGCTGTTGTTGGTGCTATGTTGTCTGAAAACTTTACTAGTTACGGCTTTTCTAATGTTGTAG
AAATGCCGAAATTTTTCTATGCGTCCAATGGCACTTATAATTGCACAGACGCTGTTTTAACTTATTCTAGTTTTGGCGT
TTGTGCAGATGGTTCTATAATTGCTGTTCAACCACGTAATGTTTCATATGATAGTGTTTCAGCTATCGTCACAGCTAAT
TTGTCTATACCCTCCAATTGGACCACTTCGGTCCAGGTTGAGTATTTACAAATTACAAGTACACCTATCGTAGTTGATT
GCTCCACTTATGTTTGCAATGGTAATGTGCGCTGTGTTGAATTGCTTAAGCAGTATACTTCTGCTTGTAAAACTATTGA
AGACGCCTTAAGAAATAGCGCCATGCTGGAGTCTGCAGATGTTAGTGAGATGCTCACTTTTGACAAGAAAGCGTTTACA
CTTGCTAATGTTAGTAGTTTTGGTGACTACAACCTTAGCAGTGTTATACCTAGCTTGCCCAGAAGTGGTAGTAGAGTGG
CTGGTCGCAGTGCCATAGAAGACATACTTTTTAGCAAACTTGTTACGTCTGGACTTGGCACTGTGGACGCAGACTACAA
AAAGTGCACTAAGGGTCTTTCCATTGCTGACTTGGCTTGTGCCCAATATTATAATGGCATTATGGTTTTGCCTGGCGTC
GCTGATGCTGAACGAATGGCCATGTATACAGGTTCTTTAATTGGTGGAATTGCTTTAGGAGGTCTTACATCAGCCGCTT
CAATACCATTTTCATTAGCAATTCAGTCACGTTTAAATTATGTTGCATTGCAGACTGATGTTTTACAAGAAAATCAGAA
AATTCTTGCTGCATCTTTTAATAAAGCAATGACCAACATAGTAGATGCCTTTACTGGTGTTAATGATGCTATTACACAA
ACTTCACAAGCTCTACAAACAGTTGCTACTGCACTTAATAAGATCCAGGATGTTGTTAATCAACAAGGCAACTCATTGA

ACCATTTAACTTCTCAGTTGAGGCAGAATTTTCAAGCCATCTCTAGCTCTATTCAGGCTATCTATGACAGACTTGATAT
TATTCAGGCTGATCAACAAGTAGATAGGCTGATTACTGGTAGATTGGCTGCTCTGAATGTATTCGTTTCTCATACATTG
ACTAAGTACACTGAAGTTCGTGCTTCCAGACAGCTTGCACAACAAAAAGTGAATGAGTGTGTCAAATCCCAGTCTAAGC
GGTATGGCTTCTGTGGAAATGGCACGCACATTTTCTCACTTGTTAATGCTGCTCCTGAGGGGCTTGTTTTTCTCCATAC
TGTCTTGTTGCCGACACAATATAAGGATGTTGAAGCGTGGTCTGGGTTGTGCGTTGATGGTAGAAACGGTTATGTGTTG
AGACAACCTAATCTTGCTCTTTACAAAGAAGGCAATTATTATAGAATTACATCTCGCATAATGTTTGAACCACGTATTC
CTACCATAGCAGATTTTGTCCAAATTGAAAATTGCAATGTCACATTTGTTAACATTTCTCGCTCTGAGTTGCAAACTAT
TGTGCCAGAGTATATTGATGTTAATAAGACGCTGCAAGAATTAAGTTACAAATTGCCAAATTACACTGTTCCAGACCTA
GTTGTCGAACAGTACAACCAGACTATTTTGAATTTGACCAGTGAAATTAGCACCCTTGAAAATAAATCTGCGGAGCTTA
ATTACACTGTTCAAAAACTGCAAACTTTGATTGACAACATAAATAGCACATTAGTTGACTTAAAGTGGCTCAACCGGGT
TGAGACTTATATCAAGTGGCCGTGGTGGGTGTGGTTGTGCATTTCAGTCGTGCTCATCTTTGTGGTGAGTATGTTGCTA
TTATGTTGTTGTTCTACTGGTTGCTGTGGCTTCTTTAGTTGTTTTGCATCTTCTATTAGAGGTTGTTGTGAATCAACTA
AACTTCCTTATTACGACGTTGAAAAGATCCACATACAGTAA

>gi|82780476|gb|DQ243975.1|_Human_coronavirus_229E_isolate_HCoV229E12/5/92_spike_glycoprotein_(S)_gene_complete_cds

ATGTTTGTTTTACTTGTTGCATATGCCTTGTTGCATATTGCTGGTTGTCAAACTACAAATGGGATGAACACTAGT
CACTCTGTTTGCAACGGCTGTGTTGGTCATTCAGAAAATGTATTTGCTGTTGAGAGTGGTGGTTATATACCCTCCGACT
TTGCATTTAATAATTGGTTCCTCTTAACTAATACCTCATCTGTTGTAGATGGTGTTGTGAGGAGTTTTCAGCCTTTGTT
GCTTAATTGCTTATGGTCTGTTTCTGGCTCGCGGTTTACTACTGGTTTTGTCTATTTTAATGGTACTGGGAGAGGTGAT
TGTAAAGGTTTTTATTCAAATGCTTCGTCTGATGTCATACGTTACAACATCAATTTTGAAGAAAACCTTAGACGTGGAA
CCATTTTGTTTAAAACATCTTATGGTGCTGTTGTGTTTTATTGTACCAACAACACTTTGGTTTCAGGTGATGCTCACAT
ACCATCTGGTACAGTTTTGGGCAATTTTTATTGCTTTGTAAATACTACTATTGGCAATGAAACTACGTCTGCTTTTGTG
GGTGCACTACCTAAGACAGTTCGTGAGTTTGTTATTTCACGCACAGGACATTTCTATATTAATGGCTATCGCTATTTCA
CTTTAGGTAATGTAGAAGCCGTTAATTTCAATGTCACTAATGCAGAAACCACTGTTTGTACTGTTGCGTTAGCTTCTTA
TGCTGACGTTTTGGTTAATGTGTCACAAACTGCTATTGCTAATATAATTTATTGCAACTCTGTTATTAACAGACTGAGA
TGTGACCAGTTGTCCTTTGATGTACCAGATGGTTTTTACTCCACAAGCCCTATTCAATCCGTTGAGCTACCTGTGTCTA
TTGTGTCGCTACCTGTTTATCACAAACACACGTTTATTGTGTTGTATGTTAACTTCAAACTTCGGAGTGGCGTTGGCAG
GTGCTATAACTGTCGTCCTGCTGTTGTTAATATTACACTGGCCAATTTTAATGAAACTAAAGGGCCTTTGTGTGTTGAC
ACATCACACTTCACTACCCAATTCGTTGGTGCCAAGTTTGATAGGTGGAGTGCTAGTATTAACACGGGAAATTGCCCTT
TTTCTTTTGGCAAAGTTAATAACTTTGTTAAATTTGGCAGTGTATGTTTTTCGCTAAAGGATATACCCGGTGGTTGCGC
AATGCCTATAATGGCTAATTTGGCTAATCTTAATTCTCATACTATAGGCTCATTGTATGTTTCTTGGAGTGATGGTGAT
GTAATTACTGGCGTCCCAAAACCTGTTGAGGGTGTTAGTTCCTTTATGAATGTTACATTGAATAAATGTACTAAATATA
ACATTTATGATGTATCTGGTGTGGGTGTTATTCGCATTAGCAATGACACCTTTCTTAATGGAATTACGTACACATCAAC
TTCAGGTAACCTTCTGGGTTTTAAAGATGTTACTAATGGCACCATCTACTCTATCACTCCTTGTAACCCACCAGATCAG
CTTGTTGTTTATCAGCAAGCTGTTGTTGGTGCTATGTTGTCTGAAAACTTTACTAGTTACGGCTTTTCTAATGTTGTAG
AAATGCCGAAATTTTTCTATGCGTCCAATGGCACTTATAATTGCACAGACGCTGTTTTAACTTATTCTAGTTTTGGCGT
TTGTGCAGATGGTTCTATAATTGCTGTTCAACCACGTAATGTTTCATATGATAGTGTTTCAGCTATCGTCACAGCTAAT
TTGTCTATACCCTCCAATTGGACCACTTCGGTCCAGGTTGAGTATTTACAAATTACAAGTACACCTATCGTAGTTGATT
GCTCCACTTATGTTTGCAATGGTAATGTGCGCTGTGTTGAATTGCTTAAGCAGTATACTTCTGCTTGTAAAACTATTGA
AGACGCCTTAAGAAATAGCGCCATGCTGGAGTCTGCAGATGTTAGTGAGATGCTCACTTTTGACAAGAAAGCGTTTACA
CTTGCTAATGTTAGTAGTTTTGGTGACTACAACCTTAGCAGTGTTATACCTAGCTTGCCCAGAAGTGGTAGTAGAGTGG

CTGGTCGCAGTGCCATAGAAGACATACTTTTTAGCAAACTTGTTACGTCTGGACTTGGCACTGTGGACGCAGACTACAA
AAAGTGCACTAAGGGTCTTTCCATTGCTGACTTGGCTTGTGCCCAATATTATAATGGCATTATGGTTTTGCCTGGCGTC
GCTGATGCTGAACGAATGGCCATGTATACAGGTTCTTTAATTGGTGGAATTGCTTTAGGAGGTCTTACATCAGCCGCTT
CAATACCATTTTCATTAGCAATTCAGTCACGTTTAAATTATGTTGCATTGCAGACTGATGTTTTACAAGAAAATCAGAA
AATTCTTGCTGCATCTTTTAATAAAGCAATGACCAACATAGTAGATGCCTTTACTGGTGTTAATGATGCTATTACACAA
ACTTCACAAGCTCTACAAACAGTTGCTACTGCACTTAATAAGATCCAGGATGTTGTTAATCAACAAGGCAACTCATTGA
ACCATTTAACTTCTCAGTTGAGGCAGAATTTTCAAGCCATCTCTAGCTCTATTCAAGCTATCTATGACAGACTTGATAT
TATTCAGGCTGATCAACAAGTAGATAGGCTGATTACTGGTAGATTGGCTGCTCTGAATGTATTCGTTTCTCATACATTG
ACTAAGTACACTGAAGTTCGTGCTTCCAGACAGCTTGCACAACAAAAAGTGAATGAGTGTGTCAAATCCCAGTCTAAGC
GGTATGGCTTCTGTGGAAATGGCACGCACATTTTCTCACTTGTTAATGCTGCTCCTGAGGGGCTTGTTTTTCTCCATAC
TGTCTTGTTGCCGACACAATATAAGGATGTTGAAGCGTGGTCTGGGTTGTGCGTTGATGGTAGAAACGGTTATGTGTTG
AGACAACCTAATCTTGCTCTTTACAAAGAAGGCAATTATTATAGAATTACATCTCGCATAATGTTTGAACCACGTATTC
CTACCATAGCAGATTTTGTCCAAATTGAAAATTGCAATGTCACATTTGTTAACATTTCTCGCTCTGAGTTGCAAACTAT
TGTGCCAGAGTATATTGATGTTAATAAGACGCTGCAAGAATTAAGTTACAAATTGCCAAATTACACTGTTCCAGACCTA
GTTGTCGAACAGTACAACCAGACTATTTTGAATTTGACCAGTGAAATTAGCACCCTTGAAAATAAATCTGCGGAGCTTA
ATTACACTGTTCAAAAACTGCAAACTTTGATTGACAACATAAATAGCACATTAGTTGACTTAAAGTGGCTCAACCGGGT
TGAGACTTATATCAAGTGGCCGTGGTGGGTGTGGTTGTGCATTTCAGTCGTGCTCATCTTTGTGGTGAGTATGTTGCTA
TTATGTTGTTGTTCTACTGGTTGCTGTGGCTTCTTTAGTTGTTTTGCATCTTCTATTAGAGGTTGTTGTGAATCAACTA
AACTTCCTTATTACGACGTTGAAAAGATCCACATACAGTAA

>gi|82780478|gb|DQ243976.1|_Human_coronavirus_229E_isolate_HCoV229E17/6/92_spike_glycoprotein_(S)_gene_complete_cds

ATGTTTGTTTTACTTGTTGCATATGCCTTGTTGCATATTGCTGGTTGTCAAACTACAAATGGGATGAACACTAGT
CACTCTGTTTGCAACGGCTGTGTTGGTCATTCAGAAAATGTATTTGCTGTTGAGAGTGGTGGTTATATACCCTCCGACT
TTGCATTTAATAATTGGTTCCTCTTAACTAATACCTCATCTGTTGTAGATGGTGTTGTGAGGAGTTTTCAGCCTTTGTT
GCTTAATTGCTTATGGTCTGTTTCTGGCTCGCGGTTTACTACTGGTTTTGTCTATTTTAATGGTACTGGGAGAGGTGAT
TGTAAAGGTTTTTATTCAAATGCTTCGTCTGATGTCATACGTTACAACATCAATTTTGAAGAAAACCTTAGACGTGGAA
CCATTTTGTTTAAAACATCTTATGGTGCTGTTGTGTTTTATTGTACCAACAACACTTTGGTTTCAGGTGATGCTCACAT
ACCATCTGGTACAGTTTTGGGCAATTTTTATTGCTTTGTAAATACTACTATTGGCAATGAAACTACGTCTGCTTTTGTG
GGTGCACTACCTAAGACAGTTCGTGAGTTTGTTATTTCACGCACAGGACATTTCTATATTAATGGCTATCGCTATTTCA
CTTTAGGTAATGTAGAAGCCGTTAATTTCAATGTCACTAATGCAGAAACCACTGTTTGTACTGTTGCGTTAGCTTCTTA
TGCTGACGTTTTGGTTAATGTGTCACAAACTGCTATTGCTAATATAATTTATTGCAACTCTGTTATTAACAGACTGAGA
TGTGACCAGTTGTCCTTTGATGTACCAGATGGTTTTTATTCCACAAGCCCTATTCAATCCGTTGAGCTACCTGTGTCTA
TTGTGTCGCTACCTGTTTATCACAAACACACGTTTATTGTGTTGTATGTTAACTTCAAACTTCGGAGTGGCGTTGGCAG
GTGCTATAACTGTCGTCCTGCTGTTGTTAATATTACACTGGCCAATTTTAATGAAACTAAAGGGCCTTTGTGTGTTGAC
ACATCACACTTCACTACCCAATTCGTTGGTGCCAAGTTTGATAGGTGGAGTGCTAGTATTAACACGGGAAATTGCCCTT
TTTCTTTTGGCAAAGTTAATAACTTTGTTAAATTTGGCAGTGTATGTTTTTCGCTAAAGGATATACCCGGTGGTTGCGC
AATGCCTATAATGGCTAATTTGGCTAATCTTAATTCTCATACTATAGGCTCATTGTATGTTTCTTGGAGTGATGGTGAT
GTAATTACTGGCGTCCCAAAACCTGTTGAGGGTGTTAGTTCCTTTATGAATGTTACATTGAATAAATGTACTAAATATA
ACATTTATGATGTATCTGGTGTGGGTGTTATTCGCATTAGCAATGACACCTTTCTTAATGGAATTACGTACACATCAAC
TTCAGGTAACCTTCTGGGTTTTAAAGATGTTACTAATGGCACCATCTACTCTATCACTCCTTGTAACCCACCAGATCAG
CTTGTTGTTTATCAGCAAGCTGTTGTTGGTGCTATGTTGTCTGAAAACTTTACTAGTTACGGCTTTTCTAATGTTGTAG

AAATGCCGAAATTTTTCTATGCGTCCAATGGCACTTATAATTGCACAGACGCTGTTTTAACTTATTCTAGTTTTGGCGT
TTGTGCAGATGGTTCTATAATTGCTGTTCAACCACGTAATGTTTCATATGATAGTGTTTCAGCTATCGTCACAGCTAAT
TTGTCTATACCCTCCAATTGGACCACTTCGGTCCAGGTTGAGTATTTACAAATTACAAGTACACCTATCGTAGTTGATT
GCTCCACTTATGTTTGCAATGGTAATGTGCGCTGTGTTGAATTGCTTAAGCAGTATACTTCTGCTTGTAAAACTATTGA
AGACGCCTTAAGAAATAGCGCCATGCTGGAGTCTGCAGATGTTAGTGAGATGCTCACTTTTGACAAGAAAGCGTTTACA
CTTGCTAATGTTAGTAGTTTTGGTGACTACAACCTTAGCAGTGTTATACCTAGCTTGCCCAGAAGTGGTAGTAGAGTGG
CTGGTCGCAGTGCCATAGAAGACATACTTTTTAGCAAACTTGTTACGTCTGGACTTGGCACTGTGGACGCAGACTACAA
AAAGTGCACTAAGGGTCTTTCCATTGCTGACTTGGCTTGTGCCCAATATTATAATGGCATTATGGTTTTGCCTGGCGTC
GCTGATGCTGAACGAATGGCCATGTATACAGGTTCTTTAATTGGTGGAATTGCTTTAGGAGGTCTTACATCAGCCGCTT
CAATACCATTTTCATTAGCAATTCAGTCACGTTTAAATTATGTTGCATTGCAGACTGATGTTTTACAAGAAAATCAGAA
AATTCTTGCTGCATCTTTTAATAAAGCAATGACCAACATAGTAGATGCCTTTACTGGTGTTAATGATGCTATTACACAA
ACTTCACAAGCTCTACAAACAGTTTCTACTGCACTTAATAAGATCCAGGATGTTGTTAATCAACAAGGCAACTCATTGA
ACCATTTAACTTCTCAGTTGAGGCAGAATTTTCAAGCCATCTCTAGCTCTATTCAGGCTATCTATGACAGACTTGATAT
TATTCAGGCTGATCAACAAGTAGATAGGCTGATTACTGGTAGATTGGCTGCTCTGAATGTATTCGTTTCTCATACATTG
ACTAAGTACACTGAAGTTCGTGCTTCCAGACAGCTTGCACAACAAAAAGTGAATGAGTGTGTCAAATCCCAGTCTAAGC
GGTATGGCTTCTGTGGAAATGGCACGCACATTTTCTCACTTGTTAATGCTGCTCCTGAGGGGCTTGTTTTTCTCCATAC
TGTCTTGTTGCCGACACAATATAAGGATGTTGAAGCGTGGTCTGGGTTGTGCGTTGATGGTAGAAACGGTTATGTGTTG
AGACAACCTAATCTTGCTCTTTACAAAGAAGGCAATTATTATAGAATTACATCTCGCATAATGTTTGAACCACGTATTC
CTACCATAGCAGATTTTGTCCAAATTGAAAATTGCAATGTCACATTTGTTAACATTTCTCGCTCTGAGTTGCAAACTAT
TGTGCCAGAGTATATTGATGTTAATAAGACGCTGCAAGAATTAAGTTACAAATTGCCAAATTACACTGTTCCAGACCTA
GTTGTCGAACAGTACAACCAGACTATTTTGAATTTGACCAGTGAAATTAGCACCCTTGAAAATAAATCTGCGGAGCTTA
ATTACACTGTTCAAAAACTGCAAACTTTGATTGACAACATAAATAGCACATTAGTTGACTTAAAGTGGCTCAACCGGGT
TGAGACTTATATCAAGTGGCCGTGGTGGGTGTGGTTGTGCATTTCAGTCGTGCTCATCTTTGTGGTGAGTATGTTGCTA
TTATGTTGTTGTTCTACTGGTTGCTGTGGCTTCTTTAGTTGTTTTGCATCTTCTATTAGAGGTTGTTGTGAATCAACTA
AACTTCCTTATTACGACGTTGAAAAGATCCACATACAGTAA

>gi|82780480|gb|DQ243977.1|_Human_coronavirus_229E_strain_HCoV229E8/8/01_spike_glycoprotein_(S)_gene_complete_cds

ATGTTTGTTTTACTTGTTGCATATGCCTTGTTGCATATTGCTGGTTGTCAAACTACAAATGGGATGAACACTAGT
CAGTCTGTTTGCAACGGCTGTGTTGGTCATTCGGAAAATGTATTTGCTGTTGAGAGTGGTGGTTATATACCCTCCAACT
TTGCATTTAATAATTGGTTCCTCTTAACTAATACCTCATCTGTTGTAGATGGTGTTGTGAGGAGTTTTCAGCCTTTGTT
GCTTAATTGCTTATGGTCTGTTTCTGGCTCGCGGCTTACTACTGGTTTTGTCTATTTTAATGGTACTGGGAGAGGTGAT
TGTAAAGGTTTTTATTCAAATGCTTCGTCTGATGTCATACGTTACAACATCAATTTTGAAGAAAACCTTAGACGTGGAA
CCATTTTGTTTAAAACATCTTATGGTGCTGTTGTGTTTTATTGTACCAACAACACTTTGGTTTCAGGTGATGCTCACAT
ACCATCTGGTACAGTTTTGGGCAATTTTTATTGCTTTGTAAATACTACTATTGGCAATGAAACTACGTCTGCTTTTGTG
GGTGCACTACCTAAGACAGTTCGTGAGTTTGTTATTTCACGCACAGGACATTTCTATATTAATGGCTATCGCTATTTCT
CTTTAGGTAATGTAGAAGCCGTTAATTTCAATGTCACTAATGCTGCAACCACTGTTTGTACTGTTGCGTTAGCTTCTTA
TGCTGACGTTTTGGTTAATGTGTCACAAACTGCTATTGCTAATATAATTTATTGCAACTCTGTTATTAACAGACTGAGA
TGTGACCAGTTGTCCTTTGATGTACCAGATGGTTTTTATTCCACAAGCCCTATTCAATCCGTTGAGCTACCTGTGTCTA
TTGTGTCGCTACCTGTTTATCACAAACACACGTTTATTGTGTTGTATGTTAACTTCGAACTTCGGCGTGGCCCTGGCAG
GTGCTATAACTGTCGTCCTGCTGTTGTTAATATTACACTGGCCAATTTTAATGAAACTAAAGGGCCTTTGTGTGTTGAC
ACATCACACTTCACCACCCAATTCGTTGGTGTCAAGTTTGATAGGTGGAGTGCTAGTATTAACACGGGAAATTGCCCTT

TTTCTTTTGGCAAAGTTAATAACTTTGTTAAATTTGGCAGTGTATGTTTTTCGCTAAAGGATATACCCGGTGGTTGCGC
AATGCCTATAATGGCTAATTTGGCTAATCTTAATTCTCATAATATAGGCTCATTGTATGTTTCTTGGAGTGATGGTGAT
GTAATTACTGGCGTCCCAAAACCTGTTGAGGGTGTTAGTTCCTTTATGAATGTTACATTGAATAAATGTACTAAATATA
ACATTTATGATGTATCTGGTGTGGGTGTTATTCGCATTAGCAATGACACCTTTCTTAATGGAATTACGTACACATCAAC
TTCAGGTAACCTTCTGGGTTTTAAAGATGTTACTAATGGCACCATCTACTCTATCACTCCTTGTAACCCACCAGATCAG
CTTGTTGTTTATCAGCAAGCTGTTGTTGGTGCTATGTTGTCTGAAAACTTTACTAGTTACGGCTTTTCTAATGTTGTAG
AAATGCCGAAATTTTTCTATGCGTCCAATGGCACTTATAATTGCACAGACGCTGTTTTAACTTATTCTAGTTTTGGCGT
TTGTGCAGATGGTTCTATAATTGCTGTTCAACCACGTAATGTTTCATATGATAGTGTTTCAGCTATTGTCACAGCTAAT
TTGTCTATACCCTCCAATTGGACCACTTCGGTCCAGGTTGAGTATTTACAAATTACAAGTACACCTATCGTAGTTGATT
GCTCCACTTATGTTTGCAATGGTAATGTGCGCTGTGTTGAATTGCTTAAGCAGTATACTTCTGCTTGTAAAACTATTGA
AGACGCCTTAAGAAATAGCGCCATGCTGGAGTCTGCAGATGTTAGTGAGATGCTCACTTTTGACAAGAAAGCGTTTACA
CTTGCTAATGTTAGTAGTTTTGGTGACTACAACCTTAGCAGTGTTATACCTAGCTTGCCCAGAAGTGGTAGTAGAGTGG
CTGGTCGCAGTGCCATAGAAGACATACTTTTTAGCAAACTTGTTACGTCTGGACTTGGCACTGTGGACGCAGACTACAA
AAAGTGTACTAAGGGTCTTTCCATTGCTGACTTGGCTTGTGCCCAATATTATAATGGCATTATGGTTTTGCCTGGCGTC
GCTGATGCTGAACGTATGGCCATGTATACAGGTTCTTTAATTGGTGGAATTGCTTTAGGAGGTCTTACATCAGCCGCTT
CAATACCATTTTCATTAGCAATTCAGTCACGTTTAAATTATGTTGCATTGCAGACTGATGTTTTACAAGAAAATCAGAA
AATTCTTGCTGCATCTTTTAATAAAGCAATGACCAACATAGTAGATGCCTTTACTGGTGTTAATGATGCTATTACACAA
ACTTCACAAGCTCTACAAACAGTTGCTACTGCACTTAATAAGATCCAGGATGTTGTTAATCAACAAGGCAACTCATTGA
ACCATTTAACTTTTCAGTTGAGGCAGAATTTTCAAGCCATTTCTAGCTCTATTCAGGCTATCTATGACAGACTTGATAT
TATTCAGGCTGATCAACAAGTAGATAGGCTGATTACTGGTAGATTGGCTGCTCTGAATGTATTCGTTTCTCATACATTG
ACTAAGTACACTGAAGTTCGTGCTTCCAGACAGCTTGCACAACAAAAAGTGAATGAGTGTGTCAAATCCCAGTCTAAGC
GGTATGGCTTCTGTGGAAATGGCACGCACATTTTCTCACTTGTTAATGCTGCTCCTGAGGGGCTTGTTTTTCTCCATAC
TGTCTTGTTGCCGACACAATATAAGGATGTTGAAGCGTGGTCTGGGTTGTGCGTTGATGGTAGAAACGGTTATGTGTTG
AGACAACCTAATCTTGCTCTTTACAAAGAAGGCAATTATTATAGAATTACATCTCGCATAATGTTTGAACCACGTATTC
CTACCATAGCAGATTTTGTCCAAATTGAAAATTGCAATGTCACATTTGTTAACATTTCTCGCTCTGAGTTGCAAACTAT
TGTGCCAGAGTATATTGATGTTAATAAGACGCTGCAAGAATTAAGTTACAAATTGCCAAATTACACTGTTCCAGACCTA
GTTGTCGAACAGTACAACCAGACTATTTTGAATTTGACCAGTGAAATTAGCACCCTTGAAAATAAATCTGCGGAGCTTA
ATTACACTGTTCAAAAACTGCAAACTTTGATCGACAACATAAATAGCACATTAGTTGACTTAAAGTGGCTCAACCGTGT
TGAGACTTATATCAAGTGGCCGTGGTGGGTGTGGTTGTGCATTTCAGTCGTGCTCATCTTTGTGGTGAGTATGTTGCTA
TTATGTTGTTGTTCTACTGGTTGCTGCGGCTTCTTTAGTTGTTTTGCATCTTCTATTAGAGGTTGTTGTGAATCAACTA
AACTTCCTTATTACGACGTTGAAAAGATCCACATACAGTAA

>gi|82780482|gb|DQ243978.1|_Human_coronavirus_229E_strain_HCoV229E27/8/01_spike_glycoprotein_(S)_gene_complete_cds

ATGTTTGTTTTACTTGTTGCATATGCCTTGTTGCATATTGCTGGTTGTCAAACTACAAATGGGATGAACACTAGT
CAGTCTGTTTGCAACGGCTGTGTTGGTCATTCGGAAAATGTATTTGCTGTTGAGAGTGGTGGTTATATACCCTCCAACT
TTGCATTTAATAATTGGTTCCTCTTAACTAATACCTCATCTGTTGTAGATGGTGTTGTGAGGAGTTTTCAGCCTTTGTT
GCTTAATTGCTTATGGTCTGTTTCTGGCTCGCGGCTTACTACTGGTTTTGTCTATTTTAATGGTACTGGGAGAGGTGAT
TGTAAAGGTTTTTATTCAAATGCTTCGTCTGATGTCATACGTTACAACATCAATTTTGAAGAAAACCTTAGACGTGGAA
CCATTTTGTTTAAAACATCTTATGGTGCTGTTGTGTTTTATTGTACCAACAACACTTTGGTTTCAGGTGATGCTCACAT
ACCATCTGGTACAGTTTTGGGCAATTTTTATTGCTTTGTAAATACTACTATTGGCAATGAAACTACGTCTGCTTTTGTG
GGTGCACTACCTAAGACAGTTCGTGAGTTTGTTATTTCACGCACAGGACATTTCTATATTAATGGCTATCGCTATTTCT

CTTTAGGTAATGTAGAAGCCGTTAATTTCAATGTCACTAATGCTGCAACCACTGTTTGTACTGTTGCGTTAGCTTCTTA
TGCTGACGTTTTGGTTAATGTGTCACAAACTGCTATTGCTAATATAATTTATTGCAACTCTGTTATTAACAGACTGAGA
TGTGACCAGTTGTCCTTTGATGTACCAGATGGTTTTTATTCCACAAGCCCTATTCAATCCGTTGAGCTACCTGTGTCTA
TTGTGTCGCTACCTGTTTATCACAAACACACGTTTATTGTGTTGTATGTTAACTTCGAACTTCGGCGTGGCCCTGGCAG
GTGCTATAACTGTCGTCCTGCTGTTGTTAATATTACACTGGCCAATTTTAATGAAACTAAAGGGCCTTTGTGTGTTGAC
ACATCACACTTCACTACCCAATTCGTTGGTGTCAAGTTTGATAGGTGGAGTGCTAGTATTAACACGGGAAATTGCCCTT
TTTCTTTTGGCAAAGTTAATAACTTTGTTAAATTTGGCAGTGTATGTTTTTCGCTAAAGGATATACCCGGTGGTTGCGC
AATGCCTATAATGGCTAATTTGGCTAATCTTAATTCTCATAATATAGGCTCATTGTATGTTTCTTGGAGTGATGGTGAT
GTAATTACTGGCGTCCCAAAACCTGTTGAGGGTGTTAGTTCCTTTATGAATGTTACATTGAATAAATGTACTAAATATA
ACATTTATGATGTATCTGGTGTGGGTGTTATTCGCATTAGCAATGACACCTTTCTTAATGGAATTACGTACACATCAAC
TTCAGGTAACCTTCTGGGTTTTAAAGATGTTACTAATGGCACCATCTACTCTATCACTCCTTGTAACCCACCAGATCAG
CTTGTTGTTTATCAGCAAGCTGTTGTTGGTGCTATGTTGTCTGAAAACTTTACTAGTTACGGCTTTTCTAATGTTGTAG
AAATGCCGAAATTTTTCTATGCGTCCAATGGCACTTATAATTGCACAGACGCTGTTTTAACTTATTCTAGTTTTGGCGT
TTGTGCAGATGGTTCTATAATTGCTGTTCAACCACGTAATGTTTCATATGATAGTGTTTCAGCTATTGTCACAGCTAAT
TTGTCTATACCCTCCAATTGGACCACTTCGGTCCAGGTTGAGTATTTACAAATTACAAGTACACCTATCGTAGTTGATT
GCTCCACTTATGTTTGCAATGGTAATGTGCGCTGTGTTGAATTGCTTAAGCAGTATACTTCTGCTTGTAAAACTATTGA
AGACGCCTTAAGAAATAGCGCCATGCTGGAGTCTGCAGATGTTAGTGAGATGCTCACTTTTGACAAGAAAGCGTTTACA
CTTGCTAATGTTAGTAGTTTTGGTGACTACAACCTTAGCAGTGTTATACCTAGCTTGCCCAGAAGTGGTAGTAGAGTGG
CTGGTCGCAGTGCCATAGAAGACATACTTTTTAGCAAACTTGTTACGTCTGGACTTGGCACTGTGGACGCAGACTACAA
AAAGTGTACTAAGGGTCTTTCCATTGCTGACTTGGCTTGTGCCCAATATTATAATGGCATTATGGTTTTGCCTGGCGTC
GCTGATGCTGAACGTATGGCCATGTATACAGGTTCTTTAATTGGTGGAATTGCTTTAGGAGGTCTTACATCAGCCGCTT
CAATACCATTTTCATTAGCAATTCAGTCACGTTTAAATTATGTTGCATTGCAGACTGATGTTTTACAAGAAAATCAGAA
AATTCTTGCTGCATCTTTTAATAAAGCAATGACCAACATAGTAGATGCCTTTACTGGTGTTAATGATGCTATTACACAA
ACTTCACAAGCTCTACAAACAGTTGCTACTGCACTTAATAAGATCCAGGATGTTGTTAATCAACAAGGCAACTCATTGA
ACCATTTAACTTTTCAGTTGAGGCAGAATTTTCAAGCCATTTCTAGCTCTATTCAGGCTATCTATGACAGACTTGATAT
TATTCAGGCTGATCAACAAGTAGATAGGCTGATTACTGGTAGATTGGCTGCTCTGAATGTATTCGTTTCTCATACATTG
ACTAAGTACACTGAAGTTCGTGCTTCCAGACAGCTTGCACAACAAAAAGTGAATGAGTGTGTCAAATCCCAGTCTAAGC
GGTATGGCTTCTGTGGAAATGGCACGCACATTTTCTCACTTGTTAATGCTGCTCCTGAGGGGCTTGTTTTTCTCCATAC
TGTCTTGTTGCCGACACAATATAAGGATGTTGAAGCGTGGTCTGGGTTGTGCGTTGATGGTAGAAACGGTTATGTGTTG
AGACAACCTAATCTTGCTCTTTACAAAGAAGGCAATTATTATAGAATTACATCTCGCATAATGTTTGAACCACGTATTC
CTACCATAGCAGATTTTGTCCAAATTGAAAATTGCAATGTCACATTTGTTAACATTTCTCGCTCTGAGTTGCAAACTAT
TGTGCCAGAGTATATTGATGTTAATAAGACGCTGCAAGAATTAAGTTACAAATTGCCAAATTACACTGTTCCAGACCTA
GTTGTCGAACAGTACAACCAGACTATTTTGAATTTGACCAGTGAAATTAGCACCCTTGAAAATAAATCTGCGGAGCTTA
ATTACACTGTTCAAAAACTGCAAACTTTGATCGACAACATAAATAGCACATTAGTTGACTTAAAGTGGCTCAACCGTGT
TGAGACTTATATCAAGTGGCCGTGGTGGGTGTGGTTGTGCATTTCAGTCGTGCTCATCTTTGTGGTGAGTATGTTGCTA
TTATGTTGTTGTTCTACTGGTTGCTGCGGCTTCTTTAGTTGTTTTGCATCTTCTATTAGAGGTTGTTGTGAATCAACTA
AACTTCCTTATTACGACGTTGAAAAGATCCACATACAGTAA

>gi|82780484|gb|DQ243979.1|_Human_coronavirus_229E_strain_HCoV229E21/6/02_spike_glycoprotein_(S)_gene_complete_cds

ATGTTTGTTTTACTTGTTGCATATGCCTTGTTGCATATTGCTGGTTGTCAAACTACAAATGGGATGAACACTAGT
CACTCTGTTTGCAACGGCTGTGTTGGTCATTCGGAAAATGTATTTGATGTTGAGAGTGGTGGTTATATACCATCCAACT

TTGCATTTAATAATTGGTTCCTCTTAACTAATACCTCATCTGTTGTAGATGGTGTTGTGAGGAGTTTTCAGCCTTTGTT
GCTTAATTGCTTATGGTCTGTTTCTGGCTCGCGGCTTACTACTGGTTTTGTCTATTTTAATGGTACTGGGAGAGGTGAT
TGTAAAGGTTTTTATTCAAATGCTTCGTCTGATGTCATACGTTACAACATCAATTTTGAAGAAAACCTTAGACGTGGAA
CCATTTTGTTTAAAACATCTTATGGTGCTGTTGTGTTTTATTGTACCAACAACACTTTGGTTTCAGGTGATGCTCACAT
ACCATCTGGTACAGTTTTGGGCAATTTTTATTGCTTTGTAAATACTACTATTGGCAATGAAACTACGTCTGCTTTTGTG
GGTGCACTACCTAAGACAGTTCGTGAGTTTGTTATTTCACGCACAGGACATTTCTATATTAATGGCTATCGCTATTTCT
CTTTAGGTAATGTAGAAGCCGTTAATTTCAATGTCACTAATGCTGCAACCACTGTTTGTACTGTTGCGTTAGCTTCTTA
TGCTGACGTTTTGGTTAATGTGTCACAAACTGCTATTGCTAATATAATTTATTGCAATTCTGTTATTAACAGACTGAGA
TGTGACCAGTTGTCCTTTGATGTACCAGATGGTTTTTATTCCACAAGCCCTATTCAATCCGTTGAGCTACCTGTGTCTA
TTGTGTCGCTACCTGTTTATCACAAACACACGTTTATTGTGTTGTATGTTAACTTCGAACTTCGGCGTGGCCCTGGCAG
GTGCTATAACTGTCGTCCTGCTGTTGTTAATATTACACTGGCCAATTTTAATGAAACTAAAGGGCCCTTGTGTGTTGAC
ACATCACACTTCACTACCCAATTCGTTGGTGTCAAGTTTGATAGGTGGAGTGCTAGTATTAACACGGGAAATTGCCCTT
TTTCTTTTGGCAAAGTTAATAACTTTGTTAAATTTGGCAGTGTATGTTTTTCGCTAAAGGATATACCCGGTGGTTGCGC
AATGCCTATAATGGCTAATTTGGCTAATCTTAATTCTCATAATATAGGCTCATTGTATGTTTCTTGGAGTGATGGTGAT
GTAATTACTGGCGTCCCGAAACCTGTTGAGGGTGTTAGTTCCTTTATGAATGTTACATTGAATAAATGTACTAAATATA
ACATTTATGATGTATCTGGTGTGGGTGTTATTCGCATTAGCAATGAAACCTTTCTTAATGGAATTACGTACACATCAAC
TTCAGGTAACCTTCTGGGTTTTAAAGATGTTACTAATGGCACCATCTACTCTATCACTCCTTGTAACCCACCAGATCAG
CTTGTTGTTTATCAGCAAGCTGTTGTTGGTGCTATGTTGTCTGAAAACTTTACTAGTTACGGCTTTTCTAATGTTGTAG
AAATGCCGAAATTTTTCTATGCGTCCAATGGCACTTATAATTGCACAGACGCTGTTTTAACTTATTCTAGTTTTGGCGT
TTGTGCAGATGGTTCTATAATTGCTGTTCAACCACGTAATGTTTCATATGATAGTGTTTCAGCTATTGTCACAGCTAAT
TTGTCTATACCCTCCAATTGGACCACTTCGGTCCAGGTTGAGTATTTACAAATTACAAGTACACCTATCGTAGTTGATT
GCTCCACTTATGTTTGCAATGGTAATGTGCGCTGTGTTGAATTGCTTAAGCAGTATACTTCTGCTTGTAAAACTATTGA
AGACGCCTTAAGAAATAGCGCCATGCTGGAGTCTGCAGATGTTAGTGAGATGCTCACTTTTGACAAGAAAGCGTTTACA
CTTGCTAATGTTAGTAGTTTTGGTGACTACAACCTTAGCAGTGTTATACCTAGCTTGCCCAGAAGTGGTAGTAGAGTGG
CTGGTCGCAGTGCCATAGAAGACATACTTTTTAGCAAACTTGTTACGTCTGGACTTGGCACTGTGGACGCAGACTACAA
AAAGTGTACTAAGGGTCTTTCCATTGCTGACTTGGCTTGTGCCCAATATTATAATGGCATTATGGTTTTGCCTGGCGTC
GCTGATGCTGAACGTATGGCCATGTATACAGGTTCTTTAATTGGTGGAATTGCTTTAGGAGGTCTTACATCAGCCGCTT
CAATACCATTTTCATTAGCAATTCAGTCACGTTTAAATTATGTTGCATTGCAGACTGATGTTTTACAAGAAAATCAGAA
AATTCTTGCTGCATCTTTTAATAAAGCAATGACCAACATAGTAGATGCCTTTACTGGTGTTAATGATGCTATTACACAA
ACTTCACAAGCTCTACAAACAGTTGCTACTGCACTTAATAAGATCCAGGATGTTGTTAATCAACAAGGCAACTCATTGA
ACCATTTAACTTCTCAGTTGAGGCAGAATTTTCAAGCCATTTCTAGCTCTATTCAGGCTATCTATGACAGACTTGATAT
TATTCAGGCTGATCAACAAGTAGATAGGCTGATTACTGGTAGATTGGCTGCTCTGAATGTATTCGTTTCTCATACATTG
ACTAAGTACACTGAAGTTCGTGCTTCCAGACAGCTTGCACAACAAAAAGTGAATGAGTGTGTCAAATCCCAGTCTAAGC
GGTATGGCTTCTGTGGAAATGGCACGCACATTTTCTCACTTGTTAATGCTGCTCCTGAGGGGCTTGTTTTTCTCCATAC
TGTCTTGTTGCCGACACAATATAAGGATGTTGAAGCGTGGTCTGGGTTGTGCGTTGATGGTAGAAACGGTTATGTGTTG
AGACAACCTAATCTTGCTCTTTACAAAGAAGGCAATTATTATAGAATTACATCTCGCATAATGTTTGAACCACGTATTC
CTACCATAGCAGATTTTGTCCAAATTGAAAATTGCAATGTCACATTTGTTAACATTTCTCGCTCTGAGTTGCAAACTAT
TGTGCCAGAGTATATTGATGTTAATAAGACGCTGCAAGAATTAAGTTACAAATTGCCAAATTACACTGTTCCAGACCTA
GTTGTCGAACAGTACAACCAGACTATTTTGAATTTGACCAGTGAAATTAGCACCCTTGAAAATAAATCTGCGGAGCTTA
ATTACACTGTTCAAAAACTGCAAACTTTGATCGACAACATAAATAGCACATTAGTTGACTTAAAGTGGCTCAACCGTGT
TGAGACTTATATCAAGTGGCCGTGGTGGGTGTGGTTGTGCATTTCAGTCGTGCTCATCTTTGTGGTGAGTATGTTGCTA
TTATGTTGTTGTTCTACTGGTTGCTGCGGCTTCTTTAGTTGTTTTGCATCTTCTATTAGAGGTTGTTGTGAATCAACTA
AACTTCCTTATTACGACGTTGAAAAGATCCACATACAGTAA

>gi|82780486|gb|DQ243980.1|_Human_coronavirus_229E_strain_HCoV229E6/1/03_spike_glycoprotein_(S)_gene_complete_cds

ATGTTTGTTTTACTTGTTGCATATGCCTTGTTGCATATTGCTGGTTGTCAAACTACAAATGGGATGAACACTAGT
CACTCTGTTTGCAACGGCTGTGTTGGTCATTCGGAAAATGTATTTGCTGTTGAGAGTGGTGGTTATATACCCTCCAACT
TTGCATTTAATAATTGGTTCCTCTTAACTAATACCTCATCTGTTGTGGATGGTGTTGTGAGGAGTTTTCAGCCTTTGTT
GCTTAATTGCTTATGGTCTGTTTCTGGCTCGCGGCTTACTACTGGTTTTGTCTATTTTAATGGTACTGGGAGAGGTGAT
TGTAAAGGTTTTTATTCAAATGCTTCGTCTGATGTCATACGTTACAACATCAATTTTGAAGAAAACCTTAGACGTGGAA
CCATTTTGTTTAAAACATCTTATGGTGCTGTTGTGTTTTATTGTACCAACAACACTTTGGTTTCAGGTGATGCTCACAT
ACCATCTGGTACAGTTTTGGGCAATTTTTATTGCTTTGTAAATACTACTATTGGCAATGAAACTACGTCTGCTTTTGTG
GGTGCACTACCTAAGACAGTTCGTGAGTTTGTTATTTCACGCACAGGACATTTCTATATTAATGGCTATCGCTATTTCT
CTTTAGGTAATGTAGAAGCCGTTAATTTCAATGTCACTAATGCTGCAACCACTGTTTGTACTGTTGCGTTAGCTTCTTA
TGCTGACGTTTTGGTTAATGTGTCACAAACTGCTATTGCTAATATAATTTATTGCAATTCTGTTATTAACAGACTGAGA
TGTGACCAGTTGTCCTTTGATGTACCAGATGGTTTTTATTCCACAAGCCCTATTCAATCCGTTGAGCTACCTGTGTCTA
TTGTGTCGCTACCTGTTTATCACAAACACACGTTTATTGTGTTGTATGTTAACTTCGAACTTCGGCGTGGCCCTGGCAG
GTGCTATAACTGTCGTCCTGCTGTTGTTAATATTACACTGGCCAATTTTAATGAAACTAAAGGGCCCTTGTGTGTTGAC
ACATCACACTTCACTACCCAATTTGTTGGTGTCAAGTTTGATAGGTGGAGTGCTAGTATTAACACGGGAAATTGCCCTT
TTTCTTTTGGCAAAGTTAATAACTTTGTTAAATTTGGCAGTGTATGTTTTTCGCTAAAGGATATACCCGGTGGTTGCGC
AATGCCTATAATGGCTAATTTGGCTAATCTTAATTCTCATAATATAGGCTCATTGTATGTTTCTTGGAGTGATGGTGAT
GTAATTACTGGCGTCCCGAAACCTGTTGAGGGTGTTAGTTCCTTTATGAATGTTACATTGAATAAATGTACTAAATATA
ACATTTATGATGTATCTGGTGTGGGTGTTATTCGCATTAGCAATGACACCTTTCTTAATGGAATTACGTACACATCAAC
TTCAGGTAACCTTCTGGGTTTTAAAGATGTTACTAATGGCACCATCTACTCTATCACTCCTTGTAACCCACCAGATCAG
CTTGTTGTTTATCAGCAAGCTGTTGTTGGTGCTATGTTGTCTGAAAACTTTACTAGTTACGGCTTTTCTAATGTTGTAG
AAATGCCGAAATTTTTCTATGCGTCCAATGGCACTTATAATTGCACAGACGCTGTTTTAACTTATTCTAGTTTTGGCGT
TTGTGCAGATGGTTCTATAATTGCTGTTCAACCACGTAATGTTTCATATGATAGTGTTTCAGCTATTGTCACAGCTAAT
TTGTCTATACCCTCCAATTGGACCACTTCGGTCCAGGTTGAGTATTTACAAATTACAAGTACACCTATCGTAGTTGATT
GCTCCACTTATGTTTGCAATGGTAATGTGCGCTGTGTTGAATTGCTTAAGCAGTATACTTCTGCTTGTAAAACTATTGA
AGACGCCTTAAGAAATAGCGCCATGCTGGAGTCTGCAGATGTTAGTGAGATGCTCACTTTTGACAAGAAAGCGTTTACA
CTTGCTAATGTTAGTAGTTTTGGTGACTACAACCTTAGCAGTGTTATACCTAGCTTGCCCAGAAGTGGTAGTAGAGTGG
CTGGTCGCAGTGCCATAGAAGACATACTTTTTAGCAAACTTGTTACGTCTGGGCTTGGCACTGTGGACGCAGACTACAA
AAAGTGTACTAAGGGTCTTTCCATTGCTGACTTGGCTTGTGCCCAATATTATAATGGCATTATGGTTTTGCCTGGCGTC
GCTGATGCTGAACGTATGGCCATGTATACAGGTTCTTTAATTGGTGGAATTGCTTTAGGAGGTCTTACATCAGCCGCTT
CAATACCATTTTCATTAGCAATTCAGTCACGTTTAAATTATGTTGCATTGCAGACTGATGTTTTACAAGAAAATCAGAA
AATTCTTGCTGCATCTTTTAATAAAGCAATGACCAACATAGTAGATGCCTTTACTGGTGTTAATGATGCTATTACACAA
ACTTCACAAGCTCTACAAACAGTTGCTACTGCACTTAATAAGATCCAGGATGTTGTTAATCAACAAGGCAACTCATTGA
ACCATTTAACTTCTCAGTTGAGGCAGAATTTTCAAGCCATTTCTAGCTCTATTCAGGCTATCTATGACAGACTTGATAT
TATTCAGGCTGATCAACAAGTAGATAGGCTGATTACTGGTAGATTGGCTGCTCTGAATGTATTCGTTTCTCATACATTG
ACTAAGTACACTGAAGTTCGTGCTTCCAGACAGCTTGCACAACAAAAAGTGAATGAGTGTGTCAAATCCCAGTCTGAGC
GGTATGGCTTCTGTGGAAATGGCACGCACATTTTCTCACTTGTTAATGCTGCTCCTGAGGGGCTTGTTTTTCTCCATAC
TGTCTTGTTGCCGACACAATATAAGGATGTTGAAGCGTGGTCTGGGTTGTGCGTTGATGGTAGAAACGGTTATGTGTTG
AGACAACCTAATCTTGCTCTTTACAAAGAAGGCAATTATTATAGAATTACATCTCGCATAATGTTTGAACCACGTATTC
CTACCATAGCAGATTTTGTCCAAATTGAAAATTGCAATGTCACATTTGTTAACATTTCTCGCTCTGAGTTGCAAACTAT
TGTGCCAGAGTATATTGATGTTAATAAGACGCTGCAAGAATTAAGTTACAAATTGCCAAATTATACTGTTCCAGACCTA

GTTGTCGAACAGTACAACCAGACTATTTTGAATTTGACCAGTGAAATTAGCACCCTTGAAAATAAATCTGCGGAGCTTA
ATTACACTGTTCAAAAACTGCAAACTTTGATCGACAACATAAATAGCACATTAGTTGACTTAAAGTGGCTCAACCGTGT
TGAGACTTATATCAAGTGGCCGTGGTGGGTGTGGTTGTGCATTTCAGTCGTGCTCATCTTTGTGGTGAGTATGTTGCTA
TTATGTTGTTGTTCTACTGGTTGCTGCGGCTTCTTTAGTTGTTTTGCATCTTCTATTAGAGGTTGTTGTGAATCAACTA
AACTTCCTTATTACGACGTTGAAAAGATCCACATACAGTAA

>gi|82780492|gb|DQ243983.1|_Human_coronavirus_229E_strain_HCoV229E30/7/03_s
pike_glycoprotein_(S)_gene_complete_cds

ATGTTTGTTTTACTTGTTGCATATGCCTTGTTGCATATTGCTGGTTGTCAAACTACAAATGGGATGAACACTAGT
CACTCTGTTTGCAACGGCTGTGTTGGTCATTCGGAAAATGTATTTGCTGTTGAGAGTGGTGGTTATATACCCTCCAACT
TTGCATTTAATAATTGGTTCCTCTTAACTAATACCTCATCTGTTGTAGATGGTGTTGTGAGGAGTTTTCAGCCTTTGTT
GCTTAATTGCTTATGGTCTGTTTCTGGCTCGCGGCTTACTACTGGTTTTGTCTATTTTAATGGTACTGGGAGAGGTGAT
TGTAAAGGTTTTTATTCAAATGCTTCGTCTGATGTCATACGTTACAACATCAATTTTGAAGAAAACCTTAGACGTGGAA
CCATTTTGTTTAAAACATCTTATGGTGCTGTTGTGTTTTATTGTACCAACAACACTTTGGTTTCAGGTGATGCTCACAT
ACCATCTGGTACAGTTTTGGGCAATTTTTATTGCTTTGTAAATACTACTATTGGCAATGAAACTACGTCTGCTTTTGTT
GGTGCACTACCTAAGACAGTTCGTGAGTTTGTTATTTCACGCACAGGACATTTTTATATTAATGGCTATCGCTATTTCT
CTTTAGGTAATGTAGAAGCCGTTAATTTCAATGTCACTAATGCTGCAACCACTGTTTGTACTGTTGCGTTAGCTTCTTA
TGCTGACGTTTTGGTTAATGTGTCACAAACTGCTATTGCTAATATAATTTATTGCAATTCTGTTATTAACAGACTGAGA
TGTGACCAGTTGTCCTTTGATGTACCAGATGGTTTTTATTCCACAAGCCCTATTCAATCCGTTGAGCTACCTGTGTCTA
TTGTGTCGCTACCTGTTTATCACAAACACACGTTTATTGTGTTGTATGTTAACTTCGAACTTCGGCGTGGCCCTGGCAG
GTGCTATAACTGTCGTCCTGCTGTTGTTAATATTACACTGGCCAATTTTAATGAAACTAAAGGGCCCTTGTGTGTTGAC
ACATCACACTTCACTACCCAATTCGTTGGTGTCAAGTTTGATAGGTGGAGTGCTAGTATTAACACGGGAAATTGCCCTT
TTTCTTTTGGCAAAGTTAATAACTTTGTTAAATTTGGCAGTGTATGTTTTTCGCTAAAGGACATACCCGGTGGTTGCGC
AATGCCTATAATGGCTAATTTGGCTAATCTTAATTCTCATAATATAGGCTCATTGTATGTTTCTTGGAGTGATGGTGAT
GTAATTACTGGCGTCCCGAAACCTGTTGAGGGTGTTAGTTCCTTTATGAATGTTACATTGAATAAATGTACTAAATATA
ACATTTATGATGTATCTGGTGTGGGTGTTATTCGCATTAGCAATGACACCTTTCTTAATGGAATTACGTACACATCAAC
TTCAGGTAACCTTCTGGGTTTTAAAGATGTTACTAATGGCACCATCTACTCTATCACTCCTTGTAACCCACCAGATCAG
CTTGTTGTTTATCAGCAAGCTGTTGTTGGTGCTATGTTGTCTGAAAACTTTACTAGTTACGGCTTTTCTAATGTTGTAG
AAATGCCGAAATTTTTCTATGCGTCCAATGGCACTTATAATTGCACAGACGCTGTTTTAACTTATTCTAGTTTTGGCGT
TTGTGCAGATGGTTCTATAATTGCTGTTCAACCACGTAATGTTTCATATGATAGTGTTTCAGCTATTGTCACAGCTAAT
TTGTCTATACCCTCCAATTGGACCACTTCGGTCCAGGTTGAGTATTTACAAATTACAAGTACACCTATCGTAGTTGATT
GCTCCACTTATGTTTGCAATGGTAATGTGCGCTGTGTTGAATTGCTTAAGCAGTATACTTCTGCTTGTAAAACTATTGA
AGACGCCTTAAGAAATAGCGCCATGCTGGAGTCTGCAGATGTTAGTGAGATGCTCACTTTTGACAAGAAAGCGTTTACA
CTTGCTAATGTTAGTAGTTTTGGTGACTACAACCTTAGCAGTGTTATACCTAGCTTGCCCAGAAGTGGTAGTAGAGTGG
CTGGTCGCAGTGCCATAGAAGACATACTTTTTAGCAAACTTGTTACGTCTGGACTTGGCACTGTGGACGCAGACTACAA
AAAGTGTACTAAGGGTCTTTCCATTGCTGACTTGGCTTGTGCCCAATATTATAATGGCATTATGGTTTTGCCTGGCGTC
GCTGATGCTGAACGTATGGCCATGTATACAGGTTCTTTAATTGGTGGAATTGCTTTAGGAGGTCTTACATCAGCCGCTT
CAATACCATTTTCATTAGCAATTCAGTCACGTTTAAATTATGTTGCATTGCAGACTGATGTTTTACAAGAAAATCAGAA
AATTCTTGCTGCATCTTTTAATAAAGCAATGACCAACATAGTAGATGCCTTTACTGGTGTTAATGATGCTATTACACAA
ACTTCACAAGCTCTACAAACAGTTGCTACTGCACTTAATAAGATCCATGATGTTGTTAATCAACAAGGCAACTCATTGA
ACCATTTAACTTCTCAGTTGAGGCAGAATTTCCAAGCCATTTCTAGCTCTATTCAGGCTATCTATGACAGACTTGATAT
TATTCAGGCTGATCAACAAGTAGATAGGCTGATTACTGGTAGATTGGCTGCTCTGAATGTATTCGTTTCTCATACATTG

ACTAAGTACACTGAAGTTCGTGCTTCCAGACAGCTTGCACAACAAAAAGTGAATGAGTGTGTCAAGTCCCAGTCTAAGC
GGTATGGCTTCTGTGGAAATGGCACGCACATTTTCTCACTTGTTAATGCTGCTCCTGAGGGGCTTGTTTTTCTCCATAC
TGTCTTGTTGCCGACACAATATAAGGATGTTGAAGCGTGGTCTGGGTTGTGCGTTGATGGTAGAAACGGTTATGTGTTG
AGACAACCTAATCTTGCTCTTTACAAAGAAGGCAATTATTATAGAATTACATCTCGCATAATGTTTGAACCACGTATTC
CTACCATAGCAGATTTTGTCCAAATTGAAAATTGCAATGTCACATTTGTTAACATTTCTCGCTCTGAGTTGCAAACTAT
TGTGCCAGAGTATATTGATGTTAATAAGACGCTGCAAGAATTAAGTTACAAATTGCCAAATTACACTGTTCCAGACCTA
GTTGTCGAACAGTACAACCAGACTATTTTGAATTTGACCAGTGAAATTAGCACCCTTGAAAATAAATCTGCGGAGCTTA
ATTACACTGTTCAAAAACTGCAAACTTTGATCGACAACATAAATAGCACATTAGTTGACTTAAAGTGGCTCAACCGTGT
TGAGACTTATATCAAGTGGCCGTGGTGGGTGTGGTTGTGCATTTCAGTCGTGCTCATCTTTGTGGTGAGTATGTTGCTA
TTATGTTGTTGTTCTACTGGTTGCTGCGGCTTCTTTAGTTGTTTTGCATCTTCTATTAGAGGTTGTTGTGAATCAACTA
AACTTCCTTATTACGACGTTGAAAAGATCCACATACAGTAA

>gi|82780494|gb|DQ243984.1|_Human_coronavirus_229E_strain_HCoV229E14/8/03_spike_glycoprotein_(S)_gene_complete_cds

ATGTTTGTTTTACTTGTTGCATATGCCTTGTTGCATATTGCTGGTTGTCAAACTACAAATGGGATGAACACTAGT
CACTCTGTTTGCAACGGCTGTGTTGGTTATTCGGAAAATGTATTTGCTGTTGAGAGTGGTGGTTATATACCATCCAACT
TTGCATTTAATAATTGGTTCCTCTTAACTAATACCTCATCTGTTGTAGATGGTGTTGTGAGGAGTTTTCAGCCTTTGTT
GCTTAATTGCTTATGGTCTGTTTCTGGCTCGCGGCTTACTACTGGTTTTGTCTATTTTAATGGTACTGGGAGAGGTGCT
TGTAAAGGTTTTTATTCAAATGCTTCGTCTGATGTCATACGTTACAACATCAATTTTGAAGAAAACCTTAGACGTGGAA
CCATTTTGTTTAAAACATCTTATGGTGCTGTTGTGTTTTATTGTACCAACAACACTTTGGTTTCAGGTGATGCTCACAT
ACCATCTGGTACAGTTTTGGGCAATTTTTATTGCTTTGTAAATACTACTATTGGCAATGAAACTACGTCTGCTTTTGTG
GGTGCACTACCTAAGACAGTTCGTGAGTTTGTTATTTCACGCACAGGACATTTCTATATTAATGGCTATCGCTATTTCT
CTTTAGGTAATGTAGAAGCCGTTAATTTCAATGTCACTAATGCTGCAACCACTGTTTGTACTGTTGCGTTAGCTTCTTA
TGCTGACGTTTTGGTTAATGTGTCACAAACTGCTATTGCTAATATAATTTATTGCAATTCTGTTATTAACAGACTGAGA
TGTGACCAGTTGTCCTTTGATGTACCAGATGGTTTTTATTCCACAAGCCCTATTCAATCCGTTGAGCTACCTGTGTCTA
TTGTGTCGCTACCTGTTTATCACAAACACACGTTTATTGTGTTGTATGTTAACTTCGAACTTCGGCGTGGCCCTGGCAG
GTGCTATAACTGTCGTCCTGCTGTTGTTAATATTACACTGGCCAATTTTAATGAAACTAAAGGGCCCTTGTGTGTTGAC
ACATCACACTTCACTACCCAATTCGTTGGTGTCAAGTTTGATAGGTGGAGTGCTAGTATTAACACGGGAAATTGCCCTT
TTTCTTTTGGCAAAGTTAATAACTTTGTTAAATTTGGCAGTGTATGTTTTTCGCTAAAGGATATACCCGGTGGTTGCGC
AATGCCTATAATGGCTAATTTGGCTAATCTTAATTCTCATAATATAGGCTCATTGTATGTTTCTTGGAGTGATGGTGAT
GTAATTACTGGCGTCCCGAAACCTGTTGAGGGTGTTAGTTCCTTTATGAATGTTACATTGAATAAATGTACTAAATATA
ACATTTATGATGTATCTGGTGTGGGTGTTATTCGCATTAGCAATGAAACCTTTCTTAATGGAATTACGTACACATCAAC
TTCAGGTAACCTTCTGGGTTTTAAAGATGTTACTAATGGCACCATCTACTCTATCACTCCTTGTAACCCACCAGATCAG
CTTGTTGTTTATCAGCAAGCTGTTGTTGGTGCTATGTTGTCTGAAAACTTTACTAGTTACGGCTTTTCTAATGTTGTAG
AAATGCCGAAATTTTTCTATGCGTCCAATGGCACTTATAATTGCACAGACGCTGTTTTAACTTATTCTAGTTTTGGCGT
TTGTGCAGATGGTTCTATAATTGCTGTTCAACCACGTAATGTTTCATATGATAGTGTTTCAGCTATTGTCACAGCTAAT
TTGTCTATACCCTCCAATTGGACCACTTCGGTCCAGGTTGAGTATTTACAAATTACAAGTACACCTATCGTAGTTGATT
GCTCCACTTATGTTTGCAATGGTAATGTGCGCTGTGTTGAATTGCTTAAGCAGTATACTTCTGCTTGTAAAACTATTGA
AGACGCCTTAAGAAATAGCGCCATGCTGGAGTCTGCAGATGTTAGTGAGATGCTCACTTTTGACAAGAAAGCGTTTACA
CTTGCTAATGTTAGTAGTTTTGGTGACTACAACCTTAGCAGTGTTATACCTAGCTTGCCCAGAAGTGGTAGTAGAGTGG
CTGGTCGCAGTGCCATAGAAGACATACTTTTTAGCAAACTTGTTACGTCTGGACTTGGCACTGTGGACGCAGACTACAA
AAAGTGTACTAAGGGTCTTTCCATTGCTGACTTGGCTTGTGCCCAATATTATAATGGCATTATGGTTTTGCCTGGCGTC

GCTGATGCTGAACGTATGGCCATGTATACAGGTTCTTTAATTGGTGGAATTGCTTTAGGAGGTCTTACATCAGCCGCTT
CAATACCATTTTCATTAGCAATTCAGTCACGTTTAAATTATGTTGCATTGCAGACTGATGTTTTACAAGAAAATCAGAA
AATTCTTGCTGCATCTTTTAATAAAGCAATGACCAACATAGTAGATGCCTTTACTGGTGTTAATGATGCTATTACACAA
ACTTCACAAGCTCTACAAACAGTTGCTACTGCACTTAATAAGATCCAGGATGTTGTTAATCAACAAGGCAACTCATTGA
ACCATTTAACTTCTCAGTTGAGGCAGAATTTTCAAGCCATTTCTAGCTCTATTCAGGCTATCTATGACAGACTTGATAT
TATTCAGGCTGATCAACAAGTAGATAGGCTGATTACTGGTAGATTGGCTGCTCTTAATGTATTCGTTTCTCATACATTG
ACTAAGTACACTGAAGTTCGTGCTTCCAGACAGCTTGCACAACAAAAAGTGAATGAGTGTGTCAAATCCCAGTCTAAGC
GGTATGGCTTCTGTGGAAATGGCACGCACATTTTCTCACTTGTTAATGCTGCTCCTGAGGGGCTTGTTTTTCTCCATAC
TGTCTTGTTGCCGACACAATATAAGGATGTTGAAGCGTGGTCTGGGTTGTGCGTTGATGGTAGAAACGGTTATGTGTTG
AGACAACCTAATCTTGCTCTTTACAAAGAAGGCAATTATTATAGAATTACATCTCGCATAATGTTTGAACCACGTATTC
CTACCATAGCAGATTTTGTCCAAATTGAAAATTGCAATGTCACATTTGTTAACATTTCTCGCTCTGAGTTGCAAACTAT
TGTGCCAGAGTATATTGATGTTAATAAGACGCTGCAAGAATTAAGTTACAAATTGCCAAATTACACTGTTCCAGACCTA
GTTGTCGAACAGTACAACCAGACTATTTTGAATTTGACCAGTGAAATTAGCACCCTTGAAAATAAATCTGCGGAGCTTA
ATTACACTGTTCAAAAACTGCAAACTTTGATCGACAACATAAATAGCACATTAGTTGACTTAAAGTGGCTCAACCGTGT
TGAGACTTATATCAAGTGGCCGTGGTGGGTGTGGTTGTGCATTTCAGTCGTGCTCATCTTTGTGGTGAGTATGTTGCTA
TTATGTTGTTGTTCTACTGGTTGCTGCGGCTTCTTTAGTTGTTTTGCATCTTCTATTAGAGGTTGTTGTGAATCAACTA
AACTTCCTTATTACGACGTTGAAAAGATCCACATACAGTAA

>gi|82780496|gb|DQ243985.1|_Human_coronavirus_229E_strain_HCoV229E19/8/03_spike_glycoprotein_(S)_gene_complete_cds

ATGTTTGTTTTACTTGTTGCATATGCCTTGTTGCATATTGCTGGTTGTCAAACTACAAATGGGATGAACACTAGT
CACTCTGTTTGCAACGGCTGTGTTGGTCATTCGGAAAATGTATTTGCTGTTGAGAGTGGTGGTTATATACCCTCCAACT
TTGCATTTAATAATTGGTTCCTCTTAACTAATACCTCATCTGTTGTAGATGGTGTTGTGAGGAGTTTTCAGCCTTTGTT
GCTTAATTGCTTATGGTCTGTTTCTGGCTCGCGGCTTACTACTGGTTTTGTCTATTTTAATGGTACTGGGAGAGGTGCT
TGTAAAGGTTTTTATTCAAATGCTTCGTCTGATGTCATACGTTACAACATCAATTTTGAAGAAAACCTTAGACGTGGAA
CCATTTTGTTTAAAACATCTTATGGTGCTGTTGTGTTTTATTGTACCAACAACACTTTGGTTTCAGGTGATGCTCATAT
ACCATCTGGTACAGTTTTGGGCAATTTTTATTGCTTTGTAAATACTACTATTGGCAATGAAACTACGTCTGCTTTTGTG
GGTGCACTACCTAAGACAGTTCGTGAGTTTGTTATTTCACGCACAGGACATTTCTATATTAATGGCTATCGCTATTTCT
CTTTAGGTAATGTAGAAGCCGTTAATTTCAATGTCACTAATGCTGCAACCACTGTTTGTACTGTTGCGTTAGCTTCTTA
TGCTGACGTTTTGGTTAATGTGTCACAAACTGCTATTGCTAATATAATTTATTGCAATTCTGTTATTAACAGACTGAGA
TGTGACCAGTTGTCCTTTGATGTACCAGATGGTTTTTATTCCACAAGCCCTATTCAATCCGTTGAGCTACCTGTGTCTA
TTGTGTCGCTACCTGTTTATCATAAACACACGTTTATTGTGTTGTATGTTAACTTCGAACTTCGGCGTGGCCCTGGCAG
GTGCTATAACTGTCGTCCTGCTGTTGTTAATATTACACTGGCCAATTTTAATGAAACTAAAGGGCCCTTGTGTGTTGAC
ACATCACACTTCACTACCCAATTTGTTGGTGTCAAGTTTGATAGGTGGAGTGCTAGTATTAACACGGGAAATTGCCCTT
TTTCTTTTGGCAAAGTTAATAACTTTGTTAAATTTGGCAGTGTATGTTTTTCGCTAAAGGATATACCCGGTGGTTGCGC
AATGCCTATAATGGCTAATTTGGCTAATCTTAATTCTCATAATATAGGCTCATTGTATGTTTCTTGGAGTGATGGTGAT
GTAATTACTGGCGTCCCGAAACCTGTTGAGGGTGTTAGTTCCTTTATGAATGTTACATTGAATAAATGTACTAAATATA
ACATTTATGATGTATCTGGTGTGGGTGTTATTCGCATTAGCAATGACACCTTTCTTAATGGAATTACGTACACATCAAC
TTCAGGTAACCTTCTGGGTTTTAAAGATGTTACTAATGGCACCATCTACTCTATCACTCCTTGTAACCCACCAGATCAG
CTTGTTGTTTATCAGCAAGCTGTTGTTGGTGCTATGTTGTCTGAAAACTTTACTAGTTACGGCTTTTCTAATGTTGTAG
AAATGCCGAAATTTTTCTATGCGTCCAATGGCACTTATAATTGCACAGACGCTGTCTTAACTTATTCTAGTTTTGGCGT
TTGTGCAGATGGTTCTATAATTGCTGTTCAACCACGTAATGTTTCATATGATAGTGTTTCAGCTATTGTCACAGCTAAT

TTGTCTATACCCTCCAATTGGACCACTTCGGTCCAGGTTGAGTATTTACAAATTACAAGTACACCTATCGTAGTTGATT
GCTCCACTTATGTTTGCAATGGTAATGTGCGCTGTGTTGAATTGCTTAAGCAGTATACTTCTGCTTGTAAAACTATTGA
AGACGCCTTAAGAAATAGCGCCATGCTGGAGTCTGCAGATGTTAGTGAGATGCTCACTTTTGACAAGAAAGCGTTTACA
CTTGCTAATGTTAGTAGTTTTGGTGACTACAACCTTAGCAGTGTTATACCTAGCTTGCCCAGAAGTGGTAGTAGAGTGG
CTGGTCGCAGTGCCATAGAAGACATACTTTTTAGCAAACTTGTTACGTCTGGACTTGGCACTGTGGACGCAGACTACAA
AAAGTGTACTAAGGGTCTTTCCATTGCTGACTTGGCTTGTGCCCAATATTATAATGGCATTATGGTTTTGCCTGGCGTC
GCTGATGCTGAACGTATGGCCATGTATACAGGTTCTTTAATTGGTGGAATTGCTTTAGGAGGTCTTACATCAGCCGCTT
CAATACCATTTTCATTAGCAATTCAGTCACGTTTAAATTATGTTGCATTGCAGACTGATGTTTTACAAGAAAATCAGAA
AATTCTTGCTGCATCTTTTAATAAAGCAATGACCAACATAGTAGATGCCTTTACTGGTGTTAATGATGCTATTACACAA
ACTTCACAAGCTCTACAAACAGTTGCTACTGCACTTAATAAGATCCAGGATGTTGTTAATCAACAAGGCAACTCATTGA
ACCATTTAACTTCTCAGTTGAGGCAGAATTTTCAAGCCATTTCTAGCTCTATTCAGGCTATCTATGACAGACTTGATAT
TATTCAGGCTGATCAACAAGTAGATAGGCTGATTACTGGTAGATTGGCTGCTCTGAATGTATTCGTTTCTCATACATTG
ACTAAGTACACTGAAGTTCGTGCTTCCAGACAGCTTGCACAACAAAAAGTGAATGAGTGTGTCGAATCCCAGTCTAAGC
GGTATGGCTTCGGTGGAAATGGCACGCACATTTTCTCACTTGTTAATGCTGCTCCTGAGGGGCTTGTTTTTCTTCATAC
TGTCTTGTTGCCGACACAATATAAGGATGTTGAAGCGTGGTCTGGGTTGTGCGTTGATGGTAGAAACGGTTATGTGTTG
AGACAACCTAATCTTGCTCTTTACAAAGAAGGCAATTATTATAGAATTACATCTCGCATAATGTTTGAACCACGTATTC
CTACCATAGCAGATTTTGTCCAAATTGAAAATTGCAATGTCACATTTGTTAACATTTCTCGCTCTGAGTTGCAAACTAT
TGTGCCAGAGTATATTGATGTTAATAAGACGCTGCAAGAATTAAGTTACAAATTGCCAAATTACACTGTTCCAGACCTA
GTTGTCGAACAGTACAACCAGACTATTTTGAATTTGACCAGTGAAATTAGCACCCTTGAAAATAAATCTGCGGAGCTTA
ATTACACTGTTCAAAAACTGCAAACTTTGATCGACAACATAAATAGCACATTAGTTGACTTAAAGTGGCTCAACCGTGT
TGAGACTTATATTAAGTGGCCGTGGTGGGTGTGGTTGTGCATTTCAGTCGTGCTCATCTTTGTGGTGAGTATGTTGCTA
TTATGTTGTTGTTCTACTGGTTGCTGCGGCTTCTTTAGTTGTTTTGCATCTTCTATTAGAGGTTGTTGTGAATCAACTA
AACTTCCTTATTACGACGTTGAAAAGATCCACATACAGTAA

>gi|82780498|gb|DQ243986.1|_Human_coronavirus_229E_strain_HCoV229E25/8/03_spike_glycoprotein_(S)_gene_complete_cds

ATGTTTGTTTTACTTGTTGCATATGCCTTGTTGCATATTGCTGGTTGTCAAACTACAAATGGGATGAACACTAGT
CACTCTGTTTGCAACGGCTGTGTTGGTCATTCGGAAAATGTATTTGCTGTTGAGAGTGGTGGTTATATACCCTCCAACT
TTGCATTTAATAATTGGTTCCTCTTAACTAATACCTCATCTGTTGTAGATGGTGTTGTGAGGAGTTTTCAGCCTTTGTT
GCTTAATTGCTTATGGTCTGTTTCTGGCTCGCGGCTTACTACTGGTTTTGTCTATTTTAATGGTACTGGGAGAGGTGCT
TGTAAAGGTTTTTATTCAAATGCTTCGTCTGATGTCATACGTTACAACATCAATTTTGAAGAAAACCTTAGACGTGGAA
CCATTTTGTTTAAAACATCTTATGGTGCTGTTGTGTTTTATTGTACCAACAACACTTTGGTTTCAGGTGATGCTCATAT
ACCATCTGGTACAGTTTTGGGCAATTTTTATTGCTTTGTAAATACTACTATTGGCAATGAAACTACGTCTGCTTTTGTG
GGTGCACTACCTAAGACAGTTCGTGAGTTTGTTATTTCACGCACAGGACATTTCTATATTAATGGCTATCGCTATTTCT
CTTTAGGTAATGTAGAAGCCGTTAATTTCAATGTCACTAATGCTGCAACCACTGTTTGTACTGTTGCGTTAGCTTCTTA
TGCTGACGTTTTGGTTAATGTGTCACAAACTGCTATTGCTAATATAATTTATTGCAATTCTGTTATTAACAGACTGAGA
TGTGACCAGTTGTCCTTTGATGTACCAGATGGTTTTTATTCCACAAGCCCTATTCAATCCGTTGAGCTACCTGTGTCTA
TTGTGTCGCTACCTGTTTATCATAAACACACGTTTATTGTGTTGTATGTTAACTTCGAACTTCGGCGTGGCCCTGGCAG
GTGCTATAACTGTCGTCCTGCTGTTGTTAATATTACACTGGCCAATTTTAATGAAACTAAAGGGCCCTTGTGTGTTGAC
ACATCACACTTCACTACCCAATTCTTTGGTGTCAAGTTTGATAGGTGGAGTGCTAGTATTAACACGGGAAATTGCCCTT
TTTCTTTTGGCAAAGTTAATAACTTTGTTAAATTTGGCAGTGTATGTTTTTCGCTAAAGGATATACCCGGTGGTTGCGC
AATGCCTATAATGGCTAATTTGGCTAATCTTAATTCTCATAATATAGGCTCATTGTATGTTTCTTGGAGTGATGGTGAT

GTAATTACTGGCGTCCCGAAACCTGTTGAGGGTGTTAGTTCCTTTATGAATGTTACATTGAATAAATGTACTAAATATA
ACATTTATGATGTATCTGGTGTGGGTGTTATTCGCATTAGCAATGACACCTTTCTTAATGGAATTACGTACACATCAAC
TTCAGGTAACCTTCTGGGTTTTAAAGATGTTACTAATGGCACCATCTACTCTATCACTCCTTGTAACCCACCAGATCAG
CTTGTTGTTTATCAGCAAGCTGTTGTTGGTGCTATGTTGTCTGAAAACTTTACTAGTTACGGCTTTTCTAATGTTGTAG
AAATGCCGAAATTTTTCTATGCGTCCAATGGCACTTATAATTGCACAGACGCTGTCTTAACTTATTCTAGTTTTGGCGT
TTGTGCAGATGGTTCTATAATTGCTGTTCAACCACGTAATGTTTCATATGATAGTGTTTCAGCTATTGTCACAGCTAAT
TTGTCTATACCCTCCAATTGGACCACTTCGGTCCAGGTTGAGTATTTACAAATTACAAGTACACCTATTGTAGTTGATT
GCTCCACTTATGTTTGCAATGGTAATGTGCGCTGTGTTGAATTGCTTAAGCAGTATACTTCTGCTTGTAAAACTATTGA
AGACGCCTTAAGAAATAGCGCCATGCTGGAGTCTGCAGATGTTAGTGAGATGCTCACTTTTGACAAGAAAGCGTTTACA
CTTGCTAATGTTAGTAGTTTTGGTGACTACAACCTTAGCAGTGTTATACCTAGCTTGCCCAGAAGTGGTAGTAGAGTGG
CTGGTCGCAGTGCCATAGAAGACATACTTTTTAGCAAACTTGTTACGTCTGGACTTGGCACTGTGGACGCAGACTACAA
AAAGTGTACTAAGGGTCTTTCCATTGCTGACTTGGCTTGTGCCCAATATTATAATGGCATTATGGTTTTGCCTGGCGTC
GCTGATGCTGAACGTATGGCCATGTATACAGGTTCTTTAATTGGTGGAATTGCTTTAGGAGGTCTTACATCAGCCGCTT
CAATACCATTTTCATTAGCAATTCAGTCACGTTTAAATTATGTTGCATTGCAGACTGATGTTTTACAAGAAAATCAGAA
AATTCTTGCTGCATCTTTTAATAAAGCAATGACCAACATAGTAGATGCCTTTACTGGTGTTAATGATGCTATTACACAA
ACTTCACAAGCTCTACAAACAGTTGCTACTGCACTTAATAAGATCCAGGATGTTGTTAATCAACAAGGCAACTCATTGA
ACCATTTAACTTCTCAGTTGAGGCAGAATTTTCAAGCCATTTCTAGCTCTATTCAGGCTATCTATGACAGACTTGATAT
TATTCAGGCTGATCAACAAGTAGATAGGCTGATTACTGGTAGATTGGCTGCTCTGAATGTATTCGTTTCTCATACATTG
ACTAAGTACACTGAAGTTCGTGCTTCCAGACAGCTTGCACAACAAAAAGTGAATGAGTGTGTCAAATCCCAGTCTAAGC
GGTATGGCTTCTGTGGAAATGGCACGCACATTTTCTCACTTGTTAATGCTGCTCCTGAGGGGCTTGTTTTTCTCCATAC
TGTCTTGTTGCCGACACAATATAAGGATGTTGAAGCGTGGTCTGGGTTGTGCGTTGATGGTAGAAACGGTTATGTGTTG
AGACAACCTAATCTGGCTCTTTACAAAGAAGGCAATTATTATAGAATTACATCTCGCATAATGTTTGAACCACGTATTC
CTACCATAGCAGATTTTGTCCAAATTGAAAATTGCAATGTCACATTTGTTAACATTTCTCGCTCTGAGTTGCAAACTAT
TGTGCCAGAGTATATTGATGTTAATAAGACGCTGCAAGAATTAAGTTACAAATTGCCAAATTACACTGTTCCAGACCTA
GTTGTCGAACAGTACAACCAGACTATTTTGAATTTGACCAGTGAAATTAGCACCCTTGAAAATAAATCTGCGGAGCTTA
ATTACACTGTTCAAAAACTGCAAACTTTGATCGACAACATAAATAGCACATTAGTTGACTTAAAGTGGCTCAACCGTGT
TGAGACTTATATTAAGTGGCCGTGGTGGGTGTGGTTGTGCATTTCAGTCGTGCTCATCTTTGTGGTGAGTATGTTGCTA
TTATGTTGTTGTTCTACTGGTTGCTGCGGCTTCTTTAGTTGTTTTGCATCTTCTATTAGAGGTTGTTGTGAATCAACTA
AACTTCCTTATTACGACGTTGAAAAGATCCACATACAGTAA

>gi|85541020|gb|DQ339101.1| Human coronavirus HKU1 strain N5P8 genotype A/B recombinant, complete genome

ATGTTTTTAATTATTTTTATTTTGCCTACAACACTAGCTGTTATAGGTGATTTTAATTGTACTAACTCTTTTATT
AATGATTATAATAAAACCATTCCGCGTATAAGCGAGGATGTTGTTGATGTATCTCTTGGTTTGGGCACATATTATGTTC
TTAACCGTGTTTATTTAAATACTACCTTGTTATTTACGGGTTATTTTCCTAAATCTGGTGCTAACTTTAGAGACTTGGC
TTTAAAGGGTTCTATATATTTGAGTACTCTCTGGTATAAACCACCTTTTCTGTCAGATTTTAATAATGGTATTTTTTCT
AAGGTTAAGAATACTAAGTTATATGTTAATAATACTTTGTATAGTGAATTTAGTACTATAGTTATAGGTAGTGTTTTTG
TTAATACTTCTTATACTATTGTTGTTCAACCTCATAATGGTATTTTGGAGATTACAGCTTGTCAGTATACTATGTGTGA
ATATCCTCACACTGTTTGTAAGTCTAAGGGTAGTATTCGTAATGAATCTTGGCACATTGATTCTTCGGAACCTTTATGC
TTGTTTAAGAAAAATTTTACTTATAATGTTTCTGCAGATTGGCTGTATTTTCATTTTTATCAAGAACGTGGTGTTTTTT
ATGCATATTATGCAGATGTAGGTATGCCTACCACTTTCTTATTTAGTCTATATTTAGGTACTATTTTATCTCATTATTA
TGTTATGCCTTTGACTTGTAATGCTATATCTTCAAATACTGACAATGAAACTTTAGAATATTGGGTTACACCGCTATCT

AGACGTCAGTATCTTCTTAATTTTGATGAGCACGGTGTTATTACTAATGCCGTTGATTGTTCAAGTAGTTTTCTTAGTG
AGATTCAATGTAAAACTCAATCTTTTGCACCTAATACTGGTGTTTATGATTTGTCTGGTTTTACTGTAAAGCCTGTTGC
AACTGTTTATCGTCGGATTCCTAATTTACCTGATTGTGACATTGACAACTGGCTTAATAATGTTAGTGTACCTTCACCT
CTTAATTGGGAACGTAGAATTTTTTCTAATTGTAACTTTAACTTAAGCACTTTACTTCGTCTAGTTCATGTTGATTCTT
TTTCTTGTAATAATCTTGATAAATCTAAAATTTTTGGTAGTTGCTTTAATAGTATTACTGTTGACAAGTTTGCTATACC
TAATCGCAGACGAGATGATTTGCAATTGGGCAGTTCTGGCTTTTTGCAATCATCTAATTACAAAATAGATATTTCTTCT
AGTTCTTGTCAATTGTATTATAGTTTACCTTTAGTTAATGTTACTATTAATAACTTTAATCCATCTTCTTGGAATAGGA
GGTATGGTTTTGGTAGTTTTAATTTGTCTTCTTATGACGTTGTTTATTCTGATCATTGTTTTTCTGTTAACAGCGACTT
TTGCCCTTGTGCAGATCCGTCTGTTGTTAATTCTTGTGCTAAATCTAAGCCTCCTTCTGCCATTTGTCCTGCTGGTACT
AAATATCGTCATTGCGACTTGGATACTACTCTTTATGTTAAAAACTGGTGTAGATGTTCTTGTCTACCTGACCCCATTT
CTACTTATTCTCCTAACACATGTCCTCAAAAGAAGGTCGTTGTTGGTATAGGTGAACATTGTCCAGGTCTTGGTATTAA
TGAGGAAAAATGTGGTACACAATTAAATCATAGTTCCTGTTTTTGTAGTCCTGATGCCTTTTTGGGTTGGTCTTTTGAT
AGTTGTATTAGTAATAATCGTTGCAATATTTTTTCTAATTTTATTTTTAATGGAATTAATAGTGGCACCACTTGTTCTA
ATGATTTGTTATATTCTAACACTGAAATTTCTACTGGTGTTTGTGTTAATTATGATCTTTATGGCATCACAGGCCAAGG
TATTTTTAAAGAAGTTTCTGCGGCTTATTATAATAATTGGCAGAATCTTTTGTATGATTCTAATGGTAATATTATTGGT
TTTAAAGATTTTTTGACTAATAAAACTTACACTATACTTCCTTGTTATTCTGGTAGAGTGTCTGCTGCATTTTATCAAA
ATTCTTCCTCACCAGCTTTGCTTTATCGTAATTTAAAGTGTAGTTATGTTTTGAATAATATTTCTTTTATCTCACAACC
ATTTTATTTCGATAGTTATCTTGGTTGTGTTTTGAATGCTGTTAATTTAACTAGCTATTCTGTATCCTCTTGTGATTTG
CGTATGGGTAGTGGATTTTGTATTGATTATGCTTTACCCTCTTCTCGGCGTAAGCGTAGAGGTATTTCTTCTCCTTATC
GCTTTGTAACTTTTGAACCCTTTAATGTTAGTTTTGTTAACGATAGTGTTGAAACTGTTGGTGGTTTATTTGAGATTCA
GATTCCTACTAACTTTACCATAGCTGGTCATGAAGAATTTATTCAGACTAGTTCTCCTAAAGTTACTATTGATTGTTCA
GCTTTTGTTTGCTCTAACTATGCTGCTTGTCATGATTTATTGTCGGAATATGGCACTTTTTGCGATAATATTAATAGTA
TTTTAAATGAAGTCAATGATTTACTTGATATTACTCAGTTGCAGGTTGCTAATGCTCTAATGCAAGGTGTTACACTTAG
TTCTAATCTTAATACTAATCTACACTCTGATGTTGATAATATAGATTTTAAATCTCTTCTAGGTTGTTTAGGTTCACAA
TGTGGTTCTTCGTCTAGATCTTTGTTAGAGGATTTATTATTCAACAAAGTCAAACTTTCAGATGTAGGTTTTGTTGAAG
CTTATAATAATTGCACTGGTGGTAGTGAAATTAGAGATCTTCTCTGTGTGCAATCTTTTAATGGTATTAAAGTATTACC
TCCCATTTTATCTGAGACTCAAATTTCTGGCTATACTACAGCTGCTACTGTGGCGGCTATGTTTCCGCCATGGTCTGCT
GCTGCTGGTGTACCATTTTCTCTTAATGTACAATATAGAATTAATGGTTTGGGTGTTACTATGGATGTTCTTAATAAGA
ATCAAAAGTTAATAGCTAATGCTTTTAATAAAGCTCTTCTTTCTATCCAGAATGGTTTTACTGCTACTAACTCTGCTCT
TGCTAAAATTCAAAGTGTCGTTAATGCTAATGCTCAAGCACTTAATAGTTTGTTACAACAATTATTTAATAAATTTGGT
GCTATTAGTTCTTCTTTACAAGAAATTTTGTCTCGCCTTGATAATTTAGAAGCTCAGGTTCAGATTGATAGGCTCATTA
ATGGTCGTTTGACTGCTTTAAATGCTTATGTTTCTCAACAGCTTAGTGATATTACACTTATTAAGGCTGGAGCTTCTCG
TGCTATTGAGAAGGTTAATGAGTGTGTTAAAAGTCAATCCCCTCGTATAAATTTTTGTGGCAATGGTAACCACATTTTA
TCATTGGTTCAAAATGCTCCTTATGGTTTGCTTTTCATTCATTTTAGTTATAAACCTACTTCTTTTAAAACTGTCTTAG
TAAGTCCAGGTTTATGTTTATCCGGTGATAGAGGTATTGCACCTAAGCAAGGTTATTTTATTAAACAAAATGATTCCTG
GATGTTTACTGGTAGTTCCTATTATTATCCAGAACCAATTTCAGATAAAAATGTTGTTTTCATGAATAGTTGCTCTGTT
AATTTTACTAAAGCTCCATTTATTTATCTTAATAATTCTATACCAAATTTGTCTGATTTTGAAGCCGAGTTATCTCTTT
GGTTTAAAAATCATACTTCTATAGCACCTAATTTAACCTTTAATTCTCATATTAATGCTACTTTTTTAGATCTGTATTA
TGAAATGAATGTTATTCAGGAATCTATTAAATCTTTGAACAGTAGTTTTATTAATCTTAAAGAAATAGGTACTTATGAA
ATGTATGTTAAATGGCCTTGGTACATTTGGTTGTTAATTGTCATTTTATTTATAATTTTTCTTATGATACTTTTCTTTA
TATGCTGCTGTACTGGTTGTGGTTCAGCATGTTTTAGTAAATGTCATAATTGTTGTGATGAGTATGGGGGTCACAATGA
TTTTGTTATTAAAGCATCTCATGATGATTAG

>gi|89515460|gb|DQ415902.1| Human coronavirus HKU1 strain N25 genotype B, complete genome

ATGTTTTTAATTATTTTTATTTTGCCTACAACACTAGCTGTTATAGGTGATTTTAATTGTACTAACTCTTTTATT
AATGATTATAATAAAACCATTCCGCGTATAAGCGAGGATGTTGTTGATGTATCTCTTGGTTTGGGCACATATTATGTTC
TTAACCGTGTTTATTTAAATACTACCTTGTTATTTACAGGTTATTTTCCTAAATCTGGTGCTAATTTTAGAGACTTGGC
TTTAAAGGGTTCTATATATTTGAGTACTCTCTGGTATAAACCACCTTTTCTGTCAGATTTTAATAATGGTATTTTTTCT
AAGGTTAAGAATACTAAGTTATATGTTAATAATACTTTGTATAGTGAATTTAGTACTATAGTTATAGGTAGTGTTTTTG
TTAATACGTCTTATACTATTGTTGTTCAACCTCACAATGGTATTTTGGAGATTACAGCTTGTCAGTATACTATGTGTGA
ATATCCTCACACTGTTTGTAAGTCTAAGGGTAGTATTCGTAATGAATCTTGGCACATTGATTCTTCGGAACCTTTATGC
TTGTTTAAGAAAAATTTTACTTATAATGTTTCTGCAGATTGGCTGTATTTTCATTTTTATCAAGAACGTGGTGTTTTTT
ATGCATATTATGCAGATGTAGGTATGCCTACCACTTTCTTATTTAGTTTATATTTAGGTACTATTTTATCTCATTATTA
TGTTATGCCTTTGACTTGTAATGCTATATCTTCAAATACTGACAATGAAACTTTAGAATATTGGGTTACACCGCTATCT
AGACGTCAGTATCTTCTCAATTTTGATGAACACGGTGTTATTACTAATGCCGTTGATTGTTCAAGTAGTTTTCTTAGTG
AGATTCAATGTAAAACTCAATCTTTTGCACCTAATACTGGTGTTTATGATTTGTCTGGTTTTACTGTAAAGCCTGTTGC
AACTGTTTATCGTCGGATTCCTAATTTACCTGATTGTGACATTGACAACTGGCTTAATAATGTTAGTGTACCTTCACCT
CTTAATTGGGAACGTAGAATTTTTTCTAATTGTAACTTCAATTTAAGCACTTTACTTCGTCTAGTTCATGTTGATTCTT
TTTCTTGTAATAATCTTGATAAATCTAAAATTTTTGGTAGTTGCTTTAATAGTATTACTGTTGACAAGTTTGCTATACC
TAATCGCAGACGAGATGATTTGCAATTGGGCAGTTCTGGCTTTTTGCAATCATCTAATTACAAAATAGATATTTCTTCT
AGTTCTTGTCAATTGTATTATAGTTTACCTTTAGTTAATGTTACTATTAATAACTTTAATCCATCTTCTTGGAATAGGA
GGTATGGTTTTGGTAGTTTTAATGTGTCTTCTTATGACGTTGTTTATTCTGATCATTGTTTTTCTGTTAACAGCGACTT
TTGCCCTTGTGCAGATCGGTCTGTTGTTAATTCTTGTGTTAAATCTAAGCCTCCTTCTGCCATTTGTCCTGCTGGTACT
AAATATCGTCATTGCGACTTGGATACTACTCTTTATGTTAAAAACTGGTGTAGATGTTCTTGTCTACCTGACCCCATTT
CTACTTATTCTCCTAACACATGTCCTCAAAAGAAGGTCGTTGTTGGTATAGGTGAACATTGTCCAGGTCTTGGTATTAA
TGAGGAAAAATGTGGTACACAATTAAATCATAGTTCCTGTTCTTGTAGTCCTGATGCCTTTTTGGGTTGGTCTTTTGAT
AGTTGTATTAGTAATAATCGTTGCAATATTTTTTCTAATTTTATTTTTAATGGAATTAATAGTGGCACCACTTGTTCTA
ATGATTTGTTATATTCTAACACTGAAGTTTCTACTGGTGTTTGTGTTAATTATGATCTTTATGGCATCACAGGCCAAGG
TATTTTTAAAGAAGTTTCTGCGGCTTATTATAATAATTGGCAGAATCTTTTGTATGATTCTAATGGTAATATTATTGGT
TTTAAAGATTTTTTGACTAATAAAACTTACACTATACTTCCTTGTTATTCTGGTAGAGTGTCTGCTGCATTTTATCAAA
ATTCTTCCTCACCAGCTTTGCTTTATCGTAATTTAAAGTGTAGTTATGTTTTGAATAATATTTCTTTTATCTCACAACC
ATTTTATTTTGATAGTTATCTTGGTTGTGTTTTGAATGCTGTTAATTTAACTAGCTATTCTGTATCCTCTTGTGATTTG
CGTATGGGTAGTGGGTTTTGTATTGATTATGCTTTACCCTCTTCTCGGCGTAAGCGTAGAGGTATTTCTTCTCCTTATC
GCTTTGTAACTTTTGAACCCTTTAATGTTAGTTTTGTTAACGATAGTGTTGAAACTGTTGGTGGTTTATTTGAGATTCA
GATTCCTACTAACTTTACCATAGCTGGTCATGAAGAATTTATTCAGACTAGTTCTCCTAAAGTTACTATTGATTGTTCA
GCTTTTGTTTGCTCTAATTATGCTGCTTGTCATGATTTATTGTCGGAATATGGCACTTTTTGCGATAATATTAATAGTA
TTTTAAATGAAGTCAATGACTTACTTGATATTACTCAGTTGCAGGTTGCTAATGCTTTAATGCAAGGTGTTACACTTAG
TTCTAATCTTAATACTAATCTACACTCTGATGTTGATAATATAGATTTTAAATCTCTTCTAGGTTGTTTAGGTTCACAA
TGTGGTTCTTCGTCTAGATCTTTGTTAGAGGATTTATTATTCAACAAGGTCAAACTTTCAGATGTAGGTTTTGTTGAAG
CTTATAATAATTGCACTGGTGGTAGTGAAATTAGAGATCTTCTCTGTGTGCAATCTTTTAATGGTATTAAAGTATTACC
TCCCATTTTATCTGAGACTCAAATTTCTGGCTATACTACAGCTGCTACTGTGGCGGCTATGTTTCCGCCATGGTCTGCT
GCTGCTGGTGTACCATTTTCTCTTAATGTACAATATAGAATTAATGGTTTGGGTGTTACTATGGATGTTCTTAATAAGA
ATCAAAAGTTAATAGCTAATGCTTTTAATAAAGCTCTTCTTTCTATCCAGAATGGTTTTACTGCTACTAACTCTGCTCT
TGCTAAAATTCAAAGTGTCGTTAATGCTAATGCTCAAGCACTTAATAGTTTGTTACAACAATTATTTAATAAATTTGGT
GCTATTAGTTCTTCTTTACAAGAAATTTTGTCTCGCCTTGATAATTTAGAAGCTCAGGTTCAGATTGATAGGCTCATTA

ATGGTCGTTTGACTGCTTTAAATGCTTATGTTTCTCAACAGCTTAGTGATATTACACTTATTAAGGCTGGAGCTTCTCG
TGCTATTGAGAAGGTTAATGAGTGTGTTAAAAGTCAATCCCCTCGTATAAATTTTTGTGGCAATGGTAACCACATTTTA
TCATTGGTTCAAAATGCTCCTTATGGTTTGCTTTTCATTCATTTTAGTTATAAACCTACTTCTTTTAAAACTGTCTTAG
TAAGTCCAGGTTTATGTTTATCCGGTGATAGAGGTATTGCACCTAAGCAAGGTTATTTTATTAAACAAAATGATTCCTG
GATGTTTACTGGTAGTTCCTATTATTACCCAGAACCAATTTCAGATAAAAATGTTGTTTTCATGAATAGTTGCTCTGTT
AATTTTACTAAAGCTCCATTTATTTATCTTAATAATTCTATACCAAATTTGTCTGATTTTGAAGCCGAGTTTTCTCTTT
GGTTTAAAAATCATACTTCTATAGCACCTAATTTAACCTTCAATTCTCATATTAATGCTACTTTTTAGATCTGTATTA
TGAAATGAATGTTATTCAGGAATCTATTAAATCTTTGAACAGTAGTTTTATTAATCTTAAAGAAATAGGTACTTATGAA
ATGTATGTTAAATGGCCTTGGTACATTTGGTTGTTAATTGTCATTTTATTTATAATTTTTCTTATGATACTTTTCTTTA
TATGCTGCTGTACTGGTTGCGGTTCAGCATGTTTTAGTAAATGTCATAATTGTTGTGATGAGTATGGGGGTCACAATGA
TTTTGTTATTAAAGCATCTCATGATGATTAG

>gi|89515469|gb|DQ415903.1| Human coronavirus HKU1 strain N3 genotype A, complete genome

ATGTTATTAATTATTTTTATTTTGCCTACAACATTAGCTGTTATAGGTGATTTTAATTGTACTAATTTTGCTATT
AATGATTTAAACACCACAGTTCCTCGCATAAGTGAGTATGTTGTGGATGTTTCTTATGGTTTGGGTACATATTATATAC
TTGATCGTGTTTATTTAAATACTACTATATTATTTACTGGTTATTTCCCTAAATCTGGTGCCAATTTTAGGGATCTATC
TTTAAAAGGTACTACATATTTGAGTACTCTTTGGTATCAGAAACCCTTTTTATCTGATTTTAATAATGGTATTTTTTCT
AGAGTTAAGAATACTAAGTTGTATGTTAATAAAACTTTGTATAGTGAGTTTAGTACTATAGTTATAGGTAGTGTTTTTA
TTAACAACTCTTATACTATTGTTGTTCAACCTCATAATGGTGTTTTGGAGATTACAGCTTGTCAATACACTATGTGTGA
GTATCCTCATACTATTTGTAAATCTAAAGGTAGTTCTCGTAATGAATCTTGGCATTTTGATAAATCTGAACCTTTGTGT
CTGTTCAAGAAAAATTTTACTTATAATGTTTCTACAGATTGGTTGTATTTTCATTTTTATCAAGAACGTGGCACTTTTT
ATGCTTATTATGCTGATTCTGGCATGCCTACTACTTTTTTATTTAGTTTGTATCTTGGTACTCTTTTATCTCATTATTA
TGTTTTGCCTTTGACTTGTAATGCTATATCTTCTAATACTGATAATGAGACTTTACAATATTGGGTTACACCTTTGTCT
AAACGCCAATATCTTCTTAAATTTGACAACCGTGGTGTTATTACTAATGCTGTTGATTGTTCTAGTAGTTTCTTTAGCG
AGATTCAATGTAAAACTAAATCTTTATTACCTAATACTGGTGTTTATGACTTATCTGGTTTTACTGTTAAGCCTGTTGC
AACTGTACATCGTCGTATTCCTGATTTACCTGATTGTGACATTGATAAATGGCTTAACAATTTTAATGTACCCTCACCT
CTTAATTGGGAACGTAAAATTTTTTCTAATTGCAACTTTAATTTGAGTACTTTGCTTCGTTTAGTTCATACTGATTCTT
TTTCTTGTAATAATTTTGATGAATCTAAGATATATGGTAGTTGTTTTAAGAGTATTGTTTTAGATAAATTTGCCATACC
CAACTCCAGACGATCTGATTTGCAGTTGGGCAGTTCTGGTTTTCTGCAATCTTCTAATTATAAAATTGACACTACTTCT
AGTTCTTGTCAATTGTATTATAGTTTGCCTGCAATTAATGTTACTATTAATAATTATAATCCTTCTTCTTGGAATAGAA
GGTATGGTTTTAATAATTTTAATTTGAGCTCTCATAGTGTTGTTTACTCACGTTATTGTTTTTCTGTTAATAATACTTT
TTGTCCTTGTGCTAAACCTTCTTTTGCTTCAAGTTGCAAGAGTCATAAACCACCTTCTGCTTCCTGTCCTATTGGTACT
AATTATCGTTCTTGTGAGAGTACTACTGTACTCGACCACACTGACTGGTGTAGGTGTTCTTGTTTACCTGATCCTATAA
CTGCTTATGACCCTAGGTCTTGTTCTCAAAAAAAGTCTCTGGTTGGTGTTGGTGAACATTGTGCAGGGTTCGGTGTTGA
TGAAGAAAAGTGTGGTGTATTGGATGGATCATATAATGTTTCTTGTCTTTGTAGTACTGATGCCTTTCTAGGTTGGTCT
TATGACACTTGCGTCAGTAACAACCGTTGTAATATTTTTTCTAATTTTATTTTAAATGGTATCAATAGTGGTACCACTT
GTTCTAATGATTTATTGCAGCCTAATACTGAAGTTTTTACTGATGTTTGTGTTGATTACGACCTTTATGGTATTACAGG
ACAAGGTATTTTTAAAGAAGTTTCTGCTGTTTATTATAATAGTTGGCAAAATCTTTTGTATGATTCTAATGGCAACATT
ATTGGTTTTAAAGATTTTGTTACTAATAAAACATATAATATTTTCCCTTGTTATGCAGGAAGAGTTTCTGCTGCTTTTC
ATCAAAATGCTTCCTCTTTGGCTTTACTTTATCGTAATTTAAAATGTAGCTATGTTTTGAATAATATTTCTTTAGCTAC
TCAGCCATATTTTGATAGTTATCTTGGTTGCGTTTTTAATGCTGATAATTTAACTGATTATTCTGTTTCTTCTTGTGCT

CTTCGCATGGGTAGTGGTTTTTGTGTTGATTATAACTCACCTTCTTCTTCCTCTTCGCGTCGTAAACGTAGAAGTATTT
CTGCTTCTTATCGTTTTGTTACTTTTGAACCCTTTAATGTCAGTTTTGTTAATGACAGTATTGAGTCTGTGGGTGGTCT
TTATGAGATCAAAATTCCCACTAACTTTACTATAGTTGGTCAAGAGGAATTTATTCAAACTAATTCTCCTAAAGTTACT
ATTGATTGTTCTTTATTTGTCTGTTCTAATTATGCAGCTTGCCATGACTTATTGTCAGAGTATGGCACTTTTTGTGATA
ATATTAATAGTATTTTAGATGAAGTTAATGGTTTACTTGATACTACTCAATTGCATGTAGCTGATACTCTTATGCAAGG
TGTCACACTTAGCTCCAATCTTAATACTAATTTGCATTTTGATGTTGATAATATTAATTTTAAATCCCTAGTTGGATGT
TTAGGTCCACACTGCGGTTCTTCTTCTCGTTCTTTTTTTGAAGATTTATTGTTTGACAAAGTTAAACTTTCAGATGTTG
GTTTTGTTGAAGCTTATAACAATTGTACTGGTGGTAGTGAAATTAGAGATCTTCTTTGTGTACAATCCTTTAATGGTAT
TAAAGTTTTGCCTCCTATTTTGTCTGAATCTCAAATTTCTGGTTACACCACAGCCGCTACTGTTGCTGCTATGTTTCCA
CCATGGTCAGCAGCAGCTGGCATACCATTTTCTCTTAATGTACAATATAGAATTAATGGTTTGGGTGTTACTATGGATG
TTCTTAATAAAAATCAAAAGTTGATAGCTACTGCTTTTAATAATGCTCTTCTTTCTATTCAGAATGGTTTTAGTGCTAC
CAACTCTGCACTTGCTAAAATACAAAGTGTTGTTAATTCTAATGCTCAAGCACTTAATAGTTTGTTACAGCAATTATTT
AATAAATTTGGTGCAATTAGTTCTTCTTTACAAGAAATTTTATCTCGTCTCGATGTTTTAGAGGCTCAGGTTCAGATTG
ATAGGCTTATTAATGGTCGTTTAACTGCTTTAAATGCTTATGTCTCTCAACAGCTTAGTGATATTTCTCTTGTAAAATT
TGGTGCTGCTTTAGCTATGGAGAAGGTTAATGAGTGTGTTAAAAGTCAATCTCCTCGTATTAATTTTTGTGGTAATGGT
AATCATATTTTGTCATTAGTTCAAAATGCTCCTTATGGTTTGTTGTTTATGCATTTTAGTTATAAACCTATTTCTTTTA
AAACTGTTTTAGTAAGTCCTGGTTTGTGTATATCAGGTGATGTAGGTATTGCACCTAAACAAGGGTATTTTATTAAACA
TAATGATCATTGGATGTTCACTGGTAGTTCTTACTATTATCCTGAACCAATTTCAGATAAAAATGTTGTTTTTATGAAT
ACTTGTTCTGTTAATTTTACTAAAGCGCCTCTTGTTTATTTGAATCATTCTGTACCAAAATTGTCTGATTTTGAATCTG
AGTTATCTCATTGGTTTAAAAATCAAACATCCATTGCGCCTAATTTGACTTTAAATCTTCATACTATTAATGCTACTTT
TTTAGATTTGTATTATGAGATGAATCTTATTCAAGAGTCTATTAAGTCTTTGAATAATAGTTATATCAATCTTAAAGAT
ATAGGTACATATGAAATGTATGTAAAATGGCCTTGGTATGTTTGGCTACTAATTTCTTTTTCATTTATAATATTCCTTG
TATTGCTCTTTTTTATATGTTGTTGTACTGGTTGTGGTTCTGCATGTTTTAGTAAATGTCATAATTGTTGTGATGAGTA
TGGTGGTCATCATGATTTTGTTATCAAAACATCTCATGATGATTAG

>gi|89515487|gb|DQ415905.1| Human coronavirus HKU1 strain N7 genotype A, complete genome

ATGTTATTAATTATTTTTATTTTGCCTACAACATTAGCTGTTATAGGTGATTTTAATTGTACTAATTTTGCTATT
AATGATTTAAACACCACAGTTCCTCGCATAAGTGAGTATGTTGTGGATGTTTCTTATGGTTTGGGTACATATTATATAC
TTGATCGTGTTTATTTAAATACTACTATATTATTTACTGGTTATTTCCCTAAATCTGGTGCCAATTTTAGGGATCTATC
TTTAAAAGGTACTACATATTTGAGTACTCTTTGGTATCAGAAACCCTTTTTATCTGATTTTAATAATGGTATTTTTTCT
AGAGTTAAGAATACTAAGTTGTATGTTAATAAAACTTTGTATAGTGAGTTTAGTACTATAGTTATAGGTAGTGTTTTTA
TTAACAACTCTTATACTATTGTTGTTCAACCTCATAATGGTGTTTTGGAGATTACAGCTTGTCAATACACTATGTGTGA
GTATCCTCATACTATTTGTAAATCTAAAGGTAGTTCTCGTAATGAATCTTGGCATTTTGATAAATCTGAACCTTTGTGT
CTGTTCAAGAAAAATTTTACTTATAATGTTTCTACAGATTGGTTGTATTTTCATTTTTATCAAGAACGTGGCACTTTTT
ATGCTTATTATGCTGATTCTGGCATGCCTACTACTTTTTTATTTAGTTTGTATCTTGGTACTCTTTTATCTCATTATTA
TGTTTTGCCTTTGACTTGTAATGCTATATCTTCTAATACTGATAATGAGACTTTACAATATTGGGTCACACCTTTGTCT
AAACGCCAATATCTTCTTAAATTTGACAACCGTGGTGTTATTACTAATGCTGTTGATTGTTCTAGTAGTTTCTTTAGCG
AGATTCAATGTAAAACTAAATCTTTATTACCTAATACTGGTGTTTATGACTTATCTGGTTTTACTGTTAAGCCTGTTGC
AACTGTACATCGTCGTATTCCTGATTTACCTGATTGTGACATTGATAAATGGCTTAACAATTTTAATGTACCCTCACCT
CTTAATTGGGAACGTAAAATTTTTTCTAATTGCAACTTTAATTTGAGTACTTTGCTTCGTTTAGTTCATACTGATTCTT
TTTCTTGTAATAATTTTGATGAATCTAAGATATATGGTAGTTGTTTTAAGAGTATTGTTTTAGATAAATTTGCTATACC

CAACTCCAGACGATCTGATTTGCAGTTGGGCAGTTCTGGTTTTCTGCAATCTTCTAATTATAAAATTGACACTACTTCT
AGTTCTTGTCAATTGTATTATAGTTTGCCTGCAATTAATGTTACTATTAATAATTATAATCCTTCTTCTTGGAATAGAA
GGTATGGTTTTAATAATTTTAATTTGAGCTCTCATAGTGTTGTTTACTCACGTTATTGTTTTTCTGTTAATAATACTTT
TTGTCCTTGTGCTAAACCTTCTTTTGCTTCAAGTTGCAAGAGTCATAAACCACCTTCTGCTTCCTGTCCTATTGGTACT
AATTATCGTTCTTGTGAGAGTACTACTGTACTCGACCACACTGACTGGTGTAGGTGTTCTTGTTTACCTGATCCTATAA
CTGCTTATGACCCTAGGTCTTGTTCTCAAAAAAAGTCTCTGGTTGGTGTTGGTGAACATTGTGCAGGGTTCGGTGTTGA
TGAAGAAAAGTGTGGTGTATTGGATGGATCATATAATGTTTCTTGTCTTTGTAGTACTGATGCCTTTCTAGGTTGGTCT
TATGACACTTGCGTCAGTAACAACCGTTGTAATATTTTTTCTAATTTTATTTTAAATGGTATCAATAGTGGTACCACTT
GTTCTAATGATTTATTGCAGCCTAATACTGAAGTTTTTACTGATGTTTGTGTTGATTACGACCTTTATGGTATTACAGG
ACAAGGTATTTTTAAAGAAGTTTCTGCTGTTTATTATAATAGTTGGCAAAATCTTTTGTATGATTCTAATGGCAACATT
ATTGGTTTTAAAGATTTTGTTACTAATAAAACATATAATATTTTCCCTTGTTATGCAGGAAGAGTTTCTGCTGCTTTTC
ATCAAAATGCTTCCTCTTTGGCTTTACTTTATCGTAATTTAAAATGTAGCTATGTTTTGAATAATATTTCTTTAGCTAC
TCAGCCATATTTTGATAGTTATCTTGGTTGCGTTTTTAATGCTGATAATTTAACTGATTATTCTGTTTCTTCTTGTGCT
CTTCGCATGGGTAGTGGTTTTTGTGTTGATTATAACTCACCTTCTTCTTCCTCTTCGCGTCGTAAACGTAGAAGTATTT
CTGCTTCTTATCGTTTTGTTACTTTTGAACCCTTTAATGTCAGTTTTGTTAATGACAGTATTGAGTCTGTGGGTGGTCT
TTATGAGATCAAAATTCCCACTAACTTTACTATAGTTGGTCAAGAGGAATTTATTCAAACTAATTCTCCTAAAGTTACT
ATTGATTGTTCTTTATTTGTCTGTTCTAATTATGCAGCTTGCCATGACTTATTGTCAGAGTATGGCACTTTTTGTGATA
ATATTAATAGTATTTTAGATGAAGTTAATGGTTTACTTGATACTACTCAATTGCATGTAGCTGATACTCTTATGCAAGG
TGTCACACTTAGCTCCAATCTTAATACTAATTTGCATTTTGATGTTGATAATATTAATTTTAAATCCCTAGTTGGATGT
TTAGGTCCACACTGCGGTTCTTCTTCTCGTTCTTTTTTTGAAGATTTATTGTTTGACAAAGTTAAACTTTCAGATGTTG
GTTTTGTTGAAGCTTATAACAATTGTACTGGTGGTAGTGAAATTAGAGATCTTCTTTGTGTACAATCCTTTAATGGTAT
TAAAGTTTTGCCTCCTATTTTGTCTGAATCTCAAATTTCTGGTTACACCACAGCCGCTACTGTTGCTGCTATGTTTCCA
CCATGGTCAGCAGCAGCTGGCATACCATTTTCTCTTAATGTACAATATAGAATTAATGGTTTGGGTGTTACTATGGATG
TTCTTAATAAAAATCAAAAGTTGATAGCTACTGCTTTTAATAATGCTCTTCTTTCTATTCAGAATGGTTTTAGTGCTAC
CAACTCTGCACTTGCTAAAATACAAAGTGTTGTTAATTCTAATGCTCAAGCACTTAATAGTTTGTTACAGCAATTATTT
AATAAATTTGGTGCAATTAGTTCTTCTTTACAAGAAATTTTATCTCGTCTCGATGCTTTAGAGGCTCAGGTTCAGATTG
ATAGGCTTATTAATGGTCGTTTAACTGCTTTAAATGCTTATGTCTCTCAACAGCTTAGTGATATTTCTCTTGTAAAATT
TGGTGCTGCTTTAGCTATGGAGAAGGTTAATGAGTGTGTTAAAAGTCAATCTCCTCGTATTAATTTTTGTGGTAATGGT
AATCATATTTTGTCATTAGTTCAAAATGCTCCTTATGGTTTGTTGTTTATGCATTTTAGTTATAAACCTATTTCTTTTA
AAACTGTTTTAGTAAGTCCTGGTTTGTGTATATCAGGTGATGTAGGTATTGCACCTAAACAAGGGTATTTTATTAAACA
TAATGATCATTGGATGTTCACTGGTAGTTCTTACTATTATCCTGAACCAATTTCAGATAAAAATGTTGTTTTTATGAAT
ACTTGTTCTGTTAATTTTACTAAAGCGCCTCTTGTTTATTTGAATCATTCTGTACCAAAATTGTCTGATTTTGAATCTG
AGTTATCTCATTGGTTTAAAAATCAAACATCCATTGCGCCTAATTTGACTTTAAATCTTCATACTATTAATGCTACTTT
TTTAGATTTGTATTATGAGATGAATCTTATTCAAGAGTCTATTAAGTCTTTGAATAATAGTTATATCAATCTTAAAGAT
ATAGGTACATATGAAATGTATGTAAAATGGCCTTGGTATGTTTGGCTACTAATTTCTTTTTCATTTATAATATTCCTTG
TATTGCTCTTTTTTATATGTTGTTGTACTGGTTGTGGTTCTGCATGTTTTAGTAAATGTCATAATTGTTGTGATGAGTA
TGGTGGTCATCATGATTTTGTTATCAAAACATCTCATGATGATTAG

>gi|89515496|gb|DQ415906.1| Human coronavirus HKU1 strain N9 genotype A, complete genome

ATGTTATTAATTATTTTTATTTTGCCTACAACATTAGCTGTTATAGGTGATTTTAATTGTACTAATTTTGCTATT
AATGATTTAAACACCACAGTTCCTCGCATAAGTGAGTATGTTGTGGATGTTTCTTATGGTTTGGGTACATATTATATAC

TTGATCGTGTTTATTTAAATACTACTATATTATTTACTGGTTATTTCCCTAAATCTGGTGCCAATTTTAGGGATCTATC
TTTAAAAGGTACTACATATTTGAGTACTCTTTGGTATCAGAAACCCTTTTTATCTGATTTTAATAATGGTATTTTTTCT
AGAGTTAAGAATACTAAGTTGTATGTTAATAAAACTTTGTATAGTGAGTTTAGTACTATAGTTATAGGTAGTGTTTTTA
TTAACAACTCTTATACTATTGTTGTTCAACCTCATAATGGTGTTTTGGAGATTACAGCTTGTCAATACACTATGTGTGA
GTATCCTCATACTATTTGTAAATCTAAAGGTAGTTCTCGTAATGAATCTTGGCATTTTGATAAATCTGAACCTTTGTGT
CTGTTCAAGAAAAAATTTACTTATAATGTTTCTACAGATTGGTTGTATTTTCATTTTTATCAAGAACGTGGCACTTTTT
ATGCTTATTATGCTGATTCTGGCATGCCTACTACTTTTTTATTTAGTTTGTATCTTGGTACTCTTTTATCTCATTATTA
TGTTTTGCCTTTGACTTGTAATGCTATATCTTCTAATACTGATAATGAGACTTTACAATATTGGGTCACACCTTTGTCT
AAACGCCAATATCTTCTTAAATTTGACAACCGTGGTGTTATTACTAATGCTGTTGATTGTTCTAGTAGTTTCTTTAGCG
AGATTCAATGTAAAACTAAATCTTTATTACCTAATACTGGTGTTTATGACTTATCTGGTTTTACTGTTAAGCCTGTTGC
AACTGTACATCGTCGTATTCCTGATTTACCTGATTGTGACATTGATAAATGGCTTAACAATTTTAATGTACCCTCACCT
CTTAATTGGGAACGTAAAATTTTTTCTAATTGCAACTTTAATTTGAGTACTTTGCTTCGTTTAGTTCATACTGATTCTT
TTTCTTGTAATAATTTTGATGAATCTAAGATATATGGTAGTTGTTTTAAGAGTATTGTTTTAGATAAATTTGCCATACC
CAACTCCAGACGATCTGATTTGCAGTTGGGCAGTTCTGGTTTTCTGCAATCTTCTAATTATAAAATTGACACTACTTCT
AGTTCTTGTCAATTGTATTATAGTTTGCCTGCAATTAATGTTACTATTAATAATTATAATCCTTCTTCTTGGAATAGAA
GGTATGGTTTTAATAATTTTAATTTGAGCTCTCATAGTGTTGTTTACTCACGTTATTGTTTTTCTGTTAATAATACTTT
TTGTCCTTGTGCTAAACCTTCTTTTGCTTCAAGTTGCAAGAGTCATAAACCACCTTCTGCTTCCTGTCCTATTGGTACT
AATTATCGTTCTTGTGAGAGTACTACTGTACTCGACCACACTGACTGGTGTAGGTGTTCTTGTTTACCTGATCCTATAA
CTGCTTATGACCCTAGGTCTTGTTCTCAAAAAAAGTCTCTGGTCGGTGTTGGTGAACATTGTGCAGGGTTCGGTGTTGA
TGAAGAAAAGTGTGGTGTATTGGATGGATCATATAATGTTTCTTGTCTTTGTAGTACTGATGCCTTTCTAGGTTGGTCT
TATGACACTTGCGTCAGTAACAACCGTTGTAATATTTTTTCTAATTTTATTTTAAATGGTATCAATAGTGGTACCACTT
GTTCTAATGATTTATTGCAGCCTAATACTGAAGTTTTTACTGATGTTTGTGTTGATTACGACCTTTATGGTATTACAGG
ACAAGGTATTTTTAAAGAAGTTTCTGCTGTTTATTATAATAGTTGGCAAAATCTTTTGTATGATTCTAATGGCAACATT
ATTGGTTTTAAAGATTTGTTACTAATAAAACATATAATATTTTCCCTTGTTATGCAGGAAGAGTTTCTGCTGCTTTTC
ATCAAAATGCTTCCTCTTTGGCTTTACTTTATCGTAATTTAAAATGTAGCTATGTTTTGAATAATATTTCTTTAGCTAC
TCAGCCATATTTTGATAGTTATCTTGGTTGCGTTTTTAATGCTGATAATTTAACTGATTATTCTGTTTCTTCTTGTGCT
CTTCGCATGGGTAGTGGTTTTTGTGTTGATTATAACTCACCTTCTTCTTCCTCTTCGCGTCGTAAACGTAGAAGTATTT
CTGCTTCTTATCGTTTTGTTACTTTTGAACCCTTTAATGTCAGTTTTGTTAATGACAGTATTGAGTCTGTGGGTGGTCT
TTATGAGATCAAAATTCCCACTAACTTTACTATAGTTGGTCAAGAGGAATTTATTCAAACTAATTCTCCTAAAGTTACT
ATTGATTGTTCTTTATTTGTCTGTTCTAATTATGCAGCTTGCCATGACTTATTGTCAGAGTATGGCACTTTTTGTGATA
ATATTAATAGTATTTTAGATGAAGTTAATGGTTTACTTGATACTACTCAATTGCATGTAGCTGATACTCTTATGCAAGG
TGTCACACTTAGCTCCAATCTTAATACTAATTTGCATTTTGATGTTGATAATATTAATTTTAAATCCCTAGTTGGATGT
TTAGGTCCACACTGCGGTTCTTCTTCTCGTTCTTTTTTTGAAGATTTATTGTTTGACAAAGTTAAACTTTCAGATGTTG
GTTTTGTTGAAGCTTATAACAATTGTACTGGTGGTAGTGAAATTAGAGATCTTCTTTGTGTACAATCCTTTAATGGTAT
TAAAGTTTTGCCTCCTATTTTGTCTGAATCTCAAATTTCTGGTTACACCACAGCCGCTACTGTTGCTGCTATGTTTCCA
CCATGGTCAGCAGCAGCTGGCATACCATTTTCTCTTAATGTACAATATAGAATTAATGGTTTGGGTGTTACTATGGATG
TTCTTAATAAAAATCAAAAGTTGATAGCTACTGCTTTTAATAATGCTCTTCTTTCTATTCAGAATGGTTTTAGTGCTAC
CAACTCTGCACTTGCTAAAATACAAAGTGTTGTTAATTCTAATGCTCAAGCACTTAATAGTTTGTTACAGCAATTATTT
AATAAATTTGGTGCAATTAGTTCTTCTTTACAAGAAATTTTATCTCGTCTCGATGCTTTAGAGGCTCAGGTTCAGATTG
ATAGGCTTATTAATGGTCGTTTAACTGCTTTAAATGCTTATGTCTCTCAACAGCTTAGTGATATTTCTCTTGTAAAATT
TGGTGCTGCTTTAGCTATGGAGAAGGTTAATGAGTGTGTTAAAAGTCAATCTCCTCGTATTAATTTTTGTGGTAATGGT
AATCATATTTTGTCATTAGTTCAAAATGCTCCTTATGGTTTGTTGTTTATGCATTTTAGTTATAAACCTATTTCTTTTA
AAACTGTTTTAGTAAGTCCTGGTTTGTGTATATCAGGTGATGTAGGTATTGCACCTAAACAAGGGTATTTTATTAAACA
TAATGATCATTGGATGTTCACTGGTAGTTCTTACTATTATCCTGAACCAATTTCAGATAAAAATGTTGTTTTTATGAAT

ACTTGTTCTGTTAATTTTACTAAAGCGCCTCTTGTTTATTTGAATCATTCTGTACCAAAATTGTCTGATTTTGAATCTG
AGTTATCTCATTGGTTTAAAAATCAAACATCCATTGCGCCTAATTTGACTTTAAATCTTCATACTATTAATGCTACTTT
TTTAGATTTGTATTATGAGATGAATCTTATTCAAGAGTCTATTAAGTCTTTGAATAATAGTTATATCAATCTTAAAGAT
ATAGGTACATATGAAATGTATGTAAAATGGCCTTGGTATGTTTGGCTACTAATTTCTTTTTCATTTATAATATTCCTTG
TATTGCTCTTTTTTATATGTTGTTGTACTGGTTGTGGTTCTGCATGTTTTAGTAAATGTCATAATTGTTGTGATGAGTA
TGGTGGTCATCATGATTTTGTTATCAAAACATCTCATGATGATTAG

>gi|89515505|gb|DQ415907.1| Human coronavirus HKU1 strain N10 genotype A, complete genome

ATGTTATTAATTATTTTTATTTTGCCTACAACATTAGCTGTTATAGGTGATTTTAATTGTACTAATTTTGCTATT
AATGATTTAAACACCACAGTTCCTCGCATAAGTGAGTATGTTGTGGATGTTTCTTATGGTTTGGGTACATATTATATAC
TTGATCGTGTTTATTTAAATACTACTATATTATTTACTGGTTATTTCCCTAAATCTGGTGCCAATTTTAGGGATCTATC
TTTAAAAGGTACTACATATTTGAGTACTCTTTGGTATCAGAAACCCTTTTTATCTGATTTTAATAATGGTATTTTTTCT
AGAGTTAAGAATACTAAGTTGTATGTTAATAAAACTTTGTATAGTGAGTTTAGTACTATAGTTATAGGTAGTGTTTTTA
TTAACAACTCTTATACTATTGTTGTTCAACCTCATAATGGTGTTTTGGAGATTACAGCTTGTCAATACACTATGTGTGA
GTATCCTCATACTATTTGTAAATCTAAAGGTAGTTCTCGTAATGAATCTTGGCATTTTGATAAATCTGAACCTTTGTGT
CTGTTCAAGAAAAATTTTACTTATAATGTTTCTACAGATTGGTTGTATTTTCATTTTTATCAAGAACGTGGCACTTTTT
ATGCTTATTATGCTGATTCTGGCATGCCTACTACTTTTTTATTTAGTTTGTATCTTGGTACTCTTTTATCTCATTATTA
TGTTTTGCCTTTGACTTGTAATGCTATATCTTCTAATACTGATAATGAGACTTTACAATATTGGGTCACACCTTTGTCT
AAACGCCAATATCTTCTTAAATTTGACAACCGTGGTGTTATTACTAATGCTGTTGATTGTTCTAGTAGTTTCTTTAGCG
AGATTCAATGTAAAACTAAATCTTTATTACCTAATACTGGTGTTTATGACTTATCTGGTTTTACTGTTAAGCCTGTTGC
AACTGTACATCGTCGTATTCCTGATTTACCTGATTGTGACATTGATAAATGGCTTAACAATTTTAATGTACCCTCACCT
CTTAATTGGGAACGTAAAATTTTTTCTAATTGCAACTTTAATTTGAGTACTTTGCTTCGTTTAGTTCATACTGATTCTT
TTTCTTGTAATAATTTTGATGAATCTAAGATATATGGTAGTTGTTTTAAGAGTATTGTTTTAGATAAATTTGCCATACC
CAACTCCAGACGATCTGATTTGCAGTTGGGCAGTTCTGGTTTTCTGCAATCTTCTAATTATAAAATTGACACTACTTCT
AGTTCTTGTCAATTGTATTATAGTTTGCCTGCAATTAATGTTACTATTAATAATTATAATCCTTCTTCTTGGAATAGAA
GGTATGGTTTTAATAATTTTAATTTGAGCTCTCATAGTGTTGTTTACTCACGTTATTGTTTTTCTGTTAATAATACTTT
TTGTCCTTGTGCTAAACCTTCTTTTGCTTCAAGTTGCAAGAGTCATAAACCACCTTCTGCTTCCTGTCCTATTGGTACT
AATTATCGTTCTTGTGAGAGTACTACTGTACTCGACCACACTGACTGGTGTAGGTGTTCTTGTTTACCTGATCCTATAA
CTGCTTATGACCCTAGGTCTTGTTCTCAAAAAAAGTCTCTGGTTGGTGTTGGTGAACATTGTGCAGGGTTCGGTGTTGA
TGAAGAAAAGTGTGGTGTATTGGATGGATCATATAATGTTTCTTGTCTTTGTAGTACTGATGCCTTTCTAGGTTGGTCT
TATGACACTTGCGTCAGTAACAACCGTTGTAATATTTTTTCTAATTTTATTTTAAATGGTATCAATAGTGGTACCACTT
GTTCTAATGATTTATTGCAGCCTAATACTGAAGTTTTTACTGATGTTTGTGTTGATTACGACCTTTATGGTATTACAGG
ACAAGGTATTTTTAAAGAAGTTTCTGCTGTTTATTATAATAGTTGGCAAAATCTTTTGTATGATTCTAATGGCAACATT
ATTGGTTTTAAAGATTTTGTTACTAATAAAACATATAATATTTTCCCTTGTTATGCAGGAAGAGTTTCTGCTGCTTTTC
ATCAAAATGCTTCCTCTTTGGCTTTACTTTATCGTAATTTAAAATGTAGCTATGTTTTGAATAATATTTCTTTAGCTAC
TCAGCCATATTTTGATAGTTATCTTGGTTGCGTTTTTAATGCTGATAATTTAACTGATTATTCTGTTTCTTCTTGTGCT
CTTCGCATGGGTAGTGGTTTTTGTGTTGATTATAACTCACCTTCTTCTTCCTCTTCGCGTCGTAAACGTAGAAGTATTT
CTGCTTCTTATCGTTTTGTTACTTTTGAACCCTTTAATGTCAGTTTTGTTAATGACAGTATTGAGTCTGTGGGTGGTCT
TTATGAGATCAAAATTCCCACTAACTTTACTATAGTTGGTCAAGAGGAATTTATTCAAACTAATTCTCCTAAAGTTACT
ATTGATTGTTCTTTATTTGTCTGTTCTAATTATGCAGCTTGCCATGACTTATTGTCAGAGTATGGCACTTTTTGTGATA
ATATTAATAGTATTTTAGATGAAGTTAATGGTTTACTTGATACTACTCAATTGCATGTAGCTGATACTCTTATGCAAGG

TGTCACACTTAGCTCCAATCTTAATACTAATTTGCATTTTGATGTTGATAATATTAATTTTAAATCCCTAGTTGGATGT
TTAGGTCCACACTGCGGTTCTTCTTCTCGTTCTTTTTTTGAAGATTTATTGTTTGACAAAGTTAAACTTTCAGATGTTG
GTTTTGTTGAAGCTTATAACAATTGTACTGGTGGTAGTGAAATTAGAGATCTTCTTTGTGTACAATCCTTTAATGGTAT
TAAAGTTTTGCCTCCTATTTTGTCTGAATCTCAAATTTCTGGTTACACCACAGCCGCTACTGTTGCTGCTATGTTTCCA
CCATGGTCAGCAGCAGCTGGCATACCATTTTCTCTTAATGTACAATATAGAATTAATGGTTTGGGTGTTACTATGGATG
TTCTTAATAAAAATCAAAAGTTGATAGCTACTGCTTTTAATAATGCTCTTCTTTCTATTCAGAATGGTTTAGTGCTAC
CAACTCTGCACTTGCTAAAATACAAAGTGTTGTTAATTCTAATGCTCAAGCACTTAATAGTTTGTTACAGCAATTATTT
AATAAATTTGGTGCAATTAGTTCTTCTTTACAAGAAATTTTATCTCGTCTCGATGCTTTAGAGGCTCAGGTTCAGATTG
ATAGGCTTATTAATGGTCGTTTAACTGCTTTAAATGCTTATGTCTCTCAACAGCTTAGTGATATTTCTCTTGTAAAATT
TGGTGCTGCTTTAGCTATGGAGAAGGTTAATGAGTGTGTTAAAAGTCAATCTCCTCGTATTAATTTTTGTGGTAATGGT
AATCATATTTTGTCATTAGTTCAAAATGCTCCTTATGGTTTGTTGTTTATGCATTTTAGTTATAAACCTATTTCTTTTA
AAACTGTTTTAGTAAGTCCTGGTTTGTGTATATCAGGTGATGTAGGTATTGCACCTAAACAAGGGTATTTTATTAAACA
TAATGATCATTGGATGTTCACTGGTAGTTCTTACTATTATCCTGAACCAATTTCAGATAAAAATGTTGTTTTTATGAAT
ACTTGTTCTGTTAATTTTACTAAAGCGCCTCTTGTTTATTTGAATCATTCTGTACCAAAATTGTCTGATTTTGAATCTG
AGTTATCTCATTGGTTTAAAAATCAAACATCCATTGCGCCTAATTTGACTTTAAATCTTCATACTATTAATGCTACTTT
TTTAGATTTGTATTATGAGATGAATCTTATTCAAGAGTCTATTAAGTCTTTGAATAATAGTTATATCAATCTTAAAGAT
ATAGGTACATATGAAATGTATGTAAAATGGCCTTGGTATGTTTGGCTACTAATTTCTTTTTCATTTATAATATTCCTTG
TATTGCTCTTTTTTATATGTTGTTGTACTGGTTGTGGTTCTGCATGTTTTAGTAAATGTCATAATTGTTGTGATGAGTA
TGGTGGTCATCATGATTTTGTTATCAAAACATCTCATGATGATTAG

>gi|89515532|gb|DQ415910.1| Human coronavirus HKU1 strain N14 genotype A, complete genome

ATGTTATTAATTATTTTTATTTTGCCTACAACATTAGCTGTTATAGGTGATTTTAATTGTACTAATTTTGCTATT
AATGATTTAAACACCACAGTTCCTCGCATAAGTGAGTATGTTGTGGATGTTTCTTATGGTTTGGGTACATATTATATAC
TTGATCGTGTTTATTTAAATACTACTATATTATTTACTGGTTATTTCCCTAAATCTGGTGCCAATTTTAGGGATCTATC
TTTAAAAGGTACTACATATTTGAGTACTCTTTGGTATCAGAAACCCTTTTTATCTGATTTTAATAATGGTATTTTTTCT
AGAGTTAAGAATACTAAGTTGTATGTTAATAAAACTTTGTATAGTGAGTTTAGTACTATAGTTATAGGTAGTGTTTTTA
TTAACAACTCTTATACTATTGTTGTTCAACCTCATAATGGTGTTTTGGAGATTACAGCTTGTCAATACACTATGTGTGA
GTATCCTCATACTATTTGTAAATCTAAAGGTAGTTCTCGTAATGAATCTTGGCATTTTGATAAATCTGAACCTTTGTGT
CTGTTCAAGAAAAATTTTACTTATAATGTTTCTACAGATTGGTTGTATTTTCATTTTTATCAAGAACGTGGCACTTTTT
ATGCTTATTATGCTGATTCTGGCATGCCTACTACTTTTTTATTTAGTTTGTATTTTGGTACTCTTTTATCTCATTATTA
TGTTTTGCCTTTGACTTGTAATGCTATATCTTCTAATACTGATAATGAGACTTTACAATATTGGGTCACACCTTTGTCT
AAACGCCAATATCTTCTTAAATTTGACAACCGTGGTGTTATTACTAATGCTGTTGATTGTTCTAGTAGTTTCTTTAGCG
AGATTCAATGTAAAACTAAATCTTTATTACCTAATACTGGTGTTTATGACTTATCTGGTTTTACTGTTAAGCCTGTTGC
AACTGTACATCGTCGTATTCCTGATTTACCTGATTGTGACATTGATAAATGGCTTAACAATTTTAATGTACCCTCACCT
CTTAATTGGGAACGTAAAATTTTTTCTAATTGCAACTTTAATTTGAGTACTTTGCTTCGTTTAGTTCATACTGATTCTT
TTTCTTGTAATAATTTTGATGAATCTAAGATATATGGTAGTTGTTTTAAGAGTATTGTTTTAGATAAATTTGCCATACC
CAACTCCAGACGATCTGATTTGCAGTTGGGCAGTTCTGGTTTTCTGCAATCTTCTAATTATAAAATTGACACTACTTCT
AGTTCTTGTCAATTGTATTATAGTTTGCCTGCAATTAATGTTACTATTAATAATTATAATCCTTCTTTTTGGAATAGAA
GGTATGGTTTTAATAATTTTAATTTGAGCTCTCATAGTGTTGTTTACTCACGTTATTGTTTTTCTGTTAATAATACTTT
TTGTCCTTGTGCTAAACCTTCTTTTGCTTCAAGTTGCAAGAGTCATAAACCACCTTCTGCTTCCTGTCCTATTGGTACT
AATTATCGTTCTTGTGAGAGTACTACTGTACTCGACCACACTGACTGGTGTAGGTGTTCTTGTTTACCTGATCCTATAA

CTGCTTATGACCCTAGGTCTTGTTCTCAAAAAAAGTCTCTGGTTGGTGTTGGTGAACATTGTGCAGGGTTCGGTGTTGA
TGAAGAAAAGTGTGGTGTATTGGATGGATCATATAATGTTTCTTGTCTTTGTAGTACTGATGCCTTTCTAGGTTGGTCT
TATGACACTTGCGTCAGTAACAACCTTTGTAATATTTTTTCTAATTTTATTTTAAATGGTATCAATAGTGGTACCACTT
GTTCTAATGATTTATTGCAGCCTAATACTGAAGTTTTTACTGATGTTTGTGTTGATTACGACCTTTATGGTATTACAGG
ACAAGGTATTTTTAAAGAAGTTTCTGCTGTTTATTATAATAGTTGGCAAAATCTTTTGTATGATTCTAATGGCAACATT
ATTGGTTTTAAAGATTTTGTTACTAATAAAACATATAATATTTTCCCTTGTTATGCAGGAAGAGTTTCTGCTGCTTTTC
ATCAAAATGCTTCCTCTTTGGCTTTACTTTATCGTAATTTAAAATGTAGCTATGTTTTGAATAATATTTCTTTAACTAC
TCAGCCATATTTTGATAGTTATCTTGGTTGCGTTTTTAATGCTGATAATTTAACTGATTATTCTGTTTCTTCTTGTGCT
CTTCGCATGGGTAGTGGTTTTTGTGTTGATTATAACTCACCTTCTTCTTCCTCTTCGCGTCGTAAACGTAGAAGTATTT
CTGCTTCTTATCGTTTTGTTACTTTTGAACCCTTTAATGTCAGTTTTGTTAATGACAGTATTGAGTCTGTGGGTGGTCT
TTATGAGATCAAAATTCCCACTAACTTTACTATAGTTGGTCAAGAGGAATTTATTCAAACTAATTCTCCTAAAGTTACT
ATTGATTGTTCTTTATTTGTCTGTTCTAATTATGCAGCTTGCCATGACTTATTGTCAGAGTATGGCACTTTTTGTGATA
ATATTAATAGTATTTTAGATGAAGTTAATGGTTTACTTGATACTACTCAATTGCATGTAGCTGATACTCTTATGCAAGG
TGTCACACTTAGCTCCAATCTTAATACTAATTTGCATTTTGATGTTGATAATATTAATTTTAAATCCCTAGTTGGATGT
TTAGGTCCACACTGCGGTTCTTCTTCTCGTTCTTTTTTTGAAGATTTATTGTTTGACAAAGTTAAACTTTCAGATGTTG
GTTTTGTTGAAGCTTATAACAATTGTACTGGTGGTAGTGAAATTAGAGATCTTCTTTGTGTACAATCCTTTAATGGTAT
TAAAGTTTTGCCTCCTATTTTGTCTGAATCTCAAATTTCTGGTTACACCACAGCCGCTACTGTTGCTGCTATGTTTCCA
CCATGGTCAGCAGCAGCTGGCATACCATTTTCTCTTAATGTACAATATAGAATTAATGGTTTGGGTGTTACTATGGATG
TTCTTAATAAAAATCAAAAGTTGATAGCTACTGCTTTTAATAATGCTCTTCTTTCTATTCAGAATGGTTTTAGTGCTAC
CAACTCTGCACTTGCTAAAATACAAAGTGTTGTTAATTCTAATGCTCAAGCACTTAATAGTTTGTTACAGCAATTATTT
AATAAATTTGGTGCAATTAGTTCTTCTTTACAAGAAATTTTATCTCGTCTCGATGCTTTAGAGGCTCAGGTTCAGATTG
ATAGGCTTATTAATGGTCGTTTAACTGCTTTAAATGCTTATGTCTCTCAACAGCTTAGTGATATTTCTCTTGTAAAATT
TGGTGCTGCTTTAGCTATGGAGAAGGTTAATGAGTGTGTTAAAAGTCAATCTCCTCGTATTAATTTTTGTGGTAATGGT
AATCATATTTTGTCATTAGTTCAAAATGCTCCTTATGGTTTGTTGTTTATGCATTTTAGTTATAAACCTATTTCTTTTA
AAACTGTTTTAGTAAGTCCTGGTTTGTGTATATCAGGTGATGTAGGTATTGCACCTAAACAAGGGTATTTTATTAAACA
TAATGATCATTGGATGTTCACTGGTAGTTCTTACTATTATCCTGAACCAATTTCAGATAAAAATGTTGTTTTTATGAAT
ACTTGTTCTGTTAATTTTACTAAAGCGCCTCTTGTTTATTTGAATCATTCTGTACCAAAATTGTCTGATTTTGAATCTG
AGTTATCTCATTGGTTTAAAAATCAAACATCCATTGCGCCTAATTTGACTTTAAATCTTCATACTATTAATGCTACTTT
TTTAGATTTGTATTATGAGATGAATCTTATTCAAGAGTCTATTAAGTCTTTGAATAATAGTTATATCAATCTTAAAGAT
ATAGGTACATATGAAATGTATGTAAAATGGCCTTGGTATGTTTGGCTATTAATTTCTTTTTCATTTATAATATTCCTTG
TATTGCTCTTTTTTATATGTTGTTGTACTGGTTGTGGTTCTGCATGTTTTAGTAAATGTCATAATTGTTGTGATGAGTA
TGGTGGTCATCATGATTTTGTTATCAAAACATCTCATGATGATTAG

>gi|90655318|gb|DQ437607.1|_Human_coronavirus_HKU1_strain_N11_spike_glycoprotein_(S)_gene_complete_cds

ATGTTATTAATTATTTTTATTTTGCCTACAACATTAGCTGTTATAGGTGATTTTAATTGTACTAATTTTGCTATT
AATGATTTAAACACCACAGTTCCTCGCATAAGTGAGTATGTTGTGGATGTTTCTTATGGTTTGGGTACATATTATATAC
TTGATCGTGTTTATTTAAATACTACTATATTATTTACTGGTTATTTCCCTAAATCTGGTGCCAATTTTAGGGATCTATC
TTTAAAAGGTACTACATATTTGAGTACTCTTTGGTATCAGAAACCCTTTTTATCTGATTTTAATAATGGTATTTTTTCT
AGAGTTAAGAATACTAAGTTGTATGTTAATAAAACTTTGTATAGTGAGTTTAGTACTATAGTTATAGGTAGTGTTTTTA
TTAACAACTCTTATACTATTGTTGTTCAACCTCATAATGGTGTTTTGGAGATTACAGCTTGTCAATACACTATGTGTGA
GTATCCTCATACTATTTGTAAATCTAAAGGTAGTTCTCGTAATGAATCTTGGCATTTTGATAAATCTGAACCTTTGTGT

CTGTTCAAGAAAAATTTTACTTATAATGTTTCTACAGATTGGTTGTATTTTCATTTTTATCAAGAACGTGGCACTTTTT
ATGCTTATTATGCTGATTCTGGCATGCCTACTACTTTTTTATTTAGTTTGTATCTTGGTACTCTTTTATCTCATTATTA
TGTTTTGCCTTTGACTTGTAATGCTATATCTTCTAATACTGATAATGAGACTTTACAATATTGGGTCACACCTTTGTCT
AAACGCCAATATCTTCTTAAATTTGACAACCGTGGTGTTATTACTAATGCTGTTGATTGTTCTAGTAGTTTCTTTAGCG
AGATTCAATGTAAAACTAAATCTTTATTACCTAATACTGGTGTTTATGACTTATCTGGTTTTACTGTTAAGCCTGTTGC
AACTGTATATCGTCGTATTCCTGATTTACCTGATTGTGACATTGATAAATGGCTTAACAATTTTAATGTACCCTCACCT
CTTAATTGGGAACGTAAAATTTTTTCTAATTGCAACTTTAATTTGAGTACTTTGCTTCGTTTAGTTCATACTGATTCTT
TTTCTTGTAATAATTTTGATGAATCTAAGATATATGGTAGTTGTTTTAAGAGTATTGTTTTAGATAAATTTGCCATACC
CAACTCCAGACGATCTGATTTGCAGTTGGGCAGTTCTGGTTTTCTGCAATCTTCTAATTATAAAATTGACACTACTTCT
AGTTCTTGTCAATTGTATTATAGTTTGCCTGCAATTAATGTTACTATTAATAATTATAATCCTTCTTCTTGGAATAGAA
GGTATGGTTTTAATAATTTTAATTTGAGCTCTCATAGTGTTGTTTACTCACGTTATTGTTTTTCTGTTAATAATACTTT
TTGTCCTTGTGCTAAACCTTCTTTTGCTTCAAGTTGCAAGAGTCATAAACCACCTTCTGCTTCCTGTCCTATTGGTACT
AATTATCGTTCTTGTGAGAGTACTACTGTACTCGACCACACTGACTGGTGTAGGTGTTCTTGTTTACCTGATCCTATAA
CTGCTTATGACCCTAGGTCTTGTTCTCAAAAAAAGTCTCTGGTTGGTGTTGGTGAACATTGTGCAGGGTTCGGTGTTGA
TGAAGAAAAGTGTGGTGTATTGGATGGATCATATAATGTTTCTTGTCTTTGTAGTACTGATGCCTTTCTAGGTTGGTCT
TATGACACTTGCGTCAGTAACAACCGTTGTAATATTTTTTCTAATTTTATTTTAAATGGTATCAATAGTGGTACCACTT
GTTCTAATGATTTATTGCAGCCTAATACTGAAGTTTTTACTGATGTTTGTGTTGATTACGACCTTTATGGTATTACAGG
ACAAGGTATTTTTAAAGAAGTTTCTGCTGTTTATTATAATAGTTGGCAAAATCTTTTGTATGATTCTAATGGCAACATT
ATTGGTTTTAAAGATTTTGTTACTAATAAAACATATAATATTTTTCCTTGTTATGCAGGAAGAGTTTCTGCTGCTTTTC
ATCAAAATGCTTCCTCTTTGGCTTTACTTTATCGTAATTTAAAATGTAGCTATGTTTTGAATAATATTTCTTTAGCTAC
TCAGCCATATTTTGATAGTTATCTTGGTTGCGTTTTTAATGCTGATAATTTAACTGATTATTCTGTTTCTTCTTGTGCT
CTTCGCATGGGTAGTGGTTTTTGTGTTGATTATAACTCACCTTCTTCTTCCTCTTCGCGTCGTAAACGTAGAAGTATTT
CTGCTTCTTATCGTTTTGTTACTTTTGAACCCTTTAATGTCAGTTTTGTTAATGACAGTATTGAGTCTGTGGGTGGTCT
TTATGAGATCAAAATTCCCACTAACTTTACTATAGTTGGTCAAGAGGAATTTATTCAAACTAATTCTCCTAAAGTTACT
ATTGATTGTTCTTTATTTGTCTGTTCTAATTATGCAGCTTGCCATGACTTATTGTCAGAGTATGGCACTTTTTGTGATA
ATATTAATAGTATTTTAGATGAAGTTAATGGTTTACTTGATACTACTCAATTGCATGTAGCTGATACTCTTATGCAAGG
TGTCACACTTAGCTCCAATCTTAATACTAATTTGCATTTTGATGTTGATAATATTAATTTTAAATCCCTAGTTGGATGT
TTAGGTCCACACTGCGGTTCTTCTTCTCGTTCTTTTTTTGAAGATTTATTGTTTGACAAAGTTAAACTTTCAGATGTTG
GTTTTGTTGAAGCTTATAACAATTGTACTGGTGGTAGTGAAATTAGAGATCTTCTTTGTGTACAATCCTTTAATGGTAT
TAAAGTTTTGCCTCCTATTTTGTCTGAATCTCAAATTTCTGGTTACACCACAGCCGCTACTGTTGCTGCTATGTTTCCA
CCATGGTCAGCAGCAGCTGGCATACCATTTTCTCTTAATGTACAATATAGAATTAATGGTTTGGGTGTTACTATGGATG
TTCTTAATAAAAATCAAAAGTTGATAGCTACTGCTTTTAATAATGCTCTTCTTTCTATTCAGAATGGTTTTAGTGCTAC
CAACTCTGCACTTGCTAAAATACAAAGTGTTGTTAATTCTAATGCTCAAGCACTTAATAGTTTGTTACAGCAATTATTT
AATAAATTTGGTGCAATTAGTTCTTCTTTACAAGAAATTTTATCTCGTCTCGATGCTTTAGAGGCTCAGGTTCAGATTG
ATAGGCTTATTAATGGTCGTTTAACTGCTTTAAATGCTTATGTCTCTCAACAGCTTAGTGATATTTCTCTTGTAAAATT
TGGTGCTGCTTTAGCTATGGAGAAGGTTAATGAGTGTGTTAAAAGTCAATCTCCTCGTATTAATTTTTGTGGTAATGGT
AATCATATTTTGTCATTAGTTCAAAATGCTCCTTATGGTTTGTTGTTTATGCATTTTAGTTATAAACCTATTTCTTTTA
AAACTGTTTTAGTAAGTCCTGGTTTGTGTATATCAGGTGATGTAGGTATTGCACCTAAACAAGGGTATTTTATTAAACA
TAATGATCATTGGATGTTCACTGGTAGTTCTTACTATTATCCTGAACCAATTTCAGATAAAAATGTTGTTTTTATGAAT
ACTTGTTCTGTTAATTTTACTAAAGCGCCTCTTGTTTATTTGAATCATTCTGTACCAAAATTGTCTGATTTTGAATCTG
AGTTATCTCATTGGTTTAAAAATCAAACATCCATTGCGCCTAATTTGACTTTAAATCTTCATACTATTAATGCTACTTT
TTTAGATTTGTATTATGAGATGAATCTTATTCAAGAGTCTATTAAGTCTTTGAATAATAGTTATATCAATCTTAAAGAT
ATAGGTACATATGAAATGTATGTAAAATGGCCTTGGTATGTTTGGCTACTAATTTCTTTTTCATTTATAATATTCCTTG
TATTGCTCTTTTTTATATGTTGTTGTACTGGTTGTGGTTCTGCATGTTTTAGTAAATGTCATAATTGTTGTGATGAGTA

TGGTGGTCATCATGATTTTGTTATCAAAACATCTCATGATGATTAG

>gi|90655320|gb|DQ437608.1|_Human_coronavirus_HKU1_strain_N13_spike_glycoprotein_(S)_gene_complete_cds

ATGTTATTAATTATTTTTATTTTGCCTACAACATTAGCTGTTATAGGTGATTTTAATTGTACTAATTTTGCTATT
AATGATTTAAACACCACAGTTCCTCGCATAAGTGAGTATGTTGTGGATGTTTCTTATGGTTTGGGTACATATTATATAC
TTGATCGTGTTTATTTAAATACTACTATATTATTTACTGGTTATTTCCCTAAATCTGGTGCCAATTTTAGGGATCTATC
TTTAAAAGGTACTACATATTTGAGTACTCTTTGGTATCAGAAACCCTTTTTATCTGATTTTAATAATGGTATTTTTTCT
AGAGTTAAGAATACTAAGTTGTATGTTAATAAAACTTTGTATAGTGAGTTTAGTACTATAGTTATAGGTAGTGTTTTTA
TTAACAACTCTTATACTATTGTTGTTCAACCTCATAATGGTGTTTTGGAGATTACAGCTTGTCAATACACTATGTGTGA
GTATCCTTATACTATTTGTAAATCTAAAGGTAGTTCTCGTAATGAATCTTGGCATTTTGATAAATCTGAACCTTTGTGT
CTGTTCAAGAAAAATTTTACTTATAATGTTTCTACAGATTGGTTGTATTTTCATTTTTATCAAGAACGTGGCACTTTTT
ATGCTTATTATGCTGATTCTGGCATGCCTACTACTTTTTTATTTAGTTTGTATCTTGGTACTCTTTTATCTCATTATTA
TGTTTTGCCTTTGACTTGTAATGCTATATCTTCTAATACTGATAATGAGACTTTACAATATTGGGTCACACCTTTGTCT
AAACGCCAATATCTTCTTAAATTTGACAACCGTGGTGTTATTACTAATGCTGTTGATTGTTCTAGTAGTTTCTTTAGCG
AGATTCAATGTAAAACTAAATCTTTATTACCTAATACTGGTGTTTATGACTTATCTGGTTTTACTGTTAAGCCTGTTGC
AACTGTACATCGTCGTATTCCTGATTTACCTGATTGTGACATTGATAAATGGCTTAACAATTTTAATGTACCCTCACCT
CTTAATTGGGAACGTAAAATTTTTTCTAATTGCAACTTTAATTTGAGTACTTTGCTTCGTTTAGTTCATACTGATTCTT
TTTCTTGTAATAATTTTGATGAATCTAAGATATATGGTAGTTGTTTTAAGAGTATTGTTTTAGATAAATTTGCCATACC
CAACTCCAGACGATCTGATTTGCAGTTGGGCAGTTCTGGTTTTCTGCAATCTTCTAATTATAAAATTGACACTACTTCT
AGTTCTTGTCAATTGTATTATAGTTTGCCTGCAATTAATGTTACTATTAATAATTATAATCCTTCTTCTTGGAATAGAA
GGTATGGTTTTAATAATTTTAATTTGAGCTCTCATAGTGTTGTTTACTCACGTTATTGTTTTTCTGTTAATAATACTTT
TTGTCCTTGTGCTAAACCTTCTTTTGCTTCAAGTTGCAAGAGTCATAAACCACCTTCTGCTTCCTGTCCTATTGGTACT
AATTATCGTTCTTGTGAGAGTACTACTGTACTCGACCACACTGACTGGTGTAGGTGTTCTTGTTTACCTGATCCTATAA
CTGCTTATGACCCTAGGTCTTGTTCTCAAAAAAAGTCTCTGGTTGGTGTTGGTGAACATTGTGCAGGGTTCGGTGTTGA
TGAAGAAAAGTGTGGTGTATTGGATGGATCATATAATGTTTCTTGTCTTTGTAGTACTGATGCCTTTCTAGGTTGGTCT
TATGACACTTGCGTCAGTAACAACCGTTGTAATATTTTTTCTAATTTTATTTTAAATGGTATCAATAGTGGTACCACTT
GTTCTAATGATTTATTGCAGCCTAATACTGAAGTTTTTACTGATGTTTGTGTTGATTACGACCTTTATGGTATTACAGG
ACAAGGTATTTTTAAAGAAGTTTCTGCTGTTTATTATAATAGTTGGCAAAATCTTTTGTATGATTCTAATGGCAACATT
ATTGGTTTTAAAGATTTTGTTACTAATAAAACATATAATATTTTCCCTTGTTATGCAGGAAGAGTTTCTGCTGCTTTTC
ATCAAAATGCTTCCTCTTTGGCTTTACTTTATCGTAATTTAAAATGTAGCTATGTTTTGAATAATATTTCTTTAGCTAC
TCAGCCATATTTTGATAGTTATCTTGGTTGCGTTTTTAATGCTGATAATTTAACTGATTATTCTGTTTCTTCTTGTGCT
CTTCGCATGGGTAGTGGTTTTTGTGTTGATTATAACTCACCTTCTTCTTCCTCTTCGCGTCGTAAACGTAGAAGTATTT
CTGCTTCTTATCGTTTTGTTACTTTTGAACCCTTTAATGTCAGTTTTGTTAATGACAGTATTGAGTCTGTGGGTGGTCT
TTATGAGATCAAAATTCCCACTAACTTTACTATAGTTGGTCAAGAGGAATTTATTCAAACTAATTCTCCTAAAGTTACT
ATTGATTGTTCTTTATTTGTCTGTTCTAATTATGCAGCTTGCCATGACTTATTGTCAGAGTATGGCACTTTTTGTGATA
ATATTAATAGTATTTTAGATGAAGTTAATGGTTTACTTGATACTACTCAATTGCATGTAGCTGATACTCTTATGCAAGG
TGTCACACTTAGCTCCAATCTTAATACTAATTTGCATTTTGATGTTGATAATATTAATTTTAAATCCTTAGTTGGATGT
TTAGGTCCACACTGCGGTTCTTCTTCTCGTTCTTTTTTTGAAGATTTATTGTTTGACAAAGTTAAACTTTCAGATGTTG
GTTTTGTTGAAGCTTATAACAATTGTACTGGTGGTAGTGAAATTAGAGATCTTCTTTGTGTACAATCCTTTAATGGTAT
TAAAGTTTTGCCTCCTATTTTGTCTGAATCTCAAATTTCTGGTTACACCACAGCCGCTACTGTTGCTGCTATGTTTCCA
CCATGGTCAGCAGCAGCTGGCATACCATTTTCTCTTAATGTACAATATAGAATTAATGGTTTGGGTGTTACTATGGATG

TTCTTAATAAAAATCAAAAGTTGATAGCTACTGCTTTTAATAATGCTCTTCTTTCTATTCAGAATGGTTTTAGTGCTAC
CAACTCTGCACTTGCTAAAATACAAAGTGTTGTTAATTCTAATGCTCAAGCACTTAATAGTTTGTTACAGCAATTATTT
AATAAATTTGGTGCAATTAGTTCTTCTTTACAAGAAATTTTATCTCGTCTCGATGCTTTAGAGGCTCAGGTTCAGATTG
ATAGGCTTATTAATGGTCGTTTAACTGCTTTAAATGCTTATGTCTCTCAACAGCTTAGTGATATTTCTCTTGTAAAATT
TGGTGCTGCTTTAGCTATGGAGAAGGTTAATGAGTGTGTTAAAAGTCAATCTCCTCGTATTAATTTTTGTGGTAATGGT
AATCATATTTTGTCATTAGTTCAAAATGCTCCTTATGGTTTGTTGTTTATGCATTTTAGTTATAAACCTATTTCTTTTA
AAACTGTTTTAGTAAGTCCTGGTTTGTGTATATCAGGTGATGTAGGTATTGCACCTAAACAAGGGTATTTTATTAAACA
TAATGATCATTGGATGTTCACTGGTAGTTCTTACTATTATCCTGAACCAATTTCAGATAAAAATGTTGTTTTTATGAAT
ACTTGTTCTGTTAATTTTACTAAAGCGCCTCTTGTTTATTTGAATCATTCTGTACCAAAATTGTCTGATTTTGAATCTG
AGTTATCTCATTGGTTTAAAAATCAAACATCCATTGCGCCTAATTTGACTTTAAATCTTCATACTATTAATGCTACTTT
TTTAGATTTGTATTATGAGATGAATCTTATTCAAGAGTCTATTAAGTCTTTGAATAATAGTTATATCAATCTTAAAGAT
ATAGGTACATATGAAATGTATGTAAAATGGCCTTGGTATGTTTGGCTACTAATTTCTTTTTCATTTATAATATTCCTTG
TATTGCTCTTTTTTATATGTTGTTGTACTGGTTGTGGTTCTGCATGTTTTAGTAAATGTCATAATTGTTGTGATGAGTA
TGGTGGTCATCATGATTTTGTTATCAAAACATCTCATGATGATTAG

>gi|90655322|gb|DQ437609.1|_Human_coronavirus_HKU1_strain_N14_spike_glycoprotein_(S)_gene_complete_cds

ATGTTATTAATTATTTTTATTTTGCCTACAACATTAGCTGTTATAGGTGATTTTAATTGTACTAATTTTGCTATT
AATGATTTAAACACCACAGTTCCTCGCATAAGTGAGTATGTTGTGGATGTTTCTTATGGTTTGGGTACATATTATATAC
TTGATCGTGTTTATTTAAATACTACTATATTATTTACTGGTTATTTCCCTAAATCTGGTGCCAATTTTAGGGATCTATC
TTTAAAAGGTACTACATATTTGAGTACTCTTTGGTATCAGAAACCCTTTTTATCTGATTTTAATAATGGTATTTTTTCT
AGAGTTAAGAATACTAAGTTGTATGTTAATAAAACTTTGTATAGTGAGTTTAGTACTATAGTTATAGGTAGTGTTTTTA
TTAACAACTCTTATACTATTGTTGTTCAACCTCATAATGGTGTTTTGGAGATTACAGCTTGTCAATACACTATGTGTGA
GTATCCTCATACTATTTGTAAATCTAAAGGTAGTTCTCGTAATGAATCTTGGCATTTTGATAAATCTGAACCTTTGTGT
CTGTTCAAGAAAAATTTTACTTATAATGTTTCTACAGATTGGTTGTATTTTCATTTTTATCAAGAACGTGGCACTTTTT
ATGCTTATTATGCTGATTCTGGCATGCCTACTACTTTTTTATTTAGTTTGTATTTTGGTACTCTTTTATCTCATTATTA
TGTTTTGCCTTTGACTTGTAATGCTATATCTTCTAATACTGATAATGAGACTTTACAATATTGGGTCACACCTTTGTCT
AAACGCCAATATCTTCTTAAATTTGACAACCGTGGTGTTATTACTAATGCTGTTGATTGTTCTAGTAGTTTCTTTAGCG
AGATTCAATGTAAAACTAAATCTTTATTACCTAATACTGGTGTTTATGACTTATCTGGTTTTACTGTTAAGCCTGTTGC
AACTGTACATCGTCGTATTCCTGATTTACCTGATTGTGACATTGATAAATGGCTTAACAATTTTAATGTACCCTCACCT
CTTAATTGGGAACGTAAAATTTTTTCTAATTGCAACTTTAATTTGAGTACTTTGCTTCGTTTAGTTCATACTGATTCTT
TTTCTTGTAATAATTTTGATGAATCTAAGATATATGGTAGTTGTTTTAAGAGTATTGTTTTAGATAAATTTGCCATACC
CAACTCCAGACGATCTGATTTGCAGTTGGGCAGTTCTGGTTTTCTGCAATCTTCTAATTATAAAATTGACACTACTTCT
AGTTCTTGTCAATTGTATTATAGTTTGCCTGCAATTAATGTTACTATTAATAATTATAATCCTTCTTTTTGGAATAGAA
GGTATGGTTTTAATAATTTTAATTTGAGCTCTCATAGTGTTGTTTACTCACGTTATTGTTTTTCTGTTAATAATACTTT
TTGTCCTTGTGCTAAACCTTCTTTTGCTTCAAGTTGCAAGAGTCATAAACCACCTTCTGCTTCCTGTCCTATTGGTACT
AATTATCGTTCTTGTGAGAGTACTACTGTACTCGACCACACTGACTGGTGTAGGTGTTCTTGTTTACCTGATCCTATAA
CTGCTTATGACCCTAGGTCTTGTTCTCAAAAAAAGTCTCTGGTTGGTGTTGGTGAACATTGTGCAGGGTTCGGTGTTGA
TGAAGAAAAGTGTGGTGTATTGGATGGATCATATAATGTTTCTTGTCTTTGTAGTACTGATGCCTTTCTAGGTTGGTCT
TATGACACTTGCGTCAGTAACAACCTTTGTAATATTTTTTCTAATTTTATTTTAAATGGTATCAATAGTGGTACCACTT
GTTCTAATGATTTATTGCAGCCTAATACTGAAGTTTTTACTGATGTTTGTGTTGATTACGACCTTTATGGTATTACAGG
ACAAGGTATTTTTAAAGAAGTTTCTGCTGTTTATTATAATAGTTGGCAAAATCTTTTGTATGATTCTAATGGCAACATT

ATTGGTTTTAAAGATTTTGTTACTAATAAAACATATAATATTTTCCCTTGTTATGCAGGAAGAGTTTCTGCTGCTTTTC
ATCAAAATGCTTCCTCTTTGGCTTTACTTTATCGTAATTTAAAATGTAGCTATGTTTTGAATAATATTTCTTTAACTAC
TCAGCCATATTTTGATAGTTATCTTGGTTGCGTTTTTAATGCTGATAATTTAACTGATTATTCTGTTTCTTCTTGTGCT
CTTCGCATGGGTAGTGGTTTTTGTGTTGATTATAACTCACCTTCTTCTTCCTCTTCGCGTCGTAAACGTAGAAGTATTT
CTGCTTCTTATCGTTTTGTTACTTTTGAACCCTTTAATGTCAGTTTTGTTAATGACAGTATTGAGTCTGTGGGTGGTCT
TTATGAGATCAAAATTCCCACTAACTTTACTATAGTTGGTCAAGAGGAATTTATTCAAACTAATTCTCCTAAAGTTACT
ATTGATTGTTCTTTATTTGTCTGTTCTAATTATGCAGCTTGCCATGACTTATTGTCAGAGTATGGCACTTTTTGTGATA
ATATTAATAGTATTTTAGATGAAGTTAATGGTTTACTTGATACTACTCAATTGCATGTAGCTGATACTCTTATGCAAGG
TGTCACACTTAGCTCCAATCTTAATACTAATTTGCATTTTGATGTTGATAATATTAATTTTAAATCCCTAGTTGGATGT
TTAGGTCCACACTGCGGTTCTTCTTCTCGTTCTTTTTTTGAAGATTTATTGTTTGACAAAGTTAAACTTTCAGATGTTG
GTTTTGTTGAAGCTTATAACAATTGTACTGGTGGTAGTGAAATTAGAGATCTTCTTTGTGTACAATCCTTTAATGGTAT
TAAAGTTTTGCCTCCTATTTTGTCTGAATCTCAAATTTCTGGTTACACCACAGCCGCTACTGTTGCTGCTATGTTTCCA
CCATGGTCAGCAGCAGCTGGCATACCATTTTCTCTTAATGTACAATATAGAATTAATGGTTTGGGTGTTACTATGGATG
TTCTTAATAAAAATCAAAAGTTGATAGCTACTGCTTTTAATAATGCTCTTCTTTCTATTCAGAATGGTTTTAGTGCTAC
CAACTCTGCACTTGCTAAAATACAAAGTGTTGTTAATTCTAATGCTCAAGCACTTAATAGTTTGTTACAGCAATTATTT
AATAAATTTGGTGCAATTAGTTCTTCTTTACAAGAAATTTTATCTCGTCTCGATGCTTTAGAGGCTCAGGTTCAGATTG
ATAGGCTTATTAATGGTCGTTTAACTGCTTTAAATGCTTATGTCTCTCAACAGCTTAGTGATATTTCTCTTGTAAAATT
TGGTGCTGCTTTAGCTATGGAGAAGGTTAATGAGTGTGTTAAAAGTCAATCTCCTCGTATTAATTTTTGTGGTAATGGT
AATCATATTTTGTCATTAGTTCAAAATGCTCCTTATGGTTTGTTGTTTATGCATTTTAGTTATAAACCTATTTCTTTTA
AAACTGTTTTAGTAAGTCCTGGTTTGTGTATATCAGGTGATGTAGGTATTGCACCTAAACAAGGGTATTTTATTAAACA
TAATGATCATTGGATGTTCACTGGTAGTTCTTACTATTATCCTGAACCAATTTCAGATAAAAATGTTGTTTTTATGAAT
ACTTGTTCTGTTAATTTTACTAAAGCGCCTCTTGTTTATTTGAATCATTCTGTACCAAAATTGTCTGATTTTGAATCTG
AGTTATCTCATTGGTTTAAAAATCAAACATCCATTGCGCCTAATTTGACTTTAAATCTTCATACTATTAATGCTACTTT
TTTAGATTTGTATTATGAGATGAATCTTATTCAAGAGTCTATTAAGTCTTTGAATAATAGTTATATCAATCTTAAAGAT
ATAGGTACATATGAAATGTATGTAAAATGGCCTTGGTATGTTTGGCTACTAATTTCTTTTTCATTTATAATATTCCTTG
TATTGCTCTTTTTTATATGTTGTTGTACTGGTTGTGGTTCTGCATGTTTTAGTAAATGTCATAATTGTTGTGATGAGTA
TGGTGGTCATCATGATTTTGTTATCAAAACATCTCATGATGATTAG

>gi|90655324|gb|DQ437610.1|_Human_coronavirus_HKU1_strain_N15_spike_glycoprotein_(S)_gene_complete_cds

ATGTTTTTAATTATTTTTATTTTGCCTACAACACTAGCTGTTATAGGTGATTTTAATTGTACTAACTCTTTTATT
AATGATTATAATAAAACCATTCCGCGTATAAGCGAGGATGTTGTTGATGTATCTCTTGGTTTGGGCACATATTATGTTC
TTAACCGTGTTTATTTAAATACTACCTTGTTATTTACAGGTTATTTTCCTAAATCTGGTGCTAATTTTAGAGACTTGGC
TTTAAAGGGTTCTATATATTTGAGTACTCTCTGGTATAAACCACCTTTTCTGTCAGATTTTAATAATGGTATTTTTTCT
AAGGTTAAGAATACTAAGTTATATGTTAATAATACTTTGTATAGTGAATTTAGTACTATAGTTATAGGTAGTGTTTTTG
TTAATACGTCTTATACTATTGTTGTTCAACCTCACAATGGTATTTTGGAGATTACAGCTTGTCAGTATACTATGTGTGA
ATATCCTCACACTGTTTGTAAGTCTAAGGGTAGTATTCGTAATGAATCTTGGCACATTGATTCTTCGGAACCTTTATGC
TTGTTTAAGAAAAATTTTACTTATAATGTTTCTGCAGATTGGCTGTATTTTCATTTTTATCAAGAACGTGGTGTTTTTT
ATGCATATTATGCAGATGTAGGTATGCCTACCACTTTCTTATTTAGTTTATATTTAGGTACTATTTTATCTCATTATTA
TGTTATGCCTTTGACTTGTAATGCTATATCTTCAAATACTGACAATGAAACTTTAGAATATTGGGTTACACCGCTATCT
AGACGTCAGTATCTTCTCAATTTTGATGAGCACGGTGTTATTACTAATGCTGTTGATTGTTCAAGTAGTTTTCTTAGTG
AGATTCAATGTAAAACTCAATCTTTTGCACCTAATACTGGTGTTTATGATTTGTCTGGTTTTACTGTAAAGCCTGTTGC

AACTGTTTATCGTCGGATTCCTAATTTACCTGATTGTGACATTGACAACTGGCTTAATAATGTTAGTGTACCTTCACCT
CTTAATTGGGAACGTAGAATTTTTTCTAATTGTAACTTCAATTTAAGCACTTTACTTCGTCTAGTTCATGTTGATTCTT
TTTCTTGTAATAATCTTGATAAATCTAAAATTTTTGGTAGTTGCTTTAATAGTATTACTGTTGACAAGTTTGCTATACC
TAATCGCAGACGAGATGATTTGCAATTGGGCAGTTCTGGCTTTTTGCAATCATCTAATTACAAAATAGATATTTCTTCT
AGTTCTTGTCAATTGTATTATAGTTTACCTTTACTTAATGTTACTATTAATAACTTTAATCCATCTTCTTGGAATAGGA
GGTATGGTTTTGGTAGTTTTAATGTGTCTTCTTATGATGTTGTTTATTCTGATCATTGTTTTTCTGTTAACAGCGACTT
TTGCCCTTGTGCAGATCCGTCTGTTGTTAATTCTTGTGTTAAATCTAAGCCTCTTTCTGCCATTTGTCCTGCTGGTACT
AAATATCGTCATTGCGACTTGGATACTACTCTTTATGTTAAAAACTGGTGTAGATGTTCTTGTCTACCTGACCCCATTT
CTACTTATTCTCCTAACACATGTCCTCAAAAGAAGGTCGTTGTTGGTATAGGTGAACATTGTCCAGGTCTTGGTATTAA
TGAGGAAAAATGTGGTACACAATTAAATCATAGTTCCTGTTCTTGTAGTCCTCATGCCTTTTTGGGTTGGTCTTTTGAT
AGTTGTATTAGTAATAATCGTTGCAATATTTTTTCTAATTTTATTTTTAATGGAATTAATAGTGGCACCACTTGTTCTA
ATGATTTGTTATATTCTAACACTGAAGTTTCTACTGGTGTTTGTGTTAATTATGATCTTTATGGCATCACAGGCCAAGG
TATTTTTAAAGAAGTTTCTGCGGCTTATTATAATAATTGGCAGAATCTTTTGTATGATTCTAATGGTAATATTATTGGT
TTTAAAGATTTTTTGACTAATAAAACTTACACTATACTTCCTTGTTATTCTGGTAGAGTGTCTGCTGCATTTTATCAAA
ATTCTTCCTCACCAGCTTTGCTTTATCGTAATTTAAAGTGTAGTTATGTTTTGAATAATATTTCTTTTATCTCACAACC
ATTTTATTTTGATAGTTATCTTGGTTGTGTTTTGAATGCTGTTAATTTAACTAGCTATTCTGTATCCTCTTGTGATTTG
CGTATGGGTAGTGGGTTTTGTATTGATTATGCTTTACCCTCTTCTCGGCGTAAGCGTAGAGGTATTTCTTCTCCTTATC
GCTTTGTAACTTTTGAACCCTTTAATGTTAGTTTTGTTAACGATAGTGTTGAAACTGTTGGTGGTTTATTTGAGATTCA
GATTCCTACTAACTTTACCATAGCTGGTCATGAAGAATTTATTCAGACTAGTTCTCCTAAAGTTACTATTGATTGTTCA
GCTTTTGTTTGCTCTAATTATGCTGCTTGTCATGATTTATTGTCGGAATATGGCACTTTTTGCGATAATATTAATAGTA
TTTTAAATGAAGTCAATGACTTACTTGATATTACTCAGTTGCAGGTTGCTAATGCTTTAATGCAAGGTGTTACACTTAG
TTCTAATCTTAATACTAATCTACACTCTGATGTTGATAATATAGATTTAAATCTCTTCTAGGTTGTTTAGGTTCACAA
TGTGGTTCTTCGTCTAGATCTTTGTTAGAGGATTTATTATTCAACAAGGTCAAACTTTCAGATGTAGGTTTTGTTGAAG
CTTATAATAATTGCACTGGTGGTAGTGAAATTAGAGATCTTCTCTGTGTGCAATCTTTTAATGGTATTAAAGTATTACC
TCCCATTTTATCTGAGACTCAAATTTCTGGCTATACTACAGCTGCTACTGTGGCGGCTATGTTTCCGCCATGGTCTGCT
GCTGCTGGTGTACCATTTTCTCTTAATGTACAATATAGAATTAATGGTTTGGGTGTTACTATGGATGTTCTTAATAAGA
ATCAAAAGTTAATAGCTAATGCTTTTAATAAAGCTCTTCTTTCTATCCAGAATGGTTTTACTGCTACTAACTCTGCTCT
TGCTAAAATTCAAAGTGTCGTTAATGCTAATGCTCAAGCACTTAATAGTTTGTTACAACAATTATTTAATAAATTTGGT
GCTATTAGTTCTTCTTTACAAGAAATTTTGTCTCGCCTTGATAATTTAGAAGCTCAGGTTCAGATTGATAGGCTCATTA
ATGGTCGTTTGACTGCTTTAAATGCTTATGTTTCTCAACAGCTTAGTGATATTACACTTATTAAGGCTGGAGCTTCTCG
TGCTATTGAGAAGGTTAATGAGTGTGTTAAAAGTCAATCCCCTCGTATAAATTTTTGTGGCAATGGTAACCACATTTTA
TCATTGGTTCAAAATGCTCCTTATGGTTTGCTTTTCATTCATTTTAGTTATAAACCTACTTCTTTTAAAACTGTCTTAG
TAAGTCCAGGTTTATGTTTATCCGGTGATAGAGGTATTGCACCTAAGCAAGGTTATTTTATTAAACAAAATGATTCCTG
GATGTTTACTGGTAGTTCCTATTATTACCCAGAACCAATTTCAGATAAAAATGTTGTTTTCATGAATAGTTGCTCTGTT
AATTTTACTAAAGCTCCATTTATTTATCTTAATAATTCTATACCAAATTTGTCTGATTTTGAAGCCGAGTTTTCTCTTT
GGTTTAAAAATCATACTTCTATAGCACCTAATTTAACCTTTAATTCTCATATTAATGCTACTTTTTTAGATCTGTATTA
TGAAATGAATGTTATTCAGGAATCTATTAAATCTTTGAACAGTAGTTTTATTAATCTTAAAGAAATAGGTACTTATGAA
ATGTATGTTAAATGGCCTTGGTACATTTGGTTGTTAATTGTCATTTTATTTATAATTTTTCTTATGATACTTTTCTTTA
TATGCTGCTGTACTGGTTGCGGTTCAGCATGTTTTAGTAAATGTCATAATTGTTGTGATGAGTATGGGGGTCACAATGA
TTTGTTATTAAAGCATCTCATGATGATTAG

>gi|90655326|gb|DQ437611.1|_Human_coronavirus_HKU1_strain_N16_spike_glycoprotein_(S)_gene_complete_cds

ATGTTTTTAATTATTTTTATTTTGCCTACAACACTAGCTGTTATAGGTGATTTTAATTGTACTAACTCTTTTATT
AATCATTATAATAAAACCATTCCGCGTATAAGCGAGGATGTTGTTGATGTATCTCTTGGTTTGGGCACATATTATGTTC
TTAACCGTGTTTATTTAAATACTACCTTGTTATTTACGGGTTATTTTCCTAAATCTGGTGCTAACTTTAGAGACTTGGC
TTTAAAGGGTTCTATATATTTGAGTACTCTCTGGTATAAACCACCTTTTCTGTCAGATTTTAATAATGGTATTTTTTCT
AAGGTTAAGAATACTAAGTTATATGTTAATAATACTTTGTATAGTGAATTTAGTACTATAGTTATAGGTAGTGTTTTTG
TTAATACTTCTTATACTATTGTTGTTCAACCTCATAATGGTATTTTGGAGATTACAGCTTGTCAGTATACTATGTGTGA
ATATCCTCACACTGTTTGTAAGTCTAAGGGTAGTATTCGTAATGAATCTTGGCACATTGATTCTTCGGAACCTTTATGC
TTGTTTAAGAAAAATTTTACTTATAATGTTTCTGCAGATTGGCTGTATTTTCATTTTTATCAAGAACGTGGTGTTTTTT
ATGCATACTATGCAGATGTAGGTATGCCTACCACTTTCTTATTTAGTCTATATTTAGGTACTATTTTATCTCATTATTA
TGTTATGCCTTTGACTTGTAATGCTATATCTTCAAATACTGACAATGAAACTTTAGAATATTGGGTTACACCGCTATCT
AGACGTCAGTATCTTCTTAATTTTGATGAGCACGGTGTTATTACTAATGCCGTTGATTGTTCAAGTAGTTTTCTTAGTG
AGATTCAATGTAAAACTCAATCTTTTGCACCTAATACTGGTGTTTATGATTTGTCTGGTTTTACTGTAAAGCCTGTTGC
AACTGTTTATCGTCGGATTCCTAATTTACCTGATTGTGACATTGACAACTGGCTTAATAATGTTAGTGTACCTTCACCT
CTTAATTGGGAACGTAGAATTTTTTCTAATTGTAACTTTAACTTAAGCACTTTACTTCGTCTAGTTCATGTTGATTCTT
TTTCTTGTAATAATCTTGATAAATCTAAAATTTTTGGTAGTTGCTTTAATAGTATTACTGTTGACAAGTTTGCTATACC
TAATCGCAGACGAGATGATTTGCAATTGGGCAGTTCTGGCTTTTTGCAATCATCTAATTACAAAATAGATATTTCTTCT
AGTTCTTGTCAATTGTATTATAGTTTACCTTTAGTTAATGTTACTATTAATAACTTTAATCCATCTTCTTGGAATAGGA
GGTATGGTTTTGGTAGTTTTAATTTGTCTTCTTATGACGTTGTTTATTCTGATCATTGTTTTTCTGTTAACAGCGACTT
TTGCCCTTGTGCAGATCCGTCTGTTGTTAATTCTTGTGTTAAATCTAAGCCTCCTTCTGCCATTTGTCCTGCTGGTACT
AAATATCGTCATTGCGACTTGGATACTACTCTTTATGTTAAAAACTGGTGTAGATGTTCTTGTCTACCTGACCCCATTT
CTACTTATTCTCCTAACACATGTCCTCAAAAGAAGGTCGTTGTTGGTATAGGTGAACATTGTCCAGGTCTTGGTATTAA
TGAGGAAAAATGTGGTACACAATTAAATCATAGTTCCTGTTTTTGTAGTCCTGATGCCTTTTTGGGTTGGTCTTTTGAT
AGTTGTATTAGTAATAATCGTTGCAATATTTTTTCTAATTTTATTTTTAATGGAATTAATAGTGGCACCACTTGTTCTA
ATGATTTGTTATATTCTAACACTGAAATTTCTACTGGTGTTTGTGTTAATTATGATCTTTATGGCATCACAGGCCAAGG
TATTTTTAAAGAAGTTTCTGCGGCTTATTATAATAATTGGCAGAATCTTTTGTATGATTCTAATGGTAATATTATTGGT
TTTAAAGATTTTTTGACTAATAAAACTTACACTATACTTCCTTGTTATTCTGGTAGAGTGTCTGCTGCATTTTATCAAA
ATTCTTCCTCACCAGCTTTGCTTTATCGTAATTTAAAGTGTAGTTATGTTTTGAATAATATTTCTTTTATCTCACAACC
ATTTTATTTCGATAGTTATCTTGGTTGTGTTTTGAATGCTGTTAATTTAACTAGCTATTCTGTATCCTCTTGTGATTTG
CGTATGGGTAGTGGATTTTGTATTGATTATGCTTTACCCTCTTCTCGGCGTAAGCGTAGAGGTATTTCTTCTCCTTATC
GCTTTGTAACTTTTGAACCCTTTAATGTTAGTTTTGTTAACGATAGTGTTGAAACTGTTGGTGGTTTATTTGAGATTCA
GATTCCTACTAACTTTACCATAGCTGGTCATGAAGAATTTATTCAGACTAGTTCTCCTAAAGTTACTATTGATTGTGCA
GCTTTTGTTTGCTCTAACTATGCTGCTTGTCATGATTTATTGTCGGAATATGGCACTTTTTGCGATAATATTAATAGTA
TTTTAAATGAAGTCAATGATTTACTTGATATTACTCAGTTGCAGGTTGCTAATGCTCTAATGCAAGGTGTTACACTTAG
TTCTAATCTTAATACTAATCTACACTCTGATGTTGATAATATAGATTTAAATCTCTTCTAGGTTGTTTAGGTTCACAA
TGTGGTTCTTCGTCTAGATCTTTGTTAGAGGATTTATTATTCAACAAAGTCAAACTTTCAGATGTAGGTTTTGTTGAAG
CTTATAATAATTGCACTGGTGGTAGTGAAATTAGAGATCTTCTCTGTGTGCAATCTTTTAATGGTATTAAAGTATTACC
TCCCATTTTATCTGAGACTCAAATTTCTGGCTATACTACAGCTGCTACTGTGGCGGCTATGTTTCCGCCATGGTCTGCT
GCTGCTGGTGTACCATTTTCTCTTAATGTACAATATAGAATTAATGGTTTGGGTGTTACTATGGATGTTCTTAATAAGA
ATCAAAAGTTAATAGCTAATGCTTTTAATAAAGCTCTTCTTTCTATCCAGAATGGTTTTACTGCTACTAACTCTGCTCT
TGCTAAAATTCAAAGTGTCGTTAATGCTAATGCTCAAGCACTTAATAGTTTGTTACAACAATTATTTAATAAATTTGGT
GCTATTAGTTCTTCTTTACAAGAAATTTTGTCTCGCCTTGATAATTTAGAAGCTCAGGTTCAGATTGATAGGCTCATTA
ATGGTCGTTTGACTGCTTTAAATGCTTATGTTTCTCAACAGCTTAGTGATATTACACTTATTAAGGCTGGAGCTTCTCG
TGCTATTGAGAAGGTTAATGAGTGTGTTAAAAGTCAATCCCCTCGTATAAATTTTTGTGGCAATGGTAACCACATTTTA
TCATTGGTTCAAAATGCTCCTTATGGTTTGCTTTTCATTCATTTTAGTTATAAACCTACTTCTTTTAAAACTGTCTTAG

TAAGTCCAGGTTTATGTTTATCCGGTGATAGAGGTATTGCACCTAAGCAAGGTTATTTTATTAAACAAAATGATTCCTG
GATGTTTACTGGTAGTTCCTATTATTACCCAGAACCAATTTCAGATAAAAATGTTGTTTTCATGAATAGTTGCTCTGTT
AATTTTACTAAAGCTCCATTTATTTATCTTAATAATTCTATACCAAATTTGTCTGATTTTGAAGCCGAGTTTTCTCTTT
GGTTTAAAAATCATACTTCTATAGCACCTAATTTAACCTTTAATTCTCATATTAATGCTACTTTTTAGATCTGTATTA
TGAAATGAATGTTATTCAGGAATCTATTAAATCTTTGAACAGTAGTTTTATTAATCTTAAAGAAATAGGTACTTATGAA
ATGTATGTTAAATGGCCTTGGTACATTTGGTTGTTAATTGTCATTTTATTTATAATTTTTCTTATGATACTTTTCTTTA
TATGCTGCTGTACTGGTTGCGGTTCAGCATGTTTTAGTAAATGTCATAATTGTTGTGATGAGTATGGGGGTCACAATGA
TTTTGTTATTAAAGCATCTCATGATGATTAG

>gi|90655332|gb|DQ437614.1|_Human_coronavirus_HKU1_strain_N19_spike_glycoprotein_(S)_gene_complete_cds

ATGTTATTAATTATTTTTATTTTGCCTACAACATTAGCTGTTATAGGTGATTTTAATTGTACTAATTTTGCTATT
AATGATTTAAACACCACAGTTCCTCGCATAAGTGAGTATGTTGTGGATGTTTCTTATGGTTTGGGTACATATTATATAC
TTGATCGTGTTTATTTAAATACTACTATATTATTTACTGGTTATTTCCCTAAATCTGGTGCCAATTTTAGGGATCTATC
TTTAAAAGGTACTACATATTTGAGTACTCTTTGGTATCAGAAACCCTTTTTATCTGATTTTAATAATGGTATTTTTTCT
AGAGTTAAGAATACTAAGTTGTATGTTAATAAAACTTTGTATAGTGAGTTTAGTACTATAGTTATAGGTAGTGTTTTTA
TTAACAACTCTTATACTATTGTTGTTCAACCTCATAATGGTGTTTTGGAGATTACAGCTTGTCAATACACTATGTGTGA
GTATCCTCATACTATTTGTAAATCTAAAGGTAGTTCTCGTAATGAATCTTGGCATTTTGATAAATCTGAACCTTTGTGT
CTGTTCAAGAAAAATTTTACTTATAATGTTTCTACAGATTGGTTGTATTTTCATTTTTATCAAGAACGTGGCACTTTTT
ATGCTTATTATGCTGATTCTGGCATGCCTACTACTTTTTTATTTAGTTTGTACCTTGGTACTCTTTTATCTCATTATTA
TGTTTTGCCTTTGACTTGTAATGCTATATCTTCTAATACTGATAATGAGACTTTACAATATTGGGTCACACCTTTGTCT
AAACGCCAATATCTTCTTAAATTTGACAACCGTGGTGTTATTACTAATGCTGTTGATTGTTCTAGTAGTTTCTTTAGCG
AGATTCAATGTAAAACTAAATCTTTATTACCTAATACTGGTGTTTATGACTTATCTGGTTTTACTGTTAAGCCTGTTGC
AACTGTACATCGTCGTATTCCTGATTTACCTGATTGTGACATTGATAAATGGCTTAACAATTTTAATGTACCCTCACCT
CTTAATTGGGAACGTAAAATTTTTTCTAATTGCAACTTTAATTTGAGTACTTTGCTTCGTTTAGTTCATACTGATTCTT
TTTCTTGTAATAATTTTGATGAATCTAAGATATATGGTAGTTGTTTTAAGAGTATTGTTTTAGATAAATTTGCCATACC
CAACTCCAGACGATCTGATTTGCAGTTGGGCAGTTCTGGTTTTCTGCAATCTTCTAATTATAAAATTGACACTACTTCT
AGTTCTTGTCAATTGTATTATAGTTTGCCTGCAATTAATGTTACTATTAATAATTATAATCCTTCTTCTTGGAATAGAA
GGTATGGTTTTAATAATTTTAATTTGAGCTCTCATAGTGTTGTTTACTCACGTTATTGTTTTTCTGTTAATAATACTTT
TTGTCCTTGTGCTAAACCTTCTTTTGCTTCAAGTTGTAAGAGTCATAAACCACCTTCTGCTTCCTGTCCTATTGGTACT
AATTATCGTTCTTGTGAGAGTACTACTGTACTCGACCACACTGACTGGTGTAGGTGTTCTTGTTTACCTGATCCTATAA
CTGCTTATGACCCTAGGTCTTGTTCTCAAAAAAAGTCTCTGGTTGGTGTTGGTGAACATTGTGCAGGGTTCGGTGTTGA
TGAAGAAAAGTGTGGTGTATTGGATGGATCATATAATGTTTCTTGTCTTTGTAGTACTGATGCCTTTCTAGGTTGGTCT
TATGACACTTGCGTCAGTAACAACCGTTGTAATATTTTTTCTAATTTTATTTTAAATGGTATCAATAGTGGTACCACTT
GTTCTAATGATTTATTGCAGCCTAATACTGAAGTTTTTACTGATGTTTGTGTTGATTACGACCTTTATGGTATTACAGG
ACAAGGTATTTTTAAAGAAGTTTCTGCTGTTTATTATAATAGTTGGCAAAATCTTTTGTATGATTCTAATGGCAACATT
ATTGGTTTTAAAGATTTTGTTACTAATAAAACATATAATATTTTCCCTTGTTATGCAGGAAGAGTTTCTGCTGCTTTTC
ATCAAAATGCTTCCTCTTTGGCTTTACTTTATCGTAATTTAAAATGTAGCTATGTTTTGAATAATATTTCTTTAGCTAC
TCAGCCATATTTTGATAGTTATCTTGGTTGCGTTTTTAATGCTGATAATTTAACTGATTATTCTGTTTCTTCTTGTGCT
CTTCGCATGGGTAGTGGTTTTTGTGTTGATTATAACTCACCTTCTTCTTCCTCTTCGCGTCGTAAACGTAGAAGTATTT
CTGCTTCTTATCGTTTTGTTACTTTTGAACCCTTTAATGTCAGTTTTGTTAATGACAGTATTGAGTCTGTGGGTGGTCT
TTATGAGATCAAAATTCCCACTAACTTTACTATAGTTGGTCAAGAGGAATTTATTCAAACTAATTCTCCTAAAGTTACT

ATTGATTGTTCTTTATTTGTCTGTTCTAATTATGCAGCTTGCCATGACTTATTGTCAGAGTATGGCACTTTTTGTGATA
ATATTAATAGTATTTTAGATGAAGTTAATGGTTTACTTGATACTACTCAATTGCATGTAGCTGATACTCTTATGCAAGG
TGTCACACTTAGCTCCAATCTTAATACTAATTTGCATTTTGATGTTGATAATATTAATTTTAAATCCCTAGTTGGATGT
TTAGGTCCACACTGCGGTTCTTCTTCTCGTTCTTTTTTTGAAGATTTATTGTTTGACAAAGTTAAACTTTCAGATGTTG
GTTTTGTTGAAGCTTATAACAATTGTACTGGTGGTAGTGAAATTAGAGATCTTCTTTGTGTACAATCCTTTAATGGTAT
TAAAGTTTTGCCTCCTATTTTGTCTGAATCTCAAATTTCTGGTTACACCACAGCCGCTACTGTTGCTGCTATGTTTCCA
CCATGGTCAGCAGCAGCTGGCATACCATTTTCTCTTAATGTACAATATAGAATTAATGGTTTGGGTGTTACTATGGATG
TTCTTAATAAAAATCAAAAGTTGATAGCTACTGCTTTTAATAATGCTCTTCTTTCTATTCAGAATGGTTTTAGTGCTAC
CAACTCTGCACTTGCTAAAATACAAAGTGTTGTTAATTCTAATGCTCAAGCACTTAATAGTTTGTTACAGCAATTATTT
AATAAATTTGGTGCAATTAGTTCTTCTTTACAAGAAATTTTATCTCGTCTCGATGCTTTAGAGGCTCAGGTTCAGATTG
ATAGGCTTATTAATGGTCGTTTAACTGCTTTAAATGCTTATGTCTCTCAACAGCTTAGTGATATTTCTCTTGTAAAATT
TGGTGCTGCTTTAGCTATGGAGAAGGTTAATGAGTGTGTTAAAAGTCAATCTCCTCGTATTAATTTTTGTGGTAATGGT
AATCATATTTTGTCATTAGTTCAAAATGCTCCTTATGGTTTGTTGTTTATGCATTTTAGTTATAAACCTATTTCTTTTA
AAACTGTTTTAGTAAGTCCTGGTTTGTGTATATCAGGTGATGTAGGTATTGCACCTAAACAAGGGTATTTTATTAAACA
TAATGATCATTGGATGTTCACTGGTAGTTCTTACTATTATCCTGAACCAATTTCAGATAAAAATGTTGTTTTTATGAAT
ACTTGTTCTGTTAATTTTACTAAAGCGCCTCTTGTTTATTTGAATCATTCTGTACCAAAATTGTCTGATTTTGAATCTG
AGTTATCTCATTGGTTTAAAAATCAAACATCCATTGCGCCTAATTTGACTTTAAATCTTCATACTATTAATGCTACTTT
TTTAGATTTGTATTATGAGATGAATCTTATTCAAGAGTCTATTAAGTCTTTGAATAATAGTTATATCAATCTTAAAGAT
ATAGGTACATATGAAATGTATGTAAAATGGCCTTGGTATGTTTGGCTACTAATTTCTTTTTCATTTATAATATTCCTTG
TATTGCTCTTTTTTATATGTTGTTGTACTGGTTGTGGTTCTGCATGTTTTAGTAAATGTCATAATTGTTGTGATGAGTA
TGGTGGTCATCATGATTTTGTTATCAAAACATCTCATGATGATTAG

>gi|90655334|gb|DQ437615.1|_Human_coronavirus_HKU1_strain_N20_spike_glycoprotein_(S)_gene_complete_cds

ATGTTTTTAATTATTTTTATTTTGCCTACAACACTAGCTGTTATAGGTGATTTTAATTGTACTAACTCTTTTATT
AATCATTATAATAAAACCATTCCGCGTATAAGCGAGGATGTTGTTGATGTATCTCTTGGTTTGGGCACATATTATGTTC
TTAACCGTGTTTATTTAAATACTACCTTGTTATTTACGGGTTATTTTCCTAAATCTGGTGCTAACTTTAGAGACTTGGC
TTTAAAGGGTTCTATATATTTGAGTACTCTCTGGTATAAACCACCTTTTCTGTCAGATTTTAATAATGGTATTTTTTCT
AAGGTTAAGAATACTAAGTTATATGTTAATAATACTTTGTATAGTGAATTTAGTACTATAGTTATAGGTAGTGTTTTTG
TTAATACTTCTTATACTATTGTTGTTCAACCTCATAATGGTATTTTGGAGATTACAGCTTGTCAGTATACTATGTGTGA
ATATCCTCACACTGTTTGTAAGTCTAAGGGTAGTATTCGTAATGAATCTTGGCACATTGATTCTTCGGAACCTTTATGC
TTGTTTAAGAAAAATTTTACTTATAATGTTTCTGCAGATTGGCTGTATTTTCATTTTTATCAAGAACGTGGTGTTTTT
ATGCATACTATGCAGATGTAGGTATGCCTACCACTTTCTTATTTAGTCTATATTTAGGTACTATTTTATCTCATTATTA
TGTTATGCCTTTGACTTGTAATGCTATATCTTCAAATACTGACAATGAAACTTTAGAATATTGGGTTACACCGCTATCT
AGACGTCAGTATCTTCTTAATTTTGATGAGCACGGTGTTATTACTAATGCCGTTGATTGTTCAAGTAGTTTTCTTAGTG
AGATTCAATGTAAAACTCAATCTTTTGCACCTAATACTGGTGTTTATGATTTGTCTGGTTTTACTGTAAAGCCTGTTGC
AACTGTTTATCGTCGGATTCCTAATTTACCTGATTGTGACATTGACAACTGGCTTAATAATGTTAGTGTACCTTCACCT
CTTAATTGGGAACGTAGAATTTTTTCTAATTGTAACTTTAACTTAAGCACTTTACTTCGTCTAGTTCATGTTGATTCTT
TTTCTTGTAATAATCTTGATAAATCTAAAATTTTTGGTAGTTGCTTTAATAGTATTACTGTTGACAAGTTTGCTATACC
TAATCGCAGACGAGATGATTTGCAATTGGGCAGTTCTGGCTTTTTGCAATCATCTAATTACAAAATAGATATTTCTTCT
AGTTCTTGTCAATTGTATTATAGTTTACCTTTAGTTAATGTTACTATTAATAACTTTAATCCATCTTCTTGGAATAGGA
GGTATGGTTTTGGTAGTTTTAATTTGTCTTCTTATGACGTTGTTTATTCTGATCATTGTTTTTCTGTTAACAGCGACTT

TTGCCCTTGTGCAGATCCGTCTGTTGTTAATTCTTGTGTTAAATCTAAGCCTCCTTCTGCCATTTGTCCTGCTGGTACT
AAATATCGTCATTGCGACTTGGATACTACTCTTTATGTTAAAAACTGGTGTAGATGTTCTTGTCTACCTGACCCCATTT
CTACTTATTCTCCTAACACATGTCCTCAAAAGAAGGTCGTTGTTGGTATAGGTGAACATTGTCCAGGTCTTGGTATTAA
TGAGGAAAAATGTGGTACACAATTAAATCATAGTTCCTGTTTTTGTAGTCCTGATGCCTTTTTGGGTTGGTCTTTTGAT
AGTTGTATTAGTAATAATCGTTGCAATATTTTTTCTAATTTTATTTTTAATGGAATTAATAGTGGCACCACTTGTTCTA
ATGATTTGTTATATTCTAACACTGAAATTTCTACTGGTGTTTGTGTTAATTATGATCTTTATGGCATCACAGGCCAAGG
TATTTTTAAAGAAGTTTCTGCGGCTTATTATAATAATTGGCAGAATCTTTTGTATGATTCTAATGGTAATATTATTGGT
TTTAAAGATTTTTTGACTAATAAAACTTACACTATACTTCCTTGTTATTCTGGTAGAGTGTCTGCTGCATTTTATCAAA
ATTCTTCCTCACCAGCTTTGCTTTATCGTAATTTAAAGTGTAGTTATGTTTTGAATAATATTTCTTTTATCTCACAACC
ATTTTATTTCGATAGTTATCTTGGTTGTGTTTTGAATGCTGTTAATTTAACTAGCTATTCTGTATCCTCTTGTGATTTG
CGTATGGGTAGTGGATTTTGTATTGATTATGCTTTACCCTCTTCTCGGCGTAAGCGTAGAGGTATTTCTTCTCCTTATC
GCTTTGTAACTTTTGAACCCTTTAATGTTAGTTTTGTTAACGATAGTGTTGAAACTGTTGGTGGTTTATTTGAGATTCA
GATTCCTACTAACTTTACCATAGCTGGTCATGAAGAATTTATTCAGACTAGTTCTCCTAAAGTTACTATTGATTGTGCA
GCTTTTGTTTGCTCTAACTATGCTGCTTGTCATGATTTATTGTCGGAATATGGCACTTTTTGCGATAATATTAATAGTA
TTTTAAATGAAGTCAATGATTTACTTGATATTACTCAGTTGCAGGTTGCTAATGCTCTAATGCAAGGTGTTACACTTAG
TTCTAATCTTAATACTAATCTACACTCTGATGTTGATAATATAGATTTTAAATCTCTTCTAGGTTGTTTAGGTTCACAA
TGTGGTTCTTCGTCTAGATCTTTGTTAGAGGATTTATTATTCAACAAAGTCAAACTTTCAGATGTAGGTTTTGTTGAAG
CTTATAATAATTGCACTGGTGGTAGTGAAATTAGAGATCTTCTCTGTGTGCAATCTTTTAATGGTATTAAAGTATTACC
TCCCATTTTATCTGAGACTCAAATTTCTGGCTATACTACAGCTGCTACTGTGGCGGCTATGTTTCCGCCATGGTCTGCT
GCTGCTGGTGTACCATTTTCTCTTAATGTACAATATAGAATTAATGGTTTGGGTGTTACTATGGATGTTCTTAATAAGA
ATCAAAAGTTAATAGCTAATGCTTTTAATAAAGCTCTTCTTTCTATCCAGAATGGTTTTACTGCTACTAACTCTGCTCT
TGCTAAAATTCAAAGTGTCGTTAATGCTAATGCTCAAGCACTTAATAGTTTGTTACAACAATTATTTAATAAATTTGGT
GCTATTAGTTCTTCTTTACAAGAAATTTTGTCTCGCCTTGATAATTTAGAAGCTCAGGTTCAGATTGATAGGCTCATTA
ATGGTCGTTTGACTGCTTTAAATGCTTATGTTTCTCAACAGCTTAGTGATATTACACTTATTAAGGCTGGAGCTTCTCG
TGCTATTGAGAAGGTTAATGAGTGTGTTAAAAGTCAATCCCCTCGTATAAATTTTTGTGGCAATGGTAACCACATTTTA
TCATTGGTTCAAAATGCTCCTTATGGTTTGCTTTTCATTCATTTTAGTTATAAACCTACTTCTTTTAAAACTGTCTTAG
TAAGTCCAGGTTTATGTTTATCCGGTGATAGAGGTATTGCACCTAAGCAAGGTTATTTTATTAAACAAAATGATTCCTG
GATGTTTACTGGTAGTTCCTATTATTATCCAGAACCAATTTCAGATAAAAATGTTGTTTTCATGAATAGTTGCTCTGTT
AATTTTACTAAAGCTCCATTTATTTATCTTAATAATTCTATACCAAATTTGTCTGATTTTGAAGCCGAGTTATCTCTTT
GGTTTAAAAATCATACTTCTATAGCACCTAATTTAACCTTTAATTCTCATATTAATGCTACTTTTTAGATCTGTATTA
TGAAATGAATGTTATTCAGGAATCTATTAAATCTTTGAACAGTAGTTTTATTAATCTTAAAGAAATAGGTACTTATGAA
ATGTATGTTAAATGGCCTTGGTACATTTGGTTGTTAATTGTCATTTTATTTATAATTTTTCTTATGATACTTTTCTTTA
TATGCTGCTGTACTGGTTGTGGTTCAGCATGTTTTAGTAAATGTCATAATTGTTGTGATGAGTATGGGGGTCACAATGA
TTTTGTTATTAAAGCATCTCATGATGATTAG

>gi|90655336|gb|DQ437616.1|_Human_coronavirus_HKU1_strain_N21_spike_glycoprotein_(S)_gene_complete_cds

ATGTTTTTAATTATTTTTATTTTGCCTACAACACTAGCTGTTATAGGTGATTTTAATTGTACTAACTCTTTTATT
AATGATTATAATAAAACCATTCCGCGTATAAGCGAGGATGTTGTTGATGTATCTCTTGGTTTGGGCACATATTATGTTC
TTAACCGTGTTTATTTAAATACTACCTTGTTATTTACGGGTTATTTTCCTAAATCTGGTGCTAATTTTAGAGACTTGGC
TTTAAAGGGTTCTATATATTTGAGTACTCTCTGGTATAAACCACCTTTTCTGTCAGATTTTAATAATGGTATTTTTTCT
AAGGTTAAGAATACTAAGTTATATGTTAATAATACTTTGTATAGTGAATTTAGTACTATAGTTATAGGTAGTGTTTTTG

TTAATACTTCTTATACTATTGTTGTTCAACCTCATAATGGTATTTTGGAGATTACAGCTTGTCAGTATACTATGTGTGA
ATATCCTCACACTGTTTGTAAGTCTAAGGGTAGTATTCGTAATGAATCTTGGCACATTGATTCTTCGGAACCTTTATGC
TTGTTTAAGAAAAATTTTACTTATAATGTTTCTGCAGATTGGCTGTATTTTCATTTTTATCAAGAACGTGGTGTTTTTT
ATGCATATTATGCAGATGTAGGTATGCCTACCACTTTCTTATTTAGTCTATATTTAGGTACTATTTTATCTCATTATTA
TGTTATGCCTTTGACTTGTAATGCTATATCTTCAAATACTGACAATGAAACTTTAGAATATTGGGTTACACCGCTATCT
AGACGTCAGTATCTTCTTAATTTTGATGAGCACGGTGTTATTACTAATGCCGTTGATTGTTCAAGTAGTTTTCTTAGTG
AGATTCAATGTAAAACTCAATCTTTTGCACCTAATACTGGTGTTTATGATTTGTCTGGTTTTACTGTAAAGCCTGTTGC
AACTGTTTATCGTCGGATTCCTAATTTACCTGATTGTGACATTGACAACTGGCTTAATAATGTTAGTGTACCTTCACCT
CTTAATTGGGAACGTAGAATTTTTTCTAATTGTAACTTTAACTTAAGCACTTTACTTCGTCTAGTTCATGTTGATTCTT
TTTCTTGTAATAATCTTGATAAATCTAAAATTTTTGGTAGTTGCTTTAATAGTATTACTGTTGACAAGTTTGCTATACC
TAATCGCAGACGAGATGATTTGCAATTGGGCAGTTCTGGCTTTTTGCAATCATCTAATTACAAAATAGATATTTCTTCT
AGTTCTTGTCAATTGTATTATAGTTTACCTTTAGTTAATGTTACTATTAATAACTTTAATCCATCTTCTTGGAATAGGA
GGTATGGTTTTGGTAGTTTTAATTTGTCTTCTTATGACGTTGTTTATTCTGATCATTGTTTTTCTGTTAACAGCGACTT
TTGCCCTTGTGCAGATCCGTCTGTTGTTAATTCTTGTGTTAAATCTAAGCCTCCTTCTGCCATTTGTCCTGCTGGTACT
AAATATCGTCATTGCGACTTGGATACTACTCTTTATGTTAAAAACTGGTGTAGATGTTCTTGTCTACCTGACCCCATTT
CTACTTATTCTCCTAACACATGTCCTCAAAAGAAGGTCGTTGTTGGTATAGGTGAACATTGTCCAGGTCTTGGTATTAA
TGAGGAAAAATGTGGTACACAATTAAATCATAGTTCCTGTTTTTGTAGTCCTGATGCCTTTTTGGGTTGGTCTTTTGAT
AGTTGTATTAGTAATAATCGTTGCAATATTTTTTCTAATTTTATTTTTAATGGAATTAATAGTGGCACCACTTGTTCTA
ATGATTTGTTATATTCTAACACTGAAATTTCTACTGGTGTTTGTGTTAATTATGATCTTTATGGCATCACAGGCCAAGG
TATTTTTAAAGAAGTTTCTGCGGCTTATTATAATAATTGGCAGAATCTTTTGTATGATTCTAATGGTAATATTATTGGT
TTTAAAGATTTTTTGACTAATAAAACTTACACTATACTTCCTTGTTATTCTGGTAGAGTGTCTGCTGCATTTTATCAAA
ATTCTTCCTCACCAGCTTTGCTTTATCGTAATTTAAAGTGTAGTTATGTTTTGAATAATATTTCTTTTATCTCACAACC
ATTTTATTTCGATAGTTATCTTGGTTGTGTTTTGAATGCTGTTAATTTAACTAGCTATTCTGTATCCTCTTGTGATTTG
CGTATGGGTAGTGGATTTTGTATTGATTATGCTTTACCCTCTTCTCGGCGTAAGCGTAGAGGTATTTCTTCTCCTTATC
GCTTTGTAACTTTTGAACCCTTTAATGTTAGTTTTGTTAACGATAGTGTTGAAACTGTTGGTGGTTTATTTGAGATTCA
GATTCCTACTAACTTTACCATAGCTGGTCATGAAGAATTTATTCAGACTAGTTCTCCTAAAGTTACTATTGATTGTTCA
GCTTTTGTTTGCTCTAACTATGCTGCTTGTCATGATTTATTGTCGGAATATGGCACTTTTTGCGATAATATTAATAGTA
TTTTAAATGAAGTCAATGATTTACTTGATATTACTCAGTTGCAGGTTGCTAATGCTCTAATGCAAGGTGTTACACTTAG
TTCTAATCTTAATACTAATCTACACTCTGATGTTGATAATATAGATTTAAATCTCTTCTAGGTTGTTTAGGTTCACAA
TGTGGTTCTTCGTCTAGATCTTTGTTAGAGGATTTATTATTCAACAAAGTCAAACTTTCAGATGTAGGTTTTGTTGAAG
CTTATAATAATTGCACTGGTGGTAGTGAAATTAGAGATCTTCTCTGTGTGCAATCTTTTAATGGTATTAAAGTATTACC
TCCCATTTTATCTGAGACTCAAATTTCTGGCTATACTACAGCTGCTACTGTGGCGGCTATGTTTCCGCCATGGTCTGCT
GCTGCTGGTGTACCATTTTCTCTTAATGTACAATATAGAATTAATGGTTTGGGTGTTACTATGGATGTTCTTAATAAGA
ATCAAAAGTTAATAGCTAATGCTTTTAATAAAGCTCTTCTTTCTATCCAGAATGGTTTTACTGCTACTAATTCTGCTCT
TGCTAAAATTCAAAGTGTCGTTAATGCTAATGCTCAAGCACTTAATAGTTTGTTACAACAATTATTTAATAAATTTGGT
GCTATTAGTTCTTCTTTACAAGAAATTTTGTCTCGCCTTGATAATTTAGAAGCTCAGGTTCAGATTGATAGGCTCATTA
ATGGTCGTTTGACTGCTTTAAATGCTTATGTTTCTCAACAGCTTAGTGATATTACACTTATTAAGGCTGGAGCTTCTCG
TGCTATTGAGAAGGTTAATGAGTGTGTTAAAAGTCAATCCCCTCGTATAAATTTTTGTGGCAATGGTAACCACATTTTA
TCATTGGTTCAAAATGCTCCTTATGGTTTGCTTTTCATTCATTTTAGTTATAAACCTACTTCTTTTAAAACTGTCTTAG
TAAGTCCAGGTTTATGTTTATCCGGTGATAGAGGTATTGCACCTAAGCAAGGTTATTTTATTAAACAAAATGATTCCTG
GATGTTTACTGGTAGTTCCTATTATTATCCAGAACCAATTTCAGATAAAAATGTTGTTTTCATGAATAGTTGCTCTGTT
AATTTTACTAAAGCTCCATTTATTTATCTTAATAATTCTATACCAAATTTGTCTGATTTTGAAGCCGAGTTATCTCTTT
GGTTTAAAAATCATACTTCTATAGCACCTAATTTAACCTTTAATTCTCATATTAATGCTACTTTTTTAGATCTGTATTA
TGAAATGAATGTTATTCAGGAATCTATTAAATCTTTGAACAGTAGTTTTATTAATCTTAAAGAAATAGGTACTTATGAA

ATGTATGTTAAATGGCCTTGGTACATTTGGTTGTTAATTGTCATTTTATTTATAATTTTTCTTATGATACTTTTCTTTA
TATGCTGCTGTACTGGTTGTGGTTCAGCATGTTTTAGTAAATGTCATAATTGTTGTGATGAGTATGGGGGTCACAATGA
TTTTGTTATTAAAGCATCTCATGATGATTAG

>gi|90655338|gb|DQ437617.1|_Human_coronavirus_HKU1_strain_N22_spike_glycoprotein_(S)_gene_complete_cds

ATGTTTTTAATTATTTTTATTTTGCCTACAACACTAGCTGTTATAGGTGATTTTAATTGTACTAACTCTTTTATT
AATCATTATAATAAAACCATTCCGCGTATAAGCGAGGATGTTGTTGATGTATCTCTTGGTTTGGGCACATATTATGTTC
TTAACCGTGTTTATTTAAATACTACCTTGTTATTTACGGGTTATTTTCCTAAATCTGGTGCTAACTTTAGAGACTTGGC
TTTAAAGGGTTCTATATATTTGAGTACTCTCTGGTATAAACCACCTTTTCTGTCAGATTTTAATAATGGTATTTTTTCT
AAGGTTAAGAATACTAAGTTATATGTTAATAATACTTTGTATAGTGAATTTAGTACTATAGTTATAGGTAGTGTTTTTG
TTAATACTTCTTATACTATTGTTGTTCAACCTCATAATGGTATTTTGGAGATTACAGCTTGTCAGTATACTATGTGTGA
ATATCCTCACACTGTTTGTAAGTCTAAGGGTAGTATTCGTAATGAATCTTGGCACATTGATTCTTCGGAACCTTTATGC
TTGTTTAAGAAAAATTTTACTTATAATGTTTCTGCAGATTGGCTGTATTTTCATTTTTATCAAGAACGTGGTGTTTTTT
ATGCATATTATGCAGATGTAGGTATGCCTACCACTTTCTTATTTAGTCTATATTTAGGTACTATTTTATCTCATTATTA
TGTTATGCCTTTGACTTGTAATGCTATATCTTCAAATACTGACAATGAAACTTTAGAATATTGGGTTACACCGCTATCT
AGACGTCAGTATCTTCTTAATTTTGATGAGCACGGTGTTATTACTAATGCCGTTGATTGTTCAAGTAGTTTTCTTAGTG
AGATTCAATGTAAAACTCAATCTTTTGCACCTAATACTGGTGTTTATGATTTGTCTGGTTTTACTGTAAAGCCTGTTGC
AACTGTTTATCGTCGGATTCCTAATTTACCTGATTGTGACATTGACAACTGGCTTAATAATGTTAGTGTACCTTCACCT
CTTAATTGGGAACGTAGAATTTTTTCTAATTGTAACTTTAACTTAAGCACTTTACTTCGTCTAGTTCATGTTGATTCTT
TTTCTTGTAATAATCTTGATAAATCTAAAATTTTTGGTAGTTGCTTTAATAGTATTACTGTTGACAAGTTTGCTATACC
TAATCGCAGACGAGATGATTTGCAATTGGGCAGTTCTGGCTTTTTGCAATCATCTAATTACAAAATAGATATTTCTTCT
AGTTCTTGTCAATTGTATTATAGTTTACCTTTAGTTAATGTTACTATTAATAACTTTAATCCATCTTCTTGGAATAGGA
GGTATGGTTTTGGTAGTTTTAATTTGTCTTCTTATGACGTTGTTTATTCTGATCATTGTTTTTCTGTTAACAGCGACTT
TTGCCCTTGTGCAGATCCGTCTGTTGTTAATTCTTGTGTTAAATCTAAGCCTCCTTCTGCCATTTGTCCTGCTGGTACT
AAATATCGTCATTGCGACTTGGATACTACTCTTTATGTTAAAAACTGGTGTAGATGTTCTTGTCTACCTGACCCCATTT
CTACTTATTCTCCTAACACATGTCCTCAAAAGAAGGTCGTTGTTGGTATAGGTGAACATTGTCCAGGTCTTGGTATTAA
TGAGGAAAAATGTGGTACACAATTAAATCATAGTTCCTGTTTTTGTAGTCCTGATGCCTTTTTGGGTTGGTCTTTTGAT
AGTTGTATTAGTAATAATCGTTGCAATATTTTTTCTAATTTTATTTTTAATGGAATTAATAGTGGCACCACTTGTTCTA
ATGATTTGTTATATTCTAACACTGAAATTTCTACTGGTGTTTGTGTTAATTATGATCTTTATGGCATCACAGGCCAAGG
TATTTTTAAAGAAGTTTCTGCGGCTTATTATAATAATTGGCAGAATCTTTTGTATGATTCTAATGGTAATATTATTGGT
TTTAAAGATTTTTTGACTAATAAAACTTACACTATACTTCCTTGTTATTCTGGTAGAGTGTCTGCTGCATTTTATCAAA
ATTCTTCCTCACCAGCTTTGCTTTATCGTAATTTAAAGTGTAGTTATGTTTTGAATAATATTTCTTTTATCTCACAACC
ATTTTATTTCGATAGTTATCTTGGTTGTGTTTTGAATGCTGTTAATTTAACTAGCTATTCTGTATCCTCTTGTGATTTG
CGTATGGGTAGTGGATTTTGTATTGATTATGCTTTACCCTCTTCTCGGCGTAAGCGTAGAGGTATTTCTTCTCCTTATC
GCTTTGTAACTTTTGAACCCTTTAATGTTAGTTTTGTTAACGATAGTGTTGAAACTGTTGGTGGTTTATTTGAGATTCA
GATTCCTACTAACTTTACCATAGCTGGTCATGAAGAATTTATTCAGACTAGTTCTCCTAAAGTTACTATTGATTGTGCA
GCTTTTGTTTGCTCTAACTATGCTGCTTGTCATGATTTATTGTCGGAATATGGCACTTTTTGCGATAATATTAATAGTA
TTTTAAATGAAGTCAATGATTTACTTGATATTACTCAGTTGCAGGTTGCTAATGCTCTAATGCAAGGTGTTACACTTAG
TTCTAATCTTAATACTAATCTACACTCTGATGTTGATAATATAGATTTTAAATCTCTTCTAGGTTGTTTAGGTTCACAA
TGTGGTTCTTCGTCTAGATCTTTGTTAGAGGATTTATTATTCAACAAAGTCAAACTTTCAGATGTAGGTTTTGTTGAAG
CTTATAATAATTGCACTGGTGGTAGTGAAATTAGAGATCTTCTCTGTGTGCAATCTTTTAATGGTATTAAAGTATTACC

TCCCATTTTATCTGAGACTCAAATTTCTGGCTATACTACAGCTGCTACTGTGGCGGCTATGTTTCCGCCATGGTCTGCT
GCTGCTGGTGTACCATTTTCTCTTAATGTACAATATAGAATTAATGGTTTGGGTGTTACTATGGATGTTCTTAATAAGA
ATCAAAAGTTAATAGCTAATGCTTTTAATAAAGCTCTTCTTTCTATCCAGAATGGTTTTACTGCTACTAACTCTGCTCT
TGCTAAAATTCAAAGTGTCGTTAATGCTAATGCTCAAGCACTTAATAGTTTGTTACAACAATTATTTAATAAATTTGGT
GCTATTAGTTCTTCTTTACAAGAAATTTTGTCTCGCCTTGATAATTTAGAAGCTCAGGTTCAGATTGATAGGCTCATTA
ATGGTCGTTTGACTGCTTTAAATGCTTATGTTTCTCAACAGCTTAGTGATATTACACTTATTAAGGCTGGAGCTTCTCG
TGCTATTGAGAAGGTTAATGAGTGTGTTAAAAGTCAATCCCCTCGTATAAATTTTTGTGGCAATGGTAACCACATTTTA
TCATTGGTTCAAAATGCTCCTTATGGTTTGCTTTTCATTCATTTTAGTTATAAACCTACTTCTTTTAAAACTGTCTTAG
TAAGTCCAGGTTTATGTTTATCCGGTGATAGAGGTATTGCACCTAAGCAAGGTTATTTTATTAAACAAAATGATTCCTG
GATGTTTACTGGTAGTTCCTATTATTATCCAGAACCAATTTCAGATAAAAATGTTGTTTTCATGAATAGTTGCTCTGTT
AATTTTACTAAAGCTCCATTTATTTATCTTAATAATTCTATACCAAATTTGTCTGATTTTGAAGCCGAGTTATCTCTTT
GGTTTAAAAATCATACTTCTATAGCACCTAATTTAACCTTTAATTCTCATATTAATGCTACTTTTTAGATCTGTATTA
TGAAATGAATGTTATTCAGGAATCTATTAAATCTTTGAACAGTAGTTTTATTAATCTTAAAGAAATAGGTACTTATGAA
ATGTATGTTAAATGGCCTTGGTACATTTGGTTGTTAATTGTCATTTTATTTATAATTTTTCTTATGATACTTTTCTTTA
TATGCTGCTGTACTGGTTGTGGTTCAGCATGTTTTAGTAAATGTCATAATTGTTGTGATGAGTATGGGGGTCACAATGA
TTTGTTATTAAAGCATCTCATGATGATTAG

>gi|90655342|gb|DQ437619.1|_Human_coronavirus_HKU1_strain_N24_spike_glycoprotein_(S)_gene_complete_cds

ATGTTATTAATTATTTTTATTTTGCCTACAACATTAGCTGTTATAGGTGATTTTAATTGTACTAATTTTGCTATT
AATGATAAAAACACCACAGTTCCTCGCATAAGTGAGTATGTTGTGGATGTTTCTTATGGTTTGGGTACATATTATATAC
TTGATCGTGTTTATTTAAATACTACTATATTATTTACTGGTTATTTCCCTAAATCTGGTGCCAATTTTAGGGATCTATC
TTTAAAAGGTACTACATATTTGAGTACTCTTTGGTATCAGAAACCCTTTTTATCTGATTTTAATAATGGTATTTTTTCT
AGAGTTAAGAATACTAAGTTGTATGTTAATAAAACTTTGTATAGTGAGTTTAGTACTATAGTTATAGGTAGTGTTTTTA
TTAACAACTCTTATACTATTGTTGTTCAACCTCATAATGGTGTTTTGGAGATTACAGCTTGTCAATACACTATGTGTGA
GTATCCTCATACTATTTGTAAATCTAAAGGTAGTTCTCGTAATGAATCTTGGCATTTTGATAAATCTGAACCTTTGTGT
CTGTTCAAGAAAAATTTTACTTATAATGTTTCTACAGATTGGTTGTATTTTCATTTTTATCAAGAACGTGGCACTTTTT
ATGCTTATTATGCTGATTCTGGCATGCCTACTACTTTTTTATTTAGTTTGTATCTTGGTACTCTTTTATCTCATTATTA
TGTTTTGCCTTTGACTTGTAATGCTATATCTTCTAATACTGATAATGAGACTTTACAATATTGGGTCACACCTTTGTCT
AAACGCCAATATCTTCTTAAATTTGACAACCGTGGTGTTATTACTAATGCTGTTGATTGTTCTAGTAGTTTCTTTAGCG
AGATTCAATGTAAAACTAAATCTTTATTACCTAATACTGGTGTTTATGACTTATCTGGTTTTACTGTTAAGCCTGTTGC
AACTGTACATCGTCGTATTCCTGATTTACCTGATTGTGACATTGATAAATGGCTTAACAATTTTAATGTACCCTCACCT
CTTAATTGGGAACGTAAAATTTTTTCTAATTGCAACTTTAATTTGAGTACTTTGCTTCGTTTAGTTCATACTGATTCTT
TTTCTTGTAATAATTTTGATGAATCTAAGATATATGGTAGTTGTTTTAAGAGTATTGTTTTAGATAAATTTGCCATACC
CAACTCCAGACGATCTGATTTGCAGTTGGGCAGTTCTGGTTTTCTGCAATCTTCTAATTATAAAATTGACACTACTTCT
AGTTCTTGTCAATTGTATTATAGTTTGCCTGCAATTAATGTTACTATTAATAATTATAATCCTTCTTCTTGGAATAGAA
GGTATGGTTTTAATAATTTTAATTTGAGCTCTCATAGTGTTGTTTACTCACGTTATTGTTTTTCTGTTAATAATACTTT
TTGTCCTTGTGCTAAACCTTCTTTTGCTTCAAGTTGCAAGAGTCATAAACCACCTTCTGCTTCCTGTCCTATTGGTACT
AATTATCGTTCTTGTGAGAGTACTACTGTACTCGACCACACTGACTGGTGTAGGTGTTCTTGTTTACCTGATCCTATAA
CTGCTTATGACCCTAGGTCTTGTTCTCAAAAAAAGTCTCTGGTTGGTGTTGGTGAACATTGTGCAGGGTTCGGTGTTGA
TGAAGAAAAGTGTGGTGTATTGGATGGATCATATAATGTTTCTTGTCTTTGTAGTACTGATGCCTTTCTAGGTTGGTCT
TATGACACTTGCGTCAGTAACAACCGTTGTAATATTTTTTCTAATTTTATTTTAAATGGTATCAATAGTGGTACCACTT

GTTCTAATGATTTATTGCAGCCCAATACTGAAGTTTTTACTGATGTTTGTGTTGATTACGACCTTTATGGTATTACAGG
ACAAGGTATTTTTAAAGAAGTTTCTGCTGTTTATTATAATAGTTGGCAAAATCTTTTGTATGATTCTAATGGCAACATT
ATTGGTTTTAAAGATTTTGTTACTAATAAAACATATAATATTTTCCCTTGTTATGCAGGAAGAGTTTCTGCTGCTTTTC
ATCAAAATGCTTCCTCTTTGGCTTTACTTTATCGTAATTTAAAATGTAGCTATGTTTTGAATAATATTTCTTTAGCTAC
TCAGCCATATTTTGATAGTTATCTTGGTTGCGTTTTTAATGCTGATAATTTAACTGATTATTCTGTTTCTTCTTGTGCT
CTTCGCATGGGTAGTGGTTTTTGTGTTGATTATAACTCACCTTCTTCTTCCTCTTCGCGTCGTAAACGTAGAAGTATTT
CTGCTTCTTATCGTTTTGTTACTTTTGAACCCTTTAATGTCAGTTTTGTTAATGACAGTATTGAGTCTGTGGGTGGTCT
TTATGAGATCAAAATTCCCACTAACTTTACTATAGTTGGTCAAGAGGAATTTATTCAAACTAATTCTCCTAAAGTTACT
ATTGATTGTTCTTTATTTGTCTGTTCTAATTATGCAGCTTGCCATGACTTATTGTCAGAGTATGGCACTTTTTGTGATA
ATATTAATAGTATTTTAGATGAAGTTAATGGTTTACTTGATACTACTCAATTGCATGTAGCTGATACTCTTATGCAAGG
TGTCACACTTAGCTCCAATCTTAATACTAATTTGCATTTTGATGTTGATAATATTAATTTTAAATCCCTAGTTGGATGT
TTAGGTCCACACTGCGGTTCTTCTTCTCGTTCTTTTTTTGAAGATTTATTGTTTGACAAAGTTAAACTTTCAGATGTTG
GTTTTGTTGAAGCTTATAACAATTGTACTGGTGGTAGTGAAATTAGAGATCTTCTTTGTGTACAATCCTTTAATGGTAT
TAAAGTTTTGCCTCCTATTTTGTCTGAATCTCAAATTTCTGGTTACACCACAGCCGCTACTGTTGCTGCTATGTTTCCA
CCATGGTCAGCAGCAGCTGGCATACCATTTTCTCTTAATGTACAATATAGAATTAATGGTTTGGGTGTTACTATGGATG
TTCTTAATAAAAATCAAAAGTTGATAGCTACTGCTTTTAATAATGCTCTTCTTTCTATTCAGAATGGTTTAGTGCTAC
CAACTCTGCACTTGCTAAAATACAAAGTGTTGTTAATTCTAATGCTCAAGCACTTAATAGTTTGTTACAGCAATTATTT
AATAAATTTGGTGCAATTAGTTCTTCTTTACAAGAAATTTTATCTCGTCTCGATGCTTTAGAGGCTCAGGTTCAGATTG
ATAGGCTTATTAATGGTCGTTTAACTGCTTTAAATGCTTATGTCTCTCAACAGCTTAGTGATATTTCTCTTGTAAAATT
TGGTGCTGCTTTAGCCATGGAGAAGGTTAATGAGTGTGTTAAAAGTCAATCTCCTCGTATTAATTTTTGTGGTAATGGT
AATCATATTTTGTCATTAGTTCAAAATGCTCCTTATGGTTTGTTGTTTATGCATTTTAGTTATAAACCTATTTCTTTTA
AAACTGTTTTAGTAAGTCCTGGTTTGTGTATATCAGGTGATGTAGGTATTGCACCTAAACAAGGGTATTTTATTAAACA
TAATGATCATTGGATGTTCACTGGTAGTTCTTACTATTATCCTGAACCAATTTCAGATAAAAATGTTGTTTTTATGAAT
ACTTGTTCTGTTAATTTTACTAAAGCGCCTCTTGTTTATTTGAATCATTCTGTACCAAAATTGTCTGATTTTGAATCTG
AGTTATCTCATTGGTTTAAAAATCAAACATCCATTGCGCCTAATTTGACTTTAAATCTTCATACTATTAATGCTACTTT
TTTAGATTTGTATTATGAGATGAATCTTATTCAAGAGTCTATTAAGTCTTTGAATAATAGTTATATCAATCTTAAAGAT
ATAGGTACATATGAAATGTATGTAAAATGGCCTTGGTATGTTTGGCTACTAATTTCTTTTTCATTTATAATATTCCTTG
TATTGCTCTTTTTTATATGTTGTTGTACTGGTTGTGGTTCTGCATGTTTTAGTAAATGTCATAATTGTTGTGATGAGTA
TGGTGGTCATCATGATTTTGTTATCAAAACATCTCATGATGATTAG

>gi|545299226|gb|KF686340.1| Human coronavirus HKU1 strain HKU1/human/USA/HKU1-5/2009, complete genome

ATGTTATTAATTATTTTTATTTTGCCTACAACATTAGCTGTTATAGGTGATTTTAATTGTACTAATTTTGCTATT
AACGATAAAAACACCACAGTTCCTCGCATAAGTGAGTATGTTGTGGATGTTTCTTATGGTTTGGGTACATATTATATAC
TTGATCGTGTTTATTTAAATACTACTATATTATTTACTGGTTATTTCCCTAAATCTGGTGCCAATTTTAGGGATCTATC
TTTAAAAGGTACTACATATTTGAGTACTCTTTGGTATCAGAAACCCTTTTTATCTGATTTTAATAATGGTATTTTTTCT
AGAGTTAAGAATACTAAGTTGTATGTTAATAAAACTTTGTATAGTGAGTTTAGTACTATAGTTATAGGTAGTGTTTTTA
TTAACAACTCTTATACTATTGTTGTTCAACCTCATAATGGTGTTTTGGAGATTACAGCTTGTCAATACACTATGTGTGA
GTATCCTCATACTATTTGTAAATCTAAAGGTAGTTCTCGTAATGAATCTTGGCATTTTGATAAATCTGAACCTTTGTGT
CTGTTCAAGAAAAATTTTACTTATAATGTTTCTACAGATTTCTTGTATTTTCATTTTTATCAAGAACGTGGCACTTTTT
ATGCTTATTATGCTGACTCTGGCATGCCTACTACTTTTTTATTTAGTTTGTATCTTGGTACTCTTTTATCTCATTATTA
TGTTTTGCCTTTGACTTGTAATGCTATATCTTCTAATACTGATAATGAGACTTTACAATATTGGGTCACACCTTTGTCT

AAACGCCAATATCTTCTTAAATTTGACAACCGTGGTGTTATTACTAATGCTGTTGATTGTTCTAGTAGTTTCTTTAGTG
AGATTCAATGTAAAACTAAATCTTTATTACCTAATACTGGTGTTTATGACTTATCTGGTTTTACTGTTAAGCCTGTTGC
AACTGTACATCGTCGTATTCCTGATTTACCTGATTGTGACATTGATAAATGGCTTAACAATTTTAATGTACCCTCACCT
CTTAATTGGGAACGTAAAATTTTTTCTAATTGCAACTTTAATTTGAGTACTTTGCTTCGTTTAGTTCATACTGATTCTT
TTTCTTGTAATAATTTTGATGAATCTAAGATATATGGTAGTTGTTTTAAGAGTATTGTTTTAGATAAATTTGCCATACC
CAACTCCAGACGATCTGATTTGCAGTTGGGCAGTTCTGGTTTTCTGCAATCTTCTAATTATAAAATTGACACTACTTCT
AGTTCTTGTCAATTGTATTATAGTTTGCCTGCAATTAATGTTACTATTAATAATTATAATCCTTCTTCTTGGAATAGAA
GGTATGGTTTTAATAATTTTAATTTGAGCTCTCATAGTGTTGTTTACTCACGTTATTGTTTTTCTGTTAATAATACTTT
TTGTCCTTGTGCTAAACCTTCTTTTGCTTCAAGTTGCAAGAGTCATAAACCACCTTCTGCTTCCTGTCCTATTGGTACT
AATTATCGTTCTTGTGAGAGTACTACTGTACTCGACCACACTGACTGGTGTAGGTGTTCTTGTTTACCTGATCCTATAA
CTGCTTATGACCCTAGGTCTTGTTCTCAAAAAAAGTCTCTGGTTGGTGTTGGTGAACATTGTGCAGGGTTCGGTGTTGA
TGAAGAAAAGTGTGGTGTATTGGATGGATCATATAATGTTTCTTGTCTTTGTAGTACTGATGCCTTTCTAGGTTGGTCT
TATGACACTTGCGTCAGTAACAACCGTTGTAATATTTTTTCTAATTTTATTTTAAATGGTATCAATAGTGGTACCACTT
GTTCCAATGATTTATTGCAGCCTAATACTGAAGTTTATACTGATGTTTGTGTTGATTACGACCTTTATGGTATTACAGG
ACAAGGTATTTTTAAAGAAGTTTCTGCTGTTTATTATAATAGTTGGCAAAATCTTTTGTATGATTCTAATGGCAACATT
ATTGGTTTTAAAGATTTTGTTACTAATAAAACATATAATATTTTCCCTTGTTATGCAGGAAGAGTTTCTGCTGCTTTTC
ATCAAAATGCTTCCTCTTTGGCTTTACTTTATCGTAATTTAAAATGTAGCTATGTTTTGAATAATATTTCTTTAGCTAC
TCAGCCATATTTTGATAGTTATCTTGGTTGCGTTTTTAATGCTGATAATTTAACTGATTATTCTGTTTCTTCTTGTGCT
CTTCGCATGGGTAGTGGTTTTTGTGTTGATTATAACTCACCTTCTTTTTCCTCTTCGCGTCGTAAACGTAGAAGTATTT
CTGCTTCTTATCGGTTTGTTACTTTTGAACCCTTTAATGTCAGTTTTGTTAATGACAGTATTGAGTCTGTGGGTGGTCT
TTATGAGATCAAAATTCCCACTAACTTTACTATAGTTGGTCAAGAGGAATTTATTCAAACTAATTCTCCTAAAGTTACT
ATTGATTGTTCTTTATTTGTCTGTTCTAATTATGCAGCTTGCCATGACTTATTGTCAGAGTATGGCACTTTTTGTGATA
ATATTAATAGTATTTTAGATGAAGTTAATGGTTTACTTGATACTACTCAATTGCATGTAGCTGATACTCTTATGCAAGG
TGTCACACTTAGCTCCAATCTTAATACTAATTTGCATTTTGATGTTGATAATATTAATTTTAAATCCCTAGTTGGATGT
TTAGGTCCACACTGCGGTTCTTCTTCTCGTTCTTTTTTTGAAGATTTATTGTTTGACAAAGTTAAACTTTCAGATGTTG
GTTTTGTTGAAGCTTATAACAATTGTACTGGTGGTAGTGAAATTAGAGATCTTCTTTGTGTACAATCCTTTAATGGTAT
TAAAGTTTTGCCTCCTATTTTGTCTGAATCTCAAATTTCTGGTTACACCACAGCCGCTACTGTTGCTGCTATGTTTCCA
CCATGGTCAGCAGCAGCTGGCATACCATTTTCTCTTAATGTACAATATAGAATTAATGGTTTGGGTGTTACTATGGATG
TTCTTAATAAAAATCAAAAGTTGATAGCTACTGCTTTTAATAATGCTCTTCTTTCTATTCAGAATGGTTTTAGTGCTAC
CAACTCTGCACTTGCTAAAATACAAAGTGTTGTTAATTCTAATGCTCAAGCACTTAATAGTTTGTTACAGCAATTATTT
AATAAATTTGGTGCAATTAGTTCTTCTTTACAAGAAATTTTATCTCGTCTCGATGCTTTAGAGGCTCAGGTTCAGATTG
ATAGGCTTATTAATGGTCGTTTAACTGCTTTAAATGCTTATGTCTCTCAACAACTTAGTGATATTTCTCTTGTAAAATT
TGGTGCTGCTTTAGCTATGGAGAAGGTTAATGAGTGTGTTAAAAGTCAATCTCCTCGTATTAATTTTTGTGGTAATGGT
AATCATATTTTGTCATTAGTTCAAAATGCTCCTTATGGTTTGTTGTTTATGCATTTTAGTTATAAACCTATTTCTTTTA
AAACTGTTTTAGTAAGTCCTGGTTTGTGTATATCAGGTGATGTAGGTATTGCACCCAAACAAGGGTATTTTATTAAACA
CAATGATCATTGGATGTTCACTGGTAGTTCTTACTATTATCCTGAACCAATTTCAGATAAAAATGTTGTTTTTATGAAT
ACTTGTTCTGTTAATTTTACTAAAGCGCCTCTTGTTTATTTGAATCATTCTGTACCAAAATTGTCTGATTTTGAATCTG
AGTTATCTCATTGGTTTAAAAATCAAACATCCATTGCGCCTAATTTGACTTTAAATCTTCATACTATTAATGCTACTTT
TTTAGATTTGTATTATGAGATGAATCTTATTCAAGAGTCTATTAAGTCTTTGAATAATAGTTATATCAATCTTAAAGAT
ATAGGTACATATGAAATGTATGTAAAATGGCCTTGGTATGTTTGGCTACTAATTTCTTTTTCATTTATAATATTCCTTG
TATTGCTCTTTTTTATATGTTGTTGTACTGGTTGTGGTTCTGCATGTTTTAGTAAATGTCATAATTGTTGTGATGAGTA
TGGTGGTCATCATGATTTTGTTATCAAAACATCTCATGATGATTAG

>gi|545299236|gb|KF686341.1| Human coronavirus HKU1 strain HKU1/human/USA/HKU1-10/2010, complete genome

ATGTTATTAATTATTTTTATTTTGCCTACAACATTAGCTGTTATAGGTGATTTTAATTGTACTAATTTTGCTATT
AATGATAAAAACACCACAGTTCCTCGCATAAGTGAGTATGTTGTGGATGTTTCTTATGGTTTGGGTACATATTATATAC
TTGATCGTGTTTATTTAAATACTACTATATTATTTACTGGTTATTTCCCTAAATCTGGTGCCAATTTTAGGGATCTATC
TTTAAAAGGTACTACATATTTGAGTACTCTTTGGTATCAGAAACCCTTTTTATCTGATTTTAATAATGGTATTTTTTCT
AGAGTTAAGAATACTAAGTTGTATGTTAATAAAACTTTGTATAGTGAGTTTAGTACTATAGTTATAGGTAGTGTTTTTA
TTAACAACTCTTATACTATTGTTGTTCAACCTCATAATGGTGTTTTGGAGATTACAGCTTGTCAATACACTATGTGTGA
GTATCCTCATACTATTTGTAAATCTAAAGGTAGTTCTCGTAATGAATCTTGGCATTTTGATAAATCTGAACCTTTGTGT
CTGTTCAAGAAAAATTTTACTTATAATGTTTCTACAGATTTCTTGTATTTTCATTTTTATCAAGAACGTGGCACTTTTT
ATGCTTATTATGCTGACTCTGGCATGCCTACTACTTTTTTATTTAGTTTGTATCTTGGTACTCTTTTATCTCATTATTA
TGTTTTGCCTTTGACTTGTAATGCTATATCTTCTAATACTGATAATGAGACTTTACAATATTGGGTCACACCTTTGTCT
AAACGCCAATATCTTCTTAAATTTGACAACCGTGGTGTTATTACTAATGCTGTTGATTGTTCTAGTAGTTTCTTTAGTG
AGATTCAATGTAAAACTAAATCTTTATTACCTAATACTGGTGTTTATGACTTATCTGGTTTTACTGTTAAGCCTGTTGC
AACTGTACATCGTCGTATTCCTGATTTACCTGATTGTGACATTGATAAATGGCTTAACAATTTTAATGTACCCTCACCT
CTTAATTGGGAACGTAAAATTTTTTCTAATTGCAACTTTAATTTGAGTACTTTGCTTCGTTTAGTTCATACTGATTCTT
TTTCTTGTAATAATTTTGATGAATCTAAGATATATGGTAGTTGTTTTAAGAGTATTGTTTTAGATAAATTTGCCATACC
CAACTCCAGACGATCTGATTTGCAGTTGGGCAGTTCTGGTTTTCTGCAATCTTCTAATTATAAAATTGACACTACTTCT
AGTTCTTGTCAATTGTATTATAGTTTGCCTGCAATTAATGTTACTATTAATAATTATAATCCTTCTTCTTGGAATAGAA
GGTATGGTTTTAATAATTTTAATTTGAGCTCTCATAGTGTTGTTTACTCACGTTATTGTTTTTCTGTTAATAATACTTT
TTGTCCTTGTGCTAAACCTTCTTTTGCTTCAAGTTGCAAGAGTCATAAACCACCTTCTGCTTCCTGTCCTATTGGTACT
AATTATCGTTCTTGTGAGAGTACTACTGTACTCGACCACACTGACTGGTGTAGGTGTTCTTGTTTACCTGATCCTATAA
CTGCTTATGACCCTAGGTCTTGTTCTCAAAAAAAGTCTCTGGTTGGTGTTGGTGAACATTGTGCAGGGTTCGGTGTTGA
TGAAGAAAAGTGTGGTGTATTGGATGGATCATATAATGTTTCTTGTCTTTGTAGTACTGATGCCTTTCTAGGTTGGTCT
TATGACACTTGCGTCAGTAACAACCGTTGTAATATTTTTTCTAATTTTATTTTAAATGGTATCAATAGTGGTACCACTT
GTTCTAATGATTTATTGCAGCCTAATACTGAAGTTTATACTGATGTTTGTGTTGATTACGACCTTTATGGTATTACAGG
ACAAGGTATTTTTAAAGAAGTTTCTGCTGTTTATTATAATAGTTGGCAAAATCTTTTGTATGATTCTAATGGCAACATT
ATTGGTTTTAAAGATTTTGTTACTAATAAAACATATAATATTTTCCCTTGTTATGCAGGAAGAGTTTCTGCTGCTTTTC
ATCAAAATGCTTCCTCTTTGGCTTTACTTTATCGTAATTTAAAATGTAGCTATGTTTTGAATAATATTTCTTTAGCTAC
TCAGCCATATTTTGATAGTTATCTTGGTTGCGTTTTTAATGCTGATAATTTAACTGATTATTCTGTTTCTTCTTGTGCT
CTTCGCATGGGTAGTGGTTTTTGTGTTGATTATAACTCACCTTCTTTTTCCTCTTCGCGTCGTAAACGTAGAAGTATTT
CTGCTTCTTATCGGTTTGTTACTTTTGAACCCTTTAATGTCAGTTTTGTTAATGACAGTATTGAGTCTGTGGGTGGTCT
TTATGAGATCAAAATTCCCACTAACTTTACTATAGTTGGTCAAGAGGAATTTATTCAAACTAATTCTCCTAAAGTTACT
ATTGATTGTTCTTTATTTGTCTGTTCTAATTATGCAGCTTGCCATGACTTATTGTCAGAGTATGGCACTTTTTGTGATA
ATATTAATAGTATTTTAGATGAAGTTAATGGTTTACTTGATACTACTCAATTGCATGTAGCTGATACTCTTATGCAAGG
TGTCACACTTAGCTCCAATCTTAATACTAATTTGCATTTTGATGTTGATAATATTAATTTTAAATCCCTAGTTGGATGT
TTAGGTCCACACTGCGGTTCTTCTTCTCGTTCTTTTTTTGAAGATTTATTGTTTGACAAAGTTAAACTTTCAGATGTTG
GTTTTGTTGAAGCTTATAACAATTGTACTGGTGGTAGTGAAATTAGAGATCTTCTTTGTGTACAATCCTTTAATGGTAT
TAAAGTTTTGCCTCCTATTTTGTCTGAATCTCAAATTTCTGGTTACACCACAGCCGCTACTGTTGCTGCTATGTTTCCA
CCATGGTCAGCAGCAGCTGGCATACCATTTTCTCTTAATGTACAATATAGAATTAATGGTTTGGGTGTTACTATGGATG
TTCTTAATAAAAATCAAAAGTTGATAGCTACTGCTTTTAATAATGCTCTTCTTTCTATTCAGAATGGTTTTAGTGCTAC
CAACTCTGCACTTGCTAAAATACAAAGTGTTGTTAATTCTAATGCTCAAGCACTTAATAGTTTGTTACAGCAATTATTT
AATAAATTTGGTGCAATTAGTTCTTCTTTACAAGAAATTTTATCTCGTCTCGATGCTTTAGAGGCTCAGGTTCAGATTG

ATAGGCTTATTAATGGTCGTTTAACTGCTTTAAATGCTTATGTCTCTCAACAACTTAGTGATATTTCTCTTGTAAAATT
TGGTGCTGCTTTAGCTATGGAGAAGGTTAATGAGTGTGTTAAAAGTCAATCTCCTCGTATTAATTTTTGTGGTAATGGT
AATCATATTTTGTCATTAGTTCAAAATGCTCCTTATGGTTTGTTGTTTATGCATTTTAGTTATAAACCTATTTCTTTTA
AAACTGTTTTAGTAAGTCCTGGTTTGTGTATATCAGGTGATGTAGGTATTGCACCTAAACAAGGGTATTTTATTAAACA
CAATGATCATTGGATGTTCACTGGTAGTTCTTACTATTATCCTGAACCAATTTCAGATAAAAATGTTGTTTTTATGAAT
ACTTGTTCTGTTAATTTTACTAAAGCGCCTCTTGTTTATTTGAATCATTCTGTACCAAAATTGTCTGATTTTGAATCTG
AGTTATCTCATTGGTTTAAAAATCAAACATCCATTGCGCCTAATTTGACTTTAAATCTTCATACTATTAATGCTACTTT
TTTAGATTTGTATTATGAGATGAATCTTATTCAAGAGTCTATTAAGTCTTTGAATAATAGTTATATCAATCTTAAAGAT
ATAGGTACATATGAAATGTATGTAAAATGGCCTTGGTATGTTTGGCTACTAATTTCTTTTTCATTTATAATATTCCTTG
TATTGCTCTTTTTTATATGTTGTTGTACTGGTTGTGGTTCTGCATGTTTTAGTAAATGTCATAATTGTTGTGATGAGTA
TGGTGGTCATCATGATTTTGTTATCAAAACATCTCATGATGATTAG

>gi|545299256|gb|KF686343.1| Human coronavirus HKU1 strain HKU1/human/USA/HKU1-13/2010, complete genome

ATGTTATTAATTATTTTTATTTTGCCTACAACATTAGCTGTTATAGGTGATTTTAATTGTACTAATTTTGCTATT
AATGATAAAAACACCACAGTTCCTCGCATAAGTGAGTATGTTGTGGATGTTTCTTATGGTTTGGGTACATATTATATAC
TTGATCGTGTTTATTTAAATACTACTATATTATTTACTGGTTATTTCCCTAAATCTGGTGCCAATTTTAGGGATCTATC
TTTAAAAGGTACTACATATTTGAGTACTCTTTGGTATCAGAAACCCTTTTTATCTGATTTTAATAATGGTATTTTTTCT
AGAGTTAAGAATACTAAGTTGTATGTTAATAAAACTTTGTATAGTGAGTTTAGTACTATAGTTATAGGTAGTGTTTTTA
TTAACAACTCTTATACTATTGTTGTTCAACCTCATAATGGTGTTTTGGAGATTACAGCTTGTCAATACACTATGTGTGA
GTATCCTCATACTATTTGTAAATCTAAAGGTAGTTCTCGTAATGAATCTTGGCATTTTGATAAATCTGAACCTTTGTGT
CTGTTCAAGAAAAATTTTACTTATAATGTTTCTACAGATTTCTTGTATTTTCATTTTTATCAAGAACGTGGCACTTTTT
ATGCTTATTATGCTGACTCTGGCATGCCTACTACTTTTTTATTTAGTTTGTATCTTGGTACTCTTTTATCTCATTATTA
TGTTTTGCCTTTGACTTGTAATGCTATATCTTCTAATACTGATAATGAGACTTTACAATATTGGGTCACACCTTTGTCT
AAACGCCAATATCTTCTTAAATTTGACAACCGTGGTGTTATTACTAATGCTGTTGATTGTTCTAGTAGTTTCTTTAGTG
AGATTCAATGTAAAACTAAATCTTTATTACCTAATACTGGTGTTTATGACTTATCTGGTTTTACTGTTAAGCCTGTTGC
AACTGTACATCGTCGTATTCCTGATTTACCTGATTGTGACATTGATAAATGGCTTAACAATTTTAATGTACCCTCACCT
CTTAATTGGGAACGTAAAATTTTTTCTAATTGCAACTTTAATTTGAGTACTTTGCTTCGTTTAGTTCATACTGATTCTT
TTTCTTGTAATAATTTTGATGAATCTAAGATATATGGTAGTTGTTTTAAGAGTATTGTTTTAGATAAATTTGCCATACC
CAACTCCAGACGATCTGATTTGCAGTTGGGCAGTTCTGGTTTTCTGCAATCTTCTAATTATAAAATTGACACTACTTCT
AGTTCTTGTCAATTGTATTATAGTTTGCCTGCAATTAATGTTACTATTAATAATTATAATCCTTCTTCTTGGAATAGAA
GGTATGGTTTTAATAATTTTAATTTGAGCTCTCATAGTGTTGTTTACTCACGTTATTGTTTTTCTGTTAATAATACTTT
TTGTCCTTGTGCTAAACCTTCTTTTGCTTCAAGTTGCAAGAGTCATAAACCACCTTCTGCTTCCTGTCCTATTGGTACT
AATTATCGTTCTTGTGAGAGTACTACTGTACTCGACCACACTGACTGGTGTAGGTGTTCTTGTTTACCTGATCCTATAA
CTGCTTATGACCCTAGGTCTTGTTCTCAAAAAAAGTCTTTGGTTGGTGTTGGTGAACATTGTGCAGGGTTCGGTGTTGA
TGAAGAAAAGTGTGGTGTATTGGATGGATCATATAATGTTTCTTGTCTTTGTAGTACTGATGCCTTTCTAGGTTGGTCT
TATGACACTTGCGTCAGTAACAACCGTTGTAATATTTTTTCTAATTTTATTTTAAATGGTATCAATAGTGGTACCACTT
GTTCTAATGATTTATTGCAGCCTAATACTGAAGTTTATACTGATGTTTGTGTTGATTACGACCTTTATGGTATTACAGG
ACAAGGTATTTTTAAAGAAGTTTCTGCTGTTTATTATAATAGTTGGCAAAATCTTTTGTATGATTCTAATGGCAACATT
ATTGGTTTTAAAGATTTTGTTACTAATAAAACATATAATATTTTCCCTTGTTATGCAGGAAGAGTTTCTGCTGCTTTTC
ATCAAAATGCTTCCTCTTTGGCTTTACTTTATCGTAATTTAAAATGTAGCTATGTTTTGAATAATATTTCTTCAGCTAC
TCAGCCATATTTTGATAGTTATCTTGGTTGCGTTTTTAATGCTGATAATTTAACTGATTATTCTGTTTCTTCTTGTGCT

CTTCGCATGGGTAGTGGTTTTTGTGTTGATTATAACTCACCTTCTTTTTCCTCTTCGCGTCGTAAACGTAGAAGTATTT
CTGCTTCTTATCGGTTTGTTACTTTTGAACCCTTTAATGTCAGTTTTGTTAATGACAGTATTGAGTCTGTGGGTGGTCT
TTATGAGATCAAAATTCCCACTAACTTTACTATAGTTGGTCAAGAGGAATTTATTCAAACTAATTCTCCTAAAGTTACT
ATTGATTGTTCTTTATTTGTCTGTTCTAATTATGCAGCTTGCCATGACTTATTGTCAGAGTATGGCACTTTTTGTGATA
ATATTAATAGTATTTTAGATGAAGTTAATGGTTTACTTGATACTACTCAATTGCATGTAGCTGATACTCTTATGCAAGG
TGTCACACTTAGCTCCAATCTTAATACTAATTTGCATTTTGATGTTGATAATATTAATTTTAAATCCCTAGTTGGATGT
TTAGGTCCACACTGCGGTTCTTCTTCTCGTTCTTTTTTTGAAGATTTATTGTTTGACAAAGTTAAACTTTCAGATGTTG
GTTTTGTTGAAGCTTATAACAATTGTACTGGTGGTAGTGAAATTAGAGATCTTCTTTGTGTACAATCCTTTAATGGTAT
TAAAGTTTTGCCTCCTATTTTGTCTGAATCTCAAATTTCTGGTTACACCACAGCCGCTACTGTTGCTGCTATGTTTCCA
CCATGGTCAGCAGCAGCTGGCATACCATTTTCTCTTAATGTACAATATAGAATTAATGGTTTGGGTGTTACTATGGATG
TTCTTAATAAAAATCAAAAGTTGATAGCTACTGCTTTTAATAATGCTCTTCTTTCTATTCAGAATGGTTTTAGTGCTAC
CAACTCTGCACTTGCTAAAATACAAAGTGTTGTTAATTCTAATGCTCAAGCACTTAATAGTTTGTTACAGCAATTATTT
AATAAATTTGGTGCAATTAGTTCTTCTTTACAAGAAATTTTATCTCGTCTCGATGCTTTAGAGGCTCAGGTTCAGATTG
ATAGGCTTATTAATGGTCGTTTAACTGCTTTAAATGCTTATGTCTCTCAACAACTTAGTGATATTTCTCTTGTAAAATT
TGGTGCTGCTTTAGCTATGGAGAAGGTTAATGAGTGTGTTAAAAGTCAATCTCCTCGTATTAATTTTTGTGGTAATGGT
AATCATATTTTGTCATTAGTTCAAAATGCTCCTTATGGTTTGTTGTTTATGCATTTTAGTTATAAACCTATTTCTTTTA
AAACTGTTTTAGTAAGTCCTGGTTTGTGTATATCAGGTGATGTAGGTATTGCACCTAAACAAGGGTATTTTATTAAACA
CAATGATCATTGGATGTTCACTGGTAGTTCTTACTATTATCCTGAACCAATTTCAGATAAAAATGTTGTTTTTATGAAT
ACTTGTTCTGTTAATTTTACTAAAGCGCCTCTTGTTTATTTGAATCATTCTGTACCAAAATTGTCTGATTTTGAATCTG
AGTTATCTCATTGGTTTAAAAATCAAACATCCATTGCTCCTAATTTGACTTTAAATCTTCATACTATTAATGCTACTTT
TTTAGATTTGTATTATGAGATGAATCTTATTCAAGAGTCTATTAAGTCTTTGAATAATAGTTATATCAATCTTAAAGAT
ATAGGTACATATGAAATGTATGTAAAATGGCCTTGGTATGTTTGGCTACTAATTTCTTTTTCATTTATAATATTCCTTG
TATTGCTCTTTTTTATATGTTGTTGTACTGGTTGTGGTTCTGCATGTTTTAGTAAATGTCATAATTGTTGTGATGAGTA
TGGTGGTCATCATGATTTTGTTATCAAAACATCTCATGATGATTAG

>gi|545299266|gb|KF686344.1| Human coronavirus HKU1 strain HKU1/human/USA/HKU1-15/2009, complete genome

ATGTTATTAATTATTTTTATTTTGCCTACAACATTAGCTGTTATAGGTGATTTTAATTGTACTAATTTTGCTATT
AATGATAAAAACACCACAGTTCCTCGCATAAGTGAGTATGTTGTGGATGTTTCTTATGGTTTGGGTACATATTATATAC
TTGATCGTGTTTATTTAAATACTACTATATTATTTACTGGTTATTTCCCTAAAGCTGGTGCTAATTTTAGGGATCTATC
TTTAAAAGGTACTACATATTTGAGTACTCTTTGGTATCAGAAACCCTTTTTATCTGATTTTAATAATGGTATTTTTTCT
AGAGTTAAGAATACTAAGTTGTATGTTAATAAAACTTTGTATAGTGAGTTTAGTACTATAGTTATAGGTAGTGTTTTTA
TTAACAACTCTTATACTATTGTTGTTCAACCTCATAATGGTGTTTTGGATATTACAGCTTGTCAATACACTATGTGTGA
GTATCCTCATACTATTTGTAAATCTAAAGGTAGTTCTCGTAATGAATCTTGGCATTTTGATAAATCTGAACCTTTGTGT
CTGTTCAAGAAAAATTTTACTTATAATGTTTCTACAGATTGGTTGTATTTTCATTTTTATCAAGAACGTGGCACTTTTT
ATGCTTATTATGCTGATTCTGGCATGCCTACTACTTTTTTATTTAGTTTGTATCTTGGTACTCTTTTATCTCATTATTA
TGTTTTGCCTTTGACTTGTAATGCTATATCTTCTAATACTGATAATGAGACTTTACAATATTGGGTCACACCTTTGTCT
AAACGCCAATATCTTCTTAAATTTGACAACCGTGGTGTTATTACTAATGCTGTTGATTGTTCTAGTAGTTTCTTTAGCG
AGATTCAATGTAAAACTAAATCTTTATTACCTAATACTGGTGTTTATGACTTATCTGGTTTTACTGTTAAGCCTGTTGC
AACTGTACATCGTCGTATTCCTGATTTACCTGATTGTGACATTGATAAATGGCTTAACAATTTTAATGTACCCTCACCT
CTTAATTGGGAACGTAAAATTTTTTCTAATTGCAACTTTAATTTGAGTACTTTGCTTCGTTTAGTTCATACTGATTCTT
TTTCTTGTAATAATTTTGATGAATCTAAGATATATGGTAGTTGTTTTAAGAGTATTATTTTAGATAAATTTGCCATACC

CAACTCCAGACGATCTGATTTGCAGTTGGGCAGTTCTGGTTTTCTGCAATCTTCTAATTATAAAATTGACACTACTTCT
AGTTCTTGTCAATTGTATTATAGTTTGCCTGCAATTAATGTTACTATTAATAATTATAATCCTTCTTCTTGGAATAGAA
GGTATGGTTTTAATAATTTTAATTTGAGCTCTCATAGTGTTGTTTACTCACGTTATTGTTTTTCTGTTAATAATACTTT
TTGTCCTTGTGCTAAACCTTCTTTTGCTTCAAGTTGCAAGAGTCATAAACCACCTTCTGCTTCCTGTCCTATTGGTACT
AATTATCGTTCTTGTGAGAGTACTACTGTACTCGACCGCACTGACTGGTGTAGGTGTTCTTGTTTACCTGATCCTATAA
CTGCTTATGACCCTAGGTCTTGTTCTCAAAAAAAGTCTCTGGTTGGTGTTGGTGAACATTGTGCAGGGTTCGGTGTTGA
TGAAGAAAAGTGTGGTGTATTGGATGGATCATATAATGCTTCTTGTCTTTGTAGTACTGATGCCTTTCTAGGTTGGTCT
TATGACACTTGCGTCAGTAACAACCGTTGTAATATTTTTTCTAATTTTATTTTAAATGGTATCAATAGTGGTACCACTT
GTTCTAATGATTTATTGCAGCCCAATACTGAAGTTTTTACTGATGTTTGTGTTGATTACGACCTTTATGGTATTACAGG
ACAAGGTATTTTTAAAGAAGTTTCTGCTGTTTATTATAATAGTTGGCAAAATCTTTTGTATGATTCTAATGGCAACATT
ATTGGTTTTAAAGATTTTGTTACTAATAAAACATATAATATTTTCCCTTGTTATGCAGGAAGAGTTTCTGCTGCTTTTC
ATCAAAATGCTTCCTCTTTGGCTTTACTTTATCGTAATTTAAAATGTAGCTATGTTTTGAATAATATTTCTTTAGCTAC
TCAGCCATATTTTGATAGTTATCTTGGTTGCGTTTTTAATGCTGATAATTTAACTGATTATTCTGTTTCTTCTTGTGCT
CTTCGCATGGGTAGTGGTTTTTGTGTTGATTATAACTCACCTTCTTCTTCCTCTTCGCGTCGTAAACGTAGAAGTATTT
CTGCTTCTTATCGTTTTGTTACTTTTGAACCCTTTAATGTCAGTTTTGTTAATGACAGTATTGAGTCTGTGGGTGGTCT
TTATGAGATCAAAATTCCCACTAACTTTACTATAGTTGGTCAAGAGGAATTTATTCAAACTAATTCTCCTAAAGTTACT
ATTGATTGTTCTTTATTTGTCTGTTCTAATTATGCAGCTTGCCATGACTTATTGTCAGAGTATGGCACTTTTTGTGATA
ATATTAATAGTATTTTAGATGAAGTTAATGGTTTACTTGATACTACTCAATTGCATGTAGCTGATACTCTTATGCAAGG
TGTCACACTTAGCTCCAATCTTAATACTAATTTGCATTTTGATGTTGATAATATTAATTTTAAATCCCTAGTTGGATGT
TTAGGTCCACACTGCGGTTCCTCTTCTCGTTCCTTTTTTGAAGATTTATTGTTTGACAAAGTTAAACTTTCAGATGTTG
GTTTTGTTGAAGCTTATAACAATTGTACTGGTGGTAGTGAAATTAGAGATCTTCTTTGTGTACAATCCTTTAATGGTAT
TAAAGTTTTGCCTCCTATTTTGTCTGAATCTCAAATTTCCGGTTACACCACAGCCGCTACTGTTGCTGCTATGTTTCCA
CCATGGTCAGCAGCAGCTGGCATACCATTTTCTCTTAATGTACAATATAGAATTAATGGTTTGGGTGTTACTATGGATG
TTCTTAATAAAAATCAAAAGTTGATAGCTACTGCTTTTAATAATGCTCTTCTTTCTATTCAGAATGGTTTTAGTGCTAC
CAACTCTGCACTTGCTAAAATACAAAGTGTTGTTAATTCTAATGCTCAAGCACTTAATAGTTTGTTACAGCAATTATTT
AATAAATTTGGTGCAATTAGTTCTTCTTTACAAGAAATTTTATCTCGTCTCGATGCTTTAGAGGCTCAGGTTCAGATTG
ATAGGCTTATTAATGGTCGTTTAACTGCTTTAAATGCTTATGTCTCTCAACAGCTTAGTGATATTTCTCTTGTAAAATT
TGGTGCTGCTTTAGCCATGGAGAAGGTTAATGAGTGTGTTAAAAGTCAATCTCCTCGTATTAATTTTTGTGGTAATGGT
AATCATATTTTGTCATTAGTTCAAAATGCTCCTTATGGTTTGTTGTTTATGCATTTTAGTTATAAACCTATTTCTTTTA
AAACTGTTTTAGTAAGTCCTGGTTTGTGTATATCAGGTGATGTAGGTATTGCACCTAAACAAGGGTATTTTATTAAACA
TAATGATCATTGGATGTTCACTGGTAGTTCTTACTATTATCCTGAACCAATTTCAGATAAAAATGTTGTTTTTATGAAT
ACTTGTTCTGTTAATTTTACTAAAGCGCCTCTTGTTTATTTGAATCATTCTGTACCAAAATTGTCTGATTTTGAATCTG
AGTTATCTCATTGGTTTAAAAATCAAACATCCATTGCGCCTAATTTGACTTTAAATCTTCATACTATTAATGCTACTTT
TTTAGATTTGTATTATGAGATGAATCTTATTCAAGAGTCTATTAAGTCTTTGAATAATAGTTATATCAATCTTAAAGAT
ATAGGTACATATGAAATGTATGTAAAATGGCCTTGGTATGTTTGGCTACTAATTTCTTTTTCATTTATAATATTCCTTG
TATTGCTCTTTTTTATATGTTGTTGTACTGGTTGTGGTTCTGCATGTTTTAGTAAATGTCATAATTGTTGTGATGAGTA
TGGTGGTCATCATGATTTTGTTATCAAAACATCTCATGATGATTAG

>gi|545299276|gb|KF686346.1| Human coronavirus HKU1 strain HKU1/human/USA/HKU1-12/2010, complete genome

ATGTTATTAATTATTTTTATTTTGCCTACAACATTAGCTGTTATAGGTGATTTTAATTGTACTAATTTTGCTATT
AATGATAAAAACACCACAGTTCCTCGCATAAGTGAGTATGTTGTGGATGTTTCTTATGGTTTGGGTACATATTATATAC

TTGATCGTGTTTATTTAAATACTACTATATTATTTACTGGTTATTTCCCTAAATCTGGTGCCAATTTTAGGGATCTATC
TTTAAAAGGTACTACATATTTGAGTACTCTTTGGTATCAGAAACCCTTTTTATCTGATTTTAATAATGGTATTTTTTCT
AGAGTTAAGAATACTAAGTTGTATGTTAATAAAACTTTGTATAGTGAGTTTAGTACTATAGTTATAGGTAGTGTTTTTA
TTAACAACTCTTATACTATTGTTGTTCAACCTCATAATGGTGTTTTGGAGATTACAGCTTGTCAATACACTATGTGTGA
GTATCCTCATACTATTTGTAAATCTAAAGGTAGTTCTCGTAATGAATCTTGGCATTTTGATAAATCTGAACCTTTGTGT
CTGTTCAAGAAAAAATTTTACTTATAATGTTTCTACAGATTTCTTGTATTTTCATTTTTATCAAGAACGTGGCACTTTTT
ATGCTTATTATGCTGACTCTGGCATGCCTACTACTTTTTTATTTAGTTTGTATCTTGGTACTCTTTTATCTCATTATTA
TGTTTTGCCTTTGACTTGTAATGCTATATCTTCTAATACTGATAATGAGACTTTACAATATTGGGTCACACCTTTGTCT
AAACGCCAATATCTTCTTAAATTTGACAACCGTGGTGTTATTACTAATGCTGTTGATTGTTCTAGTAGTTTCTTTAGTG
AGATTCAATGTAAAACTAAATCTTTATTACCTAATACTGGTGTTTATGACTTATCTGGTTTTACTGTTAAGCCTGTTGC
AACTGTACATCGTCGTATTCCTGATTTACCTGATTGTGACATTGATAAATGGCTTAACAATTTTAATGTACCCTCACCT
CTTAATTGGGAACGTAAAATTTTTTCTAATTGCAACTTTAATTTGAGTACTTTGCTTCGTTTAGTTCATACTGATTCTT
TTTCTTGTAATAATTTTGATGAATCTAAGATATATGGTAGTTGTTTTAAGAGTATTGTTTTAGATAAATTTGCCATACC
CAACTCCAGACGATCTGATTTGCAGTTGGGCAGTTCTGGTTTTCTGCAATCTTCTAATTATAAAATTGACACTACTTCT
AGTTCTTGTCAATTGTATTATAGTTTGCCTGCAATTAATGTTACTATTAATAATTATAATCCTTCTTCTTGGAATAGAA
GGTATGGTTTTAATAATTTTAATTTGAGCTCTCATAGTGTTGTTTACTCACGTTATTGTTTTTCTGTTAATAATACTTT
TTGTCCTTGTGCTAAACCTTCTTTTGCTTCAAGTTGCAAGAGTCATAAACCACCTTCTGCTTCCTGTCCTATTGGTACT
AATTATCGTTCTTGTGAGAGTACTACTGTACTCGACCACACTGACTGGTGTAGGTGTTCTTGTTTACCTGATCCTATAA
CTGCTTATGACCCTAGGTCTTGTTCTCAAAAAAAGTCTTTGGTTGGTGTTGGTGAACATTGTGCAGGGTTCGGTGTTGA
TGAAGAAAAGTGTGGTGTATTGGATGGATCATATAATGTTTCTTGTCTTTGTAGTACTGATGCCTTTCTAGGTTGGTCT
TATGACACTTGCGTCAGTAACAACCGTTGTAATATTTTTTCTAATTTTATTTTAAATGGTATCAATAGTGGTACCACTT
GTTCTAATGATTTATTGCAGCCTAATACTGAAGTTTATACTGATGTTTGTGTTGATTACGACCTTTATGGTATTACAGG
ACAAGGTATTTTTAAAGAAGTTTCTGCTGTTTATTATAATAGTTGGCAAAATCTTTTGTATGATTCTAATGGCAACATT
ATTGGTTTTAAAGATTTTGTTACTAATAAAACATATAATATTTTCCCTTGTTATGCAGGAAGAGTTTCTGCTGCTTTTC
ATCAAAATGCTTCCTCTTTGGCTTTACTTTATCGTAATTTAAAATGTAGCTATGTTTTGAATAATATTTCTTTAGCTAC
TCAGCCATATTTTGATAGTTATCTTGGTTGCGTTTTTAATGCTGATAATTTAACTGATTATTCTGTTTCTTCTTGTGCT
CTTCGCATGGGTAGTGGTTTTTGTGTTGATTATAACTCACCTTCTTTTTCCTCTTCGCGTCGTAAACGTAGAAGTATTT
CTGCTTCTTATCGGTTTGTTACTTTTGAACCCTTTAATGTCAGTTTTGTTAATGACAGTATTGAGTCTGTGGGTGGTCT
TTATGAGATCAAAATTCCCACTAACTTTACTATAGTTGGTCAAGAGGAATTTATTCAAACTAATTCTCCTAAAGTTACT
ATTGATTGTTCTTTATTTGTCTGTTCTAATTATGCAGCTTGCCATGACTTATTGTCAGAGTATGGCACTTTTTGTGATA
ATATTAATAGTATTTTAGATGAAGTTAATGGTTTACTTGATACTACTCAATTGCATGTAGCTGATACTCTTATGCAAGG
TGTCACACTTAGCTCCAATCTTAATACTAATTTGCATTTTGATGTTGATAATATTAATTTTAAATCCCTAGTTGGATGT
TTAGGTCCACACTGCGGTTCTTCTTCTCGTTCTTTTTTTGAAGATTTATTGTTTGACAAAGTTAAACTTTCAGATGTTG
GTTTTGTTGAAGCTTATAACAATTGTACTGGTGGTAGTGAAATTAGAGATCTTCTTTGTGTACAATCCTTTAATGGTAT
TAAAGTTTTGCCTCCTATTTTGTCTGAATCTCAAATTTCTGGTTACACCACAGCCGCTACTGTTGCTGCTATGTTTCCA
CCATGGTCAGCAGCAGCTGGCATACCATTTTCTCTTAATGTACAATATAGAATTAATGGTTTGGGTGTTACTATGGATG
TTCTTAATAAAAATCAAAAGTTGATAGCTACTGCTTTTAATAATGCTCTTCTTTCTATTCAGAATGGTTTTAGTGCTAC
CAACTCTGCACTTGCTAAAATACAAAGTGTTGTTAATTCTAATGCTCAAGCACTTAATAGTTTGTTACAGCAATTATTT
AATAAATTTGGTGCAATTAGTTCTTCTTTACAAGAAATTTTATCTCGTCTCGATGCTTTAGAGGCTCAGGTTCAGATTG
ATAGGCTTATTAATGGTCGTTTAACTGCTTTAAATGCTTATGTCTCTCAACAACTTAGTGATATTTCTCTTGTAAAATT
TGGTGCTGCTTTAGCTATGGAGAAGGTTAATGAGTGTGTTAAAAGTCAATCTCCTCGTATTAATTTTTGTGGTAATGGT
AATCATATTTTGTCATTAGTTCAAAATGCTCCTTATGGTTTGTTGTTTATGCATTTTAGTTATAAACCTATTTCTTTTA
AAACTGTTTTAGTAAGTCCTGGTTTGTGTATATCAGGTGATGTAGGTATTGCACCTAAACAAGGGTATTTTATTAAACA
CAATGATCATTGGATGTTCACTGGTAGTTCTTACTATTATCCTGAACCAATTTCAGATAAAAATGTTGTTTTTATGAAT

ACTTGTTCTGTTAATTTTACTAAAGCGCCTCTTGTTTATTTGAATCATTCTGTACCAAAATTGTCTGATTTTGAATCTG
AGTTATCTCATTGGTTTAAAAATCAAACATCCATTGCTCCTAATTTGACTTTAAATCTTCATACTATTAATGCTACTTT
TTTAGATTTGTATTATGAGATGAATCTTATTCAAGAGTCTATTAAGTCTTTGAATAATAGTTATATCAATCTTAAAGAT
ATAGGTACATATGAAATGTATGTAAAATGGCCTTGGTATGTTTGGCTACTAATTTCTTTTTCATTTATAATATTCCTTG
TATTGCTCTTTTTTATATGTTGTTGTACTGGTTGTGGTTCTGCATGTTTTAGTAAATGTCATAATTGTTGTGATGAGTA
TGGTGGTCATCATGATTTTGTTATCAAAACATCTCATGATGATTAG

>gi|85372431|gb|AY884001.1| Human coronavirus HKU1 genotype B, complete genome

ATGTTTTTAATTATTTTTATTTTGCCTACAACACTAGCTGTTATAGGTGATTTTAATTGTACTAACTCTTTTATT
AATGATTATAATAAAACCATTCCGCGTATAAGCGAGGATGTTGTTGATGTATCTCTTGGTTTGGGCACATATTATGTTC
TTAACCGTGTTTATTTAAATACTACCTTGTTATTTACAGGTTATTTTCCTAAATCTGGTGCTAATTTTAGAGACTTGGC
TTTAAAGGGTTCTAAATATTTGAGTACTCTCTGGTATAAACCACCTTTTCTGTCAGATTTTAATAATGGTATTTTTTCT
AAGGTTAAGAATACTAAGTTATATGTTAATAATACTTTGTATAGTGAATTTAGTACTATAGTTATAGGTAGTGTTTTTG
TTAATACTTCTTATACTATTGTTGTTCAACCTCACAATGGTATTTTGGAGATTACAGCTTGTCAGTATACTATGTGTGA
ATATCCTCACACTGTTTGTAAGTCTAAGGGTAGTATTCGTAATGAATCTTGGCACATTGATTCTTCGGAACCTTTATGC
TTGTTTAAGAAAAATTTTACTTATAATGTTTCTGCAGATTGGCTGTATTTTCATTTTTATCAAGAACGTGGTGTTTTTT
ATGCATATTATGCAGATGTAGGTATGCCTACCACTTTCTTATTTAGTTTATATTTAGGTACTATTTTATCTCATTATTA
TGTTATGCCTTTGACTTGTAAGGCTATATCTTCAAATACTGACAATGAAACTTTAGAATATTGGGTTACACCGCTATCT
AGACGTCAGTATCTTCTTAATTTTGATGAGCACGGTGTTATTACTAATGCCGTTGATTGTTCAAGTAGTTTTCTTAGTG
AGATTCAATGTAAAACTCAATCTTTTGCACCTAATACTGGTGTTTATGATTTGTCTGGTTTTACTGTAAAGCCTGTTGC
AACTGTTTATCGTCGGATTCCTAATTTACCTGATTGTGACATTGACAACTGGCTTAATAATGTTAGTGTACCTTCACCT
CTTAATTGGGAACGTAGAATTTTTTCTAATTGTAACTTCAATTTAAGCACTTTACTTCGTCTAGTTCATGTTGATTCTT
TTTCTTGTAATAATCTTGATAAATCTAAAATTTTTGGTAGTTGCTTTAATAGTATTACTGTTGACAAGTTTGCTATACC
TAATCGCAGACGAGATGATTTGCAATTGGGCAGTTCTGGCTTTTTGCAATCATCTAATTACAAAATAGATATTTCTTCT
AGTTCTTGTCAATTGTATTATAGTTTACCTTTAGTTAATGTTACTATTAATAACTTTAATCCATCTTCTTGGAATAGGA
GGTATGGTTTTGGTAGTTTTAATGTGTCTTCTTATGACGTTGTTTATTCTGATCATTGTTTTTCTGTTAACAGCGACTT
TTGCCCTTGTGCAGATCCGTCTGTTGTTAATTCTTGTGTTAAATCTAAGCCTCTTTCTGCCATTTGTCCTGCTGGTACT
AAATATCGTCATTGCGACTTGGATACTACTCTTTATGTTAATAACTGGTGTAGATGTTCTTGTCTACCTGACCCCATTT
CTACTTATTCTCCTAACACATGTCCTCAAAAGAAGGTCGTTGTTGGTATAGGTGAACATTGTCCAGGTCTTGGTATTAA
TGAGGAAAAATGTGGTACACAATTAAATCATAGTTCCTGTTCTTGTAGTCCTGATGCCTTTTTGGGTTGGTCTTTTGAT
AGTTGTATTAGTAATAATCGTTGCAATATTTTTTCTAATTTTATTTTTAATGGAATTAATAGTGGCACCACTTGTTCTA
ATGATTTGTTATATTCTAACACTGAAGTTTCTACTGGTGTTTGTGTTAATTATGATCTTTATGGCATCACAGGCCAAGG
TATTTTTAAAGAAGTTTCTGCGGCTTATTATAATAATTGGCAGAATCTTTTGTATGATTCTAATGGTAATATTATTGGT
TTTAAAGATTTTTTGACTAATAAAACTTACACTATACTTCCTTGTTATTCTGGTAGAGTGTCTGCTGCATTTTATCAAA
ATTCTTCTTCACCAGCTTTGCTTTATCGTAATTTAAAGTGTAGTTATGTTTTGAATAATATTTCTTTTATCTCACAACC
ATTTTATTTTGATAGTTATCTTGGTTGTGTTTTGAATGCTGTTAATTTAACTAGCTATTCTGTATCCTCTTGTGATTTG
CGTATGGGTAGTGGGTTTTGTATTGATTATGCTTTACCCTCTTCTCGGCGTAAGCGTAGAGGTATTTCTTCTCCTTATC
GCTTTGTAACTTTTGAACCCTTTAATGTTAGTTTTGTTAACGATAGTGTTGAAACTGTTGGTGGTTTATTTGAGATTCA
GATTCCTACTAACTTTACCATAGCTGGTCATGAAGAATTTATTCAGACTAGTTCTCCTAAAGTTACTATTGATTGTTCA
GCTTTTGTTTGCTCTAATTATGCTGCTTGTCATGATTTATTGTCGGAATATGGCACTTTTTGCGATAATATTAATAGTA
TTTTAAATGAAGTCAATGATTTACTTGATATTACTCAGTTGCAGGTTGCTAATGCTTTAATGCAAGGTGTTACACTTAG
TTCTAATCTTAATACTAATCTACACTCTGATGTTGATAATATAGATTTTAAATCTCTTCTAGGTTGTTTAGGTTCACAA

TGTGGTTCTTCGTCTAGATCTTTGTTAGAGGATTTATTATTCAACAAGGTCAAACTTTCAGATGTAGGTTTTGTTGAAG
CTTATAATAATTGCACTGGTGGTAGTGAAATTAGAGATCTTCTCTGTGTGCAATCTTTTAATGGTATTAAAGTATTACC
TCCCATTTTATCTGAGACTCAAATTTCTGGCTATACTACAGCTGCTACTGTGGCGGCTATGTTTCCGCCATGGTCTGCT
GCTGCTGGTGTACCATTTTCTCTTAATGTACAATATAGAATTAATGGTTTGGGTGTTACTATGGATGTTCTTAATAAGA
ATCAAAAGTTAATAGCTAATGCTTTTAATAAAGCTCTTCTTTCTATCCAGAATGGTTTTACTGCTACTAACTCTGCTCT
TGCTAAAATTCAAAGTGTCGTTAATGCTAATGCTCAAGCACTTAATAGTTTGTTACAACAATTATTTAATAAATTTGGT
GCTATTAGTTCTTCTTTACAAGAAATTTTGTCTCGCCTTGATAATTTAGAAGCTCAGGTTCAGATTGATAGGCTCATTA
ATGGTCGTTTGACTGCTTTAAATGCTTATGTTTCTCAACAGCTTAGTGATATTACACTTATTAAGGCTGGAGCTTCTCG
TGCTATTGAGAAGGTTAATGAGTGTGTTAAAAGTCAATCCCCTCGTATAAATTTTTGTGGCAATGGTAACCACATTTTA
TCATTGGTTCAAAATGCTCCTTATGGTTTGCTTTTCATTCATTTTAGTTATAAACCTACTTCTTTTAAAACTGTCTTAG
TAAGTCCAGGTTTATGTTTATCCGGTGATAGAGGTATTGCACCTAAGCAAGGTTATTTTATTAAACAAAATGATTCCTG
GATGTTTACTGGTAGTTCCTATTATTACCCAGAACCAATTTCAGATAAAAATGTTGTTTTCATGAATAGTTGCTCTGTT
AATTTTACTAAAGCTCCATTTATTTATCTTAATAATTCTATACCAAATTTGTCTGATTTTGAAGCCGAGTTTTCTCTTT
GGTTTAAAAATCATACTTCTATAGCACCTAATTTAACCTTTAATTCTCATATTAATGCTACTTTTTAGATCTGTATTA
TGAAATGAATGTTATTCAGGAATCTATTAAATCTTTGAACAGTAGTTTTATTAATCTTAAAGAAATAGGTACTTATGAA
ATGTATGTTAAATGGCCTTGGTACATTTGGTTGTTAATTGTCATTTTATTTATAATTTTTCTTATGATACTTTTCTTTA
TATGCTGCTGTACTGGTTGTGGTTCAGCATGTTTTAGTAAATGTCATAATTGTTGTGATGAGTATGGGGGTCACAATGA
TTTTGTTATTAAAGCATCTCATGATGATTAG

>gi|306569684|gb|HM034837.1| Human coronavirus HKU1 isolate Caen1, complete genome

ATGTTATTAATTATTTTTATTTTGCCTACAACATTAGCTGTTATAGGTGATTTTAATTGTACTAATTTTGCTATT
AATGATTTAAACACCACAATTCCTCGCATAAGTGAGTATGTTGTGGATGTTTCTTATGGTTTGGGTACATATTATATAC
TTGATCGTGTTTATTTAAATACTACTATATTATTTACTGGTTATTTCCCTAAATCTGGTGCCAATTTTAGGGATCTATC
TTTAAAAGGTACTACAAAATTGAGTACTCTTTGGTATCAGAAACCCTTTTTATCTGATTTTAATAATGGTATTTTTTCT
AGAGTTAAGAATACTAAGTTGTATGTTAATAAAACTTTGTATAGTGAGTTTAGTACTATAGTTATAGGTAGTGTTTTTA
TTAACAACTCTTATACTATTGTTGTTCAACCTCATAATGGTGTTTTGGAGATTACAGCTTGTCAATACACTATGTGTGA
GTATCCTCATACTATTTGTAAATCTATAGGTAGTTCTCGTAATGAATCTTGGCATTTTGATAAATCTGAACCTTTGTGT
CTGTTCAAGAAAAATTTTACTTATAATGTTTCTACAGATTGGTTGTATTTTCATTTTTATCAAGAACGTGGCACTTTTT
ATGCTTATTATGCTGATTCTGGCATGCCTACTACTTTTTTATTTAGTTTGTATCTTGGTACTCTTTTATCTCATTATTA
TGTTTTGCCTTTGACTTGTAATGCTATATCTTCTAATACTGATAATGAGACTTTACAATATTGGGTCACACCTTTGTCT
AAACGCCAATATCTTCTTAAATTTGACGACCGTGGTGTTATTACTAATGCTGTTGATTGTTCTAGTAGTTTCTTTAGCG
AGATTCAATGTAAAACTAAATCTTTATTACCTAATACTGGTGTTTATGACTTATCTGGTTTTACTGTTAAGCCTGTTGC
AACTGTACATCGTCGTATTCCTGATTTACCTGATTGTGACATTGATAAATGGCTTAACAATTTTAATGTACCCTCACCT
CTTAATTGGGAACGTAAAATTTTTTCTAATTGCAACTTTAATTTGAGTACTTTGCTTCGTTTAGTTCATACTGATTCTT
TTTCTTGTAATAATTTTGATGAATCTAAGATATATGGTAGTTGTTTTAAGAGTATTGTTTTAGATAAATTTGCCATACC
CAACTCCAGACGATCTGATTTGCAGTTGGGCAGTTCTGGTTTTCTGCAATCTTCTAATTATAAAATTGACACTACTTCT
AGTTCTTGTCAATTGTATTATAGTTTGCCTGCAATTAATGTTACTATTAATAATTATAATCCTTCTTCTTGGAATAGAA
GGTATGGTTTTAATAATTTTAATTTGAGTTCTCATAGTGTTGTTTACTCACGTTATTGTTTTTCTGTTAATAATACTTT
TTGTCCTTGTGCTAAACCTTCTTTTGCTTCAAGTTGCAAGAGTCATAAACCACCTTCTGCTTCTTGTCCTATTGGTACT
AATTATCGTTCTTGTGAGAGTACTACTGTACTCGACCACACTGACTGGTGTAGGTGTTCTTGTTTACCTGATCCTATAA
CTGCTTATGACCCTAGGTCTTGTTCTCAAAAAAAGTCTCTGGTTGGTGTTGGTGAACATTGTGCAGGGTTCGGTGTTGA

TGAAGAAAAGTGTGGTGTATTGGATGGATCATATAATGTTTCTTGTCTTTGTAGTACTGATGCCTTTCTAGGTTGGTCT
TATGACACTTGCGTCAGTAACAACCGTTGTAATATTTTTTCTAATTTTATTTTAAATGGTATCAATAGTGGTACCACTT
GTTCTAATGATTTATTGCAGCCTAATACTGAAGTTTTTACTGATGTTTGTGTTGATTACGACCTTTATGGTATTACAGG
ACAAGGTATTTTTAAAGAAGTTTCTGCTGTTTATTATAATAGTTGGCAAAATCTTTTGTATGATTTTAATGGCAACATT
ATTGGTTTTAAAGATTTTGTTACTAATAAAACATATAATATTTTCCCTTGTTATGCAGGAAGAGTTTCTGCTGCTTTTC
ATCAAAATGCTTCCTCTTTGGCTTTACTTTATCGTAATTTAAAATGTAGCTATGTTTTGAATAATATTTCTTTAGCTAC
TCAGCCATATTTTGATAGTTATCTTGGTTGCGTTTTTAATGCTGATAATTTAACTGATTATTCTGTTTCTTCTTGTGCT
CTTCGCATGGGTAGTGGTTTTTGTGTTGATTATAACTCACCTTCTTCTTCCTCTTCGCGTCGTAAACGTAGAAGTATTT
CTGCTTCTTATCGGTTTGTTACTTTTGAACCCTTTAATGTCAGTTTTGTTAATGACAGTATTGAGTCTGTGGGTGGTCT
TTATGAGATCAAAATTCCCACTAACTTTACTATAGTTGGTCAAGAGGAATTTATTCAAACTAATTCTCCTAAAGTTACT
ATTGATTGTTCTTTATTTGTCTGTTCTAATTATGCAGCTTGCCATGACTTATTGTCAGAGTATGGCACTTTTTGTGATA
ATATTAATAGTATTTTAGATGAAGTTAATGGTTTACTTGATACTACTCAATTGCATGTAGCTGATACTCTTATGCAAGG
TGTCACACTTAGCTCCAATCTTAATACTAATTTGCATTTTGATGTTGATAATATTAATTTTAAATCCCTAGTTGGATGT
TTAGGTCCACACTGCGGTTCTTCTTCTCGTTCTTTTTTTGAAGATTTATTGTTTGACAAAGTTAAACTTTCAGATGTTG
GTTTTGTTGAAGCTTATAACAATTGTACTGGTGGTAGTGAAATTAGAGATCTTCTTTGTGTACAATCCTTTAATGGTAT
TAAAGTTTTGCCTCCTATTTTGTCTGAATCTCAAATTTCTGGTTACACCACAGCCGCTACTGTTGCTGCTATGTTTCCA
CCATGGTCAGCAGCAGCTGGCATACCATTTTCTCTTAATGTACAATATAGAATTAATGGTTTGGGTGTTACTATGGATG
TTCTTAATAAAAATCAAAAGTTGATAGCTACTGCTTTTAATAATGCTCTTCTTTCTATTCAGAATGGTTTTAGTGCTAC
CAACTCTGCACTTGCTAAAATACAAAGTGTTGTTAATTCTAATGCTCAAGCACTTAATAGTTTGTTACAGCAATTATTT
AATAAATTTGGTGCAATTAGTTCTTCTTTACAAGAAATTTTATCTCGTCTCGATGCTTTAGAGGCTCAGGTTCAGATTG
ATAGGCTTATTAATGGTCGTTTAACTGCTTTAAATGCTTATGTTTCTCAACAGCTTAGTGATATTTCTCTTGTAAAACT
TGGTGCTGCTTTAGCTATGGAGAAGGTTAATGAGTGTGTTAAAAGTCAATCTCCTCGTATTAATTTTTGTGGTAATGGT
AATCATATTTTGTCATTAGTTCAAAATGCTCCTTATGGTTTGTTGTTTATGCATTTTAGTTATAAACCTATTTCTTTTA
AAACTGTTTTAGTAAGTCCTGGTTTATGTATATCAGGTGATGTAGGTATTGCACCTAAACAAGGGTATTTTATTAAACA
TAATGATCATTGGATGTTTACTGGTAGTTCTTACTATTATCCTGAACCAATTTCAGATAAAAATGTTGTTTTTATGAAT
ACTTGTTCTGTTAATTTTACTAAAGCGCCTCTTGTTTATTTGAATCATTCTGTACCAAAATTGTCTGATTTTGAATCTG
AGTTATCTCATTGGTTTAAAAATCAAACATCCATTGCGCCTAATTTGACTTTAAATCTTCATACTATTAATGCTACTTT
TTTAGATTTGTATTATGAGATGAATCTTATTCAAGAGTCTATTAAGTCTTTGAATAATAGTTATATCAATCTTAAAGAT
ATAGGTACATATGAAATGTATGTAAAATGGCCTTGGTATGTTTGGCTACTAATTTCTTTTTCATTTATAATATTCCTTG
TATTGCTCTTTTTTATATGTTGTTGTACTGGTTGTGGTTCTGCATGTTTTAGTAAATGTCATAATTGTTGTGATGAGTA
TGGTGGTCATCATGATTTTGTTATCAAAACATCTCATGATGATTAG

>gi|530291047|gb|KF430201.1| Human coronavirus HKU1 strain HKU1/human/USA/HKU1-18/2010, complete genome

ATGTTATTAATTATTTTTATTTTGCCTACAACATTAGCTGTTATAGGTGATTTTAATTGTACTAATTTTGCTATT
AATGATAAAAACACCACAGTTCCTCGCATAAGTGAGTATGTTGTGGATGTTTCTTATGGTTTGGGTACATATTATATAC
TTGATCGTGTTTATTTAAATACTATTATATTATTTACTGGTTATTTCCCTAAATCTGGTGCCAATTTTAGGGATCTATC
TTTAAAAGGTACTACATATTTGAGTACTCTTTGGTATCAGAAACCCTTTTTATCTGATTTTAATAATGGTATTTTTTCT
AGAGTTAAGAATACTAAGTTGTATGTTAATAAAACTTTGTATAGTGAGTTTAGTACTATAGTTATAGGTAGTGTTTTTA
TTAACAACTCTTATACTATTGTTGTTCAACCTCATAATGGTGTTTTGGAGATTACAGCTTGTCAATACACTATGTGTGA
GTATCCTCATACTATTTGTAAATCTAAAGGTAGTTCTCGTAATGAATCTTGGCATTTTGATAAATCTGAACCTTTGTGT
CTGTTCAAGAAAAATTTTACTTATAATGTTTCTACAGATTTCTTGTATTTTCATTTTTATCAAGAACGTGGCACTTTTT

ATGCTTATTATGCTGACTCTGGCATGCCTACTACTTTTTTATTTAGTTTGTATCTTGGTACTCTTTTATCTCATTATTA
TGTTTTGCCTTTGACTTGTAATGCTATATCTTCTAATACTGATAATGAGACTTTACAATATTGGGTCACACCTTTGTCT
AAACGCCAATATCTTCTTAAATTTGACAACCGTGGTGTTATTACTAATGCTGTTGATTGTTCTAGTAGTTTCTTTAGTG
AGATTCAATGTAAAACTAAATCTTTATTACCTAATACTGGTGTTTATGACTTATCTGGTTTTACTGTTAAGCCTGTTGC
AACTGTACATCGTCGTATTCCTGATTTACCTGATTGTGACATTGATAAATGGCTTAACAATTTTAATGTACCCTCACCT
CTTAATTGGGAACGTAAAATTTTTTCTAATTGCAACTTTAATTTGAGTACTTTGCTTCGTTTAGTTCATACTGATTCTT
TTTCTTGTAATAATTTTGATGAATCTAAGATATATGGTAGTTGTTTTAAGAGTATTGTTTTAGATAAATTTGCCATACC
CAACTCCAGACGATCTGATTTGCAGTTGGGCAGTTCTGGTTTTCTGCAATCTTCTAATTATAAAATTGACACTACTTCT
AGTTCTTGTCAATTGTATTATAGTTTGCCTGCAATTAATGTTACTATTAATAATTATAATCCTTCTTCTTGGAATAGAA
GGTATGGTTTTAATAATTTTAATTTGAGCTCTCATAGTGTTGTTTACTCACGTTATTGTTTTTCTGTTAATAATACTTT
TTGTCCTTGTGCTAAATCTTCTTTTGCTTCAAGTTGCAAGAGTCATAAACCACCTTCTGCTTCCTGTCCTATTGGTACT
AATTATCGTTCTTGTGAGAGTACTACTGTACTCGACCACACTGACTGGTGTAGGTGTTCTTGTTTACCTGATCCTATAA
CTGCTTATGACCCTAGGTCTTGTTCTCAAAAAAAGTCTCTGGTTGGTGTTGGTGAACATTGTGCAGGGTTCGGTGTTGA
TGAAGAAAAGTGTGGTGTATTGGATGGATATAATGTTTCTTGTCTTTGTAGTACTGATGCCTTTCTAGGTTGGTCTTAT
GACACTTGCGTCAGTAACAACCGTTGTAATATTTTTTCTAATTTTATTTTAAATGGTATCAATAGTGGTACCACTTGTT
CTAATGATTTATTGCAGCCTAATACTGAAGTTTATACTGATGTTTGTGTTGATTACGACCTTTATGGTATTACAGGACA
AGGTATTTTTAAAGAAGTTTCTGCTGTTTATTATAATAGTTGGCAAAATCTTTTGTATGATTCTAATGGCAACATTATT
GGTTTTAAAGATTTTGTTACTAATAAAACATATAATATTTTCCCTTGTTATGCAGGAAGAGTTTCTGCTGCTTTTCATC
AAAATGCTTCCTCTTTGGCTTTACTTTATCGTAATTTAAAATGTAGCTATGTTTTGAATAATATTTCTTTAGCTACTCA
GCCATATTTTGATAGTTATCTTGGTTGCGTTTTTAATGCTGATAATTTAACTGATTATTCTGTTTCTTCTTGTGCTCTT
CGCATGGGTAGTGGTTTTTGTGTTGATTATAACTCACCTTCTTTTTCCTCTTCGCGTCGTAAACGTAGAAGTATTTCTG
CTTCTTATCGGTTTGTTACTTTTGAACCCTTTAATGTCAGTTTTGTTAATGACAGTATTGAGTCTGTGGGTGGTCTTTA
TGAGATCAAAATTCCCACTAACTTTACTATAGTTGGTCAAGAGGAATTTATTCAAACTAATTCTCCTAAAGTTACTATT
GATTGTTCTTTATTTGTCTGTTCTAATTATGCAGCTTGCCATGACTTATTGTCAGAGTATGGCACTTTTTGTGATAATA
TTAATAGTATTTTAGATGAAGTTAATGGTTTACTTGATACTACTCAATTGCATGTAGCTGATACTCTTATGCAAGGTGT
CACACTTAGCTCCAATCTTAATACTAATTTGCATTTTGATGTTGATAATATTAATTTTAAATCCCTAGTTGGATGTTTA
GGTCCACACTGCGGTTCTTCTTCTCGTTCTTTTTTTGAAGATTTATTGTTTGACAAAGTTAAACTTTCAGATGTTGGTT
TTGTTGAAGCTTATAACAATTGTACTGGTGGTAGTGAAATTAGAGATCTTCTTTGTGTACAATCCTTTAATGGTATTAA
AGTTTTGCCTCCTATTTTGTCTGAATCTCAAATTTCTGGTTACACCACAGCCGCTACTGTTGCTGCTATGTTTCCACCA
TGGTCAGCAGCAGCTGGCATACCATTTTCTCTTAATGTACAATATAGAATTAATGGTTTGGGTGTTACTATGGATGTTC
TTAATAAAAATCAAAAGTTGATAGCTACTGCTTTTAATAATGCTCTTCTTTCTATTCAGAATGGTTTTAGTGCTACCAA
CTCTGCACTTGCTAAAATACAAAGTGTTGTTAATTCTAATGCTCAAGCACTTAATAGTTTGTTACAGCAATTATTTAAT
AAATTTGGTGCAATTAGTTCTTCTTTACAAGAAATTTTATCTCGTCTCGATGCTTTAGAGGCTCAGGTTCAGATTGATA
GGCTTATTAATGGTCGTTTAACTGCTTTAAATGCTTATGTCTCTCAACAACTTAGTGATATTTCTCTTGTAAAATTTGG
TGCTGCTTTAGCTATGGAGAAGGTTAATGAGTGTGTTAAAAGTCAATCTCCTCGTATTAATTTTTGTGGTAATGGTAAT
CATATTTTGTCATTAGTTCAAAATGCTCCTTATGGTTTGTTGTTTATGCATTTTAGTTATAAACCTATTTCTTTTAAAA
CTGTTTTAGTAAGTCCTGGTTTGTGTATATCAGGTGATGTAGGTATTGCACCTAAACAAGGGTATTTTATTAAACACAA
TGATCATTGGATGTTCACTGGTAGTTCTTACTATTATCCTGAACCAATTTCAGATAAAAATGTTGTTTTTATGAATACT
TGTTCTGTTAATTTTACTAAAGCGCCTCTTGTTTATTTGAATCATTCTGTACCAAAATTGTCTGATTTTGAATCTGAGT
TATCTCATTGGTTTAAAAATCAAACATCCATTGCGCCTAATTTGACTTTAAATCTTCATACTATTAATGCTACTTTTTT
AGATTTGTATTATGAGATGAATCTTATTCAAGAGTCTATTAAGTCTTTGAATAATAGTTATATCAATCTTAAAGATATA
GGTACATATGAAATGTATGTAAAATGGCCTTGGTATGTTTGGCTACTAATTTCTTTTTCATTTATAATATTCCTTGTAT
TGCTCTTTTTTATATGTTGTTGTACTGGTTGTGGTTCTGCATGTTTTAGTAAATGTCATAATTGTTGTGATGAGTATGG
TGGTCATCATGATTTTGTTATCAAAACATCTCATGATGATTAG

>gi|85667876|ref|NC_006577.2| Human coronavirus HKU1, complete genome

ATGTTATTAATTATTTTTATTTTGCCTACAACATTAGCTGTTATAGGTGATTTTAATTGTACTAATTTTGCTATT
AATGATTTAAACACCACAGTTCCTCGCATAAGTGAGTATGTTGTGGATGTTTCTTATGGTTTGGGTACATATTATATAC
TTGATCGTGTTTATTTAAATACTACTATATTATTTACTGGTTATTTCCCTAAATCTGGTGCCAATTTTAGGGATCTATC
TTTAAAAGGTACTACATATTTGAGTACTCTTTGGTATCAGAAACCCTTTTTATCTGATTTTAATAATGGTATTTTTTCT
AGAGTTAAGAATACTAAGTTGTATGTTAATAAAACTTTGTATAGTGAGTTTAGTACTATAGTTATAGGTAGTGTTTTTA
TTAACAACTCTTATACTATTGTTGTTCAACCTCATAATGGTGTTTTGGAGATTACAGCTTGTCAATACACTATGTGTGA
GTATCCTCATACTATTTGTAAATCTAAAGGTAGTTCTCGTAATGAATCTTGGCATTTTGATAAATCTGAACCTTTGTGT
CTGTTCAAGAAAAATTTTACTTATAATGTTTCTACAGATTGGTTGTATTTTCATTTTTATCAAGAACGTGGCACTTTTT
ATGCTTATTATGCTGATTCTGGCATGCCTACTACTTTTTTATTTAGTTTGTATCTTGGTACTCTTTTATCTCATTATTA
TGTTTTGCCTTTGACTTGTAATGCTATATCTTCTAATACTGATAATGAGACTTTACAATATTGGGTCACACCTTTGTCT
AAACGCCAATATCTTCTTAAATTTGACAACCGTGGTGTTATTACTAATGCTGTTGATTGTTCTAGTAGTTTCTTTAGCG
AGATTCAATGTAAAACTAAATCTTTATTACCTAATACTGGTGTTTATGACTTATCTGGTTTTACTGTTAAGCCTGTTGC
AACTGTACATCGTCGTATTCCTGATTTACCTGATTGTGACATTGATAAATGGCTTAACAATTTTAATGTACCCTCACCT
CTTAATTGGGAACGTAAAATTTTTTCTAATTGCAACTTTAATTTGAGTACTTTGCTTCGTTTAGTTCATACTGATTCTT
TTTCTTGTAATAATTTTGATGAATCTAAGATATATGGTAGTTGTTTTAAGAGTATTGTTTTAGATAAATTTGCCATACC
CAACTCCAGACGATCTGATTTGCAGTTGGGCAGTTCTGGTTTTCTGCAATCTTCTAATTATAAAATTGACACTACTTCT
AGTTCTTGTCAATTGTATTATAGTTTGCCTGCAATTAATGTTACTATTAATAATTATAATCCTTCTTCTTGGAATAGAA
GGTATGGTTTTAATAATTTTAATTTGAGCTCTCATAGTGTTGTTTACTCACGTTATTGTTTTTCTGTTAATAATACTTT
TTGTCCTTGTGCTAAACCTTCTTTTGCTTCAAGTTGCAAGAGTCATAAACCACCTTCTGCTTCCTGTCCTATTGGTACT
AATTATCGTTCTTGTGAGAGTACTACTGTACTCGACCACACTGACTGGTGTAGGTGTTCTTGTTTACCTGATCCTATAA
CTGCTTATGACCCTAGGTCTTGTTCTCAAAAAAAGTCTCTGGTTGGTGTTGGTGAACATTGTGCAGGGTTCGGTGTTGA
TGAAGAAAAGTGTGGTGTATTGGATGGATCATATAATGTTTCTTGTCTTTGTAGTACTGATGCCTTTCTAGGTTGGTCT
TATGACACTTGCGTCAGTAACAACCGTTGTAATATTTTTTCTAATTTTATTTTAAATGGTATCAATAGTGGTACCACTT
GTTCTAATGATTTATTGCAGCCTAATACTGAAGTTTTTACTGATGTTTGTGTTGATTACGACCTTTATGGTATTACAGG
ACAAGGTATTTTTAAAGAAGTTTCTGCTGTTTATTATAATAGTTGGCAAAATCTTTTGTATGATTCTAATGGCAACATT
ATTGGTTTTAAAGATTTTGTTACTAATAAAACATATAATATTTTCCCTTGTTATGCAGGAAGAGTTTCTGCTGCTTTTC
ATCAAAATGCTTCCTCTTTGGCTTTACTTTATCGTAATTTAAAATGTAGCTATGTTTTGAATAATATTTCTTTAACTAC
TCAGCCATATTTTGATAGTTATCTTGGTTGCGTTTTTAATGCTGATAATTTAACTGATTATTCTGTTTCTTCTTGTGCT
CTTCGCATGGGTAGTGGTTTTTGTGTTGATTATAACTCACCTTCTTCTTCCTCTTCGCGTCGTAAACGTAGAAGTATTT
CTGCTTCTTATCGTTTTGTTACTTTTGAACCCTTTAATGTCAGTTTTGTTAATGACAGTATTGAGTCTGTGGGTGGTCT
TTATGAGATCAAAATTCCCACTAACTTTACTATAGTTGGTCAAGAGGAATTTATTCAAACTAATTCTCCTAAAGTTACT
ATTGATTGTTCTTTATTTGTCTGTTCTAATTATGCAGCTTGCCATGACTTATTGTCAGAGTATGGCACTTTTTGTGATA
ATATTAATAGTATTTTAGATGAAGTTAATGGTTTACTTGATACTACTCAATTGCATGTAGCTGATACTCTTATGCAAGG
TGTCACACTTAGCTCCAATCTTAATACTAATTTGCATTTTGATGTTGATAATATTAATTTTAAATCCCTAGTTGGATGT
TTAGGTCCACACTGCGGTTCTTCTTCTCGTTCTTTTTTTGAAGATTTATTGTTTGACAAAGTTAAACTTTCAGATGTTG
GTTTTGTTGAAGCTTATAACAATTGTACTGGTGGTAGTGAAATTAGAGATCTTCTTTGTGTACAATCCTTTAATGGTAT
TAAAGTTTTGCCTCCTATTTTGTCTGAATCTCAAATTTCTGGTTACACCACAGCCGCTACTGTTGCTGCTATGTTTCCA
CCATGGTCAGCAGCAGCTGGCATACCATTTTCTCTTAATGTACAATATAGAATTAATGGTTTGGGTGTTACTATGGATG
TTCTTAATAAAAATCAAAAGTTGATAGCTACTGCTTTTAATAATGCTCTTCTTTCTATTCAGAATGGTTTTAGTGCTAC
CAACTCTGCACTTGCTAAAATACAAAGTGTTGTTAATTCTAATGCTCAAGCACTTAATAGTTTGTTACAGCAATTATTT
AATAAATTTGGTGCAATTAGTTCTTCTTTACAAGAAATTTTATCTCGTCTCGATGCTTTAGAGGCTCAGGTTCAGATTG

ATAGGCTTATTAATGGTCGTTTAACTGCTTTAAATGCTTATGTCTCTCAACAGCTTAGTGATATTTCTCTTGTAAAATT
TGGTGCTGCTTTAGCTATGGAGAAGGTTAATGAGTGTGTTAAAAGTCAATCTCCTCGTATTAATTTTTGTGGTAATGGT
AATCATATTTTGTCATTAGTTCAAAATGCTCCTTATGGTTTGTTGTTTATGCATTTTAGTTATAAACCTATTTCTTTTA
AAACTGTTTTAGTAAGTCCTGGTTTGTGTATATCAGGTGATGTAGGTATTGCACCTAAACAAGGGTATTTTATTAAACA
TAATGATCATTGGATGTTCACTGGTAGTTCTTACTATTATCCTGAACCAATTTCAGATAAAAATGTTGTTTTTATGAAT
ACTTGTTCTGTTAATTTTACTAAAGCGCCTCTTGTTTATTTGAATCATTCTGTACCAAAATTGTCTGATTTTGAATCTG
AGTTATCTCATTGGTTTAAAAATCAAACATCCATTGCGCCTAATTTGACTTTAAATCTTCATACTATTAATGCTACTTT
TTTAGATTTGTATTATGAGATGAATCTTATTCAAGAGTCTATTAAGTCTTTGAATAATAGTTATATCAATCTTAAAGAT
ATAGGTACATATGAAATGTATGTAAAATGGCCTTGGTATGTTTGGCTACTAATTTCTTTTTCATTTATAATATTCCTTG
TATTGCTCTTTTTTATATGTTGTTGTACTGGTTGTGGTTCTGCATGTTTTAGTAAATGTCATAATTGTTGTGATGAGTA
TGGTGGTCATCATGATTTTGTTATCAAAACATCTCATGATGATTAG

>gi|530802159|gb|KF530060.1| Human coronavirus OC43 strain OC43/human/USA/851-15/1985, complete genome

ATGTTTTTGATACTTTTAATTTCCTTACCAATGGCTTTTGCTGTTATAGGAGATTTAAAGTGTACTTCAGATACG
AGTTATATTAATGATGTTGACACCGGTGTTCCTCCTATAAGTACTGATACTGTTGATGTTACTAATGGTTTGGGTACTT
ATTATGTTTTAGATCGTGTGTATTTAAATACTACTTTGTTTCTTAATGGTTACTATCCTACTTCAGGTTCCACATATCG
TAATATGGCACTGAAGGGAAGTGTACTGTTGAGCACACTATGGTTTAAACCACCATTTCTTTCTGATTTTATTAATGGT
ATTTTTGCTAAGGTCAAAAATACCAAGGTTATTAAAGATGATGTACTGTACAGTGAGTTCCCTGCTATAACTATAGGTA
GTACTTTTGTAAATACATCCTATAGTGTGGTAGTACAACCACGTACAATCAATTTAGATAATAAATTACAAGGTCTTTT
AGAGATCTCTGTTTGCCAGTATAATATGTGCGAGTACCCACATACGATTTGTCATCCTAACTTGGGTAATCATCGCACA
GAACTATGGCATTTGGATACAGGTGTTGTTTCCTGTTTATATAAGCGTAATTTCACATATGATGTGAATGCTGATTATT
TGTATTTTCATTTTTATCAAGAAGGTGGTACTTTTTATGCATATTTTACAGACACTGGTGTTGTTACTAAGTTTTTGTT
TAATGTTTATTTAGGCATGGCGCTTTCACACTATTATGTCATGCCTCTGACTTGTAATAGTAAGCGTAAGGATGGTTTT
ACTTTAGAATATTGGGTTACACCTCTCACTTCTAGACAATATTTACTCGCTTTCAATCAAGATGGTATTATTTTTAATG
CTGTTGATTGCATGAGTGATTTTATGAGTGAGATTAAGTGTAAAACACAATCTATAGCACCACCTACTGGTGTTTATGA
ATTAAACGGTTACACTGTTCAGCCAATCGCAGATGTTTACCGACGTAAACCTAATCTTCCCAATTGTAATATAGAAGCT
TGGCTTAATGATAAGTCGGTGCCATCTCCATTAAATTGGGAACGTAAGACATTTTCAAATTGTAATTTTAATATGAGCA
GCCTGATGTCCTTTATTCAGGCAGACTCATTTACTTGTAATAATATTGATGCTGCTAAGATATATGGTATGTGTTTTTC
CAGCATAACTATAGATAAGTTTGCTATACCCAATGGCAGGAAGGTTGACCTACAATTGGGTAATTTGGGCTATTTGCAG
TCATTTAACTATAGAATTGATACTACTGCAACAAGTTGTCAGTTGTATTATAATTTACCTGCTGCTAATGTTTCTGTTA
GCAGGTTTAATCCTTCTACTTGGAATAAGAGATTCGGTTTTATAGAAGATTCTGTTTTTAAGCCTCAACCTGCAGGTGT
TTTTACCAATCATGATGTAGTTTATGCCCAACATTGTTTCAAAGCTCCCAAAAATTTCTGTCCGTGTAAATTGAATGGT
TCTCTGTGTGTAGGTAGTGGTCCTGGTAAAAATAATGGTATAGGCACTTGTCCTGCAGGTACTAATTATTTAACTTGTC
ATAATTTGTGCAATCCTGATCCTATTACATTTACAGGTCCTTATAAGTGCCCCCAAACTAAATCTTTAGTTGGTATAGG
TGAGCACTGTTCAGGTCTTGCTGTTAAAAGTGATTATTGTGGAGGCAATCCTTGCACTTGCCAACCACAAGCATTTTTG
GGTTGGTCTGCAGACTCTTGTTTACAAGGGGACAAGTGTAATATTTTTGCTAATTTAATTTTGCATGATGTTAATAGTG
GTCTTACTTGTTCTACTGATTTACAAAAAGCTAACACAGACATAATTCTTGGTGTTTGTGTTAATTATGACCTTTATGG
TATTTCAGGCCAAGGCATTTTTGTTGAGGTTAATGCGACTTATTATAATAGTTGGCAGAACCTTTTATATGATTCTAAT
GGTAATCTCTATGGTTTTAGAGACTACATAACAAACAGAACTTTTATGATTCGTAGTTGCTATAGCGGTCGTGTTTCTG
CGGCCTTTCACGCTAACTCTTCCGAACCAGCATTGCTATTTCGGAATATTAAATGCAACTACGTTTTTAATAATAGTCT
TACACGACAGCTGCAACCCATTAACTATTTTGATAGTTATCTTGGTTGTGTTGTCAATGCTTATAATAGTACTGCTATT

TCTGTTCAAACATGTGATCTCACAGTAGGTAGTGGTTACTGTGTGGATTACTCTAAAAACAGACGAAGTCGTAGAGCGA
TTACCACTGGTTATCGGTTTACTAATTTTGAGCCATTTACTGTTAATTCAGTAAATGATAGTTTAGAACCTGTAGGTGG
TTTGTACGAAATTCAAATACCTTCAGAGTTTACTATAGGTAATATGGAGGAGTTTATTCAAACAAGCTCTCCTAAAGTT
ACTATTGATTGTGCTGCATTTGTCTGTGGTGATTATGCAGCATGTAAATCACAGTTGGTTGAATATGGTAGTTTCTGTG
ATAACATTAATGCCATACTCACAGAAGTAAATGAACTACTTGACACTACACAGTTGCAAGTAGCTAATAGTTTAATGAA
TGGTGTTACTCTTAGCACTAAGCTTAAAGATGGCGTTAATTTCAATGTAGACGACATCAATTTTTCCCCTGTATTAGGT
TGTCTAGGCAGCGAATGTAGTAAAGCTTCCAGTAGATCTGCTATAGAGGATTTACTTTTTGATAAAGTAAAGTTATCTG
ATGTCGGTTTCGTTGAGGCTTATAATAATTGTACTGGAGGTGCCGAAATTAGGGACCTCATTTGTGTGCAAAGTTATAA
AGGTATCAAAGTGTTGCCTCCACTGCTCTCAGAAAATCAGATCAGTGGATACACTTTGGCTGCCACTTCTGCTAGTCTG
TTTCCTCCTTGGACAGCAGCAGCAGGTGTACCATTTTATTTAAATGTTCAGTATCGCATTAATGGGCTTGGTGTCACCA
TGGATGTGCTAAGTCAAAATCAAAAGCTTATTGCTAATGCATTTAACAATGCCCTTGATGCTATTCAGGAAGGGTTCGA
TGCAACTAATTCTGCTTTAGTTAAAATTCAAGCTGTTGTTAATGCAAATGCTGAAGCTCTTAATAACTTATTGCAACAA
CTCTCTAATAGATTTGGTGCTATAAGTTCTTCTTTACAAGAAATTCTATCTAGACTTGATGCTCTTGAAGCGGAAGCTC
AGATAGATAGACTTATTAATGGTCGTCTTACCGCTCTTAATGCTTATGTTTCTCAACAGCTTAGTGATTCTACACTGGT
AAAATTTAGTGCAGCACAAGCTATGGAGAAGGTTAATGAATGTGTCAAAAGCCAATCATCTAGGATAAATTTCTGTGGT
AATGGTAACCATATTATATCATTAGTGCAGAATGCTCCATATGGTTTGTATTTTATCCACTTTAGTTATGTCCCTACTA
AGTATGTCACAGCGAAGGTTAGTCCTGGTCTGTGCATTGCTGGTGATAGAGGTATAGCTCCTAAGAGTGGTTATTTTGT
TAATGTAAATAATACTTGGATGTACACTGGTAGTGGTTACTACTACCCTGAACCTATAACTGAAAATAATGTCGTTGTT
ATGAGTACCTGCGCTGTTAATTATACTAAAGCGCCGTATGTAATGCTGAACACTTCAACACCCAACCTTCCTGATTTTA
GGGAAGAGTTGGATCAATGGTTTAAAAATCAAACATCAGTGGCACCAGATCTGTCACTTGATTATATAAATGTTACATT
CTTGGACCTACAAGTTGAAATGAATAGGTTACAGGAGGCAATAAAAGTCTTAAATCAGAGCTACATCAATCTCAAGTAC
ATTGGTACATATGAATATTATGTAAAATGGCCTTGGTATGTATGGCTTTTAATCGGCCTTGCTGGTGTAGCTATGCTTG
TTTTACTATTCTTCATATGCTGTTGTACAGGATGTGGGACTAGTTGTTTTAAGAAATGTGGTGGTTGTTGTGATGATTA
TACTGGATACCAGGAGTTAGTAATCAAAACTTCACATGACGACTAA

>gi|530802170|gb|KF530061.1| Human coronavirus OC43 strain OC43/human/USA/901-43/1990, complete genome

ATGTTTTTGATACTTTTAATTTCCTTACCAATGGCTTTTGCTGTTATAGGAGATTTAAAGTGTACTTCAGATACG
AGTTATATTAATGATGTTGACACCGGTGTTCCTCCTATAAGTACTGATACTGTTGATGTTACTAATGGTTTGGGTACTT
ATTATGTTTTAGATCGTGTGTATTTAAATACTACTTTGTTTCTTAATGGTTACTATCCTACTTCAGGTTCCACATATCG
TAATATGGCACTGAAGGGAAGTGTACTGTTGAGCACACTATGGTTTAAACCACCATTTCTTTCTGATTTTATTAATGGT
ATTTTTGCTAAGGTCAAAAATACCAAGGTTATTAAAGATGATGTACTGTACAGTGAGTTCCCTGCTATAACTATAGGTA
GTACTTTTGTAAATACATCCTATAGTGTGGTAGTACAACCACGTACAATCAATTTAGATAATAAATTACAAGGTCTTTT
AGAGATCTCTGTTTGCCAGTATAATATGTGCGAGTACCCACATACGATTTGTCATCCTAACTTGGGTAATCATCGCACA
GAACTATGGCATTTGGATACAGGTGTTGTTTCCTGTTTATATAAGCGTAATTTCACATATGATGTGAATGCTGATTATT
TGTATTTTCATTTTTATCAAGAAGGTGGTACTTTTTATGCATATTTTACAGACACTGGTGTTGTTACTAAGTTTTTGTT
TAATGTTTATTTAGGCATGGCGCTTTCACACTATTATGTCATGCCTCTGACTTGTAATAGTAAGCGTAAGGATGGTTTT
ACTTTAGAATATTGGGTTACACCTCTCACTTCTAGACAATATTTACTCGCTTTCAATCAAGATGGTATTATTTTTAATG
CTGTTGATTGCATGAGTGATTTTATGAGTGAGATTAAGTGTAAAACACAATCTATAGCACCACCTACTGGTGTTTATGA
ATTAAACGGTTACACTGTTCAGCCAATTGCAGATGTTTACCGACGTAAACCTAATCTTCCCAATTGCAATATAGAAGCT
TGGCTTAATGATAAGTCGGTGCCATCTCCATTAAATTGGGAACGTAAGACATTTTCAAATTGTAATTTTAATATGAGCA
GCCTGATGTCCTTTATTCAGGCAGACTCATTTACTTGTAATAATATTGATGCTGCTAAGATATATGGTATGTGTTTTTC

CAGCATAACTATAGATAAGTTTGCTATACCCAATGGCAGGAAGGTTGACCTACAATTGGGTAATTTGGGCTATTTGCAG
TCATTTAACTATAGAATTGATACTACTGCAACAAGTTGTCAGTTGTATTATAATTTACCTGCTGCTAATGTTTCTGTTA
GCAGGTTTAATCCTTCTACTTGGAATAAGAGATTCGGTTTTATAGAAAATTCTGTTTTTAAGCCTCAACCTGCAGGTGT
TTTTACCAATCATGATGTAGTTTATGCCCAACATTGTTTTAAAGCTCCCAAAAATTTCTGTCCGTGTAAATTGAATGGT
TCTTTGTGTGTAGGTAGTGGTCCTGGTAAAAATAATGGTATAGGCACTTGCCCTGCAGGTACTAATTATTTAACTTGTC
ATAATTTGTGCAATCCTGATCCTATTAGATTTACAGGTCCTTATAAGTGCCCCCAAACTAAATCTTTAGTTGGTATAGG
TGAGCACTGTTCAGGTCTTGCTGTTAAAAGTGATTATTGTGGAGGCAATCCTTGCACTTGCCAACCACAAGCATTTTTG
GGTTGGTCTGCAGACTCTTGTTTACAAGGGGACAAGTGTAATATTTTTGCTAATTTAATTTTGCATGATGTTAATAGTG
GTCTTACTTGTTCTACTGATTTACAAAAAGCTAACACAGACATAATTCTTGGTGTTTGTGTTAATTATGACCTTTATGG
TATTTCAGGCCAGGGCATTTTTGTTGAGGTTAATGCGACTTATTATAATAGTTGGCAGAACCTTTTATATGATTCTAAT
GGTAATCTCTATGGTTTTAGAGACTACATAACAAACAGAACTTTTATGATTCGTAGTTGCTATAGCGGTCGTGTTTCTG
CGGCCTTTCACGCTAACTCTTCCGAACCAGCATTGCTATTTCGGAATATTAAATGCAACTACGTTTTTAATAATAGTCT
TACACGACAGCTGCAACCCATTAACTATTTTGATAGTTATCTTGGTTGTGTTGTCAATGCTTATAATAGTACTGCTATT
TCTGTTCAAACATGTGATCTCACAGTAGGTAGTGGTTACTGTGTGGATTACTCTAAAAACAGACGAAGTCGTAGAGCGA
TTACCACTGGTTATCGGTTTACTAATTTTGAGCCATTTACTGTTAATTCAGTAAATGATAGTTTAGAACCTGTAGGTGG
TTTGTACGAAATTCAAATACCTTCAGAGTTTACTATAGGTAATATGGAGGAGTTTATTCAAACAAGCTCTCCTAAAGTT
ACTATTGATTGTGCTGCATTTGTCTGTGGTGATTATGCAGCATGTAAATCACAGTTGGTTGAATATGGTAGTTTCTGTG
ATAACATTAATGCCATACTCACAGAAGTAAATGAACTACTTGACACTACACAGTTGCAAGTAGCTAATAGTTTAATGAA
TGGTGTTACTCTTAGCACTAAGCTTAAAGATGGCGTTAATTTCAATGTAGACGACATCAATTTTTCCCCTGTATTAGGT
TGTCTAGGCAGCGAATGTAGTAAAGCTTCCAGTAGATCTGCTATAGAGGATTTACTTTTTGATAAAGTAAAGTTATCTG
ATGTCGGTTTCGTTGAGGCTTATAATAATTGTACTGGAGGTGCCGAAATTAGGGACCTCATTTGTGTGCAAAGTTATAA
AGGTATCAAAGTGTTGCCTCCACTGCTCTCAGAAAATCAGATCAGTGGATACACTTTGGCTGCCACTTCTGCTAGTCTG
TTTCCTCCTTGGACAGCAGCAGCAGGTGTACCATTTTATTTAAATGTTCAGTATCGCATTAATGGGCTTGGTGTCACCA
TGGATGTGCTAAGTCAAAATCAAAAGCTTATTGCTAATGCATTTAACAATGCCCTTGATGCTATTCAGGAAGGGTTCGA
TGCAACTAATTCTGCTTTAGTTAAAATTCAAGCTGTTGTTAATGCAAATGCTGAAGCTCTTAATAACTTATTGCAACAA
CTCTCTAATAGATTTGGTGCTATAAGTTCTTCTCTACAAGAAATTCTATCTAGACTTGATGCTCTTGAAGCGGAAGCTC
AGATAGATAGACTTATTAATGGTCGTCTTACCGCTCTTAATGCTTATGTTTCTCAACAGCTTAGTGATTCTACACTGGT
AAAATTTAGTGCAGCACAAGCTATGGAGAAGGTTAATGAATGTGTCAAAAGCCAATCATCTAGGATAAATTTCTGTGGT
AATGGTAACCATATTATATCATTAGTGCAGAATGCTCCATATGGTTTGTATTTTATCCACTTTAGTTATGTCCCTACTA
AGTATGTCACAGCGAAGGTTAGTCCTGGTCTGTGCATTGCTGGTGATAGAGGTATAGCTCCTAAGAGTGGTTATTTTGT
TAATGTAAATAATACTTGGATGTACACTGGTAGTGGTTACTACTACCCTGAACCTATAACTGAAAATAATGTCGTTGTT
ATGAGTACCTGCGCTGTTAATTATACTAAAGCGCCGTATGTAATGCTGAACACTTCAACACCCAACCTTCCTGATTTTA
GGGAAGAGTTGGATCAATGGTTTAAAAATCAAACATCAGTGGCACCAGATCTGTCACTTGATTATATAAATGTTACATT
CTTGGACCTACAAGTTGAAATGAATAGGTTACAGGAGGCAATAAAAGTTTTAAATCAGAGCTACATCAATCTCAAGGAC
ATTGGTACATATGAATATTATGTAAAATGGCCTTGGTATGTATGGCTTTTAATCGGCCTTGCTGGTGTAGCTATGCTTG
TTTTACTATTCTTCATATGCTGTTGTACAGGATGTGGGACTAGTTGTTTTAAGAAATGTGGTGGTTGTTGTGATGATTA
TACTGGATACCAGGAGTTAGTAATCAAAACTTCACATGACGACTAA

>gi|530802191|gb|KF530063.1| Human coronavirus OC43 strain OC43/human/USA/9612-48/1996, complete genome

ATGTTTTTGATACTTTTAATTTCCTTACCAACGGCTTTTGCTGTTATAGGAGATTTAAAGTGTCCTTTAGATACT
AGTCGTAAAGGTAGCTTTAATAATAAAGACACCGGTCCTCCTTTTATAAGTACTGATACTGTTGATGTTACTAATGGTT

TGGGTACTTATTATGTTTTAGATCGTGTGTATTTAAATACTACGTTGTTTCTTAATGGTTATTACCCTACTTCAGGTTC
CACATATCGTAATATGGCACTGAAGGGAACTGACAAATTGAGCACACTATGGTTTAAACCACCATTTCTTTCTGATTTT
ATTAATGGTATTTTTGCTAAGGTCAAAAATACCAAGGTTTTTAAAGATGGTGTAATGTATAGTGAGTTCCCTGCTATAA
CTATAGGTAGTACTTTTGTAAATACATCCTATAGTGTGGTAGTACAACCACGTACAATTAATTCAACACAGGATGGTGT
TAATAAATTACAAGGTCTTTTAGAGGTCTCTGTTTGCCAGTATAATATGTGCGAGTACCCACATACGATTTGTCATCCT
AAACTGGGTAATCACTTCAAAGAACTATGGCATTTGGATACAGGTGTTGTTTCCTGTTTATATAAGCGTAATTTCACAT
ATGATGTGAATGCTACTTATTTGTATTTTCATTTTTATCAAGAAGGTGGTACTTTTTATGCATATTTTACAGACACTGG
TGTTGTTACTAAGTTTTTGTTTAATGTTTATTTAGGCATGGCACTTTCACACTATTATGTCATGCCTCTGACTTGTATT
AGTAGGCGTGATATTGGTTTTACTTTAGAATATTGGGTTACACCCCTCACTTCTAGACAATATTTACTCGCTTTCAATC
AAGATGGTATTATTTTTAATGCTGTTGATTGTATGAGTGATTTTATGAGTGAGATTAAGTGTAAAACACAATCTATAGC
ACCACCTACTGGTGTTTATGAATTAAACGGTTACACTGTTCAGCCAATCGCAGATGTTTACCGACGCAAACCTGATCTT
CCCAATTGCAATATAGAAGCTTGGCTTAATGATAAGTCGGTGCCCTCTCCATTAAATTGGGAACGTAAGACATTTTCAA
ATTGTAATTTTAATATGAGCAGCCTGATGTCTTTTATTCAGGCAGACTCATTTACTTGTAATAATATTGATGCTGCTAA
GATATATGGTATGTGTTTTTCCAGCATAACTATAGACAAGTTTGCTATACCCAATGGCAGGAAGGTTGACCTACAATTG
GGCAATTTGGGCTATTTGCAGTCATCCAACTATAGAATTGATACTACTGCAACAAGTTGTCAGTTGTATTATAATTTAC
CTGCTGCTAATGTCTCTGTTAGCAGGTTTAATCCTTCTACTTGGAATAAGAGATTTGGTTTTATAGAAGATTCTGTTTT
TGTGCCTCAACCTACAGGTGTTTTTACTAATCACAGTGTAGTTTATGCACAACACTGTTTCAAAGCCCCTAAAAATTTC
TGTCCGTGTAAATTGAATGGTTCGTGTCCTGATAAAAATAATGGTATAGGCACTTGTCCTGCAGGTACTAATTATTTAA
CTTGTGATAATTTGTGTACTCTTGATCCTATTACATTTAAAGCTCCAGGTACTTATAAGTGCCCCCAAACTAAATCTTT
AGTTGGCATAGGTGAGCACTGTTCGGGTCTTGCTGTTAAAAGTGATTATTGTAGAGGCAATTCTTGTATTTGCCAACCA
CAAGCATTTTTGGGTTGGTCTGCAGACTCTTGTTTACAAGGAGACAAGTGTAATATTTTTGCTAATCTTATTTTGCATG
ATGTTAATAGTGGTCTTACTTGTTCTACTGATTTACAAAAAGCTAACACAGACATAATTCTTGGTGTTTGTGTTAATTA
TGACCTCTATGGTATTTCAGGCCAAGGCATTTTTGTTGAGGTTAATGCGACTTATTATAATAGTTGGCAGAACCTTTTA
TATGATTCTAATGGTAATCTCTACGGTTTTAGAGACTACATAACAAACAGAACTTTTATGATTCGTAGTTGCTATAGCG
GTCGTGTTTCTGCGGCCTTTCATGCTAACTCTTCCGAACCAGCATTGCTATTTCGGAATATTAAATGCAACTACGTTTT
TAATAATAGTCTTACACGACAGCTGCAACCCATTAACTATTCGTTTGATAGTTATCTTGGTTGTGTTGTCAATGCTTAT
AATAGTACTGCTATTTCTGTTCAAACATGTGATCTCACAGTAGGTAGTGGTTACTGTGTGGAGTACTCTAAAAACAGAC
GAAGTCGTAGAGCGATTACCACTGGTTATCGGTTTACTAATTTTGAGCCATTTACTGTTAATTCAGTAAATGATAGTTT
GGAACCTGTAGGTGGTTTGTATGAAATTCAAATACCTTCAGAGTTTACTATAGGTAATATGGAGGAGTTTATTCAAACA
AGCTCTCCCAAAGTTACTATTGATTGTGCTGCATTTGTCTGTGGTGATTATGCAGCATGTAAATTACAGTTGGTTGAAT
ATGGTAGTTTCTGTGATAACATTAATGCCATACTCACAGAAGTAAATGAACTACTTGATACCACACAGTTGCAAGTAGC
TAATAGTTTAATGAATGGTGTTACTCTTAGCACTAAGCTTAAAGATGGTGTTAATTTCAATGTAGACGACATCAATTTC
TCCCCTGTATTAGGTTGTCTAGGCAGCGAATGTAGTAAAGCTTCCAGTAGATCTGCTATAGAGGATTTACTTTTTGATA
AAGTAAAGTTATCTGATGTCGGTTTCGTTGAGGCTTATAATAATTGTACAGGAGGTGCCGAAATTAGGGACCTCATTTG
TGTGCAAAGTTATAAAGGCATCAAAGTGTTGCCCCCACTGCTCTCAGAAAATCAGATCAGTGGATACACTTTGGCTGCC
ACCTCTGCTAGTCTATTTCCTCCTTGGACAGCAGCAGCAGGTGTACCATTTTATTTAAATGTTCAGTATCGCATTAATG
GGCTTGGTGTCACCATGGATGTGCTAAGTCAAAATCAAAAGCTTATTGCTAATGCATTTAACAATGCCCTTTATGCTAT
TCAGCAAGGGTTCGATGCAACTAATTCTGCTTTAGTTAAAATTCAAGCTGTTGTTAATGCAAATGCTGAAGCTCTTAAT
AACTTATTGCAACAACTCTCTAATAGATTTGGTGCTATAAGTGCTTCTTTACAAGAAATTCTATCTAGACTTGATGCTC
TTGAAGCGGAAGCTCAGATAGATAGACTTATTAATGGTCGTCTCACCGCTCTTAATGCTTATGTTTCTCAACAGCTTAG
TGATTCTACACTGGTAAAATTTAGTGCAGCACAAGCTATGGAGAAGGTTAATGAATGTGTCAAAAGCCAATCATCTAGG
ATAAATTTCTGTGGTAATGGTAATCATATTATATCATTAGTGCAGAATGCTCCATATGGTTTGTATTTTATCCACTTTA
ATTATGTCCCTACTAAGTATGTCACAGCGAAGGTTAGTCCTGGTCTGTGCATTGCTGGTAATAGAGGTATAGCTCCTAA
GAGTGGTTATTTTGTTAATGTAAATAATACTTGGATGTACACTGGTAGTGGTTACTACTACCCTGAACCTATAACTGAA

AATAATGTTGTTGTTATGAGTACCTGCGCTGTTAATTATACTAAAGCGCCGTATGTAATGCTGAACACTTCAATACCCA
ACCTTCCTGATTTTAAGGAAGAGTTGGATCAATGGTTTAAAAATCAAACATCAGTGGCACCAGATTTGTCACTTGATTA
TATAAATGTTACATTCTTGGACCTACAAGTTGAAATGAATAGGTTACAGGAGGCAATAAAAGTCTTAAATCATAGCTAC
ATCAATCTCAAGGACATTGGTACATATGAATATTATGTAAAATGGCCTTGGTATGTATGGCTTTTAATCTGCCTTGCTG
GTGTAGCTATGCTTGTTTTACTATTCTTCATATGCTGTTGTACAGGATGTGGGACTAGTTGTTTTAAGAAATGTGGTGG
TTGTTGTGATGATTATACTGGATACCAGGAGTTAGTAATCAAAACTTCACATGACGACTAA

>gi|530802202|gb|KF530064.1| Human coronavirus OC43 strain OC43/human/USA/9612-9/1996, complete genome

ATGTTTTTGATACTTTTAATTTCCTTACCAATGGCTTTTGCTGTTATAGGAGATTTAAAGTGTACTTCAGATACG
AGTTATATTAATGATGTTGACACCGGTGTTCCTCCTATAAGTACTGATACTGTTGATGTTACTAATGGTTTGGGTACTT
ATTATGTTTTAGATCGTGTGTATTTAAATACTACTTTGTTTCTTAATGGTTACTATCCTACTTCAGGTTCCACATATCG
TAATATGGCACTGAAGGGAAGTGTACTGTTGAGCACACTATGGTTTAAACCACCATTTCTTTCTGATTTTATTAATGGT
ATTTTTGCTAAGGTCAAAAATACCAAGGTTATTAAAGATGATGTACTGTACAGTGAGTTCCCTGCTATAACTATAGGTA
GTACTTTTGTAAATACATCCTATAGTGTGGTAGTACAACCACGTACAATCAATTTAGATAATAAATTACAAGGTCTTTT
AGAGATCTCTGTTTGCCAGTATAATATGTGCGAGTACCCACATACGATTTGTCATCCTAATTTGGGTAATCATCGCACA
GAACTATGGCATTTGGATACAGGTGTTGTTTCCTGTTTATATAAGCGTAATTTCACATATGATGTGAATGCTGATTATT
TGTATTTTCATTTTTATCAAGAAGGTGGTACTTTTTATGCATATTTTACAGACACTGGTGTTGTTACTAAGTTTTTGTT
TAATGTTTATTTAGGCATGGCGCTTTCACACTATTATGTCATGCCTCTGACTTGTAATAGTAAGCGTAAGGATGGTTTT
ACTTTAGAATATTGGGTTACACCTCTCACTTCTAGACAATATTTACTCGCTTTCAATCAAGATGGTATTATTTTTAATG
CTGTTGATTGCATGAGTGATTTTATGAGTGAGATTAAGTGTAAAACACAATCTATAGCACCACCTACTGGTGTTTATGA
ATTAAACGGTTACACTGTTCAGCCAATTGCAGATGTTTACCGACGTAAACCTAATCTTCCCAATTGTAATATAGAAGCT
TGGCTTAATGATAAGTCGGTACCATCTCCATTAAATTGGGAACGTAAGACATTTTCAAATTGTAATTTTAATATGAGCA
GCCTGATGTCGTTTATTCAGGCAGACTCATTTACTTGTAATAATATTGATGCTGCTAAGATATATGGTATGTGTTTTTC
CAGCATAACTATAGATAAGTTTGCTATACCCAATGGCAGGAAGGTTGACCTACAATTGGGTAATTTGGGCTATTTGCAG
TCATTTAACTATAGAATTGATACTACTGCAACAAGTTGTCAGTTGTATTATAATTTACCTGCTGCTAATGTTTCTGTTA
GCAGGTTTAATCCTTCTACTTGGAATAAGAGATTTGGTTTTATAGAAAATTCTGTTTTTAAGCCTCAACCTGCAGGTGT
TTTTACCAATCATGATGTAGTTTATGCCCAACATTGTTTTAAAGCTCCCAAAAATTTCTGTCCGTGTAAATTGAATGGT
TCTTTGTGTGTAGGTAGTGGTCCTGGTAAAAATAATGGTATAGGCACTTGTCCTGCAGGTACTAATTATTTAACTTGTC
ATAATTTGTGTAATCCTGATCCTATTACATTTGCAGGTCCTTATAAGTGCCCCCAAACTAAATCTTTAGTTGGTATAGG
TGAGCACTGTTCAGGTCTTGCTGTTAAAAGTGATTATTGTGGAGGCAATCCTTGTACTTGCCAACCACAAGCATTTTTG
GGTTGGTCTGCAGACTCTTGTTTACAAGGGGACAAGTGTAATATTTTTGCTAATTTAATTTTGCATGATGTTAATAGTG
GTCTTACTTGTTCTACTGATTTACAAAAAGCTAACACAGACATAATTCTTGGTGTTTGTGTTAATTATGACCTTTATGG
TATTTCAGGCCAAGGCATTTTTGTTGAGGTTAATGCGACTTATTATAATAGTTGGCAGAACCTTTTATATGATTCTAAT
GGTAATCTCTATGGTTTTAGAGACTACATAACAAATAGAACTTTTATGATTCGTAGTTGCTATAGCGGTCGTGTTTCTG
CGGCCTTTCACGCTAACTCTTCCGAACCAGCATTGCTATTTCGGAATATTAAATGCAACTACGTTTTTAATAATAGTCT
TACACGACAGCTGCAACCCATTAACTATTTTGATAGTTATCTTGGTTGTGTTGTCAATGCTTATAATAGTACTGCTATT
TCTGTTCAAACATGTGATCTCACAGTAGGTAGTGGTTACTGTGTGGATTACTCTAAAAACAGACGAAGTCGTAGAGCGA
TTACCACTGGTTATCGGTTTACTAATTTTGAGCCATTTACTGTTAATTCAGTAAATGATAGTTTAGAACCTGTAGGTGG
TTTGTACGAAATTCAAATACCTTCAGAGTTTACTATAGGTAATATGGAGGAGTTTATTCAAACAAGCTCTCCTAAAGTT
ACTATTGATTGTGCTGCATTTGTCTGTGGTGATTATGCAGCATGTAAATCACAGTTGGTTGAATATGGTAGTTTCTGTG
ATAACATTAATGCCATACTCACAGAAGTAAATGAACTACTTGACACTACACAGTTGCAAGTAGCTAATAGTTTAATGAA

TGGTGTTACTCTTAGCACTAAGCTTAAAGATGGCGTTAATTTCAATGTAGACGACATCAATTTTTCCTCTGTATTAGGT
TGTCTAGGCAGCGAATGTAGTAAAGCTTCCAGTAGATCTGCTATAGAGGATTTACTTTTTGATAAAGTAAAGTTATCTG
ATGTCGGTTTCGTTGAGGCTTATAATAATTGTACTGGAGGTGCCGAAATTAGGGACCTCATTTGTGTGCAAAGTTATAA
AGGTATCAAAGTGTTGCCTCCACTGCTCTCAGAAAATCAGATCAGTGGATACACTTTGGCTGCCACTTCTGCTAGTCTG
TTTCCTCCCTGGACAGCAGCAGCAGGTGTACCATTTTATTTAAATGTTCAGTATCGCATTAATGGGCTTGGTGTCACCA
TGGATGTGCTAAGTCAAAATCAAAAGCTTATTGCTAATGCATTTAACAATGCCCTTGATGCTATTCAGGAAGGGTTCGA
TGCAACTAATTCTGCTTTAGTTAAAATTCAAGCTGTTGTTAATGCAAATGCTGAAGCTCTTAATAACTTATTGCAACAA
CTCTCTAATAGATTTGGTGCTATAAGTTCTTCTCTACAAGAAATTCTATCTAGACTTGATGCTCTTGAAGCGGAAGCTC
AGATAGATAGACTTATTAATGGTCGTCTTACCGCTCTTAATGCTTATGTTTCTCAACAGCTTAGTGATTCTACACTGGT
AAAATTTAGTGCAGCACAAGCTATGGAGAAGGTTAATGAATGTGTCAAAAGCCAATCATCTAGGATAAATTTCTGTGGT
AATGGTAACCATATTATATCATTAGTGCAGAATGCTCCATATGGTTTGTATTTTATCCACTTTAGTTATGTCCCTACTA
AGTATGTCACAGCGAAGGTTAGTCCTGGTCTGTGCATTGCTGGTGATAGAGGTATAGCTCCTAAGAGTGGTTATTTTGT
TAATGTAAATAATACTTGGATGTACACTGGTAGTGGTTACTACTACCCTGAACCTATAACTGAAAATAATGTTGTTGTT
ATGAGTACCTGCGCTGTTAATTATACTAAAGCGCCGTATGTAATGCTGAACACTTCAACACCCAACCTTCCTGATTTTA
GGGAAGAGTTGGATCAATGGTTTAAAAATCAAACATCAGTGGCACCAGATCTGTCACTTGATTATATAAATGTTACATT
CTTGGACCTACAAGTTGAAATGAATAGGTTACAGGAGGCAATAAAAGTTTTAAATCAGAGCTACATCAATCTCAAGGAC
ATTGGTACATATGAATATTATGTAAAATGGCCTTGGTATGTATGGCTTTTAATCGGCCTTGCTGGTGTAGCTATGCTTG
TTTTACTATTCTTCATATGCTGTTGTACAGGATGTGGGACTAGTTGTTTTAAGAAATGTGGTGGTTGTTGTGATGATTA
TACTGGATACCAGGAGTTAGTAATCAAAACTTCACATGACGACTAA

>gi|530802213|gb|KF530065.1| Human coronavirus OC43 strain OC43/human/USA/901-41/1990, complete genome

ATGTTTTTGATACTTTTAATTTCCTTACCAATGGCTTTTGCTGTTATAGGAGATTTAAAGTGTACTTCAGATACG
AGTTATATTAATGATGTTGACACCGGTGTTCCTCCTATAAGTACTGATACTGTTGATGTTACTAATGGTTTGGGTACTT
ATTATGTTTTAGATCGTGTGTATTTAAATACTACTTTGTTTCTTAATGGTTACTATCCTACTTCAGGTTCCACATATCG
TAATATGGCACTGAAGGGAAGTGTACTGTTGAGCACACTATGGTTTAAACCACCATTTCTTTCTGATTTTATTAATGGT
ATTTTTGCTAAGGTCAAAAATACCAAGGTTATTAAAGATGATGTACTGTACAGTGAGTTCCCTGCTATAACTATAGGTA
GTACTTTTGTAAATACATCCTATAGTGTGGTAGTACAACCACGTACAATCAATTTAGATAATAAATTACAAGGTCTTTT
AGAGATCTCTGTTTGCCAGTATAATATGTGCGAGTACCCACATACGATTTGTCATCCTAACTTGGGTAATCATCGCACA
GAACTATGGCATTTGGATACAGGTGTTGTTTCCTGTTTATATAAGCGTAATTTCACATATGATGTGAATGCTGATTATT
TGTATTTTCATTTTTATCAAGAAGGTGGTACTTTTTATGCATATTTTACAGACACTGGTGTTGTTACTAAGTTTTTGTT
TAATGTTTATTTAGGCATGGCGCTTTCACACTATTATGTCATGCCTCTGACTTGTAATAGTAAGCGTAAGGATGGTTTT
ACTTTAGAATATTGGGTTACACCTCTCACTTCTAGACAATATTTACTCGCTTTCAATCAAGATGGTATTATTTTTAATG
CTGTTGATTGCATGAGTGATTTTATGAGTGAGATTAAGTGTAAAACACAATCTATAGCACCACCTACTGGTGTTTATGA
ATTAAACGGTTACACTGTTCAGCCAATTGCAGATGTTTACCGACGTAAACCTAATCTTCCCAATTGCAATATAGAAGCT
TGGCTTAATGATAAGTCGGTGCCATCTCCATTAAATTGGGAACGTAAGACATTTTCAAATTGTAATTTTAATATGAGCA
GCCTGATGTCCTTTATTCAGGCAGACTCATTTACTTGTAATAATATTGATGCTGCTAAGATATATGGTATGTGTTTTTC
CAGCATAACTATAGATAAGTTTGCTATACCCAATGGCAGGAAGGTTGACCTACAATTGGGTAATTTGGGCTATTTGCAG
TCATTTAACTATAGAATTGATACTACTGCAACAAGTTGTCAGTTGTATTATAATTTACCTGCTGCTAATGTTTCTGTTA
GCAGGTTTAATCCTTCTACTTGGAATAAGAGATTCGGTTTTATAGAAAATTCTGTTTTTAAGCCTCAACCTGCAGGTGT
TTTTACCAATCATGATGTAGTTTATGCCCAACATTGTTTTAAAGCTCCCAAAAATTTCTGTCCGTGTAAATTGAATGGT
TCTTTGTGTGTAGGTAGTGGTCCTGGTAAAAATAATGGTATAGGCACTTGCCCTGCAGGTACTAATTATTTAACTTGTC

ATAATTTGTGCAATCCTGATCCTATTACATTTACAGGTCCTTATAAGTGCCCCCAAACTAAATCTTTAGTTGGTATAGG
TGAGCACTGTTCAGGTCTTGCTGTTAAAAGTGATTATTGTGGAGGCAATCCTTGCACTTGCCAACCACAAGCATTTTTG
GGTTGGTCTGCAGACTCTTGTTTACAAGGGGACAAGTGTAATATTTTTGCTAATTTAATTTTGCATGATGTTAATAGTG
GTCTTACTTGTTCTACTGATTTACAAAAAGCTAACACAGACATAATTCTTGGTGTTTGTGTTAATTATGACCTTTATGG
TATTTCAGGCCAGGGCATTTTTGTTGAGGTTAATGCGACTTATTATAATAGTTGGCAGAACCTTTTATATGATTCTAAT
GGTAATCTCTATGGTTTTAGAGACTACATAACAAACAGAACTTTTATGATTCGTAGTTGCTATAGCGGTCGTGTTTCTG
CGGCCTTTCACGCTAACTCTTCCGAACCAGCATTGCTATTTCGGAATATTAAATGCAACTACGTTTTTAATAATAGTCT
TACACGACAGCTGCAACCCATTAACTATTTTGATAGTTATCTTGGTTGTGTTGTCAATGCTTATAATAGTACTGCTATT
TCTGTTCAAACATGTGATCTCACAGTAGGTAGTGGTTACTGTGTGGATTACTCTAAAAACAGACGAAGTCGTAGAGCGA
TTACCACTGGTTATCGGTTTACTAATTTTGAGCCATTTACTGTTAATTCAGTAAATGATAGTTTAGAACCTGTAGGTGG
TTTGTACGAAATTCAAATACCTTCAGAGTTTACTATAGGTAATATGGAGGAGTTTATTCAAACAAGCTCTCCTAAAGTT
ACTATTGATTGTGCTGCATTTGTCTGTGGTGATTATGCAGCATGTAAATCACAGTTGGTTGAATATGGTAGTTTCTGTG
ATAACATTAATGCCATACTCACAGAAGTAAATGAACTACTTGACACTACACAGTTGCAAGTAGCTAATAGTTTAATGAA
TGGTGTTACTCTTAGCACTAAGCTTAAAGATGGCGTTAATTTCAATGTAGACGACATCAATTTTTCCCCTGTATTAGGT
TGTCTAGGCAGCGAATGTAGTAAAGCTTCCAGTAGATCTGCTATAGAGGATTTACTTTTTGATAAAGTAAAGTTATCTG
ATGTCGGTTTCGTTGAGGCTTATAATAATTGTACTGGAGGTGCCGAAATTAGGGACCTCATTTGTGTGCAAAGTTATAA
AGGTATCAAAGTGTTGCCTCCACTGCTCTCAGAAAATCAGATCAGTGGATACACTTTGGCTGCCACTTCTGCTAGTCTG
TTTCCTCCTTGGACAGCAGCAGCAGGTGTACCATTTTATTTAAATGTTCAGTATCGCATTAATGGGCTTGGTGTCACCA
TGGATGTGCTAAGTCAAAATCAAAAGCTTATTGCTAATGCATTTAACAATGCCCTTGATGCTATTCAGGAAGGGTTCGA
TGCAACTAATTCTGCTTTAGTTAAAATTCAAGCTGTTGTTAATGCAAATGCTGAAGCTCTTAATAACTTATTGCAACAA
CTCTCTAATAGATTTGGTGCTATAAGTTCTTCTCTACAAGAAATTCTATCTAGACTTGATGCTCTTGAAGCGGAAGCTC
AGATAGATAGACTTATTAATGGTCGTCTTACCGCTCTTAATGCTTATGTTTCTCAACAGCTTAGTGATTCTACACTGGT
AAAATTTAGTGCAGCACAAGCTATGGAGAAGGTTAATGAATGTGTCAAAAGCCAATCATCTAGGATAAATTTCTGTGGT
AATGGTAACCATATTATATCATTAGTGCAGAATGCTCCATATGGTTTGTATTTTATCCACTTTAGTTATGTCCCTACTA
AGTATGTCACAGCGAAGGTTAGTCCTGGTCTGTGCATTGCTGGTGATAGAGGTATAGCTCCTAAGAGTGGTTATTTTGT
TAATGTAAATAATACTTGGATGTACACTGGTAGTGGTTACTACTACCCTGAACCTATAACTGAAAATAATGTCGTTGTT
ATGAGTACCTGCGCTGTTAATTATACTAAAGCGCCGTATGTAATGCTGAACACTTCAACACCCAACCTTCCTGATTTTA
GGGAAGAGTTGGATCAATGGTTTAAAAATCAAACATCAGTGGCACCAGATCTGTCACTTGATTATATAAATGTTACATT
CTTGGACCTACAAGTTGAAATGAATAGGTTACAGGAGGCAATAAAAGTTTTAAATCAGAGCTACATCAATCTCAAGGAC
ATTGGTACATATGAATATTATGTAAAATGGCCTTGGTATGTATGGCTTTTAATCGGCCTTGCTGGTGTAGCTATGCTTG
TTTTACTATTCTTCATATGCTGTTGTACAGGATGTGGGACTAGTTGTTTTAAGAAATGTGGTGGTTGTTGTGATGATTA
TACTGGATACCAGGAGTTAGTAATCAAAACTTCACATGACGACTAA

>gi|530802224|gb|KF530066.1| Human coronavirus OC43 strain OC43/human/USA/901-33/1990, complete genome

ATGTTTTTGATACTTTTAATTTCCTTACCAATGGCTTTTGCTGTTATAGGAGATTTAAAGTGTACTTCAGATACG
AGTTATATTAATGATGTTGACACCGGTGTTCCTCCTATAAGTACTGATACTGTTGATGTTACTAATGGTTTGGGTACTT
ATTATGTTTTAGATCGTGTGTATTTAAATACTACTTTGTTTCTTAATGGTTACTATCCTACTTCAGGTTCCACATATCG
TAATATGGCACTGAAGGGAAGTGTACTGTTGAGCACACTATGGTTTAAACCACCATTTCTTTCTGATTTTATTAATGGT
ATTTTTGCTAAGGTCAAAAATACCAAGGTTATTAAAGATGATGTACTGTACAGTGAGTTCCCTGCTATAACTATAGGTA
GTACTTTTGTAAATACATCCTATAGTGTGGTAGTACAACCACGTACAATCAATTTAGATAATAAATTACAAGGTCTTTT
AGAGATCTCTGTTTGCCAGTATAATATGTGCGAGTACCCACATACGATTTGTCATCCTAACTTGGGTAATCATCGCACA

GAACTATGGCATTTGGATACAGGTGTTGTTTCCTGTTTATATAAGCGTAATTTCACATATGATGTGAATGCTGATTATT
TGTATTTTCATTTTTATCAAGAAGGTGGTACTTTTTATGCATATTTTACAGACACTGGTGTTGTTACTAAGTTTTTGTT
TAATGTTTATTTAGGCATGGCGCTTTCACACTATTATGTCATGCCTCTGACTTGTAATAGTAAGCGTAAGGATGGTTTT
ACTTTAGAATATTGGGTTACACCTCTCACTTCTAGACAATATTTACTCGCTTTCAATCAAGATGGTATTATTTTTAATG
CTGTTGATTGCATGAGTGATTTTATGAGTGAGATTAAGTGTAAAACACAATCTATAGCACCACCTACTGGTGTTTATGA
ATTAAACGGTTACACTGTTCAGCCAATTGCAGATGTTTACCGACGTAAACCTAATCTTCCCAATTGCAATATAGAAGCT
TGGCTTAATGATAAGTCGGTGCCATCTCCATTAAATTGGGAACGTAAGACATTTTCAAATTGTAATTTTAATATGAGCA
GCCTGATGTCCTTTATTCAGGCAGACTCATTTACTTGTAATAATATTGATGCTGCTAAGATATATGGTATGTGTTTTTC
CAGCATAACTATAGATAAGTTTGCTATACCCAATGGCAGGAAGGTTGACCTACAATTGGGTAATTTGGGCTATTTGCAG
TCATTTAACTATAGAATTGATACTACTGCAACAAGTTGTCAGTTGTATTATAATTTACCTGCTGCTAATGTTTCTGTTA
GCAGGTTTAATCCTTCTACTTGGAATAAGAGATTCGGTTTTATAGAAAATTCTGTTTTTAAGCCTCAACCTGCAGGTGT
TTTTACCAATCATGATGTAGTTTATGCCCAACATTGTTTTAAAGCTCCCAAAAATTTCTGTCCGTGTAAATTGAATGGT
TCTTTGTGTGTAGGTAGTGGTCCTGGTAAAAATAATGGTATAGGCACTTGCCCTGCAGGTACTAATTATTTAACTTGTC
ATAATTTGTGCAATCCTGATCCTATTACATTTACAGGTCCTTATAAGTGCCCCCAAACTAAATCTTTAGTTGGTATAGG
TGAGCACTGTTCAGGTCTTGCTGTTAAAAGTGATTATTGTGGAGGCAATCCTTGCACTTGCCAACCACAAGCATTTTTG
GGTTGGTCTGCAGACTCTTGTTTACAAGGGGACAAGTGTAATATTTTTGCTAATTTAATTTTGCATGATGTTAATAGTG
GTCTTACTTGTTCTACTGATTTACAAAAAGCTAACACAGACATAATTCTTGGTGTTTGTGTTAATTATGACCTTTATGG
TATTTCAGGCCAGGGCATTTTTGTTGAGGTTAATGCGACTTATTATAATAGTTGGCAGAACCTTTTATATGATTCTAAT
GGTAATCTCTATGGTTTTAGAGACTACATAACAAACAGAACTTTTATGATTCGTAGTTGCTATAGCGGTCGTGTTTCTG
CGGCCTTTCACGCTAACTCTTCCGAACCAGCATTGCTATTTCGGAATATTAAATGCAACTACGTTTTTAATAATAGTCT
TACACGACAGCTGCAACCCATTAACTATTTTGATAGTTATCTTGGTTGTGTTGTCAATGCTTATAATAGTACTGCTATT
TCTGTTCAAACATGTGATCTCACAGTAGGTAGTGGTTACTGTGTGGATTACTCTAAAAACAGACGAAGTCGTAGAGCGA
TTACCACTGGTTATCGGTTTACTAATTTTGAGCCATTTACTGTTAATTCAGTAAATGATAGTTTAGAACCTGTAGGTGG
TTTGTACGAAATTCAAATACCTTCAGAGTTTACTATAGGTAATATGGAGGAGTTTATTCAAACAAGCTCTCCTAAAGTT
ACTATTGATTGTGCTGCATTTGTCTGTGGTGATTATGCAGCATGTAAATCACAGTTGGTTGAATATGGTAGTTTCTGTG
ATAACATTAATGCCATACTCACAGAAGTAAATGAACTACTTGACACTACACAGTTGCAAGTAGCTAATAGTTTAATGAA
TGGTGTTACTCTTAGCACTAAGCTTAAAGATGGCGTTAATTTCAATGTAGACGACATCAATTTTTCCCCTGTATTAGGT
TGTCTAGGCAGCGAATGTAGTAAAGCTTCCAGTAGATCTGCTATAGAGGATTTACTTTTTGATAAAGTAAAGTTATCTG
ATGTCGGTTTCGTTGAGGCTTATAATAATTGTACTGGAGGTGCCGAAATTAGGGACCTCATTTGTGTGCAAAGTTATAA
AGGTATCAAAGTGTTGCCTCCACTGCTCTCAGAAAATCAGATCAGTGGATACACTTTGGCTGCCACTTCTGCTAGTCTG
TTTCCTCCTTGGACAGCAGCAGCAGGTGTACCATTTTATTTAAATGTTCAGTATCGCATTAATGGGCTTGGTGTCACCA
TGGATGTGCTAAGTCAAAATCAAAAGCTTATTGCTAATGCATTTAACAATGCCCTTGATGCTATTCAGGAAGGGTTCGA
TGCAACTAATTCTGCTTTAGTTAAAATTCAAGCTGTTGTTAATGCAAATGCTGAAGCTCTTAATAACTTATTGCAACAA
CTCTCTAATAGATTTGGTGCTATAAGTTCTTCTCTACAAGAAATTCTATCTAGACTTGATGCTCTTGAAGCGGAAGCTC
AGATAGATAGACTTATTAATGGTCGTCTTACCGCTCTTAATGCTTATGTTTCTCAACAGCTTAGTGATTCTACATTGGT
AAAATTTAGTGCAGCACAAGCTATGGAGAAGGTTAATGAATGTGTCAAAAGCCAATCATCTAGGATAAATTTCTGTGGT
AATGGTAACCATATTATATCATTAGTGCAGAATGCTCCATATGGTTTGTATTTTATCCACTTTAGTTATGTCCCTACTA
AGTATGTCACAGCGAAGGTTAGTCCTGGTCTGTGCATTGCTGGTGATAGAGGTATAGCTCCTAAGAGTGGTTATTTTGT
TAATGTAAATAATACTTGGATGTACACTGGTAGTGGTTACTACTACCCTGAACCTATAACTGAAAATAATGTCGTTGTT
ATGAGTACCTGCGCTGTTAATTATACTAAAGCGCCGTATGTAATGCTGAACACTTCAACACCCAACCTTCCTGATTTTA
GGGAAGAGTTGGATCAATGGTTTAAAAATCAAACATCAGTGGCACCAGATCTGTCACTTGATTATATAAATGTTACATT
CTTGGACCTACAAGTTGAAATGAATAGGTTACAGGAGGCAATAAAAGTTTTAAATCAGAGCTACATCAATCTCAAGGAC
ATTGGTACATATGAATATTATGTAAAATGGCCTTGGTATGTATGGCTTTTAATCGGCCTTGCTGGTGTAGCTATGCTTG
TTTTACTATTCTTCATATGCTGTTGTACAGGATGTGGGACTAGTTGTTTTAAGAAATGTGGTGGTTGTTGTGATGATTA

TACTGGATACCAGGAGTTAGTAATCAAAACTTCACATGACGACTAA

>gi|530802235|gb|KF530067.1| Human coronavirus OC43 strain OC43/human/USA/912-10/1991, complete genome

ATGTTTTTGATACTTTTAATTTCCTTACCAACGGCTTTTGCTGTTATAGGAGATTTAAAGTGTCCTTTAGATACT
CGTTATAAAGGTACATTTAATAATAAAGACACCGGTCCTCCTTTTATAAGTACTGATACTGTTGATGTTACTAATGGTT
TGGGTACTTATTATGTTTTAGATCGTGTGTATTTAAATACTACGTTGTTTCTTAATGGTTATTACCCTACTTCAGGTTC
CACATATCGTAATATGGCACTGAAGGGAACTGACAAATTGAGCACACTATGGTTTAAACCACCATTTCTTTCTGATTTT
ATTAATGGTATTTTTGCTAAGGTCAAAAATACCAAGGTTTTTAAAGATGGTGTAATGTATAGTGAGTTCCCTGCTATAA
CTATAGGTAGTACTTTTGTAAATACATCCTATAGTGTGGTAGTACAACCACGTACAATTAATTCAACACAGGATGGTGA
TAATAAATTACAAGGTCTTTTAGAGGTCTCTGTTTGCCAGTATAATATGTGCGAGTACCCACATACGACTTGTCATCCT
AAACTGGGTAATCACTTCAAAGAACTATGGCATTTGGATACAGGTGTTGTTTCCTGTTTATATAAGCGTAATTTCACAT
ATGATGTGAATGCTAATTATTTGTATTTTCATTTTTATCAAGAAGGTGGTACTTTTTATGCATATTTTACAGACACTGG
TGTTGTTACTAAGTTTTTGTTTAATGTTTATTTAGGCATGGCACTTTCACACTATTATGTCATGCCTCTGACTTGTATT
AGTAGGCGTGATATTGGTTTTACTTTAGAATATTGGGTTACACCCCTCACTTCTAGACAATATTTACTCGCTTTCAATC
AAGATGGTATTATTTTTAATGCTGTTGATTGTATGAGTGATTTTATGAGTGAGATTAAGTGTAAAACACAATCTATAGC
ACCACCTACTGGTGTTTATGAATTAAACGGTTACACTGTTCAGCCAATCGCAGATGTTTACCGACGCAAACCTGATCTT
CCCAATTGCAATATAGAAGCTTGGCTTAATGATAAGTCGGTGCCCTCTCCATTAAATTGGGAACGTAAGACATTTTCAA
ATTGTAATTTTAATATGAGCAGCCTGATGTCTTTTATTCAGGCAGACTCATTTACTTGTAATAATATTGATGCTGCTAA
GATATATGGTATGTGTTTTTCCAGCATAACTATAGATAAGTTTGCTATACCCAATGGCAGGAAGGTTGACCTACAATTG
GGTAATTTGGGCTATTTGCAGTCATCCAACTATAGAATTGATACTACTGCAACAAGTTGTCAGTTGTATTATAATTTAC
CTGCTGCTAATGTCTCTGTTAGCAGGTTTAATCCTTCTACTTGGAATAAGAGATTTGGTTTTATAGAAGATTCTGCTTT
TGTGCCTCAACCTACAGGTGTTTTTACTAATCACAGTGTAGTTTATGCACAACACTGTTTCAAAGCCCCTAAAAATTTC
TGTCCGTGTAAATTGAATGGTTCGTGTCCTGGTAAAAATAATGGTATAGGCACTTGTCCTGCAGGTACTAATTATTTAA
CTTGTGATAATTTGTGTACTCTTGATCCTATTACATTTAAAGCTCCAGGTACTTATAAGTGCCCCCAAACTAAATCTTT
AGTTGGCATAGGTGAGCACTGTTCGGGTCTTGCTGTTAAAAGTGATTATTGTGGAGGCAATTCTTGTACTTGCCAACCA
CAAGCATTCTTGGGTTGGTCTGCAGACTCTTGTTTACAAGGAGACAAGTGTAATATTTTTGCTAATCTTATTTTGCATG
ATGTTAATAGTGGTCTTACTTGTTCTACTGATTTACAAAAAGCTAACACAGACATAATTCTTGGTGTTTGTGTTAATTA
TGACCTCTATGGTATTTCAGGCCAAGGCATTTTTGTTGAGGTTAATGCGACTTATTATAATAGTTGGCAGAACCTTTTA
TATGATTCTAATGGTAATCTCTACGGTTTTAGAGACTACATAACAAACAGAACTTTTATGATTCGTAGTTGCTATAGCG
GTCGTGTTTCTGCGGCCTTTCATGCTAACTCTTCCGAACCAGCATTGATATTTCGGAATATTAAATGCAACTACGTTTT
TAATAATAGTCTTACACGACAGCTGCAACCCATTAACTATTCGTTTGATAGTTATCTTGGTTGTGTTGTCAATGCTTAT
AATAGTACTGCTATTTCTGTTCAAACATGTGATCTCACAGTAGGTAGTGGTTACTGTGTGGATTACTCTAAAAACAGAC
GAAGTCGTAGAGCGATTACCACTGGTTATCGGTTTACTAATTTTGAGCCATTTACTGTTAATTCAGTAAATGATAGTTT
GGAACCTGTAGGTGGTTTGTATGAAATTCAAATACCTTCAGAGTTTACTATAGGTAATATGGAGGAGTTTATTCAAACA
AGCTCTCCCAAAGTTACTATTGATTGTGCTGCATTTGTCTGTGGTGATTATGCAGCATGTAAATTACAGTTGGTTGAAT
ATGGTAGTTTCTGTGATAACATTAATGCCATACTCACAGAAGTAAATGAACTACTTGATACTACTCAGTTGCAAGTAGC
TAATAGTTTAATGAATGGTGTTACTCTTAGCACTAAGCTTAAAGATGGTGTTAATTTCAATGTAGACGACATCAATTTT
TCCCCTGTATTAGGTTGTCTAGGCAGCGAATGTAGTAAAGCTTCCAGTAGATCTGCTATAGAGGATTTACTTTTTGATA
AAGTAAAGTTATCTGATGTCGGTTTCGTTGAGGCTTATAATAATTGTACAGGAGGTGCCGAAATTAGGGACCTCATTTG
TGTGCAAAGTTATAAAGGCATCAAAGTGTTGCCCCCACTGCTCTCAGAAAATCAGATCAGTGGATACACTTTGGCTGCC
ACCTCTGCTAGTCTATTTCCTCCTTGGACAGCAGCAGCAGGTGTACCATTTTATTTAAATGTTCAGTATCGCATTAATG

GGCTTGGTGTCACCATGGATGTGCTAAGTCAAAATCAAAAGCTTATTGCTAATGCATTTAACAATGCCCTTTATGCTAT
TCAGCAAGGGTTCGATGCAACTAATTCTGCTTTAGTTAAAATTCAAGCTGTTGTTAATGCAAATGCTGAAGCTCTTAAT
AACTTATTGCAACAACTCTCTAATAGATTTGGTGCTATAAGTGCTTCTTTACAAGAAATTCTATCTAGACTTGATGCTC
TTGAAGCGGAAGCTCAGATAGATAGACTTATTAATGGTCGTCTCACCGCTCTTAATGCTTATGTTTCTCAACAGCTTAG
TGATTCTACACTGGTAAAATTTAGTGCAGCACAAGCTATGGAGAAGGTTAATGAATGTGTCAAAAGCCAATCATCTAGG
ATAAATTTCTGTGGTAATGGTAATCATATTATATCATTAGTGCAGAATGCTCCATATGGTTTGTATTTTATCCACTTTA
ATTATGTCCCTACTAAGTATGTCACAGCGAAGGTTAGTCCTGGTCTGTGCATTGCTGGTAATAGAGGTATAGCTCCTAA
GAGTGGTTATTTTGTTAATGTAAATAATACTTGGATGTACACTGGTAGTGGTTACTACTACCCTGAACCTATAACTGAA
AATAATGTTGTTGTTATGAGTACCTGCGCTGTTAATTATACTAAAGCGCCGTATGTAATGCTGAACACTTCAATACCCA
ACCTTCCTGATTTTAAGGAAGAGTTGGATCAATGGTTTAAAAATCAAACATCAGTGGCACCAGATTTGTCACTTGATTA
TATAAATGTTACATTCTTGGACCTACAAGTTGAAATGAATAGGTTACAGGAGGCAATAAAAGTCTTAAATCATAGCTAC
ATCAATCTCAAGGACATTGGTACATATGAATATTATGTAAAATGGCCTTGGTATGTATGGCTCTTAATCTGCCTTGCTG
GTGTAGCTATGCTTGTTTTACTATTCTTCATATGCTGTTGTACAGGATGTGGGACTAGTTGTTTTAAGAAATGTGGTGG
TTGTTGTGATGATTATACTGGATACCAGGAGTTAGTAATCAAAACTTCACATGACGACTAA

>gi|530802246|gb|KF530068.1| Human coronavirus OC43 strain OC43/human/USA/007-11/2000, complete genome

ATGTTTTTGATACTTTTAATTTCCTTACCAACGGCTTTTGCTGTTATAGGAGATTTAAAGTGTCCTTTAGATACT
AGTCTTAAAGGTAGCTTTAATAATAGAGACACCGGTTCTCCTTCTATAAGTACTGATACTGTTGATGTTACTAATGGTT
TGGGTACTTATTATGTTTTAGATCGTGTGTATTTAAATACTACGTTGTTTCTTAATGGTTATTACCCCACTTCAGGTTC
CACATATCGTAATATGGCACTGAAGGGAACTGACAAATTGAGCACATTATGGTTTAAACCACCATTTCTTTCTGATTTT
ATTAATGGTATTTTTGCTAAGGTCAAAAATACCAAGGTTTTTAAAGATGGTGTAATGTATAGTGAGTTCCCTGCTATAA
CTATAGGTAGTACTTTTGTAAATACATCCTATAGTGTGGTAGTACAACCACGTACAATTAATTCAACACAGGATGGTGT
TAATAAATTACAAGGTCTTTTAGAGGTCTCTGTTTGCCAGTATAATATGTGCGAGTACCCACATACGATTTGTCATCCT
AAACTGGGTAACCACTTCAAAGAACTATGGCATTTGGATACAGGTGTTGTTTCCTGTTTATATAAGCGTAATTTCACAT
ATGATGTGAATGCTACTTATTTGTATTTTCATTTTTATCAAGAAGGTGGTACTTTTTATGCATATTTTACAGACACTGG
TTTTGTTACTAAGTTTTTGTTTAATGTTTATTTAGGCATGGCACTTTCACACTATTATGTCATGCCTCTGACTTGTATT
AGTAGGCGTGATATTGGTTTTACTTTAGAATATTGGGTTACACCCCTCACTTCTAGACAATATTTACTCGCTTTCAATC
AAGATGGTATTATTTTTAATGCTGTTGATTGTATGAGTGATTTTATGAGTGAGATTAAGTGTAAAACACAATCTATAGC
ACCACCTACTGGTGTTTATGAATTAAACGGTTACACTGTTCAGCCAATCGCAGATGTTTACCGACGCAAACCTGATCTT
CCCAATTGCAATATAGAAGCTTGGCTTAATGATAAGTCGGTGCCCTCTCCATTAAATTGGGAACGTAAGACATTTTCAA
ATTGTAATTTTAATATGAGCAGCCTGATGTCTTTTATTCAGGCAGACTCATTTACTTGTAATAATATTGATGCTGCTAA
GATATATGGTATGTGTTTTTCCAGCATAACTATAGATAAGTTTGCTATACCCAATGGCAGGAAGGTTGACCTACAATTG
GGCAATTTGGGCTATTTGCAGTCATCCAACTATAGAATTGATACTACTGCAACAAGTTGTCAGTTGTATTATAATTTAC
CTGCTGCTAATGTCTCTGTTAGCAGGTTTAATCCTTCTACTTGGAATAAGAGATTTGGTTTTATAGAAGATTCTGTTTT
TGTGCCTCAACCTACAGGTGTTTTTACTAATCACAGTGTAGTTTATGCACAACACTGTTTCAAAGCCCCTAAAAATTTC
TGTCCGTGTAAATTGAATGGTTCGTGTCCTGATAAAAATAATGGTATAGGCACTTGTCCTGCAGGTACTAATTATTTAA
CTTGTGATAATTTGTGTACTCTTGATCCTATTACATTTAAAGCTCCAGGTACTTATAAGTGCCCCCAAACTAAATCTTT
AGTTGGCATAGGTGAGCACTGTTCGGGTCTTGCTGTTAAAAGTGATTATTGTAGAGGCAATTCTTGTACTTGCCAACCA
CAAGCATTTTTGGGTTGGTCTGCAGACTCTTGTTTACAAGGAGACAAGTGTAATATTTTTGCTAATCTTATTTTGCATG
ATGTTAATAGTGGTCTTACTTGTTCTACTGATTTACAAAAAGCTAACACAGACATAATTCTTGGTGTTTGTGTTAATTA
TGACCTCTATGGTATTTCAGGTCAAGGCATTTTTGTTGAGGTTAATGCGACTTATTATAATAGTTGGCAGAACCTTTTA

TATGATTCTAATGGTAATCTCTACGGTTTTAGAGACTACATAACAAACAGAACTTTTATGATTCGTAGTTGCTATAGCGGTCGTGTTTCTGCGGCCTTTCATGCTAACTCTTCCGAACCAGCATTGCTATTTCGGAATATTAAATGCAACTACGTTTTTAATAATAGTCTTACACGACAGCTGCAACCCATTAACTATTCGTTTGATAGTTATCTTGGTTGTGTTGTCAATGCTTATAATAGTACTGCTATTTCTGTTCAAACATGTGATCTCACAGTAGGTAGCGGTTACTGTGTGGAGTACTCTAAAAACAGACGAAGTCGTAGAGCGATTACCACTGGTTATCGGTTTACTAATTTTGAGCCATTTACTGTTAATTCAGTAAATGATAGTTTGGAACCTGTAGGTGGTTTGTATGAAATTCAAATACCTTCAGAGTTTACTATAGGTAATATGGAGGAGTTTATTCAAACAAGCTCTCCCAAAGTTACTATTGATTGTGCTGCATTTGTCTGTGGTGATTATGCAGCATGTAAATTACAGTTGGTTGAATATGGTAGTTTCTGTGATAACATTAATGCCATACTCACAGAAGTAAATGAACTACTTGATACTACACAGTTGCAAGTAGCTAATAGTTTAATGAATGGTGTTACTCTTAGCACTAAGCTTAAAGATGGTGTTAATTTCAATGTAGACGACATCAATTTTTCCCCTGTATTAGGTTGTCTAGGCAGCGAATGTAGTAAAGCTTCCAGTAGATCTGCTATAGAGGATTTACTTTTTGATAAAGTAAAGTTATCTGATGTCGGTTTCGTTGAGGCTTATAATAATTGTACAGGAGGTGCCGAAATTAGGGACCTCATTTGTGTGCAAAGTTATAAAGGCATCAAAGTGTTGCCCCCACTGCTCTCAGAAAATCAGATCAGTGGATACACTTTGGCTGCCACCTCTGCTAGTCTATTTCCTCCTTGGACAGCAGCAGCAGGTGTACCATTTTATTTAAATGTTCAGTATCGCATTAATGGGCTTGGTGTCACCATGGATGTGCTAAGTCAAAATCAAAAGCTTATTGCTAATGCATTTAACAATGCCCTTAATGCTATTCAGCAAGGGTTCGATGCAACTAATTCTGCTTTAGTTAAAATTCAAGCTGTTGTTAATGCAAATGCTGAAGCTCTTAATAACTTATTGCAACAACTCTCTAATAGATTTGGTGCTATAAGTGCTTCTTTACAAGAAATTCTATCTAGACTTGATGCTCTTGAAGCGGAAGCTCAGATAGATAGACTTATTAATGGTCGTCTCACCGCTCTTAATGCTTATGTTTCTCAACAGCTTAGTGATTCTACACTGGTAAAATTTAGTGCAGCGCAAGCTATGGAGAAGGTTAATGAATGTGTCAAAAGCCAATCATCTAGGATAAATTTCTGTGGTAATGGTAATCATATTATATCATTAGTGCAGAATGCTCCATATGGTTTGTATTTTATCCACTTTAATTATGTCCCTACTAAGTATGTCACAGCGAAGGTTAGTCCTGGTCTGTGCATTGCTGGTAATAGAGGTATAGCTCCTAAGAGTGGTTATTTTGTTAATGTAAATAATACTTGGATGTACACTGGTAGTGGTTACTACTACCCTGAACCTATAACTGAAAATAATGTTGTTGTTATGAGTACCTGCGCTGTTAATTATACTAAAGCGCCGTATGTAATGCTGAACACTTCAATACCCAACCTTCCTGATTTTAAGGAAGAGTTGGATCAATGGTTTAAAAATCAAACATCAGTGGCACCAGATTTGTCACTTGATTATATAAATGTTACATTCTTGGACCTACAAGTTGAAATGAATAGGTTACAGGAGGCAATAAAAGTCTTAAATCATAGCTACATCAATCTCAAGGACATTGGTACATATGAATATTATGTAAAATGGCCTTGGTATGTATGGCTTTTAATCTGCCTTGCTGGTGTAGCTATGCTTGTTTTACTATTCTTCATATGCTGTTGTACAGGATGTGGGACTAGTTGTTTTAAGAAATGTGGTGGTTGTTGTGATGATTATACTGGATACCAGGAGTTAGTAATCAAAACTTCACATGACGACTAA

>gi|530802257|gb|KF530069.1| Human coronavirus OC43 strain OC43/human/USA/982-4/1998, complete genome

ATGTTTTTGATACTTTTAATTTCCTTACCAACGGCTTTTGCTGTTATAGGAGATTTAAAGTGTCCTTTAGATACTAGTCGTAAAGGTAGCTTTAATAATAAAGACACCGGTCCTCCTTTTATAAGTACTGATACTGTTGATGTTACTAATGGTTTGGGTACTTATTATGTTTTAGATCGTGTGTATTTAAATACCACGTTGTTTCTTAATGGTTATTACCCTACTTCAGGTTCCACATATCGTAATATGGCACTGAAGGGAACTGACAAATTGAGCACACTATGGTTTAAACCACCATTTCTTTCTGATTTTATTAATGGTATTTTTGCTAAGGTCAAAAATACCAAGGTTTTTAAAGATGGTGTAATGTATAGTGAGTTCCCTGCTATAACTATAGGTAGTACTTTTGTAAATACATCCTATAGTGTGGTAGTACAACCACGTACAATTAATTCAACACAGGATGGTGTTAATAAATTACAAGGTCTTTTAGAGGTCTCTGTTTGCCAGTATAATATGTGTGAGTACCCACATACGATTTGTCATCCTAAACTGGGTAATCACTTCAAAGAACTATGGCATTTGGATACAGGTGTTGTTTCCTGTTTATATAAGCGTAATTTCACATATGATGTGAATGCTACTTATTTGTATTTTCATTTTTATCAAGAAGGTGGTACTTTTTATGCATATTTTACAGACACTGGTGTTGTTACTAAGTTTTTGTTCAATGTTTATTTAGGCATGGCACTTTCACACTATTATGTCATGCCTCTGACTTGTATTAGTAGGCGTGATATTGGTTTTACTTTAGAATATTGGGTTACACCCCTCACTTCTAGACAATATTTACTCGCTTTCAATCAAGATGGTATTATTTTTAATGCTGTTGATTGTATGAGTGATTTTATGAGTGAGATTAAGTGTAAAACACAATCTATAGC

ACCACCTACTGGTGTTTATGAATTAAACGGTTACACTGTTCAGCCAATCGCAGATGTTTACCGACGCAAACCTGATCTT
CCCAATTGCAATATAGAAGCTTGGCTTAATGATAAGTCGGTGCCCTCTCCATTAAATTGGGAACGTAAGACATTTTCAA
ATTGTAATTTTAATATGAGCAGCCTGATGTCTTTTATTCAGGCAGACTCATTTACTTGTAATAATATTGATGCTGCTAA
GATATATGGTATGTGTTTTTCCAGCATAACTATAGACAAGTTTGCTATACCCAATGGCAGGAAGGTTGACCTACAATTG
GGCAATTTGGGCTATTTGCAGTCATCCAACTATAGAATTGATACTACTGCAACAAGTTGTCAGTTGTATTATAATTTAC
CTGCTGCTAATGTCTCTGTTAGCAGGTTTAATCCTTCTACTTGGAATAAGAGATTTGGTTTTATAGAAGATTCTGTTTT
TGTGCCTCAACCTACAGGTGTTTTTACTAATCACAGTGTAGTTTATGCACAACACTGTTTCAAAGCCCCTAAAAATTTC
TGTCCGTGTAAATTGAATGGTTCGTGTCCTGATAAAAATAATGGTATAGGCACTTGTCCTGCAGGTACTAATTATTTAA
CTTGTGATAATTTGTGTACTCTTGATCCTATTACATTTAAAGCTCCAGGTACTTATAAGTGCCCCCAAACTAAATCTTT
AGTTGGCATAGGTGAGCACTGTTCGGGTCTTGCTGTTAAAAGTGATTATTGTAGAGGCAATTCTTGTACTTGCCAACCA
CAAGCATTTTTGGGTTGGTCTGCAGACTCTTGTTTACAAGGAGACAAGTGTAATATTTTTGCTAATCTTATTTTGCATG
ATGTTAATAGTGGTCTTACTTGTTCTACTGATTTACAAAAAGCTAACACAGACATAATTCTTGGTGTTTGTGTTAATTA
TGACCTCTATGGTATTTCAGGCCAAGGCATTTTTGTTGAGGTTAATGCGACTTATTATAATAGTTGGCAGAACCTTTTA
TATGATTCTAATGGTAATCTCTACGGTTTTAGAGACTACATAACAAACAGAACTTTTATGATTCGTAGTTGCTATAGCG
GTCGTGTTTCTGCGGCCTTTCATGCTAACTCTTCCGAACCAGCATTGCTATTTCGGAATATTAAATGCAACTACGTTTT
TAATAATAGTCTTACACGACAGCTGCAACCCATTAACTATTCGTTTGATAGTTATCTTGGTTGTGTTGTCAATGCTTAT
AATAGTACTGCTATTTCTGTTCAAACATGTGATCTCACAGTAGGTAGTGGTTACTGTGTGGAGTACTCTAAAAACAGAC
GAAGTCGTAGAGCGATTACCACTGGTTATCGGTTTACTAATTTTGAGCCATTTACTGTTAATTCAGTAAATGATAGTTT
GGAACCTGTAGGTGGTTTGTATGAAATTCAAATACCTTCAGAGTTTACTATAGGTAATATGGAGGAGTTTATTCAAACA
AGCTCTCCCAAAGTTACTATTGATTGTGCTGCATTTGTCTGCGGTGATTATGCAGCATGTAAATTACAGTTGGTTGAAT
ATGGTAGTTTCTGTGATAACATTAATGCCATACTCACAGAAGTAAATGAACTACTTGATACCACACAGTTGCAAGTAGC
TAATAGTTTAATGAATGGTGTTACTCTTAGCACTAAGCTTAAAGATGGTGTTAATTTCAATGTAGACGACATCAATTTT
TCCCCTGTATTAGGTTGTCTAGGCAGCGAATGTAGTAAAGCTTCCAGTAGATCTGCTATAGAGGATTTACTTTTTGATA
AAGTAAAGTTATCTGATGTCGGTTTCGTTGAGGCTTATAATAATTGTACAGGAGGTGCCGAAATTAGGGACCTCATTTG
TGTGCAAAGTTATAAAGGCATCAAAGTGTTGCCCCCACTGCTCTCAGAAAATCAGATCAGTGGATACACTTTGGCTGCC
ACCTCTGCTAGTCTATTTCCTCCTTGGACAGCAGCAGCAGGTGTACCATTTTATTTAAATGTTCAGTATCGCATTAATG
GGCTTGGTGTCACCATGGATGTGCTAAGTCAAAATCAAAAGCTTATTGCTAATGCATTTAACAATGCCCTTCATGCTAT
TCAGCAAGGGTTCGATGCAACTAATTCTGCTTTAGTTAAAATTCAAGCTGTTGTTAATGCAAATGCTGAAGCTCTTAAT
AACTTATTGCAACAACTCTCTAATAGATTTGGTGCTATAAGTGCTTCTTTACAAGAAATTCTATCTAGACTTGATGCTC
TTGAAGCGGAAGCTCAGATAGATAGACTTATTAATGGTCGTCTCACCGCTCTTAATGCTTATGTTTCTCAACAGCTTAG
TGATTCTACACTGGTAAAATTTAGTGCAGCACAAGCTATGGAGAAGGTTAATGAATGTGTCAAAAGCCAATCATCTAGG
ATAAATTTCTGTGGTAATGGTAATCATATTATATCATTAGTGCAGAATGCTCCATATGGTTTGTATTTTATCCACTTTA
ATTATGTCCCTACTAAGTATGTCACAGCGAAGGTTAGTCCTGGTCTGTGCATTGCTGGTAATAGAGGTATAGCTCCTAA
GAGTGGTTATTTTGTTAATGTAAATAATACTTGGATGTACACTGGTAGTGGTTACTACTACCCTGAACCTATAACTGAA
AATAATGTTGTTGTTATGAGTACCTGCGCTGTTAATTATACTAAAGCGCCGTATGTAATGCTGAACACTTCAATACCCA
ACCTTCCTGATTTTAAGGAAGAGTTGGATCAATGGTTTAAAAATCAAACATCAGTGGCACCAGATTTGTCACTTGATTA
TATAAATGTTACATTCTTGGACCTACAAGTTGAAATGAATAGGTTACAGGAGGCAATAAAAGTCTTAAATCATAGCTAC
ATCAATCTCAAGGACATTGGTACATATGAATATTATGTAAAATGGCCTTGGTATGTATGGCTTTTAATCTGCCTTGCTG
GTGTAGCTATGCTTGTTTTACTATTCTTCATATGCTGTTGTACAGGATGTGGGACTAGTTGTTTTAAGAAATGTGGTGG
TTGTTGTGATGATTATACTGGATACCAGGAGTTAGTAATCAAAACTTCACATGACGACTAA

>gi|530802268|gb|KF530070.1| Human coronavirus OC43 strain OC43/human/USA/991-19/1999, complete genome

ATGTTTTTGATACTTTTAATTTCCTTACCAACGGCTTTTGCTGTTATAGGAGATTTAAAGTGTCCTTTAGATACT AGTCTTAAAGGTAGCTTTAATAATAGAGACACCGGTTCTCCTTCTATAAGTACTGATACTGTTGATGTTACTAATGGTT TGGGTACTTATTATGTTTTAGATCGTGTGTATTTAAATACTACGTTGTTTCTTAATGGTTATTACCCCACTTCAGGTTC CACATATCGTAATATGGCACTGAAGGGAACTGACAAATTGAGCACATTATGGTTTAAACCACCATTTCTTTCTGATTTT ATTAATGGTATTTTTGCTAAGGTCAAAAATACCAAGGTTTTTAAAGATGGTGTAATGTATAGTGAGTTCCCTGCTATAA CTATAGGTAGTACTTTTGTAAATACATCCTATAGTGTGGTAGTACAACCACGTACAATTAATTCAACACAGGATGGTGT TAATAAATTACAAGGTCTTTTAGAGGTCTCTGTTTGCCAGTATAATATGTGCGAGTACCCACATACGATTTGTCATCCT AAACTGGGTAACCACTTCAAAGAACTATGGCATTTGGATACAGGTGTTGTTTCCTGTTTATATAAGCGTAATTTCACAT ATGATGTGAATGCTACTTATTTGTATTTTCATTTTTATCAAGAAGGTGGTACTTTTTATGCATATTTTACAGACACTGG TTTTGTTACTAAGTTTTTGTTTAATGTTTATTTAGGCATGGCACTTTCACACTATTATGTCATGCCTCTGACTTGTATT AGTAGGCGTGATATTGGTTTTACTTTAGAATATTGGGTTACACCCCTCACTTCTAGACAATATTTACTCGCTTTCAATC AAGATGGTATTATTTTTAATGCTGTTGATTGTATGAGTGATTTTATGAGTGAGATTAAGTGTAAAACACAATCTATAGC ACCACCTACTGGTGTTTATGAATTAAACGGTTACACTGTTCAGCCAATCGCAGATGTTTACCGACGCAAACCTGATCTT CCCAATTGCAATATAGAAGCTTGGCTTAATGATAAGTCGGTGCCCTCTCCATTAAATTGGGAACGTAAGACATTTTCAA ATTGTAATTTTAATATGAGCAGCCTGATGTCTTTTATTCAGGCAGACTCATTTACTTGTAATAATATTGATGCTGCTAA GATATATGGTATGTGTTTTTCCAGCATAACTATAGATAAGTTTGCTATACCCAATGGCAGGAAGGTTGACCTACAATTG GGCAATTTGGGCTATTTGCAGTCATCCAACTATAGAATTGATACTACTGCAACAAGTTGTCAGTTGTATTATAATTTAC CTGCTGCTAATGTCTCTGTTAGCAGGTTTAATCCTTCTACTTGGAATAAGAGATTTGGTTTTATAGAAGATTCTGTTTT TGTGCCTCAACCTACAGGTGTTTTTACTAATCACAGTGTAGTTTATGCACAACACTGTTTCAAAGCCCCTAAAAATTTC TGTCCGTGTAAATTGAACGGTTCGTGTCCTGATAAAAATAATGGTATAGGCACTTGTCCTGCAGGTACTAATTATTTAA CTTGTGATAATTTGTGTACTCTTGATCCTATTACATTTAAAGCTCCAGGTACTTATAAGTGCCCCCAAACTAAATCTTT AGTTGGCATAGGTGAGCACTGTTCGGGTCTTGCTGTTAAAAGTGATTATTGTAGAGGCAATTCTTGTACTTGCCAACCA CAAGCATTTTTGGGTTGGTCTGCAGACTCTTGTTTACAAGGAGACAAGTGTAATATTTTTGCTAATCTTATTTTGCATG ATGTTAATAGTGGTCTTACTTGTTCTACTGATTTACAAAAAGCTAACACAGACATAATTCTTGGTGTTTGTGTTAATTA TGACCTCTATGGTATTTCAGGCCAAGGCATTTTTGTTGAGGTTAATGCGACTTATTATAATAGTTGGCAGAACCTTTTA TATGATTCTAATGGTAATCTCTACGGTTTTAGAGACTACATAACAAACAGAACTTTTATGATTCGTAGTTGCTATAGCG GTCGTGTTTCTGCGGCCTTTCATGCTAACTCTTCCGAACCAGCATTGCTATTTCGGAATATTAAATGCAACTACGTTTT TAATAATAGTCTTACACGACAGCTGCAACCCATTAACTATTCGTTTGATAGTTATCTTGGTTGTGTTGTCAATGCTTAT AATAGTACTGCTATTTCTGTTCAAACATGTGATCTCACAGTAGGTAGCGGTTACTGTGTGGAGTACTCTAAAAACAGAC GAAGTCGTAGAGCGATTACCACTGGTTATCGGTTTACTAATTTTGAGCCATTTACTGTTAATTCAGTAAATGATAGTTT GGAACCTGTAGGTGGTTTGTATGAAATTCAAATACCTTCAGAGTTTACTATAGGTAATATGGAGGAGTTTATTCAAACA AGCTCTCCCAAAGTTACTATTGATTGTGCTGCATTTGTCTGTGGTGATTATGCAGCATGTAAATTACAGTTGGTTGAAT ATGGTAGTTTCTGTGATAACATTAATGCCATACTCACAGAAGTAAATGAACTACTTGATACTACACAGTTGCAAGTAGC TAATAGTTTAATGAATGGTGTTACTCTTAGCACTAAGCTTAAAGATGGTGTTAATTTCAATGTAGACGACATCAATTTT TCCCCTGTATTAGGTTGTCTAGGCAGCGAATGTAGTAAAGCTTCCAGTAGATCTGCTATAGAGGATTTACTTTTTGATA AAGTAAAGTTATCTGATGTCGGTTTCGTTGAGGCTTATAATAATTGTACAGGAGGTGCCGAAATTAGGGACCTCATTTG TGTGCAAAGTTATAAAGGCATCAAAGTGTTGCCCCCACTGCTCTCAGAAAATCAGATCAGTGGATACACTTTGGCTGCC ACCTCTGCTAGTCTATTTCCTCCTTGGACAGCAGCAGCAGGTGTACCATTTTATTTAAATGTTCAGTATCGCATTAATG GGCTTGGTGTCACCATGGATGTGCTAAGTCAAAATCAAAAGCTTATTGCTAATGCATTTAACAATGCCCTTTATGCTAT TCAGCAAGGGTTCGATGCAACTAATTCTGCTTTAGTTAAAATTCAAGCTGTTGTTAATGCAAATGCTGAAGCTCTTAAT AACTTATTGCAACAACTCTCTAATAGATTTGGTGCTATAAGTGCTTCTTTACAAGAAATTCTATCTAGACTTGATGCTC TTGAAGCGGAAGCTCAGATAGATAGACTTATTAATGGTCGTCTCACCGCTCTTAATGCTTATGTTTCTCAACAGCTTAG TGATTCTACACTGGTAAAATTTAGTGCAGCACAAGCTATGGAGAAGGTTAATGAATGTGTCAAAAGCCAATCATCTAGG ATAAATTTCTGTGGTAATGGTAATCATATTATATCATTAGTGCAGAATGCTCCATATGGTTTGTATTTTATCCACTTTA

ATTATGTCCCTACTAAGTATGTCACAGCGAAGGTTAGTCCTGGTCTGTGCATTGCTGGTAATAGAGGTATAGCTCCTAA
GAGTGGTTATTTTGTTAATGTAAATAATACTTGGATGTACACTGGTAGTGGTTACTACTACCCTGAACCTATAACTGAA
AATAATGTTGTTGTTATGAGTACCTGCGCTGTTAATTATACTAAAGCGCCGTATGTAATGCTGAACACTTCAATACCCA
ACCTTCCTGATTTTAAGGAAGAGTTGGATCAATGGTTTAAAAATCAAACATCAGTGGCACCAGATTTGTCACTTGATTA
TATAAATGTTACATTCTTGGACCTACAAGTTGAAATGAATAGGTTACAGGAGGCAATAAAAGTCTTAAATCATAGCTAC
ATCAATCTCAAGGACATTGGTACATATGAATATTATGTAAAATGGCCTTGGTATGTATGGCTTTTAATCTGCCTTGCTG
GTGTAGCTATGTTTGTTTTACTATTCTTCATATGCTGTTGTACAGGATGTGGGACTAGTTGTTTTAAGAAATGTGGTGG
TTGTTGTGATGATTATACTGGATACCAGGAGTTAGTAATCAAAACTTCACATGACGACTAA

>gi|530802279|gb|KF530071.1| Human coronavirus OC43 strain OC43/human/USA/925-1/1992, complete genome

ATGTTTTTGATACTTTTAATTTCCTTACCAACGGCTTTTGCTGTTATAGGAGATTTAAAGTGTCCTTTAGATACT
AGTTATAAAGGTACATTTAATAATAAAGACACCGGTCCTCCTTTTATAAGTACTGATACTGTTGATGTTACTAATGGTT
TGGGTACTTATTATGTTTTAGATCGTGTGTATTTAAATACTACGTTGTTTCTTAATGGTTATTACCCTACTTCAGGTTC
CACATATCGTAATATGGCACTGAAGGGAACTGACAAATTGAGCACACTATGGTTTAAACCACCATTTCTTTCTGATTTT
ATTAATGGTATTTTTGCTAAGGTCAAAAATACCAAGGTTTTTAAAGATGGTGTAATGTATAGTGAGTTCCCTGCTATAA
CTATAGGTAGTACTTTTGTAAATACATCCTATAGTGTGGTAGTACAACCACGTACAATTAATTCACAGGATGGTGATAA
TAAATTACAAGGTCTTTTAGAGGTCTCTGTTTGCCAGTATAATATGTGCGAGTACCCACATACGATTTGTCATCCTAAA
CTGGGTAATCACTTCAAAGAACTATGGCATTTGGATACAGGTGTTGTTTCCTGTTTATATAAGCGTAATTTCACATATG
ATGTGAATGCTAATTATTTGTATTTTCATTTTTATCAAGAAGGTGGTACTTTTTATGCATATTTTACAGACTCTGGTGT
TGTTACTAAGTTTTTGTTTAATGTTTATTTAGGCATGGCACTTTCACACTATTATGTCATGCCTCTGACTTGTATTAGT
AGGCGTGATATTGGTTTTACTTTAGAATATTGGGTTACACCCCTCACTTCTAGACAATATTTACTCGCTTTCAATCAAG
ATGGTATTATTTTTAATGCTGTTGATTGTATGAGTGATTTTATGAGTGAGATTAAGTGTAAAACACAATCTATAGCACC
ACCTACTGGTGTTTATGAATTAAACGGTTACACTGTTCAGCCAATCGCAGATGTTTACCGACGCAAACCTGATCTTCCC
AATTGCAATATAGAAGCTTGGCTTAATGATAAGTCGGTGCCCTCTCCATTAAATTGGGAACGTAAGACATTTTCAAATT
GTAATTTTAATATGAGCAGCCTGATGTCTTTTATTCAGGCAGACTCATTTACTTGTAATAATATTGATGCTGCTAAGAT
ATATGGTATGTGTTTTTCCAGCATAACTATAGATAAGTTTGCTATACCCAATAGCAGGAAGGTTGACCTACAATTGGGT
AATTTGGGCTATTTGCAGTCATCCAACTATAGAATTGATACTACTGCAACAAGTTGTCAGTTGTATTATAATTTACCTG
CTGCTAATGTCTCTGTTAGCAGGTTTAATCCTTCTACTTGGAATAAGAGATTTGGTTTTATAGAAGATTCTGTTTTTGT
GCCTCAACCTACAGGTGTTTTTACTAATCACAGTGTAGTTTATGCACAACACTGTTTCAAAGCCCCTAAAAATTTCTGT
CCGTGTAAATTGAATGGTTCGTGTCCTGGTAAAAATAATGGTATAGGCACTTGTCCTGCAGGTACTAATTATTTAACTT
GTGATAATTTGTGTACTCTTGATCCTATTACATTTAAAGCTCCAGGTACTTATAAGTGCCCCCAAACTAAATCTTTAGT
TGGCATAGGTGAGCACTGTTCGGGTCTTGCTGTTAAAAGTGATTATTGTGGAGGCAATTCTTGTACTTGCCAACCACAA
GCATTTTTGGGTTGGTCTGCAGACTCTTGTTTACAAGGAGACAAGTGTAATATTTTTGCTAATCTTATTTTGCATGATG
TTAATAGTGGTCTTACTTGTTCTACTGATTTACAAAAAGCTAACACAGACATAATTCTTGGTGTTTGTGTTAATTATGA
CCTCTATGGTATTTCAGGCCAAGGCATTTTTGTTGAGGTTAATGCGACTTATTATAATAGTTGGCAGAACCTTTTATAT
GATTCTAATGGTAATCTCTACGGTTTTAGAGACTACATAACAAACAGAACTTTTATGATTCGTAGTTGCTATAGCGGTC
GTGTTTCTGCGGCCTTTCATGCTAACTCTTCCGAACCAGCATTGCTATTTCGGAATATTAAATGCAACTACGTTTTTAA
TAATAGTCTTACACGACAGCTGCAACCCATTAACTATTCGTTTGATAGTTATCTTGGTTGTGTTGTCAATGCTTATAAT
AGTACTGCTATTTCTGTTCAAACATGTGATCTCACAGTAGGTAGTGGTTACTGTGTGGAGTACTCTAAAAACAGACGAA
GTCGTAGAGCGCTTACCACTGGTTATCGGTTTACTAATTTTGAGCCATTTACTGTTAATTCAGTAAATGATAGTTTGGA
ACCTGTAGGTGGTTTGTATGAAATTCAAATACCTTCAGAGTTTACTATAGGTAATATGGAGGAGTTTATTCAAACAAGC

TCTCCCAAAGTTACTATTGATTGTGCTGCATTTGTCTGTGGTGATTATGCAGCATGTAAATTACAGTTGGTTGAATATG
GTAGTTTCTGTGATAACATTAATGCCATACTCACAGAAGTAAATGAACTACTTGATACTACACAGTTGCAAGTAGCTAA
TAGTTTAATGAATGGTGTTACTCTTAGCACTAAGCTTAAAGATGGTGTTAATTTCAATGTAGACGACATCAATTTTTCC
CCTGTATTAGGTTGTCTAGGCAGCGAATGTAGTAAAGCTTCCAGTAGATCTGCTATAGAGGATTTACTTTTTGATAAAG
TAAAGTTATCTGATGTCGGTTTCGTTGAGGCTTATAATAATTGTACAGGAGGTGCCGAAATTAGGGACCTCATTTGTGT
GCAAAGTTATAAAGGCATCAAAGTGTTGCCCCCACTGCTCTCAGAAAATCAGATCAGTGGATACACTTTGGCTGCCACC
TCTGCTAGTCTATTTCCTCCTTGGACAGCAGCAGCAGGTGTACCATTTTATTTAAATGTTCAGTATCGCATTAATGGGC
TTGGTGTCACCATGGATGTGCTAAGTCAAAATCAAAAGCTTATTGCTAATGCATTTAACAATGCCCTTTATGCTATTCA
GCAAGGGTTCGATGCAACTAATTCTGCTTTAGTTAAAATTCAAGCTGTTGTTAATGCAAATGCTGAAGCTCTTAATAAC
TTATTGCAACAACTCTCTAATAGATTTGGTGCTATAAGTGCTTCTTTACAAGAAATTCTATCTAGACTTGATGCTCTTG
AAGCGGAAGCTCAGATAGATAGACTTATTAATGGTCGTCTCACCGCTCTTAATGCTTATGTTTCTCAACAGCTTAGTGA
TTCTACACTGGTAAAATTTAGTGCAGCACAAGCTATGGAGAAGGTTAATGAATGTGTCAAAAGCCAATCATCTAGGATA
AATTTCTGTGGTAATGGTAATCATATTATATCATTAGTGCAGAATGCTCCATATGGTTTGTATTTTATCCACTTTAATT
ATGTCCCTACTAAGTATGTCACAGCGAAGGTTAGTCCTGGTCTGTGCATTGCTGGTAATAGAGGTATAGCTCCTAAGAG
TGGTTATTTTGTTAATGTAAATAATACTTGGATGTACACTGGTAGTGGTTACTACTACCCTGAACCTATAACTGAAAAT
AATGTTGTTGTTATGAGTACCTGCGCTGTTAATTATACTAAAGCGCCGTATGTAATGCTGAACACTTCAATACCCAACC
TTCCTGATTTTAAGGAAGAGTTGGATCAATGGTTTAAAAATCAAACATCAGTGGCACCAGATTTGTCACTTGATTATAT
AAATGTTACATTCTTGGACCTACAAGCTGAAATGAATAGGTTACAGGAGGCAATAAAAGTCTTAAATCATAGCTACATC
AATCTCAAGGACATTGGTACATATGAATATTATGTAAAATGGCCTTGGTATGTATGGCTCTTAATCTGCCTTGCTGGTG
TAGCTATGCTTGTTTTACTATTCTTCATATGCTGTTGTACAGGATGTGGGACTAGTTGTTTTAAGAAATGTGGTGGTTG
TTGTGATGATTATACTGGATACCAGGAGTTAGTAATCAAAACTTCACATGACGACTAA

>gi|530802290|gb|KF530072.1| Human coronavirus OC43 strain OC43/human/USA/9712-13/1997, complete genome

ATGTTTTTGATACTTTTAATTTCCTTACCAATGGCTTTTGCTGTTATAGGAGATTTAAAGTGTACTTCAGATACG
AGTTATATTAATGATGTTGACACCGGTGTTCCTCCTATAAGTACTGATACTGTTGATGTTACTAATGGTTTGGGTACTT
ATTATGTTTTAGATCGTGTGTATTTAAATACTACTTTGTTTCTTAATGGTTACTATCCTACTTCAGGTTCCACATATCG
TAATATGGCACTGAAGGGAAGTGTACTGTTGAGCACACTATGGTTTAAACCACCATTTCTTTCTGATTTTATTAATGGT
ATTTTTGCTAAGGTCAAAAATACCAAGGTTATTAAAGATGATGTACTGTACAGTGAGTTCCCTGCTATAACTATAGGTA
GTACTTTTGTAAATACATCCTATAGTGTGGTAGTACAACCACGTACAATCAATTTAGATAATAAATTACAAGGTCTTTT
AGAGATCTCTGTTTGCCAGTATAATATGTGCGAGTACCCACATACGATTTGTCATCCTAATTTGGGTAATCATCGCACA
GAACTATGGCATTTGGATACAGGTGTTGTTTCCTGTTTATATAAGCGTAATTTCACATATGATGTGAATGCTGATTATT
TGTATTTTCATTTTTATCAAGAAGGTGGTACTTTTTATGCATATTTTACAGACACTGGTGTTGTTACTAAGTTTTTGTT
TAATGTTTATTTAGGCATGGCGCTTTCACACTATTATGTCATGCCTCTGACTTGTAATAGTAAGCGTAAGGATGGTTTT
ACTTTAGAATATTGGGTTACACCTCTCACTTCTAGACAATATTTACTCGCTTTCAATCAAGATGGCATTATTTTTAATG
CTGTTGATTGCATGAGTGATTTTATGAGTGAGATTAAGTGTAAAACACAATCTATAGCACCACCTACTGGTGTTTATGA
ATTAAACGGTTACACTGTTCAGCCAATTGCAGATGTTTACCGACGTAAACCTAATCTTCCCAATTGTAATATAGAAGCT
TGGCTTAATGATAAGTCGGTACCATCTCCATTAAATTGGGAACGTAAGACATTTTCAAATTGTAATTTTAATATGAGCA
GCCTGATGTCCTTTATTCAGGCAGACTCATTTACTTGTAATAATATTGATGCTGCTAAGATATATGGTATGTGTTTTTC
CAGCATAACTATAGATAAGTTTGCTATACCCAATGGCAGGAAGGTTGACCTACAATTGGGTAATTTGGGCTATTTGCAG
TCATTTAACTATAGAATTGATACTACTGCAACAAGTTGTCAGTTGTATTATAATTTACCTGCTGCTAATGTTTCTGTTA
GCAGGTTTAATCCTTCTACTTGGAATAAGAGATTTGGTTTTATAGAAAATTCTGTTTTTAAGCCTCAACCTGCAGGTGT

TTTTACCAATCATGATGTAGTTTATGCCCAACATTGTTTTAAAGCTCCCAAAAATTTCTGTCCGTGTAAATTGAATGGT
TCTTTGTGTGTAGGTAGTGGTCCTGGTAAAAATAATGGTATAGGCACTTGTCCTGCAGGTACTAATTATTTAACTTGTC
ATAATTTGTGTAATCCTGATCCTATTACATTTGCAGGTCCTTATAAGTGCCCCCAAACTAAATCTTTAGTTGGTATAGG
TGAGCACTGTTCAGGTCTTGCTGTTAAAAGTGATTATTGTGGAGGCAATCCTTGTACTTGCCAACCACAAGCATTTTTG
GGTTGGTCTGCAGACTCTTGTTTACAAGGGGACAAGTGTAATATTTTTGCTAATTTAATTTTGCATGATGTTAATAGTG
GTCTTACTTGTTCCACTGATTTACAAAAAGCTAACACAGACATAATTCTTGGTGTTTGTGTTAATTATGACCTTTATGG
TATTTCAGGCCAAGGCATTTTTGTTGAGGTTAATGCGACTTATTATAATAGTTGGCAGAACCTTTTATATGATTCTAAT
GGTAATCTCTATGGTTTTAGAGACTACATAACAAATAGAACTTTTATGATTCGTAGTTGCTATAGCGGTCGTGTTTCTG
CGGCCTTTCACGCTAACTCTTCCGAACCAGCATTGCTATTTCGGAATATTAAATGCAACTACGTTTTTAATAATAGTCT
TACACGACAGCTGCAACCCATTAACTATTTTGATAGTTATCTTGGTTGTGTTGTCAATGCTTATAATAGTACTGCTATT
TCTGTTCAAACATGTGATCTCACAGTAGGTAGTGGTTACTGTGTGGATTACTCTAAAAACAGACGAAGTCGTAGAGCGA
TTACCACTGGTTATCGGTTTACTAATTTTGAGCCATTTACTGTTAATTCAGTAAATGATAGTTTAGAACCTGTAGGTGG
TTTGTACGAAATTCAAATACCTTCAGAGTTTACTATAGGTAATATGGAGGAGTTTATTCAAACAAGCTCTCCTAAAGTT
ACTATTGATTGTGCTGCATTTGTCTGTGGTGATTATGCAGCATGTAAATCACAGTTGGTTGAATATGGTAGTTTCTGTG
ATAACATTAATGCCATACTCACAGAAGTAAATGAACTACTTGACACTACACAGTTGCAAGTAGCTAATAGTTTAATGAA
TGGTGTTACTCTTAGCACTAAGCTTAAAGATGGCGTTAATTTCAATGTAGACGACATCAATTTTTCCTCTGTATTAGGT
TGTCTAGGCAGCGAATGTAGTAAAGCTTCCAGTAGATCTGCTATAGAGGATTTACTTTTTGATAAAGTAAAGTTATCTG
ATGTCGGTTTCGTTGAGGCTTATAATAATTGTACTGGAGGTGCCGAAATTAGGGACCTCATTTGTGTGCAAAGTTATAA
AGGTATCAAAGTGTTGCCTCCACTGCTCTCAGAAAATCAGATCAGTGGATACACTTTGGCTGCCACTTCTGCTAGTCTG
TTTCCTCCCTGGACAGCAGCAGCAGGTGTACCATTTTATTTAAATGTTCAGTATCGCATTAATGGGCTTGGTGTCACCA
TGGATGTGCTAAGTCAAAATCAAAAGCTTATTGCTAATGCATTTAACAATGCCCTTGATGCTATTCAGGAAGGGTTCGA
TGCAACTAATTCTGCTTTAGTTAAAATTCAAGCTGTTGTTAATGCAAATGCTGAAGCTCTTAATAACTTATTGCAACAA
CTCTCTAATAGATTTGGTGCTATAAGTTCTTCTCTACAAGAAATTCTATCTAGACTTGATGCTCTTGAAGCGGAAGCTC
AGATAGATAGACTTATTAATGGTCGTCTTACCGCTCTTAATGCTTATGTTTCTCAACAGCTTAGTGATTCTACACTGGT
AAAATTTAGTGCAGCACAAGCTATGGAGAAGGTTAATGAATGTGTCAAAAGCCAATCATCTAGGATAAATTTCTGTGGT
AATGGTAACCATATTATATCATTAGTGCAGAATGCTCCATATGGTTTGTATTTTATCCACTTTAGTTATGTCCCTACTA
AGTATGTCACAGCGAAGGTTAGTCCTGGTCTGTGCATTGCTGGTGATAGAGGTATAGCTCCTAAGAGTGGTTATTTTGT
TAATGTAAATAATACTTGGATGTACACTGGTAGTGGTTACTATTACCCTGAACCTATAACTGAAAATAATGTTGTTGTT
ATGAGTACCTGCGCTGTTAATTATACTAAAGCGCCGTATGTAATGCTGAACACTTCAACACCCAACCTTCCTGATTTTA
GGGAAGAGTTGGATCAATGGTTTAAAAATCAAACATCAGTGGCACCAGATCTGTCACTTGATTATATAAATGTTACATT
CTTGGACCTACAAGTTGAAATGAATAGGTTACAGGAGGCAATAAAAGTTTTAAATCAGAGCTACATCAATCTCAAGGAC
ATTGGTACATATGAATATTATGTAAAATGGCCTTGGTATGTATGGCTTTTAATCGGCCTTGCTGGTGTAGCTATGCTTG
TTTTACTATTCTTCATATGCTGTTGTACAGGATGTGGGACTAGTTGTTTTAAGAAATGTGGTGGTTGTTGTGATGATTA
TACTGGATACCAGGAGTTAGTAATCAAAACTTCACATGACGACTAA

>gi|530802301|gb|KF530073.1| Human coronavirus OC43 strain OC43/human/USA/8912-37/1989, complete genome

ATGTTTTTGATACTTTTAATTTCCTTACCAATGGCTTTTGCTGTTATAGGAGATTTAAAGTGTACTTCAGATACG
AGTTATATTAATGATGTTGACACCGGTGTTCCTCCTATAAGTACTGATACTGTTGATGTTACTAATGGTTTGGGTACTT
ATTATGTTTTAGATCGTGTGTATTTAAATACTACTTTGTTTCTTAATGGTTACTATCCTACTTCAGGTTCCACATATCG
TAATATGGCACTGAAGGGAAGTGTACTGTTGAGCACACTATGGTTTAAACCACCATTTCTTTCTGATTTTATTAATGGT
ATTTTTGCTAAGGTCAAAAATACCAAGGTTATTAAAGATGATGTACTGTACAGTGAGTTCCCTGCTATAACTATAGGTA

GTACTTTTGTAAATACATCCTATAGTGTGGTAGTACAACCACGTACAATCAATTTAGATAATAAATTACAAGGTCTTTT
AGAGATCTCTGTTTGCCAGTATAATATGTGCGAGTACCCACATACGATTTGTCATCCTAACTTGGGTAATCATCGCACA
GAACTATGGCATTTGGATACAGGTGTTGTTTCCTGTTTATATAAGCGTAATTTCACATATGATGTGAATGCTGATTATT
TGTATTTTCATTTTTATCAAGAAGGTGGTACTTTTTATGCATATTTTACAGACACTGGTGTTGTTACTAAGTTTTTGTT
TAATGTTTATTTAGGCATGGCGCTTTCACACTATTATGTCATGCCTCTGACTTGTAATAGTAAGCGTAAGGATGGTTTT
ACTTTAGAATATTGGGTTACACCTCTCACTTCTAGACAATATTTACTCGCTTTCAATCAAGATGGTATTATTTTTAATG
CTGTTGATTGCATGAGTGATTTTATGAGTGAGATTAAGTGTAAAACACAATCTATAGCACCACCTACTGGTGTTTATGA
ATTAAACGGTTACACTGTTCAGCCAATTGCAGATGTTTACCGACGTAAACCTAATCTTCCCAATTGCAATATAGAAGCT
TGGCTTAATGATAAGTCGGTGCCATCTCCATTAAATTGGGAACGTAAGACATTTTCAAATTGTAATTTTAATATGAGCA
GCCTGATGTCCTTTATTCAGGCAGACTCATTTACTTGTAATAATATTGATGCTGCTAAGATATATGGTATGTGTTTTTC
CAGCATAACTATAGATAAGTTTGCTATACCCAATGGCAGGAAGGTTGACCTACAATTGGGTAATTTGGGCTATTTGCAG
TCATTTAACTATAGAATTGATACTACTGCAACAAGTTGTCAGTTGTATTATAATTTACCTGCTGCTAATGTTTCTGTTA
GCAGGTTTAATCCTTCTACTTGGAATAAGAGATTCGGTTTTATAGAAAATTCTGTTTTTAAGCCTCAACCTGCAGGTGT
TTTTACCAATCATGATGTAGTTTATGCCCAACATTGTTTTAAAGCTCCCAAAAATTTCTGTCCGTGTAAATTGAATGGT
TCTTTGTGTGTAGGTAGTGGTCCTGGTAAAAATAATGGTATAGGCACTTGCCCTGCAGGTACTAATTATTTAACTTGTC
ATAATTTGTGCAATCCTGATCCTATTACATTTACAGGTCCTTACAAGTGCCCCCAAACTAAATCTTTAGTTGGTATAGG
TGAGCACTGTTCAGGTCTTGCTGTTAAAAGTGATTATTGTGGAGGCAATCCTTGCACTTGCCAACCACAAGCATTTTTG
GGTTGGTCTGCAGACTCTTGTTTACAAGGGGACAAGTGTAATATTTTTGCTAATTTAATTTTGCATGATGTTAATAGTG
GTCTTACTTGTTCTACTGATTTACAAAAAGCTAACACAGACATAATTCTTGGTGTTTGTGTTAATTATGACCTTTATGG
TATTTCAGGCCAGGGCATTTTTGTTGAGGTTAATGCGACTTATTATAATAGTTGGCAGAACCTTTTATATGATTCTAAT
GGTAATCTCTATGGTTTTAGAGACTACATAACAAACAGAACTTTTATGATTCGTAGTTGCTATAGCGGTCGTGTTTCTG
CGGCCTTTCACGCTAACTCTTCCGAACCAGCATTGCTATTTCGGAATATTAAATGCAACTACGTTTTTAATAATAGTCT
TACACGACAGCTGCAACCCATTAACTATTTTGATAGTTATCTTGGTTGTGTTGTCAATGCTTATAATAGTACTGCTATT
TCTGTTCAAACATGTGATCTCACAGTAGGTAGTGGTTACTGTGTGGATTACTCTAAAAACAGACGAAGTCGTAGAGCGA
TTACCACTGGTTATCTGTTTACTAATTTTGAGCCATTTACTGTTAATTCAGTAAATGATAGTTTAGAACCTGTAGGTGG
TTTGTACGAAATTCAAATACCTTCAGAGTTTACTATAGGTAATATGGAGGAGTTTATTCAAACAAGCTCTCCTAAAGTT
ACTATTGATTGTGCTGCATTTGTCTGTGGTGATTATGCAGCATGTAAATCACAGTTGGTTGAATATGGTAGTTTCTGTG
ATAACATTAATGCCATACTCACAGAAGTAAATGAACTACTTGACACTACACAGTTGCAAGTAGCTAATAGTTTAATGAA
TGGTGTTACTCTTAGCACTAAGCTTAAAGATGGCGTTAATTTCAATGTAGACGACATCAATTTTTCCCCTGTATTAGGT
TGTCTAGGCAGCGAATGTAGTAAAGCTTCCAGTAGATCTGCTATAGAGGATTTACTTTTTGATAAAGTAAAGTTATCTG
ATGTCGGTTTCGTTGAGGCTTATAATAATTGTACTGGAGGTGCCGAAATTAGGGACCTCATTTGTGTGCAAAGTTATAA
AGGTATCAAAGTGTTGCCTCCACTGCTCTCAGAAAATCAGATCAGTGGATACACTTTGGCTGCCACTTCTGCTAGTCTG
TTTCCTCCTTGGACAGCAGCAGCAGGTGTACCATTTTATTTAAATGTTCAGTATCGCATTAATGGGCTTGGTGTCACCA
TGGATGTGCTAAGTCAAAATCAAAAGCTTATTGCTAATGCATTTAACAATGCCCTTGATGCTATTCAGGAAGGGTTCGA
TGCAACTAATTCTGCTTTAGTTAAAATTCAAGCTGTTGTTAATGCAAATGCTGAAGCTCTTAATAACTTATTGCAACAA
CTCTCTAATAGATTTGGTGCTATAAGTTCTTCTCTACAAGAAATTCTATCTAGACTTGATGCTCTTGAAGCGGAAGCTC
AGATAGATAGACTTATTAATGGTCGTCTTACCGCTCTTAATGCTTATGTTTCTCAACAGCTTAGTGATTCTACACTGGT
AAAATTTAGTGCAGCACAAGCTATGGAGAAGGTTAATGAATGTGTCAAAAGCCAATCATCTAGGATAAATTTCTGTGGT
AATGGTAACCATATTATATCATTAGTGCAGAATGCTCCATATGGTTTGTATTTTATCCACTTTAGTTATGTCCCTACTA
AGTATGTCACAGCGAAGGTTAGTCCTGGTCTGTGCATTGCTGGTGATAGAGGTATAGCTCCTAAGAGTGGTTATTTTGT
TAATGTAAATAATACTTGGATGTACACTGGTAGTGGTTACTACTACCCTGAACCTATAACTGAAAATAATGTCGTTGTT
ATGAGTACCTGCGCTGTTAATTATACTAAAGCGCCGTATGTAATGCTGAACACTTCAACACCCAACCTTCCTGATTTTA
GGGAAGAGTTGGATCAATGGTTTAAAAATCAAACATCAGTGGCACCAGATCTGTCACTTGATTATATAAATGTTACATT
CTTGGACCTACAAGTTGAAATGAATAGGTTACAGGAGGCAATAAAAGTTTTAAATCAGAGCTACATCAATCTCAAGGAC

ATTGGTACATATGAATATTATGTAAAATGGCCTTGGTATGTATGGCTTTTAATCGGCCTTGCTGGTGTAGCTATGCTTGTTTTACTATTCTTCATATGCTGTTGTACAGGATGTGGGACTAGTTGTTTTAAGAAATGTGGTGGTTGTTGTGATGATTATACTGGATACCAGGAGTTAGTAATCAAAACTTCACATGACGACTAA

>gi|530802323|gb|KF530075.1| Human coronavirus OC43 strain OC43/human/USA/953-23/1995, complete genome

ATGTTTTTGATACTTTTAATTTCCTTACCAATGGCTTTTGCTGTTATAGGAGATTTAAAGTGTACTTCAGATACTAGTTATATTAATGATGTTGACACCGGTGTTCCTCCTATAAGTACTGATACTGTTGATGTTACTAATGGTTTGGGTACTTATTATGTTTTAGATCGTGTGTATTTAAATACTACTTTGTTTCTTAATGGTTACTATCCTACTTCAGGTTCCACATATCGTAATATGGCACTGAAGGGAAGTGTACTGTTGAGCACACTATGGTTTAAACCACCATTTCTTTCTGATTTTATTAATGGTATTTTTGCTAAGGTCAAAAATACCAAGGTTATTAAAGATGATGTACTGTACAGTGAGTTCCCTGCTATAACTATAGGTAGTACTTTTGTAAATACATCCTATAGTGTGGTAGTACAACCACGTACAATCAATTTAGATAATAAATTACAAGGTCTTTTAGAGATCTCTGTTTGCCAGTATAATATGTGCGAGTACCCACATACGATTTGTCATCCTAACTTGGGTAATCATCGCACAGAACTATGGCATTTGGATACAGGTGTTGTTTCCTGTTTATATAAGCGTAATTTCACATATGATGTGAATGCTGATTATTTGTATTTTCATTTTTATCAAGAAGGTGGTACTTTTTATGCATATTTTACAGACACTGGTGTTGTTACTAAGTTTTTGTTTAATGTTTATTTAGGCATGGCGCTTTCACACTATTATGTCATGCCTCTGACTTGTAATAGTAAGCGTAAGGATGGTTTTACTTTAGAATATTGGGTTACACCTCTCACTTCTAGACAATATTTACTCGCTTTCAATCAAGATGGTATTATTTTTAATGCTGTTGATTGCATGAGTGATTTTATGAGTGAGATTAAGTGTAAAACACAATCTATAGCACCACCTACTGGTGTTTATGAATTAAACGGTTACACTGTTCAGCCAATTGCAGATGTTTACCGACGTAAACCTAATCTTCCCAATTGCAATATAGAAGCTTGGCTTAATGATAAGTCGGTGCCATCTCCATTAAATTGGGAACGTAAGACATTTTCAAATTGTAATTTTAATATGAGCAGCCTGATGTCCTTTATTCAGGCAGACTCATTTACTTGTAATAATATTGATGCTGCTAAGATATATGGTATGTGTTTTTCCAGCATAACTATAGATAAGTTTGCTATACCCAATGGCAGGAAGGTTGACCTACAATTGGGTAATTTGGGCTATTTGCAGTCATTTAACTATAGAATTGATACTACCGCAACAAGTTGTCAGTTGTATTATAATTTACCTGCTGCTAATGTTTCTGTTAGCAGGTTTAATCCTTCTACTTGGAATAAGAGATTTGGTTTTATAGAAAATTCTGTTTTTAAGCCTCAACCTGCAGGTGTTTTTACCAATCATGATGTAGTTTATGCCCAACATTGTTTTAAAGCTCCCAAAAATTTCTGTCCTTGTAAATTGAATGGTTCTTTGTGTGTAGGTAGTGGTCCTGGTAAAAATAATGGTATAGGCACTTGTCCTGCAGGTACTAATTATTTAACTTGTCATAATTTGTGCAATCCTGATCCTATTACATTTACAGGTCCTTATAAGTGCCCCCAATCTAAATCTTTAGTTGGTATAGGTGAGCACTGTTCAGGTCTTGCTGTTAAAAGTGATTATTGTGGAGGCAATCCTTGCACTTGCCAACCACAAGCATTTTTGGGTTGGTCTGCAGACTCTTGTTTACAAGGGGACAAGTGTAATATTTTTGCTAATTTAATTTTGCATGATGTTAATAGTGGTCTTACTTGTTCTACTGATTTACAAAAAGCTAACACAGACATAATTCTTGGTGTTTGTGTTAATTATGATCTTTATGGTATTTCAGGCCAAGGCATTTTTGTTGAGGTTAATGCGACTTATTATAATAGTTGGCAGAACCTTTTATATGATTCTAATGGTAATCTCTATGGTTTTAGAGACTACATAACAAACAGAACTTTTATGATTCGTAGTTGCTATAGCGGTCGTGTTTCTGCGGCCTTTCACGCTAACTCTTCCGAACCAGCATTGCTATTTCGGAATATTAAATGCAACTACGTTTTTAATAATAGTCTTACACGACAGCTGCAACCCATTAACTATTTTGATAGTTATCTTGGTTGTGTTGTCAATGCTTATAATAGTACTGCTATTTCTGTTCAAACATGTGATCTCACAGTAGGTAGTGGTTACTGTGTGGATTACTCTAAAAACAGACGAAGTCGTAGAGCGATTACCACTGGTTATCGGTTTACTAATTTTGAGCCATTTACTGTTAATTCAGTAAATGATAGTTTAGAACCTGTAGGTGGTTTGTACGAAATTCAAATACCTTCAGAGTTTACTATAGGTAATATGGAGGAGTTTATTCAAACAAGCTCTCCTAAAGTTACTATTGATTGTGCTGCATTTGTCTGTGGTGATTATGCAGCATGTAAATCACAGTTGGTTGAATATGGTAGTTTCTGTGATAACATTAATGCCATACTCACAGAAGTAAATGAACTACTTGACACTACACAGTTGCAAGTAGCTAATAGTTTAATGAATGGTGTTACTCTTAGCACTAAGCTTAAAGATGGCGTTAATTTCAATGTAGACGACATCAATTTTTCCTCTGTATTAGGTTGTCTAGGCAGCGAATGTAGTAAAGCTTCCAGTAGATCTGCTATAGAGGATTTACTTTTTGATAAAGTAAAGTTATCTGATGTCGGTTTCGTTGAGGCTTATAATAATTGTACTGGAGGTGCCGAAATTAGGGACCTCATTTGTGTGCAAAGTTATAA

AGGTATCAAAGTGTTGCCTCCACTGCTCTCAGAAAATCAGATCAGTGGATACACTTTGGCTGCCACTTCTGCTAGTCTGTTTCCTCCTTGGACAGCAGCAGCAGGTGTACCATTTTATTTAAATGTTCAGTATCGCATTAATGGGCTTGGTGTCACCATGGATGTGCTAAGTCAAAATCAAAAGCTTATTGCTAATGCATTTAACAATGCCCTTGATGCTATTCAGGAAGGGTTCGATGCAACTAATTCTGCTTTAGTTAAAATTCAAGCTGTTGTTAATGCAAATGCTGAAGCTCTTAATAACTTATTGCAACAACTCTCTAATAGATTTGGTGCTATAAGTTCTTCTCTACAAGAAATTCTATCTAGACTTGATGCTCTTGAAGCGGAAGCTCAGATAGATAGACTTATTAATGGTCGTCTTACCGCTCTTAATGCTTATGTTTCTCAACAGCTTAGTGATTCTACACTGGTAAAATTTAGTGCAGCACAAGCTATGGAGAAGGTTAATGAATGTGTCAAAAGCCAATCATCTAGGATAAATTTCTGTGGTAATGGTAACCATATTATATCATTAGTGCAGAATGCTCCATATGGTTTGTATTTTATCCACTTTAGTTATGTCCCTACTAAGTATGTCACAGCGAAGGTTAGTCCTGGTCTGTGCATTGCTGGTGATAGAGGTATAGCTCCTAAGAGTGGTTATTTTGTTAATGTAAATAATACTTGGATGTACACTGGTAGTGGTTACTACTACCCTGAACCTATAACTGAAAATAATGTCGTTGTTATGAGTACCTGCGCTGTTAATTATACTAAAGCGCCGTATGTAATGCTGAACACTTCAACACCCAACCTTCCTGATTTAGGGAAGAGTTGGATCAATGGTTTAAAAATCAAACATCAGTGGCACCAGATCTGTCACTTGATTATATAAATGTTACATTCTTGGACCTACAAGTTGAAATGAATAGGTTACAGGAGGCAATAAAAGTTTTAAATCAGAGCTACATCAATCTCAAGGACATTGGTACATATGAATATTATGTAAAATGGCCTTGGTATGTATGGCTTTTAATCGGCCTTGCTGGTGTAGCTATGCTTGTTTTACTATTCTTCATATGCTGTTGCACAGGATGTGGGACTAGTTGTTTTAAGAAATGTGGTGGTTGTTGTGATGATTATACTGGATACCAGGAGTTAGTAATCAAAACTTCACATGACGACTAA

>gi|530802334|gb|KF530076.1| Human coronavirus OC43 strain OC43/human/USA/911-11/1991, complete genome

ATGTTTTTGATACTTTTAATTTCCTTACCAACGGCCTTTGCTGTTATAGGAGATTTAAAGTGTCCTTTAGATACTAGTTATAAAGGTACATTTAATAATAAAGACACCGGTCCTCCTTTTATAAGTACTGATACTGTTGATGTTACTAATGGTTTGGGTACTTATTATGTTTTAGATCGTGTGTATTTAAATACTACGTTGTTTCTTAATGGTTATTACCCTACTTCAGGTTCCACATATCGTAATATGGCACTGAAGGGAACTGACAAATTGAGCACACTATGGTTTAAACCACCATTTCTTTCTGATTTTATTAATGGTATTTTTGCTAAGGTCAAAAATACCAAGGTTTTTAAAGATGGTGTAATGTATAGTGAGTTCCCTGCTATAACTATAGGTAGTACTTTTGTAAATACATCCTATAGTGTGGTAGTACAACCACGTACAATTAATTCACAGGATGGTGATAATAAATTACAAGGTCTTTTAGAGGTCTCTGTTTGCCAGTATAATATGTGCGAGTACCCACATACGATTTGTCATCCTAAACTGGGTAATCACTTCAAAGAACTATGGCATTTGGATACAGGTGTTGTTTCCTGTTTATATAAGCGTAATTTCACATATGATGTGAATGCTAATTATTTGTATTTTCATTTTTATCAAGAAGGTGGTACTTTTTATGCATATTTTACAGACACTGGTGTTGTTACTAAGTTTTTGTTTAATGTTTATTTAGGCATGGCACTTTCACACTATTATGTCATGCCTCTGACTTGTATTAGTAGGCGTGATATTGGTTTTACTTTAGAATATTGGGTTACACCCCTCACTTCTAGACAATATTTACTCGCTTTCAATCAAGATGGTATTATTTTTAATGCTGTTGATTGTATGAGTGATTTTATGAGTGAGATTAAGTGTAAAACACAATCTATAGCACCACCTACTGGTGTTTATGAATTAAACGGTTACACTGTTCAGCCAATCGCAGATGTTTACCGACGCAAACCTGATCTTCCCAATTGTAATATAGAAGCTTGGCTTAATGATAAGTCGGTGCCCTCTCCATTAAATTGGGAACGTAAGACATTTTCAAATTGTAATTTTAATATGAGCAGCCTGATGTCTTTTATTCAGGCAGACTCATTTACTTGTAATAATATTGATGCTGCTAAGATATATGGTATGTGTTTTTCCAGCATAACTATAGATAAGTTTGCTATACCCAATAGCAGGAAGGTTGACCTACAATTGGGTAATTTGGGCTATTTGCAGTCATCCAACTATAGAATTGATACTACTGCAACAAGTTGTCAGTTGTATTATAATTTACCTGCTGCTAATGTCTCTGTTAGCAGGTTTAATCCTTCTACTTGGAATAAGAGATTTGGTTTTATAGAAGATTCTGTTTTTGTGCCTCAACCTACAGGTGTTTTTACTAATCACAGTGTAGTTTATGCACAACACTGTTTCAAAGCCCCTAAAAATTTCTGTCCGTGTAAATTGAATGGTTCGTGTCCTGGTAAAAATAATGGTATAGGCACTTGTCCTGCAGGTACTAATTACTTAACTTGTGATAATTTGTGTACTCTTGATCCTATTACATTTAAAGCTCCAGGTACTTATAAGTGCCCCCAAACTAAATCTTTAGTTGGCATAGGTGAGCACTGTTCGGGTCTTGCTGTTAAAAGTGATTATTGTGGAGGCAATTCTTGTACTTGCCAACCACAAGCATTTTTGGGTTGGTCTGCAGACTCTTGTTTACAAGGAGACAAGTGTAATATTTTTGCTAATCTTATTTTGCATGATG

TTAATAGTGGTCTTACTTGTTCTACTGATTTACAAAAAGCTAACACAGACATAATTCTTGGTGTTTGTGTTAATTATGA
CCTCTATGGTATTTCAGGCCAAGGCATTTTTGTTGAGGTTAATGCGACTTATTATAATAGTTGGCAGAACCTTTTATAT
GATTCTAATGGTAATCTCTACGGTTTTAGAGACTACATAACAAACAGAACTTTTATGATTCGTAGTTGCTATAGCGGTC
GTGTTTCTGCGGCCTTTCATGCTAACTCTTCCGAACCAGCATTGCTATTTCGGAATATTAAATGCAACTACGTTTTTAA
TAATAGTCTTACACGACAGCTGCAACCCATTAACTATTCGTTTGATAGTTATCTTGGTTGTGTTGTCAATGCTTATAAT
AGTACTGCTATTTCTGTTCAAACATGTGATCTCACAGTAGGTAGTGGTTACTGTGTGGAGTACTCTAAAAACAGACGAA
GTCGTAGAGCGCTTACCACTGGTTATCGGTTTACTAATTTTGAGCCATTTACTGTTAATTCAGTAAATGATAGTTTGGA
ACCTGTAGGTGGTTTGTATGAAATTCAAATACCTTCAGAGTTTACTATAGGTAATATGGAGGAGTTTATTCAAACAAGC
TCTCCCAAAGTTACTATTGATTGTGCTGCATTTGTCTGTGGTGATTATGCAGCATGTAAATTACAGTTGGTTGAATATG
GTAGTTTCTGTGATAACATTAATGCCATACTCACAGAAGTAAATGAACTACTTGATACTACACAGTTGCAAGTAGCTAA
TAGTTTAATGAATGGTGTTACTCTTAGCACTAAGCTTAAAGATGGTGTTAATTTCAATGTAGACGACATCAATTTTTCC
CCTGTATTAGGTTGTCTAGGCAGCGAATGTAGTAAAGCTTCCAGTAGATCTGCTATAGAGGATTTACTTTTTGATAAAG
TAAAGTTATCTGATGTCGGTTTCGTTGAGGCTTATAATAATTGTACAGGAGGTGCCGAAATTAGGGACCTCATTTGTGT
GCAAAGTTATAAAGGCATCAAAGTGTTGCCCCCACTGCTCTCAGAAAATCAGATCAGTGGATACACTTTGGCTGCCACC
TCTGCTAGTCTATTTCCTCCTTGGACAGCAGCAGCAGGTGTACCATTTTATTTAAATGTTCAGTATCGCATTAATGGGC
TTGGTGTCACCATGGATGTGCTAAGTCAAAATCAAAAGCTTATTGCTAATGCATTTAACAATGCCCTTTATGCTATTCA
GCAAGGGTTCGATGCAACTAATTCTGCTTTAGTTAAAATTCAAGCTGTTGTTAATGCAAATGCTGAAGCTCTTAATAAC
TTATTGCAACAACTCTCTAATAGATTTGGTGCTATAAGTGCTTCTTTACAAGAAATTCTATCTAGACTTGATGCTCTTG
AAGCGGAAGCTCAGATAGATAGACTTATTAATGGTCGTCTCACCGCTCTTAATGCTTATGTTTCTCAACAGCTTAGTGA
TTCTACACTGGTAAAATTTAGTGCAGCACAAGCTATGGAGAAGGTTAATGAATGTGTCAAAAGCCAATCATCTAGGATA
AATTTCTGTGGTAATGGTAATCATATTATATCATTAGTGCAGAATGCTCCATATGGTTTGTATTTTATCCACTTTAATT
ATGTCCCTACTAAGTATGTCACAGCGAAGGTTAGTCCTGGTCTGTGCATTGCTGGTAATAGAGGTATAGCTCCTAAGAG
TGGTTATTTTGTTAATGTAAATAATACTTGGATGTACACTGGTAGTGGTTACTACTACCCTGAACCTATAAATGAAAAT
AATGTTGTTGTTATGAGTACTTGCGCTGTTAATTATACTAAAGCGCCGTATGTAATGCTGAACACTTCAATACCCAACC
TTCCTGATTTTAAGGAAGAGTTGGATCAATGGTTTAAAAATCAAACATCAGTGGCACCAGATTTGTCACTTGATTATAT
AAATGTTACATTCTTGGACCTACAAGTTGAAATGAATAGGTTACAGGAGGCAATAAAAGTCTTAAATCATAGCTACATC
AATCTCAAGGACATTGGTACATATGAATATTATGTAAAATGGCCTTGGTATGTATGGCTCTTAATCTGCCTTGCTGGTG
TAGCTATGCTTGTTTTACTATTCTTCATATGCTGTTGTACAGGATGTGGGACTAGTTGTTTTAAGAAATGTGGTGGTTG
TTGTGATGATTATACTGGATACCAGGAGTTAGTAATCAAAACTTCACATGACGACTAA

>gi|530802344|gb|KF530077.1| Human coronavirus OC43 strain OC43/human/USA/873-16/1987, complete genome

ATGTTTTTGATACTTTTAATTTCCTTACCAATGGCTTTTGCTGTTATAGGAGATTTAAAGTGTACTTCAGATACG
AGTTATATTAATGATGTTGACACCGGTGTTCCTCCTATAAGTACTGATACTGTTGATGTTACTAATGGTTTGGGTACTT
ATTATGTTTTAGATCGTGTGTATTTAAATACTACTTTGTTTCTTAATGGTTACTATCCTACTTCAGGTTCCACATATCG
TAATATGGCACTGAAGGGAAGTGTACTGTTGAGCACACTATGGTTTAAACCACCATTTCTTTCTGATTTTATTAATGGT
ATTTTTGCTAAGGTCAAAAATACCAAGGTTATTAAAGATGATGTACTGTACAGTGAGTTCCCTGCTATAACTATAGGTA
GTACTTTTGTAAATACATCCTATAGTGTGGTAGTACAACCACGTACAATCAATTTAGATAATAAATTACAAGGTCTTTT
AGAAATCTCTGTTTGCCAGTATAATATGTGCGAGTACCCACATACGATTTGTCATCCTAACTTGGGTAATCATCGCACA
GAACTATGGCATTTGGATACAGGTGTTGTTTCCTGTTTATATAAGCGTAATTTCACATATGATGTGAATGCTGATTATT
TGTATTTTCATTTTTATCAAGAAGGTGGTACTTTTTATGCATATTTTACAGACACTGGTGTTGTTACTAAGTTTTTGTT
TAATGTTTATTTAGGCATGGCGCTTTCACACTATTATGTCATGCCTCTGACTTGTAATAGTAAGCGTAAGGATGGTTTT

ACTTTAGAATATTGGGTTACACCTCTCACTTCTAGACAATATTTACTCGCTTTCAATCAAGATGGTATTATTTTTAATG
CTGTTGATTGCATGAGTGATTTTATGAGTGAGATTAAGTGTAAAACACAATCTATAGCACCACCTACTGGTGTTTATGA
ATTAAACGGTTACACTGTTCAGCCAATCGCAGATGTTTACCGACGTAAACCTAATCTTCCCAATTGCAATATAGAAGCT
TGGCTTAATGATAAGTCGGTGCCATCTCCATTAAATTGGGAACGTAAGACATTTTCAAATTGTAATTTTAATATGAGCA
GCCTGATGTCCTTTATTCAGGCAGACTCATTTACTTGTAATAATATTGATGCTGCTAAGATATATGGTATGTGTTTTTC
CAGCATAACTATAGATAAGTTTGCTATACCCAATGGCAGGAAGGTTGACCTACAATTGGGTAATTTGGGCTATTTGCAG
TCATTTAACTATAGAATTGATACTACTGCAACAAGTTGTCAGTTGTATTATAATTTACCTGCTGCTAATGTTTCTGTTA
GCAGGTTTAATCCTTCTACTTGGAATAAGAGATTCGGTTTTATAGAAGATTCTGTCTTTAAGCCTCAACCTGCAGGTGT
TTTTACCAATCATGATGTAGTTTATGCCCAACATTGTTTCAAAGCTCCCAAAAATTTCTGTCCGTGTAAATTGAATGGT
TCTCTGTGTGTAGGTAGTGGTCCTGGTAAAAATAATGGTATAGGCACTTGTCCTGCAGGTACTAATTATTTAACTTGTC
ATAATTTGTGCAATCCTGATCCTATTACATTTACAGGTCCTTATAAGTGCCCCCAAACTAAATCTTTAGTTGGTATAGG
TGAGCACTGTTCAGGTCTTGCTGTTAAAAGTGATTATTGTGGAGGCAATCCTTGCACTTGCCAACCACAAGCATTTTTG
GGTTGGTCTGCAGACTCTTGTTTACAAGGGGACAAGTGTAATATTTTTGCTAATTTAATTTTGCATGATGTTAATAGTG
GTCTTACTTGTTCTACTGATTTACAAAAAGCTAACACAGACATAATTCTTGGTGTTTGTGTTAATTATGACCTTTATGG
TATTTCAGGCCAAGGCATTTTTGTTGAGGTTAATGCGACTTATTATAATAGTTGGCAGAACCTTTTATATGATTCTAAT
GGTAATCTCTATGGTTTTAGAGACTACATAACAAACAGAACTTTTATGATTCGTAGTTGCTATAGCGGTCGTGTTTCTG
CGGCCTTTCACGCTAACTCTTCCGAACCAGCATTGCTATTTCGGAATATTAAATGCAACTACGTTTTTAATAATAGTCT
TACACGACAGCTGCAACCCATTAACTATTTTGATAGTTATCTTGGTTGTGTTGTCAATGCTTATAATAGTACTGCTATT
TCTGTTCAAACATGTGATCTCACAGTAGGTAGTGGTTACTGTGTGGATTACTCTAAAAACAGACGAAGTCGTAGAGCGA
TTACCACTGGTTATCGGTTTACTAATTTTGAGCCATTTACTGTTAATTCAGTAAATGATAGTTTAGAACCTGTAGGTGG
TTTGTACGAAATTCAAATACCTTCAGAGTTTACTATAGGTAATATGGAGGAGTTTATTCAAACAAGCTCTCCTAAAGTT
ACTATTGATTGTGCTGCATTTGTCTGTGGTGATTATGCAGCATGTAAATCACAGTTGGTTGAATATGGTAGTTTCTGTG
ATAACATTAATGCCATACTCACAGAAGTAAATGAACTACTTGACACTACACAGTTGCAAGTAGCTAATAGTTTAATGAA
TGGTGTTACTCTTAGCACTAAGCTTAAAGATGGCGTTAATTTCAATGTAGACGACATCAATTTTTCCCCTGTATTAGGT
TGTCTAGGCAGCGAATGTAGTAAAGCTTCCAGTAGATCTGCTATAGAGGATTTACTTTTTGATAAAGTAAAGTTATCTG
ATGTCGGTTTCGTTGAGGCTTATAATAATTGTACTGGAGGTGCCGAAATTAGGGACCTCATTTGTGTGCAAAGTTATAA
AGGTATCAAAGTGTTGCCTCCACTGCTCTCAGAAAATCAGATCAGTGGATACACTTTGGCTGCCACTTCTGCTAGTCTG
TTTCCTCCTTGGACAGCAGCAGCAGGTGTACCATTTTATTTAAATGTTCAGTATCGCATTAATGGGCTTGGTGTCACCA
TGGATGTGCTAAGTCAAAATCAAAAGCTTATTGCTAATGCATTTAACAATGCCCTTGATGCTATTCAGGAAGGGTTCGA
TGCAACTAATTCTGCTTTAGTTAAAATTCAAGCTGTTGTTAATGCAAATGCTGAAGCTCTTAATAACTTATTGCAACAA
CTCTCTAATAGATTTGGTGCTATAAGTTCTTCTTTACAAGAAATTCTATCTAGACTTGATGCTCTTGAAGCGGAAGCTC
AGATAGATAGACTTATTAATGGTCGTCTTACCGCTCTTAATGCTTATGTTTCTCAACAGCTTAGTGATTCTACACTGGT
AAAATTTAGTGCAGCACAAGCTATGGAGAAGGTTAATGAATGTGTCAAAAGCCAATCATCTAGGATAAATTTCTGTGGT
AATGGTAACCATATTATATCATTAGTGCAGAATGCTCCATATGGTTTGTATTTTATCCACTTTAGTTATGTCCCTACTA
AGTATGTCACAGCGAAGGTTAGTCCTGGTCTGTGCATTGCTGGTGATAGAGGTATAGCTCCTAAGAGTGGTTATTTTGT
TAATGTAAATAATACTTGGATGTACACTGGTAGTGGTTACTACTACCCTGAACCTATAACTGAAAATAATGTCGTTGTT
ATGAGTACCTGCGCTGTTAATTATACTAAAGCGCCGTATGTAATGCTGAACACTTCAACACCCAACCTTCCTGATTTTA
GGGAAGAGTTGGATCAATGGTTTAAAAATCAAACATCAGTGGCACCAGATCTGTCACTTGATTATATAAATGTTACATT
CTTGGACCTACAAGTTGAAATGAATAGGTTACAGGAGGCAATAAAAGTCTTAAATCAGAGCTACATCAATCTCAAGTAC
ATTGGTACATATGAATATTATGTAAAATGGCCTTGGTATGTATGGCTTTTAATCGGCCTTGCTGGTGTAGCTATGCTTG
TTTTACTATTCTTCATATGCTGTTGTACAGGATGTGGGACTAGTTGTTTTAAGAAATGTGGTGGTTGTTGTGATGATTA
TACTGGATACCAGGAGTTAGTAATCAAAACTTCACATGACGACTAA

>gi|530802355|gb|KF530078.1| Human coronavirus OC43 strain OC43/human/USA/9612-29/1996, complete genome

ATGTTTTTGATACTTTTAATTTCCTTACCAATGGCTTTTGCTGTTATAGGAGATTTAAAGTGTACTTCAGATACG
AGTTATATTAATGATGTTGACACCGGTGTTCCTCCTATAAGTACTGATACTGTTGATGTTACTAATGGTTTGGGTACTT
ATTATGTTTTAGATCGTGTGTATTTAAATACTACTTTGTTTCTTAATGGTTACTATCCTACTTCAGGTTCCACATATCG
TAATATGGCACTGAAGGGAAGTGTACTGTTGAGCACACTATGGTTTAAACCACCATTTCTTTCTGATTTTATTAATGGT
ATTTTTGCTAAGGTCAAAAATACCAAGGTTATTAAAGATGATGTACTGTACAGTGAGTTCCCTGCTATAACTATAGGTA
GTACTTTTGTAAATACATCCTATAGTGTGGTAGTACAACCACGTACAATCAATTTAGATAATAAATTACAAGGTCTTTT
AGAGATCTCTGTTTGCCAGTATAATATGTGCGAGTACCCACATACGATTTGTCATCCTAATTTGGGTAATCATCGCACA
GAACTATGGCATTTGGATACAGGTGTTGTTTCCTGTTTATATAAGCGTAATTTCACATATGATGTGAATGCTGATTATT
TGTATTTTCATTTTTATCAAGAAGGTGGTACTTTTTATGCATATTTTACAGACACTGGTGTTGTTACTAAGTTTTTGTT
TAATGTTTATTTAGGCATGGCGCTTTCACACTATTATGTCATGCCTCTGACTTGTAATAGTAAGCGTAAGGATGGTTTT
ACTTTAGAATATTGGGTTACACCTCTCACTTCTAGACAATATTTACTCGCTTTCAATCAAGATGGTATTATTTTTAATG
CTGTTGATTGCATGAGTGATTTTATGAGTGAGATTAAGTGTAAAACACAATCTATAGCACCACCTACTGGTGTTTATGA
ATTAAACGGTTACACTGTTCAGCCAATTGCAGATGTTTACCGACGTAAACCTAATCTTCCCAATTGTAATATAGAAGCT
TGGCTTAATGATAAGTCGGTACCATCTCCATTAAATTGGGAACGTAAGACATTTTCAAATTGTAATTTTAATATGAGCA
GCCTGATGTCCTTTATTCAGGCAGACTCATTTACTTGTAATAATATTGATGCTGCTAAGATATATGGTATGTGTTTTTC
CAGCATAACTATAGATAAGTTTGCTATACCCAATGGCAGGAAGGTTGACCTACAATTGGGTAATTTGGGCTATTTGCAG
TCATTTAACTATAGAATTGATACTACTGCAACAAGTTGTCAGTTGTATTATAATTTACCTGCTGCTAATGTTTCTGTTA
GCAGGTTTAATCCTTCTACTTGGAATAAGAGATTTGGTTTTATAGAAAATTCTGTTTTTAAGCCTCAACCTGCAGGTGT
TTTTACCAATCATGATGTAGTTTATGCCCAACATTGTTTTAAAGTTCCCAAAAATTTCTGTCCGTGTAAATTGAATGGT
TCTTTGTGTGTAGGTAGTGGTCCTGGTAAAAATAATGGTATAGGCACTTGTCCTGCAGGTACTAATTATTTAACTTGTC
ATAATTTGTGTAATCCTGATCCTATTACATTTGCAGGTCCTTATAAGTGCCCCCAAACTAAATCTTTAGTTGGTATAGG
TGAGCACTGTTCAGGTCTTGCTGTTAAAAGTGATTATTGTGGAGGCAATCCTTGTACTTGCCAACCACAAGCATTTTTG
GGTTGGTCTGCAGACTCTTGTTTACAAGGGGACAAGTGTAATATTTTTGCTAATTTAATTTTGCATGATGTTAATAGTG
GTCTTACTTGTTCTACTGATTTACAAAAAGCTAACACAGACATAATTCTTGGTGTTTGTGTTAATTATGACCTTTATGG
TATTTCAGGCCAAGGCATTTTTGTTGAGGTTAATGCGACTTATTATAATAGTTGGCAGAACCTTTTATATGATTCTAAT
GGTAATCTCTATGGTTTTAGAGACTACATAACAAATAGAACTTTTATGATTCGTAGTTGCTATAGCGGTCGTGTTTCTG
CGGCCTTTCACGCTAACTCTTCCGAACCAGCATTGCTATTTCGGAATATTAAATGCAACTACGTTTTTAATAATAGTCT
TACACGACAGCTGCAACCCATTAACTATTTTGATAGTTATCTTGGTTGTGTTGTCAATGCTTATAATAGTACTGCTATT
TCTGTTCAAACATGTGATCTCACAGTAGGTAGTGGTTACTGTGTGGATTACTCTAAAAACAGACGAAGTCGTAGAGCGA
TTACCACTGGTTATCGGTTTACTAATTTTGAGCCATTTACTGTTAATTCAGTAAATGATAGTTTAGAACCTGTAGGTGG
TTTGTACGAAATTCAAATACCTTCAGAGTTTACTATAGGTAATATGGAGGAGTTTATTCAAACAAGCTCTCCTAAAGTT
ACTATTGATTGTGCTGCATTTGTCTGTGGTGATTATGCAGCATGTAAATCACAGTTGGTTGAATATGGTAGTTTCTGTG
ATAACATTAATGCCATACTCACAGAAGTAAATGAACTACTTGACACTACACAGTTGCAAGTAGCTAATAGTTTAATGAA
TGGTGTTACTCTTAGCACTAAGCTTAAAGATGGCGTTAATTTCAATGTAGACGACATCAATTTTTCCTCTGTATTAGGT
TGTCTAGGCAGCGAATGTAGTAAAGCTTCCAGTAGATCTGCTATAGAGGATTTACTTTTTGATAAAGTAAAGTTATCTG
ATGTCGGTTTCGTTGAGGCTTATAATAATTGTACTGGAGGTGCCGAAATTAAGGACCTCATTTGTGTGCAAAGTTATAA
AGGTATCAAAGTGTTGCCTCCACTGCTCTCAGAAAATCAGATCAGTGGATACACTTTGGCTGCCACTTCTGCTAGTCTG
TTTCCTCCCTGGACAGCAGCAGCAGGTGTACCATTTTATTTAAATGTTCAGTATCGCATTAATGGGCTTGGTGTCACCA
TGGATGTGCTAAGTCAAAATCAAAAGCTTATTGCTAATGCATTTAACAATGCCCTTGATGCTATTCAGGAAGGGTTCGA
TGCAACTAATTCTGCTTTAGTTAAAATTCAAGCTGTTGTTAATGCAAATGCTGAAGCTCTTAATAACTTATTGCAACAA
CTCTCTAATAGATTTGGTGCTATAAGTTCTTCTCTACAAGAAATTCTATCTAGACTTGATGCTCTTGAAGCGGAAGCTC

AGATAGATAGACTTATTAATGGTCGTCTTACCGCTCTTAATGCTTATGTTTCTCAACAGCTTAGTGATTCTACACTGGT
AAAATTTAGTGCAGCACAAGCTATGGAGAAGGTTAATGAATGTGTCAAAAGCCAATCATCTAGGATAAATTTCTGTGGT
AATGGTAACCATATTATATCATTAGTGCAGAATGCTCCATATGGTTTGTATTTTATCCACTTTAGTTATGTCCCTACTA
AGTATGTCACAGCGAAGGTTAGTCCTGGTCTGTGCATTGCTGGTGATAGAGGTATAGCTCCTAAGAGTGGTTATTTTGT
TAATGTAAATAATACTTGGATGTACACTGGTAGTGGTTACTACTACCCTGAACCTATAACTGAAAATAATGTTGTTGTT
ATGAGTACCTGCGCTGTTAATTATACTAAAGCGCCGTATGTAATGCTGAACACTTCAACACCCAACCTTCCTGATTTTA
GGGAAGAGTTGGATCAATGGTTTAAAAATCAAACATCAGTGGCACCAGATCTGTCACTTGATTATATAAATGTTACATT
CTTGGACCTACAAGTTGAAATGAATAGGTTACAGGAGGCAATAAAAGTTTTAAATCAGAGCTACATCAATCTCAAGGAC
ATTGGTACATATGAATATTATGTAAAATGGCCTTGGTATGTATGGCTTTTAATCGGCCTTGCTGGTGTAGCTATGCTTG
TTTTACTATTCTTCATATGCTGTTGTACAGGATGTGGGACTAGTTGTTTTAAGAAATGTGGTGGTTGTTGTGATGATTA
TACTGGATACCAGGAGTTAGTAATCAAAACTTCACATGACGACTAA

>gi|530802366|gb|KF530079.1| Human coronavirus OC43 strain OC43/human/USA/913-29/1991, complete genome

ATGTTTTTGATACTTTTAATTTCCTTACCAACGGCTTTTGCTGTTATAGGAGATTTAAAGTGTCCTTTAGATACT
AGTTATAAAGGTACATTTAATAATAAAGACACCGGTCCTCCTTTTATAAGTACTGATACTGTTGATGTTACTAATGGTT
TGGGTACTTATTATGTTTTAGATCGTGTGTATTTAAATACTACGTTGTTTCTTAATGGTTATTACCCTACTTCAGGTTC
CACATATCGTAATATGGCACTGAAGGGAACTGACAAATTGAGCACACTATGGTTTAAACCACCATTTCTTTCTGATTTT
ATTAATGGTATTTTTGCTAAGGTCAAAAATACCAAGGTTTTTAAAGATGGTGTAATGCATAGTGAGTTCCCTGCTATAA
CTATAGGTAGTACTTTTGTAAATACATCCTATAGTGTGGTAGTACAACCACGTACAATTAATTCAACACAGGACGGTGA
TAATAAATTACAAGGTCTTTTAGAGGTCTCTGTTTGCCAGTATAATATGTGCGAGTACCCACATACGAGTTGTCATCCT
AAACTGGGTAATCACTTCAAAGAACTATGGCATTTGGATACAGGTGTTGTTTCCTGTTTATATAAGCGTAATTTCACAT
ATGATGTGAATGCTAATTATTTGTATTTTCATTTTTATCAAGAAGGTGGTACTTTTTATGCATATTTTACAGACACTGG
TGTTGTTACTAAGTTTTTGTTTAATGTTTATTTAGGCATGGCACTTTCACACTATTATGTCATGCCTCTGACTTGTATT
AGTAGGCGTGATATTGGTTTTACTTTAGAATATTGGGTTACACCCCTCACTTCTAGACAATATTTACTCGCTTTCAATC
AAGATGGTATTATTTTTAATGCTGTTGATTGTATGAGTGATTTTATGAGTGAGATTAAGTGTAAAACACAATCTATAGC
ACCACCTACTGGTGTTTATGAATTAAACGGTTACACTGTTCAGCCAATCGCAGATGTTTACCGACGCAAACCTGATCTT
CCCAATTGCAATATAGAAGCTTGGCTTAATGATAAGTCGGTGCCTTCTCCATTAAATTGGGAACGTAAGACATTTTCAA
ATTGTAATTTTAATATGAGCAGCCTGATGTCTTTTATTCAGGCAGACTCATTTACTTGTAATAATATTGATGCTGCTAA
GATATATGGTATGTGTTTTTCCAGCATAACTATAGATAAGTTTGCTATACCCAATGGCAGGAGGGTTGACCTACAATTG
GGTAATTTGGGCTATTTGCAGTCATCCAACTATAGAATTGATACTACTGCAACAAGTTGTCAGTTGTATTATAATTTAC
CTGCTGCTAATGTCTCTGTTAGCAGGTTTAATCCTTCTACTTGGAATAAGAGATTTGGTTTTATAGAAGATTCTGTTTT
TGTGCCTCAACCTACAGGTGTTTTTACTAATCACAGTGTAGTTTATGCACAACATTGTTTCAAAGCCCCTAAAAATTTC
TGTCCGTGTAAATTGAATGGTTCGTGTCCTGGTAAAAATAATGGTATAGGCACTTGTCCTGCAGGTACTAATTATTTAA
CTTGTGATAATTTGTGTACTCTTGATCCTATTACATTTAAAGCTCCAGGTACTTATAAGTGCCCCCAAACTAAATCTTT
AGTTGGCATAGGTGAGCACTGTTCGGGTCTTGCTGTTAAAAGTGATTATTGTAGAGGCAATTTTTGTACTTGCCAACCA
CAAGCATTTTTGGGTTGGTCTGCAGACTCTTGTTTACAAGGAGATAAGTGTAATATTTTTGCTAATCTTATTTTGCATG
ATGTTAATAGTGGTCTTACTTGTTCTACTGATTTACAAAAAGCTAACACAGACATAATTCTTGGTGTTTGTGTTAATTA
TGACCTCTATGGTATTTCAGGCCAAGGCATTTTTGTTGAGGTTAATGCGACTTATTATAATAGTTGGCAGAACCTTTTA
TATGATTCTAATGGTAATCTCTACGGTTTTAGAGACTACATAACAAACAGAACTTTTATGATTCGTAGTTGCTATAGCG
GTCGTGTTTCTGCGGCCTTTCATGCTAACTCTTCCGAACCAGCATTGCTATTTCGGAATATTAAATGCAACTACGTTTT
TAATAATAGTCTTACACGACAGCTGCAACCCATTAACTATTCGTTTGATAGTTATCTTGGTTGTGTTGTCAATGCTTAT

AATAGTACTGCTATTTCTGTTCAAACATGTGATCTCACAGTAGGTAGTGGTTACTGTGTGGATTACTCTAAAAACAGAC
GAAGTCGTAGAGCGATTACCACTGGTTATCGGTTTACTAATTTTGAGCCATTTACTGTTAATTCAGTAAATGATAGTTT
GGAACCTGTAGGTGGTTTGTATGAAATTCAAATACCTTCAGAGTTTACTATAGGTAATATGGAGGAGTTTATTCAAACA
AGCTCTCCCAAAGTTACTATTGATTGTGCTGCATTTGTTTGTGGTGATTATGCAGCATGTAAATTACAGTTGGTTGAAT
ATGGTAGTTTCTGTGATAACATTAATGCCATACTCACAGAAGTAAATGAACTACTTGATACTACACAGTTGCAAGTAGC
TAATAGTTTAATGAATGGTGTTACTCTTAGCACTAAGCTTAAAGATGGTGTTAATTTCAATGTAGACGACATCAATTTT
TCCCCTGTATTAGGTTGTCTAGGCAGCGAATGTAGTAAAGCTTCCAGTAGATCTGCTATAGAGGATTTACTTTTTGATA
AAGTAAAGTTATCTGATGTCGGTTTCGTTGAGGCTTATAATAATTGTACAGGAGGTGCCGAAATTAGGGACCTCATTTG
TGTGCAAAGTTATAAAGGCATCAAAGTGTTGCCCCCACTGCTCTCAGAAAATCAGATCAGTGGATACACTTTGGCTGCC
ACCTCTGCTAGTCTATTTCCTCCTTGGACAGCAGCAGCAGGTGTACCATTTTATTTAAATGTTCAGTATCGCATTAATG
GGCTTGGTGTCACTATGGATGTGCTAAGTCAAAATCAAAAGCTTATTGCTAATGCATTTAACAATGCCCTTTATGCTAT
TCAGCAAGGGTTCGATGCAACTAATTCTGCTTTAGTTAAAATTCAAGCTGTTGTTAATGCAAATGCTGAAGCTCTTAAT
AACTTATTGCAACAACTCTCTAATAGATTTGGTGCTATAAGTGCTTCTTTACAAGAAATTCTATCTAGACTTGATGCTC
TTGAAGCGGAAGCTCAGATAGATAGACTTATTAATGGTCGTCTCACCGCTCTTAATGCTTATGTTTCTCAACAGCTTAG
TGATTCTACACTGGTAAAATTTAGTGCAGCACAAGCTATGGAGAAGGTTAATGAATGTGTCAAAAGCCAATCATCTAGG
ATAAATTTCTGTGGTAATGGTAATCATATTATATCATTAGTGCAGAATGCTCCATATGGTTTGTATTTTATCCACTTTA
ATTATGTCCCTACTAAGTATGTCACAGCGAAGGTTAGTCCTGGTCTGTGCATTGCTGGTAATAGAGGTATAGCTCCTAA
GAGTGGTTATTTTGTTAATGTAAATAATACTTGGATGTACACTGGTAGTGGTTACTACTACCCTGAACCTATAACTGAA
AATAATGTTGTTGTTATGAGTACCTGCGCTGTTAATTATACTAAAGCGCCGTATGTAATGCTGAACACTTCAATACCCA
ACCTTCCTGATTTTAAGGAAGAGTTGGATCAATGGTTTAAAAATCAAACATCAGTGGCACCAGATTTGTCACTTGATTA
TATAAATGTTACATTCTTGGACCTACAAGTTGAAATGAATAGGTTACAGGAGGCAATAAAAGTCTTAAATCATAGCTAC
ATCAATCTCAAGGACATTGGTACATATGAATATTATGTAAAATGGCCTTGGTATGTATGGCTCTTAATCTGCCTTGCTG
GTGTAGCTATGCTTGTTTTACTATTCTTCATATGCTGTTGTACAGGATGTGGGACTAGTTGTTTTAAGAAATGTGGTGG
TTGTTGTGATGATTATACTGGATACCAGGAGTTAGTAATCAAAACTTCACATGACGACTAA

>gi|530802377|gb|KF530080.1| Human coronavirus OC43 strain OC43/human/USA/9712-31/1997, complete genome

ATGTTTTTGATACTTTTAATTTCCTTACCAATGGCTTTTGCTGTTATAGGAGATTTAAAGTGTACTTCAGATACG
AGTTATATTAATGATGTTGACACCGGTGTTCCTCCTATAAGTACTGATACTGTTGATGTTACTAATGGTTTGGGTACTT
ATTATGTTTTAGATCGTGTGTATTTAAATACTACTTTGTTTCTTAATGGTTACTATCCTACTTCAGGTTCCACATATCG
TAATATGGCACTGAAGGGAAGTGTACTGTTGAGCACACTATGGTTTAAACCACCATTTCTTTCTGATTTTATTAATGGT
ATTTTTGCTAAGGTCAAAAATACCAAGGTTATTAAAGATGATGTACTGTACAGTGAGTTCCCTGCTATAACTATAGGTA
GTACTTTTGTAAATACATCCTATAGTGTGGTAGTACAACCACGTACAATCAATTTAGATAATAAATTACAAGGTCTTTT
AGAGATCTCTGTTTGCCAGTATAATATGTGCGAGTACCCACATACGATTTGTCATCCTAATTTGGGTAATCATCGCACA
GAACTATGGCATTTGGATACAGGTGTTGTTTCCTGTTTATATAAGCGTAATTTCACATATGATGTGAATGCTGATTATT
TGTATTTTCATTTTTATCAAGAAGGTGGTACTTTTTATGCATATTTTACAGACACTGGTGTTGTTACTAAGTTTTTGTT
TAATGTTTATTTAGGCATGGCGCTTTCACACTATTATGTCATGCCTCTGACTTGTAATAGTAAGCGTAAGGATGGTTTT
ACTTTAGAATATTGGGTTACACCTCTCACTTCTAGACAATATTTACTCGCTTTCAATCAAGATGGTATTATTTTTAATG
CTGTTGATTGCATGAGTGATTTTATGAGTGAGATTAAGTGTAAAACACAATCTATAGCACCACCTACTGGTGTTTATGA
ATTAAACGGTTACACTGTTCAGCCAATTGCAGATGTTTACCGACGTAAACCTAATCTTCCCAATTGTAATATAGAAGCT
TGGCTTAATGATAAGTCGGTACCATCTCCATTAAATTGGGAACGTAAGACATTTTCAAATTGTAATTTTAATATGAGCA
GCCTGATGTCCTTTATTCAGGCAGACTCATTTACTTGTAATAATATTGATGCTGCTAAGATATATGGTATGTGTTTTTC

CAGCATAACTATAGATAAGTTTGCTATACCCAATGGCAGGAAGGTTGACCTACAATTGGGTAATTTGGGCTATTTGCAG
TCATTTAACTATAGAATTGATACTACTGCAACAAGTTGTCAGTTGTATTATAATTTACCTGCTGCTAATGTTTCTGTTA
GCAGGTTTAATCCTTCTACTTGGAATAAGAGATTTGGTTTTATAGAAAATTCTGTTTTTAAGCCTCAACCTGCAGGTGT
TTTTACCAATCATGATGTAGTTTATGCCCAACATTGTTTTAAAGCTCCCAAAAATTTCTGTCCGTGTAAATTGAATGGT
TCTTTGTGTGTAGGTAGTGGTCCTGGTAAAAATAATGGTATAGGCACTTGTCCTGCAGGTACTAATTATTTAACTTGTC
ATAATTTGTGTAATCCTGATCCTATTACATTTGCAGGTCCTTATAAGTGCCCCCAAACTAAATCTTTAGTTGGTATAGG
TGAGCACTGTTCAGGTCTTGCTGTTAAAAGTGATTATTGTGGAGGCAATCCTTGTACTTGCCAACCACAAGCATTTTTG
GGTTGGTCTGCAGACTCTTGTTTACAAGGGGACAAGTGTAATATTTTTGCTAATTTAATTTTGCATGATGTTAATAGTG
GTCTTACTTGTTCTACTGATTTACAAAAAGCTAACACAGACATAATTCTTGGTGTTTGTGTTAATTATGACCTTTATGG
TATTTCAGGCCAAGGCATTTTTGTTGAGGTTAATGCGACTTATTATAATAGTTGGCAGAACCTTTTATATGATTCTAAT
GGTAATCTCTATGGTTTTAGAGACTACATAACAAATAGAACTTTTATGATTCGTAGTTGCTATAGCGGTCGTGTTTCTG
CGGCCTTTCACGCTAACTCTTCCGAACCAGCATTGCTATTTCGGAATATTAAATGCAACTACGTTTTTAATAATAGTCT
TACACGACAGCTGCAACCCATTAACTATTTTGATAGTTATCTTGGTTGTGTTGTCAATGCTTATAATAGTACTGCTATT
TCTGTTCAAACATGTGATCTCACAGTAGGTAGTGGTTACTGTGTGGATTACTCTAAAAACAGACGAAGTCGTAGAGCGA
TTACCACTGGTTATCGGTTTACTAATTTTGAGCCATTTACTGTTAATTCAGTAAATGATAGTTTAGAACCTGTAGGTGG
TTTGTACGAAATTCAAATACCTTCAGAGTTTACTATAGGTAATATGGAGGAGTTTATTCAAACAAGCTCTCCTAAAGTT
ACTATTGATTGTGCTGCATTTGTCTGTGGTGATTATGCAGCATGTAAATCACAGTTGGTTGAATATGGTAGTTTCTGTG
ATAACATTAATGCCATACTCACAGAAGTAAATGAACTACTTGACACTACACAGTTGCAAGTAGCTAATAGTTTAATGAA
TGGTGTTACTCTTAGCACTAAGCTTAAAGATGGCGTTAATTTCAATGTAGACGACATCAATTTTTCCTCTGTATTAGGT
TGTCTAGGCAGCGAATGTAGTAAAGCTTCCAGTAGATCTGCTATAGAGGATTTACTTTTTGATAAAGTAAAGTTATCTG
ATGTCGGTTTCGTTGAGGCTTATAATAATTGTACTGGAGGTGCCGAAATTAGGGACCTCATTTGTGTGCAAAGTTATAA
AGGTATCAAAGTGTTGCCTCCACTGCTCTCAGAAAATCAGATCAGTGGATACACTTTGGCTGCCACTTCTGCTAGTCTG
TTTCCTCCCTGGACAGCAGCAGCAGGTGTACCATTTTATTTAAATGTTCAGTATCGCATTAATGGGCTTGGTGTCACCA
TGGATGTGCTAAGTCAAAATCAAAAGCTTATTGCTAATGCATTTAACAATGCCCTTGATGCTATTCAGGAAGGGTTCGA
TGCAACTAATTCTGCTTTAGTTAAAATTCAAGCTGTTGTTAATGCAAATGCTGAAGCTCTTAATAACTTATTGCAACAA
CTCTCTAATAGATTTGGTGCTATAAGTTCTTCTCTACAAGAAATTCTATCTAGACTTGATGCTCTTGAAGCGGAAGCTC
AGATAGATAGACTTATTAATGGTCGTCTTACCGCTCTTAATGCTTATGTTTCTCAACAGCTTAGTGATTCTACACTGGT
AAAATTTAGTGCAGCACAAGCTATGGAGAAGGTTAATGAATGTGTCAAAAGCCAATCATCTAGGATAAATTTCTGTGGT
AATGGTAACCATATTATATCATTAGTGCAGAATGCTCCATATGGTTTGTATTTTATCCACTTTAGTTATGTCCCTACTA
AGTATGTCACAGCGAAGGTTAGTCCTGGTCTGTGCATTGCTGGTGATAGAGGTATAGCTCCTAAGAGTGGTTATTTTGT
TAATGTAAATAATACTTGGATGTACACTGGTAGTGGTTACTATTACCCTGAACCTATAACTGAAAATAATGTTGTTGTT
ATGAGTACCTGCGCTGTTAATTATACTAAAGCGCCGTATGTAATGCTGAACACTTCAACACCCAACCTTCCTGATTTTA
GGGAAGAGTTGGATCAATGGTTTAAAAATCAAACATCAGTGGCACCAGATCTGTCACTTGATTATATAAATGTTACATT
CTTGGACCTACAAGTTGAAATGAATAGGTTACAGGAGGCAATAAAAGTTTTAAATCAGAGCTACATCAATCTCAAGGAC
ATTGGTACATATGAATATTATGTAAAATGGCCTTGGTATGTATGGCTTTTAATCGGCCTTGCTGGTGTAGCTATGCTTG
TTTTACTATTCTTCATATGCTGTTGTACAGGATGTGGGACTAGTTGTTTTAAGAAATGTGGTGGTTGTTGTGATGATTA
TACTGGATACCAGGAGTTTGTAATCAAAACTTCACATGACGACTAA

>gi|530802388|gb|KF530081.1| Human coronavirus OC43 strain OC43/human/USA/991-5/1999, complete genome

ATGTTTTTGATACTTTTAATTTCCTTACCAACGGCTTTTGCTGTTATAGGAGATTTAAAGTGTCCTTTAGATACT
AGTCTTAAAGGTAGCTTTAATAATAGAGACACCGGTTCTCCTTCTATAAGTACTGATACTGTTGATGTTACTAATGGTT

TGGGTACTTATTATGTTTTAGATCGTGTGTATTTAAATACTACGTTGTTTCTTAATGGTTATTACCCCACTTCAGGTTC
CACATATCGTAATATGGCACTGAAGGGAACTGACAAATTGAGCACATTATGGTTTAAACCACCATTTCTTTCTGATTTT
ATTAATGGTATTTTTGCTAAGGTCAAAAATACCAAGGTTTTTAAAGATGGTGTAATGTATAGTGAGTTCCCTGCTATAA
CTATAGGTAGTACTTTTGTAAATACATCCTATAGTGTGGTAGTACAACCACGTACAATTAATTCAACACAGGATGGTGT
TAATAAATTACAAGGTCTTTTAGAGGTCTCTGTTTGCCAGTATAATATGTGCGAGTACCCACATACGATTTGTCATCCT
AAACTGGGTAACCACTTCAAAGAACTATGGCATTTGGATACAGGTGTTGTTTCCTGTTTATATAAGCGTAATTTCACAT
ATGATGTGAATGCTACTTATTTGTATTTCATTTTTATCAAGAAGGTGGTACTTTTTATGCATATTTTACAGACACTGG
TTTTGTTACTAAGTTTTTGTTTAATGTTTATTTAGGCATGGCACTTTCACACTATTATGTCATGCCTCTGACTTGTATT
AGTAGGCGTGATATTGGTTTTACTTTAGAATATTGGGTTACACCCCTCACTTCTAGACAATATTTACTCGCTTTCAATC
AAGATGGTATTATTTTTAATGCTGTTGATTGTATGAGTGATTTTATGAGTGAGATTAAGTGTAAAACACAATCTATAGC
ACCACCTACTGGTGTTTATGAATTAAACGGTTACACTGTTCAGCCAATCGCAGATGTTTACCGACGCAAACCTGATCTT
CCCAATTGCAATATAGAAGCTTGGCTTAATGATAAGTCGGTGCCCTCTCCATTAAATTGGGAACGTAAGACATTTTCAA
ATTGTAATTTTAATATGAGCAGCCTGATGTCTTTTATTCAGGCAGACTCATTTACTTGTAATAATATTGATGCTGCTAA
GATATATGGTATGTGTTTTTCCAGCATAACTATAGATAAGTTTGCTATACCCAATGGCAGGAAGGTTGACCTACAATTG
GGCAATTTGGGCTATTTGCAGTCATCCAACTATAGAATTGATACTACTGCAACAAGTTGTCAGTTGTATTATAATTTAC
CTGCTGCTAATGTCTCTGTTAGCAGGTTTAATCCTTCTACTTGGAATAAGAGATTTGGTTTTATAGAAGATTCTGTTTT
TGTGCCTCAACCTACAGGTGTTTTTACTAATCACAGTGTAGTTTATGCACAACACTGTTTCAAAGCCCCTAAAAATTTC
TGTCCGTGTAAATTGAATGGTTCGTGTCCTGATAAAAATAATGGTATAGGCACTTGTCCTGCAGGTACTAATTATCTAA
CTTGTGATAATTTGTGTACTCTTGATCCTATTACATTTAAAGCTCCAGGTACTTATAAGTGCCCCCAAACTAAATCTTT
AGTTGGCATAGGTGAGCACTGTTCGGGTCTTGCTGTTAAAAGTGATTATTGTAGAGGCAATTCTTGTACTTGCCAACCA
CAAGCATTTTTGGGTTGGTCTGCAGACTCTTGTTTACAAGGAGACAAGTGTAATATTTTTGCTAATCTTATTTTGCATG
ATGTTAATAGTGGTCTTACTTGTTCTACTGATTTACAAAAAGCTAACACAGACATAATTCTTGGTGTTTGTGTTAATTA
TGACCTCTATGGTATTTCAGGTCAAGGCATTTTTGTTGAGGTTAATGCGACTTATTATAATAGTTGGCAGAACCTTTTA
TATGATTCTAATGGTAATCTCTACGGTTTTAGAGACTACATAACAAACAGAACTTTTATGATTCGTAGTTGCTATAGCG
GTCGTGTTTCTGCGGCCTTTCATGCTAACTCTTCCGAACCAGCATTGCTATTTCGGAATATTAAATGCAACTACGTTTT
TAATAATAGTCTTACACGACAGCTGCAACCCATTAACTATTCGTTTGATAGTTATCTTGGTTGTGTTGTCAATGCTTAT
AATAGTACTGCTATTTCTGTTCAAACATGTGATCTCACAGTAGGTAGCGGTTACTGTGTGGAGTACTCTAAAAACAGAC
GAAGTCGTAGAGCGATTACCACTGGTTATCGGTTTACTAATTTTGAGCCATTTACTGTTAATTCAGTAAATGATAGTTT
GGAACCTGTAGGTGGTTTGTATGAAATTCAAATACCTTCAGAGTTTACTATAGGTAATATGGAGGAGTTTATTCAAACA
AGCTCTCCCAAAGTTACTATTGATTGTGCTGCATTTGTCTGTGGTGATTATGCAGCATGTAAATTACAGTTGGTTGAAT
ATGGTAGTTTCTGTGATAACATTAATGCCATACTCACAGAAGTAAATGAACTACTTGATACTACACAGTTGCAAGTAGC
TAATAGTTTAATGAATGGTGTTACTCTTAGCACTAAGCTTAAAGATGGTGTTAATTTCAATGTAGACGACATCAATTTT
TCCCCTGTATTAGGTTGTCTAGGCAGCGAATGTAGTAAAGCTTCCAGTAGATCTGCTATAGAGGATTTACTTTTTGATA
AAGTAAAGTTATCTGATGTCGGTTTCGTTGAGGCTTATAATAATTGTACAGGAGGTGCCGAAATTAGGGACCTCATTTG
TGTGCAAAGTTATAAAGGCATCAAAGTGTTGCCCCCACTGCTCTCAGAAAATCAGATCAGTGGATACACTTTGGCTGCC
ACCTCTGCTAGTCTATTTCCTCCTTGGACAGCAGCAGCAGGTGTACCATTTTATTTAAATGTTCAGTATCGCATTAATG
GGCTTGGTGTCACCATGGATGTGCTAAGTCAAAATCAAAAGCTTATTGCTAATGCATTTAACAATGCCCTTTATGCTAT
TCAGCAAGGGTTCGATGCAACTAATTCTGCTTTAGTTAAAATTCAAGCTGTTGTTAATGCAAATGCTGAAGCTCTTAAT
AACTTATTGCAACAACTCTCTAATAGATTTGGTGCTATAAGTGCTTCTTTACAAGAAATTCTATCTAGACTTGATGCTC
TTGAAGCGGAAGCTCAGATAGATAGACTTATTAATGGTCGTCTCACCGCTCTTAATGCTTATGTTTCTCAACAGCTTAG
TGATTCTACACTGGTAAAATTTAGTGCAGCACAAGCTATGGAGAAGGTTAATGAATGTGTCAAAAGCCAATCATCTAGG
ATAAATTTCTGTGGTAATGGTAATCATATTATATCATTAGTGCAGAATGCTCCATATGGTTTGTATTTTATCCACTTTA
ATTATGTCCCTACTAAGTATGTCACAGCGAAGGTTAGTCCTGGTCTGTGCATTGCTGGTAATAGAGGTATAGCTCCTAA
GAGTGGTTATTTTGTTAATGTAAATAATACTTGGATGTACACTGGTAGTGGTTACTACTACCCTGAACCTATAACTGAA

AATAATGTTGTTGTTATGAGTACCTGCGCTGTTAATTATACTAAAGCGCCGTATGTAATGCTGAACACTTCAATACCCA
ACCTTCCTGATTTTAAGGAAGAGTTGGATCAATGGTTTAAAAATCAAACATCAGTGGCACCAGATTTGTCACTTGATTA
TATAAATGTTACATTCTTGGACCTACAAGTTGAAATGAATAGGTTACAGGAGGCAATAAAAGTCTTAAATCATAGCTAC
ATCAATCTCAAGGACATTGGTACATATGAATATTATGTAAAATGGCCTTGGTATGTATGGCTTTTAATCTGCCTTGCTG
GTGTAGCTATGCTTGTTTTACTATTCTTCATATGCTGTTGTACAGGATGTGGGACTAGTTGTTTTAAGAAATGTGGTGG
TTGTTGTGATGATTATACTGGATACCAGGAGTTAGTAATCAAAACTTCACATGACGACTAA

>gi|530802421|gb|KF530084.1| Human coronavirus OC43 strain OC43/human/USA/951-18/1995, complete genome

ATGTTTTTGATACTTTTAATTTCCTTACCAACGGCTTTTGCTGTTATAGGAGATTTAAAGTGTCCTTTAGATACT
AGTCGTAAAGGTAGCTTTAATAATAAAGACACCGGTCCTCCTTTTATAAGTACTGATACTGTTGATGTTACTAATGGTT
TGGGTACTTATTATGTTTTAGATCGTGTGTATTTAAATACTACGTTGTTTCTTAATGGTTATTACCCTACTTCAGGTTC
CACATATCGTAATATGGCACTGAAGGGAACTGACAAATTGAGCACACTATGGTTTAAACCACCATTTCTTTCTGATTTT
ATTAATGGTATTTTTGCTAAGGTCAAAAATACCAAGGTTTTTAAAGAAGGTGTAATGTACAGTGAGTTCCCTGCTATAA
CTATAGGTAGTACTTTTGTAAATACATCCTATAGTGTGGTAGTACAACCACGTACAATTAATTCAACACAGGATGGTGT
TAATAAATTACAAGGTCTTTTAGAGGTCTCTGTTTGCCAGTATAATATGTGCGAGTACCCACATACGATTTGTCATCCT
AAACTGGGTAATCACTTCAAAGAACTATGGCATTTGGATACAGGTGTTGTTTCCTGTTTATATAAGCGTAATTTCACAT
ATGATGTGAATGCTGATTATTTGTATTTTCATTTTTATCAAGAAGGTGGTACTTTTTATGCATATTTTACAGACACTGG
TGTTGTTACTAAGTTTTTGTTTAATGTTTATTTAGGCATGGCACTTTCACACTATTATGTCATGCCTTTGACTTGTATT
AGTAGGCGTGATATTGGTTTTACTTTAGAATATTGGGTTACACCCCTCACTTCTAGACAATATTTACTCGCTTTCAATC
AAGATGGTATTATTTTTAATGCTGTTGATTGTATGAGTGATTTTATGAGTGAGATTAAGTGTAAAACACAATCTATAGC
ACCACCTACTGGTGTTTATGAATTAAACGGTTACACTGTTCAGCCAATCGCAGATGTTTACCGACGCAAACCTGATCTT
CCCAATTGCAATATAGAAGCTTGGCTTAATGATAAGTCGGTGCCCTCTCCATTAAATTGGGAACGTAAGACATTTTCAA
ATTGTAATTTTAATATGAGCAGCCTGATGTCTTTTATTCAGGCAGACTCATTTACTTGTAATAATATTGATGCTGCTAA
GATATATGGTATGTGTTTTTCCAGCATAACTATAGATAAGTTTGCTATACCCAATGGCAGGAAGGTTGACCTACAATTG
GGTAATTTGGGCTATTTGCAGTCATCCAACTATAGAATTGATACTACTGCAACAAGTTGTCAGTTGTATTATAATTTAC
CTGCTGCTAATGTCTCTGTTAGCAGGTTTAATCCTTCTACTTGGAATAAGAGATTTGGTTTTATAGAAGATTCTGTTTT
TGTGCCTCAACCTACAGGTGTTTTTACTAATCACAGTGTAGTTTATGCACAACACTGTTTCAAAGCCCCTAAAAATTTC
TGTCCGTGTAAATTGAATGGTTCGTGTCCTGATAAAAACAATGGTATAGGCACTTGTCCTGCAGGTACTAATTATTTAA
CTTGTGATAATTTGTGTACTCTTGATCCTATTACATTTAAAGCTCCAGGTACTTATAAGTGCCCCCAAACTAAATCTTT
AGTTGGCATAGGTGAGCACTGTTCGGGTCTTGCTGTTAAAAGTGATTATTGTAGAGGCAATTCTTGTACTTGCCAACCA
CAAGCATTTTTGGGTTGGTCTGCAGACTCTTGTTTACAAGGAGACAAGTGTAATATTTTTGCTAATCTTATTTTGCATG
ATGTTAATAGTGGTCTTACTTGTTCTACTGATTTACAAAAAGCTAACACAGACATAATTCTTGGTGTTTGTGTTAATTA
TGACCTTTATGGTATTTCAGGCCAAGGCATTTTTGTTGAGGTTAATGCGACTTATTATAATAGTTGGCAGAACCTTTTA
TATGATTCTAATGGTAATCTCTACGGTTTTAGAGACTACATAACAAACAGAACTTTTATGATTCGTAGTTGCTATAGCG
GTCGTGTTTCTGCGGCCTTTCATGCTAACTCTTCCGAACCAGCATTGCTATTTCGGAATATTAAATGCAACTACGTTTT
TAATAATAGTCTTACACGACAGCTGCAACCCATTAACTATTCGTTTGATAGTTATCTTGGTTGTGTTGTCAATGCTTAT
AATAGTACTGCTATTTCTGTTCAAACATGTGATCTCACAGTAGGTAGTGGTTACTGTGTGGAGTACTCTAAAAACAGAC
GAAGTCGTAGAGCGATTACCACTGGTTATCGGTTTACTAATTTTGAGCCATTTACTGTTAATTCAGTAAATGATAGTTT
GGAACCTGTAGGTGGTTTGTATGAAATTCAAATACCTTCAGAGTTTACTATAGGTAATATGGAGGAGTTTATTCAAACA
AGCTCTCCCAAAGTTACTATTGATTGTGCTGCATTTGTCTGTGGTGATTATGCAGCATGTAAATTACAGTTGGTTGAAT
ATGGTAGTTTCTGTGATAACATTAATGCCATACTCACAGAAGTAAATGAACTACTTGATACTACACAGTTGCAAGTAGC

TAATAGTTTAATGAATGGTGTTACTCTTAGCACTAAGCTTAAAGATGGTGTTAATTTCAATGTAGACGACATCAATTTT
TCCCCTGTATTAGGTTGTCTAGGCAGCGAATGTAGTAAAGCTTCCAGTAGATCTGCTATAGAGGATTTACTTTTTGATA
AAGTAAAGTTATCTGATGTCGGTTTCGTTGAGGCTTATAATAATTGTACAGGAGGTGCCGAAATTAGGGACCTCATTTG
TGTGCAAAGTTATAAAGGCATCAAAGTGTTGCCCCCACTGCTCTCAGAAAATCAGATCAGTGGATACACTTTGGCTGCC
ACCTCTGCTAGTCTATTTCCTCCTTGGACAGCAGCAGCAGGTGTACCATTTTATTTAAATGTTCAGTATCGCATTAATG
GGCTTGGTGTCACCATGGATGTGCTAAGTCAAAATCAAAAGCTTATTGCTAATGCATTTAACAATGCCCTTTATGCTAT
TCAGCAAGGGTTCGATGCAACTAATTCTGCTTTAGTTAAAATTCAAGCTGTTGTTAATGCAAATGCTGAAGCTCTTAAT
AACTTATTGCAACAACTCTCTAATAGATTTGGTGCTATAAGTGCTTCTTTACAAGAAATTCTATCTAGACTTGATGCTC
TTGAAGCGGAAGCTCAGATAGATAGACTTATTAATGGTCGTCTCACCGCTCTTAATGCTTATGTTTCTCAACAGCTTAG
TGATTCTACACTGGTAAAATTTAGTGCAGCACAAGCTATGGAGAAGGTTAATGAATGTGTCAAAAGCCAATCATCTAGG
ATAAATTTCTGTGGTAATGGTAATCATATTATATCATTAGTGCAGAATGCTCCATATGGTTTGTATTTTATCCACTTTA
ATTATGTCCCTACTAAGTATGTCACAGCGAAGGTTAGTCCTGGTCTGTGCATTGCTGGTAATAGAGGTATAGCTCCTAA
GAGTGGTTATTTTGTTAATGTAAATAATACTTGGATGTACACTGGTAGTGGTTACTACTACCCTGAACCTATAACTGAA
AATAATGTTGTTGTTATGAGTACCTGCGCTGTTAATTATACTAAAGCGCCGTATGTAATGCTGAACACTTCAATACCCA
ACCTTCCTGATTTTAAGGAAGAGTTGGATCAATGGTTTAAAAATCAAACATCAGTGGCACCAGATTTGTCACTTGATTA
TATAAATGTTACATTCTTGGACCTACAAGTTGAAATGAATAGGTTACAGGAGGCAATAAAAGTCTTAAATCATAGCTAC
ATCAATCTCAAGGACATTGGTACATATGAATATTATGTAAAATGGCCTTGGTATGTATGGCTTTTAATCTGCCTTGCTG
GTGTAGCTATGCTTGTTTTACTATTCTTCATATGCTGTTGTACAGGATGTGGGACTAGTTGTTTTAAGAAATGTGGTGG
TTGTTGTGATGATTATACTGGATACCAGGAGTTAGTAATCAAAACTTCACATGACGACTAA

>gi|530802432|gb|KF530085.1| Human coronavirus OC43 strain OC43/human/USA/871-25/1987, complete genome

ATGTTTTTGATACTTTTAATTTCCTTACCAATGGCTTTTGCTGTTATAGGAGATTTAAAGTGTACTTCAGATACG
AGTTATATTAATGATGTTGACACCGGTGTTCCTCCTATAAGTACTGATACTGTTGATGTTACTAATGGTTTGGGTACTT
ATTATGTTTTAGATCGTGTGTATTTAAATACTACTTTGTTTCTTAATGGTTACTATCCTACTTCAGGTTCCACATATCG
TAATATGGCACTGAAGGGAAGTGTACTGTTGAGCACACTATGGTTTAAACCACCATTTCTTTCTGATTTTATTAATGGT
ATTTTTGCTAAGGTCAAAAATACCAAGGTTATTAAAGATGATGTATTGTACAGTGAGTTCCCTGCTATAACTATAGGTA
GTACTTTTGTAAATACATCCTATAGTGTGGTAGTACAACCACGTACAATCAATTTAGATAATAAATTACAAGGTCTTTT
AGAGATCTCTGTTTGCCAGTATAATATGTGCGAGTACCCACATACGATTTGTCATCCTAACTTGGGTAATCATCGCACA
GAACTATGGCATTTGGATACAGGTGTTGTTTCCTGTTTATATAAGCGTAATTTCACATATGATGTGAATGCTGATTATT
TGTATTTTCATTTTTATCAAGAAGGTGGTACTTTTTATGCATATTTTACAGACACTGGTGTTGTTACTAAGTTTTTGTT
TAATGTTTATTTAGGCATGGCGCTTTCACACTATTATGTCATGCCTCTGACTTGTAATAGTAAGCGTAAAGATGGTTTT
ACTTTAGAATATTGGGTTACACCTCTCACTTCTAGACAATATTTACTCGCTTTCAATCAAGATGGTATTATTTTTAATG
CTGTTGATTGCATGAGTGATTTTATGAGTGAGATTAAGTGTAAAACACAATCTATAGCACCACCTACTGGTGTTTATGA
ATTAAACGGTTACACTGTTCAGCCAATTGCAGATGTTTACCGACGTAAACCTAATCTTCCCAATTGCAATATAGAAGCT
TGGCTTAATGATAAGTCGGTGCCATCTCCATTAAATTGGGAACGTAAGACATTTTCAAATTGTAATTTTAATATGAGCA
GCCTGATGTCCTTTATTCAGGCAGACTCATTTACTTGTAATAATATTGATGCTGCTAAGATATATGGTATGTGTTTTTC
CAGCATAACTATAGATAAGTTTGCTATACCCAATGGCAGGAAGGTTGACCTACAATTGGGTAATTTGGGCTATTTGCAG
TCATTTAACTATAGAATTGATACTACTGCAACAAGTTGTCAGTTGTATTATAATTTACCTGCTGCTAATGTTTCTGTTA
GCAGGTTTAATCCTTCTACTTGGAATAAGAGATTCGGTTTTATAGAAAATTCTGTTTTTAAGCCTCAACCTGCAGGTGT
TTTTACCAATCATGATGTAGTTTATGCCCAACATTGTTTTAAAGCTCCCAAAAATTTCTGTCCGTGTAAATTGAATGGT
TCTTTGTGTGTAGGTAGTGGTCCTGGTAAAAATAATGGTATAGGCACTTGTCCTGCAGGTACTAATTATTTAACTTGTC

ATAATTTGTGCAATCCTGATCCTATTACATTTACAGGTCCTTATAAGTGCCCCCAAACTAAATCTTTAGTTGGTATAGG
TGAGCACTGTTCAGGTCTTGCTGTTAAAAGTGATTATTGTGGAGGCAATCCTTGCACTTGCCAACCACAAGCATTTTTG
GGTTGGTCTGCAGACTCTTGTTTACAAGGGGACAAGTGTAATATTTTTGCTAATTTAATTTTGCATGATGTTAATAGTG
GTCTTACTTGTTCTACTGATTTACAAAAAGCTAACACAGACATAATTCTTGGTGTTTGTGTTAATTATGACCTTTATGG
TATTTCAGGCCAAGGCATTTTTGTTGAGGTTAATGCGACTTATTATAATAGTTGGCAGAACCTTTTATATGATTCTAAT
GGTAATCTCTATGGTTTTAGAGACTACATAACAAACAGAACTTTTATGATTCGTAGTTGCTATAGCGGTCGTGTTTCTG
CGGCCTTTCACGCTAACTCTTCCGAACCAGCATTGCTATTTCGGAATATTAAATGCAACTACGTTTTTAATAATAGTCT
TACACGACAGCTGCAACCCATTAACTATTTTGATAGTTATCTTGGTTGTGTTGTCAATGCTTATAATAGTACTGCTATT
TCTGTTCAAACATGTGATCTCACAGTAGGTAGTGGTTACTGTGTGGATTACTCTAAAAACAGACGAAGTCGTAGAGCGA
TTACCACTGGTTATCGGTTTACTAATTTTGAGCCATTTACTGTTAATTCAGTAAATGATAGTTTAGAACCTGTAGGTGG
TTTGTACGAAATTCAAATACCTTCAGAGTTTACTATAGGTAATATGGAGGAGTTTATTCAAACAAGCTCTCCTAAAGTT
ACTATTGATTGTGCTGCATTTGTCTGTGGTGATTATGCAGCATGTAAATCACAGTTGGTTGAATATGGTAGTTTCTGTG
ATAACATTAATGCCATACTCACAGAAGTAAATGAACTACTTGACACTACACAGTTGCAAGTAGCTAATAGTTTAATGAA
TGGTGTTACTCTTAGCACTAAGCTTAAAGATGGCGTTAATTTCAATGTAGACGACATCAATTTTTCCCCTGTATTAGGT
TGTCTAGGCAGCGAATGTAGTAAAGCTTCCAGTAGATCTGCTATAGAGGATTTACTTTTTGATAAAGTAAAGTTATCTG
ATGTCGGTTTCGTTGAGGCTTATAATAATTGTACTGGAGGTGCCGAAATTAGGGACCTCATTTGTGTGCAAAGTTATAA
AGGTATCAAAGTGTTGCCTCCACTGCTCTCAGAAAATCAGATCAGTGGATACACTTTGGCTGCCACTTCTGCTAGTCTG
TTTCCTCCTTGGACAGCAGCAGCAGGTGTACCATTTTATTTAAATGTTCAGTATCGCATTAATGGGCTTGGTGTCACCA
TGGATGTGCTAAGTCAAAATCAAAAGCTTATTGCTAATGCATTTAACAATGCCCTTGATGCTATTCAGGAAGGGTTCGA
TGCAACTAATTCTGCTTTAGTTAAAATTCAAGCTGTTGTTAATGCAAATGCTGAAGCTCTTAATAACTTATTGCAACAA
CTCTCTAATAGATTTGGTGCTATAAGTTCTTCTTTACAAGAAATTCTATCTAGACTTGATGCTCTTGAAGCGGAAGCTC
AGATAGATAGACTTATTAATGGTCGTCTTACCGCTCTTAATGCTTATGTTTCTCAACAGCTTAGTGATTCTACATTGGT
AAAATTTAGTGCAGCACAAGCTATGGAGAAGGTTAATGAATGTGTCAAAAGCCAATCATCTAGGATAAATTTCTGTGGT
AATGGTAACCATATTATATCATTAGTGCAGAATGCTCCATATGGTTTGTATTTTATCCACTTTAGTTATGTCCCTACTA
AGTATGTCACAGCGAAGGTTAGTCCTGGTCTGTGCATTGCTGGTGATAGAGGTATAGCTCCTAAGAGTGGTTATTTTGT
TAATGTAAATAATACTTGGATGTACACTGGTAGTGGTTACTACTACCCTGAACCTATAACTGAAAATAATGTCGTTGTT
ATGAGTACCTGCGCTGTTAATTATACTAAAGCGCCGTATGTAATGCTGAACACTTCAACACCCAACCTTCCTGATTTTA
GGGAAGAGTTGGATCAATGGTTTAAAAATCAAACATCAGTGGCACCAGATCTGTCACTTGATTATATAAATGTTACATT
CTTGGACCTACAAGTTGAAATGAATAGGTTACAGGAGGCAATAAAAGTCTTAAATCAGAGCTACATCAATCTCAAGGAC
ATTGGTACATATGAATATTATGTAAAATGGCCTTGGTATGTATGGCTTTTAATTGGCCTTGCTGGTGTAGCTATGCTTG
TTTTACTATTCTTCATATGCTGTTGTACAGGATGTGGGACTAGTTGTTTTAAGAAATGTGGTGGTTGTTGTGATGATTA
TACTGGATACCAGGAGTTAGTAATCAAAACTTCACATGACGACTAA

>gi|530802443|gb|KF530086.1| Human coronavirus OC43 strain OC43/human/USA/872-5/1987, complete genome

ATGTTTTTGATACTTTTAATTTCCTTACCAATGGCTTTTGCTGTTATAGGAGATTTAAAGTGTACTTCAGATACG
AGTTATATTAATGATGTTGACACCGGTGTTCCTCCTATAAGTACTGATACTGTTGATGTTACTAATGGTTTGGGTACTT
ATTATGTTTTAGATCGTGTGTATTTAAATACTACTTTGTTTCTTAATGGTTACTATCCTACTTCAGGTTCCACATATCG
TAATATGGCACTGAAGGGAAGTGTACTGTTGAGCACACTATGGTTTAAACCACCATTTCTTTCTGATTTTATTAATGGT
ATTTTTGCTAAGGTCAAAAATACCAAGGTTATTAAAGATGATGTACTGTACAGTGAGTTCCCTGCTATAACTATAGGTA
GTACTTTTGTAAATACATCCTATAGTGTGGTAGTACAACCACGTACAATCAATTTAGATAATAAATTACAAGGTCTTTT
AGAGATCTCTGTTTGCCAGTATAATATGTGCGAGTACCCACATACGATTTGTCATCCTAACTTGGGTAATCATCGCACA

GAACTATGGCATTTGGATACAGGTGTTGTTTCCTGTTTATATAAGCGTAATTTCACATATGATGTGAATGCTGATTATT
TGTATTTTCATTTTTATCAAGAAGGTGGTACTTTTTATGCATATTTTACAGACACTGGTGTTGTTACTAAGTTTTTGTT
TAATGTTTATTTAGGCATGGCGCTTTCACACTATTATGTCATGCCTCTGACTTGTAATAGTAAGCGTAAGGATGGTTTT
ACTTTAGAATATTGGGTTACACCTCTCACTTCTAGACAATATTTACTCGCTTTCAATCAAGATGGTATTATTTTTAATG
CTGTTGATTGCATGAGTGATTTTATGAGTGAGATTAAGTGTAAAACACAATCTATAGCACCACCTACTGGTGTTTATGA
ATTAAACGGTTACACTGTTCAGCCAATCGCAGATGTTTACCGACGTAAACCTAATCTTCCCAATTGCAATATAGAAGCT
TGGCTTAATGATAAGTCGGTGCCATCTCCATTAAATTGGGAACGTAAGACATTTTCAAATTGTAATTTTAATATGAGCA
GCCTGATGTCCTTTATTCAGGCAGACTCATTTACTTGTAATAATATTGATGCTGCTAAGATATATGGTATGTGTTTTTC
CAGCATAACTATAGATAAGTTTGCTATACCCAATGGCAGGAAGGTTGACCTACAATTGGGTAATTTGGGCTATTTGCAG
TCATTTAACTATAGAATTGATACTACTGCAACAAGTTGTCAGTTGTATTATAATTTACCTGCTGCTAATGTTTCTGTTA
GCAGGTTTAATCCTTCTACTTGGAATAAGAGATTCGGTTTTATAGAAGATTCTGTCTTTAAGCCTCAACCTGCAGGTGT
TTTTACCAATCATGATGTAGTTTATGCCCAACATTGTTTCAAAGCTCCCAAAAATTTCTGTCCGTGTAAATTGAATGGT
TCTCTGTGTGTAGGTAGTGGTCCTGGTAAAAATAATGGTATAGGCACTTGTCCTGCAGGTACTAATTATTTAACTTGTC
ATAATTTGTGCAATCCTGATCCTATTACATTTACAGGTCCTTATAAGTGCCCCCAAACTAAATCTTTAGTTGGTATAGG
TGAGCACTGTTCAGGTCTTGCTGTTAAAAGTGATTATTGTGGAGGCAATCCTTGCACTTGCCAACCACAAGCATTTTTG
GGTTGGTCTGCAGACTCTTGTTTACAAGGGGACAAGTGTAATATTTTTGCTAATTTAATTTTGCATGATGTTAATAGTG
GTCTTACTTGTTCTACTGATTTACAAAAAGCTAACACAGACATAATTCTTGGTGTTTGTGTTAATTATGACCTTTATGG
TATTTCAGGCCAAGGCATTTTTGTTGAGGTTAATGCGACTTATTATAATAGTTGGCAGAACCTTTTATATGATTCTAAT
GGTAATCTCTATGGTTTTAGAGACTACATAACAAACAGAACTTTTATGATTCGTAGTTGCTATAGCGGTCGTGTTTCTG
CGGCCTTTCACGCTAACTCTTCCGAACCAGCATTGCTATTTCGGAATATTAAATGCAACTACGTTTTTAATAATAGTCT
TACACGACAGCTGCAACCCATTAACTATTTTGATAGTTATCTTGGTTGTGTTGTCAATGCTTATAATAGTACTGCTATT
TCTGTTCAAACATGTGATCTCACAGTAGGTAGTGGTTACTGTGTGGATTACTCTAAAAACAGACGAAGTCGTAGAGCGA
TTACCACTGGTTATCGGTTTACTAATTTTGAGCCATTTACTGTTAATTCAGTAAATGATAGTTTAGAACCTGTAGGTGG
TTTGTACGAAATTCAAATACCTTCAGAGTTTACTATAGGTAATATGGAGGAGTTTATTCAAACAAGCTCTCCTAAAGTT
ACTATTGATTGTGCTGCATTTGTCTGTGGTGATTATGCAGCATGTAAATCACAGTTGGTTGAATATGGTAGTTTCTGTG
ATAACATTAATGCCATACTCACAGAAGTAAATGAACTACTTGACACTACACAGTTGCAAGTAGCTAATAGTTTAATGAA
TGGTGTTACTCTTAGCACTAAGCTTAAAGATGGCGTTAATTTCAATGTAGACGACATCAATTTTTCCCCTGTATTAGGT
TGTCTAGGCAGCGAATGTAGTAAAGCTTCCAGTAGATCTGCTATAGAGGATTTACTTTTTGATAAAGTAAAGTTATCTG
ATGTCGGTTTCGTTGAGGCTTATAATAATTGTACTGGAGGTGCCGAAATTAGGGACCTCATTTGTGTGCAAAGTTATAA
AGGTATCAAAGTGTTGCCTCCACTGCTCTCAGAAAATCAGATCAGTGGATACACTTTGGCTGCCACTTCTGCTAGTCTG
TTTCCTCCTTGGACAGCAGCAGCAGGTGTACCATTTTATTTAAATGTTCAGTATCGCATTAATGGGCTTGGTGTCACCA
TGGATGTGCTAAGTCAAAATCAAAAGCTTATTGCTAATGCATTTAACAATGCCCTTGATGCTATTCAGGAAGGGTTCGA
TGCAACTAATTCTGCTTTAGTTAAAATTCAAGCTGTTGTTAATGCAAATGCTGAAGCTCTTAATAACTTATTGCAACAA
CTCTCTAATAGATTTGGTGCTATAAGTTCTTCTTTACAAGAAATTCTATCTAGACTTGATGCTCTTGAAGCGGAAGCTC
AGATAGATAGACTTATTAATGGTCGTCTTACCGCTCTTAATGCTTATGTTTCTCAACAGCTTAGTGATTCTACACTGGT
AAAATTTAGTGCAGCACAAGCTATGGAGAAGGTTAATGAATGTGTCAAAAGCCAATCATCTAGGATAAATTTCTGTGGT
AATGGTAACCATATTATATCATTAGTGCAGAATGCTCCATATGGTTTGTATTTTATCCACTTTAGTTATGTCCCTACTA
AGTATGTCACAGCGAAGGTTAGTCCTGGTCTGTGCATTGCTGGTGATAGAGGTATAGCTCCTAAGAGTGGTTATTTTGT
TAATGTAAATAATACTTGGATGTACACTGGTAGTGGTTACTACTACCCTGAACCTATAACTGAAAATAATGTCGTTGTT
ATGAGTACCTGCGCTGTTAATTATACTAAAGCGCCGTATGTAATGCTGAACACTTCAACACCCAACCTTCCTGATTTTA
GGGAAGAGTTGGATCAATGGTTTAAAAATCAAACATCAGTGGCACCAGATCTGTCACTTGATTATATAAATGTTACATT
CTTGGACCTACAAGTTGAAATGAATAGGTTACAGGAGGCAATAAAAGTCTTAAATCAGAGCTACATCAATCTCAAGTAC
ATTGGTACATATGAATATTATGTAAAATGGCCTTGGTATGTATGGCTTTTAATCGGCCTTGCTGGTGTAGCTATGCTTG
TTTTACTATTCTTCATATGCTGTTGTACAGGATGTGGGACTAGTTGTTTTAAGAAATGTGGTGGTTGTTGTGATGATTA

TACTGGATACCAGGAGTTAGTAATCAAAACTTCACATGACGACTAA

>gi|530802454|gb|KF530087.1| Human coronavirus OC43 strain OC43/human/USA/873-6/1987, complete genome

ATGTTTTTGATACTTTTAATTTCCTTACCAATGGCTTTTGCTGTTATAGGAGATTTAAAGTGTACTTCAGATACG
AGTTATATTAATGATGTTGACACCGGTGTTCCTCCTATAAGTACTGATACTGTTGATGTTACTAATGGTTTGGGTACTT
ATTATGTTTTAGATCGTGTGTATTTAAATACTACTTTGTTTCTTAATGGTTACTATCCTACTTCAGGTTCCACATATCG
TAATATGGCACTGAAGGGAAGTGTACTGTTGAGCACACTATGGTTTAAACCACCATTTCTTTCTGATTTTATTAATGGT
ATTTTTGCTAAGGTCAAAAATACCAAGGTTATTAAAGATGATGTACTGTACAGTGAGTTCCCTGCTATAACTATAGGTA
GTACTTTTGTAAATACATCCTATAGTGTGGTAGTACAACCACGTACAATCAATTTAGATAATAAATTACAAGGTCTTTT
AGAGATCTCTGTTTGCCAGTATAATATGTGCGAGTACCCACATACGATTTGTCATCCTAACTTGGGTAATCATCGCACA
GAACTATGGCATTTGGATACAGGTGTTGTTTCCTGTTTATATAAGCGTAATTTCACATATGATGTGAATGCTGATTATT
TGTATTTTCATTTTTATCAAGAAGGTGGTACTTTTTATGCATATTTTACAGACACTGGTGTTGTTACTAAGTTTTTGTT
TAATGTTTATTTAGGCATGGCGCTTTCACACTATTATGTCATGCCTCTGACTTGTAATAGTAAGCGTAAGGATGGTTTT
ACTTTAGAATATTGGGTTACACCTCTCACTTCTAGACAATATTTACTCGCTTTCAATCAAGATGGTATTATTTTTAATG
CTGTTGATTGCATGAGTGATTTTATGAGTGAGATTAAGTGTAAAACACAATCTATAGCACCACCTACTGGTGTTTATGA
ATTAAACGGTTACACTGTTCAGCCAATCGCAGATGTTTACCGACGTAAACCTAATCTTCCCAATTGCAATATAGAAGCT
TGGCTTAATGATAAGTCGGTGCCATCTCCATTAAATTGGGAACGTAAGACATTTTCAAATTGTAATTTTAATATGAGCA
GCCTGATGTCGTTTATTCAGGCAGACTCATTTACTTGTAATAATATTGATGCTGCTAAGATATATGGTATGTGTTTTTC
CAGCATAACTATAGATAAGTTTGCTATACCCAATGGCAGGAAGGTTGACCTACAATTGGGTAATTTGGGCTATTTGCAG
TCATTTAACTATAGAATTGATACTACTGCAACAAGTTGTCAGTTGTATTATAATTTACCTGCTGCTAATGTTTCTGTTA
GCAGGTTTAATCCTTCTACTTGGAATAAGAGATTCGGTTTTATAGAAGATTCTGTTTTTAAGCCTCAACCTGCAGGTGT
TTTTACCAATCATGATGTAGTTTATGCCCAACATTGTTTTAAAGCTCCCAAAAATTTCTGTCCGTGTAAATTGAATGGT
TCTCTGTGTGTAGGTAGTGGTCCTGGTAAAAATAATGGTATAGGCACTTGTCCTGCAGGTACTAATTATTTAACTTGTC
ATAATTTGTGTAATCCTGATCCTATTACATTTACAGGTCCTTATAAGTGCCCCCAAACTAAATCTTTAGTTGGTATAGG
TGAGCACTGTTCAGGTCTTGCTGTTAAAAGTGATTATTGTGGAGGCAATCCTTGCACTTGCCAACCACAAGCATTTTTG
GGTTGGTCTGCAGACTCTTGTTTACAAGGGGACAAGTGTAATATTTTTGCTAATTTAATTTTGCATGATGTTAATAGTG
GTCTTACTTGTTCTACTGATTTACAAAAAGCTAACACAGACATAATTCTTGGTGTTTGTGTTAATTATGACCTTTATGG
TATTTCAGGCCAAGGCATTTTTGTTGAGGTTAATGCGACTTATTATAATAGTTGGCAGAACCTTTTATATGATTCTAAT
GGTAATCTCTATGGTTTTAGAGACTACATAACAAACAGAACTTTTATGATTCGTAGTTGCTATAGCGGTCGTGTTTCTG
CGGCCTTTCACGCTAACTCTTCCGAACCAGCATTGCTATTTCGGAATATTAAATGCAACTACGTTTTTAATAATAGTCT
TACACGACAGCTGCAACCCATTAACTATTTTGATAGTTATCTTGGTTGTGTTGTCAATGCTTATAATAGTACTGCTATT
TCTGTTCAAACATGTGATCTCACAGTAGGTAGTGGTTACTGTGTGGATTACTCTAAAAATAGACGAAGTCGTAGAGCGA
TTACCACTGGTTATCGGTTTACTAATTTTGAGCCATTTACTGTTAATTCAGTAAATGACAGTTTAGAACCTGTAGGTGG
TTTGTACGAAATTCAAATACCTTCAGAGTTTACTATAGGTAATATGGAGGAGTTTATTCAAACAAGCTCTCCTAAAGTT
ACTATTGATTGTGCTGCATTTGTCTGTGGTGATTATGCAGCATGTAAATCACAGTTGGTTGAATATGGTAGTTTCTGTG
ATAACATTAATGCCATACTCACAGAAGTAAATGAACTACTTGACACTACACAGTTGCAAGTAGCTAATAGTTTAATGAA
TGGTGTTACTCTTAGCACTAAGCTTAAAGATGGCGTTAATTTCAATGTAGACGACATCAATTTTTCCCCTGTATTAGGT
TGTCTAGGCAGCGAATGTAGTAAAGCTTCCAGTAGATCTGCTATAGAGGATTTACTTTTTGATAAAGTAAAGTTATCTG
ATGTCGGTTTCGTTGAGGCTTATAATAATTGTACTGGAGGTGCCGAAATTAGGGACCTCATTTGTGTGCAAAGTTATAA
AGGTATCAAAGTGTTGCCTCCACTGCTCTCAGAAAATCAGATCAGTGGATACACTTTGGCTGCCACTTCTGCTAGTCTG
TTTCCTCCTTGGACAGCAGCAGCAGGTGTACCATTTTATTTAAATGTTCAGTATCGCATTAATGGGCTTGGTGTCACCA

TGGATGTGCTAAGTCAAAATCAAAAGCTTATTGCTAATGCATTTAACAATGCCCTTGATGCTATTCAGGAAGGGTTCGA TGCAACTAATTCTGCTTTAGTTAAAATTCAAGCTGTTGTTAATGCAAATGCTGAAGCTCTTAATAACTTATTGCAACAA CTCTCTAATAGATTTGGTGCTATAAGTTCTTCTCTACAAGAAATTCTATCTAGACTTGATGCTCTTGAAGCGGAAGCTC AGATAGATAGACTTATTAATGGTCGTCTTACCGCTCTTAATGCTTATGTTTCTCAACAGCTTAGTGATTCTACACTGGT AAAATTTAGTGCAGCACAAGCTATGGAGAAGGTTAATGAATGTGTCAAAAGCCAATCATCTAGGATAAATTTCTGTGGT AATGGTAACCATATTATATCATTAGTGCAGAATGCTCCATATGGTTTGTATTTTATCCACTTTAGTTATGTCCCTACTA AGTATGTCACAGCGAAGGTTAGTCCTGGTCTGTGCATTGCTGGTGATAGAGGTATAGCTCCTAAGAGTGGTTATTTTGT TAATGTAAATAATACTTGGATGTACACTGGTAGTGGTTACTACTACCCTGAACCTATAACTGAAAATAATGTCGTTGTT ATGAGTACCTGCGCTGTTAATTATACTAAAGCGCCGTATGTAATGCTGAACACTTCAACACCCAACCTTCCTGATTTTA GGGAAGAGTTGGATCAATGGTTTAAAAATCAAACATCAGTGGCACCAGATCTGTCACTTGATTATATAAATGTTACATT CTTGAACCTACAAGTTGAAATGAATAGGTTACAGGAGGCAATAAAAGTCTTAAATCAGAGCTACATCAATCTCAAGGAC ATTGGTACATATGAATATTATGTAAAATGGCCTTGGTATGTATGGCTTTTAATCGGCCTTGCTGGTGTAGCTATGCTTG TTTTACTATTCTTCATATGCTGTTGTACAGGATGTGGGACTAGTTGTTTTAAGAAATGTGGTGGTTGTTGTGATGATTA TACTGGATACCAGGAGTTAGTAATCAAAACTTCACATGACGACTAA

>gi|530802465|gb|KF530088.1| Human coronavirus OC43 strain OC43/human/USA/901-54/1990, complete genome

ATGTTTTTGATACTTTTAATTTCCTTACCAACGGCTTTTGCTGTTATAGGAGATTTAAAGTGTCCTTTAGATACT AGTTATAAAGGTACATTTAATAATAAAGACACCGGTCCTCCTTTTATAAGTACTGATACTGTTGATGTTACTAATGGTT TGGGTACTTATTATGTTTTAGATCGTGTGTATTTAAATACTACGTTGTTTCTTAATGGTTATTACCCTACTTCAGGTTC CACATATCGTAATATGGCACTGAAGGGAACTGACAAATTGAGCACACTATGGTTTAAACCACCATTTCTTTCTGATTTT ATTAATGGTATTTTTGCTAAGGTCAAAAATACCAAGGTTTTTAAAGATGGTGTAATGTATAGTGAGTTCCCTGCTATAA CTATAGGTAGTACTTTTGTAAACACATCCTATAGTGTGGTAGTACAACCACGTACAATTAATTCACAGGATGGTGATAA TAAATTACAAGGTCTTTTAGAGGTCTCTGTTTGCCAGTATAATATGTGCGAGTACCCACATACGATTTGTCATCCTAAA CTGGGTAATCACTTCAAAGAACTATGGCATTTGGATACAGGTGTTGTTTCCTGTTTATATAAGCGTAATTTCACATATG ATGTGAATGCTAATTATTTGTATTTTCATTTTTATCAAGAAGGTGGTACTTTTTATGCATATTTTACAGACACTGGTGT TGTTACTAAGTTTTTGTTTAATGTTTATTTAGGCATGGCACTTTCACACTATTATGTCATGCCTCTGACTTGTATTAGT AGGCGTGATATTGGTTTTACTTTAGAATATTGGGTTACACCCCTCACTTCTAGACAATATTTACTCGCTTTCAATCAAG ATGGTATTATTTTTAATGCTGTTGATTGTATGAGTGATTTTATGAGTGAGATTAAGTGTAAAACACAATCTATAGCACC ACCTACTGGTGTTTATGAATTAAACGGTTACACTGTTCAGCCAATCGCAGATGTTTACCGACGCAAACCTGATCTTCCC AATTGCAATATAGAAGCTTGGCTTAATGATAAGTCGGTGCCCTCTCCATTAAATTGGGAACGTAAGACATTTTCAAATT GTAATTTTAATATGAGCAGCCTGATGTCTTTTATTCAGGCAGACTCATTTACTTGTAATAATATTGATGCTGCTAAGAT ATATGGTATGTGTTTTTCCAGCATAACTATAGATAAGTTTGCTATACCCAATAGCAGGAAGGTTGACCTACAATTGGGT AATTTGGGCTATTTGCAGTCATCCAACTATAGAATTGATACTACTGCAACAAGTTGTCAGTTGTATTATAATTTACCTG CTGCTAATGTCTCTGTTAGCAGGTTTAATCCTTCTACTTGGAATAAGAGATTTGGTTTTATAGAAGATTCTGTTTTTGT GCCTCAACCTACAGGTGTTTTTACTAATCACAGTGTAGTTTATGCACAACACTGTTTCAAAGCCCCTAAAAATTTCTGT CCGTGTAAATTGAATGGTTCGTGTCCTGGTAAAAATAATGGTATAGGCACTTGTCCTGCAGGTACTAATTATTTAACTT GTGATAATTTGTGTACTCTTGATCCTATTACATTTAAAGCTCCAGGTACTTATAAGTGCCCCCAAACTAAATCTTTAGT TGGCATAGGTGAGCACTGTTCGGGTCTTGCTGTTAAAAGTGATTATTGTGGAGGCAATTCTTGTACTTGCCAACCACAA GCATTTTTGGGTTGGTCTGCAGACTCTTGTTTACAAGGAGACAAGTGTAATATTTTTGCTAATCTTATTTTGCATGATG TTAATAGTGGTCTTACTTGTTCTACTGATTTACAAAAAGCTAACACAGACATAATTCTTGGTGTTTGTGTTAATTATGA CCTCTATGGTATTTCAGGCCAAGGCATTTTTGTTGAGGTTAATGCGACTTATTATAATAGTTGGCAGAACCTTTTATAT

GATTCTAATGGTAATCTCTACGGTTTTAGAGACTACATAACAAACAGAACTTTTATGATTCGTAGTTGCTATAGCGGTC
GTGTCTCTGCGGCCTTTCATGCTAACTCTTCCGAACCAGCATTGCTATTTCGGAATATTAAATGCAACTACGTTTTTAA
TAATAGTCTTACACGACAGCTGCAACCCATTAACTATTCGTTTGATAGTTATCTTGGTTGTGTTGTCAATGCTTATAAT
AGTACTGCTATTTCTGTTCAAACATGTGATCTCACAGTAGGTAGTGGTTACTGTGTGGATTACTCTAAAAACAGACGAA
GTCGTAGAGCGCTTACCACTGGTTATCGGTTTACTAATTTTGAGCCATTTACTGTTAATTCAGTAAATGATAGTTTGGA
ACCTGTAGGTGGTTTGTATGAAATTCAAATACCTTCAGAGTTTACTATAGGTAATATGGAGGAGTTTATTCAAACAAGC
TCTCCCAAAGTTACTATTGATTGTGCTGCATTTGTCTGTGGTGATTATGCAGCATGTAAATTACAGTTGGTTGAATATG
GTAGTTTCTGTGATAACATTAATGCCATACTCACAGAAGTAAATGAACTACTTGATACTACACAGTTGCAAGTAGCTAA
TAGTTTAATGAATGGTGTTACTCTTAGCACTAAGCTTAAAGATGGTGTTAATTTCAATGTAGACGACATCAATTTTTCC
CCTGTATTAGGTTGTCTAGGCAGCGAATGTAGTAAAGCTTCCAGTAGATCTGCTATAGAGGATTTACTTTTTGATAAAG
TAAAGTTATCTGATGTCGGTTTCGTTGAGGCTTATAATAATTGTACAGGAGGTGCCGAAATTAGGGACCTCATTTGTGT
GCAAAGTTATAAAGGCATCAAAGTGTTGCCCCCACTGCTCTCAGAAAATCAGATCAGTGGATACACTTTGGCTGCCACC
TCTGCTAGTCTATTTCCTCCTTGGACAGCAGCAGCAGGTGTACCATTTTATTTAAATGTTCAGTATCGCATTAATGGGC
TTGGTGTCACCATGGATGTGCTAAGTCAAAATCAAAAGCTTATTGCTAATGCATTTAACAATGCCCTTTATGCTATTCA
GCAAGGGTTCGATGCAACTAATTCTGCTTTAGTTAAAATTCAAGCTGTTGTTAATGCAAATGCTGAAGCTCTTAATAAC
TTATTGCAACAACTCTCTAATAGATTTGGTGCTATAAGTGCTTCTTTACAAGAAATTCTATCTAGACTTGATGCTCTTG
AAGCGGAAGCTCAGATAGATAGACTTATTAATGGTCGTCTCACCGCTCTTAATGCTTATGTTTCTCAACAGCTTAGTGA
TTCTACACTGGTAAAATTTAGTGCAGCACAAGCTATGGAGAAGGTTAATGAATGTGTCAAAAGCCAATCATCTAGGATA
AATTTCTGTGGTAATGGTAATCATATTATATCATTAGTGCAGAATGCTCCATATGGTTTGTATTTTATCCACTTTAATT
ATGTCCCTACTAAGTATGTCACAGCGAAGGTTAGTCCTGGTCTGTGCATTGCTGGTAATAGAGGTATAGCTCCTAAGAG
TGGTTATTTTGTTAATGTAAATAATACTTGGATGTACACTGGTAGTGGTTACTACTACCCTGAACCTATAACTGAAAAT
AATGTTGTTGTTATGAGTACCTGCGCTGTTAATTATACTAAAGCGCCGTATGTAATGCTGAACACTTCAATACCCAACC
TTCCTGATTTTAAGGAAGAGTTGGATCAATGGTTTAAAAATCAAACATCAGTGGCACCAGATTTGTCACTTGATTATAT
AAATGTTACATTCTTGGACCTACAAGTTGAAATGAATAGGTTACAGGAGGCAATAAAAGTCTTAAATCATAGCTACATT
AATCTCAAGGACATTGGTACATATGAATATTATGTAAAATGGCCTTGGTATGTATGGCTCTTAATCTGCCTTGCTGGTG
TAGCTATGCTTGTTTTACTATTCTTCATATGCTGTTGTACAGGATGTGGGACTAGTTGTTTTAAGAAATGTGGTGGTTG
TTGTGATGATTATACTGGATACCAGGAGTTAGTAATCAAAACTTCACATGACGACTAA

>gi|530802476|gb|KF530089.1| Human coronavirus OC43 strain OC43/human/USA/911-66/1991, complete genome

ATGTTTTTGATACTTTTAATTTCCTTACCAACGGCTTTTGCTGTTATAGGAGATTTAAAGTGTCCTTTAGATACT
AGTTATAAAGGTACATTTAATAATAAAGACACCGGTCCTCCTTTTATAAGTACTGATACTGTTGATGTTACTAATGGTT
TGGGTACTTATTATGTTTTAGATCGTGTGTATTTAAATACTACGTTGTTTCTTAATGGTTATTACCCTACTTCAGGTTC
CACATATCGTAATATGGCACTGAAGGGAACTGACAAATTGAGCACACTATGGTTTAAACCACCATTTCTTTCTGATTTT
ATTAATGGTATTTTTGCTAAGGTCAAAAATACCAAGGTTTTTAAAGATGGTGTAATGTATAGTGAGTTCCCTGCTATAA
CTATAGGTAGTACTTTTGTAAATACATCCTATAGTGTGGTAGTACAACCACGTACAATTAATTCAACACAGGATGGTGA
TAATAAATTACAAGGTCTTTTAGAGGTCTCTGTTTGCCAGTATAATATGTGCGAGTACCCACATACGAGTTGTCATCCT
AAACTGGGTAATCACTTCAAAGAACTATGGCATTTGGATACAGGTGTTGTTTCCTGTTTATATAAGCGTAATTTCACAT
ATGATGTGAATGCTAATTATTTGTATTTTCATTTTTATCAAGAAGGTGGTACTTTTTATGCATATTTTACAGACACTGG
TGTTGTTACTAAGTTTTTGTTTAATGTTTATTTAGGCATGGCACTTTCACACTATTATGTCATGCCTCTGACTTGTATT
AGTAGGCGTGATATTGGTTTTACTTTAGAATATTGGGTTACACCCCTCACTTCTAGACAATATTTACTCGCTTTCAATC
AAGATGGTATTATTTTTAATGCTGTTGATTGTATGAGTGATTTTATGAGTGAGATTAAGTGTAAAACACAATCTATAGC

ACCACCTACTGGTGTTTATGAATTAAACGGTTACACTGTTCAGCCAATCGCAGATGTTTACCGACGCAAACCTGATCTT
CCCAATTGCAATATAGAAGCTTGGCTTAATGATAAGTCGGTGCCCTCTCCATTAAATTGGGAACGTAAGACATTTTCAA
ATTGTAATTTTAATATGAGCAGCCTGATGTCTTTTATTCAGGCAGACTCATTTACTTGTAATAATATTGATGCTGCTAA
GATATATGGTATGTGTTTTTCCAGCATAACTATAGATAAGTTTGCTATACCCAATGGCAGGAAGGTTGACCTACAATTG
GGTAATTTGGGCTATTTGCAGTCATCCAACTATAGAATTGATACTACTGCAACAAGTTGTCAGTTGTATTATAATTTAC
CTGCTGCTAATGTCTCTGTTAGCAGGTTTAATCCTTCTACTTGGAATAAGAGATTTGGTTTTATAGAAGATTCTGTTTT
TGTGCCTCAACCTACAGGTGTTTTTACTAATCACAGTGTAGTTTATGCACAACACTGTTTCAAAGCCCCTAAAAATTTC
TGTCCGTGTAAATTGAATGGTTCGTGTCCTGGTAAAAATAATGGTATAGGCACTTGTCCTGCAGGTACTAATTATTTAA
CTTGTGATAATTTGTGTACTCTTGATCCTATTACATTTAAAGCTCCAGGTACTTATAAGTGCCCCCAAACTAAATCTTT
AGTTGGCATAGGTGAGCACTGTTCGGGTCTTGCTGTTAAAAGTGATTATTGTAGAGGCAATTCTTGTACTTGCCAACCA
CAAGCATTTTTGGGTTGGTCTGCAGACTCTTGTTTACAAGGAGACAAGTGTAATATTTTTGCTAATCTTATTTTGCATG
ATGTTAATAGTGGTCTTACTTGTTCTACTGATTTACAAAAAGCTAACACAGACATAATTCTTGGTGTTTGTGTTAATTA
TGACCTCTATGGTATTTCAGGCCAAGGCATTTTTGTTGAGGTTAATGCGACTTATTATAATAGTTGGCAGAACCTTTTA
TATGATTCTAATGGTAATCTCTACGGTTTTAGAGACTACATAACAAACAGAACTTTTATGATTCGTAGTTGCTATAGCG
GTCGTGTTTCTGCGGCCTTTCATGCTAACTCTTCCGAACCAGCATTGCTATTTCGGAATATTAAATGCAACTACGTTTT
TAATAATAGTCTTACACGACAGCTGCAACCCATTAACTATTCGTTTGATAGTTATCTTGGTTGTGTTGTCAATGCTTAT
AATAGTACTGCTATTTCTGTTCAAACATGTGATCTCACAGTAGGTAGTGGTTACTGTGTGGATTACTCTAAAAACAGAC
GAAGTCGTAGAGCGATTACCACTGGTTATCGGTTTACTAATTTTGAGCCATTTACTGTTAATTCAGTAAATGATAGTTT
GGAACCTGTAGGTGGTTTGTATGAAATTCAAATACCTTCAGAGTTTACTATAGGTAATATGGAGGAGTTTATTCAAACA
AGCTCTCCCAAAGTTACTATTGATTGTGCTGCATTTGTTTGTGGTGATTATGCAGCATGTAAATTACAGTTGGTTGAAT
ATGGTAGTTTCTGTGATAACATTAATGCCATACTCACAGAAGTAAATGAACTACTTGATACTACACAGTTGCAAGTAGC
TAATAGTTTAATGAATGGTGTTACTCTTAGCACTAAGCTTAAAGATGGTGTTAATTTCAATGTAGATGACATCAATTTT
TCCCCTGTATTAGGTTGTCTAGGCAGCGAATGTAGTAAAGCTTCCAGTAGATCTGCTATAGAGGATTTACTTTTTGATA
AAGTAAAGTTATCTGATGTCGGTTTCGTTGAGGCTTATAATAATTGTACAGGAGGTGCCGAAATTAGGGACCTCATTTG
TGTGCAAAGTTATAAAGGCATCAAAGTGTTGCCCCCACTGCTCTCAGAAAATCAGATCAGTGGATACACTTTGGCTGCC
ACCTCTGCTAGTCTATTTCCTCCTTGGACAGCAGCAGCAGGTGTACCATTTTATTTAAATGTTCAGTATCGCATTAATG
GGCTTGGTGTCACTATGGATGTGCTAAGTCAAAATCAAAAGCTTATTGCTAATGCATTTAACAATGCCCTTTATGCTAT
TCAGCAAGGGTTCGATGCAACTAATTCTGCTTTAGTTAAAATTCAAGCTGTTGTTAATGCAAATGCTGAAGCTCTTAAT
AACTTATTGCAACAACTCTCTAATAGATTTGGTGCTATAAGTGCTTCTTTACAAGAAATTCTATCTAGACTTGATGCTC
TTGAAGCGGAAGCTCAGATAGATAGACTTATTAATGGTCGTCTCACCGCTCTTAATGCTTATGTTTCTCAACAGCTTAG
TGATTCTACACTGGTAAAATTTAGTGCAGCACAAGCTATGGAGAAGGTTAATGAATGTGTCAAAAGCCAATCATCTAGG
ATAAATTTCTGTGGTAATGGTAATCATATTATATCATTAGTGCAGAATGCTCCATATGGTTTGTATTTTATCCACTTTA
ATTATGTCCCTACTAAGTATGTCACAGCGAAGGTTAGTCCTGGTCTGTGCATTGCTGGTAATAGAGGTATAGCTCCTAA
GAGTGGTTATTTTGTTAATGTAAATAATACTTGGATGTACACTGGTAGTGGTTACTACTACCCTGAACCTATAACTGAA
AATAATGTTGTTGTTATGAGTACCTGCGCTGTTAATTATACTAAAGCGCCGTATGTAATGCTGAACACTTCAATACCCA
ACCTTCCTGATTTTAAGGAAGAGTTGGATCAATGGTTTAAAAATCAAACATCAGTGGCACCAGATTTGTCACTTGATTA
TATAAATGTTACATTCTTGGACCTACAAGTTGAAATGAATAGGTTACAGGAGGCAATAAAAGTCTTAAATCATAGCTAC
ATCAATCTCAAGGACATTGGTACATATGAATATTATGTAAAATGGCCTTGGTATGTATGGCTCTTAATCTGCCTTGCTG
GTGTAGCTATGCTTGTTTTACTATTCTTCATATGCTGTTGTACAGGATGTGGGACTAGTTGTTTTAAGAAATGTGGTGG
TTGTTGTGATGATTATACTGGATACCAGGAGTTTGTAATCAAAACTTCACATGACGACTAA

>gi|530802498|gb|KF530091.1| Human coronavirus OC43 strain OC43/human/USA/911-58/1991, complete genome

ATGTTTTTGATACTTTTAATTTCCTTACCAACGGCTTTTGCTGTTATAGGAGATTTAAAGTGTCCTTTAGATACT AGTTATAAAGGTACATTTAATAATAAAGACACCGGTCCTCCTTTTATAAGTACTGATACTGTTGATGTTACTAATGGTT TGGGTACTTATTATGTTTTAGATCGTGTGTATTTAAATACTACGTTGTTTCTTAATGGTTATTACCCTACTTCAGGTTC CACATATCGTAATATGGCACTGAAGGGAACTGACAAATTGAGCACACTATGGTTTAAACCACCATTTCTTTCTGATTTT ATTAATGGTATTTTTGCTAAGGTCAAAAATACCAAGGTTTTTAAAGATGGTGTAATGTATAGTGAGTTCCCTGCTATAA CTATAGGTAGTACTTTTGTAAATACATCCTATAGTGTGGTAGTACAACCACGTACAATTAATTCACAGGATGGTGATAA TAAATTACAAGGTCTTTTAGAGGTCTCTGTTTGCCAGTATAATATGTGCGAGTACCCACATACGATTTGTCATCCTAAA CTGGGTAATCACTTCAAAGAACTATGGCATTTGGATACAGGTGTTGTTTCCTGTTTATATAAGCGTAATTTCACATATG ATGTGAATGCTAATTATTTGTATTTTCATTTTTATCAAGAAGGTGGTACTTTTTATGCATATTTTACAGACACTGGTGT TGTTACTAAGTTTTTGTTTAATGTTTATTTAGGCATGGCACTTTCACACTATTATGTCATGCCTCTGACTTGTATTAGT AGGCGTGATATTGGTTTTACTTTAGAATATTGGGTTACACCCCTCACTTCTAGACAATATTTACTCGCTTTCAATCAAG ATGGTATTATTTTTAATGCTGTTGATTGTATGAGTGATTTTATGAGTGAGATTAAGTGTAAAACACAATCTATAGCACC ACCTACTGGTGTTTATGAATTAAACGGTTACACTGTTCAGCCAATCGCAGATGTTTACCGACGCAAACCTGATCTTCCC AATTGCAATATAGAAGCTTGGCTTAATGATAAGTCGGTGCCCTCTCCATTAAATTGGGAACGTAAGACATTTTCAAATT GTAATTTTAATATGAGCAGCCTGATGTCTTTTATTCAGGCAGACTCATTTACTTGTAATAATATTGATGCTGCTAAGAT ATATGGTATGTGTTTTTCCAGCATAACTATAGATAAGTTTGCTATACCCAATAGCAGGAAGGTTGACCTACAATTGGGT AATTTGGGCTATTTGCAGTCATCCAACTATAGAATTGATACTACTGCAACAAGTTGTCAGTTGTATTATAATTTACCTG CTGCTAATGTCTCTGTTAGCAGGTTTAATCCTTCTACTTGGAATAAGAGATTTGGTTTTATAGAAGATTCTGTTTTTGT GCCTCAACCTACAGGTGTTTTTACTAATCACAGTGTAGTTTATGCACAACACTGTTTCAAAGCCCCTAAAAATTTCTGT CCGTGTAAATTGAATGGTTCGTGTCCTGGTAAAAATAATGGTATAGGCACTTGTCCTGCAGGTACTAATTACTTAACTT GTGATAATTTGTGTACTCTTGATCCTATTACATTTAAAGCTCCAGGTACTTATAAGTGCCCCCAAACTAAATCTTTAGT TGGCATAGGTGAGCACTGTTCGGGTCTTGCTGTTAAAAGTGATTATTGTGGAGGCAATTCTTGTACTTGCCAACCACAA GCATTTTTGGGTTGGTCTGCAGACTCTTGTTTACAAGGAGACAAGTGTAATATTTTTGCTAATCTTATTTTGCATGATG TTAATAGTGGTCTTACTTGTTCTACTGATTTACAAAAAGCTAACACAGACATAATTCTTGGTGTTTGTGTTAATTATGA CCTCTATGGTATTTCAGGCCAAGGCATTTTTGTTGAGGTTAATGCGACTTATTATAATAGTTGGCAGAACCTTTTATAT GATTCTAATGGTAATCTCTACGGTTTTAGAGACTACATAACAAATAGAACTTTTATGATTCGTAGTTGCTATAGCGGTC GTGTTTCTGCGGCCTTTCATGCTAACTCTTCCGAACCAGCATTGCTATTTCGGAATATTAAATGCAACTACGTTTTTAA TAATAGTCTTACACGACAGCTGCAACCCATTAACTATTCGTTTGATAGTTATCTTGGTTGTGTTGTCAATGCTTATAAT AGTACTGCTATTTCTGTTCAAACATGTGATCTCACAGTAGGTAGTGGTTACTGTGTGGATTACTCTAAAAACAGACGAA GTCGTAGAGCGCTTACCACTGGTTATCGGTTTACTAATTTTGAGCCATTTACTGTTAATTCAGTAAATGATAGTTTGGA ACCTGTAGGTGGTTTGTATGAAATTCAAATACCTTCAGAGTTTACTATAGGTAATATGGAGGAGTTTATTCAAACAAGC TCTCCCAAAGTTACTATTGATTGTGCTGCATTTGTCTGTGGTGATTATGCAGCATGTAAATTACAGTTGGTTGAATATG GTAGTTTCTGTGATAACATTAATGCCATACTCACAGAAGTAAATGAACTACTTGATACTACACAGTTGCAAGTAGCTAA TAGTTTAATGAATGGTGTTACTCTTAGCACTAAGCTTAAAGATGGTGTTAATTTCAATGTAGACGACATCAATTTTTCC CCTGTATTAGGTTGTCTAGGCAGCGAATGTAGTAAAGCTTCCAGTAGATCTGCTATAGAGGATTTACTTTTTGATAAAG TAAAGTTATCTGATGTCGGTTTCGTTGAGGCTTATAATAATTGTACAGGAGGTGCCGAAATTAGGGACCTCATTTGTGT GCAAAGTTATAAAGGCATCAAAGTGTTGCCCCCACTGCTCTCAGAAAATCAGATCAGTGGATACACTTTGGCTGCCACC TCTGCTAGTCTATTTCCTCCTTGGACAGCAGCAGCAGGTGTACCATTTTATTTAAATGTTCAGTATCGCATTAATGGGC TTGGTGTCACCATGGATGTGCTAAGTCAAAATCAAAAGCTTATTGCTAATGCATTTAACAATGCCCTTTATGCTATTCA GCAAGGGTTCGATGCAACTAATTCTGCTTTAGTTAAAATTCAAGCTGTTGTTAATGCAAATGCTGAAGCTCTTAATAAC TTATTGCAACAACTCTCTAATAGATTTGGTGCTATAAGTGCTTCTTTACAAGAAATTCTATCTAGACTTGATGCTCTTG AAGCGGAAGCTCAGATAGATAGACTTATTAATGGTCGTCTCACCGCTCTTAATGCTTATGTTTCTCAACAGCTTAGTGA TTCTACACTGGTAAAATTTAGTGCAGCACAAGCTATGGAGAAGGTTAATGAATGTGTCAAAAGCCAATCATCTAGGATA AATTTCTGTGGTAATGGTAATCATATTATATCATTAGTGCAGAATGCTCCATATGGTTTGTATTTTATCCACTTTAATT

ATGTCCCTACTAAGTATGTCACAGCGAAGGTTAGTCCTGGTCTGTGCATTGCTGGTAATAGAGGTATAGCTCCTAAGAG
TGGTTATTTTGTTAATGTAAATAATACTTGGATGTACACTGGTAGTGGTTACTACTACCCTGAACCTATAACTGAAAAT
AATGTTGTTGTTATGAGTACCTGCGCTGTTAATTATACTAAAGCGCCGTATGTAATGCTGAACACTTCAATACCCAACC
TTCCTGATTTTAAGGAAGAGTTGGATCAATGGTTTAAAAATCAAACATCAGTGGCACCAGATTTGTCACTTGATTATAT
AAATGTTACATTCTTGGACCTACAAGTTGAAATGAATAGGTTACAGGAGGCAATAAAAGTCTTAAATCATAGCTACATC
AATCTCAAGGACATTGGTACATATGAATATTATGTAAAATGGCCTTGGTATGTATGGCTCTTAATCTGCCTTGCTGGTG
TAGCTATGCTTGTTTTACTATTCTTCATATGCTGTTGTACAGGATGTGGGACTAGTTGTTTTAAGAAATGTGGTGGTTG
TTGTGATGATTATACTGGATACCAGGAGTTAGTAATCAAAACTTCACATGACGACTAA

>gi|530802509|gb|KF530092.1| Human coronavirus OC43 strain OC43/human/USA/008-5/2000, complete genome

ATGTTTTTGATACTTTTAATTTCCTTACCAATGGCTTTTGCTGTTATAGGAGATTTAAAGTGTACTTCAGATACG
AGTTATATTAATGATGTTGACACCGGTGTTCCTCCTATAAGTACTGATACTGTTGATGTTACTAATGGTTTGGGTACTT
ATTATGTTTTAGATCGTGTGTATTTAAATACTACTTTGTTTCTTAATGGTTACTATCCTACTTCAGGTTCCACATATCG
TAATATGGCACTGAAGGGAAGTGTACTGTTGAGCACACTATGGTTTAAACCACCATTTCTTTCTGATTTTATTAATGGT
ATTTTTGCTAAGGTCAAAAATACCAAGGTTATTAAAGATGATGTACTGTACAGTGAGTTCCCTGCTATAACTATAGGTA
GTACTTTTGTAAATACATCCTATAGTGTGGTAGTACAACCACGTACAATCAATTTAGATAATAAATTACAAGGTCTTTT
AGAGATCTCTGTTTGCCAGTATAATATGTGCGAGTACCCACATACGATTTGTCATCCTAATTTGGGTAATCATCGCACA
GAACTATGGCATTTGGATACAGGTGTTGTTTCCTGTTTATATAAGCGTAATTTCACATATGATGTGAATGCTGATTATT
TGTATTTTCATTTTTATCAAGAAGGTGGTACTTTTTATGCATATTTTACGGACACTGGTGTTGTTACTAAGTTTTTGTT
TAATGTTTATTTAGGCATGGCGCTTTCACACTATTATGTCATGCCTCTGACTTGTAATAGTAAGCGTAAGGATGGTTTT
ACTTTAGAATATTGGGTTACACCTCTCACTTCTAGACAATATTTACTCGCTTTCAATCAAGATGGTATTATTTTTAATG
CTGTTGATTGCATGAGTGATTTTATGAGTGAGATTAAGTGTAAAACACAATCTATAGCACCACCTACTGGTGTTTATGA
ATTAAACGGTTACACTGTTCAGCCAATTGCAGATGTTTACCGACGTAAACCTAATCTTCCCAATTGTAATATAGAAGCT
TGGCTTAATGATAAGTCGGTACCATCTCCATTAAATTGGGAACGTAAGACATTTTCAAATTGTAATTTTAATATGAGCA
GCCTGATGTCCTTTATTCAGGCAGACTCATTTACTTGTAATAATATTGATGCTGCTAAGATATATGGTATGTGTTTTTC
CAGCATAACTATAGATAAGTTTGCTATACCCAATGGCAGGAAGGTTGACCTACAATTGGGTAATTTGGGCTATTTGCAG
TCATTTAACTATAGAATTGATACTACTGCAACAAGTTGTCAGTTGTATTATAATTTACCTGCTGCTAATGTTTCTGTTA
GCAGGTTTAATCCTTCTACTTGGAATAAGAGATTTGGTTTTATAGAAAATTCTGTTTTTAAGCCTCAACCTGCAGGTGT
TTTTACCAATCATGATGTAGTTTATGCCCAACATTGTTTTAAAGCTCCCAAAAATTTCTGTCCGTGTAAATTGAATGGT
TCTTTGTGTGTAGGTAGTGGTCCTGGTAAAAATAATGGTATAGGCACTTGTCCTGCAGGTACTAATTATTTAACTTGTC
ATAATTTGTGTAATCCTGATCCTATTACATTTGCAGGTCCTTATAAGTGCCCCCAAACTAAATCTTTAGTTGGTATAGG
TGAGCACTGTTCAGGTCTTGCTGTTAAAAGTGATTATTGTGGAGGCAATCCTTGTACTTGCCAACCACAAGCATTTTTG
GGTTGGTCTGCAGACTCTTGTTTACAAGGGGACAAGTGTAATATTTTTGCTAATTTAATTTTGCATGATGTTAATAGTG
GTCTTACTTGTTCTACTGATTTACAAAAAGCTAACACAGACATAATTCTTGGTGTTTGTGTTAATTATGACCTTTATGG
TATTTCAGGCCAAGGCATTTTTGTTGAGGTTAATGCGACTTATTATAATAGTTGGCAGAACCTTTTATATGATTCTAAT
GGTAATCTCTATGGTTTTAGAGACTACATAACAAATAGAACTTTTATGATTCGTAGTTGCTATAGCGGTCGTGTTTCTG
CGGCCTTTCACGCTAACTCTTCCGAACCAGCATTGCTATTTCGGAATATTAAATGCAACTACGTTTTTAATAATAGTCT
TACACGACAGCTGCAACCCATTAACTATTTTGATAGTTATCTTGGTTGTGTTGTCAATGCTTATAATAGTACTGCTATT
TCTGTTCAAACATGTGATCTCACAGTAGGTAGTGGTTACTGTGTGGATTACTCTAAAAACAGACGAAGTCGTAGAGCGA
TTACCACTGGTTATCGGTTTACTAATTTTGAGCCATTTACTGTTAATTCAGTAAATGATAGTTTAGAACCTGTAGGTGG
TTTGTACGAAATTCAAATACCTTCAGAGTTTACTATAGGTAATATGGAGGAGTTTATTCAAACAAGCTCTCCTAAAGTT

ACTATTGATTGTGCTGCATTTGTCTGTGGTGATTATGCAGCATGTAAATCACAGTTGGTTGAATATGGTAGTTTCTGTG
ATAACATTAATGCCATACTCACAGAAGTAAATGAACTACTTGACACTACACAGTTGCAAGTAGCTAATAGTTTAATGAA
TGGTGTTACTCTTAGCACTAAGCTTAAAGATGGCGTTAATTTCAATGTAGACGACATCAATTTTTCCTCTGTATTAGGT
TGTCTAGGCAGCGAATGTAGTAAAGCTTCCAGTAGATCTGCTATAGAGGATTTACTTTTTGATAAAGTAAAGTTATCTG
ATGTCGGTTTCGTTGAGGCTTATAATAATTGTACTGGAGGTGCCGAAATTAGGGACCTCATTTGTGTGCAAAGTTATAA
AGGTATCAAAGTGTTGCCTCCACTGCTCTCAGAAAATCAGATCAGTGGATACACTTTGGCTGCCACTTCTGCTAGTCTG
TTTCCTCCCTGGACAGCAGCAGCAGGTGTACCATTTTATTTAAATGTTCAGTATCGCATTAATGGGCTTGGTGTCACCA
TGGATGTGCTAAGTCAAAATCAAAAGCTTATTGCTAATGCATTTAACAATGCCCTTGATGCTATTCAGGAAGGGTTCGA
TGCAACTAATTCTGCTTTAGTTAAAATTCAAGCTGTTGTTAATGCAAATGCTGAAGCTCTTAATAACTTATTGCAACAA
CTCTCTAATAGATTTGGTGCTATAAGTTCTTCTCTACAAGAAATTCTATCTAGACTTGATGCTCTTGAAGCGGAAGCTC
AGATAGATAGACTTATTAATGGTCGTCTTACCGCTCTTAATGCTTATGTTTCTCAACAGCTTAGTGATTCTACACTGGT
AAAATTTAGTGCAGCACAAGCTATGGAGAAGGTTAATGAATGTGTCAAAAGCCAATCATCTAGGATAAATTTCTGTGGT
AATGGTAACCATATTATATCATTAGTGCAGAATGCTCCATATGGTTTGTATTTTATCCACTTTAGTTATGTCCCTACTA
AGTATGTCACAGCGAAGGTTAGTCCTGGTCTGTGCATTGCTGGTGATAGAGGTATAGCTCCTAAGAGTGGTTATTTTGT
TAATGTAAATAATACTTGGATGTACACTGGTAGTGGTTACTATTACCCTGAACCTATAACTGAAAATAATGTTGTTGTT
ATGAGTACCTGCGCTGTTAATTATACTAAAGCGCCGTATGTAATGCTGAACACTTCAACACCCAACCTTCCTGATTTTA
GGGAAGAGTTGGATCAATGGTTTAAAAATCAAACATCAGTGGCACCAGATCTGTCACTTGATTATATAAATGTTACATT
CTTGGACCTACAAGTTGAAATGAATAGGTTACAGGAGGCAATAAAAGTTTTAAATCAGAGCTACATCAATCTCAAGGAC
ATTGGTACATATGAATATTATGTAAAATGGCCTTGGTATGTATGGCTTTTAATCGGCCTTGCTGGTGTAGCTATGCTTG
TTTTACTATTCTTCATATGCTGTTGTACAGGATGTGGGACTAGTTGTTTTAAGAAATGTGGTGGTTGTTGTGATGATTA
TACTGGATACCAGGAGTTAGTAATCAAAACTTCACATGACGACTAA

>gi|530802531|gb|KF530094.1| Human coronavirus OC43 strain OC43/human/USA/912-36/1991, complete genome

ATGTTTTTGATACTTTTAATTTCCTTACCAACGGCTTTTGCTGTTATAGGAGATTTAAAGTGTCCTTTAGATACT
AGTTATAAAGGTACATTTAATAATAAAGACACCGGTCCTCCTTTTATAAGTACTGATACTGTTGATGTTACTAATGGTT
TGGGTACTTATTATGTTTTAGATCGTGTGTATTTAAATACTACGTTGTTTCTTAATGGTTATTACCCTACTTCAGGTTC
CACATATCGTAATATGGCACTGAAGGGAACTGACAAATTGAGCACACTATGGTTTAAACCACCATTTCTTTCTGATTTT
ATTAATGGTATTTTTGCTAAGGTCAAAAATACCAAGGTTTTTAAAGATGGTGTAATGTATAGTGAGTTCCCTGCTATAA
CTATAGGTAGTACTTTTGTAAATACATCCTATAGTGTGGTAGTACAACCACGTACAATTAATTCAACACAGGATGGTGA
TAATAAATTACAAGGTCTTTTAGAGGTCTCTGTTTGCCAGTATAATATGTGCGAGTACCCACATACGAGTTGTCATCCT
AAACTGGGTAATCACTTCAAAGAACTATGGCATTTGGATACAGGTGTTGTTTCCTGTTTATATAAGCGTAATTTCACAT
ATGATGTGAATGCTAATTATTTGTATTTTCATTTTTATCAAGAAGGTGGTACTTTTTATGCATATTTTACAGACACTGG
TGTTGTTACTAAGTTTTTGTTTAATGTTTATTTAGGCATGGCACTTTCACACTATTATGTCATGCCTCTGACTTGTATT
AGTAGGCGTGATATTGGTTTTACTTTAGAATATTGGGTTACACCCCTCACTTCTAGACAATATTTACTCGCTTTCAATC
AAGATGGTATTATTTTTAATGCTGTTGATTGTATGAGTGATTTTATGAGTGAGATTAAGTGTAAAACACAATCTATAGC
ACCACCTACTGGTGTTTATGAATTAAACGGTTACACTGTTCAGCCAATCGCAGATGTTTACCGACGCAAACCTGATCTT
CCCAATTGCAATATAGAAGCTTGGCTTAATGATAAGTCGGTGCCCTCTCCATTAAATTGGGAACGTAAGACATTTTCAA
ATTGTAATTTTAATATGAGCAGCCTGATGTCTTTTATTCAGGCAGACTCATTTACTTGTAATAATATTGATGCTGCTAA
GATATATGGTATGTGTTTTTCCAGCATAACTATAGATAAGTTTGCTATACCCAATGGCAGGAAGGTTGACCTACAATTG
GGTAATTTGGGCTATTTGCAGTCATCCAACTATAGAATTGATACTACTGCAACAAGTTGTCAGTTGTATTATAATTTAC
CTGCTGCTAATGTCTCTGTTAGCAGGTTTAATCCTTCTACTTGGAATAAGAGATTTGGTTTTATAGAAGATTCTGTTTT

TGTGCCTCAACCTACAGGTGTTTTTACTAATCACAGTGTAGTTTATGCACAACACTGTTTCAAAGCCCCTAAAAATTTC
TGTCCGTGTAAATTGAATGGTTCGTGTCCTGGTAAAAATAATGGTATAGGCACTTGTCCTGCAGGTACTAATTATTTAA
CTTGTGATAATTTGTGTACTCTTGATCCTATTACATTTAAAGCTCCAGGTACTTATAAGTGCCCCCAAACTAAATCTTT
AGTTGGCATAGGTGAGCACTGTTCGGGTCTTGCTGTTAAAAGTGATTATTGTAGAGGCAATTCTTGTACTTGCCAACCA
CAAGCATTTTTGGGTTGGTCTGCAGACTCTTGTTTACAAGGAGACAAGTGTAATATTTTTGCTAATCTTATTTTGCATG
ATGTTAATAGTGGTCTTACTTGTTCTACTGATTTACAAAAAGCTAACACAGACATAATTCTTGGTGTTTGTGTTAATTA
TGACCTCTATGGTATTTCAGGCCAAGGCATTTTTGTTGAGGTTAATGCGACTTATTATAATAGTTGGCAGAACCTTTTA
TATGATTCTAATGGTAATCTCTACGGTTTTAGAGACTACATAACAAACAGAACTTTTATGATTCGTAGTTGCTATAGCG
GTCGTGTTTCTGCGGCCTTTCATGCTAACTCTTCCGAACCAGCATTGCTATTTCGGAATATTAAATGCAACTACGTTTT
TAATAATAGTCTTACACGACAGCTGCAACCCATTAACTATTCGTTTGATAGTTATCTTGGTTGTGTTGTCAATGCTTAT
AATAGTACTGCTATTTCTGTTCAAACATGTGATCTCACAGTAGGTAGTGGTTACTGTGTGGATTACTCTAAAAACAGAC
GAAGTCGTAGAGCGATTACCACTGGTTATCGGTTTACTAATTTTGAGCCATTTACTGTTAATTCAGTAAATGATAGTTT
GGAACCTGTAGGTGGTTTGTATGAAATTCAAATACCTTCAGAGTTTACTATAGGTAATATGGAGGAGTTTATTCAAACA
AGCTCTCCCAAAGTTACTATTGATTGTGCTGCATTTGTTTGTGGTGATTATGCAGCATGTAAATTACAGTTGGTTGAAT
ATGGTAGTTTCTGTGATAACATTAATGCCATACTCACAGAAGTAAATGAACTACTTGATACTACACAGTTGCAAGTAGC
TAATAGTTTAATGAATGGTGTTACTCTTAGCACTAAGCTTAAAGATGGTGTTAATTTCAATGTAGATGACATCAATTTT
TCCCCTGTATTAGGTTGTCTAGGCAGCGAATGTAGTAAAGCTTCCAGTAGATCTGCTATAGAGGATTTACTTTTTGATA
AAGTAAAGTTATCTGATGTCGGTTTCGTTGAGGCTTATAATAATTGTACAGGAGGTGCCGAAATTAGGGACCTCATTTG
TGTGCAAAGTTATAAAGGCATCAAAGTGTTGCCCCCACTGCTCTCAGAAAATCAGATCAGTGGATACACTTTGGCTGCC
ACCTCTGCTAGTCTATTTCCTCCTTGGACAGCAGCAGCAGGTGTACCATTTTATTTAAATGTTCAGTATCGCATTAATG
GGCTTGGTGTCACTATGGATGTGCTAAGTCAAAATCAAAAGCTTATTGCTAATGCATTTAACAATGCCCTTTATGCTAT
TCAGCAAGGGTTCGATGCAACTAATTCTGCTTTAGTTAAAATTCAAGCTGTTGTTAATGCAAATGCTGAAGCTCTTAAT
AACTTATTGCAACAACTCTCTAATAGATTTGGTGCTATAAGTGCTTCTTTACAAGAAATTCTATCTAGACTTGATGCTC
TTGAAGCGGAAGCTCAGATAGATAGACTTATTAATGGTCGTCTCACCGCTCTTAATGCTTATGTTTCTCAACAGCTTAG
TGATTCTACACTGGTAAAATTTAGTGCAGCACAAGCTATGGAGAAGGTTAATGAATGTGTCAAAAGCCAATCATCTAGG
ATAAATTTCTGTGGTAATGGTAATCATATTATATCATTAGTGCAGAATGCTCCATATGGTTTGTATTTTATCCACTTTA
ATTATGTCCCTACTAAGTATGTCACAGCGAAGGTTAGTCCTGGTCTGTGCATTGCTGGTAATAGAGGTATAGCTCCTAA
GAGTGGTTATTTTGTTAATGTAAATAATACTTGGATGTACACTGGTAGTGGTTACTACTACCCTGAACCTATAACTGAA
AATAATGTTGTTGTTATGAGTACCTGCGCTGTTAATTATACTAAAGCGCCGTATGTAATGCTGAACACTTCAATACCCA
ACCTTCCTGATTTTAAGGAAGAGTTGGATCAATGGTTTAAAAATCAAACATCAGTGGCACCAGATTTGTCACTTGATTA
TATAAATGTTACATTCTTGGACCTACAAGTTGAAATGAATAGGTTACAGGAGGCAATAAAAGTCTTAAATCATAGCTAC
ATCAATCTCAAGGACATTGGTACATATGAATATTATGTAAAATGGCCTTGGTATGTATGGCTCTTAATCTGCCTTGCTG
GTGTAGCTATGCTTGTTTTACTATTCTTCATATGCTGTTGTACAGGATGTGGGACTAGTTGTTTTAAGAAATGTGGTGG
TTGTTGTGATGATTATACTGGATACCAGGAGTTAGTAATCAAAACTTCACATGACGACTAA

>gi|530802553|gb|KF530096.1| Human coronavirus OC43 strain OC43/human/USA/911-38/1991, complete genome

ATGTTTTTGATACTTTTAATTTCCTTACCAACGGCTTTTGCTGTTATAGGAGATTTAAAGTGTCCTTTAGATACT
AGTTATAAAGGTACATTTAATAATAAAGACACCGGTCCTCCTTTTATAAGTACTGATACTGTTGATGTTACTAATGGTT
TGGGTACTTATTATGTTTTAGATCGTGTGTATTTAAATACTACGTTGTTTCTTAATGGTTATTACCCTACTTCAGGTTC
CACATATCGTAATATGGCACTGAAGGGAACTGACAAATTGAGCACACTATGGTTTAAACCACCATTTCTTTCTGATTTT
ATTAATGGTATTTTTGCTAAGGTCAAAAATACCAAGGTTTTTAAAGATGGTGTAATGTATAGTGAGTTCCCTGCTATAA

CTATAGGTAGTACTTTTGTAAATACATCCTATAGTGTGGTAGTACAACCACGTACAATTAATTCACAGGATGGTGATAA
TAAATTACAAGGTCTTTTAGAGGTCTCTGTTTGCCAATATAATATGTGCGAGCACCCACATACGATTTGTCATCCTAAA
CTGGGTAATCACTTCAAAGAACTATGGCATTTGGATACAGGTGTTGTTTCCTGTTTATATAAGCGTAATTTCACATATG
ATGTGAATGCTAATTATTTGTATTTTCATTTTTATCAAGAAGGTGGTACTTTTTATGCATATTTTACAGACACTGGTGT
TGTTACTAAGTTTTTGTTTAATGTTTATTTAGGCATGGCACTTTCACACTATTATGTCATGCCTCTGACTTGTATTAGT
AGGCGTGATATTGGTTTTACTTTAGAATATTGGGTTACACCCCTCACTTCTAGACAATATTTACTCGCTTTCGATCAAG
ATGGTATTATTTTTAATGCTGTTGATTGTATGAGTGATTTTATGAGTGAGATTAAGTGTAAAACACAATCTATAGCACC
ACCTACTGGTGTTTATGAATTAAACGGTTACACTGTTCAGCCAATCGCAGATGTTTACCGACGCAAACCTGATCTTCCC
AATTGCAATATAGAAGCTTGGCTTAATGATAAGTCGGTGCCCTCTCCATTAAATTGGGAACGTAAGACATTTTCAAATT
GTAATTTTAATATGAGCAGCCTGATGTCTTTTATTCAGGCAGACTCATTTACTTGTAATAATATTGATGCTGCTAAGAT
ATATGGTATGTGTTTTTCCAGCATAACTATAGATAAGTTTGCTATACCCAATGGCAGGAAGGTTGACCTACAATTGGGT
AATTTGGGCTATTTGCAGTCATCCAACTATAGAATTGATACTACTGCAACAAGTTGTCAGTTGTATTATAATTTACCTG
CTGCTAATGTCTCTGTTAGCAGGTTTAATCCTTCTACTTGGAATAAGAGATTTGGTTTTATAGAAGATTCTGTTTTTGT
GCCTCAACCTACAGGTGTTTTTACTAATCACAGTGTAGTTTATGCACAACACTGTTTCAAAGCCCCTAAAAATTTCTGT
CCGTGTAAATTGAATGGTTCGTGTCCTGGTAAAAATAATGGTATAGGCACTTGTCCTGCAGGTACTAATTATTTAACTT
GTGATAATTTGTGTACTCTTGATCCTATTACATTTAAAGCTCCAGGTACTTATAAGTGCCCCCAAACTAAATCTTTAGT
TGGCATAGGTGAGCACTGTTCGGGTCTTGCTGTTAAAAGTGATTATTGTAGAGGCAATTCTTGTACTTGCCAACCACAA
GCATTTTTGGGTTGGTCTGCAGACTCTTGTTTACAAGGAGACAAGTGTAATATTTTTGCTAATCTTATTTTGCATGATG
TTAATAGTGGTCTTACTTGTTCTACTGATTTACAAAAAGCTAACACAGACATAATTCTTGGTGTTTGTGTTAATTATGA
CCTCTATGGTATTTCAGGCCAAGGCATTTTTGTTGAGGTTAATGCGACTTATTATAATAGTTGGCAGAACCTTTTATAT
GATTCTAATGGTAATCTCTACGGTTTTAGAGACTACATAACAAACAGAACTTTTATGATTCGTAGTTGCTATAGCGGTC
GTGTTTCTGCGGCCTTTCATGCTAACTCTTCCGAACCAGCATTGCTATTTCGGAATATTAAATGCAACTACGTTTTTAA
TAATAGTCTTACACGACAGCTGCAACCCATTAACTATTCGTTTGATAGTTATCTTGGTTGTGTTGTCAATGCTTATAAT
AGTACTGCTATTTCTGTTCAAACATGTGATCTCACAGTAGGTAGTGGTTACTGTGTGGATTACTCTAAAAACAGACGAA
GTCGTAGAGCGATTACCACTGGTTATCGGTTTACTAATTTTGAGCCATTTACTGTTAATTCAGTAAATGATAGTTTGGA
ACCTGTAGGTGGTTTGTATGAAATTCAAATACCTTCAGAGTTTACTATAGGTAATATGGAGGAGTTTATTCAAACAAGC
TCTCCCAAAGTTACTATTGATTGTGCTGCATTTGTCTGTGGTGATTATGCAGCATGTAAATTACAGTTGGTTGAATATG
GTAGTTTCTGTGATAACATTAATGCCATACTCACAGAAGTAAATGAACTACTTGATACTACACAGTTGCAAGTAGCTAA
TAGTTTAATGAATGGTGTTACTCTTAGCACTAAGCTTAAAGATGGTGTTAATTTCAATGTAGACGACATCAATTTTTCC
CCTGTATTAGGTTGTCTAGGCAGCGAATGTAGTAAAGCTTCCAGTAGATCTGCTATAGAGGATTTACTTTTTGATAAAG
TAAAGTTATCTGATGTCGGTTTCGTTGAGGCTTATAATAATTGTACAGGAGGTGCCGAAATTAGGGACCTCATTTGTGT
GCAAAGTTATAAAGGCATCAAAGTGTTGCCCCCACTGCTCTCAGAAAATCAGATCAGTGGATACACTTTGGCTGCCACC
TCTGCTAGTCTATTTCCTCCTTGGACAGCAGCAGCAGGTGTACCATTTTATTTAAATGTTCAGTATCGCATTAATGGGC
TTGGTGTCACCATGGATGTGCTAAGTCAAAATCAAAAGCTTATTGCTAATGCATTTAACAATGCCCTTTATGCTATTCA
GCAAGGGTTCGATGCAACTAATTCTGCTTTAGTTAAAATTCAAGCTGTTGTTAATGCAAATGCTGAAGCTCTTAATAAC
TTATTGCAACAACTCTCTAATAGATTTGGTGCTATAAGTGCTTCTTTACAAGAAATTCTATCTAGACTTGATGCTCTTG
AAGCGGAAGCTCAGATAGATAGACTTATTAATGGTCGTCTCACCGCTCTTAATGCTTATGTTTCTCAACAGCTTAGTGA
TTCTACACTGGTAAAATTTAGTGCAGCACAAGCTATGGAGAAGGTTAATGAATGTGTCAAAAGCCAATCATCTAGGATA
AATTTCTGTGGTAATGGTAATCATATTATATCATTAGTGCAGAATGCTCCATATGGTTTGTATTTTATCCACTTTAATT
ATGTCCCTACTAAGTATGTCACAGCGAAGGTTAGTCCTGGTCTGTGCATTGCTGGTAATAGAGGTATAGCTCCTAAGAG
TGGTTATTTTGTTAATGTAAATAATACTTGGATGTACACTGGTAGTGGTTACTACTACCCTGAACCTATAACTGAAAAT
AATGTTGTTGTTATGAGTACCTGCGCTGTTAATTATACTAAAGCGCCGTATGTAATGCTGAACACTTCAATACCCAACC
TTCCTGATTTTAAGGAAGAGTTGGATCAATGGTTTAAAAATCAAACATCAGTGGCACCAGATTTGTCACTTGATTATAT
AAATGTTACATTCTTGGACCTACAAGTTGAAATGAATAGGTTACAGGAGGCAATAAAAGTCTTAAATCATAGCTACATC

AATCTCAAGGACATTGGTACATATGAATATTATGTAAAATGGCCTTGGTATGTATGGCTCTTAATCTGCCTTGCTGGTG
TAGCTATGCTTGTTTTACTATTCTTCATATGCTGTTGTACAGGATGTGGGACTAGTTGTTTTAAGAAATGTGGTGGTTG
TTGTGATGATTATACTGGATACCAGGAGTTAGTAATCAAAACTTCACATGACGACTAA

>gi|530802564|gb|KF530097.1| Human coronavirus OC43 strain OC43/human/USA/9211-43/1992, complete genome

ATGTTTTTGATACTTTTAATTTCCTTACCAATGGCTTTTGCTGTTATAGGAGATTTAAAGTGTACTTCAGATACG
AGTTATATTAATGATGTTGACACCGGTGTTCCTCCTATAAGTACTGATACTGTTGATGTTACTAATGGTTTGGGTACTT
ATTATGTTTTAGATCGTGTGTATTTAAATACTACTTTGTTTCTTAATGGTTACTATCCTACTTCAGGTTCCACATATCG
TAATATGGCACTGAAGGGAAGTGTACTGTTGAGCACACTATGGTTTAAACCACCATTTCTTTCTGATTTTATTAATGGT
ATTTTTGCTAAGGTCAAAAATACCAAGGTTATTAAAGATGATGTACTGTACAGTGAGTTCCCTGCTATAACTATAGGTA
GTACTTTTGTAAATACATCCTATAGTGTGGTAGTACAACCACGTACAATCAATTTAGATAATAAATTACAAGGTCTTTT
AGAGATCTCTGTTTGCCAGTATAATATGTGCGAGTACCCACATACGATTTGTCATCCTAACTTGGGTAATCATCGCACA
GAACTATGGCATTTGGATACAGGTGTTGTTTCCTGTTTATATAAGCGTAATTTCACATATGATGTGAATGCTGATTATT
TGTATTTTCATTTTTATCAAGAAGGTGGTACTTTTTATGCATACTTTACAGACACTGGTGTTGTTACTAAGTTTTTGTT
TAATGTTTATTTAGGCATGGCGCTTTCACACTATTATGTCATGCCTCTGACTTGTAATAGTAAGCGTAAGGATGGTTTT
ACTTTAGAATATTGGGTTACACCTCTCACTTCTAGACAATATTTACTCGCTTTCAATCAAGATGGTATTATTTTTAATG
CTGTTGATTGCATGAGTGATTTTATGAGTGAGATTAAGTGTAAAACACAATCTATAGCACCACCTACTGGTGTTTATGA
ATTAAACGGTTACACTGTTCAGCCAATTGCAGATGTTTACCGACGTAAACCTAATCTTCCCAATTGCAATATAGAAGCT
TGGCTTAATGATAAGTCGGTGCCATCTCCATTAAATTGGGAACGTAAGACATTTTCAAATTGTAATTTTAATATGAGCA
GCCTGATGTCCTTTATTCAGGCAGACTCATTTACTTGTAATAATATTGATGCTGCTAAGATATATGGTATGTGTTTTTC
CAGCATAACTATAGATAAGTTTGCTATACCCAATGGCAGGAAGGTTGACCTACAATTGGGTAATTTGGGCTATTTGCAG
TCATTTAACTATAGAATTGATACTACTGCAACAAGTTGTCAGTTGTATTATAATTTACCTGCTGCTAATGTTTCTGTTA
GCAGGTTTAATCCTTCTACTTGGAATAAGAGATTCGGTTTTATAGAAAATTCTGTTTTTAAGCCTCAACCTGCAGGTGT
TTTTACCAATCATGATGTAGTTTATGCCCAACATTGTTTTAAAGCTCCCAAAAATTTCTGTCCGTGTAAATTGAATGGT
TCTTTGTGTGTAGGTAGTGGTCCTGGTAAAAACAATGGTATAGGCACTTGTCCTGCAGGTACTAATTATTTAACTTGTC
ATAATTTGTGCAATCCTGATCCTATTACATTTACAGGTCCTTATAAGTGCCCCCAAACTAAATCTTTAGTTGGTATAGG
TGAGCACTGTTCAGGTCTTGCTGTTAAAAGTGATTATTGTGGAGGCAATCCTTGCACTTGCCAACCACAAGCATTTTTG
GGTTGGTCTGCAGACTCTTGTTTACAAGGGGACAAGTGTAATATTTTTGCTAATTTAATTTTGCATGATGTTAATAGTG
GTCTTACTTGTTCTACTGATTTACAAAAAGCTAACACAGACATAATTCTTGGTGTTTGTGTTAATTATGACCTTTATGG
TATTTCAGGCCAAGGCATTTTTGTTGAGGTTAATGCGACTTATTATAATAGTTGGCAGAACCTTTTATATGATTCTAAT
GGTAATCTCTATGGTTTTAGAGACTACATAACAAACAGAACTTTTATGATTCGTAGCTGCTATAGCGGTCGTGTTTCTG
CGGCCTTTCACGCTAACTCTTCCGAACCAGCATTGCTATTTCGGAATATTAAATGCAACTACGTTTTTAATAATAGTCT
TACACGACAGCTGCAACCCATTAACTATTTTGATAGTTATCTTGGTTGTGTTGTCAATGCTTATAATAGTACTGCTATT
TCTGTTCAAACATGTGATCTCACAGTAGGTAGTGGTTACTGTGTGGATTACTCTAAAAACAGACGAAGTCGTAGAGCGA
TTACCACTGGTTATCGGTTTACTAATTTTGAGCCATTTACTGTTAATTCAGTAAATGATAGTTTAGAACCTGTAGGTGG
TTTGTACGAAATTCAAATACCTTCAGAGTTTACTATAGGTAATATGGAGGAGTTTATTCAAACAAGCTCTCCTAAAGTT
ACTATTGATTGTGCTGCATTTGTCTGTGGTGATTATGCAGCATGTAAATCACAGTTGGTTGAATATGGTAGTTTCTGTG
ATAACATTAATGCCATACTCACAGAAGTAAATGAACTACTTGACACTACACAGTTGCAAGTAGCTAATAGTTTAATGAA
TGGTGTTACTCTTAGCACTAAGCTTAAAGATGGCGTTAATTTCAATGTAGACGACATCAATTTTTCCTCTGTATTAGGT
TGTCTAGGCAGCGAATGTAGTAAAGCTTCCAGTAGATCTGCTATAGAGGATTTACTTTTTGATAAAGTAAAGTTATCTG
ATGTCGGTTTCGTTGAGGCTTATAATAATTGTACTGGAGGTGCCGAAATTAGGGACCTCATTTGTGTGCAAAGTTATAA

AGGTATCAAAGTGTTGCCTCCACTGCTCTCAGAAAATCAGATCAGTGGATACACTTTGGCTGCCACTTCTGCTAGTCTG
TTTCCTCCTTGGACAGCAGCAGCAGGTGTACCATTTTATTTAAATGTTCAGTATCGCATTAATGGGCTTGGTGTCACCA
TGGATGTGCTAAGTCAAAATCAAAAGCTTATTGCTAATGCATTTAACAATGCCCTTGATGCTATTCAGGAAGGGTTCGA
TGCAACTAATTCTGCTTTAGTTAAAATTCAAGCTGTTGTTAATGCAAATGCTGAAGCTCTTAATAACTTATTGCAACAA
CTCTCTAATAGATTTGGTGCTATAAGTTCTTCTCTACAAGAAATTCTATCTAGACTTGATGCTCTTGAAGCGGAAGCTC
AGATAGATAGACTTATTAATGGTCGTCTTACCGCTCTTAATGCTTATGTTTCTCAACAGCTTAGTGATTCTACACTGGT
AAAATTTAGTGCAGCACAAGCTATGGAGAAGGTTAATGAATGTGTTAAAAGCCAATCATCTAGGATAAATTTCTGTGGT
AATGGTAACCATATTATATCATTAGTGCAGAATGCTCCATATGGTTTGTATTTTATCCACTTTAGTTATGTCCCTACTA
AGTATGTCACAGCGAAGGTTAGTCCTGGTCTGTGCATTTCTGGTGATAGAGGTATAGCTCCTAAGAGTGGTTATTTTGT
TAATGTAAATAATACTTGGATGTACACTGGTAGTGGTTACTACTACCCTGAACCTATAACTGAAAATAATGTCGTTGTT
ATGAGTACCTGCGCTGTTAATTATACTAAAGCGCCGTATGTAATGCTGAACACTTCAACACCCAACCTTCCTGATTTTA
GGGAAGAGTTGGATCAATGGTTTAAAAATCAAACATCAGTGGCACCAGATCTGTCACTTGATTATATAAATGTTACATT
CTTGGACCTACAAGTTGAAATGAATAGGTTACAGGAGGCAATAAAAGTTTTAAATCAGAGCTACATCAATCTCAAGGAC
ATTGGTACATATGAATATTATGTAAAATGGCCTTGGTATGTATGGCTTTTAATTGGCCTTGCTGGTGTAGCTATGCTTG
TTTTACTATTCTTCATATGCTGTTGTACAGGATGTGGGACTAGTTGTTTTAAGAAATGTGGTGGTTGTTGTGATGATTA
TACTGGATACCAGGAGTTAGTAATCAAAACTTCACATGACGACTAA

>gi|530802575|gb|KF530098.1| Human coronavirus OC43 strain OC43/human/USA/965-6/1996, complete genome

ATGTTTTTGATACTTTTAATTTCCTTACCAACGGCTTTTGCTGTTATAGGAGATTTAAAGTGTCCTTTAGATACT
AGTCGTAAAGGTAGCTTTAATAATAAAGACACCGGTCCTCCTTTTATAAGTACTGATACTGTTGATGTTACTAATGGTT
TGGGTACTTATTATGTTTTAGATCGTGTGTATTTAAATACTACGTTGTTTCTTAATGGTTATTACCCTACTTCAGGTTC
CACATATCGTAATATGGCACTGAAGGGAACTGACAAATTGAGCACACTATGGTTTAAACCACCATTTCTTTCTGATTTT
ATTAATGGTATTTTTGCTAAGGTCAAAAATACCAAGGTTTTTAAAGATGGTGTAATGTATAGTGAGTTTCCTGCTATAA
CTATAGGTAGTACTTTTGTAAATACATCCTATAGTGTGGTAGTACAACCACGTACAATTAATTCAACACAGGATGGTGT
TAATAAATTACAAGGTCTTTTAGAGGTCTCTGTTTGCCAGTATAATATGTGCGAGTACCCACATACGATTTGTCATCCT
AAACTGGGTAATCACTTCAAAGAACTATGGCATTTGGATACAGGTGTTGTTTCCTGTTTATATAAGCGTAATTTCACAT
ATGATGTGAATGCTACTTATTTGTATTTTCATTTTTATCAAGAAGGTGGTACTTTTTATGCATATTTTACAGACACTGG
TGTTGTTACTAAGTTTTTGTTTAATGTTTATTTAGGCATGGCACTTTCACACTATTATGTCATGCCTCTGACTTGTATT
AGTAGGCGTGATATTGGTTTTACTTTAGAATATTGGGTTACACCCCTCACTTCTAGACAATATTTACTCGCTTTCAATC
AAGATGGTATTATTTTTAATGCTGTTGATTGTATGAGTGATTTTATGAGTGAGATTAAGTGTAAAACACAATCTATAGC
ACCACCTACTGGTGTTTATGAATTAAACGGTTACACTGTTCAGCCAATCGCAGATGTTTACCGACGCAAACCTGATCTT
CCCAATTGCAATATAGAAGCTTGGCTTAATGATAAGTCGGTGCCCTCTCCATTAAATTGGGAACGTAAGACATTTTCAA
ATTGTAATTTTAATATGAGCAGCCTGATGTCTTTTATTCAGGCAGACTCATTTACTTGTAATAATATTGATGCTGCTAA
GATATATGGTATGTGTTTTTCCAGCATAACTATAGATAAGTTTGCTATACCCAATAGCAGGAAGGTTGACCTACAATTA
GGCAATTTGGGCTATTTGCAGTCATCCAACTATAGAATTGATACTACTGCAACAAGTTGTCAGTTGTATTATAATTTAC
CTGCTGCTAATGTCTCTGTTAGCAGGTTTAATCCTTCTACTTGGAATAAGAGATTTGGTTTTATAGAAGATTCTGTTTT
TGTGCCTCAACCTACAGGTGTTTTTACTAATCACAGTGTAGTTTATGCACAACACTGTTTCAAAGCCCCTAAAAATTTC
TGTCCGTGTAAATTGAATGGTTCGTGTCCTGATAAAAATAATGGTATAGGCACTTGTCCTGCAGGTACTAATTATTTAA
CTTGTGATAATTTGTGTACTCTTGATCCCATTACATTTAAAGCTCCAGGTACTTATAAGTGCCCCCAAACTAAATCTTT
AGTTGGCATAGGTGAGCACTGTTCGGGTCTTGCTGTTAAAAGTGATTATTGTAGAGGCAATTCTTGTACTTGCCAACCA
CAAGCATTTTTGGGTTGGTCTGCAGACTCTTGTTTACAAGGAGACAAGTGTAATATTTTTGCTAATCTTATTTTGCATG

ATGTTAATAGTGGTCTTACTTGTTCTACTGATTTACAAAAAGCTAACACAGACATAATTCTTGGTGTTTGTGTTAATTA
TGACCTCTATGGTATTTCAGGCCAAGGCATTTTTGTTGAGGTTAATGCGACTTATTATAATAGTTGGCAGAACCTTTTA
TATGATTCTAATGGTAATCTCTATGGTTTTAGAGACTACATAACAAACAGAACTTTTATGATTCGTAGTTGCTATAGCG
GTCGTGTTTCTGCGGCCTTTCATGCTAACTCTTCCGAACCAGCATTGCTATTTCGGAATATTAAATGCAACTACGTTTT
TAATAATAGTCTTACACGACAGCTGCAACCCATTAACTATTCGTTTGATAGTTATCTTGGTTGTGTTGTCAATGCTTAT
AATAGTACTGCTATTTCTGTTCAAACATGTGATCTCACAGTAGGTAGTGGTTACTGTGTGGAGTACTCTAAAAACAGAC
GAAGTCGTAGAGCGATTACCACTGGTTATCGGTTTACTAATTTTGAGCCATTTACTGTTAATTCAGTAAATGATAGTTT
GGAACCTGTAGGTGGTTTGTATGAAATTCAAATACCTTCAGAGTTTACTATAGGTAATATGGAGGAGTTTATTCAAACA
AGCTCTCCCAAAGTTACTATTGATTGTGCTGCATTTGTCTGTGGTGATTATGCAGCATGTAAATTACAGTTGGTTGAAT
ATGGTAGTTTCTGTGATAACATTAATGCCATACTCACAGAAGTAAATGAACTACTTGATACTACACAGTTGCAAGTAGC
TAATAGTTTAATGAATGGTGTTACTCTTAGCACTAAGCTTAAAGATGGTGTTAATTTCAATGTAGACGACATCAATTTT
TCCCCTGTATTAGGTTGTCTAGGCAGCGAATGTAGTAAAGCTTCCAGTAGATCTGCTATAGAGGATTTACTTTTTGATA
AAGTAAAGTTATCTGATGTCGGTTTCGTTGAGGCTTATAATAATTGTACAGGAGGTGCCGAAATTAGGGACCTCATTTG
TGTGCAAAGTTATAAAGGCATCAAAGTGTTGCCCCCACTGCTCTCAGAAAATCAGATCAGTGGATACACTTTGGCTGCC
ACCTCTGCTAGTCTATTTCCTCCTTGGACAGCAGCAGCAGGTGTACCATTTTATTTAAATGTTCAGTATCGCATTAATG
GGCTTGGTGTCACCATGGATGTGCTAAGTCAAAATCAAAAGCTTATTGCTAATGCATTTAACAATGCCCTTTATGCTAT
TCAGCAAGGGTTCGATGCAACTAATTCTGCTTTAGTTAAAATTCAAGCTGTTGTTAATGCAAATGCTGAAGCTCTTAAT
AACTTATTGCAACAACTCTCTAATAGATTTGGTGCTATAAGTGCTTCTTTACAAGAAATTCTATCTAGACTTGATGCTC
TTGAAGCGGAAGCTCAGATAGATAGACTTATTAATGGTCGTCTCACCGCTCTTAATGCTTATGTTTCTCAACAGCTTAG
TGATTCTACACTGGTAAAATTTAGTGCAGCACAAGCTATGGAGAAGGTTAATGAATGTGTCAAAAGCCAATCATCTAGG
ATAAATTTCTGTGGTAATGGTAATCATATTATATCATTAGTGCAGAATGCTCCATATGGTTTGTATTTTATCCACTTTA
ATTATGTCCCTACTAAGTATGTCACAGCGAAGGTTAGTCCTGGTCTGTGCATTGCTGGTAATAGAGGTATAGCTCCTAA
GAGTGGTTATTTTGTTAATGTAAATAATACTTGGATGTACACTGGTAGTGGTTACTACTACCCTGAACCTATAACTGAA
AATAATGTTGTTGTTATGAGTACCTGCGCTGTTAATTATACTAAAGCGCCGTATGTAATGCTGAACACTTCAATACCCA
ACCTTCCTGATTTTAAGGAAGAGTTGGATCAATGGTTTAAAAATCAAACATCAGTGGCACCAGATTTTTCACTTGATTA
TATAAATGTTACATTCTTGGACCTACAAGTTGAAATGAATAGGTTACAGGAGGCAATAAAAGTCTTAAATCATAGCTAC
ATCAATCTCAAGGACATTGGTACATATGAATATTATGTAAAATGGCCTTGGTATGTATGGCTTTTAATCTGCCTTGCTG
GTGTAGCTATGCTTGTTTTACTATTCTTCATATGCTGTTGTACAGGATGTGGGACTAGTTGTTTTAAGAAATGTGGTGG
TTGTTGTGATGATTATACTGGATACCAGGAGTTAGTAATCAAAACTTCACATGACGACTAA

>gi|530802586|gb|KF530099.1| Human coronavirus OC43 strain OC43/human/USA/971-5/1997, complete genome

ATGTTTTTGATACTTTTAATTTCCTTACCAACGGCTTTTGCTGTTATAGGAGATTTAAAGTGTCCTTTAGATACT
AGTCGTAAAGGTAGCTTTAATAATAAAGACACCGGTCCTCCTTTTATAAGTACTGATACTGTTGATGTTACTAATGGTT
TGGGTACTTATTATGTTTTAGATCGTGTGTATTTAAATACTACGTTGTTTCTTAATGGTTATTACCCTACTTCAGGTTC
CACATATCGTAATATGGCACTGAAGGGAACTGACAAATTGAGCACACTATGGTTTAAACCACCATTTCTTTCTGATTTT
ATTAATGGTATTTTTGCTAAGGTCAAAAATACCAAGGTTTTTAAAGATGGTGTAATGTATAGTGAGTTCCCTGCTATAA
CTATAGGTAGTACTTTTGTAAATACATCCTATAGTGTGGTAGTACAACCACGTACAATTAATTCAACACAGGATGGTGT
TAATAAATTACAAGGTCTTTTAGAGGTCTCTGTTTGCCAGTATAATATGTGCGAGTACCCACATACGATTTGTCATCCT
AAACTGGGTAATCACTTCAAAGAACTATGGCATTTGGATACAGGTGTTGTTTCCTGTTTATATAAGCGTAATTTCACAT
ATGATGTGAATGCTACTTATTTGTATTTTCATTTTTATCAAGAAGGTGGTACTTTTTATGCATATTTTACAGACACTGG
TGTTGTTACTAAGTTTTTGTTTAATGTTTATTTAGGCATGGCACTTTCACACTATTATGTCATGCCTCTGACTTGTATT

AGTAGGCGTGATATTGGTTTTACTTTAGAATATTGGGTTACACCCCTCACTTCTAGACAATATTTACTCGCTTTCAATC
AAGATGGTATTATTTTTAATGCTGTTGATTGTATGAGTGATTTTATGAGTGAGATTAAGTGTAAAACACAATCTATAGC
ACCACCTACTGGTGTTTATGAATTAAACGGTTACACTGTTCAGCCAATCGCAGATGTTTACCGACGCAAACCTGATCTT
CCCAATTGCAATATAGAAGCTTGGCTTAATGATAAGTCGGTGCCCTCTCCATTAAATTGGGAACGTAAGACATTTTCAA
ATTGTAATTTTAATATGAGCAGCCTGATGTCTTTTATTCAGGCAGACTCATTTACTTGTAATAATATTGATGCTGCTAA
GATATATGGTATGTGTTTTTCCAGCATAACTATAGACAAGTTTGCTATACCCAATGGCAGGAAGGTTGACCTACAATTG
GGCAATTTGGGCTATTTGCAGTCATCCAACTATAGAATTGATACTACTGCAACAAGTTGTCAGTTGTATTATAATTTAC
CTGCTGCTAATGTCTCTGTTAGCAGGTTTAATCCTTCTACTTGGAATAAGAGATTTGGTTTTATAGAAGATTCTGTTTT
TGTGCCTCAACCTACAGGTGTTTTTACTAATCACAGTGTAGTTTATGCACAACACTGTTTCAAAGCCCCTAAAAATTTC
TGTCCGTGTAAATTGAATGGTTCGTGTCCTGATAAAAATAATGGTATAGGCACTTGTCCTGCAGGTACTAATTATTTAA
CTTGTGATAATTTGTGTACTCTTGATCCTATTACATTTAAAGCTCCAGGTACTTATAAGTGCCCCCAAACTAAATCTTT
AGTTGGCATAGGTGAGCACTGTTCGGGTCTTGCTGTTAAAAGTGATTATTGTAGAGGCAATTCTTGTATTTGCCAACCA
CAAGCATTTTTGGGTTGGTCTGCAGACTCTTGTTTACAAGGAGACAAGTGTAATATTTTTGCTAATCTTATTTTGCATG
ATGTTAATAGTGGTCTTACTTGTTCTACTGATTTACAAAAAGCTAACACAGACATAATTCTTGGTGTTTGTGTTAATTA
TGACCTCTATGGTATTTCAGGCCAAGGCATTTTTGTTGAGGTTAATGCGACTTATTATAATAGTTGGCAGAACCTTTTA
TATGATTCTAATGGTAATCTCTACGGTTTTAGAGACTACATAACAAACAGAACTTTTATGATTCGTAGTTGCTATAGCG
GTCGTGTTTCTGCGGCCTTTCATGCTAACTCTTCCGAACCAGCATTGCTATTTCGGAATATTAAATGCAACTACGTTTT
TAATAATAGTCTTACACGACAGCTGCAACCCATTAACTATTCGTTTGATAGTTATCTTGGTTGTGTTGTCAATGCTTAT
AATAGTACTGCTATTTCTGTTCAAACATGTGATCTCACAGTAGGTAGTGGTTACTGTGTGGAGTACTCTAAAAACAGAC
GAAGTCGTAGAGCGCTTACCACTGGTTATCGGTTTACTAATTTTGAGCCATTTACTGTTAATTCAGTAAATGATAGTTT
GGAACCTGTAGGTGGTTTGTATGAAATTCAAATACCTTCAGAGTTTACTATAGGTAATATGGAGGAGTTTATTCAAACA
AGCTCTCCCAAAGTTACTATTGATTGTGCTGCATTTGTCTGTGGTGATTATGCAGCATGTAAATTACAGTTGGTTGAAT
ATGGTAGTTTCTGTGATAACATTAATGCCATACTCACAGAAGTAAATGAACTACTTGATACTACACAGTTGCAAGTAGC
TAATAGTTTAATGAATGGTGTTACTCTTAGCACTAAGCTTAAAGATGGTGTTAATTTCAATGTAGACGACATCAATTTT
TCCCCTGTATTAGGTTGTCTAGGCAGCGAATGTAGTAAAGCTTCCAGTAGATCTGCTATAGAGGATTTACTTTTTGATA
AAGTAAAGTTATCTGATGTCGGTTTCGTTGAGGCTTATAATAATTGTACAGGAGGTGCCGAAATTAGGGACCTCATTTG
TGTGCAAAGTTATAAAGGCATCAAAGTGTTGCCCCCACTGCTCTCAGAAAATCAGATCAGTGGATACACTTTGGCTGCC
ACCTCTGCTAGTCTATTTCCTCCTTGGACAGCAGCAGCAGGTGTACCATTTTATTTAAATGTTCAGTATCGCATTAATG
GGCTTGGTGTCACCATGGATGTGCTAAGTCAAAATCAAAAGCTTATTGCTAATGCATTTAACAATGCCCTTTATGCTAT
TCAGCAAGGGTTCGATGCAACTAATTCTGCTTTAGTTAAAATTCAAGCTGTTGTTAATGCAAATGCTGAAGCTCTTAAT
AACTTATTGCAACAACTCTCTAATAGATTTGGTGCTATAAGTGCTTCTTTACAAGAAATTCTATCTAGACTTGATGCTC
TTGAAGCGGAAGCTCAGATAGATAGACTTATTAATGGTCGTCTCACCGCTCTTAATGCTTATGTTTCTCAACAGCTTAG
TGATTCTACACTGGTAAAATTTAGTGCAGCACAAGCTATGGAGAAGGTTAATGAATGTGTCAAAAGCCAATCATCTAGG
ATAAATTTCTGTGGTAATGGTAATCATATTATATCATTAGTGCAGAATGCTCCATATGGTTTGTATTTTATCCACTTTA
ATTATGTCCCTACTAAGTATGTCACAGCGAAGGTTAGTCCTGGTCTGTGCATTGCTGGTAATAGAGGTATAGCTCCTAA
GAGTGGTTATTTTGTTAATGTAAATAATACTTGGATGTACACTGGTAGTGGTTACTACTACCCTGAACCTATAAATGAA
AATAATGTTGTTGTTATGAGTACTTGCGCTGTTAATTATACTAAAGCGCCGTATGTAATGCTGAACACTTCAATACCCA
ACCTTCCTGATTTTAAGGAAGAGTTGGATCAATGGTTTAAAAATCAAACATCAGTGGCACCAGATTTGTCACTTGATTA
TATAAATGTTACATTCTTGGACCTACAAGTTGAAATGAATAGGTTACAGGAGGCAATAAAAGTCTTAAATCATAGCTAC
ATCAATCTCAAGGACATTGGTACATATGAATATTATGTAAAATGGCCTTGGTATGTATGGCTCTTAATCTGCCTTGCTG
GTGTAGCTATGCTTGTTTTACTATTCTTCATATGCTGTTGTACAGGATGTGGGACTAGTTGTTTTAAGAAATGTGGTGG
TTGTTGTGATGATTATACTGGATACCAGGAGTTAGTAATCAAAACTTCACATGACGACTAA

>gi|62528985|gb|AY903454.1|_Human_coronavirus_OC43_strain_89996_Belgium_2003_spike_glycoprotein_(S)_gene_complete_cds

ATGTTTTTGATACTTTTAATTTCCTTACCAACGGCTTTTGCTGTTATAGGAGATTTAAAGTGTCCTTTAGATACT
AGTCTTAAAGGTAGCTTTAATAATAGAGACACCGGTTCTCCTTCTATAAGTACTGATACTGTTGATGTTACTAATGGTT
TGGGTACTTATTATGTTTTAGATCGTGTGTATTTAAATACTACGTTGTTTCTTAATGGTTATTACCCCACTTCAGGTTC
CACATATCGTAATATGGCACTGAAGGGAACTGACAAATTGAGCACACTATGGTTTAAACCACCATTTCTTTCTGATTTT
ATTAATGGTATTTTTGCTAAGGTCAAAAATACCAAGGTTTTTAAAGATGGTGTAATGTATAGTGAGTTCCCTGCTATAA
CTATAGGTAGTACTTTTGTAAATACATCCTATAGTGTAGTAGTACAACCACGTACAATTAATTCAACACAGGATGGTGT
TAATAAATTACAAGGTCTTTTAGAGGTCTCTGTTTGCCAGTATAATATGTGCGAGTACCCACATACGATTTGTCATCCT
AAACTGGGTAACCACTTCAAAGAACTATGGCATTTGGATACAGGTGTTGTTTCCTGTTTATATAAGCGTAATTTCACAT
ATGATGTGAATGCTACTTATTTGTATTTTCATTTTTATCAAGAAGGTGGTACTTTTTATGCATATTTCACAGACACTGG
TTTTGTTACTAAGTTTTTGTTTAATGTTTATTTAGGCATGGCACTTTCACACTATTATGTCATGCCTCTGACTTGTATT
AGTAGGCGTGATATTGGTTTTACTTTAGAATATTGGGTTACACCCCTCACTTCTAGACAATATTTACTCGCTTTCAATC
AAGATGGTATTATTTTTAATGCTGTTGATTGTATGAGTGATTTTATGAGTGAGATTAAGTGTAAAACACAATCTATAGC
ACCACCTACTGGTGTTTATGAATTAAACGGTTACACTGTTCAGCCAATTGCAGATGTTTACCGACGCAAACCTGATCTT
CCCAATTGCAATATAGAAGCTTGGCTTAATGATAAGTCGGTGCCCTCTCCATTAAATTGGGAACGTAAGACATTTTCAA
ATTGTAATTTTAATATGAGCAGCCTGATGTCTTTTATTCAGGCAGACTCATTTACTTGTAATAATATTGATGCTGCTAA
GATATATGGTATGTGTTTTTCCAGCATAACTATAGATAAGTTTGCTATACCCAATGGCAGGAAGGTTGACCTACAATTG
GGTAATTTGGGCTATTTGCAGTCATTTAACTATAGAATTGATACTACTGCAACAAGTTGTCAGTTGTATTATAATTTAC
CTGCTGCTAATGTTTCTGTTAGCAGGTTTAATCCTTCTACTTGGAATAAGAGATTCGGTTTTATAGAAAATTCTGTTTT
TAAGCCTCAACCTGCAGGTGTTTTTACCAATCATGATGTAGTTTATGCCCAACATTGTTTTAAAGCTCCCAAAAATTTT
TGTCCGTGTAAATTGAACGGTTCTTTGTGTGTAGGTAGTGGTCCTGGTAAAAATAATGGTATAGGCACTTGTCCTGCAG
GTACTAATTATTTAACTTGTCACAATTTGTGCAATCCTGATCCTATTACATTTACAGGTCCTTATAAGTGCCCCCAAAC
TAAATCTTTAGTTGGTATAGGTGAGCACTGTTCAGGTCTTGCTGTTAAAAGTGATTATTGTGGAGGCAATCCTTGCACT
TGCCAACCACAAGCATTTTTGGGTTGGTCTGCAGACTCTTGTTTACAAGGGGACAAGTGTAATATTTTTGCTAATTTAA
TTTTGCATGATGTTAATAGTGGTCTTACTTGTTCTACTGATTTACAAAAAGCTAACACAGACATAATTCTTGGTGTTTG
TGTTAATTATGACCTTTATGGTATTTCAGGCCAAGGCATTTTTGTTGAGGTTAATGCGACTTATTATAATAGTTGGCAG
AACCTTTTATATGATTCTAATGGTAATCTCTATGGTTTTAGAGACTACATAACAAACAGAACTTTTATGATTCGTAGTT
GCTATAGCGGTCGTGTTTCTGCGGCCTTTCACGCTAACTCTTCCGAACCAGCATTGCTATTTCGGAATATTAAATGCAA
CTACGTTTTTAATAATAGTCTTACACGACAGCTGCAACCCATTAACTATTTTGATAGTTATCTTGGTTGTGTTGTCAAT
GCTTATAATAGTACTGCTATTTCTGTTCAAACATGTGATCTCACAGTAGGTAGTGGTTACTGTGTGGATTACTCTAAAA
ACAGACGAAGTCGTAGAGCGATTACCACTGGTTATCGGTTTACTAATTTTGAGCCATTTACTGTTAATTCAGTAAATGA
TAGTTTAGAACCTGTAGGTGGTTTGTACGAAATTCAAATACCTTCAGAGTTTACTATAGGTAATATGGAGGAGTTTATT
CAAACAAGTTCTCCTAAAGTTACTATTGATTGTGCTGCATTTGTCTGTGGTGATTATGCAGCATGTAAATCACAGTTGG
TTGAATATGGTAGTTTCTGTGATAACATTAATGCCATACTCACAGAAGTAAATGAACTACTTGACACTACACAGTTGCA
AGTAGCTAATAGTTTAATGAATGGTGTCACTCTTAGCACTAAGCTTAAAGATGGCGTTAATTTCAATGTAGACGACATC
AATTTTTCCTCTGTATTAGGTTGTCTAGGCAGCGAATGTAGTAAAGCTTCCAGTAGATCTGCTATAGAGGATTTACTTT
TTGATAAAGTAAAGTTATCTGATGTCGGTTTCGTTGCGGCTTATAATAATTGTACTGGAGGTGCCGAAATTAGGGACCT
CATTTGTGTGCAAAGTTATAAAGGTATCAAAGTGTTGCCTCCACTGCTCTCAGAAAATCAGATCAGTGGATACACTTTG
GCTGCCACTTCTGCTAGTCTGTTTCCTCCTTGGACAGCAGCAGCAGGTGTACCATTTTATTTAAATGTTCAGTATCGCA
TTAATGGGCTTGGTGTCACCATGGATGTGCTAAGTCAAAATCAAAAGCTTATTGCTAATGCATTTAACAATGCCCTTGA
TGCTATTCAGGAAGGGTTCGATGCAACTAATTCTGCTTTAGTTAAAATTCAAGCTGTTGTTAATGCAAATGCTGAAGCT
CTTAATAACTTATTGCAACAACTCTCTAATAGATTTGGTGCTATAAGTTCTTCTCTACAAGAAATTCTATCTAGACTTG

ATGCTCTTGAAGCGGAAGCTCAGATAGATAGACTTATTAATGGTCGTCTTACCGCTCTTAATGCTTATGTTTCTCAACA
GCTTAGTGATTCTACACTGGTAAAATTTAGTGCAGCACAAGCTATGGAGAAGGTTAATGAATGTGTCAAAAGCCAATCA
TCTAGGATAAATTTCTGTGGTAATGGTAACCATATTATATCATTAGTGCAGAATGCTCCATATGGTTTGTATTTTATCC
ACTTTAGTTATGTCCCTACTAAGTATGTCACAGCGAAGGTTAGTCCTGGTCTGTGCATTGCTGGTGATAGAGGTATAGC
TCCTAAGAGTGGTTATTTTGTTAATGTAAATAATACTTGGATGTACACTGGTAGTGGTTACTACTACCCTGAACCTATA
ACTGAAAATAATGTCGTTGTTATGAGTACATGCGCTGTTAATTATACTAAAGCGCCGTATGTAATGCTGAACACTTCAA
TACCCAACCTTCCTGATTTTAGGGAAGAGTTGGATCAATGGTTTAAAAATCAAACATCAGTGGCACCAGATTTGTCACT
TGATTATATAAATGTTACATTCTTGGACCTACAAGTTGAAATGAATAGGTTACAGGAGGCAATAAAAGTTTTAAATCAG
AGCTACATCAATCTCAAGGACATTGGTACATATGAATATTATGTAAAATGGCCTTGGTATGTATGGCTTTTAATCTGCC
TTGCTGGTGTAGCTATGCTTGTTTTACTATTCTTCATATGCTGTTGTACAGGATGTGGGACTAGTTGTTTTAAGAAATG
TGGTGGTTGTTGTGATGATTATACTGGATACCAGGAGTTAGTAATCAAAACTTCACATGACGACTAA

>gi|62528987|gb|AY903455.1|_Human_coronavirus_OC43_strain_34364_Belgium_2004_spike_glycoprotein_(S)_gene_complete_cds

ATGTTTTTGATACTTTTAATTTCCTTACCAACGGCTTTTGCTGTTATAGGAGATTTAAATTGTCCTTTAGATACT
AGGCTTAAAGGTAGCTTTAATAATAGAGACACCGGTCCTCCTTCTATAAGTACTGATACTGTTGATGTTACTAATGGTT
TGGGTACTTATTATGTTTTAGATCGTGTGTATTTAAATACTACGTTGTTTCTTAATGGTTATTACCCCACTTCAGGTTC
CACATATCGTAATATGGCACTGAAGGGAACTGACAAATTGAGCACATTATGGTTTAAACCACCATTTCTTTCTGATTTT
ATTAATGGTATTTTTGCTAAGGTCAAAAATACCAAGGTTTTTAAAGATGGTGTAATGTATAGTGAGTTCCCTGCTATAA
CTATAGGTAGTACTTTTGTAAATACATCCTATAGTGTGGTAGTACAACCACGTACAATTAATTCAACACAGGATGGTGT
TAATAAATTACAAGGTCTTTTAGAGGTCTCTGTTTGCCAGTATAATATGTGTGAGTACCCACATACGATTTGTCATCCT
AAATTGGGTAACCACTTCAAAGAACTATGGCATTTGGATACAGGTGTTGTTTCCTGTTTATATAAGCGTAATTTCACAT
ATGATGTGAATGCTACTTATTTGTATTTTCATTTTTATCAAGAAGGTGGTACTTTTTATGCATATTTTACAGACACTGG
TTTTGTTACTAAGTTTTTGTTTAATGTTTATTTAGGCATGGCACTTTCACACTATTATGTCATGCCTCTGACTTGTATT
AGTAGGCTTGATATTGGTTTTACTTTAGAATATTGGGTTACACCCCTCACTTCTAGACAATATTTACTCGCTTTCAATC
AAGATGGTATTATTTTTAATGCTGTTGATTGTATGAGTGATTTTATGAGTGAGATTAAGTGTAAAACACAATCTATAGC
ACCACCTACTGGTGTTTATGAATTAAACGGTTACACTGTTCAGCCAATCGCAGATGTTTACCGACGCAAACCTGATCTT
CCCAATTGCAATATAGAAGCTTGGCTTAATGATAAGTCGGTGCCCTCTCCATTAAATTGGGAACGTAAGACATTTTCAA
ATTGTAATTTTAATATGAGCAGCCTGATGTCTTTTATTCAGGCAGACTCATTTACTTGTAATAATATTGATGCTGCTAA
GATATATGGTATGTGTTTTTCCAGCATAACTATAGATAAGTTTGCTATACCCAATCGCAGGAAGGTTGACCTACAATTG
GGCAATTTGGGCTATTTGCAGTCATCCAACTATAGAATTGATACTACTGCAACAAGTTGTCAGTTGTATTATAATTTAC
CTGCTGCTAATGTCTCTGTTAGCAGGTTTAATCCTTCTACTTGGAATAAGAGATTTGGTTTTATAGAAGATTCTGTTTT
TGTGCCTCAACCTACAGGTGTTTTTACTAATCACAGTGTAGTTTATGCACAACACTGTTTCAAAGCCCCTAAAAATTTC
TGTCCGTGTAAATTGAATGGTTCCTGTCCTGGTAAAAATAATGGTATAGGCACTTGTCCTGCAGGTACTAATTATTTAA
CTTGTGATAATTTATGTACGCTTGATCCTATTACATTTAAAGCTCCAGGTACTTATAAGTGCCCCCAAACTAAATCTTT
AGTTGGTATAGGTGAGCACTGTTCGGGTCTTGCTGTTAAAAGTGATTATTGTGGAAACAATTCTTGTACTTGCCAACCA
CAAGCATTTTTGGGTTGGTCTGCAGACTCTTGTTTACAAGGAGACAAGTGTAATATTTTTGCTAATTTCATTTTACATG
ATGTTAATAATGGTCTTACTTGTTCTACTGATTTACAAAAAGCTAACACAGAAATAGAACTTGGTGTTTGTGTTAATTA
TGACCTCTATGGTATTTCAGGCCAAGGCATTTTTGTTGAGGTTAATGCGACTTATTATAATAGTTGGCAGAACCTTTTA
TATGATTCTAATGGTAACCTCTACGGTTTTAGAGACTACATAACAAACAGAACTTTTATGATTCGTAGTTGCTATAGCG
GTCGTGTTTCTGCGGCCTATCATGCCAACTCTTCCGAACCAGCATTGCTATTTCGGAATATTAAATGCAACTACGTTTT
TAATAATAGTCTTACACGACAGCTGCAACCCATTAACTATTCGTTTGATAGTTATCTTGGTTGTGTTGTCAATGCTTAT

AATAGTACTGCTATTTCTGTTCAAACATGTGATCTCACAGTAGGTAGTGGTTACTGTGTGGATTACTCTAAAAACAGAC
GAAGTCGTAGAGCGATCACCACTGGTTATCGGTTTACTAATTTTGAGCCATTTACTGTTAATTCAGTAAATGATAGTTT
GGAACCTGTAGGTGGTTTGTATGAAATTCAAATACCTTCAGAGTTTACTATAGGTAATATGGAGGAGTTTATTCAAACA
AGCTCTCCCAAAGTTACTATTGATTGTGCTGCATTTGTCTGTGGTGATTATGCAGCATGTAAATTACAGTTGGTTGAAT
ATGGTAGTTTCTGTGATAACATTAATGCCATACTCACAGAAGTAAATGAACTACTTGATACTACACAGTTGCAAGTAGC
TAATAGTTTAATGAATGGTGTTACTCTTAGCACTAAGCTTAAAGATGGTGTTAATTTCAATGTAGACGACATCAATTTT
TCCCCTGTATTAGGTTGTCTAGGCAGCGAATGTAGTAAAGCTTCCAGTAGATCTGCTATAGAGGATTTACTTTTTGATA
AAGTAAAGTTATCTGATGTCGGTTTCGTTGAGGCTTATAATAATTGTACAGGAGGTGCCGAAATTAGGGACCTCATTTG
TGTGCAAAGTTATAAAGGCATCAAAGTGTTGCCCCCACTGCTCTCAGAAAATCAGATCAGTGGATATACTTTGGCTGCC
ACCTCTGCTAGTCTATTTCCTCCTTGGACAGCAGCAGCAGGTGTACCATTTTATTTAAATGTTCAGTATCGCATTAATG
GGCTTGGTGTCACCATGGATGTGCTAAGTCAAAATCAAAAGCTTATTGCTAATGCATTTAACAATGCCCTTCATGCTAT
TCAGCAAGGGTTCGATGCAACTAATTCTGCTTTAGTTAAAATTCAAGCTGTTGTTAATGCAAATGCTGAAGCTCTTAAT
AACTTATTGCAACAACTCTCTAATAGATTTGGTGCTATAAGTGCTTCTTTACAAGAAATTCTATCTAGACTTGATGCTC
TTGAAGCGGAAGCTCAGATAGATAGACTTATTAATGGTCGTCTCACCGCTCTTAATGCTTATGTTTCTCAACAGCTTAG
TGATTCTACACTGGTAAAATTTAGTGCAGCACAAGCTATGGAGAAGGTTAATGAATGTGTCAAAAGCCAATCATCTAGG
ATAAATTTCTGTGGTAATGGTAATCATATTATATCATTAGTGCAGAATGCTCCATATGGTTTGTATTTTATCCACTTTA
ATTATGTCCCTACTAAGTATGTCACAGCGAAGGTTAGTCCTGGTCTGTGCATTGCTGGTAATAGAGGTATAGCTCCTAA
GAGTGGTTATTTTGTTAATGTAAATAATACTTGGATGTACACTGGTAGTGGTTACTACTACCCTGAACCTATAACTGAA
AATAATGTTGTTGTTATGAGTACCTGCGCTGTTAATTATACTAAAGCGCCGTATGTAATGCTGAACACTTCAATACCCA
ACCTTCCTGATTTTAAGGAAGAGTTGGACCAATGGTTCAAAAATCAAACATCAGTGGCACCAGATTTGTCACTTGATTA
TATAAATGTTACATTCTTGGACCTACAAGTTGAAATGAATAGGTTACAGGAGGCAATAAAAGTCTTAAATCATAGCTAC
ATCAATCTCAAGGACATTGGTACATATGAATATTATGTAAAATGGCCTTGGTATGTATGGCTTTTAATCTGCCTTGCTG
GTGTAGCTATGCTTGTTTTACTATTCTTCATATGCTGTTGTACAGGATGTGGGACTAGTTGTTTTAAGAAATGTGGTGG
TTGTTGTGATGATTATACTGGATACCAGGAGTTAGTAATCAAAACTTCACATGACGACTAA

>gi|62528989|gb|AY903456.1|_Human_coronavirus_OC43_strain_84020_Belgium_2003_spike_glycoprotein_(S)_gene_complete_cds

ATGTTTTTGATACTTTTAATTTCCTTACCAACGGCTTTTGCTGTTATAGGAGATTTAAAGTGTCCTTTAGATACT
AGTCTTAAAGGTAGCTTTAATAATAGAGACACCGGTTCTCCTTCTATAAGTACTGATACTGTTGATGTTACTAATGGTT
TGGGTACTTATTATGTTTTAGATCGTGTGTATTTAAATACTACGTTGTTTCTTAATGGTTATTACCCCACTTCAGGTTC
CACATATCGTAATATGGCACTGAAGGGAACTGACAAATTGAGCACACTATGGTTTAAACCACCATTTCTTTCTGATTTT
ATTAATGGTATTTTTGCTAAGGTCAAAAATACCAAGGTTTTTAAAGATGGTGTAATGTATAGTGAGTTCCCTGCTATAA
CTATAGGTAGTACTTTTGTAAATACATCCTATAGTGTAGTAGTACAACCACGTACAATTAATTCAACACAGGATGGTGT
TAATAAATTACAAGGTCTTTTAGAGGTCTCTGTTTGCCAGTATAATATGTGCGAGTACCCACATACGATTTGTCATCCT
AAACTGGGTAACCACTTCAAAGAACTATGGCATTTGGATACAGGTGTTGTTTCCTGTTTATATAAGCGTAATTTCACAT
ATGATGTGAATGCTACTTATTTGTATTTTCATTTTTATCAAGAAGGTGGTACTTTTTATGCATATTTCACAGACACTGG
TTTTGTTACTAAGTTTTTGTTTAATGTTTATTTAGGCATGGCACTTTCACACTATTATGTCATGCCTCTGACTTGTATT
AGTAGGCGTGATATTGGTTTTACTTTAGAATATTGGGTTACACCCCTCACTTCTAGACAATATTTACTCGCTTTCAATC
AAGATGGTATTATTTTTAATGCTGTTGATTGTATGAGTGATTTTATGAGTGAGATTAAGTGTAAAACACAATCTATAGC
ACCACCTACTGGTGTTTATGAATTAAATGGTTACACTGTTCAGCCAATTGCAGATGTTTACCGACGCAAACCTGATCTT
CCCAATTGCAATATAGAAGCTTGGCTTAATGATAAGTCGGTGCCCTCTCCATTAAATTGGGAACGTAAGACATTTTCAA
ATTGTAATTTTAATATGAGCAGCCTGATGTCTTTTATTCAGGCAGACTCATTTACTTGTAATAATATTGATGCTGCTAA

GATATATGGTATGTGTTTTTCCAGCATAACTATAGATAAGTTTGCTATACCCAATGGCAGGAAGGTTGACCTACAATTG
GGTAATTTGGGCTATTTGCAGTCATTTAACTATAGAATTGATACTACTGCAACAAGTTGTCAGTTGTATTATAATTTAC
CTGCTGCTAATGTTTCTGTTAGCAGGTTTAATCCTTCTACTTGGAATAAGAGATTCGGTTTTATAGAAAATTCTGTTTT
TAAGCCTCAACCTGCAGGTGTTTTTACCAATCATGATGTAGTTTATGCCCAACATTGTTTTAAAGCTCCTAAAAATTTT
TGTCCGTGTAAATTGAACGGTTCTTTGTGTGTAGGTAGTGGTCCTGGTAAAAATAATGGTATAGGCACTTGTCCTGCAG
GTACTAATTATTTAACTTGTCACAATTTGTGCAATCCTGATCCTATTACATTTACAGGTCCTTATAAGTGCCCCCAAAC
TAAATCTTTAGTTGGTATAGGTGAGCACTGTTCAGGTCTTGCTGTTAAAAGTGATTATTGTGGAGGCAATCCTTGCACT
TGCCAACCACAAGCATTTTTGGGTTGGTCTGCAGACTCTTGTTTACAAGGGGACAAGTGTAATATTTTTGCTAATTTAA
TTCTGCATGATGTTAATAGTGGTCTTACTTGTTCTACTGATTTACAAAAAGCTAACACAGACATAATTCTTGGTGTTTG
TGTTAATTATGACCTTTATGGTATTTCAGGCCAAGGCATTTTTGTTGAGGTTAATGCGACTTATTATAATAGTTGGCAG
AACCTTTTATATGATTCTAATGGTAATCTCTATGGTTTTAGAGACTACATAACAAACAGAACTTTTATGATTCGTAGTT
GCTATAGCGGTCGTGTTTCTGCGGCCTTTCACGCTAACTCTTCCGAACCAGCATTGCTATTTCGGAATATTAAATGCAA
CTACGTTTTTAATAATAGTCTTACACGACAGCTGCAACCCATTAACTATTTTGATAGTTATCTTGGTTGTGTTGTCAAT
GCTTATAATAGTACTGCTATTTCTGTTCAAACATGTGATCTCACAGTAGGTAGTGGTTACTGTGTGGATTACTCTAAAA
ACAGACGAAGTCGTAGAGCGATTACCACTGGTTATCGGTTTACTAATTTTGAGCCATTTACTGTTAATTCAGTAAATGA
TAGTTTAGAACCTGTAGGTGGTTTGTACGAAATTCAAATACCTTCAGAGTTTACTATAGGTAATATGGAGGAGTTTATT
CAAACAAGCTCTCCTAAAGTTACTATTGATTGTGCTGCATTTGTCTGTGGTGATTATGCAGCATGTAAATCACAGTTGG
TTGAATATGGTAGTTTCTGTGATAACATTAATGCCATACTCACAGAAGTAAATGAACTACTTGACACTACACAGTTGCA
AGTAGCTAATAGTTTAATGAATGGTGTCACTCTTAGCACTAAGCTTAAAGATGGCGTTAATTTCAATGTAGACGACATC
AATTTTTCCTCTGTATTAGGTTGTCTAGGCAGCGAATGTAGTAAAGCTTCCAGTAGATCTGCTATAGAGGATTTACTTT
TTGATAAAGTAAAGTTATCTGATGTCGGTTTCGTTGCGGCTTATAATAATTGTACTGGAGGTGCCGAAATTAGGGACCT
CATTTGTGTGCAAAGTTATAAAGGTATCAAAGTGTTGCCTCCATTGCTCTCAGAAAATCAGATCAGTGGATACACTTTG
GCTGCCACTTCTGCTAGTCTGTTTCCTCCTTGGACAGCAGCAGCAGGTGTACCATTTTATTTAAATGTTCAGTATCGCA
TTAATGGGCTTGGTGTCACCATGGATGTGCTAAGTCAAAATCAAAAGCTTATTGCTAATGCATTTAACAATGCCCTTGA
TGCTATTCAGGAAGGGTTCGATGCAACTAATTCTGCTTTAGTTAAAATTCAAGCTGTTGTTAATGCAAATGCTGAAGCT
CTTAATAACTTATTGCAACAACTCTCTAATAGATTTGGTGCTATAAGTTCTTCTCTACAAGAAATTCTATCTAGACTTG
ATGCTCTTGAAGCGGAAGCTCAGATAGATAGACTTATTAATGGTCGTCTTACCGCTCTTAATGCTTATGTTTCTCAACA
GCTTAGTGATTCTACACTGGTAAAATTTAGTGCAGCACAAGCTATGGAGAAGGTTAATGAATGTGTCAAAAGCCAATCA
TCTAGGATAAATTTCTGTGGTAATGGTAACCATATTATATCATTAGTGCAGAATGCTCCATATGGTTTGTATTTTATCC
ACTTTAGTTATGTCCCTACTAAGTATGTCACAGCGAAGGTTAGTCCTGGTCTGTGCATTGCTGGTGATAGAGGTATAGC
TCCTAAGAGTGGTTATTTTGTTAATGTAAATAATACTTGGATGTACACTGGTAGTGGTTACTACTACCCTGAACCTATA
ACTGAAAATAATGTCGTTGTTATGAGTACATGCGCTGTTAATTATACTAAAGCGCCGTATGTAATGCTGAACACTTCAA
TACCCAACCTTCCTGATTTTAGGGAAGAGTTGGATCAATGGTTTAAAAATCAAACATCAGTGGCACCAGATTTGTCACT
TGATTATATAAATGTTACATTCTTGGACCTACAAGTTGAAATGAATAGGTTACAGGAGGCAATAAAAGTTTTAAATCAG
AGCTACATCAATCTCAAGGACATTGGTACATATGAATATTATGTAAAATGGCCTTGGTATGTATGGCTTTTAATCTGCC
TTGCTGGTGTAGCTATGCTTGTTTTACTATTCTTCATATGCTGTTGTACAGGATGTGGGACTAGTTGTTTTAAGAAATG
TGGTGGTTGTTGTGATGATTATACTGGATACCAGGAGTTAGTAATCAAAACTTCACATGACGACTAA

>gi|62528991|gb|AY903457.1|_Human_coronavirus_OC43_strain_37767_Belgium_2003_spike_glycoprotein_(S)_gene_complete_cds

ATGTTTTTGATACTTTTAATTTCCTTACCAACGGCTTTTGCTGTTATAGGAGATTTAAAGTGTCCTTTAGATACT
AGTCTTAAAGGTAGCTTTAATAATAGAGACACCGGTTCTCCTTCTATAAGTACTGATACTGTTGATGTTACTAATGGTT

TGGGTACTTATTATGTTTTAGATCGTGTGTATTTAAATACAACGTTGTTTCTTAATGGTTATTACCCCACTTCAGGTTC
CACATATCGTAATATGGCACTGAAGGGAACTGACAAATTGAGCACACTATGGTTTAAACCACCATTTCTTTCTGATTTT
ATTAATGGTATTTTTGCTAAGGTCAAAAATACCAAGGTTTTTAAAGATGGTGTAATGTATAGTGAGTTCCCTGCTATAA
CTATAGGTAGTACTTTTGTAAATACATCCTATAGTGTAGTAGTACAACCACGTACAATTAATTCAACACAGGATGGTGT
TAATAAATTACAAGGTCTTTTAGAGGTCTCTGTTTGCCAGTATAATATGTGCGAGTACCCACATACGATTTGTCATCCT
AAACTGGGTAACCACTTCAAAGAACTATGGCATTTGGATACAGGTGTTGTTTCCTGTTTATATAAGCGTAATTTCACAT
ATGATGTGAATGCTACTTATTTGTATTTTCATTTTTATCAAGAAGGTGGTACTTTTTATGCATATTTCACAGACACTGG
TTTTGTTACTAAGTTTTTGTTTAATGTTTATTTAGGCATGGCACTTTCACACTATTATGTCATGCCTCTGACTTGTATT
AGTAGGCGTGATATTGGTTTTACTTTAGAATATTGGGTTACACCCCTCACTTCTAGACAATATTTACTCGCTTTCAATC
AAGATGGTATTATTTTTAATGCTGTTGATTGTATGAGTGATTTTATGAGTGAGATTAAGTGTAAAACACAATCTATAGC
ACCACCTACTGGTGTTTATGAATTAAACGGTTACACTGTTCAGCCAATTGCAGATGTTTACCGACGCAAACCTGATCTT
CCCAATTGCAATATAGAAGCTTGGCTTAATGATAAGTCGGTGCCCTCTCCATTAAATTGGGAACGTAAGACATTTTCAA
ATTGTAATTTTAATATGAGCAGCCTGATGTCTTTTATTCAGGCAGACTCATTTACTTGTAATAATATTGATGCTGCTAA
GATATATGGTATGTGTTTTTCCAGCATAACTATAGATAAGTTTGCTATACCCAATGGCAGGAAGGTTGACCTACAATTG
GGTAATTTGGGCTATTTGCAGTCATTTAACTATAGAATTGATACTACTGCAACAAGTTGTCAGTTGTATTATAATTTAC
CTGCTGCTAATGTTTCTGTTAGCAGGTTTAATCCTTCTACTTGGAATAAGAGATTCGGTTTTATAGAAAATTCTGTTTT
TAAGCCTCAACCTGCAGGTGTTTTTACCAATCATGATGTAGTTTATGCCCAACATTGTTTTAAAGCTCCCAAAAATTTT
TGTCCGTGTAAATTGAACGGTTCTTTGTGTGTAGGTAGTGGTCCTGGTAAAAATAATGGTATAGGCACTTGTCCTGCAG
GTACTAATTATTTAACTTGTCACAATTTGTGCAATCCTGATCCTATTACATTTACAGGTCCTTATAAGTGCCCCCAAAC
TAAATCTTTAGTTGGTATAGGTGAGCACTGTTCAGGTCTTGCTGTTAAAAGTGATTATTGTGGAGGCAATCCTTGCACT
TGCCAACCACAAGCATTTTTGGGTTGGTCTGCAGACTCTTGTTTACAAGGGGACAAGTGTAATATTTTTGCTAATTTAA
TTTTGCATGATGTTAATAGTGGTCTTACTTGTTCTACTGATTTACAAAAAGCTAACACAGACATAATTCTTGGTGTTTG
TGTTAATTATGACCTTTATGGTATTTCAGGCCAAGGCATTTTTGTTGAGGTTAATGCGACTTATTATAATAGTTGGCAG
AACCTTTTATATGATTCTAATGGTAATCTCTATGGTTTTAGAGACTACATAACAAACAGAACTTTTATGATTCGTAGTT
GCTATAGCGGTCGTGTTTCTGCGGCCTTTCACGCTAACTCTTCCGAACCAGCATTGCTATTTCGGAATATTAAATGCAA
CTACGTTTTTAATAATAGTCTTACACGACAGCTGCAACCCATTAACTATTTTGATAGTTATCTTGGTTGTGTTGTCAAT
GCTTATAATAGTACTGCTATTTCTGTTCAAACATGTGATCTCACAGTAGGTAGTGGTTACTGTGTGGATTACTCTAAAA
ACAGACGAAGTCGTAGAGCGATTACCACTGGTTATCGGTTTACTAATTTTGAGCCATTTACTGTTAATTCAGTAAATGA
TAGTTTAGAACCTGTAGGTGGTTTGTACGAAATTCAAATACCTTCAGAGTTTACTATAGGTAATATGGAGGAGTTTATT
CAAACAAGCTCTCCTAAAGTTACTATTGATTGTGCTGCATTTGTCTGTGGTGATTATGCAGCATGTAAATCACAGTTGG
TTGAATATGGTAGTTTCTGTGATAACATTAATGCCATACTCACAGAAGTAAATGAACTACTTGACACTACACAGTTGCA
AGTAGCTAATAGTTTAATGAATGGTGTCACTCTTAGCACTAAGCTTAAAGATGGCGTTAATTTCAATGTAGACGACATC
AATTTTTCCTCTGTATTAGGTTGTCTAGGCAGCGAATGTAGTAAAGCTTCCAGTAGATCTGCTATAGAGGATTTACTTT
TTGATAAAGTAAAGTTATCTGATGTCGGTTTCGTTGCGGCTTATAATAATTGTACTGGAGGTGCCGAAATTAGGGACCT
CATTTGTGTGCAAAGTTATAAAGGTATCAAAGTGTTGCCTCCACTGCTCTCAGAAAATCAGATCAGTGGATACACTTTG
GCTGCCACTTCTGCTAGTCTGTTTCCTCCTTGGACAGCAGCAGCAGGTGTACCATTTTATTTAAATGTTCAGTATCGCA
TTAATGGGCTTGGTGTCACCATGGATGTGCTAAGTCAAAATCAAAAGCTTATTGCTAATGCATTTAACAATGCCCTTGA
TGCTATTCAGGAAGGGTTCGATGCAACTAATTCTGCTTTAGTTAAAATTCAAGCTGTTGTTAATGCAAATGCTGAAGCT
CTTAATAACTTATTGCAACAACTCTCTAATAGATTTGGTGCTATAAGTTCTTCTCTACAAGAAATTCTATCTAGACTTG
ATGCTCTTGAAGCGGAAGCTCAGATAGATAGACTTATTAATGGTCGTCTTACCGCTCTTAATGCTTATGTTTCTCAACA
GCTTAGTGATTCTACACTGGTAAAATTTAGTGCAGCACAAGCTATGGAGAAGGTTAATGAATGTGTCAAAAGCCAATCA
TCTAGGATAAATTTCTGTGGTAATGGTAACCATATTATATCATTAGTGCAGAATGCTCCATATGGTTTGTATTTTATCC
ACTTTAGTTATGTCCCTACTAAGTATGTCACAGCGAAGGTTAGTCCTGGTCTGTGCATTGCTGGTGATAGAGGTATAGC
TCCTAAGAGTGGTTATTTTGTTAATGTAAATAATACTTGGATGTACACTGGTAGTGGTTACTACTACCCTGAACCTATA

ACTGAAAATAATGTCGTTGTTATGAGTACATGCGCTGTTAATTATACTAAAGCGCCGTATGTAATGCTGAACACTTCAA
TACCCAACCTTCCTGATTTTAGGGAAGAGTTGGATCAATGGTTTAAAAATCAAACATCAGTGGCACCAGATTTGTCACT
TGATTATATAAATGTTACATTCTTGGACCTACAAGTTGAAATGAATAGGTTACAGGAGGCAATAAAAGTTTTAAATCAG
AGCTACATCAATCTCAAGGACATTGGTACATATGAATATTATGTAAAATGGCCTTGGTATGTATGGCTTTTAATCTGCC
TTGCTGGTGTAGCTATGCTTGTTTTACTATTCTTCATATGCTGTTGTACAGGATGTGGGACTAGTTGTTTTAAGAAATG
TGGTGGTTGTTGTGATGATTATACTGGATACCAGGAGTTAGTAATCAAAACTTCACATGACGACTAA

>gi|62528993|gb|AY903458.1|_Human_coronavirus_OC43_strain_36638_Belgium_2004_spike_glycoprotein_(S)_gene_complete_cds

ATGTTTTTGATACTTTTAATTTCCTTACCAACGGCTTTTGCTGTTATAGGAGATTTAAATTGTCCTTTAGATACT
AGGCTTAAAGGTAGCTTTAATAATAGAGACACCGGTCCTCCTTCTATAAGTACTGATACTGTTGATGTTACTAATGGTT
TGGGTACTTATTATGTTTTAGATCGTGTGTATTTAAATACTACGTTGTTTCTTAATGGTTATTACCCCACTTCAGGTTC
CACATATCGTAATATGGCACTGAAGGGAACTGACAAATTGAGCACATTATGGTTTAAACCACCATTTCTTTCTGATTTT
ATTAATGGTATTTTTGCTAAGGTCAAAAATACCAAGGTTTTTAAAGATGGTGTAATGTATAGTGAGTTCCCTGCTATAA
CTATAGGTAGTACTTTTGTAAATACATCCTATAGTGTGGTAGTACAACCACGTACAATTAATTCAACACAGGATGGTGT
TAATAAATTACAAGGTCTTTTAGAGGTCTCTGTTTGCCAGTATAATATGTGTGAGTACCCACATACGATTTGTCATCCT
AAATTGGGTAACCACTTCAAAGAACTATGGCATTTGGATACAGGTGTTGTTTCCTGTTTATATAAGCGTAATTTCACAT
ATGATGTGAATGCTACTTATTTGTATTTTCATTTTTATCAAGAAGGTGGTACTTTTTATGCATATTTTACAGACACTGG
TTTTGTTACTAAGTTTTTGTTTAATGTTTATTTAGGCATGGCACTTTCACACTATTATGTCATGCCTCTGACTTGTATT
AGTAGGCTTGATATTGGTTTTACTTTAGAATATTGGGTTACACCCCTCACTTCTAGACAATATTTACTCGCTTTCAATC
AAGATGGTATTATTTTTAATGCTGTTGATTGTATGAGTGATTTTATGAGTGAGATTAAGTGTAAAACACAATCTATAGC
ACCACCTACTGGTGTTTATGAATTAAACGGTTACACTGTTCAGCCAATCGCAGATGTTTACCGACGCAAACCTGATCTT
CCCAATTGCAATATAGAAGCTTGGCTTAATGATAAGTCGGTGCCCTCTCCATTAAATTGGGAACGTAAGACATTTTCAA
ATTGTAATTTTAATATGAGCAGCCTGATGTCTTTTATTCAGGCAGACTCATTTACTTGTAATAATATTGATGCTGCTAA
GATATATGGTATGTGTTTTTCCAGCATAACTATAGATAAGTTTGCTATACCCAATCGCAGGAAGGTTGACCTACAATTG
GGCAATTTGGGCTATTTGCAGTCATCCAACTATAGAATTGATACTACTGCAACAAGTTGTCAGTTGTATTATAATTTAC
CTGCTGCTAATGTCTCTGTTAGCAGGTTTAATCCTTCTACTTGGAATAAGAGATTTGGTTTTATAGAAGATTCTGTTTT
TGTGCCTCAACCTACAGGTGTTTTTACTAATCACAGTGTAGTTTATGCACAACACTGTTTCAAAGCCCCTAAAAATTTC
TGTCCGTGTAAATTGAATGGTTCCTGTCCTGGTAAAAATAATGGTATAGGCACTTGTCCTGCAGGTACTAATTATTTAA
CTTGTGATAATTTATGTACGCTTGATCCTATTACATTTAAAGCTCCAGGTACTTATAAGTGCCCCCAAACTAAATCTTT
AGTTGGTATAGGTGAGCACTGTTCGGGTCTTGCTGTTAAAAGTGATTATTGTGGAAACAATTCTTGTACTTGCCAACCA
CAAGCATTTTTGGGTTGGTCTGCAGACTCTTGTTTACAAGGAGACAAGTGTAATATTTTTGCTAATTTCATTTTACATG
ATGTTAATAATGGTCTTACTTGTTCTACTGATTTACAAAAAGCTAACACAGAAATAGAACTTGGTGTTTGTGTTAATTA
TGACCTCTATGGTATTTCAGGCCAAGGCATTTTTTTTGAGGTTAATGCGACTTATTATAATAGTTGGCAGAACCTTTTA
TATGATTCTAATGGTAACCTCTACGGTTTTAGAGACTACATAACAAACAGAACTTTTATGATTCGTAGTTGCTATAGCG
GTCGTGTTTCTGCGGCCTATCATGCCAACTCTTCCGAACCAGCATTGCTATTTCGGAATATTAAATGCAACTACGTTTT
TAATAATAGTCTTACACGACAGCTGCAACCCATTAACTATTCGTTTGATAGTTATCTTGGTTGTGTTGTCAATGCTTAT
AATAGTACTGCTATTTCTGTTCAAACATGTGATCTCACAGTAGGTAGTGGTTACTGTGTGGATTACTCTAAAAACAGAC
GAAGTCGTAGAGCGATCACCACTGGTTATCGGTTTACTAATTTTGAGCCATTTACTGTTAATTCAGTAAATGATAGTTT
GGAACCTGTAGGTGGTTTGTATGAAATTCAAATACCTTCAGAGTTTACTATAGGTAATATGGAGGAGTTTATTCAAACA
AGCTCTCCCAAAGTTACTATTGATTGTGCTGCATTTGTCTGTGGTGATTATGCAGCATGTAAATTACAGTTGGTTGAAT
ATGGTAGTTTCTGTGATAACATTAATGCCATACTCACAGAAGTAAATGAACTACTTGATACTACACAGTTGCAAGTAGC

TAATAGTTTAATGAATGGTGTTACTCTTAGCACTAAGCTTAAAGATGGTGTTAATTTCAATGTAGACGACATCAATTTT
TCCCCTGTATTAGGTTGTCTAGGCAGCGAATGTAGTAAAGCTTCCAGTAGATCTGCTATAGAGGATTTACTTTTTGATA
AAGTAAAGTTATCTGATGTCGGTTTCGTTGAGGCTTATAATAATTGTACAGGAGGTGCCGAAATTAGGGACCTCATTTG
TGTGCAAAGTTATAAAGGCATCAAAGTGTTGCCCCCACTGCTCTCAGAAAATCAGATCAGTGGATATACTTTGGCTGCC
ACCTCTGCTAGTCTATTTCCTCCTTGGACAGCAGCAGCAGGTGTACCATTTTATTTAAATGTTCAGTATCGCATTAATG
GGCTTGGTGTCACCATGGATGTGCTAAGTCAAAATCAAAAGCTTATTGCTAATGCATTTAACAATGCCCTTCATGCTAT
TCAGCAAGGGTTCGATGCAACTAATTCTGCTTTAGTTAAAATTCAAGCTGTTGTTAATGCAAATGCTGAAGCTCTTAAT
AACTTATTGCAACAACTCTCTAATAGATTTGGTGCTATAAGTGCTTCTTTACAAGAAATTCTATCTAGACTTGATGCTC
TTGAAGCGGAAGCTCAGATAGATAGACTTATTAATGGTCGTCTCACCGCTCTTAATGCTTATGTTTCTCAACAGCTTAG
TGATTCTACACTGGTAAAATTTAGTGCAGCACAAGCTATGGAGAAGGTTAATGAATGTGTCAAAAGCCAATCATCTAGG
ATAAATTTCTGTGGTAATGGTAATCATATTATATCATTAGTGCAGAATGCTCCATATGGTTTGTATTTTATCCACTTTA
ATTATGTCCCTACTAAGTATGTCACAGCGAAGGTTAGTCCTGGTCTGTGCATTGCTGGTAATAGAGGTATAGCTCCTAA
GAGTGGTTATTTTGTTAATGTAAATAATACTTGGATGTACACTGGTAGTGGTTACTACTACCCTGAACCTATAACTGAA
AATAATGTTGTTGTTATGAGTACCTGCGCTGTTAATTATACTAAAGCGCCGTATGTAATGCTGAACACTTCAATACCCA
ACCTTCCTGATTTTAAGGAAGAGTTGGACCAATGGTTCAAAAATCAAACATCAGTGGCACCAGATTTGTCACTTGATTA
TATAAATGTTACATTCTTGGACCTACAAGTTGAAATGAATAGGTTACAGGAGGCAATAAAAGTCTTAAATCATAGCTAC
ATCAATCTCAAGGACATTGGTACATATGAATATTATGTAAAATGGCCTTGGTATGTATGGCTTTTAATCTGCCTTGCTG
GTGTAGCTATGCTTGTTTTACTATTCTTCATATGCTGTTGTACAGGATGTGGGACTAGTTGTTTTAAGAAATGTGGTGG
TTGTTGTGATGATTATACTGGATACCAGGAGTTAGTAATCAAAACTTCACATGACGACTAA

>gi|62530888|gb|AY903459.1| Human coronavirus OC43 strain 87309 Belgium 2003, complete genome

ATGTTTTTGATACTTTTAATTTCCTTACCAACGGCTTTTGCTGTTATAGGAGATTTAAAGTGTCCTTTAGATACT
AGTCTTAAAGGTAGCTTTAATAATAGAGACACCGGTTCTCCTTCTATAAGTACTGATACTGTTGATGTTACTAATGGTT
TGGGTACTTATTATGTTTTAGATCGTGTGTATTTAAATACTACGTTGTTTCTTAATGGTTATTACCCCACTTCAGGTTC
CACATATCGTAATATGGCACTGAAGGGAACTGACAAATTGAGCACACTATGGTTTAAACCACCATTTCTTTCTGATTTT
ATTAATGGTATTTTTGCTAAGGTCAAAAATACCAAGGTTTTTAAAGATGGTGTAATGTATAGTGAGTTCCCTGCTATAA
CTATAGGTAGTACTTTTGTAAATACATCCTATAGTGTAGTAGTACAACCACGTACAATTAATTCAACACAGGATGGTGT
TAATAAATTACAAGGTCTTTTAGAGGTCTCTGTTTGCCAGTATAATATGTGCGAGTACCCACATACGATTTGTCATCCT
AAACTGGGTAACCACTTCAAAGAACTATGGCATTTGGATACAGGTGTTGTTTCCTGTTTATATAAGCGTAATTTCACAT
ATGATGTGAATGCTACTTATTTGTATTTTCATTTTTATCAAGAAGGTGGTACTTTTTATGCATATTTCACAGACACTGG
TTTTGTTACTAAGTTTTTGTTTAATGTTTATTTAGGCATGGCACTTTCACACTATTATGTCATGCCTCTGACTTGTATT
AGTAGGCGTGATATTGGTTTTACTTTAGAATATTGGGTTACACCCCTCACTTCTAGACAATATTTACTCGCTTTCAATC
AAGATGGTATTATTTTTAATGCTGTTGATTGTATGAGTGATTTTATGAGTGAGATTAAGTGTAAAACACAATCTATAGC
ACCACCTACTGGTGTTTATGAATTAAACGGTTACACTGTTCAGCCAATTGCAGATGTTTACCGACGCAAACCTGATCTT
CCCAATTGCAATATAGAAGCTTGGCTTAATGATAAGTCGGTGCCCTCTCCATTAAATTGGGAACGTAAGACATTTTCAA
ATTGTAATTTTAATATGAGCAGCCTGATGTCTTTTATTCAGGCAGACTCATTTACTTGTAATAATATTGATGCTGCTAA
GATATATGGTATGTGTTTTTCCAGCATAACTATAGATAAGTTTGCTATACCCAATGGCAGGAAGGTTGACCTACAATTG
GGTAATTTGGGCTATTTGCAGTCATTTAACTATAGAATTGATACTACTGCAACAAGTTGTCAGTTGTATTATAATTTAC
CTGCTGCTAATGTTTCTGTTAGCAGGTTTAATCCTTCTACTTGGAATAAGAGATTCGGTTTTATAGAAAATTCTGTTTT
TAAGCCTCAACCTGCAGGTGTTTTTACCAATCATGATGTAGTTTATGCCCAACATTGTTTTAAAGCTCCCAAAAATTTT
TGTCCGTGTAAATTGAACGGTTCTTTGTGTGTAGGTAGTGGTCCTGGTAAAAATAATGGTATAGGCACTTGTCCTGCAG

GTACTAATTATTTAACTTGTCACAATTTGTGCAATCCTGATCCTATTACATTTACAGGTCCTTATAAGTGCCCCCAAAC
TAAATCTTTAGTTGGTATAGGTGAGCACTGTTCAGGTCTTGCTGTTAAAAGTGATTATTGTGGAGGCAATCCTTGCACT
TGCCAACCACAAGCATTTTTGGGTTGGTCTGCAGACTCTTGTTTACAAGGGGACAAGTGTAATATTTTTGCTAATTTAA
TTTTGCATGATGTTAATAGTGGTCTTACTTGTTCTACTGATTTACAAAAAGCTAACACAGACATAATTCTTGGTGTTTG
TGTTAATTATGACCTTTATGGTATTTCAGGCCAAGGCATTTTTGTTGAGGTTAATGCGACTTATTATAATAGTTGGCAG
AACCTTTTATATGATTCTAATGGTAATCTCTATGGTTTTAGAGACTACATAACAAACAGAACTTTTATGATTCGTAGTT
GCTATAGCGGTCGTGTTTCTGCGGCCTTTCACGCTAACTCTTCCGAACCAGCATTGCTATTTCGGAATATTAAATGCAA
CTACGTTTTTAATAATAGTCTTACACGACAGCTGCAACCCATTAACTATTTTGATAGTTATCTTGGTTGTGTTGTCAAT
GCTTATAATAGTACTGCTATTTCTGTTCAAACATGTGATCTCACAGTAGGTAGTGGTTACTGTGTGGATTACTCTAAAA
ACAGACGAAGTCGTAGAGCGATTACCACTGGTTATCGGTTTACTAATTTTGAGCCATTTACTGTTAATTCAGTAAATGA
TAGTTTAGAACCTGTAGGTGGTTTGTACGAAATTCAAATACCTTCAGAGTTTACTATAGGTAATATGGAGGAGTTTATT
CAAACAAGTTCTCCTAAAGTTACTATTGATTGTGCTGCATTTGTCTGTGGTGATTTTGCAGCATGTAAATCACAGTTGG
TTGAATATGGTAGTTTCTGTGATAACATTAATGCCATACTCACAGAAGTAAATGAACTACTTGACACTACACAGTTGCA
AGTAGCTAATAGTTTAATGAATGGTGTCACTCTTAGCACTAAGCTTAAAGATGGCGTTAATTTCAATGTAGACGACATC
AATTTTTCCTCTGTATTAGGTTGTCTAGGCAGCGAATGTAGTAAAGCTTCCAGTAGATCTGCTATAGAGGATTTACTTT
TTGATAAAGTAAAGTTATCTGATGTCGGTTTCGTTGCGGCTTATAATAATTGTACTGGAGGTGCCGAAATTAGGGACCT
CATTTGTGTGCAAAGTTATAAAGGTATCAAAGTGTTGCCTCCACTGCTCTCAGAAAATCAGATCAGTGGATACACTTTG
GCTGCCACTTCTGCTAGTCTGTTTCCTCCTTGGACAGCAGCAGCAGGTGTACCATTTTATTTAAATGTTCAGTATCGCA
TTAATGGGCTTGGTGTCACCATGGATGTGCTAAGTCAAAATCAAAAGCTTATTGCTAATGCATTTAACAATGCCCTTGA
TGCTATTCAGGAAGGGTTCGATGCAACTAATTCTGCTTTAGTTAAAATTCAAGCTGTTGTTAATGCAAATGCTGAAGCT
CTTAATAACTTATTGCAACAACTCTCTAATAGATTTGGTGCTATAAGTTCTTCTCTACAAGAAATTCTATCTAGACTTG
ATGCTCTTGAAGCGGAAGCTCAGATAGATAGACTTATTAATGGTCGTCTTACCGCTCTTAATGCTTATGTTTCTCAACA
GCTTAGTGATTCTACACTGGTAAAATTTAGTGCAGCACAAGCTATGGAGAAGGTTAATGAATGTGTCAAAAGCCAATCA
TCTAGGATAAATTTCTGTGGTAATGGTAACCATATTATATCATTAGTGCAGAATGCTCCATATGGTTTGTATTTTATCC
ACTTTAGTTATGTCCCTACTAAGTATGTCACAGCGAAGGTTAGTCCTGGTCTGTGCATTGCTGGTGATAGAGGTATAGC
TCCTAAGAGTGGTTATTTTGTTAATGTAAATAATACTTGGATGTACACTGGTAGTGGTTACTACTACCCTGAACCTATA
ACTGAAAATAATGTCGTTGTTATGAGTACATGCGCTGTTAATTATACTAAAGCGCCGTATGTAATGCTGAACACTTCAA
TACCCAACCTTCCTGATTTTAGGGAAGAGTTGGATCAATGGTTTAAAAATCAAACATCAGTGGCACCAGATTTGTCACT
TGATTATATAAATGTTACATTCTTGGACCTACAAGTTGAAATGAATAGGTTACAGGAGGCAATAAAAGTTTTAAATCAG
AGCTACATCAATCTCAAGGACATTGGTACATATGAATATTATGTAAAATGGCCTTGGTATGTATGGCTTTTAATCTGCC
TTGCTGGTGTAGCTATGCTTGTTTTACTATTCTTCATATGCTGTTGTACAGGATGTGGGACTAGTTGTTTTAAGAAATG
TGGTGGTTGTTGTGATGATTATACTGGATACCAGGAGTTAGTAATCAAAACTTCACATGACGACTAA

>gi|62530898|gb|AY903460.1| Human coronavirus OC43 strain 19572 Belgium 2004, complete genome

ATGTTTTTGATACTTTTAATTTCCTTACCAACGGCTTTTGCTGTTATAGGAGATTTAAATTGTCCTTTAGATACT
AGGCTTAAAGGTAGCTTTAATAATAGAGACACCGGTCCTCCTTCTATAAGTACTGATACTGTTGATGTTACTAATGGTT
TGGGTACTTATTATGTTTTAGATCGTGTGTATTTAAATACTACGTTGTTTCTTAATGGTTATTACCCCACTTCAGGTTC
CACATATCGTAATATGGCACTGAAGGGAACTGACAAATTGAGCACATTATGGTTTAAACCACCATTTCTTTCTGATTTT
ATTAATGGTATTTTTGCTAAGGTCAAAAATACCAAGGTTTTTAAAGATGGTGTAATGTATAGTGAGTTCCCTGCTATAA
CTATAGGTAGTACTTTTGTAAATACATCCTATAGTGTGGTAGTACAACCACGTACAATTAATTCAACACAGGATGGTGT
TAATAAATTACAAGGTCTTTTAGAGGTCTCTGTTTGCCAGTATAATATGTGTGAGTACCCACATACGATTTGTCATCCT

AAATTGGGTAACCACTTCAAAGAACTATGGCATTTGGATACAGGTGTTGTTTCCTGTTTATATAAGCGTAATTTCACAT
ATGATGTGAATGCTACTTATTTGTATTTTCATTTTTATCAAGAAGGTGGTACTTTTTATGCATATTTTACAGACACTGG
TTTTGTTACTAAGTTTTTGTTTAATGTTTATTTAGGCATGGCACTTTCACACTATTATGTCATGCCTCTGACTTGTATT
AGTAGGCTTGATATTGGTTTTACTTTAGAATATTGGGTTACACCCCTCACTTCTAGACAATATTTACTCGCTTTCAATC
AAGATGGTATTATTTTTAATGCTGTTGATTGTATGAGTGATTTTATGAGTGAGATTAAGTGTAAAACACAATCTATAGC
ACCACCTACTGGTGTTTATGAATTAAACGGTTACACTGTTCAGCCAATCGCAGATGTTTACCGACGCAAACCTGATCTT
CCCAATTGCAATATAGAAGCTTGGCTTAATGATAAGTCGGTGCCCTCTCCATTAAATTGGGAACGTAAGACATTTTCAA
ATTGTAATTTTAATATGAGCAGCCTGATGTCTTTTATTCAGGCAGACTCATTTACTTGTAATAATATTGATGCTGCTAA
GATATATGGTATGTGTTTTTCCAGCATAACTATAGATAAGTTTGCTATACCCAATCGCAGGAAGGTTGACCTACAATTG
GGCAATTTGGGCTATTTGCAGTCATCCAACTATAGAATTGATACTACTGCAACAAGTTGTCAGTTGTATTATAATTTAC
CTGCTGCTAATGTCTCTGTTAGCAGGTTTAATCCTTCTACTTGGAATAAGAGATTTGGTTTTATAGAAGATTCTGTTTT
TGTGCCTCAACCTACAGGTGTTTTTACTAATCACAGTGTAGTTTATGCACAACACTGTTTCAAAGCCCCTAAAAATTTC
TGTCCGTGTAAATTGAATGGTTCCTGTCCTGGTAAAAATAATGGTATAGGCACTTGTCCTGCAGGTACTAATTATTTAA
CTTGTGATAATTTATGTACGCTTGATCCTATTACATTTAAAGCTCCAGGTACTTATAAGTGCCCCCAAACTAAATCTTT
AGTTGGTATAGGTGAGCACTGTTCGGGTCTTGCTGTTAAAAGTGATTATTGTGGAAACAATTCTTGTACTTGCCAACCA
CAAGCATTTTTGGGTTGGTCTGCAGACTCTTGTTTACAAGGAGACAAGTGTAATATTTTTGCTAATTTCATTTTGCATG
ATGTTAATAATGGTCTTACTTGTTCTACTGATTTACAAAAAGCTAACACAGAAATAGAACTTGGTGTTTGTGTTAATTA
TGACCTCTATGGTATTTCAGGCCAAGGCATTTTTGTTGAGGTTAATGCGACTTATTATAATAGTTGGCAGAACCTTTTA
TATGATTCTAATGGTAACCTCTACGGTTTTAGAGACTACATAACAAACAGAACTTTTATGATTCGTAGTTGCTATAGCG
GTCGTGTTTCTGCGGCCTATCATGCCAACTCTTCCGAACCAGCATTGCTATTTCGGAATATTAAATGCAACTACGTTTT
TAATAATAGTCTTACACGACAGCTGCAACCCATTAACTATTCGTTTGATAGTTATCTTGGTTGTGTTGTCAATGCTTAT
AATAGTACTGCTATTTCTGTTCAAACATGTGATCTCACAGTAGGTAGTGGTTACTGTGTGGATTACTCTAAAAACAGAC
GAAGTCGTAGAGCGATCACCACTGGTTATCGGTTTACTAATTTTGAGCCATTTACTGTTAATTCAGTAAATGATAGTTT
GGAACCTGTAGGTGGTTTGTATGAAATTCAAATACCTTCAGAGTTTACTATAGGTAATATGGAGGAGTTTATTCAAACA
AGCTCTCCCAAAGTTACTATTGATTGTGCTGCATTTGTCTGTGGTGATTATGCAGCATGTAAATTACAGTTGGTTGAAT
ATGGTAGTTTCTGTGATAACATTAATGCCATACTCACAGAAGTAAATGAACTACTTGATACTACACAGTTGCAAGTAGC
TAATAGTTTAATGAATGGTGTTACTCTTAGCACTAAGCTTAAAGATGGTGTTAATTTCAATGTAGACGACATCAATTTT
TCCCCTGTATTAGGTTGTCTAGGCAGCGAATGTAGTAAAGCTTCCAGTAGATCTGCTATAGAGGATTTACTTTTTGATA
AAGTAAAGTTATCTGATGTCGGTTTCGTTGAGGCTTATAATAATTGTACAGGAGGTGCCGAAATTAGGGACCTCATTTG
TGTGCAAAGTTATAAAGGCATCAAAGTGTTGCCCCCACTGCTCTCAGAAAATCAGATCAGTGGATATACTTTGGCTGCC
ACCTCTGCTAGTCTATTTCCTCCTTGGACAGCAGCAGCAGGTGTACCATTTTATTTAAATGTTCAGTATCGCATTAATG
GGCTTGGTGTCACCATGGATGTGCTAAGTCAAAATCAAAAGCTTATTGCTAATGCATTTAACAATGCCCTTCATGCTAT
TCAGCAAGGGTTCGATGCAACTAATTCTGCTTTAGTTAAAATTCAAGCTGTTGTTAATGCAAATGCTGAAGCTCTTAAT
AACTTATTGCAACAACTCTCTAATAGATTTGGTGCTATAAGTGCTTCTTTACAAGAAATTCTATCTAGACTTGATGCTC
TTGAAGCGGAAGCTCAGATAGATAGACTTATTAATGGTCGTCTCACCGCTCTTAATGCTTATGTTTCTCAACAGCTTAG
TGATTCTACACTGGTAAAATTTAGTGCAGCACAAGCTATGGAGAAGGTTAATGAATGTGTCAAAAGCCAATCATCTAGG
ATAAATTTCTGTGGTAATGGTAATCATATTATATCATTAGTGCAGAATGCTCCATATGGTTTGTATTTTATCCACTTTA
ATTATGTCCCTACTAAGTATGTCACAGCGAAGGTTAGTCCTGGTCTGTGCATTGCTGGTAATAGAGGTATAGCTCCTAA
GAGTGGTTATTTTGTTAATGTAAATAATACTTGGATGTACACTGGTAGTGGTTACTACTACCCTGAACCTATAACTGAA
AATAATGTTGTTGTTATGAGTACCTGCGCTGTTAATTATACTAAAGCGCCGTATGTAATGCTGAACACTTCAATACCCA
ACCTTCCTGATTTTAAGGAAGAGTTGGACCAATGGTTCAAAAATCAAACATCAGTGGCACCAGATTTGTCACTTGATTA
TATAAATGTTACATTCTTGGACCTACAAGTTGAAATGAATAGGTTACAGGAGGCAATAAAAGTCTTAAATCATAGCTAC
ATCAATCTCAAGGACATTGGTACATATGAATATTATGTAAAATGGCCTTGGTATGTATGGCTTTTAATCTGCCTTGCTG
GTGTAGCTATGCTTGTTTTACTATTCTTCATATGCTGTTGTACAGGATGTGGGACTAGTTGTTTTAAGAAATGTGGTGG

TTGTTGTGATGATTATACTGGATACCAGGAGTTAGTAATCAAAACTTCACATGACGACTAA

>gi|344332697|gb|JN129834.1| Human coronavirus OC43 strain HK04-01, complete genome

ATGTTTTTGATACTTTTAATTTCCTTACCAACGGCTTTTGCTGTTATAGGAGATTTAAATTGTCCTTTAGATACTAGGCTTAAAGGTAGCTTTAATAATAGAGACACCGGTCCTCCTTCTATAAGTACTGATACTGTTGATGTTACTAATGGTTTGGGTACTTATTATGTTTTAGATCGTGTGTATTTAAATACTACGTTGTTTCTTAATGGTTATTACCCCACTTCAGGTTCCACATATCGTAATATGGCACTGAAGGGAACTGACAAATTGAGCACATTATGGTTTAAACCACCATTTCTTTCTGATTTTATTAATGGTATTTTTGCTAAGGTCAAAAATACCAAGGTTTTTAAAGATGGTGTAATGTATAGTGAGTTCCCTGCTATAACTATAGGTAGTACTTTTGTAAATACATCCTATAGTGTGGTAGTACAACCACGTACAATTAATTCAACACAGGATGGTGTTAATAAATTACAAGGTCTTTTAGAGGTCTCTGTTTGCCAGTATAATATGTGTGAGTACCCACATACGATTTGTCATCCTAAATTGGGTAACCACTTCAAAGAACTATGGCATTTGGATACAGGTGTTGTTTCCTGTTTATATAAGCGTAATTTCACATATGATGTGAATGCTACTTATTTGTATTTTCATTTTTATCAAGAAGGTGGTACTTTTTATGCATATTTTACAGACACTGGTTTTGTTACTAAGTTTTTGTTTAATGTTTATTTAGGCATGGCACTTTCACACTATTATGTCATGCCTCTGACTTGTATTAGTAGGCTTGATATTGGTTTTACTTTAGAATATTGGGTTACACCCCTCACTTCTAGACAATATTTACTCGCTTTCAATCAAGATGGTATTATTTTTAATGCTGTTGATTGTATGAGTGATTTTATGAGTGAGATTAAGTGTAAAACACAATCTATAGCACCACCTACTGGTGTTTATGAATTAAACGGTTACACTGTTCAGCCAATCGCAGATGTTTACCGACGCAAACCTGATCTTCCCAATTGCAATATAGAAGCTTGGCTTAATGATAAGTCGGTGCCCTCTCCATTAAATTGGGAACGTAAGACATTTTCAAATTGTAATTTTAATATGAGCAGCCTGATGTCTTTTATTCAGGCAGACTCATTTACTTGTAATAATATTGATGCTGCTAAGATATATGGTATGTGTTTTTCCAGCATAACTATAGATAAGTTTGCTATACCCAATCGCAGGAAGGTTGACCTACAATTGGGCAATTTGGGCTATTTGCAGTCATCCAACTATAGAATTGATACTACTGCAACAAGTTGTCAGTTGTATTATAATTTACCTGCTGCTAATGTCTCTGTTAGCAGGTTTAATCCTTCTACTTGGAATAAGAGATTTGGTTTTATAGAAGATTCTGTTTTTGTGCCTCAACCTACAGGTGTTTTTACTAATCACAGTGTAGTTTATGCACAACACTGTTTCAAAGCCCCTAAAAATTTTTGTCCGTGTAAATCGAATGGTTCCTGTCCTGGTAAAAATAATGGTATAGGCACTTGTCCTGCAGGTACTAATTATTTAACTTGTGATAATTTATGTACTCTTGATCCTATTACATTTAAAGCTCCAGGTACTTATAAGTGCCCCCAAACTAAATCTTTAGTTGGTATAGGTGAGCACTGTTCGGGTCTTGCTGTTAAAAGTGATTATTGTGGAAACAATTCTTGTACTTGCCAACCACAAGCATTTTTGGGTTGGTCTGCAGACTCTTGTTTACAAGGAGACAAGTGTAATATTTTTGCTAATTTCATTTTGCATGATGTTAATAATGGTCTTACTTGTTCTACTGATTTACAAAAAGCTAACACAGAAATAGAACTTGGTGTTTGTGTTAATTATGACCTCTATGGTATTTCAGGCCAAGGCATTTTTGTTGAGGTTAATGCGACTTATTATAATAGTTGGCAGAACCTTTTATATGATTCTAATGGTAACCTCTACGGTTTTAGAGACTACATAACAAACAGAACTTTTATGATTCGTAGTTGCTATAGCGGTCGTGTTTCTGCGGCCTATCATGCCAACTCTTCCGAACCAGCATTGCTATTTCGGAATATTAAATGCAACTACGTTTTTAATAATAGTCTTACACGACAGCTGCAACCCATTAACTATTCGTTTGATAGTTATCTTGGTTGTGTTGTCAATGCTTATAATAGTACTGCTATTTCTGTTCAAAAATGTGATCTCACAGTAGGTAGTGGTTACTGTGTGGATTACTCTAAAAACAGACGAAGTCGTAGAGCGATCACCACTGGTTATCGGTTTACTAATTTTGAGCCATTTACTGTTAATTCAGTAAATGATAGTTTGGAACCTGTAGGTGGTTTGTATGAAATTCAAATACCTTCAGAGTTTACTATAGGTAATATGGAGGAGTTTATTCAAACAAGCTCTCCCAAAGTTACTATTGATTGTGCTGCATTTGTCTGTGGTGATTATGCAGCATGTAAATTACAGTTGGTTGAATATGGTAGTTTCTGTGATAACATTAATGCCATACTCACAGAAGTAAATGAACTACTTGATACTACACAGTTGCAAGTAGCTAATAGTTTAATGAATGGTGTTACTCTTAGCACTAAGCTTAAAGATGGTGTTAATTTCAATGTAGACGACATCAATTTTTCCCCTGTATTAGGTTGTCTAGGCAGCGAATGTAGTAAAGCTTCCAGTAGATCTGCTATAGAGGATTTACTTTTTGATAAAGTAAAGTTATCTGATGTCGGTTTCGTTGAGGCTTATAATAATTGTACAGGAGGTGCCGAAATTAGGGACCTCATTTGTGTGCAAAGTTATAAAGGCATCAAAGTGTTGCCCCCACTGCTCTCAGAAAATCAGATCAGTGGATATACTTTGGCTGCCACCTCTGCTAGTCTATTTCCTCCTTGGACAGCAGCAGCAGGTGTACCATTTTATTTAAATGTTCAGTATCGCATTAATG

GGCTTGGTGTCACCATGGATGTGCTAAGTCAAAATCAAAAGCTTATTGCTAATGCATTTAACAATGCCCTTCATGCTAT
TCAGCAAGGGTTCGATGCAACTAATTCTGCTTTAGTTAAAATTCAAGCTGTTGTTAATGCAAATGCTGAAGCTCTTAAT
AACTTATTGCAACAACTCTCTAATAGATTTGGTGCTATAAGTGCTTCTTTACAAGAAATTCTATCTAGACTTGATGCTC
TTGAAGCGGAAGCTCAGATAGATAGACTTATTAATGGTCGTCTCACCGCTCTTAATGCTTATGTTTCTCAACAGCTTAG
TGATTCTACACTGGTAAAATTTAGTGCAGCACAAGCTATGGAGAAGGTTAATGAATGTGTCAAAAGCCAATCATCTAGG
ATAAATTTCTGTGGTAATGGTAATCATATTATATCATTAGTGCAGAATGCTCCATATGGTTTGTATTTTATCCACTTTA
ATTATGTCCCTACTAAGTATGTCACAGCGAAGGTTAGTCCTGGTCTGTGCATTGCTGGTAATAGAGGTATAGCTCCTAA
GAGTGGTTATTTTGTTAATGTAAATAATACTTGGATGTACACTGGTAGTGGTTACTACTACCCTGAACCTATAACTGAA
AATAATGTTGTTGTTATGAGTACCTGCGCTGTTAATTATACTAAAGCGCCGTATGTAATGCTGAACACTTCAATACCCA
ACCTTCCTGATTTTAAGGAAGAGTTGGACCAATGGTTCAAAAATCAAACATCAGTGGCACCAGATTTGTCACTTGATTA
TATAAATGTTACATTCTTGGACCTACAAGTTGAAATGAATAGGTTACAGGAGGCAATAAAAGTCTTAAATCATAGCTAC
ATCAATCTCAAGGACATTGGTACATATGAATATTATGTAAAATGGCCTTGGTATGTATGGCTTTTAATCTGCCTTGCTG
GTGTAGCTATGCTTGTTTTACTATTCTTCATATGCTGTTGTACAGGATGTGGGACTAGTTGTTTTAAGAAATGTGGTGG
TTGTTGTGATGATTATACTGGATACCAGGAGTTAGTAATCAAAACTTCACATGACGACTAA

>gi|344332706|gb|JN129835.1| Human coronavirus OC43 strain HK04-02, complete genome

ATGTTTTTGATACTTTTAATTTCCTTACCAACGGCTTTTGCTGTTATAGGAGATTTAAATTGTCCTTTAGATACT
AGGCTTAAAGGTAGCTTTAATAATAGAGACACCGGTCCTCCTTCTATAAGTACTGATACTGTTGATGTTACTAATGGTT
TGGGTACTTATTATGTTTTAGATCGTGTGTATTTAAACACTACGTTGTTTCTTAATGGTTATTACCCCACTTCAGGTTC
CACATATCGTAATATGGCACTGAAGGGAACTGACAAATTGAGCACATTATGGTTTAAACCACCATTTCTTTCTGATTTT
ATTAATGGTATTTTTGCTAAGGTCAAAAATACCAAGGTTTTTAAAGATGGTGTAATGTATAGTGAGTTCCCTGCTATAA
CTATAGGTAGTACTTTTGTAAATACATCCTATAGTGTGGTAGTACAACCACGTACAATTAATTTAACACAGGATGGTGT
TAATAAATTACAAGGTCTTTTAGAGGTCTCTGTTTGCCAGTATAATATGTGTGAGTACCCACATACGATTTGTCATCCT
AAATTGGGTAACCACTTCAAAGAACTATGGCATTTGGATACAGGTGTTGTTTCCTGTTTATATAAGCGTAATTTCACAT
ATGATGTGAATGCTACTTATTTGTATTTTCATTTTTATCAAGAAGGTGGTACTTTTTATGCATATTTTACAGACACTGG
TTTTGTTACTAAGTTTTTGTTTAATGTTTATTTAGGCATGGCACTTTCACACTATTATGTCATGCCTCTGACTTGTATT
AGTAGGCTTGATATTGGTTTTACTTTAGAATATTGGGTTACACCCCTCACTTCTAGACAATATTTACTCGCTTTCAATC
AAGATGGTATTATTTTTAATGCTGTTGATTGTATGAGTGATTTTATGAGTGAGATTAAGTGTAAAACACAATCTATAGC
ACCACCTACTGGTGTTTATGAATTAAACGGTTACACTGTTCAGCCAATCGCAGATGTTTACCGACGCAAACCTGATCTT
CCCAATTGCAATATAGAAGCTTGGCTTAATGATAAGTCGGTGCCCTCTCCATTAAATTGGGAACGTAAGACATTTTCAA
ATTGTAATTTTAATATGAGCAGCCTGATGTCTTTTATTCAGGCAGACTCATTTACTTGTAATAATATTGATGCTGCTAA
GATATATGGTATGTGTTTTTCCAGCATAACTATAGATAAGTTTGCTATACCCAATCGCAGGAAGGTTGACCTACAATTG
GGCAATTTGGGCTATTTGCAGTCATCCAACTATAGAATTGATACTACTGCAACAAGTTGTCAGTTGTATTATAATTTAC
CTGCTGCTAATGTCTCTGTTAGCAGGTTTAATCCTTCTACTTGGAATAAGAGATTTGGTTTTATAGAAGATTCTGTTTT
TGTGCCTCAACCTACAGGTGTTTTTACTAATCACAGTGTAGTTTATGCACAACACTGTTTCAAAGCCCCTAAAAATTTC
TGTCCGTGTAAATTGAATGGTTCCTGTCCTGGTAAAAATAATGGTATAGGCACTTGTCCTGCAGGTACTAATTATTTAA
CTTGTGATAATTTATGTACGCTTGATCCTATTACATTTAAAGCTCCAGGTACTTATAAGTGCCCCCAAACTAAATCTTT
AGTTGGTATAGGTGAGCACTGTTCGGGTCTTGCTGTTAAAAGTGATTATTGTGGAAACAATTCTTGTACTTGCCAACCA
CAAGCATTTTTGGGTTGGTCTGCAGACTCTTGTTTACAAGGAGACAAGTGTAATATTTTTGCTAATTTCATTTTGCATG
ATGTTAATAATGGTCTTACTTGTTCTACTGATTTACAAAAAGCTAACACAGAAATAGAACTTGGTGTTTGTGTTAATTA
TGACCTCTATGGTATTTCAGGCCAAGGCATTTTTGTTGAGGTTAATGCGACTTATTATAATAGTTGGCAGAACCTTTTA

TATGATTCTAATGGTAACCTCTACGGTTTTAGAGACTACATAACAAACAGAACTTTTATGATTCGTAGTTGCTATAGCG
GTCGTGTTTCTGCGGCCTATCATGCCAACTCTTCCGAACCAGCATTGCTATTTCGGAATATTAAATGCAACTACGTTTT
TAATAATAGTCTTACACGACAGCTGCAACCCATTAACTATTCGTTTGATAGTTATCTTGGTTGTGTTGTCAATGCTTAT
AATAGTACTGCTATTTCTGTTCAAACATGTGATCTCACAGTAGGTAGTGGTTACTGTGTGGATTACTCTAAAAACAGAC
GAAGTCGTAGAGCGATCACCACTGGTTATCGGTTTACTAATTTTGAGCCATTTACTGTTAATTCAGTAAATGATAGTTT
GGAACCTGTAGGTGGTTTGTATGAAATTCAAATACCTTCAGAGTTTACTATAGGTAATATGGAGGAGTTTATTCAAACA
AGCTCTCCCAAAGTTACTATTGATTGTGCTGCATTTGTCTGTGGTGATTATGCAGCATGTAAATTACAGTTGGTTGAAT
ATGGTAGTTTCTGTGATAACATTAATGCCATACTCACAGAAGTAAATGAACTACTTGATACTACACAGTTGCAAGTAGC
TAATAGTTTAATGAATGGTGTTACTCTTAGCACTAAGCTTAAAGATGGTGTTAATTTCAATGTAGACGACATCAATTTT
TCCCCTGTATTAGGTTGTCTAGGCAGCGAATGTAGTAAAGCTTCCAGTAGATCTGCTATAGAGGATTTACTTTTTGATA
AAGTAAAGTTATCTGATGTCGGTTTCGTTGAGGCTTATAATAATTGTACAGGAGGTGCCGAAATTAGGGACCTCATTTG
TGTGCAAAGTTATAAAGGCATCAAAGTGTTGCCCCCACTGCTCTCAGAAAATCAGATCAGTGGATATACTTTGGCTGCC
ACCTCTGCTAGTCTATTTCCTCCTTGGACAGCAGCAGCAGGTGTACCATTTTATTTAAATGTTCAGTATCGCATTAATG
GGCTTGGTGTCTCCATGGATGTGCTAAGTCAAAATCAAAAGCTTATTGCTAATGCATTTAACAATGCCCTTCATGCTAT
TCAGCAAGGGTTCGATGCAACTAATTCTGCTTTAGTTAAAATTCAAGCTGTTGTTAATGCAAATGCTGAAGCTCTTAAT
AACTTATTGCAACAACTCTCTAATAGATTTGGTGCTATAAGTGCTTCTTTACAAGAAATTCTATCTAGACTTGATGCTC
TTGAAGCGGAAGCTCAGATAGATAGACTTATTAATGGTCGTCTCACCGCTCTTAATGCTTATGTTTCTCAACAGCTTAG
TGATTCTACACTGGTAAAATTTAGTGCAGCACAAGCTATGGAGAAGGTTAATGAATGTGTCAAAAGCCAATCATCTAGG
ATAAATTTCTGTGGTAATGGTAATCATATTATATCATTAGTGCAGAATGCTCCATATGGTTTGTATTTTATCCACTTTA
ATTATGTCCCTACTAAGTATGTCACAGCGAAGGTTAGTCCTGGTCTGTGCATTGCTGGTAATAGAGGTATAGCTCCTAA
GAGTGGTTATTTTGTTAATGTAAATAATACTTGGATGTACACTGGTAGTGGTTACTACTACCCTGAACCTATAACTGAA
AATAATGTTGTTGTTATGAGTACCTGCGCTGTTAATTATACTAAAGCGCCGTATGTAATGCTGAACACCTCAATACCCA
ACCTTCCTGATTTTAAGGAAGAGTTGGACCAATGGTTCAAAAATCAAACATCAGTGGCACCAGATTTGTCACTTGATTA
TATAAATGTCACATTCTTGGACCTACAAGTTGAAATGAATAGGTTACAGGAGGCAATAAAAGTCTTAAATCATAGCTAC
ATCAATCTCAAGGACATTGGTACATATGAATATTATGTAAAATGGCCTTGGTATGTATGGCTTTTAATCTGCCTTGCTG
GTGTAGCTATGCTTGTTTTACTATTCTTCATATGCTGTTGTACAGGATGTGGGACTAGTTGTTTTAAGAAATGTGGTGG
TTGTTGTGATGATTATACTGGATACCAGGAGTTAGTAATCAAAACTTCACATGACGACTAA

>gi|38018022|ref|NC_005147.1| Human coronavirus OC43, complete genome

ATGTTTTTGATACTTTTAATTTCCTTACCAACGGCTTTTGCTGTTATAGGAGATTTAAAGTGTACTTCAGATACT
AGTTATATTAATGATAAAGACACCGGTCCTCCTCCTATAAGTACTGATACTGTTGATGTTACTAATGGTTTGGGTACTT
ATTATGTTTTAGATCGTGTGTATTTAAATACTACGTTGTTTCTTAATGGTTATTACCCTACTTCAGGTTCCACATATCG
TAATATGGCACTGAAGGGAAGTGTACTATTGAGCAGACTATGGTTTAAACCACCATTTCTTTCTGATTTTATTAATGGT
ATTTTTGCTAAGGTCAAAAATACCAAGGTTATTAAAGATCGTGTAATGTATAGTGAGTTCCCTGCTATAACTATAGGTA
GTACTTTTGTAAATACATCCTATAGTGTGGTAGTACAACCACGTACAATCAATTCAACACAGGATGGTTATAATAAATT
ACAAGGTCTTTTAGAGGTCTCTGTTTGCCAGTATAATATGTGCGAGTACCCACAAACGATTTGTCATCCTAACCTGGGT
AATCATCGCAAAGAACTATGGCATTTGGATACAGGTGTTGTTTCCTGTTTATATAAGCGTAATTTCACATATGATGTGA
ATGCTGATTATTTGTATTTTCATTTTTATCAAGAAGGTGGTACTTTTTATGCATATTTTACAGACACTGGTGTTGTTAC
TAAGTTTTTGTTTAATGTTTATTTAGGCATGGCGCTTTCACACTATTATGTCATGCCTCTGACTTGTAATAGTAAGGTT
AAGAATGGTTTTACTTTAGAATATTGGGTTACACCTCTCACTTCTAGACAATATTTACTCGCTTTCAATCAAGATGGTA
TTATTTTTAATGCTGTTGATTGTATGAGTGATTTTATGAGTGAGATTAAGTGTAAAACACAATCTATAGCACCACCTAC
TGGTGTTTATGAATTAAACGGTTACACTGTTCAGCCAATCGCAGATGTTTACCGACGTAAACTTAATCTTCCCAATTGC

AATATAGAAGCTTGGCTTAATGATAAGTCGGTGCCCTCTCCATTAAATTGGGAACGTAAGACATTTTCAAATTGTAATT
TTAATATGAGCAGCCTGATGTCTTTTATTCAGGCAGACTCATTTACTTGTAATAATATTGATGCTGCTAAGATATATGG
TATGTGTTTTTCCAGCATAACTATAGATAAGTTTGCTATACCCAATGGCAGGAAGGTTGACCTACAATTGGGTAATTTG
GGCTATTTGCAGTCATTTAACTATAGAATTGATACTACTGCAACAAGTTGTCAGTTGTATTATAATTTACCTGCTGCTA
ATGTTTCTGTTAGCAGGTTTAATCCTTCTACTTGGAATAAGAGATTTGGTTTTATAGAAGATTCTGTTTTTAAGCCTCG
ACCTGCAGGTGTTCTTACTAATCATGATGTAGTTTATGCACAACACTGTTTCAAAGCTCCTAAAAATTTCTGTCCGTGT
AAATTGAATGGTTCGTGTGTAGGTAGTGGTCCTGGTAAAAATAATGGTATAGGCACTTGTCCTGCAGGTACTAATTATT
TAACTTGTGATAATTTGTGCACTCCTGATCCTATTACATTTAAAGCTACAGGTACTTATAAGTGCCCCCAAACTAAATC
TTTAGTTGGCATAGGTGAGCACTGTTCGGGTCTTGCTGTTAAAAGTGATTATTGTGGAGGCAATTCTTGTACTTGCCGA
CCACAAGCATTTTTGGGTTGGTCTGCAGACTCTTGTTTACAAGGAGACAAGTGTAATATTTTTGCTAATTTTATTTTGC
ATGATGTTAATAGTGGTCTTACTTGTTCTACTGATTTACAAAAAGCTAACACAGACATAATTCTTGGTGTTTGTGTTAA
TTATGACCTCTATGGTATTTTAGGCCAAGGCATTTTTGTTGAGGTTAATGCGACTTATTATAATAGTTGGCAGAACCTT
TTATATGATTCTAATGGTAATCTCTACGGTTTTAGAGACTACATAACAAACAGAACTTTTATGATTCGTAGTTGCTATA
GCGGTCGTGTTTCTGCGGCCTTTCACGCTAACTCTTCCGAACCAGCATTGCTATTTCGGAATATTAAATGCAACTACGT
TTTTAATAATAGTCTTACACGACAGCTGCAACCCATTAACTATTTTGATAGTTATCTTGGTTGTGTTGTCAATGCTTAT
AATAGTACTGCTATTTCTGTTCAAACATGTGATCTCACAGTAGGTAGTGGTTACTGTGTGGATTACTCTAAAAACAGAC
GAAGTCGTGGAGCGATTACCACTGGTTATCGGTTTACTAATTTTGAGCCATTTACTGTTAATTCAGTAAACGATAGTTT
AGAACCTGTAGGTGGTTTGTATGAAATTCAAATACCTTCAGAGTTTACTATAGGTAATATGGAGGAGTTTATTCAAACA
AGCTCTCCTAAAGTTACTATTGATTGTGCTGCATTTGTCTGTGGTGATTATGCAGCATGTAAATCACAGTTGGTTGAAT
ATGGTAGTTTCTGTGATAACATTAATGCCATACTCACAGAAGTAAATGAACTACTTGACACTACACAGTTGCAAGTAGC
TAATAGTTTAATGAATGGTGTTACTCTTAGCACTAAGCTTAAAGATGGCGTTAATTTCAATGTAGACGACATCAATTTT
TCCCCTGTATTAGGTTGTCTAGGCAGCGAATGTAGTAAAGCTTCCAGTAGATCTGCTATAGAGGATTTACTTTTTGATA
AAGTAAAGTTATCTGATGTCGGTTTTGTTGAGGCTTATAATAATTGTACAGGAGGTGCCGAAATTAGGGACCTCATTTG
TGTGCAAAGTTATAAAGGCATCAAAGTGTTGCCTCCACTGCTCTCAGAAAATCAGATCAGTGGATACACTTTGGCTGCC
ACCTCTGCTAGTCTATTTCCTCTTTGGACAGCAGCAGCAGGTGTACCATTTTATTTAAATGTTCAGTATCGCATTAATG
GGCTTGGTGTCACCATGGATGTGCTAAGTCAAAATCAAAAGCTTATTGCTAATGCATTTAACAATGCCCTTTATGCTAT
TCAGGAAGGGTTCGATGCAACTAATTCTGCTTTAGTTAAAATTCAAGCTGTTGTTAATGCAAATGCTGAAGCTCTTAAT
AACTTATTGCAACAACTCTCTAATAGATTTGGTGCTATAAGTGCTTCTTTACAAGAAATTCTATCTAGACTTGATGCTC
TTGAAGCGGAAGCTCAGATAGATAGACTTATTAATGGTCGTCTTACCGCTCTTAATGCTTATGTTTCTCAACAGCTTAG
TGATTCTACACTGGTAAAATTTAGTGCAGCACAAGCTATGGAGAAGGTTAATGAATGTGTCAAAAGCCAATCATCTAGG
ATAAATTTCTGTGGTAATGGTAATCATATTATATCATTAGTGCAGAATGCTCCATATGGTTTGTATTTTATCCACTTTA
GTTATGTCCCTACTAAGTATGTCACAGCGAGGGTTAGTCCTGGTCTGTGCATTGCTGGTGATAGAGGTATAGCTCCTAA
GAGTGGTTATTTTGTTAATGTAAATAATACTTGGATGTACACTGGTAGTGGTTACTACTACCCTGAACCTATAACTGAA
AATAATGTTGTTGTTATGAGTACCTGCGCTGTTAATTATACTAAAGCGCCGTATGTAATGCTGAACACTTCAATACCCA
ACCTTCCTGATTTTAAGGAAGAGTTGGATCAATGGTTTAAAAATCAAACATCAGTGGCACCAGATTTGTCACTTGATTA
TATAAATGTTACATTCTTGGACCTACAAGTTGAAATGAATAGGTTACAGGAGGCAATAAAAGTCTTAAATCAGAGCTAC
ATCAATCTCAAGGACATTGGTACATATGAATATTATGTAAAATGGCCTTGGTATGTATGGCTTTTAATCTGCCTTGCTG
GTGTAGCTATGCTTGTTTTACTATTCTTCATATGCTGTTGTACAGGATGTGGGACTAGTTGTTTTAAGAAATGTGGTGG
TTGTTGTGATGATTATACTGGATACCAGGAGTTAGTAATCAAAACTTCACATGACGACTAA

>gi|50844468|gb|AY585228.1| Human coronavirus OC43 strain ATCC VR-759, complete genome

ATGTTTTTGATACTTTTAATTTCCTTACCAACGGCTTTTGCTGTTATAGGAGATTTAAAGTGTACTTCAGATAATATTAATGATAAAGACACCGGTCCTCCTCCTATAAGTACTGATACTGTTGATGTTACTAATGGTTTGGGTACTTATTATGTTTTAGATCGTGTGTATTTAAATACTACGTTGTTTCTTAATGGTTATTACCCTACTTCAGGTTCCACATATCGTAATATGGCACTGAAGGGAAGTGTACTATTGAGCAGACTATGGTTTAAACCACCATTTCTTTCTGATTTTATTAATGGTATTTTTGCTAAGGTCAAAAATACCAAGGTTATTAAAGATCGTGTAATGTATAGTGAGTTCCCTGCTATAACTATAGGTAGTACTTTTGTAAATACATCCTATAGTGTGGTAGTACAACCACGTACAATCAATTCAACACAGGATGGTGATAATAAATTACAAGGTCTTTTAGAGGTCTCTGTTTGCCAGTATAATATGTGCGAGTACCCACAAACGATTTGTCATCCTAACCTGGGTAATCATCGCAAAGAACTATGGCATTTGGATACAGGTGTTGTTTCCTGTTTATATAAGCGTAATTTCACATATGATGTGAATGCTGATTATTTGTATTTTCATTTTTATCAAGAAGGTGGTACTTTTTATGCATATTTTACAGACACTGGTGTTGTTACTAAGTTTTTGTTTAATGTTTATTTAGGCATGGCGCTTTCACACTATTATGTCATGCCTCTGACTTGTAATAGTAAGCTTACTTTAGAATATTGGGTTACACCTCTCACTTCTAGACAATATTTACTCGCTTTCAATCAAGATGGTATTATTTTTAATGCTGTTGATTGTATGAGTGATTTTATGAGTGAGATTAAGTGTAAAACACAATCTATAGCACCACCTACTGGTGTTTATGAATTAAACGGTTACACTGTTCAGCCAATCGCAGATGTTTACCGACGTAAACCTAATCTTCCCAATTGCAATATAGAAGCTTGGCTTAATGATAAGTCGGTGCCCTCTCCATTAAATTGGGAACGTAAGACATTTTCAAATTGTAATTTTAATATGAGCAGCCTGATGTCTTTTATTCAGGCAGACTCATTTACTTGTAATAATATTGATGCTGCTAAGATATATGGTATGTGTTTTTCCAGCATAACTATAGATAAGTTTGCTATACCCAATGGCAGGAAGGTTGACCTACAATTGGGTAATTTGGGCTATTTGCAGTCATTTAACTATAGAATTGATACTACTGCAACAAGTTGTCAGTTGTATTATAATTTACCTGCTGCTAATGTTTCTGTTAGCAGGTTTAATCCTTCTACTTGGAATAAGAGATTTGGTTTTATAGAAGATTCTGTTTTTAAGCCTCGACCTGCAGGTGTTCTTACTAATCATGATGTAGTTTATGCACAACACTGTTTCAAAGCTCCTAAAAATTTCTGTCCGTGTAAATTGAATGGTTCGTGTGTAGGTAGTGGTCCTGGTAAAAATAATGGTATAGGCACTTGTCCTGCAGGTACTAATTATTTAACTTGTGATAATTTGTGCACTCCTGATCCTATTACATTTACAGGTACTTATAAGTGCCCCCAAACTAAATCTTTAGTTGGCATAGGTGAGCACTGTTCGGGTCTTGCTGTTAAAAGTGATTATTGTGGAGGCAATTCTTGTACTTGCCGACCACAAGCATTTTTGGGTTGGTCTGCAGACTCTTGTTTACAAGGAGACAAGTGTAATATTTTTGCTAATTTTATTTTGCATGATGTTAATAGTGGTCTTACTTGTTCTACTGATTTACAAAAAGCTAACACAGACATAATTCTTGGTGTTTGTGTTAATTATGACCTCTATGGTATTTTAGGCCAAGGCATTTTTGTTGAGGTTAATGCGACTTATTATAATAGTTGGCAGAACCTTTTATATGATTCTAATGGTAATCTCTACGGTTTTAGAGACTACATAACAAACAGAACTTTTATGATTCGTAGTTGCTATAGCGGTCGTGTTTCTGCGGCCTTTCACGCTAACTCTTCCGAACCAGCATTGCTATTTCGGAATATTAAATGCAACTACGTTTTTAATAATAGTCTTACACGACAGCTGCAACCCATTAACTATTTTGATAGTTATCTTGGTTGTGTTGTCAATGCTTATAATAGTACTGCTATTTCTGTTCAAACATGTGATCTCACAGTAGGTAGTGGTTACTGTGTGGATTACTCTAAAAACAGACGAAGTCGTGGAGCGATTACCACTGGTTATCGGTTTACTAATTTTGAGCCATTTACTGTTAATTCAGTAAACGATAGTTTAGAACCTGTAGGTGGTTTGTATGAAATTCAAATACCTTCAGAGTTTACTATAGGTAATATGGTGGAGTTTATTCAAACAAGCTCTCCTAAAGTTACTATTGATTGTGCTGCATTTGTCTGTGGTGATTATGCAGCATGTAAATCACAGTTGGTTGAATATGGTAGTTTCTGTGATAACATTAATGCCATACTCACAGAAGTAAATGAACTACTTGACACTACACAGTTGCAAGTAGCTAATAGTTTAATGAATGGTGTTACTCTTAGCACTAAGCTTAAAGATGGCGTTAATTTCAATGTAGACGACATCAATTTTTCCCCTGTATTAGGTTGTCTAGGCAGCGAATGTAGTAAAGCTTCCAGTAGATCTGCTATAGAGGATTTACTTTTTGATAAAGTAAAGTTATCTGATGTCGGTTTTGTTGAGGCTTATAATAATTGTACAGGAGGTGCCGAAATTAGGGACCTCATTTGTGTGCAAAGTTATAAAGGCATCAAAGTGTTGCCTCCACTGCTCTCAGAAAATCAGATCAGTGGATACACTTTGGCTGCCACCTCTGCTAGTCTATTTCCTCCTTGGACAGCAGCAGCAGGTGTACCATTTTATTTAAATGTTCAGTATCGCATTAATGGGCTTGGTGTCACCATGGATGTGCTAAGTCAAAATCAAAAGCTTATTGCTAATGCATTTAACAATGCCCTTTATGCTATTCAGGAAGGGTTCGATGCAACTAATTCTGCTTTAGTTAAAATTCAAGCTGTTGTTAATGCAAATGCTGAAGCTCTTAATAACTTATTGCAACAACTCTCTAATAGATTTGGTGCTATAAGTGCTTCTTTACAAGAAATTCTATCTAGACTTGATGCTCTTGAAGCGGAAGCTCAGATAGATAGACTTATTAATGGTCGTCTTACCGCTCTTAATGCTTATGTTTCTCAACAGCTTAGTGATTCTACACTGGTAAAATTTAGTGCAGCACAAGCTATGGAGAAGGTTAATGAATGTGTCAAAAGCCAATCATCTAGGATAAATTTCTGTGGTAATGGTAATCATATTATATCATTAGTGCAGAATGCTCCATATGGTTTGTATTTTATCCACTTTAGTTATGTCCCTACTAAGTATGTCA

CAGCGAGGGTTAGTCCTGGTCTGTGCATTGCTGGTGATAGAGGTATAGCTCCTAAGAGTGGTTATTTTGTTAATGTAAA
TAATACTTGGATGTACACTGGTAGTGGTTACTACTACCCTGAACCTATAACTGAAAATAATGTTGTTGTTATGAGTACC
TGCGCTGTTAATTATACTAAAGCGCCGTATGTAATGCTGAACACTTCAATACCCAACCTTCCTGATTTTAAGGAAGAGT
TGGATCAATGGTTTAAAAATCAAACATCAGTGGCACCAGATTTGTCACTTGATTATATAAATGTTACATTCTTGGACCT
ACAAGTTGAAATGAATAGGTTACAGGAGGCAATAAAAGTCTTAAATCAGAGCTACATCAATCTCAAGGACATTGGTACA
TATGAATATTATGTAAAATGGCCTTGGTATGTATGGCTTTTAATCTGCCTTGCTGGTGTAGCTATGCTTGTTTTACTAT
TCTTCATATGCTGTTGTACAGGATGTGGGACTAGTTGTTTTAAGAAATGTGGTGGTTGTTGTGATGATTATACTGGATA
CCAGGAGTTAGTAATCAAAACTTCACATGACGACTAA

>gi|50844477|gb|AY585229.1| Human coronavirus OC43 serotype OC43-Paris, complete genome

ATGTTTTTGATACTTTTAATTTCCTTACCAACGGCTTTTGCTGTTATAGGAGATTTAAAGTGTACTTCAGATAAT
ATTAATGATAAAGACACCGGTCCTCCTCCTATAAGTACTGATACTGTTGATGTTACTAATGGTTTGGGTACTTATTATG
TTTTAGATCGTGTGTATTTAAATACTACGTTGTTTCTTAATGGTTATTACCCTACTTCAGGTTCCACATATCGTAATAT
GGCACTGAAGGGAAGTGTACTATTGAGCAGACTATGGTTTAAACCACCATTTCTTTCTGATTTTATTAATGGTATTTTT
GCTAAGGTCAAAAATACCAAGGTTATTAAAGATCGTGTAATGTATAGTGAGTTCCCTGCTATAACTATAGGTAGTACTT
TTGTAAATACATCCTATAGTGTGGTAGTACAACCACGTACAATCAATTCAACACAGGATGGTGATAATAAATTACAAGG
TCTTTTAGAGGTCTCTGTTTGCCAGTATAATATGTGCGAGTACCCACAAACGATTTGTCATCCTAACCTGGGTAATCAT
CGCAAAGAACTATGGCATTTGGATACAGGTGTTGTTTCCTGTTTATATAAGCGTAATTTCACATATGATGTGAATGCTG
ATTATTTGTATTTTCATTTTTATCAAGAAGGTGGTACTTTTTATGCATATTTTACAGACACTGGTGTTGTTACTAAGTT
TTTGTTTAATGTTTATTTAGGCATGGCGCTTTCACACTATTATGTCATGCCTCTGACTTGTAATAGTAAGCTTACTTTA
GAATATTGGGTTACACCTCTCACTTCTAGACAATATTTACTCGCTTTCAATCAAGATGGTATTATTTTTAATGCTGTTG
ATTGTATGAGTGATTTTATGAGTGAGATTAAGTGTAAAACACAATCTATAGCACCACCTACTGGTGTTTATGAATTAAA
CGGTTACACTGTTCAGCCAATCGCAGATGTTTACCGACGTAAACCTAATCTTCCCAATTGCAATATAGAAGCTTGGCTT
AATGATAAGTCGGTGCCCTCTCCATTAAATTGGGAACGTAAGACATTTTCAAATTGTAATTTTAATATGAGCAGCCTGA
TGTCTTTTATTCAGGCAGACTCATTTACTTGTAATAATATTGATGCTGCTAAGATATATGGTATGTGTTTTTCCAGCAT
AACTATAGATAAGTTTGCTATACCCAATGGCAGGAAGGTTGACCTACAATTGGGTAATTTGGGCTATTTGCAGTCATTT
AACTATAGAATTGATACTACTGCAACAAGTTGTCAGTTGTATTATAATTTACCTGCTGCTAATGTTTCTGTTAGCAGGT
TTAATCCTTCTACTTGGAATAAGAGATTTGGTTTTATAGAAGATTCTGTTTTTAAGCCTCGACCTGCAGGTGTTCTTAC
TAATCATGATGTAGTTTATGCACAACACTGTTTCAAAGCTCCTAAAAATTTCTGTCCGTGTAAATTGAATGGTTCGTGT
GTAGGTAGTGGTCCTGGTAAAAATAATGGTATAGGCACTTGTCCTGCAGGTACTAATTATTTAACTTGTGATAATTTGT
GCACTCCTGATCCTATTACATTTACAGGTACTTATAAGTGCCCCCAAACTAAATCTTTAGTTGGCATAGGTGAGCACTG
TTCGGGTCTTGCTGTTAAAAGTGATTATTGTGGAGGCAATTCTTGTACTTGCCGACCACAAGCATTTTTGGGTTGGTCT
GCAGACTCTTGTTTACAAGGAGACAAGTGTAATATTTTTGCTAATTTTATTTTGCATGATGTTAATAGTGGTCTTACTT
GTTCTACTGATTTACAAAAAGCTAACACAGACATAATTCTTGGTGTTTGTGTTAATTATGACCTCTATGGTATTTTAGG
CCAAGGCATTTTTGTTGAGGTTAATGCGACTTATTATAATAGTTGGCAGAACCTTTTATATGATTCTAATGGTAATCTC
TACGGTTTTAGAGACTACATAACAAACAGAACTTTTATGATTCGTAGTTGCTATAGCGGTCGTGTTTCTGCGGCCTTTC
ACGCTAACTCTTCCGAACCAGCATTGCTATTTCGGAATATTAAATGCAACTACGTTTTTAATAATAGTCTTACACGACA
GCTGCAACCCATTAACTATTTTGATAGTTATCTTGGTTGTGTTGTCAATGCTTATAATAGTACTGCTATTTCTGTTCAA
ACATGTGATCTCACAGTAGGTAGTGGTTACTGTGTGGATTACTCTAAAAACAGACGAAGTCGTGGAGCGATTACCACTG
GTTATCGGTTTACTAATTTTGAGCCATTTACTGTTAATTCAGTAAACGATAGTTTAGAACCTGTAGGTGGTTTGTATGA
AATTCAAATACCTTCAGAGTTTACTATAGGTAATATGGTGGAGTTTATTCAAACAAGCTCTCCTAAAGTTACTATTGAT

TGTGCTGCATTTGTCTGTGGTGATTATGCAGCATGTAAATCACAGTTGGTTGAATATGGTAGTTTCTGTGATAACATTA
ATGCCATACTCACAGAAGTAAATGAACTACTTGACACTACACAGTTGCAAGTAGCTAATAGTTTAATGAATGGTGTTAC
TCTTAGCACTAAGCTTAAAGATGGCGTTAATTTCAATGTAGACGACATCAATTTTTCCCCTGTATTAGGTTGTCTAGGC
AGCGAATGTAGTAAAGCTTCCAGTAGATCTGCTATAGAGGATTTACTTTTTGATAAAGTAAAGTTATCTGATGTCGGTT
TTGTTGAGGCTTATAATAATTGTACAGGAGGTGCCGAAATTAGGGACCTCATTTGTGTGCAAAGTTATAAAGGCATCAA
AGTGTTGCCTCCACTGCTCTCAGAAAATCAGTTCAGTGGATACACTTTGGCTGCCACCTCTGCTAGTCTATTTCCTCCT
TGGACAGCAGCAGCAGGTGTACCATTTTATTTAAATGTTCAGTATCGCATTAATGGGCTTGGTGTCACCATGGATGTGC
TAAGTCAAAATCAAAAGCTTATTGCTAATGCATTTAACAATGCCCTTTATGCTATTCAGGAAGGGTTCGATGCAACTAA
TTCTGCTTTAGTTAAAATTCAAGCTGTTGTTAATGCAAATGCTGAAGCTCTTAATAACTTATTGCAACAACTCTCTAAT
AGATTTGGTGCTATAAGTGCTTCTTTACAAGAAATTCTATCTAGACTTGATGCTCTTGAAGCGGAAGCTCAGATAGATA
GACTTATTAATGGTCGTCTTACCGCTCTTAATGCTTATGTTTCTCAACAGCTTAGTGATTCTACACTGGTAAAATTTAG
TGCAGCACAAGCTATGGAGAAGGTTAATGAATGTGTCAAAAGCCAATCATCTAGGATAAATTTCTGTGGTAATGGTAAT
CATATTATATCATTAGTGCAGAATGCTCCATATGGTTTGTATTTTATCCACTTTAGTTATGTCCCTACTAAGTATGTCA
CAGCGAGGGTTAGTCCTGGTCTGTGCATTGCTGGTGATAGAGGTATAGCTCCTAAGAGTGGTTATTTTGTTAATGTAAA
TAATACTTGGATGTACACTGGTAGTGGTTACTACTACCCTGAACCTATAACTGAAAATAATGTTGTTGTTATGAGTACC
TGCGCTGTTAATTATACTAAAGCGCCGTATGTAATGCTGAACACTTCAATACCCAACCTTCCTGATTTTAAGGAAGAGT
TGGATCAATGGTTTAAAAATCAAACATCAGTGGCACCAGATTTGTCACTTGATTATATAAATGTTACATTCTTGGACCT
ACAAGTTGAAATGAATAGGTTACAGGAGGCAATAAAAGTCTTAAATCAGAGCTACATCAATCTCAAGGACATTGGTACA
TATGAATATTATGTAAAATGGCCTTGGTATGTATGGCTTTTAATCTGCCTTGCTGGTGTAGCTATGCTTGTTTTACTAT
TCTTCATATGCTGTTGTACAGGATGTGGGACTAGTTGTTTTAAGAAATGTGGTGGTTGTTGTGATGATTATACTGGATA
CCAGGAGTTAGTAATCAAAACTTCACATGACGACTAA

>gi|306155|gb|L14643.1|HOBOC43S Human coronavirus OC43 spike protein, complete cds

ATGTTTTTGATACTTTTAATTTCCTTACCAACGGCTTTTGCTGTTATAGGAGATTTAAAGTGTACTTCAGATAAT
ATTAATGATAAAGACACCGGTCCTCCTCCTATAAGTACTGATACTGTTGATGTTACTAATGGTTTGGGTACTTATTATG
TTTTAGATCGTGTGTATTTAAATACTACGTTGTTTCTTAATGGTTATTACCCTACTTCAGGTTCCACATATCGTAATAT
GGCACTGAAGGGAAGTGTACTATTGAGCAGACTATGGTTTAAACCACCATTTCTTTCTGATTTTATTAATGGTATTTTT
GCTAAGGTCAAAAATACCAAGGTTATTAAAGATCGTGTAATGTATAGTGAGTTCCCTGCTATAACTATAGGTAGTACTT
TTGTAAATACATCCTATAGTGTGGTAGTACAACCACGTACAATCAATTCAACACAGGATGGTGATAATAAATTACAAGG
TCTTTTAGAGGTCTCTGTTTGCCAGTATAATATGTGCGAGTACCCACAAACGATTTGTCATCCTAACCTGGGTAATCAT
CGCAAAGAACTATGGCATTTGGATACAGGTGTTGTTTCCTGTTTATATAAGCGTAATTTCACATATGATGTGAATGCTG
ATTATTTGTATTTTCATTTTTATCAAGAAGGTGGTACTTTTTATGCATATTTTACAGACACTGGTGTTGTTACTAAGTT
TTTGTTTAATGTTTATTTAGGCATGGCGCTTTCACACTATTATGTCATGCCTCTGACTTGTAATAGTAAGCTTACTTTA
GAATATTGGGTTACACCTCTCACTTCTAGACAATATTTACTCGCTTTCAATCAAGATGGTATTATTTTTAATGCTGAAG
ATTGTATGAGTGATTTTATGAGTGAGATTAAGTGTAAAACACAATCTATAGCGCCACCTACTGGTGTTTATGAATTAAA
CGGTTACACTGTTCAGCCAATCGCAGATGTTTACCGACGTAAACCTAATCTTCCCAATTGCAATATAGAAGCTTGGCTT
AATGATAAGTCGGTGCCCTCTCCATTAAATTGGGAACGTAAGACATTTTCAAATTGTAATTTTAATATGAGCAGCCTGA
TGTCTTTTATTCAGGCAGACTCATTTACTTGTAATAATATTGATGCTGCTAAGATATATGGTATGTGTTTTTCCAGCAT
AACTATAGATAAGTTTGCTATACCCAATGGCAGGAAGGTTGACCTACAATTGGGTAATTTGGGCTATTTGCAGTCATTT
AACTATAGAATTGATACTACTGCAACAAGTTGTCAGTTGTATTATAATTTACCTGCTGCTAATGTTTCTGTTAGCAGGT
TTAATCCTTCTACTTGGAATAAGAGATTTGGTTTTATAGAAGATTCTGTTTTTAAGCCTCGACCTGCAGGTGTTCTTAC

TAATCATGATGTAGTTTATGCACAACACTGTTTCAAAGCTCCTAAAAATTTCTGTCCGTGTAAATTGAATGGTTCGTGT
GTAGGTAGTGGTCCTGGTAAAAATAATGGTATAGGCACTTGTCCTGCAGGTACTAATTATTTAACTTGTGATAATTTGT
GCACTCCTGATCCTATTACATTTACAGGTACTTATAAGTGCCCCCAAACTAAATCTTTAGTTGGCATAGGTGAGCACTG
TTCGGGTCTTGCTGTTAAAAGTGATTATTGTGGAGGCAATTCTTGTACTTGCCGACCACAAGCATTTTTGGGTTGGTCT
GCAGACTCTTGTTTACAAGGAGACAAGTGTAATATTTTTGCTAATTTTATTTTGCATGATGTTAATAGTGGTCTTACTT
GTTCTACTGATTTACAAAAAGCTAACACAGACATAATTCTTGGTGTTTGTGTTAATTATGACCTCTATGGTATTTTAGG
CCAAGGCATTTTTGTTGAGGTTAATGCGACTTATTATAATAGTTGGCAGAACCTTTTATATGATTCTAATGGTAATCTC
TACGGTTTTAGAGACTACATAATAAACAGAACTTTTATGATTCGTAGTTGCTATAGCGGTCGTGTTTCTGCGGCCTTTC
ACGCTAACTCTTCCGAACCAGCATTGCTATTTCGGAATATTAAATGCAACTACGTTTTTAATAATAGTCTTACACGACA
GCTGCAACCCATTAACTATTTTGATAGTTATCTTGGTTGTGTTGTCAATGCTTATAATAGTACTGCTATTTCTGTTCAA
ACATGTGATCTCACAGTAGGTAGTGGTTACTGTGTGGATTACTCTAAAAACAGACGAAGTCGTGGAGCGATTACCACTG
GTTATCGGTTTACTAATTTTGAGCCATTTACTGTTAATTCAGTAAACGATAGTTTAGAACCTGTAGGTGGTTTGTATGA
AATTCAAATACCTTCAGAGTTTACTATAGGTAATATGGTGGAGTTTATTCAAACAAGCTCTCCTAAAGTTACTATTGAT
TGTGCTGCATTTGTCTGTGGTGATTATGCAGCATGTAAATCACAGTTGGTTGAATATGGTAGTTTCTGTGATAACATTA
ATGCCATACTCACAGAAGTAAATGAACTACTTGACACTACACAGTTGCAAGTAGCTAATAGTTTAATGAATGGTGTTAC
TCTTAGCACTAAGCTTAAAGATGGCGTTAATTTCAATGTAGACGACATCAATTTTTCCCCTGTATTAGGTTGTCTAGGC
AGCGAATGTAGTAAAGCTTCCAGTAGATCTGCTATAGAGGATTTACTTTTTGATAAAGTAAAGTTATCTGATGTCGGTT
TTGTTGAGGCTTATAATAATTGTACAGGAGGTGCCGAAATTAGGGACCTCATTTGTGTGCAAAGTTATAAAGGCATCAA
AGTGTTGCCTCCACTGCTCTCAGAAAATCAGATCAGTGGATACACTTTGGCTGCCACCTCTGCTAGTCTATTTCCTCCT
TGGACAGCAGCAGCAGGTGTACCATTTTATTTAAATGTTCAGTATCGCATTAATGGGCTTGGTGTCACCATGGATGTGC
TAAGTCAAAATCAAAAGCTTATTGCTAATGCATTTAACAATGCCCTTTATGCTATTCAGGAAGGGTTCGATGCAACTAA
TTCTGCTTTAGTTAAAATTCAAGCTGTTGTTAATGCAAATGCTGAAGCTCTTAATAACTTATTGCAACAACTCTCTAAT
AGATTTGGTGCTATAAGTGCTTCTTTACAAGAAATTCTATCTAGACTTGATGCTCTTGAAGCGGAAGCTCAGATAGATA
GACTTATTAATGGTCGTCTTACCGCTCTTAATGCTTATGTTTCTCAACAGCTTAGTGATTCTACACTGGTAAAATTTAG
TGCAGCACAAGCTATGGAGAAGGTTAATGAATGTGTCAAAAGCCAATCATCTAGGATAAATTTCTGTGGTAATGGTAAT
CATATTATATCATTAGTGCAGAATGCTCCATATGGTTTGTATTTTATCCACTTTAGTTATGTCCCTACTAAGTATGTCA
CAGCGAGGGTTAGTCCTGGTCTGTGCATTGCTGGTGATAGAGGTATAGCTCCTAAGAGTGGTTATTTTGTTAATGTAAA
TAATACTTGGATGTACACTGGTAGTGGTTACTACTACCCTGAACCTATAACTGAAAATAATGTTGTTGTTATGAGTACC
TGCGCTGTTAATTATACTAAAGCGCCGTATGTAATGCTGAACACTTCAATACCCAACCTTCCTGATTTTAAGGAAGAGT
TGGATCAATGGTTTAAAAATCAAACATCAGTGGCACCAGATTTGTCACTTGATTATATAAATGTTACATTCTTGGACCT
ACAAGTTGAAATGAATAGGTTACAGGAGGCAATAAAAGTCTTAAATCAGAGCTACATCAATCTCAAGGACATTGGTACA
TATGAATATTATGTAAAATGGCCTTGGTATGTATGGCTTTTAATCTGCCTTGCTGGTGTAGCTATGCTTGTTTTACTAT
TCTTCATATGCTGTTGTACAGGATGTGGGACTAGTTGTTTTAAGAAATGTGGTGGTTGTTGTGATGATTATACTGGATA
CCAGGAGTTAGTAATCAAAACTTCACATGACGACTAA

>gi|49035964|gb|AY567487.2| Human Coronavirus NL63, complete genome

ATGAAACTTTTCTTGATTTTGCTTGTTTTGCCCCTGGCCTCTTGCTTTTTCACATGTAATAGTAATGCTAATCTC
TCTATGTTACAATTAGGTGTTCCTGACAATTCTTCAACTATTGTTACGGGTTTATTGCCAACTCATTGGTTTTGTGCTA
ATCAGAGTACATCTGTTTACTCAGCCAATGGTTTCTTTTATATTGATGTTGGTAATCACCGTAGTGCTTTTGCGCTCCA
TACTGGTTATTATGATGCTAATCAGTATTATATTTATGTTACTAATGAAATAGGCTTAAATGCTTCTGTTACTCTTAAG
ATTTGTAAGTTTAGTAGAAACACTACTTTTGATTTTTTAAGTAATGCTTCTAGTTCTTTTGACTGTATAGTTAATTTGT
TATTTACAGAACAGTTAGGTGCGCCTTTGGGCATAACTATATCTGGTGAAACTGTGCGTCTGCATTTATATAATGTAAC

TCGTACTTTTTATGTGCCAGCAGCTTATAAACTTACTAAACTTAGTGTTAAATGTTACTTTAACTATTCCTGTGTTTTT
AGTGTTGTCAACGCCACCGTTACTGTGAATGTCACCACACATAATGGCCGTGTAGTTAACTACACTGTTTGTGATGATT
GTAATGGTTATACTGATAACATATTTTCTGTTCAACAGGATGGCCGCATTCCTAATGGTTTCCCTTTTAATAATTGGTT
TTTGTTAACTAATGGTTCCACACTAGTGGACGGGGTCTCTAGACTTTATCAACCACTCCGTTTAACTTGTTTATGGCCT
GTACCTGGTCTTAAATCTTCAACTGGTTTTGTTTATTTTAATGCCACTGGTTCTGATGTTAATTGTAACGGCTATCAAC
ATAATTCTGTTGTTGATGTTATGCGTTACAATCTTAACTTCAGTGCTAATTCTTTGGACAATCTCAAGAGTGGTGTTAT
AGTTTTTAAAACTTTACAGTACGATGTTTTGTTTTATTGTAGTAATTCTTCCTCAGGTGTTCTTGACACCACAATACCT
TTTGGCCCGTCCTCTCAACCTTATTACTGTTTTATAAACAGCACTATCAACACTACTCATGTTAGCACTTTTGTGGGTA
TTTTACCACCCACTGTGCGTGAAATTGTTGTTGCTAGAACTGGCCAGTTTTATATTAATGGTTTTAAGTATTTCGATTT
GGGTTTCATAGAAGCTGTCAATTTTAATGTCACGACTGCTAGCGCCACAGATTTTTGGACGGTTGCATTTGCTACTTTT
GTTGATGTTTTGGTTAATGTTAGTGCAACTAACATTCAAAACTTACTTTATTGCGATTCTCCATTTGAAAAGTTGCAGT
GTGAGCACTTGCAGTTTGGATTGCAGGATGGTTTTTATTCTGCAAATTTTCTTGATGATAATGTTTTGCCTGAGACTTA
TGTTGCACTCCCCATTTATTATCAACACACGGACATAAATTTTACTGCAACTGCATCTTTTGGTGGTTCTTGTTATGTT
TGTAAACCACACCAGGTTAATATATCTCTTAATGGTAACACTTCAGTGTGTGTTAGAACATCTCATTTTTCAATTAGGT
ATATTTATAACCGCGTTAAGAGTGGTTCACCAGGTGACTCTTCATGGCACATTTATTTAAAGAGTGGCACTTGTCCATT
TTCTTTTTCTAAGTTAAATAATTTTCAAAAGTTCAAGACTATTTGTTTCTCAACCGTCGAAGTGCCTGGTAGTTGTAAT
TTTCCGCTTGAAGCCACCTGGCATTACACTTCTTATACTATTGTTGGTGCTTTGTATGTTACTTGGTCTGAAGGTAATT
CTATTACTGGTGTACCTTATCCTGTCTCTGGTATTCGTGAGTTTAGTAATTTAGTTTTAAATAATTGTACCAAATATAA
TATTTATGATTATGTTGGTACTGGAATTATACGTTCTTCAAACCAGTCACTTGCTGGTGGTATTACATATGTTTCTAAC
TCTGGTAATTTACTTGGTTTTAAAAATGTTTCCACTGGTAACATTTTTATTGTGACACCATGTAACCAACCAGACCAAG
TAGCTGTTTATCAACAAAGCATTATTGGTGCCATGACCGCTGTTAATGAGTCTAGATATGGCTTGCAAAACTTACTACA
GTTACCTAACTTTTATTATGTTAGTAATGGTGGTAACAATTGCACTACGGCCGTTATGACTTATTCTAATTTTGGTATT
TGTGCTGATGGTTCTTTGATTCCTGTTCGTCCGCGTAATTCTAGTGATAATGGTATTTCAGCCATAATCACTGCTAATT
TATCCATTCCTTCTAACTGGACTACTTCAGTTCAAGTTGAGTACCTCCAAATTACTAGTACTCCAATAGTTGTTGATTG
TGCTACTTATGTGTGTAATGGTAACCCTCGCTGTAAGAATCTACTTAAGCAGTATACTTCTGCTTGTAAAACTATTGAA
GATGCCTTACGACTTAGTGCTCATTTGGAAACTAATGATGTTAGTAGTATGCTAACTTTCGATAGCAATGCTTTTAGTT
TGGCTAATGTTACTAGTTTTGGAGATTATAACCTTTCTAGTGTTTTACCTCAGAGAAACATTCGTTCAAGCCGTATAGC
AGGACGTAGTGCTTTGGAAGATTTGTTGTTTAGCAAAGTTGTTACATCTGGTTTGGGTACTGTTGATGTTGACTATAAG
TCTTGTACTAAAGGTCTTTCTATTGCTGACCTTGCTTGTGCTCAGTACTACAATGGCATAATGGTTTTGCCAGGTGTTG
CTGATGCTGAACGTATGGCCATGTACACAGGTTCTCTTATAGGTGGCATGGTGCTCGGAGGTCTTACATCAGCAGCCGC
CATACCTTTTTCTTTGGCACTGCAAGCACGACTTAACTATGTTGCTTTACAAACTGATGTGCTTCAAGAAAATCAGAAA
ATTTTGGCTGCATCATTTAATAAGGCTATTAATAATATTGTTGCTTCTTTTAGTAGCGTTAATGATGCTATTACACAAA
CTGCAGAGGCTATACATACTGTTACTATTGCACTTAATAAGATTCAGGATGTTGTTAATCAACAGGGTAGTGCTCTTAA
CCATCTCACTTCACAATTGAGACATAATTTTCAGGCCATTTCTAATTCAATTCAGGCTATTTATGACCGGCTTGATTCA
ATTCAAGCCGATCAACAAGTTGACAGATTAATTACTGGACGGCTTGCAGCTTTGAATGCATTTGTTTCCCAAGTTTTGA
ATAAATATACTGAAGTTCGTGGTTCAAGACGCTTAGCACAGCAGAAGATTAATGAATGTGTCAAGTCACAATCTAATAG
ATATGGTTTTTGTGGCAATGGCACTCACATCTTTTCAATCGTCAACTCAGCTCCAGATGGTTTGCTTTTTCTTCATACT
GTTTTGCTGCCAACTGATTACAAGAATGTAAAGGCGTGGTCTGGTATCTGTGTTGATGGCATTTATGGCTATGTTCTGC
GTCAACCTAACTTGGTTCTTTATTCTGATAATGGTGTCTTTCGTGTAACTTCCAGGGTCATGTTTCAACCTCGCTTACC
TGTTTTGTCTGATTTTGTGCAAATATATAATTGTAATGTTACTTTTGTTAACATATCTCGTGTTGAGTTACATACTGTC
ATACCTGACTACGTTGATGTTAATAAAACATTACAAGAGTTTGCACAAAACTTACCAAAGTATGTTAAGCCTAATTTTG
ACTTGACTCCTTTTAATTTAACATATCTTAATTTGAGTTCTGAGTTGAAGCAACTCGAAGCTAAAACTGCTAGTCTTTT
TCAAACTACTGTTGAATTACAAGGTCTTATTGATCAGATTAACAGTACATATGTTGATTTGAAGTTGCTTAATAGGTTT
GAAAATTATATCAAATGGCCTTGGTGGGTTTGGCTCATTATTTCTGTTGTTTTTGTTGTATTGTTGAGTCTTCTTGTGT

TTTGTTGTCTTTCTACAGGTTGTTGTGGTTGTTGCAATTGTTTAACTTCATCAATGCGAGGCTGTTGTGATTGTGGTTC
AACTAAACTTCCTTATTACGAATTTGAAAAGGTCCACGTTCAATAA

>gi|93004445|gb|DQ445911.1| Human coronavirus NL63 isolate Amsterdam 057, complete genome

ATGAAACTTTTCTTGATTTTGCTTATTTTGCCCCTGGTTTCTTGCTTTTCTACATGTAACAGTAATGCTAGTATT
TCTATGTTACAATTAGGTGTTCCTGATAACTCTTCAACTATTGTCACAGGTTTGTTGCCAGTCCATTGGATTTGTGCTA
ACCAGAGCACATCTAGTTACCCAGCCAATGGCTTTTTCTATATTGATGTCGGTAAACACCGTAGTGCTTTTGCACTCCA
TAGTGGTTATTATGATGCTAACCAGTATTATATTTATCTCACTAATAAAATACATTTAAATGCCTCTGTCACTCTGAAG
ATTTGTAAGTTTGGAAACACTTCTTTTGATTTTTTAAGTAATGTTTCTACTTCTCATCATTGTATAGTTAATTCGTCAT
TCACAGAACAGTTAGGTGTGCCTTTGGGCATAACTATATCGGGTGAAACTGTACGTTTGCATTTATATAATGCAACTCG
CACTTTTTATGTGCCGGCCGCTTATAAACTTACTAAACTTAGTGTTAAATGTTATTTTAGTGAATCCTGTGTTTTTAGT
GTTGTCAATGCTACCGTTACTGTTAATGTCACCACACTTAATGGCCGTATAGTTAATTACACTGTTTGTGATGATTGTA
ATGGTTACACTGATAACATATTTTCTGTTCAAGAGGATGGCCGTATTCCTAATGATTTTTCTTTTAATAATTGGTTTTT
GTTAACTAATGGTTCTACACTAGTAGACGGTGTCTCTAGACTTTACCAACCACTTCGTTTAACTTGTTTATGGCCTGTA
CCTGGTCTTAAATCTTCGACTGGTTTTGTTTATTTTAACGCCACTGGTTCTGATGTTAATTGTAATGGTTATCAACATC
ATTCTTTTGCTGATGTTATGCGTTACAATCTTAACTTCAGTTCTAATTCTGTGGATAATCTCAAGAGTGGTGTTATAGT
TTTTAAAACTCTACAGTACGATGTTTTGTTTTACTGTAGTAATTCCTCTTCGGGCGTTCTAGACACCACAATACCTTTT
GGCCCTTCCTCTCAACCTTATTACTGTTTTATAAATAGTACCATCAACACCACTCATGTTAGCACTTTTGTGGGTGTCC
TACCACCCACTGTGCGTGAGATTGTTGTTGCTAGAACTGGTCAGTTTTATATTAATGGTTTTAAGTATTTTGATTTGGG
TTTTATCGAAGCTGTCAATTTTAATGTCACAACTGCTAGTGCCACAGATTTTTGGACGGTTGCATTTGCTACTTTTGTT
GATGTTTTGGTTAATGTTAGTGCAACTAAGATTCAAAACTTACTTTATTGCGATTCTCCATTTGAAAAGTTGCAGTGTG
AGCACTTGCAGTTTGGATTGCAAGATGGTTTTTATTCTGCAAATTTTCTTGATGATAATGTTTTGCCTGAGACTTATGT
TGCACTCCCCATTTATTATCAACACACGGACATAAATTTTACTGCAACTGCATCTTTTGGTGGTTCTTGTTATGTTTGT
AAACCACACCAGGTTAATATATCTCTTAATGGTAACACTTCAGTGTGTGTTAGAACATCTCATTTTTCAATTAGGTATA
TTTATAACCGCGTTAAGAGTGTTTCACCAGGTGACTCTTCATGGCACATTTATTTAAAGAGTGGCACTTGTCCATTTTC
TTTTTCTAAGTTAAATAATTTTCAAAAGTTCAAGACTATTTGTTTCTCAACCGTCGAAGTGCCTGGTAGTTGTAATTTT
CCACTTGAAGCCACCTGGCATTACACTTCTTATACTATTGTTGGTGCTTTGTATGTTACTTGGTCTGAAGGTAATTCCA
TTACTGGTGTACCTTATCCTGTCTCTGGTATTCGTGAGTTTAGTAATTTAGTTTTAAATAATTGTACCAAATATAATAT
TTATGATTATGTTGGTACTGGAATTATACGTTCTTCAAACCAGTCACTTGCTGGTGGTATTACATATGTTTCTAACTCT
GGTAATTTACTTGGTTTTAAAAATGTTTCCACTGGTAACATTTTTATTGTGACACCATGTAACCAACCAGATCAAGTAG
CTGTTTATCAACAAAGCATTATTGGTGCCATGACCGCTGTTAATGAGTCTAGATATGGCTTGCAAAACTTACTACAGTT
ACCTAACTTTTATTACGTTAGTAATGGTGGTAACAATTGCACTACGGCTGTTATGACTTATTCTAATTTTGGTATTTGT
GCTGATGGTTCTTTAATTCCTGTTCGTCCGCGTAATTCTAGTGATAATGGTATTTCAGCCATAATCACTGCCAATTTAT
CCATTCCCTCTAACTGGACTACTTCAGTTCAAGTTGAGTACCTCCAAATTACTAGTACTCCAATAGTTGTTGATTGTGC
TACTTATGTGTGTAATGGTAACCCTCGCTGTAAGAATTTACTTAAGCAGTATACTTCTGCTTGTAAAACTATTGAAGAT
GCCTTACGACTTAGTGCTCATTTGGAAACTAATGATGTTAGTAGTATGCTAACTTTCGATAGCAATGCTTTTAGTTTGG
CTAATGTTACTAGTTTTGGAGATTATAACCTTTCTAGTGTTTTACCTCAGAGAAACATTCGTTCAAGCCGTATAGCAGG
ACGTAGTGCTTTAGAAGATTTGTTGTTTAGCAAAGTTGTTACATCTGGTTTGGGTACTGTTGATGTTGACTATAAGTCT
TGTACTAAAGGTCTTTCTATTGCTGACCTTGCTTGTGCTCAGTACTACAATGGCATAATGGTTTTGCCAGGTGTTGCTG
ATGCTGAACGTATGGCCATGTACACAGGTTCTCTTATAGGTGGCATGGTGCTCGGAGGTCTTACATCAGCAGCCGCCAT
ACCTTTTTCTTTGGCACTGCAAGCACGACTTAACTATGTTGCTTTACAAACTGATGTGCTTCAAGAAAATCAGAAAATT

TTGGCTGCATCATTTAATAAGGCTATTAATAATATTGTTGCTTCTTTTAGTAGCGTTAATGATGCTATTACACAAACTG
CAGAGGCTATACATACTGTTACTATTGCACTTAATAAGATTCAGGATGTTGTTAATCAACAGGGTAGTGCTCTTAACCA
TCTCACTTCACAATTGAGACATAATTTTCAGGCCATTTCTAATTCAATTCAGGCTATTTATGACCGGCTTGATTCAATT
CAAGCCGATCAACAAGTTGACAGATTAATTACTGGACGGCTTGCAGCTTTGAATGCATTTGTTTCCCAAGTTTTGAATA
AATATACTGAAGTTCGTGGTTCCAGACGCTTAGCACAGCAGAAGATTAATGAATGTGTCAAGTCACAATCTAATAGATA
TGGTTTTTGTGGCAATGGCACTCACATCTTTTCAATCGTCAACTCAGCTCCAGATGGTTTGCTTTTTCTTCATACTGTT
TTGCTGCCAACTGATTACAAGAATGTAAAGGCGTGGTCTGGTATCTGTGTTGATGGCATTTACGGCTATGTTCTGCGTC
AACCTAACTTGGTTCTTTATTCTGATAATGGTGTCTTTCGTGTAACTTCCAGGGTCATGTTTCAACCTCGCCTACCTGT
TTTGTCTGATTTTGTGCAAATATATAATTGTAATGTTACTTTTGTTAACATATCTCGTGTCGAGTTACATACTGTCATA
CCTGACTACGTTGATGTTAATAAAACATTACAAGAGTTTGCACAAAACTTACCAAAGTATGTTAAGCCTAATTTTGACT
TGACTCCTTTTAATTTAACATATCTTAATTTGAGTTCTGAGTTGAAGCAACTCGAAGCTAAAACTGCTAGTCTTTTTCA
AACTACTGTTGAATTACAAGGTCTTATTGATCAGATTAACAGTACATATGTTGATTTGAAGTTGCTTAATAGGTTTGAA
AATTATATCAAATGGCCTTGGTGGGTTTGGCTCATTATTTCTGTTGTTTTTGTTGTATTGTTGAGTCTTCTTGTGTTTT
GTTGTCTTTCTACAGGTTGTTGTGGTTGTTGCAATTGTTTAACTTCATCAATGCGAGGCTGTTGTGATTGTGGTTCAAC
TAAACTTCCTTATTACGAATTTGAAAAGGTCCACGTTCAATAA

>gi|93004452|gb|DQ445912.1| Human coronavirus NL63 isolate Amsterdam 496, complete genome

ATGAAACTTTTCTTGATTTTGCTTATTTTGCCCCTGGTTTCTTGCTTTTCTACATGTAACAGTAATGCTAGTATT
TCTATGTTACAATTAGGTGTTCCTGATAACTCTTCAACTATTGTCACAGGTTTGTTGCCAGTCCATTGGATTTGTGCTA
ATCAGAGCACATCTAGTTACCCAGCCAATGGCTTTTTCTATATTGATGTCGGTAAACACCGTAGTGCTTTTGCACTCCA
TAGTGGTTATTATGATGCTAACCAGTATTATATTTATCTCACTAATAAAATACATTTAAATGCTCCTGTCACTCTGAAG
ATTTGTAAGTTTGGAAACACTTCTTTTGATTTTTTAAGTAATGTTTCTACTTCTCATCATTGTATAGTTAATTCGTCAT
TCACAGAACAGTTAGGTGTGCCTTTGGGCATAACTATATCGGGTGAAACTGTACGTTTGCATTTATATAATGCAACTCG
CACCTTTTATGTGCCAGCCGCTTATAAACTTACTAAACTTAGTGTTAAATGTTACTTTAGTGAATCCTGTGTTTTTAGT
GTTGTCAATGCTACCGTTACTGTTAATGTCACCACACTTAATGGCCGTATAGTTAATTACACTGTTTGTGATGATTGTA
ATGGTTACACTGATAACATATTTTCTGTTCAACAGGATGGCCGCATTCCTAATGGTTTTTCTTTTAATAATTGGTTTTT
GTTAACTAATGGTTCTACACTAGTGGACGGTGTCTCTAGACTTTACCAACCACTTCGTTTAACTTGTTTATGGCCTGTA
CCTGGTCTTAAATCTTCGACTGGTTTTGTTTATTTTAACGCCACTGGTTCTGATGTTAATTGTAATGGTTATCAACATC
ATTCTGTTGCTGATGTTATGCGTTACAATCTTAACTTCAGTTCTAATTCTGTGGATAATCTCAAGAGTGGTGTTATAGT
TTTTAAAACTCTACAGTACGATGTTTTGTTTTACTGTAGTAATTCTTCTTCGGGCGTTCTAGACACCACAATACCTTTT
GGCCCTTCCTCTCAACCTTATTACTGTTTTATAAATAGTACCATCAACACCACTCATGTTAGCACTTTTGTGGGTGTCC
TACCACCCATTGTGCGTGAGATTGTTGTTGCTAGAACTGGTCAGTTTTATATTAATGGTTTTAAGTATTTTGATTTGGG
TTTTATCGAAGCTGTCAATTTTAATGTCACGACAGCTAGTGCTACAGATTTTTGGACGGTTGCATTTGCTACTTTTGTT
GATGTTTTGGTTAATGTTAGTGCAACTAACATTCAAAACTTACTTTATTGCGATTCTCCATTTGAAAAGTTGCAGTGTG
AGCACTTGCAGTTTGGATTGCAAGATGGTTTTTATTCTGCAAATTTTCTTGATGATAATGTTTTGCCTGATACTTATGT
TGCACTTCCTATTTATTATCAACATACTGATATAAATTTTACTGCGACTGCATCTTTTGGTGGTTCTTGTTATGTTTGC
AAACCACATCAGGTTAATATATCTCTTAATGGTAATACTTCAGTGTGTGTTAGAACATCTCATTTTTCAATTAGGTATA
TTTATAACCGCGTTAAGAGTGGTTCACCAGGTGACTCTTCATGGCACATTTATTTAAAGAGTGGCACTTGTCCATTTTC
TTTTTCTAAGTTAAATAATTTTCAAAAGTTTAAGACTATTTGTTTCTCAACCGTCGCAGTGCCTGGTAGTTGTAATTTT
CCACTTGAAGCCACCTGGCATTACACTTCTTATACTATTGTTGGTGCTTTGTATGTTACTTGGTCTGAAGGTAATTCCA
TTACTGGTGTACCTTATCCTGTCTCTGGTATTCGTGAGTTTAGTAATTTAGTTTTAAATAATTGTACCAAATATAATAT

TTATGATTATGTTGGTACTGGAATTATACGTTCTTCAAACCAGTCACTTGCTGGTGGTATTACATATGTTTCTAACTCT
GGTAATTTACTTGGTTTTAAAAATGTTTCCACTGGTAACATTTTTATTGTGACACCATGTAACCAACCAGACCAAGTAG
CTGTTTATCAACAAAGCATTATTGGTGCCATGACTGCTGTTAATGAGTCTAGATATGGCTTGCAAAACTTACTACAGTT
ACCTAACTTTTATTATGTTAGTAATGGTGGTAACAATTGCACTACGGCCGTTATGACTTATTCTAATTTTGGTATTTGT
GCTGATGGTTCTTTAATTCCTGTTCGTCCGCGTAATTCTAGTGATAATGGTATTTCAGCCATAATCACTGCTAATTTAT
CCATTCCCTCTAACTGGACTACTTCAGTTCAAGTTGAGTACCTCCAAATTACTAGTACTCCAATAGTTGTTGATTGTGC
TACTTATGTGTGTAATGGTAACCCTCGCTGTAAGAATCTACTTAAGCAGTATACTTCTGCTTGTAAAACTATTGAAGAT
GCCTTACGACTTAGTGCTCATTTGGAAACTAATGATGTTAGTAGTATGCTAACTTTCGATAGCAATGCTTTTAGTTTGG
CTAATGTTACTAGTTTTGGAGATTATAACCTTTCTAGTGTTTTACCTCAGAGAAACATTCATTCAAGCCGTATAGCAGG
ACGTAGTGCTTTGGAAGATTTGTTGTTTAGCAAAGTTGTTACATCTGGTTTGGGTACTGTTGATGTTGACTATAAGTCT
TGTACTAAAGGTCTTTCTATTGCTGACCTTGCTTGTGCGCAGTACTACAATGGCATAATGGTTTTGCCAGGTGTTGCTG
ATGCTGAACGTATGGCCATGTACACAGGTTCTCTTATAGGTGGCATGGTGCTCGGAGGTCTTACATCAGCAGCCGCCAT
ACCTTTTTCTTTGGCACTGCAAGCACGACTTAACTATGTTGCTTTACAAACTGATGTGCTTCAAGAAAATCAGAAAATT
TTGGCTGCATCATTTAATAAGGCTATTAATAACATTGTTGCTTCTTTTAGTAGCGTTAATGATGCTATTACACAAACTG
CAGAGGCTATACATACTGTTACTATTGCACTTAATAAGATTCAGGATGTTGTTAATCAACAGGGTAGTGCTCTTAACCA
TCTCACTTCACAATTGAGACATAATTTTCAGGCCATTTCTAATTCAATTCAAGCTATTTATGACCGGCTTGATTCAATT
CAAGCCGATCAACAAGTTGACAGATTAATTACTGGACGGCTTGCAGCTTTGAATGCATTTGTTTCCCAAGTTTTGAATA
AATATACTGAAGTTCGTAGTTCCAGACGCTTAGCACAGCAGAAGATTAATGAATGTGTCAAATCACAATCTAATAGATA
TGGTTTTTGTGGCAATGGCACCCATATCTTTTCAATCGTCAACTCAGCTCCAGATGGTTTGCTTTTTCTTCATACTGTT
TTGCTGCCAACCGACTACAAGAATGTAAAGGCGTGGTCGGGTATCTGTGTTGATGGCATTTACGGCTATGTTCTGCGTC
AACCTAACTTGGTTCTTTATTCTGATAATGGTGTCTTTCGTGTAACTTCCAGGGTCATGTTTCAACCTCGTTTACCTGT
TTTGTCTGATTTTGTGCAAATATATAATTGTAATGTTACTTTTGTTAACATATCTCGTGTCGAGTTACATACTGTCATA
CCTGACTACGTTGATGTTAATAAAACATTACAAGAGTTTGCACAAAACTTACCAAAGTATGTTAAGCCTAATTTTGACT
TGACTCCTTTTAATTTAACATATCTTAATTTGAGTTCTGAGTTGAAGCAACTCGAAGCTAAAACTGCTAGTCTTTTCCA
AACTACTGTTGAATTACAAGGTCTTATTGATCAGATTAACAGTACATATGTTGATTTGAAGTTGCTTAATAGGTTTGAA
AATTATATTAAATGGCCTTGGTGGGTTTGGCTCATTATTTCTGTTGTTTTTGTTGTATTGTTGAGTCTTCTTGTGTTTT
GTTGTCTTTCTACAGGTTGTTGTGGTTGTTGCAATTGTTTAACTTCATCAATGCGAGGTTGTTGTGATTGTGGTTCAAC
TAAACTTCCTTATTACGAATTTGAAAAGGTCCACGTTCAATAA

>gi|380710182|gb|JQ765563.1| Human coronavirus NL63 strain NL63/DEN/2009/9, complete genome

ATGAAACTTTTCTTGATTTTGCTTATTTTGCCCCTGGTTTCTTGCTTTTCTACATGTAACAGTAATGCTAGTATT
TCTATGTTACAATTAGGTGTTCCTGATAACTCTTCAACTATTGTCACAGGTTTGTTGCCAGTCCATTGGATTTGTGCTA
ATCAGAGCACATCTAGTTACCCAGCCAATGGCTTTTTCTATATTGATGTCGGTAAACACCGTAGTGCTTTTGCACTCCA
TAGTGGTTATTATGATGCTAACCAGTATTATATTTATCTCACTAATAAAATACATTTAAATGCTCCTGTCACTCTGAAG
ATTTGTAAGTTTGGAAACACTTCTTTTGATTTTTTAAGTAATGTTTCTACTTCTCATCATTGTATAGTTAATTCGTCAT
TCACAGAACAGTTAGGTGTGCCTTTGGGCATAACTATATCGGGTGAAACTGTACGTTTGCATTTATATAATGCAACTCG
CACCTTTTATGTGCCTGCCGCTTATAAACTTACTAAACTTAGTGTTAAATGTTACTTTAGTGAATCCTGTGTTTTTAGT
GTTGTCAATGCTACCGTTACTGTTAATGTCACCACACTTAATGGCCGTATAGTTAATTACACTGTTTGTGATGATTGTA
ATGGTTACACTGATAACATATTTTCTGTTCAACAGGATGGCCGCATTCCTAATGGTTTTTCTTTTAATAATTGGTTTTT
GTTAACTAATGGTTCTACACTAGTGGACGGTGTCTCTAGACTTTACCAACCACTTCGTTTAACTTGTTTATGGCCTGTA
CCTGGTCTTAAATCTTCGACTGGTTTTGTTTATTTTAACGCCACTGGTTCTGATGTTAATTGTAATGGTTATCAACATC

ATTCTGTTGCTGATGTTATGCGTTACAATCTTAACTTCAGTTCTAATTCTGTGGATAATCTCAAGAGTGGTGTTATAGT
TTTTAAAACTCTACAGTACGATGTTTTGTTTTACTGTAGTAATTCTTCTTCGGGCGTTCTAGACACCACAATACCTTTT
GGCCCTTCCTCTCAACCTTATTACTGTTTTATAAATAGTACCATCAACACCACTCATGTTAGCACTTTTGTGGGTGTCC
TACCACCCATTGTGCGTGAGATTGTTGTTGCTAGAACTGGTCAGTTTTATATTAATGGTTTTAAGTATTTTGATTTGGG
TTTTATCGAAGCTGTTAATTTTAATGTCACGACAGCTAGTGCTACAGATTTTTGGACGGTTGCATTTGCTACTTTTGTT
GATGTTTTGGTTAATGTTAGTGCAACTAACATTCAAAACTTACTTTATTGCGATTCTCCATTTGAAAAGTTGCAGTGTG
AGCACTTGCAGTTTGGATTGCAAGATGGTTTTTATTCTGCAAATTTTCTTGATGATAATGTTTTGCCTGATACTTATGT
TGCACTTCCTATTTATTATCAACATACTGATATAAATTTTACTGCGACTGCATCTTTTGGTGGTTCTTGTTATGTTTGC
AAACCACATCAGGTTAATATATCTCTTAATGGTAATACTTCAGTGTGTGTTAGAACATCTCATTTTTCAATTAGGTATA
TTTATAACCGCGTTAAGAGTGGTTCACCAGGTGACTCTTCATGGCACATTTATTTAAAGAGTGGCACTTGTCCATTTTC
TTTTTCTAAGTTAAATAATTTTCAAAAGTTTAAGACTATTTGTTTCTCAACCGTCGTAGTGCCTGGTAGTTGTAATTTT
CCACTTGAAGCCACCTGGCATTACACTTCTTATACTATTGTTGGTGCTTTGTATGTTACTTGGTCTGAAGGTAATTCCA
TTACTGGTGTACCTTATCCTGTCTCTGGTATTCGTGAGTTTAGTAATTTAGTTTTAAATAATTGTACCAAATATAATAT
TTATGATTATGTTGGTACTGGAATTATACGTTCTTCAAACCAGTCACTTGCTGGTGGTATTACATATGTTTCTAACTCT
GGTAATTTACTTGGTTTTAAAAATGTTTCCACTGGTAACATTTTTATTGTGACACCATGTAACCAACCAGACCAAGTAG
CTGTTTATCAACAAAGCATTATTGGTGCCATGACTGCTGTTAATGAGTCTAGATATGGCTTGCAAAACTTACTACAGTT
ACCTAACTTTTATTATGTTAGTAATGGTGGTAACAATTGCACTACGGCCGTTATGACTTATTCTAATTTTGGTATTTGT
GCTGATGGTTCTTTAATTCCTGTTCGTCCGCGTAATTCTAGTGATAATGGTATTTCAGCCATAGTCACTGCTAATTTAT
CCATTCCCTCTAACTGGACTACTTCAGTTCAAGTTGAGTACCTCCAAATTACTAGTACTCCAATAGTTGTTGATTGTGC
TACTTATGTGTGTAATGGTAACCCTCGCTGTAAGAATCTACTTAAGCAGTATACTTCTGCTTGTAAAACTATTGAAGAT
GCCTTACGACTTAGTGCTCATTTGGAAACTAATGATGTTAGTAGTATGCTAACTTTTGATAGCAATGCTTTTAGTTTGG
CTAATGTTACTAGTTTTGGAGATTATAACCTTTCTAGTGTTTTACCTCAGAGAAACATTCATTCAAGCCGTATAGCAGG
ACGTAGTGCTTTGGAAGATTTGTTGTTTAGCAAAGTTGTTACATCTGGTTTGGGTACTGTTGATGTTGACTATAAGTCT
TGTACTAAAGGTCTTTCTATTGCTGACCTTGCTTGTGCTCAGTACTACAATGGCATAATGGTTTTGCCAGGTGTTGCTG
ATGCTGAACGTATGGCCATGTACACAGGTTCTCTTATAGGTGGCATGGTGCTCGGAGGTCTTACATCAGCAGCCGCCAT
ACCTTTTTCTTTGGCACTGCAAGCACGACTTAACTATGTTGCTTTACAAACTGATGTGCTTCAAGAAAATCAGAAAATT
TTGGCTGCATCATTTAATAAGGCTATTAATAACATTGTTGCTTCTTTTAGTAGCGTTAATGATGCTATTACACAAACTG
CAGAGGCTATACATACTGTTACTATTGCACTTAATAAGATTCAGGATGTTGTTAATCAACAGGGTAGTGCTCTTAACCA
TCTCACTTCACAATTGAGACATAATTTTCAGGCCATTTCTAATTCAATTCAAGCTATTTATGACCGGCTTGATTCAATT
CAAGCCGATCAACAAGTTGACAGATTAATTACTGGACGGCTTGCAGCTTTGAATGCATTTGTTTCCCAAGTTTTGAATA
AATATACTGAAGTTCGTAGTTCCAGACGCTTAGCACAGCAGAAGATTAATGAATGTGTCAAATCACAATCTAATAGATA
TGGTTTTTGTGGCAATGGCACCCATATCTTTTCAATCGTCAACTCAGCTCCAGATGGTTTGCTTTTTCTTCATACTGTT
TTGCTGCCAACCGACTACAAGAATGTAAAGGCGTGGTCGGGTATCTGTGTTGATGGCATTTACGGCTATGTTCTGCGTC
AACCTAACTTGGTTCTTTATTCTGATAACGGTGTCTTTCGTGTAACTTCCAGGGTCATGTTTCAACCTCGTTTACCTGT
TTTGTCTGATTTTGTGCAAATATATAATTGTAATGTTACTTTTGTTAACATATCTCGTGTCGAGTTACATACTGTCATA
CCTGACTACGTTGATGTTAATAAAACATTACAAGAGTTTGCACAAAACTTACCAAAGTATGTTAAGCCTAATTTTGACT
TGACTCCTTTTAATTTAACATATCTTAATTTGAGTTCTGAGTTGAAGCAACTCGAAGCTAAAACTGCTAGTCTTTTCCA
AACTACTGTTGAATTACAAGGTCTTATTGATCAGATTAACAGTACATATGTTGATTTGAAGTTGCTTAATAGGTTTGAA
AATTATATTAAATGGCCTTGGTGGGTTTGGCTCATTATTTCTGTTGTTTTTGTTGTATTGTTGAGTCTTCTTGTGTTTT
GTTGTCTTTCTACAGGTTGTTGTGGTTGTTGCAATTGTTTAACTTCATCAATGCGAGGTTGTTGTGATTGTGGTTCAAC
TAAACTTCCTTATTACGAATTTGAAAAGGTCCACGTTCAATAA

>gi|380710190|gb|JQ765564.1| Human coronavirus NL63 strain NL63/DEN/2009/14,

complete genome

ATGAAACTTTTCTTGATTTTGCTTATTTTGCCCCTGGTTTCTTGCTTTTCTACATGTAACAGTAATGCTAGTATT
TCTATGTTACAATTAGGTGTTCCTGATAACTCTTCAACTATTGTCACAGGTTTGTTGCCAGTCCATTGGATTTGTGCTA
ATCAGAGCACATCTAGTTACCCAGCCAATGGCTTTTTCTATATTGATGTCGGTAAACACCGTAGTGCTTTTGCACTCCA
TAGTGGTTATTATGATGCTAACCAGTATTATATTTATCTCACTAATAAAATACATTTAAATGCTCCTGTCACTCTGAAG
ATTTGTAAGTTTGGAAACACTTCTTTTGATTTTTTAAGTAATGTTTCTACTTCTCATCATTGTATAGTTAATTCGTCAT
TCACAGAACAGTTAGGTGTGCCTTTGGGCATAACTATATCGGGTGAAACTGTACGTTTGCATTTATATAATGCAACTCG
CACCTTTTATGTGCCAGCCGCTTATAAACTTACTAAACTTAGTGTTAAATGTTACTTTAGTGAATCCTGTGTTTTTAGT
GTTGTCAATGCTACCGTTACTGTTAATGTCACCACACTTAATGGCCGTATAGTTAATTACACTGTTTGTGATGATTGTA
ATGGTTACACTGATAACATATTTTCTGTTCAACAGGATGGCCGCATTCCTAATGGTTTTTCTTTTAATAATTGGTTTTT
GTTAACTAATGGTTCTACACTAGTGGACGGTGTCTCTAGACTTTACCAACCACTTCGTTTAACTTGTTTATGGCCTGTA
CCTGGTCTTAAATCTTCGACTGGTTTTGTTTATTTTAACGCCACTGGTTCTGATGTTAATTGTAATGGTTATCAACATC
ATTCTGTTGCTGATGTTATGCGTTACAATCTTAACTTCAGTTCTAATTCTGTGGATAATCTCAAGAGTGGCGTTATAGT
TTTTAAAACTCTACAGTACGATGTTTTGTTTTACTGTAGTAATTCTTCTTCGGGCGTTCTAGACACCACAATACCTTTT
GGCCCTTCCTCTCAACCTTATTACTGTTTTATAAATAGTACCATCAACACCACTCATGTTAGCACTTTTGTGGGTGTCC
TACCACCCATTGTGCGTGAGATTGTTGTTGCTAGAACTGGTCAGTTTTATATTAATGGTTTTAAGTATTTTGATTTGGG
TTTTATCGAAGCTGTCAATTTTAATGTCACGACAGCTAGTGCTACAGATTTTTGGACGGTTGCATTTGCTACTTTTGTT
GATGTTTTGGTTAATGTTAGTGCAACTAACATTCAAAACTTACTTTATTGCGATTCTCCATTTGAAAAGTTGCAGTGTG
AGCACTTGCAGTTTGGATTGCAAGATGGTTTTTATTCTGCAAATTTTCTTGATGATAATGTTTTGCCTGATACTTATGT
TGCACTTCCTATTTATTATCAACATACTGATATAAATTTTACTGCGACTGCATCTTTTGGTGGTTCTTGTTATGTTTGC
AAACCACATCAGGTTAATATATCTCTTAATGGTAATACTTCAGTGTGTGTTAGAACATCTCATTTTTCAATTAGGTATA
TTTATAACCGCGTTAAGAGTGGTTCACCAGGTGACTCTTCATGGCACATTTATTTAAAGAGTGGCACTTGTCCATTTTC
TTTTTCTAAGTTAAATAATTTTCAAAAGTTTAAGACTATTTGTTTCTCAACCGTCGCAGTGCCTGGTAGTTGTAATTTT
CCACTTGAAGCCACCTGGCATTACACTTCTTATACTATTGTTGGTGCTTTGTATGTTACTTGGTCTGAAGGTAATTCCA
TTACTGGTGTACCTTATCCTGTCTCTGGTATTCGTGAGTTTAGTAATTTAGTTTTAAATAATTGTACCAAATATAATAT
TTATGATTATGTTGGTACTGGAATTATACGTTCTTCAAACCAGTCACTTGCTGGTGGTATTACATATGTTTCTAACTCT
GGTAATTTACTTGGTTTTAAAAATGTTTCCACTGGTAACATTTTTATTGTGACACCATGTAACCAACCAGACCAAGTAG
CTGTTTATCAACAAAGCATTATTGGTGCCATGACTGCTGTTAATGAGTCTAGATATGGCTTGCAAAACTTACTACAGTT
ACCTAACTTTTATTATGTTAGTAATGGTGGTAACAATTGCACTACGGCCGTTATGACTTATTCTAATTTTGGTATTTGT
GCTGATGGTTCTTTAATCCCTGTTCGTCCGCGTAATTCTAGTGATAATGGTATTTCAGCCATAATCACTGCTAATTTAT
CCATTCCCTCTAACTGGACTACTTCAGTTCAAGTTGAGTACCTCCAAATTACTAGTACTCCAATAGTTGTTGATTGTGC
TACTTATGTGTGTAATGGTAACCCTCGCTGTAAGAATCTACTTAAGCAGTACACTTCTGCTTGTAAAACTATTGAAGAT
GCCTTACGACTTAGTGCTCATTTGGAAACTAATGATGTTAGTAGTATGCTAACTTTCGATAGCAATGCTTTTAGTTTGG
CTAATGTTACTAGTTTTGGAGATTATAACCTTTCTAGTGTTTTACCTCAGAGAAACATTCATTCAAGCCGTATAGCAGG
ACGTAGTGCTTTGGAAGATTTGTTGTTTAGCAAAGTTGTTACATCTGGTTTGGGTACTGTTGATGTTGACTATAAGTCT
TGTACTAAAGGTCTTTCTATTGCTGACCTTGCTTGTGCTCAGTACTACAATGGCATAATGGTTTTGCCAGGTGTTGCTG
ATGCTGAACGTATGGCCATGTACACAGGTTCTCTTATAGGTGGCATGGTGCTCGGAGGTCTTACATCAGCAGCCGCCAT
ACCTTTTTCTTTGGCACTGCAAGCACGACTTAACTATGTTGCTTTACAAACTGATGTGCTTCAAGAAAATCAGAAAATT
TTGGCTGCATCATTTAATAAGGCTATTAATAACATTGTTGCTTCTTTTAGTAGCGTTAATGATGCTATTACACAAACTG
CAGAGGCTATACATACTGTTACTATTGCACTTAATAAGATTCAGGATGTTGTTAATCAACAGGGTAGTGCTCTTAACCA
TCTCACTTCACAATTGAGACATAATTTTCAGGCCATTTCTAATTCAATTCAAGCTATTTATGACCGGCTTGATTCAATT
CAAGCCGATCAACAAGTTGACAGATTAATTACTGGACGGCTTGCAGCTTTGAATGCATTTGTTTCCCAAGTTTTGAATA
AATATACTGAAGTTCGTAGTTCCAGACGCTTAGCACAGCAGAAGATTAATGAATGTGTCAAATCACAATCTAATAGATA

TGGTTTTTGTGGCAATGGCACCCATATCTTTTCAATCGTCAACTCAGCTCCAGATGGTTTGCTTTTTCTTCATACTGTT
TTGCTGCCAACCGACTACAAGAATGTAAAGGCGTGGTCGGGTATCTGTGTTGATGGCATTTACGGCTATGTTCTGCGTC
AACCTAACTTGGTTCTTTATTCTGATAATGGTGTCTTTCGTGTAACTTCCAGGGTCATGTTTCAACCTCGTTTACCTGT
TTTGTCTGATTTTGTGCAAATATATAATTGTAATGTTACTTTTGTTAACATATCTCGTGTCGAGTTACATACTGTCATA
CCTGACTACGTTGATGTTAATAAAACATTACAAGAGTTTGCACAAAACTTACCAAAGTATGTTAAGCCTAATTTTGACT
TGACTCCTTTTAATTTAACATATCTTAATTTGAGTTCTGAGTTGAAGCAACTCGAAGCTAAAACTGCTAGTCTTTTCCA
AACTACTGTTGAATTACAAGGTCTTATTGATCAGATTAACAGTACATATGTTGATTTGAAGTTGCTTAATAGGTTTGAA
AATTATATTAAATGGCCTTGGTGGGTTTGGCTCATTATTTCTGTTGTTTTTGTTGTATTGTTGAGTCTTCTTGTGTTTT
GTTGTCTTTCTACAGGTTGTTGTGGTTGTTGCAATTGTTTAACTTCATCAATGCGAGGTTGTTGTGATTGTGGTTCAAC
TAAACTTCCTTATTACGAATTTGAAAAGGTCCACGTTCAATAA

>gi|380710198|gb|JQ765565.1| Human coronavirus NL63 strain NL63/DEN/2009/15, complete genome

ATGAAACTTTTCTTGATTTTGCTTATTTTGCCCCTGGTTTCTTGCTTTTCTACATGTAACAGTAATGCTAGTATT
TCTATGTTACAATTAGGTGTTCCTGATAACTCTTCAACTATTGTCACAGGTTTGTTGCCAGTCCATTGGATTTGTGCTA
ATCAGAGCACATCTAGTTACCCAGCCAATGGCTTTTTCTATATTGATGTCGGTAAACACCGTAGTGCTTTTGCACTCCA
TAGTGGTTATTATGATGCTAACCAGTATTATATTTATCTCACTAATAAAATACATTTAAATGCTCCTGTCACTCTGAAG
ATTTGTAAGTTTGGAAACACTTCTTTTGATTTTTTAAGTAATGTTTCTACTTCTCATCATTGTATAGTTAATTCGTCAT
TCACAGAACAGTTAGGTGTGCCTTTGGGCATAACTATATCGGGTGAAACTGTACGTTTGCATTTATATAATGCAACTCG
CACCTTTTATGTGCCAGCCGCTTATAAACTTACTAAACTTAGTGTTAAATGTTACTTTAGTGAATCCTGTGTTTTTAGT
GTTGTCAATGCTACCGTTACTGTTAATGTCACCACACTTAATGGCCGTATAGTTAATTACACTGTTTGTGATGATTGTA
ATGGTTACACTGATAACATATTTTCTGTTCAACAGGATGGCCGCATTCCTAATGGTTTTTCTTTTAATAATTGGTTTTT
GTTAACTAATGGTTCTACACTAGTGGACGGTGTCTCTAGACTTTACCAACCACTTCGTTTAACTTGTTTATGGCCTGTA
CCTGGTCTTAAATCTTCGACTGGTTTTGTTTATTTTAACGCCACTGGTTCTGATGTTAATTGTAATGGTTATCAACATC
ATTCTGTTGCTGATGTTATGCGTTACAATCTTAACTTCAGTTCTAATTCTGTGGATAATCTCAAGAGTGGTGTTATAGT
TTTTAAAACTCTACAGTACGATGTTTTGTTTTACTGTAGTAATTCTTCTTCGGGCGTTCTAGACACCACAATACCTTTT
GGCCCTTCCTCTCAACCTTATTACTGTTTTATAAATAGTACCATCAACACCACTCATGTTAGCACTTTTGTGGGTGTCC
TACCACCCATTGTGCGTGAGATTGTTGTTGCTAGAACTGGTCAGTTTTATATTAATGGTTTTAAGTATTTTGATTTGGG
TTTTATCGAAGCTGTCAATTTTAATGTCACGACAGCTAGTGCTACAGATTTTTGGACGGTTGCATTTGCTACTTTTGTT
GATGTTTTGGTTAATGTTAGTGCAACTAACATTCAAAACTTACTTTATTGCGATTCTCCATTTGAAAAGTTGCAGTGTG
AGCACTTGCAGTTTGGATTGCAAGATGGTTTTTATTCTGCAAATTTTCTTGATGATAATGTTTTGCCTGATACTTATGT
TGCACTTCCTATTTATTATCAACATACTGATATAAATTTTACTGCGACTGCATCTTTTGGTGGTTCTTGTTATGTTTGC
AAACCACATCAGGTTAATATATCTCTTAATGGTAATACTTCAGTGTGTGTTAGAACATCTCATTTTTCAATTAGGTATA
TTTATAACCGCGTTAAGAGTGGTTCACCAGGTGACTCTTCATGGCACATTTATTTAAAGAGTGGCACTTGTCCATTTTC
TTTTTCTAAGTTAAATAATTTTCAAAAGTTTAAGACTATTTGTTTCTCAACCGTCGCAGTGCCTGGTAGTTGTAATTTT
CCACTTGAAGCCACCTGGCATTACACTTCTTATACTATTGTTGGTGCTTTGTATGTTACTTGGTCTGAAGGTAATTCCA
TTACTGGTGTACCTTATCCTGTCTCTGGTATTCGTGAGTTTAGTAATTTAGTTTTAAATAATTGTACCAAATATAATAT
TTATGATTATGTTGGTACTGGAATTATACGTTCTTCAAACCAGTCACTTGCTGGTGGTATTACATATGTTTCTAACTCT
GGTAATTTACTTGGTTTTAAAAATGTTTCCACTGGTAACATTTTTATTGTGACACCATGTAACCAACCAGACCAAGTAG
CTGTTTATCAACAAAGCATTATTGGTGCCATGACTGCTGTTAATGAGTCTAGATATGGCTTGCAAAATTTACTACAGTT
ACCTAACTTTTATTATGTTAGTAATGGTGGTAACAATTGCACTACGGCCGTTATGACTTATTCTAATTTTGGTATTTGT
GCTGATGGTTCTTTAATTCCTGTTCGTCCGCGTAATTCTAGTGATAATGGTATTTCAGCCATAATCACTGCTAATTTAT

CCATTCCCTCTAACTGGACTACTTCAGTTCAAGTTGAGTACCTCCAAATTACTAGTACTCCAATAGTTGTTGATTGTGC
TACTTATGTGTGTAATGGTAACCCTCGCTGTAAGAATCTACTTAAGCAGTATACTTCTGCTTGTAAAACTATTGAAGAT
GCCTTACGACTTAGTGCTCATTTGGAAACTAATGATGTTAGTAGTATGCTAACTTTCGATAGCAATGCTTTTAGTTTGG
CTAATGTTACTAGTTTTGGAGATTATAACCTTTCTAGTGTTTTACCTCAGAGAAACATTCATTCAAGCCGTATAGCAGG
ACGTAGTGCTTTGGAAGATTTGTTGTTTAGCAAAGTTGTTACATCTGGTTTGGGTACTGTTGATGTTGACTATAAGTCT
TGTACTAAAGGTCTTTCTATTGCTGACCTTGCTTGTGCTCAGTACTACAATGGCATAATGGTTTTGCCAGGTGTTGCTG
ATGCTGAACGTATGGCCATGTACACAGGTTCTCTTATAGGTGGCATGGTGCTCGGAGGTCTTACATCAGCAGCCGCCAT
ACCTTTTTCTTTGGCACTGCAAGCACGACTTAACTATGTTGCTTTACAAACTGATGTGCTTCAAGAAAATCAGAAAATT
TTGGCTGCATCATTTAATAAGGCTATTAATAACATTGTTGCTTCTTTTAGTAGCGTTAATGATGCTATTACACAAACTG
CAGAGGCTATACATACTGTTACTATTGCACTTAATAAGATTCAGGATGTTGTTAATCAACAGGGTAGTGCTCTTAATCA
TCTCACTTCACAATTGAGACATAATTTTCAGGCCATTTCTAATTCAATTCAAGCTATTTATGACCGGCTTGATTCAATT
CAAGCCGATCAACAAGTTGACAGATTAATTACTGGACGGCTTGCAGCTTTGAATGCATTTGTTTCCCAAGTTTTGAATA
AATATACTGAAGTTCGTAGTTCCAGACGCTTAGCACAGCAGAAGATTAATGAATGTGTCAAATCACAATCTAATAGATA
TGGTTTTTGTGGTAATGGCACCCATATCTTTTCAATCGTCAACTCAGCTCCAGATGGTTTGCTTTTTCTTCATACTGTT
TTGCTGCCAACCGACTACAAGAATGTAAAGGCGTGGTCGGGTATTTGTGTTGATGGCATTTACGGCTATGTTCTGCGTC
AACCTAACTTGGTTCTTTATTCTGATAATGGTGTCTTTCGTGTAACTTCCAGGGTCATGTTTCAACCTCGTTTACCTGT
TTTGTCTGATTTTGTGCAAATATATAATTGTAATGTTACTTTTGTTAACATATCTCGTGTCGAGTTACATACTGTCATA
CCTGACTACGTTGATGTTAATAAAACATTACAAGAGTTTGCACAAAACTTACCAAAGTATGTTAAGCCTAATTTTGACT
TGACTCCTTTTAATTTAACATATCTTAATTTGAGTTCTGAGTTGAAGCAACTCGAAGCTAAAACTGCTAGTCTTTTCCA
AACTACTGTTGAATTACAAGGTCTTATTGATCAGATTAACAGTACATATGTTGATTTGAAGTTGCTTAATAGGTTTGAA
AATTATATTAAATGGCCTTGGTGGGTTTGGCTCATTATTTCTGTTGTTTTTGTTGTATTGTTGAGTCTTCTTGTGTTTT
GTTGTCTTTCTACAGGTTGTTGTGGTTGTTGCAATTGTTTAACCTCATCAATGCGAGGTTGTTGTGATTGTGGTTCAAC
TAAACTTCCTTATTACGAATTTGAAAAGGTCCACGTTCAATAA

>gi|380710206|gb|JQ765566.1| Human coronavirus NL63 strain NL63/DEN/2008/16, complete genome

ATGAAACTTTTCTTGATTTTGCTTGTTTTGCCCTTGGCCTCTTGCTTTTTCACATGTAATAGTAATGCTAATCTC
TCTATGTTACAATTAGGTGTTCCTGACAATTCTTCAACTATTGTCACGGGTTTATTGCCAACTCATTGGATTTGTGCTA
ATCAGAGTACATCTGTTTACTCAGCCAATGGTTTCTTTTATATTGATGTTGGTAATCACCGTAGTGCTTTTGCGCTCCA
TACTGGTTATTATGATGTTAATCAGTATTATATTTATGTTACTAATGAAATAGGCTTAAATGCTTCTGTTACTCTTAAG
ATTTGTAAGTTTGGTATAAACACTACTTTTGATTTTTTAAGTAATTCTTCTAGTTCTTTTGACTGTATAGTTAATTTGT
TATTTACAGAACAGTTAGGTGCGCCTTTGGGCATAACTATATCTGGTGAAACTGTGCGTCTGCATTTATATAATGTAAC
TCGTACTTTTTATGTGCCAGCAGCTTATAAACTTACTAAACTTAGTGTTAAATGTTACTTTAACTATTCCTGTGTTTTT
AGTGTTGTCAACGCCACCGTTACTGTGAATGTCACCACACATAATGGCCGTGTAGTTAACTACACTGTTTGTGATGATT
GTAATGGTTATACTGATAACATATTTTCTGTTCAACAGGATGGCCGCATTCCTAATGGTTTCCCTTTTAATAATTGGTT
TTTGCTAACTAATGGTTCCACACTAGTGGACGGGGTCTCTAGACTTTATCAACCACTCCGTTTAACTTGTTTATGGCCT
GTACCTGGTCTTAAATCTTCAACTGGTTTTGTTTATTTTAATGCCACTGGTTCTGATGTTAATTGTAACGGCTATCAAC
ATAATTCTGTTGCTGATGTTATGCGTTACAATCTTAACTTCAGTGCTAATTCTGTGGATAATCTCAAGAGTGGTGTTAT
AGTTTTTAAAACTCTACAGTACGATGTTTTGTTTTACTGTAGTAATTCCTCTTCGGGCGTTCTAGATACCACAATACCT
TTTGGCCCTTCCTCTCAACCTTATTACTGTTTTATAAATAGTACCATCAACACCACTCATGTTAGCACTTTTGTGGGTG
TCCTACCACCCACTGTGCGTGAGATTGTTGTTGCTAGAACTGGTCAGTTTTATATTAATGGTTTTAAGTATTTTGATTT
GGGTTTTATCGAAGCTGTCAATTTTAATGTCACAACTGCTAGTGCCACAGATTTTTGGACGGTTGCATTTGCTACTTTT

GTCGATGTTTTGGTTAATGTTAGTGCAACTAAGATTCAAAACTTACTTTATTGCGATTCTCCATTTGAAAAGTTGCAGT
GTGAGCACTTGCAGTTTGGATTGCAAGATGGTTTTTATTCTGCAAATTTTCTTGATGATAATGTTTTGCCTGAGACTTA
TGTTGCACTCCCCATTTATTATCAACACACGGACATAAATTTTACTGCAACTGCATCTTTTGGTGGTTCTTGTTATGTT
TGTAAACCACACCAGGTTAATATATCTCTTAATGGTAACACTTCAGTGTGTGTTAGAACATCTCATTTTTCAATTAGGT
ATATTTATAACCGCGTTAAGAGTGGTTCACCAGGTGACTCTTCATGGCACATTTATTTAAAGAGTGGCACTTGTCCATT
TTCTTTTTCTAAGTTAAATAATTTTCAAAAGTTTAAGACTATTTGTTTTTCAACCGTCGCAGTGCCTGGTAGTTGTAAT
TTTCCACTTGAAGCCACCTGGCATTACACTTCTTATACTATTGTTGGTGCTTTGTATGTTACTTGGTCTGAAGGTAATT
CCATTACTGGTGTACCTTATCCTGTCTCTGGTATTCGTGAGTTTAGTAATTTAGTTTTAAATAATTGTACCAAATATAA
TATTTATGATTATGTTGGTACTGGAATTATACGTTCTTCAAACCAGTCACTTGCTGGTGGTATTACATATGTTTCTAAC
TCTGGTAATTTACTTGGTTTTAAAAATGTTTCCACTGGTAACATTTTTATTGTGACACCATGTAACCAACCAGACCAAG
TAGCTGTTTATCAACAAAGCATTATTGGTGCCATGACTGCTGTTAATGAGTCTAGATATGGCTTGCAAAACTTACTACA
GTTACCTAACTTTTATTATGTTAGTAATGGTAGTAACAATTGCACTACGGCCGTTATGACTTATTCTAATTTTGGTATT
TGTGCTGATGGTTCTTTAATTCCTGTTCGTCTGCGTAATTCTAGTGATAATGGTATTTCAGCCATAATCACTGCTAATT
TATCCATTCCCTCTAACTGGACTACTTCAGTTCAAGTTGAGTACCTCCAAATTACTAGTACTCCAATAGTTGTTGATTG
TGCTACTTATGTGTGTAATGGTAACCCTCGCTGTAAGAATCTACTTAAGCAGTATACTTCTGCTTGTAAAACTATTGAA
GATGCCTTACGACTTAGTGCTCATTTGGAAACTAATGATGTTAGTAGTATGCTAACTTTCGATAGCAATGCTTTTAGTT
TGGCTAATGTTACTAGTTTTGGAGATTATAACCTTTCTAGTGTTTTACCTCAGAGAAACATTCATTCAAGCCGTATAGC
AGGACGTAGTGCTTTGGAAGATTTGTTGTTTAGCAAAGTTGTTACATCTGGTTTGGGTACTGTTGATGTTGACTATAAG
TCTTGTACTAAAGGTCTTTCTATTGCTGACCTTGCTTGTGCTCAGTACTACAATGGCATAATGGTTTTGCCAGGTGTTG
CTGATGCTGAACGTATGGCCATGTACACAGGTTCTCTTATAGGTGGCATGGTGCTCGGAGGTCTTACATCAGCAGCCGC
CATACCTTTTTCTTTGGCACTGCAAGCACGACTTAACTATGTTGCTTTACAAACTGATGTGCTTCAAGAAAATCAGAAA
ATTTTGGCTGCATCATTTAATAAGGCTATTAATAACATTGTTGCTTCTTTTAGTAGCGTTAATGATGCTATTACACAAA
CTGCAGAGGCTATACATACTGTTACTATTGCACTTAATAAGATTCAGGATGTTGTTAATCAACAGGGTAGTGCTCTTAA
CCATCTCACTTCACAATTGAGACATAATTTTCAGGCCATTTCTAATTCAATTCAAGCTATTTATGACCGGCTTGATTCA
ATTCAAGCCGATCAACAAGTTGACAGATTAATTACTGGACGGCTTGCAGCTTTGAATGCATTTGTTTCCCAAGTTTTGA
ATAAATATACTGAAGTTCGTAGTTCCAGACGCTTAGCACAGCAGAAGATTAATGAATGTGTCAAATCACAATCTAATAG
ATATGGTTTTTGTGGCAATGGCACCCATATCTTTTCAATCGTCAACTCAGCTCCAGATGGTTTGCTTTTTCTTCATACT
GTTTTGCTGCCAACCGACTACAAGAATGTAAAGGCGTGGTCGGGTATCTGTGTTGATGGCATTTACGGCTATGTTCTGC
GTCAATCTAACTTGGTTCTTTATTCTGATAATGGTGTTTTTCGTGTAACTTCCAGGGTCATGTTTCAACCTCGTTTACC
TGTTTTGTCTGATTTTGTGCAAATATATAATTGTAATGTTACTTTTGTTAACATATCTCGTGTCGAGTTACATACTGTC
ATACCTGACTACGTTGATGTTAATAAAACATTACAAGAGTTTGCACAAAACTTACCAAAGTATGTTAAGCCTAATTTTG
ACTTGACTCCTTTTAATTTAACATATCTTAATTTGAGTTCTGAGTTGAAGCAACTCGAAGTTAAAACTGCTAGTCTTTT
CCAAACTACTGTTGAATTACAAGGTCTTATTGATCAGATTAACAGTACATATGTTGATTTGAAGTTGCTTAATAGGTTT
GAAAATTATATTAAATGGCCTTGGTGGGTTTGGCTCATTATTTCTGTTGTTTTTGTTGTATTGTTGAGTCTTCTTGTGT
TTTGTTGTCTTTCTACAGGTTGTTGTGGTTGTTGCAATTGTTTAACTTCATCAATGCGAGGTTGTTGTGATTGTGGTTC
AACTAAACTTCCTTATTACGAATTTGAAAAGGTCCACGTTCAATAA

>gi|380710222|gb|JQ765568.1| Human coronavirus NL63 strain NL63/DEN/2005/193, complete genome

ATGAAACTTTTCTTGATTTTGCTTGTTTTGCCCCTGGCCTCTTGCTTTTTCACATGTAATAGTAATGCTAATCTC
TCTATGTTACAATTAGGTGTTCCTGACAATTCTTCAACTATTGTTACGGGTTTATTGCCAACTCATTGGTTTTGTGCTA
ATCAGAGTACATCTGTTTACTCAGCCAATGGTTTCTTTTATATTGATGTTGGTAATCACCGTAGTGCTTTTGCGCTCCA

TACTGGTTATTATGATGCTAATCAGTATTATATTTATGTTACTAATGAAATAGGCTTAAATGCTTCTGTTACTCTTAAG
ATTTGTAAGTTTAGTAGAAACACTACTTTTGATTTTTTAAGTAATGCTTCTAGTTCTTTTGACTGTATAGTTAATTTGT
TATTTACAGAACAGTTAGGTGCGCCTTTGGGCATAACTATATCTGGTGAAACTGTGCGTCTGCATTTATATAATGTAAC
TCGTACTTTTTATGTGCCAGCAGCTTATAAACTTACTAAACTTAGTGTTAAATGTTACTTTAACTATTCCTGTGTTTTT
AGTGTTGTCAACGCCACCGTTACTGTGAATGTCACCACACATAATGGCCGTGTAGTTAACTACACTGTTTGTGATGATT
GTAATGGTTATACTGATAACATATTTTCTGTTCAACAGGATGGCCGCATTCCTAATGGTTTCCCTTTTAATAATTGGTT
TTTGTTAACTAATGGTTCCACACTAGTGGACGGGGTCTCTAGACTTTATCAACCACTCCGTCTAACTTGTTTATGGCCT
GTACCTGGTCTTAAATCTTCAACTGGTTTTGTTTATTTTAATGCCACTGGTTCTGATGTTAATTGTAACGGCTATCAAC
ATAATTCTGTTGCTGATGTTATGCGTTACAATCTTAACTTCAGTGCTAATTCTGTGGACAATCTCAAGAGTGGTGTTAT
AGTTTTTAAAACTTTACAGTACGATGTTTTGTTTTATTGTAGTAACTCTTCCTCAGGTGTTCTTGACACCCACAATACCT
TTTGGCCCTTCCTCTCAACCTTATTACTGTTTTATAAACAGCACTATCAACACTACTCATGTTAGCACTTTTGTGGGTA
TTTTACCACCCACTGTGCGTGAAATTGTTGTTGCTAGAACTGGTCAGTTTTATATTAATGGTTTTAAGTATTTCGATTT
GGGTTTCATAGAAGCTGTCAATTTTAATGTCACGACTGCTAGTGCCACAGATTTTTGGACGGTTGCATTTGCTACTTTT
GTTGATGTTTTGGTTAATGTTAGTGCAACTAACATTCAAAACTTACTTTATTGCGATTCTCCATTTGAAAAGTTGCAGT
GTGAGCACTTGCAGTTTGGATTGCAGGATGGTTTTTATTCTGCAAATTTTCTTGATGATAATGTTTTGCCTGAGACTTA
TGTTGCACTCCCCATTTATTATCAACACACGGACATAAATTTTACTGCAACTGCATCTTTTGGTGGTTCTTGTTATGTT
TGTAAACCACACCAGGTTAATATATCTCTTAATGGTAACACTTCAGTGTGTGTTAGAACATCTCATTTTTCAATTAGGT
ATATTTATAACCGCGTTAAGAGTGGTTCACCAGGTGACTCTTCATGGCACATTTATTTAAAGAGTGGCACTTGTCCATT
TTCTTTTTCTAAGTTAAATAATTTTCAAAAGTTCAAGACTATTTGTTTCTCAACCGTCGAAGTGCCTGGTAGTTGTAAT
TTTCCGCTTGAAGCCACCTGGCATTACACTTCTTATACTATTGTTGGTGCTTTGTATGTTACTTGGTCTGAAGGTAATT
CTATTACTGGTGTACCTTATCCTGTCTCTGGTATTCGTGAGTTTAGTAATTTAGTTTTAAATAATTGTACCAAATATAA
TATTTATGATTATGTTGGTACTGGAATTATACGTTCTTCAAACCAGTCACTTGCTGGTGGTATTACATATGTTTCTAAC
TCTGGTAATTTACTTGGTTTTAAAAATGTTTCCACTGGTAACATTTTTATTGTGACACCATGTAACCAACCAGACCAAG
TAGCTGTTTATCAACAAAGCATTATTGGTGCCATGACCGCTGTTAATGAGTCTAGATATGGCTTGCAAAACTTACTACA
GTTACCTAACTTTTATTATGTTAGTAATGGTGGTAACAATTGCACTACGGCCGTTATGACTTATTCTAATTTTGGTATT
TGTGCTGATGGTTCTTTGATTCCTGTTCGTCCGCGTAATTCTAGTGATAATGGTATTTCAGCCATAATCACTGCTAATT
TATCCATTCCCTCTAACTGGACTACTTCAGTTCAAGTTGAGTACCTCCAAATTACTAGTACTCCAATAGTTGTTGATTG
TGCTACTTATGTGTGTAATGGTAACCCTCGCTGTAAGAATCTACTTAAGCAGTATACTTCTGCTTGTAAAACTATTGAA
GATGCCTTACGACTTAGTGCTCATTTGGAAACTAATGATGTTAGTAGTATGCTAACTTTCGATAGCAATGCTTTTAGTT
TGGCTAATGTTACTAGTTTTGGAGACTATAACCTTTCTAGTGTTTTACCTCAGAGAAACATTCGTTCAAGCCGTATAGC
AGGACGTAGTGCTTTGGAAGATTTGTTGTTTAGCAAAGTTGTTACATCTGGTTTGGGTACTGTTGATGTTGACTATAAG
TCTTGTACTAAAGGTCTTTCTATTGCTGACCTTGCTTGTGCTCAGTACTACAATGGCATAATGGTTTTGCCAGGTGTTG
CTGATGCTGAACGTATGGCCATGTACACAGGTTCTCTTATAGGTGGCATGGTGCTCGGAGGTCTTACATCAGCAGCCGC
CATACCTTTTTCTTTGGCACTGCAAGCACGACTTAACTATGTTGCTTTACAAACTGATGTGCTTCAAGAAAATCAGAAA
ATTTTGGCTGCATCATTTAATAAGGCTATTAATAATATTGTTGCTTCTTTTAGTAGCGTTAATGATGCTATTACACAAA
CTGCAGAGGCTATACATACTGTTACTATTGCACTTAATAAGATTCAGGATGTTGTTAATCAACAGGGTAGTGCTCTTAA
CCATCTCACTTCACAATTGAGACATAATTTTCAGGCCATTTCTAATTCAATTCAGGCTATTTATGACCGGCTTGATTCA
ATTCAAGCCGATCAACAAGTTGACAGATTAATTACTGGACGGCTTGCAGCTTTGAATGCATTTGTTTCCCAAGTTTTGA
ATAAATATACTGAAGTTCGTGGTTCAAGACGCTTAGCACAGCAGAAGATTAATGAATGTGTCAAGTCACAATCTAATAG
ATATGGTTTTTGTGGCAATGGCACTCACATCTTTTCAATCGTCAACTCAGCTCCAGATGGTTTGCTTTTTCTTCATACT
GTTTTGCTGCCAACTGATTACAAGAATGTAAAGGCGTGGTCTGGTATCTGTGTTGATGGCATTTATGGCTATGTTCTGC
GTCAACCTAACTTGGTTCTTTATTCTGATAATGGTGTCTTTCGTGTAACTTCCAGGATCATGTTTCAACCTCGCTTACC
TGTTTTGTCTGATTTTGTGCAAATATATAATTGTAATGTTACTTTTGTTAACATATCTCGTGTTGAGTTACATACTGTC
ATACCTGACTACGTTGATGTTAATAAAACATTACAAGAGTTTGCACAAAACTTACCAAAGTATGTTAAGCCTAATTTTG

ACTTGACTCCTTTTAATTTAACATATCTTAATTTGAGTTCTGAGTTGAAGCAACTCGAAGCTAAAACTGCTAGTCTTTTTCAAACTACTGTTGAATTACAAGGTCTTATTGATCAGATTAACAGTACATATGTTGATTTGAAGTTGCTTAATAGGTTTGAAAATTATATCAAATGGCCTTGGTGGGTTTGGCTCATTATTTCTGTTGTTTTTGTTGTATTGTTGAGTCTTCTTGTGTTTTGTTGTCTTTCTACAGGTTGTTGTGGTTGTTGCAATTGTTTAACTTCTTCAATGCGAGGCTGTTGTGATTGTGGTTCAACTAAACTTCCTTATTACGAATTTGAAAAGGTCCACATTCAATAA

>gi|380710230|gb|JQ765569.1| Human coronavirus NL63 strain NL63/DEN/2005/232, complete genome

ATGAAACTTTTCTTGATTTTGCTTATTTTGCCCCTGGTTTCTTGCTTTTCTACATGTAACAGTAATGCTAGTATTTCTATGTTACAATTAGGTGTTCCTGATAACTCTTCAACTATTGTCACAGGTTTGTTGCCAGTCCATTGGATTTGTGCTAATCAGAGCACATCTAGTTACCCAGCCAATGGCTTTTTCTATATTGATGTCGGTAAACACCGTAGTGCTTTTGCACTCCATAGTGGTTATTATGATGCTAACCAGTATTATATTTATCTCACTAATAAAATACATTTAAATGCTCCTGTCACTCTGAAGATTTGTAAGTTTGGAAACACTTCTTTTGATTTTTTAAGTAATGTTTCTACTTCTCATCATTGTATAGTTAATTCGTCATTCACAGAACAGTTAGGTGTGCCTTTGGGCATAACTATATCGGGTGAAACTGTACGTTTGCATTTATATAATGCAACTCGCACCTTTTATGTGCCAGCCGCTTATAAACTTACTAAACTTAGTGTTAAATGTTACTTTAGTGAATCCTGTGTTTTTAGTGTTGTCAATGCTACCGTTACTGTTAATGTCACCACACTTAATGGCCGTATAGTTAATTACACTGTTTGTGATGATTGTAATGGTTACACTGATAACATATTTTCTGTTCAACAGGATGGCCGCATTCCTAATGGTTTTTCTTTTAATAATTGGTTTTTGTTAACTAATGGTTCTACACTAGTGGACGGTGTCTCTAGACTTTACCAACCACTTCGTTTAACTTGTTTATGGCCTGTACCTGGTCTTAAATCTTCGACTGGTTTTGTTTATTTTAACGCCACTGGTTCTGATGTTAATTGTAATGGTTATCAACATCATTCTGTTGCTGATGTTATGCGTTACAATCTTAACTTCAGTTCTAATTCTGTGGATAATCTCAAGAGTGGTGTTATAGTTTTTAAAACTCTACAGTACGATGTTTTGTTTTACTGTAGTAATTCTTCTTCGGGCGTTCTAGACACCACAATACCTTTTGGCCCTTCCTCTCAACCTTATTACTGTTTTATAAATAGTACCATCAACACCACTCATGTTAGCACTTTTGTGGGTGTCCTACCACCCATTGTGCGTGAGATTGTTGTTGCTAGAACTGGTCAGTTTTATATTAATGGTTTTAAGTATTTTGATTTGGGTTTTATCGAAGCTGTCAATTTTAATGTCACGACAGCTAGTGCTACAGATTTTTGGACGGTTGCATTTGCTACTTTTGTTGATGTTTTGGTTAATGTTAGTGCAACTAACATTCAAAACTTACTTTATTGCGATTCTCCATTTGAAAAGTTGCAGTGTGAGCACTTGCAGTTTGGATTGCAAGATGGTTTTTATTCTGCAAATTTTCTTGATGATAATGTTTTGCCTGATACTTATGTTGCACTTCCTATTTATTATCAACATACTGATATAAATTTTACTGCGACTGCATCTTTTGGTGGTTCTTGTTATGTTTGCAAACCACATCAGGTTAATATATCTCTTAATGGTAATACTTCAGTGTGTGTTAGAACATCTCATTTTTCAATTAGGTATATTTATAACCGCGTTAAGAGTGGTTCACCAGGTGACTCTTCATGGCACATTTATTTAAAGAGTGGCACTTGTCCATTTTCTTTTTCTAAGTTAAATAATTTTCAAAAGTTTAAGACTATTTGTTTCTCAACCGTCGCAGTGCCTGGTAGTTGTAATTTTCCACTTGAAGCCACCTGGCATTACACTTCTTATACTATTGTTGGTGCTTTGTATGTTACTTGGTCTGAAGGTAATTCCATTACTGGTGTACCTTATCCTGTCTCTGGTATTCGTGAGTTTAGTAATTTAGTTTTAAATAATTGTACCAAATATAATATTTATGATTATGTTGGTACTGGAATTATACGTTCTTCAAACCAGTCACTTGCTGGTGGTATTACATATGTTTCTAACTCTGGTAATTTACTTGGTTTTAAAAATGTTTCCACTGGTAACATTTTTATTGTGACACCATGTAACCAACCAGACCAAGTAGCTGTTTATCAACAAAGCATTATTGGTGCCATGACTGCTGTTAATGAGTCTAGATATGGCTTGCAAAACTTACTACAGTTACCTAACTTTTATTATGTTAGTAATGGTGGTAACAATTGCACTACGGCCGTTATGACTTATTCTAATTTTGGTATTTGTGCTGATGGTTCTTTAATTCCTGTTCGTCCGCGTAATTCTAGTGATAATGGTATTTCAGCCATAATCACTGCTAATTTATCCATTCCCTCTAACTGGACTACTTCAGTTCAAGTTGAGTACCTCCAAATTACTAGTACTCCAATAGTTGTTGATTGTGCTACTTATGTGTGTAATGGTAACCCTCGCTGTAAGAATTTACTTAAGCAGTATACTTCTGCTTGTAAAACTATTGAAGATGCCTTACGACTTAGTGCTCATTTGGAAACTAATGATGTTAGTAGTATGCTAACTTTCGATAGCAATGCTTTTAGTTTGGCTAATGTTACTAGTTTTGGAGATTATAACCTTTCTAGTGTTTTACCTCAGAGAAACATTCATTCAAGCCGTATAGCAGGACGTAGTGCTTTGGAAGATTTGTTGTTTAGCAAAGTTGTTACATCTGGTTTGGGTACTGTTGATGTTGACTATAAGTCT

TGTACTAAAGGTCTTTCTATTGCTGACCTTGCTTGTGCTCAGTACTACAATGGCATAATGGTTTTGCCAGGTGTTGCTG
ATGCTGAACGTATGGCCATGTACACAGGTTCTCTTATAGGTGGCATGGTGCTCGGAGGTCTTACATCAGCAGCCGCCAT
ACCTTTTTCTTTGGCACTGCAAGCACGACTTAACTATGTTGCTTTACAAACTGATGTGCTTCAAGAAAATCAGAAAATT
TTGGCTGCATCATTTAATAAGGCTATTAATAACATTGTTGCTTCTTTTAGTAGCGTTAATGATGCTATTACACAAACTG
CAGAGGCTATACATACTGTTACTATTGCACTTAATAAGATTCAGGATGTTGTTAATCAACAGGGTAGTGCTCTTAACCA
TCTCACTTCACAATTGAGACATAATTTTCAGGCCATTTCTAATTCAATTCAAGCTATTTATGACCGGCTTGATTCAATT
CAAGCCGATCAACAAGTTGACAGATTAATTACTGGACGGCTTGCAGCTTTGAATGCATTTGTTTCCCAAGTTTTGAATA
AATATACTGAAGTTCGTAGTTCCAGACGCTTAGCACAGCAGAAGATTAATGAATGTGTCAAATCACAATCTAATAGATA
TGGTTTTTGTGGCAATGGCACCCATATCTTTTCAATCGTCAACTCAGCTCCAGATGGTTTGCTTTTTCTTCATACTGTT
TTGCTGCCAACCGACTACAAGAATGTAAAGGCGTGGTCGGGTATCTGTGTTGATGGCATTTACGGCTATGTTCTGCGTC
AACCTAACTTGGTTCTTTATTCTGATAATGGTGTCTTTCGTGTAACTTCCAGGGTCATGTTTCAACCTCGTTTACCTGT
TTTGTCTGATTTTGTGCAAATATATAATTGTAATGTTACTTTTGTTAACATATCTCGTGTCGAGTTACATACTGTCATA
CCTGACTACGTTGATGTTAATAAAACATTACAAGAGTTTGCACAAAACTTACCAAAGTATGTTAAGCCTAATTTTGACT
TGACTCCTTTTAATTTAACATATCTTAATTTGAGTTCTGAGTTGAAGCAACTCGAAGCTAAAACTGCTAGTCTTTTCCA
AACTACTGTTGAATTACAAGGTCTTATTGATCAGATTAACAGTACATATGTTGATTTGAAGTTGCTTAATAGGTTTGAA
AATTATATTAAATGGCCTTGGTGGGTTTGGCTCATTATTTCTGTTGTTTTTGTTGTATTGTTGAGTCTTCTTGTGTTTT
GTTGTCTTTCTACAGGTTGTTGTGGTTGTTGCAATTGTTTAACTTCATCAATGCGAGGTTGTTGTGATTGTGGTTCAAC
TAAACTTCCTTATTACGAATTTGAAAAGGTCCACGTTCAATAA

>gi|380710238|gb|JQ765570.1| Human coronavirus NL63 strain NL63/DEN/2005/235, complete genome

ATGAAACTTTTCTTGATTTTGCTTATTTTGCCCCTGGTTTCTTGCTTTTCTACATGTAACAGTAATGCTAGTATT
TCTATGTTACAATTAGGTGTTCCTGATAACTCTTCAACTATTGTCACAGGTTTGTTGCCAGTCCATTGGATTTGTGCTA
ATCAGAGCACATCTAGTTACCCAGCCAATGGCTTTTTCTATATTGATGTCGGTAAACACCGTAGTGCTTTTGCACTCCA
TAGTGGTTATTATGATGCTAACCAGTATTATATTTATCTCACTAATAAAATACATTTAAATGCTCCTGTCACTCTGAAG
ATTTGTAAGTTTGGAAACACTTCTTTTGATTTTTTAAGTAATGTTTCTACTTCTCATCATTGTATAGTTAATTCGTCAT
TCACAGAACAGTTAGGTGTGCCTTTGGGCATAACTATATCGGGTGAAACTGTACGTTTGCATTTATATAATGCAACTCG
CACCTTTTATGTGCCAGCCGCTTATAAACTTACTAAACTTAGTGTTAAATGTTACTTTAGTGAATCCTGTGTTTTTAGT
GTTGTCAATGCTACCGTTACTGTTAATGTCACCACACTTAATGGCCGTATAGTTAATTACACTGTTTGTGATGATTGTA
ATGGTTACACTGATAACATATTTTCTGTTCAACAGGATGGCCGCATTCCTAATGGTTTTTCTTTTAATAATTGGTTTTT
GTTAACTAATGGTTCTACACTAGTGGACGGTGTCTCTAGACTTTACCAACCACTTCGTTTAACTTGTTTATGGCCTGTA
CCTGGTCTTAAATCTTCGACTGGTTTTGTTTATTTTAACGCCACTGGTTCTGATGTTAATTGTAATGGTTATCAACATC
ATTCTGTTGCTGATGTTATGCGTTACAATCTTAACTTCAGTTCTAATTCTGTGGATAATCTCAAGAGTGGTGTTATAGT
TTTTAAAACTCTACAGTACGATGTTTTGTTTTACTGTAGTAATTCTTCTTCGGGCGTTCTAGACACCACAATACCTTTT
GGCCCTTCCTCTCAACCTTATTACTGTTTTATAAATAGTACCATCAACACCACTCATGTTAGCACTTTTGTGGGTGTCC
TACCACCCATTGTGCGTGAGATTGTTGTTGCTAGAACTGGTCAGTTTTATATTAATGGTTTTAAGTATTTTGATTTGGG
TTTTATCGAAGCTGTCAATTTTAATGTCACGACAGCTAGTGCTACAGATTTTTGGACGGTTGCATTTGCTACTTTTGTT
GATGTTTTGGTTAATGTTAGTGCAACTAACATTCAAAACTTACTTTATTGCGATTCTCCATTTGAAAAGTTGCAGTGTG
AGCACTTGCAGTTTGGATTGCAAGATGGTTTTTATTCTGCAAATTTTCTTGATGATAATGTTTTGCCTGATACTTATGT
TGCACTTCCTATTTATTATCAACATACTGATATAAATTTTACTGCGACTGCATCTTTTGGTGGTTCTTGTTATGTTTGC
AAACCACATCAGGTTAATATATCTCTTAATGGTAATACTTCAGTGTGTGTTAGAACATCTCATTTTTCAATTAGGTATA
TTTATAACCGCGTTAAGAGTGGTTCACCAGGTGACTCTTCATGGCACATTTATTTAAAGAGTGGCACTTGTCCATTTTC

TTTTTCTAAGTTAAATAATTTTCAAAAGTTTAAGACTATTTGTTTTTCAACCGTCGCAGTGCCTGGTAGTTGTAATTTT
CCACTTGAAGCCACCTGGCATTACACTTCTTATACTATTGTTGGTGCTTTGTATGTTACTTGGTCTGAAGGTAATTCCA
TTACTGGTGTACCTTATCCTGTCTCTGGTATTCGTGAGTTTAGTAATTTAGTTTTAAATAATTGTACCAAATATAATAT
TTATGATTATGTTGGTACTGGAATTATACGTTCTTCAAACCAGTCACTTGCTGGTGGTATTACATATGTTTCTAACTCT
GGTAATTTACTTGGTTTTAAAAATGTTTCCACTGGTAACATTTTTATTGTGACACCATGTAACCAACCAGACCAAGTAG
CTGTTTATCAACAAAGCATTATTGGTGCCATGACTGCTGTTAATGAGTCTAGATATGGCTTGCAAAACTTACTACAGTT
ACCTAACTTTTATTATGTTAGTAATGGTGGTAACAATTGCACTACGGCCGTTATGACTTATTCTAATTTTGGTATTTGT
GCTGATGGTTCTTTAATTCCTGTTCGTCCGCGTAATTCTAGTGATAATGGTATTTCAGCCATAATCACTGCTAATTTAT
CCATTCCCTCTAACTGGACTACTTCAGTTCAAGTTGAGTACCTCCAAATTACTAGTACTCCAATAGTTGTTGATTGTGC
TACTTATGTGTGTAATGGTAACCCTCGCTGTAAGAATCTACTTAAGCAGTATACTTCTGCTTGTAAAACTATTGAAGAT
GCCTTACGACTTAGTGCTCATTTGGAAACTAATGATGTTAGTAGTATGCTAACTTTCGATAGCAATGCTTTTAGTTTGG
CTAATGTTACTAGTTTTGGAGATTATAACCTTTCTAGTGTTTTACCTCAGAGAAACATTCATTCAAGCCGTATAGCAGG
ACGTAGTGCTTTGGAAGATTTGTTGTTTAGCAAAGTTGTTACATCTGGTTTGGGTACTGTTGATGTTGACTATAAGTCT
TGTACTAAAGGTCTTTCTATTGCTGACCTTGCTTGTGCGCAGTACTACAATGGCATAATGGTTTTGCCAGGTGTTGCTG
ATGCTGAACGTATGGCCATGTACACAGGTTCTCTTATAGGTGGCATGGTGCTCGGAGGTCTTACATCAGCAGCCGCCAT
ACCTTTTTCTTTGGCACTGCAAGCACGACTTAACTATGTTGCTTTACAAACTGATGTGCTTCAAGAAAATCAGAAAATT
TTGGCTGCATCATTTAATAAGGCTATTAATAACATTGTTGCTTCTTTTAGTAGCGTTAATGATGCTATTACACAAACTG
CAGAGGCTATACATACTGTTACTATTGCACTTAATAAGATTCAGGATGTTGTTAATCAACAGGGTAGTGCTCTTAACCA
TCTCACTTCACAATTGAGACATAATTTTCAGGCCATTTCTAATTCAATTCAAGCTATTTATGACCGGCTTGATTCAATT
CAAGCCGATCAACAAGTTGACAGATTAATTACTGGACGGCTTGCAGCTTTGAATGCATTTGTTTCCCAAGTTTTGAATA
AATATACTGAAGTTCGTAGTTCCAGACGCTTAGCACAGCAGAAGATTAATGAATGTGTCAAATCACAATCTAATAGATA
TGGTTTTTGTGGCAATGGCACCCATATCTTTTCAATCGTCAACTCAGCTCCAGATGGTTTGCTTTTTCTTCATACTGTT
TTGCTGCCAACCGACTACAAGAATGTAAAGGCGTGGTCGGGTATCTGTGTTGATGGCATTTACGGCTATGTTCTGCGTC
AACCTAACTTGGTTCTTTATTCTGATAATGGTGTCTTTCGTGTAACTTCCAGGGTCATGTTTCAACCTCGTTTACCTGT
TTTGTCTGATTTTGTGCAAATATATAATTGTAATGTTACTTTTGTTAACATATCTCGTGTCGAGTTACATACTGTCATA
CCTGACTACGTTGATGTTAATAAAACATTACAAGAGTTTGCACAAAACTTACCAAAGTATGTTAAGCCTAATTTTGACT
TGACTCCTTTTAATTTAACATATCTTAATTTGAGTTCTGAGTTGAAGCAACTCGAAGCTAAAACTGCTAGTCTTTTCCA
AACTACTGTTGAATTACAAGGTCTTATTGATCAGATTAACAGTACATATGTTGATTTGAAGTTGCTTAATAGGTTTGAA
AATTATATTAAATGGCCTTGGTGGGTTTGGCTCATTATTTCTGTTGTTTTTGTTGTATTGTTGAGTCTTCTTGTGTTTT
GTTGTCTTTCTACAGGTTGTTGTGGTTGTTGCAATTGTTTAACTTCATCAATGCGAGGTTGTTGTGATTGTGGTTCAAC
TAAACTTCCTTATTACGAATTTGAAAAGGTCCACGTTCAATAA

>gi|380710246|gb|JQ765571.1| Human coronavirus NL63 strain NL63/DEN/2005/271, complete genome

ATGAAACTTTTCTTGATTTTGCTTGTTTTGCCCCTGGCCTCTTGCTTTTTCACATGTAATAGTAATGCTAATCTC
TCTATGTTACAATTAGGTGTTCCTGACAATTCTTCAACTATTGTTACGGGTTTATTGCCAACTCATTGGTTTTGTGCTA
ATCAGAGTACATCTGTTTACTCAGCCAATGGTTTCTTTTATATTGATGTTGGTAATCACCGTAGTGCTTTTGCGCTCCA
TACTGGTTATTATGATGCTAATCAGTATTATATTTATGTTACTAATGAAATAGGCTTAAATGCTTCTGTTACTCTTAAG
ATTTGTAAGTTTAGTAGAAACACTACTTTTGATTTTTTAAGTAATGCTTCTAGTTCTTTTGACTGTATAGTTAATTTGT
TATTTACAGAACAGTTAGGTGCGCCTTTGGGCATAACTATATCTGGTGAAACTGTGCGTCTGCATTTATATAATGTAAC
TCGTACTTTTTATGTGCCAGCAGCTTATAAACTTACTAAACTTAGTGTTAAATGTTACTTTAACTATTCCTGTGTTTTT
AGTGTTGTCAACGCCACCGTTACTGTGAATGTCACCACACATAATGGCCGTGTAGTTAACTACACTGTTTGTGATGATT

GTAATGGTTATACTGATAACATATTTTCTGTTCAACAGGATGGTCGCATTCCTAATGGTTTCCCTTTTAATAATTGGTT
TTTGTTAACTAATGGTTCCACACTAGTGGACGGGGTCTCTAGACTTTATCAACCACTCCGTTTAACTTGTTTATGGCCT
GTACCTGGTCTTAAATCTTCAACTGGTTTTGTTTATTTTAATGCCACTGGTTCTGATGTTAATTGTAACGGCTATCAAC
ATAATTCTGTTGCTGATGTTATGCGTTACAATCTTAACTTCAGTGCTAATTCTGTGGACAATCTCAAGAGTGGTGTTAT
AGTTTTTAAAACTTTACAGTACGATGTTTTGTTTTATTGTAGTAATTCTTCTTCAGGTGTTCTTGACACCACAATACCT
TTTGGCCCTTCCTCTCAACCTTATTACTGTTTTATAAACAGCACTATCAACACTACTCATGTTAGCACTTTTGTGGGTA
TTTTACCACCCACTGTGCGTGAAATTGTTGTTGCTAGAACTGGTCAGTTTTATATTAATGGTTTTAAGTATTTCGATTT
GGGTTTCATAGAAGCTGTCAATTTTAATGTCACGACTGCTAGTGCCACAGATTTTTGGACGGTTGCATTTGCTACTTTT
GTTGATGTTTTGGTTAATGTTAGTGCAACTAACATTCAAAACTTACTTTATTGCGATTCTCCATTTGAAAAGTTGCAGT
GTGAGCACTTGCAGTTTGGATTGCAGGATGGTTTTTATTCTGCAAATTTTCTTGATGATAATGTTTTGCCTGAGACTTA
TGTTGCACTCCCCATTTATTATCAACACACGGACATAAATTTTACTGCAACTGCATCTTTTGGTGGTTCTTGTTATGTT
TGTAAACCACACCAGGTTAATATATCTCTTAATGGTAACACTTCAGTGTGTGTTAGAACATCTCATTTTTCAATTAGGT
ATATTTATAACCGCGTTAAGAGTGGTTCACCAGGTGACTCTTCTTGGCACATTTATTTAAAGAGTGGCACTTGTCCATT
TTCTTTTTCTAAGTTAAATAATTTTCAAAAGTTCAAGACTATTTGTTTCTCAACCGTCGAAGTGCCTGGTAGTTGTAAT
TTTCCACTTGAAGCCACCTGGCATTACACTTCTTATACTATTGTTGGTGCTTTGTATGTTACTTGGTCTGAAGGTAATT
CTATTACTGGTGTACCTTATCCTGTCTCTGGTATTCGTGAGTTTAGTAATTTAGTTTTAAATAATTGTACCAAATATAA
TATTTATGATTATGTTGGTACTGGAATTATACGTTCTTCAAACCAGTCACTTGCTGGTGGTATTACATATGTTTCTAAC
TCTGGTAATTTACTTGGTTTTAAAAATGTTTCCACTGGTAACATTTTTATTGTGACACCATGTAACCAACCAGACCAAG
TAGCTGTTTATCAACAAAGCATTATTGGTGCCATGACCGCTGTTAATGAGTCTAGATATGGCTTGCAAAACTTACTACA
GTTACCTAACTTTTATTATGTTAGTAATGGTGGTAACAATTGCACTACGGCCGTTATGACTTATTCTAATTTTGGTATT
TGTGCTGATGGTTCTTTGATTCCTGTTCGTCCGCGTAATTCTAGTGATAATGGTATTTCAGCCATAATCACTGCTAATT
TATCCATTCCCTCTAACTGGACTACTTCAGTTCAAGTTGAGTACCTCCAAATTACTAGTACTCCAATAGTTGTTGATTG
TGCCACTTATGTGTGTAATGGTAACCCTCGCTGTAAGAATCTACTTAAGCAGTATACTTCTGCTTGTAAAACTATTGAA
GATGCCTTACGACTTAGTGCTCATTTGGAAACTAATGATGTTAGTAGTATGCTAACTTTCGATAGCAATGCTTTTAGTT
TGGCTAATGTTACTAGTTTTGGAGATTATAACCTTTCTAGTGTTTTACCTCAGAGAAACATTCGTTCAAGCCGTATAGC
AGGACGTAGTGCTTTGGAAGATTTGTTGTTTAGCAAAGTTGTTACATCTGGTTTGGGTACTGTTGATGTTGACTATAAG
TCTTGTACTAAAGGTCTTTCTATTGCTGACCTTGCTTGTGCTCAGTACTACAATGGCATAATGGTTTTGCCAGGTGTTG
CTGATGCTGAACGTATGGCCATGTACACAGGTTCTCTTATAGGTGGCATGGTGCTCGGAGGTCTTACATCAGCAGCCGC
CATACCTTTTTCTTTGGCACTGCAAGCACGACTTAACTATGTTGCTTTACAAACTGATGTGCTTCAAGAAAATCAGAAA
ATTTTGGCTGCATCATTTAATAAGGCTATTAATAATATTGTTGCTTCTTTTAGTAGCGTTAATGATGCTATTACACAAA
CTGCAGAGGCTATACATACTGTTACTATTGCACTTAATAAGATTCAGGATGTTGTTAATCAACAGGGTAGTGCTCTTAA
CCATCTCACTTCACAATTGAGACATAATTTTCAGGCCATTTCTAATTCAATTCAGGCTATTTATGACCGGCTTGATTCA
ATTCAAGCCGATCAACAAGTTGACAGATTAATTACTGGACGGCTTGCAGCTTTGAATGCATTTGTTTCTCAAGTTTTGA
ATAAATATACTGAAGTTCGTGGTTCCAGACGCTTAGCACAGCAGAAGATTAATGAATGTGTCAAGTCACAATCTAATAG
ATATGGTTTTTGTGGCAATGGCACTCACATCTTTTCAATCGTCAACTCAGCTCCAGATGGTTTGCTTTTTCTTCATACT
GTTTTGCTGCCAACTGATTACAAGAATGTAAAGGCGTGGTCTGGTATCTGTGTTGATGGTATTTATGGCTATGTTCTGC
GTCAACCTAACTTGGTTCTTTATTCTGATAATGGTGTCTTTCGTGTAACTTCCAGGGTCATGTTTCAACCTCGCTTACC
TGTTTTGTCTGATTTTGTGCAAATATATAATTGTAATGTTACTTTTGTTAACATATCTCGTGTCGAGTTACATACTGTC
ATACCTGACTACGTTGATGTTAATAAAACATTACAAGAGTTTGCACAAAACTTACCAAAGTATGTTAAGCCTAATTTTG
ACTTGACTCCTTTTAATTTAACATATCTTAATTTGAGTTCTGAGTTGAAGCAACTCGAAGCTAAAACTGCTAGTCTTTT
TCAAACTACTGTTGAATTACAAGGTCTTATTGATCAGATTAACAGTACATATGTTGATTTGAAGTTGCTTAATAGGTTT
GAAAATTATATCAAATGGCCTTGGTGGGTTTGGCTCATTATTTCTGTTGTTTTTGTTGTATTGTTGAGTCTTCTTGTGT
TTTGTTGTCTTTCTACAGGTTGTTGTGGTTGTTGCAATTGTTTAACTTCATCAATGCGAGGCTGTTGTGATTGTGGTTC
AACTAAACTTCCTTATTACGAATTTGAAAAGGTCCACGTTCAATAA

>gi|380710254|gb|JQ765572.1| Human coronavirus NL63 strain NL63/DEN/2005/347, complete genome

ATGAAACTTTTCTTGATTTTGCTTGTTTTGCCCCTGGCCTCTTGCTTTTTCACATGTAATAGTAATGCTAATCTC
TCTATGTTACAATTAGGTGTTCCTGACAATTCTTCAACTATTGTTACGGGTTTATTGCCAACTCATTGGTTTTGTGCTA
ATCAGAGTACATCTGTTTACTCAGCCAATGGTTTCTTTTATATTGATGTTGGTAATCACCGTAGTGCTTTTGCGCTCCA
TACTGGTTATTATGATGCTAATCAGTATTATATTTATGTTACTAATGAAATAGGCTTAAATGCTTCTGTTACTCTTAAG
ATTTGTAAGTTTAGTAGAAACACTACTTTTGATTTTTTAAGTAATGCTTCTAGTTCTTTTGACTGTATAGTTAATTTGT
TATTTACAGAACAGTTAGGTGCGCCTTTGGGCATAACTATATCTGGTGAAACTGTGCGTCTGCATTTATATAATGTAAC
TCGTACTTTTTATGTGCCAGCAGCTTATAAACTTACTAAACTTAGTGTTAAATGTTACTTTAACTATTCCTGTGTTTTT
AGTGTTGTCAACGCCACCGTTACTGTGAATGTCACCACACATAATGGCCGTGTAGTTAACTACACTGTTTGTGATGATT
GTAATGGTTATACTGATAACATATTTTCTGTTCAACAAGATGGTCGCATTCCTAATGGTTTCCCTTTTAATAATTGGTT
TTTGTTAACTAATGGTTCCACACTAGTGGACGGGGTCTCTAGACTTTATCAACCACTCCGTTTAACTTGTTTATGGCCT
GTACCTGGTCTTAAATCTTCAACTGGTTTTGTTTATTTTAATGCCACTGGTTCTGATGTTAATTGTAACGGCTATCAAC
ATAATTCTGTTGCTGATGTTATGCGTTACAATCTTAACTTCAGTGCTAATTCTGTGGACAATCTCAAGAGTGGTGTTAT
AGTTTTTAAAACTTTACAGTACGATGTTTTGTTTTATTGTAGTAATTCTTCTTCAGGTGTTCTTGACACCACAATACCT
TTTGGCCCTTCCTCTCAACCTTATTACTGTTTTATAAACAGCACTATCAACACTACTCATGTTAGCACTTTTGTGGGTA
TTTTACCACCCACTGTGCGTGAAATTGTTGTTGCTAGAACTGGTCAGTTTTATATTAATGGTTTTAAGTATTTCGATTT
GGGTTTCATAGAAGCTGTCAATTTTAATGTCACGACTGCTAGTGCCACAGATTTTTGGACGGTTGCATTTGCTACTTTT
GTTGATGTTTTGGTTAATGTTAGTGCAACTAACATTCAAAACTTACTTTATTGCGATTCTCCATTTGAAAAGTTGCAGT
GTGAGCACTTGCAGTTTGGATTGCAGGATGGTTTTTATTCTGCAAATTTTCTTGATGATAATGTTTTGCCTGAGACTTA
TGTTGCACTCCCCATTTATTATCAACACACGGACATAAATTTTACTGCAACTGCATCTTTTGGTGGTTCTTGTTATGTT
TGTAAACCACACCAGGTTAATATATCTCTTAATGGTAACACTTCAGTGTGTGTTAGAACATCTCATTTTTCAATTAGGT
ATATTTATAACCGCGTTAAGAGTGGTTCACCAGGTGACTCTTCATGGCACATTTATTTAAAGAGTGGCACTTGTCCATT
TTCTTTTTCTAAGTTAAATAATTTTCAAAAGTTCAAGACTATTTGTTTCTCAACCGTCGAAGTGCCTGGTAGTTGTAAT
TTTCCACTTGAAGCCACCTGGCATTACACTTCTTATACTATTGTTGGTGCTTTGTATGTTACTTGGTCTGAAGGTAATT
CTATTACTGGTGTACCTTATCCTGTCTCTGGTATTCGTGAGTTTAGTAATTTAGTTTTAAATAATTGTACCAAATATAA
TATTTATGATTATGTTGGTACTGGAATTATACGTTCTTCAAACCAGTCACTTGCTGGTGGTATTACATATGTTTCTAAC
TCTGGTAATTTACTTGGTTTTAAAAATGTTTCCACTGGTAACATTTTTATTGTGACACCATGTAACCAACCAGACCAAG
TAGCTGTTTATCAACAAAGCATTATTGGTGCCATGACCGCTGTTAATGAGTCTAGATATGGCTTGCAAAACTTACTACA
GTTACCTAACTTTTATTATGTTAGTAATGGTGGTAACAATTGCACTACGGCCGTTATGACTTATTCTAATTTTGGTATT
TGTGCTGATGGTTCTTTGATTCCTGTTCGTCCGCGTAATTCTAGTGATAATGGTATTTCAGCCATAATCACTGCTAATT
TATCCATTCCCTCTAACTGGACTACTTCAGTTCAAGTTGAGTACCTCCAAATTACTAGTACTCCAATAGTTGTTGATTG
TGCCACTTATGTGTGTAATGGTAACCCTCGCTGTAAGAATCTACTTAAGCAGTATACTTCTGCTTGTAAAACTATTGAA
GATGCCTTACGACTTAGTGCTCATTTGGAAACTAATGATGTTAGTAGTATGCTAACTTTCGATAGCAATGCTTTTAGTT
TGGCTAATGTTACTAGTTTTGGAGATTATAACCTTTCTAGTGTTTTACCTCAGAGAAACATTCGTTCAAGCCGTATAGC
AGGACGTAGTGCTTTGGAAGATTTGTTGTTTAGCAAAGTTGTTACATCTGGTTTGGGTACTGTTGATGTTGACTATAAG
TCTTGTACTAAAGGTCTTTCTATTGCTGACCTTGCTTGTGCTCAGTACTACAATGGCATAATGGTTTTGCCAGGTGTTG
CTGATGCTGAACGTATGGCCATGTACACAGGTTCTCTTATAGGTGGCATGGTGCTCGGAGGTCTTACATCAGCAGCTGC
CATACCTTTTTCTTTGGCACTGCAAGCACGACTTAACTATGTTGCTTTACAAACTGATGTGCTTCAAGAAAATCAGAAA
ATTTTGGCTGCATCATTTAATAAGGCTATTAATAATATTGTTGCTTCTTTTAGTAGCGTTAATGATGCTATTACACAAA
CTGCAGAGGCTATACATACTGTTACTATTGCACTTAATAAGATTCAGGATGTTGTTAATCAACAGGGTAGTGCTCTTAA
CCATCTCACTTCACAATTGAGACATAATTTTCAGGCCATTTCTAATTCAATTCAGGCTATTTATGACCGGCTTGATTCA

ATTCAAGCCGATCAACAAGTTGACAGATTAATTACTGGACGGCTTGCAGCTTTGAATGCATTTGTTTCCCAAGTTTTGA
ATAAATATACTGAAGTTCGTGGTTCCAGACGCTTAGCACAGCAGAAGATTAATGAATGTGTCAAGTCACAATCTAATAG
ATATGGTTTTTGTGGCAATGGCACTCACATCTTTTCAATCGTCAACTCAGCTCCAGATGGTTTGCTTTTTCTTCATACT
GTTTTGCTGCCAACTGATTACAAGAATGTAAAGGCGTGGTCTGGTATCTGTGTTGATGGTATTTATGGCTATGTTCTGC
GTCAACCTAACTTGGTTCTTTATTCTGATAATGGTGTCTTTCGTGTAACTTCCAGGGTCATGTTTCAACCTCGCTTACC
TGTTTTGTCTGATTTTGTGCAAATATATAATTGTAATGTTACTTTTGTTAACATATCTCGTGTCGAGTTACATACTGTC
ATACCTGACTACGTTGATGTTAATAAAACATTACAAGAGTTTGCACAAAACTTACCAAAGTATGTTAAGCCTAATTTTG
ACTTGACTCCTTTTAATTTAACATATCTTAATTTGAGTTCTGAGTTGAAGCAACTCGAAGCTAAAACTGCTAGTCTTTT
TCAAACTACTGTTGAATTACAAGGTCTTATTGATCAGATTAACAGTACATATGTTGATTTGAAGTTGCTTAATAGGTTT
GAAAATTATATCAAATGGCCTTGGTGGGTTTGGCTCATTATTTCTGTTGTTTTTGTTGTATTGTTGAGTCTTCTTGTGT
TTTGTTGTCTTTCTACAGGTTGTTGTGGTTGTTGCAATTGTTTAACTTCATCAATGCGAGGCTGTTGTGATTGTGGTTC
AACTAAACTTCCTTATTACGAATTTGAAAAGGTCCACGTTCAATAA

>gi|380710264|gb|JQ765573.1| Human coronavirus NL63 strain NL63/DEN/2005/1062, complete genome

ATGAAACTTTTCTTGATTTTGCTTGTTTTGCCCCTGGCCTCTTGCTTTTTCACATGTAATAGTAATGCTAATCTC
TCTATGTTACAATTAGGTGTTCCTGACAATTCTTCAACTATTGTTACGGGTTTATTGCCAACTCATTGGTTTTGTGCTA
ATCAGAGTACATCTGTTTACTCAGCCAATGGTTTCTTTTATATTGATGTTGGTAATCACCGTAGTGCTTTTGCGCTCCA
TACTGGTTATTATGATGCTAATCAGTATTATATTTATGTTACTAATGAAATAGGCTTAAATGCTTCTGTTACTCTTAAG
ATTTGTAAGTTTAGTAGAAACACTACTTTTGATTTTTTAAGTAATGCTTCTAGTTCTTTTGACTGTATAGTTAATTTGT
TATTTACAGAACAGTTAGGTGCGCCTTTGGGCATAACTATATCTGGTGAAACTGTGCGTCTGCATTTATATAATGTAAC
TCGTACTTTTTATGTACCAGCAGCTTATAAACTTACTAAACTTAGTGTTAAATGTTACTTTAACTATTCCTGTGTTTTT
AGTGTTGTCAACGCCACCGTTACTGTGAATGTCACCACACATAATGGCCGTGTAGTTAACTACACTGTTTGTGATGATT
GTAATGGTTATACTGATAACATATTTTCTGTTCAACAGGATGGCCGCATTCCTAATGGTTTCCCTTTTAATAATTGGTT
TTTGTTAACTAATGGTTCCACACTAGTGGACGGGGTCTCTAGACTTTATCAACCACTCCGTCTAACTTGTTTATGGCCT
GTACCTGGTCTTAAATCTTCAACTGGTTTTGTTTATTTTAATGCCACTGGTTCTGATGTTAATTGTAACGGCTATCAAC
ATAATTCTGTTGCTGATGTTATGCGTTACAATCTTAACTTCAGTGCTAATTCTGTGGACAATCTCAAGAGTGGTGTTAT
AGTTTTTAAAACTTTACAGTACGATGTTTTGTTTTATTGTAGTAACTCTTCCTCAGGTGTTCTTGACACCACAATACCT
TTTGGCCCTTCCTCTCAACCTTATTACTGTTTTATAAACAGCACTATCAACACTACTCATGTTAGCACTTTTGTGGGTA
TTTTACCACCCACTGTGCGTGAAATTGTTGTTGCTAGAACTGGTCAGTTTTATATTAATGGTTTTAAGTATTTCGATTT
GGGTTTCATAGAAGCTGTCAATTTTAATGTCACGACTGCTAGTGCCACAGATTTTTGGACGGTTGCATTTGCTACTTTT
GTTGATGTTTTGGTTAATGTTAGTGCAACTAACATTCAAAACTTACTTTATTGCGATTCTCCATTTGAAAAGTTGCAGT
GTGAGCACTTGCAGTTTGGATTGCAGGATGGTTTTTATTCTGCAAATTTTCTTGATGATAATGTTTTGCCTGAGACTTA
TGTTGCACTCCCCATTTATTATCAACACACGGACATAAATTTTACTGCAACTGCATCTTTTGGTGGTTCTTGTTATGTT
TGTAAACCACACCAGGTTAATATATCTCTTAATGGTAACACTTCAGTGTGTGTTAGAACATCTCATTTTTCAATTAGGT
ATATTTATAACCGCGTTAAGAGTGGTTCACCAGGTGACTCTTCATGGCACATTTATTTAAAGAGTGGCACTTGTCCATT
TTCTTTTTCTAAGTTAAATAATTTTCAAAAGTTCAAGACTATTTGTTTCTCAACCGTCGAAGTGCCTGGTAGTTGTAAT
TTTCCGCTTGAAGCCACCTGGCATTACACTTCTTATACTATTGTTGGTGCTTTGTATGTTACTTGGTCTGAAGGTAATT
CTATTACTGGTGTACCTTATCCTGTCTCTGGTATTCGTGAGTTTAGTAATTTAGTTTTAAATAATTGTACCAAATATAA
TATTTATGATTATGTTGGTACTGGAATTATACGTTCTTCAAACCAGTCACTTGCTGGTGGTATTACATATGTTTCTAAC
TCTGGTAATTTACTTGGTTTTAAAAATGTTTCCACTGGTAACATTTTTATTGTGACACCATGTAACCAACCAGACCAAG
TAGCTGTTTATCAACAAAGCATTATTGGTGCCATGACCGCTGTTAATGAGTCTAGATATGGCTTGCAAAACTTACTACA

GTTACCTAACTTTTATTATGTTAGTAATGGTGGTAACAATTGCACTACGGCCGTTATGACTTATTCTAATTTTGGTATT
TGTGCTGATGGTTCTTTGATTCCTGTTCGTCCGCGTAATTCTAGTGATAATGGTATTTCAGCCATAATCACTGCTAATT
TATCCATTCCCTCTAACTGGACTACTTCAGTTCAAGTTGAGTACCTCCAAATTACTAGTACTCCAATAGTTGTTGATTG
TGCTACTTATGTGTGTAATGGTAACCCTCGCTGTAAGAATCTACTTAAGCAGTATACTTCTGCTTGTAAAACTATTGAA
GATGCCTTACGACTTAGTGCTCATTTGGAAACTAATGATGTTAGTAGTATGCTAACTTTCGATAGCAATGCTTTTAGTT
TGGCTAATGTTACTAGTTTTGGAGACTATAACCTTTCTAGTGTTTTACCTCAGAGAAACATTCGTTCAAGCCGTATAGC
AGGACGTAGTGCTTTGGAAGATTTGTTGTTTAGCAAAGTTGTTACATCTGGTTTGGGTACTGTTGATGTTGACTATAAG
TCTTGTACTAAAGGTCTTTCTATTGCTGACCTTGCTTGTGCTCAGTACTACAATGGCATAATGGTTTTGCCAGGTGTTG
CTGATGCTGAACGTATGGCCATGTACACAGGTTCTCTTATAGGTGGCATGGTGCTCGGAGGTCTTACATCAGCAGCCGC
CATACCTTTTTCTTTGGCACTGCAAGCACGACTTAACTATGTTGCTTTACAAACTGATGTGCTTCAAGAAAATCAGAAA
ATTTTGGCTGCATCATTTAATAAGGCTATTAATAATATTGTTGCTTCTTTTAGTAGCGTTAATGATGCTATTACACAAA
CTGCAGAGGCTATACATACTGTTACTATTGCACTTAATAAGATTCAGGATGTTGTTAATCAACAGGGTAGTGCTCTTAA
CCATCTCACTTCACAATTGAGACATAATTTTCAGGCCATTTCTAATTCAATTCAGGCTATTTATGACCGGCTTGATTCA
ATTCAAGCCGATCAACAAGTTGACAGATTAATTACTGGACGGCTTGCAGCTTTGAATGCATTTGTTTCCCAAGTTTTGA
ATAAATATACTGAAGTTCGTGGTTCAAGACGCTTAGCACAGCAGAAGATTAATGAATGTGTCAAGTCACAATCTAATAG
ATATGGTTTTTGTGGCAATGGCACTCACATCTTTTCAATCGTCAACTCAGCTCCAGATGGTTTGCTTTTTCTTCATACT
GTTTTGCTGCCAACTGATTACAAGAATGTAAAGGCGTGGTCTGGTGTCTGTGTTGATGGTATTTATGGCTATGTTCTGC
GTCAACCTAACTTGGTTCTTTATTCTGATAATGGTGTCTTTCGTGTAACTTCCAGGGTCATGTTTCAACCTCGCTTACC
TGTTTTGTCTGATTTTGTGCAAATATATAATTGTAATGTTACTTTTGTTAACATATCTCGTGTTGAGTTACATACTGTC
ATACCTGACTACGTTGATGTTAATAAAACATTACAAGAGTTTGCACAAAACTTACCAAAGTATGTTAAGCCTAATTTTG
ACTTGACTCCTTTTAATTTAACATATCTTAATTTGAGTTCTGAGTTGAAGCAACTCGAAGCTAAAACTGCTAGTCTTTT
TCAAACTACTGTTGAATTACAAGGTCTTATTGATCAGATTAACAGTACATATGTTGATTTGAAGTTGCTTAATAGGTTT
GAAAATTATATCAAATGGCCTTGGTGGGTTTGGCTCATTATTTCTGTTGTTTTTGTTGTATTGTTGAGTCTTCTTGTGT
TTTGTTGTCTTTCTACAGGTTGTTGTGGTTGTTGCAATTGTTTAACTTCTTCAATGCGAGGCTGTTGTGATTGTGGTTC
AACTAAACTTCCTTATTACGAATTTGAAAAGGTCCACATTCAATAA

>gi|380710272|gb|JQ765574.1| Human coronavirus NL63 strain NL63/DEN/2005/1862, complete genome

ATGAAACTTTTCTTGATTTTGCTTGTTTTGCCCTTGGCCTCTTGCTTTTTCACATGTAATAGTAATGCTAATCTC
TCTATGTTACAATTAGGTGTTCCTGACAATTCTTCAACTATTGTCACGGGTTTATTGCCAACTCATTGGATTTGTGCTA
ATCAGAGTACATCTGTTTACTCAGCCAATGGTTTCTTTTATATTGATGTTGGTAATCACCGTAGTGCTTTTGCGCTCCA
TACTGGTTATTATGATGTTAATCAGTATTATATTTATGTTACTAATGAAATAGGCTTAAATGCTTCTGTTACTCTTAAG
ATTTGTAAGTTTGGTACAAACACTACTTTTGATTTTTTAAGTAATTCTTCTAGTTCTTTTGACTGTATAGTTAATTTGT
TATTTACAGAACAGTTAGGTGCGCCTTTGGGCATAACTATATCTGGTGAAACTGTGCGTCTGCATTTATATAATGTAAC
TCGTACTTTTTATGTGCCAGCAGCTTATAAACTTACTAAACTTAGTGTTAAATGTTACTTTAACTATTCCTGTGTTTTT
AGTGTTGTCAACGCCACCGTTACTGTGAATGTCACCACACATAATGGCCGTGTAGTTAACTACACTGTTTGTGATGATT
GTAATGGTTATACTGATAACATATTTTCTGTTCAACAGGATGGCCGCATTCCTAATGGTTTCCCTTTTAATAATTGGTT
TTTGCTAACTAATGGTTCCACACTAGTGGACGGGGTCTCTAGACTTTATCAACCACTCCGTTTAACTTGTTTATGGCCT
GTACCTGGTCTTAAATCTTCAACTGGTTTTGTTTATTTTAATGCCACTGGTTCTGATGTTAATTGTAACGGCTATCAAC
ATAATTCTGTTGCTGATGTTATGCGTTACAATCTTAACTTCAGTGCTAATTCTGTGGACAATCTCAAGAGTGGTGTTAT
AGTTTTTAAAACTTTACAGTACGATGTTTTGTTTTATTGTAGTAATTCTTCCTCAGGTGTTCTTGACACCACAATACCT
TTTGGCCCTTCCTCTCAACCTTATTACTGTTTTATAAACAGCACTATCAACACTACTCATGTTAGCACTTTTGTGGGTA

TCTTACCACCCACTGTGCGTGAAATTGTTGTTGCTAGAACTGGTCAGTTTTATATTAATGGTTTTAAGTATTTCGATTT
GGGTTTCATAGAAGCTGTCAATTTTAATGTCACGACTGCTAGTGCCACAGATTTTTGGACGGTTGCATTTGCTACTTTT
GTTGATGTTTTGGTTAATGTTAGTGCAACTAACATTCAAAACTTACTTTATTGCGATTCTCCATTTGAAAAGTTGCAGT
GTGAGCACTTGCAGTTTGGATTGCAAGATGGTTTTTATTCTGCAAATTTTCTTGATGATAATGTTTTGCCTGAGACTTA
TGTTGCACTCCCCATTTATTATCAACACACAGACATAAATTTTACTGCAACTGCATCTTTTGGTGGTTCTTGTTATGTT
TGTAAACCACGTCAGGTTAATATATCTCTTAATGGTAACACTTCAGTGTGTGTTAGAACATCTCATTTTTCAATTAGGT
ATATTTATAACCGCGTCAAGAGTGGTTCACCAGGTGACTCTTCATGGCACATTTATTTAAAGAGTGGCACTTGTCCATT
TTCTTTTTCTAAGTTAAATAATTTTCAAAAGTTCAAGACTATTTGTTTCTCAACCGTCGAAGTGCCTGGTAGTTGTAAT
TTTCCACTTGAAGCCACCTGGCATTACACTTCTTATACTATTGTTGGTGCTTTGTATGTTACTTGGTCTGAAGGTAATT
CCATTACTGGTGTACCTTATCCTGTCTCTGGTATTCGTGAGTTTAGTAATTTAGTTTTAAATAATTGTACCAAATATAA
TATTTATGATTATGTTGGTACTGGAATTATACGTTCTTCAAACCAGTCACTTGCTGGTGGTATTACATATGTTTCTAAC
TCTGGTAATTTACTTGGTTTTAAAAATGTTTCCACTGGTAACATTTTTATTGTGACACCATGTAACCAACCAGACCAAG
TAGCTGTTTATCAACAAAGCATTATTGGTGCCATGACCGCTGTTAATGAGTCTAGATATGGCTTGCAAAACTTACTACA
GTTACCTAACTTTTATTATGTTAGTAATGGTGGTAACAATTGCACTACGGCTGTTATGACTTATTCTAATTTTGGTATT
TGTGCTGATGGTTCTTTAATTCCTGTTCGTCCGCGTAATTCTAGTGATAATGGTATTTCAGCCATAATCACTGCTAATT
TATCCATTCCCTCTAACTGGACTACTTCAGTTCAAGTTGAGTACCTCCAAATTACTAGTACTCCAATAGTTGTTGATTG
TGCTACTTACGTGTGTAATGGTAACCCTCGCTGTAAGAATCTACTTAAGCAGTATACTTCTGCTTGTAAAACTATTGAA
GATGCCTTACGACTTAGTGCTCATTTGGAAACTAATGATGTTAGTAGTATGCTAACTTTCGATAGCAATGCTTTTAGTT
TGGCTAATGTTACTAGTTTTGGAGATTATAACCTTTCTAGTGTTTTACCTCAGAGAAACATTCGTTCAAGCCGTATAGC
AGGACGTAGTGCTTTGGAAGATTTGTTGTTTAGCAAAGTTGTTACATCTGGTTTGGGTACTGTTGATGTTGACTATAAG
TCTTGTACTAAAGGTCTTTCTATTGCTGACCTTGCTTGTGCTCAGTATTATAATGGCATAATGGTTTTGCCAGGTGTTG
CTGATGCTGAACGTATGGCCATGTACACAGGTTCTCTTATAGGTGGCATGGTGCTCGGAGGTCTTACATCAGCAGCCGC
CATACCTTTTTCTTTGGCACTGCAAGCACGACTTAACTATGTTGCTTTACAAACTGATGTGCTTCAAGAAAATCAGAAA
ATTTTGGCTGCATCATTTAATAAGGCTATTAATAATATTGTTGCTTCTTTTAGTAGCGTTAATGATGCTATTACACAAA
CTGCAGAGGCTATACATACTGTTACTATTGCACTTAATAAGATTCAGGATGTTGTTAATCAACAGGGTAGTGCTCTTAA
CCATCTCACTTCACAATTGAGACATAATTTTCAGGCCATTTCTAATTCAATTCAGGCTATTTATGACCGGCTTGATTCA
ATTCAAGCCGATCAACAAGTTGACAGATTAATTACTGGACGGCTTGCAGCTTTGAATGCATTTGTTTCCCAAGTTTTGA
ATAAATATACTGAAGTTCGTGGTTCCAGACGCTTAGCACAGCAGAAGATTAATGAATGTGTCAAGTCACAATCTAATAG
ATATGGTTTTTGTGGCAATGGCACTCACATCTTTTCAATCGTCAACTCAGCTCCAGATGGTTTGCTTTTTCTTCATACT
GTTCTGCTGCCAACTGATTACAAGAATGTAAAGGCGTGGTCTGGTATCTGTGTTGATGGCATTTATGGCTATGTTCTGC
GTCAACCTAACTTGGTTCTTTATTCTGATAATGGTGTCTTTCGTGTAACTTCCAGGGTCATGTTTCAACCTCGCTTACC
TGTTTTGTCTGATTTTGTGCAAATATATAATTGTAATGTTACTTTTGTTAACATATCTCGTGTCGAGTTACATACTGTC
ATACCTGACTACGTTGATGTTAATAAAACATTACAAGAGTTTGCACAAAACTTACCAAAGTATGTTAAGCCTAATTTTG
ACTTGACTCCTTTTAATTTAACATATCTTAATTTGAGTTCTGAGTTGAAGCAACTCGAAGCTAAAACTGCTAGTCTTTT
TCAAACTACTGTTGAATTACAAGGTCTTATTGATCAGATTAACAGTACATATGTTGATTTGAAGTTGCTTAATAGGTTT
GAAAATTATATTAAATGGCCTTGGTGGGTTTGGCTCATTATTTCTGCTGTTTTTGTTGTATTGTTGAGTCTTCTTGTGT
TTTGTTGTCTTTCTACAGGCTGTTGTGGTTGTTGCAATTGTTTAACTTCATCAATGCGAGGCTGTTGTGATTGTGGTTC
AACTAAACTTCCTTATTACGAATTTGAAAAGGTCCACGTTCAATAA

>gi|380710280|gb|JQ765575.1| Human coronavirus NL63 strain NL63/DEN/2005/1876, complete genome

ATGAAACTTTTCTTGATTTTGCTTGTTTTGCCCCTGGCCTCTTGCTTTTTCACATGTAATAGTAATGCTAATCTC

TCTATGTTACAATTAGGTGTTCCTGACAATTCTTCAACTATTGTTACGGGTTTATTGCCAACTCATTGGTTTTGTGCTA
ATCAGAGTACATCTGTTTACTCAGCCAATGGTTTCTTTTATATTGATGTTGGTAATCACCGTAGTGCTTTTGCGCTCCA
TACTGGTTATTATGATGCTAATCAGTATTATATTTATGTTACTAATGAAATAGGCTTAAATGCTTCTGTTACTCTTAAG
ATTTGTAAGTTTAGTAGAAACACTACTTTTGATTTTTTAAGTAATGCTTCTAGTTCTTTTGACTGTATAGTTAATTTGT
TATTTACAGAACAGTTAGGTGCGCCTTTGGGCATAACTATATCTGGTGAAACTGTGCGTCTGCATTTATATAATGTAAC
TCGTACTTTTTATGTGCCAGCAGCTTATAAACTTACTAAACTTAGTGTTAAATGTTACTTTAACTATTCCTGTGTTTTT
AGTGTTGTCAACGCCACCGTTACTGTGAATGTCACCACACATAATGGCCGTGTAGTTAACTACACTGTTTGTGATGATT
GTAATGGTTATACTGATAACATATTTTCTGTTCAACAGGATGGCCGCATTCCTAATGGTTTCCCTTTTAATAATTGGTT
TTTGTTAACTAATGGTTCCACACTAGTAGACGGGGTCTCTAGACTTTACCAACCACTCCGTCTAACTTGTTTATGGCCT
GTACCTGGTCTTAAATCTTCAACTGGTTTTGTTTATTTTAATGCCACTGGTTCTGATGTTAATTGTAACGGCTATCAAC
ATAATTCTGTTGCTGATGTTATGCGTTACAATCTTAACTTCAGTGCTAATTCTGTGGACAATCTCAAGAGTGGTGTTAT
AGTTTTTAAAACTTTACAGTACGATGTTTTGTTTTATTGTAGTAACTCTTCCTCAGGTGTTCTTGACACCACAATACCT
TTTGGCCCTTCCTCTCAACCTTATTACTGTTTTATAAACAGCACTATCAACACTACTCATGTTAGCACTTTTGTGGGTA
TTTTACCACCCACTGTGCGTGAAATTGTTGTTGCTAGAACTGGTCAGTTTTATATTAATGGTTTTAAGTATTTCGATTT
GGGTTTCATAGAAGCTGTCAATTTTAATGTCACGACTGCTAGTGCCACAGATTTTTGGACGGTTGCATTTGCTACTTTT
GTTGATGTTTTGGTTAATGTTAGTGCAACTAACATTCAAAACTTACTTTATTGCGATTCTCCATTTGAAAAGTTGCAGT
GTGAGCACTTGCAGTTTGGATTGCAGGATGGTTTTTATTCTGCAAATTTTCTTGATGATAATGTTTTGCCTGAGACTTA
TGTTGCACTCCCCATTTATTATCAACACACGGACATAAATTTTACTGCAACTGCATCTTTTGGTGGTTCTTGTTATGTT
TGTAAACCACACCAGGTTAATATATCTCTTAATGGTAACACTTCAGTGTGTGTTAGAACATCTCATTTTTCAATTAGGT
ATATTTATAACCGCGTTAAGAGTGGTTCACCAGGTGACTCTTCATGGCACATTTATTTAAAGAGTGGCACTTGTCCATT
TTCTTTTTCTAAGTTAAATAATTTTCAAAAGTTCAAGACTATTTGTTTCTCAACCGTCGAAGTGCCTGGTAGTTGTAAT
TTTCCGCTTGAAGCCACCTGGCATTATACTTCTTATACTATTGTTGGTGCTTTGTATGTTACTTGGTCTGAAGGTAATT
CTATTACTGGTGTACCTTATCCTGTCTCTGGTATTCGTGAGTTTAGTAATTTAGTTTTAAATAATTGTACCAAATATAA
TATTTATGATTATGTTGGTACTGGAATTATACGTTCTTCAAACCAGTCACTTGCTGGTGGTATTACATATGTTTCTAAC
TCTGGTAATTTACTTGGTTTTAAAAATGTTTCCACTGGTAACATTTTTATTGTGACACCATGTAACCAACCAGACCAAG
TAGCTGTTTATCAACAAAGCATTATTGGTGCCATGACCGCTGTTAATGAGTCTAGATATGGCTTGCAAAACTTACTACA
GTTACCTAACTTTTATTATGTTAGTAATGGTGGTAACAATTGCACTACGGCCGTTATGACTTATTCTAATTTGGTATT
TGTGCTGATGGTTCTTTGATTCCTGTTCGTCCGCGTAATTCTAGTGATAATGGTATTTCAGCCATAATCACTGCTAATT
TATCCATTCCCTCTAACTGGACTACTTCAGTTCAAGTTGAGTACCTCCAAATTACTAGTACTCCAATTGTTGTTGATTG
TGCTACTTATGTGTGTAATGGTAACCCTCGCTGTAAGAATCTACTTAAGCAGTATACTTCTGCTTGTAAAACTATTGAA
GATGCCTTACGACTTAGTGCTCATTTGGAAACTAATGATGTTAGTAGTATGCTAACTTTCGATAGCAATGCTTTTAGTT
TGGCTAATGTTACTAGTTTTGGAGACTATAACCTTTCTAGTGTTTTACCTCAGAGAAACATTCGTTCAAGCCGTATAGC
AGGACGTAGTGCTTTGGAAGATTTGTTGTTTAGCAAAGTTGTTACATCTGGTTTGGGTACTGTTGATGTTGACTATAAG
TCTTGTACTAAAGGTCTTTCTATTGCTGACCTTGCTTGTGCTCAGTACTACAATGGCATAATGGTTTTGCCAGGTGTTG
CTGATGCTGAACGTATGGCCATGTACACAGGTTCTCTTATAGGTGGCATGGTGCTCGGAGGTCTTACATCAGCAGCCGC
CATACCTTTTTCTTTGGCACTGCAAGCACGACTTAACTATGTTGCTTTACAAACTGATGTGCTTCAAGAAAATCAGAAA
ATTTTGGCTGCATCATTTAATAAGGCTATTAATAATATTGTTGCTTCTTTTAGTAGCGTTAATGATGCTATTACACAAA
CTGCAGAGGCTATACATACTGTTACTATTGCACTTAATAAGATTCAGGATGTTGTTAATCAACAGGGTAGTGCTCTTAA
CCATCTCACTTCACAATTGAGACATAATTTTCAGGCCATTTCTAATTCAATTCAGGCTATTTATGACCGGCTTGATTCA
ATTCAAGCCGATCAACAAGTTGACAGATTAATTACTGGACGGCTTGCAGCTTTGAATGCATTTGTTTCCCAAGTTTTGA
ATAAATATACTGAAGTTCGTGGTTCAAGACGCTTAGCACAGCAGAAGATTAATGAATGTGTCAAGTCACAATCTAATAG
ATATGGTTTTTGTGGCAATGGCACTCACATCTTTTCAATCGTCAACTCAGCTCCAGATGGTTTGCTTTTTCTTCATACT
GTTTTGCTGCCAACTGATTACAAGAATGTAAAGGCGTGGTCTGGTATCTGTGTTGATGGCATTTATGGCTATGTTCTGC
GTCAACCTAACTTGGTTCTTTATTCTGATAATGGTGTCTTTCGTGTAACTTCCAGGGTCATGTTTCAACCTCGCTTACC

TGTTTTGTCTGATTTTGTGCAAATATATAATTGTAATGTTACTTTTGTTAACATATCTCGTGTTGAGTTACATACTGTC
ATACCTGACTACGTTGATGTTAATAAAACATTACAAGAGTTTGCACAAAACTTACCAAAGTATGTTAAGCCTAATTTTG
ACTTGACTCCTTTTAATTTAACATATCTTAATTTGAGTTCTGAGTTGAAGCAACTCGAAGCTAAAACTGCTAGTCTTTT
TCAAACTACTGTTGAATTACAAGGTCTTATTGATCAGATTAACAGTACATATGTTGATTTGAAGTTGCTTAATAGGTTT
GAAAATTATATCAAATGGCCTTGGTGGGTTTGGCTCATTATTTCTGTTGTTTTTGTTGTATTGTTGAGTCTTCTTGTGT
TTTGTTGTCTTTCTACAGGTTGTTGTGGTTGTTGCAATTGTTTAACTTCTTCAATGCGAGGCTGTTGTGATTGTGGTTC
AACTAAACTTCCTTATTACGAATTTGAAAAGGTCCACATTCAATAA

>gi|398255449|gb|JX104161.1| Human coronavirus NL63 isolate CBJ 037, complete genome

ATGAAACTTTTCTTGATTTTGCTTGTTTTGCCCCTGGCCTCTTGCTTTTTCACATGTAATAGTAATGCTAATCTC
TCTATGTTACAATTAGGTGTTCCTGACAATTCTTCAACTATTGTCACGGGTTTATTGCCAACTCATTGGATTTGTGCTA
ATCAGAGTACATCTGTTTACTCAGCCAATGGTTTCTTTTATATTGATGTTGGTAATCACCGTAGTGCTTTTGCTCTCCA
TACTGGTTATTATGATGTTAATCAGTATTATATTTATGTTACTAATGAAATAGGCTTAAATGCTTCTGTTACTCTTAAG
ATTTGTAAGTTTGGTATAAACACTACTTTTGATTTTTTAAGTAATTCTTCTAGTTCTTTTGACTGTATAGTTAATTTGT
TATTTACAGAACAGTTAGGTGCGCCTTTGGGCATAACTATATCTGGTGAAACTGTGCGTCTGCATTTATATAATGTAAC
TCGTACTTTTTATGTGCCAGCAGCTTATAAACTTACTAAACTTAGTGTTAAATGTTACTTTAACTATTCCTGTGTTTTT
AGTGTTGTCAACGCCACCGTTACTGTGAATGTCACCACACATAATGGCCGTGTAGTTAACTACACTGTTTGTGATGATT
GTAATGGTTATACTGATAACATATTTTCTGTTCAACAGGATGGCCGCATTCCTAATGGTTTCCCTTTTAATAATTGGTT
TTTGCTAACTAATGGTTCCACACTAGTGGACGGGGTCTCTAGACTTTATCAACCACTCCGTTTAACTTGTTTATGGCCT
GTACCTGGTCTTAAATCTTCAACTGGTTTTGTTTATTTTAATGCCACTGGTTCTGATGTTAATTGTAACGGCTATCAAC
ATAATTCTGTTGCTGATGTTATGCGTTACAATCTTAACTTCAGTGCTAATTCTGTGGATAATCTCAAGAGTGGTGTTAT
AGTTTTTAAAACTCTACAGTACGATGTTTTGTTTTACTGTAGTAATTCCTCTTCGGGCGTTCTAGACACGACAATACCT
TTTGGCCCTTCCTCTCAACCTTATTACTGTTTTATAAATAGTACCATCAACACCACTCATGTTAGCACTTTTGTGGGTG
TCCTACCACCCACTGTGCGTGAGATTGTTGTTGCTAGAACTGGTCAGTTTTATATTAATGGTTTTAAGTATTTTGATTT
GGGTTTTATCGAAGCTGTCAATTTTAATGTCACAACTGCTAGTGCCACAGATTTTTGGACGGTTGCATTTGCTACTTTT
GTTGATGTTTTGGTTAATGTTAGTGCAACTAAGATTCAAAACTTACTTTATTGCGATTCTCCATTTGAAAAGTTGCAGT
GTGAGCACTTGCAGTTTGGATTGCAAGATGGTTTTTATTCTGCAAATTTTCTTGATGATAATGTTTTGCCTGAGACTTA
TGTTGCACTCCCCATTTATTATCAACACACGGACATAAATTTTACTGCAACTGCATCTTTTGGTGGTTCTTGTTATGTT
TGTAAACCACACCAGGTTAATATATCTCTTAATGGTAACACTTCAGTGTGTGTTAGAACATCTCATTTTTCAATTAGGT
ATATTTATAACCGCGTTAAGAGTGGTTCACCAGGTGACTCTTCATGGCACATTTATTTAAAGAGTGGCACTTGTCCATT
TTCTTTTTCTAAGTTAAATAATTTTCAAAAGTTTAAGACTATTTGTTTCTCAACCGTCGCAGTGCCTGGTAGTTGTAAT
TTTCCACTTGAAGCCACCTGGCATTACACTTCTTATACTATTGTTGGTGCTTTGTATGTTACTTGGTCTGAAGGTAATT
CCATTACTGGTGTACCTTATCCTGTCTCTGGTATTCGTGAGTTTAGTAATTTAGTTTTAAATAATTGTACCAAATATAA
TATTTATGATTATGTTGGTACTGGAATTATACGTTCTTCAAACCAGTCACTTGCTGGTGGTATTACATATGTTTCTAAT
TCTGGTAATTTACTTGGTTTTAAAAATGTTTCCACTGGTAACATTTTTATTGTGACACCATGTAACCAACCAGACCAAG
TAGCTGTTTATCAACAAAGCATTATTGGTGCCATGACTGCTGTTAATGAGTCTAGATATGGCTTGCAAAACTTACTACA
GTTACCTAACTTTTATTATGTTAGTAATGGTAGTAACAATTGCACTACGGCCGTTATGACTTATTCTAATTTTGGTATT
TGTGCTGATGGTTCTTTAATTCCTGTTCGTCTGCGTAATTCTAGTGATAATGGTATTTCAGCCATAATCACTGCTAATT
TATCCATTCCCTCTAACTGGACTACTTCAGTTCAAGTTGAGTACCTCCAAATTACTAGTACTCCAATAGTTGTTGATTG
TGCTACTTATGTGTGTAATGGTAACCCTCGCTGTAAGAATCTACTTAAGCAGTATACTTCTGCTTGTAAAACTATTGAA
GATGCCTTACGACTTAGTGCTCATTTGGAAACTAATGATGTTAGTAGTATGCTAACTTTCGATAGCAATGCTTTTAGTT

TGGCTAATGTTACTAGTTTTGGAGATTATAACCTTTCTAGTGTTTTACCTCAGAGAAACATTCATTCAAGCCGTATAGC
AGGACGTAGTGCTTTGGAAGATTTGTTGTTTAGCAAAGTTGTTACATCTGGTTTGGGTACTGTTGATGTTGACTATAAG
TCTTGTACTAAAGGTCTTTCTATTGCTGACCTTGCTTGTGCTCAGTACTACAATGGCATAATGGTTTTGCCAGGTGTTG
CTGATGCTGAACGTATGGCCATGTACACAGGTTCTCTTATAGGTGGCATGGTGCTCGGAGGTCTTACATCAGCAGCCGC
CATACCTTTTTCTTTGGCACTGCAAGCACGACTTAACTATGTTGCTTTACAAACTGATGTGCTTCAAGAAAATCAGAAA
ATTTTGGCTGCATCATTTAATAAGGCTATTAATAACATTGTTGCTTCTTTTAGTAGCGTTAATGATGCTATTACACAAA
CTGCAGAGGCTATACATACTGTTACTATTGCACTTAATAAGATTCAGGATGTTGTTAATCAACAGGGTAGTGCTCTTAA
CCATCTCACTTCACAATTGAGACATAATTTTCAGGCCATTTCTAATTCAATTCAAGCTATTTATGACCGGCTTGATTCA
ATTCAAGCCGATCAACAAGTTGACAGATTAATTACTGGACGGCTTGCAGCTTTGAATGCATTTGTTTCCCAAGTTTTGA
ATAAATATACTGAAGTTCGTAGTTCCAGACGCTTAGCACAGCAGAAGGTTAATGAATGTGTCAAATCACAATCTAATAG
ATATGGTTTTTGTGGCAATGGCACCCATATCTTTTCAATCGTCAACTCAGCTCCAGATGGTTTGCTTTTTCTTCATACT
GTTTTGCTGCCAACTGATTACAAGAATGTAAAGGCATGGTCGGGTATCTGTGTTGATGGCATTTATGGCTATGTTCTGC
GTCAACCTAACTTGGTTCTTTATTCTGATAATGGTGTCTTTCGTGTAACTTCCAGGGTCATGTTTCAACCTCGTTTACC
TGTTTTGTCTGATTTTGTGCAAATATATAATTGTAATGTTACTTTTGTTAACATATCTCGTGTCGAGTTACATACTGTC
ATACCTGACTACGTTGATGTTAATAAAACATTACAAGAGTTTGCACAAAATTTACCAAAGTATGTTAAGCCTAATTTTG
ACTTGACTCCTTTTAATTTAACATATCTTAATTTGAGTTCTGAGTTGAAGCAACTCGAAGCTAAAACTGCTAGTCTTTT
CCAAACTACTGTTGAATTACAAGGTCTTATTGATCAGATTAACAGTACATATGTTGACTTGAAGTTGCTTAATAGGTTT
GAAAATTATATTAAATGGCCTTGGTGGGTTTGGCTCATTATTTCTGTTGTTTTTGTCGTATTGTTGAGTCTTCTTGTGT
TTTGTTGTCTTTCTACAGGTTGTTGTGGTTGTTGCAATTGTTTAACTTCATCAATGCGAGGTTGTTGTGATTGTGGTTC
AACTAAACTTCCTTATTACGAATTTGAAAAGGTTCACGTTCAATAA

>gi|410032027|gb|JX504050.1| Human coronavirus NL63 isolate NL63/RPTEC/2004, complete genome

ATGAAACTTTTCTTGATTTTGCTTGTTTTGCCCCTGGCCTCTTGCTTTTTCACATGTAATAGTAATGCTAATCTC
TCTATGTTACAATTAGGTGTTCCTGACAATTCTTCAACTATTGTTACGGGTTTATTGCCAACTCATTGGTTTTGTGCTA
ATCAGAGTACATCTGTTTACTCAGCCAATGGTTTCTTTTATATTGATGTTGGTAATCACCGTAGTGCTTTTGCGCTCCA
TACTGGTTATTATGATGCTAATCAGTATTATATTTATGTTACTAATGAAATAGGCTTAAATGCTTCTGTTACTCTTAAG
ATTTGTAAGTTTAGTAGAAACACTACTTTTGATTTTTTAAGTAATGCTTCTAGTTCTTTTGACTGTATAGTTAATTTGT
TATTTACAGAACAGTTAGGTGCGCCTTTGGGCATAACTATATCTGGTGAAACTGTGCGTCTGCATTTATATAATGTAAC
TCGTACTTTTTATGTGCCAGCAGCTTATAAACTTACTAAACTTAGTGTTAAATGTTACTTTAACTATTCCTGTGTTTTT
AGTGTTGTCAACGCCACCGTTACTGTGAATGTCACCACACATAATGGCCGTGTAGTTAACTACACTGTTTGTGATGATT
GTAATGGTTATACTGATAACATATTTTCTGTTCAACAGGATGGCCGCATTCCTAATGGTTTCCCTTTTAATAATTGGTT
TTTGTTAACTAATGGTTCCACACTAGTAGACGGGGTCTCTAGACTTTATCAACCACTCCGTTTAACTTGTTTATGGCCT
GTACCTGGTCTTAAATCTTCAACTGGTTTTGTTTATTTTAATGCCACTGGTTCTGATGTTAATTGTAACGGCTATCAAC
ATAATTCTGTTGTTGATGTTATGCGTTACAATCTTAACTTCAGTGCTAATTCTTTGGACAATCTCAAGAGTGGTGTTAT
AGTTTTTAAAACTTTACAGTACGATGTTTTGTTTTATTGTAGTAATTCTTCCTCAGGTGTTCTTGACACCACAATACCT
TTTGGCCCGTCCTCTCAACCTTATTACTGTTTTATAAACAGCACTATCAACACTACTCATGTTAGCACTTTTGTGGGTA
TTTTACCACCCACTGTGCGTGAAATTGTTGTTGCTAGAACTGGTCAGTTTTATATTAATGGTTTTAAGTATTTCGATTT
GGGTTTCATAGAAGCTGTCAATTTTAATGTCACGACTGCTAGTGCCACAGATTTTTGGACGGTTGCATTTGCTACTTTT
GTTGATGTTTTGGTTAATGTTAGTGCAACTAACATTCAAAACTTACTTTATTGCGATTCTCCATTTGAAAAGTTGCAGT
GTGAGCACTTGCAGTTTGGATTGCAGGATGGTTTTTATTCTGCAAATTTTCTTGATGATAATGTTTTGCCTGAGACTTA
TGTTGCACTCCCCATTTATTATCAACACACGGACATAAATTTTACTGCAACTGCATCTTTTGGTGGTTCTTGTTATGTT

TGTAAACCACACCAGGTTAATATATCTCTTAATGGTAACACTTCAGTGTGTGTTAGAACATCTCATTTTTCAATTAGGT
ATATTTATAACCGCGTTAAGAGTGGTTCACCAGGTGACTCTTCATGGCACATTTATTTAAAGAGTGGCACTTGTCCATT
TTCTTTTTCTAAGTTAAATAATTTTCAAAAGTTCAAGACTATTTGTTTCTCAACCGTCGAAGTGCCTGGTAGTTGTAAT
TTTCCGCTTGAAGCCACCTGGCATTACACTTCTTATACTATTGTTGGTGCTTTGTATGTTACTTGGTCTGAAGGTAATT
CTATTACTGGTGTACCTTATCCTGTCTCTGGTATTCGTGAGTTTAGTAATTTAGTTTTAAATAATTGTACCAAATATAA
TATTTATGATTATGTTGGTACTGGAATTATACGTTCTTCAAACCAGTCACTTGCTGGTGGTATTACATATGTTTCTAAC
TCTGGTAATTTACTTGGTTTTAAAAATGTTTCCACTGGTAACATTTTTATTGTGACACCATGTAACCAACCAGACCAAG
TAGCTGTTTATCAACAAAGCATTATTGGTGCCATGACCGCTGTTAATGAGTCTAGATATGGCTTGCAAAACTTACTACA
GTTACCTAACTTTTATTATGTTAGTAATGGTGGTAACAATTGCACTACGGCCGTTATGACTTATTCTAATTTTGGTATT
TGTGCTGATGGTTCTTTGATTCCTGTTCGTCCGCGTAATTCTAGTGATAATGGTATTTCAGCCATAATCACTGCTAATT
TATCCATTCCTTCTAACTGGACTACTTCAGTTCAAGTTGAGTACCTCCAAATTACTAGTACTCCAATAGTTGTTGATTG
TGCTACTTATGTGTGTAATGGTAACCCTCGCTGTAAGAATCTACTTAAGCAGTATACTTCTGCTTGTAAAACTATTGAA
GATGCCTTACGACTTAGTGCTCATTTGGAAACTAATGATGTTAGTAGTATGCTAACTTTCGATAGCAATGCTTTTAGTT
TGGCTAATGTTACTAGTTTTGGAGATTATAACCTTTCTAGTGTTTTACCTCAGAGAAACATTCGTTCAAGCCGTATAGC
AGGACGTAGTGCTTTGGAAGATTTGTTGTTTAGCAAAGTTGTTACATCTGGTTTGGGTACTGTTGATGTTGACTATAAG
TCTTGTACTAAAGGTCTTTCTATTGCTGACCTTGCTTGTGCTCAGTACTACAATGGCATAATGGTTTTGCCAGGTGTTG
CTGATGCTGAACGTATGGCCATGTACACAGGTTCTCTTATAGGTGGCATGGTGCTCGGAGGTCTTACATCAGCAGCCGC
CATACCTTTTTCTTTGGCACTGCAAGCACGACTTAACTATGTTGCTTTACAAACTGATGTGCTTCAAGAAAATCAGAAA
ATTTTGGCTGCATCATTTAATAAGGCTATTAATAATATTGTTGCTTCTTTTAGTAGCGTTAATGATGCTATTACACAAA
CTGCAGAGGCTATACATACTGTTACTATTGCACTTAATAAGATTCAGGATGTTGTTAATCAACAGGGTAGTGCTCTTAA
CCATCTCACTTCACAATTGAGACATAATTTTCAGGCCATTTCTAATTCAATTCAGGCTATTTATGACCGGCTTGATTCA
ATTCAAGCCGATCAACAAGTTGACAGATTAATTACTGGACGGCTTGCAGCTTTGAATGCATTTGTTTCCCAAGTTTTGA
ATAAATATACTGAAGTTCGTGGTTCAAGACGCTTAGCACAGCAGAAGATTAATGAATGTGTCAAGTCACAATCTAATAG
ATATGGTTTTTGTGGCAATGGCACTCACATCTTTTCAATCGTCAACTCAGCTCCAGATGGTTTGCTTTTTCTTCATACT
GTTTTGCTGCCAACTGATTACAAGAATGTAAAGGCGTGGTCTGGTATCTGTGTTGATGGCATTTATGGCTATGTTCTGC
GTCAACCTAACTTGGTTCTTTATTCTGATAATGGTGTCTTTCGTGTAACTTCCAGGATCATGTTTCAACCTCGCTTACC
TGTTTTGTCTGATTTTGTGCAAATATATAATTGTAATGTTACTTTTGTTAACATATCTCGTGTTGAGTTACATACTGTC
ATACCTGACTACGTTGATGTTAATAAAACATTACAAGAGTTTGCACAAAACTTACCAAAGTATGTTAAGCCTAATTTTG
ACTTGACTCCTTTTAATTTAACATATCTTAATTTGAGTTCTGAGTTGAAGCAACTCGAAGCTAAAACTGCTAGTCTTTT
TCAAACTACTGTTGAATTACAAGGTCTTATTGATCAGATTAACAGTACATATGTTGATTTGAAGTTGCTTAATAGGTTT
GAAAATTATATCAAATGGCCTTGGTGGGTTTGGCTCATTATTTCTGTTGTTTTTGTTGTATTGTTGAGTCTTCTTGTGT
TTTGTTGTCTTTCTACAGGTTGTTGTGGTTGTTGCAATTGTTTAACTTCATCAATGCGAGGCTGTTGTGATTGTGGTTC
AACTAAACTTCCTTATTACGAATTTGAAAAGGTCCACGTTCAATAA

>gi|402479281|gb|JX524171.1| Human coronavirus NL63 isolate CBJ123, complete genome

ATGAAACTTTTCTTGATTTTGCTTGTTTTGCCCCTGGCCTCTTGCTTTTTCACATGTAATAGTAATGCTAATCTC
TCTATGTTACAATTAGGTGTTCCTGACAATTCTTCAACTATTGTCACGGGTTTATTGCCAACTCATTGGATTTGTGCTA
ATCAGAGTACATCTGTTTACTCAGCCAATGGTTTCTTTTATATTGATGTTGGTAATCACCGTAGTGCTTTTGCTCTCCA
TACTGGTTATTATGATGTTAATCAGTATTATATTTATGTTACTAATGAAATAGGCTTAAATGCTTCTGTTACTCTTAAG
ATTTGTAAGTTTGGTATAAACACTACTTTTGATTTTTTAAGTAATTCTTCTAGTTCTTTTGACTGTATAGTTAATTTGT
TATTTACAGAACAGTTAGGTGCGCCTTTGGGCATAACTATATCTGGTGAAACTGTGCGTCTGCATTTATATAATGTAAC

TCGTACTTTTTATGTGCCAGCAGCTTATAAACTTACTAAACTTAGTGTTAAATGTTACTTTAACTATTCCTGTGTTTTT
AGTGTTGTCAACGCCACCGTTACTGTGAATGTCACCACACATAATGGCCGTGTAGTTAACTACACTGTTTGTGATGATT
GTAATGGTTATACTGATAACATATTTTCTGTTCAACAGGATGGCCGCATTCCTAATGGTTTCCCTTTTAATAATTGGTT
TTTGCTAACTAATGGTTCCACACTAGTGGACGGGGTCTCTAGACTTTATCAACCACTCCGTTTAACTTGTTTATGGCCT
GTACCTGGTCTTAAATCTTCAACTGGTTTTGTTTATTTTAATGCCACTGGTTCTGATGTTAATTGTAACGGCTATCAAC
ATAATTCTGTTGCTGATGTTATGCGTTACAATCTTAACTTCAGTGCTAATTCTGTGGATAATCTCAAGAGTGGTGTTAT
AGTTTTTAAAACTCTACAGTACGATGTTTTGTTTTACTGTAGTAATTCCTCTTCGGGCGTTCTAGACACCACAATACCT
TTTGGCCCTTCCTCTCAACCTTATTACTGTTTTATAAATAGTACCATCAACACCACTCATGTTAGCACTTTTGTGGGTG
TCCTACCACCCACTGTGCGTGAGATTGTTGTTGCTAGAACTGGTCAGTTTTATATTAATGGTTTTAAGTATTTTGATTT
GGGTTTTATCGAAGCTGTCAATTTTAATGTCACAACTGCTAGTGCCACAGATTTTTGGACGGTTGCATTTGCTACTTTT
GTTGATGTTTTGGTTAATGTTAGTGCAACTAAGATTCAAAACTTACTTTATTGCGATTCTCCATTTGAAAAGTTGCAGT
GTGAGCACTTACAGTTTGGATTGCAAGATGGTTTTTATTCTGCAAATTTTCTTGATGATAATGTTTTGCCTGAGACTTA
TGTTGCACTCCCCATTTATTATCAACACACGGACATAAATTTTACTGCAACTGCATCTTTTGGTGGTTCTTGTTATGTT
TGTAAACCACACCAGGTTAATATATCTCTTAATGGTAACACTTCAGTGTGTGTTAGAACATCTCATTTTTCAATTAGGT
ATATTTATAACCGCGTTAAGAGTGGTTCACCAGGTGACTCTTCATGGCACATTTATTTAAAGAGTGGCACTTGTCCATT
TTCTTTTTCTAAGTTAAATAATTTTCAAAAGTTTAAGACTATTTGTTTCTCAACCGTCGCAGTGCCTGGTAGTTGTAAT
TTTCCACTTGAAGCCACCTGGCATTACACTTCTTATACTATTGTTGGTGCTTTGTATGTTACTTGGTCTGAAGGTAATT
CCATTACTGGTGTACCTTATCCTGTCTCTGGTATTCGTGAGTTTAGTAATTTAGTTTTAAATAATTGTACCAAATATAA
TATTTATGATTATGTTGGTACTGGAATTATACGTTCTTCAAACCAGTCACTTGCTGGTGGTATTACATATGTTTCTAAC
TCTGGTAATTTACTTGGTTTTAAAAATGTTTCCACTGGTAACATTTTTATTGTGACACCATGTAACCAACCAGACCAAG
TAGCTGTTTATCAACAAAGCATTATTGGTGCCATGACTGCTGTTAATGAGTCTAGATATGGCTTGCAAAACTTACTACA
GTTACCTAACTTTTATTATGTTAGTAATGGTAGTAACAATTGCACTACGGCCGTTATGACTTATTCTAATTTTGGTATT
TGTGCTGATGGTTCTTTAATTCCTGTTCGTCTGCGTAATTCTAGTGATAATGGTATTTCAGCCATAATCACTGCTAATT
TATCCATTCCCTCTAACTGGACTACTTCAGTTCAAGTTGAGTACCTCCAAATTACTAGTACTCCAATAGTTGTTGATTG
TGCTACTTATGTGTGTAATGGTAACCCTCGCTGTAAGAATCTACTTAAGCAGTATACTTCTGCTTGTAAAACTATTGAA
GATGCCTTACGACTTAGTGCTCATTTGGAAACTAATGATGTTAGTAGTATGCTAACTTTCGATAGCAATGCTTTTAGTT
TGGCTAATGTTACTAGTTTTGGAGATTATAACCTTTCTAGTGTTTTACCTCAGAGAAACATTCATTCAAGCCGTATAGC
AGGACGTAGTGCTTTGGAAGATTTGTTGTTTAGCAAAGTTGTTACATCTGGTTTGGGTACTGTTGATGTTGACTATAAG
TCTTGTACTAAAGGTCTTTCTATTGCTGACCTTGCTTGTGCTCAGTACTACAATGGCATAATGGTTTTGCCAGGTGTTG
CTGATGCTGAACGTATGGCCATGTACACAGGTTCTCTTATAGGTGGCATGGTGCTCGGAGGTCTTACATCAGCAGCCGC
CATACCTTTTTCTTTGGCACTGCAAGCACGACTTAACTATGTTGCTTTACAAACTGATGTGCTTCAAGAAAATCAGAAA
ATTTTGGCTGCATCATTTAATAAGGCTATTAATAACATTGTTGCTTCTTTTAGTAGCGTTAATGATGCTATTACACAAA
CTGCAGAGGCTATACATACTGTTACTATTGCACTTAATAAGATTCAGGATGTTGTTAATCAACAGGGTAGTGCTCTTAA
CCATCTCACTTCACAATTGAGACATAATTTTCAGGCCATTTCTAATTCAATTCAAGCTATTTATGACCGGCTTGATTCA
ATTCAAGCCGATCAACAAGTTGACAGATTAATTACTGGACGGCTTGCAGCTTTGAATGCATTTGTTTCCCAAGTTTTGA
ATAAAATATACTGAAGTTCGTAGTTCCAGACGCTTAGCACAGCAGAAGGTTAATGAATGTGTCAAATCACAATCTAATAG
ATATGGTTTTTGTGGCAATGGCACCCATATCTTTTCAATCGTCAACTCAGCTCCAGATGGTTTGCTTTTTCTTCATACT
GTTTTGCTGCCAACTGATTACAAGAATGTAAAGGCATGGTCGGGTATCTGTGTTGATGGCATTTATGGCTATGTTCTGC
GTCAACCTAACTTGGTTCTTTATTCTGATAATGGTGTCTTTCGTGTAACTTCCAGGGTCATGTTTCAACCTCGTTTACC
TGTTTTGTCTGATTTTGTGCAAATATATAATTGTAATGTTACTTTTGTTAACATATCTCGTGTCGAGTTACATACTGTC
ATACCTGACTACGTTGATGTTAATAAAACATTACAAGAGTTTGCACAAAATTTACCAAAGTATGTTAAGCCTAATTTTG
ACTTGACTCCTTTTAATTTAACATATCTTAATTTGAGTTCTGAGTTGAAGCAACTCGAAGCTAAAACTGCTAGTCTTTT
CCAAACTACTGTTGAATTACAAGGTCTTATTGATCAGATTAACAGTACATATGTTGACTTGAAGTTGCTTAATAGGTTT
GAAAATTATATTAAATGGCCTTGGTGGGTTTGGCTCATTATTTCTGTTGTTTTTGTCGTATTGTTGAGTCTTCTTGTGT

TTTGTTGTCTTTCTACAGGTTGTTGTGGTTGTTGCAATTGTTTAACTTCATCAATGCGAGGTTGTTGTGATTGTGGTTC
AACTAAACTTCCTTATTACGAATTTGAAAAGGTTCACGTTCAATAA

>gi|530802069|gb|KF530105.1| Human coronavirus NL63 strain NL63/human/USA/012-31/2001, complete genome

ATGAAACTTTTCTTGATTTTGCTTGTTTTGCCCTTGGCCTCTTGCTTTTTCACATGTAATAGTAATGCTAATCTC
TCTATGTTACAATTAGGTGTTCCTGACAATTCTTCAACTATTGTCACGGGTTTATTGCCAACTCATTGGATTTGTGCTA
ATCAGAGTACATCTGTTTACTCAGCCAATGGTTTCTTTTATATTGATGTTGGTAATCACCGTAGTGCTTTTGCGCTCCA
TACTGGTTATTATGATGTTAATCAGTATTATATTTATGTTACTAATGAAATAGGCTTAAATGCTTCTGTTACTCTTAAG
ATTTGTAAGTTTGGTACAAACACTACTTTTGATTTTTTAAGTAATGCTTCTAGTTCTTTTGACTGTATAGTTAATTTGT
TATTTACAGAACAGTTAGGTGCGCCTTTGGGCATAACTATATCTGGTGAAACTGTGCGTCTGCATTTATATAATGTAAC
TCGTACTTTTTATGTGCCAGCAGCTTATAAACTTACTAAACTTAGTGTTAAATGTTACTTTAACTATTCCTGTGTTTTT
AGTGTTGTCAACGCCACCGTTACTGTGAATGTCACCACACATAATGGCCGTGTAGTTAACTACACTGTTTGTGATGATT
GTAATGGTTATACTGATAACATATTTTCTGTTCAACAGGATGGCCGCATTCCTAATGGTTTTCCTTTTAATAATTGGTT
TTTGCTAACTAATGGTTCCACACTAGTGGACGGGGTCTCTAGACTTTATCAACCACTCCGTTTAACTTGTTTATGGCCT
GTACCTGGTCTTAAATCTTCAACTGGTTTTGTTTATTTTAATGCCACTGGTTCTGATGTTAATTGTAACGGCTATCAAC
ATAATTCTGTTGCTGATGTTATGCGTTACAATCTTAACTTCAGTGCTAATTCTGTGGACAATCTCAAGAGTGGTGTTAT
AGTTTTTAAAACTTTACAGTACGATGTTTTGTTTTATTGTAGTAATTCTTCCTCAGGTGTTCTTGACACCACAATACCT
TTTGGCCCTTCCTCTCAACCTTATTACTGTTTTATAAACAGCACTATCAACACTACTCATGTTAGCACTTTTGTGGGTA
TTTTACCACCCACTGTGCGTGAAATTGTTGTTGCTAGAACTGGTCAGTTTTATATTAATGGTTTTAAGTATTTCGATTT
GGGTTTCATAGAAGCTGTCAATTTTAATGTCACGACTGCTAGTGCCACAGATTTTTGGACGGTTGCATTTGCTACTTTT
GTTGATGTTTTGGTTAATGTTAGTGCAACTAACATTCAAAACTTACTTTATTGCGATTCTCCATTTGAAAAGTTGCAGT
GTGAGCACTTGCAGTTTGGATTGCAAGATGGTTTTTATTCTGCAAATTTTCTTGATGATAATGTTTTGCCTGAGACTTA
TGTTGCACTCCCCATTTATTATCAACACACGGACATAAATTTTACTGCAACTGCATCTTTTGGTGGTTCTTGTTATGTT
TGTAAACCACGTCAGGTTAATATATCTCTTAATGGTAACACTTCAGTGTGTGTTAGAACATCTCATTTTTCAATTAGGT
ATATTTATAACCGCGTTAAGAGTGGTTCACCAGGTGACTCTTCATGGCACATTTATTTAAAGAGTGGCACTTGTCCATT
TTCTTTTTCTAAGTTAAATAATTTTCAAAAGTTCAAGACTATTTGTTTCTCAACCGTCGAAGTGCCTGGTAGTTGTAAT
TTTCCACTTGAAGCCACCTGGCATTACACTTCTTATACTATTGTTGGTGCTTTGTATGTTACTTGGTCTGAAGGTAATT
CCATTACTGGTGTACCTTATCCTGTCTCTGGTATTCGTGAGTTTAGTAATTTAGTTTTAAATAATTGTACCAAATATAA
TATTTATGATTATGTTGGTACTGGAATTATACGTTCTTCAAACCAGTCACTTGCTGGTGGTATTACATATGTTTCTAAC
TCTGGTAATTTACTTGGTTTTAAAAATGTTTCCACTGGTAACATTTTTATTGTGACACCATGTAACCAACCAGACCAAG
TAGCTGTTTATCAACAAAGCATTATTGGTGCCATGACCGCTGTTAATGAGTCTAGATATGGCTTGCAAAACTTACTACA
GTTACCTAACTTTTATTATGTTAGTAATGGTGGTAACAATTGCACTACGGCTGTTATGACTTATTCTAATTTTGGTATT
TGTGCTGATGGTTCTTTAATTCCTGTTCGTCCGCGTAATTCTAGTGATAATGGTATTTCAGCCATAATCACTGCTAATT
TATCCATTCCCTCTAACTGGACTACTTCAGTTCAAGTTGAGTACCTCCAAATTACTAGTACTCCAATAGTTGTTGATTG
TGCTACTTACGTGTGTAATGGTAACCCTCGCTGTAAGAATCTACTTAAGCAGTATACTTCTGCTTGTAAAACTATTGAA
GATGCCTTACGACTTAGTGCTCATTTGGAAACTAATGATGTTAGTAGTATGCTAACTTTCGATAGCAATGCTTTTAGTT
TGGCTAATGTTACTAGTTTTGGAGATTATAACCTTTCTAGTGTTTTACCTCAGAGAAACATTCGTTCAAGCCGTATAGC
AGGACGTAGTGCTTTGGAAGATTTGTTGTTTAGCAAAGTTGTTACATCTGGTTTGGGTACTGTTGATGTTGACTATAAG
TCTTGTACTAAAGGTCTTTCTATTGCTGACCTTGCTTGTGCTCAGTATTACAATGGCATAATGGTTTTGCCAGGTGTTG
CTGATGCTGAACGTATGGCCATGTACACAGGTTCTCTTATAGGTGGCATGGTGCTCGGAGGTCTTACATCAGCAGCCGC
CATACCTTTTTCTTTGGCACTGCAAGCACGACTTAACTATGTTGCTTTACAAACTGATGTGCTTCAAGAAAATCAGAAA

ATTTTGGCTGCATCATTTAATAAGGCTATTAATAATATTGTTGCTTCTTTTAGTAGCGTTAATGATGCTATTACACAAA
CTGCAGAGGCTATACATACTGTTACTATTGCACTTAATAAGATTCAGGATGTTGTTAATCAACAGGGTAGTGCTCTTAA
CCATCTCACTTCACAATTGAGACATAATTTTCAGGCCATTTCTAATTCAATTCAGGCTATTTATGACCGGCTTGATTCA
ATTCAAGCCGATCAACAAGTTGACAGATTAATTACTGGACGGCTTGCAGCTTTGAATGCATTTGTTTCCCAAGTTTTGA
ATAAATATACTGAAGTTCGTGGTTCCAGACGCTTAGCACAGCAGAAGATTAATGAATGTGTCAAGTCACAATCTAATAG
ATATGGTTTTTGTGGCAATGGCACTCACATCTTTTCAATCGTCAACTCAGCTCCAGATGGTTTGCTTTTTCTTCATACT
GTTTTGCTGCCAACTGATTACAAGAATGTAAAGGCGTGGTCTGGTATCTGTGTTGATGGCATTTATGGCTATGTTCTGC
GTCAACCTAACTTGGTTCTTTATTCTGATAATGGTGTCTTTCGTGTAACTTCCAGGGTCATGTTTCAACCTCGCTTACC
TGTTTTGTCTGATTTTGTGCAAATATATAATTGTAATGTTACTTTTGTTAACATATCTCGTGTCGAGTTACACACTGTC
ATACCTGACTACGTTGATGTTAATAAAACATTACAAGAGTTTGCACAAAACTTACCAAAGTATGTTAAGCCTAATTTTG
ACTTGACTCCTTTTAATTTAACATATCTTAATTTGAGTTCTGAGTTGAAGCAACTCGAAGCTAAAACTGCTAGTCTTTT
TCAAACTACTGTTGAATTACAAGGTCTTATTGATCAGATTAACAGTACATATGTTGATTTGAAGTTGCTTAATAGGTTT
GAAAATTATATTAAATGGCCTTGGTGGGTTTGGCTCATTATTTCTGTTGTTTTTGTTGTATTGTTGAGTCTTCTTGTGT
TTTGTTGTCTTTCTACAGGCTGTTGTGGTTGTTGCAATTGTTTAACTTCATCAATGCGAGGCTGTTGTGATTGTGGTTC
AACTAAACTTCCTTATTACGAATTTGAAAAGGTCCACGTTCAATAA

>gi|530802077|gb|KF530106.1| Human coronavirus NL63 strain NL63/human/USA/8712-17/1987, complete genome

ATGAAACTTTTCTTGATTTTGCTTGTTTTGCCCCTGGCCTCTTGCTTTTCCACATGTAATAGTAATGCTAATATC
TCTATGTTACAATTAGGTGTTCCTGACAATTCTTCAACTATTGTTACGGGTTTATTGCCAACTCATTGGATTTGTGCTA
ATCAGAGTACATCTGTTTACTCAGCCAAAGGTTTCTTTTATATTGATGTTGGTAATCACCGTAGTGCTTTTGCGCTCCA
TACTGGTTATTATGATGCTAATCAGTATTATATTTATGTTACTAATGAAATAGGCTTAAATGCTTCTGTTACTCTTAAG
ATTTGTAAGTTTAGAAACACTACTTTTGATTTTTTAAGTAATGCTTCTAGTTCTTTTGACTGTATAGTTAATTTGTTAT
TTACAGAACAGTTAGGTGCGCCTTTGGGTATAACTATATCTGGTGAAACTGTACGTCTACATTTATATAATGTAACTCG
TACTTTTTATGTGCCAGCAGCTTATAAACTTACTAAACTTAGTGTTAAATGTTACTTCAATTATTCCTGTGTTTTTAGT
GTTGTCAACGCCACCGTTACTGTGAATGTCACCACACATAATGGCCGTGTAGTTAACTACACTGTTTGTGATGATTGTA
ATGGTTATACTGATAACATATTTTCTGTTCAACAGGATGGCCGCATTCCTAATGGTTTCCCTTTTAATAATTGGTTTTT
GTTAACTAATGGTTCCACATTAGTGGACGGGGTCTCTAGACTTTATCAACCACTCCGTTTAACTTGTTTATGGCCTGTA
CCTGGTCTTAAATCTTCAACTGGTTTTGTTTATTTTAATGCCACTGGTTCTGATGTTAATTGTAACGGCTATCAACATA
ATTCTGTTGCTGATGTTATGCGTTACAATCTTAACTTCAGTGCTAATTCTGTGGACAATCTCAAGAGTGGTGTTATAGT
TTTTAAAACTTTACAGTACGATGTTTTGTTTTATTGTAGTAATTCTTCTTCAGGTGTTCTTGACACCACAATACCTTTT
GGCCCTTCCTCTCAACCTTATTACTGTTTTATAAACAGTACTATCAACACTACTCATGTTAGCACTTTTGTGGGTATTT
TACCACCCACTGTGCGTGAAATTGTTGTTGCTAGAACTGGTCAGTTTTATATTAATGGTTTTAAGTATTTCGATTTGGG
TTTCATAGAAGCTGTCAATTTTAATGTCACGACTGCTAGTGCCACAGATTTTTGGACGGTTGCATTTGCTACTTTTGTT
GATGTTTTGGTTAATGTTAGTGCAACTAACATTCAAAACTTACTTTATTGCGATTCTCCATTTGAAAAGTTGCAGTGTG
AGCACTTGCAGTTTGGATTGCAAGATGGTTTTTATTCTGCAAATTTTCTTGATGATAATGTTTTGCCTGAGACTTATGT
TGCACTCCCCATTTATTATCAACATACGGACATAAATTTTACTGCAACTGCATCTTTTGGTGGTTCTTGTTATGTTTGT
AAACCACGCCAGGTTAATATATCTCTTAATGGTAACACTTCAGTGTGTGTTAGAACATCTCATTTTTCAATTAGGTATA
TTTATAACCGCGTTAAGAGTGGTTCACCAGGTGACTCTTCATGGCATATTTATTTAAAGAGTGGCACTTGTCCATTTTC
TTTTTCTAAGTTAAATAATTTTCAAAAGTTTAAGACTATTTGTTTCTCAACCGTCGAAGTGCCTGGTAGTTGTAATTTT
CCACTTGAAGCTACCTGGCATTACACTTCTTATACTATTGTTGGTGCTTTGTATGTTACTTGGTCTGAAGGTAATTCCA
TTACTGGTGTACCTTATCCTGTCTCTGGTATTCGTGAGTTTAGTAATTTAGTTTTAAATAATTGTACCAAATATAATAT

TTATGATTATGTTGGTACTGGAATTATACGTTCTTCAAACCAGTCACTTGCTGGTGGTATTACATATGTTTCTAACTCT
GGTAATTTACTTGGTTTTAAAAATGTTTCCACTGGTAACATTTTTATTGTGACACCATGTAACCAACCAGATCAAGTAG
CTGTTTATCAACAAAGCATTATTGGTGCCATGACCGCTGTTAATGAGTCTAGATATGGCTTGCAAAACTTACTACAGTT
ACCTAACTTTTATTATGTTAGTAATGGTGGTAACAATTGCACTACGGCTGTTATGACTTATTCTAATTTTGGTATTTGT
GCTGATGGTTCTTTAATTCCTGTTCGTCCGCGTAATTCTAGTGATAATGGTATTTCAGCCATAATCACTGCTAATTTAT
CCATTCCCTCTAACTGGACTACTTCAGTTCAAGTTGAGTACCTCCAAATTACTAGTACTCCAATAGTTGTTGATTGTGC
TACTTATGTGTGTAATGGTAACCCTCGCTGTAAGAATCTACTTAAGCAGTATACTTCTGCTTGTAAAACTATTGAAGAT
GCCTTACGACTTAGTGCTCATTTGGAAACTAATGATGTTAGTAGTATGCTAACTTTCGATAGCAATGCTTTTAGTTTGG
CTAATGTTACTAGTTTTGGAGATTATAACCTTTCTAGTGTTTTACCTCAGAGAAACATTCATTCAAGCCGTATAGCAGG
ACGTAGTGCTTTGGAAGATTTGTTGTTTAGCAAAGTTGTTACATCTGGTTTGGGTACTGTTGATGTTGACTATAAGTCT
TGTACTAAAGGTCTTTCTATTGCTGACCTTGCTTGTGCTCAGTACTACAATGGCATAATGGTTTTGCCAGGTGTTGCTG
ATGCTGAACGTATGGCCATGTACACAGGTTCTCTTATAGGTGGCATGGTGCTCGGAGGTCTTACATCAGCAGCCGCCAT
ACCTTTTTCTTTGGCACTGCAAGCACGACTTAACTATGTTGCTTTACAAACTGATGTGCTTCAAGAAAATCAGAAAATT
TTGGCTGCATCATTTAATAAGGCTATTAATAATATTGTTGCTTCTTTTAGTAGCGTTAATGATGCTATTACACAAACTG
CAGAGGCTATACATACTGTTACTATTGCACTTAATAAGATTCAGGATGTTGTTAATCAACAGGGTAGTGCTCTTAACCA
TCTCACTTCACAATTGAGACATAATTTTCAGGCCATTTCTAATTCAATTCAGGCTATTTATGACCGGCTTGATTCAATT
CAAGCCGATCAACAAGTTGACAGATTAATTACTGGACGGCTTGCAGCTTTGAATGCATTTGTTTCCCAAGTTTTGAATA
AATATACTGAAGTTCGTGGTTCCAGACGCTTAGCACAGCAGAAGATTAATGAATGTGTCAAGTCACAATCTAATAGATA
TGGTTTTTGTGGCAATGGCACTCACATCTTTTCAATCGTCAACTCAGCTCCAGATGGTTTGCTTTTTCTTCATACTGTT
TTGCTGCCAACTGATTACAAGAATGTAAAGGCGTGGTCTGGTATCTGTGTTGATGGCATTTATGGCTATGTTCTGCGTC
AACCTAACTTGGTTCTTTATTCTGATAATGGTGTCTTTCGTGTAACTTCCAGGGTCATGTTTCAACCTCGTTTACCTGT
TTTGTCTGATTTTGTGCAAATATATAATTGTAATGTTACTTTTGTTAACATATCTCGTGTCGAGTTACATACTGTCATA
CCTGACTACGTTGATGTTAATAAAACATTACAAGAGTTTGCACAAAACTTACCAAAGTATGTTAAGCCTAATTTTGACT
TGACTCCTTTTAATTTAACATATCTTAATTTGAGTTCTGAGTTGAAGCAACTCGAAGCTAAAACTGCTAGTCTTTTCCA
AACTACTGTTGAATTACAAGGTCTTATTGATCAGATTAACAGTACATATGTTGATTTGAAGTTGCTTAATAGGTTTGAA
AATTATATCAAATGGCCTTGGTGGGTTTGGCTCATTATTTCTGTTGTTTTTGTTGTATTGTTGAGTCTTCTTGTCTTTT
GTTGTCTTTCTACAGGTTGTTGTGGTTGTTGCAATTGTTTAACTTCATCAATGCGAGGCTGTTGTGATTGTGGTTCAAC
TAAACTTCCTTATTACGAATTTGAAAAGGTCCACGTTCAATAA

>gi|530802085|gb|KF530107.1| Human coronavirus NL63 strain NL63/human/USA/911-56/1991, complete genome

ATGAAACTTTTCTTGATTTTGCTTGTTTTGCCCCTGGCCTCTTGCTTTTCCACATGTAATAGTAATGCTAATATC
TCTATGTTACAATTAGGTGTTCCTGACAATTCTTCAACTATTGTTACGGGTTTATTGCCAACTCATTGGATTTGTGCTA
ATCAGAGTACATCTGTTTACTCAGCCAAAGGTTTCTTTTATATTGATGTTGGTAATCACCGTAGTGCTTTTGCGCTCCA
TACTGGTTATTATGATGCTAATCAGTATTATATTTATGTTACTAATGAAATAGGCTTAAATGCTTCTGTTACTCTTAAG
ATTTGTAAGTTTAGAAACACTACTTTTGATTTTTTAAGTAATGCTTCTAGTTCTTTTGACTGTATAGTTAATTTGTTAT
TTACAGAACAGTTAGGTGCGCCTTTGGGCATAACTATATCTGGTGAAACTGTACGTCTGCATTTATATAATGTAACTCG
TACTTTTTATGTGCCAGCAGCTTATAAACTTACTAAACTTAGTGTTAAATGTTACTTTAATTATTCCTGTGTTTTTAGT
GTTGTCAACGCCACCGTTACTGTCAATGTCACCACACATAATGGCCGTGTAGTTAACTACACTGTTTGTGATGATTGTA
ATGGTTATACTGATAACATATTTTCTGTTCAACAGGATGGCCGCATTCCTAATGGTTTCCCTTTTAATAATTGGTTTTT
GTTAACTAATGGTTCCACACTAGTGGATGGAGTCTCTAGACTTTATCAACCACTCCGTTTAACTTGTTTATGGCCTGTA
CCTGGTCTTAAATCTTCAACTGGTTTTGTTTATTTTAATGCCACTGGTTCTGATGTTAATTGTAACGGCTACCAACATA

ATTCTGTTGCTGATGTTATGCGTTACAATCTTAACTTCAGTGCTAATTCTGTGGACAATCTCAAGAGTGGTGTTATAGT
TTTTAAAACTTTACAGTACGATGTTTTGTTTTATTGTAGTAATTCTTCTTCAGGTGTTCTTGACACCACAATACCTTTT
GGCCCTTCCTCTCAACCTTATTACTGTTTTATAAACAGTACTATCAACACTACTCATGTTAGCACTTTTGTGGGTATTT
TACCACCCACTGTGCGTGAAATTGTTGTTGCTAGAACTGGTCAGTTTTATATTAATGGTTTTAAGTATTTCGATTTGGG
TTTCATAGAAGCTGTCAATTTTAATGTCACGACTGCTAGTGCCACAGATTTTTGGACGGTTGCATTTGCTACTTTTGTT
GATGTTTTGGTTAATGTTAGTGCAACTAACATTCAAAACTTACTTTATTGTGATTCTCCATTTGAAAAGTTGCAGTGTG
AGCACTTGCAGTTTGGGTTGCAAGATGGTTTTTATTCTGCAAATTTTCTTGATGATAATGTTTTGCCTGAGACTTATGT
TGCACTCCCCATTTATTATCAACATACGGACATAAATTTTACTGCAACTGCATCTTTTGGTGGTTCTTGTTATGTTTGT
AAACCACGCCAGGTTAATATATCTCTTAATGGTAACACTTCAGTGTGTGTTAGAACATCTCATTTTTCAATTAGGTATA
TTTATAACCGCGTTAAGAGTGGTTCACCAGGTGACTCTTCATGGCACATTTATTTAAAGAGTGGCACTTGTCCATTTTC
TTTTTCTAAGTTAAATAATTTTCAAAAGTTTAAGACTATTTGTTTCTCAACCGTCGAAGTGCCTGGTAGTTGTAATTTT
CCACTTGAAGCCACCTGGCATTACACTTCTTATACTATTGTTGGTGCTTTGTATGTTACTTGGTCTGAAGGTAATTCTA
TTACTGGTGTACCTTATCCTGTCTCTGGTATTCGTGAGTTTAGTAATTTAGTTTTAAATAATTGTACCAAATATAATAT
TTATGATTATGTTGGTACTGGAATTATACGTTCTTCAAACCAGTCACTTGCTGGTGGTATTACATATGTTTCTAACTCT
GGTAATTTACTTGGTTTTAAAAATGTTTCCACTGGTAACATTTTTATTGTGACACCATGTAACCAACCAGATCAAGTAG
CTGTTTATCAACAAAGCATTATTGGTGCCATGACCGCTGTTAATGAGTCTAGATATGGCTTGCAAAACTTACTACAGTT
ACCTAACTTTTATTATGTTAGTAATGGTGGTAACAATTGCACTACAGCTGTTATGACTTATTCTAATTTTGGTATTTGT
GCTGATGGTTCTTTAATTCCTGTTCGTCCACGTAATTCTAGTGATAATGGTATTTCAGCCATAATCACTGCTAATTTAT
CTATTCCCTCTAACTGGACTACTTCAGTTCAAGTTGAGTATCTCCAAATTACTAGTACTCCAATAGTTGTTGATTGTGC
TACTTATGTGTGTAATGGTAACCCTCGCTGTAAGAATCTACTTAAGCAGTATACTTCTGCTTGTAAAACTATTGAAGAT
GCCTTACGACTTAGTGCTCATTTGGAAACTAATGATGTTAGTAGTATGCTAACTTTCGATAGCAATGCTTTTAGTTTGG
CTAATGTTACTAGTTTTGGAGATTATAACCTTTCTAGTGTTTTACCTCAGAGAAACATTCATTCAAGCCGTATAGCAGG
ACGTAGTGCTTTGGAAGATTTGTTGTTTAGCAAAGTTGTTACATCTGGTTTGGGTACTGTTGATGTTGACTATAAGTCT
TGTACTAAAGGTCTTTCTATTGCTGACCTTGCTTGTGCTCAGTACTACAATGGTATAATGGTTTTGCCAGGTGTTGCTG
ATGCTGAACGTATGGCCATGTACACAGGTTCTCTTATAGGTGGCATGGTGCTCGGAGGTCTTACATCAGCAGCCGCCAT
ACCTTTTTCTTTGGCACTGCAAGCACGACTTAACTATGTTGCTTTACAAACTGATGTGCTTCAAGAAAATCAGAAAATT
TTGGCTGCATCATTTAATAAGGCTATTAATAATATTGTTGCTTCTTTTAGTAGCGTTAATGATGCTATTACACAAACTG
CAGAGGCTATACATACTGTTACTATTGCACTTAATAAGATTCAGGATGTTGTTAATCAACAGGGTAGTGCTCTTAACCA
TCTCACTTCACAATTGAGACATAATTTTCAGGCCATTTCTAATTCAATTCAGGCTATTTATGACCGGCTTGATTCAATT
CAAGCCGATCAACAAGTTGACAGATTAATTACTGGACGGCTTGCAGCTTTGAATGCATTTGTTTCCCAAGTTTTGAATA
AATATACTGAAGTTCGTGGTTCCAGACGCTTAGCACAGCAGAAGATTAATGAATGTGTCAAGTCACAATCTAATAGATA
TGGTTTTTGTGGCAATGGCACTCACATCTTTTCAATCGTCAACTCAGCTCCAGATGGTTTGCTTTTTCTTCATACTGTT
TTGCTGCCAACTGATTACAAGAATGTAAAGGCGTGGTCTGGTATCTGTGTTGATGACATTTATGGCTATGTTCTGCGTC
AACCTAACTTGGTTCTTTATTCTGATAATGGTGTCTTTCGTGTAACTTCCAGGGTCATGTTTCAACCTCGCTTACCTGT
TTTGTCTGATTTTGTGCAAATATATAATTGTAATGTTACTTTTGTTAACATATCTCGTGTCGAGTTACATACTGTCATA
CCTGACTACGTTGATGTTAATAAAACATTACAAGAGTTTGCACAAAACTTACCAAAGTATGTTAAGCCTAATTTTGACT
TGACTCCTTTTAATTTAACATATCTTAATTTGAGTTCTGAGTTGAAGCAACTCGAAGCTAAAACTGCTAGTCTTTTTCA
AACTACTGTTGAATTACAAGGTCTTATTGATCAGATTAACAGTACATATGTTGATTTGAAGTTGCTTAATAGGTTTGAA
AATTATATCAAATGGCCTTGGTGGGTTTGGCTCATTATTTCTGTTGTTTTTGTTGTATTGTTGAGTCTTCTTGTGTTTT
GTTGTCTTTCTACAGGTTGTTGTGGTTGTTGCAATTGTTTAACTTCATCAATGCGAGGCTGTTGTGATTGTGGTTCAAC
TAAACTTCCTTATTACGAATTTGAAAAGGTCCACGTTCAATAA

>gi|530802101|gb|KF530109.1| Human coronavirus NL63 strain

NL63/human/USA/903-28/1990, complete genome

ATGAAACTTTTCTTGATTTTGCTTGTTTTGCCCCTGGCCTCTTGCTTTTCCACATGTAATAGTAATGCTAATATC
TCTATGTTACAATTAGGTGTTCCTGACAATTCTTCAACTATTGTTACGGGTTTATTGCCAACTCATTGGATTTGTGCTA
ATCAGAGTACATCTGTTTACTCAGCCAAAGGTTTCTTTTATATTGATGTTGGTAATCACCGTAGTGCTTTTGCGCTCCA
TACTGGTTATTATGATGCTAATCAGTATTATATTTATGTTACTAATGAAATAGGCTTAAATGCTTCTGTTACTCTTAAG
ATTTGTAAGTTTAGAAACACTACTTTTGATTTTTTAAGTAATGCTTCTAGTTCTTTTGACTGTATAGTTAATTTGTTAT
TTACAGAACAGTTAGGTGCGCCTTTGGGCATAACTATATCTGGTGAAACTGTACGTCTGCATTTATATAATGTAACTCG
TACTTTTTATGTGCCAGCAGCTTATAAACTTACTAAACTTAGTGTTAAATGTTACTTTAATTATTCCTGTGTTTTTAGT
GTTGTCAACGCCACCGTTACTGTCAATGTCACCACACATAATGGCCGTGTAGTTAACTACACTGTTTGTGATGATTGTA
ATGGTTATACTGATAACATATTTTCTGTTCAACAGGATGGCCGCATTCCTAATGGTTTCCCTTTTAATAATTGGTTTTT
GTTAACTAATGGTTCCACACTAGTGGATGGAGTCTCTAGACTTTATCAACCACTCCGTTTAACTTGTTTATGGCCTGTA
CCTGGTCTTAAATCTTCAACTGGTTTTGTTTATTTTAATGCCACTGGTTCTGATGTTAATTGTAACGGCTACCAACATA
ATTCTGTTGCTGATGTTATGCGTTACAATCTTAACTTCAGTGCTAATTCTGTGGACAATCTCAAGAGTGGTGTTATAGT
TTTTAAAACTTTACAGTACGATGTTTTGTTTTATTGTAGTAATTCTTCTTCAGGTGTTCTTGACACCACAATACCTTTT
GGCCCTTCCTCTCAACCTTATTACTGTTTTATAAACAGTACTATCAACACTACTCATGTTAGCACTTTTGTGGGTATTT
TACCACCCACTGTGCGTGAAATTGTTGTTGCTAGAACTGGTCAGTTTTATATTAATGGTTTTAAGTATTTCGATTTGGG
TTTCATAGAAGCTGTCAATTTTAATGTCACGACTGCTAGTGCCACAGATTTTTGGACGGTTGCATTTGCTACTTTTGTT
GATGTTTTGGTTAATGTTAGTGCAACTAACATTCAAAACTTACTTTATTGTGATTCTCCATTTGAAAAGTTGCAGTGTG
AGCACTTGCAGTTTGGGTTGCAAGATGGTTTTTATTCTGCAAATTTTCTTGATGATAATGTTTTGCCTGAGACTTATGT
TGCACTCCCCATTTATTATCAACATACGGACATAAATTTTACTGCAACTGCATCTTTTGGTGGTTCTTGTTATGTTTGT
AAACCACGCCAGGTTAATATATCTCTTAATGGTAACACTTCAGTGTGTGTTAGAACATCTCATTTTTCAATTAGGTATA
TTTATAACCGCGTTAAGAGTGGTTCACCAGGTGACTCTTCATGGCACATTTATTTAAAGAGTGGCACTTGTCCATTTTC
TTTTTCTAAGTTAAATAATTTTCAAAAGTTTAAGACTATTTGTTTCTCAACCGTCGAAGTGCCTGGTAGTTGTAATTTT
CCACTTGAAGCCACCTGGCATTACACTTCTTATACTATTGTTGGTGCTTTGTATGTTACTTGGTCTGAAGGTAATTCTA
TTACTGGTGTACCTTATCCTGTCTCTGGTATTCGTGAGTTTAGTAATTTAGTTTTAAATAATTGTACCAAATATAATAT
TTATGATTATGTTGGTACTGGAATTATACGTTCTTCAAACCAGTCACTTGCTGGTGGTATTACATATGTTTCTAACTCT
GGTAATTTACTTGGTTTTAAAAATGTTTCCACTGGTAACATTTTTATTGTGACACCATGTAACCAACCAGATCAAGTAG
CTGTTTATCAACAAAGCATTATTGGTGCCATGACCGCTGTTAATGAGTCTAGATATGGCTTGCAAAACTTACTACAGTT
ACCTAACTTTTATTATGTTAGTAATGGTGGTAACAATTGCACTACAGCTGTTATGACTTATTCTAATTTTGGTATTTGT
GCTGATGGTTCTTTAATTCCTGTTCGTCCACGTAATTCTAGTGATAATGGTATTTCAGCCATAATCACTGCTAATTTAT
CTATTCCCTCTAACTGGACTACTTCAGTTCAAGTTGAGTATCTCCAAATTACTAGTACTCCAATAGTTGTTGATTGTGC
TACTTATGTGTGTAATGGTAACCCTCGCTGTAAGAATCTACTTAAGCAGTATACTTCTGCTTGTAAAACTATTGAAGAT
GCCTTACGACTTAGTGCTCATTTGGAAACTAATGATGTTAGTAGTATGCTAACTTTCGATAGCAATGCTTTTAGTTTGG
CTAATGTTACTAGTTTTGGAGATTATAACCTTTCTAGTGTTTTACCTCAGAGAAACATTCATTCAAGCCGTATAGCAGG
ACGTAGTGCTTTGGAAGATTTGTTGTTTAGCAAAGTTGTTACATCTGGTTTGGGTACTGTTGATGTTGACTATAAGTCT
TGTACTAAAGGTCTTTCTATTGCTGACCTTGCTTGTGCTCAGTACTACAATGGTATAATGGTTTTGCCAGGTGTTGCTG
ATGCTGAACGTATGGCCATGTACACAGGTTCTCTTATAGGTGGCATGGTGCTCGGAGGTCTTACATCAGCAGCCGCCAT
ACCTTTTTCTTTGGCACTGCAAGCACGACTTAACTATGTTGCTTTACAAACTGATGTGCTTCAAGAAAATCAGAAAATT
TTGGCTGCATCATTTAATAAGGCTATTAATAATATTGTTGCTTCTTTTAGTAGCGTTAATGATGCTATTACACAAACTG
CAGAGGCTATACATACTGTTACTATTGCACTTAATAAGATTCAGGATGTTGTTAATCAACAGGGTAGTGCTCTTAACCA
TCTCACTTCACAATTGAGACATAATTTTCAGGCCATTTCTAATTCAATTCAGGCTATTTATGACCGGCTTGATTCAATT
CAAGCCGATCAACAAGTTGACAGATTAATTACTGGACGGCTTGCAGCTTTGAATGCATTTGTTTCCCAAGTTTTGAATA
AATATACTGAAGTTCGTGGTTCCAGACGCTTAGCACAGCAGAAGATTAATGAATGTGTCAAGTCACAATCTAATAGATA

TGGTTTTTGTGGCAATGGCACTCACATCTTTTCAATCGTCAACTCAGCTCCAGATGGTTTGCTTTTTCTTCATACTGTT
TTGCTGCCAACTGATTACAAGAATGTAAAGGCGTGGTCTGGTATCTGTGTTGATGGCATTTATGGCTATGTTCTGCGTC
AACCTAACTTGGTTCTTTATTCTGATAATGGTGTCTTTCGTGTAACTTCCAGGGTCATGTTTCAACCTCGCTTACCTGT
TTTGTCTGATTTTGTGCAAATATATAATTGTAATGTTACTTTTGTTAACATATCTCGTGTCGAGTTACATACTGTCATA
CCTGACTACGTTGATGTTAATAAAACATTACAAGAGTTTGCACAAAACTTACCAAAGTATGTTAAGCCTAATTTTGACT
TGACTCCTTTTAATTTAACATATCTTAACTTGAGTTCTGAGTTGAAGCAACTCGAAGCTAAAACTGCTAGTCTTTTTCA
AACTACTGTTGAATTACAAGGTCTTATTGATCAGATTAACAGTACATATGTTGATTTGAAGTTGCTTAATAGGTTTGAA
AATTATATCAAATGGCCTTGGTGGGTTTGGCTCATTATTTCTGTTGTTTTTGTTGTATTGTTGAGTCTTCTTGTGTTTT
GTTGTCTTTCTACAGGTTGTTGTGGTTGTTGCAATTGTTTAACTTCATCAATGCGAGGCTGTTGTGATTGTGGTTCAAC
TAAACTTCCTTATTACGAATTTGAAAAGGTCCACGTTCAATAA

>gi|530802109|gb|KF530110.1| Human coronavirus NL63 strain NL63/human/USA/838-9/1983, complete genome

ATGAAACTTTTCTTGATTTTGCTTGTTTTGCCCCTGGCCTCTTGCTTTTTCACATGTAATAGTAATGCTAATATC
TCTATGTTACAATTAGGTGTTCCTGACAATTCTTCAACTATTGTCACGGGTTTATTGCCAACTCATTGGATTTGTGCTA
ATCAGAGTACATCTGTTTACTCAGCCAATGGTTTCTTTTATATTGATGTTGGTAATCACCGTAGTGCTTTTGCGCTCCA
TACTGGTTATTATGATGCTAATCAGTATTATATTTATGTTACTAATGAAATAGGCTTAAATGCTTCTGTTACTCTTAAG
ATTTGTAAGTTTAGTAGAAACACTACTTTTGATTTTTTAAGTAATGCTTCTAGTTCTTTTGACTGTATAGTTAATTTGT
TATTTACAGAACAGTTAGGTGCGCCTTTGGGCATAACTATATCTGGTGAAACTGTACGTCTACATTTATATAATGTAAC
TCGTACTTTTTATGTGCCAGCAGCTTATAAACTTACTAAACTTAGTGTTAAATGTTACTTCAATTATTCCTGTGTTTTT
AGTGTTGTCAACGCCACCGTTACTGTGAATGTCACCACACATAATGGCCGTGTAGTTAACTACACTGTTTGTGATGATT
GTAATGGTTATACTGATAACATATTTTCTGTTCAACAGGATGGCCGCATTCCTAATGGTTTCCCTTTTAATAATTGGTT
TTTGTTAACTAATGGTTCCACATTAGTGGACGGGGTCTCTAGACTTTATCAACCACTCCGTTTAACTTGTTTATGGCCT
GTACCTGGTCTTAAATCTTCAACTGGTTTTGTTTATTTTAATGCCACTGGTTCTGATGTTAATTGTAACGGCTATCAAC
ATAATTCTGTTGCTGATGTTATGCGTTACAATCTTAACTTCAGTGCTAATTCTGTGGACAATCTCAAGAGTGGTGTTAT
AGTTTTTAAAACTTTACAGTACGATGTTTTGTTTTATTGTAGTAATTCTTCTTCAGGTGTTCTTGACACCACAATACCT
TTTGGTCCTTCCTCTCAACCTTATTACTGTTTTATAAACAGTACTATCAACACTACTCATGTTAGCACTTTTGTGGGTA
TTTTACCACCCACTGTGCGTGAAATTGTTGTTGCTAGAACTGGTCAGTTTTATATTAATGGTTTTAAGTATTTCGATTT
GGGTTTCATAGAAGCTGTCAATTTTAATGTCACGACTGCTAGTGCCACAGATTTTTGGACGGTTGCATTTGCTACTTTT
GTTGATGTTTTGGTTAATGTTAGTGCAACTAACATTCAAAACTTACTTTATTGCGATTCTCCATTTGAAAAGTTGCAGT
GTGAGCACTTGCAGTTTGGATTGCAAGATGGTTTTTATTCTGCAAATTTTCTTGATGATAATGTTTTGCCTGAGACTTA
TGTTGCACTCCCCATTTATTATCAACATACGGACATAAATTTTACTGCAACTGCATCTTTTGGTGGTTCTTGTTATGTT
TGTAAACCACGCCAGGTTAATATATCTCTTAATGGTAACACTTCAGTGTGTGTTAGAACATCTCATTTTTCAATTAGGT
ATATTTATAACCGCGTTAAGAGTGGTTCACCAGGTGACTCTTCATGGCATATTTATTTAAAGAGTGGCACTTGTCCATT
TTCTTTTTCTAAGTTAAATAATTTTCAAAAGTTTAAGACTATTTGTTTCTCAACCGTCGAAGTGCCTGGTAGTTGTAAT
TTTCCACTTGAAGCCACCTGGCATTACACTTCTTATACTATTGTTGGTGCTTTGTATGTTACTTGGTCTGAAGGTAATT
CCATTACTGGTGTACCTTATCCTGTCTCTGGTATTCGTGAGTTTAGTAATTTAGTTTTAAATAATTGTACCAAATATAA
TATTTATGATTATGTTGGTACTGGAATTATACGTTCTTCAAACCAGTCACTTGCTGGTGGTATTACATATGTTTCTAAC
TCTGGTAATTTACTTGGTTTTAAAAATGTTTCCACTGGTAACATTTTTATTGTGACACCATGTAACCAACCAGATCAAG
TAGCTGTTTATCAACAAAGCATTATTGGTGCCATGACCGCTGTTAATGAGTCTAGATATGGCTTGCAAAACTTACTACA
GTTACCTAACTTTTATTATGTTAGTAATGGTGGTAACAATTGCACTACGGCTGTTATGACTTATTCTAATTTTGGTATT
TGTGCTGATGGTTCTTTAATTCCTGTTCGTCCGCGTAATTCTAGTGATAATGGTATTTCAGCCATAATCACTGCTAATT

TATCCATTCCCTCTAACTGGACTACTTCAGTTCAAGTTGAGTACCTCCAAATTACTAGTATTCCAATAGTTGTTGATTG
TGCTACTTATGTGTGTAATGGTAACCCTCGCTGTAAGAATCTACTTAAGCAGTATACTTCTGCTTGTAAAACTATTGAA
GATGCCTTACGACTTAGTGCTCATTTGGAAACTAATGATGTTAGTAGTATGCTAACTTTCGATAGCAATGCTTTTAGTT
TGGCTAATGTTACTAGTTTTGGAGATTATAACCTTTCTAGTGTTTTACCTCAGAGAAACATTCATTCAAGCCGTATAGC
AGGACGTAGTGCTTTGGAAGATTTGTTGTTTAGCAAAGTTGTTACATCTGGTTTGGGTACTGTTGATGTTGACTATAAG
TCTTGTACTAAAGGTCTTTCTATTGCTGACCTTGCTTGTGCTCAGTACTACAATGGCATAATGGTTTTGCCAGGTGTTG
CTGATGCTGAACGTATGGCCATGTACACAGGTTCTCTTATAGGTGGCATGGTGCTCGGAGGTCTTACATCAGCAGCCGC
CATACCTTTTTCTTTGGCACTGCAAGCACGACTTAACTATGTTGCTTTACAAACTGATGTGCTTCAAGAAAATCAGAAA
ATTTTGGCTGCATCATTTAATAAGGCTATTAATAATATTGTTGCTTCTTTTAGTAGCGTTAATGATGCTATTACACAAA
CTGCAGAGGCTATACATACTGTTACTATTGCACTTAATAAGATTCAGGATGTTGTTAATCAACAGGGTAGTGCTCTTAA
CCATCTCACTTCACAATTGAGACATAATTTTCAGGCCATTTCTAATTCAATTCAGGCTATTTATGACCGGCTTGATTCA
ATTCAAGCCGATCAACAAGTTGACAGATTAATTACTGGACGGCTTGCAGCTTTGAATGCATTTGTTTCCCAAGTTTTGA
ATAAATATACTGAAGTTCGTGGTTCCAGACGCTTAGCACAGCAGAAGATTAATGAATGTGTCAAGTCACAATCTAATAG
ATATGGTTTTTGTGGCAATGGCACTCACATCTTTTCAATCGTCAACTCAGCTCCAGATGGTTTGCTTTTTCTTCATACT
GTTTTGCTGCCAACTGATTACAAGAATGTAAAGGCGTGGTCTGGTATCTGTGTTGATGGCATTTATGGCTATGTTCTGC
GTCAACCTAACTTGGTTCTTTATTCTGATAATGGTGTCTTTCGTGTAACTTCCAGGGTCATGTTTCAACCTCGTTTACC
TGTTTTGTCTGATTTTGTGCAAATATATAATTGTAATGTTACTTTTGTTAACATATCTCGTGTCGAGTTACATACTGTC
ATACCTGACTACGTTGATGTTAATAAAACATTACAAGAGTTTGCACAAAACTTACCAAAGTATGTTAAGCCTAATTTTG
ACTTGACTCCTTTTAATTTAACATATCTTAATTTGAGTTCTGAGTTGAAGCAACTCGAAGTTAAAACTGCTAGTCTTTT
CCAAACTACTGTTGAATTACAAGGTCTTATTGATCAGATTAACAGTACATATGTTGATTTGAAGTTGCTTAATAGGTTT
GAAAATTATATCAAATGGCCTTGGTGGGTTTGGCTCATTATTTCTGTTGTTTTTGTTGTATTGTTGAGTCTTCTTGTGT
TTTGTTGTCTTTCTACAGGTTGTTGTGGTTGTTGCAATTGTTTAACTTCATCAATGCGAGGCTGTTGTGATTGTGGTTC
AACTAAACTTCCTTATTACGAATTTGAAAAGGTCCACGTTCAATAA

>gi|530802117|gb|KF530111.1| Human coronavirus NL63 strain NL63/human/USA/901-24/1990, complete genome

ATGAAACTTTTCTTGATTTTGCTTGTTTTGCCCCTGGCCTCTTGCTTTTCCACATGTAATAGTAATGCTAATATC
TCTATGTTACAATTAGGTGTTCCTGACAATTCTTCAACTATTGTTACGGGTTTATTGCCAACTCATTGGATTTGTGCTA
ATCAGAGTACATCTGTTTACTCAGCCAAAGGTTTCTTTTATATTGATGTTGGTAATCACCGTAGTGCTTTTGCGCTCCA
TACTGGTTATTATGATGCTAATCAGTATTATATTTATGTTACTAATGAAATAGGCTTAAATGCTTCTGTTACTCTTAAG
ATTTGTAAGTTTAGAAACACTACTTTTGATTTCTTAAGTAATGCTTCTAGTTCTTTTGACTGTATAGTTAATTTGTTAT
TTACAGAACAGTTAGGTGCGCCTTTGGGCATAACTATATCTGGTGAAACTGTACGTCTGCATTTATATAATGTAACTCG
TACTTTTTATGTGCCAGCAGCTTATAAACTTACTAAACTTAGTGTTAAATGTTACTTTAATTATTCCTGTGTTTTTAGT
GTTGTCAACGCCACCGTTACTGTCAATGTCACCACACATAATGGCCGTGTAGTTAACTACACTGTTTGTGATGATTGTA
ATGGTTATACTGATAACATATTTTCTGTTCAACAGGATGGCCGCATTCCTAATGGTTTCCCTTTTAATAATTGGTTTTT
GTTAACTAATGGTTCCACACTAGTGGATGGAGTCTCTAGACTTTATCAACCACTCCGTTTAACTTGTTTATGGCCTGTA
CCTGGTCTTAAATCTTCAACTGGTTTTGTTTATTTTAATGCCACTGGTTCTGATGTTAATTGTAACGGCTACCAACATA
ATTCTGTTGCTGATGTTATGCGTTACAATCTTAACTTCAGTGCTAATTCTGTGGACAATCTCAAGAGTGGTGTTATAGT
TTTTAAAACTTTACAGTACGATGTTTTGTTTTATTGTAGTAATTCTTCTTCAGGTGTTCTTGACACCACAATACCTTTT
GGCCCTTCCTCTCAACCTTATTACTGTTTTATAAACAGTACTATCAACACTACTCATGTTAGCACTTTTGTGGGTATTT
TACCACCCACTGTGCGTGAAATTGTTGTTGCTAGAACTGGTCAGTTTTATATTAATGGTTTTAAGTATTTCGATTTGGG
TTTCATAGAAGCTGTCAATTTTAATGTCACGACTGCTAGTGCCACAGATTTTTGGACGGTTGCATTTGCTACTTTTGTT

GATGTTTTGGTTAATGTTAGTGCAACTAACATTCAAAACTTACTTTATTGTGATTCTCCATTTGAAAAGTTGCAGTGTG
AGCACTTGCAGTTTGGGTTGCAAGATGGTTTTTATTCTGCAAATTTTCTTGATGATAATGTTTTGCCTGAGACTTATGT
TGCACTCCCCATTTATTATCAACATACGGACATAAATTTTACTGCAACTGCATCTTTTGGTGGTTCTTGTTATGTTTGT
AAACCACGCCAGGTTAATATATCTCTTAATGGTAACACTTCAGTGTGTGTTAGAACATCTCATTTTTCAATTAGGTATA
TTTATAACCGCGTTAAGAGTGGTTCACCAGGTGACTCTTCATGGCACATTTATTTAAAGAGTGGCACTTGTCCATTTTC
TTTTTCTAAGTTAAATAATTTTCAAAAGTTTAAGACTATTTGTTTCTCAACCGTCGAAGTGCCTGGTAGTTGTAATTTT
CCACTTGAAGCCACCTGGCATTACACTTCTTATACTATTGTTGGTGCTTTGTATGTTACTTGGTCTGAAGGTAATTCTA
TTACTGGTGTACCTTATCCTGTCTCTGGTATTCGTGAGTTTAGTAATTTAGTTTTAAATAATTGTACCAAATATAATAT
TTATGATTATGTTGGTACTGGAATTATACGTTCTTCAAACCAGTCACTTGCTGGTGGTATTACATATGTTTCTAACTCT
GGTAATTTACTTGGTTTTAAAAATGTTTCCACTGGTAACATTTTTATTGTGACACCATGTAACCAACCAGATCAAGTAG
CTGTTTATCAACAAAGCATTATTGGTGCCATGACCGCTGTTAATGAGTCTAGATATGGCTTGCAAAACTTACTACAGTT
ACCTAACTTTTATTATGTTAGTAATGGTGGTAATAATTGCACTACAGCTGTTATGACTTATTCTAATTTTGGTATTTGT
GCTGATGGTTCTTTAATTCCTGTTCGTCCACGTAATTCTAGTGATAATGGTATTTCAGCCATAATCACTGCTAATTTAT
CTATTCCCTCTAACTGGACTACTTCAGTTCAAGTTGAGTATCTCCAAATTACTAGTACTCCAATAGTTGTTGATTGTGC
TACTTATGTGTGTAATGGTAACCCTCGCTGTAAGAATCTACTTAAGCAGTATACTTCTGCTTGTAAAACTATTGAAGAT
GCCTTACGACTTAGTGCTCATTTGGAAACTAATGATGTTAGTAGTATGCTAACTTTCGATAGCAATGCTTTTAGTTTGG
CTAATGTTACTAGTTTTGGAGATTATAACCTTTCTAGTGTTTTACCTCAGAGAAACATTCATTCAAGCCGTATAGCAGG
ACGTAGTGCTTTGGAAGATTTGTTGTTTAGCAAAGTTGTTACATCTGGTTTGGGTACTGTTGATGTTGACTATAAGTCT
TGTACTAAAGGTCTTTCTATTGCTGACCTTGCTTGTGCTCAGTACTACAATGGTATAATGGTTTTGCCAGGTGTTGCTG
ATGCTGAACGTATGGCCATGTACACAGGTTCTCTTATAGGTGGCATGGTGCTCGGAGGTCTTACATCAGCAGCCGCCAT
ACCTTTTTCTTTGGCACTGCAAGCACGACTTAACTATGTTGCTTTACAAACTGATGTGCTTCAAGAAAATCAGAAAATT
TTGGCTGCATCATTTAATAAGGCTATTAATAATATTGTTGCTTCTTTTAGTAGCGTTAATGATGCTATTACACAAACTG
CAGAGGCTATACATACTGTTACTATTGCACTTAATAAGATTCAGGATGTTGTTAATCAACAGGGTAGTGCTCTTAACCA
TCTCACTTCACAATTGAGACATAATTTTCAGGCCATTTCTAATTCAATTCAGGCTATTTATGACCGGCTTGATTCAATT
CAAGCCGATCAACAAGTTGACAGATTAATTACTGGACGGCTTGCAGCTTTGAATGCATTTGTTTCCCAAGTTTTGAATA
AATATACTGAAGTTCGTGGTTCCAGACGCTTAGCACAGCAGAAGATTAATGAATGTGTCAAGTCACAATCTAATAGATA
TGGTTTTTGTGGCAATGGCACTCACATCTTTTCAATCGTCAACTCAGCTCCAGATGGTTTGCTTTTTCTTCATACTGTT
TTGCTGCCAACTGATTACAAGAATGTAAAGGCGTGGTCTGGTATCTGTGTTGATGGCATTTATGGCTATGTTCTGCGTC
AACCTAACTTGGTTCTTTATTCTGATAATGGTGTCTTTCGTGTAACTTCCAGGGTCATGTTTCAACCTCGCTTACCTGT
TTTGTCTGATTTTGTGCAAATATATAATTGTAATGTTACTTTTGTTAACATATCTCGTGTCGAGTTACATACTGTCATA
CCTGACTACGTTGATGTTAATAAAACATTACAAGAGTTTGCACAAAACTTACCAAAGTATGTTAAGCCTAATTTTGACT
TGACTCCTTTTAATTTAACATATCTTAATTTGAGTTCTGAGTTGAAGCAACTCGAAGCTAAAACTGCTAGTCTTTTTCA
AACTACTGTTGAATTACAAGGTCTTATTGATCAGATTAACAGTACATATGTTGATTTGAAGTTGCTTAATAGGTTTGAA
AATTATATCAAATGGCCTTGGTGGGTTTGGCTCATTATTTCTGTTGTTTTTGTTGTATTGTTGAGTCTTCTTGTGTTTT
GTTGTCTTTCTACAGGTTGTTGTGGTTGTTGCAATTGTTTAACTTCATCAATGCGAGGCTGTTGTGATTGTGGTTCAAC
TAAACTTCCTTATTACGAATTTGAAAAGGTCCACGTTCAATAA

>gi|530802125|gb|KF530112.1| Human coronavirus NL63 strain NL63/human/USA/0111-25/2001, complete genome

ATGAAACTTTTCTTGATTTTGCTTATTTTGCCCCTGGTTTCTTGCTTTTCTACATGTAACAGTAATGCTAGTATT
TCTATGTTACAATTAGGTGTTCCTGATAACTCTTCAACTATTGTCACAGGTTTGTTGCCAGTCCATTGGATTTGTGCTA
ATCAGAGCACATCTAGTTACTCGGCCAATGGCTTTTTCTATATTGATGTCGGTAAACACCGTAGTGCTTTTGCACTCCA

TAGTGGTTATTATGATGCTAACCAGTATTATATTTATCTCACTAATAAAATACATTTAAATGCTCCTGTCACTCTGAAG
ATTTGTAAGTTTGGAAACACTTCTTTTGATTTTTTAAGTAATGTTTCTACTTCTCATCATTGTATAGTTAATTCGTCAT
TCACAGAACAGTTAGGTGTGCCTTTGGGCATAACTATATCGGGTGAAACTGTACGTTTGCATTTATATAATGCAACTCG
CACCTTTTATGTGCCGGCCGCTTATAAACTTACTAAACTTAGTGTTAAATGTTACTTTAGTGAATCCTGTGTTTTTAGT
GTTGTCAATGCTACCGTTACTGTTAATGTCACCACACTTAATGGCCGTATAGTTAATTACACTGTTTGTGATGATTGTA
ATGGTTACACTGATAACATATTTTCTGTTCAACAGGATGGCCGCATTCCTAATGGTTTTTCTTTTAATAATTGGTTTTT
GTTAACTAATGGTTCTACACTAGTGGACGGTGTCTCTAGACTTTACCAACCACTTCGTTTAACTTGTTTATGGCCTGTA
CCTGGTCTTAAATCTTCGACTGGTTTTGTTTATTTTAACGCCACTGGTTCTGATGTTAATTGTAATGGTTATCAACATC
ATTCTGTTGCTGATGTTATGCGTTACAATCTTAACTTCAGTTCTAATTCTGTGGATAATCTCAAGAGTGGTGTTATAGT
TTTTAAAACTCTACAGTACGATGTTTTGTTTTACTGTAGTAATTCTTCTTCGGGCGTTCTAGACACCACAATACCTTTT
GGCCCTTCCTCTCAACCTTATTACTGTTTTATAAATAGTACCATCAACACCACTCATGTTAGCACTTTTGTGGGTGTCC
TACCACCCACTGTGCGTGAGATTGTTGTTGCTAGAACTGGTCAGTTTTATATTAATGGTTTTAAGTATTTTGATTTGGG
TTTTATCGAAGCTGTCAATTTTAATGTCACGACAGCTAGTGCTACAGATTTTTGGACGGTTGCATTTGCTACTTTTGTT
GATGTTTTGGTTAATGTTAGTGCAACTAACATTCAAAACTTACTTTATTGCGATTCTCCATTTGAAAAGTTGCAGTGTG
AGCACTTGCAGTTTGGATTGCAAGATGGTTTTTATTCTGCAAATTTTCTTGATGATAATGTTTTGCCTGATACTTATGT
TGCACTTCCTATTTATTATCAACATACTGATATAAATTTTACTGCGACTGCATCTTTTGGTGGTTCTTGTTATGTTTGC
AAACCACATCAGGTTAATATATCTCTTAATGGTAATACTTCAGTGTGTGTTAGAACATCTCATTTTTCAATTAGGTATA
TTTATAACCGCGTTAAGAGTGGTTCACCAGGTGACTCTTCATGGCACATTTATTTAAAGAGTGGCACTTGTCCATTTTC
TTTTTCTAAGTTAAATAATTTTCAAAAGTTTAAGACTATTTGTTTCTCAACCGTCGCAGTGCCTGGTAGTTGTAATTTT
CCACTTGAAGCCACCTGGCATTACACTTCTTATACTATTGTTGGTGCTTTGTATGTTACTTGGTCTGAAGGTAATTCCA
TTACTGGTGTACCTTATCCTGTCTCTGGTATTCGTGAGTTTAGTAATTTAGTTTTAAATAATTGTACCAAATATAATAT
TTATGATTATGTTGGTACTGGAATTATACGTTCTTCAAACCAGTCACTTGCTGGTGGTATTACATATGTTTCTAACTCT
GGTAATTTACTTGGTTTTAAAAATGTTTCCACTGGTAACATTTTTATTGTGACACCATGTAACCAACCAGACCAAGTAG
CTGTTTATCAACAAAGCATTATTGGTGCCATGACTGCTGTTAATGAGTCTAGATATGGCTTGCAAAACTTACTACAGTT
ACCTAACTTTTATTATGTTAGTAATGGTGGTAACAATTGCACTACGGCCGTTATGACTTATTCTAATTTTGGTATTTGT
GCTGATGGTTCTTTAATTCCTGTTCGTCCGCGTAATTCTAGTGATAATGGTATTTCAGCCATAATCACTGCTAATTTAT
CCATTCCCTCTAACTGGACTACTTCAGTTCAAGTTGAGTACCTCCAAATTACTAGTACTCCAATAGTTGTTGATTGTGC
TACTTATGTGTGTAATGGTAACCCTCGCTGTAAGAATTTACTTAAGCAGTATACTTCTGCTTGTAAAACTATTGAAGAT
GCCTTACGACTTAGTGCTCATTTGGAAACTAATGATGTTAGTAGTATGCTAACTTTCGATAGCAATGCTTTTAGTTTGG
CTAATGTTACTAGTTTTGGAGATTATAACCTTTCTAGTGTTTTACCTCAGAGAAACATTCATTCAAGCCGTATAGCAGG
ACGTAGTGCTTTGGAAGATTTGTTGTTTAGCAAAGTTGTTACATCTGGTTTGGGTACTGTTGATGTTGACTATAAGTCT
TGTACTAAAGGTCTTTCTATTGCTGACCTTGCTTGTGCTCAGTACTACAATGGCATAATGGTTTTGCCAGGTGTTGCTG
ATGCTGAACGTATGGCCATGTACACAGGTTCTCTTATAGGTGGCATGGTGCTCGGAGGTCTTACATCAGCAGCCGCCAT
ACCTTTTTCTTTGGCACTGCAAGCACGACTTAACTATGTTGCTTTACAAACTGATGTGCTTCAAGAAAATCAGAAAATT
TTGGCTGCATCATTTAATAAGGCTATTAATAACATTGTTGCTTCTTTTAGTAGCGTTAATGATGCTATTACACAAACTG
CAGAGGCTATACATACTGTTACTATTGCACTTAATAAGATTCAGGATGTTGTTAATCAACAGGGTAGTGCTCTTAACCA
TCTCACTTCACAATTGAGACATAATTTTCAGGCCATTTCTAATTCAATTCAAGCTATTTATGACCGGCTTGATTCAATT
CAAGCCGATCAACAAGTTGACAGATTAATTACTGGACGGCTTGCAGCTTTGAATGCATTTGTTTCCCAAGTTTTGAATA
AATATACTGAAGTTCGTAGTTCCAGACGCTTAGCACAGCAGAAGATTAATGAATGTGTCAAATCACAATCTAATAGATA
TGGTTTTTGTGGCAATGGCACCCATATCTTTTCAATCGTCAACTCAGCTCCAGATGGTTTGCTTTTTCTTCATACTGTT
TTGCTGCCAACCGACTACAAGAATGTAAAGGCGTGGTCGGGTATCTGTGTTGATGGCATTTACGGCTATGTTCTGCGTC
AACCTAACTTGGTTCTTTATTCTGATAATGGTGTCTTTCGTGTAACTTCCAGGGTCATGTTTCAACCTCGTTTACCTGT
TTTGTCTGATTTTGTGCAAATATATAATTGTAATGTTACTTTTGTTAACATATCTCGTGTCGAGTTACATACTGTCATA
CCTGACTACGTTGATGTTAATAAAACATTACAAGAGTTTGCACAAAACTTACCAAAGTATGTTAAGCCTAATTTTGACT

TGACTCCTTTTAATTTAACATATCTTAATTTGAGTTCTGAGTTGAAGCAACTCGAAGCTAAAACTGCTAGTCTTTTCCA
AACTACTGTTGAATTACAAGGTCTTATTGATCAGATTAACAGTACATATGTTGATTTGAAGTTGCTTAATAGGTTTGAA
AATTATATTAAATGGCCTTGGTGGGTTTGGCTCATTATTTCTGTTGTTTTTGTTGTATTGTTGAGTCTTCTTGTGTTTT
GTTGTCTTTCTACAGGTTGTTGTGGTTGTTGCAATTGTTTAACTTCATCAATGCGAGGTTGTTGTGATTGTGGTTCAAC
TAAACTTCCTTATTACGAATTTGAAAAGGTCCACGTTCAATAA

>gi|530802133|gb|KF530113.1| Human coronavirus NL63 strain NL63/human/USA/905-25/1990, complete genome

ATGAAACTTTTCTTGATTTTGCTTGTTTTGCCCCTGGCCTCTTGCTTTTCCACATGTAATAGTAATGCTAATATC
TCTATGTTACAATTAGGTGTTCCTGACAATTCTTCAACTATTGTTACGGGTTTATTGCCAACTCATTGGATTTGTGCTA
ATCAGAGTACATCTGTTTACTCAGCCAAAGGTTTCTTTTATATTGATGTTGGTAATCACCGTAGTGCTTTTGCGCTCCA
TACTGGTTATTATGATGCTAATCAGTATTATATTTATGTTACTAATGAAATAGGCTTAAATGCTTCTGTTACTCTTAAG
ATTTGTAAGTTTAGAAACACTACTTTTGATTTTTTAAGTAATGCTTCTAGTTCTTTTGACTGTATAGTTAATTTGTTAT
TTACAGAACAGTTAGGTGCGCCTTTGGGCATAACTATATCTGGTGAAACTGTACGTCTGCATTTATATAATGTAACTCG
TACTTTTTATGTGCCAGCAGCTTATAAACTTACTAAACTTAGTGTTAAATGTTACTTTAATTATTCCTGTGTTTTTAGT
GTTGTCAACGCCACCGTTACTGTCAATGTCACCACACATAATGGCCGTGTAGTTAACTACACTGTTTGTGATGATTGTA
ATGGTTATACTGATAACATATTTTCTGTTCAACAGGATGGCCGCATTCCTAATGGTTTCCCTTTTAATAATTGGTTTTT
GTTAACTAATGGTTCCACACTAGTGGATGGAGTCTCTAGACTTTATCAACCACTCCGTTTAACTTGTTTATGGCCTGTA
CCTGGTCTTAAATCTTCAACTGGTTTTGTTTATTTTAATGCCACTGGTTCTGATGTTAATTGTAACGGCTACCAACATA
ATTCTGTTGCTGATGTTATGCGTTACAATCTTAACTTCAGTGCTAATTCTGTGGACAATCTCAAGAGTGGTGTTATAGT
TTTTAAAACTTTACAGTACGATGTTTTGTTTTATTGTAGTAATTCTTCTTCAGGTGTTCTTGACACCACAATACCTTTT
GGCCCTTCCTCTCAACCTTATTACTGTTTTATAAACAGTACTATCAACACTACTCATGTTAGCACTTTTGTGGGTATTT
TACCACCCACTGTGCGTGAAATTGTTGTTGCTAGAACTGGTCAGTTTTATATTAATGGTTTTAAGTATTTCGATTTGGG
TTTCATAGAAGCTGTCAATTTTAATGTCACGACTGCTAGTGCCACAGATTTTTGGACGGTTGCATTTGCTACTTTTGTT
GATGTTTTGGTTAATGTTAGTGCAACTAACATTCAAAACTTACTTTATTGTGATTCTCCATTTGAAAAGTTGCAGTGTG
AGCACTTGCAGTTTGGGTTGCAAGATGGTTTTTATTCTGCAAATTTTCTTGATGATAATGTTTTGCCTGAGACTTATGT
TGCACTCCCCATTTATTATCAACATACGGACATAAATTTTACTGCAACTGCATCTTTTGGTGGTTCTTGTTATGTTTGT
AAACCACGCCAGGTTAATATATCTCTTAATGGTAACACTTCAGTGTGTGTTAGAACATCTCATTTTTCAATTAGGTATA
TTTATAACCGCGTTAAGAGTGGTTCACCAGGTGACTCTTCATGGCACATTTATTTAAAGAGTGGCACTTGTCCATTTTC
TTTTTCTAAGTTAAATAATTTTCAAAAGTTTAAGACTATTTGTTTCTCAACCGTCGAAGTGCCTGGTAGTTGTAATTTT
CCACTTGAAGCCACCTGGCATTACACTTCTTATACTATTGTTGGTGCTTTGTATGTTACTTGGTCTGAAGGTAATTCTA
TTACTGGTGTACCTTATCCTGTCTCTGGTATTCGTGAGTTTAGTAATTTAGTTTTAAATAATTGTACCAAATATAATAT
TTATGATTATGTTGGTACTGGAATTATACGTTCTTCAAACCAGTCACTTGCTGGTGGTATTACATATGTTTCTAACTCT
GGTAATTTACTTGGTTTTAAAAATGTTTCCACTGGTAACATTTTTATTGTGACACCATGTAACCAACCAGATCAAGTAG
CTGTTTATCAACAAAGCATTATTGGTGCCATGACCGCTGTTAATGAGTCTAGATATGGCTTGCAAAACTTACTACAGTT
ACCTAACTTTTATTATGTTAGTAATGGTGGTAACAATTGCACTACAGCTGTTATGACTTATTCTAATTTTGGTATTTGT
GCTGATGGTTCTTTAATTCCTGTTCGTCCACGTAATTCTAGTGATAATGGTATTTCAGCCATAATCACTGCTAATTTAT
CTATTCCCTCTAACTGGACTACTTCAGTTCAAGTTGAGTATCTCCAAATTACTAGTACTCCAATAGTTGTTGATTGTGC
TACTTATGTGTGTAATGGTAACCCTCGCTGTAAGAATCTACTTAAGCAGTATACTTCTGCTTGTAAAACTATTGAAGAT
GCCTTACGACTTAGTGCTCATTTGGAAACTAATGATGTTAGTAGTATGCTAACTTTTGATAGCAATGCTTTTAGTTTGG
CTAATGTTACTAGTTTTGGAGATTATAACCTTTCTAGTGTTTTACCTCAGAGAAACATTCATTCAAGCCGTATAGCAGG
ACGTAGTGCTTTGGAAGATTTGTTGTTTAGCAAAGTTGTTACATCTGGTTTGGGTACTGTTGATGTTGACTATAAGTCT

TGTACTAAAGGTCTTTCTATTGCTGACCTTGCTTGTGCTCAGTACTACAATGGTATAATGGTTTTGCCAGGTGTTGCTG
ATGCTGAACGTATGGCCATGTACACAGGTTCTCTTATAGGTGGCATGGTGCTCGGAGGTCTTACATCAGCAGCCGCCAT
ACCTTTTTCTTTGGCACTGCAAGCACGACTTAACTATGTTGCTTTACAAACTGATGTGCTTCAAGAAAATCAGAAAATT
TTGGCTGCATCATTTAATAAGGCTATTAATAATATTGTTGCTTCTTTTAGTAGCGTTAATGATGCTATTACACAAACTG
CAGAGGCTATACATACTGTTACTATTGCACTTAATAAGATTCAGGATGTTGTTAATCAACAGGGTAGTGCTCTTAACCA
TCTCACTTCACAATTGAGACATAATTTTCAGGCCATTTCTAATTCAATTCAGGCTATTTATGACCGGCTTGATTCAATT
CAAGCCGATCAACAAGTTGACAGATTAATTACTGGACGGCTTGCAGCTTTGAATGCATTTGTTTCCCAAGTTTTGAATA
AATATACTGAAGTTCGTGGTTCCAGACGCTTAGCACAGCAGAAGATTAATGAATGTGTCAAGTCACAATCTAATAGATA
TGGTTTTTGTGGCAATGGCACTCACATCTTTTCAATCGTCAACTCAGCTCCAGATGGTTTGCTTTTTCTTCATACTGTT
TTGCTGCCAACTGATTACAAGAATGTAAAGGCGTGGTCTGGTATCTGTGTTGATGGCATTTATGGCTATGTTCTGCGTC
AACCTAACTTGGTTCTTTATTCTGATAATGGTGTCTTTCGTGTAACTTCCAGGGTCATGTTTCAACCTCGCTTACCTGT
TTTGTCTGATTTTGTGCAAATATATAATTGTAATGTTACTTTTGTTAACATATCTCGTGTCGAGTTACATACTGTCATA
CCTGACTACGTTGATGTTAATAAAACATTACAAGAGTTTGCACAAAACTTACCAAAGTATGTTAAGCCTAATTTTGACT
TGACTCCTTTTAATTTAACATATCTTAATTTGAGTTCTGAGTTGAAGCAACTCGAAGCTAAAACTGCTAGTCTTTTTCA
AACTACTGTTGAATTACAAGGTCTTATTGATCAGATTAACAGTACATATGTTGATTTGAAGTTGCTTAATAGGTTTGAA
AATTATATCAAATGGCCTTGGTGGGTTTGGCTCATTATTTCTGTTGTTTTTGTTGTATTGTTGAGTCTTCTTGTGTTTT
GTTGTCTTTCTACAGGTTGTTGTGGTTGTTGCAATTGTTTAACTTCATCAATGCGAGGCTGTTGTGATTGTGGTTCAAC
TAAACTTCCTTATTACGAATTTGAAAAGGTCCACGTTCAATAA

>gi|530802141|gb|KF530114.1| Human coronavirus NL63 strain NL63/human/USA/891-4/1989, complete genome

ATGAAACTTTTCTTGATTTTGCTTGTTTTGCCCCTGGCCTCTTGCTTTTCCACATGTAATAGTAATGCTAATATC
TCTATGTTACAATTAGGTGTTCCTGACAATTCTTCAACTATTGTTACGGGTTTATTGCCAACTCATTGGATTTGTGCTA
ATCAGAGTACATCTGTTTACTCAGCCAAAGGTTTCTTTTATATTGATGTTGGTAATCACCGTAGTGCTTTTGCGCTCCA
TACTGGTTATTATGATGCTAATCAGTATTATATTTATGTTACTAATGAAATAGGCTTAAATGCTTCTGTTACTCTTAAG
ATTTGTAAGTTTAGAAACACTACTTTTGATTTTTTAAGTAATGCTTCTAGTTCTTTTGACTGTATAGTTAATTTGTTAT
TTACAGAACAGTTAGGTGCGCCTTTGGGCATAACTATATCTGGTGAAACTGTACGTCTGCATTTATATAATGTAACTCG
TACTTTTTATGTGCCAGCAGCTTATAAACTTACTAAACTTAGTGTTAAATGTTACTTTAATTATTCCTGTGTTTTTAGT
GTTGTCAACGCCACCGTTACTGTCAATGTCACCACACATAATGGCCGTGTAGTTAACTACACTGTTTGTGATGATTGTA
ATGGTTATACTGATAACATATTTTCTGTTCAACAGGATGGCCGCATTCCTAATGGTTTCCCTTTTAATAATTGGTTTTT
GTTAACTAATGGTTCCACACTAGTGGATGGAGTCTCTAGACTTTATCAACCACTCCGTTTAACTTGTTTATGGCCTGTA
CCTGGTCTTAAATCTTCAACTGGTTTTGTTTATTTTAATGCCACTGGTTCTGATGTTAATTGTAACGGCTACCAACATA
ATTCTGTTGCTGATGTTATGCGTTACAATCTTAACTTCAGTGCTAATTCTGTGGACAATCTCAAGAGTGGTGTTATAGT
TTTTAAAACTTTACAGTACGATGTTTTGTTTTATTGTAGTAATTCTTCTTCAGGTGTTCTTGACACCACAATACCTTTT
GGCCCTTCCTCTCAACCTTATTACTGTTTTATAAACAGTACTATCAACACTACTCATGTTAGCACTTTTGTGGGTATTT
TACCACCCACTGTGCGTGAAATTGTTGTTGCTAGAACTGGTCAGTTTTATATTAATGGTTTTAAGTATTTCGATTTGGG
TTTCATAGAAGCTGTCAATTTTAATGTCACGACTGCTAGTGCCACAGATTTTTGGACGGTTGCATTTGCTACTTTTGTT
GATGTTTTGGTTAATGTTAGTGCAACTAACATTCAAAACTTACTTTATTGTGATTCTCCATTTGAAAAGTTGCAGTGTG
AGCACTTGCAGTTTGGGTTGCAAGATGGTTTTTATTCTGCAAATTTTCTTGATGATAATGTTTTGCCTGAGACTTATGT
TGCACTCCCCATTTATTATCAACATACGGACATAAATTTTACTGCAACTGCATCTTTTGGTGGTTCTTGTTATGTTTGT
AAACCACGCCAGGTTAATATATCTCTTAATGGTAACACTTCAGTGTGTGTTAGAACATCTCATTTTTCAATTAGGTATA
TTTATAACCGCGTTAAGAGTGGTTCACCAGGTGACTCTTCATGGCACATTTATTTAAAGAGTGGCACTTGTCCATTTTC

TTTTTCTAAGTTAAATAATTTTCAAAAGTTTAAGACTATTTGTTTCTCAACCGTCGAAGTGCCTGGTAGTTGTAATTTT
CCACTTGAAGCCACCTGGCATTACACTTCTTATACTATTGTTGGTGCTTTGTATGTTACTTGGTCTGAAGGTAATTCTA
TTACTGGTGTACCTTATCCTGTCTCTGGTATTCGTGAGTTTAGTAATTTAGTTTTAAATAATTGTACCAAATATAATAT
TTATGATTATGTTGGTACTGGAATTATACGTTCTTCAAACCAGTCACTTGCTGGTGGTATTACATATGTTTCTAACTCT
GGTAATTTACTTGGTTTTAAAAATGTTTCCACTGGTAACATTTTTATTGTGACACCATGTAACCAACCAGATCAAGTAG
CTGTTTATCAACAAAGCATTATTGGTGCCATGACCGCTGTTAATGAGTCTAGATATGGCTTGCAAAACTTACTACAGTT
ACCTAACTTTTATTATGTTAGTAATGGTGGTAACAATTGCACTACAGCTGTTATGACTTATTCTAATTTTGGTATTTGT
GCTGATGGTTCTTTAATTCCTGTTCGTCCACGTAATTCTAGTGATAATGGTATTTCAGCCATAATCACTGCTAATTTAT
CTATTCCCTCTAACTGGACTACTTCAGTTCAAGTTGAGTATCTCCAAATTACTAGTACTCCAATAGTTGTTGATTGTGC
TACTTATGTGTGTAATGGTAACCCTCGCTGTAAGAATCTACTTAAGCAGTATACTTCTGCTTGTAAAACTATTGAAGAT
GCCTTACGACTTAGTGCTCATTTGGAAACTAATGATGTTAGTAGTATGCTAACTTTCGATAGCAATGCTTTTAGTTTGG
CTAATGTTACTAGTTTTGGAGATTATAACCTTTCTAGTGTTTTACCTCAGAGAAACATTCATTCAAGCCGTATAGCAGG
ACGTAGTGCTTTGGAAGATTTGTTGTTTAGCAAAGTTGTTACATCTGGTTTGGGTACTGTTGATGTTGACTATAAGTCT
TGTACTAAAGGTCTTTCTATTGCTGACCTTGCTTGTGCTCAGTACTACAATGGTATAATGGTTTTGCCAGGTGTTGCTG
ATGCTGAACGTATGGCCATGTACACAGGTTCTCTTATAGGTGGCATGGTGCTCGGAGGTCTTACATCAGCAGCCGCCAT
ACCTTTTTCTTTGGCACTGCAAGCACGACTTAACTATGTTGCTTTACAAACTGATGTGCTTCAAGAAAATCAGAAAATT
TTGGCTGCATCATTTAATAAGGCTATTAATAATATTGTTGCTTCTTTTAGTAGCGTTAATGATGCTATTACACAAACTG
CAGAGGCTATACATACTGTTACTATTGCACTTAATAAGATTCAGGATGTTGTTAATCAACAGGGTAGTGCTCTTAACCA
TCTCACTTCACAATTGAGACATAATTTTCAGGCCATTTCTAATTCAATTCAGGCTATTTATGACCGGCTTGATTCAATT
CAAGCCGATCAACAAGTTGACAGATTAATTACTGGACGGCTTGCAGCTTTGAATGCATTTGTTTCCCAAGTTTTGAATA
AATATACTGAAGTTCGTGGTTCCAGACGCTTAGCACAGCAGAAGATTAATGAATGTGTCAAGTCACAATCTAATAGATA
TGGTTTTTGTGGCAATGGCACTCACATCTTTTCAATCGTCAACTCAGCTCCAGATGGTTTGCTTTTTCTTCATACTGTT
TTGCTGCCAACTGATTACAAGAATGTAAAGGCGTGGTCTGGTATCTGTGTTGATGGCATTTATGGCTATGTTCTGCGTC
AACCTAACTTGGTTCTTTATTCTGATAATGGTGTCTTTCGTGTAACTTCCAGGGTCATGTTTCAACCTCGCTTACCTGT
TTTGTCTGATTTTGTGCAAATATATAATTGTAATGTTACTTTTGTTAACATATCTCGTGTCGAGTTACATACTGTCATA
CCTGACTACGTTGATGTTAATAAAACATTACAAGAGTTTGCACAAAACTTACCAAAGTATGTTAAGCCTAATTTTGACT
TGACTCCTTTTAATTTAACATATCTTAATTTGAGTTCTGAGTTGAAGCAACTCGAAGCTAAAACTGCTAGTCTTTTTCA
AACTACTGTTGAATTACAAGGTCTTATTGATCAGATTAACAGTACATATGTTGATTTGAAGTTGCTTAATAGGTTTGAA
AATTATATCAAATGGCCTTGGTGGGTTTGGCTCATTATTTCTGTTGTTTTTGTTGTATTGTTGAGTCTTCTTGTGTTTT
GTTGTCTTTCTACAGGTTGTTGTGGTTGTTGCAATTGTTTAACTTCATCAATGCGAGGCTGTTGTGATTGTGGTTCAAC
TAAACTTCCTTATTACGAATTTGAAAAGGTCCACGTTCAATAA

>gi|46369870|gb|AY518894.1| Human group 1 coronavirus associated with pneumonia, complete genome

ATGAAACTTTTCTTGATTTTGCTTATTTTGCCCCTGGTTTCTTGCTTTTCTACATGTAACAGTAATGCTAGTATT
TCTATGTTACAATTAGGTGTTCCTGATAACTCTTCAACTATTGTCACAGGTTTGTTGCCAGTCCATTGGATTTGTGCTA
ATCAGAGTACATCTAGTTACCCAGCCAACGGCTTTTTCTATATTGATGTTGGTAAACACCGTAGTGCCTTTGCACTCCA
TAGTGGTTATTATGATGCTAACCAGTATTATATTTATCTCACTAATAAAATACATTTAAATGCTCCTGTCACTCTGAAG
ATTTGTAAGTTTGGAAACACTTCTTTTGATTTTTTAAGTAATGTTTCTACTTCTCATGATTGTATAGTTAATTTGTCAT
TCACAGAACAGTTAGGTGTGCCTTTGGGCATAACTATATCGGGTGAAACTGTACGTTTGCATTTATATAATGCAACTCG
TACTTTTTATGTGCCGGCCGCTTATAAACTTACTAAACTTAGTGTTAAATGTTACTTTAGTGAATCCTGTGTTTTTAGT
GTTGTCAATGCCACCATTACTGTTAATGTCACCACACTTAATGGCCGTATAGTTAACTACACTGTTTGTGATGATTGTA

ATGGTTATACTGATAACATATTTTCTGTTCAACAGGATGGCCGCATTCCTAATGGTTTCCCTTTTAATAATTGGTTTTT
GTTAACTAATGGTTCCACATTAGTGGACGGGGTCTCTAGACTTTATCAACCACTCCGTTTAACTTGTTTATGGCCTGTA
CCTGGTCTTAAATCTTCAACTGGTTTTGTTTATTTTAATGCCACTGGTTCTGATGTTAATTGTAACGGCTATCAACATA
ATTCTGTTGCTGATGTTATGCGTTACAATCTTAACCTCAGTGCTAATTCTGTGGACAATCTTAAGAGTGGTGTTATAGT
TTTTAAAACTTTACAGTACGATGTTTTGTTTTATTGTAGTAATTCTTCTTCAGGTGTTCTTGACACCACAATACCTTTT
GGCCCTTCCTCTCAACCTTATTACTGTTTTATAAACAGTACTATCAACACTACTCATGTTAGCACTTTTGTGGGTATTT
TACCACCCACTGTGCGTGAAATTGTTGTTGCTAGAACTGGTCAGTTTTATATTAATGGTTTTAAGTATTTCGATTTGGG
TTTCATAGAAGCTGTCAATTTTAATGTCACGACTGCTAGTGCCACAGATTTTTGGACGGTTGCATTTGCTACTTTTGTT
GATGTTTTGGTTAATGTTAGTGCAACTAACATTCAAAACTTACTTTATTGCGATTCTCCATTTGAAAAGTTGCAGTGTG
AGCACTTGCAGTTTGGATTGCAAGATGGTTTTTATTCTGCAAATTTTCTTGATGATAATGTTTTGCCTGAGACTTATGT
TGCACTCCCCATTTATTATCAACATACGGACATAAATTTTACTGCAACTGCATCTTTTGGTGGTTCTTGTTATGTTTGT
AAACCACGCCAGGTTAATATATCTCTTAATGGTAACACTTCAGTGTGTGTTAGAACATCTCATTTTTCAATTAGGTATA
TTTATAACCGCGTTAAGAGTGGTTCACCAGGTGACTCTTCATGGCATATTTATTTAAAGAGTGGCACTTGTCCATTTTC
TTTTTCTAAGTTAAATAATTTTCAAAAGTTTAAGACTATTTGTTTCTCAACCGTCGAAGTGCCTGGTAGTTGTAATTTT
CCACTTGAAGCCACCTGGCATTACACTTCTTATACTATTGTTGGTGCTTTGTATGTTACTTGGTCTGAAGGTAATTCCA
TTACTGGTGTACCTTATCCTGTCTCTGGTATTCGTGAGTTTAGTAATTTAGTTTTAAATAATTGTACCAAATATAATAT
TTATGATTATGTTGGTACTGGAATTATACGTTCTTCAAACCAGTCACTTGCTGGTGGTATTACATATGTTTCTAACTCT
GGTAATTTACTTGGTTTTAAAAATGTTTCCACTGGTAACATTTTTATTGTGACACCATGTAACCAACCAGATCAAGTAG
CTGTTTATCAACAAAGCATTATTGGTGCCATGACCGCTGTTAATGAGTCTAGATATGGCTTGCAAAACTTACTACAGTT
ACCTAACTTTTATTATGTTAGTAATGGTGGTAACAATTGCACTACGGCTGTTATGATTTATTCTAATTTTGGTATTTGT
GCTGATGGTTCTTTAATTCCTGTTCGTCCGCGTAATTCTAGTGATAATGGTATTTCAGCCATAATCACTGCTAATTTAT
CCATTCCCTCTAACTGGACTACTTCAGTTCAAGTTGAGTACCTCCAAATTACTAGTACTCCAATAGTTGTTGATTGTGC
TACTTATGTGTGTAATGGTAACCCTCGTTGTAAGAATCTACTTAAGCAGTATACTTCTGCTTGTAAAACTATTGAAGAT
GCCTTACGACTTAGTGCTCATTTGGAAACTAATGATGTTAGTAGTATGCTAACTTTCGATAGCAATGCTTTTAGTTTGG
CTAATGTTACTAGTTTTGGAGATTATAACCTTTCTAGTGTTTTACCTCAGAGAAACATTCATTCAAGCCGTATAGCAGG
ACGTAGTGCTTTGGAAGATTTGTTGTTTAGCAAAGTTGTTACATCTGGTTTGGGTACTGTTGATGTTGACTATAAGTCT
TGTACTAAAGGTCTTTCTATTGCTGACCTTGCTTGTGCTCAGTACTACAATGGCATAATGGTTTTGCCAGGTGTTGCTG
ATGCTGAACGTATGGCCATGTACACAGGTTCTCTTATAGGTGGCATGGTGCTCGGAGGTCTTACATCAGCAGCCGCCAT
ACCTTTTTCTTTGGCACTGCAAGCACGACTTAACTATGTTGCTTTACAAACTGATGTGCTTCAAGAAAATCAGAAAATT
TTGGCTGCATCATTTAATAAGGCTATTAATAATATTGTTGCTTCTTTTAGTAGCGTTAATGATGCTATTACACATACTG
CAGAGGCTATACATACTGTTACTATTGCACTTAATAAGATTCAGGATGTTGTTAATCAACAGGGTAGTGCTCTTAACCA
TCTCACTTCACAATTGAGACATAATTTTCAGGCCATTTCTAATTCAATTCATGCTATTTATGACCGGCTTGATTCAATT
CAAGCCGATCAACAAGTTGACAGATTAATTACTGGACGGCTTGCAGCTTTGAATGCATTTGTTTCCCAAGTTTTGAATA
AATATACTGAAGTTCGTGGTTCCAGACGCTTAGCACAGCAGAAGATTAATGAATGTGTCAAGTCACAATCTAATAGATA
TGGTTTTTGTGGCAATGGCACTCACATCTTTTCAATCGTCAACTCAGCTCCAGATGGTTTGCTTTTTCTTCATACTGTT
TTGCTGCCAACTGATTACAAGAATGTAAAGGCGTGGTCTGGTATCTGTGTTGATGGCATTTATGGCTATGTTCTGCGTC
AACCTAACTTGGTTCTTTATTCTGATAATGGTGTCTTTCGTGTAACTTCCAGGGTCATGTTTCAACCTCGTTTACCTGT
TTTGTCTGATTTTGTGCAAATATATAATTGTAATGTTACTTTTGTTAACATATCTCGTGTCGAGTTACATACTGTCATA
CCTGACTACGTTGATGTTAATAAAACATTACAAGAGTTTGCACAAAACTTACCAAAGTATGTTAAGCCTAATTTTGACT
TGACTCCTTTTAATTTAACATATCTTAATTTGAGTTCTGAGTTGAAGCAACTCGAAGCTAAAACTGCTAGTCTTTTCCA
AACTACTGTTGAATTACAAGGTCTTATTGATCAGATTAACAGTACATATGTTGATTTGAAGTTGCTTAATAGGTTTGAA
AATTATATCAAATGGCCTTGGTGGGTTTGGCTCATTATTTCTGTTGTTTTTGTTGTATTGTTGAGTCTTCTTGTGTTTT
GTTGTCTTTCTACAGGTTGTTGTGGTTGTTGCAATTGTTTAACTTCATCAATGCGAGGCTGTTGTGATTGTGGTTCAAC
TAAACTTCCTTATTATGAATTTGAAAAGGTCCACGTTCAATAA

>gi|31581502|gb|AY291315.1| SARS coronavirus Frankfurt 1, complete genome

ATGTTTATTTTCTTATTATTTCTTACTCTCACTAGTGGTAGTGACCTTGACCGGTGCACCACTTTTGATGATGTT
CAAGCTCCTAATTACACTCAACATACTTCATCTATGAGGGGGGTTTACTATCCTGATGAAATTTTTAGATCAGACACTC
TTTATTTAACTCAGGATTTATTTCTTCCATTTTATTCTAATGTTACAGGGTTTCATACTATTAATCATACGTTTGGCAA
CCCTGTCATACCTTTTAAGGATGGTATTTATTTTGCTGCCACAGAGAAATCAAATGTTGTCCGTGGTTGGGTTTTTGGT
TCTACCATGAACAACAAGTCACAGTCGGTGATTATTATTAACAATTCTACTAATGTTGTTATACGAGCATGTAACTTTG
AATTGTGTGACAACCCTTTCTTTGCTGTTTCTAAACCCATGGGTACACAGACACATACTATGATATTCGATAATGCATT
TAATTGCACTTTCGAGTACATATCTGATGCCTTTTCGCTTGATGTTTCAGAAAAGTCAGGTAATTTTAAACACTTACGA
GAGTTTGTGTTTAAAAATAAAGATGGGTTTCTCTATGTTTATAAGGGCTATCAACCTATAGATGTAGTTCGTGATCTAC
CTTCTGGTTTTAACACTTTGAAACCTATTTTTAAGTTGCCTCTTGGTATTAACATTACAAATTTTAGAGCCATTCTTAC
AGCCTTTTCACCTGCTCAAGACATTTGGGGCACGTCAGCTGCAGCCTATTTTGTTGGCTATTTAAAGCCAACTACATTT
ATGCTCAAGTATGATGAAAATGGTACAATCACAGATGCTGTTGATTGTTCTCAAAATCCACTTGCTGAACTCAAATGCT
CTGTTAAGAGCTTTGAGATTGACAAAGGAATTTACCAGACCTCTAATTTCAGGGTTGTTCCCTCAGGAGATGTTGTGAG
ATTCCCTAATATTACAAACTTGTGTCCTTTTGGAGAGGTTTTTAATGCTACTAAATTCCCTTCTGTCTATGCATGGGAG
AGAAAAAAAATTTCTAATTGTGTTGCTGATTACTCTGTGCTCTACAACTCAACATTTTTTTCAACCTTTAAGTGCTATG
GCGTTTCTGCCACTAAGTTGAATGATCTTTGCTTCTCCAATGTCTATGCAGATTCTTTTGTAGTCAAGGGAGATGATGT
AAGACAAATAGCGCCAGGACAAACTGGTGTTATTGCTGATTATAATTATAAATTGCCAGATGATTTCATGGGTTGTGTC
CTTGCTTGGAATACTAGGAACATTGATGCTACTTCAACTGGTAATTATAATTATAAATATAGGTATCTTAGACATGGCA
AGCTTAGGCCCTTTGAGAGAGACATATCTAATGTGCCTTTCTCCCCTGATGGCAAACCTTGCACCCCACCTGCTCTTAA
TTGTTATTGGCCATTAAATGATTATGGTTTTTACACCACTACTGGCATTGGCTACCAACCTTACAGAGTTGTAGTACTT
TCTTTTGAACTTTTAAATGCACCGGCCACGGTTTGTGGACCAAAATTATCCACTGACCTTATTAAGAACCAGTGTGTCA
ATTTTAATTTTAATGGACTCACTGGTACTGGTGTGTTAACTCCTTCTTCAAAGAGATTTCAACCATTTCAACAATTTGG
CCGTGATGTTTCTGATTTCACTGATTCCGTTCGAGATCCTAAAACATCTGAAATATTAGACATTTCACCTTGCTCTTTT
GGGGGTGTAAGTGTAATTACACCTGGAACAAATGCTTCATCTGAAGTTGCTGTTCTATATCAAGATGTTAACTGCACTG
ATGTTTCTACAGCAATTCATGCAGATCAACTCACACCAGCTTGGCGCATATATTCTACTGGAAACAATGTATTCCAGAC
TCAAGCAGGCTGTCTTATAGGAGCTGAGCATGTCGACACTTCTTATGAGTGCGACATTCCTATTGGAGCTGGCATTTGT
GCTAGTTACCATACAGTTTCTTTATTACGTAGTACTAGCCAAAAATCTATTGTGGCTTATACTATGTCTTTAGGTGCTG
ATAGTTCAATTGCTTACTCTAATAACACCATTGCTATACCTACTAACTTTTCAATTAGCATTACTACAGAAGTAATGCC
TGTTTCTATGGCTAAAACCTCCGTAGATTGTAATATGTACATCTGCGGAGATTCTACTGAATGTGCTAATTTGCTTCTC
CAATATGGTAGCTTTTGCACACAACTAAATCGTGCACTCTCAGGTATTGCTGCTGAACAGGATCGCAACACACGTGAAG
TGTTCGCTCAAGTCAAACAAATGTACAAAACCCCAACTTTGAAATATTTTGGTGGTTTTAATTTTTCACAAATATTACC
TGACCCTCTAAAGCCAACTAAGAGGTCTTTTATTGAGGACTTGCTCTTTAATAAGGTGACACTCGCTGATGCTGGCTTC
ATGAAGCAATATGGCGAATGCCTAGGTGATATTAATGCTAGAGATCTCATTTGTGCGCAGAAGTTCAATGGACTTACAG
TGTTGCCACCTCTGCTCACTGATGATATGATTGCTGCCTACACTGCTGCTCTAGTTAGTGGTACTGCCACTGCTGGATG
GACATTTGGTGCTGGCGCTGCTCTTCAAATACCTTTTGCTATGCAAATGGCATATAGGTTCAATGGCATTGGAGTTACC
CAAAATGTTCTCTATGAGAACCAAAAACAAATCGCCAACCAATTTAACAAGGCGATTAGTCAAATTCAAGAATCACTTA
CAACAACATCAACTGCATTGGGCAAGCTGCAAGACGTTGTTAACCAGAATGCTCAAGCATTAAACACACTTGTTAAACA
ACTTAGCTCTAATTTTGGTGCAATTTCAAGTGTGCTAAATGATATCCTTTCGCGACTTGATAAAGTCGAGGCGGAGGTA
CAAATTGACAGGTTAATTACAGGCAGACTTCAAAGCCTTCAAACCTATGTAACACAACAACTAATCAGGGCTGCTGAAA
TCAGGGCTTCTGCTAATCTTGCTGCTACTAAAATGTCTGAGTGTGTTCTTGGACAATCAAAAAGAGTTGACTTTTGTGG
AAAGGGCTACCACCTTATGTCCTTCCCACAAGCAGCCCCGCATGGTGTTGTCTTCCTACATGTCACGTATGTGCCATCC
CAGGAGAGGAACTTCACCACAGCGCCAGCAATTTGTCATGAAGGCAAAGCATACTTCCCTCGTGAAGGTGTTTTTGTGT

TTAATGGCACTTCTTGGTTTATTACACAGAGGAACTTCTTTTCTCCACAAATAATTACTACAGACAATACATTTGTCTC
AGGAAATTGTGATGTCGTTATTGGCATCATTAACAACACAGTTTATGATCCTCTGCAACCTGAGCTTGACTCATTCAAA
GAAGAGCTGGACAAGTACTTCAAAAATCATACATCACCAGATGTTGATTTTGGCGACATTTCAGGCATTAACGCTTCTG
TCGTCAACATTCAAAAAGAAATTGACCGCCTCAATGAGGTCGCTAAAAATTTAAATGAATCACTCATTGACCTTCAAGA
ATTGGGAAAATATGAGCAATATATTAAATGGCCTTGGTATGTTTGGCTCGGCTTCATTGCTGGACTAATTGCCATCGTC
ATGGTTACAATCTTGCTTTGTTGCATGACTAGTTGTTGCAGTTGCCTCAAGGGTGCATGCTCTTGTGGTTCTTGCTGCA
AGTTTGATGAGGATGACTCTGAGCCAGTTCTCAAGGGTGTCAAATTACATTACACATAA

>gi|92698732|dbj|AB257344.1| SARS coronavirus Frankfurt 1 genomic RNA, nearly complete genome, clone: persistent virus #21

ATGTTTATTTTCTTATTATTTCTTACTCTCACTAGTGGTAGTGACCTTGACCGGTGCACCACTTTTGATGATGTT
CAAGCTCCTAATTACACTCAACATACTTCATCTATGAGGGGGGTTTACTATCCTGATGAAATTTTTAGATCAGACACTC
TTTATTTAACTCAGGATTTATTTCTTCCATTTTATTCTAATGTTACAGGGTTTCATACTATTAATCATACGTTTGGCAA
CCCTGTCATACCTTTTAAGGATGGTATTTATTTTGCTGCCACAGAGAAATCAAATGTTGTCCGTGGTTGGGTTTTTGGT
TCTACCATGAACAACAAGTCACAGTCGGTGATTATTATTAACAATTCTACTAATGTTGTTATACGAGCATGTAACTTTG
AATTGTGTGACAACCCTTTCTTTGCTGTTTCTAAACCCATGGGTACACAGACACATACTATGATATTCGATAATGCATT
TAATTGCACTTTCGAGTACATATCTGATGCCTTTTCGCTTGATGTTTCAGAAAAGTCAGGTAATTTTAAACACTTACGA
GAGTTTGTGTTTAAAAATAAAGATGGGTTTCTCTATGTTTATAAGGGCTATCAACCTATAGATGTAGTTCGTGATCTAC
CTTCTGGTTTTAACACTTTGAAACCTATTTTTAAGTTGCCTCTTGGTATTAACATTACAAATTTTAGAGCCATTCTTAC
AGCCTTTTCACCTGCTCAAGACATTTGGGGCACGTCAGCTGCAGCCTATTTTGTTGGCTATTTAAAGCCAACTACATTT
ATGCTCAAGTATGATGAAAATGGTACAATCACAGATGCTGTTGATTGTTCTCAAAATCCACTTGCTGAACTCAAATGCT
CTGTTAAGAGCTTTGAGATTGACAAAGGAATTTACCAGACCTCTAATTTCAGGGTTGTTCCCTCAGGAGATGTTGTGAG
ATTCCCTAATATTACAAACTTGTGTCCTTTTGGAGAGGTTTTTAATGCTACTAAATTCCCTTCTGTCTATGCATGGGAG
AGAAAAAAAATTTCTAATTGTGTTGCTGATTACTCTGTGCTCTACAACTCAACATTTTTTTCAACCTTTAAGTGCTATG
GCGTTTCTGCCACTAAGTTGAATGATCTTTGCTTCTCCAATGTCTATGCAGATTCTTTTGTAGTCAAGGGAGATGATGT
AAGACAAATAGCGCCAGGACAAACTGGTGTTATTGCTGATTATAATTATAAATTGCCAGATGATTTCATGGGTTGTGTC
CTTGCTTGGAATACTAGGAACATTGATGCTACTTCAACTGGTAATTATAATTATAAATATAGGTGTCTTAGACATGGCA
AGCTTAGGCCCTTTGAGAGAGACATATCTAATGTGCCTTTCTCCCCTGATGGCAAACCTTGCACCCCACCTGCTTTTAA
TTGTTATTGGCCATTAAATGATTATGGTTTTTACACCACTACTGGCATTGGCTACCAACCTTACAGAGTTGTAGTACTT
TCTTTTGAACTTTTAAATGCACCGGCCACGGTTTGTGGACCAAAATTATCCACTGACCTTATTAAGAACCAGTGTGTCA
ATTTTAATTTTAATGGACTCACTGGTACTGGTGTGTTAACTCCTTCTTCAAAGAGATTTCAACCATTTCAACAATTTGG
CCGTGATGTTTCTGATTTCACTGATTCCGTTCGAGATCCTAAAACATCTGAAATATTAGACATTTCACCTTGCTCTTTT
GGGGGTGTAAGTGTAATTACACCTGGAACAAATGCTTCATCTGAATTTGCTGTTCTATATCAAGATGTTAACTGCACTG
ATGTTTCTACAGCAATTCATGCAGATCAACTCACACCAGCTTGGCGCATATATTCTACTGGAAACAATGTATTCCAGAC
TCAAGCAGGCTGTCTTATAGGAGCTGAGTATGTCGACACTTCTTATGAGTGCGACATTCCTATTGGAGCTGGCATTTGT
GCTAGTTACCATACAGTTTCTTTATTACGTAGTACTAGCCAAAAATCTATTGTGGCTTATACTATGTCTTTAGGTGCTG
ATAGTTCAATTGCTTACTCTAATAACACCATTGCTATACCTACTAACTTTTCAATTAGCATTACTACAGAAGTAATGCC
TGTTTCTATGGCTAAAACCTCCGTAGATTGTAATATGTACATCTGCGGAGATTCTACTGAATGTGCTAATTTGCTTCTC
CAATATGGTAGCTTTTGCACACAACTAAATCGTGCACTCTCAGGTATTGCTGCTGAACAGGATCGCAACACACGTGAAG
TGTTCGCTCAAGTCAAACAAATGTACAAAACCCCAACTTTGAAATATTTTGGTGGTTTTAATTTTTCACAAATATTACC
TGACCCTCTAAAGTCAACTAAGAGGTCTTTTATTGAGGACTTGCTCTTTAATAAGGTGACACTCGCTGATGCTGGCTTC
ATGAAGCAATATGGCGAATGCCTAGGTGATATTAATGCTAGAGATCTCATTTGTGCGCAGAAGTTCAATGGACTTACAG

TGTTGCCACCTCTGCTCACTGATGATATGATTGCTGCCTACACTGCTGCTCTAGTTAGTGGTACTGCCACTGCTGGATG
GACATTTGGTGCTGGCGCTGCTCTTCAAATACCTTTTGCTATGCAAATGGCATATAGGTTCAATGGCATTGGAGTTACC
CAAAATGTTCTCTATGAGAACCAAAAACAAATCGCCAACCAATTTAACAAGGCGATTAGTCAAATTCAAGAATCACTTA
CAACAACATCAACTGCATTGGGCAAGCTGCAAGACGTTGTTAACCAGAATGCTCAAGCATTAAACACACTTGTTAAACA
ACTTAGCTCTAATTTTGGTGCAATTTCAAGTGTGCTAAATGATATCCTTTCGCGACTTGATAAAGTCGAGGCGGAGGTA
CAAATTGACAGGTTAATTACAGGCAGACTTCAAAGCCTTCAAACCTATGTAACACAACAACTAATCAGGGCTGCTGAAA
TCAGGGCTTCTGCTAATCTTGCTGCTACTAAAATGTCTGAGTGTGTTCTTGGACAATCAAAAAGAGTTGACTTTTGTGG
AAAGGGCTACCACCTTATGTCCTTCCCACAAGCAGCCCCGCATGGTGTTGTCTTCCTACATGTCACGTATGTGCCATCC
CAGGAGAGGAACTTCACCACAGCGCCAGCAATTTGTCATGAAGGCAAAGCATACTTCCCTCGTGAAGGTGTTTTTGTGT
TTAATGGCACTTCTTGGTTTATTACACAGAGGAACTTCTTTTCTCCACAAATAATTACTACAGACAATACATTTGTCTC
AGGAAATTGTGATGTCGTTATTGGCATCATTAACAACACAGTTTATGATCCTCTGCAACCTGAGCTTGACTCATTCAAA
GAAGAGCTGGACAAGTACTTCAAAAATCATACATCACCAGATGTTGATTTTGGCGACATTTCAGGCATTAACGCTTCTG
TCGTCAACATTCAAAAAGAAATTGACCGCCTCAATGAGGTCGCTAAAAATTTAAATGAATCACTCATTGACCTTCAAGA
ATTGGGAAAATATGAGCAATATATTAAATGGCCTTGGTATGTTTGGCTCGGCTTCATTGCTGGACTAATTGCCATCGTC
ATGGTTACAATCTTGCTTTGTTGCATGACTAGTTGTTGCAGTTGCCTCAAGGGTGCATGCTCTTGTGGTTCTTGCTGCA
AGTTTGATGAGGATGACTCTGAGCCAGTTCTCAAGGGTGTCAAATTACATTACACATAA

>gi|89474485|gb|DQ412575.1|_SARS_coronavirus_isolate_CUHKcs04ST_spike_glycoprotein_(S)_gene_complete_cds

ATGTTTATTTTCTTATTATTTCTTACTCTCACTAGTGGTAGTGACCTTGACCGGTGCACCACTTTTGATGATGTT
CAAGCTCCTAATTACACTCAACATACTTCATCTATGAGGGGGGTTTACTATCCTGATGAAATTTTTAGATCAGACACTC
TTTATTTAACTCAGGATTTATTTCTTCCATTTTATTCTAATGTTACAGGGTTTCATACTATTAATCATACGTTTGGCAA
CCCTGTCATACCTTTTAAGGATGGTATTTATTTTGCTGCCACAGAGAAATCAAATGTTGTCCGTGGTTGGGTTTTTGGT
TCTACCATGAACAACAAGTCACAGTCGGTGATTATTATTAACAATTCTACTAATGTTGTTATACGAGCATGTAACTTTG
AATTGTGTGACAACCCTTTCTTTGCTGTTTCTAAACCCATGGGTACACAGACACATACTATGATATTCGATAATGCATT
TAATTGCACTTTCGAGTACATATCTGATGCCTTTTCGCTTGATGTTTCAGAAAAGTCAGGTAATTTTAAACACTTACGA
GAGTTTGTGTTTAAAAATAAAGATGGGTTTCTCTATGTTTATAAGGGCTATCAACCTATAGATGTAGTTCGTGATCTAC
CTTCTGGTTTTAACACTTTGAAACCTATTTTTAAGTTGCCTCTTGGTATTAACATTACAAATTTTAGAGCCATTCTTAC
AGCCTTTTCACCTGCTCAAGACATTTGGGGCACGTCAGCTGCAGCCTATTTTGTTGGCTATTTAAAGCCAACTACATTT
ATGCTCAAGTATGATGAAAATGGTACAATCACAGATGCTGTTGATTGTTCTCAAAATCCACTTGCTGAACTCAAATGCT
CTGTTAAGAGCTTTGAGATTGACAAAGGAATTTACCAGACCTCTAATTTCAGGGTTGTTCCCTCAGGAGATGTTGTGAG
ATTCCCTAATATTACAAACTTGTGTCCTTTTGGAGAGGTTTTTAATGCTACTAAATTCCCTTCTGTCTATGCATGGGAG
AGAAAAAAAATTTCTAATTGTGTTGCTGATTACTCTGTGCTCTACAACTCAACATTTTTTTCAACCTTTAAGTGCTATG
GCGTTTCTGCCACTAAGTTGAATGATCTTTGCTTCTCCAATGTCTATGTAGATTCTTTTGTAGTCAAGGGAGATGATGT
AAGACAAATAGCGCCAGGACAAACTGGTGTTATTGCTGATTATAATTATAAATTGCCAGATGATTTCATGGGTTGTGTC
CTTGCTTGGAATACTAGGAACATTGATGCTACTTCAACTGGTAATTATAATTATAAATATAGGTATCTTAGACATGGCA
AGCTTAGGCCCTTTGAGAGAGACATATCTAATGTGCCTTTCTCCCCTGATGGCAAACCTTGCACCCCACCTGCTCTTAA
TTGTTATTGGCCATTAAATGATTATGGTTTTTACACCACTACTGGCATTGGCTACCAACCTTACAGAGTTGTAGTACTT
TCTTTTGAACTTTTAAATGCACCGGCCACGGTTTGTGGACCAAAATTATCCACTGACCTTATTAAGAACCAGTGTGTCA
ATTTTAATTTTAATGGACTCACTGGTACTGGTGTGTTAACTCCTTCTTCAAAGAGATTTCAACCATTTCAACAATTTGG
CCGTGATGTTTCTGATTTCACTGATTCCGTTCGAGATCCTAAAACATCTGAAATATTAGACATTTCACCTTGCTCTTTT
GGGGGTGTAAGTGTAATTACACCTGGAACAAATGCTTCATCTGAAGTTGCTGTTCTATATCAAGATGTTAACTGCACTG

ATGTTTCTACAGCAATTCATGCAGATCAACTCACACCAGCTTGGCGCATATATTCTACTGGAAACAATGTATTCCAGAC
TCAAGCAGGCTGTCTTATAGGAGCTGAGCATGTCGACACTTCTTATGAGTGCGACATTCCTATTGGAGCTGGCATTTGT
GCTAGTTACCATACAGTTTCTTTATTACGTAGTACTAGCCAAAAATCTATTGTGGCTTATACTATGTCTTTAGGTGCTG
ATAGTTCAATTGCTTACTCTAATAACACCATTGCTATACCTACTAACTTTTCAATTAGCATTACTACAGAAGTAATGCC
TGTTTCTATGGCTAAAACCTCCGTAGATTGTAATATGTACATCTGCGGAGATTCTACTGAATGTGCTAATTTGCTTCTC
CAATATGGTAGCTTTTGCACACAACTAAATCGTGCACTCTCAGGTATTGCTGCTGAACAGGATCGCAACACACGTGAAG
TGTTCGCTCAAGTCAAACAAATGTACAAAACCCCAACTTTGAAATATTTTGGTGGTTTTAATTTTTCACAAATATTACC
TGACCCTCTAAAGCCAACTAAGAGGTCTTTTATTGAGGACTTGCTCTTTAATAAGGTGACACTCGCTGATGCTGGCTTC
ATGAAGCAATATGGCGAATGCCTAGGTGATATTAATGCTAGAGATCTCATTTGTGCGCAGAAGTTCAATGGACTTACAG
TGTTGCCACCTCTGCTCACTGATGATATGATTGCTGCCTACACTGCTGCTCTAGTTAGTGGTACTGCCACTGCTGGATG
GACATTTGGTGCTGGCGCTGCTCTTCAAATACCTTTTGCTATGCAAATGGCATATAGGTTCAATGGCATTGGAGTTACC
CAAAATGTTCTCTATGAGAACCAAAAACAAATCGCCAACCAATTTAACAAGGCGATTAGTCAAATTCAAGAATCACTTA
CAACAACATCAACTGCATTGGGCAAGCTGCAAGACGTTGTTAACCAGAATGCTCAAGCATTAAACACACTTGTTAAACA
ACTTAGCTCTAATTTTGGTGCAATTTCAAGTGTGCTAAATGATATCCTTTCGCGACTTGATAAAGTCGAGGCGGAGGTA
CAAATTGACAGGTTAATTACAGGCAGACTTCAAAGCCTTCAAACCTATGTAACACAACAACTAATCAGGGCTGCTGAAA
TCAGGGCTTCTGCTAATCTTGCTGCTACTAAAATGTCTGAGTGTGTTCTTGGACAATCAAAAAGAGTTGACTTTTGTGG
AAAGGGCTACCACCTTATGTCCTTCCCACAAGCAGCCCCGCATGGTGTTGTCTTCCTACATGTCACGTATGTGCCATCC
CAGGAGAGGAACTTCACCACAGCGCCAGCAATTTGTCATGAAGGCAAAGCATACTTCCCTCGTGAAGGTGTTTTTGTGT
TTAATGGCACTTCTTGGTTTATTACACAGAGGAACTTCTTTTCTCCACAAATAATTACTACAGACAATACATTTGTCTC
AGGAAATTGTGATGTCGTTATTGGCATCATTAACAACACAGTTTATGATCCTCTGCAACCTGAGCTTGACTCATTCAAA
GAAGAGCTGGACAAGTACTTCAAAAATCATACATCACCAGATGTTGATCTTGGCGACATTTCAGGCATTAACGCTTCTG
TCGTCAACATTCAAAAAGAAATTGACCGCCTCAATGAGGTCGCTAAAAATTTAAATGAATCACTCATTGACCTTCAAGA
ATTGGGAAAATATGAGCAATATATTAAATGGCCTTGGTATGTTTGGCTCGGCTTCATTGCTGGACTAATTGCCATCGTC
ATGGTTACAATCTTGCTTTGTTGCATGACTAGTTGTTGCAGTTGCCTCAAGGGTGCATGCTCTTGTGGTTCTTGCTGCA
AGTTTGATGAGGATGACTCTGAGCCAGTTCTCAAGGGTGTCAAATTACATTACACATAA

>gi|89474491|gb|DQ412578.1|_SARS_coronavirus_isolate_CUHKcs52UR_spike_glycoprotein_(S)_gene_complete_cds

ATGTTTATTTTCTTATTATTTCTTACTCTCACTAGTGGTAGTGACCTTGACCGGTGCACCACTTTTGATGATGTT
CAAGCTCCTAATTACACTCAACATACTTCATCTATGAGGGGGGTTTACTATCCTGATGAAATTTTTAGATCAGACACTC
TTTATTTAACTCAGGATTTATTTCTTCCATTTTATTCTAATGTTACAGGGTTTCATACTATTAATCATACGTTTGGCAA
CCCTGTCATACCTTTTAAGGATGGTATTTATTTTGCTGCCACAGAGAAATCAAATGTTGTCCGTGGTTGGGTTTTTGGT
TCTACCATGAACAACAAGTCACAGTCGGTGATTATTATTAACAATTCTACTAATGTTGTTATACGAGCATGTAACTTTG
AATTGTGTGACAACCCTTTCTTTGCTGTTTCTAAACCCATGGGTACACAGACACATACTATGATATTCGATAATGCATT
TAATTGCACTTTCGAGTACATATCTGATGCCTTTTCGCTTGATGTTTCAGAAAAGTCAGGTAATTTTAAACACTTACGA
GAGTTTGTGTTTAAAAATAAAGATGGGTTTCTCTATGTTTATAAGGGCTATCAACCTATAGATGTAGTTCGTGATCTAC
CTTCTGGTTTTAACACTTTGAAACCTATTTTTAAGTTGCCTCTTGGTATTAACATTACAAATTTTAGAGCCATTCTTAC
AGCCTTTTCACCTGCTCAAGACATTTGGGGCACGTCAGCTGCAGCCTATTTTGTTGGCTATTTAAAGCCAACTACATTT
ATGCTCAAGTATGATGAAAATGGTACAATCACAGATGCTGTTGATTGTTCTCAAAATCCACTTGCTGAACTCAAATGCT
CTGTTAAGAGCTTTGAGATTGACAAAGGAATTTACCAGACCTCTAATTTCAGGGTTGTTCCCTCAGGAGATGTTGTGAG
ATTCCCTAATATTACAAACTTGTGTCCTTTTGGAGAGGTTTTTAATGCTACTAAATTCCCTTCTGTCTATGCATGGGAG
AGAAAAAAAATTTCTAATTGTGTTGCTGATTACTCTGTGCTCTACAACTCAACATTTTTTTCAACCTTTAAGTGCTATG

GCGTTTCTGCCACTAAGTTGAATGATCTTTGCTTCTCCAATGTCTATGCAGATTCTTTTGTAGTCAAGGGAGATGATGT
AAGACAAATAGCGCCAGGACAAACTGGTGTTATTGCTGATTATAATTATAAATTGCCAGATGATTTCATGGGTTGTGTC
CTTGCTTGGAATACTAGGAACATTGATGCTACTTCAACTGGTAATTATAATTATAAATATAGGTATCTTAGACATGGCA
AGCTTAGGCCCTTTGAGAGAGACATATCTAATGTGCCTTTCTCCCCTAATGGCAAACCTTGCACCCCACCTGCTCTTAA
TTGTTATTGGCCATTAAATGATTATGGTTTTTACACCACTACTGGCATTGGCTACCAACCTTACAGAGTTGTAGTACTT
TCTTTTGAACTTTTAAATGCACCGGCCACGGTTTGTGGACCAAAATTATCCACTGACCTTATTAAGAACCAGTGTGTCA
ATTTTAATTTTAATGGACTCACTGGTACTGGTGTGTTAACTCCTTCTTCAAAGAGATTTCAACCATTTCAACAATTTGG
CCGTGATGTTTCTGATTTCACTGATTCCGTTCGAGATCCTAAAACATCTGAAATATTAGACATTTCACCTTGCTCTTTT
GGGGGTGTAAGTGTAATTACACCTGGAACAAATGCTTCATCTGAAGTTGCTGTTCTATATCAAGATGTTAACTGCACTG
ATGTTTCTACAGCAATTCATGCAGATCAACTCACACCAGCTTGGCGCATATATTCTACTGGAAACAATGTATTCCAGAC
TCAAGCAGGCTGTCTTATAGGAGCTGAGCATGTCGACACTTCTTATGAGTGCGACATTCCTATTGGAGCTGGCATTTGT
GCTAGTTACCATACAGTTTCTTTATTACGTAGTACTAGCCAAAAATCTATTGTGGCTTATACTATGTCTTTAGGTGCTG
ATAGTTCAATTGCTTACTCTAATAACACCATTGCTATACCTACTAACTTTTCAATTAGCATTACTACAGAAGTAATGCC
TGTTTCTATGGCTAAAACCTCCGTAGATTGTAATATGTACATCTGCGGAGATTCTACTGAATGTGCTAATTTGCTTCTC
CAATATGGTAGCTTTTGCACACAACTAAATCGTGCACTCTCAGGTATTGCTGCTGAACAGGATCGCAACACACGTGAAG
TGTTCGCTCAAGTCAAACAAATGTACAAAACCCCAACTTTGAAATATTTTGGTGGTTTTAATTTTTCACAAATATTACC
TGACCCCCTAAAGCCAACTAAGAGGTCTTTTATTGAGGACTTGCTCTTTAATAAGGTGACACTCGCTGATGCTGGCTTC
ATGAAGCAATATGGCGAATGCCTAGGTGATATTAATGCTAGAGATCTCATTTGTGCGCAGAAGTTCAATGGACTTACAG
TGTTGCCACCTCTGCTCACTGATGATATGATTGCTGCCTACACTGCTGCTCTAGTTAGTGGTACTGCCACTGCTGGATG
GACATTTGGTGCTGGCGCTGCTCTTCAAATACCTTTTGCTATGCAAATGGCATATAGGTTCAATGGCATTGGAGTTACC
CAAAATGTTCTCTATGAGAACCAAAAACAAATCGCCAACCAATTTAACAAGGCGATTAGTCAAATTCAAGAATCACTTA
CAACAACATCAACTGCATTGGGCAAGCTGCAAGACGTTGTTAACCAGAATGCTCAAGCATTAAACACACTTGTTAAACA
ACTTAGCTCTAATTTTGGTGCAATTTCAAGTGTGCTAAATGATATCCTTTCGCGACTTGATAAAGTCGAGGCGGAGGTA
CAAATTGACAGGTTAATTACAGGCAGACTTCAAAGCCTTCAAACCTATGTAACACAACAACTAATCAGGGCTGCTGAAA
TCAGGGCTTCTGCTAATCTTGCTGCTACTAAAATGTCTGAGTGTGTTCTTGGACAATCAAAAAGAGTTGACTTTTGTGG
AAAGGGCTATCACCTTATGTCCTTCCCACAAGCAGCCCCGCATGGTGTTGTCTTCCTACATGTCACGTATGTGCCATCC
CAGGAGAGGAACTTCACCACAGCGCCAGCAATTTGTCATGAAGGCAAAGCATACTTCCCTCGTGAAGGTGTTTTTGTGT
TTAATGGCACTTCTTGGTTTATTACACAGAGGAACTTCTTTTCTCCACAAATAATTACTACAGACAATACATTTGTCTC
AGGAAATTGTGATGTCGTTATTGGCATCATTAACAACACAGTTTATGATCCTCTGCAACCTGAGCTTGACTCATTCAAA
GAAGAGCTGGACAAGTACTTCAAAAATCATACATCACCAGATGTTGATCTTGGCGACATTTCAGGCATTAACGCTTCTG
TCGTCAACATTCAAAAAGAAATTGACCGCCTCAATGAGGTCGCTAAAAATTTAAATGAATCACTCATTGACCTTCAAGA
ATTGGGAAAATATGAGCAATATATTAAATGGCCTTGGTATGTTTGGCTCGGCTTCATTGCTGGACTAATTGCCATCGTC
ATGGTTACAATCTTGCTTTGTTGCATGACTAGTTGTTGCAGTTGCCTCAAGGGTGCATGCTCTTGTGGTTCTTGCTGCA
AGTTTGATGAGGATGACTCTGAGCCAGTTCTCAAGGGTGTCAAATTACATTACACATAA

>gi|89474508|gb|DQ412588.1|_SARS_coronavirus_isolate_CUHKtc04NP_spike_glycoprotein_(S)_gene_complete_cds

ATGTTTATTTTCTTATTATTTCTTACTCTCACTAGAGGTAGTGACCTTGACCGGTGCACCACTTTTGATGATGTT
CAAGCTCCTAATTACACTCAACATACTTCATCTATGAGGGGGGTTTACTATCCTGATGAAATTTTTAGATCAGACACTC
TTTATTTAACTCAGGATTTATTTCTTCCATTTTATTCTAATGTTACAGGGTTTCATACTATTAATCATACGTTTGGCAA
CCCTGTCATACCTTTTAAGGATGGTATTTATTTTGCTGCCACAGAGAAATCAAATGTTGTCCGTGGTTGGGTTTTTGGT
TCTACCATGAACAACAAGTCACAGTCGGTGATTATTATTAACAATTCTACTAATGTTGTTATACGAGCATGTAACTTTG

AATTGTGTGACAACCCTTTCTTTGCTGTTTCTAAACCCATGGGTACACAGACACATACTATGATATTCGATAATGCATT
TAATTGCACTTTCGAGTACATATCTGATGCCTTTTCGCTTGATGTTTCAGAAAAGTCAGGTAATTTTAAACACTTACGA
GAGTTTGTGTTTAAAAATAAAGATGGGTTTCTCTATGTTTATAAGGGCTATCAACCTATAGATGTAGTTCGTGATCTAC
CTTCTGGTTTTAACACTTTGAAACCTATTTTTAAGTTGCCTCTTGGTATTAACATTACAAATTTTAGAGCCATTCTTAC
AGCCTTTTCACCTGCTCAAGACATTTGGGGCACGTCAGCTGCAGCCTATTTTGTTGGCTATTTAAAGCCAACTACATTT
ATGCTCAAGTATGATGAAAATGGTACAATCACAGATGCTGTTGATTGTTCTCAAAATCCACTTGCTGAACTCAAATGCT
CTGTTAAGAGCTTTGAGATTGACAAAGGAATTTACCAGACCTCTAATTTCAGGGTTGTTCCCTCAGGAGATGTTGTGAG
ATTCCCTAATATTACAAACTTGTGTCCTTTTGGAGAGGTTTTTAATGCTACTAAATTCCCTTCTGTCTATGCATGGGAG
AGAAAAAAAAATTTCTAATTGTGTTGCTGATTACTCTGTGCTCTACAACTCAACATTTTTTTCAACCTTTAAGTGCTATG
GCGTTTCTGCCACTAAGTTGAATGATCTTTGCTTCTCCAATGTCTATGCAGATTCTTTTGTAGTCAAGGGAGATGATGT
AAGACAAATAGCGCCAGGACAAACTGGTGTTATTGCTGATTATAATTATAAATTGCCAGATGATTTCATGGGTTGTGTC
CTTGCTTGGAATACTAGGAACATTGATGCTACTTCAACTGGTAATTATAATTATAAATATAGGTATCTTAGACATGGCA
AGCTTAGGCCCTTTGAGAGAGACATATCTAATGTGCCTTTCTCCCCTGATGGCAAACCTTGCACCCCACCTGCTCTTAA
TTGTTATTGGCCATTAAATGATTATGGTTTTTACACCACTACTGGCATTGGCTACCAACCTTACAGAGTTGTAGTACTT
TCTTTTGAACTTTTAAATGCACCGGCCACGGTTTGTGGACCAAAATTATCCACTGACCTTATTAAGAACCAGTGTGTCA
ATTTTAATTTTAATGGACTCACTGGTACTGGTGTGTTAACTCCTTCTTCAAAGAGATTTCAACCATTTCAACAATTTGG
CCGTGATGTTTCTGATTTCACTGATTCCGTTCGAGATCCTAAAACATCTGAAATATTAGACATTTCACCTTGCTCTTTT
GGGGGTGTAAGTGTAATTACACCTGGAACAAATGCTTCATCTGAAGTTGCTGTTCTATATCAAGATGTTAACTGCACTG
ATGTTTCTACAGCAATTCATGCAGATCAACTCACACCAGCTTGGCGCATATATTCTACTGGAAACAATGTATTCCAGAC
TCAAGCAGGCTGTCTTATAGGAGCTGAGCATGTCGACACTTCTTATGAGTGCGACATTCCTATTGGAGCTGGCATTTGT
GCTAGTTACCATACAGTTTCTTTATTACGTAGTACTAGCCAAAAATCTATTGTGGCTTATACTATGTCTTTAGGTGCTG
ATAGTTCAATTGCTTACTCTAATAACACCATTGCTATACCTACTAACTTTTCAATTAGCATTACTACAGAAGTAATGCC
TGTTTCTATGGCTAAAACCTCCGTAGATTGTAATATGTACATCTGCGGAGATTCTACTGAATGTGCTAATTTGCTTCTC
CAATATGGTAGCTTTTGCACACAACTAAATCGTGCACTCTCAGGTATTGCTGCTGAACAGGATCGCAACACACGTGAAG
TGTTCGCTCAAGTCAAACAAATGTACAAAACCCCAACTTTGAAATATTTTGGTGGTTTTAATTTTTCACAAATATTACC
TGACCCTCTAAAGCCAACTAAGAGGTCTTTTATTGAGGACTTGCTCTTTAATAAGGTGACACTCGCTGATGCTGGCTTC
ATGAAGCAATATGGCGAATGCCTAGGTGATATTAATGCTAGAGATCTCATTTGTGCGCAGAAGTTCAATGGACTTACAG
TGTTGCCACCTCTGCTCACTGATGATATGATTGCTGCCTACACTGCTGCTCTAGTTAGTGGTACTGCCACTGCTGGATG
GACATTTGGTGCTGGCGCTGCTCTTCAAATACCTTTTGCTATGCAAATGGCATATAGGTTCAATGGCATTGGAGTTACC
CAAAATGTTCTCTATGAGAACCAAAAACAAATCGCCAACCAATTTAACAAGGCGATTAGTCAAATTCAAGAATCACTTA
CAACAACATCAACTGCATTGGGCAAGCTGCAAGACGTTGTTAACCAGAATGCTCAAGCATTAAACACACTTGTTAAACA
ACTTAGCTCTAATTTTGGTGCAATTTCAAGTGTGCTAAATGATATCCTTTCGCGACTTGATAAAGTCGAGGCGGAGGTA
CAAATTGACAGGTTAATTACAGGCAGACTTCAAAGCCTTCAAACCTATGTAACACAACAACTAATCAGGGCTGCTGAAA
TCAGGGCTTCTGCTAATCTTGCTGCTACTAAAATGTCTGAGTGTGTTCTTGGACAATCAAAAAGAGTTGACTTTTGTGG
AAAGGGCTACCACCTTATGTCCTTCCCACAAGCAGCCCCGCATGGTGTTGTCTTCCTACATGTCACGTATGTGCCATCC
CAGGAGAGGAACTTCACCACAGCGCCAGCAATTTGTCATGAAGGCAAAGCATACTTCCCTCGTGAAGGTGTTTTTGTGT
TTAATGGCACTTCTTGGTTTATTACACAGAGGAACTTCTTTTCTCCACAAATAATTACTACAGACAATACATTTGTCTC
AGGAAATTGTGATGTCGTTATTGGCATCATTAACAACACAGTTTATGATCCTCTGCAACCTGAGCTTGACTCATTCAAA
GAAGAGCTGGACAAGTACTTCAAAAATCATACATCACCAGATGTTGATCTTGGCGACATTTCAGGCATTAACGCTTCTG
TCGTCAACATTCAAAAAGAAATTGACCGCCTCAATGAGGTCGCTAAAAATTTAAATGAATCACTCATTGACCTTCAAGA
ATTGGGAAAATATGAGCAATATATTAAATGGCCTTGGTATGTTTGGCTCGGCTTCATTGCTGGACTAATTGCCATCGTC
ATGGTTACAATCTTGCTTTGTTGCATGACTAGTTGTTGCAGTTGCCTCAAGGGTGCATGCTCTTGTGGTTCTTGCTGCA
AGTTTGATGAGGATGACTCTGAGCCAGTTCTCAAGGGTGTCAAATTACATTACACATAA

>gi|89474512|gb|DQ412590.1|_SARS_coronavirus_isolate_CUHKtc06NP_spike_glycoprotein_(S)_gene_complete_cds

ATGTTTATTTTCTTATTATTTCTTACTCTCACTAGTGGTAGTGACCTTGACCGGTGCACCACTTTTGATGATGTT
CAAGCTCCTAATTACACTCAACATACTTCATCTATGAGGGGGGTTTACTATCCTGATGAAATTTTTAGATCAGACACTC
TTTATTTAACTCAGGATTTATTTCTTCCATTTTATTCTAATGTTACAGGGTTTCATACTATTAATCATACGTTTGGCAA
CCCTGTCATACCTTTTAAGGATGGTATTTATTTTGCTGCCACAGAGAAATCAAATGTTGTCCGTGGTTGGGTTTTTGGT
TCTACCATGAACAACAAGTCACAGTCGGTGATTATTATTAACAATTCTACTAATGTTGTTATACGAGCATGTAACTTTG
AATTGTGTGACAACCCTTTCTTTGCTGTTTCTAAACCCATGGGTACACAGACACATACTATGATATTCGATAATGCATT
TAATTGCACTTTCGAGTACATATCTGATGCCTTTTCGCTTGATGTTTCAGAAAAGTCAGGTAATTTTAAACACTTACGA
GAGTTTGTGTTTAAAAATAAAGATGGGTTTCTCTATGTTTATAAGGGCTATCAACCTATAGATGTAGTTCGTGATCTAC
CTTCTGGTTTTAACACTTTGAAACCTATTTTTAAGTTGCCTCTTGGTATTAACATTACAAATTTTAGAGCCATTCTTAC
AGCCTTTTCACCTGCTCAAGACATTTGGGGCACGTCAGCTGCAGCCTATTTTGTTGGCTATTTAAAGCCAACTACATTT
ATGCTCAAGTATGATGAAAATGGTACAATCACAGATGCTGTTGATTGTTCTCAAAATCCACTTGCTGAACTCAAATGCT
CTGTTAAGAGCTTTGAGATTGACAAAGGAATTTACCAGACCTCTAATTTCAGGGTTGTTCCCTCAGGAGATGTTGTGAG
ATTCCCTAATATTACAAACTTGTGTCCTTTTGGAGAGGTTTTTAATGCTACTAAATTCCCTTCTGTCTATGCATGGGAG
AGGAAAAAAATTTCTAATTGTGTTGCTGATTACTCTGTGCTCTACAACTCAACATTTTTTTCAACCTTTAAGTGCTATG
GCGTTTCTGCCACTAAGTTGAATGATCTTTGCTTCTCCAATGTCTATGCAGATTCTTTTGTAGTCAAGGGAGATGATGT
AAGACAAATAGCGCCAGGACAAACTGGTGTTATTGCTGATTATAATTATAAATTGCCAGATGATTTCATGGGTTGTGTC
CTTGCTTGGAATACTAGGAACATTGATGCTACTTCAACTGGTAATTATAATTATAAATATAGGTATCTTAGACATGGCA
AGCTTAGGCCCTTTGAGAGAGACATATCTAATGTGCCTTTCTCCCCTGATGGCAAACCTTGCACCCCACCTGCTCTTAA
TTGTTATTGGCCATTAAATGATTATGGTTTTTACACCACTACTGGCATTGGCTACCAACCTTACAGAGTTGTAGTACTT
TCTTTTGAACTTTTAAATGCACCGGCCACGGTTTGTGGACCAAAATTATCCACTGACCTTATTAAGAACCAGTGTGTCA
ATTTTAATTTTAATGGACTCACTGGTACTGGTGTGTTAACTCCTTCTTCAAAGAGATTTCAACCATTTCAACAATTTGG
CCGTGATGTTTCTGATTTCACTGATTCCGTTCGAGATCCTAAAACATCTGAAATATTAGACATTTCACCTTGCTCTTTT
GGGGGTGTAAGTGTAATTACACCTGGAACAAATGCTTCATCTGAAGTTGCTGTTCTATATCAAGATGTTAACTGCACTG
ATGTTTCTACAGCAATTCATGCAGATCAACTCACACCAGCTTGGCGCATATATTCTACTGGAAACAATGTATTCCAGAC
TCAAGCAGGCTGTCTTATAGGAGCTGAGCATGTCGACACTTCTTATGAGTGCGACATTCCTATTGGAGCTGGCATTTGT
GCTAGTTACCATACAGTTTCTTTATTACGTAGTACTAGCCAAAAATCTATTGTGGCTTATACTATGTCTTTAGGTGCTG
ATAGTTCAATTGCTTACTCTAATAACACCATTGCTATACCTACTAACTTTTCAATTAGCATTACTACAGAAGTAATGCC
TGTTTCTATGGCTAAAACCTCCGTAGATTGTAATATGTACATCTGCGGAGATTCTACTGAATGTGCTAATTTGCTTCTC
CAATATGGTAGCTTTTGCACACAACTAAATCGTGCACTCTCAGGTATTGCTGCTGAACAGGATCGCAACACACGTGAAG
TGTTCGCTCAAGTCAAACAAATGTACAAAACCCCAACTTTGAAATATTTTGGTGGTTTTAATTTTTCACAAATATTACC
TGACCCTCTAAAGCCAACTAAGAGGTCTTTTATTGAGGACTTGCTCTTTAATAAGGTGACACTCGCTGATGCTGGCTTC
ATGAAGCAATATGGCGAATGCCTAGGTGATATTAATGCTAGAGATCTCATTTGTGCGCAGAAGTTCAATGGACTTACAG
TGTTGCCACCTCTGCTCACTGATGATATGATTGCTGCCTACACTGCTGCTCTAGTTAGTGGTACTGCCACTGCTGGATG
GACATTTGGTGCTGGCGCTGCTCTTCAAATACCTTTTGCTATGCAAATGGCATATAGGTTCAATGGCATTGGAGTTACC
CAAAATGTTCTCTATGAGAACCAAAAACAAATCGCCAACCAATTTAACAAGGCGATTAGTCAAATTCAAGAATCACTTA
CAACAACATCAACTGCATTGGGCAAGCTGCAAGACGTTGTTAACCAGAATGCTCAAGCATTAAACACACTTGTTAAACA
ACTTAGCTCTAATTTTGGTGCAATTTCAAGTGTGCTAAATGATATCCTTTCGCGACTTGATAAAGTCGAGGCGGAGGTA
CAAATTGACAGGTTAATTACAGGCAGACTTCAAAGCCTTCAAACCTATGTAACACAACAACTAATCAGGGCTGCTGAAA
TCAGGGCTTCTGCTAATCTTGCTGCTACTAAAATGTCTGAGTGTGTTCTTGGACAATCAAAAAGAGTTGACTTTTGTGG
AAAGGGCTACCACCTTATGTCCTTCCCACAAGCAGCCCCGCATGGTGTTGTCTTCCTACATGTCACGTATGTGCCATCC

CAGGAGAGGAACTTCACCACAGCGCCAGCAATTTGTCATGAAGGCAAAGCATACTTCCCTCGTGAAGGTGTTTTTGTGT
TTAATGGCACTTCTTGGTTTATTACACAGAGGAACTTCTTTTCTCCACAAATAATTACTACAGACAATACATTTGTCTC
AGGAAATTGTGATGTCGTTATTGGCATCATTAACAACACAGTTTATGATCCTCTGCAACCTGAGCTTGACTCATTCAAA
GAAGAGCTGGACAAGTACTTCAAAAATCATACATCACCAGATGTTGATCTTGGCGACATTTCAGGCATTAACGCTTCTG
TCGTCAACATTCAAAAAGAAATTGACCGCCTCAATGAGGTCGCTAAAAATTTAAATGAATCACTCATTGACCTTCAAGA
ATTGGGAAAATATGAGCAATATATTAAATGGCCTTGGTATGTTTGGCTCGGCTTCATTGCTGGACTAATTGCCATCGTC
ATGGTTACAATCTTGCTTTGTTGCATGACTAGTTGTTGCAGTTGCCTCAAGGGTGCATGCTCTTGTGGTTCTTGCTGCA
AGTTTGATGAGGATGACTCTGAGCCAGTTCTCAAGGGTGTCAAATTACATTACACATAA

>gi|89474517|gb|DQ412594.1|_SARS_coronavirus_isolate_CUHKtc10NP_spike_glycoprotein_(S)_gene_complete_cds

ATGTTAATTTTCTTATTATTTCTTACTCTCACTAGTGGTAGTGACCTTGACCGGTGCACCACTTTTGATGATGTT
CAAGCTCCTAATTACACTCAACATACTTCATCTATGAGGGGGGTTTACTATCCTGATGAAATTTTTAGATCAGACACTC
TTTATTTAACTCAGGATTTATTTCTTCCATTTTATTCTAATGTTACAGGGTTTCATACTATTAATCATACGTTTGGCAA
CCCTGTCATACCTTTTAAGGATGGTATTTATTTTGCTGCCACAGAGAAATCAAATGTTGTCCGTGGTTGGGTTTTTGGT
TCTACCATGAACAACAAGTCACAGTCGGTGATTATTATTAACAATTCTACTAATGTTGTTATACGAGCATGTAACTTTG
AATTGTGTGACAACCCTTTCTTTGCTGTTTCTAAACCCATGGGTACACAGACACATACTATGATATTCGATAATGCATT
TAATTGCACTTTCGAGTACATATCTGATGCCTTTTCGCTTGATGTTTCAGAAAAGTCAGGTAATTTTAAACACTTACGA
GAGTTTGTGTTTAAAAATAAAGATGGGTTTCTCTATGTTTATAAGGGCTATCAACCTATAGATGTAGTTCGTGATCTAC
CTTCTGGTTTTAACACTTTGAAACCTATTTTTAAGTTGCCTCTTGGTATTAACATTACAAATTTTAGAGCCATTCTTAC
AGCCTTTTCACCTGCTCAAGACATTTGGGGCACGTCAGCTGCAGCCTATTTTGTTGGCTATTTAAAGCCAACTACATTT
ATGCTCAAGTATGATGAAAATGGTACAATCACAGATGCTGTTGATTGTTCTCAAAATCCACTTGCTGAACTCAAATGCT
CTGTTAAGAGCTTTGAGATTGACAAAGGAATTTACCAGACCTCTAATTTCAGGGTTGTTCCCTCAGGAGATGTTGTGAG
ATTCCCTAATATTACAAACTTGTGTCCTTTTGGAGAGGTTTTTAATGCTACTAAATTCCCTTCTGTCTATGCATGGGAG
AGAAAAAAAATTTCTAATTGTGTTGCTGATTACTCTGTGCTCTACAACTCAACATTTTTTTCAACCTTTAAGTGCTATG
GCGTTTCTGCCACTAAGTTGAATGATCTTTGCTTCTCCAATGTCTATGCAGATTCTTTTGTAGTCAAGGGAGATGATGT
AAGACAAATAGCGCCAGGACAAACTGGTGTTATTGCTGATTATAATTATAAATTGCCAGATGATTTCATGGGTTGTGTC
CTTGCTTGGAATACTAGGAACATTGATGCTACTTCAACTGGTAATTATAATTATAAATATAGGTATCTTAGACATGGCA
AGCTTAGGCCCTTTGAGAGAGACATATCTAATGTGCCTTTCTCCCCTGATGGCAAACCTTGCACCCCACCTGCTCTTAA
TTGTTATTGGCCATTAAATGATTATGGTTTTTACACCACTACTGGCATTGGCTACCAACCTTACAGAGTTGTAGTACTT
TCTTTTGAACTTTTAAATGCACCGGCCACGGTTTGTGGACCAAAATTATCCACTGACCTTATTAAGAACCAGTGTGTCA
ATTTTAATTTTAATGGACTCACTGGTACTGGTGTGTTAACTCCTTCTTCAAAGAGATTTCAACCATTTCAACAATTTGG
CCGTGATGTTTCTGATTTCACTGATTCCGTTCGAGATCCTAAAACATCTGAAATATTAGACATTTCACCTTGCTCTTTT
GGGGGTGTAAGTGTAATTACACCTGGAACAAATGCTTCATCTGAAGTTGCTGTTCTATATCAAGATGTTAACTGCACTG
ATGTTTCTACAGCAATTCATGCAGATCAACTCACACCAGCTTGGCGCATATATTCTACTGGAAACAATGTATTCCAGAC
TCAAGCAGGCTGTCTTATAGGAGCTGAGCATGTCGACACTTCTTATGAGTGCGACATTCCTATTGGAGCTGGCATTTGT
GCTAGTTACCATACAGTTTCTTTATTACGTAGTACTAGCCAAAAATCTATTGTGGCTTATACTATGTCTTTAGGTGCTG
ATAGTTCAATTGCTTACTCTAATAACACCATTGCTATACCTACTAACTTTTCAATTAGCATTACTACAGAAGTAATGCC
TGTTTCTATGGCTAAAACCTCCGTAGATTGTAATATGTACATCTGCGGAGATTCTACTGAATGTGCTAATTTGCTTCTC
CAATATGGTAGCTTTTGCACACAACTAAATCGTGCACTCTCAGGTATTGCTGCTGAACAGGATCGCAACACACGTGAAG
TGTTCGCTCAAGTCAAACAAATGTACAAAACCCCAACTTTGAAATATTTTGGTGGTTTTAATTTTTCACAAATATTACC
TGACCCTCTAAAGCCAACTAAGAGGTCTTTTATTGAGGACTTGCTCTTTAATAAGGTGACACTCGCTGATGCTGGCTTC

ATGAAGCAATATGGCGAATGCCTAGGTGATATTAATGCTAGAGATCTCATTTGTGCGCAGAAGTTCAATGGACTTACAG
TGTTGCCACCTCTGCTCACTGATGATATGATTGCTGCCTACACTGCTGCTCTAGTTAGTGGTACTGCCACTGCTGGATG
GACATTTGGTGCTGGCGCTGCTCTTCAAATACCTTTTGCTATGCAAATGGCATATAGGTTCAATGGCATTGGAGTTACC
CAAAATGTTCTCTATGAGAACCAAAAACAAATCGCCAACCAATTTAACAAGGCGATTAGTCAAATTCAAGAATCACTTA
CAACAACATCAACTGCATTGGGCAAGCTGCAAGACGTTGTTAACCAGAATGCTCAAGCATTAAACACACTTGTTAAACA
ACTTAGCTCTAATTTTGGTGCAATTTCAAGTGTGCTAAATGATATCCTTTCGCGACTTGATAAAGTCGAGGCGGAGGTA
CAAATTGACAGGTTAATTACAGGCAGACTTCAAAGCCTTCAAACCTATGTAACACAACAACTAATCAGGGCTGCTGAAA
TCAGGGCTTCTGCTAATCTTGCTGCTACTAAAATGTCTGAGTGTGTTCTTGGACAATCAAAAAGAGTTGACTTTTGTGG
AAAGGGCTACCACCTTATGTCCTTCCCACAAGCAGCCCCGCATGGTGTTGTCTTCCTACATGTCACGTATGTGCCATCC
CAGGAGAGGAACTTCACCACAGCGCCAGCAATTTGTCATGAAGGCAAAGCATACTTCCCTCGTGAAGGTGTTTTTGTGT
TTAATGGCACTTCTTGGTTTATTACACAGAGGAACTTCTTTTCTCCACAAATAATTACTACAGACAATACATTTGTCTC
AGGAAATTGTGATGTCGTTATTGGCATCATTAACAACACAGTTTATGATCCTCTGCAACCTGAGCTTGACTCATTCAAA
GAAGAGCTGGACAAGTACTTCAAAAATCATACATCACCAGATGTTGATCTTGGCGACATTTCAGGCATTAACGCTTCTG
TCGTCAACATTCAAAAAGAAATTGACCGCCTCAATGAGGTCGCTAAAAATTTAAATGAATCACTCATTGACCTTCAAGA
ATTGGGAAAATATGAGCAATATATTAAATGGCCTTGGTATGTTTGGCTCGGCTTCATTGCTGGACTAATTGCCATCGTC
ATGGTTACAATCTTGCTTTGTTGCATGACTAGTTGTTGCAGTTGCCTCAAGGGTGCATGCTCTTGTGGTTCTTGCTGCA
AGTTTGATGAGGATGACTCTGAGCCAGTTCTCAAGGGTGTCAAATTACATTACACATAA

>gi|89474521|gb|DQ412596.1|_SARS_coronavirus_isolate_CUHKtc14NP_spike_glycoprotein_(S)_gene_complete_cds

ATGTTTATTTTCATATTATTTCTTACTCTCACAAGTGGTAGTGACCTTGACCGGTGCACCACTTTTGATGATGTT
CAAGCTCCTAATTACACTCAACATACTTCATCTATGAGGGGGGTTTACTATCCTGATGAAATTTTTAGATCAGACACTC
TTTATTTAACTCAGGATTTATTTCTTCCATTTTATTCTAATGTTACAGGGTTTCATACTATTAATCATACGTTTGGCAA
CCCTGTCATACCTTTTAAGGATGGTATTTATTTTGCTGCCACAGAGAAATCAAATGTTGTCCGTGGTTGGGTTTTTGGT
TCTACCATGAACAACAAGTCACAGTCGGTGATTATTATTAACAATTCTACTAATGTTGTTATACGAGCATGTAACTTTG
AATTGTGTGACAACCCTTTCTTTGCTGTTTCTAAACCCATGGGTACACAGACACATACTATGATATTCGATAATGCATT
TAATTGCACTTTCGAGTACATATCTGATGCCTTTTCGCTTGATGTTTCAGAAAAGTCAGGTAATTTTAAACACTTACGA
GAGTTTGTGTTTAAAAATAAAGATGGGTTTCTCTATGTTTATAAGGGCTATCAACCTATAGATGTAGTTCGTGATCTAC
CTTCTGGTTTTAACACTTTGAAACCTATTTTTAAGTTGCCTCTTGGTATTAACATTACAAATTTTAGAGCCATTCTTAC
AGCCTTTTCACCTGCTCAAGACATTTGGGGCACGTCAGCTGCAGCCTATTTTGTTGGCTATTTAAAGCCAACTACATTT
ATGCTCAAGTATGATGAAAATGGTACAATCACAGATGCTGTTGATTGTTCTCAAAATCCACTTGCTGAACTCAAATGCT
CTGTTAAGAGCTTTGAGATTGACAAAGGAATTTACCAGACCTCTAATTTCAGGGTTGTTCCCTCAGGAGATGTTGTGAG
ATTCCCTAATATTACAAACTTGTGTCCTTTTGGAGAGGTTTTTAATGCTACTAAATTCCCTTCTGTCTATGCATGGGAG
AGAAAAAAAATTTCTAATTGTGTTGCTGATTACTCTGTGCTCTACAACTCAACATTTTTTTCAACCTTTAAGTGCTATG
GCGTTTCTGCCACTAAGTTGAATGATCTTTGCTTCTCCAATGTCTATGCAGATTCTTTTGTAGTCAAGGGAGATGATGT
AAGACAAATAGCGCCAGGACAAACTGGTGTTATTGCTGATTATAATTATAAATTGCCAGATGATTTCATGGGTTGTGTC
CTTGCTTGGAATACTAGGAACATTGATGCTACTTCAACTGGTAATTATAATTATAAATATAGGTATCTTAGACATGGCA
AGCTTAGGCCCTTTGAGAGAGACATATCTAATGTGCCTTTCTCCCCTGATGGCAAACCTTGCACCCCACCTGCTCTTAA
TTGTTATTGGCCATTAAATGATTATGGTTTTTACACCACTACTGGCATTGGCTACCAACCTTACAGAGTTGTAGTACTT
TCTTTTGAACTTTTAAATGCACCGGCCACGGTTTGTGGACCAAAATTATCCACTGACCTTATTAAGAACCAGTGTGTCA
ATTTTAATTTTAATGGACTCACTGGTACTGGTGTGTTAACTCCTTCTTCAAAGAGATTTCAACCATTTCAACAATTTGG
CCGTGATGTTTCTGATTTCACTGATTCCGTTCGAGATCCTAAAACATCTGAAATATTAGACATTTCACCTTGCTCTTTT

GGGGGTGTAAGTGTAATTACACCTGGAACAAATGCTTCATCTGAAGTTGCTGTTCTATATCAAGATGTTAACTGCACTG
ATGTTTCTACAGCAATTCATGCAGATCAACTCACACCAGCTTGGCGCATATATTCTACTGGAAACAATGTATTCCAGAC
TCAAGCAGGCTGTCTTATAGGAGCTGAGCATGTCGACACTTCTTATGAGTGCGACATTCCTATTGGAGCTGGCATTTGT
GCTAGTTACCATACAGTTTCTTTATTACGTAGTACTAGCCAAAAATCTATTGTGGCTTATACTATGTCTTTAGGTGCTG
ATAGTTCAATTGCTTACTCTAATAACACCATTGCTATACCTACTAACTTTTCAATTAGCATTACTACAGAAGTAATGCC
TGTTTCTATGGCTAAAACCTCCGTAGATTGTAATATGTACATCTGCGGAGATTCTACTGAATGTGCTAATTTGCTTCTC
CAATATGGTAGCTTTTGCACACAACTAAATCGTGCACTCTCAGGTATTGCTGCTGAACAGGATCGCAACACACGTGAAG
TGTTCGCTCAAGTCAAACAAATGTACAAAACCCCAACTTTGAAATATTTTGGTGGTTTTAATTTTTCACAAATATTACC
TGACCCTCTAAAGCCAACTAAGAGGTCTTTTATTGAGGACTTGCTCTTTAATAAGGTGACACTCGCTGATGCTGGCTTC
ATGAAGCAATATGGCGAATGCCTAGGTGATATTAATGCTAGAGATCTCATTTGTGCGCAGAAGTTCAATGGACTTACAG
TGTTGCCACCTCTGCTCACTGATGATATGATTGCTGCCTACACTGCTGCTCTAGTTAGTGGTACTGCCACTGCTGGATG
GACATTTGGTGCTGGCGCTGCTCTTCAAATACCTTTTGCTATGCAAATGGCATATAGGTTCAATGGCATTGGAGTTACC
CAAAATGTTCTCTATGAGAACCAAAAACAAATCGCCAACCAATTTAACAAGGCGATTAGTCAAATTCAAGAATCACTTA
CAACAACATCAACTGCATTGGGCAAGCTGCAAGACGTTGTTAACCAGAATGCTCAAGCATTAAACACACTTGTTAAACA
ACTTAGCTCTAATTTTGGTGCAATTTCAAGTGTGCTAAATGATATCCTTTCGCGACTTGATAAAGTCGAGGCGGAGGTA
CAAATTGACAGGTTAATTACAGGCAGACTTCAAAGCCTTCAAACCTATGTAACACAACAACTAATCAGGGCTGCTGAAA
TCAGGGCTTCTGCTAATCTTGCTGCTACTAAAATGTCTGAGTGTGTTCTTGGACAATCAAAAAGAGTTGACTTTTGTGG
AAAGGGCTACCACCTTATGTCCTTCCCACAAGCAGCCCCGCATGGTGTTGTCTTCCTACATGTCACGTATGTGCCATCC
CAGGAGAGGAACTTCACCACAGCGCCAGCAATTTGTCATGAAGGCAAAGCATACTTCCCTCGTGAAGGTGTTTTTGTGT
TTAATGGCACTTCTTGGTTTATTACACAGAGGAACTTCTTTTCTCCACAAATAATTACTACAGACAATACATTTGTCTC
AGGAAATTGTGATGTCGTTATTGGCATCATTAACAACACAGTTTATGATCCTCTGCAACCTGAGCTTGACTCATTCAAA
GAAGAGCTGGACAAGTACTTCAAAAATCATACATCACCAGATGTTGATCTTGGCGACATTTCAGGCATTAACGCTTCTG
TCGTCAACATTCAAAAAGAAATTGACCGCCTCAATGAGGTCGCTAAAAATTTAAATGAATCACTCATTGACCTTCAAGA
ATTGGGAAAATATGAGCAATATATTAAATGGCCTTGGTATGTTTGGCTCGGCTTCATTGCTGGACTAATTGCCATCGTC
ATGGTTACAATCTTGCTTTGTTGCATGACTAGTTGTTGCAGTTGCCTCAAGGGTGCATGCTCTTGTGGTTCTTGCTGCA
AGTTTGATGAGGATGACTCTGAGCCAGTTCTCAAGGGTGTCAAATTACATTACACATAA

>gi|89474538|gb|DQ412609.1|_SARS_coronavirus_isolate_CUHKtc32NP_spike_glycoprotein_(S)_gene_complete_cds

ATGTTTATTTTCTTATTATTTCTTACTCTCACTAATGGTAGTGACCTTGACCGGTGCACCACTTTTGATGATGTT
CAAGCTCCTAATTACACTCAACATACTTCATCTATGAGGGGGGTTTACTATCCTGATGAAATTTTTAGATCAGACACTC
TTTATTTAACTCAGGATTTATTTCTTCCATTTTATTCTAATGTTACAGGGTTTCATACTATTAATCATACGTTTGGCAA
CCCTGTCATACCTTTTAAGGATGGTATTTATTTTGCTGCCACAGAGAAATCAAATGTTGTCCGTGGTTGGGTTTTTGGT
TCTACCATGAACAACAAGTCACAGTCGGTGATTATTATTAACAATTCTACTAATGTTGTTATACGAGCATGTAACTTTG
AATTGTGTGACAACCCTTTCTTTGCTGTTTCTAAACCCATGGGTACACAGACACATACTATGATATTCGATAATGCATT
TAATTGCACTTTCGAGTACATATCTGATGCCTTTTCGCTTGATGTTTCAGAAAAGTCAGGTAATTTTAAACACTTACGA
GAGTTTGTGTTTAAAAATAAAGATGGGTTTCTCTATGTTTATAAGGGCTATCAACCTATAGATGTAGTTCGTGATCTAC
CTTCTGGTTTTAACACTTTGAAACCTATTTTTAAGTTGCCTCTTGGTATTAACATTACAAATTTTAGAGCCATTCTTAC
AGCCTTTTCACCTGCTCAAGACATTTGGGGCACGTCAGCTGCAGCCTATTTTGTTGGCTATTTAAAGCCAACTACATTT
ATGCTCAAGTATGATGAAAATGGTACAATCACAGATGCTGTTGATTGTTCTCAAAATCCACTTGCTGAACTCAAATGCT
CTGTTAAGAGCTTTGAGATTGACAAAGGAATTTACCAGACCTCTAATTTCAGGGTTGTTCCCTCAGGAGATGTTGTGAG
ATTCCCTAATATTACAAACTTGTGTCCTTTTGGAGAGGTTTTTAATGCTACTAAATTCCCTTCTGTCTATGCATGGGAG

AGAAAAAAAATTTCTAATTGTGTTGCTGATTACTCTGTGCTCTACAACTCAACATTTTTTTCAACCTTTAAGTGCTATG
GCGTTTCTGCCACTAAGTTGAATGATCTTTGCTTCTCCAATGTCTATGCAGATTCTTTTGTAGTCAAGGGAGATGATGT
AAGACAAATAGCGCCAGGACAAACTGGTGTTATTGCTGATTATAATTATAAATTGCCAGATGATTTCATGGGTTGTGTC
CTTGCTTGGAATACTAGGAACATTGATGCTACTTCAACTGGTAATTATAATTATAAATATAGGTATCTTAGACATGGCA
AGCTTAGGCCCTTTGAGAGAGACATATCTAATGTGCCTTTCTCCCCTGATGGCAAACCTTGCACCCCACCTGCTCTTAA
TTGTTATTGGCCATTAAATGATTATGGTTTTTACACCACTACTGGCATTGGCTACCAACCTTACAGAGTTGTAGTACTT
TCTTTTGAACTTTTAAATGCACCGGCCACGGTTTGTGGACCAAAATTATCCACTGACCTTATTAAGAACCAGTGTGTCA
ATTTTAATTTTAATGGACTCACTGGTACTGGTGTGTTAACTCCTTCTTCAAAGAGATTTCAACCATTTCAACAATTTGG
CCGTGATGTTTCTGATTTCACTGATTCCGTTCGAGATCCTAAAACATCTGAAATATTAGACATTTCACCTTGCTCTTTT
GGGGGTGTAAGTGTAATTACACCTGGAACAAATGCTTCATCTGAAGTTGCTGTTCTATATCAAGATGTTAACTGCACTG
ATGTTTCTACAGCAATTCATGCAGATCAACTCACACCAGCTTGGCGCATATATTCTACTGGAAACAATGTATTCCAGAC
TCAAGCAGGCTGTCTTATAGGAGCTGAGCATGTCGACACTTCTTATGAGTGCGACATTCCTATTGGAGCTGGCATTTGT
GCTAGTTACCATACAGTTTCTTTATTACGTAGTACTAGCCAAAAATCTATTGTGGCTTATACTATGTCTTTAGGTGCTG
ATAGTTCAATTGCTTACTCTAATAACACCATTGCTATACCTACTAACTTTTCAATTAGCATTACTACAGAAGTAATGCC
TGTTTCTATGGCTAAAACCTCCGTAGATTGTAATATGTACATCTGCGGAGATTCTACTGAATGTGCTAATTTGCTTCTC
CAATATGGTAGCTTTTGCACACAACTAAATCGTGCACTCTCAGGTATTGCTGCTGAACAGGATCGCAACACACGTGAAG
TGTTCGCTCAAGTCAAACAAATGTACAAAACCCCAACTTTGAAATATTTTGGTGGTTTTAATTTTTCACAAATATTACC
TGACCCTCTAAAGCCAACTAAGAGGTCTTTTATTGAGGACTTGCTCTTTAATAAGGTGACACTCGCTGATGCTGGCTTC
ATGAAGCAATATGGCGAATGCCTAGGTGATATTAATGCTAGAGATCTCATTTGTGCGCAGAAGTTCAATGGACTTACAG
TGTTGCCACCTCTGCTCACTGATGATATGATTGCTGCCTACACTGCTGCTCTAGTTAGTGGTACTGCCACTGCTGGATG
GACATTTGGTGCTGGCGCTGCTCTTCAAATACCTTTTGCTATGCAAATGGCATATAGGTTCAATGGCATTGGAGTTACC
CAAAATGTTCTCTATGAGAACCAAAAACAAATCGCCAACCAATTTAACAAGGCGATTAGTCAAATTCAAGAATCACTTA
CAACAACATCAACTGCATTGGGCAAGCTGCAAGACGTTGTTAACCAGAATGCTCAAGCATTAAACACACTTGTTAAACA
ACTTAGCTCTAATTTTGGTGCAATTTCAAGTGTGCTAAATGATATCCTTTCGCGACTTGATAAAGTCGAGGCGGAGGTA
CAAATTGACAGGTTAATTACAGGCAGACTTCAAAGCCTTCAAACCTATGTAACACAACAACTAATCAGGGCTGCTGAAA
TCAGGGCTTCTGCTAATCTTGCTGCTACTAAAATGTCTGAGTGTGTTCTTGGACAATCAAAAAGAGTTGACTTTTGTGG
AAAGGGCTACCACCTTATGTCCTTCCCACAAGCAGCCCCGCATGGTGTTGTCTTCCTACATGTCACGTATGTGCCATCC
CAGGAGAGGAACTTCACCACAGCGCCAGCAATTTGTCATGAAGGCAAAGCATACTTCCCTCGTGAAGGTGTTTTTGTGT
TTAATGGCACTTCTTGGTTTATTACACAGAGGAACTTCTTTTCTCCACAAATAATTACTACAGACAATACATTTGTCTC
AGGAAATTGTGATGTCGTTATTGGCATCATTAACAACACAGTTTATGATCCTCTGCAACCTGAGCTTGACTCATTCAAA
GAAGAGCTGGACAAGTACTTCAAAAATCATACATCACCAGATGTTGATCTTGGCGACATTTCAGGCATTAACGCTTCTG
TCGTCAACATTCAAAAAGAAATTGACCGCCTCAATGAGGTCGCTAAAAATTTAAATGAATCACTCATTGACCTTCAAGA
ATTGGGAAAATATGAGCAATATATTAAATGGCCTTGGTATGTTTGGCTCGGCTTCATTGCTGGACTAATTGTCATCGTC
ATGGTTACAATCTTGCTTTGTTGCATGACTAGTTGTTGCAGTTGCCTCAAGGGTGCATGCTCTTGTGGTTCTTGCTGCA
AGTTTGATGAGGATGACTCTGAGCCAGTTCTCAAGGGTGTCAAATTACATTACACATAA

>gi|89474547|gb|DQ412614.1|_SARS_coronavirus_isolate_CUHKtc38NP_spike_glycoprotein_(S)_gene_complete_cds

ATGTTTATTTTCTTATTATTTCTTACTCTCACTAGAGGTAGTGACCTTGACCGGTGCACCACTTTTGATGATGTT
CAAGCTCCTAATTACACTCAACATACTTCATCTATGAGGGGGGTTTACTATCCTGATGAAATTTTTAGATCAGACACTC
TTTATTTAACTCAGGATTTATTTCTTCCATTTTATTCTAATGTTACAGGGTTTCATACTATTAATCATACGTTTGGCAA
CCCTGTCATACCTTTTAAGGATGGTATTTATTTTGCTGCCACAGAGAAATCAAATGTTGTCCGTGGTTGGGTTTTTGGT

TCTACCATGAACAACAAGTCACAGTCGGTGATTATTATTAACAATTCTACTAATGTTGTTATACGAGCATGTAACTTTG
AATTGTGTGACAACCCTTTCATTGCTGTTTCTAAACCCATGGGTACACAGACACATACTATGATATTCGATAATGCATT
TAATTGCACTTTCGAGTACATATCTGATGCCTTTTCGCTTGATGTTTCAGAAAAGTCAGGTAATTTTAAACACTTACGA
GAGTTTGTGTTTAAAAATAAAGATGGGTTTCTCTATGTTTATAAGGGCTATCAACCTATAGATGTAGTTCGTGATCTAC
CTTCTGGTTTTAACACTTTGAAACCTATTTTTAAGTTGCCTCTTGGTATTAACATTACAAATTTTAGAGCCATTCTTAC
AGCCTTTTCACCTGCTCAAGACATTTGGGGCACGTCAGCTGCAGCCTATTTTGTTGGCTATTTAAAGCCAACTACATTT
ATGCTCAAGTATGATGAAAATGGTACAATCACAGATGCTGTTGATTGTTCTCAAAATCCACTTGCTGAACTCAAATGCT
CTGTTAAGAGCTTTGAGATTGACAAAGGAATTTACCAGACCTCTAATTTCAGGGTTGTTCCCTCAGGAGATGTTGTGAG
ATTCCCTAATATTACAAACTTGTGTCCTTTTGGAGAGGTTTTTAATGCTACTAAATTCCCTTCTGTCTATGCATGGGAG
AGAAAAAAAATTTCTAATTGTGTTGCTGATTACTCTGTGCTCTACAACTCAACATTTTTTTCAACCTTTAAGTGCTATG
GCGTTTCTGCCACTAAGTTGAATGATCTTTGCTTCTCCAATGTCTATGCAGATTCTTTTGTAGTCAAGGGAGATGATGT
AAGACAAATAGCGCCAGGACAAACTGGTGTTATTGCTGATTATAATTATAAATTGCCAGATGATTTCATGGGTTGTGTC
CTTGCTTGGAATACTAGGAACATTGATGCTACTTCAACTGGTAATTATAATTATAAATATAGGTATCTTAGACATGGCA
AGCTTAGGCCCTTTGAGAGAGACATATCTAATGTGCCTTTCTCCCCTGATGGCAAACCTTGCACCCCACCTGCTCTTAA
TTGTTATTGGCCATTAAATGATTATGGTTTTTACACCACTACTGGCATTGGCTACCAACCTTACAGAGTTGTAGTACTT
TCTTTTGAACTTTTAAATGCACCGGCCACGGTTTGTGGACCAAAATTATCCACTGACCTTATTAAGAACCAGTGTGTCA
ATTTTAATTTTAATGGACTCACTGGTACTGGTGTGTTAACTCCTTCTTCAAAGAGATTTCAACCATTTCAACAATTTGG
CCGTGATGTTTCTGATTTCACTGATTCCGTTCGAGATCCTAAAACATCTGAAATATTAGACATTTCACCTTGCTCTTTT
GGGGGTGTAAGTGTAATTACACCTGGAACAAATGCTTCATCTGAAGTTGCTGTTCTATATCAAGATGTTAACTGCACTG
ATGTTTCTACAGCAATTCATGCAGATCAACTCACACCAGCTTGGCGCATATATTCTACTGGAAACAATGTATTCCAGAC
TCAAGCAGGCTGTCTTATAGGAGCTGAGCATGTCGACACTTCTTATGAGTGCGACATTCCTATTGGAGCTGGCATTTGT
GCTAGTTACCATACAGTTTCTTTATTACGTAGTACTAGCCAAAAATCTATTGTGGCTTATACTATGTCTTTAGGTGCTG
ATAGTTCAATTGCTTACTCTAATAACACCATTGCTATACCTACTAACTTTTCAATTAGCATTACTACAGAAGTAATGCC
TGTTTCTATGGCTAAAACCTCCGTAGATTGTAATATGTACATCTGCGGAGATTCTACTGAATGTGCTAATTTGCTTCTC
CAATATGGTAGCTTTTGCACACAACTAAATCGTGCACTCTCAGGTATTGCTGCTGAACAGGATCGCAACACACGTGAAG
TGTTCGCTCAAGTCAAACAAATGTACAAAACCCCAACTTTGAAATATTTTGGTGGTTTTAATTTTTCACAAATATTACC
TGACCCTCTAAAGCCAACTAAGAGGTCTTTTATTGAGGACTTGCTCTTTAATAAGGTGACACTCGCTGATGCTGGCTTC
ATGAAGCAATATGGCGAATGCCTAGGTGATATTAATGCTAGAGATCTCATTTGTGCGCAGAAGTTCAATGGACTTACAG
TGTTGCCACCTCTGCTCACTGATGATATGATTGCTGCCTACACTGCTGCTCTAGTTAGTGGTACTGCCACTGCTGGATG
GACATTTGGTGCTGGCGCTGCTCTTCAAATACCTTTTGCTATGCAAATGGCATATAGGTTCAATGGCATTGGAGTTACC
CAAAATGTTCTCTATGAGAACCAAAAACAAATCGCCAACCAATTTAACAAGGCGATTAGTCAAATTCAAGAATCACTTA
CAACAACATCAACTGCATTGGGCAAGCTGCAAGACGTTGTTAACCAGAATGCTCAAGCATTAAACACACTTGTTAAACA
ACTTAGCTCTAATTTTGGTGCAATTTCAAGTGTGCTAAATGATATCCTTTCGCGACTTGATAAAGTCGAGGCGGAGGTA
CAAATTGACAGGTTAATTACAGGCAGACTTCAAAGCCTTCAAACCTATGTAACACAACAACTAATCAGGGCTGCTGAAA
TCAGGGCTTCTGCTAATCTTGCTGCTACTAAAATGTCTGAGTGTGTTCTTGGACAATCAAAAAGAGTTGACTTTTGTGG
AAAGGGCTACCACCTTATGTCCTTCCCACAAGCAGCCCCGCATGGTGTTGTCTTCCTACATGTCACGTATGTGCCATCC
CAGGAGAGGAACTTCACCACAGCGCCAGCAATTTGTCATGAAGGCAAAGCATACTTCCCTCGTGAAGGTGTTTTTGTGT
TTAATGGCACTTCTTGGTTTATTACACAGAGGAACTTCTTTTCTCCACAAATAATTACTACAGACAATACATTTGTCTC
AGGAAATTGTGATGTCGTTATTGGCATCATTAACAACACAGTTTATGATCCTCTGCAACCTGAGCTTGACTCATTCAAA
GAAGAGCTGGACAAGTACTTCAAAAATCATACATCACCAGATGTTGATCTTGGCGACATTTCAGGCATTAACGCTTCTG
TCGTCAACATTCAAAAAGAAATTGACCGCCTCAATGAGGTCGCTAAAAATTTAAATGAATCACTCATTGACCTTCAAGA
ATTGGGAAAATATGAGCAATATATTAAATGGCCTTGGTATGTTTGGCTCGGCTTCATTGCTGGACTAATTGCCATCGTC
ATGGTTACAATCTTGCTTTGTTGCATGACTAGTTGTTGCAGTTGCCTCAAGGGTGCATGCTCTTGTGGTTCTTGCTGCA
AGTTTGATGAGGATGACTCTGAGCCAGTTCTCAAGGGTGTCAAATTACATTACACATAA

>gi|89474549|gb|DQ412615.1|_SARS_coronavirus_isolate_CUHKtc39TI_spike_glycoprotein_(S)_gene_complete_cds

ATGTTTATTTTCTTATTATTTCTTACTCTCACTAGTGGTAGTGACCTTGACCGGTGCACCACTTTTGATGATGTTCAAGCTCCTAATTACACTCAACATACTTCATCTATGAGGGGGGTTTACTATCCTGATGAAATTTTTAGATCAGACACTCTTTATTTAACTCAGGATTTATTTCTTCCATTTTATTCTAATGTTACAGGGTTTCATACTATTAATCATACGTTTGGCAACCCTGTCATACCTTTTAAGGATGGTATTTATTTTGCTGCCACAGAGAAATCAAATGTTGTCCGTGGTTGGGTTTTTGGTTCTACCATGAACAACAAGTCACAGTCGGTGATTATTATTAACAATTCTACTAATGTTGTTATACGAGCATGTAACTTTGAATTGTGTGACAACCCTTTCATTGCTGTTTCTAAACCCATGGGTACACAGACACATACTATGATATTCGATAATGCATTTAATTGCACTTTCGAGTACATATCTGATGCCTTTTCGCTTGATGTTTCAGAAAAGTCAGGTAATTTTAAACACTTACGAGAGTTTGTGTTTAAAAATAAAGATGGGTTTCTCTATGTTTATAAGGGCTATCAACCTATAGATGTAGTTCGTGATCTACCTTCTGGTTTTAACACTTTGAAACCTATTTTTAAGTTGCCTCTTGGTATTAACATTACAAATTTTAGAGCCATTCTTACAGCCTTTTCACCTGCTCAAGACATTTGGGGCACGTCAGCTGCAGCCTATTTTGTTGGCTATTTAAAGCCAACTACATTTATGCTCAAGTATGATGAAAATGGTACAATCACAGATGCTGTTGATTGTTCTCAAAATCCACTTGCTGAACTCAAATGCTCTGTTAAGAGCTTTGAGATTGACAAAGGAATTTACCAGACCTCTAATTTCAGGGTTGTTCCCTCAGGAGATGTTGTGAGATTCCCTAATATTACAAACTTGTGTCCTTTTGGAGAGGTTTTTAATGCTACTAAATTCCCTTCTGTCTATGCATGGGAGAGAAAAAAAATTTCTAATTGTGTTGCTGATTACTCTGTGCTCTACAACTCAACATTTTTTTCAACCTTTAAGTGCTATGGCGTTTCTGCCACTAAGTTGAATGATCTTTGCTTCTCCAATGTCTATGCAGATTCTTTTGTAGTCAAGGGAGATGATGTAAGACAAATAGCGCCAGGACAAACTGGTGTTATTGCTGATTATAATTATAAATTGCCAGATGATTTCATGGGTTGTGTCCTTGCTTGGAATACTAGGAACATTGATGCTACTTCAACTGGTAATTATAATTATAAATATAGGTATCTTAGACATGGCAAGCTTAGGCCCTTTGAGAGAGACATATCTAATGTGCCTTTCTCCCCTGATGGCAAACCTTGCACCCCACCTGCTCTTAATTGTTATTGGCCATTAAATGATTATGGTTTTTACACCACTACTGGCATTGGCTACCAACCTTACAGAGTTGTAGTACTTTCTTTTGAACTTTTAAATGCACCGGCCACGGTTTGTGGACCAAAATTATCCACTGACCTTATTAAGAACCAGTGTGTCAATTTTAATTTTAATGGACTCACTGGTACTGGTGTGTTAACTCCTTCTTCAAAGAGATTTCAACCATTTCAACAATTTGGCCGTGATGTTTCTGATTTCACTGATTCCGTTCGAGATCCTAAAACATCTGAAATATTAGACATTTCACCTTGCTCTTTTGGGGGTGTAAGTGTAATTACACCTGGAACAAATGCTTCATCTGAAGTTGCTGTTCTATATCAAGATGTTAACTGCACTGATGTTTCTACAGCAATTCATGCAGATCAACTCACACCAGCTTGGCGCATATATTCTACTGGAAACAATGTATTCCAGACTCAAGCAGGCTGTCTTATAGGAGCTGAGCATGTCGACACTTCTTATGAGTGCGACATTCCTATTGGAGCTGGCATTTGTGCTAGTTACCATACAGTTTCTTTATTACGTAGTACTAGCCAAAAATCTATTGTGGCTTATACTATGTCTTTAGGTGCTGATAGTTCAATTGCTTACTCTAATAACACCATTGCTATACCTACTAACTTTTCAATTAGCATTACTACAGAAGTAATGCCTGTTTCTATGGCTAAAACCTCCGTAGATTGTAATATGTACATCTGCGGAGATTCTACTGAATGTGCTAATTTGCTTCTCCAATATGGTAGCTTTTGCACACAACTAAATCGTGCACTCTCAGGTATTGCTGCTGAACAGGATCGCAACACACGTGAAGTGTTCGCTCAAGTCAAACAAATGTACAAAACCCCAACTTTGAAATATTTTGGTGGTTTTAATTTTTCACAAATATTACCTGACCCTCTAAAGCCAACTAAGAGGTCTTTTATTGAGGACTTGCTCTTTAATAAGGTGACACTCGCTGATGCTGGCTTCATGAAGCAATATGGCGAATGCCTAGGTGATATTAATGCTAGAGATCTCATTTGTGCGCAGAAGTTCAATGGACTTACAGTGTTGCCACCTCTGCTCACTGATGATATGATTGCTGCCTACACTGCTGCTCTAGTTAGTGGTACTGCCACTGCTGGATGGACATTTGGTGCTGGCGCTGCTCTTCAAATACCTTTTGCTATGCAAATGGCATATAGGTTCAATGGCATTGGAGTTACCCAAAATGTTCTCTATGAGAACCAAAAACAAATCGCCAACCAATTTAACAAGGCGATTAGTCAAATTCAAGAATCACTTACAACAACATCAACTGCATTGGGCAAGCTGCAAGACGTTGTTAACCAGAATGCTCAAGCATTAAACACACTTGTTAAACAACTTAGCTCTAATTTTGGTGCAATTTCAAGTGTGCTAAATGATATCCTTTCGCGACTTGATAAAGTCGAGGCGGAGGTACAAATTGACAGGTTAATTACAGGCAGACTTCAAAGCCTTCAAACCTATGTAACACAACAACTAATCAGGGCTGCTGAAATCAGGGCTTCTGCTAATCTTGCTGCTACTAAAATGTCTGAGTGTGTTCTTGGACAATCAAAAAGAGTTGACTTTTGTGGAAAGGGCTACCACCTTATGTCCTTCCCACAAGCAGCCCCGCATGGTGTTGTCTTCCTACATGTCACGTATGTGCCATCC

CAGGAGAGGAACTTCACCACAGCGCCAGCAATTTGTCATGAAGGCAAAGCATACTTCCCTCGTGAAGGTGTTTTTGTGT
TTAATGGCACTTCTTGGTTTATTACACAGAGGAACTTCTTTTCTCCACAAATAATTACTACAGACAATACATTTGTCTC
AGGAAATTGTGATGTCGTTATTGGCATCATTAACAACACAGTTTATGATCCTCTGCAACCTGAGCTTGACTCATTCAAA
GAAGAGCTGGACAAGTACTTCAAAAATCATACATCACCAGATGTTGATCTTGGCGACATTTCAGGCATTAACGCTTCTG
TCGTCAACATTCAAAAAGAAATTGACCGCCTCAATGAGGTCGCTAAAAATTTAAATGAATCACTCATTGACCTTCAAGA
ATTGGGAAAATATGAGCAATATATTAAATGGCCTTGGTATGTTTGGCTCGGCTTCATTGCTGGACTAATTGCCATCGTC
ATGGTTACAATCTTGCTTTGTTGCATGACTAGTTGTTGCAGTTGCCTCAAGGGTGCATGCTCTTGTGGTTCTTGCTGCA
AGTTTGATGAGGATGACTCTGAGCCAGTTCTCAAGGGTGTCAAATTACATTACACATAA

>gi|89474553|gb|DQ412617.1|_SARS_coronavirus_isolate_CUHKtc42NP_spike_glycoprotein_(S)_gene_complete_cds

ATGTTTATTTTCTTATTATTTCTTACTCTCACTAGAGGTAGTGACCTTGACCGGTGCACCACTTTTGATGATGTT
CAAGCTCCTAATTACACTCAACATACTTCATCTATGAGGGGGGTTTACTATCCTGATGAAATTTTTAGATCAGACACTC
TTTATTTAACTCAGGATTTATTTCTTCCATTTTATTCTAATGTTACAGGGTTTCATACTATTAATCATACGTTTGGCAA
CCCTGTCATACCTTTTAAGGATGGTATTTATTTTGCTGCCACAGAGAAATCAAATGTTGTCCGTGGTTGGGTTTTTGGT
TCTACCATGAACAACAAGTCACAGTCGGTGATTATTATTAACAATTCTACTAATGTTGTTATACGAGCATGTAACTTTG
AATTGTGTGACAACCCTTTCTTTGCTGTTTCTAAACCCATGGGTACACAGACACATACTATGATATTCGATAATGCATT
TAATTGCACTTTCGAGTACATATCTGATGCCTTTTCGCTTGATGTTTCAGAAAAGTCAGGTAATTTTAAACACTTACGA
GAGTTTGTGTTTAAAAATAAAGATGGGTTTCTCTATGTTTATAAGGGCTATCAACCTATAGATGTAGTTCGTGATCTAC
CTTCTGGTTTTAACACTTTGAAACCTATTTTTAAGTTGCCTCTTGGTATTAACATTACAAATTTTAGAGCCATTCTTAC
AGCCTTTTCACCTGCTCAAGACATTTGGGGCACGTCAGCTGCAGCCTATTTTGTTGGCTATTTAAAGCCAACTACATTT
ATGCTCAAGTATGATGAAAATGGTACAATCACAGATGCTGTTGATTGTTCTCAAAATCCACTTGCTGAACTCAAATGCT
CTGTTAAGAGCTTTGAGATTGACAAAGGAATTTACCAGACCTCTAATTTCAGGGTTGTTCCCTCAGGAGATGTTGTGAG
ATTCCCTAATATTACAAACTTGTGTCCTTTTGGAGAGGTTTTTAATGCTACTAAATTCCCTTCTGTCTATGCATGGGAG
AGAAAAAAAATTTCTAATTGTGTTGTTGATTACTCTGTGCTCTACAACTCAACATTTTTTTCAACCTTTAAGTGCTATG
GCGTTTCTGCCACTAAGTTGAATGATCTTTGCTTCTCCAATGTCTATGCAGATTCTTTTGTAGTCAAGGGAGATGATGT
AAGACAAATAGCGCCAGGACAAACTGGTGTTATTGCTGATTATAATTATAAATTGCCAGATGATTTCATGGGTTGTGTC
CTTGCTTGGAATACTAGGAACATTGATGCTACTTCAACTGGTAATTATAATTATAAATATAGGTATCTTAGACATGGCA
AGCTTAGGCCCTTTGAGAGAGACATATCTAATGTGCCTTTCTCCCCTGATGGCAAACCTTGCACCCCACCTGCTCTTAA
TTGTTATTGGCCATTAAATGATTATGGTTTTTACACCACTACTGGCATTGGCTACCAACCTTACAGAGTTGTAGTACTT
TCTTTTGAACTTTTAAATGCACCGGCCACGGTTTGTGGACCAAAATTATCCACTGACCTTATTAAGAACCAGTGTGTCA
ATTTTAATTTTAATGGACTCACTGGTACTGGTGTGTTAACTCCTTCTTCAAAGAGATTTCAACCATTTCAACAATTTGG
CCGTGATGTTTCTGATTTCACTGATTCCGTTCGAGATCCTAAAACATCTGAAATATTAGACATTTCACCTTGCTCTTTT
GGGGGTGTAAGTGTAATTACACCTGGAACAAATGCTTCATCTGAAGTTGCTGTTCTATATCAAGATGTTAACTGCACTG
ATGTTTCTACAGCAATTCATGCAGATCAACTCACACCAGCTTGGCGCATATATTCTACTGGAAACAATGTATTCCAGAC
TCAAGCAGGCTGTCTTATAGGAGCTGAGCATGTCGACACTTCTTATGAGTGCGACATTCCTATTGGAGCTGGCATTTGT
GCTAGTTACCATACAGTTTCTTTATTACGTAGTACTAGCCAAAAATCTATTGTGGCTTATACTATGTCTTTAGGTGCTG
ATAGTTCAATTGCTTACTCTAATAACACCATTGCTATACCTACTAACTTTTCAATTAGCATTACTACAGAAGTAATGCC
TGTTTCTATGGCTAAAACCTCCGTAGATTGTAATATGTACATCTGCGGAGATTCTACTGAATGTGCTAATTTGCTTCTC
CAATATGGTAGCTTTTGCACACAACTAAATCGTGCACTCTCAGGTATTGCTGCTGAACAGGATCGCAACACACGTGAAG
TGTTCGCTCAAGTCAAACAAATGTACAAAACCCCAACTTTGAAATATTTTGGTGGTTTTAATTTTTCACAAATATTACC
TGACCCTCTAAAGCCAACTAAGAGGTCTTTTATTGAGGACTTGCTCTTTAATAAGGTGACACTCGCTGATGCTGGCTTC

ATGAAGCAATATGGCGAATGCCTAGGTGATATTAATGCTAGAGATCTCATTTGTGCGCAGAAGTTCAATGGACTTACAG
TGTTGCCACCTCTGCTCACTGATGATATGATTGCTGCCTACACTGCTGCTCTAGTTAGTGGTACTGCCACTGCTGGATG
GACATTTGGTGCTGGCGCTGCTCTTCAAATACCTTTTGCTATGCAAATGGCATATAGGTTCAATGGCATTGGAGTTACC
CAAAATGTTCTCTATGAGAACCAAAAACAAATCGCCAACCAATTTAACAAGGCGATTAGTCAAATTCAAGAATCACTTA
CAACAACATCAACTGCATTGGGCAAGCTGCAAGACGTTGTTAACCAGAATGCTCAAGCATTAAACACACTTGTTAAACA
ACTTAGCTCTAATTTTGGTGCAATTTCAAGTGTGCTAAATGATATCCTTTCGCGACTTGATAAAGTCGAGGCGGAGGTA
CAAATTGACAGGTTAATTACAGGCAGACTTCAAAGCCTTCAAACCTATGTAACACAACAACTAATCAGGGCTGCTGAAA
TCAGGGCTTCTGCTAATCTTGCTGCTACTAAAATGTCTGAGTGTGTTCTTGGACAATCAAAAAGAGTTGACTTTTGTGG
AAAGGGCTACCACCTTATGTCCTTCCCACAAGCAGCCCCGCATGGTGTTGTCTTCCTACATGTCACGTATGTGCCATCC
CAGGAGAGGAACTTCACCACAGCGCCAGCAATTTGTCATGAAGGCAAAGCATACTTCCCTCGTGAAGGTGTTTTTGTGT
TTAATGGCACTTCTTGGTTTATTACACAGAGGAACTTCTTTTCTCCACAAATAATTACTACAGACAATACATTTGTCTC
AGGAAATTGTGATGTCGTTATTGGCATCATTAACAACACAGTTTATGATCCTCTGCAACCTGAGCTTGACTCATTCAAA
GAAGAGCTGGACAAGTACTTCAAAAATCATACATCACCAGATGTTGATCTTGGCGACATTTCAGGCATTAACGCTTCTG
TCGTCAACATTCAAAAAGAAATTGACCGCCTCAATGAGGTCGCTAAAAATTTAAATGAATCACTCATTGACCTTCAAGA
ATTGGGAAAATATGAGCAATATATTAAATGGCCTTGGTATGTTTGGCTCGGCTTCATTGCTGGACTAATTGCCATCGTC
ATGGTTACAATCTTGCTTTGTTGCATGACTAGTTGTTGCAGTTGCCTCAAGGGTGCATGCTCTTGTGGTTCTTGCTGCA
AGTTTGATGAGGATGACTCTGAGCCAGTTCTCAAGGGTGTCAAATTACATTACACATAA

>gi|89474563|gb|DQ412624.1|_SARS_coronavirus_isolate_CUHKtc49NP_spike_glycoprotein_(S)_gene_complete_cds

ATGTTTATTTTCTTATTATTTCTTACTCTCACTAGTGGTAGTGACCTTGACCGGTGCACCACTTTTGATGATGTT
CAAGCTCCTAATTACACTCAACATACTTCATCTATGAGGGGGGTTTACTATCCTGATGAAATTTTTAGATCAGACACTC
TTTATTTAACTCAGGATTTATTTCTTCCATTTTATTCTAATGTTACAGGGTTTCATACTATTAATCATACGTTTGGCAA
CCCTGTCATACCTTTTAAGGATGGTATTTATTTTGCTGCCACAGAGAAATCAAATGTTGTCCGTGGTTGGGTTTTTGGT
TCTACCATGAACAACAAGTCACAGTCGGTGATTATTATTAACAATTCTACTAATGTTGTTATACGAGCATGTAACTTTG
AATTGTGTGACAACCCTTTCTTTGCTGTTTCTAAACCCATGGGTACACAGACACATACTATGATATTCGATAATGCATT
TAATTGCACTTTCGAGTACATATCTGATGCCTTTTCGCTTGATGTTTCAGAAAAGTCAGGTAATTTTAAACACTTACGA
GAGTTTGTGTTTAAAAATAAAGATGGGTTTCTCTATGTTTATAAGGGCTATCAACCTATAGATGTAGTTCGTGATCTAC
CTTCTGGTTTTAACACTTTGAAACCTATTTTTAAGTTGCCTCTTGGTATTAACATTACAAATTTTAGAGCCATTCTTAC
AGCCTTTTCACCTGCTCAAGACATTTGGGGCACGTCAGCTGCAGCCTATTTTGTTGGCTATTTAAAGCCAACTACATTT
ATGCTCAAGTATGATGAAAATGGTACAATCACAGATGCTGTTGATTGTTCTCAAAATCCACTTGCTGAACTCAAATGCT
CTGTTAAGAGCTTTGAGATTGACAAAGGAATTTACCAGACCTCTAATTTCAGGGTTGTTCCCTCAGGAGATGTTGTGAG
ATTCCCTAATATTACAAACTTGTGTCCTTTTGGAGAGGTTTTTAATGCTACTAAATTCCCTTCTGTCTATGCATGGGAG
AGAAAAAAAATTTCTAATTGTGTTGCTGATTACTCTGTGCTCTACAACTCAACATTTTTTTCAACCTTTAAGTGCTATG
GCGTTTCTGCCACTAAGTTGAATGATCTTTGCTTCTCCAATGTCTATGCAGATTCTTTTGTAGTCAAGGGAGATGATGT
AAGACAAATAGCGCCAGGACAAACTGGTGTTATTGCTGATTATAATTATAAATTGCCAGATGATTTCATGGGTTGTGTC
CTTGCTTGGAATACTAGGAACATTGATGCTACTTCAACTGGTAATTATAATTATAAATATAGGTATCTTAGACATGGCA
AGCTTAGGCCCTTTGAGAGAGACATATCTAATGTGCCTTTCTCCCCTGATGGCAAACCTTGCACCCCACCTGCTCTTAA
TTGTTATTGGCCATTAAATGATTATGGTTTTTACACCACTACTGGCATTGGCTACCAACCTTACAGAGTTGTAGTACTT
TCTTTTGAACTTTTAAATGCACCGGCCACGGTTTGTGGACCAAAATTATCCACTGACCTTATTAAGAACCAGTGTGTCA
ATTTTAATTTTAATGGACTCACTGGTACTGGTGTGTTAACTCCTTCTTCAAAGAGATTTCAACCATTTCAACAATTTGG
CCGTGATGTTTCTGATTTCACTGATTCCGTTCGAGATCCTAAAACATCTGAAATATTAGACATTTCACCTTGCTCTTTT

GGGGGTGTAAGTGTAATTACACCTGGAACAAATGCTTCATCTGAAGTTGCTGTTCTATATCAAGATGTTAACTGCACTG
ATGTTTCTACAGCAATTCATGCAGATCAACTCACACCAGCTTGGCGCATATATTCTACTGGAAACAATGTATTCCAGAC
TCAAGCAGGCTGTCTTATAGGAGCTGAGCATGTCGACACTTCTTATGAGTGCGACATTCCTATTGGAGCTGGCATTTGT
GCTAGTTACCATACAGTTTCTTTATTACGTAGTACTAGCCAAAAATCTATTGTGGCTTATACTATGTCTTTAGGTGCTG
ATAGTTCAATTGCTTACTCTAATAACACCATTGCTATACCTACTAACTTTTCAATTAGCATTACTACAGAAGTAATGCC
TGTTTCTATGGCTAAAACCTCCGTAGATTGTAATATGTACATCTGCGGAGATTCTACTGAATGTGCTAATTTGCTTCTC
CAATATGGTAGCTTTTGCACACAACTAAATCGTGCACTCTCAGGTATTGCTGCTGAACAGGATCGCAACACACGTGAAG
TGTTCGCTCAAGTCAAACAAATGTACAAAACCCCAACTTTGAAATATTTTGGTGGTTTTAATTTTTCACAAATATTACC
TGACCCTCTAAAGCCAACTAAGAGGTCTTTTATTGAGGACTTGCTCTTTAATAAGGTGACACTCGCTGATGCTGGCTTC
ATGAAGCAATATGGCGAATGCCTAGGTGATATTAATGCTAGAGATCTCATTTGTGCGCAGAAGTTCAATGGACTTACAG
TGTTGCCACCTCTGCTCACTGATGATATGATTGCTGCCTACACTGCTGCTCTAGTTAGTGGTACTGCCACTGCTGGATG
GACATTTGGTGCTGGCGCTGCTCTTCAAATACCTTTTGCTATGCAAATGGCATATAGGTTCAATGGCATTGGAGTTACC
CAAAATGTTCTCTATGAGAACCAAAAACAAATCGCCAACCAATTTAACAAGGCGATTAGTCAAATTCAAGAATCACTTA
CAACAACATCAACTGCATTGGGCAAGCTGCAAGACGTTGTTAACCAGAATGCTCAAGCATTAAACACACTTGTTAAACA
ACTTAGCTCTAATTTTGGTGCAATTTCAAGTGTGCTAAATGATATCCTTTCGCGACTTGATAAAGTCGAGGCGGAGGTA
CAAATTGACAGGTTAATTACAGGCAGACTTCAAAGCCTTCAAACCTATGTAACACAACAACTAATCAGGGCTGCTGAAA
TCAGGGCTTCTGCTAATCTTGCTGCTACTAAAATGTCTGAGTGTGTTCTTGGACAATCAAAAAGAGTTGACTTTTGTGG
AAAGGGCTACCACCTTATGTCCTTCCCACAAGCAGCCCCGCATGGTGTTGTCTTCCTACATGTCACGTATGTGCCATCC
CAGGAGAGGAACTTCACCACAGCGCCAGCAATTTGTCATGAAGGCAAAGCATACTTCCCTCGTGAAGGTGTTTTTGTGT
TTAATGGCACTTCTTGGTTTATTACACAGAGGAACTTCTTTTCTCCACAAATAATTACTACAGACAATACATTTGTCTC
AGGAAATTGTGATGTCGTTATTGGCATCATTAACAACACAGTTTATGATCCTCTGCAACCTGAGCTTGACTCATTCAAA
GAAGAGCTGGACAAGTACTTCAAAAATCATACATCACCAGATGTTGATCTTGGCGACATTTCAGGCATTAACGCTTCTG
TCGTCAACATTCAAAAAGAAATTGACCGCCTCAATGAGGTCGCTAAAAATTTAAATGAATCACTCATTGACCTTCAAGA
ATTGGGAAAATATGAGCAATATATTAAATGGCCTTGGTATGTTTGGCTCGGCTTCATTGCTGGACTAATTGCCATCGTC
ATGGTTACAATCTTGCTTTGTTGCATGACTAGTTGTTGCAGTTGCCTCAAGGGTGCATGCTCTTGTGGTTCTTGCTGCA
AGTTTGATGAGGATGACTCTGAGCCAGTTCTCAAGGGTGTCAAATTACATTACACATAA

>gi|89474565|gb|DQ412625.1|_SARS_coronavirus_isolate_CUHKtc50NP_spike_glycoprotein_(S)_gene_complete_cds

ATGTTTATTTTCTTATTATTTCTTACTCTCACTAGTGGTAGTGACCTTGACCGGTGCACCACTTTTGATGATGTT
CAAGCTCCTAATTACACTCAACATACTTCATCTATGAGGGGGGTTTACTATCCTGATGAAATTTTTAGATCAGACACTC
TTTATTTAACTCAGGATTTATTTCTTCCATTTTATTCTAATGTTACAGGGTTTCATACTATTAATCATACGTTTGGCAA
CCCTGTCATACCTTTTAAGGATGGTATTTATTTTGCTGCCACAGAGAAATCAAATGTTGTCCGTGGTTGGGTTTTTGGT
TCTACCATGAACAACAAGTCACAGTCGGTGATTATTATTAACAATTCTACTAATGTTGTTATACGAGCATGTAACTTTG
AATTGTGTGACAACCCTTTCTTTGCTGTTTCTAAACCCATGGGTACACAGACACATACTATGATATTCGATAATGCATT
TAATTGCACTTTCGAGTACATATCTGATGCCTTTTCGCTTGATGTTTCAGAAAAGTCAGGTAATTTTAAACACTTACGA
GAGTTTGTGTTTAAAAATAAAGATGGGTTTCTCTATGTTTATAAGGGCTATCAACCTATAGATGTAGTTCGTGATCTAC
CTTCTGGTTTTAACACTTTGAAACCTATTTTTAAGTTGCCTCTTGGTATTAACATTACAAATTTTAGAGCCATTCTTAC
AGCCTTTTCACCTGCTCAAGACATTTGGGGCACGTCAGCTGCAGCCTATTTTGTTGGCTATTTAAAGCCAACTACATTT
ATGCTCAAGTATGATGAAAATGGTACAATCACAGATGCTGTTGATTGTTCTCAAAATCCACTTGCTGAACTCAAATGCT
CTGTTAAGAGCTTTGAGATTGACAAAGGAATTTACCAGACCTCTAATTTCAGGGTTGTTCCCTCAGGAGATGTTGTGAG
ATTCCCTAATATTACAAACTTGTGTCCTTTTGGAGAGGTTTTTAATGCTACTAAATTCCCTTCTGTCTATGCATGGGAG

AGAAAAAAAATTTCTAATTGTGTTGCTGATTACTCTGTGCTCTACAACTCAACATTTTTTTCAACCTTTAAGTGCTATG
GCGTTTCTGCCACTAAGTTGAATGATCTTTGCTTCTCCAATGTCTATGCAGATTCTTTTGTAGTCAAGGGAGATGATGT
AAGACAAATAGCGCCAGGACAAACTGGTGTTATTGCTGATTATAATTATAAATTGCCAGATGATTTCATGGGTTGTGTC
CTTGCTTGGAATACTAGGAACATTGATGCTACTTCAACTGGTAATTATAATTATAAATATAGGTATCTTAGACATGGCA
AGCTTAGGCCCTTTGAGAGAGACATATCTAATGTGCCTTTCTCCCCTGATGGCAAACCTTGCACCCCACCTGCTCTTAA
TTGTTATTGGCCATTAAATGATTATGGTTTTTACACCACTACTGGCATTGGCTACCAACCTTACAGAGTTGTAGTACTT
TCTTTTGAACTTTTAAATGCACCGGCCACGGTTTGTGGACCAAAATTATCCACTGACCTTATTAAGAACCAGTGTGTCA
ATTTTAATTTTAATGGACTCACTGGTACTGGTGTGTTAACTCCTTCTTCAAAGAGATTTCAACCATTTCAACAATTTGG
CCGTGATGTTTCTGATTTCACTGATTCCGTTCGAGATCCTAAAACATCTGAAATATTAGACATTTCACCTTGCTCTTTT
GGGGGTGTAAGTGTAATTACACCTGGAACAAATGCTTCATCTGAAGTTGCTGTTCTATATCAAGATGTTAACTGCACTG
ATGTTTCTACAGCAATTCATGCAGATCAACTCACACCAGCTTGGCGCATATATTCTACTGGAAACAATGTATTCCAGAC
TCAAGCAGGCTGTCTTATAGGAGCTGAGCATGTCGACACTTCTTATGAGTGCGACATTCCTATTGGAGCTGGCATTTGT
GCTAGTTACCATACAGTTTCTTTATTACGTAGTACTAGCCAAAAATCTATTGTGGCTTATACTATGTCTTTAGGTGCTG
ATAGTTCAATTGCTTACTCTAATAACACCATTGCTATACCTACTAACTTTTCAATTAGCATTACTACAGAAGTAATGCC
TGTTTCTATGGCTAAAACCTCCGTAGATTGTAATATGTACATCTGCGGAGATTCTACTGAATGTGCTAATTTGCTTCTC
CAATATGGTAGCTTTTGCACACAACTAAATCGTGCACTCTCAGGTATTGCTGCTGAACAGGATCGCAACACACGTGAAG
TGTTCGCTCAAGTCAAACAAATGTACAAAACCCCAACTTTGAAATATTTTGGTGGTTTTAATTTTTCACAAATATTACC
TGACCCTCTAAAGCCAACTAAGAGGTCTTTTATTGAGGACTTGCTCTTTAATAAGGTGACACTCGCTGATGCTGGCTTC
ATGAAGCAATATGGCGAATGCCTAGGTGATATTAATGCTAGAGATCTCATTTGTGCGCAGAAGTTCAATGGACTTACAG
TGTTGCCACCTCTGCTCACTGATGATATGATTGCTGCCTACACTGCTGCTCTAGTTAGTGGTACTGCCACTGCTGGATG
GACATTTGGTGCTGGCGCTGCTCTTCAAATACCTTTTGCTATGCAAATGGCATATAGGTTCAATGGCATTGGAGTTACC
CAAAATGTTCTCTATGAGAACCAAAAACAAATCGCCAACCAATTTAACAAGGCGATTAGTCAAATTCAAGAATCACTTA
CAACAACATCAACTGCATTGGGCAAGCTGCAAGACGTTGTTAACCAGAATGCTCAAGCATTAAACACACTTGTTAAACA
ACTTAGCTCTAATTTTGGTGCAATTTCAAGTGTGCTAAATGATATCCTTTCGCGACTTGATAAAGTCGAGGCGGAGGTA
CAAATTGACAGGTTAATTACAGGCAGACTTCAAAGCCTTCAAACCTATGTAACACAACAACTAATCAGGGCTGCTGAAA
TCAGGGCTTCTGCTAATCTTGCTGCTACTAAAATGTCTGAGTGTGTTCTTGGACAATCAAAAAGAGTTGACTTTTGTGG
AAAGGGCTACCACCTTATGTCCTTCCCACAAGCAGCCCCGCATGGTGTTGTCTTCCTACATGTCACGTATGTGCCATCC
CAGGAGAGGAACTTCACCACAGCGCCAGCAATTTGTCATGAAGGCAAAGCATACTTCCCTCGTGAAGGTGTTTTTGTGT
TTAATGGCACTTCTTGGTTTATTACACAGAGGAACTTCTTTTCTCCACAAATAATTACTACAGACAATACATTTGTCTC
AGGAAATTGTGATGTCGTTATTGGCATCATTAACAACACAGTTTATGATCCTCTGCAACCTGAGCTTGACTCATTCAAA
GAAGAGCTGGACAAGTACTTCAAAAATCATACATCACCAGATGTTGATCTTGGCGACATTTCAGGCATTAACGCTTCTG
TCGTCAACATTCAAAAAGAAATTGACCGCCTCAATGAGGTCGCTAAAAATTTAAATGAATCACTCATTGACCTTCAAGA
ATTGGGAAAATATGAGCAATATATTAAATGGCCTTGGTATGTTTGGCTCGGCTTCATTGCTGGACTAATTGTCATCGTC
ATGGTTACAATCTTGCTTTGTTGCATGACTAGTTGTTGCAGTTGCCTCAAGGGTGCATGCTCTTGTGGTTCTTGCTGCA
AGTTTGATGAGGATGACTCTGAGCCAGTTCTCAAGGGTGTCAAATTACATTACACATAA

>gi|89474567|gb|DQ412626.1|_SARS_coronavirus_isolate_CUHKtc51L_spike_glycoprotein_(S)_gene_complete_cds

ATGTTTATTTTCTTATTATTTCTTACTCTCACTAGTGGTAGTGACCTTGACCGGTGCACCACTTTTGATGATGTT
CAAGCTCCTAATTACACTCAACATACTTCATCTATGAGGGGGGTTTACTATCCTGATGAAATTTTTAGATCAGACACTC
TTTATTTAACTCAGGATTTATTTCTTCCATTTTATTCTAATGTTACAGGGTTTCATACTATTAATCATACGTTTGGCAA
CCCTGTCATACCTTTTAAGGATGGTATTTATTTTGCTGCCACAGAGAAATCAAATGTTGTCCGTGGTTGGGTTTTTGGT

TCTACCATGAACAACAAGTCACAGTCGGTGATTATTATTAACAATTCTACTAATGTTGTTATACGAGCATGTAACTTTG
AATTGTGTGACAACCCTTTCTTTGCTGTTTCTAAACCCATGGGTACACAGACACATACTATGATATTCGATAATGCATT
TAATTGCACTTTCGAGTACATATCTGATGCCTTTTCGCTTGATGTTTCAGAAAAGTCAGGTAATTTTAAACACTTACGA
GAGTTTGTGTTTAAAAATAAAGATGGGTTTCTCTATGTTTATAAGGGCTATCAACCTATAGATGTAGTTCGTGATCTAC
CTTCTGGTTTTAACACTTTGAAACCTATTTTTAAGTTGCCTCTTGGTATTAACATTACAAATTTTAGAGCCATTCTTAC
AGCCTTTTCACCTGCTCAAGACATTTGGGGCACGTCAGCTGCAGCCTATTTTGTTGGCTATTTAAAGCCAACTACATTT
ATGCTCAAGTATGATGAAAATGGTACAATCACAGATGCTGTTGATTGTTCTCAAAATCCACTTGCTGAACTCAAATGCT
CTGTTAAGAGCTTTGAGATTGACAAAGGAATTTACCAGACCTCTAATTTCAGGGTTGTTCCCTCAGGAGATGTTGTGAG
ATTCCCTAATATTACAAACTTGTGTCCTTTTGGAGAGGTTTTTAATGCTACTAAATTCCCTTCTGTCTATGCATGGGAG
AGAAAAAAAAATTTCTAATTGTGTTGCTGATTACTCTGTGCTCTACAACTCAACATTTTTTTCAACCTTTAAGTGCTATG
GCGTTTCTGCCACTAAGTTGAATGATCTTTGCTTCTCCAATGTCTATGCAGATTCTTTTGTAGTCAAGGGAGATGATGT
AAGACAAATAGCGCCAGGACAAACTGGTGTTATTGCTGATTATAATTATAAATTGCCAGATGATTTCATGGGTTGTGTC
CTTGCTTGGAATACTAGGAACATTGATGCTACTTCAACTGGTAATTATAATTATAAATATAGGTATCTTAGACATGGCA
AGCTTAGGCCCTTTGAGAGAGACATATCTAATGTGCCTTTCTCCCCTGATGGCAAACCTTGCACCCCACCTGCTCTTAA
TTGTTATTGGCCATTAAATGATTATGGTTTTTACACCACTACTGGCATTGGCTACCAACCTTACAGAGTTGTAGTACTT
TCTTTTGAACTTTTAAATGCACCGGCCACGGTTTGTGGACCAAAATTATCCACTGACCTTATTAAGAACCAGTGTGTCA
ATTTTAATTTTAATGGACTCACTGGTACTGGTGTGTTAACTCCTTCTTCAAAGAGATTTCAACCATTTCAACAATTTGG
CCGTGATGTTTCTGATTTCACTGATTCCGTTCGAGATCCTAAAACATCTGAAATATTAGACATTTCACCTTGCTCTTTT
GGGGGTGTAAGTGTAATTACACCTGGAACAAATGCTTCATCTGAAGTTGCTGTTCTATATCAAGATGTTAACTGCACTG
ATGTTTCTACAGCAATTCATGCAGATCAACTCACACCAGCTTGGCGCATATATTCTACTGGAAACAATGTATTCCAGAC
TCAAGCAGGCTGTCTTATAGGAGCTGAGCATGTCGACACTTCTTATGAGTGCGACATTCCTATTGGAGCTGGCATTTGT
GCTAGTTACCATACAGTTTCTTTATTACGTAGTACTAGCCAAAAATCTATTGTGGCTTATACTATGTCTTTAGGTGCTG
ATAGTTCAATTGCTTACTCTAATAACACCATTGCTATACCTACTAACTTTTCAATTAGCATTACTACAGAAGTAATGCC
TGTTTCTATGGCTAAAACCTCCGTAGATTGTAATATGTACATCTGCGGAGATTCTACTGAATGTGCTAATTTGCTTCTC
CAATATGGTAGCTTTTGCACACAACTAAATCGTGCACTCTCAGGTATTGCTGCTGAACAGGATCGCAACACACGTGAAG
TGTTCGCTCAAGTCAAACAAATGTACAAAACCCCAACTTTGAAATATTTTGGTGGTTTTAATTTTTCACAAATATTACC
TGACCCTCTAAAGCCAACTAAGAGGTCTTTTATTGAGGACTTGCTCTTTAATAAGGTGACACTCGCTGATGCTGGCTTC
ATGAAGCAATATGGCGAATGCCTAGGTGATATTAATGCTAGAGATCTCATTTGTGCGCAGAAGTTCAATGGACTTACAG
TGTTGCCACCTCTGCTCACTGATGATATGATTGCTGCCTACACTGCTGCTCTAGTTAGTGGTACTGCCACTGCTGGATG
GACATTTGGTGCTGGCGCTGCTCTTCAAATACCTTTTGCTATGCAAATGGCATATAGGTTCAATGGCATTGGAGTTACC
CAAAATGTTCTCTATGAGAACCAAAAACAAATCGCCAACCAATTTAACAAGGCGATTAGTCAAATTCAAGAATCACTTA
CAACAACATCAACTGCATTGGGCAAGCTGCAAGACGTTGTTAACCAGAATGCTCAAGCATTAAACACACTTGTTAAACA
ACTTAGCTCTAATTTTGGTGCAATTTCAAGTGTGCTAAATGATATCCTTTCGCGACTTGATAAAGTCGAGGCGGAGGTA
CAAATTGACAGGTTAATTACAGGCAGACTTCAAAGCCTTCAAACCTATGTAACACAACAACTAATCAGGGCTGCTGAAA
TCAGGGCTTCTGCTAATCTTGCTGCTACTAAAATGTCTGAGTGTGTTCTTGGACAATCAAAAAGAGTTGACTTTTGTGG
AAAGGGCTACCACCTTATGTCCTTCCCACAAGCAGCCCCGCATGGTGTTGTCTTCCTACATGTCACGTATGTGCCATCC
CAGGAGAGGAACTTCACCACAGCGCCAGCAATTTGTCATGAAGGCAAAGCATACTTCCCTCGTGAAGGTGTTTTTGTGT
TTAATGGCACTTCCTGGTTTATTACACAGAGGAACTTCTTTTCTCCACAAATAATTACTACAGACAATACATTTGTCTC
AGGAAATTGTGATGTCGTTATTGGCATCATTAACAACACAGTTTATGATCCTCTGCAACCTGAGCTTGACTCATTCAAA
GAAGAGCTGGACAAGTACTTCAAAAATCATACATCACCAGATGTTGATCTTGGCGACATTTCAGGCATTAACGCTTCTG
TCGTCAACATTCAAAAAGAAATTGACCGCCTCAATGAGGTCGCTAAAAATTTAAATGAATCACTCATTGACCTTCAAGA
ATTGGGAAAATATGAGCAATATATTAAATGGCCTTGGTATGTTTGGCTCGGCTTCATTGCTGGACTAATTGTCATCGTC
ATGGTTACAATCTTGCTTTGTTGCATGACTAGTTGTTGCAGTTGCCTCAAGGGTGCATGCTCTTGTGGTTCTTGCTGCA
AGTTTGATGAGGATGACTCTGAGCCAGTTCTCAAGGGTGTCAAATTACATTACACATAA

>gi|30271926|ref|NC_004718.3| SARS coronavirus, complete genome

ATGTTTATTTTCTTATTATTTCTTACTCTCACTAGTGGTAGTGACCTTGACCGGTGCACCACTTTTGATGATGTT
CAAGCTCCTAATTACACTCAACATACTTCATCTATGAGGGGGGTTTACTATCCTGATGAAATTTTTAGATCAGACACTC
TTTATTTAACTCAGGATTTATTTCTTCCATTTTATTCTAATGTTACAGGGTTTCATACTATTAATCATACGTTTGGCAA
CCCTGTCATACCTTTTAAGGATGGTATTTATTTTGCTGCCACAGAGAAATCAAATGTTGTCCGTGGTTGGGTTTTTGGT
TCTACCATGAACAACAAGTCACAGTCGGTGATTATTATTAACAATTCTACTAATGTTGTTATACGAGCATGTAACTTTG
AATTGTGTGACAACCCTTTCTTTGCTGTTTCTAAACCCATGGGTACACAGACACATACTATGATATTCGATAATGCATT
TAATTGCACTTTCGAGTACATATCTGATGCCTTTTCGCTTGATGTTTCAGAAAAGTCAGGTAATTTTAAACACTTACGA
GAGTTTGTGTTTAAAAATAAAGATGGGTTTCTCTATGTTTATAAGGGCTATCAACCTATAGATGTAGTTCGTGATCTAC
CTTCTGGTTTTAACACTTTGAAACCTATTTTTAAGTTGCCTCTTGGTATTAACATTACAAATTTTAGAGCCATTCTTAC
AGCCTTTTCACCTGCTCAAGACATTTGGGGCACGTCAGCTGCAGCCTATTTTGTTGGCTATTTAAAGCCAACTACATTT
ATGCTCAAGTATGATGAAAATGGTACAATCACAGATGCTGTTGATTGTTCTCAAAATCCACTTGCTGAACTCAAATGCT
CTGTTAAGAGCTTTGAGATTGACAAAGGAATTTACCAGACCTCTAATTTCAGGGTTGTTCCCTCAGGAGATGTTGTGAG
ATTCCCTAATATTACAAACTTGTGTCCTTTTGGAGAGGTTTTTAATGCTACTAAATTCCCTTCTGTCTATGCATGGGAG
AGAAAAAAAATTTCTAATTGTGTTGCTGATTACTCTGTGCTCTACAACTCAACATTTTTTTCAACCTTTAAGTGCTATG
GCGTTTCTGCCACTAAGTTGAATGATCTTTGCTTCTCCAATGTCTATGCAGATTCTTTTGTAGTCAAGGGAGATGATGT
AAGACAAATAGCGCCAGGACAAACTGGTGTTATTGCTGATTATAATTATAAATTGCCAGATGATTTCATGGGTTGTGTC
CTTGCTTGGAATACTAGGAACATTGATGCTACTTCAACTGGTAATTATAATTATAAATATAGGTATCTTAGACATGGCA
AGCTTAGGCCCTTTGAGAGAGACATATCTAATGTGCCTTTCTCCCCTGATGGCAAACCTTGCACCCCACCTGCTCTTAA
TTGTTATTGGCCATTAAATGATTATGGTTTTTACACCACTACTGGCATTGGCTACCAACCTTACAGAGTTGTAGTACTT
TCTTTTGAACTTTTAAATGCACCGGCCACGGTTTGTGGACCAAAATTATCCACTGACCTTATTAAGAACCAGTGTGTCA
ATTTTAATTTTAATGGACTCACTGGTACTGGTGTGTTAACTCCTTCTTCAAAGAGATTTCAACCATTTCAACAATTTGG
CCGTGATGTTTCTGATTTCACTGATTCCGTTCGAGATCCTAAAACATCTGAAATATTAGACATTTCACCTTGCGCTTTT
GGGGGTGTAAGTGTAATTACACCTGGAACAAATGCTTCATCTGAAGTTGCTGTTCTATATCAAGATGTTAACTGCACTG
ATGTTTCTACAGCAATTCATGCAGATCAACTCACACCAGCTTGGCGCATATATTCTACTGGAAACAATGTATTCCAGAC
TCAAGCAGGCTGTCTTATAGGAGCTGAGCATGTCGACACTTCTTATGAGTGCGACATTCCTATTGGAGCTGGCATTTGT
GCTAGTTACCATACAGTTTCTTTATTACGTAGTACTAGCCAAAAATCTATTGTGGCTTATACTATGTCTTTAGGTGCTG
ATAGTTCAATTGCTTACTCTAATAACACCATTGCTATACCTACTAACTTTTCAATTAGCATTACTACAGAAGTAATGCC
TGTTTCTATGGCTAAAACCTCCGTAGATTGTAATATGTACATCTGCGGAGATTCTACTGAATGTGCTAATTTGCTTCTC
CAATATGGTAGCTTTTGCACACAACTAAATCGTGCACTCTCAGGTATTGCTGCTGAACAGGATCGCAACACACGTGAAG
TGTTCGCTCAAGTCAAACAAATGTACAAAACCCCAACTTTGAAATATTTTGGTGGTTTTAATTTTTCACAAATATTACC
TGACCCTCTAAAGCCAACTAAGAGGTCTTTTATTGAGGACTTGCTCTTTAATAAGGTGACACTCGCTGATGCTGGCTTC
ATGAAGCAATATGGCGAATGCCTAGGTGATATTAATGCTAGAGATCTCATTTGTGCGCAGAAGTTCAATGGACTTACAG
TGTTGCCACCTCTGCTCACTGATGATATGATTGCTGCCTACACTGCTGCTCTAGTTAGTGGTACTGCCACTGCTGGATG
GACATTTGGTGCTGGCGCTGCTCTTCAAATACCTTTTGCTATGCAAATGGCATATAGGTTCAATGGCATTGGAGTTACC
CAAAATGTTCTCTATGAGAACCAAAAACAAATCGCCAACCAATTTAACAAGGCGATTAGTCAAATTCAAGAATCACTTA
CAACAACATCAACTGCATTGGGCAAGCTGCAAGACGTTGTTAACCAGAATGCTCAAGCATTAAACACACTTGTTAAACA
ACTTAGCTCTAATTTTGGTGCAATTTCAAGTGTGCTAAATGATATCCTTTCGCGACTTGATAAAGTCGAGGCGGAGGTA
CAAATTGACAGGTTAATTACAGGCAGACTTCAAAGCCTTCAAACCTATGTAACACAACAACTAATCAGGGCTGCTGAAA
TCAGGGCTTCTGCTAATCTTGCTGCTACTAAAATGTCTGAGTGTGTTCTTGGACAATCAAAAAGAGTTGACTTTTGTGG
AAAGGGCTACCACCTTATGTCCTTCCCACAAGCAGCCCCGCATGGTGTTGTCTTCCTACATGTCACGTATGTGCCATCC
CAGGAGAGGAACTTCACCACAGCGCCAGCAATTTGTCATGAAGGCAAAGCATACTTCCCTCGTGAAGGTGTTTTTGTGT

TTAATGGCACTTCTTGGTTTATTACACAGAGGAACTTCTTTTCTCCACAAATAATTACTACAGACAATACATTTGTCTC
AGGAAATTGTGATGTCGTTATTGGCATCATTAACAACACAGTTTATGATCCTCTGCAACCTGAGCTTGACTCATTCAAA
GAAGAGCTGGACAAGTACTTCAAAAATCATACATCACCAGATGTTGATCTTGGCGACATTTCAGGCATTAACGCTTCTG
TCGTCAACATTCAAAAAGAAATTGACCGCCTCAATGAGGTCGCTAAAAATTTAAATGAATCACTCATTGACCTTCAAGA
ATTGGGAAAATATGAGCAATATATTAAATGGCCTTGGTATGTTTGGCTCGGCTTCATTGCTGGACTAATTGCCATCGTC
ATGGTTACAATCTTGCTTTGTTGCATGACTAGTTGTTGCAGTTGCCTCAAGGGTGCATGCTCTTGTGGTTCTTGCTGCA
AGTTTGATGAGGATGACTCTGAGCCAGTTCTCAAGGGTGTCAAATTACATTACACATAA

>gi|30027617|gb|AY278741.1| SARS coronavirus Urbani, complete genome

ATGTTTATTTTCTTATTATTTCTTACTCTCACTAGTGGTAGTGACCTTGACCGGTGCACCACTTTTGATGATGTT
CAAGCTCCTAATTACACTCAACATACTTCATCTATGAGGGGGGTTTACTATCCTGATGAAATTTTTAGATCAGACACTC
TTTATTTAACTCAGGATTTATTTCTTCCATTTTATTCTAATGTTACAGGGTTTCATACTATTAATCATACGTTTGGCAA
CCCTGTCATACCTTTTAAGGATGGTATTTATTTTGCTGCCACAGAGAAATCAAATGTTGTCCGTGGTTGGGTTTTTGGT
TCTACCATGAACAACAAGTCACAGTCGGTGATTATTATTAACAATTCTACTAATGTTGTTATACGAGCATGTAACTTTG
AATTGTGTGACAACCCTTTCTTTGCTGTTTCTAAACCCATGGGTACACAGACACATACTATGATATTCGATAATGCATT
TAATTGCACTTTCGAGTACATATCTGATGCCTTTTCGCTTGATGTTTCAGAAAAGTCAGGTAATTTTAAACACTTACGA
GAGTTTGTGTTTAAAAATAAAGATGGGTTTCTCTATGTTTATAAGGGCTATCAACCTATAGATGTAGTTCGTGATCTAC
CTTCTGGTTTTAACACTTTGAAACCTATTTTTAAGTTGCCTCTTGGTATTAACATTACAAATTTTAGAGCCATTCTTAC
AGCCTTTTCACCTGCTCAAGACATTTGGGGCACGTCAGCTGCAGCCTATTTTGTTGGCTATTTAAAGCCAACTACATTT
ATGCTCAAGTATGATGAAAATGGTACAATCACAGATGCTGTTGATTGTTCTCAAAATCCACTTGCTGAACTCAAATGCT
CTGTTAAGAGCTTTGAGATTGACAAAGGAATTTACCAGACCTCTAATTTCAGGGTTGTTCCCTCAGGAGATGTTGTGAG
ATTCCCTAATATTACAAACTTGTGTCCTTTTGGAGAGGTTTTTAATGCTACTAAATTCCCTTCTGTCTATGCATGGGAG
AGAAAAAAAATTTCTAATTGTGTTGCTGATTACTCTGTGCTCTACAACTCAACATTTTTTTCAACCTTTAAGTGCTATG
GCGTTTCTGCCACTAAGTTGAATGATCTTTGCTTCTCCAATGTCTATGCAGATTCTTTTGTAGTCAAGGGAGATGATGT
AAGACAAATAGCGCCAGGACAAACTGGTGTTATTGCTGATTATAATTATAAATTGCCAGATGATTTCATGGGTTGTGTC
CTTGCTTGGAATACTAGGAACATTGATGCTACTTCAACTGGTAATTATAATTATAAATATAGGTATCTTAGACATGGCA
AGCTTAGGCCCTTTGAGAGAGACATATCTAATGTGCCTTTCTCCCCTGATGGCAAACCTTGCACCCCACCTGCTCTTAA
TTGTTATTGGCCATTAAATGATTATGGTTTTTACACCACTACTGGCATTGGCTACCAACCTTACAGAGTTGTAGTACTT
TCTTTTGAACTTTTAAATGCACCGGCCACGGTTTGTGGACCAAAATTATCCACTGACCTTATTAAGAACCAGTGTGTCA
ATTTTAATTTTAATGGACTCACTGGTACTGGTGTGTTAACTCCTTCTTCAAAGAGATTTCAACCATTTCAACAATTTGG
CCGTGATGTTTCTGATTTCACTGATTCCGTTCGAGATCCTAAAACATCTGAAATATTAGACATTTCACCTTGCTCTTTT
GGGGGTGTAAGTGTAATTACACCTGGAACAAATGCTTCATCTGAAGTTGCTGTTCTATATCAAGATGTTAACTGCACTG
ATGTTTCTACAGCAATTCATGCAGATCAACTCACACCAGCTTGGCGCATATATTCTACTGGAAACAATGTATTCCAGAC
TCAAGCAGGCTGTCTTATAGGAGCTGAGCATGTCGACACTTCTTATGAGTGCGACATTCCTATTGGAGCTGGCATTTGT
GCTAGTTACCATACAGTTTCTTTATTACGTAGTACTAGCCAAAAATCTATTGTGGCTTATACTATGTCTTTAGGTGCTG
ATAGTTCAATTGCTTACTCTAATAACACCATTGCTATACCTACTAACTTTTCAATTAGCATTACTACAGAAGTAATGCC
TGTTTCTATGGCTAAAACCTCCGTAGATTGTAATATGTACATCTGCGGAGATTCTACTGAATGTGCTAATTTGCTTCTC
CAATATGGTAGCTTTTGCACACAACTAAATCGTGCACTCTCAGGTATTGCTGCTGAACAGGATCGCAACACACGTGAAG
TGTTCGCTCAAGTCAAACAAATGTACAAAACCCCAACTTTGAAATATTTTGGTGGTTTTAATTTTTCACAAATATTACC
TGACCCTCTAAAGCCAACTAAGAGGTCTTTTATTGAGGACTTGCTCTTTAATAAGGTGACACTCGCTGATGCTGGCTTC
ATGAAGCAATATGGCGAATGCCTAGGTGATATTAATGCTAGAGATCTCATTTGTGCGCAGAAGTTCAATGGACTTACAG
TGTTGCCACCTCTGCTCACTGATGATATGATTGCTGCCTACACTGCTGCTCTAGTTAGTGGTACTGCCACTGCTGGATG

GACATTTGGTGCTGGCGCTGCTCTTCAAATACCTTTTGCTATGCAAATGGCATATAGGTTCAATGGCATTGGAGTTACC
CAAAATGTTCTCTATGAGAACCAAAAACAAATCGCCAACCAATTTAACAAGGCGATTAGTCAAATTCAAGAATCACTTA
CAACAACATCAACTGCATTGGGCAAGCTGCAAGACGTTGTTAACCAGAATGCTCAAGCATTAAACACACTTGTTAAACA
ACTTAGCTCTAATTTTGGTGCAATTTCAAGTGTGCTAAATGATATCCTTTCGCGACTTGATAAAGTCGAGGCGGAGGTA
CAAATTGACAGGTTAATTACAGGCAGACTTCAAAGCCTTCAAACCTATGTAACACAACAACTAATCAGGGCTGCTGAAA
TCAGGGCTTCTGCTAATCTTGCTGCTACTAAAATGTCTGAGTGTGTTCTTGGACAATCAAAAAGAGTTGACTTTTGTGG
AAAGGGCTACCACCTTATGTCCTTCCCACAAGCAGCCCCGCATGGTGTTGTCTTCCTACATGTCACGTATGTGCCATCC
CAGGAGAGGAACTTCACCACAGCGCCAGCAATTTGTCATGAAGGCAAAGCATACTTCCCTCGTGAAGGTGTTTTTGTGT
TTAATGGCACTTCTTGGTTTATTACACAGAGGAACTTCTTTTCTCCACAAATAATTACTACAGACAATACATTTGTCTC
AGGAAATTGTGATGTCGTTATTGGCATCATTAACAACACAGTTTATGATCCTCTGCAACCTGAGCTCGACTCATTCAAA
GAAGAGCTGGACAAGTACTTCAAAAATCATACATCACCAGATGTTGATCTTGGCGACATTTCAGGCATTAACGCTTCTG
TCGTCAACATTCAAAAAGAAATTGACCGCCTCAATGAGGTCGCTAAAAATTTAAATGAATCACTCATTGACCTTCAAGA
ATTGGGAAAATATGAGCAATATATTAAATGGCCTTGGTATGTTTGGCTCGGCTTCATTGCTGGACTAATTGCCATCGTC
ATGGTTACAATCTTGCTTTGTTGCATGACTAGTTGTTGCAGTTGCCTCAAGGGTGCATGCTCTTGTGGTTCTTGCTGCA
AGTTTGATGAGGATGACTCTGAGCCAGTTCTCAAGGGTGTCAAATTACATTACACATAA

>gi|38505482|gb|AY485277.1| SARS coronavirus Sino1-11, complete genome

ATGTTTATTTTCTTATTATTTCTTACTCTCACTAGTGGTAGTGACCTTGACCGGTGCACCACTTTTGATGATGTT
CAAGCTCCTAATTACACTCAACATACTTCATCTATGAGGGGGGTTTACTATCCTGATGAAATTTTTAGATCAGACACTC
TTTATTTAACTCAGGATTTATTTCTTCCATTTTATTCTAATGTTACAGGGTTTCATACTATTAATCATACGTTTGGCAA
CCCTGTCATACCTTTTAAGGATGGTATTTATTTTGCTGCCACAGAGAAATCAAATGTTGTCCGTGGTTGGGTTTTTGGT
TCTACCATGAACAACAAGTCACAGTCGGTGATTATTATTAACAATTCTACTAATGTTGTTATACGAGCATGTAACTTTG
AATTGTGTGACAACCCTTTCTTTGCTGTTTCTAAACCCATGGGTACACAGACACATACTATGATATTCGATAATGCATT
TAATTGCACTTTCGAGTACATATCTGATGCCTTTTCGCTTGATGTTTCAGAAAAGTCAGGTAATTTTAAACACTTACGA
GAGTTTGTGTTTAAAAATAAAGATGGGTTTCTCTATGTTTATAAGGGCTATCAACCTATAGATGTAGTTCGTGATCTAC
CTTCTGGTTTTAACACTTTGAAACCTATTTTTAAGTTGCCTCTTGGTATTAACATTACAAATTTTAGAGCCATTCTTAC
AGCCTTTTCACCTGCTCAAGACATTTGGGGCACGTCAGCTGCAGCCTATTTTGTTGGCTATTTAAAGCCAACTACATTT
ATGCTCAAGTATGATGAAAATGGTACAATCACAGATGCTGTTGATTGTTCTCAAAATCCACTTGCTGAACTCAAATGCT
CTGTTAAGAGCTTTGAGATTGACAAAGGAATTTACCAGACCTCTAATTTCAGGGTTGTTCCCTCAGGAGATGTTGTGAG
ATTCCCTAATATTACAAACTTGTGTCCTTTTGGAGAGGTTTTTAATGCTACTAAATTCCCTTCTGTCTATGCATGGGAG
AGAAAAAAAATTTCTAATTGTGTTGCTGATTACTCTGTGCTCTACAACTCAACATTTTTTTCAACCTTTAAGTGCTATG
GCGTTTCTGCCACTAAGTTGAATGATCTTTGCTTCTCCAATGTCTATGCAGATTCTTTTGTAGTCAAGGGAGATGATGT
AAGACAAATAGCGCCAGGACAAACTGGTGTTATTGCTGATTATAATTATAAATTGCCAGATGATTTCATGGGTTGTGTC
CTTGCTTGGAATACTAGGAACATTGATGCTACTTCAACTGGTAATTATAATTATAAATATAGGTCTCTTAGACATGGCA
AGCTTAGGCCCTTTGAGAGAGACATATCTAATGTGCCTTTCTCCCCTGATGGCAAACCTTGCACCCCACCTGCTCTTAA
TTGTTATTGGCCATTAAATGATTATGGTTTTTACACCACTACTGGCATTGGCTACCAACCTTACAGAGTTGTAGTACTT
TCTTTTGAACTTTTAAATGCACCGGCCACGGTTTGTGGACCAAAATTATCCACTGACCTTATTAAGAACCAGTGTGTCA
ATTTTAATTTTAATGGACTCACTGGTACTGGTGTGTTAACTCCTTCTTCAAAGAGATTTCAACCATTTCAACAATTTGG
CCGTGATGTTTCTGATTTCACTGATTCCGTTCGAGATCCTAAAACATCTGAAATATTAGACATTTCACCTTGCTCTTTT
GGGGGTGTAAGTGTAATTACACCTGGAACAAATGCTTCATCTGAAGTTGCTGTTCTATATCAAGATGTTAACTGCACTG
ATGTTTCTACAGCAATTCATGCAGATCAACTCACACCAGCTTGGCGCATATATTCTACTGGAAACAATGTATTCCAGAC
TCAAGCAGGCTGTCTTATAGGAGCTGAGCATGTCGACACTTCTTATGAGTGCGACATTCCTATTGGAGCTGGCATTTGT

GCTAGTTACCATACAGTTTCTTTATTACGTAGTACTAGCCAAAAATCTATTGTGGCTTATACTATGTCTTTAGGTGCTG
ATAGTTCAATTGCTTACTCTAATAACACCATTGCTATACCTACTAACTTTTCAATTAGCATTACTACAGAAGTAATGCC
TGTTTCTATGGCTAAAACCTCCGTAGATTGTAATATGTACATCTGCGGAGATTCTACTGAATGTGCTAATTTGCTTCTC
CAATATGGTAGCTTTTGCACACAACTAAATCGTGCACTCTCAGGTATTGCTGCTGAACAGGATCGCAACACACGTGAAG
TGTTCGCTCAAGTCAAACAAATGTACAAAACCCCAACTTTGAAATATTTTGGTGGTTTTAATTTTTCACAAATATTACC
TGACCCTCTAAAGCCAACTAAGAGGTCTTTTATTGAGGACTTGCTCTTTAATAAGGTGACACTCGCTGATGCTGGCTTC
ATGAAGCAATATGGCGAATGCCTAGGTGATATTAATGCTAGAGATCTCATTTGTGCGCAGAAGTTCAATGGACTTACAG
TGTTGCCACCTCTGCTCACTGATGATATGATTGCTGCCTACACTGCTGCTCTAGTTAGTGGTACTGCCACTGCTGGATG
GACATTTGGTGCTGGCGCTGCTCTTCAAATACCTTTTGCTATGCAAATGGCATATAGGTTCAATGGCATTGGAGTTACC
CAAAATGTTCTCTATGAGAACCAAAAACAAATCGCCAACCAATTTAACAAGGCGATTAGTCAAATTCAAGAATCACTTA
CAACAACATCAACTGCATTGGGCAAGCTGCAAGACGTTGTTAACCAGAATGCTCAAGCATTAAACACACTTGTTAAACA
ACTTAGCTCTAATTTTGGTGCAATTTCAAGTGTGCTAAATGATATCCTTTCGCGACTTGATAAAGTCGAGGCGGAGGTA
CAAATTGACAGGTTAATTACAGGCAGACTTCAAAGCCTTCAAACCTATGTAACACAACAACTAATCAGGGCTGCTGAAA
TCAGGGCTTCTGCTAATCTTGCTGCTACTAAAATGTCTGAGTGTGTTCTTGGACAATCAAAAAGAGTTGACTTTTGTGG
AAAGGGCTACCACCTTATGTCCTTCCCACAAGCAGCCCCGCATGGTGTTGTCTTCCTACATGTCACGTATGTGCCATCC
CAGGAGAGGAACTTCACCACAGCGCCAGCAATTTGTCATGAAGGCAAAGCATACTTCCCTCGTGAAGGTGTTTTTGTGT
TTAATGGCACTTCTTGGTTTATTACACAGAGGAACTTCTTTTCTCCACAAATAATTACTACAGACAATACATTTGTCTC
AGGAAATTGTGATGTCGTTATTGGCATCATTAACAACACAGTTTATGATCCTCTGCAACCTGAGCTTGACTCATTCAAA
GAAGAGCTGGACAAGTACTTCAAAAATCATACATCACCAGATGTTGATCTTGGCGACATTTCAGGCATTAACGCTTCTG
TCGTCAACATTCAAAAAGAAATTGACCGCCTCAATGAGGTCGCTAAAAATTTAAATGAATCACTCATTGACCTTCAAGA
ATTGGGAAAATATGAGCAATATATTAAATGGCCTTGGTATGTTTGGCTCGGCTTCATTGCTGGACTAATTGCCATCGTC
ATGGTTACAATCTTGCTTTGTTGCATGACTAGTTGTTGCAGTTGCCTCAAGGGTGCATGCTCTTGTGGTTCTTGCTGCA
AGTTTGATGAGGATGACTCTGAGCCAGTTCTCAAGGGTGTCAAATTACATTACACATAA

>gi|52546959|gb|AY714217.1| SARS Coronavirus CDC#200301157, complete genome

ATGTTTATTTTCTTATTATTTCTTACTCTCACTAGTGGTAGTGACCTTGACCGGTGCACCACTTTTGATGATGTT
CAAGCTCCTAATTACACTCAACATACTTCATCTATGAGGGGGGTTTACTATCCTGATGAAATTTTTAGATCAGACACTC
TTTATTTAACTCAGGATTTATTTCTTCCATTTTATTCTAATGTTACAGGGTTTCATACTATTAATCATACGTTTGGCAA
CCCTGTCATACCTTTTAAGGATGGTATTTATTTTGCTGCCACAGAGAAATCAAATGTTGTCCGTGGTTGGGTTTTTGGT
TCTACCATGAACAACAAGTCACAGTCGGTGATTATTATTAACAATTCTACTAATGTTGTTATACGAGCATGTAACTTTG
AATTGTGTGACAACCCTTTCTTTGCTGTTTCTAAACCCATGGGTACACAGACACATACTATGATATTCGATAATGCATT
TAATTGCACTTTCGAGTACATATCTGATGCCTTTTCGCTTGATGTTTCAGAAAAGTCAGGTAATTTTAAACACTTACGA
GAGTTTGTGTTTAAAAATAAAGATGGGTTTCTCTATGTTTATAAGGGCTATCAACCTATAGATGTAGTTCGTGATCTAC
CTTCTGGTTTTAACACTTTGAAACCTATTTTTAAGTTGCCTCTTGGTATTAACATTACAAATTTTAGAGCCATTCTTAC
AGCCTTTTCACCTGCTCAAGACATTTGGGGCACGTCAGCTGCAGCCTATTTTGTTGGCTATTTAAAGCCAACTACATTT
ATGCTCAAGTATGATGAAAATGGTACAATCACAGATGCTGTTGATTGTTCTCAAAATCCACTTGCTGAACTCAAATGCT
CTGTTAAGAGCTTTGAGATTGACAAAGGAATTTACCAGACCTCTAATTTCAGGGTTGTTCCCTCAGGAGATGTTGTGAG
ATTCCCTAATATTACAAACTTGTGTCCTTTTGGAGAGGTTTTTAATGCTACTAAATTCCCTTCTGTCTATGCATGGGAG
AGAAAAAAAAATTTCTAATTGTGTTGCTGATTACTCTGTGCTCTACAACTCAACATTTTTTTCAACCTTTAAGTGCTATG
GCGTTTCTGCCACTAAGTTGAATGATCTTTGCTTCTCCAATGTCTATGCAGATTCTTTTGTAGTCAAGGGAGATGATGT
AAGACAAATAGCGCCAGGACAAACTGGTGTTATTGCTGATTATAATTATAAATTGCCAGATGATTTCATGGGTTGTGTC
CTTGCTTGGAATACTAGGAACATTGATGCTACTTCAACTGGTAATTATAATTATAAATATAGGTATCTTAGACATGGCA

AGCTTAGGCCCTTTGAGAGAGACATATCTAATGTGCCTTTCTCCCCTGATGGCAAACCTTGCACCCCACCTGCTCTTAA
TTGTTATTGGCCATTAAATGATTATGGTTTTTACACCACTACTGGCATTGGCTACCAACCTTACAGAGTTGTAGTACTT
TCTTTTGAACTTTTAAATGCACCGGCCACGGTTTGTGGACCAAAATTATCCACTGACCTTATTAAGAACCAGTGTGTCA
ATTTTAATTTTAATGGACTCACTGGTACTGGTGTGTTAACTCCTTCTTCAAAGAGATTTCAACCATTTCAACAATTTGG
CCGTGATGTTTCTGATTTCACTGATTCCGTTCGAGATCCTAAAACATCTGAAATATTAGACATTTCACCTTGCTCTTTT
GGGGGTGTAAGTGTAATTACACCTGGAACAAATGCTTCATCTGAAGTTGCTGTTCTATATCAAGATGTTAACTGCACTG
ATGTTTCTACAGCAATTCATGCAGATCAACTCACACCAGCTTGGCGCATATATTCTACTGGAAACAATGTATTCCAGAC
TCAAGCAGGCTGTCTTATAGGAGCTGAGCATGTCGACACTTCTTATGAGTGCGACATTCCTGTTGGAGCTGGCATTTGT
GCTAGTTACCATACAGTTTCTTTATTACGTAGTACTAGCCAAAAATCTATTGTGGCTTATACTATGTCTTTAGGTGCTG
ATAGTTCAATTGCTTACTCTAATAACACCATTGCTATACCTACTAACTTTTCAATTAGCATTACTACAGAAGTAATGCC
TGTTTCTATGGCTAAAACCTCCGTAGATTGTAATATGTACATCTGCGGAGATTCTACTGAATGTGCTAATTTGCTTCTC
CAATATGGTAGCTTTTGCACACAACTAAATCGTGCACTCTCAGGTATTGCTGCTGAACAGGATCGCAACACACGTGAAG
TGTTCGCTCAAGTCAAACAAATGTACAAAACCCCAACTTTGAAATATTTTGGTGGTTTTAATTTTTCACAAATATTACC
TGACCCTCTAAAGCCAACTAAGAGGTCTTTTATTGAGGACTTGCTCTTTAATAAGGTGACACTCGCTGATGCTGGCTTC
ATGAAGCAATATGGCGAATGCCTAGGTGATATTAATGCTAGAGATCTCATTTGTGCGCAGAAGTTCAATGGACTTACAG
TGTTGCCACCTCTGCTCACTGATGATATGATTGCTGCCTACACTGCTGCTCTAGTTAGTGGTACTGCCACTGCTGGATG
GACATTTGGTGCTGGCGCTGCTCTTCAAATACCTTTTGCTATGCAAATGGCATATAGGTTCAATGGCATTGGAGTTACC
CAAAATGTTCTCTATGAGAACCAAAAACAAATCGCCAACCAATTTAACAAGGCGATTAGTCAAATTCAAGAATCACTTA
CAACAACATCAACTGCATTGGGCAAGCTGCAAGACGTTGTTAACCAGAATGCTCAAGCATTAAACACACTTGTTAAACA
ACTTAGCTCTAATTTTGGTGCAATTTCAAGTGTGCTAAATGATATCCTTTCGCGACTTGATAAAGTCGAGGCGGAGGTA
CAAATTGACAGGTTAATTACAGGCAGACTTCAAAGCCTTCAAACCTATGTAACACAACAACTAATCAGGGCTGCTGAAA
TCAGGGCTTCTGCTAATCTTGCTGCTACTAAAATGTCTGAGTGTGTTCTTGGACAATCAAAAAGAGTTGACTTTTGTGG
AAAGGGCTACCACCTTATGTCCTTCCCACAAGCAGCCCCGCATGGTGTTGTCTTCCTACATGTCACGTATGTGCCATCC
CAGGAGAGGAACTTCACCACAGCGCCAGCAATTTGTCATGAAGGCAAAGCATACTTCCCTCGTGAAGGTGTTTTTGTGT
TTAATGGCACTTCTTGGTTTATTACACAGAGGAACTTCTTTTCTCCACAAATAATTACTACAGACAATACATTTGTCTC
AGGAAATTGTGATGTCGTTATTGGCATCATTAACAACACAGTTTATGATCCTCTGCAACCTGAGCTTGACTCATTCAAA
GAAGAGCTGGACAAGTACTTCAAAAATCATACATCACCAGATGTTGATCTTGGCGACATTTCAGGCATTAACGCTTCTG
TCGTCAACATTCAAAAAGAAATTGACCGCCTCAATGAGGTCGCTAAAAATTTAAATGAATCACTCATTGACCTTCAAGA
ATTGGGAAAATATGAGCAATATATTAAATGGCCTTGGTATGTTTGGCTCGGCTTCATTGCTGGACTAATTGCCATCGTC
ATGGTTACAATCTTGCTTTGTTGCATGACTAGTTGTTGCAGTTGCCTCAAGGGTGCATGCTCTTGTGGTTCTTGCTGCA
AGTTTGATGAGGATGACTCTGAGCCAGTTCTCAAGGGTGTCAAATTACATTACACATAA

>gi|60267744|gb|AY772062.1| SARS coronavirus WH20, complete genome

ATGTTTATTTTCTTATTATTTCTTACTCTCACTAGTGGTAGTGACCTTGACCGGTGCACCACTTTTGATGATGTT
CAAGCTCCTAATTACACTCAACATACTTCATCTATGAGGGGGGTTTACTATCCTGATGAAATTTTTAGATCAGACACTC
TTTATTTAACTCAGGATTTATTTCTTCCATTTTATTCTAATGTTACAGGGTTTCATACTATTAATCATACGTTTGACAA
CCCTGTCATACCTTTTAAGGATGGTATTTATTTTGCTGCCACAGAGAAATCAAATGTTGTCCGTGGTTGGGTTTTTGGT
TCTACCATGAACAACAAGTCACAGTCGGTGATTATTATTAACAATTCTACTAATGTTGTTATACGAGCATGTAACTTTG
AATTGTGTGACAACCCTTTCTTTGCTGTTTCTAAACCCATGGGTACACAGACACATACTATGATATTCGATAATGCATT
TAATTGCACTTTCGAGTACATATCTGATGCCTTTTCGCTTGATGTTTCAGAAAAGTCAGGTAATTTTAAACACTTACGA
GAGTTTGTGTTTAAAAATAAAGATGGGTTTCTCTATGTTTATAAGGGCTATCAACCTATAGATGTAGTTCGTGATCTAC
CTTCTGGTTTTAACACTTTGAAACCTATTTTTAAGTTGCCTCTTGGTATTAACATTACAAATTTTAGAGCCATTCTTAC

AGCCTTTTCACCTGCTCAAGACACTTGGGGCACGTCAGCTGCAGCCTATTTTGTTGGCTATTTAAAGCCAACTACATTT
ATGCTCAAGTATGATGAAAATGGTACAATCACAGATGCTGTTGATTGTTCTCAAAATCCACTTGCTGAACTCAAATGCT
CTGTTAAGAGCTTTGAGATTGACAAAGGAATTTACCAGACCTCTAATTTCAGGGTTGTTCCCTCAGGAGATGTTGTGAG
ATTCCCTAATATTACAAACTTGTGTCCTTTTGGAGAGGTTTTTAATGCTACTAAATTCCCTTCTGTCTATGCATGGGAG
AGAAAAAAAATTTCTAATTGTGTTGCTGATTACTCTGTGCTCTACAACTCAACATTTTTTTCAACCTTTAAGTGCTATG
GCGTTTCTGCCACTAAGTTGAATGATCTTTGCTTCTCCAATGTCTATGCAGATTCTTTTGTAGTCAAGGGAGATGATGT
AAGACAAATAGCGCCAGGACAAACTGGTGTTATTGCTGATTATAATTATAAATTGCCAGATGATTTCATGGGTTGTGTC
CTTGCTTGGAATACTAGGAACATTGATGCTACTTCAACTGGTAATTATAATTATAAATATAGGTATCTTAGACATGGCA
AGCTTAGGCCCTTTGAGAGAGACATATCTAATGTGCCTTTCTCCCCTGATGGCAAACCTTGCACCCCACCTGCTCTTAA
TTGTTATTGGCCATTAAATGATTATGGTTTTTACACCACTACTGGCATTGGCTACCAACCTTACAGAGTTGTAGTACTT
TCTTTTGAACTTTTAAATGCACCGGCCACGGTTTGTGGACCAAAATTATCCACTGACCTTATTAAGAACCAGTGTGTCA
ATTTTAATTTTAATGGACTCACTGGTACTGGTGTGTTAACTCCTTCTTCAAAGAGATTTCAACCATTTCAACAATTTGG
CCGTGATGTTTCTGATTTCACTGATTCCGTTCGAGATCCTAAAACATCTGAAATATTAGACATTTCACCTTGCTCTTTT
GGGGGTGTAAGTGTAATTACACCTGGAACAAATGCTTCATCTGAAGTTGCTGTTCTATATCAAGATGTTAACTGCACTG
ATGTTTCTACAGCAATTCATGCAGATCAACTCACACCAGCTTGGCGCATATATTCTACTGGAAACAATGTATTCCAGAC
TCAAGCAGGCTGTCTTATAGGAGCTGAGCATGTCGACACTTCTTATGAGTGCGACATTCCTATTGGAGCTGGCATTTGT
GCTAGTTACCATACAGTTTCTTTATTACGTAGTACTAGCCAAAAATCTATTGTGGCTTATACTATGTCTTTAGGTGCTG
ATAGTTCAATTGCTTACTCTAATAACACCATTGCTATACCTACTAACTTTTCAATTAGCATTACTACAGAAGTAATGCC
TGTTTCTATGGCTAAAACCTCCGTAGATTGTAATATGTACATCTGCGGAGATTCTACTGAATGTGCTAATTTGCTTCTC
CAATATGGTAGCTTTTGCACACAACTAAATCGTGCACTCTCAGGTATTGCTGCTGAACAGGATCGCAACACACGTGAAG
TGTTCGCTCAAGTCAAACAAATGTACAAAACCCCAACTTTGAAATATTTTGGTGGTTTTAATTTTTCACAAATATTACC
TGACCCTCTAAAGCCAACTAAGAGGTCTTTTATTGAGGACTTGCTCTTTAATAAGGTGACACTCGCTGATGCTGGCTTC
ATGAAGCAATATGGCGAATGCCTAGGTGATATTAATGCTAGAGATCTCATTTGTGCGCAGAAGTTCAATGGACTTACAG
TGTTGCCACCTCTGCTCACTGATGATATGATTGCTGCCTACACTGCTGCTCTAGTTAGTGGTACTGCCACTGCTGGATG
GACATTTGGTGCTGGCGCTGCTCTTCAAATACCTTTTGCTATGCAAATGGCATATAGGTTCAATGGCATTGGAGTTACC
CAAAATGTTCTCTATGAGAACCAAAAACAAATCGCCAACCAATTTAACAAGGCGATTAGTCAAATTCAAGAATCACTTA
CAACAACATCAACTGCATTGGGCAAGCTGCAAGACGTTGTTAACCAGAATGCTCAAGCATTAAACACACTTGTTAAGCA
ACTTAGCTCTAATTTTGGTGCAATTTCAAGTGTGCTAAATGATATCCTTTCGCGACTTGATAAAGTCGAGGCGGAGGTA
CAAATTGACAGGTTAATTACAGGCAGACTTCAAAGCCTTCAAACCTATGTAACACAACAACTAATCAGGGCTGCTGAAA
TCAGGGCTTCTGCTAATCTTGCTGCTACTAAAATGTCTGAGTGTGTTCTTGGACAATCAAAAAGAGTTGACTTTTGTGG
AAAGGGCTACCACCTTATGTCCTTCCCACAAGCAGCCCCGCATGGTGTTGTCTTCCTACATGTCACGTATGTGCCATCC
CAGGAGAGGAACTTCACCACAGCGCCAGCAATTTGTCATGAAGGCAAAGCATACTTCCCTCGTGAAGGTGTTTTTGTGT
TTAATGGCACTTCTTGGTTTATTACACAGAGGAACTTCTTTTCTCCACAAATAATTACTACAGACAATACATTTGTCTC
AGGAAATTGTGATGTCGTTATTGGCATCATTAACAACACAGTTTATGATCCTCTGCAACCTGAGCTTGACTCATTCAAA
GAAGAGCTGGACAAGTACTTCAAAAATCATACATCACCAGATGTTGATCTTGGCGACATTTCAGGCATTAACGCTTCTG
TCGTCAACATTCAAAAAGAAATTGACCGCCTCAATGAGGTCGCTAAAAATTTAAATGAATCACTCATTGACCTTCAAGA
ATTGGGAAAATATGAGCAATATATTAAATGGCCTTGGTATGTTTGGCTCGGCTTCATTGCTGGACTAATGGCCATCGTC
ATGGTTACAATCTTGCTTTGTTGCATGACTAGTTGTTGCAGTTGCCTCAAGGGTGCATGCTCTTGTGGTTCTTGCTGCA
AGTTTGATGAGGATGACTCTGAGCCAGTTCTCAAGGGTGTCAAATTACATTACACATAA

>gi|229595250|gb|FJ882963.1| SARS coronavirus P2, complete genome

ATGTTTATTTTCTTATTATTTCTTACTCTCACTAGTGGTAGTGACCTTGACCGGTGCACCACTTTTGATGATGTT

CAAGCTCCTAATTACACTCAACATACTTCATCTATGAGGGGGGTTTACTATCCTGATGAAATTTTTAGATCAGACACTC
TTTATTTAACTCAGGATTTATTTCTTCCATTTTATTCTAATGTTACAGGGTTTCATACTATTAATCATACGTTTGGCAA
CCCTGTCATACCTTTTAAGGATGGTATTTATTTTGCTGCCACAGAGAAATCAAATGTTGTCCGTGGTTGGGTTTTTGGT
TCTACCATGAACAACAAGTCACAGTCGGTGATTATTATTAACAATTCTACTAATGTTGTTATACGAGCATGTAACTTTG
AATTGTGTGACAACCCTTTCTTTGCTGTTTCTAAACCCATGGGTACACAGACACATACTATGATATTCGATAATGCATT
TAATTGCACTTTCGAGTACATATCTGATGCCTTTTCGCTTGATGTTTCAGAAAAGTCAGGTAATTTTAAACACTTACGA
GAGTTTGTGTTTAAAAATAAAGATGGGTTTCTCTATGTTTATAAGGGCTATCAACCTATAGATGTAGTTCGTGATCTAC
CTTCTGGTTTTAACACTTTGAAACCTATTTTTAAGTTGCCTCTTGGTATTAACATTACAAATTTTAGAGCCATTCTTAC
AGCCTTTTCACCTGCTCAAGACATTTGGGGCACGTCAGCTGCAGCCTATTTTGTTGGCTATTTAAAGCCAACTACATTT
ATGCTCAAGTATGATGAAAATGGTACAATCACAGATGCTGTTGATTGTTCTCAAAATCCACTTGCTGAACTCAAATGCT
CTGTTAAGAGCTTTGAGATTGACAAAGGAATTTACCAGACCTCTAATTTCAGGGTTGTTCCCTCAGGAGATGTTGTGAG
ATTCCCTAATATTACAAACTTGTGTCCTTTTGGAGAGGTTTTTAATGCTACTAAATTCCCTTCTGTCTATGCATGGGAG
AGAAAAAAAAATTTCTAATTGTGTTGCTGATTACTCTGTGCTCTACAACTCAACATTTTTTTCAACCTTTAAGTGCTATG
GCGTTTCTGCCACTAAGTTGAATGATCTTTGCTTCTCCAATGTCTATGCAGATTCTTTTGTAGTCAAGGGAGATGATGT
AAGACAAATAGCGCCAGGACAAACTGGTGTTATTGCTGATTATAATTATAAATTGCCAGATGATTTCATGGGTTGTGTC
CTTGCTTGGAATACTAGGAACATTGATGCTACTTCAACTGGTAATTATAATTATAAATATAGGTATCTTAGACATGGCA
AGCTTAGGCCCTTTGAGAGAGACATATCTAATGTGCCTTTCTCCCCTGATGGCAAACCTTGCACCCCACCTGCTCTTAA
TTGTTATTGGCCATTAAATGATTATGGTTTTTACACCACTACTGGCATTGGCTACCAACCTTACAGAGTTGTAGTACTT
TCTTTTGAACTTTTAAATGCACCGGCCACGGTTTGTGGACCAAAATTATCCACTGACCTTATTAAGAACCAGTGTGTCA
ATTTTAATTTTAATGGACTCACTGGTACTGGTGTGTTAACTCCTTCTTCAAAGAGATTTCAACCATTTCAACAATTTGG
CCGTGATGTTTCTGATTTCACTGATTCCGTTCGAGATCCTAAAACATCTGAAATATTAGACATTTCACCTTGCTCTTTT
GGGGGTGTAAGTGTAATTACACCTGGAACAAATGCTTCATCTGAAGTTGCTGTTCTATATCAAGGTGTTAACTGCACTG
ATGTTTCTACAGCAATTCATGCAGATCAACTCACACCAGCTTGGCGCATATATTCTACTGGAAACAATGTATTCCAGAC
TCAAGCAGGCTGTCTTATAGGAGCTGAGCATGTCGACACTTCTTATGAGTGCGACATTCCTGTTGGAGCTGGCATTTGT
GCTAGTTACCATACAGTTTCTTTATTACGTAGTACTAGCCAAAAATCTATTGTGGCTTATACTATGTCTTTAGGTGCTG
ATAGTTCAATTGCTTACTCTAATAACACCATTGCTATACCTACTAACTTTTCAATTAGCATTACTACAGAAGTAATGCC
TGTTTCTATGGCTAAAACCTCCGTAGATTGTAATATGTACATCTGCGGAGATTCTACTGAATGTGCTAATTTGCTTCTC
CAATATGGTAGCTTTTGCACACAACTAAATCGTGCACTCTCAGGTATTGCTGCTGAACAGGATCGCAACACACGTGAAG
TGTTCGCTCAAGTCAAACAAATGTACAAAACCCCAACTTTGAAATATTTTGGTGGTTTTAATTTTTCACAAATATTACC
TGACCCTCTAAAGCCAACTAAGAGGTCTTTTATTGAGGACTTGCTCTTTAATAAGGTGACACTCGCTGATGCTGGCTTC
ATGAAGCAATATGGCGAATGCCTAGGTGATATTAATGCTAGAGATCTCATTTGTGCGCAGAAGTTCAATGGACTTACAG
TGTTGCCACCTCTGCTCACTGATGATATGATTGCTGCCTACACTGCTGCTCTAGTTAGTGGTACTGCCACTGCTGGATG
GACATTTGGTGCTGGCGCTGCTCTTCAAATACCTTTTGCTATGCAAATGGCATATAGGTTCAATGGCATTGGAGTTACC
CAAAATGTTCTCTATGAGAACCAAAAACAAATCGCCAACCAATTTAACAAGGCGATTAGTCAAATTCAAGAATCACTTA
CAACAACATCAACTGCATTGGGCAAGCTGCAAGACGTTGTTAACCAGAATGCTCAAGCATTAAACACACTTGTTAAACA
ACTTAGCTCTAATTTTGGTGCAATTTCAAGTGTGCTAAATGATATCCTTTCGCGACTTGATAAAGTCGAGGCGGAGGTA
CAAATTGACAGGTTAATTACAGGCAGACTTCAAAGCCTTCAAACCTATGTAACACAACAACTAATCAGGGCTGCTGAAA
TCAGGGCTTCTGCTAATCTTGCTGCTACTAAAATGTCTGAGTGTGTTCTTGGACAATCAAAAAGAGTTGACTTTTGTGG
AAAGGGCTACCACCTTATGTCCTTCCCACAAGCAGCCCCGCATGGTGTTGTCTTCCTACATGTCACGTATGTGCCATCC
CAGGAGAGGAACTTCACCACAGCGCCAGCAATTTGTCATGAAGGCAAAGCATACTTCCCTCGTGAAGGTGTTTTTGTGT
TTAATGGCACTTCTTGGTTTATTACACAGAGGAACTTCTTTTCTCCACAAATAATTACTACAGACAATACATTTGTCTC
AGGAAATTGTGATGTCGTTATTGGCATCATTAACAACACAGTTTATGATCCTCTGCAACCTGAGCTTGACTCATTCAAA
GAAGAGCTGGACAAGTACTTCAAAAATCATACATCACCAGATGTTGATCTTGGCGACATTTCAGGCATTAACGCTTCTG
TCGTCAACATTCAAAAAGAAATTGACCGCCTCAATGAGGTCGCTAAAAATTTAAATGAATCACTCATTGACCTTCAAGA

ATTGGGAAAATATGAGCAATATATTAAATGGCCTTGGTATGTTTGGCTCGGCTTCATTGCTGGACTAATTGCCATCGTC
ATGGTTACAATCTTGCTTTGTTGCATGACTAGTTGTTGCAGTTGCCTCAAGGGTGCATGCTCTTGTGGTTCTTGCTGCA
AGTTTGATGAGGATGACTCTGAGCCAGTTCTCAAGGGTGTCAAATTACATTACACATAA

>gi|285803917|gb|GU553363.1| SARS coronavirus HKU-39849 isolate TCVSP-HARROD-00001, complete genome

ATGTTTATTTTCTTATTATTTCTTACTCTCACTAGTGGTAGTGACCTTGACCGGTGCACCACTTTTGATGATGTT
CAAGCTCCTAATTACACTCAACATACTTCATCTATGAGGGGGGTTTACTATCCTGATGAAATTTTTAGATCAGACACTC
TTTATTTAACTCAGGATTTATTTCTTCCATTTTATTCTAATGTTACAGGGTTTCATACTATTAATCATACGTTTGGCAA
CCCTGTCATACCTTTTAAGGATGGTATTTATTTTGCTGCCACAGAGAAATCAAATGTTGTCCGTGGTTGGGTTTTTGGT
TCTACCATGAACAACAAGTCACAGTCGGTGATTATTATTAACAATTCTACTAATGTTGTTATACGAGCATGTAACTTTG
AATTGTGTGACAACCCTTTCTTTGCTGTTTCTAAACCCATGGGTACACAGACACATACTATGATATTCGATAATGCATT
TAATTGCACTTTCGAGTACATATCTGATGCCTTTTCGCTTGATGTTTCAGAAAAGTCAGGTAATTTTAAACACTTACGA
GAGTTTGTGTTTAAAAATAAAGATGGGTTTCTCTATGTTTATAAGGGCTATCAACCTATAGATGTAGTTCGTGATCTAC
CTTCTGGTTTTAACACTTTGAAACCTATTTTTAAGTTGCCTCTTGGTATTAACATTACAAATTTTAGAGCCATTCTTAC
AGCCTTTTCACCTGCTCAAGACATTTGGGGCACGTCAGCTGCAGCCTATTTTGTTGGCTATTTAAAGCCAACTACATTT
ATGCTCAAGTATGATGAAAATGGTACAATCACAGATGCTGTTGATTGTTCTCAAAATCCACTTGCTGAACTCAAATGCT
CTGTTAAGAGCTTTGAGATTGACAAAGGAATTTACCAGACCTCTAATTTCAGGGTTGTTCCCTCAGGAGATGTTGTGAG
ATTCCCTAATATTACAAACTTGTGTCCTTTTGGAGAGGTTTTTAATGCTACTAAATTCCCTTCTGTCTATGCATGGGAG
AGAAAAAAAATTTCTAATTGTGTTGCTGATTACTCTGTGCTCTACAACTCAACATTTTTTTCAACCTTTAAGTGCTATG
GCGTTTCTGCCACTAAGTTGAATGATCTTTGCTTCTCCAATGTCTATGCAGATTCTTTTGTAGTCAAGGGAGATGATGT
AAGACAAATAGCGCCAGGACAAACTGGTGTTATTGCTGATTATAATTATAAATTGCCAGATGATTTCATGGGTTGTGTC
CTTGCTTGGAATACTAGGAACATTGATGCTACTTCAACTGGTAATTATAATTATAAATATAGGTATCTTAGACATGGCA
AGCTTAGGCCCTTTGAGAGAGACATATCTAATGTGCCTTTCTCCCCTGATGGCAAACCTTGCACCCCACCTGCTCTTAA
TTGTTATTGGCCATTAAATGATTATGGTTTTTACACCACTACTGGCATTGGCTACCAACCTTACAGAGTTGTAGTACTT
TCTTTTGAACTTTTAAATGCACCGGCCACGGTTTGTGGACCAAAATTATCCACTGACCTTATTAAGAACCAGTGTGTCA
ATTTTAATTTTAATGGACTCACTGGTACTGGTGTGTTAACTCCTTCTTCAAAGAGATTTCAACCATTTCAACAATTTGG
CCGTGATGTTTCTGATTTCACTGATTCCGTTCGAGATCCTAAAACATCTGAAATATTAGACATTTCACCTTGCTCTTTT
GGGGGTGTAAGTGTAATTACACCTGGAACAAATGCTTCATCTGAAGTTGCTGTTCTATATCAAGATGTTAACTGCACTG
ATGTTTCTACAGCAATTCATGCAGATCAACTCACACCAGCTTGGCGCATATATTCTACTGGAAACAATGTATTCCAGAC
TCAAGCAGGCTGTCTTATAGGAGCTGAGCATGTCGACACTTCTTATGAGTGCGACATTCCTATTGGAGCTGGCATTTGT
GCTAGTTACCATACAGTTTCTTTATTACGTAGTACTAGCCAAAAATCTATTGTGGCTTATACTATGTCTTTAGGTGCTG
ATAGTTCAATTGCTTACTCTAATAACACCATTGCTATACCTACTAACTTTTCAATTAGCATTACTACAGAAGTAATGCC
TGTTTCTATGGCTAAAACCTCCGTAGATTGTAATATGTACATCTGCGGAGATTCTACTGAATGTGCTAATTTGCTTCTC
CAATATGGTAGCTTTTGCACACAACTAAATCGTGCACTCTCAGGTATTGCTGCTGAACAGGATCGCAACACACGTGAAG
TGTTCGCTCAAGTCAAACAAATGTACAAAACCCCAACTTTGAAATATTTTGGTGGTTTTAATTTTTCACAAATATTACC
TGACCCTCTAAAGCCAACTAAGAGGTCTTTTATTGAGGACTTGCTCTTTAATAAGGTGACACTCGCTGATGCTGGCTTC
ATGAAGCAATATGGCGAATGCCTAGGTGATATTAATGCTAGAGATCTCATTTGTGCGCAGAAGTTCAATGGACTTACAG
TGTTGCCACCTCTGCTCACTGATGATATGATTGCTGCCTACACTGCTGCTCTAGTTAGTGGTATTGCCACTGCTGGATG
GACATTTGGTGCTGGCGCTGCTCTTCAAATACCTTTTGCTATGCAAATGGCATATAGGTTCAATGGCATTGGAGTTACC
CAAAATGTTCTCTATGAGAACCAAAAACAAATCGCCAACCAATTTAACAAGGCGATTAGTCAAATTCAAGAATCACTTA
CAACAACATCAACTGCATTGGGCAAGCTGCAAGACGTTGTTAACCAGAATGCTCAAGCATTAAACACACTTGTTAAACA

ACTTAGCTCTAATTTTGGTGCAATTTCAAGTGTGCTAAATGATATCCTTTCGCGACTTGATAAAGTCGAGGCGGAGGTA
CAAATTGACAGGTTAATTACAGGCAGACTTCAAAGCCTTCAAACCTATGTAACACAACAACTAATCAGGGCTGCTGAAA
TCAGGGCTTCTGCTAATCTTGCTGCTACTAAAATGTCTGAGTGTGTTCTTGGACAATCAAAAAGAGTTGACTTTTGTGG
AAAGGGCTACCACCTTATGTCCTTCCCACAAGCAGCCCCGCATGGTGTTGTCTTCCTACATGTCACGTATGTGCCATCC
CAGGAGAGGAACTTCACCACAGCGCCAGCAATTTGTCATGAAGGCAAAGCATACTTCCCTCGTGAAGGTGTTTTTGTGT
TTAATGGCACTTCTTGGTTTATTACACAGAGGAACTTCTTTTCTCCACAAATAATTACTACAGACAATACATTTGTCTC
AGGAAATTGTGATGTCGTTATTGGCATCATTAACAACACAGTTTATGATCCTCTGCAACCTGAGCTTGACTCATTCAAA
GAAGAGCTGGACAAGTACTTCAAAAATCATACATCACCAGATGTTGATCTTGGCGACATTTCAGGCATTAACGCTTCTG
TCGTCAACATTCAAAAAGAAATTGACCGCCTCAATGAGGTCGCTAAAAATTTAAATGAATCACTCATTGACCTTCAAGA
ATTGGGAAAATATGAGCAATATATTAAATGGCCTTGGTATGTTTGGCTCGGCTTCATTGCTGGACTAATTGCCATCGTC
ATGGTTACAATCTTGCTTTGTTGCATGACTAGTTGTTGCAGTTGCCTCAAGGGTGCATGCTCTTGTGGTTCTTGCTGCA
AGTTTGATGAGGATGACTCTGAGCCAGTTCTCAAGGGTGTCAAATTACATTACACATAA

>gi|33411444|dbj|AP006560.1| SARS coronavirus TWS genomic RNA, complete genome

ATGTTTATTTTCTTATTATTTCTTACTCTCACTAGTGGTAGTGACCTTGACCGGTGCACCACTTTTGATGATGTT
CAAGCTCCTAATTATACTCAACATACTTCATCTATGAGGGGGGTTTACTATCCTGATGAAATTTTTAGATCAGACACTC
TTTATTTAACTCAGGATTTATTTCTTCCATTTTATTCTAATGTTACAGGGTTTCATACTATTAATCATACGTTTGGCAA
CCCTGTCATACCTTTTAAGGATGGTATTTATTTTGCTGCCACAGAGAAATCAAATGTTGTCCGTGGTTGGGTTTTTGGT
TCTACCATGAACAACAAGTCACAGTCGGTGATTATTATTAACAATTCTACTAATGTTGTTATACGAGCATGTAACTTTG
AATTGTGTGACAACCCTTTCTTTGCTGTTTCTAAACCCATGGGTACACAGACACATACTATGATATTCGATAATGCATT
TAATTGCACTTTCGAGTACATATCTGATGCCTTTTCGCTTGATGTTTCAGAAAAGTCAGGTAATTTTAAACACTTACGA
GAGTTTGTGTTTAAAAATAAAGATGGGTTTCTCTATGTTTATAAGGGCTATCAACCTATAGATGTAGTTCGTGATCTAC
CTTCTGGTTTTAACACTTTGAAACCTATTTTTAAGTTGCCTCTTGGTATTAACATTACAAATTTTAGAGCCATTCTTAC
AGCCTTTTCACCTGCTCAAGACATTTGGGGCACGTCAGCTGCAGCCTATTTTGTTGGCTATTTAAAGCCAACTACATTT
ATGCTCAAGTATGATGAAAATGGTACAATCACAGATGCTGTTGATTGTTCTCAAAATCCACTTGCTGAACTCAAATGCT
CTGTTAAGAGCTTTGAGATTGACAAAGGAATTTACCAGACCTCTAATTTCAGGGTTGTTCCCTCAGGAGATGTTGTGAG
ATTCCCTAATATTACAAACTTGTGTCCTTTTGGAGAGGTTTTTAATGCTACTAAATTCCCTTCTGTCTATGCATGGGAG
AGAAAAAAAATTTCTAATTGTGTTGCTGATTACTCTGTGCTCTACAACTCAACATTTTTTTCAACCTTTAAGTGCTATG
GCGTTTCTGCCACTAAGTTGAATGATCTTTGCTTCTCCAATGTCTATGCAGATTCTTTTGTAGTCAAGGGAGATGATGT
AAGACAAATAGCGCCAGGACAAACTGGTGTTATTGCTGATTATAATTATAAATTGCCAGATGATTTCATGGGTTGTGTC
CTTGCTTGGAATACTAGGAACATTGATGCTACTTCAACTGGTAATTATAATTATAAATATAGGTATCTTAGACATGGCA
AGCTTAGGCCCTTTGAGAGAGACATATCTAATGTGCCTTTCTCCCCTGATGGCAAACCTTGCACCCCACCTGCTCTTAA
TTGTTATTGGCCATTAAATGATTATGGTTTTTACACCACTACTGGCATTGGCTACCAACCTTACAGAGTTGTAGTACTT
TCTTTTGAACTTTTAAATGCACCGGCCACGGTTTGTGGACCAAAATTATCCACTGACCTTATTAAGAACCAGTGTGTCA
ATTTTAATTTTAATGGACTCACTGGTACTGGTGTGTTAACTCCTTCTTCAAAGAGATTTCAACCATTTCAACAATTTGG
CCGTGATGTTTCTGATTTCACTGATTCCGTTCGAGATCCTAAAACATCTGAAATATTAGACATTTCACCTTGCTCTTTT
GGGGGTGTAAGTGTAATTACACCTGGAACAAATGCTTCATCTGAAGTTGCTGTTCTATATCAAGATGTTAACTGCACTG
ATGTTTCTACAGCAATTCATGCAGATCAACTCACACCAGCTTGGCGCATATATTCTACTGGAAACAATGTATTCCAGAC
TCAAGCAGGCTGTCTTATAGGAGCTGAGCATGTCGACACTTCTTATGAGTGCGACATTCCTATTGGAGCTGGCATTTGT
GCTAGTTACCATACAGTTTCTTTATTACGTAGTACTAGCCAAAAATCTATTGTGGCTTATACTATGTCTTTAGGTGCTG
ATAGTTCAATTGCTTACTCTAATAACACCATTGCTATACCTACTAACTTTTCAATTAGCATTACTACAGAAGTAATGCC
TGTTTCTATGGCTAAAACCTCCGTAGATTGTAATATGTACATCTGCGGAGATTCTACTGAATGTGCTAATTTGCTTCTC

CAATATGGTAGCTTTTGCACACAACTAAATCGTGCACTCTCAGGTATTGCTGCTGAACAGGATCGCAACACACGTGAAG
TGTTCGCTCAAGTCAAACAAATGTACAAAACCCCAACTTTGAAATATTTTGGTGGTTTTAATTTTTCACAAATATTACC
TGACCCTCTAAAGCCAACTAAGAGGTCTTTTATTGAGGACTTGCTCTTTAATAAGGTGACACTCGCTGATGCTGGCTTC
ATGAAGCAATATGGCGAATGCCTAGGTGATATTAATGCTAGAGATCTCATTTGTGCGCAGAAGTTCAATGGACTTACAG
TGTTGCCACCTCTGCTCACTGATGATATGATTGCTGCCTACACTGCTGCTCTAGTTAGTGGTACTGCCACTGCTGGATG
GACATTTGGTGCTGGCGCTGCTCTTCAAATACCTTTTGCTATGCAAATGGCATATAGGTTCAATGGCATTGGAGTTACC
CAAAATGTTCTCTATGAGAACCAAAAACAAATCGCCAACCAATTTAACAAGGCGATTAGTCAAATTCAAGAATCACTTA
CAACAACATCAACTGCATTGGGCAAGCTGCAAGACGTTGTTAACCAGAATGCTCAAGCATTAAACACACTTGTTAAACA
ACTTAGCTCTAATTTTGGTGCAATTTCAAGTGTGCTAAATGATATCCTTTCGCGACTTGATAAAGTCGAGGCGGAGGTA
CAAATTGACAGGTTAATTACAGGCAGACTTCAAAGCCTTCAAACCTATGTAACACAACAACTAATCAGGGCTGCTGAAA
TCAGGGCTTCTGCTAATCTTGCTGCTACTAAAATGTCTGAGTGTGTTCTTGGACAATCAAAAAGAGTTGACTTTTGTGG
AAAGGGCTACCACCTTATGTCCTTCCCACAAGCAGCCCCGCATGGTGTTGTCTTCCTACATGTCACGTATGTGCCATCC
CAGGAGAGGAACTTCACCACAGCGCCAGCAATTTGTCATGAAGGCAAAGCATACTTCCCTCGTGAAGGTGTTTTTGTGT
TTAATGGCACTTCTTGGTTTATTACACAGAGGAACTTCTTTTCTCCACAAATAATTACTACAGACAATACATTTGTCTC
AGGAAATTGTGATGTCGTTATTGGCATCATTAACAACACAGTTTATGATCCTCTGCAACCTGAGCTTGACTCATTCAAA
GAAGAGCTGGACAAGTACTTCAAAAATCATACATCACCAGATGTTGATCTTGGCGACATTTCAGGCATTAACGCTTCTG
TCGTCAACATTCAAAAAGAAATTGACCGCCTCAATGAGGTCGCTAAAAATTTAAATGAATCACTCATTGACCTTCAAGA
ATTGGGAAAATATGAGCAATATATTAAATGGCCTTGGTATGTTTGGCTCGGCTTCATTGCTGGACTAATTGCCATCGTC
ATGGTTACAATCTTGCTTTGTTGCATGACTAGTTGTTGCAGTTGCCTCAAGGGTGCATGCTCTTGTGGTTCTTGCTGCA
AGTTTGATGAGGATGACTCTGAGCCAGTTCTCAAGGGTGTCAAATTACATTACACATAA

>gi|41323719|gb|AY390556.1| SARS coronavirus GZ02, complete genome

ATGTTTATTTTCTTATTATTTCTTACTCTCACTAGTGGTAGTGACCTTGACCGGTGCACCACTTTTGATGATGTT
CAAGCTCCTAATTACACTCAACATACTTCATCTATGAGGGGGGTTTACTATCCTGATGAAATTTTTAGATCAGACACTC
TTTATTTAACTCAGGATTTATTTCTTCCATTTTATTCTAATGTTACAGGGTTTCATACTATTAATCATACGTTTGACAA
CCCTGTCATACCTTTTAAGGATGGTATTTATTTTGCTGCCACAGAGAAATCAAATGTTGTCCGTGGTTGGGTTTTTGGT
TCTACCATGAACAACAAGTCACAGTCGGTGATTATTATTAACAATTCTACTAATGTTGTTATACGAGCATGTAACTTTG
AATTGTGTGACAACCCTTTCTTTGCTGTTTCTAAACCCATGGGTACACAGACACATACTATGATATTCGATAATGCATT
TAATTGCACTTTCGAGTACATATCTGATGCCTTTTCGCTTGATGTTTCAGAAAAGTCAGGTAATTTTAAACACTTACGA
GAGTTTGTGTTTAAAAATAAAGATGGGTTTCTCTATGTTTATAAGGGCTATCAACCTATAGATGTAGTTCGTGATCTAC
CTTCTGGTTTTAACACTTTGAAACCCATTTTTAAGTTGCCTCTTGGTATTAACATTACAAATTTTAGAGCCATTCTTAC
AGCCTTTTTACCTGCTCAAGACACTTGGGGCACGTCAGCTGCAGCCTATTTTGTTGGCTATTTAAAGCCAACTACATTT
ATGCTCAAGTATGATGAAAATGGTACAATCACAGATGCTGTTGATTGTTCTCAAAATCCACTTGCTGAACTCAAATGCT
CTGTTAAGAGCTTTGAGATTGACAAAGGAATTTACCAGACCTCTAATTTCAGGGTTGTTCCCTCAAGAGATGTTGTGAG
ATTCCCTAATATTACAAACTTGTGTCCTTTTGGAGAGGTTTTTAATGCTACTAAATTCCCTTCTGTCTATGCATGGGAG
AGGAAAAGAATTTCTAATTGTGTTGCTGATTACTCTGTGCTCTACAACTCAACATTTTTTTCAACCTTTAAGTGCTATG
GCGTTTCTGCCACTAAGTTGAATGATCTTTGCTTCTCCAATGTCTATGCAGATTCTTTTGTAGTCAAGGGAGATGATGT
AAGACAAATAGCGCCAGGACAAACTGGTGTTATTGCTGATTATAATTATAAATTGCCAGATGATTTCATGGGTTGTGTC
CTTGCTTGGAATACTAGGAACATTGATGCTACTTCAACTGGTAATTATAATTATAAATATAGGTATCTTAGACATGGCA
AGCTTAGGCCCTTTGAGAGAGACATATCTAATGTGCCTTTCTCCCCTGATGGCAAACCTTGCACCCCACCTGCTCTTAA
TTGTTATTGGCCATTAAATGATTATGGTTTTTACACCACTACTGGCATTGGCTACCAACCTTACAGAGTTGTAGTACTT
TCTTTTGAACTTTTAAATGCACCGGCCACGGTTTGTGGACCAAAATTATCCACTGACCTTATTAAGAACCAGTGTGTCA

ATTTTAATTTTAATGGACTCACTGGTACTGGTGTGTTAACTCCTTCTTCAAAGAGATTTCAACCATTTCAACAATTTGG
CCGTGATGTTTCTGATTTCACTGATTCCGTTCGAGATCCTAAAACATCTGAAATATTAGACATTTCACCTTGCTCTTTT
GGGGGTGTAAGTGTAATTACACCTGGAACAAATGCTTCATCTGAAGTTGCTGTTCTATATCAAGATGTTAACTGCACTG
ATGTTTCTACAGCAATTCATGCAGATCAACTCACACCAGCTTGGCGCATATATTCTACTGGAAACAATGTATTCCAGAC
TCAAGCAGGCTGTCTTATAGGAGCTGAGCATGTCGACACTTCTTATGAGTGCGACATTCCTATTGGAGCTGGCATTTGT
GCTAGTTACCATACAGTTTCTTTATTACGTAGTACTAGCCAAAAATCTATTGTGGCTTATACTATGTCTTTAGGTGCTG
ATAGTTCAATTGCTTACTCTAATAACACCATTGCTATACCTACTAACTTTTCAATTAGCATTACTACAGAAGTAATGCC
TGTTTCTATGGCTAAAACCTCCGTAGATTGTAATATGTACATCTGCGGAGATTCTACTGAATGTGCTAATTTGCTTCTC
CAATATGGTAGCTTTTGCACACAACTAAATCGTGCACTCTCAGGTATTGCTGCTGAACAGGATCGCAACACACGTGAAG
TGTTCGCTCAAGTCAAACAAATGTACAAAACCCCAACTTTGAAAGATTTTGGTGGTTTTAATTTTTCACAAATATTACC
TGACCCTCTAAAGCCAACTAAGAGGTCTTTTATTGAGGACTTGCTCTTTAATAAGGTGACACTCGCTGATGCTGGCTTC
ATGAAGCAATATGGCGAATGCCTAGGTGATATTAATGCTAGAGATCTCATTTGTGCGCAGAAGTTCAATGGACTTACAG
TGTTGCCACCTCTGCTCACTGATGATATGATTGCTGCCTACACTGCTGCTCTAGTTAGTGGTACTGCCACTGCTGGATG
GACATTTGGTGCTGGCGCTGCTCTTCAAATACCTTTTGCTATGCAAATGGCATATAGGTTCAATGGCATTGGAGTTACC
CAAAATGTTCTCTATGAGAACCAAAAACAAATCGCCAACCAATTTAACAAGGCGATTAGTCAAATTCAAGAATCACTTA
CAACAACATCAACTGCATTGGGCAAGCTGCAAGACGTTGTTAACCAGAATGCTCAAGCATTAAACACACTTGTTAAACA
ACTTAGCTCTAATTTTGGTGCAATTTCAAGTGTGCTAAATGATATCCTTTCGCGACTTGATAAAGTCGAGGCGGAGGTA
CAAATTGACAGGTTAATTACAGGCAGACTTCAAAGCCTTCAAACCTATGTAACACAACAACTAATCAGGGCTGCTGAAA
TCAGGGCTTCTGCTAATCTTGCTGCTACTAAAATGTCTGAGTGTGTTCTTGGACAATCAAAAAGAGTTGACTTTTGCGG
AAAGGGCTACCACCTTATGTCCTTCCCACAAGCAGCCCCGCATGGTGTTGTCTTCCTACATGTCACGTATGTGCCATCC
CAGGAGAGGAACTTCACCACAGCGCCAGCAATTTGTCATGAAGGCAAAGCATACTTCCCTCGTGAAGGTGTTTTTGTGT
TTAATGGCACTTCTTGGTTTATTACACAGAGGAACTTCTTTTCTCCACAAATAATTACTACAGACAATACATTTGTCTC
AGGAAATTGTGATGTCGTTATTGGCATCATTAACAACACAGTTTATGATCCTCTGCAACCTGAGCTTGACTCATTCAAA
GAAGAGCTGGACAAGTACTTCAAAAATCATACATCACCAGATGTTGATCTTGGCGACATTTCAGGCATTAACGCTTCTG
TCGTCAACATTCAAGAAGAAATTGACCGCCTCAATGAGGTCGCTAAAAATTTAAATGAATCACTCATTGACCTTCAAGA
ATTGGGAAAATATGAGCAATATATTAAATGGCCTTGGTATGTTTGGCTCGGCTTCATTGCTGGACTAATTGCCATCGTC
ATGGTTACAATCTTGCTTTGTTGCATGACTAGTTGTTGCAGTTGCCTCAAGGGTGCATGCTCTTGTGGTTCTTGCTGCA
AGTTTGATGAGGATGACTCTGAGCCAGTTCTCAAGGGTGTCAAATTACATTACACATAA

>gi|38016580|gb|AY429072.1|_SARS_coronavirus_BJ302_clone_1_spike_glycoprotein_gene_complete_cds

ATGTTTATTTTCTTATTATTTTTACTCTCACTAGTGGTAGTGACCTTGACCGGTGCACCACTTTTGATGATGTT
CAAGCTCCTAATTACACTCAACATACTTCATCTATGAGGGGGGTTTACTATCCTGATGAAATTTTTAGATCAGACACTC
TTTATTTAACTCAGGATTTATTTCTTCCATTTTATTCTAATGTTACAGGGTTTCATACTATTAATCATACGTTTGGCAA
CCCTGTCATACCTTTTAAGGATGGTATTTATTTTGCTGCCACAGAGAAATCAAATGTTGTCCGTGGTTGGGTTTTTGGT
TCTACCATGAACAACAAGTCACAGTCGGTGATTATTATTAACAATTCTACTAATGTTGTTATACGAGCATGTAACTTTG
AATTGTGTGACAACCCTTTCTTTGCTGTTTCTAAACCCATGGGTACACAGACACATACTATGATATTCGATAATGCATT
TAATTGCACTTTCGAGTACATATCTGATGCCTTTTCGCTTGATGTTTCAGAAAAGTCAGGTAATTTTAAACACTTACGA
GAGTTTGTGTTTAAAAATAAAGATGGGTTTCTCTATGTTTATAAGGGCTATCAACCTATAGATGTAGTTCGTGATCTAC
CTTCTGGTTTTAACACTTTGAAACCTATTTTTAAGTTGCCTCTTGGTATTAACATTACAAATTTTAGAGCCATTCTTAC
AGCCTTTTCACCTGCTCAAGACACTTGGGGCACGTCAGCTGCAGCCTATTTTGTTGGCTATTTAAAGCCAACTACATTT
ATGCTCAAGTATGATGAAAATGGTACAATCACAGATGCTGTTGATTGTTCTCAAAATCCACTTGCTGAACTCAAATGCT

CTGTTAAGAGCTTTGAGATTGACAAAGGAATTTACCAGACCTCTAATTTCAGGGTTGTTCCCTCAGGAGATGTTGTGAG
ATTCCCTAATATTACAAACTTGTGTCCTTTTGGAGAGGTTTTTAATGCTACTAAATTCCCTTCTGTCTATGCATGGGAG
AGAAAAAAAAATTTCTAATTGTGTTGCTGATTACTCTGTGCTCTACAACTCAACATTTTTTTCAACCTTTAAGTGCTATG
GCGTTTCTGCCACTAAGTTGAATGATCTTTGCTTCTCCAATGTCTATGCAGATTCTTTTGTAGTCAAGGGAGATGATGT
AAGACAAATAGCGCCAGGACAAACTGGTGTTATTGCTGATTATAATTATAAATTGCCAGATGATTTCATGGGTTGTGTC
CTTGCTTGGAATACTAGGAACATTGATGCTACTTCAACTGGTAATTATAATTATAAATATAGGTATCTTAGACATGGCA
AGCTTAGGCCCTTTGAGAGAGACATATCTAATGTGCCTTTCTCCCCTGATGGCAAACCTTGCACCCCACCTGCTCTTAA
TTGTTATTGGCCATTAAATGATTATGGTTTTTACACCACTACTGGCATTGGCTACCAACCTTACAGAGTTGTAGTACTT
TCTTTTGAACTTTTAAATGCACCGGCCACGGTTTGTGGACCAAAATTATCCACTGACCTTATTAAGAACCAGTGTGTCA
ATTTTAATTTTAATGGACTCACTGGTACTGGTGTGTTAACTCCTTCTTCAAAGAGATTTCAACCATTTCAACAATTTGG
CCGTGATGTTTCTGATTTCACTGATTCCGTTCGAGATCCTAAAACATCTGAAATATTAGACATTTCACCTTGCTCTTTT
GGGGGTGTAAGTGTAATTACACCTGGAACAAATGCTTCATCTGAAGTTGCTGTTCTATATCAAGATGTTAACTGCACTG
ATGTTTCTACAGCAATTCATGCAGATCAACTCACACCAGCTTGGCGCATATATTCTACTGGAAACAATGTATTCCAGAC
TCAAGCAGGCTGTCTTATAGGAGCTGAGCATGTCGACACTTCTTATGAGTGCGACATTCCTATTGGAGCTGGCATTTGT
GCTAGTTACCATACAGTTTCTTTATTACGTAGTACTAGCCAAAAATCTATTGTGGCTTATACTATGTCTTTAGGTGCTG
ATAGTTCAATTGCTTACTCTAATAACACCATTGCTATACCTACTAACTTTTCAATTAGCATTACTACAGAAGTAATGCC
TGTTTCTATGGCTAAAACCTCCGTAGATTGTAATATGTACATCTGCGGAGATTCTACTGAATGTGCTAATTTGCTTCTC
CAATATGGTAGCTTTTGCACACAACTAAATCGTGCACTCTCAGGTATTGCTGCTGAACAGGATCGCAACACACGTGAAG
TGTTCGCTCAAGTCAAACAAATGTACAAAACCCCAACTTTGAAATATTTTGGTGGTTTTAATTTTTCACAAATATTACC
TGACCCTCTAAAGCCAACTAAGAGGTCTTTTATTGAGGACTTGCTCTTTAATAAGGTGACACTCGCTGATGCTGGCTTC
ATGAAGCAATATGGCGAATGCCTAGGTGATATTAATGCTAGAGATCTCATTTGTGCGCAGAAGTTCAATGGACTTACAG
TGTTGCCACCTCTGCTCACTGATGATATGATTGCTGCCTACACTGCTGCTCTAGTTAGTGGTACTGCCACTGCTGGATG
GACATTTGGTGCTGGCGCTGCTCTTCAAATACCTTTTGCTATGCAAATGGCATATAGGTTCAATGGCATTGGAGTTACC
CAAAATGTTCTCTATGAGAACCAAAAACAAATCGCCAACCAATTTAACAAGGCGATTAGTCAAATTCAAGAATCACTTA
CAACAACATCAACCGCATTGGGCAAGCTGCAAGACGTTGTTAACCAGAATGCTCAAGCATTAAACACACTTGTTAAACA
ACTTAGCTCTAATTTTGGTGCAATTTCAAGTGTGCTAAATGATATCCTTTCGCGACTTGATAAAGTCGAGGCGGAGGTA
CAAATTGACAGGTTAATTACAGGCAGACTTCAAAGCCTTCAAACCTATGTAACACAACAACTAATCAGGGCTGCTGAGA
TCAGGGCTTCTGCTAATCTTGCTGCTACTAAAATGTCTGAGTGTGTTCTTGGACAATCAAAAAGAGTTGGCTTTTGTGG
AAAGGGCTACCACCTTATGTCCTTCCCACAAGCAGCCCCGCATGGTGTTGTCTTCCTACATGTCACGTATGTGCCATCC
CAGGAGAGGAACTTCACCACAGCGCCAGCAATTTGTCATGAAGGCAAAGCATACTTCCCTCGTGAAGGTGTTTTTGTGT
TTAATGGCACTTCTTGGTTTATTACACAGAGGAACTTCTTTTCTCCACAAATAATTACTACAGACAATACATTTGTCTC
AGGAAATTGTGATGTCGTTATTGGCATCATTAACAACACAGTTTATGATCCTCTGCAACCTGAGCTTGACTCATTCAAA
GAAGAGCTGGACAAGTACTTCAAAAATCATACATCACCAGATGTTGATCTTGGCGACATTTCAGGCATTAACGCTTCTG
TCGTCAACATTCAAAAAGAAATTGACCGCCTCAATGAGGTCGCTAAAAATTTAAATGAATCACTCATTGACCTTCAAGA
ATTGGGAAAATATGAGCAATATATTAAATGGCCTTGGTATGTTTGGCTCGGCTTCATTGCTGGACTAATTGCCATCGTC
ATGGTTACAATCTTGCTTTGTTGCATGACTAGTTGTTGCAGTTGCCTCAAGGGTGCATGCTCTTGTGGTTCTTGCTGCA
AGTTTGATGAGGATGACTCTGAGCCAGTTCTCAAGGGTGTCAAATTACATTACACATAA

>gi|38016582|gb|AY429073.1|_SARS_coronavirus_BJ302_clone_2_spike_glycoprotein_gene_complete_cds

ATGTTTATTTTCTTATTATTTTTTACTCTCACTAGTGGTAGTGACCTTGACCGGTGCACCACTTTTGATGATGTT
CAAGCTCCTAATTACACTCAACATACTTCATCTATGAGGGGGGTTTACTATCCTGATGAAATTTTTAGATCAGACACTC

TTTATTTAACTCAGGATTTATTTCTTCCATTTTATTCTAATGTTACAGGGTTTCATACTATTAATCATACGTTTGGCAA
CCCTGTCATACCTTTTAAGGATGGTATTTATTTTGCTGCCACAGAGAAATCAAATGTTGTCCGTGGTTGGGTTTTTGGT
TCTACCATGAACAACAAGTCACAGTCGGTGATTATTATTAACAATTCTACTAATGTTGTTATACGAGCATGTAACTTTG
AATTGTGTGACAACCCTTTCTTTGCTGTTTCTAAACCCATGGGTACACAGACACATACTATGATATTCGATAATGCATT
TAATTGCACTTTCGAGTACATATCTGATGCCTTTTCGCTTGATGTTTCAGAAAAGTCAGGTAATTTTAAACACTTACGA
GAGTTTGTGTTTAAAAATAAAGATGGGTTTCTCTATGTTTATAAGGGCTATCAACCTATAGATGTAGTTCGTGATCTAC
CTTCTGGTTTTAACACTTTGAAACCTATTTTTAAGTTGCCTCTTGGTATTAACATTACAAATTTTAGAGCCATTCTTAC
AGCCTTTTCACCTGCTCAAGACACTTGGGGCACGTCAGCTGCAGCCTATTTTGTTGGCTATTTAAAGCCAACTACATTT
ATGCTCAAGTATGATGAAAATGGTACAATCACAGATGCTGTTGATTGTTCTCAAAATCCACTTGCTGAACTCAAATGCT
CTGTTAAGAGCTTTGAGATTGACAAAGGAATTTACCAGACCTCTAATTTCAGGGTTGTTCCCTCAGGAGATGTTGTGAG
ATTCCCTAATATTACAAACTTGTGTCCTTTTGGAGAGGTTTTTAATGCTACTAAATTCCCTTCTGTCTATGCATGGGAG
AGAAAAAAAAATTTCTAATTGTGTTGCTGATTACTCTGTGCTCTACAACTCAACATTTTTTTCAACCTTTAAGTGCTATG
GCGTTTCTGCCACTAAGTTGAATGATCTTTGCTTCTCCAATGTCTATGCAGATTCTTTTGTAGTCAAGGGAGATGATGT
AAGACAAATAGCGCCAGGACAAACTGGTGTTATTGCTGATTATAATTATAAATTGCCAGATGATTTCATGGGTTGTGTC
CTTGCTTGGAATACTAGGAACATTGATGCTACTTCAACTGGTAATTATAATTATAAATATAGGTATCTTAGACATGGCA
AGCTTAGGCCCTTTGAGAGAGACATATCTAATGTGCCTTTCTCCCCTGATGGCAAACCTTGCACCCCACCTGCTCTTAA
TTGTTATTGGCCATTAAGTGATTATGGTTTTTACACCACTACTGGCATTGGCTACCAACCTTACAGAGTTGTAGTACTT
TCTTTTGAACTTTTAAATGCACCGGCCACGGTTTGTGGACCAAAATTATCCACTGACCTTATTAAGAACCAGTGTGTCA
ATTTTAATTTTAATGGACTCACTGGTACTGGTGTGTTAACTCCTTCTTCAAAGAGATTTCAACCATTTCAACAATTTGG
CCGTGATGTTTCTGATTTCACTGATTCCGTTCGAGATCCTAAAACATCTGAAATATTAGACATTTCACCTCGCTCTTTT
GGGGGTGTAAGTGTAATTACACCTGGAACAAATGCTTCATCTGAAGTTGCTGTTCTATATCAAGATGTTAACTGCACTG
ATGTTTCTACAGCAATTCATGCAGATCAACTCACACCAGCTTGGCGCATATATTCTACTGGAAACAATGTATTCCAGAC
TCAAGCAGGCTGTCTTATAGGAGCTGAGCATGTCGACACTTCTTATGAGTGCGACATTCCTATTGGAGCTGGCATTTGT
GCTAGTTACCATACAGTTTCTTTATTACGTAGTACTAGCCAAAAATCTATTGTGGCTTATACTATGTCTTTAGGTGCTG
ATAGTTCAATTGCTTACTCTAATAACACCATTGCTATACCTACTAACTTTTCAATTAGCATTACTACAGAAGTAATGCC
TGTTTCTATGGCTAAAACCTCCGTAGATTGTAATATGTACATCTGCGGAGATTCTACTGAATGTGCTAATTTGCTTCTC
CAATATGGTAGCTTTTGCACACAACTAAATCGTGCACTCTCAGGTATTGCTGCTGAACAGGATCGCAACACACGTGAAG
TGTTCGCTCAAGTCAAACAAATGTACAAAACCCCAACTTTGAAATATTTTGGTGGTTTTAATTTTTCACAAATATTACC
TGACCCTCTAAAGCCAACTAAGAGGTCTTTTATTGAGGACTTGCTCTTTAATAAGGTGACACTCGCTGATGCTGGCTTC
ATGAAGCAATATGGCGAATGCCTAGGTGATATTAATGCTAGAGATCTCATTTGTGCGCAGAAGTTCAATGGACTTACAG
TGTTGCCACCTCTGCTCACTGTTGATATGATTGCTGCCTACACTGCTGCTCTAGTTAGTGGTACTGCCACTGCTGGATG
GACATTTGGTGCTGGCGCTGCTCTTCAAATACCTTTTGCTATGCAAATGGCATATAGGTTCAATGGCATTGGAGTTACC
CAAAATGTTCTCTATGAGAACCAAAAACAAATCGCCAACCAATTTAACAAGGCGATTAGTCAAATTCAAGAATCACTTA
CAACAACATCAACCGCATTGGGCAAGCTGCAAGACGTTGTTAACCAGAATGCTCAAGCATTAAACACACTTGTTAAACA
ACTTAGCTCTAATTTTGGTGCAATTTCAAGTGTGCTAAATGATATCCTTTCGCGACTTGATAAAGTCGAGGCGGAGGTA
CAAATTGACAGGTTAATTACAGGCAGACTTCAAAGCCTTCAAACCTATGTAACACAACAACTAATCAGGGCTGCTGAGA
TCAGGGCTTCTGCTAATCTTGCTGCTACTAAAATGTCTGAGTGTGTTCTTGGACAATCAAAAAGAGTTGGCTTTTGTGG
AAAGGGCTACCACCTTATGTCCTTCCCACAAGCAGCCCCGCATGGTGTTGTCTTCCTACATGTCACGTATGTGCCATCC
CAGGAGAGGAACTTCACCACAGCGCCAGCAATTTGTCATGAAGGCAAAGCATACTTCCCTCGTGAAGGTGTTTTTGTGT
TTAATGGCACTTCTTGGTTTATTACACAGAGGAACTTCTTTTCTCCACAAATAATTACTACAGACAATACATTTGTCTC
AGGAAATTGTGATGTCGTTATTGGCATCATTAACAACACAGTTTATGATCCTCTGCAACCTGAGCTTGACTCATTCAAA
GAAGAGCTGGACAAGTACTTCAAAAATCATACATCACCAGATGTTGATCTTGGCGACATTTCAGGCATTAACGCTTCTG
TCGTCAACATTCAAAAAGAAATTGACCGCCTCAATGAGGTCGCTAAAAATTTAAATGAATCACTCATTGACCTTCAAGA
ATTGGGAAAATATGAGCAATATATTAAATGGCCTTGGTATGTTTGGCTCGGCTTCATTGCTGGACTAATTGCCATCGTC

ATGGTTACAATCTTGCTTTGTTGCATGACTAGTTGTTGCAGTTGCCTCAAGGGTGCATGCTCTTGTGGTTCTTGCTGCA
AGTTTGATGAGGATGACTCTGAGCCAGTTCTCAAGGGTGTCAAATTACATTACACATAA

>gi|38016584|gb|AY429074.1|_SARS_coronavirus_BJ302_clone_3_spike_glycoprote
in_gene_complete_cds

ATGTTTATTTTCTTATTATTTTTACTCTCACTAGTGGTAGTGACCTTGACCGGTGCACCACTTTTGATGATGTT
CAAGCTCCTAATTACACTCAACATACTTCATCTATGAGGGGGGTTTACTATCCTGATGAAATTTTTAGATCAGACACTC
TTTATTTAACTCAGGATTTATTTCTTCCATTTTATTCTAATGTTACAGGGTTTCATACTATTAATCATACGTTTGGCAA
CCCTGTCATACCTTTTAAGGATGGTATTTATTTTGCTGCCACAGAGAAATCAAATGTTGTCCGTGGTTGGGTTTTTGGT
TCTACCATGAACAACAAGTCACAGTCGGTGATTATTATTAACAATTCTACTAATGTTGTTATACGAGCATGTAACTTTG
AATTGTGTGACAACCCTTTCTTTGCTGTTTCTAAACCCATGGGTACACAGACACATACTATGATATTCGATAATGCATT
TAATTGCACTTTCGAGTACATATCTGATGCCTTTTCGCTTGATGTTTCAGAAAAGTCAGGTAATTTTAAACACTTACGA
GAGTTTGTGTTTAAAAATAAAGATGGGTTTCTCTATGTTTATAAGGGCTATCAACCTATAGATGTAGTTCGTGATCTAC
CTTCTGGTTTTAACACTTTGAAACCTATTTTTAAGTTGCCTCTTGGTATTAACATTACAAATTTTAGAGCCATTCTTAC
AGCCTTTTCACCTGCTCAAGACACTTGGGGCACGTCAGCTGCAGCCTATTTTGTTGGCTATTTAAAGCCAACTACATTT
ATGCTCAAGTATGATGAAAATGGTACAATCACAGATGCTGTTGATTGTTCTCAAAATCCACTTGCTGAACTCAAATGCT
CTGTTAAGAGCTTTGAGATTGACAAAGGAATTTACCAGACCTCTAATTTCAGGGTTGTTCCCTCAGGAGATGTTGTGAG
ATTCCCTAATATTACAAACTTGTGTCCTTTTGGAGAGGTTTTTAATGCTACTAAATTCCCTTCTGTCTATGCATGGGAG
AGAAAAAAAATTTCTAATTGTGTTGCTGATTACTCTGTGCTCTACAACTCAACATTTTTTTCAACCTTTAAGTGCTATG
GCGTTTCTGCCACTAAGTTGAATGATCTTTGCTTCTCCAATGTCTATGCAGATTCTTTTGTAGTCAAGGGAGATGATGT
AAGACAAATAGCGCCAGGACAAACTGGTGTTATTGCTGATTATAATTATAAATTGCCAGATGATTTCATGGGTTGTGTC
CTTGCTTGGAATACTAGGAACATTGATGCTACTTCAACTGGTAATTATAATTATAAATATAGGTATCTTAGACATGGCA
AGCTTAGGCCCTTTGAGAGAGACATATCTAATGTGCCTTTCTCCCCTGATGGCAAACCTTGCACCCCACCTGCTCTTAA
TTGTTATTGGCCATTAAGTGATTATGGTTTTTACACCACTACTGGCATTGGCTACCAACCTTACAGAGTTGTAGTACTT
TCTTTTGAACTTTTAAATGCACCGGCCACGGTTTGTGGACCAAAATTATCCACTGACCTTATTAAGAACCAGTGTGTCA
ATTTTAATTTTAATGGACTCACTGGTACTGGTGTGTTAACTCCTTCTTCAAAGAGATTTCAACCATTTCAACAATTTGG
CCGTGATGTTTCTGATTTCACTGATTCCGTTCGAGATCCTAAAACATCTGAAATATTAGACATTTCACCTCGCTCTTTT
GGGGGTGTAAGTGTAATTACACCTGGAACAAATGCTTCATCTGAAGTTGCTGTTCTATATCAAGATGTTAACTGCACTG
ATGTTTCTACAGCAATTCATGCAGATCAACTCACACCAGCTTGGCGCATATATTCTACTGGAAACAATGTATTCCAGAC
TCAAGCAGGCTGTCTTATAGGAGCTGAGCATGTCGACACTTCTTATGAGTGCGACATTCCTATTGGAGCTGGCATTTGT
GCTAGTTACCATACAGTTTCTTTATTACGTAGTACTAGCCAAAAATCTATTGTGGCTTATACTATGTCTTTAGGTGCTG
ATAGTTCAATTGCTTACTCTAATAACACCATTGCTATACCTACTAACTTTTCAATTAGCATTACTACAGAAGTAATGCC
TGTTTCTATGGCTAAAACCTCCGTAGATTGTAATATGTACATCTGCGGAGATTCTACTGAATGTGCTAATTTGCTTCTC
CAATATGGTAGCTTTTGCACACAACTAAATCGTGCACTCTCAGGTATTGCTGCTGAACAGGATCGCAACACACGTGAAG
TGTTCGCTCAAGTCAAACAAATGTACAAAACCCCAACTTTGAAATATTTTGGTGGTTTTAATTTTTCACAAATATTACC
TGACCCTCTAAAGCCAACTAAGAGGTCTTTTATTGAGGACTTGCTCTTTAATAAGGTGACACTCGCTGATGCTGGCTTC
ATGAAGCAATATGGCGAATGCCTAGGTGATATTAATGCTAGAGATCTCATTTGTGCGCAGAAGTTCAATGGACTTACAG
TGTTGCCACCTCTGCTCACTGATGATATGATTGCTGCCTACACTGCTGCTCTAGTTAGTGGTACTGCCACTGCTGGATG
GACATTTGGTGCTGGCGCTGCTCTTCAAATACCTTTTGCTATGCAAATGGCATATAGGTTCAATGGCATTGGAGTTACC
CAAAATGTTCTCTATGAGAACCAAAAACAAATCGCCAACCAATTTAACAAGGCGATTAGTCAAATTCAAGAATCACTTA
CAACAACATCAACCGCATTGGGCAAGCTGCAAGACGTTGTTAACCAGAATGCTCAAGCATTAAACACACTTGTTAAACA
ACTTAGCTCTAATTTTGGTGCAATTTCAAGTGTGCTAAATGATATCCTTTCGCGACTTGATAAAGTCGAGGCGGAGGTA

CAAATTGACAGGTTAATTACAGGCAGACTTCAAAGCCTTCAAACCTATGTAACACAACAACTAATCAGGGCTGCTGAGA
TCAGGGCTTCTGCTAATCTTGCTGCTACTAAAATGTCTGAGTGTGTTCTTGGACAATCAAAAAGAGTTGGCTTTTGTGG
AAAGGGCTACCACCTTATGTCCTTCCCACAAGCAGCCCCGCATGGTGTTGTCTTCCTACATGTCACGTATGTGCCATCC
CAGGAGAGGAACTTCACCACAGCGCCAGCAATTTGTCATGAAGGCAAAGCATACTTCCCTCGTGAAGGTGTTTTTGTGT
TTAATGGCACTTCTTGGTTTATTACACAGAGGAACTTCTTTTCTCCACAAATAATTACTACAGACAATACATTTGTCTC
AGGAAATTGTGATGTCGTTATTGGCATCATTAACAACACAGTTTATGATCCTCTGCAACCTGAGCTTGACTCATTCAAA
GAAGAGCTGGACAAGTACTTCAAAAATCATACATCACCAGATGTTGATCTTGGCGACATTTCAGGCATTAACGCTTCTG
TCGTCAACATTCAAAAAGAAATTGACCGCCTCAATGAGGTCGCTAAAAATTTAAATGAATCACTCATTGACCTTCAAGA
ATTGGGAAAATATGAGCAATATATTAAATGGCCTTGGTATGTTTGGCTCGGCTTCATTGCTGGACTAATTGCCATCGTC
ATGGTTACAATCTTGCTTTGTTGCATGACTAGTTGTTGCAGTTGCCTCAAGGGTGCATGCTCTTGTGGTTCTTGCTGCA
AGTTTGATGAGGATGACTCTGAGCCAGTTCTCAAGGGTGTCAAATTACATTACACATAA

>gi|38016586|gb|AY429075.1|_SARS_coronavirus_BJ302_clone_4_spike_glycoprotein_gene_complete_cds

ATGTTTATTTTCTTATTATTTCTTACTCTCACTAGTGGTAGTGACCTTGACCGGTGCACCACTTTTGATGATGTT
CAAGCTCCTAATTACACTCAACATACTTCATCTATGAGGGGGGTTTACTATCCTGATGAAATTTTTAGATCAGACACTC
TTTATTTAACTCAGGATTTATTTCTTCCATTTTATTCTAATGTTACAGGGTTTCATACTATTAATCATACGTTTGGCAA
CCCTGTCATACCTTTTAAGGATGGTATTTATTTTGCTGCCACAGAGAAATCAAATGTTGTCCGTGGTTGGGTTTTTGGT
TCTACCATGAACAACAAGTCACAGTCGGTGATTATTATTAACAATTCTACTAATGTTGTTATACGAGCATGTAACTTTG
AATTGTGTGACAACCCTTTCTTTGCTGTTTCTAAACCCATGGGTACACAGACACATACTATGATATTCGATAATGCATT
TAATTGCACTTTCGAGTACATATCTGATGCCTTTTCGCTTGATGTTTCAGAAAAGTCAGGTAATTTTAAACACTTACGA
GAGTTTGTGTTTAAAAATAAAGATGGGTTTCTCTATGTTTATAAGGGCTATCAACCTATAGATGTAGTTCGTGATCTAC
CTTCTGGTTTTAACACTTTGAAACCTATTTTTAAGTTGCCTCTTGGTATTAACATTACAAATTTTAGAGCCATTCTTAC
AGCCTTTTCACCTGCTCAAGACACTTGGGGCACGTCAGCTGCAGCCTATTTTGTTGGCTATTTAAAGCCAACTACATTT
ATGCTCAAGTATGATGAAAATGGTACAATCACAGATGCTGTTGATTGTTCTCAAAATCCACTTGCTGAACTCAAATGCT
CTGTTAAGAGCTTTGAGATTGACAAAGGAATTTACCAGACCTCTAATTTCAGGGTTGTTCCCTCAGGAGATGTTGTGAG
ATTCCCTAATATTACAAACTTGTGTCCTTTTGGAGAGGTTTTTAATGCTACTAAATTCCCTTCTGTCTATGCATGGGAG
AGAAAAAAAATTTCTAATTGTGTTGCTGATTACTCTGTGCTCTACAACTCAACATTTTTTTCAACCTTTAAGTGCTATG
GCGTTTCTGCCACTAAGTTGAATGATCTTTGCTTCTCCAATGTCTATGCAGATTCTTTTGTAGTCAAGGGAGATGATGT
AAGACAAATAGCGCCAGGACAAACTGGTGTTATTGCTGATTATAATTATAAATTGCCAGATGATTTCATGGGTTGTGTC
CTTGCTTGGAATACTAGGAACATTGATGCTACTTCAACTGGTAATTATAATTATAAATATAGGTATCTTAGACATGGCA
AGCTTAGGCCCTTTGAGAGAGACATATCTAATGTGCCTTTCTCCCCTGATGGCAAACCTTGCACCCCACCTGCTCTTAA
TTGTTATTGGCCATTAAGTGATTATGGTTTTTACACCACTACTGGCATTGGCTACCAACCTTACAGAGTTGTAGTACTT
TCTTTTGAACTTTTAAATGCACCGGCCACGGTTTGTGGACCAAAATTATCCACTGACCTTATTAAGAACCAGTGTGTCA
ATTTTAATTTTAATGGACTCACTGGTACTGGTGTGTTAACTCCTTCTTCAAAGAGATTTCAACCATTTCAACAATTTGG
CCGTGATGTTTCTGATTTCACTGATTCCGTTCGAGATCCTAAAACATCTGAAATATTAGACATTTCACCTCGCTCTTTT
GGGGGTGTAAGTGTAATTACACCTGGAACAAATGCTTCATCTGAAGTTGCTGTTCTATATCAAGATGTTAACTGCACTG
ATGTTTCTACAGCAATTCATGCAGATCAACTCACACCAGCTTGGCGCATATATTCTACTGGAAACAATGTATTCCAGAC
TCAAGCAGGCTGTCTTATAGGAGCTGAGCATGTCGACACTTCTTATGAGTGCGACATTCCTATTGGAGCTGGCATTTGT
GCTAGTTACCATACAGTTTCTTTATTACGTAGTACTAGCCAAAAATCTATTGTGGCTTATACTATGTCTTTAGGTGCTG
ATAGTTCAATTGCTTACTCTAATAACACCATTGCTATACCTACTAACTTTTCAATTAGCATTACTACAGAAGTAATGCC
TGTTTCTATGGCTAAAACCTCCGTAGATTGTAATATGTACATCTGCGGAGATTCTACTGAATGTGCTAATTTGCTTCTC

CAATATGGTAGCTTTTGCACACAACTAAATCGTGCACTCTCAGGTATTGCTGCTGAACAGGATCGCAACACACGTGAAG
TGTTCGCTCAAGTCAAACAAATGTACAAAACCCCAACTTTGAAATATTTTGGTGGTTTTAATTTTTCACAAATATTACC
TGACCCTCTAAAGCCAACTAAGAGGTCTTTTATTGAGGACTTGCTCTTTAATAAGGTGACACTCGCTGATGCTGGCTTC
ATGAAGCAATATGGCGAATGCCTAGGTGATATTAATGCTAGAGATCTCATTTGTGCGCAGAAGTTCAATGGACTTACAG
TGTTGCCACCTCTGCTCACTGTTGATATGATTGCTGCCTACACTGCTGCTCTAGTTAGTGGTACTGCCACTGCTGGATG
GACATTTGGTGCTGGCGCTGCTCTTCAAATACCTTTTGCTATGCAAATGGCATATAGGTTCAATGGCATTGGAGTTACC
CAAAATGTTCTCTATGAGAACCAAAAACAAATCGCCAACCAATTTAACAAGGCGATTAGTCAAATTCAAGAATCACTTA
CAACAACATCAACCGCATTGGGCAAGCTGCAAGACGTTGTTAACCAGAATGCTCAAGCATTAAACACACTTGTTAAACA
ACTTAGCTCTAATTTTGGTGCAATTTCAAGTGTGCTAAATGATATCCTTTCGCGACTTGATAAAGTCGAGGCGGAGGTA
CAAATTGACAGGTTAATTACAGGCAGACTTCAAAGCCTTCAAACCTATGTAACACAACAACTAATCAGGGCTGCTGAGA
TCAGGGCTTCTGCTAATCTTGCTGCTACTAAAATGTCTGAGTGTGTTCTTGGACAATCAAAAAGAGTTGGCTTTTGTGG
AAAGGGCTACCACCTTATGTCCTTCCCACAAGCAGCCCCGCATGGTGTTGTCTTCCTACATGTCACGTATGTGCCATCC
CAGGAGAGGAACTTCACCACAGCGCCAGCAATTTGTCATGAAGGCAAAGCATACTTCCCTCGTGAAGGTGTTTTTGTGT
TTAATGGCACTTCTTGGTTTATTACACAGAGGAACTTCTTTTCTCCACAAATAATTACTACAGACAATACATTTGTCTC
AGGAAATTGTGATGTCGTTATTGGCATCATTAACAACACAGTTTATGATCCTCTGCAACCTGAGCTTGACTCATTCAAA
GAAGAGCTGGACAAGTACTTCAAAAATCATACATCACCAGATGTTGATCTTGGCGACATTTCAGGCATTAACGCTTCTG
TCGTCAACATTCAAAAAGAAATTGACCGCCTCAATGAGGTCGCTAAAAATTTAAATGAATCACTCATTGACCTTCAAGA
ATTGGGAAAATATGAGCAATATATTAAATGGCCTTGGTATGTTTGGCTCGGCTTCATTGCTGGACTAATTGCCATCGTC
ATGGTTACAATCTTGCTTTGTTGCATGACTAGTTGTTGCAGTTGCCTCAAGGGTGCATGCTCTTGTGGTTCTTGCTGCA
AGTTTGATGAGGATGACTCTGAGCCAGTTCTCAAGGGTGTCAAATTACATTACACATAA

>gi|38016588|gb|AY429076.1|_SARS_coronavirus_BJ302_clone_5_spike_glycoprotein_gene_complete_cds

ATGTTTATTTTCTTATTATTTTTACTCTCACTAGTGGTAGTGACCTTGACCGGTGCACCACTTTTGATGATGTT
CAAGCTCCTAATTACACTCAACATACTTCATCTATGAGGGGGGTTTACTATCCTGATGAAATTTTTAGATCAGACACTC
TTTATTTAACTCAGGATTTATTTCTTCCATTTTATTCTAATGTTACAGGGTTTCATACTATTAATCATACGTTTGGCAA
CCCTGTCATACCTTTTAAGGATGGTATTTATTTTGCTGCCACAGAGAAATCAAATGTTGTCCGTGGTTGGGTTTTTGGT
TCTACCATGAACAACAAGTCACAGTCGGTGATTATTATTAACAATTCTACTAATGTTGTTATACGAGCATGTATCTTTG
AATTGTGTGACAACCCTTTCTTTGCTGTTTCTAAACCCATGGGTACACAGACACATACTATGATATTCGATAATGCATT
TAATTGCACTTTCGAGTACATATCTGATGCCTTTTCGCTTGATGTTTCAGAAAAGTCAGGTAATTTTAAACACTTACGA
GAGTTTGTGTTTAAAAATAAAGATGGGTTTCTCTATGTTTATAAGGGCTATCAACCTATAGATGTAGTTCGTGATCTAC
CTTCTGGTTTTAACACTTTGAAACCTATTTTTAAGTTGCCTCTTGGTATTAACATTACAAATTTTAGAGCCATTCTTAC
AGCCTTTTCACCTGCTCAAGACACTTGGGGCACGTCAGCTGCAGCCTATTTTGTTGGCTATTTAAAGCCAACTACATTT
ATGCTCAAGTATGATGAAAATGGTACAATCACAGATGCTGTTGATTGTTCTCAAAATCCACTTGCTGAACTCAAATGCT
CTGTTAAGAGCTTTGAGATTGACAAAGGAATTTACCAGACCTCTAATTTCAGGGTTGTTCCCTCAGGAGATGTTGTGAG
ATTCCCTAATATTACAAACTTGTGTCCTTTTGGAGAGGTTTTTAATGCTACTAAATTCCCTTCTGTCTATGCATGGGAG
AGAAAAAAAATTTCTAATTGTGTTGCTGATTACTCTGTGCTCTACAACTCAACATTTTTTTCAACCTTTAAGTGCTATG
GCGTTTCTGCCACTAAGTTGAATGATCTTTGCTTCTCCAATGTCTATGCAGATTCTTTTGTAGTCAAGGGAGATGATGT
AAGACAAATAGCGCCAGGACAAACTGGTGTTATTGCTGATTATAATTATAAATTGCCAGATGATTTCATGGGTTGTGTC
CTTGCTTGGAATACTAGGAACATTGATGCTACTTCAACTGGTAATTATAATTATAAATATAGGTATCTTAGACATGGCA
AGCTTAGGCCCTTTGAGAGAGACATATCTAATGTGCCTTTCTCCCCTGATGGCAAACCTTGCACCCCACCTGCTCTTAA
TTGTTATTGGCCATTAAGTGATTATGGTTTTTACACCACTACTGGCATTGGCTACCAACCTTACAGAGTTGTAGTACTT

TCTTTTGAACTTTTAAATGCACCGGCCACGGTTTGTGGACCAAAATTATCCACTGACCTTATTAAGAACCAGTGTGTCA
ATTTTAATTTTAATGGACTCACTGGTACTGGTGTGTTAACTCCTTCTTCAAAGAGATTTCAACCATTTCAACAATTTGG
CCGTGATGTTTCTGATTTCACTGATTCCGTTCGAGATCCTAAAACATCTGAAATATTAGACATTTCACCTCGCTCTTTT
GGGGGTGTAAGTGTAATTACACCTGGAACAAATGCTTCATCTGAAGTTGCTGTTCTATATCAAGATGTTAACTGCACTG
ATGTTTCTACAGCAATTCATGCAGATCAACTCACACCAGCTTGGCGCATATATTCTACTGGAAACAATGTATTCCAGAC
TCAAGCAGGCTGTCTTATAGGAGCTGAGCATGTCGACACTTCTTATGAGTGCGACATTCCTATTGGAGCTGGCATTTGT
GCTAGTTACCATACAGTTTCTTTATTACGTAGTACTAGCCAAAAATCTATTGTGGCTTATACTATGTCTTTAGGTGCTG
ATAGTTCAATTGCTTACTCTAATAACACCATTGCTATACCTACTAACTTTTCAATTAGCATTACTACAGAAGTAATGCC
TGTTTCTATGGCTAAAACCTCCGTAGATTGTAATATGTACATCTGCGGAGATTCTACTGAATGTGCTAATTTGCTTCTC
CAATATGGTAGCTTTTGCACACAACTAAATCGTGCACTCTCAGGTATTGCTGCTGAACAGGATCGCAACACACGTGAAG
TGTTCGCTCAAGTCAAACAAATGTACAAAACCCCAACTTTGAAATATTTTGGTGGTTTTAATTTTTCACAAATATTACC
TGACCCTCTAAAGCCAACTAAGAGGTCTTTTATTGAGGACTTGCTCTTTAATAAGGTGACACTCGCTGATGCTGGCTTC
ATGAAGCAATATGGCGAATGCCTAGGTGATATTAATGCTAGAGATCTCATTTGTGCGCAGAAGTTCAATGGACTTACAG
TGTTGCCACCTCTGCTCACTGTTGATATGATTGCTGCCTACACTGCTGCTCTAGTTAGTGGTACTGCCACTGCTGGATG
GACATTTGGTGCTGGCGCTGCTCTTCAAATACCTTTTGCTATGCAAATGGCATATAGGTTCAATGGCATTGGAGTTACC
CAAAATGTTCTCTATGAGAACCAAAAACAAATCGCCAACCAATTTAACAAGGCGATTAGTCAAATTCAAGAATCACTTA
CAACAACATCAACCGCATTGGGCAAGCTGCAAGACGTTGTTAACCAGAATGCTCAAGCATTAAACACACTTGTTAAACA
ACTTAGCTCTAATTTTGGTGCAATTTCAAGTGTGCTAAATGATATCCTTTCGCGACTTGATAAAGTCGAGGCGGAGGTA
CAAATTGACAGGTTAATTACAGGCAGACTTCAAAGCCTTCAAACCTATGTAACACAACAACTAATCAGGGCTGCTGAGA
TCAGGGCTTCTGCTAATCTTGCTGCTACTAAAATGTCTGAGTGTGTTCTTGGACAATCAAAAAGAGTTGGCTTTTGTGG
AAAGGGCTACCACCTTATGTCCTTCCCACAAGCAGCCCCGCATGGTGTTGTCTTCCTACATGTCACGTATGTGCCATCC
CAGGAGAGGAACTTCACCACAGCGCCAGCAATTTGTCATGAAGGCAAAGCATACTTCCCTCGTGAAGGTGTTTTTGTGT
TTAATGGCACTTCTTGGTTTATTACACAGAGGAACTTCTTTTCTCCACAAATAATTACTACAGACAATACATTTGTCTC
AGGAAATTGTGATGTCGTTATTGGCATCATTAACAACACAGTTTATGATCCTCTGCAACCTGAGCTTGACTCATTCAAA
GAAGAGCTGGACAAGTACTTCAAAAATCATACATCACCAGATGTTGATCTTGGCGACATTTCAGGCATTAACGCTTCTG
TCGTCAACATTCAAAAAGAAATTGACCGCCTCAATGAGGTCGCTAAAAATTTAAATGAATCACTCATTGACCTTCAAGA
ATTGGGAAAATATGAGCAATATATTAAATGGCCTTGGTATGTTTGGCTCGGCTTCATTGCTGGACTAATTGCCATCGTC
ATGGTTACAATCTTGCTTTGTTGCATGACTAGTTGTTGCAGTTGCCTCAAGGGTGCATGCTCTTGTGGTTCTTGCTGCA
AGTTTGATGAGGATGACTCTGAGCCAGTTCTCAAGGGTGTCAAATTACATTACACATAA

>gi|38016590|gb|AY429077.1|_SARS_coronavirus_BJ302_clone_6_spike_glycoprotein_gene_complete_cds

ATGTTTATTTTCTTATTATTTTTACTCTCACTAGTGGTAGTGACCTTGACCGGTGCACCACTTTTGATGATGTT
CAAGCTCCTAATTACACTCAACATACTTCATCTATGAGGGGGGTTTACTATCCTGATGAAATTTTTAGATCAGACACTC
TTTATTTAACTCAGGATTTATTTCTTCCATTTTATTCTAATGTTACAGGGTTTCATACTATTAATCATACGTTTGGCAA
CCCTGTCATACCTTTTAAGGATGGTATTTATTTTGCTGCCACAGAGAAATCAAATGTTGTCCGTGGTTGGGTTTTTGGT
TCTACCATGAACAACAAGTCACAGTCGGTGATTATTATTAACAATTCTACTAATGTTGTTATACGAGCATGTAACTTTG
AATTGTGTGACAACCCTTTCTTTGCTGTTTCTAAACCCATGGGTACACAGACACATACTATGATATTCGATAATGCATT
TAATTGCACTTTCGAGTACATATCTGATGCCTTTTCGCTTGATGTTTCAGAAAAGTCAGGTAATTTTAAACACTTACGA
GAGTTTGTGTTTAAAAATAAAGATGGGTTTCTCTATGTTTATAAGGGCTATCAACCTATAGATGTAGTTCGTGATCTAC
CTTCTGGTTTTAACACTTTGAAACCTATTTTTAAGTTGCCTCTTGGTATTAACATTACAAATTTTAGAGCCATTCTTAC
AGCCTTTTCACCTGCTCAAGACACTTGGGGCACGTCAGCTGCAGCCTATTTTGTTGGCTATTTAAAGCCAACTACATTT

ATGCTCAAGTATGATGAAAATGGTACAATCACAGATGCTGTTGATTGTTCTCAAAATCCACTTGCTGAACTCAAATGCT
CTGTTAAGAGCTTTGAGATTGACAAAGGAATTTACCAGACCTCTAATTTCAGGGTTGTTCCCTCAGGAGATGTTGTGAG
ATTCCCTAATATTACAAACTTGTGTCCTTTTGGAGAGGTTTTTAATGCTACTAAATTCCCTTCTGTCTATGCATGGGAG
AGAAAAAAAATTTCTAATTGTGTTGCTGATTACTCTGTGCTCTACAACTCAACATTTTTTTCAACCTTTAAGTGCTATG
GCGTTTCTGCCACTAAGTTGAATGATCTTTGCTTCTCCAATGTCTATGCAGATTCTTTTGTAGTCAAGGGAGATGATGT
AAGACAAATAGCGCCAGGACAAACTGGTGTTATTGCTGATTATAATTATAAATTGCCAGATGATTTCATGGGTTGTGTC
CTTGCTTGGAATACTAGGAACATTGATGCTACTTCAACTGGTAATTATAATTATAAATATAGGTATCTTAGACATGGCA
AGCTTAGGCCCTTTGAGAGAGACATATCTAATGTGCCTTTCTCCCCTGATGGCAAACCTTGCACCCCACCTGCTCTTAA
TTGTTATTGGCCATTAAGTGATTATGGTTTTTACACCACTACTGGCATTGGCTACCAACCTTACAGAGTTGTAGTACTT
TCTTTTGAACTTTTAAATGCACCGGCCACGGTTTGTGGACCAAAATTATCCACTGACCTTATTAAGAACCAGTGTGTCA
ATTTTAATTTTAATGGACTCACTGGTACTGGTGTGTTAACTCCTTCTTCAAAGAGATTTCAACCATTTCAACAATTTGG
CCGTGATGTTTCTGATTTCACTGATTCCGTTCGAGATCCTAAAACATCTGAAATATTAGACATTTCACCTTGCTCTTTT
GGGGGTGTAAGTGTAATTACACCTGGAACAAATGCTTCATCTGAAGTTGCTGTTCTATATCAAGATGTTAACTGCACTG
ATGTTTCTACAGCAATTCATGCAGATCAACTCACACCAGCTTGGCGCATATATTCTACTGGAAACAATGTATTCCAGAC
TCAAGCAGGCTGTCTTATAGGAGCTGAGCATGTCGACACTTCTTATGAGTGCGACATTCCTATTGGAGCTGGCATTTGT
GCTAGTTACCATACAGTTTCTTTATTACGTAGTACTAGCCAAAAATCTATTGTGGCTTATACTATGTCTTTAGGTGCTG
ATAGTTCAATTGCTTACTCTAATAACACCATTGCTATACCTACTAACTTTTCAATTAGCATTACTACAGAAGTAATGCC
TGTTTCTATGGCTAAAACCTCCGTAGATTGTAATATGTACATCTGCGGAGATTCTACTGAATGTGCTAATTTGCTTCTC
CAATATGGTAGCTTTTGCACACAACTAAATCGTGCACTCTCAGGTATTGCTGCTGAACAGGATCGCAACACACGTGAAG
TGTTCGCTCAAGTCAAACAAATGTACAAAACCCCAACTTTGAAATATTTTGGTGGTTTTAATTTTTCACAAATATTACC
TGACCCTCTAAAGCCAACTAAGAGGTCTTTTATTGAGGACTTGCTCTTTAATAAGGTGACACTCGCTGATGCTGGCTTC
ATGAAGCAATATGGCGAATGCCTAGGTGATATTAATGCTAGAGATCTCATTTGTGCGCAGAAGTTCAATGGACTTACAG
TGTTGCCACCTCTGCTCACTGTTGATATGATTGCTGCCTACACTGCTGCTCTAGTTAGTGGTACTGCCACTGCTGGATG
GACATTTGGTGCTGGCGCTGCTCTTCAAATACCTTTTGCTATGCAAATGGCATATAGGTTCAATGGCATTGGAGTTACC
CAAAATGTTCTCTATGAGAACCAAAAACAAATCGCCAACCAATTTAACAAGGCGATTAGTCAAATTCAAGAATCACTTA
CAACAACATCAACCGCATTGGGCAAGCTGCAAGACGTTGTTAACCAGAATGCTCAAGCATTAAACACACTTGTTAAACA
ACTTAGCTCTAATTTTGGTGCAATTTCAAGTGTGCTAAATGATATCCTTTCGCGACTTGATAAAGTCGAGGCGGAGGTA
CAAATTGACAGGTTAATTACAGGCAGACTTCAAAGCCTTCAAACCTATGTAACACAACAACTAATCAGGGCTGCTGAGA
TCAGGGCTTCTGCTAATCTTGCTGCTACTAAAATGTCTGAGTGTGTTCTTGGACAATCAAAAAGAGTTGGCTTTTGTGG
AAAGGGCTACCACCTTATGTCCTTCCCACAAGCAGCCCCGCATGGTGTTGTCTTCCTACATGTCACGTATGTGCCATCC
CAGGAGAGGAACTTCACCACAGCGCCAGCAATTTGTCATGAAGGCAAAGCATACTTCCCTCGTGAAGGTGTTTTTGTGT
TTAATGGCACTTCTTGGTTTATTACACAGAGGAACTTCTTTTCTCCACAAATAATTACTACAGACAATACATTTGTCTC
AGGAAATTGTGATGTCGTTATTGGCATCATTAACAACACAGTTTATGATCCTCTGCAACCTGAGCTTGACTCATTCAAA
GAAGAGCTGGACAAGTACTTCAAAAATCATACATCACCAGATGTTGATCTTGGCGACATTTCAGGCATTAACGCTTCTG
TCGTCAACATTCAAAAAGAAATTGACCGCCTCAATGAGGTCGCTAAAAATTTAAATGAATCACTCATTGACCTTCAAGA
ATTGGGAAAATATGAGCAATATATTAAATGGCCTTGGTATGTTTGGCTCGGCTTCATTGCTGGACTAATTGCCATCGTC
ATGGTTACAATCTTGCTTTGTTGCATGACTAGTTGTTGCAGTTGCCTCAAGGGTGCATGCTCTTGTGGTTCTTGCTGCA
AGTTTGATGAGGATGACTCTGAGCCAGTTCTCAAGGGTGTCAAATTACATTACACATAA

>gi|38016592|gb|AY429078.1|_SARS_coronavirus_BJ302_clone_7_spike_glycoprotein_gene_complete_cds

ATGTTTATTTTCTTATTATTTCTTACTCTCACTAGTGGTAGTGACCTTGACCGGTGCACCACTTTTGATGATGTT

CAAGCTCCTAATTACACTCAACATACTTCATCTATGAGGGGGGTTTACTATCCTGATGAAATTTTTAGATCAGACACTC
TTTATTTAACTCAGGATTTATTTCTTCCATTTTATTCTAATGTTACAGGGTTTCATACTATTAATCATACGTTTGGCAA
CCCTGTCATACCTTTTAAGGATGGTATTTATTTTGCTGCCACAGAGAAATCAAATGTTGTCCGTGGTTGGGTTTTTGGT
TCTACCATGAACAACAAGTCACAGTCGGTGATTATTATTAACAATTCTACTAATGTTGTTATACGAGCATGTAACTTTG
AATTGTGTGACAACCCTTTCTTTGCTGTTTCTAAACCCATGGGTACACAGACACATACTATGATATTCGATAATGCATT
TAATTGCACTTTCGAGTACATATCTGATGCCTTTTCGCTTGATGTTTCAGAAAAGTCAGGTAATTTTAAACACTTACGA
GAGTTTGTGTTTAAAAATAAAGATGGGTTTCTCTATGTTTATAAGGGCTATCAACCTATAGATGTAGTTCGTGATCTAC
CTTCTGGTTTTAACACTTTGAAACCTATTTTTAAGTTGCCTCTTGGTATTAACATTACAAATTTTAGAGCCATTCTTAC
AGCCTTTTCACCTGCTCAAGACACTTGGGGCACGTCAGCTGCAGCCTATTTTGTTGGCTATTTAAAGCCAACTACATTT
ATGCTCAAGTATGATGAAAATGGTACAATCACAGATGCTGTTGATTGTTCTCAAAATCCACTTGCTGAACTCAAATGCT
CTGTTAAGAGCTTTGAGATTGACAAAGGAATTTACCAGACCTCTAATTTCAGGGTTGTTCCCTCAGGAGATGTTGTGAG
ATTCCCTAATATTACAAACTTGTGTCCTTTTGGAGAGGTTTTTAATGCTACTAAATTCCCTTCTGTCTATGCATGGGAG
AGAAAAAAAATTTCTAATTGTGTTGCTGATTACTCTGTGCTCTACAACTCAACATTTTTTTCAACCTTTAAGTGCTATG
GCGTTTCTGCCACTAAGTTGAATGATCTTTGCTTCTCCAATGTCTATGCAGATTCTTTTGTAGTCAAGGGAGATGATGT
AAGACAAATAGCGCCAGGACAAACTGGTGTTATTGCTGATTATAATTATAAATTGCCAGATGATTTCATGGGTTGTGTC
CTTGCTTGGAATACTAGGAACATTGATGCTACTTCAACTGGTAATTATAATTATAAATATAGGTATCTTAGACATGGCA
AGCTTAGGCCCTTTGAGAGAGACATATCTAATGTGCCTTTCTCCCCTGATGGCAAACCTTGCACCCCACCTGCTCTTAA
TTGTTATTGGCCATTAAATGATTATGGTTTTTACACCACTACTGGCATTGGCTACCAACCTTACAGAGTTGTAGTACTT
TCTTTTGAACTTTTAAATGCACCGGCCACGGTTTGTGGACCAAAATTATCCACTGACCTTATTAAGAACCAGTGTGTCA
ATTTTAATTTTAATGGACTCACTGGTACTGGTGTGTTAACTCCTTCTTCAAAGAGATTTCAACCATTTCAACAATTTGG
CCGTGATGTTTCTGATTTCACTGATTCCGTTCGAGATCCTAAAACATCTGAAATATTAGACATTTCACCTTGCTCTTTT
GGGGGTGTAAGTGTAATTACACCTGGAACAAATGCTTCATCTGAAGTTGCTGTTCTATATCAAGATGTTAACTGCACTG
ATGTTTCTACAGCAATTCATGCAGATCAACTCACACCAGCTTGGCGCATATATTCTACTGGAAACAATGTATTCCAGAC
TCAAGCAGGCTGTCTTATAGGAGCTGAGCATGTCGACACTTCTTATGAGTGCGACATTCCTATTGGAGCTGGCATTTGT
GCTAGTTACCATACAGTTTCTTTATTACGTAGTACTAGCCAAAAATCTATTGTGGCTTATACTATGTCTTTAGGTGCTG
ATAGTTCAATTGCTTACTCTAATAACACCATTGCTATACCTACTAACTTTTCAATTAGCATTACTACAGAAGTAATGCC
TGTTTCTATGGCTAAAACCTCCGTAGATTGTAATATGTACATCTGCGGAGATTCTACTGAATGTGCTAATTTGCTTCTC
CAATATGGTAGCTTTTGCACACAACTAAATCGTGCACTCTCAGGTATTGCTGCTGAACAGGATCGCAACACACGTGAAG
TGTTCGCTCAAGTCAAACAAATGTACAAAACCCCAACTTTGAAATATTTTGGTGGTTTTAATTTTTCACAAATATTACC
TGACCCTCTAAAGCCAACTAAGAGGTCTTTTATTGAGGACTTGCTCTTTAATAAGGTGACACTCGCTGATGCTGGCTTC
ATGAAGCAATATGGCGAATGCCTAGGTGATATTAATGCTAGAGATCTCATTTGTGCGCAGAAGTTCAATGGACTTACAG
TGTTGCCACCTCTGCTCACTGATGATATGATTGCTGCCTACACTGCTGCTCTAGTTAGTGGTACTGCCACTGCTGGATG
GACATTTGGTGCTGGCGCTGCTCTTCAAATACCTTTTGCTATGCAAATGGCATATAGGTTCAATGGCATTGGAGTTACC
CAAAATGTTCTCTATGAGAACCAAAAACAAATCGCCAACCAATTTAACAAGGCGATTAGTCAAATTCAAGAATCACTTA
CAACAACATCAACTGCATTGGGCAAGCTGCAAGACGTTGTTAACCAGAATGCTCAAGCATTAAACACACTTGTTAAACA
ACTTAGCTCTAATTTTGGTGCAATTTCAAGTGTGCTAAATGATATCCTTTCGCGACTTGATAAAGTCGAGGCGGAGGTA
CAAATTGACAGGTTAATTACAGGCAGACTTCAAAGCCTTCAAACCTATGTAACACAACAACTAATCAGGGCTGCTGAAA
TCAGGGCTTCTGCTAATCTTGCTGCTACTAAAATGTCTGAGTGTGTTCTTGGACAATCAAAAAGAGTTGACTTTTGTGG
AAAGGGCTACCACCTTATGTCCTTCCCACAAGCAGCCCCGCATGGTGTTGTCTTCCTACATGTCACGTATGTGCCATCC
CAGGAGAGGAACTTCACCACAGCGCCAGCAATTTGTCATGAAGGCAAAGCATACTTCCCTCGTGAAGGTGTTTTTGTGT
TTAATGGCACTTCTTGGTTTATTACACAGAGGAACTTCTTTTCTCCACAAATAATTACTACAGACAATACATTTGTCTC
AGGAAATTGTGATGTCGTTATTGGCATCATTAACAACACAGTTTATGATCCTCTGCAACCTGAGCTTGACTCATTCAAA
GAAGAGCTGGACAAGTACTTCAAAAATCATACATCACCAGATGTTGATCTTGGCGACATTTCAGGCATTAACGCTTCTG
TCGTCAACATTCAAAAAGAAATTGACCGCCTCAATGAGGTCGCTAAAAATTTAAATGAATCACTCATTGACCTTCAAGA

ATTGGGAAAATATGAGCAATATATTAAATGGCCTTGGTATGTTTGGCTCGGCTTCATTGCTGGACTAATTGCCATCGTC
ATGGTTACAATCTTGCTTTGTTGCATGACTAGTTGTTGCAGTTGCCTCAAGGGTGCATGCTCTTGTGGTTCTTGCTGCA
AGTTTGATGAGGATGACTCTGAGCCAGTTCTCAAGGGTGTCAAATTACATTACACATAA

>gi|38016594|gb|AY429079.1|_SARS_coronavirus_BJ302_clone_8_spike_glycoprotein_gene_complete_cds

ATGTTTATTTTCTTATTATTTCTTACTCTCACTAGTGGTAGTGACCTTGACCGGTGCACCACTTTTGATGATGTT
CAAGCTCCTAATTACACTCAACATACTTCATCTATGAGGGGGGTTTACTATCCTGATGAAATTTTTAGATCAGACACTC
TTTATTTAACTCAGGATTTATTTCTTCCATTTTATTCTAATGTTACAGGGTTTCATACTATTAATCATACGTTTGGCAA
CCCTGTCATACCTTTTAAGGATGGTATTTATTTTGCTGCCACAGAGAAATCAAATGTTGTCCGTGGTTGGGTTTTTGGT
TCTACCATGAACAACAAGTCACAGTCGGTGATTATTATTAACAATTCTACTAATGTTGTTATACGAGCATGTATCTTTG
AATTGTGTGACAACCCTTTCTTTGCTGTTTCTAAACCCATGGGTACACAGACACATACTATGATATTCGATAATGCATT
TAATTGCACTTTCGAGTACATATCTGATGCCTTTTCGCTTGATGTTTCAGAAAAGTCAGGTAATTTTAAACACTTACGA
GAGTTTGTGTTTAAAAATAAAGATGGGTTTCTCTATGTTTATAAGGGCTATCAACCTATAGATGTAGTTCGTGATCTAC
CTTCTGGTTTTAACACTTTGAAACCTATTTTTAAGTTGCCTCTTGGTATTAACATTACAAATTTTAGAGCCATTCTTAC
AGCCTTTTCACCTGCTCAAGACACTTGGGGCACGTCAGCTGCAGCCTATTTTGTTGGCTATTTAAAGCCAACTACATTT
ATGCTCAAGTATGATGAAAATGGTACAATCACAGATGCTGTTGATTGTTCTCAAAATCCACTTGCTGAACTCAAATGCT
CTGTTAAGAGCTTTGAGATTGACAAAGGAATTTACCAGACCTCTAATTTCAGGGTTGTTCCCTCAGGAGATGTTGTGAG
ATTCCCTAATATTACAAACTTGTGTCCTTTTGGAGAGGTTTTTAATGCTACTAAATTCCCTTCTGTCTATGCATGGGAG
AGAAAAAAAATTTCTAATTGTGTTGCTGATTACTCTGTGCTCTACAACTCAACATTTTTTTCAACCTTTAAGTGCTATG
GCGTTTCTGCCACTAAGTTGAATGATCTTTGCTTCTCCAATGTCTATGCAGATTCTTTTGTAGTCAAGGGAGATGATGT
AAGACAAATAGCGCCAGGACAAACTGGTGTTATTGCTGATTATAATTATAAATTGCCAGATGATTTCATGGGTTGTGTC
CTTGCTTGGAATACTAGGAACATTGATGCTACTTCAACTGGTAATTATAATTATAAATATAGGTATCTTAGACATGGCA
AGCTTAGGCCCTTTGAGAGAGACATATCTAATGTGCCTTTCTCCCCTGATGGCAAACCTTGCACCCCACCTGCTCTTAA
TTGTTATTGGCCATTAAGTGATTATGGTTTTTACACCACTACTGGCATTGGCTACCAACCTTACAGAGTTGTAGTACTT
TCTTTTGAACTTTTAAATGCACCGGCCACGGTTTGTGGACCAAAATTATCCACTGACCTTATTAAGAACCAGTGTGTCA
ATTTTAATTTTAATGGACTCACTGGTACTGGTGTGTTAACTCCTTCTTCAAAGAGATTTCAACCATTTCAACAATTTGG
CCGTGATGTTTCTGATTTCACTGATTCCGTTCGAGATCCTAAAACATCTGAAATATTAGACATTTCACCTCGCTCTTTT
GGGGGTGTAAGTGTAATTACACCTGGAACAAATGCTTCATCTGAAGTTGCTGTTCTATATCAAGATGTTAACTGCACTG
ATGTTTCTACAGCAATTCATGCAGATCAACTCACACCAGCTTGGCGCATATATTCTACTGGAAACAATGTATTCCAGAC
TCAAGCAGGCTGTCTTATAGGAGCTGAGCATGTCGACACTTCTTATGAGTGCGACATTCCTATTGGAGCTGGCATTTGT
GCTAGTTACCATACAGTTTCTTTATTACGTAGTACTAGCCAAAAATCTATTGTGGCTTATACTATGTCTTTAGGTGCTG
ATAGTTCAATTGCTTACTCTAATAACACCATTGCTATACCTACTAACTTTTCAATTAGCATTACTACAGAAGTAATGCC
TGTTTCTATGGCTAAAACCTCCGTAGATTGTAATATGTACATCTGCGGAGATTCTACTGAATGTGCTAATTTGCTTCTC
CAATATGGTAGCTTTTGCACACAACTAAATCGTGCACTCTCAGGTATTGCTGCTGAACAGGATCGCAACACACGTGAAG
TGTTCGCTCAAGTCAAACAAATGTACAAAACCCCAACTTTGAAATATTTTGGTGGTTTTAATTTTTCACAAATATTACC
TGACCCTCTAAAGCCAACTAAGAGGTCTTTTATTGAGGACTTGCTCTTTAATAAGGTGACACTCGCTGATGCTGGCTTC
ATGAAGCAATATGGCGAATGCCTAGGTGATATTAATGCTAGAGATCTCATTTGTGCGCAGAAGTTCAATGGACTTACAG
TGTTGCCACCTCTGCTCACTGTTGATATGATTGCTGCCTACACTGCTGCTCTAGTTAGTGGTACTGCCACTGCTGGATG
GACATTTGGTGCTGGCGCTGCTCTTCAAATACCTTTTGCTATGCAAATGGCATATAGGTTCAATGGCATTGGAGTTACC
CAAAATGTTCTCTATGAGAACCAAAAACAAATCGCCAACCAATTTAACAAGGCGATTAGTCAAATTCAAGAATCACTTA
CAACAACATCAACCGCATTGGGCAAGCTGCAAGACGTTGTTAACCAGAATGCTCAAGCATTAAACACACTTGTTAAACA

ACTTAGCTCTAATTTTGGTGCAATTTCAAGTGTGCTAAATGATATCCTTTCGCGACTTGATAAAGTCGAGGCGGAGGTA
CAAATTGACAGGTTAATTACAGGCAGACTTCAAAGCCTTCAAACCTATGTAACACAACAACTAATCAGGGCTGCTGAGA
TCAGGGCTTCTGCTAATCTTGCTGCTACTAAAATGTCTGAGTGTGTTCTTGGACAATCAAAAAGAGTTGGCTTTTGTGG
AAAGGGCTACCACCTTATGTCCTTCCCACAAGCAGCCCCGCATGGTGTTGTCTTCCTACATGTCACGTATGTGCCATCC
CAGGAGAGGAACTTCACCACAGCGCCAGCAATTTGTCATGAAGGCAAAGCATACTTCCCTCGTGAAGGTGTTTTTGTGT
TTAATGGCACTTCTTGGTTTATTACACAGAGGAACTTCTTTTCTCCACAAATAATTACTACAGACAATACATTTGTCTC
AGGAAATTGTGATGTCGTTATTGGCATCATTAACAACACAGTTTATGATCCTCTGCAACCTGAGCTTGACTCATTCAAA
GAAGAGCTGGACAAGTACTTCAAAAATCATACATCACCAGATGTTGATCTTGGCGACATTTCAGGCATTAACGCTTCTG
TCGTCAACATTCAAAAAGAAATTGACCGCCTCAATGAGGTCGCTAAAAATTTAAATGAATCACTCATTGACCTTCAAGA
ATTGGGAAAATATGAGCAATATATTAAATGGCCTTGGTATGTTTGGCTCGGCTTCATTGCTGGACTAATTGCCATCGTC
ATAGTTACAATCTTGCTTTGTTGCATGACTAGTTGTTGCAGTTGCCTCAAGGGTGCATGCTCTTGTGGTTCTTGCTGCA
AGTTTGATGAGGATGACTCTGAGCCAGTTCTCAAGGGTGTCAAATTACATTACACATAA

>gi|49176846|gb|AY595412.1| SARS coronavirus LLJ-2004, complete genome

ATGTTTATTTTCTTATTATTTCTTACTCTCACTAGTGGTAGTGACCTTGACCGGTGCACCACTTTTGATGATGTT
CAAGCTCCTAATTACACTCAACATACTTCATCTATGAGGGGGGTTTACTATCCTGATGAAATTTTTAGATCAGACACTC
TTTATTTAACTCAGGATTTATTTCTTCCATTTTATTCTAATGTTACAGGGTTTCATACTATTAATCATACGTTTGACAA
CCCTGTCATACCTTTTAAGGATGGTATTTATTTTGCTGCCACAGAGAAATCAAATGTTGTCCGTGGTTGGGTTTTTGGT
TCTACCATGAACAACAAGTCACAGTCGGTGATTATTATTAACAATTCTACTAATGTTGTTATACGAGCATGTAACTTTG
AATTGTGTGACAACCCTTTCTTTGCTGTTTCTAAACCCATGGGTACACAGACACATACTATGATATTCGATAATGCATT
TAATTGCACTTTCGAGTACATATCTGATGCCTTTTCGCTTGATGTTTCAGAAAAGTCAGGTAATTTTAAACACTTACGA
GAGTTTGTGTTTAAAAATAAAGATGGGTTTCTCTATGTTTATAAGGGCTATCAACCTATAGATGTAGTTCGTGATCTAC
CTTCTGGTTTTAACACTTTGAAACCTATTTTTAAGTTGCCTCTTGGTATTAACATTACAAATTTTAGAGCCATTCTTAC
AGCCTTTTCACCTGCTCAAGACACTTGGGGCACGTCAGCTGCAGCCTATTTTGTTGGCTATTTAAAGCCAACTACATTT
ATGCTCAAGTATGATGAAAATGGTACAATCACAGATGCTGTTGATTGTTCTCAAAATCCACTTGCTGAACTCAAATGCT
CTGTTAAGAGCTTTGAGATTGACAAAGGAATTTACCAGACCTCTAATTTCAGGGTTGTTCCCTCAGGAGATGTTGTGAG
ATTCCCTAATATTACAAACTTGTGTCCTTTTGGAGAGGTTTTTAATGCTACTAAATTCCCTTCTGTCTATGCATGGGAG
AGAAAAAAAATTTCTAATTGTGTTGCTGATTACTCTGTGCTCTACAACTCAACATTTTTTTCAACCTTTAAGTGCTATG
GCGTTTCTGCCACTAAGTTGAATGATCTTTGCTTCTCCAATGTCTATGCAGATTCTTTTGTAGTCAAGGGAGATGATGT
AAGACAAATAGCGCCAGGACAAACTGGTGTTATTGCTGATTATAATTATAAATTGCCAGATGATTTCATGGGTTGTGTC
CTTGCTTGGAATACTAGGAACATTGATGCTACTTCAACTGGTAATTATAATTATAAATATAGGTATCTTAGACATGGCA
AGCTTAGGCCCTTTGAGAGAGACATATCTAATGTGCCTTTCTCCCCTGATGGCAAACCTTGCACCCCACCTGCTCTTAA
TTGTTATTGGCCATTAAATGATTATGGTTTTTACACCACTACTGGCATTGGCTACCAACCTTACAGAGTTGTAGTACTT
TCTTTTGAACTTTTAAATGCACCGGCCACGGTTTGTGGACCAAAATTATCCACTGACCTTATTAAGAACCAGTGTGTCA
ATTTTAATTTTAATGGACTCACTGGTACTGGTGTGTTAACTCCTTCTTCAAAGAGATTTCAACCATTTCAACAATTTGG
CCGTGATGTTTCTGATTTCACTGATTCCGTTCGAGATCCTAAAACATCTGAAATATTAGACATTTCACCTTGCTCTTTT
GGGGGTGTAAGTGTAATTACACCTGGAACAAATGCTTCATCTGAAGTTGCTGTTCTATATCAAGATGTTAACTGCACTG
ATGTTTCTACAGCAATTCATGCAGATCAACTCACACCAGCTTGGCGCATATATTCTACTGGAAACAATGTATTCCAGAC
TCAAGCAGGCTGTCTTATAGGAGCTGAGCATGTCGACACTTCTTATGAGTGCGACATTCCTATTGGAGCTGGCATTTGT
GCTAGTTACCATACAGTTTCTTTATTACGTAGTACTAGCCAAAAATCTATTGTGGCTTATACTATGTCTTTAGGTGCTG
ATAGTTCAATTGCTTACTCTAATAACACCATTGCTATACCTACTAACTTTTCAATTAGCATTACTACAGAAGTAATGCC
TGTTTCTATGGCTAAAACCTCCGTAGATTGTAATATGTACATCTGCGGAGATTCTACTGAATGTGCTAATTTGCTTCTC

CAATATGGTAGCTTTTGCACACAACTAAATCGTGCACTCTCAGGTATTGCTGCTGAACAGGATCGCAACACACGTGAAG
TGTTCGCTCAAGTCAAACAAATGTACAAAACCCCAACTTTGAAATATTTTGGTGGTTTTAATTTTTCACAAATATTACC
TGACCCTCTAAAGCCAACTAAGAGGTCTTTTATTGAGGACTTGCTCTTTAATAAGGTGACACTCGCTGATGCTGGCTTC
ATGAAGCAATATGGCGAATGCCTAGGTGATATTAATGCTAGAGATCTCATTTGTGCGCAGAAGTTCAATGGACTTACAG
TGTTGCCACCTCTGCTCACTGATGATATGATTGCTGCCTACACTGCTGCTCTAGTTAGTGGTACTGCCACTGCTGGATG
GACATTTGGTGCTGGCGCTGCTCTTCAAATACCTTTTGCTATGCAAATGGCATATAGGTTCAATGGCATTGGAGTTACC
CAAAATGTTCTCTATGAGAACCAAAAACAAATCGCCAACCAATTTAACAAGGCGATTAGTCAAATTCAAGAATCACTTA
CAACAACATCAACTGCATTGGGCAAGCTGCAAGACGTTGTTAACCAGAATGCTCAAGCATTAAACACACTTGTTAAACA
ACTTAGCTCTAATTTTGGTGCAATTTCAAGTGTGCTAAATGATATCCTTTCGCGACTTGATAAAGTCGAGGCGGAGGTA
CAAATTGACAGGTTAATTACAGGCAGACTTCAAAGCCTTCAAACCTATGTAACACAACAACTAATCAGGGCTGCTGAAA
TCAGGGCTTCTGCTAATCTTGCTGCTACTAAAATGTCTGAGTGTGTTCTTGGACAATCAAAAAGAGTTGACTTTTGTGG
AAAGGGCTACCACCTTATGTCCTTCCCACAAGCAGCCCCGCATGGTGTTGTCTTCCTACATGTCACGTATGTGCCATCC
CAGGAGAGGAACTTCACCACAGCGCCAGCAATTTGTCATGAAGGCAAAGCATACTTCCCTCGTGAAGGTGTTTTTGTGT
TTAATGGCACTTCTTGGTTTATTACACAGAGGAACTTCTTTTCTCCACAAATAATTACTACAGACAATACATTTGTCTC
AGGAAATTGTGATGTCGTTATTGGCATCATTAACAACACAGTTTATGATCCTCTGCAACCTGAGCTTGACTCATTCAAA
GAAGAGCTGGACAAGTACTTCAAAAATCATACATCACCAGATGTTGATCTTGGCGACATTTCAGGCATTAACGCTTCTG
TCGTCAACATTCAAAAAGAAATTGACCGCCTCAATGAGGTCGCTAAAAATTTAAATGAATCACTCATTGACCTTCAAGA
ATTGGGAAAATATGAGCAATATATTAAATGGCCTTGGTATGTTTGGCTCGGCTTCATTGCTGGACTAATTGCCATCGTC
ATGGTTACAATCTTGCTTTGTTGCATGACTAGTTGTTGCAGTTGCCTCAAGGGTGCATGCTCTTGTGGTTCTTGCTGCA
AGTTTGATGAGGATGACTCTGAGCCAGTTCTCAAGGGTGTCAAATTACATTACACATAA

>gi|50345907|gb|AY648300.1|_SARS_coronavirus_HHS2004_spike_protein_(S)_mRNA_complete_cds

ATGGCTATTTTCTTATTATTTCTTACTCTCACTAGTGGTAGTGACCTTGACCGGTGCACCACTTTTGATGATGTT
CAAGCTCCTAATTACACTCAACATACTTCATCTATGAGGGGGGTTTACTATCCTGATGAAATTTTTAGATCAGACACTC
TTTATTTAACTCAGGATTTATTTCTTCCATTTTATTCTAATGTTACAGGGTTTCATACTATTAATCATACGTTTGACAA
CCCTGTCATACCTTTTAAGGATGGTATTTATTTTGCTGCCACAGAGAAATCAAATGTTGTCCGTGGTTGGGTTTTTGGT
TCTACCATGAACAACAAGTCACAGTCGGTGATTATTATTAACAATTCTACTAATGTTGTTATACGAGCATGTAACTTTG
AATTGTGTGACAACCCTTTCTTTGCTGTTTCTAAACCCATGGGTACACAGACACATACTATGATATTCGATAATGCATT
TAATTGCACTTTCGAGTACATATCTGATGCCTTTTCGCTTGATGTTTCAGAAAAGTCAGGTAATTTTAAACACTTACGA
GAGTTTGTGTTTAAAAATAAAGATGGGTTTCTCTATGTTTATAAGGGCTATCAACCTATAGATGTAGTTCGTGATCTAC
CTTCTGGTTTTAACACTTTGAAACCTATTTTTAAGTTGCCTCTTGGTATTAACATTACAAATTTTAGAGCCATTCTTAC
AGCCTTTTCACCTGCTCAAGACACTTGGGGCACGTCAGCTGCAGCCTATTTTGTTGGCTATTTAAAGCCAACTACATTT
ATGCTCAAGTATGATGAAAATGGTACAATCACAGATGCTGTTGATTGTTCTCAAAATCCACTTGCTGAACTCAAATGCT
CTGTTAAGAGCTTTGAGATTGACAAAGGAATTTACCAGACCTCTAATTTCAGGGTTGTTCCCTCAGGAGATGTTGTGAG
ATTCCCTAATATTACAAACTTGTGTCCTTTTGGAGAGGTTTTTAATGCTACTAAATTCCCTTCTGTCTATGCATGGGAG
AGAAAAAAAATTTCTAATTGTGTTGCTGATTACTCTGTGCTCTACAACTCAACATTTTTTTCAACCTTTAAGTGCTATG
GCGTTTCTGCCACTAAGTTGAATGATCTTTGCTTCTCCAATGTCTATGCAGATTCTTTTGTAGTCAAGGGAGATGATGT
AAGACAAATAGCGCCAGGACAAACTGGTGTTATTGCTGATTATAATTATAAATTGCCAGATGATTTCATGGGTTGTGTC
CTTGCTTGGAATACTAGGAACATTGATGCTACTTCAACTGGTAATTATAATTATAAATATAGGTATCTTAAACATGGCA
AGCTTAGGCCCTTTGAGAGAGACATATCTAATGTGCCTTTCTCCCCTGATGGCAAACCTTGCACCCCACCTGCTCTTAA
TTGTTATTGGCCATTAAATGATTATGGTTTTTACACCACTACTGGCATTGGCTACCAACCTTACAGAGTTGTAGTACTT

TCTTTTGAACTTTTAAATGCACCGGCCACGGTTTGTGGACCAAAATTATCCACTGACCTTATTAAGAACCAGTGTGTCA
ATTTTAATTTTAATGGACTCACTGGTACTGGTGTGTTAACTCCTTCTTCAAAGAGATTTCAACCATTTCAACAATTTGG
CCGTGATGTTTCTGATTTCACTGATTCCGTTCGAGATCCTAAAACATCTGAAATATTAGACATTTCACCTTGCTCTTTT
GGGGGTGTAAGTGTAATTACACCTGGAACAAATGCTTCATCTGAAGTTGCTGTTCTATATCAAGATGTTAACTGCACTG
ATGTTTCTACAGCAATTCATGCAGATCAACTCACACCAGCTTGGCGCATATATTCTACTGGAAACAATGTATTCCAGAC
TCAAGCAGGCTGTCTTATAGGAGCTGAGCATGTCGACACTTCTTATGAGTGCGACATTCCTATTGGAGCTGGCATTTGT
GCTAGTTACCATACAGTTTCTTTATTACGTAGTACTAGCCAAAAATCTATTGTGGCTTATACTATGTCTTTAGGTGCTG
ATAGTTCAATTGCTTACTCTAATAACACCATTGCTATACCTACTAACTTTTCAATTAGCATTACTACAGAAGTAATGCC
TGTTTCTATGGCTAAAACCTCCGTAGATTGTAATATGTACATCTGCGGAGATTCTACTGAATGTGCTAATTTGCTTCTC
CAATATGGTAGCTTTTGCACACAACTAAATCGTGCACTCTCAGGTATTGCTGCTGAACAGGATCGCAACACACGTGAAG
TGTTCGCTCAAGTCAAACAAATGTACAAAACCCCAACTTTGAAATATTTTGGTGGTTTTAATTTTTCACAAATATTACC
TGACCCTCTAAAGCCAACTAAGAGGTCTTTTATTGAGGACTTGCTCTTTAATAAGGTGACACTCGCTGATGCTGGCTTC
ATGAAGCAATATGGCGAATGCCTAGGTGATATTAATGCTAGAGATCTCATTTGTGCGCAGAAGTTCAATGGACTTACAG
TGTTGCCACCTCTGCTCACTGATGATATGATTGCTGCCTACACTGCTGCTCTAGTTAGTGGTACTGCCACTGCTGGATG
GACATTTGGTGCTGGCGCTGCTCTTCAAATACCTTTTGCTATGCAAATGGCATATAGGTTCAATGGCATTGGAGTTACC
CAAAATGTTCTCTATGAGAACCAAAAACAAATCGCCAACCAATTTAACAAGGCGATTAGTCAAATTCAAGAATCACTTA
CAACAACATCAACTGCATTGGGCAAGCTGCAAGACGTTGTTAACCAGAATGCTCAAGCATTAAACACACTTGTTAAACA
ACTTAGCTCTAATTTTGGTGCAATTTCAAGTGTGCTCAATGATGTCCTTTCGCGACTTGATAAAGTCGAGGCGGAGGTA
CAAATTGACAGGTTAATTACAGGCAGACTTCAAAGCCTTCAAACCTATGTAACACAACAACTAATCAGGGCTGCTGAAA
TCAGGGCTTCTGCTAATCTTGCTGCTACTAAAATGTCTGAGTGTGTTCTTGGACAATCAAAAAGAGTTGACTTTTGTGG
AAAGGGCTACCACCTTATGTCCTTCCCACAAGCAGCCCCGCATGGTGTTGTCTTCCTACATGTCACGTATGTGCCATCC
CAGGAGAGGAACTTCACCACAGCGCCAGCAATTTGTCATGAAGGCAAAGCATACTTCCCTCGTGAAGGTGTTTTTGTGT
TTAATGGCACTTCTTGGTTTATTACACAGAGGAACTTCTTTTCTCCACAAATAATTACTACAGACAATACATTTGTCTC
AGGAAATTGTGATGTCGTTATTGGCATCATTAACAACACAGTTTATGATCCTCTGCAACCTGAGCTTGACTCATTCAAA
GAAGAGCTGGACAAGTACTTCAAAAATCATACATCACCAGATGTTGATCTTGGCGACATTTCAGGCATTAACGCTTCTG
TCGTCAACATTCAAAAAGAAATTGACCGCCTCAATGAGGTCGCTAAAAATTTAAATGAATCACTCATTGACCTTCAAGA
ATTGGGAAAATATGAGCAATATATTAAATGGCCTTGGTATGTTTGGCTCGGCTTCATTGCTGGACTAATTGCCATCGTC
ATGGTTACAATCTTGCTTTGTTGCATGACTAGTTGTTGCAGTTGCCTCAAGGGTGCATGCTCTTGTGGTTCTTGCTGCA
AGTTTGATGAGGATGACTCTGAGCCAGTTCTCAAGGGTGTCAAATTACATTACACATGA

>gi|82393565|gb|DQ231462.2|_SARS_coronavirus_ZJ02_spike_glycoprotein_gene_complete_cds

ATGTTTATTTTCTTATTATTTCTTACTCTCACTAGTGGTAGTGACCTTGACCGGTGCACCACTTTTGATGATGTT
CAAGCTCCTAATTACACTCAACATACTTCATCTATGAGGGGGGTTTACTATCCTGATGAAATTTTTAGATCAGACACTC
TTTATTTAACTCAGGATTTATTTCTTCCATTTTATTCTAATGTTACAGGGTTTCATACTATTAATCATACGTTTGGCAA
CCCTGTCATACCTTTTAAGGATGGTATTTATTTTGCTGCCACAGAGAAATCAAATGTTGTCCGTGGTTGGGTTTTTGGT
TCTACCATGAACAACAAGTCACAGTCGGTGATTATTATTAACAATTCTACTAATGTTGTTATACGAGCATGTAACTTTG
AATTGTGTGACAACCCTTTCTTTGCTGTTTCTAAACCCATGGGTACACAGACACATACTATGATATTCGATAATGCATT
TAATTGCACTTTCGAGTACATATCTGATGCCTTTTCGCTTGATGTTTCAGAAAAGTCAGGTAATTTTAAACACTTACGA
GAGTTTGTGTTTAAAAATAAAGATGGGTTTCTCTATGTTTATAAGGGCTATCAACCTATAGATGTAGTTCGTGATCTAC
CTTCTGGTTTTAACACTTTGAAACCTATTTTTAAGTTGCCTCTTGGTATTAACATTACAAATTTTAGAGCCATTCTTAC
AGCCTTTTCACCTGCTCAAGACATTTGGGGCACGTCAGCTGCAGCCTATTTTGTTGGCTATTTAAAGCCAACTACATTT

ATGCTCAAGTATGATGAAAATGGTACAATCACAGATGCTGTTGATTGTTCTCAAAATCCACTTGCTGAACTCAAATGCT
CTGTTAAGAGCTTTGAGATTGACAAAGGAATTTACCAGACCTCTAATTTCAGGGTTGTTCCCTCAGGAGATGTTGTGAG
ATTCCCTAATATTACAAACTTGTGTCCTTTTGGAGAGGTTTTTAATGCTACTAAATTCCCTTCTGTCTATGCATGGGAG
AGAAAAAAAATTTCTAATTGTGTTGCTGATTACTCTGTGCTCTACAACTCAACATTTTTTTCAACCTTTAAGTGTTATG
GCGTTTCTGCCACTAAGTTGAATGATCTTTGCTTCTCCAATGTCTATGCAGATTCTTTTGTAGTCAAGGGAGATGATGT
AAGACAAATAGCGCCAGGACAAACTGGTGTTATTGCTGATTATAATTATAAATTGCCAGATGATTTCATGGGTTGTGTC
CTTGCTTGGAATACTAGGAACATTGATGCTACTTCAACTGGTAATTATAATTATAAATATAGGTATCTTAGACATGGCA
AGCTTAGGCCCTTTGAGAGAGACATATCTAATGTGCCTTTCTCCCCTGATGGCAAACCTTGCACCCCACCTGCTCTTAA
TTGTTATTGGCCATTAAATGATTATGGTTTTTACACCACTACTGGCATTGGCTACCAACCTTACAGAGTTGTAGTACTT
TCTTTTGAACTTTTAAATGCACCGGCCACGGTTTGTGGACCAAAATTATCCACTGACCTTATTAAGAACCAGTGTGTCA
ATTTTAATTTTAATGGACTCACTGGTACTGGTGTGTTAACTCCTTCTTCAAAGAGATTTCAACCATTTCAACAATTTGG
CCGTGATGTTTCTGATTTCACTGATTCCGTTCGAGATCCTAAAACATCTGAAATATTAGACATTTCACCTTGCTCTTTT
GGGGGTGTAAGTGTAATTACACCTGGAACAAATGCTTCATCTGAAGTTGCTGTTCTATATCAAGATGTTAACTGCACTG
ATGTTTCTACAGCAATTCATGCAGATCAACTCACACCAGCTTGGCGCATATATTCTACTGGAAACAATGTATTCCAGAC
TCAAGCAGGCTGTCTTATAGGAGCTGAGCATGTCGACACTTCTTATGAGTGCGACATTCCTATTGGAGCTGGCATTTGT
GCTAGTTACCATACAGTTTCTTTATTACGTAGTACTAGCCAAAAATCTATTGTGGCTTATACTATGTCTTTAGGTGCTG
ATAGTTCAATTGCTTACTCTAATAACACCATTGCTATACCTACTAACTTTTCAATTAGCATTACTACAGAAGTAATGCC
TGTTTCTATGGCTAAAACCTCCGTAGATTGTAATATGTACATCTGCGGAGATTCTACTGAATGTGCTAATTTGCTTCTC
CAATATGGTAGCTTTTGCACACAACTAAATCGTGCACTCTCAGGTATTGCTGCTGAACAGGATCGCAACACACGTGAAG
TGTTCGCTCAAGTCAAACAAATGTACAAAACCCCAACTTTGAAATATTTTGGTGGTTTTAATTTTTCACAAATATTACC
TGACCCTCTAAAGCCAACTAAGAGGTCTTTTATTGAGGACTTGCTCTTTAATAAGGTGACACTCGCTGATGCTGGCTTC
ATGAAGCAATATGGCGAATGCCTAGGTGATATTAATGCTAGAGATCTCATTTGTGCGCAGAAGTTCAATGGACTTACAG
TGTTGCCACCTCTGCTCACTGATGATATGATTGCTGCCTACACTGCTGCTCTAGTTAGTGGTACTGCCACTGCTGGATG
GACATTTGGTGCTGGCGCTGCTCTTCAAATACCTTTTGCTATGCAAATGGCATATAGGTTCAATGGCATTGGAGTTACC
CAAAATGTTCTCTATGAGAACCAAAAACAAATCGCCAACCAATTTAACAAGGCGATTAGTCAAATTCAAGAATCACTTA
CAACAACATCAACTGCATTGGGCAAGCTGCAAGACGTTGTTAACCAGAATGCTCAAGCATTAAACACACTTGTTAAACA
ACTTAGCTCTAATTTTGGTGCAATTTCAAGTGTGCTAAATGATATCCTTTCGCGACTTGATAAAGTCGAGGCGGAGGTA
CAAATTGACAGGTTAATTACAGGCAGACTTCAAAGCCTTCAAACCTATGTAACACAACAACTAATCAGGGCTGCTGAAA
TCAGGGCTTCTGCTAATCTTGCTGCTACTAAAATGTCTGAGTGTGTTCTTGGACAATCAAAAAGAGTTGACTTTTGTGG
AAAGGGCTACCACCTTATGTCCTTCCCACAAGCAGCCCCGCATGGTGTTGTCTTCCTACATGTCACGTATGTGCCATCC
CAGGAGAGGAACTTCACCACAGCGCCAGCAATTTGTCATGAAGGCAAAGCATACTTCCCTCGTGAAGGTGTTTTTGTGT
TTAATGGCACTTCTTGGTTTATTACACAGAGGAACTTCTTTTCTCCACAAATAATTACTACAGACAATACATTTGTCTC
AGGAAATTGTGATGTCGTTATTGGCATCATTAACAACACAGTTTATGATCCTCTGCAACCTGAGCTTGACTCATTCAAA
GAAGAGCTGGACAAGTACTTCAAAAATCATACATCACCAGATGTTGATCTTGGCGACATTTCAGGCATTAACGCTTCTG
TCGTCAACATTCAAAAAGAAATTGACCGCCTCAATGAGGTCGCTAAAAATTTAAATGAATCACTCATTGACCTTCAAGA
ATTGGGAAAATATGAGCAATATATTAAATGGCCTTGGTATGTTTGGCTCGGCTTCATTGCTGGACTAATTGCCATCGTC
ATGGTTACAATCTTGCTTTGTTGCATGACTAGTTGTTGCAGTTGCCTCAAGGGTGCATGCTCTTGTGGTTCTTGCTGCA
AGTTTGATGAGGATGACTCTGAGCCAGTTCTCAAGGGTGTCAAATTACATTACACATAA

>gi|31416290|gb|AY278489.2| SARS coronavirus GD01, complete genome

ATGTTTATTTTCTTATTATTTCTTACTCTCACTAGTGGTAGTGACCTTGACCGGTGCACCACTTTTGATGATGTT
CAAGCTCCTAATTACACTCAACATACTTCATCTATGAGGGGGGTTTACTATCCTGATGAAATTTTTAGATCAGACACTC

TTTATTTAACTCAGGATTTATTTCTTCCATTTTATTCTAATGTTACAGGGTTTCATACTATTAATCATACGTTTGACAA
CCCTGTCATACCTTTTAAGGATGGTATTTATTTTGCTGCCACAGAGAAATCAAATGTTGTCCGTGGTTGGGTTTTTGGT
TCTACCATGAACAACAAGTCACAGTCGGTGATTATTATTAACAATTCTACTAATGTTGTTATACGAGCATGTAACTTTG
AATTGTGTGACAACCCTTTCTTTGCTGTTTCTAAACCCATGGGTACACAGACACATACTATGATATTCGATAATGCATT
TAATTGCACTTTCGAGTACATATCTGATGCCTTTTCGCTTGATGTTTCAGAAAAGTCAGGTAATTTTAAACACTTACGA
GAGTTTGTGTTTAAAAATAAAGATGGGTTTCTCTATGTTTATAAGGGCTATCAACCTATAGATGTAGTTCGTGATCTAC
CTTCTGGTTTTAACACTTTGAAACCCATTTTTAAGTTGCCTCTTGGTATTAACATTACAAATTTTAGAGCCATTCTTAC
AGCCTTTTTACCTGCTCAAGACACTTGGGGCACGTCAGCTGCAGCCTATTTTGTTGGCTATTTAAAGCCAACTACATTT
ATGCTCAAGTATGATGAAAATGGTACAATCACAGATGCTGTTGATTGTTCTCAAAATCCACTTGCTGAACTCAAATGCT
CTGTTAAGAGCTTTGAGATTGACAAAGGAATTTACCAGACCTCTAATTTCAGGGTTGTTCCCTCAAGAGATGTTGTGAG
ATTCCCTAATATTACAAACTTGTGTCCTTTTGGAGAGGTTTTTAATGCTACTAAATTCCCTTCTGTCTATGCATGGGAG
AGGAAAAGAATTTCTAATTGTGTTGCTGATTACTCTGTGCTCTACAACTCAACATTTTTTTCAACCTTTAAGTGCTATG
GCGTTTCTGCCACTAAGTTGAATGATCTTTGCTTCTCCAATGTCTATGCAGATTCTTTTGTAGTCAAGGGAGATGATGT
AAGACAAATAGCGCCAGGACAAACTGGTGTTATTGCTGATTATAATTATAAATTGCCAGATGATTTCATGGGTTGTGTC
CTTGCTTGGAATACTAGGAACATTGATGCTACTTCAACTGGTAATTATAATTATAAATATAGGTATCTTAGACATGGCA
AGCTTAGGCCCTTTGAGAGAGACATATCTAATGTGCCTTTCTCCCCTGATGGCAAACCTTGCACCCCACCTGCTCTTAA
TTGTTATTGGCCATTAAATGATTATGGTTTTTACACCACTACTGGCATTGGCTACCAACCTTACAGAGTTGTAGTACTT
TCTTATGAACTTTTAAATGCACCGGCCACGGTTTGTGGACCAAAATTATCCACTGACCTTATTAAGAACCAGTGTGTCA
ATTTTAATTTTAATGGACTCACTGGTACTGGTGTGTTAACTCCTTCTTCAAAGAGATTTCAACCATTTCAACAATTTGG
CCGTGATGTTTCTGATTTCACTGATTCCGTTCGAGATCCTAAAACATCTGAAATATTAGACATTTCACCTTGCTCTTTT
GGGGGTGTAAGTGTAATTACACCTGGAACAAATGCTTCATCTGAAGTTGCTGTTCTATATCAAGATGTTAACTGCACTG
ATGTTTCTACAGCAATTCATGCAGATCAACTCACACCAGCTTGGCGCATATATTCTACTGGAAACAATGTATTCCAGAC
TCAAGCAGGCTGTCTTATAGGAGCTGAGCATGTCGACACTTCTTATGAGTGCGACATTCCTATTGGAGCTGGCATTTGT
GCTAGTTACCATACAGTTTCTTTATTACGTAGTACTAGCCAAAAATCTATTGTGGCTTATACTATGTCTTTAGGTGCTG
ATAGTTCAATTGCTTACTCTAATAACACCATTGCTATACCTACTAACTTTTCAATTAGCATTACTACAGAAGTAATGCC
TGTTTCTATGGCTAAAACCTCCGTAGATTGTAATATGTACATCTGCGGAGATTCTACTGAATGTGCTAATTTGCTTCTC
CAATATGGTAGCTTTTGCACACAACTAAATCGTGCACTCTCAGGTATTGCTGCTGAACAGGATCGCAACACACGTGAAG
TGTTCGCTCAAGTCAAACAAATGTACAAAACCCCAACTTTGAAAGATTTTGGTGGTTTTAATTTTTCACAAATATTACC
TGACCCTCTAAAGTCAACTAAGAGGTCTTTTATTGAGGACTTGCTCTTTAATAAGGTGACACTCGCTGATGCTGGCTTC
ATGAAGCAATATGGCGAATGCCTAGGTGATATTAATGCTAGAGATCTCATTTGTGCGCAGAAGTTCAATGGACTTACAG
TGTTGCCACCTCTGCTCACTGATGATATGATTGCTGCCTACACTGCTGCTCTAGTTAGTGGTACTGCCACTGCTGGATG
GACATTTGGTGCTGGCGCTGCTCTTCAAATACCTTTTGCTATGCAAATGGCATATAGGTTCAATGGCATTGGAGTTACC
CAAAATGTTCTCTATGAGAACCAAAAACAAATCGCCAACCAATTTAACAAGGCGATTAGTCAAATTCAAGAATCACTTA
CAACAACATCAACTGCATTGGGCAAGCTGCAAGACGTTGTTAACCAGAATGCTCAAGCATTAAACACACTTGTTAAACA
ACTTAGCTCTAATTTTGGTGCAATTTCAAGTGTGCTAAATGATATCCTTTCGCGACTTGATAAAGTCGAGGCGGAGGTA
CAAATTGACAGGTTAATTACAGGCAGACTTCAAAGCCTTCAAACCTATGTAACACAACAACTAATCAGGGCTGCTGAAA
TCAGGGCTTCTGCTAATCTTGCTGCTACTAAAATGTCTGAGTGTGTTCTTGGACAATCAAAAAGAGTTGACTTTTGCGG
AAAGGGCTACCACCTTATGTCCTTCCCACAAGCAGCCCCGCATGGTGTTGTCTTCCTACATGTCACGTATGTGCCATCC
CAGGAGAGGAACTTCACCACAGCGCCAGCAATTTGTCATGAAGGCAAAGCATACTTCCCTCGTGAAGGTGTTTTTGTGT
TTAATGGCACTTCTTGGTTTATTACACAGAGGAACTTCTTTTCTCCACAAATAATTACTACAGACAATACATTTGTCTC
AGGAAATTGTGATGTCGTTATTGGCATCATTAACAACACAGTTTATGATCCTCTGCAACCTGAGCTTGACTCATTCAAA
GAAGAGCTGGACAAGTACTTCAAAAATCATACATCACCAGATGTTGATCTTGGCGACATTTCAGGCATTAACGCTTCTG
TCGTCAACATTCAAAAAGAAATTGACCGCCTCAATGAGGTCGCTAAAAATTTAAATGAATCACTCATTGACCTTCAAGA
ATTGGGAAAATATGAGCAATATATTAAATGGCCTTGGTATGTTTGGCTCGGCTTCATTGCTGGACTAATTGCCATCGTC

ATGGTTACAATCTTGCTTTGTTGCATGACTAGTTGTTGCAGTTGCCTCAAGGGTGCATGCTCTTGTGGTTCTTGCTGCA
AGTTTGATGAGGATGACTCTGAGCCAGTTCTCAAGGGTGTCAAATTACATTACACATAA

>gi|40795744|gb|AY508724.1| SARS coronavirus NS-1, complete genome

ATGTTTATTTTCTTATTATTTCTTACTCTCACTAGTGGTAGTGACCTTGACCGGTGCACCACTTTTGATGATGTT
CAAGCTCCTAATTACACTCAACATACTTCATCTATGAGGGGGGTTTACTATCCTGATGAAATTTTTAGATCAGACACTC
TTTATTTAACTCAGGATTTATTTCTTCCCTTTTATTCTAATGTTACAGGGTTTCATACTATTAATCATACGTTTGGCAA
CCCTGTCATACCTTTTAAGGATGGTATTTATTTTGCTGCCACAGAGAAATCAAATGTTGTCCGTGGTTGGGTTTTTGGT
TCTACCATGAACAACAAGTCACAGTCGGTGATTATTATTAACAATTCTACTAATGTTGTTATACGAGCATGTAACTTTG
AATTGTGTGACAACCCTTTCTTTGCTGTTTCTAAACCCATGGGTACACAGACACATACTATGATATTCGATAATGCATT
TAATTGCACTTTCGAGTACATATCTGATGCCTTTTCGCTTGATGTTTCAGAAAAGTCAGGTAATTTTAAACACTTACGA
GAGTTTGTGTTTAAAAATAAAGATGGGTTTCTCTATGTTTATAAGGGCTATCAACCTATAGATGTAGTTCGTGATCTAC
CTTCTGGTTTTAACACTTTGAAACCTATTTTTAAGTTGCCTCTTGGTATTAACATTACAAATTTTAGAGCCATTCTTAC
AGCCTTTTCACCTGCTCAAGACACTTGGGGCACGTCAGCTGCAGCCTATTTTGTTGGCTATTTAAAGCCAACTACATTT
ATGCTCAAGTATGATGAAAATGGTACAATCACAGATGCTGTTGATTGTTCTCAAAATCCACTTGCTGAACTCAAATGCT
CTGTTAAGAGCTTTGAGATTGACAAAGGAATTTACCAGACCTCTAATTTCAGGGTTGTTCCCTCAGGAGATGTTGTGAG
ATTCCCTAATATTACAAACTTGTGTCCTTTTGGAGAGGTTTTTAATGCTACTAAATTCCCTTCTGTCTATGCATGGGAG
AGAAAAAAAATTTCTAATTGTGTTGCTGATTACTCTGTGCTCTACAACTCAACATTTTTTTCAACCTTTAAGTGCTATG
GCGTTTCTGCCACTAAGTTGAATGATCTTTGCTTCTCCAATGTCTATGCAGATTCTTTTGTAGTCAAGGGAGATGATGT
AAGACAAATAGCGCCAGGACAAACTGGTGTTATTGCTGATTATAATTATAAATTGCCAGATGATTTCATGGGTTGTGTC
CTTGCTTGGAATACTAGGAACATTGATGCTACTTCAACTGGTAATTATAATTATAAATATAGGTATCTTAGACATGGCA
AGCTTAGGCCCTTTGAGAGAGACATATCTAATGTGCCTTTCTCCCCTGATGGCAAACCTTGCACCCCACCTGCTCTTAA
TTGTTATTGGCCATTAAATGATTATGGTTTTTACACCACTACTGGCATTGGCTACCAACCTTACAGAGTTGTAGTACTT
TCTTTTGAACTTTTAAATGCACCGGCCACGGTTTGTGGACCAAAATTATCCACTGACCTTATTAAGAACCAGTGTGTCA
ATTTTAATTTTAATGGACTCACTGGTACTGGTGTGTTAACTCCTTCTTCAAAGAGATTTCAACCATTTCAACAATTTGG
CCGTGATGTTTCTGATTTCACTGATTCCGTTCGAGATCCTAAAACATCTGAAATATTAGACATTTCACCTTGCTCTTTT
GGGGGTGTAAGTGTAATTACACCTGGAACAAATGCTTCATCTGAAGTTGCTGTTCTATATCAAGATGTTAACTGCACTG
ATGTTTCTACAGCAATTCATGCAGATCAACTCACACCAGCTTGGCGCATATATTCTACTGGAAACAATGTATTCCAGAC
TCAAGCAGGCTGTCTTATAGGAGCTGAGCATGTCGACACTTCTTATGAGTGCGACATTCCTATTGGAGCTGGCATTTGT
GCTAGTTACCATACAGTTTCTTTATTACGTAGTACTAGCCAAAAATCTATTGTGGCTTATACTATGTCTTTAGGTGCTG
ATAGTTCAATTGCTTACTCTAATAACACCATTGCTATACCTACTAACTTTTCAATTAGCATTACTACAGAAGTAATGCC
TGTTTCTATGGCTAAAACCTCCGTAGATTGTAATATGTACATCTGCGGAGATTCTACTGAATGTGCTAATTTGCTTCTC
CAATATGGTAGCTTTTGCACACAACTAAATCGTGCACTCTCAGGTATTGCTGCTGAACAGGATCGCAACACACGTGAAG
TGTTCGCTCAAGTCAAACAAATGTACAAAACCCCAACTTTGAAATATTTTGGTGGTTTTAATTTTTCACAAATATTACC
TGACCCTCTAAAGCCAACTAAGAGGTCTTTTATTGAGGACTTGCTCTTTAATAAGGTGACACTCGCTGATGCTGGCTTC
ATGAAGCAATATGGCGAATGCCTAGGTGATATTAATGCTAGAGATCTCATTTGTGCGCAGAAGTTCAATGGACTTACAG
TGTTGCCACCTCTGCTCACTGATGATATGATTGCTGCCTACACTGCTGCTCTAGTTAGTGGTACTGCCACTGCTGGATG
GACATTTGGTGCTGGCGCTGCTCTTCAAATACCTTTTGCTATGCAAATGGCATATAGGTTCAATGGCATTGGAGTTACC
CAAAATGTTCTCTATGAGAACCAAAAACAAATCGCCAACCAATTTAACAAGGCGATTAGTCAAATTCAAGAATCACTTA
CAACAACATCAACTGCATTGGGCAAGCTGCAAGACGTTGTTAACCAGAATGCTCAAGCATTAAACACACTTGTTAAACA
ACTTAGCTCTAATTTTGGTGCAATTTCAAGTGTGCTAAATGATATCCTTTCGCGACTTGATAAAGTCGAGGCGGAGGTA
CAAATTGACAGGTTAATTACAGGCAGACTTCAAAGCCTTCAAACCTATGTAACACAACAACTAATCAGGGCTGCTGAAA

TCATGGCTTCTGCTAATCTTGCTGCTACTAAAATGTCTGAGTGTGTTCTTGGACAATCAAAAAGAGTTGACTTTTGTGG
AAAGGGCTACCACCTTATGTCCTTCCCACAAGCAGCCCCGCATGGTGTTGTCTTCCTACATGTCACGTATGTGCCATCC
CAGGAGAGGAACTTCACCACAGCGCCAGCAATTTGTCATGAAGGCAAAGCATACTTCCCTCGTGAAGGTGTTTTTGTGT
TTAATGGCACTTCTTGGTTTATTACACAGAGGAACTTCTTTTCTCCACAAATAATTACTACAGACAATACATTTGTCTC
AGGAAATTGTGATGTCGTTATTGGCATCATTAACAACACAGTTTATGATCCTCTGCAACCTGAGCTTGACTCATTCAAA
GAAGAGCTGGACAAGTACTTCAAAAATCATACATCACCAGATGTTGATCTTGGCGACATTTCAGGCATTAACGCTTCTG
TCGTCAACATTCAAAAAGAAATTGACCGCCTCAATGAGGTCGCTAAAAATTTAAATGAATCACTCATTGACCTTCAAGA
ATTGGGAAAATATGAGCAATATATTAAATGGCCTTGGTATGTTTGGCTCGGCTTCATTGCTGGACTAATTGCCATCGTC
ATGGTTACAATCTTGCTTTGTTGCATGACTAGTTGTTGCAGTTGCCTCAAGGGTGCATGCTCTTGTGGTTCTTGCTGCA
AGTTTGATGAGGATGACTCTGAGCCAGTTCTCAAGGGTGTCAAATTACATTACACATAA

>gi|172043981|gb|EU371560.1| SARS coronavirus BJ182a, complete genome

ATGTTTATTTTCTTATTATTTCTTACTCTCACTAGTGGTAGTGACCTTGACCGGTGCACCACTTTTGATGATGTT
CAAGCTCCTAATTACACTCAACATACTTCATCTATGAGGGGGGTTTACTATCCTGATGAAATTTTTAGATCAGACACTC
TTTATTTAACTCAGGATTTATTTCTTCCATTTTATTCTAATGTTACAGGGTTTCATACTATTAATCATACGTTTGGCAA
CCCTATCATACCTTTTAAGGATGGTATTTATTTTGCTGCCACAGAGAAATCAAATGTTGTCCGTGGTTGGGTTTTTGGT
TCTACCATGAACAACAAGTCACAGTCGGTGATTATTATTAACAATTCTACTAATGTTGTTATACGAGCATGTAACTTTG
AATTGTGTGACAACCCTTTCTTTGCTGTTTCTAAACCCATGGGTACACAGACACATACTATGATATTCGATAATGCATT
TAATTGCACTTTCGAGTACATATCTGATGCCTTTTCGCTTGATGTTTCAGAAAAGTCAGGTAATTTTAAACACTTACGA
GAGTTTGTGTTTAAAAATAAAGATGGGTTTCTCTATGTTTATAAGGGCTATCAACCTATAGATGTAGTTCGTGATCTAC
CTTCTGGTTTTAACACTTTGAAACCTATTTTTAAGTTGCCTCTTGGTATTAACATTACAAATTTTAGAGCCATTCTTAC
AGCCTTTTCACCTGCTCAAGACATTTGGGGCACGTCAGCTGCAGCCTATTTTGTTGGCTATTTAAAGCCAACTACATTT
ATGCTCAAGTATGATGAAAATGGTACAATCACAGATGCTGTTGATTGTTCTCAAAATCCACTTGCTGAACTCAAATGCT
CTGTTAAGAGCTTTGAGATTGACAAAGGAATTTACCAGACCTCTAATTTCAGGGTTGTTCCCTCAGGAGATGTTGTGAG
ATTCCCTAATATTACAAACTTGTGTCCTTTTGGAGAGGTTTTTAATGCTACTAAATTCCCTTCTGTCTATGCATGGGAG
AGAAAAAAAATTTCTAATTGTGTTGCTGATTACTCTGTGCTCTACAACTCAACATTTTTTTCAACCTTTAAGTGTTATG
GCGTTTCTGCCACTAAGTTGAATGATCTTTGCTTCTCCAATGTCTATGCAGATTCTTTTGTAGTCAAGGGAGATGATGT
AAGACAAATAGCGCCAGGACAAACTGGTGTTATTGCTGATTATAATTATAAATTGCCAGATGATTTCATGGGTTGTGTC
CTTGCTTGGAATACTAGGAACATTGATGCTACTTCAACTGGTAATTATAATTATAAATATAGGTATCTTAGACATGGCA
AGCTTAGGCCCTTTGAGAGAGACATATCTAATGTGCCTTTCTCCCCTGATGGCAAACCTTGCACCCCACCTGCTCTTAA
TTGTTATTGGCCATTAAATGATTATGGTTTTTACACCACTACTGGCATTGGCTACCAACCTTACAGAGTTGTAGTACTT
TCTTTTGAACTTTTAAATGCACCGGCCACGGTTTGTGGACCAAAATTATCCACTGACCTTATTAAGAACCAGTGTGTCA
ATTTTAATTTTAATGGACTCACTGGTACTGGTGTGTTAACTCCTTCTTCAAAGAGATTTCAACCATTTCAACAATTTGG
CCGTGATGTTTCTGATTTCACTGATTCCGTTCGAGATCCTAAAACATCTGAAATATTAGACATTTCACCTTGCTCTTTT
GGGGGTGTAAGTGTAATTACACCTGGAACAAATGCTTCATCTGAAGTTGCTGTTCTATATCAAGATGTTAACTGCACTG
ATGTTTCTACAGCAATTCATGCAGATCAACTCACACCAGCTTGGCGCATATATTCTACTGGAAACAATGTATTCCAGAC
TCAAGCAGGCTGTCTTATAGGAGCTGAGCATGTCGACACTTCTTATGAGTGCGACATTCCTATTGGAGCTGGCATTTGT
GCTAGTTACCATACAGTTTCTTTATTACGTAGTACTAGCCAAAAATCTATTGTGGCTTATACTATGTCTTTAGGTGCTG
ATAGTTCAATTGCTTACTCTAATAACACCATTGCTATACCTACTAACTTTTCAATTAGCATTACTACAGAAGTAATGCC
TGTTTCTATGGCTAAAACCTCCGTAGATTGTAATATGTACATCTGCGGAGATTCTACTGAATGTGCTAATTTGCTTCTC
CAATATGGTAGCTTTTGCACACAACTAAATCGTGCACTCTCAGGTATTGCTGCTGAACAGGATCGCAACACACGTGAAG
TGTTCGCTCAAGTCAAACAAATGTACAAAACCCCAACTTTGAAATATTTTGATGGTTTTAATTTTTCACAAATATTACC

TGACCCTCTAAAGCCAACTAAGAGGTCTTTTATTGAGGACTTGCTCTTTAATAAGGTGACACTCGCTGATGCTGGCTTC
ATGAAGCAATATGGCGAATGCCTAGGTGATATTAATGCTAGAGATCTCATTTGTGCGCAGAAGTTCAATGGACTTACAG
TGTTGCCACCTCTGCTCACTGATGATATGATTGCTGCCTACACTGCTGCTCTAGTTAGTGGTACTGCCACTGCTGGATG
GGCATTTGGTGCTGGCGCTGCTCTTCAAATACCTTTTGCTATGCAAATGGCATATAGGTTCAATGGCATTGGAGTTACC
CAAAATGTTCTCTATGAGAACCAAAAACAAATCGCCAACCAATTTAACAAGGCGATTAGTCAAATTCAAGAATCACTTA
CAACAACATCAACTGCATTGGGCAAGCTGCAAGACGTTGTTAACCAGAATGCTCAAGCATTAAACACACTTGTTAAACA
ACTTAGCTCTAATTTTGGTGCAATTTCAAGTGTGCTAAATGATATCCTTTCGCGACTTGATAAAGTCGAGGCGGAGGTA
CAAATTGACAGGTTAATTACAGGCAGACTTCAAAGCCTTCAAACCTATGTAACACAACAACTAATCAGGGCTGCTGAAA
TCAGGGCTTCTGCTAATCTTGCTGCTACTAAAATGTCTGAGTGTGTTCTTGGACAATCAAAAAGAGTTGACTTTTGTGG
AAAGGGCTACCACCTTATGTCCTTCCCACAAGCAGCCCCGCATGGTGTTGTCTTCCTACATGTCACGTATGTGCCATCC
CAGGAGAGGAACTTCACCACAGCGCCAGCAATTTGTCATGAAGGCAAAGCATACTTCCCTCGTGAAGGTGTTTTTGTGT
TTAATGGCACTTCTTGGTTTATTACACAGAGGAACTTCTTTTCTCCACAAATAATTACTACAGACAATACATTTGTCTC
AGGAAATTGTGATGTCGTTATTGGCATCATTAACAACACAGTTTATGATCCTCTGCAACCTGAGCTTGACTCATTCAAA
GAAGAGCTGGACAAGTACTTCAAAAATCATACATCACCAGATGTTGATCTTGGCGACATTTCAGGCATTAACGCTTCTG
TCGTCAACATTCAAAAAGAAATTGACCGCCTCAATGAGGTCGCTAAAAATTTAAATGAATCACTCATTGACCTTCGAGA
ATTGGGAAAATATGAGCAATATATTAAATGGCCTTGGTATGTTTGGCTCGGCTTCATTGCTGGACTAATTGCCATCGTC
ATGGTTACAATCTTGCTTTGTTGCATGACTAGTTGTTGCAGTTGCCTCAAGGGTGCATGCTCTTGTGGTTCTTGCTGCA
AGTTTGATGAGGATGACTCTGAGCCAGTTCTCAAGGGTGTCAAATTACATTACACATAA

>gi|172044006|gb|EU371562.1| SARS coronavirus BJ182-4, complete genome

ATGTTTATTTTCTTATTATTTCTTACTCTCACTAGTGGTAGTGACCTTGACCGGTGCACCACTTTTGATGATGTT
CAAGCTCCTAATTACACTCAACATACTTCATCTATGAGGGGGGTTTACTATCCTGATGAAATTTTTAGATCAGACACTC
TTTATTTAACTCAGGATTTATTTCTTCCATTTTATTCTAATGTTACAGGGTTTCATACTATTAATCATACGTTGGGCAA
CCCTGTCATACCTTTTAAGGATGGTATTTATTTTGCTGCCACAGAGAAATCAAATGTTGTCCGTGGTTGGGTTTTTGGT
TCTACCATGAACAACAAGTCACAGTCGGTGATTATTATTAACAATTCTACTAATGTTGTTATACGAGCATGTAACTTTG
AATTGTGTGACAACCCTTTCTTTGCTGTTTCTAAACCCATGGGTACACAGACACATACTATGATATTCGATAATGCATT
TAATTGCACTTTCGAGTACATATCTGATGCCTTTTCGCTTGATGTTTCAGAAAAGTCAGGTAATTTTAAACACTTACGA
GAGTTTGTGTTTAAAAATAAAGATGGGTTTCTCTATGTTTATAAGGGCTATCAACCTATAGATGTAGTTCGTGATCTAC
CTTCTGGTTTTAACACTTTGAAACCTATTTTTAAGTTGCCTCTTGGTATTAACATTACAAATTTTAGAGCCATTCTTAC
AGCCTTTTCACCTGCTCAAGACATTTGGGGCACGTCAGCTGCAGCCTATTTTGTTGGCTATTTAAAGCCAACTACATTT
ATGCTCAAGTATGATGAAAATGGTACAATCACAGATGCTGTTGATTGTTCTCAAAATCCACTTGCTGAACTCAAATGCT
CTGTTAAGAGCTTTGAGATTGACAAAGGAATTTACCAGACCTCTAATTTCAGGGTTGTTCCCTCAGGAGATGTTGTGAG
ATTCCCTAATATTACAAACTTGTGTCCTTTTGGAGAGGTTTTTAATGCTACTAAATTCCCTTCTGTCTATGCATGGGAG
AGAAAAAAAATTTCTAATTGTGTTGCTGATTACTCTGTGCTCTACAACTCAACATTTTTTTCAACCTTTAAGTGTTATG
GCGTTTCTGCCACTAAGTTGAATGATCTTTGCTTCTCCAATGTCTATGCAGATTCTTTTGTAGTCAAGGGAGATGATGT
AAGACAAATAGCGCCAGGACAAACTGGTGTTATTGCTGATTATAATTATAAATTGCCAGATGATTTCATGGGTTGTGTC
CTTGCTTGGAATACTAGGAACATTGATGCTACTTCAACTGGTAATTATAATTATAAATATAGGTATCTTAGACATGGCA
AGCTTAGGCCCTTTGAGAGAGACATATCTAATGTGCCTTTCTCCCCTGATGGCAAACCTTGCACCCCACCTGCTCTTAA
TTGTTATTGGCCATTAAATGATTATGGTTTTTACACCACTACTGGCATTGGCTACCAACCTTACAGAGTTGTAGTACTT
TCTTTTGAACTTTTAAATGCACCGGCCACGGTTTGTGGACCAAAATTATCCACTGACCTTATTAAGAACCAGTGTGTCA
ATTTTAATTTTAATGGACTCACTGGTACTGGTGTGTTAACTCCTTCTTCAAAGAGATTTCAACCATTTCAACAATTTGG
CCGTGATGTTTCTGATTTCACTGATTCCGTTCGAGATCCTAAAACATCTGAAATATTAGACATTTCACCTTGCTCTTTT

GGGGGTGTAAGTGTAATTACACCTGGAACAAATGCTTCATCTGAAGTTGCTGTTCTATATCAAGATGTTAACTGCACTG
ATGTTTCTACAGCAATTCATGCAGATCAACTCACACCAGCTTGGCGCATATATTCTACTGGAAACAATGTATTCCAGAC
TCAAGCAGGCTGTCTTATAGGAGCTGAGCATGTCGACACTTCTTATGAGTGCGACATTCCTATTGGAGCTGGCATTTGT
GCTAGTTACCATACAGTTTCTTTATTACGTAGTACTAGCCAAAAATCTATTGTGGCTTATACTATGTCTTTAGGTGCTG
ATAGTTCAATTGCTTACTCTAATAACACCATTGCTATACCTACTAACTTTTCAATTAGCATTACTACAGAAGTAATGCC
TGTTTCTATGGCTAAAACCTCCGTAGATTGTAATATGTACATCTGCGGAGATTCTACTGAATGTGCTAATTTGCTTCTC
CAATATGGTAGCTTTTGCACACAACTAAATCGTGCACTCTCAGGTATTGCTGCTGAACAGGATCGCAACACACGTGAAG
TGTTCGCTCAAGTCAAACAAATGTACAAAACCCCAACTTTGAAATATTTTGATGGTTTAATTTTTCACAAATATTACC
TGACCCTCTAAAGCCAACTAAGAGGTCTTTTATTGAGGACTTGCTCTTTAATAAGGTGACACTCGCTGATGCTGGCTTC
ATGAAGCAATATGGCGAATGCCTAGGTGATATTAATGCTAGAGATCTCATTTGTGCGCAGAAGTTCAATGGACTTACAG
TGTTGCCACCTCTGCTCACTGATGATATGATTGCTGCCTACACTGCTGCTCTAGTTAGTGGTACTGCCACTGCTGGATG
GACATTTGGTGCTGGCGCTGCTCTTCAAATACCTTTTGCTATGCAAATGGCATATAGGTTCAATGGCATTGGAGTTACC
CAAAATGTTCTCTATGAGAACCAAAAACAAATCGCCAACCAATTTAACAAGGCGATTAGTCAAATTCAAGAATCACTTA
CAACAACATCAACTGCATTGGGCAAGCTGCAAGACGTTGTTAACCAGAATGCTCAAGCATTAAACACACTTGTTAAACA
ACTTAGCTCTAATTTTGGTGCAATTTCAAGTGTGCTAAATGATATCCTTTCGCGACTTGATAAAGTCGAGGCGGAGGTA
CAAATTGACAGGTTAATTACAGGCAGACTTCAAAGCCTTCAAACCTATGTAACACAACAACTAATCAGGGCTGCTGAAA
TCAGGGCTTCTGCTAATCTTGCTGCTACTAAAATGTCTGAGTGTGTTCTTGGACAATCAAAAAGAGTTGACTTTTGTGG
AAAGGGCTACCACCTTATGTCCTTCCCACAAGCAGCCCCGCATGGTGTTGTCTTCCTACATGTCACGTATGTGCCATCC
CAGGAGAGGAACTTCACCACAGCGCCAGCAATTTGTCATGAAGGCAAAGCATACTTCCCTCGTGAAGGTGTTTTTGTGT
TTAATGGCACTTCTTGGTTTATTACACAGAGGAACTTCTTTTCTCCACAAATAATTACTACAGACAATACATTTGTCTC
AGGAAATTGTGATGTCGTTATTGGCATCATTAACAACACAGTTTATGATCCTCTGCAACCTGAGCTTGACTCATTCAAA
GAAGAGCTGGACAAGTACTTCAAAAATCATACATCACCAGATGTTGATCTTGGCGACATTTCAGGCATTAACGCTTCTG
TCGTCAACATTCAAAAAGAAATTGACCGCCTCAATGAGGTCGCTAAAAATTTAAATGAATCACTCATTGACCTTCGAGA
ATTGGGAAAATATGAGCAATATATTAAATGGCCTTGGTATGTTTGGCTCGGCTTCATTGCTGGACTAATTGCCATCGTC
ATGGTTACAATCTTGCTTTGTTGCATGACTAGTTGTTGCAGTTGCCTCAAGGGTGCATGCTCTTGTGGTTCTTGCTGCA
AGTTTGATGAGGATGACTCTGAGCCAGTTCTCAAGGGTGTCAAATTACATTACACATAA

>gi|172044018|gb|EU371563.1| SARS coronavirus BJ182-8, complete genome

ATGTTTATTTTCTTATTATTTCTTACTCTCACTAGTGGTAGTGACCTTGACCGGTGCACCACTTTTGATGATGTT
CAAGCTCCTAATTACACTCAACATACTTCATCTATGAGGGGGGTTTACTATCCTGATGAAATTTTTAGATCAGACACTC
TTTATTTAACTCAGGATTTATTTCTTCCATTTTATTCTAATGTTACAGGGTTTCATACTATTAATCATACGTTTGGCAA
CCCTGTCATACCTTTTAAGGATGGTATTTATTTTGCTGCCACAGAGAAATCAAATGTTGTCCGTGGTTGGGTTTTTGGT
TCTACCATGAACAACAAGTCACAGTCGGTGATTATTATTAACAATTCTACTAATGTTGTTATACGAGCATGTAACTTTG
AATTGTGTGACAACCCTTTCTTTGCTGTTTCTAAACCCATGGGTACACAGACACATACTATGATATTCGATAATGCATT
TAATTGCACTTTCGAGTACATATCTGATGCCTTTTCGCTTGATGTTTCAGAAAAGTCAGGTAATTTTAAACACTTACGA
GAGTTTGTGTTTAAAAATAAAGATGGGTTTCTCTATGTTTATAAGGGCTATCAACCTATAGATGTAGTTCGTGATCTAC
CTTCTGGTTTTAACACTTTGAAACCTATTTTTAAGTTGCCTCTTGGTATTAACATTACAAATTTTAGAGCCATTCTTAC
AGCCTTTTCACCTGCTCAAGACATTTGGGGCACGTCAGCTGCAGCCTATTTTGTTGGCTATTTAAAGCCAACTACATTT
ATGCTCAAGTATGATGAAAATGGTACAATCACAGATGCTGTTGATTGTTCTCAAAATCCACTTGCTGAACTCAAATGCT
CTGTTAAGAGCTTTGAGATTGACAAAGGAATTTACCAGACCTCTAATTTCAGGGTTGTTCCCTCAGGAGATGTTGTGAG
ATTCCCTAATATTACAAACTTGTGTCCTTTTGGAGAGGTTTTTAATGCTACTAAATTCCCTTCTGTCTATGCATGGGAG
AGAAAAAAAATTTCTAATTGTGTTGCTGATTACTCTGTGCTCTACAACTCAACATTTTTTTCAACCTTTAAGTGTTATG

GCGTTTCTGCCACTAAGTTGAATGATCTTTGCTTCTCCAATGTCTATGCAGATTCTTTTGTAGTCAAGGGAGATGATGT
AAGACAAATAGCGCCAGGACAAACTGGTGTTATTGCTGATTATAATTATAAATTGCCAGATGATTTCATGGGTTGTGTC
CTTGCTTGGAATACTAGGAACATTGATGCTACTTCAACTGGTAATTATAATTATAAATATAGGTATCTTAGACATGGCA
AGCTTAGGCCCTTTGAGAGAGACATATCTAATGTGCCTTTCTCCCCTGATGGCAAACCTTGCACCCCACCTGCTCTTAA
TTGTTATTGGCCATTAAATGATTATGGTTTTTACACCACTACTGGCATTGGCTACCAACCTTACAGAGTTGTAGTACTT
TCTTTTGAACTTTTAAATGCACCGGCCACGGTTTGTGGACCAAAATTATCCACTGACCTTATTAAGAACCAGTGTGTCA
ATTTTAATTTTAATGGACTCACTGGTACTGGTGTGTTAACTCCTTCTTCAAAGAGATTTCAACCATTTCAACAATTTGG
CCGTGATGTTTCTGATTTCACTGATTCCGTTCGAGATCCTAAAACATCTGAAATATTAGACATTTCACCTTGCTCTTTT
GGGGGTGTAAGTGTAATTACACCTGGAACAAATGCTTCATCTGAAGTTGCTGTTCTATATCAAGATGTTAACTGCACTG
ATGTTTCTACAGCAATTCATGCAGATCAACTCACACCAGCTTGGCGCATATATTCTACTGGAAACAATGTATTCCAGAC
TCAAGCAGGCTGTCTTATAGGAGCTGAGCATGTCGACACTTCTTATGAGTGCGACATTCCTATTGGAGCTGGCATTTGT
GCTAGTTACCATACAGTTTCTTTATTACGTAGTACTAGCCAAAAATCTATTGTGGCTTATACTATGTCTTTAGGTGCTG
ATAGTTCAATTGCTTACTCTAATAACACCATTGCTATACCTACTAACTTTTCAATTAGCATTACTACAGAAGTAATGCC
TGTTTCTATGGCTAAAACCTCCGTAGATTGTAATATGTACATCTGCGGAGATTCTACTGAATGTGCTAATTTGCTTCTC
CAATATGGTAGCTTTTGCACACAACTAAATCGTGCACTCTCAGGTATTGCTGCTGAACAGGATCGCAACACACGTGAAG
TGTTCGCTCAAGTCAAACAAATGTACAAAACCCCAACTTTGAAATATTTTGATGGTTTTAATTTTTCACAAATATTACC
TGACCCTCTAAAGCCAACTAAGAGGTCTTTTATTGAGGACTTGCTCTTTAATAAGGTGACACTCGCTGATGCTGGCTTC
ATGAAGCAATATGGCGAATGCCTAGGTGATATTAATGCTAGAGATCTCATTTGTGCGCAGAAGTTCAATGGACTTACAG
TGTTGCCACCTCTGCTCACTGATGATATGATTGCTGCCTACACTGCTGCTCTAGTTAGTGGTACTGCCACTGCTGGATG
GACATTTGGTGCTGGCGCTGCTCTTCAAATACCTTTTGCTATGCAAATGGCATATAGGTTCAATGGCATTGGAGTTACC
CAAAATGTTCTCTATGAGAACCAAAAACAAATCGCCAACCAATTTAACAAGGCGATTAGTCAAATTCAAGAATCACTTA
CAACAACATCAACTGCATTGGGCAAGCTGCAAGACGTTGTTAACCAGAATGCTCAAGCATTAAACACACTTGTTAAACA
ACTTAGCTCTAATTTTGGTGCAATTTCAAGTGTGCTAAATGATATCCTTTCGCGACTTGATAAAGTCGAGGCGGAGGTA
CAAATTGACAGGTTAATTACAGGCAGACTTCAAAGCCTTCAAACCTATGTAACACAACAACTAATCAGGGCTGCTGAAA
TCAGGGCTTCTGCTAATCTTGCTGCTACTAAAATGTCTGAGTGTGTTCTTGGACAATCAAAAAGAGTTGACTTTTGTGG
AAAGGGCTACCACCTTATGTCCTTCCCACAAGCAGCCCCGCATGGTGTTGTCTTCCTACATGTCACGTATGTGCCATCC
CAGGAGAGGAACTTCACCACAGCGCCAGCAATTTGTCATGAAGGCAAAGCATACTTCCCTCGTGAAGGTGTTTTTGTGT
TTAATGGCACTTCTTGGTTTATTACACAGAGGAACTTCTTTTCTCCACAAATAATTACTACAGACAATACATTTGTCTC
AGGAAATTGTGATGTCGTTATTGGCATCATTAACAACACAGTTTATGATCCTCTGCAACCTGAGCTTGACTCATTCAAA
GAAGAGCTGGACAAGTACTTCAAAAATCATACATCACCAGATGTTGATCTTGGCGACATTTCAGGCATTAACGCTTCTG
TCGTCAACATTCAAAAAGAAATTGACCGCCTCAATGAGGTCGCTAAAAATTTAAATGAATCACTCATTGACCTTCGAGA
ATTGGGAAAATATGAGCAATATATTAAATGGCCTTGGTATGTTTGGCTCGGCTTCATTGCTGGACTAATTGCCATCGTC
ATGGTTACAATCTTGCTTTGTTGCATGACTAGTTGTTGCAGTTGCCTCAAGGGTGCATGCTCTTGTGGTTCTTGCTGCA
AGTTTGATGAGGATGACTCTGAGCCAGTTCTCAAGGGTGTCAAATTACATTACACATAA

>gi|172044030|gb|EU371564.1| SARS coronavirus BJ182-12, complete genome

ATGTTTATTTTCTTATTATTTCTTACTCTCACTAGTGGTAGTGACCTTGACCGGTGCACCACTTTTGATGATGTT
CAAGCTCCTAATTACACTCAACATACTTCATCTATGAGGGGGGTTTACTATCCTGATGAAATTTTTAGATCAGACACTC
TTTATTTAACTCAGGATTTATTTCTTCCATTTTATTCTAATGTTACAGGGTTTCATACTATTAATCATACGTTTGGCAA
CCCTGTCATACCTTTTAAGGATGGTATTTATTTTGCTGCCACAGAGAAATCAAATGTTGTCCGTGGTTGGGTTTTTGGT
TCTACCATGAACAACAAGTCACAGTCGGTGATTATTATTAACAATTCTACTAATGTTGTTATACGAGCATGTAACTTTG
AATTGTGTGACAACCCTTTCTTTGCTGTTTCTAAACCCATGGGTACACAGACACATACTATGATATTCGATAATGCATT

TAATTGCACTTTCGAGTACATATCTGATGCCTTTTCGCTTGATGTTTCAGAAAAGTCAGGTAATTTTAAACACTTACGA
GAGTTTGTGTTTAAAAATAAAGATGGGTTTCTCTATGTTTATAAGGGCTATCAACCTATAGATGTAGTTCGTGATCTAC
CTTCTGGTTTTAACACTTTGAAACCTATTTTTAAGTTGCCTCTTGGTATTAACATTACAAATTTTAGAGCCATTCTTAC
AGCCTTTTCACCTGCTCAAGACATTTGGGGCACGTCAGCTGCAGCCTATTTTGTTGGCTATTTAAAGCCAACTACATTT
ATGCTCAAGTATGATGAAAATGGTACAATCACAGATGCTGTTGATTGTTCTCAAAATCCACTTGCTGAACTCAAATGCT
CTGTTAAGAGCTTTGAGATTGACAAAGGAATTTACCAGACCTCTAATTTCAGGGTTGTTCCCTCAGGAGATGTTGTGAG
ATTCCCTAATATTACAAACTTGTGTCCTTTTGGAGAGGTTTTTAATGCTACTAAATTCCCTTCTGTCTATGCATGGGAG
AGAAAAAAAATTTCTAATTGTGTTGCTGATTACTCTGTGCTCTACAACTCAACATTTTTTTCAACCTTTAAGTGTTATG
GCGTTTCTGCCACTAAGTTGAATGATCTTTGCTTCTCCAATGTCTATGCAGATTCTTTTGTAGTCAAGGGAGATGATGT
AAGACAAATAGCGCCAGGACAAACTGGTGTTATTGCTGATTATAATTATAAATTGCCAGATGATTTCATGGGTTGTGTC
CTTGCTTGGAATACTAGGAACATTGATGCTACTTCAACTGGTAATTATAATTATAAATATAGGTATCTTAGACATGGCA
AGCTTAGGCCCTTTGAGAGAGACATATCTAATGTGCCTTTCTCCCCTGATGGCAAACCTTGCACCCCACCTGCTCTTAA
TTGTTATTGGCCATTAAATGATTATGGTTTTTACACCACTACTGGCATTGGCTACCAACCTTACAGAGTTGTAGTACTT
TCTTTTGAACTTTTAAATGCACCGGCCACGGTTTGTGGACCAAAATTATCCACTGACCTTATTAAGAACCAGTGTGTCA
ATTTTAATTTTAATGGACTCACTGGTACTGGTGTGTTAACTCCTTCTTCAAAGAGATTTCAACCATTTCAACAATTTGG
CCGTGATGTTTCTGATTTCACTGATTCCGTTCGAGATCCTAAAACATCTGAAATATTAGACATTTCACCTTGCTCTTTT
GGGGGTGTAAGTGTAATTACACCTGGAACAAATGCTTCATCTGAAGTTGCTGTTCTATATCAAGATGTTAACTGCACTG
ATGTTTCTACAGCAATTCATGCAGATCAACTCACACCAGCTTGGCGCATATATTCTACTGGAAACAATGTATTCCAGAC
TCAAGCAGGCTGTCTTATAGGAGCTGAGCATGTCGACACTTCTTATGAGTGCGACATTCCTATTGGAGCTGGCATTTGT
GCTAGTTACCATACAGTTTCTTTATTACGTAGTACTAGCCAAAAATCTATTGTGGCTTATACTATGTCTTTAGGTGCTG
ATAGTTCAATTGCTTACTCTAATAACACCATTGCTATACCTACTAACTTTTCAATTAGCATTACTACAGAAGTAATGCC
TGTTTCTATGGCTAAAACCTCCGTAGATTGTAATATGTACATCTGCGGAGATTCTACTGAATGTGCTAATTTGCTTCTC
CAATATGGTAGCTTTTGCACACAACTAAATCGTGCACTCTCAGGTATTGCTGCTGAACAGGATCGCAACACACGTGAAG
TGTTCGCTCAAGTCAAACAAATGTACAAAACCCCAACTTTGAAATATTTTGATGGTTTTAATTTTTCACAAATATTACC
TGACCCTCTAAAGCCAATTAAAAGGTCTTTTATTGAGGACTTGCTCTTTAATAAGGTGACACTCGCTGATGCTGGCTTC
ATGAAGCAATATGGCGAATGCCTAGGTGATATTAATGCTAGAGATCTCATTTGTGCGCAGAAGTTCAATGGACTTACAG
TGTTGCCACCTCTGCTCACTGATGATATGATTGCTGCCTACACTGCTGCTCTAGTTAGTGGTACTGCCACTGCTGGATG
GACATTTGGTGCTGGCGCTGCTCTTCAAATACCTTTTGCTATGCAAATGGCATATAGGTTCAATGGCATTGGAGTTACC
CAAAATGTTCTCTATGAGAACCAAAAACAAATCGCCAACCAATTTAACAAGGCGATTAGTCAAATTCAAGAATCACTTA
CAACAACATCAACTGCATTGGGCAAGCTGCAAGACGTTGTTAACCAGAATGCTCAAGCATTAAACACACTTGTTAAACA
ACTTAGCTCTAATTTTGGTGCAATTTCAAGTGTGCTAAATGATATCCTTTCGCGACTTGATAAAGTCGAGGCGGAGGTA
CAAATTGACAGGTTAATTACAGGCAGACTTCAAAGCCTTCAAACCTATGTAACACAACAACTAATCAGGGCTGCTGAAA
TCAGGGCTTCTGCTAATCTTGCTGCTACTAAAATGTCTGAGTGTGTTCTTGGACAATCAAAAAGAGTTGACTTTTGTGG
AAAGGGCTACCACCTTATGTCCTTCCCACAAGCAGCCCCGCATGGTGTTGTCTTCCTACATGTCACGTATGTGCCATCC
CAGGAGAGGAACTTCACCACAGCGCCAGCAATTTGTCATGAAGGCAAAGCATACTTCCCTCGTGAAGGTGTTTTTGTGT
TTAATGGCACTTCTTGGTTTATTACACAGAGGAACTTCTTTTCTCCACAAATAATTACTACAGACAATACATTTGTCTC
AGGAAATTGTGATGTCGTTATTGGCATCATTAACAACACAGTTTATGATCCTCTGCAACCTGAGCTTGACTCATTCAAA
GAAGAGCTGGACAAGTACTTCAAAAATCATACATCACCAGATGTTGATCTTGGCGACATTTCAGGCATTAACGCTCCTG
TCGTCAACATTCAAAAAGAAATTGACCGCCTCAATGAGGTCGCTAAAAATTTAAATGAATCACTCATTGACCTTCGAGA
ATTGGGAAAATATGAGCAATATATTAAATGGCCTTGGTATGTTTGGCTCGGCTTCATTGCTGGACTAATTGCCATCGTC
ATGGTTACAATCTTGCTTTGTTGCATGACTAGTTGTTGCAGTTGCCTCAAGGGTGCATGCTCTTGTGGTTCTTGCTGCA
AGTTTGATGAGGATGACTCTGAGCCAGTTCTCAAGGGTGTCAAATTACATTACACATAA

>gi|334683272|dbj|AB548618.1|_Porcine_epidemic_diarrhea_virus_S_gene_for_spike_protein_complete_cds_strain:_83P5

ATGAAGCCTTTAATTTACTTCTGGTTGTTCTTACCAGTACTTCTAACACTTAGCCTACCACAAGATGTCACTAGG
TGCCAGTCTACTATTAACTTTAGGCGGTTCTTTTCAAAATTTAATGTTCAGGCACCTGCCGTCGTCGTTTTGGGTGGTT
ATCTACCTAGTATGAACTCTTCTAGCTGGTACTGTGGCACAGGCATTGAAACTGATAGTGGCGTTCATGGTATTTTTCT
CAGTTACATCGATTCTGGTCAGGGCTTTGAGATTGGCATTTCGCAAGAGCCGTTTGATCCTAGTGGTTACCAGCTTTAT
TTACACAAGGCCACTAATGGTACCACTAATGCTATTGCACGACTGCGCATTTGCCAGTTTCCAGATAATAAAACATTGG
GCCCTACTGTTAATGATGTTACAACAGGTCGTAACTGCCTATTCAACAAAGCCATTCCAGCTTATATGCAGGATGGAAA
AAATATTGTTGTCGGCATAACATGGGATAATGATCGTGTCACTGTTTTTGCTGACAAGATCTATCATTTTTATATTAAA
AATGATTGGTCCCGTGTTGCGACAAGATGTTACAATAAAAGAAGTTGTGCCATGCAATATGTTTATACACCTACCTACT
ACATGCTTAATGTTACTAGTGCAGGTGAGGATGGCATTTACTATGAACCTTGTACAGCTAATTGCAGTGGTTACGCTGC
CAATGTATTTGCCACTGATTCCAATGGCCATATACCAGAAGGTTTTAGTTTTAATAATTGGTTTCTTTTGTCCAATGAC
TCCACTTTGTTGCATGGTAAAGTGGTTTCAAACCAACCTTTGTTGGTCAACTGCCTTTGGGCCATTCCTAAGATTTATG
GACTAGGCCAATTTTTCTCATTCAATCAAACGATGGATGGCGTTTGTAACGGAGCCGCTGCGCAGCGTGCCCCAGAGGC
TCTGAGGTTTAATATTAATGACACTTTTGTCATTCTTGCTGAAGGCTCAATTGTACTTCATACTGCTTTAGGAACAAAT
CTTTCTTTTGTTTGCAGTAATTCCTCAGATCCTCACAAAGCCATCTTTACCATACCTTTGGGTGTTACTGAAGTACCCT
ACTATTGCTTTCTTAAAGTGGATACTTACAACCCCACTGTTTATAAATTCTTGGCTGTTTTACCTCCTACTGTCAAGGA
AATTGTCATCACCAAGTACGGTGATGTTTATGTCAACGGGTTTGGCTATTTGCATCTCGGTTTGTTGGATGCTGTCACA
ATTAATTTCACTGGTCATGGCACTGACGATGACGTTTCAGGTTTCTGGACCGTAGCATCGACTAATTTTGTTGATGCAC
TCATCGAGGTTCAAGGAACTGCCATTCAGCGTATTCTTTATTGTGATGACCCTGTTAGCCAACTTAAGTGTTCTCAGGT
TTCTTTTGACCTTGATGATGGTTTTTACCCTATTTCTTCTAGAAACCTTCTGAGTCATGAACAGCCAATTTCTTTTGTT
ACTTTGCCATCATTCAATGATCATTCTTTTGTTAATATTACTGTCTCTGCGGCTTTTGGTGGTCATAGTGGTGCCAACC
TCATTGCATCTGACACTACTATCAATGGGTTTAGTTCTTTCTGTGTTGACACTAGACAATTTACCATTACACTGTTTTA
TAACGTTACAAACAGTTATGGTTATGTGTCTAAGTCACAGGATAGTAATTGCCCTTTCACCTTGCAATCTGTTAATGAT
TACCTGTCTTTTAGCAAATTTTGTGTTTCAACCAGCCTTTTGGCTGGTGCTTGTACCATAGATCTTTTTGGTTACCCTG
AGTTCGGTAGTGGTGTTAAGTTTACGTCCCTTTATTTTCAATTCACAAAGGGTGAGTTGATTACTGGCACGCCTAAACC
ACTTGAAGGTGTCACGGACGTTTCTTTTATGACTCTGGATGTGTGTACCAAGTATACTATCTATGGCTTTAAAGGTGAG
GGTATTATTACCCTTACAAATTCTAGCTTTTTGGCAGGTGTTTATTATACATCTGATTCTGGACAGTTGTTAGCCTTTA
AGAATGTCACTAGTGGTGCTGTTTATTCTGTTACGCCATGTTCTTTTTCAGAGCAGGCTGCATATGTTGATGATGATAT
AGTGGGTGTTATTTCTAGTTTGTCTAACTCCACTTTTAACAATACCAGGGAGTTGCCTGGTTTCTTCTACCATTCTAAT
GATGGCTCCAATTGTACAGAGCCTGTGTTGGTGTATAGTAACATAGGTGTCTGTAAATCTGGCAGTATTGGCTATGTCC
CACTTCAGGATGGCCAAGTCAAGATTGCACCCACGGTTACTGGGAATATTAGTATTCCCACCAACTTTAGTATGAGTAT
TAGAACAGAATATTTACAGCTTTACAACACGCCTGTTAGTGTTGATTGCGTTACATATGTTTGTAATGGTAACTCTCGT
TGTAAACAATTACTCACCCAGTACACTGCAGCATGTAAGACCATAGAGTCAGCATTACAACTCAGCGCTAGGCTTGAGT
CTGTTGAAGTTAACTCTATGCTTACTATTTCTGAAGAGGCTCTACAGTTAGCTACCATCAGTTCGTTTAATGGTGATGG
ATATAACTTTACTAATGTGCTGGGTGTTTCCGTGTACGATCCTGCAAGTGGCAGGGTGGTACAAAAAAGGTCTTTTATT
GAAGACCTGCTTTTTAATAAAGTGGTTACTAATGGCCTTGGTACTGTTGATGAAGACTATAAGCGCTGTTCTAATGGTC
GCTCTGTGGCAGATCTAGTCTGTGCGCAGTATTACTCTGGTGTCATGGTACTACCTGGCGTTGTTGACGCTGAGAAGCT
TCACATGTATAGTGCGTCTCTCATCGGTGGTATGGCGCTAGGAGGTTTTACTGCTGCAGCGGCATTGCCTTTTAGCTAT
GCTGTTCAAGCGAGGCTCAATTATCTTGCTTTACAGACGGATGTTCTACAGCGCAACCAGCAATTGCTTGCTGAGTCTT
TTAACTCTGCTATTGGTAATATAACTTCAGCCTTTGAGAGTGTTAAAGAGGCTATTAGTCAAACTTCCAATGGTTTGAA
CACTGTGGCTCATGCGCTTACTAAGGTTCAAGAGGTTGTTAATTCGCAGGGTTCAGCTTTGACCCAACTTACCATACAG
CTGCAACACAACTTCCAAGCCATTTCTAGTTCTATTGATGACATTTACTCCCGACTGGACATTCTTTCAGCCGATGTTC

AGGTTGATCGTCTCATCACCGGCAGATTATCAGCACTTAATGCTTTTGTTGCTCAAACCCTCACTAAGTATACTGAGGT
TCAGGCTAGCAGGAAGCTAGCACAGCAAAAGGTTAATGAGTGCGTCAAATCGCAATCTCAGCGTTATGGTTTTTGTGGT
GGTGATGGCGAGCACATCTTCTCTCTGGTACAGGCCGCACCTCAGGGCCTGCTGTTCTTACATACAGTACTTGTACCGG
GTGATTTTGTAAATGTTATTGCCATCGCTGGCTTATGCGTTAATGGTGATATTGCCTTGACTCTACGTGAGCCTGGCTT
AGTCTTGTTTACGCATGAACTTCAAACTTATACTGCGACGGAATATTTTGTTTCATCGCGACGTATGTTTGAACCTAGA
AAACCTACCGTTAGTGATTTTGTTCAAATTGAGAGTTGTGTGGTCACCTATGTCAATCTGACTAGCGACCAACTACCAG
ATGTAATCCCAGATTACATCGATGTTAACAAAACACTTGATGAGATTCTAGCTTCTCTGCCCAATAGAACTGGTCCTAG
TCTTCCCCTAGATGTTTTTAATGCCACTTATCTTAATCTCACTGGTGAAATTGCAGATTTAGAGCAGCGTTCAGAGTCT
CTCCGTAATACTACAGAAGAGCTCCGAAGTCTCATATATAATATCAACAACACACTTGTTGACCTTGAGTGGCTCAACC
GAGTTGAGACATATATCAAGTGGCCGTGGTGGGTTTGGTTGATTATTTTTATTGTTCTCATCTTTGTTGTGTCATTATT
AGTGTTCTGCTGCATTTCCACGGGTTGTTGTGGATGCTGCGGTTGCTGCGGTGCTTGTTTTTCAGGTTGTTGTAGGGGT
CCTAGACTTCAACCTTACGAAGCTTTTGAAAAGGTCCACGTGCAGTGA

>gi|334683274|dbj|AB548619.1|_Porcine_epidemic_diarrhea_virus_S_gene_for_spike_protein_complete_cds_strain:_83P5_34thpassaged

ATGAAGCCTTTAATTTACTTCTGGTTGTTCTTACCAGTACTTCTAACACTTAGCCTACCACAAGATGTCACTAGG
TGCCAGTCTACTATTAACTTTAGGCGGTTCTTTTCAAAATTTAATGTTCAGGCACCTGCCGTCGTCGTTTTGGGTGGTT
ATCTACCTAGTATGAACTCTTCTAGCTGGTACTGTGGCACAGGCATTGAAACTGATAGTGGCGTTCATGGTATTTTTCT
CAGTTACATCGATTCTGGTCAGGGCTTTGAGATTGGCATTTCGCAAGAGCCGTTTGATCCTAGTGGTTACCAGCTTTAT
TTACACAAGGCCACTAATGGTAACACTAGTGCTATTGCACGACTGCGCATTTGCCAGTTTCCAGATAATAAAACATTGG
GCCCTACTGTTAATGATGTTACAACAGGTCGTAACTGCCTATTCAACAAAGCCATTCCAGCTTATATGCAGGATGGAAA
AAATATTGTTGTCGGCATAACATGGGATAATGATCGTGTCACTGTTTTTGCTGACAAGATCTATCATTTTTATATTAAA
AATGATTGGTCCCGTGTTGCGACAAGATGTTACAATAAAAGAAGTTGTGCCATGCAATATGTTTATACACCTACCTACT
ACATGCTTAATGTTACTAGTGCAGGTGAGGATGGCATTTACTATGAACCTTGTACAGCTAATTGCAGTGGTTACGCTGC
CAATGTATTTGCCACTGATTCCAATGGCCATATACCAGAAGGTTTTAGTTTTAATAATTGGTTTCTTTTGTCCAATGAC
TCCACTTTGTTGCATGGTAAAGTGGTTTCAAACCAACCTTTGTTGGTCAACTGCCTTTGGGCCATTCCTAAGATTTATG
GACTAGGCCAATTTTTCTCATTCAATCAAACGATGGATGGCGTTTGTAACGGAGCCGCTGCGCAGCGTGCCCCAGAGGC
TCTGAGGTTTAATATTAATGACACTTTTGTCATTCTTGCTGAAGGCTCAATTGTACTTCATACTGCTTTAGGAACAAAT
CTTTCTTTTGTTTGCAGTAATTCCTCAGATCCTCACAAAGCCATCTTTACCATACCTTTGGGTGTTACTGAAGTACCCT
ACTATTGCTTTCTTAAAGTGGATACTTACAAATCCACTGTTTATAAATTCTTGGCTGTTTTACCTCCTACTGTCAAGGA
AATTGTCATCACCAAGTACGGTGATGTTTATGTCAACGGGTTTGGCTATTTGCATCTCGGTTTGTTGGATGCTGTCACA
ATTAATTTCACTGGTCATGGCACTGACGATGACGTTTCAGGTTTCTGGACCGTAGCATCGACTAATTTTGTTGATGCAC
TCATCGAGGTTCAAGGAACTGCCATTCAGCGTATTCTTTATTGTGATGACCCTGTTAGCCAACTTAAGTGTTCTCAGGT
TTCTTTTGACCTTGATGATGGTTTTTACCCTATTTCTTCTAGAAACCTTCTGAGTCATGAACAGCCAATTTCTTTTGTT
ACTTTGCCATCATTCAATGATCATTCTTTTGTTAATATTACTGTCTCTGCGGCTTTTGGTGGTCATAGTGGTGCCAACC
TCATTGCATCTGACACTACTATCAATGGGTTTAGTTCTTTCTGTGTTGACACTAGACAATTTACCATTACACTGTTTTA
TAACGTTACAAACAGTTATGGTTATGTGTCTAAGTCACAGGATAGTAATTGCCCTTTCACCTTGCAATCTGTTAATGAT
TACCTGTCTTTTAGCAAATTTTGTGTTTCAACCAGCCTTTTGGCTGGTGCTTGTACCATAGATCTTTTTGGTTACCCTG
AGTTCGGTAGTGGTGTTAAGTTTACGTCCCTTTATTTTCAATTCACAAAGGGTGAGTTGATTACTGGCACGCCTAAACC
ACTTGAAGGTGTCACGGACGTTTCTTTTATGACTCTGGATGTGTGTACCAAGTATACTATCTATGGCTTTAAAGGTGAG
GGTATTATTACCCTTACAAATTCTAGCTTTTTGGCAGGTGTTTATTATACATCTGATTCTGGACAGTTGTTAGCCTTTA
AGAATGTCACTAGTGGTGCTGTTTATTCTGTTACGCCATGTTCTTTTTCAGAGCAGGCTGCATATGTTGATGATGATAT

AGTGGGTGTTATTTCTAGTTTGTCTAACTCCACTTTTAACAATACCAGGGAGTTGCCTGGTTTCTTCTACCATTCTAAT
GATGGCTCCAATTGTACAGAGCCTGTGTTGGTGTATAGTAACATAGGTGTCTGTAAATCTGGCAGTATTGGCTATGTCC
CACTTCAGGATGGCCAAGTCAAGATTGCACCCACGGTTACTGGGAATATTAGTATTCCCACCAACTTTAGTATGAGTAT
TAGAACAGAATATTTACAGCTTTACAACACGCCTGTTAGTGTTGATTGCGTTACATATGTTTGTAATGGTAACTCTCGT
TGTAAACAATTACTCACCCAGTACACTGCAGCATGTAAGACCATAGAGTCAGCATTACAACTCAGCGCTAGGCTTGAGT
CTGTTGAAGTTAACTCTATGCTTACTATTTCTGAAGAGGCTCTACAGTTAGCTACCATCAGTTCGTTTAATGGTGATGG
ATATAACTTTACTAATGTGCTGGGTGTTTCCGTGTACGATCCTGCAAGTGGCAGGGTGGTACAAAAAAGGTCTTTTATT
GAAGACCTGCTTTTTAATAAAGTGGTTACTAATGGCCTTGGTACTGTTGATGAAGACTATAAGCGCTGTTCTAATGGTC
GCTCTGTGGCAGATCTAGTCTGTGCGCAGTATTACTCTGGTGTCATGGTACTACCTGGCGTTGTTGACGCTGAGAAGCT
TCACATGTATAGTGCGTCTCTCATCGGTGGTATGGCGCTAGGAGGTTTTACTGCTGCAGCGGCATTGCCTTTTAGCTAT
GCTGTTCAAGCGAGGCTCAATTATCTTGCTTTACAGACGGATGTTCTACAGCGCAACCAGCAATTGCTTGCTGAGTCTT
TTAACTCTGCTATTGGTAATATAACTTCAGCCTTTGAGAGTGTTAAAGAGGCTATTAGTCAAACTTCCAATGGTTTGAA
CACTGTGGCTCATGCGCTTACTAAGGTTCAAGAGGTTGTTAATTCGCAGGGTTCAGCTTTGACCCAACTTACCATACAG
CTGCAACACAACTTCCAAGCCATTTCTAGTTCTATTGATGACATTTACTCCCGACTGGACATTCTTTCAGCCGATGTTC
AGGTTGATCGTCTCATCACCGGCGGATTATCAGCACTTAATGCTTTTGTTGCTCAAACCCTCACTAAGTATACTGAGGT
TCAGGCTAGCAGGAAGCTAGCACAGCAAAAGGTTAATGAGTGCGTCAAATCGCAATCTCAGCGTTATGGTTTTTGTGGT
GGTGATGGCGAGCACATCTTCTCTCTGGTACAGGCCGCACCTCAGGGCCTGCTGTTCTTACATACAGTACTTGTACCGG
GTGATTTTGTAAATGTTATTGCCATCGCTGGCTTATGCGTTAATGGTGATATTGCCTTGACTCTACGTGAGCCTGGCTT
AGTCTTGTTTACGCATGAACTTCAAACTTATACTGCGACGGAATATTTTGTTTCATCGCGACGTATGTTTGAACCTAGA
AAACCTACCGTTAGTGATTTTGTTCAAATTGAGAGTTGTGTGGTCACCTATGTCAATCTGACTAGCGACCAACTACCAG
ATGTAATCCCAGATTACATCGATGTTAACAAAACACTTGATGAGATTCTAGCTTCTCTGCCCAATAGAACTGGTCCTAG
TCTTCCCCTAGATGTTTTTAATGCCACTTATCTTAATCTCACTGGTGAAATTGCAGATTTAGAGCAGCGTTCAGAGTCT
CTCCGTAATACTACAGAAGAGCTCCGAAGTCTCATATATAATATCAACAACACACTTGTTGACCTTGAGTGGCTCAACC
GAGTTGAGACATATATCAAGTGGCCGTGGTGGGTTTGGTTGATTATTTTTATTGTTCTCATCTTTGTTGTGTCATTATT
AGTGTTCTGCTGCATTTCCACGGGTTGTTGTGGATGCTGCGGTTGTTGCGGTGCTTGTTTTTCAGGTTGTTGTAGGGGT
CCTAGACTTCAACCTTACGAAGCTTTTGAAAAGGTCCACGTGCAGTGA

>gi|334683276|dbj|AB548620.1|_Porcine_epidemic_diarrhea_virus_S_gene_for_spike_protein_complete_cds_strain:_83P5_61stpassaged

ATGAAGCCTTTAATTTACTTCTGGTTGTTCTTACCAGTACTTCTAACACTTAGCCTACCACAAGATGTCACTAGG
TGCCAGTCTACTATTAACTTTAGGCGGTTCTTTTCAAAATTTAATGTTCAGGCACCTGCCGTCGTCGTTTTGGGTGGTT
ATCTACCTAGTATGAACTCTTCTAGCTGGTACTGTGGCACAGGCATTGAAACTGATAGTGGCGTTCATGGTATTTTTCT
CAGTTACATCGATTCTGGTCAGGGCTTTGAGATTGGCATTTCGCAAGAGCCGTTTGATCCTAGTGGTTACCAGCTTTAT
TTACACAAGGCCACTAATGGTAACACTAGTGCTATTGCACGACTGCGCATTTGCCAGTTTCCAGATAATAAAACATTGG
GCCCTACTGTTAATGATGTTACAACAGGTCGTAACTGCCTATTCAACAAAGCCATTCCAGCTTATATGCAGGATGGAAA
AAATATTGTTGTCGGCATAACATGGGATAATGATCGTGTCACTGTTTTTGCTGACAAGATCTATCATTTTTATATTAAA
AATGATTGGTCCCGTGTTGCGACAAGATGTTACAATAAAAGAAGTTGTGCCATGCAATATGTTTATACACCTACCTACT
ACATGCTTAATGTTACTAGTGCAGGTGAGGATGGCATTTACTATGAACCTTGTACAGCTAATTGCAGTGGTTACGCTGC
CAATGTATTTGCCACTGATTCCAATGGCCATATACCAGAAGGTTTTAGTTTTAATAATTGGTTTCTTTTGTCCAATGAC
TCCACTTTGTTGCATGGTAAAGTGGTTTCAAACCAACCTTTGTTGGTCAACTGCCTTTGGGCCATTCCTAAGATTTATG
GACTAGGCCAATTTTTCTCATTCAATCAAACGATGGATGGCGTTTGTAACGGAGCCGCTGCGCAGCGTGCCCCAGAGGC
TCTGAGGTTTAATATTAATGACACTTTTGTCATTCTTGCTGAAGGCTCAATTGTACTTCATACTGCTTTAGGAACAAAT

CTTTCTTTTGTTTGCAGTAATTCCTCAGATCCTCACAAAGCCATCTTTACCATACCTTTGGGTGTTACTGAAGTACCCT
ACTATTGCTTTCTTAAAGTGGATACTTACAAATCCACTGTTTATAAATTCTTGGCTGTTTTACCTCCTACTGTCAAGGA
AATTGTCATCACCAAGTACGGTGATGTTTATGTCAACGGGTTTGGCTATTTGCATCTCGGTTTGTTGGATGCTGTCACA
ATTAATTTCACTGGTCATGGCACTGACGATGACGTTTCAGGTTTCTGGACCGTAGCATCGACTAATTTTGTTGATGCAC
TCATCGAGGTTCAAGGAACTGCCATTCAGCGTATTCTTTATTGTGATGACCCTGTTAGCCAACTTAAGTGTTCTCAGGT
TTCTTTTGACCTTGATGATGGTTTTTACCCTATTTCTTCTAGAAACCTTCTGAGTCATGAACAGCCAATTTCTTTTGTT
ACTTTGCCATCATTCAATGATCATTCTTTTGTTAATATTACTGTCTCTGCGGCTTTTGGTGGTCATAGTGGTGCCAACC
TCATTGCATCTGACACTACTATCAATGGGTTTAGTTCTTTCTGTGTTGACACTAGACAATTTACCATTACACTGTTTTA
TAACGTTACAAACAGTTATGGTTATGTGTCTAAGTCACAGGATAGTAATTGCCCTTTCACCTTGCAATCTGTTAATGAT
TACCTGTCTTTTAGCAAATTTTGTGTTTCAACCAGCCTTTTGGCTGGTGCTTGTACCATAGATCTTTTTGGTTACCCTG
AGTTCGGTAGTGGTGTTAAGTTTACGTCCCTTTATTTTCAATTCACAAAGGGTGAGTTGATTACTGGCACGCCTAAACC
ACTTCAAGGTGTCACGGACGTTTCTTTTATGACTCTGGATGTGTGTACCAAGTATACTATCTATGGCTTTAAAGGTGAG
GGTATTATTACCCTTACAAATTCTAGCTTTTTGGCAGGTGTTTATTATACATCTGATTCTGGACAGTTGTTAGCCTTTA
AGAATGTCACTAGTGGTGCTGTTTATTCTGTTACGCCATGTTCTTTTTCAGAGCAGGCTGCATATGTTGATGATGATAT
AGTGGGTGTTATTTCTAGTTTGTCTAACTCCACTTTTAACAATACCAGGGAGTTGCCTGGTTTCTTCTACCATTCTAAT
GATGGCTCCAATTGTACAGAGCCTGTGTTGGTGTATAGTAACATAGGTGTCTGTAAATCTGGCAGTATTGGCTATGTCC
CACTTCAGGATGGCCAAGTCAAGATTGCACCCACGGTTACTGGGAATATTAGTATTCCCACCAACTTTAGTATGAGTAT
TAGAACAGAATATTTACAGCTTTACAACACGCCTGTTAGTGTTGATTGCGTTACATATGTTTGTAATGGTAACTCTCGT
TGTAAACAATTACTCACCCAGTACACTGCAGCATGTAAGACCATAGAGTCAGCATTACAACTCAGCGCTAGGCTTGAGT
CTGTTGAAGTTAACTCTATGCTTACTATTTCTGAAGAGGCTCTACAGTTAGCTACCATCAGTTCGTTTAATGGTGATGG
ATATAACTTTACTAATGTGCTGGGTGTTTCCGTGTACGATCCTGCAAGTGGCAGGGTGGTACAAAAAAGGTCTTTTATT
GAAGACCTGCTTTTTAATAAAGTGGTTACTAATGGCCTTGGTACTGTTGATGAAGACTATAAGCGCTGTTCTAATGGTC
GCTCTGTGGCAGATCTAGTCTGTGCGCAGTATTACTCTGGTGTCATGGTACTACCTGGCGTTGTTGACGCTGAGAAGCT
TCACATGTATAGTGCGTCTCTCATCGGTGGTATGGCGCTAGGAGGTTTTACTACTGCAGCGGCATTGCCTTTTAGCCAT
GCTGTTCAAGCGAGGCTCAATTATCTTGCTTTACAGACGGATGTTCTACAGCGCAACCAGCAATTGCTTGCTGAGTCTT
TTAACTCTGCTATTGGTAATATAACTTCAGCCTTTGAGAGTGTTAAAGAGGCTATTAGTCAAACTTCCAATGGTTTGAA
CACTGTGGCTCATGCGCTTACTAAGGTTCAAGAGGTTGTTAATTCGCAGGGTTCAGCTTTGACCCAACTTACCATACAG
CTGCAACACAACTTCCAAGCCATTTCTAGTTCTATTGATGACATTTACTCCCGACTGGACATTCTTTCAGCCGATGTTC
AGGTTGATCGTCTCATCACCGGCAGATTATCAGCACTTAATGCTTTTGTTGCTCAAACCCTCACTAAGTATACTGAGGT
TCAGGCTAGCAGGAAGCTAGCACAGCAAAAGGTTAATGAGTGCGTCAAATCGCAATCTCAGCGTTATGGTTTTTGTGGT
GGTGATGGCGAGCACATCTTCTCTCTGGTACAGGCCGCACCTCAGGGCCTGCTGTTCTTACATACAGTACTTGTACCGG
GTGATTTTGTAAATGTTATTGCCATCGATGGCTTATGCGTTAATGGTGATATTGCCTTGACTCTACGTGAGCCTGGCTT
AGTCTTGTTTACGCATGAACTTCAAACTTATACTGCGACGGAATATTTTGTTTCATCGCGACGTATGTTTGAACCTAGA
AAACCTACCGTTAGTGATTTTGTTCAAATTGAGAGTTGTGTGGTCACCTATGTCAATCTGACTAGCGACCAACTACCAG
ATGTAATCCCAGATTACATCGATGTTAACAAAACACTTGATGAGATTCTAGCTTCTCTGCCCAATAGAATTGGTCCTAG
TCTTCCCCTAGATGTTTTTAATGCCACTTATCTTAATCTCACTGGTGAAATTGCAGATTTAGAGCAGCGTTCAGAGTCT
CTCCGTAATACTACAGAAGAGCTCCGAAGTCTCATATATAATATCAACAACACACTTGTTGACCTTGAGTGGCTCAACC
GAGTTGAGACATATATCAAGTGGCCGTGGTGGGTTTGGTTGATTATTTTTATTGTTCTCATCTTTGTTGTGTCATTATT
AGTGTTCTGCTGCATTTCCACGGGTTGTTGTGGATGCTGCGGTTGTTGCGGTGCTTGTTTTTCAGGTTGTTGTAGGGGT
CCTAGACTTCAACCTTACGAAGCTTTTGAAAAGGTCCACGTGCAGTGA

>gi|334683278|dbj|AB548621.1|_Porcine_epidemic_diarrhea_virus_S_gene_for_spike_protein_complete_cds_strain:_83P5_100thpassaged

ATGACGCCTTTAATTTACTTCTGGTTGTTCTTACCAGTACTTCTAACACTTAGCCTACCACAAGATGTCACTAGG
TGCCAGTCTACTATTAACTTTAGGCGGTTCTTTTCAAAATTTAATGTTCAGGCACCTGCCGTCGTTGTTTTGGGTGGTT
ATCTACCTAGTATGAACTCTTCTAGCTGGTACTGTGGCACAGGCATTGAAACTGATAGTGGCGTTCATGGTATTTTTCT
CAGTTACATCGATTCTGGTCAGGGCTTTGAGATTGGCATTTCGCAAGAGCCGTTTGATCCTAGTGGTTACCAGCTTTAT
TTACACAAGGCCACTAATGGTAACACTAGTGCTATTGCACGACTGCGCATTTGCCAGTTTCCAGATAATAAAACATTGG
GCCCTACTGTTAATGATGTTACAACAGGTCGTAACTGCCTATTCAACAAAGCCATTCCAGCTTTGCAGGATGGAAAAAA
TATTGTTGTCGGCATAACATGGGATAATGATCGTGTCACTGTTTTTGCTGACAAGATCTATCATTTTTATATTAAAAAT
GATTGGTCCCGTGTTGCGACAAGATGTTACAATAAAAGAAGTTGTGCCATGCAATATGTTTATACACCTACCTACTACA
TGCTTAATGTTACTAGTGCAGGTGAGGATGGCATTTACTATGAACCTTGTACAGCTAATTGCAGTGGTTACGCTGCCAA
TGTATTTGCCACTGATTCCAATGGCCATATACCAGAAGGTTTTAGTTTTAATAATTGGTTTCTTTTGTCCAATGACTCC
ACTTTGTTGCATGGTAAAGTGGTTTCAAACCAACCTTTGTTGGTCAACTGCCTTTGGGCCATTCCTAAGATTTATGGAC
TAGGCCAATTTTTCTCATTCAATCAAACGATGGATGGCGTTTGTAACGGAGCCGCTGCGCAGCGTGCCCCAGAGGCTCT
GAGGTTTAATATTAATGACACTTTTGTCATTCTTGCTGAAGGCTCAATTGTACTTCATACTGCTTTAGGAACAAATCTT
TCTTTTGTTTGCAGTAATTCCTCAGATCCTCACAAAGCCATCTTTACCATACCTTTGGGTGTTACTGAAGTACCCTACT
ATTGCTTTCTTAAAGTGGATACTTACAAATCCACTGTTTATAAATTCTTGGCTGTTTTACCTCCTACTGTCAAGGAAAT
TGTCATCACCAAGTACGGTGATGTTTATGTCAACGGGTTTGGCTATTTGCATCTCGGTTTGTTGGATGCTGTCACAATT
AATTTCACTGGTCATGGCACTGACGATGACGTTTCAGGTTTCTGGACCGTAGCATCGACTAATTTTGTTGATGCACTCA
TCGAGGTTCAAGGAACTGCCATTCAGCGTATTCTTTATTGTGATGACCCTGTTAGCCAACTTAAGTGTTCTCAGGTTTC
TTTTGACCTTGATGATGGTTTTTACCCTATTTCTTCTAGAAACCTTCTGAGTCATGAACAGCCAATTTCTTTTGTTACT
TTGCCATCATTCAATGATCATTCTTTTGTTAATATTACTGTCTCTGCGGCTTTTGGTGGTCATAGTGGTGCCAACCTCA
TTGCATCTGACACTACTATCAATGGGTTTAGTTCTTTCTGTGTTGACACTAGACAATTTACCATTACACTGTTTTATAA
CGTTACAAACAGTTATGGTTATGTGTCTAAGTCACAGGATAGTAATTGCCCTTTCACCTTGCAATCTGTTAATGATTAC
CTGTCTTTTAGCAAATTTTGTGTTTCAACCAGCCTTTTGGCTGGTGCTTGTACCATAGATCTTTTTGGTTACCCTGAGT
TCGGTAGTGGTGTTAAGTTTACGTCCCTTTATTTTCAATTCACAAAGGGTGAGTTGATTACTGGCACGCCTAAACCACT
TCAAGGTGTCACGGACGTTTCTTTTATGACTCTGGATGTGTGTACCAAGTATACTATCTATGGCTTTAAAGGTGAGGGT
ATTATTACCCTTACAAATTCTAGCTTTTTGGCAGGTGTTTATTATACATCTGATTCTGGACAGTTGTTAGCCTTTAAGA
ATGTCACTAGTGGTGCTGTTTATTCTGTTACGCCATGTTCTTTTTCAGAGCAGGCTGCATATGTTGATGATGATATAGT
GGGTGTTATTTCTAGTTTGTCTAACTCCACTTTTAACAATACCAGGGAGTTGCCTGGTTTCTTCTACCATTCTAATGAT
GGCTCCAATTGTACAGAGCCTGTGTTGGTGTATAGTAACATAGGTGTCTGTAAATCTGGCAGTATTGGCTATGTCCCAC
TTCAGGATGGCCAAGTCAAGATTGCACCCATGGTTACTGGGAATATTAGTATTCCCACCAACTTTAGTATGAGTATTAG
AACAGAATATTTACAGCTTTACAACACGCCTGTTAGTGTTGATTGCGTTACATATGTTTGTAATGGTAACTCTCGTTGT
AAACAATTACTCACCCAGTACACTGCAGCATGTAAGACCATAGAGTCAGCATTACAACTCAGCGCTAGGCTTGAGTCTG
TTGAAGTTAACTCTATGCTTACTATTTCTGAAGAGGCTCTACAGTTAGCTACCATCAGTTCGTTTAATGGTGATGGATA
TAACTTTACTAATGTGCTGGGTGTTTCCGTGTACGACCCTGCAAGTGGCAGGGTGGTACAAAAAAGGTCTTTTATTGAA
GACCTGCTTTTTAATAAAGTGGTTACTAATGGCCTTGGTACTGTTGATGAAGACTATAAGCGCTGTTCTAATGGTCGCT
CTGTGGCAGATCTAGTCTGTGCGCAGTATTACTCTGGTGTCATGGTACTACCTGGCGTTGTTGACGCTGAGAAGCTTCA
CATGTATAGTGCGTCTCTCATCGGTGGTATGGCGCTAGGAGGTCTTACTACTGCAGCGGCATTGCCTTTTAGCCATGCT
GTTCAAGCGAGGCTCAATTATCTTGCTTTACAGACGGATGTTCTACAGCGCAACCAGCAATTGCTTGCTGAGTCTTTTA
ACTCTGCTATTGGTAATATAACTTCAGCCTTTGAGAGTGTTAAAGAGGCTATTAGTCAAACTTCCAATGGTTTGAACAC
TGTGGCTCATGCGCTTACTAAGGTTCAAGAGGTTGTTAATTCGCAGGGTTCAGCTTTGACCCAACTTACCATACAGCTG
CAACACAACTTCCAAGCCATTTCTAGTTCTATTGATGACATTTACTCCCGACTGGACATTCTTTCAGCCGATGTTCAGG
TTGATCGTCTCATCACCGGCAGATTATCAGCACTTAATGCTTTTGTTGCTCAAACCCTCACTAAGTATACTGAGGTTCA
GGCTAGCAGGAAGCTAGCACAGCAAAAGGTTAATGAGTGCGTCAAATCGCAATCTCAGCGTTATGGTTTTTGTGGTGGT
GATGGCGAGCACATCTTCTCTCTGGTACAGGCCGCACCTCAGGGCCTGCTGTTCTTACATACAGTACTTGTACCGGGTG

ATTTTGTAAATGTTATTGCCATCGATGGCTTATGCGTTAATGGTGATATTGCCTTGACTCTACGTGAGCCTGGCTTAGT
CTTGTTTACGCATGAACTTCAAACTTATACTGCGACGGAATATTTTGTTTCATCGCGACGTATGTTTGAACCTAGAAAA
CCTACCGTTAGTGATTTTGTTCAAATTGAGAGTTGTGTGGTCACCTATGTCAATCTGACTAGCGACCAACTACCAGATG
TAATCCCAGATTACATCGATGTTAACAAAACACTTGATGAGATTCTAGCTTCTCTGCCCAATAGAATTGGTCCTAGTCT
TCCCCTAGATGTTTTTAATGCCACTTATCTTAATCTCACTGGTGAAATTGCAGATTTAGAGCAGCGTTCAGAGTCTCTC
CGTAATACTACAGAAGAGCTCCGAAGTCTCATATATAATATCAACAACACACTTGTTGACCTTGAGTGGCTCAACCGAG
TTGAGACATATATCAAGTGGCCGTGGTGGGTTTGGTTGATTATTTTTATTGTTCTCATCTTTGTTGTGTCATTATTAGT
GTTCTGCTGCATTTCCACGGGTTGTTGTGGATGCTGCGGTTGTTGCGGTGCTTGTTTTTCAGGTTGTTGTAGGGGTCCT
AGACTTCAACCTTACGAAGCTTTTGAAAAGGTCCACGTGCAGTGA

>gi|334683280|dbj|AB548622.1|_Porcine_epidemic_diarrhea_virus_S_gene_for_spike_protein_complete_cds_strain:_KH

ATGAAGTTTTTAATTTATTTTTGGTTGTTCTTACCAGTACTTCCAACACTCAGCCTACCACAAGATGTCACTAGG
TGCTCAGCTAAGACTAACTTCAGGCGGGTCTTTTCAAAATTTAATGTTCAGGCGCCTGCAGTCGTTGTACTGGGCGGTT
ATCTACCTAGTGGTGAACAACAGAATGGGTCTGCACCACCTTGGTACTGTGCTGGCACACATACACCTGCTAGTGGCGT
TCATGGTATTTTTCTTAGCCATATTAAAGGTGGTCATGGCTTTGAGATTGGCATTTCGCAAGAGCCGTTTGACCCTAGT
GGTTACCAGCTTTATTTACATAAGGCCACTAACGGTAACACTAATGCTACTGCGCGACTGCGTATTTGCCAGTTTCTCA
GCAATAAAACATTGGGCCCCACTGCTAATGATGATGTTACAACAGGTCGTAACTGCCTATTTAACAAAGCCATTCCAGC
TCATATGAGTGAACATAGTGTTGTCGGCATAACATGGGATAATGATCGTGTCACTGTTTTTGCTGACAAGATCTATCAT
TTTCATCTTAAAAATGATTGGTCCCGTGTTGCGACAAAATGTTACAACAGTGGAGGTTGTGCTATGCAATATGTTTACG
AACCTATTTACTACATGCTTAATGTTACTAGTGCTGGTGAAGATGGTATTTCTTATCAACCCTGTACAGCTAATTGCAT
TGGTTACGCTGTCAATGTATTTGCCACTGAGTCCAATGGCCATATACCAGAAGGTTTTAGTTTTAATAATTGGTTTCTT
TTGTCCAATGACTCCACTTTGTTGCATGGTAAAGTGGTTTCAAACCAGCCTTTGTTGGTCAACTGCCTTTGGGCCATTC
CTAAGATTTATGGAATACGCAAATTTTTCTCATTCAATCAAACTATGGATGGCGTTTGTAACGGAGCCACTGTACAGCG
TGCCCCAGAGGCTTTGAGGTTTAATATTAATGACACCTCTGTCATTCTTGCTGAAGGCTCAATTGTACTTCATACTGCT
TTAGGAACAAATCTTTCTTTTGTTTTTAGTAATTCTTCAAATCCTCATTCAGCCATCTTTGCCATACCTCTGGGTGCTA
CTGAAGTACCCTATTACTGTTTTCTTAAAGTTGATACTTACAAATCCTCTGTTTATAAATTTTTGGCTGTTTTACCTCC
TACTGTTAAGGAAATTGTCATCACCAAGTACGGTGATGTTTATGTCAACGGTTTTGGCTATTTGCATCTCGGTTTGTTG
GATGCTGTCACAATTAATTTCACTGGTCATGGCACTGACGATGACGTTTCAGGTTTTTGGACCGTAGCATCGACTAATT
TTGTTGATGCACTCGTCGAGGTTCAGGGAACTGCCATTCAGCGTATTCTTTATTGTGATGACCCTGTTAGCGAACTTAA
GTGTTCTCAGGTTTCTTTTGACCTTGATGATGGTTTTTACTCTATTTCTTCTAGAAACCTTTTGAGTCATGAACAGCCA
ATTTCTTTTGTTACTTTGCCATCATTTAATGATCATTCTTTTGTTAATATTACTGTTTCTGCGGCTTTTGGTGGTCATA
GTGGTGGCAACCTCATTGCATCTGACACTACTATCAATGGGTTTAGTTCTTTTTGTGTTGACACTAGACAATTTACCAT
TAGACTGTTTTATAATGTTACAAACATTTATGGTTATGTGTCTAATTCACAGAGTAGCAATTGCCCTTTCACCTTGCAA
TCTGTTAATGACTACCTGTCTTTTAGCAAATTTTGTGTTTCAACCAGCATTTTGGCTGGTGCTTGTACCATAGATCTTT
TTGGTTACCCTGAGTTCGGTAGTGTTGTTAAGTTTACGTCCCTTTATTTTCAGTTCACAAAGGGTGAGTTGATTACTGG
CACGCCTAAACCACTTGAAGGTGTCACGGACGTTTCTTTTCTGACTTTGGATGTGTGTACCAAGTATACTATCTATGGC
TTTAAAGGTGAGGGTATTATTACCCTTACAAATTCTAGCTTTTTGGCAGGTGTTTATTACACATCTGATTCTGGACAGT
TGCTAGCTTTTAAGAATGTCACTAGTGGTGCTGTCTATTCTGTTACCCCATGTTCTTTTTCAGAGCAGGCCGCATATGT
TAATGATGATATAGTGGGTGTTATTTCCAGTTTGTCTAACTCCACTTTTAATAATACTAGGGAGTTGCCTGGCTTCTTC
TACCATTCTAATGATGGCTCCAATTGTACAGAGCCTGTGTTGGTGTATAGTAACATAGGTGTTTGTAAATCTGGCAGTA
TTGGCTATGTCCCACCTCAGGATGGCCAAGTCAAGATTGCACCCACGGTTACTGGGAATATTAGTATTCCCACCAACTT

TAGTATGAGTATTAGAACTGAATATTTACAGCTTTACAACACGCCTGTTAGTGTTGATTGTGCTACATATGTTTGTAAT
GGTAACTCTCGTTGTAAACAATTACTCACCCAGTACACTGCAGCATGTAAGACCATAGAGTCAGCATTACAACTCAGCG
CTAGGCTTGAGTCTGTTGAAGTTAACTCTATGCTTACTATTTCTGAAGAGGCTCTACAGTTAGCTACCATCAGTTCGTT
TAATGGTGATGGCTATAACTTTACTAATGTGCTGGGTGTTTCCGTGTACGATCATGCAAGTGGCAGGGTGGTACACAAA
AGGTCTTTTATTGAAGACCTGCTTTTTAATAAAGTGGTTACTAATGGCCTTGGTACTGTTGATGAAGACTATAAGCGCT
GTTCTAATGGTCGCTCTGTGGCTGATCTAGTCTGTGCGCAGTACTACTCTGGTGTCATGGTACTACCTGGCGTTGTTGA
CGCTGAGAAGCTTCACATGTATAGTGCTTCTCTCATCGGTGGTATGGCGCTAGGAGGTTTTACTGCTGCAGCGGCACTG
CCTTTTAGTTATGCTGTTCAAGCGCGGCTTAATTATCTTGCTTTACAGACGGATGTTCTACAGCGTAACCAGCAATTGC
TTGCTGAGTCTTTTAACTCTGCTATTGGTAATATAACTTCAGCCTTTGAGAGTGTCAAAGAGGCTATTAGTCAAACTTC
TAAGGGTTTGCATACTGTGGCTCATGCGCTTACTAAGGTTCAAGAGGTTGTTAATTCGCAGGGTTCAGCTTTGACCCAA
CTTACCGTACAGCTGCAACACAACTTCCAAGCCATTTCTAGTTCTATTGATGACATTTACTCCCGACTGGACGTTCTTG
CAGCCGATGTTCAGGTTGATCGTCTTATCACTGGCAGATTATCAGCACTTAATGCTTTTGTTGCTCAAACCCTCACTAA
GTATACTGAGGTTCAGGCTAGCAGGAGGCTAGCACAGCAAAAGGTTAATGAGTGCGTCAAATCTCAATCTCAGCGTTAT
GGTTTTTGTGGTGGTGATGGCGAGCACATTTTCTCTCTGGTACAGGCTGCACCTCAGGGCTTGTTGTTCTTACATACAG
TACTTGTACCGGGTGATTTTGTAAATGTTATTGCCATCGCTGGTTTATGCGTTAATGGTGAAATTGCCTTGACTCTACG
TGAGCCTGGCTTAGTCTTGTTTACGCATGAACTTCAAAATTATACTGCGACGGAATATTTTGTTTCATCGCGACGCATG
TTTGAACCTAGAAAACCTACCGTTAGTGATTTTGTTCAAATTGAGAGTTGTGTGGTCACCTATGTCAATCTGACTAGTG
ACCAACTACCAGATGTAATCCCAGATTACATCGACGTTAACAAAACACTTGATGAGATTTTAGCTTCTCTGCCCAATAG
AACTGGTACAAGTCTTCCCCTAGATGTTTTTAACGCCACTTATCTTAATCTCACTGGTGAAATTGCAGATTTAGAGCAG
CGTTCAGAGTCTCTCCGTAATACTACAGAAGAGCTCCGAAGTCTCATATATAATATCAACAACACACTTGTTGACCTTG
AGTGGCTCAACCGAGTTGAGACATATATCAAGTGGCCGTGGTGGGTTTGGTTGATTATTTTTATTGTTCTCATTTTTGT
TGTGTCATTATTAGTGTTCTGTTGCATTTCCACGGGTTGTTGTGGATGCTGCGGTTGCTGCGGTGCTTGTTTTTCAGGT
TGTTGTAGGGGTCCTAGACTTCAACCTTACGAAGCTTTTGAAAAGGTCCACGTGCAGTGA

>gi|334683282|dbj|AB548623.1|_Porcine_epidemic_diarrhea_virus_S_gene_for_spike_protein_complete_cds_strain:_NK

ATGAAGTCTTTAATTTATTTTTGGTTGTTCTTACCAGTACTTCCAACACTCAGCCTACCACAAGATGTCGATAGG
TGCCGGGCTAAGACTAACTTTAGGCGGTTCTTTTCAAAATTTAATGTTCAGGCGCCTGCAGTCGTTCTACTGGGCGGTT
ATCTACCTAGTGGTGAACAACAGGATGGGTCTGCACCACGTTGGTACTGTGGTGGCCAACATGAAACTGCTAGTGGCGT
TCATGGTATTTTTCTTAGCCATATTAAAGGTGGTCATGGCTTTGAGATTGGCATTTCGCAAGAGCCGTTTGACCCTAGT
GGTTACCAGCTTTATTTACATAAGGCCACTAACGGTAACACTAATGCTACTGCGCGACTGCGCATTTGCCAGTTTCCCA
GCAATAAAACATTGGGCCCCACTGCTAGTGATGATGTTACAACAGGTCGTAACTGCCTATTTAACAAAGCCATTCCAGC
TCATATGAGTGAACATAGTGTTGTCGGCATAACATGGGATAATGATCGTGTCACTGTTTTTGCTGACAAGATCTATCAT
TTTTATCTTAAAAATGATTGGTCCCGTGTTGCGACAAAATGTTACAACAGTGGAGGTTGTGCTATGCAATATGTTTACG
AACCTATTTACTACATGCTTAATGTTACTAGTGCTGGTGAGGATGGTATTTCTTATCAACCCTGTACAGCTAATTGCAT
TGGTTATGCTGCCAATGTATTTGCCACTGATTCCAATGGCCACATACCAGAAGGTTTTAGTTTTAATAATTGGTTTCTT
TTGTCCAATGATTCCACTTTGTTTCATGGTAAAGTGGTTTCCAACCAACCTTTGTTGGTCAACTGTCTTTGGGCCATTC
CTAAGATTTACGGACTAGGCCACTTTTTCTCATTTAATCAAACGATGGATGGCGTTTGTAACGGAGCTACTGCGTATCG
TGCCCCAGAGGCTCTGAGGTTTAATATTAATGACACTTCTGTCATTCTTGCCGAAGGCTCAATTGTACTTCATACTGCT
TTAGGAACAAATCTTTCTTTTGTTTGCAGTAATTCCTCAGATCCTCATTCAGCCATCTTTTCCATACCTCTGGGTGCTA
CCCAAGTACCCTACTATTGCTTTCTTAAAGTTGATACTTACAACTCCACTGTCTATAAATTTCTTGCTGTTTTACCTCT
TACTGTCAGGGAAATTGTCATCACCAAGTACGGTGATGTTTATGTCAATGGGTTTGGCTACTTGCATCTCGGTTTGTTG

GATGCTGTCACAATTAACTTCACTGGCCATGGCACTGACGATGACGTCTCAGGTTTCTGGACCATAGCATCGACTAATT
TTATTGATGCACTCATAGAGGTTCAAGCAACTGCTATTCAGCGTATTCTTTATTGTGATGATCCTGTTAGCCAACTCAA
GTGTTCTCAGGTTTCTTTTGACCTTGATGATGGTTTTTACCCTATTTCTTCTAGAAACCTTCTTAGTCATGAACAGCCA
ATTTCTTTTGTTACTTTGCCATCATTTAATGATCATTCTTTTGTTAATATTACTGTCTCTGCGGCTTTTGGTGATTCTG
GTGGTGCCAACCTCGTTGCATCTGACACTACTATCAATGGGTTCAGTTCTTTCTGTGTTGACACTAGACAATTTACCAT
TACACTGTTTTACAACGTTACAAACAGTTATGGTTATGTGTCTAAATCACAGCATAGCAATTGCCCTTTCACCTTGCAA
TCTGTTAATGATTACCTGTCTTTTAGCAAATTTTGTGTTTCAACCAGCCTTTTGGCTGGTGCTTGTACCATAGATCTTT
TTGGTTACCCTGCTTTCGGTAGTTCCTCTGGTGGTGGTGTTAAGTTTACGTCCCTCTATTTTCAATTCACAAAGGGTGA
GTTGATTACTGGCACGCCTAAACCACTTGAAGGTGTCACGGACGTTTCTTTTATGACTCTGGATGTGTGTACCAAGTAT
ACTATCTATGGCCTTAAAGGTGAGGGTATTATTACCCTTACAAATTCTAGCTTTTTGGCAGGTTTTTATTATACATCTG
ATTCTGGACAGTTGTTAGCCTTTAAGAATGTCACTAGTGGTGCTGTTTATTCTGTTACGCCATGTTCTTTTTCAGAGCA
GGCTGCTTATGTTGATGATGATATAGTGGGTGTTATTTCTAGTTTGTCTAACTCCACTTTTAACAATACTAGGGAGTTG
CCTGGTTTCTTCTACCATTCTAATGACGGCTCCAATTGTACAGAGCCTGTGTTGGTGTATAGTAACATAGGTGTCTGTA
AATCTGGTAGTATTGGCTATGTCCCACTTCAGGATGGCCAAGTCAAGATTGCACCCATGGTTACTGGGAATATTAGTAT
TCCCACCAACTTTAGTGTGAGTATTAGAACAGAATATTTACAGCTTTACAACACGCCTGTTAGTGTTGATTGTGCTACA
TATGTTTGTAATGGTAACTCTCGTTGTAAACAATTACTCACCCAGTACACTGCAGCATGTAAGACCATAGAGTCAGCAT
TAGAACTCAGCGCTAGGCTTGAGTCTGTTGAAGTTAACTCTATGCTTACTATTTCTGAAGAGGCTTTACAGTTAGCTAC
CATCAGTTCGTTTAATGGTGATGGGTATAACTTTACTAATGTGCTGGGTGTTTCTGTGTACGATTCTGAAAGTGGCAGG
GTGATACACAAAAGGTCTTTTATTGAAGACCTGCTTTTTAATAAGGTGGTTACTAATGGCCTTGGTACTGTTGATGAAG
ACTATAAGCGCTGTTCTAATGGTCGCTCTGTGGCTGATCTAGTCTGTGCGCAGTATTACTCTGGTATCATGGTACTACC
TGGCGTTGTTGACGCTGAGAAGCTTCACATGTATAGTGCGTCTCTCATCGGTGGTATGGCGCTAGGAGGTTTTACTGCC
GCAGTGGCATTGCCTTTTAGCTATGCTGTTCAAGCGAGACTCAATTATCTTGCTTTACAGACGGATGTTCTACAGCGGA
ACCAGCAACTGCTTGCTGAGTCTTTTAACTCTGCTATTGGTAATATAACCTCAGCCTTTGAGAGTGTTAAAGAGGCTAT
CAGTCAAACTTCTAAGGGTTTGAACACTGTGGCTCACGCGCTTACCAAAGTTCAAGAGGTTGTTAATTCGCAGGGTTCA
GCTTTGACTCAACTTACCATACAGCTGCAACACAACTTCCAAGCCATTTCTAGTTCTATTGATGACATTTACTCCCGAC
TGGACATTCTTTCAGCCGATGTTCAGGTTGATCGTCTCATCACCGGCAGATTATCAGCACTTAATGCTTTTGTTGCTCA
AACCCTCACTAAGTATACTGAGGTTCAGGCTAGCAGGAAGCTAGCACAGCAAAAGGTTAATGAGTGCGTCAAATCGCAA
TCTCAGCGTTATGGTTTTTGTGGTGGTGATGGCGAGCACATTTTCTCTCTTGTACAGGCCGCACCCCAGGGCCTGCTGT
TCTTACATACAGTACTTGTACCGGGTGATTTTGTAAATGTTATTGCCATCGCAGGCTTATGCGTTAATGGTGATATTGC
CTTGACTCTACGTGAGCCTGGCTTAGTCTTGTTTACGCATGAACTTCAAACTCATACTGCGACGGAATATTTTGTTTCA
TCGCGACGTATGTTTGAACCTAGAAAACCTACCGTTAGTGATTTTGTTCAAATTCAGAGTTGTGTGGTCACCTATGTCA
ATCTGACTAGCGACCAACTACCAGATGTAATCCCAGATTACATCGATGTTAACAAAACACTTGATGAGATTTTAGCTTC
TCTGCCCAATAGAACTGGTCCAAGTCTTCCTCTAGATGTTTTTAATGCCACTTATCTCAATCTCACTGGTGAAATTGCA
GATTTAGAGCAGCGTTCAGAGTCTCTCCGTAATACTACAGAAGAGCTCCGAAGTCTCATATATAATATCAACAACACAC
TTGTTGACCTTGAGTGGCTCAACAGAGTTGAGACATATATCAAGTGGCCGTGGTGGGTTTGGTTGATTATTTTTATTGT
TCTCATCTTTGTTGTGTCATTATTAGTGTTCTGCTGCATTTCCACGGGTTGTTGTGGATGCTGCGGTTGCTGCGGTGCT
TGTTTTTCAGGTTGTTGTAGGGGTCCTAGACTTCAACCTTACGAAGCTTTTGAAAAGGTCCACGTGCAGTGA

>gi|334683284|dbj|AB548624.1|_Porcine_epidemic_diarrhea_virus_S_gene_for_spike_protein_complete_cds_strain:_MK

ATGAAGTCTTTAATTTACTTCTGGTTGTTCTTACCAGTACTTTCAACACTTAGCATACCACAGGATGTCACTAGG
TGCCAGACTACTACTAACTTTAGGCGGTTCTTTTCAAAATTTAATGTTCAGGCACCTGCCGTCGTCATTTTGGGTGGTT

ATCTACCCAGTATGAACTCTTCTAGCTGGCACTGTGGCACAGGCATTGAAACTGATAGTGGCGTTCATGGTATTTTTCT
CAGTTACGTCGATTCTGGTCAGGGCTTTGAGATTGGCATTTCGCAAGAGCCGTTTGATCCTAGTGGTTACCAGCTTTAT
TTACATAAGGCTACTAATGGTAACACTAATGCTATTGCACGATTGCGCATTTGCCAGTTTCCAGATAATAAAACATTGG
GCCCTACTGCTAATGATGTTACAACAGGTCGTAACTGCCTATTCAACAAGGCCATTCCAGCTCATATGCAGGATAATAG
ACATATTGTTGTCGGCATAACATGGGATAATGATCGTGTCACTGTTTTTGCTGACAAGATCTATCATTTTTATATTAAA
AATGATTGGTCCCGTGTTGCGACAAGATGTTACAATAAAAGAAGTTGTGCCATGCAATATGTTTATACACCTACCTACT
ACATGCTTAATGTTACTAGTGCAGGTGAGGATGGCATTTACTATGAACCTTGCACAGTTGATTGCAGTGGTTACGCTGC
CAATGTATTTGCCACTGAATCCAATGGCCATATACCAGAAGGTTTTAGTTTTAATAATTGGTTTCTTTTGTCCAATGAC
TCCACTTTGTTGCATGGTAAAGTGGTTTCAAACCAACCTTTGTTGGTCAACTGCCTTTGGGCCATTCCTAAGATTTATG
GACTAGGCCAATTTTTCTCATTCAATCAAACGATGGATGGCGTTTGTAACGGAGCTTCTGCGCAGCGTGCCCCAGAGGC
TCTGAGGTTTAATATTAATGACACTTCTGTCATTCTTGCTGAAGGCTCAATTGTACTTCATACTGCTTTAGGAACAAAT
CTTTCTTTTGTTTGCAGTAACTCCTCAAATCCTCACAAAGCCATCTTTACCATACCTTTGGGTGTTACTGAAGTACCCT
ACTATTGTTTTCTTAAAGTGGATACTTACAACTCCACTGTTTATAAATTTTTGGCTGTTTTACCTCCTACTGTCAAGGA
AATTGTCATCACCAAGTACGGTGATGTTTATGTCAATGGGTTTGGCTATTTACATCTCGGTTTGTTGGATGCTGTCACA
ATTAATTTCACTGGTCATGGCACTGACGATGACGTTTCAGGTTTCTGGACCGTAGCATCGACTAATTTTGTTGATGCAC
TCATCGAGGTTAAAGGAACTGCCATTCAGCGTATTCTTTATTGTGATGACCCTGTTAGCCAACTTAAGTGTTCTCAGGT
TTCTTTTGACCTTGATGATGGTTTTTATCCTATTTCTTCTAGAAACCTTCTGAGTCATGAACAGCCAATTTCTTTTGTT
ACTTTGCCATCATTCACTGATCATTCTTTTGTTAATATTACTGTCTCCGCGGCTTTTGGTGGTCATAGTGGTGCCAACC
TCATTGCATCTGACACTACTATCAATGGGTTTAGTTCTTTCTGTGTTGATACTAGACAATTTACCATTACACTGTTTTA
TAACGTTACAAACAGTTATGGTTATGTGTCTAAATCACAGGATAGTAATTGCCCTTTCACCTTACAATCTGTTAATGAT
TACCTGTCTTTTAGCAAATTTTGTGTTTCAACCAGCCTTTTGGCTGGTGCTTGCACCATAGATCTTTTTGGTTATCCTG
AGTTCGGTAGTGGTGTTAAGCTTACGTCCCTTTATTTTCAATTCACAAAGGGTGAGTTGATTACTGGTACGCCTAAACC
ACTTGAAGGTGTCACGGATGTTTCTTTTATGACTCTGGATGTGTGTACCAAGTATACTATCTATGGCTTTAAAGGTGAG
GGTATTATTACCCTTACAAATTCTAGCTTTTTGGCAGGTGTTTATTATACATCTGATTCTGGACAGTTGTTAGCCTTTA
AGAATGTCACTAGTGGTGCTGTTTATTCTGTCACGCCATGTTCTTTCTCAGAGCAGGCTGCATACGTTGATGATGATAT
AGTGGGTGTTATTTCTAGTTTGTCTAACTCCACTTTTAACAATACCAGGGAGTTGCCTGGTTTCTTCTACCATTCTAAT
GACGGCTCTAATTGTACAGAGCCTGTGTTGGTGTACAGTAACATAGGTGTCTGTAAATCTGGCAGTATTGGCTATGTCC
CAATTCAGGATGGCCAAGTCAAGATTGCACCCACGGTTACTGGGAATATTAGTATTCCCACCAACTTTAGTATGAGTAT
TAGAACAGAATATTTACAGCTTTACAACACGCCTGTTAGTGTTGATTGCGCTACATATGTTTGTAATGGTAACTCTCGT
TGTAAACAATTACTCACCCAGTACACTGCAGCATGTAAGACCATAGAGTCAGCATTACAACTCAGCGCCAGGCTTGAGT
CTGTTGAAGTTAACTCTATGCTTACTATTTCTGAAGAGGCTCTACAGTTAGCTACCATCAGTTCGTTTAATGGTGATGG
ATATAACTTTACTAATGTGCTGGGTGTTTCTGTGTACGATCCTGCAAGTGGCAGGGTGGTACACAAAAGGTCTTTTATT
GAAGACCTGCTTTTTAATAAAGTGGTTACTAATGGCCTTGGTACTGTTGATGAAGACTATAAGCGCTGTTCTAATGGTC
GCTCTGTGGCAGATTTAGTCTGTGCGCAGTATTACTCTGGTGTTATGGTACTACCTGGCGTTGTTGACGCTGAGAAGCT
TCACATGTATAGTGCGTCTCTCATCGGTGGTATGGCGTTAGGAGGTTTTACTGCTGCAGTGGCATTGCCTTTTAGCTAT
GCTGTTCAAGCGAGGCTTAATTATCTTGCTTTACAGACGGATGTTCTACAGCGCAACCAGCAATTGCTTGCTGAGTCTT
TTAACTCTGCTATTGGTAATATAACTTTAGCCTTTGAGAGTGTTAAAGAGGCTATTAGTCAAACTTCCAAGGGTTTGAA
CACTGTGGCTCATGCGCTTAATAAGGTTCAAGAGGTTGTTAATTCGCAGGGTTCAGCTTTGACCCAACTTACCATACAG
CTGCAACACAACTTCCAAGCCATTTCTAGTTCTATTGATGACATTTACTCTCGACTGGACATTCTTTCAGCCGATGTTC
AGGTTGATCGTCTTATCACCGGCAGATTATCAGCACTTAATGCTTTTGTTGCTCAAACCCTCACTAAGTATACTGAGGT
TCAGGCTAGCAGGAAGCTAGCACAGCAAAAGGTTAATGAGTGCGTCAAATCGCAATCTCAGCGTTATGGTTTTTGTGGT
GGTGATGGCGAGCACATCTTCTCTCTGGTACAGGCCGCACCTCAGGGCCTGCTGTTCTTACATACAGTACTTGTACCGG
GTGATTTTGTAAATGTTATTGCCATCGCTGGCTTATGCGTTAATGGTGAAATTGCCTTGACTCTACGTGAGCCTGGCTT
AGTCTTGTTTACGCATGAACTTCAAACTTATACTGCGACGGAATATTTTGTTTCATCGCGACGTATGTTTGAACCTAGA

AAACCTACCGTTAGTGATTTTGTTCAAATTGAGAGTTGTGTGGTCACCTATGTCAACTTGACTAGCGACCAACTACCAG
ATGTAATCCCAGATTACATCGATGTTAACAAAACACTTGATGAGATTCTAGCTTCTCTGCCCAATAGAACTGGTCCTAG
TCTTTCTCTAGATGTTTTTAATGCCACTTACCTTAATCTCACTGGTGAAATTGCAGATTTAGAGCATCGTTCAGAGTAT
CTCCGTAATACTACAGAAGAGCTCCGAAGTCTCATATATAATATCAACAACACACTTGTTGACCTTGAGTGGCTCAACC
GAGTTGAGACATACATCAAGTGGCCGTGGTGGGTTTGGTTGATTATTTTTATTGTTCTCATCTTTGTTGTGTCATTATT
AGTATTCTGCTGCATTTCCACGGGTTGTTGTGGATGCTGCGGTTGCTGTGGTGCTTGTTTTTCAGGTTGTTGTAGGGGT
CCTAGACTTCAACCTTATGAAGCTTTTGAAAAGGTCCACGTGCAGTGA

>gi|27357178|gb|AY167585.1| Porcine epidemic diarrhea virus strain Chinju99 spike protein gene, complete cds

ATGAGGTCTTTAATTTATTTTTTGTTGTTCGTACCAGTACTTCCAACACTCAGCCTACCACAAGATGTCTATAGG
TGCTCAGCTAACACTAACTTTAGGCGGTTCTTTTCAAAATTTAATGTTCAGGCGCCTGCAGTCGTTGTACTGGGCGGTT
ATCTACCTAGTGTGAAACACGCTGGTTGGTACTGTGCTGCCGACATCCAAACTGCTAGTGGCGTTCATGGTATCTTCCT
TAGCCATATTAGAGGTGGTCATGGCTTTGAGATTGGCATTTCGCAAGAGCCGTTTGACCCTAGTGGTTACCAGCTTTAT
TTACATAAGGCCACTAACGGTAACACTAATGCTACTGCGCGACTGCGTATTTGCCAGTTTCCCAGCAGTAAAACATTGG
GCCCCACTGCTAATGATGTTACAACAGGTCGTAATTGCCTATTTAACAAAGCCATTCCAGCTCATATGAGTGATGGAAA
ACATAGTGTTGTCGGCATAACATGGGATAATGATCGTGTCACTGTTTTTGCTGACAAGATCTATCATTTTTATCTTAAA
AATGATTGGTCCCGCGTTGCGACAAACTGTTACAACAGTGGAGGTTGTGCTATGCAATATGTTTACGAACCTATTTACT
ACATGCTTAATGTTACTAGTGCTGGTGAGGATGGTATTTCTTATCAACCCTGTACAGCTAATTGCATTGGTTATGCTGC
CAATGTATTTGCCACTGAGTCCAATGGCCACATACCAGAAGGTTTTAGTTTTAATAATTGGTTTCTTTTGTCCAATGAT
TCCACTTTGTTTCATGGTAAAGTGGTTTCCAACCAACCTTTGTTGGTCAACTGTCTTTGGGCCATTCCTAAGATTTACG
GACTAGGCCACTTTTTCTCATTTAATCAAACGATGGATGGCGTTTGTAACGGAGCTACTGCGTATCGTGCCCCACAGGC
TCTGAGGTTTAATATTAATGACACTTCTGTCATTCTTGCCGAAGGCTCAATTGTACTTCATACTGCTTTAGGAACAAAT
CTTTCTTTTGTTTGCAGTAATTCCTCAGATCCTCACAAAGCCATCTTCTCCATACCTCTGGGTGCTACCCAAGTACCCT
ACTATTGCTTTCTTAAAGTTGATACTTACAACTCCACTGTCTATAAATTTCCTGCTGTTTTACCTTCTACTGTCAGGGA
AATTGTCATCACAAAGTACGGTGATGTTTATGTCAACGGGTTTGGCTACTTGCATCTCGGTTTGTTGGATGCTGTCACA
ATTAATTTCACTGGCCATGGCACTAACGATGACGTCTCAGGTTTCTGGACCATAGCATCGACTAATTTTATTGATGCAC
TCATCGAGGTTCAAGCAACTGCCATTCAGCGTATTCTTTATTGTGATGATCCTGTTAGCCAACTCAAGTGTTCTCAGGT
TTCTTTTGACCTTGATGATGGTTTTTACCCTATTTCTTCTAGAAACCTTCTGAGTCACGAACAGCCAATTTCTTTTGTT
ACTTTGCCATCATTTAATGATCATTCTTTTGTTAATATTACTGTATCTGCGGCTTTTGGTGATTCTGGTGGTGCCAACC
TCGTTGCATCTGACACTACTATCAATGGGTTTAGTTCTTTCTGTGTTGACACTAGACAATATACCATTAGACTGTTTTA
CAACGTTACAAACAGTTATGGTTATGTGTCTAAATCACAGGATAGCAATTGCCCTTTCACCTTGCAATCTGTTAATGAT
TACCTGTCTTTTAGCAAATTTTGTGTTTCAACCAGCCTTTTGGCTGGTGCTTGTACCATAGATCTTTTTGGTTACCCTG
CTTTCGGTAGTGGTGTTAATTACAGGTCCCTCTATTTTCAATTCACAAAAGGTGAGTGGATTACTTGGGCACCTAAACC
CTTAAACGGCCTTTTAAAGGGGGGTTTTATGACTCTGGATGTGTGTACCAAGTATACTATCTATGGCTTTAAAGGTGAG
GGTATTATTACCCTTACAAATTCTAGCATTTTGGCAGGTGTTTATTATACATCTGATTCTGGACAGTTGTTAGCCTTTA
AGAATGTCACTAGTGGTGCTGTTTATTCTGTCACGCCATGTTCTTTTTCAGAGCAGGCTGCATATGTTAATGATGATAT
AGTGGGTGTTATTTCTAGTTTGTCTAACTCCACTTTTAACAATACTAGGGAGTTGCCTGGTTTCTTCTACCATTCTAAT
GACGGCTCCAATTGTACAGAGCCTGTGTTGGTGTATAGTAACATAGGTGTTTGTAAATCTGGCAGTATTGGCTATGTTC
CATCTCAGTATGGCCAAGTCAAGATTGCACCCACGGTTACTGGGAATATTAGTATTCCCACCAACTTTAGTATGAGTAT
TAGAACAGAATATTTACAGCTTTACAACACGCCTGTTAGTGTTGATTGTGCTACATATGTTTGTAATGGTAACTCTCGT
TGTAAACAATTACTTACCCAGTACACTGCAGCATGTAAGACCATAGAGTCAGCATTAGAACTCAGCGCTAGGCTTGAGT

CTGTTGAAGTTAACTCTATGCTTACTATTTCTGAAGAGGCCTTACAGTTAGCTACCATCAGTTCGTTTAATGGTGATGG
GTATAACTTTACTAATGTGTTGGGTGTTTCTGTGTACGATTCTCAAAGTGGCAGGGTGATACACGAAAGGTCTTTTATT
GAAGACCTGCTTTTTAATAAGGTGGTTACTAATGGCCTTGGTACTGTTGATGAAGACTATAAGCGCTGTTCTAATGGTC
GCTCTGTGGCTGATAAAGTCTGTGCGCAGTATTACTCTGGTCTCATGGTACTACCTGGGGTTGTTGACGCTGAGAAGCT
TCACATGTATAGTGCGTCTCTCATCGGTGGTATGGCGCTAGGAGGTTTTACTGCCGCAGTGGCATTGCCTTTTAGCTAT
GCTGTTCAAGCGAGACTCAATTATCTTGCTTTACAGACGGATGTTTTACAGCGGAACCAGCAACTGCTTGCTGAGTCTT
TTAACTCTGCTATTGGTAATATAACCTCAGCCTTTGCGAGTGTTAAAGAGGCTATCAGTCAAACTTCTAAGGGTTTGAA
TACTGTGGCTCATGCGCTTACTAAAGTTCAAGAGGTTGTTAATTCGCAGGGTTCAGCTTTGACTCAACTTACCATACAG
CTGCAACATAACTTCCAAGCCATTTCTAGTTCTACTGATGACATTTACTCCCGACTGGACATTCTTTCAGCCGATGTTC
AGGTTGATCGTCTCATCACCGGCAGATTATCAGCACTTAATGCTTTTGTTGCTCAAACCCTCACTAAGTATACTGAGGT
TCAGGCTAGCAGGAAGCTAGCACAGCAAAAGGTTAATGAGTGCGTCAAATCGCAATCTCAGCGTTATGGTTTTTGTGGT
GGTGATGGCGAGCACATTTTCTCTCTTGTACAGGCCGCACCCCAGGGCCTGCTGTTCTTACACACAGTACTTGTACCGG
GTGATTTTGTAAATGTTATTGCCATCGCAGGCTTATGCGTTAATGGTGATATTGCCTTGACTCTACGTGAGCCTGGCTT
AGTCTTGGTTACGCATGAACTTCAAACTTATACTGCGACGGAATATTTTGTTTCATCGCGACGTATGTTTGAACCTAGA
AAACCTACCGTTAGTGATTTTGTTCAAATTCAGAGTTGTGTGGTCACCTATGTCAATCTGACTAGCGACCAACTACCAG
ATGTAATCCCAGATTACATCGATGTTAACAAAACACTTGATGAGATTTTAGCTTCTCTGCCCAATAGAACTGGTCCAAA
TCTTCCCCTAGATGTTTTTAATGCCACTTATCTTAATCTCACTGGTGAAATTGCAGATCTAGAGCAGCGTTCAGAGTCT
CTCCGTAATACTACAGAAGAGCTCCGAAGTCTTATATACAATATCAACAACACACTTGTTGACCTTGAGTGGCTCAACC
GAGTTGAGACTTATATCAAGTGGCCGTGGTGGGTTTGGTTGATTATTTTTATTGGTCTCATCTTTGTTGTGTCATTATT
AGTGTTCTGCTGCATTTCCACGGGTTGTTGTGGATGCTGCGGTTGCTGCGGTGCTTGTTTTTCAGGTTGTTGGAGGGGT
CCTAGACTTCAACCTTACGAAGGTTTTGAAAAGGTCCACGTGCAGTGA

>gi|50363109|gb|AY653204.1|_Porcine_epidemic_diarrhea_virus_strain_JS20042_spike_glycoprotein_(S)_gene_complete_cds

ATGAAGTCTTTAACTTACTTCTGGTTGTTATTACCAGTACTTTCAACACTCAGCCTACCACAAGATGTCACTAGG
TGCCAGTCCACTATTAACTTCAGGCGGTTCTTTTCAAAATTTAATGTGCAGGCACCTGCTGTCGTTGTTTTGGGTGGTT
ATCTACCTAGTATGAACTCTTCTAGCTGGTACTGTGGCACAGGTCTTGAAACTGCTAGTGGCGTGCATGGTATTTTCCT
CAGTTACATCGATGCTGGTCAGGGCTTTGAGATTGGCATTTCACAGGAGCCGTTTGATCCTAGTGGTTACCAGCTTTAT
TTACATAAGGCTACTAATGGTAATACTAATGCTATTGCACGACTGCGCATTTGCCAGTTTCCAGATAATAAAACATTGG
GCCCTACTGTTAATGATGTTACAACAGGTCGTAACTGCCTATTCAACAGAGCCATTCCAGCTTATATGCAGGATGGAAA
AAATATTGTTGTCGGCATAACATGGGATAATGATCGCGTCACTGTTTTTGCTGACAAGATCTATCATTTTTATCTTAAA
AATGATTGGTCCCGCGTTGCGACAAGATGTTACAATAAAAGGAGTTGTGCTATGCAATATGTTTATACACCTACCTACT
ACATGCTTAATGTTACTAGTGCAGGTGAGGATGGCATTTATTATGAACCATGTACAGCTAATTGCAGTGGTTACGCTGC
TAATATATTTGCCACTGATTCTAATGGCCATATACCAGAAGGTTTTAGTTTTAATAATTGGTTTCTTTTGTCCAATGAT
TCCACTTTGTTGCATGGTAAGGTGGTTTCCAACCAACCTTTGTTGGTCAATTGTCTTTTGGCCATTCCTAAGATTTATG
GACTAGGCCAATTTTTCTCATTCAATCAAACGATGGATGGCGTTTGTAATGGAGCTGCTGCGCAGCGTGCACCAGAGGC
TCTGAGGTTTAATATTAATGACACCTCTGTCATTCTTGCTGAAGGCTCAATTGTACTTCATACTGCTTTAGGAACAAAT
CTTTCTTTTGTTTGCAGTAATTCCTCAGATCCTCATTTAGCCACCTTCGCCATACCTCTGGGTGCTACCCAAGTACCCT
ATTATTGTTTTCTTAAAGTGGATACTTACAACTCCACTGTTTATAAATTCTTGGCTGTTTTACCTCCTACCGTCAGGGA
AATTGTCATCACCAAGTATGGTGATGTTTATGTCAATGGGTTTGGCTACTTGCATCTCGGTTTGTTGGATGCTGTCACA
ATTAATTTCACTGGTCATGGCACTGACGATGACGTTTCTGGTTTTTGGACCATAGCATCGACTAATTTTGTTGATGCAC
TCATCGAAGTTCAAGGAACTGCCATTCAGCGTATTCTTTATTGTGATGATCCTGTTAGCCAACTCAAGTGTTCTCAGGT

TGCTTTTGACCTTGACGATGGTTTTTACCCTATTTCTTCTAGAAACCTTCTGAGTCATGAACAGCCAATTTCTTTTGTT
ACTTTGCCATCATTTAATGATCATTCTTTTGTTAACATTACTGTCTCTGCGTCCTTTGGTGGTCATAGTGGTGCCAACC
TCATTGCATCTGACACTACTATCAATGGGTTTAGTTCTTTCTGTGTTGACACTAGACAATTTACCATTTCACTGTTTTA
TAACGTTACAAACAGTTATGGTTATGTGTCTAAATCACAGGACAGTAATTGCCCTTTCACCTTGCAATCTGTTAATGAT
TACCTGTCTTTTAGCAAATTTTGTGTTTCTACCAGCCTTTTGGCTAGTGCCTGTACCATAGATCTTTTTGGTTACCCTG
AGTTTGGTAGTGGTGTTAAGTTTACGTCCCTTTACTTTCAATTCACAGAGGGTGAGTTGATTACTGGCACGCCTAAACC
ACTTGAAGGTGTCACGGACGTTTCTTTTATGACTCTGGATGTGTGTACCAAGTATACTATCTATGGCTTTAAAGGTGAG
GGTATCATTACCCTTACAAATTCTAGCTTTTTGGCAGGTGTTTATTATACATCTGATTCTGGACAGTTGTTAGCCCTTA
AGAATGTCACTAGTGGTGCTGTTTATTCTGTTACGCCATGTTCTTTTTCAGAGCAGGCTGCATATGTTGATGATGATAT
AGTGGGTGTTATTTCTAGTTTGTCTAGCTCCACTTTTAACAGTACTAGGGAGTTGCCTGGTTTCTTCTACCATTCTAAT
GATGGCTCTAATTGTACAGAGCCTGTGTTGGTGTATAGTAACATAGGTGTTTGTAAATCTGGCAGTATTGGCTATGTCC
CATCTCAGTCTGGCCAAGTCAAGATTGCACCCACGGTTACTGGGAATATTAGTATTCCCACCAACTTTAGTATGAGTAT
TAGGACAGAATATTTACAGCTTTACAACACGCCTGTTAGTGTTGATTGTGCCACATATGTTTGTAATGGTAACTCTCGT
TGCAAACAATTACTCACCCAGTACACTGCAGCATGTAAGACCATAGAGTCAGCATTACAACTCAGCGCTAGGCTTGAGT
CTGTTGAAGTTAACTCTATGCTTACTATTTCTGAAGAGGCTCTACAGTTAGCTACCATCAGTTCGTTTAATGGTGATGG
ATATAATTTTACTAATGTGCTGGGTGTTTCCGTGTATGATCCTGCAAGTGGCAGGGTGGTACACAAAAGGTCTTTTATT
GAAGACCTGCTTTTTAATAAAGTGGTTACTAATGGCCTTGGTACTGTTGATGAAGACTATAAGCGCTGTTCTAATGGTC
GCTCTGTGGCAGATCTAGTCTGTGCACAGTATTACTCTGGTGTCATGGTACTACCTGGTGTTGTTGACGCTGAGAAGCT
TCACATGTATAGTGCGTCTCTCATCGGTGGTATGGCGCTAGGAGGTTTTACTGTTGCAGCGGCATTGCCTTTTAGCTAT
GCTGTTCAAGCTAGACTCAATTATCTTGCTCTACAGACGGATGTTCTACAGCGGAACCAGCAATTGCTTGCTGAGTCTT
TTAACTCTGCTATTGGTAATATAACTTCAGCCTTTGAGAGTGTTAAAGAGGCTATTAGTCAAACTTCCAAGGGTTTGAA
CACTGTGGCTCATGCGCTTACTAAGGTTCAAGAGGTTGTTAATTCGCAGGGTGCAGCTTTGACCCAACTTACCGTACAG
CTGCAACACAACTTCCAAGCCATTTCTAGTTCTATTGATGACATTTACTCCCGACTGGACATTCTTTCAGCCGATGTTC
AGGTTGACCGTCTCATCACCGGCAGATTATCAGCACTTAATGCTTTTGTTGCTCAAACCCTCACTAAGTATACTGAGGT
TCAGGCTAGCAGGAAGCTAGCACAGCAAAAGGTTAATGAGTGCGTTAAATCGCAATCTCAGCGTTATGGTTTTTGTGGT
GGTGATGGCGAGCACATTTTCTCTCTGGTACAGGCAGCACCTCAGGGCCTGCTGTTTTTACATACAGTACTTGTACCGG
GTGATTTTGTAGATGTTATTGCCATCGCTGGCTTATGCGTTAACGATGAAATTGCCTTGACTCTACGTGAGCCTGGCTT
AGTCTTGTTTACGCATGAACTTCAAAATCATACTGCGACGGAATATTTTGTTTCATCGCGACGTATGTTTGAACCTAGA
AAACCTACCGTTAGTGATTTTGTTCAAATTGAGAGTTGTGTGGTCACCTATGTCAATTTGACTAGAGACCAACTACCAG
ATGTAATCCCAGATTACATCGATGTTAACAAAACACTTGATGAGATTTTAGCTTCTCTGCCCTATAGAACGGGTCCAAG
TCTTCCTTTAGATGTTTTTAATGCCACTTATCTTAATCTCACTGGTGAAATTGCAGATTTAGAGCAGCGTTCAGAGTCT
CTCCGTAATACTACAGAAGAGCTCCAAAGTCTTATATATAATATCAACAACACACTAGTTGACCTTGAGTGGCTCAACC
GAGTTGAGACATATATCAAGTGGCCGTGGTGGGTTTGGTTGATTATTTTCATTGTTCTCATCTTTGTTGTGTCATTACT
AGTGTTCTGCTGCATTTCCACGGGTTGTTGTGGATGCTGCGGTTGCTGCTGTGCTTGTTTTTCAGGTTGTTGTAGGGGT
CCTAGACTTCAACCTTACGAAGCTTTTGAAAAGGTCCACGTGCAGTGA

>gi|110612800|gb|DQ462404.2| Porcine epidemic diarrhea virus strain DR13 spike protein (S) gene, complete cds

ATGACGCCTTTAATTTACTTCTGGTTGTTCTTACCAGTACTTCTAACACTTAGCCTACCACAAGATGTCACTAGG
TGCCAGTCTACTATTAACTTTAGGCGGTTCTTTTCAAAATTTAATGTTCAGGCACCTGCCGTCGTTGTTTTGGGTGGTT
ATCTACCTAGTATGAACTCTTCTAGCTGGTACTGTGGCACAGGCATTGAAACTGATAGTGGCGTTCATGGTATTTTTCT
CAGTTACATCGATTCTGGTCAGGGCTTTGAGATTGGCATTTCGCAAGAGCCGTTTGATCCTAGTGGTTACCAGCTTTAT

TTACACAAGGCCACTAATGGTAACACTAGCGCTATTGCACGACTGCGCATTTGCCAGTTTCCAGATAATAAAACATTGG
GCCCTACTGTTAATGATGTTACAACAGGTCGTAACTGCCTATTCAACAAAGCCATTCCAGCTTTGCAGGATGGAAAAAA
TACTGTTGTCGGCATAACATGGGATAATGATCGTGTCACTGTTTTTGCTGACAAGATCTATCATTTTTATATTAAAAAT
GATTGGTCCCGTGTTGCGACAAGATGTTACAATAAAAGAAGTTGTGCCATGCAATATGTTTATACACCTACCTACTACA
TGCTTAATGTTACTAGTGCAGGTGAGGATGGCATTTACTATGAACCTTGTACAGCTAATTGCAGTGGTTACGCTGCCAA
TGTATTTGCCACTGATTCCAATGGCCATATACCAGAAGGTTTTAGTTTTAATAATTGGTTTCTTTTGTCCAATGACTCC
ACTTTGTTGCATGGTAAAGTGGTTTCAAACCAACCTCTGTTGGTCAACTGCCTTTGGGCCATTCCTAAGATTTATGGAC
TAGGACAATTTTTCTCATTCAATCAAACGATGGATGGCGTTTGTAACGGAGCCGCTGCGCAGCGTGCCCCAGAGGCTCT
GAGGTTTAATATTAATGACACTTTTGTCATTCTTGCTGAAGGCTCAATTATACTTCATACTGCTTTAGGAACAAATCTT
TCTTTTGTTTGCAGTAATTCCTCAGATCCTCACAAAGCCATCTTTACCATACCTTTGGGTGTTACTGAAGTACCCTACT
ATTGCTTTCTTAAAGTGGATACTTACAAATCCACTGTTTATAAATTCTTGGCTGTTTTACCTCCTACTGTCAAGGAAAT
TGTCATCACCAAGTACGGTGATGTTTATGTCAACGGGTTTGGCTATTTGCATCTCGGTTTGTTGGATGCTGTCACAATT
AATTTCACTGGTCATGGCACTGACGATGACGTTTCAGGTTTCTGGACCGTAGCATCGACTAATTTTGTTGATGCACTCA
TCGAGGTTCAAGGAACTGCCATTCAGCGTATTCTTTATTGTGATGACCCTGTTAGCCAACTTAAGTGTTCTCAGGTTGC
TTTTGACCTTGATGATGGTTTTTACCCTATTTCTTCTAGAAACCTTCTGAGTCATGAACAGCCAATTTCTTTTGTTACT
TTGCCATCATTCAATGACCATTCTTTTGTTAATATTACTGTCTCTGCGGCTTTTGGTGGTCATAGTGGTGCCAACCTCA
TTGCATCTGACACTACTATCAATGGGTTTAGTTCTTTCTGTGTTGACACTAGACAATTTACCATTACACTGTTTTATAA
CGTTACAAACAGTTATGGTTATGTGTCTAAGTCACAGGATAGTAATTGCCCTTTCACCTTGCAATCTGTTAATGATTAC
CTGTCTTTTAGCAAATTTTGTGTTTCAACCAGCCTTTTGGCTGGTGCTTGTACCATAGATCTTTTTGGTTACCCTGAGT
TCGGTAGTGGTGTTAAGTTTACGTCCCTTTATTTTCAATTCACAAAGGGTGAGTCGATTACTGGCACGCCTAAACCACT
TCAAGGTGTCACGGACGTTTCTTTTATGACTCTGGATGTGTGTACCAAGTATACTATCTATGGCTTTAAAGGTGAGGGT
ATTATTACCCTTACAAATTCTAGCTTTTTGGCAGGTGTTTATTATACATCTGATTCTGGACAGTTGTTAGCCTTTAAGA
ATGTCACTAGTGGTGCTGTTTATTCTGTTACGCCATGTTCTTTTTCAGAGCAGGCTGCATATGTTGATGATGATATAGT
GGGTGTTATTTCTAGTTTGTCTAACTCCACTTTTAACAATACCAGGGAGTTGCCTGGTTTCTTCTACCATTCTAAGGAT
GGCTCCAATTGTACAGAGCCTGTGTTGGTGTATAGTAACATAGGTGTCTGTAAATCTGGCAGTATTGGCTATGTCCCAC
TTCAGGATGGCCAAGTCAAGATTGCACCCATGGTTACTGGGAATATTAGTATTCCCACCAACTTTAGTATGAGTATTAG
AACAGAATATTTACAGCTTTACAACACGCCTGTTAGTGTTGATTGCGTTACATATGTTTGTAATGGTAACTCTCGTTGT
AAACAATTACTCACCCAGTACACTGCAGCATGTAAGACCATAGAGTCAGCATTACAACTCAGCGCTAGGCTTGAGTCTG
TTGAAGTTAACTCTATGCTTACTATTTCTGAAGAGGCTCTACAGTTAGCTACCATCAGTTCGTTTAATGGTGATGGATA
TAACTTTACTAATGTGCTGGGTGTTTCCGTGTACGACCCTGCAAGTGGCAGGGTGGTACAAAAAGGGTCTTTTATTGAA
GACCTGCTTTTTAATAAAGTGGTTACTAATGGCCTTGGTACTGTTGATGAAGACTATAAGCGCTGTTCTAATGGTCGCT
CTGTGGCAGATCTAGTCTGTGCGCAGTATTACTCTGGTGTCATGGTACTACCTGGCGTTGTTGACGCTGAGAAGCTTCA
CATGTATAGTGCGTCTCTCATCGGTGGTATGGCGCTAGGAGGTCTTACTAGTGCAGCGGCATTGTCTTTTAGCCATGCT
GTTCAAGCGAGGCTCAATTATCTTGCTTTACAGACGGATGTTCTACAGCGCAACCAGCAATTGCTTGCTGAGTCTTTTA
ACTCTGCTATTGGTAATATAACTTCAGCCTTTGAGAGTGTTAAAGAGGCTATTAGTCAAACTTCCAATGGTTTGAACAC
TGTGGCTCATGCGCTTACTAAGGTTCAAGAGGTTGTTAATTCGCAGGGTTCAGCTTTGACCCAACTTACCATACAGCTG
CAACACAACTTCCAAGCCATTTCTAGTTCTATTGATGACATTTACTCCCGACTGGACATTCTTTCAGCCGATGTTCAGG
TTGATCGTCTCATCACCGGCAGATTATCAGCACTTAATGCTTTCGTTGCTCAAACCCTCACTAAGTATACTGAGGTTCA
GGCTAGCAGGAAGCTAGCACAGCAAAAGGTTAATGAGTGCGTCAAATCGCAATCTCAGCGTTATGGTTTTTGTGGTGGT
GATGGCGAGCACATCTTCTCTCTGGTACAGGCCGCACCTCAGGGCCTGCTGTTTTTACATACAGTACTTGTACCGGGTG
ATTTTGTAAATGTTATTGCCATCGATGGCCTATGCGTTAATGGTGATATTGCCTTGACTCTACGTGAGCCTGGCTTAGT
CTTGTTTACGCATGAACTTCAAACTTATACTGCGACGGAATATTTTGTTTCATCGCGACGTATGTTTGAACCTAGAAAA
CCTACCGTTAGTGATTTTGTTCAAATTGAGAGTTGTGTGGTCACCTATGTCAATCTGACTAGCGACCAACTACCAGATG
TAATCCCAGATTACATCGATGTTAACAAAACACTTGATGAGATTCTAGCTTCTCTGCCCAATAGAATTGGTCCTAGTCT

TCCCCTAGATGTTTTTAATGCCACTTATCTTAATCTCACTGGTGAAATTGCAGATTTAGAGCAGCGTTCAGAGTCTCTC
AGTAATACTACAGAAGAGCTCCGAAGCCTCATATATAATATCAACAACACACTTGTTGACCTTGAGTGGCTCAACCGAG
TTGAGACATATATCAAGTGGCCGTGGTGGGTTTGGTTGATTATTTTTATTGTTCTCATCTTTGTTGTGTCATTATTAGT
GTTCTGCTGCATTTCCACGGGTTGTTGTGGATGCTGCGGTTGTTGCGGTGCCTGTTTTTCAGGTTGTTGTAGGGGTCCT
AGACTTCAACCTTACGAAGCTTTTGAAAAGGTCCACGTGCAGTGA

>gi|113127132|gb|DQ862099.1| Porcine epidemic diarrhea virus strain DR13 spike protein gene, complete cds

ATGAGGTCTTTAATTTACTTCTGGTTGCTCTTACCAGTACTTCCAACACTCAGCCTACCACAAGATGTCACTAGG
TGCCAGTCTACTACTAACTTTAGGCGGTTCTTTTCAAAATTTAATGTTCAGGCACCTGCCGTCGTCGTTTTGGGTGGTT
ACCTACCTAGTATGAACTCTTCTAGCTGGTACTGTGGCACAGGCATTGAAACTGCTAGTGGCGTTCATGGTATTTTTCT
CAGCTACATCGATTCTGGTCAGGGCTTTGAGATTGGCATTTCGCAAGAGCCGTTTGATCCTAGTGGTTACCAGCTTTAT
TTACATAAGGCCACTAATGGTAACACTAATGCTATTGCACGACTGCGCATTTGCCAGTTTCCCGATAATAAAACATTGG
GCCCTACTGTTAATGATGTTACAACAGGTCGTAACTGCCTATTCAACAAAGCCATTCCAGCTTATATGCGTGATGGAAA
AGATATTGTTGTCGGCATAACATGGGATAATGATCGTGTCACTGTTTTTGCTGACAAGATCTATCATTTTTATCTTAAA
AATGATTGGTCCCGCGTTGCGACAAGATGTTACAATCGCAGAAGTTGTGCTATGCAATATGTTTATACACCTACCTACT
ACATGCTTAATGTTACTAGTGCAGGTGAGGATGGCATTTATTATGAACCCTGTACAGCTAATTGCACTGGTTACGCTGC
CAATGTATTTGCCACTGATTCCAATGGCCATATACCAGAAGGTTTTAGTTTTAATAATTGGTTTCTTTTATCCAATGAC
TCCACTTTGTTGCATGGTAAAGTGGTTTCCAACCAACCCTTGTTGGTCAATTGTCTTTTGGCCATTCCTAAGATTTATG
GACTAGGCCAATTTTTCTCATTCAATCACACGATGGATGGCGTTTGTAATGGAGCTGCTGTGGATCGTGCCCCAGAGGC
TCTGAGGTTTAATATTAATGACACCTCCGTCATTCTTGCTGAAGGCTCAATTGTACTTCATACTGCTTTAGGAACAAAT
CTTTCTTTTGTTTGCAGTAATTCCTCAGATCCTCATTTAGCCATCTTTGCCATACCTCTGGGTGCTACTGAAGTACCCT
ACTATTGCTTTCTTAAAGTGGATACTTACAACTCCACTGTTTATAAATTCTTGGCTGTTTTACCTCCTACTGTCAGGGA
AATTGTCATCACCAAGTATGGTGATGTTTATGTCAATGGGTTTGGCTATTTGCATCTCGGTTTGTTGGATGCTGTCACA
ATTAATTTCACTGGTCATGGCACTGACGATGACGTTTCAGGTTTCTGGACCATAGCATCGACTAATTTTGTTGATGCAC
TCATCGAGGTTCAAGGAACTTCCATTCAGCGTATTCTTTATTGTGATGATCCTGTTAGCCAACTCAAGTGTTCTCAGGT
TGCTTTTGACCTTGACGATGGTTTTTACCCCATCTCTTCTAGAAACCTTCTGAGTCACGAACAGCCAATTTCTTTTGTT
ACTTTGCCATCATTTAATGATCATTCTTTTGTTAATATTACTGTCTCTGCGGCTTTTGGTGGTCTTAGTAGTGCCAATC
TCGTTGCATCTGACACTACTATCAATGGGTTTAGTTCTTTCTGTGTTGACACTAGACAATTTACCATTACACTGTTTTA
TAATGTTACAAACAGTTATGGTTATGTGTCTAAATCACAGGATAGTAATTGTCCTTTCACCTTGCAATCTGTTAATGAT
TACCTGTCTTTTAGCAAATTTTGTGTTTCAACCAGCCTTTTGGCTGGTGCTTGTACCATAGATCTTTTTGGTTACCCTG
CGTTCGGTAGTGGTGTTAAGTTGACGTCCCTTTATTTTCAATTCACAAAAGGTGAGTTGATTACTGGCACGCCTAAACC
ACTTGAAGGTATCACAGACGTTTCTTTTATGACTCTGGATGTGTGTACCAAGTATACTATCTATGGCTTTAAAGGTGAG
GGTATTATTACCCTTACAAATTCTAGCATTTTGGCAGGTGTTTATTATACATCTGATTCTGGACAGTTGTTAGCCTTTA
AGAATGTCACTAGTGGTGCTGTTTATTCTGTCACGCCATGTTCTTTTTCAGAGCAGGCTGCATATGTTAATGATGATAT
AGTGGGTGTTATTTCTAGTTTGTCTAACTCCACTTTTAACAATACTAGGGAGTTGCCTGGTTTCTTCTACCATTCTAAT
GACGGCTCCAATTGTACAGAGCCTGTGTTGGTGTATAGTAACATAGGTGTTTGTAAATCTGGCAGTATTGGCTATGTTC
CACCTCAGTATGGCCAAGTCAAGATTGCACCCACGGTTACTGGGAATATTAGTATTCCCACCAACTTTAGTATGAGTAT
TAGAACAGAATATTTACAGCTTTACAACACGCCTGTTAGTGTTGATTGTGCTACATATGTTTGTAATGGTAACTCTCGT
TGTAAACAATTACTCACCCAGTACACTGCAGCATGTAAGACCATAGAGTCAGCATTACAACTCAGCGCTAGGCTTGAGT
CTGTTGAAGTTAACTCTATGCTTACCATTTCTGAAGAGGCTTTACAGTTAGCTACCATCAGTTCGTTTAATGGTGATGG
ATATAACTTTACTAATGTGCTGGGTGCTTCCGTGTACGATCCTGCAAGTGGCAGGGTGGTACAAAAAAGGTCTGTTATT

GAAGACTTGCTTTTTAATAAAGTGGTTACTAATGGCCTTGGTACTGTTGATGAAGACTATAAGCGCTGTTCTAATGGTC
GCTCTGTGGCTGATCTAGTCTGTGCGCAGTATTACTCTGGTGTCATGGTACTACCTGGCGTTGTTGACGCTGAGAAGCT
TCACATGTACAGTGCGTCTCTCATAGGTGGTATGGCGCTAGGAGGTATAACTGCTGCAGCGGCATTGCCTTTTAGCTAT
GCTGTTCAAGCGAGACTCAATTATCTTGCTTTACAGACGGATGTTCTACAGCGGAACCAGCAATTGCTTGCTGAGTCTT
TTAACTCTGCTATTGGTAATATAACTTCAGCCTTTGAGAGTGTTAAAGAGGCTATTAGTCAAACTTCCAAGGGTTTGAA
CACTGTGGCTCATGCGCTTACTAAGGTTCAAGAGGTTGTTAATTCGCAGGGTTCAGCTTTGAACCAACTTACCGTACAG
CTGCAACACAACTTCCAAGCCATTTCTAGTTCTATTGATGACATTTATTCCCGACTGGACATTCTTTCAGCCGATGTTC
AGGTTGATCGTCTCATCACCGGCAGATTATCAGCACTTAATGCTTTTGTTGCCCAAACCCTCACTAAGTATACTGAGGT
TCAGGCTAGCAGGAAGCTAGCACAGCAAAAGGTTAATGAGTGCGTCAAATCGCAATCTCAGCGTTACGGTTTTTGTGGT
GGTGATGGCGAGCACATTTTCTCTCTGGTACAGGCCGCACCTCAGGGCCTGCTGTTCTTACATACAGTACTTGTACCGG
GTGATTTTGTAAATGTTCTTGCCATCGCTGGCTTATGCGTTAATGGTGAAATTGCCTTGACTCTACGTGAGCCTGGCTT
AGTCTTGTTTACGCATGAACTTCAAAATTATACTGCGACGGAATATTTTGTTTCATCGCGACGTATGTTTGAACCTAGA
AAACCTACCGTTAGTGATTTTGTTCAAATTGAGAGTTGTGTGGTCACCTATGTCAATCTGACTAGCGACCAGCTACCAG
ATGTAATCCCAGATTACATCGATGTTAACAAAACACTTGATGAGATTTTAGCTTCTCTGCCCAATAGAAATGGTCCAAG
TCTTCCCCTAGATGTTTTTAATGCCACTTATCTTAATCTTACTGGTGAAATTGCAGATCTAGAGCAGCGTTCAGAGTCT
CTCCGTAATACTACAGAAGAGCTCCGAAGTCTCATTAACAACATCAACAACACACTTGTTGACCTTGAGTGGCTCAACC
GAGTTGAGACATACATCAAGTGGCCGTGGTGGGTTTGGTTGATCATTTTTATTGTTCTCATCTTTGTTGTGTCATTACT
AGTGTTCTGCTGCATTTCCACGGGTTGTTGTGGATGCTGCGGTTGCTGCGGTGCTTGTTTTTCAGGTTGTTGTAGGGGT
CCTAGACTTCAACCTTACGAAGCTTTTGAAAAGGTCCACGTGCAGTGA

>gi|116008265|gb|DQ985739.1| Porcine epidemic diarrhea virus spike gene, complete cds

ATGAAGTCTTTAACTTACTTCTGGTTGTTCTTACCAGTATTTTCAACACTCAGCCTACCACAAGATGTCACTAGG
TGCCAGTCCACTATTAACTTCAGGCGGTTCTTTTCAAAATTTAATGTGCAAGCACCTGCTGTCGTTGTTTTGGGTGGTT
ATCTACCTAGTATGAACTCTTCTAGCTGGTACTGTGGCACAGGCCTTGAAACTGCTAGTGGCGTTCATGGTATTTTTCT
TAGTTACATCGATTCTGGTCAGGGCTTTGAGATTGGCATTTCGCAAGAGCCGTTTGATCCTAGTGGTTACCAGCTTTAT
TTACATAAGGCCACCAATGGTAACACTAATGCTATTGCACGACTGCGTATTTGCCAGTTTCCAAATAATAAAACATTGG
GCCCCGCTGTTAATGATGTTACAACAGGTCGTAACTGCCTATTCAACAAAGCTATTCCAGCTCATATGCAGGATGGGAA
AAATATTGTTGTCGGCATAACATGGGACAATGATCGTGTCACTGTTTTTGCTGACAAGATCTATCATTTTTATCTTAAA
AATGATTGGTCCCGCGTTGCGACAAGATGTTACAATAAAAGGAGTTGTGCTATGCAATATGTTTATACACCCACCTACT
ACATGCTTAATGTTACTAGTGCAGGTGAGGATGGCATTTATTATGAACCCTGTACAGCTAATTGCAGTGGTTACGCTGC
CAATGTATTTGCCACTGATTCTAATGGCCATATACCAGAAGGTCTTAGTTTTAATAATTGGTTTCTTTTGTCCAATGAT
TCCACTTTGTTGCATGGTAAGGTGGTTTCCAACCAACCCTTGTTGGTCAATTGTCTTTTGGCCATTCCTAAGATTTATG
GACTAGGCCAATTTTTCTCATTCAATCAAACGATGTATGGCGTTTGTAATGGAGCTGCTGCGCAGCGTGCACCAGAGGC
TCTGAGGTTTAATATTAATGACACCTCTGTCATTCTTGCTGAAGGCTCAATTGTACTTCATACTGCTTTAGGAACAAAT
CTTTCTTTTGTTTGCAGTAATTCCTCAGATCCTCATTTAGCCACCTTCGCCATACCTCTGGGTGCTACCCAAGTACCCT
ATTATTGTTTTCTTAAAGTGGATACTTACAACTCCACTGTTTATAAATTCTTGGCTGTTTTACCTCCTACCGTCAGGGA
AATTGTCATCACCAAGTATGGTGATGTTTATGTCAATGGGTTTGGCTACTTGCATCTCGGTTTGTTGGATGCTGTCACA
ATTAATTTCACTGGTCATGGCACTGACGATGACGTTTCAGGTTTTTGGACCATAGCATCGACTAATTTTGTTGATGCAC
TCATCGAAGTTCAAGGAACTGCCATTCAGCGTATTCTTTATTGTGATGATCCTGTTAGCCAACTCAAGTGTTCTCAGGT
TGCTTTTGACCTTGACGATGGTTTTTACCCTATTTCTTCTAGAAACCTTCTGAGTCATGAACAGCCAATTTCTTTTGTT
ACTTTGCCATCGTTTAATGATCATTCTTTTGTTAACATTACTGTCTCTGCGTCCTTTGGTGGTCATAGTGGTGCCAACC

TCATTGCATCTGACACTACTATCAATGGGTTTAGTTCTTTCTGTGTTGACACTAGACAATTTACCATTTCACTGTTTTA
TAACGTTACAAACAGTTATGGTTATGTGTCCAAATCACAGGATAGTAATTGCCCTTTCACCTTGCAATCTGTTAATGAT
TACCTGTCTTTTAGCAAATTTTGTGTTTCTACCAGCCTTTTGGCTAGTGCCTGTACCATAGATCTTTTTGGTTACCCTG
AGTTCGGTAGTGGTGTTAAGTTTACGTCCCTTTACTTTCAATTCACAAAGGGTGAGTTGATTACTGGCACGCCTAAACC
ACTCGAAGGTGTCACGGACGTTTCTTTTATGACTCTGGATGTGTGTACCAAGTATACTATCTATGGCTTTAAAGGTGAG
GGTATCATTACCCTTACAAATTCTAGCTTTTTGGCAGGTGTTTATTATACATCTGATTCTGGACAGTTGTTAGCCTTTA
AGAATGTCACTAGTGGTGCTGTTTATTCTGTTACGCCATGTTCTTTTTCAGAGCAGGCTGCATATGTTGATGATGATAT
AGTGGGTGTTATTTCTAGTTTGTCTAGCTCCACTTTTAACAGTACTAGGGAGTTGCCTGGTTTCTTCTACCATTCTAAT
GATGGCTCTAATTGTACAGAGCCTGTGTTGGTGTATAGTAACATAGGTGTTTGTAAATCTGGCAGTATTGGCTATGTCC
CATCTCAGTCTGGCCAAGTCAAGATTGCACCCACGGTTACTGGGAATATCAGTATTCCCACCAACTTTAGTATGAGTAT
TAGGACAGAATATTTACAGCTTTACAACACGCCTGTTAGTGTTGATTGTGCCACATATGTTTGTAATGGTAACTCTCGT
TGTAAACAACTACTTACCCAGTACACTGCAGCATGTAAGACCATAGAGTCAGCATTACAACTCAGCGCTAGGCTTGAGT
CTGTTGAAGTCAACTCTATGCTTACTATTTCTGAAGAGGCTCTACAGTTAGCTACCGTCAGTTCGTTTAATGGTGATGG
ATATAACTTTACTAATGTGCTGGGCGTTTCCGTGTACGATCCTGCAAGTGACAGGGTGGTACAAAAAAGGTCTTTTATT
GAAGACCTGCTTTTTAACAAAGTGGTTACTAATGGCCTTGGTACTGTTGATGAAGACTATAAGCGCTGTTCTAATGGTC
GCCCTGTGGCAGATCTAGTCTGTGCGCAGTATTACTCTGGTGTCATGGTACTACCTGGTGTTGTTGACGCTGAGAAGCT
TCACATGTATAGTGCGTCTCTCATCGGTGGTATGGTGCTAGGAGGTTTTACTGCTGCAGCGGCATTGCCTTTTAGCTAT
GCTGTTCAAGCGAGACTCAATGAAATTGCTCTACAGACGGATGTTCTACAGCGGAACCAGCAATTGCTTGCTGAGTCTT
TTAACTCTGCTATTGGTAATATAACTTCAGCCTTTGAGAGTGTTAAAGAGGCTATTAGTCATACTTCCAAGGGTTTGAA
CACTGTGGCACATGCGCTTACTAAGGTTCAAGAGGTTGTTAATTCGCAGGGTGCAGCTTTGACCCAACTTACCGTACAG
CTGCAACACAACTTCCAAGCCATTTCTAGTTCTATTGATGACATTTACTCCCGACTGGACAGTCTTTCAGCTGATGTTC
AGGTTGGCCGTCTCATCACCGGCAGATTATCAGTACTTAATGCTTTTGTTGCTCAAACCCTTACTAAGTATACTGAGGT
TCAGGCTAGCAGGAAACTAGCACAGCAAAAGGTTAATGAGCGTGTTAAATCGCAATCTCAGCGTTATGGTTTTTGTGGT
GGTGATGGCGAGCACATTTTCTCTCTGGTACAGGCAGCACCTCAGGGCTTGCTGTTTTTACACACAGTACTTGTACCGG
GTGATTTTGTAGATGTTATTGCCATCGCTGGCTTATGCGTTAACGATGAAATTGCCTTGACTCTACGTGAGCCTGGCTT
AGTCTTGTTTACGCATGAACTTCAAAATTATACTGCGACGGAATATTTTGTTTCATCGCGACGTATGTTTGAACCTAGA
AAACCTACCGTTAGTGATTTTGTTCAAATTGAGAGTTGTGTGGTCACCTATGTCAATTTGACTAGAGACCAACTACCAG
ATGTAATCCCAGATTACATCGATGTTAACAAAACACTTGATGAGATATTAGCTTCTCTGCCCAATAGAACTGGTCCAAG
TCTTCCTCTAGATGCTTTTAATGCCACTTATCTTAATCTCACTGGTGAAATTGCAGATTTAGAGCAGCGTTCAGAGTCT
CTCCGTAATACTCTAGAAGAGCTCCAAAGTCTTATATATAATATCAACAACACACTTGTCGACCTTGAGTGGCTCAACC
GAGTTGAGACATATATCAAGTGGCCGTGGTGGGTTTGGTTGATTATTTTCATTGTTCTCATCTCTGTTGTGTCATTACT
AGTGTTCTGCTGCATTTCCACGGGTTGCTGTGGATGCTGCGGTTGCTGCGGTGCTTGTTTTTCAGGTTGTTGTAGGGGT
CCTAGACTTCAACCTTACGAAGCTTTTGAAAAGGTCCACGTGCAGTGA

>gi|121722565|gb|EF185992.1| Porcine epidemic diarrhea virus strain LZC from China, complete genome

ATGAGGTCTTTAATTTACTTCTGGTTGCTCTTACCAGTACTTCCAACACTCAGCCTACCACAAGATGTCACTAGG
TGCCAGTCTACTACTAACTTTAGGCGGTTCTTTTCAAAATTTAATGTTCAGGCACCTGCCGTCGTCGTTTTGGGTGGTT
ACCTACCTAGTATGAACTCTTCTAGCTGGTACTGTGGCACAGGCATTGAAACTGCTAGTGGCGTTCATGGTATTTTTCT
CAGCTACATCGATTCTGGTCAGGGCTTTGAGATTGGCATTTCGCAAGAGCCGTTTGATCCTAGTGGTTACCAGCTTTAT
TTACATAAGGCCACTAATGGTAACACTAATGCTATTGCACGACTGCGCATTTGCCAGTTTCCCGATAATAAAACATTGG
GCCCTACTGTTAATGATGTTACAACAGGTCGTAACTGCCTATTCAACTTTGCCATTCCAGCTTATATGCGTGATGGAAA

AGATATTGTTGTCGGCATAACATGGGATAATGATCGTGTCACTGTTTTTGCTGACAAGATCTATCATTTTTATCTTAAA
AATGATTGGTCCCGCGTTGCGACAAGATGTTACAATCGCAGAAGTTGTGCTATGCAATATGTTTATACACCTACCTACT
ACATGCTTAATGTTACTAGTGCAGGTGAGGATGGCATTTATTATGAACCCTGTACAGCTAAAAGCACTGGTTACGCTGC
CAATGTATTTGCCACTGATTCCAATGGCCATATACCAGAAGGTTTTAGTTTTAATAATTGGTTTCTTTTATCCAATGAC
TCCACTTTGTTGCATGGTAAAGTGGTTTCCAACCAACCCTTGTTGGTCAATTGTCTTTTGGCCATTCCTAAGATTTATG
GACTACCCCAATTTTTCTCATTCAATCACACGATGGATGGCGTTTGTAATGGAGCTGCTGTGGATCGTGCCCCAGAGGC
TCTGAGGTTTAATATTAATGACACCTCCGTCATTCTTGCTGAAGGCTCAATTGTACTTCATACTGCTTTAGGAACAAAT
CTTTCTTTTGTTTGCAGTAATTCCTCAGATCCTCATTTAGCCATCTTTGCCATACCTCTGGGTGCTACTGAAGTACCCT
ACTATTGCTTTCTTAAAGTGGATACTTACAACTCCACTGTTTATAAATTCTTGGCTGTTTTACCTCCTACTGTCAGGGA
AATTGTCATCACCAAGTATGGTGATGTTTATGTCAATGGGTTTGGCTATTTGCATCTCGGTTTGTTGGATGCTGTCACA
ATTAATTTCACTGGTCATGGCACTGACGATGACGTTTCAGGTTTCTGGACCATAGCATCGACTAATTTTGTTGATGCAC
TCATCGACCAACAAGGAACTTCCATTCAGCGTATTCTTTATTGTGATGATCCTGTTAGCCAACTCAAGTGTTCTCAGGT
TGCTTTTGACCTTGACGATGGTTTTTACCCCATCTCTTCTAGAAACCTTCTGAGTCACGAACAGCCAATTTCTTTTGTT
ACTTTGCCATCATTTAATGATCATTCTTTTGTTAATATTACTGTCTCTGCGGCTTTTGGTGGTCTTAGTAGTGCCAATC
TCGTTGCATCTGACACTACTATCAATGGGTTTAGTTCTTTCTGTGTTGACACTAGACAATTTACCATTACACTGTTTTA
TAATGTTACAAACAGTTATGGTTATGTGTCTAAATCACAGGATAGTAATTGTCCTTTCACCTTGCAATCTGTTAATGAT
TACCTGTCTTTTAGCAAATTTTGTGTTTCAACCAGCCTTTTGGCTGGTGCTTGTACCATAGATCTTTTTGGTTACCCTG
CGTTCGGTAGTGGTGTTAAGTTGACGTCCCTTTATTTTCAATTCACAAAAGGTGAGTTGATTACTGGCACGCCTAAACC
ACTTGAAGGTATCACAGACGTTTCTTTTATGACTCTGGATGTGTGTACCAAGTATACTATCTATGGCTTTAAAGGTGAG
GGTATTATTACCCTTACAAATTCTAGCATTTTGGCAGGTGTTTATTATACATCTGATTCTGGACAGTTGTTAGCCTTTA
AGAATGTCACTAGTGGTGCTCAATATTCTGTCACGCCATGTTCTTTTTCAGAGCAGGCTGCATATGTTAATGATGATAT
AGTGGGTGTTATTTCTAGTTTGTCTAACTCCACTTTTAACAATACTAGGGAGTTGCCTGGTTTCTTCTACCATTCTAAT
GACGGCTCCAATTGTACAGAGCCTGTGTTGGTGTATAGTAACATAGGTGTTTGTAAATCTGGCAGTATTGGCTATGTTC
CATCTCAGTATGGCCAAGTCAAGATTGCACCCACGGTTACTGGGAATATTAGTATTCCCACCAACTTTAGTATGAGTAT
TAGAACAGAATATTTACAGCTTTACAACACGCCTGTTAGTGTTGATTGTGCTACATATGTTTGTAATGGTAACTCTCGT
TGTAAACAATTACTCACCCAGTACACTGCAGCATGTAAGACCATAGAGTCAGCATTACAACTCAGCGCTAGGCTTGAGT
CTGTTGAAGTTAACTCTATGCTTACCATTTCTGTTCAGGCTTTACAGTTAGCTACCATCAGTTCGTTTAATGGTGATGG
ATATAACTTTACTAATGTGCTGGGTGCTTCCGTGTACGATCCTGCAAGTGGCAGGGTGGTACAAAAAAGGTCTGTTATT
GAAGACTTGCTTTTTAATAAAGTGGTTACTAATGGCCTTGGTACTGTTGATGAAGACTATAAGCGCTGTTCTAATGGTC
GCTCTGTGGCTGATCTAGTCTGTGCGCAGTATTACTCTGGTGTCATGGTACTACCTGGCGTTGTTGACGCTGAGAAGCT
TCACATGTACAGTGCGTCTCTCATAGGTGGTATGGCGCTAGGAGGTATAACTGCTGCAGCGGCATTGCCTTTTAGCTAT
GCTGTTCAAGCGAGACTCAATTATCTTGCTTTACAGACGGATGTTCTACAGCGGAACCAGCAATTGCTTGCTGAGTCTT
TTAACTCTGCTATTGGTAATATTTGTTCAGCCTTTGAGAGTGTTAAAGAGGCTATTAGTCAAACTTCCAAGGGTTTGAA
CACTGTGGCTCATGCGCTTACTAAGGTTCAAGAGGTTGTTAATTCGCAGGGTTCAGCTTTGAACCAACTTACCGTACAG
CTGCAACACAACTTCCAAGCCATTTCTAGTTCTATTGATGACATTTATTCCCGACTGGACATTCTTTCAGCCGATGTTC
AGGTTGATCGTCTCATCACCGGCAGATTATCAGCACTTAATGCTTTTGTTGCCCAAACCCTCACTAAGTATACTGAGGT
TCAGGCTAGCAGGAAGCTAGCACAGCAAAAGGTTAATGAGTGCGTCAAATCGCAATCTCAGCGTTACGGTTTTTGTGGT
GGTGATGGCGAGCACATTTTCTCTCTGGTACAGGCCGCACCTCAGGGCCTGCTGTTCTTACATACAGTACTTGTACCGG
GTGATTTTGTAAATGTTCTTGCCATCGCTGGCTTATGCGTTAATGGTGAAATTGCCTTGACTCTACGTGAGCCTGGCTT
AGTCTTGTTTACGCATGAACTTCAAACTTATACTGCGACGGAATATTTTGTTTCATCGCGACGTATGTTTGAACCTAGA
AAACCTACCGTTAGTGATTTTGTTCAAATTGAGAGTTGTGTGGTCACCTATGTCAATCTGACTAGCGACCAGCTACCAG
ATGTAATCCCAGATTACATCGATGTTAACAAAACACTTGATGAGATTTTAGCTTCTCTGCCCAATAGAACTGGTCCAAG
TCTTCCCCTAGATGTTTTAATTGCCAGTTATCTTAATCTTACTGGTGAAATTGCAGATCTAGAGCAGCGTTCAGAGTCT
CTCCGTAATACTACAGAAGAGCTCCGAAGTCTCATTAACAACATCAACAACACACTTGTTGACCTTGAGTGGCTCAACC

GAGTTGAGACATACATCAAGTGGCCGTGGTGGGTTTGGTTGATCATTGTTATTGTTCTCATCTTTGTTGTGTCATTACT
AGTGTTCTGCTGCATTTCCACGGGTTGTTGTGGATGCTGCGGTTGCTGCGGTGCTTGTTTTTCAGGTTGTTGTAGGGGT
CCTAGACTTCAACCTTACGAAGCTTTTGAAAAGGTCCACGTGCAGTGA

>gi|269927198|gb|GU180142.1| Porcine epidemic diarrhea virus isolate KNU-0801 spike protein gene, complete cds

ATGAGGTCTTTAATTTATTTTTTGTTGTTCGTACCAGTACTTCCAACACTCAGCCTACCACAAGATGTCTATAGG
TGCTCAGCTAACACTAACTTTAGGCGGTTCTTTTCAAAATTTAATGTTCAGGCGCCTGCAGTCGTTGTACTGGGCGGTT
ATCTACCTAGTGGTGAAACACAGGCTGGGTCAAGACCTTGGTACTGTGCTGGCCGACATCCAACTGCTAGTGGCGTTCA
TGGTATCTTTCTTAGCCATATTAGAGGTGGTCATGGCTTTGAGATTGGCATTTCGCAAGAGCCGTTTGACCCTAGTGGT
TACCAGCTTTATTTACATAAGGCCACTAACGGTAACACTAATGCCACTGCGCGACTGCGTATTTGCCAGTTTCCCAGCA
GTAAAACATTGGGCCCCACTGCTAATGATGATGTTACAACAGGTCGTAACTGCCTATTTAACAAAGCCATTCCAGCTCA
TATGAGTGAACATAGTGTTGTCGGCATAACATGGGATAATGATCGTGTCACTGTTTTTGCTGACAAGATCTATCATTTT
TATCTTAAAAATGATTGGTCCCGCGTTGCGACAAACTGTTACAACAGTGGAGGTTGTGCTATGCAATATGCTTACGAAC
CTATTTACTACATGCTTAATGTTACTAGTGCTGGTGAGGATGGTATTTCTTATCAACCCTGTACAGCTAATTGCATTGG
TTATGCCGCCAATGTATTTGCCACTGAGTCCAATGGCCACATACCAGAAGGTTTTAGTTTTAATAATTGGTTTCTTTTG
TCCAATGATTCCACTTTGTTTCATGGTAAAGTGGTTTCCAACCAACCTTTGTTGGTCAACTGTCTTTGGGCCATTCCTA
AGATTTACGGACTAGGCCACTTTTTCTCATTTAATCAAACGATGGATGGCGTTTGTAACGGAGCTACTGCGTATCGTGC
CCCAGAGGCTCTGAGGTTTAATATTAATGACACTTCTGTCATTCTTGCTGAAGGCTCAATTGTACTTCATACTGCTTTA
GGAACAAATCTTTCTTTTGTTTGCAGTAATTCCTCAGATCCTCACAAAGCCATCTTCTCCATACCTCTGGGTGCTACCC
AAGTACCCTACTATTGCTTTCTTAAAGTTGATACTTACAACTCCACTGTCTATAAATTTCTTGCTGTTTTACCTCCTAC
TGTCAGGGAAATTGTCATCACCAAGTACGGTGATGTTTATGTCAATGGGTTTGGCTACTTGCATCTCGGTTTGTTGGAT
GCTGTCACAATTAACTTCACTGGCCATGGCACTAACGATGACGTCTCAGGTTTCTGGACCATAGCATCGACTAATTTTA
TTGATGCACTCATCGAGGTTCAAGCAACTGCCATTCAGCGTATTCTTTATTGTGATGATCCTGTTAGCCAACTCAAGTG
TTCTCAGGTTTCTTTTGACCTTGATGATGGTTTTTACCCTATTTCTTCTAGAAACCTTCTTAGTCATGAACAGCCAATT
TCTTTTGTTACTTTGCCATCATTTAATGATCATTCTTTTGTTAATATTACTGTATCTGCGGCTTTTGGTGATTCTGGTG
GTGCCAACCTCGTTGCATCTGACACTACTATCAATGGGTTCAGTTCTTTCTGTGTTGACACTAGACAATTTACCATTAG
ACTGTTTTACAACGTTACAAACAGTTATGGTTATGTGTCTAAATCACAGGATAGCAATTGCCCTTTCACCTTGCAATCT
GTTAATGATTACCTGTCTTTTAGCAAATTTTGTGTTTCAACCAGCCTTTTGGCTGGTGCTTGTACCATAGATCTTTTTG
GTTACCCTGCTTTCGGTAGTGGTGTTAAGTTTACGTCCCTCTATTTTCAATTCACAAAGGGTGAGTTGATTACTGGCAC
GCCTAAACCACTTCAAGGTGTCACGGACGTTTCTTTTATGACTCTGGATGTGTGTACCAAGTATACTATCTATGGCTTT
AAAGGTGAGGGTATTATTACCCTTACAAATTCTAGCTTTTTGGCAGGTGTTTATTATACATCTGATTCTGGACAGTTGT
TAGCCTTTAAGAATGTCACTAGTGGTGCTGTTTATTCTGTTACGCCATGTTCTTTTTCAGAGCAGGCTGCTTATGTTGA
TGATGATATAGTGGGTGTTATTTCTAGTTTGTCTAACTCCACTTTTAACAATACTAGGGAGTTGCCCGGTTTCTTCTAC
CATTCTAATGACGGCTCCAATTGTACAGAGCCTGTGTTGGTGTATAGTAACATAGGTGTCTGTAAATCTGGTAGTATTG
GCTATGTCCCACTTCAGGATGGCCAAGTCAAAATTGCACCCACGGTTACTGGGAATATTAGTATTCCCACCAACTTTAG
TATGAGTATTAGAACAGAATATTTACAGCTTTACAACACGCCTGTTAGTGTTGATTGTGCTACATATGTTTGTAATGGT
AACTCTCGTTGTAAACAATTGCTTACCCAGTACACTGCAGCATGTAAGACCATAGAGTCAGCATTAGAACTCAGCGCTA
GGCTTGAGTCTGTTGAAGTTAACTCTATGCTTACTATTTCTGAAGAGGCCTTACAGTTAGCTACCATCAGTTCGTTTAA
TGGTGATGGGTATAACTTTACTAATGTGTTGGGTGTTTCTGTGTACGATTCTCAAAGTGGCAGGGTGATACACGAAAGG
TCTTTTATTGAAGACCTGCTTTTTAATAAGGTGGTTACTAATGGCCTTGGTACTGTTGATGAAGACTATAAGCGCTGTT
CTAATGGTCGCTCTGTGGCTGATCTAGTCTGTGCGCAGTATTACTCTGGTATCATGGTACTACCTGGCGTTGTTGACGC

TGAGAAGCTTCACATGTATAGTGCGTCTCTCATCGGTGGTATGGCGCTAGGAGGTTTTACTGCCGCAGTGGCATTGCCT
TTTAGCTATGCTGTTCAAGCGAGACTCAATTATCTTGCTTTACAGACGGATGTTTTACAGCGGAACCAGCAACTGCTTG
CTGAGTCTTTTAACTCTGCTATTGGTAATATAACCTCAGCCTTTGCGAGTGTTAAAGAGGCTATCAGTCAAACTTCTCA
AGGTTTGAATACTGTGGCTCATGCGCTTACTAAAGTTCAAGAGGTTGTTAATTCGCAGGGTTCAGCTTTGACTCAACTT
ACCATACAGCTGCAACATAACTTCCAAGCCATTTCTAGTTCTATTGATGACATTTACTCCCGACTGGACATTCTTTCAG
CCGATGTTCAGGTTGATCGTCTCATCACCGGCAGATTATCAGCACTTAATGCTTTTGTTGCTCAAACCCTCACTAAGTA
TACTGAGGTTCAGGCTAGCAGGAAGCTAGCACAGCAAAAGGTTAATGAGTGCGTCAAATCGCAATCTCAGCGTTATGGT
TTTTGTGGTGGTGATGGCGAGCACATTTTCTCTCTTGTACAGGCCGCACCCCAGGGCCTGCTGTTCTTACACACAGTAC
TTGTACCGGGTGATTTTGTAAATGTTATTGCCATCGCAGGCTTATGCGTTAATGGTGATATTGCCTTGACTCTACGTGA
GCCTGGCTTAGTCTTGTTTACGCATGAACTTCAAACTTATACTGCGACGGAATATTTTGTTTCATCGCGACGTATGTTT
GAACCTAGAAAACCTACCGTTAGTGATTTTGTTCAAATTCAGAGTTGTGTGGTCACCTATGTCAATCTGACTAGCGACC
AACTACCAGATGTAATCCCAGATTACATCGATGTTAACAAAACACTTGATGAGATTTTAGCTTCTCTGCCCAATAGAAC
TGGTCCAAATCTTCCTCTAGATGTTTTTAATGCCACTTATCTTAATCTCACTGGTGAAATTGCAGATTTAGAGCAGCGT
TCAGAGTCTCTCCGTAATACTACAGAAGAGCTCCGAAGTCTTATATACAATATCAACAACACACTTGTTGACCTTGAGT
GGCTCAACCGAGTTGAGACTTATATCAAGTGGCCGTGGTGGGTTTGGTTGATTATTTTTATTGTTCTCATCTTTGTTGT
GTCATTATTAGTGTTCTGCTGCATTTCCACGGGTTGTTGTGGATGCTGCGGTTGTTGCGGTGCTTGTTTTTCAGGTTGT
TGTAGGGGTCCTAGACTTCAACCTTACGAAGCTTTTGAAAAGGTCCACGTGCAGTGA

>gi|269927200|gb|GU180143.1| Porcine epidemic diarrhea virus isolate KNU-0802 spike protein gene, complete cds

ATGAGGTCTTTAATTTATTTTTTGTTGTTCGTACCAGTACTTCCAACACTCAGTCTACCACAAGATGTCCATAGG
TGCTCAGCTAACACTAATTTTAGGCGGTTCTTTTCAAAATTTAATGTTCAGGCGCCTGCAGTTGTTGTACTGGGCGGTT
ATCTACCTATTGGTGAAAACCAGGGTGTGAATTCAACTTGGTACTGTGCTGGCCAACATCCAACTGCTAGTGGCGTTCA
TGGTATCTTTCTTAGCCATATTAGAGGTGGTCATGGCTTTGAGATTGGCATTTCGCAAGAGCCGTTTGACCCTAGTGGT
TACCAGCTTTATTTACATAAGGCCACTAACGGTAACACTAATGCTACTGCGCGACTGCGCATTTGCCAGTTTCCCAGCA
GTAAAACATTGGGCCCCACTGCTAATGATGATGTTACAACAGGTCGTAACTGCCTATTTAACAAAGCCATCCCAGCTCA
TATGAGTGAACATAGTGTTGTCGGCATAACATGGGATAATGATCGTGTCACTGTTTTTTCTGACAAGATCTATCATTTT
TATTTTAAAAATGATTGGTCCCGTGTTGCGACAAAGTGTTACAACAGTGGAGGTTGTGCTATGCAATATGTTTACGAAC
CCACTTACTACATGCTTAATGTTACTAGTGCTGGTGAGGATGGTATTTCGTATCAACCCTGTACAGTTAATTGCATTGG
TTATGCTGCCAATGTATTTGCCACTGAGTCCAATGGCCACATACCAGAAGGTTTTAGTTTTAATAATTGGTTTCTTTTG
TCCAATGATTCCACTTTGGTGCATGGTAAGGTGGTTTCCAACCAACCTTTGTTGGTCAATTGTCTTTTGGCCATTCCTA
AGATTTATGGACTAGGCCAATTTTTCTCCTTCAATCAAACGATCGATGGTGTTTGTAATGGAGCTGCTGCGCAGCGTGC
ACCAGAGGCTCTGAGGTTTAATATTAATGACACCTCTGTCATTCTTGCTGAAGGCTCAGTTGTACTTCATACTGCTTTA
GGAACAAATTTTTCTTTTGTTTGCAGTAATTCCTCAAATCCTCATTTAGCCACCTTTGCCATACTTCTGGGTGCTACCC
AAGTACCCTATTATTGTTTTCTTAAAGTGGATACTTACAATTCCACTGTTTATAAATTCTTGGCTGTTTTACCTCCTAC
TGTCAGGGAAATTGTCATCACCAAGTATGGTGATGTTTATGTCAATGGGTTTGGTTATTTGCATCTCGGTTTGTTGGAC
GCTGTCACAATTAATTTCACTGGTCATGGCACTGACGATGACGTTTCAGGTTTCTGGACCATAGCATCGACTAATTTTG
TTGATGCTCTCATCGAAGTTCAAGGAACCGCCATTCAGCGTATTCTTTATTGTGATGATCCTGTTAGCCAACTCAAGTG
TTCTCAGGTTGCTTTTGACCTTGACGATGGTTTTTACCCTATATCTTCTAGAAACCTCCTGAGTCATGAACAGCCAACT
TCTTTTGTTACTTCGCCATCATTTAATGATCATTCTTTTGTTAACATTACTGTCTCTGCGTCTTTTGGTGGTCATAGTG
GTGCCAACCTCGTTGCATCTGACACTACTATCAATGGATTCAGTTCTTTCTGTGTTGACACTAGACAATTTACCATTAG
ACTGTTTTACAACGTTACAAACAGTTATGGTTATGTGTCTAATTCACAGGACAGTAATTGCCCTTTTACCTTGCAATCT

GTTAATGATTACCTGTCTTTTAGCAAATTTTGTGTTTCTACCAGCCTTTTGGCTAGTGCCTGTACCATAGATCTTTTTG
GTTACCCTGATTTCGGTAGTGGTGTTAAGTTTACGTCCCTTTACTTTCAATTCACAAAGGGTGAGTTGATTACTGGCAC
GCCTAAACCACTTGAAGGTGTCACGGACGTTTCTTTTATGACTCTGGATGTGTGTACCAAGTATACTATCTATGGCTTT
AAAGGTGAGGGTATCATTACCCTTACAAATTCTAGCTTTTTGGCAGGTGTTTATTATACATCTGATTCTGGACAGTTGT
TAGCCTTTAAGAATGTCACTAGTGGTGCTGTTTATTCTGTTACGCCATGTTCTTTTTCAGAGCAGGCTGCATATGTTGA
TGACGATATAGTGGGTGTTATTTCTAGTTTGTCTAACTCCACTTTTAACAATACTAGGGAGTTGCCTGGTTTCTTCTAC
CATTCTAATGACGGCTCCAATTGTACAGAGCCTGTGTTGGTGTATAGTAACATAGGTGTTTGTAAATCTGGCAGTATTG
GCTATGTCCCATCTCAGTCTGGCCAAGTCAAGATTGCACCCACGGTTACTGGGAATATTAGTATTCCCACCAACTTTAG
TATGAGTATTAGGACAGAATATTTACAGCTTTACAACACGCCTGTTAGTGTTGATTGTGCCACATATGTTTGTAATGGT
AACTCTCGTTGCAAACAATTACTCACCCAGTACACTGCAGCATGTAAGACCATAGAGTCAGCATTACAACTCAGCGCTA
GGCTTGAGTCTGTTGAAGTTAACTCTATGCTTACTACTTCTGAAGAGGCTCTACAGTTAGCTACCATCAGTTCGTTTAA
TGGTGATGGATATAATTTTACTAATGTGCTGGGTGTTTCCGTGTATGATTCTGCAAGTGGCAGGGTGGTACACAAAAGG
TCTTTCATTGAAGACCTGCTTTTTAATAAAGTGGTTACTAATGGCCTTGGTACTGTTGATGAAGACTATAAGCGCTGTT
CTAATGGCCGCTCTGTGGCTGATCTAGTCTGTGCGCAGTATTACTCTGGTGTCATGGTACTACCTGGCGTTGTTGACGC
TGAGAAGCTTCACATGTATAGTGCGTCTCTCATCGGTGGTATGGTGCTAGGAGGTTTTACTGCTGCAGCGGCATTGCCT
TTTAGCTATGCTGTTCAAGCGAGACTCAATTATCTTGCTTTACAGACGGATGTTCTACAGCGTAACCAGCAATTGCTTG
CTGAGTCTTTTAACTCTGCTATTGGTAATATAACTTCAGCCTTTGAGAGTGTTAAAGAGGCTATTAGTCAAACTTCCCA
GGGTTTGAACACTGTGGCTCATGCGCTTACTAAGGTTCAAGAGGTTGTTAATTCGCAGGGTGCAGCTTTGACCCAACTT
ACCGTACAGCTGCAACACAACTTTCAAGCCATTTCTAGTTCTATTGATGACATTTATTCCCGACTTGACATTCTTTCAG
CCGATGTTCAGGTTGACCGTCTCATCACTGGCAGATTATCAGCACTTAACGCTTTTGTTGCTCAAACCCTCACTAAGTA
TACTGAGGTTCAGGCTAGCAGGAAGCTAGCACAGCAAAAGGTTAATGAGTGCGTCAAATCGCAGTCTCAGCGTTATGGT
TTTTGTGGTGGTGATGGCGAACACATTTTTTCCCTGGTACAGGCCGCACCTCAGGGCCTGCTGTTCTTACACGCAGTAC
TTGTACCGGGTGATTTTGTAAATGTTATTGCTATCGCAGGCTTATGCGTTAATGGTGATATTGCCTTGACTCTACGTGA
GCCTGGCTTAGTCCTGTTTACGCATGAACTTCAAACTCATACGGCGACAGAATATTTTGTTTCATCGCGACGTATGTTT
GAACCTAGAAAACCTACCGTTAGTGATTTTGTTCAAATTGAGAGTTGTGTGGTCACCTATGTCAATCTGACTAGCGACC
AACTACCAGATGTAATCCCAGATTACATCGATGTTAACAAAACACTTGATGAGATTTTGGCTTCTCTGCCCAATAGAAC
TGGTCCAAGTCTTCCCCTAGATGTTTTTAATGCTACTTATCTTAATCTCACTGGTGAAATTGCAGATTTAGAGCAGCGT
TCAGAGTCTCTCCAAAATATCACAGAAGAGCTCCGAAGTCTCATATATAACATCAACAACACACTTGTTGACCTTGAGT
GGCTCAACCGCGTTGAGACATATATCAAGTGGCCGTGGTGGGTTTGGTTGATTATTTTCATTGTTTTCATCTTTGTTGT
GTCATTACTAGTGTTCTGCTGCATTTCCACGGGTTGTTGTGGATGCTGCGGCTGCTGCTGTGCTTGTTTTTCAGGTTGT
TGTAGGGGTCCTAGACTTCAACCTTACGAAGCTTTTGAAAAGGTCCACGTGCAGTGA

>gi|269927202|gb|GU180144.1| Porcine epidemic diarrhea virus isolate KNU-0901 spike protein gene, complete cds

ATGAGGTCTTTAACCTATTTCTGGTTGTTCTTACCAGTACTTCCAATACTCAGCCTACCACAAGATGTCTATAGG
TGCTCAGCTAACACTAACTTTAGGCGGTTCTTTTCAAAATTTAATGTTCAGGCGCCTGCAGTCGTTGTACTGGGCGGTT
ATCTACCTAGTGGTGAAACACAGGCCGGGTCAAGACCTTGGTACTGTGCTGGCCGACATCCAACTGCTAGTGGCGTTCA
TGGTATCTTTCTTAGCCATATTAGAGGTGGTCATGGCTTTGAGATTGGCATTTCGCAAGAGCCGTTTGACCCTAGTGGT
TACCAGCTTTATTTACATAAGGCCACTAACGGTAACACTAATGCCACTGCGCGACTGCGTATTTGCCAGTTTCCCAGCA
GTAAAACATTGGGCCCCACTGCTAATGATGATGTTACAACAGGTCGTAACTGCCTATTTAACAAAGCCATTCCAGCTCA
TATGAGTGAACATAGTGTTGTCGGCATAACATGGGATAATGATCGTGTCACTGTTTTTGCTGACAAGATCTATCATTTT
TATCTTAAAAATGATTGGTCCCGCGTTGCGACAAACTGTTACAACAGTGGAGGTTGTGCTATGCAATATGCTTACGAAC

CTATTTACTACATGCTTAATGTTACTAGTGCTGGTGAGGATGGTATTTCTTATCAACCCTGTACAGCTAATTGCATTGG
TTATGCCGCCAATGTATTTGCCACTGAGTCCAATGGCCACATACCAGAAGGTTTTAGTTTTAATAATTGGTTTCTTTTG
TCCAATGATTCCACTTTGTTTCATGGTAAAGTGGTTTCCAACCAACCTTTGTTGGTCAACTGTCTTTGGGCCATTCCTA
AGATTTACGGACTAGGCCACTTTTTCTCATTTAATCAAACGATGGATGGCGTTTGTAACGGAGCTACTGCGTATCGTGC
CCCAGAGGCTCTGAGGTTTAATATTAATGACACTTCTGTCATTCTTGCTGAAGGCTCAATTGTACTTCATACTGCTTTA
GGAACAAATCTTTCTTTTGTTTGCAGTAATTCCTCAGATCCTCACAAAGCCATCTTCTCCATACCTCTGGGTGCTACCC
AAGTACCCTACTATTGCTTTCTTAAAGTTGATACTTACAACTCCACTGTCTATAAATTTCTTGCTGTTTTACCTCCTAC
TGTCAGGGAAATTGTCATCACCAAGTACGGTGATGTTTATGTCAATGGGTTTGGCTACTTGCATCTCGGTTTGTTGGAT
GCTGTCGCAATTAACTTCACTGGCCATGGCACTAACGATGACGTCTCAGGTTTCTGGACCATAGCATCGACTAATTTA
TTGATGCACTCATCGAGGTTCAAGCAACTGCCATTCAGCGTATTCTTTATTGTGATGATCCTGTTAGCCAACTCAAGTG
TTCTCAGGTTTCTTTTGACCTTGATGATGGTTTTTACCCTATTTCTTCTAGAAACCTTCTTAGTCATGAACAGCCAATT
TCTTTTGTTACTTTGCCATCATTTAATGATCATTCTTTTGTTAATATTACTGTATCTGCGGCTTTTGGTGATTCTGGTG
GTGCCAACCTCGTTGCATCTGACACTACTATCAATGGGTTCAGTTCTTTCTGTGTTGACACTAGACAATTTACCATTAG
ACTGTTTTACAACGTTACAAACAGTTATGGTTATGTGTCTAAATCACAGGATAGCAATTGCCCTTTCACCTTGCAATCT
GTTAATGATTACCTGTCTTTTAGCAAATTTTGTGTTTCAACCAGCCTTTTGGCTGGTGCTTGTACCATAGATCTTTTTG
GTTACCCTGCTTTCGGTAGTGGTGTTAAGTTTACGTCCCTCTATTTTCAATTCACAAAGGGTGAGCTGATTACTGGCAC
GCCTAAACCACTTCAAGGTGTCACGGACGTTTCTTTTATGACTCTGGATGTGTGTACCAAGTATACTATCTATGGCTTT
AAAGGTGAGGGTATTATTACCCTTACAAATTCTAGCTTTTTGGCAGGTGTTTATTATACATCTGATTCTGGACAGTTGT
TAGCCTTTAAGAATGTCACTAGTGGTGCTGTTTATTCTGTTACGCCATGTTCTTTTTCAGAGCAGGCTGCTTATGTTGA
TGATGATATAGTGGGTGTTATTTCTAGTTTGTCTAACTCCACTTTTAACAATACTAGGGAGTTGCCCGGTTTCTTCTAC
CATTCTAATGACGGCTCCAATTGTACAGAGCCTGTGTTGGTGTATAGTAACATAGGTGTCTGTAAATCTGGTAGTATTG
GCTATGTCCCACTTCAGTATGGCCAAGTCAAGATTGCACCCACGGTTACTGGGAATATTAGTATTCCCACCAACTTTAG
TATGAGTATTAGAACAGAATATCTACAGCTTTACAACACGCCTGTTAGTGTTGATTGTGCTACATATGTTTGTAATGGT
AACTCTCGTTGTAAACAATTACTCACCCAGTACACTGCAGCATGTAAGACCATAGAGTCAGCATTACAACTCAGCGCTA
GGCTTGAGTCTGTTGAAGTTAACTCTATGCTTACTATTTCTGAAGAGGCTCTACAGTTAGCTACCATCAGTTCGTTTAA
TGGTGGTGGATATAATTTTACTAATGTGCTGGGTGTTTCCGTGTATGATCCTGCAAGTGGCAGGGTGGTACAAAAAAGG
TCTATTATTGAAGACCTGCTTTTTAATAAAGTGGTTACTAATGGCCTTGGTACTGTTGATGAAGACTATAAGCGCTGTT
CTAATGGCCGCTCTGTGGCAGATCTAGTCTGTGCGCAGTATTACTCTGGTGTCATGGTACTACCTGGTGTTGTTGACGC
TGAGAAGCTTCACATGTATAGTGCGTCTCTCGTCGGTGGTATGGTGCTAGGAGGTTTTACTGCTGCAGCGGCATTGCCT
TTTAGCTATGCTGTTCAAGCGAGACTCAATTATCTTGCTTTACAGACGGATGTTCTACAGCGTAATCAGCAATTGCTTG
CTGAGTCTTTTAACTCTGCTATTGGTAATATAACTTCAGCCTTCGAGAGTGTTAAAGAGGCTATTAGTCAAACTTCCCA
GGGTTTGAATACTGTGGCTCATGCACTTACTAAGGTTCAAGAGGTTGTTAATTCGCAGGGTGCAGCTTTGTCTCAACTC
ACTATACAGCTGCAACACAACTTCCAAGCCATTTCTAGTTCTATTGATGACATTTACTCCCGACTGGACATTCTTTCAG
CCGATGTTCAGGTTGATCGTCTCATCACCGGCAGATTATCAGCACTTAATGCTTTTGTTGCTCAAACCCTCACTAAGTA
TACTGAGGTTCAGGCTAGCAGGAAGCTAGCACAGCAAAAGGTTAATGAGTGCGTCAAATCGCAATCACAGCGTTATGGT
TTTTGTGGTGGTGATGGTGAACACATTTTTTCTCTGGTACAGGCCGCACCTCAGGGCCTGCTGTTTTTACACACAGTAC
TTGTACCGGGTGATTTTGTAAATGTTATTGCTATCGCAGGCTTATGCGTTAATGGTGATATTGCCTTGACTCTACGTGA
GCCTGGCTTAGTCTTGTTTACGCATGAACTTCAAACTCATACTGCGATGGAATATTTTGTTTCATCGCGACGTATGTTT
GAACCTAGAAAACCTACCGTTAGTGATTTTGTTCAAATTGAGAGTTGTGTGGTCACCTATGTTAATTTGACTAGCGACC
AACTACCAGATGTAATCCCAGATTACATCGATGTTAACAAAACACTTGATGAGATTTTGGCTTCTCTGCCCAATAGAAC
TGGTCCAAGTCTTCCATTAGATGTTTTTAATGCCACTTATCTTAATCTCACTGGTGAAATTGCAGATTTAGAGCAGCGT
GCAGAGTCTCTCCGTAATACCACAGAAGAGCTCCGAAGTCTTATATATAACATCAACAACACACGTGTAGACCTTGAGT
GGCTCAACCGTGTTGAGACATATATCAAGTGGCCGTGGTGGGTTTGGTTGGTTATTTTTATTGTTCTCATCTTTGTTGT
GTCATTATTAGTGTTCTGCTGCATTTCCACTGGTTGTTGTGGATGCTGCGGCTGCTGCTGTGCTTGTTTTTCAGGTTGT

TGTAGGGGTCCTAGACTTCAACCTTACGAAGCTTTTGAAAAGGTCCACGTGCAGTGA

>gi|269927204|gb|GU180145.1| Porcine epidemic diarrhea virus isolate KNU-0902 spike protein gene, complete cds

ATGAGGTCTTTAACCTACTTCTGGTTGTTCTTACCAGTACTTTCAACACTCAGCCTACCACAAGATGTCGCCAGG
TGCTCAGCTAACACTAATTTTAGGCGGTTCTTTTCAAAATTTAATGTTCAGGCGCCTGCAGTTGTTGTACTGGGCGGTT
ATCTACCTATTGGTGAAAACCACGGTGTCAATTCAACTTGGCACTGTGCTGGCCAACATCCAACTGCTAGTGGCGTTCA
TGGTATTTTTCTTAGCCATATTAGAGGTGGTCATGGCTTTGAGATTGGCATTTCGCAAGAGCCGTTTGACCCTAGCGGT
TACCAGCTTTATTTACATAAGGCCACTAACGGTAACACTAATGCTACTGCGCGACTGCGCATTTGCCAGTTTCCCAGCA
TTAAAACATTGGGCCCCACTGCTAATAATGATGTTACAACAGGTCGTAACTGCCTATTTAACAAAGCCATCCCAGCTCA
TATGAGTGAACATAGTGTTGTCGGCATAACATGGGATAATGATCGTGTCACTGTCTTTTCTGACAAGATCTATTATTTT
CATTTTAAAAATGATTGGTCCCGTGTTGCGACAAAGTGTTACAACAGTGGAGGTTGTGCTATGCAATATGTTTACGAAC
CCACTTACTACATGCTTAATGTTACTAGTGCTGGTGAGGATGGTATTCCTTACCAACCCTGTACAGCTAATTGCATTGG
TTATGCTGCCAATGTATTTGCTACTGAGCCCAATGGCCACATACCAGAAGGTTTTAGTTTTAATAATTGGTTTCTTTTG
TCCAATGATTCCACTTTGTTGCATGGTAAGATGGTTTCCAACCAACCATTGTTGGTCAATTGTCTTTTGGCCATTCCTA
AGATTTATGGACTAGGCCAATTTTTCTCATTCAATCAAACGATGGATGGCGTTTGTAATGGAGCTGCTGCGCAGCGTGC
ACCAGAGGCTCTGAGGTTTAATATTAATGACACCTCTGTCATTCTTGCTGAAGGCTCAATTGTACTTCATACTGCTTTA
GGAACAAATCTTTCTTTTGTTTGCAGTAATTCCTCAGATCCTCATTTAGCCACCTTCGCCATACCTCTGGGTGCTACCC
AAGTACCCTATTATTGTTTTCTTAAAGTGGACACTTACAACTCCACTGTTTATAAATTCTTGGCTGTTTTACCTCCTAT
CGTCAGGGAAATTGTCATCACCAAGTATGGTGATGTTTATGTCGATGGGTTTGGCTACTTGCATCTCGGTTTGTTGGAT
GCTGTCACAATTAATTTCACTGGTCATGGCACTGACGATGACGTTTCTGGTTTTTGGACCATAGCATCGACTAATTTTG
TTGATGCACTCATCGAAGTTCAAGGAACTGCCATTCAGCGTATTCTTTATTGTGATGATCCTGTTAGCCAACTCAAGTG
TTCTCAGGTTGCTTTTGACCTTGACGATGGTTTTTACCCTATTTCTTCTAGAAACCTTCTGAGTCATGAACAGCCAATT
TCTTTTGTTACTTTGCCATCATTTAATGATCATTCTTTTGTTAACATTACTGTCTCTGCGTCCTTTGGTGGTCATAGTG
GTGCCAACCTTATTGCATCTGACACTACTATCAATGGGTTTAGTTCTTTCTGTGTTGACACTAGACAATTTACCATTAG
ACTGTTTTATAACGTTACAAGCAGTTATGGTTATGTGTCTAACTCGCAGGATAGCAATTGCCCTTTCACCTTGCAATCT
GTTAATGATTACCTGTCTTTTAGCAAATTTTGTGTTTCAACCAGCCTTTTGGCTAGTGCCTGTACCATAGATCTTTTTG
GTTACCCTGATTTTGGTAGTGGTGTTAAGCTCACATCCCTTTACTTTCAATTCACAAAGGGTGAGTTGATTACTGGCAC
GCCTAAACCACTTGAAGGTGTTACGGACGTTTCTTTTTTGACTCTGGATGTGTGTACCAAGTATACTATCTATGGCTTT
AAAGGTGAGGGTATCATTACCCTTACAAATTCTAGCTTTTTGGCAGGTTTTTATTATACATCTGATTCTGGACAGTTGT
TAGCTTTTAAGAATGTCACTAGTGGTGCTGTTTATTCTGTTACGCCATGTTCTTTTTCCGAGCAGGCTGCATATGTTGA
TGATGATATAGTGGGTGTTATTTCTAGTTTGTCTAACTCCACTTTTAACAGTACTAGGGAGTTGCCTGGTTTCTTCTAC
CATTCTAATGACGGCTCTAATTGTACAGAGCCTGTGTTGGTGTATAGTAACATAGGTGTTTGTAAATCTGGCAGTATTG
GCTATGTCCCATCTCAGTCTGGCCAAGCCAAGACTGCACCCACGGTTACTGGGAATATTAGTATTCCCACCAACTTTAG
TATGAGTATTAGGACAGAATATTTACAGCTTTACAACACGCCTGTTAGTGTTGATTGTGCCACATATGTTTGTAATGGT
AACTCTCGTTGTAAACAATTACTCACCCAGTACACTGCAGCATGTAAGACCATAGAGTCAGCATTACAACTCAGCGCTA
GGCTTGAGTCTGTTGAAGTTAACTCTATGCTTACTATTTCTGAAGAGGCTCTACAGTTAGCCACCATCAGTTCGTTTAA
TGGTGATGGATATAACTTTACTAATGTGCTGGGTATTTCCGTGTACGAGCCTGCAAGTGGCAGGGTGGTACACAAAAGG
TCTTTTATTGAAGACCTGCTTTTTAACAAAGTGGTTACTAATGGCCTTGGTACTGTTGATGAAGACTATAAGCGCTGTT
CTAATGGTCGCTCTGTGGCAGATCTAGTCTGTGCACAGTATTACTCTGGTGTCATGGTACTACCTGGTGTTGTTGACGC
TGAGAAGCTTCACATGTATAGTGCGTCTCTCATCGGTGGTATGGCGCTAGGAGGTTTTACCGCTGCAGCGGCATTGCCT
TTTAGCTATGCTGTTCAAGCGAGACTCAATTATCTTGCTTTACAGACGGATGTTCTACAGCGTAACCAGCAATTGCTTG

CTGAGTCTTTTAACTCTGCTATTGGTAATATAACTTCAGCCTTCGAGAGTGTTAAAGAGGCTATTACTCAAACTTCCCA
GGGTTTGAATACTGTGGCTCATGCACTTACTAAGGTTCAAGAGGTTGTTAATTCGCAGGGTGCAGCTTTGTCTCAACTC
ACCATACAGCTGCAACACAACTTCCAAGCCATTTCTAGTTCTATTGATGATATCTACTCCCGACTGGACATTCTTTCAG
CCGATGTTCAGGTTGATCGTCTCATCACCGGCAGATTATCAGCACTTAATGCTTTTGTTGCTCAAACCCTCACTAAGTA
TACTGAGGTTCAGGCTAGCAGGAGGCTAGCGCAGCAAAAGGTTAATGAGTGCGTTAAATCGCAATCTCAGCGTTATGGT
TTTTGTGGTGGTGATGGCGAGCACATTTTCTCTCTGGTACAGGCCGCACCTCAGGGCCTGCTGTTTTTACACACAGTAC
TTGTACCGGGTGATTTTGTAAATGTTACTGCCATCGCTGGCTTATGCGTTAATGATGATATTGCCTTGACTCTGCGTGA
GCCTGGCTTAGTCTTGTTTACACATGAACTTCAAACTCATACTGCGACGGAATATTTTGTTTCATCGCGACGTATGTTT
GAACCTAGAAAACCTACCGTTAGTGATTTTGTTCAAATTGAGAGTTGTGTGGTCACCTATGTCAACCTGACTAGCGACC
AACTACCAGATGTAATCCCAGATTACATCGATGTTAACAAAACGCTTGATGAGATTTTAGCCTCTCTGCCCAATAGAAC
TGATCCAAGTTTTCCACTAGGTGTTTTTAATGCCACTTATCTTAATCTCACTGGTGAAATTGCAGATTTAGAGCAGCGT
TCAGAGTCTCTCCGCAATACCACAGAAGAGCTCCGAAGTCTCATATATAATATCAACAACACACTAGTTGACCTTGAGT
GGCTCAACCGAGTTGAGACGTATATCAAGTGGCCGTGGTGGGTTTGGTTGATTATTTCATTGTTTTCATCTTTGTTGT
GTCATTACTAGTGTTCTGCTGCATTTCCACGGGTTGTTGTGGATGCTGCGGCTGCTGCTGTGCTTGTTTTTCAGGTTGT
TGTAGGGGTCCTAGACTTCAACCTTACGAAGCTTTTGAAAAGGTCCACGTGCAGTGA

>gi|269927206|gb|GU180146.1| Porcine epidemic diarrhea virus isolate KNU-0903 spike protein gene, complete cds

ATGAGGTCTTTAACCTATTTCTGGTTGTTCTTACCAGTACTTCCAACACTCAGCCTACCACAAGATGTCAATAGG
TGCTCAGCTGTGACTAACTTTAGGCAGTTCTTTTCAAAATTTAATGTTCAGGCGCCTGCAGTCGTTGTACTGGGCGGTT
ATCTACCTAGTGGTGAAAAACAGGTTGATGCACCACGTTGGTACTGTGCTGGCCGATATGATACTGCTAGTGGCGTTCA
TGGTATCTTTCTTAGCCATATTAGAGGTGGTCATGGCTTTGAGATTGGCATTTCGCAAGAGCCGTTTGATCCTAGTGGT
TACCAGCTTTATTTACATAAGGCCACCAACGGTAACACTAATTCTACTGCGCGTCTGCGTATTTGCCAGTTTCCCAGCA
ATAAAACATTGGGCCCCACTGCTAATGATGATGTCACAACAGGTCGTAACTGCCTATTTAACAAAGCCATTCCAGCTCA
TATGAGTGAACATAGTGTTGTCGGCATAACATGGGATAATGATCGTGTCACTGTTTTTGCTGACAAGATCTATCATTTT
TATCTTAAAAATGATTGGTCCCGTGTTGCGTCAAAATGTTACAACAGTGGAGGTTGTGCTATGCAATATGTTTACAAAC
CTATTTACTACATGCTTAATGTTACTAGTGCTGGTGAGGATGGTATTTCTTATCAACTCTGTACAGCTAATTGCATTGG
TTATGCTGCCAATGTATTTGCTACTGAACCCAATGGCCACATACCAGAAAGTTTTAGTTTTAATAATTGGTTTCTTTTG
TCCAATGATTCCACTTTGCTCCATGGTAAAGTGGTTTCCAACCAACCTTTGTTGGTCAATTGTCTTTGGGCCATTCCTA
AGATTTATGGACTAGGCCAATTTTTCTCATTCAATCAAACGATGGATGGCGTTTGTAATGGAGCTGCTGCGCAGCGTGC
ACCAGAGGCTCTGAGGTTTAATATTAATGACACCTCTGTCATTCTTGCTGAAGGCTCAATTGTACTTCATACTGCTTTA
GGAACAAATCTTTCTTTTGTTTGCAGTAATTCCTCAGATCCTCATTTAGCCACCTTCGCCATACCTCTGGGTGCTACCC
AAGTACCCTATTATTGTTTTCTTAAAGTGGACACTTACAACTCCACTGTTTATAAATTCTTGGCTGTTTTACCTCCTAT
CGTCAGGGAAATTGTCATCACCAAGTATGGTGATGTTTATGTCGATGGGTTTGGCTACTTGCATCTCGGTTTGTTGGAT
GCTGTCACAATTAATTTCACTGGTCATGGCACTGACGATGACGTTTCTGGTTTTTGGACCATAGCATCGACTAATTTTG
TTGATGCACTCATCGAAGTTCAAGGAACTGCCATTCAGCGTATTCTTTATTGTGATGATCCTGTTAGCCAACTCAAGTG
TTCTCAGGTTGCTTTTGACCTTGACGATGGTTTTTACCCTATTTCTTCTAGAAACCTTCTGAGTCATGAACAGCCAATT
TCTTTTGTTACTTTGCCATCATTTAATGATCATTCTTTTGTTAACATTACTGTCTCTGCGTCCTTTGGTGGTCATAGTG
GTGCCAACCTTATTGCATCTGACACTACTATCAATGGGTTTAGTTCTTTCTGTGTTGACACTAGACAATTTACCATTAG
ACTGTTTTATAACGTTACAAGCAGTTATGGTTATGTGTCTAACTCGCAGGATAGCAATTGCCCTTTCACCTTGCAATCT
GTTAATGATTACCTGTCTTTTAGCAAATTTTGTGTTTCAACCAGCCTTTTGGCTAGTGCCTGTACCATAGATCTTTTTG
GTTACCCTGATTTTGGTGGTGCTGTTAAGTTCACATCCCTTTACTTTCAATTCACAAAGGGTGAGTTGATTACTGGCAC

GCCTAAACCACTTGAAGGTGTTACGGACGTTTCTTTTATGACTCTGTATGTGTGTACCAAGTATACTATCTATGGCTTT
AAAGGTGAGGGTGTCATTACCCCCACAAATTCTAGCTTTTTGGCAGGTATTTATTATACATCTGATTCCGGACAGTTGT
TAGCCTTTAAGAATGTTACTAGTGGTGCTGTTTATTCTGTTACGCCATGTTCTTTTTCAGAGCAGGCTGCTTATGTTGA
TGATGATATAGTGGGTGTTATTTCCAGTTTGTCTAACTCCACTTTTAACAATACTAGGGAGTTGCCTGGTTTCTTCTAC
CATTCTAATGACGGCTCTAATTGTACAGAGCCTGTGTTGGTGTATAGTAACATAGGTGTTTGTAAATCTGGCAGTATTG
GCTATGTTCCATCTCAGTCTGGCCAAGTCAAGATTGCACCCACGGTTACTGGGAATATTAGTATTCCCACCAACTTTAG
TATGAGTATTAGGACAGAATATTTACAGCTTTACAACACGCCTGTTAGTGTTGATTGTGCCAAATATGTTTGTAATGGT
AACTCTCGTTGTAAACAATTACTCACCCAGTACACTGCAGCATGTAAGACCATAGAGTCAGCATTACAACTCAGCGCTA
GGCTTGAGTCTGTTGAAGTTAACTCTATGCTTACTATTTCTGAAGAGGCTCTACAGTTAGCCACCATCAGTTCGTTTAA
TGGTGATGGCTATAACTTTACTAATGTGCTGGGTGTTTCCGTGTACGAGCCTGCAAGTGGCAGGGTGGTACACAAAAGG
TCTTTTATTGAAGACCTGCTTTTTAACAAAGTGGTTACTAATGGCCTTGGTACTGTTGATGAAGACTATAAGCGCTGTT
CTAATGGTCGCTCTGTGGCAGATCTAGTCTGTGCACAGTATTACTCTGGTGTCATGGTACTACCTGGTGTTGTTGACGC
TGAGAAGCTTCACATGTATAGTGCGTCTCTCATCGGTGGTATGGCGCTGGGAGGTTTTACTGCTGCAGCGGCATTGCCT
TTTAGCTATGCTGTTCAAGCGAGACTCAATTATCTTGCTTTACAGACGGATGTTCTACAGCGTAACCAGCAATTGCTTG
CTGAGTCTTTTAACTCTGCTATTGGTAATATAACTTCAGCCTTCGAGAGTGTTAAAGAGGCTATTAGTCAAACTTCCCA
GGGTTTGAATACTGTGGCTCATGCACTTACTAAGGTTCAAGAGGTTGTTAATTCGCAGGGTGCAGCTTTGTCTCAACTC
ACCATACAGCTGCAACACAACTTCCAAGCCATTTCTAGTTCTATTGATGATATCTACTCCCGACTGGACATTCTTTCAG
CCGATGTTCAGGTTGATCGTCTCATCACCGGCAGATTATCAGCACTTAATGCTTTTGTTGCTCAAACCCTCACTAAGTA
TACAGAGGTTCAGGCTAGCAGGAGGCTAGCGCAGCAAAAGGTTAATGAGTGCGTTAAATCGCAATCTCAGCGTTATGGT
TTTTGTGGTGGTGATGGCGAGCACATTTTCTCTCTGGTACAGGCCGCACCTCAGGGCCTGCTGTTCTTACACACAGTAC
TTGTACCGGGTGATTTTGTAAATGTTACTGCCATCGCTGGCTTATGCGTTAATGATGATATTGCCTTGACTCTGCGTGA
GCCTGGCTTAGTCTTGTTTACACATGAACTTCAAACTCATACTGCGACGGAATATTTTGTTTCATCGCGACGTATGTTT
GAACCTAGAAAACCTACCGTTAGTGATTTTGTTCAAATTGAGAGTTGTGTGGTCACCTATGTCAACCTGACTAGCGACC
AACTACCAGATGTAATCCCAGATTACATCGATGTTAACAAAACACTTGATGAGATTTTAGCCTCTCTGCCCAATAGAAC
TGATCTAAGTTTTCCACTAGATGTTTTTAATGCCACTTATCTTAATCTCACTGGTGAAATTGCAGATTTAGAGCAGCGT
TCAGAGTCTCTCCGCAATACCACAGAAGAGCTCCGAAGTCTCATATATAATATCAACAACACACTAGTTGACCTTGAGT
GGCTCAACCGAGTTGAGACGTATATCAAGTGGCCGTGGTGGGTTTGGTTGATTATTTTCATTGTTCTCATCTTTGTTGT
GTCATTACTAGTGTTCTGCTGCATTTCCACGGGTTGTTGTGGATGCTGCGGCTGCTGCTGTGCTTGTTTTTCAGGTTGT
TGTAGGGGTCCTAGACTTCAACCTTACGAAGCTTTTGAAAAGGTCCACGTGCAGTGA

>gi|269927208|gb|GU180147.1| Porcine epidemic diarrhea virus isolate KNU-0904 spike protein gene, complete cds

ATGAGGTCTTTAACCTATTTCTGGTTGTTCTTACCAGTACTTCCAATACTCAGCCTACCACAAGATGTCAATAGG
TGCTCAGCTGTGACTAACTTTAGGCAGTTCTTTTCAAAATTTAATGTTCAGGCGCCTGCAGTCGTTGTACTGGGCGGTT
ATCTACCTAGTGGTGAAAAACAGGTTGATGCACCAAGTTGGTACTGTGCTGGCCGATATGATACTGCTAGTGGCGTTCA
TGGTATCTTTCTTAGCCATATTAGAGGTGGTCATGGCTTTGAGATTGGCATTTCGCAAGAGCCGTTTGATCCTAGTGGT
TACCAGCTTTATTTACATAAGGCCACCAACGGTAACACTAATTCTACTGCGCGCCTGCGTATTTGCCAGTTTCCCAGCA
ACAAAACATTGGGCCCCACTGCTAATGATGATGTCACAACAGGTCGTAACTGCCTATTTAACAAAGCCATTCCAGCTTA
TATGAGTGAACATAGTGTTGTCGGCATAACATGGGATAATGATCGTGTCACTGTTTTTGCTGACAAGATCTATCATTTT
TATCTTAAAAATGATTGGTCCCGTGTTGCGTCAAAATGTTACAACAGTGGAGGTTGTGCTATGCAATATGTTTACAAAC
CTATTTACTACATGCTTAATGTTACTAGTGCTGGTGAGGATGGTATTTCTTATCAACTCTGTACAGCTAATTGCATTGG
TTATGCTGCCAATGTATTTGCTACTGAGCCCAATGGCCACATACCAGAAAGTTTTAGTTTTAATAATTGGTTTCTTTTG

TCCAATGATTCCACTTTGTTTCATGGTAAAGTGGTTTCCAACCAACCTTTGTTGGTCAATTGTCTTTGGGCCATTCCTA
AGATTTACGGACTAGGCCACTTTTTCTCTTTTAATCAAACGATGGATGGCGTTTGTAACGGAGCTACTGCTTATCGTGC
CCCAGAGGCTCTGAGGTTTAATATTAATGACACCTCTGTCATTCTTGCTGAAGGCTCAATTGTACTTCATACTGCTTTA
GGAACAAATCTTTCTTTTGTTTGCAGTAATTCCTCAGATCCTCATTTAGCCACCTTCGCCTTACCTCTGGGTGCTACCC
AAGTACCCTATTATTGTTTTCTTAAAGTGGACACTTACAACTCCACTGTTTATAAATTTTTGGCTGTTTTACCTCCTAC
CGTCAGGGAAATTGTCATCACCAAGTATGGTGATGTTTATGTCAATGGGTTTGGCTACTTGCATCTCGGTTTGTTGGAT
GCTGTCACAATTAATTTCACTGGTCATGGCACTGACGATGACGTTTCTGGTTTTTGGACCATAGCATCGACTAATTTTG
TTGATGCACTCATCGAAGTTCAAGGAACTGCCATTCAGCGTATTCTTTATTGTGATGATCCTGTTAGCCAACTCAAGTG
TTCTCAGGTTGCTTTTGACCTTGACGATGGTTTTTACCCTATTTCTTCTAGAAACCTCCTGAGTCATGAACAGCCAATT
TCTTTTGTTACTTTGCCATCATTTAATGATCATTCTTTTGTTAACATTACTGTCTCTGCGTCCTTTGGTGGTCATAATG
GTGCCAACCTTATTGCATCTGACACTACTATCAATGGGTTTAGTTCTTTCTGTGTTGACACTAGACAATTTACCATTAG
ACTGTTTTATAACGTTACAAGCAGTTACGGTTATGTGTCTAACTCGCAGGATAGCAATTGCCCTTTCACCTTGCGATCT
GTTAATGATTACCTGTCTTTTAGCAAATTTTGTGTTTCAACCAGCCTTTTGGCTGGTGCTTGTACCATAGATCTTTTTG
GTCACCCTGCTTTTGGTAGTAGTGTTAAGTTTACGTCCCTCTATTTTCAATTCACAAAGGGTGATTTGATTACTGGCAC
GCCTAAATCACTTGAAGGTGTCACGGACGTTTCTTTTATGACTATGGATGTGTGTACTAAGTATACTATCTATGGCTTT
AAAGGTGAGGGTATTATTACCCTTACAAATTCTAGCTTTTTGGCAGGTATTTATTATACATCTGATTCCGGACAGTTGT
TAGCCTTTAAGAATGTTACTAGTGGTGCTGTTTATTCTGTTACGCCATGTTCTTTTTCAGAGCAGGCTGCTTATGTTGA
TGATGATATAGTGGGTGTTATTTCCAGTTTGTCTAACTCCACTTTTAACAATACTAGGGAGTTGCCCGGTTTCTTCTAC
CATTCTAATGACGGCTCCAATTGTACAGAGCCTGTGTTGGTGTATAGTAACATAGGTGTTTGTAAATCTGGCAGTATTG
GCCATGTCCCTTCTCAGTCTGGCCCAGTCAAGATTGCACCCACGGTTACTGGGAATATTAGTATTCCCACCAACTTTAG
TATGAGTATTAGGACAGAATATTTACAGCTTTACAACACGCCTGTTAGTGTTGATTGTGCCACATATGTTTGTAATGGT
AACTCTCGTTGCAAACAATTACTCACCCAGTACACTGCAGCATGTAAGACCATAGAGTCAGCATTACAACTCAGCGCTA
GGCTTGAGTCTGTTGAAGTTAACTCTATGCTTACTATTTCTGAAGAGGCTCTACAGTTAGCTACCATCAGTTCGTTTAA
TGGTGATGGATATAATTTTACTAATGTGCTGGGTGTTTCCGTGTATGATCCTGCAAGTGGCAGGGTGGTACAAAAAAGG
TCTTTCATTGAAGACCTGCTTTTTAATAAAGTGGTTACTAATGGTCTTGGTACTGTTGATGAAGACTACAAGCGCTGTT
CTAATGGCCGCTCTGTGGCTGATCTAGTCTGTGCGCAGTATTACTCTGGTGTCATGGTACTACCTGGCGTTGTTGACGC
TGAGAAGCTTCACATGTATAGTGCGTCTCTCATCGGTGGTATGGTGCTAGGAGGTTTTACTGCTGCAGCGGCACTGCCT
TTTAGCTATGCTGTTCAAGCGAGACTCAATTATCTTGCTTTACAGACGGATGTTCTACAGCGTAACCAGCAACTGCTTG
CTGAGCCTTTTAACTCTGCTATTGGTAATATAACTTCAGCCTTTGAGAGTGTTAAAGAGGCTATTAGTCAAACTTCCCA
GGGTTTGAACACTGTGGCTCATGCGCTTACTAAGGTTCAAGAGGTTGTTAATTCGCAGGGTGCAGCTTTGACTCAACTT
ACCGTACAGCTGCAACACAACTTCCAAGCCATTTCTAGTTCTATTGATGACATTTATTCCCGACTTGACATTCTTTCAG
CCGATGTTCAGGTTGACCGTCTCATCACTGGCAGATTATCAGCACTTAACGCTTTTGTTGCTCAAACTCTCACTAAGTA
TACTGAGGTTCAGGCTAGCAGGAAGCTAGCACAGCAAAAGGTTAATGAGTGCGTCAAATCGCAGTCTCAGCGTTATGGT
TTTTGTGGTGGTGATGGCGAACACATTTTTTCCCTGGTACAGGCCGCACCTCAGGGCCTGCTGTTCTTACACACAGTAC
TTGTACCGGGTGATTTTGTAAATGTTATTGCTGTCGCAGGCTTATGCGTTAATGGTGATATTGCCTTGACTCTACGTGA
GCCTGGCTTAGTCCTGTTTACGCATGAACTTCAAACTCATACGGCGGCAGAATATTTTGTTTCATCGCGACGTATGTTT
GAACCTAGAAAACCTACCGTTAGTGATTTTGTTCAAATTGAGAGTTGTGTGGTCACCTATGTCAATCTGACTAGCGACC
AACTACCAGATGTAATCCCAGATTACATCGATGTTAACAAAACACTTGATGAGATTTTGGCCTCTCTGCCCAATAGAAC
TGGTCCAAGTCTTCCACTAGATGTTTTTAATGCTACTTATCTTAATCTCACTGGTGAAATTGCAGATTTACAGCAGCGT
TCAGAGTCTCTTCAAAATATCATAGAAGAGCTCCGAAGTTTCATTTATAGCATCAACAACACACGTGTTGACCTTGAGT
GGCTCAACCGCGTTGAGACATATATCAAGTGGCCGTGGTGGGTTTGGTTGATTCTTTTTATTGTTCTCATCTTTGTTGT
GTCATTACTAGTGTTCTGCTGCATTTCTACGGGTTGCTGTGGATGCTGCGGTTGCTGCGGTGCTTGTTTTTCAGGTTGT
TGTAGGGGTCCTAGACTTCAACCTTACGAAGCTTTTGAAAAGGTCCACGTGCAGTGA

>gi|269927210|gb|GU180148.1| Porcine epidemic diarrhea virus isolate KNU-0905 spike protein gene, complete cds

ATGAGGTCTTTAACTTACTTCTGGTTGTTATTACCAGTACTTCCAATACTCAGCCTACCACAAGATGTCAATAGG
TGCTCAGCTGTGACTAACTTTAGGCAGTTCTTTTCAAAATTTAATGTTCAGGCGCCTGCAGTCGTTGTACTGGGCGGTT
ATCTACCTAGTGGTGAAAAACAGGTTGATGCACCACGTTGGTACTGTGCTGGCCGATATGATACTGCTAGTGGCGTTCA
TGGTATCTTTCTTAGCCATATTAGAGGTGGTCATGGCTTTGAGATTGGCATTTCGCAAGAGCCGTTTGATCCTAGTGGT
TACCAGCTTTATTTACATAAGGCCACCAACGGTAACACTAATTCTACTGCGCGTCTGCGTATTTGCCAGTTTCCCAGCA
ATAAAACATTGGGCCCCACTGCTAATGATGATGTCACAACAGGTCGTAACTGCCTATTTAACAAAGCCATTCCAGCTCA
TATGAGTGAACATAGTGTTGTCGGCATAACATGGGATAATGATCGTGTCACTGTTTTTGCTGACAAGATCTATCATTTT
TATCTTAAAAATGATTGGTCCCGTGTTGCGTCAAAATGTTACAACAGTGGAGGTTGTGCTATGCAATATGTTTACAAAC
CTATTTACTACATGCTTAATGTTACTAGTGCTGGTGAGGATGGTATTTCTTATCAACTCTGTACAGCTAATTGCATTGG
TTATGCTGCCAATGTATTTGCTACTGAGCCCAATGGCCACATACCAGAAAGTTTTAGTTTTAATAATTGGTTTCTTTTG
TCCAATGATTCCACTTTGCTCCATGGTAAAGTGGTTTCCAACCAACCTTTGTTGGTCAATTGTCTTTGGGCCATTCCTA
AGATTTATGGACTAGGCCAATTTTTCTCATTCAATCAAACGATGGATGGCGTTTGTAATGGAGCTGCTGCGCAGCGTGC
ACCAGAGGCTCTGAGGTTTAATATTAATGACACCTCTGTCATTCTTGCTGAAGGCTCAATTGTACTTCATACTGCTTTA
GGAACAAATCTTTCTTTTGTTTGCAGTAATTCCTCAGATCCTCATTTAGCCACCTTCGCCATACCTCTGGGTGCTACCC
AAGTACCCTATTATTGTTTTCTTAAAGTGGACACTTACAACTCCACTGTTTATAAATTCTTGGCTGTTTTACCTCCTAT
CGTCAGGGAAATTGTCATCACCAAGTATGGTGATGTTTATGTCAATGGGTTTGGCTACTTGCATCTCGGTTTGTTGGAT
GCTGTCACAATTAATTTCACTGGTCATGGCACTGACGATGACGTTTCTGGTTTTTGGACCATAGCATCGACTAATTTTG
TTGAAGCACTCATCGAAGTTCAAGGAACTGCCATTCAGCGTATTCTTTATTGTGATGATCCTGTTAGCCAACTCAAGTG
TTCTCAGGTTGCTTTTGACCTTGACGATGGTTTTTACCCTATTTCTTCTAGAAACCTTCTGAGTCATGAACAGCCAATT
TCTTTTGTTACTTTGCCATCATTTAATGATCATTCTTTTGTTAACATTACTGTCTCTGCGTCCTTTGGTGGTCATAGTG
GTGCCAACCTTATTGCATCTGACACTACTATCAATGGGTTTAGTTCTTTCTGTGTTGACACTAGACAATTTACCATTAG
ACTGTTTTATAACGTTACAAGCAGTTATGGTTATATGTCTAACTCGCAAGATAGCAATTGCCCTTTCACCTTGCAATCT
GTTAATGATTACCTGTCTTTTAGCAAATTTTGTGTTTCAACCAGCCTTTTGGCTAGTGCCTGTACCATAGATCTTTTTG
GTTACCCTGATTTTGGTGGTGCTGTTAAGTTCACATCCCTTTACTTTCAATTCACAAAGGGTGAGTTGATTACTGGCAC
GCCTAAACCACTTGAAGGTGTTACGGACGTTTCTTTTATGACTCTGTATGTGTGTACCAAGTATACTATCTATGGCTTT
AAAGGTGAGGGTGTCATTACCCCCACAAATTCTAGCTTTTTGGCAGGTATTTATTATACATCTGATTCCGGACAGTTGT
TAGCCTTTAAGAATGTTACTAGTGGTGCTGTTTATTCTGTTACGCCATGTTCTTTTTCAGAGCAGGCTGCTTATGTTGA
TGATGATATAGTGGGTGTTATTTCCAGTTTGTCTAACTCCACTTTTAACAATACTAGGGAGTTGCCTGGTTTCTTCTAC
CATTCTAATGACGGCTCTAATTGTACAGAGCCTGTGTTGGTGTATAGTAACATAGGTGTTTGTAAATCTGGCAGTATTG
GCTATGTTCCATCTCAGTCTGGCCAAGTCAAGATTGCACCCACGGTTACTGGGAATATTAGTATTCCCACCAACTTTAG
TATGAGTATTAGGACAGAATATTTACAGCTTTACAACACGCCTGTTAGTGTTGATTGTGCCAAATATGTTTGTAATGGT
AACTCTCGTTGTAAACAATTACTCACCCAGTACACTGCAGCATGTAAGACCATAGAGTCAGCATTACAACTCAGCGCTA
GGCTTGAGTCTGTTGAAGTTAACTCTATGCTTACTATTTCTGAAGAGGCTCTACAGTTAGCCACCATCAGTTCGTTTAA
TGGTGATGGCTATAACTTTACTAATGTGCTGGGTGTTTCCGTGTACGAGCCTGCAAGTGGCAGGGTGGTACACAAAAGG
TCTTTTATTGAAGACCTGCTTTTTAACAAAGTGGTTACTAATGGCCTTGGTACTGTTGATGAAGACTATAAGCGCTGTT
CTAATGGTCGCTCTGTGGCAGATCTAGTCTGTGCACAGTATTACTCTGGTGTCATGGTACTACCTGGTGTTGTTGACGC
TGAGAAGCTTCACATGTATAGTGCGTCTCTCATCGGTGGTATGGCGCTGGGAGGTTTTACTGCTGCAGCGGCATTGCCT
TTTAGCTATGCTGTTCAAGCGAGACTCAATTATCTTGCTTTACAGACGGATGTTCTACAGCGTAACCAGCAATTGCTTG
CTGAGTCTTTTAACTCTGCTATTGGTAATATAACTTCAGCCTTCGAGAGTGTTAAAGAGGCTATTAGTCAAACTTCCCA
GGGTTTGAATACTGTGGCTCATGCACTTACTAAGGTTCAAGAGGTTGTTAATTCGCAGGGTGCAGCTTTGTCTCAACTC

ACCATACAGCTGCAACACAACTTCCAAGCCATTTCTAGTTCTATTGATGATATCTACTCCCGACTGGACATTCTTTCAG
CCGATGTTCAGGTTGATCGTCTCATCACCGGCAGATTATCAGCACTTAATGCTTTTGTTGCTCAAACCCTCACTAAGTA
TACAGAGGTTCAGGCTAGCAGGAGGCTAGCGCAGCAAAAGGTTAATGAGTGCGTTAAATCGCAATCTCAGCGTTATGGT
TTTTGTGGTGGTGATGGCGAGCACATTTTCTCTCTGGTACAGGCCGCACCTCAGGGCCTGCTGTTCTTACACACAGTAC
TCGTACCGGGTGATTTTGTAAATGTTACTGCCATCGCTGGCTTATGCGTTAATGATGATATTGCCTTGACTCTGCGTGA
GCCTGGCTTAGTCTTGTTTACACATGAACTTCAAACTCATACTGCGACGGAATATTTTGTTTCATCGCGACGTATGTTT
GAACCTAGAAAACCTACCGTTAGTGATTTTGTTCAAATTGAGAGTTGTGTGGTCACCTATGTCAACCTGACTAGCGACC
AACTACCAGATGTAATCCCAGATTACATCGATGTTAACAAAACACTTGATGAGATTTTAGCCTCTCTGCCCAATAGAAC
TGATCTAAGTTTTCCACTAGATGTTTTTAATGCCACTTATCTTAATCTCACTGGTGAAATTGCAGATTTAGAGCAGCGT
TCAGAGTCTCTCCGCAATACCACAGAAGAGCTCCGAAGTCTCATATATAATATCAACAACACACTAGTTGACCTTGAGT
GGCTCAACCGAGTTGAGACGTATATCAAGTGGCCGTGGTGGGTTTGGTTGATTATTTCATTGTTCTCATCTTTGTTGT
GTCATTACTAGTGTTCTGCTGCATTTCCACGGGTTGTTGTGGATGCTGCGGCTGCTGCTGTGCTTGTTTTTCAGGTTGT
TGTAGGGGTCCTAGACTTCAACCTTACGAAGCTTTTGAAAAGGTCCACGTGCAGTGA

>gi|356892495|gb|JN184634.1| Porcine epidemic diarrhea virus isolate CNU-091222-01 spike protein gene, complete cds

ATGAAGTCTTTAACCTACTTCTGGTTGTTCTTACCAGCACTTTCAACACTTAGCCTACCACAAGATGTCGCCAGG
TGCTCAGCTAACACTAATTTTAGGCGGTTCTTTTCAAAATTTAATGTTCATGCGCCTGCAGTTGTTGTACTGGGCGGTT
ATCTACCTACTGGTGAAAACCACGGTGTCAATTCAACTTGGTACTGTGCTGGCCAACATCCAACTGCTAGTGGCGTTCA
TGGTATCTTTCTTAGCCATATTAGAGGTGGTCATGGCTTTGAGATTGGCATTTCGCAAGAGCCGTTTGACCCTAGTGGT
TACCAGCTTTATTTACATAAGGCCACTAACGGTAACACTAATGCTACTGCGCGACTGCGCATTTGCCAGTTTCCCAGCA
GTAAAACATTGGGCCCTACTGCTAATAATGATGTTACAACAGGTCGTAACTGCCTATTTAACAAAGCCATCCCAGCTCA
TATGAGTGAACATAGTGTTGTCGGCATAACATGGGATAATGATCGTGTCACTGTCTTTTCTGACAAGATCTATTATTTT
CATTTTAAAAATGATTGGTCCCGTGTTGCGACAAAGTGTTACAACAGTGGAGGTTGTGCTATGCAATATGTTTACGAAC
CCACTTACTACATGCTTAATGTTACTAGTGCTGGTGAGGATGGTATTTCTTACCAACCCTGTACAGCTAATTGCATTGG
TTATGCTGCCAATGTATTTGCTACTGAGCCCAATGGCCACATACCAGAAGGTTTTAGTTTTAATAATTGGTTTCTTTTG
TCCAATGATTCCACTTTGTTGCATGGTAAGGTGGTTTCCAACCAACCATTGTTGGTCAATTGTCTTTTGGCCATTCCTA
AGATTTATGGACTAGGCCAATTTTTCTCCTTCAATCATACGATCGATGGTGTTTGTAATGGAGCTGCTGTGCAGCGTGC
ACCAGAGGCTCTGAGGTTTAATATTAATGACACCTCTGTCATTCTCGCTGAAGGCTCAATTGTACTTCATACTGCTTTA
GGAACAAATTTTTCTTTTGTTTGCAGTAATTCCTCAGATCCTGATTTAGCCACCTTCGCCATACCTTTGGGTGCTACCC
AAGTACCCTATTATTGTTTTCTTAAAGTGGATACTTACAACTCCACTGTTTATAAATTCTTGGCTGTTTTACCTCCTAC
CGTCAGGGAAATTGTCATCACCAAGTATGGTGATGTTTATGTCAATGGGTTTGGCTACTTGCATCTCGGTTTGTTGGAT
GCTGTCACAATTAATTTCACTGGTCATGGCACTGACGATGACGTTTCTGGTTTTTGGACCATAGCATCGACTAATTTTG
TTGATGCACTCGTCGAAGTTCAAGGAACTTCCATTCAGCGTATTCTTTATTGTGATGATCCTGTTAGCCAACTCAAGTG
TTCTCAGGTTGCTTTTGACCTTGAGGATGGTGTTTATCCTATTTCTCCTAGAAACCTTCTGAGTCATGAACAGCCAATT
TCTTTTGTTACTTTGCCATCATTTAATGATCATTCTTTTGTTAACATTACTGTCTCTGCGTCCTTTGGTGGTCATAGTG
GTGCCAACCTTATCGCATCTGACACTACTATCAATGGGTTTAGTTCTTTCTGTGTTGACACTAGACAATTTACCGTTTC
ACTGTTTTATAACGTTACAAACAGTTATGGTTATGTGTCTAAATCACAGGACAGTAATTGCCCTTTCACCTTGCAATCT
GTTAATGATTACCTGTCTTTTAGCAAATTTTGTGTTTCTACCAGCCTTTTGGCTAGTGCCTGTACCATAGATCTTTTTG
GTCACCCTGATTTTGGTAGTGATGTTAAGTTCACGTCCCTTTACTTTCAATTCACAAAGGGTGAGTTGATTACTGGCAC
GCCTAAACCACTTGAAGGTGTTACGGACGTTTCTTTTATGACTCTGGATGTGTGTACCAAGTATACTATCTATGGCTTT
AAAGGTGAGGGTATCATTACCCTTACAAATTCTAGCTTTTTGGCAGGTGTTTATTATACATCTGATTCTGGACAGTTGT

TAGCTTTTAAGAATGTCACTAGTGGTGCTGTTTATTCTGTTACGCCATGTTCTTTTTCAGAGCAGGCTGTATATGTTGA
TGATGATATAGTGGGTGCTATTTCTAGTTCATCTAACTCCACTTTTAACAGTACTAGGGAGTTGCCTGGTTTCTTCTAC
CATTCTAATGATGGCTCTAATTGTACAGAGCCTGTGTTGGTGTATAGTAACATAGGTGTTTGTAAATCTGGCAGTATTG
GCTATGTCCCATCTCAGTCTGGCCAAGTCAAGATTGCACCCACGGTTACTGGGAATATTAGTATTCCCACCAACTTTAG
TATGAGTATTAGGACAGAATATTTACAGCTTTACAACACGCCTGTTAGTGTTGATTGTGCCACATATGTTTGTAATGGT
AACTCTCGTTGTAAACAATTACTCACCCAGTACACTGCAGCATGTAAGACCATAGAGTCAGCTTTACAACTCAGCGCTA
GGCTTGAGTCTGTTGAAGTTAACTCTATGCTTACTATTTCTGAAGAGGCTCTACAGTTAGCCACCATCAGTTCGTTTAA
TGGTGATGGATATAACTTTACTAATGTGCTGGGTGTTTCCGTGTACGAGCCTGCAAGTGGCAGGGTGGTACACAAAAGG
TCTTTTATTGAAGACCTGCTTTTTAATAAAGTGGTTACTAATGGCCTTGGTACTGTTGATGAAGACTATAAGCGCTGTT
CTAATGGTCGCTCTGTGGCAGATCTAGTCTGTGCACAGTATTACTCTGGTGTCATGGTACTACCTGGTGTTGTTGACGC
TGAGAAGCTTCACATGTATAGTGCGTCTCTCATCGGTGGTATGGCGTTAGGAGGTTTTACTGCTGCAGCGGCATTGCCT
TTTAGCTATGCTGTTCAAGCGAGACTCAATTATCTTGCTTTACAGACGGATGTTCTACAGCGGAACCAGCAATTGACTG
CTGAGTCTTTTAACTCTGCTATTGGTAATATAACTTCAGCCTTCGAGAGTGTTAAAGAGGCTATTAGTCAAACTTCTCA
GGGTTTGAATACTGTGGCTCATGCACTTACTAAGGTTCAAGAGGTTGTTAATTCGCAGGGTGCAGCTTTGTCTCAACTC
ACCATACAGCTGCAACACAACTTCCAAGCCATTTCTAGTTCTATTGATGATATCTACTCCCGACTGGACATTCTTTCAG
CCGATGTTCAGGTTGATCGTCTCATCACCGGCAGATTATCAGCACTTAATGCTTTTGTTGCTCAAACCCTCACTAAGTA
TACAGAGGTTCAGGCTAGCAGGAGGCTAGCGCAGCAAAAGGTTAATGAGTGCGTTAAATCGCAATCTCAGCGTTATGGT
TTTTGTGGTGGTGATGGCGAGCACATTTTCTCTCTGGTACAGGCCGCACCTCAGGGCCTGCTGTTCTTACACACAGTAC
TTGTACCGGGTGATTTTGTAAATGTTACTGCCATCGCTGGCTTATGCGTTAATGATGATATTGCCTTGACTCTGCGTGA
GCCTGGCTTAGTCTTGTTTACACATGAACTTCAAACTCACACTGCGACGGAATATTTTGTTTCATCGCGACGTATGTTT
GAACCTAGAAAACCTACCGTTAGTGATTTTGTTCAAATTGAGAGTTGTGTGGTCACCTATGTCAACCTGACTAGCGACC
AACTACCAGATGTAATCCCAGATTACATCGATGTTAACAAAACACTTGATGAGATTTTAGCCTCTCTGCCCAATAGAAC
TGATCTAAGTTTTCCACTAGATGTTTTTAATGCCACTTATCTTAATCTCACTGGTGAAATTGCAGATTTAGAGCAGCGC
TCAGAGTCTCTCCGCAATACCACAGAAGAGCTCCGAAGTCTCATATATAATATCAACAACACACTAGTTGACCTTGAGT
GGCTCAACCGAGTTGAGACGTATATCAAGTGGCCGTGGTGGGTTTGGTTGATTATTTTCATTGTTCTCATCTTTGTCGT
GTCATTATTAGTGTTCTGCTGCATTTCCACGGGTTGTTGTGGATGCTGCGGCTGCTGCTGTGCTTGTTTTTCAGGTTGT
TGTAGGGGTCCTAGACTTCAACCTTACGAAGCTTTTGAAAAGGTCCACGTGCAGTGA

>gi|353331776|gb|JN315706.1|_Porcine_epidemic_diarrhea_virus_strain_CH/FJND2/2011_spike_protein_(S)_gene_complete_cds

ATGACGCCTTTAATTTACTTCTGGTTGTTCTTACCAGTACTTCTAACACTTAGCCTACCACAAGATGTCACTAGG
TGCCAGTCTACTATTAACTTTAGGCGGTTCTTTTCAAAATTTAATGTTCAGGCACCTGCCGTCGTTGTTTTGGGTGGTT
ATCTACCTAGTATGAACTCTTCTAGCTGGTACTGTGGCACAGGCATTGAAACTGATAGTGGCGTTCATGGTATTTTTCT
CAGTTACATCGATTCTGGTCAGGGCTTTGAGATTGGCATTTCGCAAGAGCCGTTTGATCCTAGTGGTTACCAGCTTTAT
TTACACAAGGCCACTAATGGTAACACTAGTGCTATTGCACGACTGCGCATTTGCCAGTTTCCAGATAATAAAACATTGG
GCCCTACTGTTAATGATGTTACAACAGGTCGTAACTGCCTATTTAACAAAGCCATCCCAGCTCATATGAGTGAACATAG
TGTTGTCGGCATAACATGGGATAATGATCGTGTCACTGTCTTTTCTGACAAGATCTACCATTTTTATTTTAAAAATGAT
TGGTCCCGTGTTGCGACAAAGTGTTACAACAGTGGAGGTTGTGCTATGCAATATGTTTACGAACCCACTTACTACATGC
TTAATGTTACTAGTGCTGGTGAGGATGGTATTTCTTATCAACCCTGTACGGCTAATTGCATTGGTTATGCTGCCAATGT
ATTTGCCACTGAGCCCAATGGCCACATACCAGAAGGTTTTAGTTTTAATAATTGGTTTCTTTTGTCCAATGATTCCACT
TTGGTGCATGGTAAGGTGGTTTCCAACCAACCATTGTTGGTCAATTGTCTTTTGGCCATGCCTAAGATTTATGGACTAG
GCCAATTTTTCTCCTTCAATCAAACGATGGATGGCGTTTGTAATGGAGCTGCTGTGCAGCGTGCACCAGAGGCTCTGAG

GTTTAATATTAATGACACCTCTGTCATTCTTGCTGAAGGCTCAATTGTACTTCATACTGCTTTTGGAACAAATCTTTCT
TTTGTTTGCAGTAATTCCTCAAATCCTCATTTAGCCACCTTCGCCATACCTTTGGGTGCTACCCAAGTACCTTATTATT
GTTTTCTTAAAGTGGATACTTACAACTCCACTGTTTATAAATTCTTGGCTGTTTTACCTCCCACCGTCAGGGAAATTGT
CATCACCAAGTATGGTGATGTTTATGTCAATGGGTTTGGCTACTTGCATCTCGGTTTGTTGGATGCTGTCACAATTAAT
TTCACTGGTCATGGCACTGACGATGACGTTTCTGGTTTTTGGACCATAGCATCGACTAATTTTGTTGATGCACTCATCG
AAGTTCAAGGAACTGCCATTCAGCGTATTCTTTATTGTGATGATCCTGTTAGCCAACTCAAGTGTTCTCAGGTTGCTTT
TGACCTTGACGATGGTTTTTACCCTATTTCTTCTAGGAACCTTTTGAGTCATGAACAGCCAATTTCTTTTGTTACTCTG
CCATCATTTAATGATCATTCTTTTGTTAACATTACTGTCTCTGCGTCTTTTGGTGGTCCTAGTGGTGCCAACCTTATTG
CATCTGACACTACTATCAATGGGTTTAGTTCTTTCTGTGTTGACACTAGACAATTTACCATTTCACTGTTTTATAACGT
TACAAACAGTTATGGTTATGTGTCTAAATCACAGGACAGTAATTGCCCTTTTACCTTGCAATCTGTTAATGATTACCTG
TCTTTTAGCAAATTTTGTGTTTCTACCAGCCTTTTGGCTAGTGCCTGTACCATAGATCTTTTTGGTTACCCTGAGTTTG
GTGGTGGTGTTAAGTTTACGTCCCTTTACTTTCAATTCACAAAGGGTGAGTTGATTACTGGCACGCCTAAACCACTTGA
AGGTGTTACGGACGTTTCTTTTATGACTCTGGATGTGTGTACCAAGTATACTATCTATGGCTTTAGAGGTGAGGGTATC
ATTACCCTTACAAATTCTAGCTTTTTGGCAGGTGTTTATTACACATCTGATTCTGGACAGTTGTTAGCTTTTAAGAATG
TCACTAGTGGTGCTGTTTATTCTGTTACGCCATGTTCTTTTTCAGAGCAGGCTGCATATGTTGATGATGATATAGTGGG
TGTTATTTCTAGTTTGTCTAACTCCACTTTTAACAGTACTAGGGAGTTGCCTGGTTTCTTCTACCATTCTAATGATGGC
TCTAATTGTACAGAACCTGTGTTGGTGTATAGTAACATAGGTGTTTGTAAATCTGGCAGTATTGGCTACGTCCCATCTC
AGTCTGGCCAAGTCAAGATTGCACCCACGGTTACTGGGAATATTAGTATTCCCACCAACTTTAGTATGAGTATTAGGAC
AGAATATTTACAGCTTTACAACACGCCTGTTAGTGTTGATTGTGCTACATATGTTTGTAATGGTAACTCTCGTTGTAAA
CAATTACTCACCCAGTACACTGCAGCATGTAAGACCATAGAGTCAGCATTACAACTCAGCGCTAGGCTTGAGTCTGTTG
AAGTTAACTCTATGCTTACTATTTCTGAAGAGGCTCTACAGTTAGCTACCATCAGTTCGTTTAATGGTGATGGATATAA
CTTTACTAATGTGCTGGGTGTTTCCGTGTACGACCCTGCAAGTGGCAGGGTGGTACAAAAAGGGTCTTTTATTGAAGAC
CTGCTTTTTAATAAAGTGGTTACTAATGGCCTTAGTACTGTTGATGAAGACTATAAGCGCTGTTCTAATGGTCGCTCTG
TGGCAGATCTAGTCTGTGCGCAGTATTACTCTGGTGTCATGGTACTACCTGGCGTTGTTGACGCTGAGAAGCTTCACAT
GTATAGTGCGTCTCTCATCGGTGGTATGGCGCTAGGAGGTCTTACTACTGCAGCGGCATTGCCTTTTAGCCATGCTGTT
CAAGCGAGGCTCAATTATCTTGCTTTACAGACGGATGTTCTACAGCGCAACCAGCAATTGCTTGCTGAGTCTTTTAACT
CTGCTATTGGTAATATAACTTCAGCCTTTGAGAGTGTTAAAGAGGCTATTAGTCAAACTTCCAATGGTTTGAACACTGT
GGCTCATGCGCTTACTAAGGTTCAAGAGGTTGTTAATTCGCAGGGTTCAGCTTTGACCCAACTTACCATACAGCTGCAA
CACAACTTCCAAGCCATTTCTAGTTCTATTGATGACATTTACTCCCGACTGGACATTCTTTCAGCCGATGTTCAGGTTG
ATCGTCTCATCACCGGCAGATTATCAGCACTTAATGCTTTTGTTGCTCAAACCCTCACTAAGTATACTGAGGTTCAGGC
TAGCAGGAAGCTAGCACAGCAAAAGGTTAATGAGTGCGTCAAATCGCAATCTCAGCGTTATGGTTTTTGTGGTGGTGAT
GGCGAGCACATCTTCTCTCTGGTACAGGCCGCACCTCAGGGCCTGCTGTTCTTACATACAGTACTTGTACCGGGTGATT
TTGTAAATGTTATTGCCATCGATGGCTTATGCATTAATGGTGATATTGCCTTGACTCTACGTGAGCCTGGCTTAGTCTT
GTTTACGCATGAACTTCAAACTTATACTGCGACGGAATATCTTGTTTCATCGCGGCGTATGTTTGAACCTAGAAAACCT
ACCGTTAGTGATTTTGTTCAAATTGAGAGTTGTGTGGTCACCTATGTCAATTTGACTAGAGACCAACTACCAGATGTAA
TCCCAGATTACATCGATGTTAACAAAACACTTGATGAGATTTTAGCTTCTCTGCCCAATAGAACTGGTCCAAGTCTTCC
TTTAGATGTTTTTAATGCCACTTATCTTAATCTCACTGGTGAAATTGCAGATTTAGAGCAGCGTTCAGAGTCTCTCCGT
AATACTACAGAAGAGCTCCGAAGTCTCATATATAATATCAACAACACACTTGTTGACCTTGAGTGGCTCAACCGAGTTG
AGACATATATCAAGTGGCCGTGGTGGGTTTGGTTGATTATTTTTATTGTTCTCATCTTTGTTGTGTCATTATTAGTGTT
CTGCTGCATTTCCACGGGTTGTTGTGGATGCTGCGGTTGTTGCGGTGCTTGTTTTTCAGGTTGTTGTAGGGGTCCTAGA
CTTCAACCTTACGAAGCTTTTGAAAAGGTCCACGTGCAGTGA

>gi|353331778|gb|JN381492.1|_Porcine_epidemic_diarrhea_virus_strain_CH/FJND

3/2011_spike_protein_(S)_gene_complete_cds

ATGAAGTCTTTAACCTACTTCTGGTTGTTCTTACCAGTACTTTCAACACTTAGCCTACCACAAGATGTCACCAGG
TGCTCAGCTAACACTAATTTTAGGCGGTTCTTTTCAAAATTTAATGTTCAGGCGCCTGCAGTTGTTGTACTGGGCGGTT
ATCTACCTATTGGTGAAAACCAGGGTGTCAATTCAACTTGGTACTGTGCTGGCCAACATCCAACTGCTAGTGGCGTTCA
TGGTATCTTTCTTAGCCATATTAGAGGTGGTCATGGCTTTGAGATTGGCATTTCGCAAGAGCCTTTTGACCCTAGTGGT
TACCAGCTTTATTTACATAAGGCTACTAATGGTAACACTAATGCTACTGCGCGATTGCGCATTTGCCAGTTTCCCAGCA
TCAAAACATTGGGCCCCACTGCTAATAATGATGTTACAACAGGTCGTAACTGCCTATTTAACAAAGCCATCCCAGCTCA
TATGAGTGAACATAGTGTTGTCGGCATAACATGGGATAATGATCGTGTCACTGTCTTTTCTGACAAGATCTACCATTTT
TATTTTAAAAATGATTGGTCCCGTGTTGCGACAAAGTGTTACAACAGTGGAGGTTGTGCTATGCAATATGTTTACGAAC
CCACTTACTACATGCTTAATGTTACTAGTGCTGGTGAGGATGGTATTTCTTATCAACCCTGTACGGCTAATTGCATTGG
TTATGCTGCCAATGTATTTGCCACTGAGCCCAATGGCCACATACCAGAAGGTTTTAGTTTTAATAATTGGTTTCTTTTG
TCCAATGATTCCACTTTGGTGCATGGTAAGGTGGTTTCCAACCAACCATTGTTGGTCAATTGTCTTTTGGCCATGCCTA
AGATTTATGGACTAGGCCAATTTTTCTCCTTCAATCAAACGATGGATGGCGTTTGTAATGGAGCTGCTGTGCAGCGTGC
ACCAGAGGCTCTGAGGTTTAATATTAATGACACCTCTGTCATTCTTGCTGAAGGCTCAATTGTACTTCATACTGCTTTT
GGAACAAATCTTTCTTTTGTTTGCAGTAATTCCTCAAATCCTCATTTAGCCACCTTCGCCATACCTTTGGGTGCTACCC
AAGTACCTTATTATTGTTTTCTTAAAGTGGATACTTACAACTCCACTGTTTATAAATTCTTGGCTGTTTTACCTCCCAC
CGTCAGGGAAATTGTCATCACCAAGTATGGTGATGTTTATGTCAATGGGTTTGGCTACTTGCATCTCGGTTTGTTGGAT
GCTGTCACAATTAATTTCACTGGTCATGGCACTGACGATGACGTTTCTGGTTTTTGGACCATAGCATCGACTAATTTTG
TTGATGCACTCATCGAAGTTCAAGGAACTGCCATTCAGCGTATTCTTTATTGTGATGATCCTGTTAGCCAACTCAAGTG
TTCTCAGGTTGCTTTTGACCTTGACGATGGTTTTTACCCTATTTCTTCTAGGAACCTTTTGGGTCATGAACAGCCAATT
TCTTTTGTTACTCTGCCATCATTTAATGATCATTCTTTTGTTAACATTACTGTCTCTGCGTCTTTTGGTGGTCCTAGTG
GTGCCAACCTTATTGCATCTGACACTACTATCAATGGGTTTAGGTCTTTCTGTGTTGACACTAGACAATTTACCATTTC
ACTGTTTTATAACGTTACAAACAGTTATGGTTATGTGTCTAAATCACAGGACGGTAATTGCCCTTTTACCTTGCAATCT
GTTAATGATTACCTGTCTTTTAGCAAATTTTGTGTTTCTACCAGCCTTTTGGCTAGTGCCTGTACCATAGATCTTTTTG
GTTACCCTGAGTTTGGTGGTGGTGTTAAGTTTACGTCCCTTTACTTTCAATTCACAAAGGGTGAGTTGATTACTGGCAC
GCCTAAACCACTTGAAGGTGTTACGGACGTTTCTTTTATGACTCTGGATGTGTGTGCCAAGTATACTATCTATGGCTTT
AGAGGTGAGGGTATCATTACCCTTACAAATTCTAGCTTTTTGGCAGGTGTTTATTACACATCTGATTCTGGACAGTTGT
TAGCTTTTAAGAATGTCACTAGTGGTGCTGTTTATTCTGTTACGCCATGTTCTTTTTCAGAGCAGGCTGCATATGTTGA
TGATGATATAGTGGGTGTTATTTCTAGTTTGTCTAACTCCACTTTTAACAGTACTAGGGAGTTGCCTGGTTTCTTCTAC
CATTCTAATGATGGCTCTAATTGTACAGAACCTGTGTTGGTGTATAGTAACATAGGTGTTTGTAAATCTGGCAGTATTG
GCTACGTCCCATCTCAGTCTGGCCAAGTCAAGATTGCACCCACGGTTACTGGGAATATTAGTATTCCCACCAACTTTAG
TATGAGTATTAGGACAGAATATTTACAGCTTTACAACACGCCTGTTAGTGTTGATTGTGCTACATATGTTTGTAATGGT
AACTCTCGTTGTAAACAATTACTCACCCAGTACACTGCAGCATGTAAGACCATAGAGTCAGCATTACAACTCAGCGCTA
GGCTTGAGTCTGTTGAAGTTAACTCTATGCTTACTATTTCTGAAGAGGCTCTACAGTTAGCTACCATCAGTTCGTTTAA
TGGTGATGGATATAACTTTACTAATGTGCTGGGTGTTTCCGTGTACGACCCTGCAAGTGGCAGGGTGGTACAAAAAGGG
TCTTTTATTGAAGACCTGCTTTTTAATAAAGTGGTTACTAATGGCCTTAGTACTGTTGATGAAGACTATAAGCGCTGTT
CTAATGGTCGCTCTGTGGCAGATCTAGTCTGTGCGCAGTATTACTCTGGTGTCATGGTACTACCTGGCGTTGTTGACGC
TGAGAAGCTTCACATGTATAGTGCGTCTCTCATCGGTGGTATGGCGCTAGGAGGTCTTACTACTGCAGCGGCATTGCCT
TTTAGCCATGCTGTTCAAGCGAGGCTCAATTATCTTGCTTTACAGACGGATGTTCTACAGCGCAACCAGCAATTGCTTG
CTGAGTCTTTTAACTCTGCTATTGGTAATATAACTTCAGCCTTTGAGAGTGTTAAAGAGGCTATTAGTCAAACTTCCAA
TGGTTTGAACACTGTGGCTCATGCGCTTACTAAGGTTCAAGAGGTTGTTAATTCGCAGGGTTCAGCTTTGACCCAACTT
ACCATACAGCTGCAACACAACTTCCAAGCCATTTCTAGTTCTATTGATGACATTTACTCCCGACTGGACATTCTTTCAG
CCGATGTTCAGGTTGATCGTCTCATCACCGGCAGATTATCAGCACTTAATGCTTTTGTTGCTCAAACCCTCACTAAGTA

TACTGAGGTTCAGGCTAGCAGGAAGCTAGCACAGCAAAAGGTTAATGAGTGCGTCAAATCGCAATCTCAGCGTTATGGT
TTTTGTGGTGGTGATGGCGAGCACATCTTCTCTCTGGTACAGGCCGCACCTCAGGGCCTGCTGTTCTTACATACAGTAC
TTGTACCGGGTGATTTTGTAAATGTTATTGCCATCGATGGCTTATGCATTAATGGTGATATTGCCTTGACTCTACGTGA
GCCTGGCTTAGTCTTGTTTACGCATGAACTTCAAACTTATACTGCGACGGAATATCTTGTTTCATCGCGGCGTATGTTT
GAACCTAGAAAACCTACCGTTAGTGATTTTGTTCAAATTGAGAGTTGTGTGGTCACCTATGTCAATTTGACTAGAGACC
AACTACCAGATGTAATCCCAGATTACATCGATGTTAACAAAACACTTGATGAGATTTTAGCTTCTCTGCCCAATAGAAC
TGGTCCAAGTCTTCCTTTAGATGTTTTTAATGCCACTTATCTTAATCTCACTGGTGAAATTGCAGATTTAGAGCAGCGT
TCAGAGTCTCTCCGTAATACTACAGAAGAGCTCCGAAGTCTCATATATAATATCAACAACACACTTGTTGACCTTGAGT
GGCTCAACCGAGTTGAGACATATATCAAGTGGCCGTGGTGGGTTTGGTTGATTATTTTTATTGTTCTCATCTTTGTTGT
GTCATTATTAGTGTTCTGCTGCATTTCCACGGGTTGTTGTGGATGCTGCGGTTGTTGCGGTGCTTGTTTTTCAGGTTGT
TGTAGGGGTCCTAGACTTCAACCTTACGAAGCTTTTGAAAAGGTCCACGTGCAGTGA

>gi|353529425|gb|JN543367.1|_Porcine_epidemic_diarrhea_virus_strain_CH/FJND1/2011_spike_protein_(S)_gene_complete_cds

ATGACGCCTTTAATTTACTTCTGGTTGTTCTTACCAGTACTTCTAACACTTAGCCTACCACAAGATGTCACTAGG
TGCCAGTCTACTATTAACTTTAGGCGGTTCTTTTCAAAATTTAATGTTCAGGCACCTGCCGTCGTTGTTTTGGGTGGTT
ATCTACCTAGTATGAACTCTTCTAGCTGGTACTGTGGCACAGGCATTGAAACTGATAGTGGCGTTCATGGTATTTTTCT
CAGTTACATCGATTCTGGTCAGGGCTTTGAGATTGGCATTTCGCAAGAGCCGTTTGATCCTAGTGGTTACCAGCTTTAT
TTACACAAGGCCACTAATGGTAACACTAGTGCTATTGCACGACTGCGCATTTGCCAGTTTCCAGATAATAAAACATTGG
GCCCTACTGTTAATGATGTTACAACAGGTCGTAACTGCCTATCCAACAAAGCCATTCCAGCTTTGCAGGATGGAAAAAA
TATTGTTGTCGGCATAACATGGGATAATGATCGTGTCACTGTTTTTGCTGACAAGATCTATCATTTTTATATTAAAAAT
GATTGGTCCCGTGTTGCGACAAGATGTTACAATAAAAGAAGTTGTGCCATGCAATATGTTTATACACCTACCTACTACA
TGCTTAATGTTACTAGTGCAGGTGAGGATGGCATTTACTATGAACCTTGTACAGCTAATTGCAGTGGTTACGCTGCCAA
TGTATTTGCCACTGATTCCAATGGCCATATACCAGAAGGTTTTAGTTTTAATAATTGGTTTCTTTTGTCCAATGATTCC
ACTTTGGTGCATGGTAAGGTGGTTTCCAACCAACCATTGTTGGTCAATTGTCTTTTGGCCATGCCTAAGATTTATGGAC
TAGGCCAATTTTTCTCCTTCAATCAAACGATGGATGGCGTTTGTAATGGAGCTGCTGTGCAGCGTGCACCAGAGGCTCT
GAGGTTTAATATTAATGACACCTCTGTCATTCTTGCTGAAGGCTCAATTGTACTTCATACTGCTTTTGGAACAAATCTT
TCTTTTGTTTGCAGTAATTCCTCAAATCCTCATTTAGCCACCTTCGCCATACCTTTGGGTGCTACCCAAGTACCTTATT
ATTGTTTTCTTAAAGTGGATACTTACAACTCCACTGTTTATAAATTCTTGGCTGTTTTACCTCCCACCGTCAGGGAAAT
TGTCATCACCAAGTATGGTGATGTTTATGTCAATGGGTTTGGCTACTTGCATCTCGGTTTGTTGGATGCTGTCACAATT
AATTTCACTGGTCATGGCACTGACGATGACGTTTCTGGTTTTTGGACCATAGCATCGACTAATTTTGTTGATGCACTCA
TCGAAGTTCAAGGAACTGCCATTCAGCGTATTCTTTATTGTGATGATCCTGTTAGCCAACTCAAGTGTTCTCAGGTTGC
TTTTGACCTTGACGATGGTTTTTACCCTATTTCTTCTAGGAACCTTTTGAGTCATGAACAGCCAATTTCTTTTGTTACT
CTGCCATCATTTAATGATCATTCTTTTGTTAACATTACTGTCTCTGCGTCTTTTGGTGGTCCTAGTGGTGCCAACCTTA
TTGCATCTGACACTACTATCAATGGGTTTAGTTCTTTCTGTGTTGACACTAGACAATTTACCATTTCACTGTTTTATAA
CGTTACAAACAGTTATGGTTATGTGTCTAAATCACAGGACAGTAATTGCCCTTTTACCTTGCAATCTGTTAATGATTAC
CTGTCTTTTAGCAAATTTTGTGTTTCTACCAGCCTTTTGGCTAGTGCCTGTACCATAGATCTTTTTGGTTACCCTGAGT
TTGGTGGTGGTGTTAAGTTTACGTCCCTTTACTTTCAATTCACAAAGGGTGAGTTGATTACTGGCACGCCTAAACCACT
TGAAGGTGTTACGGACGTTTCTTTTATGACTCTGGATGTGTGTACCAAGTATACTATCTATGGCTTTAGAGGTGAGGGT
ATCATTACCCTTACAAATTCTAGCTTTTTGGCAGGTGTTTATTACACATCTGATTCTGGACAGTTGTTAGCTTTTAAGA
ATGTCACTAGTGGTGCTGTTTATTCTGTTACGCCATGTTCTTTTGCAGAGCAGGCTGCATATGTTGATGATGATATAGT
GGGTGTTATTTCTAGTTTGTCTAACTCCACTTTTAACAGTACTAGGGAGTTGCCTGGTTTCTTCTACCATTCTAATGAT

GGCTCTAATTGTACAGAACCTGTGTTGGTGTATAGTAACATAGGTGTTTGTAAATCTGGCAGTATTGGCTACGTCCCAT
CTCAGTCTGGCCAAGTCAAGATTGCACCCACGGTTACTGGGAATATTAGTATTCCCACCAACTTTAGTATGAGTATTAG
GACAGAATATTTACAGCTTTACAACACGCCTGTTAGTGTTGATTGTGCTACATATGTTTGTAATGGTAACTCTCGTTGT
AAACAATTACTCACCCAGTACACTGCAGCATGTAAGACCATAGAGTCAGCATTACAACTCAGCGCTAGGCTTGAGTCTG
TTGAAGTTAACTCTATGCTTACTATTTCTGAAGAGGCTCTACAGTTAGCTACCATCAGTTCGTTTAATGGTGATGGATA
TAACTTTACTAATGTGCTGGGTGTTTCCGTGTACGACCCTGCAAGTGGCAGGGTGGTACAAAAAGGGTCTTTTATTGAA
GACCTGCTTTTTAATAAAGTGGTTACTAATGGCCTTAGTACTGTTGATGAAGACTATAAGCGCTGTTCTAATGGTCGCT
CTGTGGCAGATCTAGTCTGTGCGCAGTATTACTCTGGTGTCATGGTACTACCTGGCGTTGTTGACGCTGAGAAGCTTCA
CATGTATAGTGCGTCTCTCATCGGTGGTATGGCGCTAGGAGGTCTTACTACTGCAGCGGCATTGCCTTTTAGCCATGCT
GTTCAAGCGAGGCTCAATTATCTTGCTTTACAGACGGATGTTCTACAGCGCAACCAGCAATTGCTTGCTGAGTCTTTTA
ACTCTGCTATTGGTAATATAACTTCAGCCTTTGAGAGTGTTAAAGAGGCTATTAGTCAAACTTCCAATGGTTTGAACAC
TGTGGCTCATGCGCTTACTAAGGTTCAAGAGGTTGTTAATTCGCAGGGTTCAGCTTTGACCCAACTTACCATACAGCTG
CAACACAACTTCCAAGCCATTTCTAGTTCTATTGATGACATTTACTCCCGACTGGACATTCTTTCAGCCGATGTTCAGG
TTGATCGTCTCATCACCGGCAGATTATCAGCACTTAATGCTTTTGTTGCTCAAACCCTCACTAAGTATACTGAGGTTCA
GGCTAGCAGGAAGCTAGCACAGCAAAAGGTTAATGAGTGCGTCAAATCGCAATCTCAGCGTTATGGTTTTTGTGGTGGT
GATGGCGAGCACATCTTCTCTCTGGTACAGGCCGCACCTCAGGGCCTGCTGTTCTTACATACAGTACTTGTACCGGGTG
ATTTTGTAAATGTTATTGCCATCGATGGCTTATGCATTAATGGTGATATTGCCTTGACTCTACGTGAGCCTGGCTTAGT
CTTGTTTACGCATGAACTTCAAACTTATACTGCGACGGAATATCTTGTTTCATCGCGGCGTATGTTTGAACCTAGAAAA
CCTACCGTTAGTGATTTTGTTCAAATTGAGAGTTGTGTGGTCACCTATGTCAATTTGACTAGAGACCAACTACCAGATG
TAATCCCAGATTACATCGATGTTAACAAAACACTTGATGAGATTTTAGCTTCTCTGCCCAATAGAACTGGTCCAAGTCT
TCCTTTAGATGTTTTTAATGCCACTTATCTTAATCTCACTGGTGAAATTGCAGATTTAGAGCAGCGTTCAGAGTCTCTC
CGTAATACTACAGAAGAGCTCCGAAGTCTCATATATAATATCAACAACACACTTGTTGACCTTGAGTGGCTCAACCGAG
TTGAGACATATATCAAGTGGCCGTGGTGGGTTTGGTTGATTATTTTTATTGTTCTCATCTTTGTTGTGTCATTATTAGT
GTTCTGCTGCATTTCCACGGGTTGTTGTGGATGCTGCGGTTGTTGCGGTGCTTGTTTTTCAGGTTGTTGTAGGGGTCCT
AGACTTCAACCTTACGAAGCTTTTGAAAAGGTCCACGTGCAGTGA

>gi|351630105|gb|JN547228.1| Porcine epidemic diarrhea virus strain CH/S, complete genome

ATGAAGTCTTTAACATACTTCTGGTTGTTCCTACCAGTACTTTCAACACTCAGCCTACCACAAGATGTCACTAGG
TGCCAGTCTACTACTAACTTTAGGCGGTTCTTTTCAAAATTTAATGTGCAGGCACCTGCTGTCGTTGTTTTGGGTGGTT
ATCTACCTAGTATGAACTCTTCTAGCTGGTACTGTGGCACAGGCATTGAAACTGCTAGTGGCGTTCATGGTATTTTTCT
CAGTTACATCGATTCTGGTCAGGGCTTTGAGATTGGCATTTCGCAAGAGCCGTTTGATCCTAGTGGTTACCAGCTTTAT
TTACATAAGGCCACTAATGGTAACACTAATGCTATTGCACGACTGCGCATTTGCCAGTTTCCAGATAACAAAACATTGG
GCCCCGCTGTTAATGATGTTACAACAGGTCGTAACTGCTTATTCAACAAAGCTATTCCAGCTTATTTGCAGGATGGAAA
AAATATTGTTGTCGGCATAACATGGGACAATGATCGTGTCACTGTTTTTGCTGACAAGATCTATCATTTTTATCTTAAA
AATGATTGGTCCCGTGTTGCGACAAGATGTTACAATAAAAAAAGTTGTGCTATGCAATATGTTTATACACCTACCTACT
ACATGCTTAATGTTACTAGTGCAGGTGAGGATGGCATTTATTATGAACCCTGTACAGCCAATTGCAGTGGTTATGCTGC
CAATGTATTTGCCACTGATGCCAATGGCCATATACCAGAAGGTTTTAGTTTTAATAATTGGTTTCTTTTGTCCAATGAC
TCCACTTTGTTGCATGGTAAAGTTGTTTCCAACCAACCTTTGTTGGTCAATTGTCTTTTGGCCATTCCTAAGATATATG
GACTAGGCCAATTTTTCTCATTCAATCACACTATGGATGGCGTTTGTAACGGAGCTGTTGTGAATCGTGCCCCTGAGGC
TCTGAGGTTTAATATTAATGACACTTCTGTCATTCTTGCTGAAGGCTCAATTGTACTTCATACTGCTTTAGGAACAAAT
CTTTCTTTTGTTTGCAGTAATTCCTCAGATCCTCATTCAGCCATCTTTGCCATACCTTTGGGTGCTACCCAAGTACCCT

ATTATTGTTTTCTAAAAGTTGATACTTACAACTCCACTCTCTATAAATTCTTGGCTGTTTTACCTCCTACTGTCAAAGA
AATTGTCATCACCAAGTACGGTGATGTTTATGTTAATGGGTTTGGCTATTTGCATCTCGGTTTGTTGGATGCTGTCACA
ATTAATTTCACTGATCATGGCACTGACGATGACGTTTCAGGTTTCTGGACCGTAGCATCGACTAATTTTGTTGATGCAC
TCATTGAGGTTCAAGGAACTGCCATTCAGCATATTCTTTATTGTGATGATCCTGTTAGCCAACTCAAGTGTTCTCAGGT
TTCTTTTGACCTTGATGATGGTTTTTACCCTATTTCTTCTAGAAACCTTCTGAGTCATGAACAGCCAGTTTCTTTTGTT
ACTTTGCCATCATTTAATGATCATTCTTTTGTTAATATTACTGTCTCTGCGGCTTTTGGTGGTTCTAGTGGTGCCAACC
TCGTCGCATCTGACACTACTATTAATGGGTTTAGTTCTTTCTGTGTTGACACTAGACAATTTACCATTACACTGTTTTA
TAACGTTACAAACAGTTATGGTTATGTGTCTAACAAACAGGATAGTAATTGCCCTTTCACCTTGCAATCTGTTAATGAT
TACCTGTCTTTTAGCAAATTTTGTGTCTCAACCAGCCTTTTGGCTGGTGCTTGTACCATAGATCTTTTTGGCTACCCTG
AGTTCGGCAGTAGTGTTAAGTTTACGTCCCTTTATTTTCAATTCACAAAGGGTGAGTTGATTACTGGCACGCCTAAACC
ACTTGAAGGTGTCACGGACGTTTCTTTTATTACTCTGGATGTGTGTACCAAGTATACTATCTATGGCTTTAAAGGTGAG
GGTATTATTACCCTTACAAATTCTAGCTTTTTGGCAGGTGTTTATTATACATCTGATTCTGGACAGTTGTTAGCCTTTA
AGAATGTCACTAGTGGTGCTGTTTATTCTGTTACGCCATGTTCTTTTTCAGAGCAGGCTGCATATGTTGATGATGATAT
AGTGGGTGTTATTTCTAGTTTGTCTAACTCCACTTTTAACAATACTAGGGAGTTGCCTGGTTTCTTCTACCATTCTAAT
GACGGCTCCAATTGCACAGAGCCTGTGTTGGTGTATAGTAACATAGGTGTTTGTAAATCTGGCAGTATTGGCTATGTCC
CATCTCAGTCTGGCCAAGTCAAGATTGCACCCATGGTTACTGGGAATATTAGTATTCCCACCAACTTTAGTATGAGTAT
TAGAACAGAATATTTACAGCTTTACAACACGCCTGTTAGTGTTGATTGTTTCACATATGTTTGTAATGGCAACTCTCGT
TGTAAACAATTACTCACCCAGTACACTGCAGCATGTAAGACCATAGAGTCAGCATTACAACTCAGCGCTAGGCTTGAGT
CTGTTGAAGTTAACTCTATGCTTACTATTTCTGAAGAGGCTCTACAGTTAGCTACCATCAGTTCGTTTAATGGTGATGG
ATATAATTTTACTAATGTGCTGGGTCTTTCCGTGTATGATCCTGCAAGTGGCAGGGTGGTACACAAAAGGTCTTTCATT
GAAGACCTGCTTTTTAATAAAGTGGTTACTAATGGCCTTGGTACTGTTGATGAAGACTATAAGCGCTGTTCTAATGGTC
GCTCTGTGGCAGATCTAGTCTGTGCGCAGTATTACTCTGGTGTCATGGTACTACCTGGCGTTGTTGACGCTGAGAAGCT
TCACATGTATAGTGCTTCTCTTATCGGTGGTATGGCGCTAGGAGGTTTTACTGCTGCAGCGGCATTGCCTTTCAGCTAT
GCTGTTCAAGCGAGACTCAATTATCTTGCTTTACAGACGGATGTTCTACAGCGTAACCAGCAATTGCTTGCTGAGTCTT
TTAACTCTGCTATTGGTAATATAACTTCAGCCTTCGAGAGTGTTAAAGAGGCTATTAGTCAAACTTCCCAGGGTTTGAA
TACTGTGGCTCATGCACTTACTAAGGTTCAAGAGGTTGTTAATCCGCAGGGTGCAGCTTTGTCTCAACTTACCATACAG
CTGCAACACAACTTCCAAGCCATTTCTAGTTCTATTGATGACATTTACTCCCGACTGGACAGTCTTTCAGCCGATGTTC
AGGTTGATCGTCTCATCACTGGCAGATTATCAGCACTTAATGCTTTTGTTGCTCAAACCCTCACTAAGTATACTGAGGT
TCAGGCTAGCAGGAAGCTAGCGCAGCAAAAGGTTAATGAGTGCGTTAAATCGCAATCTCAGCGTTATGGTTTTTGTGGT
GGTGATGGCGAGCACATTTTCTCTCTGGTACAGGCCGCACCTCAGGGCCTGCTGTTCTTACATACAGTACTTGTACCGG
GTGATTTTGTAAATGTTACTGCCATCGCTGGCTTATGCGTTAATGGTGATATTGCCTTGACTCTACGTGAGCCTGGCTT
AGTCTTGTTTACGCATGAACTTCAAACTCATACTGCGACGGAATATTTTGTTTCATCGCGACGTATGTTTGAACCTAGA
AAACCTACCGTTAGTGATTTTGTTCAAATTGAGAGTTGTGTGGTCACCTATGTCAACCTGACTAGCGACCAACTACCAG
ATGTAATCCCAGATTACATCGATGTTAACAAAACACTTGATGAGATTTTAGCTTCTCTGCCCAATAGAACTGGTCCAAG
TCTTCCACTAGATGTTTTTAACGCCACTTATCTTAATCTCACTGGTGAAATTGCAGATTTAGAGCAGCGTTCAGAGTCT
CTCCGCAATACTACAGAAGAGCTCCGAAGTCTCATATACAACATCAACAACACACTTGTTGACCTTGAGTGGCTCAACC
GAGTTGAGACATATATCAAGTGGCCGTGGTGGGTTTGGTTGATTATTTTTATTGTTCTCATCTTTGTTGTGTCATTATT
AGTGTTCTGCTGCATTTCCACGGGTTGTTGTGGATGCTGCGGTTGCTGCGGTGCTTGTTTTTCAGGTTGTTGTAGGGGT
CCTAGACTTCAACCTTACGAAGCTTTTGAAAAGGTCCACGTGCAGTGA

>gi|372349655|gb|JN599150.1|_Porcine_epidemic_diarrhea_virus_strain_CV777_spike_protein_(S)_gene_complete_cds

ATGACGCCTTTAATTTACTTCTGGTTGTTCTTACCAGTACTTCTAACACTTAGCCTACCACAAGATGTCACTAGG
TGCCAGTCTACTATTAACTTTAGGCGGTTCTTTTCAAAATTTAATGTTCAGGCACCTGCCGTCGTTGTTTTGGGTGGTT
ATCTACCTAGTATGAACTCTTCTAGCTGGTACTGTGGCACAGGCATTGAAACTGATAGTGGCGTTCATGGTATTTTTCT
CAGTTACATCGATTCTGGTCAGGGCTTTGAGATTGGCATTTCGCAAGAGCCGTTTGATCCTAGTGGTTACCAGCTTTAT
TTACACAAGGCCACTAATGGTAACACTAGTGCTATTGCACGACTGCGCATTTGCCAGTTTCCAGATAATAAAACATTGG
GCCCTACTGTTAATGATGTTACAACAGGTCGTAACTGCCTATCCAACAAAGCCATTCCAGCTTTGCAGGATGGAAAAAA
TATTGTTGTCGGCATAACATGGGATAATGATCGTGTCACTGTTTTTGCTGACAAGATCTATCATTTTTATATTAAAAAT
GATTGGTCCCGTGTTGCGACAAGATGTTACAATAAAAGAAGTTGTGCCATGCAATATGTTTATACACCTACCTACTACA
TGCTTAATGTTACTAGTGCAGGTGAGGATGGCATTTACTATGAACCTTGTACAGCTAATTGCAGTGGTTACGCTGCCAA
TGTATTTGCCACTGATTCCAATGGCCATATACCAGAAGGTTTTAGTTTTAATAATTGGTTTCTTTTGTCCAATGACTCC
ACTTTGTTGCATGGTAAAGTGGTTTCAAACCAACCTTTGTTGGTCAACTGCCTTTGGGCCATTCCTAAGATTTATGGAC
TAGGCCAATTTTTCTCATTCAATCAAACGATGGATGGCGTTTGTAACGGAGCCGCTGCGCAGCGTGCCCCAGAGGCTCT
GAGGTTTAATATTAATGACACTTTTGTCATTCTTGCTGAAGGCTCAATTGTACTTCATACTGCTTTAGGAACAAATCTT
TCTTTTGTTTGCAGTAATTCCTCAGATCCTCACAAAGCCATCTTTACCATACCTTTGGGTGTTACTGAAGTACCCTACT
ATTGCTTTCTTAAAGTGGATACTTACAAATCCACTGTTTATAAATTCTTGGCTGTTTTACCTCCTACTGTCAAGGAAAT
TGTCATCACCAAGTACGGTGATGTTTATGTCAACGGGTTTGGCTATTTGCATCTCGGTTTGTTGGATGCTGTCACAATT
AATTTCACTGGTCATGGCACTGACGATGACGTTTCAGGTTTCTGGACCGTAGCATCGACTAATTTTGTTGATGCACTCA
TCGAGGTTCAAGGAACTGCCATTCAGCGTATTCTTTATTGTGATGACCCTGTTAGCCAACTTAAGTGTTCTCAGGTTTC
TTTTGACCTTGATGATGGTTTTTACCCTATTTCTTCTAGAAACCTTCTGAGTCATGAACAGCCAATTTCTTTTGTTACT
TTGCCATCATTCAATGATCATTCTTTTGTTAATATTACTGTCTCTGCGGCTTTTGGTGGTCATAGTGGTGCCAACCTCA
TTGCATCTGACACTACTATCAATGGGTTTAGTTCTTTCTGTGTTGACACTAGACAATTTACCATTACACTGTTTTATAA
CGTTACAAACAGTTATGGTTATGTGTCTAAGTCACAGGATAGTAATTGCCCTTTCACCTTGCAATCTGTTAATGATTAC
CTGTCTTTTAGCAAATTTTGTGTTTCAACCAGCCTTTTGGCTGGTGCTTGTACCATAGATCTTTTTGGTTACCCTGAGT
TCGGTAGTGGTGTTAAGTTTACGTCCCTTTATTTTCAATTCACAAAGGGTGAGTTGATTACTGGCACGCCTAAACCACT
TCAAGGTGTCACGGACGTTTCTTTTATGACTCTGGATGTGTGTACCAAGTATACTATCTATGGCTTTAAAGGTGAGGGT
ATTATTACCCTTACAAATTCTAGCTTTTTGGCAGGTGTTTATTATACATCTGATTCTGGACAGTTGTTAGCCTTTAAGA
ATGTCACTAGTGGTGCTGTTTATTCTGTTACGCCATGTTCTTTTTCAGAGCAGGCTGCATATGTTGATGATGATATAGT
GGGTGTTATTTCTAGTTTGTCTAACTCCACTTTTAACAATACCAGGGAGTTGCCTGGTTTCTTCTACCATTCTAATGAT
GGCTCCAATTGTACAGAGCCTGTGTTGGTGTATAGTAACATAGGTGTCTGTAAATCTGGCAGTATTGGCTATGTCCCAC
TTCAGGATGGCCAAGTCAAGATTGCACCCATGGTTACTGGGAATATTAGTATTCCCACCAACTTTAGTATGAGTATTAG
AACAGAATATTTACAGCTTTACAACACGCCTGTTAGTGTTGATTGCGTTACATATGTTTGTAATGGTAACTCTCGTTGT
AAACAATTACTCACCCAGTACACTGCAGCATGTAAGACCATAGAGTCAGCATTACAACTCAGCGCTAGGCTTGAGTCTG
TTGAAGTTAACTCTATGCTTACTATTTCTGAAGAGGCTCTACAGTTAGCTACCATCAGTTCGTTTAATGGTGATGGATA
TAACTTTACTAATGTGCTGGGTGTTTCCGTGTACGACCCTGCAAGTGGCAGGGTGGTACAAAAAGGGTCTTTTATTGAA
GACCTGCTTTTTAATAAAGTGGTTACTAATGGCCTTGGTACTGTTGATGAAGACTATAAGCGCTGTTCTAATGGTCGCT
CTGTGGCAGATCTAGTCTGTGCGCAGTATTACTCTGGTGTCATGGTACTACCTGGCGTTGTTGACGCTGAGAAGCTTCA
CATGTATAGTGCGTCTCTCATCGGTGGTATGGCGCTAGGAGGTCTTACTACTGCAGCGGCATTGCCTTTTAGCCATGCT
GTTCAAGCGAGGCTCAATTATCTTGCTTTACAGACGGATGTTCTACAGCGCAACCAGCAATTGCTTGCTGAGTCTTTTA
ACTCTGCTATTGGTAATATAACTTCAGCCTTTGAGAGTGTTAAAGAGGCTATTAGTCAAACTTCCAATGGTTTGAACAC
TGTGGCTCATGCGCTTACTAAGGTTCAAGAGGTTGTTAATTCGCAGGGTTCAGCTTTGACCCAACTTACCATACAGCTG
CAACACAACTTCCAAGCCATTTCTAGTTCTATTGATGACATTTACTCCCGACTGGACATTCTTTCAGCCGATGTTCAGG
TTGATCGTCTCATCACCGGCAGATTATCAGCACTTAATGCTTTTGTTGCTCAAACCCTCACTAAGTATACTGAGGTTCA
GGCTAGCAGGAAGCTAGCACAGCAAAAGGTTAATGAGTGCGTCAAATCGCAATCTCAGCGTTATGGTTTTTGTGGTGGT
GATGGCGAGCACATCTTCTCTCTGGTACAGGCCGCACCTCAGGGCCTGCTGTTCTTACATACAGTACTTGTACCGGGTG

ATTTTGTAAATGTTATTGCCATCGATGGCTTATGCGTTAATGGTGATATTGCCTTGACTCTACGTGAGCCTGGCTTAGTCTTGTTTACGCATGAACTTCAAACTTATACTGCGACGGAATATCTTGTTTCATCGCGACGTATGTTTGAACCTAGAAAACCTACCGTTAGTGATTTTGTTCAAATTGAGAGTTGTGTGGTCACCTATGTCAATCTGACTAGCGACCAACTACCAGATGTAATCCCAGATTACATCGATGTTAACAAAACACTTGATGAGATTCTAGCTTCTCTGCCCAATAGAATTGGTCCTAGTCTTCCCCTAGATGTTTTTAATGCCACTTATCTTAATCTCACTGGTGAAATTGCAGATTTAGAGCAGCGTTCAGAGTCTCTCCGTAATACTACAGAAGAGCTCCGAAGTCTCATATATAATATCAACAACACACTTGTTGACCTTGAGTGGCTCAACCGAGTTGAGACATATATCAAGTGGCCGTGGTGGGTTTGGTTGATTATTTTTATTGTTCTCATCTTTGTTGTGTCATTATTAGTGTTCTGCTGCATTTCCACGGGTTGTTGTGGATGCTGCGGTTGTTGCGGTGCTTGTTTTTCAGGTTGTTGTAGGGGTCCTAGACTTCAACCTTACGAAGCTTTTGAAAAGGTCCACGTGCAGTGA

>gi|377657487|gb|JN825706.1|_Porcine_epidemic_diarrhea_virus_isolate_BJ20112_spike_protein_(S)_mRNA_complete_cds

ATGAAGTCTTTAACCTACTTCTGGTTGTTCTTACCAGTACTTTCAACACTTAGCCTACCACAAGATGTCACCAGGTGCTCAGCTAACACTAATTTTAGGCGGTTCTTTTCAAAATTTAATGTTCAGGCGCCTGCAGTTGTTGTACTGGGCGGTTATCTACCTATTGGTGAAAACCAGGGTGTCAATTCAACTTGGTACTGTGCTGGCCAACATCCAACTGCTAGTGGCGTTCATGGTATCTTTGTTAGCCATATTAGAGGTGGTCATGGCTTTGAGATTGGCATTTCGCAAGAGCCTTTTGACCCTAGTGGTTACCAGCTTTATTTACATAAGGCTACTAACGGTAACACTAATGCTACTGCGCGACTGCGCATTTGCCAGTTTCCTAGCATTAAAACATTGGGCCCCACTGCTAATAATGATGTTACAACAGGTCGTAATTGCCTATTTAACAAAGCCATCCCAGCTCATATGAGTGAACATAGTGTTGTCGGCATAACATGGGATAATGATCGTGTCACTGTCTTTTCTGACAAGATCTATTATTTTTATTTTTAAAAATGATTGGTCCCGTGTTGCGACAAAGTGTTACAACAGTGGAGGTTGTGCTATGCAATATGTTTACGAACCCACCTATTACATGCTTAATGTTACTAGTGCTGGTGAGGATGGTATTTCTTATCAACCCTGTACAGCTAATTGCATTGGTTATGCTGCCAATGTATTTGCTACTGAGCCCAATGGCCACATACCAGAAGGTTTTAGTTTTAATAATTGGTTTCTTTTGTCCAATGATTCCACTTTGGTGCATGGTAAGGTGGTTTCCAACCAACCATTGTTGGTCAATTGTCTTTTGGCCATTCCTAAGATTTATGGACTAGGCCAATTTTTCTCCTTTAATCAAACGATCGATGGTGTTTGTAATGGAGCTGCTGTGCAGCGTGCACCAGAGGCTCTGAGGTTTAATATTAATGACACCTCTGTCATTCTTGCTGAAGGCTCAATTGTACTTCATACTGCTTTAGGAACAAATTTTTCTTTTGTTTGCAGTAATTCCTCAGATCCTCACTTAGCCACCTTCGCCATACCTCTGGGTGCTATCCAAGTACCCTATTATTGTTTTCTTAAAGTGGATACTTACAACTCCACTGTTTATAAATTCTTGGCTGTTTTACCTCCTACCGTCAGGGAAATTGTCATCACCAAGTATGGTGATGTTTATGTCAATGGGTTTGGCTACTTGCATCTCGGTTTGTTGGATGCTGTCACAATTAATTTCACTGGTCTTGGCACTGACGATGACGTTTCAGGTTTCTGGACCATAGCATCGACTAATTTTGTTGATGCACTTATCGAAGTTCAAGGAACTGCCATTCAGCGTATTCTTTATTGTGATGATCCTGTTAGCCAACTCAAGTGTTCTCAGGTTTCTTTTGACCTTGACGATGGTTTTTACCCTATTTCCTCTACAAACCTTCTGAGTCATGAACAGCCAACTTCTTTTGTTACTTTGCCATCATTTAATGATCATTCTTTTGTTAATATTACTGTCTCTGCTGCTTTTGGTGGTCATAGTGGTGCCAACCTTATTGTATCTGACACTACTATCAATGGGTTTAGTTCTTTCTGTGTTGACACTAGACAATTTACCATTTCACTGTTTTATAATGTTACAAACAGTTATGGTTATGTGTCTAACTCACAGGACAGTAATTGCCCTTTCACCTTGCAATCTGTTAATGATTACCTGTCTTTTAGCAAATTTTGTGTTTCTACCAGCCTTTTGGCTAGTGCCTGTACCATAGATCTTTTTGGTTACCCTGAGTTTGGTAGTGGTGTTAAGTTCACGTCCCTTTACTTTCAATTCACAAAGGGTGAGTTGATTACTGGCACGCCTAAACCACTTGAAGGTGTTACGGACGTTTCTTTTATGACTCTGGATGTGTGTACCAAGTATACTATCTATGGCTTTAAAGGTGAGGGTATCATTACCCTTACAAATTCTAGCTTTTTGGCAGGTGTTTATTATACATCTGATTCTGGACAGTTGTTAGCTTTTAAGAATGTCACTAGTGGTGCTGTTTATTCTGTTACGCCATGTTCTTTTTCAGAGCAGGCTGCATATGTTGATGATGATATAGTGGGTGTTATTTCTAGTTTGTCTAGCTCCACTTTTAACAGTACTAGGGAGTTGCCTGGTTTCTTCTACCATTCTAATGATGGCTCTAATTGTACAGAGCCTGTGTTGGTGTATAGTAACATAGGTGTTTGTAAATCTGGCAGTATTGGCTATGTCCCATCTCAGTCTGGCCAAGTCAAGATTGCACCCACGGTTACTGGGAATATTAGTATTCCCACCAACTTTAG

TATGAGTATTAGGACAGAATATTTACAGCTTTACAACACGCCTGTTAGTGTTGATTGTGCTACATATGTTTGTAATGGT
AACTCTCGTTGTAAACAATTACTCACCCAGTACACTGCAGCATGTAAGACCATAGAGTCAGCATTACAACTCAGCGCTA
GGCTTGAGTCTGCTGAAGTCAACTCTATGCTTACTATTTCTGAAGAGGCTCTACAGTTAGCTACCATCAGTTCGTTTAA
TGGTGATGGATATAATTTTACTAATGTGCTGGGTGTTTCCGTTCGTGATCCTGCAAGTGGCAGGGTGGTACAAAAAAGG
TCTTTTATTGAAGACCTGCTTTTTAATAAAGTGGTTACTAATGGCCTTGGTACTGTTGATGAAGACTATAAGCGCTGTT
CTAATGGCCGCTCTGTGGCAGATTTAGTCTGTGCGCAGTATTACTCTGGTGTCATGGTACTACCTGGTGTTGTTGACGC
TGAGAAGCTTCATATGTATAGTGCGTCTCTCATCGGTGGTATGGTGCTAGGAGGTTTTACTGCAGCAGCGGCATTGCCT
TTTAGCTATGCTGTTCAAGCGAGACTCAATTATCTTGCTCTACAGACGGATGTTCTACAGCGGAACCAGCAATTGCTTG
CTGAGTCTTTTAACTCTGCTATTGGTAATATAACTTCAGCCTTTGAGAGTGTTAAAGAGGCTATTAGTCAAACTTCTAA
GGGTTTGAACACTGTGGCTCATGCGCTTACTAAGGTTCAAGAGGTTGTTAATTCGCAGGGTGCAGCTTTGACCCAACTT
ACCGTACAGCTGCAACACAACTTCCAAGCCATTTCTAGTTCTATTGATGACATTTATTCCCGACTGGACATTCTTTCAG
CCGATGTTCAGGTTGACCGTCTCATCACCGGCAGATTATCAGCACTTAATGCTTTTGTTGCTCAAACCCTCACTAAGTA
TACTGAGGTTCAGGCTAGCAGGAAGCTAGCACAGCAAAAGGTTAATGAGTGCGTTAAATCGCAATCTCAGCGTTATGGT
TTTTGTGGTGGTGATGGCGAGCACATTTTCTCTCTGGTACAGGCAGCACCTCAGGGCCTGCTGTTTTTACATACAGTAC
TTGTACCGGGTGACTTTGTAGATGTTATTGCCATCGCTGGCTTATGCGTTAACGATGAAATTGCCTTGACTCTACGTGA
GCCTGGCTTAGTCTTGTTTACGCATGAACTTCAAAATCATACTGCGACGGAATATTTTGTTTCATCGCGACGTATGTTT
GAACCTAGAAAACCTACCGTTAGTGATTTTGTTCAAATTGAGAGTTGTGTGGTCACCTATGTCAATTTGACTAGAGACC
AACTACCAGATGTAATCCCAGATTACATCGATGTTAACAAAACACTTGATGAGATTTTAGCTTCTCTGCCCAATAGAAC
TGGTCCAAGTCTTCCTTTAGATGTTTTTAATGCCACTTATCTTAATCTCACTGGTGAAATTGCAGATTTAGAGCAGCGT
TCAGAGTCTCTCCGTAATACTACAGAGGAGCTCCAAAGTCTTATATATAATATCAACAACACACTAGTTGACCTTGAGT
GGCTCAACCGAGTTGAGACATATATCAAGTGGCCGTGGTGGGTTTGGTTGATTATTTTCATTGTTCTCATCTTTGTTGT
GTCATTACTAGTGTTCTGCTGCATTTCCACTGGTTGTTGTGGATGCTGCGGCTGCTGCTGTGCTTGTTTTTCAGGTTGT
TGTAGGGGTCCTAGACTTCAACCTTACGAAGTTTTTGAAAAGGTCCACGTGCAGTGA

>gi|377657489|gb|JN825707.1|_Porcine_epidemic_diarrhea_virus_isolate_HB2011
1_spike_protein_(S)_mRNA_complete_cds

ATGAAGTCTTTAACCTACTTCTGGTTGTTCTTACCAGTACTTTCAACACTTAGCCTACCACAAGATGTCACCAGG
TGCTCAGCTAACACTAATTTTAGGCGGTTCTTTTCAAAATTTAATGTTCAGGCGCCTGCAGTTGTTGTACTGGGCGGTT
ATCTACCTATTGGTGAAAACCAGGGTGTCAATTCAACTTGGTACTGTGCTGGCCAACATCCAACTGCTAGTGGCGTTCA
TGGTATCTTTGTTAGCCATATTAGAGGTGGTCATGGCTTTGAGATTGGCATTTCGCAAGAGCCTTTTGACCCTAGTGGT
TACCAGCTTTATTTACATAAGGCTACTAACGGTAACACTAATGCTACTGCGCGACTGCGCATTTGCCAGTTTCCTAGCA
TTAAAACATTGGGCCCCACTGCTAATAATGATGTTACAACAGGTCGTAATTGCCTATTTAACAAAGCCATCCCAGCTCA
TATGAGTGAACATAGTGTTGTCGGCATAACATGGGATAATGATCGTGTCACTGTCTTTTCTGACAAGATCTATTATTTT
TATTTTAAAAATGATTGGTCCCGTGTTGCGACAAAGTGTTACAACAGTGGAGGTTGTGCTATGCAATATGTTTACGAAC
CCACCTATTACATGCTTAATGTTACTAGTGCTGGTGAGGATGGTATTTCTTATCAACCCTGTACAGCTAATTGCATTGG
TTATGCTGCCAATGTATTTGCTACTGAGCCCAATGGCCACATACCAGAAGGTTTTAGTTTTAATAATTGGTTTCTTTTG
TCCAATGATTCCACTTTGGTGCATGGTAAGGTGGTTTCCAACCAACCATTGTTGGTCAATTGTCTTTTGGCCATTCCTA
AGATTTATGGACTAGGCCAATTTTTCTCCTTTAATCAAACGATCGATGGTGTTTGTAATGGAGCTGCTGTGCAGCGTGC
ACCAGAGGCTCTGAGGTTTAATATTAATGACACCTCTGTCATTCTTGCTGAAGGCTCAATTGTACTTCATACTGCTTTA
GGAACAAATTTTTCTTTTGTTTGCAGTAATTCCTCAGATCCTCACTTAGCCACCTTCGCCATACCTCTGGGTGCTATCC
AAGTACCCTATTATTGTTTTCTTAAAGTGGATACTTACAACTCCACTGTTTATAAATTCTTGGCTGTTTTACCTCCTAC
CGTCAGGGAAATTGTCATCACCAAGTATGGTGATGTTTATGTCAATGGGTTTGGCTACTTGCATCTCGGTTTGTTGGAT

GCTGTCACAATTAATTTCACTGGTCATGGCACTGACGATGACGTTTCAGGTTTCTGGACCATAGCATCGACTAATTTTG
TTGATGCACTTATCGAAGTTCAAGGAACTGCCATTCAGCGTATTCTTTATTGTGATGATCCTGTTAGCCAACTCAAGTG
TTCTCAGGTTTCTTTTGACCTTGACGATGGTTTTTACCCTATTTCCTCTACAAACCTTCTGAGTCATGAACAGCCAACT
TCTTTTGTTACTTTGCCATCATTTAATGATCATTCTTTTGTTAATATTACTGTCTCTGCTGCTTTTGGTGGTCATAGTG
GTGCCAACCTTATTGCATCTGACACTACTATCAATGGGTTTAGTTCTTTCTGTGTTGACACTAGACAATTTACCATTTC
ACTGTTTTATAATGTTACAAACAGTTATGGTTATGTGTCTAACTCACAGGACAGTAATTGCCCTTTCACCTTGCAATCT
GTTAATGATTACCTGTCTTTTAGCAAATTTTGTGTTTCTACCAGCCTTTTGGCTAGTGCCTGTACCATAGATCTTTTTG
GTTACCCTGAGTTTGGTAGTGGTGTTAAGTTCACGTCCCTTTACTTTCAATTCACAAAGGGTGAGTTGATTACTGGCAC
GCCTAAACCACTTGAAGGTGTTACGGACGTTTCTTTTATGACTCTGGATGTGTGTACCAAGTATACTATCTATGGCTTT
AAAGGTGAGGGTATCATTACCCTTACAAATTCTAGCTTTTTGGCAGGTGTTTATTATACATCTGATTCTGGACAGTTGT
TAGCTTTTAAGAATGTCACTAGTGGTGCTGTTTATTCTGTTACGCCATGTTCTTTTTCAGAGCAGGCTGCATATGTTGA
TGATGATATAGTGGGTGTTATTTCTAGTTTGTCTAGCTCCACTTTTAACAGTACTAGGGAGTTGCCTGGTTTCTTCTAC
CATTCTAATGATGGCTCTAATTGTACAGAGCCTGTGTTGGTGTATAGTAACATAGGTGTTTGTAAATCTGGCAGTATTG
GCTATGTCCCATCTCAGTCTGGCCAAGTCAAGATTGCACCCACGGTTACTGGGAATATTAGTATTCCCACCAACTTTAG
TATGAGTATTAGGACAGAATATTTACAGCTTTACAACACGCCTTTTAGTGTTGATTGTGCTACATATGTTTGTAATGGT
AACTCTCGTTGTAAACAATTACTCACCCAGTACACTGCAGCATGTAAGACCATAGAGTCAGCATTACAACTCAGCGCTA
GGCTTGAGTCTGCTGAAGTCAACTCTATGCTTACTATTTCTGAAGAGGCTCTACAGTTAGCTACCATCAGTTCGTTTAA
TGGTGATGGATATAATTTTACTAATGTGCTGGGTGTTTCCGTTCGTGATCCTGCAAGTGGCAGGGTGGTACAAAAAAGG
TCTTTTATTGAAGACCTGCTTTTTAATAAAGTGGTTACTAATGGCCTTGGTACTGTTGATGAAGACTATAAGCGCTGTT
CTAATGGCCGCTCTGTGGCAGATTTAGTCTGTGCGCAGTATTACTCTGGTGTCATGGTACTACCTGGTGTTGTTGACGC
TGAGAAGCTTCATATGTATAGTGCGTCTCTCATCGGTGGTATGGTGCTAGGAGGTTTTACTGCAGCAGCGGCATTGCCT
TTTAGCTATGCTGTTCAAGCGAGACTCAATTATCTTGCTCTACAGACGGATGTTCTACAGCGGAACCAGCAATTGCTTG
CTGAGTCTTTTAACTCTGCTATTGGTAATATAACTTCAGCCTTTGAGAGTGTTAAAGGGGCTATTAGTCAAACTTCTAA
GGGTTTGAACACTGTGGCTCATGCGCTTACTAAGGTTCAAGAGGTTGTTAATTCGCAGGGTGCAGCTTTGACCCAACTT
ACCGTACAGCTGCAACACAACTTCCAAGCCATTTCTAGTTCTATTGATGACATTTATTCCCGACTGGACATTCTTTCAG
CCGATGTTCAGGTTGACCGTCTCATCACCGGCAGATTATCAGCACTTAATGCTTTTGTTGCTCAAACCCTCACTAAGTA
TACTGAGGTTCAGGCTAGCAGGAAGCTAGCACAGCAAAAGGTTAATGAGTGCGTTAAATCGCAATCTCAGCGTTATGGT
TTTTGTGGTGGTGATGGCGAGCACATTTTCTCTCTGGTACAGGCAGCACCTCAGGGCCTGCTGTTTTTACATACAGTAC
TTGTACCGGGTGACTTTGTAGATGTTATTGCCATCGCTGGCTTATGCGTTAACGATGAAATTGCCTTGACTCTACGTGA
GCCTGGCTTAGTCTTGTTTACGCATGAACTTCAAAATCATACTGCGACGGAATATTTTGTTTCATCGCGACGTATGTTT
GAACCTAGAAAACCTACCGTTAGTGATTTTGTTCAAATTGAGAGTTGTGTGGTCACCTATGTCAATTTGACTAGAGACC
AACTACCAGATGTAATCCCAGATTACATCGATGTTAACAAAACACTTGATGAGATTTTAGCTTCTCTGCCCAATAGAAC
TGGTCCAAGTCTTCCTTTAGATGTTTTTAATGCCACTTATCTTAATCTCACTGGTGAAATTGCAGATTTAGAGCAGCGT
TCAGAGTCTCTCCGTAATACTACAGAGGAGCTCCAAAGTCTTATATATAATATCAACAACACACTAGTTGACCTTGAGT
GGCTCAACCGAGTTGAGACATATATCAAGTGGCCGTGGTGGGTTTGGTTGATTATTTTCATTGTTCTCATCTTTGTTGT
GTCATTACTAGTGTTCTGCTGCATTTCCACTGGTTGTTGTGGATGCTGCGGCTGCTGCTGTGCTTGTTTTTCAGGTTGT
TGTAGGGGTCCTAGACTTCAACCTTACGAAGTTTTTGAAAAGGTCCACGTGCAGTGA

>gi|377657491|gb|JN825708.1|_Porcine_epidemic_diarrhea_virus_isolate_HB2011
2_spike_protein_(S)_mRNA_complete_cds

ATGAAGTCTTTAACCTACTTCTGGTTGTTCTTACCAGTACTTTCAACACTTAGCCTACCACAAGATGTCACCAGG
TGCTCAGCTAACACTAATTTTAGGCGGTTCTTTTCAAAATTTAATGTTCAGGCGCCTGCAGTTGTTGTACTGGGCGGTT

ATCTACCTATTGGTGAAAACCAGGGTGTCAATTCAACTTGGTACTGTGCTGGCCAACATCCAACTGCTAGTGGCGTTCA
TGGTATCTTTGTTAGCCATATTAGAGGTGGTCATGGCTTTGAGATTGGCATTTCGCAAGAGCCTTTTGACCCTAGTGGT
TACCAGCTTTATTTACATAAGGCTACTAACGGTAACACTAATGCTACTGCGCGACTGCGCATTTGCCAGTTTCCTAGCA
TTAAAACATTGGGCCCCACTGCTAATAATGATGTTACAACAGGTCGTAATTGCCTATTTAACAAAGCCATCCCAGCTCA
TATGAGTGAACATAGTGTTGTCGGCATAACATGGGATAATGATCGTGTCACTGTCTTTTCTGACAAGATCTATTATTTT
TATTTTAAAAATGATTGGTCCCGTGTTGCGACAAAGTGTTACAACAGTGGAGGTTGTGCTATGCAATATGTTTACGAAC
CCACCTATTACATGCTTAATGTTACTAGTGCTGGTGAGGATGGTATTTCTTATCAACCCTGTACAGCTAATTGCATTGG
TTATGCTGCCAATGTATTTGCTACTGAGCCCAATGGCCACATACCAGAAGGTTTTAGTTTTAATAATTGGTTTCTTTTG
TCCAATGATTCCACTTTGGTGCATGGTAAGGTGGTTTCCAACCAACCATTGTTGGTCAATTGTCTTTTGGCCATTCCTA
AGATTTATGGACTAGGCCAATTTTTCTCCTTTAATCAAACGATCGATGGTGTTTGTAATGGAGCTGCTGTGCAGCGTGC
ACCAGAGGCTCTGAGGTTTAATATTAATGACACCTCTGTCATTCTTGCTGAAGGCTCAATTGTACTTCATACTGCTTTA
GGAACAAATTTTTCTTTTGTTTGCAGTAATTCCTCAGATCCTCACTTAGCCACCTTCGCCATACCTCTGGGTGCTATCC
AAGTACCCTATTATTGTTTTCTTAAAGTGGATACTTACAACTCCACTGTTTATAAATTCTTGGCTGTTTTACCTCCTAC
CGTCAGGGAAATTGTCATCACCAAGTATGGTGATGTTTATGTCAATGGGTTTGGCTACTTGCATCTCGGTTTGTTGGAT
GCTGTCACAATTAATTTCACTGGTCATGGCACTGACGATGACGTTTCAGGTTTCTGGACCATAGCATCGACTAATTTTG
TTGATGCACTTATCGAAGTTCAAGGAACTGCCATTCAGCGTATTCTTTATTGTGATGATCCTGTTAGCCAACTCAAGTG
TTCTCAGGTTTCTTTTGACCTTGACGATGGTTTTTACCCTATTTCTTCTACAAACCTTCTGAGTCATGAACAGCCAACT
TCTTTTGTTACTTTGCCATCATTTAATGATCATTCTTTTGTTAATATTACTGTCTCTGCTGCTTTTGGTGGTCATAGTG
GTGCCAACCTTATTGCATCTGACACTACTATCAATGGGTTTAGTTCTTTCTGTGTTGACACTAGACAATTTACCATTTC
ACTGTTTTATAACGTTACAAACAGTTATGGTTATGTGTCTAACTCACAGGACAGTAATTGCCCTTTCACCTTGCAATCT
GTTAATGATTACCTGTCTTTTAGCAAATTTTGTGTTTCTACCAGCCTTTTGGCTAGTGCCTGTACCATAGATCTTTTTG
GTTACCCTGAGTTTGGTAGTGGTGTTAAGTTTACGTCCCTTTACTTTCAATTCACAAAGGGTGAGTTGATTACTGGCAC
GCCTAAACCACTTGAAGGTGTTACGGACGTTTCTTTTATGACTCTGGATGTGTGTACCAAGTATACTATCTATGGCTTT
AAAGGTGAGGGTATCATTACCCTTACAAATTCTAGCTTTTTGGCAGGTGTTTATTATACATCTGATTCTGGACAGTTGT
TAGCTTTTAAGAATGTCACTAGTGGTGCTGTTTATTCTGTTACGCCATGTTCTTTTTCAGAGCAGGCTGCATATGTTGA
TGATGATATAGTGGGTGTTATTTCTAGTTTGTCTAGCTCCACTTTTAACAGTACTAGGGAGTTGCCTGGTTTCTTCTAC
CATTCTAATGATGGCTCTAATTGTACAGAGCCTGTGTTGGTGTATAGTAACATAGGTGTTTGTAAATCTGGCAGTATTG
GCTATGTCCCATCTCAGTCTGGCCAAGTCAAGATTGCACCCACGGTTACTGGGAATATTAGTATTCCCACCAACTTTAG
TATGAGTATTAGGACAGAATATTTACAGCTTTACAACACGCCTGTTAGTGTTGATTGTGCTACATATGTTTGTAATGGT
AACTCTCGTTGTAAACAATTACTCACCCAGTACACTGCAGCATGTAAGACCATAGAGTCAGCATTACAACTCAGCGCTA
GGCTTGAGTCTGCTGAAGTCAACTCTATGCTTACTATTTCTGAAGAGGCTCTACAGTTAGCTACCATCAGTTCGTTTAA
TGGTGATGGATATAATTTTACTAATGTGCTGGGTGTTTCCGTTCGTGATCCTGCAAGTGGCAGGGTGGTACAAAAAAGG
TCTTTTATTGAAGACCTGCTTTTTAATAAAGTGGTTACTAATGGCCTTGGTACTGTTGATGAAGACTATAAGCGCTGTT
CTAATGGCCGCTCTGTGGCAGATTTAGTCTGTGCGCAGTATTACTCTGGTGTCATGGTACTACCTGGTGTTGTTGACGC
TGAGAAGCTTCATATGTATAGTGCGTCTCTCATCGGTGGTATGGTGCTAGGAGGTTTTACTGCAGCAGCGGCATTGCCT
TTTAGCTATGCTGTTCAAGCGAGACTCAATTATCTTGCTCTACAGACGGATGTTCTACAGCGGAACCAGCAATTGCTTG
CTGAGTCTTTTAACTCTGCTATTGGTAATATAACTTCAGCCTTTGAGAGTGTTAAAGAGGCTATTAGTCAAACTTCTAA
GGGTTTGAACACTGTGGCTCATGCGCTTACTAAGGTTCAAGAGGTTGTTAATTCGCAGGGTGCAGCTTTGACCCAACTT
ACCGTACAGCTGCAACACAACTTCCAAGCCATTTCTAGTTCTATTGATGACATTTATTCCCGACTGGACATTCTTTCAG
CCGATGTTCAGGTTGACCGTCTCATCACCGGCAGATTATCAGCACTTAATGCTTTTGTTGCTCAAACCCTCACTAAGTA
TACTGAGGTTCAGGCTAGCAGGAAGCTAGCACAGCAAAAGGTTAATGAGTGCGTTAAATCGCAATCTCAGCGTTATGGT
TTTTGTGGTGGTGATGGCGAGCACATTTTCTCTCTGGTACAGGCAGCACCTCAGGGCCTGCTGTTTTTACATACAGTAC
TTGTACCGGGTGACTTTGTAGATGTTATTGCCATCGCTGGCTTATGCGTTAACGATGAAATTGCCTTGACTCTACGTGA
GCCTGGCTTAGTCTTGTTTACGCATGAACTTCAAAATCATACTGCGACGGAATATTTTGTTTCATCGCGACGTATGTTT

GAACCTAGAAAACCTACCGTTAGTGATTTTGTTCAAATTGAGAGTTGTGTGGTCACCTATGTCAATTTGACTAGAGACC
AACTACCAGATGTAATCCCAGATTACATCGATGTTAACAAAACACTTGATGAGATTTTAGCTTCTCTGCCCAATAGAAC
TGGTCCAAGTCTTCCTTTAGATGTTTTTAATGCCACTTATCTTAATCTCACTGGTGAAATTGCAGATTTAGAGCAGCGT
TCAGAGTCTCTCCGTAATACTACAGAGGAGCTCCAAAGTCTTATATATAATATCAACAACACACTAGTTGACCTTGAGT
GGCTCAACCGAGTTGAGACATATATCAAGTGGCCGTGGTGGGTTTGGTTGATTATTTTCATTGTTCTCATCTTTGTTGT
GTCATTACTAGTGTTCTGCTGCATTTCCACTGGTTGTTGTGGATGCTGCGGCTGCTGCTGTGCTTGTTTTTCAGGTTGT
TGTAGGGGTCCTAGACTTCAACCTTACGAAGTTTTTGAAAAGGTCCACGTGCAGTGA

>gi|377657493|gb|JN825709.1|_Porcine_epidemic_diarrhea_virus_isolate_HB2011
3_spike_protein_(S)_mRNA_complete_cds

ATGAAGTCTTTAACCTACTTCTGGTTGTTCTTACCAGTACTTTCAACACTTAGCCTACCACAAGATGTCACCAGG
TGCTCAGCTAACACTAATTTTAGGCGGTTCTTTTCAAAATTTAATGTTCAGGCGCCTGCAGTTGTTGTACTGGGCGGTT
ATCTACCTATTGGTGAAAACCAGGGTGTCAATTCAACTTGGTACTGTGCTGGCCAACATCCAACTGCTAGTGGCGTTCA
TGGTATCTTTGTTAGCCATATTAGAGGTGGTCATGGCTTTGAGATTGGCATTTCGCAAGAGCCTTTTGACCCTAGTGGT
TACCAGCTTTATTTACATAAGGCTACTAACGGTAACACTAATGCTACTGCGCGACTGCGCATTTGCCAGTTTCCTAGCA
TTAAAACATTGGGCCCCACTGCTAATAATGATGTTACAACAGGTCGTAATTGCCTATTTAACAAAGCCATCCCAGCTCA
TATGAGTGAACATAGTGTTGTCGGCATAACATGGGATAATGATCGTGTCACTGTCTTTTCTGACAAGATCTATTATTTT
TATTTTAAAAATGATTGGTCCCGTGTTGCGACAAAGTGTTACAACAGTGGAGGTTGTGCTATGCAATATGTTTACGAAC
CCACCTATTACATGCTTAATGTTACTAGTGCTGGTGAGGATGGTATTTCTTATCAACCCTGTACAGCTAATTGCATTGG
TTATGCTGCCAATGTATTTGCTACTGAGCCCAATGGCCACATACCAGAAGGTTTTAGTTTTAATAATTGGTTTCTTTTG
TCCAATGATTCCACTTTGGTGCATGGTAAGGTGGTTTCCAACCAACCATTGTTGGTCAATTGTCTTTTGGCCATTCCTA
AGATTTATGGACTAGGCCAATTTTTCTCCTTTAATCAAACGATCGATGGTGTTTGTAATGGAGCTGCTGTGCAGCGTGC
ACCAGAGGCTCTGAGGTTTAATATTAATGACACCTCTGTCATTCTTGCTGAAGGCTCAATTGTACTTCATACTGCTTTA
GGAACAAATTTTTCTTTTGTTTGCAGTAATTCCTCAGATCCTCACTTAGCCACCTTCGCCATACCTCTGGGTGCTATCC
AAGTACCCTATTATTGTTTTCTTAAAGTGGATACTTACAACTCCACTGTTTATAAATTCTTGGCTGTTTTACCTCCTAC
CGTCAGGGAAATTGTCATCACCAAGTATGGTGATGTTTATGTCAATGGGTTTGGCTACTTGCATCTCGGTTTGTTGGAT
GCTGTCACAATTAATTTCACTGGTCATGGCACTGACGATGACGTTTCAGGTTTCTGGACCATAGCATCGACTAATTTTG
TTGATGCACTTATCGAAGTTCAAGGAACTGCCATTCAGCGTATTCTTTATTGTGATGATCCTGTTAGCCAACTCAAGTG
TTCTCAGGTTGCTTTTGACCTTGACGATGGTTTTTACCCTATTTCTTCTACAAACCTTCTGAGTCATGAACAGCCAACT
TCTTTTGTTACTTTGCCATCATTTAATGATCATTCTTTTGTTAATATTACTGTCTCTGCGGCTTTTGGTGGTCATAGTG
GTGCCAACCTTATTGCATCTGACACTACTATCAATGGGTTTAGTTCTTTCTGTGTTGACACTAGACAATTTACCATTTC
ACTGTTTTATAATGTTACAAACAGTTATGGTTATGTGTCTAACTCACAGGACAGTAATTGCCCTTTCACCTTGCAATCT
GTTAATGATTACCTGTCTTTTAGCAAATTTTGTGTTTCTACCAGCCTTTTGGCTAGTGCCTGTACCATAGATCTTTTTG
GTTACCCTGAGTTTGGTAGTGGTGTTAAGTTCACGTCCCTTTACTTTCAATTCACAAAGGGTGAGTTGATTACTGGCAC
GCCTAAACCACTTGAAGGTGTTACGGACGTTTCTTTTATGACTCTGGATGTGTGTACCAAGTATACTATCTATGGCTTT
AAAGGTGAGGGTATCATTACCCTTACAAATTCTAGCTTTTTGGCAGGTGTTTATTATACATCTGATTCTGGACAGTTGT
TAGCCTTTAAGAATGTCACTAGTGGTGCTGTTTATTCTGTTACGCCATGTTCTTTTTCAGAGCAGGCTGCATATGTTGA
TGATGATATAGTGGGTGTTATTTCTAGTTTGTCTAGCTCCACTTTTAACAGTACTAGGGAGTTGCCTGGTTTCTTCTAC
CATTCTAATGATGGCTCTAATTGTACAGAGCCTGTGTTGGTGTATAGTAACATAGGTGTTTGTAAATCTGGCAGTATTG
GCTATGTCCCATCTCAGTCTGGCCAAGTCAAGATTGCACCCACGGTTACTGGGAATATTAGTATTCCCACCAACTTTAG
TATGAGTATTAGGACAGAATATTTACAGCTTTACAACACGCCTGTTAGTGTTGATTGTGCTACATATGTTTGTAATGGT
AACTCTCGTTGTAAACAATTACTCACCCAGTACACTGCAGCATGTAAGACCATAGAGTCAGCATTACAACTCAGCGCTA

GGCTTGAGTCTGCTGAAGTCAACTCTATGCTTACTATTTCTGAAGAGGCTCTACAGTTAGCTACCATCAGTTCGTTTAA
TGGTGATGGATATAATTTTACTAATGTGCTGGGTGTTTCCGTTCGTGATCCTGCAAGTGGCAGGGTGGTACAAAAAAGG
TCTTTTATTGAAGACCTGCTTTTTAATAAAGTGGTTACTAATGGCCTTGGTACTGTTGATGAAGACTATAAGCGCTGTT
CTAATGGCCGCTCTGTGGCAGATTTAGTCTGTGCGCAGTATTACTCTGGTGTCATGGTACTACCTGGTGTTGTTGACGC
TGAGAAGCTTCATATGTATAGTGCGTCTCTCATCGGTGGTATGGTGCTAGGAGGTTTTACTGCAGCAGCGGCATTGCCT
TTTAGCTATGCTGTTCAAGCGAGACTCAATTATCTTGCTCTACAGACGGATGTTCTACAGCGGAACCAGCAATTGCTTG
CTGAGTCTTTTAACTCTGCTATTGGTAATATAACTTCAGCCTTTGAGAGTGTTAAAGAGGCTATTAGTCAAACTTCTAA
GGGTTTGAACACTGTGGCTCATGCGCTTACTAAGGTTCAAGAGGTTGTTAATTCGCAGGGTGCAGCTTTGACCCAACTT
ACCGTACAGCTGCAACACAACTTCCAAGCCATTTCTAGTTCTATTGATGACATTTATTCCCGACTGGACATTCTTTCAG
CCGATGTTCAGGTTGACCGTCTCATCACCGGCAGATTATCAGCACTTAATGCTTTTGTTGCTCAAACCCTCACTAAGTA
TACTGAGGTTCAGGCTAGCAGGAAGCTAGCACAGCAAAAGGTTAATGAGTGCGTTAAATCGCAATCTCAGCGTTATGGT
TTTTGTGGTGGTGATGGCGAGCACATTTTCTCTCTGGTACAGGCAGCACCTCAGGGCCTGCTGTTTTTACATACAGTAC
TTGTACCGGGTGACTTTGTAGATGTTATTGCCATCGCTGGCTTATGCGTTAACGATGAAATTGCCTTGACTCTACGTGA
GCCTGGCTTAGTCTTGTTTACGCATGAACTTCAAAATCATACTGCGACGGAATATTTTGTTTCATCGCGACGTATGTTT
GAACCTAGAAAACCTACCGTTAGTGATTTTGTTCAAATTGAGAGTTGTGTGGTCACCTATGTCAATTTGACTAGAGACC
AACTACCAGATGTAATCCCAGATTACATCGATGTTAACAAAACACTTGATGAGATTTTAGCTTCTCTGCCCAATAGAAC
TGGTCCAAGTCTTCCTTTAGATGTTTTTAATGCCACTTATCTTAATCTCACTGGTGAAATTGCAGATTTAGAGCAGCGT
TCAGAGTCTCTCCGTAATACTACAGAGGAGCTCCAAAGTCTTATATATAATATCAACAACACACTAGTTGACCTTGAGT
GGCTCAACCGAGTTGAGACATATATCAAGTGGCCGTGGTGGGTTTGGTTGATTATTTTCATTGTTCTCATCTTTGTTGT
GTCATTACTAGTGTTCTGCTGCATTTCCACGGGTTGTTGTGGATGCTGCGGCTGCTGCTGTGCTTGTTTTTCAGGTTGT
TGTAGGGGTCCTAGACTTCAACCTTACGAAGTTTTTGAAAAGGTCCACGTGCAGTGA

>gi|377657495|gb|JN825710.1|_Porcine_epidemic_diarrhea_virus_isolate_ZJ20111_spike_protein_(S)_mRNA_complete_cds

ATGAAGTCTTTAACCTACTTCTGGTTGTTCTTACCAGTACTTTCAACACTTAGCCTACCACAAGATGTCACCAGG
TGCTCAGCTAACACTAATTTTAGGCGGTTCTTTTCAAAATTTAATGTTCAGGCGCCTGCAGTTGTTGTACTGGGCGGTT
ATCTACCTATTGGTGAAAACCAGGGTGTCAATTCAACTTGGTACTGTGCTGGCCAACATCCAACTGCTAGTGGCGTTCA
TGGTATCTTTGTTAGCCATATTAGAGGTGGTCATGGCTTTGAGATTGGCATTTCGCAAGAGCCTTTTGACCCTAGTGGT
TACCAGCTTTATTTACATAAGGCTACTAACGGTAACACTAATGCTACTGCGCGACTGCGCATTTGCCAGTTTCCTAGCA
TTAAAACATTGGGCCCCACTGCTAATAATGATGTTACAACAGGTCGTAATTGCCTATTTAACAAAGCCATCCCAGCTCA
TATGAGTGAACATAGTGTTGTCGGCATAACATGGGATAATGATCGTGTCACTGTCTTTTCTGACAAGATCTATTATTTT
TATTTTAAAAATGATTGGTCCCGTGTTGCGACAAAGTGTTACAACCGTGGAGGTTGTGCTATGCAATATGTTTACGAAC
CCACCTATTACATGCTTAATGTTACTAGTGCTGGTGAGGATGGTATTTCTTATCAACCCTGTACAGCTAATTGCATTGG
TTATGCTGCCAATGTATTTGCTACTGAGCCCAATGGCCACATACCAGAAGGTTTTAGTTTTAATAATTGGTTTCTTTTG
TCCAATGATTCCACTTTGGTGCATGGTAAGGTGGTTTCCAACCAACCATTGTTGGTCAATTGTCTTTTGGCCATTCCTA
AGATTTATGGACTAGGCCAATTTTTCTCCTTTAATCAAACGATCGATGGTGTTTGTAATGGAGCTGCTGTGCAGCGTGC
ACCAGAGGCTCTGAGGTTTAATATTAATGACACCTCTGTCATTCTTGCTGAAGGCTCAATTGTACTTCATACTGCTTTA
GGAACAAATTTTTCTTTTGTTTGCAGTAATTCCTCAGATCCTCACTTAGCCACCTTCGCCATACCTCTGGGTGCTATCC
AAGTACCCTATTATTGTTTTCTTAAAGTGGATACTTACAACTCCACTGTTTATAAATTCTTGGCTGTTTTACCTCCTAC
CGTCAGGGAAATTGTCATCACCAAGTATGGTGATGTTTATGTCAATGGGTTTGGCTACTTGCATCTCGGTTTGTTGGAT
GCTGTCACAATTAATTTCACTGGTCATGGCACTGACGATGACGTTTCAGGTTTCTGGACCATAGCATCGACTAATTTTG
TTGATGCACTTATCGAAGTTCAAGGAACTGCCATTCAGCGTATTCTTTATTGTGATGATTCTGTTAGCCAACTCAAGTG

TTCTCAGGTTTCTTTTGACCTTGACGATGGTTTTTACCCTATTTCCTCTACAAACCTTCTGAGTCATGAACAGCCAACT
TCTTTTGTTACTTTGCCATCATTTAATGATCATTCTTTTGTTAATATTACTGTCTCTGCTGCTTTTGGTGGTCATAGTG
GTGCCAACCTTATTGCATCTGACACTACTATCAATGGGTTTAGTTCTTTCTGTGTTGACACTAGACAATTTACCATTTC
ACTGTTTTATAATGTTACAAACAGTTATGGTTATGTGTCTAACTCACAGGACAGTAATTGCCCTTTCACCTTGCAATCT
GTTAATGATTACCTGTCTTTTAGCAAATTTTGTGTTTCTACCAGCCTTTTGGCTAGTGCCTGTACCATAGATCTTTTTG
GTTACCCTGAGTTTGGTAGTGGTGTTAAGTTCACGTCCCTTTACTTTCAATTCACAAAGGGTGAGTTGATTACTGGCAC
GCCTAAACCACTTGAAGGTGTTACGGACGTTTCTTTTATGACTCTGGATGTGTGTACCAAGTATACTATCTATGGCTTT
AAAGGTGAGGGTATCATTACCCTTACAAATTCTAGCTTTTTGGCAGGTGTTTATTATACATCTGATTCTGGACAGTTGT
TAGCTTTTAAGAATGTCACTAGTGGTGCTGTTTATTCTGTTACGCCATGTTCTTTTTCAGAGCAGGCTGCATATGTTGA
TGATGATATAGTGGGTGTTATTTCTAGTTTGTCTAGCTCCACTTTTAACAGTACTAGGGAGTTGCCTGGTTTCTTCTAC
CATTCTAATGATGGCTCTAATTGTACAGAGCCTGTGTTGGTGTATAGTAACATAGGTGTTTGTAAATCTGGCAGTATTG
GCTATGTCCCATCTCAGTCTGGCCAAGTCAAGATTGCACCCACGGTTACTGGGAATATTAGTATTCCCACCAACTTTAG
TATGAGTATTAGGACAGAATATTTACAGCTTTACAACACGCCTGTTAGTGTTGATTGTGCTACATATGTTTGTAATGGT
AACTCTCGTTGTAAACAATTACTCACCCAGTACACTGCAGCATGTAAGACCATAGAGTCAGCATTACTACTCAGCGCTA
GGCTTGAGTCTGCTGAAGTCAACTCTATGCTTACTATTTCTGAAGAGGCTCTACAGTTAGCTACCATCAGTTCGTTTAA
TGGTGATGGATATAATTTTACTAATGTGCTGGGTGTTTCCGTTCGTGATCCTGCAAGTGGCAGGGTGGTAAAAAAAAGG
TCTTTTATTGAAGACCTGCTTTTTAATAAAGTGGTTACTAATGGCCTTGGTACTGTTGATGAAGACTATAAGCGCTGTT
CTAATGGCCGCTCTGTGGCAGATTTAGTCTGTGCGCAGTATTACTCTGGTGTCATGGTACTACCTGGTGTTGTTGACGC
TGAGAAGCTTCATATGTATAGTGCGTCTCTCATCGGTGGTATGGTGCTAGGAGGTTTTACTGCAGCAGCGGCATTGCCT
TTTAGCTATGCTGTTCAAGCGAGACTCAATTATCTTGCTCTACAGACGGATGTTCTACAGCGGAACCAGCAATTGCTTG
CTGAGTCTTTTAACTCTGCTATTGGTAATATAACTTCAGCCTTTGAGAGTGTTAAAGAGGCTATTAGTCAAACTTCTAA
GGGTTTGAACACTGTGGCTCATGCGCTTACTAAGGTTCAAGAGGTTGTTAATTCGCAGGGTGCAGCTTTGACCCAACTT
ACCGTACAGCTGCAACACAACTTCCAAGCCATTTCTAGTTCTATTGATGACATTTATTCCCGACTGGACATTCTTTCAG
CCGATGTTCAGGTTGACCGTCTCATCACCGGCAGATTATCAGCACTTAATGCTTTTGTTGCTCAAACCCTCACTAAGTA
TACTGAGGTTCAGGCTAGCAGGAAGCTAGCACAGCAAAAGGTTAATGAGTGCGTTAAATCGCAATCTCAGCGTTATGGT
TTTTGTGGTGGTGATGGCGAGCACATTTTCTCTCTGGTACAGGCAGCACCTCAGGGCCTGCTGTTTTTACATACAGTAC
TTGTACCGGGTGACTTTGTAGATGTTATTGCCATCGCTGGCTTATGCGTTAACGATGAAATTGCCTTGACTCTACGTGA
GCCTGGCTTAGTCTTGTTTACGCATGAACTTCAAAATCATACTGCGACGGAATATTTTGTTTCATCGCGACGTATGTTT
GAACCTAGAAAACCTACCGTTAGTGATTTTGTTCAAATTGAGAGTTGTGTGGTCACCTATGTCAATTTGACTAGAGACC
AACTACCAGATGTAATCCCAGATTACATCGATGTTAACAAAACACTTGATGAGATTTTAGCTTCTCTGCCCAATAGAAC
TGGTCCAAGTCTTCCTTTAGATGTTTTTAATGCCACTTATCTTAATCTCACTGGTGAAATTGCAGATTTAGAGCAGCGT
TCAGAGTCTCTCCGTAATACTACAGAGGAGCTCCAAAGTCTTATATATAATATCAACAACACACTAGTTGACCTTGAGT
GGCTCAACCGAGTTGAGACATATATCAAGTGGCCGTGGTGGGTTTGGTTGATTATTTTCATTGTTCTCATCTTTGTTGT
GTCATTACTAGTGTTCTGCTGCATTTCCACTGGTTGTTGTGGATGCTGCGGCTGCTGCTGTGCTTGTTTTTCAGGTTGT
TGTAGGGGTCCTAGACTTCAACCTTACGAAGTTTTTGAAAAGGTCCACGTGCAGTGA

>gi|377657497|gb|JN825711.1|_Porcine_epidemic_diarrhea_virus_isolate_ZJ20112_spike_protein_(S)_mRNA_complete_cds

ATGAAGTCTTTGACCTACTTCTGGTTGTTCTTACCAGTACTTTCAACACTTAGCCTACCACAAGATGTCACCAGG
TGCTCAGCTAACACTAATTTTAGGCGGTTCTTTTCAAAATTTAATGTTCAGGCGCCTGCAGTTGTTGTACTGGGCGGTT
ATCTACCTATTGGTGAAAACCAGGGTGTCAATTCAACTTGGTACTGTGCTGGCCAACATCCAACTGCTAGTGGCGTTCA
TGGTATCTTTGTTAGCCATATTAGAGGTGGTCATGGCTTTGAGATTGGCACTACGCAAGAGCCTTTTGACCCTAGTGGT

TACCAGCTTTATTTACATAAGGCTACTAACGGTAACACTAATGCTACTGCGCGACTGCGCATTTGCCAGTTTCCTAGCA
TTAAAACATTGGGCCCCACTGCTAATAATGATGTTACAACAGGTCGTAATTGCCTATTTAACAAAGCCATCCCAGCTCA
TATGAGTGAACATAGTGTTGTCGGCATAACATGGGATAATGATCGTGTCACTGTCTTTTCTGACAAGATCTATTATTTT
TATTTTAAAAATGATTGGTCCCGTGTTGCGACAAAGTGTTACAACAGTGGAGGTTGTGCTATGCAATATGTTTACGAAC
CCACCTATTACATGCTTAATGTTACTAGTGCTGGTGAGGATGGTATTTCTTATCAACCCTGTACAGCTAATTGCATTGG
TTATGCTGCCAATGTATTTGCTACTGAGCCCAATGGCCACATACCAGAAGGTTTTAGTTTTAATAATTGGTTTCTTTTG
TCCAATGATTCCACTTTGGTGCATGGTAAGGTGGTTTCCAACCAACCATTGTTGGTCAATTGTCTTTTGGCCATTCCTA
AGATTTATGGACTAGGCCAATTTTTCTCCTTTAATCAAACGATCGATGGTGTTTGTAATGGAGCTGCTGTGCAGCGTGC
ACCAGAGGCTCTGAGGTTTAATATTAATGACACCTCTGTCATTCTTGCTGAAGGCTCAATTGTACTTCATACTGCTTTA
GGAACAAATTTTTCTTTTGTTTGCAGTAATTCCTCAGATCCTCACTTAGCCACCTTCGCCATACCTCTGGGTGCTATCC
AAGTACCCTATTATTGTTTTCTTAAAGTGGATACTTACAACTCCACTGTTTATAAATTCTTGGCTGTTTTACCTCCTAC
CGTCAGGGAAATTGTCATCACCAAGTATGGTGATGTTTATGTCAATGGGTTTGGCTACTTGCATCTCGGTTTGTTGGAT
GCTGTCACAATTAATTTCACTGGTCATGGCACTGACGATGACGTTTCAGGTTTCTGGACCATAGCATCGACTAATTTTG
TTGATGCACTTATCGAAGTTCAAGGAACTGCCATTCAGCGTATTCTTTATTGTGATGATCCTGTTAGCCAACTCAAGTG
TTCTCAGGTTTCTTTTGACCTTGACGATGGTTTTTACCCTATTTCCTCTAGAAACCTTCTGAGTCATGAACAGCCAATT
TCTTTTGTTACTTTGCCATCATTTAATGATCATTCTTTTGTTAATATTACTGTCTCTGCTGCTTTTGGTGGTCTTAGTG
GTGCCAACCTTATTGCATCTGACACTACTATCAATGGGTTTAGTTCTTTCTGTGTTGACACTAGACAATTTACCATTTC
ACTGTTTTATAATGTTACAAACAGTTATGGTTATGTGTCTAACTCACAGGACAGTAATTGCCCTTTCACCTTGCAATCT
GTTAATGATTACCTGTCTTTTAGCAAATTTTGTGTTTCTACCAGCCTTTTGGCTAGTGCCTGTACCATAGATCTTTTTG
GTTACCCTGAGTTTGGTAGTGGTGTTAAGTTCACGTCCCTTTACTTTCAATTCACAAAGGGTGAGTTGATTACTGGCAC
GCCTAAACCACTTGAAGGTGTCACGGACGTTTCTTTTATGACTCTGGATGTGTGTACCAAGTATACTATCTATGGCTTT
AAAGGTGAGGGTATCATTACCCTTACAAATTCTAGCTTTTTGGCAGGTGTTTATTATACATCTGATTCTGGACAGTTGT
TAGCTTTTAAGAATGTCACTAGTGGTGCTGTTTATTCTGTTACGCCATGTTCTTTTTCAGAGCAGGCTGCATATGTTGA
TGATGATATAGTGGGTGTTATTTCTAGTTTGTCTAGCTCCACTTTTAACAGTACTAGGGAGTTGCCTGGTTTCTTCTAC
CATTCTAATGATGGCTCTAATTGTACAGAGCCTGTGTTGGTGTATAGTAACATAGGTGTTTGTAAATCTGGCAGTATTG
GCTATGTCCCATCTCAGTCTGGCCAAGTCAAGATTGCACCCACGGTTACTGGGAATATTAGTATTCCCACCAACTTTAG
TATGAGTATTAGGACAGAATATTTACAGCTTTACAACACGCCTTTTAGTGTTGATTGTGCTACATATGTTTGTAATGGT
AACTCTCGTTGTAAACAATTACTCACCCAGTACACTGCAGCATGTAAGACCATAGAGTCAGCATTACAACTCAGCGCTA
GGCTTGAGTCTGCTGAAGTCAACTCTATGCTTACTATTTCTGAAGAGGCTCTACAGTTAGCTACCATCAGTTCGTTTAA
TGGTGATGGATATAATTTTACTAATGTGCTGGGTGTTTCCGTTCGTGATCCTGCAAGTGGCAGGGTGGTACAAAAAAGG
TCTTTTATTGAAGACCTGCTTTTTAATAAAGTGGTTACTAATGGCCTTGGTACTGTTGATGAAGACTATAAGCGCTGTT
CTAATGGCCGCTCTGTGGCAGATTTAGTCTGTGCGCAGTATTACTCTGGTGTCATGGTACTACCTGGTGTTGTTGACGC
TGAGAAGCTTCATATGTATAGTGCGTCTCTCATCGGTGGTATGGTGCTAGGAGGTTTTACTGCAGCAGCGGCATTGCCT
TTTAGCTATGCTGTTCAAGCGAGACTCAATTATCTTGCTCTACAGACGGATGTTCTACAGCGGAACCAGCAATTGCTTG
CTGAGTCTTTTAACTCTGCTATTGGTAATATAACTTCAGCCTTTGAGAGTGTTAAAGAGGCTATTAGTCAAACTTCTAA
GGGTTTGAACACTGTGGCTCATGCGCTTACTAAGGTTCAAGAGGTTGTTAATTCGCAGGGTGCAGCTTTGACCCAACTT
ACCGTACAGCTGCAACACAACTTCCAAGCCATTTCTAGTTCTATTGATGACATTTATTCCCGACTGGACATTCTTTCAG
CCGATGTTCAGGTTGACCGTCTCATCACCGGCAGATTATCAGCACTTAATGCTTTTGTTGCTCAAACCCTCACTAAGTA
TACTGAGGTTCAGGCTAGCAGGAAGCTAGCACAGCAAAAGGTTAATGAGTGCGTTAAATCGCAATCTCAGCGTTATGGT
TTTTGTGGTGGTGATGGCGAGCACATTTTCTCTCTGGTACAGGCAGCACCTCAGGGCCTGCTGTTTTTACATACAGTAC
TTGTACCGGGTGACTTTGTAGATGTTATTGCCATCGCTGGCTTATGCGTTAACGATGAAATTGCCTTGACTCTACGTGA
GCCTGGCTTAGTCTTGTTTACGCATGAACTTCAAAATCATACTGCGACGGAATATTTTGTTTCATCGCGACGTATGTTT
GAACCTAGAAAACCTACCGTTAGTGATTTTGTTCAAATTGAGAGTTGTGTGGTCACCTATGTCAATTTGACTAGAGACC
AACTACCAGATGTAATCCCAGATTACATCGATGTTAACAAAACACTTGATGAGATTTTAGCTTCTCTGCCCAATAGAAC

TGGTCCAAGTCTTCCTTTAGATGTTTTTAATGCCACTTATCTTAATCTCACTGGTGAAATTGCAGATTTAGAGCAGCGT
TCAGAGTCTCTCCGTAATACTACAGAGGAGCTCCAAAGTCTTATATATAATATCAACAACACACTAGTTGACCTTGAGT
GGCTCAACCGAGTTGAGACATATATCAAGTGGCCGTGGTGGGTTTGGTTGATTATTTTCATTGTTCTCATCTTTGTTGT
GTCATTACTAGTGTTCTGCTGCATTTCCACTGGTTGTTGTGGATGCTGCGGCTGCTGCTGTGCTTGTTTTTCAGGTTGT
TGTAGGGGTCCTAGACTTCAACCTTACGAAGTTTTTGAAAAGGTCCACGTGCAGTGA

>gi|379319276|gb|JN825712.1| Porcine epidemic diarrhea virus isolate BJ-2011-1, complete genome

ATGAAGTCTTTAACCTACTTCTGGTTGTTCTTACCAGTACTTTCAACACTTAGCCTACCACAAGATGTCACCAGG
TGCTCAGCTAACACTAATTTTAGGCGGTTCTTTTCAAAATTTAATGTTCAGGCGCCTGCAGTTGTTGTACTGGGCGGTT
ATCTACCTATTGGTGAAAACCAGGGTGTCAATTCAACTTGGTACTGTGCTGGCCAACATCCAACTGCTAGTGGCGTTCA
TGGTATCTTTGTTAGCCATATTAGAGGTGGTCATGGCTTTGAGATTGGCATTTCGCAAGAGCCTTTTGACCCTAGTGGT
TACCAGCTTTATTTACATAAGGCTACTAACGGTAACACTAATGCTACTGCGCGACTGCGCATTTGCCAGTTTCCTAGCA
TTAAAACATTGGGCCCCACTGCTAATAATGATGTTACAACAGGTCGTAATTGCCTATTTAACAAAGCCATCCCAGCTCA
TATGAGTGAACATAGTGTTGTCGGCATAACATGGGATAATGATCGTGTCACTGTCTTTTCTGACAAGATCTATTATTTT
TATTTTAAAAATGATTGGTCCCGTGTTGCGACAAAGTGTTACAACAGTGGAGGTTGTGCTATGCAATATGTTTACGAAC
CCACCTATTACATGCTTAATGTTACTAGTGCTGGTGAGGATGGTATTTCTTATCAACCCTGTACAGCTAATTGCATTGG
TTATGCTGCCAATGTATTTGCTACTGAGCCCAATGGCCACATACCAGAAGGTTTTAGTTTTAATAATTGGTTTCTTTTG
TCCAATGATTCCACTTTGGTGCATGGTAAGGTGGTTTCCAACCAACCATTGTTGGTCAATTGTCTTTTGGCCATTCCTA
AGATTTATGGACTAGGCCAATTTTTCTCCTTTAATCAAACGATCGATGGTGTTTGTAATGGAGCTGCTGTGCAGCGTGC
ACCAGAGGCTCTGAGGTTTAATATTAATGACACCTCTGTCATTCTTGCTGAAGGCTCAATTGTACTTCATACTGCTTTA
GGAACAAATTTTTCTTTTGTTTGCAGTAATTCCTCAGATCCTCACTTAGCCACCTTCGCCATACCTCTGGGTGCTATCC
AAGTACCCTATTATTGTTTTCTTAAAGTGGATACTTACAACTCCACTGTTTATAAATTCTTGGCTGTTTTACCTCCTAC
CGTCAGGGAAATTGTCATCACCAAGTATGGTGATGTTTATGTCAATGGGTTTGGCTATTTGCATCTCGGTTTGTTGGAC
GCTGTCACAATTAATTTCACTGGTCATGGCACTGACGATGACGTTTCAGGTTTCTGGACCATAGCATCGACTAATTTTG
TTGATGCACTTATCGAAGTTCAAGGAACTGCCATTCAGCGTATTCTTTATTGTGATGATCCTGTTAGCCAACTCAAGTG
TTCTCAGGTTGCTTTTGACCTTGACGATGGTTTCTACCCTATTTCTTCTAGAAACCTTCTGAGTCATGAACAGCCAATT
TCTTTTGTTACTTTGCCATCATTTAATGATCATTCTTTTGTTAACATTACTGTCTCTGCGTCCTTTGGTGGTCTTAGTG
GTGCCAACCTTATTGCATCTGACACTACTATCAATGGGTTTAGTTCTTTCTGTGTTGACACTAGACAATTTACCATTTC
ACTGTTTTATAACGTTACAAACAGTTATGGTTATGTGTCTAAATCACAGGACAGTAATTGCCCTTTCACCTTGCAATCT
GTTAATGATTACCTGTCTTTTAGTAAATTTTGTGTTTCCACCAGCCTTTTGGCTAGTGCCTGTACCATAGATCTTTTTG
GTTACCCTGAGTTTGGTAGTGGTGTTAAGTTTACGTCCCTTTACTTTCAATTCACAAAGGGTGAGTTGATTACTGGCAC
GCCTAAACCACTTGAAGGTGTCACGGACGTTTCTTTTATGACTCTGGATGTGTGTACCAAGTATACTATCTATGGCTTT
AAAGGTGAGGGTATCATTACCCTTACAAATTCTAGCTTTTTGGCAGGTTTTTATTACACATCTGATTCTGGACAGTTGT
TAGCCTTTAAGAATGTCACTAGTGGTGCTGTTTATTCTGTTACGCCATGTTCTTTTTCAGAGCAGGCTGCATATGTTGA
TGATGATATAGTGGGTGTTATTTCTAGTTTGTCTAGCTCCACTTTTAACAGTACTAGGGAGTTGCCTGGTTTCTTCTAC
CATTCTAATGATGGCTCTAATTGTACAGAGCCTGTGTTGGTGTATAGTAACATAGGTGTTTGTAAATCTGGCAGTATTG
GCTACGTCCCATCTCAGTCTGGCCAAGTCAAGATTGCACCCACGGTTACTGGGAATATTAGTATTCCCACCAACTTTAG
TATGAGTATTAGGACAGAATATTTACAGCTTTACAACACGCCTGTTAGTGTTGATTGTGCCACATATGTTTGTAATGGT
AACTCTCGTTGTAAACAATTACTCACCCAGTACACTGCAGCATGTAAGACCATAGAGTCAGCATTACAACTCAGCGCTA
GGCTTGAGTCTGTTGAAGTTAACTCTATGCTTACTATTTCTGAAGAGGCTCTACAGTTAGCTACCATCAGTTCGTTTAA
TGGTGATGGATATAATTTTACTAATGTGCTGGGTGTTTCTGTGTATGATCCTGCAAGTGGCAGGGTGGTACAAAAAAGG

TCTTTTATTGAAGACCTGCTTTTTAATAAAGTGGTTACTAATGGCCTTGGTACTGTTGATGAAGACTATAAGCGCTGTT
CTAATGGTCGCTCTGTGGCAGATCTAGTCTGTGCACAGTATTACTCTGGTGTCATGGTACTACCTGGTGTTGTTGACGC
TGAGAAGCTTCACATGTATAGTGCGTCTCTCATCGGTGGTATGGTGCTAGGAGGTTTTACTTCTGCAGCGGCATTGCCT
TTTAGCTATGCTGTTCAAGCTAGACTCAATTATCTTGCTCTACAGACGGATGTTCTACAGCGGAACCAGCAATTGCTTG
CTGAGTCTTTTAACTCTGCTATTGGTAATATAACTTCAGCCTTTGAGAGTGTTAAAGAGGCTATTAGTCAAACTTCCAA
GGGTTTGAACACTGTGGCTCATGCGCTTACTAAGGTTCAAGAGGTTGTTAACTCGCAGGGTGCAGCTTTGACTCAACTT
ACCGTACAGCTGCAACACAACTTCCAAGCCATTTCTAGTTCTATTGATGACATTTATTCCCGACTGGACATTCTTTCAG
CCGATGTTCAGGTTGACCGTCTCATCACCGGCAGATTATCAGCACTTAATGCTTTTGTTGCTCAAACCCTCACTAAGTA
TACTGAGGTTCAGGCTAGCAGGAAGCTAGCACAGCAAAAGGTTAATGAGTGCGTTAAATCGCAATCTCAGCGTTATGGT
TTTTGTGGTGGTGATGGCGAGCACATTTTCTCTCTGGTACAGGCAGCACCTCAGGGCCTGCTGTTTTTACATACAGTAC
TTGTACCGGGTGACTTTGTAGATGTTATTGCCATCGCTGGCTTATGCGTTAACGATGAAATTGCCTTGACTCTACGTGA
GCCTGGCTTAGTCTTGTTTACGCATGAACTTCAAAATCATACTGCGACGGAATATTTTGTTTCATCGCGACGTATGTTT
GAACCTAGAAAACCTACCGTTAGTGATTTTGTTCAAATTGAGAGTTGTGTGGTCACCTATGTCAATTTGACTAGAGACC
AACTACCAGATGTAATCCCAGATTACATCGATGTTAACAAAACACTTGATGAGATTTTAGCTTCTCTGCCCAATAGAAC
TGGTCCAAGTCTTCCTTTAGATGTTTTTAATGCCACTTATCTTAATCTCACTGGTGAAATTGCAGATTTAGAGCAGCGT
TCAGAGTCTCTCCGTAATACTACAGAGGAGCTCCAAAGTCTTATATATAATATCAACAACACACTAGTTGACCTTGAGT
GGCTCAACCGAGTTGAGACATATATCAAGTGGCCGTGGTGGGTTTGGTTGATTATTTTCATTGTTCTCATCTTTGTTGT
GTCATTACTAGTGTTCTGCTGCATTTCCACGGGTTGTTGTGGATGCTGCGGCTGCTGCTGTGCTTGTTTTTCAGGTTGT
TGTAGGGGTCCTAGACTTCAACCTTACGAAGTTTTTGAAAAGGTCCACGTGCAGTGA

>gi|363413019|gb|JN980698.1|_Porcine_epidemic_diarrhea_virus_isolate_CHGD01_spike_protein_(S)_gene_complete_cds

ATGAGGTCTTTAATTTACTTCTGGTTGTTCTTACCAGTACTTTCAACACTTAGCCTACCACAAGATGTCACCAGG
TGCTCAGCTAACACTAATTTTAGGCGGTTCTTTTCAAAATTTAATGTTCAGGCGCCTGCAGTTGTTGTACTGGGCGGTT
ATCTGCCTATTGGTGAAAACCAGGGTGTCAATTCAACTTGGTACTGTGCTGGCCAACATCCAACTGCTAGTGGCGTTCA
TGGTATCTTTCTTAGCCATATTAGAGGTGGTCATGGCTTTGAGATTGGCATTTCGCAAGAGCCTTTTGACCCTAGTGGT
TACCAGCTTTATTTACATAAGGCTACTAACGGTAACACTAATGCTACTGCGCGACTGCGCATTTGCCAGTTTCCCAGCA
TTAAAACATTGGGCCCCACTGCTAATAATGATGTTACGACAGGTCGTAACTGCCTATTTAACAAAGCCATCCCAGCTCA
TATGAGTGAGCATAGTGTTGTCGGCATAACATGGGATAATGATCGTGTCACTGTCTTTTCTGACAAGATCTATTATTTT
TATTTTAAAAATGATTGGTCCCGTGTTGCGACAAAGTGTTACAACAGTGGAGGTTGTGCTATGCAATATGTTTACGAAC
CCACTTACTACATGCTTAATGTTACTAGTGCTGGTGAGGATGGTATTTCTTATCAACCCTGTACAGCTAATTGCATTGG
TTATGCTGCCAATGTATTTGCTACTGAGCCCAATGGCCACATACCAGAAGGTTTTAGTTTTAATAATTGGTTTCTTTTG
TCCAATGATTCCACTTTGGTGCATGGTAAGGTGGTTTCCAACCAACCATTGTTGGTCAATTGTCTTTTGGCCATTCCTA
AGATTTATGGACTAGGCCAATTTTTCTCCTTTAATCAAACGATCGATGGTGTTTGTAATGGAGCTGCTGTGCAGCGTGC
ACCAGAGGCTCTGAGGTTTAATATTAATGACACCTCTGTCATTCTTGCTGAAGGCTCAATTGTACTTCATACTGCTTTA
GGAACAAATTCTTCTTTTGTTTGCAGTAATTCCTCAGATCCTCACTTAGCCACCTTTGCCATACCTCTGGGTGCTATCC
AAGTACCCTATTATTGTTTTCTTAAAGTGGATACTTACAACTCCACTGTTTATAAATTCTTGGCTGTTTTACCTCCTAC
CGTCAGGGAAATTGTCATCACCAAGTATGGTGATGTTTATGTCAATGGGTTTGGCTACTTGCATCTCGGTTTGTTGGAT
GCTGTCACAATTAATTTCACTGGTCATGGCACTGACGATGACGTTTCTGGTTTTTGGACCATAGCATCGACTAATTTTG
TTGATGCACTTATCGAAGTTCAAGGGACTGCCATTCAGCGTATTCTTTATTGTGATGATCCTGTTAGCCAACTCAAGTG
TTCTCAGGTTGCTTTTGACCTTGACGATGGTTTTTACCCTATTTCCTCTACAAACCTTCTGAGTCATGAACAGCCAACT
TCTTTTGTTACTTTGCCATCATTTAATGATCATTCTTTTGTTAATATTACTGTCTCTGCTGCTTTTGGTGGTCATAGTG

GTGCCAACCTTATTGCATCTGACACTACTATCAATGGGTTTAGTTCTTTCTGTGTTGACACTAGACAATTTACCATTTC
ACTGTTTTATAACGTTACAAACAGTTATGGTTACGTGTCTAAATCACAGGACAGTAATTGCCCTTTTACCTTGCAATCT
GTTAATGATTACCTGTCTTTTAGCAAATTTTGTGTTTCTACCAGCCTTTTGGCTAGTGCCTGTACCATAGATCTTTTTG
GTTATCCTGAGTTTGGTAGTGGTGTTAAGTTCACGTCCCTTTACTTTCAATTCACAAAGGGTGAGTTGATTACTGGCAC
GCCTAAACCACTTGAAGGTGTTACGGACGTTTCTTTTATGACTCTGGATGTGTGTACCGAGTATACTATCTATGGCTTT
AAAGGTGAGGGTATCATTACCCTTACAAATTCTAGCTTTTTGGCAGGTGTTTATTATACATCTGATTCTGGACAGTTGT
TAGCTTTTAAGAATGTCACTAGTGGTGCTGTTTATTCTGTTACGCCATGTTCTTTTTCAGAGCAGGCTGCATATGTTGA
TGATGATATAGTGGGTGTTATTTCTAGTTTGTCTAGCTCCACTTTTAACAGTACTAGGGAGTTGCCTGGTTTCTTCTAC
CATTCTAATGATGGCTCTAATTGTACAGAGCCTGTGTTGGTGTATAGTAACATAGGTGTTTGTAAATCTGGCAGTATTG
GCTATGTCCGATCTCAGTCTGGCCAAGTCAAGATTGCACCCACGGTTACTGGGAATATTAGTATTCCCACCAACTTTAG
TATGAGTATTAGGACAGAATATTTACAGCTTTACAACACGCCTGTTAGTGTTGATTGTGCTACATATGTTTGTAATGGT
AACTCTCGTTGTAAACAATTACTCACCCAGTACACTGCAGCATGTAAGACCATAGAGTCAGCATTACAACTCAGCGCTA
GGCTTGAGTCTGCTGAAGTCAACTCTATGCTTACTATTTCTGAAGAGGCTCTACAGTTAGCTACCATCAGTTCGTTTAA
TGGTGATGGATATAATTTTACTAATGTGCTGGGTGTTTCCGTGTATGACCCTGCAAGTGGCAGGGTGGTACAAAAAAGG
TCTTTCATTGAAGACCTGCTTTTTAATAAAGTGGCTACTAATGGCCTTGGTACTGTTGATGAAGACTATAAGCGCTGTT
CTAATGGCCGCTCTGTGGCAGATTTAGTCTGTGCGCAGTATTACTCTGGTGTCATGGTACTACCTGGTGTTGTTGACGC
TGAGAAGCTTCATATGTATAGTGCGTCTCTCATCGGTGGTATGGTGCTAGGAGGTTTTACTGCAGCAGCGGCATTGCCT
TTTAGCTATGCTGTTCAAGCGAGACTGAATTATCTTGCTCTACAGACGGATGTTCTACAGCGGAACCAGCAATTGCTTG
CTGAGTCTTTTAATTCTGCTATTGGTAGTATAACTTCAGCCTTTGAGAGTGTTAAAGAGGCTATTAGTCAAACTTCTAA
GGGTTTGAACACTGTGGCTCATGCGCTTACCAAGGTCCAAGAGGTTGTTAATTCGCAGGGTGCAGCTTTGACCCAACTT
ACCGTACAGCTGCAACACAACTTCCAAGCCATTTCTAGTTCTATTGATGACGTTTACTCCCGACTTGACATTCTTTCAG
CCGATGTTCAGGTAGACCGTCTCATCACCGGCAGATTATCAGCACTTAATGCTTTTGTTGCTCAAACCCTTACTAAGTA
TACTGAGGTTCAGGCTAGCAGGAAACTAGCACAGCAAAAGGTTAATGAGTGCGTTAAATCGCAATCTCAGCGTTATGGT
TTTTGTGGTGGTGATGGCGAGCACATTTTCTCTCTGGTACAGGCCGCACCTCAAGGCCTGCTGTTTTTACACACAGTAC
TTGTACCGGGTGACTTTGTAAATGTTATTGCCATCGCTGGCTTATGTGTTAACGATGAAATTGCCTTGACTCTACGTGA
GCCTGGTTTAGTCTTGTTTACGCATGAACTTCAAGATACTGCGACGGAATATTTTGTTTCATCGCGACGTATGTATGAA
CCTAGAAAACCTACCGTTGGTGATTTTGTTCAAATTGAGAGTTGTGTGGTCACCTATGTCAATTTGACTAGAGACCAAC
TACCAGAAGTAATCCCAGATTACATCGATGTTAACAAAACACTTGATGAGATTTTAGCCTCTCTGCCCAATAGAACTGG
TCCAAGTCTTTCTCTAGATGTTTTTAATGCCACTTATCTTAATCTCACTGGTGAAATTGCAGATTTAGAGCAGCGTTCA
GAGTCTCTCCGTAATACTACAGAAGAGCTCCAAAGTCTTATATATAATATCAACAACACACTAGTTGACCTTGAGTGGC
TCAACCGAGTTGAGACATATATCAAGTGGCCGTGGTGGGTTTGGTTGATTATTTTTATTATTCTCATCTTTGTTGTGTC
ATTACTAGTGTTCTGCTGCATTTCCACGGGTTGTTGTGGATGCTGCGGCTGCTGTGGTGCTTGTTTTTCAGGTTGTTGT
AGGGGTCCTAGACTTCAACCTTACGAAGCTTTTGAAAAGGTCCACGTGCAGTGA

>gi|389828682|gb|JQ239429.1|_Porcine_epidemic_diarrhea_virus_strain_CH1_spike_protein_(S)_gene_complete_cds

ATGAAGTCTTTAACCTACTTCTGGTTGTTCTTACCAGTACTTTCAACACTTAGCCTACCACAAGATGTTACCAGG
TGCTCAGCTAACACTAACTTTAGGCGGTTCTTTTCAAAATTTAATGTTCAGGCGCCTGCAGTTGTTGTACTGGGCGGTT
ATCTACCTATTGGTGAAAACCAGGGTGTCAATTCAACTTGGTACTGTGCTGGCCAACATCCAACTGCTAGTGGCGTTCA
TGGTATCTTTCTTAGCCATATTAGAGGTGGTCATGGCTTTGAGATTGGCATTTCGCAAGAGCCTTTTGACCCTAGTGGT
TACCAGCTTTATTTACATAAGGCTACTAACGGTAACACTAATGCTACTGCGCGACTGCGCATTTGCCAGTTTCCCAGCA
TTAAAACATTGGGCCCCACTGCTAATAATGATGTTACAACAGGTCGTAACTGCCTATTTAACAAAGCCATCCCAGCTCA

TATGAGTGAACATAGTGTTGTCGGCATAACATGGGATAATAATCGTGTCACTGTCTTTTCTGACAAGATCTATTATTTT
TATTTTAAAAATGATTGGTCCCGTGTTGCGACAAAGTGTTACAACAGTGGAGGTTGTGCTATGCAATATGTTTACGAAC
CCACTTACTACACGCTTAATGTTACTAGTGCTGGTGAGGATGGTATTTCTTATCAACCCTGTACAGCTAATTGCATTGG
TTATGCTGCCAATGTATTTGCTACTGAGCCCAATGGCCACATACCAGAAGGTTTTAGTTTTAATAATTGGTTTCTTTTG
TCCAATGATTCCACTTTGGTGCATGGTAAGGTGGTTTCCAACCAACCTTTGTTGGTCAATTGTCTTTTGGCCATTCCTA
AGATTTATGGACTAGGCCAATTTTTCTCATTCAATCAAACGATGGATGGCGTTTGTAATGGAGCTGCTTTGCAGCGTGC
ACCAGAGGCTCTGAGGTTTAATATTAATGACACCTCTGTCATTCTTGCTGAAGGCTCAATTGTACTTCATACTGCTTTA
GGAACAAATCTTTCTTTTGTTTGCAGTAATTCCTCAGATCCTCATTTAGCCACCTTCGCCATACCTTTGGGTGCTACAC
AAGTACCCTATTATTGTTTTCTTAAAGTGGATACTTACAACTCCACTGTTTATAAATTCTTGGCTGTTTTACCTCCAAC
CGTCAGGGAAATTGTCATCACCAAGTATGGTGATGTTTATGTCAATGGGTTTGGCTACTTGCATCTCGGTTTGTTGGAT
GCTGTCACAATTAATTTCACTGGTCATGGCACTGACGATGACGTTTCTGGTTTCTGGACCGTAGCATCGACTAATTTTG
TTGATGCACTTATCGAAGTTCAAGGAACTGCCATTCAGCGTATTCTTTATTGTGATGACCCTGTTAGCCAACTCAAGTG
TTCTCAGGTTGCTTTTGACCTTGACGATGGTTTCTACCCTATTTCTTCTAGAAACCTCCTGAGTCATGAACAGCCAACT
TCTTTTGTTACTTTGCCATCATTTAATGATCATTCTTTTGTTAACATTACTGTCTCTGCGTCCTTTGGTGGTCATAGTG
GTGCCAACCTTATTGCATCTGACACTACTATCAATGGGTTTAGTTCTTTCTGTGTTGACACTAGACAATTTACCATTTC
ACTGTTTTATAACGTTACAAACAGTTATGGTTATGTGTCTAAATCACAGGACAGTAATTGCCCTTTCACCTTGCAATCT
GTTAATGATTACCTGTCTTTTAGCAAATTTTGTGTTTCCACCAGCCTTTTGGCTAGTGCCTGTACCATAGATCTTTTTG
GTTACCCTGAGTTTGGTAGTGGTGTTAAGTTTACGTCCCTTTACTTTCAATTCACAAAGGGTGAGTTGATTACTGGCAC
GCCTAAACCACTTGAAGGTGTCACGGACGTTTCTTTTATGACTCTGGATGTGTGTACCAAGTATACTATCTATGGCTTT
AAAGGTGAGGGTATCATTACCCTTACAAATTCTAGCTTTTTGGCAGGTGTTTATTATACATCTGATTCTGGACAGTTGT
TAGCTTTTAAGAATGTCACTAGTGGTGCTGTTTATTCTGTTACGCCATGTTCTTTTTCAGAGCAGGCTGCATATGTTGA
TGATGATATAGTGGGTGTTATTTCTAGTTTGTCTAGCTCCACTTTTAACAGTACTAGGGAGTTGCCTGGTTTCTTCTAC
CATTCTAATGATGGCTCTAATTGTACAGAGCCTGTGTTGGTGTATAGTAACATAGGTGTTTGTAAATCCGGCAGTATTG
GCTATGTCCCACTTCAGGATGGCCAAGTCAAGATTGTACCCACGGTTACTGGGAATATTAGTATTCCCACCAACTTTAG
TATGAGTATTAGGACAGAATATTTACAGCTTTACAACACGCCTGTTAGTGTTGATTGTGCTACATATGTTTGTAATGGT
AACTCTCGTTGTAAACAATTACTCACCCAGTACACTGCAGCATGTAAGACCATAGAGTCAGCATTACAACTCAGCGCTA
GGCTTGAGTCTGCTGAAGTCAACTCTATGCTTACTATTTCTGAAGAGGCTCTACAGTTAGCTACCATCAGTTCGTTTAA
TGGTGATGGATATAATTTTACTAATGTGCTGGGTGTTTCCGTGTATGACCCTGCAAGTGGCAGGGTGGTACAAAAAAGG
TCTTTCATTGAAGACCTGCTTTTTAATAAAGTGGTTACTAATGGCCTTGGTACTGTTGATGAAGACTATAAGCGCTGTT
CTAATGGCCGCTCTGTGGCAGATTTAGTCTGTGCGCAGTATTACTCTGGTGTCATGGTACTACCTGGTGTTGTTGACGC
TGAGAAGCTTCATATGTACAGTGCGTCTCTCATCGGTGGTATGGTGCTAGGAGGTTTTACTGCAGCAGCGGCATTGCCT
TTTAGCTATGCTGTTCAAGCGAGACTGAATTATCTTGCTCTACAGACGGATGTTCTACAGCGGAACCAGCAATTGCTTG
CTGAGTCTTTTAATTCTGCTATTGGTAATATAACTTCAGCCTTTGAGAGTGTTAAAGAGGCTATTAGTCAAACTTCTAA
GGGTTTGAACACTGTGGCTCATGCGCTTACCAAGGTCCAAGAGGTTGTTAATTCGCAGGGTGCAGCTTTGACCCAACTT
ACCGTACAGCTGCAACACAACTTCAAAGCCATTTCTAGTTCTATTGATGACATTTACTCCCGACTTGACATTCTTTCAG
CCGATGTTCAGGTAGACCGTCTCATCACCGGCAGATTATCAGCACTTAATGCTTTTGTTGCTCAAACCCTTACTAAGTA
TACTGAGGTTCAGGCTAGCAGGAAACTAGCACAGCAAAAGGTTAATGAGTGCGTTAAATCGCAATCTCAGCGTTATGGT
TTTTGTGGTGGTGATGGCGAGCACATTTTCTCCCTGGTACAGGCCGCACCTCAAGGCCTGCTGTTTTTACACACAGTAC
TTGTACCGGGTGACTTTGTAAATGTTATTGCCATCGCTGGCTTATGTGTTAACGATGAAATTGCCTTGACTCTACGTGA
GCCTGGTTTAGTCTTGTTTACGCATGAACTTCAAGATACTGCGACGGAATATTCTGTTACATCGCGACGTATGTATGAA
CCTAGAAAACCTACCGTTGGTGATTTTGTTCAAATTGAGAGTTGTGTGGTCACCTATGTCAATTTGACTAGAGACCAAC
TACCAGAAGTAATCCCAGATTACATCGATGTTAACAAAACACTTGATGAGATTTTAGCCTCTCTGCCCAATAGAACTGG
TCCAAGTCTTTCTCTAGATGTTTTTAATGCCACTTATCTTAATCTCACTGGTGAAATTGCAGATTTAGAGCAGCGTTCA
GAGTCTCTCCGTAATAGTACAGAAGAGCTCCAAAGTCTTATATATAATATCAACAACACACTAGTTGACCTTGAGTGGC

TCAACCGAGTTGAGACATATATCAAGTGGCCGTGGTGGGTTTGGTTGATTATTTTTATTGTTCTCATCTTTGTTGTGTC
ATTACTAGTGTTCTGCTGCATTTCCACGGGTTGTTGTGGATGCTGCGGCTGCTGTGGTGCTTGTTTTTCAGGTTGTTGT
AGGGGTCCTAGACTTCAACCTTACGAAGTTTTTGAAAAGGTCCACGTGCAGTGA

>gi|389828686|gb|JQ239430.1|_Porcine_epidemic_diarrhea_virus_strain_CH2_spike_protein_(S)_gene_complete_cds

ATGAAGTCTTTAACTTACTTCTGGTTGTTATTACCAGTACTTTCAACACTCAGCCTACCACAAGATGTCACTAGG
TGCCAGTCCACTATTAACTTCAGGCGGTTCTTTTCAAAATTTAATGTGCAATCACCTGCTGTCGTTGTTTTGGGTGGTT
ATCTACCTAGTATGAACTCTTCTAGCTGGTACTGTGGCACAGGCCTTGAAACTGCTAGTGGCGTTCATGGTATTTTCCT
TAGTTACATCGATTCTGGTCAGGGCTTTGAGATTGGCATTTCACAGGAGCCGTTTGATCCTAGTGGTTACCAGCTTTAT
CTACATAAGGCCACTAATGGTAACACTAATGCTATCGCACGATTGCGCATTTGCCAGTTTCCAGATAATAAAACATTGG
GCCCTACTGTTAATGATGTTACAACAGGTCGTAACTGCCTATTCAACAGAGCCATTCCAGCTTATATGCAGGATGGAAA
AAATATTGTTGTCGGCATAACATGGGATAATGATCGCGTCACTGTTTTTGCTGACAAGATCTATCATTTTTATCTTAAA
AATGATTGGTCCCGCGTTGTGACAAGATGTTACAATAAAAGGAGTTGTGCTATGCAATATGTTTATACACCTACCTACT
ACATGCTTAATGTTACTAGTGCAGGTGAGGATGGCATTTATTATGAACCATGTACAGCTAATTGCAGTGGTTACGCTGC
CAATGTATTTGCCACTGATTCTAATGGCCATATACCAGAAGGTTTTAGTTTTAATAATTGGTTTCTTTTGTCCAATGAT
TCCACTTTGTTGCATGGTAAGGTGGTTTCCAACCAACCTTTGTTGGTCAATTGTCTTTTGGCCATTCCTAAGATTTATG
GACTAGGCCAATTTTTCTCATTCAATCAAACGATGGATGGCGTTTGTAATGGAGCTGCTGTGCAGCGTGCACCAGAGGC
TCTGAGGTTTAATATTAATGACACCTCTGTCATTCTTGCTGAAGGCTCAATTGTACTTCATACTGCTTTAGGAACAAAT
CTTTCTTTTGTTTGCAGTAATTCCTCAAATCCTCATTTAGCCACCTTCGCCATACCTTTGGGTGCTACCCAAGTACCCT
ATTATTGTTTTCTTAAAGTGGATACTTACAACTCCACTGTTTATAAATTCTTGGCTGTTTTACCTCCTACCGTCAGGGA
AATTGTCATCACTAAGTATGGTGATGTTTATGTCAATGGGTTTGGCTACTTGCATCTCGGTTTGTTGGATGCTGTCACA
ATCAATTTCACTGGTCATGGCACTGACGATGACGTTTCTGGTTTCTGGACCGTAGCATCGACTAATTTTGTTGATGCAC
TCATCGAAGTTCAAGGAACTGCCATTCAGCGTATTCTTTATTGTGATGACCCTGTTAGCCAACTCAAGTGTTCTCAGGT
TGCTTTTGACCTTGACGATGGTTTTTACCCTATCTCTTCTAGGAACCTTCTGAGTCATGAACAGCCAATTTCTTTTGTT
ACTCTGCCATCATTTAATGATCATTCTTTTGTTAACATTACTGTCTCTGCGTCCTTTGGTGGTCATAGTGGTGCCAACC
TCATTGCATCTGACACTACTATCAATGGATTTAGTTCTTTCTGTGTTGACACTAGACAATTTACCATTTCACTGTTTTA
TAACGTTACAAACAGTTATGGTTATGTGTCTAACTCACAGGACACTAATTGCCCCTTTCACCTTGCAATCTGTTAATGAT
TACCTGTCTTTTAGCAAATTTTGTGTTTCTACCAGCCTTTTGGCTAGTGCCTGTACCATAGATCTTTTTGGTTACCCTG
CGTTTGGTAGTGTTGTTAAGTTTACGTCCCTTTACTTTCAATTCACAAAGGGTGAGTTGATTACTGGCACGCCTAAACC
ACTTGAAGGTGTTACGGACGTTTCTTTTATGACTCTGGATGTGTGTACCAAGTATACTATCTATGGCTTCAAAGGTGAG
GGTATCATTACCCTTACAAATTCTAGCTTTTTGGCAGGTGTTTATTATACATCTGATTCTGGACAGTTGTTAGCCTTTA
AGAATGTCACTAGTGGTGCTGTTTATTCTGTTACGCCATGTTCTTTTTCAGAGCAGGCTGCATATGTTGATGATGATAT
AGTGGGTGTTATTTCTAGTTTGTCTAGCTCCACTTTTAGCAGTACTAGGGAGTTGCCTGGTTTCTTCTACCATTCTAAT
GATGGCTCTAATTGTACAGAGCCTGTGTTGGTGTATAGTAACATAGGTGTTTGTAAATCTGGCAGTATTGGCTATGTCC
CACTTCAGGATGGCCAAGTCAAGATTGCACCCACGGTTACTGGGAATATTAGTATTCCCACCAGCTTTAGTATGAGTAT
TAGGACAGAATATGTACAGCTTTACAACACGCCTGTTAGTGTTGATTGTGCCACATATGTTTGTAATGGTAACTCTCGT
TGTAAACAATTACTCACCCAGTACACTGCAGCATGTAAGACCATAGAGTCAGCATTACAACTCAGCGCTAGGCTTGAGT
CTGTTGAAGTTAACTCTATGCTTACTATTTCTGAAGAGGCTCTACAGTTAGCTACCATCAGTTCGTTTAATGGTGATGG
ATATAATTTTACTAATGTGCTGGGTGTTTCTGCGTATGATCCTGCAAGTGGCAGGGTGGTACAAAAAAGGTCTTTTATT
GAAGACCTGCTTTTTAATAAAGTGGTTACTAATGGCCTTGGTACTGTTGATGAAGACTATAAGCGCTGTTCTAATGGTC
GTTCTGTGGCGGATCTAGTCTGTGCACAGTATTACTCTGGTGTCATGGTACTACCTGGTGTTGTTGACGCTGAGAAGCT

TCACATGTATAGTGCGTCTCTCATCGGTGGTATGGTGCTAGGAGGTTTTACTTCTGCAGCGGCATTGCCTTTTAGCTAT
GCTGTTCAAGCTAGACTCAATTATCTTGCTCTACAGACGGATGTTCTACAGCGGAACCAGCAGTTGCTTGCTGAGTCTT
TTAACTCTGCTATTGGTAATATAACTTCAGCCTTTGAGAGTGTTAAAGAGGCTATTAGTCAAACTTCTAAGGGTTTGAA
CACTGTGGCTCATGCGCTTACTAAGGTTCAAGAGGTTGTTAACTCGCAGGGTACAGCTTTGACTCAACTTACCGTACAG
CTGCAACACAACTTCCAAGCCATTTCTAGTTCTATTGATGACATTTACTCCCGACTGGACATTCTTTCAGCCGATGTTC
AGGTTGACCGTCTCATCACCGGCAGATTATCAGCACTTAATGCTTTTGTTGCTCAAACCCTCACTAAGTATACTGAGGT
TCAGGCTAGCAGGAAGCTAGCACAGCAAAAGGTTAATGAGTGCGTTAAATCGCAATCTCAGCGTTATGGTTTTTGTGGT
GGTGATGGCGAGCACATTTTCTCTCTGGTACAGGCAGCACCTCAGGGCCTGCTGTTTTTACATACAGTACTTGTACCGG
GTGATTTTGTAGATGTTATTGCCATCGCTGGCTTATGCGTTAACGATGAAATCGCCTTGACTCTACGTGAGCCTGGCTT
AGTCTTGTTTACGCATGAACTTCAAAATCATACTGCGACGGAATATTTTGTTTCATCGCGACGTATGTTTGAACCTAGA
AAACCTACCGTTAGTGATTTTGTTCAAATTGAGAGTTGTGTGGTCACCTACGTCAATTTGACTAGAGACCAACTACCAG
ATGTAATCCCAGATTACATCGATGTTAACAAAACACTTGATGAGATTTTAGCTTCTCTGCCCAATAGAACTGGCCCAAG
TCTTCCTTTAGATGTTTTTAATGCCACTTATCTCAATCTCACTGGTGAAATTGCAGATTTAGAGCAGCGTTCAGAGTCT
CTCCGTAATACTACAGAGGAGCTCCAAAGTCTTATATATAATATCAACAACACACTAGTTGACCTTGAGTGGCTCAACC
GAGTTGAGACATATATCAAGTGGCCGTGGTGGGTTTGGTTGGTTATTTTTATTGTTCTCATCTTTGTTGTGTCATTACT
AGTGTTCTGCTGCGTTTCCACGGGTTGTTGTGGATGCTGCGGCTGCTGCTGTGCTTGTTTTTCAGGTTGTTGTAGGGGT
CCCAGACTTCAACCTTACGAAGTTTTTGAAAAGGTCCACGTGCAGTGA

>gi|389828690|gb|JQ239431.1|_Porcine_epidemic_diarrhea_virus_strain_CH3_spike_protein_(S)_gene_complete_cds

ATGACGCCTTTAATTTACTTCTGGTTGTTCTTACCAGTACTTCTAACACTTAGCCTACCACAAGATGTCACTAGG
TGCCAGTCTACTATTAACTTTAGGCGGTTCTTTTCAAAATTTAATGTTCAGGCACCTGCCGTCGTTGTTTTGGGTGGTT
ATCTACCTAGTATGAACTCTTCTAGCTGGTACTGTGGCACAGGCATTGAAACTGATAGTGGCGTTCATGGTATTTTTCT
CAGTTACATCGATTCTGGTCAGGGCTTTGAGATTGGCATTTCGCAAGAGCCGTTTGATCCTAGTGGTTACCAGCTTTAT
TTACACAAGGCCACTAATGGTAACACTAGTGCTATTGCACGACTGCGCATTTGCCAGTTTCCAGATAATAAAACATTGG
GCCCTACTGTTAATGATGTTACAACAGGTCGTAACTGCCTATTCAACAAAGCCATTCCAGCTTTGCAGGATGGAAAAAA
TATTGTTGTCGGCATAACATGGGATAATGATCGTGTCACTGTTTTTGCTGACAAGATCTATCATTTTTATATTAAAAAT
GATTGGTCCCGTGTTGCGACAAGATGTTACAATAAAAGAAGTTGTGCCATGCAATATGTTTATACACCTACCTACTACA
TGCTTAATGTTACTAGTGCAGGTGAGGATGGCATTTACTATGAACCTTGTACAGCTAATTGCAGTGGTTACGCTGCCAA
TGTATTTGCCACTGATTCCAATGGCCATATACCAGAAGGTTTTAGTTTTAATAATTGGTTTCTTTTGTCCAATGACTCC
ACTTTGTTGCATGGTAAAGTGGTTTCAAACCAACCTTTGTTGGTCAACTGCCTTTGGGCCATTCCTAAGATTTATGGAC
TAGGCCAATTTTTCTCATTCAATCAAACGATGGATGGCGTTTGTAACGGAGCCGCTGCGCAGCGTGCCCCAGAGGCTCT
GAGGTTTAATATTAATGACACTTTTGTCATTCTTGCTGAAGGCTCAATTGTACTTCATACTGCTTTAGGAACAAATCTT
TCTTTTGTTTGCAGTAATTCCTCAGATCCTCACAAAGCCATCTTTACCATACCTTTGGGTGTTACTGAAGTACCCTACT
ATTGCTTTCTTAAAGTGGATACTTACAAATCCACTGTTTATAAATTCTTGGCTGTTTTACCTCCTACTGTCAAGGAAAT
TGTCATCACCAAGTACGGTGATGTTTATGTCAACGGGTTTGGCTATTTGCATCTCGGTTTGTTGGATGCTGTCACAATT
AATTTCACTGGTCATGGCACTGACGATGACGTTTCAGGTTTCTGGACCGTAGCATCGACTAATTTTGTTGATGCACTCA
TCGAGGTTCAAGGAACTGCCATTCAGCGTATTCTTTATTGTGATGACCCTGTTAGCCAACTTAAGTGTTCTCAGGTTTC
TTTTGACCTTGATGATGATTTTTACCCTATTTCTTCTAGAAACCTTCTGAGTCATGAACAGCCAATTTCTTTTGTTACT
TTGCCATCATTCAATGATCATTCTTTTGTTAATATTACTGTCTCTGCGGCTTTTGGTGGTCATAGTGGTGCCAACCTCA
TTGCATCTGACACTACTATCAATGGGTTTAGTTCTTTCTGTGTTGACACTAGACAATTTACCATTACACTGTTTTATAA
CGTTACAAACAGTTATGGTTATGTGTCTAAGTCACAGGATAGTAATTGCCCTTTCACCTTGCAATCTGTTAATGATTAC

CTGTCTTTTAGCAAATTTTGTGTTTCAACCAGCCTTTTGGCTGGTGCTTGTACCATAGATCTTTTTGGTTACCCTGAGT
TCGGTAGTGGTGTTAAGTTTACGTCCCTTTATTTTCAATTCACAAAGGGTGAGTTGATTACTGGCACGCCTAAACCACT
TCAAGGTGTCACGGACGTTTCTTTTATGACTCTGGATGTGTGTACCAAGTATACTATCTATGGCTTTAAAGGTGAGGGT
ATTATTACCCTTACAAATTCTAGCTTTTTGGCAGGTGTTTATTATACATCTGATTCTGGACAGTTGTTAGCCTTTAAGA
ATGTCACTAGTGGTGCTGTTTATTCTGTTACGCCATGTTCTTTTTCAGAGCAGGCTGCATATGTTGATGATGATATAGT
GGGTGTTATTTCTAGTTTGTCTAACTCCACTTTTAACAATACCAGGGAGTTGCCTGGTTTCTTCTACCATTCTAATGAT
GGCTCCAATTGTACAGAGCCTGTGTTGGTGTATAGTAACATAGGTGTCTGTAAATCTGGCAGTATTGGCTATGTCCCAC
TTCAGGATGGCCAAGTCAAGATTGCACCCATGGTTACTGGGAATATTAGTATTCCCACCAACTTTAGTATGAGTATTAG
AACAGAATATTTACAGCTTTACAACACGCCTGTTAGTGTTGATTGCGTTACATATGTTTGTAATGGTAACTCTCGTTGT
AAACAATTACTCACCCAGTACACTGCAGCATGTAAGACCATAGAGTCAGCATTACAACTCAGCGCTAGGCTTGAGTCTG
TTGAAGTTAACTCTATGCTTACTATTTCTGAAGAGGCTCTACAGTTAGCTACCATCAGTTCGTTTAATGGTGATGGATA
TAACTTTACTAATGTGCTGGGTGTTTCCGTGTACGACCCTGCAAGTGGCAGGGTGGTACAAAAAGGGTCTTTTATTGAA
GACCTGCTTTTTAATAAAGTGGTTACTAATGGCCTTGGTACTGTTGATGAAGACTATAAGCGCTGTTCTAATGGTCGCT
CTGTGGCAGATCTAGTCTGTGCGCAGTATTACTCTGGTGTCATGGTACTACCTGGCGTTGTTGACGCTGAGAAGCTTCA
CATGTATAGTGCGTCTCTCATCGGTGGTATGGCGCTAGGAGGTCTTACTACTGCAGCGGCATTGCCTTTTAGCCATGCT
GTTCAAGCGAGGCTCAATTATCTTGCTTTACAGACGGATGTTCTACAGCGCAACCAGCAATTGCTTGCTGAGTCTTTTA
ACTCTGCTATTGGTAATATAACTTCAGCCTTTGAGAGTGTTAAAGAGGCTATTAGTCAAACTTCCAATGGTTTGAACAC
TGTGGCTCATGCGCTTACTAAGGTTCAAGAGGTTGTTAATTCGCAGGGTTCAGCTTTGACCCAACTTACCATACAGCTG
CAACACAACTTCCAAGCCATTTCTAGTTCTATTGATGACATTTACTCCCGACTGGACATTCTTTCAGCCGATGTTCAGG
TTGATCGTCTCATCACCGGCAGATTATCAGCACTTAATGCTTTTGTTGCTCAAACCCTCACTAAGTATACTGAGGTTCA
GGCTAGCAGGAAGCTAGCACAGCAAAAGGTTAATGAGTGCGTCAAATCGCAATCTCAGCGTTATGCTTTTTGTGGTGGT
GATGGCGAGCACATCTTCTCTCTGGTACAGGCCGCACCTCAGGGCCTGCTGTTCTTACACACAGTACTTGTACCGGGTG
ATTTTGTAAATGTTATTGCCATCGATGGCTTATGCGTTAATGGTGATATTGCCTTGACTCTACGTGAGCCTGGCTTAGT
CTTGTTTACGCATGAACTTCAAACTTATACTGCGACGGAATATTTTGTTTCATCGCGACGTATGTTTGAACCTAGAAAA
CCTACCGTTAGTGATTTTGTTCAAATTGAGAGTTGTGTGGTCACCTATGTCAATCTGACTAGCGACCAACTACCAGATG
TAATCCCAGATTACATCGATGTTAACAAAACACTTGATGAGATTCTAGCTTCTCTGCCCAATAGAATTGGTCCTAGTCT
TCCCCTAGATGTTTTTAATGCCACTTATCTTAATCTCACTGGTGAAATTGCAGATTTAGAGCAGCGTCCAGAGTCTCTC
CGTAATACTACAGAAGAGCTCCGAAGTCTCATATATAATATCAACAACACACTTGTTGACCTTGAGTGGCTCAACCGAG
TTGAGACATATATCAAGTGGCCGTGGTGGGTTTGGTTGATTATTTTTATTGTTCTCATCTTTGTTGTGTCATTATTAGT
GTTCTGCTGCATTTCCACGGGTTGTTGTGGATGCTGCGGTTGTTGCGGTGCTTGTTTTTCAGGTTGTTGTAGGGGTCCT
AGACTTCAACCTTACGAAGCTTTTGAAAAGGTCCACGTGCAGTGA

>gi|389828695|gb|JQ239432.1|_Porcine_epidemic_diarrhea_virus_strain_CH4_spike_protein_(S)_gene_complete_cds

ATGACGCCTTTAATTTACTTCTGGTTGTTCTTACCAGTACTTCTAACACTTAGCCTACCACAAGATGTCACTAGG
TGCCAGTCTACTATTAACTTTAGGCGGTTCTTTTCAAAATTTAATGTTCAGGCACCTGCCGTCGTTGTTTTGGGTGGTT
ATCTACCTAGTATGAACTCTTCTAGCTGGTACTGTGGCACAGGCATTGAAACTGATAGTGGCGTTCATGGTATTTTTCT
CAGTTACATCGATTCTGGTCAGGGCTTTGAGATTGGCATTTCGCAAGAGCCGTTTGATCCTAGTGGTTACCAGCTTTAT
TTACACAAGGCCACTAATGGTAACACTAGTGCTATTGCACGACTGCGCATTTGCCAGTTTCCAGATAATAAAACATTGG
GCCCTACTGTTAATGATGTTACAACAGGTCGTAACTGCCTATTCAACAAAGCCATTCCAGCTTTGCAGGATGGAAAAAA
TATTGTTGTCGGCATAACATGGGATAATGATCGTGTCACTGTTTTTGCTGACAAGATCTATCATTTTTATATTAAAAAT
GATTGGTCCCGTGTTGCGACAAGATGTTACAATAAAAGAAGTTGTGCCATGCAATATGTTTATACACCTACCTACTACA

TGCTTAATGTTACTAGTGCAGGTGAGGATGGCATTTACTATGAACCTTGTACAGCTAATTGCAGTGGTTACGCTGCCAA
TGTATTTGCCACTGATTCCAATGGCCATATACCAGAAGGTTTTAGTTTTAATAATTGGTTTCTTTTGTCCAATGACTCC
ACTTTGTTGCATGGTAAAGTGGTTTCAAACCAACCTTTGTTGGTCAACTGCCTTCGGGCCATTCCTAAGATTTATGGAC
TAGGCCAATTTTTCTCATTCAATCAAACGATGGATGGCGTTTGTAACGGAGCCGCTGCGCAGCGTGCCCCAGAGGCTCT
GAGGTTTAATATTAATGACACTTTTGTCATTCTTGCTGAAGGCTCAATTGTACTTCATACTGCTTTAGGAACAAATCTT
TCTTTTGTTTGCAGTAATTCCTCAGATCCTCACAAAGCCATCTTTACCATACCTTTGGGTGTTACTGAAGTACCCTACT
ATTGCTTTCTTAAAGTGGATACTTACAAATCCACTGTTTATAAATTCTTGGCTGTTTTACCTCCTACTGTCAAGGAAAT
TGTCATCACCAAGTACGGTGATGTTTATGTCAACGGGTTTGGCTATTTGCATCTCGGTTTGTTGGATGCTGTCACAATT
AATTTCACTGGTCATGGCACTGACGATGACGTTTCAGGTTTCTGGACCGTAGCATCGACTAATTTTGTTGATGCACTCA
TCGAGGTTCAAGGAACTGCCATTCAGCGTATTCTTTATTGTGATGACCCTGTTAGCCAACTTAAGTGTTCTCAGGTTTC
TTTTGACCTTGATGATGGTTTTTACCCCATTTCTTCTAGAAACCTTCTGAGTCATGAACAGCCAATTTCTTTTGTTACT
TTGCCATCATTCAATGATCATTCTTTTGTTAATATTACTGTCTCTGCGGCTTTTGGTGGTCATAGTGGTGCCAACCTCA
TTGCATCTGACACTACTACCAATGGGTTTAGTTCTTTCTGTGTTGACACTAGACAATTTACCATTACACTGTTTTATAA
CGTTACAAACAGTTATGGTTATGTGTCTAAGTCACAGGATAGTAATTGCCCTTTCACCTTGCAATCTGTTAATGATTAC
CTGTCTTTTAGCAAATTTTGTGTTTCAACCAGCCTTTTGGCTGGTGCTTGTACCATAGATCTTTTTGGTTACCCTGAGT
TCGGTAGTGGTGTTAAGTTTACGTCCCTTTATTTTCAATTCACAAAGGGTGAGTTGATTACTGGCACGCCTAAACCACT
TCAAGGTGTCACGGACGTTTCTTTTATGACTCTGGATGTGTGTACCAAGTATACTATCTATGGCTTTAAAGGTGAGGGT
ATTATTACCCTTACAAATTCTAGCTTTTTGGCAGGTGTTTATTATACATCTGATTCTGGACAGTTGTTAGCCTTTAAGA
ATGTCACTAGTGGTGCTGTTTATTCTGTTACGCCATGTTCTTTTTCAGAGCAGGCTGCATATGTTGATGATGATATAGT
GGGTGTTATTTCTAGTTTGTCTAACTCCACTTTTAACAATACCAGGGAGTTGCCTGGTTTCTTCTACCATTCTAATGAT
GGCTCCAATTGTACAGAGCCTGTGTTGGTGTATAGTAACATAGGTGTCTGTAAATCTGGCAGTATTGGCTATGTCCCAC
TTCAGGATGGCCAAGTCAAGATTGCACCCATGGTTACTGGGAATATTAGTATTCCCACCAACTTTAGTATGAGTATTAG
AACAGAATATTTACAGCTTTACAACACGCCTGTTAGTGTTGATTGCGTTACATATGTTTGTAATGGTAACTCTCGTTGT
AAACAATTACTCACCCAGTACACTGCAGCATGTAAGACCATAGAGTCAGCATTACAACTCAGCGCTAGGCTTGAGTCTG
TTGAAGTTAACTCTATGCTTACTATTTCTGAAGAGGCTCTACAGTTAGCTACCATCAGTTCGTTTAATGGTGATGGATA
TAACTTTACTAATGTGCTGGGTGTTTCCGTGTACGACCCTGCAAGTGGCAGGGTGGTACAAAAAGGGTCTTTTATTGAA
GACCTGCTTTTTAATAAAGTGGTTACTAATGGCCTTGGTACTGTTGATGAAGACTATAAGCGCTGTTCTAATGGTCGCT
CTGTGGCAGATCTAGTCTGTGCGCAGTATTACTCTGGTGTCATGGTACTACCTGGCGTTGTTGACGCTGAGAAGCTTCA
CATGTATAGTGCGTCTCTCCTCGGTGGTATGGCGCTAGGAGGTCTTACTACTGCAGCGGCATTGCCTTTTAGCAATGCT
GTTCAAGCGAGGCTCAATTATCTTGCTTTACAGACGGATGTTCTACAGCGCAACCAGCAATTGCTTGCTGAGTCTTTTA
ACTCTGCTATTGGTAATATAACTTCAGCCTTTGAGAGTGTTAAAGAGGCTATTAGTCAAACTTCCAATGGTTTGAACAC
TGTGGCTCATGCGCTTACTAAGGTTCAAGAGGTTGTTAAGTCGCAGGGTTCAGCTTTGACCCAACTTACCATACAGCTG
CAACACAACTTCCAAGCCATTTCTAGTTCTATTGATGACATTTACTCCCGACTGGACATTCTTTCAGCCGATGTTCAGG
TTGATCGTCTCATCACCGGCAGATTATCAGCACTTAATGCTTTTGTTGCTCAAACCCTCACTAAGTATACTGAGGTTCA
GGCTAGCAGGAAGCTAGCACAGCAAAAGGTTAATGAGTGCGTCAAATCGCAATCTCAGCGTTATGGTTTTTGTGGTGGT
GATGGCGAGCACATCTTCTCTCTGGTACAGGCCGCACCTCAGGGCCTGCTGTTCTTACATACAGTACTTGTACCGGGTG
ATTTTGTAAATGTTATTGCCATCGATGGCTTATGCGTTAATGGCGATATTGCCTTGACTCTACGTGAGCCTGGCTTAGT
CTTGTTTACGCATGAACTTCAAACTTATACTGCGACGGAATATTTTGTTTCATCGCGACGTATGTTTGAACCTAGAAAA
CCTACCGTTAGTGATTTTGTTCAAATTGAGAGTTGTGTGGTCACCTATGTCAATCTGACTAGCGACCAACTACCAGATG
TAATCCCAGATTACATCGATGTTAACAAAACACTTGATGAGATTCTAGCTTCTCTGCCCAATAGAATTGGTCCTAGTCT
TCCCCTAGATGTTTTTAATGCCACTTATCTTAATCTCACTGGTGAAATTGCAGATTTAGAGCAGCGTTCAGAGTCTCTC
CGTAATACTACAGAAGAGCTCCGAAGTCTCATATATAATATCAACAACACACTTGTTGACCTTGAGTGGCTCAACCGAG
TTGAGACATATATCAAGTGGCCGTGGTGGGTTTGGTTGATTATTTTTATTGTTCTCATCTTTGTTGTGTCATTATTAGT
GTTCTGCTGCATTTCCACGGGTTGTTGTGGATGCTGCGGTTGTTGCGGTGCTTGTTTTTCAGGTTGTTGTAGGGGTCCT

AGACTTCAACCTTACGAAGCTTTTGAAAAGGTCCACGTGCAGTGA

>gi|389828700|gb|JQ239433.1|_Porcine_epidemic_diarrhea_virus_strain_CH5_spike_protein_(S)_gene_complete_cds

ATGAAGTCTTTAACTTACTTCTGGTTGTTATTACCAGTACTTTCAACACTCAGCCTACCACAAGATGTCACTAGG
TGCCAGTCCACTATTAACTTCAGGCGGTTCTTTTCAAAATTTAATGTGCAATCACCTGCTGTCGTTGTTTTGGGTGGTT
ATCTACCTAGTATGAACTCTTCTAGCTGGTACTGTGGCACAGGCCTTGAAACTGCTAGTGGCGTTCATGGTATTTTCCT
TAGTTACATCGATTCTGGTCAGGGCTTTGAGATTGGCATTTCACAGGAGCCGTTTGATCCTAGTGGTTACCAGCTTTAT
CTACATAAGGCCACTAATGGTAACACTAATGCTATCGCACGATTGCGCATTTGCCAGTTTCCAGATAATAAAACATTGG
GCCCTACTGTTAATGATGTTACAACAGGTCGTAACTGCCTATTCAACAGAGCCATTCCAGCTTATATGCAGGATGGAAA
AAATATTGTTGTCGGCATAACATGGGATAATGATCGCGTCACTGTTTTTGCTGACAAGATCTATCATTTTTATCTTAAA
AATGATTGGTCCCGCGTTGTGACAAGATGTTACAATAAAAGGAGTTGTGCTATGCAATATGTTTATACACCTACCTACT
ACATGCTTAATGTTACTAGTGCAGGTGAGGATGGCATTTATTATGAACCATGTACAGCTAATTGCAGTGGTTACGCTGC
CAATGTATTTGCCACTGATTCTAATGGCCATATACCAGAAGGTTTTAGTTTTAATAATTGGTTTCTTTTGTCCAATGAT
TCCACTTTGTTGCATGGTAAGGTGGTTTCCAACCAACCTTTGTTGGTCAATTGTCTTTTGGCCATTCCTAAGATTTATG
GACTAGGCCAATTTTTCTCATTCAATCAAACGATGGATGGCGTTTGTAATGGAGCTGCTGTGCAGCGTGCACCAGAGGC
TCTGAGGTTTAATATTAATGACACCTCTGTCATTCTTGCTGAAGGCTCAATTGTACTTCATACTGCTTTAGGAACAAAT
CTTTCTTTTGTTTGCAGTAATTCCTCAAATCCTCATTTAGCCACCTTCGCCATACCTTTGGGTGCTACCCAAGTACCCT
ATTATTGTCTTCTTAAAGTGGATACTTACAACTCCACTGTTTATAAATTCTTGGCTGTTTTACCTCCTACCGTCAGGGA
AATTGTCATCACCAAGTATGGTGATGTTTATGTCAATGGGTTTGGCTACTTGCATCTCGGTTTGTTGGATGCTGTCACA
ATCAGTTTCACTGGTCATGGCACTGACGATGACGTTTCTGGTTTCTGGACCGTAGCATCGACTAATTTTGTTGATGCAC
TCATCGAAGTTCAAGGAACTGCCATTCAGCGTATTCTTTATTGTGATGACCCTGTTAGCCAACTCAAGTGTTCTCAGGT
TGCTTTTGACCTTGACGACGGTTTTTACCCTATCTCTTCTAGGAACCTTCTGAGTCATGAACAGCCAATTTCTTTTGTT
ACTCTGCCATCATTTAATGATCATTCTTTTGTTAACATTACTGTCTCTGCGTCCTTTGGTGGTCATAGTGGTGCCAACC
TCATTGCATCTGACACTACTATCAATGGATTTAGTTCTTTCTGTGTTGACACTAGACAATTTACCATTTCACTGTTTTA
TAACGTTACAAACAGTTATGGTTATGTGTCTAACGCACAGGACACTAATTGCCCTTTCACCTTGCAATCTGTTAATGAT
TACCTGTCTTTTAGCAAATTTTGTGTTTCTACCAGCCTTTTGGCTAGTGCCTGTACCATAGATCTTTTTGGTTACCCTG
CGTTTGGTAGTGTTGTTAAGTTTACGTCCCTTTACTTTCAATTCACAAAGGGTGAGTTGATTACTGGCACGCCTAAACC
ACTTGAAGGTGTTACGGACGTTTCTTTTATGACTCTGGATGTGTGTACCAAGTATACTATCTATGGCTTTAAAGGTGAG
GGTATCATTACCCTTACAAATTCTAGCTTTTTGGCAGGTGTTTATTATACATCTGATTCTGGACAGTTGTTAGCCTTTA
AGAATGTCACTAGTGGTGCTGTTTATTCTGTTACGCCATGTTCTTTTTCAGAGCAGGCTGCATATGTTGATGATGATAT
AGTGGGTGTTATTTCTAGTTTGTCTAGCTCCACTTTTAACAGTACTAGGGAGTTGCCTGGTTTCTTCTACCATTCTAAT
GATGGCTCTAATTGTACAGAGCCTGTGTTGGTGTATAGTAACATAGGTGTTTGTAAATCTGGCAGTATTGGCTATGTCC
CACTTCAGGATGGCCAAGTCAAGATTGCACCCACGGTTACTGGGAATATTAGTATTCCCACCAACTTTAGTATGAGTAT
TAGGACAGAATATGTACAGCTTTACAACACGCCTGTTAGTGTTGATTGTGCCACATATGTTTGTAATGGTAACTCTCGT
TGTAAACAATTACTCACCCAGTACACTGCAGCATGTAAGACCATAGAGTCAGCATTACAACTCAGCGCTAGGCTTGAGT
CTGTTGAAGTTAACTCTATGCTTACTATTTCTGAAGAGGCTCTACAGTTAGCTACCATCAGTTCGTTTAATGGTGATGG
ATATAATTTTACTAATGTGCTGGGTGTTTCTGCGTATGATCCTGCAAGTGACAGGGTGGTACAAAAAAGGTCTTTTATT
GAAGACCTGCTTTTTAATAAAGTGGTTACTAATGGCCTTGGTACTGTTGATGAAGGCTATAAGCGCTGTTCTAATGGTC
GTTCTGTGGCGGATCTAGTCTGTGCACAGTATTACTCTGGTGTCATGGTACTACCTGGTGTTGTTGACGCTGAGAAGCT
TCACATGTATAGTGCGTCTCTCATCGGTGGTATGGTGCTAGGAGGTTTTACTTCTGCAGCGGCATTGCCTTTTAGCTAT
GCTGTTCAAGCTAGACTCAATTATCTTGCTCTACAGACGGATGTTCTACAGCGGAACCAGCAATTGCTTGCTGAGTCTT

TTAACTCTGCTATTGGTAATATAACTTCAGCCTTTGAGAGTGTTAAAGAGGCTATTAGTCAAACTTCTAAGGGTTTGAA
CACTGTGGCTCATGCGCTTACTAAGGTTCAAGAGGTTGTTAACTCGCAGGGTACAGCTTTGACTCAACTTACCGTACAG
CTGCAACACAACTTCCAAGCCATTTCTAGTTCTATTGATGACATTTACTCCCGACTGGACATTCTTTCAGCCGATGTTC
AGGTTGACCGTCTCATCACCGGCAGATTATCAGCACTTAATGCTTTTGTTGCTCAAACCCTCACTAAGTATACTGAGGT
TCAGGCTAGCAGGAAGCTAGCACAGCAAAAGGTTAATGAGTGCGTTAAATCGCAATCTCAGCGTTATGGTTTTTGTGGT
GGTGATGGCGAGCACATTTTCTCTCTGGTACAGGCAGCACCTCAGGGCCTGCTGTTTTTACATACAGTACTTGTACCGA
GTGATTTTGTAGATGTTACTGCCATCGCTGGCTTATGCGTTAACGATGAAATTGCCTTGACTCTACGTGAGCCTGGCTT
AGTCTTGTTTACGCATGAACTTCAAAATCATACTGCGACGGAATATTTTGTTTCATCGCGACGTATGTTTGAACCTAGA
AAACCTACCGTTAGTGATTTTGTTCAAATTGAGAGTTGTGTGGTCACCTACGTCAATTTGACTAGAGACCAACTACCAG
ATGTAATCCCAGATTACATCGATGTTAACAAAACACTTGATGAGATTTTAGCTTCTCCGCCCAATAGAACTGGTCCAAG
TCTTCCTTTAAATGTTTTTAATGCCACTTATCTCAATCTCACTGGTGAAATTGCAGATTTAGAGCAGCGTTCAGAGTCT
CTCCGTAATACTACAGAGGAGCTCCAAAGTCTTATATATAATATCAACAACACACTAGTTGACCTTGAGTGGCTCAACC
GAGTTGAGACATATATCAAGTGGCCGTGGTGGGTTTGGTTGGTTATTTTTATTGTTCTCATCTTTGTTGTGTCATTACT
AGTGTTCTGCTGCATTTCCACGGGTTGTTGTGGATGCTGCGGCTGCTGCTGTGCTTGTTTTTCAGGTTGTTGTAGGGGT
CCTAGACTTCAACCTTACGAAGTTTTTGAAAAGGTCCACGTGCAGTGA

>gi|389828703|gb|JQ239434.1|_Porcine_epidemic_diarrhea_virus_strain_CH6_spike_protein_(S)_gene_complete_cds

ATGAAGTCTTTAACTTACTTCTGGTTGTTATTACCAGTACTTCCAACACTCAGCCTACCACAAGATGTCACTAGG
TGCCAGTCCACTATTAACTTCAGGCGGTTCTTTTCAAAATTTAATGTGCAATCACCTGCTGTCGTTGTTTTGGGTGGTT
ATCTACCTAGTATGAACTCTTCTAGCTGGTACTGTGGCACAGGCCTTGAAACTGCTAGTGGCGTTCATGGTATTTTCCT
TAGTTACATCGATTCTGGTCAGGGCTTTGAGATTGGCATTTCACAGGAGCCGTTTGATCCTAGTGGTTACCAGCTTTAT
CTACATAAGGCCACTAATGGTAACACTAATGCTATCGCACGATTGCGCATTTGCCAGTTTCCAGATAATAAAACATTGG
GCCCTACTGTTAATGATGTTACAACAGGTCGTAACTGCCTATTCAACAGAGCCATTCCAGCTTATATGCAGGATGGAAA
AAATATTGTTGTCGGCATAACATGGGATAATGATCGCGTCACTGTTTTTGCTGACAAGATCTATCATTTTTATCTTAAA
AATGATTGGTCCCGCGTTGTGACAAGATGTTACAATAAAAGGAGTTGTGCTATGCAATATGTTTATACACCTACCTACT
ACATGCTTAATGTTACTAGTGCAGGTGAGGATGGCATTTATTATGAACCATGTACAGCTAATTGCAGTGGTTACGCTGC
CAATGTATTTGCCACTGATTCTAATGGCCATATACCAGAAGGTTTTAGTTTTAATAATTGGTTTCTTTTGTCCAATGAT
TCCACTTTGTTGCATGGTAAGGTGGTTTCCAACCAACCTTTGTTGGTCAATTGTCTTTTGGCCATTCCTAAGATTTATG
GACTAGGCCAATTTTTCTCATTCAATCAAACGATGGATGGCGTTTGTAATGGAGCTGCTGTGCGGCGTGCGCCAGAGGC
TCTGAGGTTTAATATTAATGACACCTCTGTCATTCTTGCTGAAGGCTCAATTGTACTTCATACTGCTTTAGGAACAAAT
CTTTCTTTTGTTTGCAGTAATTCCTCAAATCCTCATTTAGCCACCTTCGCCATACCTTTGGGTGCTACCCAAGTACCCT
ATTATTGTTTTCTTAAAGTGGATACTTACAACTCCACTGTTTATAAATTCTTGGCTGTTTTACCTCCTACCGTCAGGGA
AATTGTCATCACCAAGTATGGTGATGTTTATGTCAATGGGTTTGGCTACTTGCATCTCGGTTTGTTGGATGCTGTCACA
ATCAATTTCACTGGTCATGGCACTGACGATGACGTTTCTGGTTTCTGGACCGTAGCATCGACTAATTTTGTTGATGCAC
TCATCGAAGTTCAAGGAACTGCCATTCAGCGTATTCTTTATTGTGATGATCCTGTTAGCCAACTCAAGTGTTCTCAGGT
TGCTTTTGACCTTGACGATGGTTTTTACCCTATCTCTTCTAGGAACCTTCTGAGTCATGAACAGCCAATTTCTTTTGTT
ACTCTGCCATCATTTAATGATCATTCTTTTGTTAACATTACTGTCTCTGCGTCCTTTGGTGGTCATAGTGGTGCCAACC
TCATTGCATCTGACACTACTATCAATGGATTTAGTTCTTTCTGTGTTGACACTAGACAATTTACCATTTCACTGTCTTA
TAACGTTACAAACAGTTATGGTTATGTGTCTAACTCACAGGACACTAATTGCCCTTTCACCTTGCAATCTGTTAATGAT
TACCTGTCTTTTAGCAAATTTTGTGTTTCTACCAGCCTTTTGGCTAGTGCCTGTACCATAGATCTTTTTGGTTACCCTG
CGTTTGGTAGTGTTGTTAAGTTTACGTCCCTTTACTTTCAATTCACAAAGGGTGAGTTGATTACTGGCACGCCTAAACC

ACTTGAAGGTGTTACGGACGTTTCTTTTATGACTCTGGATGTGTGTACCAAGTATACTATCTATGGCTTTAAAGGTGAG
GGTATCATTACCCTTACAAATTCTAGCTTTTTGGCAGGTGTTTATTATACATCTGATTCTGGACAGTTGTTAGCCTTTA
AGAATGTCACTAGTGGTGCTGTTTATTCTGTTACGCCATGTTCCTTTTCAGAGCAGGCTGCATATGTTGATGATGATAT
AGTGGGTGTTATTTCTAGTTTGTCTAGCTCCACTTTTAACAGTACTAGGGAGTTGCCTGGTTTCTTCTACCATTCTAAT
GATGGCTCTAATTGTACAGAGCCTGTGTTGGTGTATAGTAACATAGGTGTTTGTAAATCTGGCAGTATTGGCTATGTCC
CACTTCAGGATGGCCAAGTCAAGATTGCACCCACGGTTACTGGGAATATTAGTATTCCCACCAACTTTAGTATGAGTAT
TAGGACAGAATATGTACAGCTTTACAACACGCCTGTTAGTGTTGATTGTGCCACATATGTTTGTAATGGTAACTCTCGT
TGTAAACAATTACTCACCCAGTACACTGCAGCATGTAAGACCATAGAGTCAGCATTACAACTCAGCGCTAGGCTTGAGT
CTGTTGAAGTTAACTCTATGCTTACTATTTCTGAAGAGGCTCTACAGTTAGCTACCATCAGTTCGTTTAATGGTGATGG
ATATAATTTTACTAATGTGCTGGGTGTTTCTGCGTATGATCCTGCAAGTGGCAGGGTGGTACAAAAAAGGTCTTTTATT
GAAGACCTGCTTTTTAATAAAGTGGTTACTAATGGCCTTGGTACTGTTGATGAAGACTATAAGCGCTGTTCTAATGGTC
GTTCTGTGGCGGATCTAGTCTGTGCACAGTATTACTCTGGTGTCATGGTACTACCTGGTGTTGTTGACGCTGAGAAGCT
TCACATGTATAGTGCGTCTCTCATCGGTGGTATGGTGCTAGGAGGTTTTACTTCTGCAGCGGCATTGCCTTTTAGCTAT
GCTGTTCAAGCTAGACTCAATTATCTTGCTCTACAGACGGATGTTCTACAGCGGAACCAGCAATTGCTTGCTGAGTCTT
TTAACTCTGCTATTGGTAATATAACTTCAGCCTTTGAGAGTGTTAAAGAGGCTATTAGTCAAACTTCTAAGGGTTTGAA
CACTGTGGCTCATGCGCTTACTAAGGTTCAAGAGGTTGTTAACTCGCAGGGTACAGCTTTGACTCAACTTACCGTACAG
CTGCAACACAACTTCCAAGCCATTTCTAGTTCTATTGATGACATTTACTCCCGACTGGACATTCTTTCAGCCGATGTTC
AGGTTGACCGTCTCATCACCGGCAGATTATCAGCACTTAATGCTTTTGTTGCTCAAACCCTCACTAAGTATACTGAGGT
TCAGGCTAGCAGGAAGCTAGCACAGCAAAAGGTTAATGAGTGCGTTAAATCGCAATCTCAGCGTTATGGTTTTTGTGGT
GGTGACGGCGAGCACATTTTCTCTCTGGTACAGGCAGCACCTCAGGGCCTGCTGTTTTTACATACAGTACTTGTACCGG
GTGATTTTGTAGATGTTATTGCCATCGCTGGCTTATGCGTTAACGATGAAATTGCCTTGACTCTACGTGAGCCTGGCTT
AGTCTTGTTTACGCATGAACTTCAAAATCATACTGCGACGGAATATTTTGTTTCATCGCGACGTATGTTTGAACCTAGA
AAACCTACCGTTAGTGATTTTGTTCAAATTGAGAGTTGTGTGGTCACCTACGTCAATTTGACTAGAGACCAACTACCAG
ATGTAATCCCAGATTACATCGATGTTAACAAAACACTTGATGAGATTTTAGCTTCTCTGCCCAATAGAACTGGTCCAAG
TCTTCCTTTAGATGTTTTTAATGCCACTTATCTCAATCTCACTGGTGAAATTGCAGATTTAGAGCAGCGTTCAGAGTCT
CTCCGTAATACTACAGAGGAGCTCCAAAGTCTTATATATAATATCAACAACACACTAGTTGACCTTGAGTGGCTCAACC
GAGTTGAGACATATATCAAGTGGCCGTGGTGGGTTTGGTTGGTTATTTTTATTGTTCTCATCTTTGTTGTGTCATTACT
AGTGTTCTGCTGCATTTCCACGGGTTGTTGTGGATGCTGCGGCTGCTGCTGTGCTTGTTTTTCAGGTTGTTGTAGGGGT
CCTAGACTTCAACCTTACGAAGTTTTTGAAAAGGTCCACGTGCAGTGA

>gi|389828707|gb|JQ239435.1|_Porcine_epidemic_diarrhea_virus_strain_CH7_spike_protein_(S)_gene_complete_cds

ATGAAGTCTTTAACTTACTTCTGGTTGTTATTACCAGTACTTTCAACACTTAGCCTACCACAAGATGTCACTAGG
TGCCAGTCCACTATTAACTTCAGGCGGTTCTTTTCAAAATTTAATGTGCAAGCACCTGCTGTCGTTGTTTTGGGTGGTT
ATCTACCTAGTATGAACTCTTCTAGCTGGTACTGTGGCACAGGCCTTGAAACTGCTAGTGGCGTTCATGGTATTTTCCT
CAGTTACATCGATTCTGGTCAGGGCTTTGAGATTGGCATTTCACAGGAGCCGTTTGATCCTAGTGGTTACCAGCTTTAT
CTACATAAGGCCACTAATGGTAACACTAATGCTATTGCACGACTGCGCATTTGCCAGTTTCCAGATAACAAAACATTGG
GCCCTACTGTTAATGATGTTACAACAGGTCGTAACTGCCTATTCGACAGAGCCATTCCAGCTTATATGCAAGATGGAAA
AAATATTGTTGTCGGCATAACATGGGATAATGATCGTGTCACTGTTTTTGCTGACAAGATCTATCATTTTTATCTTAAA
AATGATTGGTCCCGCGTTGCGACAAGATGTTATAATAAAAGGAGTTGTGCTATGCAATATGTTTATACACCTACCTACT
ACATGCTTAATGTTACTAGTGCAGGTGAGGATGGCATTTATTATGAACCATGTACAGCTAATTGCAGTGGTTACGCTGC
CAATGTATTTGCCACTGATTCTAATGGCCATATACCAGAAGGTTTTAGTTTTAATAATTGGTTTCTTTTGTCCAATGAT

TCCACTTTGTTGCATGGTAAGGTGGTTTCCAACCAACCTTTGTTGGTCAATTGTCTTTTGGCCATTCCTAAGATTTATG
GACTAGGCCAATTTTTCTCATTCAATCAAACGATGGATGGCGTTTGTAATGGAGCTGCTGCGCAGCGTGCACCAGAGGC
TCTGAGGTTTAATATTAATGACACCTCTGTCATTCTTGCTGAAGGCTCAATTGTACTTCATACTGCTTTAGGAACAAAT
CTTTCCTTTGTTTGCAGTAATTCCTCAGAACCTCGTCTAGCCACCTTCGCCATACCTTTGGGTGCCACCCAAGTACCCT
ATTATTGTTTTCTTAAAGTGGATACTTACAACTCCACTGTTTATAAATTCTTGGCTGTTTTACCTCCTACCGTCAGGGA
AATTGTCATCACCAAGTATGGTGATGTTTATGTCAATGGGTTTGGCTACTTGCATCTCGGTTTGTTGGATGCTGTCACA
ATTAATTTCACTGGTCATGGCACTGACGATGACGTTTCTGGTTTCTGGACCGTAGCATCGACTAATTTTGTTGATGCAC
TCATCGAAGTTCAAGGAACTGCCATTCAGCGTATTCTTTATTGTGATGACCCTGTTAGCCAACTCAAGTGTTCTCAGGT
TGCTTTTGGCCTTGACGATGGTTTTTACCCTATTTCTTCTAGAAACCCTCTGAGTCATGAACAGCCAATTTCTTTTGTT
ACTCTGCCATCATTTAATGATCATTCTTTTGTTAACATTACTGTCGCTGCGTCCTTTGGTGGTCATAGTGGTGCCAACC
TCATTGCATCTGACACTACTATCAATGGGTTTAGTTCTTTCTGTGTTGACACTAGACAATTTACCATTTTACTGTTTTA
TAACGTTACTAACAGTTATGGTTATGTGCCTAAATCACAGGACAGTAATTGCCCTTTCACCTTGCAATCTGTTAATGAT
TACCTGTCTTTTAGCAAATTTTGTGTTTCTACCAGCCTTTTGGCTAGTGCCTGTACCATAGATCTTTTTGGTTACCCTG
AGTTTGGTAGTGCTGTTAAGTTTACGTCCCTTTACTTTCAATTCACAAAGGGTGAGTTGATTACTGGCACGCCTAAACC
ACTTGAAGGTGTCACGGACGTTTCTTTTATGACTCTGGGTGTGTGTACCAAGTATACTATCTATGGCTTTAAAGGTGAG
GGTGTCATTACCCTTACAAATTCTAGCTTTTTGGCAGGTGTTTATTACACATCTGATTCTGGACAGTTGTTAGCCTTTA
AGAATGTCACTAGTGGTGCTGTTTATTCTGTTACGCCATGTTCTTTTTCAGAGCAGGCTGCGTATGTTGATGATGATAT
AGTGGGTGTTATTTCTAGTTTGTCTAGCTCCACTTTTAACAGTACTAGGGAGTTGCCTGGTTTCTTCTACCATTCTAAT
GATGGCTCTAATTGTACAGAGCCTGTGTTGGTGTATAGTAACATAGGTGTTTGTAAATCTGGCAGTATTGGCTATGTCC
CACTTCAGGATGGCCAAGTCAAGATTGCACCCACGGTTACTGGGAATATTAGTATTCCCACCAACTTTAGTATGAGTAT
TAGGACAGAATATTTACAGCTTTACAACACGCCTGTTAGTGTTGATTGTGCTACATATGTTTGTAATGGTAACTCTCGT
TGTAAACAATTACTCACCCAGTACACTGCAGCATGTAAGACCATAGAGTCAGCATTACAACTCAGCGCTAGGCTTGAGT
CTGCTGAAGTCAACTCTATGCTTACTATTTCTGAAGAGGCTCTACAGTTAGCTACCATCAGTTCGTTTAATGGTGATGG
ATATAATTTTACTAATGTGCTGGGTGTTTCCGTGTATGACCCTGCAAGTGGCAGGGTGGTACAAAAAAGGTCTTTCATT
GAAGACCTGCTTTTTAATAAAGTGGTTACTAATGGCCTTGGTACTGTTGATGAAGACTATAAGCGCTGTTCTAATGGCC
GCTCTGTGGCAGATTTAGTCTGTGCGCAGTATTACTCTGGTGTCATGGTACTACCTGGTGTTGTTGACGCTGAGAAGCT
TCATATGTATAGTGCGTCTCTCATCGGTGGTATGGTGCTAGGAGGTTTTACTGCAGCAGCGGCATTGCCTTTTAGCTAT
GCTGTTCAAGCGAGACTGAATTATCTTGCTCTACAGACGGATGTTCTACAGCGGAACCAGCAATTGCTTGCTGAGTCTT
TTAATTCTGCTATTGGTAATATAACTTCAGCCTTTGAGAGTGTTAAAGAGGCTATTAGTCAAACTTCTAAGGGTTTGAA
CACTGTGGCTCATGCGCTTACCAAGGTCCAAGAGGTTGTTAATTCGCAGGGTGCAGCTTTGACCCAACTTACCGTACAG
CTGCAACACAACTTCAAAGCCATTTCTAGTTCTATTGATGACATTTACTCCCGACTTGACATTCTTTCAGCCGATGTTC
AGGTAGACCGTCTCATCACCGGCAGATTATCAGCACTTAATGCTTTTGTTGCTCAAACCCTTACTAAGTATACTGAGGT
TCAGGCTAGCAGGAAACTAGCACAGCAAAAGGTTAATGAGTGCGTTAAATCGCAATCTCAGCGTTATGGTTTTTGTGGT
GGTGATGGCGAGCACATTTTCTCCCTGGTACAGGCCGCACCTCAAGGCCTGCTGTTTTTACACACAGTACTTGTACCGG
GTGACTTTGTAAATGTTATTGCCATCGCTGGCTTATGTGTTAACGATGAAATTGCCTTGACTCTACGTGAGCCTGGTTT
AGTCTTGTTTACGCATGAACTTCAAGATACTGCGACGGAATATTTTGTTTCATCGCGACGTATGTATGAACCTAGAAAA
CCTACCGTTGGTGATTTTGTTCAAATTGAGAGTTGTGTGGTCACCTATGTCAATTTGACTAGAGACCAACTACCAGAAG
TAATCCCAGATTACATCGATGTTAACAAAACACTTGATGAGATTTTAGCCTCTCTGCCCAATAGAACTGGTCCAAGTCT
TTCTCTAGATGTTTTTAATGCCACTTATCTTAATCTCACTGGTGAAATTGCAGATTTAGAGCAGCGTTCAGAGTCTCTC
CGTAATAGTACAGAAGAGCTCCAAAGTCTTATATATAATATCAACAACACACTAGTTGACCTTGAGTGGCTCAACCGAG
TTGAGACATATATCAAGTGGCCGTGGTGGGTTTGGTTGATTATTTTTATTGTTCTCATCTTTGTTGTGTCATTACTAGT
GTTCTGCTGCATTTCCACGGGTTGTTGTGGATGCTGCGGCTGCTGTGGTGCTTGTTTTTCAGGTTGTTGTAGGGGTCCT
AGACTTCAACCTTACGAAGTTTTTGAAAAGGTCCACGTGCAGTGA

>gi|389828711|gb|JQ239436.1|_Porcine_epidemic_diarrhea_virus_strain_CH8_spike_protein_(S)_gene_complete_cds

ATGAAGTCTTTAACCTACTTCTGGTTGTTCTTACCAGTACTTTCAACACTTAGCCTACCACAAGATGTTACCAGG
TGCTCAGCTAACACTAACTTTAGGCGGTTCTTTTCAAAATTTAATGTTCAGGCGCCTGCAGTTGTTGTACTGGGCGGTT
ATCTACCTATTGGTGAAAACCAGGGTGTCAATTCAACTTGGTACTGTGCTGGCCAACATCCAACTGCTAGTGGCGTTCA
TGGTATCTTTCTTAGCCATATTAGAGGTGGTCATGGCTTTGAGATTGGCATATCGCAAGAGCCTTTTGACCCTAGTGGT
TACCAGCTTTATTTACATAAGGCTACTAACGGTAACACTAATGCTACTGCGCGACTGCGCATTTGCCAGTTTCCCAGCA
TTAAAACATTGGGCCCCACTGCTAATAATGATGTTACAACAGGTCGTAACTGCCTATCTAACAAAGCCATCCCAGCTCA
TATGAGTGAACATAGTGTTGTCGGCATAACATGGGATAATGATCGTGTCACTGTCTTTCCTGACAAGATCTATTATTTT
TATTTTAAAAATGATTGGTCCCGTGTTGCGACAAAGTGTTACAACAGTGGAGGTTGTGCTATGCAATATGTTTACGAAC
CCACTTACTACACGCTTAATGTTACTAGTGCTGGTGAGGATGGTATTTCTTATCAACCCTGTACAGCTAATTGCATTGG
TTATGCTGCCAATGTATTTGCTACTGAGCCCAATGGCCACATACCAGAAGGTTTTAGTTTTAATAATTGGTTTCTTTTG
TCCAATGATTCCACTTTGGTGCATGGTAAGGTGGTTTCCAACCAACCTTTGTTGGTCAATTGTCTTTTGGCCATTCCTA
AGATTTATGGACTAGGCCAATTTTTCTCATTCAATCAAACGATGGATGGTGTTTGTAATGGAGCTGCTTTGCAGCGTGC
ACCAGAGGCTCTGAGGTTTAATATTAATGACACCTCTGTCATTCTTGCTGAAGGCTCAATTGTACTTCATACTGCTTTA
GGAACAAATCTTTCTTTTGTTTGCAGTAATTCCTCAGATCCTCATTTAGCCACCTTCGCCATACCTTTGGGTGCTACCC
AAGTACCCTATTATTGTTTTCTTAAAGTGGATACTTACAACTCCACTGTTTATAAATTCTTGGCTGTTTTACCTCCAAC
CGTCAGGGAAATTGTCATCACCAAGTATGGTGATGTTTATGTCAATGGGTTTGGCTACTTGCATCTCGGTTTGTTGGAT
GCTGTCACAATTAATTTCACTGGTCATGGCACTGACGATGACGTTTCTGGTTTCTGGACCGTAGCATCGACTAATTTTG
TTGATGCACTTATCGAAGTTCAAGGAACTGCCATTCAGCGTATTCTTTATTGTGATGACCCTGTTAGCCAACTCAAGTG
TTCTCAGGTTGCTTTTGACCTTGACGATGGTTTCTACCCTATTTCTTCTAGAAACCTTCTGAGTCATGAACAGCCAACT
TCTTTTGTTACTTTGCCATCATTTAATGATCATTCTTTTGTTAACATTACTGTCTCTGCGTCCTTTGGTGGTCATAGTG
GTGCCAACCTTATTGCATCTGACACTACTATCAATGGGTTTAGTTCTTTCTGTGTTGACACTAGACAATTTACCATTTC
ACTGTTTTATAACGTTACAAACGGTTATGGTTATGTGTCTAAATCACAGGACAGTAATTGCCCTTTCACCTTGCAATCT
GTTAATGATTACCTGTCTTTTAGCAAATTTTGTGTTTCCACCAGCCTTTTGGCTAGTGCCTGTACCATAGATCTTTTTG
GTTACCCTGAGTTTGGTAGTGGTGTTAAGTTTACGTCCCTTTACTTTCAATTCACAAAGGGTGAGTTGATTACTGGCAC
GCCTAAACCACTTGAAGGTGTCACGGACGTTTCTTTTATGACTCTGGATGTGTGTACCAAGTATACTATCTATGGCTTT
AAAGGTGAGGGTATCATTACCCTTACAAATTCTAGCTTTTTGGCAGGTGTTTATTATACATCTGATTCTGGACAGTTGT
TAGCTTTTAAGAATGTCACTAGTGGTGCTGTTTATTCTGTTACGCCATGTTCTTTTTCAGAGCAGGCTGCATATGTTGA
TGATGATATAGTGGGTGTTATTTCTAGTTTGTCTAGCTCCACTTTTAACAGTACTAGGGAGTTGCCTGGTTTCTTCTAC
CATTCTAATGATGGCTCTAATTGTACAGAGCCTGTGTTGGTGTATAGTAACATAGGTGTTTGTAAATCTGGCAGTATTG
GCTATGTCCCACTTCAGGATGGCCAAGTCAAGATTGTACCCACGGTTACTGGGAATATTAGTATTCCCACCAACTTTAG
TATGAGTATTAGGACAGAATATTTACAGCTTTACAACACGCCTGTTAGTGTTGATTGTGCCACATATGTTTGTAATGGT
AACTCTCGTTGCAAACAATTACTCACCCAGTACACTGCAGCATGTAAGACCATAGAGTCAGCATTACAACTCAGCGCTA
GGCTTGAGTCTGTTGAAGTTAACTCTATGCTTACTATTTCTGAGGAGGCTCTACAGTTAGCTACCATCAGTTCGTTTAA
TGGTGATGGATATAATTTTACCAATGTGCTGGGTGTTTCTGTGTATGATCCTGCAAGTGGCAGGGTGGTACAAAAAAGG
TCTTTTATTGAAGACCTTCTTTTTAATAAAGTGGTTACTAATGGCCTTGGTACTGTTGATGAAGACTATAAGCGCTGTT
CTAATGGTCGCTCTGTGGCAGATCTAGTCTGTGCACAGTATTACTCTGGTGTCATGGTACTACCTGGTGTTGTTGACGC
TGAGAAGCTTCACATGTATAGTGCGTCTCTCATCGGTGGTATGGTGCTAGGAGGTTTTACTGCTGCAGCGGCATTGCCT
TTTAGCTATGCTGTTCAAGCTAGACTTAATTATCTTGCTCTACAGACGGATGTTCTACAGCGGAACCAGCAATTGCTTG
CTGAGTCTTTTAACTCTGCTATTGGTAATATAACTTCAGCCTTTGAGAGTGTTAAAGAGGCTATTAGTCAAACTTCCAA
GGGTTTGGACACTGTGGCTCATGCGCTTACTAAGGTTCAAGAGGTTGTTAATTCGCAGGGTGCAGCTTTGACCCAACTT

ACCGTACAGCTGCAACACAACTTCCAAGCCATTTCTAGTTCTATTGATGACATTTACTCCCGACTGGACATTCTTTCAG
CCGATGTTCAGGTTGACCGTCTCATCACCGGCAGATTATCAGCACTTAATGCTTTTGTTGCTCAAACCCTCACTAAGTA
TACTGAGGTTCAGGCTAGCAGGAAGCTAGCACAGCAAAAGGTTAATGAGTGCGTTAAATCGCAATCTCAGCGTTATGGT
TTTTGTGGTGGTGATGGCGAGCACATTTTCTCTCTGGTACAGGCAGCACCTCAGGGCCTGCTGTTTTTACATACAGTAC
TTGTACCGGGTGGTTTTGTAGATGTTATTGCCATCGCTGGCTTATGCGTTAACGATGAAATTGCCTTGACTCTACGTGA
GCCCGGCTTAGTCTTGTTTACGCATGAACTTCAAAATCATACTGCGACGGAATATGTTGTTTCATCGCGACGTATGTTT
GAACCTAGAAAACCTACCGTTAGTGATTTTGTTCAAATTGAGAGTTGTGTGGTCACCTATGTCAATTTGACTAGAGACC
AACTACCAGATGTAATCCCAGATTACATCGATGTTAACAAAACACTTGATGAGATTTTAGCTTCTCTGCCCAATAGAAC
TGGTCCAAGTCTTCCTTTAGATGTTTTTAATGCCACTTATCTTAATCTCACTGGTGAAATTGCAGATTTAAAGCAGCGT
TCGGAGTCTCTCCGTAATACTACAGAGGAGCTCCAAAGTCTTATATATAATATCAACAACACACTAGTTGACCTTGAGT
GGCTCAACCGAGTTGAGACATATATCAAGTGGCCGTGGTGGGTTTGGTTGATTATTTTTATTGTTCTCATCTTTGTTGT
GTTATTACTAGTGTTCTGCTGCATTTCCACGGGTTGTTGTGGATGCTGCGGCTGCTGCTGTGCTTGTTTTTCAGGTTGT
TGTAGGGGTCCTAGACTTCAACCTTACGAAGTTTTTGAAAAGGTCCACGTGCAGTGA

>gi|363498866|gb|JQ257005.1|_Porcine_epidemic_diarrhea_virus_strain_CH/KF/11_spike_protein_gene_complete_cds

ATGAAGTCTTTAACCTACTTCTGGTTGTTCTTACCAGTACTTTCAACACTTAGCCTACCACAAGATGTCACCAGG
TGCTCAGCTAACACTAATTTTAGGCGGTTCTTTTCAAAATTTAATGTTCAGGCGCCTGCAGTTGTTGTACTGGGCGGTT
ATCTACCTATTGGTGAAAACCAGGGTGTCAATTCAACTTGGTACTGTGCTGGCCAACATCCAACTGCTAGTGGCGTTCA
TGGTATCTTTGTTAGCCATATTAGAGGTGGTCATGGCTTTGAGATTGGCATTTCGCAAGAGCCTTTTGACCCTAGTGGT
TACCAGCTTTATTTACATAAGGCTACTAACGGTAACACTAATGCTACTGCGCGACTGCGCATTTGCCAGTTTCCTAGCA
TTAAAACATTGGGCCCCACTGCTAATAATGATGTTACAACAGGTCGTAATTGCCTATTTAACAAAGCCATCCCAGCTCA
TATGAGTGAACATAGTGTTGTCGGCATAACATGGGATAATGATCGTGTCACTGTCTTTTCTGACAAGATCTATTATTTT
TATTTTAAAAATGATTGGTCCCGTGTTGCGACAAAGTGTTACAACAGTGGAGGTTGTGCTATGCAATATGTTTACGAAC
CCACCTATTACATGCTTAATGTTACTAGTGCTGGTGAGGATGGTACTTCTTATCAACCCTGTACAGCTAATTGCATTGG
TTATGCTGCCAATGTATTTGCTACTGAGCCCAATGGCCACATACCAGAAGGTTTTAGTTTTAATAATTGGTTTCTTTTG
TCCAATGATTCCACTTTGGTGCATGGTAAGGTGGTTTCCAACCAACCATTGTTGGTCAATTGTCTTTTGGCCATTCCTA
AGATTTATGGACTAGGCCAATTTTTCTCCTTTAATCAAACGATCGATGGTGTTTGTAATGGAGCTGCTGTGCAGCGTGC
ACCAGAGGCTCTGAGGTTTAATATTAATGACACCTCTGTCATTCTTGCTGAAGGCTCAATTGTACTTCATACTGCTTTA
GGAACAAATTTTTCTTTTGTTCGCAGTAATTCCTCAGATCCTCACTTAGCCACCTTCGCCATACCTCTGGGTGCTATCC
AAGTACCCTATTATTGTTTTCTTAAAGTGGATACTTACAACTCCACTGTTTATAAATTCTTGGCTGTTTTACCTCCTAC
CGTCAGGGAAATTGTCATCACCAAGTATGGTGATGTTTATGTCAATGGGTTTGGCTACTTGCATCTCGGTTTGTTGGAT
GCTGTCACAATTAATTTCACTGGTCATGGCATTGACGATGACGTTTCTGGTTTTTGGACCATAGCATCGACTAATTTTG
TTGATGCACTTATCGAAGTTCAAGGAACTGCCATTCAGCTTATTCTTTATTGTGATGATCCTGTTAGCCAACTCAAGTG
TTCTCAGGTTGCTTTTGACCTTGACGATGGTTTCTACCCTATTTCTTCTAGAAACCTTCTGAGTCATGAACAGCCAATT
TCTTTTGTTACTTTGCCATCATTTAATGATCATTCTTTTGTTAACATTACTGTCTCTGCGTCCTTTGGTGGTCATAGTG
GTGCCAACCTTATTGCATCTGACACTACTATCAATGGGTTTAGTTCTTTCTGTGTTGACACTAGACAATTTACCATTTC
ACTGTTTTATAACGTTACAAACAGTTATGGTTATGTGTCTAACTCACAGGACAGTAATTGCCCTTTCACCTTGCAATCT
GTTAATGATTACCTGTCTTTTAGTAAATTTTGTGTTTCCACCAGCCTTTTGGCTAGTGCCTGTACCATAGATCTTTTTG
GTTACCCTGAGTTTGGTAGTGGTGTTAAGTTTACGTCCCTTTACTTTCAATTCACAAAGGGTGAGTTGATTACTGGCAC
GCCTAAACCACTTGAAGGTGTCACGGACGTTTCTTTTATGACTCTGGATGTGTGTACCGAGTATACTATCTATGGCTTT
AAAGGTGAGGGTATCATTACCCTTACAAATTCTAGCTTTTTGGCAGGTGTTTATTACACATCTGATTCTGGACAGTTGT

TAGCCTTTAAGAATGTCACTAGTGGTGCTGTTTATTCTGTTACGCCATGTTCTTTTTCAGAGCAGGCTGCATATGTTGA
TGATGATATAGTGGGTGTTATTTCTAGTTTGTCTAATTCCACTTTTAACAGTACTAGGGAGTTGCCTGGTTTCTTCTAC
CATTCTAATGATGGCTCTAATTGTACAGAGCCTGTGTTGGTGTATAGTAACATAGGTGTTTGTAAATCTGGCAGTATTG
GCTACGTCCCATCTCAGTCTGGCCAAGTCAAGATTGCACCCACGGTTACTGGGAATATTAGTATTCCCACCAACTTTAG
TATGAGTATTAGGACAGAATATTTACAGCTTTACAACACGCCTGTTAGTGTTGATTGTGCCACATATGTTTGTAATGGT
AACTCTCGTTGTAAACAATTACTCACCCAGTACACTGCAGCATGTAAGACCATAGAGTCAGCATTACAACTCAGCGCTA
GGCTTGAGTCTGTTGAAGTTAACTCTATGCTTACTATTTCTGAAGAGGCTCTACAGTTAGCTACCATCAGTTCGTTTAA
TGGTGATGGATATAATTTTACTAATGTGCTGGGTGTTTCTGTGTATGATCCTGCAAGTGGCAGGGTGGTACAAAAAAGG
TCTTTTATTGAAGACCTGCTTTTTAATAAAGTGGTTACTAATGGCCTTGGTACTGTTGATGAAGACTATAAGCGCTGTT
CTAATGGTCGCTCTGTGGCAGATCTAGTCTGTGCACAGTATTACTCTGGTGTCATGGTACTACCTGGTGTTGTTGACGC
TGAGAAGCTTCACATGTATAGTGCGTCTCTCGTCGGTGGTATGGTGCTAGGAGGTTTTACTTCTGCAGCGGCATTGCCT
TTTAGCTATGCTGTTCAAGCTAGACTCAATTATCTTGCTCTACAGACGGATGTTCTACAGCGGAACCAGCAATTGCTTG
CTGAGTCTTTTAACTCTGCTATTGGTAATATAACTTCAGCCTTTGAGAGTGTTAAAGAGGCTATTAGTCAAACTTCCAA
GGGTTTGAACACTGTGGCTCATGCGCTTACTAAGGTTCAAGAGGTTGTTAACTCGCAGGGTGCAGCTTTGACTCAACTT
ACCGTACAGCTGCAACACAACTTCCAAGCCATTTCTAGTTCTATTGATGACATTTACTCTCGACTGGACATTCTTTCAG
CCGATGTTCAGGTTGACCGTCTCATCACCGGCAGATTATCAGCACTTAATGCTTTTGTTGCTCAAACCCTCACTAAGTA
TACTGAGGTTCAGGCTAGCAGGAAGCTAGCACAGCAAAAGGTTAATGAGTGCGTTAAATCGCAATCTCAGCGTTATGGT
TTTTGTGGTGGTGATGGCGAGCACATTTTCTCTCTGGTACAGGCAGCACCTCAGGGCCTGCTGTTTTTACATACAGTAC
TTGTACCGGGTGATTTTGTAGATGTTATTGCCATCGCTGGCTTATGCGTTAACGATGAAATTGCCTTGACTCTACGTGA
GCCTGGCTTAGTCTTGTTTACGCATGAACTTCAAAATCATACTGCGACGGAATATTTTGTTTCATCGCGACGTATGTTC
GAACCTAGAAAACCTACCGTTAGTGATTTTGTTCAAATTGAGAGTTGTGTGGTCACCTATGTCAATTTGACTAGAGACC
AACTACCAGATGTAATCCCAGATTACATCGATGTTAACAAAACACTTGATGAGATTTTAGCTTCTCTGCCCAATAGAAC
TGGTCCAAGTCTTCCTTTAGATGTTTTTAATGCCGCTTATCTTAATCTCACTGGTGAAATTGCAGATTTAGAGCAGCGT
TCAGAGTCTCTCCGTAATACTACAGAGGAGCTCCAAAGTCTTATATATAATATCAACAACACACTAGTTGACCTTGAGT
GGCTCAACCGAGTTGAGACATATATCAAGTGGCCGTGGTGGGTTTGGTTGATTATTTTCATTGTTCTCATCTTTGTTGT
GTCATTACTAGTGTTCTGCTGCATTTCCACGGGTTGTTGTGGATGCTGCGGCTGCTGCTGTGCTTGTTTTTCAGGTTGT
TGTAGGGGTCCTAGACTTCAACCTTACGAAGTTTTTGAAAAGGTCCACGTGCAGTGA

>gi|363498868|gb|JQ257006.1|_Porcine_epidemic_diarrhea_virus_strain_CH/YY/11_spike_protein_gene_complete_cds

ATGAAGTCTTTAACCTACTTCTGGTTGTTCTTACCAGTACTTTCAACACTTAGCCTACCACAAGATGTCACCAGG
TGCTCAGCTAACACTAATTTTAGGCGGTTCTTTTCAAAATTTAATGTTCAGGCGCCTGCAGTTGTTGTACTGGGCGGTT
ATCTACCTATTGGTGAAAACCAGGGTGTCAATTCAACTTGGTACTGTGCTGGCCAACATCCAACTGCTAGTGGCGTTCA
TGGTATCTTTGTTAGCCATATTAGAGGTGGTCATGGCTTTGAGATTGGCATTTCGCAAGAGCCTTTTGACCCTAGTGGT
TACCAGCTTTATTTACATAAGGCTACTAACGGTAACACTAATGCTACTGCGCGACTGCGCATTTGCCAGTTTCCTAGCA
TTAAAACATTGGGCCCCACTGCTAATAATGATGTTACAACAGGTCGTAATTGCCTATTTAACAAAGCCATCCCAGCTCA
TATGAGTGAACATAGTGTTGTCGGCATAACATGGGATAATGATCGTGTCACTGTCTTTTCTGACAAGATCTATTATTTT
TATTTTAAAAATGATTGGTCCCGTGTTGCGACAAAGTGTTACAACAGTGGAGGTTGTGCTATGCAATATGTTTACGAAC
CCACCTATTACATGCTTAATGTTACTAGTGCTGGTGAGGATGGTATTTCTTATCAACCCTGTACAGCTAATTGCATTGG
TTATGCTGCCAATGTATTTGCTACTGAGCCCAATGGCCACATACCAGAAGGTTTTAGTTTTAATAATTGGTTTCTTTTG
TCCAATGATTCCACTTTGGTGCATGGTAAGGTGGTTTCCAACCAACCATTGTTGGTCAATTGTCTTTTGGCCATTCCTA
AGATTTATGGACTAGGCCAATTTTTCTCCTTTAATCAAACGATCGATGGTGTTTGTAATGGAGCTGCTGTGCAGCGTGC

ACCAGAGGCTCTGAGGTTTAATATTAATGACACCTCTGTCATTCTTGCTGAAGGCTCAATTGTACTTCATACTGCTTTA
GGAACAAATTTTTCTTTTGTTTGCAGTAATTCCTCAGATCCTCATTTAGCTACCTTCGCCATACCTCTGGGTGCTACCC
AAGTACCCTATTATTGTTTTCTTAAAGTGGATACTTACAACTCCACTGTTTATAAATTCTTGGCTGTTTTACCTCCTAC
CGTCAGGGAAATTGTCATCACCAAGTATGGTGATGTTTATGTCAATGGGTTTGGATACTTGCATCTCGGTTTGTTGGAT
GCTGTCACAATTAATTTCACTGGTCATGGCACTGACGATGATGTTTCTGGTTTTTGGACCATAGCATCGACTAATTTTG
TTGATGCACTCATCGAAGTTCAAGGAACTGCCATTCAGCGTATTCTTTATTGTGATGATCCTGTTAGCCAACTCAAGTG
TTCTCAGGTTGCTTTTGACCTTGACGATGGTTTTTACCCTATTTCTTCTAGAAACCTTCTGAGTCATGAACAGCCAATT
TCTTTTGTTACTCTGCCATCATTTAATGATCATTCTTTTGTTAGCATTACTGTCTCTGCTTCCTTTGGTGGTCTTAGTA
GTGCCAACCTTATTGCATCTGACACTACTATCAATGGGTTTAGTTCTTTCTGTGTTGACACTAGACAATTTACCATTTC
ACTGTTTTATAACGTTACAAACAGTTATGGTTATGTGTCTAACTCACAGGACAGTAATTGCCCTTTCACCTTGCAATCT
GTTAATGATTACCTGTCTTTTAGTAAATTTTGTGTTTCCACCAGCCTTTTGGCTAGTGCCTGTACCATAGATCTTTTTG
GTTACCCTGAGTTTGGTAGTGGTGTTAAGTTTACGTCCCTTTACTTTCAATTCACAAAGGGTGAGTTGATTACTGGCAC
GCCTAAACCACTTGAAGGTGTCACGGACGTTTCTTTTATGACTCTGGATGTGTGTACCGAGTATACTATCTATGGCTTT
AAAGGTGAGGGTATCATTACCCTTACAAATTCTAGCTTTTTGGCAGGTGTTTATTACACATCTGATTCTGGACAGTTGT
TAGCCTTTAAGAATGTCACTAGTGGTGCTGTTTATTCTGTTACGCCATGTTCTTTTTCAGAGCAGGCTGCATATGTTGA
TGATGATATAGTGGGTGTTATTTCTAGTTTGTCTAATTCCACTTTTAACAGTACTAGGGAGTTGCCTGGTTTCTTCTAC
CATTCTAATGATGGCTCTAATTGTACAGAGCCTGTGTTGGTGTATAGTAACATAGGTGTTTGTAAATCTGGCAGTATTG
GCTACGTCCCATCTCAGTCTGGCCAAGTCAAGATTGCACCCACGGTTACTGGGAATATTAGTATTCCCACCAACTTTAG
TATGAGTATTAGGACAGAATATTTACAGCTTTACAACACGCCTGTTAGTGTTGATTGTGCCACATATGTTTGTAATGGT
AACTCTCGTTGTAAACAATTACTCACCCAGTACACTGCAGCATGTAAGACCATAGAGTCAGCATTACAACTCAGCGCTA
GGCTTGAGTCTGTTGAAGTTAACTCTATGCTTACTATTTCTGAAGAGGCTCTACAGTTAGCTACCATCAGTTCGTTTAA
TGGTGATGGATATAATTTTACTAATGTGCTGGGTGTTTCTGTGTATGATCCTGCAAGTGGCAGGGTGGTACAAAAAAGG
TCTTTTATTGAAGACCTGCTTTTTAATAAAGTGGTTACTAATGGCCTTGGTACTGTTGATGAAGACTATAAGCGCTGTT
CTAATGGTCGCTCTGTGGCAGATCTAGTCTGTGCACAGTATTACTCTGGTGTCATGGTACTACCTGGTGTTGTTGACGC
TGAGAAGCTTCACATGTATAGTGCGTCTCTCGTCGGTGGTATGGTGCTAGGAGGTTTTACTTCTGCAGCGGCATTGCCT
TTTAGCTATGCTGTTCAAGCTAGACTCAATTATCTTGCTCTACAGACGGATGTTCTAAAGCGGAACCAGCAATTGCTTG
CTGAGTCTTTTAACTCTGCTATTGGTAATATAACTTCAGCCTTTGAGAGTGTTAAAGAGGCTATTAGTCAAACTTCCAA
GGGTTTGAACACTGTGGCTCATGCGCTTACTAAGGTTCAAGAGGTTGTTAACTCGCAGGGTGCAGCTTCGACTCAACTT
ACCGTACAGCTGCAACACAACTTCCAAGCCATTTCTAGTTCTATTGATGACATTTACTCTCGACTGGACATTCTTTCAG
CCGATGTTCAGGTTGACCGTCTCGTCACCGGCAGATTATCAGCACTTAATGCTTTTGTTGCTCAAACCCTCACTAAGTA
TACTGAGGTTCAGGCTAGCAGGAAGCTAGCACAGCAAAAGGTTAATGAGTGCGTTAAACCGCAATCTCAGCGTTATGGT
TTTTGTGGTGGTGATGGCGAGCACATTTTCTCTCTGGTACAGGCAGCACCTCAGGGCCTGCTGTTTTTACATACAGTAC
TTGTACCGGGTGACTTTGTAGATGTTATTGCCATCGCTGGCTTATGCGTTAACGATGAAATTGCCTTGACTCTACGTGA
GCCTGGCTTAGTCTTGTTTACGCATGAACTTCAAAATCATACTGCGACGGAATATCTTGTTTCATCGCGACGTATGTTT
GAACCTAGAAAACCTACCGTTAGTGATTTTGTTCAAATTGAGAGTTGTGTGGTCACCTATGTCAATTTGACTAGAGACC
AACTACCAGATGTAATCCCAGATTACATCGATGTTAACAAAACACTTGATGAGATTTTAGCTTCTCTGCCCAATAGAAC
TGGTCCAAGTCTTCCTTTAGATGTTTTTAATGCCACTTATCTTAATCTCACTGGTGAAATTGCAGATTTAGAGCAGCGT
TCAGAGTCTCTCCGTAATACTACAGAGGAGCTCCAAAGTCTTATATATAATATCAACAACACACTAGTTGACCTTGAGT
GGCTCAACCGAGTTGAGACATATATCAAGTGGCCGTGGTGGGTTTGGTTGATTATTTCATTGTTCTCATCTTTGTTGT
GTCATTACTAGTGTTCTGCTGCATTTCCACTGGTCGTTGTGGATGCTGCGGCTGCTGCTGTGCTTGTTTTTCAGGTTGT
TGTAGGGGTCCTAGACTTCAACCTTACGAAGTTTTTGAAAAGGTCCACGTGCAGTGA

>gi|363498870|gb|JQ257007.1|_Porcine_epidemic_diarrhea_virus_strain_CH/ZY/1

1_spike_protein_gene_complete_cds

ATGAAGTCTTTAACCTACTTCTGGTTGTTCTTACCAGTACTTTCAACACTTAGCCTACCACAAGATGTCACCAGG
TGCTCAGCTAACACTAATTTTAGGCGGTTCTTTTCAAAATTTAATGTTCAGGCGCCTGCAGTTGTTGTACTGGGCGGTT
ATCTACCTATTGGTGAAAACCAGGGTGTCAATTCAACTTGGTACTGTGCTGGCCGACATCCAACTGCTAGTGGCGTTCA
TGGTATCTTTGTTAGCCATATTAGAGGCGGTCATGGCTTTGAGATTGGCATTTCGCGAGAGCCTTTTGACCCTAGTGGT
TACCAGCTTTATTTACATAAGGCTACTAACGGTAACACTAATGCTACTGCGCGACTGCGCATTTGCCAGTTTCCTAGCA
TTAAAACATTGGGCCCCACTGCTAATAATGATGTTACAACAGGTCGTAATTGCCTATTTAACAAAGCCATCCCAGCTCA
TATGAATGAACATAGTGTTGTCGGCATAACATGGGATAATGATCGTGTCACTGTCTTTTCTGACAAGATCTATTATTTT
TATTTTAAAAATGATTGGTCCCGTGTTGCGACAAAGTGTTACAACAGTGGAGGTTGTGCTATGCAATATGTTTACGAAC
CCACCTATTACATGCTTAATGTTACTAGTGCTGGTGAGGATGGTATTTCTTATCAACCCTGTACAGCTAATTGCATTGG
TTATGCTGCCAATGTATTTGCTACTGAGCCCAATGGCCACATACCAGAAGGTTTTAGTTTTAATAATTGGTTTCTTTTG
TCCAATGATTCCACTTTGGTGCATGGTAAGGTGGTTTCCAACCAACCATTGTTGGTCAATTGTCTTTTGGCCATCCCTA
AGATTTATGGACTAGGCCAATTTTTCTCCTTTAATCAAACGATCGATGGTGTTTGTAATGGAGCTGCTGTGCAGCGTGC
ACCAGAGGCTCTGAGGTTTAATATTAATGACACCTCTGTCATTCTTGCCGAAGGCTCAATTGTACTTCATACTGCTTTA
GGAACAAATTTTTCTTTTGTTTGCAGTAATTCCTCAGATCCTCATTTAGCTACCTTCGCCATACCTCTGGGTGCTACCC
AAGTACCCTATTATTGTTTTCTTAAAGTGGATACTTACAACTCCACTGTTTATAAATTCTTGGCTGTTTTACCTCCTAC
CGTCAGGGAAATTGTCATCACCAAGTATGGTGATGTTTATGTCAATGGGTTTGGATACTTGCATCTCGGTTTGTTGGAT
GCTGTCACAATTAATTTCACTGGTCATGGCACTGACGATGATGTTTCTGGTTTTTGGACCATAGCATCGACTAATTTTG
TTGATGCACTCATCGAAGTTCAAGGAACTGCCATTCAGCGTATTCTTTATTGTGATGATCCTGTTAGCCAACTCAAGTG
TTCTCAGGTTGCTTTTGACCTTGACGATGGTTTTTACCCTATTTCTTCTAGAAACCTTCTGAGTCATGGACAGCCAATT
TCTTTTGTTACTCTGCCATCATTTAATGATCATTCTTTTGTTAACATTACTGTCTCTGCTTCCTTTGGTGGTCATAGTG
GTGCCAACCTTACTGCATCTGACACTACTATCAATGGGTTTAGTTCTTTCTGTGTTGACACTAGACAATTTACCATTTC
ACTGTTTTATAACGTTACAAACAGTTATGGTTATGTGTCTAAATCACAGGACAGTAATTGCCCTTTCACCTTGCAATCT
GTTAATGATTACCTGTCTTTTAGCAACTTTTGTGTTTCCACCAGCCTTTTGGCTAGTGCCTGTACCATAGATCTTTTTG
GTTACCCTGAGTTTGGTAGTGGTGTTAAGTTTACGTCCCTTTACTTTCAATTCACAAAGGGTGAGTTGATTACTGGCAC
GCCTAAACCACTTGAAGGTGTCACGGACGTTTCTTTTATGACTCTGGATGTGTGTACCGAGTATACTATCTATGGCTTT
AAAGGTGAGGGTATCATTACCCTTACAAATTCTAGCTTTTTGGCAGGTGTTTATTACACATCTGATTCTGGACAGTTGT
TAGCCTTTAAGAATGTCACTAGTGGTGCTGTTTATTCTGTTACGCCATGTTCTTTTTCAGAGCAGGCTGCATATGTTGA
TGATGATATAGTGGGTGTTATTTCTAGTTTGTCTAGCTCCACTTTTAACAGTACTAGGGAGTTGCCTGGTTTCTTCTAC
CATTCTAATGATGGCTCTAATTGTACAGAGCCTGTGTTGGTGTATAGTAACATAGGTGTTTGTAAATCTGGCAGTATTG
GCTACGTCCCATCTCAGTCTGGCCAAGTCAAGATTGCACCCACGGTTACTGGGAATATTAGTATTCCCACCAACTTTAG
TATGAGTATTAGGACAGAATATTTACAGCTTTACAACACGCCTGTTAGTGTTGATTGTGCCACATATGTTTGTAATGGT
AACTCTCGTTGTAAACAATTACTCACCCAGTACACTGCAGCATGTAAGACCATAGAGTCAGCATTACAACTCAGCGCTA
GGCTTGAGTCTGTTGAAGTTAACTCTATGCTTACTATTTCTGAAGAGGCTCTACAGTTAGCTACCATTAGTTCGTTTAA
TGGTGATGGGTATAATTTTACTAATGTGATGGGTGTTTCTGTGTATGATCCTGCAAGTGGCAGGGTGGTACAAAAAAGG
TCTTTTATTGAAGACCTGCTTTTTAATAAAGTGGTTACTAATGGCCTTGGTACTGTTGATGAAGACTATAAGCGCTGTT
CTAATGGTCGCTCTGTGGCAGATCTAGTCTGTGCACAGTATTACTCTGGTGTCATGGTACTACCTGGTGTTGTTGACGC
TGAGAAGCTTCACATGTATAGTGCGTCTCTCATCGGTGGTATGGTGCTAGGAGGTTTTACTTCTGCAGCGGCATTGCCT
TTTAGCTATGCTGTTCAAGCTAGACTCAATTATCTTGCTCTACAGACGGATGTTCTACAGCGGAACCAGCAATTGCTTG
CTGAGTCTTTTAACTCTGCTATTGGTAATATAACTTCAGCCTTTGAGAGTGTTAAAGAGGCTATTAGTCAAACTTCCAA
GGGTTTGAACACTGTGGCTCATGCGCTTACTAAGGTTCAAGAGGTTGTTAACTCGCAGGGTGCAGCTTTGACTCAACTT
ACCGTACAGCTGCAACACAACTTCCAAGCCATTTCTAGTTCTATTGATGACATTTACTCTCGACTGGACATTCTTTCAG
CCGATGTTCAGGTTGACCGTCTCATCACCGGCAGATTATCAGCACTTAATGCTTTTGTTGCTCAAACCCTCACTAAGTA

TACTGAGGTTCAGGCTAGCAGGAAGCTAGCACAGCAAAAGGTTAATGAGTGCGTTAAGTCGCAATCTCAGCGTTATGGT
TTTTGTGGTGGTGATGGCGAGCACATTTTCTCTCTGGTACAGGCAGCACCTCAGGGCCTGCTGTTTTTACATACAGTAC
TTGTACCGGGTGATTTTGTAGATGTTATTGCCATCGCTGGCTTATGCGTTAACGATGAAATTGCCTTGACTCTACGTGA
GCCTGGCTTAGTCTTGTTTACGCATGAACTTCAAAATCATACTGCGACGGAATATTTTGTTTCATCGCGACGTATGTTT
GAACCTAGAAAACCTACCGTTAGTGATTTTGTTCAAATTGAGAGTTGTGTGGTCACCTATGTCAATTTGACTAGAGACC
AACTACCAGATGTAATCCCAGATTACATCGATGTTAACAAAACACTTGATGAGATTTTAGCTTCTCTGCCCAATAGAAC
TGGTCCAAGTCTTCCTTTAGATGTTTTTAATGCCACTTATCTTAATCTCACTGGTGAAATTGCAGATTTAGAGCAGCGT
TCAGAGTCTCTCCGTAATACTACAGAGGAGCTCCAAAGTCTTATATATAATATCAACAACACACTAGTTGACCTTGAGT
GGCTCAACCGAGTTGAGACATATATCAAGTGGCCGTGGTGGGTTTGGTTGATTATTTTCATTGTTCTCATCTTTGTTGT
GTCATTACTAGTGTTCTGCTGCATTTCCACGGGTTGTTGTGGATGCTGCGGCTGCTGCTGTGCTTGTTCTTCAGGTTGT
TGTAGGGGTCCTAGACTTCAACCTTACGAAGTTTTTGAAAAGGTCCACGTGCAGTGA

>gi|385863474|gb|JQ517274.1|_Porcine_epidemic_diarrhea_virus_isolate_HuN_spike_protein_(S)_gene_complete_cds

ATGAGGTCTTTAACCTACTTCTGGTTGTTCTTACCAGTACTTTCAACACTTAGCCTACCACAAGATGTCACCAGG
TGCTCAGCTAACACTAATTTTAGGCGGTTCTTTTCAAAATTTAATGTTCAGGCGCCTGCAGTTGTTGTACTGGGCGGTT
ATCTACCTATTGGTGAAAACCAGGGTGTCAATTCAACTTGGTACTGTGCTGGCCAACATCCAACTGCTAGTGGCGTTCA
TGGTATCTTTGTTAGCCATATTAGAGGTGGTCATGGCTTTGAGATTGGCATTTCGCAAGAGCCTTTTGACCCTAGTGGT
TACCAGCTTTATTTACATAAGGCTACTAACGGTAACACTAATGCTACTGCGCGACTGCGCATTTGCCAGTTTCCTAGCA
GTAAAACATTGGGCCCCACTGCTAATAATGATGTTACAACAGGTCGTAATTGCCTATTTAACAAAGCCATCCCAGCTCA
TATGAGTGAACATAGTGTTGTCGGCATAACATGGGATAATGATCGTGTCACTGTCTTTTCTGACAAGATCTATTATTTT
TATTTTAAAAATGATTGGTCCCGTGTTGCGACAAAGTGTTACAACAGTGGAGGTTGTGCTATGCAATATGTTTACGAAC
CCACCTATTACATGCTTAATGTTACTAGTGCTGGTGAGGATGGTATTTCTTATCAACTCTGTACAGCTAATTGCATTGG
TTATGCAGCCAATGTATTTGCTACTCAGCCCAATGGCCACATACCAGAAGGTTTTAGTTTTAATAATTGGTTTCTTTTG
TCCAATGATTCCACTTTGGTGCATGGTAAGGTGGTTTCCAACCAACCATTGTTGGTCAATTGTCTTTTGGCCATTCCTA
AGATTTATGGACTAGGCCAATTTTTCTCCTTTAATCAAACGATCGATGGTGTTTGTAATGGAGCTGCTGTGCAGCGTGC
ACCAGAGGCTCTGAGGTTTAATATTAATGACACCTCTGTCATTCTTGCTGAAGGCTCAATTGTACTTCATACTGCTTTA
GGAACAAATTTTTCTTTTGTTTGCAGTAATTCCTCAGATCCTCACTTAGCCACCTTCGCCATACCTCTGGGTGCTATCC
AAGTACCCTATTATTGTTTTCTTAAAGTGGATACTTACAACTCCACTGTTTATAAATTCTTGGCTGTTTTACCTCCTAC
CGTCAGGGAAATTGTCATCACCAAGTATGGTGACGTTTATGTCAATGGGTTTGGCTACTTGCATCTCGGTTTGTTGGAT
GCTGTCACAATTAATTTCACTGGTCATGGCACTGACGATGACGTTTCTGGTTTTTGGACCATAGCATCGACTAATTTTG
TTGATGCACTTATCGAAGTTCAAGGAACTGCCATTCAGCGTATTCTTTATTGTGATGATCCTGTTAGCCAACTCAAGTG
TTCTCAGGTTTCTTTTGACCTTGACGATGGTTTTTACCCTATTTCCTCTACAAACCTTCTGAGTCATGAACAGCCAACT
TCTTTTGTTACTTTGCCATCATTTAATGATCATTCTTTTGTTAATATTACTGTCTCTGCTGCTTTTGGTGGTTATAGTG
GTGCCAACCTTATTGCATCTGACACTACTATCAATGGGTTTAGTTCTTTCTGTGTTGACACTAGACAATTTACCATTTC
ACTGTTTTATAATGTTACAAACAGTTATGGTTATGTGTCTAAATCACAGGACAGTAATTGCCCTTTCACCTTGCAATCT
GTTAATGATTACCTGTCTTTTAGCAAATTTTGTGTTTCTACCAGCCTTTTGGCTAGTGCCTGTACCATAGATCTTTTTG
GTTACCCTGAGTTTGGTAGTGGTGTTAAGTTCACGTCCCTTTACTTTCAATTCACAAAGGGTGAGTTGATTACTGGCAC
GCCTAAACCACTTGAAGGTGTTACGGACGTTTCTTTTATGACTCTGGATGTGTGTACCAAGTATACTATCTATGGCTTT
AAAGGTGAGGGTATCATTACCCTTACAAATTCTAGCTTTTTGGCAGGTGTTTATTATACATCTGATTCTGGACAGTTGT
TAGCTTTTAAGAATGTCACTAGTGGTGCTGTTTATTCTGTTACGCCATGTTCTTTTTCAGAGCAGGCTGCATATGTTGA
TGATGATATAGTGGGTGTTATTTCTAGTTTGTCTAACTCCACTTTTAACAGTACTAGGGAGTTGCCTGGTTTCTTCTAC

CATTCTAATGATGGCTCTAATTGTACAGAGCCTGTGTTGGTGTATAGTAACATAGGTGTTTGTAAATCTGGCAGTATTG
GCTATGTCCCATCTCAGTCTGGCCAAGTCAAGATTGCACCCACGGTTACTGGGAATATTAGTATTCCCACCAACTTTAG
TATGAGTATTAGGACAGAATATTTACAGCTTTACAACACGCCTTTTAGTGTTGATTGTGCTACATATGTTTGTAATGGT
AACTCTCGTTGTAAACAATTACTCACCCAGTACACTGCAGCATGTAAGACCATAGAGTCAGCATTACAACTCAGCGCCA
GGCTTGAGTCTGCTGAAGTCAACTCTATGCTTACTATTTCTGAAGAGGCTCTACAGTTAGCTACCATCAGTTCGTTTAA
TGGTGATGGATATAATTTTACTAATGTGCTGGGTGTTTCCGTGCGTGATCCTGCAAGTGGCAGGGTGGTACAAAAAAGG
TCTTTTATTGAAGACCTGCTTTTTAATAAAGTGGTTACTAATGGCCTTGGTACTGTTGATGAAGACTATAAGCGCTGTT
CTAATGGCCGCTCTGTGGCAGATTTAGTCTGTGCGCAGTATTACTCTGGTGTCATGGTACTACCTGGGGTTGTTGACGC
TGAGAAGCTTCATATGTATAGTGCGTCTCTCATCGGTGGTATGGTGCTAGGAGGTTTTACTGCAGCAGCGGCATTGCCT
TTTAGCTATGCTGTTCAAGCGAGACTCAATTATCTTGCTCTACAGACGGATGTTCTACAGCGGAACCAGCAATTGCTTG
CTGAGTCTTTTAACTCTGCTATTGGTAATATAACTTCAGCCTTTGAGAGTGTTAAAGAGGCTATTAGTCAAACTTCTAA
GGGTTTGAACACTGTGGCTCATGCGCTTACTAAGGTTCAAGAGGTTGTTAATTCGCAGGGTGCAGCTTTGACCCAACTT
ACCGTACAGCTGCAACACAACTTCCAAGCCATTTCTAGTTCTATTGATGACATTTACTCCCGACTGGACATTCTCTCAG
CTGATGTTCAGGTTGACCGTCTCATCACCGGCAGATCATCAGCACTTAATGCTTTTGTTGCTCAAACCCTCACTAAGTA
TACTGAGGTTCAGGCTAGCAGGAAGCTAGCACAGCAAAAGGTTAATGAGTGCGTTAAATCGCAATCTCAGCGTTATGGT
TTTTGCGGTGGTGATGGCGAGCACATTTTCTCTCTGGTACAGGCAGCACCTCAGGGCCTGCTGTTTTTACATACAGTAC
TTGTACCGGGTGATTTTGTAGATGTTATTGCCATCGCTGGCTTATGCGTTAACGATGAAATTGCCTTGACTCTACGTGA
GCCTGGCCTAGTCTTGTTTACGCATGAACTTCAAAATCATACTGCGACGGAATATTTTGTTTCATCGCGACGTATGTTT
GAACCTAGAAAACCTACCGTTAGTGATTTTGTTCAAATTGAGAGTTGTGTGGTCACCTATGTCAATTTGACTAGAGACC
AACTACCAGATGTAATCCCAGATTACATCGATGTTAACAAAACACTTGATGAGATTTTAGCTTCTCTGCCCAATAGAAC
TGGTCCAAGTCTTCCTTTAGATGTTTTTAATGCCACTTATCTTAATCTCACTGGTGAAATTGCAGATTTAGAGCAGCGT
TCAGAGTCTCTCCGTAATACTACAGAAGAGCTCCAAAGTCTTATATATAATATCAACAACACACTAGTTGACCTTGAGT
GGCTCAACCGAGTTGAGACATATATCAAGTGGCCGTGGTGGGTTTGGTTGATTATTTTTATTGTTCTCATCTTTGTTGT
GTCATTATTAGTGTTCTGCTGCATTTCCACGGGTTGTTGTGGATGCTGCGGCTGCTGTGGTGCTTGTTTTTCAGGTTGT
TGTAGGGGTCCTAGACTTCAACCTTACGAAGCTTTTGAAAAGGTCCACGTGCAGTGA

>gi|386785695|gb|JQ627653.1|_Porcine_epidemic_diarrhea_virus_strain_CH/AY/11_spike_protein_gene_complete_cds

ATGAAGTCTTTAACCTACTTCTGGTTGTTCTTACCAGTACTTTCAACACTTAGCCTACCACAAGACGTCACCAGG
TGCTCAGCTAACACTAATTTTAGGCGGTTCTTTTCAAAATTTAATGTTCAGGCGCCTGCAGTTGTTGTACTGGGCGGTT
ATCTACCTATTGGTGAAAACCAGGGTGTCAATTCAACTTGGTATTGTACTGGCCAACATCCAACTGCTAGTGGCGTTCA
TGGTATCTTTGTTAGCCATATTAGAGGTGGTCATGGCTTTGAGATTGGCATTTCGCAAGAGCCTTTTGACCCTAGTGGT
TACCAGCTTTATTTACATAAGGCTACTAACGGTAACACTAATGCTACTGCGCGACTGCGCATTTGCCAGTTTCCTAGCA
TTAAAACATTGGGCCCCACTGCTAATAATGATGTTACAACAGGTCGTAATTGCCTATTTAACAAAGCCATCCCAGCTCA
TATGAGTGAACATAGTGTTGTCGGCATAACATGGGATAATGATCGTGTCACTGTCTTTTCTGACAAGATCTATTATTTT
TATTTTAAAAATGATTGGTCCCGTGTTGCGACAAAGTGTTACAACAGTGGAGGTTGTGCTATGCAATATGTTTACGAAC
CCACCTATTACATGCTTAATGTTACTAGTGCTGGTGAGGATGGTATTTCTTATCAACCCTGTACAGCTAATTGCATTGG
TTATGCTGCCAATGTATTTGCTACTGAGCCCAATGGCCACATACCAGAAGGTTTTAGTTTTAATAATTGGTTTCTTTTG
TCCAATGATTCCACTTTGGTGCATGGTAAGGTGGTTTCCAACCAACCATTGTTGGTCAATTGTCTTTTGGCCATTCCTA
AGATTTATGGACTAGGCCAATTTTTCTCCTTTAATCAAACGATCGATGGTGTTTGTAATGGAGCTGCTGTGCAGCGTGC
ACCAGAGGCTCTGAGGTTTAATATTAATGACACCTCTGTCATTCTTGCTGAAGGCTCAATTGTACTTCATACTGCTTTA
GGAACAAATTTTTCTTTTGTTTGCAGTAATTCCTCAGATCCTCACTTAGCCACCTTCGCCACACCTCTGGGTGCTACCC

AAGTACCCTATTATTGTTTTCTTAAAGTGGATACTTACAACTCCACTGTTTATAAATTCTTGGCTGTTTTACCTCCTAC
CGTCAGGGAAATTGTCATCACCAAGTATGGTGATGTTTATGTCAATGGGTTTGGCTACTTGCATCTCGGTTTGTTGGAT
GCTGTCACAATTAATTTCACTGGTCATGGCACTGACGATGACGTTTCTGGTTTCTGGACCATGGCATCGACTAATTTTG
TCGTTGCACTTATCGAAGTTCAAGGGACTGCCATTCAGCGTATTCTTTATTGTGATGATCCTGTTAGCCAACTCAAGTG
TTCTCAGGTTTCTTTTGACCTTGACGATGGTTTTTACCCTATTTCCTCTACAAACCTTCTGAGTCATGAACAGCCAACT
TCTTTTGTTACTTTGCCATCATTTAATGATCATTCTTTTGTTAATATTACTGTCTCTGCTGCTTTTGGTGGTCATAGTG
GTGCCAACCTTATTGCATCTGACACTACTATCAATGGGTTTAGTTCTTTCTGTGTTGACACTAGACAATTTACCATTTC
ACTGTTTTATAATGTTACAAACAGTTATGGTTATGTGTCTAACTCACAGGACAGTAATTGCCCTTTCACCTTGCAATCT
GTTAATGATTACCTGTCTTTTAGCAAATTTTGTGTTTCTACCAGCCTTTTGGCTAGTGCCTGTACCATAGATCTTTTTG
GTTACCCTGAGTTTGGTAGTGGTGTTAAGTTCACGTCCCTTTACTTTCAATTCACAAAGGGTGAGTTGATTACTGGCAC
GCCTAAACCACTTGAAGGTGTTACGGACGTTTCTTTTATGACTCTGGATGTGTGTACCAAGTATACTATCTATGGCTTT
AAAGGTGAGGGTATCATTACCCTTACAAATTCTAGCTTTTTGGCAGGTGTTTATTATACATCTGATTCTGGACAGTTGT
TAGCTTTTAAGAATGTCACTAGTGGTGCTGTTTATTCTGTTACGCCATGTTCTTTTTCAGAGCAGGCTGCATATGTTGA
TGATGATATAGTGGGTGTTATTTCTAGTTTGTCTAACTCCACTTTTAACAGTACTAGGGAGTTGCCTGGTTTCTTCTAC
CATTCTAATGATGGCTCTAATTGTACAGAGCCTGTGTTGGTGTATAGTAACATTGGTGTTTGTAAATCTGGCAGTATTG
GCTATGTCCCATCTCAGTCTGGCCAAGTCAAGATTGCACCCACGGTTACTGGGAATATTAGTATTCCCACCAACTTTAG
TATGAGTATTAGGACAGAATATTTACAGCTTTACAACACGCCTGTTAGTGTTGATTGTGCTACATATGTTTGTAATGGT
AACTCTCGTTGTAAACAATTACTCACCCAGTACACTGCAGCATGTAAGACCATAGAGTCAGCATTACAACTCAGCGCTA
GGCTTGAGTCTGCTGAAGTCAACTCTATGCTTACTATTTCTGAAGAGGCTCTACAGTTAGCTACCATCAGTTCGTTTAA
TGGTGATGGATATAATTTTACTAATGTGCTGGGTGTTTCCGTTCGTGATCCTGCAAGTGGCAGGGTGGTACAAAAAAGG
TCTTTTATTGAAGACCTGCTTTTTAATAAAGTGGTTACTAATGGCCTTGGTACTGTTGATGAAGACTATAAGCGCTGTT
CTAATGGCCGCTCTGTGGCAGATTTAGTCTGTGCGCAGTATTACTCTGGTGTCATGGTACTACCTGGTGTTGTTGACGC
TGAGAAGCTTCATATGTATAGTGCGTCTCTCATCGGTGGTATGGTGCTAGGAGGTTTTACTGCAGCAGCGGCATTGCCT
TTTAGCTATGCTGTTCAAGCGAGACTCAATTATCTTGCTCTACAGACGGATGTTCTACAGCGGAACCAGCAATTGCTTG
CTGAGTCTTTTAACTCTGCTATTGGTAATATAACTTCAGCCTTTGAGAGTGTTAAAGAGGCTATTAGTCAAACTTCTAA
GGGTTTGAACACTGTGGCTCATGCGCTTACTAAGGTTCAAGAGGTTGTTAATTCGCAGGGTGCAGCTTTGACCCAACTT
ACCGTACAGCTGCAACACAACTTCCAAGCCATTTCTAGTTCTATTGATGACATTTATTCCCGACTGGACATTCTTTCAG
CCGATGTTCAGGTTGACCGCCTCATCACCGGCAGATTATCAGCACTTAATGCTTTTGTTGCTCAAACCCTCACTAAGTA
TACTGAGGTTCGGGCTAGCAGGAAGCTAGCACAGCAAAAGGTTAATGAGTGCGTTAAATCGCAATCTCAGCGTTATGGT
TTTTGCGGTGGTGATGGCGAGCACATTTTCTCTCTGGTACAGGCAGCACCTCAGGGCCTGCTGTTTTTACATACAGTAC
TTGTACCGGGTGATTTTGTAGATGTTATTGCCATCGCTGGCTTATGCGTTAACGATGAAATTGCCTTGACTCTACGTGA
GCCTGGCTTAGTCTTGTTTACGCATGAACTTCAAAATCATACTGCGACGGAATATTTTGTTTCATCGCGACGTATGTTT
GAACCTAGAAAACCTACCGTTAGTGATTTTGTTCAAATTGAGAGTTGTGTGGTCACCTATGTCAATTTGACTAGAGACC
AACTACCAGATGTAATCCCAGATTACATCGATGTTAACAAAACACTTGATGAGATTTTAGCTTCTCTGCCCAATAGAAC
TGGTCCAAGTCTTCCTTTAGATGTTTTTAATGCCACTTATCTTAATCTCACTGGTGAAATTGCAGATTTAGAGCAGCGT
TCAGAGTCTCTCCGTAATACTACAGAAGAGCTCCAAAGTCTTATATATAATATCAACAACACACTAGTTGACCTTGAGT
GGCTCAACCGAGTTGAGACATATATCAAGTGGCCGTGGTGGGTTTGGTTGATTATTTTTATTGTTCTCATCTTTGTTGT
GTCATTATTAGTGTTCTGCTGCATTTCCACGGGTTGTTGTGGATGCTGCGGCTGCTGTGGTGCTTGTTTTTCAGGTTGT
TGTAGGGGTCCTAGACTTCAACCTTACGAAGCTTTTGAAAAGGTCCACGTGCAGTGA

>gi|386785697|gb|JQ627654.1|_Porcine_epidemic_diarrhea_virus_strain_CH/CG/11_spike_protein_gene_complete_cds

ATGAAGTCTTTAACCTACTTCTGGTTGTTCTTACCAGTACTTTCAACACTTAGCCTACCACAAGATGTCACCAGG
TGCTCAGCTAACACTAATTTTAGGCGGTTCTTTTCAAAATTTAATGTTCAGGCGCCTGCAGTTGTTGTACTGGGCGGTT
ATCTACCTATTGGTGAAAACCAGGGTGTCAATTCAACTTGGTACTGTGCTGGCCAACATCCAACTGCTAGTGGCGTTCA
TGGTATCTTTCTTAGCCATATTAGAGGTGGTCATGGCTTTGAGATTGGCATTTCGCAAGAGCCTTTTGACCCTAGTGGT
TACCAGCTTTATTTACATAAGGCTACTAATGGTAACACTAATGCTACTGCGCGATTGCGCATTTGCCAGTTTCCCAGCA
TTAAAACATTGGGCCCCACTGCTAATAATGATGTTACAACAGGTCGTAACTGCCTATTTAACAAAGCCATCCCAGCTCA
TATGAGTGAACATAGTGTTGTCGGCATAACATGGGATAATGATCGTGTCACTGTCTTTTCTGACAAGATCTATTATTTT
TATTTTAAAAATGATTGGTCCCGTGTTGCGACAAAGTGTTACAACAGTGGAGGTTGTGCTACGCAATATGTTTACGAAC
CCACTTACTACATGCTTAATGTTACTAGTGCTGGTGAGGATGGTATTTCTTATCAACCCTGTACAGCTAATTGCATTGG
TTATGCTGCCAATGTATTTGCTACTGAGCCCAATGGCCACATACCAGAAGGTGTTAGTTTTAATAATTGGTTTCTTTTG
TCCAATGATTCCACTTTGGTGCATGGTAAGGTGGTTTCCAACCAACCATTGTTGGTCAATTGTCTTTTGGCCATGCCTA
AGATTTATGGACTAGGCCAATTTTTCTCATTCAATCAAACGATGGATGGCGTTTGTAATGGAGCTGCTGCGCAGCGTGC
ACCAGAGGCTCTGAGGTTTAATATTAATGACACCTCTGTCATTCTTGCTGAAGGCTCAATTGTACTTCATACTGCTTTA
GGAACAAATCTTTCCTTTGTTTGCAGTAATTCCTCAGATCCTCATTTAGCCACCTTCGCCATACCTCTGGGTGCTACCC
AAGTACCCTATTATTGTTTTCTTAAAGTGGATACTTATAATTCCACTGTTTATAAATTCTTGGCTGTTTTACCTCCTAC
CGTCAGGGAAATTGTCATCACCAAGTATGGTGATGTTTATGTCAATGGGTTTGGCTACTTGCATCTCGGTTTATTGGAT
GCTGTCACAATTAACTTCACTGGTCATGGCACTGACGATGACGTTTCTGGTTTTTGGACCATAGCATCGACTAATTTTG
TTGATGCACTCATCGAAGTTCAAGGAACTGCCATTCAGCGTATTCTTTATTGTGATGATCCTGTTAGCCAACTCAAGTG
TTCTCAAGTTGCTTTCGACCTTGACGATGGTTTTTACCCTATTTCTTCTAGAAACCTTCTGGGTCATGAGCAGCCAATT
TCTTTTGTTACTTTGCCATCATTTAATGATCATTCTTTTGTTAACATTACTGTCTCTGCGTCCTTTGGTGGTCATAGTG
GTGCCAACCCTATTTCATCTGACACTACTATCAATGGGTTTAGTTCTTTCTGTGTTGACACTAGACAATTTACCATTTC
ACTGTTTTATAACGTTACAAACAGTTATGGTTATGTGTCTAAATCACAGGACAGTAATTGCCCTTTCACCTTGCAATCT
GTTAATGATTACCTGTCTTTTAGCAAATTTTGTGTTTCCACCAGCCTTTTGGCTAGTGCCTGTACCATAGATCTTTTTG
GTTACCCTGAGTTTGGTAGTGGTGTTAAGTTTACGTCCCTTTACTTTCAATTCACAAAGGGTGAGTTGATTACTGGCAC
GCCTAAACCACTTGAAGGTGTCACGGACGTTTCTTCTATGACTCTGGATGTGTGTACCAAGTATACTATCTATGGCTTT
AAAGGTGAGGGTATCATTACCCTTACAAATTCTAGCTTTTTGGCAGGTGTTTATCACACATCTGATTCTGGACAGTTGT
TAGCCTTTAAGAATGTCACTAGTGGTGCTGTTTATTCTGTTACGCCATGTTCTTTTTCAGAGCAGGCTGCATATGTTGA
TGATGTTATAGTGGGTGTTATTTCTAGTTTGTCTAGCTCCACTTTTAACAGTACTAGGGAGTTGCCTGGTTTCTTCTAC
CATTCTAATGATGGCTCTAATTGTACAGAGCCTGTGTTGGTGTATAGTAACATAGGTGTTTGTAAATCTGGCAGTATTG
GCTACGTCCCATCTCAGTCTGGCCAAGTCAAGATTGCACCCACGGTTACTGGGAATATTAGTATTCCCACCAACTTTAG
TATGAGTATTAGGACAGAATATTTACAGCTTTACAACACGCCTGTTAGTGTTGATTGTGCCACATATGTTTGTAATGGT
AACTCTCGTTGTAAACAATTACTCACCCAGTACACTGCAGCATGTAAGACCATAGAGTCAGCATTACAACTCAGCGCTA
GGCTTGAGTCTGTTGAAGTTAACTCTATGCTTACTATTTCTGAAGAGGCTCTACAGTTAGCTACCATCAGTTCGTTTAA
TGGTGATGGATATAATTTTACTAATGTGCTGGGTGTTTCTGTGTATGATCCTGCAAGTGGCAGGGTGGTACAAAAAAGG
TCTTTTATTGAAGACCTGCTTTTTAATAAAGTGGTTACTAATGGCCTTGGTACTGTTGATGAAGACTATAAGCGCTGTT
CTAATGGTCGCTCTGTGGCAGATCTAGTCTGTGCACAGTATTACTCTGGTGTCATGGTACTACCTGGTGTTGTTGACGC
TGAGAAGCTTCACATGTATAGTGCGTCTCTCATCGGTGGTATGGTGCTAGGAGGTTTTACTTCTGCAGCGGCATTGCCT
TTTAGCTATGCTGTTCAAGCTAGACTCAATTATCTTGCTCTACAGACGGATGTTCTACAGCGGAACCAGCAATTGCTTG
CTGAGTCTTTTAACTCTGCTATTGGTAATATAACTTCAGCCTTTGAGAGTGTTAAAGAGGCTATTAGTCAAACTTCCAA
GGGTTTGAACACTGTGGCTCATGCGCTTACTAAGGTTCAAGAGGTTGTTAACTCGCAGGGTGCAGCTTTGACTCAACTT
ACCGTACAGCTGCAACACAACTTCCAAGCCATTTCTAGTTCTATTGATGACATTTACTCCCGACTGGACATTCTTTCAG
CCGATGTTCAGGTTGACCGTCTCATCACCGGCAGATTATCAGCACTTAATGCTTTTGTTGCTCAAACCCTCACTAAGTA
TACTGAGGTTCAGGCTAGCAGGAAGCTAGCACAGCAAAAGGTTAATGAGTGCGTTAAATCGCAATCTCAGCGTTATGGT
TTTTGTGGTGGTGATGGCGAGCACATTTTCTCTCTGGTACAGGCAGCACCTCAGGGCCTGCTGTTTTTACATACAGTAC

TTGTACCGGGTGATTTTGTAGATGTTATTGCCATCGCTGGCTTATGCGTTAACGATGAAATTGCCTTGACTCTACGTGA
GCCTGGCTTAGTCTTGTTTACGCATGAACTTCAAAATCATACTGCGACGGAATATTTTGTTTCATCGCGACGTATGTTT
GAACCTAGAAAACCTACCGTTAGTGATTTTGTTCAAATTGAGAGTTGTGTGGTCACCTATGTCAATTTGACTAGAGACC
AACTACCAGATGTAATCCCAGATTACATCGATGTTAACAAAACACTTGATGAGATTTTAGCTTCTCTGCCCAATAGAAC
TGGTCCAAGTCTTCCTTTAGATGTTTTTAATGCCACTTATCTTAATCTCACTGGTGAAATTGCAGATTTAGAGCAGCGT
TCAGAGTCTCTCCGTAATACTACAGAGGAGCTCCAAAGTCTTATATATAATATCAACAACACACTAGTTGACCTTGAGT
GGCTCAACCGAGTTGAGACATATATCAAGTGGCCGTGGTGGGTTTGGTTGATTATTTTCATTGTTCTCATCTTTGTTGT
GTCATTACTAGTGTTCTGCTGCATTTCCACGGGTTGTTGTGGATGCTGCGGCTGCTGCTGTGCTTGTTTTTCAGGTTGT
TGTAGGGGTCCTAGACTTCAACCTTACGAAGTTTTTGAAAAGGTCCACGTGCAGTGA

>gi|392301330|gb|JQ979287.1|_Porcine_epidemic_diarrhea_virus_strain_CH9FJ_spike_protein_(S)_gene_complete_cds

ATGAAGTCTTTAACTTACTTCTGGTTGTTATTACCAGTACTTTCAACACTCAGCCTACCACAAGATGTCACTAGG
TGCCAGTCCACTATTAACTTCAGGCGGTTCTTTTCAAAATTTAATGTGCAATCACCTGCTGTCGTTGTTTTGGGTGGTT
ATCTACCTAGTATGAACTCTTCTAGCTGGTACTGTGGCACAGGCCTTGAAACTGCTAGTGGCGTTCATGGTATTTTCCT
TAGTTACATCGATTCTGGTCAGGGCTTTGAGATCGGCATTTCACAGGAGCCGTTTGATCCTAGTGGTTACCAGCTTTAT
CTACATAAGGCCACTAATGGTAACACTAATGCTATCGCACGATTGCGCATTTGCCAGTTTCCAGATAATAAAACATTGG
GCCCTACTGTTAATGATGTTACAACAGGTCGTAACTGCCTATTCAACAGAGCCATTCCAGCTTATATGCAGGATGGAAA
AAATATTGTTGTCGGCATAACATGGGATAATGATCGCGTCACTGTTTTTGCTGACAAGATCTATCATTTTTATCTTAAA
AATGATTGGTCCCGCGTTGTGACAAGATGTTACAATAAAAGGAGTTGTGCTATGCAATATGTTTATACACCTACCTACT
ACATGCTTAATGTTACTAGTGCAGGTGAGGATGGCATTTATTATGAACCATGTACAGCTAATTGCAGTGGTTACGCTGC
CAATGTATTTGCCACTGATTCTAATGGCCATATACCAGAAGGTTTTAGTTTTAATAATTGGTTTCTTTTGTCCAATGAT
TCCACTTTGTTGCATGGTAAGGTGGTTTCCAACCAACCTCTGTTGGTCAATTGTCTTTTGGCCATTCCTAAGATTTATG
GACTAGGCCAATTTTTCTCATTCAATCAAACGATGGATGGCGTTTGTAATGGAGCTGCTGTGCAGCGTGCACCAGAGGC
TCTGAGGTTTAATATTAATGACACCTCTGTCATTCTTGCTGAAGGCTCAATTGTACTTCATACTGCTTTAGGAACAAAT
CTTTCTTTTGTTTGCAGTAATTCCTCAAATCCTCATTTAGCCACCTTCGCCATACCTTTGGGTGCTACCCAAGTACCCT
ATTATTGTTTTCTTAAAGTGGATACTTACAACTCCACTGTTTATAAATTCTTGGCTGTTTTACCTCCTACCGTCAGGGA
AATTGTCATCACCAAGTATGGTGATGTTTATGTCAATGGGTTTGGCTACTTGCATCTCGGTTTGTTGGATGCTGTCACA
ATCAATTTCACTGGTCATGGCACTGACGATGACGTTTCTGGTTTTTGGACCATAGCATCGACTAATTTTGTTGATGCAC
TCATCGAAGTTCAAGGAACTGCCATTCAGCGTATTCTTTATTGTGATGATCCTGTTAGCCAACTCAAGTGTTCTCAGGT
TGCTTTTGACCTTGACGATGGTTTTTACCCTATCTCTTCTAGGAACCTTCTGAGTCATGAACAGCCAATTTCTTTTGTT
ACTCTGCCATCATTTAATGATCATTCTTTTGTTAACATTACTGTCTCTGCGTCCTTTGGTGGTCATAGTGGTGCCAACC
TCATTGCATCTGACACTACTATCAATGGATTTAGTTCTTTCTGTGTTGACACTAGACAATTTACCATTTCACTGTTTTA
TAACGTTACAAACAGTTATGGTTATGTGTCTAACTCACAGGACACTAATTGCCCTTTCACCTTGCAATCTGTTAATGAT
TACCTGTCTTTTAGCAAATTTTGTGTTTCTACCAGCCTTTTGGCTAGTGCCTGTACCATAGATCTTTTTGGTTACCCTG
CGTTTGGTAGTGTTGTTAAGTTTACGTCCCTTTACTTTCAATTCACAAAGGGTGAGTTGATTACTGGCACGCCTAAACC
ACTTGAAGGTGTTACGGACGTTTCTTTTATGACTCTGGATGTGTGTACCAAGTATACTATCTATGGCTTTAAAGGTGAG
GGTATCATTACCCTTACAAATTCTAGCTTTTTGGCAGGTGTTTATTATACATCTGATTCTGGACAGTTGTTAGCCTTTA
AGAATGTCACTAGTGGTGCTGTTTATTCTGTTACGCCATGTTCTTTTTCAGAGCAGGCTGCATATGTTGATGATGATAT
AGTGGGTGTTATTTCTAGTTTGTCTAGCTCCACTTTTAACAGTACTAGGGAGTTGCCTGGTTTCTTCTACCATTCTAAT
GATGGCTCTAATTGTACAGAGCCTGTGTTGGTGTATAGTAACATAGGTGTTTGTAAATCCGGCAGTATTGGCTATGTCC
CATCTCAGTCTGGCCAAGTCAAGATTGCACCCACGGTTACTGGGAATATTAGTATTCCCACCAACTTTAGTATGAGTAT

TAGGACAGAATATGTACAGCTTTACAACACGCCTGTTAGTGTTGATTGTGCCACATATGTTTGTAATGGTAACTCTCGT
TGTAAACAATTACTCACCCAGTACACTGCAGCATGTAAGACCATAGAGTCAGCATTACAACTCAGCGCTAGGCTTGAGT
CTGTTGAAGTTAACTCTATGCTTACTATTTCTGAAGAGGCTCTACAGTTAGCTACCATCAGTTCGTTTAATGGTGATGG
ATATAATTTTACTAATGTGCTGGGTGTTTCTGCGTATGATCCTGCAAGTGGCAGGGTGGTACAAAAAAGGTCTTTTATT
GAAGACCTGCTTTTTAATAAAGTGGTTACTAATGGCCTTGGTACTGTTGATGAAGACTATAAGCGCTGTTCTAATGGTC
GTTCTGTGGCGGATCTAGTCTGTGCACAGTATTACTCTGGTGTCATGGTACTACCTGGTGTTGTTGACGCTGAGAAGCT
TCACATGTATAGTGCGTCTCTCATCGGTGGTATGGTGCTAGGAGGTTTTACTTCTGCAGCGGCATTGCCTTTTAGCTAT
GCTGTTCAAGCTAGACTCAATTATCTTGCTCTACAGACGGATGTTCTACAGCGGAACCAGCAATTGCTTGCTGAGTCTT
TTAACTCTGCTATTGGTAATATAACTTCAGCCTTTGAGAGTGTTAAAGAGGCTATTAGTCAAACTTCTAAGGGTTTGAA
CACTGTGGCTCATGCGCTTACTAAGGTTCAAGAGGTTGTTAACTCGCAGGGTACAGCTTTGACTCAACTTACCGTACAG
CTGCAACACAACTTCCAAGCCATTTCTAGTTCTATTGATGACATTTACTCCCGACTGGACATTCTTTCAGCCGATGTTC
AGGTTGACCGTCTCATCACCGGCAGATTATCAGCACTTAATGCTTTTGTTGCTCAAACCCTCACTAAGTATACTGAGGT
TCAGGCTAGCAGGAAGCTAGCACAGCAAAAGGTTAATGAGTGCGTTAAATCGCAATCTCAGCGTTATGGTTTTTGTGGT
GGTGATGGCGAGCACATTTTCTCTCTGGTACGGGCAGCACCTCAGGGCCTGCTGTTTTTACATACAGTACTTGTACCGG
GTGATTTTGTAGATGTTATTGCCATCGCTGGCTTATGCGTTAACGATGAAATTGCCTTGACTCTACGTGAGCCTGGCTT
AGTCTTGTTTACGCATGAACTTCAAAATCATACTGCGACGGAATATTTTGTTTCATCGCGACGTATGTTTGAACCTAGA
AAACCTACCGTTAGTGATTTTGTTCAAATTGAGAGTTGTGTGGTCACCTACGTCAATTTGACTAGAGACCAACTACCAG
ATGTAATCCCAGATTACATCGATGTTAACAAAACACTTGATGAGATTTTAGCTTCTCTGCCCAATAGAACTGGTCCAAG
TCTTCCTTTAGATGTTTTTAATGCCACTTATCTCAATCTCACTGGTGAAATTGCAGATTTAGAGCAGCGTTCAGAGTCT
CTCCGTAATACTACAGAGGAGCTCCAAAGTCTTATATATAATATCAACAACACACTAGTTGACCTTGAGTGGCTCAACC
GAGTTGAGACATATATCAAGTGGCCGTGGTGGGTTTGGTTGGTTATTTTTATTGTTCTCATCTTTATTGTGTCATTACT
AGTGTTCTGCTGCATTTCCACGGGTTGTTGTGGATGCTGCGGCTGCTGCTGTGCTTGTTTTTCAGGTTGTTGTAGGGGT
CCTAGACTTCAACCTTACGAAGTTTTTGAAAAGGTCCACGTGCAGTGA

>gi|392301332|gb|JQ979288.1|_Porcine_epidemic_diarrhea_virus_strain_CH13GX_spike_protein_(S)_gene_complete_cds

ATGAAGTCTTTAACTTACTTCTGGTTGTTATTACCAGTACTTTCAACACTTAGCCTACCACAAGATGTCACTAGG
TGCCAGTCCACTATTAACTTCAGGCGGTTCTTTTCAAAATTTAATGTGCAAGCACCTGCTGTCGTTGTTTTGGGTGGTT
ATCTACCTATTATGAACTCTTCTAGCTGGTACTGTGGCACAGGTCTTGAAACTGCTAGTGGCGTTCATGGTATTTTCCT
CAGTTACATCGATTCTGGTCAGGGCTTTGAGATTGGCATTTCACAGGAGCCGTTTGATCCTAGTGGTTACCAGCTTTAT
CTACATAAGGCCACTAATGGTAACACTAATGCTATTGCACGACTGCGCATTTGCCAGTTTCCAGATAACAAAACATTGG
GCCCTACTGTTAATGATGTTACAACAGGTCGTAACTGCCTATTCGACAGAGCCATTCCAGCTTATATGCAAGATGGAAA
AAATATTGTTGTCGGCATAACATGGGATAATGATCGTGTCACTGTTTTTGCTGACAAGATCTATCATTTTTATCTTAAA
AATGATTGGTCCCGCGTTGCGACAAGATGTTATAATAAAAGGAGTTGTGCTATGCAATATGTTTATACACCTACCTACT
ACATGCTTAATGTTACTAGTGCAGGTGAGGATGGCATTTATTATGAACCATGTACAGCTAATTGCAGTGGTTACGCTGC
CAATGTATTTGCCACTGATTCTAATGGCCATATACCAGAAGGTTTTAGTTTTAATAATTGGTTTCTTTTGTCCAATGAT
TCCACTTTGTTGCATGGTAAGGTGGTTTCCAACCAACCTTTGTTGGTCAATTGTCTTTTGGCCATTCCTAAGATTTATG
GACTAGGCCAATTTTTCTCATTCAATCAAACGATGGATGGCGTTTGTAATGGAGCTGCTGCGCAGCGTGCACCAGAGGC
TCTGAGGTTTAATATTAATGACACCTCTGTCATTCTTGCTGAAGGCTCAATTGTACTTCATACTGCTTTAGGAACAAAT
CTTTCCTTTGTTTGCAGTAATTCCTCAGATCCTCATCTAGCCACCTTCGCCATACCTTTGGGTGCCACCCAAGTACCCT
ATTATTGTTTTCTTAAAGTGGATACTTACAACTCCACTGTTTATAAATTCTTGGCTGTTTTACCTCCTACCGTCAGGGA
AATTGTCATCACCAAGTATGGTGATGTTTATGTCAATGGGTTTGGCTACTTGCATCTCGGTTTGTTGGATGCTGTCACA

ATTAATTTCACTGGTCATGGCACTGACGATGACGTTTCTGGTTTTTGGACCATAGCATCGACTAATTTTGTTGATGCAC
TCATCGAAGTTCAAGGAACTGCCATTCAGCGTATTCTTTATTGTGATGATCCTGTTAGCCAACTCAAGTGTTCTCAGGT
TGCTTTTGACCTTGACGATGGTTTTTACCCTATTTCTTCTAGAAACCCTCTGAGTCATGAACAGCCAATTTCTTTTGTT
ACTCTGCCATCATTTAATGATCATTCTTTTGTTAACATTACTGTCGCTGCGTCCTTTGGTGGTCATAGTGGTGCCAACC
TTATTGCATCTGACACTACTATCAATGGGTTTAGTTCTTTCTGTGTTGACACTAGACAATTTACCATTTTACTGTTTTA
TAACGTTACTAACAGTTATGGTTATGTGCCTAAATCACAGGACAGTAATTGCCCTTTCACCTTGCAATCTGTTAATGAT
TACCTGTCTTTTAGCAAATTTTGTGTTTCTACCAGCCTTTTGGCTAGTGCCTGTACCATAGATCTTTTTGGTTACCCTG
AGTTTGGTAGTGCTGTTAAGTTTACGTCCCTTTACTTTCAATTCACAAAGGGTGAGTTGATTACTGGCACGCCTAAACC
ACTTGAAGGTGTCACGGACGTTTCTTTTATGACTCTGGATGTGTGTACCAAGTATACTATCTATGGCTTTAAAGGTGAG
GGTGTCATTACCCTTACAAATTCTAGCTTTTTGGCAGGTGTTTATTACACATCTGATTCTGGACAGTTGTTAGCCTTTA
AGAATGTCACTAGTGGTGCTGTTTATTCTGTTACGCCATGTTCTTTTTCAGAGCAGGCTGCATATGTTGATGATGATAT
AGTGGGTGTTATTTCTAGTTTGTCTAGCTCCACTTTTAACAGTACTAGGGAGTTGCCTGGTTTTTTCTACCATTCTAAT
GATGGCTCTAATTGTACAGAGCCTGTGTTGGTGTATAGTAACATAGGCGTTTGTAAATCTGGCAGTATTGGCTATGTCC
CATCTCAGTCTGGCCAAGTCAAGATTGTACCCACGGTTACTGGGAATATTAGTATTCCCACCAACTTTAGTATGAGTAT
TAGGACAGAATATTTACAGCTTTACAACACGCCTGTTAGTGTTGATTGTGCCACATATGTTTGTAATGGTAACTCTCGT
TGCAAACAATTACTCACCCAGTACACTGCAGCATGTAAGACCATAGAGTCAGCATTACAACTCAGCGCTAGGCTTGAGT
CTGTTGAAGTTAACTCTATGCTTACTATTTCTGAAGAGGCTCTACAGTTAGCTACCATCAGTTCGTTTAATGGTGATGG
ATATAATTTTACCAATGTGCTGGGTGTTTCTGTGTATGATCCTGCAAGTGGCAGGGTGGTACAAAAAAGGTCTTTTATT
GAAGACCTGCTTTTTAATAAAGTGGTTACTAATGGCCTTGGTACTGTTGATGAAGACTATAAGCGCTGTTCTAATGGTC
GCTCTGTGGCAGATCTAGTCTGTGCACAGTATTACTCTGGTGTCATGGTACTACCTGGTGTTGTTGACGCTGAGAAGCT
TCACATGTATAGTGCGTCTCTCATCGGTGGTATGGTGCTAGGAGGTTTTACTGCTGCAGCGGCATTGCCTTTTAGCTAT
GCTGTTCAAGCTAGACTTAATTATCTTGCTCTACAGACGGATGTTCTACAGCGGAACCAGCAATTGCTTGCTGAGTCTT
TTAACTCTGCTATTGGTAATATAACTTCAGCCTTTGAGAGTGTTAAAGAGGCTATTAGTCAAACTTCCAAGGGTTTGAA
CACTGTGGCTCATGCGCTTACTAAGGTTCAAGAGGTTGTTAATTCGCAGGGTGCAGCTTTGACCCAACTTACCGTACAG
CTGCAACACAACTTCCAAGCCATTTCTAGTTCTATTGATGACATTTACTCCCGACTGGACATTCTTTCAGCCGATGTTC
AGGTTGACCGTCTCATCACCGGCAGATTATCAGCACTTAATGCTTTTGTTGCTCAAACCCTCACTAAGTATACTGAGGT
TCAGGCTAGCAGGAAGCTAGCACAGCAAAAGGTTAATGAGTGCGTTAAATCGCAATCTCAGCGTTATGGTTTTTGTGGT
GGTGATGGCGAGCACATTTTCTCTCTGGTACAGGCAGCACCTCAGGGCCTGCTGTTTTTACATACAGTACTTGTACCGG
GTGGTTTTGTAGATGTTATTGCCATCGCTGGCTTATGCGTTAACGATGAAATTGCCTTGACTCTACGTGAGCCTGGCTT
AGTCTTGTTTACGCATGAACTTCAAAATCATACTGCGACGGAATATTTTGTTTCATCGCGACGTATGTTTGAACCTAGA
AAACCTACCGTTAGTGATTTTGTTCAAATTGAGAGTTGTGTGGTCACCTATGTCAATTTGACTAGAGACCAACTACCAG
ATGTAATCCCAGATTACATCGATGTTAACAAAACACTTGATGAGATTTTAGCTTCTCTGCCCAATAGAACTGGTCCAAG
TCTTCCTTTAGATGTTTTTAATGCCACTTATCTTAATCTCACTGGTGAAATTGCAGATTTAAAGCAGCGTTCGGAGTCT
CTCCGTAATACTACAGAGGAGCTCCAAAGTCTTATATATAATATCAACAACACACTAGTTGACCTTGAGTGGCTCAACC
GAGTTGAGACATATATCAAGTGGCCGTGGTGGGTTTGGTTGATTATTTTTATTGTTCTCATCTTTGTTGTGTTATTACT
AGTGTTCTGCTGCATTTCCACGGGTTGTTGTGGATGCTGCGGCTGCTGCTGTGCTTGTTTTTCAGGTTGTTGTAGGGGT
CCTAGACTTCAACCTTACGAAGTTTTTGAAAAGGTCCACGTGCAGTGA

>gi|392301334|gb|JQ979289.1|_Porcine_epidemic_diarrhea_virus_strain_CH17GZ_spike_protein_(S)_gene_complete_cds

ATGAAGTCTTTAACCTACTTCTGGTTGTTCTTACCAGTACTTACAACACTTAGCCTACCACAAGATGTCACCAGG
TGCTCAGCTAACACTAATTTTAGGCGGTTCTTTTCAAAATTTAATGTTCAGGCGCCTGCAGTTGTTGTACTGGGCGGTT

ATCTACCTATTGGTGAAAACCAGGGTGTCAATTCAACTTGGTACTGTGCTGGCCAACATCCAACTGCTAGTGGCGTTCA
TGGTATCTTTGTTAGCCATATTAGAGGTGGTCATGGCTTTGAGATTGGCATTTCGCAAGAGCCTTTTGACCCTAGTGGT
TACCAGCTTTATTTACATAAGGCTACTAACGGTAACACTAATGCTACTGCGCGACTGCGCATTTGCCAGTTTCCTAGCA
TTAAAACATTGGGCCCCACTGCTAATAATGATGTTACAACAGGTCGTAATTGCCTATTTAACAAAGCCATCCCAGCTCA
TATGAGTGAACATAGTGTTGTCGGAATAACATGGGATAATGATCGTGTCACTGTCTTTTCTGACAAGATCTATTATTTT
TATTTTAAAAATGATTGGTCCCGTGTTGCGACAAAGTGTTACAACAGTGGAGGTTGTGCTATGCAATATGTTTACGAAC
CCACCTATTACATGCTTAATGTTACTAGTGCTGGTGAGGATGGTATTTCTTATCAACCCTGTACAGCTAATTGCATTGG
TTATGCTGCCAATGTATTTGCTACTGAGCCCAATGGCCACATACCAGAAGGTTTTAGTTTTAATAATTGGTTTCTTTTG
TCCAATGATTCCACTTTGGTGCATGGTAAGGTGGTTTCCAACCAACCATTGTTGGTCAATTGTCTTTTGGCCATTCCTA
AGATTTATGGACTAGGCCAATTTTTCTCCTTTAATCAAACGATCGATGGTGTTTGTAATGGAGCTGCTGTGCAGCGTGC
ACCAGAGGCTCTGAGGTTTAATATTAATGACACCTCTGTCATTCTTGCTGAAGGCTCAATTGTACTTCATACTGCTTTA
GGAACAAATTTTTCTTTTGTTTGCAGTAATTCCTCAGATCCTCACTTAGCCACCTTCGCCATACCTCTGGGTGCTATCC
AAGTACCCTATTATTGTTTTCTTAAAGTGGATACTTACAACTCCACTGTTTATAAATTCTTGGCTGTTTTACCTCCTAC
CGTCAGGGAAATTGTCATCACCAAGTATGGTGATGTTTATGTCAATGGGTTTGGCTATTTGCATCTCGGTTTGTTGGAC
GCTGTCACAATTAATTTCACTGGTCATGGCACTGACGATGACGTTTCAGGTTTCTGGACCGTAGCATCGACTAATTTTG
TTGATGCACTTATCGAAGTTCAAGGAACTGCCATTCAGCGTATTCTTTATTGTGATGATCCTGTTAGCCAACTCAAGTG
TTCTCAGGTTGCTTTTGACCTTGACGATGGTTTCTACCCTATTTCTTCTAGAAACCTTCTGAGTCATGAACAGCCAATT
TCTTTTGTTACTTTGCCATCATTTAATGATCATTCTTTTGTTAACATTACTGTCTCTGCGTCCTTTGGTGGTCATAGTG
GTGCCAACCTTATTGCATCTGACACTACTATCAATGGGTTTAGTTCTTTCTGTGTTGACACTAGACAATTTACCATTTC
ACTGTTTTATAACGTTACAAACAGTTATGGTTATGTGTCTAAATCACAGGACAGTAATTGCCCTTTCACCTTGCAATCT
GTTAATGATTACCTGTCTTTTAGTAAATTTTGTGTTTCCACCAGCCTTTTGGCTAGTGCCTGTACCATAGATCTTTTTG
GTTACCCTGAGTTTGGTAGTGGTGTTAAGTTTACGTCCCTTTACTTTCAATTCACAAAGGGTGAGTTGATTACTGGCAC
GCCTAAACCACTTGAAGGTGTCACGGACGTTTCTTTTATGACTCTGGATGTGTGTACCGAGTATACTATCTATGGCTTT
AAAGGTGAGGGTATCATTACCCTTACAAATTCTAGCTTTTTGGCAGGTGTTTATTACACATCTGATTCTGGACAGTTGT
TAGCCTTTAAGAATGTCACTAGTGGTGCTGTTTATTCTGTTACGCCATGTTCTTTTTCAGAGCAGGCTGCATATGTTGA
TGATGATATAGTGGGTGTTATTTCTAGTTTGTCTAGCTCCACTTTTAACAGTACTAGGGAGTTGCCTGGTTTCTTCTAC
CATTCTAATGATGGCTCTAATTGTACAGAGCCTGTGTTGGTGTATAGTAACATAGGTGTTTGTAAATCTGGCAGTATTG
GCTACGTCCCATCTCAGTCTGGCCAAGTCAAGATTGCACCCACGGTTACTGGGAATATTAGTATTCCCACCAACTTTAG
TATGAGTATTAGGACAGAATATTTACAGCTTTACAACACGCCTGTTAGTGTTGATTGTGCCACATATGTTTGTAATGGT
AACTCTCGTTGTAAACAATTACTCACCCAGTACACTGCAGCATGTAAGACCATAGAGTCAGCATTACAACTCAGCGCTA
GGCTTGAGTCTGTTGAAGTTAACTCTATGCTTACTATTTCTGAAGAGGCTCTACAGTTAGCTACCATCAGTTCGTTTAA
TGGTGATGGATATAATTTTACTAATGTGCTGGGTGTTTCTGTGTATGATCCTGCAAGTGGCAGGGTGGTACAAAAAAGG
TCTTTTATTGAAGACCTGCTTTTTAATAAAGTGGTTACTAATGGCCTTGGTACTGTTGATGAAGACTATAAGCGCTGTT
CTAATGGTCGCTCTGTGGCAGATCTAGTCTGTGCACAGTATTACTCTGGTGTCATGGTACTACCTGGTGTTGTTGACGC
TGAGAAGCTTCACATGTATAGTGCGTCTCTCATCGGTGGTATGGTGCTAGGAGGTTTTACTTCTGCAGCGGCATTGCCT
TTTAGCTATGCTGTTCAAGCTAGACTCAATTATCTTGCTCTACAGACGGATGTTCTACAGCGGAACCAGCAATTGCTTG
CTGAGTCTTTTAACTCTGCTATTGGTAATATAACTTCAGCCTTTGAGAGTGTTAAAGAGGCTATTAGTCAAACTTCCAA
GGGTTTGAACACTGTGGCTCATGCGCTTACTAAGGTTCAAGAGGTTGTTAACTCGCAGGGTGCAGCTTTGACTCAACTT
ACCGTACAGCTGCAACACAACTTCCAAGCCATTTCTAGTTCTATTGATGACATTTACTCTCGACTGGACATTCTTTCAG
CCGATGTTCAGGTTGACCGTCTCATCACCGGCAGATTATCAGCACTTAATGCTTTTGTTGCTCAAACCCTCACTAAGTA
TACTGAGGTTCAGGCTAGCAGGAAGCTAGCACAGCAAAAGGTTAATGAGTGCGTTAAATCGCAATCTCAGCGTTATGGT
TTTTGTGGTGGTGATGGCGAGCACATTTTCTCTCTGGTACAGGCAGCACCTCAGGGCCTGCTGTTTTTACATACAGTAC
TTGTACCGGGTGACTTTGTAGATGTTATTGCCATCGCTGGCTTATGCGTTAACGATGAAATTGCCTTGACTCTACGTGA
GCCTGGCTTAGTCTTGTTTACGCATGAACTTCAAAATCATACTGCGACGGAATATTTTGTTTCATCGCGACGTATGTTT

GAACCTAGAAAACCTACCGTTAGTGATTTTGTTCAAATTGAGAGTTGTGTGGTCACCTATGTCAATTTGACTAGAGACC
AACTACCAGATGTAATCCCAGATTACATCGATGTTAACAAAACACTTGATGAGATTTTAGCTTCTCTGCCCAATAGAAC
TGGTCCAAGTCTTCCTTTAGATGTTTTTAATGCCACTTATCTTAATCTCACTGGTGAAATTGCAGATTTAGAGCAGCGT
TCAGAGTCTCTCCGTAATACTACAGAGGAGCTCCAAAGTCTTATATATAATATCAACAACACACTAGTTGACCTTGAGT
GGCTCAACCGAGTTGAGACATATATCAAGTGGCCGTGGTGGGTTTGGTTGATTATTTTCATTGTTCTCATCTTTGTTGT
GTCATTACTAGTGTTCTGCTGCATTTCCACTGGTTGTTGTGGATGCTGCGGCTGCTGCTGTGCTTGTTTTTCAGGTTGT
TGTAGGGGTCCTAGACTTCAACCTTACGAAATTTTTGAAAAGGTCCACGTGCAGTGA

>gi|392301338|gb|JQ979291.1|_Porcine_epidemic_diarrhea_virus_strain_CH18Hainan_spike_protein_(S)_gene_complete_cds

ATGAAGTCTTTAACCTACTTCTGGTTGTTCTTACCAGTACTTTCAACACTTAGCCTACCACAAGATGTCACCAGG
TGCTCAGCTAACACTAATTTTAGGCGGTTCTTTTCAAAATTTAATGTTCAGGCGCCTGCAGTTGTTGTACTGGGCGGTT
ATCTACCTATTGGTGAAAACCAGGGTGTCAATTCAACTTGGTACTGTGCTGGCCAACATCCAACTGCTAGTGGCGTTCA
TGGTATCTTTGTTAGCCATATTAGAGGTGGTCATGGCTTTGAGATTGGCATTTCGCAAGAGCCTTTTGACCCTAGTGGT
TACCAGCTTTATTTACATAAGGCTACTAACGGTAACACTAATGCTACTGCGCGACTGCGCATTTGCCAGTTTCCTAGCA
TTAAAACATTGGGCCCCACTGCTAATAATGATGTTACAACAGGTCGTAATTGCCTATTTAACAAAGCCATCCCAGCTCA
TATGAGTGAACATAGTGTTGTCGGAATAACATGGGATAATGATCGTGTCACTGTCTTTTCTGACAAGATCTATTATTTT
TATTTTAAAAATGATTGGTCCCGTGTTGCGACAAAGTGTTACAACAGTGGAGGTTGTGCTATGCAATATGTTTACGAAC
CCACCTATTACATGCTTAATGTTACTAGTGCTGGTGAGGATGGTATTTCTTATCAACCCTGTACAGCTAATTGCATTGG
TTATGCTGCCAATGTATTTGCTACTGAGCCCAATGGCCACATACCAGAAGGTTTTAGTTTTAATAATTGGTTTCTTTTG
TCCAATGATTCCACTTTGGTGCATGGTAAGGTGGTTTCCAACCAACCATTGTTGGTCAATTGTCTTTTGGCCATTCCTA
AGATTTATGGACTAGGCCAATTTTTCTCCTTTAATCAAACGATCGATGGTGTTTGTAATGGAGCTGCTGTGCAGCGTGC
ACCAGAGGCTCTGAGGTTTAATATTAATGACACCTCTGTCATTCTTGCTGAAGGCTCAATTGTACTTCATACTGCTTTA
GGAACAAATTTTTCTTTTGTTTGCAGTAATTCCTCAGATCCTCACTTAGCCACCTTCGCCATACCTCTGGGTGCTATCC
AAGTACCCTATTATTGTTTTCTTAAAGTGGATACTTACAACTCCACTGTTTATAAATTCTTGGCTGTTTTACCTCCTAC
CGTCAGGGAAATTGTCATCACCAAGTATGGTGATGTTTATGTCAATGGGTTTGGCTATTTGCATCTCGGTTTGTTGGAC
GCTGTCACAATTAATTTCACTGGTCATGGCACTGACGATGACGTTTCAGGTTTCTGGACCATAGCATCGACTAATTTTG
TTGATGCACTCATCGAAGTTCAAGGAACTGCCATTCAGCGTATTCTTTATTGTGATGATCCTGTTAGCCAACTCAAGTG
TTCTCAGGTTGCTTTTGACCTTGACGATGGTTTCTACCCTATTTCTTCTAGAAACCTTCTGAGTCATGAACAGCCAATT
TCTTTTGTTACTTTGCCATCATTTAATGATCATTCTTTTGTTAACATTACTGTCTCTGCGTCCTTTGGTGGTCATAGTG
GTGCCAACCTTATTGCATCTGACACTACTATCAATGGGTTTAGTTCTTTCTGTGTTGACACTAGACAATTTACCATTTC
ACTGTTTTATAACGTTACAAACAGTTATGGTTATGTGTCTAAATCACAGGACAGTAATTGCCCTTTCACCTTGCAATCT
GTTAATGATTACCTGTCTTTTAGTAAATTTTGTGTTTCCACCAGCCTTTTGGCTAGTGCCTGTACCATAGATCTTTTTG
GTTACCCTGAGTTTGGTAGTGGTGTTAAGTTTACGTCCCTTTACTTTCAATTCACAAAGGGTGAGTTGATTACTGGCAC
GCCTAAACCACTTGAAGGTGTCACGGACGTTTCTTTTATGACTCTGGATGTGTGTACCGAGTATACTATCTATGGCTTT
AAAGGTGAGGGTATCATTACCCTTACAAATTCTAGCTTTTTGGCAGGTGTTTATTACACATCTGATTCTGGACAGTTGT
TAGCCTTTAAGAATGTCACTAGTGGTGCTGTTTATTCTGTTACGCCATGTTCTTTTTCAGAGCAGGCTGCATATGTTGA
TGATGATATAGTGGGTGTTATTTCTAGTTTGTCTAGCTCCACTTTTAACAGTACTAGGGAGTTGCATGGTTTCTTCTAC
CATTCTAATGATGGCTCTAATTGTACAGAGCCTGTGTTGGTGTATAGTAACATAGGTGTTTGTAAATCTGGCAGTATTG
GCTACGTCCCATCTCAGTCTGGCCAAGTCAAGATTGCACCCACGGTTACTGGGAATATTAGTATTCCCACCAACTTTAG
TATGAGTATTAGGACAGAATATTTACAGCTTTACAACACGCCTGTTAGTGTTGATTGTGCCACATATGTTTGTAATGGT
AACTCTCGTTGTAAACAATTACTCACCCAGTACACTGCAGCATGTAAGACCATAGAGTCAGCATTACAACTCAGCGCTA

GGCTTGAGTCTGTTGAAGTTAACTCTATGCTTACTATTTCTGAAGAGGCTCTACAGTTAGCTACCATCAGTTCGTTTAA
TGGTGATGGATATAATTTTACTAATGTGCTGGGTGTTTCTGTGTATGATCCTGCAAGTGGCAGGGTGGTACAAAAAAGG
TCTTTTATTGAAGACCTGCTTTTTAATAAAGTGGTTACTAATGGCCTTGGTACTGTTGATGAAGACTATAAGCGCTGTT
CTAATGGTCGCTCTGTGGCAGATCTAGTCTGTGCACAGTATTACTCTGGTGTCATGGTACTACCTGGTGTTGTTGACGC
TGAGAAGCTTCACATGTATAGTGCGTCTCTCATCGGTGGTATGGTGCTAGGAGGTTTTACTTCTGCAGCGGCATTGCCT
TTTAGCTATGCTGTTCAAGCTAGACTCAATTATCTTGCTCTACAGACGGATGTTCTACAGCGGAACCAGCAATTGCTTG
CTGAGTCTTTTAACTCTGCTATTGGTAATATAACTTCAGCCTTTGAGAGTGTTAAAGAGGCTATTAGTCAAACTTCCAA
GGGTTTGAACACTGTGGCTCATGCGCTTACTAAGGTTCAAGAGGTTGTTAACTCGCAGGGTGCAGCTTTGACTCAACTT
ACCGTACAGCTGCAACACAACTTCCAAGCCATTTCTAGTTCTATTGATGACATTTACTCTCGACTGGACATTCTTTCAG
CCGATGTTCAGGTTGACCGTCTCATCACCGGCAGATTATCAGCACTTAATGCTTTTGTTGCTCAAACCCTCACTAAGTA
TACTGAGGTTCAGGCTAGCAGGAAGCTAGCACAGCAAAAGGTTAATGAGTGCGTTAAATCGCAATCTCAGCGTTATGGT
TTTTGTGGTGGTGATGGCGAGCACATTTTCTCTCTGGTACAGGCAGCACCTCAGGGCCTGCTGTTTTTACATACAGTAC
TTGTACCGGGTGACTTTGTAGATGTTATTGCCATCGCTGGCTTATGCGTTAACGATGAAATTGCCTTGACTCTACGTGA
GCCTGGCTTAGTCTTGTTTACGCATGAACTTCAAAATCATACTGCGACGGAATATTTTGTTTCATCGCGACGTATGTTT
GAACCTAGAAAACCTACCGTTAGTGATTTTGTTCAAATTGAGAGTTGTGTGGTCACCTATGTCAATTTGACTAGAGACC
AACTACCAGATGTAATCCCAGATTACATCGATGTTAACAAAACACTTGATGAGATTTTAGCTTCTCTGCCCAATAGAAC
TGGTCCAAGTCTTCCTTTAGATGTTTTTAATGCCACTTATCTTAATCTCACTGGTGAAATTGCAGATTTAGAGCAGCGT
TCAGAGTCTCTCCGTAATACTACAGAGGAGCTCCAAAGTCTTATATATAATATCAACAACACACTAGTTGACCTTGAGT
GGCTCAACCGAGTTGAGACATATATCAAGTGGCCGTGGTGGGTTTGGTTGATTATTTTCATTGTTCTCATCTTTGTTGT
GTCATTACTAGTGTTCTGCTGCATTTCCACTGGTTGTTGTGGATGCTGCGGCTGCTGCTGTGCTTGTTTTTCAGGTTGT
TGTAGGGGTCCTAGACTTCAACCTTACGAAATTTTTGAAAAGGTCCACGTGCAGTGA

>gi|397328493|gb|JX018179.1|_Porcine_epidemic_diarrhea_virus_isolate_CH/GXNN/2012_spike_protein_gene_complete_cds

ATGAAGTCTTTAACTTACTTCTGGTTGTTCTTACCAGTACTTTCAACACTTAGCCTACCACAAGATCTCACCAGG
TGCTCAGCTAACACTAATTTTAGGCGGTTCTTTTCAAAATTTAATGTTCAGGCGCCTGCAGTTGTTGTACTGGGCGGTT
ATCTACCTACTGGTGAAAACCAGGGTGTTAATTCAACTTGGTACTGTGCTGGCCAACATCCAACTGCTAGTGGCGTTCA
TGGTATCTTTCTTAGCCATATTAGAGGTGGTCATGGCTTTGAGATTGGCATTTCGCAAGAGCCTTTTGACCCTAGTGGT
TACCAGCTTTATTTACATAAGGCTACTAATGGTAACACTAATGCTACTGCGCGATTGCGCATTTGCCAGTTTCCCAGCA
TTAAAACATTGGGCCCCACTGCTAATAATGATGTTACAACAGGTCGTAACTGCCTATTTAACAAAGCCATCCCAGCTCA
TATGAGTGAACATAGTGTTGTCGGCATAACATGGGATAATGATCGTGTCACTGTCTTTTCTGACAAGATCTATCATTTT
TATTTTAAAAATGATTGGTCCCGTGTTGCGACAAAGTGTTACAACAGTGGAGGTTGTGCTATGCAATATGTTTACGAAC
CCACTTACTACATGCTTAATGTTACTAGTGCTGGTGAGGATGGTATTTCTTATCAACCCTGTACAGCTAATTGCATTGG
TTATGCTGCCAATGTATTTGCTACTGAGCCCAATGGCCACATACCAGAAGGTTTTAGTTTTAATAATTGGTTTCTTTTG
TCCAATGATTCCACTTTGGTGCATGGTAAGGTGGTTTCCAACCAACCATTGTTGGTCAATTGTCTTTTGGTCATGCCTA
AGATTTATGGACTAGGCCAATTTTTCTCCTTCAATCAAACGATCGATGGTGTTTGTAATGGAGCTGCTGTGCAGCGTGC
ACCAGAGGCTCTGAGGTTTAATATTAATGACACCTCTGTCATTCTTGCTGAAGGCTCAATTGTACTTCATACTGCTTTA
GGAACAAATTTTTCTTTTGTTTGCAGTAATTCCTCAGATCCTCACTTAGCCACCTTTGCCATACCTCTGGGTGCTATCC
AAGTACCCTATTATTGTTTTCTTAAAGTGGATACTTACAACTCCACTGTTTATAAATTCTTGGCTGTTTTACCTCCTAC
CGTCAGGGAAATTGTCATCACCAAGTATGGTGATGTTTATGTCAATGGGTTTGGCTACTTGCATCTCGGTTTGTTGGAT
GCTGTCACAATTAATTTCACTGGTCATGGCACTGACGATGACGTTTCTGGTTTTTGGACCATAGCATCGACTAATTTTG
TTGATGCACTTATTGAAGTTCAAGGAACTGCCATTCAGCGTATTCTTTATTGTGATGATCCTGTTAGCCAACTCAAGTG

TTCTCAGGTTGCTTTTGACCTTGACGATGGTTTTTACCCTATTTCCTCTACAAACCTTTTGAGTCATGAACAGCCAACT
TCTTTTGTTACTTTGCCATCATTTAATGATCATTCTTTTGTTAATATTACTGTCTCTGCTGCTTTTGGTGGTCATAGTG
GTGCCAACCTTATTGCATCTGACACTACTATCAATGGGTTTAGTTCTTTCTGTGTTGACACTAGACAATTTACCATTTC
ACTGTTTTATAACGTTACAAACAGTTATGGTTACGTGTCTAAATCACAGGACAGTAATTGCCCTTTTACCTTGCAATCT
GTTAATGATTACCTGTCTTTTAGCAAATTTTGTGTTTCTACCAGCCTTTTGGCTAGTGCCTGTACCATAGATCTTTTTG
GTTACCCTGAGTTTGGTAGTGGTGTTAAGTTCACGTCCCTTTACTTTCAATTCACAAAGGGTGAGTTGATTACTGGCAC
GCCTAAACCACTTGAAGGTGTTACGGACGTTTCTTCTATGACTCTGGATGTGTGTACCAAGTATACTATCTATGGCTTT
AAAGGTGAGGGTATCATTACCCTTACAAATTCTAGCTTTTTGGCAGGTGTTTATTATACATCTGATTCTGGACAGTTGT
TAGCTTTTAAGAATGTCACTAGTGGTGCTGTTTATTCTGTTACGCCATGTTCTTTTTCAGAGCAGGCTGCATATGTTGA
TGATGATATAGTGGGTGTTATTTCTAGTTTGTCTAGCTCCACTTTTAACAGTACTAGGGAGTTGCCTGGTTTCTTCTAC
CATTCTAATGATGGCTCTAATTGTACAGAGCCTGTGTTGGTGTATAGTAACATAGGTGTTTGTAAATCTGGCAGTATTG
GCTATGTCCCATCTCAGTCTGGCCAAGTCAAGATTGCACCCACGGTTACTGGGAATATTAGTATTCCCACCAACTTTAG
TATGAGTATTAGGACAGAATATTTACAGCTTTACAACACGCCTGTTAGTGTTGATTGTGCTACATATGTTTGTAATGGT
AACTCTCGTTGTAAACAATTACTCACCCAGTACACTGCAGCATGTAAGACCATAGAGTCAGCATTACAACTCAGCGCTA
GGCTTGAGTCTGCTGAAGTCAACTCTATGCTTACTATTTCTGAAGAGGCTCTACAGTTAGCTACCATCAGTTCGTTTAA
TGGTGATGGATATAATTTTACTAATGTGCTGGGTGTTTCCGTGTATGACCCTGCAAGTGGCAGGGTGGTACAAAAAAGG
TCTTTTATTGAAGACCTGCTTTTTAATAAAGTGGTTACTAATGGCCTTGGTACTGTTGATGAAGACTATAAGCGCTGTT
CTAATGGCCGCTCTGTGGCAGATTTAGTCTGTGCGCAGTATTACTCTGGTGTCATGGTACTACCTGGTGTTGTTGACGC
TGAGAAGCTTCATATGTATAGTGCGTCTCTCATCGGTGGTATGGTGCTAGGAGGTTTTACTGCAGCAGCGGCATTGCCT
TTTAGCTATGCTGTTCAAGCGAGACTGAATTATCTTGCTCTACAGACGGATGTTCTACAGCGGAACCAGCAATTGCTTG
CTGAGTCTTTTAATTCTGCTATTGGTAATATAACTTCAGCCTTTGAGAGTGTTAAAGAGGCTATTAGTCAAACTTCTAA
GGGTTTGAACACTGTGGCTCATGCGCTTACCAAGGTCCAAGAGGTTGTTAATTCGCAGGGTGCAGCTTTGACCCAACTT
ACCGTACAGCTGCAACACAACTTCCAAGCCATTTCTAGTTCTATTGATGACATTTACTCCCGACTTGACATTCTTTCAG
CCGATGTTCAGGTAGACCGTCTCATCACCGGCAGATTATCAGCACTTAATGCTTTTGTTGCTCAAACCCTTACTAAGTA
TACTGAGGTTCAGGCTAGCAGGAAACTAGCACAGCAAAAGGTTAATGAGTGCGTTAAATCGCAATCTCAGCGTTATGGT
TTTTGTGGTGGTGATGGCGAGCACATTTTCTCTCTGGTACAGGCCGCACCTCAAGGCCTGCTGTTTTTACACACAGTAC
TTGTACCGGGTGACTTTGTAAATGTTATTGCCATCGCTGGCTTATGTGTTAACGATGAAATTGCCTTGACTCTACGTGA
GCCTGGTTTAGTCTTGTTTACGCATGAACTTCAAGATACTGCGACGGAATATTTTGTTTCATCGCGACGTATGTATGAA
CCTAGAAAACCTACCGTTGGTGATTTTGTTCAAATTGAGAGTTGTGTGGTCACCTATGTCAATTTGACTAGAGACCAAC
TACCAGAAGTAATCCCAGATTACATCGATGTTAACAAAACACTTGATGAGATTTTAGCCTCTCTGCCCAATAGAACTGG
TCCAAGTCTTTCTTTAGATGTTTTTAATGCCACTTATCTTAATCTCACTGGTGAAATTGCAGATTTAGAGCAGCGTTCA
GAGTCTCTCCGTAATACTACAGAAGAGCTCCAAAGTCTTATATATAATATCAACAACACACTAGTTGACCTTGAGTGGC
TCAACCGAGTTGAGACATATATCAAGTGGCCGTGGTGGGTTTGGTTGATTATTTTTATTGTTCTCATCTTTGTTGTGTC
ATTACTAGTGTTCTGCTGCATTTCCACGGGTTGTTGTGGATGCTGCGGCTGCTGTGGTGCTTGTTTTTCAGGTTGTTGT
AGGGGTCCTAGACTTCAACCTTACGAAGCTTTTGAAAAGGTCCACGTTCAGTGA

>gi|397328495|gb|JX018180.1|_Porcine_epidemic_diarrhea_virus_isolate_CH/YNKM/2012_spike_protein_gene_complete_cds

ATGACGCCTTTAATTAACTTCTGGTTGTTCTTACCAGTACTTCTAACACTTAGCCTACCACAAGATGTCACTAGG
TGCCAGTCTACTATTAACTTTAGGCGGTTCTTTTCAAAATTTAATGTTCAGGCACCTGCCGTCGTTGTTTTGGGTGGTT
ATCTACCTAGTATGAACTCTTCTAGCTGGTACTGTGGTACAGGCATTGAAACTGATAGTGGCGCTCATGGTATTTTTCT
CAGTTACATCGATTCTGGTCAGGGCTTTGAGATTGGCATTTCGCAAGAGCCGTTTGATCCTAGTGGTTACCAGCTTTAT

TTACACAAGGCCACTAATGGTAACACTAGTGCTATTGCACGACTGCGCATTTGCCAGTTTCCAGATAATAAAACATTGG
GCCCTACTGTTAATGATGTTACAACAGGTCGTAACTGCCTATCCAACAAAGCCATTCCAGCTTTACAGGATGGAAAAAA
TATTGTTGTCGGCATAACATGGGATAATGATCGTGTCACTGTTTTTGCTGACAAGATCTATCATTTTTATATTAAAAAT
GATTGGTCCCGTGTTGCGACAAGATGTTACAATAAAAGAAGTTGTGCCATGCAATATGTTTATACACCTACCTACTACA
TGCTTAATGTTACTAGTGCAGGTGAGGATGGCATTTACTATGAACCTTGTACAGCTAATTGCAGTGGTTACGCTGCCAA
TGTATTTGCCACTGATTCCAATGGCCATATACCAGAAGGTTTTAGTTTTAATAATTGGTTTCTTTTGTCCAATGACTCC
ACTTTGTTGCATGGTAAAGTGGTTTCAAACCAACCTTTGTTGGTCAACTGCCTTTGGGCCATTCCTAAGATTTATGGAC
TAGGCCAATTTTTCTCATTCAATCAAACGATGGATGGCGTTTGTAACGGAGCCGCTGCGCAGCGTGCCCCAGAGGCTCT
GAGGTTTAATATTAATGACACTTTTGTCATTCTTGCTGAAGGCTCAATTGTACTTCATACTGCTTTAGGAACAAATCTT
TCTTTTGTTTGCAGTAATTCCTCAGATCCTCACAAAGCCATCTTTACCATACCTTTGGGTGTTACTGAAGTACCCTACT
ATTGCTTTCTTAAAGTGGATACTTACAAATCCACTGTTTATAAATTCTTGGCTGTTTTACCTCCTACTGTCAAGGAAAT
TGTCATCACCAAGTACGGTGATGTTTATGTCAACGGGTTTGGCTATTTGCATCTCGGTTTGTTGGATGCTGTCACAATT
AATTTCACTGGTCATGGCACTGACGATGACGTTTCAGGTTTCTGGACCGTAGCATCGACTAATTTTGTTGATGCACTCA
TCGAGGTTCAAGGAACTGCCATTCAGCGTATTCTTTATTGTGATGACCCTGTTAGCCAACTTAAGTGTTCTCAGGTTTC
TTTTGACCTTGATGATGGTTTTTACCCTATTTCTTCTAGAAACCTTCTGAGTCATGAACAGCCAATTTCTTTTGTTACT
TTGCCATCATTCAATGATCATTCTTTTGTTAATATTACTGTCTCTGCGGCTTTTGGTGGTCATAGTGGTGCCAACCTCA
TTGCATCTGACACTACTATCAATGGGTTTAGTTCTTTCTGTGTTGACACTAGACAATTTACCATTACACTGTTTTATAA
CGTTACAAACAGTTATGGTTATGTGTCTAAGTCACAGGATAGTAATTGCCCTTTCACCTTGCAATCTGTTAATGATTAC
CTGTCTTTTAGCAAATTTTGTGTTTCAACCAGCCTTTTGGCTGGTGCTTGTACCATAGATCTTTTTGGTTACCCTGAGT
TCGGTAGTGGTGTTAAGTTTACGTCCCTTTATTTTCAATTCACAAAGGGTGAGTTGATTACTGGCACGCCTAAACCACT
TCAAGGTGTCACGGACGTTTCTTTTATGACTCTGGATGTGTGTACCAAGTATACTATCTATGGCTTTAAAGGTGAGGGT
ATTATTACCCTTACAAATTCTAGCTTTTTGGCAGGTGTTTATTATACATCTGATTCTGGACAGTTGTTAGCCTTTAAGA
ATGTCACTAGTGGTGCTGTTTATTCTGTTACGCCATGTTCTTTTTCAGAGCAGGCTGCATATGTTGATGATGATATAGT
GGGTGTTATTTCTAGTTTGTCTAACTCCACTTTTAACAATACCAGGGAGTTGCCTGGTTTCTTCTACCATTCTAATGAT
GGCTCCAATTGTACAGAGCCTGTGTTGGTGTATAGTAACATAGGTGTTTGTAAATCTGGCAGTATTGGCTACGTCCCAT
CTCAGTCTGGCCAAGTCAAGATTGCACCCACGGTTACTGGGAATATTAGTATTCCCACCAACTTTAGTATGAGTATTAG
GACAGAATATTTACAGCTTTACAACACGCCTGTTAGTGTTGATTGTGCCACATATGTTTGTAATGGTAACTCTCGTTGT
AAACAATTACTCACCCAGTACACTGCAGCATGTAAGACCATAGAGTCAGCATTACAACTCAGCGCTAGGCTTGAGTCTG
TTGAAGTTAACTCTATGCTTACTATTTCTGAAGAGGCTCTACAGTTAGCTACCATCAGTTCGTTTAATGGTGATGGATA
TAATTTTACTAATGTGCTGGGTGTTTCTGTGTATGATCCTGCAAGTGGCAGGGTGGTACAAAAAAGGTCTTTTATTGAA
GACCTGCTTTTTAATAAAGTGGTTACTAATGGCCTTGGTACTGTTGATGAAGACTATAAGCGCTGTTCTAATGGTCGCT
CTGTGGCAGATCTAGTCTGTGCACAGTATTACTCTGGTGTCATGGTACTACCTGGTGTTGTTGACGCTGAGAAGCTTCA
CATGTATAGTGCGTCTCTCATCGGTGGTATGGTGCTAGGAGGTTTTACTTCTGCAGCGGCATTGCCTTTTAGCTATGCT
GTTCAAGCTAGACTCAATTATCTTGCTCTACAGACGGATGTTCTACAGCGGAACCAGCAATTGCTTGCTGAGTCTTTTA
ACTCTGCTATTGGTAATATAACTTCAGCCTTTGAGAGTGTTAAAGAGGCTATTAGTCAAACTTCCAAGGGTTTGAACAC
TGTGGCTCATGCGCTTACTAAGGTTCAAGAGGTTGTTAACTCGCAGGGTGCAGCTTTGACTCAACTTACCGTACAGCTG
CAACACAACTTCCAAGCCATTTCTAGTTCTATTGATGACATTTACTCTCGACTGGACATTCTTTCAGCCGATGTTCAGG
TTGACCGTCTCATCACCGGCAGATTATCAGCACTTAATGCTTTTGTTGCTCAAACCCTCACTAAGTATACTGAGGTTCA
GGCTAGCAGGAAGCTAGCACAGCAAAAGGTTAATGAGTGCGTTAAATCGCAATCTCAGCGTTATGGTTTTTGTGGTGGT
GATGGCGAGCACATTTTCTCTCTGGTACAGGCAGCACCTCAGGGCCTGCTGTTTTTACATACAGTACTTGTACCGGGTG
ACTTTGTAGATGTTATTGCCATCGCTGGCTTATGCGTTAACGATGAAATTGCCTTGACTCTACGTGAGCCTGGCTTAGT
CTTGTTTACGCATGAACTTCAAAATCATACTGCGACGGAATATTTTGTTTCATCGCGACGTATGTTTGAACCTAGAAAA
CCTACCGTTAGTGATTTTGTTCAAATTGAGAGTTGTGTGGTCACCTATGTCAATTTGACTAGAGACCAACTACCAGATG
TAATCCCAGATTACATCGATGTTAACAAAACACTTGATGAGATTTTAGCTTCTCTGCCCAATAGAACTGGTCCAAGTCT

TCCTTTAGATGTTTTTAATGCCACTTATCTTAATCTCACTGGTGAAATTGCAGATTTAGAGCAGCGTTCAGAGTCTCTC
CGTAATACTACAGAGGAGCTCCAAAGTCTTATATATAATATCAACAACACACTAGTTGACCTTGAGTGGCTCAACCGAG
TTGAGACATATATCAAGTGGCCGTGGTGGGTTTGGTTGATTATTTTCATTGTTCTCATCTTTGTTGTGTCATTACTAGT
GTTCTGCTGCATTTCCACGGGTTGTTGTGGATGCTGCGGCTGCTGCTGTGCTTGTTTTTCAGGTTGTTGTAGGGGTCCT
AGACTTCAACCTTACGAAGTTTTTGAAAAGGTCCACGTGCAGTGA

>gi|397328497|gb|JX018181.1|_Porcine_epidemic_diarrhea_virus_isolate_CH/AHHF/2012_spike_protein_gene_complete_cds

ATGACGCCTTTAATTAACTTCTGGTTGTTCTTACCAGTACTTCTAACACTTAGCCTACCACAAGATGTCACTAGG
TGCCAGTCTACTATTAACTTTAGGCGGTTCTTTTCAAAATTTAATGTTCAGGCACCTGCCGTCGTTGTTTTGGGTGGTT
ATCTACCTAGTATGAACTCTTCTAGCTGGTACTGTGGCACAGGCATTGAAACTGATAGTGGCGTTCATGGTATTTTTCT
CAGTTACATCGATTCTGGTCAGGGCTTTGAGATTGGCATTTCGCAAGAGCCGTTTGATCCTAGTGGTTACCAGCTTTAT
TTACACAAGGCCACTAATGGTAGCACTAGTGCTATTGCACGACTGCGCATTTGCCAGTTTCCAGATAATAAAACATTGG
GCCCTACTGTTAATGATGTTACAACAGGTCGTAACTGCCTATCCAACAAAGCCATTCCAGCTTTGCAGGATGGAAAAAA
TATTGTTGTCGGCATAACATGGGATAATGATCGTGTCACTGTTTTTGCTGACAAGATCTATCATTTTTATATTAAAAAT
GATTGGTCCCGTGTTGCGACAAGATGTTACAACAAAAGAGGTTGTGCCATGCAATATGTTTATACACCTACCTACTACA
TGCTTAATGTTACTAGTGCAGGTGAGGATGGCATTTACTATGAACCTTGTACAGCTAATTGCAGTGGTTACGCTGCCAA
TGTATTTGCCACTGATTCCAATGGCCATATACCAGAAGGTTTTAGTTTTAATAATTGGTTTCTTTTGTCCAATGACTCC
ACTTTGTTGCATGGTAAGGTGGTTTCCAACCAACCATTGTTGGTCAATTGTCTTTTGGCCATGCCTAAGATTTATGGAC
TAGGCCAATTTTTCTCCTTCAATCAAACGATCGATGGTGTTTGTAATGGAGCTGCTGTGCAGCGTGCACCAGAGGCTCT
GAGGTTTAATATTAATGACACCTCTGTCATTCTTGCTGAAGGCTCAATTGTACTTCATACTGCTTTAGGAACAAATCTT
TCTTTTGTTTGCAGTAATTCTTCAGATCCTCATTTAGCTACCTTCGCCATACCTCTGGGTGCTACCCAAGTACCCTATT
ATTGTTTTCTTAAAGTGGATACTTACAACTCCACTGTTTATAAATTTCTGGCTGTTTTACCTCCTACCGTCAGGGAAAT
TGTCATCACCAAGTATGGTGATGTTTATGTTAATGGGTTTGGATACTTGCATCTCGGTTTGTTGGATGCTGTCACAATT
AATTTCACTGGTCATGGCACTGACGATGATGTTTCTGGTTTTTGGACCATAGCATCGACTAATTTTGTTGATGCACTCA
TCGAAGTTCAAGGAACTGCCATTCAGCGTATTCTTTATTGTGATGATCCTGTTAGCCAACTCAAGTGTTCTCAGGTTGC
TTTTGACCTTGACGATGGTTTTTACCCTATCTCTTCTAGGAACCTTCTGAGTCATGAACAGCCAATTTCTTTTGTTACT
CTGCCATCATTTAATGATCATTCTTTTGTTAACATTACTGTCTCTGCGTCCTTTGGTGGTCATAGTGGTGCCAACCTCA
TTGCATCTGACACTACTATCAATGGATTTAGTTCTTTCTGTGTTGACACTAGACAATTTACCATTTCACTGTTTTATAA
CGTTACAAACAGTTATGGTTATGTGTCTAACTCACAGAACACTAATTGCCCTTTCACCTTGCAATCTGTTAATGATTAC
CTGTCTTTTAGCAAATTTTGTGTTTCTACCAGCCTTTTGGCTAGTGCCTGTACCATAGATCTTTTTGGTTACCCTGAGT
TTGGTAGTGGTGTTAAGTTTACGTCCCTTTATTTTCAATTCACAAAGGGTGAGTTGATTACTGGCACGCCTAAACCACT
TGAAGGTGTTACGGACGTTTCTTTTATGACTCTGGATGTGTGTACCAAGTATACTATCTATGGCTTTAAAGGTGAGGGT
ATTATTACCCTTACAAATTCTAGCTTTTTGGCAGGTGTTTATTACACATCTGATTCTGGACAGTTGTTAGCCTTTAAGA
ATGTCACTAGTGGTGCTGTTTATTCTGTTACGCCATGTTCTTTTTCAGAGCAGGCTGCATATGTTGATGATGATATAGT
GGGTGTTATTTCTAGTTTGTCTAACTCCACTTTTAACAGTACTAGGGAGTTGCCTGGTTTCTTCTACCATTCTAATGAT
GGCTCTAATTGTACAGAGCCTGTGTTGGTGTATAGTAACATAGGTGTTTGTAAATCTGGCAGTATTGGCTACGTCCCAT
CTCAGTCTGGCCAAGTCAAGATTGCACCCATGGTTACTGGGAATATTAGTATTCCCACCAACTTTAGTATGAGTATTAG
GACAGAATATTTACAGCTTTACAACACGCCTGTTAGTGTTGATTGTGCCACATATGTTTGTAATGGTAACTCTCGTTGT
AAACAATTACTCACCCAGTACACTGCAGCATGTAAGACCATAGAGTCAGCATTACAACTCAGCGCTAGGCTTGAGTCTG
TTGAAGTTAACTCTATGCTTACTATTTCTGAAGAGGCTCTACAGTTAGCTACCATTAGCTCGTTTAATGGTGATGGATA
TAATTTTACTAATGTGCTGGGTGTTTCTGTGTATGATCCTGCAAGTGGCAGGGTGGTACAAAAAAGGTCTTTTATTGAA

GACCTGCTTTTTAATAAAGTGGTTACTAATGGCCTTGGTACTGTTGATGAAGACTATAAGCGCTGTTCTAATGGTCGCT
CTGTGGCAGATCTAGTCTGTGCACAGTATTACTCTGGTGTCATGGTACTACCTGGTGTTGTTGACGCTGAGAAGCTTCA
CATGTATAGTGCGTCTCTCATCGGTGGTATGGTGCTAGGAGGTTTTACTTCTGCAGCGGCATTGCCTTTTAGCTATGCT
GTTCAAGCTAGACTCAATTATCTTGCTCTACAGACGGATGTTCTACAGCGGAACCAGCAATTGCTTGCTGAGTCTTTTA
ACTCTGCTATTGGTAATATAACTTCAGCCTTTGAGAGTGTTAAAGAGGCTATTAGTCAAACTTCCAAGGGTTTGAACAC
TGTGGCTCATGCGCTTACTAAGGTTCAAGAGGTTGTTAACTCGCAGGGTGCAGCTTTGACTCAACTTACCGTACAGCTG
CAACACAACTTCCAAGCCATTTCTAGTTCTATTGATGACATTTACTCTCGACTGGACATTCTTTTAGCCGATGTTCAGG
TTGACCGTCTCATCACCGGCAGATTATCAGCACTTAATGCTTTTGTTGCTCAAACCCTCACTAAGTACACTGAGGTTCA
GGCTAGCAGGAAGCTAGCACAGCAAAAGGTTAATGAGTGCGTTAAATCGCAATCTCAGCGTTATGGTTTTTGTGGTGGT
GATGGCGAGCACATTTTCTCTCTGGTACAGGCAGCACCTCAGGGCCTGCTGTTTTTACATACAGTACTTGTACCGGGTG
ATTTTGTAGATGTTATTGCCATCGCTGGCTTATGCGTTAACGATGAAATTGCCTTGACTCTACGTGAGCCTGGCTTAGT
CTTGTTTACGCATGAACTTCAAAATCATACTGCGACGGAATATTTTGTTTCATCGCGACGTATGTTTGAACCTAGAAAA
CCTACCGTTAGTGATTTTGTTCAAATTGAGAGTTGTGTGGTCACCTATGTCAATTTGACTAGGGACCAACTACCAGATG
TAATCCCAGATTACATCGATGTTAACAAAACACTTGATGAGATTTTAGCTTCTCTGCCCAATAGAACTGGTCCAAGTCT
TCCTTTAGATGTTTTTAATGCTACTTATCTTAATCTCACTGGTGAAATTGCAGATTTAGAGCAGCGTTCAGAGTCTCTC
CGTAATACTACAGAAGAGCTCCAAAGTCTTATATATAATATCAACAACACACTAGTTGACCTTGAGTGGCTCAACCGAG
TTGAGACATATATCAAGTGGCCGTGGTGGGTTTGGTTGATTATTTTCATTGTTCTCATCTTTGTTGTGTCATTACTAGT
GTTCTGCTGCATTTCCACGGGTTGTTGTGGATGCTGCGGCTGCTGCTGTGCTTGTTTTTCAGGTTGTTGTAGGGGTCCT
AGACTTCAACCTTACGAAGCTTTTGAAAAGGTCCACGTGCAGTGA

>gi|397328499|gb|JX018182.1|_Porcine_epidemic_diarrhea_virus_isolate_CH/AHHF2/2012_spike_protein_gene_complete_cds

ATGAAGTCTTTAACTTACTTCTGGTTGCTCTTACCAGTACTATCAACACTTAGCCTACCACAAGATGTCACCAGG
TGCTCAGCTAACACTAATTTTAGGCGGTTCTTTTCAAAATTTAATGTTCAGGCGCCTGCAGTTGTTGTACTGGGCGGTT
ATCTACCTATTGGTGAAAACCAGGGTGTCAATTCAACTTGGTACTGTGCTGGCCAACATCCAACTGCTAGTGGCGTTCA
TGGTATCTTTCTTAGCCATATTAGAGGTGGTCATGGCTTTGAGATTGGCATTTCGCAAGAGCCTTTTGACCCTAGTGGT
TACCAGCTTTATTTACATAAGGCTACTAATGGTAACACTAATGCTACTGCGCGATTGCGCATTTGCCAGTTCCCCAGCA
TTAAAACATTGGGCCCCACTGCTAATAATGATGTTACAACAGGTCGTAACTGCCTATTTAACAAAGCCATCCCAGCTCA
TATGAGTGAACATAGTGTTGTCGGCATAACATGGGATAATGATCGTGTCACTGTCTTTTCTGACAAGATCTATCATTTT
TATTTTAAAAATGATTGGTCCCGTGTTGCGACAAAGTGTTACAACAGTGGAGGTTGTGCTATGCAATATGTTTACGAAC
CCACTTACTACATGCTTAATGTTACTAGCGCTGGTGAGGATGGTATTTCTTATCAACCCTGTACAGCTAATTGCATTGG
TTATGCTGCCAATGTATTTGCTACTGAGCGCAATGGCCGCATACCAGAAGGTTTTAGTTTTAATAATTGGTTTCTTTTG
TCCAATGATTCCACTTTGGTGCATGGTAAGGTGGTTTCCAACCAACCATTGCTGGTCAATTGTCTTTTGGCCATGCCTA
AGATTTATGGACTAGGCCAATTTTTCTCCTTCAATCAAACGATCGATGGTGTTTGTAATGGAGCTGCTGTGCAGCGTGC
ACCAGAGGCTCTGAGGTTTAATATTAATGACACCTCTGTCATTCTTGCTGAAGGCTCAATTGTACTTCATACTGCTTTA
GGAACAAATCTTTCTTTTGTTTGCAGTAATTCTTCAGATCCTCATTTAGCTACCTTCGCCATACCTCTGGGTGCTACCC
AAGTACCCTATTATTGTTTTCTTAAAGTGGATACTTACAACTCCACTGTTTATAAATTTCTGGCTGTTTTACCTCCTAC
CGTCAGGGAAATTGTCATCACCAAGTATGGTGATGTTTATGTTAATGGGTTTGGATACTTGCATCTCGGTTTGTTGGAT
GCTGTCACAATTAATTTCACTGGTCATGGCACTGACGATGATGTTTCTGGTTTTTGGACCATAGCATCGACTAATTTTG
TTGATGCACTCATCGAAGTTCAAGGAACTGCCATTCAGCGTATTCTTTATTGTGATGATCCTGTTAGCCAACTCAAGTG
TTCTCAGGTTGCTTTTGACCTTGACGATAGTTTTTACCCTATCTCTTCTAGGAACCTTCTGAGTCATGAACAGCCAATT
TCTTTTGTTACTCTGCCATCATTTAATGATCATTCTTTTGTTAACATTACTGTCTCTGCGTCCTTTGGTGGTCATAGTG

GTGCCAACCTCATTGCATCTGACACTACTATCAATGGATTTAGTTCTTTCTGTGTTGACACTAGACAATTTACCATTTC
ACTGTTTTATAACGTTACAAACAGTTATGGTTATGTGTCTAACTCACAGAACACTAATTGCCCTTTCACCTTGCAATCT
GTTAATGATTACCTGTCTTTTAGCAAATTTTGTGTTTCTACCAGCCTTTTGGCTAGTGCCTGTACCATAGATCTTTTTG
GTTACCCTGAGTTTGGTAGTGGTGTTAAGTTTACGTCCCTTTATTTTCAATTCACAAAGGGTGAGTTGATTACTGGCAC
GCCTAAACCACTTGAAGGTGTTACGGACGTTTCTTTTATGACTCTGGATGTGTGTACCAAGTATACTATCTATGGCTTT
AAAGGTGAGGGTATTATTACCCTTACAAATTCTAGCTTTTTGGCAGGTGTTTATTACACATCTGATTCTGGACAGTTGT
TAGCCTTTAAGAATGTCACTAGTGGTGCTGTTTATTCTGTTACGCCATGTTCTTTTTCAGAGAAGGCTGCATATGTTGA
TGATGATATAGTGGGTGTTATTTCTAGTTTGTCTAACTCCACTTTTAACAGTACTAGGGAGTTGCCTGGTTTCTTCTAC
CATTCTAATGATGGCTCTAATTGTACAGAGCCTGTGTTGGTGTATAGTAACATAGGTGTCTGTAAATCTGGCAGTATTG
GCTACGTCCCATCTCAGTCTGGCCAAGTCAAGATTGCACCCATGGTTACTGGGAATATTAGTATTCCCACCAACTTTAG
TATGAGTATTAGGACAGAATATTTACAGCTTTACAACACGCCTGTTAGTGTTGATTGTGCCACATATGTTTGTAATGGT
AACTCTCGTTGTAAACAATTACTCACCCAGTACACTGCAGCATGTAAGACCATAGAGTCAGCATTACAACTCAGCGCTA
GGCTTGAGTCTGTTGAAGTTAACTCTATGCTTACTATTTCTGAAGAGGCTCTACAGTTAGCTACCATTAGCTCGTTTAA
TGGTGATGGATATAATTTTACTAATGTGCTGGGTGTTTCTGTGTATGATCCTGCAAGTGGCAGGGTGGTACAAAAAAGG
TCTTTTATTGAAGACCTGCTTTTTAATAAAGTGGTTACTAATGGCCTTGGTACTGTTGATGAAGACTATAAGCGCTGTT
CTAATGGTCGCTCTGTGGCAGATCTAGTCTGTGCACAGTATTACTCTGGTGTCATGGTACTACCTGGTGTTGTTGACGC
TGAGAAGCTTCACATGTATAGTGCGTCTCTCATCGGTGGTATGGTGCTAGGAGGTTTTACTTCTGCAGCGGCATTGCCT
TTTAGCTATGCTGTTCAAGCTAGACTCAATTATCTTGCTCTACAGACGGATGTTCTACAGCGGAACCAGCAATTGCTTG
CTGAGTCTTTTAACTCTGCTATTGGTAATATAACTTCAGCCTTTGAGAGTGTTAAAGAGGCTATTAGTCAAACTTCCAA
GGGTTTGAACACTGTGGCTCATGCGCTTACTAAGGTTCAAGAGGTTGTTAACTCGCAGGGTGCAGCTTTGACTCAACTT
ACCGTACAGCTGCAACACAACTTCCAAGCCATTTCTAGTTCTATTGATGACATTTACTCTCGACTGGACATTCTTTTAG
CCGATGTTCAGGTTGACCGTCTCATCACCGGCAGATTATCAGCACTTAATGCTTTTGTTGCTCAAACCCTCACTAAGTA
CACTGAGGTTCAGGCTAGCAGGAAGCTAGCACAGCAAAAGGTTAATGAGTGCGTTAAATCGCAATCTCAGCGTTATGGT
TTTTGTGGTGGTGATGGCGAGCACATTTTCTCTCTGGTACAGGCAGCACCTCAGGGCCTGCTGTTTTTACATACAGTAC
TTGTACCGGGTGATTTTGTAGATGTTATTGCCATCGCTGGCTTATGCGTTAACGATGAAATTGCCTTGACTCTACGTGA
GCCTGGCTTAGTCTTGTTTACGCATGAACTTCAAAATCATACTGCGACGGAATATTTTGTTTCATCGCGACGTATGTTT
GAACCTAGAAAACCTACCGTTAGTGATTTTGTTCAAATTGAGAGTTGTGTGGTCACCTATGTCAATTTGACTAGGGACC
AACTACCAGATGTAATCCCAGATTACATCGATGTTAACAAAACACTTGATGAGATTTTAGCTTCTCTGCCCAATAGAAC
TGGTCCAAGTCTTCCTTTAGATGTTTTTAATGCTACTTATCTTAATCTCACTGGTGAAATTGCAGATTTAGAGCAGCGT
TCAGAGTCTCTCCGTAATACTACAGAAGAGCTCCAAAGTCTTATATATAATATCAACAACACACTAGTTGACCTTGAGT
GGCTCAACCGAGTTGAGACATATATCAAGTGGCCGTGGTGGGTTTGGTTGATTATTTTCATTGTTCTCATCTTTGTTGT
GTCATTACTAGTGTTCTGCTGCATTTCCACGGGTTGTTGTGGATGCTGCGGCTGCTGCTGTGCTTGTTTTTCAGGTTGT
TGTAGGGGTCCTAGACTTCAACCTTACGAAGCTTTTGAAAAGGTCCACGTGCAGTGA

>gi|397328501|gb|JX018183.1|_Porcine_epidemic_diarrhea_virus_isolate_CH/HBSN/2012_spike_protein_gene_complete_cds

ATGAAGTCTTTAACCTACTTCTGGTTGTTCTTACCAGTACTTTCAACACTTAGCCTACCACAAGATGTCACCAGG
TGCTCAGCTAACACTAATTTTAGGCGGTTCTTTTCAAAATTTAATGTTCAGGCGCCTGCAGTTGTTGTACTGGGCGGTT
ATCTACCTATTGGTGAAAACCAGGGTGTCAATTCAACTTGGTACTGTGCTGGCCAACATCCAACTGCTAGTGGCGTTCA
TGGTATCTTTCTTAGCCATATTAGAGGTGGTCATGGCTTTGAGATTGGCATTTCGCAAGAGCCTTTTGACCCTAGTGGT
TACCAGCTTTATTTACATAAGGCTACTAATGGTAACACTAATGCTACTGCGCGATTGCGCATTTGCCAGTTTCCCAGCA
TTAAAACATTGGGCCCCACTGCTAATAATGATGTTACAACAGGTCGTAACTGCCTATTTAACAAAGCCATCCCAGCTCA

TATGAGTGAACATAGTGTCGTCGGCATAACATGGGATAATGATCGTGTCACTGTCTTTTCTGACAAGATCTATCATTTT
TATTTTAAAAATGATTGGTCCCGTGTTGCGACAAAGTGTTACAACAGTGGAGGTTGTGCTACGCAATATGTTTACGAAC
CCACTTACTACATGCTTAATGTTACTAGTGCTGGTGAGGATGGTATTTCTTATCAACCCTGTACAGCTAATTGCATTGG
TTATGCTGCCAATGTATTTGCTACTGAGCCCAATGGCCACATACCAGAAGGTTTTAGTTTTAATAATTGGTTTCTTTTG
TCCAATGATTCCACTTTGGTGCATGGTAAGGTGGTTTCCAACCAACCATTGTTGGTTAATTGTCTTTTGGCCATGCCTA
AGATTTATGGACTAGGCCAATTTTTCTCCTTCAATCAAACGATCGATGGTGTTTGTAATGGAGCTGCTGTGCAGCGTGC
ACCAGAGGCTCTGAGGTTTAATATTAATGACACCTCTGTCATTCTTGCTGAAGGCTCTATTGTACTTCATACTGCTTTA
GGAACAAATCTTTCCTTTGTTTGCAGTAATTCCTCAAATCCTCATTTAGCCACCTTCGCCATACCTCTGGGTGCTACCC
AAGTACCCTATTATTGTTTTCTTAAAGTGGATACTTATAATTCCACTGTTTATAAATTCTTGGCTGTTTTACCTCCTAC
CGTCAGGGAAATTGTCATCACCAAGTATGGTGATGTTTATGTCAATGGGTTTGGCTACTTGCATCTCGGTTTATTGGAT
GCTGTCACAATTAACTTCACTGGTCATGGCACTGACGATGACGTTTCTGGTTTTTGGACCATAGCATCGACTAATTTTG
TTGATGCACTCATCGAAGTTCAAGGAACTGCCATTCAGCGTATTCTTTATTGTGATGATCCTGTTAGCCAACTCAAGTG
TTCTCAAGTTGCTTTCGACCTTGACGATGGTTTTTACCCTATTTCTTCTAGAAACCTTCTGAGTCATGAACAGCCAATT
TCTTTTGTTACTTTGCCATCATTTAATGATCATTCTTTTGTTAACATTACTGTCTCTGCGTCCTTTGGTGGTCATAGTG
GTGCCAACCTTATTGCATCTGACACTACTATCAATGGGTTTAGTTCTTTCTGTGTTGACACTAGACAATTCACCATTTC
ACTGTTTTATAATGTTACAAACAGTTATGGTTATGTGTCTAAATCACAGGACAGTAATTGCCCTTTCACCTTGCAATCT
GTTAATGATTACCTGTCTTTTAGCAAATTTTGTGTTTCTACCAGCCTTTTGGCTAGTGCCTGTACTATAGATCTTTTTG
GTTACCCTGAGTTTGGTAGTGGTGTTAAGTTTACGTCCCTTTACTTTCAATTCACAAAGGGTGAGTTGATTACTGGCAC
GCCTAAACCACTTGAAGGTGTCACGGACGTTTCTTTTATGACTCTGGATGTGTGTACCAAGTATACTATCTATGGTTTT
AAAGGTGAGGGTATCATTACCCTTACAAATTCTAGCTTTTTGGCAGGTGTTTATTACACATCTGATTCTGGACAGTTGT
TAGCTTTTAAGAATGTCACTAGTGGTGCTGTTTATTCTGTTACGCCATGTTCTTTTTCAGAGCAGGCTGCATATGTTGA
TGATGATATAGTGGGTGTTATTTCTAGTTTGTCTAACTCCACTTTTAACAGTACTAGGGAGTTGCCTGGTTTCTTCTAC
CATTCTAATGATGGCTCTAATTGTACAGAGCCTGTGTTGGTGTATAGTAACATAGGTGTTTGTAAATCTGGCAGTATTG
GCTACGTCCCATCTCAGTCTGGCCAAGTCAAGATTGCACCCACGGTTACTGGGAATATTAGTATTCCCACCAACTTTAG
TATGAGTATTAGGACAGAATATTTACAGCTTTACAACACGCCTGTTAGTGTTGATTGTGCCACATATGTTTGTAATGGT
AACTCTCGTTGTAAACAATTACTCACCCAGTACACTGCAGCATGTAAGACCATAGAGTCAGCATTACAACTCAGCGCTA
GGCTTGAGTCTGTTGAAGTTAACTCTATGCTTACTATTTCTGAAGAGGCTCTACAGTTAGCTACCATCAGTTCGTTTAA
TGGTGATGGATATAATTTTACTAATGTGCTGGGTGTTTCTGTGTATGATCCTGCAAGTGGCAGGGTGGTACAAAAAAGG
TCTTTTATTGAAGACCTGCTTTTTAATAAAGTGGTTACTAATGGCCTTGGTACTGTTGATGAAGACTATAAGCGCTGTT
CTAATGGTCGCTCTGTGGCAGATCTAGTCTGTGCACAGTATTACTCTGGTGTCATGGTACTACCTGGTGTTGTTGACGC
TGAGAAGCTTCACATGTATAGTGCGTCTCTCATCGGTGGTATGGTGCTAGGAGGTTTTACTTCTGCAGCGGCATTGCCT
TTTAGCTATGCTGTTCAAGCTAGACTCAATTATCTTGCTCTACAGACGGATGTTCTACAGCGGAACCAGCAATTGCTTG
CTGAGTCTTTTAACTCTGCTATTGGTAATATAACTTCAGCCTTTGAGAGTGTTAAAGAGGCTATTAGTCAAACTTCCAA
GGGTTTGAACACTGTGGCTCATGCGCTTACTAAGGTTCAAGAGGTTGTTAACTCGCAGGGTGCAGCTTTGACTCAACTT
ACCGTACAGCTGCAACACAACTTCCAAGCCATTTCTAGTTCTATTGATGACATTTACTCTCGACTGGACATTCTTTCAG
CCGATGTTCAGGTTGACCGTCTCATCACCGGCAGATTATCAGCACTTAATGCTTTTGTTGCTCAAACCCTCACTAAGTA
TACTGAGGTTCAGGCTAGCAGGAAGCTAGCACAGCAAAAGGTTAATGAGTGCGTTAAATCGCAATCTCAGCGTTATGGT
TTTTGTGGTGGTGATGGCGAGCACATTTTCTCTCTGGTACAGGCAGCACCTCAGGGCCTGCTGTTTTTACATACAGTAC
TTGTACCGGGTGACTTTGTAGATGTTATTGCCATCGCTGGCTTATGCGTTAACGATGAAATTGCCTTGACTCTACGTGA
GCCTGGCTTAGTCTTGTTTACGCATGAACTTCAAAATCATACTGCGACGGAATATTTTGTTTCATCGCGACGTATGTTT
GAACCTAGAAAACCTACTGTTAGTGATTTTGTTCAAATTGAGAGTTGTGTGGTCACCTATGTCAATTTGACTAGAGACC
AACTACCAGATGTAATCCCAGATTACATCGATGTTAACAAAACACTTGATGAGATTTTAGCTTCTCTGCCCAATAGAAC
TGGTCCAAGTCTTCCTTTAGATGTTTTTAATGCCACTTATCTTAATCTCACTGGTGAAATTGCAGATTTAGAGCAGCGT
TCAGAGTCTCTCCGTAATACTACAGAGGAGCTCCAAAGTCTTATATATAATATCAACAACACACTAGTTGACCTTGAGT

GGCTCAACCGAGTTGAGACATATATCAAGTGGCCGTGGTGGGTTTGGTTGATTATTTTCATTGTTCTCATCTTTGTTGT
GTCATTACTAGTGTTCTGCTGCATTTCCACGGGTTGTTGTGGATGCTGCGGCTGCTGCTGTGCTTGTTTTTCAGGTTGT
TGTAGGGGTCCTAGACTTCAACCTTACGAAGTTTTTGAAAAGGTCCACGTGCAGTGA

>gi|399146002|gb|JX070671.1|_Porcine_epidemic_diarrhea_virus_isolate_CH/FJXM1/2012_spike_protein_gene_complete_cds

ATGAAGTCTTTAACCTACTTCTGGTTGTTCTTACCAGTACTTTCAACACTTAGCCTACCACAAGATGTCACCAGG
TGCTCAGCTAACACTAATTTTAGGCGGTTCTTTTCAAAATTTAATGTTCAGGCGCCTGCAGTTGTTGTACTGGGCGGTT
ATCTACCTATTGGTGAAAACCAGGGTGTCAATTCAACTTGGTACTGTGCTGGCCAACATCCAACTGCTAGTGGCGTTCA
TGGTATCTTTGTTAGCCATATTAGAGGTGGTCATGGCTTTGAGATTGGCATTTCGCAAGAGCCTTTTGACCCTAGTGGT
TACCAGCTTTATTTACATAAGGCTACTAACGGTAACACTAATGCTACTGCGCGACTGCGCATTTGCCAGTTTCCTAGCA
TTAAAACATTGGGCCCCACTGCTAATAATGATGTTACAACAGGTCGTAATTGCCTATTTAACAAAGCCATCCCAGCTCA
TATGAGTGAACATAGTGTTGTCGGCATAACATGGGATAATGATCGTGTCACTGTCTTTTCTGACAAGATCTATTATTTT
TATTTTAAAAATGATTGGTCCCGTGTTGCGACAAAGTGTTACAACAGTGGAGGTTGTGCTATGCAATATGTTTACGAAC
CCACCTATTACATGCTTAATGTTACTAGTGCTGGTGAGGATGGTATTTCTTATCAACCCTGTACAGCTAATTGCATTGG
TTATGCTGCCAATGTATTTGCTACTGAGCCCAATGGCCACATACCAGAAGGTTTTAGTTTTAATAATTGGTTTCTTTTG
TCCAATGATTCCACTTTGGTGCATGGTAAGGTGGTTTCCAACCAACCATTGTTGGTCAATTGTCTTTTGGCCATTCCTA
AGATTTATGGACTAGGCCAATTTTTCTCCTTTAATCAAACGATCGATGGTGTTTGTAATGGAGCTGCTGTGCAGCGTGC
ACCAGAGGCCCTGAGGTTTAATATTAATGACACCTCTGTCATTCTTGCTGAAGGCTCAATTGTACTTCATACTGCTTTA
GGAACAAATTTTTCTTTTGTTTGCAGTAATTCCTCAGATCCTCACTTAGCCACCTTCGCCATACCTCTGGGTGCTATCC
AAGTACCCTATTATTGTTTTCTTAAAGTGGATACTTACAACTCCACTGTTTATAAATTCCTGGCTGTTTTACCTCCTAC
CGTCAGGGAAATTGTCATCACCAAGTATGGTGATGTTTATGTCAATGGGTTTGGCTACTTGCATCTCGGTTTGTTGGAT
GCTGTCACAATTAATTTCACTGGTCATGTCACTGACGATGACGTTTCTGGTTTTTGGACCATAGCATCGACTAATTTTG
TTGATGCACTTATCGAAGTTCAAGGAACTGCCATTCAGCGTATTCTTTATTGTGATGATCCTGTTAGCCAACTCAAGTG
TTCTCAGGTTTCTTTTGACCTTGACGATGGTTTTTACCCTATTTCCTCTACAAACCTTCTGAGTCATGAACAGCCAACT
TCTTTTGTTACTTTGCCATCATTTAATGATCATTCTTTTGTTAATATTACTGTCTCTGCTGCTTTTGGTGGTCATAGTG
GTGCCAACCTTATTGCATCTGACACTACTATCAATGGGTTTAGTTCTTTCTGTGTTGACACTAGACAATTTACCATTTC
ACTGTTTTATAATGTTACAAACAGTTATGGTTATGTGTCTAAATCACAGGACAGTAATTGCCCTTTCACCTTGCAATCT
GTTAATGATTACCTGTCTTTTAGCAAATTTTGTGTTTCTACCAGCCTTTTGGCTAGTGCCTGTACCATAGATCTTTTTG
GTTACCCTGAGTTTGGTAGTGGTGTTAAGTTCACGTCCCTTTACTTTCAATTCACAAAGGGTGAGTTGATTACTGGCAC
GCCTAAACCACTTGAAGGTGTTACGGACGTTTCTTTTATGACTCTGGATGTGTGTACCGAGTATACTATCTATGGCTTT
AAAGGTGAGGGTATCATTACCCTTACAAATTCTAGCTTTTTGGCAGGTGTTTATTATACATCTGATTCTGGACAGTTGT
TAGCTTTTAAGAATGTCACTAGTGGTGCTGTTTATTCTGTTACGCCATGTTCTTTTTCAGAGCAGGCTGCATATGTTGA
TGATGATATAGTGGGTGTTATTTCTAGTTTGTCTAGCTCCACTTTTAACAGTACTAGGGAGTTGCCTGGTTTCTTCTAC
CATTCTAATGATGGCTCTAATTGTACAGAGCCTGTGTTGGTGTATAGTAACATAGGTGTTTGTAAATCTGGCAGTATTG
GCTACGTCCCATCTCAGTCTGGCCAAGTCAAGATTGCACCCACGGTTACTGGGAATATTAGTATTCCCACCAACTTTAG
TATGAGTATTAGGACAGAATATTTACAGCTTTACAACACGCCTGTTAGTGTTGATTGTGCCACATATGTTTGTAATGGT
AACTCTCGTTGTAAACAATTACTCACCCAGTACACTGCAGCATGTAAGACCATAGAGTCAGCATTACAACTCAGCGCTA
GGCTTGAGTCTGTTGAAGTTAACTCTATGCTTACTATTTCTGAAGAGGCTCTACAGTTAGCTACCATCAGTTCGTTTAA
TGGTGATGGATATAATTTTACTAATGTGCTGGGTGTTTCTGTGTATGATCCTGCAAGTGGCAGGGTGGTACAAAAAAGG
TCTTTTATTGAAGACCTGCTTTTTAATAAAGTGGTTACTAATGGCCTTGGTACTGTTGATGAAGACTATAAGCGCTGTT
CTAATGGTCGCTCTGTGGCAGATCTAGTCTGTGCACAGTATTACTCTGGTGTCATGGTACTACCTGGTGTTGTTGACGC

TGAGAAGCTTCACATGTATAGTGCGTCTCTCATCGGTGGTATGGTGCTAGGAGGTTTTACTTCTGCAGCGGCATTGCCT
TTTAGCTATGCTGTTCAAGCTAGACTCAATTATCTTGCTCTACAGACGGATGTTCTACAGCGGAACCAGCAATTGCTTG
CTGAGTCTTTTAACTCTGCTATTGGTAATATAACTTCAGCCTTTGAGAGTGTTAAAGAGGCTATTAGTCAAACTTCCAA
GGGTTTGAACACTGTGGCTCATGCGCTTACTAAGGTTCAAGAGGTTGTTAACTCGCAGGGTGCAGCTTTGACTCAACTT
ACCGTACAGCTGCAACACAACTTCCAAGCCATTTCTAGTTCTATTGATGACATTTACTCTCGACTGGACATTCTTTCAG
CCGATGTTCAGGTTGACCGTCTCATCACCGGCAGATTATCAGCACTTAATGCTTTTGTTGCTCAAACCCTCACTAAGTA
TACTGAGGTTCAGGCTAGCAGGAAGCTAGCACAGCAAAAGGTTAATGAGTGCGTTAAATCGCAATCTCAGCGTTATGGT
TTTTGTGGTGGTGATGGCGATCACATTTTCTCTCTGGTACAGGCAGCACCTCAGGGCCTGCTGTTTTTACATACAGTAC
TTGTACCGGGTGATTTTGTAGATGTTATTGCCATCGCTGGTTTATGCGTTAACGATGAAATTGCCTTGACTCTACGTGA
GCCTGGCTTAGTCTTGTTTACGCATGAACTTCAAAATCATACTGCGACGGAATATTTTGTTTCATCGCGACGTATGTTT
GAACCTAGAAAACCTACCGTTAGTGATTTTGTTCAAATTGAGAGTTGTGTGGTCACCTATGTCAATTTGACTAGAGACC
AACTACCAGATGTAATCCCAGATTACATCGATGTTAACAAAACACTTGATGAGATTTTAGCTTCTCTGCCCAATAGAAC
TGGTCCAAGTCTTCCTTTAGATGTTTTTAATGCCACTTATCTTAATCTCACTGGTGAAATTGCAGATTTAGAGCAGCGT
TCAGAGTCTCTCCGTAATACTACAGAGGAGCTCCAAAGTCTTATATATAATATCAACAACACACTAGTTGACCTTGAAT
GGCTCAACCGAGTTGAGACATATATCAAGTGGCCGTGGTGGGTTTGGTTGATTATTTTCATTGTTCTCATCTTTGTTGT
GTCATTACTAGTGTTCTGCTGCATTTCCACGGGTTGTTGTGGATGCTGCGGCTGCTGCTGTGCTTGTTTTTCAGGTTGT
TGTAGGGGTCCTAGACTTCAACCTTACGAAGTTTTTGAAAAGGTCCACGTGCAGTGA

>gi|399146004|gb|JX070672.1|_Porcine_epidemic_diarrhea_virus_isolate_CH/FJXM2/2012_spike_protein_gene_complete_cds

ATGAAGTCTTTAACTTACTTCTGGTTGTTATTACCAGTACTTTCAACACTCAGCCTACCACAAGATGTCACTAGG
TGCCAGTCCACTATTAACTTCAGGCGGTTCTTTTCAAAATTTAATGTGCAAGCACCTGCTGTCGTTGTTTTGGGTGGTT
ATCTACCTAGTATGAACTCTTCTAGCTGGTACTGTGGCACAGGCCTTGAAACTGCTAGTGGCGTTCATGGTATTTTCCT
CAGTTACATCGATGCTGGTCAGGGCTTTGAGATTGGCATTTCACAGGAGCCGTTTGATCCTAGTGGTTACCAGCTTTAT
CTACATAAGGCCACTAATGGTAACACTAATGCTATTGCACGACTGCGCATTTGCCAGTTTCCAGATAATAAAACATTGG
GCCCTACTGTTAATGATGTTACAACAGGTCGTAACTGCCTATTCAACAAAGCCATTCCAGCTTATATGCAGGACAGAAA
AAATATCGTTGTCGGCATAACATGGGACAATGATCGTGTCACTGTTTTTGCTGACAAGATCTATCATTTTTATCTTAAA
AATGATTGGTCCCGTGTTGCGACAAGATGTTACAATAAAAGAAGTTGTGCTATGCAATATGTTTATACACCTACCTACT
ACATGCTTAATGTTACTAGTGCAGGTGAGGATGGCATTTATTATGAACCATGTACAGCTAATTGCAGTGGTTACGCTGC
CAATGTGTTTGCCACTGATTCTAATGGCCACATACCAGAAGGTTTTAGTTTTAATAATTGGTTTCTTTTGTCCAATGAT
TCCACTTTGTTGCATGGTAAGGTGGTTTCCAACCAACCTTTGTTGGTCAATTGTCTTTTGGCCATTCCTAAGATTTATG
GACTAGGCCAATTTTTCTCATTCAATCAAACGATGGATGGCGTTTGTAATGGAGCTGCTGCGCAGCGTGCACCAGAGGC
CCTGAGGTTTAATATTAATGACACCTCTGTCATTCTTGCTGAAGGCTCAATTGTACTTCATACTGCTTTAGGAACAAAT
CTTTCTTTTGTTTGCAGTAATTCTTCAAATCCTCATTTAGCTACCTTCGCCATACCTCTGGGTGCTACCCAAGTACCCT
ATTATTGTTTTCTTAAAGTGGATACTTACAATTCCACTGTTTATAAATTTTTGGCTGTTTTACCTCCTACCGTCAAGGA
AATTGTCATCACCAAGTATGGTGATGTTTATGTCAATGGGTTTGGATATTTGCATCTCGGTTTGTTGGATGCTGTCACA
ATTAATTTCACTGGTCATGGCACTGACGATGATGTTTCTGGTTTTTGGACCATAGCATCGACTAATTTTGTTGATGCAC
TCATCGAAGTTCAAGGAACTGCCATTCAGCGTGTTCTTTATTGTGATGATCCTGTTAGCCAACTCAAGTGTTCTCAGGT
TGCTTTTGACCTTGACGATGGTTTTTACCCTATTTCTTCTAGAAACCTTCTGAGTCATGAACAGCCAATTTCTTTTGTT
ACTCTGCCATCATTTAATGATCATTCTTTTGTTAACATTACTGTCTCTGCTTCCTTTGGTGGTCATAGTGGTGCCAACC
TTATTGTATCTGACACTACTATCAATGGGTTTAGTTCTTTCTGTGTTGACACTAGACAATTTACCATTTCACTGTTTTA
TAACGTTACAAACAGTTATGGTTATGTGTCTAAATCACAGGACAGTAATTGCCCTTTCACCTTGCAATCTGTTAATGAT

TACCTGTCTTTTAGCAAATTTTGTGTTTCCACCAGCCTTTTGGCTAGTGCCTGTACCATAGATCTTTTTGGTTACCCTG
AGTTTGGTAGTGGTGTTAAGTTTACGTCCCTTTACTTTCAATTCACAAAGGGTGAGTTGATTACTGGCACGCCTAAACC
ACTTGAAGGTGTCACGGACGTTTCTTTCATGACTCTGGATGTGTGTACCAAGTATACTATCTATGGCTTTAAAGGTGAG
GGTATCATTACCCTTACAAATTCTAGCTTTTTGGCAGGTGTTTATTACACATCTGATTCTGGACAGTTGTTAGCCTTTA
AGAATGTCACTAGTGGTGCTGTTTATTCTGTTACGCCATGTTCTTTTTCAGAGCAGGCTGCATATGTTGATGATGATAT
AGTGGGTGTTATTTCTAGTTTGTCTAGCTCCACTTTTAACAGTACTAGGGAGTTGCCTGGTTTCTTCTACCATTCTAAT
GATGGCTCTAATTGTACAGAGCCTGTGTTGGTGTATAGTAACATAGGTGTTTGTAAATCTGGCAGTATTGGCTACGTCC
CATCTCAGTCTGGCCAAGTCAAGATTGCACCCACGGTTACTGGGAATATTAGTATTCCCACCAACTTTAGTATGAGTAT
TAGGACAGAATATTTACAGCTTTACAACACGCCTGTTAGTGTTGATTGTGCCACATATGTTTGTAATGGTAACTCTCGT
TGTAAACAATTACTCACCCAGTACACTGCAGCATGTAAGACCATAGAGTCAGCATTACAACTCAGCGCTAGGCTTGAGT
CTGTTGAAGTTAACTCTATGCTTACTATTTCTGAAGAGGCTCTACAGTTAGCTACCATCAGTTCGTTTAATGGTGATGG
ATATAATTTTACTAATGTGCTGGGTGTTTCTGTGTATGATCCTGCAAGTGGCAGGGTGGTACAAAAAAGGTCTTTTATT
GAAGACCTGCTTTTTAATAAAGTGGTTACTAATGGCCTTGGTACTGTTGATGAAGACTATAAGCGCTGTTCTAATGGTC
GCTCTGTGGCAGATCTAGTCTGTGCACAGTATTACTCTGGTGTCATGGTACTACCTGGTGTTGTTGACGCTGAGAAGCT
TCACATGTATAGTGCGTCTCTCATCGGTGGTATGGTGCTAGGAGGTTTTACTTCTGCAGCGGCATTGCCTTTTAGCTAT
GCTGTTCAAGCTAGACTCAATTATCTTGCTCTACAGACGGATGTTCTACAGCGGAACCAGCAATTGCTTGCTGAGTCTT
TTAACTCTGCTATTGGTAATATAACTTCAGCCTTTGAGAGTGTTAAAGAGGCTATTAGTCAAACTTCCAAGGGTTTGAA
CACTGTGGCTCATGCGCTTACTAAGGTTCAAGAGGTTGTTAACTCGCAGGGTGCAGCTTTGACTCAACTTACCGTACAG
CTGCAACACAACTTCCAAGCCATTTCTAGTTCTATTGATGACATTTACTCTCGACTGGACATTCTTTCAGCCGATGTTC
AGGTTGACCGTCTCATCACCGGCAGATTATCAGCACTTAATGCTTTTGTTGCTCAAACCCTCACTAAGTATACTGAGGT
TCAGGCTAGCAGGAAGCTAGCACAGCAAAAGGTTAATGAGTGCGTTAAATCGCAATCTCAGCGTTATGGTTTTTGTGGT
GGTGATGGCGATCACATTTTCTCTCTGGTACAGGCAGCACCTCAGGGCCTGCTGTTTTTACATACAGTACTTGTACCGG
GTGATTTTGTAGATGTTATTGCCATCGCTGGTTTATGCGTTAACGATGAAATTGCCTTGACTCTACGTGAGCCTGGCTT
AGTCTTGTTTACGCATGAACTTCAAAATCATACTGCGACGGAATATTTTGTTTCATCGCGACGTATGTTTGAACCTAGA
AAACCTACCGTTAGTGATTTTGTTCAAATTGAGAGTTGTGTGGTCACCTATGTCAATTTGACTAGAGACCAACTACCAG
ATGTAATCCCAGATTACATCGATGTTAACAAAACACTTGATGAGATTTTAGCTTCTCTGCCCAATAGAACTGGTCCAAG
TCTTCCTTTAGATGTTTTTAATGCCACTTATCTTAATCTCACTGGTGAAATTGCAGATTTAGAGCAGCGTTCAGAGTCT
CTCCGTAATACTACAGAGGAGCTCCAAAGTCTTATATATAATATCAACAACACACTAGTTGACCTTGAATGGCTCAACC
GAGTTGAGACATATATCAAGTGGCCGTGGTGGGTTTGGTTGATTATTTCATTGTTCTCATCTTTGTTGTGTCATTACT
AGTGTTCTGCTGCATTTCCACGGGTTGTTGTGGATGCTGCGGCTGCTGCTGTGCTTGTTTTTCAGGTTGTTGTAGGGGT
CCTAGACTTCAACCTTACGAAGTTTTTGAAAAGGTCCACGTGCAGTGA

>gi|400294498|gb|JX112709.1| Porcine epidemic diarrhea virus strain GD-A, complete genome

ATGAAGTCTTTAACCTACTTCTGGTTGTTCTTACCAGTACTTTCAACACTTAGCCTACCACAAGATGTCACCAGG
TGCTCAGCTAACACTAATTTTAGGCGGTTCTTTTCAAAATTTAATGTTCAGGCGCCTGCAGTTGTTGTACTGGGCGGTT
ATCTACCTATTGGTGAAAACCAGGGTGTCAATTCAACTTGGTACTGTGCTGGCCAACATCCAACTGCTAGTGGCGTTCA
TGGTATCTTTCTTAGCCATATTAGAGGTGGTCATGGCTTTGAGATTGGCATTTCGCAAGAGCCTTTTGACCCTAGTGGT
TACCAGCTTTATTTACATAAGGCTACTAACGGTAACACTAATGCTACTGCGCGACTGCGCATTTGCCAGTTTCCCAGCA
TTAAAACATTGGGCCCCACTGCTAATAATGATGTTACAACAGGTCGTAACTGCCTATTTAACAAAGCCATCCCAGCTCA
TATGAGTGAACATAGTGTTGTCGGCATAACATGGGATAATGATCGTGTCACTGTCTTTTCTGACAAGATCTATTATTTT
TATTTTAAAAATGATTGGTCCCGTGTTGCGACAAAGTGTTACAACAGTGGAGGTTGTGCTATGCAATATGTTTACGAAC

CCACTTACTACATTCTTAATGTTACTAGTGCTGGTGAGGATGGTATTTCTTATCAACCCTGTACAGCTAATTGCATTGG
TTATGCTGCCAATGTATTTGCTACTGAGCCCAATGGCCACATACCAGAAGGTTTTAGTTTTAATAATTGGTTTCTTTTG
TCCAATGATTCCACTTTGGTGCATGGTAAGGTGGTTTCCAACCAACCATTGTTGGTCAATTGTCTTTTGGCCATTCCTA
AGATTTATGGACTAGGCCAATTTTTCTCCTTTAATCAAACGATCGATGGTGTTTGTAATGGAGCTGCTGTGCAGCGTGC
ACCAGAGGCTCTGAGGTTTAATATTAATGACACCTCTGTCATTCTTGCTGAAGGCTCAATTGTACTTCATACTGCTTTA
GGAACAAATTTTTCTTTTGTTTGCAGTAATTCCTCAGATCCTCACTTAGCCACCTTTGCCATACCTCTGGGTGCTATCC
AAGTACCCTATTATTGTTTTCTTAAAGTGGATACTTACAACTCCACTGTTTATAAATTCTTGGCTGTTTTACCTCCTAC
CGTCAGGGAAATTGTCATCACCAAGTATGGTGATGTTTATGTCAATGGGTTTGGCTACTTGCATCTCGGTTTGTTGGAT
GCTGTCACAATTAATTTCACTGGTCATGGCACTGACGATGACGTTTCTGGTTTTTGGACCATAGCATCGACTAATTTTG
TTGATGCACTTATCGAAGTTCAAGGAACTGCCATTCAGCGTATTCTTTATTGTGATGATCCTGTTAGCCAACTCAAGTG
TTCTCAGGTTGCTTTTGACCTTGACGATGGTTTTTACCGTATTTCCTCTACAAACCTTCTGAGTCATGAACAGCCAACT
TCTTTTGTTACTTTGCCATCATTTAATGATCATTCTTTTGTTAATATTACTGTCTCTGCTGCTTTTGGTGGTCATAGTG
GTGCCAACCTTATTGCATCTGACACTACTATCAATGGGTTTAGTTCTTTCTGTGTTGACACTAGACAATTTACCATTTC
ACTGTTTTATAACGTTACAAACAGTTATGGTTACGTGTCTAAATCACAGGACAGTAATTGCCCTTTTACCTTGCAATCT
GTTAATGATTACCTGTCTTTTAGCAAATTTTGTGTTTCTACCAGCCTTTTGGCTAGTGCCTGTACCATAGATCTTTTTG
GTTACCCTGAGTTTGGTAGTGGTGTTAAGTTCACGTCCCTTTACTTTCAATTCACAAAGGGTGAGTTGATTACTGGCAC
GCCTAAACCACTTGAAGGTGTTACGGACGTTTCTTTTATGACTCTGGATGTGTGTACCAAGTATACTATCTATGGCTTT
AAAGGTGAGGGTATCATTACCCTTACAAATTCTAGCTTTTTGGCAGGTGTTTATTATACATCTGATTCTGGACAGTTGT
TAGCTTTTAAGAATGTCACTAGTGGTGCTGTTTATTCTGTTACGCCATGTTCTTTTTCAGAGCAGGCTGCATATGTTGA
TGATGATATAGTGGGTGTTATTTCTAGTTTGTCTAGCTCCACTTTTAACAGTACTAGGGAGTTGCCTGGTTTCTTCTAC
CATTCTAATGATGGCTCTAATTGTACAGAGCCTGTGTTGGTGTATAGTAACATAGGTGTTTGTAAATCTGGCAGTATTG
GCTATGTCCGATCTCAGTCTGGCCAAGTCAAGATTGCACCCACGGTTACTGGGAATATTAGTATTCCCACCAACTTTAG
TATGAGTATTAGGACAGAATATTTACAGCTTTACAACACGCCTGTTAGTGTTGATTGTGCTACATATGTTTGTAATGGT
AACTCTCGTTGTAAACAATTACTCACCCAGTACACTGCAGCATGTAAGACCATAGAGTCAGCATTACAACTCAGCGCTA
GGCTTGAGTCTGCTGAAGTCAACTCTATGCTTACTATTTCTGAAGAGGCTCTACAGTTAGCTACCATCAGTTCGTTTAA
TGGTGATGGATATAATTTTACTAATGTGCTGGGTGTTTCCGTGTATGACCCTGCAAGTGGCAGGGTGGTACAAAAAAGG
TCTTTCATTGAAGACCTGCTTTTTAATAAAGTGGTTACTAATGGCCTTGGTACTGTTGATGAAGACTATAAGCGCTGTT
CTAATGGCCGCTCTGTGGCAGATTTAGTCTGTGCGCAGTATTACTCTGGTGTCATGGTACTACCTGGTGTTGTTGACGC
TGAGAAGCTTCATATGTATAGTGCGTCTCTCATCGGTGGTATGGTGCTAGGAGGTTTTACTGCAGCAGCGGCATTGCCT
TTTAGCTATGCTGTTCAAGCGAGACTGAATTATCTTGCTCTACAGACGGATGTTCTACAGCGGAACCAGCAATTGCTTG
CTGAGTCTTTTAATTCTGCTATTGGTAATATAACTCCAGCCTTTGAGAGTGTTAAAGAGGCTATTAGTCAAACTTCTAA
GGGTTTGAACACTGTGGCTCATGCGCTTACCAAGGTCCAAGAGGTTGTTAATTCGCAGGGTGCAGCTTTGACCCAACTT
ACCGTACAGCTGCAACACAACTTCCAAGCCATTTCTAGTTCTATTGATGACATTTACTCCCGACTTGACATTCTTTCAG
CCGATGTTCAGGTAGACCGTCTCATCACCGGCAGATTATCAGCACTTAATGCTTTTGTTGCTCAAACCCTTACTAAGTA
TACTGAGGTTCAGGCTAGCAGGAAACTAGCACAGCAAAAGGTTAATGAGTGCGTTAAATCGCAATCTCAGCGTTATGGT
TTTTGTGGTGGTGATGGCGAGCACATTTTCTCTCTGGTACAGGCCGCACCTCAAGGCCTGCTGTTTTTACACACAGTAC
TTGTACCGGGTGACTTTGTAAATGTTATTGCCATCGCTGGCTTATGTGTTAACGATGAAATTGCCTTGACTCTACGTGA
GCCTGGTTTAGTCTTGTTTACGCATGAACTTCAAGATACTGCGACGGAATATTTTGTTTCATCGCGACGTATGTATGAA
CCTAGAAAACCTACCGTTGGTGATTTTGTTCAAATTGAGAGTTGTGTGGTCACCTATGTCAATTTGACTAGAGACCAAC
TACCAGAAGTAATCCCAGATTACATCGATGTTAACAAAACACTTGATGAGATTTTAGCCTCTCTGCCCAATAGAACTGG
TCCAAGTCTTTCTCTAGATGTTTTTAATGCCACTTATCTTAATCTCACTGGTGAAATTGCAGATTTAGAGCAGCGTTCA
GAGTCTCTCCGTAATACTACAGAAGAGCTCCAAAGTCTTATATATAATATCAACAACACACTAGTTGACCTTGAGTGGC
TCAACCGAGTTGAGACATATATCAAGTGGCCGTGGTGGGTTTGGTTGATTATTTTTATTGTTCTCATCTTTGTTGTGTC
ATTACTAGTGTTCTGCTGCATTTCCACGGGTTGTTGTGGATGCTGCGGCTGCTGTGGTGCTTGTTTTTCAGGTTGTTGT

AGGGGTCCTAGACTTCAACCTTACGAAGCTTTTGAAAAGGTCCACGTGCAGTGA

>gi|402234660|gb|JX163294.1| Porcine epidemic diarrhea virus isolate HBMC2012 S protein (S) gene, complete cds

ATGAAGTCTTTAACCTACTTCTGGTTGTTCTTACCAGTACTTTCAACACTTAGCCTACCACAAGATGTCACCAGG
TGCTCAGCTAACACTAATTTTAGGCGGTTCTTTTCAAAATTTAATGTTCAGGCGCCTGCAGTTGTTGTACTGGGCGGTT
ATCTACCTATTGGTGAAAACCAGGGTGTCAATTCAACTTGGTACTGTGCTGGCCGACATCCAACTGCTAGTGGCGTTCA
TGGTATCTTTGTTAGCCATATTAGAGGTGGTCATGGCTTTGAGATTGGCATTTCGCAAGAGCCTTTTGACCCTAGTGGT
TACCAGCTTTATTTACATAAGGCTACTAACGGTAACACTAATGCTACTGCGCGACTGCGCATTTGCCAGTTTCCTAGCA
TTAAAACATTGGGCCCCACTGCTAATAATGATGTTACAACAGGTCGTAATTGCCTATTTAACAAAGCCATCCCAGCTCA
TATGAGTGAACATAGTGTTGTCGGCATAACATGGGATAATGATCGTGTCACTGTCTTTTCTGACAAGATCTATTATTTT
TATTTTAAAAATGATTGGTCCCGTGTTGCGACAAAGTGTTACAACAGTGGAGGTTGTGCTATGCAATATGTTTACGAAC
CCACCTACTACATGCTTAATGTTACTAGTGCTGGTGAGGATGGTATTTCTTATCAACCCTGTACAGCTAATTGCATTGG
TTATTCTGCCAATGTATTTGCTACTGAGCCCAATGGCCACATACCAGAAGGTTTTAGTTTTAATAATTGGTTTCTTTTG
TCCAATGATTCCACTTTGGTGCATGGTAAGGTGGTTTCCAACCAACCATTGTTGGTCAATTGTCTTTTGGCCATTCCTA
AGATTTATGGACTAGGCCAATTTTTTTCCTTTAATCAAACGATCGATGGTGTTTGTAATGGAGCTGCTGTGCAGCGTGC
ACCAGAGGCTCTGAGGTTTAATATTAATGACACCTCTGTCATTCTTGCTGAAGGCTCAATTGTACTTCATACTGCTTTA
GGAACAAATTTTTCTTTTGTTTGCAGTAATTCCTCAGATCCTCACTTAGCCACCTTCGCCATACCTCTGGGTGCTATCC
AAGTACCCTATTATTGTTTTCTTAAAGTGGATACTTACAACTCCACTGTTTATAAATTCTTGGCTGTTTTACCTCCTAC
CGTCAGGGAAATTGTCATCACCAAGTATGGTGATGTTTATGTCAATGGGTTTGGCTATTTGCATCTCGGTTTGTTGGAT
GCTGTCACAATTAATTTCACTGGTCATGGCACTGACGATGACGTTTCTGGTTTTTGGACCATAGCATCGACTAATTTTG
TTGATGCACTTATCGAAGTTCAAGGAACTGCCATTCATCGTATTCTTTATTGTGATGATCCTGTTAGCCAACTCAAGTG
TTCTCAGGTTGCTTTTGACCTTGACGATGGTTTCTACCCTATTTCTTCTAGAAACCTTCTGAGTCATGAACAGCCAATT
TCTTTTGTTACTTTGCCATCATTTAATGATCATTCTTTTGTTAACATTACTGTCTCTGCGTCCTTTGGTGGTCGTAGTG
GTGCCAACCTTATTGCATCTGACACTACTATCAATGGGTTTAGTTCTTTCTGTGTTGACACTAGACAATTTACCATTTC
ACTGTTTTATAACGTTACAAACAGTTATGGTTATGTGTCTAAATCACAGGACAGTAATTGCCCTTTCACCTTGCAATCT
GTTAATGATTACCTGTCTTTTAGTAAATTTTGTGTTTCCACCAGCCTTTTGGCTAGTGCCTGTACCATAGATCTTTTTG
GTTACCCTGAGTTTGGTAGTGGTGTTAAGTTTACGTCCCTTTACTTTCAATTCACAAAGGGTGAGTTGATTACTGGCAC
GCCTAAACCACTTGAAGGTGTCACGGACGTTTCTTTTATGACTCTGGATGTGTGTACCAAGTATACTATCTATGGCTTT
AGAGGTGAGGGTATCATTACCCTTACAAATTCTAGCTTTTTGGCAGGTGTTTATTACACATCTGATTCTGGACAGTTGT
TAGCCTTTAAGAATGTCACTAGTGGTGCTGTTTATTCTGTTACGCCATGTTCTTTTTCAGAGCAGGCTGCATATGTTGA
TGATGATATAGTGGGTGTTATTTCTAGTTTGTCTAGCTCCACTTTTAACAGTACTAGGGAGTTGCCTGGTTTCTTCTAC
CATTCTAATGATGGCTCTAATTGTACAGAGCCTGTGTTGGTGTATAGTAACATAGGTGTTTGTAAATCTGGCAGTATTG
GCTATGTCCCATCTCAGTCTGGCCAAGTCAAGATTGCACCCACGGTTACTGGGAATATTGGTATTCCCACCAACTTTAG
TATGAGTATTAGGACAGAATATTTACAGCTTTACAACACGCCTGTTAGTGTTGATTGTGCCACATATGTTTGTAATGGT
AACTCTCGTTGTAAACAATTACTCACCCAGTACACTGCAGCATGTAAGACCATAGAGTCAGCATTACAACTCAGCGCTA
GGCTTGAGTCTGTTGAAGTTAACTCTATGCTTACTATTTCTGAGGAGGCTCTACAGTTAGCTACCATCAGTTCGTTTAA
TGGTGATGGATATAATTTTACTAATGTGCTGGGTGTTTCTGTGTATGATCCTGCAAGTGGCAGGGTGGTACAAAAAAGG
TCTTTTATTGAAGACCTGCTTTTTAATAAGGTGGTTACTAATGGCCTTGGTACTGTTGATGAAGACTATAAGCGCTGTT
CTAATGGTCGCTCTGTGGCAGATCTAGTCTGTGCACAGTATTACTCTGGTGTCATGGTACTACCTGGTGTTGTTGACGC
TGAGAAGCTTCACATGTATAGTGCGTCTCTCATCGGTGGTATGGTGCTAGGAGGTTTTACTTCTGCAGCGGCATTGCCT
TTTAGCTATGCTGTTCAAGCTAGACTCAATTATCTTGCTCTACAGACGGATGTTCTACAGCGGAACCAGCAATTGCTTG

CTGAGTCTTTTAACTCTGCTATTGGTAATATAACTTCAGCCTTTGAGAGTGTTAAAGAGGCTATTAGTCAAACTTCCAA
GGGTTTGAACACTGTGGCTCATGCGCTTACTAAGGTTCAAGAGGTTGTTAACTCGCAGGGTGCAGCTTTGACTCAACTT
ACCGTACAGCTGCAACACAACTTCCAAGCCATTTCTAGTTCTATTGATGACATTTACTCTCGACTGGACATTCTTTCAG
CCGATGTTCAGGTTGACCGTCTCATCACCGGCAGATTATCAGCACTTAATGCTTTTGTTGCTCAAACCCTCACTAAGTA
TACTGAGGTTCAGGCTAGCAGGAAGCTAGCACAGCAAAAGGTTAATGAGTGCGTTAAATCGCAATCTCAGCGTTATGGT
TTTTGTGGTGGTGATGGCGAGCACATTTTCTCTCTGGTACAGGCAGCACCTCAGGGCCTGCTGTTTTTACATACAGTAC
TTGTACCGGGTGATTTTGTAGATGTTATTGCCATCGCTGGTTTATGCGTTAACGATGAAATTGCCTTGACTCTACGTGA
GCCTGGCTTAGTCTTGTTTACGCATGAACTTCAAAATCATACTTCGACGGAATATTTTGTTTCATCGCGACGTATGTTT
GAACCTAGAAAACCTACCGTTAGTGATTTTGTTCAAATTGAGAGTTGTGTGGTCACCTATGTCAATTTGACTAGAGACC
AACTACCAGATGTAATCCCAGATTACATCGATGTTAACAAAACACTTGATGAGATTTTAGCTTCTCTGCCCAATAGAAC
TGGTCCAAGTCTTCCTTTAGATGTTTTTAATGCCACTTATCTTAATCTCACTGGTGAAATTGCAGATTTAGAGCAGCGT
TCAGAGTCTCTCCGTAATACTACAGAGGAGCTCCAAAGTCTTATATATAATATCAACAACACACTAGTTGACCTTGAAT
GGCTCAACCGAGTTGAGACATATATCAAGTGGCCGTGGTGGGTTTGGTTGATTATTTTCATTGTTCTCATCTTTGTTGT
GTCATTATTAGTGTTCTGCTGCATTTCCACGGGTTGTTGTGGATGCTGCGGCTGCTGCTGTGCTTGTTTTTCAGGTTGT
TGTAGGGGTCCTAGACTTCAACCTTACGAAGTTTTTGAAAAGGTCCACGTGCAGTGA

>gi|402235146|gb|JX188454.1| Porcine epidemic diarrhea virus strain AJ1102, complete genome

ATGAAGTCTTTAACCTACTTCTGGTTGTTCTTACCAGTACTTTCAACACTTAGCCTACCACAAGATGTCACCAGG
TGCTCAGCTAACACTAATTTTAGGCGGTTCTTTTCAAAATTTAATGTTCAGGCGCCTGCAGTTGTTGTACTGGGCGGTT
ATCTACCTATTGGTGAAAACCAGGGTGTCAATTCAACTTGGTACTGTGCTGGCCAACATCCAACTGCTAGTGGCGTTCA
TGGTATCTTTCTTAGCCATATTAGAGGTGGTCATGGCTTTGAGATTGGCATTTCGCAAGAGCCTTTTGACCCTAGTGGT
TACCAGCTTTATTTACATAAGGCTACTAACGGTAACACTAATGCTACTGCGCGACTGCGCATTTGCCAGTTTCCTAGCA
TTAAAACATTGGGCCCCACTGCTAATAATGATGTTACAACAGGTCGTAACTGCCTATTTAACAAAGCCATCCCAGCTCA
TATGAGTGAACATAGTGTTGTCGGCATAACATGGGATAATGATCGTGTCACTGTCTTTTCTGACAAGATCTATTATTTT
TATTTTAAAAATGATTGGTCCCGTGTTGCGACAAAGTGTTACAACAGTGGAGGTTGTGCTATGCAATATGTTTACGAAC
CCACCTATTACATGCTTAATGTTACTAGTGCTGGTGAGGATGGTATTTCTTATCAACCCTGTACAGCTAATTGCATTGG
TTATGCTGCCAATGTATTTGCTACTGAGCCCAATGGCCACATACCAGAAGGTTTTAGTTTTAATAATTGGTTTCTTTTG
TCCAATGATTCCACTTTGGTGCATGGTAAGGTGGTTTCCAACCAACCATTGTTGGTCAATTGTCTTTTGGCCATTCCTA
AGATTTATGGACTAGGCCAATTTTTCTCCTTTAATCAAACGATCGATGGTGTTTGTAATGGAGCTGCTGTGCAGCGTGC
ACCAGAGGCTCTGAGGTTTAATATTAATGACACCTCTGTCATTCTTGCTGAAGGCTCAATTGTACTTCATACTGCTTTA
GGAACAAATTTTTCTTTTGTTTGCAGTAATTCCTCAGATCCTCACTTAGCCACCTTCGCCATACCTCTGGGTGCTATCC
AAGTACCCTATTATTGTTTTCTTAAAGTGGATACTTACAACTCCACTGTTTATAAATTCTTGGCTGTTTTACCTCCTAC
CGTCAGGGAAATTGTCATCACCAAGTATGGTGATGTTTATGTCAATGGGTTTGGCTACTTGCATCTTGGTTTGTTGGAT
GCTGTCACAGTTAATTTCACTGGTCATGGCACTGACGATGACGTTTCTGGTTTTTGGACCATAGCATCGACTAATTTTG
TTGATGCACTTATCGAAGTTCAAGGAACTGCCATTCAGCGTATTCTTTATTGTGATGATCCTGTTAGCCAACTCAAGTG
TTCTCAGGTTTCATTTGACCTTGACGATGGTTTTTACCCCATTTCCTCTACAAACCTTCTGAGTCATGAACAGCCAACT
TCTTTTGTTACTTTGCCATCATTTAATGATCATTCTTTTGTTAATATTACTGTCTCTGCTGCTTTTGGTGGTCATAGTG
GTGCCAACCTTATTGCATCTGACACTACTATCAATGGGTTTAGTTCTTTCTGTGTTGACACTAGACAATTTACCATTTC
ACTGTTTTATAATGTTACAAACAGTTATGGTTATGTGTCTAAATCACAGGACAGTAATTGCCCTTTCACCTTGCAATCT
GTTAATGATTACCTGTCTTTTAGCAAATTTTGTGTTTCTACCAGCCTTTTGGCTAGTGCCTGTACCATAGATCTCTTTG
GTTACCCTGAGTTTGGTAGTGGTGTTAAGTTCACGTCCCTTTACTTTCAATTCACAAAGGGTGAGTTGATTACTGGCAC

GCCTAAACCACTTGAAGGTGTTACGGACGTTTCTTTTATGACTCTGGATGTGTGTACCAAGTATACTATCTATGGCTTT
AAAGGTGAGGGTATCATTACCCTTACAAATTCTAGCTTTTTGGCAGGTTTTTATTATACATCTGATTCTGGACAGTTGT
TAGCTTTTAAGAATGTCACTAGTGGTGCTGTTTATTCTGTTACGCCATGTTCTTTTTCAGAGCAGGCTGCATATGTTGA
TGATGATATAGTGGGTGTTATTTCTAGTTTGTCTAGCTCCACTTTTAACAGTACTAGGGAGTTGCCTGGTTTCTTCTAC
CATTCTAATGATGGCTCTAATTGTACAGAGCCTGTGTTGGTGTATAGTAACATAGGTGTTTGTAAATCTGGCAGTATTG
GCTACGTCCCATCTCAGTCTGGCCAAGTCAAGATTGCACCCACGGTTACTGGGAATATTAGTATTCCCACCAACTTTAG
TATGAGTATTAGGACAGAATATTTACAGCTTTACAACACGCCTGTTAGTGTTGATTGTGCTACATATGTTTGTAATGGT
AACTCTCGTTGTAAACAATTACTCACCCAGTACACTGCAGCATGTAAGACCATAGAGTCAGCATTACAACTCAGCGCTA
GGCTTGAGTCTGCTGAAGTCAACTCTATGCTTACTATTTCTGAAGAGGCTCTACAGTTAGCTACCATCAGTTCGTTTAA
TGGTGATGGATATAATTTTACTAATGTGCTGGGTGTTTCCGTGTATGACCCTGCAAGTGGCAGGGTGGTACAAAAAAGG
TCTTTCATTGAAGACCTGCTTTTTAATAAAGTGGTTACTAATGGCCTTGGTACTGTTGATGAAGACTATAAGCGCTGTT
CTAATGGCCGCTCTGTGGCAGATTTAGTCTGTGCGCAGTATTACTCTGGTGTCATGGTACTACCTGGTGTTGTTGACGC
TGAGAAGCTTCATATGTATAGTGCGTCTCTCATCGGTGGTATGGTGCTAGGAGGTTTTACTGCAGCAGCGGCATTGCCT
TTTAGCTATGCTGTTCAAGCGAGACTCAATTATCTTGCTTTACAGACGGATGTTCTACAGCGGAACCAGCAATTGCTTG
CTGAGTCTTTTAATTCTGCTATTGGTAATATAACTTCAGCCTTTGAGAGTGTTAAAGAGGCTATTAGTCAAACTTCTAA
GGGTTTGAACACTGTGGCTCATGCGCTTACTAAGGTCCAAGAGGTTGTTAATTCGCAGGGTGCAGCTTTGACCCAACTT
ACCGTACAGCTGCAACACAACTTCCAAGCCATTTCTAGTTCTATTGATGACATTTACTCCCGACTTGACATTCTTTCAG
CCGATGTTCAGGTAGACCGTCTCATCACCGGCAGATTATCAGCACTTAATGCTTTTGTTGCTCAAACCCTTACTAAGTA
TACTGAGGTTCAGGCTAGCAGGAAACTAGCACAGCAAAAGGTTAATGAGTGCGTTAAATCGCAATCTCAGCGTTACGGT
TTTTGTGGTGGTGATGGCGAGCACATTTTCTCTCTGGTACAGGCCGCACCTCAAGGCCTGCTGTTCTTACACACAGTAC
TTGTACCGGGTGATTTTGTAAATGTTATTGCCATCGCTGGCTTATGTGTTAACGATGAAATTGCCCTGACTCTACGTGA
GCCTGGCTTAGTCTTGTTTACGCATGAACTTCAAAATACTGCGACGGAATATTTTGTTTCATCGCGACGTATGTATGAA
CCTAGAAAACCTACCGTTGGTGATTTTGTTCAAATTGAGAGTTGTGTGGTCACCTATGTCAATTTGACTAGAGACCAAC
TACCAGATGTAATCCCAGATTACATCGATGTTAACAAAACACTTGATGAGATTTTAGCTTCTCTGCCCAATAGAACTGG
TCCAAGTCTTTCTCTAGATGTTTTTAATGCCACTTATCTTAATCTTACTGGTGAAATTGCAGATTTAGAGCAGCGTTCA
GAGTCTCTCCGTAATACTACAGAAGAGCTTCAAAGTCTTATATATAATATCAACAACACACTAGTTGACCTTGAGTGGC
TCAACCGAGTTGAGACATATATCAAGTGGCCGTGGTGGGTTTGGTTGATTATTTTTATTGTTCTCATCTTTGTTGTGTC
ATTACTAGTGTTCTGCTGCATTTCCACGGGTTGTTGTGGATGCTGCGGCTGCTGTGGTGCTTGTTTTTCAGGTTGTTGT
AGGGGTCCTAGACTTCAACCTTACGAAGCTTTTGAAAAGGTCCACGTGCAGTGA

>gi|402914093|gb|JX242462.1|_Porcine_epidemic_diarrhea_virus_isolate_CHHKC082011_spike_protein_gene_complete_cds

ATGAAGTCTTTAACTTACTTCTGGTTGTTATTACCAGTACTTTCAACACTCAGCCTACCACAAGATGTCACTAGG
TGCCAGTCCACTATTAACTTCAGGCGGTTCTTTTCAAAATTTAATGTGCAAGCACCTGCTGTCGTTGTTTTGGGTGGTT
ATCTACCTAGTATGAACTCTTCTAGCTGGTACTGTGGCACAGGCCTTGAAACTGCTAGTGGCGTTCATGGTATTTTCCT
CAGTTACATCGATTCTGGTCAGGGCTTTGAGATTGGCATTTCACAGGAGCCGTTTGATCCTAGTGGTTACCAGCTTTAT
CTACATAAGGCCACTAATGGTAACACTAATGCTATTGCACGACTGCGCATTTGCCAGTTTCCAGATAATAAAACATTGG
GCCCTACTGTTAATGATGTTACAACAGGTCGTAACTGCCTATTCAACAAAGCCATTCCAGCTTATATGCAGGACGGAAA
AAATATCGTTGTCGGCATAACATGGGACAATGATCGTGTCACTGTTTTTGCTGACAAGATCTATCATTTTTATCTTAAA
AATGATTGGTCCCGTGTTGCGACAAGATGTTACAATAAAAGAAGTTGTGCTATGCAATATGTTTATACACCTACCTACT
ACATGCTTAATGTTACTAGTGCAGGTGAGGATGGCATTTATTATGAACCATGTACAGCTAATTGCAGTGGTTACGCTGC
CAATGTATTTGCCACTGATTCTAATGGCCATATACCAGAAGGTTTTAGTTTTAATAATTGGTTTCTTTTGTCCAATGAT

TCCACTTTGTTGCATGGTAAGGTGGTTTCCAATCAACCTTTGTTGGTCAATTGTCTTTTGGCCATTCCTAAGATCTATG
GACTAGGCCAATTTTTCTCATTCAATCAAACGATGGATGGCGTTTGTAATGGAGCTGCTGCGCAGCGTGCACCAGAGGC
TCTGAGGTTTAATATTAATGACACCTCTGTCATTCTTGCTGAAGGCTCAATTGTACTTCATACTGCTTTAGGAACAAAT
CTTTCTTTTGTTTGCAGTAATTCCACAGATCCTCATTTAGCCACCTTCGTCATACCTCTGGGTGCTACCCAAGTACCCT
ATTATTGTTTTCTTAAAGTGGATACTTACAACTCCACTGTTTATAAATTCTTGGCTGTTTTACCTCCAACCGTCAGGGA
AATTGTCATCACCAAGTATGGTGATGTTTATGTCAATGGGTTTGGCTACTTGCATCTCGGTTTGTTGGATGCTGTCACA
ATTAATTTCACTGGTCATGGCACTGACGATGACGTTTCTGGTTTTTGGACCATAGCATCGACTAATTTTGTTGATGCAC
TTATCGAAGTTCAAGGAACTGCCATTCAGCGTATTCTTTATTGTGATGATCCTGTTAGCCAACTCAAGTGTTCTCAGGT
TGCTTTTGACCTTGACGATGGTTTCTACCCTATTTCTTCTAGAAACCTTCTGAGTCATGAACAGCCAATTTCTTTTGTT
ACTTTGCCATCATTTAATGATCATTCTTTTGTTAACATTACTGTCTCTGCGTCCTTTGGTGGTCATAGTGGTGCCAACC
TTATTGCATCTGACACTACTATCAATGGGTTTAGTTCTTTCTGTGTTGACACTAGACAATTTACCATTTCACTGTTTTA
TAACGTTACAAACAGTTATGGTTATGTGTCTAACTCACAGGACAGTAATTGCCCCTTTCACCTTGCAATCTGTTAATGAT
TACCTGTCTTTTAGCAAATTTTGTGTTTCCACCAGCCTTTTGGCTAGTGCCTGTACCATAGATATTTTTGGTTACCCTG
AGTTTGGTAGTGGTGTTAAGTTTACGTCCCTTTACTTTCAATTCACAAAGGGTGAGTTGATTACTGGCACGCCTAAACC
ATTTGAAGGTGTCACGGACGTTTCTTTTATGACTCTGGATGTGTGTACCAAGTATACTATCTATGGCTTTAAAGGTGAG
GGTATCATTACCCTTACAAATTCTAGCTTTTTGGCAGGTGTTTATTATACATCTGATTCTGGACAGTTGTTAGCCTTTA
AGAATGTCACTAGTGGTGCTGTTTATTCTGTTACGCCATGTTCTTTTTCAGAGCAGGCTGCATATGTTGATGATGATAT
AGTGGGTGTTATTTCTAGTTTGTCTAGCTCCACTTTTAACAGTACTAGGGAGTTGCCTGGTTTCTTCTACCATTCCAAT
GATGGCTCTAATTGTACAGAGCCTGTGTTGGTGTATAGTAACATAGGTGTTTGTAAATCTGGCAGTATTGGCTACGTCT
CATCTCAGTCTGGCCAAGTCAAGATTGCACCCACGGTTACTGGGAATATTAGTATTCCCACCAACTTTAGTATGAGTAT
TAGGACAGAATATTTACAGCTTTACAACACGCCTGTTAGTGTTGATTGTGCCACATATGTTTGTAATGGTAACTCTCGT
TGTAAACAATTACTCACCCAGTACACTGCAGCATGTAGGACCATAGAGTCAGCATTACAACTCAGCGCTAGGCTTGAGT
CTGTTGAAGTTAACTCTATGCTTACTATTTCTGAAGAGGCTCTACAGTTAGCTACCATCAGTTCGTTTAATGGTGATGG
ATATAATTTTACTAATGTGCTGGGTGTTTCTGTGTATGATCCTGCAAGTGGCAGGGTGGTACAAAAAAGGTCTTTTATT
GAAGACCTGCTTTTTAATAAAGTGGTTACTAATGGCCTTGGTACTGTTGATGAAGACTATAAGCGCTGTTCTAATGGTC
GCTCTGTGGCAGATCTAGTCTGTGCACAGTATTACTCTGGTGTCATGGTACTACCTGGTGTTGTTGACGCTGAGAAGCT
TCACATGTATAGTGCGTCTCTCATCGGTGGTATGGTGCTAGGAGGTTTTACTTCTGCAGCGGCATTGCCTTTTAGCTAT
GCTGTTCAAGCTAGACTCAATTATCTTGCTCTACAGACGGATGTTCTACAGCGGAACCAGCAATTGCTTGCTGAGTCTT
TTAACTCTGCTATTGGTAATATAACTTCAGCCTTTGAGAGTGTTAAAGATGCTATTAGTCAAACTTCCAAGGGTTTGAA
CACTGTGGCTCATGCGCTTACTAAGGTTCAAGAGGTTGTTAACTCGCAGGGTGCAGCTTTGACTCAACTTACCGTACAG
CTGCAACACAACTTCCAAGCCATTTCTAGTTCTATTGATGACATTTACTCTCGACTGGACATTCTTTCAGCCGATGTTC
AGGTTGACCGTCTCATCACCGGCAGATTATCAGCACTTAATGCTTTTGTTGCTCAAACCCTCACTAAGTATACTGAGGT
TCAGGCTAGCAGGAAGCTAGCACAGCAAAAGGTTAATGAGTGCGTTAAATCGCAATCTCAGCGTTATGGTTTTTGTGGT
GGTGATGGCGAGCACATTTTCTCCCTGGTACAGGCAGCACCTCAGGGCCTGCTGTTTTTACATACAGTACTTGTACCGG
GTGATTTTGTAGATGTTATTGCCATCGCTGGCTTATGCGTTAACGATGAAATTGCCTTGACTCTACGTGAGCCTGGCTT
AGTCTTGTTTACGCATGAACTTCAAAATCATACTGCGACGGAATATTTTGTTTCATCGCGACGTATGTTTGAACCTAGA
AAACCTACCGTTAGTGATTTTGTTCAAATTGAGAGTTGTGTGGTCACCTATGTCAATTTGACTAGAGACCAACTACCAG
ATGTAATCCCAGATTACATCGATGTTAACAAAACACTTGATGAGATTTTAGCTTCTCTGCCCAATAGAACTGGTCCAAG
TCTTCCTTTAGATGTTTTTAATGCCACTTATCTTAATCTCACTGGTGAAATTGCAGATTTAGAGCAGCGTTCAGAGTCT
CTCCGTAATACTACAGAGGAGCTCCAAAGTCTTATATATAATATCAACAACACACTAGTTGACCTTGAGTGGCTCAACC
GAGTTGAGACATATATCAAGTGGCCGTGGTGGGTTTGGTTGATTATTTTCATTGTTCTCATCTTTGTTGTGTCATTACT
AGTGTTCTGCTGCATTTCCACGGGTTGTTGTGGATGCTGCGGCTGCTGCTGTGCTTGTTTTTCAGGTTGTTGTAGGGGT
CCTAGACTTCAACCTTACGAAGTTTTTGAAAAGGTCCACGTGCAGTGA

>gi|402914097|gb|JX242464.1|_Porcine_epidemic_diarrhea_virus_isolate_CHSHT122011_spike_protein_gene_complete_cds

ATGAAGTCTTTAACTTACTTCTGGTTGTTATTACCAGTACTTTCAACACTCAGCCTACCACAAGATGTCACTAGG
TGCCAGTCCACTATTAACTTCAGGCGGTTCTTTTCGAAATTTAATGTGCAAGCACCTGCTGTCGTTGTTTTGGGTGGTT
ATCTACCTAGTATGAACTCTTCTAGCTGGTACTGTGGCACAGGCCTTGAAACTGCTAGTGGCGTTCATGGTATTTTCCT
CAGTTACATCGATTCTGGTCAGGGCTTTGAGATTGGCATTTCACAGGAGCCGTTTGATCCTAGTGGTTACCAGCTTTAT
CTACATAAGAACACTAATGGTAACACTAATGCTATTGCACGACTGCGCATTTGCCAGTTTCCAGATAATAAAACATTGG
GCCCTACTGTTAATGATGTTACAACAGGTCGTAACTGCCTATTCAACAGAGCCATTCCAGCTTATATGCAGGATAGTAA
AAATACTGTTGTCGGCATAACATGGGATAATGATCGCGTCACTGTTTTCGCTGACAAGATCTATCATTTTTATCTTAAA
AATGATTGGTCCCGCGTTGCGACAAGATGTTATAATAAAAGGAGTTGTGCTATGCAATATGTTTATACACCTACCTACT
ACATGCTTAATGTTACTAGTGCAGGTGAGGATGGCATTTATTATGAACCATGTGCAGCTAATTGCAGTGGTTACGCTGC
CAATGTATTTGCCACTGATTCTAATGGCCATATACCAGAGGGTTTTAGTTTTAATAATTGGTTTCTTTTGTCCAATGAT
TCCACTTTGTTGCATGGTAAGGTGGTTTCCAACCAACCTTTGTTGGTCAATTGTCTTTTGGCCATTCCTAAGATTTATG
GACTAGGCCAATTTTTCTCATTCAATCAAACGATGGATGGCGTTTGTAATGGAGCTGCTGCGCAGCGTGCACCAGAGGC
TCTGAGGTTTAATATTAATGACACCTCTGTCATTCTTGCTGAAGGCTCAATTGTACTTCATACTGCTTTAGGAACAAAT
CTTTCTTTTGTTTGCAGTAATTCCTCAGATCCTCATTTAGCCACCTTCGCCATACCTCTGGGTGCTACCCAAGTACCCT
ATTATTGTTTTCTTAAAGTGGATACTTACAACTCCACTGTTTATAAATTCTTGGCTGTTTTACCTCCTACCGTCAGGGA
AATTGTCATCACCAAGTATGGTGATGTTTATGTCAATGGGTTTGGCTACTTGCATCTCGGTCTGCTGGATGCTGTCACA
ATTAATTTCACTGGTCATGGCACTGACGATGACGTTTCTGGTTTTTGGACCATAGCATCGACTAATTTTGTTGATGCAC
TCATCGAAATTCAAGGAACTGCCATTCAGCGTATTCTTTATTGTGATGATCCTGTTAGCCAACTCAAGTGTTCTCAGGT
TGCTTTTGACCTTGACGATGGTTTTTACCCTATTTCTTCTAGAAACCTTCTTAGTCATGAACAGCCAATTTCTTTTGTT
ACTCTGCCATCATTTAATGATCATTCTTTTGTTAACATTACTGTCTCTGCGTCTTTTGGTGGTCATAGTGGTGCCAACC
TCATTGCATCTGACACTACTATCAATGGGTTTAGTTCTTTCTGTGTTGATACTAGACAATTTACCATTTCACTGTTTTA
TAACGTTACAAACAGTTATGGTTATGTGTCTAAATCACAGGACACTAATTGCCCTTTCACCTTGCAATCTGTTAATGAT
TACCTGTCTTTTAGCAAATTTTGTGTTTCTACCAGCCTTTTGGCTAGTGCCTGTACCATAGATCTTTTTGGTTACCCTG
AGTTTGGTAGTGGTGTTAAGTTCACGTCCCTTTACTTTCAATTCACAAAGGGTGAGTTGATTACTGGCACGCCTAAACC
ACTTGAAGGTGTTACGGACGTTTCTTTTATGACTCTGGATGTGTGTACCGAGTATACTATCTATGGCTTTAAAGGTGAG
GGTGTCATTACCCTTACAAATTCTAGCTTTTTGGCAGGTGTTTATTATACATCTGATTCTGGACAGTTGTTAGCCTTTA
AGAATGTCACTAGTGGTGCTGTTTATTCTGTTACGCCATGTTCTTTTTCAGAGCAGGCTGCATATGTTGATGATGATAT
AGTGGGTGTTATTTCTAGTTTGTCTAGCTCCACTTTTAACAGTACTAGGGAGTTGCCTGGTTTTTTTTACCATTCTAAT
GATGGCTCTAATTGTACAGAGCCTGTGTTGGTGTATAGTAACATAGGTGTTTGTAAATCTGGCAGTATTGGCTATGTCC
CATCTCAGTCTGGCCAAGTCAAGATTGCACCCACGGTTACTGGGAATATTAGTATTCCCACCAACTTTAGTATGAGTAT
TAGGACAGAATATTTACAGCTTTACAACACGCCTGTTAGTGTTGATTGTGCCACATATGTTTGTAATGGTAACTCTCGT
TGTAAACAATTACTCACCCAGTACACTGCAGCATGTAAGACCATAGAGTCAGCATTACAACTCAGCGCTAGGCTTGAGT
CTGTTGAAGTTAACTCTATGCTTACTATTTCTGAAGAGGCTCTACAGTTAGCTACCATCAGTTCGTTTAATGGTGATGG
ATATAATTTTACTAATGTGCTGGGTGTTTCTGTGTATGATCCTGCAAGTGGCAGGGTGGTACAAAAAAGGTCTTTTATT
GAAGACCTGCTTTTTAATAAAGTGGTTACTAATGGCCTTGGTACTGTTGATGAAGACTATAAGCGCTGTTCTAATGGTC
GCTCTGTGGCAGATCTAGTCTGTGCACAGTATTACTCTGGTGTCATGGTACTACCTGGTGTTGTTGACGCTGAGAAGCT
TCACATGTATAGTGCGTCTCTCATCGGTGGTATGGTGCTAGGAGGTTTTACTTCTGCAGCGGCATTGCCTTTTAGCTAT
GCTGTTCAAGCCAGACTCAATTATCTTGCTCTACAGACGGATGTTCTACAGCGGAACCAGCAATTGCTTGCTGAGTCTT
TTAACTCTGCTATTGGTAATATAACTTCAGCCTTTGAGAGTGTTAAAGAGGCTATTAGTCAAACTTCCAAGGGTTTGAA
TACTGTGGCTCATGCGCTTACTAAGGTTCAAGAGGTTGTTAACTCGCAGGGTGCAGCTTTGACTCAACTTACCGTACAG

CTGCAACACAACTTCCAAGCTATTTCTAGTTCTATTGATGACATTTACTCCCGACTGGACATTCTTTCAGCCGATGTTC
AGGTTGATCGTCTCATCACCGGCAGATTATCAGCACTTAATGCTTTTGTTTCTCAAACCCTCACTAAGTATACTGAGGT
TCAGGCTAGCAGGAAGCTAGCACAGCAAAAGGTTAATGAGTGCGTTAAATCGCAATCTCAGCGTTATGGTTTTTGTGGT
GGTGATGGCGAGCACATTTTCTCTCTGGTACAGGCAGCACCTCAGGGCCTGCTGTTTTTACATACAGTACTTGTACCGG
GTGATTTTGTAGATGTTATTGCCATCGCTGGCTTATGCGTTAACGATGAAATTGCCTTGACTCTACGTGAGCCTGGCCT
AGTCTTGTTTACGCATGAACTTCAAAATCATACTGCGACGGAATATTTTGTTTCATCGCGACGTATGTTTGAACCTAGA
AAACCTACCGTTAGTGATTTTGTTCAAATTGAGAGTTGTGTGGTCACCTATGTCAATTTGACTAGGGACCAACTACCAG
ATGTAATCCCAGATTACATCGATGTTAACAAAACACTTGATGAGATTTTAGCTTCTCTGCCCAATAGAACTGGTCCAAG
TCTTCCTTTAGATGTTTTTAATGCCACTTATCTTAATCTCACTGGTGAAATTGCAGATTTAGAGCAGCGTTCAGAGTCT
CTCCGTAATACTACAGAGGAGCTCCAAAGTCTTATATATAATATCAACAACACACTAGTTGACCTTGAGTGGCTCAACC
GAGTTGAGACATATATCAAGTGGCCGTGGTGGGTTTGGTTGATTATTTTCATTGTTCTCATCTTTGTTGTGTCATTACT
AGTGTTCTGCTGCATTTCCACGGGTTGTTGTGGATGCTGCGGCTGCTGCTGTGCTTGTTTTTCAGGTTGTTGTAGGGGT
CCTAGACTTCAACCTTACGAAGTTTTTGAAAAGGTCCACGTGCAGTGA

>gi|402695537|gb|JX258672.1|_Porcine_epidemic_diarrhea_virus_strain_FQ/FJ/2012_spike_protein_gene_complete_cds

ATGAAGTCTTTAACCTACTTCTGGTTGTTCTTACCAGTACTTTTCACACTTAGCCTACCACAAGATGTCACCAGG
TGCTCAGCTAACACTAATTTTAGGCGGTTCTTTTCAAAATTTAATGTTCAGGCGCCTGCAGTTGTTGTACTGGGCGGTT
ATCTACCTATTGGTGAAAACCAGGGTGTCAATTCAACTTGGTACTGTGCTGGCCAACATCCAACTGCTAGTGGCGTTCA
TGGTATCTTTGTTAGCCATATTAGAGGTGGTCATGGCTTTGAGATTGGCATTTCGCAAGAGCCTTTTGACCCTAGTGGT
TACCAGCTTTATTTACATAAGGCTACTAACGGTAACACTAATGCTACTGCGCGACTGCGCATTTGCCAGTTTCCTAGCA
TTAAAACATTGGGCCCCACTGCTAATAATGATGTTACAACAGGTCGTAATTGCCTATTTAACAAAGCCATCCCAGCTCA
TATGAGTGAACATAGTGTTGTCGGCATAACATGGGATAATGATCGTGTCACTGTCTTTTCTGACAAGATCTATTATTTT
TATTTTAAAAATGATTGGTCCCGTGTTGCGACAAAGTGTTACAACAGTGGAGGTTGTGCTATGCAATATGTTTACGAAC
CCACCTATTACATGCTTAATGTTACTAGTGCTGGTGAGGATGGTATTTCTTATCAACCCTGTACAGCTAATTGCATTGG
TTATGCTGCCAATGTATTTGCTACTGAGCTCAATGGCCACATACCAGAAGGTTTTAGTTTTAATAATTGGTTTCTTTTG
TCCAATGATTCCACTTTGGTGCATGGTAAGGTGGTTTCCAACCAACCATTGTTGGTCAATTGTCTTTTGGCCATTCCTA
AGATTTATGGACTAGGCCAATTTTTCTCCTTTAATCAAACGATCGATGGTGTTTGTAATGGAGCTGCTGTGCAGCGTGC
ACCAGAGGCCCTGAGGTTTAATATTAATGACACCTCTGTCATTCTTGCTGAAGGCTCAATTGTACTTCATACTGCTTTA
GGAACAAATTTTTCTTTTGTTTGCAGTAATTCCTCAGATCCTCACTTAGCCACCTTCGCCATACCTCTGGGTGCTATCC
AAGTACCCTATTATTGTTTTCTTAAAGTGGATACTTACAACTCCACTGTTTATAAATTCCTGGCTGTTTTACCTCCTAC
CGTCAGGGAAATTGTCATCACCAAGTATGGTGATGTTTATGTCAATGGGTTTGGCTACTTGCATCTCGGTTTGTTGGAT
GCTGTCACAATTAATTTCACTGGTCATGGCACTGACGATGACGTTTCTGGTTTTTGGACCATAGCATCGACTAATTTTG
TTGATGCACTTATCGAAGTTCAAGGAACTGCCATTCAGCGTATTCTTTATTGTGATGATCCTGTTAGCCAACTCAAGTG
TTCTCAGGTTTCTTTTGACCTTGACGATGGTTTTTACCCTATTTCTTCTACAAACCTTCTGAGTCATGAACAGCCAACT
TCTTTTGTTACTTTGCCATCATTTAATGATCATTCTTTTGTTAATATTACTGTCTTTGCTGCTTTTGGTGGTCATAGTG
GTGCCAACCTTATTGCATCTGACACTACTATCAATGGGTTTAGTTCTTTCTGTGTTGACACTAGACAATTTACCATTTC
ACTGTTTTATAATGTTACAAACAGTTATGGTTATGTGTCTAACTCACAGGACAGTAATTGCCCTTTCACCTTGCAATCT
GTTAATGATTACCTGTCTTTTAGCAAATTTTGTGTTTCTACCAGCCTTTTGGCTAGTGCCTGTACCATAGATCTTTTTG
GTTACCCTGAGTTTGGTAGTGGTGTTAAGTTCACGTCCCTTTACTTTCAATTCACAAAGGGTGAGTTGATTACTGGCAC
GCCTAAACCACTTGAAGGTGTTACGGACGTTTCTTTTATGACTCTGGATGTGTGTACCAAGTATACTATCTATGGCTTT
AAAGGTGAGGGTATCATTACCCTTACAAATTCTAGCTTTTTGGCAGGTGTTTATTATACATCTGATTCTGGACAGTTGT

TAGCCTTTAAGAATGTCACTAGTGGTGCTGTTTATTCTGTTACGCCATGTTCTTTTTCAGAGCAGGCTGCATATGTTGA
TGATGATATAGTGGGTGTTATTTCTAGTTTGTCTAGCTCCACTTTTAACAGTACTAGGGAGTTGCCTGGTTTCTTCTAC
CATTCTAATGATGGCTCTAATTGTACAGAGCCTGTGTTGGTGTATAGTAACATAGGTGTTTGTAAATCTGGCAGTATTG
GCTATGTTCCATCTCAGTCTGGCCAAGTCAAGATTGCACCCACGGTTACTGGGAATATTAGTATTCCCACCAACTTTAG
TATGAGTATTAGGACAGAATATTTACAGCTTTACAACACGCCTGTTAGTGTTGATTGTGCTACATATGTTTGCAATGGT
AACTCTCGTTGTAAACAATTACTCACCCAGTACACTTCAGCATGTAAGACCATAGAGTCAGCATTACAACTCAGCGCTA
GGCTTGAGTCTGCTGAAGTCAACTCTATGCTTACTATTTCTGAAGAGGCTCTACAGTTAGCTACCATCAGTTCGTTTAA
TGGTGATGGATATAATTTTACTAATGTGCTGGGTGTTTCCGTTCGTGATCCTGCAAGTGGCAGGGTGGTACAAAAAAGG
TCTTTTATTGAAGACCTGCTTTTTAATAAAGTGGTTACTAATGGCCTTGGTACTGTTGATGAAGACTATAAGCGCTGTT
CTAATGGCCGTTCTGTGGCAGATTTAGTCTGTGCGCAGTATTACTCTGGTGTCATGGTACTACCTGGTGTTGTTGACGC
TGAGAAGCTTCATATGTATAGTGCGTCTCTCATCGGTGGTATGGTGCTAGGAGGTTTTACTGCAGCAGCGGCATTGCCT
TTTAGCTATGCTGTTCAAGCGAGACTCAATTATCTTGCTCTACAGACGGATGTTCTACAGCGGAACCAGCAATTGCTTG
CTGAGTCTTTTAACTCTGCTATTGGTAATATAACTTCAGCCTTTGAGAGTGTTAAAGAGGCTATTAGTCAAACTTCTAA
GGGTTTGAACACTGTGGCTCATGCGCTTACTAAGGTTCAAGAGGTTGTTAATTCGCAGGGTGCAGCTTTGACCCAACTT
ACCGTACAGCTGCAACACAACTTCCAAGCCATTTCTAGTTCTATTGATGACATTTATTCCCGACTGGACATTCTTTCAG
CCGATGTTCAGGTTGACCGTCTCATCACCGGCAGATTATCAGCACTTAATGCTTTTGTTGCTCAAACCCTCACTAAGTA
TACTGAGGTTCAGGCTAGCAGGAAGCTAGCACAGCAAAAGGTTAATGAGTGCGTTAAATCGCAATCTCAGCGTTATGGT
TTTTGCGGTGGTGATGGCGAGCACATTTTCTCTCTGGTACAGGCAGCACCTCAGGGCCTGCTGTTTTTACATACAGTAC
TTGTACCGGGTGATTTTGTAGATGTTATTGCCATCGCTGGCTTATGCGTTAACGATGAAATTGCCTTGACTCTACGTGA
GCCTGGCTTAGTCTTGTTTACGCATGAACTTCAAAATCATACTGCGACGGAATATTTTGTTTCATCGCGACGTATGTTT
GAACCTAGAAAACCTACCGTTAGTGACTTTGTTCAAATTGAGAGTTGTGTGGTCACCTATGTCAATTTGACTAGAGACC
AACTACCAGATGTAATCCCGGATTACATCGATGTTAACAAAACACTTGATGAGATTTTAGCTTCTCTGCCCAATAGAAC
TGGTCCAAGTCTTCCTTTAGATGTTTTTAATGCCACTTATCTTAATCTCACTGGTGAAATTGCAGATTTAGAGCAGCGT
TCAGAGTCTCTCCGTAATACTACAGAAGAGCTCCAAAGTCTTATATATAATATCAACAACACACTAGTTGACCTTGAGT
GGCTCAACCGAGTTGAGACATATATCAAGTGGCCGTGGTGGGTTTGGTTGATTATTTTTATTGTTCTCATCTTTGTTGT
GTCATTATTAGTGTTCTGCTGCATTTCCACGGGTTGTTGTGGATGCTGCGGCTGCTGTGGTGCTTGTTTTTCAGGTTGT
TGTAGGGGTCCTAGACTCAAACCTTACGAAGTTTTGAAAAGGTCCACGTGCAGTGA

>gi|404256436|gb|JX261936.1| Porcine epidemic diarrhea virus strain CHGD-01, complete genome

ATGAAGTCTTTAACCTACTTCTGGTTGTTCTTACCAGTACTTTCAACACTTAGCCTACCACAAGATGTCACCAGG
TGCTCAGCTAACACTAATTTTAGGCGGTTCTTTTCAAAATTTAATGTTCAGGCGCCTGCAGTTGTTGTACTGGGCGGTT
ATCTACCTATTGGTGAAAACCAGGGTGTCAATTCAACTTGGTACTGTGCTGGCCAACATCCAACTGCTAGTGGCGTTCA
TGGTATCTTTCTTAGCCATATTAGAGGTGGTCATGGCTTTGAGATTGGCATTTCGCAAGAGCCTTTTGACCCTAGTGGT
TACCAGCTTTATTTACATAAGGCTACTAACGGTAACACTAATGCTACTGCGCGACTGCGCATTTGCCAGTTTCCCAGCA
TTAAAACATTGGGCCCCACTGCTAATAATGATGTTACAACAGGTCGTAACTGCCTATTTAACAAAGCCATCCCAGCTCA
TATGAGTGAACATAGTGTTGTCGGCATAACATGGGATAATGATCGTGTCACTGTCTTTTCTGACAAGATCTATTATTTT
TATTTTAAAAATGATTGGTCCCGTGTTGCGACAAAGTGTTACAACAGTGGAGGTTGTGCTATGCAATATGTTTACGAAC
CCACTTACTACATTCTTAATGTTACTAGTGCTGGTGAGGATGGTATTTCTTATGAACCCTGTACAGCTAATTGCATTGG
TTATGCTGCCAATGTATTTGCTACTGAGCCCAATGGCCACATACCAGAAGGTTTTAGTTTTAATAATTGGTTTCTTTTG
TCCAATGATTCCACTTTGGTGCATGGTAAGGTGGTTTCCAACCAACCATTGTTGGTCAATTGTCTTTTGGCCATTCCTA
AGATTTATGGACTAGGCCAATTTTTCTCCTTTAATCAAACGATCGATGGTGTTTGTAATGGAGCTGCTGTGCAGCGTGC

ACCAGAGGCTCTGAGGTTTAATATTAATGACACCTCTGTCATTCTTGCTGAAGGCTCAATTGTACTTCATACTGCTTTA
GGAACAAATTTTTCTTTTGTTTGCAGTAATTCCTCAGAACCTCACTTAGCCACCTTTGCCATACCTCTGGGTGCTATCC
AAGTACCCTATTATTGTTTTCTTAAAGTGGATACTTACAACTCCACTGTTTATAAATTCTTGGCTGTTTTACCTCCTAC
CGTCAGGGAAATTGTCATCACCAAGTATGGTGATGTTTATGTCAATGGGTTTGGCTACTTGCATCTCGGTTTGTTGGAT
GCTGTCACAATTAATTTCACTGGTCATGGCACTGACGATGACGTTTCTGGTTTTTGGACCATAGCATCGACTAATTTTG
TTGATGCACTTATCGAAGTTCAAGGAACTGCCATTCAGCGTATTCTTTATTGTGATGATCCTGTTAGCCAACTCAAGTG
TTCTCAGGTTGCTTTTGACCTTGACGATGGTTTTTACCGTATTTCCTCTACAAACCTTCTGAGTCATGAACAGCCAACT
TCTTTTGTTACTTTGCCATCATTTAATGATCATTCTTTTGTTAATATTACTGTCTCTGCTGCTTTTGGTGGTCATAGTG
GTGCCAACCTTATTGCATCTGACACTACTATCAATGGGTTTAGTTCTTTCTGTGTTGACACTAGACAATTTACCATTTC
ACTGTTTTATAACGTTACAAACAGTTATGGTTACGTGTCTAAATCACAGGACAGTAATTGCCCTTTTACCTTGCAATCT
GTTAATGATTACCTGTCTTTTAGCAAATTTTGTGTTTCTACCAGCCTTTTGGCTAGTGCCTGTACCATAGATCTTTTTG
GTTACCCTGAGTTTGGTAGTGGTGTTAAGTTCACGTCCCTTTACTTTCAATTCACAAAGGGTGAGTTGATTACTGGCAC
GCCTAAACCACTTGAAGGTGTTACGGACGTTTCTTTTATGACTCTGGATGTGTGTACCAAGTATACTATCTATGGCTTT
AAAGGTGAGGGTATCATTACCCTTACAAATTCTAGCTTTTTGGCAGGTGTTTATTATACATCTGATTCTGGACAGTTGT
TAGCTTTTAAGAATGTCACTAGTGGTGCTGTTTATTCTGTTACGCCATGTTCTTTTTCAGAGCAGGCTGCATATGTTGA
TGATGATATAGTGGGTGTTATTTCTAGTTTGTCTAGCTCCACTTTTAACAGTACTAGGGAGTTGCCTGGTTTCTTCTAC
CATTCTAATGATGGCTCTAATTGTACAGAGCCTGTGTTGGTGTATAGTAACATAGGTGTTTGTAAATCTGGCAGTATTG
GCTATGTCCGATCTCAGTCTGGCCAAGTCAAGATTGCACCCACGGTTACTGGGAATATTAGTATTCCCACCAACTTTAG
TATGAGTATTAGGACAGAATATTTACAGCTTTACAACACGCCTGTTAGTGTTGATTGTGCTACATATGTTTGTAATGGT
AACTCTCGTTGTAAACAATTACTCACCCAGTACACTGCAGCATGTAAGACCATAGAGTCAGCATTACAACTCAGCGCTA
GGCTTGAGTCTGCTGAAGTCAACTCTATGCTTACTATTTCTGAAGAGGCTCTACAGTTAGCTACCATCAGTTCGTTTAA
TGGTGATGGATATAATTTTACTAATGTGCTGGGTGTTTCCGTGTATGACCCTGCAAGTGGCAGGGTGGTACAAAAAAGG
TCTTTCATTGAAGACCTGCTTTTTAATAAAGTGGTTACTAATGGCCTTGGTACTGTTGATGAAGACTATAAGCGCTGTT
CTAATGGCCGCTCTGTGGCAGATTTAGTCTGTGCGCAGTATTACTCTGGTGTCATGGTACTACCTGGTGTTGTTGACGC
TGAGAAGCTTCACATGTATAGTGCGTCTCTCATCGGTGGTATGGTGCTAGGAGGTTTTACTGCAGCAGCGGCATTGCCT
TTTAGCTATGCTGTTCAAGCGAGACTGAATTATCTTGCTCTACAGACGGATGTTCTACAGCGGAACCAGCAATTGCTTG
CTGAGTCTTTTAATTCTGCTATTGGTAATATAACTCCAGCCTTTGAGAGTGTTAAAGAGGCTATTAGTCAAACTTCTAA
GGGTTTGAACACTGTGGCTCATGCGCTTACCAAGGTCCAAGAGGTTGTTAATTCGCAGGGTGCAGCTTTGACCCAACTT
ACCGTACAGCTGCAACACAACTTCCAAGCCATTTCTAGTTCTATTGATGACATTTACTCCCGACTTGACATTCTTTCAG
CCGATGTTCAGGTAGACCGTCTCATCACCGGCAGATTATCAGCACTTAATGCTTTTGTTGCTCAAACCCTTACTAAGTA
TACTGAGGTTCAGGCTAGCAGGAAACTAGCACAGCAAAAGGTTAATGAGTGCGTTAAATCGCAATCTCAGCGTTATGGT
TTTTGTGGTGGTGATGGCGAGCACATTTTCTCTCTGGTACAGGCCGCACCTCAAGGCCTGCTGTTCTTACACACAGTAC
TTGTACCGGGTGATTTTGTAAATGTTATTGCCATCGCTGGCTTATGTGTTAACGATGAAATTGCCCTGACTCTACGTGA
GCCTGGCTTAGTCTTGTTTACGCATGAACTTCAAAATACTGCGACGGAATATTTTGTTTCATCGCGACGTATGTATGAA
CCTAGAAAACCTACCGTTGGTGATTTTGTTCAAATTGAGAGTTGTGTGGTCACCTATGTCAATTTGACTAGAGACCAAC
TACCAGATGTAATCCCAGATTACATCGATGTTAACAAAACACTTGATGAGATTTTAGCTTCTCTGCCCAATAGAACTGG
TCCAAGTCTTTCTCTAGATGTTTTTAATGCCACTTATCTTAATCTTACTGGTGAAATTGCAGATTTAGAGCAGCGTTCA
GAGTCTCTCCGTAATACTACAGAAGAGCTTCAAAGTCTTATATATAATATCAACAACACACTAGTTGACCTTGAGTGGC
TCAACCGAGTTGAGACATATATCAAGTGGCCGTGGTGGGTTTGGTTGATTATTTTTATTGTTCTCATCTTTGTTGTGTC
ATTACTAGTGTTCTGCTGCATTTCCACGGGTTGTTGTGGATGCTGCGGCTGCTGTGGTGCTTGTTTTTCAGGTTGTTGT
AGGGGTCCTAGACTTCAACCTTACGAAGCTTTTGAAAAGGTCCACGTGCAGTGA

>gi|407830448|gb|JX435298.1|_Porcine_epidemic_diarrhea_virus_isolate_BJ2011

3_spike_protein_(S)_mRNA_complete_cds

ATGAAGTCTTTAACCTACTTCTGGTTGTTCTTACCAGTACTTTCAACACTTAGCCTACCACAAGATGTCACCAGG
TGCTCAGCTAACACTAATTTTAGGCGGTTCTTTTCAAAATTTAATGTTCAGGCGCCTGCAGTTGTTGTACTGGGCGGTT
ATCTACCTATTGGTGAAAACCAGGGTGTCAATTCAACTTGGTACTGTGCTGGCCAACATCCAACTGCTAGTGGCGTTCA
TGGTATCTTTGTTAGCCATATTAGAGGTGGTCATGGCTTTGAGATTGGCATTTCGCAAGAGCCTTTTGACCCTAGTGGT
TACCAGCTTTATTTACATAAGGCTACTAACGGTAACACTAATGCTACTGCGCGACTGCGCATTTGCCAGTTTCCTAGCA
TTAAAAGCTTGGGCCCCACTGCTAATAATGATGTTACAACAGGTCGTAATTGCCTATTTAACAAAGCCATCCCAGCTCA
TATGAGTGAACATAGTGTTGTCGGCATAACATGGGATAATGATCGTGTCACTGTCTTTTCTGACAAGATCTATTATTTT
TATTTTAAAAATGATTGGTCCCGTGTTGCGACAAAGTGTTACAACAGTGGAGGTTGTGCTATGCAATATGTTTACGAAC
CCACCTATTACATGCTTAATGTTACTAGTGCTGGTGAGGATGGTATTTCTTATCAACCCTGTACAGCTAATTGCATTGG
TTATGCTGCCAATGTATTTGCTACTGAGCCCAATGGCCACATACCAGAAGGTTTTAGTTTTAATAATTGGTTTCTTTTG
TCCAATGATTCCACTTTGGTGCATGGTAAGGTGGTTTCCAACCAACCATTGTTGGTCAATTGTCTTTTGGCCATTCCTA
AGATTTATGGACTAGGCCAATTTTTCTCCTTTAATCAAACGATCGATGGTGTTTGTAATGGAGCTGCTGTGCAGCGTGC
ACCAGAGGCTCTGAGGTTTAATATTAATGACACCTCTGTCATTCTTGCTGAAGGCTCAATTGTACTTCATACTGCTTTA
GGAACAAATTTTTCTTTTGTTTGCAGTAATTCCTCAGATCCTCACTTAGCCACCTTCGCCATACCTCTGGGTGCTATCC
AAGTACCCTATTATTGTTTTCTTAAAGTGGATACTTACAACTCCACTGTTTATAAATTCTTGGCTGTTTTACCTCCTAC
CGTCAGGGAAATTGTCATCACCAAGTATGGTGATGTTTATGTCAATGGGTTTGGCTATTTGCATCTCGGTTTGTTGGAC
GCTGTCACAATTAATTTCACTGGTCATGGCACTGACGATGACGTTTCAGGTTTCTGGACCATAGCATCGACTAATTTTG
TTGATGCACTTATCGAAGTTCAAGGAACTGCCATTCAGCGTATTCTTTATTGTGATGATCCTGTTAGCCAACTCAAGTG
TTCTCAGGTTGCTTTTGACCTTGACGATGGTTTCTACCCTATTTCTTCTAGAAACCTTCTGAGTCATGAACAGCCAATT
TCTTTTGTTACTTTGCCATCATTTAATGATCATTCTTTTGTTAACATTACTGTCTCTGCGTCCTTTGGTGGTCATAGTG
GTGCCAACCTTATTGCATCTGACACTACTATCAATGGGTTTAGTTCTTTCTGTGTTGACACTAGACAATTTACCATTTC
ACTGTTTTATAACGTTACAAACAGTTATGGTTATGTGTCTAAATCACAGGACAGTAATTGCCCTTTCACCTTGCAATCT
GTTAATGATTACCTGTCTTTTAGTAAATTTTGTGTTTCCACCAGCCTTTTGGCTAGTGCCTGTACCATAGATCTTTTTG
GTTACCCTGAGTTTGGTAGTGGTGTTAAGTTTACGTCCCTTTACTTTCAATTCACAAAGGGTGAGTTGATTACTGGCAC
GCCTAAACCACTTGAAGGTGTCACGGACGTTTCTTTTATGACTCTGGATGTGTGTACCGAGTATACTATCTATGGCTTT
AAAGGTGAGGGTATCATTACCCTTACAAATTCTAGCTTTTTGGCAGGTGTTTATTACACATCTGATTCTGGACAGTTGT
TAGCCTTTAAGAATGTCACTAGTGGTGCTGTTTATTCTGTTACGCCATGTTCTTTTTCAGAGCAGGCTGCATATGTTGA
TGATGATATAGTGGGTGTTATTTCTAGTTTGTCTAGCTCCACTTTTAACAGTACTAGGGAGTTGCCTGGTTTCTTCTAC
CATTCTAATGATGGCTCTAATTGTACAGAGCCTGTGTTGGTGTATAGTAACATAGGTGTTTGTAAATCTGGCAGTATTG
GCTACGTCCCATCTCAGTCTGGCCAAGTCAAGATTGCACCCACGGTTACTGGGAATATTAGTATTCCCACCAACTTTAG
TATGAGTATTAGGACAGAATATTTACAGCTTTACAACACGCCTGTTAGTGTTGATTGTGCCACATATGTTTGTAATGGT
AACTCTCGTTGTAAACAATTACTCACCCAGTACACTGCAGCATGTAAGACCATAGAGTCAGCATTACAACTCAGCGCTA
GGCTTGAGTCTGTTGAAGTTAACTCTATGCTTACTATTTCTGAAGAGGCTCTACAGTTAGCTACCATCAGTTCGTTTAA
TGGTGATGGATATAATTTTACTAATGTGCTGGGTGTTTCTGTGTATGATCCTGCAAGTGGCAGGGTGGTACAAAAAAGG
TCTTTTATTGAAGACCTGCTTTTTAATAAAGTGGTTACTAATGGCCTTGGTACTGTTGATGAAGACTATAAGCGCTGTT
CTAATGGTCGCTCTGTGGCAGATCTAGTCTGTGCACAGTATTACTCTGGTGTCATGGTACTACCTGGTGTTGTTGACGC
TGAGAAGCTTCACATGTATAGTGCGTCTCTCATCGGTGGTATGGTGCTAGGAGGTTTTACTTCTGCAGCGGCATTGCCT
TTTAGCTATGCTGTTCAAGCTAGACTCAATTATCTTGCTCTACAGACGGATGTTCTACAGCGGAACCAGCAATTGCTTG
CTGAGTCTTTTAACTCTGCTATTGGTAATATAACTTCAGCCTTTGAGAGTGTTAAAGAGGCTATTAGTCAAACTTCCAA
GGGTTTGAACACTGTGGCTCATGCGCTTACTAAGGTTCAAGAGGTTGTTAACTCGCAGGGTGCAGCTTTGACTCAACTT
ACCGTACAGCTGCAACACAACTTCCAAGCCATTTCTAGTTCTATTGATGACATTTACTCTCGACTGGACATTCTTTCAG
CCGATGTTCAGGTTGACCGTCTCATCACCGGCAGATTATCAGCACTTAATGCTTTTGTTGCTCAAACCCTCACTAAGTA

TACTGAGGTTCAGGCTAGCAGGAAGCTAGCACAGCAAAAGGTTAATGAGTGCGTTAAATCGCAATCTCAGCGTTATGGT
TTTTGTGGTGGTGATGGCGAGCACATTTTCTCTCTGGTACAGGCAGCACCTCAGGGCCTGCTGTTTTTACATACAGTAC
TTGTACCGGGTGACTTTGTAGATGTTATTGCCATCGCTGGCTTATGCGTTAACGATGAAATTGCCTTGACTCTACGTGA
GCCTGGCTTAGTCTTGTTTACGCATGAACTTCAAAATCATACTGCGACGGAATATTTTGTTTCATCGCGACGTATGTTT
GAACCTAGAAAACCTACCGTTAGTGATTTTGTTCAAATTGAGAGTTGTGTGGTCACCTATGTCAATTTGACTAGAGACC
AACTACCAGATGTAATCCCAGATTACATCGATGTTAACAAAACACTTGATGAGATTTTAGCTTCTCTGCCCAATAGAAC
TGGTCCAAGTCTTCCTTTAGATGTTTTTAATGCCACTTATCTTAATCTCACTGGTGAAATTGCAGATTTAGAGCAGCGT
TCAGAGTCTCTCCGTAATACTACAGAGGAGCTCCAAAGTCTTATATATAATATCAACAACACACTAGTTGACCTTGAGT
GGCTCAACCGAGTTGAGACATATATCAAGTGGCCGTGGTGGGTTTGGTTGATTATTTTCATTGTTCTCATCTTTGTTGT
GTCATTACTAGTGTTCTGCTGCATTTCCACTGGTTGTTGTGGATGCTGCGGCTGCTGCTGTGCTTGTTTTTCAGGTTGT
TGTAGGGGTCCTAGACTTCAACCTTACGAAGTTTTTGAAAAGGTCCACGTGCAGTGA

>gi|407830450|gb|JX435299.1|_Porcine_epidemic_diarrhea_virus_isolate_BJ2012
1_spike_protein_(S)_mRNA_complete_cds

ATGAAGTCTTTAACCTACTTCTGGTTGTTCTTACCAGTACTTTCAACACTTAGCCTACCACAAGATGTCACCAGG
TGCTCAGCTAACACTAATTTTAGGCGGTTCTTTTCAAAATTTAATGTTCAGGCGCCTGCAGTTGTTGTACTGGGCGGTT
ATCTACCTATTGGTGAAAACCAGGGTGTCAATTCAACTTGGTACTGTGCTGGCCAACATCCAACTGCTAGTGGCGTTCA
TGGTATCTTTGTTAGCCATATTAGAGGTGGTCATGGCTTTGAGATTGGCATTTCGCAAGAGCCTTTTGACCCTAGTGGT
TACCAGCTTTATTTACATAAGGCTACTAACGGTAACACTAATGCTACTGCGCGACTGCGCATTTGCCAGTTTCCTAGCA
TTAAAACATTGGGCCCCACTGCTAATAATGATGTTACAACAGGTCGTAATTGCCTATTTAACAAAGCCATCCCAGCTCA
TATGAGTGAACATAGTGTTGTCGGCATAACATGGGATAATGATCGTGTCACTGTCTTTTCTGACAAGATCTATTATTTT
TATTTTAAAAATGATTGGTCCCGTGTTGCGACAAAGTGTTACAACAGTGGAGGTTGTGCTATGCAATATGTTTACGAAC
CCACCTATTACATGCTTAATGTTACTAGTGCTGGTGAGGATGGTATTTCTTATCAACCCTGTACAGCTAATTGCATTGG
TTATGCTGCCAATGTATTTGCTACTGAGCTCAATGGCCACATACCAGAAGGTTTTAGTTTTAATAATTGGTTTCTTTTG
TCCAATGATTCCACTTTGGTGCATGGTAAGGTGGTTTCCAACCAACCATTGTTGGTCAATTGTCTTTTGGCCATTCCTA
AGATTTATGGACTAGGCCAATTTTTCTCCTTTAATCAAACGATCGATGGTGTTTGTAATGGAGCTGCTGTGCAGCGTGC
ACCAGAGGCCCTGAGGTTTAATATTAATGACACCTCTGTCATTCTTGCTGAAGGCTCAATTGTACTTCATACTGCTTTA
GGAACAAATTTTTCTTTTGTTTGCAGTAATTCCTCAGATCCTCACTTAGCCACCTTCGCCATACCTCTGGGTGCTATCC
AAGTACCCTATTATTGTTTTCTTAAAGTGGATACTTACAACTCCACTGTTTATAAATTCCTGGCTGTTTTACCTCCTAC
CGTCAGGGAAATTGTCATCACCAAGTATGGTGATGTTTATGTCAATGGGTTTGGCTACTTGCATCTCGGTTTGTTGGAT
GCTGTCACAATTAATTTCACTGGTCATGGCACTGACGATGACGTTTCTGGTTTTTGGACCATAGCATCGACTAATTTTG
TTGATGCACTTATCGAAGTTCAAGGAACTGCCATTCAGCGTATTCTTTATTGTGATGATCCTGTTAGCCAACTCAAGTG
TTCTCAGGTTTCTTTTGACCTTGACGATGGTTTTTATCCTATTTCCTCTACAAACCTTCTGAGTCATGAACAGCCAACT
TCTTTTGTTACTTTGCCATCATTTAATGATCATTCTTTTGTTAATATTACTGTCTCTGCTGCTTTTGGTGGTCATAGTG
GTGCCAACCTTATTGCATCTGACACTACTATCAATGGGTTTAGTTCTTTCTGTGTTGACACTAGACAATTTACCATTTC
ACTGTTTTATAATGTTACAAACAGTTATGGTTATGTGTCTAAATCACAGTACAGTAATTGCCCTTTCACCTTGCAATCT
GTTAATGATTACCTGTCTTTTAGCAAATTTTGTGTTTCTACCAGCCTTTTGGCTAGTGCCTGTACCATAGATCTTTTTG
GTTACCCTGAGTTTGGTAGTGGTGTTAAGTTCACGTCCCTTTACTTTCAATTCACAAAGGGTGAGTTGATTACTGGCAC
GCCTAAACCACTTGAAGGTGTTACGGACGTTTCTTTTATGACTCTGGATGTGTGTACCAAGTATACTATCTATGGCTTT
AAAGGTGAGGGTATCATTACCCTTACAAATTCTAGCTTTTTGGCAGGTGTTTATTATACATCTGATTCTGGACAGTTGT
TAGCTTTTAAGAATGTCACTAGTGGTGCTGTTTATTCTGTTACGCCATGTTCTTTTTCAGAGCAGGCTGCATATGTTGA
TGATGATATAGTGGGTGTTATTTCTAGTTTGTCTAGCTCCACTTTTAACAGTACTAGGGAGTTGCCTGGTTTCTTCTAC

CATTCTAATGATGGCTCTAATTGTACAGAGCCTGTGTTGGTGTATAGTAACATAGGTGTTTGTAAATCTGGCAGTATTG
GCTATGTTCCATCTCAGTCTGGCCAAGTCAAGATTGCACCCACGGTTACTGGGAATATTAGTATTCCCACCAACTTTAG
TATGAGTATTAGGACAGAATATTTACAGCTTTACAACACGCCTGTTAGTGTTGATTGTGCTACATATGTTTGCAATGGT
AACTCTCGTTGTAAACAATTACTCACCCAGTACACTGCAGCATGTAAGACCATAGAGTCAGCATTACAACTCAGCGCTA
GGCTTGAGTCTGCTGAAGTCAACTCTATGCTTACTATTTCTGAAGAGGCCCTACAGTTAGCTACCATCAGTTCGTTTAA
TGGTGATGGATATAATTTTACTAATGTGCTGGGTGTTTCCGTTCGTGATCCTGCAAGTGGCAGGGTGGTACAAAAAAGG
TCTTTTATTGAAGACCTGCTTTTTAATAAAGTGGTTACTAATGGCCTTGGTACTGTTGATGAAGACTATAAGCGCTGTT
CTAATGGCCGCTCTGTGGCAGATTTAGTCTGTGCGCAGTATTACTCTGGTGTCATGGTACTACCTGGTGTTGTTGACGC
TGAGAAGCTTCATATGTATAGTGCGTCTCTCATCGGTGGTATGGTGCTAGGAGGTTTTACTGCAGCAGCGGCATTGCCT
TTTAGCTATGCTGTTCAAGCGAGACTCAATTATCTTGCTCTACAGACGGATGTTCTACAGCGGAACCAGCAATTGCTTG
CTGAGTCTTTTAACTCTGCTATTGGTAATATAACTTCAGCCTTTGAGAGTGTTAAAGAGGCTATTAGTCAAACTTCTAA
GGGTTTGAACACTGTGGCTCATGCGCTTACTAAGGTTCAAGAGGTTGTTAACTCGCAGGGTGCAGCTTTGACTCAACTT
ACCGTACAGCTGCAACACAACTTCCAAGCCATTTCTAGTTCTATTGATGACATTTACTCTCGACTGGACATTCTTTCAG
CCGATGTTCAGGTTGACCGTCTCATCACCGGCAGATTATCAGCACTTAATGCTTTTGTTGCTCAAACCCTCACTAAGTA
TACTGAGGTTCAGGCTAGCAGGAAGCTAGCACAGCAAAAGGTTAATGAGTGCGTTAAATCGCAATCTCAGCGTTATGGT
TTTTGTGGTGGTGATGGCGAGCACATTTTCTCTCTGGTACAGGCAGCACCTCAGGGCCTGCTGTTTTTACATACAGTAC
TTGTACCGGGTGACTTTGTAGATGTTATTGCCATCGCTGGCTTATGCGTTAACGATGAAATTGCCTTGACTCTACGTGA
GCCTGGCTTAGTCTTGTTTACGCATGAACTTCAAAATCATACTGCGACGGAATATTTTGTTTCATCGCGACGTATGTTT
GAACCTAGAAAACCTACCGTTAGTGATTTTGTTCAAATTGAGAGTTGTGTGGTCACCTATGTCAATTTGACTAGAGACC
AACTACCAGATGTAATCCCAGATTACATCGATGTTAACAAAACACTTGATGAGATTTTAGCTTCTCTGCCCAATAGAAC
TGGTCCAAGTCTTCCTTTAGATGTTTTTAATGCCACTTATCTTAATCTCACTGGTGAAATTGCAGATTTAGAGCAGCGT
TCAGAGTCTCTCCGTAATACTACAGAGGAGCTCCAAAGTCTTATATATAATATCAACAACACACTAGTTGACCTTGAGT
GGCTCAACCGAGTTGAGACATATATCAAGTGGCCGTGGTGGGTTTGGTTGATTATTTTCATTGTTCTCATCTTTGTTGT
GTCATTACTAGTGTTCTGCTGCATTTCCACTGGTTGTTGTGGATGCTGCGGCTGCTGCTGTGCTTGTTTTTCAGGTTGT
TGTAGGGGTCCTAGACTTCAACCTTACGAAGTTTTTGAAAAGGTCCACGTGCAGTGA

>gi|407830452|gb|JX435300.1|_Porcine_epidemic_diarrhea_virus_isolate_BJ2012
2_spike_protein_(S)_mRNA_complete_cds

ATGAAGTCTTTAACCTACTTCTGGTTGTTCTTACCAGTACTTTCAACACTTAGCCTACCACAAGATGTCACCAGG
TGCTCAGCTAACACTAATTTTAGGCGGTTCTTTTCAAAATTTAATGTTCAGGCGCCTGCAGTTGTTGTACTGGGCGGTT
ATCTACCTATTGGTGAAAACCAGGGTGTCAATTCAACTTGGTACTGTGCTGGCCAACATCCAACTGCTAGTGGCGTTCA
TGGTATCTTTGTTAGCCATATTAGAGGTGGTCATGGCTTTGAGATTGGCATTTCGCAAGAGCCTTTTGACCCTAGTGGT
TACCAGCTTTATTTACATAAGGCTACTAACGGTAACACTAATGCTACTGCGCGACTGCGCATTTGCCAGTTTCCTAGCA
TTAAAACATTGGGCCCCACTGCTAATAATGATGTTACAACAGGTCGTAATTGCCTATTTAACAAAGCCATCCCAGCTCA
TATGAGTGAACATAGTGTTGTCGGCATAACATGGGATAATGATCGTGTCACTGTCTTTTCTGACAAGATCTATTATTTT
TATTTTAAAAATGATTGGTCCCGTGTTGCGACAAAGTGTTACAACAGTGGAGGTTGTGCTATGCAATATGTTTACGAAC
CCACCTATTACATGCTTAATGTTACTAGTGCTGGTGAGGATGGTATTTCTTATCAACCCTGTACAGCTAATTGCATTGG
TTATGCTGCCAATGTATTTGCTACTGAGCCCAATGGCCACATACCAGAAGGTTTTAGTTTTAATAATTGGTTTCTTTTG
TCCAATGATTCCACTTTGGTGCATGGTAAGGTGGTTTCCAACCAACCATTGTTGGTCAATTGTCTTTTGGCCATTCCTA
AGATTTATGGACTAGGCCAATTTTTCTCCTTTAATCAAACGATCGATGGTGTTTGTAATGGAGCTGCTGTGCAGCGTGC
ACCAGAGGCTCTGAGGTTTAATATTAATGACACCTCTGTCATTCTTGCTGAAGGCTCAATTGTACTTCATACTGCTTTA
GGAACAAATTTTTCTTTTGTTTGCAGTAATTCCTCAGATCCTCACTTAGCCACCTTCGCCATACCTCTGGGTGCTATCC

AAGTACCCTATTATTGTTTTCTTAAAGTGGATACTTACAACTCCACTGTTTATAAATTCTTGGCTGTTTTACCTCCTAC
CGTCAGGGAAATTGTCATCACCAAGTATGGTGATGTTTATGTCAATGGGTTTGGCTATTTGCATCTCGGTTTGTTGGAC
GCTGTCACAATTAATTTCACTGGTCATGGCACTGACGATGACGTTTCAGGTTTCTGGACCATAGCATCGACTAATTTTG
TTGATGCACTTATCGAAGTTCAAGGAACTGCCATTCAGCGTATTCTTTATTGTGATGATCCTGTTAGCCAACTCAAGTG
TTCTCAGGTTGCTTTTGACCTTGACGATGGTTTCTACCCTATTTCTTCTAGAAACCTTCTGAGTCATGAACAGCCAATT
TCTTTTGTTACTTTGCCATCATTTAATGATCATTCTTTTGTTAACATTACTGTCTCTGCGTCCTTTGGTGGTCATAGTG
GTGCCAACCTTATTGCATCTGACACTACTATCAATGGGTTTAGTTCTTTCTGTGTTGACACTAGACAATTTACCATTTC
ACTGTTTTATAACGTTACAAACAGTTATGGTTATGTGTCTAAATCACAGGACAGTAATTGCCCTTTCACCTTGCAATCT
GTTAATGATTACCTGTCTTTTAGTAAATTTTGTGTTTCCACCAGCCTTTTGGCTAGTGCCTGTACCATAGATCTTTTTG
GTTACCCTGAGTTTGGTAGTGGTGTTAAGTTTACGTCCCTTTACTTTCAATTCACAAAGGGTGAGTTGATTACTGGCAC
GCCTAAACCACTTGAAGGTGTCACGGACGTTTCTTTTATGACTCTGGATGTGTGTACCGAGTATACTATCTATGGCTTT
AAAGGTGAGGGTATCATTACCCTTACAAATTCTAGCTTTTTGGCAGGTGTTTATTACACATCTGATTCTGGACAGTTGT
TAGCCTTTAAGAATGTCACTAGTGGTGCTGTTTATTCTGTTACGCCATGTTCTTTTTCAGAGCAGGCTGCATATGTTGA
TGATGATATAGTGGGTGTTATTTCTAGTTTGTCTAGCTCCACTTTTAACAGTACTAGGGAGTTGCCTGGTTTCTTCTAC
CATTCTAATGATGGCTCTAATTGTACAGAGCCTGTGTTGGTGTATAGTAACATAGGTGTTTGTAAATCTGGCAGTATTG
GCTACGTCCCATCTCAGTCTGGCCAAGTCAAGATTGCACCCACGGTTACTGGGAATATTAGTATTCCCACCAACTTTAG
TATGAGTATTAGGACAGAATATTTACAGCTTTACAACACGCCTGTTAGTGTTGATTGTGCCACATATGTTTGTAATGGT
AACTCTCGTTGTAAACAATTACTCACCCAGTACACTGCAGCATGTAAGACCATAGAGTCAGCATTACAACTCAGCGCTA
GGCTTGAGTCTGTTGAAGTTAACTCTATGCTTACTATTTCTGAAGAGGCTCTACAGTTAGCTACCATCAGTTCGTTTAA
TGGTGATGGATATAATTTTACTAATGTGCTGGGTGTTTCTGTGTATGATCCTGCAAGTGGCAGGGTGGTACAAAAAAGG
TCTTTTATTGAAGACCTGCTTTTTAATAAAGTGGTTACTAATGGCCTTGGTACTGTTGATGAAGACTATAAGCGCTGTT
CTAATGGTCGCTCTGTGGCAGATCTAGTCTGTGCACAGTATTACTCTGGTGTCATGGTACTACCTGGTGTTGTTGACGC
TGAGAAGCTTCACATGTATAGTGCGTCTCTCATCGGTGGTATGGTGCTAGGAGGTTTTACTTCTGCAGCGGCATTGCCT
TTTAGCTATGCTGTTCAAGCTAGACTCAATTATCTTGCTCTACAGACGGATGTTCTACAGCGGAACCAGCAATTGCTTG
CTGAGTCTTTTAACTCTGCTATTGGTAATATAACTTCAGCCTTTGAGAGTGTTAAAGAGGCTATTAGTCAAACTTCCAA
GGGTTTGAACACTGTGGCTCATGCGCTTACTAAGGTTCAAGAGGTTGTTAACTCGCAGGGTGCAGCTTTGACTCAACTT
ACCGTACAGCTGCAACACAACTTCCAAGCCATTTCTAGTTCTATTGATGACATTTACTCTCGACTGGACATTCTTTCAG
CCGATGTTCAGGTTGACCGTCTCATCACCGGCAGATTATCAGCACTTAATGCTTTTGTTGCTCAAACCCTCACTAAGTA
TACTGAGGTTCAGGCTAGCAGGAAGCTAGCACAGCAAAAGGTTAATGAGTGCGTTAAATCGCAATCTCAGCGTTATGGT
TTTTGTGGTGGTGATGGCGAGCACATTTTCTCTCTGGTACAGGCAGCACCTCAGGGCCTGCTGTTTTTACATACAGTAC
TTGTACCGGGTGACTTTGTAGATGTTATTGCCATCGCTGGCTTATGCGTTAACGATGAAATTGCCTTGACTCTACGTGA
GCCTGGCTTAGTCTTGTTTACGCATGAACTTCAAAATCATACTGCGACGGAATATTTTGTTTCATCGCGACGTATGTTT
GAACCTAGAAAACCTACCGTTAGTGATTTTGTTCAAATTGAGAGTTGTGTGGTCACCTATGTCAATTTGACTAGAGACC
AACTACCAGATGTAATCCCAGATTACATCGATGTTAACAAAACACTTGATGAGATTTTAGCTTCTCTGCCCAATAGAAC
TGGTCCAAGTCTTCCTTTAGATGTTTTTAATGCCACTTATCTTAATCTCACTGGTGAAATTGCAGATTTAGAGCAGCGT
TCAGAGTCTCTCCGCAATACTACAGAGGAGCTCCAAAGTCTTATATATAATATCAACAACACACTAGTTGACCTTGAGT
GGCTCAACCGAGTTGAGACATATATCAAGTGGCCGTGGTGGGTTTGGTTGATTATTTTCATTGTTCTCATCTTTGTTGT
GTCATTACTAGTGTTCTGCTGCATTTCCACTGGTTGTTGTGGATGCTGCGGCTGCTGCTGTGCTTGTTTTTCAGGTTGT
TGTAGGGGTCCTAGACTTCAACCTTACGAAGTTTTTGAAAAGGTCCACGTGCAGTGA

>gi|407830454|gb|JX435301.1|_Porcine_epidemic_diarrhea_virus_isolate_HB2011
4_spike_protein_(S)_mRNA_complete_cds

ATGAAGTCTTTAACCTACTTCTGGTTGTTCTTACCAGTACTTTCAACACTTAGCCTACCACAAGATGTCACCAGG
TGCTCAGCTAACACTAATTTTAGGCGGTTCTTTTCAAAATTTAATGTTCAGGCGCCTGCAGTTGTTGTACTGGGCGGTT
ATCTACCTATTGGTGAAAACCAGGGTGTCAATTCAACTTGGTACTGTGCTGGCCAACATCCAACTGCTAGTGGCGTTCA
TGGTATCTTTGTTAGCCATATTAGAGGTGGTCATGGCTTTGAGATTGGCATTTCGCAAGAGCCTTTTGACCCTAGTGGT
TACCAGCTTTATTTACATAAGGCTACTAACGGTAACACTAATGCTACTGCGCGACTGCGCATTTGCCAGTTTCCTAGCA
TTAAAAGCTTGGGCCCCACTGCTAATAATGATGTTACAACAGGTCGTAATTGCCTATTTAACAAAGCCATCCCAGCTCA
TATGAGTGAACATAGTGTTGTCGGCATAACATGGGATAATGATCGTGTCACTGTCTTTTCTGACAAGATCTATTATTTT
TATTTTAAAAATGATTGGTCCCGTGTTGCGACAAAGTGTTACAACAGTGGAGGTTGTGCTATGCAATATGTTTACGAAC
CCACCTATTACATGCTTAATGTTACTAGTGCTGGTGAGGATGGTATTTCTTATCAACCCTGTACAGCTAATTGCATTGG
TTATGCTGCCAATGTATTTGCTACTGAGCCCAATGGCCACATACCAGAAGGTTTTAGTTTTAATAATTGGTTTCTTTTG
TCCAATGATTCCACTTTGGTGCATGGTAAGGTGGTTTCCAACCAACCATTGTTGGTCAATTGTCTTTTGGCCATTCCTA
AGATTTATGGACTAGGCCAATTTTTCTCCTTTAATCAAACGATCGATGGTGTTTGTAATGGAGCTGCTGTGCAGCGTGC
ACCAGAGGCTCTGAGGTTTAATATTAATGACACCTCTGTCATTCTTGCTGAAGGCTCAATTGTACTTCATACTGCTTTA
GGAACAAATTTTTCTTTTGTTTGCAGTAATTCCACAGATCCTCATTTAGCCACCTTCGCCATACCTCTGGGTGCTACCC
AAGTACCCTATTATTGTTTTCTTAAAGTGGATATTTACAACTCCACTGTTTATAAATTCTTGGCTGTTTTACCTCCAAC
CGTCAGGGAAATTGTCATCACCAAGTATGGTGATGTTTATGTCAATGGGTTTGGCTATTTGCATCTCGGTTTGTTGGAC
GCTGTCACAATTAATTTCACTGGTCATGGCACTGACGATGACGTTTCAGGTTTCTGGACCATAGCATCGACTAATTTTG
TTGATGCACTTATCGAAGTTCAAGGAACTGCCATTCAGCGTATTCTTTATTGTGATGATCCTGTTAGCCAACTCAAGTG
TTCTCAGGTTGCTTTTGACCTTGACGATGGTTTCTACCCTATTTCTTCTAGAAACCTTCTGAGTCATGAACAGCCAATT
TCTTTTGTTACTTTGCCATCATTTAATGATCATTCTTTTGTTAACATTACTGTCTCTGCGTCCTTTGGTGGTCGTAGTG
GTGCCGACCTTATTGCATCTGACACTACTATCAATGGGTTTAGTTCTTTCTGTGTTGACACTAGACAATTTACCATTTC
ACTGTTTTATAACGTTACAAACAGTTATGGTTATGTGTCTAACTCACAGGACAGTAATTGCCCTTTCACCTTGCAATCT
GTTAATGATTACCTGTCTTTTAGTAAATTTTGTGTTTCCACCAGCCTTTTGGCTAGTGCCTGTACCATAGATCTTTTTG
GTTACCCTGAGTTTGGTAGTGGTGTTAAGTTTACGTCCCTTTACTTTCAATTCACAAAGGGTGAGTTGATTACTGGCAC
GCCTAAACCACTTGAAGGTGTCACGGACGTTTCTGTTATGACTCTGGATGTGTGTACCAAGTATACTATCTATGGCTTT
AAAGGTGAGGGTATCATTACCCTTACAAACTCTAGCTTTTTGGCAGGTGTTTATTACACATCTGATTCTGGACAGTTGT
TAGCCTTTAAGAATGTCACTAGTGGTGCTGTTTATTCTGTTACGCCATGTTCTTTTTCAGAGCAGGCTGCATATGTTGA
TGATGATATAGTGGGTATTATTTCTAGTTTGTCTAGCTCCACTTTTAACAGTACTAGGGAGTTGCCTGGTTTCTTCTAC
CATTCTAATGATGGCTCTAATTGTACAGAGCCTGTGTTGGTGTATAGTAACATAGGTGTTTGTAAATCTGGCAGTATTG
GCTACGTCCCATCTCAGTCTGGCCAAGTCAAGATTGCACCCACGGTTACTGGGAATATTAGTGTTCCCACCAACTTTAG
TATGAGTATTAGGACAGAATATTTACAGCTTTACAACACGCCTGTTAGTGTTGATTGTGCCACATATGTTTGTAATGGT
AACTCTCGTTGTAAACAATTACTCACCCAGTACACTGCAGCATGTAAGACCATAGAGTCAGCATTACAACTCAGCGCTA
GGCTTGAGTCTGTTGAAGTTAACTCTATGCTTACTATTTCTGAAGAGGCTCTACAGTTAGCTACCATCAGTTCGTTTAA
TGGTGATGGATATAATTTTACTAATGTGCTGGGTGTTTCTGTGTATGACCCTGCAAGTGGCAGGGTGGTACAAAAAAGG
TCTTTTATTGAAGACCTGCTTTTTAATAAAGTGGTTACTAATGGCCTTGGTACTGTTGATGAAGACTATAAGCGCTGTT
CTAATGGTCGCTCTGTGGCAGATCTAGTCTGTGCACAGTATTACTCTGGTGTCATGGTACTACCTGGTGTTGTTGACGC
TGAGAAGCTTCACATGTATAGTGCGTCTCTCATCGGTGGTATGGTGCTAGGAGGTTTTACTTCTGCAGCGGCATTGCCT
TTTAGCTATGCTGTTCAAGCTAGACTCAATTATCTTGCTCTACAGACGGATGTTCTACAGCGGAACCAGCAATTGCTTG
CTGAGTCTTTTAACTCTGCTATTGGTAATATAACTTCAGCCTTTGAGAGTGTTAAAGAGGCTATTAGTCAAACTTCCAA
GGGTTTGAACACTGTGGCTCATGCGCTTACTAAGGTTCAAGAGGTTGTTAACTCGCAGGGTGCAGCTTTGACTCAACTT
ACCGTACAGCTGCAACACAACTTCCAAGCCATTTCTAGTTCTATTGATGACATTTACTCTCGACTGGACATTCTTTCAG
CCGATGTTCAGGTTGACCGTCTCATCACCGGCAGATTATCAGCACTTAATGCTTTTGTTGCTCAAACCCTCACTAAGTA
TACTGAGGTTCAGGCTAGCAGGAAGCTAGCACAGCAAAAGGTTAATGAGTGCGTTAAATCGCAATCTCAGCGTTATGGT
TTTTGTGGTGGTGATGGCGAGCACATTTTCTCTCTGGTACAGGCAGCACCTCAGGGCCTGCTGTTTTTACATACAGTAC

TTGTACCGGGTGACTTTGTAGATGTTATTGCCATCGCTGGCTTATGCGTTAACGATGAAATTGCCTTGACTCTACGTGA
GCCTGGCTTAGTCTTGTTTACGCATGAACTTCAAAATCATACTGCGACGGAATATTTTGTTTCATCGCGACGTATGTTT
GAACCTAGAAAACCTACCGTTAGTGATTTTGTTCAAATTGAGAGTTGTGTGGTCACCTATGTCAATTTGACTAGAGACC
AACTACCAGATGTAATCCCAGATTACATCGATGTTAACAAAACACTTGATGAGATTTTAGCTTCTCTGCCCAATAGAAC
TGGTCCAAGTCTTCCTTTAGATGTTTTTAATGCCACTTATCTTAATCTCACTGGTGAAATTGCAGATTTAGAGCAGCGT
TCAGAGTCTCTCCGTAATACTACAGAGGAGCTCCAAAGTCTTATATATAATATCAACAACACACTAGTTGACCTTGAGT
GGCTCAACCGAGTTGAGACATATATCAAGTGGCCGTGGTGGGTTTGGTTGATTATTTTCATTGTTCTCATCTTTGTTGT
GTCATTACTAGTGTTCTGCTGCATTTCCACTGGTTGTTGTGGATGCTGCGGCTGCTGCTGTGCTTGTTTTTCAGGTTGT
TGTAGGGGTCCTAGACTTCAACCTTACGAAGTTTTTGAAAAGGTCCACGTGCAGTGA

>gi|407830456|gb|JX435302.1|_Porcine_epidemic_diarrhea_virus_isolate_HB2012
1_spike_protein_(S)_mRNA_complete_cds

ATGAAGTCTTTAACCTACTTCTGGTTGTTCTTACCAGTACTTTCAACACTTAGCCTACCACAAGATGTCACCAGG
TGCTCAGCTAACACTAATTTTAGGCGGTTCTTTTCAAAATTTAATGTTCAGGCGCCTGCAGTTGTTGTACTGGGCGGTT
ATCTACCTATTGGTGAAAACCAGGGTGTCAATTCAACTTGGTACTGTGCTGGCCAACATCCAACTGCTAGTGGCGTTCA
TGGTATCTTTGTTAGCCATATTAGAGGTGGTCATGGCTTTGAGATTGGCATTTCGCAAGAGCCTTTTGACCCTAGTGGT
TACCAGCTTTATTTACATAAGGCTACTAACGGTAATACTAATGCTACTGCGCGACTGCGCATTTGCCAGTTTCCTAGCA
TTAAAACATTGGGCCCCACTGCTAATAATGATGTTACAACAGGTCGTAATTGCCTATTTAACAAAGCCATCCCAGCTCA
TATGAGTGAACATAGTGTTGTCGGCATAACATGGGATAATGATCGTGTCACTGTCTTTTCTGACAAGATCTATTATTTT
TATTTTAAAAATGATTGGTCCCGTGTTGCGACAAAGTGTTACAACAGTGGAGGTTGTGCTATGCAATATGTTTACGAAC
CCACCTATTACATGCTTAATGTTACTAGTGCTGGTGAGGATGGTATCTCTTATCAACCCTGTACAGCTAATTGCATTGG
TTATGCTGCCAATGTATTTGCTACTGAGCCCAATGGCCACATACCAGAAGGTTTTAGTTTTAATAATTGGTTTCTTTTG
TCCAATGATTCCACTTTGGTGCATGGTAAGGTGGTTTCCAACCAACCATTGTTGGTCAATTGTCTTTTGGCCATTCCTA
AGATTTATGGACTAGGCCAATTTTTCTCCTTTAATCAAACGATCGATGGTGTTTGTAATGGAGCTGCTGTGCAGCGTGC
ACCAGAGGCTCTGAGGTTTAATATTAATGACACCTCTGTCATTCTTGCTGAAGGCTCAATTGTACTTCATACTGCTTTA
GGAACAAATTTTTCTTTTGTTTGCAGTAATTCCTCAGATCCTCACTTAGCCACCTTCGCCATACCTCTGGGTGCTATCC
AAGTACCCTATTATTGTTTTCTTAAAGTGGATACTTACAACTCCACTGTTTATAAATTCTTGGCTGTTTTACCTCCTAC
CGTCAGGGAAATTGTCATCACCAAGTATGGTGATGTTTATGTCAATGGGTTTGGCTATTTGCATCTCGGTTTGTTGGAC
GCTGTCACAATTAATTTCACTGGTCATGGCACTGACGATGACGTTTCAGGTTTCTGGACCATAGCATCGACTAATTTTG
TTGATGCACTTATCGAAGTTCAAGGAACTGCCATTCAGCGTATTCTTTATTGTGATGATCCTGTTAGCCAACTCAAGTG
TTCTCAGGTTGCTTTTGACCTTGACGATGGTTTCTACCCTATTTCTTCTAGAAACCTTCTGAGTCATGAACAGCCAATT
TCTTTTGTTACTTTGCCATCATTTAATGATCATTCTTTTGTTAACATTACTGTCTCTGCGTCCTTTGGTGGTCATAGTG
GTGCCAACCTTATTGCATCTGACACTACTATCAATGGGTTTAGTTCTTTCTGTGTTGACACTAGACAATTTACCATTTC
ACTGTTTTATAACGTTACAAACAGTTATGGTTATGTGTCTAAATCACAGGACAGTAATTGCCCTTTCACCTTGCAATCT
GTTAATGATTACCTGTCTTTTAGTAAATTTTGTGTTTCCACCAGCCTTTTGGCTAGTGCCTGTACCATAGATCTTTTTG
GTTACCCTGAGTTTGGTAGTGGTGTTAAGTTTACGTCCCTTTACTTTCAATTCACAAAGGGTGAGTTGATTACTGGCAC
GCCTAAACCACTTGAAGGTGTCACGGACGTTTCTTTTATGACTCTGGATGTGTGTACCATGTATACTATCTATGGCTTT
AAAGGTGAGGGTATCATTACCCTTACAAATTCTAGCTTTTTGGCAGGTGTTTATTACACATCTGATTCTGGACAGTTGT
TAGCCTTTAAGAATGTCACTAGTGGTGCTGTTTATTCTGTTACGCCATGTTCTTTTTCAGAGCAGGCTGCATATGTTGA
TGATGATATAGTGGGTGTTATTTCTAGTTTGTCTAGCTCCACTTTTAACAGTACTAGGGAGTTGCCTGGTTTCTTCTAC
CATTCTAATGATGGCTCTAATTGTACAGAGCCTGTGTTGGTGTATAGTAACATAGGTGTTTGTAAATCTGGCAGTATTG
GCTACGTCCCATCTCAGTCTGGCCAAGTCAAGATTGCACCCACGGTTACTGGGAATATTAGTATTCCCACCAACTTTAG

TATGAGTATTAGGACAGAATATTTACAGCTTTACAACACGCCTGTTAGTGTTGATTGTGCCACATATGTTTGTAATGGT
AACTCTCGTTGTAAACAATTACTCACCCAGTACACTGCAGCATGTAAGACCATAGAGTCAGCATTACAACTCAGCGCTA
GGCTTGAGTCTGTTGAAGTTAACTCTATGCTTACTATTTCTGAAGAGGCTCTACAGTTAGCTACCATCAGTTCGTTTAA
TGGTGATGGATATAATTTTACTAATGTGCTGGGTGTTTCTGTGTATGATCCTGCAAGTGGCAGGGTGGTACAAAAAAGG
TCTTTTATTGAAGACCTGCTTTTTAATAAAGTGGTTACTAATGGCCTTGGTACTGTTGATGAAGACTATAAGCGCTGTT
CTAATGGTCGCTCTGTGGCAGATCTAGTCTGTGCACAGTATTACTCTGGTGTCATGGTACTACCTGGTGTTGTTGACGC
TGAGAAGCTTCACATGTATAGTGCGTCTCTCATCGGTGGTATGGTGCTAGGAGGTTTTACTTCTGCAGCGGCATTGCCT
TTTAGCTATGCTGTTCAAGCTAGACTCAATTATCTTGCTCTACAGACGGATGTTCTACAGCGGAACCAGCAATTGCTTG
CTGAGTCTTTTAACTCTGCTATTGGTAATATAACTTCAGCCTTTGAGAGTGTTAAAGAGGCTATTAGTCAAACTTCCAA
GGGTTTGAACACTGTGGCTCATGCGCTTACTAAGGTTCAAGAGGTTGTTAACTCGCAGGGTGCAGCTTTGACTCAACTT
ACCGTACAGCTGCAACACAACTTCCAAGCCATTTCTAGTTCTATTGATGACATTTACTCTCGACTGGACATTCTTTCAG
CCGATGTTCAGGTTGACCGTCTCATCACCGGCAGATTATCAGCACTTAATGCTTTTGTTGCTCAAACCCTCACTAAGTA
TACTGAGGTTCAGGCTAGCAGGAAGCTAGCACAGCAAAAGGTTAATGAGTGCGTTAAATCGCAATCTCAGCGTTATGGT
TTTTGTGGTGGTGATGGCGAGCACATTTTCTCTCTGGTACAGGCAGCACCTCAGGGCCTGCTGTTTTTACATACAGTAC
TTGTACCGGGTGACTTTGTAGATGTTATTGCCATCGCTGGCTTATGCGTTAACGATGAAATTGCCTTGACTCTACGTGA
GCCTGGCTTAGTCTTGTTTACGCATGAACTTCAAAATCATACTGCGACGGAATATTTTGTTTCATCGCGACGTATGTTT
GAACCTAGAAAACCTACCGTTAGTGATTTTGTTCAAATTGAGAGTTGTGTGGTCACCTATGTCAATTTGACTAGAGACC
AACTACCAGATGTAATCCCAGATTACATCGATGTTAACAAAACACTTGATGAGATTTTAGCTTCTCTGCCCAATAGAAC
TGGTCCAAGTCTTCCTTTAGATGTTTTTAATGCCACTTATCTTAATCTCACTGGTGAAATTGCAGATTTAGAGCAGCGT
TCAGAGTCTCTCCGTAATACTACAGAGGAGCTCCAAAGTCTTATATATAATATCAACAACACACTAGTTGACCTTGAGT
GGCTCAACCGAGTTGAGACATATATCAAGTGGCCGTGGTGGGTTTGGTTGATTATTTTCATTGTTCTCATCTTTGTTGT
GTCATTACTAGTGTTCTGCTGCATTTCCACTGGTTGTTGTGGATGCTGCGGCTGCTGCTGTGCTTGTTTTTCAGGTTGT
TGTAGGGGTCCTAGACTTCAACCTTACGAAGTTTTTGAAAAGGTCCACGTGCAGTGA

>gi|407830458|gb|JX435303.1|_Porcine_epidemic_diarrhea_virus_isolate_HB2012
2_spike_protein_(S)_mRNA_complete_cds

ATGAAGTCTTTAACCTACTTCTGGTTGTTCTTACCAGTACTTTCAACACTTAGCCTACCACAAGATGTCACCAGG
TGCTCAGCTAACACTAATTTTAGGCGGTTCTTTTCAAAATTTAATGTTCAGGCGCCTGCAGTTGTTGTACTGGGCGGTT
ATCTACCTATTGGTGAAAACCAGGGTGTCAATTCAACTTGGTACTGTGCTGGCCAACATCCAACTGCTAGTGGCGTTCA
TGGTATCTTTGTTAGCCATATTAGAGGTGGTCATGGCTTTGAGATTGGCATTTCGCAAGAGCCTTTTGACCCTAGTGGT
TACCAGCTTTATTTACATAAGGCTACTAACGGTAACACTAATGCCACTGCGCGACTGCGCATTTGCCAGTTTCCTAGCA
TTAAAACATTGGGCCCCACTGCTAATAATGATGTTACAACAGGTCGTAATTGCCTATTTAACAAAGCCATCCCAGCTCA
TATGAGTGAACATAGTGTTGTCGGCATAACATGGGATAATGATCGTGTCACTGTCTTTTCTGACAAGATCTATTATTTT
TATTTTAAAAATGATTGGTCCCGTGTTGCGACAAAGTGTTACAACAGTGGAGGTTGTGCTATGCAATATGTTTACGAAC
CCACCTATTACATGCTTAATGTTACTAGTGCTGGTGAGGATGGTATTTCTTATCAACCCTGTACAGCTAATTGCATTGG
TTATTCTGCCAATGTATTTGCTACTGAGCCCAATGGCCACATACCAGAAGGTTTTAGTTTTAATAATTGGTTTCTTTTG
TCCAATGATTCCACTTTGGCGCATGGTAAGGTGGTTTCTAACCAACCATTGTTGGTCAATTGTCTTTTGGCCATTCCTA
AGATTTATGGACTAGGCCAATTTTTCTCCTTTAATCAAACGATCGATGGTGTTTGTAATGGAGCTGCTGTGCAGCGTGC
ACCAGAGGCTCTGAGGTTTAATATTAATGACACCTCTGTCATTCTTGCTGAAGGCTCAATTGTACTTCATACTGCTTTA
GGAACAAATTTTTCTTTTGTTTGCAGTAATTCCTCAGATCCTCACTTAGCCACCTTCGCCATACCTCTGGGTGCTATCC
AAGTACCCTATTATTGTTTTCTTAAAGTGGATACTTACAACTCCACTGTTTATAAATTCTTGGCTGTTTTACCTCCTAC
CGTCAGGGAAATTGTCATCACCAAGTATGGTGATGTTTATGTCAATGGGTTTGGCTACTTGCATCTCGGTTTGTTGGAT

GCTGTCACAATTAATTTCACTGGTCATGGCACTGACGATGACGTTTCTGGTTTTTGGACCATAGCATCGACTAATTTTG
TTGATGCACTTATCGAAGTTCAAGGAACTGCCATTCAGCGTATTCTTTATTGTGATGATCCTGTTAGCCAACTCAAGTG
TTCTCAGGTTGCTTTTGACCTTGACGATGGTTTCTACCCTATTTCTTCTAGAAACCTTCTGAGTCATGAACAGCCAATT
TCTTTTGTTACTTTGCCATCATTTAATGATCATTCTTTTGTTAACATTACTGTCTCTGCGTCCTTTGGTGGTCATAGTG
GTGCCAACCTTATTGCATCTGACACTACTATCAATGGGTTTAGTTCTTTCTGTGTTGACACTAGACAATTTACCATTTC
ACTGTTTTATAACGTTACAAACAGTTATGGTTATGTGTCTAAATCACAGGACAGTAATTGCCCTTTCACCTTGCAATCT
GTTAATGATTACCTGTCTTTTAGTAAATTTTGTGTTTCCACCAGCCTTTTGGCTAGTGCCTGTACCATAGATCTTTTTG
GTTACCCTGAGTTTGGTAGTGGTGTTAAGTTTACGTCCCTTTACTTTCAATTCACAAAGGGTGAGTTGATTACTGGCAC
GCCTAAACCACTTGTAGGTGTCACGGACGTTTCTTTTATGACTCTGGATGTGTGTACCAAGTATACTATCTATGGCTTT
AAAGGTGAGGGTATCATTACCCTTACAAATTCTAGCTTTTTGGCAGGTGTTTATTACACATCTGATTCTGGACAGTTGT
TAGCCTTTAAGAATGTCACTAGTGGTGCTGTTTATTCTGTTACGCCATGTTCTTTTTCAGAGCAGGCTGCATATGTTGA
TGATGATATAGTGGGTGTTATTTCTAGTTTGTCTAGCTCCACTTTTAACAGTACTAGGGAGTTGCCTGGTTTCTTCTAC
CATTCTAATGATGGCTCTAATTGTACAGAGCCTGTGTTGGTGTATAGTAACATAGGTGTTTGTAAATCTGGCAGTATTG
GCTACGTCCCATCTCAGTCTGGCCAAGTCAAGATTGCACCCACGGTTACTGGGAATATTAGTATTCCCACCAACTTTAG
TATGAGTATTAGGACAGAATATTTACAGCTTTACAACACGCCTGTTAGTGTTGATTGTGCCACATATGTTTGTAATGGT
AACTCTCGTTGTAAACAATTACTCACCCAGTACACTGCAGCATGTAAGACCATAGAGTCAGCATTACAACTCAGCGCTA
GGCTTGAGTCTGTTGAAGTTAACTCTATGCTTACTATTTCTGAAGAGGCTCTACAGTTAGCTACCATCAGTTCGTTTAA
TGGTGATGGATATAATTTTACTAATGTGCTGGGTGTTTCTGTGTATGATCCTGCAAGTGGCAGGGTGGTACAAAAAAGG
TCTTTTATTGAAGACCTGCTTTTTAATAAGGTGGTTACTAATGGCCTTGGTACTGTTGATGAAGACTATAAGCGCTGTT
CTAATGGTCGCTCTGTGGCAGATCTAGTCTGTGCACAGTATTACTCTGGTGTCATGGTACTACCTGGTGTTGCTGACGC
TGAGAAGCTTCACATGTATAGTGCGTCTCTCATCGGTGGTATGGTGCTAGGAGGTTTTACTTCTGCAGCGGCATTGCCT
TTTAGCTATGCTGTTCAAGCTAGACTCAATTATCTTGCTCTACAGACGGATGTTCTACAGCGGAACCAGCAATTGCTTG
CTGAGTCTTTTAACTCTGCTATTGGTAATATAACTTCAGCCTTTGAGAGTGTTAAAGAGGCTATTAGTCAAACTTCCAA
GGGTTTGAACACTGTGGCTCATGCGCTTACTAAGGTTCAAGAGGTTGTTAACTCGCAGGGTGCAGCTTTGACTCAACTT
ACCGTACAGCTGCAACACAACTTCCAAGCCATTTCTAGTTCTATTGATGACATTTACTCTCGACTGGACATTCTTTCAG
CCGATGTTCAGGTTGACCGTCTCATCACCGGCAGATTATCAGCACTTAATGCTTTTGTTGCTCAAACCCTCACTAAGTA
TACTGAGGTTCAGGCTAGCAGGAAGCTAGCACAGCAAAAGGTTAATGAGTGCGTTAAATCGCAATCTCAGCGTTATGGT
TTTTGTGGTGGTGATGGCGAGCACATTTTCTCTCTGGTACAGGCAGCACCTCAGGGCCTGCTGTTTTTACATACAGTAC
TTGTACCGGGTGACTTTGTAGATGTTATTGCCATCGCTGGCTTATGCGTTAACGATGAAATTGCCTTGACTCTACGTGA
GCCTGGCTTAGTCTTGTTTACGCATGAACTTCAAAATCATACTGCGACGGAATATTTTGTTTCATCGCGACGTATGTTT
GAACCTAGAAAACCTACCGTTAGTGATTTTGTTCAAATTGAGAGTTGTGTGGTCACCTATGTCAATTTGACTAGAGACC
AACTACCAGATGTAATCCCAGATTACATCGATGTTAACAAAACACTTGATGAGATTTTAGCTTCTCTGCCCAATAGAAC
TGGTCCAAGTCTTCCTTTAGATGTTTTTAATGCCACTTATCTTAATCTCACTGGTGAAATTGCAGATTTAGAGCAGCGT
TCAGAGTCTCTCCGTAATACTACAGAGGAGCTCCAAAGTCTTATATATAATATCAACAACACACTAGTTGACCTTGAGT
GGCTCAACCGAGTTGAGACATATATCAAGTGGCCGTGGTGGGTTTGGTTGATTATTTTCATTGTTCTCATCTTTGTTGT
GTCATTACTAGTGTTCTGCTGCATTTCCACTGGTTGTTGTGGATGCTGCGGCTGCTGCTGTGCTTGTTTTTCAGGTTGT
TGTAGGGGTCCTAGACTTCAACCTTACGAAGTTTTTGAAAAGGTCCACGTGCAGTGA

>gi|407830460|gb|JX435304.1|_Porcine_epidemic_diarrhea_virus_isolate_HB2012
3_spike_protein_(S)_mRNA_complete_cds

ATGAAGTCTTTAACCTACTTCTGGTTGTTCTTACCAGTACTTTCAACACTTAGCCTACCACAAGATGTCACCAGG
TGCTCAGCTAACACTAATTTTAGGCGGTTCTTTTCAAAATTTAATGTTCAGGCGCCTGCAGTTGTTGTACTGGGCGGTT

ATCTACCTATTGGTGAAAACCAGGGTGTCAATTCAACTTGGTACTGTGCTGGCCAACATCCAACTGCTAGTGGCGTTCA
TGGTATCTTTGTTAGCCATATTAGAGGTGGTCATGGCTTTGAGATTGGCATTTCGCAAGAGCCTTTTGACCCTAGTGGT
TACCAGCTTTATTTACATAAGGCTACTAACGGTAACACTAATGCTACTGCGCGACTGCGCATTTGCCAGTTTCCTAGCA
TTAAAAGCTTGGGCCCCACTGCTAATAATGATGTTACAACAGGTCGTAATTGCCTATTTAACAAAGCCATCCCAGCTCA
TATGAGTGAACATAGTGTTGTCGGCATAACATGGGATAATGATCGTGTCACTGTCTTTTCTGACAAGATCTATTATTTT
TATTTTAAAAATGATTGGTCCCGTGTTGCGACAAAGTGTTACAACAGTGGAGGTTGTGCTATGCAATATGTTTACGAAC
CCACCTATTACATGCTTAATGTTACTAGTGCTGGTGAGGATGGTATTTCTTATCAACCCTGTACAGCTAATTGCATTGG
TTATGCTGCCAATGTATTTGCTACTGAGCCCAATGGCCACATACCAGAAGGTTTTAGTTTTAATAATTGGTTTCTTTTG
TCCAATGATTCCACTTTGGTGCATGGTAAGGTGGTTTCCAACCAACCATTGTTGGTCAATTGTCTTTTGGCCATTCCTA
AGATTTATGGACTAGGCCAATTTTTCTCCTTTAATCAAACGATCGATGGTGTTTGTAATGGAGCTGCTGTGCAGCGTGC
ACCAGAGGCTCTGAGGTTTAATATTAATGACACCTCTGTCATTCTTGCTGAAGGCTCAATTGTACTTCATACTGCTTTA
GGAACAAATTTTTCTTTTGTTTGCAGTAATTCCACAGATCATCATTTAGCCACCTTCGCCATACCTCTGGGTGCTACCC
AAGTACCCTATTATTGTTTTCTTAAAGTGGATATTTACAACTCCACTGTTTATAAATTCTTGGCTGTTTTACCTCCAAC
CGTCAGGGAAATTGTCATCACCAAGTATGGTGATGTTTATGTCAATGGGTTTGGCTATTTGCATCTCGGTTTGTTGGAC
GCTGTCACAATTAATTTCACTGGTCATGGCACTGACGATGACGTTTCAGGTTTCTGGACCATAGCATCGACTAATTTTG
TTGATGCACTTATCGAAGTTCAAGGAACTGCCATTCAGCGTATTCTTTATTGTGATGATCCTGTTAGCCAACTCAAGTG
TTCTCAGGTTGCTTTTGACCTTGACGATGGTTTCTACCCTATTTCTTCTAGAAACCTTCTGAGTCATGAACAGCCAATT
TCTTTTGTTACTTTGCCATCATTTAATGATCATTCTTTTGTTAACATTACTGTCTCTGCGTCCTTTGGTGGTCGTAGTG
GTGCCGACCTTATTGCATCTGACACTACTATCAATGGGTTTAGTTCTTTCTGTGTTGACACTAGACAATTTACCATTTC
ACTGTTTTATAACGTTACAAACAGTTATGGTTATGTGTCTAACTCACAGGACAGTAATTGCCCTTTCACCTTGCAATCT
GTTAATGATTACCTGTCTTTTAGTAAATTTTGTGTTTCCACCAGCCTTTTGGCTAGTGCCTGTACCATAGATCTTTTTG
GTTACCCTGAGTTTGGTAGTGGTGTTAAGTTTACGTCCCTTTACTTTCAATTCACAAAGGGTGAGTTGATTACTGGCAC
GCCTAAACCACTTGAAGGTGTCACGGACGTTTCTGTTATGACTCTGGATGTGTGTACCAAGTATACTATCTATGGCTTT
AAAGGTGAGGGTATCATTACCCTTACAAACTCTAGCTTTTTGGCAGGTGTTTATTACACATCTGATTCTGGACAGTTGT
TAGCCTTTAAGAATGTCACTAGTGGTGCTGTTTATTCTGTTACGCCATGTTCTTTTTCAGAGCAGGCTGCATATGTTGA
TGATGATATAGTGGGTATTATTTCTAGTTTGTCTAACTCCACTTTTAACAGTACTAGGGAGTTGCCTGGTTTCTTCTAC
CATTCTAATGATGGCTCTAATTGTACAGAGCCTGTGTTGGTGTATAGTAACATAGGTGTTTGTAAATCTGGCAGTATTG
GCTACGTCCCATCTCAGTCTGGCCAAGTCAAGATTGCACCCACGGTTACTGGGAATATTAGTATTCCCACCAACTTTAG
TATGAGTATTAGGACAGAATATTTACAGCTTTACAACACGCCTGTTAGTGTTGATTGTGCCACATATGTTTGTAATGGT
AACTCTCGTTGTAAACAATTACTCACCCAGTACACTGCAGCATGTAAGACCATAGAGTCAGCATTACAACTCAGCGCTA
GGCTTGAGTCTGTTGAAGTTAACTCTATGCTTACTATTTCTGAAGAGGCTCTACAGTTAGCTACCATCAGTTCGTTTAA
TGGTGATGGATATAATTTTACTAATGTGCTGGGTGTTTCTGTGTATGATCCTGCAAGTGGCAGGGTGGTAAAAAAAAGG
TCTTTTATTGAAGACCTGCTTTTTAATAAAGTGGTTACTAATGGCCTTGGTACTGTTGATGAAGACTATAAGCGCTGTT
CTAATGGTCGTTCTGTGGCAGATCTAGTCTGTGCACAGTATTACTCTGGTGTCATGGTACTACCTGGTGTTGTTGACGC
TGAGAAGCTTCACATGTATAGTGCGTCTCTCATCGGTGGTATGGTGCTAGGAGGTTTTACTTCTGCAGCGGCATTGCCT
TTTAGCTATGCTGTTCAAGCTAGACTCAATTATCTTGCTCTACAGACGGATGTTCTACAGCGGAACCAGCAATTGCTTG
CTGAGTCTTTTAACTCTGCTATTGGTAATATAACTTCAGCCTTTGAGAGTGTTAAAGAGGCTATTAGTCAAACTTCCAA
GGGTTTGAACACTGTGGCTCATGCGCTTACTAAGGTTCAAGAGGTTGTTAACTCGCAGGGTGCAGCTTTGACTCAACTT
ACCGTACAGCTGCAACACAACTTCCAAGCCATTTCTAGTTCTATTGATGACATTTACTCTCGACTGGACATTCTTTCAG
CCGATGTTCAGGTTGACCGTCTCATCACCGGCAGATTATCAGCACTTAATGCTTTTGTTGCTCAAACCCTCACTAAGTA
TACTGAGGTTCAGGCTAGCAGGAAGCTAGCACAGCAAAAGGTTAATGAGTGCGTTAAATCGCAATCTCAGCGTTATGGT
TTTTGTGGTGGTGATGGCGAGCACATTTTCTCTCTGGTACAGGCAGCACCTCAGGGCCTGCTGTTTTTACATACAGTAC
TTGTACCGGGTGACTTTGTAGATGTTATTGCCATCGCTGGCTTATGCGTTAACGATGAAATTGCCTTGACTCTACGTGA
GCCTGGCTTAGTCTTGTTTACGCATGAACTTCAAAATCATACTGCGACGGAATATTTTGTTTCATCGCGACGTATGTTT

GAACCTAGAAAACCTACCGTTAGTGATTTTGTTCAAATTGAGAGTTGTGTGGTCACCTATGTCAATTTGACTAGAGACC
AACTACCAGATGTAATCCCAGATTACATCGATGTTAACAAAACACTTGATGAGATTTTAGCTTCTCTGCCCAATAGAAC
TGGTTCAAGTCTTCCTTTAGATGTTTTTAATGCCACTTATCTTAATCTCACTGGTGAAATTGCAGATTTAGAGCAGCGT
TCAGAGTCTCTCCGTAATACTACAGAGGAGCTCCAAAGTCTTATATATAATATCAACAACACACTAGTTGACCTTGAGT
GGCTCAACCGAGTTGAGACATATATCAAGTGGCCGTGGTGGGTTTGGTTGATTATTTTCATTGTGCTCATCTTTGTTGT
GTCATTACTAGTGTTCTGCTGCATTTCCACTGGTTGTTGTGGATGCTGCGGCTGCTGCTGTGCTTGTTTTTCAGGTTGT
TGTAGGGGTCCTAGACTTCAACCTTACGAAGTTTTTGAAAAGGTCCACGTGCAGTGA

>gi|407830462|gb|JX435305.1|_Porcine_epidemic_diarrhea_virus_isolate_HB2012
4_spike_protein_(S)_mRNA_complete_cds

ATGAAGTCTTTAACCTACTTCTGGTTGCTCTTACCAGTACTTTCAACACTTAGCCTACCACAAGATGTCACCAGG
TGCTCAGCTAACACTAATTTTAGGCGGTTCTTTTCAAAATTTAATGTTCAGGCGCCTGCAGTTGTTGTACTGGGCGGTT
ATCTACCTATTGGTGAAAACCAGGGTGTCAATTCAACTTGGTACTGTGCTGGCCAACATCCAACTGCTAGTGGCGTTCA
TGGTATCTTTGTTAGCCATATTAGAGGTGGTCATGGCTTTGAGATTGGCATTTCGCAAGAGCCTTTTGACCCTAGTGGT
TACCAGCTTTATTTACATAAGGCTACTAACGGTAACACTAATGCTACTGCGCGACTGCGCATTTGCCAGTTTCCTAGCA
TTAAAACATTGGGCCCCACTGCTAATAATGATGTTACAACAGGTCGTAATTGCCTATTTAACAAAGCCATCCCAGCTCA
TATGAGTGAACATAGTGTTGTCGGCATAACATGGGATAATGATCGTGTCACTGTCTTTTCTGACAAGATCTATTATTTT
TATTTTAAAAATGATTGGTCCCGTGTTGCGACAAAGTGTTACAACAGTGGAGGTTGTGCTATGCAATATGTTTACGAAC
CCACCTATTACATGCTTAATGTTACTAGTGCTGGTGAGGATGGTATTTCTTATCAACCCTGTACAGCTAATTGCATTGG
TTATGCTGCCAATGTATTTGCTACTGAGCCCAATGGCCACATACCAGAAGGTTTTAGTTTTAATAATTGGTTTCTTTTG
TCCAATGATTCCACTTTGGTGCATGGTAAGGTGGTTTCCAACCAACCATTGTTGGTCAATTGTCTTTTGGCCATTCCTA
AGATTTATGGACTAGGCCAATTTTTCTCCTTTAATCAAACGATCGATGGTGTTTGTAATGGAGCTGCTGTGCAGCGTGC
ACCAGAGGCTCTGAGGTTTAATATTAATGACACCTCTGTCATTCTTGCTGAAGGCTCAATTGTACTTCATACTGCTTTA
GGAACAAATTTTTCTTTTGTTTGCAGTAATTCCTCAGATCTTCACTTAGCCACCTTCGCCATACCTCTGGGTGCTATCC
AAGTACCCTATTATTGTTTTCTTAAAGTGGATACTTACAACTCCACTGTTTATAAATTCTTGGCTGTTTTACCTCCTAC
CGTCAGGGAAATTGTCATCACCAAGTATGGTGATGTTTATGTCAATGGGTTTGGCTATTTGCATCTCGGTTTGTTGGAC
GCTGTCACAATTAATTTCACTGGTCATGGCACTGACGATGACGTTTCAGGTTTCTGGACCATAGCATCGACTAATTTTG
TTGATGCACTTATCGAAGTTCAAGGAACTTCCATTCAGCGTATTCTTTATTGTGATGATCCTGTTAGCCAACTCAAGTG
TTCTCAGGTTGCTTTTGACCTTGACGATGGTTTCTACCCTATTTCTTCTAGAAACCTTCTGAGTCATGAACAGCCAATT
TCTTTTGTTACTTTGCCATCATTTAATGATCATTCTTTTGTTAACATTACTGTCTCTGCGTCCTTTGGTGGTCATAGTG
GTGCCAACCTTATTGCATCTGACACTACTATCAATGGGTTTAGTTCTTTCTGTGTTGACACTAGACAATTTACCATTTC
ACTGTTTTATAACGTTACAAACAGTTATGGTTATGTGTCTAAATCACAGGACAGTAATTGCCCTTTCACCTTGCAATCT
GTTAATGATTATCTGTCTTTTAGTAAATTTTGTGTTTCCACCAGCCTTTTGGCTAGTGCTTGTACCATAGATCTTTTTG
GTTACCCTGAGTTTGGTAGTGGTGTTAAGTTTACGTCCCTTTACTTTCAATTCACAAAGGGTGAGTTGATTACTGGCAC
GCCTAAACCACTTGAAGGTGTCACGGACGTTTCTTCTATGACTCTGGATGTGTGTACCAAGTATACTATCTATGGCTTT
AAAGGTGAGGGTATCATTACCCTTACAAATTCTAGCTTTTTGGCAGGTGTTTATTACACATCTGATTCTGGACAGTTGT
TAGCCTTTAAGAATGTCACTAGTGGTGCTGTTTATTCTGTTACGCCATGTTCTTTTTCAGAGCAGGCTGCATATGTTGA
TGATGATATAGTGGGTGTTATTTCTAGTTTGTCTAGCTCCACTTTTAACAGTACTAGGGAGTTGCCTGGTTTCTTCTAC
CATTCTAATGATGGCTCTAATTGTACAGAGCCTGTGTTGGTGTATAGTAACATAGGTGTTTGTAAATCTGGCAGTATTG
GCTACGTCCCATCTCAGTCTGGCCAAGTCAAGATTGCACCCACGGTTACTGGGAATATTAGTATTCCCACCAACTTTAG
TATGAGTATTAGGACAGAATATTTACAGCTTTACAACACGCCTGTTAGTGTTGATTGTGCCACATATGTTTGTAATGGT
AACTCTCGTTGTAAACAATTACTCACCCAGTACACTGCAGCATGTAAGACCATAGAGTCAGCATTACAACTCAGCGCTA

GGCTTGAGTCTGTTGAAGTTAACTCTATGCTTACTATTTCTGAAGAGGCTCTACAGTTAGCTACCATCAGTTCGTTTAA
TGGTGATGGATATAATTTTACTAATGTGCTGGGTGTTTCTGTGTATGATCCTGCAAGTGGCAGGGTGGTACAAAAAAGG
TCTTTTATTGAAGACCTGCTTTTTAATAAAGTGGTTACTAATGGCCTTGGTACTGTTGATGAAGACTATAAGCGCTGTT
CTAATGGTCGCTCTGTGGCAGATCTAGTCTGTGCACAGTATTACTCTGGTGTCATGGTACTACCTGGTGTTGTTGACGC
TGAGAAGCTTCACATGTATAGTGCGTCTCTCATCGGTGGTATGGTGCTAGGAGGTTTTACTTCTGCAGCGGCATTGCCT
TTTAGCTATGCTGTTCAAGCTAGACTCAATTATCTTGCTCTACAGACGGATGTTCTACAGCGGAACCAGCAATTGCTTG
CTGAGTCTTTTAACTCTGCTATTGGTAATATAACTTCAGCCTTTGAGAGTGTTAAAGAGGCTATTAGTCAAACTTCCAA
GGGTTTGAACACTGTGGCTCATGCGCTTACTAAGGTTCAAGAGGTTGTTAACTCGCAGGGTGCAGCTTTGACTCAACTT
ACCGTACAGCTGCAACACAACTTCCAAGCCATTTCTAGTTCTATTGATGACATTTACTCTCGACTGGACATTCTTTCAG
CCGATGTTCAGGTTGACCGTCTCATCACCGGCAGATTATCAGCACTTAATGCTTTTGTTGCTCAAACCCTCACTAAGTA
TACTGAGGTTCAGGCTAGCAGGAAGCTAGCACAGCAAAAGGTTAATGAGTGCGTTAAATCGCAATCTCAGCGTTATGGT
TTTTGTGGTGGTGATGGCGAGCACATTTTCTCTCTGGTACAGGCAGCACCTCAGGGCCTGCTGTTTTTACATACAGTAC
TTGTACCGGGTGACTTTGTAGATGTTATTGCCATCGCTGGCTTATGCGTTAACGATGAAATTGCCTTGACTCTACGTGA
GCCTGGCTTAGTCTTGTTTACGCATGAACTTCAAAATCATACTGCGACGGAATATTTTGTTTCATCGCGACGTATGTTT
GAACCTAGAAAACCTACCGTTAGTGATTTTGTTCAAATTGAGAGTTGTGTGGTCACCTATGTCAATTTGACTAGAGACC
AACTACCAGATGTAATCCCAGATTACATCGATGTTAACAAAACACTTGATGAGATTTTAGCTTCTCTGCCCAATAGAAC
TGGTCCAAGTCTTCCTTTAGATGTTTTTAATGCCACTTATCTTAATCTCACTGGTGAAATTGCAGATTTAGAGCAGCGT
TCAGAGTCTCTCCGTAATACTACAGAGGAGCTCCAAAGTCTTATATATAATATCAACAACACACTAGTTGACCTTGAGT
GGCTCAACCGAGTTGAGACATATATCAAGTGGCCGTGGTGGGTTTGGTTGATTATTTTCATTGTTCTCATCTTTGTTGT
GTCATTACTAGTGTTCTGCTGCATTTCCACTGGTTGTTGTGGATGCTGCGGCTGCTGCTGTGCTTGTTTTTCAGGTTGT
TGTAGGGGTCCTAGACTTCAACCTTACGAAGTTTTTGAAAAGGTCCACGTGCAGTGA

>gi|409034597|gb|JX489155.1| Porcine epidemic diarrhea virus strain LC, complete genome

ATGAAGTCTTTAACCTACTTCTGGTTGTTCTTACCAGTACTTTCAACACTTAGCCTACCACAAGATGTCACCAGG
TGCTCAGCTAACACTAATTTTAGGCGGTTCTTTTCAAAATTTAATGTTCAGGCGCCTGCAGTTGTTGTACTGGGCGGTT
ATCTACCTATTGGTGAAAACCAGGGTGTCAATTCAACTTGGTACTGTGCTGGCCAACATCCAACTGCTAGTGGCGTTCA
TGGTATCTTTCTTAGCCATATTAGAGGTGGTCATGGCTTTGAGATTGGCATTTCGCAAGAGCCTTTTGACCCTAGTGGT
TACCAGCTTTATTTACATAAGGCTACTAACGGTAACACTAATGCTACTGCGCGACTGCGCATTTGCCAGTTTCCTAGCA
TTAAAACATTGGGCCCCACTGCTAATAATGATGTTACAACAGGTCGTAACTGCCTATTTAACAAAGCCATCCCAGCTCA
TATGAGTGAACATAGTGTTGTCGGCATAACATGGGATAATGATCGTGTCACTGTCTTTTCTGACAAGATCTATTATTTT
TATTTTAAAAATGATTGGTCCCGTGTTGCGACAAAGTGTTACAACAGTGGAGGTTGTGCTATGCAATATGTTTACGAAC
CCACCTATTACATGCTTAATGTTACTAGTGCTGGTGAGGATGGTATTTCTTATCAACCCTGTACAGCTAATTGCATTGG
TTATGCTGCCAATGTATTTGCTACTGAGCCCAATGGCCACATACCAGAAGGTTTTAGTTTTAATAATTGGTTTCTTTTG
TCCAATGATTCCACTTTGGTGCATGGTAAGGTGGTTTCCAACCAACCATTGTTGGTCAATTGTCTTTTGGCCATTCCTA
AGATTTATGGACTAGGCCAATTTTTCTCCTTTAATCAAACGATCGATGGTGTTTGTAATGGAGCTGCTGTGCAGCGTGC
ACCAGAGGCTCTGAGGTTTAATATTAATGACACCTCTGTCATTCTTGCTGAAGGCTCAATTGTACTTCATACTGCTTTA
GGAACAAATTTTTCTTTTGTTTGCAGTAATTCCTCAGATCCTCACTTAGCCACCTTCGCCATACCTCTGGGTGCTATCC
AAGTACCCTATTATTGTTTTCTTAAAGTGGATACTTACAACTCCACTGTTTATAAATTCTTGGCTGTTTTACCTCCTAC
CGTCAGGGAAATTGTCATCACCAAGTATGGTGATGTTTATGTCAATGGGTTTGGCTACTTGCATCTTGGTTTGTTGGAT
GCTGTCACAGTTAATTTCACTGGTCATGGCACTGACGATGACGTTTCTGGTTTTTGGACCATAGCATCGACTAATTTTG
TTGATGCACTTATCGAAGTTCAAGGAACTGCCATTCAGCGTATTCTTTATTGTGATGATCCTGTTAGCCAACTCAAGTG

TTCTCAGGTTTCATTTGACCTTGACGATGGTTTTTACCCCATTTCCTCTACAAACCTTCTGAGTCATGAACAGCCAACT
TCTTTTGTTACTTTGCCATCATTTAATGATCATTCTTTTGTTAATATTACTGTCTCTGCTGCTTTTGGTGGTCATAGTG
GTGCCAACCTTATTGCATCTGACACTACTATCAATGGGTTTAGTTCTTTCTGTGTTGACACTAGACAATTTACCATTTC
ACTGTTTTATAATGTTACAAACAGTTATGGTTATGTGTCTAAATCACAGGACAGTAATTGCCCTTTCACCTTGCAATCT
GTTAATGATTACCTGTCTTTTAGCAAATTTTGTGTTTCTACCAGCCTTTTGGCTAGTGCCTGTACCATAGATCTCTTTG
GTTACCCTGAGTTTGGTAGTGGTGTTAAGTTCACGTCCCTTTACTTTCAATTCACAAAGGGTGAGTTGATTACTGGCAC
GCCTAAACCACTTGAAGGTGTTACGGACGTTTCTTTTATGACTCTGGATGTGTGTACCAAGTATACTATCTATGGCTTT
AAAGGTGAGGGTATCATTACCCTTACAAATTCTAGCTTTTTGGCAGGTTTTTATTATACATCTGATTCTGGACAGTTGT
TAGCTTTTAAGAATGTCACTAGTGGTGCTGTTTATTCTGTTACGCCATGTTCTTTTTCAGAGCAGGCTGCATATGTTGA
TGATGATATAGTGGGTGTTATTTCTAGTTTGTCTAGCTCCACTTTTAACAGTACTAGGGAGTTGCCTGGTTTCTTCTAC
CATTCTAATGATGGCTCTAATTGTACAGAGCCTGTGTTGGTGTATAGTAACATAGGTGTTTGTAAATCTGGCAGTATTG
GCTACGTCCCATCTCAGTCTGGCCAAGTCAAGATTGCACCCACGGTTACTGGGAATATTAGTATTCCCACCAACTTTAG
TATGAGTATTAGGACAGAATATTTACAGCTTTACAACACGCCTGTTAGTGTTGATTGTGCTACATATGTTTGTAATGGT
AACTCTCGTTGTAAACAATTACTCACCCAGTACACTGCAGCATGTAAGACCATAGAGTCAGCATTACAACTCAGCGCTA
GGCTTGAGTCTGCTGAAGTCAACTCTATGCTTACTATTTCTGAAGAGGCTCTACAGTTAGCTACCATCAGTTCGTTTAA
TGGTGATGGATATAATTTTACTAATGTGCTGGGTGTTTCCGTGTATGACCCTGCAAGTGGCAGGGTGGTACAAAAAAGG
TCTTTCATTGAAGACCTGCTTTTTAATAAAGTGGTTACTAATGGCCTTGGTACTGTTGATGAAGACTATAAGCGCTGTT
CTAATGGCCGCTCTGTGGCAGATTTAGTCTGTGCGCAGTATTACTCTGGTGTCATGGTACTACCTGGTGTTGTTGACGC
TGAGAAGCTTCACATGTATAGTGCGTCTCTCATCGGTGGTATGGTGCTAGGAGGTTTTACTGCAGCAGCGGCATTGCCT
TTTAGCTATGCTGTTCAAGCGAGACTCAATTATCTTGCTCTACAGACGGATGTTCTACAGCGGAACCAGCAATTGCTTG
CTGAGTCTTTTAATTCTGCTATTGGTAATATAACTTCAGCCTTTGAGAGTGTTAAAGAGGCTATTAGTCAAACTTCTAA
GGGTTTGAACACTGTGGCTCATGCGCTTACTAAGGTCCAAGAGGTTGTTAATTCGCAGGGTGCAGCTTTGACCCAACTT
ACCGTACAGCTGCAACACAACTTCCAAGCCATTTCTAGTTCTATTGATGACATTTACTCCCGACTTGACATTCTTTCAG
CCGATGTTCAGGTAGACCGTCTCATCACCGGCAGATTATCAGCACTTAATGCTTTTGTTGCTCAAACCCTTACTAAGTA
TACTGAGGTTCAGGCTAGCAGGAAACTAGCACAGCAAAAGGTTAATGAGTGCGTTAAATCGCAATCTCAGCGTTACGGT
TTTTGTGGTGGTGATGGCGAGCACATTTTCTCTCTGGTACAGGCCGCACCTCAAGGCCTGCTGTTCTTACACACAGTAC
TTGTACCGGGTGATTTTGTAAATGTTATTGCCATCGCTGGCTTATGTGTTAACGATGAAATTGCCCTGACTCTACGTGA
GCCTGGCTTAGTCTTGTTTACGCATGAACTTCAAAATACTGCGACGGAATATTTTGTTTCATCGCGACGTATGTATGAA
CCTAGAAAACCTACCGTTGGTGATTTTGTTCAAATTGAGAGTTGTGTGGTCACCTATGTCAATTTGACTAGAGACCAAC
TACCAGATGTAATCCCAGATTACATCGATGTTAACAAAACACTTGATGAGATTTTAGCTTCTCTGCCCAATAGAACTGG
TCCAAGTCTTTCTCTAGATGTTTTTAATGCCACTTATCTTAATCTTACTGGTGAAATTGCAGATTTAGAGCAGCGTTCA
GAGTCTCTCCGTAATACTACAGAAGAGCTTCAAAGTCTTATATATAATATCAACAACACACTAGTTGACCTTGAGTGGC
TCAACCGAGTTGAGACATATATCAAGTGGCCGTGGTGGGTTTGGTTGATTATTTTTATTGTTCTCATCTTTGTTGTGTC
ATTACTAGTGTTCTGCTGCATTTCCACGGGTTGTTGTGGATGCTGCGGCTGCTGTGGTGCTTGTTTTTCAGGTTGTTGT
AGGGGTCCTAGACTTCAACCTTACGAAGCTTTTGAAAAGGTCCACGTGCAGTGA

>gi|410072477|gb|JX501317.1|_Porcine_epidemic_diarrhea_virus_isolate_CH/CY/12_spike_glycoprotein_gene_complete_cds

ATGAAGTCTTTAACCTACTTCTGGTTGTTCTTACCAGTACTTTCAACACTTAGCCTACCACAAGATGTCACCAGG
TGCTCAGCTAGCACTAATTTTAGGCGGTTCTTTTCAAAATTTAATGTTCAGGCGCCTGCAGTTGTTGTACTGGGCGGTT
ATCTTCCTATTGGTGAAAACCAGGGTTTCAATTCAACTTGGTACTGTGCTGGCCAACATCCAACTGCTAGTGGCGTTCA
TGGTATCTTTCTTAGCCATATTAGAGGTGGTCATGGCTTTGAGATTGGCATTTCGCAAGAGCCTTTTGACCCTAGTGGT

TACCAGCTTTATTTACATAAGGCTACTAATGGTAACACTAATGCTACTGCGCGATTGCGCATTTGCCAGTTTCCCAGCA
TTAAAACATTGGGCCCCACTGCTAATAATGATGTTACAACAGGTCGTAACTGCCTATTTAACAAAGCCATCCCAGCTCA
TATGAGTGAACATAGTGTTGTCGGCATAACATGGGATAATGATCGTGTCACTGTCTTTTCTGACAAGATCTATCATTTT
TATTTTAAAAATGATTGGTCCCGTGTTGCGACAAAGTGTTACAACAGTGGAGGTTGTGCTACGCAATATGTTTACGAAC
CCACTTACTACACGCTTAATGTTACTAGTGCTGGTGAGGATGGTATTTCTTATCAACCCTGTACAGCTAATTGCATTGG
TTATGCTGCCAATGTATTTGCTACTGAGCCCAATGGCCACATACCAGAAGGTTTTAGTTTTAATAATTGGTTTCTTTTG
TCCAATGATTCCACTTTGGTGCATGGTAAGGTGGTTTCCAACCAACCATTGTTGGTCAATTGTCTTTTGGCCATGCCTA
AGATTTATGGACTAGGCCAATTTTTCTCATTCAATCAAACGATGGATGGCGTTTGTAATGGAGCTGCTGCGCAGCGTGC
ACCAGAGGCTCTGAGGTTTAATATTAATGACACCTCTGTCATTCTTGCTGAAGGCTCAATTGTACTTCATACTGCTTTA
GGAACAAATCTTTCCTTTGTTTGCAGTAATTCCTCAGATCCTCATTTAGCCACCTTCGCCATACCTCTGGGTGCTACCC
AAGTACCCTATTATTGTTTTCTTAAAGTGGATACTTATAATTCCACTGTTTATAAATTCTTGGCTGTTTTACCTCCTAC
CGTCAGGGAAATTGTCATCACCAAGTATGGTGATGTTTATGTCAATGGGTTTGGCTACTTGCATCTCGGTTTATTGGAT
GCTGTCACAATTAACTTCACTGGTCATGGCACTGACGATGACGTTTCTGGTTTTTGGACCATAGCATCGACTAATTTTG
TTGATGCACTCATCGAAGTTCAAGGAACTGCCATTCAGCGTATTCTTTATTGTGATGATCCTGTTAGCCAACTCAAGTG
TTCTCAAGTTGCTTTCGACCTTGACGATGGTTTTTACCCTATTTCTTCTAGAAACCTTCTGAGTCATGAACAGCCAATT
TCTTTTGTTACTTTGCCATCATTTAATGATCATTCTTTTGTTAACATTACTGTCTCTGCGTCCTTTGGTGGTCATAGTG
GTGCCAACCTTATTTCATCTGACACTACTATCAATGGGTTTAGTTCTTTCTGTGTTGACACTAGACAATTTACCATTTC
ACTGTTTTATAACGTTACAAACAGTTATGGTTATGTGTCTAAATCACAGGACAGTAATTGCCCTTTCACCTTACAATCT
GTTAATGATTACCTGTCTTTTAGCAAATTTTGTGTTTCCACCAGCCTTTTGGCTAGTGCCTGTACCATAGATCTTTTTG
GTTACCCTGAGTTTGGTAGTGGTGTTAAGTTTACGTCCCTTTACTTTCAATTCACAAAGGGTGAGTTGATTACTGGCAC
GCCTAAACCACTTGAAGGTGTCACGGACGTTTCTTCTATGACTCTGGATGTGTGTACCAAGTATACTATCTATGGCTTT
AAAGGTGAGGGTATCATTACCCTTACAAATTCTAGCTTTTTGGCAGGTGTTTATTACACATCTGATTCTGGACAGTTGT
TAGCCTTTAAGAATGTCACTAGTGGTGCTGTTTATTCTGTTACGCCATGTTCTTTTTCAGAGCAGGCTGCATATGTTGA
TGATGTTATAGTGGGTGTTATTTCTAGTTTGTCTAGCTCCACTTTTAACAGTACTAGGGAGTTGCCTGGTTTCTTCTAC
CATTCTAATGATGGCTCTAATTGTACAGAGCCTGTGTTGGTGTATAGTAACATAGGTGTTTGTAAATCTGGCAGTATTG
GCTACGTCCCATCTCAGTCTGGCCAAGTCAAGATTGCACCCACGGTTACTGGGAATATTAGTATTCCCACCAACTTTAG
TATGAGTATTAGGACAGAATATTTACAGCTTTACAACACGCCTGTTAGTGTTGATTGTGCCACATATGTTTGTAATGGT
AACTCTCGTTGTAAACAATTACTCACCCAGTACACTGCAGCATGTAAGACCATAGAGTCAGCATTACAACTCAGCGCTA
GGCTTGAGTCTGTTGAAGTTAACTCTATGCTTACTATTTCTGAAGAGGCTCTACAGTTAGCTACCATCAGTTCGTTTAA
TGGTGATGGATATAATTTTACTAATGTGCTGGGTGTTTCTGTGTATGATCCCGCAAGTGGCAGGGTGGTACAAAAAAGG
TCTTTTATTGAAGACCTGCTTTTTAATAAAGTGGTTACTAATGGCCTTGGTACTGTTGACGAAGACTATAAGCGCTGTT
CTAATGGTCGCTCTGTGGCAGATCTAGTCTGTGCACAGTATTACTCTGGTGTCATGGTACTACCTGGTGTTGTTGACGC
TGAGAAGCTTCACATGTATAGTGCGTCTCTCATCGGTGGTATGGTGCTAGGAGGTTTTACTTCTGCAGCGGCATTGCCT
TTTAGCTATGCTGTTCAAGCTAGACTCAATTATCTTGCTCTACAGACGGATGTTCTACAGCGGAACCAGCAATTGCTTG
CTGAGTCTTTTAACTCTGCTATTGGTAATATAACTTCAGCCTTTGAGAGTGTTAAAGAGGCTATTAGTCAAACTTCCAA
GGGTTTGAACACTGTGGCTCATGCGCTTACTAAGGTTCAAGAGGTTGTTAACTCGCAGGGTGCAGCTTTGACTCAACTT
ACCGTACAGCTGCAACACAACTTCCAAGCCATTTCTAGTTCTATTGATGACATTTACTCCCGACTGGACATTCTTTCAG
CCGATGTTCAGGTTGACCGTCTCATCACCGGCAGATTATCAGCACTTAATGCTTTTGTTGCTCAAACCCTCACTAAGTA
TACTGAGGTTCAGGCTAGCAGGAAGCTAGCACAGCAAAAGGTTAATGAGTGCGTTAAATCGCAATCTCAGCGTTATGGT
TTTTGTGGTGGTGATGGCGAGCACATTTTCTCTCTGGTACAGGCAGCACCTCAGGGCCTGCTGTTTTTACATACAGTAC
TTGTACCGGGTGATTTTGTAGATGTTATTGCCATCGCTGGCTTATGCGTTAACGATGAAATTGCCTTGACTCTACGTGA
GCCTGGCTTAGTCTTGTTTACGCATGAACTTCAAAATCATACTGCGACGGAATATTTTGTTTCATCGCGACGTATGTTT
GAACCTAGAAAACCTACCGTTAGTGATTTTGTTCAAATTGAGAGTTGTGTGGTCACCTATGTCAATTTGACTAGAGACC
AACTACCAGATGTAATCCCAGATTACATCGATGTTAACAAAACACTTGATGAGATTCTAGCTTCTCTGCCCAATAGAAC

TGGTCCAAGTCTTTCTTTAGATGTTTTTAATGCCACTTATCTTAATCTCACTGGTGAAATTGCAGATTTAGAGCAGCGT
TCAGAGTCTCTCCGTAATACTACAGAGGAGCTCCAAAGTCTTATATATAATATCAACAACACACTAGTTGACCTTGAGT
GGCTCAACCGAGTTGAGACATATATCAAGTGGCCGTGGTGGGTTTGGTTGATTATTTTCATTGTTCTCATCTTTGTTGT
GTCATTACTAGTGTTCTGCTGCATTTCCACGGGTTGTTGTGGATGCTGCGGCTGCTGCTGTGCTTGTTTTTCAGGTTGT
TGTAGGGGTCCTAGACTTCAACCTTACGAAGTTTTTGAAAAGGTCCACGTGCAGTGA

>gi|410072479|gb|JX501318.1|_Porcine_epidemic_diarrhea_virus_isolate_CH/HBQX/10_spike_glycoprotein_gene_complete_cds

ATGAAGTCTTTAAATTACTTCTGGTTGTTCTTACCATTACTTTCAACACTCAGCCTACCACAAGATGTCACTAGG
TGCCAGTCCACTATTAACTTCAGGCGGTTCTTTTCAAAATTTAATGTGCAGGCACCTGCTGTCGTTGTGTTGGGTGGTT
ATCTACCTAGTATGAACTCCTCTAGCTGGTACTGTGGCACAGGTCTTGAAACTGCTAGTGGCGTGCATGGTATTTTCCT
CAGTTACATCGATGCTGGTCAGGGCTTTGAGATTGGCATTTCACAGGAGCCGTTTGATCCTAGTGGTTACCAGCTTTAT
TTACATAAGGCCACTAATGGTAACCATAATGCTATTGCACGACTGCGCATTTGCCAGTTTCCAGATAATAAAACATTGG
GCCCTACTGTTAATGATGTTACAACAGGTCGTAACTGCCTATTCAATAAAGCCATTCCAGCTTATATGCAGGATGGAAA
AAATATCGTTGTCGGCATAACATGGGACAATGATCGTGTCACTGTTTTTGCTGACAAGATCTATCATTTTTATCTTAAA
AATGATTGGTCCCGTGTCGCGACAAGATGTTACAATAAAAGAAGTTGTGCTATGCAATATGTTTATACACCTACCTACT
ACATGCTTAATGTTACTAGTGCAGGTGAGGATGGCATTTATTATGAACCATGTACAGCTAATTGCAGTGGTTACGCTGC
CAATGTGTTTGCCACTGATTCTAATGGCCACATACCAGAAGGTTTTAGTTTTAATAATTGGTTTCTTTTGTCCAATGAT
TCCACTTTGTTGCATGGTAAGGTGGTTTCCAACCAACCTTTGTTGGTCAATTGTCTTTTGGCCATTCCTAAGATTTATG
GACTAGGCCAATTTTTCTCATTCAATCAAACGATGGATGGCGTTTGTAATGGAGCTGCTGCGCAGCGTGCACCAGAGGC
TCTGAGGTTTAATATTAATGACACCTCTGTCATTCTTGCTGAAGGCTCAATTGTACTTCATACTGCTTTAGGAACAAAT
CTTTCTTTTGTTTGCAGTAATTCTTCAGATCCTCATTTAGCTACCTTCGCCATACCTCTGGGTGCTACCCAAGTACCCT
ATTATTGTTTTCTTAAAGTGGATACTTACAACTCCACTGTTTATAAATTTTTGGCTGTTTTACCTCCTACCGTCAGGGA
AATTGTCATCACCAAGTATGGTGATGTTTATGTCAATGGGTTTGGATACTTGCATCTCGGTTTGTTGGATGCTGTCACA
ATTAATTTCACTGGTCATGGCACTGACGATGATGTTTCTGGTTTTTGGACCATAGCATCGACTAATTTTGTTGATGCAC
TCATCGAAGTTCAAGGAACTGCCATTCAGCGTATTCTTTATTGTGATGATCCTGTTAGCCAACTCAAGTGTTCTCAGGT
TGCTTTTGACCTTGACGATGGTTTTTACCCTATTTCTTCTAGAAACCTTCTGAGTCATGAACAGCCAATTTCTTTTGTT
ACTCTGCCATCATTTAATGATCATTCTTTTGTTAACATTACTGTCTCTGCTTCCTTTGGTGGTCATAGTGGTGCCAACC
TTATTGCATCTGACACTACTATCAATGGGTTTAGTTCTTTCTGTGTTGACACTAGACAATTTACCATTTCACTGTTTTA
TAACGTTACAAACAGTTATGGTTATGTGTCTAAATCACAGGACAGTAATTGCCCTTTCACCTTGCAATCTGTTAATGAT
TACCTGTCTTTTAGCAAATTTTGTGTTTCCACCAGCCTTTTGGCTAGTGCCTGTACCATAGATCTTTTTGGTTACCCTG
AGTTTGGTAGTGGTGTTAAGTTTACGTCCCTTTACTTTCAATTCACAAAGGGTGAGTTGATTACTGGCACGCCTAAACC
ACTTGAAGGTGTCACGGACGTTTCTTCTATGACTCTGGATGTGTGTACCAAGTATACTATCTATGGCTTTAAAGGTGAG
GGTATCATTACCCTTACAAATTCTAGCTTTTTGGCAGGTGTTTATCACACATCTGATTCTGGACAGTTGTTAGCCTTTA
AGAATGTCACTAGTGGTGCTGTTTATTCTGTTACGCCATGTTCTTTTTCAGAGCAGGCTGCATATGTTGATGATGTTAT
AGTGGGTGTTATTTCTAGTTTGTCTAGCTCCACTTTTAACAGTACTAGGGAGTTGCCTGGTTTCTTCTACCATTCTAAT
GATGGCTCTAATTGTACAGAGCCTGTGTTGGTGTATAGTAACATAGGTGTTTGTAAATCTGGCAGTATTGGCTACGTCC
CATCTCAGTCTGGCCAAGTCAAGATTGCACCCACGGTTACTGGGAATATTAGTATTCCCACCAACTTTAGTATGAGTAT
TAGGACAGAATATTTACAGCTTTACAACACGCCTGTTAGTGTTGATTGTGCCACATATGTTTGTAATGGTAACTCTCGT
TGTAAACAATTACTCACCCAGTACACTGCAGCATGTAAGACCATAGAGTCAGCATTACAACTCAGCGCTAGGCTTGAGT
CTGTTGAAGTTAACTCTATGCTTACTATTTCTGAAGAGGCTCTACAGTTAGCTACCATCAGTTCGTTTAATGGTGATGG
ATATAATTTTACTAATGTGCTGGGTGTTTCTGTGTATGATCCTGCAAGTGGCAGGGTGGTACAAAAAAGGTCTTTTATT

GAAGACCTGCTTTTTAATAAAGTGGTTACTAATGGCCTTGGTACTGTTGATGAAGACTATAAGCGCTGTTCTAATGGTC
GCTCTGTGGCAGATCTAGTCTGTGCACAGTATTACTCTGGTGTCATGGTACTACCTGGTGTTGTTGACGCTGAGAAGCT
TCACATGTATAGTGCGTCTCTCATCGGTGGTATGGTGCTAGGAGGTTTTACTTCTGCAGCGGCATTGCCTTTTAGCTAT
GCTGTTCAAGCTAGACTCAATTATCTTGCTCTACAGACGGATGTTCTACAGCGGAACCAGCAATTGCTTGCTGAGTCTT
TTAACTCTGCTATTGGTAATATAACTTCAGCCTTTGAGAGTGTTAAAGAGGCTATTAGTCAAACTTCCAAGGGTTTGAA
CACTGTGGCTCATGCGCTTACTAAGGTTCAAGAGGTTGTTAACTCGCAGGGTGCAGCTTTGACTCAACTTACCGTACAG
CTGCAACACAACTTCCAAGCCATTTCTAGTTCTATTGATGACATTTACTCTCGACTGGACATTCTTTCAGCCGATGTTC
AGGTTGACCGTCTCATCACCGGCAGATTATCAGCACTTAATGCTTTTGTTGCTCAAACCCTCACTAAGTATACTGAGGT
TCAGGCTAGCAGGAAGCTAGCACAGCAAAAGGTTAATGAGTGCGTTAAATCGCAATCTCAGCGTTATGGTTTTTGTGGT
GGTGATGGCGAGCACATTTTCTCTCTGGTACAGGCAGCACCTCAGGGCCTGCTGTTTTTACATACAGTACTTGTACCGG
GTGATTTTGTAGATGTTATTGCCATCGCTGGCTTATGCGTTAACGATGAAATTGCCTTGACTCTACGTGAGCCTGGCTT
AGTCTTGTTTACGCATGAACTTCAAAATCATACTGCGACGGAATATTTTGTTTCATCGCGACGTATGTTTGAACCTAGA
AAACCTACCGTTAGTGATTTTGTTCAAATTGAGAGTTGTGTGGTCACCTATGTCAATTTGACTAGAGACCAACTACCAG
ATGTAATCCCAGATTACATCGATGTTAACAAAACACTTGATGAGATTTTAGCTTCTCTGCCCAATAGAACTGGTCCAAG
TCTTCCTTTAGATGTTTTTAATGCCACTTATCTTAATCTCACTGGTGAAATTGCAGATTTAGAGCAGCGTTCAGAGTCT
CTCCGTAATACTACAGAGGAGCTCCAAAGTCTTATATATAATATCAACAACACACTAGTTGACCTTGAGTGGCTCAACC
GAGTTGAGACATATATCAAGTGGCCGTGGTGGGTTTGGTTGATTATTTTCATTGTTCTCATCTTTGTTGTGTCATTACT
AGTGTTCTGCTGCATTTCCACGGGTTGTTGTGGATGCTGCGGCTGCTGCTGTGCTTGTTTTTCAGGTTGTTGTAGGGGT
CCTAGACTTCAACCTTACGAAGTTTTTGAAAAGGTCCACGTGCAGTGA

>gi|410072481|gb|JX501319.1|_Porcine_epidemic_diarrhea_virus_isolate_CH/HBXX2/11_spike_glycoprotein_gene_complete_cds

ATGAAGTCTTTAACCTACTTCTGGTTGTTCTTACCAGTACTTTCAACACTTAGCCTACCACAAGATGTCACCAGG
TGCTCAGCTAACACTAATTTTAGGCGGTTCTTTTCAAAATTTAATGTTCAGGCGCCTGCAGTTGTTGTACTGGGCGGTT
ATCTACCTATTGGTGAAAACCAGGGTGTCAATTCAACTTGGTACTGTGCTGGCCAACATCCAACTGCTAGTGGCGTTCA
TGGTATCTTTCTTAGCCATATTAGAGGTGGTCATGGCTTTGAGATTGGCATTTCGCAAGAGCCTTTTGACCCTAGTGGT
TACCAGCTTTATTTACATAAGGCTACTAATGGTAACACTAATGCTACTGCGCGATTGCGCATTTGCCAGTTTCCCAGCA
TTAAAACATTGGGCCCCACTGCTAATAATGATGTTACAACAGGTCGTAACTGCCTATTTAACAAAGCCATCCCAGCTCA
TATGAGTGAACATAGTGTTGTCGGCATAACATGGGATAATGATCGTGTCACTGTCTTTTCTGACAAGATCTATCATTTT
TATTTTAAAAATGATTGGTCCCGTGTTGCGACAAAGTGTTACAACAGTGGAGGTTGTGCTACGCAATATGTTTACGAAC
CCACTTACTACATGCTTAATGTTACTAGTGCTGGTGAGGATGGTATTTCTTATCAACCCTGTACAGCTAATTGCATTGG
TTATGCTGCCAATGTATTTGCTACTGAGCCCAATGGCCACATACCGGAAGGTTTTAGTTTTAATAATTGGTTTCTTTTG
TCCAATGATTCCACTTTGGTGCATGGTAAGGTGGTTTCCAACCAACCATTGTTGGTCAATTGTCTTTTGGCCATGCCTA
AGATTTATGGACTAGGCCAATTTTTCTCATTCAATCAAACGATGGATGGTGTTTGTAATGGAGCTGCTGCGCAGCGTGC
ACCAGAGGCTCTGAGGTTTAATATTAATGACACCTCTGTCATTCTTGCTGAAGGCTCAATTGTACTTCATACTGCTTTA
GGAACAAATCTTTCCTTTGTTTGCAGTAATTCCTCAGATCCTCATTTAGCCACCTTCGCCATACCTCTGGGTGCTACCC
AAGTACCCTATTATTGTTTTCTTAAAGTGGATACTTATAATTCCACTGTTTATAAATTCTTGGCTGTTTTACCTCCTAC
CGTCAGGGAAATTGTCATCACCAAGTATGGTGATGTTTATGTCAATGGGTTTGGCTACTTGCATCTCGGTTTATTGGAT
GCTGTCACAATTAACTTCACTGGTCATGGCACTGACGATGACGTTTCTGGTTTTTGGACCATAGCATCGACTAATTTTG
TTGATGCACTCATCGAAGTTCAAGGAACTGCCATTCAGCGTATTCTTTATTGTGATGATCCTGTTAGCCAACTCAAGTG
TTCTCAAGTTGCTTTCGACCTTGACGATGGTTTTTACCCTATTTCTTCTAGAAACCTTCTGAGTCATGAACAGCCAATT
TCTTTTGTTACTTTGCCATCATTTAATGATCATTCTTTTGTTAACATTACTGTCTCTGCGTCCTTTGGTGGTCATAGTG

GTGCCAACCTTATTTCATCTGACACTACTATCAATGGGTTTAGTTCTTTCTGTGTTGACACTAGACAATTTACCATTTC
ACTGTTTTATAACGTTACAAACAGTTATGGTTATGTGTCTAAATCACAGGACAGTAATTGCCCTTTCACCTTGCAATCT
GTTAATGATTACCTGTCTTTTAGCAAATTTTGTGTTTCCACCAGCCTTTTGGCTAGTGCCTGTACCATAGATCTTTTTG
GTTACCCTGAGTTTGGTAGTGGTGTTAAGTTTACGTCCCTTTACTTTCAATTCACAAAGGGTGAGTTGATTACTGGCAC
GCCTAAACCACTTGAAGGTGTCACGGACGTTTCTTCTATGACTCTGGATGTGTGTACCAAGTATACTATCTATGGCTTT
AAAGGTGAGGGTATCATTACCCTTACAAATTCTAGCTTTTTGGCAGGTGTTTATTACACATCTGATTCTGGACAGTTGT
TAGCCTTTAAGAATGTCACTAGTGGTGCTGTTTATTCTGTTACGCCATGTTCTTTTTCAGAGCAGGCTGCATATGTTGA
TGATGTTATAGTGGGTGTTATTTCTAGTTTGTCTAGCTCCACTTTTAACAGTACTAGGGAGTTGCCTGGTTTCTTCTAC
CATTCTAATGATGGCTCTAATTGTACAGAGCCTGTGTTGGTGTATAGTAACATAGGTGTTTGTAAATCTGGCAGTATTG
GCTACGTCCCATCTCAGTCTGGCCAAGTCAAGATTGCACCCACGGTTACTGGGAATATTAGTATTCCCACCAACTTTAG
TATGAGTATTAGGACAGAATATTTACAGCTTTACAACACGCCTGTTAGTGTTGATTGTGCCACATATGTTTGTAATGGT
AACTCTCGTTGTAAACAATTACTCACCCAGTACACTGCAGCATGTAAGACCATAGAGTCAGCATTACAACTCAGCGCTA
GGCTTGAGTCTGTTGAAGTTAACTCTATGCTTACTATTTCTGAAGAGGCTCTACAGTTAGCTACCATCAGTTCGTTTAA
TGGTGATGGATATAATTTTACTAATGTGCTGGGTGTTTCTGTGTATGATCCTGCAAGTGGCAGGGTGGCACAAAAAAGG
TCTTTTATTGAAGACCTGCTTTTTAATAAAGTGGTTACTAATGGCCTTGGTACTGTTGATGAAGACTATAAGCGCTGTT
CTAATGGTCGCTCTGTGGCAGATCTAGTCTGTGCACAGTATTACTCTGGTGTCATGGTACTACCTGGTGTTGTTGACGC
TGAGAAGCTTCACATGTATAGTGCGTCTCTCATCGGTGGTATGGTGCTAGGAGGTTTTACTTCTGCAGCGGCATTGCCT
TTTAGCTATGCTGTTCAAGCTAGACTCAATTATCTTGCTCTACAGACGGATGTTCTACAGCGGAACCAGCAATTGCTTG
CTGAGTCTTTTAACTCTGCTATTGGTAATATAACTTCAGCCTTTGAGAGTGTTAAAGAGGCTATTAGTCAAACTTCCAA
GGGTTTGAACACTGTGGCTCATGCGCTTACTAAGGTTCAAGAGGTTGTTAACTCGCAGGGTGCAGCTTTGACTCAACTT
ACCGTACAGCTGCAACACAACTTCCAAGCCATTTCTAGTTCTATTGATGACATTTACTCCCGACTGGACATTCTTTCAG
CCGATGTTCAGGTTGACCGTCTCATCACCGGCAGATTATCAGCACTTAATGCTTTTGTTGCTCAAACCCTCACTAAGTA
TACTGAGGTTCAGGCTAGCAGGAAGCTAGCACAGCAAAAGGTTAATGAGTGCGTTAAATCGCAATCTCAGCGTTATGGT
TTTTGTGGTGGTGATGGCGAGCACATTTTCTCTCTGGTACAGGCAGCACCTCAGGGCCTGCTGTTTTTACATACAGTAC
TTGTACCGGGTGATTTTGTAGATGTTATTGCCATCGCTGGCTTATGCGTTAACGATGAAATTGCCTTGACTCTACGTGA
GCCTGGCTTAGTCTTGTTTACGCATGAACTTCAAAATCATACTGCGACGGAATATTTTGTTTCATCGCGACGTATGTTT
GAACCTAGAAAACCTACCGTTAGTGATTTTGTTCAAATTGAGAGTTGTGTGGTCACCTATGTCAATTTGACTAGAGACC
AACTACCAGATGTAATCCCAGATTACATCGATGTTAACAAAACACTTGATGAGATTTTAGCTTCTCTGCCCAATAGAAC
TGGTCCAAGTCTTCCTTTAGATGTTTTTAATGCCACTTATCTTAATCTCACTGGTGAAATTGCAGATTTAGAGCAGCGT
TCAGAGTCTCTCCGTAATACTACAGAGGAGCTCCAAAGTCTTATATATAATATCAACAACACACTAGTTGACCTTGAGT
GGCTCAACCGAGTTGAGACATATATCAAGTGGCCGTGGTGGGTTTGGTTGATTATTTTCATTGTTCTCATCTTTGTTGT
GTCATTACTAGTGTTCTGCTGCATTTCCACGGGTTGTTGTGGATGCTGCGGCTGCTGCTGTGCTTGTTTTTCAGGTTGT
TGTAGGGGTCCTAGACTTCAACCTTACGAAGTTTTTGAAAAGGTCCACGTGCAGTGA

>gi|410072483|gb|JX501320.1|_Porcine_epidemic_diarrhea_virus_isolate_CH/HBXX3/11_spike_glycoprotein_gene_complete_cds

ATGAAGTCTTTAACCTACTTCTGGTTGTTCTTACCAGTACTTTCAACACTTAGCCTACCACAAGATGTCACCAGG
TGCTCAGCTAACACTAATTTTAGGCGGTTCTTTTCAAAATTTAATGTTCAGGCGCCTGCAGTTGTTGTACTGGGCGGTT
ATCTACCTATTGGTGAAAACCAGGGTGTCAATTCAACTTGGTACTGTGCTGGCCAACATCCAACTGCTAGTGGCGTTCA
TGGTATCTTTCTTAGCCATATTAGAGGTGGTCATGGCTTTGAGATTGGCATTTCGCAAGAGCCTTTTGACCCTAGTGGT
TACCAGCTTTATTTACATAAGGCTACTAATGGTAACACTAATGCTACTGCGCGATTGCGCATTTGCCAGTTTCCCAGCA
TTAAAACATTGGGCCCCACTGCTAATAATGATGTTACAACAGGTCGTAACTGCCTATTTAACAAAGCCATCCCAGCTCA

TATGAGTGAACATAGTGTTGTCGGCATAACATGGGATAATGATCGTGTCACTGTCTTTTCTGACAAGATCTATCATTTT
TATTTTAAAAATGATTGGTCCCGTGTTGCGACAAAGTGTTACAACAGTGGAGGTTGTGCTACGCAATATGTTTACGAAC
CCACTTACTACATGCTTAATGTTACTAGTGCTGGTGAGGATGGTATTTCTTATCAACCCTGTACAGCTAATTGCATTGG
TTATGCTGCCAATGTATTTGCTACTGAGCCCAATGGCCACATACCAGAAGGTTTTAGTTTTAATAATTGGTTTCTTTTG
TCCAATGATTCCACTTTGGTGCATGGTAAGGTGGTTTCCAACCAACCATTGTTGGTCAATTGTCTTTTGGCCATGCCTA
AGATTTATGGACTAGGCCAATTTTTCTCATTCAATCAAACGATGGATGGCGTTTGTAATGGAGCTGCTGCGCAGCGTGC
ACCAGAGGCTCTGAGGTTTAATATTAATGACACCTCTGTCATTCTTGCTGAAGGCTCAATTGTACTTCATACTGCTTTA
GGAACAAATCTTTCCTTTGTTTGCAGTAATTCCTCAGATCCTCATTTAGCCACCTTCGCCATACCTCTGGGTGCTACCC
AAGTACCCTATTATTGTTTTCTTAAAGTGGATACTTATAATTCCACTGTTTATAAATTCTTGGCTGTTTTACCTCCTAC
CGTCAGGGAAATTGTCATCACCAAGTATGGTGATGTTTATGTCAATGGGTTTGGCTACTTGCATCTCGGTTTATTGGAT
GCTGTCACAATTAACTTCACTGGTCATGGCACTGACGATGACGTTTCTGGTTTTTGGACCATAGCATCGACTAATTTTG
TTGATGCACTCATCGAAGTTCAAGGAACTGCCATTCAGCGTATTCTTTATTGTGATGATCCTGTTAGCCAACTCAAGTG
TTCTCAAGTTGCTTTCGACCTTGACGATGGTTTTTACCCTATTTCTTCTAGAAACCTTCTGAGTCATGAACAGCCAATT
TCTTTTGTTACTTTGCCATCATTTAATGATCATTCTTTTGTTAACATTACTGTCTCTGCGTCCTTTGGTGGTCATAGTG
GTGCCAACCTTATTTCATCTGACACTACTATCAATGGGTTTAGTTCTTTCTGTGTTGACACTAGACAATTTACCATTTC
ACTGTTTTATAACGTTACAAACAGTTATGGTTATGTGTCTAAATCACAGGACAGTAATTGCCCTTTCACCTTGCAATCT
GTTAATGATTACCTGTCTTTTAGCAAATTTTGTGTTTCCACCAGCCTTTTGGCTAGTGCCTGTACCATAGATCTTTTTG
GTTACCCTGAGTTTGGTAGTGGTGTTAAGTTTACGTCCCTTTACTTTCAATTCACAAAGGGTGAGTTGATTACTGGCAC
GCCTAAACCACTTGTAGGTGTCACGGACGTTTCTTTTATGACTCTGGATGTGTGTACCAAGTATACTATCTATGGCTTT
AAAGGTGAGGGTATCATTACCCTTACAAATTCTAGCTTTTTGGCAGGTGTTTATTACACATCTGATTATGGACAGTTGT
TAGCCTTTAAGAATGTCACTAGTGGTGCTGTTTATTCTGTTACGCCATGTTCTTTTTCAGAGCAGGCTGCATATGTTGA
TGATGTTATAGTGGGTGTTATTTCTAGTTTGTCTAGCTCCACTTTTAACAGTACTAGGGAGTTGCCTGGTTTCTTCTAC
CATTCTAATGATGGCTCTAATTGTACAGAGCCTGTGTTGGTGTATAGTAACATAGGTGTTTGTAAATCTGGCAGTATTG
GCTACGTCCCATCTCAGTCTGGCCAAGTCAAGATTGCACCCACGGTTACTGGGAATATTAGTATTCCCACCAACTTTAG
TATGAGTATTAGGACAGAATATTTACAGCTTTACAACACGCCTGTTAGTGTTGATTGTGCCACATATGTTTGTAATGGT
AACCCTCGTTGTAAACAATTACTCACCCAGTACACTGCAGCATGTAAGACCATAGAGTCAGCATTACAACTCAGCGCTA
GGCTTGAGTCTGTTGAAGTTAACTCTATGCTTACTATTTCTGAAGAGGCTCTACAGTTAGCTACCATCAGTTCGTTTAA
TGGTGATGGATATAATTTTACTAATGTGCTGGGTGTTTCTGTGTATGATCCTGCGAGTGGCAGGGTGGTACAAAAAAGG
TCTTTTATTGAAGACCTGTTTTTTAATAAAGTGGTTACTAATGGCCTTGGTACTGTTGATGAAGACTATAAGCGCTGTT
CTAATGGTCGCTCTGTGGCAGATCTAGTCTGTGCACAGTATTACTCTGGTGTCATGGTACTACCTGGTGTTGTTGACGC
TGAGAAGCTTCACATGTATAGTGCGTCTCTCATCGGTGGTATGGTGCTAGGAGGTTTTACTTCTGCAGCGGCATTGCCT
TTTAGCTATGCTGTTCAAGCTAGACTCAATTATCTTGCTCTACAGACGGATGTTCTACAGCGGAACCAGCAATTGCTTG
CTGAGTCTTTTAACTCTGCTATTGGTAATATAACTTCAGCCTTTGAGAGTGTTAAAGAGGCTATTAGTCAAACTTCCAA
GGGTTTGAACACTGTGGCTCATGCGCTTACTAAGGTTCAAGAGGTTGTTAACTCGCAGGGTGCAGCTTTGACTCAACTT
ACCGTACAGCTGCAACACAACTTCCAAGCCATTTCTAGTTCTATTGATGACATTTACTCCCGACTGGACATTCTTTCAG
CCGATGTTCAGGTTGACCGTCTCATCACCGGCAGATTATCAGCACTTAATGCTTTTGTTGCTCAAACCCTCACTAAGTA
TACTGAGGTTCAGGCTAGCAGGAAGCTAGCACAGCAAAAGGTTAATGAGTGCGTTAAATCGCAATCTCAGCGTTATGGT
TTTTGTGGTGGTGATGGCGAGCACATTTTCTCTCTGGTACAGGCAGCACCTCAGGGCCTGCTGTTTTTACATACAGTAC
TTGTACCGGGTGATTTTGTAGATGTTATTGCCATCGCTGGCTTATGCGTTAACGATGAAATTGCCTTGACTCTACGTGA
GCCTGGCTTAGTCTTGTTTACGCATGAACTTCAAAATCATACTGCGACGGAATATTTTGTTTCATCGCGACGTATGTTT
GAACCTAGAAAACCTACCGTTAGTGATTTTGTTCAAATTGAGAGTTGTGTGGTCACCTATGTCAATTTGACTAGAGACC
AACTACCAGATGTAATCCCAGATTACATCGATGTTAACAAAACACTTGATGAGATTTTAGCTTCTCTGCCCAATAGAAC
TGGTCCAAGTCTTCCTTTAGATGTTTTTAATGCCACTTATCTTAATCTCACTGGTGAAATTGCAGATTTAGAGCAGCGT
TCAGAGTCTCTCCGTAATACTACAGAGGAGCTCCAAAGTCTTATATATAATATCAACAACACACTAGTTGACCTTGAGT

GGCTCAACCGAGTTGAGACATATATCAAGTGGCCGTGGTGGGTTTGGTTGATTATTTTCATTGTTCTCATCTTTGTTGT
GTCATTACTAGTGTTCTGCTGCATTTCCACGGGTTGTTGTGGATGCTGCGGCTGCTGCTGTGCTTGTTTTTCAGGTTGT
TGTAGGGGTCCTAGACTTCAACCTTACGAAGTTTTTGAAAAGGTCCACGTGCAGTGA

>gi|410072485|gb|JX501321.1|_Porcine_epidemic_diarrhea_virus_isolate_CH/TY/12_spike_glycoprotein_gene_complete_cds

ATGAAGTCTTTAACCTACTTCTGGTTATTCTTACCAGTACTTTCAACACTTAGCCTACCACAAGATGTCACCAGG
TGCTCAGCTAACACTAATTTTAGGCGGTTCTTTTCAAAATTTAATGTTCAGGCGCCTGCAGTTGTTGTACTGGGCGGTT
ATCTACCTATTGGTGAAAACCAGGGTGTCAATTCAACTTGGTACTGTGCTGGCCAACATCCAACTGCTAGTGGCGTTCA
TGGTATCTTTGTTAGCCATATTAGAGGTGGTCATGGCTTTGAGATTGGCATTTCGCAAGAGCCTTTTGACCCTAGTGGT
TACCAGCTTTATTTACATAAGGCTACTAACGGTAACACTAATGCTACTGCGCGACTGCGCATTTGCCAGTTTCCTAGCA
TTAAAACATTGGGCCCCACTGCTAATAATGATGTTACAACAGGTCGTAATTGCCTATTTAACAAAGCCATCCCAGCTCA
TATGAGTGAACATAGTGTTGTCGGCATAACATGGGATAATGATCGTGTCACTGTCTTTTCTGACAAGATCTATTATTTT
TATTTTAAAAATGATTGGTCCCGTGTTGCGACAAAGTGTTACAACAGTGGAGGTTGTGCTATGCAATATGTTTACGAAC
CCACCTATTACATGCTTAATGTTACTAGTGCTGGTGAGGATGGTATTTCTTATCAACCCTGCACAGCCAATTGCATTGG
TTATGCTGCCAATGTATTTGCTACTGAGCCCAATGGCCACATACCAGAAGGTTTTAGTTTTAATAATTGGTTTCTTTTG
TCCAATGATTCCACTTTGGTGCATGGTAAGGTGGCTTCCAACCAACCATTGTTGGTCAATTGTCTTTTGGCCATTCCTA
AGATTTATGGACTAGGCCAATTTTTCTCCTTTAATCAAACGATCGATGGTGTTTGTAATGGAGCTGCTGTGCAGCGTGC
ACCAGAGGCTCTGAGGTTTAATATTAATGACACCTCTGTCATTCTTGCTGAAGGCTCAATTGTACTTCATACTGCTTTA
GGAACAAATTTTTCTTTTGTTTGCAGTAATTCCACAGATCCTCATTTAGCCACCTTCGCCATACCTCTGGGTGCTACCC
AAGTACCCTATTATTGTTTTCTTAAAGTGGATACTTACAACTCCACTGTTTATAAATTCTTGGCTGTTTTACCTCCAAC
CGTCAGGGAAATTGTCATCACCAAGTATGGTGATGTTTATGTCAATGGGTTTGGCTATTTGCATCTCGGTTTGTTGGAC
GCTGTCACAATTAATTTCACTGGTCATGGCACTGACGATGACGTTTCAGGTTTCTGGACCATAGCATCGACTAATTTTG
TTGATGCACTTATCGAAGTTCAAGGAACTGCCATTCAGCGTATTCTTTATTGTGATGATCCTGTTAGCCAACTCAAGTG
TTCTCAGGTTGCTTTTGACCTTGACGATGGTTTCTATCCTATTTCTTCTAGAAACCATCTGAGTCATGAACAGCCAATT
TCTTTTGTTGCTTTGCCATCATTTAATGATCATTCTTTTGTTAACATTACTGTCTCTGCGTCCTTTGGTGGTCATAGTG
GTGCCAATCTTATTGCATCTGACACTACTATCAATGGGTTTAGTTCTTTCTGTGTTGACACTAGACAATTTACCATTTC
ACTGTTTCATAACGTTACAAACAGTTATGGTTATGTGTCTAAATCACAGGACAGTAATTGCCCTTTCACCTTGCAATCT
GTTAATGATTACCTGTCTTTTAGTAAATTTTGTGTTTCCACCAGCCTTTTGGCTAGTGCCTGTACCATAGATCTTTTTG
GTTACCCTGAGTTTGGTAGTGGTGTTAAGTTTACGTCCCTTTACTTTCAATTCACAAAGGGTGAGTTGATTACTGGCAC
GCCTAAACCACTTGAAGGTGTCACGGACGTTTCTTTTATGACTCTGGATGTGTGTACCACGTATACTATCTATGGCTTT
AAAGGTGAGGGTATCATTACCCTTACAAATTCTAGCTTTTTGGCAGGTGTTTATTACACATCTGATTCTGGACAGTTGT
TAGCCTTTAAGAATGTCACTAGTGGTGCTGTTTATTCTGTTACGCCATGTTCTTTTTCAGAGCAGGCTGCATATGTTGA
TGATGATATAGTGGGTGTTATTTCTAGTTTGTCTAGCTCCACTTTTAACAGTACTAGGGAGTTGCCTGGTTTCTTCTAC
CATTCTAATGATGGCTCTAATTGTACAGAGCCTGTGTTGGTGTATAGTAACATAGGTGTTTGTAAATCTGGCAGTATTG
GCTACGTCCCATCTCAGTCTGGCCAAGTCAAGATTGCACCCACGGTTACTGGGAATATTAGTATTCCCACCAACTTTAG
TATGAGTATTAGGACAGAATATTTACAGCTTTACAACACGCCTGTTAGTGTTGATTGTGCCACATATGTTTGTAATGGT
AACTCTCGTTGTAAACAATTACTCACCCAGTACACTGCAGCATGTAAGACCATAGAGTCAGCATTACAACTCAGCGCTA
GGCTTGAGTCTGTTGAAGTTAACTCTATGCTTACTATTTCTGAAGAGGCTCTACAGTTAGCTACCATCAGTTCGTTTAA
TGGTGATGGATATAATTTTACTAATGTGCTGGGTGTTTCTGTGTATGATCCTGCAAGTGGCAGGGTGGTACAAAAAAGG
TCTTTTATTGAAGACCTGCTTTTTAATAAAGTGGTTACTAATGGCCTTGGTACTGTTGATGAAGACTATAAGCGCTGTT
CTAATGGTCGCTCTGTGGCAGATCTAGTCTGTGCACAGTATTACTCTGGTGTCATGGTACTACCTGGTGTTGTTGACGC

TGAGAAGCTTCACATGTATAGTGCGTCTCTCATCGGTGGTATGGTGCTAGGAGGTTTTACTTCTGTAGCGGCATTGCCT
TTTAGCTATGCTGTTCAAGCTAGACTCAATTATCTTGCTCTACAGACGGATGTTCTACAGCGGAACCAGCAATTGCTTG
CTGAGTCTTTTAACTCTGCTATTGGTAATATAACTTCAGCCTTTGAGAGTGTTAAAGAGGCTATTAGTCAAACTTCCAA
GGGTTTGAACACTGTGGCTCATGCGCTTACTAAGGTTCAAGAGGTTGTTAACTCGCAGGGTGCAGCTTTGACTCAACTT
ACCGTACAGCTGCAACACAACTTCCAAGCCATTTCTAGTTCTATTGATGACATTTACTCTCGACTGGACATTCTTTCAG
CCGATGTTCAGGTTGACCGTCTCATCACCGGCAGATTATCAGCACTTAATGCTTTTGTTGCCCAAACCCTCACTAAGTA
TACTGAGGTTCAGGCTAGCAGGAAGCTAGCACAGCAAAAGGTTAATGAGTGCGTTAAATCGCAATCTCAGCGTTATGGT
TTTTGTGGTGGTGATGGCGAGCACATTTTCTCTCTGGTACAGGCAGCACCTCAGGGCCTGCTGTTTTTACATACAGTAC
TTGTACCGGGTGACTTTGTAGATGTTATTGCCATCGCTGGCTTATGCGTTAACGATGAAATTGCCTTGACTCTACGTGA
GCCTGGCTTAGTCTTGTTTACGCATGAACTTCAAAATCATACTGCGACGGAATATTTTGTTTCATCGCGACGTATGTTT
GAACCTAGAAAACCTACCGTTAGTGATTTTGTTCAAATTGAGAGTTGTGTGGTCACCTATGTCAATTTGACTAGAGACC
AACTACCAGATGTAATCCCAGATTACATCGATGTTAACAAAACACTTGATGAGATTTTAGCTTCTCTGCCCAATAGAAC
TGGTCCAAGTCTTCCTTTAGATGTTTTTAATGCCACTTATCTTAATCTCACTGGTGAAATTGCAGATCTAGAGCAGCGT
TCAGAGTCTCTCCGTAATACTACAGAGGAGCTCCAAAGTCTTATATATAATATCAACAACACACTAGTTGACCTTGAGT
GGCTCAACCGAGTTGAGACATATATCAAGTGGCCGTGGTGGGTTTGGTTGATTATTTTCATTGTTCTCATCTTTGTTGT
GTCATTACTAGTGTTCTGCTGCATTTCCACTGGTTGTTGTGGATGCTGCGGCTGCTGCTGTGCTTGTTTTTCAGGTTGT
TGTAGGGGTCCTAGACTTCAACCTTACGAAGTTTTTGAAAAGGTCCACGTGCAGTGA

>gi|410072487|gb|JX501322.1|_Porcine_epidemic_diarrhea_virus_isolate_CH/XC/12_spike_glycoprotein_gene_complete_cds

ATGAAGTCTTTAACCTACTTCTGGTTGTTCTTACCAGTACTTTCAACACTTAGCCTACCACAAGATGTCACCAGG
TGCTCAGCTAACACTAATTTTAGGCGGTTCTTTTCAAAATTTAATGTTCAGGCGCCTGCAGTTGTTGTACTGGGCGGTT
ATCTACCTATTGGTGAAAACCAGGGTGTCAATTCAACTTGGTACTGTGCTGGCCAACATCCAACTGCTAGTGGCGTTCA
TGGTATCTTTGTTAGCCATATTAGAGGTGGTCATGGCTTTGAGATTGGCATTTCGCAAGAGCCTTTTGACCCTAGTGGT
TACCAGCTTTATTTACATAAGGCTACTAACGGTAACACTAATGCTACTGCGCGACTGCGCATTTGCCAGTTTCCTAGCA
TTAAAACATTGGGCCCCACTGCTAATAATGATGTTACAACAGGTCGTAATTGCCTATTTAACAAAGCCATCCCAGCTCA
TATGAGTGAACATAGTGTTGTCGGCATAACATGGGATAATGATCGTGTCACTGTCTTTTCTGACAAGATCTATTATTTT
TATTTTAAAAATGATTGGTCCCGTGTTGCGACAAAGTGTTACAACAGTGGAGGTTGTGCTATGCAATATGTTTACGAAC
CCACCTATTACATGCTTAATGTTACTAGTGCTGGTGAGGATGGTATTTCTTATCAACCCTGTACAGCTAATTGCATTGG
TTATTCTGCCAATGTATTTGCTACTGAGCCCAATGGCCACATACCAGAAGGTTTTAGTTTTAATAATTGGTTTCTTTTG
TCCAATGATTCCACTTTGGTGCATGGTAAGGTGGTTTCCAACCAACCATTGTTGGTCAACTGTCTTTTGGCCATTCCTA
AGATTTATGGACTAGGCCAATTTTTCTCCTTTAATCAAACGATCGATGGTGTTTGTAATGGAGCTGCTGTGCAGCGTGC
ACCAGAGGCTCTGAGGTTTAATATTAATGACACCTCTGTCATTCTTGCTGAAGGCTCAATTGTACTTCATACTGCTTTA
GGAACAAATTTTTCTTTTGTTTGCAGTAATTCCTCAGGTCCTCACTTAGCCACCTTCGCCATACCTCTGGGTGCTATCC
AAGTACCCTATTATTGTTTTCTTAAAGTGGATACTTACAACTCCACTGTTTATAAATTCTTGGCTGTTTTACCTCCTAC
CGTCAGGGAAATTGTCATCACCAAGTATGGTGATGTTTATGTCAATGGGTTTGGCTACTTGCATCTCGGTTTGTTGGAT
GCTGTCACAATTAATTTCACTGGTCATGGCACTGACGATGACGTTTCTGGTTTTTGGACCATAGCATCGACTAATTTTG
TTGATGCACTTATCGAAGTTCAAGGAACTGCCATTCAGCGTATTCTTTATTGTGATGATCCTGTTAGCCAACTCAAGTG
TTCTCAGGTTGCTTTTGACCTTGACGATGGTTTCTACCCTATTTCTTCTAGAAACCTTCTGAGTCATGAACAGCCAATT
TCTTTTGTTACTTTGCCATCATTTAATGATCATTCTTTTGTTAACATTACTGTCTCTGCGTCCTTCGGTGGTCATAGTG
GTGCCAACCTTATTGCATCTGACACTACTATCAATGGGTTTAGTTCTTTCTGTGTTGACACTAGACAATTTACCATTTC
ACTGTTTTATAACGTTACAAACAGTTATGGTTATGTGTCTAACTCACAGGACAGTAATTGCCCTTTCACCTTGCAATCT

GTTAATGATTACCTGTCTTTTAGTAAATTTTGTGTTTCCACCAGCCTTTTGGCTAGTGCCTGTACCATAGATCTTTTTG
GTTACCCTGAGTTTGGTAGTGGTGTTAAGTTTACGTCCCTTTACTTTCAATTCACAAAGGGTGAGTTGATTACTGGCAC
GCCTAAACCACTTGAAGGTGTCACGGACGTTTCTTTTATGACTCTGGATGTGTGTACCAAGTATACTATCTATGGCTTT
AAAGGTGAGGGTATCATTACCCTTATAAATTCTAGCTTTTTGGCAGGTGTTTATTACACATCTGATTCTGGACAGTTGT
TAGCCTTTAAGAATGTCACTAGTGGTGCTGTTTATTCTGTTACGCCATGTTCTTTTTCAGAGCAGGCTGCATATGTTGA
TGATGATATAGTGGGTGTTATTTCTAGTTTGTCTAACTCCACTTTTAACAGTACTAGGGAGTTGCCTGGTTTCTTCTAC
CATTCTAATGATGGCTCTAATTGTACAGAGCCTGTGTTGGTGTATAGTAACATAGGTGTTTGTAAATCTGGCAGTATTG
GCTACGTCCCATCTCAGTCTGGCCAAGTCAAGATTGCACCCATGGTTACTGGGAATATTAGTATTCCCACCAACTTTAG
TATGAGTATTAGGACAGAATATTTACAGCTTTACAACACGCCTGTTAGTGTTGATTGTGCCACATATGTTTGTAATGGT
AACTCTCGTTGTAAACAATTACTCACCCAGTACACTGCAGCATGTAAGACCATAGAGTCAGCATTACAACTCAGCGCTA
GGCTTGAGTCTGTTGAAGTTAACTCTATGCTTACTATTTCTGAAGAGGCTCTACAGTTAGCTACCATCAGTTCGTTTAA
TGGTGATGGATATAATTTTACTAATGTGCTGGGTGTTTCTGTGTATGACCCTGCAAGTGGCAGGGTGGTACAAAAAAGG
TCTTTTATTGAAGACCTGCTTTTTAATAAGGTGGTTACTAATGGCCTTGGTACTGTTGATGAAGACTATAAGCGCTGTT
CTAATGGTCGCTCTGTGGCAGATCTAGTCTGTGCACAGTATTACTCTGGTGTCATGGTACTACCTGGTGTTGTTGACGC
TGAGAAGCTTCACATGTATAGTGCGTCTCTCATCGGTGGTATGGTGCTAGGAGGTTTTACTTCTGCAGCGGCATTGCCT
TTTAGCTATGCTGTTCAAGCTAGTCTCAATTATCTTGCTCTACAGACGGATGTTCTACAGCGGAACCAGCAATTGCTTG
CTGAGTCTTTTAACTCTGCTATTGGTAATATAACTTCAGCCTTTGAGAGTGTTAAAGAGGCTATTAGTCAAACTTCCAA
GGGTTTGAACACTGTGGCTCATGCGCTTACTAAGGTTCAAGAGGTTGTTAACTCGCAGGGTGCAGCTTTGACTCAACTT
ACCGTACAGCTGCAACACAATTTCCAAGCCATTTCTAGTTCTATTGATGACATTTACTCTCGACTGGACATTCTTTCAG
CCGATGTTCAGGTTGACCGTCTCATCACCGGCAGATTATCAGCACTTAATGCTTTTGTTGCTCAAACCCTCACTAAGTA
TACTGAGGTTCAGGCTAGCAGGAAGCTAGCACAGCAAAAGGTTAATGAGTGCGTTAAATCGCAATCTCAGCGTTATGGT
TCTTGTGGTGGTGATGGCGAGCACATTTTCTCTCTGGTACAGGCAGCACCTCAGGGCCTGCTGTTTTTACATACAGTAC
TTGTACCGGGTGATTTTGTAGATGTTATTGCCATCGCTGGTTTATGCGTTAACGATGAAATTGCCTTGACTCTACGTGA
GCCTGGCTTAGTCTTGTTTACGCATGAACTTCAAAATCATACTGCGACGGAATATTTTGTTTCATCGCGACGTATGTTT
GAACCTAGAAAACCTACCGTTAGTGATTTTGTTCAAATTGAGAGTTGTGTGGTCACCTATGTCAATTTGACTAGAGACC
AACTACCAGATGTAATCCCAGATTACATCGATGTTAACAAAACACTTGATGAGATTTTAGCTTCTCTGCCCAATAGAAC
TGGTCCAAGTCTTCCTTTAGATGTTTTTAATGCCACTTATCTTAATCTCACTGGTGAAATTGCAGATTTAGAGCAGCGT
TCAGAGTCTCTCCGTAATACTACAGAGGAGCTCCAAAGTCTTATATATAATATCAACAACACACTAGTTGACCTTGAAT
GGCTCAACCGAGTTGAGACATATATCAAGTGGCCGTGGTGGGTTTGGTTGATTATTTTCATTGTTCTCATCTTTGTTGT
GTCATTACTAGTGTTCTGCTGCATTTCCACGGGTTGTTGTGGATGCTGCGGCTGCTGCTGTGCTTGTTTTTCAGGTTGT
TGTAGGGGTCCTAGACTTCAACCTTACGAAGTTTTTGAAAAGGTCCACGTGCAGTGA

>gi|410072489|gb|JX501323.1|_Porcine_epidemic_diarrhea_virus_isolate_CH/XCYL/11_spike_glycoprotein_gene_complete_cds

ATGAAGTCTTTAACCTACTTCTGGTTGTTCTTACCAGTACTTTCAACACTTAGCCTACCACAAGATGTCACCAGG
TGCTCAGCTAACACTAATTTTAGGCGGTTCTTTTCAAAATTTAATGTTCAGGCGCCTGCAGTTGTTGTACTGGGCGGTT
ATCTACCTATTGGTGAAAACCAGGGTGTCAATTCAACTTGGTACTGTGCTGGCCAACATCCAACTGCTAGTGGCGTTCA
TGGTATCTTTGTTAGCCATATTAGAGGTGGTCATGGCTTTGAGATTGGCATTTCGCAAGAGCCTTTTGACCCTAGTGGT
TACCAGCTTTATTTACATAAGGCTACTAACGGTAACACTAATGCTACTGCGCGACTGCGCATTTGCCAGTTTCCTAGCA
TTAAAACATTGGGCCCCACTGCTAATAATGATGTTACAACAGGTCGTAATTGCCTATTTAACAAAGCCATCCCAGCTCA
TATGAGTGAACATAGTGTTGTCGGCATAACATGGGATAATGATCGTGTCACTGTCCTTTCTGACAAGATCTATTATTTT
TATTTTAAAAATGATTGGTCCCGTGTTGCGACAAAGTGTTACAACAGTGGAGGTTGTGCTATGCAATATGTTTACGAAC

CCACCTATTACATGCTTAATGTTACTAGTGCTGGTGAGGATGGTATTTCTTATCAACCCTGTACAGCTAATTGCATTGG
TTATGCTGCCAATGTATTTGCTACTGAGCCCAATGGCCACATACCAGAAGGTTTTAGTTTTAATAATTGGTTTCTTTTG
TCCAATGATTCCACTTTGGTGCATGGTAAGGTGGTTTCCAACCAACCATTGTTGGTCAATTGTCTTTTGGCCATTCCTA
AGATTTATGGACTAGGCCAATTTTTCTCCTTTAATCAAACGATCGATGGTGTTTGTAATGGAGCTGCTGTGCAGCGTGC
ACCAGAGGCTCTGAGGTTTAATATTAATGACACCTCTGTCATTCTTGCTGAAGGCTCAATTGTACTTCATACTGCTTTA
GGAACAAATTTTTCTTTTGTTTGCAGTAATTCCTCAGATCCTCACTTAGCCACCTTCGCCATACCTCTGGGTGCTATCC
AAGTACCCTATTATTGTTTTCTTAAAGTGGATACTTACGACTCCACTGTTTATAAATTCTTGGCTGTTTTACCTCCTAC
CGTCAGGGAAATTGTCATCACCAAGTATGGTGATGTTTATGTCAATGGGTTTGGCTATTTGCATCTCGGTTTGTTGGAC
GCTGTCACAATTAATTTCACTGGTCATGGCACTGACGATGACGTTTCAGGTTTCTGGACCATAGCATCGACTAATTTTG
TTGATGCACTTATCGAAGTTCAAGGAACTGCCATTCAGCGTATTCTTTATTGTGATGATCCTGTTAGCCAACTCAAGTG
TTCTCAGGTTGCTTTTGACCTTGACGATGGTTTCTACCCTATTTCTTCTAGAAACCTTCTGAGTCATGAACAGCCAATT
TCTTTTGTTACTTTGCCATCATTTAATGATCATTCTTTTGTTAGCATTACTGTCTCTGCGTCCTTTGGTGGTCATAGTG
GTGCCAACCTTATTGCATCTGACACTACTATCAATGGGTTTAGTTCTTTCTGTGTTGACACTAGACAATTTACCATTTC
ACTGTTTTATAACGTTACAAACAGTTATGGTTATGTGTCTAAATCACAGGACAGTAATTGCCCTTTCACCTTGCAATCT
GTTAATGATTACCTGTCTTTTAGTAAATTTTGTGTTTCCACCAGCCTTTTGGCTAGTGCCTGTACCATAGATCTTTTTG
GTTACCCTGAGTTTGGTAGTGGTGTTAAGTTTACGTCCCTTTACTTTCAATTCACAGAGGGTGAGTTGATTACTGGCAC
GCCTAAACCACTTGAAGGTGTCACGGACGTTTCTTTTATGACTCTGGATGTGTGTACCAAGTATACTATCTATGGCTTT
AAAGGTGAGGGTATCATTACCCTTACAAATTCTAGCTTTTTGGCAGGTATTTATTACACATCTGATTCTGGACAGTTGT
TAGCCTTTAAGAATGTCACTAGTGGTGCTGTTTATTCTGTTACGCCATGTTCTTTTTCAGAGCAGGCTGCATATGTTGA
TGATGATATAGTGGGTGTTATTTCTAGTTTGTCTAACTCCACTTTTAACAGTACTAGGGAGTTGCCTGGTTTCTTCTAC
CATTCTAATGATGGCTCTAATTGTACAGAGCCTGTGTTGGTGTATAGTAACATAGGTGTTTGTAAATCTGGCAGTATTG
GCTACGTCCCGTCTCAGTCTGGCCGAGTCAAGATTGCACCCACGGTTACTGGGAATATTAGTATTCCCACCAACTTTAG
TATGAGTATTAGGACAGAATATTTACAGCTTTACAACACGCCTGTTAGTGTTGATTGTGCCACATATGTTTGTAATGGT
AACTCTCGTTGTAAACAATTACTCACCCAGTACACTGCAGCATGTAAGACCATAGAGTCAGCATTACAACTCAGCGCTA
GGCTTGAGTCTGTTGAAGTTAACTCTATGCTTACTATTTCTGAAGAGGCTCTACAGTTAGCTACCATCAGTTCGTTTAA
TGGTGATGGATATAATTTTACTAATGTGCTGGGTGTTTCTGTGTATGATCCTGCAAGTGGCAGGGTGGTACAAAAAAGG
TCTTTTATTGAAGACCTGCTTTTTAATAAAGTGGTTACTAATGGCCTTGGTACTGTTGATGAAGACTATAAGCGCTGTT
CTAATGGTCGCTCTGTGGCAGATCTAGTCTGTGCACAGTATTACTCTGGTGTCATGGTACTACCTGGTGTTGTTGACGC
TGAGAAGCTTCACATGTATAGTGCGTCTCTCATCGGTGGTATGGTGCTAGGAGGTTTTACTTCTGCAGCGGCATTGCCT
TTTAGCTATGCTATTCAAGCTAGACTCAATTATCTTGCTCTACAGACGGATGTTCTACAGCGGAACCAGCAATTGCTTG
CTGAGTCTTTTAACTCTGCTATTGGTAATATAACTTCAGCCTTTGAGAGTGTTAAAGAGGCTATTAGTCAAACTTCCAA
GGGTTTGAACACTGTGGCTCATGCGCTTACTAAGGTTCAAGAGGTTGTTAACTCGCAGGGTGCAGCTTTGACTCAACTT
ACCGTACAGCTGCAACACAACTTCCAAGCCATTTCTAGTTCTATTGATGACATTTACTCTCGACTGGACATTCTTTCAG
CCGATGTTCAGGTTGACCGTCTCATCACCGGCAGATTATCAGCACTTAATGCTTTTGTTGCTCAAACCCTCACTAAGTA
TACTGAGGTTCAGGCTAGCAGGAAGCTAGCACAGCAAAAGGTTAATGAGTGCGTTAAATCGCAATCTCAGCGTTATGGT
TTTTGTGGTGGTGATGGCGAGCACATTTTCTCTCTGGTACAGGCAGCACCTCAGGGCTTGCTGTTTTTACATACAGTAC
TTGTATCGGGTGACTTTGTAGATGTTATTGCCATCGCTGGCTTATGCGTTAACGATGAAATTGCCTTGACTCTACGTGA
GCCTGGCTTAGTCTTGTTTACGCATGAACTTCAAAATCATACTGCGACGGAATATTTTGTTTCATCGCGACGTATGTTT
GAACCTAGAAAACCTACCGTTAGTGATTTTGTTCAAATTGAGAGTTGTGTGGTCACCTATGTCAATTTGACTAGAGACC
AACTACCAGATGTAATCCCAGATTACATCGATGTTAACAAAACACTTGATGAGATTTTAGCTTCTCTGCCCAATAGAAC
TGGTCCAAGTCTTCCTTTAGATGTTTTTAATGCCACTTATCTTAATCTCACTGGTGAAATTGCAGATTTAGAGCAGCGT
TCAGAGTCTCTCCGTAATACTACAGAGGAGCTCCAAAGTCTTATATATAATATCAACAACACACTAGATGACCTTGAGT
GGCTCAACCGAGTTGAGACATATATCAAGTGGCCGTGGTGGGTTTGGTTGATTATTTTCATTGTTCTCATCTTTGTTGT
GTCATTACTAGTGTTCTGCTGCATTTCCACTGGTTGTTGTGGATGCTGCGGCTGCTGCTGTGCTTGTTTTTCAGGTTGT

TGTAGGGGTCCTAGACTTCAACCTTACGAAGTTTTTGAAAAGGTCCACGTGCAGTGA

>gi|406828976|gb|JX512907.1|_Porcine_epidemic_diarrhea_virus_strain_HLJ2012_spike_protein_gene_complete_cds

ATGAAGTCTTTAACCTACTTCTGGTTGTTCTTACCAGTACTTTCAACACTTAGCCTACCACAAGATGTCACCAGG
TGCTCAGCTAACACTAATTTTAGGCGGTTCTTTTCAAAATTTAATGTTCAGGCGCCTGCAGTTGTTGTACTGGGCGGTT
ATCTACCTATTGGTGAAAACCAGGGTGTCAATTCAACTTGGTACTGTGCTGGCCAACATCCAACTGCTAGTGGCGTTCA
TGGTATCTTTGTTAGCCATATTAGAGGTGGTCATGGCTTTGAGATTGGCATTTCGCAAGAGCCTTTTGACCCTAGTGGT
TACCAGCTTTATTTACATAAGGCTACTAACGGTAACACTAATGCTACTGCGCGACTGCGCATTTGCCAGTTTCCTAGCA
TTAAAACATTGGGCCCCACTGCTAATAATGATGTTACAACAGGTCGTAATTGCCTATTTAACAAAGCCATCCCAGCTCA
TATGAGTGAACATAGTGTTGTCGGCATAACATGGGATAATGATCGTGTCACTGTCTTTTCTGACAAGATCTATTATTTT
TATTTTAAAAATGATTGGTCCCGTGTTGCGACAAAGTGTTACAACAGTGGAGGTTGTGCTATGCAATATGTTTACGAAC
CCACCTATTACATGCTTAATGTTACTAGTGCTGGTGAGGATGGTATTTCTTATCAACCCTGTACAGCTAATTGCATTGG
TTATGCTGCCAATGTATTTGCTACTGAGCCCAATGGCCACATACCAGAAGGTTTTAGTTTTAATAATTGGTTTCTTTTG
TCCAATGATTCCACTTTGGTGCATGGTAAGGTGGTTTCCAACCAACCATTGTTGGTCAATTGTCTTCTGGCCATTCCTA
AGATTTATGGACTAGGCCAATTTTTCTCCTTTAATCAAACGATCGATGGTGTTTGTAATGGAGCTGCTGTGCAGCGTGC
ACCAGAGGCTCTGAGGTTTAATATTAATGACACCTCTGTCATTCTTGCTGAAGGCTCAATTGTACTTCATACTGCTTTA
GGAACAAATTTTTCTTTTGTTTGCAGTAATTCCTCAGATCCTCACTTAGCCACCTTCGCCATACCTCTGGGTGCTATCC
AAGTACCCTATTATTGTTTTCTTAAAGTGGATACTTACAACTCCACTGTTTATAAATTCTTGGCTGTTTTACCTCCTAC
CGTCAGGGAAATTGTCATCACCAAGTATGGTGATGTTTATGTCAATGGGTTTGGCTATTTGCATCTCGGTTTGTTGGAC
GCTGTCACAATTAATTTCACTGGTCATGGCACTGACGATGACGTTTCAGGTTTCTGGACCATAGCATCGACTAATTTTG
TTGATGCACTTATCGAAGTTCAAGGAACTGCCATTCAGCGTATTCTTTATTGTGATGATCCTGTTAGCCAACTCAAGTG
TTCTCAGGTTGCTTTTGACCTTGACGATGGTTTCTACCCTATTTCTTCTAGAAACCTTCTGAGTCATGAACAGCCAATT
TCTTTTGTTACTTTGCCATCATTTAATGATCATTCTTTTGTTAACATTACTGTCTCTGCGTCCTTTGGTGGTCATAGTG
GTGCCAACCTTATTGCATCTGACACTACTATCAATGGGTTTAGTTCTTTCTGTGTTGACACTAGACAATTTACCATTTC
ACTGTTTTATAACGTTACAAACAGTTATGGTTATGTGTCTAAATCACAGGACAGTAATTGCCCTTTCACCTTGCAATCT
GTTAATGATTACCTGTCTTTTAGTAAATTTTGTGTTTCCACCAGCCTTTTGGCTAGTGCCTGTACCATAGATCTTTTTG
GTTACCCTGAGTTTGGTAGTGGTGTTAAGTTTACGTCCCTTTACTTTCAATTCACAAAGGGTGAGTTGATTACTGGCAC
GCCTAAACCACTTGAAGGTGTCACGGACGTTTCTTTTATGACTCTGGATGTGTGTACCAAGTATACTATCTATGGCTTT
AAAGGTGAGGGTATCATTACCCTTACAAATTCTAGCTTTTTGGCAGGTGTTTATTACACATCTGATTCTGGACAGTTGT
TAGCCTTTAAGAATGTCACTAGTGGTGCTGTTTATTCTGTTACGCCATGTTCTTTTTCAGAGCAGGCTGCATATGTTGA
TGATGATATAGTGGGTGTTATTTCTAGTTTGTCTAGCTCCACTTTTAACAGTACTAGGGAGTTGCCTGGTTTCTTCTAC
CATTCTAATGATGGCTCTAATTGTACAGAGCCTGTGTTGGTGTATAGTAACATAGGTGTTTGTAAATCTGGCAGTATTG
GCTACGTCCCATCTCAGTCTGGCCAAGTCAAGATTGCACCCACGGTTACTGGGAATATTAGTATTCCCACCAACTTTAG
TATGAGTATTAGGACAGAATATTTACAGCTTTACAACACGCCTGTTAGTGTTGATTGTGCCACATATGTTTGTAATGGT
AACTCTCGTTGTAAACAATTACTCACCCAGTACACTGCAGCATGTAAGACCATAGAGTCAGCATTACAACTCAGCGCTA
GGCTTGAGTCTGTTGAAGTTAACTCTATGCTTACTATTTCTGAAGAGGCTCTACAGTTAGCTACCATCAGTTCGTTTAA
TGGTGATGGATATAATTTTACTAATGTGCTGGGTGTTTCTGTGTATGATCCTGCAAGTGGCAGGGTGGTACAAAAAAGG
TCTTTTATTGAAGACCTGCTTTTTAATAAAGTGGTTACTAATGGCCTTGGTACTGTTGATGAAGACTATAAGCGCTGTT
CTAATGGTCGCTCTGTGGCAGATCTAGTCTGTGCACAGTATTACTCTGGTGTCATGGTACTACCTGGTGTTGTTGACGC
TGAGAAGCTTCACATGTATAGTGCGTCTCTCATCGGTGGTATGGTGCTAGGAGGTTTTACTTCTGCAGCGGCATTGCCT
TTTAGCTATGCTGTTCAAGCGAGACTCAATTATCTTGCTCTACAGACGGATGTTCTACAGCGGAACCAGCAATTGCTTG

CTGAGTCTTTTAACTCTGCTATTGGTAATACAACTTCAGCCTTTGAGAGTGTTAAAGAGGCTATTAGTCAAACTTCCAA
GGGTTTGAACACTGTGGCTCATGCGCTTACTAAGGTTCAAGAGGTTGTTAACTCGCAGGGTGCAGCTTTGACTCAACTT
ACCGTACAGCTGCAACACAACTTCCAAGCCATTTCTAGTTCTATTGATGACATTTACTCTCGACTGGACATTCTTTCAG
CCGATGTTCAGGTTGACCGTCTCATCACCGGCAGATTATCAGCACTTAATGCTTTTGTTGCTCAAACCCTCACTAAGTA
TACTGAGGTTCAGGCTAGCAGGAAGCTAGCACAGCAAAAGGTTAATGAGTGCGTTAAATCGCAATCTCAGCGTTATGGT
TTTTGTGGTGGTGATGGCGAGCACATTTTCTCTCTGGTACAGGCAGCACCTCAGGGCCTGCTGTTTTTACATACAGTAC
TTGTACCGGGTGACTTTGTAGATGTTATTGCCATCGCTGGCTTATGCGTTAACGATGAAATTGCCTTGACTCTACGTGA
GCCTGGCTTAGTCTTGTTTACGCATGAACTTCAAAATCATACTGCGACGGAATATTTTGTTTCATCGCGACGTATGTTT
GAACCTAGAAAACCTACCGTTAGTGATTTTGTTCAAATTGAGAGTTGTGTGGTCACCTATGTCAATTTGACTAGAGACC
AACTACCAGATGTAATCCCAGATTACATCGATGTTAACAAAACACTTGATGAGATTTTAGCTTCTCTGCCCAATAGAAC
TGGTCCAAGTCTTCCTTTAGATGTTTTTAATGCCACTTATCTTAATCTCACTGGTGAAATTGCAGATTTAGAGCAGCGT
TCAGAGTCTCTCCGTAATACTACAGAGGAGCTCCAAAGTCTTATATATAATATCAACAACACACTAGTTGACCTTGAGT
GGCTCAACCGAGTTGAGACATATATCAAGTGGCCGTGGTGGGTTTGGTTGATTATTTTCATTGTTCTCATCTTTGTTGT
GTCATTACTAGTGTTCTGCTGCATTTCCACTGGTTGTTGTGGATGCTGCGGCTGCTGCTGTGCTTGTTTTTCAGGTTGT
TGTAGGGGTCCTAGACTTCAACCTTACGAAGTTTTTGAAAAGGTCCACGTGCAGTGA

>gi|409898552|gb|JX524137.1| Porcine epidemic diarrhea virus strain ZJCZ4, complete genome

ATGAAGTCTTTAACCTACTTCTGGTTGTTCTTACCAGTACTTTCAACACTTAGCCTACCACAAGATGTCACCAGG
TGCTCAGCTAACACTAATTTTAGGCGGTTCTTTTCAAAATTTAATGTTCAGGCGCCTGCAGTTGTTGTACTGGGCGGTT
ATCTACCTATTGGTGAAAACCAGGGTGTCAATTCAACTTGGTACTGTGCTGGCCAACATCCAACTGCTAGTGGCGTTCA
TGGTATCTTTGTTAGCCATATTAGAGGTGGTCATGGCTTTGAGATTGGCATTTCGCAAGAGCCTTTTGACCCTAGTGGT
TACCAGCTTTATTTACATAAGGCTACTAACGGTAACACTAATGCTACTGCGCGACTGCGCATTTGCCAGTTTCCTAGCA
TTAAAACATTGGGCCCCACTGCTAATAATGATGTTACAACAGGTCGTAATTGCCTATTTAACAAAGCCATCCCAGCTCA
TATGAGTGAACATAGTGTTGTCGGCATAACATGGGATAATGATCGTGTCACTGTCTTTTCTGACAAGATCTATTATTTT
TATTTTAAAAATGATTGGTCCCGTGTTGCGACAAAGTGTTACAACAGTGGAGGTTGTGCTATGCAATATGTTTACGAAC
CCACCTATTACATGCTTAATGTTACTAGTGCTGGTGAGGATGGTATTTCTTATCAACCCTGTACAGCTAATTGCATTGG
TTATGCTGCCAATGTATTTGCTACTGAGCCCAATGGCCACATACCAGAAGGTTTTAGTTTTAATAATTGGTTTCTTTTG
TCCAATGATTCCACTTTGGTGCATGGTAAGGTGGTTTCCAACCAACCATTGTTGGTCAATTGTCTTTTGGCCATTCCTA
AGATTTATGGACTAGGCCAATTTTTCTCCTTTAATCAAACGATCGATGGTGTTTGTAATGGAGCTGCTGTGCAGCGTGC
ACCAGAGGCTCTGAGGTTTAATATTAATGACACCTCTGTCATTCTTGCTGAAGGCTCAATTGTACTTCATACTGCTTTA
GGAACAAATTTTTCTTTTGTTTGCAGTAATTCCTCAGATCCTCACTCAGCCACCTTCGCCATACCTCTGGGTGCTACCC
AAGTACCCTATTATTGTTTTCTTAAAGTGGATACTTACAACTCCACTGTTTATAAATTCTTGGCTGTTTTACCTCCTAC
CGTCAGGGAAATTGTCATCACCAAGTATGGTGATGTTTATGTCAATGGGTTTGGCTACTTGCATCTCGGTTTGTTGGAT
GCTGTCACAATTAATTTCACTGGTCATGGCACTGACGATGACGTTTCTGGTTTTTGGACCATAGCATCGACTAATTTTG
TTGATGCACTTATCGAAGTTCAAGGAACTGCCATTCAGCGTATTCTTTATTGTGATGATCCTGTTAGCCAACTCAAGTG
TTCTCAGGTTTCTTTTGACCTTGACGATGGTTTTTACCCTATTTCCTCTACAAACCTTCTGAGTCATGAACAGCCAACT
TCTTTTGTTACTTTGCCATCATTTAATGATCATTCTTTTGTTAATATTACTGTCTCTGCTGCTTTTGGTGGTCATAGTG
GTGCCAACCTTATTGCATCTGACACTACTATCAATGGGTTTAGTTCTTTCTGTGTTGACACTAGACAATTTACCATTTC
ACTGTTTTATAATGTTACAAACAGTTATGGTTATGTGTCTAACTCACAGGACAGTAATTGCCCTTTCACCTTGCAATCT
GTTAATGATTACCTGTCTTTTAGCAAATTTTGTGTTTCTACCAGCCTTTTGGCTAGTGCCTGTACCATAGATCTTTTTG
GTTACCCTGAGTTTGGTAGTGGTGTTAAGTTCACGTCCCTTTACTTTCAATTCACAAAGGGTGAGTTGATTACTGGCAC

GCCTAAACCACTTGAAGGTGTTACGGACGTTTCTTTTATGACTCTGGATGTGTGTACCAAGTATACTATCTATGGCTTT
AAAGGTGAGGGTATCATTACCCTTATAAATTCTAGCTTTTTGGCAGGTGTTTATTATACATCTGATTCTGGACAGTTGT
TAGCTTTTAAGAATGTCACTAGTGGTGCTGTTTATTCTGTTACGCCATGTTCTTTTTCAGAGCAGGCTGCATATGTTGA
TGATGATATAGTGGGTGTTATTTCTAGTTTGTCTAGCTCCACTTTTAACAGTACTAGGGAGTTGCCTGGTTTCTTCTAC
CATTCTAATGATGGCTCTAATTGTACAGAGCCTGTGTTGGTGTATAGTAACATTGGTGTTTGTAAATCTGGCAGTATTG
GCTATGTCCCATCTCAGTCTGGCCAAGTCAAGATTGCACCCACGGTTACTGGGAATATTAGTATTCCCACCAACTTTAG
TATGAGTATTAGGACAGAATATTTACAGCTTTACAACACGCCTGTTAGTGTTGATTGTGCTACATATGTTTGTAATGGT
AACTCTCGTTGTAAACAATTACTCACCCAGTACACTGCAGCATGTAAGACCATAGAGTCAGCATTACAACTCAGCGCTA
GGCTTGAGTCTGCTGAAGTCAACTCTATGCTTACTATTTCTGAAGAGGCTCTACAGTTAGCTACCATCAGTTCGTTTAA
TGGTGATGGATATAATTTTACTAATGTGCTGGGTGTTTCCGTTCGTGATCCTGCAAGTGGCAGGGTGGTACAAAAAAGG
TCTTTTATTGAAGACCTGCTTTTTAATAAAGTGGTTACTAATGGCCTTGGTACTGTTGATGAAGACTATAAGCGCTGTT
CTAATGGCCGCTCTGTGGCAGATTTAGTCTGTGCGCAGTATTACTCTGGTGTCATGGTACTACCTGGTGTTGTTGACGC
TGAGAAGCTTCATATGTATAGTGCGTCTCTCATCGGTGGTATGGTGCTAGGAGGTTTTACTGCAGCAGCGGCATTGCCT
TTTAGCTATGCTGTTCAAGCGAGACTCAATTATCTTGCTCTACAGACGGATGTTCTACAGCGGAACCAGCAATTGCTTG
CTGAGTCTTTTAACTCTGCTATTGGTAATATAACTTCAGCCTTTGAGAGTGTTAAAGAGGCTATTAGTCAAACTTCTAA
GGGTTTGAACACTGTGGCTCATGCGCTTACTAAGGTTCAAGAGGTTGTTAATTCGCAGGGTGCAGCTTTGACCCAACTT
ACCGTACAGCTGCAACACAACTTCCAAGCCATTTCTAGTTCTATTGATGACATTTATTCCCGACTGGACATTCTTTCAG
CCGATGTTCAGGTTGACCGTCTCATCACCGGCAGATTATCAGCACTTAATGCTTTTGTTGCTCAAACCCTCACTAAGTA
TACTGAGGTTCAGGCTAGCAGGAAGCTAGCACAGCAAAAGGTTAATGAGTGCGTTAAATCGCAATCTCAGCGTTATGGT
TTTTGCGGTGGTGATGGCGAGCACATTTTCTCTCTGGTACAGGCAGCACCTCAGGGCCTGCTGTTTTTACATACAGTAC
TTGTACCGGGTGATTTTGTAGATGTTATTGCCATCGCTGGCTTATGCGTTAACGATGAAATTGCCTTGACTCTACGTGA
GCCTGGCTTAGTCTTGTTTACGCATGAACTTCAAAATCATACTGCGACGGAATATTTTGTTTCATCGCGACGTATGTTT
GAACCTAGAAAACCTACCGTTAGTGATTTTGTTCAAATTGAGAGTTGTGTGGTCACCTATGTCAATTTGACTAGAGACC
AACTACCAGATGTAATCCCAGATTACATCGATGTTAACAAAACACTTGATGAGATTTTAGCTTCTCTGCCCAATAGAAC
TGGTCCAAGTCTTCCTTTAGATGTTTTTAATGCCACTTATCTTAATCTCACTGGTGAAATTGCAGATTTAGAGCAGCGT
TCAGAGTCTCTCCGTAATACTACAGAAGAGCTCCAAAGTCTTATATATAATATCAACAACACACTAGTTGACCTTGAGT
GGCTCAACCGAGTTGAGACATATATCAAGTGGCCGTGGTGGGTTTGGTTGATTATTTTTATTGTTCTCATCTTTGTTGT
GTCATTATTAGTGTTCTGCTGCATTTCCACGGGTTGTTGTGGATGCTGCGGCTGCTGTGGTGCTTGTTTTTCAGGTTGT
TGTAGGGGTCCTAGACTTCAACCTTACGAAGCTTTTGAAAAGGTCCACGTGCAGTGA

>gi|425856767|gb|JX560761.1| Porcine epidemic diarrhea virus strain SD-M, complete genome

ATGACGCCTTTAATTTACTTCTGGTTGTTCTTACCAGTACTTCTAACACTTAGCCTACCACAAGATGTCACTAGG
TGCCAGTCTACTATTAACTTTAGGCGGTTCTTTTCAAAATTTAATGTTCAGGCACCTGCCGTCGTTGTTTTGGGTGGTT
ATCTACCTAATATGAACTCTTCTAGCTGGTACTGTGGCACAGGCATTGAAACTGATAGTGGCGTTCATGGTATTTTTCT
CAGTTACATCGATTCTGGTCAGGGCTTTGAGATTGGCATTTCGCAAGAGCCGTTTGATCCTAGTGGTTACCAGCTTTAT
TTACACAAGGCCACTAATGGTAACACTAGTGCTATTGCACGACTGCGCATTTGCCAGTTTCCAGATAATAAAACATTGG
GCCCTACTGTTAATGATGTTACAACAGGTCGTAACTGCCTATTCAACAAAGCCATTCCAGCTTTGCAGGATGGAAAAAA
TATTGTTGTCGGCATAACATGGGATAATGATCGTGTCACTGTTTTTGCTGACAAGATCTATCATTTTTATATTAAAAAT
GATTGGTCCCGTGTTGCGACAAGATGTTACAATAAAAGAAGTTGTGCCATGCAATATGTTTATACACCTACCTACTACA
TGCTTAATGTTACTAGTGCAGGTGAGGATGGCATTTACTATGAACCTTGTACAGCTAATTGCAGTGGTTACGCTGCCAA
TGTATTTGCCACTGATTCCAATGGCCATATACCAGAAGGTTTTAGTTTTAATAATTGGTTTCTTTTGTCCAATGACTCC

ACTTTGTTGCATGGTAAAGTGGTTTCAAACCAACCTTTGTTGGTCAACTGCCTTTGGGCCATTCCTAAGATTTATGGAC
TAGGCCAATTTTTCTCATTCAATCAAACGATGGATGGCGTTTGTAACGGAGCCGCTGCGCAGCGTGCCCCAGAGGCTCT
GAGGTTTAATATTAATGACACTTTTGTCATTCTTGCTGAAGGCTCAATTGTACTTCATACTGCTTTAGGAACAAATCTT
TCTTTTGTTTGCAGTAATTCCTCAGATCCTCACAAAGCCATCTTTACCATACCTTTGGGTGTTACTGAAGTACCCTACT
ATTGCTTTCTTAAAGTGGATACTTACAAATCCACTGTTTATAAATTCTTGGCTGTTTTACCTCCTACTGTCAAGGAAAT
TGTCATCACCAAGTACGGTGATGTTTATGTCAACGGGTTTGGCTATTTGCATCTCGGTTTGTTGGATGCTGTCACAATT
AATTTCACTGGTCATGGCACTGACGATGACGTTTCAGGTTTCTGGACCGTAGCATCGACTAATTTTGTTGATGCACTCA
TCGAGGTTCAAGGAACTGCCATTCAGCGTATTCTTTATTGTGATGACCCTGTTAGCCAACTTAAGTGTTCTCAGGTTTC
TTTTGACCTTGATGATGGTTTTTACCCTATTTCTTCTAGAAACCTTCTGAGTCATGAACAGCCAATTTCTTTTGTTACT
TTGCCATCATTCAATGATCATTCTTTTGTTAATATTACTGTCTCTGCGGCTTTTGGTGGTCATAGTGGTGCCAACCTCA
TTGCATCTGACACTACTATCAATGGGTTTAGTTCTTTCTGTGTTGACACTAGACAATTTACCATTACACTGTTTTATAA
CGTTACAAACAGTTATGGTTATGTGTCTAAGTCACAGGATAGTAATTGCCCTTTCACCTTGCAATCTGTTAATGATTAC
CTGTCTTTTAGCAAATTTTGTGTTTCAACCAGCCTTTTGGCTGGTGCTTGTACCATAGATCTTTTTGGTTACCCTGAGT
TCGGTAGTGGTGTTAAGTTTACGTCCCTTTATTTTCAATTCACAAAGGGTGAGTTGATTACTGGCACGCCTAAACCACT
TCAAGGTGTCACGGACGTTTCTTTTATGACTCTGGATGTGTGTACCAAGTATACTATCTATGGCTTTAAAGGTGAGGGT
ATTATTACCCTTACAAATTCTAGCTTTTTGGCAGGTGTTTATTATACATCTGATTCTGGACAGTTGTTAGCCTTTAAGA
ATGTCACTAGTGGTGCTGTTTATTCTGTTACGCCATGTTCTTTTTCAGAGCAGGCTGCATATGTTGATGATGATATAGT
GGGTGTTATTTCTAGTTTGTCTAACTCCACTTTTAACAATACCAGGGAGTTGCCTGGTTTCTTCTACCATTCTAATGAT
GGCTCCAATTGTACAGAGCCTGTGTTGGTGTATAGTAACATAGGTGTCTGTAAATCTGGCAGTATTGGCTATGTCCCAC
TTCAGGATGGCCAAGTCAAGATTGCACCCATGGTTACTGGGAATATTAGTATTCCCACCAACTTTAGTATGAGTATTAG
AACAGAATATTTACAGCTTTACAACACGCCTGTTAGTGTTGATTGCGTTACATATGTTTGTAATGGTAACTCTCGTTGT
AAACAATTACTCACCCAGTACACTTCAGCATGTAAGACCATAGAGTCAGCATTACAACTCAGCGCTAGGCTTGAGTCTG
TTGAAGTTAACTCTATGCTTACTATTTCTGAAGAGGCTCTACAGTTAGCTACCATCAGTTCGTTTAATGGTGATGGATA
TAACTTTACTAATGTGCTGGGTGTTTCCGTGTACGACCCTGCAAGTGGCAGGGTGGTACAAAAAGGGTCTTTTATTGAA
GACCTGCTTTTTAATAAAGTGGTTACTAATGGCCTTGGTACTGTTGATGAAGACTATAAGCGCTGTTCTAATGGTCGCT
CTGTGGCAGATCTAGTCTGTGCGCAGTATTACTCTGGTGTCATGGTACTACCTGGCGTTGTTGACGCTGAGAAGCTTCA
CATGTATAGTGCGTCTCTCCTCGGTGGTATGGCGCTAGGAGGTCTTACTACTGCAGCGGCATTGCCTTTTAGCAATGCT
GTTCAAGCGAGGCTCAATTATCTTGCTTTACAGACGGATGTTCTACAGCGCAACCAGCAATTGCTTGCTGAGTCTTTTA
ACTCTGCTATTGGTAATATAACTTCAGCCTTTGAGAGTGTTAAAGAGGCTATTAGTCAAACTTCCAATGGTTTGAACAC
TGTGGCTCATGCGCTTACTAAGGTTCAAGAGGTTGTTAAGTCGCAGGGTTCAGCTTTGACCCAACTTACCATACAGCTG
CAACACAACTTCCAAGCCATTTCTAGTTCTATTGATGACATTTACTCCCGACTGGACATTCTTTCAGCCGATGTTCAGG
TTGATCGTCTCATCACCGGCAGATTATCAGCACTTAATGCTTTTGTTGCTCAAACCCTCACTAAGTATACTGAGGTTCA
GGCTAGCAGGAAGCTAGCACAGCAAAAGGTTAATGAGTGCGTCAAATCGCAATCTCAGCGTTATGGTTTTTGTGGTGGT
GATGGCGAGCACATCTTCTCTCTGGTACAGGCCGCACCTCAGGGCCTGCTGTTCTTACATACAGTACTTGTACCGGGTG
ATTTTGTAAATGTTATTGCCATCGATGGCTTATGCGTTAATGGTGATATTGCCTTGACTCTACGTGAGCCTGGCTTAGT
CTTGTTTACGCATGAACTTCAAACTTATACTGCGACGGAATATTTTGTTTCATCGCGACGTATGTTTGAACCTAGAAAA
CCTACCGTTAGTGATTTTGTTCAAATTGAGAGTTGTGTGGTCACCTATGTCAATCTGACTAGCGACCAACTACCAGATG
TAATCCCAGATTACATCGATGTTAACAAAACACTTGATGAGATTCTAGCTTCTCTGCCCAATAGAATTGGTCCTAGTCT
TCCCCTAGATGTTTTTAATGCCACTTATCTTAATCTCACTGGTGAAATTGCAGATTTAGAGCAGCGTTCAGAGTCTCTC
CGTAATACTACAGAAGAGCTCCGAAGTCTCATATATAATATCAACAACACACTTGTTGACCTTGAGTGGCTCAACCGAG
TTGAGACATATATCAAGTGGCCGTGGTGGGTTTGGTTGATTATTTTTATTGTTCTCATCTTTGTTGTGTCATTATTAGT
GTTCTGCTGCATTTCCACGGGTTGTTGTGGATGCTGCGGTTGTTGCGGTGCTTGTTTTTCAGGTTGTTGTAGGGGTCCT
AGACTTCAACCTTACGAAGCTTTTGAAAAGGTCCACGTGCAGTGA

>gi|414151650|gb|JX647847.1| Porcine epidemic diarrhea virus strain GD-1, complete genome

ATGAAGTCTTTAACCTACTTCTGGTTGTTCTTACCAGTACTTTCAACACTTAGCCTACCACAAGATGTCACCAGG
TGCTCAGCTAACACTAATTTTAGGCGGTTCTTTTCAAAATTTAATGTTCAGGCGCCTGCAGTTGTTGTACTGGGCGGTT
ATCTACCTATTGGTGAAAACCAGGGTGTCAATTCAACTTGGTACTGTGCTGGCCAACATCCAACTGCTAGTGGCGTTCA
TGGTATCTTTCTTAGCCATATTAGAGGTGGTCATGGCTTTGAGATTGGCATTTCGCAAGAGCCTTTTGACCCTAGTGGT
TACCAGCTTTATTTACATAAGGCTACTAACGGTAACACTAATGCTACTGCGCGACTGCGCATTTGCCAGTTTCCCAGCA
TTAAAACATTGGGCCCCACTGCTAATAATGATGTTACAACAGGTCGTAACTGCCTATTTAACAAAGCCATCCCAGCTCA
TATGAGTGAACATAGTGTTGTCGGCATAACATGGGATAATGATCGTGTCACTGTCTTTTCTGACAAGATCTATTATTTT
TATTTTAAAAATGATTGGTCCCGTGTTGCGACAAAGTGTTACGACAGTGGAGGTTGTGCTATGCAATATGTTTACGAAC
CCACTTACTACATTCTTAATGTTACTAGTGCTGGTGAGGATGGTATTTCTTATCAACCCTGTACAGCTAATTGCATTGG
TTATGCTGCCAATGTATTTGCTACTGAGCCCAATGGCCACATACCAGAAGGTTTTAGTTTTAATAATTGGTTTCTTTTG
TCCAATGATTCCACTTTGGTGCATGGTAAGGTGGTTTCCAACCAACCATTGTTGGTCAATTGTCTTTTGGCCATTCCTA
AGATTTATGGACTAGGCCAATTTTTCTCCTTTAATCAAACGATCGATGGTGTTTGTAATGGAGCTGCTGTGCAGCGTGC
ACCAGAGGCTCTGAGGTTTAATATTAATGACACCTCTGTCATTCTTGCTGAAGGCTCAATTGTACTTCATACTGCTTTA
GGAACAAATTTTTCTTTTGTTTGCAGTAATTCCTCAGATCCTCACTTAGCCACCTTTGCCATACCTCTGGGTGCTATCC
AAGTACCCTATTATTGTTTTCTTAAAGTGGATACTTACAACTCCACTGTTTATAAATTCTTGGCTGTTTTACCTCCTAC
CGTCAGGGAAATTGTCATCACCAAGTATGGTGATGTTTATGTCAATGGGTTTGGCTACTTGCATCTCGGTTTGTTGGAT
GCTGTCACAATTAATTTCACTGGTCATGGCACTGACGATGACGTTTCTGGTTTTTGGACCATAGCATCGACTAATTTTG
TTGATGCACTTATCGAAGTTCAAGGAACTGCCATTCAGCGTATTCTTTATTGTGATGATCCTGTTAGCCAACTCAAGTG
TTCTCAGGTTGCTTTTGACCTTGACGATGGTTTTTACCGTATTTCCTCTACAAACCTTCTGAGTCATGAACAGCCAACT
TCTTTTGTTACTTTGCCATCATTTAATGATCATTCTTTTGTTAATATTACTGTCTCTGCTGCTTTTGGTGGTCATAGTG
GTGCCAACCTTATTGCATCTGACACTACTATCAATGGGTTTAGTTCTTTCTGTGTTGACACTAGACAATTTACCATTTC
ACTGTTTTATAACGTTACAAACAGTTATGGTTACGTGTCTAAATCACAGGACAGTAATTGCCCTTTTACCTTGCAATCT
GTTAATGATTACCTGTCTTTTAGCAAATTTTGTGTTTCTACCAGCCTTTTGGCTAGTGCCTGTACCATAGATCTTTTTG
GTTACCCTGAGTTTGGTAGTGGTGTTAAGTTCACGTCCCTTTACTTTCAATTCACAAAGGGTGAGTTGATTACTGGCAC
GCCTAAACCACTTGAAGGTGTTACGGACGTTTCTTTTATGACTCTGGATGTGTGTACCAAGTATACTATCTATGGCTTT
AAAGGTGAGGGTATCATTACCCTTACAAATTCTAGCTTTTTGGCAGGTGTTTATTATACATCTGATTCTGGACAGTTGT
TAGCTTTTAAGAATGTCACTAGTGGTGCTGTTTATTCTGTTACGCCATGTTCTTTTTCAGAGCAGGCTGCATATGTTGA
TGATGATATAGTGGGTGTTATTTCTAGTTTGTCTAGCTCCACTTTTAACAGTACTAGGGAGTTGCCTGGTTTCTTCTAC
CATTCTAATGATGGCTCTAATTGTACAGAGCCTGTGTTGGTGTATAGTAACATAGGTGTTTGTAAATCTGGCAGTATTG
GCTATGTCCGATCTCAGTCTGGCCAAGTCAAGATTGCACCCACGGTTACTGGGAATATTAGTATTCCCACCAACTTTAG
TATGAGTATTAGGACAGAATATTTACAGCTTTACAACACGCCTGTTAGTGTTGATTGTGCTACATATGTTTGTAATGGT
AACTCTCGTTGTAAACAATTACTCACCCAGTACACTGCAGCATGTAAGACCATAGAGTCAGCATTACAACTCAGCGCTA
GGCTTGAGTCTGCTGAAGTCAACTCTATGCTTACTATTTCTGAAGAGGCTCTACAGTTAGCTACCATCAGTTCGTTTAA
TGGTGATGGATATAATTTTACTAATGTGCTGGGTGTTTCCGTGTATGACCCTGCAAGTGGCAGGGTGGTACAAAAAAGG
TCTTTCATTGAAGACCTGCTTTTTAATAAAGTGGTTACTAATGGCCTTGGTACTGTTGATGAAGACTATAAGCGCTGTT
CTAATGGCCGCTCTGTGGCAGATTTAGTCTGTGCGCAGTATTACTCTGGTGTCATGGTACTACCTGGTGTTGTTGACGC
TGAGAAGCTTCATATGTATAGTGCGTCTCTCATCGGTGGTATGGTGCTAGGAGGTTTTACTGCAGCAGCGGCATTGCCT
TTTAGCTATGCTGTTCAAGCGAGACTGAATTATCTTGCTCTACAGACGGATGTTCTACAGCGGAACCAGCAATTGCTTG
CTGAGTCTTTTAATTCTGCTATTGGTAATATAACTCCAGCCTTTGAGAGTGTTAAAGAGGCTATTAGTCAAACTTCTAA
GGGTTTGAACACTGTGGCTCATGCGCTTACCAAGGTCCAAGAGGTTGTTAATTCGCAGGGTGCAGCTTTGACCCAACTT

ACCGTACAGCTGCAACACAACCTCCAAGCCATTTCTAGTTCTATTGATGACATTTACTCCCGACTTGACATTCTTTCAG
CCGATGTTCAGGTAGACCGTCTCATCACCGGCAGATTATCAGCACTTAATGCTTTTGTTGCTCAAACCCTTACTAAGTA
TACTGAGGTTCAGGCTAGCAGGAAACTAGCACAGCAAAAGGTTAATGAGTGCGTTAAATCGCAATCTCAGCGTTATGGT
TTTTGTGGTGGTGATGGCGAGCACATTTTCTCTCTGGTACAGGCCGCACCTCAAGGCCTGCTGTTTTTACACACAGTAC
TTGTACCGGGTGACTTTGTAAATGTTATTGCCATCGCTGGCTTATGTGTTAACGATGAAATTGCCTTGACTCTACGTGA
GCCTGGTTTAGTCTTGTTTACGCATGAACTTCAAGATACTGCGACGGAATATTTTGTTTCATCGCGACGTATGTATGAA
CCTAGAAAACCTACCGTTGGTGATTTTGTTCAAATTGAGAGTTGTGTGGTCACCTATGTCAATTTGACTAGAGACCAAC
TACCAGAAGTAATCCCAGATTACATCGATGTTAACAAAACACTTGATGAGATTTTAGCCTCTCTGCCCAATAGAACTGG
TCCAAGTCTTTCTCTAGATGTTTTTAATGCCACTTATCTTAATCTCACTGGTGAAATTGCAGATTTAGAGCAGCGTTCA
GAGTCTCTCCGTAATACTACAGAAGAGCTCCAAAGTCTTATATATAATATCAACAACACACTAGTTGACCTTGAGTGGC
TCAACCGAGTTGAGACATATATCAAGTGGCCGTGGTGGGTTTGGTTGATTATTTTTATTGTTCTCATCTTTGTTGTGTC
ATTACTAGTGTTCTGCTGCATTTCCACGGGTTGTTGTGGATGCTGCGGCTGCTGTGGTGCTTGTTTTTCAGGTTGTTGT
AGGGGTCCTAGACTTCAACCTTACGAAGCTTTTGAAAAGGTCCACGTGCAGTGA

>gi|431878534|gb|KC196276.1| Porcine epidemic diarrhea virus strain CH/ZMDZY/11, complete genome

ATGAAGTCTTTAACCTACTTCTGGTTGTTCTTACCAGTACTTTCAACACTTAGCCTACCACAAGATGTCACCAGG
TGCTCAGCTAACACTAATTTTAGGCGGTTCTTTTCAAAATTTAATGTTCAGGCGCCTGCAGTTGTTGTACTGGGCGGTT
ATCTACCTATTGGTGAAAACCAGGGTGTCAATTCAACTTGGTACTGTGCTGGCCGACATCCAACTGCTAGTGGCGTTCA
TGGTATCTTTGTTAGCCATATTAGAGGCGGTCATGGCTTTGAGATTGGCATTTCGCGAGAGCCTTTTGACCCTAGTGGT
TACCAGCTTTATTTACATAAGGCTACTAACGGTAACACTAATGCTACTGCGCGACTGCGCATTTGCCAGTTTCCTAGCA
TTAAAACATTGGGCCCCACTGCTAATAATGATGTTACAACAGGTCGTAATTGCCTATTTAACAAAGCCATCCCAGCTCA
TATGAATGAACATAGTGTTGTCGGCATAACATGGGATAATGATCGTGTCACTGTCTTTTCTGACAAGATCTATTATTTT
TATTTTAAAAATGATTGGTCCCGTGTTGCGACAAAGTGTTACAACAGTGGAGGTTGTGCTATGCAATATGTTTACGAAC
CCACCTATTACATGCTTAATGTTACTAGTGCTGGTGAGGATGGTATTTCTTATCAACCCTGTACAGCTAATTGCATTGG
TTATGCTGCCAATGTATTTGCTACTGAGCCCAATGGCCACATACCAGAAGGTTTTAGTTTTAATAATTGGTTTCTTTTG
TCCAATGATTCCACTTTGGTGCATGGTAAGGTGGTTTCCAACCAACCATTGTTGGTCAATTGTCTTTTGGCCATCCCTA
AGATTTATGGACTAGGCCAATTTTTCTCCTTTAATCAAACGATCGATGGTGTTTGTAATGGAGCTGCTGTGCAGCGTGC
ACCAGAGGCTCTGAGGTTTAATATTAATGACACCTCTGTCATTCTTGCCGAAGGCTCAATTGTACTTCATACTGCTTTA
GGAACAAATTTTTCTTTTGTTTGCAGTAATTCCTCAGATCCTCATTTAGCTACCTTCGCCATACCTCTGGGTGCTACCC
AAGTACCCTATTATTGTTTTCTTAAAGTGGATACTTACAACTCCACTGTTTATAAATTCTTGGCTGTTTTACCTCCTAC
CGTCAGGGAAATTGTCATCACCAAGTATGGTGATGTTTATGTCAATGGGTTTGGATACTTGCATCTCGGTTTGTTGGAT
GCTGTCACAATTAATTTCACTGGTCATGGCACTGACGATGATGTTTCTGGTTTTTGGACCATAGCATCGACTAATTTTG
TTGATGCACTCATCGAAGTTCAAGGAACTGCCATTCAGCGTATTCTTTATTGTGATGATCCTGTTAGCCAACTCAAGTG
TTCTCAGGTTGCTTTTGACCTTGACGATGGTTTTTACCCTATTTCTTCTAGAAACCTTCTGAGTCATGGACAGCCAATT
TCTTTTGTTACTCTGCCATCATTTAATGATCATTCTTTTGTTAACATTACTGTCTCTGCTTCCTTTGGTGGTCGTAGTG
GTGCCAACCTTACTGCATCTGACACTACTATCAATGGGTTTAGTTCTTTCTGTGTTGACACTAGACAATTTACCATTTC
ACTGTTTTATAACGTTACAAACAGTTATGGTTATGTGTCTAAATCACAGGACAGTAATTGCCCTTTCACCTTGCAATCT
GTTAATGATTACCTGTCTTTTAGCAACTTTTGTGTTTCCACCAGCCTTTTGGCTAGTGCCTGTACCATAGATCTTTTTG
GTTACCCTGAGTTTGGTAGTGGTGTTAAGTTTACGTCCCTTTACTTTCAATTCACAAAGGGTGAGTTGATTACTGGCAC
GCCTAAACCACTTGAAGGTGTCACGGACGTTTCTTTTATGACTCTGGATGTGTGTACCGAGTATACTATCTATGGCTTT
AAAGGTGAGGGTATCATTACCCTTACAAATTCTAGCTTTTTGGCAGGTGTTTATTACACATCTGATTCTGGACAGTTGT

TAGCCTTTAAGAATGTCACTAGTGGTGCTGTTTATTCTGTTACGCCATGTTCTTTTTCAGAGCAGGCTGCATATGTTGA
TGATGATATAGTGGGTGTTATTTCTAGTTTGTCTAGCTCCACTTTTAACAGTACTAGGGAGTTGCCTGGTTTCTTCTAC
CATTCTAATGATGGCTCTAATTGTACAGAGCCTGTGTTGGTGTATAGTAACATAGGTGTTTGTAAATCTGGCAGTATTG
GCTACGTCCCATCTCAGTCTGGCCAAGTCAAGATTGCACCCACGGTTACTGGGAATATTAGTATTCCCACCAACTTTAG
TATGAGTATTAGGACAGAATATTTACAGCTTTACAACACGCCTGTTAGTGTTGATTGTGCCACATATGTTTGTAATGGT
AACTCTCGTTGTAAACAATTACTCACCCAGTACACTGCAGCATGTAAGACCATAGAGTCAGCATTACAACTCAGCGCTA
GGCTTGAGTCTGTTGAAGTTAACTCTATGCTTACTATTTCTGAAGAGGCTCTACAGTTAGCTACCATTAGTTCGTTTAA
TGGTGATGGGTATAATTTTACTAATGTGATGGGTGTTTCTGTGTATGATCCTGCAAGTGGCAGGGTGGTACAAAAAAGG
TCTTTTATTGAAGACCTGCTTTTTAATAAAGTGGTTACTAATGGCCTTGGTACTGTTGATGAAGACTATAAGCGCTGTT
CTAATGGTCGCTCTGTGGCAGATCTAGTCTGTGCACAGTATTACTCTGGTGTCATGGTACTACCTGGTGTTGTTGACGC
TGAGAAGCTTCACATGTATAGTGCGTCTCTCATCGGTGGTATGGTGCTAGGAGGTTTTACTTCTGCAGCGGCATTGCCT
TTTAGCTATGCTGTTCAAGCTAGACTCAATTATCTTGCTCTACAGACGGATGTTCTACAGCGGAACCAGCAATTGCTTG
CTGAGTCTTTTAACTCTGCTATTGGTAATATAACTTCAGCCTTTGAGAGTGTTAAAGAGGCTATTAGTCAAACTTCCAA
GGGTTTGAACACTGTGGCTCATGCGCTTACTAAGGTTCAAGAGGTTGTTAACTCGCAGGGTGCAGCTTTGACTCAACTT
ACCGTACAGCTGCAACACAACTTCCAAGCCATTTCTAGTTCTATTGATGACATTTACTCTCGACTGGACATTCTTTCAG
CCGATGTTCAGGTTGACCGTCTCATCACCGGCAGATTATCAGCACTTAATGCTTTTGTTGCTCAAACCCTCACTAAGTA
TACTGAGGTTCAGGCTAGCAGGAAGCTAGCACAGCAAAAGGTTAATGAGTGCGTTAAGTCGCAATCTCAGCGTTATGGT
TTTTGTGGTGGTGATGGCGAGCACATTTTCTCTCTGGTACAGGCAGCACCTCAGGGCCTGCTGTTTTTACATACAGTAC
TTGTACCGGGTGATTTTGTAGATGTTATTGCCATCGCTGGCTTATGCGTTAACGATGAAATTGCCTTGACTCTACGTGA
GCCTGGCTTAGTCTTGTTTACGCATGAACTTCAAAATCATACTGCGACGGAATATTTTGTTTCATCGCGACGTATGTTT
GAACCTAGAAAACCTACCGTTAGTGATTTTGTTCAAATTGAGAGTTGTGTGGTCACCTATGTCAATTTGACTAGAGACC
AACTACCAGATGTAATCCCAGATTACATCGATGTTAACAAAACACTTGATGAGATTTTAGCTTCTCTGCCCAATAGAAC
TGGTCCAAGTCTTCCTTTAGATGTTTTTAATGCCACTTATCTTAATCTCACTGGTGAAATTGCAGATTTAGAGCAGCGT
TCAGAGTCTCTCCGTAATACTACAGAGGAGCTCCAAAGTCTTATATATAATATCAACAACACACTAGTTGACCTTGAGT
GGCTCAACCGAGTTGAGACATATATCAAGTGGCCGTGGTGGGTTTGGTTGATTATTTTCATTGTTCTCATCTTTGTTGT
GTCATTACTAGTGTTCTGCTGCATTTCCACGGGTTGTTGTGGATGCTGCGGCTGCTGCTGTGCTTTTTCTTCAGGTTGT
TGTAGGGGTCCTAGACTTCAACCTTACGAAGTTTTTGAAAAGGTCCACGTGCAGTGA

>gi|455475446|gb|KC242898.1|_Porcine_epidemic_diarrhea_virus_isolate_HeN1220122_spike_protein_gene_complete_cds

ATGAAGTCTTTAACCTACTTCTGGTTGTTCTTACCAGTACTTTCAACACTTAGCCTACCACAAGATGTCACCAGG
TGCTCAGCTAACACTAATTTTAGGCGGTTCTTTTCAAAATTTAATGTTCAGGCGCCTGCAGTTGTTGTACTGGGCGGTT
ATCTACCTATTGGTGAAAACCAGGGTGTCAATTCAACTTGGTACTGTGCTGGCCAACATCCAACTGCTAGTGGCGTTCA
TGGTATCTTTGTTAGCCATATTAGAGGTGGTCATGGCTTTGAGATTGGCATTTCGCAAGAGCCTTTTGACCCTAGTGGT
TACCAGCTTTATTTACATAAGGCTACTAACGGTAACACTAATGCTACTGCGCGACTGCGCATTTGCCAGTTTCCTAGCA
TTAAAACATTGGGCCCCACTGCTAATAATGGTGTTACAACAGGTCGTAATTGCCTATTTAACAAAGCCATCCCAGCTCA
TATGAGTGAACATAGTGTTGTCGGCATAACATGGGATAATGATCGTGTCACTGTCTTTTCTGACAAGATCTATTATTTT
TATTTTAAAAATGATTGGTCCCGTGTTGCGACAAAGTGTTACAACAGTGGAGGTTGTGCTATGCAATATGTTTACGAAC
CCACCTATTACATGCTTAATGTTACTAGTGCTGGTGAGGATGGTATTTCTTATCAACCCTGTACAGCTAATTGCATTGG
TTATGCTGCCAATGTATTTGCTACTGAGCCCAATGGCCACATACCAGAAGGTTTTAGTTTTAATAATTGGTTTCTTTTG
TCCAATGATTCCACTTTGGTGCATGGTAAGGTGGTTTCCAACCAACCATTGTTGGTCAATTGTCTTTTGGCCATTCCTA
AGATTTATGGACTAGGCCAATTTTTCTCCTTTAATCAAACGATCGATGGTGTTTGTAATGGAGCTGCTGTGCAGCGTGC

ACCAGAGGCTCTGAGGTTTAATATTAATGACACCTCTGTCATTCTTGCTGAAGGCTCAATTGTACTTCATACTGCTTTA
GGAACAAATTTTTCTTTTGTTTGCAGTAATTCCTCAAATCCTCACTTAGCCACCTTCGCCATACCTCTGGGTGCTACCC
AAGTACCCTATTATTGTTTTCTTAAAGTGGATACTTACAACTCCACTGTTTATAAATTCTTGGCTGTTTTACCTCCTAC
CGTCAGGGAAATTGTCATCACCAAGTATGGTGATGTTTATGTCAATGGGTTTGGCTATTTGCATCTCGGTTTGTTGGAC
GCTGTCACAATTAATTTCACTGGTCATGGCACTGACGATGACGTTTCAGGTTTCTGGACCATAGCATCGACTAATTTTG
TTGATGCACTTATCGAAGTTCAAGGAACTGCCATTCAGCGTATTCTTTATTGTGATGATCCTGTTAGCCAACTCAAGTG
TTCTCAGGTTGCTTTTGACCTTGACGATGGTTTCTACCCTATTTCTTCTAGAAACCTTCTGAGTCATGAACAGCCAACT
TCTTTTGTTACTTTGCCATCATTTAATGATCATTCTTTTGTTAATATTACTGTCTCTGCTGCTTTTGGTGGTCATAGTG
GTGCCAACCTTATTGCATCTGACACTACTATCAATGGGTTTAGTTCTTTCTGTGTTGACACTAGACAATTTACCATTTC
ACTGTTTTATAATGTTACAAACAGTTATGGTTATGTGTCTAACTCACAGGACAGTAATTGCCCTTTCACCTTGCAATCT
GTTAATGATTACCTGTCTTTTAGCAAATTTTGTGTTTCTACCAGCCTTTTGGCTAGTGCCTGTACCATAGATCTTTTTG
GTTACCCTGAGTTTGGTAGTGGTGTTAAATTCACGTCCCTTTACTTTCAATTCACAAAGGGTGAGTTGATTACTGGCAC
GCCTAAACCACTTGAAGGTGTTACGGACGTTTCTTTTATGACTCTGGATGTGTGTACCAAGTATACTATCTATGGCTTT
AAAGGTGAGGGTATCATTACCCTTACAAATTCTAGCTTTTTGGCAGGTGTTTATTATACATCTGATTCTGGACAGTTGT
TAGCTTTTAAGAATGTCACTAGTGGTGCTGTTTATTCTGTTACGCCATGTTCTTTTTCAGAGCAGGCTGCATATGTTGA
TGATGATATAGTGGGTGTTATTTCTAGTTTGTCTAGCTCCACTTTTAACAGTACTAGGGAGTTGCCTGGTTTCTTCTAC
CATTCTAATGATGACTCTAATTGTACAGAGCCTGTGTTGGTGTATAGTAACATTGGTGTTTGTAAATCTGGCAGTATTG
GCTATGTCCCATCTCAGTCTGGCCAAGTCAAGATTGCACCCATGGTTACTGGGAATATTAGTATTCCCACCAACTTTAG
TATGAGTATTAGGACAGAATATTTACAGCTTTACAACACGCCTGTTAGTGTTGATTGTGCTACATATGTTTGTAATGGT
AACTCTCGTTGTAAACAATTACTCACCCAGTACATTGCAGCATGTAAGACTATAGAGTCAGCATTACAACTCAGCGCTA
GGCTTGAGTCTGCTGAAGTTAACTCTATGCTTACTATTTCTGAAGAGGCTCTACAGTTAGCTACCATCAGTTCGTTTAA
TGGTGATGGATATAATTTTACTAATGTGCTGGGTGTTTCCGTTCGTGATCCTGCAAGTGGCAGGATGGTACAAAAAAGG
TCTTTTATTGAAGACCCGCTTTTTAATAAAGTGGTTACTAATGGCCTTGGTACTGTTGATGAAGACTATAAGCGCTGTT
CTAATGGCCGCTCTGTGGCAGATTTAGTCTGTGCGCAGTATTACTCTGGTGTCATGGTACTACCTGGTGTTGTTGACGC
TGAGAAGCTTCATATGTATAGTGCGTCTCTCATCGGTGGTATGGTGCTAGGAGGTTTTACTTCTGCAGCGGCATTGCCT
TTTAGCTATGCTGTTCAAGCTAGACTCAATTATCTTGCTCTACAGACGGATGTTCTACAGCGGAACCAGCAATTGCTTG
CTGAGTCTTTTAACTCTGCTATTGGTAATATAACTTCAGCCTTTGAGAGTGTTAAAGAGGCTATTAGTCAAACTTCCAA
GGGTTTGAACACTGTGGCTCATGCGCTTACTAAGGTTCAAGAGGTTGTTAACTCGCATGGTGCAGCTTTGACTCAACTT
ACCGTACAGCTGCAACACAACTTCCAAGCCATTTCTAGTTCTATTGATGACATTTACTCTCGACTGGACATTCTTTCAG
CCGATGTTCAGGTTGACCGTCTCATCACCGGCAGATTATCAGCACTTAATGCTTTTGTTGCTCAAACCCTCACTAAGTA
TACTGAGGTTCAGGCTAGCAGGAAGCTAGCACAGCAAAAGGTTAATGAGTGCGTTAAATCGCAATCTCAGCGTTATGGT
TTTTGTGGTGGTGATGGCGAGCACATTTTCTCTCTGGTACAGGCAGCACCTCAGGGCCTGCTGTTTTTACATACAGTAC
TTGTACCGGGTGACTTTGTAGATGTTATTGCCATCGCTGGCTTATGCGTTAACGATGAAATTGCCTTGACTCTACGTGA
GCCTGGCTTAGTCTTGTTTACGCATGAACTTCAAAATCATACTGCGACGGAATATTTTGTTTCATCGCGACGTATGTTT
GAACCTAGAAAACCTACCGTTAGTGATTTTGTTCAAATTGAGAGTTGTGTGGTCACCTATGTCAATTTGACTAGAGACC
AACTACCAGATGTAATCCCAGATTACATCGATGTTAACAAAACACTTGATGAGATTTTAGCTTCTCTGCCCAATAGAAC
TGGTCCAAGTCTTCCTTTAGATGTTTTTAATGCCACTTATCTTAATCTCACTGGTGAAATTGCAGATTTAGAGCAGCGT
TCAGAGTCTCTCCGTAATACTACAGAGGAGCTCCAAAGTCTTATATATAATATCAACAACACACTAGTTGACCTTGAGT
GGCTCAACCGAGTTGAGACATATATCAAGTGGCCGTGGTGGGTTTGGTTGATTATTTCATTGTTCTCATCTTTGTTGT
GTCATTACTAGTGTTCTGCTGCATTTCCACTGGTTGTTGTGGATGCTGCGGCTGCTGCTGTGCTCGTTTTTCAGGTTGT
TGTAGGGGTCCTAGACTTCAACCTTACGAAGTTTTTGAAAAGGTCCACGTGCAGTGA

>gi|455475448|gb|KC242899.1|_Porcine_epidemic_diarrhea_virus_isolate_HB1201

22_spike_protein_gene_complete_cds

ATGAAGTCTTTAACCTACTTCTGGTTGTCATTACCAGTACTTTCAACACTTAGCCTACCACAAGATGTCACCAGG
TGCTCAGCTAACACTAATTTTAGGCGGTTCTTTTCAAAATTTAATGTTCAGGCGCCTGCAGTTGTTGTACTGGGCGGTT
ATCTACCTATTGGTGAAAACCAGGGTGTTAATTCAACCTGGTACTGTGCTGGCCAACATCCAACTGCTAGTGGCGTTCA
TGGTATCTTTGTTAGCCATATTCGAGGTGGTCATGGCTTTGAGATTGGCATTTCGCAAGAGCCTTTTGACCCTAGTGGT
TACCAGCTTTATTTACATAAGGCTACTAACGGTAACACTAATGCTACTGCGCGACTGCGCATTTGCCAGTTTCCTAGCA
TTAAAACATTGGGCCCCACTGCTAATAATGATGTTACAACAGGTCGTAATTGCCTATTTAACAAAGCCATCCCAGCTCA
TATGAGTGAACATAGTGTTGTCGGCATAACATGGGATAATGATCGTGTCACTGTCTTTTCTGACAAGATCTATTATTTT
TATTTTAAAAATGATTGGTCCCGTGTTGCGACAAAGTGTTACAACAGTGGAGGTTGTGCTATGCAATATGTTTACGAAC
CCACCTATTACATGCTTAATGTTACTAGTGCTGGTGAGGATGGTATTTCTTATCAACCCTGTACAGCTAATTGCATTGG
TTATGCTGCCAATGTATTTGCTACTGAGCCCAATGGCCACATACCAGAAGGTTTTAGTTTTAATAATTGGTTTCTTTTG
TCCAATGATTCCACTTTGGTGCGTGGTAAGGTGGTTTCCAACCAACCATTGCTGGTCAATTGTCTTTTGGCCATTCCTA
AGATTTATGGACTAGGCCAATTTTTCTCCTTTAATCAAACGATCGATGGTGTTTGTAATGGAGCTGCTGTGCAGCGTGC
ACCAGAGGCTCTGAGGTTTAATATTAATGACACCTCTGTCATTCTTGCTGAAGGCTCAATTGTACTTCATACTGCCTTA
GGAACAAATTTTTCTTTTGTTTGCAGTAATTCCTCAAATCCTCACTTAGCCACCTTCGCCATACCTCTGGGTGCTACCC
AAGTACCCTATTATTGTTTTCTTAAAGTGGATACTTACAACTCCACTGTTTATAAATTCTTGGCTGTTTTACCTCCTAC
CGTCAGGGAAATTGTCATCACCAAGTATGGTGATGTTTATGTCAATGGGTTTGGTTACTTGCATCTCGGTTTGTTGGAT
GCTGTCACAATTAATTTCACTGGTCATGGCACTGACGATGACGTTTCAGGTTTTTGGACCATAGCATCGACTAATTTTG
TTGATGCACTTATCGAAGTTAAAGGAACTGCCATTCAGCGTATTCTTTATTGTGATGATCCCGTTAGCCAACTCAAGTG
TTCTCAGGTTTCTTTTGACCTTGACGATGGTTTTTACCCTATTTCTTCTACAAACCTTCTGAGTCATGAACAGCCAATT
TCTTTTGTTACTTTGCCATCATTTAATGATCATTCTTTTGTTAACATTACTGTCTCTGCGTCCTTTGGTGGTCATAGTG
GTGCCAACCTTATTGCATCTGACACTACTATCAATGGGTTTAGTTCTTTCTGTGTTGACACTAGACAATTTACCATTTC
ACTGTTTTATAACGTTACAAACAGTTATGGTTATGTGTCTAACTCACAGGACAGTAATTGCCCTTTCACCTTGCAATCT
GTTAATGATTACCTGTCTTTTAGTAAATTTTGTGTTTCCACCAGCCTTTTGGCTAGTGCCTGTACCATAGATCTTTTTG
GTTACCCTGAGTTTGGTAGTAGTGTTAAGTTTACGTCCCTTTACTTTCAATTCACAAAGGGTGAGTTGATTACTGGCAC
GCCTAAACCACTTGAAGGTGTCACGGACGTTTCTTTTATGACTCTGGATGTGTGTACCAAGTATACTATCTATGGCTTT
AAAGGTGAGGGTATCATTACCCTTATAAATTCTAGCTTTTTGGCAGGTGTTTATTACACATCTGATTCTGGACAGTTGT
TAGCCTTTAAGAATGTCACTAGTGGTGCTGTTTATTCTGTTACGCCATGTTCTTTTTCAGAGCAGGCTGCATATGTTGA
TGATGATGTAGTGGGTGTTATTTCTAGTTTGTCTAGCTCCACTTTTAACAGTACTAGGGAGTTGCCTGGTTTCTTCTAC
CATTCTAATGATGGCTCTAATTGTACAGAGCCTGTGTTGGTGTATAGTAACATAGGTGTTTGTAAATCTGGCAGTATTG
GCTACGTCCCATCTCAGTCTGGCCAAGTCAAGATTGCACCCACGGTTACTGGGAATATTAGTATTCCCACCAACTTTAG
TATGAGTATTAGGACAGAATATTTACAGCTTTACAACACGCCTGTTAGTGTTGATTGTGCCACATATGTTTGTAATGGT
AACTCTCGTTGTAAACAATTACTCACCCAGTACACTGCAGCATGTAAGACCATAGAGTCAGCATTACAACTCAGCGCTA
GGCTTGAGTCTGTTGAAGTTAACTCTATGCTTACTATTTCTGAAGAGGCTCTACAGTTAGCTACCATCAGTTCGTTTAA
TGGTGATGGATATAATTTTACTAATGTGCTGGGTGTTTCTGTGTATGATCCTGCAAGTGGCAGGGTGGTACAAAAAAGG
TCTTTTATTGAAGACCTGCTTTTTAATAAAGTGGTTACTAATGGCCTTGGTACTGTTGATGAAGACTATAAGCGCTGTT
CTAATGGTCGCTCTGTGGCAGATCTAGTCTGTGCACAGTATTACTCTGGTGTCATGGTACTACCTGGTGTTGTTGACGC
TGAGAAGCTTCACATGTATAGTGCGTCTCTCATCGGTGGTATGGTGCTAGGAGGTTTTACTTCTGCAGCGGCATTGCCT
TTTAGCTATGCTGTTCAAGCTAGACTCAATTATCTTGCTCTACAGACGGATGTTCTACAGCGGAACCAGCAATTGCTTG
CTGAGTCTTTTAACTCTGCTATTGGTAATATAACTTCAGCCTTTGAGAGTGTTAAAGAGGCTATTAGTCAAACTTCCAA
GGGTTTGAACACTGTGGCTCATGCGCTTACTAAGGTTCAAGAGGTTGTTAACTCGCATGGTGCAGCTTTGACTCAACTT
ACCGTACAGCTGCAACACAACTTCCAAGCCATTTCTAGTTCTATTGATGACATTTACTCTCGACTGGACATTCTTTCAG
CCGATGTTCAGGTTGACCGTCTCATCACCGGCAGATTATCAGCACTTAATGCTTTTGTTGCTCAAACCCTCACTAAGTA

TACTGAGGTTCAGGCTAGCAGGAAGCTAGCACAGCAAAAGGTTAATGAGTGCGTTAAATCGCAATCTCAGCGTTATGGT
TTTTGTGGTGGTGATGGCGAGCACATTTTCTCTCTGGTACAGGCAGCACCTCAGGGCCTGCTGTTTTTACATACAGTAC
TTGTACCGGGTGACTTTGTAGATGTTATTGCCATCGCTGGCTTATGCGTTAACGATGAAATTGCCTTGACTCTACGTGA
GCCTGGCTTAGTCTTGTTTACGCATGAACTTCAAAATCATACTGCGACGGAATATTTTGTTTCATCGCGACGTATGTTT
GAACCTAGAAAACCTACCGTTAGTGATTTTGTTCAAATTGAGAGTTGTGTGGTCACCTATGTCAATTTGACTAGAGACC
AACTACCAGATGTAATCCCAGATTACATCGATGTTAACAAAACACTTGATGAGATTTTAGCTTCTCTGCCCAATAGAAC
TGGTCCAAGTCTTCCTTTAGATGTTTTTAATGCCACTTATCTTAATCTCACTGGTGAAATTGCAGATTTAGAGCAGCGT
TCAGAGTCTCTCCGTAATACTACAGAGGAGCTCCAAAGTCTTATATATAATATCAACAACACACTAGTTGACCTTGAGT
GGCTCAACCGAGTTGAGACATATATCAAGTGGCCGTGGTGGGTTTGGTTGATTATTTTCATTGTTCTCATCTTTGTTGT
GTCATTACTAGTGTTCTGCTGCATTTCCACTGGTTGTTGTGGATGCTGCGGCTGCTGCTGTGCTCGTTTTTCAGGTTGT
TGTAGGGGTCCTAGACTTCAACCTTACGAAGTTTTTGAAAAGGTCCACGTGCAGTGA

>gi|455475450|gb|KC242900.1|_Porcine_epidemic_diarrhea_virus_isolate_HB2201
22_spike_protein_gene_complete_cds

ATGAAGTCTTTAACCTACTTCTGGTTGTTCTTACCAGTACTTTCAACACTTAGCCTACCACAAGATGTCACCAGG
TGCTCAGCTAACACTAATTTTAGGCGGTTCTTTTCAAAATTTAATGTTCAGGCGCCTGCAGTTGTTGTACTGGGCGGTT
ATCTACCTATTGGTGAAAACCAGGGTGTCAATTCAACTTGGTACTGTGCTGGCCAACATCCAACTGCTAGTGGCGTTCA
TGGTATCTTTGTTAGCCATATTAGAGGTGGTCATGGCTTTGAGATTGGCATTTCGCAAGAGCCTTTTGACCCTAGTGGT
TACCAGCTTTATTTACATAAGGCTACTAACGGTAACACTAATGCTACTGCGCGACTGCGCATTTGCCAGTTTCCTAGCA
TTAAAACATTGGGCCCCACTGCTAATAATGGTGTTACAACAGGTCGTAATTGCCTATTTAACAAAGCCATCCCAGCTCA
TATGAGTGAACATAGTGTTGTCGGCATAACATGGGATAATGATCGTGTCACTGTCTTTTCTGACAAGATCTATTATTTT
TATTTTAAAAATGATTGGTCCCGTGTTGCGACAAAGTGTTACAACAGTGGAGGTTGTGCTATGCAATATGTTTACGAAC
CCACCTATTACATGCTTAATGTTACTAGTGCTGGTGAGGATGGTATTTCTTATCAACCCTGTACAGCTAATTGCATTGG
TTATGCTGCCAATGTATTTGCTACTGAGCCCAATGGCCACATACCAGAAGGTTTTAGTTTTAATAATTGGTTTCTTTTG
TCCAATGATTCCACTTTGGTGCATGGTAAGGTGGTTTCCAACCAACCATTGTTGGTCAATTGTCTTTTGGCCATTCCTA
AGATTTATGGACTAGGCCAATTTTTCTCCTTTAATCAAACGATCGATGGTGTTTGTAATGGAGCTGCTGTGCAGCGTGC
ACCAGAGGCTCTGAGGTTTAATATTAATGACACCTCTGTCATTCTTGCTGAAGGCTCAATTGTACTTCATACTGCTTTA
GGAACAAATTTTTCTTTTGTTTGCGGTAATTCCTCAAATCCTCACTTAGCCACCTTCGCCATACCTCTGGGTGCTACCC
AAGTACCCTATTATTGTTTTCTTAAAGTGGATACTTACAACTCCACTGTTTATAAATTCTTGGCTGTTTTACCTCCTAC
CGTCAGGGAAATTGTCATCACCAAGTATGGTGATGTTTATGTCAATGGGTTTGGCTATTTGCATCTCGGTTTGTTGGAC
GCTGTCACAATTAATTTCACTGGTCATGGCACTGACGATGACGTTTCAGGTTTCTGGACCATAGCATCGACTAATTTTG
TTGATGCACTTATCGAAGTTCAAGGAACTGCCATTCAGCGTATTCTTTATTGTGATGATCCTGTTAGCCAACTCAAGTG
TTCTCAGGTTGCTTTTGACCTTGACGATGGTTTCTACCCTATTTCTTCTAGAAACCTTCTGAGTCATGAACAGCCAACT
TCTTTTGTTACTTTGCCATCATTTAATGATCATTCTTTTGTTAATATTACTGTCTCTGCTGCTTTTGGTGGTCATAGTG
GTGCCAACCTTATTGCATCTGACACTACTATCAATGGGTTTAGTTCTTTCTGTGTTGACACTAGACAATTTACCATTTC
ACTGTTTTATAATGTTACAAACAGTTATGGTTATGTGTCTAACTCACAGGACAGTAATTGCCCTTTCACCTTGCAATCT
GTTAATGATTACCTGTCTTTTAGCAAATTTTGTGTTTCTACCAGCCTTTTGGCTAGTGCCTGTACCATAGATCTTTTTG
GTTACCCTGAGTTTGGTAGTGGTGTTAAATTCACGTCCCTTTACTTTCAATTCACAAAGGGTGAGTTGATTACTGGCAC
GCCTAAACCACTTGAAGGTGTTACGGACGTTTCTTTTATGACTCTGGATGTGTGTACCAAGTATACTATCTATGGCTTT
AAAGGTGAGGGTATCATTACCCTTACAAATTCTAGCTTTTTGGCAGGTGTTTATTATACATCTGATTCTGGACAGTTGT
TAGCTTTTAAGAATGTCACTAGTGGTGCTGTTTATTCTGTTACGCCATGTTCTTTTTCAGAGCAGGCTGCATATGTTGA
TGATGATATAGTGGGTGTTATTTCTAGTTTGTCTAGCTCCACTTTTAACAGTACTAGGGAGTTGCCTGGTTTCTTCTAC

CATTCTAATGATGACTCTAATTGTACAGAGCCTGTGTTGGTGTATAGTAACATTGGTGTTTGTAAATCTGGCAGTATTG
GCTATGTCCCATCTCAGTCTGGCCAAGTCAAGATTGCACCCATGGTTACTGGGAATATTAGTATCCCCACCAACTTTAG
TATGAGTATTAGGACAGAATATTTACAGCTTTACAACACGCCTGTTAGTGTTGATTGTGCTACATATGTTTGTAATGGT
AACTCTCGTTGTAAACAATTACTCACCCAGTACATTGCAGCATGTAAGACTATAGAGTCAGCATTACAACTCAGCGCTA
GGCTTGAGTCTGCTGAAGTTAACTCTATGCTTACTATTTCTGAAGAGGCTCTACAGTTAGCTACCATCAGTTCGTTTAA
TGGTGATGGATATAATTTTACTAATGTGCTGGGTGTTTCCGTTCGTGATCCTGCAAGTGGCAGGATGGTACAAAAAAGG
TCTTTTATTGAAGACCTGCTTTTTAATAAAGTGGTTACTAATGGCCTTGGTACTGTTGATGAAGACTATAAGCGCTGTT
CTAATGGCCGCTCTGTGGCAGATTTAGTCTGTGCGCAGTATTACTCTGGTGTCATGGTACTACCTGGTGTTGTTGACGC
TGAGAAGCTTCACATGTATAGTGCGTCTCTCATCGGTGGTATGGTGCTAGGAGGTTTTACTTCTGCAGCGGCATTGCCT
TTTAGCTATGCTGTTCAAGCTAGACTCAATTATCTTGCTCTACAGACGGATGTTCTACAGCGGAACCAGCAATTGCTTG
CTGAGTCTTTTAACTCTGCTATTGGTAATATAACTTCAGCCTTTGAGAGTGTTAAAGAGGCTATTAGTCAAACTTCCAA
GGGTTTGAACACTGTGGCTCATGCGCTTACTAAGGTTCAAGAGGTTGTTAACTCGCATGGTGCAGCTTTGACTCAACTT
ACCGTACAGCTGCAACACAACTTCCAAGCCATTTCTAGTTCTATTGATGACATTTACTCTCGACTGGACATTCTTTCAG
CCGATGTTCAGGTTGACCGTCTCATCACCGGCAGATTATCAGCACTTAATGCTTTTGTTGCTCAAACCCTCACTAAGTA
TACTGAGGTTCAGGCTAGCAGGAAGCTAGCACAGCAAAAGGTTAATGAGTGCGTTAAATCGCAATCTCAGCGTTATGGT
TTTTGTGGTGGTGATGGCGAGCACATTTTCTCTCTGGTACAGGCAGCACCTCAGGGCCTGCTGTTTTTACATACAGTAC
TTGTACCGGGTGACTTTGTAGATGTTATTGCCATCGCTGGCTTATGCGTTAACGATGAAATTGCCTTGACTCTACGTGA
GCCTGGCTTAGTCTTGTTTACGCATGAACTTCAAAATCATACTGCGACGGAATATTTTGTTTCATCGCGACGTATGTTT
GAACCTAGAAAACCTACCGTTAGTGATTTTGTTCAAATTGAGAGTTGTGTGGTCACCTATGTCAATTTGACTAGAGACC
AACTACCAGATGTAATCCCAGATTACATCGATGTTAACAAAACACTTGATGAGATTTTAGCTTCTCTGCCCAATAGAAC
TGGTCCAAGTCTTCCTTTAGATGTTTTTAATGCCACTTATCTTAATCTCACTGGTGAAATTGCAGATTTAGAGCAGCGT
TCAGAGTCTCTCCGTAATACTACAGAGGAGCTCCAAAGTCTTATATATAATATCAACAACACACTAGTTGACCTTGAGT
GGCTCAACCGAGTTGAGACATATATCAAGTGGCCGTGGTGGGTTTGGTTGATTATTTTCATTGTTCTCATCTTTGTTGT
GTCATTACTAGTGTTCTGCTGCATTTCCACTGGTTGTTGTGGATGCTGCGGCTGCTGCTGTGCTTGTTTTTCAGGTTGT
TGTAGGGGTCCTAGACTTCAACCTTACGAAGTTTTTGAAAAGGTCCACGTGCAGTGA

>gi|455475454|gb|KC242902.1|_Porcine_epidemic_diarrhea_virus_isolate_HB320123_spike_protein_gene_complete_cds

ATGAAGTCTTTAACCTACTTCTGGTTGTTCTTACCAGTACTTTCAACACTTAGCCTACCACAAGATGTCACCAGG
TGCTCAGCTAACACTAATTTTAGGCGGTTCTTTTCAAAATTTAATGTTCAGGCGCCTGCAGTTGTTGTACTGGGCGGTT
ATCTACCTATTGGTGAAAACCAGGGTGTCAATTCAACTTGGTACTGTGCTGGCCAACATCCAACTGCTAGTGGCGTTCA
TGGTATCTTTGTTAGCCATATTAGAGGTGGTCATGGCTTTGAGATTGGCATTTCGCAAGAGCCTTTTGACCCTAGTGGT
TACCAGCTTTATTTACATAAGGCTACTAACGGTAACACTAATGCTACTGCGCGACTGCGCATTTGCCAGTTTCCTAGCA
TTAAAACATTGGGCCCCACTGCTAATAATGGTGTTACAACAGGTCGTAATTGCCTATTTAACAAAGCCATCCCAGCTCA
TATGAGTGAACATAGTGTTGTCGGCATAACATGGGATAATGATCGTGTCACTGTCTTTTCTGACAAGATCTATTATTTT
TATTTTAAAAATGATTGGTCCCGTGTTGCGACAAAGTGTTACAACAGTGGAGGTTGTGCTATGCAATATGTTTACGAAC
CCACCTATTACATGCTTAATGTTACTAGTGCTGGTGAGGATGGTATTTCTTATCAACCCTGTACAGCTAATTGCATTGG
TTATGCTGCCAATGTATTTGCTACTGAGCCCAATGGCCACATACCAGAAGGTTTTAGTTTTAATAATTGGTTTCTTTTG
TCCAATGATTCCACTTTGGTGCATGGTAAGGTGGTTTCCAACCAACCATTGTTGGTCAATTGTCTTTTGGCCATTCCTA
AGATTTATGGACTAGGCCAATTTTTCTCCTTTAATCAAACGATCGATGGTGTTTGTAATGGAGCTGCTGTGCAGCGTGC
ACCAGAGGCTCTGAGGTTTAATATTAATGACACCTCTGTCATTCTTGCTGAAGGCTCAATTGTACTTCATACTGCTTTA
GGAACAAATTTTTCTTTTGTTTGCAGTAATTCCTCAAATCCTCACTTAGCCACCTTCGCCATACCTCTGGGTGCTACCC

AAGTACCCTATTATTGTTTTCTTAAAGTGGATACTTACAACTCCACTGTTTATAAATTCTTGGCTGTTTTACCTCCTAC
CGTCAGGGAAATTGTCATCACCAAGTATGGTGATGTTTATGTCAATGGGTTTGGCTATTTGCATCTCGGTTTGTTGGAC
GCTGTCACAATTAATTTCACTGGTCATGGCACTGACGATGACGTTTCAGGTTTCTGGACCATAGCATCGACTAATTTTG
TTGATGCACTTATCGAAGTTCAAGGAACTGCCATTCAGCGTATTCTTTATTGTGATGATCCTGTTAGCCAACTCAAGTG
TTCTCAGGTTGCTTTTGACCTTGACGATGGTTTCTACCCTATTTCTTCTAGAAACCTTCTGAGTCATGAACCGCCAACT
TCTTTTGTTACTTTGCCATCATTTAATGATCATTCTTTTGTTAATATTACTGTCTCTGCTGCTTTTGGTGGTCATAGTG
GTGCCAACCTTATTGCATCTGACACTACTATCAATGGGTTTAGTTCTTTCTGTGTTGACACTAGACAATTTACCATTTC
ACTGTTTTATAATGTTACAAACAGTTATGGTTATGTGTCTAACTCACAGGACAGTAATTGCCCTTTCACCTTGCAATCT
GTTAATGATTACCTGTCTTTTAGCAAATTTTGTGTTTCTACCAGCCTTTTGGCTAGTGCCTGTACCATAGATCTTTTTG
GTTACCCTGAGTTTGGTAGTGGTGTTAAATTCACGTCCCTTTACTTTCAATTCACAAAGGGTGAGTTGATTACTGGCAC
GCCTAAACCACTTGAAGGTGTTACGGACGTTTCTTTTATGACTCTGGATGTGTGTACCAAGTATACTATCTATGGCTTT
AAAGGTGAGGGTATCATTACCCTTACAAATTCTAGCTTTTTGGCAGGTGTTTATTATACATCTGATTCTGGACAGTTGT
TAGCTTTTAAGAATGTCACTAGTGGTGCTGTTTATTCTGTTACGCCATGTTCTTTTTCAGAGCAGGCTGCATATGTTGA
TGATGATATAGTGGGTGTTATTTCTAGTTTGTCTAGCTCCACTTTTAACAGTACTAGGGAGTTGCCTGGTTTCTTCTAC
CATTCTAATGATGACTCTAATTGTACAGAGCCTGTGTTGGTGTATAGTAACATTGGTGTTTGTAAATCTGGCAGTATTG
GCTATGTCCCATCTCAGTCTGGCCAAGTCAAGATTGCACCCATGGTTACTGGGAATATTAGTATTCCCACCAACTTTAG
TATGAGTATTAGGACAGAATATTTACAGCTTTACAACACGCCTGTTAGTGTTGATTGTGCTACATATGTTTGTAATGGT
AACTCTCGTTGTAAACAATTACTCACCCAGTACATTGCAGCATGTAAGACTATAGAGTCAGCATTACAACTCAGCGCTA
GGCTTGAGTCTGCTGAAGTTAACTCTATGCTTACTATTTCTGAAGAGGCTCTACAGTTAGCTACCATCAGTTCGTTTAA
TGGTGATGGATATAATTTTACTAATGTGCTGGGTGTTTCCGTTCGTGATCCTGCAAGTGGCAGGATGGTACAAAAAAGG
TCTTTTATTGAAGACCTGCTTTTTAATAAAGTGGTTACTAATGGCCTTGGTACTGTTGATGAAGACTATAAGCGCTGTT
CTAATGGCCGCTCTGTGGCAGATTTAGTCTGTGCGCAGTATTACTCTGGTGTCATGGTACTACCTGGTGTTGTTGACGC
TGAGAAGCTTCATATGTATAGTGCGTCTCTCATCGGTGGTATGGTGCTAGGAGGTTTTACTTCTGCAGCGGCATTGCCT
TTTAGCTATGCTGTTCAAGCTAGACTCAATTATCTTGCTCTACAGACGGATGTTCTACAGCGGAACCAGCAATTGCTTG
CTGAGTCTTTTAACTCTGCTATTGGTAATATAACTTCAGCCTTTGAGAGTGTTAAAGAGGCTATTAGTCAAACTTCCAA
GGGTTTGAACACTGTGGCTCATGCGCTTACTAAGGTTCAAGAGGTTGTTAACTCGCATGGTGCAGCTTTGACTCAACTT
ACCGTACAGCTGCAACACAACTTCCAAGCCATTTCTAGTTCTATTGATGACATTTACTCTCGACTGGACATTCTTTCAG
CCGATGTTCAGGTTGACCGTCTCATCACCGGCAGATTATCAGCACTTAATGCTTTTGTTGCTCAAACCCTCACTAAGTA
TACTGAGGTTCAGGCTAGCAGGAAGCTAGCACAGCAAAAGGTTAATGAGTGCGTTAAATCGCAATCTCAGCGTTATGGT
TTTTGTGGTGGTGATGGCGAGCACATTTTCTCTCTGGTACAGGCAGCACCTCAGGGCCTGCTGTTTTTACATACAGTAC
TTGTACCGGGTGACTTTGTAGATGTTATTGCCATCGCTGGCTTATGCGTTAACGATGAAATTGCCTTGACTCTACGTGA
GCCTGGCTTAGTCTTGTTTACGCATGAACTTCAAAATCATACTGCGACGGAATATTTTGTTTCATCGCGACGTATGTTT
GAACCTAGAAAACCTACCGTTAGTGATTTTGTTCAAATTGAGAGTTGTGTGGTCACCTATGTCAATTTGACTAGAGACC
AACTACCAGATGTAATCCCAGATTACATCGATGTTAACAAAACACTTGATGAGATTTTAGCTTCTCTGCCCAATAGAAC
TGGTCCAAGTCTTCCTTTAGATGTTTTTAATGCCACTTATCTTAATCTCACTGGTGAAATTGCAGATTTAGAGCAGCGT
TCAGAGTCTCTCCGTAATACTACAGAGGAGCTCCAAAGTCTTATATATAATATCAACAACACACTAGTTGACCTTGAGT
GGCTCAACCGAGTTGAGACATATATCAAGTGGCCGTGGTGGGTTTGGTTGATTATTTTCATTGTTCTCATCTTTGTTGT
GTCATTACTAGTGTTCTGCTGCATTTCCACTGGTTGTTGTGGATGCTGCGGCTGCTGCTGTGCTCGTTTTTCAGGTTGT
TGTAGGGGTCCTAGACTTCAACCTTACGAAGTTTTTGAAAAGGTCCACGTGCAGTGA

>gi|455475456|gb|KC242903.1|_Porcine_epidemic_diarrhea_virus_isolate_ZJ1720125_spike_protein_gene_complete_cds

ATGAAGTCTTTAACCTACTTCTGGTTGTCATTACCAGTACTTTCAACACTTAGCCTACCACAAGATGTCACCAGG
TGCTCAGCTAACACTAATTTTAGGCGGTTCTTTTCAAAATTTAATGTTCAGGCGCCTGCAGTTGTTGTACTGGGCGGTT
ATCTACCTATTGGTGAAAACCAGGGTGTTAATTCAACCTGGTACTGTGCTGGCCAACATCCAACTGCTAGTGGCGTTCA
TGGTATCTTTGTTAGCCATATTAGAGGTGGTCATGGCTTTGAGATTGGCATTTCGCAAGAGCCTTTTGACCCTAGTGGT
TACCAGCTTTATTTACATAAGGCTACTAACGGTAACACTAATGCTACTGCGCGACTGCGCATTTGCCAGTTTCCTAGCA
TTAAAACATTGGGCCCCACTGCTAATAATGATGTTACAACAGGTCGTAATTGCCTATTTAACAAAGCCATCCCAGCTCA
TATGAGTGAACATAGTGTTGTCGGCATAACATGGGATAATGATCGTGTCACTGTCTTTTCTGACAAGATCTATTATTTT
TATTTTAAAAATGATTGGTCCCGTGTTGCGACAAAGTGTTACAACAGTGGAGGTTGTGCTATGCAATATGTTTACGAAC
CCACCTATTACATGCTTAATGTTACTAGTGCTGGTGAGGATGGTATTTCTTATCAACCCTGTACAGCTAATTGCATTGG
TTATGCTGCCAATGTATTTGCTACTGAGCCCAATGGCCACATACCAGAAGGTTTTAGTTTTAATAATTGGTTTCTTTTG
TCCAATGATTCCACTTTGGTGCATGGTAAGGTGGTTTCCAACCAACCATTGCTGGTCAATTGTCTTTTGGCCATTCCTA
AGATTTATGGACTAGGCCAATTTTTCTCCTTTAATCAAACGATCGATGGTGTTTGTAATGGAGCTGCTGTGCAGCGTGC
ACCAGAGGCTCTGAGGTTTAATATTAATGACACCTCTGTCATTCTTGCTGAAGGCTCAATTGTACTTCATACTGCTTTA
GGAACAAATTTTTCTTTTGTTTGCAGTAATTCCTCAAATCCTCACTTAGCCACCTTCGCCATACCTCTGGGTGCTACCC
AAGTACCCTATTATTGTTTTCTTAAAGTGGATACTTACAACTCCACTGTTTATAAATTCTTGGCTGTTTTACCTCCTAC
CGTCAGGGAAATTGTCATCACCAAGTATGGTGATGTTTATGTCAATGGGTTTGGTTACTTGCATCTCGGTTTGTTGGAT
GCTGTCACAATTAATTTCACTGGTCATGGCACTGACGATGACGTTTCTGGTTTTCGGACCATAGCATCGACTAATTTTG
TTGATGCACTTATCGAAGTTAAAGGAACTGCCATTCAGCGTATTCTTTATTGTGATGATCCCGTTAGCCAACTCAAGTG
TTCTCAGGTTTCTTTTGACCTTGACGATGGTTTTTACCCTATTTCTTCTACAAACCTTCTGAGTCATGAACAGCCAACT
TCTTTTGTTACTTTGCCATCATTTAATGATCATTCTTTTGTTAATATTACTGTCTCTGCTGCTTTTGGTGGTCATAGTG
GTGCCAACCTTATTGCATCTGACACTACTATCAATGGGTTTAGTTCTTTCTGTGTTGACACTAGACAATTTACCATTTC
ACTGTTTTATAATGTTACAAACAGTTATGGTTATGTGTCTAACTCACAGGACAGTAATTGCCCTTTCACCTTGCAATCT
GTTAATGATTACCTGTCTTTTAGCAAATTTTGTGTTTCTACCAGCCTTTTGGCTAGTGCCTGTACCATAGATCTTTTTG
GTTACCCTGAGTTTGGTAGTGGTGTTAAATTCACGTCCCTTTACTTTCAATTCACAAAGGGTGAGTTGATTACTGGCAC
GCCTAAACCACTTGAAGGTGTTACGGACGTTTCTTTTATGACTCTGGATGTGTGTACCAAGTATACTATCTATGGCTTT
AAAGGTGAGGGTATCATTACCCTTACAAATTCTAGCTTTTTGGCAGGTGTTTATTATACATCTGATTCTGGACAGTTGT
TAGCTTTTAAGAATGTCACTAGTGGTGCTGTTTATTCTGTTACGCCATGTTCTTTTTCAGAGCAGGCTGCATATGTTGA
TGATGATATAGTGGGTGTTATTTCTAGTTTGTCTAGCTCCACTTTTAACAGTACTAGGGAGTTGCCTGGTTTCTTCTAC
CATTCTAATGATGACTCTAATTGTACAGAGCCTGTGTTGGTGTATAGTAACATTGGTGTTTGTAAATCTGGCAGTATTG
GCTATGTCCCATCTCAGTCTGGCCAAGTCAAGATTGCACCCATGGTTACTGGGAATATTAGTATTCCCACCAACTTTAG
TATGAGTATTGGGACAGAATATTTACAGCTTTACAACACGCCTGTTAGTGTTGATTGTGCTACATATGTTTGTAATGGT
AACTCTCGTTGTAAACAATTACTCACCCAGTACATTGCAGCATGTAAGGCTATAGAGTCAGCATTACAACTCAGCGCTA
GGCTTGAGTCTGCTGAAGTTAACTCTATGCTTACTATTTCTGAAGAGGCTCTACAGTTAGCTACCATCAGTTCGTTTAA
TGGTGATGGATATAATTTTACTAATGTGCTGGGTGTTTCCGTTCGTGATCCTGCAAGTGGCAGGATGGTACAAAAAAGG
TCTTTTATTGAAGACCTGCTTTTTAATAAAGTGGTTACTAATGGCCTTGGTACTGTTGATGAAGACTATAAGCGCTGTT
CTAATGGCCGCTCTGTGGCAGATTTAGTCTGTGCGCAGTATTACTCTGGTGTCATGGTACTACCTGGTGTTGTTGACGC
TGAGAAGCTTCATATGTATAGTGCGTCTCTCATCGGTGGTATGGTGCTAGGAGGTTTTACTGCAGCAGCGGCATTGCCT
TTTAGCTATGCTGTTCAAGCGAGACTCAATTATCTTGCTCTACAGACGGATGTTCTACAGCGGAACCAGCAATTGCTTG
CTGAGTCTTTTAACTCTGCTATTGGTAATATAACCTCAGCCTTTGAGAGTGTTAAAGAGGCTATTAGTCAAACTTCTAA
GGGTTTGAACACTGTGGCTCATGCGCTTACTAAGGTTCAAGAGGTTGTTAATTCGCAGGGTGCAGCTTTGACCCAACTT
ACCGTACAGCTGCAACACAACTTCCAAGCCATTTCTAGTTCTATTGATGACATTTATTCCCGACTGGACATTCTTTCAG
CCGATGTTCAGGTTGACCGTCTCATCACCGGCAGATTATCAGCACTTAATGCTTTTGTTGCTCAAACCCTCACTAAGTA
TACTGAGGTTCAGGCTAGCAGGAAGCTAGCACAGCAAAAGGTTAATGAGTGCGTTAAATCGCAATCTCAGCGTTATGGT
TTTTGCGATGGTGATGGCGAGCACATTTTCTCTCTGGTACAGGCAGCACCTCAGGGCCTGCTGTTTTTACATACAGTAC

TTGTACCGGGCGATTTTGTAGATGTTATTGCCATCGCTGGCTTATGCGTTAACGATGAAATTGCCTTGACTCTACGTGA
GCCTGGCTTAGTCTTGTTTACGCATGAACTTCAAAATCATACTGCGACGGAATATTTTGTTTCAACGCGACGTATGTTT
GAACCTAGAAAACCTACCGTTAGTGATTTTGTTCAAATTGAGAGTTGTGTGGTCACCTATGTCAATTTGACTAGGGACC
AACTACCAGATGTAATCCCAGATTACATCGATGTTAACAAAACACTTGATGAGATTTTAGCTTCTCTGCCCAATAGAAC
TGGTCCAAGCCTTCCTTTAGATGTTTTTAATGCCACTTATCTTAATCTCACTGGTGAAATTGCAGATTTAGAGCAGCGT
TCAGAGTCTCTCCGTAATACTACAGAAGAGCTCCAAAGTCTTATATATAATATCAACAACACACTAGTTGACCTTGAGT
GGCTCAACCGAGTTGAGACATATATCAAGTGGCCGTGGTGGGTTTGGTTGATTATTTTTATTGTTCTCATCTTTGTTGT
GTCATTATTAGTGTTCTGCTGCATTTCCACGGGTTGTTGTGGATGCTGCGGCTGCTGTGGTGCTTGTTTTTCAGGTTGT
TGTAGGGGTCCTAGACTTCAACCTTACGAAGCTTTTGAAAAGGTCCACGTGCAGTGA

>gi|455475458|gb|KC242904.1|_Porcine_epidemic_diarrhea_virus_isolate_YN220127_spike_protein_gene_complete_cds

ATGAAGTCTTTAACCTACTTCTGGTTGTTCTTACCAGTACTTTCAACACTTAGCCTACCACAAGATGTCACCAGG
TGCTCAGCTAACACTAATTTTAGGCGGTTCTTTTCAAAATTTAATGTTCAGGCGCCTGCAGTTGTTGTACTGGGCGGTT
ATCTACCTATTGGTGAAAACCAGGGTGTCAATTCAACTTGGTACTGTGCTGGCCAACATCCAACTGCTAGTGGCGTTCA
TGGTATCTTTGTTAGCCATATTAGAGGTGGTCATGGCTTTGAGATTGGCATTTCGCAAGAGCCTTTTGACCCTAGTGGT
TACCAGCTTTATTTACATAAGGCTACTAACGGTAACACTAATGCTACTGCGCGACTGCGCATTTGCCAGTTTCCTAGCA
TTAAAACATTGGGCCCCACTGCTAATAATGGTGTTACAACAGGTCGTAATTGCCTATTTAACAAAGCCATCCCAGCTCA
TATGAGTGAACATAGTGTTGTCGGCATAACATGGGATAATGATCGTGTCACTGTCTTTTCTGACAAGATCTATTATTTT
TATTTTAAAAATGATTGGTCCCGTGTTGCGACAAAGTGTTACAACAGTGGAGGTTGTGCTATGCAATATGTTTACGAAC
CCACCTATTACATGCTTAATGTTACTAGTGCTGGTGAGGATGGTATTTCTTATCAACCCTGTACAGCTAATTGCATTGG
TTATGCTGCCAATGTATTTGCTACTGAGCCCAATGGCCACATACCAGAAGGTTTTAGTTTTAATAATTGGTTTCTTTTG
TCCAATGATTCCACTTTGGTGCATGGTAAGGTGGTTTCCAACCAACCATTGTTGGTCAATTGTCTTTTGGCCATTCCTA
AGATTTATGGACTAGGCCAATTTTTCTCCTTTAATCAAACGATCGATGGTGTTTGTAATGGAGCTGCTGTGCAGCGTGC
ACCAGAGGCTCTGAGGTTTAATATTAATGACACCTCTGTCATTCTTGCTGAAGGCTCAATTGTACTTCATACTGCTTTA
GGAACAAATTTTTCTTTTGTTTGCAGTAATTCCTCAAATCCTCACTTAGCCACCTTCGCCATACCTCTGGGTGCTACCC
AAGTACCCTATTATTGTTTTCTTAAAGTGGATACTTACAACTCCACTGTTTATAAATTCTTGGCTGTTTTACCTCCTAC
CGTCAGGGAAATTGTCATCACCAAGTATGGTGATGTTTATGTCAATGGGTTTGGCTATTTGCATCTCGGTTTGTTGGAC
GCTGTCACAATTAATTTCACTGGTCATGGCACTGACGATGACGTTTCAGGTTTCTGGACCATAGCATCGACTAATTTTG
TTGATGCACTTATCGAAGTTCAAGGAACTGCCATTCAGCGTATTCTTTATTGTGATGATCCTGTTAGCCAACTCAAGTG
TTCTCAGGTTGCTTTTGACCTTGACGATGGTTTCTACCCTATTTCTTCTAGAAACCTTCTGAGTCATGAACAGCCAATT
TCTTTTGTTACTTTGCCATCATTTAATGATCATTCTTTTGTTAACATTACTGTCTCTGCGTCCTTTGGTGGTCATAGTG
GTGCCAACCTTATTGCATCTGACACTACTATCAATGGGTTTAGTTCTTTCTGTGTTGACACTAGACAATTTACCATTTC
ACTGTTTTATAACGTTACAAACAGTTATGGTTATGTGTCTAACTCACAGGACAGTAATTGCCCTTTCACCTTGCAATCT
GTTAATGATTACCTGTCTTTTAGTAAATTTTGTGTTTCCACCAGCCTTTTGGCTAGTGCCTGTACCATAGATCTTTTTG
GTTACCCTGAGTTTGGTAGTGGTGTTAAGTTTACGTCCCTTTACTTTCAATTCACAAAGGGTGAGTTGATTACTGGCAC
GCCTAAACCACTTGAAGGTGTCACGGACGTTTCTTTTATGACTCTGGATGTGTGTACCAAGTATACTATCTATGGCTTT
AAAGGTGAGGGTATCATTACCCTTATAAATTCTAGCTTTTTGGCAGGTGTTTATTACACATCTGATTCTGGACAGTTGT
TAGCCTTTAAGAATGTCACTAGTGGTGCTGTTTATTCTGTTACGCCATGTTCTTTTTCAGAGCAGGCTGCATATGTTGA
TGATGATGTAGTGGGTGTTATTTCTAGTTTGTCTAGCTCCACTTTTAACAGTACTAGGGAGTTGCCTGGTTTCTTCTAC
CATTCTAATGATGGCTCTAATTGTACAGAGCCTGTGTTGGTGTATAGTAACATAGGTGTTTGTAAATCTGGCAGTATTG
GCTACGTCCCATCTCAGTCTGGCCAAGTCAAGATTGCACCCACGGTTACTGGGAATATTAGTATTCCCACCAACTTTAG

TATGAGTATTAGGACAGAATATTTACAGCTTTACAACACGCCTGTTAGTGTTGATTGTGCCACATATGTTTGTAATGGT
AACTCTCGTTGTAAACAATTACTCACCCAGTACACTGCAGCATGTAAGACCATAGAGTCAGCATTACAACTCAGCGCTA
GGCTTGAGTCTGTTGAAGTTAACTCTATGCTTACTATTTCTGAAGAGGCTCTACAGTTAGCTACCATCAGTTCGTTTAA
TGGTGATGGATATAATTTTACTAATGTGCTGGGTGTTTCTGTGTATGATCCTGCAAGTGGCAGGGTGGTACAAAAAAGG
TCTTTTATTGAAGACCTGCTTTTTAATAAAGTGGTTACTAATGGCCTTGGTACTGTTGATGAAGACTATAAGCGCTGTT
CTAATGGTCGCTCTGTGGCAGATCTAGTCTGTGCACAGTATTACTCTGGTGTCATGGTACTACCTGGTGTTGTTGACGC
TGAGAAGCTTCACATGTATAGTGCGTCTCTCATCGGTGGTATGGTGCTAGGAGGTTTTACTTCTGCAGCGGCATTGCCT
TTTAGCTATGCTGTTCAAGCTAGACTCAATTATCTTGCTCTACAGACGGATGTTCTACAGCGGAACCAGCAATTGCTTG
CTGAGTCTTTTAACTCTGCTATTGGTAATATAACTTCAGCCTTTGAGAGTGTTAAAGAGGCTATTAGTCAAACTTCCAA
GGGTTTGAACACTGTGGCTCATGCGCTTACTAAGGTTCAAGAGGTTGTTAACTCGCATGGTGCAGCTTTGACTCAACTT
ACCGTACAGCTGCAACACAACTTCCAAGCCATTTCTAGTTCTATTGATGACATTTACTCTCGACTGGACATTCTTTCAG
CCGATGTTCAGGTTGACCGTCTCATCACCGGCAGATTATCAGCACTTAATGCTTTTGTTGCTCAAACCCTCACTAAGTA
TACTGAGGTTCAGGCTAGCAGGAAGCTAGCACAGCAAAAGGTTAATGAGTGCGTTAAATCGCAATCTCAGCGTTATGGT
TTTTGTGGTGGTGATGGCGAGCACATTTTCTCTCTGGTACAGGCAGCACCTCAGGGCCTGCTGTTTTTACATACAGTAC
TTGTACCGGGTGACTTTGTAGATGTTATTGCCATCGCTGGCTTATGCGTTAACGATGAAATTGCCTTGACTCTACGTGA
GCCTGGCTTAGTCTTGTTTACGCATGAACTTCAAAATCATACTGCGACGGAATATTTTGTTTCATCGCGACGTATGTTT
GAACCTAGAAAACCTACCGTTAGTGATTTTGTTCAAATTGAGAGTTGTGTGGTCACCTATGTCAATTTGACTAGAGACC
AACTACCAGATGTAATCCCAGATTACATCGATGTTAACAAAACACTTGATGAGATTTTAGCTTCTCTGCCCAATAGAAC
TGGTCCAAGTCTTCCTTTAGATGTTTTTAATGCCACTTATCTTAATCTCACTGGTGAAATTGCAGATTTAGAGCAGCGT
TCAGAGTCTCTCCGTAATACTACAGAGGAGCTCCAAAGTCTTATATATAATATCAACAACACACTAGTTGACCTTGAGT
GGCTCAACCGAGTTGAGACATATATCAAGTGGCCGTGGTGGGTTTGGTTGATTATTTTCATTGTTCTCATCTTTGTTGT
GTCATTACTAGTGTTCTGCTGCATTTCCACTGGTTGTTGTGGATGCTGCGGCTGCTGCTGTGCTTGTTTTTCAGGTTGT
TGTAGGGGTCCTAGACTTCAACCTTACGAAGTTTTTGAAAAGGTCCACGTGCAGTGA

>gi|455475460|gb|KC242905.1|_Porcine_epidemic_diarrhea_virus_isolate_FJ520128_spike_protein_gene_complete_cds

ATGAAGTCTTTAACCTACTTCTGGTTGTCATTACCAGTACTTTCAACACTTAGCCTACCACAAGATGTCACCAGG
TGCTCAGCTAACACTAATTTTAGGCGGTTCTTTTCAAAATTTAATGTTCAGGCGCCTGCAGTTGTTGTACTGGGCGGTT
ATCTACCTATTGGTGAAAACCAGGGTGTTAATTCAACCTGGTACTGTGCTGGCCAACATCCAACTGCTAGTGGCGTTCA
TGGTATCTTTGTTAGCCATATTAGAGGAGGTCATGGCTTTGAGATTGGCATTTCGCAAGAGCCTTTTGACCCTAGTGGT
TACCAGCTTTATTTACATAAGGCTACTAACGGTAACACTAATGCTACTGCGCGACTGCGCATTTGCCAGTTTCCTAGCA
TTAAAACATTGGGCCCCACTGCTAATAATGATGTTACAACAGGTCGTAATTGCCTATTTAACAAAGCCATCCCAGCTCA
TATGAGTGAACATAGTGTTGTCGGCATAACATGGGATAATGATCGTGTCACTGTCTTTTCTGACAAGATCTATTATTTT
TATTTTAAAAATGATTGGTCCCGTGTTGCGACAAAGTGTTACAACAGTGGAGGTTGTGCTATGCAATATGTTTACGAAC
CCACCTATTACATGCTTAATGTTACTAGTGCTGGTGAGGATGGTATTTCTTATCAACCCTGTACAGCTAATTGCATTGG
TTATGCTGCCAATGTATTTGCTACTGAGCCCAATGGCCACATACCAGAAGGTTTTAGTTTTAATAATTGGTTTCTTTTG
TCCAATGATTCCACTTTGGTGCATGGTAAGGTGGTTTCCAACCAACCATTGCTGGTCAATTGTCTTTTGGCCATTCCTA
AGATTTATGGACTAGGCCAATTTTTCTCCTTTAATCAAACGATCGATGGTGTTTGTAATGGAGCTGCTGTGCAGCGTGC
ACCAGAGGCTCTGAGGTTTAATATTAATGACACCTCTGTCATTCTTGCTGAAGGCTCAATTGTACTTCATACTGCTTTA
GGAACAAATTTTTCTTTTGTTTGCAGTAATTCCTCAAATCCTCACTTAGCCACCTTCGCCATACCTCTGGGTGCTACCC
AAGTACCCTATTATTGTTTTCTTAAAGTGGATACTTACAACTCCACTGTTTATAAATTCTTGGCTGTTTTACCTCCTAC
CGTCAGGGAAATTGTCATCACCAAGTATGGTGATGTTTATGTCAATGGGTTTGGTTACTTGCATCTCGGTTTGTTGGAT

GCTGTCACAATTAATTTCACTGGTCATGGCACTGACGATGACGTTTCTGGTTTTTGGACCATAGCATCGACTAATTTTG
TTGATGCACTTATTGAAGTTAAAGGAACTGCCATTCAGCGTATTCTTTATTGTGATGATCCCGTTAGCCAACTCAAGTG
TTCTCAGGTTTCTTTTGACCTTGACGATGGTTTTTACCCTATTTCTTCTACAAACCTTCTGAGTCATGAACAGCCAACT
TCTTTTGTTACTTTGCCATCATTTAATGATCATTCTTTTGTTAATATTACTGTCTCTGCTGCTTTTGGTGGTCATAGTG
GTGCCAACCTTATTGCATCTGACACTACTATCAATGGGTTTAGTTCTTTCTGTGTTGACACTAGACAATTTACCATTTC
ACTGTTTTATAATGTTACAAACAGTTATGGTTATGTGTCTAACTCACAGGACAGTAATTGCCCTTTCACCTTGCAATCT
GTTAATGATTACCTGTCTTTTAGCAAATTTTGTGTTTCTACCAGCCTTTTGGCTAGTGCCTGTACCATAGATCTTTTTG
GTTACCCTGAGTTTGGTAGTGGTGTTAAATTCACGTCCCTTTACTTTCAATTCACAAAGGGTGAGTTGATTACTGGCAC
GCCTAAACCACTTGAAGGTGTTACGGACGTTTCTTTTATGACTCTGGATGTGTGTACCAAGTATACTATCTATGGCTTT
AAAGGTGAGGGTATCATTACCCTTACAAATTCTAGCTTTTTGGCAGGTGTTTATTATACATCTGATTCTGGACAGTTGC
TAGCTTTTAAGAATGTCACTAGTGGTGCTGTTTATTCTGTTACGCCATGTTCTTTTTCAGAGCAGGCTGCATATGTTGA
TGATGATATAGTGGGTGTTATTTCTAGTTTGTCTAGCTCCACTTTTAACAGTACTAGGGAGTTGCCTGGTTTCTTCTAC
CATTCTAATGATGACTCTAATTGTACAGAGCCTGTGTTGGTGTATAGTAACATTGGTGTTTGTAAATCTGGCAGTATTG
GCTATGTCCCATCTCAGTCTGGCCAAGTCAAGATTGCACCCATGGTTACTGGGAATATTAGTATTCCCACCAACTTTAG
TATGAGTATTAGGACAGAATATTTACAGCTTTACAACACGCCTGTTAGTGTTGATTGTGCTACATATGTTTGTAATGGT
AACTCTCGTTGTAAACAATTACTCACCCAGTACATTGCAGCATGTAAGACTATAGAGTCAGCATTACAACTCAGCGCTA
GGCTTGAGTCTGCTGAAGTTAACTCTATGCTTACTATTTCTGAAGAGGCTCTACAGTTAGCTACCATCAGTTCGTTTAA
TGGTGATGGATATAATTTTACTAATGTGCTGGGTGTTTCCGTTCGTGATCCTGCAAGTGGCAGGATGGTACAAAAAAGG
TCTTTTATTGAAGACCTGCTTTTTAATAAAGTGGTTACTAATGGCCTTGGTACTGTTGATGAAGACTATAAGCGCTGTT
CTAATGGCCGCTCTGTGGCAGATTTAGTCTGTGCGCAGTATTACTCTGGTGTCATGGTACTACCTGGTGTTGTTGACGC
TGAGAAGCTTCATATGTATAGTGCGTCTCTCATCGGTGGTATGGTGCTAGGAGGTTTTACTGCAGCAGCGGCATTGCCT
TTTAGCTATGCTGTTCAAGCGAGACTCAATTATCTTGCTCTACAGACGGATGTTCTACAGCGGAACCAGCAATTGCTTG
CTGAGTCTTTTAACTCTGCTATTGGTAATATAACCTCAGCCTTTGAGAGTGTTAAAGAGGCTATTAGTCAAACTTCTAA
GGGTTTGAACACTGTGGCTCATGCGCTTACTAAGGTTCAAGAGGTTGTTAATTCGCAGGGTGCAGCTTTGACCCAACTT
ACCGTACAGCTGCAACACAACTTCCAAGCCATTTCTAGTTCTATTGATGACATTTATTCCCGACTGGACATTCTTTCAG
CCGATGTTCAGGTTGACCGTCTCATCACCGGCAGATTATCAGCACTTAATGCTTTTGTTGCTCAAACCCTCACTAAGTA
TACTGAGGTTCAGGCTAGCAGGAAGCTAGCACAGCAAAAGGTTAATGAGTGCGTTAAATCGCAATCTCAGCGTTATGGT
TTTTGCGGTGGTGATGGCGAGCACATTTTCTCTCTGGTACAGGCAGCACCTCAGGGCCTGCTGTTTTTACATACAGTAC
TTGTACCGGGCGATTTTGTAGATGTTATTGCCATCGCTGGCTTATGCGTTAACGATGAAATTGCCTTGACTCTACGTGA
GCCTGGCTTAGTCTTGTTTACGCATGAACTTCAAAATCATACTGCGACGGAATATTTTGTTTCAACGCGACGTATGTTT
GAACCTAGAAAACCTACCGTTAGTGATTTTGTTCAAATTGAGAGTTGTGTGGTCACCTATGTCAATTTGACTAGGGACC
AACTACCAGATGTAATCCCAGATTACATCGATGTTAACAAAACACTTGATGAGATTTTAGCTTCTCTGCCCAATAGAAC
TGGTCCAAGCCTTCCTTTAGATGTTTTTAATGCCACTTATCTTAATCTCACTGGTGAAATTGCAGATTTAGAGCAGCGT
TCAGAGTCTCTCCGTAATACTACAGAAGAGCTCCAAAGTCTTATATATAATATCAACAACACACTAGTTGACCTTGAGT
GGCTCAACCGAGTTGAGACATATATCAAGTGGCCGTGGTGGGTTTGGTTGATTATTTTTATTGTTCTCATCTTTGTTGT
GTCATTATTAGTGTTCTGCTGCATTTCCACGGGTTGTTGTGGATGCTGCGGCTGCTGTGGTGCTTGTTTTTCAGGTTGT
TGTAGGGGTCCTAGACTTCAACCTTACGAAGCTTTTGAAAAGGTCCACGTGCAGTGA

>gi|455475462|gb|KC242906.1|_Porcine_epidemic_diarrhea_virus_isolate_FJZZ92
0128_spike_protein_gene_complete_cds

ATGAAGTCTTTAACCTACTTCTGGTTGTCATTACCAGTACTTTCAACACTTAGCCTACCACAAGATGTCACCAGG
TGCTCAGCTAACACTAATTTTAGGCGGTTCTTTTCAAAATTTAATGTTCAGGCGCCTGCAGTTGTTGTACTGGGCGGTT

ATCTACCTATTGGTGAAAACCAGGGTGTTAATTCAACCTGGTACTGTGCTGGCCAACATCCAACTGCTAGTGGCGTTCA
TGGTATCTTTGTTAGCCATATTAGAGGTGGTCATGGCTTTGAGATTGGCATTTCGCAAGAGCCTTTTGACCCTAGTGGT
TACCAGCTTTATTTACATAAGGCTACTAACGGTAACACTAATGCTACTGCGCGACTGCGCATTTGCCAGTTTCCTAGCA
TTAAAACATTGGGCCCCACTGCTAATAATGATGTTACAACAGGTCGTAATTGCCTATTTAACAAAGCCATCCCAGCTCA
TATGAGTGAACATAGTGTTGTCGGCATAACATGGGATAATGATCGTGTCACTGTCTTTTCTGACAAGATCTATTATTTT
TATTTTAAAAATGATTGGTCCCGTGTTGCGACAAAGTGTTACAACAGTGGAGGTTGTGCTATGCAATATGTTTACGAAC
CCACCTATTACATGCTTAATGTTACTAGTGCTGGTGAGGATGGTATTTCTTATCAACCCTGTACAGCTAATTGCATTGG
TTATGCTGCCAATGTATTTGCTACTGAGCCCAATGGCCACATACCAGAAGGTTTTAGTTTTAATAATTGGTTTCTTTTG
TCCAATGATTCCACTTTGGTGCATGGTAAGGTGGTTTCCAACCAACCATTGCTGGTCAATTGTCTTTTGGCCATTCCTA
AGATTTATGGACTAGGCCAATTTTTCTCCTTTAATCAAACGATCGATGGTGTTTGTAATGGAGCTGCTGTGCAGCGTGC
ACCAGAGGCTCTGAGGTTTAATATTAATGACACCTCTGTCATTCTTGCTGAAGGCTCAATTGTACTTCATACTGCTTTA
GGAACAAATTTTTCTTTTGTTTGCAGTAATTCCTCAAATCCTCACTTAGCCACCTTCGCCATACCTCTGGGTGCTACCC
AAGTACCCTATTATTGTTTTCTTAAAGTGGATACTTACAACTCCACTGTTTATAAATTCTTGGCTGTTTTACCTCCTAC
CGTCAGGGAAATTGTCATCACCAAGTATGGTGATGTTTATGTCAATGGGTTTGGTTACTTGCATCTCGGTTTGTTGGAT
GCTGTCACAATTAATTTCACTGGTCATGGCACTGACGATGACGTTTCTGGTTTTTGGACCATAGCATCGACTAATTTTG
TTGATGCACTTATCGAAGTTAAAGGAACTGCCATTCAGCGTATTCTTTATTGTGATGATCCCGTTAGCCAACTCAAGTG
TTCTCAGGTTTCTTTTGACCTTGACGATGGTTTTTACCCTATTTCTTCTACAAACCTTCTGAGTCATGAACAGCCAACT
TCTTTTGTTACTTTGCCATCATTTAATGATCATTCTTTTGTTAATATTACTGTCTCTGCTGCTTTTGGTGGTCATAGTG
GTGCCAACCTTATTGCATCTGACACTACTATCAATGGGTTTAGTTCTTTCTGTGTTGACACTAGACAATTTACCATTTC
ACTGTTTTATAATGTTACAAACAGTTATGGTTATGTGTCTAACTCACAGGACAGTAATTGCCCTTTCACCTTGCAATCT
GTTAATGATTACCTGTCTTTTAGCAAATTTTGTGTTTCTACCAGCCTTTTGGCTAGTGCCTGTACCATAGATCTTTTTG
GTTACCCTGAGTTTGGTAGTGGTGTTAAATTCACGTCCCTTTACTTTCAATTCACAAAGGGTGAGTTGATTACTGGCAC
GCCTAAACCACTTGAAGGTGTTACGGACGTTTCTTTTATGACTCTGGATGTGTGTACCAAGTATACTATCTATGGCTTT
AAAGGTGAGGGTATCATTACCCTTACAAATTCTAGCTTTTTGGCAGGTGTTTATTATACATCTGATTCTGGACAGTTGT
TAGCTTTTAAGAATGTCACTAGTGGTGCTGTTTATTCTGTTACGCCATGTTCTTTTTCAGAGCAGGCTGCATATGTTGA
TGATGATATAGTGGGTGTTATTTCTAGTTTGTCTAGCTCCACTTTTAACAGTACTAGGGAGTTGCCTGGTTTCTTCTAC
CATTCTAATGATGACTCTAATTGTACAGAGCCTGTGTTGGTGTATAGTAACATTGGTGTTTGTAAATCTGGCAGTATTG
GCTATGTCCCATCTCAGTCTGGCCAAGTCAAGATTGCACCCATGGTTACTGGGAATATTAGTATTCCCACCAACTTTAG
TATGAGTATTAGGACAGAATATTTACAGCTTTACAACACGCCTGTTAGTGTTGATTGTGCTACATATGTTTGTAATGGT
AACTCTCGTTGTAAACAATTACTCACCCAGTACATTGCAGCATGTAAGACTATAGAGTCAGCATTACAACTCAGCGCTA
GGCTTGAGTCTGCTGAAGTTAACTCTATGCTTACTATTTCTGAAGAGGCTCTACAGTTAGCTACCATCAGTTCGTTTAA
TGGTGATGGATATAATTTTACTAATGTGCTGGGTGTTTCCGTTCGTGATCCTGCAAGTGGCAGGATGGTACAAAAAAGG
TCTTTTATTGAAGACCTGCTTTTTAATAAAGTGGTTACTAATGGCCTTGGTACTGTTGATGAAGACTATAAGCGCTGTT
CTAATGGCCGCTCTGTGGCAGATTTAGTCTGTGCGCAGTATTACTCTGGTGTCATGGTACTACCTGGTGTTGTTGACGC
TGAGAAGCTTCATATGTATAGTGCGTCTCTCATCGGTGGTATGGTGCTAGGAGGTTTTACTGCAGCAGCGGCATTGCCT
TTTAGCTATGCTGTTCAAGCGAGACTCAATTATCTTGCTCTACAGACGGATGTTCTACAGCGGAACCAGCAATTGCTTG
CTGAGTCTTTTAACTCTGCTATTGGTAATATAACCTCAGCCTTTGAGAGTGTTAAAGAGGCTATTAGTCAAACTTCTAA
GGGTTTGAACACTGTGGCTCATGCGCTTACTAAGGTTCAAGAGGTTGTTAATTCGCAGGGTGCAGCTTTGACCCAACTT
ACCGTACAGCTGCAACACAACTTCCAAGCCATTTCTAGTTCTATTGATGACATTTATTCCCGACTGGACATTCTTTCAG
CCGATGTTCAGGTTGACCGTCTCATCACCGGCAGATTATCAGCACTTAATGCTTTTGTTGCTCAAACCCTCACTAAGTA
TACTGAGGTTCAGGCTAGCAGGAAGCTAGCACAGCAAAAGGTTAATGAGTGCGTTAAATCGCAATCTCAGCGTTATGGT
TTTTGCGGTGGTGATGGCGAGCACATTTTCTCTCTGGTACAGGCAGCACCTCAGGGCCTGCTGTTTTTACATACAGTAC
TTGTACCGGGCGATTTTGTAGATGTTATTGCCATCGCTGGCTTATGCGTTAACGATGAAATTGCCTTGACTCTACGTGA
GCCTGGCTTAGTCTTGTTTACGCATGAACTTCAAAACCATACTGCGACGGAATATTTTGTTTCAACGCGACGTATGTTT

GAACCTAGAAAACCTACCGTTAGTGATTTTGTTCAAATTGAGAGTTGTGTGGTCACCTATGTCAATTTGACTAGGGACC
AACTACCAGATGTAATCCCAGATTACATCGATGTTAACAAAACACTTGATGAGATTTTAGCTTCTCTGCCCAATAGAAC
TGGTCCAAGCCTTCCTTTAGATGTTTTTAATGCCACTTATCTTAATCTCACTGGTGAAATTGCAGATTTAGAGCAGCGT
TCAGAGTCTCTCCGTAATACTACAGAAGAGCTCCAAAGTCTTATATATAATATCAACAACACACTAGTTGACCTTGAGT
GGCTCAACCGAGTTGAGACATATATCAAGTGGCCGTGGTGGGTTTGGTTGATTATTTTTATTGTTCTCATCTTTGTTGT
GTCATTATTAGTGTTCTGCTGCATTTCCACGGGTTGTTGTGGATGCTGCGGCTGCTGTGGTGCTTGTTTTTCAGGTTGT
TGTAGGGGTCCTAGACTTCAACCTTACGAAGCTTTTGAAAAGGTCCACGTGCAGTGA

>gi|455475464|gb|KC242907.1|_Porcine_epidemic_diarrhea_virus_isolate_JX420129_spike_protein_gene_complete_cds

ATGAAGTCTTTAACCTACTTCTGGTTGCTCTTACCAGTACTTTCAACACTTAGCCTACCACAAGATGTCACCAGG
TGCTCAGCTAACACTAATTTTAGGCGGTTCTTTTCAAAATTTAATGTTCAGGCGCCTGCAGTTGTTGTACTGGGCGGTT
ATCTACCTATTGGTGAAAACCAGGGTGTCAATTCAACTTGGTACTGTGCTGGCCAACATCCAACTGCTAGTGGCGTTCA
TGGTATCTTTGTTAGCCATATTAGAGGTGGTCATGGCTTTGAGATTGGCATTTCGCAAGAGCCTTTTGACCCTAGTGGT
TACCAGCTTTATTTACATAAGGCTACTAATGGTAACACTAATGCTACTGCGCGACTGCGCATTTGCCAGTTTCCTAGCA
TTAAAACATTGGGCCCCACTGCTAATAATGACGTTACAACAGGTCGTAATTGCCTATTTAACAAAGCCATCCCAGCTCA
TATGAGTGAACATAGTGTTGTCGGCATAACATGGGATAATGATCGTGTCACTGTCTTTTCTGACAAGATCTATTACTTT
TATTTTAAAAATGATTGGTCCCGTGTTGCGACAAAGTGTTACAACAGTGGAGGTTGTGCTATGCAATATGTTTACGAAC
CCACCTATTACATGCTTAATGTTACTAGTGCTGGTGAGGATGGTATTTCTTATCAACCCTGTACAGCTAATTGCATTGG
TTATGCTGCCAATGTATTTGCTACTGAGCCCAATGGCCACATACCAGAAGGTTTTAGTTTTAATAATTGGTTTCTTTTG
TCCAATGATTCCACTTTGGTGCATGGTAAGGTGGTTTCCAACCAACCATTGTTGGTCAATTGTCTTTTGGCCATTCCTA
AGATTTATGGACTAGGCCAATTTTTCTCCTTTAATCAAACGATCGATGGTGTTTGTAATGGAGCTGCTGTGCAGCGTGC
ACCAGAGGCTCTGAGGTTTAATATTAATGACACCTCTGTCATTCTTGCTGAAGGCTCAATTGTACTTCATACTGCTTTA
GGAACAAATTTTTCTTTTGTTTGCAGTAATTCCTCAAATCCTCACTTAGCCACCTTCGCCATACCTCTGGGTGCTATCC
AAGTACCCTATTATTGTTTTCTTAAAGTGGATACTTACAACTCCACTGTTTATAAATTCTTGGCTGTTTTACCTCCTAC
CGTCAGGGAAATTGTCATCACCAAGTATGGTGATGTTTATGTCAATGGGTTTGGCTACTTGCATCTCGGTTTGTTGGAT
GCTGTCACAATTAATTTCACTGGTCATGGCACTGACGATGACCTTTCTGGTTTTTGGACCATAGCATCGACTAATTTTG
TTGATGCACTTATCGAAGTTCAAGGAACTGCCATTCAGCGTATTCTTTATTGTGATGATTCTGTTAGCCAACTCAAGTG
TTCTCAGGTTTCTTTTGACCTTGACGATGGTTTTTACCCTATTTCTTCTACAAACCTTCTGAGTCATGAACAGCCAACT
TCTTTTGTTACTTTGCCATCATTTAATGATCATTCTTTTGTTAATATTACTGTCTCTGCTGCTTTTGGTGGTCGTAGTG
GTGCCAACCTTATTGCATCTGACACTACTATCAATGGGTTTAGTTCTTTCTGTGTTGACACTAGACAATTTACCATTTC
ACTGTTTTATAATGTTACAAACAGTTATGGTTATGTGTCTAAATCACAGGACAGTAATTGCCCTTTCACCTTGCAGTCT
GTTAATGATTACCTGTCTTTTAGTAAATTTTGTGTTTCCACCAGCCTTTTGGCTAGTGCTTGCACCATAGATCTTTTTG
GTTACCCTGAGTTTGGTAGTGGTGTTAAGTTTACGTCCCTTTACTTTCAATTCACAAAGGGTGAGTTGATTACTGGCAC
GCCTAAACCACTTGAAGGTGTCAAGGACGTTTCTTTTATGACTCTGGATGTGTGTACCAAGTATACTATCTATGGCTTT
AAAGGTGAGGGTATCATCACCCTTACAAATTCTAGCTTTTTGGCAGGTGTTTATTACACATCTGATTCTGGACAGTTGT
TAGCCTTTAAGAATGTCACTAGTGGTGCTGTTTATTCTGTTACGCCATGTTCTTTTTCAGAGCAGGCTGCATACGTTGA
TGATGATATAGTGGGTGTTATTTCTAGTTTGTCTAGCTCCACTTTTAACAGTACTAGGGAGTTGCCTGGTTTCTTCTAC
CATTCTAATGATGGCTCTAATTGTACAGAGCCTGTTTTGGTGTATAGTAACATAGGTGTTTGTAAATCTGGCAGTATTG
GCCACGTCCCATCTCAGTCTGGCCAAGTCAAGATTGCACCCACGGTTACTGGGAATATTAGTATTCCCACCAACTTTAG
TATGAGTATTAGGACAGAATATTTACAGCTTTACAACACGCCTGTTAGTGTTGATTGTGCCACATATGTTTGTAATGGT
AACTCTCGTTGTAAACAATTACTCACCCAGTACACTTCAGCATGTAAGACCATAGAGTCAGCATTACAACTCAGCGCTA

GGCTTGAGTCTGTTGAAGTTAACTCTATGCTTACTATTTCTGAAGAGGCTCTACAGTTAGCTACCATCAGTTCGTTTAA
TGGTGATGGATATAATTTTACTAATGTGCTGGGTGTTTCTGTGTATGATCCTGCAAGTGGCAGGGTGGTACAAAAAAGG
TCTTTTATTGAAGACCTGCTTTTTAATAAAGTGGTTACTAATGGCCTTGGTACTGTTGATGAAGACTATAAGCGCTGTT
CTAATGGTCGCTCTGTGGCAGATCTAATCTGTGCACAGTATTACTCTGGTGTCATGGTACCACCTGGTGTCGTTGACGC
TGAGAAGCTTCACATGTATAGTGCGTCTCTCATCGGTGGTATGGTGCTAGGAGGTTTTACTTCTGCAGCGGCATTGCCT
TTTAGCTATGCTGTTCAAGCTAGACTCAATTATCTTGCTCTACAGACGGATGTTCTACAGCGGAACCAGCAATTGCTTG
CTGAGTCTTTTAACTCTGCTATTGGTAATATAACTTCAGCCTTTGAGAGTGTTAAAGAGGCTATTAGTCAAACTTCCAA
GGGTTTGAACACTGTGGCTCATGCGCTTACTAAGGTTCAAGAGGTTGTTAACTCGCAGGGTGCAGCTTTGACTCAACTT
ACCGTACAGCTGCAACACAACTTCCAAGCCATTTCTAGTTCTATTGATGACATTTACTCTCGACTGGACATTCTTTCAG
CCGATGTTCAGGTTGACCGTCTCATCACCGGCAGATTATCAGCACTTAATGCTTTTGTTGCTCAAACCCTCACTAAGTA
TACTGAGGTTCAGGCTAGCAGGAAGCTAGCACAGCAAAAGGTTAATGAGTGCGTTAAATCGCAATCTCAGCGTTATGGT
TTTTGTGGTGGTGATGGCGAGCACATTTTCTCTCTGGTACAGGCAGCACCTCAGGGCCCGCTGTTTTTACATACAGTAC
TTGTACCGGGTGACTTTGTAGATGTTATTGCCATCGCTGGCTTATGCGTTAACGATGAAATTGCCTTGACTCTACGTGA
GCCTGGCTTAGTCTTGTTTACGCATGAACTTCAAAATCATACTGCGACGGAATATTTTGTTTCATCGCGACGTATGTTT
GAACCTAGAAAACCTACCGTTAGTGATTTTGTTCAAATTGAGAGTTGTGTGGTCACCTATGTCAATTTGACTAGAGACC
AACTACCAGATGTAATCCCAGATTACATCGATGTTAACAAAACACTTGATGAGATTTTAGCTTCTCTGCCCAATAGAAC
TGGTCCAAGTCTTCCTTTAGATGTTTTTAATGCCACTTATCTTAATCTCACTGGTGAAATTGCAGATTTAGAGCAGCGT
TCAGAGTCTCTCCGTAATACTACAGAGGAGCTCCAAAGTCTTATATATAATATCAACAACACACTAGTTGACCTTGAGT
GGCTCAACCGAGTTGAGACATATATCAAGTGGCCGTGGTGGGTTTGGTTGATTATTTTCATTGTTCTCATCTTTGTTGT
GTCATTACTAGTGTTCTGCTGCATTTCCACTGGTTGTTGTGGTTGCTGCGGCTGCTGCTGTGCTTGTTTTTCAGGTTGT
TGTAGGGGTCCTAGACTTCAACCTTACGAAGTTTTTGAAAAGGTCCACGTGCAGTGA

>gi|455475466|gb|KC242908.1|_Porcine_epidemic_diarrhea_virus_isolate_HN420129_spike_protein_gene_complete_cds

ATGAAGTCTTTAACTTACTTCTGGTTGTTCTTACCAGTACTTTCAACACTTAGCCTACCACAAGATGTCACCAGG
TGCTCAGCTAACACTAATTTTAGGCGGTTCTTTTCAAAATTTAATGTTCAGGCGCCTGCAGTTGTTGTACTGGGCGGTT
ATCTACCTATTGGTGAAAACCAGGGTGTCAATTCAACTTGGTACTGTGCTGGCCAACATCCAACTGCTAGTGGCGTTCA
TGGTATCTTTGTTAGCCATATTAGAGGTGGTCATGGCTTTGAGATTGGCATTTCGCAAGAGCCTTTTGACCCTAGTGGT
TACCAGCTTTATTTACATAAGGCTACTAACGGTAACACTAATGCTACTGCGCGACTGCGCATTTGCCAGTTTCCTAGCA
TTAAAACATTGGGCCCCACTGCTAATAATGATGTTACAACAGGTCGTAATTGCCTATTTAACAAAGCCATCCCAGCTCA
TATGAGTGAACATAGTGTTGTCGGCATAACATGGGATAATGATCGTGTCACTGTCTTTTCTGACAAGATCTATTATTTT
TATTTTAAAAATGATTGGTCCCGTGTTGCGACAAAGTGTTACAACAGTGGAGGTTGTGCTATGCAATATGTTTACGAAC
CCACCTATTACATGCTTAATGTTACTAGTGCTGGTGAGGATGGTATTTCTTATCAACCCTGTACAGCTAATTGCATTGG
TTATTCTGCCAATGTATTTGCTACTGAGCCCAATGGCCACATACCAGAAGGTTTTAGTTTTAATAATTGGTTTCTTTTG
TCCAATGATTCCACTTTGGTGCATGGTAAGGTGGTTTCCAACCAACCATTGTTGGTCAATTGTCTTTTGGCCATTCCTA
AGATTTATGGACTAGGCCAATTTTTCTCCTTTAATCAAACGATCGATGGTGTTTGTAATGGAGCTGCTGTGCAGCGTGC
ACCAGAGGCTCTGAGGTTTAATATTAATGACACCTCTGTCATTCTTGCTGAAGGCTCAATTGTACTTCACACTGCTTTA
GGAACAAATTTTTCTTTTGTTTGCAGTAATTCCTCAAATCCTCACTTAGCCACCTTCGCCATACCTCTGGGTGCTATCC
AAGTACCCTATTATTGTTTTCTTAAAGTGGATACTTACAACTCCACTGTTTATAAATTCTTGGCTGTTTTACCTCCTAC
CGTCAGGGAAATTGTCATCACCAAGTATGGTGATGTTTATGTCAATGGGTTTGGCTACTTGCATCTCGGTTTGTTGGAT
GCTGTCACAATTAATTTCACTGGCCATGGCACTGACGATGACGTTTCTGGTTTTTGGACCATAGCATCGACTAATTTTG
TTGATGCACTTATCGAAGTTCAAGGAACTGCCATTCAGCGTATTCTTTATTGTGATGATCCTGTTAGCCAACTCAAGTG

TTCTCAGGTTGCTTTTGACCTTGACGATGGTTTCTACCCTATTTCTTCTAGAAACCCTCTGAGTCATGAACAGCCAATT
TCTTTTGTTACTTTGCCATCATTTAATGATCATTCTTTTGTTAACATTACTGTCTCTGCGTCCTTTGGTGGTCATAGTG
GTGCCAACCTCATTGCATCTGACATTACTATCAATGGGTTTAGTTCTTTCTGTGTTGACACTAGACAATTTACCATTTC
ACTGTTTTATAACGTTACAAACAGTTATGGTTATGTGTCTAAATCACAGGACACTAATTGCCCTTTCACCTTGCAATCT
GTTAATGATTACCTGTCTTTTAGTAAATTTTGTGTTTCTACCAGCCTTTTGGCTAGTGCCTGTACCATAGATTTTTTTG
GTTACCCTGAGTTTGGTAGTGGTGTTAAGTTCACGTCCCTTTACTTTCAATTCACAAAGGGTGAGTTGATTACTGGCAC
GCCTAAACCACTTGAAGGTGTTACGGACGTTTCTTTTATGACTCTGGATGTGTGTACCAAGTATACTATCTATGGCTTT
AAAGGTGAGGGTGTCATTACCCTTACAAATTCTAGCCTTTTGGCAGGTGTTTATTACACATCTGATTCTGGACAGTTGT
TAGCCTTTAAGAATGTCACTAGTGGTGCTGTTTATTCTGTTACGCCATGTTCTTTTTCAGAGCAGGCTGCATATGTTGA
TGATGATATAGTGGGTGTTATTTCTAGTTTGTCTAGCTCCACTTTTAACAGTACTAGGGAGTTGCCTGGTTTCTTCTAC
CATTCTAATGATGGCTCTAATTGTACAGAGCCTGTGTTGGTGTATAGTAACATAGGTGTTTGTAAATCTGGCAGTATTG
GCTATGTCCCATCTCAGTCTGGCCAAGTCAAGATTGCACCCACGGTTACTGGGAATATTAGTATTCCCACCAACTTTAG
TATGAGTATTAGGACAGAATATTTACAGCTTTACAACACGCCTGTTAGTGTTGATTGTGCCATATATGTTTGTAATGGT
AACTCTCGTTGTAAACAATTACTCACCCAGTACACTGCAGCATGTAAGACCATAGAGTCAGCATTACAACTCAGCGCTA
GGCTTGAGTCTGTTGAAGTTAACTCTATGCTTACTATCTCTGAAGAGGCTCTACAGTTAGCTACCATCAGTTCGTTTAA
TGGTGATGGATATAATTTTACTAATTTGCTGGGTGTTTCTGTGTATGATCCTGCAAGTGGCAGGGTGGTACAAAAAAGG
TCTTTTATTGAAGACCTGCTTTTTAATAAAGTGGTTACTAATGGCCTTGGTACTGTTGATGAAGACTATAAGCGCTGTT
CTAATGGTCGCTCTGTGGCAGATCTAGTCTGTGCACAGTATTACTCTGGTGTCATGGTACTACCTGGTGTTGTTGACGC
TGAGAAGCTTCACATGTATAGTGCGTCTCTCATCGGTGGTATGGTGCTAGGAGGTTTTACTTCTGCAGCGGCATTGCCT
TTTAGCTATGCTGTTCAAGCTAGACTCAATTATCTTGCTCTACAGACGGATGTTCTACAGCGGAACCAGCAATTGCTTG
CTGAGTCTTTTAACTCTGCTATTGGTAATATAACTTCAGCCTTTGAGAGTGTTAAAGAGGCTATTAGTCAAACTTCCAA
GGGTTTGAACACTGTGGCTCAAGCGCTTACTAAGGTTCAAGAGGTTGTTAATTCGCAGGGTGCAGCTTTGACTCAACTT
ACCGTACAGCTGCAACACAACTTCCAAGCTATTTCTAGTTCTATTGATGACATTTACTCCCGACTGGACATTCTTTCAG
CCGATGTTCAGGTTGACCGTCTCATCACCGGCAGATTATCAGCACTTAATGCTTTTGTTGCTCAAACCCTCACTAAGTA
CACTGAGGTTCAGGCTAGCAGGAAGCTAGCACAGCAAAAGGTTAATGAGTGCGTTAAATCGCAATCTCAGCGTTATGGT
TTTTGTGGTGGTGATGGCGAGCACATTTTCTCTCTGGTACAGGCAGCACCTCAGGGCCTGCTGTTTTTACATACAGTAC
TTGTACCGGGTGATTTTGTAGATGTTATTGCCATCGCTGGCTTATGCGTTAACGATGAAATTGCCTTGACTCTACGTGA
GCCTGGCTTAGTCTTGTTTACGCATGAACTTCAAAATCATACTGCGACGGAATATTTTGTTTCATCGCGACGTATGTTT
GAACCTAGAAAACCTACCGTTAGTGATTTTGTTCAAATTGAGAGTTGTGTGGTCACCTATGTCAATTTGACTAGGGACC
AACTACCAGATGTAATCCCAGATTACATCGATGTTAACAAAACACTTGATGAGATTTTAGCTTCTCTGCCCAATAGAAC
TGGTCCAAGTCTTCCTTTAGATGTTTTTAATGCTACTTATCTTAATCTCACTGGTGAAATTGCAGATTTAGAGCAGCGT
TCAGAGTCTCTCCGTAATACTACAGAAGAGCTCCAAAGTCTTATATATAATATCAACAACACACTAGTTGACCTTGAGT
GGCTCAACCGAGTTGAGACATATATCAAGTGGCCGTGGTGGGTTTGGTTGATTATTTTCATTGTTCTCATCTTTGTTGT
GTCATTACTAGTGTTCTGTTGCATTTCCACGGGTTGTTGTGGATGCTGCGGCTGCTGCTGTGCTTGTTTTTCAGGTTGT
TGTAGGGGTCCTAGACTTCAACCTTACGAAGCTTTTGAAAAGGTCCACGTGCAGTGA

>gi|455475468|gb|KC242909.1|_Porcine_epidemic_diarrhea_virus_isolate_GD520129_spike_protein_gene_complete_cds

ATGAAGTCTTTAACCTACTTCTGGTTGTCATTACCAGTACTTTCAACACTTAGCCTACCACAAGATGTCACCAGG
TGCTCAGCTAACACTAATTTTAGGCGGTTCTTTTCAAAATTTAATGTTCAGGCGCCTGCAGTTGTTGTACTGGGCGGTT
ATCTACCTATTGGTGAAAACCAGGGTGTTAATTCAACCTGGTACTGTGCTGGCCAACATCCAACTGCTAGTGGCGTTCA
TGGTATCTTTGTTAGCCATATTAGAGGTGGTCATGGCTTTGAGATTGGCATTTCGCAAGAGCCTTTTGACCCTAGTGGT

TACCAGCTTTATTTACATAAGGCTACTAACGGTAACACTAATACTACTGCGCGACTGCGCATTTGCCAGTTTCCTAGCA
TTAAAACATTGGGCCCCACTGCTAATAATGATGTTACAACAGGTCGTAATTGCCTATTTAACAAAGCCATCCCAGCTCA
TATGAGTGAACATAGTGTTGTCGGCATAACATGGGATAATGATCGTGTCACTGTCTTTTCTGACAAGATCTATTATTTT
TATTTTAAAAATGATTGGTCCCGTGTTGCGACAAAGTGTTACAACAGTGGAGGTTGTGCTATGCAATATGTTTACGAAC
CCACCTATTACATGCTTAATGTTACTAGTGCTGGTGAGGATGGTATTTCTTATCAACCCTGTACAGCTAATTGCATTGG
TTATGCTGCCAATGTATTTGCTACTGAGCCCAATGGCCACATACCAGAAGGTTTTAGTTTTAATAATTGGTTTCTTTTG
CCCAATGATTCCACTTTGGTGCATGGTAAGGTGGTTTCCAACCAACCATTGCTGGTCAATTGTCTTTTGGCCATTCCTA
AGATTTATGGACTAGGCCAATTTTTCTCCTTTAATCAAACGATCGATGGTGTTTGTAATGGAGCTGCTGTGCAGCGTGC
ACCAGAGGCTCTGAGGTTTAATATTAATGACACCTCTGTCATTCTTGCTGAAGGCTCAATTGTACTTCATACTGCTTTA
GGAACAAATTTTTCTTTTGTTTGCAGTAATTCCTCAAATCCTCACTTAGCCACCTTCGCCATACCTCTGGGTGCTACCC
AAGTACCCTATTATTGTTTTCTTAAAGTGGATACTTACAACTCCACTGTTTATAAATTCTTGGCTGTTTTACCTCCTAC
CGTCAGGGAAATTGTCATCACCAAGTATGGTGATGTTTATGTCAATGGGTTTGGTTACTTGCATCTCGGTTTGTTGGAT
GCTGTCACAATTAATTTCACTGGTCATGGCACTGACGATGACGTTTCTGGTTTTTGGACCATAGCATCGACTAATTTTG
TTGATGCACTTATCGAAGTTAAAGGAACTGCCATTCAGCGTATTCTTTATTGTGATGATCCCGTTAGCCAACTCAAGTG
TTCTCAGGTTTCTTTTGACCTTGACGATGGTTTTTACCCTATTTCTTCTACAAACCTTCTGAGTCATGAACAGCCAACT
TCTTTTGTTACTTTGCCATCATTTAATGATCATTCTTTTGTTAATATTACTGTCTCTGCTGCTTTTGGTGGTCATAGTG
GTGCCAACCTTATTGCATCTGACACTACTATCAATGGGTTTAGTTCTTTCTGTGTTGACACTAGACAATTTACCATTTC
ACTGTTTTATAATGTTACAAACAGTTATGGTTATGTGTCTAACTCACAGGACAGTAATTGCCCTTTCACCTTGCAATCT
GTTAATGATTACCTGTCTTTTAGCAAATTTTGTGTTTCTACCAGCCTTTTGGCTAGTGCCTGTACCATAGATCTTTTTG
GTTACCCTGAGTTTGGTAGTGGTGTTAAATTCACGTCCCTTTACTTTCAATTCACAAAGGGTGAGTTGATTACTGGCAC
ACCTAAACCACTTGAAGGTGTTACGGACGTTTCTTTTATGACTCTGGATGTGTGTACCAAGTATACTATCTATGGCTTT
AAAGGTGAGGGTATCATTACCCTTACAAATTCTAGCTTTTTGGCAGGTGTTTATTATACATCTGATTCTGGACAGTTGT
TAGCTTTTAAGAATGTCACTAGTGGTGCTGTTTATTCTGTTACGCCATGTTCTTTTTCAGAGCAGGCTGCATATGTTGA
TGATGATATAGTGGGTGTTATTTCTAGTTTGTCTAGCTCCACTTTTAACAGTACTAGGGAGTTGCCTGGTTTCTTCTAC
CATTCTAATGATGACTCTAATTGTACAGAGCCTGTGTTGGTGTATAGTAACATTGGTGTTTGTAAATCTGGCAGTATTG
GCTATGTCCCATCTCAGTCTGGCCAAGTCAAGATTGCACCCATGGTTACTGGGAATATTAGTATTCCCACCAACTTTAG
TATGAGTATTAGGACAGAATATTTACAGCTTTACAACACGCCTGTTAGTGTTGATTGTGCTACATATGTTTGTAATGGT
AACTCTCGTTGTAAACAATTACTCACCCAGTACATTGCAGCATGTAAGACTATAGAGTCAGCATTACAACTCAGCGCTA
GGCTTGAGTCTGCTGAAGTTAACTCTATGCTTACTATTTCTGAAGAGGCTCTACAGTTAGCTACCATCAGTTCGTTTAA
TGGTGATGGATATAATTTTACTAATGTGCTGGGTGTTTCCGTTCGTGATCCTGCAAGTGGCAGGATGGTACAAAAAAGG
TCTTTTATTGAAGACCTGCTTTTTAATAAAGTGGTTACTAATGGCCTTGGTACTGTTGATGAAGACTATAAGCGCTGTT
CTAATGGCCGCTCTGTGGCAGATTTAGTCTGTGCGCAGTATTACTCTGGTGTCATGGTACTACCTGGTGTTGTTGACGC
TGAGAAGCTTCATATGTATAGTGCGTCTCTCATCGGTGGTATGGTGCTAGGAGGTTTTACTGCAGCAGCGGCATTGCCT
TTTAGCTATGCTGTTCAAGCGAGACTCAATTATCTTGCTCTACAGACGGATGTTCTACAGCGGAACCAGCAATTGCTTG
CTGAGTCTTTTAACTCTGCTATTGGTAATATAACCTCAGCCTTTGAGAGTGTTAAAGAGGCTATTAGTCAAACTTCTAA
GGGTTTGAACACTGTGGCTCATGCGCTTACTAAGGTTCAAGAGGTTGTTAATTCGCAGGGTGCAGCTTTGACCCAACTT
ACCGTACAGCCGCAACACAACTTCCAAGCCATTTCTAGTTCTATTGATGACATTTATTCCCGACTGGACATTCTTTCAG
CCGATGTTCAGGTTGACCGTCTCATCACCGGCAGATTATCAGCACTTAATGCTTTTGTTGCTCAAACCCTCACTAAGTA
TACTGAGGTTCAGGCTAGCAGGAAGCTAGCACAGCAAAAGGTTAATGAGTGCGTTAAATCGCAATCTCAGCGTTATGGT
TTTTGCGGTGGTGATGGCGAGCACATTTTCTCTCTGGTACAGGCAGCACCTCAGGGCCTGCTGTTTTTACATACAGTAC
TTGTACCGGGCGATTTTGTAGATGTTATTGCCATCGCTGGCTTATGCGTTAACGATGAAATTGCCTTGACTCTACGTGA
GCCTGGCTTAGTCTTGTTTACGCATGAACTTCAAAATCATACTGCGACGGAATATTTTGTTTCAACGCGACGTATGTTT
GAACCTAGAAAACCTACCGTTAGTGATTTTGTTCAAATTGAGAGTTGTGTGGTCACCTATGTCAATTTGACTAGGGACC
AACTACCAGATGTAATCCCAGATTACATCGATGTTAACAAAACACTTGATGAGATTTTAGCTTCTCTGCCCAATAGAAC

TGGTCCAAGCCTTCCTTTAGATGTTTTTAATGCCACTTATCTTAATCTCACTGGTGAAATTGCAGATTTAGAGCAGCGT
TCAGAGTCTCTCCGTAATACTACAGAAGAGCTCCAAAGTCTTATATATAATATCAACAACACACTAGTTGACCTTGAGT
GGCTCAACCGAGTTGAGACATATATCAAGTGGCCGTGGTGGGTTTGGTTGATTATTTTTATTGTTCTCATCTTTGTTGT
GTCATTATTAGTGTTCTGCTGCATTTCCACGGGTTGTTGTGGATGCTGCGGCTGCTGTGGTGCTTGTTTTTCAGGTTGT
TGTAGGGGTCCTAGACTTCAACCTTACGAAGCTTTTGAAAAGGTCCACGTGCAGTGA

>gi|455475470|gb|KC242910.1|_Porcine_epidemic_diarrhea_virus_isolate_GD820129_spike_protein_gene_complete_cds

ATGAAGTCTTTAACCTACTTCTGGTTGTCATTACCAGTACTTTCAACACTTAGCCTACCACAAGATGTCACCAGG
TGCTCAGCTAACACTAATTTTAGGCGGTTCTTTTCAAAATTTAATGTTCAGGCGCCTGCAGTTGTTGTACTGGGCGGTT
ATCTACCTATTGGTGAAAACCAGGGTGTTAATTCAACCTGGTACTGTGCTGGCCAACATCCAACTGCTAGTGGCGTTCA
TGGTATCTTTGTTAGCCATATTAGAGGTGGTCATGGCTTTGAGATTGGCATTTCGCAAGAGCCTTTTGACCCTAGTGGT
TACCAGCTTTATTTACATAAGGCTACTAACGGTAACACTAATGCTACTGCGCGACTGCGCATTTGCCAGTTTCCTAGCA
TTAAAACATTGGGCCCCACTGCTAATAATGATGTTACAACAGGTCGTAATTGCCTATTTAACAAAGCCATCCCAGCTCA
TATGAGTGAACATAGTGTTGTCGGCATAACATGGGATAATGATCGTGTCACTGTCTTTTCTGACAAGATCTATTATTTT
TATTTTAAAAATGATTGGTCCCGTGTTGCGACAAAGTGTTACAACAGTGGAGGTTGTGCTATGCAATATGTTTACGAAC
CCACCTATTACATGCTTAATGTTACTAGTGCTGGTGAGGATGGTATTTCTTATCAACCCTGTACAGCTAATTGCATTGG
TTATGCTGCCAATGTATTTGCTACTGAGCCCAATGGCCACATACCAGAAGGTTTTAGTTTTAATAATTGGTTTCTTTTG
TCCAATGATTCCACTTTGGTGCATGGTAAGGTGGTTTCCAACCAACTATTGCTGGTCAATTGTCTTTTGGCCATTCCTA
AGATTTATGGACTAGGCCAATTTTTCTCCTTTAATCAAACGATCGATGGTGTTTGTAATGGAGCTGCTGTGCAGCGTGC
ACCAGAGGCTCTGAGGTTTAATATTAATGACACCTCTGTCATTCTTGCTGAAGGCTCAATTGTACTTCATACTGCTTTA
GGAACAAATTTTTCTTTTGTTTGCAGTAATTCCTCAAATCCTCACTTAGCCACCTTCGCCATACCTCTGGGTGCTACCC
AAGTACCCTATTATTGTTTTCTTAAAGTGGATACTTACAACTCCACTGTTTATAAATTCTTGGCTGTTTTACCTCCTAC
CGTCAGGGAAATTGTCATCACCAAGTATGGTGATGTTTATGTCAATGGGTTTGGTTACTTGCATCTCGGTTTGTTGGAT
GCTGTCACAATTAATTTCACTGGTCATGGCACTGACGATGACGTTTCTGGTTTTTGGACCATAGCATCGACTAATTTTG
TTGATGCACTTATCGAAGTTAAAGGAACTGCCATTCAGCGTATTCTTTATTGTGATGATCCCGTTAGCCAACTCAAGTG
TTCTCAGGTTTCTTTTGACCTTGACGATGGTTTTTACCCTATTTCTTCTACAAACCTTCTGAGTCATGAACAGCCAACT
TCTTTTGTTACTTTGCCATCATTTAATGATCATTCTTTTGTTAATATTACTGTCTCTGCTGCTTTTGGTGGTCATAGTG
GTGCCAACCTTATTGCATCTGACACTACTATCAATGGGTTTAGTTCTTTCTGTGTTGACACTAGACAATTTACCATTTC
ACTGTTTTATAATGTTACAAACAGTTATGGTTATGTGTCTAACTCACAGGACAGTAATTGCCCTTTCACCTTGCAATCT
GTTAATGATTACCTGTCTTTTAGCAAATTTTGTGTTTCTACCAGCCTTTTGGCTAGTGCCTGTACCATAGATCTTTTTG
GTTACCCTGAGTTTGGTAGTGGTGTTAAATTCACGTCCCTTTACTTTCAATTCACAAAGGGTGAGTTGATTACTGGCAC
GCCTAAACCACTTGAAGGTGTTACGGACGTTTCTTTTATGACTCTGGATGTGTGTACCAAGTATACTATCTATGGCTTT
AAAGGTGAGGGTATCATTACCCTTACAAATTCTAGCTTTTTGGCAGGTGTTTATTATACATCTGATTCTGGACAGTTGT
TAGCTTTTAAGAATGTCACTAGTGGTGCTGTTTATTCTGTTACGCCATGTTCTTTTTCAGAGCAGGCTGCATATGTTGA
TGATGATATAGTGGGTGTTATTTCTAGTTTGTCTAGCTCCACTTTTAACAGTACTAGGGAGTTGCCTGGTTTCTTCTAC
CATTCTAATGATGACTCTAATTGTACAGAGCCTGTGTTGGTGTATAGTAACATTGGTGTTTGTAAATCTGGCAGTATTG
GCTATGTCCCATCTCAGTCTGGCCAAGTCAAGATTGCACCCATGGTTACTGGGAATATTAGTATTCCCACCAACTTTAG
TATGAGTATTAGGACAGAATATTTACAGCTTTACAACACGCCTGTTAGTGTTGATTGTGCTACATATGTTTGTAATGGT
AACTCTCGTTGTAAACAATTACTCACCCAGTACATTGCAGCATGTAAGACTATAGAGTCAGCATTACAACTCAGCGCTA
GGCTTGAGTCTGCTGAAGTTAACTCTATGCTTACTATTTCTGAAGAGGCTCTACAGTTAGCTACCATCAGTTCGTTTAA
TGGTGATGGATATAATTTTACTAATGTGCTGGGTGTTTCCGTTCGTGATCCTGCAAGTGGCAGGATGGTACAAAAAAGG

TCTTTTATTGAAGACCTGCTTTTTAATAAAGTGGTTACTAATGGCCTTGGTACTGTTGATGAAGACTATAAGCGCTGTT
CTAATGGCCGCTCTGTGGCAGATTTAGTCTGTGCGCAGTATTACTCTGGTGTCATGGTACTACCTGGTGTTGTTGACGC
TGAGAAGCTTCATATGTATAGTGCGTCTCTCATCGGTGGTATGGTGCTAGGAGGTTTTACTGCAGCAGCGGCATTGCCT
TTTAGCTATGCTGTTCAAGCGAGACTCAATTATCTTGCTCTACAGACGGATGTTCTACAGCGGAACCAGCAATTGCTTG
CTGAGTCTTTTAACTCTGCTATTGGTAATATAACCTCAGCCTTTGAGAGTGTTAAAGAGGCTATTAGTCAAACTTCTAA
GGGTTTGAACACTGTGGCTCATGCGCTTACTAAGGTTCAAGAGGTTGTTAATTCGCAGGGTGCAGCTTTGACCCAACTT
ACCGTACAGCTGCAACACAACTTCCAAGCCATTTCTAGTTCTATTGATGACATTTATTCCCGACTGGACATTCTTTCAG
CCGATGTTCAGGTTGACCGTCTCATCACCGGCAGATTATCAGCACTTAATGCTTTTGTTGCTCAAACCCTCACTAAGTA
TACTGAGGTTCAGGCTAGCAGGAAGCTAGCACAGCAAAAGGTTAATGAGTGCGTTAAATCGCAATCTCAGCGTTATGGT
TTTTGCGGTGGTGATGGCGAGCACATTTTCTCTCTGGTACAGGCAGCACCTCAGGGCCTGCTGTTTTTACATACAGTAC
TTGTACCGGGCGATTTTGTAGATGTTATTGCCATCGCTGGCTTATGCGTTAACGATGAAATTGCCTTGACTCTACGTGA
GCCTGGCTTAGTCTTGTTTACGCATGAACTTCAAAATCATACTGCGACGGAATATTTTGTTTCAACGCGACGTATGTTT
GAACCTAGAAAACCTACCGTTAGTGATTTTGTTCAAATTGAGAGTTGTGTGGTCACCTATGTCAATTTGACTAGGGACC
AACTACCAGATGTAATCCCAGATTACATCGATGTTAACAAAACACTTGATGAGATTTTAGCTTCTCTGCCCAATAGAAC
TGGTCCAAGCCTTCCTTTAGATGTTTTTAATGCCACTTATCTTAATCTCACTGGTGAAATTGCAGATTTAGAGCAGCGT
TCAGAGTCTCTCCGTAATACTACAGAAGAGCTCCAAAGTCTTATATATAATATCAACAACACACTAGTTGACCTTGAGT
GGCTCAACCGAGTTGAGACATATATCAAGTGGCCGTGGTGGGTTTGGTTGATTATTTTTATTGTTCTCATCTTTGTTGT
GTCATTATTAGTGTTCTGCTGCATTTCCACGGGTTGTTGTGGATGCTGCGGCTGCTGTGGTGCTTGTTTTTCAGGTTGT
TGTAGGGGTCCTAGACTTCAACCTTACGAAGCTTTTGAAAAGGTCCACGTGCAGTGA

>gi|455475472|gb|KC242911.1|_Porcine_epidemic_diarrhea_virus_isolate_HN6201211_spike_protein_gene_complete_cds

ATGAAGTCTTTAACTTACTTCTGGTTGTTCTTACCAGTACTTTCAACACTTAGCCTACCACAAGATGTCACCAGG
TGCTCAGCTAACACTAATTTTAGGCGGTTCTTTTCAAAATTTAATGTTCAGGCGCCTGCAGTTGTTGTACTGGGCGGTT
ATCTACCTATTGGTGAAAACCAGGGTGTCAATTCAACTTGGTACTGTGCTGGCCAACATCCAACTGCTAGTGGCGTTCA
TGGTATCTTTGTTAGCCATATTAGAGGTGGTCATGGCTTTGAGATTGGCATTTCGCAAGAGCCTTTTGACCCTAGTGGT
TACCAGCTTTATTTACATAAGGCTACTAACGGTAACACTAATGCTACTGCGCGACTGCGCATTTGCCAGTTTCCTAGCA
TTAAAACATTGGGCCCCACTGCTAATAATGATGTTACAACAGGTCGTAATTGCCTATTTAACAAAGCCATCCCAGCTCA
TATGAGTGAACATAGTGTTGTCGGCATAACATGGGATAATGATCGTGTCACTGTCTTTTCTGACAAGATCTATTATTTT
TATTTTAAAAATGATTGGTCCCGTGTTGCGACAAAGTGTTACAACAGTGGAGGTTGTGCTATGCAATATGTTTACGAAC
CCACCTATTACATGCTTAATGTTACTAGTGCTGGTGAGGATGGTATTTCTTATCAACCCTGTACAGCTAATTGCATTGG
TTATTCTGCCAATGTATTTGCTACTGAGCCCAATGGCCACATACCAGAAGGTTTTAGTTTTAATAATTGGTTTCTTTTG
TCCAATGATTCCACTTTGGTGCATGGTAAGGTGGTTTCCAACCAACCATTGTTGGTCAATTGTCTTTTGGCCATTCCTA
AGATTTATGGACTAGGCCAATTTTTCTCCTTTAATCAAACGATCGATGGTGTTTGTAATGGAGCTGCTGTGCAGCGTGC
ACCAGAGGCTCTGAGGTTTAATATTAATGACACCTCTGTCATTCTTGCTGAAGGCTCAATTGTACTTCACACTGCTTTA
GGAACAAATTTTTCTTTTGTTTGCAGTAATTCCTCAAATCCTCACTTAGCCACCTTCGCCATACCTCTGGGTGCTATCC
AAGTACCCTATTATTGTTTTCTTAAAGTGGATACTTACAACTCCACTGTTTATAAATTCTTGGCTGTTTTACCTCCTAC
CGTCAGGGAAATTGTCATCACCAAGTATGGTGATGTTTATGTCAATGGGTTTGGCTACTTGCATCTCGGTTTGTTGGAT
GCTGTCACAATTAATTTCACTGGCCATGGCACTGACGATGACGTTTCTGGTTTTTGGACCATAGCATCGACTAATTTTG
TTGATGCACTTATCGAAGTTCAAGGAACTGCCATTCAGCGTATTCTTTATTGTGATGATCCTGTTAGCCAACTCAAGTG
TTCTCAGGTTGCTTTTGACCTTGACGATGGTTTCTACCCTATTTCTTCTAGAAACCCTCTGAGTCATGAACAGCCAATT
TCTTTTGTTACTTTGCCATCATTTAATGATCATTCTTTTGTTAACATTACTGTCTCTGCGTCCTTTGGTGGTCATAGTG

GTGCCAACCTCATTGCATCTGACATTACTATCAATGGGTTTAGTTCTTTCTGTGTTGACACTAGACAATTTACCATTTC
ACTGTTTTATAACGTTACAAACAGTTATGGTTATGTGTCTAAATCACAGGACACTAATTGCCCTTTCACCTTGCAATCT
GTTAATGATTACCTGTCTTTTAGTAAATTTTGTGTTTCTACCAGCCTTTTGGCTAGTGCCTGTACCATAGATCTTTTTG
GTTACCCTGAGTTTGGTAGTGGTGTTAAGTTCACGTCCCTTTACTTTCAATTCACAAAGGGTGAGTTGATTACTGGCAC
GCCTAAACCACTTGAAGGTGTTACGGACGTTTCTTTTATGACTCTGGATGTGTGTACCAAGTATACTATCTATGGCTTT
AAAGGTGAGGGTGTCATTACCCTTACAAATTCTAGCCTTTTGGCAGGTGTTTATTACACATCTGATTCTGGACAGTTGT
TAGCCTTTAAGAATGTCACTAGTGGTGCTGTTTATTCTGTTACGCCATGTTCTTTTTCAGAGCAGGCTGCATATGTTGA
TGATGATATAGTGGGTGTTATTTCTAGTTTGTCTAGCTCCACTTTTAACAGTACTAGGGAGTTGCCTGGTTTCTTCTAC
CATTCTAATGATGGCTCTAATTGTACAGAGCCTGTGTTGGTGTATAGTAACATAGGTGTTTGTAAATCTGGCAGTATTG
GCTATGTCCCATCTCAGTCTGGCCAAGTCAAGATTGCACCCACGGTTACTGGGAATATTAGTATTCCCACCAACTTTAG
TATGAATATTAGGACAGAATATTTACAGCTTTACAACACGCCTGTTAGTGTTGATTGTGCCATATATGTTTGTAATGGT
AACTCTCGTTGTAAGCAATTACTCACCCAGTACACTGCAGCATGTAAGACCATAGAGTCAGCATTACAACTCAGCGCTA
GGCTTGAGTCTGTTGAAGTTAACTCTATGCTTACTATCTCTGAAGAGGCTCTACAGTTAGCTACCATCAGTTCGTTTAA
TGGTGATGGATATAATTTTACTAATTTGCTGGGTGTTTCTGTGTATGATCCTGCAAGTGGCAGGGTGGTACAAAAAAGG
TCTTTTATTGAAGACCTGCTTTTTAATAAAGTGGTTACTAATGGCCTTGGTACTGTTGATGAAGACTATAAGCGCTGTT
CTAATGGTCGCTCTGTGGCAGATCTAGTCTGTGCACAGTATTACTCTGGTGTCATGGTACTACCTGGTGTTGTTGACGC
TGAAAAGCTTCACATGTATAGTGCGTCTCTCATCGGTGGTATGGTGCTAGGAGGTTTTACTTCTGCAGCGGCATTGCCT
TTTAGCTATGCTGTTCAAGCTAGACTCAATTATCTTGCTCTACAGACGGATGTTCTACAGCGGAACCAGCAATTGCTTG
CTGAGTCTTTTAACTCTGCTATTGGTAATATAACTTCAGCCTTTGAGAGTGTTAAAGAGGCTATTAGTCAAACTTCCAA
GGGTTTGAACACTGTGGCTCATGCGCTTACTAAGGTTCAAGAGGTTGTTAATTCGCAGGGTGCAGCTTTGACTCAACTT
ACCGTACAGCTGCAACACAACTTCCAAGCTATTTCTAGTTCTATTGATGACATTTACTCCCGACTGGACATTCTTTCAG
CCGATGTTCAGGTTGACCGTCTCATCACCGGCAGATTATCAGCACTTAATGCTTTTGTTGCTCAAACCCTCACTAAGTA
CACTGAGGTTCAGGCTAGCAGGAAGCTAGCACAGCAAAAGGTTAATGAGTGCGTTAAATCGCAATCTCAGCGTTATGGT
TTTTGTGGTGGTGATGGCGAGCACATTTTCTCTCTGGTACAGGCAGCACCTCAGGGCCTGCTGTTTTTACATACAGTAC
TTGTACCGGGTGATTTTGTAGATGTTATTGCCATCGCTGGCTTATGCGTTAACGATGAAATTGCCTTGACTCTACGTGA
GCCTGGCTTAGTCTTGTTTACGCATGAACTTCGAAATCATACTGCGACGGAATATTTTGTTTCATCGCGACGTATGTTT
GAACCTAGAAAACCTACCGTTAGTGATTTTGTTCAAATTGAGAGTTGTGTGGTCACCTATGTCAATTTGACTAGGGACC
AACTACCAGATGTAATCCCAGATTACATCGATGTTAACAAAACACTTGATGAGATTTTAGCTTCTCTGCCCAATAGAAC
TGGTCCAAGTCTTCCTTTAGATGTTTTTAATGCTACTTATCTTAATCTCACTGGTGAAATTGCAGATTTAGAGCAGCGT
TCAGAGTCTCTCCGTAATACTACAGAAGAGCTCCAAAGTCTTATATATAATATCAACAACACACTAGTTGACCTTGAGT
GGCTCAACCGAGTTGAGACATATATCAAGTGGCCGTGGTGGGTTTGGTTGATTATTTTCATTGTTCTCATCTTTGTTGT
GTCATTACTAGTGTTCTGCTGCATTTCCACGGGTTGTTGTGGATGCTGCGGCTGCTGCTGTGCTTGTTTTTCAGGTTGT
TGTAGGGGTCCTAGACTTCAACCTTACGAAGCTTTTGAAAAGGTCCACGTGCAGTGA

>gi|455475474|gb|KC242912.1|_Porcine_epidemic_diarrhea_virus_isolate_HN8201211_spike_protein_gene_complete_cds

ATGAAGTCTTTAACTTACTTCTGGTTGTTCTTACCAGTACTTTCAACACTTAGCCTACCACAAGATGTCACCAGG
TGCTCAGCTAACACTAATTTTAGGCGGTTCTTTTCAAAATTTAATGTTCAGGCGCCTGCAGTTGTTGTACTGGGCGGTT
ATCTACCTATTGGTGAAAACCAGGGTGTCAATTCAACTTGGTACTGTGCTGGCCAACATCCAACTGCTAGTGGCGTTCA
TGGTATCTTTGTTAGCCATATTAGAGGTGGTCATGGCTTTGAGATTGGCATTTCGCAAGAGCCTTTTGACCCTAGTGGT
TACCAGCTTTATTTAAATAAGGCTACTAACGGTAACACTAATGCTACTGCGCGACTGCGCATTTGCCAGTTTCCTAGCA
TTAAAACATTGGGCCCCACTGCTAATAATGATGTTACAACAGGTCGTAATTGCCTATTTAACAAAGCCATCCCAGCTCA

TATGAGTGAACATAGTGTTGTCGGCATAACATGGGATAATGATCGTGTCACTGTCTTTTCTGACAAGATCTATTATTTT
TATTTTAAAAATGATTGGTCCCGTGTTGCGACAAAGTGTTACAACAGTGGAGGTTGTGCTATGCAATATGTTTACGAAC
CCACCTATTACATGCTTAATGTTACTAGTGCTGGTGAGGATGGTATTTCTTATCAACCCTGTACAGCTAATTGCATTGG
TTATTCTGCCAATGTATTTGCTACTGAGCCCAATGGCCACATACCAGAAGGTTTTAGTTTTAATAATTGGTTTCTTTTG
TCCAATGATTCCACTTTGGTGCATGGTAAGGTGGTTTCCAACCAACCATTGTTGGTCAATTGTCTTTTGGCCATTCCTA
AGATTTATGGACTAGGCCAATTTTTCTCCTTTAATCAAACGATCGATGGTGTTTGTAATGGAGCTGCTGTGCAGCGTGC
ACCAGAGGCTCTGAGGTTTAATATTAATGACACCTCTGTCATTCTTGCTGAAGGCTCAATTGTACTTCACACTGCTTTA
GGAACAAATTTTTCTTTTGTTTGCAGTAATTCCTCAAATCCTCACTTAGCCACCTTCGCCATACCTCTGGGTGCTATCC
AAGTACCCTATTATTGTTTTCTTAAAGTGGATATTTACAACTCCACTGTTTATAAATTCTTGGCTGTTTTACCTCCTAC
CGTCAGGGAAATTGTCATCACCAAGTATGGCGATGTTTATGTCAATGGGTTTGGCTACTTGCATCTCGGTTTGTTGGAT
GCTGTCACAATTAATTTCACTGGCCATGGCACTGACGATGACGTTTCTGGTTTTTGGACCATAGCATCGACTAATTTTG
TTGATGCACTTATCGAAGTTCAAGGAACTGCCATTCAGCGTATTCTTTATTGTGATGATCCTGTTAGCCAACTCAAGTG
TTCTCAGGTTGCTTTTGACCTTGACGATGGTTTCTACCCTATTTCTTCTAGAAACCCTCTGAGTCATGAACAGCCAATT
TCTTTTGTTACTTTGCCATCATTTAATGATCATTCTTTTGTTAACATTACTGTCTCTGCGTCCTTTGGTGGTCATAGTG
GTGCCAACCTCATTGCATCTGACATTACTATCAATGGGTTTAGTTCTTTCTGTGTTGACACTAGACAATTTACCATTTC
ACTGTTTTATAACGTTACAAACAGTTATGGTTATGTGTCTAAATCACAGGACACTAATTGCCCTTTCACCTTGCAATCT
GTTAATGATTACCTGTCTTTTAGTAAATTTTGTGTTTCTACCAGCCTTTTGGCTAGTGCCTGTACCATAGATCTTTTTG
GTTACCCTGAGTTTGGTAGTGGTGTTAAGTTCACGTCCCTTTACTTTCAATTCACAAAGGGTGAGTTGATTACTGGCAC
GCCTAAACCACTTGAAGGTGTTACGGACGTTTCTTTTATGACTCTGGATGTGTGTACCAAGTATACTATCTATGGCTTT
AAAGGTGAGGGTGTCATTACCCTTACAAATTCTAGCCTTTTGGCAGGTGTTTATTACACATCTGATTCTGGACAGTTGT
TAGCCTTTAAGAATGTCACTAGTGGTGCTGTTTATTCTGTTACGCCATGTTCTTTTTCAGAGCAGGCTGCATATGTTGA
TGATGATATAGTGGGTGTTATTTCTAGTTTGTCTAGCTCCACTTTTAACAGTACTAGGGAGTTGCCTGGTTTCTTCTAC
CATTCTAATGATGGCTCTAATTGTACAGAGCCTGTGTTGGTGTATAGTAACATAGGTGTTTGTAAATCTGGCAGTATTG
GCTATGTCCCATCTCAGTCTGGCCCAGTCAAGATTGCACCCACGGTTACTGGGAATATTAGTATTCCCACCAACTTTAG
TATGAGTATTAGGACAGAATATTTACAGCTTTACAACACGCCTGTTAGTGTTGATTGTGCCATATATGTTTGTAATGGT
AACTCTCGTTGTAAACAATTACTCACCCAGTACACTGCAGCATGTAAGACCATAGAGTCGGCATTACAACTCAGCGCTA
GGCTTGAGTCTGTTGAAGTTAACTCTATGCTTACTATCTCTGAAGAGGTTCTACAGTTAGCTACCATCAGTTCGTTTAA
TGGTGATGGATATAATTTTACTAATTTGCTGGGTGTTTCTGTGTATGATCCTGCAAGTGGCAGGGTGGTACAAAAAAGG
TCTTTTATTGAAGACCTGCTTTTTAATAAAGTGGTTACTAATGGCCTTGGTACTGTTGATGAAGACTATAAGCGCTGTT
CTAATGGTCGCTCTGTGGCAGATCTAGTCTGTGCACAGTATTACTCTGGTGTCATGGTACTACCTGGTGTTGTTGACGC
TGAGAAGCTTCACATGTATAGTGCGTCTCTCATCGGTGGTATGGTGCTAGGAGGTTTTACTTCTGCAGCGGCATTGCCT
TTTAGCTATGCTGTTCAAGCTAGACTCAATTATCTTGCTCTACAGACGGATGTTCTACAGCGGAACCAGCAATTGCTTG
CTGAGTCTTTTAACTCTGCTATTGGTAATATAACTTCAGCCTTTGAGAGTGTTAAAGAGGCTATTAGTCAAACTTCCAA
GGGTTTGAACACTGTGGCTCAAGCGCTTACTAAGGTTCAAGAGGTTGTTAATTCGCAGGGTGCAGCTTTGAATCAACTT
ACCGTACAGCTGCAACACAACTTCCAAGCTATTTCTAGTTCTATTGATGACATTTACTCCCGACTGGACATTCTTTCAG
CCGATGTTCAGGTTGACCGTCTCATCACCGGCAGACTATCAGCACTTAATGCTTTTGTTGCTCAAACCCTCACTAAGTA
CACTGAGGTTCAGGCTAGCAGGAAGCTAGCACAGCAAAAGGTTAATGAGTGCGTTAAATCGCAATCTCAGCGTTATGGT
TTTTGTGGTGGTGATGGCGAGCACATTTTCTCTCTGGTACAGGCAGCACCTCAGGGCCTGCTGTTTTTACATACAGTAC
TTGTACCGGGTGATTTTGTAGATGTTATTGCCATCGCTGGCTTATGCGTTAACGATGAAATTGCCTTGACTCTACGTGA
GCCTGGCTTAGTCTTGTTTACGCATGAACTTCAAAATCATACTGCGACGGAATATTTTGTTTCATCGCGACGTATGTTT
GAACCTAGAAAACCTACCGTTAGTGATTTTGTTCAAATTGAGAGTTGTGTGGTCACCTATGTCAATTTGACTAGGGACC
AACTACCAGATGTAATCCCAGATTACATCGATGTTAACAAAACACTTGATGAGATTTTAGCTTCTCTGCCCAATAGAAC
TGGTCCAAGTCTTCCTTTAGATGTTTTTAATGCTACTTATCTTAATCTCACTGGTGAAATTGCAGATTTAGAGCAGCGT
TCAGAGTCTCTCCGTAATACTACAGAAGAGCTCCAAAGTCTTATATATAATATCAACAACACACTAGTTGACCTTGAGT

GGCTCAACCGAGTTGAGACATATATCAAGTGGCCGTGGTGGGTTTGGTTGATTATTTTCATTGTTCTCATCTTTGTTGT
GTCATTACTAGTGTTCTGCTGCATTTCCACGGGTTGTTGTGGATGCTGCGGCTGCTGCTGTGCTTGTTTTTCAGGTTGT
TGTAGGGGTCCTAGACTTCAACCTTACGAAGCTTTTGAAAAGGTCCACGTGCAGTGA

>gi|514833073|gb|KC787537.1|_Porcine_epidemic_diarrhea_virus_isolate_CHDLC012013_spike_protein_gene_complete_cds

ATGAAGTCTCTAACCTACTTCTGGTTGTTCTTACCAGTACTTTCAACACTTAGCCTACCACAAGATGTCACCAGG
TGCTCAGCTAACACTAATTTTAGGCGGTTCTTTTCAAAATTTAATGTTCAGGCGCCTGCAGTTGTTGTACTGGGCGGTT
ATCTACCTATTGGTGAAAACCAGGGTGTCAATTCAACTTGGTACTGTGCTGGCCAACATCCAACTGCTAGTGGCGTTCA
TGGTATCTTTGTTAGCCATATTAGAGGTGGTCATGGCTTTGAGATTGGCATTTCGCAAGAGCCTTTTGACCCTAGTGGT
TACCAGCTTTATTTACATAAGGCTACTAACGGTAACACTAATGCTACTGCGCGACTGCGCATTTGCCAGTTTCCTAGCA
TTAAAACATTGGGCCCCACTGCTAATAATGATGTTACAACAGGTCGTAATTGCCTATTTAACAAAGCCATCCCAGCTCA
TATGAGTGAACATAGTGTTGTCGGCATAACATGGGATAATGATCGTGTCACTGTCTTTTCTGACAAGATCTATTATTTT
TATTTTAAAAATGATTGGTCCCGTGTTGCGACAAAGTGTTACAACAGTGGAGGTTGTGCTATGCAATATGTTTACGAAC
CCACCTATTACATGCTTAATGTTACTAGTGCTGGTGAGGATGGTATTTCTTATCAACCCTGTACAGCTAATTGCATTGG
TTATTCTGCCAATGTATTTGCTACTGAGCCCAATGGCCACATACCAGAAGGTTTTAGTTTTAATAATTGGTTTCTTTTG
TCCAATGATTCCACTTTGGTGCATGGTAAGGTGGTTTCCAACCAACCATTGTTGGTCAATTGTCTTTTGGCCATTCCTA
AGATTTTTGGACTAGGCCAATTTTTCTCCTTTAATCAAACGATCGATGGTGTTTGTAATGGAGCTGCTGTGCAGCGTGC
ACCAGAGGCTCTGAGGTTTAATATTAATGACACCTCTGTCATTCTTGCTGAAGGCTCAATTGTACTTCATACTGCTTTA
GGAACAAATTTTTCTTTTGTTTGCAGTAATTCCTTAAATCCTCATTTAGCCACCTTTGCCATACCTCTGGGTGCTACCC
AAGTACCCTATTATTGTTTTCTTAAAGTGGATACTTACAACTCCCCTGTTTATAAATTCTTGGCTGTTTTACCTCCTAC
CGTCAGGGAAATTGTCATCACCAAGTATGGTGATGTTTATGTCAATGGGTTTGGCTACTTGCATCTCGGTTTGTTGGAT
GCTGTCACAATTAATTTCACTGGTCATGGCACTGACGATGACGTTTCTGGTTTTTGGACCATAGCATCGACTAATTTTG
TTGATGCACTTATCGAAGTTCAAGGAACTGCCATTCAGCGTATTCTTTATTGTGATGATCCTGTTAGCCAACTCAAGTG
TTCTCAGGTTGCTTTTGACCTTGACGATGGTTTCTACCCTATTTCTTCTAGAAACCTTCTGAGTCATGAACAGCCAATT
TCTTTTGTTACTTTGCCATCATTTAATGATCATTCTTTTGTTAACATTACTGTCTCCGCGTCCTTTGGTGATCATAGTG
GTGCCAACCTTATTGCATCTGACACTACTATCAATGGGTTTAGTTCTTTCTGTGTTGACACTAGACAATTTACCATTTC
ACTGTTTTATAACGTTACAAACAGTTATGGTTATGTGTCTAAATCACAGGACAGTAATTGCCCTTTCACCTTGCAATCT
GTTAATGATTACCTGTCTTTTAGTAAATTTTGTGTTTCCACCAGCCTTTTGGCTAGTGCCTGTACCATAGATCTTTTTG
GTTACCCTGAGTTTGGTAGTGGTGTTAAGTTTACGTCCCTTTACTTTCAATTCACAAAGGGTGAGTTGATTACTGGCAC
GCCTAAACCACTTGAAGGTGTCACGGACGCTTCTTTTATGACTCTGGATGTGTGTACCAAGTATACTATCTATGGCTTT
AAAGGTGAGGGTATCATTACCCTTACAAATTCTAGCTTTTTGGCAGGTGTTTATTACACATCTGATTCTGGACAGTTGT
TAGCCTTTAAGAATGTCACTAGTGGTGCTGTTTATTCTGTTACGCCATGTTCTTTTTCAGAGCAGGCTGCATATGTTGA
TGATGATATAGTGGGTGTTATTTCTAGTTTGTCTAACTCCACTTTTAACAGTACTAGGGAGTTGCCTGGTTTCTTCTAC
CATTCTAATGATGGCTCTAATTGTACAGAGCCTGTGTTGGTGTATAGTAACATAGGTGTTTGTAAATCTGGCAGTATTG
GCTACGTCCCATCTCAGTCAGGCCAAGTCAAGATTGCACCCATGGTTACTGGGAATATTAGTATTCCCACCAACTTTAG
TATGAGTATTAGGACAGAATATTTACAGCTTTACAACACGCCTGTTAGTGTTGATTGTGCCACATATGTTTGTAATGGT
AACTCTCGTTGTAAACAATTACTCACCCAGTACACTGCAGCATGTAAGACCATAGAGTCAGCATTACAACTCAGCGCTA
GGCTTGAGTCTGTTGAAGTTAACTCTATGCTTACTATTTCTGAAGAGGCTCTACAGTTAGCTACCATCAGTTCGTTTAA
TGGTGATGGATATAATTTTACTAATGTGCTGGGTGTTTCTGTGTATGATCCTGCAAGTGGCAGGGTGGTACAAAAAAGG
TCTTTTATTGAAGACCTGCTTTTTAATAAGGTGGTTACTAATGGCCTTGGTACTGTTGATGAAGACTATAAGCGCTGTT
CTAATGGTCGCTCTGTGGCAGATCTAGTCTGTGCACAGTATTACTCTGGTGTCATGGTACTACCTGGTGTTGTTGACGC

TGAGAAGCTTCACATGTATAGTGCGTCTCTCATCGGTGGTATGGTGCTAGGAGGTTTTACTTCTGCAGCGGCATTGCCT
TTTAGCTATGCTGTTCAAGCTAGACTCAATTATCTTGCTCTACAGACGGATGTTCTACAGCGGAACCAGCAATTGCTTG
CTGAGTCTTTTAACTCTGCTATTGGTAATATAACTTCAGCCTTTGAGAGTGTTAAAGAGGCTATTAGTCAAACTTCCAA
GGGTTTGAACACTGTGGCTCATGCGCTTACTAAGGTTCAAGAGGTTGTTAACTCGCAGGGTGCAGCTTTGACTCAACTT
ACCGTACAGCTGCAACACAACTTCCAAGCCATTTCTAGTTCTATTGATGACATTTACTCTCGACTGGACATTCTTTCAG
CCGATGTTCAGGTTGACCGTCTCATCACCGGCAGATTATCAGCACTTAATGCTTTTGTTGCTCAAACCCTCACTAAGTA
TACTGAGGTTCAGGCTAGCAGGAAGCTAGCACAGCAAAAGGTTAATGAGTGCGTTAAATCGCAATCTCAGCGTTATGGT
TTTTGTGGTGGTGATGGCGAGCACATTTTCTCTCTGGTACAGGCAGCACCTCAGGGCCTGCTGTTTTTACATACAGTAC
TTGTACCGGGTGATTTTGTAGATGTTATTGCCATCGCTGGCTTATGCGTTAACGATGAAATTGCCTTGACTCTACGTGA
GCCTGGCTTAGTCTTGTTTACGCATGAACTTCAAAATCATACTGCGACGGAATATTTTGTTTCATCGCGACGTATGTTT
GAACCTAGAAAACCTACCGTTAGTGATTTTGTTCAAATTGAGAGTTGTGTGGTCACCTATGTCAATTTGACTAGAGACC
AACTACCAGATGTAATCCCAGATTACATCGATGTTAACAAAACACTTGATGAGATTTTAGCTTCTCTGCCCAATAGAAC
TGGTCCAAGTCTTCCTTTAGATGTTTTTAATGCCACTTATCTTAATCTCACTGGTGAAATTGCAGATTTAGAGCAGCGT
TCAGAGTCTCTCCGTAATACTACAGAGGAGCTTCAAAGTCTTATATATAATATCAACAACACACTAGTTGACCTTGAGT
GGCTCAACCGAGTTGAGACATATATCAAGTGGCCGTGGTGGGTTTGGTTGATTATTTTCATTGTTCTCATCTTTGTTGT
GTCATTACTAGTGTTCTGCTGCATTTCCACGGGTTGTTGTGGATGCTGCGGCTGCTGCTGTGCTTGTTTTTCAGGTTGT
TGTAGGGGTCCTAGACTTCAACCTTACGAAGTTTTTGAAAAGGTCCACGTGCAGTGA

>gi|514833080|gb|KC787540.1|_Porcine_epidemic_diarrhea_virus_isolate_CHHGC012013_spike_protein_gene_complete_cds

ATGAAGTCTTTAACCTACTTCTGGTTGTTCTTACCAGTACTTTCAACACTTAGCCTACCACAAGATGTCACCAGG
TGCTCAGCTAACACTAATTTTAGGCGGTTCTTTTCAAAATTTAATGTTCAGGCGCCTGCAGTTGTTGTACTGGGCGGTT
ATCTACCTATTGGTGAAAACCAGGGTGTCAATTCAACTTGGTACTGTGCTGGCCAACATCCAACTGCTAGTGGCGTTCA
TGGTATCTTTGTTAGCCACATTAGAGGTGGTCATGGCTTTGAGATTGGCATTTCGCAAGAGCCTTTTGACCCTAGTGGT
TACCAGCTTTATTTACATAAGGCTACTAACGGTAATACTAATGCTACTGCGCGACTGCGCATTTGCCAGTTTCCTAGCA
TTAAAACATTGGGCCCCACTGCTAATAATGATGTTACAACAGGTCGTAATTGCCTATTTAACAAAGCCATCCCAGCTCA
TATGAGTGAACATAGTGTTGTCGGCATAACATGGGATAATGATCGTGTCACTGTCTTTTCAGACAAGATTTATTATTTT
TATTTTAAAAATGATTGGTCCCGTGTTGCGACAAAGTGTTACAACAGTGGAGGTTGTGCTGTGCAATATGTTTACGAAC
CCACCTATTACATGCTTAATGTTACTAGTGCTGGTGAGGATGGTATTTCTTATCAACTCTGTACAGCTAATTGCATTGG
TTATGCTGCCAATGTATTTGCTACTGAGCCCAATGGCCACATACCAGAAGGTTTTAGTTTTAATAATTGGTTTCTTTTG
TCCAATGATTCCACTTTGGTGCATGGTAAGGTGGTTTCCAACCAACCATTGTTGGTCAATTGTCTTTTGGCCATTCCTA
AGATTTATGGACTAGGCCAATTTTTCTCCTTTAATCAAACGACCGATGGTGTTTGTAATGGAGCTGCTGTGCAGCGTGC
ACCAGAGGCTCTGAGGTTTAATATTAATGACACCTCTGTCATTCTTGCTGAAGGCTCAATTGTACTTCATACTGCTTTA
GGAACAAATTTTTCTTTTGTTTGCAGTAATTCTTCAGATCCTCATTTAGCTACCTTCGCCATACCTCTGGGTGCTACCC
AAGTACCCTATTATTGTTTTCTTAAATTGGATACTTACAACTCCACTGTTTATAAATTTTTGGCTGTTTTACCTCCTAC
CGTCAGGGAAATTGTCATCACCAAGTATGGTGATGTTTATGTTAATGGGTTTGGATACTTGCATCTCGGTTTGTTGGAT
GCTGTCACAATTAATTTCACTGGTCATGGCACTGACGATGATGTTTCTGGTTTTTGGACCATAGCATCGACTAATTTTG
TTGATGCACTCATCGAAGTTCAAGGAACTGCCATTCAGCGTATTCTTTATTGTGATGATCCTGTTAGCCAACTCAAGTG
TTCTCAGGTTGCTTTTGACCTTGATGATGGTTTTTACCCTATTTCTTCTAGAAACCTTCTGAGTCATGAACAGCCAATT
TCTTTTGTTACTCTGCCATCATTTAATGATCATTCTTTTGTTAACATTACTGTCTCTGCTTCCTTTGGTGGTCATAGTG
GTGCCAACCTTATTGCATCTGACACTACTATCAATGGGTTTAGTTCTTTCTGTGTTGACACTAAACAATTTACCATTTC
GCTGTTTTATAACGTTACAAACAGTTATGGTTATGTGTCTAAATCACAGGACAGTAATTGCCCTTTCACCTTGCAATCT

GTTAATGATTACCTGTCTTTTAGCAAATTTTGTGTTTCCACCAGCCTTTTGGCTAGTGCCTGTACCATAGATCTTTTTG
GTTACCCTGAGTTTGGTAGTGGTGTTAAGTTTACGTCCCTTTACTTTCAATTCACAAAGGGTGAGTTGATTACTGGCAC
GCCTAAACCACTTGAAGGTGTCACGGACGTTTCTTTTATGACTCTGGATGTGTGTACCAAGTATACTATCTATGGCTTT
AAAGGTGAGGGTATCATTACCCTTACAAATTCTAGCTTTTTGGCAGGTGTTTATTACACATCTGATTCTGGACAGTTGT
TAGCCTTTAAGAATGTCACTAGTGGTGCTGTTTATTCTGTTACGCCATGTTCTTTTTCAGAGCAGGCTGCATATGTTGA
TGATGATATAGTGGGTGTTATTTCTAGTTTGTCTAGCTCCACTTTTAACAGTACTAGGGAGTTGCCTGGTTTCTTCTAC
CATTCTAATGATGGCTCTAATTGTACAGAGCCTGTGTTGGTGTATAGTAACATAGGTGTTTGTAAATCTGGCAGTATTG
GCTACGTCCCATCCCAGTCTGGCCAAGTCAAGATTGCACCCACGGTTACTGGGAATATTAGTATTCCCACCAACTTTAG
TATGAGTATTAGGACAGAATATTTACAGCTTTACAACACGCCTGTTAGTGTTGATTGTGCCACATATGTTTGTAATGGT
AACTCTCGTTGTAAACAATTACTCACCCAGTACACTGCAGCATGTAAGACCATAGAGTCAGCATTACAACTCAGCGCTA
GGCTTGAGTCTGTTGAAGTTAACTCTATGCTTACCATTTCTGAAGAGGCTCTACAGTTAGCTACCATTAGTTCGTTTAA
TGGTGATGGATATAATTTTACTAATGTGCTGGGTGTTTCTGTGTATGATCCTGCAAGTGGCAGGGTGGTACAAAAAAGG
TCTTTTATTGAAGACCTGCTTTTTAATAAAGTGGTTACTAATGGCCTTGGTACTGTTGATGAAGACTATAAGCGCTGTT
CTAATGGTCGCTCTGTGGCAGATCTAGTCTGTGCACAGTATTACTCTGGTGTCATGGTACTACCTGGTGTTGTTGACGC
AGAGAAGCTTCACATGTATAGTGCGTCTCTCATCGGTGGTATGGTGCTAGGAGGTTTTACTTCTGCAGCGGCATTGCCT
TTTAGCTATGCTGTTCAAGCTAGACTCAATTATCTTGCTCTACAGACGGATGTTCTACAGCGGAACCAGCAATTGCTTG
CTGAGTCTTTTAACTCTGCTATTGGTAATATAACTTCAGCCTTTGAGAGTGTTAAAGAGGCTATTAGTCAAACTTCCAA
GGGTTTGAACACTGTGGCTCATGCGCTTACTAAGGTTCAAGAGGTTGTTAACTCGCAGGGTGCAGCTTTGACTCAACTT
ACCGTACAGCTGCAACACAACTTCCAAGCCATTTCTAGTTCTATTGATGACATTTACTCTCGACTGGACATTCTTTCAG
CCGATGTTCAGGTTGACCGTCTCATCACCGGCAGATTATCAGCACTTAATGCTTTTGTTGCTCAAACCCTCACTAAGTA
TACTGAGGTTCAGGCTAGCAGGAAGCTAGCACAGCAAAAGGTTAATGAGTGCGTTAAATCGCAATCTCAGCGTTATGGT
TTTTGTGGTGGTGATGGCGAGCACATTTTCTCTCTGGTACAGGCAGCACCTCAGGGCCTGCTGTTTTTACATACAGTAC
TTGTACCGGGTGATTTTGTAGATGTTATTGCCATCGCTGGTTTATGCGTTAACGATGAAATTGCCTTGACTCTACGTGA
GCCTGGCTTAGTCTTGTTTACGCATGAACTTCAAAATCATACTGCGATGGAATATTTTGTTTCATCGCGACGTATGTTT
GAACCTAGAAAACCTACCGTTAGTGATTTTGTTCAAATTGAGAGTTGTGTGGTCACCTATGTCAATTTGACTAGAGACC
AACTACCAGATGTAATCCCAGATTACATCGATGTTAACAAAACACTTGATGAGATTTTAGCTTCTCTGCCCAATAGAAC
TGGTCCAAGTCTTCCTTTAGATGTTTTTAATGCCACTTATCTTAATCTCACTGGTGAAATTGCAGATTTAGAGCAGCGT
TCAGAGTCTCTCCGTAATACTACAGAGGAGCTCCAAAGTCTTATATATAATATCAACAACACACTAGTTGACCTTGAGT
GGCTCAACCGAGTTGAGACATATATCAAGTGGCCGTGGTGGGTTTGGTTGATTATTTTCATTGTTCTCATCTTTGTTGT
GTCATTACTAGTGTTCTGCTGCATTTCCACGGGTTGTTGTGGATGCTGCGGCTGCTGCTGTGCTTGTTTTTCAGGTTGT
TGTAGGGGTCCTAGACTTCAACCTTACGAAGTTTTTGAAAAGGTCCACGTGCAGTGA

>gi|514833086|gb|KC787543.1|_Porcine_epidemic_diarrhea_virus_isolate_CHSTC122012_spike_protein_gene_complete_cds

ATGAAGTCTTTAACTTACTTCTGGTTGTTCTTACCAGTACTTTCAACACTCAGCCTACCACAAGATGTCACTAGG
TGCCATTCCACTATTAACTTCAGGCGGTTCTTTTCAAAATTTAATGTGCAAGCACCTGCTGTCGTTGTTTTGGGTGGTT
ATCTACCTAGTATGAACTCTTCTAGCTGGTACTGTGGCACAGGCCTTGAAACTGCTAGTGGCGTTCATGGTATTTTCCT
CAGTTACATCGATGCTGGTCAGGGCTTTGAGATTGGCATTTCACAGGAGCCGTTTGATCCTAGTGGTTACCAGCTTTAT
CTACATAAGGCCACTAATGGTAACACTAATGCTATTGCACGACTGCGGATTTGCCAGTTTCCAGATAATAAAACATTGG
GCCCTACTGTTAATGATGTTACAACAGGTCGTAACTGCCTATTCAACAAAGCCATTCCAGCTTATATGCAGGATGGAAA
AAATATCGTTGTCGGCATAACATGGGACAATGATCGTGTCACTGTTTTTGCTGACAAGATCTATCATTTTTATCTTAAA
AATGATTGGTCCCGTGTTGCGACAAGATGTTACAATAAAAGAAGTTGTGCTATGCAATATGTTTACACACCCACCTACT

ACATGCTTAATGTTACTAGTGCAGGTGAGGATGGCATTTATTATGAACCATGTACAGCTAATTGCAGTGGTTACGCTGC
CAATGTATTTGCCACTGATTCTAATGGCCATATACCAGAAGGTTTTAGTTTTAATAATTGGTTTCCTTTGTCCAATGAC
TCCACTTTGTTGCATGGTAAGGTGGTTTCCAACCAACCTTTGTTGGTCAATTGTCTTTTGGCCATTCCTAAGATTTATG
GACTAGGCCAATTTTTCTCATTCAATCAAACGATGGATGGCGTTTGTAATGGAGCTGCTGTGCAGCGTGCACCAGAGGC
TCTGAGGTTTAATATTAATGACACCTCTGTCATTCTTGCTGAAGGCTCAATTGTACTTCATACTGCTTTAGGAACAAAT
CTTTCTTTTGTTTGCAGTAATTCCTCAGATCCTCATTTAGCCACCTTCGCCATACCTTTGGGTGCTACCCAAGTACCCT
ATTATTGTTTTCTTAAAGTGGATACTTACAACTCCACTGTTTATAAATTCTTGGCTGTTTTACCTCCTACCGTCAGGGA
AATTGTCATCACCAAGTATGGTGATGTTTATGTCAATGGGTTTGGCTACTTGCATCTCGGTTTGTTGGATGCTGTCACA
ATTAATTTCACTGGTCATGGCACTGACGATGACGTTTCTGGTTTTTGGACCATAGCATCGACTAATTTTGTTGATGCAC
TTATCGAAGTTCAAGGAACTGCCATTCAGCGTATTCTTTATTGTGATGATCCTGTTAGCCAACTCAAGTGTTCTCAGGT
TGCTTTTGACCTTGACGATGGTTTCTACCCTATTTCTTCTAGAAACCTTTTGAGTCATGAACAGCCAATTTCTTTTGTT
ACTTTGCCATCATTTAATGATCATTCTTTTGTTAACATTACTGTCTCTGCGTCCTTTGGTGATCATAGTGGTGCCAACC
TTATTGCATCTGACACTACTATCAATGGGTTTAGTTCTTTCTGTGTTGACACTAGACAATTTACCATTTCACTGTTTTA
TAACGTTACAAACAGTTATGGTTATGTGTCTAAATCACAGGAGAGTAATTGCCCTTTCACCTTGCAATCTGTTAATGAT
TACCTGTCTTTTAGCAAATTTTGTGTTTCCACCAGCCTTTTGGCTAGTGCCTGTACCATAGATCTTTTTGGTTACCCTG
AGTTTGGTAGTGGTGTTAAGTTTACGTCCCTTTACTTTCAATTCACAAAGGGTGAGTTGATTACTGGCACGCCTAAACC
ACTTGAAGGTGTCACGGACGTTTCTTTTATGACTTTGGATGTGTGTACCAAGTATACTATCTATGGCTTTAAAGGTGAG
GGTATCATTACCCTTACAAATTCTAGCTTTTTGGCAGGTGTTTATTACACATCTGATTCTGGACAGTTGTTAGCCTTTA
AGAATGTCACTAGTGGTGCTGTTTATTCTGTTACGCCATGTTCTTTTTCAGAGCAGGCTGCATATGTTGATGATGATAT
AGTGGGTGTTATTTCTAGTTTGTCTAGCTCCACTTTTAACAGTACTAGGGAGTTGCCTGGTTTCTTCTACCATTCTAAT
GATGGCTCTAATTGTACAGAGCCTGTGTTGGTGTATAGTAACATAGGTGTTTGTAAATCTGGCAGTATTGGCTACGTCC
CATCTCAGTCTGGCCAAGTCAAGATTGCACCCACGGTTACTGGGAATATTAGTATTCCCACCAACTTTAGTATGAGTAT
TAGGACAGAATATTTACAGCTTTACAACACGCCTGTTAGTGTTGATTGTGCCACATATGTTTGTAATGGTAACTCTCGT
TGTAAACAATTACTCACCCAGTACACTGCAGCTTGTAAGACCATAGAGTCAGCATTACAACTCAGCGCTAGGCTTGAGT
CTGTTGAAGTTAACTCTATGCTTACTATTTCTGAAGAGGCTCTACAGTTAGCTACCATCAGTTCGTTTAATGGTGACGG
ATATAATTTTACTAATGTGCTGGGTGTTTCTGTGTATGATCCTGCAAGTGGCAGGGTGGTACAAAAAAGGTCTTTTATT
GAAGACCTGCTTTTTAATAAAGTGGTTACTAATGGCCTTGGTACTGTTGATGAAGACTATAAGCGCTGTTCTAATGGTC
GCTCTGTGGCAGATCTAGTCTGTGCACAGTATTACTCTGGTGTCATGGTACTACCTGGTGTTGTTGACGCTGAGAAGCT
TCACATGTATAGTGCGTCTCTCATCGGTGGTATGGTGCTAGGAGGTTTTACTTCTGCAGCGGCATTGCCTTTTAGCTAT
GCTGTTCAAGCTAGACTCAATTATCTTGCTCTACAGACGGATGTTCTACAGCGGAACCAGCAATTGCTTGCTGAGTCTT
TTAACTCTGCTATTGGTAATATAACTTCAGCCTTTGAGAGTGTTAAAGAGGCTATTAGTCAAACTTCCAAGGGTTTGAA
CACTGTGGCTCATGCGCTTACTAAGGTTCAAGAGGTTGTTAACTCGCAGGGTGCAGCTTTGACTCAACTTACCGTACAG
CTGCAACACAACTTCCAAGCCATTTCTAGTTCTATTGATGACATTTACTCTCGACTGGACAGTCTTTCAGCCGATGTTC
AGGTTGACCGTCTCATCACCGGCAGATTATCAGCACTTAATGCTTTTGTTGCTCAAACCCTCACTAAGTATACTGAGGT
TCAGGCTAGCAGGAAGCTAGCACAGCAAAAGGTTAATGAGTGCGTTAAATCGCAATCTCAGCGTTATGGTTTTTGTGGT
GGTGATGGCGAGCACATTTTCTCTCTGGTACAGGCAGCACCTCAGGGCCTGCTGTTTTTACATACAGTACTTGTACCGG
GTGACTTTGTAGATGTTATTGCCATCGCTGGCTTATGCGTTAACGATGAAATTGCCTTGACTCTACGTGAGCCTGGCTT
AGTCTTGTTTACGCATGAACTTCAAAATCATACTGCGACGGAATATTTTGTTTCATCGCGACGTATGTTTGAACCTAGA
AAACCTACCGTTAGTGATTTTGTTCAAATTGAGAGTTGTGTGGTCACCTATGTCAATCTGACTAGAGACCAATTACCAG
ATGTAATCCCAGATTACATCGATGTTAACAAAACACTTGATGAGATTTTAGCTTCTCTGCCCAATAGAACTGGTCCAAG
TCTTTCTTTAGATGTTTTTAATGCCACTTATCTTAATCTCACTGGTGAAATTGCAGATTTAGAGCAGCGTTCAGAGTCT
CTCCGTAATACTACAGAGGAGCTCCAAAGTCTTATATATAATATCAACAACACACTAGTTGACCTTGAGTGGCTCAACC
GAGTTGAGACATATATCAAGTGGCCGTGGTGGGTTTGGTTGATTATTTTCATTGTTCTCATCTTTGTTGTGTCATTACT
AGTGTTCTGCTGCATTTCCACGGGTTGTTGTGGATGCTGCGGCTGCTGCTGTGCTTGTTTTTCAGGTTGTTGTAGGAGT

CCTAGACTTCAACCTTACGAAGTTTTTGAAAAGGTCCACGTGCAGTGA

>gi|527461756|gb|KC879275.1|_Porcine_epidemic_diarrhea_virus_strain_AS01_spike_protein_(S)_gene_complete_cds

ATGAAGTCTTTAACCTATTTCTGGTTGTTCTTACCAGTACTTCCAATACTCAGCCTACCACAAGATGTCAATAGG
TGCTCAGCTGTGACTAACTTTAGGCAGTTCTTTTCAAAATTTAATGTTCAGGCGCCTGCAGTCGTTGTACTGGGCGGTT
ATCTACCTAGTGGTGAAAAACAGGTTGATGCACCAAGTTGGTACTGTGCTGGCCGATATGATACTGCTAGTGGCGTTCA
TGGTATCTTTCTTAGCCATATTAGAGGTGGTCATGGCTTTGAGATTGGCATTTCGCAAGAGCCGTTTGATCCTAGTGGT
TACCAGCTTTATTTACATAAGGCCACCAACGGTAACACTAATTCTACTGCGCGCCTGCGTATTTGCCAGTTTCCCAGCA
ACAAAACATTGGGCCCCACTGCTAATGATGATGTCACAACAGGTCGTAACTGCCTATTTAACAAAGCCATTCCAGCTTA
TATGAGTGAACATAGTGTTGTCGGCATAACATGGGATAATGATCGTGTCACTGTTTTTGCTGACAAGATCTATCATTTT
TATCTTAAAAATGATTGGTCCCGTGTTGCGTCAAAATGTTACAACAGTGGAGGTTGTGCTATGCAATATGTTTACAAAC
CTATTTACTACATGCTTAATGTTACTAGTGCTGGTGAGGATGGTATTTCTTATCAACTCTGTACAGCTAATTGCATTGG
TTATGCTGCCAATGTATTTGCTACTGAGCCCAATGGCCACATACCAGAAAGTTTTAGTTTTAATAATTGGTTTCTTTTG
TCCAATGATTCCACTTTGTTTCATGGTAAAGTGGTTTCCAACCAACCTTTGTTGGTCAATTGTCTTTGGGCCATTCCTA
AGATTTACGGACTAGGCCACTTTTTCTCTTTTAATCAAACGATGGATGGCGTTTGTAACGGAGCTACTGCTTATCGTGC
CCCAGAGGCTCTGAGGTTTAATATTAATGACACCTCTGTCATTCTTGCTGAAGGCTCAATTGTACTTCATACTGCTTTA
GGAACAAATCTTTCTTTTGTTTGCAGTAATTCCTCAGATCCTCATTTAGCCACCTTCGCCTTACCTCTGGGTGCTACCC
AAGTACCCTATTATTGTTTTCTTAAAGTGGACACTTACAACTCCACTGTTTATAAATTTTTGGCTGTTTTACCTCCTAC
CGTCAGGGAAATTGTCATCACCAAGTATGGTGATGTTTATGTCAATGGGTTTGGCTACTTGCATCTCGGTTTGTTGGAT
GCTGTCACAATTAATTTCACTGGTTATGGCACTGACGATGACGTTTCTGGTTTTTGGACCATAGCATCGACTAATTTTG
TTGATGCACTCATCGAAGTTCAAGGAACTGCCATTCAGCGTATTCTTTATTGTGATGATCCTGTTAGCCAACTCAAGTG
TTCTCAGGTTGCTTTTGACCTTGACGATGGTTTTTACCCTATTTCTTCTAGAAACCTTCTGAGTCATGAACAGCCAATT
TCTTTTGTTACTTTGCCATCATTTAATGATCATTCTTTTGTTAACATTACTGTCTCTGCGTCCTTTGGTGGTCATAGTG
GTGCCAACCTTATTGCATCTGACACTACTATCAATGGGTTTAGTTCTTTCTGTGTTGACACTAGACAATTTACCATTAG
ACTGTTTTATAACGTTACAAGCAGTTACGGTTATGTGTCTAACTCGCAGGATAGCAATTGCCCTTTCACCTTGCGATCT
GTTAATGATTACCTGTCTTTTAGCAAATTTTGTGTTTCAACCAGCCTTTTGGCTGGTGCTTGTACCATAGATCTTTTTG
GTCACCCTGCTTTTGGTAGTAGTGTTAAGTTTACGTCCCTCTATTTTCAATTCACAAAGGGTGATTTGATTACTGGCAC
GCCTAAATCACTTGAAGGTGTCACGGACGTTTCTTTTATGACTAAGGATGTGTGTACTAAGTATACTATCTATGGCTTT
AAAGGTGAGGGTATTATTACCCTTACAAATTCTAGCTTTTTGGCAGGTATTTATTATACATCTGATTCCGGACAGTTGT
TAGCCTTTAAGAATGTTACTAGTGGTGCTGTTTATTCTGTTACGCCATGTTCTTTTTCAGAGCAGGCTGCTTATGTTGA
TGATGATATAGTGGGTGTTATTTCCAGTTTGTCTAACTCCACTTTTAACAATACTAGGGAGTTGCCCGGTTTCTTCTAC
CATTCTAATGACGGCTCCAATTGTACAGAGCCTGTGTTGGTGTATAGTAACATAGGTGTCTGTAAATCTGGTAGTATTG
GCTATGTCCCATCTCAGTATGGCCAAGTCAAGATTGCACCCACGGTTACTGGGAATATTAGTATTCCCACCAACTTTAG
TATGAGTGTTAGAACAGAATATCTACAGCTTTACAACACGCCTGTTAGTGTTGATTGTGCTACATATGTTTGTAATGGT
AACTCTCGTTGTAAACAATTACTCACCCAGTACACTGCAGCATGTAAGACCATAGAGTCAGCATTACAACTCAGCGCTA
GGCTTGAGTCTGTTGAAGTTAACTCTATGCTTACTATTTCTGAAGAGGCTCTACAGTTAGCTACCATCAGTTCGTTTAA
TGGTGGTGGATATAATTTTACTAATGTGCTGGGTGTTTCCGTGTATGATCCTGCAAGTGGCAGGGTGGTACAAAAAAGG
TCTATTATTGAAGACCTGCTTTTTAATAAAGTGGTTACTAATGGCCTTGGTACTGTTGATGAAGACTATAAGCGCTGTT
CTAATGGCCGCTCTGTGGCAGATCTAGTCTGTGCGCAGTATTACTCTGGTGTCATGGTACTACCTGGTGTTGTTGACGC
TGAGAAGCTTCACATGTATAGTGCGTCTCTCGTCGGTGGTATGGTGCTAGGGGGTTTTACTGCTGCAGCGGCATTGCCT
TTTAGCTATGCTGTTCAAGCGAGACTCAATTATCTTGCTTTACAGACGGATGTTCTACAGCGTAATCAGCAATTGCTTG

CTGAGTCTTTTAACTCTGCTATTGGTAATATAACTTCAGCCTTCGAGAGTGTTAAAGAGGCTATTAGTCAAACTTCCCA
GGGTTTGAATACTGTGGCTCATGCACTTACTAAGGTTCAAGAGGTTGTTAATTCGCAGGGTGCAGCTTTGTCTCAACTC
ACTATACAGCTGCAACACAACTTCCGAGCCATTTCTAGTTCTATTGATGACATTTACTCCCGACTGGACATTCTTTCAG
CCGATGTTCAGGTTGATCGTCTCATCACCGGCAGATTATCAGCACTTAATGCTTTTGTTGCTCAAACCCTCACTAAGTA
TACTGAGGTTCAGGCTAGCAGGAAGCTAGCACAGCAAAAGGTTAATGAGTGCGTCAAATCGCAATCTCAGCGTTATGGT
TTTTGTGGTGGTGATGGTGAACACATTTTTCTCTGGTACAGGCCGCACCTCAGGGCCTGCTGTTTTACACACAGTAC
TTGTACCGGGTGATTTTGTAAATGTTATTGCTATCGCAGGCTTATGCGTTAATGGTGATATTGCCTTGACTCTACGTGA
GCCTGGCTTAGTCTTGTTTACGCATGAACTTCAAACTCATACTGCGATGGAATATTTTGTTTCATCGCGACGTATGTTT
GAACCTAGAAAACCTACCGTTAGTGATTTTGTTCAAATTGAGAGTTGTGTGGTCACTTATGTTAATTTGACTAGCGACC
AACTACCAGATGTAATCCCAGATTACATCGATGTTAACAAAACACTTGATGAGATTTTAGCTTCTCTGCCCAATAGAAC
TGGTCCAAGTCTTCCATTAGATGTTTTTAATGCCACTTATCTTAATCTCACTGGTGAAATTGCAGATTTAGAGCAGCGT
GCAGAGTCTCTCCGTAATACCACAGAAGAGCTCCGAAGTCTTATATATAACATCAACAACACACGTGTAGACCTTGAGT
GGCTCAACCGTGTTGAGACATATATCAAGTGGCCGTGGTGGGTTTGGTTGGTTATTTTTATTGTTCTCATCTTTGTTGT
GTCATTATTAGTGTTCTGCCGCATTTCCACGGGTTGTTGTGGATGCTGCGGCTGCTGCTGTGCTTGTTTTTCAGGTTGT
TGTAGGGGTCCTAGACTTCAACCTTACGAAGCTTTTGAAAAGGTCCACGTGCAGTGA

>gi|527461758|gb|KC879276.1|_Porcine_epidemic_diarrhea_virus_strain_AS02_spike_protein_(S)_gene_complete_cds

ATGAAGTCTTTAACCTATTTCTGGTTGTTCTTACCAGTACTTCCAATACTCAGCCTACCACAAGATGTCAATAGG
TGCTCAGCTGTGACTAACTTTAGGCAGTTCTTTTCAAAATTTAATGTTCAGGCGCCTGCAGTCGTTGTACTGGGCGGTT
ATCTACCTAGTGGTGAAAAACAGGTTGATGCACCAAGTTGGTACTGTGCTGGCCGATATGATACTGCTAGTGGCGTTCA
TGGTATCTTTCTTAGCCATATTAGAGGTGGTCATGGCTTTGAGATTGGCATTTCGCAAGAGCCGTTTGATCCTAGTGGT
TACCAGCTTTATTTACATAAGGCCACCAACGGTAACACTAATTCTACTGCGCGCCTGCGTATTTGCCAGTTTCCCAGCA
ACAAAACATTGGGCCCCACTGCTAATGATGATGTCACAACAGGTCGTAACTGCCTATTTAACAAAGCCATTCCAGCTTA
TATGAGTGAACATAGTGTTGTCGGCATAACATGGGATAATGATCGTGTCACTGTTTTTGCTGACAAGATCTATCATTTT
TATCTTAAAAATGATTGGTCCCGTGTTGCGTCAAAATGTTACAACAGTGGAGGTTGTGCTATGCAATATGTTTACAAAC
CTATTTACTACATGCTTAATGTTACTAGTGCTGGTGAGGATGGTATTTCTTATCAACTCTGTACAGCTAATTGCATTGG
TTATGCTGCCAATGTATTTGCTACTGAGCCCAATGGCCACATACCAGAAAGTTTTAGTTTTAATAATTGGTTTCTTTTG
TCCAATGATTCCACTTTGTTTCATGGTAAAGTGGTTTCCAACCAACCTTTGTTGGTCAATTGTCTTTGGGCCATTCCTA
AGATTTACGGACTAGGCCACTTTTTCTCTTTTAATCAAACGATGGATGGCGTTTGTAACGGAGCTACTGCTTATCGTGC
CCCAGAGGCTCTGAGGTTTAATATTAATGACACCTCTGTCATTCTTGCTGAAGGCTCAATTGTACTTCATACTGCTTTA
GGAACAAATCTTTCTTTTGTTTGCAGTAATTCCTCAGATCCTCATTTAGCCACCTTCGCCTTACCTCTGGGTGCTACCC
AAGTACCCTATTATTGTTTTCTTAAAGTGGACACTTACAACTCCACTGTTTATAAATTTTTGGCTGTTTTACCTCCTAC
CGTCAGGGAAATTGTCATCACCAAGTATGGTGATGTTTATGTCAATGGGTTTGGCTACTTGCATCTCGGTTTGTTGGAT
GCTGTCACAATTAATTTCACTGGTTATGGCACTGACGATGACGTTTCTGGTTTTTGGACCATAGCATCGACTAATTTTG
TTGATGCACTCATCGAAGTTCAAGGAACTGCCATTCAGCGTATTCTTTATTGTGATGATCCTGTTAGCCAACTCAAGTG
TTCTCAGGTTGCTTTTGACCTTGACGATGGTTTTTACCCTATTTCTTCTAGAAACCTTCTGAGTCATGAACAGCCAATT
TCTTTTGTTACTTTGCCATCATTTAATGATCATTCTTTTGTTAACATTACTGTCTCTGCGTCCTTTGGTGGTCATAGTG
GTGCCAACCTTATTGCATCTGACACTACTATCAATGGGTTTAGTTCTTTCTGTGTTGACACTAGACAATTTACCATTAG
ACTGTTTTATAACGTTACAAGCAGTTACGGTTATGTGTCTAACTCGCAGGATAGCAATTGCCCTTTCACCTTGCGATCT
GTTAATGATTACCTGTCTTTTAGCAAATTTTGTGTTTCAACCAGCCTTTTGGCTGGTGCTTGTACCATAGATCTTTTTG
GTCACCCTGCTTTTGGTAGTAGTGTTAAGTTTACGTCCCTCTATTTTCAATTCACAAAGGGTGATTTGATTACTGGCAC

GCCTAAATCACTTGAAGGTGTCACGGACGTTTCTTTTATGACTAAGGATGTGTGTACTAAGTATACTATCTATGGCTTT
AAAGGTGAGGGTATTATTACCCTTACAAATTCTAGCTTTTTGGCAGGTATTTATTATACATCTGATTCCGGACAGTTGT
TAGCCTTTAAGAATGTTACTAGTGGTGCTGTTTATTCTGTTACGCCATGTTCTTTTTCAGAGCAGGCTGCTTATGTTGA
TGATGATATAGTGGGTGTTATTTCCAGTTTGTCTAACTCCACTTTTAACAATACTAGGGAGTTGCCCGGTTTCTTCTAC
CATTCTAATGACGGCTCCAATTGTACAGAGCCTGTGTTGGTGTATAGTAACATAGGTGTCTGTAAATCTGGTAGTATTG
GCTATGTCCCATCTCAGTATGGCCAAGTCAAGATTGCACCCACGGTTACTGGGAATATTAGTATTCCCACCAACTTTAG
TATGAGTGTTAGAACAGAATATCTACAGCTTTACAACACGCCTGTTAGTGTTGATTGTGCTACATATGTTTGTAATGGT
AACTCTCGTTGTAAACAATTACTCACCCAGTACACTGCAGCATGTAAGACCATAGAGTCAGCATTACAACTCAGCGCTA
GGCTTGAGTCTGTTGAAGTTAACTCTATGCTTACTATTTCTGAAGAGGCTCTACAGTTAGCTACCATCAGTTCGTTTAA
TGGTGGTGGATATAATTTTACTAATGTGCTGGGTGTTTCCGTGTATGATCCTGCAAGTGGCAGGGTGGTACAAAAAAGG
TCTATTATTGAAGACCTGCTTTTTAATAAAGTGGTTACTAATGGCCTTGGTACTGTTGATGAAGACTATAAGCGCTGTT
CTAATGGCCGCTCTGTGGCAGATCTAGTCTGTGCGCAGTATTACTCTGGTGTCATGGTACTACCTGGTGTTGTTGACGC
TGAGAAGCTTCACATGTATAGTGCGTCTCTCGTCGGTGGTATGGTGCTAGGGGGTTTTACTGCTGCAGCGGCATTGCCT
TTTAGCTATGCTGTTCAAGCGAGACTCAATTATCTTGCTTTACAGACGGATGTTCTACAGCGTAATCAGCAATTGCTTG
CTGAGTCTTTTAACTCTGCTATTGGTAATATAACTTCAGCCTTCGAGAGTGTTAAAGAGGCTATTAGTCAAACTTCCCA
GGGTTTGAATACTGTGGCTCATGCACTTACTAAGGTTCAAGAGGTTGTTAATTCGCAGGGTGCAGCTTTGTCTCAACTC
ACTATACAGCTGCAACACAACTTCCGAGCCATTTCTAGTTCTATTGATGACATTTACTCCCGACTGGACATTCTTTCAG
CCGATGTTCAGGTTGATCGTCTCATCACCGGCAGATTATCAGCACTTAATGCTTTTGTTGCTCAAACCCTCACTAAGTA
TACTGAGGTTCAGGCTAGCAGGAAGCTAGCACAGCAAAAGGTTAATGAGTGCGTCAAATCGCAATCTCAGCGTTATGGT
TTTTGTGGTGGTGATGGTGAACACATTTTTTCTCTGGTACAGGCCGCACCTCAGGGCCTGCTGTTTTTACACACAGTAC
TTGTACCGGGTGATTTTGTAAATGTTATTGCTATCGCAGGCTTATGCGTTAATGGTGATATTGCCTTGACTCTACGTGA
GCCTGGCTTAGTCTTGTTTACGCATGAACTTCAAACTCATACTGCGATGGAATATTTTGTTTCATCGCGACGTATGTTT
GAACCTAGAAAACCTACCGTTAGTGATTTTGTTCAAATTGAGAGTTGTGTGGTCACTTATGTTAATTTGACTAGCGACC
AACTACCAGATGTAATCCCAGATTACATCGATGTTAACAAAACACTTGATGAGATTTTGGCTTCTCTGCCCAATAGAAC
TGGTCCAAGTCTTCCATTAGATGTTTTTAATGCCACTTATCTTAATCTCACTGGTGAAATTGCAGATTTAGAGCAGCGT
GCAGAGTCTCTCCGTAATACCACAGAAGAGCTCCGAAGTCTTATATATAACATCAACAACACACGTGTAGACCTTGAGT
GGCTCAACCGTGTTGAGACATATATCAAGTGGCCGTGGTGGGTTTGGTTGGTTATTTTTATTGTTCTCATCTTTGTTGT
GTCATTATTAGTGTTCTGCTGCATTTCCACTGGTTGTTGTGGATGCTGCGGCTGCTGCTGTGCTTGTTTTTCAGGTTGT
TGTAGGGGTCCTAGACTTCAACCTTACGAAGCTTTTGAAAAGGTCCACGTGCAGTGA

>gi|527461760|gb|KC879277.1|_Porcine_epidemic_diarrhea_virus_strain_AS03_spike_protein_(S)_gene_complete_cds

ATGAAGTCTTTAACCTATTTCTGGTTGTTCTTACCAGTACTTCCAATACTCAGCCTACCACAAGATGTCAATAGG
TGCTCAGCTGTGACTAACTTTAGGCAGTTCTTTTCAAAATTTAATGTTCAGGCGCCTGCAGTCGTTGTACTGGGCGGTT
ATCTACCTAGTGGTGCAAAACAGGTTGATGCACCAAGTTGGTACTGTGCTGGCCGATATGATACTGCTAGTGGCGTTCA
TGGTATCTTTCTTAGCCATATTAGAGGTGGTCATGGCTTTGAGATTGGCATTTCGCAAGAGCCGTTTGATCCTAGTGGT
TACCAGCTTTATTTACATAAGGCCACCAACGGTAACACTAATTCTACTGCGCGCCTGCGTATTTGCCAGTTTCCCAGCA
ACAAAACATTGGGCCCCACTGCTAATGATGATGTCACAACAGGTCGTAACTGCCTATTTAACAAAGCCATTCCAGCTTA
TATGAGTGAACATAGTGTTGTCGGCATAACATGGGATAATGATCGTGTCACTGTTTTTGCTGACAAGATCTATCATTTT
TATCTTAAAAATGATTGGTCCCGTGTTGCGTCAAAATGTTACAACAGTGGAGGTTGTGCTATGCAATATGTTTACAAAC
CTATTTACTACATGCTTAATGTTACTAGTGCTGGTGAGGATGGTATTTCTTATCAACTCTGTACAGCTAATTGCATTGG
TTATGCTGCCAATGTATTTGCTACTGAGCCCAATGGCCACATACCAGAAAGTTTTAGTTTTAATAATTGGTTTCTTTTG

TCCAATGATTCCACTTTGTTTCATGGTAAAGTGGTTTCCAACCAACCTTTGTTGGTCAATTGTCTTTGGGCCATTCCTA
AGATTTACGGACTAGGCCACTTTTTCTCTTTTAATCAAACGATGGATGGCGTTTGTAACGGAGCTACTGCTTATCGTGC
CCCAGAGGCTCTGAGGTTTAATATTAATGACACCTCTGTCATTCTTGCTGAAGGCTCAATTGTACTTCATACTGCTTTA
GGAACAAATCTTTCTTTTGTTTGCAGTAATTCCTCAGATCCTCATTTAGCCACCTTCGCCTTACCTCTGGGTGCTACCC
AAGTACCCTATTATTGTTTTCTTAAAGTGGACACTTACAACTCCACTGTTTATAAATTTTTGGCTGTTTTACCTCCTAC
CGTCAGGGAAATTGTCATCACCAAGTATGGTGATGTTTATGTCAATGGGTTTGGCTACTTGCATCTCGGTTTGTTGGAT
GCTGTCACAATTAATTTCACTGGTTATGGCACTGACGATGACGTTTCTGGTTTTTGGACCATAGCATCGACTAATTTTG
TTGATGCACTCATCGAAGTTCAAGGAACTGCCATTCAGCGTATTCTTTATTGTGATGATCCTGTTAGCCAACTCAAGTG
TTCTCAGGTTGCTTTTGACCTTGACGATGGTTTTTACCCTATTTCTTCTAGAAACCTTCTGAGTCATGAACAGCCAATT
TCTTTTGTTACTTTGCCATCATTTAATGATCATTCTTTTGTTAACATTACTGTCTCTGCGTCCTTTGGTGGTCATAGTG
GTGCCAACCTTATTGCATCTGACACTACTATCAATGGGTTTAGTTCTTTCTGTGTTGACACTAGACAATTTACCATTAG
ACTGTTTTATAACGTTACAAGCAGTTACGGTTATGTGTCTAACTCGCAGGATAGCAATTGCCCTTTCACCTTGCGATCT
GTTAATGATTACCTGTCTTTTAGCAAATTTTGTGTTTCAACCAGCCTTTTGGCTGGTGCTTGTACCATAGATCTTTTTG
GTCACCCTGCTTTTGGTAGTAGTGTTAAGTTTACGTCCCTCTATTTTCAATTCACAAAGGGTGATTTGATTACTGGCAC
GCCTAAATCACTTGAAGGTGTCACGGACGTTTCTTTTATGACTAAGGATGTGTGTACTAAGTATACTATCTATGGCTTT
AAAGGTGAGGGTATTATTACCCTTACAAATTCTAGCTTTTTGGCAGGTATTTATTATACATCTGATTCCGGACAGTTGT
TAGCCTTTAAGAATGTTACTAGTGGTGCTGTTTATTCTGTTACGCCATGTTCTTTTTCAGAGCAGGCTGCTTATGTTGA
TGATGATATAGTGGGTGTTATTTCCAGTTTGTCTAACTCCACTTTTAACAATACTAGGGAGTTGCCCGGTTTCTTCTAC
CATTCTAATGACGGCTCCAATTGTACAGAGCCTGTGTTGGTGTATAGTAACATAGGTGTCTGTAAATCTGGTAGTATTG
GCTATGTCCCATCTCAGTATGGCCAAGTCAAGATTGCACCCACGGTTACTGGGAATATTAGTATTCCCACCAACTTTAG
TATGAGTGTTAGAACAGAATATCTACAGCTTTACAACACGCCTGTTAGTGTTGATTGTGCTACATATGTTTGTAATGGT
AACTCTCGTTGTAAACAATTACTCACCCAGTACACTGCAGCATGTAAGACCATAGAGTCAGCATTACAACTCAGCGCTA
GGCTTGAGTCTGTTGAAGTTAACTCTATGCTTACTATTTCTGAAGAGGCTCTACAGTTAGCTACCATCAGTTCGTTTAA
TGGTGGTGGATATAATTTTACTAATGTGCTGGGTGTTTCCGTGTATGATCCTGCAAGTGGCAGGGTGGTACAAAAAAGG
TCTATTATTGAAGACCTGCTTTTTAATAAAGTGGTTACTAATGGCCTTGGTACTGTTGATGAAGACTATAAGCGCTGTT
CTAATGGCCGCTCTGTGGCAGATCTAGTCTGTGCGCAGTATTACTCTGGTGTCATGGTACTACCTGGTGTTGTTGACGC
TGAGAAGCTTCACATGTATAGTGCGTCTCTCGTCGGTGGTATGGTGCTAGGGGGTTTTACTGCTGCAGCGGCATTGCCT
TTTAGCTATGCTGTTCAAGCGAGACTCAATTATCTTGCTTTACAGACGGATGTTCTACAGCGTAATCAGCAATTGCTTG
CTGAGTCTTTTAACTCTGCTATTGGTAATATAACTTCAGCCTTCGAGAGTGTTAAAGAGGCTATTAGTCAAACTTCCCA
GGGTTTGAATACTGTGGCTCATGCACTTACTAAGGTTCAAGAGGTTGTTAATTCGCAGGGTGCAGCTTTGTCTCAACTC
ACTATACAGCTGCAACACAACTTCCGAGCCATTTCTAGTTCTATTGATGACATTTACTCCCGACTGGACATTCTTTCAG
CCGATGTTCAGGTTGATCGTCTCATCACCGGCAGATTATCAGCACTTAATGCTTTTGTTGCTCAAACCCTCACTAAGTA
TACTGAGGTTCAGGCTAGCAGGAAGCTAGCACAGCAAAAGGTTAATGAGTGCGTCAAATCGCAATCTCAGCGTTATGGT
TTTTGTGGTGGTGATGGTGAACACATTTTTTCTCTGGTACAGGCCGCACCTCAGGGCCTGCTGTTTTTGCACACAGTAC
TTGTACCGGGTGATTTTGTAAATGTTATTGCTATCGCAGGCTTATGCGTTAATGGTGATATTGCCTTGACTCTACGTGA
GCCTGGCTTAGTCTTGTTTACGCATGAACTTCAAACTCATACTGCGATGGAATATTTTGTTTCATCGCGACGTATGTTT
GAACCTAGAAAACCTACCGTTAGTGATTTTGTTCAAATTGAGAGTTGCGTGGTCACTTATGTTAATTTGACTAGCGACC
AACTACCAGATGTAATCCCAGATTACATCGATGTTAACAAAACACTTGATGAGATTTTGGCTTCTCTGCCCAATAGAAC
TGGTCCAAGTCTTCCATTAGATGTTTTTAATGCCACTTATCTTAATCTCACTGGTGAAATTGCAGATTTAGAGCAGCGT
GCAGAGTCTCTCCGTAATACCACAGAAGGGCTCCGAAGTCTTATATATAACATCAACAACACACGTGTAGACCTTGAGT
GGCTCAACCGTGTTGAGACATATATCAAGTGGCCGTGGTGGGTTTGGTTGGTTATTTTTATTGTTCTCATCTTTGTTGT
GTCATTATTAGTGTTCTGCTGCATTTCCACTGGTTGTTGTGGATGCTGCGGCTGCTGCTGTGCTTGTTTTTCAGGTTGT
TGTAGGGGTCCTAGACTTCAACCTTACGAAGCTTTTGAAAAGGTCCACGTGCAGTGA

>gi|527461762|gb|KC879278.1|_Porcine_epidemic_diarrhea_virus_strain_NJ01_spike_protein_(S)_gene_complete_cds

ATGAGGTCTTTAACTTACTTCTGGTTGTTATTACCAGTACTTCCAATACTCAGCCTACCACAAGATGTCAATAGG
TGCTCAGCTGTGACTAACTTTAGGCAGTTCTTTTCAAAATTTAATGTTCAGGCGCCTGCAGTCGTTGTACTGGGCGGTT
ATCTACCTAGTGGTGAAAAACAGGTTGATGCACCACGTTGGTACTGTGCTGGCCGATATGATACTGCTAGTGGCGTTCA
TGGTATCTTTCTTAGCCATATTAGAGGTGGTCATGGCTTTGAGATTGGCATTTCGCAAGAGCCGTTAGATCCTAGTGGT
TACCAGCTTTATTTACATAAGGACACCAACGGTAACACTAATTCTACTGCGCGCCTGCGTATTTGCCAGTTTCCCAGCA
ATAAAACATTGGGCCCCACTGCTAATGATGATGTCACAACAGGTCGTAACTGCCTATTTAACAAAGCCATTCCAGCTCA
TATGAGTGAACATAGTGTTGTCGGCATAACATGGGATAATGATCGTGTCACTGTTTTTGCTGACAAGATCTATCATTTT
TATCTTAAAAATGATTGGTCCCGTGTTGCGTCAAAATGTTACAACAGTGGAGGTTGTGCTATGCAATATGTTTACAAAC
CTACCTACTACATGCTTAATGTTACTAGTGTAGGTGAGGATGGCATTTATTATGAGCCATGTACAGCTAATTGCATTGG
TTACGCTGGCAATGTGTTTGCCGTTGAGTCCAATGGCCACATACCAGAAGGTTTTAGTTTTAATAATTGGTTTCTTTTG
TCCAATGATTCCACTTTGGTGCATGGTAAGGTGGTTTCCAACCAACCTTTGTTGGTCAATTGTCTTTTGGCCATTCCTA
AGATTTATGGACTAGGCCAATTTTTCTCCTTCAATCAAACGATCGATGGTGTTTGTAATGGAGCTGTTGCGCGGCGTGC
ACCAGAGGCTTTGAGGTTTAATATTAATGACACTTCTGTCATTCTTGCTGAAGGCTCAATTGTACTTCATACTGCTTTA
GGAACAAATTTTTCTTTTGTTTGCAGTAATTCCTCAACTCCTCATTCAGCCACCTTTGCTATACCTCTGGGTGCCACCC
AAGTACCCTATTATTGTTTTCTTAAAGTGGACACTTATAATTCCACTGTTTATAAATTCTTGGCTGTTTTACCTCTTAC
TGTCAGGGAAATTGTCATCACCAAGTATGGTGATGTTTATGTCAATGGGTTTGGCTATTTGCATCTCGGTTTGTTGGAC
GCTGTCACAATTAATTTCACTGGTCATGGCACTGACGATGACGTTTCAGGTTTCTGGACCATAGCATCGACTAATTTTG
TTGATGCACTCATCGAAGTTCAAGGAACTGCCATTCAGCGTATTCTTTATTGTGATGATCCTGTTAGCCAACTCAAGTG
TTCTCAGATTGCTTTTGACCTTGACGATGGTTTTTACCCTATATCTTCTAGAAACCTCCTGAGTCATGAACAGCCAGTT
TCTTTTGTTACTTTGCCATCATTTAATGATCATTCTTTTGTTAACATTACTGTCTCTGCGTCTTTTGGTGGTCATAGTG
GTGCAAACCTCGTTGCATCTGACACTACTATCAATGGGTTCAGTTTTTTCTGTGTTGAAACTAGACAATTTACCATTAG
ACTGTTTTACAACGTTACAAACAGTTTTGGTTATGTGTCTAACTCACAGGATAGCAATTGCCCTTTTACCTTGCAATCT
GTTAATGATTACCTGTCTTTTAGCAAATTTTGTGTTTCAACCAGCCTTTTGGCTGGTGCCTGTACCATAGATCTTTTTG
GTTACCCTGAGTTTGGTAGTAGTGTTAAGTTTACGTCCCTTTACTTTCAATTCACAAAGGGTGAGTTGATTACTGGCAT
GCCTAAACCACTTGAAGGTGTCACGGACGTTTCTTTTATGACTCTGGATGTGTGTACCAAGTATACTATCTATGGCTTT
AAAGGTGAGGGTATCATTACCCTTACAAATTCTAGCTTTTTGGCAGGTGTTTATTATACATCTGATTCTGGACAGTTGT
TAGCCTTTAAGAATGTCACTAGTAGTGCTGTTTATTCTGTTACGCCATGTTCTTTCTCAGAGCAGGCTGCATATGTTGA
TGATGGTATAGTGGGTGTTATTTCTAGTTTGTCTAACTCCACTTTTAACAATACTAGGGAGTTGCCTGGTTTTTTCTAC
CATTCTAATGACGGCTCCAATTGTACAGAGCCTGTGTTGGTGTATAGTAACATAGGTGTTTGTAAATCTGGCAGTATTG
GCCATGTCCCTTCTCAGTCTGGCCCAGTCAAGATTGCACCCACGGTTACTGGGAATATTAGTATTCCCACCAACTTTAG
TGTGAGTATTAGGACAGAATATTTACAGCTTTACAACACGCCTGTTAGTGTTGATTGTGCCACATACGTTTGTAATGGT
AACTCTCGTTGCAAACAATTACTCACCCAGTACACTGCAGCATGTAAGACCATAGAGTCAGCATTACAACTCAGCGCTA
GGCTTGAGTCTGTTGAAGTTAACTCTATGCTTACTATTTCTGAAGAGGCTCTACAGTTAGCTACCATCAGTTCGTTTAA
TGGTGATGGATATAATTTTACTAATGTGCTGGGTGTTTCCGTGTATGATCCTGCAAGTGGCAGGGTGGTACAAAAAAGG
TCTTTCATTGAAGACCTGCTTTTTAATAAAGTGGTTACTAATGGTCTTGGTACTGTTGATGAAGACTACAAGCGCTGTT
CTAATGGCCGCTCTGTGGCTGATCTAGTCTGTGCGCAGTATTACTCTGGTGTCATGGTACTACCTGGCGTTGTTGACGC
TGAGAAGCTTCACATGTATAGTGCGTCTCTCATCGGTGGTATGGTGCTAGGAGGTTTTACTGCTGCAGCGGCACTGCCT
TTTAGCTATGCTGTTCAAGCGAGACTCAATTATCTTGCTTTACAGACGGATGTTCTACAGCGTAACCAGCAACTGCTTG
CTGAGTCTTTTAACTCTGCTATTGGTAATATAACTTCAGCCTTTGAGAGTGTTAAAGAGGCTATTAGTCAAACTTCCCA
GGGTTTGAACACTGTGGCTCATGCGCTTACTAAGGTTCAAGAGGTTGTTAATTCGCAGGGTGCAGCTTTGACTCAACTT

ACCGTACAGCTGCAACGCAACTTCCAAGCCATTTCTAGTTCTATTGATGACATTTATTCCCGACTTGACATTCTTTCAG
CCGATGTTCAGGTTGACCGTCTCATCACTGGCAGATTATCAGCACTTAACGCTTTTGTTGCTCAAACCCTCACTAAGTA
TACTGAGGTTCAGGCTAGCAGGAAGCTAGCACAGCAAAAGGTTAATGAGTGCGTCAAATCGCAGTCTCAGCGTTATGGT
TTTTGTGGTGGTGATGGCGAACACATTTTTTCCCTGGTACAGGCCGCACCTCAGGGCCTGCTGTTCTTACACACAGTAC
TTGTACCGGGTGATTTTGTAAATGTTATTGCTGTCGCAGGCTTATGCGTTAATGGTGATATTGCCTTGACTCTACGTGA
GCCTGGCTTAGTCCTGTTTACGCATGAACTTCAAACTCATACGGCGGCAGAATATTTTGTTTCATCGCGACGTATGTTT
GAACCTAGAAAACCTACCGTTAGTGATTTTGTTCAAATTGAGAGTTGTGTGGTCACCTATGTCAATCTGACTAGCGACC
AACTACCAGATGTAATCCCAGATTACATCGATGTTAACAAAACACTTGATGAGATTTTGGCTTCTCTGCCCAATAGAAC
TGGTCCAAGTCTTCCACTAGATGTTTTTAATGCTACTTATCTTAATCTCACTGGTGAAATTGCAGATTTACAGCAGCGT
TCAGAGTCTCTTCAAAATACTATAGAAGAGCTCCGAAGTCTTATTTATAGCATCAACAACACACGTGTTGACCTTGAGT
GGCTCAACCGCGTTGAGACATATATCAAGTGGCCGTGGTGGGTTTGGTTGATTCTTTTTATTGTTCTCATCTTTGTTGT
GTCATTACTAGTGTTCTGCTGCATTTCTACGGGTTGCTGTGGATGCTGCGGTTGCTGCGGTGCTTGTTTTTCAGGTTGT
TGTAGGGGTCCTAGACTTCAACCTTACGAAGCTTTTGAAAAGGTCCACGTGCAGTGA

>gi|527461764|gb|KC879279.1|_Porcine_epidemic_diarrhea_virus_strain_NJ02_spike_protein_(S)_gene_complete_cds

ATGAGGTCTTTAACTTACTTCTGGTTGTTATTACCAGTACTTCCAATACTCAGCCTACCACAAGATGTCAATAGG
TGCTCAGCTGTGACTAACTTTAGGCAGTTCTTTTCAAAATTTAATGTTCAGGCGCCTGCAGTCGTTGTACTGGGCGGTT
ATCTACCTAGTGGTGAAAAACAGGTTGATGCACCACGTTGGTACTGTGCTGGCCGATATGATACTGCTAGTGGCGTTCA
TGGTGTCTTTCTTAGCCATATTAGAGGTGGTCATGGCTTTGAGATTGGCATTTCGCAAGAGCCGTTAGATCCTAGTGGT
TACCAGCTTTATTTACATAAGGACACCAACGGTAACATTAATTCTACTGCGCGCCTGCGTATTTGCCAGTTTCCCAGCA
ATAAAACATTGGGCCCCACTGCTAATGATGTCACAACAGGTCGTAACTGCCTATTTAACAAAGCCATTCCAGCTCATAT
GAGTGAACATAGTGTTGTCGGCATAACATGGGATAATGATCGTGTCACTGTTTTTGCTGACAAGATCTATCATTTTTAT
CTTAAAAATGATTGGTCCCGTGTTGCGTCAAAATGTTACAACAGTGGAGGTTGTGCTATGCAATATGTTTACAAATTTA
CCTACTACGTGCTTAATGTTACTAGTGTAGGTGAGGATGGCATTTATTATGAGCCATGTACAGCTAATTGCATTGGTTA
CGCTGGCAATGTGTTTGCCGTTGAGTCCAATGGCCACATACCAGAAGGTTTTAGTTTTAATAATTGGTTTCTTTTGTCC
AATGATTCCACTTTGGTGCATGGTAAGGTGGTTTCCAACCAACCTTTGTTGGTCAATTGTCTTTTGGCCATTCCTAAGA
TTTATGGACTAGGCCAATTTTTCTCCTTCAATCAAACGATCGATGGTGTTTGTAATGGAGCTGTTGCGCGGCGTGCACC
AGAGGCTTTGAGGTTTAATATTAATGACACTTCTGTCATTCTTGCTGAAGGCTCAATTGTACTTCATACTGCTTTAGGA
ACAAATTTTTCTTTTGTTTGCAGTAATTCCTCAACTCCTCATTCAGCCACCTTTGCTATACCTCTGGGTGCCACCCAAG
TACCCTATTATTGTTTTCTTAAAGTGGACACTTATAATTCCACTGTTTATAAATTCTTGGCTGTTTTACCTCTTACTGT
CAGGGAAATTGTCATCACCAAGTATGGTGATGTTTATGTCAATGGGTTTGGCTATTTGCATCTCGGTTTGTTGGACGCT
GTCACAATTAATTTCACTGGTCATGGCACTGACGATGACGTTTCAGGTTTCTGGACCATAGCATCGACTAATTTTGTTG
ATGCACTCATCGAAGTTCAAGGAACTGCCATTCAGCGTATTCTTTATTGTGATGATCCTGTTAGCCAACTCAAGTGTTC
TCAGATTGCTTTTGACCTTGACGATGGTTTTTACCCTATATCTTCTAGAAACCTCCTGAGTCATGAACAGCCAGTTTCT
TTTGTTACTTTGCCATCATTTAATGATCATTCTTTTGTTAACATTACTGTCTCTGCGTCTTTTGGTGGTCATAGTGGTG
CAAACCTCGTTGCATCTGACACTACTATCAATGGGTTCAGTTTTTTCTGTGTTGAAACTAGACAATTTACCATTAGACT
GTTTTACAACGTTACAAACAGTTTTGGTTATGTGTCTAACTCACAGGATAGCAATTGCCCTTTTACCTTGCAATCTGTT
AATGATTACCTGTCTTTTAGCAAATTTTGTGTTTCAACCAGCCTTTTGGCTGGTGCCTGTACCATAGATCTTTTTGGTT
ACCCTGAGTTTGGTAGTAGTGTTAAGTTTACGTCCCTTTACTTTCAATTCACAAAGGGTGAGTTGATTACTGGCATGCC
TAAACCACTTGAAGGTGTCACGGACGTTTCTTTTATGACTCTGGATGTGTGTACCAAGTATACTATCTATGGCTTTAAA
GGTGAGGGTATCATTACCCTTACAAATTCTAGCTTTTTGGCAGGTGTTTATTATACATCTGATTCTGGACAGTTGTTAG

CCTTTAAGAATGTCACTAGTAGTGCTGTTTATTCTGTTACGCCATGTTCTTTCTCAGAGCAGGCTGCATATGTTGATGA
TGGTATAGTGGGTGTTATTTCTAGTTTGTCTAACTCCACTTTTAACAATACTAGGGAGTTGCCTGGTTTTTTCTACCAT
TCTAATGACGGCTCCAATTGTACAGAGCCTGTGTTGGTGTATAGTAACATAGGTGTTTGTAAATCTGGCAGTATTGGCC
ATGTCCCTTCTCAGTCTGGCCCAGTCAAGATTGCACCCACGGTTACTGGGAATATTAGTATTCCCACCAACTTTAGTGT
GAGTATTAGGACAGAATATTTACAGCTTTACAACACGCCTGTTAGTGTTGATTGTGCCACATACGTTTGTAATGGTAAC
TCTCGTTGCAAACAATTACTCACCCAGTACACTGCAGCATGTAAGACCATAGAGTCAGCATTACAACTCAGCGCTAGGC
TTGAGTCTGTTGAAGTTAACTCTATGCTTACTATTTCTGAAGAGGCTCTACAGTTAGCTACCATCAGTTCGTTTAATGG
TGATGGATATAATTTTACTAATGTGCTGGGTGTTTCCGTGTATGATCCTGCAAGTGGCAGGGTGGTACAAAAAAGGTCT
TTCATTGAAGACCTGCTTTTTAATAAAGTGGTTACTAATGGTCTTGGTACTGTTGATGAAGACTACAAGCGCTGTTCTA
ATGGCCGCTCTGTGGCTGATCTAGTCTGTGCGCAGTATTACTCTGGTGTCATGGTACTACCTGGCGTTGTTGACGCTGA
GAAGCTTCACATGTATAGTGCGTCTCTCATCGGTGGTATGGTGCTAGGAGGTTTTACTGCTGCAGCGGCACTGCCTTTT
AGCTATGCTGTTCAAGCGAGACTCAATTATCTTGCTTTACAGACGGATGTTCTACAGCGTAACCAGCAACTGCTTGCTG
AGTCTTTTAACTCTGCTATTGGTAATATAACTTCAGCCTTTGAGAGTGTTAAAGAGGCTATTAGTCAAACTTCCCAGGG
TTTGAACACTGTGGCTCATGCGCTTACTAAGGTTCAAGAGGTTGTTAATTCGCAGGGTGCAGCTTTGACTCAACTTACC
GTACAGCTGCAACGCAACTTCCAAGCCATTTCTAGTTCTATTGATGACATTTATTCCCGACTTGACATTCTTTCAGCCG
ATGTTCAGGTTGACCGTCTCATCACTGGCAGATTATCAGCACTTAACGCTTTTGTTGCTCAAACCCTCACTAAGTATAC
TGAGGTTCAGGCTAGCAGGAAGCTAGCACAGCAAAAGGTTAATGAGTGCGTCAAATCGCAGTCTCAGCGTTATGGTTTT
TGTGGTGGTGATGGCGAACACATTTTTTCCCTGGTACAGGCCGCACCTCAGGGCCTGCTGTTCTTACACACAGTACTTG
TACCGGGTGATTTTGTAAATGTTATTGCTGTCGCAGGCTTATGCGTTAATGATGATATTGCCTTGACTCTGCGTGAGCC
TGGCTTAGTCTTGTTTACACATGAACTTCAAACTCATACTGCGACGGAATATTTTGTTTCATCGCGACGTATGTTTGAA
CCTAGAAAACCTACCGTTAGTGATTTTGTTCAAATTGAGAGTTGTGTGGTCACCTATGTCAACCTGACTAGCGACCAAC
TACCAGATGTAATCCCAGATTACATCGATGTTAACAAAACACTTGATGAGATTTTAGCCTCTCTGCCCAATAGAACTGA
TCTAAATTTTCCACTAGATGTTTTTAATGCCACTTATCTTAATCTCACTGGTGAAATTGCAGATTTAGAGCAGCGTTCA
GAGTCTCTCCACAATACCACAGAAGAGCTCCGAAGTCTCATATATAATATCAACAACACACTAGTTGACCTTGAGTGGC
TCAACCGAGTTGAGACGTATATCAAGTGGCCGTGGTGGGTTTGGTTGATTATTTTCATCGTTCTCATCTTTGTTGTGTC
ATTACTAGTGTTCTGCTGCATTTCCACGGGTTGTTGTGGATGCTGCGGCTGCTGCTGTGCTTGTTTTTCAGGTTGTTGT
AGGGGTCCTAGACTTCAACCTTACGAAGCTTTTGAAAAGGTCCACGTGCAGTGA

>gi|527461766|gb|KC879280.1|_Porcine_epidemic_diarrhea_virus_strain_AD01_spike_protein_(S)_gene_complete_cds

ATGAGGTCTTTAATTTACTTTTTGTTGTTCGTACCAGTACTTCCAACACTCAGTCTACCACAAGATGTCCATAGG
TGCTCAGCTAACACTAATTTTAGGCGGTTCTTTTCAAAATTTAATGTTCAGGCGCCTGCAGTTGTTGTACTGGGCGGTT
ATCTACCTATTGGTGAAAACCAGGGTGTCAATTCAACTTGGTACTGTGCTGGCCAACATCCAACTGCTAGTGGCGTTCA
TGGTATCTTTCTTAGCCATATTAGAGGTGGTCATGGCTTTGAGATTGGCATTTCGCAAGAGCCGTTTGACCCTAGTGGT
TACCAGCTTTATTTACATAAGGCCACTAACGGTAACACTAATGCTACTGCGCGACTGCGCATTTGCCAGTTTCCCAGCA
GTAAAACATTGGGCCCCACTGCTAATGATGATGTTACAACAGGTCGTAACTGCCTATTTAACAAAGCCATCCCAGCTCA
TATGAGTGAACATAGTGTTGTCGGCATAACATGGGATAATGATCGTGTCACTGTTTTTTCTGACAAGATCTATCATTTT
CATTTTAAAAATGATTGGTCCCGTGTTGCGACAAAGTGTTACAACAGTGGAGGTTGTGCTATGCAATATGTTTACGAAC
CCACTTACTACATGCTTAATGTTACTAGTGCTGGTGAGGATGGTATTTCTTATCAACCCTGTACAGCTAATTGCATTGG
TTATGCTGCCAATGTATTTGCCACTGAGTCCAATGGCCACATACCAGAAGGTTTTAGTTTTAATAATTGGTTTCTTTTG
TCCAATGATTCCACTTTGGTGCATGGTAAGGTGGTTTCCAACCAACCTTTGTTGGTCAATTGTCTTTTGGCCATTCCTA
AGATTTATGGACTAGGCCAATTTTTCTCCTTCAATCAAACGATCGATGGTGTTTGTAATGGAGCTGCTGCGCAGCGTGC

ACCAGAGGCTCTGAGGTTTAATATTAATGACACCTCTGTCATTCTTGCTGAAGGCTCAATTGTACTTCATACTGCTTTA
GGAACAAATTTTTCTTTTGTTTGCAGTAATTCCTCAAATCCTCATTTAGCCACCTTTGCCATACCTCTGGGTGCTACCC
AAGTACCCTATTATTGTTTTCTTAAAGTGGATACTTACAATTCCACTGTTTATAAATTCTTGGCTGTTTTACCTCCTAC
TGTCAGGGAAATTGTCATCACCAAGTATGGTGATGTTTATGTCAATGGGTTTGGCTATTTGCATCTCGGTTTGTTGGAC
GCTGTCACAATTAATTTCACTGGTCATGGCACTGACGATGACGTTTCAGGTTTCTGGGCCATAGCATCGACTAATTTTG
TTGATGCACTCATCGAAGTTCAAGGAACTGCCATTCAGCGTATTCTTTATTGTGATGATCCTGTTAGCCAACTCAAGTG
TTCTCAGGTTGCTTTTGACCTTGACGATGGTTTTTACCCTATATCTTCTAGAAACCTCCTGAGTCATGAACAGCCAACT
TCTTTTGTTACTTTGCCATCATTTAATGATCATTCTTTTGTTAACATTACTGTCTCTGCGTCTTTTGGTGATCATAGTG
GTGCCAACCTCGTTGCATCTGACACTACTATCAATGGGTTCAGTTCTTTCTGTGTTGACACTAGACAATTTACCATTAG
ACTGTTTTACAACGTTACAAACAGTTATGGTTATGTGTCTAAATCACAGGACAGTAATTGCCCTTTTACCTTGCAATCT
GTTAATGATTACCTGTCTTTTAGCAAATTTTGTGTTTCAACCAGCCTTTTGGCTAGTTCCTGTACCATAGATCTTTTTG
GTTACCCTGATTTCGGTAGTGGTGTTAAGTTTACGTCCCTTTACTTTCAATTCACAGAGGGTGAGTTGATTACTGGCAC
ACCTAAACCACTTGAAGGTGTCACGGACGTTTCTTTTATGACTCTGGATGTGTGTACCAAGTATACTATCTATGGCTTT
AAAGGTGAGGGTATCATTACCCTTACAAATTCTAGCTTTTTGGCAGGTGTTTATTATACATCTGATTCTGGACAGTTGT
TAGCCTTTAAGAATGTCACTAGTGGTGCTGTTTATTCTGTTACGCCATGTTCTTTTTCAGAGCAGGCTGCATATGTTGA
TGACGATATAGTGGGTGTTATTTCTAGTTTGTCTAACTCCACTTTTAACAATACTAGGGAGTTGCCTGGTTTCTTCTAC
CATTCTAATGACGGCTCCAATTGTACAGAGCCTGTGTTGGTGTATAGTAACATAGGTGTTTGTAAATCTGGCAGTATTG
GCTATGTCCCATCTCAGTCTGGCCAAGTCAAGATTGCACCCACGGTTACTGGGAATATTAGTATTCCCACCAACTTTAG
TATGAGTATTAGGACAGAATATTTACAGCTTTACAACACGCCTGTTAGTGTTGATTGTGCCACATATGTTTGTAATGGT
AACTCTCGTTGCAAACAATTACTCACCCAGTACACTGCAGCATGTAAGACCATAGAGTCAGCATTACAACTCAGCGCTA
GGCTTGAGTCTGTTGAAGTTAACTCTATGCTTACTACTTCTGAAGAGGCTCTACAGTTAGCTACCATCAGTTCGTTTAA
TGGTGATGGATATAATTTTACTAATGTGCTGGGTGTTTCCGTGTATGATCCTGCAAGTGGCAGGGTGGTACACAAAAGG
TCTTTCATTGAAGGCCTGCTTTTTAATAAAGTGGTTACTAATGGCCTTGGTACTGTTGATGAAGACTATAAGCGCTGTT
CTAATGGCCTCTCTGTGGCTGATCTAGTCTGTGCGCAGTATTACTCTGGTGTCATGGTACTACCTGGCGTTGTTGACCC
TGAGAAGCTTCACATGTATAGTGCGTCTCTCATCGGTGGTATGGTGCTAGGAGGTTTTACTGCTGCAGCGGCATTGCCT
TTTAGCTATGCTGTTCAAGCGAGACTCAATTATCTTGCTTTACAGACGGATGTTCTACAGCGTAACCAGCAATTGCTTG
CTGAGTCTTTTAACTCTGCTATTGGTAATATAACTTCAGCCTTTGAGAGTGTTAAAGAGGCTATTAGTCAAACTTCCCA
GGGTTTGAACACTGTGGCTCATGCGCTTACTAAGGTTCAAGAGGTTGTTAATTCGCAGGGTGCAGCTTTGACCCAACTT
ACCGTACAGCTGCAACACAACTTCCAAGCCATTTCTAGTTCTATTGATGACATTTATTCCCGACTTGACATTCTTTCAG
CCGATGTTCAGGTTGACCGTCTCATCACTGGCAGATTATCAGCACTTAACGCTTTTGTTGCTCAAACCCTCACTAAGTA
TACTGAGGTTCAGGCTAGCAGGAAGCTAGCACAGCAAAAGGTTAATGAGTGCGTCAAATCGCAGTCTCAGCGTTATGGT
TTTTGTGGTGGTGATGGCGAACACATTTTTTCCCTGGTACAGGCCGCACCTCAGGGCCTGCTGTTCTTACACACAGTAC
TTGTACCGGGTGATTTTGTAAATGTTATTGCTATCGCAGGCTTATGCGTTAATGGTGATATTGCCTTGACTCTACGTGA
GCCTGGCTTAGTCCTGTTTACGCATGAACTTCAAACTCATACGGCGACAGAATATTTTGTTTCATCGCGACGTATGTTT
GAACCTAGAAAACCTACCGTTAGTGATTTTGTTCAAATTGAGAGTTGTGTGGTCACCTATGTCAATCTGACTAGCGACC
AACTACCAGATGTAATCCCAGATTACATCGATGTTAACAAAACACTTGATGAGATTTTGGCTTCTCTGCCCAATAGAAC
TGGTCCAAGTCTTCCACTAGATGTTTTTAATGCTACTTATCTTAATCTCACTGGTGAAATTGCAGATTTAGAGCAGCGT
TCAGAGTCTCTCCAAAATATCACAGAAGAGCTCCGAAGTCTCATATATAACATCAACAACACACTTGTTGACCTTGAGT
GGCTCAACCGCGTTGAGACATATATCAAGTGGCCGTGGTGGGTTTGGTTGATTATTTTTATTGTTCTCATCTTTGTTGT
GTCATTACTAGTGTTCTGCTGCATTTCTACGGGTTGCTGTGGATGCTGCGGTTGCTGCGGTGCTTGTTTTTCAGGTTGT
TGTAGGGGTCCTAGACTTCAACCTTACGAAGCTTTTGAAAAGGTCCACGTGCAGTGA

>gi|527461768|gb|KC879281.1|_Porcine_epidemic_diarrhea_virus_strain_AD02_sp

ike_protein_(S)_gene_complete_cds

ATGAGGTCTTTAATTTACTTTTTGTTGTTCGTACCAGTACTTCCAACACTCAGTCTACCACAAGATGTCCATAGG
TGCTCAGCTAACACTAATTTTAGGCGGTTCTTTTCAAAATTTAATGTTCAGGCGCCTGCAGTTGTTGTACTGGGCGGTT
ATCTACCTATTGGTGAAAACCAGGGTGTTAATTCAACTTGGTACTGTGCTGGCCAACATCCAACTGCTAGTGGCGTTCA
TGGTATCTTTCTTAGCCATATTAGAGGTGGTCATGGCTTTGAGATTGGCATTTCGCAAGAGCCGTTTGACCCTAGTGGT
TACCAGCTTTATTTACATAAGGCCACTAACGGTAACACTAATGCTACTGCGCGACTGCGCATTTGCCAGTTTCCCAGCA
GTAAAACATTGGGCCCCACTGCTAATGATGATGTTACAACAGGTCGTAACTGCCTATTTAACAAAGCCATCCCAGCTCA
TATGAGTGAACATAGTGTTGTCGGCATAACATGGGATAATGATCGTGTCACTGTTTTTCTAACAAGATCTATCATTTT
CATTTTAAAAATGATTGGTCCCGTGTTGCGACAAAGTGTTACAACAGTGGAGGTTGTGCTATGCAATATGTTTACGAAC
CCACTTACTACATGCTTAATGTTACTAGTGCTGGTGAGGATGGTATTTCTTATCAACCCTGTACAGCTAATTGCATTGG
TTATGCTGCCAATGTATTTGCCACTGAGTCCAATGGCCACATACCAGAAGGTTTTAGTTTTAATAATTGGTTTCTTTTG
TCCAATGATTCCACTTTGGTGCATGGTAAGGTGGTTTCCAACCAACCTTTGTTGGTCAATTGTCTTTTGGCCATTCCTA
AGATTTATGGACTAGGCCAATTTTTCTCCTTCAATCAAACGATCGATGGTGTTTGTAATGGAGCTGCTGCGCAGCGTGC
ACCAGAGGCTCTGAGGTTTAATATTAATGACACCTCTGTCATTCTTGCTGAAGGCTCAATTGTACTTCATACTGCTTTA
GGAACAAATTTTTCTTTTGTTTGCAGTAATTCCTCAAATCCTCATTTAGCCACCTTTGCCATACCTCTGGGTGCTACCC
AAGTACCCTATTATTGTTTTCTTAAAGTGGATACTTACAATTCCACTGTTTATAAATTCTTGGCTGTTTTACCTCCTAC
TGTCAGGGAAATTGTCATCACCAAGTATGGTGATGTTTATGTCAATGGGTTTGGCTATTTGCATCTCGGTTTGTTGGAC
GCTGTCACAATTAATTTCACTGGTCATGGCACTGACGATGGCGTTTCAGGTTTCTGGACCATAGCATCGACTAATTTTG
TTGATGCACTCATCGAAGTTCAAGGAACTGCCATTCAGCGTATTCTTTATTGTGATGATCCTGTTAGCCAACTCAAGTG
TTCTCAGGTTGCTTTTGACCTTGACGATGGTTTTTACCCTATATCTTCTAGAAACCTCCTGAGTCATGAACAGCCAACT
TCTTTTGTTACTTTGCCATCATTTAATGATCATTCTTTTGTTAACATTACTGTCTCTGCGTCTTTTGGTGATCATAGTG
GTGCCAACCCCGTTGCATCTGACACTACTATCAATGGGTTCAGTTCTTTCTGTGTTGACACTAGACAATTTACCATTAG
ACTGTTTTACAACGTTACAAACAGTTATGGTTATGTGTCTAAATCACAGGACAGTAATTGCCCTTTTACCTTGCAATCT
GTTAATGATTACCTGTCTTTTAGCAAATTTTGTGTTTCTACCAGCCTTTTGGCTAGTTCCTGTACCATAGATCTTTTTG
GTTACCCTGATTTCGGTAGTGGTGTTAAGTTTACGTCCCTTTACTTTCAATTCACAGAGGGTGAGTTGATTACTGGCAC
ACCTAAACCACTTGAAGGTGTCACGGACGTTTCTTTTATGACTCTGGATGTGTGTACCAAGTATACTATCTATGGCTTT
AAAGGTGAGGGTATCATTACCCTTACAAATTCTAGCTTTTTGGCAGGTGTTTATTATACATCTGATTCTGGACAGTTGT
TAGCCTTTAAGAATGTCACTAGTGGTGCTGTTTATTCTGTTACGCCATGTTCTTTTTCAGAGCAGGCTGCATATGTTGA
TGACGATATAGTGGGTGTTATTTCTAGTTTGTCTAACTCCACTTTTAACAATACTAGGGAGTTGCCTGGTTTCTTCTAC
CATTCTAATGACGGCTCCAATTGTACAGAGCCTGTGTTGGTGTATAGTAACATAGGTGTTTGTAAATCTGGCTGTATTG
GCTATGTCCCATCTCAGTCTGGCCAAGTCAAGATTGCACCCACGGTTACTGGGAATATTAGTATTCCCACCAACTTTAG
TATGAGTATTAGGACAGAATATTTACAGCTTTACAACACGCCTGTTAGTGTTGATTGTGCCACATATGTTTGTAATGGT
AACTCTCGTTGCAAACAATTACTCACCCAGTACACTGCAGCATGTAAGACCATAGAGTCAGCATTACAACTCAGCGCTA
GGCTTGAGTCTGTTGAAGTTAACTCTATGCTTACTACTTCTGAAGAGGCTCTACAGTTAGCTACCATCAGTTCGTTTAA
TGGTGATGGATATAATTTTACTAATGTGCTGGGTGTTTCCGTGTATGATCCTGCAAGTGGCAGGGTGGTACACAAAAGG
TCTTTCATTGAAGACCTGCTTTTTAATAAAGTGGTTACTAATGGCCTTGGTACTGTTGATGAAGACTATAAGCGCTGTT
CTAATGGCCTCTCTGTGGCTGATCTAGTCTGTGCGCAGTATTACTCTGGTGTCATGGTACTACCTGGCGTTGTTGACGC
TGAGAAGCTTCACATGTATAGTGCGTCTCTCATCGGTGGTATGGTGCTAGGAGGTTTTACTGCTGCAGCGGCATTGCCT
TTTAGCTATGCTGTTCAAGCAAGACTCAATTATCTTGCTTTACAGACGGATGTTCTACAGCGTAACCAGCAATTGCTTG
CTGAGTCTTTTAACTCTGCTATTGGTAATATAACTTCAGCCTTTGAGAGTGTTAAAGAGGCTATTAGTCAAACTTCCCA
GGGTTTGAACACTGTGGCTCATGCGCTTACTAAGGTTCAAGAGGTTGTTAATTCGCAGGGTGCAGCTTTGACCCAACTT
ACCGTACAGCTGCAACACAACTTCCAAGCCATTTCTAGTTCTATTGATGACATTTATTCCCGACTTGACATTCTTTCAG
CCGATGTTCAGGTTGACCGTCTCATCACTGGCAGACTATCAGCACTTAACGCTTTTGTTGCTCAAACCCTCACTAAGTA

TACTGAGGTTCAGGCTAGCAGGAAGCTAGCACAGCAAAAGGTTAATGAGTGCGTCAAATCGCGGTCTCAGCGTTATGGTTTTTGTGGTGGTGATGGCGAACACATTTTTTCCCTGGTACAGGCCGCACCTCAGGGCCTGCTGTTCTTACACACAGTACTTGTACCGGGTGATTTTGTAAATGTTATTGCTATCGCAGGCTTATGCGTTAATGGTGATATTGCCTTGACTCTACGTGAGCCTGGCTTAGTCCTGTTTACGCATGAACTTCAAACTCATACGGCGACAGAATATTTTGTTTCATCGCGACGTATGTTTGAACCTAGAAAACCTACCGTTAGTGATTTTGTTCAAATTGAGAGTTGTGTGGTCACCTATGTCAATCTGACTAGCGACCAACTACCAGATGTAATCCCAGATTACATCGATGTTAACAAAACACTTGATGAGATTTTGGCTTCTCTGCCCAATAGAACTGGTCCAAGTCTTCCACTAGATGTTTTTAATGCTACTTATCTTAATCTCACTGGTGAAATTGCAGATTTAGAGCAGCGTTCAGAGTCTCTCCAAAATATCACAGAAGAGCTCCGAAGTCTCATATATAACATCAACAACACACTTGTTGACCTTGAGTGGCTCAACCGCGTTGAGACATATATCAAGTGGCCGTGGTGGGTTTGGTTGATTATTTTTATTGTTCTCATCTTTGTTGTGTCATTACTAGTGTTCTGCTGCATTTCTACGGGTTGCTGTGGATGCTGCGGTTGCTGCGGTGCTTGTTTTTCAGGTTGTTGTAGGGGTCCTAGACTTCAACCTTACGAAGCTTTTGAAAAGGTCCACGTGCAGTGA

>gi|527461770|gb|KC879282.1|_Porcine_epidemic_diarrhea_virus_strain_AD03_spike_protein_(S)_gene_complete_cds

ATGAGGTCTTTAATTTACTTTTTGTTGTTCGTACCAGTACTTCCAACACTCAGTCTACCACAAGATGTCCATAGGTGCTCAGCTAACACTAATTTTAGGCGGTTCTTTTCAAAATTTAATGTTCAGGCGCCTGCAGTTGTTGTACTGGGCGGTTATCTACCTATTGGTGAAAACCAGGGTGTCAATTCAACTTGGTACTGTGCTGGCCAACATCCAACTGCTAGTGGCGTTCATGGTATCTTTCTTAGCCATATTAGAGGTGGTCATGGCTTTGAGATTGGCATTTCGCAAGAGCCGTTTGACCCTAGTGGTTACCAGCTTTATTTACATAAGGCCACTAACGGTAACACTAATGCTACTGCGCGACTGCGCATTTGCCAGTTTCCCAGCAGTAAAACATTGGGCCCCACTGCTAATGATGATGTTACAACAGGTCGTAACTGCCTATTTAACAAAGCCATCCCAGCTCATATGAGTGAACATAGTGTTGTCGGCATAACATGGGATAATGATCGTGTCACTGTTTTTTCTGACAAGATCTATCATTTTCATTTTAAAAATGATTGGTCCCGTGTTGCGACAAAGTGTTACAACAGTGGAGGTTGTGCTATGCAATATGTTTACGAACCCACTTACTACATGCTTAATGTTACTAGTGCTGGTGAGGATGGTATTTCTTATCAACCCTGTACAGCTAATTGCATTGGTTATGCTGCCAATGTATTTGCCACTGAGTCCAATGGCCACATACCAGAAGGTTTTAGTTTTAATAATTGGTTTCTTTTGTCCAATGATTCCACTTTGGTGCATGGTAAGGTGGTTTCCAACCAACCTTTGTTGGTCAATTGTCTCTTGGCCATCCCTAAGATTTATGGACTAGGCCAATTTTTCTCCTTCAATCAAACGATCGATGGTGTTTGTAATGGAGCTGCTGCGCAGCGTGCACCAGAGGCTCTGAGGTTTAATATTAATGACACCTCTGTCATTCTTGCTGAAGGCTCAATTGTACTTCATACTGCTTTAGGAACAAATTTTTCTTTTGTTTGCAGTAATTCCTCAAATCCTCATTTAGCCACCTTTGCCATACCTCTGGGTGCTACCCAAGTACCCTATTATTGTTTTCTTAAAGTGGATACTTACAATTCCACTGTTTATAAATTCTTGGCTGTTTTACCTCCTACTGTCAGGGAAATTGTCATCACCAAGTATGGTGATGTTTATGTCAATGGGTTTGGCTATTTGCATCTCGGTTTGTTGGACGCTGTCACAATTAATTTCACTGGTCATGGCACTGACGATGACGTTTCAGGTTTCTGGACCATAGCATCGACTAATTTTGTTGATGCACTCATCGAAGTTCAAGGAACTGCCATTCAGCGTATTCTTTATTGTGATGATCCTGTTAGCCAACTCAAGTGTTCTCAGGTTGCTTTTGACCTTGACGATGGTTTTTACCCTATATCTTCTAGAAACCTCCTGAGTCATGAACAGCCAACTTCTTTTGTTACTTTGCCATCATTTAATGATCATTCTTTTGTTAACATTACTGTCTCTGCGTCTTTTGGTGATCATAGTGGTGCCAACCTCGTTGCATCTGACACTACTATCAATGGGTTCAGTTCTTTCTGTGTTGACACTAGACAATTTACCATTAGACTGTTTTACAACGTTACAAACAGTTATGGTTATGTGTCTAAATCACAGGACAGTAATTGCCCTTTTACCTTGCAATCTGTTAATGATTACCTGTCTTTTAGCAAATTTTGTGTTTCTACCAGCCTTTTGGCTAGCTCCTGTACCATAGATCTTTTTGGTTACCCTGATTTCGGTAGTGGTGTTAAGTTTACGTCCCTTTACTTTCAATTCACAGAGGGTGAGTTGATTACTGGCACACCTAAACCACTTGAAGGTGTCACGGACGTTTCTTTTATGACTCTGGATGTGTGTACCAAGTATACTATCTATGGCTTTAAAGGTGAGGGTATCATTACCCTTACAAATTCTAGCTTTTTGGCAGGTGTTTATTATACATCTGATTCTGGACAGTTGTTAGCCTTTAAGAATGTCACTAGTGGTGCTGTTTATTCTGTTACGCCATGTTCTTTTTCAGAGCAGGCTGCATATGTTGATGACGATATAGTGGGTGTTATTTCTAGTTTGTCTAACTCCACTTTTAACAATACTAGGGAGTTGCCTGGTTTCTTCTAC

CATTCTAATGACGGCTCCAATTGTACAGAGCCTGTGTTGGTGTATAGTAACATAGGTGTTTGTAAATCTGGCAGTATTG
GCTATGTCCCATCTCAGTCTGGCCAAGTCAAGATTGCACCCACGGTTACTGGGAATATTAGTATTCCCACCAACTTTAG
TATGAGTATTAGGACAGAATATTTACAGCTTTACAACACGCCTGTTAGTGTTGATTGTGCCACATATGTTTGTAATGGT
AACTCTCGTTGCAAACAATTACTCACCCAGTACACTGCAGCATGTAAGACCATAGAGTCAGCATTACAACTCAGCGCTA
GGCTTGAGTCTGTTGAAGTTAACTCTATGCTTACTACTTCTGAAGAGGCTCTACAGTTAGCTACCATCAGTTCGTTTAA
TGGTGATGGATATAATTTTACTAATGTGCTGGGTGTTTCCGTGTATGATCCTGCAAGTGGCAGGGTGGTACACAAAAGG
TCTTTCATTGAAGACCTGCTTTTTAATAAAGTGGTTACTAATGGCCTTGGTACTGTTGATGAAGACTATAAGCGCTGTT
CTAATGGCCTCTCTGTGGCTGATCTAGTCTGTGCGCAGTATTACTCTGGTGTCATGGTACTACCTGGCGTTGTTGGCGC
TGAGAAGCTTCACATGTATAGTGCGTCTCTCATCGGTGGTATGGTGCTAGGAGGTTTTACTGCTGCAGCGGCATTGCCT
TTTAGCTATGCTGTTCAAGCGAGACTCAATTATCTTGCTTTACAGACGGATGTTCTACAGCGTAACCAGCAATTGCTTG
CTGAGTCTTTTAACTCTGCTATTGGTAATATAACTTCAGCCTTTGAGAGTGTTAAAGAGGCTATTAGTCAAACTTCCCA
GGGTTTGAACACTGTGGCTCATGCGCTTACTAAGGTTCAAGAGGTTGTTAATTCGCAGGGTGCAGCTTTGACCCAACTT
ACCGTACAGCTGCAACACAACTTCCAAGCCATTTCTAGTTCTATTGATGACATTTATTCCCGACTTGACATTCTTTCAG
CCGATGTTCAGGTTGACCGTCTCATCACTGGCAGATTATCAGCACTTAACGCTTTTGTTGCTCAAACCCTCACTAAGTA
TACTGAGGTTCAGGCTAGCAGGAAGCTAGCACAGCAAAAGGTTAATGAGTGCGTCAAATCGCAGTCTCAGCGTTATGGT
TTTTGTGGTGGTGATGGCGAACACATTTTTTCCCTGGTACAGGCCGCACCTCAGGGCCTGCTGTTCTTACACACAGTAC
TTGTACCGGGTGATCTTGTAAATGTTATTGCTATCGCAGGCTTATGCGTTAATGGTGATATTGCCTTGACTCTACGTGA
GCCTGGCTTAGTCCTGTTTACGCATGAACTTCAAACTCATACGGCGACAGAATATTTTGTTTCATCGCGACGTATGTTT
GAACCTAGAAAACCTACCGTTAGTGATTTTGTTCAAATTGAGAGTTGTGTGGTCACCTATGTCAATCTGACTAGCGACC
AACTACCAGATGTAATCCCAGATTACATCGATGTTAACAAAACACTTGATGAGATTTTGGCTTCTCTGCCCAATAGAAC
TGGTCCAAGTCTTCCACTAGATGTTTTTAATGCTACTTATCTTAATCTCACTGGTGAAATTGCAGATTTAGAGCAGCGT
TCAGAGTCTCTCCAAAATATCACAGAAGAGCTCCGAAGTCTCATATATAACATCAACAACACACTTGTTGACCCTGAGT
GGCTCAACCGCGTTGAGACATATATCAAGTGGCCGTGGTGGGTTTGGTTGATTATTTTTATTGTTCTCATCTTTGTTGT
GTCATTACTAGTGTTCTGCTGCATTTCTACGGGTTGCTGTGGATGCTGCGGTTGCTGCGGTGCTTGTTTTTCAGGTTGT
TGTAGGGGTCCTAGACCTCAACCTTACGAAGCTTTTGAAAAGGTCCACGTGCAGTGA

>gi|543335530|gb|KF177256.1|_Porcine_epidemic_diarrhea_virus_isolate_JY7C_spike_protein_(S)_gene_complete_cds

ATGAAGTCTTTAACCTACTTCTGGTTGTTCTTACCAGTACTTTCAACACTTAGCCTACCACAAGATGTCACCAGG
TGCTCAGCTAACACTAATTTTAGGCGGTTCTTTTCAAAATTTAATGTTCAGGCGCCTGCAGTTGTTGTACTGGGCGGTT
ATCTACCTATTGGTGAAAACCAGGGTGTCAATTCAACTTGGTACTGTGCTGGCCAACATCCAACTGCTAGTGGCGTTCA
TGGTATCTTTGTTAGCCATATTAGAGGTGGTCATGGCTTTGAGATTGGCATTTCGCAAGAGCCTTTTGACCCTAGTGGT
TACCAGCTTTATTTACATAAGGCTACTAACGGTAACACTAATGCTACTGCGCGACTGCGCATTTGCCAGTTTCCTAGCA
TTAAAACATTGGGCCCCACTGCTAATAATGATGTTACAACAGGTCGTAATTGCCTATTTAACAAAGCCATCCCAGCTCA
TATGAGTGAACATAGTGTTGTCGGCATAACATGGGATAATGATCGTGTCACTGTCTTTTCTGACAAGATCTATTATTTT
TATTTTAAAAATGATTGGTCCCGTGTTGCGACAAAGTGTTACAACAGTGGAGGTTGTGCTATGCAATATGTTTACGAAC
CCACCTATTACATGCTTAATGTTACTAGTGCTGGTGAGGATGGTATTTCTTATCAACCCTGTACAGCTAATTGCATTGG
TTATTCTGCCAATGTATTTGCTACTGAGCCCAATGGCCACATACCAGAAGGTTTTAGTTTTAATAATTGGTTTCTTTTG
TCCAATGATTCCACTTTGTTGCATGGTAAGGTGGTTTCCAACCAACCATTGTTGGTCAATTGTCTTTTGGCCATTCCTA
AGATTTATGGACTAGGCCAATTTTTCTCCTTTAATCAAACGATCGATGGTGTTTGTAATGGAGCTGCTGTGCAGCGTGC
ACCAGAGGCTCTGAGGTTTAATATTAATGACACCTCTGTCATTCTTGCTGAAGGCTCAATTGTACTTCATACTGCTTTA
GGAACAAATTTTTCTTTTGTTTGCAGTAATTCCTCAGATCCTCACTTAGCCACCTTCGCCATACCTCTGGGTGCTATCC

AAGTACCCTATTATTGTTTTCTTAAAGTGGATACTTACAACTCCACTGTTTATAAATTCTTGGCTGTTTTACCTCCTAC
CGTCAGGGAAATTGTCATCACCAAGTATGGTGATGTTTATGTCAATGGGTTTGGCTACTTGCATCTCGGTTTGTTGGAT
GCTGTCACACTTAATTTCACTGGTCATGGCACTGACGATGACGTTTCTGGTTTTTGGACCATAGCATCGACTAATTTTG
TTGATGCACTTATCGAAGTTCAAGGAACTTCCATTCAGCGTATTCTTTATTGTGATGATCCTGTTAGCCAACTCAAGTG
TTCTCAGGTTGCTTTTGACCTTGACGATGGTTTCTACCCTATTTCTTCTAGAAACCTTCTGAGTCATGAACAGCCAATT
TCTTTTGTTACTTTGCCATCATTTAATGATCATTCTTTTGTTAACATTACTGTCTCTGCGTCCTTTGGTGGTCATAGTG
GTGCCAACCTTATTGCATCTGACACTACTATCAATGGGTTTAGTTCTTTCTGTGTTGACACTAGACAATTTACCATTTC
ACTGTTTTATAACGTTACAAACAGTTATGGTTATGTGTCTAAATCACAGGACAGTAATTGCCCTTTCACCTTGCAATCT
GTTAATGATTACCTGTCTTTTAGTAAATTTTGTGTTTCCACCAGCCTTTTGGCTAGTGCCTGTACCATAGATCTTTTTG
GTTACCCTGAGTTTGGTAGTGGTGTTAAGTTTACGTCCCTTTACTTTCAATTCACAAAGGGTGAGTTGATTACTGGCAC
GCCTAAACCACTTGAAGGTGTCACGGACGTTTCTTCTATGACTCTGGATGTGTGTACCAAGTATACTATCTATGGCTTT
AAAGGTGAGGGTATCATTACCCTTACAAATTCTAGCTTTTTGGCAGGTGTTTATTACACATCTGATTCTGGACAGTTGT
TAGCCTTTAAGAATGTCACTAGTGGTGCTGTTTATTCTGTTACGCCATGTTCTTTTTCAGAGCAGGCTGCATATGTTGA
TGATGATATAGTGGGTGTTATTTCTAGTTTGTCTAGCTCCACTTTTAACAGTACTAGGGAGTTGCCTGGTTTCTTTTAC
CATTCTAATGATGGCTCTAATTGCACAGAGCCTGTGTTGGTGTATAGTAACATAGGTGTTTGTAAATCTGGCAGTATTG
GCTACGTCCCATCTCAGTCTGGCCAAGTCAAGATTGCACCCACGGTTACTGGGAATATTAGTATTCCCACCAACTTTAG
TATGAGTATTAGGACAGAATATTTACAGCTTTACAACACGCCTGTTAGTGTTGATTGTGCCACATATGTTTGTAATGGT
AACTCTCGTTGTAAACAATTACTCACCCAGTACACTGCAGCATGTAAGACCATAGAGTCAGCATTACAACTCAGCGCTA
GGCTTGAGTCTGTTGAAGTTAACTCTATGCTTACTATTTCTGAAGAGGCTCTACAGTTAGCTACCATCAGTTCGTTTAA
TGGTGATGGATATAATTTTACTAATGTGCTGGGTGTTTCTGTGTATGATCCTGCAAGTGGCAGGGTGGTACAAAAAAGG
TCTTTTATTGAAGACCTGCTTTTTAATAAGGTGGTTACTAATGGCCTTGGTACTGTTGATGAAGACTATAAGCGCTGTT
CTAATGGTCGCTCTGTGGCAGATCTAGTCTGTGCACAGTATTACTCTGGTGTCATGGTACTACCTGGTGTTGTTGACGC
TGAGAAGCTTCACATGTATAGTGCGTCTCTCATCGGTGGTATGGTGCTAGGAGGTTTTACTTCTGCAGCGGCATTGCCT
TTTAGCTATGCTGTTCAAGCTAGACTCAATTATCTTGCTCTACAGACGGATGTTCTACAGCGGAACCAGCAATTGCTTG
CTGAGTCTTTTAACTCTGCTATTGGTAATATAACTTCAGCCTTTGAGAGTGTTAAAGAGGCTATTAGTCAAACTTCCAA
GGGTTTGAACACTGTGGCTCATGCGCTTACTAAGGTTCAAGAGGTTGTTAACTCGCAGGGTGCAGCTTTGACTCAACTT
ACCGTACAGCTGCAACACAACTTCCAAGCCATTTCTAGTTCTATTGATGACATTTACTCTCGACTGGACATTCTTTCAG
CCGATGTTCAGGTTGACCGTCTTATCACCGGCAGATTATCAGCACTTAATGCTTTTGTTGCTCAAACCCTCACTAAGTA
TACTGAGGTTCAGGCTAGCAGGAAGCTAGCACAGCAAAAGGTTAATGAGTGCGTTAAATCGCAATCTCAGCGTTATGGT
TTTTGTGGTGGTGATGGCGAGCACATTTTCTCTCTGGTACAGGCAGCACCTCAGGGCCTGCTGTTTTTACATACAGTAC
TTGTACCGGGTGATTTTGTAGATGTTATTGCCATCGCTGGTTTATGCGTTAACGATGAAATTGCCTTGACTCTACGTGA
GCCTGGCTTAGTCTTGTTTACGCATGAACTTCAAAATCATACTGCGACGGAATATTTTGTTTCATCGCGACGTATGTTT
GAACCTAGAAAACCTACCGTTAGTGATTTTGTTCAAATTGAGAGTTGTGTGGTCACCTATGTCAATTTGACTAGAGACC
AACTACCAGATGTAATCCCAGATTACATCGATGTTAACAAAACACTTGATGAGATTTTAGCTTCTCTGCCCAATAGAAC
TGGTCCAAGTCTTTCTTTAGATGTTTTTAATGCCACTTATCTTAATCTCACTGGTGAAATTGCAGATTTAGAGCAGCGT
TCAGAGTCTCTCCGTAATACTACAGAGGAGCTCCAAAGTCTTATATATAATATCAACAACACACTAGTTGACCTTGAAT
GGCTCAACCGAGTTGAGACATATATCAAGTGGCCGTGGTGGGTTTGGTTGATCATTTTCATTGTTCTCATCTTTGTTGT
GTCATTACTAGTGTTCTGCTGCATTTCCACGGGTTGTTGTGGATGCTGCGGCTGCTGCTGTGCTTGTTTTTCAGGTTGT
TGTAGGGGTCCTAGACTTCAACCTTACGAAGTTTTTGAAAAGGTCCACGTGCAGTGA

>gi|543335532|gb|KF177257.1|_Porcine_epidemic_diarrhea_virus_isolate_YJ3F_spike_protein_(S)_gene_complete_cds

ATGAAGTCTTTAACCTACTTCTGGTTGTTCTTACCAGTACTTTCAACACTTAGCCTACCACAAGATGTCACCAGG
TGCTCAGCTAACACTAATTTTAGGCGGTTCTTTTCAAAATTTAATGTTCAGGCGCCTGCAGTTGTTGTACTGGGCGGTT
ATCTACCTATTGGTGAAAACCAGGGTGTCAATTCAACTTGGTACTGTGCTGGCCAACATCCAACTGCTAGTGGCGTTCA
TGGTATCTTTGTTAGCCATATTAGAGGTGGTCATGGCTTTGAGATTGGCATTTCGCAAGAGCCTTTTGACCCTAGTGGT
TACCAGCTTTATTTACATAAGGCTACTAACGGTAACACTAATGCTACTGCGCGACTGCGCATTTGCCAGTTTCCTAGCA
TTAAAACATTGGGCCCCACTGCTAATAATGATGTTACAACAGGTCGTAATTGCCTATTTAACAAAGCCATCCCAGCTCA
TATGAGTGAACATAGTGTTGTCGGCATAACATGGGATAATGATCGTGTCACTGTCTTTTCTGACAAGATCTATCATTTT
TATTTTAAAAATGATTGGTCCCGTGTTGCGACAAAGTGTTACAACAGTGGAGGTTGTGCTATGCAATATGTTTACGAAC
CCACCTATTACATGCTTAATGTTACTAGTGCTGGTGAGGATGGTATTTCTTATCAACCCTGTACAGCTAATTGCATTGG
TTATGCTGCCAATGTATTTGCTACTGAGCCCAATGGCCACATACCAGAAGGTTTTAGTTTTAATAATTGGTTTCTTTTG
TCCAATGATTCCACTTTGGTGCATGGTAAGGTGGTTTCCAACCAACCATTGTTGGTCAGTTGTCTTTTGGCCACTCCTA
AGATTTATGGACTAGGCCAATTTTTCTCCTTTAATCAAACGATCGATGGTGTTTGTAATGGAGCTGCTGTGCAGCGTGC
ACCAGAGGCTCTGAGGTTTAATATTAATGACACCTCTGTCATTCTTGCTGAAGGCTCAATTGTACTTCATACTGCTTTA
GGAACAAATTTTTCTTTTGTTTGCAGTAATTCCTCAGATCCTCACTTAGCCACCTTCGCCATACCTCTGGGTGCTATCC
AAGTACCCTATTATTGTTTTCTTAAAGTGGATACTTACAACTCCACTGTTTATAAATTCTTGGCTGTTTTACCTCCTAC
CGTCAGGGAAATTGTCATCACCAAGTATGGTGATGTTTATGTCAATGGGTTTGGCTATTTGCATCTCGGTTTGTTGGAC
GCTGTCACAATTAATTTCACTGGTCATGGCACTGACGATGACGTTTCAGGTTTCTGGACCATAGCATCGACTAATTTTG
TTGATGCACTTATCGAAGTTCAAGGAACTGCCATTCAGCGTATTCTTTATTGTGATGATCCTGTTAGCCAACTCAAGTG
TTCTCAGGTTGCTTTTGACCTTGACGATGGTTTCTACCCTATTTCTTCTAGAAACCTTCTGAGTCATGAACAGCCAATT
TCTTTTGTTACTTTGCCATCATTTAATGATCATTCTTTTGTTAACATTACTGTCTCTGCGTCCTTTGGTGGTCTTAGTG
GTGCCAACCTTATTGCATCTGACACTACTATCAATGGGTTTAGTTCTTTCTGTGTTGACACTAGACAATTTACCATTTC
ACTGTTTTATAACGTTACAAACAGTTATGGTTATGTGTCTAAATCACAGGACAGTAATTGCCCTTTCACCTTGCAATCT
GTTAATGATTACCTGTCTTTTAGTAAATTTTGTGTTTCCACCAGCCTTTTGGCTAGTGCCTGTACCATAGATCTTTTTG
GTTACCCTGAGTTTGGTAGTGGTGTTAAGTTTACGTCCCTTTACTTTCAATTCACAAAGGGTGAGTTGATTACTGGCAC
GCCTAAACCACTTGAAGGTGTCACGGACGTTTCTTTTATGACTCTGGATGTGTGTACCAAGTATACTATCTATGGCTTT
AAAGGTGAGGGTATCATTACCCTTATAAATTCTAGCTTTTTGGCAGGTGTTTATTACACATCTGATTCTGGACAGTTGT
TAGCCTTTAAGAATGTCACTAGTGGTGCTGTTTATTCTGTTACGCCATGTTCTTTTTCAGAGCAGGCTGCATATGTTGA
TGATGATATAGTGGGTGTTATTTCTAGTTTGTCTAGCTCCACTTTTAACAGTACTAGGGAGTTGCCTGGTTTCTTCTAC
CATTCTAATGATGGCTCTAATTGTACAGAGCCTGTGTTGGTGTATAGTAACATAGGTGTTTGTAAATCTGGCAGTATTG
GCTACGTCCCATCCCAGTCTGGCCAAGTCAAGATTGCACCCATGGTTACTGGGAATATTAGTATTCCCACCAACTTTAG
TATGAGTATTAGGACAGAATATTTACAGCTTTACAACACGCCTGTTAGTGTTGATTGTGCCACATATGTTTGTAATGGT
AACTCTCGTTGTAAACAATTACTCACCCAGTACACTGCAGCATGTAAGACCATAGAGTCAGCATTACAACTCAGCGCTA
GGCTTGAGTCTGTTGAAGTTAACTCTATGCTTACTATTTCTGAAGAGGCTCTACAGTTAGCTACCATCAGTTCGTTTAA
TGGTGATGGATATAATTTTACTAATGTGCTGGGTGTTTCTGTGTATGATCCTGCAAGTGGCAGGGTGGTACAAAAAAGG
TCTTTTATTGAAGACCTGCTTTTTAATAAAGTGGTTACTAATGGCCTTGGTACTGTTGATGAAGACTATAAGCGCTGTT
CTAATGGTCGCTCTGTGGCAGATCTAGTCTGTGCACAGTATTACTCTGGTGTCATGGTACTACCTGGTGTTGTTGACGC
TGAGAAGCTTCACATGTATAGTGCGTCTCTCATCGGTGGTATGGTGCTAGGAGGTTTTACTTCTGCAGCGGCATTGCCT
TTTAGCTATGCTGTTCAAGCTAGACTCAATTATCTTGCTCTACAGACGGATGTTCTACAGCGGAACCAGCAATTGCTTG
CTGAGTCTTTTAACTCTGCTATTGGTAATATAACTTCAGCCTTTGAGAGTGTTAAAGAGGCTATTAGTCAAACTTCCAA
GGGTTTGAACACTGTGGCTCATGCGCTTACTAAGGTTCAAGAGGTTGTTAACTCGCAGGGTGCAGCTTTGACTCAACTT
ACCGTACAGCTGCAACACAACTTCCAAGCCATTTCTAGTTCTATTGATGACGTTTACTCTCGACTGGACATTCTTTCAG
CCGATGTTCAGGTTGACCGTCTCATCACCGGCAGATTATCAGCACTTAATGCTTTTGTTTCTCAAACCCTCACTAAGTA
TACTGAGGTTCAGGCTAGCAGGAAGCTAGCACAGCAAAAGGTTAATGAGTGCGTTAAATCGCAATCTCAGCGTTATGGT
TTTTGTGGTGGTGATGGCGAGCACATTTTCTCTCTGGTACAGGCAGCACCTCAGGGCCTGCTGTTTTTACATACAGTAC

TTGTACCGGGTGACTTTGTAGATGTTATTGCCATCGCTGGCTTATGCGTTAACGATGAAATTGCCTTGACTCTACGTGA
GCCTGGCTTAGTCTTGTTTACGCATGAACTTCAAAATCATACTGCGACGGAATATTTTGTTTCATCGCGACGTATGTTT
GAACCTAGAAAACCTACCGTTAGTGATTTTGTTCAAATTGAGAGTTGTGTGGTCACCTATGTCAATTTGACTAGAGACC
AACTACCAGATGTAATCCCAGATTACATCGATGTTAACAAAACACTTGATGAGATTTTAGCTTCTCTGCCCAATAGAAC
TGGTCCAAGTCTTCCTTTAGATGTTTTTAATGCCACTTATCTTAATCTCACTGGTGAAATTGCAGATTTAGAGCAGCGT
TCAGAGTCTCTCCGTAATACTACAGAGGAGCTCCAAAGTCTTATATATAATATCAACAACACACTAGTTGACCTTGAGT
GGCTCAACCGAGTTGAGACATATATCAAGTGGCCGTGGTGGGTTTGGTTGATTATTTTCATTGTTCTCATCTTTGTTGT
GTCATTACTAGTGTTCTGCTGCATTTCCACTGGTTGTTGTGGATGCTGCGGCTGCTGCTGTGCTTGTTTTTCAGGTTGT
TGTAGGGGTCCTAGACTTCAACCTTACGAAGTTTTTGAAAAGGTCCACGTGCAGTGA

>gi|543335534|gb|KF177258.1|_Porcine_epidemic_diarrhea_virus_isolate_YJ7C_spike_protein_(S)_gene_complete_cds

ATGAAGTCTTTAACCTACTTCTGGTTGTTCTTACCAGTACTTTCAACACTTAGCCTACCACAAGATGTCACCAGG
TGCTCAGCTAACACTAATTTTAGGCGGTTCTTTTCAAAATTTAATGTTCAGGCGCCTGCAGTTGTTGTACTGGGCGGTT
ATCTACCTATTGGTGAAAACCAGGGTGTCAATTCAACTTGGTACTGTGCTGGCCAACATCCAACTGCTAGTGGCGTTCA
TGGTATCTTTGTTAGCCATATTAGAGGTGGTCATGGCTTTGAGATTGGCATTTCGCAAGAGCCTTTTGACCCTAGTGGT
TACCAGCTTTATTTACATAAGGCTACTAACGGTAACACTAATGCTACTGCGCGACTGCGCATTTGCCAGTTTCCTAGCA
TTAAAACATTGGGCCCCACTGCTAATAATGATGTTACAACAGGTCGTAATTGCCTATTTAACAAAGCCATCCCAGCTCA
TATGAGTGAACATAGTGTTGTCGGCATAACATGGGATAATGATCGTGTCACTGTCTTTTCTGACAAGATCTATCATTTT
TATTTTAAAAATGATTGGTCCCGTGTTGCGACAAAGTGTTACAACAGTGGAGGTTGTGCTATGCAATATGTTTACGAAC
CCACCTATTACATGCTTAATGTTACTAGTGCTGGTGAGGATGGTATTTCTTATCAACCCTGTACAGCTAATTGCATTGG
TTATGCTGCCAATGTATTTGCTACTGAGCCCAATGGCCACATACCAGAAGGTTTTAGTTTTAATAATTGGTTTCTTTTG
TCCAATGATTCCACTTTGGTGCATGGTAAGGTGGTTTCCAACCAACCATTGTTGGTCAATTGTCTTTTGGCCACTCCTA
AGATTTATGGACTAGGCCAATTTTTCTCCTTTAATCAAACGATCGATGGTGTTTGTAATGGAGCTGCTGTGCAGCGTGC
ACCAGAGGCTCTGAGGTTTAATATTAATGACACCTCTGTCATTCTTGCTGAAGGCTCAATTGTACTTCATACTGCTTTA
GGAACAAATTTTTCTTTTGTTTGCAGTAATTCCTCAGATCCTCACTTAGCCACCTTCGCCATACCTCTGGGTGCTATCC
AAGTACCCTATTATTGTTTTCTTAAAGTGGATACTTACAACTCCACTGTTTATAAATTCTTGGCTGTTTTACCTCCTAC
CGTCAGGGAAATTGTCATCACCAAGTATGGTGATGTTTATGTCAATGGGTTTGGCTATTTGCATCTCGGTTTGTTGGAC
GCTGTCACAATTAATTTCACTGGTCATGGCACTGACGATGACGTTTCAGGTTTCTGGACCATAGCATCGACTAATTTTG
TTGATGCACTTATCGAAGTTCAAGGAACTGCCATTCAGCGTATTCTTTATTGTGATGATCCTGTTAGCCAACTCAAGTG
TTCTCAGGTTGCTTTTGACCTTGACGATGGTTTCTACCCTATTTCTTCTAGAAACCTTCTGAGTCATGAACAGCCAATT
TCTTTTGTTACTTTGCCATCATTTAATGATCATTCTTTTGTTAACATTACTGTCTCTGCGTCCTTTGGTGGTCTTAGTG
GTGCCAACCTTATTGCATCTGACACTACTATCAATGGGTTTAGTTCTTTCTGTGTTGACACTAGACAATTTACCATTTC
ACTGTTTTATAACGTTACAAACAGTTATGGTTATGTGTCTAAATCACAGGACAGTAATTGCCCTTTCACCTTGCAATCT
GTTAATGATTACCTGTCTTTTAGTAAATTTTGTGTTTCCACCAGCCTTTTGGCTAGTGCCTGTACCATAGATCTTTTTG
GTTACCCTGAGTTTGGTAGTGGTGTTAAGTTTACGTCCCTTTACTTTCAATTCACAAAGGGTGAGTTGATTACTGGCAC
GCCTAAACCACTTGAAGGTGTCACGGACGTTTCTTTTATGACTCTGGATGTGTGTACCAAGTATACTATCTATGGCTTT
AAAGGTGAGGGTATCATTACCCTTATAAATTCTAGCTTTTTGGCAGGTGTTTATTACACATCTGATTCTGGACAGTTGT
TAGCCTTTAAGAATGTCACTAGTGGTGCTGTTTATTCTGTTACGCCATGTTCTTTTTCAGAGCAGGCTGCATATGTTGA
TGATGATATAGTGGGTGTTATTTCTAGTTTGTCTAACTCCACTTTTAACAGTACTAGGGAGTTGCCTGGTTTCTTCTAC
CATTCTAATGATGGCTCTAATTGTACAGAGCCTGTGTTGGTGTATAGTAACATAGGTGTTTGTAAATCTGGCAGTATTG
GCTACGTCCCATCCCAGTCTGGCCAAGTCAAGATTGCACCCATGGTTACTGGGAATATTAGTATTCCCACCAACTTTAG

TATGAGTATTAGGACAGAATATTTACAGCTTTACAACACGCCTGTTAGTGTTGATTGTGCCACATATGTTTGTAATGGT
AACTCTCGTTGTAAACAATTACTCACCCAGTACACTGCAGCATGTAAGACCATAGAGTCAGCATTACAACTCAGCGCTA
GGCTTGAGTCTGTTGAAGTTAACTCTATGCTTACTATTTCTGAAGAGGCTCTACAGTTAGCTACCATCAGTTCGTTTAA
TGGTGATGGATATAATTTTACTAATGTGCTGGGTGTTTCTGTGTATGATCCTGCAAGTGGCAGGGTGGTACAAAAAAGG
TCTTTTATTGAAGACCTGCTTTTTAATAAAGTGGTTACTAATGGCCTTGGTACTGTTGATGAAGACTATAAGCGCTGTT
CTAATGGTCGCTCTGTGGCAGATCTAGTCTGTGCACAGTATTACTCTGGTGTCATGGTACTACCTGGTGTTGTTGACGC
TGAGAAGCTTCACATGTATAGTGCGTCTCTCATCGGTGGTATGGTGCTAGGAGGTTTTACTTCTGCAGCGGCATTGCCT
TTTAGCTATGCTGTTCAAGCTAGACTCAATTATCTTGCTCTACAGACGGATGTTCTACAGCGGAACCAGCAATTGCTTG
CTGAGTCTTTTAACTCTGCTATTGGTAATATAACTTCAGCCTTTGAGAGTGTTAAAGAGGCTATTAGTCAAACTTCCAA
GGGTTTGAACACTGTGGCTCATGCGCTTACTAAGGTTCAAGAGGTTGTTAACTCGCAGGGTGCAGCTTTGACTCAACTT
ACCGTACAGCTGCAACACAACTTCCAAGCCATTTCTAGTTCTATTGATGACGTTTACTCTCGACTGGACATTCTTTCAG
CCGATGTTCAGGTTGACCGTCTCATCACCGGCAGATTATCAGCACTTAATGCTTTTGTTTCTCAAACCCTCACTAAGTA
TACTGAGGTTCAGGCTAGCAGGAAGCTAGCACAGCAAAAGGTTAATGAGTGCGTTAAATCGCAATCTCAGCGTTATGGT
TTTTGTGGTGGTGATGGCGAGCACATTTTCTCTCTGGTACAGGCAGCACCTCAGGGCCTGCTGTTTTTACATACAGTAC
TTGTACCGGGTGACTTTGTAGATGTTATTGCCATCGCTGGCTTATGCGTTAACGATGAAATTGCCTTGACTCTACGTGA
GCCTGGCTTAGTCTTGTTTACGCATGAACTTCAAAATCATACTGCGACGGAATATTTTGTTTCATCGCGACGTATGTTT
GAACCTAGAAAACCTACCGTTAGTGATTTTGTTCAAATTGAGAGTTGTGTGGTCACCTATGTCAATTTGACTAGAGACC
AACTACCAGATGTAATCCCAGATTACATCGATGTTAACAAAACACTTGATGAGATTTTAGCTTCTCTGCCCAATAGAAC
TGGTCCAAGTCTTCCTTTAGATGTTTTTAATGCCACTTATCTTAATCTCACTGGTGAAATTGCAGATTTAGAGCAGCGT
TCAGAGTCTCTCCGTAATACTACAGAGGAGCTCCAAAGTCTTATATATAATATCAACAACACACTAGTTGACCTTGAGT
GGCTCAACCGAGTTGAGACATATATCAAGTGGCCGTGGTGGGTTTGGTTGATTATTTTCATTGTTCTCATCTTTGTTGT
GTCATTACTAGTGTTCTGCTGCATTTCCACTGGTTGTTGTGGATGCTGCGGCTGCTGCTGTGCTTGTTTTTCAGGTTGT
TGTAGGGGTCCTAGACTTCAACCTTACGAAGTTTTTGAAAAGGTCCACGTGCAGTGA

>gi|525470002|gb|KF384500.1| Porcine epidemic diarrhea virus strain CH/GDGZ/2012, complete genome

ATGAAGTCTTTAACCTACTTCTGGTTGTTCTTACCAGTACTTTCAACACTTAGCCTACCACAAGATGTCACCAGG
TGCTCAGCTAACACTAATTTTAGGCGGTTCTTTTCAAAATTTAATGTTCAGGCGCCTGCAGTTGTTGTACTGGGCGGTT
ATCTACCTATTGGTGAAAACCAGGGTGTCAATTCAACTTGGTACTGTGCTGGCCAACATCCAACTGCTAGTGGCGTTCA
TGGTATCTTTCTTAGCCATATTAGAGGTGGTCATGGCTTTGAGATTGGCATTTCGCAAGAGCCTTTTGACCCTAGTGGT
TACCAGCTTTATTTACATAAGGCTACTAACGGTAACACTAATGCTACTGCGCGACTGCGCATTTGCCAGTTTCCCGATA
ATAAAACATTGGGCCCCACTGCTAATAATGATGTTACAACAGGTCGTAACTGCCTATTTAACAAAGCCATCCCAGCTCA
TATGAGTGAACATAGTGTTGTCGGCATAACATGGGATAATGATCGTGTCACTGTCCTTTCTGACAAGATCTATTATTTT
TATTTTAAAAATGATTGGTCCCGTGTTGCGACAAAGTGTTACAACAGTGGAGGTTGTGCTATGCAATATGTTTACGAAC
CCACTTACTACATGCTTAATGTTACTAGTGCTGGTGAGGATGGTATTTCTTATCAACCCTGTACAGCTAATTGCATTGG
TTATGCTGCCAATGTATTTGCTACTGAGCCCAATGGCCACATACCAGAAGGTTTTAGTTTTAATAATTGGTTTCTTTTG
TCCAATGATTCCACTTTGGTGCATGGTAAGGTGGTTTCCAACCAACCATTGTTGGTCAATTGTCTTTTGGCCATTCCTA
AGATTTATGGACTAGGCCAATTTTTCTCCTTTAATCAAACGATCGATGGTGTTTGTAATGGAGCTGCTGTGCAGCGTGC
ACCAGAGGCTCTGAGGTTTAATATTAATGACACCTCTGTCATTCTTGCTGAAGGCTCAATTGTACTTCATACTGCTTTA
GGAACAAATTTTTCTTTTGTTTGCAGTAATTCCTCAGATCCTCACTTAGCCACCTTTGCCATACCTCTGGGTGCTATCC
AAGTACCCTATTATTGTTTTCTTAAAGTGGATACTTACAACTCCACTGTTTATAAATTCTTGGCTGTTTTACCTCCTAC
CGTCAGGGAAATTGTCATCACCAAGTATGGTGATGTTTATGTCAATGGGTTTGGCTACTTGCATCTCGGTTTGTTGGAT

GCTGTCACAATTAATTTCACTGGTCATGGCACTGACGATGACGTTTCTGGTTTTTGGACCATAGCATCGACTAATTTTG
TTGATGCACTTATCGAAGTTCAAGGAACTGCCATTCAGCGTATTCTTTATTGTGATGATCCTGTTAGCCAACTCAAGTG
TTCTCAGGTTGCTTTTGACCTTGACGATGGTTTTTACCCTATTTCCTCTACAAACCTTCTGAGTCATGAACAGCCAACT
TCTTTTGTTACTTTGCCATCATTTAATGATCATTCTTTTGTTAATATTACTGTCTCTGCTGCTTTTGGTGGTCATAGTG
GTGCCAACCTTATTGCATCTGACACTACTATCAATGGGTTTAGTTCTTTCTGTGTTGACACTAGACAATTTACCATTTC
ACTGTTTTATAACGTTACAAACAGTTATGGTTACGTGTCTAAATCACAGGACAGTAATTGCCCTTTTACCTTGCAATCT
GTTAATGATTACCTGTCTTTTAGCAAATTTTGTGTTTCTACCAGCCTTTTGGCTAGTGCCTGTACCATAGATCTTTTTG
GTTACCCTGAGTTTGGTAGTGGTGTTAAGTTCACGTCCCTTTACTTTCAATTCACAAAGGGTGAGTTGATTACTGGCAC
GCCTAAACCACTTGAAGGTGTTACGGACGTTTCTTTTATGACTCTGGATGTGTGTACCAAGTATACTATCTATGGCTTT
AAAGGTGAGGGTATCATTACCCTTACAAATTCTAGCTTTTTGGCAGGTGTTTATTATACATCTGATTCTGGACAGTTGT
TAGCTTTTAAGAATGTCACTAGTGGTGCTGTTTATTCTGTTACGCCATGTTCTTTTTCAGAGCCGGCTGCATATGTTGA
TGATGATATAGTGGGTGTTATTTCTAGTTTGTCTAACTCCACTTTTAACAGTACTAGGGAGTTGCCTGGTTTCTTCTAC
CATTCTAATGATGGCTCTAATTGTACAGAGCCTGTGTTGGTGTATAGTAACATAGGTGTTTGTAAATCTGGCAGTATTG
GCTATGTCCCATCTCAGTCTGGCCAAGTCAAGATTGCACCCACGGTTACTGGGAATATTAGTATTCCCACCAACTTTAG
TATGAGTATTAGGACAGAATATTTACAGCTTTACAACACGCCTGTTAGTGTTGATTGTGCTACATATGTTTGTAATGGT
AACTCTCGTTGTAAACAATTACTCACCCAGTACACTGCAGCATGTAAGACCATAGAGTCAGCATTACAACTCAGCGCTA
GGCTTGAGTCTGCTGAAGTCAACTCTATGCTTACTATTTCTGAAGAGGCTCTACAGTTAGCTACCATCAGTTCGTTTAA
TGGTGATGGATATAATTTTACTAATGTGCTGGGTGTTTCCGTGTATGACCCTGCAAGTGGCAGGGTGGTACAAAAAAGG
TCTTTCATTGAAGACCTGCTTTTTAATAAAGTGGTTACTAATGGCCTTGGTACTGTTGATGAAGACTATAAGCGCTGTT
CTAATGGCCGCTCTGTGGCAGATTTAGTCTGTGCGCAGTATTACTCTGGTGTCATGGTACTACCTGGTGTTGTTGACGC
CGAGAAGCTTCATATGTATAGTGCGTCTCTCATCGGTGGTATGGTGCTAGGAGGTTTTACTGCAGCAGCGGCATTGCCT
TTTAGCTATGCTGTTCAAGCGAGACTGAATTATCTTGCTCTACAGACGGATGTTCTACAGCGGAACCAGCAATTGCTTG
CTGAGTCTTTTAATTCTGCTATTGGTAATATAACTTCAGTCTTTGAGAGTGTTAAAGAGGCTATTAGTCAAACTTCTAA
GGGTTTGAACACTGTGGCTCATGCGCTTACCAGGGTTCAAGAGGTTGTTAATTCGCAAGGTGCAGCTTTGACCCAACTT
ACCGTACAGCTGCAACACGACTTCCAAGCCATTTCTAGTTCTATTGATGACATTTACTTTCGACTTGACATTCATTCAG
CCGATGTTCAGTTAGACGGTCTCATCCCTGGCGAATTATCAGCACTTAATGCTTTTGTTGCTCAAACCCTTACTAAGTA
TACTGAGGTTCAGGCTAGCAGGAAACTAGCACAGCAAAAGGTTAATGAGTGCGTTAAATCGCAATCTCAGCGTTATGGT
TTTTGTGGTGGTGATGGCGAGCACATTTTCTCTCTGGTACAGGCCGCACCTCAAGGCCTGCTGTTTTTACACACAGTAC
TTGTACCGGGTGACTTTGTAAATGTTATTGCCATCGCTGGCTTATGTGTTAACGATGAAATTGCCTTGACTCTACGTGA
GCCTGGTTTAGTCTTGTTTACGCATGAACTTCAAGATACTGCGACGGAATATTTTGTTTCATCGCGACGTATGTATGAA
CCTAGAAAACCTACCGTTGGTGATTTTGTTCAAATTGAGAGTTGTGTGGTCACCTATGTCAATTTGACTAGAGACCAAC
TACCAGAAGTAATCCCAGATTACATCGATGTTAACAAAACACTTGATGAGATTTTAGCCTCTCTGCCCAATAGAACTGG
TCCAAGTCTTTCTTTAGATGTTTTTAATGCCACTTATCTTAATCTCACTGGTGAAATTGCAGATTTAGAGCAGCGTTCA
GAGTCTCTCCGTAATACTACAGAAGAGCTCCAAAGTCTTATATATAATATCAACAACACACTAGTTGACCTTGAGTGGC
TCAACCGAGTCGAGACATATATCAAGTGGCCGTGGTGGGTTTGGTTGATTATTTTTATTGTTCTCATCTTTGTTGTGTC
ATTACTAGTGTTCTGCTGCATTTCCACGGGTTGTTGTGGATGCTGCGGCTGCTGTGGTGCTTGTTTTTCAGGTTGTTGT
AGGGGTCCTAGACTTCAACCTTACGAAGCTTTTGAAAAGGTCCACGTGCAGTGA

>gi|410505|emb|Z25483.1| Porcine epidemic diarrhea virus spike protein mRNA, complete CDS

ATGAGGTCTTTAATTTACTTCTGGTTGCTCTTACCAGTACTTCCAACACTCAGCCTACCACAAGATGTCACTAGG
TGCCAGTCTACTACTAACTTTAGGCGGTTCTTTTCAAAATTTAATGTTCAGGCACCTGCCGTCGTCGTTTTGGGTGGTT

ACCTACCTAGTATGAACTCTTCTAGCTGGTACTGTGGCACAGGCATTGAAACTGCTAGTGGCGTTCATGGTATTTTTCT
CAGCTACATCGATTCTGGTCAGGGCTTTGAGATTGGCATTTCGCAAGAGCCGTTTGATCCTAGTGGTTACCAGCTTTAT
TTACATAAGGCCACTAATGGTAACACTAATGCTACTGCACGACTGCGCATTTGCCAGTTTCCCGATAATAAAACATTGG
GCCCTACTGTTAATGATGTTACAACAGGTCGTAACTGCCTATTCAACAAAGCCATTCCAGCTTATATGCGTGATGGAAA
AGATATTGTTGTCGGCATAACATGGGATAATGATCGTGTCACTGTTTTTGCTGACAAGATCTATCATTTTTATCTTAAA
AATGATTGGTCCCGCGTTGCGACAAGATGTTACAATCGCAGAAGTTGTGCTATGCAATATGTTTATACACCTACCTACT
ACATGCTTAATGTTACTAGTGCAGGTGAGGATGGCATTTATTATGAACCCTGTACAGCTAATTGCACTGGTTACGCTGC
CAATGTATTTGCCACTGATTCCAATGGCCATATACCAGAAGGTTTTAGTTTTAATAATTGGTTTCTTTTATCCAATGAC
TCCACTTTGTTGCATGGTAAAGTGGTTTCCAACCAACCCTTGTTGGTCAATTGTCTTTTGGCCATTCCTAAGATTTATG
GACTAGGCCAATTTTTCTCATTCAATCACACGATGGATGGCGTTTGTAATGGAGCTGCTGTGGATCGTGCCCCAGAGGC
TCTGAGGTTTAATATTAATGACACCTCCGTCATTCTTGCTGAAGGCTCAATTGTACTTCATACTGCTTTAGGAACAAAT
CTTTCTTTTGTTTGCAGTAATTCCTCAGATCCTCATTTAGCCATCTTTGCCATACCTCTGGGTGCTACTGAAGTACCCT
ACTATTGCTTTCTTAAAGTGGATACTTACAACTCCACTGTTTATAAATTCTTGGCTGTTTTACCTTCTACTGTCAGGGA
AATTGTCATCACCAAGTATGGTGATGTTTATGTCAATGGGTTTGGCTATTTGCATCTCGGTTTGTTGGATGCTGTCACA
ATTTATTTCACTGGTCATGGCACTGACGATGACGTTTCAGGTTTCTGGACCATAGCATCGACTAATTTTGTTGATGCAC
TCATCGAGGTTCAAGGAACTTCCATTCAGCGTATTCTTTATTGTGATGATCCTGTTAGCCAACTCAAGTGTTCTCAGGT
TGCTTTTGACCTTGACGATGGTTTTTACCCCATCTCTTCTAGAAACCTTCTGAGTCACGAACAGCCAATTTCTTTTGTT
ACTTTGCCATCATTTAATGATCATTCTTTTGTTAATATTACTGTCTCTGCGGCTTTTGGTGGTCTTAGTAGTGCCAATC
TCGTTGCATCTGACACTACTATCAATGGGTTTAGTTCTTTCTGTGTTGACACTAGACAATTTACCATTACACTGTTTTA
TAATGTTACAAACAGTTATGGTTATGTGTCTAAATCACAGGATAGTAATTGTCCTTTCACCTTGCAATCTGTTAATGAT
TACCTGTCTTTTAGCAAATTTTGTGTTTCAACCAGCCTTTTGGCTGGTGCTTGTACCATAGATCTTTTTGGTTACCCTG
CGTTCGGTAGTGGTGTTAAGTTGACGTCCCTTTATTTTCAATTCACAAAAGGTGAGTTGATTACTGGCACGCCTAAACC
ACTTGAAGGTATCACAGACGTTTCTTTTATGACTCTGGATGTGTGTACCAAGTATACTATCTATGGCTTTAAAGGTGAG
GGTATTATTACCCTTACAAATTCTAGCATTTTGGCAGGTGTTTATTATACATCTGATTCTGGACAGTTGTTAGCCTTTA
AGAATGTCACTAGTGGTGCTGTTTATTCTGTCACGCCATGTTCTTTTTCAGAGCAGGCTGCATATGTTAATGATGATAT
AGTGGGTGTTATTTCTAGTTTGTCTAACTCCACTTTTAACAATACTAGGGAGTTGCCTGGTTTCTTCTACCATTCTAAT
GACGGCTCCAATTGTACAGAGCCTGTGTTGGTGTATAGTAACATAGGTGTTTGTAAATCTGGCAGTATTGGCTATGTTC
CATCTCAGTATGGCCAAGTCAAGATTGCACCCACGGTTACTGGGAATATTAGTATTCCCACCAACTTTAGTATGAGTAT
TAGAACAGAATATTTACAGCTTTACAACACGCCTGTTAGTGTTGATTGTGCTACATATGTTTGTAATGGTAACTCTCGT
TGTAAACAATTACTCACCCAGTACACTGCAGCATGTAAGACCATAGAGTCAGCATTACAACTCAGCGCTAGGCTTGAGT
CTGTTGAAGTTAACTCTATGCTTACCATTTCTGAAGAGGCTTTACAGTTAGCTACCATCAGTTCGTTTAATGGTGATGG
ATATAACTTTACTAATGTGCTGGGTGCTTCCGTGTACGATCCTGCAAGTGGCAGGGTGGTACAAAAAAGGTCTGTTATT
GAAGACTTGCTTTTTAATAAAGTGGTTACTAATGGCCTTGGTACTGTTGATGAAGACTATAAGCGCTGTTCTAATGGTC
GCTCTGTGGCTGATCTAGTCTGTGCGCAGTATTACTCTGGTGTCATGGTACTACCTGGCGTTGTTGACGCTGAGAAGCT
TCACATGTACAGTGCGTCTCTCATAGGTGGTATGGCGCTAGGAGGTATAACTGCTGCAGCGGCATTGCCTTTTAGCTAT
GCTGTTCAAGCGAGACTCAATTATCTTGCTTTACAGACGGATGTTCTACAGCGGAACCAGCAATTGCTTGCTGAGTCTT
TTAACTCTGCTATTGGTAATATAACTTCAGCCTTTGAGAGTGTTAAAGAGGCTATTAGTCAAACTTCCAAGGGTTTGAA
CACTGTGGCTCATGCGCTTACTAAGGTTCAAGAGGTTGTTAATTCGCAGGGTTCAGCTTTGAACCAACTTACCGTACAG
CTGCAACACAACTTCCAAGCCATTTCTAGTTCTATTGATGACATTTATTCCCGACTGGACATTCTTTTAGCCGATGTTC
AGGTTGATCGTCTCATCACCGGCAGATTATCAGCACTTAATGCTTTTGTTGCCCAAACCCTCACTAAGTATACTGAGGT
TCAGGCTAGCAGGAAGCTAGCACAGCAAAAGGTTAATGAGTGCGTCAAATCGCAATCTCAGCGTTACGGTTTTTGTGGT
GGTGATGGCGAGCACATTTTCTCTCTGGTACAGGCCGCACCTCAGGGCCTGCTGTTCTTACATACAGTACTTGTACCGG
GTGATTTTGTAAATGTTCTTGCCATCGCTGGCTTATGCGTTAATGGTGAAATTGCCTTGACTCTACGTGAGCCTGGCTT
AGTCTTGTTTACGCATGAACTTCAAACTTATACTGCGACGGAATATTTTGTTTCATCGCGACGTATGTTTGAACCTAGA

AAACCTACCGTTAGTGATTTTGTTCAAATTGAGAGTTGTGTGGTCACCTATGTCAATCTGACTAGCGACCAGCTACCAG
ATGTAATCCCAGATTACATCGATGTTAACAAAACACTTGATGAGATTTTAGCTTCTCTGCCCAATAGAACTGGTCCAAG
TCTTCCCCTAGATGTTTTTAATGCCACTTATCTTAATCTTACTGGTGAAATTGCAGATCTAGAGCAGCGTTCAGAGTCT
CTCCGTAATACTACAGAAGAGCTCCGAAGTCTCATTAACAACATCAACAACACACTTGTTGACCTTGAGTGGCTCAACC
GAGTTGAGACATACATCAAGTGGCCGTGGTGGGTTTGGTTGATCATTGTTATTGTTCTCATCTTTGTTGTGTCATTACT
AGTGTTCTGCTGCATTTCCACGGGTTGTTGTGGATGCTGCGGTTGCTGCGGTGCTTGTTTTTCAGGTTGTTGTAGGGGT
CCTAGACTTCAACCTTACGAAGCTTTTGAAAAGGTCCACGTGCAGTGA

>gi|13752444|gb|AF353511.1| Porcine epidemic diarrhea virus strain CV777, complete genome

ATGAGGTCTTTAATTTACTTCTGGTTGCTCTTACCAGTACTTCCAACACTCAGCCTACCACAAGATGTCACTAGG
TGCCAGTCTACTACTAACTTTAGGCGGTTCTTTTCAAAATTTAATGTTCAGGCACCTGCCGTCGTCGTTTTGGGTGGTT
ACCTACCTAGTATGAACTCTTCTAGCTGGTACTGTGGCACAGGCATTGAAACTGCTAGTGGCGTTCATGGTATTTTTCT
CAGCTACATCGATTCTGGTCAGGGCTTTGAGATTGGCATTTCGCAAGAGCCGTTTGATCCTAGTGGTTACCAGCTTTAT
TTACATAAGGCCACTAATGGTAACACTAATGCTATTGCACGACTGCGCATTTGCCAGTTTCCCGATAATAAAACATTGG
GCCCTACTGTTAATGATGTTACAACAGGTCGTAACTGCCTATTCAACAAAGCCATTCCAGCTTATATGCGTGATGGAAA
AGATATTGTTGTCGGCATAACATGGGATAATGATCGTGTCACTGTTTTTGCTGACAAGATCTATCATTTTTATCTTAAA
AATGATTGGTCCCGCGTTGCGACAAGATGTTACAATCGCAGAAGTTGTGCTATGCAATATGTTTATACACCTACCTACT
ACATGCTTAATGTTACTAGTGCAGGTGAGGATGGCATTTATTATGAACCCTGTACAGCTAATTGCACTGGTTACGCTGC
CAATGTATTTGCCACTGATTCCAATGGCCATATACCAGAAGGTTTTAGTTTTAATAATTGGTTTCTTTTATCCAATGAC
TCCACTTTGTTGCATGGTAAAGTGGTTTCCAACCAACCCTTGTTGGTCAATTGTCTTTTGGCCATTCCTAAGATTTATG
GACTAGGCCAATTTTTCTCATTCAATCACACGATGGATGGCGTTTGTAATGGAGCTGCTGTGGATCGTGCCCCAGAGGC
TCTGAGGTTTAATATTAATGACACCTCCGTCATTCTTGCTGAAGGCTCAATTGTACTTCATACTGCTTTAGGAACAAAT
CTTTCTTTTGTTTGCAGTAATTCCTCAGATCCTCATTTAGCCATCTTTGCCATACCTCTGGGTGCTACTGAAGTACCCT
ACTATTGCTTTCTTAAAGTGGATACTTACAACTCCACTGTTTATAAATTCTTGGCTGTTTTACCTCCTACTGTCAGGGA
AATTGTCATCACCAAGTATGGTGATGTTTATGTCAATGGGTTTGGCTATTTGCATCTCGGTTTGTTGGATGCTGTCACA
ATTAATTTCACTGGTCATGGCACTGACGATGACGTTTCAGGTTTCTGGACCATAGCATCGACTAATTTTGTTGATGCAC
TCATCGAGGTTCAAGGAACTTCCATTCAGCGTATTCTTTATTGTGATGATCCTGTTAGCCAACTCAAGTGTTCTCAGGT
TGCTTTTGACCTTGACGATGGTTTTTACCCCATCTCTTCTAGAAACCTTCTGAGTCACGAACAGCCAATTTCTTTTGTT
ACTTTGCCATCATTTAATGATCATTCTTTTGTTAATATTACTGTCTCTGCGGCTTTTGGTGGTCTTAGTAGTGCCAATC
TCGTTGCATCTGACACTACTATCAATGGGTTTAGTTCTTTCTGTGTTGACACTAGACAATTTACCATTACACTGTTTTA
TAATGTTACAAACAGTTATGGTTATGTGTCTAAATCACAGGATAGTAATTGTCCTTTCACCTTGCAATCTGTTAATGAT
TACCTGTCTTTTAGCAAATTTTGTGTTTCAACCAGCCTTTTGGCTGGTGCTTGTACCATAGATCTTTTTGGTTACCCTG
CGTTCGGTAGTGGTGTTAAGTTGACGTCCCTTTATTTTCAATTCACAAAAGGTGAGTTGATTACTGGCACGCCTAAACC
ACTTGAAGGTATCACAGACGTTTCTTTTATGACTCTGGATGTGTGTACCAAGTATACTATCTATGGCTTTAAAGGTGAG
GGTATTATTACCCTTACAAATTCTAGCATTTTGGCAGGTGTTTATTATACATCTGATTCTGGACAGTTGTTAGCCTTTA
AGAATGTCACTAGTGGTGCTGTTTATTCTGTCACGCCATGTTCTTTTTCAGAGCAGGCTGCATATGTTAATGATGATAT
AGTGGGTGTTATTTCTAGTTTGTCTAACTCCACTTTTAACAATACTAGGGAGTTGCCTGGTTTCTTCTACCATTCTAAT
GACGGCTCCAATTGTACAGAGCCTGTGTTGGTGTATAGTAACATAGGTGTTTGTAAATCTGGCAGTATTGGCTATGTTC
CATCTCAGTATGGCCAAGTCAAGATTGCACCCACGGTTACTGGGAATATTAGTATTCCCACCAACTTTAGTATGAGTAT
TAGAACAGAATATTTACAGCTTTACAACACGCCTGTTAGTGTTGATTGTGCTACATATGTTTGTAATGGTAACTCTCGT
TGTAAACAATTACTCACCCAGTACACTGCAGCATGTAAGACCATAGAGTCAGCATTACAACTCAGCGCTAGGCTTGAGT

CTGTTGAAGTTAACTCTATGCTTACCATTTCTGAAGAGGCTTTACAGTTAGCTACCATCAGTTCGTTTAATGGTGATGG
ATATAACTTTACTAATGTGCTGGGTGCTTCCGTGTACGATCCTGCAAGTGGCAGGGTGGTACAAAAAAGGTCTGTTATT
GAAGACTTGCTTTTTAATAAAGTGGTTACTAATGGCCTTGGTACTGTTGATGAAGACTATAAGCGCTGTTCTAATGGTC
GCTCTGTGGCTGATCTAGTCTGTGCGCAGTATTACTCTGGTGTCATGGTACTACCTGGCGTTGTTGACGCTGAGAAGCT
TCACATGTACAGTGCGTCTCTCATAGGTGGTATGGCGCTAGGAGGTATAACTGCTGCAGCGGCATTGCCTTTTAGCTAT
GCTGTTCAAGCGAGACTCAATTATCTTGCTTTACAGACGGATGTTCTACAGCGGAACCAGCAATTGCTTGCTGAGTCTT
TTAACTCTGCTATTGGTAATATAACTTCAGCCTTTGAGAGTGTTAAAGAGGCTATTAGTCAAACTTCCAAGGGTTTGAA
CACTGTGGCTCATGCGCTTACTAAGGTTCAAGAGGTTGTTAATTCGCAGGGTTCAGCTTTGAACCAACTTACCGTACAG
CTGCAACACAACTTCCAAGCCATTTCTAGTTCTATTGATGACATTTATTCCCGACTGGACATTCTTTCAGCCGATGTTC
AGGTTGATCGTCTCATCACCGGCAGATTATCAGCACTTAATGCTTTTGTTGCCCAAACCCTCACTAAGTATACTGAGGT
TCAGGCTAGCAGGAAGCTAGCACAGCAAAAGGTTAATGAGTGCGTCAAATCGCAATCTCAGCGTTACGGTTTTTGTGGT
GGTGATGGCGAGCACATTTTCTCTCTGGTACAGGCCGCACCTCAGGGCCTGCTGTTCTTACATACAGTACTTGTACCGG
GTGATTTTGTAAATGTTCTTGCCATCGCTGGCTTATGCGTTAATGGTGAAATTGCCTTGACTCTACGTGAGCCTGGCTT
AGTCTTGTTTACGCATGAACTTCAAACTTATACTGCGACGGAATATTTTGTTTCATCGCGACGTATGTTTGAACCTAGA
AAACCTACCGTTAGTGATTTTGTTCAAATTGAGAGTTGTGTGGTCACCTATGTCAATCTGACTAGCGACCAGCTACCAG
ATGTAATCCCAGATTACATCGATGTTAACAAAACACTTGATGAGATTTTAGCTTCTCTGCCCAATAGAACTGGTCCAAG
TCTTCCCCTAGATGTTTTTAATGCCACTTATCTTAATCTTACTGGTGAAATTGCAGATCTAGAGCAGCGTTCAGAGTCT
CTCCGTAATACTACAGAAGAGCTCCGAAGTCTCATTAACAACATCAACAACACACTTGTTGACCTTGAGTGGCTCAACC
GAGTTGAGACATACATCAAGTGGCCGTGGTGGGTTTGGTTGATCATTGTTATTGTTCTCATCTTTGTTGTGTCATTACT
AGTGTTCTGCTGCATTTCCACGGGTTGTTGTGGATGCTGCGGTTGCTGCGGTGCTTGTTTTTCAGGTTGTTGTAGGGGT
CCTAGACTTCAACCTTACGAAGCTTTTGAAAAGGTCCACGTGCAGTGA

>gi|326486529|gb|GU937797.1| Porcine epidemic diarrhea virus isolate SM98, complete genome

ATGAGGTCTTTAATTTACTTCTGGTTGCTCTTACCAGTACTTCCAACACTCAGCCTACCACAAGATGTCACTAGG
TGCCAGTCTACTACTAACTTTAGGCGGTTCTTTTCAAAATTTAATGTTCAGGCACCTGCCGTCGTCGTTTTGGGTGGTT
ACCTACCTAGTATGAACTCTTCTAGCTGGTACTGTGGCACAGGCATTGAAACTGCTAGTGGCGTTCATGGTATTTTTCT
CAGCTACATCGATTCTAGTCAGGGCTTTGAGATTGGCATTTCGCAAGAGCCGTTTGATCCTAGTGGTTACCAGCTTTAT
TTACATAAGGCCACTAATGAGAGTCAGAATGGTAACACTAATGCTATTGCACGACTGCGCATTTGCCAGTTTCCCGATA
ATAAAACATTGGGCCCTACTGTTAATGATGTTACAACAGGTCGTAACTGCCTATTCAACAAAGCCATTCCAGCTTATAT
GCGTGATGGAAAAGATATTGTTGTCGGCATAACATGGGATAATGATCGTGTCACTGTTTTTGCTGACAAGATCTATCAT
TTTTATCTTAAAAATGATTGGTCCCGCGTTGCGACAAGATGTTACAATCGCAGAAGTTGTGCTATGCAATATGTTTATA
CACCTACCTACTACATGCTTAATGTTACTAGTGCAGGTGAGGATGGCATTTATTATGAACCCTGTACAGCTAATTGCAC
TGGTTACGCTGCCAATGTATTTGCCACTGATTCCAATGGCCATATACCAGAATGTTTTAGTTTTAATAATTGGTTTCTT
TTATCCAATGACTCCACTTTGTTGCATGGTAAAGTGGTTTCCAACCAACCCTTGTTGGTCAATTGTCTTTTGGCCATTC
CTAAGATTTATGGACTAGGCCAATTTTTCTCATTCAATCACACGATGGATGGCGTTTGTAATGGAGCTGCTGTGGATCG
TGCCCCAGAGGCTCTGAGGTTTAATATTAATGACACCTCCGTCATTCTTGCTGAAGGCTCAATTGTACTTCATACTGCT
TTAGGAACAAATCTTTCTTTTGTTTGCAGTAATTCCTCAGATCCTCATTTAGCCATCTTTGCCATACCTCTGGGTGCTA
CTGAAGTACCCTACTATTGCTTTCTTAAAGTGGATACTTACAACTCCACTGTTTATAAATTCTTGGCTGTTTTACCTCC
TACTGTCAGGGAAATTGTCATCACCAAGTATGGTGATGTTTATGTCAATGGGTTTGGCTATTTGCATCTCGGTTTGTTG
GATGCTGTCACAATTAATTTCACTGGTCATGGCACTGACGATGACGTTTCAGGTTTCTGGACCATAGCATCGACTAATT
TTGTTGATGCACTCATCGAGGTTCAAGGAACTTCCATTCAGCGTATTCTTTATTGTGATGATCCTGTTAGCCAACTCAA

GTGTTCTCAGGTTGCTTTTGACCTTGACGATGGTTTTACCCCATCTCTTCTAGAAACCTTCTGAGTCACGAACAGCCA
ATTTCTTTTGTTACTTTGCCATTATTTAATGATCATTCTTTTGTTAATATTACTGTCTCTGCGGCTTTTGGTGGTCTTA
GTAGTGCCAATCTCGTTGCATCTGACACTACTATCAATGGGTTTAGTTCTTTCTGTGTTGACACTAGACAATTTACCAT
TACACTGTTTTATAATGTTACAAACAGTTATGGTTATGTGTCTAAATCACAGGATAGTAATTGTCCTTTCACCTTGCAA
TCTGTTAATGATTACCTGTCTTTTAGCAAATTTTGTGTTTCAACCAGCCTTTTGGCTGGTGCTTGTACCATAGATCTTT
TTGGTTACCCTGCGTTCGGTAGTGGTGTTAAGTTGACGTCCCTTTATTTTCAATTCACAAAAGGTGAGTTGATTACTGG
CACGCCTAAACCACTTGAAGGTATCACAGACGTTTCTTTTATGACTCTGGATGTGTGTACCAAGTATACTATCTATGGC
TTTAAAGGTGAGGGTATTATTACCCTTACAAATTCTAGCATTTTGGCAGGTGTTTATTATACATCTGATTCTGGACAGT
TGTTAGCCTTTAAGAATGTCACTAGTGGTGCTGTTTATTCTGTCACGCCATGTTCTTTTTCAGAGCAGGCTGCATATGT
TAATGATGATATAGTGGGTGTTATTTCTAGTTTGTCTAACTCCACTTTTAGCAATACTAGGGAGTTGCCTGGTTTCTTC
TACCATTCTAATGACGGCTCCAATTGTACAGAGCCTGTGTTGGTGTATAGTAACATAGGTGTTTGTAAATCTGGCAGTA
TTGGCTATGTTCCACCTCAGTATGGCCAAGTCAAGATTGCACCCACGGTTACTGGGAATATTAGTATTCCCACCAACTT
TAGTATGAGTATTAGAACAGAATATTTACAGCTTTACAACACGCTTGTTAGTGTTGATTGTGCTACATATGTTTGTAAT
GGTAACTCTCGTTGTAAACAATTACTCACCCAGTACACTGCAGCATGTAAGACCATAGAGTCAGCATTACAACTCAGCG
CTAGGCTTGAGTCTGTTGAAGTTAACTCTATGCTTACCATTTCTGAAGAGGCTTTACAGTTAGCTACCATCAGTTCGTT
TAATGGTGATGGATATAACTTTACTAATGTGCTGGGTGCTTCCGTGTACGATCCTGCAAGTGGCAGGGTGGTACAAAAA
AGGTCTGTTATTGAAGACTTGCTTTTTAATAAAGTGGTTACTAATGGCCTTGGTACTGTTGATGAAGACTATAAGCGCT
GTTCTAATGGTCGCTCTGTGGCTGATCTAGTCTGTGCGCAGTATTACTCTGGTGTCATGGTACTACCTGGCGTTGTTGA
CGCTGAGAAGCTTCACATGTACAGTGCGTCTCTCATAGGTGGTATGGCGCTAGGAGGTATAACTGCTGCAACGGCATTG
CCTTTTAGCTATGCTGTTCAAGCGAGACTCAATTATCTTGCTTTACAGACGGATGTTCTACAGCGGAACCAGCAATTGC
TTGCTGAGTCTTTTAACTCTGCTATTGGTAATATAACTTCAGCCTTTGAGAGTGTTAAAGAGGCTATTAGTCAAACTTC
CAAGGGTTTGAACACTGTGGCTCATGCGCTTACTAAGGTTCAAGAGGTTGTTAATTCGCAGGGTTCAGCTTTGAACCAA
CTTACCGTACAGCTGCAACACAACTTCCAAGCCATTTCTAGTTCTATTGATGACATTTATTCCCGACTGGACATTCTTT
CAGCCGATGTTCAGGTTGATCGTCTCATCAACGGCAGATTATCAGCACTTAATGCTTTTGTTGCCCAAACCCTCACTAA
GTATACTGAGGTTCAGGCTAGCAGGAAGCTAGCACAGCAAAAGGTTAATGAGTGCGTCAAATCGCAATCTCAGCGTTAC
GGTTTTTGTGGTGGTGATGGCGAGCACATTTTCTCTCTGGTACAGGCCGCACCTCAGGGCCTGCTGTTCTTACATACAG
TACTTGTACCGGGTGATTTTGTAAATGTTCTTGCCATCGCTGGCTTATGCGTTAATGGTGAAATTGCCTTGACTCTACG
CGAGCCTGGCTTAGTCTTGTTTACGCATGAACTTCAAAATTATACTGCGACGGAATATTTTGTTTCATCGCGACGTATG
TTTGAACCTAGAAAACCTACCGTTAGTGATTTTGTTCAAATTGAGAGTTGTGTGGTCACCTATGTCAATCTGACTAGCG
ACCAGCTACCAGATGTAATCCCAGATTACATCGATGTTAACAAAACACTTGATGAGATTTTAGCTTCTCTGCCCAATAG
AAATGGTCCAAGTCTTTCCCTAGATGTTTTTAATGCCACTTATCCTAATCTTACTGGTGAAATTGCAGATCTAGAGCAG
CGTTCAGAGTCTCTCCGTAATACTACAGAAGAGCTCCGAAGTCTCATTAACAACATCAACAACACACTTGTTGACCTTG
AGTGGCTCAACCGAGTTGAGACATACATCAAGTGGCCGTGGTGGGTTTGGTTGATCATTGTTATTGTTCTCATCTTTGT
TGTGTCATTACTAGTGTTCTGCTGCATTTCCACGGGTTGTTGTGGATGCTGCGGTTGCTGCGGTGCTTGTTTTTCAGGT
TGTTGTAGGGGTCCTAGACTTCAACCTTACGAAGCTTGA

>gi|380851043|gb|JQ023161.1| Porcine epidemic diarrhea virus strain virulent DR13, complete genome

ATGAGGTCTTTAATTTACTTCTGGTTGTTCTTACCAGTACTTCTAACACTTAGCCTACCACAAGATGTCACTAGG
TGCCAGTCTACTATTAACTTTAGGCGGTTCTTTTCAAAATTTAATGTTCAGGCACCTGCCGTCGTTGTTTTGGGTGGTT
ATCTACCTAGTATGAACTCTTCTAGCTGGTACTGTGGCACAGGCATTGAAACTGATAGTGGCGTTCATGGTATTTTTCT
CAGTTACATCGATTCTGGTCAGGGCTTTGAGATTGGCATTTCGCAAGAGCCGTTTGATCCTAGTGGTTACCAGCTTTAT

TTACATAAGGCCACTAATGGTAACACTAATGCTATTGCACGACTGCGCATTTGCCAGTTTCCAGATAATAAAACATTGG
GCCCTACTGTTAATGATGTTACAACAGGTCGTAACTGCCTATTCAACAAAGCCATTCCAGCTTATTTGCAGGATGGAAA
AAATATTGTTGTCGGCATAACATGGGATAATGATCGTGTCACTGTTTTTGCTGACAAGATCTATCATTTTTATCTTAAA
AATGATTGGTCCCGTGTTGCGACAAGATGTTACAATAAAAGAAGTTGTGCTATGCAATATGTTTATACACCTACCTACT
ACATGCTTAATGTTACTAGTGCAGGTGAGGATGGCATTTATTATGAACCCTGTACAGCTAATTGCAGTGGTTACGCTGC
CAATGTATTTGCCACTGATTCCAATGGCCATATACCAGAAGGTTTTAGTTTTAATAATTGGTTTCTTTTGTCCAATGAC
TCCACTTTGTTGCATGGTAAAGTGGTTTCCAACCAACCTTTGTTGGTCAATTGTCTTTTGGCCATTCCTAAGATTTATG
GACTAGGCCAATTTTTCTCATTCAATCAAACGATGGATGGCGTTTGTAATGGAGCTGCTGCGCAGCGTGCCCCAGAGGC
TCTGAGGTTTAATATTAATGACACCTCCGTCATTCTTGCTGAAGGCTCAATTGTACTTCATACTGCTTTAGGAACAAAT
CTTTCTTTTGTTTGCAGTAATTCCTCAGATCCTCATTTAGCCATCTTTGCCATACCTCTGGGTGCTACTGAAGTACCCT
ACTATTGCTTTCTTAAAGTGGATACTTACAACTCCACTGTTTATAAATTCTTGGCTGTTTTACCTCCTACTGTCAGGGA
AATTGTCATCACCAAGTATGGTGATGTTTATGTCAATGGGTTTGGCTATTTGCATCTCGGTTTGTTGGATGCTGTCACA
ATTAATTTCACTGGTCATGGCACTGACGATGACGTTTCAGGTTTCTGGACCATAGCATCGACTAATTTTGTTGATGCAC
TCATCGAGGTTCAAGGAACTGCCATTCAGCGTATTCTTTATTGTGATGATCCTGTTAGCCAACTCAAGTGTTCTCAGGT
TGCTTTTGACCTTGACGATGGTTTTTACCCTATATCTTCTAGAAACCTTCTGAGTCATGAACAGCCAATTTCTTTTGTT
ACTTTGCCATCATTTAATGATCATTCTTTTGTTAATATTACTGTCTCTGCGGCTTTTGGTGGTCATAGTGGTGCCAACC
TCATTGCATCTGACACTACTATCAATGGGTTTAGTTCTTTCTGTGTTGACACTAGACAATTTACCATTACACTGTTTTA
TAACGTTACAAACAGTTATGGTTATGTGTCTAAATCACAGGATAGTAATTGCCCTTTCACCTTGCAATCTGTTAATGAT
TACCTGTCTTTTAGCAAATTTTGTGTTTCAACCAGCCTTTTGGCTGGTGCTTGTACCATAGATCTTTTTGGTTACCCTG
AGTTCGGTAGTGGTGTTAAGTTTACGTCCCTTTATTTTCAATTCACAAAGGGTGAGTTGATTACTGGCACGCCTAAACC
ACTTGAAGGTGTCACGGACGTTTCTTTTATGACTCTGGATGTGTGTACCAAGTATACTATCTATGGCTTTAAAGGTGAG
GGTATTATTACCCTTACAAATTCTAGCTTTTTGGCAGGTGTTTATTATACATCTGATTCTGGACAGTTGTTAGCCTTTA
AGAATGTCACTAGTGGTGCTGTTTATTCTGTTACGCCATGTTCTTTTTCAGAGCAGGCTGCATATGTTGATGATGATAT
AGTGGGTGTTATTTCTAGTTTGTCTAACTCCACTTTTAACAATACTAGGGAGTTGCCTGGTTTCTTCTACCATTCTAAT
GACGGCTCCAATTGTACAGAGCCTGTGTTGGTGTATAGTAACATAGGTGTTTGTAAATCTGGCAGTATTGGCTATGTCC
CATCTCAGTATGGCCAAGTCAAGATTGCACCCACGGTTACTGGGAATATTAGTATTCCCACCAACTTTAGTATGAGTAT
TAGAACAGAATATTTACAGCTTTACAACACGCCTGTTAGTGTTGATTGTGCTACATATGTTTGTAATGGTAACTCTCGT
TGTAAACAATTACTCACCCAGTACACTGCAGCATGTAAGACCATAGAGTCAGCATTACAACTCAGCGCTAGGCTTGAGT
CTGTTGAAGTTAACTCTATGCTTACTATTTCTGAAGAGGCTCTACAGTTAGCTACCATCAGTTCGTTTAATGGTGATGG
ATATAACTTTACTAATGTGCTGGGTGTTTCCGTGTACGATCCTGCAAGTGGCAGGGTGGTACAAAAAAGGTCTTTTATT
GAAGACCTGCTTTTTAATAAAGTGGTTACTAATGGCCTTGGTACTGTTGATGAAGACTATAAGCGCTGTTCTAATGGTC
GCTCTGTGGCTGATCTAGTCTGTGCGCAGTATTACTCTGGTGTCATGGTACTACCTGGCGTTGTTGACGCTGAGAAGCT
TCACATGTATAGTGCGTCTCTCATCGGTGGTATGGCGCTAGGAGGTATTACTGCTGCAGCGGCATTGCCTTTTAGCTAT
GCTGTTCAAGCGAGACTCAATTATCTTGCTTTACAGACGGATGTTCTACAGCGGAACCAGCAATTGCTTGCTGAGTCTT
TTAACTCTGCTATTGGTAATATAACTTCAGCCTTTGAGAGTGTTAAAGAGGCTATTAGTCAAACTTCCAAGGGTTTGAA
CACTGTGGCTCATGCGCTTACTAAGGTTCAAGAGGTTGTTAATTCGCAGGGTTCAGCTTTGACCCAACTTACCGTACAG
CTGCAACACAACTTCCAAGCCATTTCTAGTTCTATTGATGACATTTATTCCCGACTGGACATTCTTTCAGCCGATGTTC
AGGTTGATCGTCTCATCACCGGCAGATTATCAGCACTTAATGCTTTTGTTGCTCAAACCCTCACTAAGTATACTGAGGT
TCAGGCTAGCAGGAAGCTAGCACAGCAAAAGGTTAATGAGTGCGTCAAATCGCAATCTCAGCGTTATGGTTTTTGTGGT
GGTGATGGCGAGCACATTTTCTCTCTGGTACAGGCCGCACCTCAGGGCCTGCTGTTCTTACATACAGTACTTGTACCGG
GTGATTTTGTAAATGTTATTGCCATCGCTGGCTTATGCGTTAATGGTGATATTGCCTTGACTCTACGTGAGCCTGGCTT
AGTCTTGTTTACGCATGAACTTCAAACTTATACTGCGACGGAATATTTTGTTTCATCGCGACGTATGTTTGAACCTAGA
AAACCTACCGTTAGTGATTTTGTTCAAATTGAGAGTTGTGTGGTCACCTATGTCAATCTGACTAGCGACCAGCTACCAG
ATGTAATCCCAGATTACATCGATGTTAACAAAACACTTGATGAGATTTTAGCTTCTCTGCCCAATAGAACTGGTCCAAG

TCTTCCCCTAGATGTTTTTAATGCCACTTATCTTAATCTCACTGGTGAAATTGCAGATTTAGAGCAGCGTTCAGAGTCT
CTCCGTAATACTACAGAAGAGCTCCGAAGTCTCATATATAACATCAACAACACACTTGTTGACCTTGAGTGGCTCAACC
GAGTTGAGACATATATCAAGTGGCCGTGGTGGGTTTGGTTGATCATTTTTATTGTTCTCATCTTTGTTGTGTCATTACT
AGTGTTCTGCTGCATTTCCACGGGTTGTTGTGGATGCTGCGGTTGCTGCGGTGCTTGTTTTTCAGGTTGTTGTAGGGGT
CCTAGACTTCAACCTTACGAAGCTTTTGAAAAGGTCCACGTGCAGTGA

>gi|380851050|gb|JQ023162.1| Porcine epidemic diarrhea virus strain attenuated DR13, complete genome

ATGACGCCTTTAATTTACTTCTGGTTGTTCTTACCAGTACTTCTAACACTTAGCCTACCACAAGATGTCACTAGG
TGCCAGTCTACTATTAACTTTAGGCGGTTCTTTTCAAAATTTAATGTTCAGGCACCTGCCGTCGTTGTTTTGGGTGGTT
ATCTACCTAGTATGAACTCTTCTAGCTGGTACTGTGGCACAGGCATTGAAACTGATAGTGGCGTTCATGGTATTTTTCT
CAGTTACATCGATTCTGGTCAGGGCTTTGAGATTGGCATTTCGCAAGAGCCGTTTGATCCTAGTGGTTACCAGCTTTAT
TTACACAAGGCCACTAATGGTAACACTAGTGCTATTGCACGACTGCGCATTTGCCAGTTTCCAGATAATAAAACATTGG
GCCCTACTGTTAATGATGTTACAACAGGTCGTAACTGCCTATTCAACAAAGCCATTCCAGCTTTGCAGGATGGAAAAAA
TATTGTTGTCGGCATAACATGGGATAATGATCGTGTCACTGTTTTTGCTGACAAGATCTATCATTTTTATATTAAAAAT
GATTGGTCCCGTGTTGCGACAAGATGTTACAATAAAAGAAGTTGTGCCATGCAATATGTTTATACACCTACCTACTACA
TGCTTAATGTTACTAGTGCAGGTGAGGATGGCATTTACTATGAACCTTGTACAGCTAATTGCAGTGGTTACGCTGCCAA
TGTATTTGCCACTGATTCCAATGGCCATATACCAGAAGGTTTTAGTTTTAATAATTGGTTTCTTTTGTCCAATGACTCC
ACTTTGTTGCATGGTAAAGTGGTTTCAAACCAACCTTTGTTGGTCAACTGCCTTTGGGCCATTCCTAAGATTTATGGAC
TAGGCCAATTTTTCTCATTCAATCAAACGATGGATGGCGTTTGTAACGGAGCCGCTGCGCAGCGTGCCCCAGAGGCTCT
GAGGTTTAATATTAATGACACTTTTGTCATTCTTGCTGAAGGCTCAATTGTACTTCATACTGCTTTAGGAACAAATCTT
TCTTTTGTTTGCAGTAATTCCTCAGATCCTCACAAAGCCATCTTTACCATACCTTTGGGTGTTACTGAAGTACCCTACT
ATTGCTTTCTTAAAGTGGATACTTACAAATCCACTGTTTATAAATTCTTGGCTGTTTTACCTCCTACTGTCAAGGAAAT
TGTCATCACCAAGTACGGTGATGTTTATGTCAACGGGTTTGGCTATTTGCATCTCGGTTTGTTGGATGCTGTCACAATT
AATTTCACTGGTCATGGCACTGACGATGACGTTTCAGGTTTCTGGACCGTAGCATCGACTAATTTTGTTGATGCACTCA
TCGAGGTTCAAGGAACTGCCATTCAGCGTATTCTTTATTGTGATGACCCTGTTAGCCAACTTAAGTGTTCTCAGGTTGC
TTTTGACCTTGATGATGGTTTTTACCCTATTTCTTCTAGAAACCTTCTGAGTCATGAACAGCCAATTTCTTTTGTTACT
TTGCCATCATTCAATGATCATTCTTTTGTTAATATTACTGTCTCTGCGGCTTTTGGTGGTCATAGTGGTGCCAACCTCA
TTGCATCTGACACTACTATCAATGGGTTTAGTTCTTTCTGTGTTGACACTAGACAATTTACCATTACACTGTTTTATAA
CGTTACAAACAGTTATGGTTATGTGTCTAAGTCACAGGATAGTAATTGCCCTTTCACCTTGCAATCTGTTAATGATTAC
CTGTCTTTTAGCAAATTTTGTGTTTCAACCAGCCTTTTGGCTGGTGCTTGTACCATAGATCTTTTTGGTTACCCTGAGT
TCGGTAGTGGTGTTAAGTTTACGTCCCTTTATTTTCAATTCACAAAGGGTGAGTTGATTACTGGCACGCCTAAACCACT
TCAAGGTGTCACGGACGTTTCTTTTATGACTCTGGATGTGTGTACCAAGTATACTATCTATGGCTTTAAAGGTGAGGGT
ATTATTACCCTTACAAATTCTAGCTTTTTGGCAGGTGTTTATTATACATCTGATTCTGGACAGTTGTTAGCCTTTAAGA
ATGTCACTAGTGGTGCTGTTTATTCTGTTACGCCATGTTCTTTTTCAGAGCAGGCTGCATATGTTGATGATGATATAGT
GGGTGTTATTTCTAGTTTGTCTAACTCCACTTTTAACAATACCAGGGAGTTGCCTGGTTTCTTCTACCATTCTAATGAT
GGCTCCAATTGTACAGAGCCTGTGTTGGTGTATAGTAACATAGGTGTCTGTAAATCTGGCAGTATTGGCTATGTCCCAC
TTCAGGATGGCCAAGTCAAGATTGCACCCATGGTTACTGGGAATATTAGTATTCCCACCAACTTTAGTATGAGTATTAG
AACAGAATATTTACAGCTTTACAACACGCCTGTTAGTGTTGATTGCGTTACATATGTTTGTAATGGTAACTCTCGTTGT
AAACAATTACTCACCCAGTACACTGCAGCATGTAAGACCATAGAGTCAGCATTACAACTCAGCGCTAGGCTTGAGTCTG
TTGAAGTTAACTCTATGCTTACTATTTCTGAAGAGGCTCTACAGTTAGCTACCATCAGTTCGTTTAATGGTGATGGATA
TAACTTTACTAATGTGCTGGGTGTTTCCGTGTACGACCCTGCAAGTGGCAGGGTGGTACAAAAAGGGTCTTTTATTGAA

GACCTGCTTTTTAATAAAGTGGTTACTAATGGCCTTGGTACTGTTGATGAAGACTATAAGCGCTGTTCTAATGGTCGCT
CTGTGGCAGATCTAGTCTGTGCGCAGTATTACTCTGGTGTCATGGTACTACCTGGCGTTGTTGACGCTGAGAAGCTTCA
CATGTATAGTGCGTCTCTCATCGGTGGTATGGCGCTAGGAGGTCTTACTAGTGCAGCGGCATTGCCTTTTAGCCATGCT
GTTCAAGCGAGGCTCAATTATCTTGCTTTACAGACGGATGTTCTACAGCGCAACCAGCAATTGCTTGCTGAGTCTTTTA
ACTCTGCTATTGGTAATATAACTTCAGCCTTTGAGAGTGTTAAAGAGGCTATTAGTCAAACTTCCAATGGTTTGAACAC
TGTGGCTCATGCGCTTACTAAGGTTCAAGAGGTTGTTAATTCGCAGGGTTCAGCTTTGACCCAACTTACCATACAGCTG
CAACACAACTTCCAAGCCATTTCTAGTTCTATTGATGACATTTACTCCCGACTGGACATTCTTTCAGCCGATGTTCAGG
TTGATCGTCTCATCACCGGCAGATTATCAGCACTTAATGCTTTTGTTGCTCAAACCCTCACTAAGTATACTGAGGTTCA
GGCTAGCAGGAAGCTAGCACAGCAAAAGGTTAATGAGTGCGTCAAATCGCAATCTCAGCGTTATGGTTTTTGTGGTGGT
GATGGCGAGCACATCTTCTCTCTGGTACAGGCCGCACCTCAGGGCCTGCTGTTTTTACATACAGTACTTGTACCGGGTG
ATTTTGTAAATGTTATTGCCATCGATGGCTTATGCGTTAATGGTGATATTGCCTTGACTCTACGTGAGCCTGGCTTAGT
CTTGTTTACGCATGAACTTCAAACTTATACTGCGACGGAATATTTTGTTTCATCGCGACGTATGTTTGAACCTAGAAAA
CCTACCGTTAGTGATTTTGTTCAAATTGAGAGTTGTGTGGTCACCTATGTCAATCTGACTAGCGACCAACTACCAGATG
TAATCCCAGATTACATCGATGTTAACAAAACACTTGATGAGATTCTAGCTTCTCTGCCCAATAGAATTGGTCCTAGTCT
TCCCCTAGATGTTTTTAATGCCACTTATCTTAATCTCACTGGTGAAATTGCAGATTTAGAGCAGCGTTCAGAGTCTCTC
CGTAATACTACAGAAGAGCTCCGAAGTCTCATATATAATATCAACAACACACTTGTTGACCTTGAGTGGCTCAACCGAG
TTGAGACATATATCAAGTGGCCGTGGTGGGTTTGGTTGATTATTTTTATTGTTCTCATCTTTGTTGTGTCATTATTAGT
GTTCTGCTGCATTTCCACGGGTTGTTGTGGATGCTGCGGTTGTTGCGGTGCTTGTTTTTCAGGTTGTTGTAGGGGTCCT
AGACTTCAACCTTACGAAGCTTTTGAAAAGGTCCACGTGCAGTGA

>gi|399227061|gb|JX088695.1| Porcine epidemic diarrhea virus strain GD-B, complete genome

ATGAAGTCTTTAACCTACTTCTGGTTGTTCTTACCAGTACTTTCAACACTTAGCCTACCACAAGATGTCACCAGG
TGCTCAGCTAACACTAATTTTAGGCGGTTCTTTTCAAAATTTAATGTTCAGGCGCCTGCAGTTGTTGTACTGGGCGGTT
ATCTACCTATTGGTGAAAACCAGGGTGTCAATTCAACTTGGTACTGTGCTGGCCAACATCCAACTGCTAGTGGCGTTCA
TGGTATCTTTGTTAGCCATATTAGAGGTGGTCATGGCTTTGAGATTGGCATTTCGCAAGAGCCTTTTGACCCTAGTGGT
TACCAGCTTTATTTACATAAGGCTACTAACGGTAACACTAATGCTACTGCGCGACTGCGCATTTGCCAGTTTCCTAGCA
TTAAAACATTGGGCCCCACTGCTAATAATGATGTTACAACAGGTCGTAATTGCCTATTTAACAAAGCCATCCCAGCTCA
TATGAGTGAACATAGTGTTGTCGGCATAACATGGGATAATGATCGTGTCACTGTCTTTTCTGACAAGATCTATTATTTT
TATTTTAAAAATGATTGGTCCCGTGTTGCGACAAAGTGTTACAACAGTGGAGGTTGTGCTATGCAATATGTTTACGAAC
CCACCTATTACATGCTTAATGTTACTAGTGCTGGTGAGGATGGTATCTCTTATCAACCCTGTACAGCTAATTGCATTGG
TTATGCTGCCAATGTATTTGCTACTGAGCCCAATGGCCACATACCAGAAGGTTTTAGTTTTAATAATTGGTTTCTTTTG
TCCAATGATTCCACTTTGGTGCATGGTAAGGTGGTTTCCAACCAACCATTGTTGGTCAATTGTCTTTTGGCCATTCCTA
AGATTTATGGACTAGGCCAATTTTTCTCCTTTAATCAAACGATCGATGGTGTTTGTAATGGAGCTGCTGTGCAGCGTGC
ACCAGAGGCTCTGAGGTTTAATATTAATGACACCTCTGTCATTCTTGCTGAAGGCTCAATTGTACTTCATACTGCTTTA
GGAACAAATTTTTCTTTTGTTTGCAGTAATTCCTCAGATCCTCACTTAGCCACCTTCGCCATACCTCTGGGTGCTATCC
AAGTACCCTATTATTGTTTTCTTAAAGTGGATACTTACAACTCCACTGTTTATAAATTCTTGGCTGTTTTACCTCCTAC
CGTCAGGGAAATTGTCATCACCAAGTATGGTGATGTTTATGTCAATGGGTTTGGCTATTTGCATCTCGGTTTGTTGGAC
GCTGTCACAATTAATTTCACTGGTCATGGCACTGACGATGACGTTTCAGGTTTCTGGACCATAGCATCGACTAATTTTG
TTGATGCACTTATCGAAGTTCAAGGAACTGCCATTCAGCGTATTCTTTATTGTGATGATCCTGTTAGCCAACTCAAGTG
TTCTCAGGTTGCTTTTGACCTTGACGATGGTTTCTACCCTATTTCTTCTAGAAACCTTCTGAGTCATGAACAGCCAATT
TCTTTTGTTACTTTGCCATCATTTAATGATCATTCTTTTGTTAACATTACTGTCTCTGCGTCCTTTGGTGGTCGTAGTG

GTGCCAACCTTATTGCATCTGACACTACTATCAATGGGTTTAGTTCTTTCTGTGTTGACACTAGACAATTTACCATTTC
ACTGTTTTATAACGTTACAAACAGTTATGGTTATGTGTCTAAGTCACAGGACAGTAATTGCCCTTTCACCTTGCAATCT
GTTAATGATTACCTGTCTTTTAGTAAATTTTGTGTTTCCACCAGCCTTTTGGCTAGTGCCTGTACCATAGATCTTTTTG
GTTACCCTGATTTTGGTAGTGGTGTTAAGTTTACGTCCCTTTACTTTCAATTCACAACGGGTGAGTTGATTACTGGCAC
GCCTAAACCACTTGAAGGTGTCACGGACGTTTCTTTTATGACTCTGGATGTGTGTACCAAGTATACTATCTATGGCTTT
AAAGGTGAGGGTATCATTACCCTTATAAATTCTAGCTTTTTGGCAGGTGTTTATTACACATCTGATTCTGGACAGTTGT
TAGCCTTTAAGAATGTCACTAGTGGTGCTGTTTATTCTGTTACGCCATGTTCTTTTTCAGAGCACGCTGCATATGTTGA
TGATGATATAGTGGGTGTTATTTCTAGTTTGTCTAGCTCCACTTTTAACAGTACTAGGGAGTTGCCTGGTTTCTTCTAC
CATTCTAATGATGGCTCTAATTGTACAGAGCCTGTGTTGGTGTATAGTAACATAGGTGTTTGTAAATCTGGCAGTATTG
GCTACGTCCCATCTCAGTCTGGCCAAGTCAAGATTGCACCCACGGTTACTGGGAATATTAGTATTCCCACCAACTTTAG
TATGAGTATTAGGACAGAATATTTACAGCTTTACAACACGCCTGTTAGTGTTGATTGTGCCACATATGTTTGTAATGGT
AACTCTCGTTGTAAACAATTACTCACCCAGTACACTGCAGCATGTAAGACCATAGAGTCAGCATTACAACTCAGCGCTA
GGCTTGAGTCTGTTGAAGTTAACTCTATGCTTACTATTTCTGAAGAGGCTCTACAGTTAGCTACCATCAGTTCGTTTAA
TGGTGATGGATATAATTTTACTAATGTGCTGGGTGTTTCTGTGTATGATCCTGCAAGTGGCAGGGTGGTACAAAAAAGG
TCTTTTATTGAAGACCTGCTTTTTAATAAAGTGGTTACTAATGGCCTTGGTACTGTTGATGAAGACTATAAGCGCTGTT
CTAATGGTCGCTCTGTGGCAGATCTAGTCTGTGCACAGTATTACTCTGGTGTCATGGTACTACCTGGTGTTGTTGACGC
TGAGAAGCTTCACATGTATAGTGCGTCTCTCATCGGTGGTATGGTGCTAGGAGGTTTTACTTCTGCAGCGGCATTGCCT
TTTAGCTATGCTGTTCAAGCTAGACTCAATTATCTTGCTCTACAGACGGATGTTCTACAGCGGAACCAGCAATTGCTTG
CTGAGTCTTTTAACTCTGCTATTGGTAATATAACTTCAGCCTTTGAGAGTGTTAAAGAGGCTATTAGTCAAACTTCCAA
GGGTTTGAACACTGTGGCTCATGCGCTTACTAAGGTTCAAGAGGTTGTTAACTCGCAGGGTGCAGCTTTGACTCAACTT
ACCGTACAGCTGCAACACAACTTCCAAGCCATTTCTAGTTCTATTGATGACATTTACTCTCGACTGGACATTCTTTCAG
CCGATGTTCAGGTTGACCGTCTCATCACCGGCAGATTATCAGCACTTAATGCTTTTGTTGCTCAAACCCTCACTAAGTA
TACTGAGGTTCAGGCTAGCAGGAAGCTAGCACAGCAAAAGGTTAATGAGTGCGTTAAATCGCAATCTCAGCGTTATGGT
TTTTGTGGTGGTGATGGCGAGCACATTTTCTCTCTGGTACAGGCAGCACCTCAGGGCCTGCTGTTTTTACATACAGTAC
TTGTACCGGGTGACTTTGTAGATGTTATTGCCATCGCTGGCTTATGCGTTAACGATGAAATTGCCTTGACTCTACGTGA
GCCTGGCTTAGTCTTGTTTACGCATGAACTTCAAAATCATACTGCGACGGAATATTTTGTTTCATCGCGACGTATGTTT
GAACCTAGAAAACCTACCGTTAGTGATTTTGTTCAAATTGAGAGTTGTGTGGTCACCTATGTCAATTTGACTAGAGACC
AACTACCAGATGTAATCCCAGATTACATCGATGTTAACAAAACACTTGATGAGATTTTAGCTTCTCTGCCCAATAGAAC
TGGTCCAAGTCTTCCTTTAGATGTTTTTAATGCCACTTATCTTAATCTCACTGGTGAAATTGCAGATTTAGAGCAGCGT
TCAGAGTCTCTCCGTAATACTACAGAGGAGCTCCAAAGTCTTATATATAATATCAACAACACACTAGTTGACCTTGAGT
GGCTCAACCGAGTTGAGACATATATCAAGTGGCCGTGGTGGGTTTGGTTGATTATTTTCATTGTTCTCATCTTTGTTGT
GTCATTACTAGTGTTCTGCTGCATTTCCACTGGTTGTTGTGGATGCTGCGGCTGCTGCTGTGCTTGTTTTTCAGGTTGT
TGTAGGGGTCCTAGACTTCAACCTTACGAAGTTTTTGAAAAGGTCCACGTGCAGTGA

>gi|444300615|gb|KC109141.1| Porcine epidemic diarrhea virus isolate JS2008, complete genome

ATGACGCCTTTAATTTACTTCTGGTTGTTCTTACCAGTACTTCTAACACTTAGCCTACCACAAGATGTCACTAGG
TGCCAGTCTACTATTAACTTTAGGCGGTTCTTTTCAAAATTTAATGTTCAGGCACCTGCCGTCGTTGTTTTGGGTGGTT
ATCTACCTAGTATGAACTCTTCTAGCTGGTACTGTGGCACAGGCATTGAAACTGATAGTGGCGTTCATGGTATTTTTCT
CAGTTACATCGATTCTGGTCAGGGCTTTGAGATTGGCATTTCGCAAGAGCCGTTTGATCCTAGTGGTTACCAGCTTTAT
TTACACAAGGCCACTAATGGTAACACTAGTGCTATTGCACGACTGCGCATTTGCCAGTTTCCAGATAATAAAACATTGG
GCCCTACTGTTAATGATGTTACAACAGGTCGTAACTGCCTATTCAACAAAGCCATTCCAGCTTTGCAGGATGGAAAAAA

TATTGTTGTCGGCATAACATGGGATAATGATCGTGTCACTGTTTTTGCTGACAAGATCTATCATTTTTATATTAAAAAT
GATTGGTCCCGTGTTGCGACAAGATGTTACAATAAAAGAAGTTGTGCCATGCAATATGTTTATACACCTACCTACTACA
TGCTTAATGTTACTAGTGCAGGTGAGGATGGCATTTACTATGAACCTTGTACAGCTAATTGCAGTGGTTACGCTGCCAA
TGTATTTGCCACTGATTCCAATGGCCATATACCAGAAGGTTTTAGTTTTAATAATTGGTTTCTTTTGTCCAATGACTCC
ACTTTGTTGCATGGTAAAGTGGTTTCAAACCAACCTTTGTTGGTCAACTGCCTTTGGGCCATTCCTAAGATTTATGGAC
TAGGCCAATTTTTCTCATTCAATCAAACGATGGATGGCGTTTGTAATGGAGCTGCTGTGCAGCGTGCACCAGAGGCTCT
GAGGTTTAATATTAATGACACTTTTGTCATTCTTGCTGAAGGCTCAATTGTACTTCATACTGCTTTAGGAACAAATCTT
TCTTTTGTTTGCAGTAATTCCTCAGATCCTCACAAAGCCATCTTTACCATACCTTTGGGTGTTACTGAAGTACCCTACT
ATTGCTTTCTTAAAGTGGATACTTACAAATCCACTGTTTATAAATTCTTGGCTGTTTTACCTCCTACTGTCAAGGAAAT
TGTCATCACCAAGTACGGTGATGTTTATGTCAACGGGTTTGGCTATTTGCATCTCGGTTTGTTGGATGCTGTCACAATT
AATTTCACTGGTCATGGCACTGACGATGACGTTTCAGGTTTCTGGACCGTAGCATCGACTAATTTTGTTGATGCACTCA
TCGAGGTTCAAGGAACTGCCATTCAGCGTATTCTTTATTGTGATGACCCTGTTAGCCAACTTAAGTGTTCTCAGGTTTC
TTTTGACCTTGATGATGGTTTTTACCCTATTTCTTCTAGAAACCTTCTGAGTCATGAACAGCCAATTTCTTTTGTTACT
TTGCCATCATTCAATGATCATTCTTTTGTTAATATTACTGTCTCTGCGGCTTTTGGTGGTCATAGTGGTGCCAACCTCA
TTGCATCTGACACTACTATCAATGGGTTTAGTTCTTTCTGTGTTGACACTAGACAATTTACCATTACACTGTTTTATAA
CGTTACAAACAGTTATGGTTATGTGTCTAAGTCACAGGATAGTAATTGCCCTTTCACCTTGCAATCTGTTAATGATTAC
CTGTCTTTTAGCAAATTTTGTGTTTCAACCAGCCTTTTGGCTGGTGCTTGTACCATAGATCTTTTTGGTTACCCTGAGT
TCGGTAGTGGTGTTAAGTTTACGTCCCTTTATTTTCAATTCACAAAGGGTGAGTTGATTACTGGCACGCCTAAACCACT
TCAAGGTGTCACGGACGTTTCTTTTATGACTCTGGATGTGTGTACCAAGTATACTATCTATGGCTTTAAAGGTGAGGGT
ATTATTACCCTTACAAATTCTAGCTTTTTGGCAGGTGTTTATTATACATCTGATTCTGGACAGTTGTTAGCCTTTAAGA
ATGTCACTAGTGGTGCTGTTTATTCTGTTACGCCATGTTCTTTTTCAGAGCAGGCTGCATATGTTGATGATGATATAGT
GGGTGTTATTTCTAGTTTGTCTAACTCCACTTTTAACAATACCAGGGAGTTGCCTGGTTTCTTCTACCATTCTAATGAT
GGCTCCAATTGTACAGAGCCTGTGTTGGTGTATAGTAACATAGGTGTCTGTAAATCTGGCAGTATTGGCTATGTCCCAT
CTCAGTCTGGCCAAGTCAAGATTGCACCCACGGTTACTGGGAATATTAGTATTCCCACCAACTTTAGTATGAGTATTAG
GACAGAATATTTACAGCTTTACAACACGCCTGTTAGTGTTGATTGCGTTACATATGTTTGTAATGGTAACTCTCGTTGT
AAACAATTACTCACCCAGTACACTGCAGCATGTAAGACCATAGAGTCAGCATTACAACTCAGCGCTAGGCTTGAGTCTG
TTGAAGTTAACTCTATGCTTACTATTTCTGAAGAGGCTCTACAGTTAGCTACCATCAGTTCGTTTAATGGTGATGGATA
TAACTTTACTAATGTGCTGGGTGTTTCCGTGTACGACCCTGCAAGTGGCAGGGTGGTACAAAAAGGGTCTTTTATTGAA
GACCTGCTTTTTAATAAAGTGGTTACTAATGGCCTTGGTACTGTTGATGAAGACTATAAGCGCTGTTCTAATGGTCGCT
CTGTGGCAGATCTAGTCTGTGCGCAGTATTACTCTGGTGTCATGGTACTACCTGGCGTTGTTGACGCTGAGAAGCTTCA
CATGTATAGTGCGTCTCTCATCGGTGGTATGGCGCTAGGAGGTCTTACTACTGCAGCGGCATTGCCTTTTAGCCATGCT
GTTCAAGCGAGGCTCAATTATCTTGCTTTACAGACGGATGTTCTACAGCGCAACCAGCAATTGCTTGCTGAGTCTTTTA
ACTCTGCTATTGGTAATATAACTTCAGCCTTTGAGAGTGTTAAAGAGGCTATTAGTCAAACTTCCAATGGTTTGAACAC
TGTGGCTCATGCGCTTACTAAGGTTCAAGAGGTTGTTAATTCGCAGGGTTCAGCTTTGACCCAACTTACCATACAGCTG
CAACACAACTTCCAAGCCATTTCTAGTTCTATTGATGACATTTACTCCCGACTGGACATTCTTTCAGCCGATGTTCAGG
TTGATCGTCTCATCACCGGCAGATTATCAGCACTTAATGCTTTTGTTGCTCAAACCCTCACTAAGTATACTGAGGTTCA
GGCTAGCAGGAAGCTAGCACAGCAAAAGGTTAATGAGTGCGTCAAATCGCAATCTCAGCGTTATGGTTTTTGTGGTGGT
GATGGCGAGCACATCTTCTCTCTGGTACAGGCCGCACCTCAGGGCCTGCTGTTCTTACATACAGTACTTGTACCGGGTG
ATTTTGTAAATGTTATTGCCATCGATGGCTTATGCGTTAATGGTGATATTGCCTTGACTCTACGTGAGCCTGGCTTAGT
CTTGTTTACGCATGAACTTCAAAATCATACTGCGACGGAATATTTTGTTTCATCGCGACGTATGTTTGAACCTAGAAAA
CCTACCGTTAGTGATTTTGTTCAAATTGAGAGGTGTGTGGTCACCTATGTCAATCTGACTAGCGACCAACTACCAGATG
TAATCCCAGATTACATCGATGTTAACAAAACACTTGATGAGATTCTAGCTTCTCTGCCCAATAGAATTGGTCCTAGTCT
TCCCCTAGATGTTTTTAATGCCACTTATCTTAATCTCACTGGTGAAATTGCAGATTTAGAGCAGCGTTCAGAGTCTCTC
CGTAATACTACAGAAGAGCTCCGAAGTCTCATATATAATATCAACAACACACTTGTTGACCTTGAGTGGCTCAACCGAG

TTGAGACATATATCAAGTGGCCGTGGTGGGTTTGGTTGATTATTTTTATTGTTCTCATCTTTGTTGTGTCATTATTAGT
GTTCTGCTGCATTTCCACGGGTTGTTGTGGATGCTGCGGTTGTTGCGGTGCTTGTTTTTCAGGTTGTTGTAGGGGTCCT
AGACTTCAACCTTACGAAGCTTTTGAAAAGGTCCACGTGCAGTGA

>gi|486138040|gb|KC140102.1| Porcine epidemic diarrhea virus strain CH/FJZZ-9/2012, complete genome

ATGAAGTCTTTAACTTACTTCTGGTTGTCATTACCAGTACTTTCAACACTTAGCCTACCACAAGATGTCACCAGG
TGCTCAGCTAACACTAATTTTAGGCGGTTCTTTTCAAAATTTAATGTTCAGGCGCCTGCAGTTGTTGTACTGGGCGGTT
ATCTACCTATTGGTGAAAACCAGGGTGTTAATTCAACCTGGTACTGTGCTGGCCAACATCCAACTGCTAGTGGCGTTCA
TGGTATCTTTGTTAGCCATATTAGAGGAGGTCATGGCTTTGAGATTGGCATTTCGCAAGAGCCTTTTGACCCTAGTGGT
TACCAGCTTTATTTACATAAGGCTACTAACGGTAACACTAATGCTACTGCGCGACTGCGCATTTGCCAGTTTCCTAGCA
TTAAAACATTGGGCCCCACTGCTAATAATGATGTTACAACAGGTCGTAATTGCCTATTTAACAAAGCCATCCCAGCTCA
TATGAGTGAACATAGTGTTGTCGGCATAACATGGGATAATGATCGTGTCACTGTCTTTTCTGACAAGATCTATTATTTT
TATTTTAAAAATGATTGGTCCCGTGTTGCGACAAAGTGTTACAACAGTGGAGGTTGTGCTATGCAATATGTTTACGAAC
CCACCTATTACATGCTTAATGTTACTAGTGCTGGTGAGGATGGTATTTCTTATCAACCCTGTACAGCTAATTGCATTGG
TTATGCTGCCAATGTATTTGCTACTGAGCCCAATGGCCACATACCAGAAGGTTTTAGTTTTAATAATTGGTTTCTTTTG
TCCAATGATTCCACTTTGGTGCATGGTAAGGTGGTTTCCAACCAACCATTGCTGGTCAATTGTCTTTTGGCCATTCCTA
AGATTTATGGACTAGGCCAATTTTTCTCCTTTAATCAAACGATCGATGGTGTTTGTAATGGAGCTGCTGTGCAGCGTGC
ACCAGAGGCTCTGAGGTTTAATATTAATGACACCTCTGTCATTCTTGCTGAAGGCTCAATTGTACTTCATACTGCTTTA
GGAACAAATTTTTCTTTTGTTTGCAGTAATTCCTCAAATCCTCACTTAGCCACCTTCGCCATACCTCTGGGTGCTACCC
AAGTACCCTATTATTGTTTTCTTAAAGTGGATACTTACAACTCCACTGTTTATAAATTCTTGGCTGTTTTACCTCCTAC
CGTCAGGGAAATTGTCATCACCAAGTATGGTGATGTTTATGTCAATGGGTTTGGTTACTTGCATCTCGGTTTGTTGGAT
GCTGTCACAATTAATTTCACTGGTCATGGCACTGACGATGACGTTTCTGGTTTTTGGACCATAGCATCGACTAATTTTG
TTGATGCACTTATTGAAGTTAAAGGAACTGCCATTCAGCGTATTCTTTATTGTGATGATCCCGTTAGCCAACTCAAGTG
TTCTCAGGTTTCTTTTGACCTTGACGATGGTTTTTACCCTATTTCTTCTACAAACCTTCTGAGTCATGAACAGCCAACT
TCTTTTGTTACTTTGCCATCATTTAATGATCATTCTTTTGTTAATATTACTGTCTCTGCTGCTTTTGGTGGTCATAGTG
GTGCCAACCTTATTGCATCTGACACTACTATCAATGGGTTTAGTTCTTTCTGTGTTGACACTAGACAATTTACCATTTC
ACTGTTTTATAATGTTACAAACAGTTATGGTTATGTGTCTAACTCACAGGACAGTAATTGCCCTTTCACCTTGCAATCT
GTTAATGATTACCTGTCTTTTAGCAAATTTTGTGTTTCTACCAGCCTTTTGGCTAGTGCCTGTACCATAGATCTTTTTG
GTTACCCTGAGTTTGGTAGTGGTGTTAAATTCACGTCCCTTTACTTTCAATTCACAAAGGGTGAGTTGATTACTGGCAC
GCCTAAACCACTTGAAGGTGTTACGGACGTTTCTTTTATGACTCTGGATGTGTGTACCAAGTATACTATCTATGGCTTT
AAAGGTGAGGGTATCATTACCCTTACAAATTCTAGCTTTTTGGCAGGTGTTTATTATACATCTGATTCTGGACAGTTGC
TAGCTTTTAAGAATGTCACTAGTGGTGCTGTTTATTCTGTTACGCCATGTTCTTTTTCAGAGCAGGCTGCATATGTTGA
TGATGATATAGTGGGTGTTATTTCTAGTTTGTCTAGCTCCACTTTTAACAGTACTAGGGAGTTGCCTGGTTTCTTCTAC
CATTCTAATGATGACTCTAATTGTACAGAGCCTGTGTTGGTGTATAGTAACATTGGTGTTTGTAAATCTGGCAGTATTG
GCTATGTCCCATCTCAGTCTGGCCAAGTCAAGATTGCACCCATGGTTACTGGGAATATTAGTATTCCCACCAACTTTAG
TATGAGTATTAGGACAGAATATTTACAGCTTTACAACACGCCTGTTAGTGTTGATTGTGCTACATATGTTTGTAATGGT
AACTCTCGTTGTAAACAATTACTCACCCAGTACATTGCAGCATGTAAGACTATAGAGTCAGCATTACAACTCAGCGCTA
GGCTTGAGTCTGCTGAAGTTAACTCTATGCTTACTATTTCTGAAGAGGCTCTACAGTTAGCTACCATCAGTTCGTTTAA
TGGTGATGGATATAATTTTACTAATGTGCTGGGTGTTTCCGTTCGTGATCCTGCAAGTGGCAGGATGGTACAAAAAAGG
TCTTTTATTGAAGACCTGCTTTTTAATAAAGTGGTTACTAATGGCCTTGGTACTGTTGATGAAGACTATAAGCGCTGTT
CTAATGGCCGCTCTGTGGCAGATTTAGTCTGTGCGCAGTATTACTCTGGTGTCATGGTACTACCTGGTGTTGTTGACGC

TGAGAAGCTTCATATGTATAGTGCGTCTCTCATCGGTGGTATGGTGCTAGGAGGTTTTACTGCAGCAGCGGCATTGCCT
TTTAGCTATGCTGTTCAAGCGAGACTCAATTATCTTGCTCTACAGACGGATGTTCTACAGCGGAACCAGCAATTGCTTG
CTGAGTCTTTTAACTCTGCTATTGGTAATATAACCTCAGCCTTTGAGAGTGTTAAAGAGGCTATTAGTCAAACTTCTAA
GGGTTTGAACACTGTGGCTCATGCGCTTACTAAGGTTCAAGAGGTTGTTAATTCGCAGGGTGCAGCTTTGACCCAACTT
ACCGTACAGCTGCAACACAACTTCCAAGCCATTTCTAGTTCTATTGATGACATTTATTCCCGACTGGACATTCTTTCAG
CCGATGTTCAGGTTGACCGTCTCATCACCGGCAGATTATCAGCACTTAATGCTTTTGTTGCTCAAACCCTCACTAAGTA
TACTGAGGTTCAGGCTAGCAGGAAGCTAGCACAGCAAAAGGTTAATGAGTGCGTTAAATCGCAATCTCAGCGTTATGGT
TTTTGCGGTGGTGATGGCGAGCACATTTTCTCTCTGGTACAGGCAGCACCTCAGGGCCTGCTGTTTTTACATACAGTAC
TTGTACCGGGCGATTTTGTAGATGTTATTGCCATCGCTGGCTTATGCGTTAACGATGAAATTGCCTTGACTCTACGTGA
GCCTGGCTTAGTCTTGTTTACGCATGAACTTCAAAATCATACTGCGACGGAATATTTTGTTTCAACGCGACGTATGTTT
GAACCTAGAAAACCTACCGTTAGTGATTTTGTTCAAATTGAGAGTTGTGTGGTCACCTATGTCAATTTGACTAGGGACC
AACTACCAGATGTAATCCCAGATTACATCGATGTTAACAAAACACTTGATGAGATTTTAGCTTCTCTGCCCAATAGAAC
TGGTCCAAGCCTTCCTTTAGATGTTTTTAATGCCACTTATCTTAATCTCACTGGTGAAATTGCAGATTTAGAGCAGCGT
TCAGAGTCTCTCCGTAATACTACAGAAGAGCTCCAAAGTCTTATATATAATATCAACAACACACTAGTTGACCTTGAGT
GGCTCAACCGAGTTGAGACATATATCAAGTGGCCGTGGTGGGTTTGGTTGATTATTTTTATTGTTCTCATCTTTGTTGT
GTCATTATTAGTGTTCTGCTGCATTTCCACGGGTTGTTGTGGATGCTGCGGCTGCTGTGGTGCTTGTTTTTCAGGTTGT
TGTAGGGGTCCTAGACTTCAACCTTACGAAGCTTTTGAAAAGGTCCACGTGCAGTGA

>gi|509265008|gb|KC189944.1| Porcine epidemic diarrhea virus, complete genome

ATGACGCCTTTAATTTACTTCTGGTTGTTCTTACCAGTACTTCTAACACTTAGCCTACCACAAGATGTCACTAGG
TGCCAGTCTACTATTAACTTTAGGCGGTTCTTTTCAAAATTTAATGTTCAGGCACCTGCCGTCGTTGTTTTGGGTGGTT
ATCTACCTAGTATGAACTCTTCTAGCTGGTACTGTGGCACAGGCATTGAAACTGATAGTGGCGTTCATGGTATTTTTCT
CAGTTACATCGATTCTGGTCAGGGCTTTGAGATTGGCATTTCGCAAGAGCCGTTTGATCCTAGTGGTTACCAGCTTTAT
TTACACAAGGCCACTAATGGTAACACTAGTGCTATTGCACGACTGCGCATTTGCCAGTTTCCAGATAATAAAACATTGG
GCCCTACTGTTAATGATGTTACAACAGGTCGTAACTGCCTATTCAACAAAGCCATTCCAGCTTTGCAGGATGGAAAAAA
TATTGTTGTCGGCATAACATGGGATAATGATCGTGTCACTGTTTTTGCTGACAAGATCTGTCATTTTTATATTAAAAAT
GATTGGTCCCGTGTTGCGACAAGATGTTACAATAAAAGAAGTTGTGCCATGCAATATGTTTATACACCTACCTACTACA
TGCTTAATGTTACTAGTGCAGGTGAGGATGGCATTTACTATGAACCTTGTACAGCTAATTGCAGTGGTTACGCTGCCAA
TGTATTTGCCACTGATTCCAATGGCCATATACCAGAAGGTTTTAGTTTTAATAATTGGTTTCTTTTGTCCAATGACTCC
ACTTTGTTGCATGGTAAAGTGGTTTCAAACCAACCTTTGTTGGTCAACTGCCTTTGGGCCATTCCTAAGATTTATGGAC
TAGGCCAATTTTTCTCATTCAATCAAACGATGGATGGCGTTTGTAACGGAGCCGCTGCGCAGCGTGCCCCAGAGGCTCT
GAGGTTTAATATTAATGACACTTTTGTCATTCTTGCTGAAGGCTCAATTGTACTTCATACTGCTTTAGGAACAAATCTT
TCTTTTGTTTGCAGTAATTCCTCAGATCCTCACAAAGCCATCTTTACCATACCTTTGGGTGTTACTGAAGTACCCTACT
ATTGCTTTCTTAAAGTGGATACTTACAAATCCACTGTTTATAAATTCTTGGCTGTTTTACCTCCTACTGTCAAGGAAAT
TGTCATCACCAAGTACGGTGATGTTTATGTCAACGGGTTTGGCTATTTGCATCTCGGTTTGTTGGATGCTGTCACAATT
AATTTCACTGGTCATGGCACTGACGATGACGTTTCAGGTTTCTGGACCGTAGCATCGACTAATTTTGTTGATGCACTCA
TCGAGGTTCAAGGAACTGCCATTCAGCGTATTCTTTATTGTGATGACCCTGTTAGCCAACTTAAGTGTTCTCAGGTTTC
TTTTGACCTTGATGATGGTTTTTACCCTATTTCTTCTAGAAACCTTCTGAGTCATGAACAGCCAATTTCTTTTGTTACT
TTGCCATCATTCAATGATCATTCTTTTGTTAATATTACTGTCTCTGCGGCTTTTGGTGGTCATAGTGGTGCCAACCTCA
TTGCATCTGACACTACTATCAATGGGTTTAGTTCTTTCTGTGTTGACACTAGACAATTTACCATTACACTGTTTTATAA
CGTTACAAACAGTTATGGTTATGTGTCTAAGTCACAGGATAGTAATTGCCCTTTCACCTTGCAATCTGTTAATGATTAC
CTGTCTTTTAGCAAATTTTGTGTTTCAACCAGCCTTTTGGCTGGTGCTTGTACCATAGATCTTTTTGGTTACCCTGAGT

TCGGTAGTGGTGTTAAGTTTACGTCCCTTTATTTTCAATTCACAAAGGGTGAGTTGATTACTGGCACGCCTAAACCACT
TCAAGGTGTCACGGACGTTTCTTTTATGACTCTGGATGTGTGTACCAAGTATACTATCTATGGCTTTAAAGGTGAGGGT
ATTATTACCCTTACGAATTCTAGCATTTTGGCAGGTGTTTATTATACATCTGATTCTGGACAGTTGTTAGCCTTTAAGA
ATGTCACTAGTGGTGCTGTTTATTCTGTTACGCCATGTTCTTTTTCAGAGCAGGCTGCATATGTTGATGATGATATAGT
GGGTGTTATTTCTAGTTTGTCTAACTCCACTTTTAACAATACCAGGGAGTTGCCTGGTTTCTTCTACCATTCTAATGAT
GGCTCCAATTGTACAGAGCCTGTGTTGGTGTATAGTAACATAGGTGTCTGTAAATCTGGCAGTATTGGCTATGTCCCAC
TTCAGGATGGCCAAGTCAAGATTGCACCCATGGTTACTGGGAATATTAGTATTCCCACCAACTTTAGTATGAGTATTAG
AACAGAATATTTACAGCTTTACAACACGCCTGTTAGTGTTGATTGCGTTACATATGTTTGTAATGGTAACTCTCGTTGT
AAACAATTACTCACCCAGTACACTGCAGCATGTAAGACCATAGAGTCAGCATTACAACTCAGCGCTAGGCTTGAGTCTG
TTGAAGTTAACTCTATGCTTACTATTTCTGAAGAGGCTCTACAGTTAGCTACCATCAGTTCGTTTAATGGTGATGGATA
TAACTTTACTAATGTGCTGGGTGTTTCCGTGTACGACCCTGCAAGTGGCAGGGTGGTACAAAAAGGGTCTTTTATTGAA
GACCTGCTTTTTAATAAAGTGGTTACTAATGGCCTTGGTACTGTTGATGAAGACTATAAGCGCTGTTCTAATGGTCGCT
CTGTGGCAGATCTAGTCTGTGCGCAGTATTACTCTGGTGTCATGGTACTACCTGGCGTTGTTGACGCTGAGAAGCTTCA
CATGTATAGTGCGTCTCTCCTCGGTGGTATGGCGCTAGGAGGTCTTACTACTGCAGCGGCATTGCCTTTTAGCAATGCT
GTTCAAGCGAGGCTCAATTATCTTGCTTTACAGACGGATGTTCTACAGCGCAACCAGCAATTGCTTGCTGAGTCTTTTA
ACTCTGCTATTGGTAATATAACTTCAGCCTTTGAGAGTGTTAAAGAGGCTATTAGTCAAACTTCCAATGGTTTGAACAC
TGTGGCTCATGCGCTTACTAAGGTTCAAGAGGTTGTTAAGTCGCAGGGTTCAGCTTTGACCCAACTTACCATACAGCTG
CAACACAACTTCCAAGCCATTTCTAGTTCTATTGATGACATTTACTCCCGACTGGACATTCTTTCAGCCGATGTTCAGG
TTGATCGTCTCATCACCGGCAGATTATCAGCACTTAATGCTTTTGTTGCTCAAACCCTCACTAAGTATACTGAGGTTCA
GGCTAGCAGGAAGCTAGCACAGCAAAAGGTTAATGAGTGCGTCAAATCGCAATCTCAGCGTTATGGTTTTTGTGGTGGT
GATGGCGAGCACATCTTCTCTCTGGTACAGGCCGCACCTCAGGGCCTGCTGTTCTTACATACAGTACTTGTACCGGGTG
ATTTTGTAAATGTTATTGCCATCGATGGCTTATGCGTTAATGGTGATATTGCCTTGACTCTACGTGAGCCTGGCTTAGT
CTTGTTTACGCATGAACTTCAAACTTATACTGCGACGGAATATTTTGTTTCATCGCGACGTATGTTTGAACCTAGAAAA
CCTACCGTTAGTGATTTTGTTCAAATTGAGAGTTGTGTGGTCACCTATGTCAATCTGACTAGCGACCAACTACCAGATG
TAATCCCAGATTACATCGATGTTAACAAAACACTTGATGAGATTCTAGCTTCTCTGCCCAATAGAATTGGTCCTAGTCT
TCCCCTAGATGTTTTTAATGCCACTTATCTTAATCTCACTGGTGAAATTGCAGATTTAGAGCAGCGTTCAGAGTCTCTC
CGTAATACTACAGAAGAGCTCCGAAGTCTCATATATAATATCAACAACACACTTGTTGACCTTGAGTGGCTCAACCGAG
TTGAGACATATATCAAGTGGCCGTGGTGGGTTTGGTTGATTATTTTTATTGTTCTCATCTTTGTTGTGTCATTATTAGT
GTTCTGCTGCATTTCCACGGGTTGTTGTGGATGCTGCGGTTGTTGCGGTGCTTGTTTTTCAGGTTGTTGTAGGGGTCCT
AGACTTCAACCTTACGAAGCTTTTGAAAAGGTCCACGTGCAGTGA

>gi|459357901|gb|KC210145.1| Porcine epidemic diarrhea virus strain AH2012, complete genome

ATGAAGTCTTTAACCTACTTCTGGTTGTTCTTACCAGTACTTTCAACACTTAGCCTACCACAAGATGTCACCAGG
TGCTCAGCTAACACTAATTTTAGGCGGTTCTTTTCAAAATTTAATGTTCAGGCGCCTGCAGTTGTTGTACTGGGCGGTT
ATCTACCTATTGGTGAAAACCAGGGTGTCAATTCAACTTGGTACTGTGCTGGCCAACATCCAACTGCTAGTGGCGTTCA
TGGTATCTTTGTTAGCCATATTAGAGGTGGTCATGGCTTTGAGATTGGCATTTCGCAAGAGCCTTTTGACCCTAGTGGT
TACCAGCTTTATTTACATAAGGCTACTAACGGTAACACTAATGCTACTGCGCGACTGCGCATTTGCCAGTTTCCTAGCA
TTAAAACATTGGGCCCCACTGCTAATAATGATGTTACAACAGGTCGTAATTGCCTATTTAACAAAGCCATCCCAGCTCA
TATGAGTGAACATAGTGTTGTCGGCATAACATGGGATAATGATCGTGTCACTGTCTTTTCTGACAAGATCTATTATTTT
TATTTTAAAAATGATTGGTCCCGTGTTGCGACAAAGTGTTACAACAGTGGAGGTTGTGCTATGCAATATGTTTACGAAC
CCACCTATTACATGCTTAATGTTACTAGTGCTGGTGAGGATGGTATTTCTTATCAACCCTGTACAGCTAATTGCATTGG

TTATGCTGCCAATGTATTTGCTACTGAGCCCAATGGCCACATACCAGAAGGTTTTAGTTTTAATAATTGGTTTCTTTTG
TCCAATGATTCCACTTTGGTGCATGGTAAGGTGGTTTCCAACCAACCATTGTTGGTCAATTGTCTTTTGGCCATTCCTA
AGATTTATGGACTAGGCCAATTTTTCTCCTTTAATCAAACGATCGATGGTGTTTGTAATGGAGCTGCTGTGCAGCGTGC
ACCAGAGGCTCTGAGGTTTAATATTAATGACACCTCTGTCATTCTTGCTGAAGGCTCAATTGTACTTCATACTGCTTTA
GGAACAAATTTTTCTTTTGTTTGCAGTAATTCCTCAAATCCTCACTTAGCCACCTTCGCCATACCTTTGGGTGCTACCC
AAGTACCCTATTATTGTTTTCTTAAAGTGGATACTTACAACTCCACTGTTTATAAATTCTTGGCTGTTTTACCTCCAAC
CGTCAGGGAAATTGTCATCACCAAGTATGGTGATGTTTATGTCAATGGGTTTGGCTATTTGCATCTCGGTTTGTTGGAC
GCTGTCACAATTAATTTCACTGGTCATGGCACTGACGATGACGTTTCAGGTTTCTGGACCATAGCATCGACTAATTTTG
TTGATGCACTTATCGAAGTTCAAGGAACTGCCATTCAGCGTATTCTTTATTGTGATGATCCTGTTAGCCAACTCAAGTG
TTCTCAGGTTGCTTTTGACCTTGACGATGGTTTCTACCCTATTTCTTCTAGAAACCTCTTGAGTCATGAACAGCCAATT
TCTTTTGTTACTTTGCCATCATTTAATGATCATTCTTTTGTTAACATTACTGTCTCTGCGTCCTTTGGTGGTCATAGTG
GTGCCAACCTTATTGCATCTGACACTACTATCAATGGGTTTAGTTCTTTCTGTGTTGACACTAGACAATTTACCATTTC
ACTGTTTTATAACGTTACAAACAGTTATGGTTATGTGTCTAACTCACAGGACAGTAATTGCCCTTTCACCTTGCAATCT
GTTAATGATTACCTGTCTTTTAGTAAATTTTGTGTTTCCACCAGCCTTTTGGCTAGTGCCTGTACCATAGATCTTTTTG
GTTACCCTGATTTTGGTAGTGGTGTTAAGTTTACGTCCCTTTACTTTCAATTCACAAAGGGTGAGTTGATTACTGGCAC
GCCTAAACCACTTGAAGGTGTCACGGACGTTTCTTTTATGACTCTGGATGTGTGTACCAAGTATACTATCTATGGCTTT
AAAGGTGAGGGTATCATTACCCTTACAAATTCTAGCTTTTTGGCAGGTGTTTATTACACATCTGATTCTGGACAGTTGT
TAGCCTTTAAGAATGTCACTAGTGGTGCTGTTTATTCTGTTACGCCATGTTCTTTTTCAGAGCAGGCTGCATATGTTGA
TGATGATATAGTGGGTGTTATTTCTAGTTTGTCTAATTCCACTTTTAACAGTACTAGGGAGTTGCCTGGTTTCTTCTAC
CATTCTAATGATGGCTCTAATTGTACAGAGCCTGTGTTGGTGTATAGTAACATAGGTGTTTGTAAATCTGGCAGTATTG
GCTACGTCCCATCTCAGTCTGGCCAAGTCAAGATTGCACCCACGGTTACTGGGAATATTAGTATTCCCACCAACTTTAG
TATGAGTATTAGGACAGAATATTTACAGCTTTACAACACGCCTGTTAGTGTTGATTGTGCCACATATGTTTGTAATGGT
AACTCTCGTTGTAAACAATTACTCACCCAGTACACTGCAGCATGTAAGACCATAGAGTCAGCATTACAACTCAGCGCTA
GGCTTGAGTCTGTTGAAGTTAACTCTATGCTTACTATTTCTGAAGAGGCTCTACAGTTAGCTACCATCAGTTCGTTTAA
TGGTGATGGATATAATTTTACTAATGTGCTGGGTGTTTCTGTGTATGATCCTGCAAGTGGCAGGGTGGTACAAAAAAGG
TCTTTTATTGAAGACCTGCTTTTTAATAAAGTGGTTACTAATGGCCTTGGTACTGTTGATGAAGACTATAAGCGCTGTT
CTAATGGTCGCTCTGTGGCAGATCTAGTCTGTGCACAGTATTACTCTGGTGTCATGGTACTACCTGGTGTTGTTGACGC
TGAGAAGCTTCACATGTATAGTGCGTCTCTCATCGGTGGTATGGTGCTAGGAGGTTTTACTTCTGCAGCGGCATTGCCT
TTTAGCTATGCTGTTCAAGCTAGACTCAATTATCTTGCTCTACAGACGGATGTTCTACAGCGGAACCAGCAATTGCTTG
CTGAGTCTTTTAACTCTGCTATTGGTAATATAACTTCAGCCTTTGAGAGTGTTAAAGAGGCTATTAGTCAAACTTCCAA
GGGTTTGAACACTGTGGCTCATGCGCTTACTAAGGTTCAAGAGGTTGTTAACTCGCAGGGTGCAGCTTTGACTCAACTT
ACCGTACAGCTGCAACACAACTTCCAAGCCATTTCTAGTTCTATTGATGACATTTACACCCGACTGGACATTCTTTCAG
CCGATGTTCAGGTTGACCGTCTCATCACCGGCAGATTATCAGCACTTAATGCTTTTGTTGCTCAAACCCTCACTAAGTA
TACTGAGGTTCAGGCTAGCAGGAAGCTAGCACAGCAAAAGGTTAATGAGTGCGTTAAATCGCAATCTCAGCGTTATGGT
TTTTGTGGTGGTGATGGCGATCACATTTTCTCTCTGGTACAGGCAGCACCTCAGGGCCTGCTGTTTTTACATACAGTAC
TTGTACCGGGTGATTTTGTAGATGTTATTGCCATCGCTGGCTTATGCGTTAACGATGAAATTGCCTTGACTCTACGTGA
GCCTGGCTTAGTCTTGTTTACGCATGAACTTCAAAATCATACTGCGACGGAATATTTTGTTTCATCGCGACGTATGTTT
GAACCTAGAAAACCTACCGTTAGTGATTTTGTTCAAATTGAGAGTTGTGTGGTCACCTATGTCAATTTGACTAGAGACC
AACTACCAGATGTAATCCCAGATTACATCGATGTTAACAAAACACTTGATGAGATTTTAGCTTCTCTGCCCAATAGAAC
TGGTCCAAGTCTTCCTTTAGATGTTTTTAATGCCACTTATCTCAATCTCACTGGTGAAATTGCAAATTTAGAGCAGCGT
TCAGAGTCTCTCCGTAATACTACAGAGGAGCTCCAAAGTCTTATACATAATATCAACAACACACTAGTTGACCTTGAGT
GGCTCAACCGAGTTGAGACATATATCAAGTGGCCGTGGTGGGTTTGGTTGGTTATTTTTATTGTTCTCATCTTTGTTGT
GTCATTACTAGTGTTCTGCTGCATTTCCACGGGTTGTTGTGGATGCTGCGGCTGCTGCTGTGCTTGTTTTTCAGGTTGT
TGTAGGGGTCCTAGACTTCAACCTTACGAAGTTTTTGAAAAGGTCCACGTGCAGTGA

>gi|459357915|gb|KC210147.1| Porcine epidemic diarrhea virus strain JS-HZ2012, complete genome

ATGAAGTCTTTAACCTACTTCTGGTTGTTCTTACCAGTACTTTCAACACTTAGCCTACCACAAGATGTCACCAGG
TGCTCAGCTAACACTAATTTTAGGCGGTTCTTTTCAAAATTTAATGTTCAGGCGCCTGCAGTTGTTGTACTGGGCGGTT
ATCTACCTATTGGTGAAAACCAGGGTGTCAATTCAACTTGGTACTGTGCTGGCCAACATCCAACTGCTAGTGGCGTTCA
TGGTATCTTTGTTAGCCATATTAGAGGTGGTCATGGCTTTGAGATTGGCATTTCGCAAGAGCCTTTTGACCCTAGTGGT
TACCAGCTTTATTTACATAAGGCTACTAACGGTAACACTAATGCTACTGCGCGACTGCGCATTTGCCAGTTTCCTAGCA
TTAAAACATTGGGCCCCACTGCTAATAATGATGTTACAACAGGTCGTAATTGCCTATTTAACAAAGCCATCCCAGCTCA
TATGAGTGAACATAGTGTTGTCGGCATAACATGGGATAATGATCGTGTCACTGTCTTTTCTGACAAGATCTATTATTTT
TATTTTAAAAATGATTGGTCCCGTGTTGCGACAAAGTGTTACAACAGTGGAGGTTGTGCTATGCAATATGTTTACGAAC
CCACCTATTACATGCTTAATGTTACTAGTGCTGGTGAGGATGGTATTTCTTATCAACCCTGTACAGCTAATTGCATTGG
TTATGCTGCCAATGTATTTGCTACTGAGCCCAATGGCCACATACCAGAAGGTTTTAGTTTTAATAATTGGTTTCTTTTG
TCCAATGATTCCACTTTGGTGCATGGTAAGGTGGTTTCCAACCAACCATTGTTGGTCAATTGTCTTTTGGCCATTCCTA
AGATTTATGGACTAGGCCAATTTTTCTCCTTCAATCAAACGATCGATGGTGTTTGTAATGGAGCTGCTGTGCAGCGTGC
ACCAGAGGCTCTGAGGTTTAATATTAATGACACCTCTGTCATTCTTGCTGAAGGCTCAATTGTACTTCATACTGCTTTA
GGAACAAATTTTTCTTTTGTTTGCAGTAATTCCTCAGATCCTCACTTAGCCACCTTCGCCATACCTCTGGGTGCTATCC
AAGTACCCTATTATTGTTTTCTTAAAGTGGATACTTACAACTCCACTGTTTATAAATTCTTGGCTGTTTTACCTCCTAC
CGTCAGGGAAATTGTCATCACCAAGTATGGTGATGTTTATGTCAATGGGTTTGGCTATTTGCATCTCGGTTTGTTGGAC
GCTGTCACAATTAATTTCACTGGTCATGGCACTGACGATGACGTTTCAGGTTTCTGGACCATAGCATCGACTAATTTTG
TTGATGCACTTATCGAAGTTCAAGGAACTGCCATTCAGCGTATTCTTTATTGTGATGATCCTGTTAGCCAACTCAAGTG
TTCTCAGGTTGCTTTTGACCTTGACGATGGTTTCTACCCTATTTCTTCTAGAAACCTTCTGAGTCATGAACAGCCAATT
TCTTTTGTTACTTTGCCATCATTTAATGATCATTCTTTTGTTAACATTACTGTCTCTGCGTCCTTTGGTGGTCATAGTG
GTGCCAACCTTATTGCATCTGACACTACTATCAATGGGTTTAGTTCTTTCTGTGTTGACACTAGACAATTTACCATTTC
ACTGTTTTATAACGTTACAAACAGTTATGGTTATGTGTCTAAATCACAGGACAGTAATTGCCCTTTCACCTTGCAATCT
GTTAATGATTACCTGTCTTTTAGTAAATTTTGTGTTTCCACCAGCCTTTTGGCTAGTGCCTGTACCATAGATCTTTTTG
GTTACCCTGAGTTTGGTAGTGGTGTTAAGTTTACGTCCCTTTACTTTCAATTCACAAAGGGTGAGTTGATTACTGGCAC
GCCTAAACCACTTGAAGGTGTCACGGACGTTTCTTTTATGACTCTGGATGTGTGTACCAAGTATACTATCTATGGCTTT
AAAGGTGAGGGTATCATTACCCTTACAAATTCTAGCTTTTTGGCAGGTGTTTATTACACATCTGATTCTGGACAGTTGT
TAGCCTTTAAGAATGTCACTAGTGGTGCTGTTTATTCTGTTACGCCATGTTCTTTTTCAGAGCAGGCTGCATATGTTGA
TGATGATATAGTGGGTGTTATTTCTAGTTTGTCTAGCTCCACTTTTAACAGTACTAGGGAGTTGCCTGGTTTCTTCTAC
CATTCTAATGATGGCTCTAATTGTACAGAGCCTGTGTTGGTGTATAGTAACATAGGTGTTTGTAAATCTGGCAGTATTG
GCTACGTCCCATCTCAGTCTGGCCAAGTCAAGATTGCACCCACGGTTACTGGGAATATTAGTATTCCCACCAACTTTAG
TATGAGTATTAGGACAGAATATTTACAGCTTTACAACACGCCTGTTAGTGTTGATTGTGCCACATATGTTTGTAATGGT
AACTCTCGTTGTAAACAATTACTCACCCAGTACACTGCAGCATGTAAGACCATAGAGTCAGCATTACAACTCAGCGCTA
GGCTTGAGTCTGTTGAAGTTAACTCTATGCTTACTATTTCTGAAGAGGCTCTACAGTTAGCTACCATCAGTTCGTTTAA
TGGTGATGGATATAATTTTACTAATGTGCTGGGTGTTTCTGTGTATGATCCTGCAAGTGGCAGGGTGGTACAAAAAAGG
TCTTTTATTGAAGACCTGCTTTTTAATAAAGTGGTTACTAATGGCCTTGGTACTGTTGATGAAGACTATAAGCGCTGTT
CTAATGGTCGCTCTGTGGCAGATCTAGTCTGTGCACAGTATTACTCTGGTGTCATGGTACTACCTGGTGTTGTTGACGC
TGAGAAGCTTCACATGTATAGTGCGTCTCTCATCGGTGGTATGGTGCTAGGAGGTTTTACTTCTGCAGCGGCATTGCCT
TTTAGCTATGCTGTTCAAGCTAGACTCAATTATCTTGCTCTACAGACGGATGTTCTACAGCGGAACCAGCAATTGCTTG
CTGAGTCTTTTAACTCTGCTATTGGTAATATAACTTCAGCCTTTGAGAGTGTTAAAGAGGCTATTAGTCAAACTTCCAA
GGGTTTGAACACTGTGGCTCATGCGCTTACTAAGGTTCAAGAGGTTGTTAACTCGCAGGGTGCAGCTTTGACTCAACTT

ACCGTACAGCTGCAACACAACTTCCAAGCCATTTCTAGTTCTATTGATGACATTTACTCTCGACTGGACATTCTTTCAG
CCGATGTTCAGGTTGACCGTCTCATCACCGGCAGATTATCAGCACTTAATGCTTTTGTTGCTCAAACCCTCACTAAGTA
TACTGAGGTTCAGGCTAGCAGGAAGCTAGCACAGCAAAAGGTTAATGAGTGCGTTAAATCGCAATCTCAGCGTTATGGT
TTTTGTGGTGGTGATGGCGAGCACATTTTCTCTCTGGTACAGGCAGCACCTCAGGGCCTGCTGTTTTTACATACAGTAC
TTGTACCGGGTGACTTTGTAGATGTTATTGCCATCGCTGGCTTATGCGTTAACGATGAAATTGCCTTGACTCTACGTGA
GCCTGGCTTAGTCTTGTTTACGCATGAACTTCAAAATCATACTGCGACGGAATATTTTGTTTCATCGCGACGTATGTTT
GAACCTAGAAAACCTACCGTTAGTGATTTTGTTCAAATTGAGAGTTGTGTGGTCACCTATGTCAATTTGACTAGAGACC
AACTACCAGATGTAATCCCAGATTACATCGATGTTAACAAAACACTTGATGAGATTTTAGCTTCTCTGCCCAATAGAAC
TGGTCCAAGTCTTCCTTTAGATGTTTTTAATGCCACTTATCTTAATCTCACTGGTGAAATTGCAGATTTAGAGCAGCGT
TCAGAGTCTCTCCGTAATACTACAGAGGAGCTCCAAAGTCTTATATATAATATCAACAACACACTAGTTGACCTTGAGT
GGCTCAACCGAGTTGAGACATATATCAAGTGGCCGTGGTGGGTTTGGTTGATTATTTTCATTGTTCTCATCTTTGTTGT
GTCATTACTAGTGTTCTGCTGCATTTCCACTGGTTGTTGTGGATGCTGCGGCTGCTGCTGTGCTTGTTTTTCAGGTTGT
TGTAGGGGTCCTAGACTTCAACCTTACGAAGTTTTTGAAAAGGTCCACGTGCAGTGA

>gi|527290413|gb|KF267450.1| Porcine epidemic diarrhea virus isolate 13-019349, complete genome

ATGAAGTCTTTAACCTACTTCTGGTTGTTCTTACCAGTACTTTCAACACTTAGCCTACCACAAGATGTCACCAGG
TGCTCAGCTAACACTAATTTTAGGCGGTTCTTTTCAAAATTTAATGTTCAGGCGCCTGCAGTTGTTGTACTGGGCGGTT
ATCTACCTATTGGTGAAAACCAGGGTGTCAATTCAACTTGGTACTGTGCTGGCCAACATCCAACTGCTAGTGGCGTTCA
TGGTATCTTTGTTAGCCATATTAGAGGTGGTCATGGCTTTGAGATTGGCATTTCGCAAGAGCCTTTTGACCCTAGTGGT
TACCAGCTTTATTTACATAAGGCTACTAACGGTAACACTAATGCTACTGCGCGACTGCGCATTTGCCAGTTTCCTAGCA
TTAAAACATTGGGCCCCACTGCTAATAATGATGTTACAACAGGTCGTAATTGCCTATTTAACAAAGCCATCCCAGCTCA
TATGAGTGAACATAGTGTTGTCGGCATAACATGGGATAATGATCGTGTCACTGTCTTTTCTGACAAGATCTATTATTTT
TATTTTAAAAATGATTGGTCCCGTGTTGCGACAAAGTGTTACAACAGTGGAGGTTGTGCTATGCAATATGTTTACGAAC
CCACCTATTACATGCTTAATGTTACTAGTGCTGGTGAGGATGGTATTTCTTATCAACCCTGTACAGCTAATTGCATTGG
TTATGCTGCCAATGTATTTGCTACTGAGCCCAATGGCCACATACCAGAAGGTTTTAGTTTTAATAATTGGTTTCTTTTG
TCCAATGATTCCACTTTGGTGCATGGTAAGGTGGTTTCCAACCAACCATTGTTGGTCAATTGTCTTTTGGCCATTCCTA
AGATTTATGGACTAGGCCAATTTTTCTCCTTTAATCAAACGATCGATGGTGTTTGTAATGGAGCTGCTGTGCAGCGTGC
ACCAGAGGCTCTGAGGTTTAATATTAATGACATCTCTGTCATTCTTGCTGAAGGCTCAATTGTACTTCATACTGCTTTA
GGAACAAATTTTTCTTTTGTTTGCAGTAATTCCTCAAATCCTCACTTAGCCACCTTCGCCATACCTCTGGGTGCTACCC
AAGTACCTTATTATTGTTTTCTTAAAGTGGATACTTACAACTCCACTGTTTATAAATTTTTGGCTGTTTTACCTCCTAC
CGTCAGGGAAATTGTCATCACCAAGTATGGTGATGTTTATGTCAATGGGTTTGGATACTTGCATCTCGGTTTGTTGGAT
GCTGTCACAATTAATTTCACTGGTCATGGCACTGACGATGATGTTTCTGGTTTTTGGACCATAGCATCGACTAATTTTG
TTGATGCACTCATCGAAGTTCAAGGAACCGCCATTCAGCGTATTCTTTATTGTGATGATCCTGTTAGCCAACTCAAGTG
TTCTCAGGTTGCTTTTGACCTTGACGATGGTTTTTACACTATTTCTTCTAGAAACCTTCTGAGTCATGAACAGCCAATT
TCTTTTGTTACTCTGCCATCATTTAATGATCATTCTTTTGTTAACATTACTGTATCTGCTTCCTTTGGTGGTCATAGTG
GTGCCAACCTTATTGCATCTGACACTACTATCAATGGGTTTAGTTCTTTCTGTGTTGACACTAGACAATTTACCATTTC
ACTGTTTTATAACGTTACAAACAGTTATGGTTATGTGTCTAAATCACAGGACAGTAATTGCCCTTTCACCTTGCAATCT
GTTAATGATTACCTGTCTTTTAGCAAATTTTGTGTTTCCACCAGCCTTTTGGCTAGTGCCTGTACCATAGATCTTTTTG
GTTACCCTGAGTTTGGTAGTGGTGTTAAGTTTACGTCCCTTTACTTTCAATTCACAAAGGGTGAGTTGATTACTGGCAC
GCCTAAACCATTTGAAGGTGTCACGGACGTTTCTTTTATGACTCTGGATGTGTGTACCAAGTATACTATCTATGGCTTT
AAAGGTGAGGGTATCATTACCCTTACAAATTCTAGCTTTTTGGCAGGTGTTTATTACACATCTGATTCTGGACAGTTGT

TAGCCTTTAAGAATGTCACTAGTGGTGCTGTTTATTCTGTTACGCCATGTTCTTTTTCAGAGCAGGCTGCATATGTTGA
TGATGATATAGTGGGTGTTATTTCTAGTTTGTCTAGCTCCACTTTTAACAGTACTAGGGAGTTGCCTGGTTTCTTCTAC
CATTCTAATGATGGCTCTAATTGTACAGAGCCTGTGTTGGTGTATAGTAACATAGGTGTTTGTAAATCTGGCAGTATTG
GCTACGTCCCATCTCAGTCTGGCCAAGTCAAGATTGCACCCACGGTTACTGGGAATATTAGTATTCCCACCAACTTTAG
TATGAGTATTAGGACAGAATATTTACAGCTTTACAACACGCCTGTTAGTGTTGATTGTGCCACATATGTTTGTAATGGT
AACTCTCGTTGTAAACAATTACTCACCCAGTACACTGCAGCATGTAAGACCATAGAGTCAGCATTACAACTCAGCGCTA
GGCTTGAGTCTGTTGAAGTTAACTCTATGCTTACTATTTCTGATGAGGCTCTACAGTTAGCTACCATTAGTTCGTTTAA
TGGTGATGGATATAATTTTACTAATGTGCTGGGTGTTTCTGTGTATGATCCTGCAAGTGGCAGGGTGGTACAAAAAAGG
TCTTTTATTGAAGACCTGCTTTTTAATAAAGTGGTTACTAATGGCCTTGGTACTGTTGATGAAGACTATAAGCGCTGTT
CTAATGGTCGCTCTGTGGCAGATCTAGTCTGTGCACAGTATTACTCTGGTGTCATGGTACTACCTGGTGTTGTTGACGC
TGAGAAGCTTCACATGTATAGTGCGTCTCTCATCGGTGGTATGGTGCTAGGAGGTTTTACTTCTGCAGCGGCATTGCCT
TTTAGCTATGCTGTTCAAGCTAGACTCAATTATCTTGCTCTACAGACGGATGTTCTACAGCGGAACCAGCAATTGCTTG
CTGAGTCTTTTAACTCTGCTATTGGTAATATAACTTCAGCCTTTGAGAGTGTTAAAGAGGCTATTAGTCAAACTTCCAA
GGGTTTGAACACTGTGGCTCATGCGCTTACTAAGGTTCAAGAGGTTGTTAACTCGCAGGGTGCAGCTTTGACTCAACTT
ACCGTACAGCTGCAACACAACTTCCAAGCCATTTCTAGTTCTATTGATGACATTTACTCTCGACTGGACATTCTTTCAG
CCGATGCTCAGGTTGACCGTCTCATCACCGGCAGATTATCAGCACTTAATGCTTTTGTTGCTCAAACCCTCACTAAGTA
TACTGAGGTTCAGGCTAGCAGGAAGTTAGCACAGCAAAAGGTTAATGAGTGCGTTAAATCGCAATCTCAGCGTTATGGT
TTTTGTGGTGGTGATGGCGAGCACATTTTCTCTCTGGTACAGGCAGCACCTCAGGGCCTGCTGTTTTTACATACAGTAC
TTGTACCGAGTGATTTTGTAGATGTTATTGCCATCGCTGGCTTATGCGTTAACGATGAAATTGCCTTGACTCTACGTGA
GCCTGGCTTAGTCTTGTTTACGCATGAACTTCAAAATCATACTGCGACGGAATATTTTGTTTCATCGCGACGTATGTTT
GAACCTAGAAAACCTACCGTTAGTGATTTTGTTCAAATTGAGAGTTGTGTGGTCACCTATGTCAATTTGACTAGAGACC
AACTACCAGATGTAATCCCAGATTACATCGATGTTAACAAAACACTTGATGAGATTTTAGCTTCTCTGCCCAATAGAAC
TGGTCCAAGTCTTCCTTTAGATGTTTTTAATGCCACTTATCTTAATCTCACTGGTGAAATTGCAGATTTAGAGCAGCGT
TCAGAGTCTCTCCGTAATACTACAGAGGAGCTCCAAAGTCTTATATATAATATCAACAACACACTAGTTGACCTTGAGT
GGCTCAACCGAGTTGAGACATATATCAAGTGGCCGTGGTGGGTTTGGTTGATTATTTTCATTGTTCTCATCTTTGTTGT
GTCATTACTAGTGTTCTGCTGCATTTCCACGGGTTGTTGTGGATGCTGCGGCTGCTGCTGTGCTTGTTTCTCAGGTTGT
TGTAGGGGTCCTAGACTTCAACCTTACGAAGTTTTTGAAAAGGTCCACGTGCAGTGA

>gi|514483276|gb|KF272920.1| Porcine epidemic diarrhea virus strain USA/Colorado/2013, complete genome

ATGAAGTCTTTAACCTACTTCTGGTTGTTCTTACCAGTACTTTCAACACTTAGCCTACCACAAGATGTCACCAGG
TGCTCAGCTAACACTAATTTTAGGCGGTTCTTTTCAAAATTTAATGTTCAGGCGCCTGCAGTTGTTGTACTGGGCGGTT
ATCTACCTATTGGTGAAAACCAGGGTGTCAATTCAACTTGGTACTGTGCTGGCCAACATCCAACTGCTAGTGGCGTTCA
TGGTATCTTTGTTAGCCATATTAGAGGTGGTCATGGCTTTGAGATTGGCATTTCGCAAGAGCCTTTTGACCCTAGTGGT
TACCAGCTTTATTTACATAAGGCTACTAACGGTAACACTAATGCTACTGCGCGACTGCGCATTTGCCAGTTTCCTAGCA
TTAAAACATTGGGCCCCACTGCTAATAATGATGTTACAACAGGTCGTAATTGCCTATTTAACAAAGCCATCCCAGCTCA
TATGAGTGAACATAGTGTTGTCGGCATAACATGGGATAATGATCGTGTCACTGTCTTTTCTGACAAGATCTATTATTTT
TATTTTAAAAATGATTGGTCCCGTGTTGCGACAAAGTGTTACAACAGTGGAGGTTGTGCTATGCAATATGTTTACGAAC
CCACCTATTACATGCTTAATGTTACTAGTGCTGGTGAGGATGGTATTTCTTATCAACCCTGTACAGCTAATTGCATTGG
TTATGCTGCCAATGTATTTGCTACTGAGCCCAATGGCCACATACCAGAAGGTTTTAGTTTTAATAATTGGTTTCTTTTG
TCCAATGATTCCACTTTGGTGCATGGTAAGGTGGTTTCCAACCAACCATTGTTGGTCAATTGTCTTTTGGCCATTCCTA
AGATTTATGGACTAGGCCAATTTTTCTCCTTTAATCAAACGATCGATGGTGTTTGTAATGGAGCTGCTGTGCAGCGTGC

ACCAGAGGCTCTGAGGTTTAATATTAATGACACCTCTGTCATTCTTGCTGAAGGCTCAATTGTACTTCATACTGCTTTA
GGAACAAATTTTTCTTTTGTTTGCAGTAATTCCTCAAATCCTCACTTAGCCACCTTCGCCATACCTCTGGGTGCTACCC
AAGTACCTTATTATTGTTTTCTTAAAGTGGATACTTACAACTCCACTGTTTATAAATTTTTGGCTGTTTTACCTCCTAC
CGTCAGGGAAATTGTCATCACCAAGTATGGTGATGTTTATGTCAATGGGTTTGGATACTTGCATCTCGGTTTGTTGGAT
GCTGTCACAATTAATTTCACTGGTCATGGCACTGACGATGATGTTTCTGGTTTTTGGACCATAGCATCGACTAATTTTG
TTGATGCACTCATCGAAGTTCAAGGAACCGCCATTCAGCGTATTCTTTATTGTGATGATCCTGTTAGCCAACTCAAGTG
TTCTCAGGTTGCTTTTGACCTTGACGATGGTTTTTACCCTATTTCTTCTAGAAACCTTCTGAGTCATGAACAGCCAATT
TCTTTTGTTACTCTGCCATCATTTAATGATCATTCTTTTGTTAACATTACTGTATCTGCTTCCTTTGGTGGTCATAGTG
GTGCCAACCTTATTGCATCTGACACTACTATCAATGGGTTTAGTTCTTTCTGTGTTGACACTAGACAATTTACCATTTC
ACTGTTTTATAACGTTACAAACAGTTATGGTTATGTGTCTAAATCACAGGACAGTAATTGCCCTTTCACCTTGCAATCT
GTTAATGATTACCTGTCTTTTAGCAAATTTTGTGTTTCCACCAGCCTTTTGGCTAGTGCCTGTACCATAGATCTTTTTG
GTTACCCTGAGTTTGGTAGTGGTGTTAAGTTTACGTCCCTTTACTTTCAATTCACAAAGGGTGAGTTGATTACTGGCAC
GCCTAAACCACTTGAAGGTGTCACGGACGTTTCTTTTATGACTCTGGATGTGTGTACCAAGTATACTATCTATGGCTTT
AAAGGTGAGGGTATCATTACCCTTACAAATTCTAGCTTTTTGGCAGGTGTTTATTACACATCTGATTCTGGACAGTTGT
TAGCCTTTAAGAATGTCACTAGTGGTGCTGTTTATTCTGTTACGCCATGTTCTTTTTCAGAGCAGGCTGCATATGTTGA
TGATGATATAGTGGGTGTTATTTCTAGTTTGTCTAGCTCCACTTTTAACAGTACTAGGGAGTTGCCTGGTTTCTTCTAC
CATTCTAATGATGGCTCTAATTGTACAGAGCCTGTGTTGGTGTATAGTAACATAGGTGTTTGTAAATCTGGCAGTATTG
GCTACGTCCCATCTCAGTCTGGCCAAGTCAAGATTGCACCCACGGTTACTGGGAATATTAGTATTCCCACCAACTTTAG
TATGAGTATTAGGACAGAATATTTACAGCTTTACAACACGCCTGTTAGTGTTGATTGTGCCACATATGTTTGTAATGGT
AACTCTCGTTGTAAACAATTACTCACCCAGTACACTGCAGCATGTAAGACCATAGAGTCAGCATTACAACTCAGCGCTA
GGCTTGAGTCTGTTGAAGTTAACTCTATGCTTACTATTTCTGAAGAGGCTCTACAGTTAGCTACCATTAGTTCGTTTAA
TGGTGATGGATATAATTTTACTAATGTGCTGGGTGTTTCTGTGTATGATCCTGCAAGTGGCAGGGTGGTACAAAAAAGG
TCTTTTATTGAAGACCTGCTTTTTAATAAAGTGGTTACTAATGGCCTTGGTACTGTTGATGAAGACTATAAGCGCTGTT
CTAATGGTCGCTCTGTGGCAGATCTAGTCTGTGCACAGTATTACTCTGGTGTCATGGTACTACCTGGTGTTGTTGACGC
TGAGAAGCTTCACATGTATAGTGCGTCTCTCATCGGTGGTATGGTGCTAGGAGGTTTTACTTCTGCAGCGGCATTGCCT
TTTAGCTATGCTGTTCAAGCTAGACTCAATTATCTTGCTCTACAGACGGATGTTCTACAGCGGAACCAGCAATTGCTTG
CTGAGTCTTTTAACTCTGCTATTGGTAATATAACTTCAGCCTTTGAGAGTGTTAAAGAGGCTATTAGTCAAACTTCCAA
GGGTTTGAACACTGTGGCTCATGCGCTTACTAAGGTTCAAGAGGTTGTTAACTCGCAGGGTGCAGCTTTGACTCAACTT
ACCGTACAGCTGCAACACAACTTCCAAGCCATTTCTAGTTCTATTGATGACATTTACTCTCGACTGGACATTCTTTCAG
CCGATGTTCAGGTTGACCGTCTCATCACCGGCAGATTATCAGCACTTAATGCTTTTGTTGCTCAAACCCTCACTAAGTA
TACTGAGGTTCAGGCTAGCAGGAAGTTAGCACAGCAAAAGGTTAATGAGTGCGTTAAATCGCAATCTCAGCGTTATGGT
TTTTGTGGTGGTGATGGCGAGCACATTTTCTCTCTGGTACAGGCAGCACCTCAGGGCCTGCTGTTTTTACATACAGTAC
TTGTACCGAGTGATTTTGTAGATGTTATTGCCATCGCTGGCTTATGCGTTAACGATGAAATTGCCTTGACTCTACGTGA
GCCTGGCTTAGTCTTGTTTACGCATGAACTTCAAAATCATACTGCGACGGAATATTTTGTTTCATCGCGACGTATGTTT
GAACCTAGAAAACCTACCGTTAGTGATTTTGTTCAAATTGAGAGTTGTGTGGTCACCTATGTCAATTTGACTAGAGACC
AACTACCAGATGTAATCCCAGATTACATCGATGTTAACAAAACACTTGATGAGATTTTAGCTTCTCTGCCCAATAGAAC
TGGTCCAAGTCTTCCTTTAGATGTTTTTAATGCCACTTATCTTAATCTCACTGGTGAAATTGCAGATTTAGAGCAGCGT
TCAGAGTCTCTCCGTAATACTACAGAGGAGCTCCAAAGTCTTATATATAATATCAACAACACACTAGTTGACCTTGAGT
GGCTCAACCGAGTTGAGACATATATCAAGTGGCCGTGGTGGGTTTGGTTGATTATTTTCATTGTTCTCATCTTTGTTGT
GTCATTACTAGTGTTCTGCTGCATTTCCACGGGTTGTTGTGGATGCTGCGGCTGCTGCTGTGCTTGTTTCTCAGGTTGT
TGTAGGGGTCCTAGACTTCAACCTTACGAAGTTTTTGAAAAGGTCCACGTGCAGTGA

>gi|564760327|gb|KF452322.1| Porcine epidemic diarrhea virus strain

USA/Iowa/16465/2013, complete genome

ATGAAGTCTTTAACCTACTTCTGGTTGTTCTTACCAGTACTTTCAACACTTAGCCTACCACAAGATGTCACCAGG
TGCTCAGCTAACACTAATTTTAGGCGGTTCTTTTCAAAATTTAATGTTCAGGCGCCTGCAGTTGTTGTACTGGGCGGTT
ATCTACCTATTGGTGAAAACCAGGGTGTCAATTCAACTTGGTACTGTGCTGGCCAACATCCAACTGCTAGTGGCGTTCA
TGGTATCTTTGTTAGCTACATCGATTCTGGTCATGGCTTTGAGATTGGCATTTCGCAAGAGCCTTTTGACCCTAGTGGT
TACCAGCTTTATTTACATAAGGCTACTAACGGTAACACTAATGCTACTGCGCGACTGCGCATTTGCCAGTTTCCCAACA
ATAAAACATTGGGCCCCACTGCTAATAATGATGTTACAACAGGTCGTAATTGCCTATTTAACAAAGCCATCCCAGCTCA
TATGAGTGAACATAGTGTTGTCGGCATAACATGGGATAATGATCGTGTCACTGTCTTTTCTGACAAGATCTATTATTTT
TATTTTAAAAATGATTGGTCCCGTGTTGCGACAAAGTGTTACAACAGTGGAGGTTGTGCTATGCAATATGTTTACGAAC
CCACCTATTACATGCTTAATGTTACTAGTGCTGGTGAGGATGGTATTTCTTATCAACCCTGTACAGCTAATTGCATTGG
TTATGCTGCCAATGTATTTGCTACTGAGCCCAATGGCCACATACCAGAAGGTTTTAGTTTTAATAATTGGTTTCTTTTG
TCCAATGATTCCACTTTGGTGCATGGTAAGGTGGTTTCCAACCAACCATTGTTGGTCAATTGTCTTTTGGCCATTCCTA
AGATTTATGGACTAGGCCAATTTTTCTCCTTTAATCAAACGATCGATGGTGTTTGTAATGGAGCTGCTGTGCAGCGTGC
ACCAGAGGCTCTGAGGTTTAATATTAATGACACCTCTGTCATTCTTGCTGAAGGCTCAATTGTACTTCATACTGCTTTA
GGAACAAATTTTTCTTTTGTTTGCAGTAATTCCTCAAATCCTCACTTAGCCACCTTCGCCATACCTTTGGGTGCTACCC
AAGTACCCTATTATTGTTTTCTTAAAGTGGATACTTACAACTCCACTGTTTATAAATTCTTGGCTGTTTTACCTCCAAC
CGTCAGGGAAATTGTCATCACCAAGTATGGTGATGTTTATGTCAATGGGTTTGGATACTTGCATCTCGGTTTGTTGGAT
GCTGTCACAATTAATTTCACTGGTCATGGCACTGACGATGATGTTTCTGGTTTTTGGACCATAGCATCGACTAATTTTG
TTGATGCACTCATCGAAGTTCAAGGAACCGCCATTCAGCGTATTCTTTATTGTGATGATCCTGTTAGCCAACTCAAGTG
TTCTCAGGTTGCTTTTGACCTTGACGATGGTTTTTACCCTATTTCTTCTAGAAACCTTCTGAGTCATGAACAGCCAATT
TCTTTTGTTACTCTGCCATCATTTAATGATCATTCTTTTGTTAACATTACTGTATCTGCTTCCTTTGGTGGTCATAGTG
GTGCCAACCTTATTGCATCTGACACTACTATCAATGGGTTTAGTTCTTTCTGTGTTGACACTAGACAATTTACCATTTC
ACTGTTTTATAACGTTACAAACAGTTATGGTTATGTGTCTAAATCACAGGACAGTAATTGCCCTTTCACCTTGCAATCT
GTTAATGATTACCTGTCTTTTAGCAAATTTTGTGTTTCCACCAGCCTTTTGGCTAGTGCCTGTACCATAGATCTTTTTG
GTTACCCTGAGTTTGGTAGTGGTGTTAAGTTTACGTCCCTTTACTTTCAATTCACAAAGGGTGAGTTGATTACTGGCAC
GCCTAAACCACTTGAAGGTGTCACGGACGTTTCTTTTATGACTCTGGATGTGTGTACCAAGTATACTATCTATGGCTTT
AAAGGTGAGGGTATCATTACCCTTACAAATTCTAGCTTTTTGGCAGGTGTTTATTACACATCTGATTCTGGACAGTTGT
TAGCCTTTAAGAATGTCACTAGTGGTGCTGTTTATTCTGTTACGCCATGTTCTTTTTCAGAGCAGGCTGCATATGTTGA
TGATGATATAGTGGGTGTTATTTCTAGTTTGTCTAGCTCCACTTTTAACAGTACTAGGGAGTTGCCTGGTTTCTTCTAC
CATTCTAATGATGGCTCTAATTGTACAGAGCCTGTGTTGGTGTATAGTAACATAGGTGTTTGTAAATCTGGCAGTATTG
GCTACGTCCCATCTCAGTCTGGCCAAGTCAAGATTGCACCCACGGTTACTGGGAATATTAGTATTCCCACCAACTTTAG
TATGAGTATTAGGACAGAATATTTACAGCTTTACAACACGCCTGTTAGTGTTGATTGTGCCACATATGTTTGTAATGGT
AACTCTCGTTGTAAACAATTACTCACCCAGTACACTGCAGCATGTAAGACCATAGAGTCAGCATTACAACTCAGCGCTA
GGCTTGAGTCTGTTGAAGTTAACTCTATGCTTACTATTTCTGAAGAGGCTCTACAGTTAGCTACCATTAGTTCGTTTAA
TGGTGATGGATATAATTTTACTAATGTGCTGGGTGTTTCTGTGTATGATCCTGCAAGTGGCAGGGTGGTACAAAAAAGG
TCTTTTATTGAAGACCTGCTTTTTAATAAAGTGGTTACTAATGGCCTTGGTACTGTTGATGAAGACTATAAGCGCTGTT
CTAATGGTCGCTCTGTGGCAGATCTAGTCTGTGCACAGTATTACTCTGGTGTCATGGTACTACCTGGTGTTGTTGACGC
TGAGAAGCTTCACATGTATAGTGCGTCTCTCATCGGTGGTATGGTGCTAGGAGGTTTTACTTCTGCAGCGGCATTGCCT
TTTAGCTATGCTGTTCAAGCTAGACTCAATTATCTTGCTCTACAGACGGATGTTCTACAGCGGAACCAGCAATTGCTTG
CTGAGTCTTTTAACTCTGCTATTGGTAATATAACTTCAGCCTTTGAGAGTGTTAAAGAGGCTATTAGTCAAACTTCCAA
GGGTTTGAACACTGTGGCTCATGCGCTTACTAAGGTTCAAGAGGTTGTTAACTCGCAGGGTGCAGCTTTGACTCAACTT
ACCGTACAGCTGCAACACAACTTCCAAGCCATTTCTAGTTCTATTGATGACATTTACTCTCGACTGGACATTCTTTCAG
CCGATGTTCAGGTTGACCGTCTCATCACCGGCAGATTATCAGCACTTAATGCTTTTGTTGCTCAAACCCTCACTAAGTA

TACTGAGGTTCAGGCTAGCAGGAAGTTAGCACAGCAAAAGGTTAATGAGTGCGTTAAATCGCAATCTCAGCGTTATGGT
TTTTGTGGTGGTGATGGCGAGCACATTTTCTCTCTGGTACAGGCAGCACCTCAGGGCCTGCTGTTTTTACATACAGTAC
TTGTACCGAGTGATTTTGTAGATGTTATTGCCATCGCTGGCTTATGCGTTAACGATGAAATTGCCTTGACTCTACGTGA
GCCTGGCTTAGTCTTGTTTACGCATGAACTTCAAAATCATACTGCGACGGAATATTTTGTTTCATCGCGACGTATGTTT
GAACCTAGAAAACCTACCGTTAGTGATTTTGTTCAAATTGAGAGTTGTGTGGTCACCTATGTCAATTTGACTAGAGACC
AACTACCAGATGTAATCCCAGATTACATCGATGTTAACAAAACACTTGATGAGATTTTAGCTTCTCTGCCCAATAGAAC
TGGTCCAAGTCTTCCTTTAGATGTTTTTAATGCCACTTATCTTAATCTCACTGGTGAAATTGCAGATTTAGAGCAGCGT
TCAGAGTCTCTCCGTAATACTACAGAGGAGCTCCAAAGTCTTATATATAATATCAACAACACACTAGTTGACCTTGAGT
GGCTCAACCGAGTTGAGACATATATCAAGTGGCCGTGGTGGGTTTGGTTGATTATTTTCATTGTTCTCATCTTTGTTGT
GTCATTACTAGTGTTCTGCTGCATTTCCACGGGTTGTTGTGGATGCTGCGGCTGCTGCTGTGCTTGTTTCTCAGGTTGT
TGTAGGGGTCCTAGACTTCAACCTTACGAAGTTTTTGAAAAGGTCCACGTGCAGTGA

>gi|564760356|gb|KF452323.1| Porcine epidemic diarrhea virus strain USA/Indiana/17846/2013, complete genome

ATGAAGTCTTTAACCTACTTCTGGTTGTTCTTACCAGTACTTTCAACACTTAGCCTACCACAAGATGTCACCAGG
TGCTCAGCTAACACTAATTTTAGGCGGTTCTTTTCAAAATTTAATGTTCAGGCGCCTGCAGTTGTTGTACTGGGCGGTT
ATCTACCTATTGGTGAAAACCAGGGTGTCAATTCAACTTGGTACTGTGCTGGCCAACATCCAACTGCTAGTGGCGTTCA
TGGTATCTTTGTTAGCCATATTAGAGGTGGTCATGGCTTTGAGATTGGCATTTCGCAAGAGCCTTTTGACCCTAGTGGT
TACCAGCTTTATTTACATAAGGCTACTAACGGTAACACTAATGCTACTGCGCGACTGCGCATTTGCCAGTTTCCTAGCA
TTAAAACATTGGGCCCCACTGCTAATAATGATGTTACAACAGGTCGTAATTGCCTATTTAACAAAGCCATCCCAGCTCA
TATGAGTGAACATAGTGTTGTCGGCATAACATGGGATAATGATCGTGTCACTGTCTTTTCTGACAAGATCTATTATTTT
TATTTTAAAAATGATTGGTCCCGTGTTGCGACAAAGTGTTACAACAGTGGAGGTTGTGCTATGCAATATGTTTACGAAC
CCACCTATTACATGCTTAATGTTACTAGTGCTGGTGAGGATGGTATTTCTTATCAACCCTGTACAGCTAATTGCATTGG
TTATGCTGCCAATGTATTTGCTACTGAGCCCAATGGCCACATACCAGAAGGTTTTAGTTTTAATAATTGGTTTCTTTTG
TCCAATGATTCCACTTTGGTGCATGGTAAGGTGGTTTCCAACCAACCATTGTTGGTCAATTGTCTTTTGGCCATTCCTA
AGATTTATGGACTAGGCCAATTTTTCTCCTTTAATCAAACGATCGATGGTGTTTGTAATGGAGCTGCTGTGCAGCGTGC
ACCAGAGGCTCTGAGGTTTAATATTAATGACACCTCTGTCATTCTTGCTGAAGGCTCAATTGTACTTCATACTGCTTTA
GGAACAAATTTTTCTTTTGTTTGCAGTAATTCCTCAAATCCTCACTTAGCCACCTTCGCCATACCTTTGGGTGCTACCC
AAGTACCCTATTATTGTTTTCTTAAAGTGGATACTTACAACTCCACTGTTTATAAATTCTTGGCTGTTTTACCTCCAAC
CGTCAGGGAAATTGTCATCACCAAGTATGGTGATGTTTATGTCAATGGGTTTGGATACTTGCATCTCGGTTTGTTGGAT
GCTGTCACAATTAATTTCACTGGTCATGGCACTGACGATGATGTTTCTGGTTTTTGGACCATAGCATCGACTAATTTTG
TTGATGCACTCATCGAAGTTCAAGGAACCGCCATTCAGCGTATTCTTTATTGTGATGATCCTGTTAGCCAACTCAAGTG
TTCTCAGGTTGCTTTTGACCTTGACGATGGTTTTTACCCTATTTCTTCTAGAAACCTTCTGAGTCATGAACAGCCAATT
TCTTTTGTTACTCTGCCATCATTTAATGATCATTCTTTTGTTAACATTACTGTATCTGCTTCCTTTGGTGGTCATAGTG
GTGCCAACCTTATTGCATCTGACACTACTATCAATGGGTTTAGTTCTTTCTGTGTTGACACTAGACAATTTACCATTTC
ACTGTTTTATAACGTTACAAACAGTTATGGTTATGTGTCTAAATCACAGGACAGTAATTGCCCTTTCACCTTGCAATCT
GTTAATGATTACCTGTCTTTTAGCAAATTTTGTGTTTCCACCAGCCTTTTGGCTAGTGCCTGTACCATAGATCTTTTTG
GTTACCCTGAGTTTGGTAGTGGTGTTAAGTTTACGTCCCTTTACTTTCAATTCACAAAGGGTGAGTTGATTACTGGCAC
GCCTAAACCACTTGAAGGTGTCACGGACGTTTCTTTTATGACTCTGGATGTGTGTACCAAGTATACTATCTATGGCTTT
AAAGGTGAGGGTATCATTACCCTTACAAATTCTAGCTTTTTGGCAGGTGTTTATTACACATCTGATTCTGGACAGTTGT
TAGCCTTTAAGAATGTCACTAGTGGTGCTGTTTATTCTGTTACGCCATGTTCTTTTTCAGAGCAGGCTGCATATGTTGA
TGATGATATAGTGGGTGTTATTTCTAGTTTGTCTAGCTCCACTTTTAACAGTACTAGGGAGTTGCCTGGTTTCTTCTAC

CATTCTAATGATGGCTCTAATTGTACAGAGCCTGTGTTGGTGTATAGTAACATAGGTGTTTGTAAATCTGGCAGTATTG
GCTACGTCCCATCTCAGTCTGGCCAAGTCAAGATTGCACCCACGGTTACTGGGAATATTAGTATTCCCACCAACTTTAG
TATGAGTATTAGGACAGAATATTTACAGCTTTACAACACGCCTGTTAGTGTTGATTGTGCCACATATGTTTGTAATGGT
AACTCTCGTTGTAAACAATTACTCACCCAGTACACTGCAGCATGTAAGACCATAGAGTCAGCATTACAACTCAGCGCTA
GGCTTGAGTCTGTTGAAGTTAACTCTATGCTTACTATTTCTGAAGAGGCTCTACAGTTAGCTACCATTAGTTCGTTTAA
TGGTGATGGATATAATTTTACTAATGTGCTGGGTGTTTCTGTGTATGATCCTGCAAGTGGCAGGGTGGTACAAAAAAGG
TCTTTTATTGAAGACCTGCTTTTTAATAAAGTGGTTACTAATGGCCTTGGTACTGTTGATGAAGACTATAAGCGCTGTT
CTAATGGTCGCTCTGTGGCAGATCTAGTCTGTGCACAGTATTACTCTGGTGTCATGGTACTACCTGGTGTTGTTGACGC
TGAGAAGCTTCACATGTATAGTGCGTCTCTCATCGGTGGTATGGTGCTAGGAGGTTTTACTTCTGCAGCGGCATTGCCT
TTTAGCTATGCTGTTCAAGCTAGACTCAATTATCTTGCTCTACAGACGGATGTTCTACAGCGGAACCAGCAATTGCTTG
CTGAGTCTTTTAACTCTGCTATTGGTAATATAACTTCAGCCTTTGAGAGTGTTAAAGAGGCTATTAGTCAAACTTCCAA
GGGTTTGAACACTGTGGCTCATGCGCTTACTAAGGTTCAAGAGGTTGTTAACTCGCAGGGTGCAGCTTTGACTCAACTT
ACCGTACAGCTGCAACACAACTTCCAAGCCATTTCTAGTTCTATTGATGACATTTACTCTCGACTGGACATTCTTTCAG
CCGATGTTCAGGTTGACCGTCTCATCACCGGCAGATTATCAGCACTTAATGCTTTTGTTGCTCAAACCCTCACTAAGTA
TACTGAGGTTCAGGCTAGCAGGAAGTTAGCACAGCAAAAGGTTAATGAGTGCGTTAAATCGCAATCTCAGCGTTATGGT
TTTTGTGGTGGTGATGGCGAGCACATTTTCTCTCTGGTACAGGCAGCACCTCAGGGCCTGCTGTTTTTACATACAGTAC
TTGTACCGAGTGATTTTGTAGATGTTATTGCCATCGCTGGCTTATGCGTTAACGATGAAATTGCCTTGACTCTACGTGA
GCCTGGCTTAGTCTTGTTTACGCATGAACTTCAAAATCATACTGCGACGGAATATTTTGTTTCATCGCGACGTATGTTT
GAACCTAGAAAACCTACCGTTAGTGATTTTGTTCAAATTGAGAGTTGTGTGGTCACCTATGTCAATTTGACTAGAGACC
AACTACCAGATGTAATCCCAGATTACATCGATGTTAACAAAACACTTGATGAGATTTTAGCTTCTCTGCCCAATAGAAC
TGGTCCAAGTCTTCCTTTAGATGTTTTTAATGCCACTTATCTTAATCTCACTGGTGAAATTGCAGATTTAGAGCAGCGT
TCAGAGTCTCTCCGTAATACTACAGAGGAGCTCCAAAGTCTTATATATAATATCAACAACACACTAGTTGACCTTGAGT
GGCTCAACCGAGTTGAGACATATATCAAGTGGCCGTGGTGGGTTTGGTTGATTATTTTCATTGTTCTCATCTTTGTTGT
GTCATTACTAGTGTTCTGCTGCATTTCCACGGGTTGTTGTGGATGCTGCGGCTGCTGCTGTGCTTGTTTCTCAGGTTGT
TGTAGGGGTCCTAGACTTCAACCTTACGAAGTTTTTGAAAAGGTCCACGTGCAGTGA

>gi|551485548|gb|KF468752.1| Porcine epidemic diarrhea virus isolate MN, complete genome

ATGAAGTCTTTAACCTACTTCTGGTTGTTCTTACCAGTACTTTCAACACTTAGCCTACCACAAGATGTCACCAGG
TGCTCAGCTAACACTAATTTTAGGCGGTTCTTTTCAAAATTTAATGTTCAGGCGCCTGCAGTTGTTGTACTGGGCGGTT
ATCTACCTATTGGTGAAAACCAGGGTGTCAATTCAACTTGGTACTGTGCTGGCCAACATCCAACTGCTAGTGGCGTTCA
TGGTATCTTTGTTAGCCATATTAGAGGTGGTCATGGCTTTGAGATTGGCATTTCGCAAGAGCCTTTTGACCCTAGTGGT
TACCAGCTTTATTTACATAAGGCTACTAACGGTAACACTAATGCTACTGCGCGACTGCGCATTTGCCAGTTTCCTAGCA
TTAAAACATTGGGCCCCACTGCTAATAATGATGTTACAACAGGTCGTAATTGCCTATTTAACAAAGCCATCCCAGCTCA
TATGAGTGAACATAGTGTTGTCGGCATAACATGGGATAATGATCGTGTCACTGTCTTTTCTGACAAGATCTATTATTTT
TATTTTAAAAATGATTGGTCCCGTGTTGCGACAAAGTGTTACAACAGTGGAGGTTGTGCTATGCAATATGTTTACGAAC
CCACCTATTACATGCTTAATGTTACTAGTGCTGGTGAGGATGGTATTTTTTATCAACCCTGTACAGCTAATTGCATTGG
TTATGCTGCCAATGTATTTGCTACTGAGCCCAATGGCCACATACCAGAAGGTTTTAGTTTTAATAATTGGTTTCTTTTG
TCCAATGATTCCACTTTGGTGCATGGTAAGGTGGTTTCCAACCAACCATTGTTGGTCAATTGTCTTTTGGCCATTCCTA
AGATTTATGGACTAGGCCAATTTTTCTCCTTTAATCAAACGATCGATGGTGTTTGTAATGGAGCTGCTGTGCAGCGTGC
ACCAGAGGCTCTGAGGTTTAATATTAATGACACCTCTGTCATTCTTGCTGAAGGCTCAATTGTACTTCATACTGCTTTA
GGAACAAATTTTTCTTTTGTTTGCAGTAATTCCTCAAATCCTCACTTAGCCACCTTCGCCATACCTTTGGGTGCTACCC

AAGTACCCTATTATTGTTTTCTTAAAGTGGATACTTACAACTCCACTGTTTATAAATTCTTGGCTGTTTTACCTCCAAC
CGTCAGGGAAATTGTCATCACCAAGTATGGTGATGTTTATGTCAATGGGTTTGGATACTTGCATCTCGGTTTGTTGGAT
GCTGTCACAATTAATTTCACTGGTCATGGCACTGACGATGATGTTTCTGGTTTTTGGACCATAGCATCGACTAATTTTG
TTGATGCACTCATCGAAGTTCAAGGAACCGCCATTCAGCGTATTCTTTATTGTGATGATCCTGTTAGCCAACTCAAGTG
TTCTCAGGTTGCTTTTGACCTTGACGATGGTTTTTACCATATTTCTTCTAGAAACCTTCTGAGTCATGAACAGCCAATT
TCTTTTGTTACTCTGCCATCATTTAATGATCATTCTTTTGTTAACATTACTGTATCTGCTTCCTTTGGTGGTCATAGTG
GTGCCAACCTTATTGCATCTGACACTACTATCAATGGGTTTAGTTCTTTCTGTGTTGACACTAGACAATTTACCATTTC
ACTGTTTTATAACGTTACAAACAGTTATGGTTATGTGTCTAAATCACAGGACAGTAATTGCCCTTTCACCTTGCAATCT
GTTAATGATTACCTGTCTTTTAGCAAATTTTGTGTTTCCACCAGCCTTTTGGCTAGTGCCTGTACCATAGATCTTTTTG
GTTACCCTGAGTTTGGTAGTGGTGTTAAGTTTACGTCCCTTTACTTTCAATTCACAAAGGGTGAGTTGATTACTGGCAC
GCCTAAACCACTTGAAGGTGTCACGGACGTTTCTTTTATGACTCTGGATGTGTGTACCAAGTATACTATCTATGGCTTT
AAAGGTGAGGGTATCATTACCCTTACAAATTCTAGCTTTTTGGCAGGTGTTTATTACACATCTGATTCTGGACAGTTGT
TAGCCTTTAAGAATGTCACTAGTGGTGCTGTTTATTCTGTTACGCCATGTTCTTTTTCAGAGCAGGCTGCATATGTTGA
TGATGATATAGTGGGTGTTATTTCTAGTTTGTCTAGCTCCACTTTTAACAGTACTAGGGAGTTGCCTGGTTTCTTCTAC
CATTCTAATGATGGCTCTAATTGTACAGAGCCTGTGTTGGTGTATAGTAACATAGGTGTTTGTAAATCTGGCAGTATTG
GCTACGTCCCATCTCAGTCTGGCCAAGTCAAGATTGCACCCACGGTTACTGGGAATATTAGTATTCCCACCAACTTTAG
TATGAGTATTAGGACAGAATATTTACAGCTTTACAACACGCCTGTTAGTGTTGATTGTGCCACATATGTTTGTAATGGT
AACTCTCGTTGTAAACAATTACTCACCCAGTACACTGCAGCATGTAAGACCATAGAGTCAGCATTACAACTCAGCGCTA
GGCTTGAGTCTGTTGAAGTTAACTCTATGCTTACTATTTCTGAAGAGGCTCTACAGTTAGCTACCATTAGTTCGTTTAA
TGGTGATGGATATAATTTTACTAATGTGCTGGGTGTTTCTGTGTATGATCCTGCAAGTGGCAGGGTGGTACAAAAAAGG
TCTTTTATTGAAGACCTGCTTTTTAATAAAGTGGTTACTAATGGCCTTGGTACTGTTGATGAAGACTATAAGCGCTGTT
CTAATGGTCGCTCTGTGGCAGATCTAGTCTGTGCACAGTATTACTCTGGTGTCATGGTACTACCTGGTGTTGTTGACGC
TGAGAAGCTTCACATGTATAGTGCGTCTCTCATCGGTGGTATGGTGCTAGGAGGTTTTACTTCTGCAGCGGCATTGCCT
TTTAGCTATGCTGTTCAAGCTAGACTCAATTATCTTGCTCTACAGACGGATGTTCTACAGCGGAACCAGCAATTGCTTG
CTGAGTCTTTTAACTCTGCTATTGGTAATATAACTTCAGCCTTTGAGAGTGTTAAAGAGGCTATTAGTCAAACTTCCAA
GGGTTTGAACACTGTGGCTCATGCGCTTACTAAGGTTCAAGAGGTTGTTAACTCGCAGGGTGCAGCTTTGACTCAACTT
ACCGTACAGCTGCAACACAACTTCCAAGCCATTTCTAGTTCTATTGATGACATTTACTCTCGACTGGACATTCTTTCAG
CCGATGTTCAGGTTGACCGTCTCATCACCGGCAGATTATCAGCACTTAATGCTTTTGTTGCTCAAACCCTCACTAAGTA
TACTGAGGTTCAGGCTAGCAGGAAGTTAGCACAGCAAAAGGTTAATGAGTGCGTTAAATCGCAATCTCAGCGTTATGGT
TTTTGTGGTGGTGATGGCGAGCACATTTTCTCTCTGGTACAGGCAGCACCTCAGGGCCTGCTGTTTTTACATACAGTAC
TTGTACCGAGTGATTTTGTAGATGTTATTGCCATCGCTGGCTTATGCGTTAACGATGAAATTGCCTTGACTCTACGTGA
GCCTGGCTTAGTCTTGTTTACGCATGAACTTCAAAATCATACTGCGACGGAATATTTTGTTTCATCGCGACGTATGTTT
GAACCTAGAAAACCTACCGTTAGTGATTTTGTTCAAATTGAGAGTTGTGTGGTCACCTATGTCAATTTGACTAGAGACC
AACTACCAGATGTAATCCCAGATTACATCGATGTTAACAAAACACTTGATGAGATTTTAGCTTCTCTGCCCAATAGAAC
TGGTCCAAGTCTTCCTTTAGATGTTTTTAATGCCACTTATCTTAATCTCACTGGTGAAATTGCAGATTTAGAGCAGCGT
TCAGAGTCTCTCCGTAATACTACAGAGGAGCTCCAAAGTCTTATATATAATATCAACAACACACTAGTTGACCTTGAGT
GGCTCAACCGAGTTGAGACATATATCAAGTGGCCGTGGTGGGTTTGGTTGATTATTTTCATTGTTCTCATCTTTGTTGT
GTCATTACTAGTGTTCTGCTGCATTTCCACGGGTTGTTGTGGATGCTGCGGCTGCTGCTGTGCTTGTTTCTCAGGTTGT
TGTAGGGGTCCTAGACTTCAACCTTACGAAGTTTTTGAAAAGGTCCACGTGCAGTGA

>gi|551485555|gb|KF468753.1| Porcine epidemic diarrhea virus isolate IA1, complete genome

ATGAAGTCTTTAACCTACTTCTGGTTGTTCTTACCAGTACTTTCAACACTTAGCCTACCACAAGATGTCACCAGG
TGCTCAGCTAACACTAATTTTAGGCGGTTCTTTTCAAAATTTAATGTTCAGGCGCCTGCAGTTGTTGTACTGGGCGGTT
ATCTACCTATTGGTGAAAACCAGGGTGTCAATTCAACTTGGTACTGTGCTGGCCAACATCCAACTGCTAGTGGCGTTCA
TGGTATCTTTGTTAGCCATATTAGAGGTGGTCATGGCTTTGAGATTGGCATTTCGCAAGAGCCTTTTGACCCTAGTGGT
TACCAGCTTTATTTACATAAGGCTACTAACGGTAACACTAATGCTACTGCGCGACTGCGCATTTGCCAGTTTCCTAGCA
TTAAAACATTGGGCCCCACTGCTAATAATGATGTTACAACAGGTCGTAATTGCCTATTTAACAAAGCCATCCCAGCTCA
TATGAGTGAACATAGTGTTGTCGGCATAACATGGGATAATGATCGTGTCACTGTCTTTTCTGACAAGATCTATTATTTT
TATTTTAAAAATGATTGGTCCCGTGTTGCGACAAAGTGCTACAACAGTGGAGGTTGTGCTATGCAATATGTTTACGAAC
CCACCTATTACATGCTTAATGTTACTAGTGCTGGTGAGGATGGTATTTCTTATCAACCCTGTACAGCTAATTGCATTGG
TTATGCTGCCAATGTATTTGCTACTGAGCCCAATGGCCACATACCAGAAGGTTTTAGTTTTAATAATTGGTTTCTTTTG
TCCAATGATTCCACTTTGGTGCATGGTAAGGTGGTTTCCAACCAACCATTGTTGGTCAATTGTCTTTTGGCCATTCCTA
AGATTTATGGACTAGGCCAATTTTTCTCCTTTAATCAAACGATCGATGGTGTTTGTAATGGAGCTGCTGTGCAGCGTGC
ACCAGAGGCTCTGAGGTTTAATATTAATGACACCTCTGTCATTCTTGCTGAAGGCTCAATTGTACTTCATACTGCTTTA
GGAACAAATTTTTCTTTTGTTTGCAGTAATTCCTCAAATCCTCACTTAGCCACCTTCGCCATACCTCTGGGTGCTACCC
AAGTACCTTATTATTGTTTTCTTAAAGTGGATACTTACAACTCCACTGTTTATAAATTTTTGGCTGTTTTACCTCCTAC
CGTCAGGGAAATTGTCATCACCAAGTATGGTGATGTTTATGTCAATGGGTTTGGATACTTGCATCTCGGTTTGTTGGAT
GCTGTCACAATTAATTTCACTGGTCATGGCACTGACGATGATGTTTCTGGTTTTTGGACCATAGCATCGACTAATTTTG
TTGATGCACTCATCGAAGTTCAAGGAACCGCCATTCAGCGTATTCTTTATTGTGATGATCCTGTTAGCCAACTCAAGTG
TTCTCAGGTTGCTTTTGACCTTGACGATGGTTTTTACCCTATTTCTTCTAGAAACCTTCTGAGTCATGAACAGCCAATT
TCTTTTGTTACTCTGCCATCATTTAATGATCATTCTTTTGTTAACATTACTGTATCTGCTTCCTTTGGTGGTCATAGTG
GTGCCAACCTTATTGCATCTGACACTACTATCAATGGGTTTAGTTCTTTCTGTGTTGACACTAGACAATTTACCATTTC
ACTGTTTTATAACGTTACAAACAGTTATGGTTATGTGTCTAAATCACAGGACAGTAATTGCCCTTTCACCTTGCAATCT
GTTAATGATTACCTGTCTTTTAGCAAATTTTGTGTTTCCACCAGCCTTTTGGCTAGTGCCTGTACCATAGATCTTTTTG
GTTACCCTGAGTTTGGTAGTGGTGTTAAGTTTACGTCCCTTTACTTTCAATTCACAAAGGGTGAGTTGATTACTGGCAC
GCCTAAACCACTTGAAGGTGTCACGGACGTTTCTTTTATGACTCTGGATGTGTGTACCAAGTATACTATCTATGGCTTT
AAAGGTGAGGGTATCATTACCCTTACAAATTCTAGCTTTTTGGCAGGTGTTTATTACACATCTGATTCTGGACAGTTGT
TAGCCTTTAAGAATGTCACTAGTGGTGCTGTTTATTCTGTTACGCCATGTTCTTTTTCAGAGCAGGCTGCATATGTTGA
TGATGATATAGTGGGTGTTATTTCTAGTTTGTCTAGCTCCACTTTTAACAGTACTAGGGAGTTGCCTGGTTTCTTCTAC
CATTCTAATGATGGCTCTAATTGTACAGAGCCTGTGTTGGTGTATAGTAACATAGGTGTTTGTAAATCTGGCAGTATTG
GCTACGTCCCATCTCAGTCTGGCCAAGTCAAGATTGCACCCACGGTTACTGGGAATATTAGTATTCCCACCAACTTTAG
TATGAGTATTAGGACAGAATATTTACAGCTTTACAACACGCCTGTTAGTGTTGATTGTGCCACATATGTTTGTAATGGT
AACTCTCGTTGTAAACAATTACTCACCCAGTACACTGCAGCATGTAAGACCATAGAGTCAGCATTACAACTCAGCGCTA
GGCTTGAGTCTGTTGAAGTTAACTCTATGCTTACTATTTCTGAAGAGGCTCTACAGTTAGCTACCATTAGTTCGTTTAA
TGGTGATGGATATAATTTTACTAATGTGCTGGGTGTTTCTGTGTATGATCCTGCAAGTGGCAGGGTGGTACAAAAAAGG
TCTTTTATTGAAGACCTGCTTTTTAATAAAGTGGTTACTAATGGCCTTGGTACTGTTGATGAAGACTATAAGCGCTGTT
CTAATGGTCGCTCTGTGGCAGATCTAGTCTGTGCACAGTATTACTCTGGTGTCATGGTACTACCTGGTGTTGTTGACGC
TGAGAAGCTTCACATGTATAGTGCGTCTCTCATCGGTGGTATGGTGCTAGGAGGTTTTACTTCTGCAGCGGCATTGCCT
TTTAGCTATGCTGTTCAAGCTAGACTCAATTATCTTGCTCTACAGACGGATGTTCTACAGCGGAACCAGCAATTGCTTG
CTGAGTCTTTTAACTCTGCTATTGGTAATATAACTTCAGCCTTTGAGAGTGTTAAAGAGGCTATTAGTCAAACTTCCAA
GGGTTTGAACACTGTGGCTCATGCGCTTACTAAGGTTCAAGAGGTTGTTAACTCGCAGGGTGCAGCTTTGACTCAACTT
ACCGTACAGCTGCAACACAACTTCCAAGCCATTTCTAGTTCTATTGATGACATTTACTCTCGACTGGACATTCTTTCAG
CCGATGTTCAGGTTGACCGTCTCATCACCGGCAGATTATCAGCACTTAATGCTTTTGTTGCTCAAACCCTCACTAAGTA
TACTGAGGTTCAGGCTAGCAGGAAGTTAGCACAGCAAAAGGTTAATGAGTGCGTTAAATCGCAATCTCAGCGTTATGGT
TTTTGTGGTGGTGATGGCGAGCACATTTTCTCTCTGGTACAGGCAGCACCTCAGGGCCTGCTGTTTTTACATACAGTAC

TTGTACCGAGTGATTTTGTAGATGTTATTGCCATCGCTGGCTTATGCGTTAACGATGAAATTGCCTTGACTCTACGTGA
GCCTGGCTTAGTCTTGTTTACGCATGAACTTCAAAATCATACTGCGACGGAATATTTTGTTTCATCGCGACGTATGTTT
GAACCTAGAAAACCTACCGTTAGTGATTTTGTTCAAATTGAGAGTTGTGTGGTCACCTATGTCAATTTGACTAGAGACC
AACTACCAGATGTAATCCCAGATTACATCGATGTTAACAAAACACTTGATGAGATTTTAGCTTCTCTGCCCAATAGAAC
TGGTCCAAGTCTTCCTTTAGATGTTTTTAATGCCACTTATCTTAATCTCACTGGTGAAATTGCAGATTTAGAGCAGCGT
TCAGAGTCTCTCCGTAATACTACAGAGGAGCTCCAAAGTCTTATATATAATATCAACAACACACTAGTTGACCTTGAGT
GGCTCAACCGAGTTGAGACATATATCAAGTGGCCGTGGTGGGTTTGGTTGATTATTTTCATTGTTCTCATCTTTGTTGT
GTCATTACTAGTGTTCTGCTGCATTTCCACGGGTTGTTGTGGATGCTGCGGCTGCTGCTGTGCTTGTTTCTCAGGTTGT
TGTAGGGGTCCTAGACTTCAACCTTACGAAGTTTTTGAAAAGGTCCACGTGCAGTGA

>gi|551485562|gb|KF468754.1| Porcine epidemic diarrhea virus isolate IA2, complete genome

ATGAAGTCTTTAACCTATTTCTGGTTGTTCTTACCAGTACTTTCAACACTTAGCCTACCACAAGATGTCACCAGG
TGCTCAGCTAACACTAATTTTAGGCGGTTCTTTTCAAAATTTAATGTTCAGGCGCCTGCAGTTGTTGTACTGGGCGGTT
ATCTACCTATTGGTGAAAACCAGGGTGTCAATTCAACTTGGTACTGTGCTGGCCAACATCCAACTGCTAGTGGCGTTCA
TGGTATCTTTGTTAGCCATATTAGAGGTGGTCATGGCTTTGAGATTGGCATTTCGCAAGAGCCTTTTGACCCTAGTGGT
TACCAGCTTTATTTACATAAGGCTACTAACGGTAACACTAATGCTACTGCGCGACTGCGCATTTGCCAGTTTCCTAGCA
TTAAAACATTGGGCCCCACTGCTAATAATGATGTTACAACAGGTCGTAATTGCCTATTTAACAAAGCCATCCCAGCTCA
TATGAGTGAACATAGTGTTGTCGGCATAACATGGGATAATGATCGTGTCACTGTCTTTTCTGACAAGATCTATTATTTT
TATTTTAAAAATGATTGGTCCCGTGTTGCGACAAAGTGTTACAACAGTGGAGGTTGTGCTATGCAATATGTTTACGAAC
CCACCTATTACATGCTTAATGTTACTAGTGCTGGTGAGGATGGTATTTCTTATCAACCCTGTACAGCTAATTGCATTGG
TTATGCTGCCAATGTATTTGCTACTGAGCCCAATGGCCACATACCAGAAGGTTTTAGTTTTAATAATTGGTTTCTTTTG
TCCAATGATTCCACTTTGGTGCATGGTAAGGTGGTTTCCAACCAACCATTGTTGGTCAATTGTCTTTTGGCCATTCCTA
AGATTTATGGACTAGGCCAATTTTTCTCCTTTAATCAAACGATCGATGGTGTTTGTAATGGAGCTGCTGTGCAGCGTGC
ACCAGAGGCTCTGAGGTTTAATATTAATGACACCTCTGTCATTCTTGCTGAAGGCTCAATTGTACTTCATACTGCTTTA
GGAACAAATTTTTCTTTTGTTTGCAGTAATTCCTCAAATCTTCACTTAGCCACCTTCGCCATACCTTTGGGTGCTACCC
AAGTACCCTATTATTGTTTTCTTAAAGTGGATACTTACAACTCCACTGTTTATAAATTCTTGGCTGTTTTACCTCCAAC
CGTCAGGGAAATTGTCATCACCAAGTATGGTGATGTTTATGTCAATGGGTTTGGATACTTGCATCTCGGTTTGTTGGAT
GCTGTCACAATTAATTTCACTGGTCATGGCACTGACGATGATGTTTCTGGTTTTTGGACCATAGCATCGACTAATTTTG
TTGATGCACTCATCGAAGTTCAAGGAACCGCCATTCAGCGTATTCTTTATTGTGATGATCCTGTTAGCCAACTCAAGTG
TTCTCAGGTTGCTTTTGACCTTGACGATGGTTTTTACCCTATTTCTTCTAGAAACCTTCTGAGTCATGAACAGCCAATT
TCTTTTGTTACTCTGCCATCATTTAATGATCATTCTTTTGTTAACATTACTGTATCTGCTTCCTTTGGTGGTCATAGTG
GTGCCAACCTTATTGCATCTGACACTACTATCAATGGGTTTAGTTCTTTCTGTGTTGACACTAGACAATTTACCATTTC
ACTGTTTTATAACGTTACAAACAGTTATGGTTATGTGTCTAAATCACAGGACAGTAATTGCCCTTTCACCTTGCAATCT
GTTAATGATTACCTGTCTTTTAGCAAATTTTGTGTTTCCACCAGCCTTTTGGCTAGTGCCTGTACCATAGATCTTTTTG
GTTACCCTGAGTTTGGTAGTGGTGTTAAGTTTACGTCCCTTTACTTTCAATTCACAAAGGGTGAGTTGATTACTGGCAC
GCCTAAACCACTTGAAGGTGTCACGGACGTTTCTTTTATGACTCTGGATGTGTGTACCAAGTATACTATCTATGGCTTT
AAAGGTGAGGGTATCATTACCCTTACAAATTCTAGCTTTTTGGCAGGTGTTTATTACACATCTGATTCTGGACAGTTGT
TAGCCTTTAAGAATGTCACTAGTGGTGCTGTTTATTCTGTTACGCCATGTTCTTTTTCAGAGCAGGCTGCATATGTTGA
TGATGATATAGTGGGTGTTATTTCTAGTTTGTCTAGCTCCACTTTTAACAGTACTAGGGAGTTGCCTGGTTTCTTCTAC
CATTCTAATGATGGCTCTAATTGTACAGAGCCTGTGTTGGTGTATAGTAACATAGGTGTTTGTAAATCTGGCAGTATTG
GCTACGTCCCATCTCAGTCTGGCCAAGTCAAGATTGCACCCACGGTTACTGGGAATATTAGTATTCCCACCAACTTTAG

TATGAGTATTAGGACAGAATATTTACAGCTTTACAACACGCCTGTTAGTGTTGATTGTGCCACATATGTTTGTAATGGT
AACTCTCGTTGTAAACAATTACTCACCCAGTACACTGCAGCATGTAAGACCATAGAGTCAGCATTACAACTCAGCGCTA
GGCTTGAGTCTGTTGAAGTTAACTCTATGCTTACTATTTCTGAAGAGGCTCTACAGTTAGCTACCATTAGTTCGTTTAA
TGGTGATGGATATAATTTTACTAATGTGCTGGGTGTTTCTGTGTATGATCCTGCAAGTGGCAGGGTGGTACAAAAAAGG
TCTTTTATTGAAGACCTGCTTTTTAATAAAGTGGTTACTAATGGCCTTGGTACTGTTGATGAAGACTATAAGCGCTGTT
CTAATGGTCGCTCTGTGGCAGATCTAGTCTGTGCACAGTATTACTCTGGTGTCATGGTACTACCTGGTGTTGTTGACGC
TGAGAAGCTTCACATGTATAGTGCGTCTCTCATCGGTGGTATGGTGCTAGGAGGTTTTACTTCTGCAGCGGCATTGCCT
TTTAGCTATGCTGTTCAAGCTAGACTCAATTATCTTGCTCTACAGACGGATGTTCTACAGCGGAACCAGCAATTGCTTG
CTGAGTCTTTTAACTCTGCTATTGGTAATATAACTTCAGCCTTTGAGAGTGTTAAAGAGGCTATTAGTCAAACTTCCAA
GGGTTTGAACACTGTGGCTCATGCGCTTACTAAGGTTCAAGAGGTTGTTAACTCGCAGGGTGCAGCTTTGACTCAACTT
ACCGTACAGCTGCAACACAACTTCCAAGCCATTTCTAGTTCTATTGATGACATTTACTCTCGACTGGACATTCTTTCAG
CCGATGTTCAGGTTGACCGTCTCATCACCGGCAGATTATCAGCACTTAATGCTTTTGTTGCTCAAACCCTCACTAAGTA
TACTGAGGTTCAGGCTAGCAGGAAGTTAGCACAGCAAAAGGTTAATGAGTGCGTTAAATCGCAATCTCAGCGTTATGGT
TTTTGTGGTGGTGATGGCGAGCACATTTTCTCTCTGGTACAGGCAGCACCTCAGGGCCTGCTGTTTTTACATACAGTAC
TTGTACCGAGTGATTTTGTAGATGTTATTGCCATCGCTGGCTTATGCGTTAACGATGAAATTGCCTTGACTCTACGTGA
GCCTGGCTTAGTCTTGTTTACGCATGAACTTCAAAATCATACTGCGACGGAATATTTTGTTTCATCGCGACGTATGTTT
GAACCTAGAAAACCTACCGTTAGTGATTTTGTTCAAATTGAGAGTTGTGTGGTCACCTATGTCAATTTGACTAGAGACC
AACTACCAGATGTAATCCCAGATTACATCGATGTTAACAAAACACTTGATGAGATTTTAGCTTCTCTGCCCAATAGAAC
TGGTCCAAGTCTTCCTTTAGATGTTTTTAATGCCACTTATCTTAATCTCACTGGTGAAATTGCAGATTTAGAGCAGCGT
TCAGAGTCTCTCCGTAATACTACAGAGGAGCTCCAAAGTCTTATATATAATATCAACAACACACTAGTTGACCTTGAGT
GGCTCAACCGAGTTGAGACATATATCAAGTGGCCGTGGTGGGTTTGGTTGATTATTTTCATTGTTCTCATCTTTGTTGT
GTCATTACTAGTGTTCTGCTGCATTTCCACGGGTTGTTGTGGATGCTGCGGCTGCTGCTGTGCTTGTTTCTCAGGTTGT
TGTAGGGGTCCTAGACTTCAACCTTACGAAGTTTTTGAAAAGGTCCACGTGCAGTGA

>gi|557844677|gb|KF650371.1| Porcine epidemic diarrhea virus isolate ISU13-19338E-IN-passage3, complete genome

ATGAAGTCTTTAACCTACTTCTGGTTGTTCTTACCAGTACTTTCAACACTTAGCCTACCACAAGATGTCACCAGG
TGCTCAGCTAACACTAATTTTAGGCGGTTCTTTTCAAAATTTAATGTTCAGGCGCCTGCAGTTGTTGTACTGGGCGGTT
ATCTACCTATTGGTGAAAACCAGGGTGTCAATTCAACTTGGTACTGTGCTGGCCAACATCCAACTGCTAGTGGCGTTCA
TGGTATCTTTGTTAGCCATATTAGAGGTGGTCATGGCTTTGAGATTGGCATTTCGCAAGAGCCTTTTGACCCTAGTGGT
TACCAGCTTTATTTACATAAGGCTACTAACGGTAACACTAATGCTACTGCGCGACTGCGCATTTGCCAGTTTCCTAGCA
TTAAAACATTGGGCCCCACTGCTAATAATGATGTTACAACAGGTCGTAATTGCCTATTTAACAAAGCCATCCCAGCTCA
TATGAGTGAACATAGTGTTGTCGGCATAACATGGGATAATGATCGTGTCACTGTCTTTTCTGACAAGATCTATTATTTT
TATTTTAAAAATGATTGGTCCCGTGTTGCGACAAAGTGTTACAACAGTGGAGGTTGTGCTATGCAATATGTTTACGAAC
CCACCTATTACATGCTTAATGTTACTAGTGCTGGTGAGGATGGTATTTCTTATCAACCCTGTACAGCTAATTGCATTGG
TTATGCTGCCAATGTATTTGCTACTGAGCCCAATGGCCACATACCAGAAGGTTTTAGTTTTAATAATTGGTTTCTTTTG
TCCAATGATTCCACTTTGGTGCATGGTAAGGTGGTTTCCAACCAACCATTGTTGGTCAATTGTCTTTTGGCCATTCCTA
AGATTTATGGACTAGGCCAATTTTTCTCCTTTAATCAAACGATCGATGGTGTTTGTAATGGAGCTGCTGTGCAGCGTGC
ACCAGAGGCTCTGAGGTTTAATATTAATGACACCTCTGTCATTCTTGCTGAAGGCTCAATTGTACTTCATACTGCTTTA
GGAACAAATTTTTCTTTTGTTTGCAGTAATTCCTCAAATCCTCACTTAGCCACCTTCGCCATACCTTTGGGTGCTACCC
AAGTACCCTATTATTGTTTTTTTAAAGTGGATACTTACAACTCCACTGTTTATAAATTCTTGGCTGTTTTACCTCCAAC
CGTCAGGGAAATTGTCATCACCAAGTATGGTGATGTTTATGTCAATGGGTTTGGATACTTGCATCTCGGTTTGTTGGAT

GCTGTCACAATTAATTTCACTGGTCATGGCACTGACGATGATGTTTCTGGTTTTTGGACCATAGCATCGACTAATTTTG
TTGATGCACTCATCGAAGTTCAAGGAACCGCCATTCAGCGTATTCTTTATTGTGATGATCCTGTTAGCCAACTCAAGTG
TTCTCAGGTTGCTTTTGACCTTGACGATGGTTTTTACCCTATTTCTTCTAGAAACCTTCTGAGTCATGAACAGCCAATT
TCTTTTGTTACTCTGCCATCATTTAATGATCATTCTTTTGTTAACATTACTGTATCTGCTTCCTTTGGTGGTCATAGTG
GTGCCAACCTTATTGCATCTGACACTACTATCAATGGGTTTAGTTCTTTCTGTGTTGACACTAGACAATTTACCATTTC
ACTGTTTTATAACGTTACAAACAGTTATGGTTATGTGTCTAAATCACAGGACAGTAATTGCCCTTTCACCTTGCAATCT
GTTAATGATTACCTGTCTTTTAGCAAATTTTGTGTTTCCACCAGCCTTTTGGCTAGTGCCTGTACCATAGATCTTTTTG
GTTACCCTGAGTTTGGTAGTGGTGTTAAGTTTACGTCCCTTTACTTTCAATTCACAAAGGGTGAGTTGATTACTGGCAC
GCCTAAACCACTTGAAGGTGTCACGGACGTTTCTTTTATGACTCTGGATGTGTGTACCAAGTATACTATCTATGGCTTT
AAAGGTGAGGGTATCATTACCCTTACAAATTCTAGCTTTTTGGCAGGTGTTTATTACACATCTGATTCTGGACAGTTGT
TAGCCTTTAAGAATGTCACTAGTGGTGCTGTTTATTCTGTTACGCCATGTTCTTTTTCAGAGCAGGCTGCATATGTTGA
TGATGATATAGTGGGTGTTATTTCTAGTTTGTCTAGCTCCACTTTTAACAGTACTAGGGAGTTGCCTGGTTTCTTCTAC
CATTCTAATGATGGCTCTAATTGTACAGAGCCTGTGTTGGTGTATAGTAACATAGGTGTTTGTAAATCTGGCAGTATTG
GCTACGTCCCATCTCAGTCTGGCCAAGTCAAGATTGCACCCACGGTTACTGGGAATATTAGTATTCCCACCAACTTTAG
TATGAGTATTAGGACAGAATATTTACAGCTTTACAACACGCCTGTTAGTGTTGATTGTGCCACATATGTTTGTAATGGT
AACTCTCGTTGTAAACAATTACTCACCCAGTACACTGCAGCATGTAAGACCATAGAGTCAGCATTACAACTCAGCGCTA
GGCTTGAGTCTGTTGAAGTTAACTCTATGCTTACTATTTCTGAAGAGGCTCTACAGTTAGCTACCATTAGTTCGTTTAA
TGGTGATGGATATAATTTTACTAATGTGCTGGGTGTTTCTGTGTATGATCCTGCAAGTGGCAGGGTGGTACAAAAAAGG
TCTTTTATTGAAGACCTGCTTTTTAATAAAGTGGTTACTAATGGCCTTGGTACTGTTGATGAAGACTATAAGCGCTGTT
CTAATGGTCGCTCTGTGGCAGATCTAGTCTGTGCACAGTATTACTCTGGTGTCATGGTACTACCTGGTGTTGTTGACGC
TGAGAAGCTTCACATGTATAGTGCGTCTCTCATCGGTGGTATGGTGCTAGGAGGTTTTACTTCTGCAGCGGCATTGCCT
TTTAGCTATGCTGTTCAAGCTAGACTCAATTATCTTGCTCTACAGACGGATGTTCTACAGCGGAACCAGCAATTGCTTG
CTGAGTCTTTTAACTCTGCTATTGGTAATATAACTTCAGCCTTTGAGAGTGTTAAAGAGGCTATTAGTCAAACTTCCAA
GGGTTTGAACACTGTGGCTCATGCGCTTACTAAGGTTCAAGAGGTTGTTAACTCGCAGGGTGCAGCTTTGACTCAACTT
ACCGTACAGCTGCAACACAACTTCCAAGCCATTTCTAGTTCTATTGATGACATTTACTCTCGACTGGACATTCTTTCAG
CCGATGTTCAGGTTGACCGTCTCATCACCGGCAGATTATCAGCACTTAATGCTTTTGTTGCTCAAACCCTCACTAAGTA
TACTGAGGTTCAGGCTAGCAGGAAGTTAGCACAGCAAAAGGTTAATGAGTGCGTTAAATCGCAATCTCAGCGTTATGGT
TTTTGTGGTGGTGATGGCGAGCACATTTTCTCTCTGGTACAGGCAGCACCTCAGGGCCTGCTGTTTTTACATACAGTAC
TTGTACCGAGTGATTTTGTAGATGTTATTGCCATCGCTGGCTTATGCGTTAACGATGAAATTGCCTTGACTCTACGTGA
GCCTGGCTTAGTCTTGTTTACGCATGAACTTCAAAATCATACTGCGACGGAATATTTTGTTTCATCGCGACGTATGTTT
GAACCTAGAAAACCTACCGTTAGTGATTTTGTTCAAATTGAGAGTTGTGTGGTCACCTATGTCAATTTGACTAGAGACC
AACTACCAGATGTAATCCCAGATTACATCGATGTTAACAAAACACTTGATGAGATTTTAGCTTCTCTGCCCAATAGAAC
TGGTCCAAGTCTTCCTTTAGATGTTTTTAATGCCACTTATCTTAATCTCACTGGTGAAATTGCAGATTTAGAGCAGCGT
TCAGAGTCTCTCCGTAATACTACAGAGGAGCTCCAAAGTCTTATATATAATATCAACAACACACTAGTTGACCTTGAGT
GGCTCAACCGAGTTGAGACATATATCAAGTGGCCGTGGTGGGTTTGGTTGATTATTTTCATTGTTCTCATCTTTGTTGT
GTCATTACTAGTGTTCTGCTGCATTTCCACGGGTTGTTGTGGATGCTGCGGCTGCTGCTGTGCTTGTTTCTCAGGTTGT
TGTAGGGGTCCTAGACTTCAACCTTACGAAGTTTTTGAAAAGGTCCACGTGCAGTGA

>gi|557844691|gb|KF650372.1| Porcine epidemic diarrhea virus isolate ISU13-19338E-IN-passage9, complete genome

ATGAAGTCTTTAACCTACTTCTGGTTGTTCTTACCAGTACTTTCAACACTTAGCCTACCACAAGATGTCACCAGG
TGCTCAGCTAACACTAATTTTAGGCGGTTCTTTTCAAAATTTAATGTTCAGGCGCCTGCAGTTGTTGTACTGGGCGGTT

ATCTACCTATTGGTGAAAACCAGGGTGTCAATTCAACTTGGTACTGTGCTGGCCAACATCCAACTGCTAGTGGCGTTCA
TGGTATCTTTGTTAGCCATATTAGAGGTGGTCATGGCTTTGAGATTGGCATTTCGCAAGAGCCTTTTGACCCTAGTGGT
TACCAGCTTTATTTACATAAGGCTACTAACGGTAACACTAATGCTACTGCGCGACTGCGCATTTGCCAGTTTCCTAGCA
TTAAAACATTGGGCCCCACTGCTAATAATGATGTTACAACAGGTCGTAATTGCCTATTTAACAAAGCCATCCCAGCTCA
TATGAGTGAACATAGTGTTGTCGGCATAACATGGGATAATGATCGTGTCACTGTCTTTTCTGACAAGATCTATTATTTT
TATTTTAAAAATGATTGGTCCCGTGTTGCGACAAAGTGTTACAACAGTGGAGGTTGTGCTATGCAATATGTTTACGAAC
CCACCTATTACATGCTTAATGTTACTAGTGCTGGTGAGGATGGTATTTCTTATCAACCCTGTACAGCTAATTGCATTGG
TTATGCTGCCAATGTATTTGCTACTGAGCCCAATGGCCACATACCAGAAGGTTTTAGTTTTAGTAATTGGTTTCTTTTG
TCCAATGATTCCACTTTGGTGCATGGTAAGGTGGTTTCCAACCAACCATTGTTGGTCAATTGTCTTTTGGCCATTCCTA
AGATTTATGGACTAGGCCAATTTTTCTCCTTTAATCAAACGATCGATGGTGTTTGTAATGGAGCTGCTGTGCAGCGTGC
ACCAGAGGCTCTGAGGTTTAATATTAATGACACCTCTGTCATTCTTGCTGAAGGCTCAATTGTACTTCATACTGCTTTA
GGAACAAATTTTTCTTTTGTTTGCAGTAATTCCTCAAATCCTCACTTAGCCACCTTCGCCATACCTTTGGGTGCTACCC
AAGTACCCTATTATTGTTTTTTTAAAGTGGATACTTACAACTCCACTGTTTATAAATTCTTGGCTGTTTTACCTCCAAC
CGTCAGGGAAATTGTCATCACCAAGTATGGTGATGTTTATGTCAATGGGTTTGGATACTTGCATCTCGGTTTGTTGGAT
GCTGTCACAATTAATTTCACTGGTCATGGCACTGACGATGATGTTTCTGGTTTTTGGACCATAGCATCGACTAATTTTG
TTGATGCACTCATCGAAGTTCAAGGAACCGCCATTCAGCGTATTCTTTATTGTGATGATCCTGTTAGCCAACTCAAGTG
TTCTCAGGTTGCTTTTGACCTTGACGATGGTTTTTACCCTATTTCTTCTAGAAACCTTCTGAGTCATGAACAGCCAATT
TCTTTTGTTACTCTGCCATCATTTAATGATCATTCTTTTGTTAACATTACTGTATCTGCTTCCTTTGGTGGTCATAGTG
GTGCCAACCTTATTGCATCTGACACTACTATCAATGGGTTTAGTTCTTTCTGTGTTGACACTAGACAATTTACCATTTC
ACTGTTTTATAACGTTACAAACAGTTATGGTTATGTGTCTAAATCACAGGACAGTAATTGCCCTTTCACCTTGCAATCT
GTTAATGATTACCTGTCTTTTAGCAAATTTTGTGTTTCCACCAGCCTTTTGGCTAGTGCCTGTACCATAGATCTTTTTG
GTTACCCTGAGTTTGGTAGTGGTGTTAAGTTTACGTCCCTTTACTTTCAATTCACAAAGGGTGAGTTGATTACTGGCAC
GCCTAAACCACTTGAAGGTGTCACGGACGTTTCTTTTATGACTCTGGATGTGTGTACCAAGTATACTATCTATGGCTTT
AAAGGTGAGGGTATCATTACCCTTACAAATTCTAGCTTTTTGGCAGGTGTTTATTACACATCTGATTCTGGACAGTTGT
TAGCCTTTAAGAATGTCACTAGTGGTGCTGTTTATTCTGTTACGCCATGTTCTTTTTCAGAGCAGGCTGCATATGTTGA
TGATGATATAGTGGGTGTTATTTCTAGTTTGTCTAGCTCCACTTTTAACAGTACTAGGGAGTTGCCTGGTTTCTTCTAC
CATTCTAATGATGGCTCTAATTGTACAGAGCCTGTGTTGGTGTATAGTAACATAGGTGTTTGTAAATCTGGCAGTATTG
GCTACGTCCCATCTCAGTCTGGCCAAGTCAAGATTGCACCCACGGTTACTGGGAATATTAGTATTCCCACCAACTTTAG
TATGAGTATTAGGACAGAATATTTACAGCTTTACAACACGCCTGTTAGTGTTGATTGTGCCACATATGTTTGTAATGGT
AACTCTCGTTGTAAACAATTACTCACCCAGTACACTGCAGCATGTAAGACCATAGAGTCAGCATTACAACTCAGCGCTA
GGCTTGAGTCTGTTGAAGTTAACTCTATGCTTACTATTTCTGAAGAGGCTCTACAGTTAGCTACCATTAGTTCGTTTAA
TGGTGATGGATATAATTTTACTAATGTGCTGGGTGTTTCTGTGTATGATCCTGCAAGTGGCAGGGTGGTACAAAAAAGG
TCTTTTATTGAAGACCTGCTTTTTAATAAAGTGGTTACTAATGGCCTTGGTACTGTTGATGAAGACTATAAGCGCTGTT
CTAATGGTCGCTCTGTGGCAGATCTAGTCTGTGCACAGTATTACTCTGGTGTCATGGTACTACCTGGTGTTGTTGACGC
TGAGAAGCTTCACATGTATAGTGCGTCTCTCATCGGTGGTATGGTGCTAGGAGGTTTTACTTCTGCAGCGGCATTGCCT
TTTAGCTATGCTGTTCAAGCTAGACTCAATTATCTTGCTCTACAGACGGATGTTCTACAGCGGAACCAGCAATTGCTTG
CTGAGTCTTTTAACTCTGCTATTGGTAATATAACTTCAGCCTTTGAGAGTGTTAAAGAGGCTATTAGTCAAACTTCCAA
GGGTTTGAACACTGTGGCTCATGCGCTTACTAAGGTTCAAGAGGTTGTTAACTCGCAGGGTGCAGCTTTGACTCAACTT
ACCGTACAGCTGCAACACAACTTCCAAGCCATTTCTAGTTCTATTGATGACATTTACTCTCGACTGGACATTCTTTCAG
CCGATGTTCAGGTTGACCGTCTCATCACCGGCAGATTATCAGCACTTAATGCTTTTGTTGCTCAAACCCTCACTAAGTA
TACTGAGGTTCAGGCTAGCAGGAAGTTAGCACAGCAAAAGGTTAATGAGTGCGTTAAATCGCAATCTCAGCGTTATGGT
TTTTGTGGTGGTGATGGCGAGCACATTTTCTCTCTGGTACAGGCAGCACCTCAGGGCCTGCTGTTTTTACATACAGTAC
TTGTACCGAGTGATTTTGTAGATGTTATTGCCATCGCTGGCTTATGCGTTAACGATGAAATTGCCTTGACTCTACGTGA
GCCTGGCTTAGTCTTGTTTACGCATGAACTTCAAAATCATACTGCGACGGAATATTTTGTTTCATCGCGACGTATGTTT

GAACCTAGAAAACCTACCGTTAGTGATTTTGTTCAAATTGAGAGTTGTGTGGTCACCTATGTCAATTTGACTAGAGACC
AACTACCAGATGTAATCCCAGATTACATCGATGTTAACAAAACACTTGATGAGATTTTAGCTTCTCTGCCCAATAGAAC
TGGTCCAAGTCTTCCTTTAGATGTTTTTAATGCCACTTATCTTAATCTCACTGGTGAAATTGCAGATTTAGAGCAGCGT
TCAGAGTCTCTCCGTAATACTACAGAGGAGCTCCAAAGTCTTATATATAATATCAACAACACACTAGTTGACCTTGAGT
GGCTCAACCGAGTTGAGACATATATCAAGTGGCCGTGGTGGGTTTGGTTGATTATTTTCATTGTTCTCATCTTTGTTGT
GTCATTACTAGTGTTCTGCTGCATTTCCACGGGTTGTTGTGGATGCTGCGGCTGCTGCTGTGCTTGTTTCTCAGGTTGT
TGTAGGGGTCCTAGACTTCAACCTTACGAAGTTTTTGAAAAGGTCCACGTGCAGTGA

>gi|557844705|gb|KF650373.1| Porcine epidemic diarrhea virus isolate ISU13-22038-IA-homogenate, complete genome

ATGAAGTCTTTAACCTACTTCTGGTTGTTCTTACCAGTACTTTCAACACTTAGCCTACCACAAGATGTCACCAGG
TGCTCAGCTAACACTAATTTTAGGCGGTTCTTTTCAAAATTTAATGTTCAGGCGCCTGCAGTTGTTGTACTGGGCGGTT
ATCTACCTATTGGTGAAAACCAGGGTGTCAATTCAACTTGGTACTGTGCTGGCCAACATCCAACTGCTAGTGGCGTTCA
TGGTATCTTTGTTAGCCATATTAGAGGTGGTCATGGCTTTGAGATTGGCATTTCGCAAGAGCCTTTTGACCCTAGTGGT
TACCAGCTTTATTTACATAAGGCTACTAACGGTAACACTAATGCTACTGCGCGACTGCGCATTTGCCAGTTTCCTAGCA
TTAAAACATTGGGCCCCACTGCTAATAATGATGTTACAACAGGTCGTAATTGCCTATTTAACAAAGCCATCCCAGCTCA
TATGAGTGAACATAGTGTTGTCGGCATAACATGGGATAATGATCGTGTCACTGTCTTTTCTGACAAGATCTATTATTTT
TATTTTAAAAATGATTGGTCCCGTGTTGCGACAAAGTGTTACAACAGTGGAGGTTGTGCTATGCAATATGTTTACGAAC
CCACCTATTACATGCTTAATGTTACTAGTGCTGGTGAGGATGGTATTTCTTATCAACCCTGTACAGCTAATTGCATTGG
TTATGCTGCCAATGTATTTGCTACTGAGCCCAATGGCCACATACCAGAAGGTTTTAGTTTTAATAATTGGTTTCTTTTG
TCCAATGATTCCACTTTGGTGCATGGTAAGGTGGTTTCCAACCAACCATTGTTGGTCAATTGTCTTTTGGCCATTCCTA
AGATTTATGGACTAGGCCAATTTTTCTCCTTTAATCAAACGATCGATGGTGTTTGTAATGGAGCTGCTGTGCAGCGTGC
ACCAGAGGCTCTGAGGTTTAATATTAATGACACCTCTGTCATTCTTGCTGAAGGCTCAATTGTACTTCATACTGCTTTA
GGAACAAATTTTTCTTTTGTTTGCAGTAATTCCTCAAATCCTCACTTAGCCACCTTCGCCATACCTTTGGGTGCTACCC
AAGTACCCTATTATTGTTTTCTTAAAGTGGATACTTACAACTCCACTGTTTATAAATTCTTGGCTGTTTTACCTCCAAC
CGTCAGGGAAATTGTCATCACCAAGTATGGTGATGTTTATGTCAATGGGTTTGGATACTTGCATCTCGGTTTGTTGGAT
GCTGTCACAATTAATTTCACTGGTCATGGCACTGACGATGATGTTTCTGGTTTTTGGACCATAGCATCGACTAATTTTG
TTGATGCACTCATCGAAGTTCAAGGAACCGCCATTCAGCGTATTCTTTATTGTGATGATCCTGTTAGCCAACTCAAGTG
TTCTCAGGTTGCTTTTGACCTTGACGATGGTTTTTACCCTATTTCTTCTAGAAACCTTCTGAGTCATGAACAGCCAATT
TCTTTTGTTACTCTGCCATCATTTAATGATCATTCTTTTGTTAACATTACTGTATCTGCTTCCTTTGGTGGTCATAGTG
GTGCCAACCTTATTGCATCTGACACTACTATCAATGGGTTTAGTTCTTTCTGTGTTGACACTAGACAATTTACCATTTC
ACTGTTTTATAACGTTACAAACAGTTATGGTTATGTGTCTAAATCACAGGACAGTAATTGCCCTTTCACCTTGCAATCT
GTTAATGATTACCTGTCTTTTAGCAAATTTTGTGTTTCCACCAGCCTTTTGGCTAGTGCCTGTACCATAGATCTTTTTG
GTTACCCTGAGTTTGGTAGTGGTGTTAAGTTTACGTCCCTTTACTTTCAATTCACAAAGGGTGAGTTGATTACTGGCAC
GCCTAAACCACTTGAAGGTGTCACGGACGTTTCTTTTATGACTCTGGATGTGTGTACCACGTATACTATCTATGGCTTT
AAAGGTGAGGGTATCATTACCCTTACAAATTCTAGCTTTTTGGCAGGTGTTTATTACACATCTGATTCTGGACAGTTGT
TAGCCTTTAAGAATGTCACTAGTGGTGCTGTTTATTCTGTTACGCCATGTTCTTTTTCAGAGCAGGCTGCATATGTTGA
TGATGATATAGTGGGTGTTATTTCTAGTTTGTCTAGCTCCACTTTTAACAGTACTAGGGAGTTGCCTGGTTTCTTCTAC
CATTCTAATGATGGCTCTAATTGTACAGAGCCTGTGTTGGTGTATAGTAACATAGGTGTTTGTAAATCTGGCAGCATTG
GCTACGTCCCATCTCAGTCTGGCCAAGTCAAGATTGCACCCACGGTTACTGGGAATATTAGTATTCCCACCAACTTTAG
TATGAGTATTAGGACAGAATATTTACAGCTTTACAACACGCCTGTTAGTGTTGATTGTGCCACATATGTTTGTAATGGT
AACTCTCGTTGTAAACAATTACTCACCCAGTACACTGCAGCATGTAAGACCATAGAGTCAGCATTACAACTCAGCGCTA

GGCTTGAGTCTGTTGAAGTTAACTCTATGCTTACTATTTCTGAAGAGGCTCTACAGTTAGCTACCATTAGTTCGTTTAA
TGGTGATGGATATAATTTTACTAATGTGCTGGGTGTTTCTGTGTATGATCCTGCAAGTGGCAGGGTGGTACAAAAAAGG
TCTTTTATTGAAGACCTGCTTTTTAATAAAGTGGTTACTAATGGCCTTGGTACTGTTGATGAAGACTATAAGCGCTGTT
CTAATGGTCGCTCTGTGGCAGATCTAGTCTGTGCACAGTATTACTCTGGTGTCATGGTACTACCTGGTGTTGTTGACGC
TGAGAAGCTTCACATGTATAGTGCGTCTCTCATCGGTGGTATGGTGCTAGGAGGTTTTACTTCTGCAGCGGCATTGCCT
TTTAGCTATGCTGTTCAAGCTAGACTCAATTATCTTGCTCTACAGACGGATGTTCTACAGCGGAACCAGCAATTGCTTG
CTGAGTCTTTTAACTCTGCTATTGGTAATATAACTTCAGCCTTTGAGAGTGTTAAAGAGGCTATTAGTCAAACTTCCAA
GGGTTTGAACACTGTGGCTCATGCGCTTACTAAGGTTCAAGAGGTTGTTAACTCGCAGGGTGCAGCTTTGACTCAACTT
ACCGTACAGCTGCAACACAACTTCCAAGCCATTTCTAGTTCTATTGATGACATTTACTCTCGACTGGACATTCTTTCAG
CCGATGTTCAGGTTGACCGTCTCATCACCGGCAGATTATCAGCACTTAATGCTTTTGTTGCTCAAACCCTCACTAAGTA
TACTGAGGTTCAGGCTAGCAGGAAGTTAGCACAGCAAAAGGTTAATGAGTGCGTTAAATCGCAATCTCAGCGTTATGGT
TTTTGTGGTGGTGATGGCGAGCACATTTTCTCTCTGGTACAGGCAGCACCTCAGGGCCTGCTGTTTTTACATACAGTAC
TTGTACCGAGTGATTTTGTAGATGTTATTGCCATCGCTGGCTTATGCGTTAACGATGAAATTGCCTTGACTCTACGTGA
GCCTGGCTTAGTCTTGTTTACGCATGAACTTCAAAATCATACTGCGACGGAATATTTTGTTTCATCGCGACGTATGTTT
GAACCTAGAAAACCTACCGTTAGTGATTTTGTTCAAATTGAGAGTTGTGTGGTCACCTATGTCAATTTGACTAGAGACC
AACTACCAGATGTAATCCCAGATTACATCGATGTTAACAAAACACTTGATGAGATTTTAGCTTCTCTGCCCAATAGAAC
TGGTCCAAGTCTTCCTTTAGATGTTTTTAATGCCACTTATCTTAATCTCACTGGTGAAATTGCAGATTTAGAGCAGCGT
TCAGAGTCTCTCCGTAATACTACAGAGGAGCTCCAAAGTCTTATATATAATATCAACAACACACTAGTTGACCTTGAGT
GGCTCAACCGAGTTGAGACATATATCAAGTGGCCGTGGTGGGTTTGGTTGATTATTTTCATTGTTCTCATCTTTGTTGT
GTCATTACTAGTGTTCTGCTGCATTTCCACGGGTTGTTGTGGATGCTGCGGCTGCTGCTGTGCTTGTTTCTCAGGTTGT
TGTAGGGGTCCTAGACTTCAACCTTACGAAGTTTTTGAAAAGGTCCACGTGCAGTGA

>gi|557844721|gb|KF650374.1| Porcine epidemic diarrhea virus isolate ISU13-22038-IA-passage3, complete genome

ATGAAGTCTTTAACCTACTTCTGGTTGTTCTTACCAGTACTTTCAACACTTAGCCTACCACAAGATGTCACCAGG
TGCTCAGCTAACACTAATTTTAGGCGGTTCTTTTCAAAATTTAATGTTCAGGCGCCTGCAGTTGTTGTACTGGGCGGTT
ATCTACCTATTGGTGAAAACCAGGGTGTCAATTCAACTTGGTACTGTGCTGGCCAACATCCAACTGCTAGTGGCGTTCA
TGGTATCTTTGTTAGCCATATTAGAGGTGGTCATGGCTTTGAGATTGGCATTTCGCAAGAGCCTTTTGACCCTAGTGGT
TACCAGCTTTATTTACATAAGGCTACTAACGGTAACACTAATGCTACTGCGCGACTGCGCATTTGCCAGTTTCCTAGCA
TTAAAACATTGGGCCCCACTGCTAATAATGATGTTACAACAGGTCGTAATTGCCTATTTAACAAAGCCATCCCAGCTCA
TATGAGTGAACATAGTGTTGTCGGCATAACATGGGATAATGATCGTGTCACTGTCTTTTCTGACAAGATCTATTATTTT
TATTTTAAAAATGATTGGTCCCGTGTTGCGACAAAGTGTTACAACAGTGGAGGTTGTGCTATGCAATATGTTTACGAAC
CCACCTATTACATGCTTAATGTTACTAGTGCTGGTGAGGATGGTATTTCTTATCAACCCTGTACAGCTAATTGCATTGG
TTATGCTGCCAATGCATTTGCTACTGAGCCCAATGGCCACATACCAGAAGGTTTTAGTTTTAATAATTGGTTTCTTTTG
TCCAATGATTCCACTTTGGTGCATGGTAAGGTGGTTTCCAACCAACCATTGTTGGTCAATTGTCTTTTGGCCATTCCTA
AGATTTATGGACTAGGCCAATTTTTCTCCTTTAATCAAACGATCGATGGTGTTTGTAATGGAGCTGCTGTGCAGCGTGC
ACCAGAGGCTCTGAGGTTTAATATTAATGACACCTCTGTCATTCTTGCTGAAGGCTCAATTGTACTTCATACTGCTTTA
GGAACAAATTTTTCTTTTGTTTGCAGTAATTCCTCAAATCCTCACTTAGCCACCTTCGCCATACCTTTGGGTGCTACCC
AAGTACCCTATTATTGTTTTCTTAAAGTGGATACTTACAACTCCACTGTTTATAAATTCTTGGCTGTTTTACCTCCAAC
CGTCAGGGAAATTGTCATCACCAAGTATGGTGATGTTTATGTCAATGGGTTTGGATACTTGCATCTCGGTTTGTTGGAT
GCTGTCACAATTAATTTCACTGGTCATGGCACTGACGATGATGTTTCTGGTTTTTGGACCATAGCATCGACTAATTTTG
TTGATGCACTCATCGAAGTTCAAGGAACCGCCATTCAGCGTATTCTTTATTGTGATGATCCTGTTAGCCAACTCAAGTG

TTCTCAGGTTGCTTTTGACCTTGACGATGGTTTTTACCCTATTTCTTCTAGAAACCTTCTGAGTCATGAACAGCCAATT
TCTTTTGTTACTCTGCCATCATTTAATGATCATTCTTTTGTTAACATTACTGTATCTGCTTCCTTTGGTGGTCATAGTG
GTGCCAACCTTATTGCATCTGACACTACTATCAATGGGTTTAGTTCTTTCTGTGTTGACACTAGACAATTTACCATTTC
ACTGTTTTATAACGTTACAAACAGTTATGGTTATGTGTCTAAATCACAGGACAGTAATTGCCCTTTCACCTTGCAATCT
GTTAATGATTACCTGTCTTTTAGCAAATTTTGTGTTTCCACCAGCCTTTTGGCTAGTGCCTGTACCATAGATCTTTTTG
GTTACCCTGAGTTTGGTAGTGGTGTTAAGTTTACGTCCCTTTACTTTCAATTCACAAAGGGTGAGTTGATTACTGGCAC
GCCTAAACCACTTGAAGGTGTCACGGACGTTTCTTTTATGACTCTGGATGTGTGTACCACGTATACTATCTATGGCTTT
AAAGGTGAGGGTATCATTACCCTTACAAATTCTAGCTTTTTGGCAGGTGTTTATTACACATCTGATTCTGGACAGTTGT
TAGCCTTTAAGAATGTCACTAGTGGTGCTGTTTATTCTGTTACGCCATGTTCTTTTTCAGAGCAGGCTGCATATGTTGA
TGATGATATAGTGGGTGTTATTTCTAGTTTGTCTAGCTCCACTTTTAACAGTACTAGGGAGTTGCCTGGTTTCTTCTAC
CATTCTAATGATGGCTCTAATTGTACAGAGCCTGTGTTGGTGTATAGTAACATAGGTGTTTGTAAATCTGGCAGCATTG
GCTACGTCCCATCTCAGTCTGGCCAAGTCAAGATTGCACCCACGGTTACTGGGAATATTAGTATTCCCACCAACTTTAG
TATGAGTATTAGGACAGAATATTTACAGCTTTACAACACGCCTGTTAGTGTTGATTGTGCCACATATGTTTGTAATGGT
AACTCTCGTTGTAAACAATTACTCACCCAGTACACTGCAGCATGTAAGACCATAGAGTCAGCATTACAACTCAGCGCTA
GGCTTGAGTCTGTTGAAGTTAACTCTATGCTTACTATTTCTGAAGAGGCTCTACAGTTAGCTACCATTAGTTCGTTTAA
TGGTGATGGATATAATTTTACTAATGTGCTGGGTGTTTCTGTGTATGATCCTGCAAGTGGCAGGGTGGTACAAAAAAGG
TCTTTTATTGAAGACCTGCTTTTTAATAAAGTGGTTACTAATGGCCTTGGTACTGTTGATGAAGACTATAAGCGCTGTT
CTAATGGTCGCTCTGTGGCAGATCTAGTCTGTGCACAGTATTACTCTGGTGTCATGGTACTACCTGGTGTTGTTGACGC
TGAGAAGCTTCACATGTATAGTGCGTCTCTCATCGGTGGTATGGTGCTAGGAGGTTTTACTTCTGCAGCGGCATTGCCT
TTTAGCTATGCTGTTCAAGCTAGACTCAATTATCTTGCTCTACAGACGGATGTTCTACAGCGGAACCAGCAATTGCTTG
CTGAGTCTTTTAACTCTGCTATTGGTAATATAACTTCAGCCTTTGAGAGTGTTAAAGAGGCTATTAGTCAAACTTCCAA
GGGTTTGAACACTGTGGCTCATGCGCTTACTAAGGTTCAAGAGGTTGTTAACTCGCAGGGTGCAGCTTTGACTCAACTT
ACCGTACAGCTGCAACACAACTTCCAAGCCATTTCTAGTTCTATTGATGACATTTACTCTCGACTGGACATTCTTTCAG
CCGATGTTCAGGTTGACCGTCTCATCACCGGCAGATTATCAGCACTTAATGCTTTTGTTGCTCAAACCCTCACTAAGTA
TACTGAGGTTCAGGCTAGCAGGAAGTTAGCACAGCAAAAGGTTAATGAGTGCGTTAAATCGCAATCTCAGCGTTATGGT
TTTTGTGGTGGTGATGGCGAGCACATTTTCTCTCTGGTACAGGCAGCACCTCAGGGCCTGCTGTTTTTACATACAGTAC
TTGTACCGAGTGATTTTGTAGATGTTATTGCCATCGCTGGCTTATGCGTTAACGATGAAATTGCCTTGACTCTACGTGA
GCCTGGCTTAGTCTTGTTTACGCATGAACTTCAAAATCATACTGCGACGGAATATTTTGTTTCATCGCGACGTATGTTT
GAACCTAGAAAACCTACCGTTAGTGATTTTGTTCAAATTGAGAGTTGTGTGGTCACCTATGTCAATTTGACTAGAGACC
AACTACCAGATGTAATCCCAGATTACATCGATGTTAACAAAACACTTGATGAGATTTTAGCTTCTCTGCCCAATAGAAC
TGGTCCAAGTCTTCCTTTAGATGTTTTTAATGCCACTTATCTTAATCTCACTGGTGAAATTGCAGATTTAGAGCAGCGT
TCAGAGTCTCTCCGTAATACTACAGAGGAGCTCCAAAGTCTTATATATAATATCAACAACACACTAGTTGACCTTGAGT
GGCTCAACCGAGTTGAGACATATATCAAGTGGCCGTGGTGGGTTTGGTTGATTATTTTCATTGTTCTCATCTTTGTTGT
GTCATTACTAGTGTTCTGCTGCATTTCCACGGGTTGTTGTGGATGCTGCGGCTGCTGCTGTGCTTGTTTCTCAGGTTGT
TGTAGGGGTCCTAGACTTCAACCTTACGAAGTTTTTGAAAAGGTCCACGTGCAGTGA

>gi|557844737|gb|KF650375.1| Porcine epidemic diarrhea virus isolate ISU13-22038-IA-passage9, complete genome

ATGAAGTCTTTAACCTACTTCTGGTTGTTCTTACCAGTACTTTCAACACTTAGCCTACCACAAGATGTCACCAGG
TGCTCAGCTAACACTAATTTTAGGCGGTTCTTTTCAAAATTTAATGTTCAGGCGCCTGCAGTTGTTGTACTGGGCGGTT
ATCTACCTATTGGTGAAAACCAGGGTGTCAATTCAACTTGGTACTGTGCTGGCCAACATCCAACTGCTAGTGGCGTTCA
TGGTATCTTTGTTAGCCATATTAGAGGTGGTCATGGCTTTGAGATTGGCATTTCGCAAGAGCCTTTTGACCCTAGTGGT

TACCAGCTTTATTTACATAAGGCTACTAACGGTAACACTAATGCTACTGCGCGACTGCGCATTTGCCAGTTTCCTAGCA
TTAAAACATTGGGCCCCACTGCTAATAATGATGTTACAACAGGTCGTAATTGCCTATTTAACAAAGCCATCCCAGCTCA
TATGAGTGAACATAGTGTTGTCGGCATAACATGGGATAATGATCGTGTCACTGTCTTTTCTGACAAGATCTATTATTTT
TATTTTAAAAATGATTGGTCCCGTGTTGCGACAAAGTGTTACAACAGTGGAGGTTGTGCTATGCAATATGTTTACGAAC
CCATCTATTACATGCTTAATGTTACTAGTGCTGGTGAGGATGGTATTTCTTATCAACCCTGTACAGCTAATTGCATTGG
TTATGCTGCCAATGCATTTGCTACTGAGCCCAATGGCCACATACCAGAAGGTTTTAGTTTTAATAATTGGTTTCTTTTG
TCCAATGATTCCACTTTGGTGCATGGTAAGGTGGTTTCCAACCAACCATTGTTGGTCAATTGTCTTTTGGCCATTCCTA
AGATTTATGGACTAGGCCAATTTTTCTCCTTTAATCAAACGATCGATGGTGTTTGTAATGGAGCTGCTGTGCAGCGTGC
ACCAGAGGCTCTGAGGTTTAATATTAATGACACCTCTGTCATTCTTGCTGAAGGCTCAATTGTACTTCATACTGCTTTA
GGAACAAATTTTTCTTTTGTTTGCAGTAATTCCTCAAATCCTCACTTAGCCACCTTCGCCATACCTTTGGGTGCTACCC
AAGTACCCTATTATTGTTTTCTTAAAGTGGATACTTACAACTCCACTGTTTATAAATTCTTGGCTGTTTTACCTCCAAC
CGTCAGGGAAATTGTCATCACCAAGTATGGTGATGTTTATGTCAATGGGTTTGGATACTTGCATCTCGGTTTGTTGGAT
GCTGTCACAATTAATTTCACTGGTCATGGCACTGACGATGATGTTTCTGGTTTTTGGACCATAGCATCGACTAATTTTG
TTGATGCACTCATCGAAGTTCAAGGAACCGCCATTCAGCGTATTCTTTATTGTGATGATCCTGTTAGCCAACTCAAGTG
TTCTCAGGTTGCTTTTGACCTTGACGATGGTTTTTACCCTATTTCTTCTAGAAACCTTCTGAGTCATGAACAGCCAATT
TCTTTTGTTACTCTGCCATCATTTAATGATCATTCTTTTGTTAACATTACTGTATCTGCTTCCTTTGGTGGTCATAGTG
GTGCCAACCTTATTGCATCTGACACTACTATCAATGGGTTTAGTTCTTTCTGTGTTGACACTAGACAATTTACCATTTC
ACTGTTTTATAACGTTACAAACAGTTATGGTTATGTGTCTAAATCACAGGACAGTAATTGCCCTTTCACCTTGCAATCT
GTTAATGATTACCTGTCTTTTAGCAAATTTTGTGTTTCCACCAGCCTTTTGGCTAGTGCCTGTACCATAGATCTTTTTG
GTTACCCTGAGTTTGGTAGTGGTGTTAAGTTTACGTCCCTTTACTTTCAATTCACAAAGGGTGAGTTGATTACTGGCAC
GCCTAAACCACTTGAAGGTGTCACGGACGTTTCTTTTATGACTCTGGATGTGTGTACCACGTATACTATCTATGGCTTT
AAAGGTGAGGGTATCATTACCCTTACAAATTCTAGCTTTTTGGCAGGTGTTTATTACACATCTGATTCTGGACAGTTGT
TAGCCTTTAAGAATGTCACTAGTGGTGCTGTTTATTCTGTTACGCCATGTTCTTTTTCAGAGCAGGCTGCATATGTTGA
TGATGATATAGTGGGTGTTATTTCTAGTTTGTCTAGCTCCACTTTTAACAGTACTAGGGAGTTGCCTGGTTTCTTCTAC
CATTCTAATGATGGCTCTAATTGTACAGAGCCTGTGTTGGTGTATAGTAACATAGGTGTTTGTAAATCTGGCAGCATTG
GCTACGTCCCATCTCAGTCTGGCCAAGTCAAGATTGCACCCACGGTTACTGGGAATATTAGTATTCCCACCAACTTTAG
TATGAGTATTAGGACAGAATATTTACAGCTTTACAACACGCCTGTTAGTGTTGATTGTGCCACATATGTTTGTAATGGT
AACTCTCGTTGTAAACAATTACTCACCCAGTACACTGCAGCATGTAAGACCATAGAGTCAGCATTACAACTCAGCGCTA
GGCTTGAGTCTGTTGAAGTTAACTCTATGCTTACTATTTCTGAAGAGGCTCTACAGTTAGCTACCATTAGTTCGTTTAA
TGGTGATGGATATAATTTTACTAATGTGCTGGGTGTTTCTGTGTATGATCCTGCAAGTGGCAGGGTGGTACAAAAAAGG
TCTTTTATTGAAGACCTGCTTTTTAATAAAGTGGTTACTAATGGCCTTGGTACTGTTGATGAAGACTATAAGCGCTGTT
CTAATGGTCGCTCTGTGGCAGATCTAGTCTGTGCACAGTATTACTCTGGTGTCATGGTACTACCTGGTGTTGTTGACGC
TGAGAAGCTTCACATGTATAGTGCGTCTCTCATCGGTGGTATGGTGCTAGGAGGTTTTACTTCTGCAGCGGCATTGCCT
TTTAGCTATGCTGTTCAAGCTAGACTCAATTATCTTGCTCTACAGACGGATGTTCTACAGCGGAACCAGCAATTGCTTG
CTGAGTCTTTTAACTCTGCTATTGGTAATATAACTTCAGCCTTTGAGAGTGTTAAAGAGGCTATTAGTCAAACTTCCAA
GGGTTTGAACACTGTGGCTCATGCGCTTACTAAGGTTCAAGAGGTTGTTAACTCGCAGGGTGCAGCTTTGACTCAACTT
ACCGTACAGCTGCAACACAACTTCCAAGCCATTTCTAGTTCTATTGATGACATTTACTCTCGACTGGACATTCTTTCAG
CCGATGTTCAGGTTGACCGTCTCATCACCGGCAGATTATCAGCACTTAATGCTTTTGTTGCTCAAACCCTCACTAAGTA
TACTGAGGTTCAGGCTAGCAGGAAGTTAGCACAGCAAAAGGTTAATGAGTGCGTTAAATCGCAATCTCAGCGTTATGGT
TTTTGTGGTGGTGATGGCGAGCACATTTTCTCTCTGGTACAGGCAGCACCTCAGGGCCTGCTGTTTTTACATACAGTAC
TTGTACCGAGTGATTTTGTAGATGTTATTGCCATCGCTGGCTTATGCGTTAACGATGAAATTGCCTTGACTCTACGTGA
GCCTGGCTTAGTCTTGTTTACGCATGAACTTCAAAATCATACTGCGACGGAATATTTTGTTTCATCGCGACGTATGTTT
GAACCTAGAAAACCTACCGTTAGTGATTTTGTTCAAATTGAGAGTTGTGTGGTCACCTATGTCAATTTGACTAGAGACC
AACTACCAGATGTAATCCCAGATTACATCGATGTTAACAAAACACTTGATGAGATTTTAGCTTCTCTGCCCAATAGAAC

TGGTCCAAGTCTTCCTTTAGATGTTTTTAATGCCACTTATCTTAATCTCACTGGTGAAATTGCAGATTTAGAGCAGCGT
TCAGAGTCTCTCCGTAATACTACAGAGGAGCTCCAAAGTCTTATATATAATATCAACAACACACTAGTTGACCTTGAGT
GGCTCAACCGAGTTGAGACATATATCAAGTGGCCGTGGTGGGTTTGGTTGATTATTTTCATTGTTCTCATCTTTGTTGT
GTCATTACTAGTGTTCTGCTGCATTTCCACGGGTTGTTGTGGATGCTGCGGCTGCTGCTGTGCTTGTTTCTCAGGTTGT
TGTAGGGGTCCTAGACTTCAACCTTACGAAGTTTTTGAAAAGGTCCACGTGCAGTGA

>gi|575502656|gb|KF761675.1| Porcine epidemic diarrhea virus strain CH/YNKM-8/2013, complete genome

ATGAAGTCTTTAACCTACTTCTGGTTGTTCTTACCAGTACTTTCAACACTTAGCCTACCACAAGATGTCACCAGG
TGCTCAGCTAACACTAATTTTAGGCGGTTCTTTTCAAAATTTAATGTTCAGGCGCCTGCAGTTGTTGTACTGGGCGGTT
ATCTACCTATTGGTGAAAACCAGGGTGTCAATTCAACTTGGTACTGTGCTGGCCAACATCCAACTGCTAGTGGCGTTCA
TGGTATCTTTCTTAGCCATATTAGAGGTGGTCATGGCTTTGAGATTGGCATTTCGCAAGAGCCTTTTGACCCTAGTGGT
TACCAGCTTTATTTACATAAGGCTACTAACGGTAACACTAATGCTACTGCGCGACTGCGCATTTGCCAGTTTCCTAGCA
TTAAAACATTGGGCCCCACTGCTAATAATGATGTTACAACAGGTCGTAACTGCCTATTTAACAAAGCCATCCCAGCTCA
TATGAGTGAACATAGTGTTGTCGGCATAACATGGGATAATGATCGTGTCACTGTCTTTTCTGACAAGATCTATTATTTT
TATTTTAAAAATGATTGGTCCCGTGTTGCGACAAAGTGTTACAACAGTGGAGGTTGTGCTATGCAATATGTTTACGAAC
CCACCTATTACATGCTTAATGTTACTAGTGCTGGTGAGGATGGTATTTCTTATCAACCCTGTACAGCTAATTGCATTGG
TTATGCTGCCAATGTATTTGCTACTGAGCCCAATGGCCACATACCAGAAGGTTTTAGTTTTAATAATTGGTTTCTGTTG
TCCAATGATTCCACTTTGGTGCATGGTAAGGTGGTTTCCAACCAACCATTGTTGGTCAATTGTCTTTTGGCCATTCCTA
AGATTTATGGACTAGGCCAATTTTTCTCCTTTAATCAAACGATCGATGGTGTTTGTAATGGAGCTGCTGTGCAGCGTGC
ACCAGAGGCTCTGAGGTTTAATATTAATGACACCTCTGTCATTCTTGCTGAAGGCTCAATTGTACTTCATACTGCTTTA
GGAACAAATTTTTCTTTTGTTTGCAGTAGTTCCTCAGATCCTCACTTAGCCACCTTCGCCATACCTCTGGGTGCTATCC
AAGTACCCTATTATTGTTTTCTTAAAGTGGATACTTACAACTCCACTGTTTATAAATTCTTGGCTGTTTTACCTCCTAC
CGTCAGGGAAATTGTCATCACCAAGTATGGTGATGTTTATGTCAATGGGTTTGGCTATTTGCATCTCGGTTTGTTGGAT
GCTGTCACAGTTAATTTCACTGGTCATGGCACTGACGATGACGTTTCTGGTTTTTGGACCATAGCATCGACTAATTTTG
TTGATGCACTTATCGAAGTTCAAGGAACTGCCATTCAGCGTATTCTTTATTGTGATGATCCTGTTAGCCAACTCAAGTG
TTCTCAGGTTTCATTTGACCTTGACGATGGTTTCTACCCTATTTCTTCTAGAAACCTTCTGAGTTATGAACAGCCAACT
TCTTTTGTTACTTTGCCATCATTTAATGATCATTCTTTTGTTAATATTACTGTCTCTGCTGCTTTTGGTGGTCATAGTG
GTGCCAACCTTATTGCATCTGACACTACTATCAATGGGTTTAGTTCTTTCTGTGTTGACACTAGACAATTTACCATTTC
ACTGTTTTATAATGTTACAAACAGTTATGGTTATGTGTCTAAATCACAGGACAGTAATTGCCCTTTCACCTTGCAATCT
GTTAATGATTACCTGTCTTTTAGCAAATTTTGTGTTTCTACCAGCCTTTTGGCTAGTGCCTGTACCATAGATCTCTTTG
GTTACCCTGAGTTTGGTAGTGGTGTTAAGTTCACGTCCCTTTACTTTCAATTCACAAAGGGTGAGTTGATTACTGGCAC
GCCTAAACCACTTGAAGGTGTTACGGACGTTTCTTTTATGACTCTGGATGTGTGTACCAAGTATACTATCTATGGCTTT
AAAGGTGAGGGTATCATTACCCTTACAAATTCTAGCTTTTTGGCAGGTTTTTATTATACATCTGATTCTGGACAGTTGT
TAGCTTTTAAGAATGTCACTAGTGGTGCTGTTTATTCTGTTACGCCATGTTCTTTTTCAGAGCAGGCTGCATATGTTGA
TGATGATATAGTGGGTGTTATTTCTAGTTTGTCTAGCTCCACTTTTAACAGTACTAGGGAGTTGCCTGGTTTCTTCTAC
CATTCTAATGATGGCTCTAATTGTACAGAGCCTGTGTTGGTGTATAGTAACATAGGTGTTTGTAAATCTGGCAGTATTG
GCTACGTCCCATCTCAGTCTGGCCAAGTCAAGATTGCACCCACGGTTACTGGGAATATTAGTATTCCCACCAACTTTAG
TATGAGTATTAGGACAGAATATTTACAGCTTTACAACACGCCTGTTAGTGTTGATTGTGCTACATATGTTTGTAATGGT
AACTCTCGTTGTAAACAATTACTCACCCAGTACACTGCAGCATGTAAGACCATAGAGTCAGCATTACAACTCAGCGCTA
GGCTTGAGTCTGCTGAAGTCAACTCTATGCTTACTATTTCTGAAGAGGCTCTACAGTTAGCTACCATCAGTTCGTTTAA
TGGTGATGGATATAATTTTACTAATGTGCTGGGTGTTTCCGTGTATGACCCTGCAAGTGGCAGGGTGGTACAAAAAAGG

TCTTTCATTGAAGACCTGCTTTTTAATAAAGTGGTTACTAATGGCCTTGGTACTGTTGATGAAGACTATAAGCGCTGTT
CTAATGGTCGCTCTGTGGCAGATCTAGTCTGTGCACAGTATTACTCTGGTGTCATGGTACTACCTGGTGTTGTTGACGC
TGAGAAGCTTCACATGTATAGTGCGTCTCTCATCGGTGGTATGGTGCTAGGAGGTTTTACTTCTGCAGCGGCATTGCCT
TTTAGCTATGCTGTTCAAGCTAGACTCAATTATCTTGCTCTACAGACGGATGTTCTACAGCGGAACCAGCAATTGCTTG
CTGAGTCTTTTAACTCTGCTATTGGTAATATAACTTCAGCCTTTGAGAGTGTTAAAGAGGCTATTAGTCAAACTTCCAA
GGGTTTGAACACTGTGGCTCATGCGCTTACTAAGGTTCAAGAGGTTGTTAACTCGCATGGTGCAGCTTTGACTCAACTT
ACCGTACAGCTGCAACACAACTTCCAAGCCATTTCTAGTTCTATTGATGACATTTACTCTCGACTGGACATTCTTTCAG
CCGATGTTCAGGTTGACCGTCTCATCACCGGCAGATTATCAGCACTTAATGCTTTTGTTGCTCAAACCCTCACTAAGTA
TACTGAGGTTCAGGCTAGCAGGAAGCTAGCACAGCAAAAGGTTAATGAGTGCGTTAAATCGCAATCTCAGCGTTATGGT
TTTTGTGGTGGTGATGGCGAGCACATTTTCTCTCTGGTACAGGCAGCACCTCAGGGCCTGCTGTTTTTACATACAGTAC
TTGTACCGGGTGACTTTGTAGATGTTATTGCCATCGCTGGCTTATGCGTTAACGATGAAATTGCCTTGACTCTACGTGA
GCCTGGCTTAGTCTTGTTTACGCATGAACTTCAAAATCATACTGCGACGGAATATTTTGTTTCATCGCGACGTATGTTT
GAACCTAGAAAACCTACCGTTAGTGATTTTGTTCAAATTGAGAGTTGTGTGGTCACCTATGTCAATTTGACTAGAGACC
AACTACCAGATGTAATCCCAGATTACATCGATGTTAACAAAACACTTGATGAGATTTTAGCTTCTCTGCCCAATAGAAC
TGGTCCAAGTCTTTCTCTAGATGTTTTTAATGCCACTTATCTTAATCTTACTGGTGAAATTGCAGATTTAGAGCAGCGT
TCAGAGTCTCTCCGTAATACTACAGAAGAGCTTCAAAGTCTTATATATAATATCAACAACACACTAGTTGACCTTGAGT
GGCTCAACCGAGTTGAGACATATATCAAGTGGCCGTGGTGGGTTTGGTTGATTATTTTTATTGTTCTCATCTTTGTTGT
GTCATTACTAGTGTTCTGCTGCATTTCCACGGGTTGTTGTGGATGCTGCGGCTGCTGTGGTGCTTGTTTTTCAGGTTGT
TGTAGGGGTCCTAGACTTCAACCTTACGAAGCTTTTGAAAAGGTCCACGTGCAGTGA

>gi|661567690|gb|KF840537.1| Porcine epidemic diarrhea virus isolate CH/ZJCX-1/2012, complete genome

ATGAAGTCTTTAACCTACTTCTGGTTGTTCTTACCAGTACTTTCAACACTTAGCCTACCACAAGATGTCACCAGG
TGCTCAGCTAACACTAATTTTAGGCGGTTCTTTTCAAAATTTAATGTTCAGGCGCCTGCAGTTGTTGTACTGGGCGGTT
ATCTACCTATTGGTGAAAACCAGGGTGTCAATTCAACTTGGTACTGTGCTGGCCAACATCCAACTGCTAGTGGCGTTCA
TGGTATCTTTGTTAGCCATATTAGAGGTGGTCATGGCTTTGAGATTGGCATTTCGCAAGAGCCTTTTGACCCTAGTGGT
TACCAGCTTTATTTACATAAGGCTACTAACGGTAACACTAATGCTACTGCGCGACTGCGCATTTGCCAGTTTCCTAGCA
TTAAAACATTGGGCCCCACTGCTAATAATGATGTTACAACAGGTCGTAACTGCCTATTTAACAAAGCCATCCCAGCTCA
TATTAGTGAACATAGTGTTGTCGGCATAACATGGGATAATGATCGTGTCACTGTCTTTTCTGACAAGATCTATTATTTT
TATTTTAAAAATGATTGGTCCCGTGTTGCGACAAAGTGTTACAACAGTGGAGGTTGTGCTATGCAATATGTTTACGAAC
CCACCTATTACATGCTTAATGTTACTAGTGCTGGTGAGGATGGTATTTCTTATCAACCCTGTACAGCTAATTGCATTGG
TTATGCTGCCAATGTATTTGCTACTGAGCCCAATGGCCACATACCAGAAGGTTTTAGTTTTAATAATTGGTTTCTTTTG
TCCAATGATTCCACTTTGGTGCATGGTAAGGTGGTTTCCAACCAACCATTGTTGGTCAATTGTCTTTTGGCCATTCCTA
AGATTTATGGACTAGGCCAATTTTTCTCCTTTAATCAAACGATCGATGGTGTTTGTAATGGAGCTGCTGTGCAGCGTGC
ACCAGAGGCTCTGAGGTTTAATATTAATGACACCTCTGTCATTCTTGCTGAAGGCTCAATTGTACTTCATACTGCTTTA
GGAACAAATTTTTCTTTTGTTTGCAGTAATTCCTCAGATCCTCACTTAGCCACCTTCGCCATACCTCTGGGTGCTATCC
AAGTACCCTATTATTGTTTTCTTAAAGTGGATACTTACAACTCCACTGTTTATAAATTCTTGGCTGTTTTACCTCCTAC
CGTCAGGGAAATTGTCATCACCAAGTATGGTGATGTTTATGTCAATGGGTTTGGCTATTTGCATCTCGGTTTGTTGGAC
GCTGTCACAATTAATTTCACTGGTCATGGCACTGACGATGACGTTTCAGGTTTCTGGACCATAGCATCGACTAATTTTG
TTGATGCACTTATCGAAGTTCAAGGAACTGCCATTCAGCGTATTCTTTATTGTGATGATCCTGTTAGCCAACTCAAGTG
TTCTCAGGTTGCTTTTGACCTTGACGATGGTTTCTACCCTATTTCTTCTAGAAACCTTCTGAGTCATGAACAGCCAATT
TCTTTTGTTACTTTGCCATCATTTAATGATCATTCTTTTGTTAACATTACTGTCTCTGCGTCCTTTGGTGGTCATAGTG

GTGCCAACCTTATTGCATCTGACACTACTATCAATGGGTTTAGTTCTTTCTGTGTTGACACTAGACAATTTACCATTTC
ACTGTTTTATAACGTTACAAACAGTTATGGTTATGTGTCTAAATCACAGGACAGTAATTGCCCTTTCACCTTGCAATCT
GTTAATGATTACCTGTCTTTTAGTAAATTTTGTGTTTCCACCAGCCTTTTGGCTAGTGCCTGTACCATAGATCTTTTTG
GTTACCCTGAGTTTGGTAGTGGTGTTAAGTTTACGTCCCTTTACTTTCAATTCACAAAGGGTGAGTTGATTACTGGCAC
GCCTAAACCACTTGAAGGTGTCACGGACGTTTCTTCTATGACTCTGGATGTGTGTACCAAGTATACTATCTATGGCTTT
AAAGGTGAGGGTATCATTACCCTTACAAATTCTAGCTTTTTGGCAGGTGTTTATTACACATCTGATTCTGGACAGTTGT
TAGCCTTTAAGAATGTCACTAGTGGTGCTGTTTATTCTGTTACGCCATGTTCTTTTTCAGAGCAGGCTGCATATGTTGA
TGATGATATAGTGGGTGTTATTTCTAGTTTGTCTAGCTCCACTTTTAACAGTACTAGGGAGTTGCCTGGTTTCTTCTAC
CATTCTAATGATGGCTCTAATTGTACAGAGCCTGTGTTGGTGTATAGTAACATAGGTGTTTGTAAATCTGGCAGTATTG
GCTACGTCCCATCTCAGTCTGGCCAAGTCAAGATTGCACCCACGGTTACTGGGAATATTAGTATTCCCACCAACTTTAG
TATGAGTATTAGGACAGAATATTTACAGCTTTACAACACGCCTGTTAGTGTTGATTGTGCCACATATGTTTGTAATGGT
AACTCTCGTTGTAAACAATTACTCACCCAGTACACTGCAGCATGTAAGACCATAGAGTCAGCATTACAACTCAGCGCTA
GGCTTGAGTCTGTTGAAGTTAACTCTATGCTTACTATTTCTGAAGAGGCTCTACAGTTAGCTACCATCAGTTCGTTTAA
TGGTGATGGATATAATTTTACTAATGTGCTGGGTGTTTCTGTGTATGATCCTGCAAGTGGCAGGGTGGTACAAAAAAGG
TCTTTTATTGAAGACCTGCTTTTTAATAAAGTGGTTACTAATGGCCTTGGTACTGTTGATGAAGACTATAAGCGCTGTT
CTAATGGTCGCTCTGTGGCAGATCTAGTCTGTGCACAGTATTACTCTGGTGTCATGGTACTACCTGGTGTTGTTGACGC
TGAGAAGCTTCACATGTATAGTGCGTCTCTCATCGGTGGTATGGTGCTAGGAGGTTTTACTTCTGCAGCGGCATTGCCT
TTTAGCTATGCTGTTCAAGCTAGACTCAATTATCTTGCTCTACAGACGGATGTTCTACAGCGGAACCAGCAATTGCTTG
CTGAGTCTTTTAACTCTGCTATTGGTAATATAACTTCAGCCTTTGAGAGTGTTAAAGAGGCTATTAGTCAAACTTCCAA
GGGTTTGAACACTGTGGCTCATGCGCTTACTAAGGTTCAAGAGGTTGTTAACTCGCAGGGTGCAGCTTTGACTCAACTT
ACCGTACAGCTGCAACACAACTTCCAAGCCATTTCTAGTTCTATTGATGACATTTACTCTCGACTGGACATTCTTTCAG
CCGATGTTCAGGTTGACCGTCTCATCACCGGCAGATTATCAGCACTTAATGCTTTTGTTGCTCAAACCCTCACTAAGTA
TACTGAGGTTCAGGCTAGCAGGAAGCTAGCACAGCAAAAGGTTAATGAGTGCGTTAAATCGCAATCTCAGCGTTATGGT
TTTTGTGGTGGTGATGGCGAGCACATTTTCTCTCTGGTACAGGCAGCACCTCAGGGCCTGCTGTTTTTACATACAGTAC
TTGTACCGGGTGACTTTGTAGATGTTATTGCCATCGCTGGCTTATGCGTTAACGATGAAATTGCCTTGACTCTACGTGA
GCCTGGCTTAGTCTTGTTTACGCATGAACTTCAAAATCATACTGCGACGGAATATTTTGTTTCATCGCGACGTATGTTT
GAACCTAGAAAACCTACCGTTAGTGATTTTGTTCAAATTGAGAGTTGTGTGGTCACCTATGTCAATTTGACTAGAGACC
AACTACCAGATGTAATCCCAGATTACATCGATGTTAACAAAACACTTGATGAGATTTTAGCTTCTCTGCCCAATAGAAC
TGGTCCAAGTCTTCCTTTAGATGTTTTTAATGCCACTTATCTTAATCTCACTGGTGAAATTGCAGATTTAGAGCAGCGT
TCAGAGTCTCTCCGTAATACTACAGAGGAGCTCCAAAGTCTTATATATAATATCAACAACACACTAGTTGACCTTGAGT
GGCTCAACCGAGTTGAGACATATATCAAGTGGCCGTGGTGGGTTTGGTTGATTATTTTCATTGTTCTCATCTTTGTTGT
GTCATTACTAGTGTTCTGCTGCATTTCCACTGGTTGTTGTGGATGCTGCGGCTGCTGCTGTGCTTGTTTTTCAGGTTGT
TGTAGGGGTCCTAGACTTCAACCTTACGAAGTTTTTGAAAAGGTCCACGTGCAGTGA

>gi|595597734|gb|KJ020932.1| Porcine epidemic diarrhea virus isolate CHYJ130330, complete genome

ATGAAGTCTTTAACCTACTTCTGGTTGTTCTTACCAGTACTTTCAACACTTAGCCTACCACAAGATGTCACCAGG
TGCTCAGCTAACACTAATTTTAGGCGGTTCTTTTCAAAATTTAATGTTCAGGCGCCTGCAGTTGTTGTACTGGGCGGTT
ATCTACCTATTGGTGAAAACCAGGGTGTCAATTCAACTTGGTACTGTGCTGGCCAACATCCAACTGCTAGTGGCGTTCA
TGGTATCTTTGTTAGCCATATTAGAGGTGGTCATGGCTTTGAGATTGGCATTTCGCAAGAGCCTTTTGACCCTAGTGGT
TACCAGCTTTATTTACATAAGGCTACTAACGGTAACACTAATGCTACTGCGCGACTGCGCATTTGCCAGTTTCCTAGCA
TTAAAACATTGGGCCCCACTGCTAATAATGATGCTACAACAGGTCGTAATTGCCTATTTAACAAAGCCATCCCAGCTCA

TATGAGTGAACATAGTGTTGTCGGCATAACATGGGATAATGATCGTGTCACTGTCTTTTCTGACAAGATCTATTATTTT
TATTTTAAAAATGATTGGTCCCGTGTTGCGACAAAGTGTTACAACAGTGGAGGTTGTGCTATGCAATATGTTTACGAAC
CCACCTATTACATGCTTAATGTTACTAGTGCTGGTGAGGATGGTATTTCTTATCAACCCTGTACAGCTAATTGCATTGG
TTATGCTGCCAATGTATTTGCTACTGAGCCCAATGGCCACATACCAGAAGGTTTTAGTTTTAATAATTGGTTTCTTTTG
TCCAATGATTCCACTTTGTTGCATGGTAAGGTGGTTTCCAACCAACCTTTGTTGGTCAATTGTCTTTTGGCCATTCCTA
AGATTTATGGATTAGGCCAATTTTTCTCATTCAATCAAACGATGGATGGCGTTTGTAATGGAGCTGCTGCGCAGCGTGC
ACCAGAGGCTCTGAGGTTTAATATTAATGACACCTCTGTCATTCTTGCTGAAGGCTCAATTGTACTTCATACTGCTTTA
GGAACAAATCTTTCTTTTGTTTTCAGTAATTCCACAAATCCTCATTTAGCCACCTTCGCCATACCTCTGGGTGCTACCC
AAGTACCCTATTATTGTTTTCTTAAAGTGGATACTTACAACTCCACTGTTTATAAATTCTTGGCTGTTTTACCTCCTAC
CGTCAGGGAAATTGTCATCACCAAGTATGGTGATGTTTATGTCAATGGGTTTGGCTATTTGCATCTCGGCTTGTTGGAC
GCTGTCACAATTAATTTCACTGGTCATGGCACTGACGATGACGTTTCAGGTTTCTGGACTATAGCATCGACTAACTTTG
TTGATGCACTTATCGAAGTTCAAGGAACTGCCATTCAGCGTATTCTTTATTGTGATGATCCTGTTAGCCAACTCAAGTG
TTCTCAGGTTGCTTTTGACCTTGACGATGGTTTCTACACTATTTCTTCTAGAAACCTTCTGAGTCATGAACAGCCAATT
TCTTTTGTTACTTTGCCATCATTTAATGATCATTCTTTTGTTAATATTACTGTCTCTGCTGCTTTTGGTGGTCAAAGTG
GTGCCAACCTTATTGCATCTGACACTATTATTAATGGGTTTAGTTCTTTCTGTGTTGACACTAGACAATTTACCATTTC
ACTGTTTTATAATGTTACAAACAGTTATGGTTATGTGTCTAAATCACAGGACAGTAATTGCCCTTTCACCTTGCAATCT
GTTAATGATTACCTGTCTTTTAGCAAATTTTGTGTTTCTACCAGCCTTTTGGCTAGTGCCTGTACCATAGATCTTTTTG
GTTACCCTGAGTTTGGTAGTGGTGTTAAGTTCACGTCCCTTTACTTTCAATTCACAAAGGGTGAGTTGATTACTGGCAC
GCCTAAACCACTTGAAGGTGTTACGGACGTTTCTTTTATGACTCTGGATGTGTGTACCAAGTATACTATCTATGGCTTT
AAAGGTGAGGGTATCATTACCCTTACAAATTCTAGCTTTTTGGCAGGTGTTTATTATACATCTGATTCTGGACAGTTGT
TAGCTTTTAAGAATGTCACTAGTGGTGCTGTTTATTCTGTTACGCCATGTTCTTTTTCAGAGCAGGCTGCATATGTTGA
TGATGATATAGTGGGTGTTATTTCTAGTTTGTCTAACTCCACTTTTAACAGTACTAGGGAGTTGCCTGGTTTCTTCTAC
CATTCTAATGATGGCTCTAATTGTACAGAGCCTGTGTTGGTGTATAGTAACATAGGTGTTTGTAAATCTGGCAGTATTG
GCTATGTTCCATCTCAGTCTGGCCAAGTCAAGATTGCACCCACGGTTACTGGGAATATTACTATTCCCACCAACTTTAG
TATGAGTATTAGGACAGAATATTTACAGCTTTACAACACGCCTGTTAGTGTTGATTGTGCTACATATGTTTGTAATGGT
AACTCTCGTTGTAAACAATTACTCACCCAGTACACTGCAGCATGTAAGACCATAGAGTCAGCATTACAACTCAGCGCTA
GGCTTGAGTCTGCTGAAGTCAACTCTATGCTTACTATTTCTGAAGAGGCTCTACAGTTAGCTACCATTAGTTCGTTTAA
TGGTGATGGATATAATTTTACTAATGTGCTGGGTGTTTCTGTGTATGATCCTGCAAGTGGCAGGGTGGTACAAAAAAGG
TCTTTTATTGAAGACCTGCTTTTTAATAAAGTGGTTACTAATGGCCTTGGTACTGTTGATGAAGACTATAAGCGCTGTT
CTAATGGTCGCTCTGTGGCAGATCTAGTCTGTGCACAGTATTACTCTGGTGTCATGGTACTACCTGGTGTTGTTGACGC
TGAGAAGCTTCACATGTATAGTGCGTCTCTCATCGGTGGTATGGTGCTAGGAGGTTTTACTTCTGCAGCGGCATTGCCT
TTTAGCTATGCTGTTCAAGCTAGACTCAATTATCTTGCTCTACAGACGGATGTTCTACAGCGGAACCAGCAATTGCTTG
CTGAGTCTTTTAACTCTGCTATTGGTAATATAACTTCAGCCTTTGAGAGTGTTAAAGAGGCTATTAGTCAAACTTCCAA
GGGTTTGAACACTGTGGCTCATGCGCTTACTAAGGTTCAAGAGGTTGTTAACTCGCAGGGTGCAGCTTTGAACCAACTT
ACCGTACAGCTGCAACACAACTTCCAAGCCATTTCTAGTTCTATTGATGACATTTACTCTCGGCTGGACATTCTTTCAG
CCGATGTTCAGGTTGACCGTCTCATCACCGGCAGATTATCAGCACTTAATGCTTTTGTTGCTCAAACCCTCACTAAGTA
TACTGAGGTTCAGGCTAGCAGGAAGCTAGCACAGCAAAAGGTTAATGAGTGCGTTAAATCGCAATCTCAGCGTTATGGT
TTTTGTGGTGGTGATGGCGAGCACATTTTCTCTCTGGTACAGGCAGCACCTCAGGGCCTGCTGTTTTTACATACAGTAC
TTGTACCGGGTGATTTTGTAGATGTTATTGCCATCGCTGGCTTATGCGTTAACGATGAAATTGCCTTGACTCTACGTGA
GCCTGGCTTAGTCTTGTTTACGCATGAACTTCAAAATCATACTGCGACGGAATATTTTGTTTCATCGCGACGTATGTTT
GAACCTAGAAAACCTACCGTTAGTGATTTTGTTCAAATTGAGAGTTGTGTGGTCACCTATGTCAATTTGACTAGAGACC
AACTACCAGATGTAATCCCAGATTACATCGATGTTAACAAAACACTTGATGAGATTTTAGCTTCTCTGCCCAATAGAAC
TGGTCCGAGTCTTCCTTTAGATGTTTTTAATGCCACTTATCTTAATCTCACTGGTGAAATTGCAGATTTAGAGCAGCGT
TCAGAGTCTCTCCGTAATACTACAGAGGAGCTCCAAAGTCTTATATATAATATCAACAACACACTAGTTGACCTTGAGT

GGCTCAACCGAGTTGAGACATATATCAAGTGGCCGTGGTGGGTTTGGTTGATTATTTTCATTGTTCTCATCTTTGTTGT
GTCATTACTAGTGTTCTGCTGCATTTCCACGGGTTGTTGCGGATGCTGCGGCTGCTGCTGTGCTTGTTTTTCAGGTTGT
TGTAGGGGTCCTAGACTTCAACCTTACGAAGTTTTTGAAAAGGTCCACGTGCAGTGA

>gi|575502846|gb|KJ158152.1| Porcine epidemic diarrhea virus strain AH-M, complete genome

ATGACGCCTTTAATTTACTTCTGGTTGTTCTTACCAGTACTTCTAACACTTAGCCTACCACAAGATGTCACTAGG
TGCCAGTCTACTATTAACTTTAGGCGGTTCTTTTCAAAATTTAATGTTCAGGCACCTGCCGTCGTTGTTTTGGGTGGTT
ATCTACCTAGTATGAACTCTTCTAGCTGGTACTGTGGCACAGGCATTGAAACTGATAGTGGCGTTCATGGTATTTTTCT
CAGTTACATCGATTCTGGTCAGGGCTTTGAGATTGGCATTTCGCAAGAGCCGTTTGATCCTAGTGGTTACCAGCTTTAT
TTACACAAGGCCACTAATGGTAACACTAGTGCTATTGCACGACTGCGCATTTGCCAGTTTCCAGATAATAAAACATTGG
GCCCTACTGTTAATGATGTTACAACAGGTCGTAACTGCCTATTCAACAAAGCCATTCCAGCTTTGCAGGATGGAAAAAA
TATTGTTGTCGGCATAACATGGGATAATGATCGTGTCACTGTTTTTGCTGACAAGATCTATCATTTTTATATTAAAAAT
GATTGGTCCCGTGTTGCGACAAGATGTTACAATAAAAGAAGTTGTGCCATGCAATATGTTTATACACCTACCTACTACA
TGCTTAATGTTACTAGTGCAGGTGAGGATGGCATTTACTATGAACCTTGTACAGCTAATTGCAGTGGTTACGCTGCCAA
TGTATTTGCCACTGATTCCAATGGCCATATACCAGAAGGTTTTAGTTTTAATAATTGGTTTCTTTTGTCCAATGACTCC
ACTTTGTTGCATGGTAAAGTGGTTTCAAACCAACCTTTGTTGGTCAACTGCCTTTGGGCCATTCCTAAGATTTATGGAC
TAGGCCAATTTTTCTCATTCAATCAAACGATGGATGGCGTTTGTAACGGAGCCGCTGCGCAGCGTGCCCCAGAGGCTCT
GAGGTTTAATATTAATGACACTTTTGTCATTCTTGCTGAAGGCTCAATTGTACTTCATACTGCTTTAGGAACAAATCTT
TCTTTTGTTTGCAGTAATTCCTCAGATCCTCACAAAGCCATCTTTACCATACCTTTGGGTGTTACTGAAGTACCCTACT
ATTGCTTTCTTAAAGTGGATACTTACAAATCCACTGTTTATAAATTCTTGGCTGTTTTACCTCCTACTGTCAAGGAAAT
TGTCATCACCAAGTACGGTGATGTTTATGTCAACGGGTTTGGCTATTTGCATCTCGGTTTGTTGGATGCTGTCACAATT
AATTTCACTGGTCATGGCACTGACGATGACGTTTCAGGTTTCTGGACCGTAGCATCGACTAATTTTGTTGATGCACTCA
TCGAGGTTCAAGGAACTGCCATTCAGCGTATTCTTTATTGTGATGACCCTGTTAGCCAACTTAAGTGTTCTCAGGTTTC
TTTTGACCTTGATGATGGTTTTTACCCTATTTCTTCTAGAAACCTTCTGAGTCATGAACAGCCAATTTCTTTTGTTACT
TTGCCATCATTCAATGATCATTCTTTTGTTAATATTACTGTCTCTGCGGCTTTTGGTGGTCATAGTGGTGCCAACCTCA
TTGCATCTGACACTACTATCAATGGGTTTAGTTCTTTCTGTGTTGACACTAGACAATTTACCATTACACTGTTTTATAA
CGTTACAAACAGTTATGGTTATGTGTCTAAGTCACAGGATAGTAATTGCCCTTTCACCTTGCAATCTGTTAATGATTAC
CTGTCTTTTAGCAAATTTTGTGTTTCAACCAGCCTTTTGGCTGGTGCTTGTACCATAGATCTTTTTGGTTACCCTGAGT
TCGGTAGTGGTGTTAAGTTTACGTCCCTTTATTTTCAATTCACAAAGGGTGAGTTGATTACTGGCACGCCTAAACCACT
TCAAGGTGTCACGGACGTTTCTTTTATGACTCTGGATGTGTGTACCAAGTATACTATCTATGGCTTTAAAGGTGAGGGT
ATTATTACCCTTACAAATTCTAGCTTTTTGGCAGGTGTTTATTATACATCTGATTCTGGACAGTTGTTAGCCTTTAAGA
ATGTCACTAGTGGTGCTGTTTATTCTGTTACGCCATGTTCTTTTTCAGAGCAGGCTGCATATGTTGATGATGATATAGT
GGGTGTTATTTCTAGTTTGTCTAACTCCACTTTTAACAATACCAGGGAGTTGCCTGGTTTCTTCTACCATTCTAATGAT
GGCTCCAATTGTACAGAGCCTGTGTTGGTGTATAGTAACATAGGTGTCTGTAAATCTGGCAGTATTGGCTATGTCCCAC
TTCAGGATGGCCAAGTCAAGATTGCACCCATGGTTACTGGGAATATTAGTATTCCCACCAACTTTAGTATGAGTATTAG
AACAGAATATTTACAGCTTTACAACACGCCTGTTAGTGTTGATTGCGTTACATATGTTTGTAATGGTAACTCTCGTTGT
AAACAATTACTCACCCAGTACACTGCAGCATGTAAGACCATAGAGTCAGCATTACAACTCAGCGCTAGGCTTGAGTCTG
TTGAAGTTAACTCTATGCTTACTATTTCTGAAGAGGCTCTACAGTTAGCTACCATCAGTTCGTTTAATGGTGATGGATA
TAACTTTACTAATGTGCTGGGTGTTTCCGTGTACGACCCTGCAAGTGGCAGGGTGGTACAAAAAGGGTCTTTTATTGAA
GACCTGCTTTTTAATAAAGTGGTTACTAATGGCCTTGGTACTGTTGATGAAGACTATAAGCGCTGTTCTAATGGTCGCT
CTGTGGCAGATCTAGTCTGTGCGCAGTATTACTCTGGTGTCATGGTACTACCTGGCGTTGTTGACGCTGAGAAGCTTCA

CATGTATAGTGCGTCTCTCATCGGTGGTATGGCGCTAGGAGGTCTTACTACTGCAGCGGCATTGCCTTTTAGCCATGCT
GTTCAAGCGAGGCTCAATTATCTTGCTTTACAGACGGATGTTCTACAGCGCAACCAGCAATTGCTTGCTGAGTCTTTTA
ACTCTGCTATTGGTAATATAACTTCAGCCTTTGAGAGTGTTAAAGAGGCTATTAGTCAAACTTCCAATGGTTTGAACAC
TGTGGCTCATGCGCTTACTAAGGTTCAAGAGGTTGTTAATTCGCAGGGTTCAGCTTTGACCCAACTTACCATACAGCTG
CAACACAACTTCCAAGCCATTTCTAGTTCTATTGATGACATTTACTCCCGACTGGACATTCTTTCAGCCGATGTTCAGG
TTGATCGTCTCATCACCGGCAGATTATCAGCACTTAATGCTTTTGTTGCTCAAACCCTCACTAAGTATACTGAGGTTCA
GGCTAGCAGGAAGCTAGCACAGCAAAAGGTTAATGAGTGCGTCAAATCGCAATCTCAGCGTTATGGTTTTTGTGGTGGT
GATGGCGAGCACATCTTCTCTCTGGTACAGGCCGCACCTCAGGGCCTGCTGTTCTTACATACAGTACTTGTACCGGGTG
ATTTTGTAAATGTTATTGCCATCGATGGCTTATGCGTTAATGGTGATATTGCCTTGACTCTACGTGAGCCTGGCTTAGT
CTTGTTTACGCATGAACTTCAAACTTATACTGCGACGGAATATTTTGTTTCATCGCGACGTATGTTTGAACCTAGAAAA
CCTACCGTTAGTGATTTTGTTCAAATTGAGAGTTGTGTGGGCACCTATGTCAATCTGACTAGCGACCAACTACCAGATG
TAATCCCAGATTACATCGATGTTAACAAAACACTTGATGAGATTCTAGCTTCTCTGCCCAATAGAATTGGTCCTAGTCT
TCCCCTAGATGTTTTTAATGCCACTTATCTTAATCTCACTGGTGAAATTGCAGATTTAGAGCAGCGTTCAGAGTCTCTC
CGTAATACTACAGAAGAGCTCCGAAGTCTCATATATAATATCAACAACACACTTGTTGACCTTGAGTGGCTCAACCGAG
TTGAGACATATATCAAGTGGCCGTGGTGGGTTTGGTTGATTATTTTTATTGTTCTCATCTTTGTTGTGTCATTATTAGT
GTTCTGCTGCATTTCCACGGGTTGTTGTGGATGCTGCGGTTGTTGCGGTGCTTGTTTTTCAGGTTGTTGTAGGGGTCCT
AGACTTCAACCTTACGAAGCTTTTGAAAAGGTCCACGTGCAGTGA

>gi|606235531|gb|KJ196348.1| Porcine epidemic diarrhea virus strain SHQP/YM/2013, complete genome

ATGAAGTCTTTAACCTACTTCTGGTTGTTCTTACCAGTACTTTCAACACTTAGCCTACCACAAGATGTCACCAGG
TGCTCAGCTAACACTAATTTTAGGCGGTTCTTTTCAAAATTTAATGTTCAGGCGCCTGCAGTTGTTGTACTGGGCGGTT
ATCTACCTATTGGTGAAAACCAGGGTGTCAATTCAACTTGGTACTGTGCTGGCCAACATCCAACTGCTAGTGGCGTTCA
TGGTATCTTTGTTAGCCATATTAGAGGTGGTCATGGCTTTGAGATTGGCATTTCGCAAGAGCCTTTTGACCCTAGTGGT
TACCAGCTTTATTTACATAAGGCTACTAACGGTAACACTAATGCTACTGCGCGACTGCGCATTTGCCAGTTTCCTAGCA
TTAAAACATTGGGTCCCACTGTTAATAATGATGTTACAACAGGTCGTAATTGCTTATTTAACAAAGCCATCCCAGCTCA
TATGAGTGAACATAGTGTTGTCGGCATAACATGGGATAATGATCGTGTCACTGTCTTTTCTGACAAGATCTATTATTTT
TATTTTAAAAATAATTGGTCCCGTGTTGCGACAAAGTGTTACAACAGTGGAGGTTGTGCTATGCAATATGTTTACGAAC
CCACCTATTACATGCTTAATGTTACTAGTGCTGGTGAGGATGGTATTTCTTATCAACCCTGTACAGCTAATTGCATTGG
TTATGCTGCCAATGTATTTGCTACTGAGCCCAATGGCCACATACCAGAAGGTTTTAGTTTTAATAATTGGTTTCTTTTG
TCCAATGATTCCACTTTGGTGCATGGTAAGGTGGTTTCCAACCAACCATTGTTGGTCAATTGTCTTTTGGCCATTCCTA
AGATTTATGGACTAGGCCAATTTTTCTCCTTTAATCAAACGATCGATGGTGTTTGTAATGGAGCTGCTGTGCAGCGTGC
ACCAGAGGCTCTGAGGTTTAATATTAATGACACCTCTGTCATTCTTGCTGAAGGCTCAATTGTACTTCATACTGCTTTA
GGAACAAATTTTTCTTTTGTTTGCAGTAATTCCTCAAATCCTCACTTAGCCACCTTCGCCATACCTCTGGGTGCTACCC
AAGTACCCTATTATTGTTTTCTTAAAGTGGATACTTACAACTCCACTGTTTATAAATTTTTGGCTGTTTTACCTCCTAC
CGTCAGGGAAATTGTCATCACCAAGTATGGTGATGTTTATGTCAATGGGTTTGGCTATTTGCATCTCGGTTTGTTGGAC
GCTGTCACAATTAATTTCACTGGTCATGGCACTGACGATGACGTTTCAGGTTTCTGGACCATAGCATCGACTAATTTTG
TTGATGCACTTATCGAAGTTCAAGGAACTGCCATTCAGCGTATTCTTTATTGTGATGATCCTGTTAGCCAACTCAAGTG
TTCTCAGGTTGTTTTTGACCTTGACGATGGTTTCTACCCTATTTCTTCTAGAAACCTTCTGAGTCATGAACAGCCAATT
TCTTTTGTTACTTTGCCATCATTTAATGATCATTCTTTTGTTAACATTACTGTCTCTGCGTCCTTTGGTGGTCATAGTG
GTGCCAACCTTATTGTATCTGACACTACTATCAATGGGTTTAGTTCTTTCTGTGTTGACACTAGACAATTTACCATTTC
ACTGTTTTATAACGTTACAAACAGTTATGGTTATGTGTCTAACTCACAGGACAGTAAGTGCCCTTTCACCTTGCAATCT

GTTAATGATTACCTGTCTTTTAGTAAATTTTGTGTTTCCACCAGCCTTTTGGCTAGTGCCTGTACCATAGATCTTTTTG
GTTACCCTGAGTTTGGTAGTGGTGTTAAGTTTACGTCCCTTTACTTTCAATTCACAAAGGGTGAGTTGATTACTGGCAC
GCCTAAACCACTTGAAGGTGTCACGGACGTTTCTTTTATGACTCTGGACGTGTGTACCAAGTATACTATCTATGGCTTT
AAAGGTGAGGGTATCATTACCCTTACAAATTCTAGCTTTTTGGCAGGTGTTTATTACACATCTGATTCTGGACAGTTGT
TAGCCTTTAAGAATGTCACTAGTGGTGCTGTTTATTCTGTTACGCCATGTTCTTTTTCAGAGCAGGCTGCATATGTTGA
TGATGATATAGTGGGTGTTATTTCTAGTTTGTCTAACTCCACTTTTAACAGTACTAGGGAGTTGCCTGGTTTCTTCTAC
CATTCTAATGATGGCTCTAATTGTACAGAGCCTGTGTTGGTGTATAGTAACATAGGTGTTTGTAAATCTGGCAGTATTG
GCTACGTCCCATCTCAGTCTGGCCAAGTCAAGATTGCACCCACGGTTACTGGGAATATTAGTATTCCCACCAACTTTAG
TATGAGTATTAGGACAGAATATTTACAGCTTTACAACACGCCTGTTAGTGTTGATTGTGCCACATATGTTTGTAATGGT
AACTCTCGTTGTAAACAATTACTCACCCAGTACACTGCAGCATGTAAGACCATAGAGTCAGCATTACAACTCAGCGCTA
GGCTTGAGTCTGTTGAAGTTAACTCTATGCTTACTATTTCTGAAGAGGCTCTACAGTTAGCTACCATCAGTTCGTTTAA
TGGTGATGGATATAATTTTACTAATGTGCTGGGTGTTTCTGTGTATGATCCTGCAAGTGGCAGGGTGGTACAAAAAAGG
TCTTTTATTGAAGACCTGCTTTTTAATAAAGTGGTTACTAATGGCCTTGGTACTGTTGATGAAGACTATAAGCGCTGTT
CTAATGGTCGCTCTGTGGCAGATCTAGTCTGTGCACAGTATTACTCTGGTGTCATGGTACTACCTGGTGTTGTTGACGC
TGAGAAGCTTCACATGTATAGTGCGTCTCTCATCGGTGGTATGGTGCTAGGAGGTTTTACTTCTGCAGCGGCATTGCCT
TTTAGCTATGCTGTTCAAGCTAGACTCAATTATCTTGCTCTACAGACGGATGTTCTACAGCGGAACCAGCAATTGCTTG
CTGAGTCTTTTAACTCTGCTATTGGTAATATAACTTCAGCCTTCGAGAGTGTTAAAGAGGCTATTAGTCAAACTTCCAA
GGGTTTGAACACTGTGGCTCATGCGCTTACTAAGGTTCAAGAGGTTGTTAACTCGCAGGGTGCAGCTTTGACTCAACTT
ACCGTACAGCTGCAACACAACTTCCAATCCATCTCTAGTTCTATTGATGACATTTACTCTCGACTGGACATTCTTTCAG
CCGATGTTCAAGTTGACCGTCTCATCACCGGCAGATTATCAGCACTTAATGCTTTTGTTGCTCAAACCCTCACTAAGTA
TACTGAGGTTCAGGCTAGCAGGAAGCTAGCACAGCAAAAGGTTAATGAGTGCGTTAAATCGCAATCTCAGCGTTATGGT
TTTTGTGGTGGTGATGGCGAGCACATTTTCTCTCTGGTACAGGCAGCACCTCAGGGCCTGCTGTTTTTACATACAGTAC
TTGTACCGGGTGACTTTGTAGATGTTATTGCCATCGCTGGCTTATGCGTTAACGATGAAATTGCCTTGACTCTACGTGA
GCCTGGCTTAGTCTTGTTTACGCATGAACTTCAAAATCATACTGCGACGGAATATTTTGTTTCATCGCGACGTATGTTT
GAACCTAGAAAACCTACCGTTAGTGATTTTGTTCAAATTGAGAGTTGTGTGGTCACCTATGTCAATTTGACTAGAGACC
AACTACCAGATGTAATCCCAGATTACATCGATGTTAACAAAACACTTGATGAGATTTTAGCTTCTCTGCCCAATAGAAC
TGGTCCAAGTCTTCCTTTAGATGTTTTTAATGCCACTTATCTTAATCTCACTGGTGAAATTGCAGATTTAGAGCAGCGT
TCAGAGTCTCTCCGTAATACTACAGAGGAGCTCCAAAGTCTTATATATAATATCAACAACACACTAGTTGACCTTGAGT
GGCTCAACCGAGTTGAGACATATATCAAGTGGCCGTGGTGGGTTTGGTTGATTATTTTTATTGTTCTCATCTTTGTTGT
GTCATTACTAGTGTTCTGCTGCATTTCCACTGGTTGTTGTGGATGCTGCGGCTGCTGCTGTGCTTGTTTTTCAGGTTGT
TGTAGGGGTCCTAGACTTCAACCTTACGAAGTTTTTGAAAAGGTCCACGTGCAGTGA

>gi|591400267|gb|KJ399978.1| Porcine epidemic diarrhea virus strain OH851, complete genome

ATGAAGTCTTTAAATTACTTCTGGTTGTTCTTACCAGTACTTTCAACACTCAGCCTACCACAAGATGTCACTAGG
TGCCAGTCCACTATTAACTTCAGGCGGTTCTTTTCAAAATTTAATGTGCAGGCACCTGCTGTCGTTGTGTTGGGTGGTT
ATCTACCTAGTATGAACTCCTCTAGCTGGTACTGTGGCACAGGTCTTGAAACTGCTAGTGGCGTGCATGGTATTTTCCT
CAGTTACATCGATGCTGGTCAGGGCTTTGAGATTGGCATTTCACAGGAGCCGTTTGATCCTAGTGGTTACCAGCTTTAT
TTACATAAGGCCACTAATGGTAACCATAATGCTATTGCACGACTGCGCATTTGCCAGTTTCCAGATAATAAAACATTGG
GCCCTACTGTTAATGATGTTACAACAGGTCGTAACTGCCTATTCAACAAAGCCATTCCAGCTTATATGCAGGATGGAAA
AAATATCGTTGTCGGCATAACATGGGACAATGATCGTGTCACTGTTTTTGCTGACAAGATCTATCATTTTTATCTTAAA
AATGATTGGTCCCGTGTTGCGACAAGATGTTACAATAAAAGAAGTTGTGCTATGCAATATGTTTATACACCTACCTACT

ACATGCTTAATGTTACTAGTGCAGGTGAGGATGGCATTTATTATGAACCATGTACAGCTAATTGCAGTGGTTACGCTGC
CAATGTGTTTGCCACTGATTCTAATGGCCACATACCAGAAGGTTTTAGTTTTAATAATTGGTTTCTTTTGTCCAATGAT
TCCACTTTGTTGCATGGTAAGGTGGTTTCCAACCAACCTTTGTTGGTCAATTGTCTTTTGGCCATTCCTAAGATTTATG
GACTAGGCCAATTTTTCTCATTCAATCAAACGATGGATGGCGTTTGTAATGGAGCTGCTGCGCAGCGTGCACCAGAGGC
TCTGAGGTTTAATATTAATGACACCTCTGTCATTCTTGCTGAAGGCTCAATTGTACTTCATACTGCTTTAGGAACAAAT
CTTTCTTTTGTTTGCAGTAATTCTTCAGATCCTCATCTAGCTACCTTCGCCATACCTCTGGGTGCTACCCAAGTACCTT
ATTATTGTTTTCTTAAAGTGGATACTTACAACTCCACTGTTTATAAATTTTTGGCTGTTTTACCTCCTACCGTCAGGGA
AATTGTCATCACCAAGTATGGTGATGTTTATGTCAATGGGTTTGGATACTTGCATCTCGGTTTGTTGGATGCTGTCACA
ATTAATTTCACTGGTCATGGCACTGACGATGATGTTTCTGGTTTTTGGACCATAGCATCGACTAATTTTGTTGATGCAC
TCATCGAAGTTCAAGGAACCGCCATTCAGCGTATTCTTTATTGTGATGATCCTGTTAGCCAACTCAAGTGTTCTCAGGT
TGCTTTTGACCTTGACGATGGTTTTTACCCTATTTCTTCTAGAAACCTTCTGAGTCATGAACAGCCAATTTCTTTTGTT
ACTCTGCCATCATTTAATGATCATTCTTTTGTTAACATTACTGTATCTGCTTCCTTTGGTGGTCATAGTGGTGCCAACC
TTATTGCATCTGACACTACTATCAATGGGTTTAGTTCTTTCTGTGTTGACACTAGACAATTTACCATTTCACTGTTTTA
TAACGTTACAAACAGTTATGGTTATGTGTCTAAATCACAGGACAGTAATTGCCCTTTCACCTTGCAATCTGTTAATGAT
TACCTGTCTTTTAGCAAATTTTGTGTTTCCACCAGCCTTTTGGCTAGTGCCTGTACCATAGATCTTTTTGGTTACCCTG
AGTTTGGTAGTGGTGTTAAGTTTACGTCCCTTTACTTTCAATTCACAAAGGGTGAGTTGATTACTGGCACGCCTAAACC
ACTTGAAGGTGTCACGGACGTTTCTTTTATGACTCTGGATGTGTGTACCAAGTATACTATCTATGGCTTTAAAGGTGAG
GGTATCATTACCCTTACAAATTCTAGCTTTTTGGCAGGTGTTTATTACACATCTGATTCTGGACAGTTGTTAGCCTTTA
AGAATGTCACTAGTGGTGATGTTTATTCTGTTACGCCATGTTCTTTTTCAGAGCAGGCTGCATATGTTGATGATGATAT
AGTGGGTGTTATTTCTAGTTTGTCTAGCTCCACTTTTAACAGTACTAGGGAGTTGCCTGGTTTCTTCTACCATTCTAAT
GATGGCTCTAATTGTACAGAGCCTGTGTTGGTGTATAGTAACATAGGTGTTTGTAAATCTGGCAGTATTGGCTACGTCC
CATCTCAGTCTGGCCAAGTCAAGATTGCACCCACGGTTACTGGGAATATTAGTATTCCCACCAACTTTAGTATGAGTAT
TAGGACAGAATATTTACAGCTTTACAACACGCCTGTTAGTGTTGATTGTGCCACATATGTTTGTAATGGTAACTCTCGT
TGTAAACAATTACTCACCCAGTACACTGCAGCATGTAAGACCATAGAGTCAGCATTACAACTCAGCGCTAGGCTTGAGT
CTGTTGAAGTTAACTCTATGCTTACTATTTCTGAAGAGGCTCTACAGTTAGCTACCATTAGTTCGTTTAATGGTGATGG
ATATAATTTTACTAATGTGCTGGGTGTTTCTGTGTATGATCCTGCAAGTGGCAGGGTGGTACAAAAAAGGTCTTTTATT
GAAGACCTGCTTTTTAATAAAGTGGTTACTAATGGCCTTGGTACTGTTGATGAAGACTATAAGCGCTGTTCTAATGGTC
GCTCTGTGGCAGATCTAGTCTGTGCACAGTATTACTCTGGTGTCATGGTACTACCTGGTGTTGTTGACGCTGAGAAGCT
TCACATGTATAGTGCGTCTCTCATCGGTGGTATGGTGCTAGGAGGTTTTACTTCTGCAGCGGCATTGCCTTTTAGCTAT
GCTGTTCAAGCTAGACTCAATTATCTTGCTCTACAGACGGATGTTCTACAGCGGAACCAGCAATTGCTTGCTGAGTCTT
TTAACTCTGCTATTGGTAATATAACTTCAGCCTTTGAGAGTGTTAAAGAGGCTATTAGTCAAACTTCCAAGGGTTTGAA
CACTGTGGCTCATGCGCTTACTAAGGTTCAAGAGGTTGTTAACTCGCAGGGTGCAGCTTTGACTCAACTTACCGTACAG
CTGCAACACAACTTCCAAGCCATTTCTAGTTCTATTGATGACATTTACTCTCGACTGGACATTCTTTCAGCCGATGTTC
AGGTTGACCGTCTCATCACCGGCAGATTATCAGCACTTAATGCTTTTGTTGCTCAAACCCTCACTAAGTATACTGAGGT
TCAGGCTAGCAGGAAGTTAGCACAGCAAAAGGTTAATGAGTGCGTTAAATCGCAATCTCAGCGTTATGGTTTTTGTGGT
GGTGATGGCGAGCACATTTTCTCTCTGGTACAGGCAGCACCTCAGGGCCTGCTGTTTTTACATACAGTACTTGTACCGA
GTGATTTTGTAGATGTTATTGCCATCGCTGGCTTATGCGTTAACGATGAAATTGCCTTGACTCTACGTGAGCCTGGCTT
AGTCTTGTTTACGCATGAACTTCAAAATCATACTGCGACGGAATATTTTGTTTCATCGCGACGTATGTTTGAACCTAGA
AAACCTACCGTTAGTGATTTTGTTCAAATTGAGAGTTGTGTGGTCACCTATGTCAATTTGACTAGAGACCAACTACCAG
ATTTAATCCCAGATTACATCGATGTTAACAAAACACTTGATGAGATTTTAGCTTCTCTGCCCAATAGAACTGGTCCAAG
TCTTCCTTTAGATGTTTTTAATGCCACTTATCTTAATCTCACTGGTGAAATTGCAGATTTAGAGCAGCGTTCAGAGTCT
CTCCGTAATACTACAGAGGAGCTCCAAAGTCTTATATATAATATCAACAACACACTAGTTGACCTTGAGTGGCTCAACC
GAGTTGAGACATATATCAAGTGGCCGTGGTGGGTTTGGTTGATTATTTTCATTGTTCTCATCTTTGTTGTGTCATTACT
AGTGTTCTGCTGCATTTCCACGGGTTGTTGTGGATGCTGCGGCTGCTGCTGTGCTTGTTTCTCAGGTTGTTGTAGGGGT

CCTAGACTTCAACCTTACGAAGTTTTTGAAAAGGTCCACGTGCAGTGA

>gi|599176356|gb|KJ408801.1| Porcine epidemic diarrhea virus strain OH1414, complete genome

ATGAAGTCTTTAACCTACTTCTGGTTGTTCTTACCAGTACTTTCAACACTTAGCCTACCACAAGATGTCACCAGG
TGCTCAGCTAACACTAATTTTAGGCGGTTCTTTTCAAAATTTAATGTTCAGGCGCCTGCAGTTGTTGTACTGGGCGGTT
ATCTACCTATTGGTGAAAACCAGGGTGTCAATTCAACTTGGTACTGTGCTGGCCAACATCCAACTGCTAGTGGCGTTCA
TGGTATCTTTGTTAGCCATATTAGAGGTGGTCATGGCTTTGAGATTGGCATTTCGCAAGAGCCTTTTGACCCTAGTGGT
TACCAGCTTTATTTACATAAGGCTACTAACGGTAACACTAATGCTACTGCGCGACTGCGCATTTGCCAGTTTCCTAGCA
TTAAAACATTGGGCCCCACTGCTAATAATGATGTTACAACAGGTCGTAATTGCCTATTTAACAAAGCCATCCCAGCTCA
TATGAGTGAACATAGTGTTGTCGGCATAACATGGGATAATGATCGTGTCACTGTCTTTTCTGACAAGATCTATTATTTT
TATTTTAAAAATGATTGGTCCCGTGTTGCGACAAAGTGTTACAACAGTGGAGGTTGTGCTATGCAATATGTTTACGAAC
CCACCTATTACATGCTTAATGTTACTAGTGCTGGTGAGGATGGTATTTCTTATCAACCCTGTACAGCTAATTGCATTGG
TTATGCTGCCAATGTATTTGCTACAGAGCCCAATGGCCACATACCAGAAGGTTTTAGTTTTAATAATTGGTTTCTTTTG
TCCAATGATTCCACTTTGGTGCATGGTAAGGTGGTTTCCAACCAACCATTGTTGGTCAATTGTCTTTTGGCCATTCCTA
AGATTTATGGACTAGGCCAATTTTTCTCCTTTAATCAAACGATCGATGGTGTTTGTAATGGAGCTGCTGTGCAGCGTGC
ACCAGAGGCTCTGAGGTTTAATATTAATGACACCTCTGTCATTCTTGCTGAAGGCTCAATTGTACTTCATACTGCTTTA
GGAACAAATTTTTCTTTTGTTTGCAGTAATTCCTCAAATCCTCACTTAGCCACCTTCGCCATACCTTTGGGTGCTACCC
AAGTACCCTATTATTGTTTTCTTAAAGTGGATACTTACAACTCCACTGTTTATAAATTCTTGGCTGTTTTACCTCCAAC
CGTCAGGGAAATTGTCATCACCAAGTATGGTGATGTTTATGTCAATGGGTTTGGATACTTGCATCTCGGTTTGTTGGAT
GCTGTCACAATTAATTTCACTGGTCATGGCACTGACGATGATGTTTCTGGTTTTTGGACCATAGCATCGACTAATTTTG
TTGATGCACTCATCGAAGTTCAAGGAACCGCCATTCAGCGTATTCTTTATTGTGATGATCCTGTTAGCCAACTCAAGTG
TTCTCAGGTTGCTTTTGACCTTGACGATGGTTTTTACCCTATTTCTTCTAGAAACCTTCTGAGTCATGAACAGCCAATT
TCTTTTGTTACTCTGCCATCATTTAATGATCATTCTTTTGTTAACATTACTGTATCTGCTTCCTTTGGTGGTCATAGTG
GTGCCAACCTTATTGCATCTGACACTACTATCAATGGGTTTAGTTCTTTCTGTGTTGACACTAGACAATTTACCATTTC
ACTGTTTTATAACGTTACAAACAGTTATGGTTATGTGTCTAAATCACAGGACAGTAATTGCCCTTTCACCTTGCAATCT
GTTAATGATTACCTGTCTTTTAGCAAATTTTGTGTTTCCACCAGCCTTTTGGCTAGTGCCTGTACCATAGATCTTTTTG
GTTACCCTGAGTTTGGTAGTGGTGTTAAGTTTACGTCCCTTTACTTTCAATTCACAAAGGGTGAGTTGATTACTGGCAC
GCCTAAACCACTTGAAGGTGTCACGGACGTTTCTTTTATGACTCTGGATGTGTGTACCAAGTATACTATCTATGGCTTT
AAAGGTGAGGGTATCATTACCCTTACAAATTCTAGCTTTTTGGCAGGTGTTTATTACACATCTGATTCTGGACAGTTGT
TAGCCTTTAAGAATGTCACTAGTGGTGCTGTTTACTCTGTTACGCCATGTTCTTTTTCAGAGCAGGCTGCATATGTTGA
TGATGATATAGTGGGTGTTATTTCTAGTTTGTCTAGCTCCACTTTTAACAGTACTAGGGAGTTGCCTGGTTTCTTCTAC
CATTCTAATGATGGCTCTAATTGTACAGAGCCTGTGTTGGTGTATAGTAACATAGGTGTTTGTAAATCTGGCAGTATTG
GCTACGTCCCATCTCAGTCTGGCCAAGTCAAGATTGCACCCACGGTTACTGGGAATATTAGTATTCCCACCAACTTTAG
TATGAGTATTAGGACAGAATATTTACAGCTTTACAACACGCCTGTTAGTGTTGATTGTGCCACATATGTTTGTAATGGT
AACTCTCGTTGTAAACAATTACTCACCCAGTACACTGCAGCATGTAAGACCATAGAGTCAGCATTACAACTCAGCGCTA
GGCTTGAGTCTGTTGAAGTTAACTCTATGCTTACTATTTCTGAAGAGGCTCTACAGTTAGCTACCATTAGTTCGTTTAA
TGGTGATGGATATAATTTTACTAATGTGCTGGGTGTTTCTGTGTATGATCCTGCAAGTGGCAGGGTGGTACAAAAAAGG
TCTTTTATTGAAGACCTGCTTTTTAATAAAGTGGTTACTAATGGCCTTGGTACTGTTGATGAAGACTATAAGCGCTGTT
CTAATGGTCGCTCTGTGGCAGATCTAGTCTGTGCACAGTATTACTCTGGTGTCATGGTACTACCTGGTGTTGTTGACGC
TGAGAAGCTTCACATGTATAGTGCGTCTCTCATCGGTGGTATGGTGCTAGGAGGTTTTACTTCTGCAGCGGCATTGCCT
TTTAGCTATGCTGTTCAAGCTAGACTCAATTATCTTGCTCTACAGACGGATGTTCTACAGCGGAACCAGCAATTGCTTG

CTGAGTCTTTTAACTCTGCTATTGGTAATATAACTTCAGCCTTTGAGAGTGTTAAAGAGGCTATTAGTCAAACTTCCAA
GGGTTTGAACACTGTGGCTCATGCGCTTACTAAGGTTCAAGAGGTTGTTAACTCGCAGGGTGCAGCTTTGACTCAACTT
ACCGTACAGCTGCAACACAACTTCCAAGCCATTTCTAGTTCTATTGATGACATTTACTCTCGACTGGACATTCTTTCAG
CCGATGTTCAGGTTGACCGTCTCATCACCGGCAGATTATCAGCACTTAATGCTTTTGTTGCTCAAACCCTCACTAAGTA
TACTGAGGTTCAGGCTAGCAGGAAGTTAGCACAGCAAAAGGTTAATGAGTGCGTTAAATCGCAATCTCAGCGTTATGGT
TTTTGTGGTGGTGATGGCGAGCACATTTTCTCTCTGGTACAGGCAGCACCTCAGGGCCTGCTGTTTTTACATACAGTAC
TTGTACCGAGTGACTTTGTAGATGTTATTGCCATCGCTGGCTTATGCGTTAACGATGAAATTGCCTTGACTCTACGTGA
GCCTGGCTTAGTCTTGTTTACGCATGAACTTCAAAATCATACTGCGACGGAATATTTTGTTTCATCGCGACGTATGTTT
GAACCTAGAAAACCTACCGTTAGTGATTTTGTTCAAATTGAGAGTTGTGTGGTCACCTATGTCAATTTGACTAGAGACC
AACTACCAGATGTAATCCCAGATTACATCGATGTTAACAAAACACTTGATGAGATTTTAGCTTCTCTGCCCAATAGAAC
TGGTCCAAGTCTTCCTTTAGATGTTTTTAATGCCACTTATCTTAATCTCACTGGTGAAATTGCAGATTTAGAGCAGCGT
TCAGAGTCTCTCCGTAATACTACAGAGGAGCTCCAAAGTCTTATATATAATATCAACAACACACTAGTTGACCTTGAGT
GGCTCAACCGAGTTGAGACATATATCAAGTGGCCGTGGTGGGTTTGGTTGATTATTTTCATTGTTCTCATCTTTGTTGT
GTCATTACTAGTGTTCTGCTGCATTTCCACGGGTTGTTGTGGATGCTGCGGCTGCTGCTGTGCTTGTTTCTCAGGTTGT
TGTAGGGGTCCTAGACTTCAACCTTACGAAGTTTTTGAAAAGGTCCACGTGCAGTGA

>gi|641452900|gb|KJ584361.1| Porcine epidemic diarrhea virus strain OH15962, complete genome

ATGAAGTCTTTAACCTACTTCTGGTTGTTCTTACCAGTACTTTCAACACTTAGCCTACCACAAGATGTCACCAGG
TGCTCAGCTAACACTAATTTTAGGCGGTTCTTTTCAAAATTTAATGTTCAGGCGCCTGCAGTTGTTGTACTGGGCGGTT
ATCTACCTATTGGTGAAAACCAGGGTGTCAATTCAACTTGGTACTGTGCTGGCCAACATCCAACTGCTAGTGGCGTTCA
TGGTATCTTTGTTAGCCATATTAGAGGTGGTCATGGCTTTGAGATTGGCATTTCGCAAGAGCCTTTTGACCCTAGTGGT
TACCAGCTTTATTTACATAAGGCTACTAACGGTAACACTAATGCTACTGCGCGACTGCGCATTTGCCAGTTTCCTAGCA
TTAAAACATTGGGCCCCACTGCTAATAATGATGTTACAACAGGTCGTAATTGCCTATTTAACAAAGCCATCCCAGCTCA
TATGAGTGAACATAGTGTTGTCGGCATAACATGGGATAATGATCGTGTCACTGTCTTTTCTGACAAGATCTATTATTTT
TATTTTAAAAATGATTGGTCCCGTGTTGCGACAAAGTGTTACAACAGTGGAGGTTGTGCTATGCAATATGTTTACGAAC
CCACCTATTACATGCTTAATGTTACTAGTGCTGGTGAGGATGGTATTTCTTATCAACCCTGTACAGCTAATTGCATTGG
TTATGCTGCCAATGTATTTGCTACTGAGCCCAATGGCCACATACCAGAAGGTTTTAGTTTTAATAATTGGTTTCTTTTG
TCCAATGATTCCACTTTGGTGCATGGTAAGGTGGTTTCCAACCAACCATTGTTGGTCAATTGTCTTTTGGCCATTCCTA
AGATTTATGGACTAGGCCAATTTTTCTCCTTTAATCAAACGATCGATGGTGTTTGTAATGGAGCTGCTGTGCAGCGTGC
ACCAGAGGCTCTGAGGTTTAATATTAATGACACCTCTGTCATTCTTGCTGAAGGCTCAATTGTACTTCATACTGCTTTA
GGAACAAATTTTTCTTTTGTTTGCAGTAATTCCTCAAATCCTCACTTAGCCACCTTCGCCATACCTTTGGGTGCTACCC
AAGTACCCTATTATTGTTTTCTTAAAGTGGATACTTACAACTCCACTGTTTATAAATTCTTGGCTGTTTTACCTCCAAC
CGTCAGGGAAATTGTCATCACCAAGTATGGTGATGTTTATGTCAATGGGTTTGGATACTTGCATCTCGGTTTGTTGGAT
GCTGTCACAATTAATTTCACTGGTCATGGCACTGACGATGATGTTTCTGGTTTTTGGACCATAGCATCGACTAATTTTG
TTGATGCACTCATCGAAGTTCAAGGAACCGCCATTCAGCGTATTCTTTATTGTGATGATCCTGTTAGCCAACTCAAGTG
TTCTCAGGTTGCTTTTGACCTTGACGATGGTTTTTACCCTATTTCTTCTAGAAACCTTCTGAGTCATGAACAGCCAATT
TCTTTTGTTACTCTGCCATCATTTAATGATCATTCTTTTGTTAACATTACTGTATCTGCTTCCTTTGGTGGTCATAGTG
GTGCCAACCTTATTGCATCTGACACTACTATCAATGGGTTTAGTTCTTTCTGTGTTGACACTAGACAATTTACCATTTC
ACTGTTTTATAACGTTACAAACAGTTATGGTTATGTGTCTAAATCACAGGACAGTAATTGCCCTTTCACCTTGCAATCT
GTTAATGATTACCTGTCTTTTAGCAAATTTTGTGTTTCCACCAGCCTTTTGGCTAGTGCCTGTACCATAGATCTTTTTG
GTTACCCTGAGTTTGGTAGTGGTGTTAAGTTTACGTCCCTTTACTTTCAATTCACAAAGGGTGAGTTGATTACTGGCAC

GCCTAAACCACTTGAAGGTGTCACGGACGTTTCTTTTATGACTCTGGATGTGTGTACCAAGTATACTATCTATGGCTTT
AAAGGTGAGGGTATCATTACCCTTACAAATTCTAGCTTTTTGGCAGGTGTTTATTACACATCTGATTCTGGACAGTTGT
TAGCCTTTAAGAATGTCACTAGTGGTGCTGTTTATTCTGTTACGCCATGTTCTTTTTCAGAGCAGGCTGCATATGTTGA
TGATGATATAGTGGGTGTTATTTCTAGTTTATCTAGCTCCACTTTTAACAGTACTAGGGAGTTGCCTGGTTTCTTCTAC
CATTCTAATGATGGCTCTAATTGTACAGAGCCTGTGTTGGTGTATAGTAACATAGGTGTTTGTAAATCTGGCAGTATTG
GCTACGTCCCATCTCAGTCTGGCCAAGTCAAGATTGCACCCACGGTTACTGGGAATATTAGTATTCCCACCAACTTTAG
TATGAGTATTAGGACAGAATATTTACAGCTTTACAACACGCCTGTTAGTGTTGATTGTGCCACATATGTTTGTAATGGT
AACTCTCGTTGTAAACAATTACTCACCCAGTACACTGCAGCATGTAAGACCATAGAGTCAGCATTACAACTCAGCGCTA
GGCTTGAGTCTGTTGAAGTTAACTCTATGCTTACTATTTCTGAAGAGGCTCTACAGTTAGCTACCATTAGTTCGTTTAA
TGGTGATGGATATAATTTTACTAATGTGCTGGGTGTTTCTGTGTATGACCCTGCAAGTGGCAGGGTGGTACAAAAAAGG
TCTTTTATTGAAGACCTGCTTTTTAATAAAGTGGTTACTAATGGCCTTGGTACTGTTGATGAAGACTATAAGCGCTGTT
CTAATGGTCGCTCTGTGGCAGATCTAGTCTGTGCACAGTATTACTCTGGTGTCATGGTACTACCTGGTGTTGTTGACGC
TGAGAAGCTTCACATGTATAGTGCGTCTCTCATCGGTGGTATGGTGCTAGGAGGTTTTACTTCTGCAGCGGCATTGCCT
TTTAGCTATGCTGTTCAAGCTAGACTCAATTATCTTGCTCTACAGACGGATGTTCTACAGCGGAACCAGCAATTGCTTG
CTGAGTCTTTTAACTCTGCTATTGGTAATATAACTTCAGCCTTTGAGAGTGTTAAAGAGGCTATTAGTCAAACTTCCAA
GGGTTTGAACACTGTGGCTCATGCGCTTACTAAGGTTCAAGAGGTTGTTAACTCGCAGGGTGCAGCTTTGACTCAACTT
ACCGTACAGCTGCAACACAACTTCCAAGCCATTTCTAGTTCTATTGATGACATTTACTCTCGACTGGACATTCTTTCAG
CCGATGTTCAGGTTGACCGTCTCATCACCGGCAGATTATCAGCACTTAATGCTTTTGTTGCTCAAACCCTCACTAAGTA
TACTGAGGTTCAGGCTAGCAGGAAGTTAGCACAGCAAAAGGTTAATGAGTGCGTTAAATCGCAATCTCAGCGTTATGGT
TTTTGTGGTGGTGATGGCGAGCACATTTTCTCTCTGGTACAGGCAGCACCTCAGGGCCTGCTGTTTTTACATACAGTAC
TTGTACCGAGTGATTTTGTAGATGTTATTGCCATCGCTGGCTTATGCGTTAACGATGAAATTGCCTTGACTCTACGTGA
GCCTGGCTTAGTCTTGTTTACGCATGAACTTCAAAATCATACCGCGACGGAATATTTTGTTTCATCGCGACGTATGTTT
GAACCTAGAAAACCTACCGTTAGTGATTTTGTTCAAATTGAGAGTTGTGTGGTCACCTATGTCAATTTGACTAGAGACC
AACTACCAGATGTAATCCCAGATTACATCGATGTTAACAAAACACTTGATGAGATTTTAGCTTCTCTGCCCAATAGAAC
TGGTCCAAGTCTTCCTTTAGATGTTTTTAATGCCACTTATCTTAATCTCACTGGTGAAATTGCAGATTTAGAGCAGCGT
TCAGAGTCTCTCCGTAATACTACAGAGGAGCTCCAAAGTCTTATATATAATATCAACAACACACTAGTTGACCTTGAGT
GGCTCAACCGAGTTGAGACATATATCAAGTGGCCGTGGTGGGTTTGGTTGATTATTTTCATTGTTCTCATCTTTGTTGT
GTCATTACTAGTGTTCTGCTGCATTTCCACGGGTTGTTGTGGATGCTGCGGCTGCTGCTGTGCTTGTTTCTCAGGTTGT
TGTAGGGGTCCTAGACTTCAACCTTACGAAGTTTTTGAAAAGGTCCACGTGCAGTGA

>gi|619728094|gb|KJ623926.1| Porcine epidemic diarrhea virus strain K14JB01, complete genome

ATGAAGTCTTTAACCTACTTCTGGTTGTTCTTACCAGTACTTTCAACACTTAGCCTACCACAAGATGTCACCAGG
TGCTCAGCTAACACTAATTTTAGGCGGTTCTTTTCAAAATTTAATGTTCAGGCGCCTGCAGTTGTTGTACTGGGCGGTT
ATCTACCTATTGGTGAAAACCAGGGTGTCAATTCAACTTGGTACTGTGCTGGCCAACATCCAACTGCTAGTGGCGTTCA
TGGTATCTTTGTTAGCCATATTAGAGGTGGTCATGGCTTTGAGATTGGCATTTCGCAAGAGCCTTTTGACCCTAGTGGT
TACCAGCTTTATTTACATAAGGCTACTAACGGTAACACTAATGCTACTGCGCGACTGCGCATTTGCCAGTTTCCTAGCA
TTAAAACATTGGGCCCCACTGCTAATAATGATGTTACAACAGGTCGTAATTGCCTATTTAACAAAGCCATCCCAGCTCA
TATGAGTGAACATAGTGTTGTCGGCATAACATGGGATAATGATCGTGTCACTGTCTTTTCTGACAAGATCTATTATTTT
TATTTTAAAAATGATTGGTCCCGTGTTGCGACAAAGTGTTACAACAGTGGAGGTTGTGCTATGCAATATGTTTACGAAC
CCACCTATTACATGCTTAATGTTACTAGTGCTGGTGAGGATGGTATTTCTTATCAACCCTGTACAGCTAATTGCATTGG
TTATGCTGCCAATGTATTTGCTACTGAGCCCAATGGCCACATACCAGAAGGTTTTAGTTTTAATAATTGGTTTCTTTTG

TCCAATGATTCCACTTTGGTGCATGGTAAGGTGGTTTCCAACCAACCATTGTTGGTCAATTGTCTTTTGGCCATTCCTA
AGATTTATGGACTAGGCCAATTTTTCTCCTTTAATCAAACGATCGATGGTGTTTGTAATGGAGCTGCTGTGCAGCGTGC
ACCAGAGGCTCTGAGGTTTAATATTAATGACACCTCTGTCATTCTTGCTGAAGGCTCAATTGTACTTCATACTGCTTTA
GGAACAAATTTTTCTTTTGTTTGCAGTAATTCCTCAAATCCTCACTTAGCCACCTTCGCCATACCTTTGGGTGCTACCC
AAGTACCCTATTATTGTTTTCTTAAAGTGGATACTTACAACTCCACTGTTTATAAATTCTTGGCTGTTTTACCTCCAAC
CGTCAGGGAAATTGTCATCACCAAGTATGGTGATGTTTATGTCAATGGGTTTGGATACTTGCATCTCGGTTTGTTGGAT
GCTGTCACAATTAATTTCACTGGTCATGGCACTGACGATGATGTTTCTGGTTTTTGGACCATAGCATCGACTAATTTTG
TTGATGCACTCATCGAAGTTCAAGGAACCGCCATTCAGCGTATTCTTTATTGTGATGATCCTGTTAGCCAACTCAAGTG
TTCTCAGGTTGCTTTTGACCTTGACGATGGTTTTTACCCTATTTCTTCTAGAAACCTTCTGAGTCATGAACAGCCAATT
TCTTTTGTTACTCTGCCATCATTTAATGATCATTCTTTTGTTAACATTACTGTATCTGCTTCCTTTGGTGGTCATAGTG
GTGCCAACCTTATTGCATCTGACACTACTATCAATGGGTTTAGTTCTTTCTGTGTTGACACTAGACAATTTACCATTTC
ACTGTTTTATAACGTTACAAACAGTTATGGTTATGTGTCTAAATCACAGGACAGTAATTGCCCTTTCACCTTGCAATCT
GTTAATGATTACCTGTCTTTTAGCAAATTTTGTGTTTCCACCAGCCTTTTGGCTAGTGCCTGTACCATAGATCTTTTTG
GTTACCCTGAGTTTGGTAGTGGTGTTAAGTTTACGTCCCTTTACTTTCAATTCACAAAGGGTGAGTTGATTACTGGCAC
GCCTAAACCACTTGAAGGTGTCACGGACGTTTCTTTTATGACTCTGGATGTGTGTACCAAGTATACTATCTATGGCTTT
AAAGGTGAGGGTGTCATTACCCTTACAAATTCTAGCTTTTTGGCAGGTGTTTATTACACATCTGATTCTGGACAGTTGT
TAGCCTTTAAGAATGTCACTAGTGGTGCTGTTTATTCTGTTACGCCATGTTCTTTTTCAGAGCAGGCTGCATATGTTGA
TGATGATATAGTGGGTGTTATTTCTAGTTTGTCTAGCTCCACTTTTAACAGTACTAGGGAGTTGCCTGGTTTCTTCTAC
CATTCTAATGATGGCTCTAATTGTACAGAGCCTGTGTTGGTGTATAGTAACATAGGTGTTTGTAAATCTGGCAGTATTG
GCTACGTCCCATCTCAGTCTGGCCAAGTCAAGATTGCACCCACGGTTACTGGGAATATTAGTATTCCCACCAACTTTAG
TATGAGTATTAGGACAGAATATTTACAGCTTTACAACACGCCTGTTAGTGTTGATTGTGCCACATATGTTTGTAATGGT
AACTCTCGTTGTAAACAATTACTCACCCAGTACACTGCAGCATGTAAGACCATAGAGTCAGCATTACAACTCAGCGCTA
GGCTTGAGTCTGTTGAAGTTAACTCTATGCTTACTATTTCTGAAGAGGCTCTACAGTTAGCTACCATTAGTTCGTTTAA
TGGTGATGGATATAATTTTACTAATGTGCTGGGTGTTTCTGTGTATGATCCTGCAAGTGGCAGGGTGGTACAAAAAAGG
TCTTTTATTGAAGACCTGCTTTTTAATAAAGTGGTTACTAATGGCCTTGGTACTGTTGATGAAGACTATAAGCGCTGTT
CTAATGGTCGCTCTGTGGCAGATCTAGTCTGTGCACAGTATTACTCTGGTGTCATGGTACTACCTGGTGTTGTTGACGC
TGAGAAGCTTCACATGTATAGTGCGTCTCTCATCGGTGGTATGGTGCTAGGAGGTTTTACTTCTGCAGCGGCATTGCCT
TTTAGCTATGCTGTTCAAGCTAGACTCAATTATCTTGCTCTACAGACGGATGTTCTACAGCGGAACCAGCAATTGCTTG
CTGAGTCTTTTAACTCTGCTATTGGTAATATAACTTCAGCCTTTGAGAGTGTTAAAGAGGCTATTAGTCAAACTTCCAA
GGGTTTGAACACTGTGGCTCATGCGCTTACTAAGGTTCTAGAGGTTGTTAACTCGCAGGGTGCAGCTTTGACTCAACTT
ACCGTACAGCTGCAACACAACTTCCAAGCCATTTCTAGTTCTATTGATGACATTTACTCTCGACTGGACATTCTTTCAG
CCGATGTTCAGGTTGACCGTCTCATCACCGGCAGATTATCAGCACTTAATGCTTTTGTTGCTCAAACCCTCACTAAGTA
TACTGAGGTTCAGGCTAGCAGGAAGTTAGCACAGCAAAAGGTTAATGAGTGCGTTAAATCGCAATCTCAGCGTTATGGT
TTTTGTGGTGGTGATGGCGAGCACATTTTCTCTCTGGTACAGGCAGCACCTCAGGGCCTGCTGTTTTTACATACAGTAC
TTGTACCGAGTGATTTTGTAGATGTTATTGCCATCGCTGGCTTATGCGTTAACGATGAAATTGCCTTGACTCTACGTGA
GCCTGGCTTAGTCTTGTTTACGCATGAACTTCAAAATCATACTGCGATGGAATATTTTGTTTCATCGCGACGTATGTTT
GAACCTAGAAAACCTACCGTTAGTGATTTTGTTCAAATTGAGAGTTGTGTGGTCACCTATGTCAATTTGACTAGAGACC
AACTACCAGATGTAATCCCAGATTACATCGATGTTAACAAAACACTTGATGAGATTTTAGCTTCTCTGCCCAATAGAAC
TGGTCCAAGTCTTCCTTTAGATGTTTTTAATGCCACTTATCTTAATCTCACTGGTGAAATTGCAGATTTAGAGCAGCGT
TCAGAGTCTCTCCGTAATACTACAGAGGAGCTCCAAAGTCTTATATATAATATCAACAACACACTAGTTGACCTTGAGT
GGCTCAACCGAGTTGAGACATATATCAAGTGGCCGTGGTGGGTTTGGTTGATTATTTTCATTGTTCTCATCTTTGTTGT
GTCATTACTAGTGTTCTGCTGCATTTCCACGGGTTGTTGTGGATGCTGCGGCTGCTGCTGTGCTTGTTTCTCAGGTTGT
TGTAGGGGTCCTAGACTTCAACCTTACGAAGTTTTTGAAAAGGTCCACGTGCAGTGA

>gi|659495319|gb|KJ662670.1| Porcine epidemic diarrhea virus isolate KNU-1305, complete genome

ATGAAGTCTTTAACCTACTTCTGGTTGTTCTTACCAGTACTTTCAACACTTAGCCTACCACAAGATGTCACCAGG
TGCTCAGCTAACACTAATTTTAGGCGGTTCTTTTCAAAATTTAATGTTCAGGCGCCTGCAGTTGTTGTACTGGGCGGTT
ATCTACCTATTGGTGAAAACCAGGGTGTCAATTCAACTTGGTACTGTGCTGGCCAACATCCAACTGCTAGTGGCGTTCA
TGGTATCTTTGTTAGCCATATTAGAGGTGGTCATGGCTTTGAGATTGGCATTTCGCAAGAGCCTTTTGACCCTAGTGGT
TACCAGCTTTATTTACATAAGGCTACTAACGGTAACACTAATGCTACTGCGCGACTGCGCATTTGCCAGTTTCCTAGCA
TTAAAACATTGGGCCCCACTGCTAATAATGATGTTACAACAGGTCGTAATTGCCTATTTAACAAAGCCATCCCAGCTCA
TATGAGTGAACATAGTGTTGTCGGCATAACATGGGATAATGATCGTGTCACTGTCTTTTCTGACAAGATCTATTATTTT
TATTTTAAAAATGATTGGTCCCGTGTTGCGACAAAGTGTTACAACAGTGGAGGTTGTGCTATGCAATATGTTTACGAAC
CCACCTATTACATGCTTAATGTTACTAGTGCTGGTGAGGATGGTATTTCTTATCAACCCTGTACAGCTAATTGCATTGG
TTATGCTGCCAATGTATTTGCTACTGAGCCCAATGGCCACATACCAGAAGGTTTTAGTTTTAATAATTGGTTTCTTTTG
TCCAATGATTCCACTTTGGTGCATGGTAAGGTGGTTTCCAACCAACCATTGTTGGTCAATTGTCTTTTGGCCATTCCTA
AGATTTATGGACTAGGCCAATTTTTCTCCTTTAATCAAACGATCGATGGTGTTTGTAATGGAGCTGCTGTGCAGCGTGC
ACCAGAGGCTCTGAGGTTTAATATTAATGACACCTCTGTCATTCTTGCTGAAGGCTCAATTGTACTTCATACTGCTTTA
GGAACAAATTTTTCTTTTGTTTGCAGTAATTCCTCAAATCCTCACTTAGCCACCTTCGCCATACCTTTGGGTGCTACCC
AAGTACCCTATTATTGTTTTCTTAAAGTGGATACTTACAACTCCACTGTTTATAAATTCTTGGCTGTTTTACCTCCAAC
CGTCAGGGAAATTGTCATCACCAAGTATGGTGATGTTTATGTCAATGGGTTTGGATACTTGCATCTCGGTTTGTTGGAT
GCTGTCACAATTAATTTCACTGGTCATGGCACTGACGATGATGTTTCTGGTTTTTGGACCATAGCATCGACTAATTTTG
TTGATGCACTCATCGAAGTTCAAGGAACCGCCATTCAGCGTATTCTTTATTGTGATGATCCTGTTAGCCAACTCAAGTG
TTCTCAGGTTGCTTTTGACCTTGACGATGGTTTTTACCCTATTTCTTCTAGAAACCTTCTGAGTCATGAACAGCCAATT
TCTTTTGTTACTCTGCCATCATTTAATGATCATTCTTTTGTTAACATTACTGTATCTGCTTCCTTTGGTGGTCATAGTG
GTGCCAACCTTATTGCATCTGACACTACTATCAATGGGTTTAGTTCTTTCTGTGTTGACACTAGACAATTTACCATTTC
ACTGTTTTATAACGTTACAAACAGTTATGGTTATGTGTCTAAATCACAGGACAGTAATTGCCCTTTCACCTTGCAATCT
GTTAATGATTACCTGTCTTTTAGCAAATTTTGTGTTTCCACCAGCCTTTTGGCTAGTGCCTGTACCATAGATCTTTTTG
GTTACCCTGAGTTTGGTAGTGGTGTTAAGTTTACGTCCCTTTACTTTCAATTCACAAAGGGTGAGTTGATTACTGGCAC
GCCTAAACCACTTGAAGGTGTCACGGACGTTTCTTTTATGACTCTGGATGTGTGTACCAAGTATACTATCTATGGCTTT
AAAGGTGAGGGTATCATTACCCTTACAAATTCTAGCTTTTTGGCAGGTGTTTATTACACATCTGATTCTGGACAGTTGT
TAGCCTTTAAGAATGTCACTAGTGGTGCTGTTTATTCTGTTACGCCATGTTCTTTTTCAGAGCAGGCTGCATATGTTGA
TGATGATATAGTGGGTGTTATTTCTAGTTTGTCTAGCTCCACTTTTAACAGTACTAGGGAGTTGCCTGGTTTCTTCTAC
CATTCTAATGATGGCTCTAATTGTACAGAGCCTGTGTTGGTGTATAGTAACATAGGTGTTTGTAAATCTGGCAGTATTG
GCTACGTCCCATCTCAGTCTGGCCAAGTCAAGATTGCACCCACGGTTACTGGGAATATTAGTATTCCCACCAACTTTAG
TATGAGTATTAGGACAGAATATTTACAGCTTTACAACACGCCTGTTAGTGTTGATTGTGCCACATATGTTTGTAATGGT
AACTCTCGTTGTAAACAATTACTCACCCAGTACACTGCAGCATGTAAGACCATAGAGTCAGCATTACAATTCAGCGCTA
GGCTTGAGTCTGTTGAAGTTAACTCTATGCTTACTATTTCTGAAGAGGCTCTACAGTTAGCTACCATTAGTTCGTTTAA
TGGTGATGGATATAATTTTACTAATGTGCTGGGTGTTTCTGTGTATGATCCTGCAAGTGGCAGGGTGGTACAAAAAAGG
TCTTTTATTGAAGACCTGCTTTTTAATAAAGTGGTTACTAATGGCCTTGGTACTGTTGATGAAGACTATAAGCGCTGTT
CTAATGGTCGCTCTGTGGCAGATCTAGTCTGTGCACAGTATTACTCTGGTGTCATGGTACTACCTGGTGTTGTTGACGC
TGAGAAGCTTCACATGTATAGTGCGTCTCTCATCGGTGGTATGGTGCTAGGAGGTTTTACTTCTGCAGCGGCATTGCCT
TTTAGCTATGCTGTTCAAGCTAGACTCAATTATCTTGCTCTACAGACGGATGTTCTACAGCGGAACCAGCAATTGCTTG
CTGAGTCTTTTAACTCTGCTATTGGTAATATAACTTCAGCCTTTGAGAGTGTTAAAGAGGCTATTAGTCAAACTTCCAA
GGGTTTGAACACTGTGGCTCATGCGCTTACTAAGGTTCAAGAGGTTGTTAACTCGCAGGGTGCAGCTTTGACTCAACTT

ACCGTACAGCTGCAACACAACTTCCAAGCCATTTCTAGTTCTATTGATGACATTTACTCTCGACTGGACATTCTTTCAG
CCGATGTTCAGGTTGACCGTCTCATCACCGGCAGATTATCAGCACTTAATGCTTTTGTTGCTCAAACCCTCACTAAGTA
TACTGAGGTTCAGGCTAGCAGGAAGTTAGCACAGCAAAAGGTTAATGAGTGCGTTAAATCGCAATCTCAGCGTTATGGT
TTTTGTGGTGGTGATGGCGAGCACATTTTCTCTCTGGTACAGGCAGCACCTCAGGGCCTGCTGTTTTTACATACAGTAC
TTGTACCGAGTGATTTTGTAGATGTTATTGCCATCGCTGGCTTATGCGTTAACGATGAAATTGCCTTGACTCTACGTGA
GCCTGGCTTAGTCTTGTTTACGCATGAACTTCAAAATCATACTGCGATGGAATATTTTGTTTCATCGCGACGTATGTTT
GAACCTAGAAAACCTACCGTTAGTGATTTTGTTCAAATTGAGAGTTGTGTGGTCACCTATGTCAATTTGACTAGAGACC
AACTACCAGATGTAATCCCAGATTACATCGATGTTAACAAAACACTTGATGAGATTTTAGCTTCTCTGCCCAATAGAAC
TGGTCCAAGTCTTCCTTTAGATGTTTTTAATGCCACTTATCTTAATCTCACTGGTGAAATTGCAGATTTAGAGCAGCGT
TCAGAGTCTCTCCGTAATACTACAGAGGAGCTCCAAAGTCTTATATATAATATCAACAACACACTAGTTGACCTTGAGT
GGCTCAACCGAGTTGAGACATATATCAAGTGGCCGTGGTGGGTTTGGTTGATTATTTCATTGTTCTCATCTTTGTTGT
GTCATTACTAGTGTTCTGCTGCATTTCCACGGGTTGTTGTGGATGCTGCGGCTGCTGCTGTGCTTGTTTCTCAGGTTGT
TGTAGGGGTCCTAGACTTCAACCTTACGAAGTTTTTGAAAAGGTCCACGTGCAGTGA

>gi|635718004|gb|KJ778615.1| Porcine epidemic diarrhea virus isolate NPL-PEDv/2013, complete genome

ATGAAGTCTTTAACCTACTTCTGGTTGTTCTTACCAGTACTTTCAACACTTAGCCTACCACAAGATGTCACCAGG
TGCTCAGCTAACACTAATTTTAGGCGGTTCTTTTCAAAATTTAATGTTCAGGCGCCTGCAGTTGTTGTACTGGGCGGTT
ATCTACCTATTGGTGAAAACCAGGGTGTCAATTCAACTTGGTACTGTGCTGGCCAACATCCAACTGCTAGTGGCGTTCA
TGGTATCTTTGTTAGCCATATTAGAGGTGGTCATGGCTTTGAGATTGGCATTTCGCAAGAGCCTTTTGACCCTAGTGGT
TACCAGCTTTATTTACATAAGGCTACTAACGGTAACACTAATGCTACTGCGCGACTGCGCATTTGCCAGTTTCCTAGCA
TTAAAACATTGGGCCCCACTGCTAATAATGATGTTACAACAGGTCGTAATTGCCTATTTAACAAAGCCATCCCAGCTCA
TATGAGTGAACATAGTGTTGTCGGCATAACATGGGATAATGATCGTGTCACTGTCTTTTCTGACAAGATCTATTATTTT
TATTTTAAAAATGATTGGTCCCGTGTTGCGACAAAGTGTTACAACAGTGGAGGTTGTGCTATGCAATATGTTTACGAAC
CCACCTATTACATGCTTAATGTTACTAGTGCTGGTGAGGATGGTATTTCTTATCAACCCTGTACAGCTAATTGCATTGG
TTATGCTGCCAATGTATTTGCTACTGAGCCCAATGGCCACATACCAGAAGGTTTTAGTTTTAATAATTGGTTTCTTTTG
TCCAATGATTCCACTTTGGTGCATGGTAAGGTGGTTTCCAACCAACCATTGTTGGTCAATTGTCTTTTGGCCATTCCTA
AGATTTATGGACTAGGCCAATTTTTCTCCTTTAATCAAACGATCGATGGTGTTTGTAATGGAGCTGCTGTGCAGCGTGC
ACCAGAGGCTCTGAGGTTTAATATTAATGACACCTCTGTCATTCTTGCTGAAGGCTCAATTGTACTTCATACTGCTTTA
GGAACAAATTTTTCTTTTGTTTGCAGTAATTCCTCAAATCCTCACTTAGCCACCTTCGCCATACCTCTGGGTGCTACCC
AAGTACCTTATTATTGTTTTCTTAAAGTGGATACTTACAACTCCACTGTTTATAAATTTTTGGCTGTTTTACCTCCTAC
CGTCAGGGAAATTGTCATCACCAAGTATGGTGATGTTTATGTCAATGGGTTTGGATACTTGCATCTCGGTTTGTTGGAT
GCTGTCACAATTAATTTCACTGGTCATGGCACTGACGATGATGTTTCTGGTTTTTGGACCATAGCATCGACTAATTTTG
TTGATGCACTCATCGAAGTTCAAGGAACCGCCATTCAGCGTATTCTTTATTGTGATGATCCTGTTAGCCAACTCAAGTG
TTCTCAGGTTGCTTTTGACCTTGACGATGGTTTTTACCCTATTTCTTCTAGAAACCTTCTGAGTCATGAACAGCCAATT
TCTTTTGTTACTCTGCCATCATTTAATGATCATTCTTTTGTTAACATTACTGTATCTGCTTCCTTTGGTGGTCATAGTG
GTGCCAACCTTATTGCATCTGACACTACTATCAATGGGTTTAGTTCTTTCTGTGTTGACACTAGACAATTTACCATTTC
ACTGTTTTATAACGTTACAAACAGTTATGGTTATGTGTCTAAATCACAGGACAGTAATTGCCCTTTCACCTTGCAATCT
GTTAATGATTACCTGTCTTTTAGCAAATTTTGTGTTTCCACCAGCCTTTTGGCTAGTGCCTGTACCATAGATCTTTTTG
GTTACCCTGAGTTTGGTAGTGGTGTTAAGTTTACGTCCCTTTACTTTCAATTCACAAAGGGTGAGTTGATTACTGGCAC
GCCTAAACCACTTGAAGGTGTCACGGACGTTTCTTTTATGACTCTGGATGTGTGTACCAAGTATACTATCTATGGCTTT
AAAGGTGAGGGTATCATTACCCTTACAAATTCTAGCTTTTTGGCAGGTGTTTATTACACATCTGATTCTGGACAGTTGT

TAGCCTTTAAGAATGTCACTAGTGGTGCTGTTTATTCTGTTACGCCATGTTCTTTTTCAGAGCAGGCTGCATATGTTGA
TGATGATATAGTGGGTGTTATTTCTAGTTTGTCTAGCTCCACTTTTAACAGTACTAGGGAGTTGCCTGGTTTCTTCTAC
CATTCTAATGATGGCTCTAATTGTACAGAGCCTGTGTTGGTGTATAGTAACATAGGTGTTTGTAAATCTGGCAGTATTG
GCTACGTCCCATCTCAGTCTGGCCAAGTCAAGATTGCACCCACGGTTACTGGGAATATTAGTATTCCCACCAACTTTAG
TATGAGTATTAGGACAGAATATTTACAGCTTTACAACACGCCTGTTAGTGTTGATTGTGCCACATATGTTTGTAATGGT
AACTCTCGTTGTAAACAATTACTCACCCAGTACACTGCAGCATGTAAGACCATAGAGTCAGCATTACAACTCAGCGCTA
GGCTTGAGTCTGTTGAAGTTAACTCTATGCTTACTATTTCTGAAGAGGCTCTACAGTTAGCTACCATTAGTTCGTTTAA
TGGTGATGGATATAATTTTACTAATGTGCTGGGTGTTTCTGTGTATGATCCTGCAAGTGGCAGGGTGGTACAAAAAAGG
TCTTTTATTGAAGACCTGCTTTTTAATAAAGTGGTTACTAATGGCCTTGGTACTGTTGATGAAGACTATAAGCGCTGTT
CTAATGGTCGCTCTGTGGCAGATCTAGTCTGTGCACAGTATTACTCTGGTGTCATGGTACTACCTGGTGTTGTTGACGC
TGAGAAGCTTCACATGTATAGTGCGTCTCTCATCGGTGGTATGGTGCTAGGAGGTTTTACTTCTGCAGCGGCATTGCCT
TTTAGCTATGCTGTTCAAGCTAGACTCAATTATCTTGCTCTACAGACGGATGTTCTACAGCGGAACCAGCAATTGCTTG
CTGAGTCTTTTAACTCTGCTATTGGTAATATAACTTCAGCCTTTGAGAGTGTTAAAGAGGCTATTAGTCAAACTTCCAA
GGGTTTGAACACTGTGGCTCATGCGCTTACTAAGGTTCAAGAGGTTGTTAACTCGCAGGGTGCAGCTTTGACTCAACTT
ACCGTACAGCTGCAACACAACTTCCAAGCCATTTCTAGTTCTATTGATGACATTTACTCTCGACTGGACATTCTTTCAG
CCGATGTTCAGGTTGACCGTCTCATCACCGGCAGATTATCAGCACTTAATGCTTTTGTTTCTCAAACCCTCACTAAGTA
TACTGAGGTTCAGGCTAGCAGGAAGTTAGCACAGCAAAAGGTTAATGAGTGCGTTAAATCGCAATCTCAGCGTTATGGT
TTTTGTGGTGGTGATGGCGAGCACATTTTCTCTCTGGTACAGGCAGCACCTCAGGGCCTGCTGTTTTTACATACAGTAC
TTGTACCGAGTGATTTTGTAGATGTTATTGCCATCGCTGGCTTATGCGTTAACGATGAAATTGCCTTGACTCTACGTGA
GCCTGGCTTAGTCTTGTTTACGCATGAACTTCAAAATCATACTGCGACGGAATATTTTGTTTCATCGCGACGTATGTTT
GAACCTAGAAAACCTACCGTTAGTGATTTTGTTCAAATTGAGAGTTGTGTGGTCACCTATGTCAATTTGACTAGAGACC
AACTACCAGATGTAATCCCAGATTACATCGATGTTAACAAAACACTTGATGAGATTTTAGCTTCTCTGCCCAATAGAAC
TGGTCCAAGTCTTCCTTTAGATGTTTTTAATGCCACTTATCTTAATCTCACTGGTGAAATTGCAGATTTAGAGCAGCGT
TCAGAGTCTCTCCGTAATACTACAGAGGAGCTCCAAAGTCTTATATATAATATCAACAACACACTAGTTGACCTTGAGT
GGCTCAACCGAGTTGAGACATATATCAAGTGGCCGTGGTGGGTTTGGTTGATTATTTTCATTGTTCTCATCTTTGTTGT
GTCATTACTAGTGTTCTGCTGCATTTCCACGGGTTGTTGTGGATGCTGCGGCTGCTGCTGTGCTTGTTTCTCAGGTTGT
TGTAGGGGTCCTAGACTTCAACCTTACGAAGTTTTTGAAAAGGTCCACGTGCAGTGA

>gi|635718011|gb|KJ778616.1| Porcine epidemic diarrhea virus isolate NPL-PEDv/2013/P10, complete genome

ATGAAGTCTTTAACCTACTTCTGGTTGTTCTTACCAGTACTTTCAACACTTAGCCTACCACAAGATGTCACCAGG
TGCTCAGCTAACACTAATTTTAGGCGGTTCTTTTCAAAATTTAATGTTCAGGCGCCTGCAGTTGTTGTACTGGGCGGTT
ATCTACCTATTGGTGAAAACCAGGGTGTCAATTCAACTTGGTACTGTGCTGGCCAACATCCAACTGCTAGTGGCGTTCA
TGGTATCTTTGTTAGCCATATTAGAGGTGGTCATGGCTTTGAGATTGGCATTTCGCAAGAGCCTTTTGACCCTAGTGGT
TACCAGCTTTATTTACATAAGGCTACTAACGGTAACACTAATGCTACTGCGCGACTGCGCATTTGCCAGTTTCCTAGCA
TTAAAACATTGGGCCCCACTGCTAATAATGATGTTACAACAGGTCGTAATTGCCTATTTAACAAAGCCATCCCAGCTCA
TATGAGTGAACATAGTGTTGTCGGCATAACATGGGATAATGATCGTGTCACTGTCTTTTCTGACAAGATCTATTATTTT
TATTTTAAAAATGATTGGTCCCGTGTTGCGACAAAGTGTTACAACAGTGGAGGTTGTGCTATGCAATATGTTTACGAAC
CCACCTATTACATGCTTAATGTTACTAGTGCTGGTGAGGATGGTATTTCTTATCAACCCTGTACAGCTAATTGCATTGG
TTATGCTGCCAATGTATTTGCTACTGAGCCCAATGGCCACATACCAGAAGGTTTTAGTTTTAATAATTGGTTTCTTTTG
TCCAATGATTCCACTTTGGTGCATGGTAAGGTGGTTTCCAACCAACCATTGTTGGTCAATTGTCTTTTGGCCATTCCTA
AGATTTATGGACTAGGCCAATTTTTCTCCTTTAATCAAACGATCGATGGTGTTTGTAATGGAGCTGCTGTGCAGCGTGC

ACCAGAGGCTCTGAGGTTTAATATTAATGACATCTCTGTCATTCTTGCTGAAGGCTCAATTGTACTTCATACTGCTTTA
GGAACAAATTTTTCTTTTGTTTGCAGTAATTCCTCAAATCCTCACTTAGCCACCTTCGCCATACCTCTGGGTGCTACCC
AAGTACCTTATTATTGTTTTTTTAAAGTGGATACTTACAACTCCACTGTTTATAAATTTTTGGCTGTTTTACCTCCTAC
CGTCAGGGAAATTGTCATCACCAAGTATGGTGATGTTTATGTCAATGGGTTTGGATACTTGCATCTCGGTTTGTTGGAT
GCTGTCACAATTAATTTCACTGGTCATGGCACTGACGATGATGTTTCTGGTTTTTGGACCATAGCATCGACTAATTTTG
TTGATGCACTCATCGAAGTTCAAGGAACCGCCATTCAGCGTATTCTTTATTGTGATGATCCTGTTAGCCAACTCAAGTG
TTCTCAGGTTGCTTTTGACCTTGACGATGGTTTTTACACTATTTCTTCTAGAAACCTTCTGAGTCATGAACAGCCAATT
TCTTTTGTTACTCTGCCATCATTTAATGATCATTCTTTTGTTAACATTACTGTATCTGCTTCCTTTGGTGGTCATAGTG
GTGCCAACCTTATTGCATCTGACACTACTATCAATGGGTTTAGTTCTTTCTGTGTTGACACTAGACAATTTACCATTTC
ACTGTTTTATAACGTTACAAACAGTTATGGTTATGTGTCTAAATCACAGGACAGTAATTGCCCTTTCACCTTGCAATCT
GTTAATGATTACCTGTCTTTTAGCAAATTTTGTGTTTCCACCAGCCTTTTGGCTAGTGCCTGTACCATAGATCTTTTTG
GTTACCCTGAGTTTGGTAGTGGTGTTAAGTTTACGTCCCTTTACTTTCAATTCACAAAGGGTGAGTTGATTACTGGCAC
GCCTAAACCACTTGAAGGTGTCACGGACGTTTCTTTTATGACTCTGGATGTGTGTACCAAGTATACTATCTATGGCTTT
AAAGGTGAGGGTATCATTACCCTTACAAATTCTAGCTTTTTGGCAGGTGTTTATTACACATCTGATTCTGGACAGTTGT
TAGCCTTTAAGAATGTCACTAGTGGTGCTGTTTATTCTGTTACGCCATGTTCTTTTTCAGAGCAGGCTGCATATGTTGA
TGATGATATAGTGGGTGTTATTTCTAGTTTGTCTAGCTCCACTTTTAACAGTACTAGGGAGTTGCCTGGTTTCTTCTAC
CATTCTAATGATGGCTCTAATTGTACAGAGCCTGTGTTGGTGTATAGTAACATAGGTGTTTGTAAATCTGGCAGTATTG
GCTACGTCCCATCTCAGTCTGGCCAAGTCAAGATTGCACCCACGGTTACTGGGAATATTAGTATTCCCACCAACTTTAG
TATGAGTATTAGGACAGAATATTTACAGCTTTACAACACGCCTGTTAGTGTTGATTGTGCCACATATGTTTGTAATGGT
AACTCTCGTTGTAAACAATTACTCACCCAGTACACTGCAGCATGTAAGACCATAGAGTCAGCATTACAACTCAGCGCTA
GGCTTGAGTCTGTTGAAGTTAACTCTATGCTTACTATTTCTGATGAGGCTCTACAGTTAGCTACCATTAGTTCGTTTAA
TGGTGATGGATATAATTTTACTAATGTGCTGGGTGTTTCTGTGTATGATCCTGCAAGTCGCAGGGTGGTACAAAAAAGG
TCTTTTATTGAAGACCTGCTTTTTAATAAAGTGGTTACTAATGGCCTTGGTACTGTTGATGAAGACTATAAGCGCTGTT
CTAATGGTCGCTCTGTGGCAGATCTAGTCTGTGCACAGTATTACTCTGGTGTCATGGTACTACCTGGTGTTGTTGACGC
TGAGAAGCTTCACATGTATAGTGCGTCTCTCATCGGTGGTATGGTGCTAGGAGGTTTTACTTCTGCAGCGGCATTGCCT
TTTAGCTATGCTGTTCAAGCTAGACTCAATTATCTTGCTCTACAGACGGATGTTCTACAGCGGAACCAGCAATTGCTTG
CTGAGTCTTTTAACTCTGCTATTGGTAATATAACTTCAGCCTTTGAGAGTGTTAAAGAGGCTATTAGTCAAACTTCCAA
GGGTTTGAACACTGTGGCTCATGCGCTTACTAAGGTTCAAGAGGTTGTTAACTCGCAGGGTGCAGCTTTGACTCAACTT
ACCGTACAGCTGCAACACAACTTCCAAGCCATTTCAAGTTCTATTGATGACATTTACTCTCGACTGGACATTCTTTCAG
CCGATGCTCAGGTTGACCGTCTCATCACCGGCAGATTATCAGCACTTAATGCTTTTGTTGCTCAAACCCTCACTAAGTA
TACTGAGGTTCAGGCTAGCAGGAAGTTAGCACAGCAAAAGGTTAATGAGTGCGTTAAATCGCAATCTCAGCGTTATGGT
TTTTGTGGTGGTGATGGCGAGCACATTTTCTCTCTGGTACAGGCAGCACCTCAGGGCCTGCTGTTTTTACATACAGTAC
TTGTACCGAGTGATTTTGTAGATGTTATTGCCATCGCTGGCTTATGCGTTAACGATGAAATTGCCTTGACTCTACGTGA
GCCTGGCTTAGTCTTGTTTACGCATGAACTTCAAAATCATACTGCGACGGAATATTTTGTTTCATCGCGACGTATGTTT
GAACCTAGAAAACCTACCGTTAGTGATTTTGTTCAAATTGAGAGTTGTGTGGTCACCTATGTCAATTTGACTAGAGACC
AACTACCAGATGTAATCCCAGATTACATCGATGTTAACAAAACACTTTATGAGATTTTAGCTTCTCTGCCCAATAGAAC
TGGTCCAAGTCTTCCTTTAGATGTTTTTAATGCCACTTATCTTAATCTCACTGGTGAAATTGCAGATTTAGAGCAGCGT
TCAGAGTCTCTCCGTAATACTACAGAGGAGCTCCAAAGTCTTATATATAATATCAACAACACACTAGTTGACCTTGAGT
GGCTCAACCGAGTTGAGACATATATCAAGTGGCCGTGGTGGGTTTGGTTGATTATTTTCATTGTTCTCATCTTTGTTGT
GTCATTACTAGTGTTCTGCTGCATTTCCACGGGTTGTTGTGGATGCTGCGGCTGCTGCTGTGCTTGTTTCTCAGGTTGT
TGTAGGGGTCCTAGACTTCAACCTTACGAAGTTTTTGAAAAGGTCCACGTGCAGTGA

>gi|429484689|gb|JQ693049.1|_Transmissible_gastroenteritis_virus_isolate_13

3_spike_glycoprotein_(S)_gene_complete_cds

ATGAAAAAACTATTTGTGGTTTTGGTCGTAATGCCATTGATTTATGGAGACAATTTTCCTTGTTCTAAATTGACT
AATAGAACTATAGGCAACCAGTGGAATCTCATTGAAACCTTCCTTCTAAACTATAGTAGTAGGTTACCACCTAATTCAG
ATGTGGTGTTAGGTGATTATTTTCCTACTGTACAACCTTGGTTTAATTGCATTCGCAATAATAGTAATGACCTTTATGT
TACACTGGAAAATCTTAAAGCATTGTATTGGGATTATGCTACAGAAAATATCACTTGGAATCACAGACAACGGTTAAAC
GTAGTCGTTAATGGATACCCATACTCCATCACAGTTACAACAACCCGCAATTTTAATTCTGCTGAAGGTGCTATTATAT
GCATTTGTAAGGGCTCACCACCTACTACCACCACAGAATCTAGTTTGACTTGCAATTGGGGTAGTGAGTGCAGGTTAAA
CCATAAGTTCCCTATATGTCCTTCTAATTCAGAGGCAAATTGTGGTAATATGCTGTATGGCCTACAATGGTTTGCAGAT
GAGGTTGTTGCTTATTTACATGGTGCTAGTTACCGTATTAGTTTTGAAAATCAATGGTCTGACACTGTCACATTTGGTG
ATATGCGTGCGACAACATTAGAAGTCGCTGGCACGCTTGTAGACCTTTGGTGGTTTAATCCTGTTTATGATGTCAGTTA
TTATAGGGTTAATAATAAAAATGGTACTACCGTAGTTTCCAATTGCACTGATCAATGTGCTAGTTATGTGGCTAATGTT
TTTACTACACAGCCAGGAGGTTTTATACCATCAGATTTTAGTTTTAATAATTGGTTCCTTCTAACTAATGGCTCCACGT
TGGTTAGTGGTAAATTAGTTACCAAACAGCCGTTATTAGTTAATTGCTTATGGCCAGTCCCTAGCTTTGAAGAAGCAGC
TTCTACATTTTGTTTTGAGGGTGCTGGCTTTGATCAATGTAATGGTGCTGTTTTAAATAATACTGTAGACGTCATTAGG
TTCAACCTTAATTTTACTACAAATGTACAATCAGGTAAGGGTGCCACAGTGTTTTCATTGAACACAACGGGTGGTGTCA
CTCCTGAAATCTCATGTTATAATGATACAGTGAGTGACTCGAGCTTTTTCAGTTACGGTGAAATTCCGTTCGGCGTAAC
TGATGGACCACGGTACTGTCACGTACTCTACAATGGCACAGCTCTTAAGTATATAGGAATATTACCACCTAGTGTCAAG
GAGATTGCTATTAGTAAGTGGGGCCATTTTTATATTAATGGTTACAATTTCTTTAGCACACTTCCTATTGATTGTATAT
CTTTTAATTTGACCACTGGTGATAGTGACGTTTTCTGGACAATAGCTTACACATCGTACACTGAAGCATTAGTACAAGT
TGAAAACACAGCTATTACAAAGGTGACGTATAGTAATAGTTACGTTAATAACATTAAATGCTCTCAACTTACTGCTAAT
TTGAATAATGGATTTTATCCTGTTTCTTCAAGTGAAGTTGGTCTTGTCAATAAGAGTGTTGTGTTACTACCTAGCTTTT
ACACACATACCATTGTTAACATAACTATTGGTCTTGGTATGAAGCGTAGTGGTTATGGTCAACCCATAGCCTCAACATT
AAGTAACATCACACTACCAATGCAGGATAACAAAACCGATGTGTACTGTATTCGTTCTGACCAATTTTCAGTTTATGTT
CATTCTACTTGCAAAAGTGCTTTATGGGACAATATTTTTAAGCGAAACTGCACGGACGTTTTACATGCCACAGCTGTTA
TAAAAACTGGTACTTGTCCTTTCTCATTTGATAAATTGAACAATTACTTAACTTTTAACAAGTTCTGTTTGTCGTTGAG
TCCTGTTGGTGCTAATTGTAAGTTTGATGTAGCTGCCCGTACAAGAACCAATGAGCAGGTTGTTAGAAGTTTGTATGTA
ATATATGAAGAAGGAGACAACATAGTGGGTGTACCGTCTGATAATAGTGGTGTGCACGATTTGTCAGTGCTACACCTAG
ATTCCTGCACAGATTACAATATATATGGTAGAACTGGTGTTGGTATTATTAGAAAAACTAACAGGACGCTACTTAGTGG
CTTATATTACACATCACTATCAGGTGATTTGTTAGGTTTTAAAAATGTTAGTGATGGTGTCATCTACTCTGTAACGCCA
TGTGATGTAAGCGCACAAGCAGCTGTTATTGATGGTACCATAGTTGGGGCTATCACTTCCATTAACAGTGAACTGTTAG
GTCTAACACATTGGACAACAACACCTAATTTTTATTACTACTCTATATATAATTACACAAATGATAGGACTCGTGGCAC
TGCAATTGACAGTAATGATGTTGATTGTGAACCTGTCATAACCTATTCTAACATAGGTGTTTGTAAAAATGGTGCTTTT
GTTTTTATTAACGTCACACATTCTGATGGAGACGTGCAACCAATTAGCACTGGTAATGTCACGATACCTACAAACTTTA
CCATATCCGTGCAAGTCGAATATATTCAGGTTTACACTACACCAGTGTCAATAGACTGTTCAAGATATGTTTGTAATGG
TAACCCTAGGTGTAACAAATTGTTAACACAATACGTTTCTGCATGTCAAACTATTGAGCAAGCACTTGCAATGGGTGCC
AGACTTGAAAACATGGAGGTTGATTCCATGTTGTTTGTTTCTGAAAATGCCCTTAAATTGGCATCTGTTGAAGCATTCA
ATAGTTCAGAAACTTTAGACCCTATTTACAAAGAATGGCCTAATATAGGTGGTTCTTGGCTAGAAGGTCTAAAATACAT
ACTTCCGTCCCATAATAGCAAACGTAAGTATCGTTCAGCTATAGAGGACTTGCTTTTTGATAAGGTTGTAACATCTGGT
TTAGGTACAGTTGATGAAGATTATAAACGTTGTACAGGTGGTTATGACATAGCTGACTTAGTATGTGCTCAATACTATA
ATGGCATCATGGTGCTACCTGGTGTGGCTAATGCTGACAAAATGACTATGTACACAGCATCCCTTGCAGGTGGTATAAC
ATTAGGTGCACTTGGTGGAGGCGCCGTGGCTATACCTTTTGCAGTAGCAGTTCAGGCTAGACTTAATTATGTTGCTCTA
CAAACTGATGTATTGAACAAAAACCAGCAGATTCTGGCTAGTGCTTTCAATCAAGCTATTGGTAACATTACACAGTCAT
TTGGTAAGGTTAATGATGCTATACATCAAACATCACGAGGTCTTGCTACTGTTGCTAAAGCATTGGCAAAAGTGCAAGA

TGTTGTCAACATACAAGGGCAAGCTTTAAGCCACCTAACAGTACAATTGCAAAATAATTTCCAAGCCATTAGTAGTTCT
ATTAGTGACATTTATAATAGGCTTGACGAATTGAGTGCTGATGCACAAGTTGACAGGCTGATCACAGGAAGACTTACAG
CACTTAATGCATTTGTGTCTCAGACTCTAACCAGACAAGCGGAGGTTAGGGCTAGTAGACAACTTGCCAAAGACAAGGT
TAATGAATGCGTTAGGTCTCAGTCTCAGAGATTCGGATTCTGTGGTAATGGTACACATTTGTTTTCACTCGCAAATGCA
GCACCAAATGGCATGATTTTCTTTCACACAGTGCTATTACCAACGGCTTATGAAACTGTGACTGCTTGGGCAGGTATTT
GTGCTTTAGATGGTGATCGCACTTTTGGACTTGTCGTTAAAGATGTCCAGTTGACTTTGTTTCGTAATCTAGATGACAA
ATTCTATTTGACCCCCAGAACTATGTATCAGCCTAGAGTTGTAACTAGTTCTGACTTTGTTCAAATTGAAGGGTGCGAT
GTGCTGTTTGTTAATGCAACTGTAAGTGATTTGCCTAGTATTATACCTGATTATATTGATATTAATCAGACTGTTCAAG
ATATATTAGAAAATTTTAGACCAAATTGGACTGTACCTGAGTTGACATTTGACATTTTTAACGCAACCTATTTAAACCT
GACTGGTGAAATTGATGACTTAGAATTTACGTCAGAAAAGCTACATAACACTACTGTAGAACTTGCCATTCTTATTGAC
AACATTAACAATACATTAGTCAATCTTGAATGGCTCAATAGAATTGAAACCTATGTAAAATGGCCTTGGTATGTGTGGC
TACTAATAGGCTTAGTAGTAATATTTTGCATACCATTACTGCTATTTTGCTGTTGTAGTACAGGTTGCTGTGGATGCAT
AGGTTGTTTAGGAAGTTGTTGTCATTCTATATGTAGTAGAAGACAATTTGAAAATTACGAACCTATTGAAAAAGTGCAC
GTCCATTAA

>gi|429484691|gb|JQ693050.1|_Transmissible_gastroenteritis_virus_isolate_DAE_spike_glycoprotein_(S)_gene_complete_cds

ATGAAAAAACTATTTGTGGTTTTGGTCGTAATGCCATTGATTTATGGAGACAATTTTCCTTGTTCTAAATTGACT
AATAGAACTATAGGCAACCAGTGGAATCTCATTGAAACCTTCCTTCTAAACTATAGTAGTAGGTTACCACCTAATTCAG
ATGTGGTGTTAGGTGATTATTTTCCTACTGTACAACCTTGGTTTAATTGCATTCGCAATAATAGTAATGACCTTTATGT
TACACTGGAAAATCTTAAAGCATTGTATTGGGATTATGCTACAGAAAATATCACTCGGAATCACAGACAACGGTTAAAC
GTAGTCGTTAATGGATACCCATACTCCATCACAGTTACAACAACCCGCAATTTTAATTCTGCTGAAGGTGCTATTATAT
GCATTTGTAAGGGCTCACCACCTACTACCACCACAGAATCTAGTTTGACTTGCAATTGGGGTAGTGAGTGCAGGTTAAA
CCATAAGTTCCCTATATGTCCTTCTAATTCAGAGGCAAATTGTGGTAATATGCTGTATGGCCTACAATGGTTTGCAGAT
GAGGTTGTTGCTTATTTACATGGTGCTAGTTACCGTATTAGTTTTGAAAATCAATGGTCTGGCACTGTCACATTTGGTG
ATATGCGTGCGACAACACTAGAAGTCTCTGGCACGCTTGTAGACCTTTGGTGGTTTAATCCTGTTTATGATGTCAGTTA
TTATAGGGTTAATAATAAAAATGGTACTACCGTAGTTTCCAATTGCACTGATCAATGTGCTAGTTATGTGGCTAATGTT
TTTACTACACAGCCAGGAGGTTTTATACCATCAGATTTTAGTTTTAATAATTGGTTCCTTCTAACTAATAGCTCCACGT
TGGTTAGTGGTAAATTAGTTACCAAACAGCCGCTATTAGTTAATTGCTTATGGCCAGTCCCTAGCTTTGAAGAAGCAGC
TTCTACATTTTGTTTTGAGGGTGCTGGCTTTGATCAATGTAATGGTGCTGTTTTAAATAATACTGTAGACGTCATTAGG
TTCAACCTTAATTTTACTACAAATGTACAATCAGGTAAGGGTGCCACAGTGTTTTCATTGAACACAACGGGTGGTGTCA
CTCTTGAAATTTCATGTTATACAGTGAGTGACTCGAGCTTTTTCAGTTACGGTGAAATTCCGTTCGGCGTAACTGATGG
ACCACGGTACTGTTACGTACACTATAATGGCACAGCTCTTAAGTATTTAGGAACATTACCACCTAGTGTCAAGGAGATT
GCTATTAGTAAGTGGGGCCATTTTTATATTAATGGTTACAATTTCTTTAGCACATTTCCTATTGATTGTATATCTTTTA
ATTTGACCACTGGTGATAGTGACGTTTTCTGGACAATAGCTTACACATCGTACACTGAAGCATTAGTACAAGTTGAAAA
CACAGCTATTACAAAGGTGACGTATTGTAATAGTCACGTTAATAATATTAAATGCTCTCAAATTACTGCTAATTTGAAT
AATGGATTTTATCCTGTTTCTTCAAGTGAAGTTGGTCTTGTCAATAAGAGTGTTGTGTTACTACCTAGCTTTTACACAC
ATACCATTGTTAACATAACTATTGGTCTTGGTATGAAGCGTAGTGGTTATGGTCAACCCATAGCCTCAACATTAAGTAA
CATCACACTACCAATGCAGGATCACAACACCGATGTGTACTGTATTCGTTCTGACCAATTTTCAGTTTATGTTCATTCT
ACTTGCAAAAGTGCTTTATGGGACAATATTTTTAAGCGAAACTGCACGGACGTTTTAGATGCCACAGCTGTTATAAAAA
CTGGTACTTGTCCTTTCTCATTTGATAAATTGAACAATTACTTAACTTTTAACAAGTTCTGTTTGTCGTTGAGTCCTGT
TGGTGCTAATTGTAAGTTTGATGTAGCTGCCCGTACAAGAACCAATGAGCAGGTTGTTAGAAGTTTGTATGTAATATAT

GAAGAAGGAGACAACATAGTGGGTGTACCGTCTGATAATAGTGGTGTGCACGATTTGTCAGTGCTACACCTAGATTCCT
GCACAGATTACAATATATATGGTAGAACTGGTGTTGGTATTATTAGAAAAACTAACAGGACGCTACTTAGTGGCTTATA
TTACACATCACTATCAGGTGATTTGTTAGGTTTTAAAAATGTTAGTGATGGTGTCATCTACTCTGTAACGCCATGTGAT
GTAAGCGCACAAGCAGCTGTTATTGATGGTACCATAGTTGGGGCTATCACTTCCATTAACAGTGAACTGTTAGGTCTAA
CACATTGGACAACAACACCTAATTTTTATTACTACTCTATATATAATTACACAAATGATAGGACTCGTGGCACTGCAAT
TGACAGTAATGATGTTGATTGTGAACCTGTCATAACCTATTCTAACATAGGTGTTTGTAAAAATGGTGCTTTTGTTTTT
ATTAACGTCACACATTCTGATGGAGACGTGCAACCAATTAGCACTGGTAATGTCACGATACCTACAAACTTTACCATAT
CCGTGCAAGTCGAATATATTCAGGTTTACACTACACCAGTGTCAATAGACTGTTCAAGATATGTTTGTAATGGTAACCC
TAGGTGTAACAAATTGTTAACACAATACGTTTCTGCATGTCAAACTATTGAGCAAGCACTTGCAATGGGTGCCAGACTT
GAAAACATGGAGGTTGATTCCATGTTGTTTGTTTCTGAAAATGCCCTTAAATTGGCATCTGTTGAAGCATTCAATAGTT
CAGAAACTTTAGACCCTATTTACAAAGAATGGCCTAATATAGGTGGTTCTTGGCTAGAAGGTCTAAAATACATACTTCC
GTCCCATAATAGCAAACGTAAGTATCGTTCAGCTATAGAGGACTTGCTTTTTGATAAGGTTGTAACATCTGGTTTAGGT
ACAGTTGATGAAGATTATAAACGTTGTACAGGTGGTTATGACATAGCTGACTTAGTATGTGCTCAATACTATAATGGCA
TCATGGTGCTACCTGGTGTGGCTAATGCTGACAAAATGACTATGTACACAGCATCCCTTGCAGGTGGTATAACATTAGG
TGCACTTGGTGGAGGCGCCGTGGCTATACCTTTTGCAGTAGCAGTTCAGGCTAGACTTAATTATGTTGCTCTACAAACT
GATGTATTGAACAAAAACCAGCAGATTCTGGCTAGTGCTTTCAATCAAGCTATTGGTAACATTACACAGTCATTTGGTA
AGGTTAATGATGCTATACATCAAACATCACGAGGTCTTGCTACTGTTGCTAAAGCATTGGCAAAAGTGCAAGATGTTGT
CAACATACAAGGGCAAGCTTTAAGCCACCTAACAGTACAATTGCAAAATAATTTCCAAGCCATTAGTAGTTCTATTAGT
GACATTTATAATAGGCTTGACGAATTGAGTGCTGATGCACAAGTTGACAGGCTGATCACAGGAAGACTTACAGCACTTA
ATGCATTTGTGTCTCAGACTCTAACCAGACAAGCGGAGGTTAGGGCTAGTAGACAACTTGCCAAAGACAAGGTTAATGA
ATGCGTTAGGTCTCAGTCTCAGAGATTCGGATTCTGTGGTAATGGTACACATTTGTTTTCACTCGCAAATGCAGCACCA
AATGGCATGATTTTCTTTCACACAGTGCTATTACCAACGGCTTATGAAACTGTGACTGCTTGGCCAGGTATTTGTGCTT
CAGATGGTGATCGCACTTTTGGACTTGTCGTTAAAGATGTCCAGTTGACTTTGTTTCGTAATCTAGATGACAAGTTCTA
TTTGACCCCCAGAACTATGTATCAGCCTAGAGTTGCAACTAGTTCTGACTTTGTTCAAATTGAAGGGTGCGATGTGCTG
TTTGTTAATGCAATTGTAAGTGATTTGCCTAGTATTATACCTGATTATATTGATATTAATCAGACTGTTCAAGACATAT
TAGAAAATTTTAGACCAAATTGGACTGTACCTGAGTTGACATTTGACATTTTTAACGCAACCTATTTAAACCTGACTGG
TGAAATTGATGACTTAGAATTTAGGTCAGAAAAGCTACATAACACCACTGTAGAACTTGCCATTCTCATTGACAACATT
AACAATACATTAGTCAATCTTGAATGGCTCAATAGAATTGAAACCTATGTAAAATGGCCTTGGTATGTGTGGCTACTAA
TAGGCTTAGTAGTAATATTTTGCATACCATTACTGCTATTTTGCTGTTGTAGTACAGGTTGCTGTGGATGCATAGGTTG
TTTAGGAAGTTGTTGTCACTCTATATGTAGTAGAAGACAATTTGAAAATTACGAACCTATTGAAAAAGTGCACGTCCAT
TAA

>gi|429484693|gb|JQ693051.1|_Transmissible_gastroenteritis_virus_isolate_KT2_spike_glycoprotein_(S)_gene_complete_cds

ATGAAAAAACTATTTGTGGTTTTGGTTGTAATGCCATTGATTTATGGAGACAATTTTCCTTGTTCTAAATTGACT
AATAGAACTATAGGTAACCATTGGAATCTCATTGAAACCTTCCTTCTAAATTATAGTAGTAGGTTACCACCTAATTCAG
ATGTGGTGTTAGGTGATTATTTTCCTACTGTACAACCTTGGTTTAATTGCATTCGCAATGATAGTAATGACCTTTATGT
TACACTGGAAAATCTTAAAGCATTGTATTGGGATTATGCTACAGAAAATATCACTTGGAATCACAGACAACGGTTACAC
GTAGTCGTTAATGGATACCCATATTCCATCACAGTTACAACAACCCGCGATTTTAATTCTGCTGAAGGTGCTATTATAT
GCATTTGTAAGGGCTCACCACCTACTACCACCACAGAATCTAGTTTGACTTGCAATTGGGGTAGTGAGTGCAGGTTAAA
CCATAAGTTCCCTATATGTCCTTCTAATTCAGAGGCAAATTGTGGTAATATGCTGTATGGCCTACAATGGTTTGCAGAT
GAGGTTGTTGCTTATTTACATGGTGCTAGTTACCGTATTAGTTTTGAAAATCAATGGTCTGGCACTGTCACACTTGGTG

ATATGCGTGCGACAACATTAGAAGTCGCTGGCACGCTTGTAGACCTTTGGTGGTTTAATCCTGTTTATGATGTTAGTTA
TTATAGAGTTAATAATAAAAATGGTACTACCGTAGTTTCCAATTGCACTGATCAATGTGCTAGTTATGTGGCTAATGTT
TTTACTACACAGCCAGGAGGTTTCATACCATCAGATTTTAGTTTTAATAATTGGTTCCTTCTAACTAATAGCTCCACGT
TGGTTAGTGGTAAATTAGTTACCAAACAGCCGTTATTAGTTAATTGCTTATGGCCAGTCCTTAGCTTTGAAGAAGCAGC
TTCTACATTTTGTTTTGAGGGTGCTGGCTTTGATCAATGTAATGGTGCTGTTTTAAATAACACTGTAGACGTCATTAGG
TTCAACCTTAATTTTACTACAAATGTACAATCAGGTAAGGGTGCCACAGTGTTTTCATTGAACACAACGGGTGGTGTCA
CTCTTGAAATCTCATGTTATAATGATACAGTGAGTGACTCGAGCTTTTTCAGTTACGGTGAAATTCCGTTCGGCGTAAC
TGATGGACCACGGTACTGTTACATACTCTACAATGGCACAGCTCTTAAGTATATAGGAATATTACCACCTAGTGTCAAG
GAGATTGCTATTAGTAAGTGGGGCCATTTTTATATTAATGGTCACAATTTCTTTAGCACACTTCCTATTGATTGTATAT
CTTTTAATTTGACCACTGGTGATAGTGACCTTTTCTGGACAATAGCTTACACATCGTACACTCAATCATTATTACAAGT
TGAAAACACAGCTATTACAAAGGTGACGTATTGTAATAGTTACGTTAATAACATTAAATGCTCTCAACTTACTGCTAAT
TTGAATAATGGATTTTATCCTGTTTCTTCAAGTGAAGTTGGTCTTGTCAATAAGAGTGTTGTGTTACTACCTAGCTTTT
ACACACATACCATTGTTAACATAACTATTGGTCTTGGTATGAAGCGTAGTGGTTATGGTCAACCCATAGCCTCAACATT
AAGTAACATCACACTACCAATGCAGGATAACAAAACCGATGTGTACTGTATTCGTTCTGACCAATTTTCAGTTTATGTT
CATTCTACTTGCAAAAGTGCTTTATGGGACAATATTTTTAAGCGAAACTGCACGGACGTTTTACATGCCACAGCTGTTA
TAAAAACTGGTACTTGTCCTTTCTCATTTGATAAATTGAACAATTACTTAACTTTTAACAAGTTCTGTTTGTCGTTGAG
CCATGTTGGTGCTAATTGTAAGTTTGATGTAGCTGCCGGTACAAGAAGCAATGAGCAGGTTGTTAGAAGTTTGTATGTA
ATATATGAAGAAGGAGACAACATAGTGGGTGTACCGTCTGATAATAGTGGTTTGCACGATTTGTCAGTGCTACACCTAG
ATTCCTGCACAGATTACAATATATATGGTAGAACTGGTGTTGGTATTATTAGACGTACTAACAGGACGCTACTTAGTGG
CTTATATTACACATCACTATCAGGTGATTTGTTAGGTTTTAAAAATGTTAGTGATGGTGTCATCTACTCTGTAACGCCA
TGTGATGTAAGCGCACAAGCAGCTGTTATTGATGGTACCATAGTTGGGGCTATCACTTCCATTAACAGTGAACTGTTAG
GTCTAACACATTGGACAACAACACCTAATTTTTATTACTACTCTATATATAATTACACAAATGATAGGGCTCGTGGCAC
TGCAATTGACAGTAATGATGTTGATTGTGAACCTGTCATAACCTATTCTAACATAGGTGTTTGTAAAAATGGTGCTTTG
GTTTTTATTAACGTCACACATTCTGATGGAGACGTGCAACCAATTAGCACTGGTAACGTCACGATACCTACAAACTTTA
CCATATCCGTGCAAGTCGAATACATTCAGGTTTACACTACACCAGTGTCAATAGACTGTTCAAGATATGTTTGTAATGG
TAACCCTAGGTGTAACAAATTGTTAACACAATACGTTTCTGCATGTCAAACTATTGAGCAAGCACTTGCAATGGGTGCC
AGACTTGAAAACATGGAGGTTGATTCCATGTTGTTTGTTTCTGAAAATGCCCTTAAATTGGCTTCTGTTGAAGCATTCA
ATAGTTCAGAAACTTTAGATCCTATTTACAAAGAATGGCCTAATATAGGTGGTTCTTGGCTAGAAGGTCTAAAATACAT
ACTTCCGTCCGATAATAGCAAACGTAAGTATCGTTCAGCTATAGAGGACTTGCTTTTTGATAAGGTTGTAACATCTGGT
TTAGGTACAGTTGATGAAGATTATAAACGTTGCACAGGTGGTTATGACATAGCTGACTTAGTATGTGCTCAATACTATA
ATGGCATCATGGTGCTACCTGGTGTGGCTAATGCTGACAAAATGACTATGTACACAGCATCCCTTGCAGGTGGTATAAC
ATTAGGTGCACTTGGTGGAGGCGCCGTGGCTATACCTTTTGCAGTAGCAGTTCAGGCTAGACTTAATTATGTTGCTCTA
CAAACTGATGTATTGAACAAAAACCAGCAGATCCTGGCTAGTGCTTTCAATCAAGCTATTGGTAACATTACACAGTCAT
TTGGTAAGGTTAATGATGCTATACATCAAACATCACGAGGTCTTGCTACTGTTGCTAAAGCATTGGCAAAAGTGCAAGA
TGTTGTCAACATACAAGGGCAAGCTTTAAGCCACCTAACAGTACAATTGCAAAATAATTTCCAAGCCATTAGTAGTTCT
ATTAGTGACATTTATAATAGGCTTGACGAATTGAGTGCTGATGCACAAGTTGACAGGCTGATCACAGGAAGACTTACAG
CACTTAATGCATTTGTGTCTCAGACTCTAACCAGACAAGCGGAGGTTAGGGCTAGTAGACAACTTGCCAAAGACAAGGT
TAATGAATGCGTTAGGTCTCAGTCTCAGAGATTCGGATTCTGTGGTAATGGTACACATTTGTTTTCACTCGCAAATGCA
GCACCAAATGGCATGATTTTCTTTCACACAGTGCTATTACCAACGGCTTATGAAACTGTGACTGCTTGGGCAGGTATTT
GTGCTTTAGATGGTGATCGCACTTTTGGACTTGTCGTTAAAGATGTCCAGTTGACTTTGTTTCGTAATCTAGATGACAA
ATTCTATTTGACCCCCAGAACTATGTATCAGCCTAGAGTTGTAACTAGTTCTGACTTTGTTCAAATTGAAGGGTGCGAT
GTGCTGTTTGTTAATGCAACTGTAAGTGATTTGCCTAGTATTATACCTGATTATATTGATATTAATCAGACTGTTCAAG
ATATATTAGAAAATTTTAGACCAAATTGGACTGTACCTGAGTTGACATTTGACATTTTTAACGCAACCTATTTAAACCT
GACTGGTGAAATTGATGACTTAGAATTTAGGTCAGAAAAGCTACATAACACTACTGTAGAACTTGCCATTCTTATTGAC

AACATTAACAATACATTAGTCAATCTTGAATGGCTCAATAGAATTGAAACCTATGTAAAATGGCCTTGGTATGTGTGGC
TACTAATAGGCTTAGTAGTAATATTTTGCATACCATTACTGCTATTTTGCTGTTGTAGTACAGGTTGCTGTGGATGCAT
AGGTTGTTTAGGAAGTTGTTGTCATTCTATATGTAGTAGAAGACAATTTGAAAATTACGAACCTATTGAAAAAGTGCAC
GTCCATTAA

>gi|429484695|gb|JQ693052.1|_Transmissible_gastroenteritis_virus_isolate_KT3_spike_glycoprotein_(S)_gene_complete_cds

ATGAAAAAACTATTTGTGGTTTTGGTTGTAATGCCATTGATTTATGGAGACAATTTTCCTTGTTCTAAATTGACT
AATAGAACTATAGGTAACCATTGGAATCTCATTGAAACCTTCCTTCTAAATTATAGTAGTAGGTTACCACCTAATTCAG
ATGTGGTGTTAGGTGATTATTTTCCTACTGTACAACCTTGGTTTAATTGCATTCGCAATGATAGTAATGACCTTTATGT
TACACTGGAAAATCTTAAAGCATTGTATTGGGATTATGCTACAGAAAATATCACTTGGAATCACAGACAACGGTTACAC
GTAGTCGTTAATGGATACCCATATTCCATCACAGTTACAACAACCCGCGATTTTAATTCTGCTGAAGGTGCTATTATAT
GCATTTGTAAGGGCTCACCACCTATTACCACCACAGAATCTAGTTTGACTTGCAATTGGGGTAGTGAGTGCAGGTTAAA
CCATAAGTTCCCTATATGTCCTTCTAATTCAGAGGCAAATTGTGGTAATATGCTGTATGGCCTACAATGGTTTGCAGAT
GAGGTTGTTGCTTATTTACATGGTGCTAGTTACCGTATTAGTTTTGAAAATCAATGGTCTGGCACTGTCACACTTGGTG
ATATGCGTGCGACAACATTAGAAGTCGCTGGCACGCTTGTAGACCTTTGGTGGTTTAATCCTGTTTATGATGTTAGTTA
TTATAGAGTTAATAATAAAAATGGTACTACCGTAGTTTCCAATTGCACTGATCAATGTGCTAGTTATGTGGCTAATGTT
TTTACTACACAGCCAGGAGGTTTCATACCATCAGATTTTAGTTTTAATAATTGGTTCCTTCTAACTAATAGCTCCACGT
TGGTTAGTGGTAAATTAGTTACCAAACAGCCGTTATTAGTTAATTGCTTATGGCCAGTCCCTAGCTTTGAAGAAGCAGC
TTCTACATTTTGTTTTGAGGGTGCTGGCTTTGATCAATGTAATGGTGCTGTTTTAAATAACACTGTAGACGTCATTAGG
TTCAACCTTAATTTTACTACAAATGTACAATCAGGTAAGGGTGCCACAGTGTTTTCATTGAACACAACGGGTGGTGTCA
CTCTTGAAATCTCATGTTATAATGATACAGTGAGTGACTCGAGCTTTTTCAGTTACGGTGAAATTCCGTTCGGCGTAAC
TGATGGACCACGGTACTGTCACATACTCTACAATGGCACAGCTCTTAAGTATATAGGAATATTACCACCTAGTGTCAAG
GAGATTGCTATTAGTAAGTGGGGCCATTTTTATATTAATGGTCACAATTTCTTTAGCTCACTTCCTATTGATTGTATAT
CTTTTAATTTGACCACTGGTGATAGTGACGTTTTCTGGACAATAGCTTACACATCGTACACTGAAGCATTAGTACAAGT
TGAAAACACGGCTATTACAAAGGTGACGTATTGTAATAGTTACGTTAATAACATTAAATGCTCTCAACTTACTGCTAAT
TTGAATAATGGATTTTATCCTGTTTCTTCAAGTGAAGTTGGTCTTGTCAATAAGAGTGTTGTGTTACTACCTAGCTTTT
ACACACATACCATTGTTAACATAACTATTGGTCTTGGTATGAAGCGTAGTGGTTATGGTCAACCCATAGCCTCAACATT
AAGTAACATCACACTACCAATGCAGGATAACAAAACCGATGTGTACTGTATTCGTTCTGACCAATTTTCAGTTTATGTT
CATTCTACTTGCAAAAGTGCTTTATGGGACAATATTTTTAAGCGAAACTGCACGGACGTTTTACATGCCACAGCTGTTA
TAAAAACTGGTACTTGTCCTTTCTCATTTGATAAATTGAACAATTACTTAACTTTTAACAAGTTCTGTTTGTCGTTGAG
TCCTGTTGGTGCTAATTGTAAGTTTGATGTAGCTGCCCGTACAAGAAGCAATGAGCAGGTTGTTAGAAGTTTGTATGTA
ATATATGAAGAAGGAGACAACATAGTGGGTGTACCGTCTGATAATAGTGGTTTGCACGATTTGTCAGTGCTACACCTAG
ATTCCTGCACAGATTACAATATATATGGTAGAACTGGTGTTGGTATTATTAGACGTACTAACAGGACGCTACTTAGTGG
CTTATATTACACATCACTATCAGGTGATTTGTTAGGTTTTAAAAATGTTAGTGATGGTGTCATCTACTCTGTAACGCCA
TGTGATGTAAGCGCACAACGAGCTGTTATTGATGGTACCATAGTTGGGGCTATCACTTCCATTAACAGTGAACTGTTAG
GTCTAACACATTGGACAACAACACCTAATTTTTATTACTACTCTATATATAATTACACAAATGATAGGACTCGTGGCAC
TGCAATTGACAGTAATGATGTTGATTGTGAACCTGTCATAACCTATTCTAACATAGGTGTTTGTAAAAATGGTGCTTTG
GTTTTTATTAACGTCACACATTCTGATGGAGACGTGCAACCAATTAGCACTGGTAACGTCACGATACCTACAAACTTTA
CCATATCCGTGCAAGTCGAATACATTCAGGTTTACACTACACCAGTGTCAATAGACTGTTCAAGATATGTTTGTAATGG
TAACCCTAGGTGTAACAAATTGTTAACACAATACGTTTCTGCATGTCAAACTATTGAGCAAGCACTTGCAATGGGTGCC
AGACTTGAAAACATGGAGGTTGATTCCATGTTGTTTGTTTCTGAAAATGCCCTTAAATTGGCTTCTGTTGAAGCATTCA

ATAGTTCAGAAACTTTAGATCCTATTTACAAAGAATGGCCTAATATAGGTGGTTCTTGGCTAGAAGGTCTAAAATACAT
ACTTCCGTCCGATAATAGCAAACGTAAGTATCGTTCAGCTATAGAGGACTTGCTTTTTGATAAGGTTGTAACATCTGGT
TTAGGTACAGTTGATGAAGATTATAAGCGTTGCACAGGTGGTTATGACATAGCTGACTTAGTATGTGCTCAATACTATA
ATGGCATCATGGTGCTACCTGGTGTGGCTAATGCTGACAAAATGACTATGTACACAGCATCCCTTGCAGGTGGTATAAC
ATTAGGTGCACTTGGTGGAGGCGCCGTGGCTATACCTTTTGCAGTAGCAGTTCAGGCTAGACTTAATTATGTTGCTCTA
CAAACTGATGTATTGAACAAAAACCAGCAGATCCTGGCTAGTGCTTTCAATCAAGCTATTGGTAACATTACACAGTCAT
TTGGTAAGGTTAATGATGCTATACATCAAACATCACGAGGTCTTGCTACTGTTGCTAAAGCATTGGCAAAAGTGCAAGA
TGTTGTCAACATACAAGGGCAAGCTTTAAGCCACCTAACAGTACAATTGCAAAATAATTTCCAAGCCATTAGTAGTTCT
ATTAGTGACATTTATAATAGGCTTGACGAATTGAGTGCTGATGCACAAGTTGACAGGCTGATCACAGGAAGACTTACAG
CACTTAATGCATTTGTGTCTCAGACTCTAACCAGACAAGCGGAGGTTAGGGCTAGTAGACAACTTGCCAAAGACAAGGT
TAATGAATGCGTTAGGTCTCAGTCTCAGAGATTCGGATTCTGTGGTAATGGTACACATTTGTTTTCACTCGCAAATGCA
GCACCAAATGGCATGATTTTCTTTCACACAGTGCTATTACCAACGGCTTATGAAACTGTGACTGCTTGGGCAGGTATTT
GTGCTTTAGATGGTGATCGCACTTTTGGACTTGTCGTTAAAGATGTCCAGTTGACTTTGTTTCGTAATCTAGATGACAA
ATTCTATTTGACCCCCAGAACTATGTATCAGCCTAGAGTTGTAACTAGTTCTGACTTTGTTCAAATTGAAGGGTGCGAT
GTGCTGTTTGTTAATGCAACTGTAAGTGATTTGCCTAGTATTATACCTGATTATATTGATATTAATCAGACTGTTCAAG
ATATATTAGAAAATTTTAGACCAAATTGGACTGTACCTGAGTTGACATTTGACATTTTTAACGCAACCTATTTAAACCT
GACTGGTGAAATTGATGACTTAGAATTTAGGTCAGAAAAGCTACATAACACTACTGTAGAACTTGCCATTCTTATTGAC
AACATTAACAATACATTAGTCAATCTTGAATGGCTCAATAGAATTGAAACCTATGTAAAATGGCCTTGGTATGTGTGGC
TACTAATAGGCTTAGTAGTAATATTTTGCATACCATTACTGCTATTTTGCTGTTGTAGTACAGGTTGCTGTGGATGCAT
AGGTTGTTTAGGAAGTTGTTGTCATTCTATATGTAGTAGAAGACAATTTGAAAATTACGAACCTATTGAAAAAGTGCAC
GTCCATTAA

>gi|33304212|gb|AY335548.1|_Transmissible_gastroenteritis_virus_spike_glycoprotein_(S)_gene_complete_cds

ATGAAAAAATTATTTGTGGTTTTGGTTGTAATGCCATTGATTTATGGAGACAATTTTCCTTGTTCTAAATTGACT
AATAGAACTATAGGTAACCATTGGAATCTCATTGAAACCTTCCTTCTAAATTATAGTAGTAGGTTATCACCTAATTCAG
ATGTGGTGTTAGGTGATTATTTTCCTACTGTACAACCTTGGTTTAATTGCATTCGCAATAATAGTAATGACCTTTATGT
TACATTGGAAAATCTTAAAGCATTGTATTGGGATTATGCTACAGAAAATATCACTTGGAATCACAAACAACGGTTAAAC
GTAGTCGTTAATGGATACCCATACTCCATCACAGTTACAACAACCCGCAATTTTAATTCTGCTGAAGGTGCTATTATAT
GCATTTGCAAGGGCTCACCACCTACTACCACCACAGAATCTAGTTTGACTTGCAATTGGGGTAGTGAGTGCAGGTTAAA
CCATAAGTTCCCTATATGTCCTTCTAATTCAGAGGCAAATTGTGGTAATATGCTGTATGGCCTACAATGGTTTGCAGAT
GCGGTTGTTGCTTATTTACATGGTGCTAGTTACCGTATTAGTTTTGAAAATCAATGGTCTGGCACTGTTACACTTGGTG
ATATGCGTGCGACTACATTAGAAACCGCTGGCACGCTTGTAGACCTTTGGTGGTTTAATCCTGTTTATGATGTCAGTTA
TTATAGAGTTAATAATAAAAATGGTACTACCGTAGTTTCCAATTGCACTGATCAATGTGCTAGTTATGTGGCTAATGTT
TTTACTACACAGCCAGGAGGCTTTATACCATCAGATTTAGTTTTAATAATTGGTTCCTTCTAACTAATAGCTCCACGT
TGGTTAGTGGTAAATTAGTTACCAAACAGCCGTTATTAGTTAATTGCTTATGGCCAGTCCCTAGCTTTGAAGAAGCAGC
TTCTACATTTTGTTTTGAAGGTGCTGGCTTTGATCAATGTAATGGTGCTGTTTTAAATAACACTGTAGACGTCATCAGG
TTTAACCTTAATTTTACTACAAATGTACAATCAGGTAAGGGTGCCACAGTGTTTTCATTGAACACAACGGGTGGTGTCA
CTCTTGAAATCTCATGTTATAATGATACAGTGAGTGACTCGAGCTTTTCCAGTTACGGTGAAATGCCGTTCGGCGTAAC
TGATGGACCACGGTACTGTTACGTACTCTATAATGGCACAGCTCTTAAGTATCTAGGAACATTACCACCTATTGTCAAG
GAGATTGCTATTAGTAAGTGGGGCCATTTTTATATTAATGGTTACAATTTCTTTAGCACATTTCCTATTGATTGTATAT
CTTTTAATTTGACCACTGGTGATAGTGACGTTTTCTGGACAATAGCTTACACATCGTACACTGAAGCATTAGTGCAAGT

TGAAAACACAGCTATTACAAAGGTGACGTATTGTAATAGTTACGTTAATAACATTAAATGCTCTCAACTTACTGCTAAT
TTGAATAATGGATTTTATCCTGTTTCTTCAAGTGAAGTTGGTCTTGTCAATAAGAGTGTTGTGTTACTACCTAGCTTTT
ACACACATACCATTGTTAACATAACTATTGGTCTTGGTATGAAGCGTAGTGGTTATGGTCAACCCATAGCCTCAACATT
AAGTAACATTACACTACCAATGCAGGATAACAACACCGATGTGTACTGTATTCGTTCTGACCAATTTTCAGTTTGTGTT
CATTCTACTTGCAAAAGTTCTTTATGGGACAATGTTTTTAAGCGAAACTGCACGGACGTTTTAGATGCCACAGCTGTTA
TAAAAACTGGTACTTGTCCTTTCTCATTTGATAAATTGAACAATTACTTAACTTTTAACAAGTTCTGTTTGTCGTTGAG
TCCTGTTGGTGCTAATTGTAAGTTTGATGTAGCTGCCCGTACAAGAACCAATGATCAGGTTGTTAGAAGTTTGTATGTA
ATATATGAAGAAGGAGACAACATAGTGGGTGTACCGTCTGATAATAGTGGTTTACACGATTTGTCAGTGCTACACCTAG
ATTCCTGCACAGATTACAATATATATGGTAGAACTGGTGTTGGTATTATTAGACAAACTAACAGGACGCTACTTAGTGG
CTTATATTACACATCACTATCAGGTGATTTGTTAGGTTTTAAAAATGTTAGTGATGGTGTCATCTACTCTGTAACGCCA
TGTGATGTAAGCGCACAAGCAGCTGTTATTGATGGTACCATAGTTGGGGCTATCACTTCCATTAACAGTGAACTGTTAG
GTCTAACACATTGGACAACAACACCTAATTTTTATTACTACTCTATATATAATTACACAAATGATAGGACTCGTGGCAC
TGCAATTGACAGTAATGATGTTGATTGTGAACCTGTCATAACCTATTCTAACATAGGTGTTTGTAAAAATGGTGCTTTG
GTTTTTATTAACGTCACACATTCTGATGGAGACGTGCAACCAATTAGCACTGGTAACGTCACGATACCTACAAACTTTA
CTATATCCGTGCAAGTCGAATATATTCAGGTTTACACTACACCAGTGTCAATGGACTGTTCAAGATATGTTTGTAATGG
CAACCCTAGGTGTAACAAATTGTTAACACAATACGTTTCTGCATGTCAAACTATTGAGCAAGCACTTGCAATGGGTGCC
AGACTTGAAAACATGGAAGTTGATTCCATGTTATTTGTTTCTGAAAATGCCCTTAAATTGGCTTCTGTCGAAGCATTCA
ATAGTTCAGAAACTTTAGATCCTATTTACAAAGAATGGCCTAATATAGGTGGCTCTTGGCTAGAAGGTCTAAAATACAT
ACTTCCGTCCGATAATAGCAAACGTAAGTATCGTTCAGCTATAGAGGACTTGCTTTTTGCTAAGGTTGTAACATCTGGT
TTAGGTACAGTTGATGAAGATTATAAACGTTGTACAGGTGGTTATGGCATAGCTGACTTAGTATGTGCTCAATACTACA
ATGGCATCATGGTGCTACCTGGTGTGGCTAATGCTGACAAAATGACTATGTACACAGCATCCCTCACAGGTGGTATAAC
ATTAGGTGCACTTGGTGGAGGCGCCGTGGCTATACCTTTTGCAGTAGCAGTTCAGGCTAGACTTAATTATGTTGCTCTA
CAAACTGATGTATTGAACAAAAACCAGCAGATCCTGGCTAGTGCTTTCAATCAAGCTATTGGTAACATTACACAGTCAT
TTGGTAAGGTTAATGATGCTATACATCAGACATCACGAGGTCTTGCAACTGTTGCTAAAGCATTGGCAAAAGTGCAAGA
TGTTGTCAACACACAAGGGCAAGCTTTAAGCCACCTAACAGTACAATTGCAAAATAATTTCCAAGCCATTAGTAGTTCT
ATTAGTGACATTTATAATAGGCTTGACGAATTGAGTGCTGATGCACAAGTTGACAGGCTGATCACAGGAAGACTTACAG
CACTTAATGCATTTGTGTCTCAGACTCTAACCAGACAAGCCGAGGTTAGGGCTAGTAGACAACTTGCCAAAGACAAGGT
TAATGAATGCGTTAGGTCTCAGTCTCAGAGATTCGGATTCTGTGGTAATGGTACACATTTGTTTTCACTCGCAAATGCA
GCACCAAATGGCATGATCTTCTTTCACACAGTGCTATTACCAACGGCTTATGAAACTGTGACTGCTTGGGCAGGTATTT
GTGCTTTAGATGGTGATCGCACTTTTGGACTTGTCGTTAAAGATGTCCAGTTGACTTTGTTTCGTAATCTAGATGACAA
GTTCTATTTGACCCCCAGAACTATGTATCAGCCTAGAGTGGCAACTAGTTCTGATTTTGTTCAAATTGAAGGGTGCGAT
GTGCTGTTTGTTAATGCAACTGCAAGTGATTTGCCTAGTATTATACCTGATTATATTGATATTAATCAGACTGTTCAAG
ACATATTAGAAAATTTTAGACCAAATTGGACTGTACCTGAGTTGACATTTGACATTTTTAACGCAACCTATTTAAACCT
GACAGGTGAAATTGATGACTTAGAATTTAGGTCAGAAAAGCTACATAACACTACTGTAGAACTTGCCATTCTTATTGAC
AACATTAACAATACATTAGTCAATCTTGAATGGCTCAATAGAATTGAAACCTATGTAAAATGGCCTTGGTATGTGTGGC
TACTAATAGGCTTAGTAGTAATATTTTGCGTACCATTACTGCTATTTTGCTGTTGTGGTACAGGTTGCTGTGGATGCAT
AGGTTGTTTAGGAAGTTGTTGTCACTCTATATGCAGTAGAAGACAATTTGAAAATTACGAACCTATTGAAAAAGTGCAC
GTCCATTAA

>gi|46561023|gb|AY587882.1|_Transmissible_gastroenteritis_virus_strain_HN2002_spike_glycoprotein_(S)_mRNA_complete_cds

ATGAAAAAATTATTTGCGGTTTTGGTTGTAATGCCATTGATTTATGGAGACAATTTTCCTTGTTCTAAATTGACT

AATAGAACTATAGGTAACCATTGGAATCTCATTGAAACCTTCCTTCTAAATTATAGTAGTAGGTTATCACCTAATTCAG
ATGTGGTGTTAGGTGATTATTTTCCTACTGTACAACCTTGGTTTAATTGCATTCGCAATAATAGTAATGACCTTTATGT
TACATTGGAAAATCTTAAAGCATTGTATTGGGATTATGCTACAGAAAATATCACTTCGAATCACAAACAACGGTTAAAC
GTAGTCGTTAATGGATACCCATACTCCATCACAGTTACAACAACCCGCAATTTTAATTCTGCTGAAGGTGCTATTATAT
GCATTTGCAAGGGCTCACCACCTACTACCACCACAGAATCTAGTTTGACTTGCAATTGGGGTAGTGAGTGCAGGTTAAA
CCATAAGTTCCCTATATGTCCTTCTAATTCAGAGGCAAATTGTGGTAATATGCTGTATGGCCTACAATGGTTTGCAGAT
GCGGTTGTTGCTTATTTACATGGTGCTAGTTACCGTATTAGTTTTGAAAATCAATGGTCTGGCACTGTTACACTTGGTG
ATATGCGTGCGACTACATTAGAAACCGCTGGCACGCTTGTAGACCTTTGGTGGTTTAATCCTGTTTATGATGTCAGTTA
TTATAGAGTTAATAATAAAAATGGTACTACCGTAGTTTCCAATTGCACTGATCAATGTGCTAGTTATGTGGCTAATGTT
TTTACTACACAGCCAGGAGGCTTTATACCATCAGATTTTAGTTTTAATAATTGGTTCCTTCTAACTAATAGCTCCACGT
TGGTTAGTGGTAAATTAGTTACCAAACAGCCGTTATTAGTTAATTGCTTATGGCCAGTCCCTAGCTTTGAAGAAGCAGC
TTCTACATTTTGTTTTGAAGGTGCTGGCTTTGATCAATGTAATGGTGCTGTTTTAAATAACACTGTAGACGTCATCAGG
TTTAACCTTAATTTTACTACAAATGTACAATCAGGTAAGGGTGCCACAGTGTTTTCATTGAACACAACGGGTGGTGTCA
CTCTTGAAATCTCATGTTATAATGATACAGTGAGTGACTCGAGCTTTTCCAGTTACGGTGAAATGCCGTTCGGCGTAAC
TGATGGACCACGGTACTGTTACGTACTCTATAATGGCACAGCTCTTAAGTATCTAGGAACATTACCACCTATTGTCAAG
GAGATTGCTATTAGTAAGTGGGGCCATTTTTATATTAATGGTTACAATTTCTTTAGCACATTTCCTATTGATTGTATAT
CTTTTAATTTGACCACTGGTGATAGTGACGTTTTCTGGACAATAGCTTACACATCGTACACTGAAGCATTAGTACAAGT
TGAAAACACAGCTATTACAAAGGTGACGTATTGTAATAGTTACGTTAATAACATTAAATGCTCTCAACTTACTGCTAAT
TTGAATAATGGATTTTATCCTGTTTCTTCAAGTGAAGTTGGTCTTGTCAATAAGAGTGTTGTGTTACTACCTAGCTTTT
ACACACATACCATTGTTAACATAACTATTGGTCTTGGTATGAAGCGTAGTGGTTATGGTCAACCCATAGCCTCAACATT
AAGTAACATTACACTACCAATGCAGGATAACAACACCGATGTGTACTGTATTCGTTCTGACCAATTTTCAGTTTATGTT
CATTCTACTTGCAAAAGTTCTTTATGGGACAATGTTTTTAAGCGAAACTGCACGGACGTTTTAGATGCCACAGCTGTTA
TAAAAACTGGTACTTGTCCTTTCTCATTTGATAAATTGAACAATTACTTAACTTTTAACAAGTTCTGTTTGTCGTTGAG
TCCTGTTGGTGCTAATTGTAAGTTTGATGTAGCTGCCCGTACAAGAACCAATGATCAGGTTGTTAGAAGTTTGTATGTA
ATATATGAAGAAGGAGACAACATAGTGGGTGTACCGTCTGATAATAGTGGTTTACACGATTTGTCAGTGCTACACCTAG
ATTCCTGCACAGATTACAATATATATGGTAGAACTGGTGTTGGTATTATTAGACAAACTAACAGGACGCTACTTAGTGG
CTTATATTACACATCACTATCAGGTGATTTGTTAGGTTTTAAAAATGTTAGTGATGGTGTCATCTACTCTGTAACGCCA
TGTGATGTAAGCGCACAAGCAGCTGTTATTGATGGTACCATAGTTGGGGCTATCACTTCCATTAACAGTGAACTGTTAG
GTCTAACACATTGGACAACAACACCTAATTTTTATTACTACTCTATATATAATTACACAAATGATAGGACTCGTGGCAC
TGCAATTGACAGTAATGATGTTGATTGTGAACCTGTCATAACCTATTCTAACATAGGTGTTTGTAAAAATGGTGCTTTG
GTTTTTATTAACGTCACACATTCTGATGGAGACGTGCAACCAATTAGCACTGGTAACGTCACGATACCTACAAACTTTA
CTATATCCGTGCAAGTCGAATATATTCAGGTTTACACTACACCAGTGTCAATAGACTGTTCAAGATATGTTTGTAATGG
CAACCCTAGGTGTAACAAATTGTTAACACAATACGTTTCCGCATGTCAAACTATTGAGCAAGCACTTGCAATGGGTGCC
AGACTTGAAAACATGGAAGTTGATTCCATGTTATTTGTTTCTGAAAATGCCCTTAAATTGGCTTCTGTCGAAGCATTCA
ATAGTTCAGAAACTTTAGATCCTATTTACAAAGAATGGCCTAATATAGGTGGCTCTTGGCTAGAAGGTCTAAAATACAT
ACTTCCGTCCGATAATAGCAAACGTAAGTATCGTTCAGCTATAGAGGACTTGCTTTTTGCTAAGGTTGTAACATCTGGT
TTAGGTACAGTTGATGAAGATTATAAACGTTGTACAGGTGGTTATGACATAGCTGACTTAGTATGTGCTCAATACTACA
ATGGCATCATGGTGCTACCTGGTGTGGCTAATGCTGACAAAATGACTATGTACACAGCATCCCTCGCAGGTGGTATAAC
ATTAGGTGCACTTGGTGGAGGCGCCGTGGCTATACCTTTTGCAGTAGCAGTTCAGGCTAGACTTAATTATGTTGCTCTA
CAAACTGATGTATTGAACAAAAACCAGCAGATCCTGGCTAGTGCTTTCAATCAAGCTATTGGTAACATTACACAGTCAT
TTGGTAAGGTTAATGATGCTATACATCAAACATCACGAGGTCTTGCAACTGTTGCTAAAGCATTGGCAAAAGTGCAAGA
TGTTGTCAACACACAAGGGCAAGCTTTAAGCCACCTAACAGTACAATTGCAAAATAATTTCCAAGCCATTAGTAGTTCT
ATTAGTGACATTTATAATAGGCTTGATGAATTGAGTGCTGATGCACAAGTTGACAGGCTGATCACAGGAAGACTTACAG
CACTTAATGCATTTGTGTCTCAGACTCTAACCAGACAAGCCGAGGTTAGGGCTAGTAGACAACTTGCCAAAGACAAGGT

TAATGAATGCGTTAGGTCTCAGTCTCAGAGATTCGGATTCTGTGGTAATGGTACACATTTGTTTTCACTCGCAAATGCA
GCACCAAATGGCATGATCTTCTTTCACACAGTGCTATTACCAACGGCTTATGAAACTGTGACTGCTTGGGCAGGTATTT
GTGCTTTAGATGGTGATCGCACTTTTGGACTTGTCGTTAAAGATGTCCAGTTGACTTTGTTTCGTAATCTAGATGACAA
GTTCTATTTGACCCCCAGAACTATGTATCAGCCTAGAGTGGCAACTAGTTCTGATTTTGTTCAAATTGAAGGGTGCGAT
GTGCTGTTTGTTAATGCAACTGTAAGTGATTTGCCTAGTATTATACCTGATTATATTGATATTAATCAGACTGTTCAAG
ACATATTAGAAAATTTTAGACCAAATTGGACTGTACCTGAGTTGACATTTGACATTTTTAACGCAACCTATTTAAACCT
GACTGGTGAAATTGATGACTTAGAATTTAGGTCAGAAAAGCTACATAACACTACTGTAGAACTTGCCATTCTTATTGAC
AACATTAACAATACATTAGTCAATCTTGAATGGCTCAATAGAATTGAAACCTATGTAAAATGGCCTTGGTATGTGTGGC
TACTAATAGGCTTAGTAGTAATATTTTGCATACCATTACTGCTATTTTGCTGTTGTAGTACAGGTTGCTGTGGATGCAT
AGGTTGTTTAGGAAGTTGTTGTCACTCTATATGCAGTAGAAGACAATTTGAAAATTACGAACCTATTGAAAAAGTGCAC
GTCCATTAA

>gi|62946551|gb|DQ001167.1|_Transmissible_gastroenteritis_virus_strain_TSX_spike_glycoprotein_(S)_gene_complete_cds

ATGAAAATACTATTTGTGGTTTTGGTTATAATGCCATTGATTTATGGAGATAATTTTCCTTGTTCTAAATTGACT
AATAGAACTATAGGTAAACATTGGAATCTCATTGATACCTTTCTTATAAATTATAGTAGCAGGTTACCACCTAATTCAG
ATGTGGTGTTAGGTGATTATTTTCCTACTGTACAACCTTGGTTTAATTGTATTCGCAATGATAGCAACGACCTTTATGT
TACATTGGAAAATCTTAAAGCATTGTATTGGGATTATGCTACAGAAAATATCACTTGGAATCACAGACAACGGTTAAAC
GTAGTCGTTAATGGACACCCATACTCCATTACAGTTACAACAACCCGCAATTTTAATTCTGCTGAAGGTGCTATTATAT
GCATTTGTAAGGGCCCACCACCTACTACCACCACCGAGTCTAGTTTGACTTGCAATTGGGGTAGTGAGTGCAGGTTAAA
CCATAAGTTCCCTATATGTCCTTCTAATTCAGAGGCAAATTGTGGTAATATGTTGTATGGCCTACAATGGTCTGCAGAT
GCGGTTGTTGCTTATTTACATGGTGCTAGTTACCGTATTAGTTTTGAAAATCAATGGTCTGGCACTGTTACACTTGGTG
ATATGCGTGCGACTACGTTAGAAACCGTTGGCACGCTTGTAGACCTTTGGTGGTTTAATCCTGTTTATGATGTCAGTTA
TTATAGAGTTAATAATAAGAACGGTACCACCATAGTTTCCAATTGCACTGATCAGTGTGCTAGTTATGTGGCTAATGTT
TTTGTTACACAGCCAGGAGGCTTTATACCATCAGATTTTAGTTTTAATAATTGGTTCCTTCTAACTAATAGCTCCACGT
TGGTTAGTGGTAAATTAGTTACCAAACAGCCATTATTAGTTAATTGCTTATGGCCAGTCCCTAGCTTTGAAGAAGCAGC
TTCTACATTTTGTTTTGAAGGTGCTGACTTTGATCAATGTAATGGTGCTGTCTTAAATAATACTGTAGATGTCATCAGG
TTTAACCTTAATTTTACTACAAATGTACAATCAGGTAAGGGTGCCACAGTGTTTTCATTGAACACAACTGGTGGTGTCA
CTCTTGAAATCTCATGTTATAATGATACAGTGAGTGACTCGAGCTTTTCCAGTTACGGTGAAATTCCGTTCGGCGTGAC
TGATGGACCACGGTACTGTTACGTACTCTATAATGGCACAGCTCTTAAGTATTTAGGAACATTACCACCTAGTGTCAAG
GAGATTGCTATTAGTAAGTGGGGCCATTTTTATATTAATGGTTACAATTTCTTTAGCACATTTCCTATTGATTGTATAT
CTTTCAATTTGACCACTGGTGATAGTGACGTTTTCTGGACAATAGCTTACACATCGTACACTGAAGCGTTAGTACAAGT
TGAAAACACAGCTATTACAAAGGTGACGTATTGTAATAGTTACGTTAATAATATTAAATGCTCTCAACTTACTGCTAAT
TTGAATAATGGATTTTACCCTGTTTCTTCAAGTGAAGTTGGTCTTGTCAATAAGAGTGTTGTGTTACTACCTAGCTTTT
ACACGCATACCATTGTTAACATAACTATTGATCTTGGTATGAAGCGTAGTGGTTATGGTCAACCCATAGCCTCAACTCT
AAGTAACACTACACTACCAATGCAGGATAACAACACCGATGTGTACTGTATTCGTTCTGATCAATTTTCAGTTTATGTT
CATTCTACTTGTAAAAGTGCTTTATGGGACAATGTTTTTAAGCGAAACTGCACGGACGTTTTAGATGCCACAGCTGTTA
TAAAAACTGGTACTTGTCCTTTCTCATTTGATAAACTGAACAATCACTTAACTTTTAACAAGTTCTGTTTTTCGTTGAG
TCCTGTTGGTGCTAATTGTAAGTTTGATGTAGTTGCCCGTACAAGAACCAATGATCAGGTTGTTAGAAGTTTGTATGTA
ATATATGAAGAAGGAGACAACATAGTGGGTGTACCGTCTGATAATAGTGGTTTGTACGATTTGTCAGTGCTACACCTAG
ATTCATGCACAGATTACAATATATATGGTAGAACTGGTGTTGGTATTATTAGACAAACTAACAGGACACTACTTAGTGG
CTTATATTACACATCACTATCAGGCGATTTGTTAGGTTTCAAAAATGTTAGTGATGGTGTTATCTACTCTGTAACGCCG

TGTGATGTAAGCGCACAAGCAGCTGTTATTGATGGTACCATAGTTGGGGCTATCACTTCTATTAACAGTGAATTGTTGG
GTCTAACGCATTGGACGACAACACCTAATTTTTATTACTACTCTATATATAATTACACAAATGATAGGATTCGTGGCAC
TGCAATTGACAGTAATGACGTTGATTGTGAACCTGTCATAACCTATTCTAACATAGGTGTTTGTAAAAACGGTGCTTTG
GTTTTTATCAACGTCACACATTCTGATGGAGACGTGCAACCAATTAGCACTGGTAACGTCACGATACCTACAAATTTTA
CTATATCCGTGCAAGTCGAATACATTCAGGTTTACACTACACCAGTGTCAATAGACTGTTCAAGATATGTTTGTAATGG
CAACCCCAGGTGTAACAAACTATTAACACAATATGTTTCTGCATGTCAAACTATTGAGCAAGCACTTGCAATGGGTGCC
AGACTTGAAAACATGGAAGTTGATTCCATGTTATTTGTTTCTGAAAATGCCCTTAAATTGGCTTCTGTTGAAGCATTCA
ATAGTTCAGAAGCTTTAGATCCTATTTACAAAGACTGGCCTAATATAGGTGGCTCTTGGCTAGAAGGTCTAAAATATAT
ACTGCCGTCCGATAATAGCAAACGTCAGTATCGTTCAGCTATAGAGGACTTGCTTTTTTCTAAAGTTGTAACATCTGGT
TTAGGCACAGTTGATGAAGATTATAAACGTTGTACAGGTGGTTATGACATAGCTGACTTAGTGTGTGCTCAATATTATA
ATGGCATCATGGTGCTACCTGGTGTGGCTAATGCTGACAAAATGACTATGTACACAGCATCCCTCGCAGGTGGTATAAC
ATTAGGTGCACTTGGTGGAGGTGCCGTAGCTATACCTTTTGCAGTAGCAGTTCAGGCTAGACTTAATTATGTTGCTCTA
CAAACTGATGTATTAAACAAAAACCAGCAGATCCTGGCTAGTGCTTTTAATCAAGCTATTGGTAACATTACACAGTCAT
TTGGTAAGGTTAATGATGCTATACATCAAACGTCACGAGGTCTTGCAACTGTTGCTAAAGCATTGGCAAAAGTGCAAGA
TGTTGTTAATACACAAGGGCAAGCTTTAAGCCACCTAACAGTACAATTGCAAAATAATTTCCAAGCCATTAGTAGCTCT
ATTAGTGACATTTATAATAGGCTTGATGAATTGAGTGCTGATGCACAAGTTGATAGGCTGATCACAGGAAGACTTACAG
CACTTAATGCATTTGTATCTCAGACTCTAACCAGACAAGCCGAGGTTAGGGCTAGTAGACAACTTGCCAAAGACAAGGT
TAATGAATGCGTTAGGTCTCAGTCCCAGAGATTCGGCTTCTGTGGTAATGGTACACATTTGTTTTCACTCGCGAATGCA
GCACCAAATGGCATGATCTTCTTTCATACAGTGCTATTACCAACGGCCTATGAAACTGTGACTGCTTGGGCAGGTATTT
GTGCTCTAGATGGTGATCGCACTTTTGGACTTGTCGTTAAAGATGTCCAGCTGACTTTGTTTCGTAATCTAGATGACAG
GTTCTATTTGACCCCCAGAACTATGTACCAGCCTAGAGTGGCAACTAGTTCTGATTTTGTACAAATTGAAGGGTGCGAT
GTGCTGTTTGTTAATGCAACTGTAAGTGATTTGCCTAGTATTATACCTGATTATATTGATATTAATCAAACTGTTCAAG
ATATACTAGAAAATTTCAGACCAAATTGGACTGTACCTGATTTGACATTTGACATTTTTAACGCAACCTATTTAAATCT
GACTGTTGAAATTGATGACTTAGAATTTAGGTCAGAAAAGCTACATAACACCACTGTAGAACTTGCCATTCTTATTGAT
AACATTAACAATACATTAGTCAATCTCGAATGGCTTAATAGAATTGAAACCTATGTAAAATGGCCTTGGTATGTGTGGC
TACTAATAGGTTTAGTAGTAATATTTTGCATACCATTATTGCTATTTTGCTGTTGTAGTACAGGTTGCTGTGGATGCAT
AGGTTGTTTAGGAAGTTGTTGTCACTCTATATGCAGTAGAAGACAATTTGAAAATTATGAACCTATTGAAAAAGTGCAC
GTCCATTAA

>gi|85680429|gb|DQ201447.1| Transmissible gastroenteritis virus strain TS, complete genome

ATGAAAAAATTATTTGTGGTTTTGGTTGTAATGCCATTGATTTATGGAGACAATTTTCCTTGTTCTAAATTGACT
AATAGAACTATAGGTAACCATTGGAATCTCATTGAAACCTTCCTTCTAAATTATAGTAGTAGGTTATCACCTAATTCAG
ATGTGGTGTTAGGTGATTATTTTCCTACTGTACAACCTTGGTTTAATGGCATTCGCAATAATAGTAATGACCTTTATGT
TACATTGGAAAATCTTAAAGCATTGTATTGGGATTATGCTACAGAAAATATCACTTGGAATCACAAACAACGGTTAAAC
GTAGTCGTTAATGGATACCCATACTCCATCACAGTTACAACAACCCGCAATTTTAATTCTGCTGAAGGTGCTATTATAT
GCATTTGCAAGGGCTCACCACCTACTACCACCACAGAATCTAGTTTGACTTGCAATTGGGGTAGTGAGTGCAGGTTAAA
CCATAAGTTCCCTATATGTCCTTCTAATTCAGAGGCAAATTGTGGTAATATGCTGTATGGCCTACAATGGTTTGCAGAT
GCGGTTGTTGCTTATTTACATGGTGCTAGTTACCGTATTAGTTTTGAAAATCAATGGTCTGGCACTGTTACACTTGGTG
ATATGCGTGCGACTACATTAGAAACCGCTGGCACGCTTGTAGACCTTTGGTGGTTTAATCCTGTTTATGATGTCAGTTA
TTATAGAGTTAATAATAAAAATGGTACTACCGTAGTTTCCAATTGCACTGATCAATGTGCTAGTTATGTGGCTAATGTT
TTTACTACACAGCCAGGAGGCTTTATACCATCAGATTTTAGTTTTAATAATTGGTTCCTTCTAACTAATAGCTCCACGT

TGGTTAGTGGTAAATTAGTTACCAAACAGCCGTTATTAGTTAATTGCTTATGGCCAGTCCCTAGCTTTGAAGAAGCAGC
TTCTACATTTTGTTTTGAAGGTGCTGGCTTTGATCAATGTAATGGTGCTGTTTTAAATAACACTGTAGACGTCATCAGG
TTTAACCTTAATTTTACTACAAATGTACAATCAGGTAAGGGTGCCACAGTGTTTTCATTGAACACAACGGGTGGTGTCA
CTCTTGAAATCTCATGTTATAATGATACAGTGAGTGACTCGAGCTTTTCCAGTTACGGTGAAATGCCGTTCGGCGTAAC
TGATGGACCACGGTACTGTTACGTACTCTATAATGGCACAGCTCTTAAGTATCTAGGAACATTACCACCTATTGTCAAG
GAGATTGCTATTAGTAAGTGGGGCCATTTTTATATTAATGGTTACAATTTCTTTAGCACATTTCCTATTGATTGTATAT
CTTTTAATTTGACCACTGGTGATAGTGACGTTTTCTGGACAATAGCTTACACATCGTACACTGAAGCATTAGTACAAGT
TGAAAACACAGCTATTACAAAGGTGACGTATTGTAATAGTTACGTTAATAACATTAAATGCTCTCAACTTACTGCTAAT
TTGAATAATGGATTTTATCCTGTTTCTTCAAGTGAAGTTGGTCTTGTCAATAAGAGTGTTGTGTTACTACCTAGCTTTT
ACACACATACCATTGTTAACATAACTATTGGTCTTGGTATGAAGCGTAGTGGTTATGGTCAACCCATAGCCTCAACATT
AAGTAACATTACACTACCAATGCAGGATAACAACACCGATGTGTACTGTATTCGTTCTGACCAATTTTCAGTTTATGTT
CATTCTACTTGCAAAAGTTCTTTATGGGACAATGTTTTTAAGCGAAACTGCACGGACGTTTTAGATGCCACAGCTGTTA
TAAAAACTGGTACTTGTCCTTTCTCATTTGATAAATTGAACAATTACTTAACTTTTAACAAGTTCTGTTTGTCGTTGAG
TCCTGTTGGTGCTAATTGTAAGTTTGATGTAGCTGCCCGTACAAGAACCAATGATCAGGTTGTTAGAAGTTTGTATGTA
ATATATGAAGAAGGAGACAACATAGTGGGTGTACCGTCTGATAATAGTGGTTTACACGATTTGTCAGTGCTACACCTAG
ATTCCTGCACAGATTACAATATATATGGTAGAACTGGTGTTGGTATTATTAGACAAACTAACAGGACGCTACTTAGTGG
CTTATATTACACATCACTATCAGGTGATTTGTTAGGTTTTAAAAATGTTAGTGATGGTGTCATCTACTCTGTAACGCCA
TGTGATGTAAGCGCACAAGCAGCTGTTATTGATGGTACCATAGTTGGGGCTATCACTTCCATTAACAGTGAACTGTTAG
GTCTAACACATTGGACAACAACACCTAATTTTTATTACTACTCTATATATAATTACACAAATGATAGGACTCGTGGCAC
TGCAATTGACAGTAATGATGTTGATTGTGAACCTGTCATAACCTATTCTAACATAGGTGTTTGTAAAAATGGTGCTTTG
GTTTTTATTAACGTCACACATTCTGATGGAGACGTGCAACCAATTAGCACTGGTAACGTCACGATACCTACAAACTTTA
CTATATCCGTGCAAGTCGAATATATTCAGGTTTACACTACACCAGTGTCAATAGACTGTTCAAGATATGTTTGTAATGG
CAACCCTAGGTGTAACAAATTGTTAACACAATACGTTTCTGCATGTCAAACTATTGAGCAAGCACTTGCAATGGGTGCC
AGACTTGAAAACATGGAAGTTGATTCCATGTTATTTGTTTCTGAAAATGCCCTTAAATTGGCTTCTGTCGAAGCATTCA
ATAGTTCAGAAACTTTAGATCCTATTTACAAAGAATGGCCTAATATAGGTGGCTCTTGGCTAGAAGGTCTAAAATACAT
ACTTCCGTCCGATAATAGCAAACGTAAGTATCGTTCAGCTATAGAGGACTTGCTTTTTGCTAAGGTTGTAACATCTGGT
TTAGGTACAGTTGATGAAGATTATAAACGTTGTACAGGTGGTTATGACATAGCTGACTTAGTATGTGCTCAATACTACA
ATGGCATCATGGTGCTACCTGGTGTGGCTAATGCTGACAAAATGACTATGTACACAGCATCCCTCGCAGGTGGTATAAC
ATTAGGTGCACTTGGTGGAGGCGCCGTGGCTATACCTTTTGCAGTAGCAGTTCAGGCTAGACTTAATTATGTTGCTCTA
CAAACTGATGTATTGAACAAAAACCAGCAGATCCTGGCTAGTGCTTTCAATCAAGCTATTGGTAACATTACACAGTCAT
TTGGTAAGGTTAATGATGCTATACATCAAACATCACGAGGTCTTGCAACTGTTGCTAAAGCATTGGCAAAAGTGCAAGA
TGTTGTCAACACACAAGGGCAAGCTTTAAGCCACCTAACAGTACAATTGCAAAATAATTTCCAAGCCATTAGTAGTTCT
ATTAGTGACATTTATAATAGGCTTGATGAATTGAGTGCTGATGCACAAGTTGACAGGCTGATCACAGGAAGACTTACAG
CACTTAATGCATTTGTGTCTCAGACTCTAACCAGACAAGCCGAGGTTAGGGCTAGTAGACAACTTGCCAAAGACAAGGT
TAATGAATGCGTTAGGTCTCAGTCTCAGAGATTCGGATTCTGTGGTAATGGTACACATTTGTTTTCACTCGCAAATGTA
GCACCAAATGGCATGATCTTCTTTCACACAGTGCTATTACCAACGGCTTATGAAACTGTGACTGCTTGGGCAGGTATTT
GTGCTTTAGATGGTGATCGCACTTTTGGACTTGTCGTTAAAGATGTCCAGTTGACTTTGTTTCGTAATCTAGATGACAA
GTTCTATTTGACCCCCAGAACTATGTATCAGCCTAGAGTGGCAACTAGTTCTGATTTTGTTCAAATTGAAGGGTGCGAT
GTGCTGTTTGTTAATGCAACTGTAAGTGATTTGCCTAGTATTATACCTGATTATATTGATATTAATCAGACTGTTCAAG
ACATATTAGAAAATTTTAGACCAAATTGGACTGTACCTGAGTTGACATTTGACATTTTTAACGCAACCTATTTAAACCT
GACTGGTGAAATTGATGACTTAGAATTTAGGTCAGAAAAGCTACATAACACTACTGTAGAACTTGCCATTCTTATTGAC
AACATTAACAATACATTAGTCAATCTTGAATGGCTCAATAGAATTGAAACCTATGTAAAATGGCCTTGGTATGTGTGGC
TACTAATAGGCTTAGTAGTAATATTTTGCATACCATTACTGCTATTTTGCTGTTGTAGTACAGGTTGCTGTGGATGCAT
AGGTTGTTTAGGAAGTTGTTGTCACTCTATATGCAGTAGAAGACAATTTGAAAATTACGAACCTATTGAAAAAGTGCAC

GTCCATTAA

>gi|90735256|gb|DQ443743.1| Transmissible gastroenteritis virus strain SC-Y, complete genome

ATGAAAAAACTATTTGTGGTTTTGGTCGTAATGCCATTGATTTATGGAGACAATTTTCCTTGTTCTAAATTGACT
AATAGAACTATAGGCAACCAGTGGAATCTCATTGAAACCTTCCTTCTAAACTATAGTAGTAGGTTACCACCTAATTCAG
ATGTGGTGTTAGGTGATTATTTTCCTACTGTACAACCTTGGTTTAATTGCATTCGCAATAATAGTAATGACCTTTATGT
TACACTGGAAAATCTTAAAGCATTGTATTGGGATTATGCTACAGAAAATATCACTTGGAATCACAGACAACGGTTAAAC
GTAGTCGTTAATGGATACCCATACTCCATCACAGTTACAACAACCCGCAATTTTAATTCTGCTGAAGGTGCTATTATAT
GCATTTGTAAGGGCTCACCACCTACTACCACCACAGAATCTAATTTGACTTGCAATTGGGGTAGTGAGTGCAGGTTAAA
CCATAAGTTCCCTATATGTCCTTCTAATTCAGAGGCAAATTGTGGTAATATGCTGTATGGCCTACAATGGTTTGCAGAT
GAGGTTGTTGCTTATTTACATGGTGCTAGTTACCGTATTAGTTTTGAAAATCAATGGTCTGGCACTGTCACATTTGGTG
ATATGCGTGCGACAACATTAGAAGTCGCTGGCACGCTTGTAGACCTTTGGTGGTTTAATCCTGTTTATGATGTCAGTTA
TTATAGGGTTAATAATAAAAATGGTACTACCGTATTTTCCAATTGCACTGATCAATGTGCTAGTTATGTGGCTAATGTT
TTTACTACACAGCCAGGAGGTTTTATACCATCAGATTTTAGCTTTAATAATTGGTTCCTTCTAACTAATAGCTCCACGT
TGGTTAGTGGTAAATTAGTTACCAAACAGCCGTTATTAGTTAATTGCTTATGGCCAGTCCCTAGCTTTGAAGAAGCAGC
TTCTACATTTTGTTTTGAGGGTGCTGGCTTTGATCAATGTAATGGTGCTGTTTTAAATAATACTGTAGACGTCATTAGG
TTCAACCTTAATTTTACTACAAATGTACAATCAGGTAAGGGTGCCACAGTGTTTTCATTGAACACAACGGGTGGTGTCA
CTCTTGAAATTTCATGTTATACAGTGAGTGACTCGAGCTTTTTCAGTTACGGTGAAATTCCGTTCGGCGTAACTGATGG
ACCACGGTACTGTTACGTACACTATAATGGCACAGCTCTTAAGTATTTAGGAACATTACCACCTAGTGTCAAGGAGATT
GCTATTAGTAAGTGGGGCCATTTTTATATTAATGGTTACAATTTCTTTAGCACATTTCCTATTGATTGTATATCTTTTA
ATTTGACCACTGGTGATAGTGACGTTTTCTGGACAATAGCTTACACATCGTACACTGAAGCATTAGTACAAGTTGAAAA
CACAGCTATTACAAAGGTGACGTATTGTAATAGTCACGTTAATAACATTAAATGCTCTCAAATTACTGCTAATTTGAAT
AATGGATTTTATCCTGTTTCTTCAAGTGAAGTTGGTCTTGTCAATAAGAGTGTTGTGTTACTACCTAGCTTTTACACAC
ATACCATTGTTAACATAACTATTGGTCTTGGTATGAAGCGTAGTGGTTATGGTCAACCCATAGCCTCAACATTAAGTAA
CATCACACTACCAATGCAGGATCACAACACCGATGTGTACTGTATTCGTTCTGACCAATTTTCAGTTTATGTTCATTCT
ACTTGCAAAAGTGCTTTATGGGACAATATTTTTAAGCGAAACTGCACGGACGTTTTAGATGCCACAGCTGTTATAAAAA
CTGGTACTTGTCCTTTCTCATTTGATAAATTGAACAATTACTTAACTTTTAACAAGTTCTGTTTGTCGTTGAGTCCTGT
TGGTGCTAATTGTAAGTTTGATGTAGCTGCCCGTACAAGAACCAATGAGCAGGTTGTCAGAAGTTTGTATGTAATATAT
GAAGAAGGAGACAACATAGTGGGTGTACCGTCTGATAATAGTGGTGTGCACGATTTGTCAGTGCTACACCTAGATTCCT
GCACAGATTACAATATATATGGTAGAACTGGTGTTGGTATTATTAGAAAAACTAACAGGACGCTACTTAGTGGCTTATA
TTACACATCACTATCAGGTGATTTGTTAGGTTTTAAAAATGTTAGTGATGGTGTCATCTACTCTGTAACGCCATGTGAT
GTAAGCGCACAAGCAGCTGTTATTGATGGTACCATAGTTGGGGCTATCACTTCCATTAACAGTGAACTGTTAGGTCTAA
CACATTGGACAACAACACCTAATTTTTATTACTACTCTATATATAATTACACAAATGATAGGACTCGTGGCACTGCAAT
TGACAGTAATGATGTTGATTGTGAACCTGTCATAACCTATTCTAACATAGGTGTTTGTAAAAATGGTGCTTTTGTTTTT
ATTAACGTCACACATTCTGATGGAGACGTGCAACCAATTAGCACTGGTAATGTCACGATACCTACAAACTTTACCATAT
CCGTGCAAGTCGAATATATTCAGGTTTACACTACACCAGTGTCAATAGACTGTTCAAGATATGTTTGTAATGGTAACCC
TAGGTGTAACAAATTGTTAACACAATACGTTTCTGCATGTCAAACTATTGAGCAAGCACTTGCAATGGGTGCCAGACTT
GAAAACATGGAGGTTGATTCCATGTTGTTTGTTTCTGAAAATGCCCTTAAATTGGCATCTGTTGAAGCATTCAATAGTT
CAGAAACTTTAGACCCTATTTACAAATAATGGCCTAATATAGGTGGTTCTTGGCTAGAAGGTCTAAAATACATACTTCC
GTCCCATAATAGCAAACGTAAGTATCGTTCAGCTATAGAGGACTTGCTTTTTGATAAGGTTGTAACATCTGGTTTAGGT
ACAGTTGATGAAGATTATAAACGTTGTACAGGTGGTTATGACATAGCTGACTTAGTATGTGCTCAATACTATAATGGCA

TCATGGTGCTACCTGGTGTGGCTAATGCTGACAAAATGACTATGTCCACAGCATCCCTTGCAGGTGGTATAACATTAGG
TGCACTTGGTGGAGGCGCCGCGGCTATACCTTTTGCAGTAGCCGTGCAGGCTAGACTTAATTATGTTGCTCTACAAACT
GATGTATTGAACAAAAACCAGCAGATTCTGGCTAGTGCTTTCAATCAAGCTATTGGTAACATTACACAGTCATTTGGTA
AGGTTAATGATGCTATACATCAAACATCACGAGGTCTTGCTACTGTTGCTAAAGCATTGGCAAAAGTGCAAGATGTTGT
CAACATACAAGGGCAAGCTTTAAGCCACCTAACAGTACAATTGCAAAATAATTTCCAAGCCATTAGTAGTTCTATTAGT
GACATTTATAATAGGCTTGACGAATTGAGTGCTGATGCACAAGTTGACAGGCTGATCACAGGAAGACTTACAGCACTTA
ATGCATTTGTGTCTCAGACTCTAACCAGACAAGCGGAGGTTAGGGCTAGTAGACAACTTGCCAAAGACAAGGTTAATGA
ATGCGTTAGGTCTCAGTCTCAGAGATTCGGATTCTGTGGTAATGGTACACATTTGTTTTCACTCGCAAATGCAGCACCA
AATGGCATGATTTTCTTTCACACAGTGCTATTACCAACGGCTTATGAAACTGTGACTGCTTGGCCAGGTATTTGTGCTT
CAGATGGTGATCGCACTTTTGGACTTGTCGTTAAAGATGTCCAGTTGACTTTGTTTCGTAATCTAGATGACAAGTTCTA
TTTGACCCCCAGAACTATGTATCAGCCTAGAGTTGCAACTAGTTCTGACTTTGTTCAAATTGAAGGGTGCGATGTGCTG
TTTGTTAATGCAACTGTAAGTGATTTGCCTAGTATTATACCTGATTATATTGATATTAATCAGACTGTTCAAGACATAT
TAGAAAATTTTAGACCAAATTGGACTGTACCTGAGTTGACATTTGACATTTTTAACGCAACCTATTTAAACCTGACTGG
TGAAATTGATGACTTAGAATTTAGGTCAGAAAAGCTACATAACACCACTGTAGAACTTTCCATTCTCATTGACAACATT
AACAATACATTAGTCAATCTTGAATGGCTCAATAGAATTGAAACCTATGTAAAATGGCCTTGGTATGTGTGGCTACTAA
TAGGCTTAGTAGTAATATTTTGCATACCATTACTGCTATTTTGCTGTTGTAGTACAGGTTGCTGTGGATGCATAGGTTG
TTTAGGAAGTTGTTGTCACTCTATATGTAGTAGAAGACAATTTGAAAATTACGAACCAATTGAAAAAGTGCACGTCCAT
TAA

>gi|256433417|gb|EU074218.2| Transmissible gastroenteritis virus strain attenuated H, complete genome

ATGAAAAAATTATTTGTGGTTTTGGTTGTAATGCCATTGATTTATGGAGACAATTTTCCTTGTTCTAAATTGACT
AATAGAACTATAGGTAACCATTGGAATCTCATTGAAACCTTCCTTCTAAATTATAGTAGTAGGTTATCACCTAATTCAG
ATGCGGTGTTAGGTGATTATTTTCCTACTGTACAACCTTGGTTTAATTGCATTCGCAATAATAGTAATGACCTTTATGT
TACATTGGAAAATCTTAAAGCATTGTATTGGGATTATGCTATAGAAAATATCACTTCGAATCACAAACAACGGTTAAAC
GTAGTCGTTAATGGATACCCATACTCCATCACAGTTACAACAACCCGCAATTTTAATTCTGCTGAAGGTGCTATTATAT
GCATTTGCAAGGGCTCACCACCTACTACCACCACAGAATCTAGTTTGACTTGCAATTGGGGTAGTGAGTGCAGGTTAAA
CCATAAGTTCCCTATATGTCCTTCTAATTCAGAGGCAAATTGTGGTAATATGCTGTATGGCCTACAATGGTTTGCAGAT
GCGGTTGTTGCTTATTTACATGGTGCTAGTTACCGTATTAGTTTTGAAAATCAATGGTCTGGCACTGTTACACTTGGTG
ATATGCGTGCGACTACATTAGAAACCGCTGGCACGCTTGTAGACCTTTGGTGGTTTAATCCTGTTTATGATGTCAGTTA
TTATAGAGTTAATAATAAAAATGGTACTACCGTAGTTTCCAATTGCACTGATCAATGTGCTAGTTATGTGGCTAATGTT
TTTACTACACAGCCAGGAGGCTTTATACCATCAGATTTTAGTTTTAATAATTGGTTCCTTCTAACTAATAGCTCCACGT
TGGTTAGTGGTAAATTAGTTACCAAACAGCCGTTATTAGTTAATTGCTTATGGCCAGTCCCTAGCTTTGAAGAAGCAGC
TTCTACATTTTGTTTTGAAGGTGCTGGCTTTGATCAATGTAATGGTGCTGTTTTAAATAACACTGTAGACGTCATCAGG
TTTAACCTTAATTTTACTACAAATGTACAATCAGGTAAGGGTGCCACAGTGTTTTCATTGAACACAACGGGTGGTGTCA
CTCTTGAAATCTCATGTTATAATGATACAGTGAGTGACTCGAGCTTTTCCAGTTACGGTGAAATGCCGTTCGGCGTAAC
TGATGGACCACGGTACTGTTACGTACTCTATAATGGCACAGCTCTTAAGTATCTAGGAACATTACCACCTGTTGTCAAG
GAGATTGCTATTAGTAAGTGGGGCCATTTTTATATTAATGGTTACAATTTCTTTAGCACATTTCCTATTGATTGTATAT
CTTTTAATTTGACCACTGGTGATAGTGACGTTTTCTGGACAATAGCTTACACATCGTACACTGAAGCATTAGTACAAGT
TGAAAACACAGCTATTACAAAGGTGACGTATTGTAATAGTTACGTTAATAACATTAAATGCTCTCAACTTACTGCTAAT
TTGAATAATGGATTTTATCCTGTTTCTTCAAGTGAAGTTGGTCCTGTCAATAAGAGTGTTGTGTTACTACCTAGCCCTT
ACACACATACCATTGTTAACATAACTATAGGTCTTGGTATGAAGCGTAGTGGTTATGGTCAACCCATAGCCTCAACATT

AAGTAACATTACACTACCAATGCAGGATGACAACACCGATGTGTACTGTATTCGTTCTGACCAATTTTCAGTTTATGTT
CATTCTACTTGCAAAAGTGCTTTATGGGACAATGTTTTTAAGCGAAACTGCACGGACGTTTTAGATGCCACAGCTGTTA
TAAAAACTGGTACTTGTCCTTTCTCATTTGCTAAATTGAACAATTACTTAACTTTTAACAAGTTCTGTTTGTCGTTGAG
TCCTGTTGGTGCTAATTGTAAGTTTGATGTAGCTGCCCGTACAAGAACCAATGATCAGGTTGTTAGAAGTTTGTATGTA
ATATATGAAGAAGGAGACAACATAGTGGGTGTACCGTCTGGTAATAGTGGTTTACACGATTTGTCAGTGCTACACCTAG
ATTCCTGCACAGATTACAATATATATGGTAGAACTGGTGTTGGTATTATTAGACAAACTAACAGGACGCTACTTAGTGG
CTTATATTACACATCACTATCAGGTGATTTGTTAGGTTTTAAAAATGTTAGTGATGGTGTCATCTACTCTGTAACGCCA
TGTGATGTAAGCGCACAAGCAGCTGTTATTGATGGTACCATAGTTGGGGCTATCACTTCCATTAACAGTGAACTGTTAG
GTCTAACACATTGGACAACAACACCTAATTTTTATTACTACTCTATATATAATTACACAAATGATAGGACTCGTGGCAC
TGCAATTGACAGTAATGATGATTGTGAACCTGTCATAACCTATTCTAACATAGGTGTTTGTAAAAATGGTGCTTTGGTT
TTTATTAACGTCACACATTCTGATGGAGACGTGCAACCAATTAGCACTGGTAACGTCACGATACCTACAAACTTTACTA
TATCCGTGCAAGTCGAATATATTCAGGTTTACACTACACCAGTGTCAATAGACTGTTCAAGATATGTTTGTAATGGCAA
CCCTAGGTGTAACAAATTGTTAACACAATACGTTTCTGCATGTCAAACTATTGAGCAAGCACTTGCAATGGGTGCCAGA
CTTGAAAACATGGAAGTTGATTCCATGTTATTTGTTTCTGAAAATGCCCTTAAATTGGCTTCTGTCGAAGCATTCAATA
GTTCAGAAACTTTAGATCCTATTTACAAAGAATGGCCTAATATAGGTGGCTCTTGGCTAGAAGGTCTAAAATACATACT
TCCGTCCGATAATAGCAAACGTAAGTATCGTTCAGCTATAGAGGACTTGCTTTTTGCTAAGGTTGTAACATCTGGTTTA
GGTACAGTTGATGAAGATTATAAACGTTGTACAGGTGGTTATGACATAGCTGACTTAGTATGTGCTCAATACTACAATG
GCATCATGGTGCTACCTGGTGTGGCTAATGCTGACAAAATGACTATGTACACAGCATCCCTCGCAGGTGGTATAACATT
AGGTGCACTTGGTGGAGGCGCCGTGGCTATACCTTTTGCAGTAGCAGTTCAGGCTAGACTTAATTATGTTGCTCTACAA
ACTGATGTATTGAACAAAAACCAGCAGATCCTGGCTAGTGCTTTCAATCAAGCTATTGGTAACATTACACAGTCATTTG
GTAAGGTTAATGATGCTATACATCAAACATCACGAGGTCTTGCAACTGTTGCTAAAGCATTGGCAAAAGTGCAAGATGT
TGTCAACGCACAAGGGCAAGCTTTAAGCCACCTAACAGTACAATTGCAAAATAATTTCCAAGCCATTAGTAGTTCTATT
AGTGACATTTATAATAGGCTTGATGAATTGAGTGCTGATGCACAAGTTGACAGGCTGATCACAGGAAGACTTACAGCAC
TTAATGCATTTGTGTCTCAGACTCTAACCAGACAAGCCGAGGTTAGGGCTAGTAGACAACTTGCCAAAGACAAGGTTAA
TGAATGCGTTAGGTCTCAGTCTCAGAGATTCGGATTCTGTGGTAATGGTACACATTTGTTTTCACTCGCAAATGCAGCA
CCAAATGGCATGATCTTCTTTCACACATTGCTATTACCAACGGCTTATGAAACTGTGACTGCTTGGGCAGGTATTTGTG
CTTTAGATGGTGATCGCACTTTTGGACTTGTCGTTAAAGATGTCCAGTTGACTTTGTTTCGTAATCTAGATGACAAGTT
CTATTTGACCCCCAGAACTATGTATCAGCCTAGAGTGGCAACTAGTTCTGATTTTGTTCAAATTGAAGGGTGCGATGTG
CTGTTTGTTAATGCAACTGTAAGTGATTTGCCTAGTATTATACCTGATTATATTGATATTAATCAGACTGTTCAAGACA
TATTAGAAAATTTTAGACCAAATTGGACTGTACCTGAGTTGACATTTGACATTTTTAACGCAACCTATTTAAACCTGAC
TGGTGAAATTGATGACTTAGAATTTAGGTCAGAAAAGCTACATAACACTACTGTAGAACTTGCCATTCTTATTGACAAC
ATTAACAATACATTAGTCAATCTTGAATGGCTTAATAGAATTGAAACCTATGTAAAATGGCCTTGGTATGTGTGGCTAC
TAATAGGCTTAGTAGTAATATTTTGCATACCATTACTGCTATTTTGCTGTTGTAGTACAGGTTGCTGTGGATGCATAGG
TTGTTTAGGAAGTTGTTGTCACTCTATATGCAGTAGAAGACAATTTGAAAATTACGAACCTATTGAAAAAGTGCACGTC
CATTAA

>gi|324497636|gb|HQ462571.1| Transmissible gastroenteritis virus strain WH-1, complete genome

ATGAAAAAAACTATTTGTGGTTTTGGTCGTAATGCCATTGATTTATGGAGACAATTTTCCTTGTTCTAAATTGACT
AATAGAACTATAGGCAACCAGTGGAATCTCATTGAAACCTTCCTTCTAAACTATAGTAGTAGGTTACCACCTAATTCAG
ATGTGGTGTTAGGTGATTATTTTCCTACTGTACAACCTTGGTTTAATTGCATTCGCAATGATAGTAATGACCTTTATGT
TACACTGGAAAATCTTAAAGCATTGTATTGGGATTATGCTACAGAAAATATCACTTGGAATCACAGACAACGGTTAAAC

GTAGTCGTTAATGGATACCCATACTCCATCACAGTTACAACAACCCGCAATTTTAATTCTGCTGAAGGTGCTATTATAT
GCATTTGTAAGGGCTCACCACCTACTACCACCACAGAATCTAGTTTGACTTGCAATTGGGGTAGTGAGTGCAGGTTAAA
CCATAAGTTCCCTATATGTCCTTCTAATTCAGAGGCAAATTGTGGTAATATGCTGTATGGCCTACAATGGTTTGCAGAT
GAGGTTGTTGCTTATTTACATGGTGCTAGTTACCGTATTAGTTTTGAAAATCAATGGTCTGGCACTGTCACATTTGGTG
ATATGCGTGCGACAACATTAGAAGTCGCTGGCACGCTTGTAGACCTTTGGTGGTTTAATCCTGTTTATGATGTCAGTTA
TTATAGGGTTAATAATAAAAATGGTACTACCGTAGTTTCCAATTGCACTGATCAATGTGCTAGTTATGTGGCTAATGTT
TTTACTACACAGCCAGGAGGTTTTATACCATCAGATTTTAGTTTTAATAATTGGTTCCTTCTAACTAATAGCTCCACGT
TGGTTAGTGGTAAATTAGTTACCAAACAGCCGTTATTAGTTAATTGCTTATGGCCAGTCCCTAGCTTTGAAGAAGCAGC
TTCTACATTTTGTTTTGAGGGTGCTGGCTTTGATCAATGTAATGGTGCTGTTTTAAATAATACTGTAGACGTCATTAGG
TTCAACCTTAATTTTACTACAAATGTACAATCAGGTAAGGGTGCCACAGTGTTTTCATTGAACACAACGGGTGGTGTCA
CTCTTGAAATTTCATGTTATACAGTGAGTGACTCGAGCTTTTTCAGTTACGGTGAAATTCCGTTCGGCGTAACTGATGG
ACCACGGTACTGTTACGTACACTATAATGGCACAGCTCTTAAGTATTTAGGAACATTACCACCTAGTGTCAAGGAGATT
GCTATTAGTAAGTGGGGCCATTTTTATATTAATGGTTACAATTTCTTTAGCACATTTCCTATTGATTGTATATCTTTTA
ATTTGACCACTGGTGATAGTGACGTTTTCTGGACAATAGCTTACACATCGTACACTGAAGCATTAGTACAAGTTGAAAA
CACAGCTATTACAAAGGTGACGTATTGTAATAGTCACGTTAATAACATTAAATGCTCTCAAATTACTGCTAATTTGAAT
AATGGATTTTATCCTGTTTCTTCAAGTGAAGTTGGTCTTGTCAATAAGAGTGTTGTGTTACTACCTAGCTTTTACACAC
ATACCATTGTTAACATAACTATTGGTCTTGGTATGAAGCGTAGTGGTTATGGTCAACCCATAGCCTCAACATTAAGTAA
CATCACACTACCAATGCAGGATCACAACACCGATGTGTACTGTATTCGTTCTGACCAATTTTCAGTTTATGTTCATTCT
ACTTGCAAAAGTGCTTTATGGGACAATATTTTTAAGCGAAACTGCACGGACGTTTTAGATGCCACAGCTGTTATAAAAA
CTGGTACTTGTCCTTTCTCATTTGATAAATTGAACAATTACTTAACTTTTAACAAGTTCTGTTTGTCGTTGAGTCCTGT
TGGTGCTAATTGTAAGTTTGATGTAGCTGCCCGTACAAGAACCAATGAGCAGGTTGTTAGAAGTTTGTATGTAATATAT
GAAGAAGGAGACAACATAGTGGGTGTACCGTCTGATAATAGTGGTGTGCACGATTTGTCAGTGCTACACCTAGATTCCT
GCACAGATTACAATATATATGGTAGAACTGGTGTTGGTATTATTAGACAAACTAACAGGACGCTACTTAGTGGCTTATA
TTACACATCACTATCAGGTGATTTGTTAGGTTTTAAAAATGTTAGTGATGGTGTCATCTACTCTGTAACGCCATGTGAT
GTAAGCGCACAAGCAGCTGTTATTGATGGTACCATAGTTGGGGCTATCACTTCCATTAACAGTGAACTGTTAGGTCTAA
CACATTGGACAACAACACCTAATTTTTATTACTACTCTATATATAATTACACAAATGATAGGACTCGTGGCACTGCAAT
TGACAGTAATGATGTTGATTGTGAACCTGTCATAACCTATTCTAACATAGGTGTTTGTAAAAATGGTGCTTTTGTTTTT
ATTAACGTCACACATTCTGATGGAGACGTGCAACCAATTAGCACTGGTAATGTCACGATACCTACAAACTTTACCATAT
CCGTGCAAGTCGAATATATTCAGGTTTACACTACACCAGTGTCAATAGACTGTTCAAGATATGTTTGTAATGGTAACCC
TAGGTGTAACAAATTGTTAACACAATACGTTTCTGCATGTCAAACTATTGAGCAAGCACTTGCAATGGGTGCCAGACTT
GAAAACATGGAGGTTGATTCCATGTTGTTTGTTTCTGAAAATGCCCTTAAATTGGCATCTGTTGAAGCATTCAATAGTT
CAGAAACTTTAGACCCTATTTACAAAGAATGGCCTAATATAGGTGGTTCTTGGCTAGAAGGTCTAAAATACATACTTCC
GTCCCATAATAGCAAACGTAAGTATCGTTCAGCTATAGAGGACTTGCTTTTTGATAAGGTTGTAACATCTGGTTTAGGT
ACAGTTGATGAAGATTATAAACGTTGTACAGGTGGTTATGACATAGCTGACTTAGTATGTGCTCAATACTATAATGGCA
TCATGGTGCTACCTGGTGTGGCTAATGCTGACAAAATGACTATGTACACAGCATCCCTTGCAGGTGGTATAACATTAGG
TGCACTTGGTGGAGGCGCCGTGGCTATACCTTTTGCAGTAGCAGTTCAGGCTAGACTTAATTATGTTGCTCTACAAACT
GATGTATTGAACAAAAACCAGCAGATTCTGGCTAGTGCTTTCAATCAAGCTATTGGTAACATTACACAGTCATTTGGTA
AGGTTAATGATGCTATACATCAAACATCACGAGGTCTTGCTACTGTTGCTAAAGCATTGGCAAAAGTGCAAGATGTTGT
CAACATACAAGGGCAAGCTTTAAGCCACCTAACAGTACAATTGCAAAATAATTTCCAAGCCATTAGTAGTTCTATTAGT
GACATTTATAATAGGCTTGACGAATTGAGTGCTGATGCACAAGTTGACAGGCTGATCACAGGAAGACTTACAGCACTTA
ATGCATTTGTGTCTCAGACTCTAACCAGACAAGCGGAGGTTAGGGCTAGTAGACAACTTGCCAAAGACAAGGTTAATGA
ATGCGTTAGGTCTCAGTCTCAGAGATTCGGATTCTGTGGTAATGGTACACATTTGTTTTCACTCGCAAATGCAGCACCA
AATGGCATGATTTTCTTTCACACAGTGCTATTACCAACGGCTTATGAAACTGTGACTGCTTGGCCAGGTATTTGTGCTT
CAGATGGTGATCGCACTTTTGGACTTGTCGTTAAAGATGTCCAGTTGACTTTGTTTCGTAATCTAGATGACAAGTTCTA

TTTGACCCCCAGAACTATGTATCAGCCTAGAGTTGCAACTAGTTCTGACTTTGTTCAAATTGAAGGGTGCGATGTGCTG
TTTGTTAATGCAACTGTAAGTGATTTGCCTAGTATTATACCTGATTATATTGATATTAATCAGACTGTTCAAGACATAT
TAGAAAATTTTAGACCAAATTGGACTGTACCTGAGTTGACATTTGACATTTTTAACGCAACCTATTTAAACCTGACTGG
TGAAATTGATGACTTAGAATTTAGGTCAGAAAAGCTACATAACACCACTGTAGAACTTGCCATTCTCATTGACAACATT
AACAATACATTAGTCAATCTTGAATGGCTCAATAGAATTGAAACCTATGTAAAATGGCCTTGGTATGTGTGGCTACTAA
TAGGCTTAGTAGTAATATTTTGCATACCATTACTGCTATTTTGCTGTTGTAGTACAGGTTGCTGTGGATGCATAGGTTG
TTTAGGAAGTTGTTGTCACTCTATATGTAGTAGAAGACAATTTGAAAATTACGAACCAATTGAAAAAGTGCACGTCCAT
TAA

>gi|258407521|gb|FJ755618.2| Transmissible gastroenteritis virus strain H16, complete genome

ATGAAAAAATTATTTGTGGTTTTGGTTGTAATGCCATTGATTTATGGAGACAATTTTCCTTGTTCTAAATTGACT
AATAGAACTATAGGTAACCATTGGAATCTCATTGAAACCTTCCTTCTAAATTATAGTAGTAGGTTACCACCTAATTCAG
ATGCGGTGTTAGGTGATTATTTTCCTACTGTACAACCTTGGTTTAATTGCATTCGCAATAATAGTAATGACCTTTATGT
TACATTGGAAAATCTTAAAGCATTGTATTGGGATTATGCTATAGAAAATATCACTTCGAATCACAAACAACGGTTAAAC
GTAGTCGTTAATGGATACCCATACTCCATCACAGTTACAACAACCCGCAATTTTAATTCTGCTGAAGGTGCTATTATAT
GCATTTGCAAGGGCTCACCACCTACTACCACCACAGAATCTAGTTTGACTTGCAATTGGGGTAGTGAGTGCAGGTTAAA
CCATAAGTTCCCTATATGTCCTTCTAATTCAGAGGCAAATTGTGGTAATATGCTGTATGGCCTACAATGGTTTGCAGAT
GCGGTTGTTGCTTATTTACATGGTGCTAGTTACCGTATTAGTTTTGAAAATCAATGGTCTGGCACTGTTACACTTGGTG
ATATGCGTGCGACTACATTAGAAACCGCTGGCACGCTTGTAGACCTTTGGTGGTTTAATCCTGTTTATGATGTCAGTTA
TTATAGAGTTAATAATAAAAATGGTACTACCGTAGTTTCCAATTGCACTGATCAATGTGCTAGTTATGTGGCTAATGTT
TTTACTACACAGCCAGGAGGCTTTATACCATCAGATTTTAGTTTTAATAATTGGTTCCTTCTAACTAATAGCTCCACGT
TGGTTAGTGGTAAATTAGTTACCAAACAGCCGTTATTAGTTAATTGCTTATGGCCAGTCCCTAGCTTTGAAGAAGCAGC
TTCTACATTTTGTTTTGAAGGTGCTGGCTTTGATCAATGTAATGGTGCTGTTTTAAATAACACTGTAGACGTCATCAGG
TTTAACCTTAATTTTACTACAAATGTACAATCAGGTAAGGGTGCCACAGTGTTTTCATTGAACACAACGGGTGGTGTCA
CTCTTGAAATCTCATGTTATAATGATACAGTGAGTGACTCGAGCTTTTCCAGTTACGGTGAAATGCCGTTCGGCGTAAC
TGATGGACCACGGTACTGTTACGTACTCTATAATGGCACAGCTCTTAAGTATCTAGGAACATTACCACCTGTTGTCAAG
GAGATTGCTATTAGTAAGTGGGGCCATTTTTATATTAATGGTTACAATTTCTTTAGCACATTTCCTATTGATTGTATAT
CTTTTAATTTGACCACTGGTGATAGTGACGTTTTCTGGACAATAGCTTACACATCGTACACTGAAGCATTAGTACAAGT
TGAAAACACAGCTATTACAAAGGTGACGTATTGTAATAGTTACGTTAATAACATTAAATGCTCTCAACTTACTGCTAAT
TTGAATAATGGATTTTATCCTGTTTCTTCAAGTGAAGTTGGTCTTGTCAATAAGAGTGTTGTGTTACTACCTAGCTTTT
ACACACATACCATTGTTAACATAACTATTGGTCTTGGTATGAAGCGTAGTGGTTATGGTCAACCCATAGCCTCAACATT
AAGTAACATTACACTACCAATGCAGGATGACAACACCGATGTGTACTGTATTCGTTCTGACCAATTTTCAGTTTATGTT
CATTCTACTTGCAAAAGTGCTTTATGGGACAATGTTTTTAAGCGAAACTGCACGGACGTTTTAGATGCCACAGCTGTTA
TAAAAACTGGTACTTGTCCTTTCTCATTTGCTAAATTGAACAATTACTTAACTTTTAACAAGTTCTGTTTGTCGTTGAG
TCCTGTTGGTGCTAATTGTAAGTTTGATGTAGCTGCCCGTACAAGAACCAATGATCAGGTTGTTAGAAGTTTGTATGTA
ATATATGAAGAAGGAGACAACATAGTGGGTGTACCGTCTGATAATAGTGGTTTACACGATTTGTCAGTGCTACACCTAG
ATTCCTGCACAGATTACAATATATATGGTAGAACTGGTGTTGGTATTATTAGACAAACTAACAGGACGCTACTTAGTGG
CTTATATTACACATCACTATCAGGTGATTTGTTAGGTTTTAAAAATGTTAGTGATGGTGTCATCTACTCTGTAACGCCA
TGTGATGTAATCGCACAAGCAGCTGTTATTGATGGTACCATAGTTGGGGCTATCACTTCCATTAACAGTGAACTGTTAG
GTCTAACACATTGGACAACAACACCTAATTTTTATTACTACTCTATATATAATTACACAAATGATAGGACTCGTGGCAC
TGCAATTGACAGTAATGATGATTGTGAACCTGTCATAACCTATTCTAACATAGGTGTTTGTAAAAATGGTGCTTTGGTT

TTTATTAACGTCACACATTCTGATGGAGACGTGCAACCAATTAGCACTGGTAACGTCACGATACCTACAAACTTTACTA
TATCCGTGCAAGTCGAATATATTCAGGTTTACACTACACCAGTGTCAATAGACTGTTCAAGATATGTTTGTAATGGCAA
CCCTAGGTGTAACAAATTGTTAACACAATACGTTTCTGCATGTCAAACTATTGAGCAAGCACTTGCAATGGGTGCCAGA
CTTGAAAACATGGAAGTTGATTCCATGTTATTTGTTTCTGAAAATGCCCTTAAATTGGCTTCTGTCGAAGCATTCAATA
GTTCAGAAACTTTAGATCCTATTTACAAAGAATGGCCTAATATAGGTGGCTCTTGGCTAGAAGGTCTAAAATACATACT
TCCGTCCGATAATAGCAAACGTAAGTATCGTTCAGCTATAGAGGACTTGCTTTTTGCTAAGGTTGTAACATCTGGTTTA
GGTACAGTTGATGAAGATTATAAACGTTGTACAGGTGGTTATGACATAGCTGACTTAGTATGTGCTCAATACTACAATG
GCATCATGGTGCTACCTGGTGTGGCTAATGCTGACAAAATGACTATGTACACAGCATCCCTCGCAGGTGGTATAACATT
AGGTGCACTTGGTGGAGGCGCCGTGGCTATACCTTTTGCAGTAGCAGTTCAGGCTAGACTTAATTATGTTGCTCTACAA
ACTGATGTATTGAACAAAAACCAGCAGATCCTGGCTAGTGCTTTCAATCAAGCTATTGGTAACATTACACAGTCATTTG
GTAAGGTTAATGATGCTATACATCAAACATCACGAGGTCTTGCAACTGTTGCTAAAGCATTGGCAAAAGTGCAAGATGT
TGTCAACGCACAAGGGCAAGCTTTAAGCCACCTAACAGTACAATTGCAAAATAATTTCCAAGCCATTAGTAGTTCTATT
AGTGACATTTATAATAGGCTTGATGAATTGAGTGCTGATGCACAAGTTGACAGGCTGATCACAGGAAGACTTACAGCAC
TTAATGCATTTGTGTCTCAGACTCTAACCAGACAAGCCGAGGTTAGGGCTAGTAGACAACTTGCCAAAGACAAGGTTAA
TGAATGCGTTAGGTCTCAGTCTCAGAGATTCGGATTCTGTGGTAATGGTACACATTTGTTTTCACTCGCAAATGCAGCA
CCAAATGGCATGATCTTCTTTCACACATTGCTATTACCAACGGCTTATGAAACTGTGACTGCTTGGGCAGGTATTTGTG
CTTTAGATGGTGATCGCACTTTTGGACTTGTCGTTAAAGATGTCCAGTTGACTTTGTTTCGTAATCTAGATGACAAGTT
CTATTTGACCCCCAGAACTATGTATCAGCCTAGAGTGGCAACTAGTTCTGATTTTGTTCAAATTGAAGGGTGCGATGTG
CTGTTTGTTAATGCAACTGTAAGTGATTTGCCTAGTATTATACCTGATTATATTGATATTAATCAGACTGTTCAAGACA
TATTAGAAAATTTTAGACCAAATTGGACTGTACCTGAGTTGACATTTGACATTTTTAACGCAACCTATTTAAACCTGAC
TGGTGAAATTGATGACTTAGAATTTAGGTCAGAAAAGCTACATAACACTACTGTAGAACTTGCCATTCTTATTGACAAC
ATTAACAATACATTAGTCAATCTTGAATGGCTCAATAGAATTGAAACCTATGTAAAATGGCCTTGGTATGTGTGGCTAC
TAATAGGCTTAGTAGTAATATTTTGCATACCATTACTGCTATTTTGCTGTTGTAGTACAGGTTGCTGTGGATGCATAGG
TTGTTTAGGAAGTTGTTGTCACTCTATATGCAGTAGAAGACAATTTGAAAATTACGAACCTATTGAAAAAGTGCACGTC
CATTAA

>gi|507864394|gb|KC962433.1| Transmissible gastroenteritis virus isolate TGEV-HX, complete genome

ATGAAAAAACTATTTGTGGTTTTGGTCGTAATGCCATTGATTTATGGAGACAATTTTCCTTGTTCTAAATTGACT
AATAGAACTATAGGCAACCAGTGGAATCTCATTGAAACCTTCCTTCTAAACTATAGTAGTAGGTTACCACCTAATTCAG
ATGTGGTGTTAGGTGATTATTTTCCTACTGTACAACCTTGGTTTAATTGCATTCGCAATGATAGTAATGACCTTTATGT
TACACTGGAAAATCTTAAAGCATTGTATTGGGATTATGCTACAGAAAATATCACTTGGAATCACAGACAACGGTTAAAC
GTAGTCGTTAATGGATACCCATACTCCATCACAGTTACAACAACCCGCAATTTTAATTCTGCTGAAGGTGCTATTATAT
GCATTTGTAAGGGCTCACCACCTACTACCACCACAGAATCTAGTTTGACTTGCAATTGGGGTAGTGAGTGCAGGTTAAA
CCATAAGTTCCCTATATGTCCTTCTAATTCAGAGGCAAATTGTGGTAATATGCTGTATGGCCTACAATGGTTTGCAGAT
GAGGTTGTTGCTTATTTACATGGTGCTAGTTACCGTATTAGTTTTGAAAATCAATGGTCTGGCACTGTCACATTTGGTG
ATATGCGTGCGACAACATTAGAAGTCGCTGGCACGCTTGTAGACCTTTGGTGGTTTAATCCTGTTTATGATGTCAGTTA
TTATAGGGTTAATAATAAAAATGGTACTACCGTAGTTTCCAATTGCACTGATCAATGTGCTAGTTATGTGGCTAATGTT
TTTACTACACAGCCAGGAGGTTTTATACCATCAGATTTTAGTTTTAATAATTGGTTCCTTCTAACTAATAGCTCCACGT
TGGTTAGTGGTAAATTAGTTACCAAACAGCCGTTATTAGTTAATTGCTTATGGCCAGTCCCTAGCTTTGAAGAAGCAGC
TTCTACATTTTGTTTTGAGGGTGCTGGCTTTGATCAATGTAATGGTGCTGTTTTAAATAATACTGTAGACGTCATTAGG
TTCAACCTTAATTTTACTACAAATGTACAATCAGGTAAGGGTGCCACAGTGTTTTCATTGAACACAACGGGTGGTGTCA

CTCTTGAAATTTCATGTTATACAGTGAGTGACTCGAGCTTTTTCAGTTACGGTGAAATTCCGTTCGGCGTAACTGATGG
ACCACGGTACTGTTACGTACACTATAATGGCACAGCTCTTAAGTATTTAGGAACATTACCACCTAGTGTCAAGGAGATT
GCTATTAGTAAGTGGGGCCATTTTTATATTAATGGTTACAATTTCTTTAGCACATTTCCTATTGATTGTATATCTTTTA
ATTTGACCACTGGTGATAGTGACGTTTTCTGGACAATAGCTTACACATCGTACACTGAAGCATTAGTACAAGTTGAAAA
CACAGCTATTACAAAGGTGACGTATTGTAATAGTCACGTTAATAACATTAAATGCTCTCAAATTACTGCTAATTTGAAT
AATGGATTTTATCCTGTTTCTTCAAGTGAAGTTGGTCTTGTCAATAAGAGTGTTGTGTTACTACCTAGCTTTTACACAC
ATACCATTGTTAACATAACTATTGGTCTTGGTATGAAGCGTAGTGGTTATGGTCAACCCATAGCCTCAACATTAAGTAA
CATCACACTACCAATGCAGGATCACAACACCGATGTGTACTGTATTCGTTCTGACCAATTTTCAGTTTATGTTCATTCT
ACTTGCAAAAGTGCTTTATGGGACAATATTTTTAAGCGAAACTGCACGGACGTTTTAGATGCCACAGCTGTTATAAAAA
CTGGTACTTGTCCTTTCTCATTTGATAAATTGAACAATTACTTAACTTTTAACAAGTTCTGTTTGTCGTTGAGTCCTGT
TGGTGCTAATTGTAAGTTTGATGTAGCTGCCCGTACAAGAACCAATGAGCAGGTTGTTAGAAGTTTGTATGTAATATAT
GAAGAAGGAGACAACATAGTGGGTGTACCGTCTGATAATAGTGGTGTGCACGATTTGTCAGTGCTACACCTAGATTCCT
GCACAGATTACAATATATATGGTAGAACTGGTGTTGGTATTATTAGACAAACTAACAGGACGCTACTTAGTGGCTTATA
TTACACATCACTATCAGGTGATTTGTTAGGTTTTAAAAATGTTAGTGATGGTGTCATCTACTCTGTAACGCCATGTGAT
GTAAGCGCACAAGCAGCTGTTATTGATGGTACCATAGTTGGGGCTATCACTTCCATTAACAGTGAACTGTTAGGTCTAA
CACATTGGACAACAACACCTAATTTTTATTACTACTCTATATATAATTACACAAATGATAGGACTCGTGGCACTGCAAT
TGACAGTAATGATGTTGATTGTGAACCTGTCATAACCTATTCTAACATAGGTGTTTGTAAAAATGGTGCTTTTGTTTTT
ATTAACGTCACACATTCTGATGGAGACGTGCAACCAATTAGCACTGGTAATGTCACGATACCTACAAACTTTACCATAT
CCGTGCAAGTCGAATATATTCAGGTTTACACTACACCAGTGTCAATAGACTGTTCAAGATATGTTTGTAATGGTAACCC
TAGGTGTAACAAATTGTTAACACAATACGTTTCTGCATGTCAAACTATTGAGCAAGCACTTGCAATGGGTGCCAGACTT
GAAAACATGGAGGTTGATTCCATGTTGTTTGTTTCTGAAAATGCCCTTAAATTGGCATCTGTTGAAGCATTCAATAGTT
CAGAAACTTTAGACCCTATTTACAAAGAATGGCCTAATATAGGTGGTTCTTGGCTAGAAGGTCTAAAATACATACTTCC
GTCCCATAATAGCAAACGTAAGTATCGTTCAGCTATAGAGGACTTGCTTTTTGATAAGGTTGTAACATCTGGTTTAGGT
ACAGTTGATGAAGATTATAAACGTTGTACAGGTGGTTATGACATAGCTGACTTAGTATGTGCTCAATACTATAATGGCA
TCATGGTGCTACCTGGTGTGGCTAATGCTGACAAAATGACTATGTACACAGCATCCCTTGCAGGTGGTATAACATTAGG
TGCACTTGGTGGAGGCGCCGTGGCTATACCTTTTGCAGTAGCAGTTCAGGCTAGACTTAATTATGTTGCTCTACAAACT
GATGTATTGAACAAAAACCAGCAGATTCTGGCTAGTGCTTTCAATCAAGCTATTGGTAACATTACACAGTCATTTGGTA
AGGTTAATGATGCTATACATCAAACATCACGAGGTCTTGCTACTGTTGCTAAAGCATTGGCAAAAGTGCAAGATGTTGT
CAACATACAAGGGCAAGCTTTAAGCCACCTAACAGTACAATTGCAAAATAATTTCCAAGCCATTAGTAGTTCTATTAGT
GACATTTATAATAGGCTTGACGAATTGAGTGCTGATGCACAAGTTGACAGGCTGATCACAGGAAGACTTACAGCACTTA
ATGCATTTGTGTCTCAGACTCTAACCAGACAAGCGGAGGTTAGGGCTAGTAGACAACTTGCCAAAGACAAGGTTAATGA
ATGCGTTAGGTCTCAGTCTCAGAGATTCGGATTCTGTGGTAATGGTACACATTTGTTTTCACTCGCAAATGCAGCACCA
AATGGCATGATTTTCTTTCACACAGTGCTATTACCAACGGCTTATGAAACTGTGACTGCTTGGCCAGGTATTTGTGCTT
CAGATGGTGATCGCACTTTTGGACTTGTCGTTAAAGATGTCCAGTTGACTTTGTTTCGTAATCTAGATGACAAGTTCTA
TTTGACCCCCAGAACTATGTATCAGCCTAGAGTTGCAACTAGTTCTGACTTTGTTCAAATTGAAGGGTGCGATGTGCTG
TTTGTTAATGCAACTGTAAGTGATTTGCCTAGTATTATACCTGATTATATTGATATTAATCAGACTGTTCAAGACATAT
TAGAAAATTTTAGACCAAATTGGACTGTACCTGAGTTGACATTTGACATTTTTAACGCAACCTATTTAAACCTGACTGG
TGAAATTGATGAATTAGAATTTAGGTCAGAAAAGCTACATAACACCACTGTAGAACTTGCCATTCTCATTGACAACATT
AACAATACATTAGTCAATCTTGAATGGCTCAATAGAATTGAAACCTATGTAAAATGGCCTTGGTATGTGTGGCTACTAA
TAGGCTTAGTAGTAATATTTTGCATACCATTACTGCTATTTTGCTGTTGTAGTACAGGTTGCTGTGGATGCATAGGTTG
TTTAGGAAGTTGTTGTCACTCTATATGTAGTAGAAGACAATTTGAAAATTACGAACCAATTGAAAAAGTGCACGTCCAT
TAA

>gi|380005466|gb|JQ065043.1| Porcine coronavirus HKU15 strain HKU15-155, complete genome

ATGCAGAGAGCTCTATTGATTATGACCTTACTTTGTCTCGTTCGAGCAAAGTTTGCTGATGATCTACTCGATTTG
CTCACCTTCCCGGGTGCACATCGCTTCTTACATAAACTCACGAGTAATTCCAGCAGTCTCTACTCGCGGGCTAATAATT
TTGATGTTGGCGTTCTTCCTGGCTACCCCACTAAGAACGTTAACCTCTTCTCACCACTTACTAACTCCACTTTGCCCAT
TAATGGCCTTCATCGGAGTTATCAACCACTCATGCTGAATTGTCTTACTAAAATAACTAACCACACTCTCAGCATGTAT
CTCCAACCTAGTGAGATACAAACCTATAGCTGCGGCGGTGCCATGGTTAAATACCAGACACATGATGCAGTTCGTATCA
TTTTAGACCTCACTGCCACTGACCACATCTCTGTTGAAGTCGTTGGCCAGCATGGTGAAAATTATGTGTTTGTTTGTAG
TGAGCAGTTTAACTATACCACTGCATTACACAACTCTACCTTCTTCTCACTTAATTCTGAGCTTTATTGCTTTACTAAT
AACACCTACTTAGGTATTCTTCCACCTGATTTAACTGACTTTACGGTCTATCGTACTGGTCAGTTCTATGCTAATGGTT
ACCTTTTAGGTACTTTACCTATTACGGTTAACTATGTTAGGTTGTATCGGGGTCACTTGGCGGCCAATAGTGCCCACTT
TGCCCTTGCAAACCTAACCGATACACTCATAACACTTACCAATACTACTATATCGCAAATCACTTATTGTGATAAGTCA
GTAGTTGATTCAATAGCATGCCAGCGCTCTTCTCACGAAGTGGAGGATGGGTTTTACTCTGACCCTAAATCTGCCGTTA
GAGCTAGGCAACGTACTATTGTTACACTACCTAAGCTCCCTGAGCTTGAAGTAGTGCAGTTAAATATTTCTGCACACAT
GGATTTTGGCGAAGCCAGACTTGACAGCGTTACCATCAATGGTAACACATCCTATTGTGTCACTAAGCCTTACTTCAGG
CTTGAAACTAACTTTATGTGTACAGGTTGCACTATGAATCTGCGCACTGATACCTGTAGTTTTGACCTGTCAGCAGTAA
ACAATGGCATGTCATTCTCTCAATTCTGTCTAAGCACTGAATCTGGTGCTTGTGAGATGAAAATTATTGTTACCTACGT
ATGGAATTACTTGCTAAGGCAGCGTTTGTATGTTACAGCTGTAGAGGGCCAGACTCACACTGGAACCACTTCAGTACAT
GCAACAGACACTTCTAGTGTAATCACTGATGTCTGCACTGACTACACTATCTATGGAGTCTCTGGCACTGGCATTATTA
AGCCATCAGATCTCTTATTGCACAATGGCATAGCATTCACCTCTCCAACAGGTGAGCTTTATGCATTTAAAAATATAAC
CACTGGCAAAACCCTTCAGGTCTTACCGTGTGAAACCCCTTCTCAACTGATTGTGATAAACAACACCGTTGTCGGTGCT
ATCACATCCAGTAATTCAACTGAAAATAATAGGTTTACTACTACTATTGTCACACCTACTTTCTTTTATTCCACAAATG
CCACCACCTTCAACTGCACCAAGCCTGTTTTGTCCTATGGACCCATCAGCGTGTGTAGTGATGGTGCAATTGCGGGAAC
ATCCACATTACAGAATACTCGACCATCCATAGTTTCACTATACGATGGCGAAGTTGAAATACCATCTGCATTTTCTCTT
TCTGTTCAGACGGAGTATTTGCAAGTTCAATCAGAGCAAGTTATAGTTGATTGTCCTCAGTATGTATGCAATGGCAACA
GCCGGTGTCTACAATTACTGGCACAATACACCTCAGCTTGCTCTAACATTGAAGCAGCTCTGCATTCCTCTGCACAGTT
GGATAGCAGAGAGATTATAAATATGTTTCAAACATCAACACAGTCCTTGCAGTTGGCTAATATTACCAACTTCAAGGGT
GACTACAATTTTAGCAGCATACTAACCACCAGACTTGGTGGCAGATCTGCTATTGAAGACCTTCTTTTTAATAAAGTTG
TTACTAGTGGCCTTGGCACTGTTGATCAGGACTACAAAGCCTGCTCTAGAGACATGGCCATCGCTGACTTAGTTTGTTC
CCAGTATTACAATGGCATCATGGTTCTACCTGGTGTTGTTGATGCTGAGAAAATGGCAATGTATACTGGCTCTCTTACT
GGAGCTATGGTATTTGGGGGACTGACTGCTGCAGCGGCAATACCATTCGCCACGGCAGTACAAGCCCGCCTTAATTATG
TCGCACTGCAAACAAATGTACTACAAGAAAACCAGAAAATTCTTGCAGAATCATTTAACCAAGCAGTTGGCAATATATC
ACTTGCACTATCTTCTGTTAATGATGCCATCCAGCAAACTTCTGAGGCTCTTAACACCGTAGCTATTGCTATTAAAAAG
ATTCAAACAGTTGTTAACCAGCAGGGTGAGGCATTATCACACCTGACTGCACAGCTGTCAAACAATTTTCAAGCAATTT
CGACTTCTATTCAAGACATTTACAACCGTCTTGAGGAAGTAGAGGCTAACCAGCAAGTTGACCGTCTCATCACAGGACG
GTTGGCTGCACTTAATGCATATGTTACTCAGTTACTCAATCAGATGTCTCAGGTTAGACAATCTCGATTGCTAGCTCAG
CAAAAGATTAATGAGTGTGTCAAATCTCAGTCGTCCAGATACGGTTTCTGTGGAAATGGCACACACATCTTCTCACTTA
CACAGACTGCACCAAATGGCATATTTTTCATGCATGCAGTGCTTGTACCCAACAAATTCACACGTGTCAACGCTTCTGC
CGGCATTTGTGTGGATAATACCAGAGGCTACTCATTGCAGCCTCAACTTATACTCTACCAGTTTAATAACTCCTGGAGA
GTTACACCTAGAAATATGTATGAACCCAGACTGCCCCGGCAAGCTGATTTCATACAATTAACTGATTGCAGCGTTACTT
TTTATAACACCACCGCTGCTAATCTTCCCAATATTATTCCTGACGTTATAGATGTCAATCAAACAGTCAGTGATATTAT
TGACAATTTACCTACAGCAACACCTCCTCAGTGGGATGTTGGTATCTATAACAACACTATTCTCAACCTTACCGTTGAG
ATTAATGATCTACAAGAGCGGTCTAAAAACCTCTCACAGATTGCAGATCGTTTACAAAATTATATTGATAATCTTAACA

ATACTCTAGTTGACCTTGAATGGCTCAACAGAGTGGAAACTTACCTTAAATGGCCGTGGTATATATGGCTTGCCATTGC
CCTGGCTCTTATTGCATTTGTGACAATCCTCATAACAATCTTTCTTTGTACTGGTTGTTGTGGTGGTTGCTTTGGTTGT
TGTGGCGGTTGTTTTGGCCTTTTCTCTAAGAAGAAAAGGTCTACCGACGACCAACCAACACCGTCCTTTAAGTTTAAGG
AATGGTAG

>gi|383081726|ref|NC_016990.1| Porcine coronavirus HKU15, complete genome

ATGCAGAGAGCTCTATTGATTATGACCTTACTTTGTCTCGTTCGAGCAAAGTTTGCTGATGATCTATTCGATTTG
CTCACCTTCCCGGGTGCACATCGCTTCTTACATAAACCCACGAGGAATTCCAGCAGTCTCTACTCGCGGGCTAATAATA
ATTTTGATGTTGGCGTTCTTCCTGGCTACCCCACTAAGAACGTTAACCTCTTCTCACCACTTACTAACTCTACTTTGCC
CATTAATGGCCTTCATCGGAGTTATCAACCACTCATGCTGAATTGTCTTACTAAAATAACTAACCACACTCTCAGCATG
TATCTCCTACCTAGTGAGATACAAACTTATAGCTGCGGCGGTGCCATGGTTAAATACCAGACACATGATGCAGTTCGTA
TCATTTTAGACCTCACTGCCACTGACCACATCTCTGTTGAAGTCGTTGGCCAACATGGTGAAAATTATGTGTTTGTTTG
TAGTGAGCAGTTTAACTATACTACTGCATTACACAACTCTACCTTTTTCTCACTTAATTCTGAGCTTTATTGCTTTACT
AATAACACCTACTTAGGTATTCTTCCACCTGATTTAACTGACTTTACGGTCTATCGTACTGGTCAGTTTTATGCTAATG
GTTACCTTTTAGGTACTTTACCTATTACTGTTAACTATGTTAGGTTGTATCGGGGTCATTTGTCGGCCAATAATGCCCA
CTTTGCCCTAGCAAACCTAACCGATACACTCATAACACTTACCAATACTACTATATCGCAAATCACTTATTGTGATAAG
TCAGTAGTTGATTCAATAGCATGCCAGCGCTCTTCTCACGAAGTGGAGGATGGGTTTTACTCTGACCCTAAATCTGCCG
TTAGAGCTAGGCAACGTACTATTGTTACACTACCTAAGCTCCCTGAGCTTGAAGTAGTGCAGTTAAATATTTCTGCACA
CATGGATTTTGGCGAAGCCAGACTTGACAGCGTTACCATCAATGGTAACACATCCTATTGTGTCACTAAGCCTTACTTC
AGGCTTGAAACTAACTTTATGTGTACAGGTTGCACTATGAATCTGCGCACTGATACCTGTAGTTTTGACCTGTCAGCAG
TAAACAATGGCATGTCATTCTCTCAATTCTGTCTAAGCACTGAATCTGGTGCTTGTGAGATGAAAATTATTGTTACCTA
CGTATGGAATTACTTGCTAAGGCAGCGTTTGTATGTTACTGCTGTAGAAGGCCAGACTCACACTGGAACCACTTCAGTA
CATGCAACAGACACTTCTAGTGTAATCACTGATGTCTGCACTGACTACACTATCTATGGAGTCTCTGGTACTGGCATTA
TTAAGCCATCAGATCTCTTATTGCACAATGGCATAGCATTCACCTCTCCAACAGGTGAGCTTTATGCATTTAAAAATAT
AACCACTGGCAAAACCCTTCAGGTCTTACCGTGTGAAACCCCTTCTCAACTGATTGTGATAAACAACACCGTTGTCGGT
GCTATCACATCCAGTAACTCAACTGAAAATAATAGGTTTACTACTACTATTGTCACACCTACTTTCTTTTATTCCACAA
ATGCCACCACCTTCAACTGCACCAAGCCTGTTTTGTCCTATGGACCCATCAGCGTGTGTAGTGATGGTGCAATTGCGGG
AACATCCACATTACAGAATACTCGACCATCCATAGTTTCACTATACGATGGCGAAGTTGAAATACCATCTGCATTTTCT
CTTTCTGTTCAGACGGAGTATTTGCAAGTTCAAGCAGAGCAAGTTATAGTTGATTGTCCTCAGTATGTATGCAATGGCA
ACAGCCGTTGTCTACAATTACTGGCACAATACACCTCAGCTTGCTCTAACATTGAAGCAGCTCTGCATTCCTCTGCACA
GTTGGATAGCAGAGAGATTATAAATATGTTTCAAACATCAACACAGTCCTTGCAGTTAGCTAATATTACCAACTTCAAG
GGTGACTACAATTTTAGCAGCATACTAACCACCAGACTTGGTGGCAGATCTGCTATTGAAGACCTTCTTTTTAATAAAG
TTGTTACTAGTGGCCTTGGCACTGTTGATCAGGACTACAAAGCCTGCTCTAGAGACATGGCCATCGCTGATTTAGTTTG
TTCCCAGTATTACAATGGCATCATGGTTCTACCTGGTGTTGTTGATGCTGAGAAAATGGCAATGTATACTGGCTCTCTT
ACTGGAGCTATGGTATTTGGGGGACTGACTGCTGCAGCGGCAATACCATTCGCTACAGCAGTACAAGCTCGCCTCAATT
ATGTCGCACTGCAAACAAATGTACTACAAGAAAACCAGAAAATTCTTGCAGAATCATTTAACCAAGCAGTTGGCAATAT
ATCACTTGCACTATCTTCTGTTAATGATGCCATCCAGCAAACTTCTGAGGCTCTTAACACCGTAGCTATTGCTATTAAA
AAGATTCAAACAGTTGTTAACCAGCAGGGTGAGGCATTATCACACCTGACTGCACAGCTGTCAAACAATTTTCAAGCAA
TTTCGACTTCTATTCAAGACATTTACAACCGTCTTGAGGAAGTAGAGGCTAACCAGCAAGTTGACCGTCTCATCACAGG
ACGGTTGGCTGCACTTAATGCATATGTTACTCAGTTACTCAATCAGATGTCTCAGATTAGACAATCTCGATTGTTAGCT
CAGCAAAAGATTAATGAGTGTGTCAAATCTCAGTCGCCCAGATACGGTTTCTGTGGAAATGGCACACACATCTTCTCAC
TTACACAGACTGCACCAAATGGCATATTTTTCATGCATGCAGTGCTTGTACCCAACAAATTCACACGTGTCAACGCTTC

TGCCGGCATTTGTGTGGATAATACGAGAGGCTACTCATTGCAGCCTCAACTTATACTCTACCAGTTTAATAACTCCTGG
AGAGTTACACCTAGAAATATGTATGAACCCAGACTGCCCCGGCAAGCTGATTTCATACAATTAACTGATTGCAGCGTTA
CTTTTTACAACACAACCGCTGCTAATCTTCCCAATATTATTCCTGACGTCATAGATGTCAATCAAACAGTCAGTGATAT
TATTGACAATTTACCTACAGCAACACCTCCTCAGTGGGATGTTGGTATTTATAACAACACTATTCTCAACCTCACCGTT
GAGATTAATGATCTACAAGAGCGGTCTAAAAACCTCTCACAGATTGCAGATCGTTTACAAAATTATATTGACAATCTTA
ACAATACTCTAGTTGACCTTGAATGGCTCAACAGAGTGGAAACTTACCTTAAATGGCCGTGGTATATATGGCTTGCCAT
TGCCCTGGCTCTTATTGCATTTGTGACAATCCTCATAACAATCTTTCTTTGTACTGGTTGTTGTGGTGGTTGCTTTGGT
TGTTGTGGCGGTTGTTTTGGCCTTTTCTCTAAGAAGAAAAGGTATACCGACGACCAACCAACACCGTCCTTTAAGTTTA
AGGAATGGTAG

>gi|597710833|gb|KJ462462.1| Porcine coronavirus HKU15 strain OH1987, complete genome

ATGCAGAGAGCTCTATTGATTATGACCTTACTTTGTCTCGTTCGAGCAAAGTTTGCTGATGATCTACTCGATTTG
CTCACCTTCCCGGGTGCACATCGCTTCTTACATAAACCCACGAGGAATTCCAGCAGTCTCTACTCGCGGGCTAATAATA
ATTTTGATGTTGGCGTTCTTCCTGGCTACCCCACTAAGAACGTTAACCTCTTCTCACCACTTACTAACTCCACTTTGCC
CATTAATGGCCTTCATCGGAGTTACCAACCACTCATGCTGAATTGTCTTACTAAAATAACTAACCACACTCTCAGCATG
TATCTCCTACCTAGTGAGATACAAACTTATAGCTGCGGCGGTGCCATGGTTAAATACCAGACACATGATGCAGTTCGTA
TCATTTTAGACCTCACTGCCACTGACCACATCTCTGTTGAAGTCGTTGGCCAACATGGTGAAAATTATGTGTTTGTTTG
CAGTGAGCAGTTTAACTACACCACTGCATTACACAACTCTACCTTCTTCTCACTTAATTCTGAGCTTTATTGCTTTACT
AATAACACCTACTTAGGTATTCTTCCACCTGATTTAACTGACTTTACTGTCTACCGTACTGGTCAGTTCTATGCTAATG
GTTACCTTTTAGGTACTTTACCTATTACGGTTAACTATGTTAGGTTGTATCGGGGTCATTTGTCTGCCAATAGTGCCCA
CTTTGCCCTTGCAAACCTAACCGATACACTCATAACACTTACCAATACTACTATATCGCAAATCACTTATTGTGATAAG
TCAGTAGTTGATTCAATAGCATGCCAGCGCTCTTCTCACGAAGTGGAGGATGGGTTTTACTCCGACCCTAAATCTGCCG
TTAGAGCTAGGCAACGTACTATTGTTACACTACCTAAGCTCCCTGAGCTTGAAGTAGTGCAGTTAAATATTTCTGCACA
CATGGATTTTGGCGAAGCCAGACTTGACAGCGTTACCATCAATGGTAACACATCCTATTGTGTCACTAAGCCTTACTTC
AGGCTTGAAACTAACTTTATGTGTACAGGTTGCACTATGAATCTGCGCACTGATACCTGTAGTTTTGACCTGTCAGCAG
TAAACAATGGCATGTCATTCTCTCAATTCTGTCTAAGCACTGAATCTGGTGCTTGTGAGATGAAAATTATTGTTACCTA
CGTATGGAATTACTTGCTAAGGCAGCGTTTGTATGTTACTGCTGTAGAGGGCCAGACTCACACTGGAACCACTTCAGTA
CATGCAACAGACACTTCTAGTGTAATCACTGATGTCTGCACTGACTACACTATCTATGGAGTCTCTGGTACTGGCATTA
TTAAGCCATCAGATCTCTTATTGCACAATGGCATAGCATTCACCTCTCCAACAGGTGAGCTTTATGCATTTAAAAATAT
AACCACTGGCAAAACCCTTCAGGTCTTACCGTGTGAAACCCCTTCTCAACTGATTGTGATAAACAACACCGTTGTCGGT
GCTATCACATCCAGTAATTCAACTGAAAATAATAGGTTTACTACTACTATTGTCACACCTACTTTCTTTTATTCCACAA
ATGCCACCACTTTCAACTGCACTAAGCCTGTTTTGTCCTATGGACCTATCAGCGTGTGTAGTGATGGTGCAATTGTGGG
AACATCCACATTACAGAATACTCGACCATCCATAGTTTCACTATACGATGGCGAAGTTGAAATACCATCTGCATTTTCT
CTTTCCGTTCAGACGGAGTACTTGCAAGTTCAAGCAGAGCAAGTTATAGTTGATTGTCCTCAGTATGTATGCAATGGCA
ACAGCCGTTGTCTACAATTACTGGCACAATACACCTCAGCTTGCTCTAACATTGAAGCAGCTCTGCATTCCTCTGCACA
GTTAGATAGCAGAGAGATTATAAATATGTTTCAAACATCAACACAGTCCTTGCAGTTAGCTAATATTACCAACTTCAAG
GGTGACTACAATTTTAGCAGCATACTAACCACCAGAATTGGTGGCAGATCTGCTATTGAAGACCTTCTTTTTAATAAAG
TTGTTACTAGTGGCCTTGGCACTGTTGATCAGGACTACAAATCCTGCTCTAGAGACATGGCCATCGCTGACTTAGTTTG
TTCCCAGTATTACAATGGCATCATGGTTCTACCTGGTGTTGTTGATGCTGAGAAAATGGCAATGTATACTGGCTCTCTT
ACTGGAGCTATGGTATTTGGAGGACTGACTGCCGCAGCGGCAATACCATTTGCCACGGCAGTACAAGCTCGCCTCAATT
ATGTCGCACTGCAAACAAATGTACTACAAGAAAACCAGAAAATTCTTGCAGAATCATTTAACCAAGCAGTTGGCAATAT

ATCACTTGCACTATCTTCTGTTAATGATGCCATCCAGCAAACTTCTGAGGCTCTTAACACCGTAGCTATTGCTATTAAA
AAGATTCAAACAGTTGTTAACCAGCAGGGCGAGGCATTATCACACCTGACTGCACAGCTGTCAAACAATTTTCAAGCAA
TTTCGACTTCTATTCAAGACATTTACAACCGTCTTGAGGAAGTAGAGGCTAACCAGCAAGTTGACCGTCTCATCACAGG
ACGGTTGGCTGCACTTAATGCATATGTTACTCAGCTACTCAATCAGATGTCTCAGATTAGACAATCTCGATTGTTAGCT
CAGCAAAAGATTAATGAGTGTGTCAAATCACAGTCGTCCAGATACGGTTTCTGTGGAAATGGCACACACATCTTCTCAC
TTACACAGACTGCACCAAATGGCATATTTTTCATGCATGCAGTGCTAGTACCCAACAAATTCACACGTGTCAACGCTTC
TGCCGGCATTTGTGTGGATAATACGAGAGGCTACTCATTGCAGCCTCAACTTATACTCTACCAGTTTAATAACTCCTGG
AGAGTTACACCTAGAAATATGTATGAACCCAGACTGCCCCGGCAGGCTGATTTCATACAATTAACTGATTGCAGCGTTA
CTTTTTACAACACCACCGCTGCTAATCTTCCCAATATTATCCCTGACATTATAGATGTCAACCAAACAGTCAGTGATAT
TATTGACAATTTACCTACAGCAACACCTCCTCAGTGGGATGTTGGTATCTATAACAACACTATTCTCAACCTCACCGTT
GAGATTAATGATCTACAAGAGCGGTCTAAAAACCTCTCACAGATTGCAGATCGTTTACAAAATTATATTGACAATCTTA
ACAATACTCTAGTTGACCTTGAATGGCTCAACAGAGTGGAAACTTACCTTAAATGGCCGTGGTATATATGGCTTGCCAT
TGCCCTGGCTCTTATTGCATTTGTGACAATCCTCATAACAATCTTTCTTTGTACTGGTTGTTGTGGTGGTTGCTTTGGT
TGTTGTGGCGGTTGTTTTGGCCTTTTCTCTAAGAAGAAAAGGTATACCGACGACCAACCAACACCGTCCTTTAAGTTTA
AGGAATGGTAG

>gi|597710841|gb|KJ569769.1| Porcine coronavirus HKU15 strain IN2847, complete genome

ATGCAGAGAGCTCTATTGATTATGACCTTACTTTGTCTCGTTCGAGCAAAGTTTGCTGATGATCTACTCGATTTG
CTCACCTTCCCGGGTGCACATCGCTTCTTACATAAACCCACGAGGAATTCCAGCAGTCTCTACTCGCGGGCTAATAATA
ATTTTGATGTTGGCGTTCTTCCTGGCTACCCCACTAAGAACGTTAACCTCTTCTCACCACTTACTAACTCCACTTTGCC
CATTAATGGCCTTCATCGGAGTTACCAACCACTCATGCTGAATTGTCTTACTAAAATAACTAACCACACTCTCAGCATG
TATCTCCTACCTAGTGAGATACAAACTTATAGCTGCGGCGGTGCCATGGTTAAATACCAGACACATGATGCAGTTCGTA
TCATTTTAGACCTCACTGCCACTGACCACATCTCTGTTGAAGTCGTTGGCCAACATGGTGAAAATTATGTGTTTGTTTG
CAGTGAGCAGTTTAACTACACCACTGCATTACACAACTCTACCTTCTTCTCACTTAATTCTGAGCTTTATTGCTTTACT
AATAACACCTACTTAGGTGTTCTTCCACCTGATTTAACTGACTTTACGGTCTACCGTACTGGTCAGTTCTATGCTAATG
GTTACCTTTTAGGTACTTTACCTATTACGGTTAACTATGTTAGGTTGTATCGGGGTCATTTGTCTGCCAATAGTGCCCA
CTTTGCCCTTGCAAACCTAACCGATACACTCATAACACTTACCAATACTACTATATCGCAAATCACTTATTGTGATAAG
TCAGTAGTTGATTCAATAGCATGCCAGCGCTCTTCTCACGAAGTGGAGGATGGGTTTTACTCCGACCCTAAATCTGCCG
TTAGAGCTAGGCAACGTACTATTGTTACACTACCTAAGCTCCCTGAGCTTGAAGTAGTGCAGTTAAATATTTCTGCACA
CATGGATTTTGGCGAAGCCAGACTTGACAGCGTTACCATCAATGGTAACACATCCTATTGTGTCACTAAGCCTTACTTC
AGGCTTGAAACTAACTTTATGTGTACAGGTTGCACTATGAATCTGCGCACTGATACCTGTAGTTTTGACCTGTCAGCAG
TAAACAATGGCATGTCATTCTCTCAATTCTGTCTAAGCACTGAATCTGGTGCTTGTGAGATGAAAATTATTGTTACCTA
CGTATGGAATTACTTGCTAAGGCAGCGTTTGTATGTTACTGCTGTAGAGGGCCAGACTCACACTGGAACCACTTCAGTA
CATGCAACAGACACTTCTAGTGTAATCACTGATGTCTGCACTGACTACACTATCTATGGAGTCTCTGGTACTGGCATTA
TTAAGCCATCAGATCTCTTATTGCACAATGGCATAGCATTCACCTCTCCAACAGGTGAGCTTTATGCATTTAAAAATAT
AACCACTGGCAAAACCCTTCAGGTCTTACCGTGTGAAACCCCTTCTCAACTGATTGTGATAAACAACACCGTTGTCGGT
GCTATCACATCCAGTAATTCAACTGAAAATAATAGGTTTACTACTACTATTGTCACACCTACTTTCTTTTATTCCACAA
ATGCCACCACTTTCAACTGCACTAAGCCTGTTTTGTCCTATGGACCTACCAGCGTGTGTAGTGATGGTGCAATTGTGGG
AACATCCACATTACAGAATACTCGACCATCCATAGTTTCACTATACGATGGCGAAGTTGAAATACCATCTGCATTTTCT
CTTTCCGTTCAGACGGAGTACTTGCAAGTTCAAGCAGAGCAAGTTATAGTTGATTGTCCTCAGTATGTATGCAATGGCA
ACAGCCGTTGTCTACAATTACTGGCACAATACACCTCAGCTTGCTCTAACATTGAAGCAGCTCTGCATTCCTCTGCACA

GTTGGATAGCAGAGAGATTATAAACATGTTTCGAACATCAACACAGTCCTTGCAGTTAGCTAATATTACCAACTTCAAG
GGTGACTACAATTTTAGCAGCATACTAACCACCAGAATTGGTGGCAGATCTGCTATTGAAGACCTTCTTTTTAATAAAG
TTGTTACTAGTGGCCTTGGCACTGTTGATCAGGACTACAAATCCTGCTCTAGAGACATGGCCATCGCTGACTTAGTTTG
TTCCCAGTATTACAATGGCATCATGGTTCTACCTGGTGTTGTTGATGCTGAGAAAATGGCAATGTATACTGGCTCTCTT
ACTGGAGCTATGGTATTTGGAGGACTGACTGCCGCAGCAGCAATACCATTTGCCACGGCAGTACAAGCTCGCCTCAATT
ATGTCGCACTGCAAACAAATGTACTACAAGAAAACCAGAAAATTCTTGCAGAATCATTTAACCAAGCAGTTGGCAATAT
ATCACTTGCACTATCTTCTGTTAATGATGCCATCCAGCAAACTTCTGAGGCTCTTAACACCGTAGCTATTGCTATTAAA
AAGATTCAAACAGTTGTTAACCAGCAGGGCGAGGCATTATCACACCTGACTGCACAGCTGTCAAACAATTTTCAAGCAA
TTTCGACTTCTATTCAAGACATTTACAACCGTCTTGAGGAAGTAGAGGCTAACCAGCAAGTTGACCGTCTCATCACAGG
ACGGTTGGCTGCACTTAATGCATATGTTACTCAGTTACTCAATCAGATGTCTCAGATTAGACAATCTCGATTGTTAGCT
CAGCAAAAGATTAATGAGTGTGTCAAATCACAGTCGTCCAGATACGGTTTCTGTGGAAATGGCACACACATCTTCTCAC
TTACACAGACTGCACCAAATGGCATATTTTTCATGCATGCAGTGCTAGTACCCAACAAATTCACACGTGTCAACGCTTC
TGCCGGCATTTGTGTGGATAATACGAGAGGCTACTCATTGCAGCCTCAACTTATACTCTACCAGTTTAATAACTCCTGG
AGAGTTACACCTAGAAATATGTATGAACCCAGACTGCCCCGGCAGGCTGATTTCATACAATTAACTGATTGCAGCGTTA
CTTTTTACAACACCACCGCTGCTAATCTTCCCAATATTATCCCTGACATTATAGATGTCAATCAAACAGTCAGTGATAT
TATTGACAATTTACCTACAGCAACACCTCCTCAGTGGGATGTTGGTATCTATAACAACACTATTCTCAACCTCACCGTT
GAGATTAATGATCTACAAGAGCGGTCTAAAAACCTCTCACAGATTGCAGATCGTTTACAAAATTATATTGACAATCTTA
ACAATACTCTAGTTGACCTTGAATGGCTCAACAGAGTGGAAACTTACCTTAAATGGCCGTGGTATATATGGCTTGCCAT
TGCCCTGGCTCTTATTGCATTTGTGACAATCCTCATAACAATCTTTCTTTGTACTGGTTGTTGTGGTGGTTGCTTTGGT
TGTTGTGGCGGTTGTTTTGGCCTTTTCTCTAAGAAGAAAAGGTATACCGACGACCAACCAACACCGTCCTTTAAGTTTA
AGGAATGGTAG

>gi|641452853|gb|KJ584355.1| Porcine coronavirus HKU15 strain IL2768, complete genome

ATGCAGAGAGCTCTATTGATTATGACCTTACTTTGTCTCGTTCGAGCAAAGTTTGCTGATGATCTACTCGATTTG
CTCACCTTCCCGGGTGCACATCGCTTCTTACATAAACCCACGAGGAATTCCAGCAGTCTCTACTCGCGGGCTAATAATA
ATTTTGATGTTGGCGTTCTTCCTGGCTACCCCACTAAGAACGTTAACCTCTTCTCACCACTTACTAACTCCACTTTGCC
CATTAATGGCCTTCATCGGAGTTACCAACCACTCATGCTGAATTGTCTTACTAAAATAACTAACCACACTCTCAGCATG
TATCTCCTACCTAGTGAGATACAAACTTATAGCTGCGGCGGTGCCATGGTTAAATACCAGACACATGATGCAGTTCGTA
TCATTTTAGACCTCACTGCCACTGACCACATCTCTGTTGAAGTCGTTGGCCAACATGGTGAAAATTATGTGTTTGTTTG
CAGTGAGCAGTTTAACTACACCACTGCATTACACAACTCTACCTTCTTCTCACTTAATTCTGAGCTTTATTGCTTTACT
AATAACACCTACTTAGGTATTCTTCCACCTGATTTAACTGACTTTACGGTCTACCGTACTGGTCAGTTCTATGCTAATG
GTTACCTTTTAGGTACTTTACCTATTACGGTTAACTATGTTAGGTTGTATCGGGGTCATTTGTCTGCCAATAGTGCCCA
CTTTGCCCTTGCAAACCTAACCGATACACTCATAACACTTACCAATACTACTATATCGCAAATCACTTATTGTGATAAG
TCAGTAGTTGATTCAATAGCATGCCAGCGCTCTTCTCACGAAGTGGAGGATGGGTTTTACTCCGACCCTAAATCTGCCG
TTAGAGCTAGGCAACGTACTATTGTTACACTACCTAAGCTCCCTGAGCTTGAAGTAGTGCAGTTAAATATTTCTGCACA
CATGGATTTTGGCGAAGCCAGACTTGACAGCGTTACCATCAATGGTAACACATCCTATTGTGTCACTAAGCCTTACTTC
AGGCTTGAAACTAACTTTATGTGTACAGGTTGCACTATGAATCTGCGCACTGATACCTGTAGTTTTGACCTGTCAGCAG
TAAACAATGGCATGTCATTCTCTCAATTCTGTCTAAGCACTGAATCTGGTGCTTGTGAGATGAAAATTATTGTTACCTA
CGTATGGAATTACTTGCTAAGGCAGCGTTTGTATGTTACTGCTGTAGAGGGCCAGACTCACACTGGAACCACTTCAGTA
CATGCAACAGACACTTCTAGTGTAATCACTGATGTCTGCACTGACTACACTATCTATGGAGTCTCTGGTACTGGCATTA
TTAAGCCATCAGATCTCTTATTGCACAATGGCATAGCATTCACCTCTCCAACAGGTGAGCTTTATGCATTTAAAAATAT

AACCACTGGCAAAACCCTTCAGGTCTTACCGTGTGAAACCCCTTCTCAACTGATTGTGATAAACAACACCGTTGTCGGT
GCTATCACATCCAGTAATTCAACTGAAAATAATAGGTTTACTACTACTATTGTCACACCTACTTTCTTTTATTCCACAA
ATGCCACCACTTTCAACTGCACTAAGCCTGTTTTGTCCTATGGACCTATCAGCGTGTGTAGTGATGGTGCAATTGTGGG
AACATCCACATTACAGAATACTCGACCATCCATAGTTTCACTATACGATGGCGAAGTTGAAATACCATCTGCATTTTCT
CTTTCCGTTCAGACGGAGTACTTGCAAGTTCAAGCAGAGCAAGTTATAGTTGATTGTCCTCAGTATGTATGCAATGGCA
ACAGCCGTTGTCTACAATTACTGGCACAATACACCTCAGCTTGCTCTAACATTGAAGCAGCTCTGCATTCCTCTGCACA
GTTGGATAGCAGAGAGATTATAAATATGTTTCAAACATCAACACAGTCCTTGCAGTTAGCTAATATTACCAACTTCAAG
GGTGACTACAATTTTAGCAGCATACTAACCACCAGAATTGGTGGCAGATCTGCTATTGAAGACCTTCTTTTTAATAAAG
TTGTTACTAGTGGCCTTGGCACTGTTGATCAGGACTACAAATCCTGCTCTAGAGACATGGCCATCGCTGACTTAGTTTG
TTCCCAGTATTACAATGGCATCATGGTTCTACCTGGTGTTGTTGATGCTGAGAAAATGGCAATGTATACTGGCTCTCTT
ACTGGAGCTATGGTATTTGGAGGACTGACTGCCGCAGCGGCAATACCATTTGCCACGGCAGTACAAGCTCGCCTCAATT
ATGTCGCACTGCAAACAAATGTACTACAAGAAAACCAGAAAATTCTTGCAGAATCATTTAACCAAGCAGTTGGCAATAT
ATCACTTGCACTATCTTCTGTTAATGATGCCATCCAGCAAACTTCTGAGGCTCTTAACACCGTAGCTATTGCTATTAAA
AAGATTCAAACAGTTGTTAACCAGCAGGGCGAGGCATTATCACACCTGACTGCACAGCTGTCAAACAATTTTCAAGCAA
TTTCGACTTCTATTCAAGACATTTACAATCGTCTTGAGGAAGTAGAGGCTAACCAGCAAGTTGACCGTCTCATCACAGG
ACGGTTGGCTGCACTTAATGCATATGTTACTCAGTTACTCAATCAGATGTCTCAGATTAGACAATCTCGATTGTTAGCT
CAGCAAAAGATTAATGAGTGTGTCAAATCACAGTCGTCCAGATACGGTTTCTGTGGAAATGGCACACACATCTTCTCAC
TTACACAGACTGCACCAAATGGCATATTTTTCATGCATGCAGTGCTAGTACCCAACAAATTCACACGTGTCAACGCTTC
TGCCGGCATTTGTGTGGATAATACGAGAGGCTACTCATTGCAGCCTCAACTTATACTCTACCAGTTTAATAACTCCTGG
AGAGTTACACCTAGAAATATGTATGAACCCAGACTGCCCCGGCAGGCTGATTTCATACAATTAACTGATTGCAGCGTTA
CTTTTTACAACACCACCGCTGCTAATCTTCCCAATATTATCCCTGACATTATAGATGTCAATCAAACAGTCAGTGATAT
TATTGACAATTTACCTACAGCAACACCTCCTCAGTGGGATGTTGGTATCTATAACAACACTATTCTCAACCTCACCGTT
GAGATTAATGATCTACAAGAGCGGTCTAAAAACCTCTCACAGATTGCAGATCGTTTACAAAATTATATTGACAATCTTA
ACAATACTCTAGTTGACCTTGAATGGCTCAACAGAGTGGAAACTTATCTTAAATGGCCGTGGTATATATGGCTTGCCAT
TGCCCTGGCTCTTATTGCATTTGTGACAATCCTCATAACAATCTTTCTTTGTACTGGTTGTTGTGGTGGTTGCTTTGGT
TGTTGTGGCGGTTGTTTTGGCCTTTTCTCTAAGAAGAAAAGGTATACCGACGACCAACCAACACCGTCCTTTAAGTTTA
AGGAATGGTAG

>gi|641452861|gb|KJ584356.1| Porcine coronavirus HKU15 strain SD3424, complete genome

ATGCAGAGAGCTCTATTGATTATGACCTTACTTTGTCTCGTTCGAGCAAAGTTTGCTGATGATCTACTCGATTTG
CTCACCTTCCCGGGTGCACATCGCTTCTTACATAAACCCACGAGGAATTCCAGCAGTCTCTACTCGCGGGCTAATAATA
ATTTTGATGTTGGCGTTCTTCCTGGCTACCCCACTAAGAACGTTAACCTCTTCTCACCACTTACTAACTCCACTTTGCC
CATTAATGGCCTTCATCGGAGTTACCAACCACTCATGCTGAATTGTCTTACTAAAATAACTAACCACACTCTCAGCATG
TATCTCCTACCTAGTGAGATACAAACTTATAGCTGCGGCGGTGCCATGGTTAAATACCAGACACATGATGCAGTTCGTA
TCATTTTAGACCTCACTGCCACTGACCACATCTCTGTTGAAGTCGTTGGCCAACATGGTGAAAATTATGTGTTTGTTTG
CAGTGAGCAGTTTAACTACACCACTGCATTACACAACTCTACCTTCTTCTCACTTAATTCTGAGCTTTATTGCTTTACT
AATAACACCTACTTAGGTATTCTTCCACCTGATTTAACTGACTTTACTGTCTACCGTACTGGTCAGTTCTATGCTAATG
GTTACCTTTTAGGTACTTTACCTATTACGGTTAACTATGTTAGGTTGTATCGGGGTCATTTGTCTGCCAATAGTGCCCA
CTTTGCCCTTGCAAACCTAACCGATACACTCATAACACTTACCAATACTACTATATCGCAAATCACTTATTGTGATAAG
TCAGTAGTTGATTCAATAGCATGCCAGCGCTCTTCTCACGAAGTGGAGGATGGGTTTTACTCCGACCCTAAATCTGCCG
TTAGAGCTAGGCAACGTACTATTGTTACACTACCTAAGCTCCCTGAGCTTGAAGTAGTGCAGTTAAATATTTCTGCACA

CATGGATTTTGGCGAAGCCAGACTTGACAGCGTTACCATCAATGGTAACACATCCTATTGTGTCACTAAGCCTTACTTC
AGGCTTGAAACTAACTTTATGTGTACAGGTTGCACTATGAATCTGCGCACTGATACCTGTAGTTTTGACCTGTCAGCAG
TAAACAATGGCATGTCATTCTCTCAATTCTGTCTAAGCACTGAATCTGGTGCTTGTGAGATGAAAATTATTGTTACCTA
CGTATGGAATTACTTGCTAAGGCAGCGTTTGTATGTTACTGCTGTAGAGGGCCAGACTCACACTGGAACCACTTCAGTA
CATGCAACAGACACTTCTAGTGTAATCACTGATGTCTGCACTGACTACACTATCTATGGAGTCTCTGGTACTGGCATTA
TTAAGCCATCAGATCTCTTATTGCACAATGGCATAGCATTCACCTCTCCAACAGGTGAGCTTTATGCATTTAAAAATAT
AACCACTGGCAAAACCCTTCAGGTCTTACCGTGTGAAACCCCTTCTCAACTGATTGTGATAAACAACACCGTTGTCGGT
GCTATCACATCCAGTAATTCAACTGAAAATAATAGGTTTACTACTACTATTGTCACACCTACTTTCTTTTATTCCACAA
ATGACACCACTTTCAACTGCACTAAGCCTGTTTTGTCCTATGGACCTATCAGCGTGTGTAGTGATGGTGCAATTGTGGG
AACATCCACATTACAGAATACTCGACCATCCATAGTTTCACTATACGATGGCGAAGTTGAAATACCATCTGCATTTTCT
CTTTCCGTTCAGACGGAGTACTTGCAAGTTCAAGCAGAGCAAGTTATAGTTGATTGTCCTCAGTATGTATGCAATGGCA
ACAGCCGTTGTCTACAATTACTGGCACAATACACCTCAGCTTGCTCTAACATTGAAGCAGCTCTGCATTCCTCTGCACA
GTTGGATAGCAGAGAGATTATAAATATGTTTAAAACATCAACACAGTCCTTGCAGTTAGCTAATATTACCAACTTCAAG
GGTGACTACAATTTTAGCAGCATACTAACCACCAGAATTGGTGGCAGATCTGCTATTGAAGACCTTCTTTTTAATAAAG
TTGTTACTAGTGGCCTTGGCACTGTTGATCAGGACTACAAATCCTGCTCTAGAGACATGGCCATCGCTGACTTAGTTTG
TTCCCAGTATTACAATGGCATCATGGTTCTACCTGGTGTTGTTGATGCTGAGAAAATGGCAATGTATACTGGCTCTCTT
ACTGGAGCTATGGTATTTGGAGGACTGACTGCCGCAGCGGCAATACCATTTGCCACGGCAGTACAAGCTCGCCTCAATT
ATGTCGCACTGCAAACAAATGTACTACAAGAAAACCAGAAAATTCTTGCAGAATCATTTAACCAAGCAGTTGGCAATAT
ATCACTTGCACTATCTTCTGTTAATGATGCCATCCAGCAAACTTCTGAGGCTCTTAACACCGTAGCTATTGCTATTAAA
AAGATTCAAACAGTTGTTAACCAGCAGGGCGAGGCATTATCACACCTGACTGCACAGCTGTCAAACAATTTTCAAGCAA
TTTCGACTTCTATTCAAGACATTTACAACCGTCTTGAGGAAGTAGAGGCTAACCAGCAAGTTGACCGTCTCATCACAGG
ACGGTTGGCTGCACTTAATGCATATGTTACTCAGTTACTCAATCAGATGTCTCAGATTAGACAATCTCGATTGTTAGCT
CAGCAAAAGATTAATGAGTGTGTCAAATCACAGTCGTCCAGATACGGTTTCTGTGGAAATGGCACACACATCTTCTCAC
TTACACAGACTGCACCAAATGGCATATTTTTCATGCATGCAGTGCTAGTACCCAACAAATTCACACGTGTCAACGCTTC
TGCCGGCATTTGTGTGGATAATACGAGAGGCTACTCATTGCAGCCTCAACTTATACTCTACCAGTTTAATAACTCCTGG
AGAGTTACACCTAGAAATATGTATGAACCCAGACTGCCCCGGCAGGCTGATTTCATACAATTAACTGATTGCAGCGTTA
CTTTTTACAACACCACCGCTGCTAATCTTCCCAATATTATCCCTGACATTATAGATGTCAACCAAACAGTCAGTGATAT
TATTGACAATTTACCTACAGCAACACCTCCTCAGTGGGATGTTGGTATCTATAACAACACTATTCTCAACCTCACCGTT
GAGATTAATGATCTACAAGAGCGGTCTAAAAACCTCTCACAGATTGCAGATCGTTTACAAAATTATATTGACAATCTTA
ACAATACTCTAGTTGACCTTGAATGGCTCAACAGAGTGGAAACTTACCTTAAATGGCCGTGGTATATATGGCTTGCCAT
TGCCCTGGCTCTTATTGCATTTGTGACAATCCTCATAACAATCTTTCTTTGTACTGGTTGTTGTGGTGGTTGCTTTGGT
TGTTGTGGCGGTTGTTTTGGCCTTTTCTCTAAGAAGAAAAGGTATACCGACGACCAACCAACACCGTCCTTTAAGTTTA
AGGAATGGTAG

>gi|641452869|gb|KJ584357.1| Porcine coronavirus HKU15 strain KY4813, complete genome

ATGCAGAGAGCTCTATTGATTATGACCTTACTTTGTCTCGTTCGAGCAAAGTTTGCTGATGATCTACTCGATTTG
CTCACCTTCCCGGGTGCACATCGCTTCTTACATAAACCCACGAGGAATTCCAGCAGTCTCTACTCGCGGGCTAATAATA
ATTTTGATGTTGGCGTTCTTCCTGGCTACCCCACTAAGAACGTTAACCTCTTCTCACCACTTACTAACTCCACTTTGCC
CATTAATGGCCTTCATCGGAGTTACCAACCACTCATGCTGAATTGTCTTACTAAAATAACTAACCACACTCTCAGCATG
TATCTCCTACCTAGTGCGATACAAACTTATAGCTGCGGCGGTGCCATGGTTAAATACCAGACACATGATGCAGTTCGTA
TCATTTTAGACCTCACTGCCACTGACCACATCTCTGTTGAAGTCGTTGGCCAACATGGTGAAAATTATGTGTTTGTTTG

CAGTGAGCAGTTTAACTACACCACTGCATTACACAACTCTACCTTCTTCTCACTTAATTCTGAGCTTTATTGCTTTACT
AATAACACCTACTTAGGTATTCTTCCACCTGATTTAACTGACTTTACGGTCTACCGTACTGGTCAGTTCTATGCTAATG
GTTACCTTTTAGGTACTTTACCTATTACGGTTAACTATGTTAGGTTGTATCGGGGTCATTTGTCTGCCAATAGTGCCCA
CTTTGCCCTTGCAAACCTAACCGATACACTCATAACACTTACCAATACTACTATATCGCAAATCACTTATTGTGATAAG
TCAGTAGTTGATTCAATAGCATGCCAGCGCTCTTCTCACGAAGTGGAGGATGGGTTTTACTCCGACCCTAAATCTGCCG
TTAGAGCTAGGCAACGTACTATTGTTACACTACCTAAGCTCCCTGAGCTTGAAGTAGTGCAGTTAAATATTTCTGCACA
CATGGATTTTGGCGAAGCCAGACTTGACAGCGTTACCATCAATGGTAACACATCCTATTGTGTCACTAAGCCTTACTTC
AGGCTTGAAACTAACTTTATGTGTACAGGTTGCACTATGAATCTGCGCACTGATACCTGTAGTTTTGACCTGTCAGCAG
TAAACAATGGCATGTCATTCTCTCAATTCTGTCTAAGCACTGAATCTGGTGCTTGTGAGATGAAAATTATTGTTACCTA
CGTATGGAATTACTTGCTAAGGCAGCGTTTGTATGTTACTGCTGTAGAGGGCCAGACTCACACTGGAACCACTTCAGTA
CATGCAACAGACACTTCTAGTGTAATCACTGATGTCTGCACTGACTACACTATCTATGGAGTCTCTGGTACTGGCATTA
TTAAGCCATCAGATCTCTTATTGCACAATGGCATAGCATTCACCTCTCCAACAGGTGAGCTTTATGCATTTAAAAATAT
AACCACTGGCAAAACCCTTCAGGTCTTACCGTGTGAAACCCCTTCTCAACTGATTGTGATAAACAACACCGTTGTCGGT
GCTATCACATCCAGTAATTCAACTGAAAATAATAGGTTTACTACTACTATTGTCACACCTACTTTCTTTTATTCCACAA
ATGCCACCACTTTCAACTGCACTAAGCCTGTTTTGTCCTATGGACCTATCAGCGTGTGTAGTGATGGTGCAATTGTGGG
AACATCCACATTACAGAATACTCGACCATCCATAGTTTCACTATACGATGGCGAAGTTGAAATACCATCTGCATTTTCT
CTTTCCGTTCAGACGGAGTACTTGCAAGTTCAAGCAGAGCAAGTTATAGTTGATTGTCCTCAGTATGTATGCAATGGCA
ACAGCCGTTGTCTACAATTACTGGCACAATACACCTCAGCTTGCTCTAACATTGAAGCAGCTCTGCATTCCTCTGCACA
GTTGGATAGCAGAGAGATTATAAATATGTTTCAAACATCAACACAGTCCTTGCAGTTAGCTAATATTACCAACTTCAAG
GGTGACTACAATTTTAGCAGCATACTAACCACCAGAATTGGTGGCAGATCTGCTATTGAAGACCTTCTTTTTAATAAAG
TTGTTACTAGTGGCCTTGGCACTGTTGATCAGGACTACAAATCCTGCTCTAGAGACATGGCCATCGCTGACTTAGTTTG
TTCCCAGTATTACAATGGCATCATGGTTCTACCTGGTGTTGTTGATGCTGAGAAAATGGCAATGTATACTGGCTCTCTT
ACTGGAGCTATGGTATTTGGAGGACTGACTGCCGCAGCGGCAATACCATTTGCCACGGCAGTACAAGCTCGCCTCAATT
ATGTCGCACTGCAAACAAATGTACTACAAGAAAACCAGAAAATTCTTGCAGAATCATTTAACCAAGCAGTTGGCAATAT
ATCACTTGCACTATCTTCTGTTAATGATGCCATCCAGCAAACTTCTGAGGCTCTTAACACCGTAGCTATTGCTATTAAA
AAGATTCAAACAGTTGTTAACCAGCAGGGCGAGGCATTATCACACCTGACTGCACAGCTGTCAAACAATTTTCAAGCAA
TTTCGACTTCTATTCAAGACATTTACAACCGTCTTGAGGAAGTAGAGGCTAACCAGCAAGTTGACCGTCTCATCACAGG
ACGGTTGGCTGCACTTAATGCATATGTTACTCAGTTACTCAATCAGATGTCTCAGATTAGACAATCTCGATTGTTAGCT
CAGCAAAAGATTAATGAGTGTGTCAAATCACAGTCGTCCAGATACGGTTTCTGTGGAAATGGCACACACATCTTCTCAC
TTACACAGACTGCACCAAATGGCATATTTTTCATGCATGCAGTGCTAGTACCCAACAAATTCACACGTGTCAACGCTTC
TGCCGGCATTTGTGTGGATAATACGAGAGGCTACTCATTGCAGCCTCAACTTATACTCTACCAGTTTAATAACTCCTGG
AGAGTTACACCTAGAAATATGTATGAACCCAGACTGCCCCGGCAGGCTGATTTCATACAATTAACTGATTGCAGCGTTA
CTTTTTACAACACCACCGCTGCTAATCTTCCCAATATTATCCCTGACATTATAGATGTCAATCAAACAGTCAGTGATAT
TATTGACAATTTACCTACAGCAACACCTCCTCAGTGGGATGTTGGTATCTATAACAACACTATTCTCAACCTCACCGTT
GAGATTAATGATCTACAAGAGCGGTCTAAAAACCTCTCACAGATTGCAGATCGTTTACAAAATTATATTGACAATCTTA
ACAATACTCTAGTTGAACTTGAATGGCTCAACAGAGTGGAAACTTACCTTAAATGGCCGTGGTATATATGGCTTGCCAT
TGCCCTGGCTCTTATTGCATTTGTGACAATCCTCATAACAATCTTTCTTTGTACTGGTTGTTGTGGTGGTTGCTTTGGT
TGTTGTGGCGGTTGTTTTGGCCTTTTCTCTAAGAAGAAAAGGTATACCGACGACCAACCAACACCGTCCTTTAAGTTTA
AGGAATGGTAG

>gi|641452877|gb|KJ584358.1| Porcine coronavirus HKU15 strain PA3148, complete genome

ATGCAGAGAGCTCTATTGATTATGACCTTACTTTGTCTCGTTCGAGCAAAGTTTGCTGATGATCTACTCGATTTG
CTCACCTTCCCGGGTGCACATCGCTTCTTACATAAACCCACGAGGAATTCCAGCAGTCTCTACTCGCGGGCTAATAATA
ATTTTGATGTTGGCGTTCTTCCTGGCTACCCCACTAAGAACGTTAACCTCTTCTCACCACTTACTAACTCCACTTTGCC
CATTAATGGCCTTCATCGGAGTTACCAACCACTCATGCTGAATTGTCTTACTAAAATAACTAACCACACTCTCAGCATG
TATCTCCTACCTAGTGAGATACAAACTTATAGCTGCGGCGGTGCCATGGTTAAATACCAGACACATGATGCAGTTCGTA
TCATTTTAGACCTCACTGCCACTGACCACATCTCTGTTGAAGTCGTTGGCCAACATGGTGAAAATTATGTGTTTGTTTG
CAGTGAGCAGTTTAACTACACCACTGCATTACACAACTCTACCTTCTTCTCACTTAATTCTGAGCTTTATTGCTTTACT
AATAACACCTACTTAGGTATTCTTCCACCTGATTTAACTGACTTTACGGTCTACCGTACTGGTCAGTTCTATGCTAATG
GTTACCTTTTAGGTACTTTACCTATTACGGTTAACTATGTTAGGTTGTATCGGGGTCATTTGTCTGCCAATAGTGCCCA
CTTTGCCCTTGCAAACCTAACCGATACACTCATAACACTTACCAATACTACTATATCGCAAATCACTTATTGTGATAAG
TCAGTAGTTGATTCAATAGCATGCCAGCGCTCTTCTCACGAAGTGGAGGATGGGTTTACTCCGACCCTAAATCTGCCG
TTAGAGCTAGGCAACGTACTATTGTTACACTACCTAAGCTCCCTGAGCTTGAAGTAGTGCAGTTAAATATTTCTGCACA
CATGGATTTTGGCGAAGCCAGACTTGACAGCGTTACCATCAATGGTAACACATCCTATTGTGTCACTAAGCCTTACTTC
AGGCTTGAAACTAACTTTATGTGTACAGGTTGCACTATGAATCTGCGCACTGATACCTGTAGTTTTGACCTGTCAGCAG
TAAACAATGGCATGTCATTCTCTCAATTCTGTCTAAGCACTGAATCTGGTGCTTGTGAGATGAAAATTATTGTTACCTA
CGTATGGAATTACTTGCTAAGGCAGCGTTTGTATGTTACTGCTGTAGAGGGCCAGACTCACACTGGAACCACTTCAGTA
CATGCAACAGACACTTCTAGTGTAATCACTGATGTCTGCACTGACTACACTATCTATGGAGTCTCTGGTACTGGCATTA
TTAAGCCATCAGATCTCTTATTGCACAATGGCATAGCATTCACCTCTCCAACAGGTGAGCTTTATGCATTTAAAAATAT
AACCACTGGCAAAACCCTTCAGGTCTTACCGTGTGAAACCCCTTCTCAACTGATTGTGATAAACAACACCGTTGTCGGT
GCTATCACATCCAGTAATTCAACTGAAAATAATAGGTTTACTACTACTATTGTCACACCTACTTTCTTTTATTCCACAA
ATGCCACCACTTTCAACTGCACTAAGCCTGTTTTGTCCTATGGACCTATCAGCGTGTGTAGTGATGGTGCAATTGTGGG
AACATCCACATTACAGAATACTCGACCATCCATAGTTTCACTATACGATGGCGAAGTTGAAATACCATCTGCATTTTCT
CTTTCCGTTCAGACGGAGTACTTGCAAGTTCAAGCAGAGCAAGTTATAGTTGATTGTCCTCAGTATGTATGCAATGGCA
ACAGCCGTTGTCTACAATTACTGGCACAATACACCTCAGCTTGCTCTAACATTGAAGCAGCTCTGCATTCCTCTGCACA
GTTGGATAGCAGAGAGATTATAAATATGTTTCAAACATCAACACAGTCCTTGCAGTTAGCTAATATTACCAACTTCAAG
GGTGACTACAATTTTAGCAGCATACTAACCACCAGAATTGGTGGCAGATCTGCTATTGAAGACCTTCTTTTTAATAAAG
TTGTTACTAGTGGCCTTGGCACTGTTGATCAGGACTACAAATCCTGCTCTAGAGACATGGCCATCGCTGACTTAGTTTG
TTCCCAGTATTACAATGGCATCATGGTTCTACCTGGTGTTGTTGATGCTGAGAAAATGGCAATGTATACTGGCTCTCTT
ACTGGAGCTATGGTATTTGGAGGACTGACTGCCGCAGCGGCAATACCATTTGCCACGGCAGTACAAGCTCGCCTCAATT
ATGTCGCACTGCAAACAAATGTACTACAAGAAAACCAGAAAATTCTTGCAGAATCATTTAACCAAGCAGTTGGCAATAT
ATCACTTGCACTATCTTCTGTTAATGATGCCATCCAGCAAACTTCTGAGGCTCTTAACACCGTAGCTATTGCTATTAAA
AAGATTCAAACAGTTGTTAACCAGCAGGGCGAGGCATTATCACACCTGACTGCACAGCTGTCAAACAATTTTCAAGCAA
TTTCGACTTCTATTCAAGACATTTACAACCGTCTTGAGGAAGTAGAGGCTAACCAGCAAGTTGACCGTCTCATCACAGG
ACGGTTGGCTGCACTTAATGCATATGTTACTCAGTTACTCAATCAGATGTCTCAGATTAGACAATCTCGATTGTTAGCT
CAGCAAAAGATTAATGAGTGTGTCAAATCACAGTCGTCCAGATACGGTTTCTGTGGAAATGGCACACACATCTTCTCAC
TTACACAGACTGCACCAAATGGCATATTTTTCATGCATGCAGTGCTAGTACCCAACAAATTCACACGTGTCAACGCTTC
TGCCGGCATTTGTGTGGATAATACGAGAGGCTACTCATTGCAGCCTCAACTTATACTCTACCAGTTTAATAACTCCTGG
AGAGTTACACCTAGAAATATGTATGAACCCAGACTGCCCCGGCAGGCTGATTTCATACAATTAACTGATTGCAGCGTTA
CTTTTTACAACACCACCGCTGCTAATCTTCCCAATATTATCCCTGACATTATAGATGTTAATCAAACAGTCAGTGATAT
TATTGACAATTTACCTACAGCAACACCTCCTCAGTGGGATGTTGGTATCTATAACAACACTATTCTCAACCTCACCGTT
GAGATTAATGATCTACAAGAGCGGTCTAAAAACCTCTCACAGATTGCAGATCGTTTACAAAATTATATTGACAATCTTA
ACAATACTCTAGTTGACCTTGAATGGCTCAACAGAGTGGAAACTTACCTTAAATGGCCGTGGTATATATGGCTTGCCAT
TGCCCTGGCTCTTATTGCATTTGTGACAATCCTCATAACAATCTTTCTTTGCACTGGTTGTTGTGGTGGTTGCTTTGGT
TGTTGTGGCGGTTGTTTTGGCCTTTTCTCTAAGAAGAAAAGGTATACCGACGACCAACCAACACCGTCCTTTAAGTTTA

AGGAATGGTAG

>gi|641452885|gb|KJ584359.1| Porcine coronavirus HKU15 strain NE3579, complete genome

ATGCAGAGAGCTCTATTGATTATGACCTTACTTTGTCTCGTTCGAGCAAAGTTTGCTGATGATCTACTCGATTTG
CTCACCTTCCCGGGTGCACATCGCTTCTTACATAAACCCACGAGGAATTCCAGCAGTCTCTACTCGCGGGCTAATAATA
ATTTTGATGTTGGCGTTCTTCCTGGCTACCCCACTAAGAACGTTAACCTCTTCTCACCACTTACTAACTCCACTTTGCC
CATTAATGGCCTTCATCGGAGTTACCAACCACTCATGCTGAATTGTCTTACTAAAATAACTAACCACACTCTCAGCATG
TATCTCCTACCTAGTGAGATACAAACTTATAGCTGCGGCGGTGCCATGGTTAAATACCAGACACATGATGCAGTTCGTA
TCATTTTAGACCTCACTGCCACTGACCACATCTCTGTTGAAGTCGTTGGCCAACATGGTGAAAATTATGTGTTTGTTTG
CAGTGAGCAGTTTAACTACACCACTGCATTACACAACTCTACCTTCTTCTCACTTAATTCTGAGCTTTATTGCTTTACT
AATAACACCTACTTAGGTATTCTTCCACCTGATTTAACTGACTTTACGGTCTACCGTACTGGTCAGTTCTATGCTAATG
GTTACCTTTTAGGTACTTTACCTATTACGGTTAACTATGTTAGGTTGTATCGGGGTCATTTGTCTGCCAATAGTGCCCA
CTTTGCCCTTGCAAACCTAACCGATACACTCATAACACTTACCAATACTACTATATCGCAAATCACTTATTGTGATAAG
TCAGTAGTTGATTCAATAGCATGCCAGCGCTCTTCTCACGAAGTGGAGGATGGGTTTTACTCCGACCCTAAATCTGCCG
TTAGAGCTAGGCAACGTACTATTGTTACACTACCTAAGCTCCCTGAGCTTGAAGTAGTGCAGTTAAATATTTCTGCACA
CATGGATTTTGGCGAAGCCAGACTTGACAGCGTTACCATCAATGGTAACACATCCTATTGTGTCACTAAGCCTTACTTC
AGGCTTGAAACTAACTTTATGTGTACAGGTTGCACTATGAATCTGCGCACCGATACCTGTAGTTTTGACCTGTCAGCAG
TAAACAATGGCATGTCATTCTCTCAATTCTGTCTAAGCACTGAATCTGGTGCTTGTGAGATGAAAATTATTGTTACCTA
CGTATGGAATTACTTGCTAAGGCAGCGTTTGTATGTTACTGCTGTAGAGGGCCAGACTCACACTGGAACCACTTCAGTA
CATGCAACAGACACTTCTAGTGTAATCACTGATGTCTGCACTGACTACACTATCTATGGAGTCTCTGGTACTGGCATTA
TTAAGCCATCAGATCTCTTATTGCACAATGGCATAGCATTCACCTCTCCAACAGGTGAGCTTTATGCATTTAAAAATAT
AACCACTGGCAAAACCCTTCAGGTCTTACCGTGTGAAACCCCTTCTCAACTGATTGTGATAAACAACACCGTTGTCGGT
GCTATCACATCCAGTAATTCAACTGAAAATAATAGGTTTACTACTACTATTGTCACACCTACTTTCTTTTATTCCACAA
ATGCCACCACTTTCAACTGCACTAAGCCTGTTTTGTCCTATGGACCTATCAGCGTGTGTAGTGATGGTGCAATTGTGGG
AACATCCACATTACAGAATACTCGACCATCCATAGTTTCACTATACGATGGCGAAGTTGAAATACCATCTGCATTTTCT
CTTTCCGTTCAGACGGAGTACTTGCAAGTTCAAGCAGAGCAAGTTATAGTTGATTGTCCTCAGTATGTATGCAATGGCA
ACAGCCGTTGTCTACAATTACTGGCACAATACACCTCAGCTTGCTCTAACATTGAAGCAGCTCTGCATTCCTCTGCACA
GTTGGATAGCAGAGAGATTATAAATATGTTTCAAACATCAACACAGTCCTTGCAGTTAGCTAATATTACCAACTTCAAG
GGTGACTACAATTTTAGCAGCATACTAACCACCAGAATTGGTGGCAGATCTGCTATTGAAGACCTTCTTTTTAATAAAG
TTGTTACTAGTGGCCTTGGCACTGTTGATCAGGACTACAAATCCTGCTCTAGAGACATGGCCATCGCTGACTTAGTTTG
TTCCCAGTATTACAATGGCATCATGGTTCTACCTGGTGTTGTTGATGCTGAGAAAATGGCAATGTATACTGGCTCTCTT
ACTGGAGCTATGGTATTTGGAGGACTGACTGCCGCAGCGGCAATACCATTTGCCACGGCAGTACAAGCTCGCCTCAATT
ATGTCGCACTGCAAACAAATGTACTACAAGAAAACCAGAAAATTCTTGCAGAATCATTTAACCAAGCAGTTGGCAATAT
ATCACTTGCACTATCTTCTGTTAATGATGCCATCCAGCAAACTTCTGAGGCTCTTAACACCGTAGCTATTGCTATTAAA
AAGATTCAAACAGTTGTTAACCAGCAGGGCGAGGCATTATCACACCTGACTGCACAGCTGTCAAACAATTTTCAAGCAA
TTTCGACTTCTATTCAAGACATTTACAATCGTCTTGAGGAAGTACAGGCTAACCAGCAAGTTGACCGTCTCATCACAGG
ACGGTTGGCTGCACTTAATGCATATGTTACTCAGTTACTCAATCAGATGTCTCAGATTAGACAATCTCGATTGTTAGCT
CAGCAAAAGATTAATGAGTGTGTCAAATCACAGTCGTCCAGATACGGTTTCTGTGGAAATGGCACACACATCTTCTCAC
TTACACAGACTGCACCAAATGGCATATTTTTCATGCATGCAGTGCTAGTACCCAACAAATTCACACGTGTCAACGCTTC
TGCCGGCATTTGTGTGGATAATACGAGAGGCTACTCATTGCAGCCTCAACTTATACTCTACCAGTTTAATAACTCCTGG
AGAGTTACACCTAGAAATATGTATGAACCCAGACTGCCCCGGCAGGCTGATTTCATACAATTAACTGATTGCAGCGTTA

CTTTTTACAACACCACCGCTGCTAATCTTCCCAATATTATCCCTGACATTATAGATGTCAATCAAACAGTCAGTGATAT
TATTGACAATTTACCTACAGCAACACCTCCTCAGTGGGATGTTGGTATCTATAACAACACTATTCTCAACCTCACCGTT
GAGATTAATGATCTACAAGAGCGGTCTAAAAACCTCTCACAGATTGCAGATCGTTTACAAAATTATATTGACAATCTTA
ACAATACTCTAGTTGACCTTGAATGGCTCAACAGAGTGGAAACTTATCTTAAATGGCCGTGGTATATATGGCTTGCCAT
TGCCCTGGCTCTTATTGCATTTGTGACAATCCTCATAACAATCTTTCTTTGTACTGGTTGTTGTGGTGGTTGCTTTGGT
TGTTGTGGCGGTTGTTTTGGCCTTTTCTCTAAGAAGAAAAGGTATACCGACGACCAACCAACACCGTCCTTTAAGTTTA
AGGAATGGTAG

>gi|643431439|gb|KJ620016.1| Porcine coronavirus HKU15 strain MI6148, complete genome

ATGCAGAGAGCTCTATTGATTATGACCTTACTTTGTCTCGTTCGAGCAAAGTTTGCTGATGATCTACTCGATTTG
CTCACCTTCCCGGGTGCACATCGCTTCTTACATAAACCCACGAGGAATTCCAGCAGTCTCTACTCGCGGGCTAATAATA
ATTTTGATGTTGGCGTTCTTCCTGGCTACCCCACTAAGAACGTTAACCTCTTCTCACCACTTACTAACTCCACTTTGCC
CATTAATGGCCTTCATCGGAGTTACCAACCACTCATGCTGAATTGTCTTACTAAAATAACTAACCACACTCTCAGCATG
TATCTCCTACCTAGTGAGATACAAACTTATAGCTGCGGCGGTGCCATGGTTAAATACCAGACACATGATGCAGTTCGTA
TCATTTTAGACCTCACTGCCACTGACCACATCTCTGTTGAAGTCGTTGGCCAACATGGTGAAAATTATGTGTTTGTTTG
CAGTGAGCAGTTTAACTACACCACTGCATTACACAACTCTACCTTCTTCTCACTTAATTCTGAGCTTTATTGCTTTACT
AATAACACCTACTTAGGTATTCTTCCACCTGATTTAACTGACTTTACGGTCTACCGTACTGGTCAGTTCTATGCTAATG
GTTACCTTTTAGGTACTTTACCTATTACGGTTAACTATGTTAGGTTGTATCGGGGTCATTTGTCTGCCAATAGTGCCCA
CTTTGCCCTTGCAAACCTAACCGATACACTCATAACACTTACCAATACTACTATATCGCAAATCACTTATTGTGATAAG
TCAGTAGTTGATTCAATAGCATGCCAGCGCTCTTCTCACGAAGTGGAGGATGGGTTTTACTCCGACCCTAAATCTGCCG
TTAGAGCTAGGCAACGTACTATTGTTACACTACCTAAGCTCCCTGAGCTTGAAGTAGTGCAGTTAAATATTTCTGCACA
CATGGATTTTGGCGAAGCCAGACTTGACAGCGTTACCATCAATGGTAACACATCCTATTGTGTCACTAAGCCTTACTTC
AGGCTTGAAACTAACTTTATGTGTACAGGTTGCACTATGAATCTGCGCACTGATACCTGTAGTTTTGACCTGTCAGCAG
TAAACAATGGCATGTCATTCTCTCAATTCTGTCTAAGCACTGAATCTGGTGCTTGTGAGATGAAAATTATTGTTACCTA
CGTATGGAATTACTTGCTAAGGCAGCGTTTGTATGTTACTGCTGTAGAGGGCCAGACTCACACTGGAACCACTTCAGTA
CATGCAACAGACACTTCTAGTGTAATCACTGATGTCTGCACTGACTACACTATCTATGGAGTCTCTGGTACTGGCATTA
TTAAGCCATCAGATCTCTTATTGCACAATGGCATAGCATTCACCTCTCCAACAGGTGAGCTTTATGCATTTAAAAATAT
AACCACTGGCAAAACCCTTCAGGTCTTACCGTGTGAAACCCCTTCTCAACTGATTGTGATAAACAACACCGTTGTCGGT
GCTATCACATCCAGTAATTCAACTGAAAATAATAGGTTTACTACTACTATTGTCACACCTACTTTCTTTTATTCCACAA
ATGCCACCACTTTCAACTGCACTAAGCCTGTTTTGTCCTATGGACCTATCAGCGTGTGTAGTGATGGTGCAATTGTGGG
AACATCCACATTACAGAATACTCGACCATCCATAGTTTCACTATACGATGGCGAAGTTGAAATACCATCTGCATTTTCT
CTTTCCGTTCAGACGGAGTACTTGCAAGTTCAAGCAGAGCAAGTTATAGTTGATTGTCCTCAGTATGTATGCAATGGCA
ACAGCCGTTGTCTACAATTACTGGCACAATACACCTCAGCTTGCTCTAACATTGAAGCAGCTCTGCATTCCTCTGCACA
GTTAGATAGCAGAGAGATTATAAATATGTTTCAAACATCAACACAGTCCTTGCAGTTAGCTAATATTACCAACTTCAAG
GGTGACTACAATTTTAGCAGCATACTAACCACCAGAATTGGTGGCAGATCTGCTATTGAAGACCTTCTTTTTAATAAAG
TTGTTACTAGTGGCCTTGGCACTGTTGATCAGGACTACAAATCCTGCTCTAGAGACATGGCCATCGCTGACTTAGTTTG
TTCCCAGTATTACAATGGCATCATGGTTCTACCTGGTGTTGTTGATGCTGAGAAAATGGCAATGTATACTGGCTCTCTT
ACTGGAGCTATGGTATTTGGAGGACTGACTGCCGCAGCGGCAATACCATTTGCCACGGCAGTACAAGCTCGCCTCAATT
ATGTCGCACTGCAAACAAATGTACTACAAGAAAACCAGAAAATTCTTGCAGAATCATTTAACCAAGCAGTTGGCAATAT
ATCACTTGCACTATCTTCTGTTAATGATGCCATCCAGCAAACTTCTGAGGCTCTTAACACCGTAGCTATTGCTATTAAA
AAGATTCAAACAGTTGTTAACCAGCAGGGCGAGGCATTATCACACCTGACTGCACAGCTGTCAAACAATTTTCAAGCAA

TTTCGACTTCTATTCAAGACATTTACAACCGTCTTGAGGAAGTAGAGGCTAACCAGCAAGTTGACCGTCTCATCACAGG
ACGGTTGGCTGCACTTAATGCATATGTTACTCAGTTACTCAATCAGATGTCTCAGATTAGACAATCTCGATTGTTAGCT
CAGCAAAAGATTAATGAGTGTGTCAAATCACAGTCGTCCAGATACGGTTTCTGTGGAAATGGCACACACATCTTCTCAC
TTACACAGACTGCACCAAATGGCATATTTTTCATGCATGCAGTGCTAGTACCCAACAAATTCACACGTGTCAACGCTTC
TGCCGGCATTTGTGTGGATAATACGAGAGGCTACTCATTGCAGCCTCAACTTATACTCTACCAGTTTAATAACTCCTGG
AGAGTTACACCTAGAAATATGTATGAACCCAGACTGCCCCGGCAGGCTGATTTCATACAATTAACTGATTGCAGCGTTA
CTTTTTACAACACCACCGCTGCTAATCTTCCCAATATTATCCCTGACATTATAGATGTTAATCAAACAGTCAGTGATAT
TATTGACAATTTACCTACAGCAACACCTCCTCAGTGGGATGTTGGTATCTATAACAACACTATTCTCAACCTCACCGTT
GAGATTAATGATCTACAAGAGCGGTCTAAAAACCTCTCACAGATTGCAGATCGTTTACAAAATTATATTGACAATCTTA
ACAATACTCTAGTTGACCTTGAATGGCTCAACAGAGTGGAAACTTACCTTAAATGGCCGTGGTATATATGGCTTGCCAT
TGCCCTGGCTCTTATTGCATTTGTGACAATCCTCATAACAATCTTTCTTTGTACTGGTTGTTGTGGTGGTTGCTTTGGT
TGTTGTGGCGGTTGTTTTGGCCTTTTCTCTAAGAAGAAAAGGTATACCGACGACCAACCAACACCGTCCTTTAAGTTTA
AGGAATGGTAG

>gi|85718614|ref|NC_007732.1| Porcine hemagglutinating encephalomyelitis virus, complete genome

ATGTTTTTTATACTTTTAATCTCCCTGCCTTCTGCTTTTGCAGTTTTAGGGGATTTAAAGTGTAATACTTCATCA
ATTAATGACGTTGACACTGGTGTGCCATCTATTAGCTCTGAAGTTGTTGATGTCACTAATGGTTTGGGGACTTTTTATG
TTTTAGATCGTGTCTATTTAAATACCACATTGTTGCTCAATGGTTATTACCCAATTTCAGGTGCTACATTTCGTAATAT
GGCTCTGAAAGGAACTCGATTATTGAGCACCTTGTGGTTTAAGCCGCCTTTTTTATCACCGTTTAATGATGGTATTTTT
GCCAAGGTTAAAAACAGCAGATTTTTCAAAGATGGTGTTATTTATAGTGAGTTTCCTGCTATTACTATAGGTAGTACTT
TTGTAAATACTTCCTATAGCATAGTAGTAGAGCCTCATACTTTACTTATTAATGGTAATTTACAAGGTTTGTTGCAAAT
CTCTGTTTGTCAATACACTATGTGTGAATACCCACATACTATTTGTCATCCTAATTTGGGTAACCAACGCATAGAATTA
TGGCATTATGACACAGATGTTGTTTCTTGTTTATACAGGCGTAATTTCACATATGATGTGAATGCTGATTATTTATATT
TTCATTTTTATCAGGAAGGTGGCACGTTTTATGCATACTTTACAGATACTGGTTTTGTGACCAAGTTTCTGTTTAAGTT
GTATTTAGGCACTGTGCTGTCACATTATTATGTTATGCCATTGACTTGTGATAGCGCTTTATCTTTAGAATATTGGGTT
ACACCTCTCACTACTAGACAATTTCTTCTAGCCTTTGACCAGGATGGTGTTTTATACCATGCTGTTGATTGTGCTAGTG
ATTTTATGAGTGAGATTATGTGTAAAACTTCTTCAATTACACCACCTACTGGTGTTTATGAACTAAACGGTTACACAGT
TCAACCTGTTGCCACTGTATATCGTAGAATACCTGATTTACCCAATTGCGATATCGAAGCTTGGCTTAATTCTAAGACC
GTTTCGTCGCCTCTTAATTGGGAACGTAAAATTTTTTCTAATTGTAATTTTAACATGGGCAGGCTGATGTCTTTCATTC
AGGCTGACTCTTTTGGTTGTAACAATATTGATGCTTCTCGCTTATATGGTATGTGTTTTGGTAGCATTACTATTGATAA
GTTTGCTATACCCAATAGTAGAAAGGTTGATCTGCAAGTGGGTAAATCTGGTTATTTACAATCTTTTAACTATAAGATT
GACACTGCTGTTAGCAGCTGTCAACTCTATTATAGTTTGCCTGCAGCAAATGTATCTGTCACTCATTATAATCCTTCAT
CTTGGAATAGAAGGTATGGGTTTAATAATCAGAGTTTTGGTTCCAGAGGCCTTCATGATGCTGTTTATTCACAGCAATG
TTTTAATACACCTAACACATATTGTCCCTGTAGAACAAGTCAATGCATAGGTGGTGCAGGTACAGGAACTTGTCCTGTA
GGAACCACTGTGCGTAAGTGTTTTGCTGCAGTTACTAACGCTACTAAGTGTACTTGCTGGTGCCAACCAGATCCTTCCA
CATATAAAGGTGTAAATGCTTGGACTTGTCCGCAATCCAAAGTTTCTATACAACCAGGTCAGCATTGCCCTGGTTTGGG
TCTTGTGGAGGATGATTGCTCTGGCAACCCTTGCACTTGTAAACCACAGGCCTTCATAGGCTGGAGCTCAGAAACTTGT
TTGCAAAATGGTAGGTGTAATATTTTTGCTAATTTTATTTTGAATGATGTTAATAGCGGTACAACCTGTTCTACTGATT
TACAACAGGGTAATACTAATATTACTACTGATGTTTGTGTTAATTATGACCTATATGGCATTACAGGCCAGGGCATACT
TATAGAAGTTAATGCCACTTATTATAATAGTTGGCAGAATCTTCTTTATGATTCTAGTGGTAATCTCTATGGCTTTAGA
GATTATTTATCAAATAGAACTTTTCTTATTCGTAGCTGCTATAGTGGAAGAGTTTCAGCAGTTTTTCATGCTAACTCTT

CTGAACCAGCTTTGATGTTTCGTAATCTTAAATGCAGCCACGTTTTTAATAATACTATTTTAAGACAAATACAGCTTGT
TAACTATTTTGATAGTTACCTTGGTTGTGTTGTTAATGCTTATAATAATACAGCTAGTGCTGTAAGTACTTGTGATTTA
ACCGTTGGCAGCGGCTATTGTGTTGATTATGTTACAGCACTTAGATCACGTAGATCTTTTACTACAGGTTATCGCTTTA
CTAATTTTGAACCATTTGCCGTTAATTTGGTAAATGATAGTATAGAACCTGTTGGTGGTTTGTATGAAATACAGATACC
TTCAGAGTTTACCATTGGTAATTTAGAAGAGTTCATTCAAACGAGCTCCCCTAAGGTTACTATAGACTGTGCTACATTT
GTTTGTGGTGACTATGCAGCATGTAGACAACAGTTAGCCGAGTATGGTAGTTTTTGTGAGAACATTAATGCTATACTCA
TAGAAGTAAATGAACTACTTGACACTACACAGTTGCAAGTAGCTAATAGTTTAATGAATGGTGTCACCCTTAGTACTAA
GATTAAGGATGGCATTAATTTCAATGTTGACGATATCAACTTCTCCCCTGTATTAGGTTGTTTAGGAAGCGAATGTAAT
AGAGCTTCCACTAGATCTGCTATAGAGGACTTACTTTTTGATAAAGTAAAATTGTCTGATGTCGGCTTTGTACAGGCCT
ATAATAACTGCACTGGAGGTGCCGAAATTAGGGATCTCATTTGTGTGCAAAGTTATAATGGTATCAAAGTGTTGCCTCC
ATTGTTATCTGAAAATCAGATCAGTGGTTACACTTTGGCAGCCACCGCTGCTAGCTTATTCCCTCCATGGACAGCTGCA
GCAGGTGTACCATTTTATTTAAATGTTCAGTATCGTATTAATGGGCTTGGCGTCACTATGGATGTGCTAAGCCAAAATC
AAAAGCTTATTGCTAGTGCATTTAACAATGCTCTTGATGCTATTCAGGAAGGGTTCGACGCAACCAATTCTGCTTTAGT
TAAAATTCAGTCTGTTGTTAATGCAAATGCTGAAGCACTTAATAACTTATTGCAGCAACTCTCTAATAGATTTGGTGCC
ATAAGTGCGTCTTTACAAGAAATTCTATCTAGGCTCGATGCTCTTGAAGCTAAAGCTCAGATAGACAGACTTATTAATG
GGCGTCTTACCGCTCTTAATGCCTATGTTTCTCAGCAGCTTAGTGATTCTACACTAGTAAAATTTAGTGCAGCACAAGC
TATGGAGAAAGTTAATGAATGTGTTAAAAGCCAATCATCCAGGATAAATTTCTGTGGTAATGGTAATCATATTATATCA
TTAGTACAGAATGCTCCATATGGTTTGTATTTTATCCACTTTAGCTATGTCCCCACCAAGTATGTTACAGCAAAGGTTA
GTCCTGGTTTGTGTATTGCTGGCGATATAGGAATATCGCCTAAGAGTGGTTATTTTATTAATGTAAATAATTCTTGGAT
GTTCACTGGTAGTGGTTATTACTACCCTGAACCTATAACCCAAAATAATGTTGTTATGATGAGTACCTGTGCTGTTAAT
TATACTAAAGCACCGGATCTAATGCTGAACACATCGACACCTAACCTTCCTGACTTCAAGGAAGAATTGTATCAATGGT
TTAAAAACCAATCTTCAGTGGCACCAGATTTGTCACTTGATTATATAAATGTTACGTTCTTGGACCTACAAGATGAAAT
GAATAGGTTACAAGAGGCTATAAAAGTCTTAAATCATAGCTACATCAATCTCAAGGACATTGGTACATATGAGTATTAT
GTGAAATGGCCTTGGTATGTATGGCTTTTAATTGGCCTTGCTGGTGTAGCTATGCTTGTTTTACTATTCTTCATATGCT
GTTGTACAGGATGTGGGACTAGTTGTTTTAAGAAATGTGGCGGTTGTTGTGATGATTATACTGGACACCAGGAGTTTGT
AATCAAAACTTCACATGACGACTAA

>gi|21734847|gb|AF481863.1| Porcine hemagglutinating encephalomyelitis virus HE glycoprotein, spike glycoprotein (S), 4.9 kDa nonstructural protein (ns4.9), 122.7 kDa nonstructural protein, small membrane protein E (E), matrix glycoprotein (M), phosphorylated nucleocapsid protein N, and N2 (N2) genes, complete cds

ATGTTTTTTATACTTTTAATCACCCTGCCTTCTGTTTTTGCAGTTATAGGGGATTTAAAGTGTAATACTTCATCA
ATTAATGACGTTGACACTGGTGTGCCATCTATTAGCTCTGAAGTTGTTGATGTCACTAATGGTTTGGGGACTTTCTATG
TTTTAGATCGTGTCTATTTAAATACCACATTGTTGCTCAATGGTTATTACCCAATTTCAGGTGCTACATTTCGTAATGT
GGCTCTGAAAGGAACTCGATTATTGAGCACCTTGTGGTTTAAGCCGCCTTTTTTATCACCTTTTAATGATGGTATTTTT
GCCAAGGTTAAAAACAGCAGATTTTCTAAACATGGTGTTATTTATAGTGAGTTTCCTGCTATTACTATAGGTAGTACTT
TTGTAAATACTTCCTATAGCATAGTAGTAAAGCCTCATACCTCATTTATTAATGGTAATTTACAAGGTTTTTTGCAAAT
TTCTGTTTGTCAATATACTATGTGTGAATACCCACAGACTATTTGTCATCCTAATTTGGGTAATCAACGCATAGAATTA
TGGCATCATGACACAGATGTTGTTTCTTGTTTATACAGGCGTAATTTCACATATGATGTGAATGCTGATTATTTATATT
TTCACTTTTATCAGGAAGGTGGCACTTTTTATGCATACTTTACAGATACTGGTTTTGTGACCAAGTTTCTGTTTAAGTT
GTATTTAGGCACTGTGCTGTCACACTATTATGTTATGCCATTGACTTGTGATAGCGCTTTATCTTTAGAATATTGGGTT
ACACCTCTCACTACTAGACAATTTCTTCTAGCCTTTGACCAGGATGGTGTTTTATACCATGCTGTTGATTGTGCTAGTG

ATTTTATGAGTGAGATTATGTGTAAAACTTCTTCAATTACACCACCTACTGGTGTTTATGAACTAAACGGTTACACAGT
TCAACCTGTTGCCACTGTGTATCGTAGAATACCTGACTTACCCAATTGCGATATCGAAGCTTGGCTTAATTCTAAGACC
GTTTCTTCGCCTCTTAATTGGGAACGTAAAATTTTTTCTAATTGTAATTTTAACATGGGCAGGCTGATGTCTTTTATTC
AGGCTGACTCTTTTGGTTGTAACAATATTGATGCTTCTCGCTTATATGGTATGTGTTTTGGTAGCATTACTATTGACAA
GTTTGCTATACCCAATAGTAGAAAGGTTGATCTGCAAGTGGGTAAATCTGGTTATTTACAATCTTTTAATTATAAGATT
GACACTGCTGTTAGCAGTTGTCAACTCTATTATAGTTTGCCTGCAGCAAACGTATCTGTCACTCATTATAATCCTTCAT
CTTGGAACAGAAGGTATGGGTTTATTAATCAGAGTTTTGGTTCCAGAGGCCTTCATGATGCTGTATATTCACAGCAATG
TTTTAATACACCTAATACATATTGTCCTTGTAGAACAAGTCAATGCATAGGTGGTGCTGGCACAGGAACTTGTCCTGTA
GGCACCACTGTGCGCAAGTGTTTTGCTGCAGTTACAAACGCTACTAAGTGTACTTGCTGGTGTCAACCAGATCCTTCCA
CATATAAAGGTGTAAATGCCTGGACTTGTCCGCAATCTAAAGTTTCTATACAACCAGGTCAGCATTGCCCTGGCTTGGG
TCTTGTGGAGGATGATTGCTCTGGTAATCCTTGCACTTGTAAACCACAGGCTTTCATAGGCTGGAGTTCAGAAACTTGT
TTGCAAAATGGTAGGTGTAATATTTTTGCTAATTTTATTTTGAATGATGTTAATAGCGGTACTACCTGTTCTACTGATT
TACAACAGGGTAATACTAATATTACTACTGATGTTTGTGTTAATTATGACCTATATGGCATTACAGGCCAGGGCATACT
TATAGAAGTTAATGCCACGTATTATAATAGTTGGCAGAATCTTCTTTATGATTCTAGTGGTAATCTCTATGGCTTTAGA
GATTATTTATCAAATAGAACCTTTCTTATTCGTAGCTGCTATAGTGGAAGAGTTTCAGCAGTCTTTCATGCTAACTCTT
CTGAACCAGCTTTGATGTTTCGTAATCTTAAATGCAGCCACGTTTTTAATTATACCATTTTAAGACAAATACAGCTTGT
TAATTATTTTGATAGTTACCTTGGTTGTGTTGTTAATGCTTATAATAATACAGCTAGTGCTGTAAGTACTTGTGATTTA
ACCGTTGGTAGCGGCTATTGTGTTGATTATGTTACAGCACTTAGATCACGTAGATCTTTTACTACAGGTTATCGCTTTA
CTAATTTTGAACCATTTGCCGCTAATTTGGTAAATGATAGTATAGAACCTGTTGGTGGTTTGTATGAAATACAGATACC
TTCAGAGTTTACCATTGGTAATTTAGAAGAATTCATTCAAACGAGTTCCCCTAAGGTTACTATAGATTGTGCTACATTT
GTTTGTGGTGACTATGCTGCATGTAGACAACAGTTAGCTGAGTATGGTAGTTTTTGTGAGAACATTAATGCTATACTCA
TAGAAGTAAATGAACTACTTGACACTACACAGTTGCAAGTAGCTAATAGTTTAATGAATGGAGTCACCCTTAGTACTAA
GATTAAGGATGGGATTAATTTCAATGTTGACGATATCAACTTCTCCTCTGTATTAGGTTGTTTAGGAAGCGAATGTAAC
AGAGCTTCCACTAGATCTGCTATAGAGGATTTACTTTTTGATAAAGTAAAATTGTCTGATGTCGGTTTTGTACAGGCCT
ATAATAACTGCACTGGAGGAGCCGAAATTAGGGATCTCATTTGTGTGCAAAGTTATAATGGTATCAAAGTGTTGCCTCC
ATTGTTATCTGAAAATCAGATTAGTGGTTACACTTCGGCAGCCACCGCTGCTAGCCTATTTCCTCCCTGGACAGCTGCA
GCAGGTGTACCATTTTATTTAAATGTTCAGTATCGTATAAATGGGCTTGGCGTCACCATGGATGTGCTAAGCCAAAACC
AAAAGCTTATTGCTAGTGCATTTAACAACGCTCTTGATTCTATCCAGGAAGGGTTCGACGCAACCAATTCTGCTTTAGT
TAAAATTCAGGCTGTTGTTAATGCAAATGCTGAAGCACTTAATAACTTATTGCAGCAACTCTCTAACAGATTTGGTGCC
ATAAGTGCCTCTTTACAAGAAATTTTATCCAGGCTCGATGCTCTTGAAGCTAAAGCTCAGATAGACAGACTTATTAATG
GGCGTCTCACCGCTCTTAATGCTTATGTTTCTCAGCAGCTTAGTGATTCTACACTAGTAAAATTTAGTGCAGCACAAGC
TATTGAGAAAGTTAATGAATGTGTTAAAAGCCAATCATCTAGGATAAATTTCTGTGGTAATGGTAATCATATTATATCA
TTAGTACAGAATGCTCCATATGGTTTGTATTTTATCCATTTTAGCTATGTCCCCACCAAGTATGTTACAGCAAAGGTTA
GTCCTGGTTTGTGCATTGCTGGCGATATAGGAATATCGCCTAAGAGTGGTTATTTTATTAATGTAAATAACTCTTGGAT
GTTCACTGGTAGTGGCTATTACTACCCTGAACCTATAACCCAAAATAATGTTGTTGTGATGAGTACGTGTGCTGTTAAT
TATACTAAAGCACCGGATCTAATGCTGAACACATCGACACCCAACCTTCCTGATTTCAAGGAAGAATTGTATCAATGGT
TTAAAAACCAATCTTCATTGGCACCAGATTTGTCATTTGATTATATTAATGTTACGTTCTTGGACCTACAAGATGAAAT
GAATAGGTTACAAGAAGCTATAAAAGTTCTAAATCATAGCTACATCAATCTCAAGGACATTGGTACATATGAGTATTAT
GTGAAATGGCCTTGGTATGTATGGCTTTTAATTTGCCTTGCTGGTGTAGTTATGCTTGTTTTACTATTCTTCATATGCT
GCTGTACAGGATGTGGGACTAGTTGTTTTAAGAAATGTGGCGGTTGTTTTGATGATTATACTGGACACCAGGAGTTTGT
AATCAAAACTTCACATGACGATTAA

>gi|23295758|gb|AY078417.1| Porcine hemagglutinating encephalomyelitis virus

hemagglutinin-esterase protein, spike glycoprotein, 4.9 kDa non-structural protein, 12.7 kDa non-structural protein, small envelope-associated protein, matrix glycoprotein, nucleocapsid protein, and N2 protein genes, complete cds

ATGTTTTTATACTTTTAATCTCCCTGCCTTCTGCTTTTGCAGTTATAGGGGATTTAAAGTGTACTACTTCATTA
ATTAATGACGTTGACACTGGTGTGCCATCTATTAGCTCTGAAGTTGTTGATGTCACTAATGGTTTGGGGACTTTCTATG
TTTTAGATCGTGTCTATTTAAATACCACATTGTTGCTCAATGGTTATTACCCAATTTCAGGTGCTACATTTCGTAATAT
GGCTCTGAAAGGAACTCGATTATTGAGCACCTTGTGGTTTAAGCCGCCTTTTTTATCACCTTTTAATGATGGTATTTTT
GCCAAGGTTAAAAACAGCAGATTTTCTAAAGATGGTGTTATTTATAGTGAGTTTCCTGCTATTACTATAGGTAGTACTT
TTGTAAATACTTCCTATAGCATAGTAGTAGAGCCTCATACCTCACTTATTAATGGTAATTTACAAGGTTTGTTGCAAAT
TTCTGTTTGTCAATACACTATGTGTGAATACCCACATACTATTTGTCATCCTAATTTGGGTAATCAACGCATAGAATTA
TGGCATTATGACACAGATGTTGTTTCTTGTTTATACAGGCGTAATTTCACATATGATGTGAATGCTGATTATTTATATT
TTCACTTTTATCAGGAAGGTGGCACTTTTTATGCATACTTTACAGATACTGGTTTTGTGACCAAGTTTCTGTTTAAGTT
GTATTTAGGCACTGTGCTGTCACATTATTATGTTATGCCATTGACTTGTAATAGCGCTTTATCTTTAGAATACTGGGTT
ACACCTCTCACTACTAGACAATTTCTTCTAGCCTTTGACCAGGATGGTGTTTTATACCATGCTGTTGATTGTGCTAGTG
ATTTTATGAGTGAGATTATGTGTAAAACTTCTTCAATTACACCACCTACTGGTGTTTATGAACTAAACGGTTACACAGT
TCAACCTGTTGCCACTGTATATCGTAGAATACCTGATTTACCCAATTGCGATATCGAAGCTTGGCTTAATTCTAAGACC
GTTTCTTCGCCTCTTAATTGGGAACGTAAAATTTTTTCTAATTGTAATTTTAACATGGGCAGGCTGATGTCTTTTATTC
AGGCTGACTCTTTTGGTTGTAACAATATTGATGCTTCTCGCTTATATGGTATGTGTTTTGGTAGCATTACTATTGATAA
GTTTGCTATACCCAATAGTAGAAAGGTTGATCTGCAAGTGGGTAAATCTGGTTATTTACAATCTTTTAATTATAAGATT
GACACTGCTGTTAGCAGTTGTCAACTCTATTATAGTTTGCCTGCAGCAAACGTATCTGTCACTCATTATAATCCTTCAT
CTTGGAATAGAAGGTATGGGTTTAATAATCAGAGTTTTGGTTCCAGAGGCCTTCATGATGCTGTTTATTCACAGCAATG
TTTTAATACACCTAACACATATTGTCCTTGTAGAACAAGTCAATGCATAGGTGGTGCAGGCACAGGAACTTGTCCTGTA
GGCACCACTGTGCGCAAGTGTTTTGCTGCAGTTACAAAAGCTACTAAGTGTACTTGCTGGTGTCAACCAGATCCTTCCA
CATATAAAGGTGTAAACGCCTGGACTTGTCCGCAATCTAAAGTTTCTATACAACCAGGTCAGCATTGCCCTGGTTTGGG
TCTTGTGGAGGATGATTGCTCTGGCAACCCTTGCACTTGTAAACCACAGGCTTTCATAGGCTGGAGTTCAGAAACTTGT
TTGCAAAATGGTAGGTGTAATATTTTTGCTAATTTTATTCTGAATGATGTTAATAGCGGTACAACCTGTTCTACTGATT
TACAACAGGGTAATACTATTATTACTACTGATGTTTGTGTTAATTATGACCTATATGGCATTACAGGCCAGGGCATACT
TATAGAAGTTAATGCCACTTATTATAATAGTTGGCAGAATCTTCTTTATGATTCTAGTGGTAATCTCTATGGCTTTAGA
GATTATTTATCAAATAGAACTTTTCTTATTCGTAGCTGCTATAGTGGAAGAGTTTCAGCAGTTTTTCATGCTAACTCAT
CTGAACCAGCTTTGATGTTTCGTAATCTTAAATGCAGCCACGTTTTTAATAATACCATTTTAAGACAAATACAGCTTGT
TAACTATTTTGATAGTTACCTTGGTTGTGTTGTTAATGCTTATAATAATACAGCTAGTGCTGTAAGTACTTGTGATTTA
ACCGTTGGTAGCGGCTATTGTGTTGATTATGTTACAGCACTTAGATCACGTAGATCTTTTACTACAGGTTATCGCTTTA
CTAATTTTGAACCATTTGCCGCTAATTTGGTAAATGATAGTATAGAACCTGTTGGTGGTTTGTATGAAATACAGATACC
TTCAGAGTTTACCATTGGTAATTTAGAAGAGTTCATTCAAACGAGATCCCCTAAGGTTACTATAGACTGTGCTACATTT
GTTTGTGGTGACTATGCAGCATGTAGACAACAGTTAGCTGAGTATGGTAGTTTTTGTGAGAACATTAATGCTATACTCA
CAGAAGTAAATGAACTACTTGACACTACACAGTTGCAAGTAGCTAATAGTTTAATGAATGGAGTCACCCTTAGTACCAA
GATTAAGGATGGCATTAATTTCAATGTTGACGATATCAACTTCTCCCCTGTATTAGGTTGTTTAGGAAGCGAATGTAAT
AGAGCTTCCACTAGATCTGCTATAGAGGATTTACTTTTTGATAAAGTAAAATTGTCTGATGTCGGCTTTGTACAGGCCT
ATAATAACTGCACTGGAGGTGCCGAAATTAGGGATCTCATTTGTGTGCAAAGTTATAATGGTATCAAAGTGTTGCCTCC
ATTGTTATCTGAAAATCAGATCAGTGGCTACACTTTGGCAGCCACCGCTGCTAGCTTATTCCCTCCTTGGACAGCTGCA
GCAGGTGTACCATTTTATTTAAATGTTCAGTATCGTATAAATGGGCTTGGCGTCACTATGGATGTGCTAAGTCAAAACC
AAAAGCTTATTGCTAGTGCATTTAACAATGCTCTTGATGCTATCCAGGAAGGGTTCGACGCAACCAATTCTGCTTTAGT
TAAAATTCAGGCTGTTGTTAATGCAAATGCTGAAGCACTTAATAACTTATTGCAGCAACTCTCTAACAGATTTGGTGCC

ATAAGTGCCTCTTTACAAGAAATTTTATCCAGGCTCGATGCTCTTGAAGCTAAAGCTCAGATAGACAGACTTATCAATG
GGCGTCTCACCGCTCTTAATGCTTATGTTTCTCAGCAGCTTAGTGATTCTACACTAGTAAAATTTAGTGCAGCACAAGC
TATTGAGAAAGTTAATGAATGTGTTAAAAGCCAATCATCTAGGATAAATTTCTGTGGTAATGGTAATCATATTATATCA
TTAGTACAGAATGCTCCATATGGTTTGTATTTTATCCATTTTAGCTATGTCCCCACCAAGTATGTTACAGCAAAGGTTA
GTCCTGGTTTGTGCATTGCTGGCGATATAGGAATATCGCCTAAGAGTGGTTATTTTATTAATGTAAATAATTCTTGGAT
GTTCACTGGTAGTAGCTATTACTACCCTGAACCTATAACCCAAAATAATGTTGTTGTGATGAGTACCTGTGCTGTTAAT
TATACTAAAGCACCGGATCTAATGCTGAACACATCGACACCCAACCTTCCTGACTTCAAGGAAGAATTGTATCAATGGT
TTAAAAACCAATCTTCAGTGGCACCAGATTTGTCACTTGATTATATTAATGTTACGTTCTTGGACCTACAAGATGAAAT
GAATAGGTTACAAGAGGCTATAAAAGTTTTAAATCAGAGCTACATCAATCTCAAGGACATTGGTACATATGAGTATTAT
GTGAAATGGCCTTGGTATGTATGGCTTTTAATTGGCCTTGCTGGTGTAGCTATGCTTGTTTTACTATTCTTCATATGCT
GCTGTACAGGATGTGGGACTAGTTGTTTTAAGAAATGTGGCGGTTGTTGTGATGATTATACTGGACACCAGGAGTTTGT
AATCAAAACTTCACATGACGATTAA

>gi|197260969|gb|EU919227.1|_Porcine_hemagglutinating_encephalomyelitis_virus_strain_HEVJT06_truncated_spike_glycoprotein_(S)_gene_complete_cds

ATGTTTTTATACTTTTAATCTCCCTGCCTTCTGCTTTTGCAGTTATAGGGGATTTAAAGTGTACTACTTCATTA
ATTAATGACGTTGACTCTGGTGTGCCATCTATTAGCTCTGAAGTTGTTGATGTCACTAATGGTTTGGGGACTTTCTATG
TTTTAGATCGTGTCTATTTAAATACCACATTGTTGCTCAATGGTTATTACCCAATTTCAGGTGCTACATTTCGTAATAT
GGCTCTGAAAGGAACTCGATTATTGAGCACCTTGTGGTTTAAGCCGCCTTTTTTATCACCTTTTAATGATGGTATTTTT
GCCAAGGTTAAAAACAGCAGATTTTCTAAAGATGGTGTTATTTATAGTGAGTTTCCTGCTATTACTATAGGTAGTACTT
TTGTAAATACTTCCTATAGCATAGTAGTAGAGCCTCATACCTCACTTATTAATGGTAATTTACAAGGTTTGTTGCAAAT
TTCTGTTTGTCAATACACTATGTGTGAATACCCACATACTATTTGTCATCCTAATTTGGGTAATCAACGCATAGAATTA
TGGCATTATGACACAGATGTTGTTTCTTGTTTATACAGGCGTAATTTCACATATGATGTGAATGCTGATTATTTATATT
TTCACTTTTATCAGGAAGGTGGCACTTTTTATGCTTACTTTACAGATACTGGTTTTGTGACCAAGTTTCTGTTTAAGTT
GCATTTAGGCACTGTGCTGTCACATTATTATGTTATGCCATTGACTTGTAATAGCGCTTTATCTTTAGAATACTGGGTT
ACACCTCTCACTACTAGACAATTTCTTCTAGCCTTTGACCAGGATGGTGTTTTATACCATGCTGTTGATTGTGCTAGTG
ATTTTATGAGTGAGATTATGTGTAAAACTTCTTCAATTACACCACCTACTGGTGTTTATGAACTAAACGGTTACACAGT
TCAACCTGTTGCCACTGTATATCGTAGAATACCTGATTTACCCAATTGCGATATCGAAGCTTGGCTTAATTCTAAGACC
GTTTCTTCGCCTCTTAATTGGGAACGTAAAATTTTTTCTAATTGTAATTTTAACATGGGCAGGCTGATGTCTTTTATTC
AGGCTGACTCTTTTGGTTGTAACAATATTGATGCTTCTCGCTTATATGGTATGTGTTTTGGTAGCATTACTATTGATAA
GTTTGCTATACCCAATAGTAGAAAGGTTGATCTGCAAGTGGGTAAATCTGGTTATTTACAATCTTTTAATTATAAGATT
GACACTGCTGTTAGCAGTTGTCAACTCTATTATAGTTTGCCTGCAGCAAACGTATCTGTCACTCATTATAATCCTTCAT
CTTGGAATAGAAGGTATGGGTCTAATAGTCAGAGTTTTGGTTCCAGAGGCCTTCATGATGCTGTTTATTCACAGCAATG
TTTTAATACACCTAACACATATTGTCCTTGTAGAACAAGTCAATGCATAGGTGGTGCAGGCACAGGAACTTGTCCTGTA
GGCACCACTGTGCGCAAGTGTTTTGCTGCAGTTACAAAAGCTACTAAGTGTACTTGCTGGTGTCAACCAGATCCTTCCA
CATATAAAGGTGTAAACGCCTGGACTTGTCCGCAATCTAAAGTTTCTATACAACCAGGTCAGCATTGCCCTGGTTTGGG
TCTTGTGGAGGATGATTGCTCTGGCAACCCTTGCACTTGTAAACCACAGGCTTTCATAGGCTGGAGTTCAGAAACTTGT
TTGCAAAATGGTAGGTGTAATATTTTTGCTAATTTTATTCTGAATGATGTTAATAGCGGTACAACCTGTTCTACTGATT
TACAACAGGGTAATACTATTATTACTACTGATGTTTGTGTTAATTATGACCTATATGGCATTACAGGCCAGGGCATACT
TATAGAAGTTAATGCCACTTATTATAATAGTTGGCAGAATCTTCTTTATGATTCTAGTGGTAATCTCTATGGCTTTAGA
GATTATTTATCAAATAGAACTTTTCTTATTCGTAGCTGCTATAGTGGAAGAGTTTCAGCAGTTTTTCATGCTCACTCAT
CTGAACCAGCTTTGATGTTTCGTAATCTTAAATGCAGCCACGTTTTTAATAATACCATTTTAAGACAAATACAGCTTGT

TAACTATTTTGATAGTTACCTTGGTTGTGTTGTTAATGCTTATAATAATACAGCTAGTGCTGTAAGTACTTGTGATTTA
ACCGTTGGTAGCGGCTATTGTGTTGATTATGTTACAGCACTTAGATCACGTAGATCTTTTACTACAGGTTATCGCTTTA
CTAATTTTGAACCATTTGCCGCTAATTTGGTAAATGATAGTATAGAACCTGTTGGTGGTTTGTATGAAATACGGATACC
TTCAGAGTTTACCATTGGTAATTTAGAAGAGTTCATTCAAACGAGATCCCCTAAGGTTACTATAGACTGTGCTACATTT
GTTTGTGGTGACTATGCAGCATGTAGACAACAGTTAGCTGAGTATGGTAGTTTTTGTGAGAACATTAATGCTATACTCA
CAGAACTAAATGAACTACTTGACACTACACAGTTGCAAGTAGTTAATAGTTTAATGAATGGAGTCACCCTTAGTACCAA
GATTAAGGATGGCATTAATTTCAATGTTGACGATTTCAACTTCTCCCCTGTATTAGGTTGTTTAGGAAGCGAATGTAAT
AGAGCTTCCACTAGATCTGCTATAGAGGATTTACTTTTTGATAAAGTAAAATTGTCTGATGTCGGCTTTGTACAGGCCT
ATAATAACTGCACTGGAGGTGCCGAAATTAGGGATCTCATTTGTGTGCAAAGTTATAATGGTATCAAAGTGTTGCCTCC
ATTGTTATCTGAAAATCAGATCAGTGGCTACACTTTGGCAGCCACCGCTGCTAGCTTATTCCCTCCTTGGACAGCTGCA
GCAGGTGTATCATGTTATTTAAATGTTCAGTATCGTATAAATGGGCTTGGCGTCACTATGGATGTGCTAAGTCAAAACC
AAAAGCTTATTGCTAGTGCATTTAACAATGCTCTTGATGCTATCCAGGAAGGGTTCGACGCAACCAATTCTGCTTTAGT
TAAAATTCAGGCTGTTGTTAATGCAAATGCTGAAGCACTTAATAACTTATTGCAGCAACTCTCTAACAGATTTGGTGCC
ATAAGTGCCTCTTTACATGAAATTTTATCCAGGCTCGATGCTCTTGAAGCTAAAGCTCAGATAGACAGACTTATCAATG
GGCGTCTCACCGCTCTTAATGCTTATGTTTCTCAGCAGCTTAGTGATTCTACACTAGTAAAATTTAGTGCAGCACAAGC
TATTGATTAAGGTAATGAATGTGTTAAAAGCCAATCATGTAGGATAAATTTCTGTGGTAATGGTAATCATATTATATCA
TTAGTACAGAATGCTCCATATGGTTTGTATTTTATCCATTTTAGCTATGTCCCCACCAAGTATGTTACAGCAAAGGTTA
GTCCTGGTTTGTGCATTGCTGGCGATATAGGAATATCGCCTAAGAGTGGTTATTTTATTAATGTAAATAATTCTTGGAT
GTTCACTGGTAGTAGCTATTAATACCCTGAACCTATAACCCAAAATAATGTAGTTGTGATGAGTACCTGTGCTGTTAAT
TATACTAAAGCACCGGATCTAATGCTGAACACATCGACACCCAACCTTCCTGACTTCAAGGAAGAATTGTATCAATGGT
TTAAAAACCAATCTTCAGTGGCACCAGATTTGTCACTTGATTATATTAATGTTACGTTCTTGGACCTACAAGATGAAAT
GAATAGGTTACAAGAGGCTATAAAAGTTTTAAATCAGAGCTACATCAATCTCAAGGACATTGGTACATATGAGTATTAT
GTGAAATGGCCTTGGTATGTATGGCTTTTAATTGGCCTTGCTGGTGTAGCTATGCTTGTTTTACTATTCTTCATATGCT
GCTGTACAGGATGTGGGACTAGTTGTTTTAAGAAATGTGGCGGTTGTTGTGATGATTATACTGGACACCAGGAGTTTGT
AATCAAAACTTCACATGACGATTAA

>gi|110746792|gb|DQ811785.1| TGEV Miller M6, complete genome

ATGAAAAAATTATTTGTGGTTTTGGTTGTAATGCCATTGATTTATGGAGACAATTTTCCTTGTTCTAAATTGACT
AATAGAACTATAGGTAACCATTGGAATCTCATTGAAACCTTCCTTCTAAATTATAGTAGTAGGTTATCACCTAATTCAG
ATGTGGTGTTAGGTGATTATTTTCCTACTGTACAACCTTGGTTTAATTGCATTCGCAATAATAGTAATGACCTTTATGT
TACATTGGAAAATCTTAAAGCATTGTATTGGGATTATGCTACAGAAAATATCACTTTGAATCACAAACAACGGTTAAAC
GTAGTCGTTAATGGATACCCATACTCCATCACAGTTACAACAACCCGCAATTTTAATTCTGCTGAAGGTGCTATTATAT
GCATTTGCAAGGGCTCACCACCTACTACCACCACAGAATCTAGTTTGACTTGCAATTGGGGTAGTGAGTGCAGGTTAAA
CCATAAGTTCCCTATATGTCCTTCTAATTCAGAGGCAAATTGTGGTAATATGCTGTATGGCCTACAATGGTTTGCAGAT
GCGGTTGTTGCTTATTTACATGGTGCTAGTTACCGTATTAGTTTTGAAAATCAATGGTCTGGCACTGTTACACTTGGTG
ATATGCGTGCGACTACATTAGAAACCGCTGGCACGCTTGTAGACCTTTGGTGGTTTAATCCTGTTTATGATGTCAGTTA
TTATAGAGTTAATAATAAAAATGGTACTACCGTAGTTTCCAATTGCACTGATCAATGTGCTAGTTATGTGGCTAATGTT
TTTACTACACAGCCAGGAGGCTTTATACCATCAGATTTTAGTTTTAATAATTGGTTCCTTCTAACTAATAGCTCCACGT
TGGTTAGTGGTAAATTAGTTACCAAACAGCCGTTATTAGTTAATTGCTTATGGCCAGTCCCTAGCTTTGAAGAAGCAGC
TTCTACACTTTGTTTTGAAGGTGCTGGCTTTGATCAATGTAATGGTGCTGTTTTAAATAACACTGTAGACGTCATCAGG
TTTAACCTTAATTTTACTACAAATGTACAATCAGGTAAGGGTGCCACAGTGTTTTCATTGAACACAACGGGTGGTGTCA
CTCTTGAAATCTCATGTTATAATGATACAGTGAGTGACTCGAGCTTTTCCAGTTACGGTGAAATGCCGTTCGGCGTAAC

TGATGGACCACGGTACTGTTACGTACTCTATAATGGCACAGCTCTTAAGTATCTAGGAACATTACCACCTAGTGTCAAG
GAGATTGCTATTAGTAAGTGGGGCCATTTTTATATTAATGGTTACAATTTCTTTAGCACATTTCCTATTGATTGTATAT
CTTTTAATTTGACCACTGGTGATAGTGACGTTTTCTGGACAATAGCTTACACATCGTACACTGAAGCATTAGTACAAGT
TGAAAACACAGCTATTACAAAGGTGACGTATTGTAATAGTTACGTTAATAACATTAAATGCTCTCAACTTACTGCTAAT
TTGAATAATGGATTTTATCCTGTTTCTTCAAGTGAAGTTGGTCTTGTCAATAAGAGTGTTGTGTTACTACCTAGCTTTT
ACACACATACCATTGTTAACATAACTATTGGTCTTGGTATGAAGCGTAGTGGTTATGGTCAACCCATAGCCTCAACATT
AAGTAACATTACACTACCAATGCAGGATAACAACACCGATGTGTACTGTATTCGTTCTGACCAATTTTCAGTTTATGTT
CATTCTACTTGCAAAAGTTCTTTATGGGACAATGTTTTTAAGCGAAACTGCACGGACGTTTTAGATGCCACAGCTGTTA
TAAAAACTGGTACTTGTCCTTTCTCATTTGATAAATTGAACAATTACTTAACTTTTAACAAGTTCTGTTTGTCGTTGAG
TCCTGTTGGTGCTAATTGTAAGTTTGATGTAGCTGCCCGTACAAGAACCAATGATCAGGTTGTTAGAAGTTTGTATGTA
ATATATGAAGAAGGAGACAACATAGTGGGTGTACCGTCTGATAATAGTGGTTTACACGATTTGTCAGTGCTACACCTAG
ATTCCTGCACAGATTACAATATATATGGTAGAACTGGTGTTGGTATTATTAGACAAACTAACAGGACGCTACTTAGTGG
CTTATATTACACATCACTATCAGGTGATTTGTTAGGTTTTAAAAATGTTAGTGATGGTGTCATCTACTCTGTAACGCCA
TGTGATGTAAGCGCACAAGCAGCTGTTATTGATGGTACCATAGTTGGGGCTATCACTTCCATTAACAGTGAACTGTTAG
GTCTAACACATTGGACAACAACACCTAATTTTTATTACTACTCTATATATAATTACACAAATGATAGGACTCGTGGCAC
TGCAATTGACAGTAATGATGTTGATTGTGAACCTGTCATAACCTATTCTAACATAGGTGTTTGTAAAAATGGTGCTTTG
GTTTTTATTAACGTCACACATTCTGATGGAGACGTGCAACCAATTAGCACTGGTAACGTCACGATACCTACAAACTTTA
CTATATCCGTGCAAGTCGAATATATTCAGGTTTACACTACACCAGTGTCAATAGACTGTTCAAGATATGTTTGTAATGG
CAACCCTAGGTGTAACAAATTGTTAACACAATACGTTTCTGCATGTCTAACTATTGAGCAAGCACTTGCAATGGGTGCC
AGACTTGAAAACATGGAAGTTGATTCCATGTTATTTGTTTCTGAAAATGCCCTTAAATTGGCTTCTGTCGAAGCATTCA
ATAGTTCAGAAACTTTAGATCCTATTTACAAAGAATGGCCTAATATAGGTGGCTCTTGGCTAGAAGGTCTAAAATACAT
ACTTCCGTCCGATAATAGCAAACGTAAGTATCGTTCAGCTATAGAGGACTTGCTTTTTGCTAAGGTTGTAACATCTGGT
TTAGGTACAGTTGATGAAGATTATAAACGTTGTACAGGTGGTTATGACATAGCTGACTTAGTATGTGCTCAATACTACA
ATGGCATCATGGTGCTACCTGGTGTGGCTAATGCTGACAAAATGACTATGTACACAGCATCCCTCGCAGGTGGTATAAC
ATTAGGTGCACTTGGTGGAGGCGCCGTGGCTATACCTTTTGCAGTAGCAGTTCAGGCTAGACTTAATTATGTTGCTCTA
CAAACTGATGTATTGAACAAAAACCAGCAGATCCTGGCTAGTGCTTTCAATCAAGCTATTGGTAACATTACACAGTCAT
TTGGTAAGGTTAATGATGCTATACATCAAACATCACGAGGTCTTGCAACTGTTGCTAAAGCATTGGCAAAAGTGCAAGA
TGTTGTCAACACACAAGGGCAAGCTTTAAGCCACCTAACAGTACAATTGCAAAATAATTTCCAAGCCATTAGTAGTTCT
ATTAGTGACATTTATAATAGGCTTGATGAATTGAGTGCTGATGCACAAGTTGACAGGCTGATCACAGGAAGACTTACAG
CACTTAATGCATTTGTGTCTCAGACTCTAACCAGACAAGCCGAGGTTAGGGCTAGTAGACAACTTGCCAAAGACAAGGT
TAATGAATGCGTTAGGTCTCAGTCTCAGAGATTCGGATTCTGTGGTAATGGTACACATTTGTTTTCACTCGCAAATGCA
GCACCAAATGGCATGATCTTCTTTCACACAGTGCTATTACCAACGGCTTATGAAACTGTGACTGCTTGGGCAGGTATTT
GTGCTTTAGATGGTGATCGCACTTTTGGACTTGTCGTTAAAGATGTCCAGTTGACTTTGTTTCGTAATCTAGATGACAA
GTTCTATTTGACCCCCAGAACTATGTATCAGCCTAGAGTGGCAACTAGTTCTGATTTTGTTCAAATTGAAGGGTGCGAT
GTGCTGTTTGTTAATGCAACTGTAAGTGATTTGCCTAGTATTATACCTGATTATATTGATATTAATCAGACTGTTCAAG
ACATATTAGAAAATTTTAGACCAAATTGGACTGTACCTGAGTTGACATTTGACATTTTTAACGCAACCTATTTAAACCT
GACTGGTGAAATTGATGACTTAGAATTTAGGTCAGAAAAGCTACATAACACTACTGTAGAACTTGCCATTCTTATTGAC
AACATTAACAATACATTAGTCAATCTTGAATGGCTCAATAGAATTGAAACCTATGTAAAATGGCCTTGGTATGTGTGGC
TACTAATAGGCTTAGTAGTAATATTTTGCATACCATTACTGCTATTTTGCTGTTGTAGTACAGGTTGCTGTGGATGCAT
AGGTTGTTTAGGAAGTTGTTGTCACTCTATATGCAGTAGAAGACAATTTGAAAATTACGAACCTATTGAAAAAGTGCAC
GTCCATTAA

>gi|331265414|gb|DQ811786.2| TGEV Miller M60, complete genome

ATGAAAAAATTATTTGTGGTTTTGGTTGTAATGCCATTGATTTATGGAGACAATTTTCCTTGTTCTAAATTGACT
AATAGAACTATAGGTAACCATTGGAATCTCATTGAAACCTTCCTTCTAAATTATAGTAGTAGGTTATCACCTAATTCAG
ATGCGGTGTTAGGTGATTATTTTCCTACTGTACAACCTTGGTTTAATTGCATTCGCAATAATAGTAATGACCTTTATGT
TACATTGGAAAATCTTAAAGCATTGTATTGGGATTATGCTACAGAAAATATCACTTCGAATTACAAACAACGGTTAAAC
GTAGTCGTTAATGGATACCCATACTCCATCACAGTTACAACAACCCGCAATTTTAATTCTGCTGAAGGTGCTATTATAT
GCATTTGCAAGGGCTCACCACCTACTACCACCACAGAATCTAGTTTGACTTGCAATTGGGGTAGTGAGTGCAGGTTAAA
CCATAAGTTCCCTATATGTCCTTCTAATTCAGAGGCAAATTGTGGTAATATGCTGTATGGCCTACAATGGTTTGCAGAT
GCGGTTGTTGCTTATTTACATGGTGCTAGTTACCGTATTAGTTTTGAAAATCAATGGTCTGGCACTGTTACACTTGGTG
ATATGCGTGCGACTACATTAGAAACCGCTGGCACGCTTGTAGACCTTTGGTGGTTTAATCCTGTTTATGATGTCAGTTA
TTATAGAGTTAATAATAAAAATGGTACTACCGTAGTTTCCAATTGCACTGATCAATGTGCTAGTTATGTGGCTAATGTT
TTTACTACACAGCCAGGAGGCTTTATACCATCAGATTTAGTTTTAATAATTGGTTCCTTCTAACTAATAGCTCCACGT
TGGTTAGTGGTAAATTAGTTACCAAACAGCCGTTATTAGTTAATTGCTTATGGCCAGTCCCTAGCTTTGAAGAAGCAGC
TTCTACATTTTGTTTTGAAGGTGCTGGCTTTGATCAATGTAATGGTGCTGTTTTAAATAACACTGTAGACGTCATCAGG
TTTAACCTTAATTTTACTACAAATGTACAATCAGGTAAGGGTGCCACAGTGTTTTCATTGAACACAACGGGTGGTGTCA
CTCTTGAAATCTCATGTTATAATGATACAGTGAGTGACTCGAGCTTTTCCAGTTACGGTGAAATGCCGTTCGGCGTAAC
TGATGGACCACGGTACTGTTACGTACTCTATAATGGCACAGCTCTTAAGTATCTAGGAACATTACCACCTATTGTCAAG
GAGATTGCTATTAGTAAGTGGGGCCATTTTTATATTAATGGTTACAATTTCTTTAGCACATTTCCTATTGATTGTATAT
CTTTTAATTTGACCACTGGTGATAGTGACGTTTTCTGGACAATAGCTTACACATCGTACACTGAAGCATTAGTACAAGT
TGAAAACACAGCTATTACAAAGGTGACGTATTGTAATAGTTACGTTAATAACATTAAATGCTCTCAACTTACTGCTAAT
TTGAATAATGGATTTTATCCTGTTTCTTCAAGTGAAGTTGGTCTTGTCAATAAGAGTGTTGTGTTACTACCTAGCTTTT
ACACACATACCATTGTTAACATAACTATTGGTCTTGGTATGAAGCGTAGTGGTTATGGTCAACCCATAGCCTCAACATT
AAGTAACATTACACTACCAATGCAGGATAACAACAACGATGTGTACTGTATTCGTTCTGACCAATTTTCAGTTTATGTT
CATTCTACTTGCAAAAGTGCTTTATGGGACAATGTTTTTAAGCGAAACTGCACGGACGTTTTAGATGCCACAGCTGTTA
TAAAAACTGGTACTTGTCCTTTCTCATTTGATAAATTGAACAATTACTTAACTTTTAACAAGTTCTGTTTGTCGTTGAG
TCCTGTTGGTGCTAATTGTAAGTTTGATGTAGCTGCCCGTACAAGAACCAATGATCAGGTTGTTAGAAGTTTGTATGTA
ATATATGAAGAAGGAGACAACATAGTGGGTGTACCGTCTGATAATAGTGGTTTACACGATTTGTCAGTGCTACACCTAG
ATTCCTGCACAGATTACAATATATATGGTAGAACTGGTGTTGGTATTATTAGACAAACTAACAGGACGCTACTTAGTGG
CTTATATTACACATCACTATCAGGTGATTTGTTAGGTTTTAAAAATGTTAGTGATGGTGTCATCTACTCTGTAACGCCA
TGTGATGTAAGCGCACAAGCAGCTGTTATTGATGGTACCATAGTTGGGGCTATCACTTCCATTAACAGTGAACTGTTAG
GTCTAACACATTGGACAACAACACCTAATTTTTATTACTACTCTATATATAATTACACAAATGATAGGACTCGTGGCAC
TGCAATTGACAGTAATGATGATTGTGAACCTGTCATAACCTATTCTAACATAGGTGTTTGTAAAAATGGTGCTTTGGTT
TTTATTAACGTCACACATTCTGATGGAGACGTGCAACCAATTAGCACTGGTAACGTCACGATACCTACAAACTTTACTA
TATCCGTGCAAGTCGAATATATTCAGGTTTACACTACACCAGTGTCAATAGACTGTTCAAGATATGTTTGTAATGGCAA
CCCTAGGTGTAACAAATTGTTAACACAATACGTTTCTGCATGTCAAACTATTGAGCAAGCACTTGCAATGGGTGCCAGA
CTTGAAAACATGGAAGTTGATTCCATGTTATTTGTTTCTGAAAATGCCCTTAAATTGGCTTCTGTCGAAGCATTCAATA
GTTCAGAAACTTTAGATCCTATTTACAAAGAATGGCCTAATATAGGTGGCTCTTGGCTAGAAGGTCTAAAATACATACT
TCCGTCCGATAATAGCAAACGTAAGTATCGTTCAGCTATAGAGGACTTGCTTTTTGCTAAGGTTGTAACATCTGGTTTA
GGTACAGTTGATGAAGATTATAAACGTTGTACAGGTGGTTATGACATAGCTGACTTAGTATGTGCTCAATACTACAATG
GCATCATGGTGCTACCTGGTGTGGCTAATGCTGACAAAATGACTATGTACACAGCATCCCTCGCAGGTGGTATAACATT
AGGTGCACTTGGTGGAGGCGCCGTGGCTATACCTTTTGCAGTAGCAGTTCAGGCTAGACTTAATTATGTTGCTCTACAA
ACTGATGTATTGAACAAAAACCAGCAGATCCTGGCTAGTGCTTTCAATCAAGCTATTGGTAACATTACACAGTCATTTG
GTAAGGTTAATGATGCTATACATCAAACATCACGAGGTCTTGCAACTGTTGCTAAAGCATTGGCAAAAGTGCAAGATGT
TGTCAACACACAAGGGCAAGCTTTAAGCCACCTAACAGTACAATTGCAAAATAATTTCCAAGCCATTAGTAGTTCTATT
AGTGACATTTATAATAGGCTTGATGAATTGAGTGCTGATGCACAAGTTGACAGGCTGATCACAGGAAGACTTACAGCAC

TTAATGCATTTGTGTCTCAGACTCTAACCAGACAAGCCGAGGTTAGGGCTAGTAGACAACTTGCCAAAGACAAGGTTAA
TGAATGCGTTAGGTCTCAGTCTCAGAGATTCGGATTCTGTGGTAATGGTACACATTTGTTTTCACTCGCAAATGCAGCA
CCAAATGGCATGATCTTCTTTCACACAGTGCTATTACCAACGGCTTATGAAACTGTGACTGCTTGGGCAGGTATTTGTG
CTTTAGATGGTGATCGCACTTTTGGACTTGTCGTTAAAGATGTCCAGTTGACTTTGTTTCGTAATCTAGATGACAAGTT
CTATTTGACCCCCAGAACTATGTATCAGCCTAGAGTGGCAACTAGTTCTGATTTTGTTCAAATTGAAGGGTGCGATGTG
CTGTTTGTTAATGCAACTGTAAGTGATTTGCCTAGTATTATACCTGATTATATTGATATTAATCAGACTGTTCAAGACA
TATTAGAAAATTTTAGACCAAATTGGACTGTACCTGAGTTGACATTTGACATTTTTAACGCAACCTATTTAAACCTGAC
TGGTGAAATTGATGACTTAGAATTTAGGTCAGAAAAGCTACATAACACTACTGTAGAACTTGCCATTCTTATTGACAAC
ATTAACAATACATTAGTCAATCTTGAATGGCTCAATAGAATTGAAACCTATGTAAAATGGCCTTGGTATGTGTGGCTAC
TAATAGGCTTAGTAGTAATATTTTGCATACCATTACTGCTATTTTGCTGTTGTAGTACAGGTTGCTGTGGATGCATAGG
TTGTTTAGGAAGTTGTTGTCACTCTATATGCAGTAGAAGACAATTTGAAAATTACGAACCTATTGAAAAAGTGCACGTC
CATTAA

>gi|110746812|gb|DQ811787.1| PRCV ISU-1, complete genome

ATGAAAACATTATTTGTGGTTTTGGTTATAATGCCATTGATTTATGGAGATAATTTTCCTTGTTCCAATTGCACT
GATCAATGTGCTAGTTATGTGGCTAATGTCTTTGTTACACAGCCAGGAGGCTTTATACCATCAGATTTTAGTTTTAATA
ATTGGTTCCTTCTAACTAATAGCTCCACGTTGGTTAGTGGTAAATTAGTTACCAAACAGCCATTATTAGTTAATTGCTT
ATGGCCAGTCCCTAGCTTTGAAGAAGCAGCTTCTACATTTTGTTTTGAAGGTGCTGACTTTGATCAATGTAATGGTGCT
GTCTTAAATAATACTGTAGATGTCATCAGGTTTAACCTTAATTTTACTACAAATGTACAATCAGGTAAGGGTGCCACAG
TGTTTTCATTGAACACAACGGGTGGTGTCACTCTTGAAATCTCATGTTATAATGATACAGGGAGTGACTCGAGCTTTTC
CAGTTACGGTGAAATTCCGTTCGGCGTGACTGATGGACCACGGTACTGTTACGTACTCTATAATGGCACAGCTCTTAAG
TATTTAGGAACATTACCACCTAGTGTCAAGGAGATTGCTATTAGTAAGTGGGGCCATTTTTATATTAATGGTTACAATT
TCTTTAGCACATTTCCTATTGATTGTATATCTTTCAATTTGACCACTGGTGATAGTGACGTTTTCTGGACAATAGCTTA
CACATCGTACACTGAAGCGTTAGTACAAGTTGAAAACACAGCTATTACAAAGGTGACGTATTGTAATAGTTACGTTAAT
AACATTAAATGCTCTCAACTTACTGCTAATTTGAATAATGGATTTTATCCTGTTTCTTCAAGTGAAGTTGGTCTTGTCA
ATAAGAGTGTTGTGTTACTACCTAGCTTTTACACGCATACCATTGTTAACATAACTATTGGTCTTGGTATGAAGCGTAG
TGGTTATGGTCAACCCATAGCCTCAACTCTAAGTAACATTACACTACCAACGCAGGATAACAACACCGATGTGTACTGT
ATTCTTTCTGATCAATTTTCAGTTTATGTTCATTCTACTTGTAAAAGTGCTTTGTGGGACAATGTTTTTAAGCGAAACT
GCACGGACGTTTTAGATGCCACAGCTGTTATAAAAACTGGTACTTGTCCTTTCTCATTTGATAAACTGAATAATTACTT
AACTTTTAACAAGTTCTGTTTGTCGTTGAGTCCTGTTGGTGCTAATTGCAAGTTTGATGTAGTTGCCCGTACAAGAACC
AATGATCAGGTTGTTAGAAGTTTGTATGTAATATATGAAGAAGGAGACAACATAGTAGGTGTACCTTCTGACAATAGTG
GTTTGCACGATTTGTCAGTGCTACACCTAGATTCATGCACAGATTACAATATATATGGTAGAACCGGTGTTGGTATTAT
TAGACAAACTAACAGGACACTACTTAGTGGCTTATATTACACATCACTATCTGGTGATTTGTTAGGTTTTAAAAATGTT
AGTGATGGTGTTATCTACTCTGTAACGCCGTGTGATGTAAGCGCACAAGCAGCTGTTATTGATGGTACCATAGTTGGGG
CTATCACTTCTATTAACAGTGAATTGTTAGGTCTAACACATTGGACGACAACACCTAATTTTTATTACTACTCTATATA
TAATTACACAAATGATAGGATTCGTGGCACTGCAATTGACAGTAATGACGTTGATTGTGAACCTGTCATAACCTATTCT
AACATAGGTGTTTGTAAAAACGGTGCTTTGGTTTTTATTAACGTTACACATTCTGATGGAGACGTGCAACCAATTAGCA
CTGGTAACGTCACGATACCTACAAATTTTACTATATCCGTGCAAGTCGAATACATTCAGGTTTACACTACACCAGTGTC
AATAGACTGTTCAAGATATGTTTGTAATGGCAACCCTAGGTGTAACAAACTATTAACACAATATGTTTCTGCATGTCAA
ACTATTGAGCAAGCACTTGCAATGAGTGCCAGACTTGAAAATATGGAAGTTGATTCCATGTTATTTGTTTCTGAAAATG
CCCTTAAATTGGCTTCTGTTGAAGCATTCAATAGTTCAGAAACTTTAGATCCTATTTACAAAGACTGGCCTAATATAGG
TGGCTCTTGGCTAGAAGGTCTAAAATATATACTGCCGTCCGATAATAGCAAACGTCAGTATCGTTCAGCTATAGAGGAC

TTGCTTTTTTCTAAGGTTGTAACATCTGGTTTAGGTACAGTTGATGAAGATTATAAACGTTGTACAGGTGGTTATGACA
TAGCTGACTTAGTGTGTGCTCAATACTATAATGGCATCATGGTGCTACCTGGTGTGGCTAATGCTGACAAAATGACTAT
GTACACAGCATCCCTCGCAGGTGGTATAACATTAGGTGCACTTGGTGGAGGCGCCGTGGCTATACCTTTTGCAGTAGCA
GTTCAGGCTAGACTTAATTATGTTGCTCTACAAACTGATGTATTAAACAAAAACCAGCAGATCCTGGCTAGTGCTTTTA
ATCAAGCTATTGGTAACATTACACAGTCATTTGGTAAGGTTAATGATGCTATACATCAAACGTCACGAGGTCTTGCAAC
TGTTGCTAAAGCATTGGCAAAAGTGCAAGATGTTGTTAATACACAAGGGCAAGCTTTAAGCCACCTAACAGTACAATTG
CAAAATAATTTCCAAGCCATTAGTAGCTCTATTAGTGACATTTATAATAGGCTTGATGAATTGAGTGCTGATGCACAAG
TTGACAGGCTGATCACAGGAAGACTTACAGCACTTAATGCATTTGTATCTCAGACTCTAACCAGACAAGCCGAGGTTAG
GGCTAGTAGACAACTTGCCAAAGACAAGGTTAATGAATGCGTTAGGTCTCAGTCTCAGAGATTCGGCTTCTGTGGTAAT
GGTACACATTTGTTTTCACTCGCAAATGCAGCACCAAATGGCATGATTTTCTTTCACACAGTGCTATTACCAACGGCCT
ATGAAACTGTGACTGCTTGGGCAGGTATTTGTGCTTTAGATGGTGACCGCACTTTTGGACTTGTCGTTAAAGATGTCCA
GCTGACTTTGTTTCGTAATCTAGATGACAAGTTCTATTTGACCCCCAGAACTATGTATCAGCCTAGAGTGGCAACTAGT
TCTGATTTTGTTCAAATTGAAGGGTGCGATGTGCTGTTTGTTAATGCAACTGTAAGTGATTTGCCTAGTATTATACCTG
ATTATATTGATATTAATCAAACTGTTCAAGACATACTAGAAAATTTCAGACCAAATTGGACTGTACCTGATTTGACATT
TGACATTTTTAACGCAACCTATTTAAACCTGACTGTTGAAATTGATGACTTAGAATTTAGGTCAGAAAAGCTACATAAC
ACTACTGTAGAACTTGCCATTCTTATTGACAACATTAACAATACATTAGTCAATCTTGAATGGCTTAATAGAATTGAAA
CCTATGTAAAATGGCCTTGGTATGTGTGGCTACTAATAGGTTTAGTAGTAATATTTTGCATACCATTATTGCTATTTTG
CTGTTGTAGTACAGGTTGCTGTGGATGCATAGGTTGTTTAGGAAGTTGTTGTCACTCTATATGCAGTAGAAGACAATTT
GAAAATTATGAACCTATTGAAAAAGTGCACGTCCATTAA

>gi|110746821|gb|DQ811788.1| TGEV Purdue P115, complete genome

ATGAAAAAACTATTTGTGGTTTTGGTCGTAATGCCATTGATTTATGGAGACAATTTTCCTTGTTCTAAATTGACT
AATAGAACTATAGGCAACCAGTGGAATCTCATTGAAACCTTCCTTCTAAACTATAGTAGTAGGTTACCACCTAATTCAG
ATGTGGTGTTAGGTGATTATTTTCCTACTGTACAACCTTGGTTTAATTGCATTCGCAATGATAGTAATGACCTTTATGT
TACACTGGAAAATCTTAAAGCAGTGTATTGGGATTATGCTACAGAAAATATCACTTGGAATCACAGACAACGGTTAAAC
GTAGTCGTTAATGGATACCCATACTCCATCACAGTTACAACAACCCGCAATTTTAATTCTGCTGAAGGTGCTATTATAT
GCATTTGTAAGGGCTCACCACCTACTACCACCACAGAATCTAGTTTGACTTGCAATTGGGGTAGTGAGTGCAGGTTAAA
CCATAAGTTCCCTATATGTCCTTCTAATTCAGAGGCAAATTGTGGTAATATGCTGTATGGCCTACAATGGTTTGCAGAT
GAGGTTGTTGCTTATTTACATGGTGCTAGTTACCGTATTAGTTTTGAAAATCAATGGTCTGGCACTGTCACATTTGGTG
ATATGCGTGCGACAACATTAGAAGTCGCTGGCACGCTTGTAGACCTTTGGTGGTTTAATCCTGTTTATGATGTCAGTTA
TTATAGGGTTAATAATAAAAATGGTACTACCGTAGTTTCCAATTGCACTGATCAATGTGCTAGTTATGTGGCTAATGTT
TTTACTACACAGCCAGGAGGTTTTATACCATCAGATTTAGTTTTAATAATTGGTTCCTTCTAACTAATAGCTCCACGT
TGGTTAGTGGTAAATTAGTTACCAAACAGCCGTTATTAGTTAATTGCTTATGGCCAGTCCCTAGCTTTGAAGAAGCAGC
TTCTACATTTTGTTTTGAGGGTGCTGGCTTTGATCAATGTAATGGTGCTGTTTTAAATAATACTGTAGACGTCATTAGG
TTCAACCTTAATTTTACTACAAATGTACAATCAGGTAAGGGTGCCACAGTGTTTTCATTGAACACAACGGGTGGTGTCA
CTCTTGAAATTTCATGTTATACAGTGAGTGACTCGAGCTTTTTCAGTTACGGTGAAATTCCGTTCGGCGTAACTGATGG
ACCACGGTACTGTTACGTACACTATAATGGCACAGCTCTTAAGTATTTAGGAACATTACCACTTAGTGTCAAGGAGATT
GCTATTAGTAAGTGGGGCCATTTTTATATTAATGGTTACAATTTCTTTAGCACATTTCCTATTGATTGTATATCTTTTA
ATTTGACCACTGGTGATAGTGACGTTTTCTGGACAATAGCTTACACATCGTACACTGAAGCATTAGTACAAGTTGAAAA
CACAGCTATTACAAAGGTGACGTATTGTAATAGTCACGTTAATAACATTAAATGCTCTCAAATTACTGCTAATTTGAAT
AATGGATTTTATCCTGTTTCTTCAAGTGAAGTTGGTCTTGTCAATAAGAGTGTTGTGTTACTACCTAGCTTTTACACAC
ATACCATTGTTAACATAACTATTGGTCTTGGTATGAAGCGTAGTGGTTATGGTCAACCCATAGCCTCAACATTAAGTAA

CATCACACTACCAATGCAGGATCACAACACCGATGTGTACTGTATTCGTTCTGACCAATTTTCAGTTTATGTTCATTCT
ACTTGCAAAAGTGCTTTATGGGACAATATTTTTAAGCGAAACTGCACGGACGTTTTAGATGCCACAGCTGTTATAAAAA
CTGGTACTTGTCCTTTCTCATTTGATAAATTGAACAATTACTTAACTTTTAACAAGTTCTGTTTGTCGTTGAGTCCTGT
TGGTGCTAATTGTAAGTTTGATGTAGCTGCCCGTACAAGAACCAATGAGCAGGTTGTTAGAAGTTTGTATGTAATATAT
GAAGAAGGAGACAACATAGTGGGTGTACCGTCTGATAATAGTGGTGTGCACGATTTGTCAGTGCTACACCTAGATTCCT
GCACAGATTACAATATATATGGTAGAACTGGTGTTGGTATTATTAGACAAACTAACAGGACGCTACTTAGTGGCTTATA
TTACACATCACTATCAGGTGATTTGTTAGGTTTTAAAAATGTTAGTGATGGTGTCATCTACTCTGTAACGCCATGTGAT
GTAAGCGCACAAGCAGCTGTTATTGATGGTACCATAGTTGGGGCTATCACTTCCATTAACAGTGAACTGTTAGGTCTAA
CACATTGGACAACAACACCTAATTTTTATTACTACTCTATATATAATTACACAAATGATAGGACTCGTGGCACTGCAAT
TGACAGTAATGATGTTGATTGTGAACCTGTCATAACCTATTCTAACATAGGTGTTTGTAAAAATGGTGCTTTTGTTTTT
ATTAACGTCACACATTCTGATGGAGACGTGCAACCAATTAGCACTGGTAATGTCACGATACCTACAAACTTTACCATAT
CCGTGCAAGTCGAATATATTCAGGTTTACACTACACCAGTGTCAATAGACTGTTCAAGATATGTTTGTAATGGTAACCC
TAGGTGTAACAAATTGTTAACACAATACGTTTCTGCATGTCAAACTATTGAGCAAGCACTTGCAATGGGTGCCAGACTT
GAAAACATGGAGGTTGATTCCATGTTGTTTGTTTCTGAAAATGCCCTTAAATTGGCATCTGTTGAAGCATTCAATAGTT
CAGAAACTTTAGACCCTATTTACAAAGAATGGCCTAATATAGGTGGTTCTTGGCTAGAAGGTCTAAAATACATACTTCC
GTCCCATAATAGCAAACGTAAGTATCGTTCAGCTATAGAGGACTTGCTTTTTGATAAGGTTGTAACATCTGGTTTAGGT
ACAGTTGATGAAGATTATAAACGTTGTACAGGTGGTTATGACATAGCTGACTTAGTATGTGCTCAATACTATAATGGCA
TCATGGTGCTACCTGGTGTGGCTAATGCTGACAAAATGACTATGTACACAGCATCCCTTGCAGGTGGTATAACATTAGG
TGCACTTGGTGGAGGCGCCGTGGCTATACCTTTTGCAGTAGCAGTTCAGGCTAGACTTAATTATGTTGCTCTACAAACT
GATGTATTGAACAAAAACCAGCAGATTCTGGCTAGTGCTTTCAATCAAGCTATTGGTAACATTACACAGTCATTTGGTA
AGGTTAATGATGCTATACATCAAACATCACGAGGTCTTGCTACTGTTGCTAAAGCATTGGCAAAAGTGCAAGATGTTGT
CAACATACAAGGGCAAGCTTTAAGCCACCTAACAGTACAATTGCAAAATAATTTCCAAGCCATTAGTAGTTCTATTAGT
GACATTTATAATAGGCTTGACGAATTGAGTGCTGATGCACAAGTTGACAGGCTGATCACAGGAAGACTTACAGCACTTA
ATGCATTTGTGTCTCAGACTCTAACCAGACAAGCGGAGGTTAGGGCTAGTAGACAACTTGCCAAAGACAAGGTTAATGA
ATGCGTTAGGTCTCAGTCTCAGAGATTCGGATTCTGTGGTAATGGTACACATTTGTTTTCACTCGCAAATGCAGCACCA
AATGGCATGATTTTCTTTCACACAGTGCTATTACCAACGGCTTATGAAACTGTGACTGCTTGGCCAGGTATTTGTGCTT
CAGATGGTGATCGCACTTTTGGACTTGTCGTTAAAGATGTCCAGTTGACTTTGTTTCGTAATCTAGATGACAAGTTCTA
TTTGACCCCCAGAACTATGTATCAGCCTAGAGTTGCAACTAGTTCTGACTTTGTTCAAATTGAAGGGTGCGATGTGCTG
TTTGTTAATGCAACTGTAAGTGATTTGCCTAGTATTATACCTGATTATATTGATATTAATCAGACTGTTCAAGACATAT
TAGAAAATTTTAGACCAAATTGGACTGTACCTGAGTTGACATTTGACATTTTTAACGCAACCTATTTAAACCTGACTGG
TGAAATTGATGACTTAGAATTTAGGTCAGAAAAGCTACATAACACCACTGTAGAACTTGCCATTCTCATTGACAACATT
AACAATACATTAGTCAATCTTGAATGGCTCAATAGAATTGAAACCTATGTAAAATGGCCTTGGTATGTGTGGCTACTAA
TAGGCTTAGTAGTAATATTTTGCATACCATTACTGCTATTTTGCTGTTGTAGTACAGGTTGCTGTGGATGCATAGGTTG
TTTAGGAAGTTGTTGTCACTCTATATGTAGTAGAAGACAATTTGAAAATTACGAACCAATTGAAAAAGTGCACGTCCAT
TAA

>gi|155369167|dbj|AB354579.1| Bovine coronavirus genomic RNA, complete genome, strain: Kakegawa

ATGTTTTTGATACTTTTAATTTCCTTACCAATGGCTTTTGCTGTTATAGGAGATTTAAAGTGTACTACGGTTTCC
ATTAATGATGTTGACACCGGTGCTCCTTCTATTAGCACTGATACTGTCGATGTTACTAATGGTTTAGGTACTTATTATG
TTTTAGATCGTGTGTATTTAAATACTACGTTGTTGCTTAATGGTTACTACCCTACTTCAGGTTCTACATATCGTAATAT
GGCACTGAAGGGAACTTTACTATTGAGCAGACTATGGTTTAAACCACCTTTTCTTTCTGATTTTATTAATGGTATTTTT

GCTAAGGTCAAAAATACCAAGGTTATTAAAAAGGGTGTAATGTATAGTGAGTTTCCTGCTATAACTATAGGTAGTACTT
TTGTAAATACATCCTATAGTGTGGTAGTACAACCACATACTACCAATTTGGATAATAAATTACAAGGTCTCTTAGAGAT
CTCTGTTTGCCAGTATACTATGTGCGAGTACCCACATACGATTTGTCATCCTAATCTGGGTAATCGACGCGTAGAACTA
TGGCATTGGGATACAGGTGTTGTTTCCTGTTTATATAAGCGTAATTTCACATATGATGTGAATGCTGATTACTTGTATT
TCCATTTTTATCAAGAAGGTGGTACTTTTTATGCATATTTTACAGACACTGGTGTTGTTACTAAGTTTCTGTTTAATGT
TTATTTAGGCACGGTGCTTTCACATTATTATGTCCTGCCTTTGACTTGTTATAGTGCTATGACTTTAGAATATTGGGTT
ACACCTCTCACTTCTAAACAATATTTACTAGCTTTCAATCAAGATGGTGTTATTTTTAATGCTGTTGATTGTAAGAGTG
ATTTTATGAGTGAGATTAAGTGTAAAACACTATCTATAGCACCATCTACTGGTGTTTATGAATTAAACGGTTACACTGT
TCAGCCAATTGCAGATGTTTACCGACGTATACCTAATCTTCCCGATTGTAATATAGAGGCTTGGCTTAATGATAAGTCG
GTGCCCTCTCCATTAAATTGGGAACGTAAGACCTTTTCAAATTGTAATTTTAATATGAGCAGCCTGATGTCTTTTATTC
AGGCAGACTCATTTACTTGTAATAATATTGATGCTGCTAAGATATATGGTATGTGTTTTTCCAGCATAACTATAGATAA
GTTTGCTATACCCAATGGTAGGAAGGTTGACCTACAATTGGGCAATTTGGGCTATTTGCAGTCTTTTAACTATAGAATT
GATACTACTGCTACAAGTTGTCAGTTGTATTATAATTTACCTGCTGCTAATGTTTCTGTTAGCAGGTTTAATCCTTCTA
CTTGGAATAGGAGATTTGGTTTTACAGAACAATCTGTTTTTAAGCCTCAACCTGTAGGTGTTTTTACTCATCATGATGT
TGTTTATGCACAACATTGTTTTAAAGCTCCCACAAATTTCTGTCCGTGTAAATTGGATGGGTCTTTGTGTGTAGGTAAT
GGTCCTGGTATAGATGCTGGTTATAAAAATAGTGGTATAGGCACTTGTCCTGCAGGTACTAATTATTTAACTTGCCATA
ATGCTGCCCAATGTGATTGTTTGTGCACTCCCGACCCCATTACATCTAAATCTACAGGGCCTTACAAGTGCCCCCAAAC
TAAATACTTAGTTGGCATAGGTGAGCACTGTTCGGGTCTTGCTATTAAAAGTGATTATTGTGGAGGTAATCCTTGTACT
TGCCAACCACAAGCATTTTTGGGTTGGTCTGTTGACTCTTGTTTACAAGGGGATAGGTGTAATATTTTTGCTAATTTTA
TTTTGCATGATGTTAATAGTGGTACTACTTGTTCTACTGATTTACAAAAATCAAACACAGACATAATTCTTGGTGTTTG
TGTTAATTATGATCTTTATGGTATTACAGGCCAAGGTATTTTTGTTGAGGTTAATGCGACTTATTATAATAGTTGGCAG
AACCTTTTATATGATTCTAATGGTAATCTCTATGGTTTTAGAGACTACTTAACAAACAGAACTTTTATGATTCGTAGTT
GCTATAGCGGTCGTGTTTCAGCGGCCTTTCATGCTAACTCTTCCGAACCAGCATTGCTATTTCGGAATATTAAATGCAA
TTACGTTTTTAATAATACTCTTTCACGACAGCTGCAACCTATTAACTATTTTGATAGTTATCTTGGTTGTGTTGTCAAT
GCTGATAATAGTACTTCTAGTGTTGTTCAAACATGTGATCTCACAGTAGGTAGTGGTTACTGTGTGGATTACTCTACAA
AAAGACGAAGTCGTAGAGCGATTACCACTGGTTATCGGTTTACTAATTTTGAGCCATTTACTGTTAATTCAGTAAATGA
TAGTTTAGAACCTGTAGGTGGTTTGTATGAAATTCAAATACCTTCAGAGTTTACTATAGGTAATATGGAGGAGTTTATT
CAAACAAGCTCTCCTAAAGTTACTATTGATTGTTCTGCTTTTGTCTGTGGTGATTATGCAGCATGTAAATCACAGTTGG
TTGAATATGGTAGCTTCTGTGACAATATTAATGCTATACTCACAGAAGTAAATGAACTACTTGACACTACACAGTTGCA
AGTAGCTAATAGTTTAATGAATGGTGTCACTCTTAGCACTAAGCTTAAAGATGGCGTTAATTTCAATGTAGACGACATC
AATTTTTCCCCTGTATTAGGTTGTTTAGGAAGCGCTTGTAGTAAAGTTTCCAGCAGATCTGCTATAGAGGATTTACTTT
TTTCTAAAGTAAAGTTATCTGATGTCGGTTTCGTTGAGGCTTATAATAATTGTACTGGAGGTGCCGAAATTAGGGACCT
CATTTGTGTGCAAAGTTATAATGGTATCAAAGTGTTGCCTCCACTGCTCTCAGTAAATCAGATCAGTGGATACACTTTG
GCTGCCACCTCTGCTAGTCTGTTTCCTCCTTGGTCAGCAGCAGCAGGTGTACCATTTTATTTAAATGTTCAGTATCGTA
TTAATGGGATTGGTGTTACCATGGATGTGTTAAGTCAAAATCAAAAGCTTATTGCTAATGCATTTAACAATGCTCTTGA
TGATATTCAGGAAGGGTTTGATGCTACCAATTCTGCTTTAGTTAAAATTCAAGCTGTTGTTAATGCAAATGCTGAAGCT
CTTAATAACTTATTGCAACAACTCTCTAATAGATTTGGTGCTATAAGTTCTTCTTTACAAGAAATTCTATCTAGACTGG
ATGCTCTTGAAGCGCAAGCTCAGATAGACAGACTTATTAATGGGCGTCTTACCGCTCTTAATGCTTATGTTTCTCAACA
GCTTAGTGATTCTACACTAGTAAAATTTAGTGCAGCACAAGCTATGGAGAAGGTTAATGAATGTGTCAAAAGCCAATCA
TCTAGGATAAATTTTTGTGGTAATGGTAATCATATTATATCATTAGTGCAGAATGCTCCATATGGTTTGTATTTTATCC
ACTTTAGCTATGTCCCTACTAAGTATGTCACTGCGAAGGTTAGTCCCGGTCTGTGCATTGCTGGTGATAGAGGTATAGC
CCCTAAGAGTGGTTATTTTGTTAATGTAAATAATACTTGGATGTTCACTGGTAGTGGTTATTACTACCCTGAACCCATA
ACTGGAAATAATGTTGTTGTTATGAGTACCTGTGCTGTTAACTATACTAAAGCGCCGGATGTAATGCTGAACATTTCAA
CACCCAACCTCCATGATTTTAAGGAAGAGTTGGATCAATGGTTTAAAAACCAAACATCAGTGGCACCAGATTTGTCACT

TGATTATATAAATGTTACATTCTTGGACCTACAAGATGAAATGAATAGGTTACAGGAGGCAATAAAAGTTTTAAATCAG
AGCTACATCAATCTCAAGGACATTGGTACATATGAGTATTATGTAAAATGGCCTTGGTATGTATGGCTTTTAATTGGCT
TTGCTGGTGTAGCTATGCTTGTTTTACTATTCTTCATATGCTGTTGTACAGGATGTGGGACTAGTTGTTTTAAGATATG
TGGTGGTTGTTGTGATGATTATACTGGACACCAGGAGTTAGTAATTAAAACATCACATGACGACTAA

>gi|18033971|gb|AF391542.1| Bovine coronavirus isolate BCoV-LUN, complete genome

ATGTTTTTGATACTTTTAATTTCCTTACCAACGGCTTTTGCTGTTATAGGAGATTTAAAGTGTACTACAGTTTCC
ATTAATGATGTTGACACTGGTGTTCCTTCTATTAGCACTGATACTGTCGATGTTACTAAAGGTTTAGGTACTTATTATG
TTTTAGATCGTGTGTATTTAAATACTACGTTGTTGCTTAATGGTTATTACCCTACTTCAGGTTCTACATATCGTAATAT
GGCACTGAAGGGAACTTTACTATTGAGCACACTGTGGTTTAAACCACCTTTTCTTTCTGATTTTACTAATGGTATTTTT
GCTAAGGTCAAAAATACCAAGGTTATTAAAGATGGTGTAATGTATAGTGAGTTTCCTGCTATAACTATAGGTAGTACTT
TTGTAAATACATCCTATAGTGTGGTAGTACAACCACATACTACCATTTTGGGTAATAAATTACAAGGTTTCTTAGAGAT
CTCTGTTTGCCAGTATACTATGTGCGAGTACCCAAATACGATTTGTAATCCTAATCTGGGTAATCGACGCGTAGAACTA
TGGCATTGGGATACAGGTGTTGTTTCCTGTTTATATAAGCGTAATTTCACATATGATGTGAATGCTGATTATTTGTATT
TCCATTTTTATCAAGAAGGTGGTACTTTTTATGCATATTTTACAGACACTGGTGTCGTTACTAAGTTTCTGTTTAATGT
TTATTTAGGCACGGTGCTCTCACATTATTATGTCATGCCTTTGACTTGTAATAGTGCTCTGACTTTAGAATATTGGGTT
ACACCCCTCACTTCTAAACAATATCTACTAGCTTTCAATCAAGATGGTGTTATTTTTAATGCTGTTGATTGTAAGAGTG
ATTTTATGAGTGAGATTAAGTGTAAAACACTATCTATAGCACCATCTACTGGTGTTTATGAATTAAACGGTTACACTGT
TCAGCCAATTGCAGATGTTTACCGACGTATACCTAATCTTCCCGATTGCAATATTGAGGCTTGGCTTAATGATAAGTCG
GTGCCCTCTCCATTAAATTGGGAACGTAAGACTTTTTCAAATTGTAATTTTAATATGAGCAGCCTGATGTCTTTTATTC
AGGCAGACTCATTTACTTGTAATAATATTGATGCTGCTAAGATATATGGTATGTGTTTTTCCAGCATAACTATAGATAA
GTTTGCAATACCCAATGGTAGGAAGGTTGACCTACAATTGGGTAATTTGGGTTATTTGCAGTCTTTTAACTATAGAATT
GATACTACTGCTACAAGTTGTCAGTTGTATTATAATTTACCTGCTGCTAATGTTTCTGTTAGCAGGTTTAATCCTTCTA
CTTGGAATAGGAGATTTGGTTTTACAGAACAATCTGTTTTTAAGCCTCAACCTGCAGGTGTTTTTACTGATCATGATGT
TGTTTATGCACAACATTGTTTTAAAGCTCCCACAAATTTCTGTCCGTGTAAATTGGATGGGTCTTTGTGTGTAGGTAAT
GGTCCTGGTATAGATGCTGGTTATAAAACTAGTGGCATAGGCACTTGTCCTGCAGGTACTAATTATTTAACTTGCCATA
ATGCTGCCCAATGTGATTGTTTGTGCACTCCAGACCCCATTACATCTAAAGCTACAGGGCCTTACAAGTGCCCCCAAAC
TAAATATTTAGTTGGCATAGGTGAGCACTGTTCGGGTCTTGCTATTAAAAGTGATCATTGTGGAGGTAACCCTTGTACT
TGCCAACCACAAGCATTTTTGGGGTGGTCTGTTGACTCTTGTTTACAAGGGGATAGGTGTAATATTTTTGCTAATTTCA
TTTTGCATGATGTTAATAGTGGTACTACTTGTTCTACTGATTTACAAAAATCAAACACAGACATAATTCTTGGTGTTTG
TGTTAATTATGATCTTTATGGTATTACTGGCCAAGGTATCTTTGTTGAGGTTAATGCGACTTATTATAATAGTTGGCAG
AACCTTTTATATGATTCTAATGGTAATCTCTATGGTTTTAGAGACTACTTAACAAATAGAACTTTTATGATTCGTAGTT
GCTATAGTGGTCGTGTTTCAGCGGCCTTTCATGCTAACTCTTCCGAACCAGCATTGCTATTTCGGAATATTAAATGCAA
TTACGTTTTTAATAATACTCTTTCACGACAGCTGCAACCTATTAACTATTTTGATAGCTATCTTGGTTGTGTTGTCAAT
GCTGATAATAGTACTTCTAGTGTTGTTCAAACATGTGATCTCACAGTAGGTAGTGGTTACTGTGTGGATTACTCTACAA
AAAGACGAAGTCGTAGATCGATTACCACTGGTTATCGGTTTACTAATTTTGAGCCATTTACTGTTAATTCAGTAAATGA
TAGCTTAGAACCTGTAGGTGGTTTGTACGAAATTCAAATACCTTCAGAGTTTACTATAGGTAATATGGAGGAGTTTATT
CAAACAAGCTCTCCTAAAGTTACTATTGATTGTTCTGCTTTTGTCTGTGGTGATTATGCAGCATGTAAATCACAGTTGG
TTGAATATGGTAGCTTCTGTGACAATATTAATGCTATACTCACAGAAGTAAATGAACTACTTGACACTACACAGTTGCA
AGTAGCTAATAGTTTAATGAATGGTGTCACTCTTAGCACTAAGCTTAAAGATGGCGTTAATTTCAATGTAGACGACATC
AATTTTTCCCCTGTATTAGGTTGTTTAGGAAGCGATTGTAATAAAGTTTCCAGTAGATCTGCTATAGAGGACTTACTTT

TTTCTAAAGTAAAGTTATCTGATGTCGGTTTCGTTGAGGCTTATAATAATTGTACTGGAGGTGCCGAAATTAGGGACCT
CATTTGTGTGCAAAGTTATAATGGTATCAAGGTGTTGCCTCCACTGCTCTCAGAAAATCAGATCAGTGGATACACTTTG
GCTGCCACCTCTGCTAGCCTGTTTCCTCCATGGTCAGCAGCAGCAGGTGTACCATTCTATTTAAATGTTCAGTATCGTA
TTAATGGTATTGGTGTTACCATGGATGTGCTAAGTCAAAATCAAAAGCTTATTGCTAATGCATTTAACAATGCTCTTGG
TGCTATTCAGGAAGGGTTTGATGCTACCAATTCTGCTTTAGTTAAAATTCAAGCTGTTGTTAATGCAAATGCTGAAACT
CTTAATAACTTATTGCAACAACTCTCTAATAGATTTGGTGCTATAAGTTCTTCTTTACAAGAAATTCTATCTAGACTGG
ATGCTCTTGAAGCGCAAGCTCAGATAGACAGACTTATTAATGGGCGTCTTACCGCTCTTAATGCTTATGTTTCTCAACA
GCTTAGTGACTCTACACTAGTAAAGTTTAGTGCAGCACAAGCTATGGAGAAGGTTAATGAATGTGTCAAAAGCCAATCA
TCTAGGATAAATTTTTGTGGTAATGGTAATCATATTATATCATTAGTGCAGAATGCTCCATATGGTTTGTATTTTATCC
ACTTTAGCTATGTCCCTACTAAGTATGTCACTGCGAAGGTTAGTCCCGGTCTGTGCATTGCTGGTGGTAGAGGTATAGC
CCCTAAGAGTGGTTATTTTGTTAATGTAAATAACACTTGGATGTTCACTGGTAGTGGTTATTACTACCCTGAACCTATA
ACTGGAAATAATGTTGTTGTTATGAGTACCTGTGCTGTTAATTATACTAAAGCGCCGGATGTAATGCTGAACATTTCAA
CACCCAACCTCCCTGATTTTAAGGAAGAGTTGGATCAATGGTTTAAAAACCAAACTTCAGTGGCACCAGATTTGTCACT
TGATTATATAAATGTTACATTCTTGGACCTACAAGATGAAATGAATAGGTTACAGGAGGCAATAAAAGTTTTAAATCAG
AGCTACATCAATCTCAAGGACATTGGTACATATGAGTATTATGTAAAATGGCCTTGGTATGTATGGCTTTTAATTGGCT
TTGCTGGTGTAGCTATGCTTGTTTTACTATTCTTCATATGCTGTTGTACAGGATGTGGGACTAGTTGTTTTAAGAAATG
TGGTGGTTGTTGTGATGATTATACTGGACACCAGGAGTTAGTAATTAAAACATCACATGAAGATTAA

>gi|61105551|gb|AY935637.1|_Bovine_coronavirus_isolate_KWD1_spike_glycoprotein_precursor_(S)_gene_complete_cds

ATGTTTTTGATACTTTTAATTTCCTTACCAACGGCTTTTGCTGTAATAGGAGATTTAAAGTGTACTACAGTTTCC
ATTAATGATGTTGACACTGGTGTTCCTTCTATTAGCACTGATACTGTCGATGTTACTAATGGTTTAGGTACTTATTATG
TTTTAGATCGTGTGTATTTAAATACTACGTTGTTGCTTAATGGTTACTACCCTACTTCAGGTTCTACATATCGTAATAT
GGCACTGAAGGGAACTTTACTATTGAGCACACTATGGTTTAAACCACCTTTTCTTTCTGATTTTACTAATGGTATTTTT
GCTAAGGTCAAAAATACCAAGGTTGTTAAAAATGGTGTAATGTATAGTGAGTTTCCTGCTATAACTATAGGTAGTACTT
TTGTAAATACATCCTATAGTGTGGTAGTACAACCACATACTACTAATTTTGATAGTAAATTACAAGGTCTCTTAGAGAT
TTCTGTTTGCCAGTATACTATGTGCGAGTACCCAAATACGATTTGTAATCCTAATCTGGGTAATCGACGCGTAGAACTA
TGGCATTGGGATACAGGTGTTGTTTCCTGTTTATATAAGCGTAATTTCACATATGATGTGAATGCTGATTATTTGTATT
TCCATTTTTATCAAGAAGGTGGTACTTTTTATGCATATTTTACAGACACTGGTGTTGTTACTAAGTTTCTGTTTAATGT
TTATTTAGGCACGGTGCTCTCACATTATTATGTCATGCCTTTGACTTGTAATAGTGCTCTGACTTTAGAATATTGGGTT
ACACCTCTCACTTCTAAACAATATCTACTAGCTTTCAATCAAGATGGTGTTATTTTTAATGCTGTTGATTGTAAGAGTG
ATTTTATGAGTGAGATTAAGTGTAAAACACTATCTATAGCACCATCTACTGGTGTTTATGAATTAAACGGTTACACTGT
TCAGCCAATTGCAGATGTTTACCGACGTATACCTAATCTTCCTGATTGCAATATTGAGGCTTGGCTTAATGATAAGTCG
GTGCCCTCTCCATTAAATTGGGAACGTAAGACCTTTTCAAATTGTAATTTTAATATGAGCAGCCTGATGTCTTTTATTC
AGGCAGACTCATTTACTTGTAATAATATTGATGCTGCTAAGATATATGGTATGTGTTTTTCCAGCATAACTATAGATAA
GTTTGCAATACCCAATGGTAGGAAGGTTGACCTACAATTGGGTAATTTGGGTTATTTGCAGTCTTTTAACTATAGAATT
GATACTACTGCTACAAGTTGTCAGTTGTATTATAATTTACCTGCTGCTAATGTTTCTGTTAGCAGGTTTAATCCTTCTA
CTTGGAATAGGAGATTTGGTTTTACAGAACAATCTGTTTTTAAGCCTCAACCTGCAGGTGTTTTTACTGATCATGATGT
TGTTTATGCACAACATTGTTTTAAAGCTCCCACAAATTTCTGTCCGTGTAAATTGGATGGGTCTTTGTGTGTAGGTAGT
GGTTCTGGTATAGATGCTGGTTATAAAAATACTGGTATAGGCACTTGTCCTGCAGGTACTAATTATTTAACTTGCCATA
ATGCTGCCCAATGTGATTGTTTGTGCACTCCAGACCCCATTACATCTAAAGCTACAGGGCCTTACAAGTGCCCCCAAAC
TAAATATTTAGTTGGTATAGGTGAGCACTGTTCGGGTCTTGCTATTAAAAGTGATTATTGTGGAGGTAATCCTTGTAGT

TGCCAACCACAAGCATTTTTGGGTTGGTCTGTTGACTCTTGTTTACAAGGGGATAGGTGTAATATTTTTGCTAATTTTA
TTTTGCATGATGTTAATAGTGGTACTACTTGTTCTATTGATTTACAAAAATCAAACACAGACATAATTCTTGGTGTTTG
TGTTAATTATGATCTTTATGGTATTACTGGCCAAGGGATTTTTGTTGAGGTTAATGCGACTTATTATAATAGTTGGCAG
AACCTTTTATATGATTCTAATGGTAATCTCTATGGTTTTAGAGACTACTTAACAAATAGAACTTTTATGATTCGTAGTT
GCTATAGTGGTCGTGTTTCAGCGGCCTTTCATGCTAACTCTTCCGAACCAGCATTGCTATTTCGGAATATCAAATGCAA
TTACGTTTTTAATAATATTCTTTCACGACAGCTGCAACCTATTAACTATTTTGATAGCTATCTTGGTTGTGTTGTCAAT
GCTGATAATAGTACTTCTAGTGTTGTTCAAACATGTGATCTCACAGTAGGTAGTGGTTACTGTGTGGATTACTCTACAA
AAAGACGAAGTCGTAGATCGATTACCACTGGTTATCGGTTTACTAATTTTGAGCCATTTACTGTTAATTCAGTAAATGA
TAGTTTAGAACCTGTAGGTGGTTTGTATGAAATTCAAATACCTTCAGAGTTTACTATAGGTAATATGGAGGAGTTTATT
CAAACAAGCTCTCCTAAAGTTACTATTGATTGTTCTGCTTTTGTCTGTGGTGATTATGCAGCATGTAAATCACAGTTGG
TTGAATATGGTAGCTTCTGTGACAATATTAATGCTATACTCACAGAAGTAAATGAACTACTTGACACTACACAGTTGCA
AGTAGCTAATAGTTTAATGAATGGTGTCACTCTTAGCACTAAGCTTAAAGATGGCGTTAATTTCAATGTAGACGACATC
AATTTTTCCCCTGTATTAGGTTGTTTAGGAAGCGATTGCAATAAAGTTTCCAGTAGATCTGCTATAGAGGACTTACTTT
TTTCTAAAGTAAAGTTATCTGATGTCGGTTTCGTTGAGGCTTATAATAATTGTACTGGAGGTGCCGAAATTAGGGACCT
CATTTGTGTGCAAAGTTATAATGGTATCAAGGTGGTGCCTCCACTGCTCTCAGAAAATCAGATCAGTGGATACACTTTG
GCTGCCACCTCTGCTAGCCTGTTTCCTCCATGGTCAGCAGCAGCAGGTGTACCATTCTATTTAAATGTTCAGTATCGTA
TTAATGGTATTGGTGTTACCATGGATGTGCTAAGTCAAAATCAAAAGCTTATTGCTAATGCATTTAACAATGCTCTTGG
TGCTATTCAGGAAGGGTTTGATGCTACCAATTCTGCTTTAGTTAAAATTCAAGCTGTTGTTAATGCAAATGCTGAAGCT
CTTAATAACTTATTGCAACAACTCTCTAATAGATTTGGTGCTATAAGTTCTTCTTTACAAGAAATTCTATCTAGACTGG
ATGCTCTTGAAGCGCAAGCTCAGATAGACAGACTTATTAATGGGCGTCTTACCGCTCTTAATGCTTATGTTTCTCAACA
GCTTAGTGACTCTACACTAGTAAAGTTTAGTGCAGCACAAGCTATGGAGAAGGTTAATGAATGTGTCAAAAGCCAATCA
TCTAGGATAAATTTTTGTGGTAATGGTAATCATATTATATCATTAGTGCAGAATGCTCCATATGGTTTGTATTTCATCC
ACTTTAGCTATGTCCCTACTAAGTATGTCACAGCGAAGGTTAGTCCCGGTCTGTGCATTGCTGGTGATAGAGGTATAGC
CCCTAAGAGTGGTTATTTTGTTAATGTAAATAATACTTGGATGTTCACTGGTAGTGGTTATTACTACCCTGAACCTATA
ACTGGAAATAATGTTGTTGTTATGAGTACCTGTGCTGTTAATTATACTAAAGCGCCGGATGTAATGCTGAACATTTCAA
CACCCAACCTCCCTGACTTTAAGGAAGAGTTGGATCAATGGTTTAAAAACCAAACATCAGTGGCACCAGATTTGTCACT
TGATTATATAAATGTTACATTCTTGGACCTACAAGATGAAATGAATAGGTTACAGGAGGCAATAAAAGTTTTAAATCAG
AGCTACATCAATCTCAAGGACATTGGTACTTATGAGTATTATGTAAAATGGCCTTGGTATGTATGGCTTTTAATTGGCT
TTGCTGGTGTAGCTATGCTTGTTTTACTATTCTTCATATGCTGTTGTACAGGATGTGGGACTAGTTGTTTTAAGAAATG
TGGTGGTTGTTGTGATGATTATACTGGACACCAGGAGTTAGTAATTAAAACATCACATGACGACTAA

>gi|61105553|gb|AY935638.1|_Bovine_coronavirus_isolate_KWD2_spike_glycoprotein_precursor_(S)_gene_complete_cds

ATGTTTTTGATACTTTTAATTTCCTTACCAACGGCTTTTGCTGTTATAGGAGATTTAAAGTGTACTACAGTTTCC
ATTAATGATGTTGACACTGGTGTTCCTTCTATTAGCACTGATACTGTCGATGTTACTAATGGTTTAGGTACTTATTATG
TTTTAGATCGTGTGTATTTAAATACTACGTTGTTGCTTAATGGTTACTACCCTACTTCAGGTTCTACATATCGTAATAT
GGCACTGAAGGGAACTTTACTATTGAGCACACTATGGTTTAAACCACCTTTTCTTTCTGATTTTACTAATGGTATTTTT
GCTAAGGTCAAAAATACCAAGGTTGTTAAAAATGGTGTAATGTATAGTGAGTTTCCTGCTATAACTATAGGTAGTACTT
TTGTAAATACATCCTATAGTGTGGTAGTACAACCACATACTACTAATTTTGATAGTAAATTACAAGGTCTCTTAGAGAT
TTCTGTTTGCCAGTATACTATGTGCGAGTACCCAAATACGATTTGTAATCCTAATCTGGGTAATCGACGCGTAGAACTA
TGGCATTGGGATACAGGTGTTGTTTCCTGTTTATATAAGCGTAATTTCACATATGATGTGAATGCTGATTATTTGTATT
TCCATTTTTATCAAGAAGGTGGTACTTTTTATGCATATTTTACAGACACTGGTGTTGTTACTAAGTTTCTGTTTAATGT

TTATTTAGGCACGGTGCTCTCACATTATTATGTCATGCCTTTGACTTGTAATAGTGCTCTGACTTTAGAATATTGGGTT
ACACCTCTCACTTCTAAACAATATCTACTAGCTTTCAATCAAGATGGTGTTATTTTTAATGCTGTTGATTGTAAGAGTG
ATTTTATGAGTGAGATTAAGTGTAAAACACTATCTATAGCACCATCTACTGGTGTTTATGAATTAAACGGTTACACTGT
TCAGCCAATTGCAGATGTTTACCGACGTATACCTAATCTTCCTGATTGCAATATTGAGGCTTGGCTTAATGATAAGTCG
GTGCCCTCTCCATTAAATTGGGAACGTAAGACCTTTTCAAATTGTAATTTTAATATGAGCAGCCTGATGTCTTTTATTC
AGGCAGACTCATTTACTTGTAATAATATTGATGCTGCTAAGATATATGGTATGTGTTTTTCCAGCATAACTATAGATAA
GTTTGCAATACCCAATGGTAGGAAGGTTGACCTACAATTGGGTAATTTGGGTTATTTGCAGTCTTTTAACTATAGAATT
GATACTACTGCTACAAGTTGTCAGTTGTATTATAATTTACCTGCTGCTAATGTTTCTGTTAGCAGGTTTAATCCTTCTA
CTTGGAATAGGAGATTTGGTTTTACAGAACAATCTGTTTTTAAGCCTCAACCTGCAGGTGTTTTTACTGATCATGATGT
TGTTTATGCACAACATTGTTTTAAAGCTCCCACAAATTTCTGTCCGTGTAAATTGGATGGGTCTTTGTGTGTAGGTAGT
GGTTCTGGTATAGATGCTGGTTATAAAAATACTGGTATAGGCACTTGTCCTGCAGGTACTAATTATTTAACTTGCCATA
ATGCTGCCCAATGTGATTGTTTGTGCACTCCAGACCCCATTACATCTAAAGCTACAGGGCCTTACAAGTGCCCCCAAAC
TAAATATTTAGTTGGTATAGGTGAGCACTGTTCGGGTCTTGCTATTAAAAGTGATTATTGTGGAGGTAATCCTTGTAGT
TGCCAACCACAAGCATTTTTGGGTTGGTCTGTTGACTCTTGTTTACAAGGGGATAGGTGTAATATTTTTGCTAATTTTA
TTTTGCATGATGTTAATAGTGGTACTACTTGTTCTATTGATTTACAAAAATCAAACACAGACATAATTCTTGGTGTTTG
TGTTAATTATGATCTTTATGGTATTACTGGCCAAGGGATTTTTGTTGAGGTTAATGCGACTTATTATAATAGTTGGCAG
AACCTTTTATATGATTCTAATGGTAATCTCTATGGTTTTAGAGACTACTTAACAAATAGAACTTTTATGATTCGTAGTT
GCTATAGTGGTCGTGTTTCAGCGGCCTTTCATGCTAACTCTTCCGAACCAGCATTGCTATTTCGGAATATCAAATGCAA
TTACGTTTTTAATAATACTCTTTCACGACAGCTGCAACCTATTAACTATTTTGATAGCTATCTTGGTTGTGTTGTCAAT
GCTGATAATAGTACTTCTAGTGTTGTTCAAACATGTGATCTCACAGTAGGTAGTGGTTACTGTGTGGATTACTCTACAA
AAAGACGAAGTCGTAGATCGATTACCACTGGTTATCGGTTTACTAATTTTGAGCCATTTACTGTTAATTCAGTAAATGA
TAGTTTAGAACCTGTAGGTGGTTTGTATGAAATTCAAATACCTTCAGAGTTTACTATAGGTAATATGGAGGAGTTTATT
CAAACAAGCTCTCCTAAAGTTACTATTGATTGTTCTGCTTTTGTCTGTGGTGATTATGCAGCATGTAAATCACAGTTGG
TTGAATATGGTAGCTTCTGTGACAATATTAATGCTATACTCACAGAAGTAAATGAACTACTTGACACTACACAGTTGCA
AGTAGCTAATAGTTTAATGAATGGTGTCACTCTTAGCACTAAGCTTAAAGATGGCGTTAATTTCAATGTAGACGACATC
AATTTTTCCCCTGTATTAGGTTGTTTAGGAAGCGATTGCAATAAAGTTTCCAGTAGATCTGCTATAGAGGACTTACTTT
TTTCTAAAGTAAAGTTTTCTGATGTCGGTTTCGTTGAGGCTTATAATAATTGTACTGGAGGTGCCGAAATTAGGGACCT
CATTTGTGTGCAAAGTTATAATGGTATCAAGGTGTTGCCTCCACTGCTCTCAGAAAATCAGATCAGTGGATACACTTTG
GCTGCCACCTCTGCTAGCCTGTTTCCTCCATGGTCAGCAGCAGCAGGTGTACCATTCTATTTAAATGTTCAGTATCGTA
TTAATGGTATTGGTGTTACCATGGATGTGCTAAGTCAAAATCAAAAGCTTATTGCTAATGCATTTAACAATGCTCTTGG
TGCTATTCAGGAAGGGTTTGATGCTACCAATTCTGCTTTAGTTAAAATTCAAGCTGTTGTTAATGCAAATGCTGAAGCT
CTTAATAACTTATTGCAACAACTCTCTAATAGATTTGGTGCTATAAGTTCTTCTTTACAAGAAATTCTATCTAGACTGG
ATGCTCTTGAAGCGCAAGCTCAGATAGACAGACTTATTAATGGGCGTCTTACCGCTCTTAATGCTTATGTTTCTCAACA
GCTTAGTGACTCTACACTAGTAAAGTTTAGTGCAGCACAAGCTATGGAGAAGGTTAATGAATGTGTCAAAAGCCAATCA
TCTAGGATAAATTTTTGTGGTAATGGTAATCATATTATATCATTAGTGCAGAATGCTCCATATGGTTTGTATTTTATCC
ACTTTAGCTATGTCCCTACTAAGTATGTCACAGCGAAGGTTAGTCCCGGTCTGTGCATTGCTGGTGATAGAGGTATAGC
CCCTAAGAGTGGTTATTTTGTTAATGTAAATAATACTTGGATGTTCACTGGTAGTGGTTATTACTACCCTGAACCTATA
ACTGGAAATAATGTTGTTGTTATGAGTACCTGTGCTGTTAATTATACTAAAGCGCCGGATGTAATGCTGAACATTTCAA
CACCCAACCTCCCTGACTTTAAGGAAGAGTTGGATCAATGGTTTAAAAACCAAACATCAGTGGCACCAGATTTGTCACT
TGATTATATAAATGTTACATTCTTGGACCTACAAGATGAAATGAATAGGTTACAGGAGGCAATAAAAGTTTTAAATCAG
AGCTACATCAATCTCAAGGACATTGGTACTTATGAGTATTATGTAAAATGGCCTTGGTATGTATGGCTTTTAATTGGCT
TTGCTGGTGTAGCTATGCTTGTTTTACTATTCTTCATATGCTGTTGTACAGGATGTGGGACTAGTTGTTTTAAGAAATG
TGGTGGTTGTTGTGATGATTATACTGGACACCAGGAGTTAGTAATTAAAACATCACATGACGACTAA

>gi|61105555|gb|AY935639.1|_Bovine_coronavirus_isolate_KWD3_spike_glycoprotein_precursor_(S)_gene_complete_cds

ATGTTTTTGATACTTTTAATTTCCTTACCAATGGCTTTTGCTGTTATAGGAGATTTAAAGTGTACTACAGTTTCC
ATTAATGATGTTGACACTGGTGTTCCTTCTATTAGCACTGATACTGTCGATGTTACTAATGGTTTAGGTACTTATTATG
TTTTAGATCGTGTGTATTTAAATACTACGTTGTTGCTTAATGGTTACTACCCTACTTCAGGTTCTACATATCGTAATAT
GGCACTGAAGGGAACTTTACTATTGAGCACACTATGGTTTAAACCACCTTTTCTTTCTGATTTTACTAATGGTATTTTT
GCTAAGGTCAAAAATACCAAGGTTGTTAAAAATGGTGTAATGTATAGTGAGTTTCCTGCTATAACTATAGGTAGTACTT
TTGTAAATACATCCTATAGTGTGGTAGTACAACCACATACTACTAATTTTGATAGTAAATTACAAGGTCTCTTAGAGAT
TTCTGTTTGCCAGTATACTATGTGCGAGTACCCAAATACGATTTGTAATCCTAATCTGGGTAATCGACGCGTAGAACTA
TGGCATTGGGATACAGGTGTTGTTTCCTGTTTATATAAGCGTAATTTCACATATGATGTGAATGCTGATTATTTGTATT
TCCATTTTTATCAAGAAGGTGGTACTTTTTATGCATATTTTACAGACACTGGTGTTGTTACTAAGTTTCTGTTTAATGT
TTATTTAGGCACGGTGCTCTCACATTATTATGTCATGCCTTTGACTTGTAATAGTGCTCTGACTTTAGAATATTGGGTT
ACACCTCTCACTTCTAAACAATATCTACTAGCTTTCAATCAAGATGGTGTTATTTTTAATGCTGTTGATTGTAAGAGTG
ATTTTATGAGTGAGATTAAGTGTAAAACACTATCTATAGCACCATCTACTGGTGTTTATGAATTAAACGGTTACACTGT
TCAGCCAATTGCAGATGTTTACCGACGTATACCTAATCTTCCTGATTGCAATATTGAGGCTTGGCTTAATGATAAGTCG
GTGCCCTCTCCATTAAATTGGGAACGTAAGACCTTTTCAAATTGTAATTTTAATATGAGCAGCCTGATGTCTTTTATTC
AGGCAGACTCATTTACTTGTAATAATATTGATGCTGCTAAGATATATGGTATGTGTTTTTCCAGCATAACTATAGATAA
GTTTGCAATACCCAATGGTAGGAAGGTTGACCTACAATTGGGTAATTTGGGTTATTTGCAGTCTTTTAACTATAGAATT
GATACTACTGCTACAAGTTGTCAGTTGTATTATAATTTACCTGCTGCTAATGTTTCTGTTAGCAGGTTTAATCCTTCTA
CTTGGAATAGGAGATTTGGTTTTACAGAACAATCTGTTTTTAAGCCTCAACCTGCAGGTGTTTTTACTGATCATGATGT
TGTTTATGCACAACATTGTTTTAAAGCTCCCACAAATTTCTGTCCGTGTAAATTGGATGGGTCTTTGTGTGTAGGTAAT
GGTTCTGGTATAGATGCTGGTTATAAAAATACTGGTATAGGCACTTGTCCTGCAGGTACTAATTATTTAACTTGCCATA
ATGCTGCCCAATGTGATTGTTTGTGCACTCCAGACCCCATTACATCTAAAGCTACAGGGCCTTACAAGTGCCCCCAAAC
TAAATATTTAGTTGGTATAGGTGAGCACTGTTCGGGTCTTGCTATTAAAAGTGATTATTGTGGAGGTAATCCTTGTAGT
TGCCAACCACAAGCATTTTTGGGTTGGTCTGTTGACTCTTGTTTACAAGGGGATAGGTGTAATATTTTTGCTAATTTTA
TTTTGCATGATGTTAATAGTGGTACTACTTGTTCTATTGATTTACAAAAATCAAACACAGACATAATTCTTGGTGTTTG
TGTTAATTATGATCTTTATGGTATTACTGGCCAAGGGATTTTTGTTGAGGTTAATGCGACTTATTATAATAGTTGGCAG
AACCTTTTATATGATTCTAATGGTAATCTCTATGGTTTTAGAGACTACTTAACAAATAGAACTTTTATGATTCGTAGTT
GCTATAGTGGTCGTGTTTCAGCGGCCTTTCATGCTAACTCTTCCGAACCAGCATTGCTATTTCGGAATATCAAATGCAA
TTACGTTTTTAATAATACTCTTTCACGACAGCTGCAACCTATAAACTATTTTGATAGCTATCTTGGTTGTGTTGTCAAT
GCTGATAATAGTACTTCTAGTGTTGTTCAAACATGTGATCTCACAGTAGGTAGTGGTTACTGTGTGGATTACTCTACAA
AAAGACGAAGTCGTAGATCGATTACCACTGGTTATCGGTTTACTAATTTTGAGCCATTTACTGTTAATTCAGTAAATGA
TAGTTTAGAACCTGTAGGTGGTTTGTATGAAATTCAAATACCTTCAGAGTTTACTATAGGTAATATGGAGGAGTTTATT
CAAACAAGCTCTCCTAAAGTTACTATTGATTGTTCTGCTTTTGTCTGTGGTGATTATGCAGCATGTAAATCACAGTTGG
TTGAATATGGTAGCTTCTGTGACAATATTAATGCTATACTCACAGAAGTAAATGAACTACTTGACACTACACAGTTGCA
AGTAGCTAATAGTTTAATGAATGGTGTCACTCTTAGCACTAAGCTTAAAGATGGCGTTAATTTCAATGTAGACGACATC
AATTTTTCCCCTGTATTAGGTTGTTTAGGAAGCGATTGCAATAAAGTTTCCAGTAGATCTGCTATAGAGGACTTACTTT
TTTCTAAAGTAAAGTTATCTGATGTCGGTTTCGTTGAGGCTTATAATAATTGTACTGGAGGTGCCGAAATTAGGGACCT
CATTTGTGTGCAAAGTTATAATGGTATCAAGGTGTTGCCTCCACTGCTCTCAGAAAATCAGATCAGTGGATACACTTTG
GCTGCCACCTCTGCTAGCCTGTTTCCTCCATGGTCAGCAGCAGCAGGTGTACCATTCTATTTAAATGTTCAGTATCGTA
TTAATGGTATTGGTGTTACCATGGATGTGCTAAGTCAAAATCAAAAGCTTATTGCTAATGCATTTAACAATGCTCTTGG
TGCTATTCAGGAAGGGTTTGATGCTACCAATTCTGCTTTAGTTAAAATTCAAGCTGTTGTTAATGCAAATGCTGAAGCT

CTTAATAACTTATTGCAACAACTCTCTAATAGATTTGGTGCTATAAGTTCTTCTTTACAAGAAATTCTATCTAGACTGG
ATGCTCTTGAAGCGCAAGCTCAGATAGACAGACTTATTAATGGGCGTCTTACCGCTCTTAATTCTTATGTTTCTCAACA
GCTTAGTGACTCTACACTAGTAAAGTTTAGTGCAGCACAAGCTATGGAGAAGGTTAATGAATGTGTCAAAAGCCAATCA
TCTAGGATAAATTTTTGTGGTAATGGTAATCATATTATATCATTAGTGCAGAATGCTCCATATGGTTTGTATTTTATCC
ACTTTAGCTATGTCCCTACTAAGTATGTCACAGCGAAGGTTAGTCCCGGTCTGTGCATTGCTGGTGATAGAGGTATAGC
CCCTAAGAGTGGTTATTTTGTTAATGTAAATAATACTTGGATGTTCACTGGTAGTGGTTATTACTACCCTGAACCTATA
ACTGGAAATAATGTTGTTGTTATGAGTACCTGTGCTGTTAATTATACTAAAGCGCCGGATGTAATGCTGAACATTTCAA
CACCCAACCTCCCTGACTTTAAGGAAGAGTTGGATCAATGGTTTAAAAACCAAACATCAGTGGCACCAGATTTGTCACT
TGATTATATAAATGTTACATTCTTGGACCTACAAGATGAAATGAATAGGTTACAGGAGGCAATAAAAGTTTTAAATCAG
AGCTACATCAATCTCAAGGACATTGGTACTTATGAGTATTATGTAAAATGGCCTTGGTATGTATGGCTTTTAATTGGCT
TTGCTGGTGTAGCTATGCTTGTTTTACTATTCTTCATATGCTGTTGTACAGGATGTGGGACTAGTTGTTTTAAGAAATG
TGGTGGTTGTTGTGATGATTATACTGGACACCAGGAGTTAGTAATTAAAACATCACATGACGACTAA

>gi|61105559|gb|AY935641.1|_Bovine_coronavirus_isolate_KWD5_spike_glycoprotein_precursor_(S)_gene_complete_cds

ATGTTTTTGATACTTTTAATTTCCTTACCAATGGCTTTTGCTGTTATAGGAGATTTAAAGTGTACTACAGTTTCC
ATTAATGATGTTGACACTGGTGTTCCTTCTATTAGCACTGATACTGTCGATGTTACTAATGGTTTAGGTACTTATTATG
TTTTAGATCGTGTGTATTTAAATACTACGTTGTTGCTTAATGGTTACTACCCTACTTCAGGTTCTACATATCGTAATAT
GGCACTGAAGGGAACTTTACTATTGAGCACACTATGGTTTAAACCACCTTTTCTTTCTGATTTTACTAATGGTATTTTT
GCTAAGGTCAAAAATACCAAGGTTGTTAAAAATGGTGTAATGTATAGTGAGTTTCCTGCTATAACTATAGGTAGTACTT
TTGTAAATACATCCTATAGTGTGGTAGTACAACCACATACTACTAATTTTGATAGTAAATTACAAGGTCTCTTAGAGAT
TTCTGTTTGCCAGTATACTATGTGCGAGTACCCAAATACGATTTGTAATCCTAATCTGGGTAATCGACGCGTAGAACTA
TGGCATTGGGATACAGGTGTTGTTTCCTGTTTATATAAGCGTAATTTCACATATGATGTGAATGCTGATTATTTGTATT
TCCATTTTTATCAAGAAGGTGGTACTTTTTATGCATATTTTACAGACACTGGTGTTGTTACTAAGTTTCTGTTTAATGT
TTATTTAGGCACGGTGCTCTCACATTATTATGTCATGCCTTTGACTTGTAATAGTGCTCTGACTTTAGAATATTGGGTT
ACACCTCTCACTTCTAAACAATATCTACTAGCTTTCAATCAAGATGGTGTTATTTTTAATGCTGTTGATTGTAAGAGTG
ATTTTATGAGTGAGATTAAGTGTAAAACACTATCTATAGCACCATCTACTGGTGTTTATGAATTAAACGGTTACACTGT
TCAGCCAATTGCAGATGTTTACCGACGTATACCTAATCTTCCTGATTGCAATATTGAGGCTTGGCTTAATGATAAGTCG
GTGCCCTCTCCATTAAATTGGGAACGTAAGACCTTTTCAAATTGTAATTTTAATATGAGCAGCCTGATGTCTTTTATTC
AGGCAGACTCATTTACTTGTAATAATATTGATGCTGCTAAGATATATGGTATGTGTTTTTCCAGCATAACTATAGATAA
GTTTGCAATACCCAATGGTAGGAAGGTTGACCTACAATTGGGTAATTTGGGTTATTTGCAGTCTTTTAACTATAGAATT
GATACTACTGCTACAAGTTGTCAGTTGTATTATAATTTACCTGCTGCTAATGTTTCTGTTAGCAGGTTTAATCCTTCTA
CTTGGAATAGGAGATTTGGTTTTACAGAACAATCTGTTTTTAAGCCTCAACCTGCAGGTGTTTTTACTGATCATGATGT
TGTTTATGCACAACATTGTTTTAAAGCTCCCACAAATTTCTGTCCGTGTAAATTGGATGGGTCTTTGTGTGTAGGTAGT
GGTTCTGGTATAGATGCTGGTTATAAAAATACTGGTATAGGCACTTGTCCTGCAGGTACTAATTATTTAACTTGCCATA
ATGCTGCCCAATGTGATTGTTTGTGCACTCCAGACCCCATTACATCTAAAGCTACAGGGCCTTACAAGTGCCCCCAAAC
TAAATATTTAGTTGGTATAGGTGAGCACTGTTCGGGTCTTGCTATTAAAAGTGATTATTGTGGAGGTAATCCTTGTAGT
TGCCAACCACAAGCATTTTTGGGTTGGTCTGTTGACTCTTGTTTACAAGGGGATAGGTGTAATATTTTTGCTAATTTTA
TTTTGCATGATGTTAATAGTGGTACTACTTGTTCTATTGATTTACAAAAATCAAACACAGACATAATTCTTGGTGTTTG
TGTTAATTATGATCTTTATGGTATTACTGGCCAAGGGATTTTTGTTGAGGTTAATGCGACTTATTATAATAGTTGGCAG
AACCTTTTATATGATTCTAATGGTAATCTCTATGGTTTTAGAGACTACTTAACAAATAGAACTTTTATGATTCGTAGTT
GCTATAGTGGTCGTGTTTCAGCGGCCTTTCATGCTAACTCTTCCGAACCAGCATTGCTATTTCGGAATATCAAATGCAA

TTACGTTTTTAATAATATTCTTTCACGACAGCTGCAACCTATTAACTATTTTGATAGCTATCTAGGTTGTGTTGTCAAT
GCTGATAATAGTACTTCTAGTGTTGTTCAAACATGTGATCTCACAGTAGGTAGTGGTTACTGTGTGGATTACTCTACAA
AAAGACGAAGTCGTAGATCGATTACCACTGGTTATCGGTTTACTAATTTTGAGCCATTTACTGTTAATTCAGTAAATGA
TAGTTTAGAACCTGTAGGTGGTTTGTATGAAATTCAAATACCTTCAGAGTTTACTATAGGTAATATGGAGGAGTTTATT
CAAACAAGCTCTCCTAAAGTTACTATTGATTGTTCTGCTTTTGTCTGTGGTGATTATGCAGCATGTAAATCACAGTTGG
TTGAATATGGTAGCTTCTGTGACAATATTAATGCTATACTCACAGAAGTAAATGAACTACTTGACACTACACAGTTGCA
AGTAGCTAATAGTTTAATGAATGGTGTCACTCTTAGCACTAAGCTTAAAGATGGCGTTAATTTCAATGTAGACGACATC
AATTTTTCCCCTGTATTAGGTTGTTTAGGAAGCGATTGCAATAAAGTTTCCAGTAGATCTGCTATAGAGGACTTACTTT
TTTCTAAAGTAAAGTTATCTGATGTCGGTTTCGTTGAGGCTTATAATAATTGTACTGGAGGTGCCGAAATTAGGGACCT
CATTTGTGTGCAAAGTTATAATGGTATCAAGGTGTTGCCTCCACTGCTCTCAGAAAATCAGATCAGTGGATACACTTTG
GCTGCCACCTCTGCTAGCCTGTTTCCTCCATGGTCAGCAGCAGCAGGTGTACCATTCTATTTAAATGTTCAGTATCGTA
TTAATGGTATTGGTGTTACCATGGATGTGCTAAGTCAAAATCAAAAGCTTATTGCTAATGCATTTAACAATGCTCTTGG
TGCTATTCAGGAAGGGTTTGATGCTACCAATTCTGCTTTAGTTAAAATTCAAGCTGTTGTTAATGCAAATGCTGAAGCT
CTTAATAACTTATTGCAACAACTCTCTAATAGATTTGGTGCTATAAGTTCTTCTTTACAAGAAATTCTATCTAGACTGG
ATGCTCTTGAAGCGCAAGCTCAGATAGACAGACTTATTAATGGGCGTCTTACCGCTCTTAATGCTTATGTTTCTCAACA
GCTTAGTGACTCTACACTAGTAAAGTTTAGTGCAGCACAAGCTATGGAGAAGGTTAATGAATGTGTCAAAAGCCAATCA
TCTAGGATAAATTTTTGTGGTAATGGTAATCATATTATATCATTAGTGCAGAATGCTCCATATGGGTTGTATTTTATCC
ACTTTAGCTATGTCCCTACTAAGTATGTCACAGCGAAGGTTAGTCCCGGTCTGTGCATTGCTGGTGATAGAGGTATAGC
CCCTAAGAGTGGTTATTTTGTTAATGTAAATAATACTTGGATGTTCACTGGTAGTGGTTATTACTACCCTGAACCTATA
ACTGGAAATAATGTTGTTGTTATGAGTACCTGTGCTGTTAATTATACTAAAGCGCCGGATGTAATGCTGAACATTTCAA
CACCCAACCTCCCTGACTTTAAGGAAGAGTTGGATCAATGGTTTAAAAACCAAACATCAGTGGCACCAGATTTGTCACT
TGATTATATAAATGTTACATTCTTGGACCTACAAGATGAAATGAATAGGTTACAGGAGGCAATAAAAGTTTTAAATCAG
AGCTACATCAATCTCAAGGACATTGGTACTTATGAGTATTATGTAAAATGGCCTTGGTATGTATGGCTTTTAATTGGCT
TTGCTGGTGTAGCTATGCTTGTTTTACTATTCTTCATATGCTGTTGTACAGGATGTGGGACTAGTTGTTTTAAGAAATG
TGGTGGGTGTTGTGATGATTATACTGGACACCAGGAGTTAGTAATTAAAACATCACATGACGACTAA

>gi|61105561|gb|AY935642.1|_Bovine_coronavirus_isolate_KWD6_spike_glycoprotein_precursor_(S)_gene_complete_cds

ATGTTTTTGATACTTTTAATTTCCTTACCAATGGCTTTTGCTGTTATAGGAGATTTAAAGTGTACTACAGTTTCC
ATTAAAGAGGTTGACACTGGTGTTCCTTCTATTAGCACTGATACTGTCGATGTTACTAATGGTTTAGGTACTTATTATG
TTTTAGATCGTGTGTATTTAAATACTACGTTGTTGCTTAATGGTTACTACCCTACTTCAGGTTCTACATATCGTAATAT
GGCACTGAAGGGAACTTTACTATTGAGCACACTATGGTTTAAACCACCTTTTCTTTCTGATTTTACTAATGGTATTTTT
GCTAAGGTCAAAAATACCAAGGTTGTTAAAAATGGTGTAATGTATAGTGAGTTTCCTGCTATAACTATAGGTAGTACTT
TTGTAAATACATCCTATAGTGTGGTAGTACAACCACATACTACTAATTTTGATAGTAAATTACAAGGTCTCTTAGAGAT
TTCTGTTTGCCAGTATACTATGTGCGAGTACCCAAATACGATTTGTAATCCTAATCTGGGTAATCGACGCGTAGAACTA
TGGCATTGGGATACAGGTGTTGTTTCCTGTTTATATAAGCGTAATTTCACATATGATGTGAATGCTGATTATTTGTATT
TCCATTTTTATCAAGAAGGTGGTACTTTTTATGCATATTTTACAGACACTGGTGTTGTTACTAAGTTTCTGTTTAATGT
TTATTTAGGCACGGTGCTCTCACATTATTATGTCATGCCTTTGACTTGTAATAGTGCTCTGACTTTAGAATATTGGGTT
ACACCTCTCACTTCTAAACAATATCTACTAGCTTTCAATCAAGATGGTGTTATTTTTAATGCTGTTGATTGTAAGAGTG
ATTTTATGAGTGAGATTAAGTGTAAAACACTATCTATAGCACCATCTACTGGTGTTTATGAATTAAACGGTTACACTGT
TCAGCCAATTGCAGATGTTTACCGACGTATACCTAATCTTCCTGATTGCAATATTGAGGCTTGGCTTAATGATAAGTCG
GTGCCCTCTCCATTAAATTGGGAACGTAAGACCTTTTCAAATTGTAATTTTAATATGAGCAGCCTGATGTCTTTTATTC

AGGCAGACTCATTTACTTGTAATAATATTGATGCTGCTAAGATATATGGTATGTGTTTTTCCAGCATAACTATAGATAA
GTTTGCAATACCCAATGGTAGGAAGGTTGACCTACAATTGGGTAATTTGGGTTATTTGCAGTCTTTTAACTATAGAATT
GATACTACTGCTACAAGTTGTCAGTTGTATTATAATTTACCTGCTGCTAATGTTTCTGTTAGCAGGTTTAATCCTTCTA
CTTGGAATAGGAGATTTGGTTTTACAGAACAATCTGTTTTTAAGCCTCAACCTGCAGGTGTTTTTACTGATCATGATGT
TGTTTATGCACAACATTGTTTTAAAGCTCCCACAAATTTCTGTCCGTGTAAATTGGATGGGTCTTTGTGTGTAGGTAGT
GGTTCTGGTATAGATGCTGGTTATAAAAATACTGGTATAGGCACTTGTCCTGCAGGTACTAATTATTTAACTTGCCATA
ATGCTGCCCAATGTGATTGTTTGTGCACTCCAGACCCCATTACATCTAAAGCTACAGGGCCTTACAAGTGCCCCCAAAC
TAAATATTTAGTTGGTATAGGTGAGCACTGTTCGGGTCTTGCTATTAAAAGTGATTATTGTGGAGGTAATCCTTGTAGT
TGCCAACCACAAGCATTTTTGGGTTGGTCTGTTGACTCTTGTTTACAAGGGGATAGGTGTAATATTTTTGCTAATTTTA
TTTTGCATGATGTTAATAGTGGTACTACTTGTTCTATTGATTTACAAAAATCAAACACAGACATAATTCTTGGTGTTTG
TGTTAATTATGATCTTTATGGTATTACTGGCCAAGGGATTTTTGTTGAGGTTAATGCGACTTATTATAATAGTTGGCAG
AACCTTTTATATGATTCTAATGGTAATCTCTATGGTTTTAGAGACTACTTAACAAATAGAACTTTTATGATTCGTAGTT
GCTATAGTGGTCGTGTTTCAGCGGCCTTTCATGCTAACTCTTCCGAACCAGCATTGCTATTTCGGAATATCAAATGCAA
TTACGTTTTTAATAATACTCTTTCACGACAGCTGCAACCTATTAACTATTTTGATAGCTATCTTGGTTGTGTTGTCAAT
GCTGATAATAGTACTTCTAGTGTTGTTCAAACATGTGATCTCACAGTAGGTAGTGGTTACTGTGTGGATTACTCTACAA
AAAGACGAAGTCGTAGATCGATTACCACTGGTTATCGGTTTACTAATTTTGAGCCATTTACTGTTAATTCAGTAAATGA
TAGTTTAGAACCTGTAGGTGGTTTGTATGAAATTCAAATACCTTCAGAGTTTACTATAGGTAATATGGAGGAGTTTATT
CAAACAAGCTCTCCTAAAGTTACTATTGATTGTTCTGCTTTTGTCTGTGGTGATTATGCAGCATGTAAATCACAGTTGG
TTGAATATGGTAGCTTCTGTGACAATATTAATGCTATACTCACAGAAGTAAATGAACTACTTGACACTACACAGTTGCA
AGTAGCTAATAGTTTAATGAATGGTGTCACTCTTAGCACTAAGCTTAAAGATGGCGTTAATTTCAATGTAGACGACATC
AATTTTTCCCCTGTATTAGGTTGTTTAGGAAGCGATTGCAATAAAGTTTCCAGTAGATCTGCTATAGAGGACTTACTTT
TTTCTAAAGTAAAGTTATCTGATGTCGGTTTCGTTGAGGCTTATAATAATTGTACTGGAGGTGCCGAAATTAGGGACCT
CATTTGTGTGCAAAGTTATAATGGTATCAAGGTGTTGCCTCCACTGCTCTCAGAAAATCAGATCAGTGGATACACTTTG
GCTGCCACCTCTGCTAGCCTGTTTCCTCCATGGTCAGCAGCAGCAGGTGTACCATTCTATTTAAATGTTCAGTATCGTA
TTAATGGTATTGGTGTTACCATGGATGTGCTAAGTCAAAATCAAAAGCTTATTGCTAATGCATTTAACAATGCTCTTGG
TGCTATTCAGGAAGGGTTTGATGCTACCAATTCTGCTTTAGTTAAAATTCAAGCTGTTGTTAATGCAAATGCTGAAGCT
CTTAATAACTTATTGCAACAACTCTCTAATAGATTTGGTGCTATAAGTTCTTCTTTACAAGAAATTCTATCTAGACTGG
ATGCTCTTGAAGCGCAAGCTCAGATAGACAGACTTATTAATGGGCGTCTTACCGCTCTTAATGCTTATGTTTCTCAACA
GCTTAGTGACTCTACACTAGTAAAGTTTAGTGCAGCACAAGCTATGGAGAAGGTTAATGAATGTGTCAAAAGCCAATCA
TCTAGGATAAATTTTTGTGGTAATGGTAATCATATTATATCATTAGTGCAGAATGCTCCATATGGTTTGTATTTTATCC
ACTTTAGCTATGTCCCTACTAAGTATGTCACAGCGAAGGTTAGTCCCGGTCTGTGCATTGCTGGTGATAGAGGTATAGC
CCCTAAGAGTGGTTATTTTGTTAATGTAAATAATACTTGGATGTTCACTGGTAGTGGTTATTACTACCCTGAACCTATA
ACTGGAAATAATGTTGTTGTTATGAGTACCTGTGCTGTTAATTATACTAAAGCGCCGGATGTAATGCTGAACATTTCAA
CACCCAACCTCCCTGACTTTAAGGAAGAGTTGGATCAATGGTTTAAAAACCAAACATCAGTGGCACCAGATTTGTCACT
TGATTATATAAATGTTACATTCTTGGACCTACAAGATGAAATGAATAGGTTACAGGAGGCAATAAAAGTTTTAAATCAG
AGCTACATCAATCTCAAGGACATTGGTACTTATGAGTATTATGTAAAATGGCCTTGGTATGTATGGCTTTTAATTGGCT
TTGCTGGTGTAGCTATGCTTGTTTTACTATTCTTCATATGCTGTTGTACAGGATGTGGGACTAGTTGTTTTAAGAAATG
TGGTGGTTGTTGTGATGATTATACTGGACACCAGGAGTTAGTAATTAAAACATCACATGACGACTAA

>gi|61105563|gb|AY935643.1|_Bovine_coronavirus_isolate_KWD7_spike_glycoprotein_precursor_(S)_gene_complete_cds

ATGTTTTTGATACTTTTAATTTCCTTACCAATGGCTTTTGCTGTTATAGGAGATTTAAAGTGTACTACAGTTTCC

ATTAATGATGTTGACACTGGTGTTCCCTCTATTAGCACTGATACTGTCGATGTTACTAATGGTTTAGGTACTTATTATG
TTTTAGATCGTGTGTATTTAAATACTACGTTGTTGCTTAATGGTTACTACCCTACTTCAGGTTCTACATATCGTAATAT
GGCACTGAAGGGAACTTTACTATTGAGCACACTATGGTTTAAACCACCTTTTCTTTCTGATTTTACTAATGGTATTTTT
GCTAAGGTCAAAAATACCAAGGTTATTAAAGATGGTGTAATGTATAGTGAGTTTCCTGCTATAACTATAGGTAGTACTT
TTGTAAATACATCCTATAGTGTGGTAGTACAACCACATACTACTAATTTTGATAGTAAATTACAAGGTCTCTTAGAGAT
TTCTGTTTGCCAGTATACTATGTGCGAGTACCCAAATACGATTTGTAATCCTAATCTGGGTAATCGACGCGTAGAACTA
TGGCATTGGGATACAGGTGTTGTTTCCTGTTTATATAAGCGTAATTTCACATATGATGTGAATGCTGATTATTTGTATT
TCCATTTTTATCAAGAAGGTGGTACTTTTTATGCATATTTTACAGACACTGGTGTTGTTACTAAGTTTCTGTTTAATGT
TTATTTAGGCACGGTGCTCTCACATTATTATGTCATGCCTTTGACTTGTAATAGTGCTCTGACTTTAGAATATTGGGTT
ACACCTCTCACTTCTAAACAATATCTACTAGCTTTCAATCAAGATGGTGTTATTTTTAATGCTGTTGATTGTAAGAGTG
ATTTTATGAGTGAGATTAAGTGTAAAACACTATCTATAGCACCATCTACTGGTGTTTATGAATTAAACGGTTACACTGT
TCAGCCAATTGCAGATGTTTACCGACGTATACCTAATCTTCCCGATTGCAATATTGAGGCTTGGCTTAATGATAAGTCG
GTGCCCTCTCCATTAAATTGGGAACGTAAGACCTTTTCAAATTGTAATTTTAATATGAGCAGCCTGATGTCTTTTATTC
AGGCAGACTCATTTACTTGTAATAATATTGATGCTGCTAAGATATATGGTATGTGTTTTTCCAGCATAACTATAGATAA
GTTTGCAATACCCAATGGTAGGAAGGTTGACCTACAATTGGGTAATTTGGGTTATTTGCAGTCTTTTAACTATAGAATT
GATACTACTGCTACAAGTTGTCAGTTGTATTATAATTTACCTGCTGCTAATGTTTCTGTTAGCAGGTTTAATCCTTCTA
CTTGGAATAGGAGATTTGGTTTTACAGAACAATCTGTTTTTAAGCCTCAACCTGCAGGTGTTTTTACTGATCATGATGT
TGTTTATGCACAACATTGTTTTAAAGCTCACACTAATTTCTGTCCGTGTAAATTGGATGGGTCTTTGTGTGTAGGTAGT
GGTTCTGGTATAGATGCTGGTTATAAAAATACTGGTATAGGCACTTGTCCTGCAGGTACTAATTATTTAACTTGCCATA
ATGCTGCCCAATGTGATTGTTTGTGCACCCCCGACCCCATTACATCTAAAGCTACAGGGCCTTACAAGTGCCCCCAAAC
TAAATATTTAGTTGGTATAGGTGAGCACTGTTCGGGTCTTGCTATTAAAAGTGATTATTGTGGAGGTAATCCTTGTAGT
TGCCAACCACAAGCATTTTTGGGTTGGTCTGTTGACTCTTGTTTACAAGGGGATAGGTGTAATATTTTTGCTAATTTTA
TTTTGCATGATGTTAATAGTGGTACTACTTGTTCTACTGATTTACAAAAATCAAACACAGACATAATTCTTGGTGTTTG
TGTTAATTATGATCTTTATGGTATTACTGGCCAAGGTATTTTTGTTGAGGTTAATGCGACTTATTATAATAGTTGGCAG
AACCTTTTATATGATTCTAATGGTAATCTCTATGGTTTTAGAGACTACTTAACAAATAGAACTTTTATGATTCGTAGTT
GCTATAGCGGTCGTGTATCAGCGGCCTTTCATGCTAACTCTTCCGAACCAGCATTGCTATTTCGGAATATTAAATGCAA
CTACGTTTTTAATAATACTCTTCCACGACAGCTGCAACCTATTAACTATTTTGATAGCTATCTTGGTTGTGTTGTCATT
GCTGATAATAGTACTTCTAGTGTTGTTCAAACATGCGATCTCACAGTAGGTAGTGGTTACTGTGTGGATTACTCTACAA
AAAGACGAAGTCGTAGATCGATTACCACTGGTTATCGGTTTACTAATTTTGAGCCATTTACTGTTAATTCAGTAAATGA
TAGTTTAGAACCTGTAGGTGGTTTGTATGAAATTCAAATACCTTCAGAGTTTACTATAGGTAATATGGAGGAGTTTATT
CAAACAAGCTCTCCTAAAGTTACTATTGATTGTTCTGCTTTTGTCTGTGGTGATTATGCAGCATGTAAATCACAGTTGG
TTGAATATGGTAGCTTCTGTGACAATATTAACGCTATACTCACAGAAGTAAATGAACTACTTGACACTACACAGTTGCA
AGTAGCTAATAGTTTAATGAATGGTGTCACTCTTAGCACTAAGCTTAAAGATGGCGTTAATTTCAATGTAGATGACATC
AATTTTTCCCCTGTATTAGGTTGTTTAGGAAGCGACTGTAATAAAGTTTCCAGTAGATCTGCTATAGAGGATTTACTTT
TTTCTAAAGTAAAGTTATCTGATGTCGGTTTTGTTGAGGCTTATAATAATTGTACTGGAGGTGCCGAAATTAGGGACCT
CATTTGTGTGCAAAGTTATAACGGTATCAAGGTGTTGCCTCCACTGCTCTCAGAAAATCAGATCAGTGGATACACTTTG
GCTGCCACCTCTGCTAGTCTGTTTCCTCCTTGGTCAGCAGCAGCAGGTGTACCATTTTATTTAAATGTTCAGTATCGCA
TTAATGGGATTGGTGTTACCATGGATGTGCTAAGTCAAAATCAAAAGCTTATTGCTAACGCATTTAACAATGCTCTTGG
TGCTATTCAGGACGGTTTTGACGCTACCAATTCTGCTTTAGTTAAAATTCAAGCTGTTGTTAATGCAAATGCTGAAGCT
CTTAATAACTTATTGCAACAACTCTCTAATAGGTTTGGTGCTATAAGTTCTTCTTTACAAGAAATTCTATCTAGACTGG
ATGCTCTTGAAGCGCAAGCTCAGATAGACAGACTTATTAATGGGCGTCTTACCGCTCTTAATGCTTATGTTTCTCAACA
GCTTAGTGACTCTACACTAGTAAAATTTAGTGCAGCACAAGCTATGGAGAAGGTTAATGAATGTGTCAAAAGCCAATCA
TCTAGGATAAATTTTTGTGGTAATGGTAATCATATTATATCATTAGTGCAGAATGCTCCATATGGTTTGTATTTCATCC
ACTTTAGCTATGTCCCTACTAAGTATGTCACAGCGAAGGTTAGTCCCGGTCTGTGCATTGCTGGTGATAGAGGTATAGC

CCCCAAGAGTGGTTATTTTGTTAATGTAAATAATACTTGGATGTTCACTGGTAGTGGTTATTACTACCCTGAACCTATA
ACTGGAAATAATGTTGTTGTTATGAGTACCTGTGCTGTTAATTATACTAAAGCGCCGGATGTAATGCTGAACATTTCAA
CACCCAACCTCCCTGATTTTAAGGAAGAGTTGGATCAATGGTTTAAAAACCAAACATCAGTGGCACCAGATTTGTCACT
TGATTATATAAATGTTACATTCTTGGACCTACAAGATGAAATGAATAGGTTACAGGAGGCAATAAAAGTTTTAAATCAG
AGCTACATCAATCTCAAGGACATTGGTACATATGAGTATTATGTAAAATGGCCTTGGTATGTATGGCTTTTAATTGGCT
TTGCTGGTGTAGCTATGCTTGTTTTACTATTTTTCATATGCTGTTGTACAGGATGTGGGACTAGTTGTTTTAAGAAATG
TGGCGGTTGTTGTGATGATTATACTGGACACCAGGAGTTAGTAATTAAAACATCACATGACGACTAA

>gi|61105565|gb|AY935644.1|_Bovine_coronavirus_isolate_KWD8_spike_glycoprotein_precursor_(S)_gene_complete_cds

ATGTTTTTGATACTTTTAATTTCCTTACCAATGGCTTTTGCTGTTATAGGAGATTTAAAGTGTACTACAGTTTCC
ATTAATGATGTTGACACTGGTGTTCCTTCTATTAGCACTGATACTGTCGATGTTACTAATGGTTTAGGTACTTATTATG
TTTTAGATCGTGTGTATTTAAATACTACGTTGTTGCTTAATGGTTACTACCCTACTTCAGGTTCTACATATCGTAATAT
GGCACTGAAGGGAACTTTACTATTGAGCACACTATGGTTTAAACCACCTTTTCTTTCTGATTTTACTAATGGTATTTTT
GCTAAGGTCAAAAATACCAAGGTTGTTAAAAATGGTGTAATGTATAGTGAGTTTCCTGCTATAACTATAGGTAGTACTT
TTGTAAATACATCCTATAGTGTGGTAGTACAACCACATACTACTAATTTTGATAGTAAATTACAAGGTCTCTTAGAGAT
TTCTGTTTGCCAGTATACTATGTGCGAGTACCCAAATACGATTTGTAATCCTAATCTGGGTAATCGACGCGTAGAACTA
TGGCATTGGGATACAGGTGTTGTTTCCTGTTTATATAAGCGTAATTTCACATATGATGTGAATGCTGATTATTTGTATT
TCCATTTTTATCAAGAAGGTGGTACTTTTTATGCATATTTTACAGACACTGGTGTTGTTACTAAGTTTCTGTTTAATGT
TTATTTAGGCACGGTGCTCTCACATTATTATGTCATGCCTTTGACTTGTAATAGTGCTCTGACTTTAGAATATTGGGTT
ACACCTCTCACTTCTAAACAATATCTACTAGCTTTCAATCAAGATGGTGTTATTTTTAATGCTGTTGATTGTAAGAGTG
ATTTTATGAGTGAGATTAAGTGTAAAACACTATCTATAGCACCATCTACTGGTGTTTATGAATTAAACGGTTACACTGT
TCAGCCAATTGCAGATGTTTACCGACGTATACCTAATCTTCCTGATTGCAATATTGAGGCTTGGCTTAATGATAAGTCG
GTGCCCTCTCCATTAAATTGGGAACGTAAGACCTTTTCAAATTGTAATTTTAATATGAGCAGCCTGATGTCTTTTATTC
AGGCAGACTCATTTACTTGTAATAATATTGATGCTGCTAAGATATATGGTATGTGTTTTTCCAGCATAACTATAGATAA
GTTTGCAATACCCAATGGTAGGAAGGTTGACCTACAATTGGGTAATTTGGGTTATTTGCAGTCTTTTAACTATAGAATT
GATACTACTGCTACAAGTTGTCAGTTGTATTATAATTTACCTGCTGCTAATGTTTCTGTTAGCAGGTTTAATCCTTCTA
CTTGGAATAGGAGATTTGGTTTTACAGAACAATCTGTTTTTAAGCCTCAACCTGCAGGTGTTTTTACTGATCATGATGT
TGTTTATGCACAACATTGTTTTAAAGCTCCCACAAATTTCTGTCCGTGTAAATTGGATGGGTCTTTGTGTGTAGGTAAT
GGTTCTGGTATAGATGCTGGTTATAAAAATACTGGTATAGGCACTTGTCCTGCAGGTACTAATTATTTAACTTGCCATA
ATGCTGCCCAATGTGATTGTTTGTGCACTCCCGACCCCATTACATCTAAAGCTACAGGGCCTTACAAGTGCCCCCAAAC
TAAATATTTAGTTGGTATAGGTGAGCACTGTTCGGGTCTTGCTATTAAAAGTGATTATTGTGGAGGTAATCCTTGTAGT
TGCCAACCACAAGCATTTTTGGGTTGGTCTGTTGACTCTTGTTTACAAGGGGATAGGTGTAATATTTTTGCTAATTTTA
TTTTGCATGATGTTAATAGTGGTACTACTTGTTCTATTGATTTACAAAAATCAAACACAGACATAATTCTTGGTGTTTG
TGTTAATTATGATCTTTATGGTATTACTGGCCAAGGGATTTTTGTTGAGGTTAATGCGACTTATTATAATAGTTGGCAG
AACCTTTTATATGATTCTAATGGTAATCTCTATGGTTTTAGAGACTACTTAACAAATAGAACTTTTATGATTCGTAGTT
GCTATAGCGGTCGTGTTTCAGCGGCCTTTCATGCTAACTCTTCCGAACCAGCATTGCTATTTCGGAATATCAAATGCAA
TTACGTTTTTAATAATATTCTTTCACGACAGCTGCAACCTATTAACTATTTTGATAGCTATCTTGGTTGTGTTGTCAAT
GCTGATAATAGTACTTCTAGTGTTGTTCAAACATGTGATCTCACAGTAGGTAGTGGTTACTGTGTGGATTACTCTACAA
AAAGACGAAGTCGTAGATCGATTACCACTGGTTATCGGTTTACTAATTTTGAGCCATTTACTGTTAATTCAGTAAATGA
TAGTTTAGAACCTGTAGGTGGTTTGTATGAAATTCAAATACCTTCAGAGTTTACTATAGGTAATATGGAGGAGTTTATT
CAAACAAGCTCTCCTAAAGTTACTATTGATTGTTCTGCTTTTGTCTGTGGTGATTATGCAGCATGTAAATCACAGTTGG

TTGAATATGGTAGCTTCTGTGACAATATTAATGCTATACTCACAGAAGTAAATGAACTACTTGACACTACACAGTTGCA
AGTAGCTAATAGTTTAATGAATGGTGTCACTCTTAGCACTAAGCTTAAAGATGGCGTTAATTTCAATGTAGACGACATC
AATTTTTCCCCTGTATTAGGTTGTTTAGGAAGCGATTGCAATAAAGTTTCCAGTAGATCTGCTATAGAGGACTTACTTT
TTTCTAAAGTAAAGTTATCTGATGTCGGTTTCGTTGAGGCTTATAATAATTGTACTGGAGGTGCCGAAATTAGGGACCT
CATTTGTGTGCAAAGTTATAATGGTATCAAGGTGTTGCCTCCACTGCTCTCAGAAAATCAGATCAGTGGATACACTTTG
GCTGCCACCTCTGCTAGCCTGTTTCCTCCATGGTCAGCAGCAGCAGGTGTACCATTCTATTTAAATGTTCAGTATCGTA
TTAATGGTATTGGTGTTACCATGGATGTGCTAAGTCAAAATCAAAAGCTTATTGCTAATGCATTTAACAATGCTCTTGG
TGCTATTCAGGAAGGGTTTGATGCTACCAATTCTGCTTTAGTTAAAATTCAAGCTGTTGTTAATGCAAATGCTGAAGCT
CTTAATAACTTATTGCAACAACTCTCTAATAGATTTGGTGCTATAAGTTCTTCTTTACAAGAAATTCTATCTAGACTGG
ATGCTCTTGAAGCGCAAGCTCAGATAGACAGACTTATTAATGGGCGTCTTACCGCTCTTAATGCTTATGTTTCTCAACA
GCTTAGTGACTCTACACTAGTAAAGTTTAGTGCAGCACAAGCTATGGAGAAGGTTAATGAATGTGTCAAAAGCCAATCA
TCTAGGATAAATTTTTGTGGTAATGGTAATCATATTATATCATTAGTGCAGAATGCTCCATATGGTTTGTATTTTATCC
ACTTTAGCTATGTCCCTACTAAGTATGTCACAGCGAAGGTTAGTCCCGGTCTGTGCATTGCTGGTGATAGAGGTATAGC
CCCTAAGAGTGGTTATTTTGTTAATGTAAATAATACTTGGATGTTCACTGGTAGTGGTTATTACTACCCTGAACCTATA
ACTGGAAATAATGTTGTTGTTATGAGTACCTGTGCTGTTAATTATACTAAAGCGCCGGATGTAATGCTGAACATTTCAA
CACCCAACCTCCCTGACTTTAAGGAAGAGTTGGATCAATGGTTTAAAAACCAAACATCAGTGGCACCAGATTTGTCACT
TGATTATATAAATGTTACATTCTTGGACCTACAAGATGAAATGAATAGGTTACAGGAGGCAATAAAAGTTTTAAATCAG
AGCTACATCAATCTCAAGGACATTGGTACTTATGAGTATTATGTAAAATGGCCTTGGTATGTATGGCTTTTAATTGGCT
TTGCTGGTGTAGCTATGCTTGTTTTACTATTCTTCATATGCTGTTGTACAGGATGTGGGACTAGTTGTTTTAAGAAATG
TGGTGGTTGTTGTGATGATTATACTGGACACCAGGAGTTAGTAATTAAAACATCACATGACGACTAA

>gi|61105569|gb|AY935646.1|_Bovine_coronavirus_isolate_KWD10_spike_glycoprotein_precursor_(S)_gene_complete_cds

ATGTTTTTGATACTTTTAATTTCCTTACCAATGGCTTTTGCTGTTATAGGAGATTTAAAGTGTACTACAGTTTCC
ATTAATGATGTTGACACTGGTGTTCCTTCTATTAGCACTGATACTGTCGATGTTACTAATGGTTTAGGTACTTATTATG
TTTTAGATCGTGTGTATTTAAATACTACGTTGTTGCTTAATGGTTACTACCCTACTTCAGGTTCTACATATCGTAATAT
GGCACTGAAGGGAACTTTACTATTGAGCACACTATGGTTTAAACCACCTTTTCTTTCTGATTTTACTAATGGTATTTTT
GCTAAGGTCAAAAATACCAAGGTTGTTAAAAATGGTGTAATGTATAGTGAGTTTCCTGCTATAACTATAGGTAGTACTT
TTGTAAATACATCCTATAGTGTGGTAGTACAACCACATACTACTAATTTTGATAGTAAATTACAAGGTCTCTTAGAGAT
TTCTGTTTGCCAGTATACTATGTGCGAGTACCCAAATACGATTTGTAATCCTAATCTGGGTAATCGACGCGTAGAACTA
TGGCATTGGGATACAGGTGTTGTTTCCTGTTTATATAAGCGTAATTTCACATATGATGTGAATGCTGATTATTTGTATT
TCCATTTTTATCAAGAAGGTGGTACTTTTTATGCATATTTTACAGACACTGGTGTTGTTACTAAGTTTCTGTTTAATGT
TTATTTAGGCACGGTGCTCTCACATTATTATGTCATGCCTTTGACTTGTAATAGTGCTCTGACTTTAGAATATTGGGTT
ACACCTCTCACTTCTAAACAATATCTACTAGCTTTCAATCAAGATGGTGTTATTTTTAATGCTGTTGATTGTAAGAGTG
ATTTTATGAGTGAGATTAAGTGTAAAACACTATCTATAGCACCATCTACTGGTGTTTATGAATTAAACGGTTACACTGT
TCAGCCAATTGCAGATGTTTACCGACGTATACCTAATCTTCCTGATTGCAATATTGAGGCTTGGCTTAATGATAAGTCG
GTGCCCTCTCCATTAAATTGGGAACGTAAGACCTTTTCAAATTGTAATTTTAATATGAGCAGCCTGATGTCTTTTATTC
AGGCAGACTCATTTACTTGTAATAATATTGATGCTGCTAAGATATATGGTATGTGTTTTTCCAGCATAACTATAGATAA
GTTTGCAATACCCAATGGTAGGAAGGTTGACCTACAATTGGGTAATTTGGGTTATTTGCAGTCTTTTAACTATAGAATT
GATACTACTGCTACAAGTTGTCAGTTGTATTATAATTTACCTGCTGCTAATGTTTCTGTTAGCAGGTTTAATCCTTCTA
CTTGGAATAGGAGATTTGGTTTTACAGAACAATCTGTTTTTAAGCCTCAACCTGCAGGTGTTTTTACTGATCATGATGT
TGTTTATGCACAACATTGTTTTAAAGCTCCCACAAATTTCTGTCCGTGTAAATTGGATGGGTCTTTGTGTGTAGGTAAG

GGTCCTGGTATAGATGCTGGTTATAAAAATACTGGTATAGGCACTTGTCCTGCAGGTACTAATTATTTAACTTGCCATA
ATGCTGCCCAATGTGATTGTTTGTGCACTCCAGACCCCATTACATCTAAAGCTACAGGGCCTTACAAGTGCCCCCAAAC
TAAATATTTAGTTGGTATAGGTGAGCACTGTTCGGGTCTTGCTATTAAAAGTGATTATTGTGGAGGTAATCCTTGTAGT
TGCCAACCACAAGCATTTTTGGGTTGGTCTGTTGACTCTTGTTTACAAGGGGATAGGTGTAATATTTTTGCTAATTTTA
TTTTGCATGATGTTAATAGTGGTACTACTTGTTCTATTGATTTACAAAAATCAAACACAGACATAATTCTTGGTGTTTG
TGTTAATTATGATCTTTATGGTATTACTGGCCAAGGGATTTTTGTTGAGGTTAATGCGACTTATTATAATAGTTGGCAG
AACCTTTTATATGATTCTAATGGTAATCTCTATGGTTTTAGAGACTACTTAACAAATAGAACTTTTATGATTCGTAGTT
GCTATAGCGGTCGTGTTTCAGCGGCCTTTCATGCTAACTCTTCCGAACCAGCATTGCTATTTCGGAATATCAAATGCAA
TTACGTTTTTAATAATATTCTTTCACGACAGCTGCAACCTATTAACTATTTTGATAGCTATCTTGGTTGTGTTGTCAAT
GCTGATAATAGTACTTCTAGTGTTGTTCAAACATGTGATCTCACAGTAGGTAGTGGTTACTGTGTGGATTACTCTACAA
AAAGACGAAGTCGTAGATCGATTACCACTGGTTATCGGTTTACTAATTTTGAGCCATTTACTGTTAATTCAGTAAATGA
TAGTTTAGAACCTGTAGGTGGTTTGTATGAAATTCAAATACCTTCAGAGTTTACTATAGGTAATATGGAGGAGTTTATT
CAAACAAGCTCTCCTAAAGTTACTATTGATTGTTCTGCTTTTGTCTGTGGTGATTATGCAGCATGTAAATCACAGTTGG
TTGAATATGGTAGCTTCTGTGACAATATTAATGCTATACTCACAGAAGTAAATGAACTACTTGACACTACACAGTTGCA
AGTAGCTAATAGTTTAATGAATGGTGTCACTCTTAGCACTAAGCTTAAAGATGGCGTTAATTTCAATGTAGACGACATC
AATTTTTCCCCTGTATTAGGTTGTTTAGGAAGCGATTGCAATAAAGTTTCCAGTAGATCTGCTATAGAGGACTTACTTT
TTTCTAAAGTAAAGTTATCTGATGTCGGTTTCGTTGAGGCTTATAATAATTGGACTGGAGGTGCCGAAATTAGGGACCT
CATTTGTGTGCAAAGTTATAATGGTATCAAGGTGTTGCCTCCACTGCTCTCACAAAATCAGATCAGTGGATACACTTTG
GCTGCCACCTCTGCTAGCCTGTTTCCTCCATGGTCAGCAGCAGCAGGTGTACCATTCTATTTAAATGTTCAGTATCGTA
TTAATGGTATTGGTGTTACCATGGATGTGCTAAGTCAAAATCAAAAGCTTATTGCTAATGCATTTAACAATGCTCTTGG
TGCTATTCAGGAAGGGTTTGATGCTACCAATTCTGCTTTAGTTAAAATTCAAGCTGTTGTTAATGCAAATGCTGAAGCT
CTTAATAACTTATTGCAACAACTCTCTAATAGATTTGGTGCTATAAGTTCTTCTTTACAAGAAATTCTATCTAGACTGG
ATGCTCTTGAAGCGCAAGCTCAGATAGACAGACTTATTAATGGGCGTCTTACCGCTCTTAATGCTTATGTTTCTCAACA
GCTTAGTGACTCTACACTAGTAAAGTTTAGTGCAGCACAAGCTATGGAGAAGGTTAATGAATGTGTCAAAAGCCAATCA
TCTAGGATAAATTTTTGTGGTAATGGTAATCATATTATATCATTAGTGCAGAATGCTCCATATGGTTTGTATTTCATCC
ACTTTAGCTATGTCCCTACTAAGTATGTCACAGCGAAGGTTAGTCCCGGTCTGTGCATTGCTGGTGATAGAGGTATAGC
CCCTAAGAGTGGTTATTTTGTTAATGTAAATAATACTTGGATGTTCACTGGTAGTGGTTATTACTACCCTGAACCTATA
ACTGGAAATAATGTTGTTGTTATGAGTACCTGTGCTGTTAATTATACTAAAGCGCCGGATGTAATGCTGAACATTTCAA
CACCCAACCTCCCTGACTTTAAGGAAGAGTTGGATCAATGGTTTAAAAACCAAACATCAGTGGCACCAGATTTGTCACT
TGATTATATAAATGTTACATTCTTGGACCTACAAGATGAAATGAATAGGTTACAGGAGGCAATAAAAGTTTTAAATCAG
AGCTACATCAATCTCAAGGACATTGGTACTTATGAGTATTATGTAAAATGGCCTTGGTATGTATGGCTTTTAATTGGCT
TTGCTGGTGTAGCTATGCTTGTTTTACTATTCTTCATATGCTGTTGTACAGGATGTGGGACTAGTTGTTTTAAGAAATG
TGGTGGTTGTTGTGATGATTATACTGGACACCAGGAGTTAGTAATTAAAACATCACATGACGACTAA

>gi|87082911|gb|DQ389632.1|_Bovine_coronavirus_isolate_KCD1_spike_glycoprotein_precursor_(S)_gene_complete_cds

ATGTTTTTGATACTTTTAATTTCCTTACCAATGGCTTTTGCTGTTATAGGAGATTTAAAGTGTACTACAGTTTCC
ATTAATGATGTTGACACTGGTGTTCCTTCTATTAGCACTGATACTGTCGATGTTACTAATGGTTTAGGTACTTATTATG
TTTTAGATCGTGTGTATTTAAATACTACGTTGTTGCTTAATGGTTACTACCCTACTTCAGGTTCTACATATCGTAATAT
GGCACTGAAGGGAACTTTACTATTGAGCACACTATGGTTTAAACCACCTTTTCTTTCTGATTTTACTAATGGTATTTTT
GCTAAGGTCAAAAATACCAAGGTTATTAAAGATGGTGTAATGTATAGTGAGTTTCCTGCTATAACTATAGGTAGTACTT
TTGTAAATACATCCTATAGTGTGGTAGTACAACCACATACTACTAATTTTGATAGTAAATTACAAGGTCTCTTAGAGAT

TTCTGTTTGCCAGTATACTATGTGCGAGTACCCAAATACGATTTGTCATCCTAATCTGGGTAATCAACGCGTAGAACTA
TGGCATTGGGATACAGGTGTTGTTTCCTGTTTATATAAGCGTAATTTCACATATGATGTGAATGCTGATTACTTGTATT
TCCATTTTTATCAAGAAGGTGGTACTTTTTATGCATATTTTACAGACACTGGTGTTGTTACTAAGTTTCTGTTTAATGT
TTATTTAGGCACGGTGCTCTCACATTATTATGTCATGCCTTTGACTTGTAATAGTGCTCTGACTTTAGAATATTGGGTT
ACACCTCTCACTTCTAAACAATATCTACTAGCTTTCAATCAAGATGGTGTTATTTTTAATGCTGTTGATTGTAAGAGTG
ATTTTATGAGTGAGATTAAGTGTAAAACACTATCTATAGCACCATCTACTGGTGTTTATGAATTAAACGGTTACACTGT
TCAGCCAATTGCAGATGTTTACCGACGTATACCTAATCTTCCCGATTGTAATATTGAGGCTTGGCTTAATGATAAGTCG
GTGCCCTCTCCATTAAATTGGGAACGTAAGACCTTTTCAAATTGTAATTTTAATATGAGTAGCCTGATGTCTTTTATTC
AGGCAGACTCATTTACTTGTAATAATATTGATGCTGCTAAGATATATGGTATGTGTTTTTCCAGCATAACTATAGATAA
GTTTGCAATACCCAATGATAGGAAGGTTGACCTACAATTGGGTAATTTGGGTTATTTGCAGTCTTTTAACTATAGAATT
GATACTACTGCTACAAGTTGTCAGTTGTATTATAATTTACCTGCTGCTAATGTTTCTGTTAGCAGGTTTAATCCTTCTA
CTTGGAATAGGAGATTTGGTTTTACAGAACAATCTGTTTTTAAGCCTCAACCTGCAGGTGTTTTTACTGATCATGATGT
TGTTTATGCACAACATTGTTTTAAAGCTCCCACAAATTTCTGTCCTTGTAAATTGGATGGGTCTTTGTGTGTAGGTAGT
GGTTCTGGTATAGATGCTGGTTATAAAAATACTGGTATAGGCACTTGTCCTGCAGGTACTAATTATTTAACTTGCCATA
ATGCTGCCCAATGTGATTGTTTGTGCACCCCAGACCCCATTACATCTAAAGCTACAGGGCCTTACAAGTGCCCCCAAAC
TAAATACTTAGTTGGTATAGGTGAGCACTGTTCGGGTCTCGCTATTAAAAGTGATTATTGTGGAGGTAATCCTTGTAGT
TGCCAACCACAAGCATTTTTGGGTTGGTCTGTTGACTCTTGTTTACAAGGGGATAGGTGTAATATTTTTGCTAATTTTA
TTTTGCATGATGTTAATAGTGGTACTACTTGTTCTACTGATTTACAAAAATCAAACACAGACATAATTCTTGGTGTTTG
TGTTAATTATGATCTTTATGGTATTACTGGCCAAGGTATTTTTGTTGAGGTTAATGCGACTTATTATAATAGTTGGCAG
AACCTTTTATATGATTCTAATGGTAATCTCTATGGTTTTAGAGACTACTTAACAAATAGAACTTTTATGATTCGTAGTT
GCTATAGCGGTCGTGTATCAGCGGCCTTTCATGCTAACTCTTCCGAACCAGCATTGCTATTTCGGAATATTAAATGCAA
CTACGTTTTTAATAATACTCTTTCACGACAGCTGCAACCTATTAACTATTTTGTTAGCTATCTTGGTTGTGTTGTCAAT
GCTGATAATAGTACTTCTAGTGTTGTTCAAACATGCGATCTCACAGTAGGTAGTGGTTACTGTGTGGATTACTCTACAA
AAAGACGAAGTCGTAGATCGATTACCACTGGTTATCGGTTTACTAATTTTGAGCCATTTACTGTTAATTCAGTAAATGA
TAGTTTAGAACCTGTAGGTGGTTTGTATGAAATTCAAATACCTTCAGAGTTTACTATAGGTAATATGGAGGAGTTTATT
CAAACAAGCTCTCCTAAAGTTACTATTGATTGTTCTGCTTTTGTCTGTGGTGATTATGCAGCATGTAAATCACAGTTGG
TTGAATATGGTAGCTTCTGTGACAATATTAACGCTATACTCACAGAAGTAAATGAACTACTTGACACTACACAGTTGCA
AGTAGCTAATAGTTTAATGAATGGTGTCACTCTTAGCACTAAGCTTAAAGATGGCGTTAATTTCAATGTAGATGACATC
AATTTTTCCCCTGTATTAGGTTGTTTAGGAAGCGACTGTAATAAAGTTTCCAGTAGATCTGCTATAGAGGATTTACTTT
TTTCTAAAGTAAAGTTATCTGATGTCGGTTTTGTTGAGGCTTATAACAATTGTACTGGAGGTGCCGAAATTAGGGACCT
CATTTGTGTGCAAAGTTATAACGGTATCAAGGTGTTGCCTCCACTGCTCTCAGAAAATCAGATCAGTGGATACACTTTG
GCTGCCACCTCTGCTAGTCTGTTTCCTCCTTGGTCAGCAGCAGCAGGTGTACCATTTTATTTAAATGTTCAGTATCGCA
TTAATGGGATTGGTGTTACCATGGATGTGCTAAGTCAAAATCAAAAGCTTATTGCTAATGCATTTAACAATGCTCTTGG
TGCTATTCAGGACGGTTTTGATGCTACCAATTCTGCTTTAGTTAAAATTCAAGCTGTTGTTAATGCAAATGCTGAAGCT
CTTAATAACTTATTGCAACAACTCTCTAATAGGTTTGGTGCTATAAGTTCTTCTTTACAAGAAATTCTATCTAGACTGG
ATGCTCTTGAAGCGCAAGCTCAGATAGACAGACTTATTAATGGGCGTCTTACCGCTCTTAATGCCTATGTTTCTCAACA
GCTTAGTGACTCTACACTAGTAAAATTTAGTGCAGCACAAGCTATGGAGAAGGTTAATGAATGTGTCAAAAGCCAATCA
TCTAGGATAAATTTTTGTGGTAATGGTAATCATATTATATCATTAGTGCAGAATGCTCCATATGGTTTGTATTTCATCC
ACTTTAGCTATGTCCCTACTAAGTATGTCACAGCGAAGGTTAGTCCCGGTCTGTGCATTGCTGGTGATAGAGGTATAGC
CCCCAAGAGTGGTTATTTTGTTAATGTAAATAATACTTGGATGTTCACTGGTAGTGGTTATTACTACCCTGAACCTATA
ACTGGAAATAATGTTGTTGTTATGAGTACCTGTGCTGTTAATTATACTAAAGCGCCGGATGTAATGCTGAACATTTCAA
CACCCAACCTCCCTGATTTTAAGGAAGAGTTGGATCAATGGTTTAAAAACCAAACATCAGTGGCACCAGATTTGTCACT
TGATTATATAAATGTTACATTCTTGGACCTACAAGATGAAATTAATAGGTTACAGGAGGCAATAAAAGTTTTAAATCAG
AGCTACATCAATCTCAAGGACATTGGTACATATGAGTATTATGTAAAATGGCCTTGGTATGTATGGCTTTTAGTTGGCT

TTGCTGGTGTAGCTATGCTTGTTTTACTATTCTTCATATGCTGTTGTACAGGATGTGGGACTAGTTGTTTTAAGAAATG
TGGCGGTTGTTGTGATGATTATACTGGACACCAGGAGTTAGTAATTAAAACATCACATGACGACTAA

>gi|87082913|gb|DQ389633.1|_Bovine_coronavirus_isolate_KCD2_spike_glycoprotein_precursor_(S)_gene_complete_cds

ATGTTTTTGATACTTTTAATTTCCTTACCAATGGCTTTAGCTGTTATAGGAGATTTAAAGTGTACTACAGTTTCC
ATTAATGATGTTGACACTGGTGTTCCCTCTATTAGCACTGATACTGTCGATGTTACTAATGGTTTAGGTACTTATTATG
TTTTAGATCGTGTGTATTTAAATACTACGTTGTTGCTTAATGGTTACTACCCTACTTCAGGTTCTACATATCGTAATAT
GGCACTGAAGGGAACTTTACTATTGAGCACACTATGGTTTAAACCACCTTTTCTTTCTGATTTTACTAATGGTATTTTT
GCTAAGGTCAAAAATACCAAGGTTATTAAAGATGGTGTAATGTATAGTGAGTTTCCTGCTATAACTATAGGTAGTACTT
TTGTAAATACATCCTATAGTGTGGTAGTACAACCACATACTACTAATTTTGATAGTAAATTACAAGGTCTCTTAGAGAT
TTCTGTTTGCCAGTATACTATGTGCGAGTACCCAAATACGATTTGTAATCCTAATCTGGGTAATCAACGCGTAGAACTA
TGGCATTGGGATACAGGTGTTGTTTCCTGTTTATATAAGCGTAATTTCACATATGATGTGAATGCTGATTATTTGTATT
TCCATTTTTATCAAGAAGGTGGTACTTTTTATGCATATTTTACAGACACTGGTGTTGTTACTAAGTTTCTGTTTAATGT
TTATTTAGGCACGGTGCTCTCACATTATTATGTCATGCCTTTGACTTGTAATAGTGCTCTGACTTTAGAATATTGGGTT
ACACCTCTCACTTCTAAACAATATCTACTAGCTTTCAATCAAGATGGTGTTATTTTTAATGCTGTTGATTGTAAGAGTG
ATTTTATGAGTGAGATTAAGTGTAAAACACTATCTATAGCACCATCTACTGGTGTTTATGAATTAAACGGTTACACTGT
TCAGCCAATTGCAGATGTTTACCGACGTATACCTAATCTTCCCGATTGTAATATTGAGGCTTGGCTTAATGATAAGTCG
GTGCCCTCTCCATTAAATTGGGAACGTAAGACCTTTTCAAATTGTAATTTTAATATGAGCAGCCTGATGTCTTTTATTC
AGGCAGACTCATTTACTTGTAATAATATTGATGCTGCTAAGATATATGGTATGTGTTTTTCCAGCATAACTATAGATAA
GTTTGCAATACCCAATGGTAGGAAGGTTGACCTACAATTGGGTAATTTGGGTTATTTGCAGTCTTTTAACTATAGAATT
GATACTACTGCTACAAGTTGTCAGTTGTATTATAATTTACCTGCTGCTAATGTTTCTGTTAGCAGGTTTAATCCTTCTA
TTTGGAATAGGAGATTTGGTTTTACAGAACAATCTGTTTTTAAGCCTCAACCTGCAGGTGTTTTTACTGATCATGATGT
TGTTTATGCACAACATTGTTTTAAAGCTCCCACAAATTTCTGTCCGTGTAAATTGGACGGGTCTTTGTGTGTAGGTACT
GGTTCTGGTATAGATGCTGGTTATAAAAATACTGGTATAGGCACTTGTCCTGCAGGTACTAATTATTTAACTTGCCATA
ATGCTGCCCAATGTGATTGTTTGTGCACCCCAGACCCCATTACATCTAAAGCTACAGGGCCTTACAAGTGCCCCCAAAC
TAAATACTTAGTTGGTATAGGTGAGCACTGTTCGGGTCTCGCTATTAAAAGTGATTATTGTGGAGGTAATCCTTGTAGT
TGCCAACCACAAGCATTTTTGGGTTGGTCTGTTGACTCTTGTTTACAAGGGGATAGGTGTAATATTTTTGCTAATTTTA
TTTTGCATGATGTTAATAGTGGTACTACTTGTTCTACTGATTTACAAAAATCAAACACAGACATAATTCTTGGTGTTTG
TGTTAATTATGATCTTTATGGTATTACTGGCCAAGGTATTTTTGTTGAGGTTAATGCGACTTATTATAATAGTTGGCAG
AACCTTTTATATGATTCTAATGGTAATCTCTATGGTTTTAGAGACTACTTAACAAATAGAACTTTTATGATTCGTAGTT
GCTATAGCGGTCGTGTATCAGCGGCCTTTCATGCTAACTCTTCCGAACCAGCATTGCTATTTCGGAATATTAAATGCAA
CTACGTTTTTAATAATACTCTTTCACGACAGCTGCAACCTATTAACTATTTTGATAGCTATCTTGGTTGTGTTGTCAAT
GCTGATAATAGTACTTCTAGTGTTGTTCAAACATGCGATCTCACAGTAGGTAGTGGTTACTGTGTGGATTACTCTACAA
AAAGACGAAGTCGTAGATCGATTACCACTGGTTATCGGTTTACTAATTTTGAGCCATTTACTGTTAATTCAGTAAATGA
TAGTTTAGAACCTGTAGGTGGTTTGTATGAAATTCAAATACCTTCAGAGTTTACTATAGGTAATATGGAGGAGTTTATT
CAAACAAGCTCTCCTAAAGTTACTATTGATTGTTCTGCTTTTGTCTGTGGTGATTATGCAGCATGTAAATCACAGTTGG
TTGAATATGGTAGCTTCTGTGACAATATTAACGCTATACTTACAGAAGTAAATGAACTACTTGACACTACACAGTTGCA
AGTAGCTAATAGTTTAATGAATGGTGTCACTCTTAGCACTAAGCTTAAAGATGGCGTTAATTTCAATGTAGACGACATC
AATTTTTCCCCTGTATTAGGTTGTTTAGGAAGCGACTGTAATAAAGTTTCCAGTAGATCTGCTATAGAGGATTTACTTT
TTTCTAAAGTAAAGTTATCTGATGTCGGTTTTGTTGAGGCTTATAATAATTGTACTGGAGGTGCCGAAATTAGGGACCT
CATTTGTGTGCAAAGTTATAATGGTATCAAGGTGTTGCCTCCACTGCTCTCAGAAAATCAGATCAGTGGATACACTTTG

GCTGCCACCTCTGCTAGTCTGTTTCCTCCTTGGTCAGCAGCAGCAGGTGTACCATTTTATTTAAATGTTCAGTATCGTA
TTAATGGGATTGGTGTTACCATGGATGTGCTAAGTCAAAATCAAAAGCTTATTGCTAATGCATTTAACAATGCTCTTGG
TGCTATTCAGGACGGTTTTGATGCTACCAATTCTGCTTTAGTTAAAATTCAAGCTGTTGTTAATGCAAATGCTGAAGCT
CTTAATAACTTATTGCAACAACTCTCTAATAGGTTTGGTGCTATAAGTTCTTCTTTACAAGAAATTCTATCTAGACTGG
ATGCTCTTGAAGCGCAAGCTCAGATAGACAGACTTATTAATGGGCGTCTTACCGCTCTTAATGCCTATGTTTCTCAACA
GCTTAGTGACTCTACACTAGTAAAATTTAGTGCAGCACAAGCTATGGAGAAGGTTAATGAATGTGTCAAAAGCCAATCA
TCTAGGATAAATTTTTGTGGTAATGGTAATCATATTATATCATTAGTGCAGAATGCTCCATATGGTTTGTATTTCATCC
ACTTTAGCTATGTCCCTACTAAGTATGTCACAGCGAAGGTTAGTCCCGGTCTGTGCATTGCTGGTGATAGAGGTATAGC
CCCCAAGAGTGGTTATTTTGTTAATGTAAATAATACTTGGATGTTCACTGGTAGTGGTTATTACTACCCTGAACCTATA
ACTGGAAATAATGTTGTTGTTATGAGTACCTGTGCTGTTAATTATACTAAAGCGCCGGATGTAATGCTGAACATTTCAA
CACCCAACCTCCCTGATTTTAAGGAAGAGTTGGATCAATGGTTTAAAAACCAAACATCAGTGGCACCAGATTTGTCACT
TGATTATATAAATGTTACATTCTTGGACCTACAAGATGAAATGAATAGGTTACAGGAGGCAATAAAAGTTTTAAATCAG
AGCTACATCAATCTCAAGGACATTGGTACATATGAGTATTATGTAAAATGGCCTTGGTATGTATGGCTTTTAATTGGCT
TTGCTGGTGTAGCTATGCTTGTTTTACTATTCTTCATATGCTGTTGTACAGGATGTGGGACTAGTTGTTTTAAGAAATG
TGGCGGTTGTTGTGATGATTATACTGGACACCAGGAGTTAGTAATTAAAACATCACATGACGACTAA

>gi|87082915|gb|DQ389634.1|_Bovine_coronavirus_isolate_KCD3_spike_glycoprotein_precursor_(S)_gene_complete_cds

ATGTTTTTGATACTTTTAATTTCCTTACCAATGGCTTTTGCTGTTATAGGAGATTTAAAGTGTACTACAGTTTCC
ATTAATGATGTTGACACTGGTGTTCCTTCTATTAGCACTGATACTGTCGATGTTACTAATGGTTTAGGTACTTATTATG
TTTTAGATCGTGTGTATTTAAATACTACGTTGTTGCTTAATGGTTACTACCCTACTTCAGGTTCTACATATCGTAATAT
GGCACTGAAGGGAACTTTACTATTGAGCACACTATGGTTTAAACCACCTTTTCTTTCTGATTTTACTAATGGTATTTTT
GCTAAGGTCAAAAATACCAAGGTTATTAAAGATGGTGTAATGTATAGTGAGTTTCCTGCTATAACTATAGGTAGTACTT
TTGTAAATACATCCTATAGTGTGGTAGTACAACCACATACTACTAATTTTGATAGTAAATTACAAGGTCTCTTAGAGAT
TTCTGTTTGCCAGTATACTATGTGCGAGTACCCAAATACGATTTGTAATCCTAATCTGGGTAATCAACGCGTAGAACTA
TGGCATTGGGATACAGGTGTTGTTTCCTGTTTATATAAGCGTAATTTCACATATGATGTGAATGCTGATTATTTGTATT
TCCATTTTTATCAAGAAGGTGGTACTTTTTATGCATATTTTACAGACACTGGTGTTGTTACTAAGTTTCTGTTTAATGT
TTATTTAGGCACGGTGCTCTCACATTATTATGTCATGCCTTTGACTTGTAATAGTGCTCTGACTTTAGAATATTGGGTT
ACACCTCTCACTTCTAAACAATATCTACTAGCTTTCAATCAAGATGGTGTTATTTTTAATGCTGTTGATTGTAAGAGTG
ATTTTATGAGTGAGATTAAGTGTAAAACACTATCTATAGCACCATCTACTGGTGTTTATGAATTAAACGGTTACACTGT
TCAGCCAATTGCAGATGTTTACCGACGTATACCTAATCTTCCCGATTGTAATATTGAGGCTTGGCTTAATGATAAGTCG
GTGCCCTCTCCATTAAATTGGGAACGTAAGACCTTTTCAAATTGTAATTTTAATATGAGTAGCCTGATGTCTTTTATTC
AGGCAGACTCATTTACTTGTAATAATATTGATGCTGCTAAGATATATGGTATGTGTTTTTCCAGCATAACTATAGATAA
GTTTGCTATACCCAATGGTTGGAAGGTTGACCTACAATTGGGCAATTTGGGACAATTGCAGTCTTTTAACTATAGAATT
GATACTACTGCTACAAGTTGTCAGTTGTATTATAATTTACCTGCTGCTAATGTTTCTGTTAGCAGGTTTAATCCTTCTA
CTTGGAATAGGAGATTTGGTTTTACAGAACAATCTGTTTTTAAGCCTCAACCTGCAGGTGTTTTTACTGATCATGATGT
TGTTTATGCACAACATTGTTTTAAAGCTCCCACAAATTTCTGTCCTTGTAAATTGGATGGGTCTTTGTGTGTAGGTAGT
GGTTCTGGTATAGATGCTGGTTATAAAAATACTGGTATAGGCACTTGTCCTGCAGGTACTAATTATTTAACTTGCCATA
ATGCTGCCCAATGTGATTGTTTGTGCACCCCAGACCCCATTACATCTAAAGCTACAGGGCCTTACAAGTGCCCCCAAAC
TAAATACTTAGTTGGTATAGGTGAGCACTGTTCGGGTCTCGCTATTAAAAGTGATTATTGTGGAGGTAATCCTTGTAGT
TGCCAACCACAAGCATTTTTGGGTTGGTCTGTTGACTCTTGTTTACAAGGGGATAGGTGTAATATTTTTGCTAATTTTA
TTTTGCATGATGTTAATAGTGGTACTACTTGTTCTACTGATTTACAAAAATCAAACACAGACATAATTCTTGGTGTTTG

TGTTAATTATGATCTTTATGGTATTACTGGCCAAGGTATTTTTGTTGAGGTTAATGCGACTTATTATAATAGTTGGCAG
AACCTTTTATATGATTCTAATGGTAATCTCTATGGTTTTAGAGACTACTTAACAAATAGAACTTTTATGATTCGTAGTT
GCTATAGCGGTCGTGTATCAGCGGCCTTTCATGCTAACTCTTCCGAACCAGCATTGCTATTTCGGAATATTAAATGCAA
CTACGTTTTTAATAATACTCTTTCACGACAGCTGCAACCTATTAACTATTTTGATAGCTATCTTGGTTGTGTTGTCAAT
GCTGATAATAGTACTTCTAGTGTTGTTCAAACATGCGATCTCACAGTACGTAGTGGTTACTGTGTGGATTACTCTACAA
AAAGACGAAGTCGTAGATCGATTACCACTGGTTATCGGTTTACTAATTTTGAGCCATTTACTGTTAATTCAGTAAATGA
TAGTTTAGAACCTGTAGGTGGTTTGTATGAAATTCAAATACCTTCAGAGTTTACTATAGGTAATATGGAGGAGTTTATT
CAAACAAGCTCTCCTAAAGTTACTATTGATTGTTCTGCTTTTGTCTGTGGTGATTATGCAGCATGTAAATCACAGTTGG
TTGAATATGGTAGCTTCTGTGACAATATTAACGCTATACTCACAGAAGTAAATGAACTACTTGACACTACACAGTTGCA
AGTAGCTAATAGTTTAATGAATGGTGTCACTCTTAGCACTAAGCTTGAAGATGGCGTTAATTTCAATGTAGACGACATC
AATTTTTCCCCTGTATTAGGTTGTTTAGGAAGCGATTGTAATAAAGTTTCCAGCAGATCTGCTATAGAGGATTTACTTT
TTTCTAAAGTAAAGTTATCTGATGTCGGTTTTGTTGAGGCTTATAACAATTGTACTGGAGGTGCCGAAATTAGGGACCT
CATTTGTGTGCAAAGTTATAACGGTATCAAGGTGTTGCCTCCACTGCTCTCAGAAAATCAGATCAGTGGATACACTTTG
GCTGCCACCTCTGCTAGTCTGTTTCCTCCTTGGTCAGCAGCAGCAGGTGTACCATTTTATTTAAATGTTCAGTATCGCA
TTAATGGGATTGGTGTTACCATGGATGTGCTAAGTCAAAATCAAAAGCTTATTGCTAATGCATTTAACAATGCTCTTGG
TGCTATTCAGGACGGTTTTGATGCTACCAATTCTGCTTTAGTTAAAATTCAAGCTGTTGTTAATGCAAATGCTGAAGCT
CTTAATAACTTATTGCAACAACTCTCTAATAGGTTTGGTGCTATAAGTTCTTCTTTACAAGAAATTCTATCTAGACTGG
ATGCTCTTGAAGCGCAAGCTCAGATAGACAGACTTATTAATGGGCGTCTTACCGCTCTTAATGCCTATGTTTCTCAACA
GCTTAGTGACTCTACACTAGTAAAATTTAGTGCAGCACAAGCTATGGAGAAGGTTAATGAATGTGTCAAAAGCCAATCA
TCTAGGATAAATTTTTGTGGTAATGGTAATCATATTATATCATTAGTGCAGAATGCTCCATATGGTTTGTATTTCATCC
ACTTTAGCTATGTCCCTACTAAGTATGTCACAGCGAAGGTTAGTCCCGGTCTGTGCATTGCTGGTGATAGAGGTATAGC
CCCCAAGAGTGGTTATTTTGTTAATGTAAATAATACTTGGATGTTCACTGGTAGTGGTTATTACTACCCTGAACCTATA
ACTGGAAATAATGTTGTTGTTATGAGTACCTGTGCTGTTAATTATACTAAAGCGCCGGATGTAATGCTGAACATTTCAA
CACCCAACCTCCCTGATTTTAAGGAAGAGTTGGATCAATGGTTTAAAAACCAAACATCAGTGGCACCAGATTTGTCACT
TGATTATATAAATGTTACATTCTTGGACCTACAAGATGAAATTAATAGGTTACAGGAGGCAATAAAAGTTTTAAATCAG
AGCTACCTCAATCTCAAGGACATTGGTACATATGAGTATTATGTAAAATGGCCTTGGTATGTATGGCTTTTAGTTGGCT
TTGCTGGTGTAGCTATGCTTGTTTTACTATTCTTCATATGCTGTTGTACAGGATGTGGGACTAGTTGTTTTAAGAAATG
TGGCGGTTGTTGTGATGATTATACTGGACACCAGGAGTTAGTAATTAAAACATCACATGACGACTAA

>gi|87082917|gb|DQ389635.1|_Bovine_coronavirus_isolate_KCD4_spike_glycoprotein_precursor_(S)_gene_complete_cds

ATGTTTTTGATACTTTTAATTTCCTTACCAATGGCTTTTGCTGTTATAGGAGATTTAAAGTGTACTACAGTTTCC
ATTAATGATGTTGACACTGGTGTTCCTTCTATTAGCACTGATACTGTCGATGTTACTAATGGTTTAGGTACTTATTATG
TTTTAGATCGTGTGTATTTAAATACTACGTTGTTGCTTAATGGTTACTACCCTACTTCAGGTTCTACATATCGTAATAT
GGCACTGAAGGGAACTTTACTATTGAGCAGACTATGGTTTAAACCACCTTTTCTTTCTGATTTTACTAATGGTATTTTT
GCTAAGGTCAAAAATACCAAGGTTATTAAAGATGGTGTAATGTATAGTGAGTTTCCTGCTATAACTATAGGTAGTACTT
TTGTAAATACATCCTATAGTGTGGTAGTACAACCACATACTACTAATTTTGATAGTAAATTACAAGGTCTCTTAGAGAT
TTCTGTTTGCCAGTATACTATGTGCGAGTACCCAAATACGATTTGTAATCCTAATCTGGGTAATCAACGCGTAGAACTA
TGGCATTGGGATACAGGTGTTGTTTCCTGTTTATATAAGCGTAATTTCACATATGATGTGAATGCTGATTATTTGTATT
TCCATTTTTATCAAGAAGGTGGTACTTTTTATGCATATTTTACAGACACTGGTGTTGTTACTAAGTTTCTGTTTAATGT
TTATTTAGGCACGGTGCTCTCACATTATTATGTCATGCCTTTGACTTGTAATAGTGCTCTGACTTTAGAATATTGGGTT
ACACCTCTCACTTCTAAACAATATCTACTAGCTTTCAATCAAGATGGTGTTATTTTTAATGCTGTTGATTGTAAGAGTG

ATTTTATGAGTGAGATTAAGTGTAAAACACTATCTATAGCACCATCTACTGGTGTTTATGAATTAAACGGTTACACTGT
TCAGCCAATTGCAGATGTTTACCGACGTATACCTAATCTTCCCGATTGTAATATTGAGGCTTGGCTTAATGATAAGTCG
GTGCCCTCTCCATTAAATTGGGAACGTAAGACCTTTTCAAATTGTAATTTTAATATGAGTAGCCTGATGTCTTTTATTC
AGGCAGACTCATTTACTTGTAATAATATTGATGCTGCTAAGATATATGGTATGTGTTTTTCCAGCATAACTATAGATAA
GTTTGCAATACCCAATGGTAGGAAGGTTGACCTACAATTGGGTAATTTGGGTTATTTGCAGTCTTTTAACTATAGAATT
GATACTACTGCTACAAGTTGTCAGTTGTATTATAATTTACCTGCTGCTAATGTTTCTGTTAGCAGGTTTAATCCTTATA
CTTGGAATAGGAGATTTGGTTTTACAGAACAATCTGTTTTTAAGCCTCAACCTGCAGGTGTTTTTACTGATCATGATGT
TGTTTATGCACAACATTGTTTTAAAGCTCCCACAAATTTCTGTCCTTGTAAATTGGATGGGTCTTTGTGTGTAGGTAGT
GGTTCTGGTATAGATGCTGGTTATAAAAATACTGGTATAGGCACTTGTCCTGCAGGTACTAATTATTTAACTTGCCATA
ATGCTGCCCAATGTGATTGTTTGTGCACCCCAGACCCCATTACATCTAAAGCTACAGGGCCTTACAAGTGCCCCCAAAC
TAAATACTTAGTTGGTATAGGTGAGCACTGTTCGGGTCTCGCTATTAAAAGTGATTATTGTGGAGGTAATCCTTGTAGT
TGCCAACCACAAGCATTTTTGGGTTGGTCTGTTGACTCTTGTTTACAAGGGGATAGGTGTAATATTTTTGCTAATTTTA
TTTTGCATGATGTTAATAGTGGTACTACTTGTTCTACTGATTTACAAAAATCAAACACAGACATAATTCTTGGTGTTTG
TGTTAATTATGATCTTTATGGTATTACTGGCCAAGGTATTTTTGTTGAGGTTAATGCGACTTATTATAATAGTTGGCAG
AACCTTTTATATGATTCTAATGGTAATCTCTATGGTTTTAGAGACTACTTAACAAATAGAACTTTTATGATTCGTAGTT
GCTATAGCGGTCGTGTATCAGCGGCCTTTCATGCTAACTCTTCCGAACCAGCATTGCTATTTCGGAATATTAAATGCAA
CTACGTTTTTAATAATACTCTTTCACGACAGCTGCAACCTATTAACTATTTTGATAGCTATCTTGGTTGTGTTGTCAAT
GCTGATAATAGTACTTCTAGTGTTGTTCAAACATGCGATCTCACAGTAGGTAGTGGTTACTGTGTGGATTACTCTACAA
AAAGACGAAGTCGTAGATCGATTACCACTGGTTATCGGTTTACTAATTTTGAGCCATTTACTGTTAATTCAGTAAATGA
TAGTTTAGAACCTGTAGGTGGTTTGTATGAAATTCAAATACCTTCAGAGTTTACTATAGGTAATATGGAGGAGTTTATT
CAAACAAGCTCTCCTAAAGTTACTATTGATTGTTCTGCTTTTGTCTGTGGTGATTATGCAGCATGTAAATCACAGTTGG
TTGAATATGGTAGCTTCTGTGACAATATTAATGCTATACTCACAGAAGTAAATGAACTACTTGACACTACACAGTTGCA
AGTAGCTAATAGTTTAATGAATGGTGTCACTCTTAGCACTAAGCTTAAAGATGGCGTTAATTTCAATGTAGATAAAATC
AATTTTTCCCCTGTATTAGGTTTTTTAGGAAGCGACTGTAATAAAGTTTCCAGTAGATCTGCTATAGAGGATTTACTTT
TTTCTAAAGTAAAGTTATCTGATGTCGGTTTTGTTGAGGCTTATAACAATTGTACTGGAGGTGCCGAAATTAGGGACCT
CATTTGTGTGCAAAGTTATAACGGTATCAAGGTGTTGCCTCCACTGCTCTCAGAAAATCAGATCAGTGGATACACTTTG
GCTGCCACCTCTGCTAGTCTGTTTCCTCCTTGGTCAGCAGCAGCAGGTGTACCATTTTATTTAAATGTTCAGTATCGCA
TTAATGGGATTGGTGTTACCATGGATGTGCTAAGTCAAAATCAAAAGCTTATTGCTAATGCATTTAACAATGCTCTTGG
TGCTATTCAGGACGGTTTTGATGCTACCAATTCTGCTTTAGTTAAAATTCAAGCTGTTGTTAATGCAAATGCTGAAGCT
CTTAATAACTTATTGCAACAACTCTCTAATAGGTTTGGTGCTATAAGTTCTTCTTTACAAGAAATTCTATCTAGACTGG
ATGCTCTTGAAGCGCAAGCTCAGATAGACAGACTTATTAATGGGCGTCTTACCGCTCTTAATGCCTATGTTTCTCAACA
GCTTAGTGACTCTACACTAGTAAAATTTAGTGCAGCACAAGCTATGGAGAAGGTTAATGAATGTGTCAAAAGCCAATCA
TCTAGGATAAATTTTTGTGGTAATGGTAATCATATTATATCATTAGTGCAGAATGCTCCATATGGTTTGTATTTCATCC
ACTTTAGCTATGTCCCTACTAAGTATGTCACAGCGAAGGTTAGTCCCGGTCTGTGCATTGCTGGTGATAGAGGTATAGC
CCCCAAGAGTGGTTATTTTGTTAATGTAAATAATACTTGGATGTTCACTGGTAGTGGTTATTACTACCCTGAACCTATA
ACTGGAAATAATGTTGTTGTTATGAGTACCTGTGCTGTTAATTATACTAAAGCGCCGGATGTAATGCTGAACATTTCAA
CACCCAACCTCCCTGATTTTAAGGAAGAGTTGGATCAATGGTTTAAAAACCAAACATCAGTGGCACCAGATTTGTCACT
TGATTATATAAATGTTACATTCTTGGACCTACAAGATGAAATTAATAGGTTACAGGAGGCAATAAAAGTTTTAAATCAG
AGCTACATCAATCTCAAGGACATTGGTACATATGAGTATTATGTAAAATGGCCTTGGTATGTATGGCTTTTAGTTGGCT
TTGCTGGTGTAGCTATGCTTGTTTTACTATTCTTCATATGCTGTTGTACAGGATGTGGGACTAGTTGTTTTAAGAAATG
TGGCGGTTGTTGTGATGATTATACTGGACACCAGGAGTTAGTAATTAAAACATCACATGACGACTAA

>gi|87082919|gb|DQ389636.1|_Bovine_coronavirus_isolate_KCD5_spike_glycoprot

ein_precursor_(S)_gene_complete_cds

ATGTTTTTGATACTTTTAATTTCCTTACCAATGGCTTTTGCTGTTATAGGAGATTTAAAGTGTACTACAGTTTCCATTAATGATGTTGACACTGGTGTTCCTTCTATTAGCACTGATACTGTCGATGTTACTAATGGTTTAGGTACTTATTATGTTTTAGATCGTGTGTATTTAAATACTACGTTGTTGCTTAATGGTTACTACCCTACTTCAGGTTCTACATATCGTAATATGGCACTGAAGGGAACTTTACTATTGAGCACACTATGGTTTAAACCACCTTTTCTTTCTGATTTTACTAATGGTATTTTTGCTAAGGTCAAAAATACCAAGGTTATTAAAGGTGGTGTAATGTATAGTGAGTTTCCTGCTATAACTATAGGTAGTACTTTTGTAAATACATCCTATAGTGTGGTAGTACAACCACATACTACTAATTTTGATAGTAAATTACAAGGTCTCTTAGAGATTTCTGTTTGCCAGTATACTATGTGCGAGTACCCAAATACGATTTGTAATCCTAATCTGGGTAATCAACGCGTAGAACTATGGCATTGGGATACAGGTGTTGTTTCCTGTTTATATAAGCGTAATTTCACATATGATGTGAATGCTGATTATTTGTATTTCCATTTTTATCAAGAAGGTGGTACTTTTTATGCATATTTTACAGACACTGGTGTTGTTACTAAGTTTCTGTTTAATGTTTATTTAGGCACGGTGCTCTCACATTATTATGTCATGCCTTTGACTTGTAATAGTGCTCTGACTTTAGAATATTGGGTTACACCTCTCACTTCTAAACAATATCTACTAGCTTTCAATCAAGATGGTGTTATTTTTAATGCTGTTGATTGTAAGAGTGATTTTATGAGTGAGATTAAGTGTAAAACACTATCTATAGCACCATCTACTGGTGTTTATGAATTAAGCGGTTACACTGTTCAGCCAATTGCAGATGTTTACCGACGTATACCTAATTTTCCCGATTGCAATATTGAGGCTTGGCTTAATGATAAGTCGGTGCCCTCTCCATTAAATTGGGAACGTAAGACCTTTTCAAATTGTAATTTTAATATGAGCAGCCTGATGTCTTTTATTCAGGCAGACTCATTTACTTGTAATAATATTGATGCTGCTAAGATATATGGTATGTGTTTTTCCAGCATAACTATAGATAAGTTTGCAATACCCAATGGTAGGAAGGTTGACCTACAATTGGGTAATTTGGGTTATTTGCAGTCTTTTAACTATAGAATTGATACTACTGCTACAAGTTGTCAGTTGTATTATAATTTACCTGCTGCTAATGTTTCTGTTAGCAGGTTTAATCCTTCTACTTGGAATAGGAGATTTGGTTTTACAGAACAATCAGTTTTTAAGCCTCAACCTGTAGGTGTTTTTACTGATCATGATGTTGTTTATGCACAACATTGTTTTAAAGCTCCCACTAATTTCTGTCCTTGTAAATTGGATGGGTCTTTGTGTGTAGGTAGTGGTTCTGGTATAGATGCTGGTTATAAAAATACTGGTATAGGCACTTGTCCTGCAGGTACTAATTATTTAACTTGCCATAATGCTGCCCAATGTGATTGTTTGTGCACCCCAGACCCCATTACATCTAAAGCTACAGGGCCTTACAAGTGCCCCCAAACTAAATACTTAGTTGGTATAGGTGAGCACTGTTCGGGTCTCGCTATTAAAAGTGATTATTGTGGAGGTAATCCTTGTAGTTGCCAACCACAAGCATTTTTGGGTTGGTCTGTTGACTCTTGTTTACAAGGGGATAGGTGTAATATTTTTGCTAATTTTATTTTGCATGATGTTAATAGTGGTACTACTTGTTCTACTGATTTACAAAAATCAAACACAGACATAATTCTTGGTGTTTGTGTTAATTATGATCTTTATGGTATTACTGGCCAAGGTATTTTTGTTGAGGTTAATGCGACTTATTATAATAGTTGGCAGAACCTTTTATATGATTCTAATGGTAATCTCTATGGTTTTAGAGACTACTTAACAAATAGAACTTTTATGATTCGTAGTTGCTATAGCGGTCGTGTATCAGCGGCCTTTCATGCTAACTCTTCCGAACCAGCATTGCTATTTCGGAATATTAAATGCAACTACGTTTTTAATAATACTCTTTCACGACAGCTGCAACCTATTAACTATTTTGATAGCTATCTTGGTTGTGTTGTCAATGCTGATAATAGTACTTCTAGTGTTGTTCAAACATGCGATCTCACAGTAGGTAGTGGTTACTGTGTGGATTACTCTACAAAAAGACGAAGTCGTAGATCGATTACCACTGGTTATCGGTTTACTAATTTTGAGCCATTTACTGTTAATTCAGTAAATGATAGTTTAGAACCTGTAGGTGGTTTGTATGAAATTCAAATACCTTCAGAGTTTACTATAGGTAATATGGAGGAGTTTATTCAAACAAGCTCTCCTAAAGTTACTATTGATTGTTCTGCTTTTGTCTGTGGTGATTATGCAGCATGTAAATCACAGTTGGTTGAATATGGTAGCTTCTGTGACAATATTAACGCTATACTCACAGAAGTAAATGAACTACTTGACACTACTCAGTTGCAAGTAGCTAATAGTTTAATGAATGGTGTCACTCTTAGCACTAAGCTTAAAGATGGCGTTAATTTCAATATAGTAGACATCAATTTTTCCCCTGTATTAGGTTGTTTAGGAAGCGATTGTAATAAAGTTTCCAGTAGATCTGCTATAGAGGATTTACTTTTTTCTAAAGTAAAGTTATCTGATGTCGGTTTTGTTGAGGCTTATAATAATTGTACTGGAGGTGCCGAAATTAGGGACCTCATTTGTGTGCAAAGTTATAACGGTATCAAGGTGTTGCCTCCACTGCTCTCAGAAAATCAGATCAGTGGATACACTTTGGCTGCCACCTCTGCTAGTCTGTTTCCTCCTTGGTCAGCAGCAGCAGGTGTACCATTTTATTTAAATGTTCAGTATCGCATTAATGGGATTGGTGTTACCATGGATGTGCTAAGTCAAAATCAAAAGCTTATTGCTAATGCATTTAACAATGCTCTTGGTGCTATTCAGGACGGTTTTGATGCTACCAATTCTGCTTTAGTTAAAATTCAAGCTGTTGTTAATGCAAATGCTGAAGCTCTTAATAACTTATTGCAACAACTCTCTAATAGGTTTGGTGCTATAAGTTCTTCTTTACAAGAAATTCTATCTAGACTGGATGCTCTTGAAGCGCAAGCTCAGATAGACAGACTTATTAATGGGCGTCTTACCGCTCTTAATGCCTATGTTTCTCAACA

GCTTAGTGACTCTACACTAGTAAAATTTAGTGCAGCACAAGCTATGGAGAAGGTTAATGAATGTGTCAAAAGCCAATCA
TCTAGGATAAATTTTTGTGGTAATGGTAATCATATTATATCATTAGTGCAGAATGCTCCATATGGTTTGTATTTCATCC
ACTTTAGCTATGTCCCTACTAAGTATGTCACAGCGAAGGTTAGTCCCGGTCTGTGCATTGCTGGTGATAGAGGTATAGC
CCCCAAGAGTGGTTATTTTGTTAATGTAAATAATACTTGGATGTTCACTGGTAGTGGTTATTACTACCCTGAACCTATA
ACTGGAAATAATGTTGTTGTTATGAGTACCTGTGCTGTTAATTATACTAAAGCGCCGGATGTAATGCTGAACATTTCTA
CACCCAACCTCCATGATTTTAAGGAAGAGTTGGATCAATGGTTTAAAAACCAAACATCAGTGGCACCATATTTGTCACT
TGATTATATAAATGTTACATTCTTGGACCTACAAGATGAAATGAATAGGTTACAGGAGGCAATAAAAGTTTTAAATCAG
AGCTACATCAATCTCAAGGACATTGGTACATATGAGTATTATGTAAAATGGCCTTGGTATGTATGGCTTTTAATTGGCT
TTGCTGGTGTAGCTATGCTTGTTTTACTATTCTTCATATGCTGTTGTACAGGATGTGGGACTAGTTGTTTTAAGAAATG
TGGCGGTTGTTGTGATGATTATACTGGACACCAGGAGTTAGTAATTAAAACATCACATGACGACTAA

>gi|87082921|gb|DQ389637.1|_Bovine_coronavirus_isolate_KCD6_spike_glycoprotein_precursor_(S)_gene_complete_cds

ATGTTTTTGATACTTTTAAATTCCTTACCAACGGCTTTTGCTGTTATAGGAGATTTAAAGTGTACTACAGTTTCC
ATTAATGATGTTGACACTGGTGTTCCTTCTATTAGCACTGATACTGTCGATGTTACTAATGGTTTAGGTACTTATTATG
TTTTAGATCGTGTGTATTTAAATACTACGTTGTTGCTTAATGGTTACTACCCTACTTCAGGTTCTACATATCGTAATAT
GGCACTGAAGGGAACTTTACTATTGAGCACACTATGGTTTAAACCACCTTTTCTTTCTGATTTTACTAATGGTATTTTT
GCTAAGGTCAAAAATACCAAGGTTATTAAAGGTGGTGTAATGTATAGTGAGTTTCCTGCTATAACTATAGGTAGTACTT
TTGTAAATACATCCTATAGTGTGGTAGTACAACCACATACTACTAATTTTGATAGTAAATTACAAGGTCTCTTAGAGAT
TTCTGTTTGCCAGTATACTATGTGCGAGTACCCAAATACGATTTGTAATCCTAATCTGGGTAATCAACGCGTAGAACTA
TGGCATTGGGATACAGGTGTTGTTTCCTGTTTATATAAGCGTAATTTCACATATGATGTGAATGCTGATTATTTGTATT
TCCATTTTTATCAAGAAGGTGGTACTTTTTATGCATATTTTACAGACACTGGTGTTGTTACTAAGTTTCTGTTTAATGT
TTATTTAGGCACGGTGCTCTCACATTATTATGTCATGCCTTTGACTTGTAATAGTGCTCTGACTTTAGAATATTGGGTT
ACACCTCTCACTTCTAAACAATATCTACTAGCTTTCAATCAAGATGGTGTTATTTTTAATGCTGTTGATTGTAAGAGTG
ATTTTATGAGTGAGATTAAGTGTAAAACACTATCTATAGCACCATCTACTGGTGTTTATGAATTAAGCGGTTACACTGT
TCAGCCAATTGCAGATGTTTACCGACGTATACCTAATTTTCCCGATTGCAATATTGAGGCTTGGCTTAATGATAAGTCG
GTGCCCTCTCCATTAAATTGGGAACGTAAGACCTTTTCAAATTGTAATTTTAATATGAGCAGCCTGATGTCTTTTATTC
AGGCAGACTCATTTACTTGTAATAATATTGATGCTGCTAAGATATATGGTATGTGTTTTTCCAGCATAACTATAGATAA
GTTTGCAATACCCAATGGTAGGAAGGTTGACCTACAATTGGGTAATTTGGGTTATTTGCAGTCTTTTAACTATAGAATT
GATACTACTGCTACAAGTTGTCAGTTGTATTATAATTTACCTGCTGCTAATGTTTCTGTTAGCAGGTTTAATCCTTCTA
CTTGGAATAGGAGATTTGGTTTTACAGAACAATCTGTTTTTAAGCCTCAACCTGCAGGTGTTTTTACTGATCATGATGT
TGTTTATGCACAACATTGTTTTAAAGCTCCCACTAATTTCTGTCCTTGTAAATTGGATGGGTCTTTGTGTGTAGGTAGT
GGTTCTGGTATAGATGCTGGTTATAAAAATACTGGTATAGGCACTTGTCCTGCAGGTACTAATTATTTAACTTGCCATA
ATGCTGCCCAATGTGATTGTTTGTGCACCCCAGACCCCATTACATCTAAAGCTACAGGGCCTTACAAGTGCCCCCAAAC
TAAATACTTAGTTGGTATAGGTGAGCACTGTTCGGGTCTCGCTATTAAAAGTGATTATTGTGGAGGTAATCCTTGTAGT
TGCCCACCACAAGCATTTTTGGGTTGGTCTGTTGACTCTTGTTTACAAGGGGATAGGTGTAATATTTTTGCTAATTTTA
TTTTGCATGATGTTAATAGTGGTACTACTTGTTCTACTGATTTACAAAAATCAAACACAGACATAATTCTTGGTGTTTG
TGTTAATTATGATCTTTATGGTATTACTGGCCAAGGTATTTTTGTTGAGGTTAATGCGACTTATTATAATAGTTGGCAG
AACCTTTTATATGATTCTAATGGTAATCTCTATGGTTTTAGAGACTACTTAACAAATAGAACTTTTATGATTCGTAGTT
GCTATAGCGGTCGTGTATCAGCGGCCTTTCATGCTAACTCTTCCGAACCAGCATTGCTATTTCGGAATATTAAATGCAA
CTACGTTTTTAATAATACTCTTTCACGACAGCTGCAACCTATTAACTATTTTGATAGCTATCTTGGTTGTGTTGTCAAT
GCTGATAATAGTACTTCTAGTGTTGTTCAAACATGCGATCTCACAGTAGGTAGTGGTTACTGTGTGGATTACTCTACAA

AAAGACGAAGTCGTAGATCGATTACCACTGGTTATCGGTTTACTAATTTTGAGCCATTTACTGTTAATTCAGTAAATGA
TAGTTTAGAACCTGTAGGTGGTTTGTATGAAATTCAAATACCTTCAGAGTTTACTATAGGTAATATGGAGGAGTTTATT
CAAACAAGCTCTCCTAAAGTTACTATTGATTGTTCTGCTTTTGTCTGTGGTGATTATGCAGCATGTAAATCACAGTTGG
TTGAATATGGTAGCTTCTGTGACAATATTAACGCTATACTCACAGAAGTAAATGAACTACTTGACACTACACAGTTGCA
AGTAGCTAATAGTTTAATGAATGGTGTCACTCTTAGCACTAAGCTTAAAGATGGCGTTAATTTCAATGTAGATGACATC
AATTTTTCCCCTGTATTAGGTTGTTTAGGAAGCGACTGTAATAAAGTTTCCAGTAGATCTGCTATAGAGGATTTACTTT
TTTCTAAAGTAAAGTTATCTGATGTCGGTTTTGTTGAGGCTTATAATAATTGTACTGGAGGTGCCGAAATTAGGGACCT
CATTTGTGTGCAAAGTTATAACGGTATCAAGGTGTTGCCTCCACTGCTCTCAGAAAATCAGATCAGTGGATACACTTTG
GCTGCCACCTCTGCTAGTCTGTTTCCTCCTTGGTCAGCAGCAGCAGGTGTACCATTTTATTTAAATGTTCAGTATCGCA
TTAATGGGATTGGTGTTACCATGGATGTGCTAAGTCAAAATCAAAAGCTTATTGCTAATGCATTTAACAATGCTCTTGG
TGCTATTCAGGACGGTTTTGATGCTACCAATTCTGCTTTAGTTAAAATTCAAGCTGTTGTTAATGCAAATGCTGAAGCT
CTTAATAACTTATTGCAACAACTCTCTAATAGGTTTGGTGCTATAAGTTCTTCTTTACAAGAAATTCTATCTAGACTGG
ATGCTCTTGAAGCGCAAGCTCAGATAGACAGACTTATTAATGGGCGTCTTACCGCTCTTAATGCCTATGTTTCTCAACA
GCTTAGTGACTCTACACTAGTAAAATTTAGTGCAGCACAAGCTATGGAGAAGGTTAATGAATGTGTCAAAAGCCAATCA
TCTAGGATAAATTTTTGTGGTAATGGTAATCATATTATATCATTAGTGCAGAATGCTCCATATGGTTTGTATTTCATCC
ACTTTAGCTATGTCCCTACTAAGTATGTCACAGCGAAGGTTAGTCCCGGTCTGTGCATTGCTGGTGATAGAGGTATAGC
CCCCAAGAGTGGTTATTTTGTTAATGTAAATAATACTTGGATGTTCACTGGTAGTGGTTATTACTACCCTGAACCTATA
ACTGGAAATAATGTTGTTGTTATGAGTACCTGTGCTGTTAATTATACTAAAGCGCCGGATGTAATGCTGAACATTTCAA
CACCCAACCTCCCTGATTTTAAGGAAGAGTTGGATCAATGGTTTAAAAACCAAACATCAGTGGCACCAGATTTGTCACT
TGATTATATAAATGTTACATTCTTGGACCTACAAGATGAAATGAATAGGTTACAGGAGGCAATAAAAGTTTTAAATCAG
AGCTACATCAATCTCAAGGACATTGGTACATATGAGTATTATGTAAAATGGCCTTGGTATGTATGGCTTTTAATTGGCT
TTGCTGGTGTAGCTATGCTTGTTTTACTATTCTTCATATGCTGTTGTACAGGATGTGGGACTAGTTGTTTTAAGAAATG
TGGCGGTTGTTGTGATGATTATACTGGACACCAGGAGTTAGTAATTAAAACATCACATGACGACTAA

>gi|87082923|gb|DQ389638.1|_Bovine_coronavirus_isolate_KCD7_spike_glycoprotein_precursor_(S)_gene_complete_cds

ATGTTTTTGATACTTTTAATTTCCTTACCAATGGCTTTTGCTGTTATAGGAGATTTAAAGTGTACTACAGTTTCC
ATTAATGATGTTGACACTGGTGTTCCTTCTATTAGCACTGATACTGTCGATGTTACTAATGGTTTAGGTACTTATTATG
TTTTAGATCGTGTGTATTTAAATACTACGTTGTTGCTTAATGGTTACTACCCTACTTCAGGTTCTACATATCGTAATAT
GGCACTGAAGGGAACTTTACTATTGAGCACACTATGGTTTAAACCACCTTTTCTTTCTGATTTTACTAATGGTATTTTT
GCTAAGGTCAAAAATACCAAGGTTATTAAAGGTGGTGTAATGTATAGTGAGTTTCCTGCTATAACTATAGGTAGTACTT
TTGTAAATACATCCTATAGTGTGGTAGTACAACCACATACTACTAATTTTGATAGTAAATTACAAGGTCTCTTAGAGAT
TTCTGTTTGCCAGTATACTATGTGCGAGTACCCAAATACGATTTGTAATCCTAATCTGGGTAATCAACGCGTAGAACTA
TGGCATTGGGATACAGGTGTTGTTTCCTGTTTATATAAGCGTAATTTCACATATGATGTGAATGCTGATTATTTGTATT
TCCATTTTTATCAAGAAGGTGGTACTTTTTATGCATATTTTACAGACACTGGTGTTGTTACTAAGTTTCTGTTTAATGT
TTATTTAGGCACGGTGCTCTCACATTATTATGTCATGCCTTTGACTTGTAATAGTGCTCTGACTTTAGAATATTGGGTT
ACACCTCTCACTTCTAAACAATATCTACTAGCTTTCAATCAAGATGGTGTTATTTTTAATGCTGTTGATTGTAAGAGTG
ATTTTATGAGTGAGATTAAGTGTAAAACACTATCTATAGCACCATCTACTGGTGTTTATGAATTAAGCGGTTACACTGT
TCAGCCAATTGCAGATGTTTACCGACGTATACCTAATTTTCCCGATTGCAATATTGAGGCTTGGCTTAATGATAAGTCG
GTGCCCTCTCCATTAAATTGGGAACGTAAGACCTTTTCAAATTGTAATTTTAATATGAGCAGCCTGATGTCTTTTATTC
AGGCAGACTCATTTACTTGTAATAATATTGATGCTGCTAAGATATATGGTATGTGTTTTTCCAGCATAACTATAGATAA
GTTTGCAATACCCAATGGTAGGAAGGTTGACCTACAATTGGGTAATTTGGGTTATTTGCAGTCTTTTAACTATAGAATT

GATACTACTGCTACAAGTTGTCAGTTGTATTATAATTTACCTGCTGCTAATGTTTCTGTTAGCAGGTTTAATCCTTCTA
CTTGGAATAGGAGATTTGGTTTTACAGAACAATCTGTTTTTAAGCCTCAACCTGCAGGTGTTTTTACTGATCATGATGT
TGTTTATGCACAACATTGTTTTAAAGCTCCCACTAATTTCTGTCCTTGTAAATTGGATGGGTCTTTGTGTGTAGGTAGT
GGTTCTGGTATAGATGCTGGTTATAAAAATACTGGTATAGGCACTTGTCCTGCAGGTACTAATTATTTAACTTGCCATA
ATGCTGCCCAATGTGATTGTTTGTGCACCCCAGACCCCATTACATCTAAAGCTACAGGGCCTTACAAGTGCCCCCAAAC
TAAATACTTAGTTGGTATAGGTGAGCACTGTTCGGGTCTCGCTATTAAAAGTGATTATTGTGGAGGTAATCCTTGTAGT
TGCCAACCACAAGCATTTTTGGGTTGGTCTGTTGACTCTTGTTTACAAGGGGATAGGTGTAATATTTTTGCTAATTTTA
TTTTGCATGATGTTAATAGTGGTACTACTTGTTCTACTGATTTACAAAAATCAAACACAGACATAATTCTTGGTGTTTG
TGTTAATTATGATCTTTATGGTATTACTGGCCAAGGTATTTTTGTTGAGGTTAATGCGACTTATTATAATAGTTGGCAG
AACCTTTTATATGATTCTAATGGTAATCTCTATGGTTTTAGAGACTACTTAACAAATAGAACTTTTATGATTCGTAGTT
GCTATAGCGGTCGTGTATCAGCGGCCTTTCATGCTAACTCTTCCGAACCAGCATTGCTATTTCGGAATATTAAATGCAA
CTACGTTTTTAATAATATTCTTTCACGACAGCTGCAACCTATTAACTATTTTGATAGCTATCTTGGTTGTGTTGTCAAT
GCTGATAATAGTACTTCTAGTGTTGTTCAAACATGCGATCTCACAGTAGGTAGTGGTTACTGTGTGGATTACTCTACAA
AAAGACGAAGTCGTAGATCGATTACCACTGGTTATCGGTTTACTAATTTTGAGCCATTTACTGTTAATTCAGTAAATGA
TAGTTTAGAACCTGTAGGTGGTTTGTATGAAATTCAAATACCTTCAAAGATTACTATAGGTAAAATGGAGGAGTTTATT
CAAACAAGCTCTCCTAAAGTTACTATTGATTGTTCTGCTTTTGTCTGTGGTGATTATGCAGCATGTAAATCACAGTTGG
TTGAATATGGTAGCTTCTGTGACAATATTAACGCTATACTCACAGAAGTAAATGAACTACTTGACACTACACAGTTGCA
AGTAGCTAATAGTTTAATGAATGGTGTCACTCTTAGCACTAAGCTTAAAGATGGCGTTAATTTCAATGTAGATGACATC
AATTTTTCCCCTGTATTAGGTTGTTTAGGAAGCGACTGTAATAAAGTTTCCAGTAGATCTGCTATAGAGGATTTACTTT
TTTCTAAAGTAAAGTTATCTGATGTCGGTTTTGTTGAGGCTTATAATAATTGTACTGGAGGTGCCGAAATTAGGGACCT
CATTTGTGTGCAAAGTTATAACGGTATCAAGGTGTTGCCTCCACTGCTCTCAGAAAATCAGATCAGTGGATACACTTTG
GCTGCCACCTCTGCTAGTCTGTTTCCTCCTTGGTCAGCAGCAGCAGGTGTACCATTTTATTTAAATGTTCAGTATCGCA
TTAATGGGATTGGTGTTACCATGGATGTGCTAAGTCAAAATCAAAAGCTTATTGCTAATGCATTTAACAATGCTCTTGG
TGCTATTCAGGACGGTTTTGATGCTACCAATTCTGCTTTAGTTAAAATTCAAGCTGTTGTTAATGCAAATGCTGAAGCT
CTTAATAACTTATTGCAACAACTCTCTAATAGGTTTGGTGCTATAAGTTCTTCTTTACAAGAAATTCTATCTAGACTGG
ATGCTCTTGAAGCGCAAGCTCAGATAGACAGACTTATTAATGGGCGTCTTACCGCTCTTAATGCCTATGTTTCTCAACA
GCTTAGTGACTCTACACTAGTAAAATTTAGTGCAGCACAAGCTATGGAGAAGGTTAATGAATGTGTCAAAAGCCAATCA
TCTAGGATAAATTTTTGTGGTAATGGTAATCATATTATATCATTAGTGCAGAATGCTCCATATGGTTTGTATTTCATCC
ACTTTAGCTATGTCCCTACTAAGTATGTCACAGCGAAGGTTAGTCCCGGTCTGTGCATTGCTGGTGATAGAGGTATAGC
CCCCAAGAGTGGTTATTTTGTTAATGTAAATAATACTTGGATGTTCACTGGTAGTGGTTATTACTACCCTGAACCTATA
ACTGGAAATAATGTTGTTGTTATGAGTACCTGTGCTGTTAATTATACTAAAGCGCCGGATGTAATGCTGAACATTTCAA
CACCCAACCTCCCTGATTTTAAGGAAGAGTTGGATCAATGGTTTAAAAACCAAACATCAGTGGCACCAGATTTGTCACT
TGATTATATAAATGTTACATTCTTGGACCTACAAGATGAAATGAATAGGTTACAGGAGGCAATAAAAGTTTTAAATCAG
AGCTACATCAATCTCAAGGACATTGGTACATATGAGTATTATGTAAAATGGCCTTGGTATGTATGGCTTTTAATTGGCT
TTGCTGGTGTAGCTATGCTTGTTTTACTATTCTTCATATGCTGTTGTACAGGATGTGGGACTAGTTGTTTTAAGAAATG
TGGCGGTTGTTGTGATGATTATACTGGATACCAGGAGTTAGTAATTAAAACATCACATGACGACTAA

>gi|87082925|gb|DQ389639.1|_Bovine_coronavirus_isolate_KCD8_spike_glycoprotein_precursor_(S)_gene_complete_cds

ATGTTTTTGATACTTTTAATTTCCTTACCAATGGCTTTTGCTGTTATAGGAGATTTAAAGTGTACTACAGTTTCC
ATTAATGATGTTGACACTGGTGTTCCTTCTATTAGCACTGATACTGTCGATGTTACTAATGGTTTAGGTACTTATTATG
TTTTAGATCGTGTGTATTTAAATACTACGTTGTTGCTTAATGGTTACTACCCTACTTCAGGTTCTACATATCGTAATAT

GGCACTGAAGGGAACTTTACTATTGAGCACACTATGGTTTAAACCACCTTTTCTTTCTGATTTTACTAATGGTATTTTT
GCTAAGGTCAAAAATACCAAGGTTATTAAAGGTGGTGTAATGTATAGTGAGTTTCCTGCTATAACTATAGGTAGTACTT
TTGTAAATACATCCTATAGTGTGGTAGTACAACCACATACTACTAATTTTGATAGTAAATTACAAGGTCTCTTAGAGAT
TTCTGTTTGCCAGTATACTATGTGCGAGTACCCAAATACGATTTGTAATCCTAATCTGGGTAATCAACGCGTAGAACTA
TGGCATTGGGATACAGGTGTTGTTTCCTGTTTATATAAGCGTAATTTCACATATGATGTGAATGCTGATTATTTGTATT
TCCATTTTTATCAAGAAGGTGGTACTTTTTATGCATATTTTACAGACACTGGTGTTGTTACTAAGTTTCTGTTTAATGT
TTATTTAGGCACGGTGCTCTCATATTATTATGTCATGCCTTTGACTTGTAATAGTGCTCTGACTTTAGAATATTGGGTT
ACACCTCTCACTTCTAAACAATATCTACTAGCTTTCAATCAAGATGGTGTTATTTTTAATGCTGTTGATTGTAAGAGTG
ATTTTATGAGTGAGATTAAGTGTAAAACACTATCTATAGCACCATCTACTGGTGTTTATGAATTAAGCGGTTACACTGT
TCAGCCAATTGCAGATGTTTACCGACGTATACCTAATCTTCCCGATTGCAATATTGAGGCTTGGCTTAATGATAAGTCG
GTGCCCTCTCCATTAAATTGGGAACGTAAGACCTTTTCAAATTGTAATTTTAATATGAGCAGCCTGATGTCTTTTATTC
AGGCAGACTCATTTACTTGTAATAATATTGATGCTGCTAAGATATATGGTATGTGTTTTTCCAGCATAACTATAGATAA
GTTTGCAATACCCAATGGTAGGAAGGTTGACCTACAATTGGGTAATTTGGGTTATTTGCAGTCTTTTAACTATAGAATT
GATACTACTGCTACAAGTTGTCAGTTGTATTATAATTTACCTGCTGCTAATGTTTCTGTTAGCAGGTTTAATCCTTCTA
CTTGGAATAGGAGATTTGGTTTTACAGAACAATCTGTTTTTAAGCCTCAACCTGCAGGTGTTTTTACTGATCATGATGT
TGTTTATGCACAACATTGTTTTAAAGCTCCCACTAATTTCTGTCCTTGTAAATTGGATGGGTCTTTGTGTGTAGGTAGT
GGTTCTGGTATAGATGCTGGTTATAAAAATACTGGTATAGGCACTTGTCCTGCAGGTACTAATTATTTAACTTGCCATA
ATGCTGCCCAATGTAATTGTTTGTGCACCCCAGACCCCATTACATCTAAAGCTACAGGGCCTTACAAGTGCCCCCAAAC
TAAATACTTAGTTGGTATAGGTGAGCACTGTTCGGGTCTCGCTATTAAAAGTGATTATTGTGGAGGTAATCCTTGTAGT
TGCCAACCACAAGCATTTTTGGGTTGGTCTGTTGACTCTTGTTTACAAGGGGATAGGTGTAATATTTTTGCTAATTTTA
TTTTGCATGATGTTAATAGTGGTACTACTTGTTCTACTGATTTACAAAAATCAAACACAGACATAATTCTTGGTGTTTG
TGTTAATTATGATCTTTATGGTATTACTGGCCAAGGTATTTTTGTTGAGGTTAATGCGACTTATTATAATAGTTGGCAG
AACCTTTTATATGATTCTAATGGTAATCTCTATGGTTTTAGAGACTACTTAACAAATAGAACTTTTATGATTCGTAGTT
GCTATAGCGGTCGTGTATCAGCGGCCTTTCATGCTAACTCTTCCGAACCAGCATTGCTATTTCGGAATATTAAATGCAA
CTACGTTTTTAATAATACTCTTTCACGACAGCTGCAACCTATTAACTATTTTGATAGCTATCTTGGTTGTGTTGTCAAT
GCTGATAATAGTACTTCTAGTGTTGTTCAAACATGCGATCTCACAGTAGGTAGTGGTTACTGTGTGGATTACTCTACAA
AAAGACGAAGTCGTAGATCGATTACCACTGGTTATCGGTTTACTAATTTTGAGCCATTTACTGTTAATTCAGTAAATGA
TAGTTTAGAACCTGTAGGTGGTTTGTATGAAATTCAAATACCTTCAGAGTTTACTATAGGTAATATGGAGGAGTTTATT
CAAACAAGCTCTCCTAAAGTTACTATTGATTGTTCTGCTTTTGTCTGTGGTGATTATGCAGCATGTAAATCACAGTTGG
TTGAATATGGTGGCTTCTGTGACAATATTAACGCTATACTCACAGAAGTAAATGAACTACTTGACACTACACAGTTGCA
AGTAGCTAATAGTTTAATGAATGGTGTCACTCTTAGCACTAAGCTTAAAGATGGCGTTAATTTCAATGTAGATGACATC
AATTTTTCCCCTGTATTAGGTTGTTTAGGAAGCGACTGTAATAAAGTTTCCAGTAGATCTGCTATAGAGGATTTACTTT
TTTCTAAAGTAAAGTTATCTGATGTCGGTTTTGTTGAGGCTTATAATAATTGTACTGGAGGTGCCGAAATTAGGGACCT
CATTTGTGTGCAAAGTTATAACGGTATCAAGGTGTTGCCTCCACTGCTCTCAGAAAATCAGATCAGTGGATACACTTTG
GCTGCCACCTCTGCTAGTCTGTTTCCTCCTTGGTCAGCAGCAGCAGGTGTACCATTTTATTTAAATGTTCAGTATCGCA
TTAATGGGATTGGTGTTACCATGGATGTGCTAAGTCAAAATCAAAAGCTTATTGCTAATGCATTTAACAATGCTCTTGG
TGCTATTCAGGACGGTTTTGATGCTACCAATTCTGCTTTAGTTAAAATTCAAGCTGTTGTTAATGCAAATGCTGAAGCT
CTTAATAACTTATTGCAACAACTCTCTAATAGGTTTGGTGCTATAAGTTCTTCTTTACAAGAAATTCTATCTAGACTGG
ATGCTCTTGAAGCGCAAGCTCAGATAGACAGACTTATTAATGGGCGTCTTACCGCTCTTAATGCCTATGTTTCTCAACA
GCTTAGTGACTCTACACTAGTAAAATTTAGTGCAGCACAAGCTATGGAGAAGGTTAATGAATGTGTCAAAAGCCAATCA
TCTAGGATAAATTTTTGTGGTAATGGTAATCATATTATATCATTAGTGCAGAATGCTCCATATGGTTTGTATTTCATCC
ACTTTAGCTATGTCCCTACTAAGTATGTCACAGCGAAGGTTAGTCCCGGTCTGTGCATTGCTGGTGATAGAGGTATAGC
CCCCAAGAGTGGTTATTTTGTTAATGTAAATAATACTTGGATGTTCACTGGTAGTGGTTATTACTACCCTGAACCTATA
ACTGGAAATAATGTTGTTGTTATGAGTACCTGTGCTGTTAATTATACTAAAGCGCCGGATGTAATGCTGAACATTTCAA

CACCCAACCTCCCTGATTTTAAGGAAGAGTTGGATCAATGGTTTAAAAACCAAACATCAGTGGCACCAGATTTGTCACT
TGATTATATAAATGTTACATTCTTGGACCTACAAGATGAAATGAATAGGTTACAGGAGGCAATAAAAGTTTTAAATCAG
AGCTACATCAATCTCAAGGACATTGGTACATATGAGTATTATGTAAAATGGCCTTGGTATGTATGGCTTTTAATTGGCT
TTGCTGGTGTAGCTATGCTTGTTTTACTATTCTTCATATGCTGTTGTACAGGATGTGGGACTAGTTGTTTTAAGAAATG
TGGCGGTTGTTGTGATGATTATACTGGATACCAGGAGTTAGTAATTAAAACATCACATGACGACTAA

>gi|87082927|gb|DQ389640.1|_Bovine_coronavirus_isolate_KCD9_spike_glycoprotein_precursor_(S)_gene_complete_cds

ATGTTTTTGATACTTTTAATTTCCTTACCAATGGCTTTTGCTGTTATAGGAGATTTAAAGTGTACTACAGTTTCC
ATTAATGATGTTGACACTGGTGTTCCTTCTATTAGCACTGATACTGTCGATGTTACTAATGGTTTAGGTACTTATTATG
TTTTAGATCGTGTGTATTTAAATACTACGTTGTTGCTTAATGGTTACTACCCTACTTCAGGTTCTACATATCGTAATAT
GGCACTGAAGGGAACTTTACTATTGAGCACACTATGGTTTAAACCACCTTTTCTTTCTGATTTTACTAATGGTATTTTT
GCTAAGGTCAAAAATACCAAGGTTATTAAAGGTGGTGTAATGTATAGTGAGTTTCCTGCTATAACTATAGGTAGTACTT
TTGTAAATACATCCTATAGTGTGGTAGTACAACCACATACTACTAATTTTGATAGTAAATTACAAGGTCTCTTAGAGAT
TTCTGTTTGCCAGTATACTATGTGCGAGTACCCAAATACGATTTGTAATCCTAATCTGGGTAATCAACGCGTAGAACTA
TGGCATTGGGATACAGGTGTTGTTTCCTGTTTATATAAGCGTAATTTCACATATGATGTGAATGCTGATTATTTGTATT
TCCATTTTTATCAAGAAGGTGGTACTTTTTATGCATATTTTACAGACACTGGTGTTGTTACTAAGTTTCTGTTTAATGT
TTATTTAGGCACGGTGCTCTCACATTATTATGTCATGCCTTTGACTTGTAATAGTGCTCTGACTTTAGAATATTGGGTT
ACACCTCTCACTTCTAAACAATATCTACTAGCTTTCAATCAAGATGGTGTTATTTTTAATGCTGTTGATTGTAAGAGTG
ATTTTATGAGTGAGATTAAGTGTAAAACACTATCTATAGCACCATCTACTGGTGTTTATGAATTAAGCGGTTACACTGT
TCAGCCAATTGCAGATGTTTACCGACGTATACCTAATTTTCCCGATTGCAATATTGAGGCTTGGCTTAATGATAAGTCG
GTGCCCTCTCCATTAAATTGGGAACGTAAGACCTTTTCAAATTGTAATTTTAATATGAGCAGCCTGATGTCTTTTATTC
AGGCAGACTCATTTACTTGTAATAATATTGATGCTGCTAAGATATATGGTATGTGTTTTTCCAGCATAACTATAGATAA
GTTTGCAATACCCAATGGTAGGAAGGTTGACCTACAATTGGGTAATTTGGGTTATTTGCAGTCTTTTAACTATAGAATT
GATACTACTGCTACAAGTTGTCAGTTGTATTATAATTTACCTGCTGCTAATGTTTCTGTTAGCAGGTTTAATCCTTATA
CTTGGAATAGGAGATTTGGTTTTACAGAACAATCTGTTTTTAAGCCTCAACCTGCAGGTGTTTTTACTGATCATGATGT
TGTTTATGCACAACATTGTTTTAAAGCTCCCACTAATTTCTGTCCTTGTAAATTGGATGGGTCTTTGTGTGTAGGTAGT
GGTTCTGGTATAGATGCTGGTTATAAAAATACTGGTATAGGCACTTGTCCTGCAGGTACTAATTATTTAACTTGCCATA
ATGCTGCCCAATGTGATTGTTTGTGCACCCCAGACCCCATTACATCTAAAGCTACAGGGCCTTACAAGTGCCCCCAAAC
TAAATACTTAGTTGGTATAGGTGAGCACTGTTCGGGTCTCGCTATTAAAAGTGATTATTGTGGAGGTAATCCTTGTAGT
TGCCAACCACAAGCATTTTTGGGTTGGTCTGTTGACTCTTGTTTACAAGGGGATAGGTGTAATATTTTTGCTAATTTTA
TTTTGCATGATGTTAATAGTGGTACTACTTGTTCTACTGATTTACAAAAATCAAACACAGACATAATTCTTGGTGTTTG
TGTTAATTATGATCTTTATGGTATTACTGGCCAAGGTATTTTTGTTGAGGTTAATGCGACTTATTATAATAGTTGGCAG
AACCTTTTATATGATTCTAATGGTAATCTCTATGGTTTTAGAGACTACTTAACAAATAGAACTTTTATGATTCGTAGTT
GCTATAGCGGTCGTGTATCAGCGGCCTTTCATGCTAACTCTTCCGAACCAGCATTGCTATTTCGGAATATTAAATGCAA
CTACGTTTTTAATAATATTCTTTCACGACAGCTGCAACCTATTAACTATTTTGATAGCTATCTTGGTTGTGTTGTCAAT
GCTGATAATAGTACTTCTAGTGTTGTTCAAACATGCGATCTCACAGTAGGTAGTGGTTACTGTGTGGATTACTCTACAA
AAAGACGAAGTCGTAGATCGATTACCACTGGTTATCGGTTTACTAATTTTGAGCCATTTACTGTTAATTCAGTAAATGA
TAGTTTAGAACCTGTAGGTGGTTTGTATGAAATTCAAATACCTTCAGAGTTTACTATAGGTAATATGGAGGAGTTTATT
CAAACAAGCTCTCCTAAAGTTACTATTGATTGTTCTGCTTTTGTCTGTGGTGATTATGCAGCATGTAAATCACAGTTGG
TTGAATATGGTAGCTTCTGTGACAATATTAACGCTATACTCACAGAAGTAAATGAACTACTTGACACTACACAGTTGCA
AGTAGCTAATAGTTTAATGAATGGTGTCACTCTTAGCAATAAGCTTAAAGATGGCGTTAATTTCAATGTAGACGACATC

AATTTTTCCCCTGTATTAGGTTGTTTAGGAAGCGACTGTAATAAAGTTTCCAGTAGATCTGCTATAGAGGATTTACTTT
TTTCTAAAGTAAAGTTATCTGATGTCGGTTTTGTTGAGGCTTATAATAATTGTACTGGAGGTGCCGAAATTAGGGACCT
CATTTGTGTGCAAAGTTATAACGGTATCAAGGTGTTGCCTCCACTGCTCTCAGAAAATCAGATCAGTGGATACACTTTG
GCTGCCACCTCTGCTAGTCTGTTTCCTCCTTGGTCAGCAGCAGCAGGTGTACCATTTTATTTAAATGTTCAGTATCGCA
TTAATGGGATTGGTGTTACCATGGATGTGCTAAGTCAAAATCAAAAGCTTATTGCTAATGCATTTAACAATGCTCTTGG
TGCTATTCAGGACGGTTTTGATGCTACCAATTCTGCTTTAGTTAAAATTCAAGCTGTTGTTAATGCAAATGCTGAAGCT
CTTAATAACTTATTGCAACAACTCTCTAATAGGTTTGGTGCTATAAGTTCTTCTTTACAAGAAATTCTATCTAGACTGG
ATGCTCTTGAAGCGCAAGCTCAGATAGACAGACTTATTAATGGGCGTCTTACCGCTCTTAATGCCTATGTTTCTCAACA
GCTTAGTGACTCTACACTAGTAAAATTTAGTGCAGCACAAGCTATGGAGAAGGTTAATGAATGTGTCAAAAGCCAATCA
TCTAGGATAAATTTTTGTGGTAATGGTAATCATATTATATCATTAGTGCAGAATGCTCCATATGGTTTGTATTTCATCC
ACTTTAGCTATGTCCCTACTAAGTATGTCACAGCGAAGGTTAGTCCCGGTCTGTGCATTGCTGGTGATAGAGGTATAGC
CCCCAAGAGTGGTTATTTTGTTAATGTAAATAATACTTGGATGTTCACTGGTAGTGGTTATTACTACCCTGAACCTATA
ACTGGAAATAATGTTGTTGTTATGAGTACCTGTGCTGTTAATTATACTAAAGCGCCGGATGTAATGCTGAACATTTCAA
CACCCAACCTCCCTGATTTTAAGGAAGAGTTGGATCAATGGTTTAAAAACCAAACATCAGTGGCACCAGATTTGTCACT
TGATTATATAAATGTTACATTCTTGGACCTACAAGATGAAATGAATAGGTTACAGGAGGCAATAAAAGTTTTAAATCAG
AGCTACATCAATCTCAAGGACATTGGTACATATGAGTATTATGTAAAATGGCCTTGGTATGTATGGCTTTTAATTGGCT
TTGCTGGTGTAGCTATGCTTGTTTTACTATTCTTCATATGCTGTTGTACAGGATGTGGGACTAGTTGTTTTAAGAAATG
TGGCGGTTGTTGTGATGATTATACTGGACACCAGGAGTTAGTAATTAAAACATCACATGACGACTAA

>gi|87082929|gb|DQ389641.1|_Bovine_coronavirus_isolate_KCD10_spike_glycoprotein_precursor_(S)_gene_complete_cds

ATGTTTTTGATACTTTTAATTTCCTTACCAATGGCTTTTGCTGTTATAGGAGATTTAAAGTGTACTACAGTTTCC
ATTAATGATGTTGACACTGGTGTTCCTTCTATTAGCACTGATACTGTCGATGTTACTAATGGTTTAGGTACTTATTATG
TTTTAGATCGTGTGTATTTAAATACTACGTTGTTGCTTAATGGTTACTACCCTACTTCAGGTTCTACATATCGTAATAT
GGCACTGAAGGGAACTTTACTATTGAGCACACTATGGTTTAAACCACCTTTTCTTTCTGATTTTACTAATGGTATTTTT
GCTAAGGTCAAAAATACCAAGGTTATTAAAGGTGGTGTAATGTATAGTGAGTTTCCTGCTATAACTATAGGTAGTACTT
TTGTAAATACATCCTATAGTGTGGTAGTACAACCACATACTACTAATTTTGATAGTAAATTACAAGGTCTCTTAGAGAT
TTCTGTTTGCCAGTATACTATGTGCGAGTACCCAAATACGATTTGTAATCCTAATCTGGGTAATCAACGCGTAGAACTA
TGGCATTGGGATACAGGTGTTGTTTCCTGTTTATATAAGCGTAATTTCACATATGATGTGAATGCTGATTATTTGTATT
TCCATTTTTATCAAGAAGGTGGTACTTTTTATGCATATTTTACAGACACTGGTGTTGTTACTAAGTTTCTGTTTAATGT
TTATTTAGGCACGGTGCTCTCACATTATTATGTCATGCCTTTGACTTGTAATAGTGCTCTGACTTTAGAATATTGGGTT
ACACCTCTCACTTCTAAACAATATCTACTAGCTTTCAATCAAGATGGTGTTATTTTTAATGCTGTTGATTGTAAGCGTG
ATTTTATGAGTGAGATTAAGTGTAAAACACTATCTATAGCACCATCTACTGGTGTTTATGAATTAAGCGGTTACACTGT
TCAGCCAATTGCAGATGTTTACCGACGTATACCTAATCTTCCCGATTGCAATATTGAGGCTTGGCTTAATGATAAGTCG
GTGCCCTCTCCATTAAATTGGGAACGTAAGACCTTTTCAAATTGTAATTTTAATATGAGCAGCCTGATGTCTTTTATTC
AGGCAGACTCATTTACTTGTAATAATATTGATGCTGCTAAGATATATGGTATGTGTTTTTCCAGCATAACTATAGATAA
GTTTGCAATACCCAATGGTAGGAAGGTTGACCTACAATTGGGTAATTTGGGTTATTTGCAGTCTTTTAACTATAGAATT
GATACTACTGCTACAAGTTGTCAGTTGTATTATAATTTACCTGCTGCTAATGTTTCTGTTAGCAGGTTTAATCCTTCTA
CTTGGAATAGGAGATTTGGTTTTACAGAACAATCTGTTTTTAAGCCTCAACCTGCAGGTGTTTTTACTGATCATGATGT
TGTTTATGCACAACATTGTTTTAAAGCTCCCACTAATTTCTGTCCTTGTAAATTGGATGGGTCTTTGTGTGTAGGTAGT
GGTTCTGGTATAGATGCTGGTTATAAAAATACTGGTATAGGCACTTGTCCTGCAGGTACTAATTATTTAACTTGCCATA
ATGCTGCCCAATGTGATTGTTTGTGCACCCCAGACCCCATTACATCTAAAGCTACAGGGCCTTACAAGTGCCCCCAAAC

TAAATACTTAGTTGGTATAGGTGAGCACTGTTCGGGTCTCGCTATTAAAAGTGATTATTGTGGAGGTAATCCTTGTAGT
TGCCAACCACAAGCATTTTTGGGTTGGTCTGTTGACTCTTGTTTACAAGGGGATAGGTGTAATATTTTTGCTAATTTTA
TTTTGCATGATGTTAATAGTGGTACTACTTGTTCTACTGATTTACAAAAATCAAACACAGACATAATTCTTGGTGTTTG
TGTTAATTATGATCTTTATGGTATTACTGGCCAAGGTATTTTTGTTGAGGTTAATGCGACTTATTATAATAGTTGGCAG
AACCTTTTATATGATTCTAATGGTAATCTCTATGGTTTTAGAGACTACTTAACAAATAGAACTTTTATGATTCGTAGTT
GCTATAGCGGTCGTGTATCAGCGGCCTTTCATGCTAACTCTTCCGAACCAGCATTGCTATTTCGGAATATTAAATGCAA
CTACGTTTTTAATAATACTCTTTCACGACAGCTGCAACCTATTAACTATTTTGATAGCTATCTTGGTTGTGTTGTCAAT
GCTGATAATAGTACTTCTAGTGTTGTTCAAACATGCGATCTCACAGTAGGTAGTGGTTACTGTGTGGATTACTCTACAA
AAAGACGAAGTCGTAGATCGATTACCACTGGTTATCGGTTTACTAATTTTGAGCCATTTACTGTTAATTCAGTAAATGA
TAGTTTAGAACCTGTAGGTGGTTTGTATGAAATTCAAATACCTTCAGAGTTTACTATAGGTAATATGGAGGAGTTTATT
CAAACAAGCTCTCCTAAAGTTACTATTGATTGTTCTGCTTTTGTCTGTGGTGATTATGCAGCATGTAAATCACAGTTGG
TTGAATATGGTAGCTTCTGTGACAATATTAACGCTATACTCACAGAAGTAAATGAACTACTTGACACTACACAGTTGCA
AGTAGCTAATAGTTTAATGAATGGTGTCACTCTTAGCACTAAGCTTAAAGATGGCGTTAATTTCAATGTAGATGACATC
AATTTTTCCCCTGTATTAGGTTGTTTAGGAAGCGACTGTAATAAAGTTTCCAGTAGATCTGCTATAGAGGATTTACTTT
TTTCTAAAGTAAAGTTATCTGATGTCGGTTTTGTTGAGGCTTATAATAATTGTACTGGAGGTGCCGAAATTAGGGACCT
CATTTGTGTGCAAAGTTATAACGGTATCAAGGTGTTGCCTCCACTGCTCTCAGAAAATCAGATCAGTGGATACACTTTG
GCTGCCACCTCTGCTAGTCTGTTTCCTCCTTGGTCAGCAGCAGCAGGTGTACCATTTTATTTAAATGTTCAGTATCGCA
TTAATGGGATTGGTGTTACCATGGATGTGCTAAGTCAAAATCAAAAGCTTATTGCTAATGCATTTAACAATGCTCTTGG
TGCTATTCAGGACGGTTTTGATGCTACCAATTCTGCTTTAGTTAAAATTCAAGCTGTTGTTAATGCAAATGCTGAAGCT
CTTAATAACTTATTGCAACAACTCTCTAATAGGTTTGGTGCTATAAGTTCTTCTTTACAAGAAATTCTATCTAGACTGG
ATGCTCTTGAAGCGCAAGCTCAGATAGACAGACTTATTAATGGGCGTCTTACCGCTCTTAATGCCTATGTTTCTCAACA
GCTTAGTGACTCTACACTAGTAAAATTTAGTGCAGCACAAGCTATGGAGAAGGTTAATGAATGTGTCAAAAGCCAATCA
TCTAGGATAAATTTTTGTGGTAATGGTAATCATATTATATCATTAGTGCAGAATGCTCCATATGGTTTGTATTTCATCC
ACTTTAGCTATGTCCCTACTAAGTATGTCACAGCGAAGGTTAGTCCCGGTCTGTGCATTGCTGGTGATAGAGGTATAGC
CCCCAAGAGTGGTTATTTTGTTAATGTAAATAATACTTGGATGTTCACTGGTAGTGGTTATTACTACCCTGAACCTATA
ACTGGAAATAATGTTGTTGTTATGAGTACCTGTGCTGTTAACTATACTAAAGCGCCGGATGTAATGCTGAACATTTCAA
CACCCAACCTCCATGATTTTAAGGAAGAGTTGGATCAATGGTTTAAAAACCAAACATCAGTGGCACCAGATTTGTCACT
TGATTATATAAATGTTACATTCTTGGACCTACAAGATGAAATGAATAGGTTACAGGAGGCAATAAAAGTTTTAAATCAG
AGCTACATCAATCTCAAGGACATTGGTACATATGAGTATTATGTAAAATGGCCTTGGTATGTATGGCTTTTAATTGGCT
TTGCTGGTGTAGCTATGCTTGTTTTACTATTCTTCATATGCTGTTGTACAGGATGTGGGACTAGTTGTTTTAAGAAATG
TGGCGGTTGTTGTGATGATTATACTGGACACCAGGAGTTAGTAATTAAAACATCACATGACGACTAA

>gi|87082951|gb|DQ389652.1|_Bovine_coronavirus_isolate_KWD11_spike_glycoprotein_precursor_(S)_gene_complete_cds

ATGTTTTTGATACTTTTAATTTCCTTACCAATGGCTTTTGCTGTTATAGGAGATTTAAAGTGTACTACAGTTTCC
ATTAATGATGTTGACACTGGTGTTCCCTCTATTAGCACTGATACTGTCGATGTTACTAATGGTTTAGGTACTTATTATG
TTTTAGATCGTGTGTATTTAAATACTACGTTGTTGCTTAATGGTTACTACCCTACTTCAGGTTCTACATATCGTAATAT
GGCACTGAAGGGAACTTTACTATTGAGCACACTATGGTTTAAACCACCTTTTCTTTCTGATTTTACTAATGGTATTTTT
GCTAAGGTCAAAAATACCAAGGTTATTAAAGATGGTGTAATGTATAGTGAGTTTCCTGCTATAACTATAGGTAGTACTT
TTGTAAATACATCCTATAGTGTGGTAGTACAACCACATACTACTAATTTTGATAGTAAATTACAAGGTCTCTTAGAGAT
TTCTGTTTGCCAGTATACTATGTGCGAGTACCCAAATACGATTTGTAATCCTAATCTGGGTAATCAACGCGTAGAACTA
TGGCATTGGGATACAGGTGTTGTTTCCTGTTTATATAAGCGTAATTTCACATATGATGTGAATGCTGATTATTTGTATT

TCCATTTTTATCAAGAAGGTGGTACTTTTTATGCATATTTTACAGACACTGGTGTTGTTACTAAGTTTCTGTTTAATGT
TTATTTAGGCACGGTGCTCTCATATTATTATGTCATGCCTTTGACTTGTAATAGTGCTCTGACTTTAGAATATTGGGTT
ACACCTCTCACTTCTAAACAATATTTACTAGCTTTCAATCAAGATGGTGTTATTTTTAATGCTGTTGATTGTAAGAGTG
ATTTTATGAGTGAGATTAAGTGTAAAACACTATCTATAGCACCATCTACTGGTGTTTATGAATTAAACGGTTACACTGT
GCAGCCAATTGCAGATGTTTACCGACGTATACCTAATCTTCCCGATTGCAATATTGAGGCTTGGCTTAATGATAAGTCG
GTGCCCTCTCCATTAAATTGGGAACGTAAGACCTTTTCAAATTGTAATTTTAATATGAGCAGCCTGATGTCTTTTATTC
AGGCAGACTCATTTACTTGTAATAATATTGATGCTGCTAAGATATATGGTATGTGTTTTTCCAGCATAACTATAGATAA
GTTTGCAATACCCAATGGTAGGAAGGTTGACCTACAATTGGGTAATTTGGGTTATTTGCAGTCTTTTAACTATAGAATT
GATACTACTGCTATAAGTTGTCAGTTGTATTATAATTTACCTGCTGCTAATGTTTCTGTTAGCAGGTTTAATCCTTCTA
CTTGGAATAGGAGATTTGGTTTTACAGAACAATCTGTTTTTAAGCCTCAACCTGCAGGTGTTTTTACTGATCATGATGT
TGTTTATGCACAACATTGTTTTAAAGCTCCCACTAATTTCTGTCCGTGTAAATTGGATGGGTCTTTGTGTGTAGGTAGT
GGTTCTGGTATAGATGCTGGTTATAAAAATACTGGTATAGGCACTTGTCCTGCAGGTACTAATTATTTAACTTGCCATA
ATGCTGCCCAATGTAATTGTTTGTGCACCCCAGACCCCATTACATCTAAAGCTACAGGGCCTTACAAGTGCCCCCAAAC
TAAATATTTAGTTGGTATAGGTGAGCACTGTTCGGGTCTTGCTATTAAAAGTGATTATTGTGGAGGTAATCCTTGTAGT
TGCCAACCACAAGCATTTTTGGGTTGGTCTGTTGACTCTTGTTTACAAGGGGATAGGTGTAATATTTTTGCTAATTTTA
TTTTGCATGATGTTAATAGTGGTACTACTTGTTCTACTGATTTACAAAAATCAAACACAGACATAATTCTTGGTGTTTG
TGTTAATTATGATCTTTATGGTATTACTGGCCAAGGTATTTTTGTTGAGGTTAATGCGACTTATTATAATAGTTGGCAG
AACCTTTTATATGATTCTAATGGTAATCTCTATGGTTTTAGAGACTACTTAACAAATAGAACTTTTATGATTCGTAGTT
GCTATAGCGGTCGTGTATCAGCGGCCTTTCATGCTAACTCTTCCGAACCAGCATTGCTATTTCGGAATATTAAATGCAA
CTACGTTTTTAATAATACTCTTCCACGACAGCTGCAACCTATTAACTATTTTGATAGCTATCTTGGTTGTGTTGTCAAT
GCTGATAATAGTACTTCTAGTGTTGTTCAAACATGCGATCTCACAGTAGGTAGTGGTTACTGTGTGGATTACTCTACAA
AAAGACGAAGTCGTAGATCGATTACCACTGGTTATCGGTTTACTAATTTTGAGCCATTTACTGTTAATTCAGTAAATGA
TAGTTTAGAACCTGTAGGTGGTTTGTATGAAATTCAAATACCTTCAGAGTTTACTATAGGTAATATGGAGGAGTTTATT
CAAACAAGCTCTCCTAAAGTTACTATTGATTGTTCTGCTTTTGTCTGTGGTGATTATGCAGCATGTAAATCACAGTTGG
TTGAATATGGTAGCTTCTGTGACAATGTTAACGCTATACTCACAGAAGTAAATGAACTACTTGACACTACACAGTTGCA
AGTAGCTAATAGTTTAATGAATGGTGTCACTCTTAGCACTAAGCTTAAAGATGGCGTTAATTTCAATGTAGATGACATC
AATTTTTCCCCTGTATTAGGTTGTTTAGGAAGCGACTGTAATAAAGTTTCCAGTAGATCTGCTATAGAGGATTTACTTT
TTTCTAAAGTAAAGTTATCTGATGTCGGTTTCGTTGAGGCTTATAATAATTGTACTGGAGGTGCCGAAATTAGGGACCT
CATTTGTGTGCAAAGTTATAACGGTATCAAGGTGTTGCCTCCACTGCTCTCAGAAAATCAGATCAGTGGATACACTTTG
GCTGCCACCTCTGCTAGTCTGTTTCCTCCTTGGTCAGCAGCAGCAGGTGTACCATTTTATTTAAATGTTCAGTATCGCA
TTAATGGGATTGGTGTTACCATGGATGTGCTAAGTCAAAATCAAAAGCTTATTGCTAACGCATTTAACAATGCTCTTGG
TGCTATTCAGGACGGTTTTGACGCTACCAATTCTGCTTTAGTTAAAATTCAAGCTGTTGTTAATGCAAATGCTGAAGCT
CTTAATAACTTATTGCAACAACTCTCTAATAGGTTTGGTGCTATAAGTTCTTCTTTACAAGAAATTCTATCTAGACTGG
ATGCTCTTGAAGCGCAAGCTCAGATAGACAGACTTATTAATGGGCGTCTTACCGCTCTTAATGCTTATGTTTCTCAACA
GCTTAGTGACTCTACACTAGTAAAATTTAGTGCAGCACAAGCTATGGAGAAGGTTAATGAATGTGTCAAAAGCCAATCA
TCTAGGATAAATTTTTGTGGTAATGGTAATCATATTATATCATTAGTGCAGAATGCTCCATATGGTTTGTATTTCATCC
ACTTTAGCTATGTCCCTACTAAGTATGTCACAGCGAAGGTTAGTCCCGGTCTGTGCATTGCTGGTGATAGAGGTATAGC
CCCCAAGAGTGGTTATTTTGTTAATGTAAATAATACTTGGATGTTCACTGGTAGTGGTTATTACTACCCTGAACCTATA
ACTGGAAATAATGTTGTTGTTATGAGTACCTGTGCTGTTAATTATACTAAAGCGCCGGATGTAATGCTGAACATTTCAA
CACCCAACCTCCCTGATTTTAAGGAAGAGTTGGATCAATGGTTTAAAAACCAAACATCAGTGGCACCAGATTTGTCACT
TGATTATATAAATGTTACATTCTTGGACCTACAAGATGAAATGAATAGGTTACAGGAGGCAATAAAAGTTTTAAATCAG
AGCTACATCAATCTCAAGGACATTGGTACATATGAGTATTATGTAAAATGGCCTTGGTATGTATGGCTTTTAATTGGCT
TTGCTGGTGTAGCTATGCTTGTTTTACTATTTTTCATATGCTGTTGTACAGGATGTGGGACTAGTTGTTTTAAGATATG
TGGTGGTTGTTGTGATGATTATGCTGGACACCAGGAGTTAGTAATTAAAACATCACATGACGACTAA

>gi|87082953|gb|DQ389653.1|_Bovine_coronavirus_isolate_KWD12_spike_glycoprotein_precursor_(S)_gene_complete_cds

ATGTTTTTGATACTTTTAATTTCCTTACCAATGGCTTTTGCTGTTATAGGAGATTTAAAGTGTACTACGGTTTCCATTAATGATGTTGACACTGGTGTTCCTTCTATTAGCACTGATACTGTCGATGTTACTAATGGTTTAGGCACTTATTATGTTTTAGATCGTGTGTATTTAAATACTACGTTGTTGCTTAATGGTTACTACCCTACTTCAGGTTCTACATATCGTAATATGGCACTGAAGGGAACTTTACTATTGAGCACACTATGGTTTAAACCACCTTTTCTTTCTGATTTTACTAATGGTATTTTTGCTAAGGTCAAAAATACCAAGGTTATTAAAGATGGTGTAATGTATAGTGAGTTTCCTGCTATAACTATAGGTAGTACTTTTGTAAATACATCCTATAGTGTGGTAGTACAACCACATACTACTAATTTTGATAGTAAATTACAAGGTCTCTTAGAGATTTCTGTTTGCCAGTATACTATGTGCGAGTACCCAAATACGATTTGTAATCCTAATCTGGGTAATCAACGCGTAGAACTATGGCATTGGGATACAGGTGTTGTTTCCTGTTTATATAAGCGTAATTTCACATATGATGTGAATGCTGATTATTTGTATTTCCATTTTTATCAAGAAGGTGGTACTTTTTATGCATATTTTACAGACACTGGTGTTGTTACTAAGTTTCTGTTTAATGTTTATTTAGGCACGGTGCTCTCATATTATTATGTCATGCCTTTGACTTGTAATAGTGCTCTGACTTTAGAATATTGGGTTACACCTCTCACTTCTAAACAATATCTACTAGCTTTCAATCAAGATGGTGTTATTTTTAATGCTGTTGATTGTAAGAGTGATTTTATGAGTGAGATTAAGTGTAAAACACTATCTATAGCACCATCTACTGGTGTTTATGAATTAAACGGTTACACTGTTCAGCCAATTGCAGATGTTTACCGACGTATACCTAATCTTCCCGATTGCAATATTGAGGCTTGGCTTAATGATAAGTCGGTGCCCTCTCCATTAAATTGGGAACGTAAGACCTTTTCAAATTGTAATTTTAATATGAGCAGCCTGATGTCTTTTATTCAGGCAGACTCATTTACTTGTAATAATATTGATGCTGCTAAGATATATGGTATGTGTTTTTCCAGCATAACTATAGATAAGTTTGCAATACCCAATGGTAGGAAGGTTGACCTACAATTGGGTAATTTGGGTTATTTGCAGTCTTTTAACTATAGAATTGATACTACTGCTACAAGTTGTCAGTTGTATTATAATTTACCTGCTGCTAATGTTTCTGTTAGCAGGTTTAATCCTTCTACTTGGAATAGGAGATTTGGTTTTACAGAACAATCTGTTTTTAAGCCTCAACCTGCAGGTGTTTTTACTGATCATGATGTTGTTTATGCACAACATTGTTTTAAAGCTCCCACTAATTTCTGTCCGTGTAAATTGGATGGGTCTTTGTGTGTAGGTAGTGGTCCTGGTATAGATGCTGGTTATAAAAATACTGGTATAGGCACTTGTCCTGCAGGTACTAATTATTTAACTTGCCATAATGCTGCCCAGTGTAATTGTTTGTGCACCCCAGACCCCATTACATCTAAAGCTACAGGGCCTTACAAGTGCCCCCAAACTAAATATTTAGTTGGTATAGGTGAGCACTGTTCGGGTCTTGCTATTAAAAGTGATTATTGTGGAGGTAATCCTTGTAGTTGCCAACCACAAGCATTTTTGGGTTGGTCTGTTGACTCTTGTTTACAAGGGGATAGGTGTAATATTTTTGCTAATTTTATTTTGCATGATGTTAATAGTGGTACTACTTGTTCTACTGATTTACAAAAATCAAACACAGACATAATTCTTGGTGTTTGTGTTAATTATGATCTTTATGGTATTACTGGCCAAGGTATTTTTGTTGAGGTTAATGCGACTTATTATAATAGTTGGCAGAACCTTTTATATGATTCTAATGGTAATCTCTATGGTTTTAGAGACTACTTAACAAATAGAACTTTTATGATTCGTAGTTGCTATAGCGGTCGTGTATCAGCGGCCTTTCATGCTAACTCTTCCGAACCAGCATTGCTATTTCGGAATATTAAATGCAACTACGTTTTTAATAATATTCTTCCACGACAGCTGCAACCTATTAACTATTTTGATAGCTATCTTGGTTGTGTTGTCAATGCTGATAATAGTACTTCTAGTGTTGTTCAAACATGCGATCTCACAGTAGGTAGTGGTTACTGTGTGGATTACTCTACAAAAAGACGAAGTCGTAGATCGATTACCACTGGTTATCGGTTTACTAATTTTGAGCCATTTACTGTTAATTCAGTAAATGATAGTTTAGAACCTGTAGGTGGTTTGTATGAAATTCAAATACCTTCAGAGTTTACTATAGGTAATATGGAGGAGTTTATTCAAACAAGCTCTCCTAAAGTTACTATTGATTGTTCTGCTTTTGTCTGTGGTGATTATGCAGCATGTAAATCACAGTTGGTTGAATATGGTAGCTTCTGTGACAATGTTAACGCTATACTCACAGAAGTAAATGAACTACTTGACACTACACAGTTGCAAGTAGCTAATAGTTTAATGAATGGTGTCACTCTTAGCACTAAGCTTAAAGATGGCGTTAATTTCAATGTAGATGACATCAATTTTTCCCCTGTATTAGGTTGTTTAGGAAGCGACTGTAATAAAGTTTCCAGTAGATCTGCTATAGAGGATTTACTTTTTTCTAAAGTAAAGTTATCTGATGTCGGTTTTGTTGAGGCTTATAATAATTGTACTGGAGGTGCCGAAATTAGGGACCTCATTTGTGTGCAAAGTTATAATGGTATCAAGGTGTTGCCTCCACTGCTCTCAGAAAATCAGATCAGTGGATACACTTTGGCTGCCACCTCTGCTAGTCTGTTTCCTCCTTGGTCAGCAGCAGCAGGTGTACCATTTTATTTAAATGTTCAGTATCGTATTAATGGGATTGGTGTTACCATGGATGTGCTAAGTCAAAATCAAAAGCTTATTGCTAATGCATTTAACAATGCTCTTGGTGCTATTCAGGACGGTTTTGACGCTACCAATTCTGCTTTAGTTAAAATTCAAGCTGTTGTTAATGCAAACGCTGAAGCC

CTTAATAACTTATTGCAACAACTCTCTAATAGGTTTGGTGCTATAAGTTCTTCTTTACAAGAAATTCTATCTAGACTGG
ATGCTCTTGAAGCGCAAGCTCAGATAGACAGACTTATTAATGGGCGTCTTACCGCTCTTAATGCTTATGTTTCTCAACA
GCTTAGTGACTCTACACTAGTAAAATTTAGTGCAGCACAAGCTATGGAGAAGGTTAATGAATGTGTCAAAAGCCAATCA
TCTAGGATAAATTTTTGTGGTAATGGTAATCATATTATATCATTAGTGCAGAATGCTCCATATGGTTTGTATTTCATCC
ACTTTAGCTATGTCCCTACTAAGTATGTCACAGCGAAGGTTAGTCCCGGTCTGTGCATTGCTGGTGATAGAGGTATAGC
CCCCAAGAGTGGTTATTTTGTTAATGTAAATAATACTTGGATGTTCACTGGTAGTGGTTATTACTACCCTGAACCTATA
ACTGGAAATAATGTTGTTGTTATGAGTACCTGTGCTGTTAATTATACTAAAGCGCCGGATGTAATGCTGAACATTTCAA
CACCCAACCTCCCTGATTTTAAGGAAGAGTTGTATCAATGGTTTAAAAACCAAACATCAGTGGCACCAGATTTGTCACT
TGATTATATAAATGTTACATTCTTGGACCTACAAGATGAAATGAATAGGTTACAGGAGGCAATAAAAGTTTTAAATCAG
AGCTACATCAATCTCAAGGACATTGGTACATATGAGTATTATGTAAAATGGCCTTGGTATGTATGGCTTTTAATTGGCT
TTGCTGGTGTAGCTATGCTTGTTTTACTATTCTTCATATGCTGTTGTACAGGATGTGGGACTAGTTGTTTTAAGAAATG
TGGCCGTTGTTGTGATGATTATGCTGGACACCAGGAGTTAGTAATTAAAACATCACATGACGACTAA

>gi|87082955|gb|DQ389654.1|_Bovine_coronavirus_isolate_KWD13_spike_glycoprotein_precursor_(S)_gene_complete_cds

ATGTTTTTGATACTTTTAATTTCCTTACCAATGGCTTTTGCTGTTATAGGAGATTTAAAGTGTACTACAGTTTCC
ATTAATGATGTTGACACTGGTGTTCCTTCTATTAGCACTGATACTGTCGATGTTACTAATGGTTTAGGTACTTATTATG
TTTTAGATCGTGTGTATTTAAATACTACGTTGTTGCTTAATGGTTACTACCCTACTTCAGGTTCTACATATCGTAATAT
GGCACTGAAGGGAACTTTACTATTGAGCACACTATGGTTTAAACCACCTTTTCTTTCTGATTTTACTAATGGTATTTTT
GCTAAGGTCAAAAATACCAAGGTTATTAAAGATGGTGTAATGTATAGTGAGTTTCCTGCTATAACTATAGGTAGTACTT
TTGTAAATACATCCTATAGTGTGGTAGTACAACCACATACTACTAATTTTGATAGTAAATTACAAGGTCTCTTAGAGAT
TTCTGTTTGCCAGTATACTATGTGCGAGTACCCAAATACGATTTGTAATCCTAATCTGGGTAATCGACGCGTAGAACTA
TGGCATTGGGATACAGGTGTTGTTTCCTGTTTATATAAGCGTAATTTCACATATGATGTGAATGCTGATTATTTGTATT
TCCATTTTTATCAAGAAGGTGGTACTTTTTATGCATATTTTACAGACACTGGTGTTGTTACTAAGTTTCTGTTTAATGT
TTATTTAGGCACGGTGCTCTCACATTATTATGTCATGCCTTTGACTTGTAATAGTGCTCTGACTTTAGAATATTGGGTT
ACACCTCTCACTTCTAAACAATATCTACTAGCTTTCAATCAAGATGGTGTTATTTTTAATGCTGTTGATTGTAAGAGTG
ATTTTATGAGTGAGATTAAGTGTAAAACACTATCTATAGCACCATCTACTGGTGTTTATGAATTAAACGGTTACACTGT
TCAGCCAATTGCAGATGTTTACCGACGTATACCTAATCTTCCCGATTGTAATATTGAGGCTTGGCTTAATGATAAGTCG
GTGCCCTCTCCATTAAATTGGGAACGTAAGACCTTTTCAAATTGTAATTTTAATATGAGCAGCCTGATGTCTTTTATTC
AGGCAGACTCATTTACTTGTAATAATATTGATGCTACTAAGATATATGGTATGTGTTTTTCCAGCATAACTATAGATAA
GTTTGCAATACCCAATGGTAGGAAGGTTGACCTACAATTGGGTAATTTGGGTTATTTGCAGTCTTTTAACTATAGAATT
GATACTACTGCTACAAGTTGTCAGTTGTATTATAATTTACCTGCTGCTAATGTTTCTGTTAGCAGGTTTAATCCTTCTA
CTTGGAATAGGAGATTTGGTTTTACAGAACAATCTGTTTTTAAGCCTCAACCTGCAGGTGTTTTTACTGATCATGATGT
TGTTTATGCACAACATTGTTTTAAAGCTCCCACAAATTTCTGTCCGTGTAAATTGGACGGGTCTTTGTGTGTAGGTACT
GGTTCTGGTATAGATGCTGGTTATAAAAATACTGGTATAGGCACTTGTCCTGCAGGTACTAATTATTTAACTTGCCATA
ATGCTGCCCAATGTGGTTGTTTGTGCACCCCAGACCCCATTACATCTAAAGCTACAGGGCCTTATAAGTGCCCCCAAAC
TAAATATTTAGTTGGTATAGGTGAGCACTGTTCGGGTCTTGCTATTAAAAGTGATTATTGTGGAGGTAATCCTTGTAGT
TGCCAACCACAAGCATTTTTGGGTTGGTCTGTTGACTCTTGTTTACAAGGGGATAGGTGTAATATTTTTGCTAATTTTA
TTTTGCATGATGTTAATAGTGGTACTACTTGTTCTACTGATTTACAAAAATCAAACACAGACATAATTCTTGGTGTTTG
TGTTAATTATGATCTTTATGGTATTACAGGCCAAGGGATTTTTGTTGAGGTTAATGCGACTTATTATAATAGTTGGCAG
AACCTTTTATATGATTCTAATGGTAATCTCTATGGTTTTAGAGACTACTTAACAAATAGAACTTTTATGATTCGTAGTT
GCTATAGCGGTCGTGTATCAGCGGCCTTTCATGCTAACTCGTCCGAACCAGCATTGCTATTTCGGAATATTAAATGCAA

CTACGTTTTTAATAATACTCTTTCACGACAGCTGCAACCTATTAACTATTTTGATAGCTATCTTGGTTGTGTTGTCAAT
GCTGATAATAGTACTTCTAGTGTTGTTCAAACATGCGATCTCACAGTAGGTAGTGGTTACTGTGTGGATTACTCTACAA
AAAGACGAAGTCGTAGATCGATTACCACTGGTTATCGGTTTACTAATTTTGAGCCATTTACTGTTAATTCAGTAAATGA
TAGTTTAGAACCTGTAGGTGGTTTGTATGAAATTCAAATACCTTCAGAGTTTACTATAGGTAATATGGAGGAGTTTATT
CAAACAAGCTCTCCTAAAGTTACTATTGATTGTTCTGCTTTTGTCTGTGGTGATTATGCAGCATGTAAATCACAGTTGG
TTGAATATGGTAGCTTCTGTGACAATATTAACGCTATACTCACAGAAGTAAATGAACTACTTGACACTACACAGTTGCA
AGTAGCTAATAGTTTAATGAATGGTGTCACTCTTAGCACTAAGCTTAAAGATGGCGTTAATTTCAATGTAGACGACATC
AATTTTTCCCCTGTATTAGGTTGTTTAGGAAGCGACTGTAATAAAGTTTCCAGTAGATCTGCTATAGAGGATTTACTTT
TTTCTAAAGTAAAGTTATCTGATGTCGGTTTTGTTGAGGCTTATAATAATTGTACTGGAGGTGCCGAAATTAGGGACCT
CATTTGTGTGCAAAGTTATAATGGTATCAAGGTGTTGCCTCCACTGCTCTCAGAAAATCAGATCAGTGGATACACTTTG
GCTGCCACCTCTGCTAGTCTGTTTCCTCCTTGGTCAGCAGCAGCAGGTGTACCATTTTATTTAAATGTTCAGTATCGTA
TTAATGGGATTGGTGTTACCATGGATGTGCTAAGTCAAAATCAAAAGCTTATTGCTAATGCATTTAACAATGCTCTTGG
TGCTATTCAGGACGGTTTTGATGCTACCAATTCTGCTTTAGTTAAAATTCAAGCTGTTGTTAATGCAAATGCTGAAGCT
CTTAATAACTTATTGCAACAACTCTCTAATAGGTTTGGTGCTATAAGTTCTTCTTTACAAGAAATTCTATCTAGACTGG
ATGCTCTTGAAGCGCAAGCTCAGATAGACAGACTTATTAATGGGCGTCTTACCGCTCTTAATGCTTATGTTTCTCAACA
GCTTAGTGACTCTACACTAGTAAAATTTAGTGCAGCACAAGCTATGGAGAAGGTTAATGAATGTGTCAAAAGCCAATCA
TCTAGGATAAATTTTTGTGGTAATGGTAATCATATTATATCATTAGTGCAGAATGCTCCATATGGTTTGTATTTCATCC
ACTTTAGCTATGTCCCTACTAAGTATGTCACAGCGAAGGTTAGTCCCGGTCTGTGCATTGCTGGTGATAGAGGTATAGC
CCCCAAGAGTGGTTATTTTGTTAATGTAAATAATACTTGGATGTTCACTGGTAGTGGTTATTACTACCCTGAACCTATA
ACTGGAAATAATGTTGTTGTTATGAGTACCTGTGCTGTTAATTATACTAAAGCGCCGGATGTAATGCTGAACATTTCAA
CACCCAACCTCCCTGATTTTAAGGAAGAGTTGGATCAATGGTTTAAAAACCAAACATCAGTGGCACCAGATTTGTCACT
TGATTATATAAATGTTACATTCTTGGACCTACAAGATGAAATGAATAGGTTACAGGAGGCAATAAAAGTTTTAAATCAG
AGCTACATCAATCTCAAGGACATTGGTACATATGAGTATTATGTAAAATGGCCTTGGTATGTATGGCTTTTAATTGGCT
TTGCTGGTGTAGCTATGCTTGTTTTACTATTCTTCATATGCTGTTGTACAGGATGTGGGACTAGTTGTTTTAAGAAATG
TGGCCGTTGTTGTGATGATTATACTGGACACCAGGAGTTAGTAATTAAAACATCACATGACGACTAA

>gi|87082957|gb|DQ389655.1|_Bovine_coronavirus_isolate_KWD14_spike_glycoprotein_precursor_(S)_gene_complete_cds

ATGTTTTTGATACTTTTAATTTCCTTACCAATGGCTTTTGCTGTTATAGGAGATTTAAAGTGTACTACAGTTTCC
ATTAATGATGTTGACACTGGTGTTCCTTCTATTAGCACTGATACTGTCGATGTTACTAATGGTTTAGGTACTTATTATG
TTTTAGATCGTGTGTATTTAAATACTACGTTGTTGCTTAATGGTTACTACCCTACTTCAGGTTCTACATATCTTAATAT
GGCACTGAAGGGAACTTTACTATTGAGCACACTATGGTTTAAACCACCTTTTCTTTCTGATTTTACTAATGGTATTTTT
GCTAAGGTCAAAAATACCAAGGTTATTAAAGATGGTGTAATGTATAGTGAGTTTCCTGCTATAACTATAGGTAGTACTT
TTGTAAATACATCCTATAGTGTGGTAGTACAACCACATACTACTAATTTTGATAGCAAATTACAAGGTCTCACAAAGAC
TTCTGTTTGCCAGTATACTATGTGCGAGTACCCAAATACGATTTGTAATCCTAATCTGGGTAATCGACGCGTAGAACTA
TGGCATTGGGATACAGGTGTTGTTTCCTGTTTATATAAGCGTAATTTCACATATGATGTGAATGCTGATTATTTGTATT
TCCATTTTTATCAAGAAGGTGGTACTTTTTATGCATATTTTACAGACACTGGTGTTGTTACTAAGTTTCTGTTTAATGT
TTATTTAGGCACGGTGCTCTCACATTATTATGTCATGCCTTTGACTTGTAATAGTGCTCTGACTTTAGAATATTGGGTT
ACACCTCTCACTTCTAAACAATATCTACTAGCTTTCAATCAAGATGGTGTTATTTTTAATGCTGTTGATTGTAAGAGTG
ATTTTATGAGTGAGATTAAGTGTAAAACACTATCTATAGCACCATCTACTGGTGTTTATGAATTAAACGGTTACACTGT
TCAGCCAATTGCAGATGTTTACCGACGTATACCTAATCTTCCCGATTGCAATATTGAGGCTTGGCTTAATGATAAGTCG
GTGCCCTCTCCATTAAATTGGGAACGTAAGACCTTTTCAAATTGTAATTTTAATATGAGCAGCCTGATGTCTTTTATTC

AGGCAGACTCATTTACTTGTAATAATATTGATGCTGCTAAGATATATGGTATGTGTTTTTCCAGCATAACTATAGATAA
GTTTGCAATACCCAATGGTAGGAAGGTTGACCTACAATTGGGTAATTTGGGTTATTTGCAGTCTTTTAACTATAGAATT
GATACTACTGCTACAAGTTGTCAGTTGTATTATAATTTACCTGCTGCTAATGTTTCTGTTAGCAGGTTTAATCCTTCTA
CTTTGAATAGGAGATTTGGTTTTACAGAACAATCTGTTTTTAAGCCTCAACCTGCAGGTGTTTTTACTGATCATGATGT
TGTTTACGCACAACATTGTTTTAAAGCTCCCACAAATTTCTGTCCGTGTAAATTGGATGGGTCTTTGTGTGTAGGTAGT
GGTTCTGGTATAGATGCTGGTTATAAAAATACTGGTATAGGCACTTGTCCTGCAGGTACTAATTATTTAACTTGCCATA
ATGCTGCCCAATGTGATTGTTTGTGCACCCCAGACCCCATTACATCTAAAGCTACAGGGCCTTATAAGTGCCCCCAAAC
TAAATATTTAGTTGGTATAGGTGAGCACTGTTCGGGTCTTGCTATTAAAAGTGATTATTGTGGAGGTAATCCTTGTAGT
TGCCAACCACAAGCATTTTTGGGTTGGTCTGTTGACTCTTGTTTACAAGGGGATAGGTGTAATATTTTTGCTAATTTTA
TTTTGCATGATGTTAATAGTGGTACTACTTGTTCTACTGATTTACAAAAATCAAACACAGACATAATTCTTGGTGTTTG
TGTTAATTATGATCTTTATGGTATTACTGGCCAAGGTATTTTTGTTGAGGTTAATGCGACTTATTATAATAGTTGGCAG
AACCTTTTATATGATTCTAATGGTAATCTCTATGGTTTTAGAGACTACTTAACAAATAGAACTTTTATGATTCGTAGTT
GCTATAGCGGTCGTGTATCAGCGGCCTTTCATGCTAACTCGTCCGAACCAGCATTGCTATTTCGGAATATTAAATGCAA
CTACGTTTTTAATAATACTCTTTCACGACAGCTGCAACCCATTAACTATTTTGATAGCTATCTTGGTTGTGTTGTCAAT
GCTGATAATAGTACTTCTAGTGTTGTTCAAACATGCGATCTCACAGTAGGTAGTGGTTACTGTGTGGATTACTCTACAA
AAAGACGAAGTCGTAGATCGATTACCACTGGTTATCGGTTTACTAATTTTGAGCCATTTACTGTTAATTCAGTAAATGA
TAGTTTAGAACCTGTAGGTGGTTTGTATGAAATTCAAATACCTTCAGAGTTTACTATAGGTAATATGGAGGAGTTTATT
CAAACAAGCTCTCCTAAAGTTACTATTGATTGTTCTGCTTTTGTCTGTGGTGATTATGCAGCATGTAAATCACAGTTGG
TTGAATATGGTAGCTTCTGTGACAATATTAACGCTATACTCACAGAAGTAAATGAACTACTTGACACTACACAGTTGCA
AGTAGCTAATAGTTTAATGAATGGTGTCACTCTTAGCACTAAGCTTAAAGATGGCGTTAATTTCAATGTAGATGACATC
AATTTTTCCCCTGTATTAGGTTGTTTAGGAAGCGACTGTAATAAAGTTCCAGGTAGATCTGCTATAGAGGATTTACTTT
TTTCTAAAGTAAAGTTATCTGATGTCGGTTTTGTTGAGGCTTATAATAATTGTACTGGAGGTGCCGAAATTAGGGACCT
CATTTGTGTGCAAAGTTATAATGGTATCAAGGTGTTGCCTCCACTGCTCTCAGAAAATCAGATCAGTGGATACACTTTG
GCTGCCACCTCTGCTAGTCTGTTTCCTCCTTGGTCAGCAGCAGCAGGTGTACCATTTTATTTAAATGTTCAGTATCGTA
TTAATGGGATTGGTGTTACCATGGATGTGCTAAGTCAAAATCAAAAGCTTATTGCTAATGCATTTAACAATGCTCTTGG
TGCTATTCAGGACGGTTTTGATGCTACCAATTCTGCTTTAGTTAAAATTCAAGCTGTTGTTAATGCAAATGCTGAAGCT
CTTAATAACTTATTGCAACAACTCTCTAATAGGTTTGGTGCTATAAGTTCTTCTTTACAAGAAATTCTATCTAGACTGG
ATGCTCTTGAAGCGCAAGCTCAGATAGACAGACTTATTAATGGGCGTCTCACCGCTCTTAATGCTTATGTTTCTCAACA
GCTTAGTGACTCTACACTAGTAAAATTTAGTGCAGCACAAGCTATGGAGAAGGTTAATGAATGTGTCAAAAGCCAATCA
TCTAGGATAAATTTTTGTGGTAATGGTAATCATATTATATCATTAGTGCAGAATGCTCCATATGGTTTGTATTTCATCC
ACTTTAGCTATGTCCCTACTAAGTATGTCACAGCGAAGGTTAGTCCCGGTCTGTGCATTGCTGGTGATAGAGGTATAGC
CCCCAAGAGTGGTTATTTTGTTAATGTAAATAATACTTGGATGTTCACTGGTAGTGGTTATTACTACCCTGAACCTATA
ACTGGAAATAATGTTGTTGTTATGAGTACCTGTGCTGTTAATTATACTAAAGCGCCGGATGTAATGCTGAACATTTCAA
CACCCAACCTCCCTGATTTTAAGGAAGAGTTGGATCAATGGTTTAAAAACCAAACATCAGTGGCACCAGATTTGTCACT
TGATTATATAAATGTTACATTCTTGGACCTACAAGATGAAATGAATAGGTTACAGGAGGCAATAAAAGTTTTAAATCAG
AGCTACATCAATCTCAAGGACATTGGTACATATGAGTATTATGTAAAATGGCCTTGGTATGTATGGCTTTTAATTGGCT
TTGCTGGTGTAGCTATGCTTGTTTTACTATTCTTCATATGCTGTTGTACAGGATGTGGGACTAGTTGTTTTAAGAAATG
TGGCGGTTGTTGTGATGATTATACCGGACACCAGGAGTTAGTAATTAAAACATCACATGACGACTAA

>gi|87082959|gb|DQ389656.1|_Bovine_coronavirus_isolate_KWD15_spike_glycoprotein_precursor_(S)_gene_complete_cds

ATGTTTTTGATACTTTTAATTTCCTTACCAATGGCTTTTGCTGTTATAGGAGATTTAAAGTGTACTACAGTTTCC

ATTAATGATGTTGACACTGGTGTTCCTTCTATTAGCACTGATACTGTCGATGTTACTAATGGTTTAGGTACTTATTATG
TTTTAGATCGTGTGTATTTAAATACTACGTTGTTGCTTAATGGTTACTACCCTACTTCAGGTTCTACATATCGTAATAT
GGCACTGAAGGGAACTTTACTATTGAGCACACTATGGTTTAAACCACCTTTTCTTTCTGATTTTACTAATGGTATTTTT
GCTAAGGTCAAAAATACCAAGGTTATTAAAGATGGTGTAATGTATAGTGAGTTTCCTGCTATAACTATAGGTAGTACTT
TTGTAAATACATCCTATAGTGTGGTAGTACAACCACATACTACTAATTTTGATAGTAAATTACAAGGTCTCTTAGAGAT
TTCTGTTTGCCAGTATACTATGTGCGAGTACCCAAATACGATTTGTAATCCTAATCTGGGTAATCGACGCGTAGAACTA
TGGCATTGGGATACAGGTGTTGTTTCCTGTTTATATAAGCGTAATTTCACATATGATGTGAATGCTGATTATTTGTATT
TCCATTTTTATCAAGAAGGTGGTACTTTTTATGCATATTTTACAGACACTGGTGTTGTTACTAAGTTTCTGTTTAATGT
TTATTTAGGCACGGTGCTCTCACATTATTATGTCATGCCTTTGACTTGTAATAGTGCTCTGACTTTAGAATATTGGGTT
ACACCTCTCACTTCTAAACAATATCTACTAGCTTTCAATCAAGATGGTGTTATTTTTAATGCTGTTGATTGTAAGAGTG
ATTTTATGAGTGAGATTAAGTGTAAAACACTATCTATAGCACCATCTACTGGTGTTTATGAATTAAACGGTTACACTGT
TCAGCCAATTGCAGATGTTTACCGACGTATACCTAATCTTCCCGATTGTAATATTGAGGCTTGGCTTAATGATAAGTCG
GTGCCCTCTCCATTAAATTGGGAACGTAAGACCTTTTCAAATTGTAATTTTAATATGAGCAGCCTGATGTCTTTTATTC
AGGCAGACTCATTTACTTGTAATAATATTGATGCTGCTAAGATATATGGTATGTGTTTTTCCAGCATAACTATAGATAA
GTTTGCAATACCCAATGGTAGGAAGGTTGACCTACAATTGGGTAATTTGGGTTATTTGCAGTCTTTTAACTATAGAATT
GATACTACTGCGACAAGTTGTCAGTTGTATTATAATTTACCTGCTGCTAATGTTTCTGTTAGCAGGTTTAATCCTTCTA
CTTGGAATAGGAGATTTGGTTTTACAGAACAATCTGTTTTTAAGCCTCAACCTGCAGGTGTTTTTACTGATCATGATGT
TGTTTATGCACAACATTGTTTTAAAGCTCCCACTAATTTCTGTCCGTGTAAATTGGATGGGTCTTTGTGTGTAGGTAGT
GGTCCTGGTATAGATGCTGGTTATAAAAATACTGGTATAGGCACTTGTCCTGCAGGTACTAATTATTTAACTTGCCATA
ATGCTGCCCAATGTGATTGTTTGTGCACCCCAGACCCCATTACATCTAAAGCTACAGGGCCTTACAAGTGCCCCCAAAC
TAAATACTTAGTTGGTATAGGTGAGCACTGTTCGGGTCTTGCTATTAAAAGTGATTATTGTGGAGGCAATCCTTGTAGT
TGCCAACCACAAGCATTTTTGGGTTGGTCTGTTGACTCTTGTTTACAAGGGGATAGGTGTAATATTTTTGCTAATTTTA
TTTTGCATGATGTTAATAGTGGTACTACTTGTTCTACTGATTTACAAAAATCAAACACAGACATAATTCTTGGTGTTTG
TGTTAATTATGATCTTTATGGTATTACTGGCCAAGGTATTTTTGTTGAGGTTAATGCGACTTATTATAATAGTTGGCAG
AACCTTTTATATGATTCTAATGGTAATCTCTATGGTTTTAGAGACTACTTAACAAATAGAACTTTTATGATTCGTAGTT
GCTATAGCGGTCGTGTATCAGCGGCCTTTCATGCTAACTCTTCCGAACCAGCATTGCTATTTCGGAATATTAAATGCAA
CTACGTTTTTAATAATATTCTTTCACGACAGCTGCAACCTATTAACTATTTTGATAGCTATCTTGGTTGTGTTGTCAAT
GCTGATAATAGTACTTCTAGTGTTGTTCAAACATGCGATCTCACAGTAGGTAGTGGTTACTGTGTGGATTACTCTACAA
AAAGACGAAGTCGTAGATCGATTACCACTGGTTATCGGTTTACTAATTTTGAGCCATTTACTGTTAATTCAGTAAATGA
TAGTTTAGAACCTGTAGGTGGTTTGTATGAAATTCAAATACCTTCAGAGTTTACTATAGGTAATATGGAGGAGTTTATT
CAAACAAGCTCTCCTAAAGTTACTATTGATTGTTCTGCTTTTGTCTGTGGTGATTATGCAGCATGTAAATCACAGTTGG
TTGAATATGGTAGCTTCTGTGACAATATCAACGCTATACTCACAGAAGTAAATGAACTACTTGACACTACACAGCTGCA
AGTAGCTAATAGTTTAATGAATGGTGCCACTCTTAGCACTAAGCTTAAAGATGGCGTTAATTTCAATGTAGATGACATC
AATTTTTCCCCTGTATTAGGTTGTTTAGGAAGCGACTGTAATAAAGTTTCCAGTAGATCTGCTATAGAGGATTTGCTTT
TTTCTAAAGTAAAGTTATCTGATGTCGGTTTTGTTGAGGCTTATAATAATTGTACTGGAGGTGCCGAAATTAGGGACCT
CATTTGTGTGCAAAGTTATAACGGTATCAAGGTGTTGCCTCCACTGCTCTCAGAAAATCAGATCAGTGGATACACTTTG
GCTGCCACCTCTGCTAGTCTGTTTCCTCCTTGGTCAGCAGCAGCAGGTGTACCATTTTATTTAAATGTTCAGTATCGTA
TTAATGGGATTGGCGTTACCATGGATGTGCTAAGTCAAAATCAAAAGCTTATTGCTAACGCATTTAAGAAGGCTGTTGG
TGCTATTCAGGAAGGTTTTGACGCTACCAATTCTGCTTTAGTTAAAATTCAAGCTGTTGTTAATGCAAATGCTGAAGCT
CTTAGTAACGTATTGCAACAACTCTGTAATAGGTTTGGTGCCATGAGTTCTTCTTTACAAGAAATTCTATCTAGACTGG
ATGCTCTTGAAGCGCAAGCTCAGATAGACAGACTTATTAATGGGCGTCTTACCGCTCTTAATGCTTATGCTTCTCAACA
GCTTAGTGACTCTACACTAGTAAAATTTAGTGCAGCACAAGCTATGGAGAAGGTTAATGAATGTGTTAAAAGCCAATCA
TCTAGGATAAATTTTTGTGGTAATGGTAATCATATTATATCATTAGTGCAGAATGCTCCATATGGTTTGTATTTCATCC
ACTTTAGCTATGTCCCTACTAAGTATGTTACAGCGAAGGTTAGTCCCGGTCTGTGCATTGCTGGTGATAGAGGTATAGC

CCCCAAGAGTGGTTATTTTGTTAATGTAAATAATACTTGGATGTTCACTGGTAGTGGTTATTACTACCCTGAACCTATA
ACTGGAAATAATGTTGTTGTTATGAGTACCTGTGCTGTTAATTATACTAAAGCGCCGGATGTAATGCTGAACATTTCAA
CACCCAACCTCCCTGATTTTAAGGAAGAGTTGGATCAATGGTTTAAAAACCAAACATCAGTGGCACCAGATTTGTCACT
TGATTATATAAATGTTACATTCTTGGACCTACAAGATGAAATGAATAGGTTACAGGAGGCAATAAAAGTTTTAAATCAG
AGCTACATCAATCTCAAGGACATTGGTACATATGAGTATTATGTAAAATGGCCTTGGTATGTATGGCTTTTAATTGGCT
TTGCTGGTGTAGCTATGCTTGTTTTACTATTCTTCATATGCTGTTGTACAGGATGTGGGACTAGTTGTTTTAAGAAATG
TGGCGGTTGTTGTGATGATTATACCGGACACCAGGAGTTAGTAATTAAAACATCACATGACGACTAA

>gi|87082961|gb|DQ389657.1|_Bovine_coronavirus_isolate_KWD16_spike_glycoprotein_precursor_(S)_gene_complete_cds

ATGTTTTTGATACTTTTAATTTCCTTACCAATGGCTTTTGCTGTTATAGGAGATTTAAAGTGTACTACAGTTTCC
ATTAATGATGTTGACACTGGTGTTCCCTCTATTAGCACTGATACTGTCGATGTTACTAATGGTTTAGGTACTTATTATG
TTTTAGATCGTGTGTATTTAAATACTACGTTGTTGCTTAATGGTTACTACCCTACTTCAGGTTCTACATATCGTAATAT
GGCACTGAAGGGAACTTTACTATTGAGCACACTATGGTTTAAACCACCTTTTCTTTCTGATTTTACTAATGGTATTTTT
GCTAAGGTCAAAAATACCAAGGTTATTAAAGATGGTGTAATGTATAGTGAGTTTCCTGCTATAACTATAGGTAGTACTT
TTGTAAATACATCCTATAGTGTGGTAGTACAACCACATACTACTAATTTTGATAGTAAATTACAAGGTCTCTTAGAGAT
TTCTGTTTGCCAGTATACTATGTGCGAGTACCCAAATACGATTTGTAATCCTAATCTGGGTAATCAACGCGTAGAACTA
TGGCATTGGGATACAGGTGTTGTTTCCTGTTTATATAAGCGTAATTTCACATATGATGTGAATGCTGATTATTTGTATT
TCCATTTTTATCAAGAAGGTGGTACTTTTTATGCATATTTTACAGACACTGGTGTTGTTACTAAGTTTCTGTTTAATGT
TTATTTAGGCACGGTGCTCTTACATTATTATGTCATGCCTTTGACTTGTAATAGTGCTCTGACTTTAGAATATTGGGTT
ACACCTCTCACTTCTAAACAATATCTACTAGCTTTCAATCAAGATGGTGTTATTTTTAATGCTGTTGATTGTAAGAGTG
ATTTTATGAGTGAGATTAAGTGTAAAACACTATCTATAGCACCATCTACTGGTGTTTATGAATTAAACGGTTACACTGT
TCAGCCAATTGCAGATGTTTACCGACGTATACCTAATCTTCCCGATTGCAATATTGAGGCTTGGCTTAATGATAAGTCG
GTGCCCTCTCCATTAAATTGGGAACGTAAGACCTTTTCAAATTGCAATTTTAATATGAGCAGCCTGATGTCTTTTATTC
AGGCAGACTCATTTACTTGTAATAATATTGATGCTGCTAAGATATATGGTATGTGTTTTTCCAGCATAACTATAGATAA
GATTGCAATACCCAATGGTAGGAAGGTTGACCTACAATTGGGTAGTTTGGGTGATTTGCAGTCTTTTAACTATAGGATT
GATACTACTGCTACAAGTTGTCAGTTGTATTATAATTTACCTGCTGCTAATGTTTCTGTTAGCAGGTTTAATCCTTCTA
CTTGGAATAGGAGATTTGGTTTTATAGAACAATCTGTTTTTAAGCCTCAACCTGCAGGTGTTTTTACTGATCATGATGT
TGTTTATGCACAACATTGTTTTAAAGCTCCCACAAATTTCTGTCCGTGTAAATTGGATGGGTCTTTGTGTGTAGGTAGT
GGTCCTGGTATAGATGCTGGTTATAAAAATACTGGTATAGGCACTTGTCCTGCAGGTACTAATTATTTAACTTGCCATA
ATGCTGCCCAATGTGATTGTTTGTGCACCCCAGACCCCATTACATCTAAAGCTACAGGGCCTTATAAGTGCCCCCAAAC
TAAATATTTAGTTGGTATAGGTGAGCACTGTTCGGGTCTTGCTATTAAAAGTGATTATTGTGGAGGTAATCCTTGTAGT
TGCCAACCACAAGCATTTTTGGGTTGGTCTGTTGACTCTTGTTTACAAGGGGATAGGTGTAATATTTTTGCTAATTTTA
TTTTGCATGATGTTAATAGTGGTACTACTTGTTCTACTGATTTACAAAAATCAAACACAGACATAATTCTTGGTGTTTG
TGTTAATTATGATCTTTATGGTATTACTGGCCAAGGTATTTTTGTTGAGGTTAATGCGACTTATTATAATAGTTGGCAG
AACCTTTTATATGATTCTAATGGTAATCTCTATGGTTTTAGAGACTACTTAACAAATAGAACTTTTATGATTCGTAGTT
GCTATAGCGGTCGTGTATCAGCGGCCTTTCATGCTAACTCGTCCGAACCAGCATTGCTATTTCGGAATATTAAATGCAA
CTACGTTTTTAATAATACTCTTTCACGACAGCTGCAACCTATTAACTATTTTGATAGCTATCTTGGTTGTGTTGTCAAT
GCTGATAATAGTACTTCTAGTGTTGGTCAAACATGCGATCTCACAGTAGGTAGTGGTTACTGTGTGGATTACTCTATAA
AAAGACGAAGTCGTAGATCGATTACCACTGGTTATCGGTTTACTAATTTTGAGCCATTTACTGTTAATTCAGTAAATGA
TAGTTTAGAACCTGTAGGTGGTTTGTATGAAATTCAAATACCTTCAGAGTTTACTATAGGTAATATGGAGGAGTTTATT
CAAACAAGCTCTCCTAAAGTTACTATTGATTGTTCTGCTTTTGTCTGTGGTGATTATGCAGCATGTAAATCACAGTTGG

TTGAATATGGTAGCTTCTGTGACAATATTAACGCTATACTCACAGAAGTAAATGAACTACTTGACACTACACAGTTGCA
AGTAGCTAATAGTTTAATGAATGGTGTCACTCTTAGCACTAAGCTTAAAGATGGCGTTAATTTCAATGTAGATGACATC
AATTTTTCCCCTGTATTAGGTTGTTTAGGAAGCGACTGTAATAAAGTTTCCAGTAGATCTGCTATAGAGGATTTACTTT
TTTCTAAAGTAAAGTTATCTGATGTCGGTTTTGTTGAGGCTTATAATAATTGTACTGGAGGTGCCGAAATTAGGGACCT
CATTTGTGTGCAAAGTTATAATGGTATCAAGGTGTTGCCTCCACTGCTCTCAGAAAATCAGATCAGTGGATACACTTTG
GCTGCCACCTCTGCTAGTCTGTTTCCTCCTTGGTCAGCAGCAGCAGGTGTACCATTTTATTTAAATGTTCAGTATCGTA
TTAATGGGATTGGTGTTACCATGGATGTGCTAAGTCAAAATCAAAAGCTTATTGCTAATGCATTTAACAATGCTCTTGG
TGCTATTCAGGACGGTTTTGATGCTACCAATTCTGCTTTAGTTAAAATTCAAGCTGTTGTTAATGCAAATGCTGAAGCT
CTTAATAACTTATTGCAACAACTCTCTAATAGGTTTGGTGCTATAAGTTCTTCTTTACAAGAAATTCTATCTAGACTGG
ATGCTCTTGAAGCGCAAGCTCAGATAGACAGACTTATTAATGGGCGTCTCACCGCTCTTAATGCTTATGTTTCTCAACA
GCTTAGTGACTCTACACTAGTAAAATTTAGTGCAGCACAAGCTATGGAGAAGGTTAATGAATGTGTCAAAAGCCAATCA
TCTAGGATAAATTTTTGTGGTAATGGTAATCATATTATATCATTAGTGCAGAATGCTCCATATGGTTTGTATTTCATCC
ACTTTAGCTATGTCCCTACTAAGTATGTCACAGCGAAGGTTAGTCCCGGTCTGTGCATTGCTGGTGATAGAGGTATAGC
CCCCAAGAGTGGTTATTTTGTTAATGTAAATAATACTTGGATGTTCACTGGTAGTGGTTATTACTACCCTGAACCTATA
ACTGGAAATAATGTTGTTGTTATGAGTACCTGTGCTGTTAATTATACTAAAGCGCCGGATGTAATGCTGAACATTTCAA
CACCCAACCCCCCTGATTTTAAGGAACAGTTGTATCAATGGTTTAAAAACCAAACATCAGTGGCACCAGATTTGTCACT
TGATTATATAAATGTTACATTCTTGGACCTACAAGATGAAATGAATAGGTTACAGGAGGCAATAAAAGTTTTAAATCAG
AGCTACATCAATCTCAAGGACATTGGTACATATGAGTATTATGTAAAATGGCCTTGGTATGTATGGCTTTTAATTGGCT
TTGCTGGTGTAGCTATGCTTGTTTTACTATTCTTCATATGCTGTTGTACAGGATGTGGGACTAGTTGTTTTAAGAAATG
TGGCGGTCGTTGTGATGATTATGCTGGACACCAGGAGTTAGTAATTAAAACATCACATGACGACTAA

>gi|87082963|gb|DQ389658.1|_Bovine_coronavirus_isolate_KWD17_spike_glycoprotein_precursor_(S)_gene_complete_cds

ATGTTTTTGATACTTTTAATTTCCTTACCAATGGCTTTTGCTGTTATAGGAGATTTAAAGTGTACTACAGTTTCC
ATTAATGATGTTGACACTGGTGTTCCCTCTATTAGCACTGATACTGTCGATGTTACTAATGGTTTAGGTACTTATTATG
TTTTAGATCGTGTGTATTTAAATACTACGTTGTTGCTTAATGGTTACTACCCTACTTCAGGTTCTACATATCGTAATAT
GGCACTGAAGGGAACTTTACTATTGAGCACACTATGGTTTAAACCACCTTTTCTTTCTGATTTTACTAATGGTATTTTT
GCTAAGGTCAAAAATACCAAGGTTATTAAAGATGGTGTAATGTATAGTGAGTTTCCTGCTATAACTATAGGTAGTACTT
TTGTAAATACATCCTATAGTGTGGTAGTACAACCACATACTACTAATTTTGATAGTAAATTACAAGGTCTCTTAGAGAT
TTCTGTTTGCCAGTATACTATGTGCGAGTACCCAAATACGATTTGTAATCCTAATCTGGGTAATCAACGCGTAGAACTA
TGGCATTGGGATACAGGTGTTGTTTCCTGTTTATATAAGCGTAATTTCACATATGATGTGAATGCTGATTATTTGTATT
TCCATTTTTATCAAGAAGGTGGTACTTTTTATGCATATTTTACAGACACTGGTGTTGTTACTAAGTTTCTGTTTAATGT
TTATTTAGGCACGGTGCTCTTACATTATTATGTCATGCCTTTGACTTGTAATAGTGCTCTGACTTTAGAATATTGGGTT
ACACCTCTCACTTCTAAACAATATCTACTAGCTTTCAATCAAGATGGTGTTATTTTTAATGCTGTTGATTGTAAGAGTG
ATTTTATGAGTGAGATTAAGTGTAAAACACTATCTATAGCACCATCTACTGGTGTTTATGAATTAAACGGTTACACTGT
TCAGCCAATTGCAGATGTTTACCGACGTATACCTAATCTTCCCGATTGCAATATTGAGGCTTGGCTTAATGATAAGTCG
GTGCCCTCTCCATTAAATTGGGAACGTAAGACCTTTTCAAATTGTAATTTTAATATGAGCAGCCTGATGTCTTTTATTC
AGGCAGACTCATTTACTTGTAATAATATTGATGCTGCTAAGATATATGGTATGTGTTTTTCCAGCATAACTATAGATAA
GTTTGCAATACCCAATGGTAGGGAGGTTGACCTACAATTGGGTAATTTGGGTCATTTGCCGACTTTTAACTATAGAATT
GAGACTACTGCTAGAAGTTGTCAGTTGTATTATAATTTACCTGCTGCTAATGTTTCTGTGAGCAGGTTTAATCCTTCTA
CTTGGAATAGGAGATTTGGTTTTATAGAACAATCTGTTTTTAAGCCTCAACCTGCAGGTGTTTTTACTGATCATGATGT
TGTTTATGCACAACATTGTTTTAAAGCTCCCACAAATTTCTGTCCGTGTAAATTGGATGGGTCTTTGTGTGTAGGTAGT

GGTTCTGGTATAGATGCTGGTTATAAAAATACTGGTATAGGCACTTGTCCTGCAGGTACTAATTATTTAACTTGCCATA
ATGCTGCCCAATGTGATTGTTTGTGCACCCCAGACCCCATTACATCTAAAGCTACAGGGCCTTATAAGTGCCCCCAAAC
TAAATATTTAGTTGGTATAGGTGAGCACTGTTCGGGTCTTGCTATTAAAAGTGATTATTGTGGAGGTAATCCTTGTAGT
TGCCAACCACAAGCATTTTTGGGTTGGTCTGTTGACTCTTGTTTACAAGGGGATAGGTGTAATATTTTTGCTAATTTTA
TTTTGCATGATGTTAATAGTGGTACTACTTGTTCTACTGATTTACAAAAATCAAACACAGACATAATTCTTGGTGTTTG
TGTTAATTATGATCTTTATGGTATTACTGGCCAAGGTATTTTTGTTGAGGTTAATGCGACTTATTATAATAGTTGGCAG
AACCTTTTATATGATTCTAATGGTAATCTCTATGGTTTTAGAGACTACTTAACAAATAGAACTTTTATGATTCGTAGTT
GCTATAGCGGTCGTGTATCAGCGGCCTTTCATGCTAACTCGTCCGAACCAGCATTGCTATTTCGGAATATTAAATGCAA
CTACGTTTTTAATAATACTCTTTCACGACAGCTGCAACCTATTAACTATTTTGATAGCTATCTTGGTTGTGTTGTCAAT
GCTGATAATAGTACTTCTAGGTGTGTCAAACCATGCGATCTCACAGTAGGTAGTGGTTACTGTGTGGATTACTCTACAA
AAAGACGAAGTCGTAGATCGATTACCACTGGTTATCGGTTTACTAATTTTGAGCCATTTACTGTTAATTCAGTAAATGA
TAGTTTAGAACCTGTAGGTGGTTTGTATGAAATTCAAATACCTTCAGAGTTTACTATAGGTAATATGGAGGAGTTTATT
CAAACAAGCTCTCCTAAAGTTACTATTGATTGTTCTGCTTTTGTCTGTGGTGATTATGCAGCATGTAAATCACAGTTGG
TTGAATATGGTAGCTTCTGTGACAATATTAACGCTATACTCACAGAAGTAAATGAACTACTTGACACTACACAGTTGCA
AGTAGCTAATAGTTTAATGAATGGTGTCACTCTTAGCACTAAGCTTAAAGATGGCGTTAATTTCAATGTAGACGACATC
AATTTTTCCCCTGTATTAGGTTGTTTAGGAAGCGACTGTAATAAAGTTTCCAGTAGATCTGCTATAGAGGATTTACTTT
TTTCTAAAGTAAAGTTATCTGATGTCGGTTTTTTTGAGGCTTATAATAATTGTACTGGAGGTGCCGAAATTAGGGACCT
CATTTGTGTGCAAAGTTATAATGGTATCAAGGTGTTGCCTCCACTGCTCTCAGAAAATCAGATCAGTGGATACACTTCG
GCTGCCACCTCTGCTAGTCTGTTTCCTCCTTGGTCAGCCGCAGCAGGTGTACCATTTTATTTAAATGTTCAGTATCGTA
TTAATGGGATTGGTGTTACCATGGATGTGCTAAGCCAAAATCAAAAGATTATTGCTAATGCATTTAACAATGCTCTTGG
TGCTATTCAGGACGGTTTTGATGCTACCAATTCTGCTTTAGTTAAAATTCAAGCTGTTGTTAATGCAAATGCTGAAGCT
CTTAATAACTTATTGCAACAACTCTCTAATAGGTTTGGTGCTATAAGTTCTTCTTTACAAGAAATTCTATCTAGACTGG
ATGCTCTTGAAGCGCAAGCTCAGATAGACAGACTTATTAATGGGCGTCTTACCGCTCTTAATGCTTATGTTTCTCAACA
GCTTAGTGACTCTACACTAGTAAAATTTAGTGCAGCACAAGCTATGGAGAAGGTTAATGAATGTGTCAAAAGCCAATCA
TCTAGGATAAATTTTTGTGGTAATGGTAATCATATTATATCATTAGTGCCGCATGCTCCATATGGTTTGTATTTCATCC
ACTTTAGCTATGTCCCTACTAAGTATGTCACAGCGAAGGTTAGTCCCGGTCTGTGCATTGCTGGTGATAGAGGTATAGC
CCCCAAGAGTGGTTATTTTGTTAATGTAAATAATACTTGGATGTTCACTGGTAGTGGTTATTACTACCCTGAACCTATA
ACTGGAAATAATGTTGTTGTTATGAGTACCTGTGCTGTTAATTATACTAAAGCGCCGGATGTAATGCTGAACATTTCAA
CACCCAACCTCCCTGATTTTAAGGAAGAGTTGGATCAATGGTTTAAAAACCGAACATCAGTGGCACCAGATTTGTCACT
TGATTATAAAAATGTCACATTCTTGGACCTCCAAGATGAAATGAATAGGTTACAGGAGGCAATAAAAGTTTTAAATCAG
AGCTACATCAATCTCAAGGACATTGGTACATATGAGTATTATGTAAAATGGCCTTGGTATGTATGGCTTTTAATTGGCT
TTGCTGGTGTAGCTATGCTTGTTTTACTATTCTTCATATGCTGTTGTACAGGATGTGGGACTAGTCGTTTTAAGATATG
CGGTGGTTGTTGTGATGATTATGCTGGACACCAGGAGTTAGTAATTAAAACATCACATGACGACTAA

>gi|87082965|gb|DQ389659.1|_Bovine_coronavirus_isolate_KWD18_spike_glycoprotein_precursor_(S)_gene_complete_cds

ATGTTTTTGATACTTTTAATTTCCTTACCAATGGCTTTTGCTGTTATAGGAGATTTAAAGTGTACTACAGTTTCC
ATTAATGATGTTGACACTGGTGTTCCCTCTATTAGCACTGATACTGTCGATGTTACTAATGGTTTAGGTACTTATTATG
TTTTAGATCGTGTGTATTTAAATACTACGTTGTTGCTTAATGGTTACTACCCTACTTCAGGTTCTACATATCGTAATAT
GGCACTGAAGGGAACTTTACTATTGAGCACACTATGGTTTAAACCACCTTTTCTTTCTGATTTTACTAATGGTATTTTT
GCTAAGGTCAAAAATACCAAGGTTATTAAAGATGGTGTAATGTATAGTGAGTTTCCTGCTATAACTATAGGTAGTACTT
TTTTAAATACATCCTATAGTGTGGTAGTACAACCACATACTACTAATTTTGATAGTAAATTACAAGGTCTCTTAGAGAT

TTCTGTTTGCCAGTATACTATGTGCGAGTACCCAAATACGATTTGTAATCCTAATCTGGGTAATCAACGCGTAGAACTA
TGGCATTGGGATACAGGTGTTGTTTCCTGTTTATATAAGCGTAATTTCACATATGATGTGAATGCTGATTATTTGTATT
TCCATTTTTATCAAGAAGGTGGTACTTTTTATGCATATTTTACAGACACTGGTGTTGTTACTAAGTTTCTGTTTAATGT
TTATTTAGGCACGGTGCTCTCACATTATTATGTCATGCCTTTGACTTGTAATAGTGCTCTGACTTTAGAATATTGGGTT
ACACCTCTCACTTCTAAACAATATCTACTAGCTTTCAATCAAGATGGTGTTATTTTTAATGCTGTTGATTGTAAGAGTG
ATTTTATGAGTGAGATTAAGTGTAAAACACTATCTATAGCACCATCTACTGGTGTTTATGAATTAAACGGTTACACTGT
TCAGCCAATTGCAGATGTTTACCGACGTATACCTAATCTTCCCGATTGCAATATTGAGGCTTGGCTTAATGATAAGTCG
GTGCCCTCTCCATTAAATTGGGAACGTAAGACCTTTTCAAATTGTAATTTTAATATGAGCAGCCTGATGTCTTTTATTC
AGGCAGACTCATTTACTTGTAATAATATTGATGCTGCTAAGATATATGGTATGTGTTTTTCCAGCATAACTATAGATAA
GTTTGCAATACCCAATGGTAGGAAGGTTGACCTACAATTGGGTAATTTGGGTTATTTGCAGTCTTTTAACTATAGAATT
GATACTACTGCTACAAGTTGTCAGTTGTATTATAATTTACCTGCTGCTAATGTTTCTGTTAGCAGGTTTAATCCTTCTA
CTTGGAATAGGAGATTTGGTTTTACAGAACAATCGGTTTTTAAGCCTCAACCTGCAGGTGTTTTTACTGATCATGATGT
TGTTTATGCACAACATTGTTTTAAAGCTCCCACTAATTTCTGTCCGTGTAAATTGGATGGGTCTTTGTGTGTAGGTAGT
GGTCCTGGTATAGATGCTGGTTATAAAAATACTGGTATAGGCACTTGTCCTGCAGGTACTAATTATTTAACTTGCCATA
ATGCTGCCCAATGTGATTGTTTGTGCACCCCAGACCCCATTACATCTAAAGCTACAGGGCCTTACAAGTGCCCCCAAAC
TAAATATTTAGTTGGTATAGGTGAGCACTGTTCGGGTCTTGCTATTAAAAGTGATTATTGTGGAGGTAATCCTTGTAGT
TGCCAACCACAAGCATTTTTGGGTTGGTCTGTTGACTCTTGTTTACAAGGGGATAGGTGTAATATTTTTGCTAATTTTA
TTTTGCATGATGTTAATAGTGGTACTACTTGTTCTACTGATTTACAAAAATCAAACACAGACATAATTCTTGGTGTTTG
TGTTAATTATGATCTTTATGGTATTACTGGCCAAGGTATTTTTGTTGAGGTTAATGCGACTTATTATAATAGTTGGCAG
AACCTTTTATATGATTCTAATGGTAATCTCTATGGTTTTAGAGACTACTTAACAAATAGAACTTTTATGATTCGTAGTT
GCTATAGCGGTCGTGTATCAGCGGCCTTTCATGCTAACTCTTCCGAACCAGCATTGCTATTTCGGAATATTAAATGCAA
CTACGTTTTTAATAATACTCTTCCACGACAGCTGCAACCTATTAACTATTTTGATAGCTATCTTGGTTGTGTTGTCAAT
GCTGATAATAGTACTTCTAGTGTTGTTCAAACATGCGATCTCACAGTAGGTAGTGGTTACTGTGTGGATTACTCTACAA
AAAGACGAAGTCGTAGATCGATTACCACTGGTTATCGGTTTACTAATTTTGAGCCATTTACTGTTAATTCAGTAAATGA
TAGTTTAGAACCTGTAGGTGGTTTGTATGAAATTCAAATACCTTCAGAGTTTACTATAGGTAATATGGAGGAGTTTATT
CAAACAAGCTCTCCTAAAGTTACTATTGATTGTTCTGCTTTTGTCTGTGGTGATTATGCAGCATGTAAATCACAGTTGG
TTGAATATGGTAGCTTCAGTGACAATATTAACGCTATACTCACAGAAGTAAACGAACTACTTGACACTACACAGTTGCA
AGTAGCTAATAGTTTAATAAATGATGACACTCTTAGCACTAAGCTTAAAGATGGCGTTAATTTCAATGTAGACGACATC
AATTTTTCCCCTGTATTAGGTTGTTTAGGAAGCGACTGTAATAAAGTTTCCAGTAGATCTGCTATAGAGGATTTACTTT
TTTCTAAAGTAAAGTTATCTGATGTCGGTTCTGTTGAGGCTTATAATAATTGTACTGGAGGTGCCGAAATTAGGGACCT
CATTTGTGTGCAAAGTTATAACGGTATCAAGGTGTTGCCTCCACTGCTCTCAGAAAATCAGATCAGTGGATACACTTTG
GCTGCCACCTCTGCTAGTCTGTTTCCTCCTTGGTCAGCAGCAGCAGGTGTACCATTTTATTTAAATGTTCAGTATCGCA
TTAATGGGATTGGTGTTACCATGGATGTGCTAAGTCAAAATCAAAAGCTTATTGCTAACGCATTTAACAATGCTCTTGG
TGCTATTCAGGACGGTTTTGACGCTACCAATTCTGCTTTAGTTAAAATTCAAGCTGTTGTTAATGCAAATGCTGAAGCT
CTTAATAACTTATTGCAACAACTCTCTAATAGGTTTGGTGCTATAAGTTCTTCTTTACAAGAAATTCTATCTAGACTGG
ATGCTCTTGAAGCGCAAGCTCAGATAGACAGACTTATTAATGGGCGTCTTACCGCTCTTAATGCTTATGCTTCTCAACA
GCTTAGTGACTCTACACTAGTAAAATTTAGTGCAGCACAAGCTATGGAGAAGGTTAATGAATGTGTCAAAAGCCAATCA
TCTAGGATAAATTTTTGTGGTAATGGTAATCATATTATATCATTAGTGCAGAATGCTCCATATGGTTTGTATTTCATCC
ACTTTAGCTATGTCCCTACTAAGTATGTCACAGCGAAGGTTAGTCCCGGTCTGTGCATTGCTGGTGATAGAGGTATAGC
CCCCAAGAGTGGTTATTTTGTTAATGTAAATAATACTTGGATGTTCACTGGTAGTGGTTATTACTACCCTGAACCTATA
ACTGGAAATAATGTTGTTGTTATGAGTACCTGTGCTGTTAATTATACTAAAGCGCCGGATGTAATGCTGAACATTTCAA
CACCCAACCTCCCTGATTTTAAGGAAGAGTTGGATCAATGGTTTAAAAACCAAACATCAGTGGCACCAGATTTGTCACT
TGATTATATAAATGTTACATTCTTGGACCTACAAGATGAAATGAATAGGTTACAGGAGGCAATAAAAGTTTTAAATCAG
AGCTACATCAATCTCAAGGACATTGGTACATATGAGTATTATGTAAAATGGCCTTGGTATGTATGGCTTTTAATTGGCT

TTGCTGGTGTAGCTATGCTTGTTTTACTATTTTTCATATGCTGTTGTACAGGATGTGGGACTAGTTGTTTTAAGAAATG
TGGCGGTTGTTGTGATGATTATACCGGACCCCAGCAGTTAGTAATTAAAACATCACATGACGACTAA

>gi|87082967|gb|DQ389660.1|_Bovine_coronavirus_isolate_KWD19_spike_glycoprotein_precursor_(S)_gene_complete_cds

ATGTTTTTGATACTTTTAATTTCCTTACCAACGGCTTTTGCTGTAATAGGAGATTTAAAGTGTACTACAGTTTCC
ATTAATGATGTTGACACTGGTGTTCCCTCTATTAGCACTGATACTGTCGATGTTACTAATGGTTTAGGTACTTATTATG
TTTTAGATCGTGTGTATTTAAATACTACGTTGTTGCTTAATGGTTACTACCCTACTTCAGGTTCTACATATCGTAATAT
GGCACTGAAGGGAACTTTACTATTGAGCACACTATGGTTTAAACCACCTTTTCTTTCTGATTTTACTAATGGTATTTTT
GCTAAGGTCAAAAATACCAAGGTTATTAAAGATGGTGTAATGTATAGTGAGTTTCCTGCTATAACTATAGGTAGTACTT
TTGTAAATACATCCTATAGTGTGGTAGTACAACCACATACTACTAATTTTGATAGTAAATTACAAGGTCTCTTAGAGAT
TTCTGTTTGCCAGTATACTATGTGCGAGTACCCAAATACGATTTGTAATCCTAATCTGGGTAATCAACGCGTAGAACTA
TGGCATTGGGATACAGGTGTTGTTTCCTGTTTATATAAGCGTAATTTCACATATGATGTGAATGCTGATTATTTGTATT
TCCATTTTTATCAAGAAGGTGGTACTTTTTATGCATATTTTACAGACACTGGTGTTGTTACTAAGTTTCTGTTTAATGT
TTATTTAGGCACGGTGCTCTTACATTATTATGTCATGCCTTTGACTTGTAATAGTGCTCTGACTTTAGAATATTGGGTT
ACACCTCTCACTTCTAAACAATATCTACTAGCTTTCAATCAAGATGGTGTTATTTTTAATGCTGTTGATTGTAAGAGTG
ATTTTATGAGTGAGATTAAGTGTAAAACACTATCTATAGCACCATCTACTGGTGTTTATGAATTAAACGGTTACACTGT
TCAGCCAATTGCAGATGTTTACCGACGTATACCTAATCTTCCCGATTGCAATATTGAGGCTTGGCTTAATGATAAGTCG
GTGCCCTCTCCATTAAATTGGGAACGTAAGACCTTTTCAAATTGTAATTTTAATATGAGCAGCCTGATGTCTTTTATTC
AGGCAGACTCATTTACTTGTAATAATATTGATGCTGCTAAGATATATGGTATGTGTTTTTCCAGCATAACTATAGATAA
GTTTGCAATACCCAATGGTAGGAAGGTTGACCTACAATTGGGTAATTTGGGTTATTTGCAGTCTTTTAACTATAGAATT
GATACTACTGCTACAAGTTGTCAGTTGTATTATAATTTACCTGCTGCTAATGTTTCTGTTAGCAGGTTTAATCCTTCTA
TTTGGAATAGGAGATTTGGTTTTATAGAACAATCTGTTTTTAAGCCTCAACCTGCAGGTGTTTTTACTGATCATGATGT
TGTTTATGCACAACATTGTTTTAAAGCTCCCACAAATTTCTGTCCGTGTAAATTGGATGGGTCTTTGTGTGTAGGTAGT
GGTTCTGGTATAGATGCTGGTTATAAAAATACTGGTATAGGCACTTGTCCTGCAGGTACTAATTATTTAACTTGCCATA
ATGCTGCCCAATGTGATTGTTTGTGCACCCCAGACCCCATTACATCTAAAGCTACAGGGCCTTATAAGTGCCCCCAAAC
TAAATATTTAGTTGGTATAGGTGAGCACTGTTCGGGTCTTGCTATTAAAAGTGATTATTGTGGAGGTAATCCTTGTAGT
TGCCAACCACAAGCATTTTTGGGTTGGTCTGTTGACTCTTGTTTACAAGGGGATAGGTGTAATATTTTTGCTAATTTTA
TTTTGCATGATGTTAATAGTGGTACTACTTGTTCTACTGATTTACAAAAATCAAACACAGACATAATTCTTGGTGTTTG
TGTTAATTATGATCTTTATGGTATTACTGGCCAAGGTATTTTTGTTGAGGTTAATGCGACTTATTATAATAGTTGGCAG
AACCTTTTATATGATTCTAATGGTAATCTCTATGGTTTTAGAGACTACTTAACAAATAGAACTTTTATGATTCGTAGTT
GCTATAGCGGTCGTGTATCAGCGGCCTTTCATGCTAACTCGTCCGAACCAGCATTGCTATTTCGGAATATTAAATGCAA
CTACGTTTTTAATAATACTCTTTCACGACAGCTGCAACCTATTAACTATTTTGATAGCTATCTTGGTTGTGTTGTCAAT
GCTGATAATAGTACTTCTAGTGTTGTTCAAACATGCGATCTCACAGTAGGTAGTGGTTACTGTGTGGATTACTCTATAA
AAAGACGAAGTCGTAGATCGATTACCACTGGTTATCGGTTTACTAATTTTGAGCCATTTACTGTTAATTCAGTAAATGA
TAGTTTAGAACCTGTAGGTGGTTTGTATGAAATTCAAATACCTTCAGAGTTTACTATAGGTAATATGGAGGAGTTTATT
CAAACAAGCTCTCCTAAAGTTACTATTGATTGTTCTGCTTTTGTCTGTGGTGATTATGCAGCATGTAAATCACAGTTGG
TTGAATATGGTAGCTTCTGTGACAATATTAATGCTATACTCACAGAAGTAAATGAACTACTTGACACTACACAGTTGCA
AGTAGCTAATAGTTTAATGAATGGTGTCACTCTTAGCACTAAGCTTAAAGATGGCGTTAATTTCAATGTAGACGACATC
AATTTTTCCCCTGTATTAGGTTGTTTAGGAAGCGATTGCAATAAAGTTTCCAGTAGATCTGCTATAGAGGATTTACTTT
TTTCTAAAGTAAAGTTATCTGATGTCGGTTTTGTTGAGGCTTATAATAATTGTACTGGAGGTGCCGAAATTAGGGACCT
CATTTGTGTGCAAAGTTATAATGGTATCAAGGTGTTGCCTCCACTGCTCTCAGAAAATCAGATCAGTGGATACACTTTG

GCTGCCACCTCTGCTAGTCTGTTTCCTCCTTGGTCAGCAGCAGCAGGTGTACCATTTTATTTAAATGTTCAGTATCGTA
TTAATGGGATTGGTGTTACCATGGATGTGCTAAGTCAAAATCAAAAGCTTATTGCTAATGCATTTAACAATGCTCTTGG
TGCTATTCAGGACGGTTTTGATGCTACCAATTCTGCTTTAGTTAAAATTCAAGCTGTTGTTAATGCAAATGCTGAAGCT
CTTAATAACTTATTGCAACAACTCTCTAATAGGTTTGGTGCTATAAGTTCTTCTTTACAAGAAATTCTATCTAGACTGG
ATGCTCTTGAAGCGCAAGCTCAGATAGACAGACTTATTAATGGGCGTCTCACCGCTCTTAATGCTTATGTTTCTCAACA
GCTTAGTGACTCTACACTAGTAAAATTTAGTGCAGCACAAGCTATGGAGAAGGTTAATGAATGTGTCAAAAGCCAATCA
TCTAGGATAAATTTTTGTGGTAATGGTAATCATATTATATCATTAGTGCAGAATGCTCCATATGGTTTGTATTTCATCC
ACTTTAGCTATGTCCCTACTAAGTATGTCACAGCGAAGGTTAGTCCCGGTCTGTGCATTGCTGGTGATAGAGGTATAGC
CCCCAAGAGGTGGTATTTTGTTAATGTAAATAATACTTGGATGTTCACTGGTAGTGGTTATTACTACCCTGAACCTATA
ACTGGAAATAATGTTGTTGTTATGAGTACCTGTGCTGTTAATTATACTAAAGCGCCGGATGTAATGCTGAACATTTCAA
CACCCAACCTCCCTGACTTTAAGGAAGAGTTGGATCAATGGTTTAAAAACCAAACATCAGTGGCACCAGATTTGTCACT
TGATTATATAAATGTTACATTCTTGGACCTACAAGATGAAATGAATAGGTTACAGGAGGCAATAAAAGTTTTAAATCAG
AGCTACATCAATCTCAAGGACATTGGTACTTATGAGTATTATGTAAAATGGCCTTGGTATGTATGGCTTTTAATTGGCT
TTGCTGGTGTAGCTATGCTTGTTTTACTATTCTTCATATGCTGTTGTACAGGATGTGGGACTAGTTGTTTTAAGAAATG
TGGTGGTTGTTGTGATGATTATACTGGACACCAGGAGTTAGTAATTAAAACATCACATGACGACTAA

>gi|122726677|gb|EF193075.1|_Bovine_coronavirus_strain_V270_spike_protein_gene_complete_cds

ATGTTTTTGATACTTTTAATTTCCTTACCAATGGCTTTTGCTGTTATAGGAGATTTAAAGTGTACTACGGTTTCC
ATTAATGATGTTGACACCGGTGCTCCTTCTATTAGCACTGATATTGTCGATGTTACTAATGGTTTAGGTACTTATTATG
TTTTAGATCGTGTGTATTTAAATACTACGTTGTTGCTTAATGGTTACTACCCTACTTCAGGTTCTACATATCGTAATAT
GGCACTGAAGGGAACTTTACTATTGAGCAGACTATGGTTTAAACCACCTTTTCTTTCTGATTTTATTAATGGTATTTTT
GCTAAGGTCAAAAATACCAAGGTTATTAAAAAGGGTGTAATGTATAGTGAGTTTCCTGCTATAACTATAGGTAGTACTT
TTGTAAATACATCCTATAGTGTGGTAGTACAACCACATACTACCAATTTGGATAATAAATTACAAGGTCTCTTAGAGAT
CTCTGTTTGCCAGTATACTATGTGCGAGTACCCACATACGATTTGTCATCCTAATCTGGGTAATCGACGCGTAGAACTA
TGGCATTGGGATACAGGTGTTGTTTCCTGTTTATATAAGCGTAATTTCACATATGATGTGAATGCTGATTACTTGTATT
TCCATTTTTATCAAGAAGGTGGTACTTTTTATGCATATTTTACAGACACTGGTGTTGTTACTAAGTTTCTGTTTAATGT
TTATTTAGGCACGGTGCTTTCACATTATTATGTCCTGCCTTTGACTTGTTCTAGTGCTATGACTTTAGAATATTGGGTT
ACACCTCTCACTTCTAAACAATATTTACTAGCTTTCAATCAAGATGGTGTTATTTTTAATGCTGTTGATTGTAAGAGTG
ATTTTATGAGTGAGATTAAGTGTAAAACACTATCTATAGCACCATCTACTGGTGTTTATGAATTAAACGGTTACACTGT
TCAGCCAATTGCAGATGTTTACCGACGTATACCTAATCTTCCCGATTGTAATATAGAGGCTTGGCTTAATGATAAGTCG
GTGCCCTCTCCATTAAATTGGGAACGTAAGACCTTTTCAAATTGTAATTTTAATATGAGCAGCCTGATGTCTTTTATTC
AGGCAGACTCATTTACTTGTAATAATATTGATGCTGCTAAGATATATGGTATGTGTTTTTCCAGCATAACTATAGATAA
GTTTGCTATACCCAATGGTAGGAAGGTTGACCTACAATTGGGCAATTTGGGCTATTTGCAGTCTTTTAACTATAGAATT
GATACTACTGCTACAAGTTGTCAGTTGTATTATAATTTACCTGCTGCTAATGTTTCTGTTAGCAGGTTTAATCCTTCTA
CTTGGAATAGGAGATTTGGTTTTACAGAACAATCTGTTTTTAAGCCTCAACCTGTAGGTGTTTTTACTCATCATGATGT
TGTTTATGCACAACATTGTTTTAAAGCTCCCACAAATTTCTGTCCGTGTAAATTGGATGGGTCTTTGTGTGTAGGTAAT
CGTCCTGGTATAGATGCTGGTTATAAAAATAGTGGTATAGGCACTTGTCCTGCAGGTACTAATTATTTAACTTGCCATA
ATGCTGCCCAATGTGATTGTTTGTGCACTCCCGACCCCATTACATCTAAATCTACAGGGCCTTACAAGTGCCCCCAAAC
TAAATACTTAGTTGGCATAGGTGAGCACTGTTCGGGTCTTGCTATTAAAAGTGATTATTGTGGAGGTAATCCTTGTACT
TGCCAACCACAAGCATTTTTGGGTTGGTCTGTTGACTCTTGTTTACAAGGGGATAGGTGTAATATTTTTGCTAATTTTA
TTTTGCATGATGTTAATAGTGGTACTACTTGTTCTACTGATTTACAAAAATCAAACACAGACATAATTCTTGGTGTTTG

TGTTAATTATGATCTTTATGGTATTACAGGCCAAGGTATTTTTGTTGAGGTTAATGCGACTTATTATAATAGTTGGCAG
AACCTTTTATATGATTCTAATGGTAATCTCTATGGTTTTAGAGACTACTTAACAAACAGAACTTTTATGATTCGTAGTT
GCTATAGCGGTCGTGTTTCAGCGGCCTTTCATGCTAACTCTTCCGAACCAGCATTGCTATTTCGGAATATTAAATGCAA
TTACGTTTTTAATAATACTCTTTCACGACAGCTGCAACCTATTAACTATTTTGATAGTTATCTTGGTTGTGTTGTCAAT
GCTGATAATAGTACTTCTAGTGTTGTTCAAACATGTGATCTCACAGTAGGTAGTGGTTACTGTGTGGATTACTCTACAA
AAAGACGAAGTCGTAGAGCGATTACCACTGGTTATCGGTTTACTAATTTTGAGCCATTTACTGTTAATTCAGTAAATGA
TAGTTTAGAACCTGTAGGTGGTTTGTATGAAATTCAAATACCTTCAGAGTTTACTATAGGTAATATGGAGGAGTTTATT
CAAACAAGCTCTCCTAAAGTTACTATTGATTGTTCTGCTTTTGTCTGTGGTGATTATGCAGCATGTAAATCACAGTTGG
TTGAATATGGTAGCTTCTGTGACAATATTAATGCTATACTCACAGAAGTAAATGAACTACTTGACACTACACAGTTGCA
AGTAGCTAATAGTTTAATGAATGGTGTCACTCTTAGCACTAAGCTTAAAGATGGCGTTAATTTCAATGTAGACGACATC
AATTTTTCCCCTGTATTAGGTTGTTTAGGAAGCTATTGTAATAAAGTTTCCAGCAGATCTGCTATAGAGGATTTACTTT
TTTCTAAAGTAAAGTTATCTGATGTCGGTTTCGTTGAGGCTTATAATAATTGTACTGGAGGTGCCGAAATTAGGGACCT
CATTTGTGTGCAAAGTTATAATGGTATCAAAGTGTTGCCTCCACTGCTCTCAGTAAATCAGATCAGTGGATACACTTTG
GCTGCCACCTCTGCTAGTCTGTTTCCTCCTTTGTCAGCAGCAGCAGGTGTACCATTTTATTTAAATGTTCAGTATCGTA
TTAATGGGATTGGTGTTACCATGGATGTGTTAAGTCAAAATCAAAAGCTTATTGCTAATGCATTTAACAATGCTCTTGA
TGCTATTCAGGAAGGGTTTGATGCTACCAATTCTGCTTTAGTTAAAATTCAAGCTGTTGTTAATGCAAATGCTGAAACT
CTTAATAACTTATTGCAACAACTCTCTAATAGATTTGGTGCTATAAGTTCTTCTTTACAAGAAATTCTATCTAGACTGG
ATGCTCTTGAAGCGCAAGCTCAGATAGACAGGCTTATTAATGGGCGTCTTACCGCTCTTAATGCTTATGTTTCTCAACA
GCTTAGTGATTCTACACTAGTAAAATTTAGTGCAGCACAAGCTATGGAGAAGGTTAATGAATGTGTCAAAAGCCAATCA
TCTAGGATAAATTTTTGTGGTAATGGTAATCATATTATATCATTAGTGCAGAATGCTCCATATGGTTTGTATTTTATCC
ACTTTAGCTATGTCCCTACTAAGTATGTCACTGCGAAGGTTAGTCCCGGTCTGTGCATTGCTGGTGATAGAGGTATAGC
CCCTAAGAGTGGTTATTTTGTTAATGTAAATAATACTTGGATGTTCACTGGTAGTGGTTATTACTACCCTGAACCCATA
ACTGGAAATAATGTTGTTGTTATGAGTACCTGTGCTGTTAACTATACTAAAGCGCCGGATGTAATGCTGAACATTTCAA
CACCCAACCTCCATGATTTTAAGGAAGAGTTGGATCAATGGTTTAAAAACCAAACATCAGTGGCACCAGATTTGTCACT
TGATTATATAAATGTTACATTCTTGGACCTACAAGATGAAATGAATAGGTTACAGGAGGCAATAAAAGTTTTAAATCAG
AGCTACATCAATCTCAAGGACATTGGTACATATGAGTATTATGTAAAATGGCCTTGGTATGTATGGCTTTTAATTGGCT
TTGCTGGTGTAGCTATGCTTGTTTTACTATTCTTCATATGCTGTTGTACAGGATGTGGGACTAGTTGTTTTAAGATATG
TGGTGGTTGTTGTGATGATTATACTGGACACCAGGAGTTAGTAATTAAAACATCACATGACGACTAA

>gi|170675006|gb|EU401986.1|_Bovine_coronavirus_isolate_0502_spike_protein_(S)_gene_complete_cds

ATGTTTTTGATACTTTTAATTTCCTTACCAATGGCTTTTGCTGTTATAGGAGATTTAAAGTGTACTACAGTTTCC
ATTAATGATGTTGACACTGGTGTTCCTTCTATTAGCACTGATACTGTCGATGTTACTAATGGTTTAGGTACTTATTATG
TTTTAGATCGTGTGTATTTAAATACTACGTTGTTGCTTAATGGTTATTACCCTACTTCAGGTTCTACATATCGTAATAT
GGCACTGAAGGGAACTTTACTATTGAGCACACTATGGTTTAAACCACCTTTTCTTTCTGATTTTACTAATGGTATTTTT
GCTAAGGTCAAAAATACCAAGGTTATTAAAGATGGTGTAATGTATAGTGAGTTTCCTGCTATAACTATAGGTAGTACTT
TTGTAAATACATCTTATAGTGTGGTAGTACAACCACATACTACTAATTTTGATAGTAAATTACAAGGTCTCTTAGAGAT
TTCTGTTTGCCAGTATACTATGTGCGAGTACCCAAATACGATTTGTAATCCTAATCTGGGTAATCAACGCGTAGAACTA
TGGCATTGGGATACAGGTGTTGTTTCCTGTTTATATAAGCGTAATTTCACATATGATGTGAATGCTGATTATTTGTATT
TCCATTTTTATCAAGAAGGTGGTACTTTTTATGCATATTTTACAGACACTGGTGTTGTTACTAAGTTTCTGTTTAATGT
TTATTTAGGCACGGTGCTCTCACATTATTATGTCATGCCTTTGACTTGTAATAGTGCTCTGACTTTAGAATATTGGGTT
ACACCTCTCACTTCTAAACAATATCTACTAGCTTTCAATCAAGATGGTGTTATTTTTAATGCTGTTGATTGTAAGAGTG

ATTTTATGAGTGAGATTAAGTGTAAAACACTATCTATAGCACCATCTACTGGTGTTTATGAATTAAACGGTTACACTGT
TCAGCCAATTGCAGATGTTTACCGACGTATACCTAATCTTCCCGATTGTAATATTGAGGCTTGGCTTAATGATAAGTCG
GTGCCCTCTCCATTAAATTGGGAACGTAAGACCTTTTCAAATTGTAATTTTAATATGAGTAGCCTGATGTCTTTTATTC
AGGCAGACTCATTTACTTGTAATAATATTGATGCTGCTAAGATATATGGTATGTGTTTTTCCAGCATAACTATAGATAA
GTTTGCAATACCCAATGGTAGGAAGGTTGACCTACAATTGGGTAATTTGGGTTATTTGCAGTCTTTTAACTATAGAATT
GATACTACTGCTACAAGTTGTCAGTTGTATTATAATTTACCTGCTGCTAATGTTTCTGTTAGCAGGTTTAATCCTTCTA
CTTGGAATAGGAGATTTGGTTTTACAGAACAATCTGTTTTTAAGCCTCAACCTGCAGGTGTTTTTACTGATCATGATGT
TGTTTATGCACAACATTGTTTTAAAGCTCCCACAAATTTCTGTCCGTGTAAATTGGACGGGTCTTTGTGTGTAGGTAAT
GGTCCTGGTATAGATGCTGGTTATAAAAATAGTGGTATAGGCACTTGTCCTGCAGGTACTAATTATTTAACTTGCCATA
ATGCTGCCCAATGTGATTGTTTGTGCACTCCCGACCCCATTACATCTAAATCTACAGGGCCTTACAAGTGCCCCCAAAC
TAAATACTTAGTTGGCATAGGTGAGCACTGTTCGGGTCTTGCTATTAAAAGTGATTATTGTGGAGGTAATCCTTGTACT
TGCCAACCACAAGCATTTTTGGGTTGGTCTGTTGACTCTTGTTTACAAGGGGATAGGTGTAATATTTTTGCTAATTTTA
TTTTGCATGATGTTAATAGTGGTACTACTTGTTCTACTGATTTACAAAAATCAAACACAGACATAATTCTTGGTGTTTG
TGTTAATTATGATCTTTATGGTATTACAGGCCAAGGTATTTTTGTTGAGGTTAATGCGACTTATTATAATAGTTGGCAG
AACCTTTTATATGATTCTAATGGTAATCTCTATGGTTTTAGAGACTACTTAACAAATAGAACTTTTATGATTCGTAGTT
GCTATAGCGGTCGTGTATCAGCGGCCTTTCATGCTAACTCTTCCGAACCAGCATTGCTATTTCGGAATATTAAATGCAA
CTACGTTTTTAATAATACTTTTTCACGACAGCTGCAACCTATTAACTATTTTGATAGTTATCTTGGTTGTGTTGTCAAT
GCTGATAATAGTACTTCTAGTGTTGTTCAAACATGCGATCTCACAGTAGGTAGTGGTTACTGTGTGGATTACTCTACAA
AAAGACGAAGTCGTAGATCGATTACCACTGGTTATCGGTTTACTAATTTTGAGCCATTTACTGTTAATTCAGTAAATGA
TAGTTTAGAACCTGTAGGTGGTTTGTATGAAATTCAAATACCTTCAGAGTTTACTATAGGTAATATGGAGGAGTTTATT
CAAACAAGCTCTCCTAAAGTTACTATTGATTGTTCTGCTTTTGTCTGTGGTGATTATGCAGCATGTAAATCACAGTTGG
TTGAATATGGTAGCTTCTGTGACAATATTAACGCTATACTCACAGAAGTAAATGAACTACTTGACACTACACAGTTGCA
AGTAGCTAATAGTTTAATGAATGGTGTCACTCTTAGCACTAAGCTTAAAGATGGCGTTAATTTCAATGTAGATGACATC
AATTTTTCCCCTGTATTAGGTTGTTTAGGAAGCGACTGTAATAAAGTTTCCAGTAGATCTGCTATAGAGGATTTACTTT
TTTCTAAAGTTAAGTTATCTGATGTCGGTTTTGTTGAGGCTTATAATAATTGTACTGGAGGTGCCGAAATTAGGGACCT
CATTTGTGTGCAAAGTTATAACGGTATCAAGGTGTTGCCTCCACTGCTCTCAGAAAATCAGATCAGTGGATACACTTTG
GCTGCCACCTCTGCTAGTCTGTTTCCTCCTTGGTCAGCAGCAGCAGGTGTACCATTTTATTTAAATGTTCAGTATCGCA
TTAATGGGATTGGTGTTACCATGGATGTGCTAAGTCAAAATCAAAAGCTTATTGCTAATGCATTTAACAATGCTCTTGG
TGCTATTCAGGACGGTTTTGATGCTACCAATTCTGCTTTAGTTAAAATTCAAGCTGTTGTTAATGCAAATGCTGAAGCT
CTTAATAACTTATTGCAACAACTCTCTAATAGGTTTGGTGCTATAAGTTCTTCTTTACAAGAAATTCTATCTAGACTGG
ATGCTCTTGAAGCGCAAGCTCAGATAGACAGACTTATTAATGGGCGTCTTACCGCTCTTAATGCCTATGTTTCTCAACA
GCTTAGTGACTCTACACTAGTAAAATTTAGTGCAGCACAAGCTATGGAGAAGGTTAATGAATGTGTCAAAAGCCAATCA
TCTAGGATAAATTTTTGTGGTAATGGTAATCATATTATATCATTAGTGCAGAATGCTCCATATGGTTTGTATTTCATCC
ACTTTAGCTATGTCCCTACTAAGTATGTCACAGCGAAGGTTAGTCCCGGTCTGTGCATTGCTGGTGATAGAGGTATAGC
CCCCAAGAGTGGTTATTTTGTTAATGTAAATAATACTTGGATGTTCACTGGTAGTGGTTATTACTACCCTGAACCTATA
ACTGGAAATAATGTTGTTGTTATGAGTACCTGTGCTGTTAATTATACTAAAGCGCCGGATGTAATGCTGAACATTTCAA
CACCCAACCTCCCTGATTTTAAGGAAGAGTTGGATCAATGGTTTAAAAACCAAACATCAGTGGCACCAGATTTGTCACT
TGATTATATAAATGTTACATTCTTGGACCTACAAGATGAAATGAATAGGTTACAGGAGGCAATAAAAGTTTTAAATCAG
AGCTACATCAATCTCAAGGACATTGGTACATATGAGTATTATGTAAAATGGCCTTGGTATGTATGGCTTTTAATTGGCT
TTGCTGGTGTAGCTATGCTTGTTTTACTATTCTTCATATGCTGTTGTACAGGATGTGGGACTAGTTGTTTTAAGAAATG
TGGCGGTTGTTGTGATGATTATACTGGACACCAGGAGTTAGTAATTAAAACATCACATGACGACTAA

>gi|170675010|gb|EU401988.1|_Bovine_coronavirus_isolate_SUN5_spike_protein_

(S)_gene_complete_cds

ATGTTTTTGATACTTTTAATTTCCTTACCAATGGCTTTTGCTGTTATAGGAGATTTAAAGTGTACTACGGTTTCC
ATTAATGATGTTGACACCGGTGCTCCTTCTATTAGCACTGATATTGTCGATGTTACTAATGGTTTAGGTACTTATTATG
TTTTAGATCGTGTGTATTTAAATACTACGTTGTTGCTTAATGGTTACTACCCTACTTCAGGTTCTACATATCGTAATAT
GGCACTGAAGGGAACTTTACTATTGAGCAGACTATGGTTTAAACCACCTTTTCTTTCTGATTTTATTAATGGTATTTTT
GCTAAGGTCAAAAATACCAAGGTTATTAAAAAGGGTGTAATGTATAGTGAGTTTCCTGCTATAACTATAGGTAGTACTT
TTGTAAATACATCCTATAGTGTGGTAGTACAACCACATACTACCAATTTGGATAATAAATTACAAGGTCTCTTAGAGAT
CTCTGTTTGCCAGTATACTATGTGCGAGTACCCACATACGATTTGTCATCCTAAGCTGGGTAATCGACGCGTAGAACTA
TGGCATTGGGATACAGGTGTTGTTTCCTGTTTATATAAGCGTAATTTCACATATGATGTGAATGCTGATTACTTGTATT
TCCATTTTTATCAAGAAGGTGGTACTTTTTATGCATATTTTACAGACACTGGTGTTGTTACTAAGTTTCTGTTTAATGT
TTATTTAGGCACGGTGCTTTCACATTATTATGTCCTGCCTTTGACTTGTTCTAGTGCTATGACTTTAGAATATTGGGTT
ACACCTCTCACTTCTAAACAATATTTACTAGCTTTCAATCAAGATGGTGTTATTTTTAATGCTGTTGATTGTAAGAGTG
ATTTTATGAGTGAGATTAAGTGTAAAACACTATCTATAGCACCATCTACTGGTGTTTATGAATTAAACGGTTACACTGT
TCAGCCAATTGCAGATGTTTACCGACGTATACCTAATCTTCCCGATTGTAATATAGAGGCTTGGCTTAATGATAAGTCG
GTGCCCTCTCCATTAAATTGGGAACGTAAGACCTTTTCAAATTGTAATTTTAATATGAGCAGCCTGATGTCTTTTATTC
AGGCAGACTCATTTACTTGTAATAATATTGATGCTGCTAAGATATATGGTATGTGTTTTTCCAGCATAACTATAGATAA
GTTTGCTATACCCAATGGTAGGAAGGTTGACCTACAATTGGGCAATTTGGGCTATTTGCAGTCTTTTAACTATAGAATT
GATACTACTGCTACAAGTTGTCAGTTGTATTATAATTTACCTGCTGCTAATGTTTCTGTTAGCAGGTTTAATCCTTCTA
CTTGGAATAGGAGATTTGGTTTTACAGAACAATCTGTTTTTAAGCCTCAACCTGTAGGTGTTTTTACTCATCATGATGT
TGTTTATGCACAACATTGTTTTAAAGCTCCCACAAATTTCTGTCCGTGTAAATTGGATGGGTCTTTGTGTGTAGGTAAT
GGTCCTGGTATAGATGCTGGTTATAAAAATAGTGGTATAGGCACTTGTCCTGCAGGTACTAATTATTTAACTTGCCATA
ATGCTGCCCAATGTGATTGTTTGTGCACTCCCGACCCCATTACATCTAAATCTACAGGGCCTTACAAGTGCCCCCAAAC
TAAATACTTAGTTGGCATAGGTGAGCACTGTTCGGGTCTTGCTATTAAAAGTGATTATTGTGGAGGTAATCCTTGTACT
TGCCAACCACAAGCATTTTTGGGTTGGTCTGTTGACTCTTGTTTACAAGGGGATAGGTGTAATATTTTTGCTAATTTTA
TTTTGCATGATGTTAATAGTGGTACTACTTGTTCTACTGATTTACAAAAATCAAACACAGACATAATTCTTGGTGTTTG
TGTTAATTATGATCTTTATGGTATTACAGGCCAAGGTATTTTTGTTGAGGTTAATGCGACTTATTATAATAGTTGGCAG
AACCTTTTATATGATTCTAATGGTAATCTCTATGGTTTTAGAGACTACTTAACAAACAGAACTTTTATGATTCGTAGTT
GCTATAGCGGTCGTGTTTCAGCGGCCTTTCATGCTAACTCTTCCGAACCAGCATTGCTATTTCGGAATATTAAATGCAA
TTACGTTTTTAATAATACTCTTTCACGACAGCTGCAACCTATTAACTATTTTGATAGTTATCTTGGTTGTGTTGTCAAT
GCTGATAATAGTACTTCTAGTGTTGTTCAAACATGTGATCTCACAGTAGGTAGTGGTTACTGTGTGGATTACTCTACAA
AAAGACGAAGTCGTAGAGCGATTACCACTGGTTATCGGTTTACTAATTTTGAGCCATTTACTGTTAATTCAGTAAATGA
TAGTTTAGAACCTGTAGGTGGTTTGTATGAAATTCAAATACCTTCAGAGTTTACTATAGGTAATATGGAGGAGTTTATT
CAAACAAGCTCTCCTAAAGTTACTATTGATTGTTCTGCTTTTGTCTGTGGTGATTATGCAGCATGTAAATCACAGTTGG
TTGAATATGGTAGCTTCTGTGACAATATTAATGCTATACTCACAGAAGTAAATGAACTACTTGACACTACACAGTTGCA
AGTAGCTAATAGTTTAATGAATGGTGTCACTCTTAGCACTAAGCTTAAAGATGGCGTTAATTTCAATGTAGACGACATC
AATTTTTCCCCTGTATTAGGTTGTTTAGGAAGCGCTTGTAGTAAAGTTTCCAGCAGATCTGCTATAGAGGATTTACTTT
TTTCTAAAGTAAAGTTATCTGATGTCGGTTTCGTTGAGGCTTATAATAATTGTACTGGAGGTGCCGAAATTAGGGACCT
CATTTGTGTGCAAAGTTATAATGGTATCAAAGTGTTGCCTCCACTGCTCTCAGTAAATCAGATCAGTGGATACACTTTG
GCTGCCACCTCTGCTAGTCTGTTTCCTCCTTGGACAGCAGCAGCAGGTGTACCATTTTATTTAAATGTTCAGTATCGTA
TTAATGGGATTGGTGTTACCATGGATGTGTTAAGTCAAAATCAAAAGCTTATTGCTAATGCATTTAACAATGCTCTTGA
TGATATTCAGGAAGGGTTTGATGCTACCAATTCTGCTTTAGTTAAAATTCAAGCTGTTGTTAATGCAAATGCTGAAGCT
CTTAATAACTTATTGCAACAACTCTCTAATAGATTTGGTGCTATAAGTTCTTCTTTACAAGAAATTCTATCTAGACTGG
ATGCTCTTGAAGCGCAAGCTCAGATAGACAGACTTATTAATGGGCGTCTTACCGCTCTTAATGCTTATGTTTCTCAACA

GCTTAGTGATTCTACACTAGTAAAATTTAGTGCAGCACAAGCTATGGAGAAGGTTAATGAATGTGTCAAAAGCCAATCATCTAGGATAAATTTTTGTGGTAATGGTAATCATATTATATCATTAGTGCAGAATGCTCCATATGGTTTGTATTTTATCCACTTTAGCTATGTCCCTACTAAGTATGTCACTGCGAAGGTTAGTACCGGTCTGTGCATTGCTGGTGATAGAGGTATAGCCCCTAAGAGTGGTTATTTTGTTAATGTAAATAATACTTGGATGTTCACTGGTAGTGGTTATTACTACCCTGAACCCATAACTGGAAATAATGTTGTTGTTATGAGTACCTGTGCTGTTAACTATACTAAAGCGCCGGATGTAATGCTGAACATTTCAACACCCAACCTCCATGATTTTAAGGAAGAGTTGGATCAATGGTTTAAAAACCAAACATTAGTGGCACCAGATTTGTCACTTGATTATATAAATGTTACATTCTTGGACCTACAAGATGAAATGAATAGGTTACAGGAGGCAATAAAAGTTTTAAATCAGAGCTACATCAATCTCAAGGACATTGGTACATATGAGTATTATGTAAAATGGCCTTGGTATGTATGGCTTTTAATTGGCTTTGCTGGTGTAGCTATGCTTGTTTTACTATTCTTCATATGCTGTTGTACAGGATGTGGGACTAGTTGTTTTAAGATATGTGGTGGTTGTTGTGATGATTATACTGGACACCAGGAGTTAGTAATTAAAACATCACATGACGACTAA

>gi|170675012|gb|EU401989.1|_Bovine_coronavirus_strain_BC94_spike_protein_(S)_gene_complete_cds

ATGTTTTTGATACTTTTAATTTCCTTACCAATGGCTTTTGCTGTTATAGGAGATTTAAAGTGTACTACGGTTGCCATTAATGATGTTGACACCGGTGCTCCTTCTATTAGCACTGATATTGTCGATGTTACTAATGGTTTAGGTACTTATTATGTTTTAGATCGTGTGTATTTAAATACTACGTTGTTGCTTAATGGTTACTACCCTACTTCAGGTTCTACATATCGTAATATGGCACTGAAGGGAACTTTACTATTGAGCAGACTATGGTTTAAACCACCTTTTCTTTCTGATTTTATTAATGGTATTTTTGCTAAGGTCAAAAATACCAAGGTTATTAAAAAGGGTGTAATGTATAGTGAGTTTCCTGCTATAACTATAGGTAGTACTTTTGTAAATACATCCTATAGTGTGGTAGTACAACCACATACTACCAATTTGGATAATAAATTACAAGGTCTCTTAGAGATCTCTGTTTGCCAGTATACTATGTGCGAGTACCCACATACGATTTGTCATCCTAAGCTGGGTAATCGACGCGTAGAACTATGGCATTGGGATACAGGTGTTGTTTCCTGTTTATATAAGCGTAATTTCACATATGATGTGAATGCTGATTACTTGTATTTCCATTTTTATCAAGAAGGTGGTACTTTTTATGCATATTTTACAGACACTGGTGTTGTTACTAAGTTTCTGTTTAATGTTTATTTAGGCACGGTGCTTTCACATTATTATGTCCTGCCTTTGACTTGTTCTAGTGCTATGACTTTAGAATATTGGGTTACACCTCTCACTTCTAAACAATATTTACTAGCTTTCAATCAAGATGGTGTTATTTTTAATGCTGTTGATTGTAAGAGTGATTTTATGAGTGAGATTAAGTGTAAAACACTATCTATAGCACCATCTACTGGTGTTTATGAATTAAACGGTTACACTGTTCAGCCAATTGCAGATGTTTACCGACGTATACCTAATCTTCCCGATTGTAATATAGAGGCTTGGCTTAATGATAAGTCGGTGCCCTCTCCATTAAATTGGGAACGTAAGACCTTTTCAAATTGTAATTTTAATATGAGCAGCCTGATGTCTTTTATTCAGGCAGACTCATTTACTTGTAATAATATTGATGCTGCTAAGATATATGGTATGTGTTTTTCCAGCATAACTATAGATAAGTTTGCTATACCCAATGGTAGGAAGGTTGACCTACAATTGGGCAATTTGGGCTATTTGCAGTCTTTTAACTATAGAATTGATACTACTGCTACAAGTTGTCAGTTGTATTATAATTTACCTGCTGCTAATGTTTCTGTTAGCAGGTTTAATCCTTCTACTTGGAATAGGAGATTTGGTTTTACAGAACAATCTGTTTTTAAGCCTCAACCTGTAGGTGTTTTTACTCATCATGATGTTGTTTATGCACAACATTGTTTTAAAGCTCCCACAAATTTCTGTCCGTGTAAATTGGATGGGTCTTTGTGTGTAGGTAATGGTCCTGGTATAGATGCTGGTTATAAAAATAGTGGTATAGGCACTTGTCCTGCAGGTACTAATTATTTAACTTGCCATAATGCTGCCCAATGTGATTGTTTGTGCACTCCCGACCCCATTACATCTAAATCTACAGGGCCTTACAAGTGCCCCCAAACTAAATACTTAGTTGGCATAGGTGAGCACTGTTCGGGTCTTGCTATTAAAAGTGATTATTGTGGAGGTAATCCTTGTACTTGCCAACCACAAGCATTTTTGGGTTGGTCTGTTGACTCTTGTTTACAAGGGGATAGGTGTAATATTTTTGCTAATTTATTTTGCATGATGTTAATAGTGGTACTACTTGTTCTACTGATTTACAAAAATCAAACACAGACATAATTCTTGGTGTTTGTGTTAATTATGATCTTTATGGTATTACAGGCCAAGGTATTTTTGTTGAGGTTAATGCGACTTATTATAATAGTTGGCAGAACCTTTTATATGATTCTAATGGTAATCTCTATGGTTTTAGAGACTACTTAACAAACAGAACTTTTATGATTCGTAGTTGCTATAGCGGTCGTGTTTCAGCGGCCTTTCATGCTAACTCTTCCGAACCAGCATTGCTATTTCGGAATATTAAATGCAATTACGTTTTTAATAATGCTCTTTCACGACAGCTGCAACCTATTAACTATTTTGATAGTTATCTTGGTTGTGTTGTCAATGCTGATAATAGTACTTCTAGTGTTGTTCAAACATGTGATCTCACAGTAGGTAGTGGTTACTGTGTGGATTACTCTACAA

AAAGACGAAGTCGTAGAGCGATTACCACTGGTTATCGGTTTACTAATTTTGAGCCATTTACTGTTAATTCAGTAAATGA
TAGTTTAGAACCTGTAGGTGGTTTGTATGAAATTCAAATACCTTCAGAGTTTACTATAGGTAATATGGAGGAGTTTATT
CAAACAAGCTCTCCTAAAGTTACTATTGATTGTTCTGCTTTTGTCTGTGGTGATTATGCAGCATGTAAATCACAGTTGG
TTGAATATGGTAGCTTCTGTGACAATATTAATGCTATACTCACAGAAGTAAATGAACTACTTGACACTACACAGTTGCA
AGTAGCTAATAGTTTAATGAATGGTGTCACTCTTAGCACTAAGCTTAAAGATGGCGTTAATTTCAATGTAGACGACATC
AATTTTTCCCCTGTATTAGGTTGTTTAGGAAGCGCTTGTAGTAAAGTTTCCAGCAGATCTGCTATAGAGGATTTACTTT
TTTCTAAAGTAAAGTTATCTGATGTCGGTTTCGTTGAGGCTTATAATAATTGTACTGGAGGTGCCGAAATTAGGGACCT
CATTTGTGTGCAAAGTTATAATGGTATCAAAGTGTTGCCTCCACTGCTCTCAGTAAATCAGATCAGTGGATACACTTTG
GCTGCCACCTCTGCTAGTCTGTTTCCTCCTTGGACAGCAGCAGCAGGTGTACCATTTTATTTAAATGTTCAGTATCGTA
TTAATGGGATTGGTGTTACCATGGATGTGTTAAGTCAAAATCAAAAGCTTATTGCTAATGCATTTAACAATGCTCTTGA
TGATATTCAGGAAGGGTTTGATGCTACCAATTCTGCTTTAGTTAAAATTCAAGCTGTTGTTAATGCAAATGCTGAAGCT
CTTAATAACTTATTGCAACAACTCTCTAATAGATTTGGTGCTATAAGTTCTTCTTTACAAGAAATTCTATCTAGACTGG
ATGCTCTTGAAGCGCAAGCTCAGATAGACAGACTTATTAATGGGCGTCTTACCGCTCTTAATGCTTATGTTTCTCAACA
GCTTAGTGATTCTACACTAGTAAAATTTAGTGCAGCACAAGCTATGGAGAAGGTTAATGAATGTGTCAAAAGCCAATCA
TCTAGGATAAATTTTTGTGGTAATGGTAATCATATTATATCATTAGTGCAGAATGCTCCATATGGTTTGTATTTTATCC
ACTTTAGCTATGTCCCTACTAAGTATGTCACTGCGAAGGTTAGTACCGGTCTGTGCATTGCTGGTGATAGAGGTATAGC
CCCTAAGAGTGGTTATTTTGTTAATGTAAATAATACTTGGATGTTCACTGGTAGTGGTTATTACTACCCTGAACCCATA
ACTGGAAATAATGTTGTTGTTATGAGTACCTGTGCTGTTAACTATACTAAAGCGCCGGATGTAATGCTGAACATTTCAA
CACCCAACCTCCATGATTTTAAGGAAGAGTTGGATCAATGGTTTAAAAACCAAACATCAGTGGCACCAGATTTGTCACT
TGATTATATAAATGTTACATTCTTGGACCTACAAGATGAAATGAATAGGTTACAGGAGGCAATAAAAGTTTTAAATCAG
AGCTACATCAATCTCAAGGACATTGGTACATATGAGTATTATGTAAAATGGCCTTGGTATGTATGGCTTTTAATTGGCT
TTGCTGGTGTAGCTATGCTTGTTTTACTATTCTTCATATGCTGTTGTACAGGATGTGGGATTAGTTGTTTTAAGATATG
TGGTGGTTGTTGTGATGATTATACTGGACACCAGGAGTTAGTAATTAAAACATCACATGACGACTAA

>gi|193795728|gb|EU814647.1|_Bovine_coronavirus_strain_438/06TN_S_protein_(S)_gene_complete_cds

ATGTTTTTGATACTTTTAATTTCCTTACCAACGGCTTTTGCTGTTATAGGAGATTTAAAGTGTACTTCGCTTTCC
ATTAATGATGTTGACACTGGTGTTCCTTCTATTAGCACTGATACTGTTGATGTTACTAATGGTTTAGGTACTTATTATG
TTTTAGATCGTGTGTATTTAAATACTACCTTGTTGCTTAATGGTTATTACCCTACTTCTGGTTCTACATATCGTAATAT
GGCACTGAAGGGAACTTTACTATTGAGCACACTATGGTTTAAACCACCTTTTCTTTCTGATTTTACTAATGGTATTTTT
GCTAAGGTCAAAAATACCAAGGTTGTTAAAAATGGTGTAATGTATAGTGAGTTTCCTGCTATAACTATAGGTAGTACTT
TTGTAAATACATCTTATAGTGTGGTAGTACAACCACATACTACCAATTTGGATAATAAATTACAAGGTCTCTTAGAGAT
CTCTGTTTGCCAGTATACTATGTGCGAGTACCCAAATACGATTTGTAATCCTAATCTGGGTAATCAACGCGTAGAACTA
TGGCATTGGGATACAGGTGTTGTTTCCTGTTTATATAAGCGTAATTTCACATATGATGTGAATGCTGATTATTTGTATT
TCCATTTTTATCAAGAAGGTGGTACTTTTTATGCATATTTTACAGACACTGGTGTTGTTACTAAGTTTCTGTTTAATGT
ATATTTAGGCACGGTGCTTTCACACTATTATGTCATGCCTTTGACTTGTAATAGTGCTTTGAATTTAGAATATTGGGTT
ACCCCTCTCACTTCTAAACAATATTTACTAGCTTTCAATCAAGATGGTGTTATTTTTAATGCTGTTGATTGTAAGAGTG
ATTTTATGAGTGAGATTAAGTGTAAAACATTATCTATAGCACCATCTACTGGTGTTTATGAATTAAACGGTTACACTGT
TCAGCCAATTGCAGATGTTTACCGACGTATACCTAATCTTCCCGATTGTAATATAGAGGCTTGGCTTAATGACAAGTCG
GTGCCCTCTCCATTAAATTGGGAACGTAAGACCTTTTCAAATTGTAATTTTAATATGAGCAGCCTGATGTCTTTTATTC
AGGCAGACTCATTTACTTGTAATAATATTGATGCTGCTAAGATATATGGTATGTGTTTTTCCAGCATAACTATAGACAA
GTTTGCTATACCCAATGGTAGGAAGGTTGACCTACAATTGGGCAATTTGGGCTATTTGCAGTCTTTTAACTATAGAATT

GATACTACTGCTACAAGTTGTCAGTTGTATTATAATTTACCTGCTGCTAATGTTTCTGTTAGCAGGTTTAATCCTTCTA
CTTGGAATAGGAGATTTGGTTTTACAGAACAATCTGTTTTTAAGCCTCAACCTGCAGGTGTCTTTACTGATCATGATGT
TGTTTATGCACAACATTGTTTCAAAGCTCCCACAAATTTCTGTCCGTGTAAATTGGATGGGTCTTTGTGTGTAGGTAGT
GGTTCTGGTATAGATGCTGGTTATAAAAATAGTGGTATAGGCACTTGTCCTGCAGGTACTAATTATTTAACTTGTTATA
ATGCTGCCCAATGTGACTGTTTGTGCACTCCAGACCCCATTACATCTAAAGCTACAGGGCCTTACAAGTGCCCCCAAAC
TAAATATTTAGTTGGCATAGGTGAGCACTGTTCAGGTCTTGCTATTAAAAGTGATCATTGTGGAGGTAATCCATGTACT
TGCCAACCACAAGCATTTTTGGGTTGGTCTGTTGACTCTTGTTTACAAGGGGATAGGTGTAATATTTTTGCTAATTTTA
TTTTGCATGGTGTTAATAGTGGTACTACTTGTTCTACTGATTTACAAAAATCAAACACAGACATAATTCTTGGTGTTTG
TGTTAATTATGATCTTTATGGTATTACTGGCCAAGGTATTTTTGTTGAGGTTAATGCGACTTATTATAATAGTTGGCAG
AACCTTTTATATGATTCTAATGGTAATCTCTATGGTTTTAGAGACTACTTAACAAACAGAACTTTCATGATTCGTAGTT
GCTATAGCGGTCGTGTTTCAGCGGCCTTTCATGCTAACTCTTCCGAACCAGCATTGCTATTTCGGAATATTAAATGCAA
CTACGTTTTTAATAATACTCTTTCACGACAGCTGCAACCTATTAACTATTTTGATAGCTATCTTGGTTGTGTTGTCAAT
GCTGATAATAGTACTTCTAGTGTTGTTCAAACATGTGATCTCACAGTAGGTAGTGGTTACTGTGTGGATTACTCTACAA
AAAGACGAAGTCGTAGAGCGATTACCACTGGTTATCGGTTTACTAATTTTGAGCCATTTACTGTTAAGTCAGTAAATGA
TAGTTTAGAACCTGTAGGTGGTTTGTATGAAATTCAAATACCTTCAGATTTTACTATAGGTAACATGGAGGAGTTTATT
CAAACAAGCTCTCCTAAAGTTACTATTGATTGTTCTACTTTTGTCTGTGGTGATTATGCAGCATGTAAATCACAGTTGG
TTGAATATGGTAGCTTTTGTGACAATATTAATGCTATACTCACAGAAGTAAATGAACTACTTGACACTACACAGTTGCA
AGTAGCTAATAGTTTAATGAATGGTGTCACTCTTAGCACTAAGCTTAAAGATGGCGTTAAGTTCAATGTAGACGACATC
AATTTTTCCCCTGTATTAGGTTGTTTAGGAAGCGATTGTAACAGAGTTTCCAGTAGATCTGCTATAGAGGATTTACTTT
TTTCTAAAGTAAAGTTAGCTGATGTCGGTTTCGTTGAGGCTTATAATAATTGTACTGGAGGTGCCGAAATTAGGGACCT
CATTTGTGTGCAAAGTTATAATGGTATCAAGGTTTTGCCTCCACTGCTCTCAGAAAATCAGATCAGTGGATATACTTTG
GCTGCGACCTCTGCTAGTTTGTTTCCTCCTTGGTCAGCAGCAGCAGGTGTACCATTTTATTTAAATGTTCAGTATCGTA
TTAATGGGATTGGTGTTACCATGGATGTGCTAAGTCAAAATCAAAAGCTTATTGCTAATGCATTTAACAATGCCCTTGG
TGCTATTCAGGAAGGGTTTGATGCTACCAATTCTGCTTTAGTTAAAATTCAAGCTGTTGTTAATGCAAATGCTGAAGCT
CTTAATAACTTATTGCAACAACTCTCTAACAGGTTTGGTGCTATAAGTTCTTCTTTACAAGAAATTCTATCTAGACTGG
ATGCTCTTGAAGCGCAAGCTCAGATAGACAGACTTATTAATGGGCGTCTTACCGCTCTTAATGCTTATGTTTCTCAACA
GCTTAGTGACTCTACACTAGTAAAATTTAGTGCAGCACAAGCTATGGAGAAGGTTAATGAATGTGTCAAAAGCCAATCA
TCTAGGATAAATTTTTGTGGTAATGGTAATCATATTATATCATTAGTGCAGAATGCTCCATATGGTTTGTATTTTATCC
ACTTTAGCTATGTCCCTACTAAGTATGTCACTGCGAAGGTTAGTCCTGGTCTGTGCATTGCTGGTGATAGAGGTATAGC
CCCCAAGAGTGGTTATTTTGTTAATGTAAATAATACTTGGATGTTCACTGGTAGTGGTTATTACTACCCTGAACCTATA
ACTGGAAATAATGTTGTTGTTATGAGTACCTGTGCTGTTAATTATACTAAAGCACCGGATGTAATGCTGAACATTTCAA
CACCCAACCTCCCTGATTTTAAGGAAGAGTTGGATCAATGGTTTAAAAACCAAACATCAGTGGCACCAAATTTGTCACT
TGATTATATAAATGTTACATTCTTGGACCTACAAGATGAAATGAATAGGTTACAGGAGGCAATAAAAGTTTTAAATCAG
AGCTACATCAATCTCAAGGACATTGGTACATATGAGTATTATGTAAAATGGCCTTGGTATGTATGGCTTTTAATTGGCT
TTGCTGGTGTAGCTATGCTTGTTTTACTATTCTTCATATGCTGTTGTACAGGATGTGGGACTAGTTGTTTTAAGAAATG
TGGCGGTTGTTGTGATGATTATACTGGACACCAGGAGTTAGTAATTAAAACATCACATGACGACTAA

>gi|193795730|gb|EU814648.1|_Bovine_coronavirus_strain_438/06TN50_S_protein_(S)_gene_complete_cds

ATGTTTTTGATACTTTTAATTTCCTTACCAACGGCTTTTGCTGTTATAGGAGATTTAAAGTGTACTTCGCGTTCC
ATTAATGATGTTGACACTGGTGTTCCTTCTACTAGCACTGCTACTGTTGATGTTACTAATGGTTTAGGTACTTATTATG
TTTTAGATCGTGTGTATTTAAATACTACCTTGTTGCTTAATGGTTATTACCCTACTTCTGGTTCTACATATCGTAATAT

GGCACTGAAGGGAACTTTACTATTGAGCACACTATGGTTTAAACCACCTTTTCTTTCTGATTTTACTAATGGTATTTTT
GCTAAGGTCAAAAATACCAAGGTTGTTAAAAAGGGTGTAATGTATAGTGAGTTTCCTGCTATAACTATAGGTAGTACTT
TTGTAAATACATCTTATAGTGTGGTAGTACAACCACATACTACCAATTTGGATAATAAATTACAAGGTCTCTTAGAGAT
CTCTGTTTGCCAGTATACTATGTGCGAGTACCCAAATACGATTTGTAATCCTAATCTGGGTAATCGACGCGTAGAACTA
TGGCATTGGGATACAGGTGTTGTTTCCTGTTTATATAAGCGTAATTTCACATATGATGTGAATGCTGATTATTTGTATT
TCCATTTTTATCAAGAAGGTGGTACTTTTTATGCATATTTTACAGACACTGGTGTTGTTACTAAGTTTCTGTTTAATGT
ATATTTAGGCACGGTGCTTTCACACTATTATGTCATGCCTTTGACTTGTAATAGTGCTTTGAATTTAGAATATTGGGTT
ACACCTCTCACTTCTAAACAATATTTACTAGCTTTCAATCAAGATGGTGTTATTTTTAATGCTGTTGATTGTAAGAGTG
ATTTTATGAGTGAGATTAAGTGTAAAACATTATCTATAGCACCATCTACTGGTGTTTATGAATTAAACGGTTACACTGT
TCAGCCAATTGCAGATGTTTACCGACGTATACCTAATCTTCCCGATTGTAATATAGAGGCTTGGCTTAATGACAAGTCG
GTGCCCTCTCCATTAAATTGGGAACGTAAGACCTTTTCAAATTGTAATTTTAATATGAGCAGCCTGATGTCTTTTATTC
AGGCAGACTCATTTACTTGTAATAATATTGATGCTGCTAAGATATATGGTATGTGTTTTTCCAGCATAACTATAGACAA
GTTTGCTATACCCAATGGTAGGAAGGTTGACCTACAATTGGGCAATTTGGGCTATTTGCAGTCTTTTAACTATAGAATT
GATACTACTGCTACAAGTTGTCAGTTGTATTATAATTTACCTGCTGCTAATGTTTCTGTTAGCAGGTTTAATCCTTCTA
CTTGGAATAGGAGATTTGGTTTTACAGAACAATCTGTTTTTAAGCCTCAACCTGCAGGTGTCTTTACTGATCATGATGT
TGTTTATGCACAACATTGTTTCAAAGCTCCCACAAATTTCTGTCCGTGTAAATTGGATGGGTCTTTGTGTGTAGGTAGT
GGTTCTGGTATAGATGCTGGTTATAAAAATAGTGGTATAGGCACTTGTCCTGCAGGTACTAATTATTTAACTTGTTATA
ATGCTGCCCAATGTGACTGTTTGTGCACTCCAGACCCCATTACATCTAAAGCTACAGGGCCTTACAAGTGCCCCCAAAC
TAAATATTTAGTTGGCATAGGTGAGCACTGTTCAGGTCTTGCTATTAAAAGTGATCATTGTGGAGGTAATCCATGTACT
TGCCAACCACAAGCATTTTTGGGTTGGTCTGTTGACTCTTGTTTACAAGGGGATAGGTGTAATATTTTTGCTAATTTTA
TTTTGCATGGTGTTAATAGTGGTACTACTTGTTCTACTGATTTACAAAAATCAAACACAGACATAATTCTTGGTGTTTG
TGTTAATTATGATCTTTATGGTATTACTGGCCAAGGTATTTTTGTTGAGGTTAATGCGACTTATTATAATAGTTGGCAG
AACCTTTTATATGATTCTAATGGTAATCTCTATGGTTTTAGAGACTACTTAACAAACAGAACTTTCATGATTCGTAGTT
GCTATAGCGGTCGTGTTTCAGCGGCCTTTCATGCTAACTCTTCCGAACCAGCATTGCTATTTCGGAATATTAAATGCAA
CTACGTTTTTAATAATACTCTTTCACGACAGCTGCAACCTATTAACTATTTTGATAGCTATCTTGGTTGTGTTGTCAAT
GCTGATAATAGTACTTCTAGTGTTGTTCAAACATGTGATCTCACAGTAGGTAGTGGTTACTGTGTGGATTACTCTACAA
AAAGACGAAGTCGTAGAGCGATTACCACTGGTTATCGGTTTACTAATTTTGAGCCATTTACTGTTAAGTCAGTAAATGA
TAGTTTAGAACCTGTAGGTGGTTTGTATGAAATTCAAATACCTTCAGATTTTACTATAGGTAACATGGAGGAGTTTATT
CAAACAAGCTCTCCTAAAGTTACTATTGATTGTTCTACTTTTGTCTGTGGTGATTATGCAGCATGTAAATCACAGTTGG
TTGAATATGGTAGCTTTTGTGACAATATTAATGCTATACTCACAGAAGTAAATGAACTACTTGACACTACACAGTTGCA
AGTAGCTAATAGTTTAATGAATGGTGTCACTCTTAGCACTAAGCTTAAAGATGGCGTTAAGTTCAATGTAGACGACATC
AATTTTTCCCCTGTATTAGGTTGTTTAGGAAGCGCTTGTAACAGAGTTTCCAGTAGATCTGCTATAGAGGATTTACTTT
TTTCTAAAGTAAAGTTAGCTGATGTCGGTTTCGTTGAGGCTTATAATAATTGTACTGGAGGTGCCGAAATTAGGGACCT
CATTTGTGTGCAAAGTTATAATGGTATCAAGGTTTTGCCTCCACTGCTCTCAGAAAATCAGATCAGTGGATATACTTTG
GCTGCGACCTCTGCTAGTTTGTTTCCTCCTTGGTCAGCAGCAGCAGGTGTACCATTTTATTTAAATGTTCAGTATCGTA
TTAATGGGATTGGTGTTACCATGGATGTGCTAAGTCAAAATCAAAAGCTTATTGCTAATGCATTTAACAATGCCCTTGG
TGCTATTCAGGAAGGGTTTGATGCTACCAATTCTGCTTTAGTTAAAATTCAAGCTGTTGTTAATGCAAATGCTGAAGCT
CTTAATAACTTATTGCAACAACTCTCTAACAGGTTTGGTGCTATAAGTTCTTCTTTACAAGAAATTCTATCTAGACTGG
ATGCTCTTGAAGCGCAAGCTCAGATAGACAGACTTATTAATGGGCGTCTTACCGCTCTTAATGCTTATGTTTCTCAACA
GCTTAGTGACTCTACACTAGTAAAATTTAGTGCAGCACAAGCTATGGAGAAGGTTAATGAATGTGTCAAAAGCCAATCA
TCTAGGATAAATTTTTGTGGTAATGGTAATCATATTATATCATTAGTGCAGAATGCTCCATATGGTTTGTATTTTATCC
ACTTTAGCTATGTCCCTACTAAGTATGTCACTGCGAAGGTTAGTCCTGGTCTGTGCATTGCTGGTGATAGAGGTATAGC
CCCCAAGAGTGGTTATTTTGTTAATGTAAATAATACTTGGATGTTCACTGGTAGTGGTTATTACTACCCTGAACCTATA
ACTGGAAATAATGTTGTTGTTATGAGTACCTGTGCTTTTAATTATACTAAAGCACCGGATGTAATGCTGAACATTTCAA

CACCCAACCTCCCTGATTTTAAGGAAGAGTTGGATCAATGGTTTAAAAACCAAACATCAGTGGCATCAAATTTGTCACT
TGATTATATAAATGTTACATTCTTGGACCTACAAGATGAAATGAATAGGTTACAGGAGGCAATAAAAGTTTTAAATCAG
AGCTACATCAATCTCAAGGACATTGGTACATATGAGTATTATGTAAAATGGCCTTGGTATGTATGGCTTTTAATTGGCT
TTGCTGGTGTAGCTATGCTTGTTTTACTATTCTTCATATGCTGTTGTACAGGATGTGGGACTAGTTGTTTTAAGATATG
TGGCGGTTGTTGTGATGATTATACTGGACACCAGGAGTTAGTAATTAAAACATCACATGACGACTAA

>gi|310769581|gb|HM573326.1|_Bovine_coronavirus_strain_Wisent_10/01_spike_glycoprotein_precursor_(S)_gene_complete_cds

ATGTTTTTGATACTTTTAATTTCCTTACCAATGGCTTTTGCTGTTATAGGAGATTTAAAGTGTACTACAGTTTCC
ATTAATGATGTTGACACTGGTGTTCCTTCTATTAGCACTGATACTGTCGATGTTACTAATGGTTTAGGTACTTATTATG
TTTTAGACCGTGTGTATTTAAATACTACTTTGTTGCTTAATGGTTATTACCCTACTTCAGGTTCTACATATCGTAATAT
GGCACTGAAGGGAACTTTACTATTGAGCACACTATGGTTTAAACCACCTTTTCTTTCTGATTTTACTAATGGTATTTTT
GCTAAGGTCAAAAATACCAAGGTTATTAAAGATGGTGTAATGTATAGTGAGTTTCCTGCTATAACTATAGGTAGTACTT
TTGTAAATACATCCTATAGTGTGGTAGTACAACCACATACTACTAATTTTGATAGTAAATTACAAGGTCTCTTAGAGAT
TTCTGTTTGCCAGTATACTATGTGCGAGTACCCAAATACGATTTGTAATCCTAATCTGGGTAATCAACGCGTAGAACTA
TGGCATTGGGATACAGGTGTTGTTTCCTGTTTATATAAGCGTAATTTCACATATGATGTGAATGCTGATTATTTGTATT
TCCATTTTTATCAAGAAGGTGGTACTTTTTATGCATATTTTACAGACACTGGTGTTGTTACTAAGTTTCTGTTTAATGT
TTATTTAGGCACGGTGCTTTCACATTATTATGTCATGCCTTTGACTTGTAATAGTGCTCTGACTTTAGAATATTGGGTT
ACACCTCTCACTTCTAAACAATATCTACTAGCTTTCAATCAAGATGGTGTTATTTTTAATGCTGTTGATTGTAAGAGTG
ATTTTATGAGTGAGATTAAGTGTAAAACACTATCTATAGCACCATCTACTGGTGTTTATGAATTAAACGGTTACACTGT
TCAGCCAATTGCAGATGTTTACCGACGTATACCTAATCTTCCCGATTGTAATATTGAGGCTTGGCTTAATGATAAGTCG
GTGCCCTCTCCATTAAATTGGGAACGTAAGACCTTTTCAAATTGTAATTTTAATATGAGTAGCCTGATGTCTTTTATTC
AGGCAGACTCATTTACTTGTAATAATATTGATGCTGCTAAGATATATGGTATGTGTTTTTCCAGCATAACTATAGATAA
GTTTGCAATACCCAATGGTAGGAAGGTTGACCTACAATTGGGTAATTTGGGTTATTTGCAGTCTTTTAACTATAGAATT
GATACTACTGCTACAAGTTGTCAGTTGTATTATAATTTACCTGCTGCTAATGTTTCTGTCAGCAGGTTTAATCCTTCTA
CTTGGAATAGGAGATTTGGTTTTACAGAACAATCTGTTTTTAAGCCTCAACCTGCAGGTGTTTTTACTGATCATGATGT
TGTTTATGCACAACATTGTTTTAAAGCTCCCACAAATTTCTGTCCGTGTAAATTGGACGGGTCTTTGTGTGTAGGTACT
GGTTCTGGTATAGATGCTGGTTATAAAAATACTGGTATAGGCACTTGTCCTGCAGGTACTAATTATTTAACTTGCCATA
ATGCTGCCCAATGTGATTGTTTGTGCACCCCAGACCCCATTACATCTAAAGCTACAGGGCCTTACAAGTGCCCCCAAAC
TAAATACTTAGTTGGTATAGGAGAGCACTGTTCGGGTCTTGCTATTAAAAGTGATTATTGTGGAGGTAATCCTTGTAGT
TGCCAACCACAAGCATTTTTGGGTTGGTCTGTTGACTCTTGTTTACAAGGGGATAGGTGTAATATTTTTGCTAATTTTA
TTTTGCATGATGTTAATAGTGGTACTACTTGTTCTACTGATTTACAAAAATCAAACACAGACATAATTCTTGGTGTTTG
TGTTAATTATGATCTTTATGGTATTACTGGCCAAGGTATTTTTGTTGAGGTTAATGCGACTTATTATAATAGTTGGCAG
AACCTTTTATATGATTCTAATGGTAATCTCTATGGTTTTAGAGACTACTTAACAAATAGAACTTTTATGATTCGTAGTT
GCTATAGCGGTCGTGTATCAGCGGCCTTTCATGCTAACTCTTCCGAACCAGCATTGCTATTTCGGAATATTAAATGCAA
CTACGTTTTTAATAATACTCTTTCACGACAGCTGCAACCTATTAACTATTTTGATAGCTATCTTGGTTGTGTTGTCAAT
GCTGATAATAGTACTTCTAGTGTTGTTCAAACATGCGATCTCACAGTAGGTAGTGGTTACTGTGTGGATTACTCTACAA
AAAGACGAAGTCGTAGATCGATTACCACTGGTTATCGGTTTACTAATTTTGAGCCATTTACTGTTAATTCAGTAAATGA
TAGTTTAGAACCTGTAGGTGGTTTGTATGAAATTCAAATACCTTCAGAGTTTACTATAGGTAATATGGAGGAGTTTATT
CAAACAAGCTCTCCTAAAGTTACTATTGATTGTTCTGCTTTTGTCTGTGGTGATTATGCAGCATGTAAATCACAGTTGG
TTGAATATGGTAGTTTCTGTGACAATATTAACGCTATACTCACAGAAGTAAATGAACTACTTGACACTACACAGTTGCA
AGTAGCTAATAGTTTAATGAATGGTGTCACTCTTAGTACTAAGCTTAAAGATGGCGTTAATTTCAATGTAGATGACATC

AATTTTTCCCCTGTATTAGGTTGCTTAGGAAGCGACTGTAATAAAGTTTCCAGTAGATCTGCTATAGAGGATTTACTTT
TTTCTAAAGTTAAGTTATCTGATGTCGGTTTTGTTGAGGCTTATAATAATTGTACTGGAGGTGCCGAAATTAGGGACCT
CATTTGTGTGCAAAGTTATAACGGTATCAAGGTGTTGCCTCCACTGCTCTCAGAAAATCAGATCAGTGGATACACTTTG
GCTGCCACCTCTGCTAGTCTGTTTCCTCCTTGGTCAGCAGCAGCAGGTGTACCATTTTATTTAAATGTTCAGTATCGCA
TTAATGGGATTGGTGTTACCATGGATGTGCTAAGTCAAAATCAAAAGCTTATTGCTAATGCATTTAACAATGCTCTTGG
TGCTATTCAGGATGGTTTTGATGCTACCAATTCTGCTTTAGTTAAAATTCAAGCTGTTGTTAATGCAAATGCTGAAGCT
CTTAATAACTTATTGCAACAACTCTCTAATAGGTTTGGTGCTATAAGTTCTTCTTTACAAGAAATTCTATCTAGACTGG
ATGCTCTTGAAGCGCAAGCTCAGATAGACAGACTTATTAATGGGCGTCTTACCGCTCTTAATGCCTATGTTTCTCAACA
GCTTAGTGACTCTACACTAGTAAAATTTAGTGCAGCACAAGCTATGGAGAAGGTTAATGAATGTGTCAAAAGCCAATCA
TCTAGGATAAATTTTTGTGGTAATGGTAATCATATTATATCATTAGTGCAGAATGCTCCATATGGTTTGTATTTCATCC
ACTTTAGCTATGTCCCTACTAAGTATGTCACAGCGAAGGTTAGTCCCGGTCTGTGCATTGCTGGTGATAGAGGTATAGC
CCCCAAGAGTGGTTATTTTGTTAATGTAAATAATACTTGGATGTTCACTGGTAGTGGTTATTACTACCCTGAACCTATA
ACTGGAAATAATGTTGTTGTTATGAGTACCTGTGCTGTTAATTATACTAAAGCGCCGGATGTAATGCTGAACATTTCAA
CACCCAACCTCCCTGATTTTAAGGAAGAGTTGGATCAATGGTTTAAAAACCAAACATCAGTGGCACCAGATTTGTCACT
TGATTATATAAATGTTACATTCTTGGACCTACAAGATGAAATGAATAGGTTACAGGAGGCAATAAAAGTTTTAAATCAG
AGCTACATCAATCTCAAGGACATTGGTACATATGAGTATTATGTAAAATGGCCTTGGTATGTATGGCTTTTAATTGGCT
TTGCTGGTGTAGCTATGCTTGTTTTACTATTCTTCATATGCTGTTGTACAGGATGTGGGACTAGTTGTTTTAAGAAATG
TGGCGGTTGTTGTGATGATTATACTGGACACCAGGAGTTAGTAATTAAAACATCACATGACGACTAA

>gi|310769589|gb|HM573330.1|_Bovine_coronavirus_strain_Nyala_10/01_spike_glycoprotein_precursor_(S)_gene_complete_cds

ATGTTTTTGATACTTTTAATTTCCTTACCAACGGCTTTTGCTGTTATAGGAGATTTAAAGTGTACTACAGTTTCC
ATTAATGATGTTGACACTGGTGTTCCTTCTATTAGCACTGATACTGTCGATGTTACTAATGGTTTAGGTACTTATTATG
TTTTAGATCGTGTGTATTTAAATACTACTTTGTTGCTTAATGGTTATTACCCTACTTCAGGTTCTACATATCGTAATAT
GGCACTGAAGGGAACTTTACTATTGAGCACACTATGGTTTAAACCACCTTTTCTTTCTGATTTTACTAATGGTATTTTT
GCTAAGGTCAAAAATACCAAGGTTATTAAAGATGGTGTAATGTATAGTGAGTTTCCTGCTATAACTATAGGTAGTACTT
TTGTAAATACATCCTATAGTGTGGTAGTACAACCACATACTACTAATTTTGATAGTAAATTACAAGGTCTCTTAGAGAT
TTCTGTTTGCCAGTATACTATGTGCGAGTACCCAAATACGATTTGTAATCCTAATCTGGGTAATCAACGCGTAGAACTA
TGGCATTGGGATACAGGTGTTGTTTCCTGTTTATATAAGCGTAATTTCACATATGATGTGAATGCTGATTATTTGTATT
TCCATTTTTATCAAGAAGGTGGTACTTTTTATGCATATTTTACAGACACTGGTGTTGTTACTAAGTTTCTGTTTAATGT
TTATTTAGGCACGGTGCTTTCACATTATTATGTCATGCCTTTGACTTGTAATAGTGCTCTGACTTTAGAATATTGGGTT
ACACCTCTCACTTCTAAACAATATCTACTAGCTTTCAATCAAGATGGTGTTATTTTTAATGCTGTTGATTGTAAGAGTG
ATTTTATGAGTGAGATTAAGTGTAAAACACTATCTATAGCACCATCTACTGGTGTTTATGAATTAAACGGTTACACTGT
TCAGCCAATTGCAGATGTTTACCGACGTATACCTAATCTTCCCGATTGTAATATTGAGGCTTGGCTTAATGATAAGTCG
GTGCCCTCTCCATTAAATTGGGAACGTAAGACCTTTTCAAATTGTAATTTTAATATGAGTAGCCTGATGTCTTTTATTC
AGGCAGACTCATTTACTTGTAATAATATTGATGCTGCTAAGATATATGGTATGTGTTTTTCCAGCATAACTATAGATAA
GTTTGCAATACCCAATGGTAGGAAGGTTGACCTACAATTGGGTAATTTGGGTTATTTGCAGTCTTTTAACTATAGAATT
GATACTACTGCTACAAGTTGTCAGTTGTATTATAATTTACCTGCTGCTAATGTTTCTGTCAGCAGGTTTAATCCTTCTA
CTTGGAATAGGAGATTTGGTTTTACAGAACAATCTGTTTTTAAGCCTCAACCTGCAGGTGTTTTTACTGATCATGATGT
TGTTTATGCACAACATTGTTTTAAAGCTCCCACAAATTTCTGTCCGTGTAAATTGGACGGGTCTTTGTGTGTAGGTACT
GGTTCTGGTATAGATGCTGGTTATAAAAATACTGGTATAGGCACTTGTCCTGCAGGTACTAATTATTTAACTTGCCATA
ATGCTGCCCAATGTGATTGTTTGTGCACCCCAGACCCCATTACATCTAAAGCTACAGGGCCTTACAAGTGCCCCCAAAC

TAAATACTTAGTTGGTATAGGAGAGCACTGTTCGGGTCTTGCTATTAAAAGTGATTATTGTGGAGGTAATCCTTGTAGT
TGCCAACCACAAGCATTTTTGGGTTGGTCTGTTGACTCTTGTTTACAAGGGGATAGGTGTAATATTTTTGCTAATTTTA
TTTTGCATGATGTTAATAGTGGTACTACTTGTTCTACTGATTTACAAAAATCAAACACAGACATAATTCTTGGTGTTTG
TGTTAATTATGATCTTTATGGTATTACTGGCCAAGGTATTTTTGTTGAGGTTAATGCGACTTATTATAATAGTTGGCAG
AACCTTTTATATGATTCTAATGGTAATCTCTATGGTTTTAGAGACTACTTAACAAATAGAACTTTTATGATTCGTAGTT
GCTATAGCGGTCGTGTATCAGCGGCCTTTCATGCTAACTCTTCCGAACCAGCATTGCTATTTCGGAATATTAAATGCAA
CTACGTTTTTAATAATACTCTTTCACGACAGCTGCAACCTATTAACTATTTTGATAGCTATCTTGGTTGTGTTGTCAAT
GCTGATAATAGTACTTCTAGTGTTGTTCAAACATGCGATCTCACAGTAGGTAGTGGTTACTGTGTGGATTACTCTACAA
AAAGACGAAGTCGTAGATCGATTACCACTGGTTATCGGTTTACTAATTTTGAGCCATTTACTGTTAATTCAGTAAATGA
TAGTTTAGAACCTGTAGGTGGTTTGTATGAAATTCAAATACCTTCAGAGTTTACTATAGGTAATATGGAGGAGTTTATT
CAAACAAGCTCTCCTAAAGTTACTATTGATTGTTCTGCTTTTGTCTGTGGTGATTATGCAGCATGTAAATCACAGTTGG
TTGAATATGGTAGTTTCTGTGACAATATTAACGCTATACTCACAGAAGTAAATGAACTACTTGACACTACACAGTTGCA
AGTAGCTAATAGTTTAATGAATGGTGTCACTCTTAGTACTAAGCTTAAAGATGGCGTTAATTTCAATGTAGATGACATC
AATTTTTCCCCTGTATTAGGTTGCTTAGGAAGCGACTGTAATAAAGTTTCCAGTAGATCTGCTATAGAGGATTTACTTT
TTTCTAAAGTTAAGTTATCTGATGTCGGTTTTGTTGAGGCTTATAATAATTGTACTGGAGGTGCCGAAATTAGGGACCT
CATTTGTGTGCAAAGTTATAACGGTATCAAGGTGTTGCCTCCACTGCTCTCAGAAAATCAGATCAGTGGATACACTTTG
GCTGCCACCTCTGCTAGTCTGTTTCCTCCTTGGTCAGCAGCAGCAGGTGTACCATTTTATTTAAATGTTCAGTATCGCA
TTAATGGGATTGGTGTTACCATGGATGTGCTAAGTCAAAATCAAAAGCTTATTGCTAATGCATTTAACAATGCTCTTGG
TGCTATTCAGGATGGTTTTGATGCTACCAATTCTGCTTTAGTTAAAATTCAAGCTGTTGTTAATGCAAATGCTGAAGCT
CTTAATAACTTATTGCAACAACTCTCTAATAGGTTTGGTGCTATAAGTTCTTCTTTACAAGAAATTCTATCTAGACTGG
ATGCTCTTGAAGCGCAAGCTCAGATAGACAGACTTATTAATGGGCGTCTTACCGCTCTTAATGCCTATGTTTCTCAACA
GCTTAGTGACTCTACACTAGTAAAATTTAGTGCAGCACAAGCTATGGAGAAGGTTAATGAATGTGTCAAAAGCCAATCA
TCTAGGATAAATTTTTGTGGTAATGGTAATCATATTATATCATTAGTGCAGAATGCTCCATATGGTTTGTATTTCATCC
ACTTTAGCTATGTCCCTACTAAGTATGTCACAGCGAAGGTTAGTCCCGGTCTGTGCATTGCTGGTGATAGAGGTATAGC
CCCCAAGAGTGGTTATTTTGTTAATGTAAATAATACTTGGATGTTCACTGGTAGTGGTTATTACTACCCTGAACCTATA
ACTGGAAATAATGTTGTTGTTATGAGTACCTGTGCTGTTAATTATACTAAAGCGCCGGATGTAATGCTGAACATTTCAA
CACCCAACCTCCCTGATTTTAAGGAAGAGTTGGATCAATGGTTTAAAAACCAAACATCAGTGGCACCAGATTTGTCACT
TGATTATATAAATGTTACATTCTTGGACCTACAAGATGAAATGAATAGGTTACAGGAGGCAATAAAAGTTTTAAATCAG
AGCTACATCAATCTCAAGGACATTGGTACATATGAGTATTATGTAAAATGGCCTTGGTATGTATGGCTTTTAATTGGCT
TTGCTGGTGTAGCTATGCTTGTTTTACTATTCTTCATATGCTGTTGTACAGGATGTGGGACTAGTTGTTTTAAGAAATG
TGGCGGTTGTTGTGATGATTATACTGGACACCAGGAGTTAGTAATTAAAACATCACATGACGACTAA

>gi|519668481|gb|KF169908.1|_Bovine_coronavirus_isolate_SWE/C/92_spike_glycoprotein_(S)_gene_complete_cds

ATGTTTTTGATACTTTTAATTTCCTTACCGACGGCTTTTGCTGTTATAGGAGATTTAAAGTGTACTACAGTTTCC
ATTAATGATGTTGACACTGGTGTTCCTTCTATTAGCACTGATACTGTCGATGTTACTAATGGTCTAGGTACTTATTATG
TTTTAGATCGTGTGTATTTAAATACTACTTTGTTGCTTAATGGTTACTATCCTACTTCAGGTTCTACATATCGTAATAT
GGCACTGAAGGGAACTTTACTATTGAGCACACTATGGTTTAAACCACCTTTTCTTTCTGATTTTACTAATGGTATTTTT
GCTAAGGTCAAAAATACCAAGGTTACTAAAGATGGTGTAATGTATAGTGAGTTTCCTGCTATAACTATAGGTAGTACTT
TTTTAAATACATCTTATAGTGTGGTAGTACAACCACATACTACCAATTTGGATAATAAATTACAAGGTCTCTTAGAGAT
CTCTGTTTGCCAGTATACTATGTGCGAGTACCCAAATACGATTTGCCATCCTAATCTGGGTAATCAACGCGTAGAACTA
TGGCATTGGGATACAGGTGTTGTTTCCTGTTTATATAAGCGTAATTTCACATATGATGTGAATGCTGATTATTTGTATT

TTCATTTTTATCAAGAAGGTGGTACTTTTTATGCATATTTTACAGACACTGGTGTTGTTACTAAGTTTCTGTTTAATGT
TTATTTAGGCACGGTGCTTTCACACTATTATGTCATGCCTTTGACTTGTAATAGTGCTTTGACTTTAGAATATTGGGTT
ACACCTCTCACTTCTAAACAATATTTACTCGCTTTCAATCAAGATGGTGTTATTTTTAATGCTGTTGATTGTAATAGTG
ATTTTATGAGTGAGATTAAGTGTAAAACACTATCTATAGCACCATCTACTGGTGTTTATGAATTAAACGGTTACACTGT
TCAGCCAATTGCAGATGTTTACCGACGTATACCTAATCTTCCCGATTGTAATATAGAGGCTTGGCTTAATGATAAGTCG
GTGCCCTCTCCATTAAATTGGGAACGTAAGACCTTTTCAAATTGTAATTTTAATATGAGCAGCCTGATGTCTTTTATTC
AGGCAGACTCATTTACTTGTAATAATATTGATGCTGCTAAGATATATGGTATGTGTTTTTCCAGCATAACTATAGATAA
GTTTGCTATACCCAATGGTAGGAAAGTTGACCTACAATTGGGCAATTTGGGCTATTTGCAGTCTTTTAACTATAGAATT
GATACTACTGCTACAAGTTGTCAGTTGTATTATAATTTACCTGCTGCTAATGTTTCTGTTAGCAGGTTTAATCCTTCTA
CTTGGAACAGGAGATTTGGTTTTACAGAACAATCTGTTTTTAAGCCTCAACCTGCAGGTGTTTTTACTGATTATGATGT
TGTTTATGCACAACATTGTTTTAAAGCTCCCACAAATTTCTGTCCGTGTAAATTGGATGGGTCTTTGTGTGTAGGTACT
GGTCCTGGTATAGATGCTGGTTATAAAACTAGTGGTATAGGCACTTGTCCTGCAGGTACTAATTATTTAACTTGCCATA
ATGCTGCCCAATGTGATTGTTTGTGCACTCCAGACCCCATCACATCTAAATCTACAGGGCCTTACAAGTGCCCCCAAAC
TAAATACTTAGTTGGCATAGGTGAGCACTGTTCGGGTCTTGCTATTAAAAGTGATCATTGTGGAGGTAATCCTTGTACT
TGCCAACCACAAGCATTTTTGGGTTGGTCTGTTGAGTCTTGTTTACAAGGGGATAGGTGTAATATTTTTGCTAATTTTA
TTTTGCATGGTGTTAATAGTGGTACTACTTGTTCTACTGATTTACAAAAATCAAACACAGACATAATTCTTGGTGTTTG
TGTTAATTATGATCTTTATGGTATTACTGGCCAAGGTATTTTTGTTGAGGTTAATGCGACTTATTATAATAGTTGGCAG
AACCTTTTATATGATTCTAATGGTAATCTTTATGGTTTTAGAGACTACTTAACAAATAGAACTTTTATGATTCGTAGTT
GCTATAGCGGTCGTGTTTCAGCGGCCTTTCATGCTAACTCTTCCGAACCAGCATTGCTATTTCGGAATATTAAATGCAA
TTACGTTTTTAATAATACTCTTTCACGACAGCTGCAACCTATTAACTATTTTGATAGCTATCTTGGTTGTGTTGTCAAT
GCTGATAATAGTACTTCTAGTGTTGTTCAAACATGTGATCTCACAGTAGGTAGTGGTTACTGTGTGGATTACTCTACAA
AAAGACGAAGTCGTAGAGCGATTACCACTGGTTATCGGTTTACTAATTTTGAGCCATTTACTGTTAACTCAGTAAATGA
TAGTTTAGAACCTGTAGGTGGTTTGTATGAAATTCAAATACCTTCAGAGTTTACTATAGGTAACATGGAGGAGTTTATT
CAAACAAGCTCTCCTAAAGTTACTATTGATTGTTCTGCTTTTGTCTGTGGTGATTATGCAGCATGTAAATCACAGTTGG
TTGAATATGGTAGCTTCTGTGACAATATTAATGCTATACTCACAGAAGTAAATGAACTACTTGACACTACACAGTTGCA
AGTAGCTAATAGTTTAATGAATGGTGTCACTCTTAGCACTAAGCTTAAAGATGGCGTCAATTTCAATGTAGACGACATC
AATTTTTCCCCTGTATTAGGTTGGTTAGGAAGCGATTGTAATAGAGTTTCCAGTAGATCTGCTATAGAGGATTTACTTT
TTTCTAAAGTAAAGTTAGCTGATGTCGGTTTCGTTGAGGCTTATAATAATTGTACTGGAGGTGCCGAAATTAGGGACCT
CATTTGTGTGCAAAGTTATAATGGTATTAAGGTGTTGCCTCCACTGCTCTCAGAAAATCAGATCAGTGGATACACTTTG
GCTGCGACCTCTGCTAGTTTGTTTCCTCCTTGGTCAGCAGCAGCAGGTGTACCATTTTATTTAAATGTTCAGTATCGTA
TTAATGGGATTGGTGTTACCATGGATGTGCTAAGTCAAAATCAAAAGCTTATTGCTAATGCATTTAACAATGCTCTTGG
TGCTATTCAGGAAGGGTTTGATGCTACCAATTCTGCTTTAGTTAAAATTCAAGCTGTTGTTAATGCAAATGCTGAAGCT
CTTAATAACTTATTGCAACAACTCTCTAATAGATTTGGTGCTATAAGTTCTTCTTTACAAGAAATTCTATCTAGACTGG
ATTCTCTTGAAGCGCAAGCTCAGATAGATAGACTTATTAATGGGCGTCTTACCGCTCTTAATGCTTATGTCTCTCAACA
GCTTAGTGACTCTACACTAGTAAAATTTAGTGCAGCACAAGCTATGGAGAAGGTTAATGAATGTGTCAAAAGCCAATCA
TCTAGGATAAATTTTTGTGGTAATGGTAATCATATTATATCATTAGTGCAGAATGCTCCATATGGTTTGTATTTTATCC
ACTTTAGCTATGTCCCTACTAAGTATGTTACTGCGAAGGTTAGTCCCGGTCTGTGCATTGCTGGTGATAGAGGTATAGC
CCCTAAGAGTGGTTATTTTGTTAATGTAAATAATACTTGGATGTTCACTGGTAGTGGTTATTACTACCCTGAACCTATA
ACTGGAAATAATGTTGTTGTTATGAGTACCTGTGCTGTTAACTATACTAAAGCACCGGATGTAATGCTGAACATTTCAA
CACCCAACCTCCCTGACTTTAAGGAAGAGTTGGATCAATGGTTTAAAAACCAAACATCAGTGGCACCAGATTTGTCACT
TGATTATATAAATGTTACATTCTTGGACCTACAAGATGAAATGAATAGGTTACAGGAGGCAATAAAAGTTTTAAATCAG
AGCTACATCAATCTCAAGGACATTGGTACTTATGAGTATTATGTAAAATGGCCTTGGTATGTATGGCTTTTAATTGGCT
TTGCTGGTGTAGCTATGCTTGTTTTACTATTCTTCATATGCTGTTGTACAGGATGTGGGACTAGTTGTTTTAAGAAATG
TGGTGGTTGTTGTGATGATTATACTGGACACCAGGAGTTAGTAATTAAAACATCCCATGACGACTAA

>gi|519668483|gb|KF169909.1|_Bovine_coronavirus_isolate_SWE/021_spike_glycoprotein_(S)_gene_complete_cds

ATGTTTTTGATACTTTTAATTTCCTTACCAACGGCTTTTGCTGTTATAGGAGATTTAAAGTGTACTACAGTTTCC
ATTAATGATGTTGACACTGGTGTTCCTTTTATTAGCACTGATACTGTCGATGTTACTAATGGTTTAGGTACTTATTATG
TTTTAGATCGTGTGTATTTAAATACTACTTTGTTGCTTAATGGTTACTATCCCACTTCAGGTTCTACATATCGTAATAT
GGCACTGAAGGGAACTTTACTATTGAGCACACTATGGTTTAAACCACCTTTTCTTTCTGATTTTACTAATGGTATTTTT
GCTAAGGTCAAAAATACCAAGGTTACTAAAGATGGTGTAATGTATAGTGAGTTTCCTGCTATAACTATAGGTAGTACTT
TTGTAAATACATCTTATAGTGTGGTAGTACAACCACATACTACCAATTTGGATAATAAATTACAAGGTCTCTTAGAGAT
CTCTGTTTGCCAGTATACTATGTGCGAGTACCCAAATACGATTTGTAATCCTAATCTGGGTAATCAACGCGTAGAACTA
TGGCATTGGGATACAGGTGTTGTTTCCTGTTTATATAAGCGTAATTTCACATATGATGTGAATGCTGATTATTTGTATT
TTCATTTTTATCAAGAAGGTGGTACTTTTTATGCATATTTTACAGACACTGGTTTTGTTACTAAGTTTCTGTTTAATGT
ATATTTAGGCACGGTGCTTTCACACTATTATGTCATGCCTTTGACTTGTAATAGTGCTTTGAATTTAGAATATTGGGTT
ACACCTCTCACTTCTAAACAATATTTACTCGCTTTCAATCAAGATGGTGTTATTTTTAATGCTGTTGATTGTAAGAGTG
ATTTTATGAGTGAGATTAAGTGTAAAACACTATCTATAGCACCATCTACTGGTGTTTATGAATTAAACGGTTACACTGT
TCAGCCAATTGCAGATGTTTACCGACGTATACCTAATCTTCCCGATTGTAATATAGAGGCTTGGCTTAATGACAAGTCG
GTGCCCTCTCCATTAAATTGGGAACGTAAGACCTTTTCAAATTGTAATTTTAATATGAGCAGCCTGATGTCTTTTATTC
AGGCAGACTCATTTACTTGTAATAATATTGATGCTGCTAAGATATATGGTATGTGTTTTTCCAGCATAACTATAGATAA
GTTTGCAATACCCAATGGTAGGAAGGTTGACCTACAATTGGGTAATTTGGGCTATTTGCAGTCTTTTAACTATAGAATT
GATACTACTGCTACAAGTTGTCAGTTGTATTATAATTTACCTGCTGCTAATGTTTCTGTTAGCAGGTTTAATCCTTCTA
TTTGGAATAGGAGATTTGGTTTTACAGAACAATCTGTTTTTAAGCCTCAACCTGCAGGTGTCTTTACTGATTATGATGT
TGTTTATGCACAACATTGTTTTAAAGCTCCCACAAATTTCTGTCCGTGTAAATTGGATGGGTCTTTGTGTGTAGGTAGT
GGTTCTGGTATAGATGCTGGTTATAAAAATAATGGTATAGGCACTTGTCCTGCAGGTACTAATTATTTAACTTGTCATA
ATGCTGCCCAATGTGACTGTTTGTGCACTCCAGACCCTATTACATCTAAAGCTACAGGGCCTTACAAGTGCCCCCAAAC
TAAATATTTAGTTGGCATAGGTGAGCACTGTTCAGGTCTTGCTATTAAAAGTGATCATTGTGGAGGTAATCCATGTACT
TGCCAACCACAAGCATTTCTGGGTTGGTCTGTTGACTCTTGTTTACAAGGGGATAGGTGTAATATTTTTGCTAATTTTA
TTTTGCATGGTGTTAATAGTGGTACTACTTGTTCTACTGATTTACAAAAATCAAACACAGACATAATTCTTGGTGTTTG
TGTTAATTATGATCTTTATGGTATTACTGGCCAAGGTATTTTTGTTGAGGTTAATGCGACTTATTATAATAGTTGGCAG
AACCTTTTATATGATTCTAATGGTAATCTCTATGGCTTTAGAGACTACTTAACAAACAGAACTTTCATGATTCGTAGTT
GCTATAGCGGTCGTGTTTCAGCGGCCTTTCATGCTAACTCTTCCGAACCAGCATTGCTATTTCGGAATATTAAATGCAA
CTACGTTTTTAATAATACTCTTTCAAGACAGCTGCAACCTATTAACTATTTTGATAGCTATCTTGGTTGTGTTGTCAAT
GCTGATAATAGTACTTCTAGTGTTGTTCAAACATGTGATCTCACAGTAGGTAGTGGTTACTGTGTGGATTATTCTACAA
AAAGACGAAGTCGTAGAGCGATTACCACTGGTTATCGGTTTACTAATTTTGAGCCATTTACTGTTAATTCAGTAAATGA
TAGTTTAGAACCTGTAGGTGGTTTGTATGAAATTCAAATACCTTCAGAGTTTACTATAGGTAACATGGAGGAGTTTATT
CAAACAAGCTCTCCTAAAGTTACTATTGATTGTTCTGCTTTTGTTTGTGGTGATTATGCAGCATGTAAATCACAGTTGG
TTGAATATGGTAGCTTCTGTGACAATATTAATGCTATACTCACAGAAGTAAATGAACTACTTGACACTACACAGTTGCA
AGTAGCTAATAGTTTAATGAATGGTGTCACTCTTAGCACTAAGCTTAAAGATGGCGTTAATTTCAATGTAGACGACATC
AATTTTTCCCCTGTATTAGGTTGTTTAGGAAGCGATTGTAATAGAGTTTCCAGTAGATCTGCTATAGAGGATTTACTTT
TTTCTAAAGTAAAGTTAGCTGATGTCGGTTTCGTTGAGGCTTATAATAATTGTACTGGAGGTGCCGAAATTAGGGACCT
CATTTGTGTGCAAAGTTATAATGGTATCAAGGTGTTGCCTCCACTGCTCTCAGAAAATCAGATCAGTGGATATACTTTG
GCTGCGACCTCTGCTAGTCTGTTTCCTCCTTGGTCAGCAGCAGCAGGTGTACCATTTTATTTAAATGTTCAGTATCGTA
TTAATGGGATTGGTGTTACCATGGATGTGCTAAGTCAAAATCAAAAGCTTATTTCTAATGCATTTAACAATGCCCTTGG
TGCTATTCAGGAAGGGTTTGATTCTACCAATTCTGCTTTAGTTAAAATTCAAGCTGTTGTTAATGCAAATGCTGAAGCT

CTTAATAACTTATTGCAACAACTCTCTAACAGGTTTGGTGCTATAAGTTCTTCTTTACAAGAAATTCTATCTAGACTGG
ATGCTCTTGAAGCGCAAGCTCAGATAGACAGACTTATTAATGGGCGTCTTACCGCTCTTAATGCTTATGTTTCTCAACA
GCTTAGTGACTCTACACTAGTAAAATTTAGTGCAGCACAAGCTATGGAGAAGGTTAATGAATGTGTCAAAAGCCAATCA
TCTAGGATAAATTTTTGTGGTAATGGTAATCATATTATATCATTAGTGCAGAATGCTCCATATGGTTTGTATTTTATCC
ACTTTAGCTATGTCCCTACTAAGTATGTCACTGCGAAGGTTAGTCCTGGTCTGTGCATAGCTGGTGATAGAGGTATAGC
CCCCAAGAGTGGTTATTTTGTTAATGTAAATAATACTTGGATGTTCACTGGTAGTGGTTATTACTACCCTGAACCTATA
ACTGGAAATAATGTTGTTGTTATGAGTACCTGTGCTGTTAACTACACCAAAGCACCGGATGTAATGCTGAACATTTCAA
CACCCAAACTCCCTGATTTTAAGGAAGAGTTGGATCAATGGTTTAAAAACCAAACATCAGTGGCACCAAATTTGTCACT
TGATTATATAAATGTTACATTCTTGGACCTACAAGACGAAATGAATAGGTTACAGGAGGCAATAAAAGTTTTAAATCAG
AGCTACATCAATCTCAAGGACATTGGTACATATGAGTATTATGTAAAATGGCCTTGGTATGTATGGCTTTTAATTGGCT
TTGCTGGTGTAGCTATGCTTGTTTTACTATTCTTCATATGCTGTTGTACAGGATGTGGGACTAGTTGTTTTAAGAAATG
TGGTGGTTGTTGTGATGATTATACTGGACACCAGGAGTTAGTAATTAAAACATCACATGACGACTAA

>gi|519668485|gb|KF169910.1|_Bovine_coronavirus_isolate_SWE/022_spike_glycoprotein_(S)_gene_complete_cds

ATGTTTTTGATACTTTTAATTTCCTTACCAACGGCTTTTGCTGTTATAGGAGATTTAAAGTGTACTACAGTTTCC
ATTAATGATGTTGACACTGGTGTTCCTTTTATTAGCACTGATACTGTCGATGTTACTAATGGTTTAGGTACTTATTATG
TTTTAGATCGTGTGTATTTAAATACTACTTTGTTGCTTAATGGTTACTATCCCACTTCAGGTTCTACATATCGTAATAT
GGCACTGAAGGGAACTTTACTATTGAGCACACTATGGTTTAAACCACCTTTTCTTTCTGATTTTACTAATGGTATTTTT
GCTAAGGTCAAAAATACCAAGGTTACTAAAGATGGTGTAATGTATAGTGAGTTTCCTGCTATAACTATAGGTAGTACTT
TTGTAAATACATCTTATAGTGTGGTAGTACAACCACATACTACCAATTTGGATAATAAATTACAAGGTCTCTTAGAGAT
CTCTGTTTGCCAGTATACTATGTGCGAGTACCCAAATACGATTTGTAATCCTAATCTGGGTAATCAACGCGTAGAACTA
TGGCATTGGGATACAGGTGTTGTTTCCTGTTTATATAAGCGTAATTTCACATATGATGTGAATGCTGATTATTTGTATT
TTCATTTTTATCAAGAAGGTGGTACTTTTTATGCATATTTTACAGACACTGGTTTTGTTACTAAGTTTCTGTTTAATGT
ATATTTAGGCACGGTGCTTTCACACTATTATGTCATGCCTTTGACTTGTAATAGTGCTTTGAATTTAGAATATTGGGTT
ACACCTCTCACTTCTAAACAATATTTACTCGCTTTCAATCAAGATGGTGTTATTTTTAATGCTGTTGATTGTAAGAGTG
ATTTTATGAGTGAGATTAAGTGTAAAACACTATCTATAGCACCATCTACTGGTGTTTATGAATTAAACGGTTACACTGT
TCAGCCAATTGCAGATGTTTACCGACGTATACCTAATCTTCCCGATTGTAATATAGAGGCTTGGCTTAATGACAAGTCG
GTGCCCTCTCCATTAAATTGGGAACGTAAGACCTTTTCAAATTGTAATTTTAATATGAGCAGCCTGATGTCTTTTATTC
AGGCAGACTCATTTACTTGTAATAATATTGATGCTGCTAAGATATATGGTATGTGTTTTTCCAGCATAACTATAGATAA
GTTTGCAATACCCAATGGTAGGAAGGTTGACCTACAATTGGGTAATTTGGGCTATTTGCAGTCTTTTAACTATAGAATT
GATACTACTGCTACAAGTTGTCAGTTGTATTATAATTTACCTGCTGCTAATGTTTCTGTTAGCAGGTTTAATCCTTCTA
TTTGGAATAGGAGATTTGGTTTTACAGAACAATCTGTTTTTAAGCCTCAACCTGCAGGTGTCTTTACTGATTATGATGT
TGTTTATGCACAACATTGTTTTAAAGCTCCCACAAATTTCTGTCCGTGTAAATTGGATGGGTCTTTGTGTGTAGGTAGT
GGTTCTGGTATAGATGCTGGTTATAAAAATAATGGTATAGGCACTTGTCCTGCAGGTACTAATTATTTAACTTGTCATA
ATGCTGCCCAATGTGACTGTTTGTGCACTCCAGACCCTATTACATCTAAAGCTACAGGGCCTTACAAGTGCCCCCAAAC
TAAATATTTAGTTGGCATAGGTGAGCACTGTTCAGGTCTTGCTATTAAAAGTGATCATTGTGGAGGTAATCCATGTACT
TGCCAACCACAAGCATTTCTGGGTTGGTCTGTTGACTCTTGTTTACAAGGGGATAGGTGTAATATTTTTGCTAATTTTA
TTTTGCATGGTGTTAATAGTGGTACTACTTGTTCTACTGATTTACAAAAATCAAACACAGACATAATTCTTGGTGTTTG
TGTTAATTATGATCTTTATGGTATTACTGGCCAAGGTATTTTTGTTGAGGTTAATGCGACTTATTATAATAGTTGGCAG
AACCTTTTATATGATTCTAATGGTAATCTCTATGGCTTTAGAGACTACTTAACAAACAGAACTTTCATGATTCGTAGTT
GCTATAGCGGTCGTGTTTCAGCGGCCTTTCATGCTAACTCTTCCGAACCAGCATTGCTATTTCGGAATATTAAATGCAA

CTACGTTTTTAATAATACTCTTTCAAGACAGCTGCAACCTATTAACTATTTTGATAGCTATCTTGGTTGTGTTGTCAAT
GCTGATAATAGTACTTCTAGTGTTGTTCAAACATGTGATCTCACAGTAGGTAGTGGTTACTGTGTGGATTATTCTACAA
AAAGACGAAGTCGTAGAGCGATTACCACTGGTTATCGGTTTACTAATTTTGAGCCATTTACTGTTAATTCAGTAAATGA
TAGTTTAGAACCTGTAGGTGGTTTGTATGAAATTCAAATACCTTCAGAGTTTACTATAGGTAACATGGAGGAGTTTATT
CAAACAAGCTCTCCTAAAGTTACTATTGATTGTTCTGCTTTTGTTTGTGGTGATTATGCAGCATGTAAATCACAGTTGG
TTGAATATGGTAGCTTCTGTGACAATATTAATGCTATACTCACAGAAGTAAATGAACTACTTGACACTACACAGTTGCA
AGTAGCTAATAGTTTAATGAATGGTGTCACTCTTAGCACTAAGCTTAAAGATGGCGTTAATTTCAATGTAGACGACATC
AATTTTTCCCCTGTATTAGGTTGTTTAGGAAGCGATTGTAATAGAGTTTCCAGTAGATCTGCTATAGAGGATTTACTTT
TTTCTAAAGTAAAGTTAGCTGATGTCGGTTTCGTTGAGGCTTATAATAATTGTACTGGAGGTGCCGAAATTAGGGACCT
CATTTGTGTGCAAAGTTATAATGGTATCAAGGTGTTGCCTCCACTGCTCTCAGAAAATCAGATCAGTGGATATACTTTG
GCTGCGACCTCTGCTAGTCTGTTTCCTCCTTGGTCAGCAGCAGCAGGTGTACCATTTTATTTAAATGTTCAGTATCGTA
TTAATGGGATTGGTGTTACCATGGATGTGCTAAGTCAAAATCAAAAGCTTATTTCTAATGCATTTAACAATGCCCTTGG
TGCTATTCAGGAAGGGTTTGATTCTACCAATTCTGCTTTAGTTAAAATTCAAGCTGTTGTTAATGCAAATGCTGAAGCT
CTTAATAACTTATTGCAACAACTCTCTAACAGGTTTGGTGCTATAAGTTCTTCTTTACAAGAAATTCTATCTAGACTGG
ATGCTCTTGAAGCGCAAGCTCAGATAGACAGACTTATTAATGAGCGTCTTACCGCTCTTAATGCTTATGTTTCTCAACA
GCTTAGTGACTCTACACTAGTAAAATTTAGTGCAGCACAAGCTATGGAGAAGGTTAATGAATGTGTCAAAAGCCAATCA
TCTAGGATAAATTTTTGTGGTAATGGTAATCATATTATATCATTAGTGCAGAATGCTCCATATGGTTTGTATTTTATCC
ACTTTAGCTATGTCCCTACTAAGTATGTCACTGCGAAGGTTAGTCCTGGTCTGTGCATAGCTGGTGATAGAGGTATAGC
CCCCAAGAGTGGTTATTTTGTTAATGTAAATAATACTTGGATGTTCACTGGTAGTGGTTATTACTACCCTGAACCTATA
ACTGGAAATAATGTTGTTGTTATGAGTACCTGTGCTGTTAACTACACCAAAGCACCGGATGTAATGCTGAACATTTCAA
CACCCAAACTCCCTGATTTTAAGGAAGAGTTGGATCAATGGTTTAAAAACCAAACATCAGTGGCACCAAATTTGTCACT
TGATTATATAAATGTTACATTCTTGGACCTACAAGACGAAATGAATAGGTTACAGGAGGCAATAAAAGTTTTAAATCAG
AGCTACATCAATCTCAAGGACATTGGTACATATGAGTATTATGTAAAATGGCCTTGGTATGTATGGCTTTTAATTGGCT
TTGCTGGTGTAGCTATGCTTGTTTTACTATTCTTCATATGCTGTTGTACAGGATGTGGGACTAGTTGTTTTAAGAAATG
TGGTGGTTGTTGTGATGATTATACTGGACACCAGGAGTTAGTAATTAAAACATCCCATGACGACTAA

>gi|519668489|gb|KF169912.1|_Bovine_coronavirus_isolate_SWE/024_spike_glycoprotein_(S)_gene_complete_cds

ATGTTTTTGATACTTTTAATTTCCTTACCAACGGCTTTTGCTGTTATAGGAGATTTAAAGTGTACTACAGTTTCC
ATTAATGATGTTGACACTGGTGTTCCTTTTATTAGCACTGATACTGTCGATGTTACTAATGGTTTAGGTACTTATTATG
TTTTAGATCGTGTGTATTTAAATACTACTTTGTTGCTTAATGGTTACTATCCCACTTCAGGTTCTACATATCGTAATAT
GGCACTGAAGGGAACTTTACTATTGAGCACACTATGGTTTAAACCACCTTTTCTTTCTGATTTTACTAATGGTATTTTT
GCTAAGGTCAAAAATACCAAGGTTACTAAAGATGGTGTAATGTATAGTGAGTTTCCTGCTATAACTATAGGTAGTACTT
TTGTAAATACATCTTATAGTGTGGTAGTACAACCACATACTACCAATTTGGATAATAAATTACAAGGTCTCTTAGAGAT
CTCTGTTTGCCAGTATACTATGTGCGAGTACCCAAATACGATTTGTAATCCTAATCTGGGTAATCAACGCGTAGAACTA
TGGCATTGGGATACAGGTGTTGTTTCCTGTTTATATAAGCGTAATTTCACATATGATGTGAATGCTGATTATTTGTATT
TTCATTTTTATCAAGAAGGTGGTACTTTTTATGCATATTTTACAGACACTGGTTTTGTTACTAAGTTTCTGTTTAATGT
ATATTTAGGCACGGTGCTTTCACACTATTATGTCATGCCTTTGACTTGTAATAGTGCTTTGAATTTAGAATATTGGGTT
ACACCTCTCACTTCTAAACAATATTTACTCGCTTTCAATCAAGATGGTGTTATTTTTAATGCTGTTGATTGTAAGAGTG
ATTTTATGAGTGAGATTAAGTGTAAAACACTATCTATAGCACCATCTACTGGTGTTTATGAATTAAACGGTTACACTGT
TCAGCCAATTGCAGATGTTTACCGACGTATACCTAATCTTCCCGATTGTAATATAGAGGCTTGGCTTAATGACAAGTCG
GTGCCCTCTCCATTAAATTGGGAACGTAAGACCTTTTCAAATTGTAATTTTAATATGAGCAGCCTGATGTCTTTTATTC

AGGCAGACTCATTTACTTGTAATAATATTGATGCTGCTAAGATATATGGTATGTGTTTTTCCAGCATAACTATAGATAA
GTTTGCAATACCCAATGGTAGGAAGGTTGACCTACAATTGGGTAATTTGGGCTATTTGCAGTCTTTTAACTATAGAATT
GATACTACTGCTACAAGTTGTCAGTTGTATTATAATTTACCTGCTGCTAATGTTTCTGTTAGCAGGTTTAATCCTTCTA
TTTGGAATAGGAGATTTGGTTTTACAGAACAATCTGTTTTTAAGCCTCAACCTGCAGGTGTCTTTACTGATTATGATGT
TGTTTATGCACAACATTGTTTTAAAGCTCCCACAAATTTCTGTCCGTGTAAATTGGATGGGTCTTTGTGTGTAGGTAGT
GGTTCTGGTATAGATGCTGGTTATAAAAATAATGGTATAGGCACTTGTCCTGCAGGTACTAATTATTTAACTTGTCATA
ATGCTGCCCAATGTGACTGTTTGTGCACTCCAGACCCTATTACATCTAAAGCTACAGGGCCTTACAAGTGCCCCCAAAC
TAAATATTTAGTTGGCATAGGTGAGCACTGTTCAGGTCTTGCTATTAAAAGTGATCATTGTGGAGGTAATCCATGTACT
TGCCAACCACAAGCATTTCTGGGTTGGTCTGTTGACTCTTGTTTACAAGGGGATAGGTGTAATATTTTTGCTAATTTTA
TTTTGCATGGTGTTAATAGTGGTACTACTTGTTCTACTGATTTACAAAAATCAAACACAGACATAATTCTTGGTGTTTG
TGTTAATTATGATCTTTATGGTATTACTGGCCAAGGTATTTTTGTTGAGGTTAATGCGACTTATTATAATAGTTGGCAG
AACCTTTTATATGATTCTAATGGTAATCTCTATGGCTTTAGAGACTACTTAACAAACAGAACTTTCATGATTCGTAGTT
GCTATAGCGGTCGTGTTTCAGCGGCCTTTCATGCTAACTCTTCCGAACCAGCATTGCTATTTCGGAATATTAAATGCAA
CTACGTTTTTAATAATACTCTTTCAAGACAGCTGCAACCTATTAACTATTTTGATAGCTATCTTGGTTGTGTTGTCAAT
GCTGATAATAGTACTTCTAGTGTTGTTCAAACATGTGATCTCACAGTAGGTAGTGGTTACTGTGTGGATTATTCTACAA
AAAGACGAAGTCGTAGAGCGATTACCACTGGTTATCGGTTTACTAATTTTGAGCCATTTACTGTTAATTCAGTAAATGA
TAGTTTAGAACCTGTAGGTGGTTTGTATGAAATTCAAATACCTTCAGAGTTTACTATAGGTAACATGGAGGAGTTTATT
CAAACAAGCTCTCCTAAAGTTACTATTGATTGTTCTGCTTTTGTTTGTGGTGATTATGCAGCATGTAAATCACAGTTGG
TTGAATATGGTAGCTTCTGTGACAATATTAATGCTATACTCACAGAAGTAAATGAACTACTTGACACTACACAGTTGCA
AGTAGCTAATAGTTTAATGAATGGTGTCACTCTTAGCACTAAGCTTAAAGATGGCGTTAATTTCAATGTAGACGACATC
AATTTTTCCCCTGTATTAGGTTGTTTAGGAAGCGATTGTAATAGAGTTTCCAGTAGATCTGCTATAGAGGATTTACTTT
TTTCTAAAGTAAAGTTAGCTGATGTCGGTTTCGTTGAGGCTTATAATAATTGTACTGGAGGTGCCGAAATTAGGGACCT
CATTTGTGTGCAAAGTTATAATGGTATCAAGGTGTTGCCTCCACTGCTCTCAGAAAATCAGATCAGTGGATATACTTTG
GCTGCGACCTCTGCTAGTCTGTTTCCTCCTTGGTCAGCAGCAGCAGGTGTACCATTTTATTTAAATGTTCAGTATCGTA
TTAATGGGATTGGTGTTACCATGGATGTGCTAAGTCAAAATCAAAAGCTTATTTCTAATGCATTTAACAATGCCCTTGG
TGCTATTCAGGAAGGGTTTGATTCTACCAATTCTGCTTTAGTTAAAATTCAAGCTGTTGTTAATGCAAATGCTGAAGCT
CTTAATAACTTATTGCAACAACTCTCTAACAGGTTTGGTGCTATAAGTTCTTCTTTACAAGAAATTCTATCTAGACTGG
ATGCTCTTGAAGCGCAAGCTCAGATAGACAGACTTATTAATGGGCGTCTTACCGCTCTTAATGCTTATGTTTCTCAACA
GCTTAGTGACTCTACACTAGTAAAATTTAGTGCAGCACAAGCTATGGAGAAGGTTAATGAATGTGTCAAAAGCCAATCA
TCTAGGATAAATTTTTGTGGTAATGGTAATCATATTATATCATTAGTGCAGAATGCTCCATATGGTTTGTATTTTATCC
ACTTTAGCTATGTCCCTACTAAGTATGTCACTGCGAAGGTTAGTCCTGGTCTGTGCATAGCTGGTGATAGAGGTATAGC
CCCCAAGAGTGGTTATTTTGTTAATGTAAATAATACTTGGATGTTCACTGGTAGTGGTTATTACTACCCTGAACCTATA
ACTGGAAATAATGTTGTTGTTATGAGTACCTGTGCTGTTAACTACACCAAAGCACCGGATGTAATGCTGAACATTTCAA
CACCCAAACTCCCTGATTTTAAGGAAGAGTTGGATCAATGGTTTAAAAACCAAACATCAGTGGCACCAAATTTGTCACT
TGATTATATAAATGTTACATTCTTGGACCTACAAGACGAAATGAATAGGTTACAGGAGGCAATAAAAGTTTTAAATCAG
AGCTACATCAATCTCAAGGACATTGGTACATATGAGTATTATGTAAAATGGCCTTGGTATGTATGGCTTTTAATTGGCT
TTGCTGGTGTAGCTATGCTTGTTTTACTATTCTTCATATGCTGTTGTACAGGATGTGGGACTAGTTGTTTTAAGAAATG
TGGTGGTTGTTGTGATGATTATACTGGACACCAGGAGTTAGTAATTAAAACATCCCATGACGACTAA

>gi|519668491|gb|KF169913.1|_Bovine_coronavirus_isolate_DEN/031_spike_glycoprotein_(S)_gene_complete_cds

ATGTTTTTGATACTTTTAATTTCCTTACCAACGGCTTTTGCTGTTATAGGAGATTTAAAGTGTACTACAGTCTCC

ATTAATGATGTTGACACTGGTGTTCCTTCTATTAGCACTGATACTGTCGATGTTACTAATGGTTTAGGTACTTATTATG
TTTTAGATCGTGTGTATTTAAATACTACTTTGTTGCTTAATGGTTACTATCCTACTTCAGGTTCTACATATCGTAATAT
GGCACTGAAGGGAACTTTACTATTGAGCACACTATGGTTTAAACCACCTTTTCTTTCTGATTTTACTAATGGTATTTTT
GCTAAGGTCAAAAATACCAAGGTTACTAAAGATCGTGTAATGTATAGTGAGTTTCCTGCTATAACTATAGGTAGTACTT
TTGTAAATACATCTTATAGTGTGGTAGTACAACCACATACTACCAATTTGGATAATAAATTACAAGGTCTCTTAGAGAT
CTCTGTTTGCCAGTATACTATGTGCGAGTACCCAAATACGATTTGTAATCCTAATCTGGGTAATCAACGCGTAGAACTA
TGGCATTGGGATACAGGTGTTGTTTCCTGTTTATATAAGCGTAATTTCACATATGATGTGAATGCTGATTATTTGTATT
TTCATTTTTATCAAGAAGGTGGTACTTTTTATGCATATTTTACAGACACTGGTGTTGTTACTAAGTTTCTGTTTAATGT
ATATTTAGGCACTGTGCTTTCACACTATTATGTCATGCCTTTGACTTGTAATAGTGCTTTGAATTTAGAATATTGGGTT
ACACCTCTCACTTCTAAACAATATTTACTCGCTTTCAATCAAGATGGTGTTATTTTTAATGCTGTTGATTGTAAGAGTG
ATTTTATGAGTGAGATTAAGTGTAAAACACTATCTATAGCACCACATACTGGTGTTTATGAATTAAACGGTTACACTGT
TCAGCCAATTGCAGATGTTTACCGACGTATACCTAATCTTCCCGATTGTAATATAGAGGCTTGGCTTAATGACAAGTCG
GTGCCCTCTCCATTAAATTGGGAACGTAAGACCTTTTCAAATTGTAATTTTAATATGAGCAGCCTGATGTCCTTTATTC
AGGCAGACTCATTTACTTGTAATAATATTGATGCTGCTAAGATATATGGTATGTGTTTTTCCAGCATAACTATAGATAA
GTTTGCTATACCCAATGGTAGGAAGGTTGACCTACAATTGGGCAATTTGGGCTATTTGCAGTCTTTTAACTATAGAATT
GATACTACTGCTACAAGTTGTCAGTTGTATTATAATTTACCTGCTGCTAATGTTTCTGTTAGCAGGTTTAATCCTTCTA
CTTGGAATAGGAGATTTGGTTTTACAGAACAATCTGTTTTTAAGCCTCAACCTGCAGGTGTCTTTACTGATCATGATGT
TGTTTATGCACAACATTGTTTCAAAGCTCCCACAAATTTCTGTCCTTGTAAATTGGATGGGTCTTTGTGTGTAGGTAGT
GGTTCTGGTATAGATGCTGGTTATAAAAATAGTGGTATAGGCACTTGTCCTGCAGGTACTAATTATTTAACTTGTTATA
ATGCTGCCCAATGTGATTGTTTGTGCACTCCAGACCCCATTATATCTAAAGCTACAGGGCCTTACAAGTGCCCCCAAAC
TAAATATTTAGTTGGCATAGGTGAGCACTGTTCAGGTCTCGCTATTAAAAGTGATCATTGTGGAGGTAATCCTTGTACT
TGCCAACCACAAGCATTTTTGGGTTGGTCTGTTGAGTCTTGTTTACAAGGGGATAGGTGTAATATTTTTGCTAATTTTA
TTTTGCATGGTGTTAATAGTGGTACTACTTGTTCTACTGATTTACAAAAATCAAACACAGACATAATTCTTGGTGTTTG
TGTTAATTATGATCTTTATGGTATTACTGGCCAAGGTATTTTTGTTGAGGTTAATGCGACTTATTATAATAGTTGGCAG
AACCTTTTATATGATTCTAATGGTAATCTCTATGGTTTTAGAGACTACTTAACAAATAGAACTTTTATGATTCGTAGTT
GCTATAGCGGTCGTGTTTCAGCGGCCTTTCATGCTAACTCTTCCGAACCAGCATTGCTATTTCGGAATATTAAATGCAA
TTACGTTTTTAATAATACTCTTTCTCGACAGCTGCGACCTATTAACTATTTTGATAGCTATCTTGGTTGTGTTGTCAAC
GCTGATAATAGTACTTCTAGTGTTGTTCAAACATGTGATCTCACAGTAGGTAGTGGTTACTGTGTGGATTACTCTACAA
AAAGACGAAGTCGTAGAGCGATTACCACTGGTTATCGGTTTACTAATTTTGAGCCATTTACTGTTAATTCAGTAAATGA
TAGTTTAGAACCTGTAGGTGGTTTGTATGAAATTCAAATACCTTCAGAGTTTACTATAGGTAACATGGAGGAGTTTATT
CAAACAAGCTCTCCTAAAGTTACTATTGATTGTTCTGCTTTTGTCTGTGGCGATTATGCAGCATGTAAATCACAGTTGG
TTGAATATGGTAGCTTCTGTGACAATATTAATGCTATACTCACAGAAGTAAATGAACTACTTGACACTACACAGTTGCA
AGTAGCTAATAGTTTAATGAATGGTGTCACTCTTAGCACTAAGCTTAAAGATGGCGTTAATTTCAATGTAGACGACATC
AATTTTTCCCCTGTATTAGGTTGTTTAGGAAGCGATTGTAATAGAGTTTCCAGTAGATCTGCTATAGAGGATTTACTTT
TTTCTAAAGTAAAGTTAGCTGATGTCGGTTTCGTTGAGGCTTATAATAATTGTACTGGAGGTGCCGAAATTAGAGACCT
CATTTGTGTGCAAAGTTATAATGGTATCAAGGTGTTGCCTCCACTGCTCTCAGAAAATCAGATCAGTGGATATACTTTG
GCTGCGACCTCTGCTAGTCTGTTTCCTCCTTGGTCAGCAGCAGCAGGTGTACCATTTTATTTAAATGTTCAGTATCGTA
TTAATGGGATTGGTGTTACCATGGATGTGCTAAGTCAAAATCAAAAGCTTATTGCTAATGCATTTAACAATGCCCTTGG
TGCTATTCAGGAAGGGTTTGATGCTACCAATTCTGCTTTAGTTAAAATTCAAGCTGTTGTTAATGCAAATGCTGAAGCT
CTTAATAACTTATTGCAACAACTCTCTAATAGGTTTGGTGCTATAAGTTCTTCTTTACAAGAAATTCTATCTAGACTGG
ATGCTCTTGAAGCGCAAGCTCAGATAGACAGACTTATTAATGGGCGTCTTACCGCTCTTAATGCTTATGTTTCTCAACA
GCTTAGTGACTCTACACTAGTAAAATTTAGTGCAGCACAAGCTATGGAGAAGGTTAATGAATGTGTCAAAAGCCAATCA
TCTAGGATAAATTTTTGTGGTAATGGTAATCATATTATATCATTAGTGCAGAATGCTCCATATGGTTTGTATTTTATCC
ACTTTAGCTATGTCCCTACTAAGTATGTCACTGCGAAGGTTAGTCCTGGTCTGTGCATTGCTGGTGATAGAGGTATAGC

CCCTAAGAGTGGTTATTTTGTTAATGTAAATAATACTTGGATGTTCACTGGTAGTGGTTATTACTACCCTGAACCTATA
ACTGGAAATAATGTTGTTGTTATGAGTACCTGTGCTGTTAATTACACTAAAGCACCGGATGTAATGCTGAACATTTCAA
CACCCAACCTCCCTGATTTTAAGGAAGAGTTGGATCAATGGTTTAAAAACCAAACATCAGTGGCACCAAATTTGTCACT
TGATTATATAAATGTTACATTCTTGGACCTACAAGACGAAATGAATAGGTTACAGGAGGCAATAAAAGTTTTAAATCAG
AGCTACATCAATCTCAAGGACATTGGTACATATGAGTATTATGTAAAATGGCCTTGGTATATATGGCTTTTAATTGGCT
TTGCTGGTGTAGCTATGCTTGTTTTACTATTCTTCATATGCTGTTGTACAGGATGTGGGACTAGTTGTTTTAAGAAATG
TGGTGGTTGTTGTGAAGATTATATTGGATACCAGGAGTTAGTAATTAAAACATCACATGACGACTAA

>gi|519668493|gb|KF169914.1|_Bovine_coronavirus_isolate_DEN/032_spike_glycoprotein_(S)_gene_complete_cds

ATGTTTTTGATACTTTTAATTTCCTTACCAACGGCTTTTGCTGTTATAGGAGATTTAAAGTGTACTTCAGTTTCC
ATTAATGATGTTGACACTGGTGTTCCTTCTATTAGCACTGATACTGTCGATGTTACTAATGGTTTAGGTACTTATTATG
TTTTAGATCGTGTGTATTTAAATACTACTTTGTTGCTTAATGGTTACTATCCTACTTCAGGTTCTACATATCGTAATAT
GGCACTGAAGGGAACTTTACTATTGAGCACACTATGGTTTAAACCACCTTTTCTTTCTGATTTTACTAATGGTATTTTT
GCTAAGGTCAAAAATACCAAGGTTACTAAAGATGGTGTAATGTATAGTGAGTTTCCTGCTATAACTATAGGTAGTACTT
TTGTAAATACATCCTATAGTGTGGTAGTACAACCATATACTACCAATTTTGATAATAAAATACAAGGTCTCTTAGAGGT
CTCTGTTTGCCAGTATACTATGTGTGAGTACCCAAACACGATTTGTAATCCTAATCTGGGTAATCAACGCGTAGAACTA
TGGCATTGGGATACAGGTGTTGTTTCCTGTTTATATAAGCGTAATTTCACATATGATGTGAATGCTGATTATTTGTATT
TCCATTTTTATCAAGAAGGTGGTACTTTTTATGCATATTTTACAGACACTGGTGTTGTTACTAAGTTTCTGTTTAATGT
ATATTTAGGCACGGTGCTTTCACACTATTATGTCATGCCTTTGACTTGTAATAGTGCTTTGAATTTAGAATATTGGGTT
ACACCTCTCACTTCTAAACAATATTTACTCGCTTTCAATCAAGATGGTGTTATTTTTAATGCTGTTGATTGTAAGAGTG
ATTTTATGAGTGAGATTAAGTGTAAAACATTATCTATAGCACCATCTACTGGTGTTTATGAATTAAACGGTTACACTGT
TCAGCCAATTGCAGATGTTTACCGACGTATACCTAATCTTCCCGATTGTAATATAGAGGCTTGGCTTAATGACAAGTCG
GTGCCCTCTCCATTAAATTGGGAACGTAACACCTTTTCAAATTGTAATTTTAATATGAGCAGCCTGATGTCTTTTATTC
AGGCAGACTCATTTACTTGTAATAATATTGATGCTGCTAAGATATATGGTATGTGTTTTTCCAGCATAACTATAGATAA
GTTTGCTATACCCAATGGTAGGAAGGTTGACCTACAATTGGGCAATTTGGGCTATTTGCAGTCTTTTAACTATAGAATT
GATACTACTGCTACAAGTTGTCAGTTGTATTATAATTTACCTGCTGCTAATGTTTCTGTTAGCAGGTTTAATCCTTCTA
CTTGGAATAGGAGATTTGGTTTTACAGAACAATCTGTTTTTAAGCCTCAACCTGCAGGTGTCTTTACTGATCATGATGT
TGTTTATGCACAACATTGTTTCAAAGCTCCCACAAATTTCTGTCCGTGTAAATTGGATGGGTCTTTGTGTGTAGGTAGT
GGTTCTGGTATAGATGCTGGTTATAAAAATAGTGGTATAGGCACTTGTCCTGCAGGTACTAATTATTTAACTTGTCATA
ATGCTGCCCAATGTGACTGTTTGTGCACTCCAGACCCCATTACATCTAAAGCTACAGGGCCTTACAAGTGCCCCCAAAC
TAAATATTTAGTTGGCATAGGTGAGCACTGTTCAGGTCTTGCTATTAAAAGTGATCATTGTGGAGGTAATCCATGTACT
TGCCAACCACAAGCATTTTTGGGTTGGTCTGTTGACTCTTGTTTACAAGGGGATAGGTGTAATATTTTTGCTAATTTTA
TTTTGCATGGTGTTAATAGTGGTACTACTTGTTCTACTGATTTACAAAAATCAAACACAGACATAATTCTTGGTGTTTG
TGTTAATTATGATCTTTATGGTATTACTGGCCAAGGTATTTTTGTTGAGGTTAATGCGACTTATTATAATAGTTGGCAG
AACCTTTTATATGATTCTAATGGTAATCTCTATGGTTTTAGAGACTACTTAACAAACAGAACTTTCATGATTCGTAGTT
GCTATAGCGGTCGTGTTTCAGCGGCCTTTCATGCTAACTCTTCCGAACCAGCATTGCTATTTCGGAATATTAAATGCAA
CTACGTTTTTAATAATACTCTTTCACGACAGCTGCAACCTATTAACTATTTTGATAGCTATCTTGGTTGTGTTGTCAAT
GCTGATAATAGTACTTCTAGTGCTGTTCAAACATGTGATCTCACAGTAGGTAGTGGTTACTGTGTGGATTACTCTACAA
AAAGACGAAGTCGTAGAGCGATTACCACTGGTTATCGGTTTACTAATTTTGAGCCATTTACTGTTAAGTCAGTAAATGA
TAGTTTAGAACCTGTAGGTGGTTTGTATGAAATTCAAATACCTTCAGATTTTACTATAGGTAACATGGAGGAGTTTATT
CAAACAAGCTCTCCTAAAGTTACTATTGATTGTTCTACTTTTGTCTGTGGTGATTATGCAGCATGTAAATCACAGTTGG

TTGAATATGGTAGCTTTTGTGACAATATTAATGCTATACTCACAGAAGTAAATGAACTACTTGACACTACACAGTTGCA
AGTAGCTAATAGTTTAATGAATGGTGTCACTCTTAGCACTAAGCTTAAAGATGGCGTTAAGTTCAATGTAGACGACATC
AATTTTTCCCCTGTATTAGGTTGTTTAGGAAGCGATTGTAATAGAGTTTCCAGTAGATCTGCTATAGAGGATTTACTTT
TTTCTAAAGTAAAGTTAGCTGATGTCGGTTTCGTTGAGGCTTATAATAATTGTACTGGAGGTGCCGAAATTAGGGACCT
CATTTGTGTGCAAAGTTATAATGGTATCAAGGTTTTGCCTCCACTGCTCTCAGAAAATCAGATCAGTGGATATACTTTG
GCTGCGACCTCTGCTAGTTTGTTTCCTCCTTGGTCAGCAGCAGCAGGTGTACCATTTTATTTAAATGTTCAGTATCGTA
TTAATGGGATTGGTGTTACCATGGATGTGCTAAGTCAAAATCAAAAGCTTATTGCTAATGCATTTAACAATGCCCTTGG
TGCTATTCAGGAAGGGTTTGATGCTACCAATTCTGCTTTAGTTAAAATTCAAGCTGTTGTTAATGCAAATGCTGAAGCT
CTTAATAACTTATTGCAACAACTCTCTAACAGGTTTGGTGCTATAAGTTCTTCTTTACAAGAAATTCTATCTAGACTGG
ATGCTCTTGAAGCGCAAGCTCAGATAGACAGACTTATTAATGGGCGTCTTACCGCTCTTAATGCTTATGTTTCTCAACA
GCTTAGTGACTCTACACTAGTAAAATTTAGTGCAGCACAAGCTATGGAGAAGGTTAATGAATGTGTCAAAAGCCAATCA
TCTAGGATAAATTTTTGTGGTAATGGTAATCATATTATATCATTAGTGCAGAATGCTCCATATGGTTTGTATTTTATCC
ACTTTAGCTATGTCCCTACTAAGTATGTCACTGCGAAGGTTAGTCCTGGTCTGTGCATTGCTGGTGATAGAGGTATAGC
CCCCAAGAGTGGTTATTTTGTTAATGTAAATAATACTTGGATGTTCACTGGTAGTGGTTATTACTACCCTGAACCTATA
ACTGGAAATAATGTTGTTGTTATGAGTACCTGTGCTGTTAATTACACTAAAGCACCGGATGTAATGCTGAACATTTCAA
CACCCAACCTCCCTGATTTTAAGGAAGAGTTGGATCAATGGTTTAAAAACCAAACATCAGTGGCACCAAATTTGTCACT
TGATTATATAAATGTTACATTCTTGGACCTACAAGATGAAATGAATAGGTTACAGGAGGCAATAAAAGTTTTAAATCAG
AGCTACATCAATCTCAAGGACATTGGTACATATGAGTATTATGTAAAATGGCCTTGGTATGTATGGCTTTTAATTGGCT
TTGCTGGTGTAGCTATGCTTGTTTTACTATTCTTCATATGCTGTTGTACAGGATGTGGGACTAGTTGTTTTAAGAAATG
TGGTGGTTGTTGTGATGATTATACTGGACACCAGGAGTTAGTAATTAAAACATCCCATGACGACTAA

>gi|519668495|gb|KF169915.1|_Bovine_coronavirus_isolate_DEN/033_spike_glycoprotein_(S)_gene_complete_cds

ATGTTTTTGATACTTTTAATTTCCTTACCAACGGCTTTTGCTGTTATAGGAGATTTAAAGTGTACTACAGTCTCC
ATTAATGATGTTGACACTGGTGTTCCTTCTATTAGCACTGATACTGTCGATGTTACTAATGGTTTAGGTACTTATTATG
TTTTAGATCGTGTGTATTTAAATACTACTTTGTTGCTTAATGGTTACTATCCTACTTCAGGTTCTACATATCGTAATAT
GGCACTGAAGGGAACTTTACTATTGAGCACACTATGGTTTAAACCACCTTTTCTTTCTGATTTTACTAATGGTATTTTT
GCTAAGGTCAAAAATACCAAGATTACTAAAGATCGTGTAATGTATAGTGAGTTTCCTGCTATAACTATAGGTAGTACTT
TTGTAAATACATCTTATAGTGTGGTAGTACAACCACATACTACCAATTTGGATAATAAATTACAAGGTCTCTTAGAGAT
CTCTGTTTGCCAGTATACTATGTGCGAGTACCCAAATACGATTTGTAATCCTAATCTGGGTAATCAACGCGTAGAACTA
TGGCATTGGGATACAGGTGTTGTTTCCTGTTTATATAAGCGTAATTTCACATATGATGTGAATGCTGATTATTTGTATT
TTCATTTTTATCAAGAAGGTGGTACTTTTTATGCATATTTTACAGACACTGGTGTTGTTACTAAGTTTCTGTTTAATGT
ATATTTAGGCACTGTGCTTTCACACTATTATGTCATGCCTTTGACTTGTAATAGTGCTTTGAATTTAGAATATTGGGTT
ACACCTCTCACTTCTAAACAATATTTACTCGCTTTCAATCAAGATGGTGTTATTTTTAATGCTGTTGATTGTAAGAGTG
ATTTTATGAGTGAGATTAAGTGTAAAACACTATCTATAGCACCATCTACTGGTGTTTATGAATTAAACGGTTACACTGT
TCAGCCAATTGCAGATGTTTACCGACGTATACCTAATCTTCCCGATTGTAATATAGAGGCTTGGCTTAATGACAAGTCG
GTGCCCTCTCCATTAAATTGGGAACGTAAGACCTTTTCAAATTGTAATTTTAATATGAGCAGCCTGATGTCCTTTATTC
AGGCAGACTCATTTACTTGTAATAATATTGATGCTGCTAAGATATATGGTATGTGTTTTTCCAGCATAACTATAGATAA
GTTTGCTATACCCAATGGTAGGAAGGTTGACCTACAATTGGGCAATTTGGGCTATTTGCAGTCTTTTAACTATAGAATT
GATACTACTGCTACAAGTTGTCAGTTGTATTATAATTTACCTGCTGCTAATGTTTCTGTTAGCAGGTTTAATCCTTCTA
CTTGGAATAGGAGATTTGGTTTTACAGAACAATCTGTTTTTAAGCCTCAACCTGCAGGTGTCTTTACTGATCATGATGT
TGTTTATGCACAACATTGTTTCAAAGCTCCCACAAATTTCTGTCCGTGTAAATTGGATGGGTCTTTGTGTGTAGGTAGT

GGTTCTGGTATAGATGCTGGTTATAAAAATAGTGGTATAGGCACTTGTCCTGCAGGTACTAATTATTTAACTTGTCATA
ATGCTGCCCAATGTGATTGTTTGTGCACTCCAGACCCCATTACATCTAAAGCTACAGGGCCTTACAAGTGCCCCCAAAC
TAAATATTTAGTTGGCATAGGTGAGCACTGTTCAGGTCTTGCTATTAAAAGTGATCATTGTGGAGGTAATCCTTGTACT
TGCCAACCACAAGCATTTTTGGGTTGGTCTGTTGAGTCTTGTTTACAAGGGGATAGGTGTAATATTTTTGCTAATTTTA
TTTTGCATGGTGTTAATAGTGGTACTACTTGTTCTACTGATTTACAAAAATCAAACACAGACATAATTCTTGGTGTTTG
TGTTAATTATGATCTTTATGGTATTACTGGCCAAGGTATTTTTGTTGAGGTTAATGCGACTTATTATAATAGTTGGCAG
AACCTTTTATATGATTCTAATGGTAATCTCTATGGTTTTAGAGACTACTTAACAAATAGAACTTTTATGATTCGTAGTT
GCTATAGCGGTCGTGTTTCAGCGGCCTTTCATGCTAACTCTTCCGAACCAGCATTGCTATTTCGGAATATTAAATGCAA
TTACGTTTTTAATAATACTCTTTCACGACAGCTGCGACCTATTAACTATTTTGATAGCTATCTTGGTTGTGTTGTCAAT
GCTGATAATAGTACTTCTAGTGTTGTTCAAACATGTGATCTCACAGTAGGTAGTGGTTACTGTGTGGATTACTCTACAA
AAAGACGAAGTCGTAGAGCGATTACCACTGGTTATCGGTTTACTAATTTTGAGCCATTTACTGTTAATTCAGTAAATGA
TAGTTTAGAACCTGTAGGTGGTTTGTATGAAATTCAAATACCTTCAGAGTTTACTATAGGTAACATGGAGGAGTTTATT
CAAACAAGCTCTCCTAAAGTTACTATTGATTGTTCTGCTTTTGTCTGTGGTGATTATGCAGCATGTAAATCACAGTTGG
TTGAATATGGTAGCTTCTGTGACAATATTAATGCTATACTCACAGAAGTAAATGAACTACTTGACACTACACAGTTGCA
AGTAGCTAATAGTTTAATGAATGGTGTCACTCTTAGCACTAAGCTTAAAGATGGCGTTTATTTCAATGTAGACGACATC
AATTTTTCCCCTGTATTAGGTTGTTTAGGAAGCGATTGTAATAGAGTTTCCAGTAGATCTGCTATAGAGGATTTACTTT
TTTCTAAAGTAAAGTTAGCTGATGTCGGTTTCGTTGAGGCTTATAATAATTGTACTGGAGGTGCCGAAATTAGAGACCT
CATTTGTGTGCAAAGTTATAATGGTATCAAGGTGTTGCCTCCACTGCTCTCAGAAAATCAGATCAGTGGATATACTTTG
GCTGCGACCTCTGCTAGTCTGTTTCCTCCTTGGTCAGCAGCAGCAGGTGTACCATTTTATTTAAATGTTCAGTATCGTA
TTAATGGGATTGGTGTTACCATGGATGTGCTAAGTCAAAATCAAAAGCTTATTGCTAATGCATTTAACAATGCCCTTGG
TGCTATTCAGGAAGGGTTTGATGCTACCAATTCTGCTTTAGTTAAAATTCAAGCTGTTGTTAATGCAAATGCTGAAGCT
CTTAATAACTTATTGCAACAACTCTCTAATAGGTTTGGTGCTATAAGTTCTTCTTTACAAGAAATTCTATCTAGACTGG
ATGCTCTTGAAGCGCAAGCTCAGATAGACAGACTTATTAATGGGCGTCTTACCGCTCTTAATGCTTATGTTTCTCAACA
GCTTAGTGACTCTACACTAGTAAAATTTAGTGCAGCACAAGCTATGGAGAAGGTTAATGAATGTGTCAAAAGCCAATCA
TCTAGGATAAATTTTTGTGGTAATGGTAATCATATTATATCAGTAGTGCAGAATGCTCCATATGGTTTGTATTTTATCC
ACTTTAGCTATGTCCCTACTAAGTATGTCACTGCGAAGGTTAGTCCTGGTCTGTGCATTGCTGGTGATAGAGGTATAGC
CCCTAAGAGTGGTTATTTTGTTAATGTAAATAATACTTGGATGTTCACTGGTAGTGGTTATTACTACCCTGAACCTATA
ACTGGAAATAATGTTGTTGTTATGAGTACCTGTGCTGTTAATTACACTAAAGCACCGGATGTAATGCTGAACATTTCAA
CACCCAACCTCCCTGATTTTAAGGAAGAGTTGGATCAATGGTTTAAAAACCAAACATCAGTGGCACCAAATTTGTCACT
TGATTATATAAATGTTACATTCTTGGACCTACAAGACGAAATGAATAGGTTACAGGAGGCAATAAAAGTTTTAAATCAG
AGCTACATCAATCTCAAGGACATTGGTACATATGAATATTATGTAAAATGGCCTTGGTATGTATGGCTTTTAATTGGCT
TTGCTGGTGTAGCTATGCTTGTTTTACTATTCTTCATATGCTGTTGTACAGGATGTGGGACTAGTTGTTTTAAGAAATG
TGGTGGTTGTTGTGATGATTATACTGGACACCAGGAGTTAGTAATTAAAACATCCCATGACGACTAA

>gi|519668499|gb|KF169917.1|_Bovine_coronavirus_isolate_DEN/052_spike_glycoprotein_(S)_gene_complete_cds

ATGTTTTTGATACTTTTAATTTCCTTACCAATGGCTTTTGCTGTTATAGGAGATTTAAAGTGTACTACAGTCTCC
ATTAATGATGTTGACACTGGTGTTCCTTCTATTAGCACTGATACTGTCGATGTTACTAATGGTTTAGGTACTTATTATG
TCTTAGATCGTGTTTATTTAAATACTACTTTGTTGCTTAATGGTTACTATCCTACTTCAGGTTCTACATATCGTAATAT
GGCACTGAAGGGAACTTTACTATTGAGCACACTATGGTTTAAACCACCTTTTCTTTCTCATTTTACTAATGGTATTTTT
GCTAAGGTCAAAAATACCAAGGTTACTAAAGATGGTGTAATGTATAGTGAGTTTCCTGCTATAACTATAGGTACTACTT
TTGTAAATACATCTTATAGTGTGGTAGTACAACCACATACTACCAATTTGGATAATAAATTACAAGGTCTCTTAGAGAT

CTCTGTTTGCCAGTATACTATGTGCGAGTACCCAAATACGATTTGTAATCCTAATCTGGGTAATAAACGCGTAGAACTA
TGGCATTGGGATACAGGTGTTGTTTCCTGTTTATATAAGCGTAATTTCACATATGATGTGAATGCTGATTATTTGTATT
TTCATTTTTATCAAAAAGGTGGTACTTTTTATGCATATTTTACAGACACTGGTGTTGTTACTAAGTTTCTGTTTAATGT
ATATTTAGGCACGGTGCTTTCACACTATTATGTCATGCCTTTGACTTGTAATAGTGCTTTGAATTTAGAATATTGGGTT
ACACCTCTCACTTCTAAACAATATTTACTCGCTTTCAATCAAGATGGTGTTATTTTTAATGCTGTTGATTGTAAGAGTG
ATTTTATGAGTGAGATTAAGTGTAAAACATTATCTATAGCACCAGCTACTGGTGTTTATGAATTAAACGGTTACACTGT
TCAGCCAATTGCAGATGTTTACCGACGTATACCTAATCTTCCCGATTGTAATATAGAGGCTTGGCTTAATGACAAATCG
GTGCCCTCTCCATTAAATTGGGAACGTAAGACCTTTTCAAATTGTAATTTTAATATGAGCAGCCTGATGTCTTTTATTC
AGGCAGACTCATTTACTTGTAATAATATTGATGCTGCTAAGATATATGGTATGTGTTTTTCCAGCATAACTATAGATAA
GTTTGCTATACCCAATGGTAGGAAGGTTGACCTACAATTGGGCAATTTGGGCTATTTGCAGTCTTTTAATTATAGAATT
GATACTACTGCTACAAGTTGTCAGTTGTATTATAATTTACCTGCTGCTAATGTTTCTGTTAGCAGGTTTAATCCTTCTA
CTTGGAATAGGAGATTTGGTTTTATAGAACAATCTGTTTTTAAGCCTCAACCTGCAGGTGTCTTTACTGATCATGATGT
TGTTTTTGCACAACACTGTTTCAAAGCTCCCACAAATTTCTGTCCGTGTAAACTGGATGGGTCTTTGTGTGTAGGTAGT
GGTTTTGGTATAGATGCTGGTTATAAAAATAGTGGTATAGGCACTTGTCCTGCAGGTACTAATTATTTAACTTGTCATA
ATGCTGCCCAATGTGATTGTTTGTGCACTCCAGACCCCATTACATCTAAAGCTACAGGGCCTTACAAGTGCCCCCAAAC
TGAATATTTAGTTGGCATAGGTGAGCACTGTTCAGGTCTTGCTATTAAAAGTGATCATTGTGGAGGTAATCCTTGTACT
TGCCAACCACAAGCATTTTTGGGTTGGTCTGTTGAGTCTTGTTTACAAGGGGATAGGTGTAATATTTTTGCTAATTTTA
TTTTGCATGGTGTTAATAGTGGTACTACTTGTTCTACTGATTTACAAAAATCAAACACAGACATAATTCTTGGTGTTTG
TGTTAATTATGATCTTTATGGTATTACTGGCCAAGGTATTTTTGTTGAGGTTAATGCGACTTATTATAATAGTTGGCAG
AACCTTTTATATGATTCTAATGGTAATCTCTATGGTTTTAGAGACTACTTAACAAATAGAACTTTTATGATTCGTAGTT
GCTATAGCGGTCGTGTTTCAGCGGCCTTTCATGCTAACTCTTCCGAACCAGCATTGCTATTTCGGAATATTAAATGCAA
TTACGTTTTTAATAATACTCTTTTACGACAGCTGCAACCTATTAACTATTTTGATAGCTATCTTGGTTGTGTTGTCAAT
GCTGATAATAGTACTTCTAGTGTTGTTCAAACATGTGATCTCACAGTAGGTAGTGGTTACTGTGTGGATTACTCTACAA
AAAGACGAAGTCGTAGAGCGATTACCACTGGTTATCGGTTTACTAATTTTGAGCCATTTACTGTTAATTCAGTAAATGA
TAGTTTAGAACCTGTAGGTGGTTTGTATGAAATTCAAATACCTTCAGAGTTTACTATAGGTAACATGGAGGAGTTTATT
CAAACAAGCTCTCCTAAAGTTACTATTGATTGTTCTGCTTTTGTCTGTGGTGATTATGCAGCATGTAAATCACAGTTGG
TTGAATATGGTAGCTTCTGTGACAATATTAATGCTATACTCACAGAAGTAAATGAACTACTTGACACTACACAGTTGCA
AGTAGCTAATAGTTTAATGAATGGTGTCACTCTTAGCACTAAGCTTAAAGATGGCGTTAATTTCAATGTAGACGACATC
AATTTTTCCCCTGTATTAGGTTGTTTAGGAAGCGATTGTTATAAAGTTTCCAGTAGATCTGCTATAGAGGATTTACTTT
TTTCTAAAGTAAAGTTAGCTGATGTCGGTTTCGTTGAGGCTTATAATAATTGTACTGGAGGTGCCGAAATTAGAGACCT
CATTTGTGTGCAAAGTTATAATGGTATCAAGGTGTTGCCTCCACTGCTCTCAGAAAATCAGATCAGTGGATATACTTTG
GCTGCGACCTCTGCTAGTCTGTTTCCTCCTTGGTCAGCAGCAGCAGGTGTACCATTTTATTTAAATGTTCAGTATCGTA
TTAATGGGATTGGTGTAACCATGGATGTGCTAAGTCAAAATCAAAAGCTTATCGCTAATGCATTTAACAATGCCCTTGG
TGCTATTCAGGAAGGGTTTGATTCTACCAATTCTGCTTTAGTTAAAATTCAAGCTGTTGTTAATGCAAATGCTGAAGCT
CTTAATAACTTATTGCAACAACTCTCTAATAGGTTTGGTGCTATAAGTTCTTCTTTACAAGAAATTCTATCTAGACTGG
ATGCTCTTGAAGCGCAAGCTCAGATAGACAGACTTATTAATGGGCGTCTTACCGCTCTTAATGCTTATGTTTCTCAACA
GCTTAGTGACTCTACACTAGTAAAATTTAGTGCAGCACAAGCTATGGAGAAGGTTAATGAATGTGTTAAAAGCCAATCA
TCTAGGATAAATTTTTGTGGTAATGGTAATCATATTATATCATTAGTGCAGAATGCTCCATATGGTTTGTATTTTATCC
ACTTTAGCTATGTCCCTACTAAGTATGTCACTGCGAAGGTTAGTCCTGGTCTGTGCATTGCTGGTGATAGAGGTATAGC
CCCTAAGAGTGGTTATTTTGTTAATGTAAATAATACTTGGATGTTCACTGGTAGTGGTTATTACTACCCTGAACCTATA
ACTGGAAATAATGTTGTTGTTATGAGTACCTGTGCTGTTAATTACACTAAAGCACCGGATGTAATGCTGAACACTTCAA
CACCCAACCTCCCTGATTTTAAGGAAGAGTTGGATCAATGGTTTAAAAACCAAACATCAGTGGCACCAAATTTGTCACT
TGATTATATAAATGTTACATTCTTGGACCTACAAGACGAAATGAATAGGTTACAGGAGGCAATAAAAGTTTTAAATCAG
AGCTACATCAATCTCAAGGACATTGGTACATATGAGTATTATGTAAAATGGCCTTGGTATGTATGGCTTTTAATTGGCT

TTGCTGGTGTAGCTATGCTTGTTTTACTATTCTTCATATGCTGTTGTACAGGATGTGGGACTAGTTGTTTTAAGAAATG
TGGTGGTTGTTGTGATGATTATACTGGACACCAGGAGTTAGTAATTAAAACATCCCATGACGACTAA

>gi|519668501|gb|KF169918.1|_Bovine_coronavirus_isolate_DEN/053_spike_glycoprotein_(S)_gene_complete_cds

ATGTTTTTGATACTTTTAATTTCCTTACCAATGGCTTTTGCTGTTATAGGAGATTTAAAGTGTACTACAGTCTCC
ATTAATGATGTTGACACTGGTGTTCCTTCTATTAGCACTGATACTGTCGATGTTACTAATGGTTTAGGTACTTATTATG
TCTTAGATCGTGTTTATTTAAATACTACTTTGTTGCTTAATGGTTACTATCCTACTTCAGGTTCTACATATCGTAATAT
GGCACTGAAGGGAACTTTACTATTGAGCACACTATGGTTTAAACCACCTTTTCTTTCTCATTTTACTAATGGTATTTTT
GCTAAGGTCAAAAATACCAAGGTTACTAAAGATGGTGTAATGTATAGTGAGTTTCCTGCTATAACTATAGGTACTACTT
TTGTAAATACATCTTATAGTGTGGTAGTACAACCACATACTACCAATTTGGATAATAAATTACAAGGTCTCTTAGAGAT
CTCTGTTTGCCAGTATACTATGTGCGAGTACCCAAATACGATTTGTAATCCTAATCTGGGTAATAAACGCGTAGAACTA
TGGCATTGGGATACAGGTGTTGTTTCCTGTTTATATAAGCGTAATTTCACATATGATGTGAATGCTGATTATTTGTATT
TTCATTTTTATCAAAAAGGTGGTACTTTTTATGCATATTTTACAGACACTGGTGTTGTTACTAAGTTTCTGTTTAATGT
ATATTTAGGCACGGTGCTTTCACACTATTATGTCATGCCTTTGACTTGTAATAGTGCTTTGAATTTAGAATATTGGGTT
ACACCTCTCACTTCTAAACAATATTTACTCGCTTTCAATCAAGATGGTGTTATTTTTAATGCTGTTGATTGTAAGAGTG
ATTTTATGAGTGAGATTAAGTGTAAAACATTATCTATAGCACCAGCTACTGGTGTTTATGAATTAAACGGTTACACTGT
TCAGCCAATTGCAGATGTTTACCGACGTATACCTAATCTTCCCGATTGTAATATAGAGGCTTGGCTTAATGACAAATCG
GTGCCCTCTCCATTAAATTGGGAACGTAAGACCTTTTCAAATTGTAATTTTAATATGAGCAGCCTGATGTCTTTTATTC
AGGCAGACTCATTTACTTGTAATAATATTGATGCTGCTAAGATATATGGTATGTGTTTTTCCAGCATAACTATAGATAA
GTTTGCTATACCCAATGGTAGGAAGGTTGACCTACAATTGGGCAATTTGGGCTATTTGCAGTCTTTTAATTATAGAATT
GATACTACTGCTACAAGTTGTCAGTTGTATTATAATTTACCTGCTGCTAATGTTTCTGTTAGCAGGTTTAATCCTTCTA
CTTGGAATAGGAGATTTGGTTTTATAGAACAATCTGTTTTTAAGCCTCAACCTGCAGGTGTCTTTACTGATCATGATGT
TGTTTTTGCACAACACTGTTTCAAAGCTCCCACAAATTTCTGTCCGTGTAAACTGGATGGGTCTTTGTGTGTAGGTAGT
GGTTTTGGTATAGATGCTGGTTATAAAAATAGTGGTATAGGCACTTGTCCTGCAGGTACTAATTATTTAACTTGTCATA
ATGCTGCCCAATGTGATTGTTTGTGCACTCCAGACCCCATTACATCTAAAGCTACAGGGCCTTACAAGTGCCCCCAAAC
TGAATATTTAGTTGGCATAGGTGAGCACTGTTCAGGTCTTGCTATTAAAAGTGATCATTGTGGAGGTAATCCTTGTACT
TGCCAACCACAAGCATTTTTGGGTTGGTCTGTTGAGTCTTGTTTACAAGGGGATAGGTGTAATATTTTTGCTAATTTTA
TTTTGCATGGTGTTAATAGTGGTACTACTTGTTCTACTGATTTACAAAAATCAAACACAGACATAATTCTTGGTGTTTG
TGTTAATTATGATCTTTATGGTATTACTGGCCAAGGTATTTTTGTTGAGGTTAATGCGACTTATTATAATAGTTGGCAG
AACCTTTTATATGATTCTAATGGTAATCTCTATGGTTTTAGAGACTACTTAACAAATAGAACTTTTATGATTCGTAGTT
GCTATAGCGGTCGTGTTTCAGCGGCCTTTCATGCTAACTCTTCCGAACCAGCATTGCTATTTCGGAATATTAAATGCAA
TTACGTTTTTAATAATACTCTTTTACGACAGCTGCAACCTATTAACTATTTTGATAGCTATCTTGGTTGTGTTGTCAAT
GCTGATAATAGTACTTCTAGTGTTGTTCAAACATGTGATCTCACAGTAGGTAGTGGTTACTGTGTGGATTACTCTACAA
AAAGACGAAGTCGTAGAGCGATTACCACTGGTTATCGGTTTACTAATTTTGAGCCATTTACTGTTAATTCAGTAAATGA
TAGTTTAGAACCTGTAGGTGGTTTGTATGAAATTCAAATACCTTCAGAGTTTACTATAGGTAACATGGAGGAGTTTATT
CAAACAAGCTCTCCTAAAGTTACTATTGATTGTTCTGCTTTTGTCTGTGGTGATTATGCAGCATGTAAATCACAGTTGG
TTGAATATGGTAGCTTCTGTGACAATATTAATGCTATACTCACAGAAGTAAATGAACTACTTGACACTACACAGTTGCA
AGTAGCTAATAGTTTAATGAATGGTGTCACTCTTAGCACTAAGCTTAAAGATGGCGTTAATTTCAATGTAGACGACATC
AATTTTTCCCCTGTATTAGGTTGTTTAGGAAGCGATTGTAATAGAGTTTCCAGTAGATCTGCTATAGAGGATTTACTTT
TTTCTAAAGTAAAGTTAGCTGATGTCGGTTTCGTTGAGGCTTATAATAATTGTACTGGAGGTGCCGAAATTAGAGACCT
CATTTGTGTGCAAAGTTATAATGGTATCAAGGTGTTGCCTCCACTGCTCTCAGAAAATCAGATCAGTGGATATACTTTG

GCTGCGACCTCTGCTAGTCTGTTTCCTCCTTGGTCAGCAGCAGCAGGTGTACCATTTTATTTAAATGTTCAGTATCGTA
TTAATGGGATTGGTGTAACCATGGATGTGCTAAGTCAAAATCAAAAGCTTATCGCTAATGCATTTAACAATGCCCTTGG
TGCTATTCAGGAAGGGTTTGATTCTACCAATTCTGCTTTAGTTAAAATTCAAGCTGTTGTTAATGCAAATGCTGAAGCT
CTTAATAACTTATTGCAACAACTCTCTAATAGGTTTGGTGCTATAAGTTCTTCTTTACAAGAAATTCTATCTAGACTGG
ATGCTCTTGAAGCGCAAGCTCAGATAGACAGACTTATTAATGGGCGTCTTACCGCTCTTAATGCTTATGTTTCTCAACA
GCTTAGTGACTCTACACTAGTAAAATTTAGTGCAGCACAAGCTATGGAGAAGGTTAATGAATGTGTTAAAAGCCAATCA
TCTAGGATAAATTTTTGTGGTAATGGTAATCATATTATATCATTAGTGCAGAATGCTCCATATGGTTTGTATTTTATCC
ACTTTAGCTATGTCCCTACTAAGTATGTCACTGCGAAGGTTAGTCCTGGTCTGTGCATTGCTGGTGATAGAGGTATAGC
CCCTAAGAGTGGTTATTTTGTTAATGTAAATAATACTTGGATGTTCACTGGTAGTGGTTATTACTACCCTGAACCTATA
ACTGGAAATAATGTTGTTGTTATGAGTACCTGTGCTGTTAATTACACTAAAGCACCGGATGTAATGCTGAACACTTCAA
CACCCAACCTCCCTGATTTTAAGGAAGAGTTGGATCAATGGTTTAAAAACCAAACATCAGTGGCACCAAATTTGTCACT
TGATTATATAAATGTTACATTCTTGGACCTACAAGACGAAATGAATAGGTTACAGGAGGCAATAAAAGTTTTAAATCAG
AGCTACATCAATCTCAAGGACATTGGTACATATGAGTATTATGTAAAATGGCCTTGGTATGTATGGCTTTTAATTGGCT
TTGCTGGTGTAGCTATGCTTGTTTTACTATTCTTCATATGCTGTTGTACAGGATGTGGGACTAGTTGTTTTAAGAAATG
TGGTGGTTGTTGTGATGATTATACTGGACACCAGGAGTTAGTAATTAAAACATCACATGACGACTAA

>gi|519668503|gb|KF169919.1|_Bovine_coronavirus_isolate_DEN/054_spike_glycoprotein_(S)_gene_complete_cds

ATGTTTTTGATACTTTTAATTTCCTTACCAATGGCTTTTGCTGTTATAGGAGATTTAAAGTGTACTACAGTCTCC
ATTAATGATGTTGACACTGGTGTTCCTTCTATTAGCACTGATACTGTCGATGTTACTAATGGTTTAGGTACTTATTATG
TCTTAGATCGTGTTTATTTAAATACTACTTTGTTGCTTAATGGTTACTATCCTACTTCAGGTTCTACATATCGTAATAT
GGCACTGAAGGGAACTTTACTATTGAGCACACTATGGTTTAAACCACCTTTTCTTTCTCATTTTACTAATGGTATTTTT
GCTAAGGTCAAAAATACCAAGGTTACTAAAGATGGTGTAATGTATAGTGAGTTTCCTGCTATAACTATAGGTACTACTT
TTGTAAATACATCTTATAGTGTGGTAGTACAACCACATACTACCAATTTGGATAATAAATTACAAGGTCTCTTAGAGAT
CTCTGTTTGCCAGTATACTATGTGCGAGTACCCAAATACGATTTGTAATCCTAATCTGGGTAATAAACGCGTAGAACTA
TGGCATTGGGATACAGGTGTTGTTTCCTGTTTATATAAGCGTAATTTCACATATGATGTGAATGCTGATTATTTGTATT
TTCATTTTTATCAAAAAGGTGGTACTTTTTATGCATATTTTACAGACACTGGTGTTGTTACTAAGTTTCTGTTTAATGT
ATATTTAGGCACGGTGCTTTCACACTATTATGTCATGCCTTTGACTTGTAATAGTGCTTTGAATTTAGAATATTGGGTT
ACACCTCTCACTTCTAAACAATATTTACTCGCTTTCAATCAAGATGGTGTTATTTTTAATGCTGTTGATTGTAAGAGTG
ATTTTATGAGTGAGATTAAGTGTAAAACATTATCTATAGCACCAGCTACTGGTGTTTATGAATTAAACGGTTACACTGT
TCAGCCAATTGCAGATGTTTACCGACGTATACCTAATCTTCCCGATTGTAATATAGAGGCTTGGCTTAATGACAAATCG
GTGCCCTCTCCATTAAATTGGGAACGTAAGACCTTTTCAAATTGTAATTTTAATATGAGCAGCCTGATGTCTTTTATTC
AGGCAGACTCATTTACTTGTAATAATATTGATGCTGCTAAGATATATGGTATGTGTTTTTCCAGCATAACTATAGATAA
GTTTGCTATACCCAATGGTAGGAAGGTTGACCTACAATTGGGCAATTTGGGCTATTTGCAGTCTTTTAATTATAGAATT
GATACTACTGCTACAAGTTGTCAGTTGTATTATAATTTACCTGCTGCTAATGTTTCTGTTAGCAGGTTTAATCCTTCTA
CTTGGAATAGGAGATTTGGTTTTATAGAACAATCTGTTTTTAAGCCTCAACCTGCAGGTGTCTTTACTGATCATGATGT
TGTTTTTGCACAACACTGTTTCAAAGCTCCCACAAATTTCTGTCCGTGTAAACTGGATGGGTCTTTGTGTGTAGGTAGT
GGTTTTGGTATAGATGCTGGTTATAAAAATAGTGGTATAGGCACTTGTCCTGCAGGTACTAATTATTTAACTTGTCATA
ATGCTGCCCAATGTGATTGTTTGTGCACTCCAGACCCCATTACATCTAAAGCTACAGGGCCTTACAAGTGCCCCCAAAC
TGAATATTTAGTTGGCATAGGTGAGCACTGTTCAGGTCTTGCTATTAAAAGTGATCATTGTGGAGGTAATCCTTGTACT
TGCCAACCACAAGCATTTTTGGGTTGGTCTGTTGAGTCTTGTTTACAAGGGGATAGGTGTAATATTTTTGCTAATTTTA
TTTTGCATGGTGTTAATAGTGGTACTACTTGTTCTACTGATTTACAAAAATCAAACACAGACATAATTCTTGGTGTTTG

TGTTAATTATGATCTTTATGGTATTACTGGCCAAGGTATTTTTGTTGAGGTTAATGCGACTTATTATAATAGTTGGCAG
AACCTTTTATATGATTCTAATGGTAATCTCTATGGTTTTAGAGACTACTTAACAAATAGAACTTTTATGATTCGTAGTT
GCTATAGCGGTCGTGTTTCAGCGGCCTTTCATGCTAACTCTTCCGAACCAGCATTGCTATTTCGGAATATTAAATGCAA
TTACGTTTTTAATAATACTCTTTTACGACAGCTGCAACCTATTAACTATTTTGATAGCTATCTTGGTTGTGTTGTCAAT
GCTGATAATAGTACTTCTAGTGTTGTTCAAACATGTGATCTCACAGTAGGTAGTGGTTACTGTGTGGATTACTCTACAA
AAAGACGAAGTCGTAGAGCGATTACCACTGGTTATCGGTTTACTAATTTTGAGCCATTTACTGTTAATTCAGTAAATGA
TAGTTTAGAACCTGTAGGTGGTTTGTATGAAATTCAAATACCTTCAGAGTTTACTATAGGTAACATGGAGGAGTTTATT
CAAACAAGCTCTCCTAAAGTTACTATTGATTGTTCTGCTTTTGTCTGTGGTGATTATGCAGCATGTAAATCACAGTTGG
TTGAATATGGTAGCTTCTGTGACAATATTAATGCTATACTCACAGAAGTAAATGAACTACTTGACACTACACAGTTGCA
AGTAGCTAATAGTTTAATGAATGGTGTCACTCTTAGCACTAAGCTTAAAGATGGCGTTAATTTCAATGTAGACGACATC
AATTTTTCCCCTGTATTAGGTTGTTTAGGAAGCGATTGTAATAGAGTTTCCAGTAGATCTGCTATAGAGGATTTACTTT
TTTCTAAAGTAAAGTTAGCTGATGTCGGTTTCGTTGAGGCTTATAATAATTGTACTGGAGGTGCCGAAATTAGAGACCT
CATTTGTGTGCAAAGTTATAATGGTATCAAGGTGTTGCCTCCACTGCTCTCAGAAAATCAGATCAGTGGATATACTTTG
GCTGCGACCTCTGCTAGTCTGTTTCCTCCTTGGTCAGCAGCAGCAGGTGTACCATTTTATTTAAATGTTCAGTATCGTA
TTAATGGGATTGGTGTAACCATGGATGTGCTAAGTCAAAATCAAAAGCTTATCGCTAATGCATTTAACAATGCCCTTGG
TGCTATTCAGGAAGGGTTTGATTCTACCAATTCTGCTTTAGTTAAAATTCAAGCTGTTGTTAATGCAAATGCTGAAGCT
CTTAATAACTTATTGCAACAACTCTCTAATAGGTTTGGTGCTATAAGTTCTTCTTTACAAGAAATTCTATCTAGACTGG
ATGCTCTTGAAGCGCAAGCTCAGATAGACAGACTTATTAATGGGCGTCTTACCGCTCTTAATGCTTATGTTTCTCAACA
GCTTAGTGACTCTACACTAGTAAAATTTAGTGCAGCACAAGCTATGGAGAAGGTTAATGAATGTGTTAAAAGCCAATCA
TCTAGGATAAATTTTTGTGGTAATGGTAATCATATTATATCATTAGTGCAGAATGCTCCATATGGTTTGTATTTTATCC
ACTTTAGCTATGTCCCTACTAAGTATGTCACTGCGAAGGTTAGTCCTGGTCTGTGCATTGCTGGTGATAGAGGTATAGC
CCCTAAGAGTGGTTATTTTGTTAATGTAAATAATACTTGGATGTTCACTGGTAGTGGTTATTACTACCCTGAACCTATA
ACTGGAAATAATGTTGTTGTTATGAGTACCTGTGCTGTTAATTACACTAAAGCACCGGATGTAATGCTGAACACTTCAA
CACCCAACCTCCCTGATTTTAAGGAAGAGTTGGATCAATGGTTTAAAAACCAAACATCAGTGGCACCAAATTTGTCACT
TGATTATATAAATGTTACATTCTTGGACCTACAAGACGAAATGAATAGGTTACAGGAGGCAATAAAAGTTTTAAATCAG
AGCTACATCAATCTCAAGGACATTGGTACATATGAGTATTATGTAAAATGGCCTTGGTATGTATGGCTTTTAATTGGCT
TTGCTGGTGTAGCTATGCTTGTTTTACTATTCTTCATATGCTGTTGTACAGGATGTGGGACTAGTTGTTTTAAGAAATG
TGGTGGTTGTTGTGATGATTATACTGGACACCAGGAGTTAGTAATTAAAACATCCCATGACGACTAA

>gi|519668505|gb|KF169920.1|_Bovine_coronavirus_isolate_SWE/N/051_spike_glycoprotein_(S)_gene_complete_cds

ATGTTTTTGATACTTTTAATTTCCTTACCAACGACTTTTGCTGTTATAGGAGATTTAAAGTGTACTACAGTTTCC
ATTAATGATGTTGACACTGGTGTTCCTTCTATTAGCACTGATACTGTCGATGTTACTAATGGTTTAGGTACTTATTATG
TTTTAGATCGTGTGTATTTAAATACTACCTTGTTGCTTAATGGTTATTACCCTACTTCAGGTTCTACATATCGTAATAT
GGCACTGAAGGGAACTTTACTATTGAGCACACTATGGTTTAAACCACCTTTCCTTTCTGATTTTACTAATGGTATTTTT
GCTAAGGTCAAAAATACCAAGGTTATTAAAAATGGTGTAATGTATAGTGAGTTTCCTGCTATAACTATAGGTAGTACTT
TTGTAAATACATCCTATAGTGTGGTAGTACAACCACATACTACCAATTTTGACAATAAAATACAAGGTCTCTTAGAGGT
CTCTGTTTGCCAGTATACTATGTGTGAGTACCCAAATACGATTTGTAATCCTAATCTGGGTAATCAACGCGTAGAACTA
TGGCATTGGGATATAGGTGTTGTTTCTTGTTTATATAAGCGTAATTTCACATATGATGTGAATGCTGATTATTTGTATT
TTCATTTTTATCAAGAAGGTGGTACTTTTTATGCATATTTTACAGACACTGGTGTTGTTACTAAGTTTCTGTTTAATGT
ATATTTAGGCACGGTGCTTTCACACTATTATGTCATGCCTTTGACTTGTAATAGTGCTTTGAAGTTAGAATATTGGGTT
ACACCTCTCACTTCTAAACAATATTTACTCGCTTTCAATCAAGATGGTGTTATTTTTAATGCTGTTGATTGTAAGAGTG

ATTTTATGAGTGAGATTAAGTGTAAAACACTATCTATAGCACCATCTACTGGTGTTTATGAATTAAACGGTTACACTGT
TCAGCCAATTGCAGATGTTTACCGACGTATACCTAATCTTCCCGATTGTAATATAGAGGCTTGGCTTAATGACAAGTCG
GTGCCCTCTCCATTAAATTGGGAACGTAAGACCTTTTCAAATTGTAATTTTAATATGAGCAGCCTGATGTCTTTTATTC
AGGCAGACTCATTTACTTGTAATAATATTGATGCTGCTAAGATATATGGTATGTGTTTTTCCAGCATAACTATAGATAA
GTTTGCTATACCCAATGGTAGGAAGGTTGACCTACAATTGGGCAATTTGGGCTATTTGCAGTCTTTTAATTATAGAATT
GATACTACTGCTACAAGTTGTCAGTTGTATTATAATTTACCTTCTGCTAATGTTTCTGTTAGCAGGTTTAATCCTTCTA
CTTGGAATAGGAGATTTGGTTTTACAGAACAATCTGTTTTTAAGCCTCAACCTGCAGGTGTTTTTACTGATCATGATGT
TGTTTATGCACAACATTGTTTCAAAGCTCCCACAAATTTCTGTCCGTGTAAATTGGATGGGTCTTTGTGTGTAGGTAGT
GGTTCTGGTATAGATGCTGGTTATAAAAATATTGGTATAGGCACTTGTCCTGCAGGTACTAATTATTTAACTTGTCATA
ATGCTGCCCAATGTGATTGTTTGTGCACTCCAGACCCCATTACATCTAAAGCTACAGGGCCTTACAAGTGCCCCCAAAC
TAAATATTTAGTTGGCATAGGTGAGCACTGTTCAGGTCTTGCTATTAAAAGTGATCATTGTGGAGGTAATCCTTGTACT
TGTCAACCACAAGCATTTTTGGGTTGGTCTGTTGAGTCTTGTTTACAAGGGGATAGGTGTAATATTTTTGCTAATTTTA
TTTTGCATGGTGTTAATAGTGGTACTACTTGTTCTACTGATTTACAAAAATCAAACACAGACATAATTCTTGGTGTTTG
TGTTAATTATGATCTTTATGGTATTACTGGTCAAGGTATTTTTGTTGAGGTTAATGCGACTTATTATAATAGTTGGCAG
AACCTTTTATATGATTCTAATGGTAATCTCTATGGTTTTAGAGACTACTTAACAAATAGAACTTTTATGATTCGTAGTT
GCTATAGCGGTCGTGTTTCAGCGGCCTTTCATGCTAACTCTTCCGAACCAGCATTGCTATTTCGGAACATTAAATGCAA
TTACGTTTTTAATAATACTCTTTCACGACAGCTGCAACCTATTAACTATTTTGATAGCTATCTTGGTTGTGTTGTCAAT
GCTGATAATAGTACTTCTAGTGTTGTTCAAACATGTGATCTCACAGTAGGTAGTGGTTACTGTGTGGATTACTCTACAA
AAAGACGAAGTCGTAGAGCGATTACCACCGGTTATCGGTTTACCAATTTTGAGCCATTTACTGTTAATTCAGTAAATGA
TAGTTTAGAACCTGTAGGTGGTTTGTATGAAATTCAAATACCTTCAGATTTTACTATAGGTAACATGGAGGAGTTTATT
CAAACAAGCTCTCCTAAAGTTACTATTGATTGTTCTGCTTTTGTCTGTGGTGATTATGCAGCATGTAAATCACAGTTGG
TTGAATATGGTAGCTTCTGTGACAATATTAATGCTATACTCACAGAAGTAAATGAACTACTTGACACTACACAGTTGCA
AGTAGCTAATAGTTTAATGAATGGTGTCACTCTTAGCACTAAGCTTAAAGATGGCGTTAATTTCAATGTAGACGACATC
AATTTTTCCCCTGTATTAGGTTGTTTAGGAAGCGATTGTAATAGAGTTTCCAGTAGATCTGCTATAGAGGATTTACTTT
TTTCTAAAGTAAAGCTAGCTGATGTCGGTTTTGTTGAGGCTTATAATAATTGTACTGGAGGTGCCGAAATTAGGGACCT
CATTTGTGTGCAAAGTTATAATGGTATCAAGGTGTTGCCTCCACTGCTCTCAGAAAATCAGATCAGTGGATATACTTTG
GCTGCGACCTCTGCTAGTCTGTTTCCTCCATGGTCAGCAGCAGCAGGTGTACCATTTTATTTAAATGTTCAGTATCGTA
TTAATGGGATTGGTGTTACCATGGATGTGCTAAGTCAAAATCAAAAGCTTATTGCTAATGCATTTAACAATGCCCTTGG
TGCTATTCAGGAAGGGTTTGATTCTACCAATTCTGCTTTAGTTAAAATTCAAGCTGTTGTTAATGCAAATGCTGAAGCT
CTTAATAACTTATTGCAACAACTCTCTAATAGGTTTGGTGCTATAAGTTCTTCTTTACAAGAAATTCTATCTAGACTGG
ATGCTCTTGAAGCGCAAGCTCAGATAGACAGACTTATTAATGGGCGTCTTACCGCTCTTAATGCTTATGTTTCTCAACA
GCTTAGTGACTCTACACTAGTAAAATTTAGTGCAGCACAAGCTATGGAGAAGGTTAATGAATGTGTCAAAAGCCAATCA
TCTAGGATAAATTTTTGTGGTAATGGCAATCATATTATATCATTAGTGCAGAATGCTCCATATGGTTTGTATTTTATCC
ACTTTAGCTATGTCCCTACTAAGTATGTCACTGCGAAGGTTAGTCCTGGTCTGTGCATTGCTGGTGATAGAGGTATAGC
CCCTAAGAGTGGTTATTTTGTTAATGTAAATAATACTTGGATGTTCACTGGTAGTGGTTATTACTACCCTGAACCTATA
ACTGGAAATAATGTTGTTGTTATGAGTACCTGTGCTGTTAACTACACCAAAGCACCGGATGTAATGCTGAACATTTCAA
CACCCAACCTCCCTGATTTTAAGGAAGAGTTGGATCAATGGTTTAAAAATCAAACATCAGTGGCACCAAATTTGTCACT
TGATTATATAAATGTTACATTCTTGGACCTACAAGACGAAATGAATAGGTTACAGGAGGCAATAAAAGTTTTAAATCAG
AGCTACATCAATCTCAAGGACATTGGTACATATGAGTATTATGTAAAATGGCCTTGGTATGTATGGCTTTTAATTGGCT
TTGCTGGTGTAGCTATGCTTGTTTTACTATTCTTCATATGCTGTTGTACAGGATGTGGGGCTAGTTGTTTTAAGAAATG
TGGTGGTTGTTGTGATGATTATACTGGACACCAGGAGTTAGTAATTAAAACATCCCATGACGACTAA

>gi|519668507|gb|KF169921.1|_Bovine_coronavirus_isolate_SWE/N/052_spike_gly

coprotein_(S)_gene_complete_cds

ATGTTTTTGATACTTTTAATTTCGTTACCAACGACTTTTGCTGTTATAGGAGATTTAAAGTGTACTACAGTTTCC
ATTAATGATGTTGACACTGGTGTTCCTTCTATTAGCACTGATACTGTCGATGTTACTAATGGTTTAGGTACTTATTATG
TTTTAGATCGTGTGTATTTAAATACTACCTTGTTGCTTAATGGTTATTACCCTACTTCAGGTTCTACATATCGTAATAT
GGCACTGAAGGGAACTTTACTATTGAGCACACTATGGTTTAAACCACCTTTCCTTTCTGATTTTACTAATGGTATTTTT
GCTAAGGTCAAAAATACCAAGGTTATTAAAAATGGTGTAATGTATAGTGAGTTTCCTGCTATAACTATAGGTAGTACTT
TTGTAAATACATCCTATAGTGTGGTAGTACAACCACATACTACCAATTTTGACAATAAAATACAAGGTCTCTTAGAGGT
CTCTGTTTGCCAGTATACTATGTGTGAGTACCCAAATACGATTTGTAATCCTAATCTGGGTAATCAACGCGTAGAACTA
TGGCATTGGGATATAGGTGTTGTTTCTTGTTTATATAAGCGTAATTTCACATATGATGTGAATGCTGATTATTTGTATT
TTCATTTTTATCAAGAAGGTGGTACTTTTTATGCATATTTTACAGACACTGGTGTTGTTACTAAGTTTCTGTTTAATGT
ATATTTAGGCACGGTGCTTTCACACTATTATGTCATGCCTTTGACTTGTAATAGTGCTTTGAAGTTAGAATATTGGGTT
ACACCTCTCACTTCTAAACAATATTTACTCGCTTTCAATCAAGATGGTGTTATTTTTAATGCTGTTGATTGTAAGAGTG
ATTTTATGAGTGAGATTAAGTGTAAAACACTATCTATAGCACCATCTACTGGTGTTTATGAATTAAACGGTTACACTGT
TCAGCCAATTGCAGATGTTTACCGACGTATACCTAATCTTCCCGATTGTAATATAGAGGCTTGGCTTAATGACAAGTCG
GTGCCCTCTCCATTAAATTGGGAACGTAAGACCTTTTCAAATTGTAATTTTAATATGAGCAGCCTGATGTCTTTTATTC
AGGCAGACTCATTTACTTGTAATAATATTGATGCTGCTAAGATATATGGTATGTGTTTTTCCAGCATAACTATAGATAA
GTTTGCTATACCCAATGGTAGGAAGGTTGACCTACAATTGGGCAATTTGGGCTATTTGCAGTCTTTTAATTATAGAATT
GATACTACTGCTACAAGTTGTCAGTTGTATTATAATTTACCTGCTGCTAATGTTTCTGTTAGCAGGTTTAATCCTTCTA
CTTGGAATAGGAGATTTGGTTTTACAGAACAATTTGTTTTTAAGCCTCAACCTGCAGGTGTTTTTACTGATCATGATGT
TGTTTATGCACAACATTGTTTCAAAGCTCCCACAAATTTCTGTCCGTGTAAATTGGATGGGTCTTTGTGTGTAGGTAGT
GGTTCTGGTATAGATGCTGGTTATAAAAATATTGGTATAGGCACTTGTCCTGCAGGTACTAATTATTTAACTTGTCATA
ATGCTGCCCAATGTGATTGTTTGTGCACTCCAGACCCCATTACATCTAAAGCTACAGGGCCTTACAAGTGCCCCCAAAC
TAAATATTTAGTTGGCATAGGTGAGCACTGTTCAGGTCTTGCTATTAAAAGTGATCATTGTGGAGGTAATCCTTGTACT
TGTCAACCACAAGCATTTTTGGGTTGGTCTGTTGAGTCTTGTTTACAAGGGGATAGGTGTAATATTTTTGCTAATTTTA
TTTTGCATGGTGTTAATAGTGGTACTACTTGTTCTACTGATTTACAAAAATCAAACACAGACATAATTCTTGGTGTTTG
TGTTAATTATGATCTTTATGGTATTACTGGTCAAGGTATTTTTGTTGAGGTTAATGCGACTTATTATAATAGTTGGCAG
AACCTTTTATATGATTCTAATGGTAATCTCTATGGTTTTAGAGACTACTTAACAAATAGAACTTTTATGATTCGTAGTT
GCTATAGCGGTCGTGTTTCAGCGGCCTTTCATGCTAACTCTTCCGAACCAGCATTGCTATTTCGGAACATTAAATGCAA
TTACGTTTTTAATAATACTCTTTCACGACAGCTGCAACCTATTAACTATTTTGATAGCTATCTTGGTTGGGTTGTCAAT
GCTGATAATAGTACTTCTAGTGTTGTTCAAACATGTGATCTCACAGTAGGTAGTGGTTACTGTGTGGATTACTCTACAA
AAAGACGAAGTCGTAGAGCGATTACCACCGGTTATCGGTTTACCAATTTTGAGCCATTTACTGTTAATTCAGTAAATGA
TAGTTTAGAACCTGTAGGTGGTTTGTATGAAATTCAAATACCTTCAGATTTTACTATAGGTAACATGGAGGAGTTTATT
CAAACAAGCTCTCCTAAAGTTACTATTGATTGTTCTGCTTTTGTCTGTGGTGATTATGCAGCATGTAAATCACAGTTGG
TTGAATATGGTAGCTTCTGTGACAATATTAATGCTATACTCACAGAAGTAAATGAACTACTTGACACTACACAGTTGCA
AGTAGCTAATAGTTTAATGAATGGTGTCACTCTTAGCACTAAGCTTAAAGATGGCGTTAATTTCAATGTAGACGACATC
AATTTTTCCCCTGTATTAGGTTGTTTAGGAAGCGATTGTAATAGAGTTTCCAGTAGATCTGCTATAGAGGATTTACTTT
TTTCTAAAGTAAAGCTAGCTGATGTCGGTTTTGTTGAGGCTTATAATAATTGTACTGGAGGTGCCGAAATTAGGGACCT
CATTTGTGTGCAAAGTTATAATGGTATCAAGGTGTTGCCTCCACTGCTCTCAGAAAATCAGATCAGTGGATATACTTTG
GCTGCGACCTCTGCTAGTCTGTTTCCTCCATGGTCAGCAGCAGCAGGTGTACCATTTTATTTAAATGTTCAGTATCGTA
TTAATGGGATTGGTGTTACCATGGATGTGCTAAGTCAAAATCAAAAGCTTATTGCTAATGCATTTAACAATGCCCTTGG
TGCTATTCAGGAAGGGTTTGATTCTACCAATTCTGCTTTAGTTAAAATTCAAGCTGTTGTTAATGCAAATGCTGAAGCT
CTTAATAACTTATTGCAACAACTCTCTAATAGGTTTGGTGCTATAAGTTCTTCTTTACAAGAAATTCTATCTAGACTGG
ATGCTCTTGAAGCGCAAGCTCAGATAGACAGACTTATTAATGGGCGTCTTACCGCTCTTAATGCTTATGTTTCTCAACA

GCTTAGTGACTCTACACTAGTAAAATTTAGTGCAGCACAAGCTATGGAGAAGGTTAATGAATGTGTCAAAAGCCAATCA
TCTAGGATAAATTTTTGTGGTAATGGCAATCATATTATATCATTAGTGCAGAATGCTCCATATGGTTTGTATTTTATCC
ACTTTAGCTATGTCCCTACTAAGTATGTCACTGCGAAGGTTAGTCCTGGTCTGTGCATTGCTGGTGATAGAGGTATAGC
CCCTAAGAGTGGTTATTTTGTTAATGTAAATAATACTTGGATGTTCACTGGTAGTGGTTATTACTACCCTGAACCTATA
ACTGGAAATAATGTTGTTGTTATGAGTACCTGTGCTGTTAACTACACCAAAGCACCGGATGTAATGCTGAACATTTCAA
CACCCAACCTCCCTGATTTTAAGGAAGAGTTGGATCAATGGTTTAAAAATCAAACATCAGTGGCACCAAATTTGTCACT
TGATTATATAAATGTTACATTCTTGGACCTACAAGACGAAATGAATAGGTTACAGGAGGCAATAAAAGTTTTAAATCAG
AGCTACATCAATCTCAAGGACATTGGTACATATGAGTATTATGTAAAATGGCCTTGGTATGTATGGCTTTTAATTGGCT
TTGCTGGTGTAGCTATGCTTGTTTTACTATTCTTCATATGCTGTTGTACAGGATGTGGGACTAGTTGTTTTAAGAAATG
TGGTGGTTGTTGTGATGATTATACTGGACACCAGGAGTTAGTAATTAAAACATCCCATGACGACTAA

>gi|519668509|gb|KF169922.1|_Bovine_coronavirus_isolate_SWE/AC/061_spike_glycoprotein_(S)_gene_complete_cds

ATGTTTTTGATACTTTTAATTTCCTTACCAACGGCTTTTGCTGTTATAGGAGATTTAAAGTGTACTTCGGTTTCC
ATTAATGATGTTGACACTGGTGTTCCTTTTATTAGCACTGATACTGTTGATGTTACTAATGGTTTAGGTACTTATTATG
TTTTAGATCGTGTGTATTTAAATACTACCTTGTTGCTTAATGGTTATTACCCTACTTCAGGTTCTACATATCGTAATAT
GGCACTGAAGGGAACTCTACTATTGAGCACACTATGGTTTAAACCACCTTTTCTTTCTGATTTTACTAATGGTATTTTT
GCTAAGGTCAAAAATACCAAGGTTATTAAAAATGGTGTAATGTATAGTGAGTTTCCTGCTATAACTATAGGTAGTACTT
TTGTAAATACATCCTATAGTGTGGTAGTACAACCATATACTACCAATTTTGATAATAAAATACAAGGTCTCTTAGAGGT
CTCTGTTTGCCAGTATACTATGTGCGAGTACCCAAATACGATTTGTAATCCTAATCTGGGTAATCAACGCGTAGAACTA
TGGCATTGGGATACAGGTGTTGTTTCCTGTTTATATAAGCGTAATTTCACATATGATGTGAATGCTGATTATTTGTATT
TCCATTTTTATCAAGAAGGTGGTACTTTTTATGCATATTTTACAGACACTGGTGTTGTTACTAAGTTTCTGTTTAATGT
ATATTTAGGCACGGTGCTTTCACACTATTATGTCATGCCTTTGACTTGTAATAGTGCTTTGAATTTAGAATATTGGGTT
ACACCTCTCACTTCTAAACAATATTTACTCGCTTTCAATCAAGATGGTGTTATTTTTAATGCTGTTGATTGTAAGAGTG
ATTTTATGAGTGAGATTAAGTGTAAAACATTATCTATAGCACCATCGACTGGTGTTTATGAATTAAACGGTTACACTGT
TCAGCCAATTGCAGATGTTTACCGACGTATACCTAATCTTCCCGATTGTAATATAGAGGCTTGGCTTAATGACAAGTCG
GTGCCTTCTCCATTAAATTGGGAACGTAAGACCTTTTCAAATTGTAATTTTAATATGAGCAGCCTGATGTCTTTTATTC
AGGCAGACTCATTTACTTGTAATAATATTGATGCTGCTAAGATATATGGTATGTGTTTTTCCAGCATAACTATAGATAA
GTTTGCTATACCCAATGGTAGGAAGGTTGACCTACAATTGGGTAATTTGGGCTATTTGCAGTCTTTTAACTATAGAATT
GATACTACTGCTACAAGTTGTCAGTTGTATTATAATTTACCTGCTGCTAATGTTTCTGTTAGCAGGTTTAATCCTTCTA
CTTGGAATAGGAGATTTGGTTTTACAGAACAATTTGTTTTTAAGCCTCAACCTGCAGGTGTCTTTACTGATCATGATGT
TGTTTATGCACAACATTGTTTTAAAGCTCCCACAAATTTTTGTCCGTGTAAATTGGATGGGTCTTTGTGTGTAGGTAGT
GGTTCTGGTATAGATGCTGGTTATAAAAATAGTGGTATAGGCACTTGTCCTGCAGGTACTAATTATTTAATTTGTTATA
ATGCTGCCCAATGTGATTGTTTGTGCACTCCAGACCCCATTACATCTAAAGCTACAGGGCCTTACAAGTGCCCCCAAAC
TAAATATTTAGTTGGTATAGGTGAGCACTGTTCAGGTCTTGCTATTAAAAGTGATCATTGTGGAGGTAATCCTTGTACT
TGCCAACCACAAGCATTTTTGGGTTGGTCTGTTGAGTCTTGTTTAGAAGGGGATAGGTGTAATATTTTTGCTAATTTTA
TTTTGCATGGTGTTAATAGTGGTACTACTTGTTCTACTGATTTACAAAAATCAAACACAGACATAATTCTTGGTGTTTG
TGTTAATTATGATCTTTATGGTATTACTGGCCAAGGTATTTTTGTTGAGGTTAATGCGACTTATTATAATAGTTGGCAG
AACCTTTTATATGATTCTAATGGTAATCTCTATGGTTTTAGAGACTACTTAACAAATAGAACTTTTATGATTCGTAGCT
GCTATAGCGGTCGTGTTTCAGCGGCCTTTCATGCTAACTCTTCCGAACCAGCATTGCTATTTCGGAATATTAAATGCAA
TTACGTTTTTAATAATACTCTTTCACGACAGCTGCAACCTATTAACTATTTTGACAGCTATCTTGGTTGTGTTGTCAAT
GCTGATAATAGTACTTCTAGTGTTGTTCAAACATGTGATCTCACAGTAGGTAGTGGTTACTGTGTAGATTACTCTACAA

AAAGACGAAGTCGTAGAGCGATTACCACTGGTTATCGGTTTACTAATTTTGAGCCATTTACTGTTAATTCAGTAAATGA
TAGTTTAGAACCTGTAGGTGGTTTGTATGAAATTCAAATACCTTCAGAGTTTACTATAGGTAACATGGAGGAGTTTATT
CAAACAAGCTCTCCTAAAGTTACTATTGATTGTTCTGCTTTTGTCTGTGGTGATTATGCAGCATGTAAATCACAGTTGG
TTGAATATGGTAGCTTCTGTGACAATATTAATGCTATACTCACAGAAGTAAATGAACTACTTGACACTACACAGTTGCA
AGTAGCTAATAGTTTAATGAATGGTGTCACTCTTAGCAGTAAGCTTAAAGATGGCGTTAATTTCAATGTAGACGACATC
AATTTTTCCCCTGTATTAGGTTGTTTAGGAAGCGATTGTAATAGAGTTTCCAGTAGATCTGCTATAGAGGATTTACTTT
TTTCTAAAGTAAAGTTAGCTGATGTCGGTTTCGTTGAGGCTTATAATAATTGTACTGGAGGTGCCGAAATTAGGGACCT
CATTTGTGTGCAAAGTTATAATGGTATCAAGGTGTTGCCTCCACTGCTCTCAGAAAACCAGATCAGTGGATATACTTTG
GCTGCGACCTCTGCTAGTCTGTTTCCTCCTTGGTCAGCAGCAGCAGGTGTACCATTTTATTTAAATGTTCAGTATCGTA
TTAATGGGATTGGTGTTACCATGGATGTGCTAAGTCAAAATCAAAAGCTTATTGCTAATGCATTTAACAATGCCCTTGG
TGCTATTCAGGAAGGGTTTGATGCTACCAATTCTGCTTTAGTTAAAATTCAAGCTGTTGTTAATGCAAATGCTGAAGCT
CTTAATAACTTATTGCAACAACTCTCTAATAGGTTTGGTGCTATAAGTTCTTCTTTACAAGAAATTCTATCTAGACTGG
ATGCTCTTGAAGCGCAAGCTCAGATAGACAGACTTATTAATGGGCGTTTTACCGCTCTTAATGCTTATGTTTCTCAACA
GCTTAGTGATTCTACACTTGTAAAATTTAGTGCAGCACAAGCTATTGAGAAGGTTAATGAATGTGTCAAAAGCCAATCA
TCTAGGATAAATTTTTGTGGTAATGGTAATCATATTATATCATTAGTGCAGAATGCTCCATATGGTTTGTATTTTATCC
ACTTTAGCTATGTCCCTACTAAGTATGTCACTGCGAAGGTTAGTCCTGGTCTGTGCATTGCTGGTGATAGAGGTATAGC
CCCCAAGAGTGGTTATTTTGTTAATGTAAATAACACTTGGATGTTCACTGGTAGTGGTTATTACTACCCTGAACCTATA
ACTGGAAATAATGTTGTTGTTATGAGTACCTGTGCTGTTAATTACACTAAAGCACCGGATGTAATGCTGAACATTTCAA
CACCCAAACTCCCTGATTTTAAGGAAGAGTTAGATCAATGGTTTAAAAATCAAACATCAGTGGCACCAAATTTGTCACT
CGATTATATAAATGTTACATTCTTGGACCTGCAAGATGAAATGAATAGGTTACAGGAGGCAATAAAAGTTTTAAATCAG
AGCTACATCAATCTCAAGGACATTGGTACATATGAGTATTATGTAAAATGGCCTTGGTATGTATGGCTTTTAATTGGCT
TTGCTGGTGTAGCTATGCTTGTTTTACTATTCTTCATATGCTGTTGTACAGGATGTGGGACTAGTTGTTTTAAGAAATG
TGGTGGTTGTTGTGATGATTATACTGGACATCAGGAGTTAGTAATTAAAACATCCCATGACGACTAA

>gi|519668511|gb|KF169923.1|_Bovine_coronavirus_isolate_SWE/M/063*_spike_glycoprotein_(S)_gene_complete_cds

ATGTTTTTGATACTTTTAATTTCCTTACCAACGACTTTTGCTGTTATAGGAGATTTAAAGTGTACTACAGTTTCC
ATTAATGATGTTGACACTGGTGTTCCTTCTATTAGCACTGATACTGTCGATGTTACTAATGGTTTAGGTACTTATTATG
TCTTAGATCGTGTGTATTTAAATACTACCTTGTTGCTTAATGGTTATTACCCTACTTCAGGTTCTACATATCGTAATAT
GGCACTGAAGGGAACTTTACTATTGAGCACACTATGGTTTAAACCACCTTTCCTTTCTGATTTTACTAATGGTATTTTT
GCTAAGGTCAAAAATACCAAGGTTATTAAAAATGGTGTAATGTATAGTGAGTTTCCTGCTATAACTATAGGTAGTACTT
TTGTAAATACATCCTATAGTGTGGTAGTACAACCACATACTACCAATTTTGACAATAAAATACAAGGTCTCTTAGAGGT
CTCTGTTTGCCAGTATACTATGTGTGAGTACCCAAATACGATTTGTAATCCTAATCTGGGTAATCAACGCGTAGAACTA
TGGCATTGGGATATAGGTGTTGTTTCCTGTTTATATAAGCGTAATTTCACATATGATGTGAATGCTGATTATTTGTATT
TTCATTTTTATCAGGAAGGTGGTACTTTTTATGCATATTTTACAGACACTGGTGTTGTTACTAAGTTTCTGTTTAATGT
ATATTTAGGCACGTTGCTTTCACACTATTATGTCATGCCTTTGACTTGTAATAGTGCTTTGAATTTAGAATATTGGGTT
ACACCTCTCACTTCTAAACAATATTTACTCGCTTTCAATCAAGATGGTGTTATTTTTAATGCTGTTGATTGTAAGAGTG
ATTTTATGAGTGAGATTAAGTGTAAAACACTATCTATAGCACCATCTACTGGTGTTTATGAATTAAACGGTTACACTGT
TCAGCCAATTGCAGATGTTTACCGACGTATACCTAATCTTCCCGATTGTAATATAGAGGCTTGGCTTAATGACAAGTCG
GTGCCCTCTCCATTAAATTGGGAACGTAAGACCTTTTCAAATTGTAATTTTAATATGAGCAGCCTGATGTCTTTTATTC
AGGCAGACTCATTTACTTGTAATAATATTGATGCTGCTAAGATATATGGTATGTGTTTTTCCAGCATAACTATAGATAA
GTTTGCTATACCCAATGGTAGGAAGGTTGACCTACAATTGGGCAATTTGGGCTATTTGCAGTCTTTTAACTATAGAATT

GATACTACTGTTACAAGTTGTCAGTTGTATTATAATTTACCTGCTGCTAATGTTTCTGTTAGCAGGTTTAATCCTTCTA
CTTGGAATAGGAGATTTGGTTTTACAGAACAATCTGTTTTTAAGCCTCAATCTGCAGGTGTTTTTACTGATCATGATGT
TGTTTATGCACAACATTGTTTCAAAGCTCCCACAAATTTCTGTCCGTGTAAATTGGATGGTTCTTTGTGTGTAGGTAGT
GGTTCTGGTATAGATGCTGGTTATAAAAATAGTGGTATAGGCACTTGTCCTGCAGGTACTAATTATTTAACTTGTCATA
ATGCTGCCCAATGTGATTGTTTGTGCACTCCAGACCCCATTACATCTAAAGCTACAGGGCCTTACAAGTGCCCCCAAAC
TAAATATTTAGTTGGCATAGGTGAGCACTGTTCAGGTCTTGCTATTAAAAGTGATCATTGTGGAGGTAATCCTTGTATT
TGTCAACCACAAGCATTTTTGGGTTGGTCTGTTGAGTCTTGTTTACAAGGGGATAGGTGTAATATTTTTGCTAATTTTA
TTTTGCATGGTGTTAATAGTGGTACTACTTGTTCTACTGATTTACAAAAATCAAACACAGACATAATTCTTGGTGTTTG
TGTTAATTATGATCTTTATGGTATTACTGGTCAAGGTATTTTTGTTGAGGTTAATGCGACTTATTATAATAGTTGGCAG
AACCTTTTATATGATTCTAATGGTAATCTCTATGGTTTTAGAGACTACTTAACAAATAGAACTTTTATGATTCGTAGTT
GCTATAGCGGTCGTGTTTCAGCGGCCTTTCATGCTAACTCTTCCGAACCAGCATTGCTATTTCGGAACATTAAATGCAA
TTACGTTTTTAATAATACTATTTCACGACAGCTGCAACCTATTAACTATTTTGATAGCTATCTTGGTTGTGTTGTCAAT
GCTGATAATAGTACTTCTAGTGTTGTTCAAACATGTGATCTCACAGTAGGTAGTGGTTACTGTGTGGATTACTCTACAA
AAAGACGAAGTCGTAGAGCGATTACCACTGGTTATCGGTTTACCAATTTTGAGCCATTTACTGTTAATTCAGTAAATGA
TAGTTTAGAACCTGTAGGTGGTTTGTATGAAATTCAAATACCTTCAGAGTTTACTATAGGTAACATGGAGGAGTTTATT
CAAACAAGCTCTCCTAAAGTTACTATTGATTGTTCTGCTTTTGTCTGTGGTGATTATGCAGCATGTAAATCACAGTTGG
TTGAATATGGTAGCTTCTGTGACAATATTAATGCTATACTCACAGAAGTAAATGAACTACTTGACACTACACAGTTGCA
AGTAGCTAATAGTTTAATGAATGGTGTCACTCTTAGCAGTAAGCTTAAAGATGGCGTTAATTTCAATGTAGACGACATC
AATTTTTCCCCTGTATTAGGTTGTTTAGGAAGCGATTGTAATAGAGTTTCCAGTAGATCTGCTATAGAGGATTTACTTT
TTTCTAAAGTAAAGTTAGCTGATGTCGGTTTTGTTGAGGCTTATAATAATTGTACTGGAGGTGCCGAAATTAGGGACCT
CATTTGTGTGCAAAGTTATAATGGTATCAAGGTGTTGCCTCCACTGCTCTCAGAAAATCAGATCAGTGGATATACTTTG
GCTGCGACCTCTGCTAGTCTGTTTCCTCCATGGTCAGCAGCAGCAGGTGTACCATTTTATTTAAATGTTCAGTATCGTA
TTAATGGGATTGGTGTTACCATGGATGTGCTAAGTCAAAATCAAAAGCTTATTGCTAATGCATTTAACAATGCCCTTGG
TGCTATTCAGGAAGGGTTTGATGCTACCAATTCTGCTTTAGTTAAAATTCAAGCTGTTGTTAATGCAAATGCTGAAGCT
CTTAATAACTTATTGCAACAACTCTCTAATAGGTTTGGTGCTATAAGTTCTTCTTTACAAGAAATTCTATCTAGACTGG
ATGCTCTTGAAGCGCAAGCTCAGATAGACAGACTTATTAATGGGCGTCTTACCGCTCTTAATGCTTATGTTTCTCAACA
GCTTAGTGACTCTACACTAGTAAAATTTAGTGCAGCACAAGCTATGGAGAAGGTTAATGAATGTGTCAAAAGCCAATCA
TCTAGGATAAATTTTTGTGGTAATGGCAATCATATTATATCATTAGTGCAGAATGCTCCATATGGTTTGTATTTTATCC
ACTTTAGCTATGTCCCTACTAAGTATGTCACTGCGAAGGTTAGTCCTGGTCTGTGCATTGCTGGTGATAGAGGTATAGC
CCCCAAGAGTGGTTATTTTGTTAATGTAAATAATACTTGGATGTTCACTGGTAGTGGTTATTACTACCCTGAACCTATA
ACTGGAAATAATGTTGTCGTTATGAGTACCTGTGCTGTTAACTACACCAAAGCACCGGATGTAATGCTGAACATTTCAA
CACCCAACCTCCCTGATTTTAAGGAAGAGTTGGATCAATGGTTTAAAAATCAAACATCAGTGGCACCAAATTTGTCACT
TGATTATATAAATGTTACATTCTTGGACCTACAAGACGAAATGAATAGGTTACAGGAGGCAATAAAAGTTTTAAATCAG
AGCTACATCAATCTCAAGGACATTGGTACATATGAGTATTATGTAAAATGGCCTTGGTATGTATGGCTTTTAATTGGCT
TTGCTGGTGTAGCTATGCTTGTTTTACTATTCTTCATATGCTGTTGTACAGGATGTGGGACTAGTTGTTTTAAGAAATG
TGGTGGTTGTTGTGATGAATATACTGGACACCAGGAGTTAGTAATTAAAACATCACATGACGACTAA

>gi|519668513|gb|KF169924.1|_Bovine_coronavirus_isolate_SWE/M/064*_spike_glycoprotein_(S)_gene_complete_cds

ATGTTTTTGATACTTTTAATTTCCTTACCAACGGCTTTTGCTGTTATAGGAGATTTAAAGTGTACTTCAGTTTCC
ATTAATGATGTTGACACTGGTGTTCCTTCTATTAGCACTGATACTGTCGATGTTACTAATGGTTTAGGTACTTATTATG
TTTTAGATCGTGTGTATTTAAATACTACTTTGTTGCTTAATGGTTACTATCCTACTTCAGGTTCTACATATCGTAATAT

GGCACTGAAGGGAACTTTACTATTGAGCACACTATGGTTTAAACCACCTTTCCTTTCTGATTTTACTAATGGTATTTTT
GCTAAGGTCAAAAATACCAAGGTTATTAAAAATGGTGTAATGTATAGTGAGTTTCCTGCTATAACTATAGGTAGTACTT
TTGTAAATACATCCTATAGTGTGGTAGTACAACCATATACTACCAATTTTGATAATAAAATACAAGGTCTCTTAGAGGT
CTCTGTTTGCCAGTATACTATGTGTGAGTACCCAAACACGATTTGTAATTCTAATCTGGGTAATCAACGCGTAGAACTA
TGGCATTGGGATACAGGTGTTGTTTCCTGTTTATATAAGCGTAATTTCACATATGATGTGAATGCTGATTATTTGTATT
TCCATTTTTATCAAGAAGGTGGTACTTTTTATGCATATTTTACAGACACTGGTGTTGTTACTAAGTTTCTGTTTAATGT
ATATTTAGGCACGGTGCTTTCACACTATTATGTCATGCCTTTGACTTGTAATAGTGCTTTGAATTTAGAATATTGGGTT
ACACCTCTCACTTCTAAACAATATTTACTCGCTTTCAATCAAGATGGTGTTATTTTTAATGCTGTTGATTGTAAGAGTG
ATTTTATGAGTGAGATTAAGTGTAAAACATTATCTATAGCACCATCTACTGGTGTTTATGAATTAAACGGTTACACTGT
TCAGCCAATTGCAGATGTTTACCGACGTATACCTAATCTTCCCGATTGTAATATAGAGGCTTGGCTTAATGACAAGTCG
GTGCCCTCTCCATTAAATTGGGAACGTAAGACCTTTTCAAATTGTAATTTTAATATGAGCAGCCTGATGTCTTTTATTC
AGGCAGACTCATTTACTTGTAATAATATTGATGCTGCTAAGATATATGGTATGTGTTTTTCCAGCATAACTATAGATAA
GTTTGCTATACCCAATGGTAGGAAGGTTGACCTACAATTGGGCAATTTGGGTTATTTGCAGTCTTTTAATTATAGAATT
GATACTACTGCTACAAGTTGTCAGTTGTATTATAATTTACCTGCTGCTAATGTTTCTGTTAGCAGGTTTAATCCTTCTA
CTTGGAATAGGAGATTTGGTTTTACAGAACAATCTGTTTTTAAGCCTAAACCTGCAGGTGTCTTTACTGATCATGATGT
TGTTTATGCACAACATTGTTTCAAAGCTCCCACAAATTTCTGTCCGTGTAAATTGGATGGGTCTGTGTGTGTAGGTAGT
GGTTTTGGTTTAGATGCTGGTTATAAAAATAGTGGTATAGGCATTTGTCCTGCAGGTACTAATTATTTAACTTGTCATA
ATGCTGCCCAATGTGATTGTTTGTGCACTCCAGACCCCATTACATCTAAAGCTACAGGGCCTTACAAGTGCCCCCAAAC
TAAATATTTAGTTGGCATAGGTGAGCACTGTTCAGGTCTTGCTATTAAAAGTGATCATTGTGGAGGTAATTCTTGTACT
TGCCAACCACAAGCATTTTTGGGTTGGTCTGTTGAGTCTTGTTTACAAGGGGATAGGTGTAATATTTTTGCTAATTTTA
TTTTGCATGGTGTTAATAGTGGTACTACTTGTTCTACTGATTTACAAAAATCAAACACAGACATAATTCTTGGTGTTTG
TGTTAATTATGATCTTTATGGTATTACTGGCCAAGGTGTTTTTGTTGAGGTTAATGCGACTTATTATAATAGTTGGCAG
AACCTTTTATATGATTCTAATGGTAATCTCTATGGTTTTAGAGACTACTTAACAAATAGAACTTTTATGATTCGTAGTT
GCTATAGCGGTCGTGTTTCAGCAGCCTTTCATGCTAACTCTTCCGAACCAGCATTGCTATTTCGGAATATTAAATGCAA
TTACGTTTTTAATAATACTCTTTCACGACAGCTGCGACCTATTAACTATTTTGATAGCTATCTTGGTTGTGTTGTCAAT
GCTGATAATAGTACTTCTAGTGTTGTTCAAACATGTGATCTCACAGTAGGTAGTGGTTACTGTGTGGATTACTCTACAA
AAAGACGAAGTCGTAGAGCGATTACCACTGGTTATCGGTTTACTAATTTTGAGCCATTTACTGTTAATTCAGTAAATGA
TAGTTTAGAACCTGTAGGTGGTTTGTATGAAATTCAAATACCTTCAGAGTTTACTATAGGTAACATGGAGGAGTTTATT
CAAACAAGCTCTCCTAAAGTTACTATTGATTGTTCTGCTTTTGTCTGTGGTGATTATGCAGCATGTAAATCACAGTTGG
TTGAATATGGTAGCTTCTGTGACAATATTAATGCTATACTCACAGAAGTAAATGAACTACTTGACACTACACAGTTGCA
AGTAGCTAATAGTTTAATGAATGGTGTCACTCTTAGCACTAAGCTTAAAGATGGCGTTAAATTCAATGTAGACGACATC
AATTTTTCCCCTGTATTAGGTTGTTTAGGAAGCGATTGTAATAGAGTTTCCAGTAGATCTGCTATAGAGGATTTACTTT
TTTCTAAAGTAAAGTTAGCTGATGTCGGTTTTGTTGAGGCTTATAATAATTGTACTGGAGGTGCCGAAATTAGGGACCT
CATTTGTGTGCAAAGTTATAATGGTATCAAGGTGTTGCCTCCACTGCTCTCAGAAAATCAGATCAGTGGATATACTTTG
GCTGCGACCTCTGCTAGTCTGTTTCCTCCATGGTCAGCAGCAGCAGGTGTACCATTTTATTTAAATGTTCAGTATCGTA
TTAATGGGATTGGTGTTACCATGGATGTGCTAAGTCAAAATCAAAAGCTTATTGCTAATGCATTTAACAATGCCCTTGG
TGCTATTCAGGAAGGGTTTGATGCTACCAATTCTGCTTTAGTTAAAATTCAAGCTGTTGTTAATGCAAATTCTGAAGCT
CTTAATAACTTATTGCAACAACTCTCTAATAGGTTTGGTGCTATAAGTTCTTCTTTACAAGAAATTCTATCTAGACTGG
ATGCTCTTGAAGCGCAAGCTCAGATAGACAGACTTATTAATGGGCGTCTTACCGCTCTTAATGCTTATGTTTCTCAACA
GCTTAGTGATTCTACACTAGTAAAATTTAGTGCAGCACAAGCTATGGAGAAGGTTAATGAATGTGTCAAAAGCCAATCA
TCTAGGATAAATTTTTGTGGTAATGGTAATCATATTATATCATTAGTGCAGAATGCTCCATATGGTTTGTATTTTATCC
ACTTTAGCTATGTCCCTACTAAGTATGTCACTGCGAAGGTTAGTCCTGGTCTGTGCATTGCTGGTGATAGAGGTATAGC
CCCCAAGAGTGGTTATTTTGTTAATGTAAATAATACTTGGATGTTCACTGGTAGTGGTTACTACTACCCTGAACCTATA
ACTGTAAATAATGTTGTTGTTATGAGTACCTGTGCTGTTAATTACACTAAAGCACCGGATGTAATGTTGAACATTTCAA

CACCCAAACTCCCTGATTTTAAGGAAGAGTTGGATCAATGGTTTAAAAACCAAACATCAGTGGCACCAAATTTGTCACT
TGATTATATAAATGTTACATTCTTGGACCTACAAGACGAAATGAATAGGTTACAGGAGGCAATAAAAGTTTTAAATCAT
AGCTACATCAATCTCAAGGACATTGGTACATATGAGTATTATGTAAAATGGCCTTGGTATGTATGGCTTTTAATTGGCT
TTGCTGGTGTAGCTATGCTTGTTTTACTATTCTTCATATGCTGTTGTACAGGATGTGGAACTAGTTGTTTTAAGAAATG
TGGTGGTTGTTGTGATGATTATACTGGACATCAGGAGTTAGTAATTAAAACATCACATGACGACTAA

>gi|519668515|gb|KF169925.1|_Bovine_coronavirus_isolate_SWE/Z/071_spike_glycoprotein_(S)_gene_complete_cds

ATGTTTTTGATACTTTTAATTTCCTTACCAACGACTTTTGCTGTTATAGGAGATTTAAAGTGTACTACAGTTTCC
ATTAATGATGTTGACACTGGTGTTCCTTCTATTAGCACTGATACTGTCGATGTTACTAATGGTTTAGGTACTTATTATG
TTTTAGATCGTGTGTATTTAAATACTACCTTGTTGCTTAATGGTTATTACCCTACTTCAGGTTCTACATATCGTAATAT
GGCACTGAAGGGAACTTTACTATTGAGCACACTATGGTTTAAACCACCTTTCCTTTCTGATTTTACTAATGGTATTTTT
GCTAAGGTCAAAAATACCAAGGTTATTAAAAATGGTGTAATGTATAGTGAGTTTCCTGCTATAACTATAGGTAGTACTT
TTGTAAATACATCCTATAGTGTGGTAGTACAACCACATACTACCAATTTTGACAATAAAATACAAGGTCTCTTAGAGGT
CTCTGTTTGCCAGTATACTATGTGTGAGTACCCAAATACGATTTGTAATCCTAATCTGGGTAATCAACGCGTAGAACTA
TGGCATTGGGATACAGGTGTTGTTTCCTGTTTATATAAGCGTAATTTCACATATGATGTGAATGCTGATTATTTGTATT
TTCATTTTTATCAGGAAGGTGGTACTTTTTATGCATATTTTACAGACACTGGTGTTGTTACTAAGTTTCTGTTTAATGT
ATATTTAGGCACGTTGCTTTCACACTATTATGTCATGCCTTTGACTTGTAATAGTGCTTTGAATTTAGAATATTGGGTT
ACACCTCTCACTTCTAAACAATATTTACTCGCTTTCAATCAAGATGGTGTTATTTTTAATGCTGTTGATTGTAAGAGTG
ATTTTATGAGTGAGATTAAGTGTAAAACACTATCTATAGCACCATCTACTGGTGTTTATGAATTAAACGGTTACACTGT
TCAGCCAATTGCAGATGTTTACCGACGTATACCTAATCTTCCCGATTGTAATATAGAGGCTTGGCTTAATGACAAGTCG
GTGCCCTCTCCATTAAATTGGGAACGTAAGACCTTTTCAAATTGTAATTTTAATATGAGCAGCCTGATGTCTTTTATTC
AGGCAGACTCATTTACTTGTAATAATATTGATGCTGCTAAGATATATGGTATGTGTTTTTCCAGCATAACTATAGATAA
GTTTGCTATACCCAATGGTAGGAAGGTTGACCTACAATTGGGCAATTTGGGCTATTTGCAGTCTTTTAACTATAGAATT
GATACTACTGCTACAAGTTGTCAGTTGTATTATAATTTACCTGCTGCTAATGTTTCTGTCAGCAGGTTTAATCCTTCTA
CTTGGAATAGGAGATTTGGTTTTACAGAACAATCTGTTTTTAAGCCTCAACCTGCAGGTGTTTTTACTGATCATGATGT
TGTTTATGCACAACATTGTTTCAAAGCTCCCACAAATTTCTGTCCGTGTAAATTGGATGGGTCTTTGTGTGTAGGTAGT
GGTTCTGGTATAGATGCTGGTTATAAAAATAGTGGTATAGGCACTTGTCCTGCAGGTACTAATTATTTAACTTGTCATA
ATGCTGCCCAATGTGATTGTTTGTGCACTCCAGACCCCATTACATCTAAAGCTACAGGGCCTTACAAGTGCCCCCAAAC
TAAATATTTAGTTGGTATAGGTGAGCACTGTTCAGGTCTTGCTATTAAAAGTGATTATTGTGGAGGTAATCCTTGTACT
TGTCAACCACAAGCATTTTTGGGTTGGTCTGTTGAGTCTTGTTTACAAGGGGATAGGTGTAATATTTTTGCTAATTTTA
TTTTGCATGGTGTTAATAGTGGTACTACTTGTTCTACTGATTTACAAAAATCAAACACAGACATAATTCTTGGTGTTTG
TGTTAATTATGATCTTTATGGTATTACTGGTCAAGGTATTTTTGTTGAGGTTAATGCGACTTATTATAATAGTTGGCAG
AACCTTTTATATGATTCTAATGGTAATCTCTATGGTTTTAGAGACTACTTAACAAATAGAACTTTTATGATTCGTAGTT
GCTATAGCGGTCGTGTTTCAGCGGCCTTTCATGCTAACTCTTCCGAACCAGCATTGCTATTTCGGAACATTAAATGCAA
TTACGTTTTTAATAATACTCTTTCACGACAGCTGCAACCTATTAACTATTTTGATAGCTATCTTGGTTGTGTTGTCAAT
GCTGATAATAGTACTTCTAGTGTTGTTCAAACATGTGATCTCACAGTAGGTAGTGGTTACTGTGTGGATTACTCTACAA
AAAGACGAAGTCGTAGAGCGATTACCACTGGTTATCGGTTTACCAATTTTGAGCCATTTACTGTTAATTCAGTAAATGA
TAGTTTAGAACCTGTAGGTGGTTTGTATGAAATTCAAATACCTTCAGAGTTTACTATAGGTAACATGGAGGAGTTTATT
CAAACAAGCTCTCCTAAAGTTACTATTGATTGTTCTGCTTTTGTCTGTGGTGATTATGCAGCATGTAAATCACAGTTGG
TTGAATATGGTAGCTTCTGTGACAATATTAATGCTATACTCACAGAAGTGAATGAACTACTTGACACTACACAGTTGCA
AGTAGCTAATAGTTTAATGAATGGTGTCACTCTTAGCACTAAGCCTAAAGATGGCGTTAATTTCAATGTAGACGACATC

AATTTTTCCCCTGTATTAGGTTGTTTAGGAAGCGATTGTAATAGAGTTTCCAGTAGATCAGCTATAGAGGATTTACTTT
TTTCTAAAGTAAAGTTAGCTGATGTCGGTTTTGTTGAGGCTTATAATAATTGTACTGGAGGTGCCGAAATTAGGGACCT
CATTTGTGTGCAAAGTTATAATGGTATCAAGGTGTTGCCTCCACTGCTCTCAGAAAATCAGATCAGTGGATATACTTTG
GCTGCGACCTCTGCTAGTCTGTTTCCTCCATGGTCAGCAGCAGCAGGTGTACCATTTTATTTAAATGTTCAGTATCGTA
TTAATGGGATTGGTGTTACCATGGATGTGCTAAGTCAAAATCAAAAGCTTATTGCTAATGCATTTAACAATGCCCTTGG
TGCTATTCAGGAAGGGTTTGATGCTACCAATTCTGCTTTAGTTAAAATTCAAGCTGTTGTTAATGCAAATGCTGAAGCT
CTTAATAACTTATTGCAACAACTCTCTAATAGGTTTGGTGCTATAAGTTCTTCTTTACAAGAAATTCTATCTAGACTGG
ATGCTCTTGAAGCGCAAGCTCAGATAGACAGACTTATTAATGGGCGTCTTACCGCTCTTAATGCTTATGTTTCTCAACA
GCTTAGTGACTCTACACTAGTAAAATTTAGTGCAGCACAAGCTATGGAGAAGGTTAATGAATGTGTCAAAAGCCAATCA
TCTAGGATAAATTTTTGTGGTAATGGCAATCATATTATATCATTAGTGCAGAATGCTCCATATGGTTTGTATTTTATCC
ACTTTAGCTATGTCCCTACTAAGTATGTCACTGCGAAGGTTAGTCCTGGTCTGTGCATTGCTGGTGATAGAGGTATAGC
CCCCAAGAGTGGTTATTTTGTTAATGTAAATAATACTTGGATGTTCACTGGTAGTGGTTATTACCACCCTGAACCTATA
ACTGGAAATAATGTTGTCGTTATGAGTACCTGTGCTGTTAACTACACCAAAGCACCGGATGTAATGCTGAACATTTCAA
CACCCAACCTCCCTGATTTTAAGGAAGAGTTGGATCAATGGTTTAAAAATCAAACATCAGTGGCACCAAATTTGTCACT
TGATTATATAAATGTTACATTCTTGGACCTACAAGACGAAATGACTAGGTTACAGGAGGCAATAAAAGTTTTAAATCAG
AGCTACATCAATCTCAAGGACATTGGTACATATGAGTATTATGTAAAATGGCCTTGGTATGTATGGCTTTTAATTGGCT
TTGCTGGTGTAGCTATGCTTGTTTTACTATTCTTCATATGCTGTTGTACAGGATGTGGGACTAGTTGTTTTAAGAAATG
TGGTGGTTGTTGTGATGATTATACTGGACACCAGGAGTTAGTAATTAAAACATCACATGACGACTAA

>gi|519668517|gb|KF169926.1|_Bovine_coronavirus_isolate_SWE/C/072_spike_glycoprotein_(S)_gene_complete_cds

ATGTTTTTGATACTTTTAATTTCCTTACCAACGACTTTTGCTGTTATAGGAGATTTAAAGTGTACTACAGTTTCC
ATTAATGATGTTAACACTGGTGTTCCTCTTATTAGCACTGATACTGTCGATGTTACTAATGGTTTAGGTACTTATTATG
TTTTAGATCGTGTGTATTTAAATACTACCTTGTTGCTTAATGGTTATTACCCTACTTCAGGTTCTACATATCGTAATAT
GGCACTGAAGGGAACTTTACTATTGAGCACACTATGGTTTAAACCACCTTTCCTTTCTGATTTTACTAATGGTATTTTT
GCTAAGGTCAAAAATACCAAGGTTATTAAAAATGGTGTAATGTATAGTGAGTTTCCTGCTATAACTATAGGTAGTACTT
TTGTAAATACATCCTATAGTGTGGTAGTACAACCACATACTACCAATTTTGACAATAAAATACAAGGTCTCTTAGAGGT
CTCTGTTTGCCAGTATACTATGTGTGAGTACCCAAATACGATTTGTAATCCTAATCTGGGTAATCAACGCGTAGAACTA
TGGCATTGGGATACAGGTGTTGTTTCCTGTTTATATAAGCGTAATTTCACATATGATGTGAATGCTGATTATTTGTATT
TTCATTTTTATCAGGAAGGTGGTACTTTTTATGCATATTTTACAGACACTGGTGTTGTTACTAAGTTTCTGTTTAATGT
ATATTTAGGCACGTTGCTTTCACACTATTATGTCATGCCTTTGACTTGTAATAGTGCTTTGAATTTAGAATATTGGGTT
ACACCTCTCACTTCTAAACAATATTTACTCGCTTTCAATCAAGATGGTGTTATTTTTAATGCTGTTGATTGTAAGAGTG
ATTTTATGAGTGAGATTAAGTGTAAAACACTATCTATAGCACCATCTACTGGTGTTTATGAATTAAACGGTTACACTGT
TCAGCCAATTGCAGATGTTTACCGACGTATACCTAATCTTCCCGATTGTAATATAGAGGCTTGGCTTAATGACAAGTCG
GTGCCCTCTCCATTAAATTGGGAACGTAAGACCTTTTCAAATTGTAATTTTAATATGAGCAGCCTGATGTCTTTTATTC
AGGCAGACTCATTTACTTGTAATAATATTGATGCTGCTAAGATATATGGTATGTGTTTTTCCAGCATAACTATAGATAA
GTTTGCTATACCCAATGGTAGGAAGGTTGACCTACAATTGGGCAATTTGGGCTATTTGCAGTCTTTTAACTATAGAATT
GATACTACTGCTACAAGTTGTCAGTTGTATTATAATTTACCTGCTGCTAATGTTTCTGTTAGCAGGTTTAATCCTTCTA
CTTGGAATAGGAGATTTGGTTTTACAGAACAATCTGTTTTTAAGCCTCAACCTGCAGGTGTTTTTACTGATCATGATGT
TGTTTATGCACAACATTGTTTCAAAGCTCCCACAAATTTCTGTCCGTGTAAATTGGATGGGTCTTTGTGTGTAGGTAGT
GGTTCTGGTATAGATGCTGGTTATAAAAATAGTGGTATAGGCACTTGTCCTGCAGGTACTAATTATTTAACTTGTTATA
ATGCTGCCCAATGTGATTGTTTGTGCACTCCAGACCCCATTACATCTAAATCTACAGGGCCTTACAAGTGCCCCCAAAC

TAAATATTTGGTTGGCATAGGTGAGCACTGTTCAGGTCTTGCTATTAAAAGTGATCATTGTGGAGGTAATTTTTGTACT
TGTCAACCACAAGCATTTTTGGGTTGGTCTGTTGAGTCTTGTTTACAAGGGGATAGGTGTAATATTTTTGCTAATTTTA
TTTTGCATGGTGTTAATAGTGGTACTACTTGTTCTACTGATTTACAAAAATCAAACACAGACATAATTCTTGGTGTTTG
TGTTAATTATGATCTTTATGGTATTACTGGTCAAGGTATTTTTGTTGAGGTTAATGCGACTTATTATAATAGTTGGCAG
AACCTTTTATATGATTCTAATGGTAATCTCTATGGTTTTAGAGACTACTTAACAAATAGAACTTTTATGATTCGTAGTT
GCTATAGCGGTCGTGTTTCAGCGGCCTTTCATGCTAACTCTTCCGAACCAGCATTGCTATTTCGGAACATTAAATGCAA
TTACGTTTTTAATAATACTCTTTCACGACAGCTGCAACCTATTAACTATTTTGATAGCTATCTTGGTTGTGTTGTCAAT
GCTGATAATAGTACTTCTAGTGTTGTTCAAACATGTGATCTCACAGTAGGTAGTGGTTACTGTGTGGATTACTCTACAA
AAAGACGAAGTCGTAGAGCGATTACCACTGGTTATCGGTTTACCAATTTTGAGCCATTTACTGTTAATTCAGTAAATGA
TAGTTTAGAACCTGTAGGTGGTTTGTATGAAATTCAAATACCTTCAGAGTTTACTATAGGTAACATGGAGGAGTTTATT
CAAACAAGCTCTCCTAAAGTTACTATTGATTGTTCTGCTTTTGTCTGTGGTGATTATGCAGCATGTAAATCACAGTTGG
TTGAATATGGTAGCTTCTGTGACAATATTAATGCTATACTCACAGAAGTGAATGAACTACTTGACACTACACAGTTGCA
AGTAGCTAATAGTTTAATGAATGGTGTCACTCTTAGCACTAAGCCTAAAGATGGCGTTAATTTCAATGTAGACGACATC
AATTTTTCCCCTGTATTAGGTTGTTTAGGAAGCGATTGTAATAGAGTTTCCAGTAGATCAGCTATAGAGGATTTACTTT
TTTCTAAAGTAAAGTTAGCTGATGTCGGTTTTGTTGAGGCTTATAATAATTGTACTGGAGGTGCCGAAATTAGGGACCT
CATTTGTGTGCAAAGTTATAATGGTATCAAGGTGTTGCCTCCACTGCTCTCAGAAAATCAGATCAGTGGATATACTTTG
GCTGCGACCTCTGCTAGTCTGTTTCCTCCATGGTCAGCAGCAGCAGGTGTACCATTTTATTTAAATGTTCAGTATCGTA
TTAATGGGATTGGTGTTACCATGGATGTGCTAAGTCAAAATCAAAAGCTTATTGCTAATGCATTTAACAATGCCCTTGG
TGCTATTCAGGAAGGGTTTGATGCTACCAATTCTGCTTTAGTTAAAATTCAAGCTGTTGTTAATGCAAATGCTGAAGCT
CTTAATAACTTATTGCAACAACTCTCTAATAGGTTTGGTGCTATAAGTTCTTCTTTACAAGAAATTCTATCTAGACTGG
ATGCTCTTGAAGCGCAAGCTCAGATAGACAGACTTATTAATGGGCGTCTTACCGCTCTTAATGCTTATGTTTCTCAACA
GCTTAGTGACTCTACACTAGTAAAATTTAGTGCAGCACAAGCTATGGAGAAGGTTAATGAATGTGTCAAAAGCCAATCA
TCTAGGATAAATTTTTGTGGTAATGGCAATCATATTATATCATTAGTGCAGAATGCTCCATATGGTTTGTATTTTATCC
ACTTTAGCTATGTCCCTACTAAGTATGTCACTGCGAAGGTTAGTCCTGGTCTGTGCATTGCTGGTGATAGAGGTATAGC
CCCCAAGAGTGGTTATTTTGTTAATGTAAATAATACTTGGATGTTCACTGGTAGTGGTTATTACCACCCTGAACCTATA
ACTGGAAATAATGTTGTCGTTATGAGTACCTGTGCTGTTAACTACACCAAAGCACCGGATGTAATGCTGAACATTTCAA
CACCCAACCTCCCTGATTTTAAGGAAGAGTTGGATCAATGGTTTAAAAATCAAACATCAGTGGCACCAAATTTGTCACT
TGATTATATAAATGTTACATTCTTGGACCTACAAGACGAAATGACTAGGTTACAGGAGGCAATAAAAGTTTTAAATCAG
AGCTACATCAATCTCAAGGACATTGGTACATATGAGTATTATGTAAAATGGCCTTGGTATGTATGGCTTTTAATTGGCT
TTGCTGGTGTAGCTATGCTTGTTTTACTATTCTTCATATGCTGTTGTACAGGATGTGGGACTAGTTGTTTTAAGAAATG
TGGTGGTTGTTGTGATGATTATACTGGACACCAGGAGTTAGTAATTAAAACATCACATGACGACTAA

>gi|519668523|gb|KF169929.1|_Bovine_coronavirus_isolate_SWE/I/075_spike_glycoprotein_(S)_gene_complete_cds

ATGTTTTTGATACTTTTAATTTCCTTACCAACGACTTTTGCTGTTATAGGAGATTTAAAGTGTACTACAGTTTCC
ATTAATGATGTTGACACTGGTGTTCCTTCTATTAGCACTGATACTGTCGATGTTACTAATGGTTTAGGTACTTATTATG
TTTTAGATCGTGTGTATTTAAATACTACCTTGTTGCTTAATGGTTATTACCCTACTTCAGGTTCTACATATCGTAATAT
GGCACTGAAGGGAACTTTACTATTGAGCACACTATGGTTTAAACCACCTTTCCTTTCTGATTTTACTAATGGTATTTTT
GCTAAGGTCAAAAATACCAAGGTTATTAAAAATGGTGTAATGTATAGTGAGTTTCCTGCTATAACTATAGGTAGTACTT
TTGTAAATACATCCTATAGTGTGGTAGTACAACCACATACTACCAATTTTGACAATAAAATACAAGGTCTCTTAGAGGT
CTCTGTTTGCCAGTATACTATGTGTGAGTACCCAAATACGATTTGTAATCCTAATCTGGGTAATCAACGCGTAGAACTA
TGGCATTGGGATATAGGTGTTGTTTCCTGTTTATATAAGCGTAATTTCACATATGATGTGAATGCTGATTATTTGTATT

TTCATTTTTATCAAGAAGGTGGTACTTTTTATGCATATTTTACAGACACTGGTGTTGTTACTAAGTTTCTGTTTAATGT
ATATTTAGGCACGGTGCTTTCACACTATTATGTCATGCCTTTGACTTGTAATAGTGCTTTGAAGTTAGAATATTGGGTT
ACACCTCTCACTTCTAAACAATATTTACTCGCTTTCAATCAAGATGGTGTTATTTTTAATGCTGTTGATTGTAAGAGTG
ATTTTATGAGTGAGATTAAGTGTAAAACACTATCTATAGCACCATCTACTGGTGTTTATGAATTAAACGGTTACACTGT
TCAGCCAATTGCAGATGTTTACCGACGTATACCTAATCTTCCCGATTGTAATATAGAGGCTTGGCTTAATGACAAGTCG
GTGCCCTCTCCATTAAATTGGGAACGTAAGACCTTTTCAAATTGTAATTTTAATATGAGCAGCCTGATGTCTTTTATTC
AGGCAGACTCATTTACTTGTAATAATATTGATGCTGCTAAGATATATGGTATGTGTTTTTCCAGCATAACTATAGATAA
GTTTGCTATACCCAATGGTAGGAAGGTTGACCTACAATTGGGCAATTTGGGCTATTTGCAGTCTTTTAACTATAGAATT
GATACTACTGCTACAAGTTGTCAGTTGTATTATAATTTACCTGCTGCTAATGTTTCTGTTAGCAGGTTTAATCCTTCTA
CTTGGAATAGGAGATTTGGTTTTACAGAACAATCTGTTTTTAAGCCTCAACCTGTAGGTGTTTTTACTGATCATGATGT
TGTCTATGCACAACATTGTTTCAAAACTCCCACAAATTTCTGTCCGTGTAAATTGGATGGGTCTTTGTGTGTAGGTAGT
GGTTCTGGTATAGATGCTGGTTATAAAAATAGTGGTATAGGCACTTGTCCTGCAGGTACTAATTATTTAACTTGTCATA
ATGCTGCCCAATGTGATTGTTTGTGCACTCCAGACCCCATTACATCTAAAGCTACAGGGCCTTACAAGTGCCCCCAAAC
TAAATATTTAGTTGGCATAGGTGAGCACTGTTCAGGTCTTGCTATTAAAAGTGATCATTGTGGAGGTAATCCTTGTACT
TGTCAACCACAAGCATTTTTGGGTTGGTCTGTTGAGTCTTGTTTACAAGGGGATAGGTGTAATATTTTTGCTAATTTTA
TTTTGCATGGTGTTAATAGTGGTACTACTTGTTCTACTGATTTACAAAAATCAAACACAGACATAATTCTTGGTGTTTG
TGTTAATTATGATCTTTATGGTATTACTGGTCAAGGTATTTTTGTTGAGGTTAATGCGACTTATTATAATAGTTGGCAG
AACCTTTTATATGATTCTAATGGTAATCTCTATGGTTTTAGAGACTACTTAACAAATAGAACTTTTATGATTCGTAGTT
GCTATAGCGGTCGTGTTTCAGCGGCCTTTCATGCTAACTCTTCCGAACCAGCATTGCTATTTCGGAACATTAAATGCAA
TTACGTTTTTAATAATACTCTTTCACGACAGCTGCAACCTATTAACTATTTTGATAGCTATCTTGGTTGTGTTGTCAAT
GCTGATAATAGTACTTCTAGTGTTGTTCAAACATGTGATCTCACAGTAGGTAGTGGTTACTGTGTGGATTACTCTACAA
AAAGACGAAGTCGTAGAGCGATTACCACTGGTTATCGGTTTACCAATTTTGAGCCATTTACTGTTAATTCAGTAAATGA
TAGTTTAGAACCTGTAGGTGGTTTGTATGAAATTCAAATACCTTCAGAGTTTACTATAGGTAACATGGAGGAGTTTATT
CAAACAAGCTCTCCTAAAGTTACTATTGATTGTTCTGCTTTTGTTTGTGGTGATTATGCAGCATGTAAATCACAGTTGG
TTGAATATGGTAGCTTCTGTGACAATATTAATGCTATACTCACAGAAGTAAATGAACTACTTGACACTACACAGTTGCA
AGTAGCTAACAGTTTAATGAATGGTGTCACTCTTAGCACTAAGCTTAAAGATGGCGTTAATTTCAATGTAGACGACATC
AATTTTTCCCCTGTATTAGGTTGTTTAGGAAGCGATTGTAATAGAGTTTCCAGTAGATCTGCTATAGAGGATTTACTTT
TTTCTAAAGTAAAGTTAGCTGATGTCGGTTTCGTTGAGGCTTATAATAATTGTACTGGAGGTGCCGAAATTAGGGACCT
CATTTGTGTGCAAAGTTATAATGGTATCAAGGTGTTGCCTCCACTGCTCTCAGAAAATCAGATCAGTGGATATACTTTG
GCTGCGACCTCTGCTAGTCTGTTTCCTCCTTGGTCAGCAGCAGCAGGTGTACCATTTTATTTAAATGTTCAGTATCGTA
TTAATGGGATTGGTGTTACCATGGATGTGCTAAGTCAAAATCAAAAGCTTATTGCTAATGCATTTAACAATGCCCTTGG
TGCTATTCAGGAAGGGTTTGATTCTACCAATTCTGCTTTAGTTAAAATTCAAGCTGTTGTTAATGCAAATGCTGAAGCT
CTTAATAACTTATTGCAACAACTCTCTAACAGGTTTGGTGCTATAAGTTCTTCTTTACAAGAAATTCTATCTAGACTGG
ATGCTCTTGAAGCGCAAGCTCAGATAGACAGACTTATTAATGGGCGTCTTACCGCTCTTAATGCTTATGTTTCTCAACA
GCTTAGTGACTCTACACTAGTAAAATTTAGTGCAGCACAAGCTATGGAGAAGGTTAATGAATGTGTCAAAAGCCAATCA
TCCAGGATAAATTTTTGTGGTAATGGTAATCATATTATATCATTAGTGCAGAATGCTCCATATGGTTTGTATTTTATCC
ACTTTAGCTATGTCCCTACTAAGTATGTCACTGCGAAGGTTAGTCCTGGTCTGTGCATTGCTGGTGATAGAGGTATAGC
CCCCAAGAGTGGTTATTTTGTTAATGTAAATAATACTTGGATGTTCACTGGTAGTGGTTATTACTACCCTGAACCTATA
ACTGGAAATAATGCTGTCGTTATGAGTACCTGTGCTGTTAACTACACCAAAGCACCGGATGTAATGCTGAACATTTCAA
TACCCAACCTCCCTGATTTTAAGGAAGAGTTGGATCAATGGTTTAAAAATCAAACATCAGTGGCACCAAATTTGTCACT
TGATTATATAAATGTTACATTCTTGGACCTACAAGATGAAATGAATAGGTTACAGGAGGCAATAAAAGTTTTAAATCAG
AGCTACATCAATCTCAAGGACATTGGTACATATGAGTATTATGTAAAATGGCCTTGGTATGTATGGCTTTTAATTGGCT
TTGCTGGTGTAGCTATGCTTGTTTTACTATTCTTCATATGCTGTTGTACAGGATGTGGGACTAGTTGTTTTAAGAAATG
TGGTGGTTGTTGTGATGATTATACTGGACACCAGGAGTTAGTAATTAAAACATCACATGACGACTAA

>gi|519668525|gb|KF169930.1|_Bovine_coronavirus_isolate_SWE/C/076_spike_glycoprotein_(S)_gene_complete_cds

ATGTTTTTGATACTTTTAATTTCCTTACCAACGACTTTTGCTGTTATAGGAGATTTAAAGTGTACTACAGTTTCC
ATTAATGATGTTGACACTGGTGTTCCTCTTATTAGCACTGATACTGTCGATGTTACTAATGGTTTAGGTACTTATTATG
TTTTAGATCGTGTGTATTTAAATACTACCTTGTTGCTTAATGGTTATTACCCTACTTCAGGTTCTACATATCGTAATAT
GGCACTGAAGGGAACTTTACTATTGAGCACACTATGGTTTAAACCACCTTTCCTTTCTGATTTTACTAATGGTATTTTT
GCTAAGGTCAAAAATACCAAGGTTATTAAAAATGGTGTAATGTATAGTGAGTTTCCTGCTATAACTATAGGTAGTACTT
TTGTAAATACATCCTATAGTGTGGTAGTACAACCACATACTACCAATTTTGACAATAAAATACAAGGTCTCTTAGAGGT
CTCTGTTTGCCAGTATACTATGTGTGAGTACCCAAATACGATTTGTAATCCTAATCTGGGTAATCAACGCGTAGAACTA
TGGCATTGGGATATAGGTGTTGTTTCCTGTTTATATAAGCGTAATTTCACATATGATGTGAATGCTGATTATTTGTATT
TTCATTTTTATCAGGAAGGTGGTACTTTTTATGCATATTTTACAGACACTGGTGTTGTTACTAAGTTTCTGTTTAATGT
ATATTTAGGCACGTTGCTTTCACACTATTATGTCATGCCTTTGACTTGTAATAGTGCTTTGAATTTAGAATATTGGGTT
ACACCTCTCACTTCTAAACAATATTTACTCGCTTTCAATCAAGATGGTGTTATTTTTAATGCTGTTGATTGTAAGAGTG
ATTTTATGAGTGAGATTAAGTGTAAAACACTATCTATAGCACCATCTACTGGTGTTTATGAATTAAACGGTTACACTGT
TCAGCCAATTGCAGATGTTTACCGACGTATACCTAATCTTCCCGATTGTAATATAGAGGCTTGGCTTAATGACAAGTCG
GTGCCCTCTCCATTAAATTGGGAACGTAAGACCTTTTCAAATTGTAATTTTAATATGAGCAGCCTGATGTCTTTTATTC
AGGCAGACTCATTTACTTGTAATAATATTGATGCTGCTAAGATATATGGAATGTGTTTTTCCAGCATAACTATAGATAA
GTTTGCTATACCCAATGGTAGGAAGGTTGACCTACAATTGGGCAATTTGGGCTATTTGCAGTCTTTTAACTATAGAATT
GATACTACTGCTACAAGTTGTCAGTTGTATTATAATTTACCTGCTGCTAATGTTTCTGTTAGCAGGTTTAATCCTTCTA
CTTGGAATAGGAGATTTGGTTTTACAGAACAATCTGTTTTTAAGCCTCAACCTGCAGGTGTTTTTACTGATCATGATGT
TGTTTATGCACAACATTGTTTCAAAGCTCCCACAAATTTCTGTCCGTGTAAATTGGATGGGTCTTTGTGTGTAGGTAGT
GGTTCTGGTATAGATGCTGGTTATAAAAATAGTGGTATAGGCACTTGTCCTGCAGGTACTAATTATTTAACTTGTTATA
ATGCTGCCCAATGTGATTGTTTGTGCACTCCAGACCCCATTACATCTAAAGCTACAGGGCCTTACAAGTGCCCCCAAAC
TAAATATTTGGTTGGCATAGGTGAGCACTGTTCAGGTCTTGCTATTAAAAGTGATCATTGTGGAGGTAATCTTTGTACT
TGTCAACCACAAGCATTTTTGGGTTGGTCTGTTGAGTCTTGTTTACAAGGGGATAGGTGTAATATTTTTGCTAATTTTA
TTTTGCATGGTGTTAATAGTGGTACTACTTGTTCTACTGATTTACAAAAATCAAACACAGACATAATTCTTGGTGTTTG
TGTTAATTATGATCTTTATGGTATTACTGGTCAAGGTATTTTTGTTGAGGTTAATGCGACTTATTATAATAGTTGGCAG
AACCTTTTATATGATTCTAATGGTAATCTCTATGGTTTTAGAGACTACTTAACAAATAGAACTTTTATGATTCGTAGTT
GCTATAGCGGTCGTGTTTCAGCGGCCTTTCATGCTAACTCTTCCGAACCAGCATTGCTATTTCGGAACATTAAATGCAA
TTACGTTTTTAATAATACTCTTTCACGACAGCTGCAACCTATTAACTATTTTGATAGCTATCTTGGTTGTGTTGTCAAT
GCTGATAATAGTACTTCTAGTGTTGTTCAAACATGTGATCTCACAGTAGGTAGTGGTTACTGTGTGGATTACTCTACAA
AAAGACGAAGTCGTAGAGCGATTACCACTGGTTATCGGTTTACTAATTTTGAGCCATTTACTGTTAATTCAGTAAATGA
TAGTTTAGAACCTGTAGGTGGTTTGTATGAAATTCAAATACCTTCAGAGTTTACTATAGGTAACATGGAGGAGTTTATT
CAAACAAGCTCTCCTAAAGTTACTATTGATTGTTCTGCTTTTGTCTGTGGTGATTATGCAGCATGTAAATCACAGTTGG
TTGAATATGGTAGCTTCTGTGACAATATTAATGCTATACTCACAGAAGTAAATGAACTACTTGACACTACACAGTTGCA
AGTAGCTAATAGTTTAATGAATGGTGTCACTCTTAGCAGTAAGCTTAAAGATGGCGTTAATTTCAATGTAGACGACATC
AATTTTTCCCCTGTATTAGGTTGTTTAGGAAGCGATTGTAATAGAGTTTCCAGTAGATCTGCTATAGAGGATTTACTTT
TTTCTAAAGTAAAGTTAGCTGATGTCGGTTTTGTTGAGGCTTATAATAATTGTACTGGAGGTGCCGAAATTAGGGACCT
CATTTGTGTGCAAAGTTATAATGGTATCAAGGTGTTGCCTCCACTGCTCTCAGAAAATCAGATCAGTGGATATACTTTG
GCTGCGACCTCTGCTAGTCTGTTTCCTCCATGGTCAGCAGCAGCAGGTGTACCATTTTATTTAAATGTTCAGTATCGTA
TTAATGGGATTGGTGTTACCATGGATGTGTTAAGTCAAAATCAAAAGCTTATTGCTAATGCATTTAACAATGCCCTTGG
TGCTATTCAGGAAGGGTTTGATGCTACCAATTCTGCTTTAGTTAAAATTCAAGCTGTTGTTAATGCAAATGCTGAAGCT

CTTAATAACTTATTGCAACAACTCTCTAATAGGTTTGGTGCTATAAGTTCTTCTTTACAAGAAATTCTATCTAGACTGG
ATGCTCTTGAAGCGCAAGCTCAGATAGACAGACTTATTAATGGGCGTCTTACCGCTCTTAATGCTTATGTTTCTCAACA
GCTTAGTGACTCTACACTAGTAAAATTTAGTGCAGCACAAGCTATGGAGAAGGTTAATGAATGTGTCAAAAGCCAATCA
TCTAGGATAAATTTTTGTGGTAATGGCAATCATATTATATCATTAGTGCAGAATGCTCCATATGGTTTGTATTTTATCC
ACTTTAGCTATGTCCCTACTAAGTATGTCACTGCGAAGGTTAGTCCTGGTCTGTGCATTGCTGGTGATAGAGGTATAGC
CCCCAAGAGTGGTTATTTTGTTAATGTAAATAATACTTGGATGTTCACTGGTAGTGGTTATTACTACCCTGAACCTATA
ACTGGAAATAATGTTGTCGTTATGAGTACCTGTGCTGTTAACTACACCAAAGCACCGGATGTAATGCTGAACATTTCAA
CACCCAACCTCCCTGATTTTAAGGAAGAGTTGGATCAATGGTTTAAAAATCAAACATCAGTGGCACCAAATTTGTCACT
TGATTATATAAATGTTACATTCTTGGACCTACAAGACGAAATGAATAGGTTACAGGAGGCAATAAAAGTTTTAAATCAG
AGCTACATCAATCTCAAGGACATTGGTACATATGAGTATTATGTAAAATGGCCTTGGTATGTATGGCTTTTAATTGGCT
TTGCTGGTGTAGCTATGCTTGTTTTACTATTCTTCATATGCTGTTGTACAGGATGTGGGACTAGTTGTTTTAAGAAATG
TGGTGGTTGTTGTGATGATTATACTGGACACCAGGAGTTAGTAATTAAAACATCCCATGACGACTAA

>gi|519668527|gb|KF169931.1|_Bovine_coronavirus_isolate_SWE/AC/081_spike_glycoprotein_(S)_gene_complete_cds

ATGTTTTTGATACTTTTAATTTCCTTACCAACGACTTTTGCTGTTATAGGAGATTTAAAGTGTACTACAGTTTCC
ATTAATGATGTTAACACTGGTGTTCCTCTTATTAGCACTGATACTGTCGATGTTACTAATGGTTTAGGTACTTATTATG
TTTTAGATCGTGTGTATTTAAATACTACCTTGTTGCTTAATGGTTATTACCCTACTTCAGGTTCTACATATCGTAATAT
GGCACTGAAGGGAACTTTACTATTGAGCACACTATGGTTTAAACCACCTTTCCTTTCTGATTTTACTAATGGTATTTTT
GCTAAGGTCAAAAATACCAAGGTTATTAAAAATGGTGTAATGTATAGTGAGTTTCCTGCTATAACTATAGGTAGTACTT
TTGTAAATACATCCTATAGTGTGGTAGTACAACCACATACTACCAATTTTGACAATAAAATACAAGGTCTCTTAGAGGT
CTCTGTTTGCCAGTATACTATGTGTGAGTACCCAAATACGATTTGTAATCCTAATCTGGGTAATCAACGCGTAGAACTA
TGGCATTGGGATACAGGTGTTGTTTCCTGTTTATATAAGCGTAATTTCACATATGATGTGAATGCTGATTATTTGTATT
TTCATTTTTATCAGGAAGGTGGTACTTTTTATGCATATTTTACAGACACTGGTGTTGTTACTAAGTTTCTGTTTAATGT
ATATTTAGGCACGTTGCTTTCACACTATTATGTCATGCCTTTGACTTGTAATAGTGCTTTGAATTTAGAATATTGGGTT
ACACCTCTCACTTCTAAACAATATTTACTCGCTTTCAATCAAGATGGTGTTATTTTTAATGCTGTTGATTGTAAGAGTG
ATTTTATGAGTGAGATTAAGTGTAAAACACTATCTATAGCACCATCTACTGGTGTTTATGAATTAAACGGTTACACTGT
TCAGCCAATTGCAGATGTTTACCGACGTATACCTAATCTTCCCGATTGTAATATAGAGGCTTGGCTTAATGACAAGTCG
GTGCCCTCTCCATTAAATTGGGAACGTAAGACCTTTTCAAATTGTAATTTTAATATGAGCAGCCTGATGTCTTTTATTC
AGGCAGACTCATTTACTTGTAATAATATTGATGCTGCTAAGATATATGGTATGTGTTTTTCCAGCATAACTATAGATAA
GTTTGCTATACCCAATGGTAGGAAGGTTGACCTACAATTGGGCAATTTGGGCTATTTGCAGTCTTTTAACTATAGAATT
GATACTACTGCTACAAGTTGTCAGTTGTATTATAATTTACCTGCTGCTAATGTTTCTGTTAGCAGGTTTAATCCTTCTA
CTTGGAATAGGAGATTTGGTTTTACAGAACAATCTGTTTTTAAGCCTCAACCTGCAGGTGTTTTTACTGATCATGATGT
TGTTTATGCACAACATTGTTTCAAAGCTCCCACAAATTTCTGTCCGTGTAAATTGGATGGGTCTTTGTGTGTAGGTAGT
GGTTCTGGTATAGATGCTGGTTATAAAAATAGTGGTATAGGCACTTGTCCTGCAGGTACTAATTATTTAACTTGTTATA
ATGCTGCCCAATGTGATTGTTTGTGCACTCCAGACCCCATTACATCTAAATTTACAGGGCCTTACAAGTGCCCCCAAAC
TAAATATTTGGTTGGCATAGGTGAGCACTGTTCAGGTCTTGCTATTAAAAGTGATCATTGTGGAGGTAATTTTTGTACT
TGTCAACCACAAGCATTTTTGGGTTGGTCTGTTGAGTCTTGTTTACAAGGGGATAGGTGTAATATTTTTGCTAATTTTA
TTTTGCATGGTGTTAATAGTGGTACTACTTGTTCTACTGATTTACAAAAATCAAACACAGACATAATTCTTGGTGTTTG
TGTTAATTATGATCTTTATGGTATTACTGGTCAAGGTATTTTTGTTGAGGTTAATGCGACTTATTATAATAGTTGGCAG
AACCTTTTATATGATTCTAATGGTAATCTCTATGGTTTTAGAGACTACTTAACAAATAGAACTTTTATGATTCGTAGTT
GCTATAGCGGTCGTGTTTCAGCGGCCTTTCATGCTAACTCTTCCGAACCAGCATTGCTATTTCGGAACATTAAATGCAA

TTACGTTTTTAATAATACTCTTTCACGACAGCTGCAACCTATTAACTATTTTGATAGCTATCTTGGTTGTGTTGTCAAT
GCTGATAATAGTACTTCTAGTGTTGTTCAAACATGTGATCTCACAGTAGGTAGTGGTTACTGTGTGGATTACTCTACAA
AAAGACGAAGTCGTAGAGCGATTACCACTGGTTATCGGTTTACCAATTTTGAGCCATTTACTGTTAATTCAGTAAATGA
TAGTTTAGAACCTGTAGGTGGTTTGTATGAAATTCAAATACCTTCAGAGTTTACTATAGGTAACATGGAGGAGTTTATT
CAAACAAGCTCTCCTAAAGTTACTATTGATTGTTCTGCTTTTGTCTGTGGTGATTATGCAGCATGTAAATCACAGTTGG
TTGAATATGGTAGCTTCTGTGACAATATTAATGCTATACTCACAGAAGTGAATGAACTACTTGACACTACACAGTTGCA
AGTAGCTAATAGTTTAATGAAGGGTGTCACTCTTAGCACTAAGCTTAAAGATGGCGTTAATTTCAATGTAGACGACATC
AATTTTTCCCCTGTATTAGGTTGTTTAGGAAGCGATTGTAATAGAGTTTCCAGTAGATCTGCTATAGAGGATTTACTTT
TTTCTAAAGTAAAGTTAGCTGATGTCGGTTTCGTTGAGGCTTATAATAATTGTACTGGAGGTGCCGAAATTAGGGACCT
CATTTGTGTGCAAAGTTATAATGGTATCAAGGTGTTGCCTCCACTGCTCTCAGAAAATCAGATCAGTGGATATACTTTG
GCTGCGACCTCTGCTAGTCTGTTTCCTCCATGGTCAGCAGCAGCAGGTGTACCATTTTATTTAAATGTTCAGTATCGTA
TTAATGGGATTGGTGTTACCATGGATGTGTTAAGTCAAAATCAAAAGCTTATTGCTAATGCATTTAACAATGCCCTTGG
TGCTATTCAGGAAGGGTTTGATGCTACCAATTCTGCTTTAGTTAAAATTCAAGCTGTTGTTAATGCAAATGCTGAAGCT
CTTAATAACTTATTGCAACAACTCTCTAATAGGTTTGGTGCTATAAGTTCTTCTTTACAAGAAATTCTATCTAGACTGG
ATGCTCTTGAAGCGCAAGCTCAGATAGACAGACTTATTAATGGGCGTCTTACCGCTCTTAATGCTTATGTTTCTCAACA
GCTTAGTGACTCTACACTAGTAAAATTTAGTGCAGCACAAGCTATGGAGAAGGTTAATGAATGTGTCAAAAGCCAATCA
TCTAGGATAAATTTTTGTGGTAATGGCAATCATATTATATCATTAGTGCAGAATGCTCCATATGGTTTGTATTTTATCC
ACTTTAGCTATGTCCCTACTAAGTATGTCACTGCGAAGGTTAGTCCTGGTCTGTGCATTGCTGGTGATAGAGGTATAGC
CCCCAAGAGTGGTTATTTTGTTAATGTAAATAATACTTGGATGTTCACTGGTAGTGGTTATTACTACCCTGAACCTATA
ACTGGAAATAATGTTGTCGTTATGAGTACCTGTGCTGTTAACTACACCAAAGCACCGGATGTAATGCTGAACATTTCAA
TACCCAACCTCCCTGATTTTAAGGAAGAGTTGGATCAATGGTTTAAAAATCAAACATCAGTGGCACCAAATTTGTCACT
TGATTATATAAATGTTACATTCTTGGACCTACAACACGAAATGAATAGGTTACAGGAGGCAATAAAAGTTTTAAATCAG
AGCTACATCAATCTCAAGGACATTGGTACATATGAGTATTATGTAAAATGGCCTTGGTATGTATGGCTTTTAATTGGCT
TTGCTGGTGTAGCTATGCTTGTTTTACTATTCTTCATATGCTGTTGTACAGGATGTGGGACTAGTTGTTTTAAGAAATG
TGGTGGTTGTTGTGATGATTATACTGGACACCAGGAGTTAGTAATTAAAACATCACATGACGACTAA

>gi|519668529|gb|KF169932.1|_Bovine_coronavirus_isolate_SWE/C/082_spike_glycoprotein_(S)_gene_complete_cds

ATGTTTTTGATACTTTTAATTTCCTTACCAACGACTTTTGCTGTTATAGGAGATTTAAAGTGTACTACAGTTTCC
ATTAATGATGTTAACACTGGTGTTCCTCTTATTAGCACTGATACTGTCGATGTTACTAATGGTTTAGGTACTTATTATG
TTTTAGATCGTGTGTATTTAAATACTACCTTGTTGCTTAATGGTTATTACCCTACTTCAGGTTCTACATATCGTAATAT
GGCACTGAAGGGAACTTTACTATTGAGCACACTATGGTTTAAACCACCTTTCCTTTCTGATTTTACTAATGGTATTTTT
GCTAAGGTCAAAAATACCAAGGTTATTAAAAATGGTGTAATGTATAGTGAGTTTCCTGCTATAACTATAGGTAGTACTT
TTGTAAATACATCCTATAGTGTGGTAGTACAACCACATACTACCAATTTTGACAATAAAATACAAGGTCTCTTAGAGGT
CTCTGTTTGCCAGTATACTATGTGTGAGTACCCAAATACGATTTGTAATCCTAATCTGGGTAATCAACGCGTAGAACTA
TGGCATTGGGATACAGGTGTTGTTTCCTGTTTATATAAGCGTAATTTCACATATGATGTGAATGCTGATTATTTGTATT
TTCATTTTTATCAGGAAGGTGGTACTTTTTATGCATATTTTACAGACACTGGTGTTGTTACTAAGTTTCTGTTTAATGT
ATATTTAGGCACGTTGCTTTCACACTATTATGTCATGCCTTTGACTTGTAATAGTGCTTTGAATTTAGAATATTGGGTT
ACACCTCTCACTTCTAAACAATATTTACTCGCTTTCAATCAAGATGGTGTTATTTTTAATGCTGTTGATTGTAAGAGTG
ATTTTATGAGTGAGATTAAGTGTAAAACACTATCTATAGCACCATCTACTGGTGTTTATGAATTAAACGGTTACACTGT
TCAGCCAATTGCAGATGTTTACCGACGTATACCTAATCTTCCCGATTGTAATATAGAGGCTTGGCTTAATGACAAGTCG
GTGCCCTCTCCATTAAATTGGGAACGTAAGACCTTTTCAAATTGTAATTTTAATATGAGCAGCCTGATGTCTTTTATTC

AGGCAGACTCATTTACTTGTAATAATATTGATGCTGCTAAGATATATGGTATGTGTTTTTCCAGCATAACTATAGATAA
GTTTGCTATACCCAATGGTAGGAAGGTTGACCTACAATTGGGCAATTTGGGCTATTTGCAGTCTTTTAACTATAGAATT
GATACTACTGCTACAAGTTGTCAGTTGTATTATAATTTACCTGCTGCTAATGTTTCTGTTAGCAGGTTTAATCCTTCTA
TTTGGAATAGGAGATTTGGTTTTACAGAACAATCTGTTTTTAAGCCTCAACCTGCAGGTGTTTTTACTGATCATGATGT
TGTTTATGCACAACATTGTTTCAAAGCTCCCACAAATTTCTGTCCGTGTAAATTGGATGGGTCTTTGTGTGTAGGTAGT
GGTTTTGGTATAGATGCTGGTTATAAAAATAGTGGTATAGGCACTTGTCCTGCAGGTACTAATTATTTAACTTGTTATA
ATGCTGCCCAATGTGATTGTTTGTGCACTCCAGACCCCATTACATCTAAATCTACAGGGCCTTACAAGTGCCCCCAAAC
TAAATATTTGGTTGGCATAGGTGAGCACTGTTCAGGTCTTGCTATTAAAAGTGATCATTGTGGAGGTAATTTTTGTACT
TGTCAACCACAAGCATTTTTGGGTTGGTCTGTTGAGTCTTGTTTACAAGGGGATAGGTGTAATATTTTTGCTAATTTTA
TTTTGCATGGTGTTAATAGTGGTACTACTTGTTCTACTGATTTACAAAAATCAAACACAGACATAATTCTTGGTGTTTG
TGTTAATTATGATCTTTATGGTATTACTGGTCAAGGTATTTTTGTTGAGGTTAATGCGACTTATTATAATAGTTGGCAG
AACCTTTTATATGATTCTAATGGTAATCTCTATGGTTTTAGAGACTACTTAACAAATAGAACTTTTATGATTCGTAGTT
GCTATAGCGGTCGTGTTTCAGCGGCCTTTCATGCTAACTCTTCCGAACCAGCATTGCTATTTCGGAACATTAAATGCAA
TTACGTTTTTAATAATACTCTTTCACGACAGCTGCAACCTATTAACTATTTTGATAGCTATCTTGGTTGTGTTGTCAAT
GCTGATAATAGTACTTCTAGTGTTGTTCAAACATGTGATCTCACAGTAGGTAGTGGTTACTGTGTGGATTACTCTACAA
AAAGACGAAGTCGTAGAGCGATTACCACTGGTTATCGGTTTACCAATTTTGAGCCATTTACTGTTAATTCAGTAAATGA
TAGTTTAGAACCTGTAGGTGGTTTGTATGAAATTCAAATACCTTCAGAGTTTACTATAGGTAACATGGAGGAGTTTATT
CAAACAAGCTCTCCTAAAGTTACTATTGATTGTTCTGCTTTTGTCTGTGGTGATTATGCAGCATGTAAATCACAGTTGG
TTGAATATGGTAGCTTCTGTGACAATATTAATGCTATACTCACAGAAGTGAATGAACTACTTGACACTACACAGTTGCA
AGTAGCTAATAGTTTAATGAAGGGTGTCACTCTTAGCACTAAGCTTAAAGATGGCGTTAATTTCAATGTAGACGACATC
AATTTTTCCCCTGTATTAGGTTGTTTAGGAAGCGATTGTAATAGAGTTTCCAGTAGATCTGCTATAGAGGATTTACTTT
TTTCTAAAGTAAAGTTAGCTGATGTCGGTTTTGTTGAGGCTTATAATAATTGTACTGGAGGTGCCGAAATTAGGGACCT
CATTTGTGTGCAAAGTTATAATGGTATCAAGGTGTTGCCTCCACTGCTCTCAGAAAATCAGATCAGTGGATATACTTTG
GCTGCGACCTCTGCTAGTCTGTTTCCTCCATGGTCAGCAGCAGCAGGTGTACCATTTTATTTAAATGTTCAGTATCGTA
TTAATGGGATTGGTGTTACCATGGATGTGTTAAGTCAAAATCAAAAGCTTATTGCTAATGCATTTAACAATGCCCTTGG
TGCTATTCAGGAAGGGTTTGATGCTACCAATTCTGCTTTAGTTAAAATTCAAGCTGTTGTTAATGCAAATGCTGAAGCT
CTTAATAACTTATTGCAACAACTCTCTAATAGGTTTGGTGCTATAAGTTCTTCTTTACAAGAAATTCTATCTAGACTGG
ATGCTCTTGAAGCGCAAGCTCAGATAGACAGACTTATTAATGGGCGTCTTACCGCTCTTAATGCTTATGTTTCTCAACA
GCTTAGTGACTCTACACTAGTAAAATTTAGTGCAGCACAAGCTATGGAGAAGGTTAATGAATGTGTCAAAAGCCAATCA
TCTAGGATAAATTTTTGTGGTAATGGCAATCATATTATATCATTAGTGCAGAATGCTCCATATGGTTTGTATTTTATCC
ACTTTAGCTATGTCCCTACTAAGTATGTCACTGCGAAGGTTAGTCCTGGTCTGTGCATTGCTGGTGATAGAGGTATAGC
CCCCAAGAGTGGTTATTTTGTTAATGTAAATAATACTTGGATGTTCACTGGTAGTGGTTATTACTACCCTGAACCTATA
ACTGGAAATAATGTTGTCGTTATGAGTACCTGTGCTGTTAACTACACCAAAGCACCGGATGTAATGCTGAACATTTCAA
TACCCAACCTCCCTGATTTTAAGGAAGAGTTGGATCAATGGTTTAAAAATCAAACATCAGTGGCACCAAATTTGTCACT
TGATTATATAAATGTTACATTCTTGGACCTACAACACGAAATGAATAGGTTACAGGAGGCAATAAAAGTTTTAAATCAG
AGCTACATCAATCTCAAGGACATTGGTACATATGAGTATTATGTAAAATGGCCTTGGTATGTATGGCTTTTAATTGGCT
TTGCTGGTGTAGCTATGCTTGTTTTACTATTCTTCATATGCTGTTGTACAGGATGTGGGACTAGTTGTTTTAAGAAATG
TGGTGGTTGTTGTGATGATTATACTGGACACCAGGAGTTAGTAATTAAAACATCACATGACGACTAA

>gi|519668531|gb|KF169933.1|_Bovine_coronavirus_isolate_SWE/I/083_spike_glycoprotein_(S)_gene_complete_cds

ATGTTTTTGATACTTTTAATTTCCTTACCAACGGCTTTTGCTGTTATAGGAGATTTAAAGTGTACTTCGCTTTCC

ATTAATGATGTTGACACTGGTGTTCCTTCTATTAGCACTGATACTGTTGATGTTACTAATGGTTTAGGTACTTATTATG
TTTTAGATCGTGTGTATTTAAATACTACCTTGTTGCTTAATGGTTATTACCCTACTTCAGGTTCTACATATCGTAATAT
GGCACTGAAGGGAACTTTACTATTGAGCACACTATGGTTTAAACCACCTTTTTTTTCTGATTTTACTAATGGTATTTTT
GCTAAGGTCAAAAATACCAAGGTTATTAAAAATGGTGTAATGTATAGTGAGTTTCCTGCTATAACTATAGGTAGTACTT
TTGTAAATACATCTTATAGTGTGGTAGTACAACCACATACTACCAATTTGGATAATAAATTACAAGGTCTCTTAGAGAT
CTCTGTTTGCCAGTATACTATGTGCGAGTACCCAAATACGATTTGTAATCCTAATCTGGGTAACCAACGCGTAGAACTA
TGGCATTGGGATACAGGTGTTGTTTCCTGTTTATATAAGCGTAATTTCACATATGATGTGAATGCTGATTATTTGTATT
TCCATTTTTATCAAGAAGGTGGTACTTTTTATGCATATTTTACAGACACTGGTGTTGTTACTAAGTTTCTGTTTAATGT
ATATTTAGGCACCGTGCTTTCACACTATTATGTCATGCCTTTGACTTGTAATAGTGCTTTGAATTTAGAATATTGGGTT
ACACCTCTCACTTCTAAACAATATTTACTAGCTTTCAATCAAGATGGTGTTATTTTTAATGCTGTTGATTGTAAGAGTG
ATTTTATGAGTGAGATTAAGTGTAAAACATTATCTATAGCACCATCTACTGGTGTTTATGAATTAAACGGTTACACTGT
TCAGCCAATTGCAGATGTTTACCGACGTATACCTAATCTTCCCGATTGTAATATAGAGGCTTGGCTTAATGACAAGTCG
GTGCCCTCTCCATTAAATTGGGAACGTAAGACCTTTTCAAATTGTAATTTTAATATGAGCAGCCTGATGTCTTTTATTC
AGGCAGACTCATTTACTTGTAATAATATTGATGCTGCTAAGATATATGGTATGTGTTTTTCCAGCATAACTATAGATAA
GTTTGCAATACCCAATGGTAGGAAGGTTGACCTACAATTGGGCAATTTGGGCTATTTGCAGTCTTTTAATTATAGAATT
GATACTACTGCTACAAGTTGTCAGTTGTATTATAATTTACCTGCTGCTAATGTTTCTGTTAGCAGGTTTAATCCTTCTA
CTTGGAATAGGAGATTTGGTTTTACAGAACAATCTGTTTTTAAGCCTCAACCTGCAGGTGTCTTTACTGATCATGATGT
TGTTTATGCACAACATTGTTTTAAAGCTCCCACAAATTTCTGTCCGTGTAAATTGGATGGGTCTTTGTGTGTAGGTAGT
GGTTCTGGTATAGATGCTGGTTATAAAAATAGTGGTATAGGCACTTGTCCTGCAGGTACTAATTATTTAATTTGTTATA
ATGCTGCCCAATGTGACTGTTTGTGCACTCCAGACCCCATTACATCTAAAGCTACAGGGCCTTACAAGTGCCCCCAAAC
TAAATATTTAGTTGGCATAGGTGAGCACTGTTCAGGTCTTGCTATTAAAAGTGATCATTGTGGAGGTAATCCATGTACT
TGCCAACCACAAGCATTTTTGGGTTGGTCTGTTGACTCTTGTTTACAAGGGGATAGGTGTAATATTTTTGCTAATTTTA
TTTTGCATGGTGTTAATAGTGGTACTACTTGTTCTACTGATTTACAAAAATCAAACACAGACATAATTCTTGGTGTTTG
TGTTAGTTATGATCTTTATGGTATTACTGGCCAAGGTATTTTTGTTGAGGTTAATGCGACTTATTATAATAGTTGGCAG
AACCTTTTATATGATTCTAATGGTAATCTCTATGGTTTTAGAGACTACTTAACAAACAGAACTTTCATGATTCGTAGTT
GCTATAGCGGTCGTGTTTCAGCGGCCTTTCATGCTAACTCTTCCGAACCAGCATTGCTATTTCGGAATATTAAATGCAA
CTACGTTTTTAATAATACTCTTTCACGACAGCTGCAACCTATTAACTATTTTGATAGCTATCTTGGTTGTGTTGTCAAT
GCTGATAATAGTACTTCTAGTGTTGTTCAAACATGTGATCTCACAGTAGGTAGTGGTTACTGTGTGGATTACTCTACAA
AAAGACGAAGTCGTAGAGCGATTACCACTGGTTATCGGTTTACTAATTTTGAGCCATTTACTGTTAAGTCAGTAAATGA
TAGTTTAGAACCTGTAGGTGGTTTGTATGAAATTCAAATACCTTCAGATTTTACTATAGGTAACATGGAGGAGTTTATT
CAAACAAGCTCTCCTAAAGTTACTATTGATTGTTCTACTTTTGTCTGTGGTGATTATGCAGCATGTAAATCACAGTTGG
TTGAATATGGTAGCTTTTGTGACAATATTAATGCTATACTCACAGAAGTAAATGAACTACTTGACACTACACAGTTGCA
AGTAGCTAATAGTTTAATGAATGGTGTCACTCTTAGCACTAAGCTTAATGATGGCGTTAAGTTCAATGTAGACGACATC
AATTTTTCCCCTGTATTAGGTTGTTTAGGAAGCGATTGTAACAGAGTTTCCAGTAGATCTGCTATAGAGGATTTACTTT
TTTCTAAAGTAAAGTTAGCTGATGTCGGTTTCGTGGAAGCTTATAATAATTGTACTGGAGGTGCCGAAATTAGGGACCT
CATTTGTGTGCAAAGTTATAATGGTATCAAGGTTTTGCCTCCACTGCTCTCAGAAAATCAGATCAGTGGATATACTTTG
GCTGCGACCTCTGCTAGTCTGTTTCCTCCTTGGTCAGCAGCAGCAGGTGTACCATTTTATTTAAATGTTCAGTATCGTA
TTAATGGGATTGGTGTTACCATGGATGTGCTAAGTCAAAATCAAAAGCTTATTGCTAATGCATTTAACAATGCCCTTGG
TGCTATCCAGGAAGGGTTTGATGCTACCAATTCTGCTTTAGTCAAAATTCAAGCTGTTGTTAATGCAAATGCTGAAGCT
CTTAATAACTTACTGCAACAACTCTCTAACAGGTTTGGTGCTATAAGTTCTTCTTTACAAGAAATTCTATCTAGACTGG
ATGCTCTTGAAGCGCAAGCTCAGATAGACAGACTTATTAATGGGCGTCTTACCGCTCTTAATGCTTATGTTTCTCAACA
GCTTAGTGACTCTACACTAGTAAAATTTAGTGCAGCACAAGCTATGGAGAAGGTTAATGAATGTGTCAAAAGCCAATCA
TCTAGGATAAATTTTTGTGGTAATGGTAATCATATTATATCATTAGTGCAGAATGCTCCATATGGTTTGTATTTTATCC
ACTTTAGCTATGTCCCTACTAAGTATGTCACCGCGAAGGTTAGTCCTGGTCTGTGCATTGCTGGTGATAGAGGTATAGC

ACCCAAGAGTGGTTATTTTGTTAATGTAAATAATACTTGGATGTTCACTGGTAGTGGTTATTACTACCCTGAACCTATA
ACTGGAAATAATGTTGTTGTTATGAGTACCTGTGCTGTTAATTATACTAAAGCACCGGATGTAATGCTGAACATTTCAA
CACCCAACCTCCCTGATTTTAAGGAAGAGTTGGATCAATGGTTTAAAAACCAAACATCAGTGGCACCAAATTTGTCACT
TGATTATATAAATGTTACATTCTTGGACCTACAAGATGAAATGAATAGGTTACAGGAGGCAATAAAAGTTTTAAATCAG
AGCTACATCAATCTCAAGGACATTGGTACATATGAGTATTATGTAAAATGGCCTTGGTATGTATGGCTTTTAATTGGCT
TTGCTGGTGTAGCTATGCTTGTTTTACTATTCTTCATATGCTGTTGCACAGGATGTGGGACTAGTTGTTTTAAGAAATG
TGGTGGTTGTTGTGATGATTATACTGGACACCAGGAGTTGGTAATTAAAACATCACATGACGACTAA

>gi|519668533|gb|KF169934.1|_Bovine_coronavirus_isolate_SWE/P/091_spike_glycoprotein_(S)_gene_complete_cds

ATGTTTTTGATACTTTTAATTTCCTTACCAACGACTTTTGCTGTTATAGGAGATTTAAAGTGTACTACAGTTTCC
ATTAATGATGTTGACACTGGTGTTCCTTTTATTAGCACTGATACTGTCGATGTTACTAATGGTTTAGGTACTTATTATG
TTTTAGATCGTGTGTATTTAAATACTACCTTGTTGCTTAATGGTTATTACCCTACTTCAGGTTCTACATATCGTAATAT
GGCACTGAAGGGAACTTTACTATTGAGCACACTATGGTTTAAACCACCTTTCCTTTCTGATTTTACTAATGGTATTTTT
GCTAAGGTCAAAAATACCAAAGTTATTAAAAATGGTGTAATGTATAGTGAGTTTCCTGCTATAACTATAGGTAGTACTT
TTGTAAATACATCCTATAGTGTGGTAGTACAACCACATACTACCAATTTTGACAATAAAAATACAAGGTCTCTTAGAGGT
CTCTGTTTGCCAGTATACTATGTGTGAGTACCCAAATACGATTTGTAATCCTAATCTGGGTAATCAACGCGTAGAACTA
TGGCATTGGGATACAGGTGTTGTTTCCTGTTTATATAAGCGTAATTTCACATATGATGTGAATGCTGATTATTTGTATT
TTCATTTTTATCAAGAAGGTGGTACTTTTTATGCATATTTTACAGACACTGGTGTTGTTACTAAGTTTCTGTTTAATGT
ATATTTAGGCACGGTGCTTTCACACTATTATGTCATGCCTTTGACTTGTAATAGTGCTTTGAAGTTAGAATATTGGGTT
ACACCTCTCACTTCTAAACAATATTTACTCGCTTTCAATCAAGATGGTGTTATTTTTAATGCTGTTGATTGTAAGAGTG
ATTTTATGAGTGAGATTAAGTGTAAAACACTATCTATAGCACCATCTACTGGTGTTTATGAATTAAACGGTTACACTGT
TCAGCCAATTGCAGATGTTTACCGACGTATACCTAATCTTCCCGATTGTAATATAGAGGCTTGGCTTAATGACAAGTCG
GTGCCCTCTCCATTAAATTGGGAACGTAAGACCTTTTCAAATTGTAATTTTAATATGAGCAGCCTGATGTCTTTTATTC
AGGCAGACTCATTTACTTGTAATAATATTGATGCTGCTAAGATATATGGTATGTGTTTTTCCAGCATAACTATAGATAA
GTTTGCTATACCCAATGGTAGGAAGGTTGACCTACAATTGGGCAATTTGGGCTATTTGCAGTCTTTTAACTATAGAATT
GATACTACTGCTACAAGTTGTCAGTTGTATTATAATTTACCTGCTGCTAATGTTTCTGTTAGCAGGTTTAATCCTTCTA
CTTGGAATAGGAGATTTGGTTTTACAGAACAATCTGTTTTTAAGCCTCAACCTGTAGGTGTTTTTACTGATCATGATGT
TGTCTATGCACAACATTGTTTCAAAACTCCCACAAATTTCTGTCCGTGTAAATTGGATGGGTCTTTGTGTGTAGGTAAT
GGTTCTGGTATAGATGCTGGTTATAAAAATAGTGGTATAGGCACTTGTCCTGCAGGTACTAATTATATAACTTGTTATA
ATGCTGCCCAATGTGATTGTTTGTGCACTCCAGACCCCATTACATCTAAAGCTACAGGGCCTTACAAGTGCCCCCAAAC
TAAATATTTAGTTGGCATAGGTGAGCACTGTTCAGGTCTTGCTATTAAAAGTGATCATTGTGGAGGTAATCCTTGTACT
TGTCAACCACAAGCATTTTTGGGTTGGTCTGTTGAGTTTTGTTTACAAGGGGATAGGTGTAATATTTTTGCTAATTTTA
TTTTGCATGGTGTTAATAGTGGTACTACTTGTTCTACTGATTTACAAAAATCAAACACAGACATAATTCTTGGTGTTTG
TGTTAATTATGATCTTTATGGTATTACTGGTCAAGGTATTTTTGTTGAGGTTAATGCGACTTATTATAACAGTTGGCAG
AACCTTTTATATGATTCTAATGGTAATCTCTATGGTTTTAGAGACTACTTAACAAATAGAACTTTTATGATTCGTAGTT
GCTATAGCGGTCGTGTTTCAGCGGCCTTTCATGCTAACTCTTCCGAACCAGCATTGCTATTTCGGAACATTAAATGCAA
TTACGTTTTTAATAATACTCTTTCACGACAGCTGCAACCTATTAACTATTTTGATAGCTATCTTGGTTGTGTTGTCAAT
GCTGATAATAGTACTTCTAGTGTTGTTCAAACATGTGATCTCACAGTAGGTAGTGGTTACTGTGTGGATTACTCTACAA
AAAGACGAAGTCGTAGAGCGATTACCACTGGTTATCGGTTTACCAATTTTGAGCCATTTACTGTTAATTCAGTAAATGA
TAGTTTAGAACCTGTAGGTGGTTTGTATGAAATTCAAATACCTTCAGAGTTTACTATAGGTAACATGGAGGAGTTTATT
CAAACAAGCTCTCCTAAAGTTACTATTGATTGTTCTGCTTTTGTTTGTGGTGATTATGCAGCATGTAAATCACAGTTGG

TTGAATATGGTAGCTTCTGTGACAATATTAATGCTATACTCACAGAAGTAAATGAACTACTTGACACTACACAGTTGCA
AGTAGCTAACAGTTTAATGAATGGTGTCACTCTTAGCACTAAGCTTAAAGATGGCGTTAATTTCAATGTAGACGACATC
AATTTTTCCCCTGTATTAGGTTGTTTAGGAAGCGATTGTAATAGAGTTTCCAGTAGATCTGCTATAGAGGATTTACTTT
TTTCTAAAGTAAAGTTAGCTGATGTCGGTTTCGTTGAGGCTTATAATAATTGTACTGGAGGTGCCGAAATTAGGGACCT
CATTTGTGTGCAAAGTTATAATGGTATCAAGGTGTTGCCTCCACTGCTCTCAGAAAATCAGATCAGTGGATATACTTTG
GCTGCGACCTCTGCTAGTCTGTTTCCTCCTTGGTCAGCAGCAGCAGGTGTACCATTTTATTTAAATGTTCAGTATCGTA
TTAATGGTATTGGTGTTACCATGGATGTGCTAAGTCAAAATCAAAAGCTTATTGCTAATGCATTTAACAATGCCCTTGG
TGCTATTCAGGAAGGGTTTGATTCTACCAATTCTGCTTTAGTTAAAATTCAAGCTGTTGTTAATGCAAATGCTGAAGCT
CTTAATAACTTATTGCAACAACTCTCTAACAGGTTTGGTGCTATAAGTTCTTCTTTACAAGAAATTCTATCTAGACTGG
ATGCTCTTGAAGCGCAAGCTCAGATAGATAGACTTATTAATGGGCGTCTTACCGCTCTTAATGCTTATGTTTCTCAACA
GCTTAGTGATTCTACACTAGTAAAATTTAGTGCAGCACAAGCTATGGAGAAGGTTAATGAATGTGTCAAAAGCCAATCA
TCCAGGATAAATTTTTGTGGTAATGGTAATCATATTATATCATTAGTGCAGAATGCTCCATATGGTTTGTATTTTATCC
ACTTTAGCTATGTCCCTACTAAGTATGTCACTGCGAAGGTTAGTCCTGGTCTGTGCATTGCTGGTGATAGAGGTATAGC
CCCCAAGAGTGGTTATTTTGTTAATGTAAATAATACTTGGATGTTCACTGGTAGTGGTTATTACTACCCTGAACCTATA
ACTGGAAATAATGTTGTCGTTATGAGTACCTGTGCTGTTAACTACACCAAAGCACCGGATGTAATGCTGAACATTTCAA
TACCCAACCTCCCTGATTTTAAGGAAGAGTTGGATCAATGGTTTAAAAATCAAACATCAGTGGCACCAAATTTGTCACT
TGATTATATAAATGTTACATTCTTGGACCTACAAGATGAAATGAATAGGTTACAGGAGGCAATAAAAGTTTTAAATCAG
AGCTACATCAATCTCAAGGACATTGGTACATATGAGTATTATGTAAAATGGCCTTGGTATGTATGGCTTTTAATTGGCT
TTGCTGGTGTAGCTATGCTTGTTTTACTATTCTTCATATGCTGTTGTACAGGATGTGGGACTAGTTGTTTTAAGAAATG
TGGTGGTTGTTGTGATGATTATACTGGACACCAGGAGTTAGTAATTAAAACATCACATGACGACTAA

>gi|519668537|gb|KF169936.1|_Bovine_coronavirus_isolate_SWE/U/093*_spike_glycoprotein_(S)_gene_complete_cds

ATGTTTTTGATACTTTTAATTTCCTTACCAACGACTTTTGCTGTTATAGGAGATTTAAAGTGTACTACAGTTTCC
ATTAATGATGTTGACACTGGTGTTCCTTCTATTAGCACTGATACTGTCGATGTTACTAATGGTTTAGGTACTTATTATG
TTTTAGATCGTGTGTATTTAAATACTACCTTGTTGCTTAATGGTTATTACCCTACTTCAGGTTCTACATATCGTAATAT
GGCACTGAAGGGAACTTTACTATTGAGCACACTATGGTTTAAACCACCTTTCCTTTCTGATTTTACTAATGGTATTTTT
GCTAAGGTCAAAAATACCAAGGTTATTAAAAATGGTGTAATGTATAGTGAGTTTCCTGCTATAACTATAGGTAGTACTT
TTGTAAATACATCCTATAGTGTGGTAGTACAACCACATACTACCAATTTTGACAATAAAATACAAGGTCTCTTAGAGGT
CTCTGTTTGCCAGTATACTATGTGTGAGTACCCAAATACGATTTGTAATCCTAATCTGGGTAATCAACGCGTAGAACTA
TGGCATTGGGATACAGGTGTTGTTTCCTGTTTATATAAGCGTAATTTCACATATGATGTGAATGCTGATTATTTGTATT
TTCATTTTTATCAGGAAGGTGGTACTTTTTATGCATATTTTACAGACACTGGTGTTGTTACTAAGTTTCTGTTTAATGT
ATATTTAGGCACGTTGCTTTCACACTATTATGTCATGCCTTTGACTTGTAATAGTGCTTTGAATTTAGAATATTGGGTT
ACACCTCTCACTTCTAAACAATATTTACTCGCTTTCAATCAAGATGGTGTTATTTTTAATGCTGTTGATTGTAAGAGTG
ATTTTATGAGTGAGATTAAGTGTAAAACACTATCTATAGCACCATCTACTGGTGTTTATGAATTAAACGGTTACACTGT
TCAGCCAATTGCAGATGTTTACCGACGTATACCTAATCTTCCCGATTGTAATATAGAGGCTTGGCTTAATGACAAGTCG
GTGCCCTCTCCATTAAATTGGGAACGTAAGACCTTTTCAAATTGTAATTTTAATATGAGCAGCCTGATGTCTTTTATTC
AGGCAGACTCATTTACTTGTAATAATATTGATGCTGCTAAGATATATGGTATGTGTTTTTCCAGCATAACTATAGATAA
GTTTGCTATACCCAATAGTAGGAAGGTTGACCTACAATTGGGCAATTTGGGCTATTTGCAGTCTTTTAACTATAGAATT
GATACTACTGCTACAAGTTGTCAGTTGTATTATAATTTACCTGCTGCTAATGTTTCTGTCAGCAGGTTTAATCCTTCTA
CTTGGAATAGGAGATTTGGTTTTACAGAACAATCTGTTTTTAAGCCTCAACCTGCAGGTGTTTTTACTGATCATGATGT
TGTTTATGCACAACATTGTTTTAAAGCTCCCACAAATTTCTGTCCGTGTAAATTGGATGGGTCTTTGTGTGTAGGTAGT

GGTTCTGGTATAGATGCTGGTTATAAAAATAGTGGTATAGGCACTTGTCCTGCAGGTACTAATTATTTAACTTGTTATA
ATGCTGCCCAATGTGATTGTTTGTGCACTCCAGACCCCATTACATTTAAAGCTACAGGGCCTTACAAGTGCCCCCAAAC
TAAATATTTAGTTGGTATAGGTGAGCACTGTTCAGGTCTTGCTATTAAAAGTGATCATTGTGGAGGTAATCCTTGTATT
TGTCAACCACAAGCATTTTTGGGTTGGTCTGTTGAGTCTTGTTTACAAGGGGATAGGTGTAATATTTTTGCTAATTTTA
TTTTGCATGGTGTTAATAGTGGTACTACTTGTTCTACTGATTTACAAAAATCAAACACAGACATAATTCTTGGTGTTTG
TGTTAATTATGATCTTTATGGTATTACTGGTCAAGGTATTTTTGTTGAGGTTAATGCGACTTATTATAATAGTTGGCAA
AACCTTTTATATGATTCTAATGGTAATCTCTATGGTTTTAGAGACTACTTAACAAATAGAACTTTTATGATTCGTAGGT
GCTATAGCGGTCGTGTTTCAGCGGCCTTTCATGCTAACTCTTCCGAACCAGCATTGCTATTTCGGAACATTAAATGCAA
TTACGTTTTTAATAATACTCTTTCACGACAGCTGCAACCTATTAACTATTTTGATAGCTATCTTGGTTGTGTTGTCAAT
GCTGATAATAGTACTTCTAGTGTTGTTCAAACATGTGATCTCACAGTAGGTAGTGGTTACTGTGTGGATTACTCTACAA
AAAGACGAAGTCGTAGAGCGATTACCACTGGTTATCGGTTTACCAATTTTGAGCCATTTACTGTTAATTCAGTAAATGA
TAGTTTAGAACCTGTAGGTGGTTTGTATGAAATTCAAATACCTTCAGAGTTTACTATAGGTAACATGGAGGAGTTTATT
CAAACAAGCTCTCCTAAAGTTACCATTGATTGTTCTGCTTTTGTCTGTGGTGATTATGCAGCATGTAAATCACAGTTGG
TTGAATATGGTAGCTTCTGTGACAATATTAATGCTATACTCACAGAAGTGAATGAACTACTTGACACTACACAGTTGCA
AGTAGCTAATAGTTTAATGAATGGTGTCACTCTTAGCACTAAGCTTAAAGATGGCGTTAATTTCAATGTAGAGGACATC
AATTTTTCCCCTGTATTAGGTTGTTTAGGAAGCGATTGTAATAGAGTTTCCAGTAGATCAGCTATAGAGGATTTACTTT
TTTCTAAAGTAAAGTTAGCTGATGTTGGTTTTGTTGAGGCTTATAATAATTGTACTGGAGGTGCCGAAATTAGGGACCT
CATTTGTGTGCAAAGTTATAATGGTATCAAGGTGTTGCCTCCACTGCTCTCAGAAAATCAGATCAGTGGATATACTTTG
GCTGCGACCTCTGCTAGTCTGTTTCCTCCATGGTCAGCAGCAGCAGGTGTACCATTTTATTTAAATGTTCAGTATCGTA
TTAATGGGATTGGTGTTACCATGGATGTGCTAAGTCAAAATCAAAAGCTTATTGCTAATGCATTTAACAATGCCCTTGG
TGCTATTCAGGAAGGGTTTGATGCTACCAATTCTGCTTTAGTTAAAATTCAAGCTGTTGTTAATGCAAATGCTGAAGCT
CTTAATAACTTATTGCAACAACTCTCTAATAGGTTTGGTGCTATAAGTTCTTCTTTACAAGAAATTCTATCTAGACTGG
GTGCTCTTGAAGCGCAAGCTCAGATAGACAGACTTATTAATGGGCGTCTTACCGCTCTTAATGCTTATGTTTCTCAACA
ACTTAGTGACTCTACACTAGTAAAATTTAGTGCAGCACAAGCTATTGAGAAGGTTAATGAATGTGTCAAAAGCCAATCA
TCTAGGATAAATTTTTGTGGTAATGGCAATCATATTATATCATTAGTGCAGAATGCTCCATATGGTTTGTATTTTATCC
ACTTTAGCTATGTCCCTACTAAGTATGTCACTGCGAAGGTTAGTCCTGGTCTGTGCATTGCTGGTGATAGAGGTATAGC
CCCCAAGAGTGGTTATTTTGTTAATGTAAATAATACTTGGATGTTCACTGGTAGTGGTTATTACTACCCTGAACCTATA
ACTGGAAATAATGTTGTCGTTATGAGTACCTGTGCTGTTAACTACACCAAAGCACCGGATGTAATGCTGAACATTTCAA
CACCCAACCTCCCTGATTTTAAGGAAGAGTTGGATCAATGGTTTAAAAATCAAACATCAGTGGCACCAAATTTGTCACT
TGATTATATAAATGTTACATTCTTGGACCTACAAGACGAAATGACTAGGTTACAGGAGGCAATAAAAGTTTTAAATCAG
AGCTACATCAATCTCAAGGACATTGGTACATATGAGTATTATGTAAAATGGCCTTGGTATGTATGGCTTTTAATTGGCT
TTGCTGGTGTAGCTATGCTTGTTTTACTATTCTTCATATGCTGTTGTACAGGATGTGGGACTAGTTGTTTTAAGAAATG
TGGTGGTTGTTGTGATGATTATACTGGACACCAGGAGTTAGTAATTAAAACATCACATGACGACTAA

>gi|519668539|gb|KF169937.1|_Bovine_coronavirus_isolate_SWE/M/101_spike_glycoprotein_(S)_gene_complete_cds

ATGTTTTTGATACTTTTAATTTCCTTACCAACGGCTTTTGCTGTTATAGGAGATTTAAAGTGTACTTCAGTTTCC
ATTAATGATGTTGACACTGGTGTTCCTTCTATTAGCACTGATACTGTTGATGTTACTAATGGTTTAGGTACTTATTATG
TTTTAGATCGTGTGTATTTAAATACTACCTTGTTGCTTAATGGTTATTACCCTACTTCAGGTTCTACATATCGTAATAT
GGCACTGAAGGGAACTCTACTATTGAGCACACTATGGTTTAAACCACCTTTTCTTTCTGATTTTACTAATGGTATTTTT
GCTAAGGTCAAAAATACCAAGTTTATTAAAAATGGTGTAATGTATAGTGAGTTTCCTGCTATAACTATAGGTAGTACTT
TTGTAAATACATCCTATAGTGTGGTAGTACAACCACATACTACCAATTTTGATAATAAAATACAAGGTCTCCTAGAGGT

CTCTGTTTGCCAGTATACTATGTGTGAGTACCCAAACACGATTTGTAATCCTAATCTGGGTAATCAACGCGTAGAACTA
TGGCATTGGGATACAGGTGTTGTTTCCTGTTTATATAAGCGTAATTTCACATATGATGTGAATGCTGATTATTTGTATT
TCCATTTTTATCAAGAAGGTGGTACTTTTTATGCATATTTTACAGACACTGGTGTTGTTACTAAGTTTCTGTTTAATGT
ATATTTAGGCACGGTGCTTTCACACTATTATGTCATGCCTTTGACTTGTAATAGTGCTTTGAATTTAGAATATTGGGTT
ACACCTCTCACTTCTAAACAATATTTACTCGCTTTCAATCAAGATGGTGTTATTTTTAATGCTGTTGATTGTAAGAGTG
GTTTTATGAGTGAGATTAAGTGTAAAACATTATCTATAGCACCATCGACTGGTGTTTATGAATTAAACGGTTACACTGT
TCAGCCAATTGGAGATGTTTACCGACGTATACCTAATCTTCCCGATTGTAATATAGAGGCTTGGCTTAATGATAAGTCG
GTGCCCTCTCCATTAAATTGGGAACGTAAGACCTTTTCAAATTGTAATTTTAATATGAGCAGCCTGATGTCTTTTATTC
AGGCAGACTCATTTACTTGTAATAATATTGATGCTGCTAAGATATATGGTATGTGTTTTTCCAGCATAACTATAGATAA
GTTTGCTATACCCAATGGTAGGAAGGTTGACCTACAATTGGGCAATTTGGGCTATTTGCAGTCTTTTAACTATAGAATT
GATACTACTGCTACAAGTTGTCAGTTGTATTATAATTTACCTGCTGCTAATGTTTCTGTTAGCAGGTTTAATCCTTCTA
CTTGGAATAGGAGATTTGGTTTTACAGAACAATCTGTTTTTAAGCCTCAACCTGCAGGTGTTTTTACTGATCATGATGT
TGTTTATGCACAACATTGCTTCAAAGTTCCCACAAATTTCTGTCCGTGTAAATTGGATGGGTCTTTGTGTGTAGGTAGT
GGTTTTGGTGTAGATGCTGGTTATAAAAATAGTGGTATAGGCACTTGTCCTGCAGGCACTAATTATTTAACTTGTCATA
ATGCTGCCCAATGTGATTGTTTGTGCACTCCAGACCCCATTACATCTAAAGCTACAGGGTCTTACAAGTGCCCCCAAAC
TAAATATTTAGTTGGCATAGGTGAGCACTGTTCAGGTCTTGCTATTAAAAGTGATCATTGTGTAGGTAATCCATGTACT
TGCCAACCACAAGCATTTTTGGGTTGGTCTGTTGACTCTTGTTTACAAGGGAATAGGTGTAATATTTTTGCTAATTTTA
TTTTGCATGGTGTTAATAGTGGTACTACTTGTTCTACTGATTTACAAAAATCAAACACAGACATAATTCTTGGTGTTTG
TGTTAATTATGATCTTTATGGTATTACTGGCCAAGGTATTTTTGTTGAGGTTAATGCGACTTATTATAATAGTTGGCAG
AACCTTTTATATGATTCTAATGGTAATCTCTATGGTTTTAGAGACTACTTAACAAATAGAACTTTCATGATTCGTAGTT
GCTATAGCGGTCGTGTTTCAGCGGCCTTTCATGCTAACTCTTCCGAACCAGCATTGCTATTTCGGAATATTAAATGCAA
CTACGTTTTTAATAATACTCTTTCACGACAGCTGCAACCTATTAACTATTTTGATAGCTATCTTGGTTGTGTTGTCAAT
GCTGATAATAGTACTTCTAGTGTTGTTCAAACATGTGATCTCACAGTAGGTAGTGGTTACTGTGTGGATTACTCTACAA
AAAGACGAAGTCGTAGAGAGATTACCACTGGTTATCGGTTTACTAATTTTGAGCCATTTATTGTTAAGTCAGTAAATGA
TAGTTTAGAACCTGTAGGTGGTTTGTATGAAATTCAAATACCTTCAGATTTTACTATAGGTAACATGGAGGAGTTTGTT
CAAACAAGCTCTCCTAAAGTTACTATTGATTGTTCTACTTTTGTCTGTGGTGATTATGCAGCATGTAAATCACAGTTGG
TTGAATATGGTAGCTTTTGTGACAATATTAATGCTATACTCACAGAAGTAAATGAACTACTTGACACTACACAGTTGCA
AGTAGCTAATAGTTTAATGAATGGTGTCACTCTTAGCACTAAGCTTAAAGATGGCGTTAAGTTCAATGTAGATGACATC
AATTTTTCCCCTGTATTAGGTTGTTTAGGAGGCGATTGTAACAGAGTTTCCAGTAGATCTGCTATAGAGGATTTACTTT
TTTCTAAAGTAAAGTTAGCTGATGTCGGTTTCGTTGAGGCTTATAATAATTGTACTGGAGGTGCCGAAATTAGGGACCT
CATTTGTGTGCAAAGTTATAATGGTATCAAGGTTTTGCCTCCACTGCTCTCAGAAAATCAGATCAGTGGATATACTTTG
GCTGCGACCTCTGCTAGTTTGTTTCCTCCTTGGTCAGCAGCAGCAGGTGTACCATTTTATTTAAATGTTCAGTATCGTA
TTAATGGGATTGGTGTTACCATGGATGTGCTAAGTCAAAATCAAAAGCTTATTGCTAATGCATTTAACAATGCCCTTGG
TGCTATTCAGGAAGGGTTTGATGCTACCAATTCTGCTTTAGTTAAAATTCAAGCTGTTGTTAATGCAAATGCTGAAGCT
CTTAATAACTTATTGCAACAACTCTCTAATAGGTTTGGTGCTATAAGTTCTTCTTTACAAGAAATTCTATCTAGACTGG
ATGCTCTTGAAGCGCAAGCTCAGATAGACAGACTTATTAATGGGCGTCTTACCGCTCTTAATGCTTATGTTTCTCAACA
GCTTAGTGACTCTACACTAGTAAAATTTAGTGCAGCACAAGCTATGGAGAAGGTTAATGAATGTGTAAAAAGCCAATCA
TCTAGGATAAATTTTTGTGGTAATGGTAATCATATTATATCATTAGTGCAGAATGCTCCATATGGTTTGTATTTTATCC
ACTTTAGCTATGTCCCTACTAAGTATGTCACTGCTAAGGTTAGTCCTGGTCTGTGCATTGCTGGTGATAGAGGTATAGC
CCCTAAGAGTGGTTATTTTGTTAATGTAAATAATACTTGGATGTTCACTGGTAGTGGTTATTACTACCCTGAACCTATA
ACTGGAAACAATGTTGTTGTTATGAGTACCTGTGCTGTTAATTACACTAAAGCACCGGATGTAATGCTGAACATTTCAA
CACCCAACCTCCCGGATTTTAAGGAAGAGTTGGATCAATGGTTTAAAAACCAAACATCAGTGGCACCAAATTTGTCACT
TGATTATATAAATGTTACATTCTTGGACCTACAAGATGAAATGAATAGGTTACAGGAGGCAATAAAAGTTTTAAATCAG
AGCTACATCAATCTCAAGGACATTGGTACATATGAGTATTATGTAAAATGGCCTTGGTATGTATGGCTTTTAATTGGCT

TTGCTGGTGTAGCTATGCTTGTTTTACTATTCTTCATATGCTGTTGTACAGGATGTGGGACTAGTTGTTTTAAGAAATG
TGGTGGTTGTTGTGATGATTATACTGGACACCAGGAGTTAGTAATTAAAACATCCCATGACGACTAA

>gi|519668541|gb|KF169938.1|_Bovine_coronavirus_isolate_SWE/M/102_spike_glycoprotein_(S)_gene_complete_cds

ATGTTTTTGATACTTTTAATTTCCTTACCAACGGCTTTTGCTGTTATAGGAGATTTAAAGTGTACTTCAGTTTCC
ATTAATGATGTTGACACTGGTGTTCCTTCTATTAGCACTGATACTGTTGATGTTACTAATGGTTTAGGTACTTATTATG
TTTTAGATCGTGTGTATTTAAATACTACCTTGTTGCTTAATGGTTATTACCCTACTTCAGGTTCTACATATCGTAATAT
GGCACTGAAGGGAACTCTACTATTGAGCACACTATGGTTTAAACCACCTTTTCTTTCTGATTTTACTAATGGTATTTTT
GCTAAGGTCAAAAATACCAAGTTTATTAAAAATGGTGTAATGTATAGTGAGTTTCCTGCTATAACTATAGGTAGTACTT
TTGTAAATACATCCTATAGTGTGGTAGTACAACCACATACTACCAATTTTGATAATAAAATACAAGGTCTCCTAGAGGT
CTCTGTTTGCCAGTATACTATGTGTGAGTACCCAAACACGATTTGTAATCCTAATCTGGGTAATCAACGCGTAGAACTA
TGGCATTGGGATACAGGTGTTGTTTCCTGTTTATATAAGCGTAATTTCACATATGATGTGAATGCTGATTATTTGTATT
TCCATTTTTATCAAGAAGGTGGTACTTTTTATGCATATTTTACAGACACTGGTGTTGTTACTAAGTTTCTGTTTAATGT
ATATTTAGGCACGGTGCTTTCACACTATTATGTCATGCCTTTGACTTGTAATAGTGCTTTGAATTTAGAATATTGGGTT
ACACCTCTCACTTCTAAACAATATTTACTCGCTTTCAATCAAGATGGTGTTATTTTTAATGCTGTTGATTGTAAGAGTG
GTTTTATGAGTGAGATTAAGTGTAAAACATTATCTATAGCACCATCGACTGGTGTTTATGAATTAAACGGTTACACTGT
TCAGCCAATTGCAGATGTTTACCGACGTATACCTAATCTTCCCGATTGTAATATAGAGGCTTGGCTTAATGATAAGTCG
GTGCCCTCTCCATTAAATTGGGAACGTAAGACCTTTTCAAATTGTAATTTTAATATGAGCAGCCTGATGTCTTTTATTC
AGGCAGACTCATTTACTTGTAATAATATTGATGCTGCTAAGATATATGGTATGTGTTTTTCCAGCATAACTATAGATAA
GTTTGCTATACCCAATGGTAGGAAGGTTGACCTACAATTGGGCAATTTGGGCTATTTGCAGTCTTTTAACTATAGAATT
GATACTACTGCTACAAGTTGTCAGTTGTATTATAATTTACCTGCTGCTAATGTTTCTGTTAGCAGGTTTAATCCTTCTA
CTTGGAATAGGAGATTTGGTTTTACAGAACAATCTGTTTTTAAGCCTCAACCTGCAGGTGTTTTTACTGATCATGATGT
TGTTTATGCACAACATTGCTTCAAAGTTCCCACAAATTTCTGTCCGTGTAAATTGGATGGGTCTTTGTGTGTAGGTAGT
GGTTCTGGTATAGATGCTGGTTATAAAAATAGTGGTATAGGCACTTGTCCTGCAGGTACTAATTATTTAACTTGTTATA
ATGCTGCCCAATGTGATTGTTTGTGCACTCCAGACCCCATTACATCTAAAGCTACAGGGCCTTACAAGTGCCCCCAAAC
TAAATATTTGGTTGGCATAGGTGAGCACTGTTCAGGTCTTGCTATTAAAAGTGATCATTGTGGAGGTAATCCATGTACT
TGCCAACCACAAGCATTTTTGGGTTGGTCTGTTGACTCTTGTTTACAAGGGGATAGGTGTAATATTTTTGCTAACTTTA
TTTTGCATGGTGTTAATAGTGGTACTACTTGTTCTACTGATTTACAAAAATCAAACACAGACATAATTCTTGGTGTTTG
TGTTAATTATGATCTTTATGGTATTACTGGCCAAGGTATTTTTGTTGAGGTTAATGCGACTTATTATAATAGTTGGCAG
AACCTTTTATATGATTCTAATGGTAATCTCTATGGTTTTAGAGACTACTTAACAAACAGAACTTTCATGATTCGTAGTT
GCTATAGCGGTCGTGTTTCAGCGGCCTTTCATGCTAACTCTTCCGAACCAGCATTGCTATTTCGGAATGTTAAATGCAA
CTACGTTTTTAATAATACTCTTTCACGACAGCTGCAACCTATTAACTATTTTGATAGCTATCTTGGTTGTATTGTCAAT
GCTGATAATAGTACTTCTAGTGTTGTTCAAACATGTGATCTCACAGTAGGTAGTGGTTACTGTGTGGATTACTCTACAA
AAAGACGAAGTCGTAGAGAGATTACCACTGGTTATCGGTTTACTAATTTTGAGCCATTTATTGTTAAGTCAGTAAATGA
TAGTTTAGAACCTGTAGGTGGTTTGTATGAAATTCAAATACCTTCAGATTTTACTATAGGTAACATGGAGGAGTTTGTT
CAAACAAGCTCTCCTAAAGTTACTATTGATTGTTCTACTTTTGTCTGTGGTGATTATGCAGCATGTAAATCACAGTTGG
TTGAATATGGTAGCTTTTGTGACAATATTAATGCTATACTCACAGAAGTAAATGAACTACTTGACACTACACAGTTGCA
AGTAGCTAATAGTTTAATGAATGGTGTCACTCTTAGCACTAAGCTTAAAGATGGTGTTAAGTTCAATGTAGATGACATC
AATTTTTCCCCTGTATTAGGTTGTTTAGGAAGCGATTGTAACAGAGTTTCCAGTAGATCTGCTATAGAGGATTTACTTT
TTTCTAAAGTAAAGTTAGCTGATGTCGGTTTCGTTGAGGCTTATAATAATTGTACTGGAGGTGCCGAAATTAGGGACCT
CATTTGTGTGCAAAGTTATAATGGTATCAAGGTTTTGCCTCCACTGCTCTCAGAAAATCAGATCAGTGGATATACTTTG

GCTGCGACCTCTGCTAGTTTGTTTCCTCCTTGGTCAGCAGCAGCAGGTGTACCATTTTATTTAAATGTTCAGTATCGTA
TTAATGGGATTGGTGTTACCATGGATGTGCTAAGTCAAAATCAAAAGCTTATTGCTAATGCATTTAACAATGCCCTTGG
TGTTATTCAGGAAGGGTTTGATGCTACCAATTCTGCTTTAGTTAAAATTCAAGCTGTTGTTAATGCAAATGCTGAAGCT
CTTAATAACTTATTGCAACAACTCTCTAATAGGTTTGGTGCTATAAGTTCTTCTTTACAAGAAATTCTATCTAGACTGG
ATGCTCTTGAAGCGCAAGCTCAGATAGACAGACTTATTAATGGGCGTCTTACCGCTCTTAATGCTTATGTTTCTCAACA
GCTTAGTGACTCTACACTAGTAAAATTTAGTGCAGCACAAGCTATGGAGAAGGTTAATGAATGTGTCAAAAGCCAATCA
TCTAGGATAAATTTTTGTGGTAATGGTAATCATATTATATCATTAGTGCAGAATGCTCCATATGGTTTGTATTTTATCC
ACTTTAGCTATGTCCCTACTAAGTATGTCACTGCGAAGGTTAGTCCTGGTCTGTGCATTGCTGGTGATAGAGGTATAGC
CCCTAAGAGTGGTTATTTTGTTAATGTAAATAATACTTGGATGTTCACTGGTAGTGGTTATTACTACCCTGAACCTATA
ACTGGAAACAATGTTGTTGTTATGAGTACCTGTGCTGTTAATTACACTAAAGCACCGGATGTAATGCTGAACATTTCAA
CACCCAACCTCCCTGATTTTAAGGAAGAGTTGGATCAATGGTTTAAAAACCAAACATCAGTGGCACCAAATTTGTCACT
TGATTATATAAATGTTACATTCTTGGACCTACAAGATGAAATGAATAGGTTACAGGAGGCAATAAAAGTTTTAAATCAG
AGCTACATCAATCTCAAGGACATTGGTACATATGAGTATTATGTAAAATGGCCTTGGTATGTATGGCTTTTAATTGGCT
TTGCTGGTGTAGCTATGCTTGTTTTACTATTCTTCATATGCTGTTGTACAGGATGTGGGACTAGTTGTTTTAAGAAATG
TGGTGGTTGTTGTGATGATTATACTGGACACCAGGAGTTAGTAATTAAAACATCCCATGACGACTAA

>gi|519668543|gb|KF169939.1|_Bovine_coronavirus_isolate_SWE/Y/103_spike_glycoprotein_(S)_gene_complete_cds

ATGTTTTTGATACTTCTTATTTCCTTACCAACGGCTTTCGCTGTTATAGGAGATTTAAAGTGTACTTCGCTTTCC
ATTAATGATGTTGACACTGGTGTTCCTTCTATTAGCACTGATACTGTTGATGTTACTAATGGTTTAGGTACTTATTATG
TTTTAGATCGTGTGTATTTAAATACTACCTTGTTGCTTAATGGTTATTACCCTACTTCAGGTTCTACATATCGTAATAT
GGCACTGAAGGGAACTCTACTATTGAGCACACTATGGTTTAAACCACCTTTTCTTTCTGATTTTACTAATGGTATTTTT
GCTAAGGTCAAAAACACCAAGGTTATTAAAAATGGTGTAATGTATAGTGAGTTTCCTGCTATAACTATAGGTAGTACTT
TTGTAAATACATCCTATAGTGTGGTAGTACAACCATATACTACCAATTTTGATAATAAAATACAAGGTCTCTTAGAGGT
CTCTGTTTGCCAGTATACTATGTGTGAGTACCCAAATACGATTTGTAATCCTAATCTGGGTAATCAACGCGTGGAACTA
TGGCATTGGGATACAGGTGTTGTTTCCTGTTTATATAAGCGTAATTTCACATATGATGTGAATGCTGATTATTTGTATT
TCCATTTTTATCAAGAAGGTGGTACTTTTTATGCATATTTTACAGACACTGGTGTTGTTACTAAGTTTCTGTTTAATGT
ATATTTAGGCACGGTGCTTTCACACTATTATGTCATGCCTTTGACTTGTAATAGTGCTTTGAATTTAGAATATTGGGTT
ACACCTCTCACTTCTAAACAATATTTACTCGCTTTCAATCAAGATGGTGTTATTTTTAATGCTGTTGATTGTAAGAGTG
ATTTTATGAGTGAGATTAAGTGTAAAACACTATCTATAGCACCATCGACTGGTGTTTATGAATTAAACGGTTACACTGT
TCAGCCAATTGCAGATGTTTACCGACGTATACCTAATCTTCCCGATTGTAATATAGAGGCTTGGCTTAATGACAAGTCG
GTGCCCTCTCCATTAAATTGGGAACGTAAGACCTTTTCAAATTGTAATTTTAATATGAGCAGCCTGATGTCTTTTATTC
AGGCAGACTCATTTACTTGTAATAATATTGATGCTGCTAAGATATATGGTATGTGTTTTTCCAGCATAACTATAGATAA
GTTTGCTATACCCAATGGTAGGAAGGTTGACCTACAATTGGGTAATTTGGGTTACTTGCAGTCTTTTAATTATAGAATT
GATACTACTGCTACAAGTTGTCAGTTGTATTATAATTTACCTGCTGCTAATGTTTCTGTTAGCAGGTTTAATCCTTCTA
CTTGGAATAGGAGATTTGGTTTTACAGAACAATCTGTTTTTAAGCCTCAACCTGCAGGTGTCTTTACTGATCATGATGT
TGTTTATGCACAACATTGTTTTAAAGCTCCCACAAATTTCTGTCCGTGTAAATTGGATGGGTCTTTGTGTGTAGGTAGT
GGTTTTGGTGTAGATGCTGGTTATAAAAATAGTGGTATAGGCACTTGTCCTGCAGGCACTAATTATTTAACTTGTCATA
ATGCTGCCCAATGTGATTGTTTGTGCACTCCAGACCCCATTACATCTAAAGCTACAGGGTCTTACAAGTGCCCCCAAAC
TAAATATTTAGTTGGCATAGGTGAGCACTGTTCAGGTCTTGCTATTAAAAGTGATCATTGTGTAGGTAATCCATGTACT
TGCCAACCACAAGCATTTTTGGGTTGGTCTGTTGACTCTTGTTTACAAGGGAATAGGTGTAATATTTTTGCTAATTTTA
TTTTGCATGGTGTTAATAGTGGTACTACTTGTTCTACTGATTTACAAAAATCAAACACAGACATAATTCTTGGTGTTTG

TGTTAATTATGATCTTTATGGTATTACTGGCCAAGGTATTTTTGTTGAGGTTAATGCGACTTATTATAATAGTTGGCAG
AACCTTTTATATGATTCTAATGGTAATCTCTATGGTTTTAGAGACTACTTAACAAATAGAACTTTCATGATTCGTAGTT
GCTATAGCGGTCGTGTTTCAGCGGCCTTTCATGCTAACTCTTCCGAACCAGCATTGCTATTTCGGAATATTAAATGCAA
CTACGTTTTTAATAATACTCTTTCACGACAGCTGCAACCTATTAACTATTTTGATAGCTATCTTGGTTGTGTTGTCAAT
GCTGATAATAGTACTTCTAGTGTTGTTCAAACATGTGATCTCACAGTAGGTAGTGGTTACTGTGTGGATTACTCTACAA
AAAAACGAAGTCGTAGAGCGATTACCACTGGTTATCGGTTTACTAATTTTGAGCCATTTACTGTTAATTCAGTAAATGA
TAGTTTAGAACCTGTAGGTGGTTTGTATGAAATTCAAATACCTTCAGATTTTACTATAGGTAACATGGAGGAGTTTATT
CAAACAAGCTCTCCTAAAGTTACTATTGATTGTTCTACTTTTGTCTGTGGTGATTATGCAGCATGTAAATCACAGTTGG
TTGAATATGGTAGCTTTTGTGACAATATTAATGCTATACTCACAGAAGTAAATGAACTACTTGACACTACACAGTTGCA
AGTAGCTAATAGTTTAATGAATGGTGTCACTCTTAGCACTAAGCTTAAAGATGGCGTTAAGTTCAATGTAGATGACATC
AATTTTTCCCCTGTATTAGGTTGTTTAGGAAGCGATTGTAATAGAGTTTCCAGTAGATCTGCTATAGAGGATTTACTTT
TTTCTAAAGTAAAGTTAGCTGATGTCGGTTTCGTTGAGGCTTATAATAATTGTACTGGAGGTGCCGAAATTAGGGACCT
CATTTGTGTGCAAAGTTATAATGGTATCAAGGTTTTGCCTCCACTGCTCTCAGAAAATCAGATCAGTGGATATACTTTG
GCTGCGACCTCTGCTAGTTTGTTTCCTCCTTGGTCAGCAGCAGCAGGTGTACCATTTTATTTAAATGTTCAGTATCGTA
TTAATGGGATTGGTGTTACCATGGATGTGCTAAGTCAAAATCAAAAGCTTATTGCTAATGCATTTAACAATGCCCTTGG
TGCTATTCAGGAAGGGTTTGATGCTACCAATTCTGCTTTAGTCAAAATTCAAGCTGTTGTTAATGCAAATGCTGAAGCT
CTTAATAATTTATTGCAACAACTCTCTAACAGGTTTGGTGCTATAAGTTCTTCTTTACAAGAAATTCTATCTAGACTGG
ATGCTCTTGAAGCGCAAGCTCAGATAGACAGACTTATTAATGGGCGTCTTACCGCTCTTAATGCTTATGTTTCTCAACA
GCTTAGTGACTCTACACTAGTAAAATTTAGTGCAGCACAAGCTATGGAGAAGGTTAATGAATGTGTCAAAAGCCAATCA
TCTAGGATAAATTTTTGTGGTAATGGTAATCATATTATATCATTAGTGCAGAATGCTCCATATGGTTTGTATTTTATCC
ACTTTAGCTATGTCCCTACTAAGTATGTCACTGCGAAGGTTAGTCCTGGTCTGTGCATTGCTGGTGATAGAGGTATAGC
CCCCAAGAGTGGTTATTTTGTTAATGTAAATAATACTTGGATGTTCACTGGTAGTGGTTATTACTACCCTGAACCTATA
ACTGGAAATAATGTTGTTGTTATGAGTACCTGTGCTGTTAATTATACTAAAGCACCGGATGTAATGCTGAACATTTCAA
TACCCAACCTCCCTGATTTTAAGGAAGAGTTGGATCAATGGTTTAAAAACCAAACATCAGTGGCACCAAATTTGTCACT
TGATTATATAAATGTTACATTCTTGGACCTACAAGATGAAATGAATAGGTTACAGGAGGCAATAAAAGTTTTAAACCAG
AGCTACATCAATCTCAAGGACATTGGTACATATGAGTATTATGTAAAATGGCCTTGGTATGTATGGCTTTTAATTGGCT
TTGCTGGTGTAGCTATGCTTGTTTTACTATTCTTCATATGCTGTTGTACAGGATGTGGGACTAGTTGTTTTAAGAAATG
TGGTGGTTGTTGTGATGATTATACTGGACACCAGGAGTTAGTAATTAAAACATCACATGACGACTAA

>gi|519668545|gb|KF169940.1|_Bovine_coronavirus_isolate_SWE/P/104_spike_glycoprotein_(S)_gene_complete_cds

ATGTTTTTGATACTTCTAATTTCCTTACCAACGGCTTTCGCTGTTATAGGAGATTTAAAGTGTACTTCGCTTTCC
ATTAATGATGTTGACACTGGTGTTCCTTCTATTAGCACTGATACTGTTGATGTTACTAATGGTTTAGGTACTTATTATG
TTTTAGATCGTGTGTATTTAAATACTACCTTGTTGCTTAATGGTTATTACCCTACTTCAGGTTCTACATATCGTAATAT
GGCACTGAAGGGAACTCTACTATTGAGCACACTATGGTTTAAACCACCTTTTCTTTCTGATTTTACTAATGGTATTTTT
GCTAAGGTCAAAAACACCAAGGTTATTAAAAATGGTGTAATGTATAGTGAGTTTCCTGCTATAACTATAGGTAGTACTT
TTGTAAATACATCCTATAGTGTGGTAGTACAACCATATACTACCAATTTTGATAATAAAATACAAGGTCTCTTAGAGGT
CTCTGTTTGCCAGTATACTATGTGTGAGTACCCAAATACGATTTGTAATCCTAATCTGGGTAATCAACGCGTGGAACTA
TGGCATTGGGATACAGGTGTTGTTTCCTGTTTATATAAGCGTAATTTCACATATGATGTGAATGCTGATTATTTGTATT
TCCATTTTTATCAAGAAGGTGGTACTTTTTATGCATATTTTACAGACACTGGTGTTGTTACTAAGTTTCTGTTTAATGT
ATATTTAGGCACGGTGCTTTCACACTATTATGTCATGCCTTTGACTTGTAATAGTGCTTTGAATTTAGAATATTGGGTT
ACACCTCTCACTTCTAAACAATATTTACTCGCTTTCAATCAAGATGGTGTTATTTTTAATGCTGTTGATTGTAAGAGTG

ATTTTATGAGTGAGATTAAGTGTAAAACACTATCTATAGCACCATCGACTGGTGTTTATGAATTAAACGGTTACACTGT
TCAGCCAATTGCAGATGTTTACCGACGTATACCTAATCTTCCCGATTGTAATATAGAGGCTTGGCTTAATGACAAGTCG
GTGCCTTCTCCATTAAATTGGGAACGTAAGACCTTTTCAAATTGTAATTTTAATATGAGCAGCCTGATGTCTTTTATTC
AGGCAGACTCATTTACTTGTAATAATATTGATGCTGCTAAGATATATGGTATGTGTTTTTCCAGCATAACTATAGATAA
GTTTGCTATACCCAATGGTAGGAAGGTTGACCTACAATTGGGTAATTTGGGTTACTTGCAGTCTTTTAATTATAGAATT
GATACTACTGCTACAAGTTGTCAGTTGTATTATAATTTACCTGCTGCTAATGTTTCTGTTAGCAGGTTTAATCCTTCTA
CTTGGAATAGGAGATTTGGTTTTACAGAACAATCTGTTTTTAAGCCTCAACCTGCAGGTGTCTTTACTGATCATGATGT
TGTTTATGCACAACATTGTTTTAAAGCTCCCACAAATTTCTGTCCGTGTAAATTGGATGGGTCTTTGTGTGTAGGTAGT
GGTTTTGGTGTAGATGCTGGTTATAAAAATAGTGGTATAGGCACTTGTCCTGCAGGCACTAATTATTTAACTTGTCATA
ATGCTGCCCAATGTGATTGTTTGTGCACTCCAGACCCCATTACATCTAAAGCTACAGGGTCTTACAAGTGCCCCCAAAC
TAAATATTTAGTTGGCATAGGTGAGCACTGTTCAGGTCTTGCTATTAAAAGTGATCATTGTGTAGGTAATCCATGTACT
TGCCAACCACAAGCATTTTTGGGTTGGTCTGTTGACTCTTGTTTACAAGGGAATAGGTGTAATATTTTTGCTAATTTTA
TTTTGCATGGTGTTAATAGTGGTACTACTTGTTCTACTGATTTACAAAAATCAAACACAGACATAATTCTTGGTGTTTG
TGTTAATTATGATCTTTATGGTATTACTGGCCAAGGTATTTTTGTTGAGGTTAATGCGACTTATTATAATAGTTGGCAG
AACCTTTTATATGATTCTAATGGTAATCTCTATGGTTTTAGAGACTACTTAACAAATAGAACTTTCATGATTCGTAGTT
GCTATAGCGGTCGTGTTTCAGCGGCCTTTCATGCTAACTCTTCCGAACCAGCATTGCTATTTCGGAATATTAAATGCAA
CTACGTTTTTAATAATACTCTTTCACGACAGCTGCAACCTATTAACTATTTTGATAGCTATCTTGGTTGTGTTGTCAAT
GCTGATAATAGTACTTCTAGTGTTGTTCAAACATGTGATCTCACAGTAGGTAGTGGTTACTGTGTGGATTACTCTACAA
AAAAACGAAGTCGTAGAGCGATTACCACTGGTTATCGGTTTACTAATTTTGAGCCATTTACTGTTAATTCAGTAAATGA
TAGTTTAGAACCTGTAGGTGGTTTGTATGAAATTCAAATACCTTCAGATTTTACTATAGGTAACATGGAGGAGTTTATT
CAAACAAGCTCTCCTAAAGTTACTATTGATTGTTCTACTTTTGTCTGTGGTGATTATGCAGCATGTAAATCACAGTTGG
TTGAATATGGTAGCTTTTGTGACAATATTAATGCTATACTCACAGAAGTAAATGAACTACTTGACACTACACAGTTGCA
AGTAGCTAATAGTTTAATGAATGGTGTCACTCTTAGCACTAAGCTTAAAGATGGCGTTAAGTTCAATGTAGATGACATC
AATTTTTCCCCTGTATTAGGTTGTTTAGGAAGCGATTGTAATAGAGTTTCCAGTAGATCTGCTATAGAGGATTTACTTT
TTTCTAAAGTAAAGTTAGCTGATGTCGGTTTCGTTGAGGCTTATAATAATTGTACTGGAGGTGCCGAAATTAGGGACCT
CATTTGTGTGCAAAGTTATAATGGTATCAAGGTTTTGCCTCCACTGCTCTCAGAAAATCAGATCAGTGGATATACTTTG
GCTGCGACCTCTGCTAGTTTGTTTCCTCCTTGGTCAGCAGCAGCAGGTGTACCATTTTATTTAAATGTTCAGTATCGTA
TTAATGGGATTGGTGTTACCATGGATGTGCTAAGTCAAAATCAAAAGCTTATTGCTAATGCATTTAACAATGCCCTTGG
TGCTATTCAGGAAGGGTTTGATGCTACCAATTCTGCTTTAGTCAAAATTCAAGCTGTTGTTAATGCAAATGCTGAAGCT
CTTAATAATTTATTGCAACAACTCTCTAACAGGTTTGGTGCTATAAGTTCTTCTTTACAAGAAATTCTATCTAGACTGG
ATGCTCTTGAAGCGCAAGCTCAGATAGACAGACTTATTAATGGGCGTCTTACCGCTCTTAATGCTTATGTTTCTCAACA
GCTTAGTGACTCTACACTAGTAAAATTTAGTGCAGCACAAGCTATGGAGAAGGTTAATGAATGTGTCAAAAGCCAATCA
TCTAGGATAAATTTTTGTGGTAATGGTAATCATATTATATCATTAGTGCAGAATGCTCCATATGGTTTGTATTTTATCC
ACTTTAGCTATGTCCCTACTAAGTATGTCACTGCGAAGGTTAGTCCTGGTCTGTGCATTGCTGGTGATAGAGGTATAGC
CCCCAAGAGTGGTTATTTTGTTAATGTAAATAATACTTGGATGTTCACTGGTAGTGGTTATTACTACCCTGAACCTATA
ACTGGAAATAATGTTGTTGTTATGAGTACCTGTGCTGTTAATTATACTAAAGCACCGGATGTAATGCTGAACATTTCAA
TACCCAACCTCCCTGATTTTAAGGAAGAGTTGGATCAATGGTTTAAAAACCAAACATCAGTGGCACCAAATTTGTCACT
TGATTATATAAATGTTACATTCTTGGACCTACAAGATGAAATGAATAGGTTACAGGAGGCAATAAAAGTTTTAAACCAG
AGCTACATCAATCTCAAGGACATTGGTACATATGAGTATTATGTAAAATGGCCTTGGTATGTATGGCTTTTAATTGGCT
TTGCTGGTGTAGCTATGCTTGTTTTACTATTCTTCATATGCTGTTGTACAGGATGTGGGACTAGTTGTTTTAAGAAATG
TGGTGGTTGTTGTGATGATTATACTGGACACCAGGAGTTAGTAATTAAAACATCCCATGACGACTAA

>gi|15081544|ref|NC_003045.1| Bovine coronavirus, complete genome

ATGTTTTTGATACTTTTAATTTCCTTACCAACGGCTTTTGCTGTTATAGGAGATTTAAAGTGTACTACAGTTTCC
ATTAATGATGTTGACACTGGTGTTCCTTCTATTAGCACTGATACTGTCGATGTTACTAATGGTTTAGGTACTTATTATG
TTTTAGATCGTGTGTATTTAAATACTACGTTGTTGCTTAATGGTTACTACCCTACTTCAGGTTCTACATATCGTAATAT
GGCACTGAAGGGAACTTTACTATTGAGCACACTGTGGTTTAAACCACCTTTTCTTTCTGATTTTACTAATGGTATTTTT
GCTAAGGTCAAAAATACCAAGGTTATTAAAGATGGTGTAATGTATAGTGAGTTTCCTGCTATAACTATAGGTAGTACTT
TTGTAAATACATCTTATAGTGTGGTAGTACAACCACATACTACCATTTTGGGTAATAAATTACAAGGTTTCTTAGAGAT
CTCTGTTTGCCAGTATACTATGTGCGAGTACCCAAATACGATTTGTAATCCTAATCTGGGTAATCAACGCGTAGAACTA
TGGCATTGGGATACAGGTGTTGTTTCCTGTTTATATAAGCGTAATTTCACATACGATGTGAATGCTGATTATTTGTATT
TCCATTTTTATCAAGAAGGTGGTACTTTTTATGCATATTTTACAGACACTGGTGTTGTTACTAAGTTTCTGTTTAATGT
TTATTTAGGCACGGTGCTCTCACATTATTATGTCATGCCTTTGACTTGTAATAGTGCTCTGACTTTAGAATATTGGGTT
ACACCCCTCACTTCTAAACAATATCTACTAGCTTTCAATCAAGATGGTGTTATTTTTAATGCTGTTGATTGTAAGAGTG
ATTTTATGAGTGAGATTAAGTGTAAAACACTATCTATAGCACCATCTACTGGTGTTTATGAATTAAACGGTTACACTGT
TCAGCCAATTGCAGATGTTTACCGACGTATACCTAATCTTCCCGATTGCAATATTGAGGCTTGGCTTAATGATAAGTCG
GTGCCCTCTCCATTAAATTGGGAACGTAAGACCTTTTCAAATTGTAATTTTAATATGAGCAGCCTGATGTCTTTTATTC
AGGCATACTCATTTACTTGTAATAATATTGATGCTGCTAAGATATATGGTATGTGTTTTTCCAGCATAACTATAGATAA
GTTTGCAATACCCAATGGTAGGAAGGTTGACCTACAATTGGGTAATTTGGGCTATTTGCAGTCTTTTAATTATAGAATT
GATACTACTGCTACAAGTTGTCAGTTGTATTATAATTTACCTGCTGCTAATGTTTCTGTTAGCAGGTTTAATCCTTCTA
CTTGGAATAGGAGATTTGGTTTTACAGAACAATCTGTTTTTAAGCCTCAACCTGCAGGTGTTTTTACTGATCATGATGT
TGTTTATGCACAACATTGTTTTAAAGCTTCCACAAATTTCTGTCCGTGTAAATTGGATGGGTCTTTGTGTGTAGGTAAT
GGTCCTGGTATAGATGCTGGTTATAAAACTAGTGGCATAGGCACTTGTCCTGCAGGTACTAATTATTTAACTTGCCATA
ATGCTGCCCAATGTGATTGTTTGTGCACCCCAGACCCCATTACATCTAAAGCTACAGGGCCTTACAAGTGCCCCCAAAC
TAAATATTTAGTTGGCATAGGTGAGCACTGTTCGGGTCTTGCTATTAAAAGTGATCATTGTGGAGGTAATCCTTGTACT
TGCCAACCACAAGCATTTTTGGGTTGGTCTGTTGACTCTTGTTTACAAGGGGATAGGTGTAATATTTTTGCTAATTTTA
TTTTGCATGATGTTAATAGTGGTACTACTTGTTCTACTGATTTACAAAAATCAAACACAGACATAATTCTTGGTGTTTG
TGTTAATTATGATCTTTATGGTATTACTGGCCAAGGTATTTTTGTTGAGGTTAATGCGACTTATTATAATAGTTGGCAG
AACCTTTTATATGATTCTAATGGTAATCTCTATGGTTTAGAGACTACTTAACAAATAGAACTTTTATGATTCGTAGTT
GCTATAGTGGTCGTGTTTCAGCGGCCTTTCATGCTAACTCTTCCGAACCAGCATTGCTATTTCGGAATATCAAATGCAA
TTACGTTTTTAATAACACTCTTTCACGACAGCTGCAACCTATTAACTATTTTGATAGCTATCTTGGTTGTGTTGTCAAT
GCTGATAATAGTACTTCTAGTGTTGTTCAAACATGTGATCTCACAGTAGGTAGTGGTTACTGTGTGGATTACTCTACAA
AAAGACGAAGTCGTAGATCGATTACCACTGGTTATCGGTTTACTAATTTTGAGCCATTTACTGTTAATTCAGTAAATGA
TAGTTTAGAACCTGTAGGTGGTTTGTATGAAATTCAAATACCTTCAGAGTTTACTATAGGTAATATGGAGGAGTTTATT
CAAACAAGCTCTCCTAAAGTTACTATTGATTGTTCTGCTTTTGTCTGTGGTGATTATGCAGCATGTAAATCACAGTTGG
TTGAATATGGTAGCTTCTGTGACAATATTAATGCTATACTCACAGAAGTAAATGAACTACTTGACACTACACAGTTGCA
AGTAGCTAATAGTTTAATGAATGGTGTCACTCTTAGCACTAAGCTTAAAGATGGCGTTAATTTCAATGTAGACGACATC
AATTTTTCCCCTGTATTAGGTTGTTTAGGAAGCGATTGCAATAAAGTTTCCAGTAGATCTGCTATAGAGGACTTACTTT
TTTCTAAAGTAAAGTTATCTGATGTCGGTTTCGTTGAGGCTTATAATAATTGTACTGGAGGTGCCGAAATTAGGGACCT
CATTTGTGTGCAAAGTTATAATGGTATCAAGGTGTTGCCTCCACTGCTCTCGGAAAATCAGATCAGTGGATACACTTTG
GCTGCCACTTCTGCTAGCCTGTTTCCTCCATGGTCAGCAGCAGCAGGTGTACCATTCTATTTAAACGTTCAGTATCGTA
TTAATGGTATTGGTGTTACCATGGATGTGTTAAGTCAAAATCAAAAGCTTATTGCTAATGCATTTAACAATGCTCTTGG
TGCTATTCAGGAAGGGTTTGATGCTACCAATTCTGCTTTAGTTAAAATTCAAGCTGTTGTTAATGCAAATGCTGAAGCT
CTTAATAACTTATTGCAACAACTCTCTAATAGATTTGGTGCTATAAGTTCTTCTTTACAAGAAATTCTATCTAGACTGG
ATGCTCTTGAAGCGCAAGCTCAGATAGACAGACTTATTAATGGGCGTCTTACCGCTCTTAATGCTTATGTTTCTCAACA
GCTTAGTGACTCTACACTAGTAAAGTTTAGTGCAGCACAAGCTATGGAGAAGGTTAATGAATGTGTCAAAAGCCAATCA
TCTAGGATAAATTTTTGTGGTAATGGTAATCATATTATATCATTAGTGCAGAATGCTCCATATGGTTTGTATTTTATCC

ACTTTAGCTATGTCCCTACTAAGTATGTCACTGCGAAGGTTAGTCCCGGCCTGTGCATTGCTGGTGATAGAGGTATAGC
CCCTAAGAGTGGTTATTTTGTTAATGTAAATAATACTTGGATGTTCACTGGTAGTGGTTATTACTACCCTGAACCTATA
ACTGGAAATAATGTTGTTGTTATGAGTACCTGTGCTGTTAATTATACTAAAGCGCCGGATGTAATGCTGAACATTTCAA
CACCCAACCTCCCTTATTTTAAGGAAGAGTTGGATCAATGGTTTAAAAACCAAACATCAGTGGCACCAGATTTGTCACT
TGATTATATAAATGTTACATTCTTGGACCTACAAGATGAAATGAATAGGTTACAGGAGGCAATAAAAGTTTTAAATCAG
AGCTACATCAATCTCAAGGACATTGGTACATATGAGTATTATGTAAAATGGCCTTGGTATGTATGGCTTTTAATTGGCT
TTGCTGGTGTAGCTATGCTTGTTTTACTATTCTTCATATGCTGTTGTACAGGATGTGGGACTAGTTGTTTTAAGAAATG
TGGTGGTTGTTGTGATGATTATACTGGACACCAGGAGTTAGTAATTAAAACATCACATGAAGACTAA

>gi|331264481|gb|DQ811784.2| Bovine coronavirus DB2, complete genome

ATGTTTTTGATACTTTTAATTTCCTTACCAACGGCTTTTGCTGTTATAGGAGATTTAAAGTGTACTACAGTTTCC
ATTAATGATGTTGACACTGGTGTTCCTTCTATTAGCACTGATACTGTCGATGTTACTAATGGTTTAGGTACTTATTATG
TTTTAGATCGTGTGTATTTAAATACTACGTTGTTGCTTAATGGTTACTACCCTACTTCAGGTTCTACATATCGTAATAT
GGCACTGAAGGGAACTTTACTATTGAGCACACTATGGTTTAAACCACCTTTTCTTTCTGATTTTACTAATGGTATTTTT
GCTAAGGTCAAAAATACCAAGGTTATTAAAAATGGTGTAATGTATAGTGAGTTTCCTGCTATAACTATAGGTAGTACTT
TTGTAAATACATCCTATAGTGTGGTAGTACAACCACATACTACCAATTTGGATAATAAATTACAAGGTCTCTTAGAGAT
CTCTGTTTGCCAGTATACTATGTGCGAGTACCCAAATACGATTTGTCATCCTACTCTGGGTAATCAACGCGTAGAACTA
TGGCATTGGGATACAGGTGTTGTTTCCTGTTTATATAAGCGTAATTTCACATATGATGTGAATGCTGATTACTTGTATT
TCCATTTTTATCAAGAAGGTGGTACTTTTTATGCATATTTTACAGACACTGGTGTTGTTACTAAGTTTCTGTTTAATGT
TTATTTAGGCACGGTGCTTTCACATTATTATGTCATGCCTTTGACTTGTGATAGTGCTATGACTTTAGAATATTGGGTT
ACACCCCTCACTTCTAAACAATATCTACTAGCTTTCAATCAAGATGGTGTTATTTTTAATGCTGTTGATTGTAAGAGTG
ATTTTATGAGTGAGATTAAGTGTAAAACACTATCTATAGCACCATCTACTGGTGTTTATGAATTAAACGGTTACACTGT
TCAGCCAATTGCAGATGTTTACCGACGTATACCTAATCTTCCCGATTGTAATATTGAGGCTTGGCTTAATGATAAGTCG
GTGCCCTCTCCATTAAATTGGGAACGTAAGACCTTTTCAAATTGTAATTTTAATATGAGCAGCCTGATGTCTTTTATTC
AGGCAGACTCATTTACTTGTAATAATATTGATGCTGCTAAGATATATGGTATGTGTTTTTCCAGCATAACTATAGATAA
GTTTGCTATACCCAATGGTAGGAAGGTTGACCTACAATTGGGTAATTTGGGCTATTTGCAGTCTTTTAATTATAGAATT
GATACTACTGCTACAAGTTGTCAGTTGTATTATAATTTACCTGCTGCTAATGTTTCTGTTAGCAGGTTTAATCCTTCTA
CTTGGAATAGGAGATTTGGTTTTACAGAACAATCTGTTTTTAAGCCTCAACCTGCAGGTGTTTTTACTGCTCATGATGT
TGTTTATGCACAACATTGTTTTAAAGCTCCCACAAATTTCTGTCCGTGTAAATTGGATGGGTCTTTGTGTGTAGGTAAT
GGTCCTGGTATAGATGCTGGTTATAAAACTAGTGGCATAGGCACTTGTCCTGCAGGTACTAATTATTTAACTTGCCATA
ATGCTGCCCAATGTGATTGTTTGTGCACTCCAGACCCCATTTCATCTAAAGCTACAGGGCCTTACAAGTGCCCCCAAAC
TAAATATTTAGTTGGCATAGGTGAGCACTGTTCGGGTCTTGCTATTAAAAGTGATTATTGTGGAGGTAATCCTTGTACT
TGCCAACCACAAGCATTTTTGGGTTGGTCTGTTGACTCTTGTTTACAAGGGGATAGGTGTAATATTTTTGCTAATTTTA
TTTTGCATGATGTTAATAGTGGTACTACTTGTTCTACTGATTTACAAAAATCAAACACAGACATAATTCTTGGTGTTTG
TGTTAATTATGATCTTTATGGTATTACTGGCCAAGGTATTTTTGTTGAGGTTAATGCGACTTATTATAATAGTTGGCAG
AACCTTTTATATGATTCTAATGGTAATCTCTATGGTTTTAGAGACTACTTAACAAATAGAACTTTTATGATTCGTAGTT
GCTATAGCGGTCGTGTTTCAGCGGCCTTTCATGCTAACTCTTCCGAACCAGCATTGCTATTTCGGAATATTAAATGCAA
TTACGTTTTTAATAATACTCTTTCACGACAGCTGCAACCTATTAACTATTTTGATAGCTATCTTGGTTGTGTTGTCAAT
GCTGATAATAGTACTTCTAGTGTTGTTCAAACATGTGATCTCACAGTAGGTAGTGGTTACTGTGTGGATTACTCTACAA
AAAGACGAAGTCGTAGATCGATTACCACTGGTTATCGGTTTACTAATTTTGAGCCATTTACTGTTAATTCAGTAAATGA
TAGTTTACAACCTGTAGGTGGTTTGTATGAAATTCAAATACCTTCAGAGTTTACTATAGGTAATATGGAGGAGTTTATT
CAAACAAGCTCTCCTAAAGTTACTATTGATTGTTCTGCTTTTGTCTGTGGTGATTATGCAGCATGTAAATCACAGTTGG

TTGAATATGGTAGCTTCTGTGACAATATTAATGCTATACTCACAGAAGTAAATGAATTACTTGACACTACACAGTTGCA
AGTAGCTAATAGTTTAATGAATGGTGTCACTCTTAGCACTAAGCTTAAAGATGGCGTTAATTTCAATGTAGACGACATC
AATTTTTCCCCTGTATTAGGTTGTTTAGGAAGCGATTGCAATAAAGTTTCCAGTAGATCTGCTATAGAGGACTTACTTT
TTTCTAAAGTAAAGTTATCTGATGTCGGTTTCGTTGAGGCTTATAATAATTGTACTGGAGGTGCCGAAATTAGGGACCT
CATTTGTGTGCAAAGTTATAATGGTATCAAGGTGTTGCCTCCACTGCTCTCAGAAAATCAGATCAGTGGATACACTTTG
GCTGCCACCTCTGCTAGTCTGTTTCCTCCTTGGTCAGCAGCAGCAGGTGTACCATTTTATTTAAATGTTCAGTATCGTA
TTAATGGGATTGGTGTTACCATGGATGTGCTAAGTCAAAATCAAAAGCTTATTGCTAATGCATTTAACAATGCTCTTGG
TGCTATTCAGGAAGGGTTTGATGCTACCAATTCTGCTTTAGTTAAAATTCAAGCTGTTGTTAATGCAAATGCTGAAGCT
CTTAATAACTTATTGCAACAACTCTCTAATAGGTTTGGTGCTATAAGCTCTTCTTTACAAGAAATTCTATCTAGACTGG
ATGCTCTTGAAGCGCAAGCTCAGATAGACAGACTTATTAATGGGCGTCTTACCGCTCTTAATGCTTATGTTTCTCAACA
GCTTAGTGATTCTACACTAGTAAAATTTAGTGCAGCACAAGCTATGGAGAAGGTTAATGAATGTGTCAAAAGCCAATCA
TCTAGGATAAATTTTTGTGGTAATGGTAATCATATTATATCATTAGTGCAGAATGCTCCATATGGTTTGTATTTTATCC
ACTTTAGCTATGTCCCTACTAAGTATGTCACTGCGAAGGTTAGTCCCGGTCTGTGCATTGCTGGTGATAGAGGTATAGC
CCCTAAGAGTGGTTATTTTGTTAATGTAAATAATACTTGGATGTTCACTGGTAGTGGTTATTACTACCCTGAACCTATA
ACTGGAAATAATGTTGTTGTTATGAGTACCTGTGCTGTTAACTATACTAAAGCGCCGGATGTAATGCTGAACATTTCAA
CACCCAACCTCCCTGATTTTAAGGAAGAGTTGGATCAATGGTTTAAAAACCAAACATCAGTGGCACCACATTTGTCACT
TGATTATATAAATGTTACATTCTTGGACCTACAAGATGAAATGAATAGGTTACAGGAGGCAATAAAAGTTTTAAATCAG
AGCTACATCAATCTCAAGGACATTGGTACTTATGAGTATTATGTAAAATGGCCTTGGTATGTATGGCTTTTAATTGGCT
TTGCTGGTGTAGCTATGCTTGTTTTACTATTCTTCATATGCTGTTGTACAGGATGTGGGACTAGTTGTTTTAAGAAATG
TGGTGGTTGTTGTGATGATTATACTGGACACCAGGAGTTAGTAATTAAAACATCACATGACGACTAA

>gi|145208884|gb|EF424615.1| Bovine coronavirus E-AH65, complete genome

ATGTTTTTGATACTTTTAATTTCCTTACCAACGGCTTTTGCTGTTATAGGAGATTTAAAGTGTACTACACTTTCC
ATTAATGATGTTGACACTGGTGTTCCTTCTATTAGCACTGATACTGTCGATGTTACTAATGGTTTAGGTACTTATTATG
TTTTAGATCGTGTGTATTTAAATACTACGTTGTTGCTTAATGGTTATTACCCTACTTCAGGTTCTACATATCGTAATAT
GGCACTGAAGGGAACTTTACTATTGAGCACACTGTGGTTTAAACCACCTTTTCTTTCTGATTTTACTAATGGTATTTTT
GCTAAGGTCAAAAATACCAAGGTTATTAAAGATGGTGTAATGTATAGTGAGTTTCCTGCTATAACTATAGGTAGTACTT
TTGTAAATACATCCTATAGTGTGGTAGTACAACCACATACTACCATTTTGGGTAATAAATTACAAGGTTTCTTAGAGAT
CTCTGTTTGCCAGTATACTATGTGCGAGTACCCAAATACGATTTGTAATCCTAATCTGGGTAATCAACGCGTAGAACTA
TGGCATTGGGATACAGGTGTTGTTTCCTGTTTATATAAGCGTAATTTCACATATGATGTGAATGCTGATTATTTGTATT
TTCATTTTTATCAAGAAGGTGGTACTTTTTATGCATATTTTACAGACACTGGTGTTGTTACTAAGTTTCTGTTTAATGT
TTATTTAGGCACGGTGCTCTCACATTATTATGTCATGCCTTTGACTTGTAATAGTGCTCTGACTTTAGAATATTGGGTT
ACACCCCTCACTTCTAAACAATATCTACTAGCTTTCAATCAAGATGGTGTTATTTTTAATGCTGTTGATTGTAAGAGTG
ATTTTATGAGTGAGATTAAGTGTAAAACACTATCTATAGCACCATCTACTGGTGTTTATGAATTAAACGGTTACACTGT
TCAGCCAATTGCAGATGTTTACCGACGTATACCTAATCTTCCCGATTGCAATATTGAGGCTTGGCTTAATGATAAGTCG
GTGCCCTCTCCATTAAATTGGGAACGTAAGACTTTTTCAAATTGTAATTTTAATATGAGCAGCCTGATGTCTTTTATTC
AGGCAGACTCATTTACTTGTAATAATATTGATGCTGCTAAGATATATGGTATGTGTTTTTCCAGCATAACTATAGATAA
GTTTGCAATACCCAATGGTAGGAAGGTTGACCTACAATTGGGTAATTTGGGTTATTTGCAGTCTTTTAACTATAGAATT
GATACTACTGCTACAAGTTGTCAGTTGTATTATAATTTACCTGCTGCTAATGTTTCTGTTAGCAGGTTTAATCCTTCTA
CTTGGAATAGGAGATTTGGTTTTACAGAACAATCTGTTTTTAAGCCTCAACCTGCAGGTGTTTTTACTGATCATGATGT
TGTTTATGCACAACATTGTTTTAAAGCTCCCACAAATTTCTGTCCGTGTAAATTGGATGGGTCTTTGTGTGTAGGTAGT
GGTTCTGGTATAGATGCTGGTTATAAAAATACTGGTATAGGCACTTGTCCTGCAGGTACTAATTATTTAACTTGCCATA

ATGCTGCCCAATGTGATTGTTTGTGCACTCCAGACCCCATTACATCTAAAGCTACAGGGCCTTACAAGTGCCCCCAAAC
TAAATACTTAGTTGGCATAGGTGAGCACTGTTCGGGTCTTGCTATTAAAAGTGATTATTGTGGAGGTAACCCTTGTAGT
TGCCAACCACAAGCATTTTTGGGTTGGTCTGTTGACTCTTGTTTACAAGGGGATAGGTGTAATATTTTTGCTAATTTTA
TTTTGCATGATGTTAATAGTGGTACTACTTGTTCTACTGATTTACAAAAATCAAACACAGACATAATTCTTGGTGTTTG
TGTTAATTATGATCTTTATGGTATTACTGGCCAAGGGATTTTTGTTGAGGTTAATGCGACTTATTATAATAGTTGGCAG
AACCTTTTATATGATTCTAATGGTAATCTCTATGGTTTTAGAGACTACTTAACAAATAGAACTTTTATGATTCGTAGTT
GCTATAGTGGTCGTGTTTCAGCGGCCTTTCATGCTAACTCTTCCGAACCAGCATTGCTATTTCGGAATATCAAATGCAA
TTACGTTTTTAATAACACTCTTTCACGACAGCTGCAACCTATTAACTATTTTGATAGCTATCTTGGTTGTGTTGTCAAT
GCTGATAATAGTACTTCTAGTGTTGTTCAAACATGTGATCTAACAGTAGGTAGTGGTTACTGTGTGGATTACTCTACAA
AAAGACGAAGTCGTAGATCGATTACCACTGGTTATCGGTTTACTAATTTTGAGCCATTTACTGTTAATTCAGTAAATGA
TAGTTTAGAACCTGTAGGTGGTTTGTATGAAATTCAAATACCTTCAGAGTTTACTATAGGTAATATGGAGGAGTTTATT
CAAACAAGCTCTCCTAAAGTTACTATTGATTGTTCTGCTTTTGTCTGTGGTGATTATGCAGCATGTAAATCACAGTTGG
TTGAATATGGTAGCTTCTGTGACAATATTAATGCTATACTCACAGAAGTAAATGAATTACTTGACACTACACAGTTGCA
AGTAGCTAATAGTTTAATGAATGGTGTCACTCTTAGCACTAAGCTTAAAGATGGCGTTAATTTCAATGTAGACGACATC
AATTTTTCCCCTGTATTAGGTTGTTTAGGAAGCGATTGCAATAAAGTTTCCAGTAGATCTGCTATAGAGGACTTACTTT
TTTCTAAAGTAAAGTTATCTGATGTCGGTTTCGTTGAGGCTTATAATAATTGTACTGGAGGTGCCGAAATTAGGGACCT
CATTTGTGTGCAAAGTTATAATGGTATCAAGGTGTTGCCTCCACTGCTCTCAGAAAATCAGATCAGTGGATACACTTTG
GCTGCCACCTCTGCTAGCCTGTTTCCTCCATGGTCAGCAGCAGCAGGTGTACCATTCTATTTAAATGTTCAGTATCGTA
TTAATGGTATTGGTGTTACCATGGATGTGCTAAGTCAAAATCAAAAGCTTATTGCTAATGCATTTAACAATGCTCTTGG
TGCTATCCAGGAAGGGTTTGATGCTACCAATTCTGCTTTAGTTAAAATTCAAGCTGTTGTTAATGCAAATGCTGAAGCT
CTTAATAACTTATTGCAACAACTCTCTAATAGATTTGGTGCTATAAGTTCTTCTTTACAAGAAATTCTATCTAGACTGG
ATGCTCTTGAAGCGCAAGCTCAGATAGACAGACTTATTAATGGGCGTCTTACCGCTCTTAATGCTTATGTTTCTCAACA
GCTTAGTGACTCTACACTAGTAAAGTTTAGTGCAGCACAAGCTATGGAGAAGGTTAATGAATGTGTCAAAAGCCAATCA
TCCAGGATAAATTTTTGTGGTAATGGTAATCATATTATATCATTAGTGCAGAATGCTCCATATGGTTTGTATTTTATCC
ACTTTAGCTATGTCCCTACTAAGTATGTCACTGCGAAGGTTAGTCCCGGCCTGTGCATTGCTGGTGATAGAGGTATAGC
CCCTAAGAGTGGTTATTTCGTTAATGTAAATAATACTTGGATGTTCACTGGTAGTGGTTATTACTACCCTGAACCTATA
ACTGGAAATAATGTTGTTGTTATGAGTACCTGTGCTGTTAATTATACTAAAGCGCCGGATGTAATGCTGAACATTTCAA
CACCCAACCTCCCTGATTTTAAGGAAGAGTTGGATCAATGGTTTAAAAACCAAACATCAGTGGCACCAGATTTGTCACT
TGATTATATAAATGTTACATTCTTGGACCTACAAGATGAAATGAATAGGTTACAGGAGGCAATAAAAGTTTTAAATCAG
AGCTACATCAATCTCAAGGACATTGGTACATATGAGTATTATGTAAAATGGCCTTGGTATGTATGGCTTTTAATTGGCT
TTGCTGGTGTAGCTATGCTTGTTTTACTATTCTTCATATGCTGTTGTACAGGATGTGGGACTAGTTGTTTTAAGAAATG
TGGCGGTTGTTGTGATGATTATACTGGACACCAGGAGTTAGTAATTAAAACATCACATGAAGACTAA

>gi|145208896|gb|EF424616.1| Bovine coronavirus E-AH65-TC, complete genome

ATGTTTTTGATACTTTTAATTTCCTTACCCACGGCTTTTGCTGTTATAGGAGATTTAAAGTGTACTACAGTCTCC
ATTAATGATGTTGACACTGGTGTTCCTTCTATTAGCACTGATACTGTCGATGTTACTAATGGTTTAGGTACTTATTATG
TTTTAGATCGTGTGTATTTAAATACTACGTTGTTGCTTAATGGTTACTACCCTACTTCAGGTTCTACATATCGTAATAT
GGCACTGAAGGGAACTTTACTATTGAGCACACTGTGGTTTAAACCACCTTTTCTTTCTGATTTTACTAATGGTATTTTT
GCTAAGGTCAAAAATACCAAGGTTGTTAAAGATGGTGTAATGTATAGTGAGTTTCCTGCTATAACTATAGGTAGTACTT
TTGTAAATACATCTTATAGTGTGGTAGTACAACCACATACTACCATTTTGGGTAATAAATTACAAGGTTTCTTAGAGAT
CTCTGTTTGCCAGTATACTATGTGCGAGTACCCAAATACGATTTGTAATCCTAATCTGGGCAATCAACGCGTAGAACTA
TGGCATTGGGATACAGGTGTTGTTTCCTGTTTATATAAGCGTAATTTCACATACGATGTGAATGCTGATTATTTGTATT

TCCATTTTTATCAAGAAGGTGGTACTTTTTATGCATATTTTACAGACACTGGTGTTGTTACTAAGTTTCTGTTTAATGT
TTATTTAGGCACGGTGCTCTCACATTATTATGTCATGCCTTTGACTTGTAATAGTGCTCTGACTTTAGAATATTGGGTT
ACACCCCTCACTTCTAAACAATATCTACTAGCTTTCAATCAAGATGGTGTTATTTTTAATGCTGTTGATTGTAAGAGTG
ATTTTATGAGTGAGATTAAGTGTAAAACACTATCTATAGCACCATCTACTGGTGTTTATGAATTAAACGGTTACACTGT
TCAGCCAATTGCAGATGTTTACCGACGTATACCTAATCTTCCCGATTGCAATATTGAGGCTTGGCTTAATGATAAGTCG
GTGCCCTCTCCATTAAATTGGGAACGTAAGACCTTTTCAAATTGTAATTTTAATATGAGCAGCCTGATGTCTTTTATTC
AGGCAGACTCATTTACTTGTAATAATATTGATGCTGCTAAGATATATGGCATGTGTTTTTCCAGCATAACTATAGATAA
GTTTGCAATACCCAATGGTAGGAAGGTTGACCTACAATTGGGTAATTTGGGTTATTTGCAGTCTTTTAACTATAGAATT
GATACTACTGCTACAAGTTGTCAGTTGTATTATAATTTACCTGCTGCTAATGTTTCTGTTAGCAGGTTTAATCCTTCTA
CTTGGAATAGGAGATTTGGTTTTACAGAACAATCTGTTTTTAAGCCTCAACCTGCAGGTGTTTTTACTGATCATGATGT
TGTTTATGCACAACATTGTTTTAAAGCTCCCACAAATTTCTGTCCGTGTAAATTGGATGGGTCTTTGTGTGTAGGTAGT
GGTTCTGGTATAGATGCTGGTTATAAAAATACTGGTATAGGCACTTGTCCTGCAGGTACTAATTATTTAACTTGCCATA
ATGCTGCCCAATGTAATTGTTTGTGCACTCCAGACCCCATTACATCTAAAGCTACAGGGCCTTACAAGTGCCCCCAAAC
TAAATATTTAGTTGGTATAGGTGAGCACTGTTCGGGTCTTGCTATTAAAAGTGATCATTGTGGAGGTAATCCTTGTAGT
TGCCAACCACAAGCATTTTTGGGTTGGTCTGTTGACTCTTGTTTACAAGGGGATAGGTGTAATATTTTTGCTAATTTTA
TTTTGCATGATGTTAATAGTGGTACTACTTGTTCTACTGATTTACAAAAATCAAACACAGACATAATTCTTGGTGTTTG
TGTTAATTATGATCTTTATGGTATTACTGGCCAAGGGATTTTTGTTGAGGTCAATGCGACTTATTATAATAGTTGGCAG
AACCTTTTATATGATTCTAATGGTAATCTCTATGGTTTTAGAGACTACTTAACAAATAGAACTTTTATGATTCGTAGTT
GCTATAGTGGTCGTGTTTCAGCGGCCTTTCATGCTAACTCTTCCGAACCAGCATTGCTATTTCGGAATATCAAATGCAA
TTACGTTTTTAATAACACTCTTTCACGACAGCTGCAACCTATTAACTATTTTGATAGCTATCTTGGTTGTGTTGTCAAT
GCTGATAATAGTACTTCTAGTGTTGTTCAAACATGTGATCTCACAGTAGGTAGTGGTTACTGTGTGGATTACTCTACAA
AAAGACGAAGTCGTAGATCGATTACCACTGGTTATCGGTTTACTAATTTTGAGCCATTTACTGTTAATTCAGTAAATGA
TAGTTTAGAACCTGTAGGTGGTTTGTATGAAATTCAAATACCTTCAGAGTTTACTATAGGTAATATGGAGGAGTTTATT
CAAACAAGCTCTCCTAAAGTTACTATTGATTGTTCTGCTTTTGTTTGTGGTGATTATGCAGCATGTAAATCACAGTTGG
TTGAATATGGTAGCTTCTGTGACAATATTAATGCTATACTCACAGAAGTAAATGAACTACTTGACACTACACAGTTGCA
AGTAGCTAATAGTTTAATGAATGGTGTCACTCTTAGCACTAAGCTTAAAGATGGCGTTAATTTCAATGTAGACGACATC
AATTTTTCCCCTGTATTAGGTTGTTTAGGAAGCGATTGCAATAAAGTTTCCAGTAGATCTGCTATAGAGGACTTACTTT
TTTCTAAAGTAAAGTTATCTGATGTCGGTTTCGTTGAGGCTTATAATAATTGTACTGGAGGTGCCGAAATTAGGGACCT
CATTTGTGTGCAAAGTTATAATGGTATCAAGGTGTTGCCTCCACTGCTCTCAGAAAATCAGATCAGTGGATACACTTTG
GCTGCCACCTCTGCTAGCCTGTTTCCTCCATGGTCAGCAGCAGCAGGTGTACCATTCTATTTAAATGTTCAGTATCGTA
TTAATGGTATTGGTGTTACCATGGATGTGCTAAGTCAAAATCAAAAGCTTATTGCTAATGCATTTAACAATGCTCTTGG
TGCTATTCAGGAAGGGTTTGATGCTACCAATTCTGCTTTAGTTAAAATTCAAGCTGTTGTTAATGCAAATGCTGAAGCT
ATTAATAACTTATTGCAACAACTCTCTAATAGATTTGGTGCTATAAGTTCTTCTTTACAAGAAATTCTATCTAGACTGG
ATGCTCTTGAAGCGCAAGCTCAGATAGACAGACTTATTAATGGGCGTCTTACCGCTCTTAATGCTTATGTTTCTCAACA
GCTTAGTGACTCTACACTAGTAAAGTTTAGTGCAGCACAAGCTATGGAGAAGGTTAATGAATGTGTCAAAAGCCAATCA
TCTAGGATAAATTTTTGTGGTAATGGTAATCATATTATATCATTAGTGCAGAATGCTCCATATGGTTTGTATTTTATCC
ACTTTAGCTATGTCCCTACTAAGTATGTCACAGCGAAGGTTAGTCCCGGTCTGTGCATTGCTGGTGGTAGAGGTATAGC
CCCTAAGAGTGGTTATTTTGTTAATGTAAATAATACTTGGATGTTCACTGGTAGTGGTTATTACTACCCTGAACCTATA
ACTGGAAATAATGTTGTTGTTATGAGTACCTGTGCTGTTAATTATACTAAAGCGCCGGATGTAATGCTGAACATTTCAA
CACCCAACCTCCCTGATTTTAAGGAAGAGTTGGATCAATGGTTTAAAAACCAAACCTCAGTGGCACCAGATTTGTCACT
TGATTATATAAATGTTACATTCTTGGACCTACAAGATGAAATGAATAGGTTACAGGAGGCAATAAAAGTTTTAAATCAG
AGCTACATCAATCTCAAGGACATTGGTACTTATGAGTATTATGTAAAATGGCCTTGGTATGTATGGCTTTTAATTGGCT
TTGCAGGTGTAGCTATGCTTGTTTTACTATTCTTCATATGCTGTTGTACAGGATGTGGGACTAGTTGTTTTAAGAAATG
TGGTGGTTGTTGTGATGATTATACAGGACACCAGGAGTTAGTAATTAAAACATCACATGAAGACTAA

>gi|145208908|gb|EF424617.1| Bovine coronavirus R-AH65, complete genome

ATGTTTTTGATACTTTTAATTTCCTTACCCACGGCTTTTGCTGTTATAGGAGATTTAAAGTGTACTACAGTCTCC
ATTAATGATGTTGACACTGGTGTTCCTTCTATTAGCACTGATACTGTCGATGTTACTAATGGTTTAGGTACTTATTATG
TTTTAGATCGTGTGTATTTAAATACTACGTTGTTGCTTAATGGTTACTACCCTACTTCAGGTTCTACATATCGTAATAT
GGCACTGAAGGGAACTTTACTATTGAGCACACTGTGGTTTAAACCACCTTTTCTTTCTGATTTTACTAATGGTATTTTT
GCTAAGGTCAAAAATACCAAGGTTGTTAAAGATGGTGTAATGTATAGTGAGTTTCCTGCTATAACTATAGGTAGTACTT
TTGTAAATACATCTTATAGTGTGGTAGTACAACCACATACTACCATTTTGGGTAATAAATTACAAGGTTTCTTAGAGAT
CTCTGTTTGCCAGTATACTATGTGCGAGTACCCAAATACGATTTGTAATCCTAATCTGGGCAATCAACGCGTAGAACTA
TGGCATTGGGATACAGGTGTTGTTTCCTGTTTATATAAGCGTAATTTCACATACGATGTGAATGCTGATTATTTGTATT
TCCATTTTTATCAAGAAGGTGGTACTTTTTATGCATATTTTACAGACACTGGTGTTGTTACTAAGTTTCTGTTTAATGT
TTATTTAGGCACGGTGCTCTCACATTATTATGTCATGCCTTTGACTTGTAATAGTGCTCTGACTTTAGAATATTGGGTT
ACACCCCTCACTTCTAAACAATATCTACTAGCTTTCAATCAAGATGGTGTTATTTTTAATGCTGTTGATTGTAAGAGTG
ATTTTATGAGTGAGATTAAGTGTAAAACACTATCTATAGCACCATCTACTGGTGTTTATGAATTAAACGGTTACACTGT
TCAGCCAATTGCAGATGTTTACCGACGTATACCTAATCTTCCCGATTGCAATATTGAGGCTTGGCTTAATGATAAGTCG
GTGCCCTCTCCATTAAATTGGGAACGTAAGACCTTTTCAAATTGTAATTTTAATATGAGCAGCCTGATGTCTTTTATTC
AGGCAGACTCATTTACTTGTAATAATATTGATGCTGCTAAGATATATGGCATGTGTTTTTCCAGCATAACTATAGATAA
GTTTGCAATACCCAATGGTAGGAAGGTTGACCTACAATTGGGTAATTTGGGTTATTTGCAGTCTTTTAACTATAGAATT
GATACTACTGCTACAAGTTGTCAGTTGTATTATAATTTACCTGCTGCTAATGTTTCTGTTAGCAGGTTTAATCCTTCTA
CTTGGAATAGGAGATTTGGTTTTACAGAACAATCTGTTTTTAAGCCTCAACCTGCAGGTGTTTTTACTGATCATGATGT
TGTTTATGCACAACATTGTTTTAAAGCTCCCACAAATTTCTGTCCGTGTAAATTGGATGGGTCTTTGTGTGTAGGTAGT
GGTTCTGGTATAGATGCTGGTTATAAAAATACTGGTATAGGCACTTGTCCTGCAGGTACTAATTATTTAACTTGCCATA
ATGCTGCCCAATGTAATTGTTTGTGCACTCCAGACCCCATTACATCTAAAGCTACAGGGCCTTACAAGTGCCCCCAAAC
TAAATATTTAGTTGGTATAGGTGAGCACTGTTCGGGTCTTGCTATTAAAAGTGATCATTGTGGAGGTAATCCTTGTAGT
TGCCAACCACAAGCATTTTTGGGTTGGTCTGTTGACTCTTGTTTACAAGGGGATAGGTGTAATATTTTTGCTAATTTTA
TTTTGCATGATGTTAATAGTGGTACTACTTGTTCTACTGATTTACAAAAATCAAACACAGACATAATTCTTGGTGTTTG
TGTTAATTATGATCTTTATGGTATTACTGGCCAAGGGATTTTTGTTGAGGTCAATGCGACTTATTATAATAGTTGGCAG
AACCTTTTATATGATTCTAATGGTAATCTCTATGGTTTTAGAGACTACTTAACAAATAGAACTTTTATGATTCGTAGTT
GCTATAGTGGTCGTGTTTCAGCGGCCTTTCATGCTAACTCTTCCGAACCAGCATTGCTATTTCGGAATATCAAATGCAA
TTACGTTTTTAATAACACTCTTTCACGACAGCTGCAACCTATTAACTATTTTGATAGCTATCTTGGTTGTGTTGTCAAT
GCTGATAATAGTACTTCTAGTGTTGTTCAAACATGTGATCTCACAGTAGGTAGTGGTTACTGTGTGGATTACTCTACAA
AAAGACGAAGTCGTAGATCGATTACCACTGGTTATCGGTTTACTAATTTTGAGCCATTTACTGTTAATTCAGTAAATGA
TAGTTTAGAACCTGTAGGTGGTTTGTATGAAATTCAAATACCTTCAGAGTTTACTATAGGTAATATGGAGGAGTTTATT
CAAACAAGCTCTCCTAAAGTTACTATTGATTGTTCTGCTTTTGTTTGTGGTGATTATGCAGCATGTAAATCACAGTTGG
TTGAATATGGTAGCTTCTGTGACAATATTAATGCTATACTCACAGAAGTAAATGAACTACTTGACACTACACAGTTGCA
AGTAGCTAATAGTTTAATGAATGGTGTCACTCTTAGCACTAAGCTTAAAGATGGCGTTAATTTCAATGTAGACGACATC
AATTTTTCCCCTGTATTAGGTTGTTTAGGAAGCGATTGCAATAAAGTTTCCAGTAGATCTGCTATAGAGGACTTACTTT
TTTCTAAAGTAAAGTTATCTGATGTCGGTTTCGTTGAGGCTTATAATAATTGTACTGGAGGTGCCGAAATTAGGGACCT
CATTTGTGTGCAAAGTTATAATGGTATCAAGGTGTTGCCTCCACTGCTCTCAGAAAATCAGATCAGTGGATACACTTTG
GCTGCCACCTCTGCTAGCCTGTTTCCTCCATGGTCAGCAGCAGCAGGTGTACCATTCTATTTAAATGTTCAGTATCGTA
TTAATGGTATTGGTGTTACCATGGATGTGCTAAGTCAAAATCAAAAGCTTATTGCTAATGCATTTAACAATGCTCTTGG
TGCTATTCAGGAAGGGTTTGATGCTACCAATTCTGCTTTAGTTAAAATTCAAGCTGTTGTTAATGCAAATGCTGAAGCT
CTTAATAACTTATTGCAACAACTCTCTAATAGATTTGGTGCTATAAGTTCTTCTTTACAAGAAATTCTATCTAGACTGG

ATGCTCTTGAAGCGCAAGCTCAGATAGACAGACTTATTAATGGGCGTCTTACCGCTCTTAATGCTTATGTTTCTCAACA
GCTTAGTGACTCTACACTAGTAAAGTTTAGTGCAGCACAAGCTATGGAGAAGGTTAATGAATGTGTCAAAAGCCAATCA
TCTAGGATAAATTTTTGTGGTAATGGTAATCATATTATATCATTAGTGCAGAATGCTCCATATGGTTTGTATTTTATCC
ACTTTAGCTATGTCCCTACTAAGTATGTCACAGCGAAGGTTAGTCCCGGTCTGTGCATTGCTGGTGGTAGAGGTATAGC
CCCTAAGAGTGGTTATTTTGTTAATGTAAATAATACTTGGATGTTCACTGGTAGTGGTTATTACTACCCTGAACCTATA
ACTGGAAATAATGTTGTTGTTATGAGTACCTGTGCTGTTAATTATACTAAAGCGCCGGATGTAATGCTGAACATTTCAA
CACCCAACCTCCCTGATTTTAAGGAAGAGTTGGATCAATGGTTTAAAAACCAAACCTCAGTGGCACCAGATTTGTCACT
TGATTATATAAATGTTACATTCTTGGACCTACAAGATGAAATGAATAGGTTACAGGAGGCAATAAAAGTTTTAAATCAG
AGCTACATCAATCTCAAGGACATTGGTACTTATGAGTATTATGTAAAATGGCCTTGGTATGTATGGCTTTTAATTGGCT
TTGCAGGTGTAGCTATGCTTGTTTTACTATTCTTCATATGCTGTTGTACAGGATGTGGGACTAGTTGTTTTAAGAAATG
TGGTGGTTGTTGTGATGATTATACAGGACACCAGGAGTTAGTAATTAAAACATCACATGAAGACTAA

>gi|145208944|gb|EF424620.1| Bovine coronavirus R-AH187, complete genome

ATGTTTTTGATACTTTTAATTTCCTTACCAACGGCTTTTGCTGTTATAGGAGATTTAAAGTGTACTACACTTTCC
ATTAATGATGTTGACACTGGTGTTCCTTCTATTAGCACTGATACTGTCGATGTTACTAATGGTTTAGGTACTTATTATG
TTTTAGATCGTGTGTATTTAAATACTACGTTGTTGCTTAATGGTTATTACCCTACTTCAGGTTCTACATATCGTAATAT
GGCACTGAAGGGAACTTTACTACTGAGCACACTGTGGTTTAAACCACCTTTTCTTTCTGATTTTACTAATGGTATTTTT
GCTAAGGTCAAAAATACCAAGGTTATTAAAGATGGTGTAATGTATAGTGAGTTTCCTGCTATAACTATAGGTAGTACTT
TTGTAAATACATCCTATAGTGTGGTAGTACAACCACATACTACCATTTTGGGTAATAAATTACAAGGTTTCTTAGAGAT
CTCTGTTTGCCAGTATACTATGTGCGAGTACCCAAATACGATTTGTAATCCTAATCTGGGTAATCAACGCGTAGAACTA
TGGCATTGGGATACAGGTGTTGTTTCCTGTTTATATAAGCGTAATTTCACATATGATGTGAATGCTGATTATTTGTATT
TCCATTTTTATCAAGAAGGTGGTACTTTTTATGCATATTTTACAGACACTGGTGTTGTTACTAAGTTTCTGTTTAATGT
TTATTTAGGCACGGTGCTCTCACATTATTATGTCATGCCTTTGACTTGTAATAGTGCTCTGACTTTAGAATATTGGGTT
ACACCCCTCACTTCTAAACAATATCTACTAGCTTTCAATCAAGATGGTGTTATTTTTAATGCTGTTGATTGTAAGAGTG
ATTTTATGAGTGAGATTAAGTGTAAAACACTATCTATAGCACCATCTACTGGTGTTTATGAATTAAACGGTTACACTGT
TCAGCCAATTGCAGATGTTTACCGACGTATACCTAATCTTCCCGATTGCAATATTGAGGCTTGGCTTAATGATAAGTCG
GTGCCCTCTCCATTAAATTGGGAACGTAAGACTTTTTCAAATTGTAATTTTAATATGAGCAGCCTGATGTCTTTTATTC
AGGCAGACTCATTTACTTGTAATAATATTGATGCTGCTAAGATATATGGTATGTGTTTTTCCAGCATAACTATAGATAA
GTTTGCAATACCCAATGGTAGGAAGGTTGACCTACAATTGGGTAATTTGGGTTATTTGCAGTCTTTTAACTATAGAATT
GATACTACTGCTACAAGTTGTCAGTTGTATTATAATTTACCTGCTGCTAATGTTTCTGTTAGCAGGTTTAATCCTTCTA
CTTGGAATAGGAGATTTGGTTTTACAGAACAATCTGTTTTTAAGCCTCAACCTGCAGGTGTTTTTACTGATCATGATGT
TGTTTATGCACAACATTGTTTTAAAGCTCCCACAAATTTCTGTCCGTGTAAATTGGATGGGTCTTTGTGTGTAGGTAAT
GGTCCTGGTATAGATGCTGGTTATAAAACTAGTGGCATAGGCACTTGTCCTGCAGGTACTAATTATTTAACTTGCCATA
ATGCTGCCCAATGTGATTGTTTGTGCACTCCAGACCCCATTACATCTAAAGCTACAGGGCCTTACAAGTGCCCCCAAAC
TAAATACTTAGTTGGCATAGGTGAGCACTGTTCGGGTCTTGCTATTAAAAGTGATTATTGTGGAGGTAACCCTTGTAGT
TGCCAACCACAAGCATTTTTGGGTTGGTCTGTTGACTCTTGTTTACAAGGGGATAGGTGTAATATTTTTGCTAATTTTA
TTTTGCATGATGTTAATAGTGGTACTACTTGTTCTACTGATTTACAAAAATCAAACACAGACATAATTCTTGGTGTTTG
TGTTAATTATGATCTTTATGGTATTACTGGCCAAGGGATTTTTGTTGAGGTTAATGCGACTTATTATAATAGTTGGCAG
AACCTTTTATATGATTCTAATGGTAATCTCTATGGTTTAGAGACTACTTAACAAATAGAACTTTTATGATTCGTAGTT
GCTATAGTGGTCGTGTTTCAGCGGCCTTTCATGCTAACTCTTCCGAACCAGCATTGCTATTTCGGAATATCAAATGCAA
TTACGTTTTTAATAACACTCTTTCACGACAGCTGCAACCTATTAACTATTTTGATAGCTATCTTGGTTGTGTTGTCAAT
GCTGATAATAGTACTTCTATTGTTGTTCAAACATGTGATCTCACAGTAGGTAATGGTTACTGTGTGGATTACTCTACAA

AAAGACGAAGTCGTAGATCGATTACCACTGGTTATCGGTTTACTAATTTTGAGCCATTTACTGTTAATTCAGTAAATGA
TAGTTTAGAACCTGTAGGTGGTTTGTATGAAATTCAAATACCTTCAGAGTTTACTATAGGTAATATGGAGGAGTTTATT
CAAACAAGCTCTCCTAAAGTTACTATTGATTGTTCTGCTTTTGTCTGTGGTGATTATGCAGCATGTAAATCACAGTTGG
TTGAATATGGTAGCTTCTGTGACAATATTAATGCTATACTCACAGAAGTAAATGAATTACTTGACACTACACAGTTGCA
AGTAGCTAATAGTTTAATGAATGGTGTCACTCTTAGCACTAAGCTTAAAGATGGCGTTAATTTCAATGTAGACGACATC
AATTTTTCCCCTGTATTAGGTTGTTTAGGAAGCGATTGCAATAAAGTTTCCAGTAGATCTGCTATAGAGGACTTACTTT
TTTCTAAAGTAAAGTTATCTGATGTCGGTTTCGTTGAGGCTTATAATAATTGTACTGGAGGTGCCGAAATTAGGGACCT
CATTTGTGTGCAAAGTTATAATGGTATCAAGGTGTTGCCTCCACTGCTCTCAGAAAATCAGATCAGTGGATACACTTTG
GCTGCCACCTCTGCTAGCCTGTTTCCTCCATGGTCAGCAGCAGCAGGTGTACCATTCTATTTAAATGTTCAGTATCGTA
TTAATGGTATTGGTGTTACCATGGATGTGCTAAGTCAAAATCAAAAGCTTATTGCTAATGCATTTAACAATGCTCTTGG
TGCTATCCAGGAAGGGTTTGATGCTACCAATTCTGCTTTAGTTAAAATTCAAGCTGTTGTTAATGCAAATGCTGAAGCT
CTTAATAACTTATTGCAACAACTCTCTAATAGATTTGGTGCTATAAGTTCTTCTTTACAAGAAATTCTATCTAGACTGG
ATGCTCTTGAAGCGCAAGCTCAGATAGACAGACTTATTAATGGGCGTCTTACCGCTCTTAATGCTTATGTTTCTCAACA
GCTTAGTGACTCTACACTAGTAAAGTTTAGTGCAGCACAAGCTATGGAGAAGGTTAATGAATGTGTCAAAAGCCAATCA
TCTAGGATAAATTTTTGTGGTAATGGTAATCATATTATATCATTAGTGCAGAATGCTCCATATGGTTTGTATTTTATCC
ACTTTAGCTATGTCCCTACTAAGTATGTCACAGCGAAGGTTAGTCCCGGTCTGTGCATTGCTGGTGGTAGAGGTATAGC
CCCTAAGAGTGGTTATTTTGTTAATGTAAATAACTCTTGGATGTTCACTGGTAGTGGTTATTACTACCCTGAACCTATA
ACTGGAAATAATGTTGTTGTTATGAGTACCTGTGCTGTTAATTATACTAAAGCGCCGGATGTAATGCTGAACATTTCAA
CACCCAACCTCCCTGATTTTAAGGAAGAGTTGGATCAATGGTTTAAAAACCAAACATCAGTGGCACCAGATTTGTCACT
TGATTATATAAATGTTACATTCTTGGACCTACAAGATGAAATGAATAGGTTACAGGAGGCAATAAAAGTTTTAAATCAG
AGCTACATCAATCTCAAGGACATTGGTACATATGAGTATTATGTAAAATGGCCTTGGTATGTATGGCTTTTAATTGGCT
TTGCTGGTGTAGCTATGCTTGTTTTACTATTCTTCATATGCTGTTGTACAGGATGTGGGACTAGTTGTTTTAAGAAATG
TGGTGGTTGTTGTGATGATTATACTGGACACCAGGAGTTAGTAATTAAAACATCACATGAAGACTAA

>gi|30061510|gb|U00735.2|BCU00735 Bovine coronavirus strain Mebus, complete genome

ATGTTTTTGATACTTTTAATTTCCTTACCAATGGCTTTTGCTGTTATAGGAGATTTAAAGTGTACTACGGTTTCC
ATTAATGATGTTGACACCGGTGCTCCTTCTATTAGCACTGATATTGTCGATGTTACTAATGGTTTAGGTACTTATTATG
TTTTAGATCGTGTGTATTTAAATACTACGTTGTTGCTTAATGGTTACTACCCTACTTCAGGTTCTACATATCGTAATAT
GGCACTGAAGGGAACTTTACTATTGAGCAGACTATGGTTTAAACCACCTTTTCTTTCTGATTTTATTAATGGTATTTTT
GCTAAGGTCAAAAATACCAAGGTTATTAAAAAGGGTGTAATGTATAGTGAGTTTCCTGCTATAACTATAGGTAGTACTT
TTGTAAATACATCCTATAGTGTGGTAGTACAACCACATACTACCAATTTGGATAATAAATTACAAGGTCTCTTAGAGAT
CTCTGTTTGCCAGTATACTATGTGCGAGTACCCACATACGATTTGTCATCCTAATCTGGGTAATAAACGCGTAGAACTA
TGGCATTGGGATACAGGTGTTGTTTCCTGTTTATATAAGCGTAATTTCACATATGATGTGAATGCTGATTACTTGTATT
TCCATTTTTATCAAGAAGGTGGTACTTTTTATGCATATTTTACAGACACTGGTGTTGTTACTAAGTTTCTGTTTAATGT
TTATTTAGGCACGGTGCTTTCACATTATTATGTCCTGCCTTTGACTTGTTCTAGTGCTATGACTTTAGAATATTGGGTT
ACACCTCTCACTTCTAAACAATATTTACTAGCTTTCAATCAAGATGGTGTTATTTTTAATGCTGTTGATTGTAAGAGTG
ATTTTATGAGTGAGATTAAGTGTAAAACACTATCTATAGCACCATCTACTGGTGTTTATGAATTAAACGGTTACACTGT
TCAGCCAATTGCAGATGTTTACCGACGTATACCTAATCTTCCCGATTGTAATATAGAGGCTTGGCTTAATGATAAGTCG
GTGCCCTCTCCATTAAATTGGGAACGTAAGACCTTTTCAAATTGTAATTTTAATATGAGCAGCCTGATGTCTTTTATTC
AGGCAGACTCATTTACTTGTAATAATATTGATGCTGCTAAGATATATGGTATGTGTTTTTCCAGCATAACTATAGATAA
GTTTGCTATACCCAATGGTAGGAAGGTTGACCTACAATTGGGCAATTTGGGCTATTTGCAGTCTTTTAACTATAGAATT

GATACTACTGCTACAAGTTGTCAGTTGTATTATAATTTACCTGCTGCTAATGTTTCTGTTAGCAGGTTTAATCCTTCTA
CTTGGAATAGGAGATTTGGTTTTACAGAACAATTTGTTTTTAAGCCTCAACCTGTAGGTGTTTTTACTCATCATGATGT
TGTTTATGCACAACATTGTTTTAAAGCTCCCTCAAATTTCTGTCCGTGTAAATTGGATGGGTCTTTGTGTGTAGGTAAT
GGTCCTGGTATAGATGCTGGTTATAAAAATAGTGGTATAGGCACTTGTCCTGCAGGTACTAATTATTTAACTTGCCATA
ATGCTGCCCAATGTAATTGTTTGTGCACTCCCGACCCCATTACATCTAAATCTACAGGGCCTTACAAGTGCCCCCAAAC
TAAATACTTAGTTGGCATAGGTGAGCACTGTTCGGGTCTTGCTATTAAAAGTGATTATTGTGGAGGTAATCCTTGTACT
TGCCAACCACAAGCATTTTTGGGCTGGTCTGTTGACTCTTGTTTACAAGGGGATAGGTGTAATATTTTTGCTAATTTTA
TTTTGCATGATGTTAATAGTGGTACTACTTGTTCTACTGATTTACAAAAATCAAACACAGACATAATTCTTGGTGTTTG
TGTTAATTATGATCTTTATGGTATTACAGGCCAAGGTATTTTTGTTGAGGTTAATGCGACTTATTATAATAGTTGGCAG
AACCTTTTATATGATTCTAATGGTAATCTCTATGGTTTTAGAGACTACTTAACAAACAGAACTTTTATGATTCGTAGTT
GCTATAGCGGTCGTGTTTCAGCGGCCTTTCATGCTAACTCTTCCGAACCAGCATTGCTATTTCGGAATATTAAATGCAA
TTACGTTTTTAATAATACTCTTTCACGACAGCTGCAACCTATTAACTATTTTGATAGTTATCTTGGTTGTGTTGTCAAT
GCTGATAATAGTACTTCTAGTGTTGTTCAAACATGTGATCTCACAGTAGGTAGTGGTTACTGTGTGGATTACTCTACAA
AAAGACGAAGTCGTAGAGCGATTACCACTGGTTATCGGTTTACTACTTTTGAGCCATTTACTGTTAATTCAGTAAATGA
TAGTTTAGAACCTGTAGGTGGTTTGTATGAAATTCAAATACCTTCAGAGTTTACTATAGGTAATATGGAGGAGTTTATT
CAAACAAGCTCTCCTAAAGTTACTATTGATTGTTCTGCTTTTGTCTGTGGTGATTATGCAGCATGTAAATCACAGTTGG
TTGAATATGGTAGCTTCTGTGACAATATTAATGCTATACTCACAGAAGTAAATGAACTACTTGACACTACACAGTTGCA
AGTAGCTAATAGTTTAATGAATGGTGTCACTCTTAGCACTAAGCTTAAAGATGGCGTTAATTTCAATGTAGACGACATC
AATTTTTCCCCTGTATTAGGTTGTTTAGGAAGCGATTGTAATAAAGTTTCCAGCAGATCTGCTATAGAGGATTTACTTT
TTTCTAAAGTAAAGTTATCTGATGTCGGTTTCGTTGAGGCTTATAATAATTGTACTGGAGGTGCCGAAATTAGGGACCT
CATTTGTGTGCAAAGTTATAATGGTATCAAAGTGTTGCCTCCACTGCTCTCAGTAAATCAGATCAGTGGATACACTTTG
GCTGCCACCTCTGCTAGTCTGTTTCCTCCTTTGTCAGCAGCAGTAGGTGTACCATTTTATTTAAATGTTCAGTATCGTA
TTAATGGGATTGGTGTTACCATGGATGTGTTAAGTCAAAATCAAAAGCTTATTGCTAATGCATTTAACAATGCTCTTGA
TGCTATTCAGGAAGGGTTTGATGCTACCAATTCTGCTTTAGTTAAAATTCAAGCTGTTGTTAATGCAAATGCTGAAGCT
CTTAATAACTTATTGCAACAACTCTCTAATAGATTTGGTGCTATAAGTTCTTCTTTACAAGAAATTCTATCTAGACTGG
ATGCTCTTGAAGCGCAAGCTCAGATAGACAGACTTATTAATGGGCGTCTTACCGCTCTTAATGTTTATGTTTCTCAACA
GCTTAGTGATTCTACACTAGTAAAATTTAGTGCAGCACAAGCTATGGAGAAGGTTAATGAATGTGTCAAAAGCCAATCA
TCTAGGATAAATTTTTGTGGTAATGGTAATCATATTATATCATTAGTGCAGAATGCTCCATATGGTTTGTATTTTATCC
ACTTTAGCTATGTCCCTACTAAGTATGTCACTGCGAAGGTTAGTCCCGGTCTGTGCATTGCTGGTGATAGAGGTATAGC
CCCTAAGAGTGGTTATTTTGTTAATGTAAATAATACTTGGATGTTCACTGGTAGTGGTTATTACTACCCTGAACCCATA
ACTGGAAATAATGTTGTTGTTATGAGTACCTGTGCTGTTAACTATACTAAAGCGCCGGATGTAATGCTGAACATTTCAA
CACCCAACCTCCATGATTTTAAGGAAGAGTTGGATCAATGGTTTAAAAACCAAACATCAGTGGCACCAGATTTGTCACT
TGATTATATAAATGTTACATTCTTGGACCTACAAGATGAAATGAATAGGTTACAGGAGGCAATAAAAGTTTTAAATCAG
AGCTACATCAATCTCAAGGACATTGGTACATATGAGTATTATGTAAAATGGCCTTGGTATGTATGGCTTTTAATTGGCT
TTGCTGGTGTAGCTATGCTTGTTTTACTATTCTTCATATGCTGTTGTACAGGATGTGGGACTAGTTGTTTTAAGATATG
TGGTGGTTGTTGTGATGATTATACTGGACACCAGGAGTTAGTAATTAAAACATCACATGACGACTAA

>gi|323355|gb|M64667.1|COBSA Bovine coronavirus spike glycoprotein (S) gene, complete cds

ATGTTTTTGATACTTTTAATTTCCTTACCAATGGCTTTTGCTGTTATAGGAGATTTAAAGTGTACTACGGTTTCC
ATTAATGATGTTGACACCGGTGCTCCTTCTATTAGCACTGATATTGTCGATGTTACTAATGGTTTAGGTACTTATTATG
TTTTAGATCGTGTGTATTTAAATACTACGTTGTTGCTTAATGGTTACTACCCTACTTCAGGTTCTACATATCGTAATAT

GGCACTGAAGGGAACTTTACTATTGAGCAGACTATGGTTTAAACCACCTTTTCTTTCTGATTTTATTAATGGTATTTTT
GCTAAGGTCAAAAATACCAAGGTTATTAAAAAGGGTGTAATGTATAGTGAGTTTCCTGCTATAACTATAGGTAGTACTT
TTGTAAATACATCCTATAGTGTGGTAGTACAACCACATACTACCAATTTGGATAATAAATTACAAGGTCTCTTAGAGAT
CTCTGTTTGCCAGTATACTATGTGCGAGTACCCACATACGATTTGTCATCCTAATCTGGGTAATAAACGCGTAGAACTA
TGGCATTGGGATACAGGTGTTGTTTCCTGTTTATATAAGCGTAATTTCACATATGATGTGAATGCTGATTACTTGTATT
TCCATTTTTATCAAGAAGGTGGTACTTTTTATGCATATTTTACAGACACTGGTGTTGTTACTAAGTTTCTGTTTAATGT
TTATTTAGGCACGGTGCTTTCACATTATTATGTCCTGCCTTTGACTTGTAATAGTGCTATGACTTTAGAATATTGGGTT
ACACCTCTCACTTCTAAACAATATTTACTAGCTTTCAATCAAGATGGTGTTATTTTTAATGCTGTTGATTGTAAGAGTG
ATTTTATGAGTGAGATTAAGTGTAAAACACTATCTATAGCACCATCTACTGGTGTTTATGAATTAAACGGTTACACTGT
TCAGCCAATTGCAGATGTTTACCGACGTATACCTAATCTTCCCGATTGTAATATAGAGGCTTGGCTTAATGATAAGTCG
GTGCCCTCTCCATTAAATTGGGAACGTAAGACCTTTTCAAATTGTAATTTTAATATGAGCTGCCTGATGTCTTTTATTC
AGGCAGACTCATTTACTTGTAATAATATTGATGCTGCTAAGATATATGGTATGTGTTTTTCCAGCATAACTATAGATAA
GTTTGCTATACCCAATGGTAGGAAGGTTGACCTACAATTGGGCAATTTGGGCTATTTGCAGTCTTTTAACTATAGAATT
GATACTACTGCTACAAGTTGTCAGTTGTATTATAATTTACCTGCTGCTAATGTTTCTGTTAGCAGGTTTAATCCTTCTA
CTTGGAATAGGAGATTTGGTTTTACAGAACAATCTGTTTTTAAGCCTCAACCTGTAGGTGTTTTTACTCATCATGATGT
TGTTTATGCACAACATTGTTTTAAAGCTCCCACAAATTTCTGTCCGTGTAAATTGGATGGGTCTTTGTGTGTAGGTAAT
GGTCCTGGTATAGATGCTGGTTATAAAAATAGTGGTATAGGCACTTGTCCTGCAGGTACTAATTATTTAACTTGCCATA
ATGCTGCCCAATGTGATTGTTTGTGCACTCCCGACCCCATTACATCTAAATCTACAGGGCCTTACAAGTGCCCCCAAAC
TAAATACTTAGTTGGCATAGGTGAGCACTGTTCGGGTCTTGCTATTAAAAGTGATTATTGTGGAGGTAATCCTTGTACT
TGCCAACCACAAGCATTTTTGGGTTGGTCTGTTGACTCTTGTTTACAAGGGGATAGGTGTAATATTTTTGCTAATTTTA
TTTTGCATGATGTTAATAGTGGTACTACTTGTTCTACTGATTTACAAAAATCAAACACAGACATAATTCTTGGTGTTTG
TGTTAATTATGATCTTTATGGTATTACAGGCCAAGGTATTTTTGTTGAGGTTAATGCGCCTTATTATAATAGTTGGCAG
AACCTTTTATATGATTCTAATGGTAATCTCTATGGTTTTAGAGACTACTTAACAAACAGAACTTTTATGATTCGTAGTT
GCTATAGCGGTCGTGTTTCAGCGGCCTTTCATGCTAACTCTTCCGAACCAGCATTGCTATTTCGGAATATTAAATGCAA
TTACGTTTTTAATAATACTCTTTCACGACAGCTGCAACCTATTAACTATTTTGATAGTTATCTTGGTTGTGTTGTCAAT
GCTGATAATAGTACTTCTAGTGTTGTTCAAACATGTGATCTCACAGTAGGTAGTGGTTACTGTGTGGATTACTCTACAA
AAAGACGAAGTCGTAGAGCGATTACCACTGGTTATCGGTTTACTAATTTTGAGCCATTTACTGTTAATTCAGTAAATGA
TAGTTTAGAACCTGTAGGTGGTTTGTATGAAATTCAAATACCTTCAGAGTTTACTATAGGTAATATGGAGGAGTTTATT
CAAACAAGCTCTCCTAAAGTTACTATTGATTGTTCTGCTTTTGTCTGTGGTGATTATGCAGCATGTAAATCACAGTTGG
TTGAATATGGTAGCTTCTGTGACAATATTAATGCTATACTCACAGAAGTAAATGAACTACTTGACACTACACAGTTGCA
AGTAGCTAATAGTTTAATGAATGGTGTCACTCTTAGCACTAAGCTTAAAGATGGCGTTAATTTCAATGTAGACGACATC
AATTTTTCCCCTGTATTAGGTTGTTTAGGAAGCGATTGTAATAAAGTTTCCAGCAGATCTGCTATAGAGGATTTACTTT
TTTCTAAAGTAAAGTTATCTGATGTCGGTTTCGTTGAGGCTTATAATAATTGTACTGGAGGTGCCGAAATTAGGGACCT
CATTTGTGTGCAAAGTTATAATGGTATCAAAGTGTTGCCTCCACTGCTCTCAGTAAATCAGATCAGTGGATACACTTTG
GCTGCCACCTCTGCTAGTCTGTTTCCTCCTTGGTCAGCAGCAGCAGGTGTACCATTTTATTTAAATGTTCAGTATCGTA
TTAATGGGATTGGTGTTACCATGGATGTGTTAAGTCAAAATCAAAAGCTTATTGCTAATGCATTTAACAATGCTCTTGA
TGCTATTCAGGAAGGGTTTGATGCTACCAATTCTGCTTTAGTTAAAATTCAAGCTGTTGTTAATGCAAATGCTGAAGCT
CTTAATAACTTATTGCAACAACTCTCTAATAGATTTGGTGCTATAAGTTCTTCTTTACAAGAAATTCTATCTAGACTGG
ATGCTCTTGAAGCGCAAGCTCAGATAGACAGACTTATTAATGGGCGTCTTACCGCTCTTAATGCTTATGTTTCTCAACA
GCTTAGTGATTCTACACTAGTAAAATTTAGTGCAGCACAAGCTATGGAGAAGGTTAATGAATGTGTCAAAAGCCAATCA
TCTAGGATAAATTTTTGTGGTAATGGTAATCATATTATATCATTAGTGCAGAATGCTCCATATGGTTTGTATTTTATCC
ACTTTAGCTATGTCCCTACTAAGTATGTCACTGCGAAGGTTAGTCCCGGTCTGTGCATTGCTGGTGATAGAGGTATAGC
CCCTAAGAGTGGTTATTTTGTTAATGTAAATAATACTTGGATGTTCACTGGTAGTGGTTATTACTACCCTGAACCCATA
ACTGGAAATAATGTTGTTGTTATGAGTACCTGTGCTGCTAACTATACTAAAGCGCCGGATGTAATGCTGAACATTTCAA

CACCCAACCTCCATGATTTTAAGGAAGAGTTGGATCAATGGTTTAAAAACCAAACATCAGTGGCACCAGATTTGTCACT
TGATTATATAAATGTTACATTCTTGGACCTACAAGATGAAATGAATAGGTTACAGGAGGCAATAAAAGTTTTAAATCAG
AGCTACATCAATCTCAAGGACATTGGTACATATGAGTATTATGTAAAATGGCCTTGGTATGTATGGCTTTTAATTGGCT
TTGCTGGTGTAGCTATGCTTGTTTTACTATTCTTCATATGCTGTTGTACAGGATGTGGGACTAGTTGTTTTAAGATATG
TGGTGGTTGTTGTGATGATTATACTGGACACCAGGAGTTAGTAATTAAAACATCACATGACGACTAA

>gi|323357|gb|M64668.1|COBSB Bovine coronavirus spike glycoprotein (S) gene, complete cds

ATGTTTTTGATACTTTTAATTTCCTTACCAATGGCTTTTGCTGTTATAGGAGATTTAAAGTGTACTACGGTTTCC
ATTAATGATGTTGACACCGGTGCTCCTTCTATTAGCACTGATACTGTCGATGTTACTAATGGTTTAGGTACTTATTATG
TTTTAGATCGTGTGTATTTAAATACTACGTTGTTGCTTAATGGTTACTACCCTACTTCAGGTTCTACATATCGTAATAT
GGCACTGAAGGGAACTTTACTATTGAGCAGACTATGGTTTAAACCACCTTTTCTTTCTGATTTTATTAATGGTATTTTT
GCTAAGGTCAAAAATACCAAGGTTATTAAAAAGGGTGTAATGTATAGTGAGTTTCCTGCTATAACTATAGGTAGTACTT
TTGTAAATACATCCTATAGTGTGGTAGTACAACCACATACTACCAATTTGGATAATAAATTACAAGGTCTCTTAGAGAT
CTCTGTTTGCCAGTATACTATGTGCGAGTACCCACATACGATTTGTCATCCTAATCTGGGTAATCAACGCGTAGAACTA
TGGCATTGGGATACAGGTGTTGTTTCCTGTTTATATAAGCGTAATTTCACATATGATGTGAATGCTGATTACTTGTATT
TCCATTTTTATCAAGAAGGTGGTACTTTTTATGCATATTTTACAGACACTGGTGTTGTTACTAAGTTTCTGTTTAATGT
TTATTTAGGCACGGTGCTTTCACATTATTATGTCCTGCCTTTGACTTGTTATAGTGCTATGACTTTAGAATATTGGGTT
ACACCTCTCACTTCTAAACAATATTTACTAGCTTTCAATCAAGATGGTGTTATTTTTAATGCTGTTGATTGTAAGAGTG
ATTTTATGAGTGAGATTAAGTGTAAAACACTATCTATAGCACCATCTACTGGTGTTTATGAATTAAACGGTTACACTGT
TCAGCCAATTGCAGATGTTTACCGACGTATACCTAATCTTCCCGATTGTAATATAGAGGCTTGGCTTAATGATAAGTCT
GTGCCCTCTCCATTAAATTGGGAACGTAAGACCTTTTCAAATTGTAATTTTAATATGAGCAGCCTGATGTCTTTTATTC
AGGCAGACTCATTTACTTGTAATAATATTGAAGCTGCTAAGATATATGGTATGTGTTTTTCCAGCATAACTATAGATAA
GTTTGCTATACCCAATGGTAGGAAGGTTGACCTACAATTGGGCAATTTGGGCTATTTGCAGTCTTTTAACTATAGAATT
GATACTACTGCTGCAAGTTGTCAGTTGTATTATAATTTACCTGCTGCTAATGTTTCTGTTAGCAGGTTTAATCCTTCTA
CTTGGAATAGGAGATTTGGTTTTACAGAACAATCTGTTTTTAAGCCTCAACCTGTAGGTGTTTTTACTCATCATGATGT
TGTTTATGCACAACATTGTTTTAAAGCTCCCACAAATTTCTGTCCGTGTAAATTGGATGGGTCTTTGTGTGTAGGTAAT
GGTCCTGGTATAGATGCTGGTTATAAAAATAGTGGTATAGGCACTTGTCCTGCAGGTACTAATTATTTAACTTGCCATA
ATGCTGCCCAATGTGATTGTTTGTGCACTCCCGACCCCATTACATCTAAATCTACAGGGCCTTACAAGTGCCCCCAAAC
TAAATACTTAGTTGGCATAGGTGAGCACTGTTCGGGTCTTGCTATTAAAAGTGATTATTGTGGAGGTAATCCTTGTACT
TGCCAACCACAAGCATTTTTGGGTTGGTCTGTTGACTCTTGTTTACAAGGGGATAGGTGTAATATTTTTGCTAATTTTA
TTTTGCATGATGTTAATAGTGGTACTACTTGTTCTACTGATTTACAAAAATCAAACACAGACATAATTCTTGGTGTTTG
TGTTAATTATGATCTTTATGGTATTACAGGCCAAGGTATTTTTGTTGAGGTTAATGCGACTTATTATAATAGTTGGCAG
AACCTTTTATATGATTCTAATGGTAATCTCTATGGTTTTAGAGACTACTTAACAAACAGAACTTTTATGATTCGTAGTT
GCTATAGCGGTCGTGTTTCAGCGGCCTTTCATGCTAACTCTTCCGAACCAGCATTGCTATTTCGGAATATTAAATGCAA
TTACGTTTTTAATAATACTCTTTCACGACAGCTGCAACCTATTAACTATTTTGATAGTTATCTTGGTTGTGTTGTCAAT
GCTGATAATAGTACTTCTAGTGTTGTTCAAACATGTGATCTCACAGTAGGTAGTGGTTACTGTGTGGATTACTCTACAA
AAAGACGAAGTCGTAGAGCGATTACCACTGGTTATCGGTTTACTAATTTTGAGCCATTTACTGTTAATTCAGTAAATGA
TAGTTTAGAACCTGTAGGTGGTTTGTATGAAATTCAAATACCTTCAGAGTTTACTATAGGTAATATGGAGGAGTTTATT
CAAACAAGCTCTCCTAAAGTTACTATTGATTGTTCTGCTTTTGTCTGTGGTGATTATGCAGCATGTAAATCACAGTTGG
TTGAATATGGTAGCTTCTGTGACAATATTAATGCTATACTCACAGAAGTAAATGAACTACTTGACACTACACAGTTGCA
AGTAGCTAATAGTTTAATGAATGGTGTCACTCTTAGCACTAAGCTTAAAGATGGCGTTAATTTCAATGTAGACGACATC

AATTTTTCCCCTGTATTAGGTTGTTTAGGAAGCGATTGTAATAAAGTTTCCAGCAGATCTGCTATAGAGGATTTACTTT
TTTCTAAAGTAAAGTTATCTGATGTCGGTTTCGTTGAGGCTTATAATAATTGTACTGGAGGTGCCGAAATTAGGGACCT
CATTTGTGTGCAAAGTTATAATGGTATCAAAGTGTTGCCTCCACTGCTCTCAGTAAATCAGATCAGTGGATACACTTTG
GCTGCCACCTCTGCTAGTCTGTTTCCTCCTTGGTCAGCAGCAGCAGGTGTACCATTTTATTTAAATGTTCAGTATCGTA
TTAATGGGATTGGTGTTACCATGGATGTGTTAAGTCAAAATCAAAAGCTTATTGCTAATGCATTTAACAATGCTCTTGA
TGCTATTCAGGAAGGGTTTGATGCTACCAATTCTGCTTTAGTTAAAATTCAAGCTGTTGTTAATGCAAATGCTGAAGCT
CTTAATAACTTATTGCAACAACTCTCTAATAGATTTGGTGCTATAAGTTCTTCTTTACAAGAAATTCTATCTAGACTGG
ATGCTCTTGAAGCGCAAGCTCAGATAGACAGACTTATTAATGGGCGTCTTACCGCTCTTAATGCTTATGTTTCTCAACA
GCTTAGTGATTCTACACTAGTAAAATTTAGTGCAGCACAAGCTATGGAGAAGGTTAATGAATGTGTCAAAAGCCAATCA
TCTAGGATAAATTTTTGTGGTAATGGTAATCATATTATATCATTAGTGCAGAATGCTCCATATGGTTTGTATTTTATCC
ACTTTAGCTATGTCCCTACTAAGTATGTCACTGCGAAGGTTAGTCCCGGTCTGTGCATTGCTGGTGATAGAGGTATAGC
CCCTAAGAGTGGTTATTTTGTTAATGTAAATAATACTTGGATGTTCACTGGTAGTGGTTATTACTACCCTGAACCCATA
ACTGGAAATAATGTTGTTGTTATGAGTACCTGTGCTGTTAACTATACTAAAGCGCCGGATGTAATGCTGAACATTTCAA
CACCCAACCTCCATGATTTTAAGGAAGAGTTGGATCAATGGTTTAAAAACCAAACATCAGTGGCACCAGATTTGTCACT
TGATTATATAAATGTTACATTCTTGGACCTACAAGATGAAATGAATAGGTTACAGGAGGCAATAAAAGTTTTAAATCAG
AGCTACATCAATCTCAAGGACATTGGTACATATGAGTATTATGTAAAATGGCCTTGGTATGTATGGCTTTTAATTGGCT
TTGCTGGTGTAGCTATGCTTGTTTTACTATTCTTCATATGCTGTTGTACAGGATGTGGGACTAGTTGTTTTAAGATATG
TGGTGGTTGTTGTGATGATTATACTGGACACCAGGAGTTAGTAATTAAAACATCACATGACGACTAA

>gi|221138|dbj|D00662.1|COBSQUE Bovine coronavirus gene for S peplomer polypeptide precursor, complete cds

ATGTTTTTGATACTTTTAATTTCCTTACCAATGGCTTTTGCTGTTATAGGAGATTTAAAGTGTACTACGGTTTCC
ATTAATGATGTTGACACCGGTGCTCCCTCTATTAGCACTGATATTGTCGATGTTACTAATGGTTTAGGTACTTATTATG
TTTTAGATCGTGTGTATTTAAATACTACGTTGTTGCTTAATGGTTACTACCCTACTTCAGGTTCTACATATCGTAATAT
GGCACTGAAGGGAACTTTACTATTGAGCAGACTATGGTTTAAACCACCTTTTCTTTCTGATTTTATTAATGGTATTTTT
GCTAAGGTCAAAAATACCAAGGTTATTAAAAAGGGTGTAATGTATAGTGAGTTTCCTGCTATAACTATAGGTAGTACTT
TTGTAAATACATCCTATAGTGTGGTAGTACAACCACATACTACCAATTTGGATAATAAATTACAAGGTCTCTTAGAGAT
CTCTGTTTGCCAGTATACTATGTGCGAGTACCCACATACGATTTGTCATCCTAAGCTGGGTAATAAACGCGTAGAACTA
TGGCATTGGGATACAGGTGTTGTTTCCTGTTTATATAAGCGTAATTTCACATATGATGTGAATGCTGATTACTTGTATT
TCCATTTTTATCAAGAAGGTGGTACTTTTTATGCATATTTTACAGACACTGGTGTTGTTACTAAGTTTCTGTTTAATGT
TTATTTAGGCACGGTGCTTTCACATTATTATGTCCTGCCTTTGACTTGTTCTAGTGCTATGACTTTAGAATATTGGGTT
ACACCTCTCACTTCTAAACAATATTTACTAGCTTTCAATCAAGATGGTGTTATTTTTAATGCTGTTGATTGTAAGAGTG
ATTTTATGAGTGAGATTAAGTGTAAAACACTATCTATAGCACCATCTACTGGTGTTTATGAATTAAACGGTTACACTGT
TCAGCCAATTGCAGATGTTTACCGACGTATACCTAATCTTCCCGATTGTAATATAGAGGCTTGGCTTAATGATAAGTCG
GTGCCCTCTCCATTAAATTGGGAACGTAAGACCTTTTCAAATTGTAATTTTAATATGAGCAGCCTGATGTCTTTTATTC
AGGCAGACTCATTTACTTGTAATAATATTGATGCTGCTAAGATATATGGTATGTGTTTTTCCAGCATAACTATAGATAA
GTTTGCTATACCCAATGGTAGGAAGGTTGACCTACAATTGGGCAATTTGGGCTATTTGCAGTCTTTTAACTATAGAATT
GATACTACTGCTACAAGTTGTCAGTTGTATTATAATTTACCTGCTGCTAATGTTTCTGTTAGCAGGTTTAATCCTTCTA
CTTGGAATAGGAGATTTGGTTTTACAGAACAATTTGTTTTTAAGCCTCAACCTGTAGGTGTTTTTACTCATCATGATGT
TGTTTATGCACAACATTGTTTTAAAGCTCCCAAAAATTTCTGTCCGTGTAAATTGGATGGGTCTTTGTGTGTAGGTAAT
GGTCCTGGTATAGATGCTGGTTATAAAAATAGTGGTATAGGCACTTGTCCTGCAGGTACTAATTATTTAACTTGCCATA
ATGCTGCCCAATGTGATTGTTTGTGCACTCCCGACCCCATTACATCTAAATCTACAGGGCCTTACAAGTGCCCCCAAAC

TAAATACTTAGTTGGCATAGGTGAGCACTGTTCGGGTCTTGCTATTAAAAGTGATTATTGTGGAGGTAATCCTTGTACT
TGCCAACCACAAGCATTTTTGGGTTGGTCTGTTGACTCTTGTTTACAAGGGGATAGGTGTAATATTTTTGCTAATTTTA
TTTTTCATGATGTTAATAGTGGTACTACTTGTTCTACTGATTTACAAAAATCAAACACAGACATAATTCTTGGTGTTTG
TGTTAATTATGATCTTTATGGTATTACAGGCCAAGGTATTTTTGTTGAGGTTAATGCGACTTATTATAATAGTTGGCAG
AACCTTTTATATGATTCTAATGGTAATCTCTATGGTTTTAGAGACTACTTAACAAACAGAACTTTTATGATTCGTAGTT
GCTATAGCGGTCGTGTTTCAGCGGCCTTTCATGCTAACTCTTCCGAACCAGCATTGCTATTTCGGAATATTAAATGCAA
TTACGTTTTTAATAATACTCTTTCACGACAGCTGCAACCTATTAACTATTTTGATAGTTATCTTGGTTGTGTTGTCAAT
GCTGATAATAGTACTTCTAGTGTTGTTCAAACATGTGATCTCACAGTAGGTAGTGGTTACTGTGTGGATTACTCTACAA
AAAGACGAAGTCGTAGAGCGATTACCACTGGTTATCGGTTTACTAATTTTGAGCCATTTACTGTTAATTCAGTAAATGA
TAGTTTAGAACCTGTAGGTGGTTTGTATGAAATTCAAATACCTTCAGAGTTTACTATAGGTAATATGGAGGAGTTTATT
CAAACAAGCTCTCCTAAAGTTACTATTGATTGTTCTGCTTTTGTCTGTGGTGATTATGCAGCATGTAAATCACAGTTGG
TTGAATATGGTAGCTTCTGTGACAATATTAATGCTATACTCACAGAAGTAAATGAACTACTTGACACTACACAGTTGCA
AGTAGCTAATAGTTTAATGAATGGTGTCACTCTTAGCACTAAGCTTAAAGATGGCGTTAATTTCAATGTAGACGACATC
AATTTTTCCCCTGTATTAGGTTGTTTAGGAAGCGCTTGTAATAAAGTTTCCAGCAGATCTGCTATAGAGGATTTACTTT
TTTCTAAAGTAAAGTTATCTGATGTCGGTTTCGTTGAGGCTTATAATAATTGTACTGGAGGTGCCGAAATTAGGGACCT
CATTTGTGTGCAAAGTTATAATGGTATCAAAGTGTTGCCTCCACTGCTCTCAGTAAATCAGATCAGTGGATACACTTTG
GCTGCCACCTCTGCTAGTCTGTTTCCTCCTTTGTCAGCAGCAGTAGGTGTACCATTTTATTTAAATGTTCAGTATCGTA
TTAATGGGATTGGTGTTACCATGGATGTGTTAAGTCAAAATCAAAAGCTTATTGCTAATGCATTTAACAATGCTCTTGA
TGCTATTCAGGAAGGGTTTGATGCTACCAATTCTGCTTTAGTTAAAATTCAAGCTGTTGTTAATGCAAATGCTGAAGCT
CTTAATAACTTATTGCAACAACTCTCTAATAGATTTGGTGCTATAAGTTCTTCTTTACAAGAAATTCTATCTAGACTGG
ATGCTCTTGAAGCGCAAGCTCAGATAGACAGACTTATTAATGGGCGTCTTACCGCTCTTAATGTTTATGTTTCTCAACA
GCTTAGTGATTCTACACTAGTAAAATTTAGTGCAGCACAAGCTATGGAGAAGGTTAATGAATGTGTCAAAAGCCAATCA
TCTAGGATAAATTTTTGTGGTAATGGTAATCATATTATATCATTAGTGCAGAATGCTCCATATGGTTTGTATTTTATCC
ACTTTAGCTATGTCCCTACTAAGTATGTCACTGCGAAGGTTAGTCCCGGTCTGTGCATTGCTGGTGATAGAGGTATAGC
CCCTAAGAGTGGTTATTTTGTTAATGTAAATAATACTTGGATGTTCACTGGTAGTGGTTATTACTACCCTGAACCCATA
ACTGGAAATAATGTTGTTGTTATGAGTACCTGTGCTGTTAACTATACTAAAGCGCCGGATGTAATGCTGAACATTTCAA
CACCCAACCTCCATGATTTTAAGGAAGAGTTGGATCAATGGTTTAAAAACCAAACATCAGTGGCACCAGATTTGTCACT
TGATTATATAAATGTTACATTCTTGGACCTACAAGATGAAATGAATAGGTTACAGGAGGCAATAAAAGTTTTAAATCAG
AGCTACATCAATCTCAAGGACATTGGTACATATGAGTATTATGTAAAATGGCCTTGGTATGTATGGCTTTTAATTGGCT
TTGCTGGTGTAGCTATGCTTGTTTTACTATTCTTCATATGCTGTTGTACAGGATGTGGGACTAGTTGTTTTAAGATATG
TGGTGGTTGTTGTGATGATTATACTGGACACCAGGAGTTAGTAATTAAAACATCACATGACGACTAA

>gi|221136|dbj|D00731.1|BECS Bovine enteric coronavirus gene for spike glycoprotein precursor, complete cds

ATGTTTTTGATACTTTTAATTTCCTTACCAATGGCTCTTGCTGTTATAGGAGATTTAAAGTGTACTACGGTTTCC
ATTAATGATGTTGACACCGGTGTTCCTTCTATTAGCACTGATACTGTCGATGTTACTAATGGTTTAGGTACTTATTATG
TTTTAGATCGTGTGTATTTAAATACTACGCTGTTGCTTAATGGTTACTACCCTACTTCAGGTTCTACATATCGTAATAT
GGCACTGAAGGGAACTTTACTATTGAGCACACTATGGTTTAAACCACCTTTTCTTTCTGATTTTATTAATGGTATTTTT
GCTAAGGTCAAAAATACCAAGGTTATTAAACATGGTGTAATGTATAGTGAGTTTCCTGCTATAACTATAGGTAGTACTT
TTGTAAATACATCCTATAGTGTGGTAGTACAACCACATACTACCAATTTAGATAATAAATTACAAGGTCTCTTAGAGAT
CTCTGTTTGCCAGTATACTATGTGCGAGTACCCAAATACGATTTGTCATCCTAATTTGGGTAATCGGCGCGTAGAACTA
TGGCATTGGGATACAGGTGTTGTTTCCTGTTTATATAAGCGTAACTTCACATATGATGTGAATGCTGATTATTTGTATT

TCCATTTTTATCAAGAAGGTGGTACTTTTTATGCATATTTTACAGATACTGGTGTTGTTACTAAGTTTCTGTTTAATGT
TTATTTAGGCACGGTGCTTTCACATTATTATGTCATGCCTTTGACTTGTAATAGTGCTATGACTTTAGAATATTGGGTT
ACACCTCTCACTTCTAAACAATATTTACTCGCTTTCAATCAAGATGGTGTTATTTTTAATGCTGTTGATTGTAAGAGTG
ATTTTATGAGTGAGATTAAGTGTAAAACACTATCTATAGCACCATCTACTGGTGTTTATGAATTAAACGGTTACACTGT
TCAGCCAATTGCAGATGTTTACCGACGTATACCTAATCTTCCCGATTGTAATATAGAGGCTTGGCTTAATGATAAGTCT
GTGCCCTCTCCATTAAATTGGGAACGTAAGACCTTTTCAAATTGTAATTTTAATATGAGCAGCCTGATGTCTTTTATCC
AGGCAGACTCATTTACTTGTAATAATATTGATGCTGCTAAGATATATGGTATGTGTTTTTCCAGCATAACTATAGATAA
GTTTGCTATACCCAATGGTAGGAAGGTTGACCTACAATTGGGCAATTTGGGCTATTTGCAGTCTTTTAACTATAGAATT
GATACTACTGCTACAAGTTGTCAGTTGTATTATAATTTACCTGCTGCTAATGTTTCTCTCAGCAGGTTTAATCCTTCTA
TTTGGAATAGGAGATTTGGTTTTACAGAACAATCTGTTTTTAAGCCTCAACCTGTAGGTGTTTTTACTGATCATGATGT
TGTTTATGCACAACATTGTTTTAAAGCTCCCACAAATTTCTGTCCGTGTAAATTGGATGGGTCTTTGTGTGTAGGTAAT
GGTCCTGGTATAGATGCTGGTTATAAAAATAGTGGTATAGGCACTTGTCCTGCAGGCACTAATTATTTAACTTGCCATA
ATGCTGCCCAATGTAATTGTTTGTGCACTCCAGACCCCATTACATCTAAATCTACAGGGCCTTACAAGTGCCCCCAAAC
TAAATACTTAGTTGGCATAGGTGAGCACTGTTCGGGTCTTGCTATTAAAAGTGATTATTGTGGAGGTAATCCTTGTACT
TGCCAACCACAAGCATTTTTGGGTTGGTCTGTTGACTCTTGTTTACAAGGGGATAGGTGTAATATTTTTGCTAATTTTA
TTTTGCATGATGTTAATAGTGGTACTACTTGTTCTACTGATTTACAAAAATCAAACACAGACATAATTCTTGGTGTTTG
TGTTAATTATGATCTTTATGGTATTACTGGCCAAGGTATTTTTGTTGAGGCTAATGCGACTTATTATAATAGTTGGCAG
AACCTTTTATATGATTCTAATGGTAATCTCTATGGTTTTAGAGACTACTTAACAAACAGAACTTTTATGATTCGTAGTT
GCTATAGCGGTCGTGTTTCAGCGGCCTTTCATGCTAACTCTTCCGAACCAGCACTGCTATTTCGGAATATTAAATGCAA
TTACGTTTTTAATAATACTCTTTCACGACAGCTGCAACCTATTAACTATTTTGATAGCTATCTTGGTTGTGTTGTCAAT
GCTGATAATAGTACTGCTAGTGCTGTTCAAACATGTGATCTCACAGTAGGTAGTGGTTACTGTGTGGATTACTCTACAA
AAAGACGAAGCGTAAGAGCGATTACCACTGGTTATCGGTTTACTAATTTTGAGCCATTTACTGTTAATTCAGTAAATGA
TAGTTTAGAACCTGTAGGTGGTTTGTATGAAATTCAAATACCTTCAGAATTTACTATAGGTAATATGGAGGAGTTTATT
CAAACAAGCTCTCCTAAAGTTACTATTGATTGTTCTGCTTTTGTCTGTGGTGATTGTGCAGCATGTAAATCACAGTTGG
TTGAATATGGTAGTTTCTGTGACAATATTAATGCTATACTCACAGAAGTAAATGAACTACTTGACACTACACAGTTGCA
AGTAGCTAATAGTTTAATGAATGGTGTCACTCTTAGCACTAAGCTTAAAGATGGCGTTAATTTCAATGTAGACGACATC
AATTTTTCCCCTGTATTAGGTTGTTTAGGAAGCGAATGTAATAAAGTTTCCAGTAGATCTGCTATAGAGGATTTACTTT
TTTCTAAAGTAAAGTTATCTGATGTCGGTTTCGTTGAGGCTTATAATAATTGTACTGGGGGTGCCGAAATCAGGGACCT
CATTTGCGTGCAAAGTTATAATGGTATCAAAGTGTTGCCTCCACTGCTCTCAGAAAATCAGATCAGTGGATACACTTTG
GCTGCTACCTCTGCTAGTCTGTTTCCTCCTTGGTCAGCAGCAGCAGGTGTACCATTTTATTTAAATGTTCAGTATCGTA
TTAATGGGATTGGTGTTACCATGGATGTGCTAAGTCAAAATCAAAAGCTTATTGCTAATGCATTTAACAATGCTCTTGA
TGCTATTCAGGAAGGGTTTGATGCTACCAATTCTGCTTTAGTTAAAATTCAAGCTGTTGTTAATGCAAATGCTGAAGCT
CTTAATAACTTATTGCAACAACTCTCTAATAGATTTGGTGCTATAAGTTCTTCTTTACAAGAAATTCTATCCAGACTTG
ATGCTCTTGAAGCGCAACGTCAGATAGACAGACTTATTAATGGGCGTTTCACCGCTCTTAATGCTTATGTTTCTCAACA
GCTTAGTGATTCTACACTAGTAAAATTTAGTGCAGCACAAGCTATGGAGAAGGTTAATGAATGTGTCAAAAGCCAATCA
TCTAGGATAAATTTTTGTGGTAATGGTAATCATATTATATCATTAGTGCAGAATGCTCCATATGGTTTGTATTTTATCC
ATTTTAGCTATGTCCCTACTAAGTATGTCACCGCGAAGGTTAGTCCCGGTCTGTGCATTGCTGGTGATAGAGGTATAGC
CCCTAAGAGTGGTTATTTTGTTAATGTAAATAACACTTGGATGTTCACTGGTAGTGGTTATTACTACCCTGAACCTATA
ACTGGAAATAATGTTGTTGTTATGAGTACCTGTGCTGTTAATTACACTAAAGCGCCGGATGTAATGCTGAACATTTCAA
CACCCAACCTCCCTGATTTTAAGGAAGAGTTGGATCAATGGTTTAAAAACCAAACATCAGTGGCACCAGATTTGTCACT
TGATTATATAAATGTTACATTCTTGGACCTACAAGATGAAATGAATAGGTTACAGGAGGCAATAAAACTTTTAAATCAG
AGCTACATCAATCTCAAGGACATTGGTACATATGAGTATTATGTAAAATGGCCTTGGTATGTATGGCTTTTAATTGGCT
TTGCTGGTGTAGCTATGCTTGTTTTACTATTCTTCATATGCTGTTGTACAGGATGTGGGACTAGTTGTTTTAAGAAATG
TGGTGGTTGTTGTGATGATTATACTGGACACCAGGAGTTAGTAATTAAAACATCACATGACGACTAA

>gi|519668535|gb|KF169935.1|_Bovine_coronavirus_isolate_SWE/C/092_spike_glycoprotein_(S)_gene_complete_cds

ATGTTTTTGATACTTTTAATTTCCTTACCAACGACTTTTGCTGTTATAGGAGATTTAAAGTGTACTACAGTTTCC
ATTAATGATGTTGACACTGGTGTTCCTTTTATTAGCACTGATACTGTCGATGTTACTAATGGTTTAGGTACTTATTATG
TTTTAGATCGTGTGTATTTAAATACTACCTTGTTGCTTAATGGTTATTACCCTACTTCAGGTTCTACATATCGTAATAT
GGCACTGAAGGGAACTTTACTATTGAGCACACTATGGTTTAAACCACCTTTCCTTTCTGATTTTACTAATGGTATTTTT
GCTAAGGTCAAAAATACCAAGTTTATTAAAAATGGTGTAATGTATAGTGAGTTTCCTGCTATAACTATAGGTAGTACTT
TTGTAAATACATCCTATAGTGTGGTAGTACAACCACATACTACCAATTTTGACAATAAAATACAAGGTCTCTTAGAGGT
CTCTGTTTGCCAGTATACTATGTGTGAGTACCCAAATACGATTTGTAATCCTAATCTGGGTAATCAACGCATAGAACTA
TGGCATTGGGATACAGGTGTTGTTTCCTGTTTATATAAGCGTAATTTCACATATGATGTGAATGCTGATTATTTGTATT
TTCATTTTTATCAAGAAGGTGGTACTTTTTATGCATATTTTACAGACACTGGTGTTGTTACTAAGTTTCTGTTTAATGT
ATATTTAGGTACGGTTCTTTCACACTATTATGTCATGCCTTTGACTTGTAATAGTGCTTTGAAGTTAGAATATTGGGTT
ACACCTCTCACTTCTAAACAATATTTACTCGCTTTCAATCAAGATGGTGTTATTTTTAATGCTGTTGATTGTAAGAGTG
ATTTTATGAGTGAGATTAAGTGTAAAACATTATCTATAGCACCATCTACTGGTGTTTATGAATTAAACGGTTACACTGT
TCAGCCAATTGCAGATGTTTACCGACGTATACCTAATCTTCCCGATTGTAATATAGAGGCTTGGCTTAATGACAAGTCG
GTGCCCTCTCCATTAAATTGGGAACGTAAGACCTTTTCAAATTGTAATTTTAATATGAGCAGCCTGATGTCTTTTATTC
AGGCAGACTCATTTACTTGTAATAATATTGATGCTGCTAAGATATATGGTATGTGTTTTTCCAGCATAACTATAGATAA
GTTTGCTATACCCAATGGTAGGAAGGTTGACCTACAATTGGGCAATTTGGGCTATTTGCAGTCTTTTAACTATAGAATT
GATACTACTGCTACAAGTTGTCAGTTGTATTATAATTTACCTGCTGCTAATGTTTCTGTTAGCAGGTTTAATCCTTCTA
TTTGGAATAGGAGATTTGGTTTTACAGAACAATCTTTTCTTAAGCCTCAACCTGCAGGTGTTTTTACTGATCATGATGT
TGTCTATGCACAACATTGTTTCAAAGCTCCCACAAATTTCTGTCCGTGTAAATTGGATGGGTCTTTGTGTGTAGGTAGT
GGTTTTGGTATAGATGCTGGTTATAAAAATAGTGGTATAGGCACTTGTCCTGCAGGTACTAATTATTTAACTTGTCATA
ATGCTGCCCAATGTGATTGTTTGTGCACTCCAGACCCCATTACATCTAAAGCTACAGGGCCTTACAAGTGCCCCCAAAC
TAAATACTTAGTTGGCATAGGTGAGCACTGTTCAGGTCTTGCTATTAAAAGTGATCATTGTGGAGGTAATCCTTGTATT
TGTCAACCACAGGCATTTTTGGGTTGGTCTGTTGAGTCTTGTTTACAAGGGGATAGGTGTAATATTTTTGCTAATTTTA
TTTTGCATGGTGTTAATAGTGGTACTACTTGTTCTACTGATTTACAAAAATTAAATACAGATATAATTCTTGGTGTTTG
TGTTAATTATGATCTTTATGGTATTACTGGTCAAGGTATTTTTGTTGAGGTTAATGCGACTTATTATAATAGTTGGCAG
AACCTTTTATATGATTCTAATGGTAATCTCTATGGTTTTAGAGATTACTTAACAAATAGAACTTTTATGATTCGTAGTT
GCTATAGCGGTCGTGTTTTAGCGGCCTTTCATGCTAACTCTTCCGAACCAGCATTGCTATTTCGGAACATTAAATGCAA
TTACGTTTTTAATAATACTCTTTTACGACAGCTGCAACCTATTAACTATTTTGATAGCTATCTTGGTTGTGTTGTCAAT
GCTGATAATAGTACTTCTAGTGTTGTTCAAACATGTGATCTCACAGTAGGTGGTGGTTACTGTGTGGATTACTCTACAA
AAAGACGAAGTCGTAGAGCGATTACCTCTGGTTATCGGTTTACCAATTTTGAGCCATTTACTGTTAATTCAGTAAATGA
TAGTTTAGAACCTGTAGGTGGTTTGTATGAAATTCAAATACCTTCAGAGTTTACTATAGGTAACATGGAGGAGTTTATT
CAAACAAGCTCTCCTAAAGTTACTATTGATTGTTCTGTTTTTGTCTGTGGTGATTATGCAGCATGTAAATCACAGTTGG
TTGAATATGGTAGCTTCTGTGACAATATTAATGCTATACTCACAGAAGTAAATGAACTACTTGACACTACACAGTTGCA
AGTAGCTAATAGTTTAATGAATGGTGTCACTCTTAGCACTAAGCTTAAAGATGGCGTTAATTTCAATGTAGACGACATC
AATTTTTCCCCTGTATTAGGTTGTTTAGGAAGCGATTGTAATAGAGTTTCCAGTAGATCTGCTATAGAGGATTTACTTT
TTTCTAAAGTAAAGTTAGCTGATGTCGGTTTTGTTGAGGCTTATAATAATTGTACTGGAGGTGCCGAAATTAGGGACCT
CATTTGTGTGCAAAGTTATAATGGTATTAAGGTGTTGCCTCCACTGCTCTCAGAAAATCAGATCAGTGGATATACTTTG
GCTGCGACCTCTGCTAGTCTGTTTCCTCCATGGTCAGCAGCAGCAGGTGTACCATTTTATTTAAATGTTCAGTATCGTA
TTAATGGGATTGGTGTTACCATGGATGTGCTAAGTCAAAATCAAAAGCTTATTGCTAATGCATTTAACAATGCCCTTGG
TGCTATTCAGGAAGGGTTTGATGCCACCAATTCTGCTTTAGTTAAAATTCAAGCTGTTGTTAATGCAAATGCTGAAGCT

CTTAATAACTTATTGCAACAACTCTCTAATAGGTTTGGTGCTATAAGTTCTTCTTTACAAGAAATTCTATCTAGACTGG
ATGCTCTTGAAGCGCAAGCTCAGATAGACAGACTTATTAATGGGCGTCTTACCGCTCTTAATGCTTATGTTTCTCAACA
GCTTAGTGACTCTACACTAGTAAAATTTAGTGCAGCACAAGCTATGGAGAAGGTTAATGAATGTGTCAAAAGCCAATCA
TCTAGGATAAATTTTTGTGGTAATGGCAATCATATTATATCATTAGTGCAGAATGCTCCATATGGTTTGTATTTTATCC
ATTTTAGCTATGTCCCTACTAAGTATGTCACAGCGAAGGTTAGTCCTGGTCTGTGCATTGCTGGTGATAGAGGTATAGC
CCCCAAGAGTGGTTATTTTGTTAATGTAAATAATACTTGGATGTTCACTGGTAGTGGTTATTACTACCCTGAACCTATA
ACTGGAAATAATGTTGTCGTTATGAGTACCTGTGCTGTTAACTACACCAAAGCACCGGATGTAATGCTGAACATTTCAA
CACCCAACCTCCCTGATTTTAAGGAAGAGTTGGATCAATGGTTTAAAAATCAAACATCAGTGGCACCAAATTTGTCACT
TGATTATATAAATGTTACATTCTTGGACCTACAAGACGAAATGAATAGGTTACAGGAGGCAATAAAAGTTTAAATCAG
AGCTACATCAATCTCAAGGACATTGGTACATATGAGTATTATGTAAAATGGCCTTGGTATGTATGGCTTTTAATTGGCT
TTGCTGGTGTAGCTATGCTTGTTTTACTATTCTTCATATGCTGTTGTACAGGATGTGGGACTAGTTGTTTTAAGAAATG
TGGTGGTTGTTGTGATGATTATACTGGACACCAGGAGTTAGTAATTAAAACATCACATGACGACTAA

>gi|124389486|gb|EF065514.1| Bat coronavirus HKU9-2, complete genome

ATGTTGGTGATATTGTTAATATTACCACTAGTCTCTGCTAGACCTTCATGCGTTACTATAGAAAATTTGAGTTAT
GTTGCACATAACTTTACTCACGTTAGTTTTAGTATGAGTAAAGTGTCACGTATTTTTCCAGATCCATTTGTGGCGTATT
CAGGTCAAACTTTGCGCCAATCATTATATATTGCTGACACGTCTAACACTACCGTCTATCCTATTACTCCACCACCTGT
TGGAGGCAACCCTGGTATTTATAACTTAACAACATTACCTGTTAATGAGGGTTTCCTGGTCCATACTTTTATGTATAGA
GACCAGCCAGTAGACATGTATTGCCAAGAGCCCTTTGGCGTGGCATTTGGCACTACTTTTCAAAATGACCTTATTGCCA
TAGTTATGATAGTGCCTGGTCAATATGGATCGTGGGCTGAGGTAAAGAGGCAAAACACTACTAATGTTCATATATTAGT
TTGTGGTAATGCAACTTTGTGTCAATACCCAGCTTTTAATCGCTGGGGACCAGCTGGTTCTATTTATAGTAGTGATGCT
TTCACTACTTATGGTGAGTCGTGTTTTATTAATAACACGTTTAGTATAAACCTTAACACGTCTAGGTTAAATTTAGGGT
TTAGGTTTAGTGATGGTAATCTTTATATTTACCATTCAAGTTGGTTGCCCATTTCAGGTTTAAACCTTATGTTAGACTA
CCCCCTCCATTATTATATGAGTGTTGGTGTAGGTTCTAATTTGCCCAATATGCAATTTTTCCAGTCGGTTTCCCGTAAG
GGAACACCTCCCGCTCAGGGTGCTAATAATAATGATGGTAGTTGTTTGGCGTTTCAGAACAATCTTTATTTAGCCTATG
TCACTCGTAGAGAACTGTTAGTTTCCTATGATGACAATGGGTTTCCCACCGCAGTAGCAGACTGTTCGTATGATGCAGG
TGATGAGTTGTACTGTGTGACGGGGTCCTTTACACCACAGGTAGGTGTTTACCCGCTTAGTAGGTACAGAGCACAGGTT
AGTGGCTATGTACAAATTACGCAGCAGGGTGATACTTGTGTATTACCGTATTCGGACATAGTTAGGCCACCACAACCTG
TTGTGTGGCGTAGATACACTGTTACTAGCTGCTCATTTGATTTTGAAGCTATTGTTAATAGACTTCCTACTTTTGAGCT
TAAATGCTTTGGCATATCTCCAGCTCGTTTGGCGCAGATGTGCTATTCTAGTGTAACTCTCGATTTGTTTCGTGCTAAT
ACAACACATCTGGCTAATATGCTAGGTGGCGTTCCAGATCTGTTTAGTAAGTATAATTATGCATTACCTTCTAATTTTT
ATGGTTGTGTACATGCGTATTATATTAATGATACTAATAAAGATTATGCTATAGCTCAGCGCTGGCCGGCCACCCCAAT
AACTCCTGGCGGGCGCCAGCCATATAGTGATTATGTTAGGACTGTCTTGAACACTCCTAACCCTAGTTGCACGACACTT
ACATGTTTTGGTGTTGTTGTTATATCACTTAAACCTGCATCTGGTAGGAAGTTAGTGTGTCCTAGCGTTAATGATACAG
ACATGCGCACCAATGAGTGCGTTAAGTATAACTTGTATGGTTATACGGGTACAGGGGTGTTTAATGTATCTACATTGAC
CATTCCTGATTCTAAGTTATTTGTTGCTAATGGTGCTGGTGACATTATAGCAGCGAGTGTTAAGGGTACAGTTTATAGT
ATAACTCCTTGTGTGTCTGTGCCCGTTTCTGTAGGGTATGACCCTAATTTTGAGCGCGCGCTATTATTTAACGGACTAG
CATGTAAAGAGCGCGCAGTAGCCGTAAGTATGCCAGCATCATTATTCTGGCGGACTGTAGTGCAGGACAACATCACAAA
TAGCGTGACAACCTTTGACACGCTGTCTGGTTGTGTGTACAATGTGTACAATGCCACTAATATAGTGGTACAGTCTTGT
TCTATGCCATTGGGCAACAGCCTATGTTTGGCTAATTTAACCACTAGTACGGTTTCTGTGAGTAGGGCCGGTAATTTGC
TAAGTCTAGTGACTTATGACCCTACCGCCATAGCGGGTGTTAAGGTTATGACACCCGTTTATTGGATTAGTATACCTAC
TAATTTTACGCTAGGTGCTGTGTCTGAATACATTCAAACGACATCTCCGAAGATTAATGTAGACTGTGTTAAATATTTG

TGTGGCGATTCTGAGCGTTGTACTACTGTTTTGTTACAATATGGCACTTTTTGTAATGATGTTAATAAGGCGTTGTCAG
AAGTTAGTGCTATTATAGATGCTAGTATGGTTTCTCTAGTGTCTGAAATTACGGCTGACGTTGTGCGTAGTGAAAATGC
TCATTTCGACACAACGTATAATTTTACTGGTCTCCTAGGCTGTGTAGGTTCCAGCTGTAGTAGTACTTACAGGTCTGCA
CTTTCAGACCTATTATACAATAAAGTTAAGGTTACGGACCCCGGCTTTATGAGTTCTTATCAGAAATGTATTTCGCAGT
GGGGTGGTGACGTTAGAGATTTGTTTTGTACGCAAAACTTTAATGGCATCTCCGTATTACCTCCTATAGTGTCACCTGG
TATGCAGGCGTTGTACACTTCGTTGTTAGTAGGGGCTGTAGCTTCGGCGGGTTTTACTTTTGGTGTCACATCTGTTGGT
GTTATACCTTTTGCAACCCAGCTCCAGTTTAGATTGAATGGTTTAGGTGTTACTACTCAGGTTTTGGTGGAAAACCAGC
AACTTATAGCTAACTCTTTTAATAAGGCTCTTGTTAGTATACAGCAGGGGTTCGATGCCACCAATGAGGCTCTTAGTAA
GATGCAGTCTGTCATTAACCAGCATGCCCAGCAACTACAGACGCTAGTTTCACAGTTGGGTAATTCATTTGGCGCCATT
TCTTCCTCTATTAATGAAATATTTAGTCGGTTAGATGGTTTGGAGGCTAATGCACAAGTCGATAGACTTATTAATGGCC
GTATGGTTGTACTTAATACTTATGTTACGCAACTGCTCATTAAGGCTTCAGAGGTGCGGGCCCAAGCGCTGCTCGCTAA
ACAGAAGATTAGCGAGTGCGTTAAGTCGCAATCTCTTCGTAATGACTTTTGTGGTAATGGTACACATGTATTTTCTGTA
CCGCAGTTAGCTCCTAATGGTATTATGTTTTTACATTATACCTACAAACCCACAGCTTATGCTGTGGTGCAAACTGCTG
CAGGCCTGTGTTTTAATAACACTGGGTATGCACCAGTTGGAGGTTTGTTTGTACTGCCTAACGACAGTATTTACTGGCA
TTTTACTAAGTTGAATTTTTATAATCCTGTCAACCTTAGTAATAGTAACACGCAGGTGCTTACGACCTGCAGTGTTAAT
TATACAGCCGTTAATTACACAGTTTTAGACCCCGTGGTCAATTCTGACTTTAATTTTACGGCTGAATTTGATAAGTGGT
ACAAAAATCACAGTTCCTACTTTAATAACACTTTCAACCCAGGAGATTTTAATTTCTCAACGGTTGATATTGAGCGAGA
GCTGTCCACCTTAACGGAGGTGGTTAGACAGCTGAATGAGTCTTTTATCGATTTGAAGAAGTTAAACGTTTATGAACAG
ACTATTAAGTGGCCGTGGTATGTATGGCTTGCTATGATAGCTGGCTTAGTTGGTTTGGCTCTAGCCGTTGTTATGCTAT
TATGTATGACTAATTGTTGTTCATGCTTTAAAGGTATGTGTGCATGTAAACCTTGCCATTATGACGAGGTCGAAGATGT
TTATCCTGCAGTTCGTGTATATAATAAACGAACAGCATGA

>gi|124389495|gb|EF065515.1| Bat coronavirus HKU9-3, complete genome

ATGTTGGTGATATTGCTAATATTGCCTTTAGTACATGCTAGACCCAGTTGTCTCACTGTGTCACCTTTACAGTAT
TTAGAACATAATATTTCTTATGTTTCCTTGTCTGTTAGTAAAGTGACACGTTTAGTGCCGGATCCTTATGTAGCGTATT
CCGGGCAGACTATACGTCAAAGTTTGTATTATGCGGACACTTCTAATGTTACTGTGTATCCCGTAACGCCGTATGCGGC
GCCTGACAAGACGAGCATATATAATACCTCCTTTGTGATGGTAAATGACGGTTTGTTTGTGCATACGTATATGTATTTG
AATCAACCGTCTAATATGTACTGTCAAGACCCGTTTGGTGTGGCCTTTGGGACCACATTTGAACAGGATCATATAGCTA
TTGTGGTGATAGCACCTGACAATAGAGGTAGCTGGACGGCTGCGTCAAAGCGGAATGTAACTACAGTTCATATATTAGT
CTGTAGTAACGCAACCCTTTGTGCGTACCCTGCATTTAATCGTTGGGGTCCTGCGAGTAGCATATACGCAAGTGATGCT
TTTGTAGACCATGGTAATTCCTGTTTTGTAAACAACAGTTTTGATATACCTATTAATACGTCTCGTATTAATTTGGCTT
TTAGGTTCTTGGATGGTAACCTATTGCTGTACCATTCTAGGTGGCTTCCAGGTTCTGGGCTTAATTTAGCCACAGATTA
CCCCCTACACTTCTTTATGAGTGTGGGTGTTGGCGCTAATTTGCCTAATGCACAGTTTTTCCAATCCGTAGTCCGCTAT
AACCGGGGTGTTGATGAAGCGCGATGTCATACTTTTCAGAATAATTTGTATATAGCACCTTTGAGTTTACGTGAAGTTT
TAGTGTCTTACAGTGATAGTGGCTTGCCACTTAAGGTGGCTGATTGTGCGGCCGATGCAGGTGATGAGCTATTTTGTGT
TACTGGCTCATTTGAACCTGCCATTGGTGTGTACCCTCTTAGTAGGTACCGTGCTCAGGTTAGTGATTATGTGCACATT
ACACAGCAATCACAGGCCTGCAAACTGCCTTACGCTGACATTGTATCACCACCTCAGCCTATTGTCTGGCGTCGATATA
CAGTGTCTAGTTGCTCATTTGACTTTGAGAGCATAGTTAATAATTTACCTACCTATGATCTCAAGTGCTATGGTGTATC
ACCCGCGCGGCTTGCACAAATGTGCTATGCGGGTGTTACTCTGGACGTTATGCGCATAAACAAGACGCATTATAGCAAT
TTAATTGGTAATGTGCCTGATTTATTTACAAAGTATAATTATGCCCTGCCCACCAATTTTTATGGTTGTGTTCACGCAT
ATTATATTAATGTTACTGATGGCCGCTATGCTTTAGCGACTCATTACCCGGCCACTGTCATTACACCAGGAGGTAGACA
GCCTTATAATAGCTATGTGGCTACTGTTTTGAATACAGTTAATAACTGGTGTACCAGAGCTAATTGTTTTGGTCTTGTA

GTTATAGGATTGAAGCCTGCATCAGGTAGGCAGTTAGTCTGCCCTAAGGCTAATGATACTGAAGTTATAGTGCAGCAAT
GTGTTAAGTATAATTTGTACGGTTACACAGGTACCGGCGTTTTAAGCCCGTCTAATTTAACCATACCAGATGGTAAGTT
GTTTGTGGCTAATAACGCAGGTAATATTGTTGCTGTTAATGCTTCTGGCACCGTATATGGCATTTCATCTTGCGTGTCA
GTTCCTGTCTCAGTAGGATATAACCCCTCATTTGAGCAGGCGCTACTATTTAATGGTTTAGCGTGTTCAGAGAGAGCAG
TTGCTGTTAATATGCCGGCGTCAACTTATTGGCTATCGGTGTCTAGAAACGCAGCCCCACCTGATGGTGGTGTGTTTGA
CACACCTTCTGGTTGTGTTTATAATGTACGCAATTTAACTACCATGGTAGTTAATTCGTGTGATATGCCTATTGGCAAC
AGCCTTTGTTTAACTAATGTAACTTATGCTAATAGGGCTACAGATAGTTTACCTAGGTTGAGTTTAGTCACTTATGATC
CAACGGCTGCTAATGCTGTCCAGTTGCTCACACCAGTTTACTGGGTTAGTATACCCACTAATTTTACTCTGGCTGCTAC
TTCTGAGTATATACAAACTAGCTCACCAAAGATCAATGTAGATTGCGCGAAGTATTTATGTGGCGACTCTGATAGGTGT
ATGAATGTGTTGCTGCAGTATGGCACATTTTGTGATGACGTTAATAAGGCGCTGGCCGAGGTCAGTGCTACAATTGACG
CATCCGTTGTGTCATTGATGTCAGAACTCACATCGGACGTTGTACGTAATGAAAACATGCAGTTTGACACTACATACAA
CTTTACCTCACTTATGGGTTGTTTGGGATCAGATTGTGATTCTAAAATGTACAGGTCCGCTTTATCGGACTTGTTGTAT
AATAAGGTCAAGGTCACGGACCCTGGCTTTATGCAGTCCTATCAGAAGTGCATTTCTCAGTGGGGTGGTGAAATTAGAG
ACCTTATGTGTACTCAAACCTTTAATGGTATTGCTGTTTTACCACCTATTGTTTCTCCAGGCATGCAAGCCTTGTACAC
ATCATTACTTGTGGGTGCGGTAGCATCTGCAGGTTATACATTTGGCATTACTTCTGTCGGGGTTGTGCCTTTTGCGACG
CAATTGCAATTTAGATTAAATGGCATAGGCGTCACTACGCAGGTGCTTGTAGAGAATCAGAAACTTATTGCTAATTCTT
TTAACAATGCCCTTACTAATATACAGAAGGGTTTTGATGCCACTAATGAGGCGATTAGTAAGATGCAGCTTGTTATTAA
CCAGCATGCTCAGCAATTGCAGACTTTGGTGAACCAGTTAGGTAATTCATTTGGTGCTATATCTGCTTCTATTAATGAA
ATATTTAGTCGTTTAGATGGTTTAGAGGCTGATGCTCAAGTGGATAGGCTTATTAATGGACGCACTGTTGTGCTTAATA
CTTATGTAACTCAGTTGCTTATTAAAGCTACAGAAGTGCGTAGTCAGGCCCTGTTAGCTAAGCAGAAAATTAGTGAGTG
CGTTAAAGCACAATCTCTTCGTAATGATTTCTGTGGCAATGGCACACATGTTTTGTCTATACCACAGTTAGCTCCTAAT
GGTATTTTGTTTTTGCATTATACCTATAAACCGACGGCTTTTGCTGTTGTGCAAACTGCTGCTGGTCTGTGTTATAATG
GCACAGGGTATGCACCTGTAGGTGGCATGTTTGTGTTACCTAATGGTACGCAGGCCTGGCACTTTACTAAAATGAATTT
TTATAATCCTGTTAGTATTACAGTTGAGAACACGCAGGTTTTATCTACATGTGGCTTGAATTATTCACATGTTAACTAC
ACTGTGGTGAACCCGTCGGTACCTAGTGATTTTGATTTTGAGGAAGAGTTTAATAAGTGGTATAAAAACCATACCTCTA
TATTTAATAATACATTCGACCCTAGTGCTTTTAATTTTTCTATGGTCGATGTAAATAAGCAACTGGCTACTCTTACAGA
TGTTGTTCAGCAGCTAAACAAGTCGTATATTGATTTGAAGCAATTAAATGTTTATGAACAGACCATTAAATGGCCGTGG
TATATATGGCTCGCTATGATAGCGGGCCTTGTTGGCTTAGCCCTTGCAGTGGTCATGCTTTTATGTATGACTAACTGTT
GTTCATGCTTCAAAGGCATGTGTTCATGCAAGCAGTGCCATTATGATGAGGTAGATGATGTATATCCTGCTGTTCGTGT
ATATAATAAACGAACAGCATGA

>gi|124389504|gb|EF065516.1| Bat coronavirus HKU9-4, complete genome

ATGTTGCTGATAGCATTCATATTGCTACCAGTGGTAACAGCGTCTAGACCTTTTTGTTTAGGTCGGGACTTTGAT
GTCTCACGTGTTTTACCTCATAATATTAGCACCGGCTCATTTGTCGTTAACAAGTTATCTACACTTTTGCCTGATCCTT
ATATAGCATATTCTGGCCAGACAGTACGACAAACTTTGTTTGTTGCAAATGCTACGGACACTACTATTTACCCGATTAC
CCCTTATAAAGAAGGTGACCGTGCTTATTACTATAATACTTCTCTGTATTTAGTTAGTCAAGGATTCTTTGTACATAGT
TATATGTTCAAGAATCAGATTGAAGGTTTATATTGTCAGCAACCATTTGGTGTGGTTTTTGGCAATACTTTTGAGCAAG
ACCGCATTGCTATAGTTATTATAGCACCAGATGTGTACGGTCGTTGGATTACTGCTACTCCACGCTCATATACTAACGT
TACTATCTTAGTTTGTAGTAATGCTACTATATGCGCTAATCCTGCTTTTAATAGATGGGGACCAGCAGGTGACTATCTA
GCTAATAATGCCTTTACTGAGCATGATGATTCTTGCTTTATAAATAACACTATGCGTATACCATATAATACATCACGTA
TTAATTTGGCATTTCGGTTCCAAGATGGTAACCTACTAATTTATTACTCAGATTGGTTACCACATGGTAGCTTAGATTT
AAGTGCGCCTTGGCCTCTTAATTTTTACACGTCTGTGGCAGTTGGTGGCAATTTGAAGTATGCTCAATTTTTCCAGTCA

GTAGTGCGTAATTATAATACGCCTACACAATCACTTACGGACGGCAATTGTGTTGGTGATTTGGTTAATTTGTATGTAG
CCCCTCTAGTGAAGCGTGAATTGCTCGTACAATATTCATTGGCGGGCAGGCCTATTAATGTAGCTGATTGTGCATTAGA
CTCAGGTGAAGAACTTTATTGTGTCACTGGCACATTTACTCCTGATGCGGGTGTTTACTCTTTGAGTAGATACCGTGCA
CAGACTGTAGGGCATGTTAGCATCACTCAACAATTTGAGACATGTGACATACCCTATGCAGCTTTGCAGACTCCACCTC
AACCCATTGCATGGCGTAGGTACGCTGTCAGTAAGTGTGGGTTTGATTTTGAAGCAGTAATTAATAGACTGCCAACTTT
TGAGCTAAAGTGTTTTGGTGTTTCACCTGCACGTTTGGCCTCTATGTGCTATGGTAAGGTTACTATAGATGTTTTTCGC
ATAAATGTGACTCACCTGGCAAACCTAATAGCTGGTGTGCCTGATGCATTTTCTAAATATAATTATGCTCTGCCAAGAG
ACTTCTATGGTTGTGTGCATGCTTTTTACGTTAATATGTCTAGTGACTATATTATCGCTGATAGCTGGCCGGCCGTTGT
TATTCAACCAGGTGGCCGACAAACTGATTCTTCACTTGTAGGGACCTGGCTTAATTCACCGGCCAAGTCCCATTGCACC
ATTCTTAGCTGCTTCGGTGTAGCTGTTATAGGTTTACAGCCTGCTTCGGGCACACAACTTGTTTGCCCGAAACAAAATG
ACACAACGCTTATAGAGGACCAGTGTGTCACTTATAATGTGTATGGCTATACTGGTACTGGTGTTTTCACCACTTCCAA
TTTGACCATTCCGGATAACAAGCTATTTTCAGCTAGTGCTACCGGTGCTATATCTGCTGTTAGTATACATAATGCTGTT
TATGCTATCAGCCCATGTGTTTCGGTACCTGTCTCTGTAGGGTTTAGTCCTAACTTCGACAAGGTGCTACTTTTTAATG
GTCTGGCATGCAAGGAACGTGCTGTTGCTGTGTCCATACCTGCTTCTGACTATTGGATGCGTGTACGTTCATCTGAGAA
TGACACAATATTTGATACGGAATCTGGTTGTGTGTATAATGCACACAATCGTACCGATTTGGTTGTTAATGAGTGCAGT
TTGCCATTAGGTAATAGCCTGTGTCTTATTAATGACACTGCGTCTACTAGACGAGAGGCTTCTAATTTATTAACACTAG
TTAGTTTTCAACCAGGTATCATAGCTAGTGTTAAGGTCCTTACACCTGTTTACTGGGTCCGTGTACCCACTAACTTTAC
TCTAACTGCTACTACCGAGTTCATTGAGACGACGGCCCCTAAAATCACAATTGATTGTGCTCGTTACTTGTGTGGTGAC
TCTAGTCGCTGTTTGGCAGTTCTAGAGCAATATGGTACATTTTGTGATGATGTCAACACAGCTCTGCAGCGTGTCAATA
CTATGTTGGACGCTGCTGTTGTGTCGCTTACCTCTCAGTTGGTTTCAGACATTGTGCCAACTGAGAATAGTAATTTTGA
TTCAACTTACAACTTTACAGCACTTATGGGCTGTTTAGGTTCGAGTTGTAATTCTAAGCGTTCTGCTATTTCTGACCTA
TTATATAATAAGGTTAAAATAGCAGACCCCGGTTTTATGGACTCTTACCAAAAGTGTATTGATTCACAGTGGGGTGGCA
ATATCAGAGATCTCATTTGCACGCAAGTGTTTAATGGCATAGCTGTTTTACCACCTATCGTTTCACCAGGAATGCAAGC
ACTCTACACCTCTCTCCTGGTGGGTGCTGTTGCTTCTTCTGGTTATACCTTTGGTATAACATCAGTGGGCGTCATACCA
TTTGCCACTCAGTTGCAGTTTAGGCTGAATGGTATAGGCGTAACTACGCAGGTGTTGGTGGATAATCAGAAGCTTATTG
CTTCGTCTTTTAATAATGCCCTAAATCAAATACAGAAGGGTTTTGATGCCACCAACTCGGCACTTTCTAAGATTCAAGC
TGTCATAAATCAACATGCCACACAGCTGCAAACTTTGGTTTTGCAGTTAGGCAATACTTTTGGGGCTATTTCATCATCT
ATTAACATTATTTTCAGTCGTCTGGAAGGTTTGGAAGCTGATGCAGAAGTCGACAGACTTATTAGTGGCCGCATGGTTG
TGCTTAATACTTACGTAACTCAGTTACTAGTACAAGCATCACGTATTAAAGCTCAAAGCGATCTGGCTTTGCAGAAAAT
TAATGAGTGCGTTAAGAGTCAGACTCTTCGCAATGAATTCTGTGGTAATGGAACACATGTCCTTAGTGTACCACAATTG
GCGCCTAATGGTATCATGTTTATCCATTACTCTTATACTCCTACACAGTATGCCACTGTGCAGACGGCTGCGGGTCTGT
GCTTTAATGGTACAGGTTATGCACCTAGAAATGGCTTATTTATTTTACCTAATAACTCTAATTTTTGGTACTTTACACA
AGCCAATTTTTATAATCCTGTTAATATTAGCAATTCTAATACTCAGGTTTTAGAGTCGTGTAGTGTTAATTATACTACT
GTTAATTACACTATTTTATCTCCACAGGAACCTCTCTACAATAACTTTGACGAGGAGTTTAATAAGTTCTATAAGAACC
TTTCTAGTGTTTTTAATAACACTTTTAATCCAGGTGCTTTCAACTTCAGTACTGTTGAATTGCAATCAGAAATTGCGAC
ACTAAATGAAGTGGTTCAACAGCTTAACAAGTCCTTCATTGATCTGAAACAAATGAATGTTTATGAACAAACCATTAAA
TGGCCTTGGTATGTTTGGCTCGCTATGATAGCGGGACTTGTAGGTTTAGCACTTGCAGTTGTTATGCTTTTATGTATGA
CAAATTGCTGCTCTTGCTTTAAAGGCATGTGTTCCTGTAGACACTGTTATTATGACGAAATAGAAGATGTTTATCCTGC
TGTACGTGTACATAATAAACGAACAGCATGA

>gi|148283148|gb|EF203065.1| Bat coronavirus HKU2 strain HKU2/HK/46/2006, complete genome

ATGAAACTTTTTATAGTTTTTGTGCTCCTTTTTAGGGTGTGTTATTGCTGTGACTATGTAGACTTCCGTTTGTTT
AATGGGATTTTTTCTACCAGTCGTGGCTTAAGTAATACAACTACTGTTATTACTGGTGCATATCCATCTACTAACAAAG
CCAAATGGTTTTGTCCTACTAATGTTGGTAGGCCTGTTGGTACAGGTGTTGGTATAGGTGTGTATGCGCAGACTGCTCA
AGCTTCCTATGAGACTGGAGGATCGGGTGCTGGTGGGTATACTTTTAGTGTATCACCCAAGCATGTCACTAATTTGACG
TGGTCATTGTGGGTACATAGACCGTGGGGTGCTAATGCTAACGTCACTGTGCGATTGTGCAGATGGTGGCAGAAGTTTT
CCTTTAATGAGACAGCCCACTTTCAACCTGCTGGTCCTAGTAGTGCCTTTGAGTGCCTTGTTAATGGTAGTTTTCCGTC
CTCACAACATAAAGGTTATATGTTTGGTGTTACTTGGTATAATGATTTTGTACGTATTATATTTCCACCCACTGTTTTT
GAGCTTCAGGTAGATGGGTTGCAATGGGAGTATGTACAGTTTACTGGTCCAGTTAATGCTGGGCGTATGACCAAATTTA
ATGTTGTGACAGAGATTTCTAGTGTTTTAGTTTTAACAGATCAAAGTGGTGCTGTTACTAGGTATTCGTATTGCGCCGA
TGGTTTTGTCAATGGTCTTCAATGTAAGTTGAGACTTTTTGATATCCCGCCTGGCGTATATTCTAATAGTGAAGTTGAA
TACCCTGTAGCACTTTACACTGTTGTTCATAATATGTCAGTATGCCCCCAGCGCCCTGAGAGTTATTGTGGGTCTAATT
ATTGTCCATTTAAAAGGGTTGTATTTTCTAATTGTGTTGTTAATTACACTAGTTGGACTAGTGGCTTGTTACGTGATTA
TCAACATCTTGTGCTGCCTAATGGCAAGTTCAACCCATTTACGGAGTGTAATGGTTTGAACCGCATTGTTGATGACTGT
GTTACAGGCTTTGTTTTAAGGGTTGGTCGTGGCACAGCTGTTAATCGTACTGTTATAACACCATATCTTAAGCCTAATG
AATGTTTTGGTTGGTCATGGAATGATTATCAAGACAGCATTTATGATTGGTGGATAGCTGATTTTGTGTCGACAGGTGC
TTTTGTTTGCGAGAAAAATCCTGATGCGCCACGAACTGGTGTTTGTATTACTTACACTATTGAGAAGGTCACGTTTCAG
GGTGTTCTCTATGAGAGTAATTTTACATTTGCCCAGTATTATAATGTACTTTATTTTGGTTCACAGCTTAAGTACGTTC
GTATTTTGGGCAAAGTTTATGAGGTTGCACCTTGTTTTGAAGCTTCCTATGATGTGCTTTTTCGTAGTAGTTCCTCCTT
TGGTTTGTTATATAGGAGTTTTGATTGTAATCAGCTACGTATTAGTGCATCACGTTTTGCCGAGCGGCTCTTACCGTCT
CATAATGGTACTGCTACTGCCCTAGGTTGTTTATTTAATGCCACCTACGCTCCTAATGATACTATGGTGAACTGTACTA
ACCCTCTTGGTGATGGGTTTTGTGCTGATTTACTTAGTAATGTCGTTGTGCGGCGTATGACATTCGAGAAACATGACAC
CACCTATGTGGCACCAGTTACCATAGAACGGTTTACTGAGTTACCACTGGATCACCAGTTAGTTTTGACAGAGCAGTTT
TTGCAAACTACCATGCCCAAATTTAGCATTAGTTGCGAAACATATATTTGTGATGTTAGCAAAGCATGCAAAAATTTGT
TATTTAGGTATGGTGGTTTTTGTCAGAAAATTGAGGCTGACATTCGGGGTGCTGGTGTTTTGCTTGACAGTGATGTTAG
TGGTCTGTATTCCACTATTGCTGCTAAGACAAGCTCTATTATGCCTACTACAGATCGCTTCAATGTGTCGCAATTTTTC
TTGCCTAAAGTGCAGTCAACTTCAGAGCGCTTTGAGTCTAGGTCTGTTATAGAGGATTTGCTATTTAGCAAAATAGAGA
CGACCGGCCCTGGTTTTTATGGTGACTATTATAATTGTAAGAAAAATGCTATACAGGACCTTACTTGTGCACAGTATCA
TAATGGTATACTTGTTATACCACCAGTTATGGATGCTGAGACATTGGGGATGTATGGCGGTATTGCAGCTGCTTCTCTT
ACACTTGGTATTTTTGGCGGTCAAGCTGGTATTACTACCTGGTCACTTGCCATGGCTGGACGCCTTAATGCATTAGGTG
TGGTTCAGAATGCATTGGTTGATGATGTTAATAAATTGGCTAATGGCTTCAATCAGTTGACTGCTAGTGTTAGTAAGCT
TGCTTTAACAACATCAAGTGCTTTGCAGGCTATTCAAGCTGTTGTTAACCAAAATGCTGCTCAAGTTGAGTCTCTTGTT
AGTGGTATCACTGAAAATTTTGGTGCTATTAGTACTAATTTTAAGGTTATATCACAGCGTCTTGACAAGCTTGAGGCTG
ATGTTCAGATGGATAGGCTTATTAATGGAAGAATGAATGTCTTACAGCTTTTTGTTACTAATTATAAGCTGAAGATCGC
CGAGCTACGTAACACACACCGTTATGTTCAATCTTTGATCAATGAGTGTGTTTATGCTCAGAGTCTAAGAAATGGCTTT
TGTGGTCAGGGCCTACATGTACTTTCCCTTATGCAGAATGCACCTAGTGGTATAATGTTTTTTCATTACTCACTTATAC
CTAATAACACTATTATTGTTAAGACCACACCAGGTCTTTGTGAGAGTGATGAATTAGGTTCTAAGTGCATTGTTGCTAA
GGATGGTGTATTAGTCTCTGCTAATTTAAGCTATTGGCAGTGGTCACCGCGTAACCTTTATAAACCAGAAAATTTGACC
TTTGCTAATGTTATTGCTGTCTCTCGTGGTGCTAATTACACTACATTGAATAGAACCTTTGATATACCTGAGTTGAATA
ACACTTTTCCAATTGAGGAGGAGTTTCGTGAATATTTTCAGAATATGTCATCTGAATTACAGGCCCTGAAAAATTTGAC
TGCTGACATGAGCAAGCTCAATATTAGTGCTGAGATTCAGCTTATTAATGAGATTGCTCACAATGTTTCTAACATGCGC
GTTGAGGTTGAGAAATTCCAGCGCTATGTTAATTATGTCAAATGGGCTTGGTGGCAGTGGCTTATTATTTTTATAGCTT
TGACTTTGCTTGCTGGTCTTATGCTTTGGTGCTGTCTTGCTACAGGCTGTTGTGGTATGTGTGGTTGCTTAGCAGCTAC
TTGTGCCTCATGTTGTGATTGTAGAGGAACTAAACTTCAATCTTACGAGATTGAAAAGGTCCATATTCAGTAA

>gi|148283166|gb|EF203067.1| Bat coronavirus HKU2 strain HKU2/HK/33/2006, complete genome

ATGAAACTTTTTATAGTTTTTGTGCTCCTTTTTAGGGTGTGTTATTGCTGTGACTATGTAGACTTCCGTTTGTTT
AATGGGATTTTTTCTACCAGTCGTGGCTTAAGTAATACAACTACTGTTATTACTGGTGCATATCCATCTACTAACAAAG
CCAAATGGTTTTGTCCTACTAATGTTGGTAGGCCTGTTGGTACAGGTGTTGGTATAGGTGTGTATGCGCAGACTGCTCA
AGCTTCCTATGAGACTGGAGGATCGGGTGCTGGTGGGTATACTTTTAGTGTATCACCCAAGCATGTCACTAATTTGACG
TGGTCATTGTGGGTACATAGACCGTGGGGTGCTAATGCTAACGTCACTGTGCGATTGTGCAGATGGTGGCAGAAGTTTT
CCTTTAATGAGACAGCCCACTTTCAACCTGCTGGTCCTAGTAGTGCCTTTGAGTGCCTTGTTAATGGTAGTTTTCCGTC
CTCACAACATAAAGGTTATATGTTTGGTGTTACTTGGTATAATGATTTTGTACGTATTATATTTCCACCCACTGTTTTT
GAGCTTCAGTTAGATGGGTTGCAATGGGAGTATGTACAGTTTACTGGTCCAGTTAATGCTGGGCGTATGACCAAATTTA
ATGTTGTGACAGAGATTTCTAGTGTTTTAGTTTTAACAGATCAAAGTGGTGCTGTTACTAGGTATTCGTATTGCGCCGA
TGGTTTTGTCAATGGTCTTCAATGTAAGTTGAGACTTTTTGATATCCCGCCTGGCGTATATTCTAATAGTGAAGTTGAA
TACCCTGTAGCACTTTACACTGTTGTTCATAATATGTCAGTATGCCCCCAGCGCCCTGAGAGTTATTGTGGGTCTAATT
ATTGTCCATTTAAAAGGGTTGTATTTTCTAATTGTGTTGTTAATTACACTAGTTGGACTAGTGGCTTGTTACGTGATTA
TCAACATCTTGTGCTGCCTAATGGCAAGTTCAACCCATTTACGGAGTGTAATGGTTTGAACCGCATTGTTGATGACTGT
GTTACAGGCTTTGTTTTAAGGGTTGGTCGTGGCACAGCTGTTAATCGTACTGTTATAACACCATATCTTAAGCTTAATG
AATGTTTTGGTTGGTCATGGAATGATTATCAAGACAGCATTTATGATTGGTGGATAGCTGATTTTGTGTCGACAGGTGC
TTTTGTTTGCGAGAAAAATCCTGATGCGCCACGAACTGGTGTTTGTATTACTTACACTATTGAGAAGGTCACGTTTCAG
GGTGTTCTCTATGAGAGTAATTTTACATTTGCCCAGTATTATAATGTACTTTATTTTGGTTCACAGCTTAAGTACGTTC
GTATTTTGGGCAAAGTTTATGAGGTTGCACCTTGTTTTGAAGCTTCCTATGATGTGCTTTTTCGTAGTAGTTCCTCCTT
TGGTTTGTTATATAGGAGTTTTGATTGTAATCAGCTACGTATTAGTGCATCACGGTTTGCCGAGCGGCTCTTACCGTCT
CATAATGGTACTGCTACTGCCCTAGGTTGTTTATTTAATGCCACCTACGCTCCTAATGATACTATGGTGAACTGTACTA
ACCCTCTTGGTGATGGGTTTTGTGCTGATTTACTTAGTAATGTCGTTGTGCGGCGTATGACATTCGAGAAACATGACAC
CACCTATGTGGCACCAGTTACCATAGAACGGTTTACTGAGTTACCACTGGATCACCAGTTAGTTTTGACAGAGCAGTTT
TTGCAAACTACCATGCCCAAATTTAGCATTAGTTGCGAAACATATATTTGTGATGTTAGCAAAGCATGCAAAAATTTGT
TATTTAGGTATGGTGGTTTTTGTCAGAAAATTGAGGCTGACATTCGGGGTGCTGGTGTTTTGCTTGACAGTGATGTTAG
TGGTCTGTATTCCACTATTGCTGCTAAGACAAGCTCTATTATGCCTACTACAGATCGCTTCAATGTGTCGCAATTTTTC
TTGCCTAAAGTGCAGTCAACTTCAGAGCGCTTTGAGTCTAGGTCTGTTATAGAGGATTTGCTATTTAGCAAAATAGAGA
CGACCGGCCCTGGTTTTTATGGTGACTATTATAATTGTAAGAAAAATGCTATACAGGACCTTACTTGTGCACAGTATCA
TAATGGTATACTTGTTATACCACCAGTTATGGATGCTGAGACATTGGGGATGTATGGCGGTATTGCAGCTGCTTCTCTT
ACACTTGGTATTTTTGGCGGTCAAGCTGGTATTACTACCTGGTCACTTGCCATGGCTGGACGCCTTAATGCATTAGGTG
TGGTTCAGAATGCATTGGTTGATGATGTTAATAAATTGGCTAATGGCTTCAATCAGTTGACTGCTAGTGTTAGTAAGCT
TGCTTTAACAACATCAAGTGCTTTGCAGGCTATTCAAGCTGTTGTTAACCAAAATGCTGCTCAAGTTGAGTCTCTTGTT
AGTGGTATCACTGAAAATTTTGGTGCTATTAGTACTAATTTTAAGGTTATATCACAGCGTCTTGACAAGCTTGAGGCTG
ATGTTCAGATGGATAGGCTTATTAATGGAAGAATGAATGTCTTACAGCTTTTTGTTACCAATTATAAGCTGAAGATCGC
CGAGCTACGTAACACACACCGTTATGTTCAATCTTTGATCAATGAGTGTGTTTATGCTCAGAGTCTAAGAAATGGCTTT
TGTGGTCAGGGCCTACATGTACTTTCCCTTATGCAGAATGCACCTAGTGGTATAATGTTTTTTCATTACTCACTTATAC
CTAATAACACTATTATTGTTAAGACCACACCAGGTCTTTGTGAGAGTGATGAATTAGGTTCTAAGTGCATTGTTGCTAA
GGATGGTGTATTAGTCTCTGCTAATTTAAGCTATTGGCAGTGGTCACCGCGTAACCTTTATAAACCAGAAAATTTGACC
TTTGCTAATGTTATTGCTGTCTCTCGTGGTGCTAATTACACTACATTGAATAGAACCTTTGATATACCTGAGTTGAATA
ACACTTTTCCAATTGAGGAGGAGTTTCGTGAATATTTTCAGAATATGTCATCTGAATTACAGGCCCTGAAAAATTTGAC
TGCTGACATGAGCAAGCTCAATATTAGTGCTGAGATTCAGCTTATTAATGAGATTGCTCACAATGTTTCTAACATGCGC

GTTGAGGTTGAGAAATTCCAGCGCTATGTTAATTATGTCAAATGGGCTTGGTGGCAGTGGCTTATTATTTTTATAGCTT
TGACTTTGCTTGCTGGTCTTATGCTTTGGTGCTGTCTTGCTACAGGCTGTTGTGGTATGTGTGGTTGCTTAGCAGCTAC
TTGTGCCTCATGTTGTGATTGTAGAGGAACTAAACTTCAATCTTACGAGATTGAAAAGGTCCATATTCAGTAA

>gi|141537146|gb|EF434381.1|_Bat_coronavirus_Shandong/977/2006_spike_protein_(S)_gene_complete_cds

ATGTTAAGTTTTGCCGTTTATTTGGCTGTTTTATTGTCCAGTTTAAAGTTTGTTGTTACAGCAGGTCATGCTACA
GGTAAAGAATGTGGTGGAGACCCTGGTTTATTGCCTAACTTGAATCTTGGTTTACCAGCTAATAGTACAGTTTTGGTTA
GTGGTTACTTGCCAGAACCAGTTAGTGTTAGTAATAATTGGATATGTCCTGTAAAACCATTAGACCAATTTGGTGTTGA
TTACTTTACTAACCTTAAAGGTATTTGGTGGTCGTATTATAGTGGTAACACTGGTGTCGAATTTGGTATTGCAGATTCC
AATTATGAACAAAACCCTAATCAGTGGGCTTTGTACATGTACCAGAATAATAATGTCACTTTCAATGGTTCGCAAGGTG
TTTTGTACTTTCGTATCTGTAAATACAGTGGTAAGTACACACCTGTAGGTGATCCTGATAAAGACAATGTTCACAAAGT
TTGTCTTATTGATAAAAAGGTCAATTTCAAATTTGTTGAACAAGCTAATCAAGTCATAGGTGTTACGTGGTCTGGTAAT
TACGTGACTTTCTATGGCATTGACAAGTCTTTTAGAATTTACTTACCTAATAAATGGAACATTGTTGCTGTTAAGTGTC
TTGTTAAAGAAACTTGTGCATTCTTTGCAGCTCACAAAATGGTCACTCTGAACATTACTACAAATGGTCAAGGTTTGAT
TAAAAATTATAGTGTTTGCAAAGAATGCAATGGCTTTCCAGCACATGTTTTTCCAGTTCTAGATGGTGGCAAAATTCCT
GGTGATTTTAGTTTCGACAATTGGTTTCTTCTCACTAATAGTTCTACTATAGTGAATGGAAAAATAGTCGACATACAAC
CTCTTAAATTGCTTTGTCTATGGCCTGTTCCAGCACTGAGTTCAGATGATGATAAAATTTATTTTAACATCACTGGTGC
AAATTGTAATGGCTTTAAAGATGAGAATGCTTATGCAGATGTGCTTCGTTTTTCACTGAATTTCACAAATAGCCAAGTT
TTTAATGGTATCCATAGTGTTACTGTTAATGTAGTTGGCGGTAATTTAAAATTTAGTTGTTCTAATACGAGCACTTATA
CTGCTTCAGATGAAGTTTTACCATTTGGACCTCTAAGTAGTGTTTATTACTGTTTTGTTTCTGGACAGCTCGGCAATGA
AACTTTCAGCAGATTTGTTGGCATATTGCCCCCTGTTGTTAAAGAAATTGTCATTAGCCGCTATGGTTCCTTTTACATG
AACGGTGTTAAGCTTTTTGAGGTTCCTTATGTGGAAAGTGTCATTTTTAATGTCACTAGTAGTGTTGGTTCTGACTTCT
GGACAGTAGCTTATGCCCAAAACACAGAAGTTCTGCTAGAAGTAAATTCGACTGACATTAAAGACATATTGTATTGCGA
CACACCTTCGAACAAATTAAAGTGTCAACAACTCAGTTTTAGCTTGGAAGATGGTTTTTACCCTGCCGCTGTTGTAGCT
GGTGTTGACGTACCGCGCACATATGTTGCTTTGCCTTACCATGCTACCCATTCATTTGTTAATTTGACAGTGCATTTGG
GTCAAGGTGTTGACAGTGCTGCTTATGCGCAGATTAATGGTGTTAACGATTCATATTGTGTCAATACTACACAGTTTAC
TACCAACTTTCAACAGTTGGATGTTAATAACAATGTGAAAGCAGTGATGCAAAATGGTGATTGCCCTTTTAATTTTGAT
AGTTTAAACAACTTCCTCAGCTTCGATAGTATTTGTTTCAATCTACAACCTGTTGGTTCTAGCTGTACAATTTCTATTA
TGAGAAGCTGGATGGGTTTTAATACACCCTGGAGAAGTATTTATGTCAGTTTTAAAAGAGGCAATCGTATTACAGGTGT
TAAAACTGCTAGTACTGGCATTTTTGATCCCAGTGTCATGGAATTAGATAATTGTACAGATTGCACTATTTATGGTGTT
TCTGGTAGAGGCATCATTCGTAAGGCAAATTCGTCTTACATTAGTGGGTTGTACTACACATCTATTGCAGGTCAGATCA
TTGGTTTCAAAAATGCAACTACTGGTGAAGTGTTTTCAGTTACACCATGTCAGCTTACTATGCAAGCTGCTGTTGTTAA
TGACGACATTGTTGGTGTAATTTCCTCTACTAACGCTTCTAGTATACCTTTTTCACACACCATTACTACGAAAACATTT
TATTACCACACCAATGTGCAACAAAATTGTTCTGAACCAGTACTTACTAATGCTAATATTGGTGTGTGTTCAGATGGTT
CAATTACTGAAGTCACTATAAGAAAAACTGAACCAGAGCCTGTTGCACCAATCACTACTGGAAATGTTAGTATACCTTC
CAACTTTACAGTTTCTGTACAAGTTGAGTACTTACAAATGTACAACAAACCAGTTAGTGTTGATTGTTCCACTTATGTT
TGCAATGGTAATCCTCGTTGTTTAAAATTATTAACTCAATATGCCACAGCCTGCAGAACAATTGAAGAATCTTTGCAGC
TTAGTGCTCGTTTAGAGTCAGTTGAAGTTGCTAACATGATCTCTGTTTCTGAAGAAGCTATTCAGTTGGCAAACGTTAG
TTATTTTGACACATATAACATTAGTGCACTTTTACCAAGAGGTCAGAGTCGCGGTAGTGTTATAGAAGACCTATTGTTT
AACAAAGTTATTACAAGTGGTCTTGGAACTGTGGATGAGGATTACAAAGCTTGCACAGGTGAAGGATTTGGTGCTGCAC
TGGCAGATGTCTTTTGTGCTCAGTATTACAATGGTATTATGGTGTTACCAGGTGTTGTAGATGAAGCAAAAATGGGATT

ATACACTGCATCACTTACTGGTGCTATGGTAATGGGTGGTATTACAGCTGCTGCAGCTATTCCATTTTCATTGGCCGTA
CAATCCAGACTTAATTACGTTGCTTTGCAAACTGATGTTTTGCAAGAAAACCAAAAGATACTTGCTTCAGCTTTCAACA
GTGCCATGAGTAACATCACTTATGCTTTCACAGAAGTTAAAAATGCCATTAAGGATACGTCCGTGGCTATTAACACAGT
TGCTCAAGCGCTTGGAAAGATTCAGAATGTTGTGAATGATCAAGGTCAAGCCTTGTCACAGCTTACTAGACAATTAGCC
TCAAACTTTCAAGCTATCTCGTCTTCTATCCAGGATATCTATAATAGATTGAACGGTTTAGAGGCAGATGCACAAGTTG
ATAGGCTTATTACTGGTAGGCTTGCTGCACTTAATGCTTTTGTGACTCAGACTTTGACTAAGTACACAGAAGTTCGTGC
TTCACGACAGCTGGCTCAACAAAAGATTAATGAGTGTGTAAAATCGCAGTCATCTCGTTATGGATTTTGCGGTAATGGC
ACTCATTTGTTCTCAATTTCTAATGCTGCACCACAAGGAATCATCTTTTTTCACACAGTTTTGCTTCCTACAGAATATG
AAACTGTTGAAGCTTGGTCTGGTGTCTGCGTCAATAGTCAATACGGTCTAGTTTTAAGAAATGTTCATGATGCGCTGTA
TAAGCGTAATGACAGTTATTACATTACTAGTCGTGACATGTATGAACCTAGAGTTCCACAAGAGTCCGATTTTGTTAGA
ATAACTGGCTGCAGTGTTGTGTATCTCAATATAACTCACACTCAAATTGGTGAGATTATACCTGAGTACATTGATGTTA
ATAAAACTCTTGAGGAGTTTCTTAGCAGCTATCCTAATTACACTGTCCCAGATTTGAACTTAGATCTTTATAACCAGAC
TGTTTTGAATTTGACTGAAGACATCAGTAAGCTGTATGAAAAAGCTGAAAGTCTCCACAATAAGACCATTATACTACAA
CAGTTGATTGATAATCTCAACAACACATATGTTGATTTAGAATGGTTGAATCGTGTGGAAACTTACATTAAGTGGCCTT
GGTATGTGTGGTTAGTCATCTTTTTGGCACTTGCAGCTTTTACGTTTTTAATGCTGTACTGTTGTATCGCAACAGGTTG
CTGCGGTTGTTTGAGTTGTATTTTCAGCTCTTGTGCTGATTGTAGAGGCAGAAGATTACAACGTTACGAAGTGGAAAAG
ATTCACATTCAATAA

>gi|255733174|gb|FJ588692.1|_Bat_SARS_Cov_Rs806/2006_spike_protein_gene_complete_cds

ATGAAAATTTTAATTCTTGCTTTCTTAGCTAACTTAGCTAAAGCACAGGAAGGATGCGGTATTATCAGTAGAAAA
CCGCAACCTAAAATGGCACAAGTCTCATCCTCTCGTCGGGGCGTTTATTATAATGATGACATCTTTCGTTCTGACATAT
TACACCTTACACAGGATTACTTCCTGCCATTTGACTCAAATTTAACACAGTACTTTTCGCTTAATGTGGATTCAGATAG
GTACACCTATTTTGACAACCCTATACTAGACTTTGGTGATGGTGTTTATTTCGCTGCCACCGAAAAGTCTAATGTAATA
AGAGGCTGGATTTTTGGTTCATCTTTTGATAACACCACCCAGTCAGCTGTCATAGTTAATAATTCAACACACATTATTA
TACGTGTGTGTAATTTTAACTTATGTAAAGAACCCATGTACACCGTTAGTCAGGGTACACAACAAAATGCCTGGGTTTA
TCAGAGTGCATTTAATTGTACATATGATCGAGTGGAAAAGAGTTTTCAACTTGACACTACTCCTAAAACTGGAAATTTT
AAAGACCTACGTGAGTATGTCTTTAAAAACAGGGATGGCTTTTTGTCTGTCTACCAAACTTATACTGCTGTTAATTTAC
CTAGAGGACTACCTGCGGGTTTTTCAGTTTTGAAACCAATCCTAAAACTGCCCTTTGGAATTAACATCACTTCTTATAG
AGTAGTTATGGCAATGTTCAGTCAAACTACTTCTAATTTCCTACCGGAAAGTGCTGCTTACTATGTTGGTAATCTTAAA
TATTCTACCTTCATGCTTAAATTTAATGAAAATGGGACCATCACGGATGCTGTAGATTGTTCCCAAAATCCTCTTGCTG
AATTAAAATGCACCATTAAAAATTTTAATGTTGATAAAGGAATCTACCAAACATCCAATTTTAGAGTTTCACCTACTCA
AGAGGTTGTTAGATTTCCTAACATTACAAATCGCTGTCCTTTCGACAAGGTTTTTAATGCTACTCGCTTTCCTAATGTT
TATGCATGGGAGAGAACAAAAATTTCTGATTGTGTTGCTGACTATACTGTCCTCTACAACTCAACCTCTTTCTCAACTT
TTAAATGTTATGGAGTGTCTCCATCTAAGTTGATTGACTTATGCTTTACAAGTGTGTATGCTGATACATTCTTGATAAG
ATCTTCTGAAGTAAGACAAGTTGCACCAGGTGAAACTGGTGTTATTGCTGACTACAACTACAAGTTGCCTGATGATTTC
ACTGGCTGTGTAATTGCTTGGAATACTGCTAAGCAGGATACTGGCAATTATTATTACAGATCTCATCGCAAGACTAAAT
TAAAGCCTTTTGAGAGAGATCTGTCTTCTGACGATGATGGTGTGTATACACTTTCAACATATGACTTTAACCCTAACGT
TCCAGTAGCATATCAGGCTACTAGGGTTGTTGTACTCTCTTTTGAACTTCTTAATGCACCTGCTACAGTTTGTGGACCT
AAATTATCCACACAACTAGTTAAGAACCAGTGTGTTAATTTCAACTTCAACGGACTTAAAGGTACTGGTGTTTTGACTC
CTTCTTCGAAAAGATTTCAGTCATTTCAACAATTTGGCCGTGACACGTCTGACTTTACAGATTCTGTACGCGACCCACA
GACTTTAGAAATACTTGACATTTCACCTTGCTCCTTTGGTGGTGTTAGTGTTATCACACCTGGAACGAACGCCTCATCA

GAGGTAGCTGTTCTCTATCAAGATGTGAATTGCACCGATGTCCCCACAGCCATTCGTGCGGATCAGTTAACACCAGCTT
GGCGCGTTTATTCCACGGGAGTAAATGTGTTTCAAACACAAGCCGGCTGTCTTATTGGAGCTGAGCATGTCAATGCATC
CTATGAGTGTGACATTCCTATTGGTGCAGGCATTTGTGCTAGTTACCATACAGCTTCTCTTCTACGTAGTACAGGCCAA
AAGTCAATTGTTGCCTATACTATGTCACTGGGTGCTGAAAATTCCATTGCATATGCTAACAATTCAATTGCCATACCTA
CAAATTTTTCAATCAGCGTCACTACAGAAGTGATGCCTGTTTCAATGGCTAAAACATCCGTGGATTGTACCATGTACAT
CTGTGGTGATTCTTTAGAGTGCAGCAACCTACTATTGCAGTATGGTAGCTTTTGCACGCAACTCAATCGTGCCCTTACT
GGCATTGCCATAGAACAGGACAAAAACACTCAGGAAGTTTTTGCCCAGGTTAAACAAATGTACAAAACACCTGCCATAA
AAGATTTTGGCGGTTTCAATTTCTCACAAATATTGCCTGACCCTTCAAAGCCAACGAAGAGATCATTTATTGAAGACTT
GCTCTTCAATAAAGTGACTCTCGCTGATGCTGGCTTTATGAAACAATATGGTGAATGCCTAGGCGATGTTAGTGCCAGA
GACCTTATCTGTGCTCAGAAGTTCAATGGACTTACTGTGCTACCACCACTGCTCACAGATGAGATGATTGCTGCATACA
CAGCTGCGCTAGTTAGTGGTACTGCTACGGCAGGCTGGACTTTTGGTGCAGGTGCGGCTCTTCAAATACCGTTTGCTAT
GCAAATGGCTTATAGGTTTAATGGCATTGGAGTTACTCAAAACGTTCTCTATGAGAACCAAAAGCTGATAGCCAATCAG
TTTAATAGTGCTATAGGCAAAATTCAAGAATCACTATCATCTACTGCAAGTGCACTAGGAAAACTGCAGGATGTGGTTA
ACCAAAATGCACAAGCTCTTAACACGCTTGTTAAACAACTCAGCTCTAATTTTGGAGCTATCTCAAGTGTGTTAAATGA
TATTCTCTCTCGCCTTGATAAAGTTGAGGCAGAAGTTCAAATTGATAGGTTGATTACAGGCAGATTGCAAAGCCTTCAA
ACCTACGTAACACAACAACTTATCAGAGCCGCTGAAATCAGAGCTTCTGCTAATCTTGCTGCTACTAAAATGTCTGAGT
GCGTTCTTGGACAATCAAAAAGAGTTGATTTCTGTGGAAAAGGCTACCATCTTATGTCTTTTCCCCAATCAGCACCTCA
CGGTGTCGTTTTTCTACATGTCACATATGTGCCATCACAAGAGAGAAACTTCACCACAGCTCCAGCCATTTGTCATGAA
GGCAAAGCTTACTTCCCTCGTGAAGGTGTTTTCGTTTCTAATGGCACTTCCTGGTTTATTACGCAGAGGAATTTTTACT
CTCCACAATTAATTACAACAGATAACACTTTTGTTTCTGGTAATTGTGATGTCGTAATCGGCATCATTAATAACACTGT
TTACGACCCTCTGCAACCTGAGCTTGACTCATTTAAAGAAGAGCTGGACAAGTACTTTAAAAATCATACATCACCAGAT
GTTGACCTTGGCGACATTTCAGGCATTAATGCTTCAGTCGTCAACATTCAAAAGGAGATCGACCGCCTCAATGAGGTTG
CCAAAAACCTAAATGAATCACTCATTGACCTCCAAGAACTTGGGAAATATGAGCAATACATCAAGTGGCCGTGGTATGT
TTGGCTCGGCTTTATTGCTGGACTAATTGCCATTGTCATGGTTACAATCTTGCTTTGTTGCATGACCAGCTGTTGCAGT
TGTCTCAAGGGTGCATGCTCTTGTGGTTCTTGCTGCAAATTTGATGAGGACGACTCTGAGCCAGTGCTCAAAGGAGTCA
AATTACATTACACATAA

>gi|76160337|gb|DQ022305.2| Bat SARS coronavirus HKU3-1, complete genome

ATGAAAATTTTAATTTTTGCTTTCTTAGCTAATTTAGCTAAAGCACAGGAAGGATGCGGTATCATCAGTAGAAAA
CCGCAACCTAAAATGGCACAAGTCTCATCTTCTCGTCGGGGCGTTTATTATAATGATGACATTTTCCGTTCTGATGTGT
TACACCTCACACAGGATTACTTCCTGCCATTTGACTCAAATTTAACTCAGTACTTTTCGCTTAATGTGGATTCAGATAG
GTATACCTATTTTGACAACCCCATACTAGACTTTGGTGATGGCGTTTATTTCGCTGCCACTGAAAAGTCTAACGTAATA
AGAGGCTGGATTTTTGGTTCATCTTTTGATAACACCACCCAGTCAGCTGTTATAGTTAATAATTCAACACACATTATTA
TACGTGTGTGTAATTTTAACTTATGTAAAGAACCCATGTATACTGTTAGTAGAGGTACACAGCAAAATGCCTGGGTTTA
TCAGAGTGCATTTAATTGTACATATGACCGAGTGGAAAAGAGTTTTCAACTTGACACTACTCCTAAAACTGGAAATTTT
AAAGACCTACGTGAGTATGTCTTTAAAAATAGGGATGGCTTTCTGTCTGTCTACCAAACTTATACTGCTGTTAATTTAC
CCAGAGGACTACCCACGGGTTTCTCAGTCTTGAAACCAATTTTAAAATTGCCCTTTGGAATTAATATCACTTCTTATAG
AGTAGTTATGGCAATGTTTAGCCAAACTACTTCTAATTTTTTACCAGAAAGTGCTGCTTATTATGTTGGTAATCTTAAA
TATTCTACCTTCATGCTCCGATTTAATGAAAATGGGACGATCACGGATGCTGTAGATTGTTCCCAAAATCCTCTTGCTG
AATTAAAATGCACCATTAAAAATTTCAATGTTGACAAAGGAATCTACCAAACATCCAACTTCAGAGTTTCACCCACTCA
AGAGGTTATTAGATTTCCTAACATTACAAATCGCTGTCCTTTTGACAAGGTTTTTAATGCTACTCGCTTTCCTAATGTT
TATGCATGGGAGAGAACAAAAATCTCTGATTGTGTTGCTGACTACACTGTTCTCTACAACTCAACCTCTTTCTCGACTT

TTAAATGTTATGGAGTGTCTCCATCTAAGTTGATTGACCTATGCTTTACAAGTGTGTATGCTGATACATTCTTGATAAG
ATCTTCTGAAGTAAGACAAGTTGCACCAGGTGAAACTGGTGTTATTGCTGACTACAATTACAAGTTGCCTGATGATTTC
ACTGGCTGTGTAATTGCTTGGAATACTGCTAAACATGATACTGGCAATTATTACTACAGATCTCATCGCAAGACTAAGT
TAAAGCCTTTTGAGAGAGACCTGTCTTCTGACGATGGTAATGGTGTGTATACACTCTCAACATATGACTTTAACCCTAA
CGTTCCAGTAGCATATCAGGCTACTAGGGTTGTTGTACTCTCTTTTGAACTTCTTAATGCACCTGCTACAGTTTGTGGA
CCTAAATTATCCACAGAACTAGTTAAGAACCAGTGTGTTAATTTCAATTTCAATGGACTTAAAGGTACTGGTGTTTTGA
CTTCTTCTTCAAAAAGATTCCAGTCATTTCAACAATTTGGTCGTGACACATCTGACTTTACGGATTCAGTACGTGACCC
ACAGACTTTAGAAATACTTGACATTTCACCTTGTTCCTTTGGTGGTGTTAGTGTTATCACACCTGGAACGAACGCCTCA
TCAGAGGTAGCTGTTCTCTATCAAGATGTAAATTGCACTGATGTCCCTACAGCAATACGTGCAGATCAATTAACACCTG
CTTGGCGCGTTTATTCCACTGGAGTAAATGTGTTTCAAACACAAGCTGGCTGTCTTATAGGAGCTGAGCATGTCAACGC
CTCTTATGAGTGTGACATTCCTATTGGTGCAGGCATTTGTGCTAGTTACCATACAGCTTCTGTTCTACGTAGTACCGGC
CAGAAATCAATTGTTGCCTATACTATGTCACTGGGTGCTGAAAATTCCATTGCATACGCTAATAATTCAATTGCCATAC
CTACAAATTTTTCAATCAGTGTCACTACAGAAGTGATGCCTGTTTCAATGGCTAAAACAGCCGTGGATTGTACTATGTA
CATCTGCGGTGATTCTTTAGAGTGCAGCAACTTACTATTGCAGTATGGAAGCTTTTGCACACAACTCAATCGTGCCCTC
ACTGGCATTGCTATAGAACAGGACAAAAACACTCAGGAGGTCTTTGCCCAGGTTAAACAAATGTACAAGACACCTGCCA
TAAAGGACTTTGGCGGTTTCAATTTTTCACAAATATTGCCTGACCCTTCAAAGCCAACGAAGAGATCATTTATTGAAGA
CTTGCTCTTCAATAAAGTGACTCTCGCTGATGCTGGCTTTATGAAACAATATGGTGACTGCCTAGGTGATGTTAGTGCT
AGAGACCTTATCTGTGCCCAGAAGTTCAATGGACTTACTGTGCTACCGCCACTGCTCACAGATGAGATGGTTGCTGCAT
ACACAGCTGCGCTAGTTAGTGGTACTGCTACGGCGGGCTGGACGTTTGGTGCAGGTGCAGCTCTTCAAATACCATTTGC
TATGCAAATGGCTTATAGGTTTAATGGCATTGGAGTTACTCAAAATGTTCTCTATGAGAACCAAAAGCTGATAGCCAAT
CAGTTTAATAGTGCTATAGGCAAAATTCAAGAATCATTATCATCTACTGCAAGTGCACTAGGAAAACTGCAGGATGTGG
TTAACCAAAATGCACAAGCTCTTAACACGCTTGTTAAACAACTCAGCTCTAATTTTGGAGCTATCTCAAGTGTGTTAAA
TGATATTCTCTCTCGCCTTGATAAAGTTGAGGCAGAAGTTCAAATTGACAGGTTGATTACAGGCAGATTGCAAAGCCTT
CAAACCTACGTAACACAACAACTTATCAGAGCTGCTGAAATCAGAGCTTCTGCTAATCTTGCTGCTACTAAAATGTCTG
AGTGCGTTCTTGGACAATCAAAAAGAGTTGATTTCTGTGGAAAAGGCTACCACCTTATGTCCTTCCCTCAATCAGCACC
TCACGGTGTCGTTTTTCTACATGTCACATATGTGCCATCACAAGAGAAAAACTTCACAACAGCTCCAGCTATTTGTCAC
GAAGGCAAAGCTTATTTCCCTCGTGAAGGTGTCTTTGTGTCTAATGGCACTTCTTGGTTTATTACGCAGAGGAATTTTT
ACTCTCCACAATTAATTACAACAGATAATACTTTTGTTTCTGGTAATTGTGATGTCGTGATCGGCATCATTAATAATAC
TGTTTATGATCCTCTGCAGCCTGAACTTGACTCGTTTAAGGAAGAGCTGGACAAGTACTTCAAAAATCATACATCACCA
GATGTTGATCTTGGCGACATTTCAGGCATTAATGCTTCAGTCGTCAACATTCAAAAGGAGATTGACCGACTCAATGAGG
TTGCCAAAAACCTAAATGAATCACTCATTGACCTCCAAGAACTTGGGAAATATGAGCAATACATCAAGTGGCCTTGGTA
TGTTTGGCTCGGCTTTATTGCTGGACTAATTGCCATCGTCATGGTTACAATCTTGCTTTGTTGCATGACCAGCTGTTGC
AGTTGTCTCAAGGGTGCATGCTCTTGTGGTTCGTGCTGCAAATTTGATGAGGACGACTCTGAGCCAGTGCTCAAAGGAG
TCAAATTACATTACACATAA

>gi|71796704|gb|DQ084199.1| bat SARS coronavirus HKU3-2, complete genome

ATGAAAATTTTAATTTTTGCTTTCTTAGCTAATTTAGCTAAAGCACAGGAAGGATGCGGTATCATCAGTAGAAAA
CCGCAACCTAAAATGGCACAAGTCTCATCTTCTCGTCGGGGCGTTTATTATAATGATGACATTTTCCGTTCTGATGTGT
TACACCTCACACAGGATTACTTCCTGCCATTTGACTCAAATTTAACTCAGTACTTTTCGCTTAATGTGGATTCAGATAG
GTATACCTATTTTGACAACCCCATACTAGACTTTGGTGATGGCGTTTATTTCGCTGCCACTGAAAAGTCTAACGTAATA
AGAGGCTGGATTTTTGGTTCATCTTTTGATAACACCACCCAGTCAGCTGTTATAGTTAATAATTCAACACACATTATTA
TACGTGTGTGTAATTTTAACTTATGTAAAGAACCCATGTATACTGTTAGTAGAGGTACACAGCAAAATGCCTGGGTTTA

TCAGAGTGCATTTAATTGTACATATGACCGAGTGGAAAAGAGTTTTCAACTTGACACTACTCCTAAAACTGGAAATTTT
AAAGACCTACGTGAGTATGTCTTTAAAAATAGGGATGGCTTTCTGTCTGTCTACCAAACTTATACTGCTGTTAATTTAC
CCAGAGGACTACCCACGGGTTTCTCAGTCTTGAAACCAATTTTAAAATTGCCCTTTGGAATTAATATCACTTCTTATAG
AGTAGTTATGGCAATGTTTAGCCAAACTACTTCTAATTTTTTACCAGAAAGTGCTGCTTATTATGTTGGTAATCTTAAA
TATTCTACCTTCATGCTCCGATTTAATGAAAATGGGACGATCACGGATGCTGTAGATTGTTCCCAAAATCCTCTTGCTG
AATTAAAATGCACCATTAAAAATTTCAATGTTGACAAAGGAATCTACCAAACATCCAACTTCAGAGTTTCACCCACTCA
AGAGGTTATTAGATTTCCTAACATTACAAATCGCTGTCCTTTTGACAAGGTTTTTAATGCTACTCGCTTTCCTAATGTT
TATGCATGGGAGAGAACAAAAATCTCTGATTGTGTTGCTGACTACACTGTTCTCTACAACTCAACCTCTTTCTCGACTT
TTAAATGTTATGGAGTGTCTCCATCTAAGTTGATTGACCTATGCTTTACAAGTGTGTATGCTGATACATTCTTGATAAG
ATCTTCTGAAGTAAGACAAGTTGCACCAGGTGAAACTGGTGTTATTGCTGACTACAATTACAAGTTGCCTGATGATTTC
ACTGGCTGTGTAATTGCTTGGAATACTGCTAAACATGATACTGGCAATTATTACTACAGATCTCATCGCAAGACTAAGT
TAAAGCCTTTTGAGAGAGACCTGTCTTCTGACGATGGTAATGGTGTGTATACACTCTCAACATATGACTTTAACCCTAA
CGTTCCAGTAGCATATCAGGCTACTAGGGTTGTTGTACTCTCTTTTGAACTTCTTAATGCACCTGCTACAGTTTGTGGA
CCTAAATTATCCACAGAACTAGTTAAGAACCAGTGTGTTAATTTCAATTTTAATGGACTTAAAGGTACTGGTGTTTTGA
CTTCTTCTTCAAAAAGATTCCAGTCATTTCAACAATTTGGTCGTGACACATCTGACTTTACGGATTCAGTACGTGACCC
ACAGACTTTAGAAATACTTGACATTTCACCTTGTTCCTTTGGTGGTGTTAGTGTTATCACACCTGGAACGAACGCCTCA
TCAGAGGTAGCTGTTCTCTATCAAGATGTAAATTGCACTGATGTCCCTACAGCAATACGTGCAGATCAATTAACACCTG
CTTGGCGCGTTTATTCCACTGGAGTAAATGTGTTTCAAACACAAGCTGGCTGTCTTATAGGAGCTGAGCATGTCAACGC
CTCTTATGAGTGTGACATTCCTATTGGTGCAGGCATTTGTGCTAGTTACCATACAGCTTTTGTTTTACGTAGTACCGGC
CAGAAATCAATTGTTGCCTATACTATGTCACTGGGTGCTGAAAATTCCATTGCATACGCTAATAATTCAATTGCCATAC
CTACAAATTTTTCAATCAGTGTCACTACAGAAGTGATGCCTGTTTCAATGGCTAAAACAGCCGTGGATTGTACTATGTA
CATCTGCGGTGATTCTTTAGAGTGCAGCAACTTACTATTGCAGTATGGAAGCTTTTGCACACAACTCAATCGTGCCCTC
ACTGGCATTGCTATAGAACAGGACAAAAACACTCAGGAGGTCTTTGCCCAGGTTAAACAAATGTACAAGACACCTGCCA
TAAAGGACTTTGGCGGTTTCAATTTTTCACAAATATTGCCTGACCCTTCAAAGCCAACGAAGAGATCATTTATTGAAGA
CTTGCTCTTCAATAAAGTGACTCTCGCTGATGCTGGCTTTATGAAACAATATGGTGACTGCCTAGGTGATGTTAGTGCT
AGAGACCTTATCTGTGCCCAGAAGTTCAATGGACTTACTGTGCTACCGCCACTGCTCACAGATGAGATGGTTGCTGCAT
ACACAGCTGCGCTAGTTAGTGGTACTGCTACGGCGGGCTGGACGTTTGGTGCAGGTGCAGCTCTTCAAATACCATTTGC
TATGCAAATGGCTTATAGGTTTAATGGCATTGGAGTTACTCAAAATGTTCTCTATGAGAACCAAAAGCTGATAGCCAAT
CAGTTTAATAGTGCTATAGGCAAAATTCAAGAATCATTATCATCTACTGCAAGTGCACTAGGAAAACTGCAGGATGTGG
TTAACCAAAATGCACAAGCTCTTAACACGCTTGTTAAACAACTCAGCTCTAATTTTGGAGCTATCTCAAGTGTGTTAAA
TGATATTCTCTCTCGCCTTGATAAAGTTGAGGCAGAAGTTCAAATTGACAGGTTGATTACAGGCAGATTGCAAAGCCTT
CAAACCTACGTAACACAACAACTTATCAGAGCTGCTGAAATCAGAGCTTCTGCTAATCTTGCTGCTACTAAAATGTCTG
AGTGCGTTCTTGGACAATCAAAAAGAGTTGATTTCTGTGGAAAAGGCTACCACCTTATGTCCTTCCCTCAATCAGCACC
TCACGGTGTCGTTTTTCTACATGTCACATATGTGCCATCACAAGAGAAAAACTTCACAACAGCTCCAGCTATTTGTCAC
GAAGGCAAAGCTTATTTCCCTCGTGAAGGTGTCTTTGTGTCTAATGGCACTTCTTGGTTTATTACGCAGAGGAATTTTT
ACTCTCCACAATTAATTACAACAGATAATACTTTTGTTTCTGGTAATTGTGATGTCGTGATCGGCATCATTAATAATAC
TGTTTATGATCCTCTGCAGCCTGAACTTGACTCGTTTAAGGAAGAGCTGGACAAGTACTTCAAAAATCATACATCACCA
GATGTTGATCTTGGCGACATTTCAGGCATTAATGCTTCAGTCGTCAACATTCAAAAGGAGATTGACCGCCTCAATGAGG
TTGCCAAAAACCTAAATGAATCACTCATTGACCTCCAAGAACTTGGGAAATATGAGCAATACATCAAGTGGCCTTGGTA
TGTTTGGCTCGGCTTTATTGCTGGACTAATTGCCATCGTCATGGTTACAATCTTGCTTTGTTGCATGACCAGCTGTTGC
AGTTGTCTCAAGGGTGCATGCTCTTGTGGTTCGTGCTGCAAATTTGATGAGGACGACTCTGAGCCAGTGCTCAAAGGAG
TCAAATTACATTACACATAA

>gi|292660135|gb|GQ153539.1| Bat SARS coronavirus HKU3-4, complete genome

ATGAAAATTTTAATTTTTGCTTTCTTAGCTAATTTAGCTAAAGCACAGGAAGGATGCGGTATCATCAGTAGAAAA
CCGCAACCTAAAATGGCACAAGTCTCATCTTCTCGTCGGGGCGTTTATTATAATGATGACATTTTCCGTTCTGATGTGT
TACACCTCACACAGGATTACTTCCTGCCATTTGACTCAAATTTAACTCAGTACTTTTCGCTTAATGTGGATTCAGATAG
GTATACCTATTTTGACAACCCCATACTAGACTTTGGTGATGGCGTTTATTTCGCTGCCACTGAAAAGTCTAACGTAATA
AGAGGCTGGATTTTTGGTTCATCTTTTGATAACACCACCCAGTCAGCTGTTATAGTTAATAATTCAACACACATTATTA
TACGTGTGTGTAACTTTAACTTATGTAAAGAACCCATGTATACTGTTAGTAGAGGTACACAGCAAAATGCCTGGGTTTA
TCAGAGTGCATTTAATTGTACATATGACCGAGTGGAAAAGAGTTTTCAACTTGACACTACTCCTAAAACTGGAAATTTT
AAAGACCTACGTGAGTATGTCTTTAAAAATAGGGATGGCTTTCTGTCTGTCTACCAAACTTATACTGCTGTTAATTTAC
CCAGAGGACTACCCACGGGTTTCTCAGTCTTGAAACCAATTTTAAAATTGCCCTTTGGAATTAATATCACTTCTTATAG
AGTAGTTATGGCAATGTTTAGCCAAACTACTTCTAATTTTTTACCAGAAAGTGCTGCTTATTATGTTGGTAATCTTAAA
TATTCTACCTTCATGCTCCGATTTAATGAAAATGGGACGATCACGGATGCTGTAGATTGTTCCCAAAATCCTCTTGCTG
AATTAAAATGCACCATTAAAAATTTCAATGTTGACAAAGGAATCTACCAAACATCCAACTTCAGAGTTTCACCCACTCA
AGAGGTTATTAGATTTCCTAACATTACAAATCGCTGTCCTTTTGACAAGGTTTTTAATGCTACTCGCTTTCCTAATGTT
TATGCATGGGAGAGAACAAAAATCTCTGATTGTGTTGCTGACTACACTGTTCTCTACAACTCAACCTCTTTCTCGACTT
TTAAATGTTATGGAGTGTCTCCATCTAAGTTGATTGACCTATGCTTTACAAGTGTGTATGCTGATACATTCTTGATAAG
ATCTTCTGAAGTAAGACAAGTTGCACCAGGTGAAACTGGTGTTATTGCTGACTACAATTACAAGTTGCCTGATGATTTC
ACTGGCTGTGTAATTGCTTGGAATACTGCTAAACATGATACTGGCAATTATTACTACAGATCTCATCGCAAGACTAAGT
TAAAGCCTTTTGAGAGAGACCTGTCTTCTGACGATGGTAATGGTGTGTATACACTCTCAACATATGACTTTAACCCTAA
CGTTCCAGTAGCATATCAGGCTACTAGGGTTGTTGTACTCTCTTTTGAACTTCTTAATGCACCTGCTACAGTTTGTGGA
CCTAAATTATCCACAGAACTAGTTAAGAACCAGTGTGTTAATTTCAATTTTAATGGACTTAAAGGTACTGGTGTTTTGA
CTTCTTCTTCAAAAAGATTCCAGTCATTTCAACAATTTGGTCGTGACACATCTGACTTTACGGATTCAGTACGTGACCC
ACAGACTTTAGAAATACTTGACATTTCACCTTGTTCCTTTGGTGGTGTTAGTGTTATCACACCTGGAACGAACGCCTCA
TCAGAGGTAGCTGTTCTCTATCAAGATGTAAATTGCACTGATGTCCCTACAGCAATACGTGCAGATCAATTAACACCTG
CTTGGCGCGTTTATTCCACTGGAGTAAATGTGTTTCAAACACAAGCTGGCTGTCTTATAGGAGCTGAGCATGTCAACGC
CTCTTATGAGTGTGACATTCCTATTGGTGCAGGCATTTGTGCTAGTTACCATACAGCTTCTGTTCTACGTAGTACCGGC
CAGAAATCAATTGTTGCCTATACTATGTCACTGGGTGCTGAAAATTCCATTGCATACGCTAATAATTCAATTGCCATAC
CTACAAATTTTTCAATCAGTGTCACTACAGAAGTGATGCCTGTTTCAATGGCTAAAACAGCCGTGGATTGTACTATGTA
CATCTGCGGTGATTCTTTAGAGTGCAGCAACTTACTATTGCAGTATGGAAGCTTTTGCACACAACTCAATCGTGCCCTC
ACTGGCATTGCTATAGAACAGGACAAAAACACTCAGGAGGTCTTTGCCCAGGTTAAACAAATGTACAAGACACCTGCCA
TAAAGGACTTTGGCGGTTTCAATTTTTCACAAATATTGCCTGACCCTTCAAAGCCAACGAAGAGATCATTTATTGAAGA
CTTGCTCTTCAATAAAGTGACTCTCGCTGATGCTGGCTTTATGAAACAATATGGTGACTGCCTAGGTGATGTTAGTGCT
AGAGACCTTATCTGTGCCCAGAAGTTCAATGGACTTACTGTGCTACCGCCACTGCTCACAGATGATATGGTTGCTGCAT
ACACAGCTGCGCTAGTTAGTGGTACTGCTACGGCGGGCTGGACGTTTGGTGCAGGTGCAGCTCTTCAAATACCATTTGC
TATGCAAATGGCTTATAGGTTTAATGGCATTGGAGTTACTCAAAATGTTCTCTATGAGAACCAAAAGCTGATAGCCAAT
CAGTTTAATAGTGCTATAGGCAAAATTCAAGAATCATTATCATCTACTGCAAGTGCACTAGGAAAACTGCAGGATGTGG
TTAACCAAAATGCACAAGCTCTTAACACGCTTGTTAAACAACTCAGCTCTAATTTTGGAGCTATCTCAAGTGTGTTAAA
TGATATTCTCTCTCGCCTTGATAAAGTTGAGGCAGAAGTTCAAATTGACAGGTTGATTACAGGCAGATTGCAAAGCCTT
CAAACCTACGTAACACAACAACTTATCAGAGCTGCTGAAATCAGAGCTTCTGCTAATCTTGCTGCTACTAAAATGTCTG
AGTGCGTTCTTGGACAATCAAAAAGAGTTGATTTCTGTGGAAAAGGCTACCACCTTATGTCCTTCCCTCAATCAGCACC
TCACGGTGTCGTTTTTCTACATGTCACATATGTGCCATCACAAGAGAAAAACTTCACAACAGCTCCAGCTATTTGTCAC
GAAGGCAAAGCTTATTTCCCTCGTGAAGGTGTCTTTGTGTCTAATGGCACTTCTTGGTTTATTACGCAGAGGAATTTTT
ACTCTCCACAATTAATTACAACAGATAATACTTTTGTTTCTGGTAATTGTGATGTCGTGATCGGCATCATTAATAATAC

TGTTTATGATCCTCTGCAGCCTGAACTTGACTCGTTTAAGGAAGAGCTGGACAAGTACTTCAAAAATCATACATCACCA
GATGTTGATCTTGGCGACATTTCAGGCATTAATGCTTCAGTCGTCAACATTCAAAAGGAGATTGACCGCCTCAATGAGG
TTGCCAAAAACCTAAATGAATCACTCATTGACCTCCAAGAACTTGGGAAATATGAGCAATACATCAAGTGGCCTTGGTA
TGTTTGGCTCGGCTTTATTGCTGGACTAATTGCCATCGTCATGGTTACAATCTTGCTTTGTTGCATGACCAGCTGTTGC
AGTTGTCTCAAGGGTGCATGCTCTTGTGGTTCGTGCTGCAAATTTGATGAGGACGACTCTGAGCCAGTGCTCAAAGGAG
TCAAATTACATTACACATAA

>gi|292660147|gb|GQ153540.1| Bat SARS coronavirus HKU3-5, complete genome

ATGAAAATTTTAATTTTTGCTTTCTTAGCTAATTTAGCTAAAGCACAGGAAGGATGCGGTATCATCAGTAGAAAA
CCGCAACCTAAAATGGCACAAGTCTCATCTTCTCGTCGGGGCGTTTATTATAATGATGACATTTTCCGTTCTGATGTGT
TACACCTCACACAGGATTACTTCCTGCCATTTGACTCAAATTTAACTCAGTACTTTTCGCTTAATGTGGATTCAGATAG
GTATACCTATTTTGACAACCCCATACTAGACTTTGGTGATGGCGTTTATTTCGCTGCCACTGAAAAGTCTAACGTAATA
AGAGGCTGGATTTTTGGTTCATCTTTTGATAACACCACCCAGTCAGCTGTTATAGTTAATAATTCAACACACATTATTA
TACGTGTGTGTAATTTTAACTTATGTAAAGAACCCATGTATACTGTTAGTAGAGGTACACAGCAAAATGCCTGGGTTTA
TCAGAGTGCATTTAATTGTACATATGACCGAGTGGAAAAGAGTTTTCAACTTGACACTACTCCTAAAACTGGAAATTTT
AAAGACCTACGTGAGTATGTCTTTAAAAATAGGGATGGCTTTCTGTCTGTCTACCAAACTTATACTGCTGTTAATTTAC
CCAGAGGACTACCCACGGGTTTCTCAGTCTTGAAACCAATTTTAAAATTGCCCTTTGGAATTAATATCACTTCTTATAG
AGTAGTTATGGCAATGTTTAGCCAAACTACTTCTAATTTTTTACCAGAAAGTGCTGCTTATTATGTTGGTAATCTTAAA
TATTCTACCTTCATGCTCCGATTTAATGAAAATGGGACGATCACGGATGCTGTAGATTGTTCCCAAAATCCTCTTGCTG
AATTAAAATGCACCATTAAAAATTTCAATGTTGACAAAGGAATCTACCAAACATCCAACTTCAGAGTTTCACCCACTCA
AGAGGTTATTAGATTTCCTAACATTACAAATCGCTGTCCTTTTGACAAGGTTTTTAATGCTACTCGCTTTCCTAATGTT
TATGCATGGGAGAGAACAAAAATCTCTGATTGTGTTGCTGACTACACTGTTCTCTACAACTCAACCTCTTTCTCGACTT
TTAAATGTTATGGAGTGTCTCCATCTAAGTTGATTGACCTATGCTTTACAAGTGTGTATGCTGATACATTCTTGATAAG
ATCTTCTGAAGTAAGACAAGTTGCACCAGGTGAAACTGGTGTTATTGCTGACTACAATTACAAGTTGCCTGATGATTTC
ACTGGCTGTGTAATTGCTTGGAATACTGCTAAACATGATACTGGCAATTATTACTACAGATCTCATCGCAAGACTAAGT
TAAAGCCTTTTGAGAGAGACCTGTCTTCTGACGATGGTAATGGTGTGTATACACTCTCAACATATGACTTTAACCCTAA
CGTTCCAGTAGCATATCAGGCTACTAGGGTTGTTGTACTCTCTTTTGAACTTCTTAATGCACCTGCTACAGTTTGTGGA
CCTAAATTATCCACAGAACTAGTTAAGAACCAGTGTGTTAATTTCAATTTTAATGGACTTAAAGGTACTGGTGTTTTGA
CTTCTTCTTCAAAAAGATTCCAGTCATTTCAACAATTTGGTCGTGACACATCTGACTTTACGGATTCAGTACGTGACCC
ACAGACTTTAGAAATACTTGACATTTCACCTTGTTCCTTTGGTGGTGTTAGTGTTATCACACCTGGAACGAACGCCTCA
TCAGAGGTAGCTGTTCTCTATCAAGATGTAAATTGCACTGATGTCCCTACAGCAATACGTGCAGATCAATTAACACCTG
CTTGGCGCGTTTATTCCACTGGAGTAAATGTGTTTCAAACACAAGCTGGCTGTCTTATAGGAGCTGAGCATGTCAACGC
CTCTTATGAGTGTGACATTCCTATTGGTGCAGGCATTTGTGCTAGTTACCATACAGCTTCTGTTCTACGTAGTACCGGC
CAGAAATCAATTGTTGCCTATACTATGTCACTGGGTGCTGAAAATTCCATTGCATACGCTAATAATTCAATTGCCATAC
CTACAAATTTTTCAATCAGTGTCACTACAGAAGTGATGCCTGTTTCAATGGCTAAAACAGCCGTGGATTGTACTATGTA
CATCTGCGGTGATTCTTTAGAGTGCAGCAACTTACTATTGCAGTATGGAAGCTTTTGCACACAACTCAATCGTGCCCTC
ACTGGCATTGCTATAGAACAGGACAAAAACACTCAGGAGGTCTTTGCCCAGGTTAAACAAATGTACAAGACACCTGCCA
TAAAGGACTTTGGCGGTTTCAATTTTTCACAAATATTGCCTGACCCTTCAAAGCCAACGAAGAGATCATTTATTGAAGA
CTTGCTCTTCAATAAAGTGACTCTCGCTGATGCTGGCTTTATGAAACAATATGGTGACTGCCTAGGTGATGTTAGTGCT
AGAGACCTTATCTGTGCCCAGAAGTTCAATGGACTTACTGTGCTACCGCCACTGCTCACAGATGATATGGTTGCTGCAT
ACACAGCTGCGCTAGTTAGTGGTACTGCTACGGCGGGCTGGACGTTTGGTGCAGGTGCAGCTCTTCAAATACCATTTGC
TATGCAAATGGCTTATAGGTTTAATGGCATTGGAGTTACTCAAAATGTTCTCTATGAGAACCAAAAGCTGATAGCCAAT

CAGTTTAATAGTGCTATAGGCAAAATTCAAGAATCATTATCATCTACTGCAAGTGCACTAGGAAAACTGCAGGATGTGG
TTAACCAAAATGCACAAGCTCTTAACACGCTTGTTAAACAACTCAGCTCTAATTTTGGAGCTATCTCAAGTGTGTTAAA
TGATATTCTCTCTCGCCTTGATAAAGTTGAGGCAGAAGTTCAAATTGACAGGTTGATTACAGGCAGATTGCAAAGCCTT
CAAACCTACGTAACACAACAACTTATCAGAGCTGCTGAAATCAGAGCTTCTGCTAATCTTGCTGCTACTAAAATGTCTG
AGTGCGTTCTTGGACAATCAAAAAGAGTTGATTTCTGTGGAAAAGGCTACCACCTTATGTCCTTCCCTCAATCAGCACC
TCACGGTGTCGTTTTTCTACATGTCACATATGTTCCATCACAAGAGAAAAACTTCACAACAGCTCCAGCTATTTGTCAC
GAAGGCAAAGCTTATTTCCCTCGTGAAGGTGTCTTTGTGTCTAATGGCACTTCTTGGTTTATTACGCAGAGGAATTTTT
ACTCTCCACAATTAATTACAACAGATAATACTTTTGTTTCTGGTAATTGTGATGTCGTGATCGGCATCATTAATAATAC
TGTTTATGATCCTCTGCAGCCTGAACTTGACTCGTTTAAGGAAGAGCTGGACAAGTACTTCAAAAATCATACATCACCA
GATGTTGATCTTGGCGACATTTCAGGCATTAATGCTTCAGTCGTCAACATTCAAAAGGAGATTGACCGCCTCAATGAGG
TTGCCAAAAACCTAAATGAATCACTCATTGACCTCCAAGAACTTGGGAAATATGAGCAATACATCAAGTGGCCTTGGTA
TGTTTGGCTCGGCTTTATTGCTGGACTAATTGCCATCGTCATGGTTACAATCTTGCTTTGTTGCATGACCAGCTGTTGC
AGTTGTCTCAAGGGTGCATGCTCTTGTGGTTCGTGCTGCAAATTTGATGAGGACGACTCTGAGCCAGTGCTCAAAGGAG
TCAAATTACATTACACATAA

>gi|292660159|gb|GQ153541.1| Bat SARS coronavirus HKU3-6, complete genome

ATGAAAATTTTAATTTTTGCTTTCTTAGCTAATTTAGCTAAAGCACAGGAAGGATGCGGTATCATCAGTAGAAAA
CCGCAACCTAAAATGGCACAAGTCTCATCTTCTCGTCGGGGCGTTTATTATAATGATGACATTTTCCGTTCTGATGTGT
TACACCTCACACAGGATTACTTCCTGCCATTTGACTCAAATTTAACTCAGTACTTTTCGCTTAATGTGGATTCAGATAG
GTATACCTATTTTGACAACCCCATACTAGACTTTGGTGATGGCGTTTATTTCGCTGCCACTGAAAAGTCTAACGTAATA
AGAGGCTGGATTTTTGGTTCATCTTTTGATAACACCACCCAGTCAGCTGTTATAGTTAATAATTCAACACACATTATTA
TACGTGTGTGTAATTTTAACTTATGTAAAGAACCCATGTATACTGTTAGTAGAGGTACACAGCAAAATGCCTGGGTTTA
TCAGAGTGCATTTAATTGTACATATGACCGAGTGGAAAAGAGTTTTCAACTTGACACTACTCCTAAAACTGGAAATTTT
AAAGACCTACGTGAGTATGTCTTTAAAAATAGGGATGGCTTTCTGTCTGTCTACCAAACTTATACTGCTGTTAATTTAC
CCAGAGGACTACCCACGGGTTTCTCAGTCTTGAAACCAATTTTAAAATTGCCCTTTGGAATTAATATCACTTCTTATAG
AGTAGTTATGGCAATGTTTAGCCAAACTACTTCTAATTTTTTACCAGAAAGTGCTGCTTATTATGTTGGTAATCTTAAA
TATTCTACCTTCATGCTCCGATTTAATGAAAATGGGACGATCACGGATGCTGTAGATTGTTCCCAAAATCCTCTTGCTG
AATTAAAATGCACCATTAAAAATTTCAATGTTGACAAAGGAATCTACCAAACATCCAACTTCAGAGTTTCACCCACTCA
AGAGGTTATTAGATTTCCTAACATTACAAATCGCTGTCCTTTTGACAAGGTTTTTAATGCTACTCGCTTTCCTAATGTT
TATGCATGGGAGAGAACAAAAATCTCTGATTGTGTTGCTGACTACACTGTTCTCTACAACTCAACCTCTTTCTCGACTT
TTAAATGTTATGGAGTGTCTCCATCTAAGTTGATTGACCTATGCTTTACAAGTGTGTATGCTGATACATTCTTGATAAG
ATCTTCTGAAGTAAGACAAGTTGCACCAGGTGAAACTGGTGTTATTGCTGACTACAATTACAAGTTGCCTGATGATTTC
ACTGGCTGTGTAATTGCTTGGAATACTGCTAAACATGATACTGGCAATTATTACTACAGATCTCATCGCAAGACTAAGT
TAAAGCCTTTTGAGAGAGACCTGTCTTCTGACGATGGTAATGGTGTGTATACACTCTCAACATATGACTTTAACCCTAA
CGTTCCAGTAGCATATCAGGCTACTAGGGTTGTTGTACTCTCTTTTGAACTTCTTAATGCACCTGCTACAGTTTGTGGA
CCTAAATTATCCACAGAACTAGTTAAGAACCAGTGTGTTAATTTCAATTTTAATGGACTTAAAGGTACTGGTGTTTTGA
CTTCTTCTTCAAAAAGATTCCAGTCATTTCAACAATTTGGTCGTGACACATCTGACTTTACGGATTCAGTACGTGACCC
ACAGACTTTAGAAATACTTGACATTTCACCTTGTTCCTTTGGTGGTGTTAGTGTTATCACACCTGGAACGAACGCCTCA
TCAGAGGTAGCTGTTCTCTATCAAGATGTAAATTGCACTGATGTCCCTACAGCAATACGTGCAGATCAATTAACACCTG
CTTGGCGCGTTTATTCCACTGGAGTAAATGTGTTTCAAACACAAGCTGGCTGTCTTATAGGAGCTGAGCATGTCAACGC
CTCTTATGAGTGTGACATTCCTATTGGTGCAGGCATTTGTGCTAGTTACCATACAGCTTCTGTTCTACGTAGTACCGGC
CAGAAATCAATTGTTGCCTATACTATGTCACTGGGTGCTGAAAATTCCATTGCATACGCTAATAATTCAATTGCCATAC

CTACAAATTTTTCAATCAGTGTCACTACAGAAGTGATGCCTGTTTCAATGGCTAAAACAGCCGTGGATTGTACTATGTA
CATCTGCGGTGATTCTTTAGAGTGCAGCAACTTACTATTGCAGTATGGAAGCTTTTGCACACAACTCAATCGTGCCCTC
ACTGGCATTGCTATAGAACAGGACAAAAACACTCAGGAGGTCTTTGCCCAGGTTAAACAAATGTACAAGACACCTGCCA
TAAAGGACTTTGGCGGTTTCAATTTTTCACAAATATTGCCTGACCCTTCAAAGCCAACGAAGAGATCATTTATTGAAGA
CTTGCTCTTCAATAAAGTGACTCTCGCTGATGCTGGCTTTATGAAACAATATGGTGACTGCCTAGGTGATGTTAGTGCT
AGAGACCTTATCTGTGCCCAGAAGTTCAATGGACTTACTGTGCTACCGCCACTGCTCACAGATGATATGGTTGCTGCAT
ACACAGCTGCGCTAGTTAGTGGTACTGCTACGGCGGGCTGGACGTTTGGTGCAGGTGCAGCTCTTCAAATACCATTTGC
TATGCAAATGGCTTATAGGTTTAATGGCATTGGAGTTACTCAAAATGTTCTCTATGAGAACCAAAAGCTGATAGCCAAT
CAGTTTAATAGTGCTATAGGCAAAATTCAAGAATCATTATCATCTACTGCAAGTGCACTAGGAAAACTGCAGGATGTGG
TTAACCAAAATGCACAAGCTCTTAACACGCTTGTTAAACAACTCAGCTCTAATTTTGGAGCTATCTCAAGTGTGTTAAA
TGATATTCTCTCTCGCCTTGATAAAGTTGAGGCAGAAGTTCAAATTGACAGGTTGATTACAGGCAGATTGCAAAGCCTT
CAAACCTACGTAACACAACAACTTATCAGAGCTGCTGAAATCAGAGCTTCTGCTAATCTTGCTGCTACTAAAATGTCTG
AGTGCGTTCTTGGACAATCAAAAAGAGTTGATTTCTGTGGAAAAGGCTACCACCTTATGTCCTTCCCTCAATCAGCACC
TCACGGTGTCGTTTTTCTACATGTCACATATGTGCCATCACAAGAGAAAAACTTCACAACAGCTCCAGCTATTTGTCAC
GAAGGCAAAGCTTATTTCCCTCGTGAAGGTGTCTTTGTGTCTAATGGCACTTCTTGGTTTATTACGCAGAGGAATTTTT
ACTCTCCACAATTAATTACAACAGATAATACTTTTGTTTCTGGTAATTGTGATGTCGTGATCGGCATCATTAATAATAC
TGTTTATGATCCTCTGCAGCCTGAACTTGACTCGTTTAAGGAAGAGCTGGACAAGTACTTCAAAAATCATACATCACCA
GATGTTGATCTTGGCGACATTTCAGGCATTAATGCTTCAGTCGTCAACATTCAAAAGGAGATTGACCGCCTCAATGAGG
TTGCCAAAAACCTAAATGAATCACTCATTGACCTCCAAGAACTTGGGAAATATGAGCAATACATCAAGTGGCCTTGGTA
TGTTTGGCTCGGCTTTATTGCTGGACTAATTGCCATCGTCATGGTTACAATCTTGCTTTGTTGCATGACCAGCTGTTGC
AGTTGTCTCAAGGGTGCATGCTCTTGTGGTTCGTGCTGCAAATTTGATGAGGACGACTCTGAGCCAGTGCTCAAAGGAG
TCAAATTACATTACACATAA

>gi|292660171|gb|GQ153542.1| Bat SARS coronavirus HKU3-7, complete genome

ATGAAAATTTTAATTTTTGCTTTCCTAGCTAGTCTAGCTAAAGCACAAGAAGGATGTGGCATTATCAGTCGAAAG
CCGCAGCCAAAAATGGCACAAGTCTCTTCTTCTCGTCGAGGTGTGTACTATAATGATGATATTTTTCGCTCTGATGTGC
TACACCTCACACAGGATTATTTCCTGCCATTCGATTCCAATCTAACACAGTACTTTTCTCTTAATGTTGACTCAGATAG
GTATACCTATTTTGACAACCCTATACTAGACTTTGGTGATGGCGTTTATTTTGCTGCCACCGAAAAGTCTAATGTAGTA
AGAGGCTGGATTTTTGGTTCGTCTTTTGATAATACCACCCAGTCAGCTGTCATAGTTAATAATTCAACACACATTATTA
TACGTGTGTGTAATTTCAACTTGTGTAAAGAACCCATGTACACTGTTAGTAGGGGTACACAACAGAATTCTTGGGTTTA
TCAAAGTGCATTTAATTGCACCTATGACAGAGTGGAGAAGAGCTTTCAGCTCGATACAGTTCCTAAAACTGGAAATTTT
AAGGACTTACGTGAGTACGTCTTCAAGAATAGGGATGGTTTCCTTAGTGTTTATCAAACCTATACTGCTGTTAATTTAC
CCAGAGGTTTGCCTGAAGGTTTTTCAGTTTTAAGACCAATTCTCAAATTACCTTTTGGAATTAATATCACTTCTTATAG
AGTAGTTATGGCAATGTTTAGCCAAACTACTTCTAATTTTCTACCAGAAAGTGCTGCTTATTATGTTGGTAATCTTAAA
TATTCTACCTTCATGCTGCGATTTAATGAAAATGGGACCATCACGGATGCTGTGGATTGTTCCCAAAACCCACTTGCTG
AATTAAAATGCACCATTAAAAATTTTAATGTTGACAAAGGAATCTACCAAACATCCAACTTTAGAGTTTCACCCACCCA
AGAGGTTATTAGATTCCCTAACATTACAAACCGCTGTCCTTTTGACAGGGTTTTTAATGCAAGCCGCTTTCCTAGTGTT
TATGCATGGGAGCGGACGAAAATTTCTGAGTGTGTTGCAGACTACACAGTTCTCTACAACTCAACCTCTTTCTCAACTT
TTAAATGTTATGGAGTGTCTCCATCTAAGTTGATTGACTTATGCTTTACAAGTGTGTATGCTGATACATTCTTGATAAG
ATCTTCTGAAGTAAGACAAGTTGCACCAGGTGAAACTGGTGTTATTGCTGACTACAATTACAAGTTGCCTGATGATTTC
ACTGGCTGTGTAATTGCTTGGAATACTGCTAAACAGGATACTGGCAATTATTACTACAGATCTCATCGCAAGACTAAGT
TAAAGCCTTTTGAGAGAGATCTGTCTTCTGACGACGGTAATGGTGTGTACACACTTTCAACATATGATTTTAACCCTAA

TGTTCCAGTAGCATATCAGGCTACTAGGGTTGTTGTACTCTCTTTTGAACTTCTTAATGCACCTGCTACAGTTTGTGGA
CCTAAATTATCCACACAACTAGTTAAGAACCAGTGTGTTAATTTCAATTTCAATGGACTTAAAGGTACTGGTGTTTTGA
CTCCTTCTTCAAAAAGATTCCAGTCATTTCAACAATTTGGTCGTGACACATCTGACTTTACGGATTCAGTACGTGACCC
ACAGACTTTAGAAATACTTGACATTTCACCTTGTTCCTTTGGTGGTGTTAGTGTTATCACACCTGGAACGAATGCCTCA
TCAGAGGTGGCTGTTCTCTATCAAGATGTAAATTGCACTGATGTCCCTACAGCGATTCGTGCAGACCAATTAACACCTG
CTTGGCGCGTTTATTCCACTGGAGTAAATGTGTTTCAAACACAAGCTGGCTGTCTTATAGGAGCTGAGCATGTCAACGC
CTCTTATGAGTGTGACATTCCTATTGGTGCAGGCATTTGTGCTAGTTACCATACAGCTTCCGTTCTACGTAGTACCGGC
CAGAAATCAATTGTTGCCTATACTATGTCACTGGGTGCTGAAAATTCCATTGCATACGCTAATAATTCAATTGCCATAC
CTACAAATTTTTCAATCAGTGTCACTATAGAAGTGATGCCTGTTTCAATGGCTAAAACATCCGTGGATTGTACAATGTA
CATCTGTGGTGACTCTTTGGAGTGCAGCAACCTACTATTGCAGTATGGAAGCTTTTGCACACAGCTCAATCGCGCCCTT
ACTGGCATTGCTATAGAACAGGACAAAAACACTCAGGAAGTCTTTGCCCAGGTTAAACAAATGTACAAGACACCTGCCA
TAAAAGATTTTGGCGGTTTCAATTTCTCACAAATATTGCCTGACCCTTCAAAGCCAACGAAGAGATCATTTATTGAAGA
CCTGCTCTTCAACAAAGTGACTCTCGCTGATGCTGGCTTTATGAAACAATATGGTGAATGCCTAGGTGATGTTAGTGCT
AGAGACCTTATCTGTGCCCAGAAGTTCAATGGACTTACTGTGCTACCACCACTGCTCACAGATGAGATGATTGCTGCAT
ATACAGCTGCGCTAGTTAGTGGTACTGCTACGGCAGGCTGGACATTCGGTGCAGGGGCAGCTCTCCAAATACCGTTCGC
TATGCAAATGGCCTATAGGTTTAATGGCATTGGAGTTACTCAAAACGTTCTCTATGAGAACCAGAAGCTGATAGCTAAT
CAGTTTAATAGTGCTATAGGCAAAATTCAAGACTCATTATCATCTACTGCGAGTGCACTTGGAAAACTTCAGGATGTTG
TTAACCAAAATGCACAAGCTCTTAACACGCTTGTTAAACAGCTTAGCTCTAATTTTGGAGCTATTTCGAGTGTGTTAAA
TGATATTCTTTCTCGACTTGATAAAGTTGAGGCGGAGGTTCAAATCGATAGGTTGATCACAGGCAGATTGCAAAGTCTT
CAAACCTATGTAACACAGCAACTTATCAGAGCTGCTGAAATCAGAGCTTCTGCTAATCTTGCTGCTACTAAAATGTCTG
AGTGTGTCCTCGGACAATCGAAAAGAGTTGATTTTTGTGGAAAAGGCTACCATCTTATGTCTTTTCCTCAATCAGCACC
ACACGGTGTCGTTTTTCTACACGTTACATACGTGCCTTCACAAGAGAGAAACTTCACCACAGCTCCAGCCATTTGTCAT
GAAGGCAAAGCTTACTTCCCTCGTGAAGGTGTTTTCGTTTCTAATGGCACTTCTTGGTTTATTACGCAGAGGAATTTTT
ACTCTCCACAATTAATTACAACAGACAATACTTTTGTTTCTGGTAATTGTGATGTCGTAATCGGCATCATTAATAACAC
TGTTTACGATCCTCTGCAACCTGAGCTTGACTCATTTAAAGAAGAGCTGGACAAGTACTTCAAAAATCATACATCACCA
GATGTTGATCTTGGCGACATTTCAGGCATTAATGCTTCAGTCGTCAATATTCAAAAGGAGATTGACCGCCTCAATGAGG
TTGCCAAAAACCTAAATGAATCACTCATTGACCTCCAAGAACTTGGGAAATATGAGCAATACATCAAGTGGCCGTGGTA
TGTTTGGCTCGGCTTTATTGCTGGACTAATTGCCATCGTCATGGTTACAATCTTGCTTTGTTGCATGACCAGCTGTTGC
AGTTGTCTCAAGGGTGCATGCTCTTGTGGTTCTTGCTGCAAATTTGATGAGGACGACTCTGAGCCAGTGCTCAAGGGAG
TCAAATTACATTACACATAA

>gi|292660183|gb|GQ153543.1| Bat SARS coronavirus HKU3-8, complete genome

ATGAAAATTTTAATTTTTGCTTTCCTAGCTAGTCTAGCTAAAGCACAAGAAGGATGTGGCATTATCAGTCGAAAG
CCGCAGCCAAAAATGGCACAAGTCTCTTCTTCTCGTCGAGGTGTGTACTATAATGATGATATTTTTCGCTCTGATGTGC
TACACCTCACACAGGATTATTTCCTGCCATTCGATTCCAATCTAACACAGTACTTTTCTCTTAATGTTGACTCAGATAG
GTATACCTATTTTGACAACCCTATACTAGACTTTGGTGATGGCGTTTATTTTGCTGCCACCGAAAAGTCTAATGTAATA
AGAGGCTGGATTTTTGGTTCGTCTTTTGATAATACCACCCAGTCAGCTGTCATAGTTAATAATTCAACACACATTATTA
TACGTGTGTGTAATTTCAACTTGTGTAAAGAACCCATGTACACTGTTAGTATGGGTACACAACAGAATTCTTGGGTTTA
TCAAAGTGCATTTAATTGCACCTATGACAGAGTGGAGAAGAGCTTTCAGCTCGATACAGTTCCTAAAACTGGAAATTTT
AAGGACTTACGTGAGTACGTCTTCAAGAATAGGGATGGTTTCCTTAGTGTTTATCAAACCTATACTGCTGTTAATTTAC
CCAGAGGTTTGCCTGAAGGTTTTTCAGTTTTAAGACCAATTCTCAAATTACCTTTTGGAATTAATATCACTTCTTATAG
AGTAGTTATGGCAATGTTTAGCCAAACTACTTCTAATTTTCTACCAGAAAGTGCTGCTTATTATGTTGGTAATCTTAAA

TATTCTACCTTCATGCTGCGATTTAATGAAAATGGGACCATCACGGATGCTGTGGATTGTTCCCAAAACCCACTTGCTG
AATTAAAATGCACCATTAAAAATTTTAATGTTGACAAAGGAATCTACCAAACATCCAACTTTAGAGTTTCACCCACCCA
AGAGGTTATTAGATTCCCTAACATTACAAACCGCTGTCCTTTTGACAGGGTTTTTAATGCAAGCCGCTTTCCTAGTGTT
TATGCATGGGAGCGGACGAAAATTTCTGAGTGTGTTGCAGACTACACAGTTCTCTACAACTCAACCTCTTTCTCAACTT
TTAAATGTTATGGAGTGTCTCCATCTAAGTTGATTGACTTATGCTTTACAAGTGTGTATGCTGATACATTCTTGATAAG
ATCTTCTGAAGTAAGACAAGTTGCACCAGGTGAAACTGGTGTTATTGCTGACTACAATTACAAGTTGCCTGATGATTTC
ACTGGCTGTGTAATTGCTTGGAATACTGCTAAACAGGATACTGGCAATTATTACTACAGATCTCATCGCAAGACTAAGT
TAAAGCCTTTTGAGAGAGATCTGTCTTCTGACGACGGTAATGGTGTGTACACACTTTCAACATATGATTTTAACCCTAA
TGTTCCAGTAGCATATCAGGCTACTAGGGTTGTTGTACTCTCTTTTGAACTTCTTAATGCACCTGCTACAGTTTGTGGA
CCTAAATTATCCACACAACTAGTTAAGAACCAGTGTGTTAATTTCAATTTCAATGGACTTAAAGGTACTGGTGTTTTGA
CTCCTTCTTCAAAAAGATTCCAGTCATTTCAACAATTTGGTCGTGACACATCTGACTTTACGGATTCAGTACGTGACCC
ACAGACTTTAGAAATACTTGACATTTCACCTTGTTCCTTTGGTGGTGTTAGTGTTATCACACCTGGAACGAATGCCTCA
TCAGAGGTGGCTGTTCTCTATCAAGATGTAAATTGCACTGATGTCCCTACAGCGATTCGTGCAGACCAATTAACACCTG
CTTGGCGCGTTTATTCCACTGGAGTAAATGTGTTTCAAACACAAGCTGGCTGTCTTATAGGAGCTGAGCATGTCAACGC
CTCTTATGAGTGTGACATTCCTATTGGTGCAGGCATTTGTGCTAGTTACCATACAGCTTCCGTTCTACGTAGTACCGGC
CAGAAATCAATTGTTGCCTATACTATGTCACTGGGTGCTGAAAATTCCATTGCATACGCTAATAATTCAATTGCCATAC
CTACAAATTTTTCAATCAGTGTCACTACAGAAGTGATGCCTGTTTCAATGGCTAAAACATCCGTGGATTGTACAATGTA
CATCTGTGGTGACTCTTTGGAGTGCAGCAACCTACTATTGCAGTATGGAAGCTTTTGCACACAGCTCAATCGTGCCCTT
ACTGGCATTGCTATAGAGCAGGACAAAAACACTCAGGAAGTCTTTGCCCAGGTTAAACAAATGTACAAGACACCTGCCA
TAAAAGATTTTGGCGGTTTCAATTTCTCACAAATATTGCCTGACCCTTCAAAGCCAACGAAGAGATCATTTATTGAAGA
CCTGCTCTTCAACAAAGTGACTCTCGCTGATGCTGGCTTTATGAAACAATATGGTGAATGCCTAGGTGATGTTAGTGCT
AGAGACCTTATCTGTGCCCAGAAGTTCAATGGACTTACTGTGCTACCACCACTGCTCACAGATGAGATGATTGCTGCAT
ATACAGCTGCGCTAGTTAGTGGTACTGCTACGGCAGGCTGGACATTCGGTGCAGGGGCAGCTCTCCAAATACCGTTCGC
TATGCAAATGGCCTATAGGTTTAATGGCATTGGAGTTACTCAAAACGTTCTCTATGAGAACCAGAAGCTGATAGCTAAT
CAGTTTAATAGTGCTATAGGCAAAATTCAAGACTCATTATCATCTACTGCGAGTGCACTTGGAAAACTTCAGGATGTTG
TTAACCAAAATGCACAAGCTCTTAACACGCTTGTTAAACAGCTTAGCTCTAATTTTGGAGCTATTTCGAGTGTGTTAAA
TGATATTCTTTCTCGACTTGATAAAGTTGAGGCGGAGGTTCAAATCGATAGGTTGATCACAGGCAGATTGCAAAGTCTT
CAAACCTATGTAACACAGCAACTTATCAGAGCTGCTGAAATCAGAGCTTCTGCTAATCTTGCTGCTACTAAAATGTCTG
AGTGTGTCCTCGGACAATCGAAAAGAGTTGATTTTTGTGGAAAAGGCTACCATCTTATGTCTTTTCCTCAATCAGCACC
ACACGGTGTCGTTTTTCTACACGTTACATACGTGCCTTCACAAGAGAGAAACTTCACCACAGCTCCAGCCATTTGTCAT
GAAGGCAAAGCTTACTTCCCTCGTGAAGGTGTTTTCGTTTCTAATGGCACTTCTTGGTTTATTACGCAGAGGAATTTTT
ACTCTCCACAATTAATTACAACAGACAATACTTTTGTTTCTGGTAATTGTGATGTCGTAATCGGCATCATTAATAACAC
TGTTTACGATCCTCTGCAACCTGAGCTTGACTCATTTAAAGAAGAGCTGGACAAGTACTTCAAAAATCATACATCACCA
GATGTTGATCTTGGCGACATTTCAGGCATTAATGCTTCAGTCGTCAATATTCAAAAGGAGATTGACCGCCTCAATGAGG
TTGCCAAAAACCTAAATGAATCACTCATTGACCTCCAAGAACTTGGGAAATATGAGCAATACATCAAGTGGCCGTGGTA
TGTTTGGCTCGGCTTTATTGCTGGACTAATTGCCATCGTCATGGTTACAATCTTGCTTTGTTGCATGACCAGCTGTTGC
AGTTGTCTCAAGGGTGCATGCTCTTGTGGTTCTTGCTGCAAATTTGATGAGGACGACTCTGAGCCAGTGCTCAAGGGAG
TCAAATTACATTACACATAA

>gi|292660221|gb|GQ153546.1| Bat SARS coronavirus HKU3-11, complete genome

ATGAAAATTTTAATTTTTGCTTTCTTAGCTAATTTAGCTAAAGCACAGGAAGGATGCGGTATCATCAGTAGAAAA
CCGCAACCTAAAATGGCACAAGTCTCATCTTCTCGTCGGGGCGTTTATTATAATGATGACATTTTCCGTTCTGATGTGT

TACACCTCACACAGGATTACTTCCTGCCATTTGACTCAAATTTAACTCAGTACTTTTCGCTTAATGTGGATTCAGATAG
GTATACCTATTTTGACAACCCCATACTAGACTTTGGTGATGGCGTTTATTTCGCTGCCACTGAAAAGTCTAACGTAATA
AGAGGCTGGATTTTTGGTTCATCTTTTGATAACACCACCCAGTCAGCTGTTATAGTTAATAATTCAACACACATTATTA
TACGTGTGTGTAATTTTAACTTATGTAAAGAACCCATGTATACTGTTAGTAGAGGTACACAGCAAAATGCCTGGGTTTA
TCAGAGTGCATTTAATTGTACATATGACCGAGTGGAAAAGAGTTTTCAACTTGACACTACTCCTAAAACTGGAAATTTT
AAAGACCTACGTGAGTATGTCTTTAAAAATAGGGATGGCTTTCTGTCTGTCTACCAAACTTATACTGCTGTTAATTTAC
CCAGAGGACTACCCACGGGTTTCTCAGTCTTGAAACCAATTTTAAAATTGCCCTTTGGAATTAATATCACTTCTTATAG
AGTAGTTATGGCAATGTTTAGCCAAACTACTTCTAATTTTTTACCAGAAAGTGCTGCTTATTATGTTGGTAATCTTAAA
TATTCTACCTTCATGCTCCGATTTAATGAAAATGGGACGATCACGGATGCTGTAGATTGTTCCCAAAATCCTCTTGCTG
AATTAAAATGCACCATTAAAAATTTCAATGTTGACAAAGGAATCTACCAAACATCCAACTTCAGAGTTTCACCCACTCA
AGAGGTTATTAGATTTCCTAACATTACAAATCGCTGTCCTTTTGACAAGGTTTTTAATGCTACTCGCTTTCCTAATGTT
TATGCATGGGAGAGAACAAAAATCTCTGATTGTGTTGCTGACTACACTGTTCTCTACAACTCAACCTCTTTCTCGACTT
TTAAATGTTATGGAGTGTCTCCATCTAAGTTGATTGACCTATGCTTTACAAGTGTGTATGCTGATACATTCTTGATAAG
ATCTTCTGAAGTAAGACAAGTTGCACCAGGTGAAACTGGTGTTATTGCTGACTACAATTACAAGTTGCCTGATGATTTC
ACTGGCTGTGTAATTGCTTGGAATACTGCTAAACATGATACTGGCAATTATTACTACAGATCTCATCGCAAGACTAAGT
TAAAGCCTTTTGAGAGAGACCTGTCTTCTGACGATGGTAATGGTGTGTATACACTCTCAACATATGACTTTAACCCTAA
CGTTCCAGTAGCATATCAGGCTACTAGGGTTGTTGTACTCTCTTTTGAACTTCTTAATGCACCTGCTACAGTTTGTGGA
CCTAAATTATCCACAGAACTAGTTAAGAACCAGTGTGTTAATTTCAATTTTAATGGACTTAAAGGTACTGGTGTTTTGA
CTTCTTCTTCAAAAAGATTCCAGTCATTTCAACAATTTGGTCGTGACACATCTGACTTTACGGATTCAGTACGTGACCC
ACAGACTTTAGAAATACTTGACATTTCACCTTGTTCCTTTGGTGGTGTTAGTGTTATCACACCTGGAACGAACGCCTCA
TCAGAGGTAGCTGTTCTCTATCAAGATGTAAATTGCACTGATGTCCCTACAGCAATACGTGCAGATCAATTAACACCTG
CTTGGCGCGTTTATTCCACTGGAGTAAATGTGTTTCAAACACAAGCTGGCTGTCTTATAGGAGCTGAGCATGTCAACGC
CTCTTATGAGTGTGACATTCCTATTGGTGCAGGCATTTGTGCTAGTTACCATACAGCTTCTGTTCTACGTAGTACCGGC
CAGAAATCAATTGTTGCCTATACTATGTCACTGGGTGCTGAAAATTCCATTGCATACGCTAATAATTCAATTGCCATAC
CTACAAATTTTTCAATCAGTGTCACTACAGAAGTGATGCCTGTTTCAATGGCTAAAACAGCCGTGGATTGTACTATGTA
CATCTGCGGTGATTCTTTAGAGTGCAGCAACTTACTATTGCAGTATGGAAGCTTTTGCACACAACTCAATCGTGCCCTC
ACTGGCATTGCTATAGAACAGGACAAAAACACTCAGGAGGTTTTTGCCCAGGTTAAACAAATGTACAAGACACCTGCCA
TAAAGGACTTTGGCGGTTTCAATTTTTCACAAATATTGCCTGACCCTTCAAAGCCAACGAAGAGATCATTTATTGAAGA
CTTGCTCTTCAATAAAGTGACTCTCGCTGATGCTGGCTTTATGAAACAATATGGTGACTGCCTAGGTGATGTTAGTGCT
AGAGACCTTATCTGTGCCCAGAAGTTCAATGGACTTACTGTGCTACCGCCACTGCTCACAGATGATATGGTTGCTGCAT
ACACAGCTGCGCTAGTTAGTGGTACTGCTACGGCGGGCTGGACGTTTGGTGCAGGTGCAGCTCTTCAAATACCATTTGC
TATGCAAATGGCTTATAGGTTTAATGGCATTGGAGTTACTCAAAATGTTCTCTATGAGAACCAAAAGCTGATAGCCAAT
CAGTTTAATAGTGCTATAGGCAAAATTCAAGAATCATTATCATCTACTGCAAGTGCACTAGGAAAACTGCAGGATGTGG
TTAACCAAAATGCACAAGCTCTTAACACGCTTGTTAAACAACTCAGCTCTAATTTTGGAGCTATCTCAAGTGTGTTAAA
TGATATTCTCTCTCGCCTTGATAAAGTTGAGGCAGAAGTTCAAATTGACAGGTTGATTACAGGCAGATTGCAAAGCCTT
CAAACCTACGTAACACAACAACTTATCAGAGCTGCTGAAATCAGAGCTTCTGCTAATCTTGCTGCTACTAAAATGTCTG
AGTGCGTTCTTGGACAATCAAAAAGAGTTGATTTCTGTGGAAAAGGCTACCACCTTATGTCCTTCCCTCAATCAGCACC
TCACGGTGTCGTTTTTCTACATGTCACATATGTTCCATCACAAGAGAAAAACTTCACAACAGCTCCAGCTATTTGTCAC
GAAGGCAAAGCTTATTTCCCTCGTGAAGGTGTCTTTGTGTCTAATGGCACTTCTTGGTTTATTACGCAGAGGAATTTTT
ACTCTCCACAATTAATTACAACAGATAATACTTTTGTTTCTGGTAATTGTGATGTCGTGATTGGCATCATTAATAATAC
TGTTTATGATCCTCTGCAGCCTGAACTTGACTCGTTTAAGGAAGAGCTGGACAAGTACTTCAAAAATCATACATCACCA
GATGTTGATCTTGGCGACATTTCAGGCATTAATGCTTCAGTCGTCAACATTCAAAAGGAGATTGACCGCCTCAATGAGG
TTGCCAAAAACCTAAATGAATCACTCATTGACCTCCAAGAACTTGGGAAATATGAGCAATACATCAAGTGGCCTTGGTA
TGTTTGGCTCGGCTTTATTGCTGGACTAATTGCCATCGTCATGGTTACAATCTTGCTTTGTTGCATGACCAGCTGTTGC

AGTTGTCTCAAGGGTGCATGCTCTTGTGGTTCGTGCTGCAAATTTGATGAGGACGACTCTGAGCCAGTGCTCAAAGGAG
TCAAATTACATTACACATAA

>gi|292660233|gb|GQ153547.1| Bat SARS coronavirus HKU3-12, complete genome

ATGAAAATTTTAATTTTTGCTTTCTTAGCTAATTTAGCTAAAGCACAGGAAGGATGCGGTATCATCAGCAGAAAA
CCGCAACCTAAAATGGCACAAGTCTCATCTTCTCGTCGGGGCGTTTATTATAATGATGACATTTTCCGTTCTGATGTGT
TACACCTCACACAGGATTACTTCCTGCCATTTGACTCAAATTTAACTCAGTACTTTTCGCTTAATGTGGATTCAGATAG
GTATACCTATTTTGACAACCCCATACTAGACTTTGGTGATGGCGTTTATTTCGCTGCCACTGAAAAGTCTAACGTAATA
AGAGGCTGGATTTTTGGTTCATCTTTTGATAACACCACCCAGTCAGCTGTTATAGTTAATAATTCAACACACATTATTA
TACGTGTGTGTAATTTTAACTTATGTAAAGAACCCATGTATACTGTTAGTAGAGGTACACAGCAAAATGCCTGGGTTTA
TCAGAGTGCATTTAATTGTACATATGACCGAGTGGAAAAGAGTTTTCAACTTGACACTACTCCTAAAACTGGAAATTTT
AAAGACCTACGTGAGTATGTCTTTAAAAATAGGGATGGCTTTCTGTCTGTCTACCAAACTTATACTGCTGTTAATTTAC
CCAGAGGACTACCCACGGGTTTCTCAGTCTTGAAACCAATTTTAAAATTGCCCTTTGGAATTAATATCACTTCTTATAG
AGTAGTTATGGCAATGTTTAGCCAAACTACTTCTAATTTTTTACCAGAAAGTGCTGCTTATTATGTTGGTAATCTTAAA
TATTCTACCTTCATGCTCCGATTTAATGAAAATGGGACGATCACGGATGCTGTAGATTGTTCCCAAAATCCTCTTGCTG
AATTAAAATGCACCATTAAAAATTTCAATGTTGACAAAGGAATCTACCAAACATCCAACTTCAGAGTTTCACCCACTCA
AGAGGTTATTAGATTTCCTAACATTACAAATCGCTGTCCTTTTGACAAGGTTTTTAATGCTACTCGCTTTCCTAATGTT
TATGCATGGGAGAGAACAAAAATTTCTGATTGTGTTGCTGACTACACTGTTCTCTACAACTCAACCTCTTTCTCGACTT
TTAAATGTTATGGAGTGTCTCCATCTAAGTTGATTGACCTATGCTTTACAAGTGTGTATGCTGATACATTCTTGATAAG
ATCTTCTGAAGTAAGACAAGTTGCACCAGGTGAAACTGGTGTTATTGCTGACTACAATTACAAGTTGCCTGATGATTTC
ACTGGCTGTGTAATTGCTTGGAATACTGCTAAACATGATACTGGCAATTATTACTACAGATCTCATCGCAAGACTAAGT
TAAAGCCTTTTGAGAGAGACCTGTCTTCTGACGATGGTAATGGTGTGTATACACTCTCAACATATGACTTTAACCCTAA
CGTTCCAGTAGCATATCAGGCTACTAGGGTTGTTGTACTCTCTTTTGAACTTCTTAATGCACCTGCTACAGTTTGTGGA
CCTAAATTATCCACAGAACTAGTTAAGAACCAGTGTGTTAATTTCAATTTTAATGGACTTAAAGGTACTGGTGTTTTGA
CTTCTTCTTCAAAAAGATTCCAGTCATTTCAACAATTTGGTCGTGACACATCTGACTTTACGGATTCAGTACGTGACCC
ACAGACTTTAGAAATACTTGACATTTCACCTTGTTCCTTTGGTGGTGTTAGTGTTATCACACCTGGAACGAACGCCTCA
TCAGAGGTAGCTGTTCTCTATCAAGATGTAAATTGCACTGATGTCCCTACAGCAATACGTGCAGATCAATTAACACCTG
CTTGGCGCGTTTATTCCACTGGAGTAAATGTGTTTCAAACACAAGCTGGCTGTCTTATAGGAGCTGAGCATGTCAACGC
CTCTTATGAGTGTGACATTCCTATTGGTGCAGGCATTTGTGCTAGTTACCATACAGCTTCTGTTCTACGTAGTACCGGC
CAGAAATCAATTGTTGCCTATACTATGTCACTGGGTGCTGAAAATTCCATTGCATACGCTAATAATTCAATTGCCATAC
CTACAAATTTTTCAATCAGTGTCACTACAGAAGTGATGCCTGTTTCAATGGCTAAAACATCCGTGGATTGTACTATGTA
CATCTGCGGTGATTCTTTAGAGTGCAGCAACTTACTATTGCAGTATGGAAGCTTCTGCACACAACTCAATCGTGCCCTC
ACTGGCATTGCTATAGAACAGGACAAAAACACTCAGGAAGTCTTTGCCCAGGTTAAACAAATGTACAAGACACCTGCCA
TAAAGGACTTTGGCGGTTTCAATTTTTCACAAATATTGCCTGACCCTTCAAAGCCAACGAAGAGATCATTTATTGAAGA
CCTGCTCTTCAATAAAGTGACTCTCGCGGATGCTGGCTTTATGAAACAATATGGTGAATGCCTAGGTGATGTTAGTGCT
AGAGACCTTATCTGTGCCCAGAAGTTCAATGGACTTACTGTGCTACCACCACTGCTTACAGATGAGATGATTGCTGCAT
ACACAGCTGCGCTAGTTAGTGGTACTGCTACGGCAGGCTGGACGTTTGGTGCAGGTGCAGCTCTTCAAATACCATTTGC
TATGCAAATGGCTTATAGGTTCAATGGCATTGGAGTTACTCAAAACGTTCTCTATGAGAATCAAAAGCTGATAGCCAAT
CAGTTTAATAGTGCTATAGGCAAAATTCAAGAATCATTATCATCTACTGCAAGTGCACTAGGAAAATTGCAGGATGTGG
TTAACCAAAATGCACAAGCTCTTAACACGCTTGTTAAACAACTTAGCTCTAATTTTGGAGCTATCTCAAGTGTGTTAAA
TGACATTCTCTCTCGCCTTGATAAAGTTGAGGCAGAAGTTCAAATTGATAGGTTGATTACAGGCAGATTGCAAAGCCTT
CAAACCTATGTAACACAACAACTTATCAGAGCTGCTGAAATCAGAGCTTCTGCTAATCTTGCTGCTACTAAAATGTCTG

AGTGCGTTCTTGGACAATCAAAAAGAGTTGATTTCTGTGGAAAAGGCTACCACCTTATGTCCTTCCCTCAATCAGCACC
TCATGGTGTCGTTTTTCTACATGTCACATATGTGCCATCACAAGAGAAAAACTTCACAACAGCCCCAGCTATTTGTCAC
GAAGGCAAAGCTTATTTCCCTCGTGAAGGTGTCTTTGTGTCTAATGGCACTTCTTGGTTTATTACGCAGAGGAATTTTT
ACTCTCCACAATTAATTACAACAGATAACACTTTTGTTTCTGGTAATTGTGATGTCGTAATCGGCATCATTAATAATAC
TGTTTATGACCCTCTGCAACCTGAGCTTGACTCGTTTAAGGAAGAGCTGGACAAGTACTTCAAAAATCATACATCACCA
GATGTTGATCTTGGCGACATTTCAGGCATTAATGCTTCAGTCGTCAACATTCAAAAGGAGATTGACCGCCTCAATGAGG
TTGCCAAAAACCTAAATGAATCACTCATTGACCTCCAAGAACTTGGGAAATATGAGCAATACATCAAGTGGCCTTGGTA
TGTTTGGCTCGGCTTTATTGCTGGACTAATTGCCATCGTCATGGTTACAATCTTGCTTTGTTGCATGACCAGCTGTTGC
AGTTGTCTCAAGGGTGCATGCTCTTGTGGTTCGTGCTGCAAATTTGATGAGGACGACTCTGAGCCAGTGCTCAAAGGAG
TCAAATTACATTACACATAA

>gi|292660245|gb|GQ153548.1| Bat SARS coronavirus HKU3-13, complete genome

ATGAAAATTTTAATTTTTGCTTTCTTAGCTAATTTAGCTAAAGCACAGGAAGGATGCGGTATCATCAGTAGAAAA
CCGCAACCTAAAATGGCACAAGTCTCATCTTCTCGTCGGGGCGTTTATTATAATGATGACATTTTCCGTTCTGATGTGT
TACACCTCACACAGGATTACTTCCTGCCATTTGACTCAAATTTAACTCAGTACTTTTCGCTTAATGTGGATTCAGATAG
GTATACCTATTTTGACAACCCCATACTAGACTTTGGTGATGGCGTTTATTTCGCTGCCACTGAAAAGTCTAACGTAATA
AGAGGCTGGATTTTTGGTTCATCTTTTGATAACACCACCCAGTCAGCTGTTATAGTTAATAATTCAACACACATTATTA
TACGTGTGTGTAATTTTAACTTATGTAAAGAACCCATGTATACTGTTAGTAGAGGTACACAGCAAAATGCCTGGGTTTA
TCAGAGTGCATTTAATTGTACATATGACCGAGTGGAAAAGAGTTTTCAACTTGACACTACTCCTAAAACTGGAAATTTT
AAAGACCTACGTGAGTATGTCTTTAAAAATAGGGATGGCTTTCTGTCTGTCTACCGAACTTATACTGCTGTTAATTTAC
CCAGAGGACTACCCACGGGTTTCTCAGTCTTGAAACCAATTTTAAAATTGCCCTTTGGAATTAATATCACTTCTTATAG
AGTAGTTATGGCAATGTTTAGCCAAACTACTTCTAATTTTTTACCAGAAAGTGCTGCTTATTATGTTGGTAATCTTAAA
TATTCTACCTTCATGCTCCGATTTAATGAAAATGGGACGATCACGGATGCTGTAGATTGTTCCCAAAATCCTCTTGCTG
AATTAAAATGCACCATTAAAAATTTCAATGTTGATAAAGGAATCTACCAAACATCCAACTTCAGAGTTTCACCCACTCA
AGAGGTTATTAGATTTCCTAACATTACAAATCGCTGTCCTTTTGACAAGGTTTTTAATGCTACTCGCTTTCCTAATGTT
TATGCATGGGAGAGAACAAAAATCTCTGATTGTGTTGCTGACTACACTGTTCTCTACAACTCAACCTCTTTCTCGACTT
TTAAATGTTATGGAGTGTCTCCATCTAAGTTGATTGACCTATGCTTTACAAGTGTGTATGCTGATACATTCTTGATAAG
ATCTTCTGAAGTAAGACAAGTTGCACCAGGTGAAACTGGTGTTATTGCTGACTACAATTACAAGTTGCCTGATGATTTC
ACTGGCTGTGTAATTGCTTGGAATACTGCTAAACATGATACTGGCAATTATTACTACAGATCTCATCGCAAGACTAAGT
TAAAGCCTTTTGAGAGAGACCTGTCTTCTGATGATGGTAATGGTGTGTATACACTCTCAACATATGACTTTAACCCTAA
CGTTCCAGTAGCATATCAGGCTACTAGGGTTGTTGTACTCTCTTTTGAACTTCTTAATGCACCTGCTACAGTTTGTGGA
CCTAAATTATCCACAGAACTAGTTAAGAACCAGTGTGTTAATTTCAATTTTAATGGACTTAAAGGTACTGGTGTTTTGA
CTTCTTCTTCAAAAAGATTCCAGTCATTTCAACAATTTGGTCGTGACACATCTGACTTTACGGATTCAGTACGTGACCC
ACAGACTTTAGAAATACTTGACATTTCACCTTGTTCCTTTGGTGGTGTTAGTGTTATCACACCTGGAACGAACGCCTCA
TCAGAGGTAGCTGTTCTCTATCAAGATGTAAATTGCACTGATGTCCCTACAGCAATACGTGCAGATCAATTAACACCTG
CTTGGCGCGTTTATTCCACTGGAGTAAATGTGTTTCAAACACAAGCTGGCTGTCTTATAGGAGCTGAGCATGTCAACGC
CTCTTATGAGTGTGACATTCCTATTGGTGCAGGCATTTGTGCTAGTTACCATACAGCTTCTGTTCTACGTAGTACCGGC
CAGAAATCAATTGTTGCCTATACTATGTCACTGGGTGCTGAAAATTCCATTGCATACGCTAATAATTCAATTGCCATAC
CTACAAATTTTTCAATCAGTGTCACTACAGAAGTGATGCCTGTTTCAATGGCTAAAACAGCCGTGGATTGTACTATGTA
CATCTGCGGTGATTCTTTAGAGTGCAGCAACTTACTATTGCAGTATGGAAGCTTTTGCACACAACTCAATCGTGCCCTC
ACTGGCATTGCTATAGAACAGGACAAAAACACTCAGGAGGTTTTTGCCCAGGTTAAACAAATGTACAAGACACCTGCCA
TAAAGGACTTTGGCGGTTTCAATTTTTCACAAATATTGCCTGACCCTTCAAAGCCAACGAAGAGATCATTTATTGAAGA

CTTGCTCTTCAATAAAGTGACTCTCGCTGATGCTGGCTTTATGAAACAATATGGTGACTGCCTAGGTGATGTTAGTGCT
AGAGACCTTATCTGTGCCCAGAAGTTCAATGGACTTACTGTGCTACCGCCACTGCTCACAGATGATATGGTTGCTGCAT
ACACAGCTGCGCTAGTTAGTGGTACTGCTACGGCGGGCTGGACGTTTGGTGCAGGTGCAGCTCTTCAAATACCATTTGC
TATGCAAATGGCTTATAGGTTTAATGGCATTGGAGTTACTCAAAATGTTCTCTATGAGAACCAAAAGCTGATAGCCAAT
CAGTTTAATAGTGCTATAGGCAAAATTCAAGAATCATTATCATCTACTGCAAGTGCACTAGGAAAACTGCAGGATGTGG
TTAACCAAAATGCACAAGCTCTTAACACGCTTGTTAAACAACTCAGCTCTAATTTTGGAGCTATCTCAAGTGTGTTAAA
TGATATTCTCTCTCGCCTTGATAAAGTTGAGGCAGAAGTTCAAATTGACAGGTTGATTACAGGCAGATTGCAAAGCCTT
CAAACCTACGTAACACAACAACTTATCAGAGCTGCTGAAATCAGAGCTTCTGCTAATCTTGCTGCTACTAAAATGTCTG
AGTGCGTTCTTGGACAATCAAAAAGAGTTGATTTCTGTGGAAAAGGCTACCACCTTATGTCCTTCCCTCAATCAGCACC
TCACGGTGTCGTTTTTCTACATGTCACATATGTTCCATCACAAGAGAAAAACTTCACAACAGCTCCAGCTATTTGTCAC
GAAGGCAAAGCTTATTTCCCTCGTGAAGGTGTCTTTGTGTCTAATGGCACTTCTTGGTTTATTACGCAGAGGAATTTTT
ACTCTCCACAATTAATTACAACAGATAATACTTTTGTTTCTGGTAATTGTGATGTCGTGATTGGCATCATTAATAATAC
TGTTTATGATCCTCTGCAGCCTGAACTTGACTCGTTTAAGGAAGAGCTGGACAAGTACTTCAAAAATCATACATCACCA
GATGTTGATCTTGGCGACATTTCAGGCATTAATGCTTCAGTCGTCAACATTCAAAAGGAGATTGACCGCCTCAATGAGG
TTGCCAAAAACCTAAATGAATCACTCATTGACCTCCAAGAACTTGGGAAATATGAGCAATACATCAAGTGGCCTTGGTA
TGTTTGGCTCGGCTTTATTGCTGGACTAATTGCCATCGTCATGGTTACAATCTTGCTTTGTTGCATGACCAGCTGTTGC
AGTTGTCTCAAGGGTGCATGCTCTTGTGGTTCGTGCTGCAAATTTGATGAGGACGACTCTGAGCCAGTGCTCAAAGGAG
TCAAATTACATTACACATAA

>gi|304435156|gb|HM211098.1| Bat coronavirus HKU9-5-1, complete genome

ATGTTGGTGATATTGCTAATATTGCCTTTAGTACATGCTAGACCCAGTTGTCTCACTGTGTCACCTTTACAGTAT
TTAGAACATAATATTTCTTATGTTTCCTTGTCTGTTAGTAAAGTGACACGTTTAGTGCCGGATCCTTATGTAGCGTATT
CCGGGCAGACTATACGTCAAAGTTTGTATTATGCGGACACTTCTAATGTTACTGTGTATCCCGTAACGCCGTATGCGGC
GCCTGACAAGACGAGCATATATAATACCTCCTTTGTGATGGTAAATGACGGTTTGTTTGTGCATACGTATATGTATTTG
AATCAACCGTCTAATATGTACTGTCAAGACCCGTTTGGTGTGGCCTTTGGGACCACATTTGAACAGGATCATATAGCTA
TTGTGGTGATAGCACCTGACAATAGAGGTAGCTGGACGGCTGCGTCAAAGCGGAATGTAACTACAGTTCATATATTAGT
CTGTAGTAACGCAACCCTTTGTGCGTACCCTGCATTTAATCGTTGGGGTCCTGCGAGTAGCATATACGCAAGTGATGCT
TTTGTAGACCATGGTAATTCCTGTTTTGTAAACAACAGTTTTGATATACCTATTAATACGTCTCGTATTAATTTGGCTT
TTAGGTTCTTGGATGGTAACCTATTGCTGTACCATTCTAGGTGGCTTCCAGGTTCTGGGCTTAATTTAGCCACAGATTA
CCCCCTACACTTCTTTATGAGTGTGGGTGTTGGCGCTAATTTGCCTAATGCACAGTTTTTCCAATCCGTAGTCCGCTAT
AACCGGGGTGTTGATGAAGCGCGATGTCATACTTTTCAGAATAATTTGTATATAGCACCTTTGAGTTTACGTGAAGTTT
TAGTGTCTTACAGTGATAGTGGCTTGCCACTTAAGGTGGCTGATTGTGCGGCCGATGCAGGTGATGAGCTATTTTGTGT
TACTGGCTCATTTGAACCTGCCATTGGTGTGTACCCTCTTAGTAGGTACCGTGCTCAGGTTAGTGATTATGTGCACATT
ACACAGCAATCACAGGCCTGCAAACTGCCTTACGCTGACATTGTATCACCACCTCAGCCTATTGTCTGGCGTCGATATA
CAGTGTCTAGTTGCTCATTTGACTTTGAGAGCATAGTTAATAATTTACCTACCTATGATCTCAAGTGCTATGGTGTATC
ACCCGCGCGGCTTGCACAAATGTGCTATGCGGGTGTTACTCTGGACGTTATGCGCATAAACAAGACGCATTATAGCAAT
TTAATTGGTAATGTGCCTGATTTATTTACAAAGTATAATTATGCCCTGCCCACCAATTTTTATGGTTGTGTTCACGCAT
ATTATATTAATGTTACTGATGGCCGCTATGCTTTAGCGACTCATTACCCGGCCACTGTCATTACACCAGGAGGTAGACA
GCCTTATAATAGCTATGTGGCTACTGTTTTGAATACAGTTAATAACTGGTGTACCAGAGCTAATTGTTTTGGTCTTGTA
GTTATAGGATTGAAGCCTGCATCAGGTAGGCAGTTAGTCTGCCCTAAGGCTAATGATACTGAAGTTATAGTGCAGCAAT
GTGTTAAGTATAATTTGTACGGTTACACAGGTACCGGCGTTTTAAGCCCGTCTAATTTAACCATACCAGATGGTAAGTT
GTTTGTGGCTAATAACGCAGGTAATATTGTTGCTGTTAATGCTTCTGGCACCGTATATGGCATTTCATCTTGCGTGTCA

GTTCCTGTCTCAGTAGGATATAACCCCTCATTTGAGCAGGCGCTACTATTTAATGGTTTAGCGTGTTCAGAGAGAGCAG
TTGCTGTTAATATGCCGGCGTCAACTTATTGGCTATCGGTGTCTAGAAACGCAGCCCCACCTGATGGTGGTGTGTTTGA
CACACCTTCTGGTTGTGTTTATAATGTACGCAATTTAACTACCATGGTAGTTAATTCGTGTGATATGCCTATTGGCAAC
AGCCTTTGTTTAACTAATGTAACTTATGCTAATAGGGCTACAGATAGTTTACCTAGGTTGAGTTTAGTCACTTATGATC
CAACGGCTGCTAATGCTGTCCAGTTGCTCACACCAGTTTACTGGGTTAGTATACCCACTAATTTTACTCTGGCTGCTAC
TTCTGAGTATATACAAACTAGCTCACCAAAGATCAATGTAGATTGCGCGAAGTATTTATGTGGCGACTCTGATAGGTGT
ATGAATGTGTTGCTGCAGTATGGCACATTTTGTGATGACGTTAATAAGGCGCTGGCCGAGGTCAGTGCTACAATTGACG
CATCCGTTGTGTCATTGATGTCAGAACTCACATCGGACGTTGTACGTAATGAAAACATGCAGTTTGACACTACATACAA
CTTTACCTCACTTATGGGTTGTTTGGGATCAGATTGTGATTCTAAAATGTACAGGTCCGCTTTATCGGACTTGTTGTAT
AATAAGGTCAAGGTCACGGACCCTGGCTTTATGCAGTCCTATCAGAAGTGCATTTCTCAGTGGGGTGGTGAAATTAGAG
ACCTTATGTGTACTCAAACCTTTAATGGTATTGCTGTTTTACCACCTATTGTTTCTCCAGGCATGCAAGCCTTGTACAC
ATCATTACTTGTGGGTGCGGTAGCATCTGCAGGTTATACATTTGGCATTACTTCTGTCGGGGTTGTGCCTTTTGCGACG
CAATTGCAATTTAGATTAAATGGCATAGGCGTCACTACGCAGGTGCTTGTAGAGAATCAGAAACTTATTGCTAATTCTT
TTAACAATGCCCTTACTAATATACAGAAGGGTTTTGATGCCACTAATGAGGCGCTTAGTAAGATGCAGCTTGTTATTAA
CCAGCATGCTCAGCAATTGCAGACTTTGGTGAACCAGTTAGGTAATTCATTTGGTGCTATATCTGCTTCTATTAATGAA
ATATTTAGTCGTTTAGATGGTTTAGAGGCTGATGCTCAAGTGGATAGGCTTATTAATGGACGCACTGTTGTGCTTAATA
CTTATGTAACTCAGTTGCTTATTAAAGCTACAGAAGTGCGTAGTCAGGCCCTGTTAGCTAAGCAGAAAATTAGTGAGTG
CGTTAAAGCACAATCTCTTCGTAATGATTTCTGTGGCAATGGCATACATGTTTTGTCTATACCACAGTTAGCTCCTAAT
GGTATTTTGTTTTTGCATTATACCTATAAACCGACGGCTTTTGCTGTTGTGCAAACTGCTGCTGGTCTGTGTTATAATG
GCACAGGGTATGCACCTGTAGGTGGCATGTTTGTGTTACCTAATGGTACGCAGGCCTGGCACTTTACTAAAATGAATTT
TTATAATCCTGTTAGTATTACAGTTGAGAACACGCAGGTTTTATCTACATGTGGCTTGAATTATTCACATGTTAACTAC
ACTGTGGTGAACCCGTCGGTACCTAGTGATTTTGATTTTGAGGAAGAGTTTAATAAGTGGTATAAAAACCATAGCTCTA
TATTTAATAATACGTTCGACCCTAGTGCTTTTAATTTTTCTATGGTCGATGTAAATAAGCAACTGGCTACTCTTACAGA
TGTTGTTCAGCAGCTAAACAAGTCGTATATTGATTTGAAGCAATTAAATGTTTATGAACAGACCATTAAATGGCCGTGG
TATATATGGCTCGCTATGATAGCGGGCCTTGTTGGCTTAGCCCTTGCAGTGGTCATGCTTTTATGTATGACTAACTGTT
GTTCATGCTTCAAAGGCATGTGTTCATGCAAGCAGTGCCATTATGATGAGGTAGATGATGTATATCCTGCTGTTCGTGT
ATATAATAAACGAACAGCATGA

>gi|304435165|gb|HM211099.1| Bat coronavirus HKU9-5-2, complete genome

ATGTTGGTGATATTGTTAATGTTACCACTAGTTTTTGCTAGACCATCTTGTGTTACTATTAGTAACTTACAGTAT
GTTGATCATAACTACACTCACAGATCTTTTGATATGAGTGCAGTTTCACGTGTATTTCCTGATCCCTTTGTAGCCTATT
CTGGTCAGACACTTAAGCAGTCCCTTTACATAGCAGATACGTCTAATACCACGGTTTATCCAGTAACACCACCTGCTGT
TAATGGAAAACCAGGTATTTATAATTTGACAATATTGCCTGTCGGCGATGGTTTCTTAGTTCAAACTTATATGTACAAG
GACCAACCCAGCGACACTTATTGTCAGGAGCCATTTGGCGTTGCGTTTGGTACGACATTTGAGTACGACCGTATCGCTA
TTGTTACTATAGTACCAGGTTATAAAGGTTCTTGGTCTGCAGTTCAGAAACAGACTACTACTAATGTCAATATTTTGGT
CTGTAGTAATGCAACTTTGTGTGCATATCCTGCATTTAACCGATGGGGTCCCGCAGGTACTATTTATACTACTAATGAT
TTTGTTGTATATGGCGATTCTTGCTTTATAAACAACACTTTCACAATATCTATTAATACTTCTAGGTTAAACCTGGGGT
TTAGGTTTAATGATGGGAACTTGTACTTGTACCATTCTAAATGGTTGCCTATGCAAGGGCTTAATTTGATGGTGGATTA
CCCTCTGCACTTTCTTATGAGTGTAGGGGTGGGTGCAAATCTACCAAATATGCAGTTTTATCAGGCTGTTGTGCGTCAA
GGTACTCCTCCAGTTACTGGCGCTGATAAAAATGATGCCAATTGTTTGGCTTTTCAGAATAATTTGTATCTAGCATATA
TAAGTAAGCGGGATTTGCTTGTGTCTTATGATGATAATGGACTTCCCATTGCTGTAGCAGATTGTTCCCAGAACGCTGG
GGATGAATTGTACTGTGTCACAGGCACTTTTAGTCCGCAGGTTGGTGTTTACCCATTAAGTAGGTATAGAGCTTTAGTT

AGTGATTATGTTCAAATATCGCAGCAAGGTAGCATTTGTAGCCTGCCTTATTCCGATATTTTGAAACCACCGCAACCTA
TAGTGTGGAAGCGTCACACAGTTACAAATTGTTCATTTGACTTTGACGCTATTGTTAATAGACTCCCTACTTTTCAGCT
TAAATGCTTTGGGGTGTCACCTGCTAAGTTGGCCCAAATGTGCTATTCCAGTGTTACACTGGACATTTTTAGAGCCAAT
ACAACTCACCTAGCTAATATGTTAGGTAAGGTTCCTGATGTTTTCAGTAAGTATAATTATGCACTTCCACCCGATTTTT
ACGGGTGTGTGCATTCGTATTATATTAATGATAGCAGCCGCATGTATGCCATAGCGCAGCAATGGCCTGCTACCGTTAT
AGCACCAGGTGGCCGCCAACCTTACAATAGTTATGTTGGCACCGTATTAAATACACCAAACCCTACGTGCACCCAGCTA
ACTTGCTTTGGTGTGGTAGTTATTTCACTTAAGCCAGCCTCCGGTAAACATCTAGTTTGTCCTAGTGCCAATGACACGG
ATTTGGTGACGCGCGAATGTGTTAAGTATAATCTTTATGGTTATACTGGTACTGGTGTATTCAATGAGAGCTCATTGGT
TATACCAGATTCTAAATTATTCGTGGCTAGTTCCACGGGTGATATTATTGCTGCTAAAGTTAGAGGCAAGGTTTATTCT
ATAACACCTTGTGTTTCGGTGCCTATATCCGTAGGTTATGACCCTTCTTTTGAGAGAGCACTACTATTTAATGGTTTAT
CTTGTAGTGAACGCTCCGTAGCTGTCAGCTTACCAGCCTCTGATTACTGGTTAGCTGCTGTTGCAGATAATGCTAGTAC
TGGTGTAGTTACATTTGACACGCTTTCGGGTTGCGTGCATAATGTTCGTAATGCCACTGATATAACTGTGCCGACATGT
TTAATGCCTTTAGGTAACAGTTTATGTTTAGCTAATATTACACAACCCTCGCAGTATGGTTTAAGTACTACTAGCAGTG
TAAACCTTTTGAGTTTAGTTACTTATGACCCCACCTTTCAGGGTGGTGTTAAAGTTATGTCACCTGTTTACTGGATTAG
CATCCCAACAAATTTTACATTGGGTGCTATTACTGAATACATTCAAACTACGTCACCTAAAGTTAATGTGGACTGCGTC
AAATATTTGTGTGGAGATTCTGAACGTTGCACTACTGTGTTGCTGCAGTATGGCACATTTTGTGATGATGTCAATAAGG
CGTTGTCAGAAGTAAGTTCTATAATTGATGCTAGCATGATATCGCTAGTTTCAGAGATTACAGCTGATGTAGTTCGTAG
TGAGAATGCTTTATTCGACACTACCTATAACTTCACTGGTTTGTTGGGTTGTGTAGGTTCTAATTGTAATGACCGCACC
ACTTATCGGTCAGCACTTTCTGACTTGTTATATAACAAGGTAAAAGTCACTGACCCAGGTTTTATGAGCTCTTACCAAA
AGTGTATATCTCAGTGGGGTGGTGATATCAGGGATTTGTTCTGTACTCAGAACTTTAATGGTATCTCTGTTTTGCCCCC
CATAGTGTCACCAGGCATGCAAGCGCTGTACACTTCCTTATTAGTGGGTGCAGTTGCGTCTGCTGGATACACATTTGGT
GTAACTTCAGTAGGCGTTATTCCTTTTGCCACTCAGCTACAATTTAGACTTAATGGTCTAGGTGTTACCACACAGGTGC
TGGTGGAGAACCAGCAGCTTATAGCTAACTCTTTTAATAAAGCGCTTGTTAGTATACAACAAGGCTTTGATGCTACGAA
CGAGGCACTTAGTAAGATGCAGTCCGTTATTAATCAACATGCACAGCAGCTGCAGACATTAGTTTCGCAGTTGGGAAAT
TCATTTGGTGCTATTTCGTCTTCTATTAATGAGATTTTCAGTCGGTTAGATGGTTTAGAAGCCAACGCGCAGGTAGACA
GACTCATTAATGGGCGCATGGTTGTACTTAATACCTATGTGACACAGCTTCTCATTAAAGCTTCTGAAGTTAAGTCGCA
GGCTCTACTCGCAAAGCAGAAAATTAGCGAGTGTGTTAAATCGCAGTCTTTGCGTAATGACTTCTGTGGTAATGGTACT
CATGTGTTTTCCGTACCGCAGTTAGCTCCTAATGGTATAATGTTTTTGCATTATACATACAAGCCTACTTCATATGCAT
TGGTACAAACAGCTGCAGGTCTTTGTTTAAATAACACTGGCTATGCACCACGTGATGGTTTGTTTGTTTTGCCCAATGG
TTCTATATACTGGCAGTTTACTAAAATGAATTTTTATAATCCTGTCAGACTTACTAATAGTAACACTCAGGTGCTTACT
ACGTGTAGTGTTAACTATACTACAGTTAATTACACTGTGTTGCCTCCTACGGACAACATGGATTTTAACTTTACTGCAG
AGTTTGAGAAGTGGTATAAAAATCACAGTTCTCAATTTAATAACACTTTCAACCCTGGGGATTTTAATTTTTCCACGGT
TGATATACAGAATGAACTTAACACCCTTAATGCTGTTGTTAAGCAGCTGAATGAGTCTTTTATTGACCTGAAGAAGTTA
AATGTTTATGAACAGACCATTAAATGGCCGTGGTATATATGGCTTGCTATGATAGCTGGCTTAGTTGGTTTAGCTCTAG
CAGTGGTTATGCTATTATGTATGACTAATTGTTGTTCATGCTTTAAAGGTATGTGTGCATGTAAACCTTGCCATTATGA
CGAGGTCGAAGATGTTTATCCTGCAGTTCGTGTATATAATAAACGAACAGCATGA

>gi|304435174|gb|HM211100.1| Bat coronavirus HKU9-10-1, complete genome

ATGTTGGTGATATTGCTAATATTGCCTTTAGTACATGCTAGACCCAGTTGTCTCACTGTGTCACCTTTACAGTAT
TTAGAACATAATATTTCTTATGTTTCCTTGTCTGTTAGTAAAGTGACACGTTTAGTGCCGGATCCTTATGTAGCGTATT
CCGGGCAGACTATACGTCAAAGTTTGTATTATGCGGACACTTCTAATGTTACTGTGTATCCCGTAACGCCGTATGCGGC
GCCTGACAAGACGAGCATATATAATACCTCCTTTGTGATGGTAAATGACGGTTTGTTTGTGCATACGTATATGTATTTG

AATCAACCGTCTAATATGTACTGTCAAGACCCGTTTGGTGTGGCCTTTGGGACCACATTTGAACAGGATCATATAGCTA
TTGTGGTGATAGCACCTGACAATAGAGGTAGCTGGACGGCTGCGTCAAAGCGGAATGTAACTACAGTTCATATATTAGT
CTGTAGTAACGCAACCCTTTGTGCGTACCCTGCATTTAATCGTTGGGGTCCTGCGAGTAGCATATACGCAAGTGATGCT
TTTGTAGACCATGGTAATTCCTGTTTTGTAAACAACAGTTTTGATATACCTATTAATACGTCTCGTATTAATTTGGCTT
TTAGGTTCTTGGATGGTAACCTATTGCTGTACCATTCTAGGTGGCTTCCAGGTTCTGGGCTTAATTTAGCCACAGATTA
CCCCCTACACTTCTTTATGAGTGTGGGTGTTGGCGCTAATTTGCCTAATGCACAGTTTTTCCAATCCGTAGTCCGCTAT
AACCGGGGTGTTGATGAAGCGCGATGTCATACTTTTCAGAATAATTTGTATATAGCACCTTTGAGTTTACGTGAAGTTT
TAGTGTCTTACAGTGATAGTGGCTTGCCACTTAAGGTGGCTGATTGTGCGGCCGATGCAGGTGATGAGCTATTTTGTGT
TACTGGCTCATTTGAACCTGCCATTGGTGTGTACCCTCTTAGTAGGTACCGTGCTCAGGTTAGTGATTATGTGCACATT
ACACAGCAATCACAGGCCTGCAAACTGCCTTACGCTGACATTGTATCACCACCTCAGCCTATTGTCTGGCGTCGATATA
CAGTGTCTAGTTGCTCATTTGACTTTGAGAGCATAGTTAATAATTTACCTACCTATGATCTCAAGTGCTATGGTGTATC
ACCCGCGCGGCTTGCACAAATGTGCTATGCGGGTGTTACTCTGGACGTTATGCGCATAAACAAGACGCATTATAGCAAT
TTAATTGGTAATGTGCCTGATTTATTTACAAAGTATAATTATGCCCTGCCCACCAATTTTTATGGTTGTGTTCACGCAT
ATTATATTAATGTTACTGATGGCCGCTATGCTTTAGCGACTCATTACCCGGCCACTGTCATTACACCAGGAGGTAGACA
GCCTTATAATAGCTATGTGGCTACTGTTTTGAATACAGTTAATAACTGGTGTACCAGAGCTAATTGTTTTGGTCTTGTA
GTTATAGGATTGAAGCCTGCATCAGGTAGGCAGTTAGTCTGCCCTAAGGCTAATGATACTGAAGTTATAGTGCAGCAAT
GTGTTAAGTATAATTTGTACGGTTACACAGGTACCGGCGTTTTAAGCCCGTCTAATTTAACCATACCAGATGGTAAGTT
GTTTGTGGCTAATAACGCAGGTAATATTGTTGCTGTTAATGCTTCTGGCACCGTATATGGCATTTCATCTTGCGTGTCA
GTTCCTGTCTCAGTAGGATATAACCCCTCATTTGAGCAGGCGCTACTATTTAATGGTTTAGCGTGTTCAGAGAGAGCAG
TTGCTGTTAATATGCCGGCGTCAACTTATTGGCTATCGGTGTCTAGAAACGCAGCCCCACCTGATGGTGGTGTGTTTGA
CACACCTTCTGGTTGTGTTTATAATGTACGCAATTTAACTACCATGGTAGTTAATTCGTGTGATATGCCTATTGGCAAC
AGCCTTTGTTTAACTAATGTAACTTATGCTAATAGGGCTACAGATAGTTTACCTAGGTTGAGTTTAGTCACTTATGATC
CAACGGCTGCTAATGCTGTCCAGTTGCTCACACCAGTTTACTGGGTTAGTATACCCACTAATTTTACTCTGGCTGCTAC
TTCTGAGTATATACAAACTAGCTCACCAAAGATCAATGTAGATTGCGCGAAGTATTTATGTGGCGACTCTGATAGGTGT
ATGAATGTGTTGCTGCAGTATGGCACATTTTGTGATGACGTTAATAAGGCGCTGGCCGAGGTCAGTGCTACAATTGACG
CATCCGTTGTGTCATTGATGTCAGAACTCACATCGGACGTTGTACGTAATGAAAACATGCAGTTTGACACTACATACAA
CTTTACCTCACTTATGGGTTGTTTGGGATCAGATTGTGATTCTAAAATGTACAGGTCCGCTTTATCGGACTTGTTGTAT
AATAAGGTCAAGGTCACGGACCCTGGCTTTATGCAGTCCTATCAGAAGTGCATTTCTCAGTGGGGTGGTGAAATTAGAG
ACCTTATGTGTACTCAAACCTTTAATGGTATTGCTGTTTTACCACCTATTGTTTCTCCAGGCATGCAAGCCTTGTACAC
ATCATTACTTGTGGGTGCGGTAGCATCTGCAGGTTATACATTTGGCATTACTTCTGTCGGGGTTGTGCCTTTTGCGACG
CAATTGCAATTTAGATTAAATGGCATAGGCGTCACTACGCAGGTGCTTGTAGAGAATCAGAAACTTATTGCTAATTCTT
TTAACAATGCCCTTACTAATATACAGAAGGGTTTTGATGCCACTAATGAGGCGCTTAGTAAGATGCAGCTTGTTATTAA
CCAGCATGCTCAGCAATTGCAGACTTTGGTGAACCAGTTAGGTAATTCATTTGGTGCTATATCTGCTTCTATTAATGAA
ATATTTAGTCGTTTAGATGGTTTAGAGGCTGATGCTCAAGTGGATAGGCTTATTAATGGACGCACTGTTGTGCTTAATA
CTTATGTAACTCAGTTGCTTATTAAAGCTACAGAAGTGCGTAGTCAGGCCCTGTTAGCTAAGCAGAAAATTAGTGAGTG
CGTTAAAGCACAATCTCTTCGTAATGATTTCTGTGGCAATGGCACACATGTTTTGTCTATACCACAGTTAGCTCCTAAT
GGTATTTTGTTTTTGCATTATACCTATAAACCGACGGCTTTTGCTGTTGTGCAAACTGCTGCTGGTCTGTGTTATAATG
GCACAGGGTATGCACCTGTAGGTGGCATGTTTGTGTTACCTAATGGTACGCAGGCCTGGCACTTTACTAAAATGAATTT
TTATAATCCTGTTAGTATTACAGTTGAGAACACGCAGGTTTTATCTACATGTGGCTTGAATTATTCACATGTTAACTAC
ACTGTGGTGAACCCGTCGGTACCTAGTGATTTTGATTTTGAGGAAGAGTTTAATAAGTGGTATAAAAACCATAGCTCTA
TATTTAATAATACGTTCGACCCTAGTGCTTTTAATTTTTCTATGGTCGATGTAAATAAGCAACTGGCTACTCTTACAGA
TGTTGTTCAGCAGCTAAACAAGTCGTATATTGATTTGAAGCAATTAAATGTTTATGAACAGACCATTAAATGGCCGTGG
TATATATGGCTCGCTATGATAGCGGGCCTTGTTGGCTTAGCCCTTGCAGTGGTCATGCTTTTATGTATGACTAACTGTT
GTTCATGCTTCAAAGGCATGTGTTCATGCAAGCAGTGCCATTATGATGAGGTAGATGATGTATATCCTGCTGTTCGTGT

ATATAATAAACGAACAGCATGA

>gi|304435183|gb|HM211101.1| Bat coronavirus HKU9-10-2, complete genome

ATGTTGGTGATATTGTTAATGTTACCACTAGTTTTTGCTAGACCATCTTGTGTTACTATTAGTAACTTACAGTAT
GTTGATCATAACTACACTCACAGATCTTTTGATATGAGTGCAGTTTCACGTGTATTTCCTGATCCCTTTGTAGCCTATT
CTGGTCAGACACTTAAGCAGTCCCTTTACCTAGCAGATACGTCTAATACCACGGTTTATCCAGTAACACCACCTGCTGT
TAATGGAAAACCAGGTATTTATAATTTGACAATATTGCCTGTCGGCGATGGTTTCTTAGTTCAAACTTATATGTACAAG
GACCAACCCAGTGACACTTATTGTCAGGAACCATTTGGCGTTGCGTTTGGTACGACATTTGAGTACGACCGTATCGCTA
TTGTTACTATAGTACCAGGTTATAAAGGTTCTTGGTCTGCAGTTCAGAAACAGACTACTACTAATGTTAATATTTTGGT
CTGTAGTAATGCAACTTTGTGTGCATATCCTGCATTTAACCGATGGGGTCCCGCAGGTACTATTTATACTACTAATGAT
TTTGTTGTATATGGCGATTCTTGCTTTATAAACAACACTTTCACAATATCTATTAATACTTCTAGGTTAAACCTGGGGT
TTAGGTTTAATGATGGGAACTTGTACTTGTACCATTCTAAATGGTTGCCTATGCAAGGGCTTAACTTGATGGTGGATTA
CCCTCTGCACTTTCTTATGAGTGTAGGGGTGGGTGCAAATCTACCAAATATGCAGTTTTATCAGGCTGTTGTGCGTCAA
GGTACTCCACCAGTTACAGGCGCTTATAAAAATGATGCCAATTGTTTGGCCTTTCAGAATAATTTGTATCTAGCATATA
TAAGTAAGCGGGATTTGCTTGTGTCTTATGATGATAATGGACTTCCCATTGCTGTAGCAGATTGTTCCCAGAACGCTGG
GGATGAATTGTACTGTGTCACAGGCACTTTTAGTCCGCAGGTTGGTGTTTACCCATTAAGTAGGTATAGAGCTTTAGTT
AGTGATTACGTTCAAATATCGCAGCAAGGTAGCATTTGTAGCCTGCCTTATTCCGATATTTTGAAACCACCGCAACCTA
TAGTGTGGAAGCGTCACACAGTTACAAATTGTTCATTTGACTTTGACGCTATTGTTAATAGACTCCCTACTTTTCAGCT
TAAATGCTTTGGGGTGTCACCTGCTAAGTTGGCTCAAATGTGTTATTCCAGTGTTACACTGGACATTTTTAGAGCCAAT
ACAACTCACCTAGCTAATATGTTAGGTAAGGTTCCTGATGTTTTCAGTAAGTATAATTATGCACTTCCACCCGATTTTT
ATGGGTGTGTGCATTCGTATTATATTAATGATACCAGCCCCATGTATGCCATAGCGCAGCAGTGGCCTGCTACCGTTAT
AGCACCAGGTGGCCGCCAACCTTACAATAGTTATGTTGGCACCGTATTAAATACACCAAACCCCAAGTGCACTCAGCTA
ACTTGCTTTGGTGTGGTAGTTATTTCACTTAAGCCGGCCTCCGGTAAACATCTAGTTTGTCCTAGTGCCAATGACACGG
ATTTGGTGACGCGCGAATGTGTTAAGTATAATCTTTATGGTTATACTGGTACTGGTGTATTCAATGAGAGTTCATTGGT
TATACCAGATTCTAAATTATTCGTGGCTAGTTCCACGGGTGATATTATTGCTGCTAAAGTTAGAGGCAAGGTTTATTCT
ATAACACCTTGTGTTTCGGTGCCTATATCCGTAGGTTATGACCCTTCTTTTGAGAGAGCACTACTATTTAATGGTTTAT
CTTGTAGTGAACGCTCCGTAGCTGTCAGCTTACCAGCCTCTGATTACTGGGTAGCTGCTGTTGCAGATAATGCTAGTAC
TGGTGTAGTTACATTTGACACGCTTTCGGGTTGCGTGCATAATGTTCGTAATGCCACTGATATAACTGTGCCGACATGT
TTAATGCCTTTAGGTAACAGTTTATGTTTAGCTAATATTACACAACCCTCGCAGTATGGTTTAAGTACTACTAGCAGTG
TAAACCTTTTGAGTTTAGTTACTTATGACCCCACCTTTCAAGGTGGTGTTAAAGTTATGTCACCTGTTTATTGGATTAG
CATCCCAACAAATTTTACATTGGGTGCTATTACTGAATACATTCAAACTACGTCACCTAAAGTTAATGTGGACTGCGTC
AAATATTTGTGTGGAGATTCTGAACGTTGCACTACTGTGTTGCTGCAGTATGGCACATTTTGTGATGATGTCAATAAGG
CGTTGTCAGAAGTAAGTTCTATAATTGATGCTAGCATGATATCGCTAGTTTCAGAGATTACAGCTGATGTAGTTCGTAG
TGAGAATGCTTTATTCGACACTACCTATAACTTCACTGGTTTGTTGGGTTGTGTAGGTTCTAATTGTAATGACCGCACC
ACTTATCGGTCAGCACTTTCTGACTTGTTATATAACAAGGTAAAAGTTACTGACCCAGGTTTTATGAGCTCTTACCAAA
AGTGTATATCTCAGTGGGGTGGTGATATCAGGGATTTGTTCTGTACTCAGAACTTTAATGGTATCTCTGTTTTGCCCCC
CATAGTGTCACCAGGCATGCAAGCGCTGTACACTTCCCTATTAGTGGGTGCAGTTGCGTCTGCTGGATACACTTTTGGT
GTAACTTCAGTAGGCGTTATTCCTTTTGCCACTCAGCTACAATTTAGACTTAATGGTCTAGGTGTTACCACACAGGTGC
TGGTGGAGAACCAGCAGCTTATAGCTAACTCTTTTAATAAAGCGCTTGTTAGTATACAACAAGGCTTTGATGCTACGAA
TGAGGCACTTAGTAAGATGCAGTCTGTTATCAATCAACATGCACAGCAGCTGCAGACATTAGTTTCGCAGTTGGGAAAT
TCATTTGGTGCTATTTCGTCTTCTATTAATGAGATTTTCAGTCGGTTAGATGGTTTAGAAGCCAACGCGCAGGTAGACA
GACTCATTAATGGGCGCATGGTTGTACTTAATACCTATGTGACACAGCTTCTCATTAAAGCTTCTGAAGTTAAGTCGCA

GGCTCTACTCGCAAAGCAGAAAATTAGTGAGTGTGTTAAATCGCAGTCTTTGCGTAATGACTTCTGTGGTAATGGTACT
CATGTGTTTTCCGTACCACAGTTAGCCCCTAATGGTATAATGTTTTTGCATTATACATACAAGCCTACTTCATATGCAT
TGGTACAAACAGCTGCAGGTCTTTGTTTAAATAACACTGGCTATGCACCACGTGATGGTTTGTTTGTTTTGCCCAATGG
CTCTATATACTGGCAGTTTACTAAAATGAATTTTTATAATCCTGTCAGACTTACTAATAGTAACACTCAGGTTCTTACT
ACGTGTAGTGTTAACTATACTACAGTTAATTATACTGTGTTGCCACCAACGGACAACATGGATTTTAACTTTACTGCAG
AGTTTGAGAAGTGGTATAAAAATCACAGTTCTCAATTTAATAATACTTTCAACCCTGGGGATTTTAATTTTTCCACGGT
TGATATACAGAATGAACTTAACACCCTTAATGCTGTTGTTAAGCAGCTGAATGAGTCTTTTATTGACCTGAAGAAGTTA
AATGTTTATGAACAGACCATTAAATGGCCGTGGTATATATGGCTTGCTATGATAGCTGGCTTAGTTGGTTTAGCTCTAG
CAGTGGTTATGCTATTATGTATGACTAATTGTTGTTCATGCTTTAAAGGTATGTGTGCATGTAAACCTTGCCATTATGA
CGAGGTCGAAGATGTTTATCCTGCAGTTCGTGTATATAATAAACGAACAGCATGA

>gi|408796052|gb|JQ989266.1| Hipposideros bat coronavirus HKU10 isolate TT3A, complete genome

ATGATTTCGCTGGCTGTGTATTTGGCTGAATTGTTGGTTCTAATTACTAACTTGCCTTATACACGTGCTACTTGC
TCCAGTGCGCAAAACCCTGCTGCATTACCTAACTTAAACCTTGGTTTGCCTGCCAACTCTAATGTTTTTGTTAGTGGTT
ATTTGCCGATTGAAAACAATTGGCGTTGTCAGACAGGTGATACTGTTTATAACAATGTCAATGCTGTTTATTGGTCATA
CTACTCACATGGCACACCTGTTGAATTGGCTATTTCTAATTCTGATCAGCTTCAAAATGCTGATCAATGGGCGCTGTAT
ATATACCAGGGTAATAATTATGGTTATATGCACTTTAAAATTTGTAAGTGGAATGGCAAGTATAGTTTTATTAATAACC
CATACCCTAGTAGAGGTGTGTGTATGTATAATAAAAAATTTCCATTTGTTTTTCCACATGCGCGTAACACTGTAATAGG
TGCTTCTTGGAGTGGTGATTATGTTACACTTTTTACTACCACTAGTGCTAGGCGTTTTTATTTAAAAAATAACTGGTCT
AGGGTTAATGTAAAATGTCAGTTAGCAGAATCATGTGCAATACAAACAGTCTCCAACATCAATACACTAAATATTACCA
CCAATCAACAAGGTTTTATACAGTCTTACAGTGTATGCACTGAGTGTGACGGTTTTCCTAAACATGTTTTTCCTGTCCT
TGATGATGGGAAAATACCTGGTGATTTTAGTTTTAGTAATTGGTTTGTTTTGACCAACTCTTCCACTGTTTTGCAGGGT
CGAATTGTCACTGTACAACCTTTAAAATTGCTTTGTTTATGGCCTGTACCTTCTCTTAACGCAAATGATAAACCTGTCT
ATTTTAACCTATCACTTAACTCAGATGTTAGATGTAATGGTCATTCTATTGCTGGTTATGCTCAAGCGTTGAGGTTTTC
GCTTAACTTTACAGCAAACCAAGTCTTGAATGGTGTGCATAGTATTAAACTTTTCTCTTCCAATCAAAACTTTACATTC
ACATGCTCAACTAATGGTACTCTAGAAAGAATACCTTTTGGTCGTGTTGAAAGTGTGTATAAATGTTTTGTTCGTATTG
ATGCAATTAATTCTACAAAAAATTTGTTTGTGGGTATTTTACCACCTGTTACAAAGGAGTTTGTTATTTCACGTTATGG
TGCTGTTTATATAAATGGCGTACATATAATGTCTTTACCTCCTTTGGAAAGTGTCATTTTTAACGTCACTAGTAGTGTG
GGTTCTGACTTTTGGACTGTTGCTTTTGCTGATGAAGCTGAAGTTTTGTTGGATTTGAATGCCACAAACATAGTAGACA
TTTTATATTGTGACACTTTGGTCAATAAAATAAAATGTCAAAATCTTGCATTTTCTTTGGAGGATGGTTTTTATGCTAC
ATCCAATCTTATAGAACGTGACATACCTAGATCTTTCATAACTTTACCCTACCATGTAACTCATTCTGTAGTGGGTTTG
GATGTTAGGATTTATTATGAAAATGAAGCTGCAAGTAATCTCACTTTGTCTCCTTCAGATCCTGTGTGTGTTGATAGCT
CACAGTTCTCAACTTATTTTAATGTCACTGCTGACACTAATGGTTTCAATGCCAGAATTATTAATGTTGACTGTCCTTT
TACTTTTGATAAGCTTAACAATTACATTAGTTTTGAATCCATTTGCTTTTCTCTGGAACCTTTTGCTGGTGGTTGTTCT
ATGCGTATTGAAAGTTTCTTTTCCAGCTATGTTAGGACTGTGGGCAGTCTTTACATTCTTTTTAAGAATGGTAATAAGA
TTACTGGTGTTACAGAAAGTTCAGCTGGCGTTTATGATACTAGTTTTCTACACACAGACATTTGCACAGATTACACTAT
TTATGGCACTACTGGTAGAGGTGTTATAACCAAGCTTAATACTACTATTATAGCTGGTTTATATTACACCTCACAATCT
GGTCAAATACTTGGTTTTAAAAATGCCACCAATGGTGACATTTATGCTGTTAGACCTTGTGACTTGTCCAGTCAAGCTG
CTGTTGTTAGTAATCAAATTGTTGCTGTTATGTCTGCTTCTGAAAACATTACTTTTGGCTTTAACAATTCTCTGGAATT
GCCTAAATTTTACCTACATTCTAATGCTGAAAATGCTACTAATTGCACAGAACCAGTACTTATCTATGGTAAAATTGGC
ATTTGCGAAGATGGTAGCATTACTGAAGTTAAACCACGTATTGCAGAACCTGAACCTTACTCGCCTATTGTTACAGCTA

ATATTACAATCCCTAGTAATTTTAGCATTTCTGTGCAGGCTGAGTATGTTCAAATGTTTTTGACACCTGTTAGTGTTGA
CTGTAATATGTATGTTTGTAATGGTAATAGGCATTGTCTTCGCCTTTTAGGTGAATATGTTACTGCTTGTAAAAATATT
GAAGCTGCACTTCAGCTTAGTGCTCGCTTGGAATCTATTGAAGTTAATAATTTCATTACTGTTTCTGAGAATGCTATAT
CTCTTGCCAACATCTCCAACTTTGATAGCTACAACCTTTCAGTTTTACTACCTAAACAGAATGGTAAGAGTGTTATTGA
AGACATTTTGTTTGATAAGGTTGTGACTAATGGTCTTGGTACCGTTGACCAGGATTATAAGGAGTGCATCAAGCGTAGT
GGAGTTAAGGATGTGGCAGATGTGGCTTGTGCTCAGTATTACAATGGAATTATGGTGCTCCCTGGTGTTGTTGATGACG
TAAAAATGGGTCTTTACACAGCTTCTTTAACTGGTGCTATGGTTATGGGCGGTTTAACTGCCGCTGCTGCTATACCTTT
TTCAATAGCTGTTCAATCACGACTCAATTATGTTGCACTTCAAACTGATGTTTTGCAGAAGAACCAGCAAATTTTAGCA
GATTCATTTAACAATGCTATGAGTAATATTACTATGGCATTTGCTCAGGTGAATGATGCCATTAAAGAAACTTCTCAAG
CCATCAGTACCGTAGCTCAAGCTTTGAGTAAGGTTCAGAGTGTTGTCAATGAACAGGGTGAAGCACTTTCACAGCTTAC
TAAACAATTGGCTTCCAATTTTCAGGCTATTTCGTCTTCCATAGAAGACATTTATAACCGCTTGGACATGATTGCCGCT
GATGCTCAAGTTGACAGACTTATCACGGGCAGAATTGGTGCTTTGAATGCTTTTGTCACACAAACACTTACAAAGTACA
CTGAAGTTCGTGCTAGTCGTCAACTTGCTTTGCAGAAAATTAATGAATGTGTCAAATCACAGTCTAGCCGATTTGGTTT
TTGTGGAAATGGCACTCATTTATTTCAATCGCTAACGCTGCTCCTAACGGTATAATGTTATTTCACACAGTCCTCATG
CCTACTGAGTATGTTGTTGTACGTGCATGGGCTGGACTTTGTATTGAAGACAAAGCTTTTGTGCTACGTGACGTTAAAA
CAACTCTTTTTAAGACTGGTGATACTTATTACATCACTACTAGAGACATGTATCAACCTAGACTTCCACAAGTTAGTGA
CTTCGTTCAAATTAAAGATTGTCAAGTTGACTATCTTAATTTGACATCAGATGAAGTTAATAATGTGATACCTGACTAT
ATTGATGTAAATAAAACTTTGGAAGATTTTGGCCAAATACTTGCTAATAATACACGACCACCAATACCTGATTTTGGTT
TAGATGTTTACAATAACACAATTCTCAACCTGACTGCTGAGATTGATGTATTGCAAAATAAATCTGAACAACTTTTGGC
CTCAACTGAAAGACTTCAACAACTTATTGACAATCTCAATAAGACTTATGTTGATCTTGAGTGGCTCAACAGGTTCGAA
CAATACATTAAGTGGCCATGGTATGTGTGGCTAGCAATTTTTCTTGCCGTTATACTATTTTCTTTCTTAATGCTTTACT
GTTGTTGTGCTACAGGTTGTTGTGGTTGTTTTAGCTGTCTTTCTAATTCTTGTGATTGTCGCGGCAAAAATTTACAACG
TTACGAAGTAGAAAAAGTGCACATTCAATAA

>gi|408796061|gb|JQ989267.1| Hipposideros bat coronavirus HKU10 isolate SL12A, complete genome

ATGATTTCGCTGGCTGTGTATTTGGCTGAATTGTTGGTTCTAATTACTAACTTGCCTTATACACGTGCTACTTGC
TCCAGTGCGCAAAACCCTGCTGCATTACCTAACTTAAACCTTGGTTTGCCTGCCAACTCTAATGTTTTTGTTAGTGGTT
ATTTGCCGATTGAAAACAATTGGCGTTGTCAGACAGGTGATACTGTTTATAACAATGTCAATGCTGTTTATTGGTCATA
CTACTCACATGGCACACCTGTTGAATTGGCTATTTCTAATTCTGATCAGCTTCAAAATGCTGATCAATGGGCGCTGTAT
ATATACCAGGGTAATAATTATGGTTATATGCACTTTAAAATTTGTAAGTGGAATGGCAAGTATAGTTTTATTAATAACC
CATACCCTAGTAGAGGTGTGTGTATGTATAATAAAAAATTTCCATTTGTTTTTCCACATGCGCGTAACACTGTAATAGG
TGCTTCTTGGAGTGGTGATTATGTTACACTTTTTACTACCACTAGTGCTAGGCGTTTTTATTTAAAAAATAACTGGTCT
AGGGTTAATGTAAAATGTCAGTTAGCAGAATCATGTGCAATACAAACAGTCTCCAACATCAATACACTAAATATTACCA
CCAATCAACAAGGTTTTATACAGTCTTACAGTGTATGCACTGAGTGTGACGGTTTTCCTAAACATGTTTTTCCTGTCCT
TGATGATGGGAAAATACCTGGTGATTTTAGTTTTAGTAATTGGTTTGTTTTGACCAACTCTTCCACTGTTTTGCAGGGT
CGAATTGTCACTGTACAACCTTTAAAATTGCTTTGTTTATGGCCTGTACCTTCTCTTAACGCAAATGATAAACCTGTCT
ATTTTAACCTATCACTTAACTCAGATGTTAGATGTAATGGTCATTCTATTGCTGGTTATGCTCAAGCGTTGAGGTTTTC
GCTTAACTTTACAGCAAACCAAGTCTTGAATGGTGTGCATAGTATTAAACTTTTCTCTTCCAATCAAAACTTTACATTC
ACATGCTCAACTAATGGTACTCTAGAAAGAATACCTTTTGGTCGTGTTGAAAGTGTGTATAAATGTTTTGTTCGTATTG
ATGCAATTAATTCTACAAAAAATTTGTTTGTGGGTATTTTACCACCTGTTACAAAGGAGTTTGTTATTTCACGTTATGG
TGCTGTTTATATAAATGGCGTACATATAATGTCTTTACCTCCTTTGGAAAGTGTCATTTTTAACGTCACTAGTAGTGTG

GGTTCTGACTTTTGGACTGTTGCTTTTGCTGATGAAGCTGAAGTTTTGTTGGATTTGAATGCCACAAACATAGTAGACA
TTTTATATTGTGACACTTTGGTCAATAAAATAAAATGTCAAAATCTTGCATTTTCTTTGGAGGATGGTTTTTATGCTAC
ATCCAATCTTATAGAACGTGACATACCTAGATCTTTCATAACTTTACCCTACCATGTAACTCATTCTGTAGTGGGTTTG
GATGTTAGGATTTATTATGAAAATGAAGCTGCAAGTAATCTCACTTTGTCTCCTTCAGATCCTGTGTGTGTTGATAGCT
CACAGTTCTCAACTTATTTTAATGTCACTGCTGACACTAATGGTTTCAATGCCAGAATTATTAATGTTGACTGTCCTTT
TACTTTTGATAAGCTTAACAATTACATTAGTTTTGAATCCATTTGCTTTTCTCTGGAACCTTTTGCTGGTGGTTGTTCT
ATGCGTATTGAAAGTTTCTTTTCCAGCTATGTTAGGACTGTGGGCAGTCTTTACATTCTTTTTAAGAATGGTAATAAGA
TTACTGGTGTTACAGAAAGTTCAGCTGGCGTTTATGATACTAGTTTTCTACACACAGACATTTGCACAGATTACACTAT
TTATGGCACTACTGGTAGAGGTGTTATAACCAAGCTTAATACTACTATTATAGCTGGTTTATATTACACCTCACAATCT
GGTCAAATACTTGGTTTTAAAAATGCCACCAATGGTGACATTTATGCTGTTAGACCTTGTGACTTGTCCAGTCAAGCTG
CTGTTGTTAGTAATCAAATTGTTGCTGTTATGTCTGCTTCTGAAAACATTACTTTTGGCTTTAACAATTCTCTGGAATT
GCCTAAATTTTACCTACATTCTAATGCTGAAAATGCTACTAATTGCACAGAACCAGTACTTATCTATGGTAAAATTGGC
ATTTGCGAAGATGGTAGCATTACTGAAGTTAAACCACGTATTGCAGAACCTGAACCTTACTCGCCTATTGTTACAGCTA
ATATTACAATCCCTAGTAATTTTAGCATTTCTGTGCAGGCTGAGTATGTTCAAATGTTTTTGACACCTGTTAGTGTTGA
CTGTAATATGTATGTTTGTAATGGTAATAGGCATTGTCTTCGCCTTTTAGGTGAATATGTTACTGCTTGTAAAAATATT
GAAGCTGCACTTCAGCTTAGTGCTCGCTTGGAATCTATTGAAGTTAATAATTTCATTACTGTTTCTGAGAATGCTATAT
CTCTTGCCAACATCTCCAACTTTGATAGCTACAACCTTTCAGTTTTACTACCTAAACAGAATGGTAAGAGTGTCATTGA
AGACATTTTGTTTGATAAGGTTGTGACTAATGGTCTTGGTACCGTTGACCAGGATTATAAGGAGTGCATCAAGCGTAGT
GGAGTTAAGGATGTGGCAGATGTGGCTTGTGCTCAGTATTACAATGGAATTATGGTGCTCCCTGGTGTTGTTGATGACG
TAAAAATGGGTCTTTACACAGCTTCTTTAACTGGTGCTATGGTTATGGGCGGTTTAACTGCCGCTGCTGCTATACCTTT
TTCAATAGCTGTTCAATCACGACTCAATTATGTTGCACTTCAAACTGATGTTTTGCAGAAGAACCAGCAAATTTTAGCA
GATTCATTTAACAATGCTATGAGTAATATTACTATGGCATTTGCTCAGGTGAATGATGCCATTAAAGAAACTTCTCAAG
CCATCAGTACCGTAGCTCAAGCTTTGAGTAAGGTTCAGAGTGTTGTCAATGAACAGGGTGAAGCACTTTCACAGCTTAC
TAAACAATTGGCTTCCAATTTTCAGGCTATTTCGTCTTCCATAGAAGACATTTATAACCGCTTGGACATGATTGCCGCT
GATGCTCAAGTTGACAGACTTATCACGGGCAGAATTGGTGCTTTGAATGCTTTTGTCACACAAACACTTACAAAGTACA
CTGAAGTTCGTGCTAGTCGTCAACTTGCTTTGCAGAAAATTAATGAATGTGTCAAATCACAGTCTAGCCGATTTGGTTT
TTGTGGAAATGGCACTCATTTATTTTCAATCGCTAACGCTGCTCCTAACGGTATAATGTTATTTCACACAGTCCTCATG
CCTACTGAGTATGTTGTTGTACGTGCATGGGCTGGACTTTGTATTGAAGACAAAGCTTTTGTGCTACGTGACGTTAAAA
CAACTCTTTTTAAGACTGGTGATACTTATTACATCACTACTAGAGACATGTATCAACCTAGACTTCCACAAGTTAGTGA
CTTCGTTCAAATTAAAGATTGTCAAGTTGACTATCTTAATTTGACATCAGATGAAGTTAATAATGTGATACCTGACTAT
ATTGATGTAAATAAAACTTTGGAAGATTTTGGCCAAATACTTGCTAATAATACACGACCACCAATACCTGATTTTGGTT
TAGATGTTTACAATAACACAATTCTCAACCTGACTGCTGAGATTGATGTATTGCAAAATAAATCTGAACAACTTTTGGC
CTCAACTGAAAGACTTCAACAACTTATTGACAATCTCAATAAGACTTATGTTGATCTTGAGTGGCTCAACAGGTTCGAA
CAATACATTAAGTGGCCATGGTATGTGTGGCTAGCAATTTTTCTTGCCGTTATACTATTTTCTTTCTTAATGCTTTACT
GTTGTTGTGCTACAGGTTGTTGTGGTTGTTTTAGCTGTCTTTCTAATTCTTGTGATTGTCGCGGCAAAAATTTACAACG
TTACGAAGTAGAAAAAGTGCACATTCAATAA

>gi|408796070|gb|JQ989268.1| Hipposideros bat coronavirus HKU10 isolate TLC1310A, complete genome

ATGATTTCGCTGGCTGTGTATTTGGCTGAATTGTTGGTTCTAATTACTAACTTGCCTTATACACGTGCTACTTGC
TCCAGTGCGCAAAACCCTGCTGCATTACCTAACTTAAACCTTGGTTTGCCTGCCAACTCTAATGTTTTTGTTAGTGGTT
ATTTGCCGATTGAAAACAATTGGCGTTGTCAGACAGGTGATACTGTTTATAACAATGTCAATGCTGTTTATTGGTCATA

CTACTCACATGGCACACCTGTTGAATTGGCTATTTCTAATTCTGATCAGCTTCAAAATGCTGATCAATGGGCGCTGTAT
ATATACCAGGGTAATAATTATGGTTATATGCACTTTAAAATTTGTAAGTGGAATGGCAAGTATAGTTTTATTAATAACC
CATACCCTAGTAGAGGTGTGTGTATGTATAATAAAAAATTTCGATTTGTTTTTCCACATGCGCGTAACACTGTAATAGG
TGCTTCTTGGAGTGGTGATTATGTTACACTTTTTACTACCACTAGTGCTAGGCGTTTTTATTTAAAAAATAACTGGTCT
AGGGTTAATGTAAAATGTCAGTTAGCAGAATCATGTGCAATACAAACAGTCTCCAACATCAGTACACTAAATATTACCA
CCAATCAACAAGGTCTTATACAGTCTTACAGTGTATGCACTGAGTGTGACGGTTTTCCTAAACATGTTTTTCCTGTCCT
TGATGATGGGAAAATACCTGGTGATTTTAGTTTTAGTAATTGGTTTGTTTTGACCAACTCTTCCACTGTTTTGCAGGGT
CGAATTGTCACTGTACAACCTTTAAAATTGCTTTGTTTATGGCCTGTACCTTCTCTTAACGCAAATGATAAACCTGTCT
ATTTTAACCTATCACTTAACTCAGATGTTAGATGTAATGGTCATTCTATTGCTGGTTATGCTCAAGCGTTGAGGTTTTC
GCTTAACTTTACAGCAAACCAAGTCTTGAATGGTGTGCATAGTATTAAACTTTTCTCTTCCAATCAAAACTTTACATTC
ACATGCTCAACTAATGGTACTCTAGAAAGAATACCTTTTGGTCGTGTTGAAAGTGTGTATAAATGTTTTGTTCGTATTG
ATGCAATTAATTCTACAAAAAATTTGTTTGTGGGTATTTTACCACCTGTTACAAAGGAGTTTGTTATTTCACGTTATGG
TGCTGTTTATATAAATGGCGTACATATAATGTCTTTACCTCCTTTGGAAAGTGTCATTTTTAACGTCACTAGTAGTGTG
GGTTCTGACTTTTGGACTGTTGCTTTTGCTGATGAAGCTGAAGTTTTGTTGGATTTGAATGCCACAAACATAGTAGACA
TTTTATATTGTGACACTTTGGTCAATAAAATAAAATGTCAAAATCTTGCATTTTCTTTGGAGGATGGTTTTTATGCTAC
TTCCAAGCTTATAGAACGTCACATACCTAGATCTTTCATAACTTTACCCTACCATGTAACTCATTCTGTAGTGGGTTTG
GATGTTAGGATTTATTATGAAAATGAAGCTGCAAGTAATCTCACTTTGTCTCCTTCAGATCCTGTGTGTGTTGATAGCT
CACAGTTCTCAACTTATTTTAATGTCACTGCTGACACTAATGGTTTCAATGCCAGAATTATTAATGTTGACTGTCCTTT
TACTTTTGATAAGCTTAACAATTACATTAGTTTTGAATCCATTTGCTTTTCTCTGGAACCTTTTGCTGGTGGTTGTTCT
ATGCGTATTGAAAGTTTCTTTTCCAGCTATGTTAGGACTGTGGGCAGTCTTTACATTCTTTTTAAGAATGGTAATAAGA
TTACTGGTGTTACAGAAAGTTCAGCTGGCGTTTATGATACTAGTTTTCTACACACAGACATTTGCACAGATTACACTAT
TTATGGCACTACTGGTAGAGGTGTTATAACCAAGCTTAATACTACTATTATAGCTGGTTTATATTACACCTCACAATCT
GGTCAAATATTTGGTTTTAAAAATGCCACCAATGGTGACATTTATGCTGTTAGACCTTGTGACTTGTCCAGTCAAGCTG
CTGTTGTTAGTAATCAAATTGTTGCTGTTATGTCTGCTTCTGAAAACATTACTTTTGGCTTTAACAATTCTCTGGAATT
GCCTAAATTTTACCTACATTCTAATGCTGAAAATGCTACTAATTGCACAGAACCAGTACTTATCTATGGTAAAATTGGC
ATTTGCGAAGATGGTAGCATTACTGAAGTTAAACCACGTATTGCAGAACCTGAACCTTACTCGCCTATTGTTACAGCTA
ATATTACAATCCCTAGTAATTTTAGCATTTCTGTGCAGGCTGAGTATGTTCAAATGTTTTTGACACCTGTTAGTGTTGA
CTGTAATATGTATGTTTGTAATGGTAATAGGCATTGTCTTCGCCTTTTAGGTGAATATGTTACTGCTTGTAAAAATATT
GAAGCTGCACTTCAGCTTAGTGCTCGCTTGGAATCTATTGAAGTTAATAATTTCATTACTGTTTCTGAGAATGCTATAT
CTCTTGCCAACATCTCCAACTTTGATAGCTACAACCTTTCAGTTTTACTACCTAAACAGAATGGTAAGAGTGTCATTGA
AGACATTTTGTTTGATAAGGTTGTGACTAATGGTCTTGGTACCGTTGACCAGGATTATAAGGAGTGCATCAAGCGTAGT
GGAGTTAAGGATGTGGCAGATGTGGCTTGTGCTCAGTATTACAATGGAATTATGGTGCTCCCTGGTGTTGTTGATGACG
TAAAAATGGGTCTTTACACAGCTTCTTTAACTGGTGCTATGGTTATGGGCGGTTTAACTGCCGCTGCTGCTATACCTTT
TTCAATAGCTGTTCAATCACGACTCAATTATGTTGCACTTCAAACTGATGTTTTGCAGAAGAACCAGCAAATTTTAGCA
GATTCATTTAACAATGCTATGAGTAATATTACTATGGCATTTGCTCAGGTGAATGATGCCATTAAAGAAACTTCTCAAG
CCATCAGTACCGTAGCTCAAGCTTTGAGTAAGGTTCAGAGTGTTGTCAATGAACAGGGTGAAGCACTTTCACAGCTTAC
TAAACAATTGGCTTCCAATTTTCAGGCTATTTCGTCTTCCATAGAAGACATTTATAACCGCTTGGACATGATTGCCGCT
GATGCTCAAGTTGACAGACTTATCACGGGCAGAATTGGTGCTTTGAATGCTTTTGTCACACAAACACTTACAAAGTACA
CTGAAGTTCGTGCTAGTCGTCAACTTGCTTTGCAGAAAATTAATGAATGTGTCAAATCACAGTCTAGCCGATTTGGTTT
TTGTGGAAATGGCACTCATTTATTTTCAATCGCTAACGCTGCTCCTAACGGTATAATGTTATTTCACACAGTCCTCATG
CCTACTGAGTATGTTGTTGTACGTGCATGGGCTGGACTTTGTATTGAAGACAAAGCTTTTGTGCTACGTGACGTTAAAA
CAACTCTTTTTAAGACTGGTGATACTTATTACATCACTACTAGAGACATGTATCAACCTAGACTTCCACAAGTTAGTGA
CTTCGTTCAAATTAAAGATTGTCAAGTTGACTATCTTAATTTGACATCAGATGAAGTTAATAATGTGATACCTGACTAT
ATTGATGTAAATAAAACTTTGGAAGATTTTGGCCAAATACTTGCTAATAATACACGACCACCAATACCTGATTTTGGTT

TAGATATTTACAATAACACAATTCTCAACCTGACTGCTGAGATTGATGTATTGCAAAATAAATCTGAACAACTTTTGGC
CTCAACTGAAAGACTTCAACAACTTATTGACAATCTCAATAAGACTTATGTTGATCTTGAGTGGCTCAACAGGTTCGAA
CAATACATTAAGTGGCCATGGTATGTGTGGCTAGCAATTTTTCTTGCCGTTATACTATTTTCTTTCTTAATGCTTTACT
GTTGTTGTGCTACAGGTTGTTGTGGTTGTTTTAGCTGTCTTTCTAATTCTTGTGATTGTCGCGGCAAAAATTTACAACG
TTACGAAGTAGAAAAAGTGCACATTCAATAA

>gi|408796080|gb|JQ989269.1| Hipposideros bat coronavirus HKU10 isolate LSH5A, complete genome

ATGATTTCGCTGGCTGTGTATTTGGCTGAATTGTTGGTTCTAATTACTAACGTGCCTTATACACGTGCTACTTGC
TCCAGTGCGCAAAACCCTGCTGCATTACCTAACTTAAACCTTGGTTTGCCTGCCAACTCTAATGTTTTTGTTAGTGGTT
ATTTGCCGATTGAAAACAATTGGCGTTGTCAGACAGGTGATACTGTTTATAACAATGTCAATGCTGTTTATTGGTCATA
CTACTCACATGGCACACCTGTTGAATTGGCTATTTCTAATTCTGATCAGCTTCAAAATGCTGATCAATGGGCGCTGTAT
ATATACCAGGGTAATAATTATGGTTATATGCACTTTAAAATTTGTAAGTGGAATGGCAAGTATAGTTTTATTAATAACC
CATACCCTAGTAGAGGTGTGTGTATGTATAATAAAAAATTTCGATTTGTTTTTCCACATGCGCGTAACACTGTAATAGG
TGCTTCTTGGAGTGGTGATTATGTTACACTTTTTACTACCACTAGTGCTAGGCGTTTTTATTTAAAAAATAACTGGTCT
AGGGTTAATGTAAAATGTCAGTTAGCAGAATCATGTGCAATACAAACAGTCTCCAACATCAATACACTAAATATTACCA
CCAATCAACAAGGTCTTATACAGTCTTACAGTGTATGCACTGAGTGTGACGGTTTTCCTAAACATGTTTTTCCTGTCCT
TGATGATGGGAAAATACCTGGTGATTTTAGTTTTAGTAATTGGTTTGTTTTGACCAACTCTTCCACTGTTTTGCAGGGT
CGAATTGTCACTGTACAACCTTTAAAATTGCTTTGTTTATGGCCTGTACCTTCTCTTAACGCAAATGATAAACCTGTCT
ATTTTAACCTATCACTTAACTCAGATGTTAGATGTAATGGTCATTCTATTGCTGGTTATGCTCAAGCGTTGAGGTTTTC
GCTTAACTTTACAGCAAACCAAGTCTTGAATGGTGTGCATAGTATTAAACTTTTCTCTTCCAATCAAAACTTTACATTC
ACATGCTCAACTAATGGTACTCTAGAAAGAATACCTTTTGGTCGTGTTGAAAGTGTGTATAAATGTTTTGTTCGTATTG
ATGCAATTAATTCTACAAAAAATTTGTTTGTGGGTATTTTACCACCTGTTACAAAGGAGTTTGTTATTTCACGTTATGG
TGCTGTTTATATAAATGGCGTACATATAATGTCTTTACCTCCTTTGGAAAGTGTCATTTTTAACGTCACTAGTAGTGTG
GGTTCTGACTTTTGGACTGTTGCTTTTGCTGATGAAGCTGAAGTTTTGTTGGATTTGAATGCCACAAACATAGTAGACA
TTTTATATTGTGACACTTTGGTCAATAAAATAAAATGTCAAAATCTTGCATTTTCTTTGGAGGATGGTTTTTATGCTAC
ATCCAATCTTATAGAACGTGACATACCTAGATCTTTCATAACTTTACCCTACCATGTAACTCATTCTGTAGTGGGTTTG
GATGTTAGGATTTATTATGAAAATGAAGCTGCAAGTAATCTCACTTTGTCTCCTTCAGATCCTGTGTGTGTTGATAGCT
CACAGTTCTCAACTTATTTTAATGTCACTGCTGACACTAATGGTTTCAATGCCAGAATTATTAATGTTGACTGTCCTTT
TACTTTTGATAAGCTTAACAATTACATTAGTTTTGAATCCATTTGCTTTTCTCTGGAACCTTTTGCTGGTGGTTGTTCT
ATGCGTATTGAAAGTTTCTTTTCCAGCTATGTTAGGACTGTGGGCAGTCTTTACATTCTTTTTAAGAATGGTAATAAGA
TTACTGGTGTTACAGAAAGTTCAGCTGGCGTTTATGATACTAGTTTTCTACACACAGACATTTGCACAGATTACACTAT
TTATGGCACTACTGGTAGAGGTGTTATAACCAAGCTTAATACTACTATTATAGCTGGTTTATATTACACCTCACAATCT
GGTCAAATACTTGGTTTTAAAAATGCCACCAATGGTGACATTTATGCTGTTAGACCTTGTGACTTGTCCAGTCAAGCTG
CTGTTGTTAGTAATCAAATTGTTGCTGTTATGTCTGCTTCTGAAAACATTACTTTTGGCTTTAACAATTCTCTGGAATT
GCCTAAATTTTACCTACATTCTAATGCTGAAAATGCTACTAATTGCACAGAACCAGTACTTATCTATGGTAAAATTGGC
ATTTGCGAAGATGGTAGCATTACTGAAGTTAAACCACGTATTGCAGAACCTGAACCTTACTCGCCTACTGTTACAGCTA
ATATTACAATCCCTAGTAATTTTAGCATTTCTGTGCAGGCTGAGTATGTTCAAATGTTTTTGACACCTGTTAGTGTTGA
CTGTAATATGTATGTTTGTAATGGTAATAGGCATTGTCTTCGCCTTTTAGGTGAATATGTTACTGCTTGTAAAAATATT
GAAGCTGCACTTCAGCTTAGTGCTCGCTTGGAATCTATTGAAGTTAATAATTTCATTACTGTTTCTGAGAATGCTATAT
CTCTTGCCAACATCTCCAACTTTGATAGCTACAACCTTTCAGTTTTACTACCTAAACAGAATGGTAAGAGTGTCATTGA
AGACATTTTGTTTGATAAGGTTGTGACTAATGGTCTTGGTACCGTTGACCAGGATTATAAGGAGTGCATCAAGCGTAGT

GGAGTTAAGGATGTGGCAGATGTGGCTTGTGCTCAGTATTACAATGGAATTATGGTGCTCCCTGGTGTTGTTGATGACG
TAAAAATGGGTCTTTACACAGCTTCTTTAACTGGTGCTATGGTTATGGGCGGTTTAACTGCCGCTGCTGCTATACCTTT
TTCAATAGCTGTTCAATCACGACTCAATTATGTTGCACTTCAAACTGATGTTTTGCAGAAGAACCAGCAAATTTTAGCA
GATTCATTTAACAATGCTATGAGTAATATTACTATGGCATTTGCTCAGGTGAATGATGCCATTAAAGAAACTTCTCAAG
CCATCAGTACCGTAGCTCAAGCTTTGAGTAAGGTTCAGAGTGTTGTCAATGAACAGGGTGAAGCACTTTCACAGCTTAC
TAAACAATTGGCTTCCAATTTTCAGGCTATTTCGTCTTCCATAGAAGACATTTATAACCGCTTGGACATGATTGCCGCT
GATGCTCAAGTTGACAGACTTATCACGGGCAGAATTGGTGCTTTGAATGCTTTTGTCACACAAACACTTACAAAGTACA
CTGAAGTTCGTGCTAGTCGTCAACTTGCTTTGCAGAAAATTAATGAATGTGTCAAATCACAGTCTAGCCGATTTGGTTT
TTGTGGAAATGGCACTCATTTATTTTCAATCGCTAACGCTGCTCCTAACGGTATAATGTTATTTCACACAGTCCTCATG
CCTACTGAGTATGTTGTTGTACGTGCATGGGCTGGACTTTGTATTGAAGACAAAGCTTTTGTGCTACGTGACGTTAAAA
CAACTCTTTTTAAGACTGGTGATACTTATTACATCACTACTAGAGACATGTATCAACCTAGACTTCCACAAGTTAGTGA
CTTCGTTCAAATTAAAGATTGTCAAGTTGACTATCTTAATTTGACATCAGATGAAGTTAATAATGTGATACCTGACTAT
ATTGATGTAAATAAAACTTTGGAAGATTTTGGCCAAATACTTGCTAATAATACACGACCACCAATACCTGATTTTGGTT
TAGATGTTTACAATAACACAATTCTCAACCTGACTGCTGAGATTGATGTATTGCAAAATAAATCTGAACAACTTTTGGC
CTCAACTGAAAGACTTCAACAACTTATTGACAATCTCAATAAGACTTATGTTGATCTTGAGTGGCTCAACAGGTTCGAA
CAATACATTAAGTGGCCATGGTATGTGTGGCTAGCAATTTTTCTTGCCGTTATACTATTTTCTTTCTTAATGCTTTACT
GTTGTTGTGCTACAGGTTGTTGTGGTTGTTTTAGCTGTCTTTCTAATTCTTGTGATTGTCGCGGCAAAAATTTACAACG
TTACGAAGTAGAAAAAGTGCACATTCAATAA

>gi|408796090|gb|JQ989270.1| Rousettus bat coronavirus HKU10 isolate 183A, complete genome

ATGATTTCTCTTGCTGTTTTTATAGCAGAGATACTTGTTATTATTACGAGATTTTCGTGTCTTGCTGATCAATGT
CAAGGTACACCTTGGTTAGCTAAGAACTTGAATCTTGGTTTGTCACCTAATTTGACTAATGTTTATGTACCAGGTTTGT
TGCCTACAAAGGACACCACAAAGTGGTATTGTCGCACAGAATCATACTTTTTTACAGGTTATAAAGGTGTGTTTCTATC
GTACCACAGTTTTGGTATGCCTTGGTCTATTGGTGTTTCCAATTCTGATCTCTCTGTTGCACGTGAAACATGGTCATTG
TATGTTTCTCATGGCAATAATAGAGGTGTTATGACCTTTAGAATTTGTAAATGGCCACGTGTGCCTGGACTAGCACAAG
AACCTACTCCTAACACTGGTGGTGTTAATTGTGTAGTAGATAAGCAATTTCCTTTCACTTTTCAACACGAGGCTAATGA
AATGGTGGGTATTACTTGGAGTTCTGATTACGTTCGTATTTATGGGCTTCAAAGTACCTATCGTTTTTATTTTCCTAAT
AAGTGGAATAAAGTTAATGTTAATTGTGCACATAAGAGTTCTTGTCAGGTCTACCCTGTTAAAGACAGTGTTACATGGG
TTGTTAATACCACAGCTGATGGACGTGTTAAAGATTACACTATTTGTGGTTCTTGTAATGGTTTTCCGCAACACGTCTT
CCCTGTTTCACCTGGTGGTTTAATACCTCCTGATTTTAGTTTTGACGGTTGGTATGTTTTAACCAACTCTTCCACTATG
GTGCAAGGTAAGTTTGTTGCATCTCAACCTTTAAAATTGTTGTGCCTTTGGCCTGTACCTGCTTTGGATTCTAGTGCTA
GTGTTGTTTATTTTAATTTATCACGTAGTAGTGCGCAATGCAATGGTGCTACTGAAAATGGTTTAGCCGATGCATTACG
TTTTTCTATGAATTTCTCTAGAAATGGTTTCAGTGGTGCTCACAGTTTGACTTTAAAAGGTGCCATTGATTATGTTTTT
AGGTGCACTAATAACACTAACGTAACAAGTTTTTATTCTATACCATTTGGTTATATTAAAACAGTTTACAGGTGTTTTG
TATCCTATAATAGCACTAATGGCACAGTGTCAGAGTTTGTGGGTGTTTTGCCACCTCTTGTTAAAGAAATTGTTGTCTC
ACGTTATGGCACCATTTATTTGAATGGTGTACGTATCTTTAAGTTACCGCCTTTAGAAGGTGTTGTGTTCAATGTAACT
AGTGATGTTGGTTCAGACTTCTGGACTGTTGCATTTGCGCAAAACGCTGAAGTTTTGTTGGAAGTTAATGCAACTAACA
TTGTTAATTTGCACTATTGTGATAATACGCTGAACAAGGTTAAGTGTCAACAACTTTCTTTTCAATTACCTGATGGTTT
TTATCCTACAACGAACCTGGTGGATAGTGTTGTTCCTAAAACATATGTTGCTTTACCCTATCATGCAACACACACGTAC
ATTGAATTAAATATAACTGTTAATTATGTACGTGATGAGATAGCATTAACTGGAGGTGGTGAGTTGACTTGTGTTAATA
CAACACAATTCACCACACTCTTCTCAACTAATGCTGCTACTGATGGGAACACTTATGAAGGTGTAGTTATTAATGTCAA

TTGTCCTTTTGACTTTGACAACCTTAATAATTACCTGTCTTTTGACTCCATTTGTTTCTCTTTGAAACCAATCGCTGGT
GGTTGCACTATGCGACTTGTTAAACAAATTCTTGTGTCATATTTTGACATAGGTGTTTTGTATGTTTCCTATACACCTG
GTTCAAAAATTACTGGTGTTTTACAACCTACAGGTGGTGTTTATGACCCTAGTATTGTTCATATGGGTGTTTGTACAGA
CTACGTTGTTTATGGTCTTACTGGTAGAGGTGTTATATCTGTCAGTAACACCACTTACATTGCTGGTTTGTACTATACA
TCATTTGCAGGACAGTTGTTAGGTTTTAAAAATTCTACTACAGGTGTTCTTTATGCTGTTGAACCATGTGACACTTCTA
GTCAAGTTGCCGTCTACGGTGATGCTATTATAGGTGCTATGACATCTTCTGCTAATGTCACTTTTGGTTTTGTTAACAC
CACAGTGCTACCATCTTTTTACTACCACACTAATGGTTTTGGTGGTTGTCAGACACCCGTGTTAGAGTATGGTTCCATG
GGTTTGTGTGCTGACGGTAGTGTTGTAGATGTTACATTACGCCAATACCAACCAGCGCCAGTTTCACCAATTGCAACAA
CAAACATTAGCATACCATTAAACTTCACAATCTCCATTCAAACAGAGTACATACAGATGTACTCACAACAAGTTTCAGT
TGATTGTAGTATGTATGTTTGTAATGGTAATGTTAGGTGTTTGCGTTTACTCACGCAGTATGCAACTGCTTGTAGGAAT
ATTGAAAGTGCTTTACAACTTAGTGCTCGTTTAGAGTCGTTAGAAGTCAGCTCAGTTGTTTCTGTTTCTGAAAATGCCT
TAAAGTTGGCAAATATCACACATTTTGATAGTTACAATTTATCAGTTTTGCTGCCTAAAAGTGATGGTAAAAGTGTTGT
TGAAGACATACTTTTTGATAAAGTTGTTACTAGTGGTCTTGGTACCGTTGACCAGGATTACAAAAATTGTGTGGGTAAA
CTTGGCGTTGCACAGGACATAGCTGACGTTGGTTGTGCACAGTATTATAACGGCATCATGGTGTTACCTGGTGTTGTGA
ATGAAGCAAAAATGGGTTTGTATACTGCATCTTTAACAGGTGCCATGGTGATGGGAGGTTTTACAGCTGCAGCTGCTAT
ACCATTTTCTTTGGCTGTACAATCTCGTCTTAATTATGTGGCCTTGCAAACTGATGTTCTGCAACAAAATCAAAAGTTG
TTGGCAGACTCTTTTAATAATGCACTTTCTAATATAACATTGGCCTTCTCCAATGTTAATGATGCTTTGCAAGATGCTT
CTGAAGCCATTAACACGGTTGCGGTGGCATTAGGCAAGGTTCAAGGTGTTGTCAATGAGCAAGGACAAGCCATAGCGCA
CCTTACACAACAATTAACGCAAAATTTTCAGGCTATTTCGCATTCTATAGCTGATATTTATAATAGACTTGATGAACTT
GCTGCTGACGCACAAACAGACCGTTTGATAACGGGTCGTCTTAGTGCGTTGAATGCTTTTGTTACTCAAACTCTAACTA
AGTCAGCTGAAGTTAAACAGAGCAGGCTTTTGGCTCAACAAAAGGTGAATGAGTGTGTTAAATCACAATCAAATCGGTT
TGGTTTTTGTGGCAATGGTACACATTTGTTTTCCATTGTTAACAATGCACCTAATGGTTTGATGTTTTTCCATACCGTT
TTAGTTCCTACTGTTTACGTCACAGTTTCTGCATGGGCTGGTGTTTGTTTTAATAACCGTGCGTACATTCTTAGAGATG
TTGAGAGTGTGCTTTTTAATAATAACGGTTATTATGTCACTAACAGGAAGATGTATGAACCACGTGTTCCTCAGTTCTC
CGACTTTGTGCAAGTTGAGAATTGTGTTGTGTCTTATTTGAATCTTTCTGATGTTGATGTTAACACTGTAATACCTGAT
TACATAGATGTTAACAAAACTCTAGAGGATTTGTTACAACAGTTACCAAATCACACACTTCCTGACCTTGGCCTAGATC
AGTATAATAATACAATTTTAAACTTGACAGCTGAAATAGACATTTTACAGAACAAGTCTGACTTGTTGTTGGCATCTAC
TGAAAGGTTGCAACAACTTATTGATAACCTCAATAAGACGTATGTGGACTTAGAGTGGCTTAATAGATTTGAACAGTAC
ATTAAGTGGCCTTGGTATGTGTGGTTGGCAATTTTGCTTGCCATCATACTATTCTCATTTTTGATGTTATATTGTTGTT
GTGCCACTGGTTGTTGTGGTTGTCTTAGCTGTTTGACCAATTCTTGTGATTGTCGCGGCAAAAATTTACAACGTTACGA
AGTAGAAAAAGTGCACATTCAATAA

>gi|408796100|gb|JQ989271.1| Rousettus bat coronavirus HKU10 isolate 175A, complete genome

ATGATTTCTCTTGCTGTTTTTATAGCAGAGATACTTGTTATTATTACGAGATTTCCGTGTCTTGCTGATCAATGT
CAAGGCACACCTTGGTTGGCCAGGAACTTGAATCTTGGTTTGTCACCTAATTTGACTAATGTTTATGTATCAGGTTTGT
TGCCTACAAAGGACACCAACAATTGGTATTGTCGCACAGCATCATACTTTTTTACAGGTTATAAAGGTGTGTTTGTATC
GTACCACAGTTTTGGTATGCCTTGGTCTATTGGTGTTTCCAATTCTGATCTCTCTGTTGCACGTGAAACATGGTCATTG
TATGTTTCTCATGGCAATAATAGAGGTGTTATGACCTTTAGAATTTGTAAATGGCCACGTGTACCTGGACTAGCACAAG
AACCTACTCCTAACACTGGTGGTGTTAATTGTGTAGTAGATAAGCAATTTCCTTTCACTTTTCAACACGAGGCTAATGA
AATGGTGGGTGTTACTTGGAGTTCTGATTACGTTCGTATTTATGGGCTTCAAAGTACCTATCGTTTTTATTTTCCTAAT
AAGTGGAATAAGGTTAATGTTAATTGTGCACATAAGAGTTCTTGTCAGGTTTACCCTGTTAAAGACAGTGTTACGTGGG

TTGTTAATACCACAGCTGATGGACGTGTTAAAGACTACTCTATTTGTGGTTCTTGTAATGGTTTTCCGCAACACGTCTT
CCCTGTTTCACCTGGTGGTTTAATACCTCCTGATTTTAGTTTTGACGGTTGGTATGTTTTAACCAACTCTTCCACTATG
GTGCAAGGTAAGTTTGTTGCATCTCAACCTTTAAAATTGTTGTGCCTTTGGCCTGTACCTGCTTTGGATTCTAGTGCTA
GTGTTGTTTATTTTAATTTATCACGTAGTAGTGCGCAATGCAACGGTGCTACTGAAAATGGTCTAGCCGATGCATTACG
TTTTGCTATGAATTTCTCTAGAAATGGTTTTAGTGGTGCTCACAGTTTGACTTTAAAAGGTGCCATTGATTATGTTTTT
AGGTGCACTAATAACACTAATGTAACAAGTTTTTATTCTATACCATTTGGTTATATTAAAACAGTTTACAGGTGTTTTG
TATCCTATAATAGCACTAATGGCACAGTGTCAGAGTTTGTGGGTGTTTTGCCACCTCTTGTTAAAGAAATTGTTGTCTC
ACGTTATGGCACCATTTATTTGAATGGTGTACGTATTTTTAAGTTACCGCCTTTAGAAGGCGTTGTGTTCAATGTAACT
AGTGATGTTGGTTCAGATTTCTGGACTGTTGCATTTGCGCAAAACGCTGAAGTTTTGTTGGAAGTTAATGCAACTAACA
TTGTTAATTTGCACTATTGTGATAATACGTTGAACAAGGTTAAGTGTCAACAACTTTCTTTTCAATTACCTGATGGTTT
TTATCCTACAACGAACCTGGTGGATAGTGTTGTTCCTAAAACATATGTTGCTTTACCCTATCATGCAACACACACGTAC
ATTGAATTAAATATAACTGTTAATTATGTACGTGATGAGATAGCATTAACTGGAGGTGGTGAGTTGACTTGTGTTAATA
CAACACAATTCACTACACTCTTCTCAACTAATGCTGCTACTGATGGGAACACTTACGAGGGTGTAGTTATTAATGTCAA
TTGTCCTTTTGACTTTGACAACCTTAATAATTACTTGTCCTTTGACTCTATTTGTTTCTCCTTGAAACCAATCGCTGGT
GGTTGCACCATGCGGCTTGTTAAACAAATTCTTGTGTCATATTTTGACATAGGTGTTTTGTATGTTTCTTATACACCTG
GTTCAAAAATTACTGGTGTTTTACAACCTACAGGTGGTGTTTATGACCCTAGTATTGTTCATATGGGTGTTTGTACGGA
CTACGTTGTTTATGGTCTTACTGGTAGAGGTGTTATATCTGTCAGTAACACCACTTACATTGCTGGTTTGTACTATACA
TCATTTGCAGGGCAGTTGTTAGGTTTTAAAAATTCCACTACAGGTGTTCTTTATGCTGTTGAACCATGTGACACTTCTA
GTCAAGTTGCCGTCTACGGTGATGCTATTATAGGTGCTATGACATCTTCTGCTAATGTCACTTTTGGTTTTGTTAACAC
CACAGTGCTACCATCTTTCTACTACCACACTAATGGTTTTGGTGGTTGTCAGACACCCGTGTTAGAGTATGGTTCCATG
GGTTTGTGTGCTGACGGTAGTGTTGTAGATGTTACATTACGCCAATACCAACCAGCGCCAGTTTCACCAATTGCAACAA
CAAACATTAGCATACCATTAAACTTCACAATCTCCATTCAAACAGAGTACATACAGATGTACTCACAACAAGTTTCAGT
TGATTGTAGTATGTATGTTTGTAATGGTAATGTTAGGTGTTTGCGTTTACTCACGCAGTATGCAACTGCTTGTAGGAAT
ATTGAAAGTGCTTTACAGCTTAGTGCTCGTTTAGAGTCGTTAGAAGTCAGCTCAGTTGTTTCTGTTTCTGAAAATGCCT
TAAAATTGGCAAACATCACACATTTTGATAGTTACAATTTATCAGTTTTGTTGCCTAAAAGTAACGGTAAAAGTGTTGT
TGAAGACATCCTTTTTGATAAAGTTGTTACTAGTGGTCTTGGTACCGTTGACCAGGATTACAAAAATTGTGTGGGCAAA
CTTGGCGTTGCACAGGACATAGCTGACGTTGGTTGTGCACAGTATTACAACGGCATCATGGTGCTACCTGGTGTCGTGA
ATGAAGCAAAAATGGGTTTGTACACTGCATCTTTAACAGGTGCTATGGTGATGGGAGGTTTTACAGCTGCAGCTGCTAT
ACCATTTTCTTTGGCTGTACAATCTCGTCTTAATTATGTGGCCTTGCAAACTGATGTCCTGCAACAAAATCAAAAATTG
TTGGCAGACTCTTTTAATAATGCACTTTCTAATATAACATTGGCTTTTTCCAATGTTAATGATGCTTTGCAAGATGCTT
CTGAAGCCATTAACACGGTTGCAGTAGCATTAGGCAAGGTTCAAGGTGTTGTCAATGAGCAAGGACAAGCCATAGCGCA
CCTTACACAACAATTAACGCAAAATTTTCAGGCTATTTCGCATTCTATAGCTGATATTTATAATAGACTTGATGAACTT
GCTGCTGACGCACAAACAGACCGTTTGATAACGGGTCGTCTTAGTGCGTTGAATGCTTTTGTTACTCAAACTCTAACTA
AGTCAGCTGAAGTTAAACAGAGCAGGCTTTTGGCCCAACAGAAGGTGAATGAGTGTGTTAAATCACAATCAACTCGGTT
TGGTTTTTGTGGCAATGGTACACATTTGTTTTCCATTGTTAACAATGCACCTAATGGTTTGATGTTTTTCCATACCGTT
TTAGTTCCTACGGTTTACGTCACAGTTTCTGCATGGGCTGGTGTTTGTTTTAATAACCGTGCGTACATTCTTAGGGATG
TTGAGAGTGTGCTTTTTAATAATAACGGTTATTATGTCACTAACAGGAAGATGTATGAACCACGTGTTCCTCAGTTCTC
CGACTTTGTGCAAGTTGAGAATTGTGTTGTGTCTTATTTGAATCTTTCTGATGTTGATGTTAACACTGTAATACCTGAT
TACATAGATGTTAACAAAACTCTTGAGGATTTGTTACAACAGTTACCAAATCACACACTTCCTGACCTTGGCCTAGATC
AGTATAATAATACAATTTTAAATTTGACAGCTGAAATAGACATTTTACAGAACAAGTCTGACTTGTTGTTGGCATCTAC
TGAAAGGTTGCAACAACTTATTGATAACCTCAATAAGACGTATGTGGACTTGGAGTGGCTTAATAGATTTGAACAGTAC
ATTAAGTGGCCTTGGTATGTGTGGTTGGCAATTTTGCTTGCCATTATACTATTCTCATTTTTAATGTTATATTGTTGTT
GTGCCACTGGTTGTTGTGGTTGTCTTAGCTGTTTGACCAATTCTTGTGATTGTCGCGGCAAAAATTTACAACGTTACGA
AGTAGAAAAAGTGCACATTCAGTAA

>gi|408796110|gb|JQ989272.1| Hipposideros bat coronavirus HKU10 isolate TLC1343A, complete genome

ATGTTTACGCTAGCTGTGTGGCTTGCTGAAATCGTTGTTATTTTAACCAGGCCTGTTTCGTCTAGTAATGGTTGTACTGGTCAACCACACTTATTGCCCAATCTCAATTTGGGTTTGCCTGCAAATTCCACGGTGTTTGTTAGTGGTTATTTACCCGTGGCGGACGGCTGGAAATGTACAACCGGTAATATGGTTTACGAAAATGTAAATGCTGTTTACTGGTCTTATTTTTCACATGCCACTCCTGTAGAAATTGCCATTTCCAACCCTGACCAACTTACTAATTCTGATCAATGGGCTTTGTACATTTACCATGGAAATAACCATGGTTTTATGTACTTTAAAATATGTAAATGGCAAGGCAAGTATAGTTTTGTAAATAACCCTTATCCAAATAGAGGTGTTTGCATGTATAATAGACAATTTCGTTTTGTTTTTCCACATGCTAAAAACATTGTTGTTGGTGCTTCATGGACAGGAGATTATGTTACATTATTTACCACTACTAGTGCCAGACGTTTTTATCTTAAAAACAATTGGTCTAGAGTTAATGTTAAATGTGACCAGGCTCCGTCTTGCGCAATACAAACAGTGTCAAATGTCACCACACTAAACATCACCACAAATAGCCAAGGTCTTATACAGTCTTACAGTGTATGTACTGAGTGTGACGGTTTTCCTAAACATGTTTTTCCTGTCCTTGACGATGGGAAAATACCTGGTGATTTTGGTTTTGATAATTGGTTCGTTTTGACCAACTCTTCCACTGTTTTGCAGGGTCGAATTGTCACTGTACAACCTTTAAAATTGCTTTGTTTATGGCCTGTACCTTCTCTTAACGCAAATGATAAACCTGTCTATTTTAACCTATCACTTAACTCAGATGTTAGATGTAATGGTTATTCTAGTGCTGGTTATGCTCAAGCGTTGAGGTTTTCGCTTAACTTTACAGCAAATCAAGTCTTGAATGGTGTGCATAGTATTAAACTTTTCTCTTCCAATCAAAACTTTACATTCACATGTTCAACTAACGGTACTCTAGAAAGAATACCTTTTGGTCGTGTTGAAAGTGTGTATAAATGTTTTGTTCGTATTGATGCAATTAATTCTACAAAAAAATTTGTTTGTGGGTATTTTACCACCTGTCACAAAGGAGTTTGTTATTTCACGTTATGGTGCTGTTTATATAAATGGCGTACATATAATGTCTTTACCTCCTTTGGAAAGTGTCATTTTTAATGTCACTAGTAGTGTGGGTTCTGACTTTTGGACTGTTGCTTTTGCTGATGAAGCTGAAGTTTTGTTGGATTTGAATGCCACAAACATAGTAGACATTTTATATTGTGACACTTTGGTCAATAAAATAAAATGTCAAAATCTTGCATTTTCTTTGGAGGATGGTTTTTATGCTACATCCAATCTTATAGAACGTGACATACCTAGATCTTTCATAACTTTACCCTACCATGTAACTCATTCTGTAGTGGGTTTGGATGTTAGGATTTATTATGAAAATGAAGCTGCAAGTAATCTCACTTTGTCTCCTTCAGATCCTGTGTGTGTTGATAGCTCACAGTTCTCAACTTATTTCAATGTCACTGCTGACGTTACTGGTTTCAATGCCAGAATTATTAATGTTGACTGTCCTTTTACTTTTGATAAGCTTAACAATTACATTAGTTTTGAATCCATTTGTTTTTCTTTGGAACCTTTTGCTGGTGGTTGTTCTATGCGTATTGAAAGTTTCTTTTCTAGCTATGTTAGGACTGTGGGCAGTCTTTACATTCTTTTTAAGAATGGTAATAAGATTACTGGTGTTACAGAAAGTTCAGCTGGCGTTTATGATACTAGTTTTCTACACACAGACATTTGCACAGATTACACTATTTATGGCACTACTGGTAGAGGTGTTATAACCAAGCTTAATACTACTATTATAGCTGGTTTATATTACACCTCACAATCTGGTCAAATACTTGGTTTTAAAAATGCCACCAATGGTGACATTTATGCTGTTAGACCTTGTGACTTGTCCAGTCAAGCTGCTGTTGTTAGTAATCAAATTGTTGCTGTTATGTCTGCTTCTGAAAACATTACCTTTGGCTTTAACAATTCTCTTGAATTGCCTAAATTTTACCTACATTCTAATGCTGAAAATGCTACTAATTGCACAGAACCAGCACTTATTTATGGTAAAATTGGCATTTGCGAAGATGGTAGCATTACTGAAGTTAAACCACGTATTGCAGAACCTGAACCTTACTCGCCTATTGTTACAGCTAATATTACAATCCCTAGTAATTTTAGCATTTCTGTGCAGGCTGAGTATGTTCAAATGTTTTTGACACCTGTTAGTGTTGACTGTAATATGTATGTTTGTAATGGTAATAGGCATTGTCTTCGCCTTTTAGGTGAATATGTTACTGCTTGTAAAAATATTGAAGCTGCACTTCAGCTTAGTGCTCGCTTGGAATCTATTGAAGTTAATAATTTCATTACTGTTTCTGAGAATGCTATATCTCTTGCCAACATCTCCAACTTTGATAGCTACAACCTTTCAGTTTTACTACCTAAACAGAATGGTAAGAGTGTCATTGAAGACATTTTGTTTGATAAGGTTGTGACTAATGGTCTTGGTACCGTTGACCAGGATTATAAGGAGTGCATCAAGCGTAGTGGAGTTAAGGATGTAGCAGATGTGGCTTGTGCTCAGTATTACAATGGAATTATGGTGCTCCCTGGTGTTGTTGATGACGTAAAAATGGGTCTTTACACAGCTTCTTTAACTGGTGCTATGGTTATGGGCGGTTTAACTGCCGCTGCTGCTATACCTTTTTCAATAGCTGTTCAATCACGACTCAATTATGTTGCACTTCAAACTGATGTTTTGCAGAAGAACCAGCAAATTTTAGCAGATTCATTTAACAATGCTATGAGTAATATTACTATGGCATTTGCTCAGTTGAATGATGCCATTAAAGAAACTTCTCAAGCCATCAGTACCGTAGCTCAAGCTTTGAGTAAGGTTCAGAGTGTTGTCAATGAACAGGGTGAAGCACTTTCACAGCTTACTAAACAATTGGCTTCCAATTTTCAGGCTATTTCGTCTTCCATAGAAGACATTTATAACCGCTTGGACATGCTTGCTGCTGATGCT

CAAGTTGACAGACTTATCACGGGCAGAATTGGTGCTTTGAATGCTTTTGTCACACAAACACTTACAAAGTACACTGAAG
TTCGTGCTAGTCGTCAACTTGCTTTGCAGAAAATTAATGAATGTGTCAAATCACAGTCTAGCCGATTTGGTTTTTGTGG
AAATGGCACTCATTTATTTTCAATCGCTAACGCTGCTCCTAACGGTATAATGTTATTTCACACAGTCCTCATGCCTACT
GAGTATGTTGTTGTACGTGCATGGGCTGGACTTTGTATTGAAGACAAAGCTTTTGTGCTACGTGACGTTAAAACAACCC
TTTTTAAGACTGGTGAAACTTATTACATCACTACTAGAGACATGTATCAACCTAGACTTCCACAAGTTAGTGACTTCGT
TCAAATTAAAGATTGTCAAGTTGATTATCTTAATTTGACATCAGATGAAGTTAATAATGTGATACCTGACTATATTGAT
GTAAATAAAACTTTGGAAGATTTTGGCCAAATACTTGCTAATAATACACGACCATCAATACCTGATTTTGGTTTAGATG
TTTACAATAACACAATTCTCAACTTGACTGCTGAGATTGATGTATTGCAAAATAAATCTGAACAACTTTTGGCTTCAAC
TGAAAGACTTCAACAACTTATTGATAATCTCAATAAGACTTATGTTGATCTTGAGTGGCTCAACAGGTTCGAACAATAC
ATTAAGTGGCCATGGTATGTGTGGCTAGCAATTTTTCTTGCCGTTATACTATTTTCTTTCTTAATGCTTTACTGTTGTT
GTGCTACAGGTTGTTGTGGTTGTTTTAGCTGTCTTTCTAATTCTTGTGATTGTCGCGGCAAAAATTTACAACGTTACGA
AGTAGAAAAAGTGCACATTCAATAA

>gi|408796120|gb|JQ989273.1| Hipposideros bat coronavirus HKU10 isolate TLC1347A, complete genome

ATGTTTACGCTAGCTGTGTGGCTTGCTGAAATCGTTGTTATTTTAACCAGGCCTGTTTCGTCTAGTAATGGTTGT
ACTGGTCAACCACACTTATTGCCCAATCTCAATTTGGGTTTGCCTGCAAATTCCACGGTGTTTGTTAGTGGTTATTTAC
CCGTGGCGGACGGCTGGAAATGTACAACCGGTAATATGGTTTACGAAAATGTAAATGCTGTTTACTGGTCTTATTTTTC
ACATGCCACTCCTGTAGAAATTGCCATTTCCAACCCTGACCAACTTACTAATTCTGATCAATGGGCTTTGTACATTTAC
CATGGAAATAACCATGGTTTTATGTACTTTAAAATATGTAAATGGCAAGGCAAGTATAGTTTTGTAAATAACCCTTATC
CAAATAGAGGTGTTTGCATGTATAATAGACAATTTCGTTTTGTTTTTCCACATGCTAAAAACATTGTTGTTGGTGCTTC
ATGGACAGGAGATTATGTTACATTATTTACCACTACTAGTGCCAGACGTTTTTATCTTAAAAACAATTGGTCTAGAGTT
AATGTTAAATGTGACCAGGCTCCGTCTTGCGCAATACAAACAGTGTCAAATGTCACCACACTAAACATCACCACAAATA
GCCAAGGTCTTATACAGTCTTACAGTGTATGTACTGAGTGTGACGGTTTTCCTAAACATGTTTTTCCTGTCCTTGACGA
TGGGAAAATACCTGGTGATTTTGGTTTTGATAATTGGTTCGTTTTGACCAACTCTTCCACTGTTTTGCAGGGTCGAATT
GTCACTGTACAACCTTTAAAATTGCTTTGTTTATGGCCTGTACCTTCTCTTAACGCAAATGATAAACCTGTCTATTTTA
ACCTATCACTTAACTCAGATGTTAGATGTAATGGTTATTCTAGTGCTGGTTATGCTCAAGCGTTGAGGTTTTCGCTTAA
CTTTACAGCAAATCAAGTCTTGAATGGTGTGCATAGTATTAAACTTTTCTCTTCCAATCAAAACTTTACATTCACATGT
TCAACTAACGGTACTCTAGAAAGAATACCTTTTGGTCGTGTTGAAAGTGTGTATAAATGTTTTGTTCGTATTGATGCAA
TTAATTCTACAAAAAATTTGTTTGTGGGTATTTTACCACCTGTCACAAAGGAGTTTGTTATTTCACGTTATGGTGCTGT
TTATATAAATGGCGTACATATAATGTCTTTACCTCCTTTGGAAAGTGTCATTTTTAATGTCACTAGTAGTGTGGGTTCT
GACTTTTGGACTGTTGCTTTTGCTGATGAAGCTGAAGTTTTGTTGGATTTGAATGCCACAAACATAGTAGACATTTTAT
ATTGTGACACTTTGGTCAATAAAATAAAATGTCAAAATCTTGCATTTTCTTTGGAGGATGGTTTTTATGCTACATCCAA
TCTTATAGAACGTGACATACCTAGATCTTTCATAACTTTACCCTACCATGTAACTCATTCTGTAGTGGGTTTGGATGTT
AGGATTTATTATGAAAATGAAGCTGCAAGTAATCTCACTTTGTCTCCTTCAGATCCTGTGTGTGTTGATAGCTCACAGT
TCTCAACTTATTTCAATGTCACTGCTGACGTTACTGGTTTCAATGCCAGAATTATTAATGTTGACTGTCCTTTTACTTT
TGATAAGCTTAACAATTACATTAGTTTTGAATCCATTTGTTTTTCTTTGGAACCTTTTGCTGGTGGTTGTTCTATGCGT
ATTGAAAGTTTCTTTTCTAGCTATGTTAGGACTGTGGGCAGTCTTTACATTCTTTTTAAGAATGGTAATAAGATTACTG
GTGTTACAGAAAGTTCAGCTGGCGTTTATGATACTAGTTTTCTACACACAGACATTTGCACAGATTACACTATTTATGG
CACTACTGGTAGAGGTGTTATAACCAAGCTTAATACTACTATTATAGCTGGTTTATATTACACCTCACAATCTGGTCAA
ATACTTGGTTTTAAAAATGCCACCAATGGTGACATTTATGCTGTTAGACCTTGTGACTTGTCCAGTCAAGCTGCTGTTG
TTAGTAATCAAATTGTTGCTGTTATGTCTGCTTCTGAAAACATTACTTTTGGCTTTAACAATTCTCTTGAATTGCCTAA

ATTTTACCTACATTCTAATGCTGAAAATGCTACTAATTGCACAGAACCAGCACTTATTTATGGTAAAATTGGCATTTGC
GAAGATGGTAGCATTACTGAAGTTAAACCACGTATTGCAGAACCTGAACCTTACTCGCCTATTGTTACAGCTAATATTA
CAATCCCTAGTAATTTTAGCATTTCTGTGCAGGCTGAGTATGTTCAAATGTTTTTGACACCTGTTAGTGTTGACTGTAA
TATGTATGTTTGTAATGGTAATAGGCATTGTCTTCGCCTTTTAGGTGAATATGTTACTGCTTGTAAAAATATTGAAGCT
GCACTTCAGCTTAGTGCTCGCTTGGAATCTATTGAAGTTAATAATTTCATTACTGTTTCTGAGAATGCTATATCTCTTG
CCAACATCTCCAACTTTGATAGCTACAACCTTTCAGTTTTACTACCTAAACAGAATGGTAAGAGTGTCATTGAAGACAT
TTTGTTTGATAAGGTTGTGACTAATGGTCTTGGTACCGTTGACCAGGATTATAAGGAGTGCATCAAGCGTAGTGGAGTT
AAGGATGTAGCAGATGTGGCTTGTGCTCAGTATTACAATGGAATTATGGTGCTCCCTGGTGTTGTTGATGACGTAAAAA
TGGGTCTTTACACAGCTTCTTTAACTGGTGCTATGGTTATGGGCGGTTTAACTGCCGCTGCTGCTATACCTTTTTCAAT
AGCTGTTCAATCACGACTCAATTATGTTGCACTTCAAACTGATGTTTTGCAGAAGAACCAGCAAATTTTAGCAGATTCA
TTTAACAATGCTATGAGTAATATTACTATGGCATTTGCTCAGGTGAATGATGCCATTAAAGAAACTTCTCAAGCCATCA
GTACCGTAGCTCAAGCTTTGAGTAAGGTTCAGAGTGTTGTCAATGAACAGGGTGAAGCACTTTCACAGCTTACTAAACA
ATTGGCTTCCAATTTTCAGGCTATTTCGTCTTCCATAGAAGACATTTATAACCGCTTGGACATGCTTGCTGCTGATGCT
CAAGTTGACAGACTTATCACGGGCAGAATTGGTGCTTTGAATGCTTTTGTCACACAAACACTTACAAAGTACACTGAAG
TTCGTGCTAGTCGTCAACTTGCTTTGCAGAAAATTAATGAATGTGTCAAATCACAGTCTAGCCGATTTGGTTTTTGTGG
AAATGGCACTCATTTATTTTCAATCGCTAACGCTGCTCCTAACGGTATAATGTTATTTCACACAGTCCTCATGCCTACT
GAGTATGTTGTTGTACGTGCATGGGCTGGACTTTGTATTGAAGACAAAGCTTTTGTGCTACGTGACGTTAAAACAACCC
TTTTTAAGACTGGTGAAACTTATTACATCACTACTAGAGACATGTATCAACCTAGACTTCCACAAGTTAGTGACTTCGT
TCAAATTAAAGATTGTCAAGTTGATTATCTTAATTTGACATCAGATGAAGTTAATAATGTGATACCTGACTATATTGAT
GTAAATAAAACTTTGGAAGATTTTGGCCAAATACTTGCTAATAATACACGACCATCAATACCTGATTTTGGTTTAGATG
TTTACAATAACACAATTCTCAACTTGACTGCTGAGATTGATGTATTGCAAAATAAATCTGAACAACTTTTGGCTTCAAC
TGAAAGACTTCAACAACTTATTGATAATCTCAATAAGACTTATGTTGATCTTGAGTGGCTCAACAGGTTCGAACAATAC
ATTAAGTGGCCATGGTATGTGTGGCTAGCAATTTTTCTTGCCGTTATACTATTTTCTTTCTTAATGCTTTACTGTTGTT
GTGCTACAGGTTGTTGTGGTTGTTTTAGCTGTCTTTCTAATTCTTGTGATTGTCGCGGCAAAAATTTACAACGTTACGA
AGTAGAAAAAGTGCACATTCAATAA

>gi|126030112|ref|NC_009019.1| Bat coronavirus HKU4-1, complete genome

ATGACACTCTTAATGTGTCTACTGATGTCCTTGTTAATTTTCGTAAGAGGCTGTGATTCACAATTTGTTGACATG
AGTCCAGCTAGTAATACTAGTGAGTGTTTGGAGTCACAAGTTGACGCAGCTGCATTTTCTAAATTAATGTGGCCTTATC
CTATTGATCCTTCCAAAGTTGATGGTATCATTTATCCACTTGGTAGGACATATTCTAATATTACATTAGCATATACTGG
TCTTTTTCCCCTACAAGGAGATTTAGGTAGCCAGTATTTATATTCTGTAAGTCATGCTGTAGGTCATGATGGTGATCCT
ACAAAAGCTTATATTTCTAATTATTCACTATTGGTGAATGATTTTGATAACGGTTTTGTTGTACGAATTGGTGCTGCAG
CTAATTCAACTGGCACCATTGTAATTTCACCGTCTGTTAATACTAAGATTAAAAAAGCTTATCCTGCTTTTATCTTAGG
TTCGTCTCTTACTAATACATCGGCTGGTCAGCCATTGTATGCTAATTACAGTTTAACCATCATTCCTGATGGTTGTGGG
ACTGTGTTGCATGCTTTTTATTGCATATTAAAACCACGTACTGTGAATCGATGTCCTAGTGGTACTGGTTATGTGTCTT
ATTTTATTTATGAAACAGTTCATAATGATTGTCAGTCTACAATTAACAGGAACGCTTCTCTTAATTCTTTTAAGAGCTT
TTTCGATCTTGTTAATTGTACTTTCTTTAATAGTTGGGATATCACTGCTGATGAAACAAAAGAGTGGTTTGGTATTACC
CAAGACACTCAAGGTGTTCATCTCTACTCTTCTAGGAAAGGTGATTTATATGGCGGAAATATGTTTCGCTTTGCTACAT
TACCTGTGTACGAGGGTATAAAATATTACACTGTTATACCCAGAAGTTTTCGGTCTAAAGCTAACAAAAGAGAGGCTTG
GGCTGCATTTTATGTGTATAAATTACATCAGCTGACTTACTTGTTAGATTTTTCTGTTGATGGTTATATACGTAGAGCT
ATAGATTGTGGACATGATGATTTGTCACAGCTCCATTGTTCTTATACATCTTTTGAAGTTGACACTGGTGTCTATTCTG
TTTCTTCATATGAAGCTTCAGCTACTGGCACCTTTATTGAACAACCTAATGCTACTGAGTGTGATTTTTCACCTATGCT

TACAGGTGTAGCTCCTCAGGTTTATAATTTTAAAAGGTTGGTTTTTTCCAATTGTAATTATAATCTTACTAAACTCCTC
TCTTTGTTTGCAGTTGATGAGTTTTCTTGTAATGGAATTTCACCTGATTCTATTGCACGTGGTTGTTATTCTACTTTAA
CAGTGGATTATTTTGCCTATCCACTTAGTATGAAGTCATATATCAGACCTGGTTCTGCAGGCAATATACCACTTTACAA
TTATAAACAGTCTTTTGCCAATCCTACGTGTAGAGTTATGGCATCTGTTCTTGCTAATGTTACTATTACTAAGCCTCAT
GCTTATGGTTATATTTCCAAGTGTTCTCGTCTGACTGGTGCAAACCAAGATGTTGAAACACCATTATATATTAACCCTG
GTGAGTACTCTATTTGTAGAGATTTTTCTCCTGGGGGTTTTTCTGAAGATGGTCAGGTCTTCAAGCGTACTCTTACCCA
ATTCGAGGGTGGTGGCTTGCTTATTGGAGTTGGCACTAGAGTACCTATGACTGATAACTTACAAATGAGTTTTATAATC
TCTGTCCAATATGGTACCGGCACCGATAGTGTTTGCCCCATGTTGGACTTAGGTGATTCTTTAACTATCACTAACCGTT
TAGGTAAGTGTGTAGATTATTCACTCTATGGTGTTACTGGTAGAGGTGTTTTTCAAAACTGTACTGCCGTTGGTGTTAA
ACAGCAGCGTTTTGTTTATGATAGCTTTGACAATTTAGTTGGTTACTATTCTGATGATGGTAACTACTATTGTGTCAGA
CCTTGTGTCAGTGTACCTGTGTCTGTTATATATGATAAAAGTACAAATTTACATGCGACTTTATTCGGTAGTGTTGCTT
GTGAGCACGTTACTACAATGATGTCGCAATTTTCACGCCTTACTCAAAGTAATTTACGTAGGCGTGATTCTAATATACC
TCTTCAAACAGCTGTTGGTTGTGTTATAGGTCTTTCTAATAACTCACTAGTAGTTAGTGATTGTAAATTGCCATTAGGC
CAGTCACTTTGTGCAGTCCCTCCTGTGTCTACTTTTAGAAGTTATAGCGCTAGCCAATTTCAGCTTGCTGTGCTTAATT
ATACTTCTCCTATTGTTGTTACTCCTATTAATTCCAGTGGTTTTACCGCTGCAATTCCAACTAATTTTTCTTTTTCAGT
TACACAGGAGTACATTGAAACATCCATTCAGAAGGTTACTGTAGATTGTAAACAGTATGTCTGTAATGGTTTTACTAGG
TGTGAAAAGCTATTGGTTGAATATGGGCAATTTTGTTCCAAAATTAATCAAGCTTTACATGGTGCTAATCTTAGACAAG
ATGAGTCTGTTTATAGTCTTTATAGTAACATTAAAACCACCTCTACCCAAACTCTTGAGTATGGTTTAAATGGTGATTT
TAATCTTACATTATTACAAGTACCACAAATTGGAGGTTCTTCCTCTAGTTATCGTAGTGCTATTGAAGACTTATTATTT
GATAAGGTAACTATTGCAGATCCCGGTTATATGCAGGGCTATGATGATTGCATGAAACAAGGACCCCAGTCTGCTAGAG
ATCTTATTTGTGCTCAATATGTGTCTGGTTACAAAGTTTTACCACCACTTTATGACCCTAATATGGAAGCTGCTTACAC
ATCTTCACTATTAGGTAGTATAGCAGGTGCTGGATGGACAGCTGGTCTTTCATCCTTTGCCGCTATACCTTTTGCACAA
AGTATGTTTTATAGGTTGAATGGCGTTGGTATTACTCAACAAGTTCTCTCTGAGAACCAAAAGTTGATTGCTAACAAGT
TTAACCAAGCCTTAGGTGCTATGCAAACTGGCTTTACAACATCTAACCTAGCTTTTAGCAAGGTTCAAGATGCTGTAAA
TGCCAATGCACAAGCACTTTCTAAGTTAGCATCAGAGTTGTCTAATACTTTTGGTGCTATTTCTTCATCTATTAGTGAC
ATCCTTGCTAGGCTTGACACTGTTGAACAGGATGCGCAAATTGATCGTTTGATCAATGGTCGCTTAATTTCTCTTAACG
CATTTGTTTCACAACAACTTGTGCGTTCTGAAACTGCTGCTAGATCTGCTCAGTTGGCTAGTGACAAAGTCAACGAGTG
TGTAAAATCACAATCCAAGAGAAATGGGTTCTGTGGGTCTGGCACTCATATAGTATCATTTGTTGTTAATGCTCCAAAT
GGTTTTTATTTCTTTCATGTTGGTTATGTTCCAACAAATTATACTAATGTTACAGCTGCTTATGGTCTCTGTAACAATA
ACAACCCTCCTTTGTGCATTGCACCAATTGACGGGTATTTCATTACTAATCAGACTACCACTTATAGTGTTGATACCGA
ATGGTATTACACTGGTAGTTCTTTTTATAAACCTGAACCTATAACTCAGGCTAATTCACGTTATGTTTCTAGTGATGTG
AAATTTGACAAACTTGAGAATAACTTACCACCTCCACTCCTTGAGAATTCTACTGATGTGGATTTTAAGGATGAATTGG
AAGAATTTTTCAAGAATGTTACCTCGCATGGTCCTAACTTTGCAGAGATATCTAAGATTAACACTACACTGCTTGACCT
TTCAGATGAGATGGCAATGTTGCAGGAAGTTGTTAAACAATTAAATGATTCCTACATTGATCTTAAAGAATTGGGAAAT
TATACTTATTATAACAAGTGGCCTTGGTACGTATGGCTAGGTTTTATTGCAGGCCTTGTTGCCTTATTATTGTGTGTCT
TTTTCCTTCTATGCTGTACTGGTTGTGGTACTAGCTGCTTGGGAAAAATGAAGTGTAAGAATTGTTGTGATTCTTATGA
AGAATATGATGTCGAAAAGATTCATGTTCATTAA

>gi|124389407|gb|EF065506.1| Bat coronavirus HKU4-2, complete genome

ATGACACTCTTAATGTGTCTACTGATGTCCTTGTTAATTTTCGTAAGAGGCTGTGATTCACAATTTGTTGACATG
AGTCCAGCTAGTAATACTAGTGAGTGTTTGGAGTCACAAGTTGACGCAGCTGCATTTTCTAAATTAATGTGGCCTTATC
CTATTGATCCTTCCAAAGTTGATGGTATCATTTATCCACTTGGTAGGACATATTCTAATATTACATTAGCATATACTGG

TCTTTTTCCCCTACAAGGAGATTTAGGTAGCCAGTATTTATATTCTGTAAGTCATGCTGTAGGTCATGATGGTGATCCT
ACAAAAGCTTATATTTCTAATTATTCACTATTGGTGAATGATTTTGATAACGGTTTTGTTGTACGAATTGGTGCTGCAG
CTAATTCAACTGGCACCATTGTAATTTCACCGTCTGTTAATACTAAGATTAAAAAAGCTTATCCTGCTTTTATCTTAGG
TTCGTCTCTTACTAATACATCGGCTGGTCAGCCATTGTATGCTAATTACAGTTTAACCATCATTCCTGATGGTTGTGGG
ACTGTGTTGCATGCTTTTTATTGCATATTAAAACCACGTACTGTGAATCGATGTCCTAGTGGTACTGGTTATGTGTCTT
ATTTTATTTATGAAACAGTTCATAATGATTGTCAGTCTACAATTAACAGGAACGCTTCTCTTAATTCTTTTAAGAGCTT
TTTCGATCTTGTTAATTGTACTTTCTTTAATAGTTGGGATATCACTGCTGATGAAACAAAAGAGTGGTTTGGTATTACC
CAAGACACTCAAGGTGTTCATCTCTACTCTTCTAGGAAAGGTGATTTATATGGCGGAAATATGTTTCGCTTTGCTACAT
TACCTGTGTACGAGGGTATAAAATATTACACTGTTATACCCAGAAGTTTTCGGTCTAAAGCTAACAAAAGAGAGGCTTG
GGCTGCATTTTATGTGTATAAATTACATCAGCTGACTTACTTGTTAGATTTTTCTGTTGATGGTTATATACGTAGAGCT
ATAGATTGTGGACATGATGATTTGTCACAGCTCCATTGTTCTTATACATCTTTTGAAGTTGACACTGGTGTCTATTCTG
TTTCTTCATATGAAGCTTCAGCTACTGGCACCTTTATTGAACAACCTAATGCTACTGAGTGTGATTTTTCACCTATGCT
TACAGGTGTAGCTCCTCAGGTTTATAATTTTAAAAGGTTGGTTTTTTCCAATTGTAATTATAATCTTACTAAACTCCTC
TCTTTGTTTGCAGTTGATGAGTTTTCTTGTAATGGAATTTCACCTGATGCTATTGCACGTGGTTGTTATTCTACTTTAA
CAGTGGATTATTTTGCCTATCCACTTAGTATGAAGTCATATATCAGACCTGGTTCTGCAGGCAATATACCACTTTACAA
TTATAAACAGTCTTTTGCCAATCCTACGTGTAGAGTTATGGCATCTGTTCTTGCTAATGTTACTATTACTAAGCCTCAT
GCTTATGGTTATATTTCCAAGTGTTCTCGTCTGACTGGTGCAAACCAAGATGTTGAAACACCATTATATATTAACCCTG
GTGAGTACTCTATTTGTAGAGATTTTTCTCCTGGGGGTTTTTCTGAAGATGGTCAGGTCTTCAAGCGTACTCTTACCCA
ATTCGAGGGTGGTGGCTTGCTTATTGGAGTTGGCACTAGAGTACCTATGACTGATAACTTACAAATGAGTTTTATAATC
TCTGTCCAATATGGTACCGGCACCGATAGTGTTTGCCCCATGTTGGACTTAGGTGATTCTTTAACTATCACTAACCGTT
TAGGTAAGTGTGTAGATTATTCACTCTATGGTGTTACTGGTAGAGGTGTTTTTCAAAACTGTACTGCCGTTGGTGTTAA
ACAGCAGCGTTTTGTTTATGATAGCTTTGACAATTTAGTTGGTTACTATTCTGATGATGGTAACTACTATTGTGTCAGA
CCTTGTGTCAGTGTACCTGTGTCTGTTATATATGATAAAAGTACAAATTTACATGCGACTTTATTCGGTAGTGTTGCTT
GTGAGCACGTTACTACAATGATGTCGCAATTTTCACGCCTTACTCAAAGTAATTTACGTAGGCGTGATTCTAATATACC
TCTTCAAACAGCTGTTGGTTGTGTTATAGGTCTTTCTAATAACTCACTAGTAGTTAGTGATTGTAAATTGCCATTAGGC
CAGTCACTTTGTGCAGTCCCTCCTGTGTCTACTTTTAGAAGTTATAGCGCTAGCCAATTTCAGCTTGCTGTGCTTAATT
ATACTTCTCCTATTGTTGTTACTCCTATTAATTCCAGTGGTTTTACCGCTGCAATTCCAACTAATTTTTCTTTTTCAGT
TACACAGGAGTACATTGAAACATCCATTCAGAAGGTTACTGTAGATTGTAAACAGTATGTCTGTAATGGTTTTACTAGG
TGTGAAAAGCTATTGGTTGAATATGGGCAATTTTGTTCCAAAATTAATCAAGCTTTACATGGTGCTAATCTTAGACAAG
ATGAGTCTGTTTATAGTCTTTATAGTAACATTAAAACCACCTCTACCCAAACTCTTGAGTATGGTTTAAATGGTGATTT
TAATCTTACATTATTACAAGTACCACAAATTGGAGGTTCTTCCTCTAGTTATCGTAGTGCTATTGAAGACTTATTATTT
GATAAGGTAACTATTGCAGATCCCGGTTATATGCAGGGCTATGATGATTGCATGAAACAAGGACCCCAGTCTGCTAGAG
ATCTTATTTGTGCTCAATATGTGTCTGGTTACAAAGTTTTACCACCACTTTATGACCCTAATATGGAAGCTGCTTACAC
ATCTTCACTATTAGGTAGTATAGCAGGTGCTGGATGGACAGCTGGTCTTTCATCCTTTGCCGCTATACCTTTTGCACAA
AGTATGTTTTATAGGTTGAATGGCGTTGGTATTACTCAACAAGTTCTCTCTGAGAACCAAAAGTTGATTGCTAACAAGT
TTAACCAAGCCTTAGGTGCTATGCAAACTGGCTTTACAACATCTAACCTAGCTTTTAGCAAGGTTCAAGATGCTGTAAA
TGCCAATGCACAAGCACTTTCTAAGTTAGCATCAGAGTTGTCTAATACTTTTGGTGCTATTTCTTCATCTATTAGTGAC
ATCCTTGCTAGGCTTGACACTGTTGAACAGGATGCGCAAATTGATCGTTTGATCAATGGTCGCTTAACTTCTCTTAACG
CATTTGTTTCACAACAACTTGTGCGTTCTGAAACTGCTGCTAGATCTGCTCAGTTGGCTAGTGACAAAGTCAACGAGTG
TGTAAAATCACAATCCAAGAGAAATGGGTTCTGTGGGTCTGGCACTCATATAGTATCATTTGTTGTTAATGCTCCAAAT
GGTTTTTATTTCTTTCATGTTGGTTATGTTCCAACAAATTATACTAATGTTACAGCTGCTTATGGTCTCTGTAACAATA
ACAACCCTCCTTTGTGCATTGCACCAATTGACGGGTATTTCATTACTAATCAGACTACCACTTATAGTGTTGATACCGA
ATGGTATTACACTGGTAGTTCTTTTTATAAACCTGAACCTATAACTCAGGCTAATTCACGTTATGTTTCTAGTGATGTG
AAATTTGACAAACTTGAGAATAACTTACCACCTCCACTCCTTGAGAATTCTACTGATGTGGATTTTAAGGATGAATTGG

AAGAATTTTTCAAGAATGTTACCTCGCATGGTCCTAACTTTGCAGAGATATCTAAGATTAACACTACACTGCTTGACCT
TTCAGATGAGATGGCAATGTTGCAGGAAGTTGTTAAACAATTAAATGATTCCTACATTGATCTTAAAGAATTGGGAAAT
TATACTTATTATAACAAGTGGCCTTGGTACGTATGGCTAGGTTTTATTGCAGGCCTTGTTGCCTTATTATTGTGTGTCT
TTTTCCTTCTATGCTGTACTGGTTGTGGTACTAGCTGCTTGGGAAAAATGAAGTGTAAGAATTGTTGTGATTCTTATGA
AGAATATGATGTCGAAAAGATTCATGTTCATTAA

>gi|124389417|gb|EF065507.1| Bat coronavirus HKU4-3, complete genome

ATGACACTCTTAATGTGTCTACTGATGTCCTTGTTAATTTTCGTAAGAGGCTGTGATTCACAATTTGTTGACATG
AGTCCAGCTAGTAATACTAGTGAGTGTTTGGAGTCACAAGTTGACGCAGCTGCATTTTCTAAATTAATGTGGCCTTATC
CTATTGATCCTTCCAAAGTTGATGGTATCATTTATCCACTTGGTAGGACATATTCTAATATTACATTAGCATATACTGG
TCTTTTTCCCCTACAAGGAGATTTAGGTAGCCAGTATTTATATTCTGTAAGTCATGCTGTAGGTCATGATGGTGATCCT
ACAAAAGCTTATATTTCTAATTATTCACTATTGGTGAATGATTTTGATAACGGTTTTGTTGTACGAATTGGTGCTGCAG
CTAATTCAACTGGCACCATTGTAATTTCACCGTCTGTTAATACTAAGATTAAAAAAGCTTATCCTGCTTTTATCTTAGG
TTCGTCTCTTACTAATACATCGGCTGGTCAGCCATTGTATGCTAATTACAGTTTAACCATCATTCCTGATGGTTGTGGG
ACTGTGTTGCATGCTTTTTATTGCATATTAAAACCACGTACTGTGAATCGATGTCCTAGTGGTACTGGTTATGTGTCTT
ATTTTATTTATGAAACAGTTCATAATGATTGTCAGTCTACAATTAACAGGAACGCTTCTCTTAATTCTTTTAAGAGCTT
TTTCGATCTTGTTAATTGTACTTTCTTTAATAGTTGGGATATCACTGCTGATGAAACAAAAGAGTGGTTTGGTATTACC
CAAGACACTCAAGGTGTTCATCTCTACTCTTCTAGGAAAGGTGATTTATATGGCGGAAATATGTTTCGCTTTGCTACAT
TACCTGTGTACGAGGGTATAAAATATTACACTGTTATACCCAGAAGTTTTCGGTCTAAAGCTAACAAAAGAGAGGCTTG
GGCTGCATTTTATGTGTATAAATTACATCAGCTGACTTACTTGTTAGATTTTTCTGTTGATGGTTATATACGTAGAGCT
ATAGATTGTGGACATGATGATTTGTCACAGCTCCATTGTTCTTATACATCTTTTGAAGTTGACACTGGTGTCTATTCTG
TTTCTTCATATGAAGCTTCAGCTACTGGCACCTTTATTGAACAACCTAATGCTACTGAGTGTGATTTTTCACCTATGCT
TACAGGTGTAGCTCCTCAGGTTTATAATTTTAAAAGGTTGGTTTTTTCCAATTGTAATTATAATCTTACTAAACTCCTC
TCTTTGTTTGCAGTTGATGAGTTTTCTTGTAATGGAATTTCACCTGTTGCTATTGCACGTGGTTGTTATTCTACTTTAA
CAGTGGATTATTTTGCCTATCCACTTAGTATGAAGTCATATATCAGACCTGGTTCTGCAGGCAATATACCACTTTACAA
TTATAAACAGTCTTTTGCCAATCCTACGTGTAGAGTTATGGCATCTGTTCTTGCTAATGTTACTATTACTAAGCCTCAT
GCTTATGGTTATATTTCCAAGTGTTCTCGTCTGACTGGTGCAAACCAAGATGTTGAAACACCATTATATATTAACCCTG
GTGAGTACTCTATTTGTAGAGATTTTTCTCCTGGGGGTTTTTCTGAAGATGGTCAGGTCTTCAAGCGTACTCTTACCCA
ATTCGAGGGTGGTGGCTTGCTTATTGGAGTTGGCACTAGAGTACCTATGACTGATAACTTACAAATGAGTTTTATAATC
TCTGTCCAATATGGTACCGGCACCGATAGTGTTTGCCCCATGTTGGACTTAGGTGATTCTTTAACTATCACTAACCGTT
TAGGTAAGTGTGTAGATTATTCACTCTATGGTGTTACTGGTAGAGGTGTTTTTCAAAACTGTACTGCCGTTGGTGTTAA
ACAGCAGCGTTTTGTTTATGATAGCTTTGACAATTTAGTTGGTTACTATTCTGATGATGGTAACTACTATTGTGTCAGA
CCTTGTGTCAGTGTACCTGTGTCTGTTATATATGATAAAAGTACAAATTTACATGCGACTTTATTCGGTAGTGTTGCTT
GTGAGCACGTTACTACAATGATGTCGCAATTTTCACGCCTTACTCAAAGTAATTTACGTAGGCGTGATTCTAATATACC
TCTTCAAACAGCTGTTGGTTGTGTTATAGGTCTTTCTAATAACTCACTAGTAGTTAGTGATTGTAAATTGCCATTAGGC
CAGTCACTTTGTGCAGTCCCTCCTGTGTCTACTTTTAGAAGTTATAGCGCTAGCCAATTTCAGCTTGCTGTGCTTAATT
ATACTTCTCCTATTGTTGTTACTCCTATTAATTCCAGTGGTTTTACCGCTGCAATTCCAACTAATTTTTCTTTTTCAGT
TACACAGGAGTACATTGAAACATCCATTCAGAAGGTTACTGTAGATTGTAAACAGTATGTCTGTAATGGTTTTACTAGG
TGTGAAAAAGCTATTGGTTGAATATGGGCAATTTTGTTCCAAAATTAATCAAGCTTTACATGGTGCTAATCTTAGACAAG
ATGAGTCTGTTTATAGTCTTTATAGTAACATTAAAACCACCTCTACCCAAACTCTTGAGTATGGTTTAAATGGTGATTT
TAATCTTACATTATTACAAGTACCACAAATTGGAGGTTCTTCCTCTAGTTATCGTAGTGCTATTGAAGACTTATTATTT
GATAAGGTAACTATTGCAGATCCCGGTTATATGCAGGGCTATGATGATTGCATGAAACAAGGACCCCAGTCTGCTAGAG

ATCTTATTTGTGCTCAATATGTGTCTGGTTACAAAGTTTTACCACCACTTTATGACCCTAATATGGAAGCTGCTTACAC
ATCTTCACTATTAGGTAGTATAGCAGGTGCTGGATGGACAGCTGGTCTTTCATCCTTTGCCGCTATACCTTTTGCACAA
AGTATGTTTTATAGGTTGAATGGCGTTGGTATTACTCAACAAGTTCTCTCTGAGAACCAAAAGTTGATTGCTAACAAGT
TTAACCAAGCCTTAGGTGCTATGCAAACTGGCTTTACAACATCTAACCTAGCTTTTAGCAAGGTTCAAGATGCTGTAAA
TGCCAATGCACAAGCACTTTCTAAGTTAGCATCAGAGTTGTCTAATACTTTTGGTGCTATTTCTTCATCTATTAGTGAC
ATCCTTGCTAGGCTTGACACTGTTGAACAGGATGCGCAAATTGATCGTTTGATCAATGGTCGCTTAATTTCTCTTAACG
CATTTGTTTCACAACAACTTGTGCGTTCTGAAACTGCTGCTAGATCTGCTCAGTTGGCTAGTGACAAAGTCAACGAGTG
TGTAAAATCACAATCCAAGAGAAATGGGTTCTGTGGGTCTGGCACTCATATAGTATCATTTGTTGTTAATGCTCCAAAT
GGTTTTTATTTCTTTCATGTTGGTTATGTTCCAACAAATTATACTAATGTTACAGCTGCTTATGGTCTCTGTAACAATA
ACAACCCTCCTTTGTGCATTGCACCAATTGACGGGTATTTCATTACTAATCAGACTACCACTTATAGTGTTGATACCGA
ATGGTATTACACTGGTAGTTCTTTTTATAAACCTGAACCTATAACTCAGGCTAATTCACGTTATGTTTCTAGTGATGTG
AAATTTGACAAACTTGAGAATAACTTACCACCTCCACTCCTTGAGAATTCTACTGATGTGGATTTTAAGGATGAATTGG
AAGAATTTTTCAAGAATGTTACCTCGCATGGTCCTAACTTTGCAGAGATATCTAAGATTAACACTACACTGCTTGACCT
TTCAGATGAGATGGCAATGTTGCAGGAAGTTGTTAAACAATTAAATGATTCCTACATTGATCTTAAAGAATTGGGAAAT
TATACTTATTATAACAAGTGGCCTTGGTACGTATGGCTAGGTTTTATTGCAGGCCTTGTTGCCTTATTATTGTGTGTCT
TTTTCCTTCTATGCTGTACTGGTTGTGGTACTAGCTGCTTGGGAAAAATGAAGTGTAAGAATTGTTGTGATTCTTATGA
AGAATATGATGTCGAAAAGATTCATGTTCATTAA

>gi|124389427|gb|EF065508.1| Bat coronavirus HKU4-4, complete genome

ATGACACTCTTAATGTGTCTACTGATGTCCTTGTTAATTTTCGTAAGAGGCTGTGATTCACAATTTGTTGACATG
AGTCCAGCTAGTAATACTAGTGAGTGTTTGGAGTCACAAGTTGACGCAGCTGCATTTTCTAAATTAATGTGGCCTTATC
CTATTGATCCTTCCAAAGTTGATGGTATCATTTATCCACTTGGTAGGACATATTCTAATATTACATTAGCATATACTGG
TCTTTTTCCCCTACAAGGAGATTTAGGTAGCCAGTATTTATATTCTGTAAGTCATGCTGTAGGTCATGATGGTGATCCT
ACAAAAGCTTATATTTCTAATTATTCACTATTGGTGAATGATTTTGATAACGGTTTTGTTGTACGAATTGGTGCTGCAG
CTAATTCAACTGGCACCATTGTAATTTCACCGTCTGTTAATACTAAGATTAAAAAAGCTTATCCTGCTTTTATCTTAGG
TTCGTCTCTTACTAATACATCGGCTGGTCAGCCATTGTATGCTAATTACAGTTTAACCATCATTCCTGATGGTTGTGGG
ACTGTGTTGCATGCTTTTTATTGCATATTAAAACCACGTACTGGGAATCGATGTCCTAGTGGTACTGGTTATGTGTCTT
ATTTTATTTATGAAACACTTCATAATGATTGTCAGTCTACAATTAACAGGAACGCTTCCCTTAATTCTTTTAAGAGCTT
TTTTGATCTTGTTAATTGTACTTTCTTTAATAGTTGGGATATCACTGCTGATGAAACAAAAGAGTGGTTTGGTATTACC
CAAGACACTCAAGGTGTTCATCTCTACTCTTCTAGGAAAGGTGATTTATATGGCGGAAATATGTTTCGCTTTGCTACAT
TACCTGTGTACGAGGGTATAAAATATTACACTGTTATACCCAGAAGTTTTCGGTCTAAAGCTAACAAAAGAGAGTCTTG
GGCTGCATTTTATGTGTATAAATTACATCAGCTGACTTACTTGTTAGATTTTTCTGTTGATGGTTATATACGTAGAGCT
ATAGATTGTGGACATGATGATTTGTCACAGCTCCATTGTTCTTATACATCTTTTGAAGTTGATACTGGTGTCTATTCTG
TTTCTTCATATGAAGCTTCAGCTACTGGCACCTTTATTGAACAACCTAATGCTACTGAGTGTGATTTTTCACCTATGCT
TACAGGTGTAGCTCCTCAGGTTTATAATTTTAAAAGGTTGGTTTTTTCCAATTGTAATTATAATCTTACTAAACTCCTC
TCTTTGTTTGCAGTTGATGAGTTTTCTTGTAATGGAATTTCACCTGATGCTATTGCACGTGGTTGTTATTCTACTTTAA
CAGTGGATTATTTTGCCTATCCACTTAGTATGAAGTCATATATTAGACCTGGTTCTGCAGGCAATATACCACTTTACAA
TTATAAACAGTCTTTTGCCAATCCTACGTGTAGAGTTATGGCATCTGTTCCTGCTAATGTTACTATTACTAAGCCTCAG
GCTTATGGTTATATTTCCAAGTGTTCTCGTCTGACTGGTGCAAACCAAGATGTTGAAACACCATTATATATTAACCCTG
GTGAGTACTCTATTTGTAGAGATTTTTCTCCTGGGGGTTTTTCTGAAGATGGTCAGGTCTTCAAGCGTACTCTTACCCA
ATTCGAGGGTGGTGGCTTGCTCATTGGAGTTGGCACTAAAGTACCTATGACCGATAACTTACAAATGAGTTTTATAATC
TCTGTCCAATATGGTACCGGCACCGATAGTGTTTGCCCCATGTTGGACTTAGGTGATTCTTTAACTATCACTAACCGTT

TAGGTAAGTGTGTAGATTATTCACTCTATGGTGTTACTGGTAGAGGTGTTTTTCAAAACTGTTCTGCCGTTGGTGTTAA
ACAGCAGCGTTTTGTTTATGATAGCTTTGACAATTTAGTTGGTTACTATTCTGATGATGGTAACTACTATTGTGTCAGA
CCTTGTGTCAGTGTACCTGTGTCTGTTATATATGATAAAAGTACAAATTTACATGCGACTTTATTCGGTAGTGTTGCTT
GTGAGCACGTTACTACAATGATGTCGCAATTTTCACGCCTTACTCAAAGTAATTTACGTATGCGTGATTCTAATATACC
TCTTCAAACAGCTGTTGGTTGTGTTATAGGTCTTTCTAATAACTCACTAGTAGTTAGTAATTGTAAATTGCCATTAGGC
CAGTCACTTTGTGCAGTCCCTCCTGTGTCTACTTTTAGAAGTTATAGCGCTAGCCAATTTCAGCTTGCTGTGCTTAATT
ATACTTCTCCTATTGTTGTTACTCCTATTAATTCCAGTGGTTTTACCGCTGCAATTCCAACTAATTTTTCTTTTTCAGT
TACACAGGAGTACATTGAAACATCCATTCAGAAGGTTACTGTAGATTGTAAACAGTATGTCTGTAATGGTTTTACTAGG
TGTGAAAAGCTATTGGTTGAATATGGGCAATTTTGTTCCAAAATTAATCAAGCTTTACATGGTGCTAATCTTAGACAAG
ATGAGTCTGTTTATAGTCTTTATAGTAACATTAAAACCACCTCTACCCAAACTCTTGAGTATGGTTTAAATGGTGATTT
TAATCTTACATTGTTACAGGTACCACAAATTGGAGGTTCATCCTCTAGTTATCGTAGTGCTATTGAAGACTTATTATTT
GATAAGGTAACTATTGCAGATCCTGGTTATATGCAGGGTTATGATGATTGCATGAAACAAGGACCCCAGTCTGCTAGGG
ATCTTATTTGTGCTCAATATGTGTCTGGTTATAAAGTTTTACCACCACTTTATGACCCTAATATGGAAGCTGCTTACAC
ATCTTCACTATTAGGTAGTATAGCAGGTGCTGGATGGACAGCTGGTCTTTCATCCTTTGCCGCTATACCTTTTGCACAA
AGTATGTTTTATAGGTTGAATGGCGTTGGTATTACTCAACAAGTTCTCTCTGAGAACCAAAAGTTGATTGCTAACAAGT
TTAACCAAGCCTTAGGTGCTATGCAAACTGGCTTTACAACATCTAACCTAGCTTTTAGCAGGGTTCAAGATGCTGTAAA
TGCCAATGCACAAGCACTTTCTAAGTTAGCATCAGAGTTGTCTAATACTTTTGGTGCTATTTCTTCATCTATTAGTGAC
ATCCTTGCTAGGCTTGACACTGTTGAACAGGATGCGCAAATTGATCGTTTGATCAATGGTCGCTTAACTTCTCTTAATG
CATTTGTTTCACAACAACTTGTGCGTTCTGAAACGGCTGCTAGATCTGCTCAGTTGGCCAGTGATAAAGTCAACGAGTG
TGTAAAATCACAATCCAAGAGAAATGGGTTTTGTGGGTCTGGCACTCATATAGTATCATTTGTTGTTAATGCTCCAAAT
GGTTTTTATTTCTTTCATGTTGGTTATGTTCCCACAAATTATACTAATGTTACAGCAGCTTATGGTCTCTGTAACAATA
ACAGCCCTCCTTTGTGCATTGCACCAATTGACGGGTATTTCATTACTAATCAGACTACCACTTATAGTATTGATACTGA
ATGGTATTACACTGGTAGTTCTTTTTTTAAACCTGAACCTATAACTCAGGTTAATTCACGTTATGTTTCTAGTGATGTG
AAATTTGAAAAACTTGAGAATAACTTACCACCTCCACTCCTTGAGAATTCTACTGATGTGGATTTTAAGGATGAATTGG
AAGAATTTTTCAAGAATGTTACCTCGCATGGTCCTAACTTTGCAGAGATATCTAAGATTAATACTACACTGCTTGACCT
CTCAGATGAGATGGCAATATTGCAGGAAGTTGTTAAACAATTAAATGATTCCTACATTGATCTTAAAGAATTGGGGAAT
TATACTTATTATAACAAGTGGCCATGGTATATATGGCTAGGTTTTATTGCAGGCCTTGTTGCCTTATTATTGTGTGCCT
TTTTCCTTCTATGCTGTACTGGTTGCGGTACTAGCTGCTTAGGAAAAATGAAGTGTAAGAATTGTTGTGATTCTTATGA
AGAATATGATGTTGAAAAGATTCATGTTCATTAA

>gi|520688470|gb|KC522049.1|_Tylonycteris_bat_coronavirus_HKU4_isolate_2S_spike_glycoprotein_gene_complete_cds

ATGACACTCTTAATGTGTCTACTGATGTCCTTGTTAATTTTCGTAAGAGGCTGTGATTCACAATTTGTTGACATG
AGTCCAGCTAGTAATACTAGTGAGTGTTTGGAGTCACAAGTTGATGCAGCTGCATTTTCTAAATTAATGTGGCCTTATC
CTATTGATCCTTCCAAAGTTGATGGTATCATTTATCCACTTGGTAGGACATATTCTAATATTACATTAGCATATACTGG
TCTTTTTCCCCTACAAGGAGATTTAGGTAGCCAGTATTTATATTCTGTAAGTCATGCTGTAGGTCATGATGGTGATCCT
ACAAAAGCTTATATTTCTAATTATTCACTATTGGTGAATGATTTTGATAACGGTTTTGTTGTACGAATTGGTGCTGCAG
CTAATTCAACTGGCACCATTGTAATTTCACCGTCTGTTAATACTAAGATTAAAAAAGCTTATCCTGCTTTTATCTTAGG
TTCGTCTCTTACTAATACATCGGCTGGTCAGCCATTGTATGCTAATTACAGTTTAACCATCATTCCTGATGGTTGTGGG
ACTGTGTTGCATGCTTTTTATTGCATATTAAAACCACGTACTGGGAATCAATGTCCTAGTGGTACTGGTTATGTGTCTT
ATTTTATTTATGAAACACTTCATAATGATTGTCAGTCTACAATTAACAGGAACGCTTCTCTTAATTCTTTTAAGAGCTT
TTTCGATCTTGTTAATTGTACTTTCTTTAATAGTTGGGATATCACTGCTGATGAAACAAAAGAGTGGTTTGGTATTACC

CAAGACACTCAAGGTGTTCATCTCTACTCTTCTAGGAAAGGTGATTTATATGGCGGAAATATGTTTCGCTTTGCTACAT
TACCTGTGTACGAGGGTATAAAATATTACACTGTTATACCCAGAAGTTTTCGGTCTAAAGCTAACAAAAGAGAGGCTTG
GGCTGCATTTTATGTGTATAAATTACATCAGCTGACTTACTTGTTAGATTTTTCTGTTGATGGCTATATACGTAGAGCT
ATAGATTGTGGACATGATGATTTGTCACAGCTCCATTGTTCTTATACATCTTTTGAAGTTGATACTGGTGTCTATTCTG
TTTCTTCATATGAAGCTTCAGCTACTGGCACCTTTATTGAACAACCTAATGCTACTGAGTGTGATTTTTCACCTATGCT
TACAGGTGTAGCTCCTCAGGTTTATAATTTTAAAAGGTTGGTTTTTTCCAATTGTAATTATAATCTTACTAAACTCCTC
TCTTTGTTTGCAGTTGATGAGTTTTCTTGTAATGGAATTTCACCTGATGCTATTGCACGTGGTTGTTATTCTACTCTAA
CAGTGGATTATTTTGCCTATCCACTTAGTATGAAGTCATATATCAGACCTGGTTCTGCAGGCAATATACCACTTTACAA
TTATAAACAGTCTTTTGCCAATCCTACGTGTAGAGTTATGGCATCTGTTCCTGCTAATGTTACTATTACTAAGCCTCAG
GCTTATGGTTATATTTCTAAGTGTTCTCGTCTGACTGGTGCAAACCAAGATGTTGAAACACCATTATATATTAACCCTG
GTGAGTACTCTATTTGTAGAGATTTTTCTCCTTGGGGTTTTTCTGAAGATGGTCAGGTCTTCAAGCGTACTCTTACCCA
GTTTGAGGGTGGTGGCTTGCTTATTGGAGTTGGCACTAGAGTACCTATGACCGATAATTTACAAATGAGTTTTATAATC
TCTGTCCAATATGGTACTGGCACAGATAGTGTGTGCCCCATGTTGGACTTAGGTGATTCTTTAACTATCACTAACCGTC
TAGGTAAGTGTGTAGATTATTCCCTCTATGGTGTTACTGGTAGAGGTGTTTTTCAAAATTGTACTGCTGTCGGTGTTAA
ACAGCAGCGTTTTGTTTATGATAGCTTTGACAATTTAGTTGGTTACTATTCTGATGATGGTAACTACTATTGTGTCAGA
CCTTGTGTCAGTGTACCTGTGTCTGTTATATATGATAAAAGTACAAATTTACATGCGACTTTATTCGGTAGTGTTGCTT
GTGAGCACGTTACTACAATGATGTCGCAATTTTCACGCCTTACTCAAAGTAATTTACGTAGGCGTGATTCTAATATACC
TCTTCAAACAGCTGTTGGTTGTGTTATAGGTCTTTCTAATAACTCACTAGTAGTTAGTGATTGTAAATTGCCATTAGGC
CAGTCACTTTGTGCAGTCCCTCCTGTGTCTACTTTTAGAAGTTATAGCGCTAGCCAATTTCAGCTTGCTGTGCTTAATT
ATACTTCTCCTATTGTTGTTACTCCTATTAATTCCAGTGGTTTTACCGCTGCAATTCCAACTAATTTTTCTTTTTCAGT
TACACAGGAGTACATTGAAACATCCATTCAGAAGGTTACTGTAGATTGTAAACAGTATGTCTGTAATGGTTTTACTAGG
TGTGAAAAAGCTATTGGTTGAATATGGGCAATTTTGTTCAAAAATTAACCAAGCTTTACATGGTGCTAATCTTAGACAAG
ATGAGTCTGTCTATAGTCTTTATAGTAACATTAAAACCACCTCCACCCAAACTCTTGAGTATGGTTTAAATGGTGATTT
TAATCTTACATTGTTACAGGTACCACAAATTGGAGGTTCATCCTCTAGTTATCGTAGTGCTATTGAAGACTTATTATTT
GATAAGGTAACTATTGCAGATCCTGGTTATATGCAGGGTTATGATGATTGCATGAAACAAGGACCCCAGTCTGCTAGGG
ATCTTATTTGTGCTCAATATGTGTCTGGTTATAAAGTTTTACCACCACTTTATGACCCTAATATGGAAGCTGCCTATAC
ATCTTCACTATTAGGTAGTATAGCTGGTGCTGGGTGGACAGCTGGTCTTTCATCTTTTGCTGCTATACCTTTTGCACAA
AGTATGTTTTATAGGTTGAATGGTGTTGGTATTACTCAACAAGTTCTCTCTGAGAATCAAAAGTTGATTGCTAACAAGT
TTAACCAAGCCTTAGGTGCTATGCAAACTGGCTTTACAACATCTAACCTAGCTTTTAGCAAAGTTCAAGATGCTGTAAA
TGCCAATGCACAAGCACTCTCTAAGTTAGCATCAGAATTATCTAATACTTTTGGTGCTATTTCTTCATCTATTAGTGAC
ATCCTTGCTAGGCTTGACACTGTTGAACAGGATGCGCAAATTGATCGTTTGATCAATGGTCGCTTAACTTCTCTTAACG
CATTTGTCTCACAACAACTTGTGCGTTCTGAAACTGCTGCTAGATCTGCTCAGTTGGCTAGTGACAAAGTCAATGAGTG
TGTAAAATCACAATCCAAAAGAAATGGGTTTTGTGGGTCTGGCACTCATATAGTATCATTTGTTGTTAACGCTCCAAAT
GGTTTTTATTTCTTTCATGTTGGTTATGTTCCAACAAATTATACTAATGTTACAGCTGCTTATGGTCTCTGTAACAATA
ACAACCCTCCTTTGTGCATTGCACCAATTGACGGGTATTTCATTACTAATCAGACTACCACTTATAGTGTTGATACCGA
ATGGTATTACACTGGTAGTTCTTTTTATAAACCTGAACCTATAACTCAGGCTAATTCACGTTATGTTTCTAGTGATGTG
AAATTTGAAAAACTTGAGAATAATTTACCACCTCCACTCCTTGAGAATTCTACTGATGTGGATTTTAAGGATGAATTGG
AAGAATTTTTCAAGAATGTTACCTCGCATGGTCCTAACTTTGCAGAGATATCTAAGATTAACACTACACTGCTTGACCT
TTCAGATGAGATGGCAATATTGCAGGAAGTTGTTAAACAATTAAATGATTCCTACATTGATCTTAAAGAATTGGGAAAT
TATACTTATTATAACAAGTGGCCTTGGTATATATGGCTAGGTTTTATTGCAGGCCTTGTTGCCTTATTATTGTGTGTCT
TTTTCCTTCTATGCTGTACTGGTTGTGGTACTAGCTGCTTGGGAAAAAATGAAGTGTAAGAATTGTTGTGATTCTTATGA
AGAATATGATGTCGAAAAGATTCATGTTCATTAA

>gi|520688472|gb|KC522050.1|_Tylonycteris_bat_coronavirus_HKU4_isolate_3S_spike_glycoprotein_gene_complete_cds

ATGACACTCTTAATGTGTCTACTGATGTCCTTGTTAATTTTCGTAAGAGGCTGTGATTCACAATTTGTTGACATG
AGTCCAGCTAGTAATACTAGTGAGTGTTTGGAGTCACAAGTTGACGCAGCTGCATTTTCTAAATTAATGTGGCCTTATC
CTATTGATCCTTCCAAAGTTGATGGTATCATTTATCCACTTGGTAGGACATATTCTAATATTACATTAGCATATACTGG
TCTTTTTCCCCTACAAGGAGATTTAGGTAGCCAGTATTTATATTCTGTAAGTCATGCTGTAGGTCATGATGGTGATCCT
ACAAAAGCTTATATTTCTAATTATTCACTATTGGTGAATGATTTTGATAACGGTTTTGTTGTACGAATTGGTGCTGCAG
CTAATTCAACTGGCACCATTGTAATTTCACCGTCTGTTAATACTAAGATTAAAAAAGCTTATCCTGCTTTTATCTTAGG
TTCGTCTCTTACTAATACATCGGCTGGTCAGCCATTGTATGCTAATTACAGTTTAACCATCATTCCTGATGGTTGTGGG
ACTGTGTTGCATGCTTTTTATTGCATATTAAAACCACGTACTGGGAATCGATGTCCTAGTGGTACTGGTTATGTGTCTT
ATTTTATTTATGAAACACTTCATAATGATTGTCAGTCTACAATTAACAGGAACGCTTCTCTTAATTCTTTTAAGAGCTT
TTTCGATCTTGTTAATTGTACTTTCTTTAATAGTTGGGATATCACTGCTGATGAAACAAAAGAGTGGTTTGGTATTACC
CAAGACACTCAAGGTGTTCATCTCTACTCTTCTAGGAAAGGTGATTTATATGGCGGAAATATGTTTCGCTTTGCTACAT
TACCTGTGTACGAGGGTATAAAATATTACACTGTTATACCCAGAAGTTTTCGGTCTAAAGCTAACAAAAGAGAGGCTTG
GGCTGCATTTTATGTGTATAAATTACATCAGCTGACTTACTTGTTAGATTTTTCTGTTGATGGTTATATACGTAGAGCT
ATAGATTGTGGACATGATGATTTGTCACAGCTCCATTGTTCTTATACATCTTTTGAAGTTGATACTGGTGTCTATTCTG
TTTCTTCATATGAAGCTTCAGCTACTGGCACCTTTATTGAACAACCTAATGCTACTGAGTGTGATTTTTCACCTATGCT
TACAGGTGTAGCTCCTCAGGTTTATAATTTTAAAAGGTTGGTTTTTTCCAATTGTAATTATAATCTTACTAAACTCCTC
TCTTTGTTTGCAGTTGATGAGTTTTCTTGTAATGGAATTTCACCTGATGCTATTGCACGTGGTTGTTATTCTACTTTAA
CAGTGGATTATTTTGCCTATCCACTTAGTATGAAGTCATATATCAGACCTGGTTCTGCAGGCAATATACCACTTTACAA
TTATAAACAGTCTTTTGCCAATCCTACGTGTAGAGTTATGGCATCTGTTCCTGCTAATGTTACTATTACTAAGCCTCAG
GCTTATGGTTATATTTCCAAGTGTTCTCGTCTGACTGGTGCAAACCAAGATGTTGAAACACCATTATATATTAACCCTG
GTGAGTACTCTATTTGTAGAGATTTTTCTCCTGGGGGTTTTTCTGAAGATGGTCAGGTCTTCAAGCGTACTCTTACCCA
ATTCGAGGGTGGTGGCTTGCTTATTGGAGTTGGCACTAGAGTACCTATGACCGATAACTTACAAATGAGTTTTATAATC
TCTGTCCAATATGGTACCGGCACCGATAGTGTTTGCCCCATGTTGGACTTAGGTGATTCTTTAACTATCACTAACCGTT
TAGGTAAGTGTGTAGATTATTCACTCTATGGTGTTACTGGTAGAGGTGTTTTTCAAAACTGTTCTGCCGTTGGTGTTAA
ACAGCAGCGTTTTGTTTATGATAGCTTTGACAATTTAGTTGGTTACTATTCTGATGATGGTAACTACTATTGTGTCAGA
CCTTGTGTCAGTGTACCTGTGTCTGTTATATATGATAAAAGTACAAATTTACATGCGACTTTATTCGGTAGTGTTGCTT
GTGAGCACGTTACTACAATGATGTCGCAATTTTCACGCCTTACTCAAAGTAATTTACGTAGGCGTGATTCTAATATACC
TCTTCAAACAGCTGTTGGTTGTGTTATAGGTCTTTCTAATAACTCACTAGTAGTTAGTGATTGTAAATTGCCATTAGGC
CAGTCACTTTGTGCAGTCCCTCCTGTGTCTACTTTTAGAAGTTATAGCGCTAGCCAATTTCAGCTTGCTGTGCTTAATT
ATACTTCTCCTATTGTTGTTACTCCTATTAATTCCAGTGGTTTTACCGCTGCAATTCCAACTAATTTTTCTTTTTCAGT
TACACAGGAGTACATTGAAACATCCATTCAGAAGGTTACTGTAGATTGTAAACAGTATGTCTGTAATGGTTTTACTAGG
TGTGAAAAGCTATTGGTTGAATATGGGCAATTTTGTTCCAAAATTAATCAAGCTTTACATGGTGCTAATCTTAGACAAG
ATGAGTCTGTTTATAGTCTTTATAGTAACATTAAAACCACCTCTACCCAAACTCTTGAGTATGGTTTAAATGGTGATTT
TAATCTTACATTATTACAAGTACCACAAATTGGAGGTTCTTCCTCTAGTTATCGTAGTGCTATTGAAGACTTATTATTT
GATAAGGTAACTATTGCAGATCCCGGTTATATGCAGGGCTATGATGATTGCATGAAACAAGGACCCCAGTCTGCTAGAG
ATCTTATTTGTGCTCAATATGTGTTTGGTTACAAAGTTTTACCACCACTTTATGACCCTAATATGGAAGCTGCTTACAC
ATCTTCACTATTAGGTAGTATAGCAGGTGCTGGATGGACAGCTGGTCTTTCATCCTTTGCCGCTATACCTTTTGCACAA
AGTATGTTTTATAGGTTGAATGGCGTTGGTATTACTCAACAAGTTCTCTCTGAGAACCAAAAGTTGATTGCTAACAAGT
TTAACCAAGCCTTAGGTGCTATGCAAACTGGCTTTACAACATCTAACCTAGCTTTTAGCAAGGTTCAAGATGCTGTAAA
TGCCAATGCACAAGCACTTTCTAAGTTAGCATCAGAGTTGTCTAATACTTTTGGTGCTATTTCTTCATCTATTAGTGAC
ATCCTTGCTAGGCTTGACACTGTTGAACAGGATGCGCAAATTGATCGTTTGATCAATGGTCGCTTAACTTCTCTTAACG

CATTTGTTTCACAACAACTTGTGCGTTCTGAAACTGCTGCTAGATCTGCTCAGTTGGCTAGTGACAAAGTCAACGAGTG
TGTAAAATCACAATCCAAGAGAAATGGGTTCTGTGGGTCTGGCACTCATATAGTATCATTTGTTGTTAATGCTCCAAAT
GGTTTTTATTTCTTTCATGTTGGTTATGTTCCAACAAATTATACTAATGTTACAGCTGCTTATGGTCTCTGTAACAATA
ACAACCCTCCTTTGTGCATTGCACCAATTGACGGGTATTTCATTACTAATCAGACTACCACTTATAGTGTTGATACCGA
ATGGTATTACACTGGTAGTTCTTTTTATAAACCTGAACCTATAACTCAGGCTAATTCACGTTATGTTTCTAGTGATGTG
AAATTTGAAAAACTTGAGAATAACTTACCACCTCCACTCCTTGAGAATTCTACTGATGTGGATTTTAAGGATGAATTGG
AAGAATTTTTCAAGAATGTTACCTCGCATGGTCCTAACTTTGCAGAGATATCAAAGATTAACACTACACTGCTTGACCT
TTCAGATGAGATGGCAATGTTGCAGGAAGTTGTTAAACAATTAAATGATTCCTACATTGATCTTAAAGAATTGGGAAAT
TATACTTATTATAACAAGTGGCCTTGGTACGTATGGCTAGGTTTTATTGCAGGCCTTGTTGCCTTATTATTGTGTGTCT
TTTTCCTTCTATGCTGTACTGGTTGTGGTACTAGCTGCTTGGGAAAAATGAAGTGTAAGAATTGTTGTGATTCTTATGA
AGAATATGATGTCGAAAAGATTCATGTTCATTAA

>gi|520688486|gb|KC522057.1|_Tylonycteris_bat_coronavirus_HKU4_isolate_10S_spike_glycoprotein_gene_complete_cds

ATGACACTCTTAATGTGTCTACTGATGTCCTTGTTAATTTTCGTAAGAGGCTGTGATTCACAATTTGTTGACATG
AGTCCAGCTAGTAATACTAGTGAGTGTTTGGAGTCACAAGTTGACGCAGCTGCATTTTCTAAATTAATGTGGCCTTATC
CTATTGATCCTTCCAAAGTTGATGGTATCATTTATCCACTTGGTAGGACATATTCTAATATTACATTAGCATATACTGG
TCTTTTTCCCCTACAAGGAGATTTAGGTAGCCAGTATTTATATTCTGTAAGTCATGCTGTAGGTCATGATGGTGATCCT
ACAAAAGCTTATATTTCTAATTATTCACTATTGGTGAATGATTTTGATAACGGTTTTGTTGTACGAATTGGTGCTGCAG
CTAATTCAACTGGCACCATTGTAATTTCACCGTCTGTTAATACTAAGATTAAAAAAGCTTATCCTGCTTTTATCTTAGG
TTCGTCTCTTACTAATACATCGGCTGGTCAGCCATTGTATGCTAATTACAGTTTAACCATCATTCCTGATGGTTGTGGG
ACTGTGTTGCATGCTTTTTATTGCATATTAAAACCACGTACTGGGAATCGATGTCCTAGTGGTACTGGTTATGTGTCTT
ATTTTATTTATGAAACACTTCATAATGATTGTCAGTCTACAATTAACAGGAACGCTTCTCTTAATTCTTTTAAGAGCTT
TTTTGATCTTGTTAATTGTACTTTCTTTAATAGTTGGGATATCACTGCTGATGAAACAAAAGAGTGGTTTGGTATTACC
CAAGACACTCAAGGTGTTCATCTCTACTCTTCTAGGAAAGGTGATTTATATGGCGGAAATATGTTTCGCTTTGCTACAT
TACCTGTGTACGAGGGTATAAAATATTACACTGTTATACCCAGAAGTTTTCGGTCTAAAGCTAACAAAAGAGAGGCTTG
GGCTGCATTTTATGTGTATAAATTACATCAGCTGACTTACTTGTTAGATTTTTCTGTTGATGGTTATATACGTAGAGCT
ATAGATTGTGGACATGATGATTTGTCACAGCTCCATTGTTCTTATACATCTTTTGAAGTTGATACTGGTGTCTATTCTG
TTTCTTCATATGAAGCTTCAGCTACTGGCACCTTTATTGAACAACCTAATGCTACTGAGTGTGATTTTTCACCTATGCT
TACAGGTGTAGCTCCTCAGGTTTATAATTTTAAAAGGTTGGTTTTTTCCAATTGTAATTATAATCTTACTAAACTCCTC
TCTTTGTTTGAAGTTGATGAGTTTTCTTGTAATGGAATTTCACCTGATGCTATTGCACGTGGTTGTTATTCTACTTTAA
CAGTGGATTATTTTGCCTATCCACTTAGTATGAAGTCATATATCAGACCTGGTTCTGCAGGCAATATACCACTTTACAA
TTATAAACAGTCTTTTGCCAATCCTACGTGTAGAGTTATGTCATCTGTTCCTGCTAATGTTACTATTACTAAGCCTCAG
GCTTATGGTTATATTTCCAAGTGTTCTCGTCTGACTGGTGCAAACCAAGATGTTGAAACACCATTATATATTAACCCTG
GTGAGTACTCTATTTGTAGAGATTTTTCTCCTGGGGGTTTTTCTGAAGATGGTCAGGTCTTCAAGCGTACTCTTACCCA
ATTCGAGGGTGGTGGCTTGCTTATTGGAGTTGGCACTAGAGTACCTATGACCGATAACTTACAAATGAGTTTTATAATC
TCTGTCCAATATGGTACCGGCACCGATAGTGTTTGCCCCATGTTGGACTTAGGTGATTCTTTAACTATCACTAACCGTT
TAGGTAAGTGTGTAGATTATTCACTCTATGGTGTTACTGGTAGAGGTGTTTTTCAAAACTGTTCTGCCGTTGGTGTTAA
ACAGCAGCGTTTTGTTTATGATAGCTTTGACAATTTAGTTGGTTACTATTCTGATGATGGTAACTACTATTGTGTCAGA
CCTTGTGTCAGTGTACCTGTGTCTGTTATATATGATAAAAGTACAAATTTACATGCGACTTTATTCGGTAGTGTTGCTT
GTGAGCACGTTACTACAATGATGTCGCAATTTTCACGCCTTACTCAAAGTAATTTACGTAGGCGTGATTCTAATATACC
TCTTCAAACAGCTGTTGGTTGTGTTATAGGTCTTTCTAATAACTCACTAGTAGTTAGTGATTGTAAATTGCCATTAGGC

CAGTCACTTTGTGCAGTCCCTCCTGTGTCTACTTTTAGAAGTTATAGCGCTAGCCAATTTCAGCTTGCTGTGCTTAATT
ATACTTCTCCTATTGTTGTTACTCCTATTAATTCCAGTGGTTTTACCGCTGCAATTCCAACTAATTTTTCTTTTTCAGT
TACACAGGAGTACATTGAAACATCCATTCAGAAGGTTACTGTAGATTGTAAACAGTATGTCTGTAATGGTTTTACTAGG
TGTGAAAAGCTATTGGTTGAATATGGGCAATTTTGTTCCAAAATTAATCAAGCTTTACATGGTGCTAATCTTAGACAAG
ATGAGTCTGTTTATAGTCTTTATAGTAACATTAAAACCACCTCTACCCAAACTCTTGAGTATGGTTTAAATGGTGATTT
TAATCTTACATTATTACAAGTACCACAAATTGGAGGTTCTTCCTCTAGTTATCGTAGTGCTATTGAAGACTTATTATTT
GATAAGGTAACTATTGCAGATCCCGGTTATATGCAGGGCTATGATGATTGCATGAAACAAGGACCCCAGTCTGCTAGAG
ATCTTATTTGTGCTCAATATGTGTTTGGTTACAAAGTTTTACCACCACTTTATGACCCTAATATGGAAGCTGCTTACAC
ATCTTCACTATTAGGTAGTATAGCAGGTGCTGGATGGACAGCTGGTCTTTCATCCTTTGCCGCTATACCTTTTGCACAA
AGTATGTTTTATAGGTTGAATGGCGTTGGTATTACTCAACAAGTTCTCTCTGAGAACCAAAAGTTGATTGCTAACAAGT
TTAACCAAGCCTTAGGTGCTATGCAAACTGGCTTTACAACATCTAACCTAGCTTTTAGCAAGGTTCAAGATGCTGTAAA
TGCCAATGCACAAGCACTTTCTAAGTTAGCATCAGAGTTGTCTAATACTTTTGGTGCTATTTCTTCATCTATTAGTGAC
ATCCTTGCTAGGCTTGACACTGTTGAACAGGATGCGCAAATTGATCGTTTGATCAATGGTCGCTTAACTTCTCTTAACG
CATTTGTTTCACAACAACTTGTGCGTTCTGAAACTGCTGCTAGATCTGCTCAGTTGGCTAGTGACAAAGTCAACGAGTG
TGTAAAATCACAATCCAAGAGAAATGGGTTCTGTGGGTCTGGCACTCATATAGTATCATTTGTTGTTAATGCTCCAAAT
GGTTTTTATTTCTTTCATGTTGGTTATGTTCCAACAAATTATACTAATGTTACAGCTGCTTATGGTCTCTGTAACAATA
ACAACCCTCCTTTGTGCATTGCACCAATTGACGGGTATTTCATTACTAATCAGACTACCACTTATAGTGTTGATACCGA
ATGGTATTACACTGGTAGTTCTTTTTATAAACCTGAACCTATAACTCAGGCTAATTCACGTTATGTTTCTAGTGATGTG
AAATTTGAAAAACTTGAGAATAACTTACCACCTCCACTCCTTGAGAATTCTACTGATGTGGATTTTAAGGATGAATTGG
AAGAATTTTTCAAGAATGTTACCTCGCATGGTCCTAACTTTGCAGAGATATCAAAGATTAACACTACACTGCTTGACCT
TTCAGATGAGATGGCAATGTTGCAGGAAGTTGTTAAACAATTAAATGATTCCTACATTGATCTTAAAGAATTGGGAAAT
TATACTTATTATAACAAGTGGCCTTGGTACGTATGGCTAGGTTTTATTGCAGGCCTTGTTGCCTTATTATTGTGTGTCT
TTTTCCTTCTATGCTGTACTGGTTGTGGTACTAGCTGCTTGGGAAAAATGAAGTGTAAGAATTGTTGTGATTCTTATGA
AGAATATGATGTCGAAAAGATTCATGTTCATTAA

>gi|520688494|gb|KC522061.1|_Tylonycteris_bat_coronavirus_HKU4_isolate_15S_spike_glycoprotein_gene_complete_cds

ATGACACTCTTAATGTGTCTACTGATGTCCTTGTTAATTTTCGTAAGAGGCTGTGATTCACAATTTGTTGACATG
AGTCCAGCTAGTAATACTAGTGAGTGTTTGGAGTCACAAGTTGACGCAGCTGCATTTTCTAAATTAATGTGGCCTTATC
CTATTGATCCTTCCAAAGTTGATGGTATCATTTATCCACTTGGTAGGACATATTCTAATATTACATTAGCATATACTGG
TCTTTTTCCCCTACAAGGAGATTTAGGTAGCCAGTATTTATATTCTGTAAGTCATGCTGTAGGTCATGATGGTGATCCT
ACAAAAGCTTATATTTCTAATTATTCACTATTGGTGAATGATTTTGATAACGGTTTTGTTGTACGAATTGGTGCTGCAG
CTAATTCAACTGGCACCATTGTAATTTCACCGTCTGTTAATACTAAGATTAAAAAAGCTTATCCTGCTTTTATCTTAGG
TTCGTCTCTTACTAATACATCGGCTGGTCAGCCATTGTATGCTAATTACAGTTTAACCATCATTCCTGATGGTTGTGGG
ACTGTGTTGCATGCTTTTTATTGCATATTAAAACCACGTACTGGGAATCGATGTCCTAGTGGTACTGGTTATGTGTCTT
ATTTTATTTATGAAACACTTCATAATGATTGTCAGTCTACAATTAACAGGAACGCTTCTCTTAATTCTTTTAAGAGCTT
TTTCGATCTTGTTAATTGTACTTTCTTTAATAGTTGGGATATCACTGCTGATGAAACAAAAGAGTGGTTTGGTATTACC
CAAGACACTCAAGGTGTTCATCTCTACTCTTCTAGGAAAGGTGATTTATATGGCGGAAATATGTTTCGCTTTGCTACAT
TACCTGTGTACGATGGTATAAAATATTACACTGTTATACCCAGAAGTTTTCGGTCTAAAGCTAACAAAAGAGAGGCTTG
GGCTGCATTTTATGTGTATAAATTACATCAGCTGACTTACTTGTTAGATTTTTCTGTTGATGGTTATATACGTAGAGCT
ATAGATTGTGGACATGATGATTTGTCACAGCTCCATTGTTCTTATACATCTTTTGAAGTTGATACTGGTGTCTATTCTG
TTTCTTCATATGAAGCTTCAGCTACTGGCACCTTTATTGAACAACCTAATGCTACTGAGTGTGATTTTTCACCTATGCT

TACAGGTGTAGCTCCTCAGGTTTATAATTTTAAAAGGTTGGTTTTTTCCAATTGTAATTATAATCTTACTAAACTCCTC
TCTTTGTTTGCAGTTGATGAGTTTTCTTGTAATGGAATTTCACCTGATGCTATTGCACGTGGTTGTTATTCTACTTTAA
CAGTGGATTATTTTGCCTATCCACTTAGTATGAAGTCATATATCAGACCTGGTTCTGCAGGCAATATACCACTTTACAA
TTATAAACAGTCTTTTGCCAATCCTACGTGTAGAGTTATGGCATCTGTTCCTGCTAATGTTACTATTACTAAGCCTGAT
GCTTATGGTTATATTTCCAAGTGTTCTCGTCTGACTGGTGCAAACCAAGATGTTGAAACACCATTATATATTAACCCTG
GTGAGTACTCTATTTGTAGAGATTTTTCTCCTGGGGGTTTTTCTGAAGATGGTCAGGTCTTCAAGCGTACTCTTACCCA
ATTCGAGGGTGGTGGCTTGCTTATTGGAGTTGGCACTAGAGTACCTATGACTGATAACTTACAAATGAGTTTTATAATC
TTTGTCCAATATGGTACCGGCACCGATAGTGTTTGCCCCATGTTGGACTTAGGTGATTCTTTAACTATCACTAACCGTT
TAGGTAAGTGTGTAGATTATTCACTCTATGGTGTTACTGGTAGAGGTGTTTTTCAAAACTGTTCTGCCGTTGGTGTTAA
ACAGCAGCGTTTTGTTTATGATAGCTTTGACAATTTAGTTGGTTACTATTCTGATGATGGTAACTACTATTGTGTCAGA
CCTTGTGTCAGTGTACCTGTGTCTGTTATATATGATAAAAGTACAAATTTACATGCGACTTTATTCGGTAGTGTTGCTT
GTGAGCACGTTACTACAATGATGTCGCAATTTTCACGCCTTACTCAAAGTAATTTACGTAGGCGTGATTCTAATATACC
TCTTCAAACAGCTGTTGGTTGTGTTATAGGTCTTTCTAATAACTCACTAGTAGTTAGTGATTGTAAATTGCCATTAGGC
CAGTCACTTTGTGCAGTCCCTCCTGTGTCTACTTTTAGAAGTTATAGCGCTAGCCAATTTCAGCTTGCTGTGCTTAATT
ATACTTCTCCTATTGTTGTTACTCCTATTAATTCCAGTGGTTTTACCGCTGCAATTCCAACTAATTTTTCTTTTTCAGT
TACACAGGAGTACATTGAAACATCCATTCAGAAGGTTACTGTAGATTGTAAACAGTATGTCTGTAATGGTTTTACTAGG
TGTGAAAAAGCTATTGGTTGAATATGGGCAATTTTGTTCCAAAATTAACCAAGCTTTACATGGTGCTAATCTTAGACAAG
ATGAGTCTGTTTATAGTCTTTATAGTAACATTAAAACCACCTCTACCCAAACTCTTGATTATGGTTTAAATGGTGATTT
TAATCTTACATTATTACAAGTACCACAAATTGGAGGTTCTTCCTCTAGTTATCGTAGTGCTATTGAAGACTTATTATTT
GATAAGGTAACTATTGCAGATCCCGGTTATATGCAGGGCTATGATGATTGCATGAAACAAGGACCCCAGTCTGCTAGAG
ATCTTATTTGTGCTCAATATGTGTCTGGTTACAAAGTTTTACCACCACTTTATGACCCTAATATGGAAGCTGCTTACAC
ATCTTCACTATTAGGTAGTATAGCAGGTGCTGGATGGACAGCTGGTCTTTCATCATTTGCCGCTATACCTTTTGCACAA
AGTATGTTTTATAGGTTGAATGGCGTTGGTATTACTCAACAAGTTCTCTCTGAGAACCAAAAGTTGATTGCTAACAAGT
TTAACCAAGCCTTAGGTGCTATGCAAACTGGCTTTACAACATCTAACCTAGCTTTTAGCAAGGTTCAAGATGCTGTAAA
TGCCAATGCACAAGCACTTTCTAAGTTAGCATCAGAGTTGTCTAATACTTTTGGTGCTATTTCTTCATCTATTAGTGAC
ATCCTTGCTAGGCTTGACACTGTTGAACAGGATGCGCAAATTGATCGTTTGATCAATGGTCGCTTAACTTCTCTTAACG
CATTTGTTTCACAACAACTTGTGCGTTCTGAAACTGCTGCTAGATCTGCTCAGTTGGCTAGTGACAAAGTCAACGAGTG
TGTAAAATCACAATCCAAGAGAAATGGGTTCTGTGGGTCTGGCACTCATATAGTATCATTTGTTGTTAATGCTCCAAAT
GGTTTTTATTTCTTTCATGTTGGTTATGTTCCAACAAATTATACTAATGTTACAGCTGCTTATGGTCTCTGTAACAATA
ACAACCCTCCTTTGTGCATTGCACCAATTGACGGGTATTTCATTACTAATCAGACTACCACTTATAGTGTTGATACCGA
ATGGTATTACACTGGTAGTTCTTTTTATAAACCTGAACCTATTACTCAGGCTAATTCACGTTATGTTTCTAGTGATGTG
AAATTTGAAAAACTTGAGAATAACCTACCACCTCCACTCCTTGAGAATTCTACCGATGTGGATTTTAAGGATGAATTGG
AAGAATTTTTCAAGAATGTTACCTCGCATGGTCCTAACTTTGCAGAGATATCTAAGATTAACACTACACTGCTTGACCT
TTCAGATGAGATGGCAATATTGCAGGAAGTTGTTAAACAATTAAATGATTCCTATATTGATCTTAAAGAATTGGGAAAT
TATACTTATTATAACAAGTGGCCTTGGTACGTATGGCTAGGTTTTATTGCAGGCCTTGTTGCCTTATTATTGTGTGTCT
TTTTCCTTCTATGCTGTACTGGTTGTGGTACTAGCTGCTTGGGAAAAAATGAAGTGTAAGAATTGTTGTGATTCTTATGA
AGAATATGATGTCGAAAAGATTCATGTTCATTAA

>gi|520688496|gb|KC522090.1|_Pipistrellus_bat_coronavirus_HKU5_isolate_16S_spike_glycoprotein_gene_complete_cds

ATGATACGCTCAGTGTTAGCACTGATGTGCTCGTTAACTTTTATAGGAAACCTCACAAGAGGTCAAAGTGTTGAT
ATGGGACCCAATGGCACCGGTTCATGTTTAGATTCTCAGGTGCAACCTGATTACTTTGAATCTGTGCGCACTACTTGGC

CCATGCCAATTGATACGAGTAAGGCTGAAGGTGTCATTTACCCTAATGGCAAGTCATACTCTAATATTTCTTTAACTTA
TACTGGACTGTATCCAAAGGCTAATGACCTTGGTAAACAGTATTTGTTTTCCGACGGACACAGTGCTCCAGGACGGCTT
AACAACTTATTTGTCAGTAATTACTCCTCACAAGTGGAGTCTTTTGATGATGGCTTTGTTGTCCGGATTGGTGCTGCTG
CCAACAAAACTGGAACCACTGTCATTTCCCAATCCACTTCTAAACCCATTAAGAAAATCTATCCAGCCTTTTTGCTTGG
ACATTCTGTGGGTAATTATACACCTTCCAATAGGTCAGGCCGTTATTTCAACCACACACTTGTCATCCTACCGGATGGT
TGTGGTACTATCTTACATGCATTCTATTGCATTTTGATACCTCGCACGCAACAGAATTGTGCTGGCGATACGAATTTCA
AATCCCTCTCTCTTTGGGATACTCCTGCATCTGATTGTAATCCTGGCTCTTATAATGAACAGGCCACGCTCGGTGCTTT
TAAAGTGTATTTTGACCTCATTAATTGCACGTTCAGATACAATTATACTATAACAGAGGATGAAAACGCTGAGTGGTTT
GGCATAACCCAAGATACACAAGGCGTTCACCTCTATTCCTCTCGAAAAGAGAACGTGTTCAGAAATAACATGTTCCACT
TTGCTACTTTACCTGTGTATCAGAAAATCCTCTATTACACAGTCATTCCGCGCAGCATCCGAAGTCCTTTCAATGACAG
AAAAGCTTGGGCTGCCTTCTACATTTATAAACTACATCCACTCACATATTTGCTAAATTTTGATGTGGAGGGCTATATA
ACCAAAGCTGTAGACTGTGGCTATGATGATTTCGCACAGCTACAATGTTCATATGAAAACTTCGATGTTGAAACTGGTG
TTTATTCCGTTTCATCGTTCGAAGCTTCTCCTAGAGGTGAGTTCATTGAACAGGCAACAACTCAAGAGTGTGATTTCAC
ACCTATGTTGACTGGTACACCTCCTCCTATATATAATTTCAAAAGGTTGGTCTTCACCAATTGCAATTACAACTTAACA
AAACTCCTTTCACTGTTTCAAGTGAGCGAGTTTTCTTGTCATCAAGTTTCACCTAGTAGTCTTGCTACCGGTTGTTACT
CTTCTCTTACAGTGGACTATTTCGCTTATTCCACTGATATGAGTTCTTACCTGCAACCTGGCTCCGCTGGAGAAATTGT
GCAGTTTAATTACAAACAAGACTTTAGCAATCCCACATGTAGAGTGCTTGCTACTGTTCCACAAAATCTTACTACAATT
ACTAAACCTAGTAATTATGCTTATCTTACAGAGTGTTATAAAACCAGTGCATATGGCAAGAATTACTTGTACAATGCGC
CTGGCGGTTACACTCCTTGCTTATCTTTAGCCTCTCGTGGGTTCTCTACTAAATACCAGTCACATAGTGATGGCGAGCT
AACCACTACTGGTTACATTTATCCTGTCACTGGAAATCTTCAAATGGCTTTCATAATTTCTGTTCAGTATGGAACTGAC
ACTAACAGTGTCTGCCCCATGCAAGCATTAAGAAATGATACTAGCATTGAAGATAAGCTAGACGTTTGTGTTGAATACT
CGCTCCATGGTATAACTGGAAGGGGGGTTTTCCACAATTGCACATCTGTTGGACTGAGAAACCAGCGGTTTGTGTATGA
TACTTTCGACAATTTAGTTGGTTACCATTCTGGCAATGGTAATTACTATTGTGTCAGACCTTGTGTCAGTGTGCCTGTC
TCTGTGATTTACGACAAGGCATCTAATTCTCATGCTACATTATTCGGAAGCGTTGCATGTTCGCACGTTACCACAATGA
TGTCGCAATTTTCACGCATGACCAAAACTAATTTGCTCGCGCGTACAACTCCAGGTCCATTGCAAACTACTGTTGGTTG
TGCAATGGGCTTTATCAATTCCTCAATGGTAGTTGACGAATGTCAACTTCCGCTTGGTCAATCACTTTGTGCTATTCCA
CCAACTACTTCTTCACGCGTTCGACGTGCTACTTCTGGTGCATCTGATGTGTTTCAAATCGCCACTCTTAATTTTACTA
GTCCATTAACACTCGCACCAATAAATTCTACTGGATTTGTTGTTGCTGTGCCGACTAATTTCACATTTGGTGTCACTCA
AGAATTCATTGAGACTACCATTCAAAAGATTACTGTCGATTGCAAGCAGTACGTTTGTAATGGTTTTAAGAAGTGCGAA
GACTTGCTCAAAGAATATGGCCAGTTTTGCTCTAAAATTAACCAGGCTCTTCATGGTGCAAACCTACGTCAAGACGAGT
CTATTGCTAATCTATTTTCAAGTATTAAAACCCAGAATACCCAGCCTCTTCAGGCGGGATTGAATGGTGATTTTAACTT
GACTATGCTTCAAATACCTCAAGTTACTACAGGTGAACGTAAGTACAGGAGTACTATCGAAGACCTTCTTTTTAATAAG
GTTACTATTGCTGATCCTGGCTATATGCAAGGCTATGATGAATGTATGCAGCAAGGTCCCCAGTCAGCTCGAGACTTGA
TCTGTGCACAATATGTTGCTGGCTACAAAGTGCTGCCACCCTTATATGACCCCTATATGGAAGCTGCTTACACTTCTTC
CCTATTGGGCAGTATTGCTGGTGCTAGTTGGACAGCAGGTCTGTCGTCTTTCGCAGCAATACCATTTGCACAAAGTATC
TTTTATCGCTTGAATGGTGTTGGCATCACTCAGCAGGTTCTTTCTGAGAATCAGAAGATCATTGCCAACAAATTCAATC
AAGCTCTTGGTGCCATGCAAACTGGCTTTACTACTACTAACCTTGCTTTCAACAAGGTTCAGGATGCAGTAAATGCTAA
TGCAATGGCTCTTTCCAAACTAGCTGCGGAATTGTCTAACACTTTCGGTGCTATTTCATCATCCATCAGTGACATCCTT
GCAAGGCTTGACACTGTTGAACAAGAGGCTCAAATTGATCGGTTGATCAATGGACGTCTTACATCCCTTAACGCATTCG
TTGCGCAACAACTCGTGCGTACTGAAGCTGCTGCTAGATCTGCCCAATTGGCTCAAGATAAGGTCAATGAGTGCGTGAA
GTCGCAATCCAAACGGAATGGATTCTGCGGAACTGGCACACATATTGTTTCATTTGCCATTAATGCTCCTAATGGCCTC
TACTTCTTCCATGTTGGTTACCAGCCAACATCCCATGTTAATGCAACTGCCGCTTATGGCCTTTGCAACACTGAAAATC
CACAAAAGTGCATTGCACCTATTGATGGATACTTCGTCTTAAACCAAACTACCAGCACTGTCGCAGACAGTGACCAACA
ATGGTATTATACTGGTAGTTCTTTCTTCCATCCTGAACCCATCACAGAAGCAAATTCTAAGTATGTGTCCATGGATGTA

AAATTCGAAAACCTCACTAATAGGCTTCCTCCACCGCTCCTTAGTAACTCAACAGATTTGGATTTCAAGGAAGAGCTGG
AAGAATTCTTCAAAAACGTCTCCTCACAAGGACCAAACTTCCAGGAGATTTCTAAGATTAACACTACACTGCTTAACCT
CAACACAGAGTTGATGGTGCTGAGTGAAGTTGTTAAACAATTAAATGAATCCTATATCGACTTGAAAGAGTTGGGGAAT
TATACTTTTTACCAAAAATGGCCATGGTATATATGGCTTGGCTTTATTGCAGGGCTTGTTGCTCTTGCTCTTTGTGTGT
TCTTCATCTTATGCTGTACTGGCTGTGGTACTAGCTGTTTGGGGAAACTAAAATGTAATCGTTGTTGTGACTCGTATGA
TGAGTATGAGGTCGAGAAGATCCATATTCATTGA

>gi|520688498|gb|KC522091.1|_Pipistrellus_bat_coronavirus_HKU5_isolate_17S_spike_glycoprotein_gene_complete_cds

ATGATACGCTCAGTGTTAGTACTGATGTGCTCATTAACTTTTATAGGAAACCTCACAAGAGGTCAAAGTGTTGAT
ATGGGACCCAATGGCACTGGTTCATGTTTAGATTCTCATGTGCAACCTGATTATTTTGAATCTGTGCGTACCACGTGGC
CCATGCCTATTGATACGAGTAAGGCTGAAGGTGTCATTTACCCTAATGGCAAGTCATACTCTAATATTTCTTTAACTTA
TACGGGACTGTATCCTAAGGCCAATGACCTTGGTAAACAGTATTTATTTTCTGATGGACATAGTGCTCCAGGAAAACTT
AATAATTTATTTGTCAGTAATTACTCCTCACAAGTGGAGTCTTTTGATGATGGCTTTGTGGTCCGGATTGGTGCTGCTG
CCAATAAAACTGGAACCACTGTCATATCTCAATCCACATTTAAACCCATTAAGAAAATCTATCCAGCCTTTTTGCTTGG
ACATTCTGTGGGTAATTATACACCGTCCAATAGGACAGGTCGTTATCTCAACCACACACTTGTCATCCTACCGGATGGT
TGTGGTACTCTTCTACATGCATTTTATTGCATTTTAATACCTCGCACGCAACAAAATTGTGCTGGCGACGTTAATTTTA
AATCCCTCTCTCTTTGGGATACTCCTGCATCTGATTGTAACCCTGGTTCTTATAATGAACAGGCTACTCTAGGTGCTTT
CAAAGTGTATTTTGACCTCATTAACTGTACGTTCAGATACAATTATACTATAACAGAGGACGAAAACGCTGAGTGGTTT
GGTATAACCCAAGATACACAAGGCGTCCACCTCTATTCCTCTCGAAAAGAGAATGTGTTCAGAAATAACATGTTCCACT
TTGCTACCTTACCTGTGTACCAGAAAATCCTCTATTACACAGTCATTCCTCGCAGTATCCGAAGTCCCTTCAATGACAG
AAAAGCTTGGGCTGCCTTCTACATTTATAAACTACATCCACTCACATATCTGTTAAATTTTGATGTGGAGGGCTATATA
ACTAAAGCAGTAGACTGTGGCTATGATGATTTCGCACAGCTACAATGTTCATATGAAAGCTTCGATGTTGAAACAGGTG
TCTATTCTGTTTCGTCTTTTGAAGCATCTCCACGTGGTATGTTTGTTGAGCAATCTACTTCTGAAGAATGTGACTTCAC
CAAGATGCTCACAGGCACACCTCCCCAGGTTTATAATTTCAATAGGATGGTTTTTACCAATTGCAATTATAACCTAACA
AAACTACTTAACCTTTTCCAGGTGAGTGAGTTTTCCTGTTACCAAGTTTCGCCCAGTAGTCTTGCTACTGGTTGCTACT
CTTCTCTCACTGTGGATTCCTTTGCGTTTCCACTAGAATTAAGTTCTTACATGCAGACTGGCTCCGCTGGACCCATTCG
GCAGTTCAACTATAATCAAGACTTTAGCAAGCCAACGTGTAGAGTTCTTGCTTCTGTTCCTTCTAATTATACTGCGCTT
AGTAAGCCCAGTAATTATGTGCACCTTACTGAGTGCTATACTCTAGGATACTCCGCTAAGCAGTATCATAATACACAAC
CTGGTCAATATACGCCATGCCTTCAATTGGCTAACCAGGGTTTCTCTCGAAGTGGCCAGTCAAATTCAGTTGAACTCCC
TTCTGGTGAGTTCAAAACTGCCACTGGCTACATCATACCCATGACTGATCAACTTCAGATGGCTTTTGTTATTTCTGTA
CAGTATGGTACTGACACTAATAGTGTTTGTCCCATGCAGGCTTTAAGAAACGATACTAGCATTCAAGATAAGCTAGACG
TCTGCGTAGAATACTCCCTTCATGGTGTTACAGGAAGGGGAGTTTTCCACAATTGCACATCTGTTGGGTTGAGAAACCA
GCGGTTTGTATATGACACTTTCGACAACCTAGTTGGTTACCATTCCGATAATGGTAATTACTATTGTGTCAGACCTTGT
GTTAGTGTACCGGTCTCTGTTATTTATGACAAGGTATCTAATTCTCATGCCACATTGTTTGGTAGTGTTGCATGTTCGC
ACGTTACCACTATGATGTCGCAATTTTCACGCATGACCAAAACTAATTTGCTCCAGCGTATAACTCCTGGCCCTTTGCA
AACTACTGTTGGTTGTGCAATGGGTTTCATCAATTCTTCAATGGTAGTTGATGAATGTCAACTTCCTCTTGGTCAATCA
CTTTGTGCTATCCCACCAAACATTTCCTCACGCGTTCGACGTGTTGCTTCTGGTGTATCTGATGTCTTTCAAATCGCCA
CTCTTAACTTTACTAGTCCACTAACACTTGCACCAATAAATTCCACTGGATTTGTTGTTGCAGTGCCGACTAATTTCAC
ATTTGGTGTTACTCAAGAATACATTGAGACTACCATTCAAAAGATTACTGTAGATTGCAAGCAGTACGTTTGTAATGGT
TTTAAGAAGTGCGAAGACTTGCTCAAAGAATACGGCCAGTTTTGCTCTAAAATTAACCAGGCTCTTCATGGTGCAAACC
TACGTCAAGATGAGTCTATTGCTAATTTATTTTCAAGTATTAAAACCCAGAACACCCAGCCTCTTCAGGCGGGATTGAA

TGGTGATTTTAACTTGACAATGCTTCAAATACCTCAAGTTACTACTGGTGAACGCAAGTACAGGAGTGCTATTGAAGAC
CTTCTTTTCAATAAGGTTACTATTGCTGATCCTGGCTATATGCAAGGCTATGATGAATGTATGCAGCAAGGTCCTCAGT
CAGCTAGAGACTTGATCTGTGCACAATACGTCGCTGGCTACAAAGTTTTGCCACCCTTGTATGACCCCTATATGGAAGC
TGCTTACACTTCTTCCCTATTGGGTAGTATTGCTGGTGCTAGTTGGACAGCAGGTCTGTCATCTTTCGCAGCAATACCC
TTTGCACAAAGTATCTTTTATCGCTTGAATGGTGTTGGCATCACTCAGCAGGTTCTGTCTGAGAATCAGAAGATTATCG
CCAACAAGTTCAATCAAGCTCTTGGTGCCATGCAAACTGGCTTTACTACTACTAACCTAGCTTTCAGCAAGGTTCAGGA
TGCAGTAAATGCTAATGCCATGGCTCTCTCTAAACTAGCTGCGGAATTGTCTAACACTTTCGGTGCTATTTCATCTTCT
ATCAGTGACATCCTTGCGAGGCTTGACACTGTTGAACAAGAGGCACAAATTGATCGGTTGATCAATGGACGTCTCACAT
CCCTTAACGCATTCGTTGCGCAACAACTTGTGCGTACTGAAGCTGCTGCCAGATCCGCTCAATTGGCCCAAGATAAGGT
CAATGAGTGCGTCAAATCGCAATCCAAACGAAATGGATTTTGCGGAACTGGCACACATATTGTTTCATTTGCCATTAAT
GCCCCTAATGGCCTCTATTTTTTCCATGTTGGTTACCAGCCAACATCCCATGTTAATGCAACCGCTGCCTACGGCCTTT
GCAACACTGAGAATCCACCAAAGTGCATTGCACCTATTGATGGCTACTTTGTTTTAAATCAAACTACCAGCACTGCAAG
AAGTAGTGAAGACCAACATTGGTATTATACTGGTAGTTCTTTCTTCCATCCTGAACCCATCACAGAAGCAAATTCTAAG
TATGTGTCCATGGATGTAAAATTCGAGAACCTCACTAATAAACTTCCTCCACCTCTCCTTAGTAATTCCACAGATTTGG
ATTTCAAGGATGAGCTGGAGGAATTTTTCAAAAACGTCTCCTCACAAGGACCAAACTTTCAGGAGATTTCTAAGATTAA
CACCACACTGCTCAACCTCAACAACGAGTTGTTGGTGCTTAGTGAAGTTGTTAAACAATTAAATGAATCCTACATCGAC
TTGAAGGAGTTGGGGAATTATACTTTTTACCAGAAATGGCCATGGTACATATGGCTTGGATTCGTTGCTGGGCTTGTTG
CCCTTGCCCTTTGTGTGTTCTTCATTCTTTGCTGTACTGGTTGCGGTACTAGCTGTATGGGGAAACTAAAATGTAATCG
TTGTTGTGACTCGTATGATGAGTATGAGGTCGAGAAGATCCATGTTCATTGA

>gi|520688500|gb|KC522092.1|_Pipistrellus_bat_coronavirus_HKU5_isolate_18S_
spike_glycoprotein_gene_complete_cds

ATGATACGCTCAGTGTTAGTACTGATGTGCTCGTTAACTTCTATAGGAAGCCTTGTAAACAGTCAAAGCGTTGAC
ATGGGTACTACTGGTAGTGGTAACTGCATAGAGTCTCAAGTCGCGCCTGACTTTTTTGAGACGGCACGTAATATATGGC
CGCTTCCCATCGATACGAGTAAGGCTGAAGGTGTCATTTATCCCAATGGCAAGTCGTACTCTAATATTTCTTTAACTTA
TACGGGACTGTATCCCAAGGCTAATGACCTTGGTAAACAGTACATATTTTCCGACGGACATAGCAGTCCTGGCACATTA
AGTCGTCTATTTGTCAGTAACTATTCTAGACAAGTAGAAACATTTGACGATGGTTTCATTGTTCGTATTGGTGCTGCCT
CCAATAAGACAGGGACCACTGTAATTTCCCAATCCACTAATAGACCAATTAAGAAAATTTACCCAGCTTTTATGCTTGG
TCATTCTGTTGGTAATTATACACCCACTAATATCACTGGGCGTTACCTTAACCACACACTTGTCATCCTGCCGGATGGT
TGTGGTACATTGGTTCATGCATTTTATTGCATCTTACAACCCCGCACACAAGCATATTGTGCTGGTGCTAGTACATTCA
CGTCTGTCACAGTGTGGGACACACCCGCATCTGATTGTGCAAACTCTCAGAGTTACAATCAGTTGGCAAATTTGAATGC
TTTCAAATTGTATTTTGACCTCATTAATTGCACGTTCAGATACAATTATACTATAACTGAGGACGAAAACGCTGAGTGG
TTTGGCATAACCCAAGATACACAAGGCGTTCACCTCTATTCATCTCGAAAAGAGAATGTGTTCAGAAATAACATGTTCC
ACTTTGCCACTTTACCTGTGTACCAGAAAATCCTCTATTACACAGTCATTCCTCGCAGCATCCGCAGTCCTTTCAATGA
CAGAAAAGCTTGGGCTGCCTTCTACATTTATAAACTTCATCCACTCACATATTTGCTAAATTTTGATGTGGAGGGCTAT
ATAACTAAAGCGGTAGACTGTGGCTATGATGATTTCACACAGCTACAATGTTCATACGAAAACTTCGATGTTGAGACAG
GTGTTTACTCCGTCTCATCGTTCGAAGCTTCCCCTAGAGGTGAGTTCATTGAACAATCAGTTACCAAAGAGTGTGACTT
TTCACCCATGCTAACTGGCACGCCTCCCCCTATATATGATTTCAAAAGATTGGTTTTCACCAATTGCAATTACAACTTA
ACAAAACTCCTTTCACTTTTTCAGGTGAGTGAGTTTTCTTGTCATCAAGTATCTCCCAGTAGTCTTGCTACTGGCTGTT
ACTCTTCTCTTACAGTGGATTACTTTGCATACTCCACTGATATGAGTTCTTACCTGCAACCTGGCTCCGCTGGAGAAAT
TGTGCAGTTTAATTACAAACAAGACTTTAGCAATCCCACCTGCAGAGTGCTTGCTACTGTTCCCACCAATCTTACTACA
ATTACTAAACCTAGTAATTATGTTCATCTTACGGAGTGTTATAAAAGCACCGCATATGGCAAGAATTACTTGTTTAATG

CACCTGGTGGTTACACTCCTTGCTTATCTTTAGCCTCTAGTGGGTTCTCACGTGATAGACAGTCTCACAGTCAGGAATT
ACCTGATGGTAGTTTTTTAACTACTACTGGATCTGTTTACTCTGTAGGCTCTAACCTTCAAATGGCTTTCATAATCTCA
GTACAGTATGGCACTGACACTAATAGTGTCTGTCCCATGCAAGCTCTAAGAAACGATACTAGCATTGAAGATAAGCTAG
ATGTTTGTGTTGAATACTCCCTCCACGGCATAACTGGAAGAGGAGTTTTCCATAATTGCACTTCTGTTGGACTGAGAAA
CCAGCGGTTTGTGTATGATACTTTCGACAATCTAGTTGGTTACCATTCACATAATGGTAATTACTATTGTGTCAGACCT
TGTGTCAGTGTACCTGTCTCTGTGATTTATGACAAGGTATCTAATTCTCATGCCACATTATTTGGTAGTGTCGCATGTT
CGCATGTTACCACTATGATGTCTCAATTTTCACGCATGACTAAAACTAATTTGCTCGCGCGTACAACTCCTGGCCCATT
GCAAACTACTGTTGGTTGTGCAATGGGCTTCATTAATTCCTCAATGGTAGTTGATGAATGTCAACTTCCTCTTGGCCAA
TCACTTTGTGCTATTCCACCAACCATTTCTACACGCTTTCGACGTGCTACTTCTGGTGTATCTGATGTGTTTCAAATCG
CCACTCTAAATTTTACTAGTCCTCTAACACTTGCACCAATAAATTCAACTGGATTTGTTGTTGCTGTGCCGACTAATTT
CACATTTGGTGTCACTCAAGAATTCATTGAGACTACCATTCAAAAGATTACTGTTGATTGTAAGCAGTACGTTTGTAAT
GGTTTTAAGAAGTGCGAAGACTTGCTCAAAGAATACGGCCAGTTTTGCTCTAAAATTAACCAGGCTCTTCATGGTGCAA
ATCTACGTCAAGATGAGTCTATTGCTAATCTATTTTCAAGTATTAAAACCCAGAACACCCAGCCTCTTCAGGCAGGATT
GAATGGTGATTTTAACTTGACAATGCTTCAAATACCTCAAGTTACTACTGGTGAACGTAAGTACAGGAGTGCTATCGAA
GACCTTCTTTTCAATAAGGTCACTATTGCTGATCCTGGCTATATGCAAGGCTATGACGAATGTATGCAGCAAGGTCCTC
AGTCAGCTCGAGACTTGATCTGTGCACAATATGTTGCTGGCTACAAAGTGTTGCCACCCCTATATGACCCCTATATGGA
AGCTGCTTATACTTCTTCCCTATTGGGCAGTATTGCTGGTGCTAGTTGGACAGCAGGTTTGTCATCTTTCGCAGCAATA
CCATTTGCACAAAGTATCTTTTATCGCTTGAATGGTGTTGGCATCACTCAGCAGGTTCTGTCTGAGAATCAAAAGATTA
TTGCCAACAAGTTCAATCAAGCTCTTGGTGCTATGCAAACTGGCTTTACTACAACTAACCTAGCTTTCAACAAGGTTCA
GGATGCAGTAAATGCTAATGCCATGGCTCTTTCCAAACTAGCTGCGGAATTGTCTAACACTTTCGGTGCTATTTCATCT
TCTATCAGTGACATCCTTGCGAGGCTTGACACTGTTGAACAAGAGGCACAAATTGATCGGTTGATCAATGGCCGTCTCA
CATCCCTTAATGCATTCGTTGCGCAACAACTTGTGCGTACTGAAGCTGCTGCCAGATCTGCCCAATTGGCCCAAGATAA
GGTCAATGAGTGCGTCAAATCGCAATCCAAACGCAATGGATTCTGCGGAACTGGTACACATATTGTCTCATTTGCCATT
AACGCTCCTAATGGCCTCTATTTCTTTCATGTTGGTTATCAGCCAACATCCCATGTTAATGCAACTGCCGCCTATGGCC
TTTGCAACACAGAAAATCCACCAAAGTGCATAGCACCCATTGATGGATATTTCGTTTTAAATCAAACTATCAGCACTGT
AGCAAACAGTGACCAGCAATGGTATTATACTGGTAGTTCTTTCTTCCATCCTGAACCCATTACAGAAGCAAATTCTAAG
TATGTGTCCATGGATGTAAAATTCGAAAACCTCACTAATAAACTTCCTCCACCTCTCCTTAGCAATTCCACAGATTTGG
ATTTCAAGGAAGAGCTGGAAGAATTCTTCAAAAACGTCTCCTCACAAGGACCAAACTTTCAGGAGATTTCTAAGATTAA
CACTACACTGCTTAACCTCAACACAGAGTTGATGGTGCTTAGTGAAGTTGTTAAACAACTAAATGAATCCTACATCGAC
TTGAAAGAGTTGGGGAATTATACTTTTTACCAGAAATGGCCATGGTACATATGGCTTGGCTTTATTGCAGGGCTTGTCG
CTCTTGCTCTTTGTGTGTTCTTCATTCTATGTTGTACTGGTTGCGGTACTAACTGCTTGGGGAAACTAAAATGTAATCG
TTGTTGTGACTCGTATGATGAGTATGAGGTCGAGAAGATCCATGTTCATTGA

>gi|520688502|gb|KC522093.1|_Pipistrellus_bat_coronavirus_HKU5_isolate_19S_spike_glycoprotein_gene_complete_cds

ATGATACGCTCAGTGTTAGTACTGATGTGCTCGTTAACTTCTATAGGAAGCCTTGTAAACAGTCAAAGCGTTGAT
ATGGGTACTACTGGCAGTGGCAACTGCATAGAGTCCCAAGTTCAGCCTGACTTTTTTGAGACGGCACGCAATACATGGC
CGTTATCAATAGATACGAGTAAGGCTGAAGGTGTCATTTACCCTAATGGCAAGTCTTACTCTAATATTACTCTAACCTA
TACGGGACTGTATCCTAAGGCCAATGACCTTGGTAAACAGTATGTTTTTTCCGATGGACATAGTAGTCCTGGCACTTTA
AGTCGTCTATTTGTCAGTAATTACTCTAGACAAGTAGAACCATTTGATAGTGGTTTCGTTGTTCGTATTGGAGCTGCTG
CCAATAAGACTGGGACCACTATAATTTCTCAATCTACTAATCGACCAATTAAGAAAATTTACCCTGCTTTTATGTTGGG
TCATTCTGTTGGTAATTACACGCCTACTAACATTACTGGGCGCTATCTTAACCACACACTTGTCATCCTACCGGATGGC

TGTGGTACCCTAGTGCATGCATTCTATTGCATTCTACAACCTCGTACACAAGCCTATTGTGCTGGTGCTAGTACATTCA
CATCTGTCACAGTGTGGGATACACCCGCATCTGATTGTGCAAACTCGCAGAGTTACAACCGGTTGGCAAATTTGAATGC
TTTTAAATTGTATTTTGACCTGATCAATTGCACGTTCAGATACAATTATACTATAACAGAGGATGAAAATGCTGAGTGG
TTTGGCATTACCCAAGATACACAAGGCGTCCACCTCTATTCATCTCGAAAAGAGAACGTGTTCAGAAACAACATGTTCC
ACTTTGCTACTTTACCTGTGTACCAGAAAATCCTCTATTACACAGTCATTCCTCGCAGTATCCGAAGTCCTTTCAATGA
CAGAAAGGCGTGGGCTGCATTCTACATTTATAAGCTACATCCACTCACGTATCTGTTAAATTTTGATGTGGAGGGCTAT
ATAACTAAAGCAGTTGACTGTGGCTATGATGATCTAGCTCAGCTGCAATGTTCCTACCAATCCTTCGACGTTGAAACAG
GTGTTTATTCCGTTTCATCTTTCGAAGCTTCTCCTAGAGGTGAGTTCATTGAACAGGCAACAACTCAAGAGTGTGATTT
CACACCTATGTTGACTGGCACACCCCCTCCTATATATAATTTCAAAAGGTTGGTTTTCACCAATTGCAATTACAACTTA
ACAAAACTCCTTTCACTGTTCCAAGTGAGTGAGTTTTCTTGTCATCAAGTTTCACCTAGTAGTCTTGCTACTGGCTGTT
ACTCTTCTCTTACAGTAGACTATTTCGCATACTCTACTGATATGAGTTCTTATCTGCAACCTGGCTCCGCTGGAGAAAT
TGTGCAGTTTAATTACAAACAAGACTTTAGCAATCCAACGTGTAGAGTGCTTGCTACTGTCCCACAAAATCTTACTACA
ATTACTAAACCTAGTAATTATGCTTATCTTACGGAGTGTTATAAAACCAGTGCATATGGCAAGAACTACTTGTACAATG
CACCTGGCGGTTACACTCCTTGCTTGTCTTTAGCCTCTCGTGGATTCTCTACTAAATATCAGTCACACAGTGATGGAGA
GTTAACCACCACTGGTTATATCTATCCAGTGACTGGCAATCTTCAAATGGCCTTCATTATTTCTGTTCAGTATGGAACT
GACACTAATAGTGTCTGCCCCATGCAAGCTTTAAGAAACGACACTAGCATTGAAGATAAGCTAGATGTTTGTGTTGAAT
ACTCCCTCCATGGCATAACTGGAAGAGGAGTTTTCCACAATTGCACATCTGTTGGACTGAGAAACCAGCGGTTTGTGTA
TGATACTTTCGACAATTTAGTTGGTTACCATTCATATAATGGTAATTACTATTGTGTCAGACCTTGTGTAAGTGTACCT
GTCTCTGTGATTTACGACAAGGTATCTAATTCTCATGCCACATTATTTGGAAGTGTTGCATGTTCGCACGTTACCACCA
TGATGTCGCAATTTTCACGCATGACCAAAACGAATTTGCTCGCGCGTACTACTCCAGGTCCTTTGCAAACTGTTGTCGG
TTGTGCAATGGGCTTCATCAATACTTCTATGGTAGTTGACGAATGTCAACTTCCACTTGGTCAATCACTTTGTGCTATT
CCACCAAATCCTTCTGCTCGTCTTGCACGTGCATCTTCTGGCGTTACTGATGTTTTTCAGATCGCTACTCTTAATTTTA
CTAGTCCATTAACACTTGCACCAATCAATTCCACTGGATTTGTTGTTGCAGTGCCGACTAATTTTACATTTGGTGTTAC
TCAAGAATACATTGAGACTACCATTCAAAAGATTACTGTCGATTGTAAGCAGTACGTTTGCAATGGTTTTAAAAAGTGT
GAAGAACTGCTCACTGAATATGGTCAGTTCTGCTCTAAAATTAACCAGGCTCTTCATGGTGCAAACCTACGTCAAGATG
AGTCTATTGCTAATCTATTTTCAAGTATTAAAACCCAGAATACCCAGCCTCTTCAGGCGGGATTGAATGGTGATTTTAA
CTTGACAATGCTTCAAATACCTCAAGTTACTACAGGTGAACGTAAGTACAGGAGTGCCATCGAAGACCTTCTCTTCAAC
AAGGTTACTATTGCTGATCCTGGCTATATGCAAGGCTATGATGAGTGTATGCAGCAAGGTCCTCAGTCAGCTCGAGACT
TGATCTGTGCACAATATGTTGCTGGCTACAAAGTGTTGCCACCCCTATATGACCCCTATATGGAAGCTGCTTACACTTC
TTCCCTATTGGGCAGTATTGCTGGCGCTAGCTGGACAGCAGGCTTGTCATCTTTCGCAGCAATACCATTTGCACAAAGT
ATCTTTTATCGCTTGAATGGTGTTGGCATCACTCAGCAGGTTTTGTCTGAGAATCAAAAGATTATTGCCAACAAGTTCA
ATCAAGCTCTTGGTGCTATGCAAACTGGCTTTACTACGACTAACCTTGCGTTTAACAAGGTTCAGGACGCAGTAAATGC
TAATGCCATGGCTCTCTCTAAACTAGCAGCGGAATTGTCCAACACTTTCGGTGCTATTTCATCATCCATCAGTGACATC
CTCGCAAGGCTTGACACTGTTGAACAGGAGGCTCAAATTGATCGCTTGATCAATGGCCGTCTCACATCCCTTAACGCAT
TCGTTGCGCAACAACTTGTGCGTACGGAAGCCGCTGCCAGATCCGCCCAGTTGGCCCAAGATAAGGTCAATGAGTGCGT
CAAATCGCAATCCAAACGGAATGGATTCTGCGGAACTGGTACACATATTGTCTCATTTGCCATTAACGCTCCTAATGGC
CTATACTTCTTCCATGTTGGTTACCAGCCAACATCCCATGTTAATGCAACTGCCGCTTATGGCCTTTGCAACACTGAAA
ATCCACCAAAGTGCATTGCACCCATTGGTGGATATTTTGTTCTTAATCAAACTACCAGCACTGTTGCAAATAGTGAGCA
ACAATGGTATTACACTGGTAGTTCTTTCTTCCATCCTGAACCCATCACAGAAGTAAATTCTAAGTATGTGTCCATGGAT
GTAAAATTTGAAAACCTCACTAATAAGCTTCCCCCACCTCTCCTTAGTAATACCACAGATTTGGATTTCAAGGAAGAGC
TGGAAGAATTTTTCAAAAACGTCTCCTCACAAGGACCAAACTTCCAGGAGATTTCTAAGATTAACACTACACTGCTTAA
CCTCAACACAGAGTTGATGGTGCTAAGTGAAGTTGTTAAACAATTAAATGAATCCTATATCGACTTGAAAGAGTTGGGG
AATTACACTTTTTACCAGAAATGGCCATGGTACATATGGCTTGGTTTTATAGCAGGGCTTGTTGCTCTTGCCCTCTGTG
TGTTCTTCATTTTATGTTGTACCGGTTGCGGTACTAGCTGTTTGGGGAAACTAAAATGTAATCGTTGTTGTGACTCGTA

TGATGAGTATGAGGTCGAGAAGCTCCATGTTCATTGA

>gi|520688504|gb|KC522094.1|_Pipistrellus_bat_coronavirus_HKU5_isolate_20S_spike_glycoprotein_gene_complete_cds

ATGATACGCTCAGTGTTAGTACTGATGTGCTCGTTAACTTCTATAGGAAGCCTTGTAAACAGTCAAAGCGTTGAC
ATGGGTACTACTGGTAGTGGTAACTGCATAGAGTCTCAAGTCGCGCCTGACTTTTTTGAGACGGCACGTAATATATGGC
CGCTTCCCATCGATACGAGTAAGGCTGAAGGTGTCATTTATCCCAATGGCAAGTCGTACTCTAATATTTCTTTAACTTA
TACGGGACTGTATCCCAAGGCTAATGACCTTGGTAAACAGTACATATTTTCCGACGGACATAGCAGTCCTGGCACATTA
AGTCGTCTATTTGTCAGTAACTATTCTAGACAAGTAGAAACATTTGACGATGGTTTCATTGTTCGTATTGGTGCTGCCT
CCAATAAGACAGGGACCACTGTAATTTCCCAATCCACTAATAGACCAATTAAGAAAATTTACCCAGCTTTTATGCTTGG
TCATTCTGTTGGTAATTATACACCCACTAATATCACTGGGCGTTACCTTAACCACACACTTGTCATCCTGCCGGATGGT
TGTGGTACATTGGTTCATGCATTTTATTGCATCTTACAACCCCGTACACAAGCATATTGTGCTGGTGCTAGTACATTCA
CGTCTGTCACAGTGTGGGACACACCCGCATCTGATTGTGCAAACTCTCAGAGTTACAATCAGTTGGCAAATTTGAATGC
TTTCAAATTGTATTTTGACCTCATTAATTGCACGTTCAGATACAATTATACTATAACTGAGGACGAAAACGCTGAGTGG
TTTGGCATAACCCAAGATACACAAGGCGTTCACCTCTATTCATCTCGAAAAGAGAATGTGTTCAGAAATAACATGTTCC
ACTTTGCCACTTTACCTGTGTACCAGAAAATCCTCTATTACACAGTCATTCCTCGCAGCATCCGCAGTCCTTTCAATGA
CAGAAAAGCTTGGGCTGCCTTCTACATTTATAAACTTCATCCACTCACATATTTGCTAAATTTTGATGTGGAGGGCTAT
ATAACTAAAGCGGTAGACTGTGGCTATGATGATTTCGCACAGCTACAATGTTCATACGAAAACTTCGATGTTGAGACAG
GTGTTTACTCCGTCTCATCGTTCGAAGCTTCCCCTAGAGGTGAGTTCATTGAACAATCAGTTACCAAAGAGTGTGACTT
TTCACCCATGCTAACTGGCACGCCTCCCCCTATATATGATTTCAAAAGATTGGTTTTCACCAATTGCAATTACAACTTA
ACAAAACTCCTTTCACTTTTTCAGGTGAGTGAGTTTTCTTGTCATCAAGTATCTCCCAGTAGTCTTGCTACTGGCTGTT
ACTCTTCTCTTACAGTGGATTACTTTGCATACTCCACTGATATGAGTTCTTACCTGCAACCTGGCTCCGCTGGAGAAAT
TGTGCAGTTTAATTACAAACAAGACTTTAGCAATCCCACCTGCAGAGTGCTTGCTACTGTTCCCACCAATCTTACTACA
ATTACTAAACCTAGTAATTATGTTCATCTTACGGAGTGTTATATAAGCACCGCATATGGCAAGAATTACTTGTTTAATG
CACCTGGTGGTTACACTCCTTGCTTATCTTTAGCCTCTAGTGGGTTCTCACGTGATAGACAGTCTCACAGTCAGGAATT
ACCTGATGGTAGTTTTTTAACTACTACTGGATCTGTTTACTCTGTAGGCTCTAACCTTCAAATGGCTTTCATAATCTCA
GTACAGTATGGCACTGACACTAATAGTGTCTGTCCCATGCAAGCTCTAAGAAACGATACTAGCATTGAAGATAAGCTAG
ATGTTTGTGTTGAATACTCCCTCCACGGCATAACTGGAAGAGGAGTTTTCCATAATTGCACTTCTGTTGGACTGAGAAA
CCAGCGGTTTGTGTATGATACTTTCGACAATCTAGTTGGTTACCATTCACATAATGGTAATTACTATTGTGTCAGACCT
TGTGTCAGTGTACCTGTCTCTGTGATTTATGACAAGGTATCTAATTCTCATGCCACATTATTTGGTAGTGTCGCATGTT
CGCATGTTACCACTATGATGTCTCAATTTTCACGCATGACTAAAACTAATTTGCTCGCGCGTACAACTCCTGGCCCATT
GCAAACTACTGTTGGTTGTGCAATGGGCTTCATTAATTCCTCAATGGTAGTTGATGAATGTCAACTTCCTCTCGGCCAA
TCACTTTGTGCTATTCCACCAACCATTTCTACACGCTTTCGACGTGCTACTTCTGGTGTATCTGATGTGTTTCAAATCG
CCACTCTAAATTTTACTAGTCCTCTAACACTTGCACCAATAAATTCAACTGGATTTGTTGTTGCTGTGCCGACTAATTT
CACATTTGGTGTCACTCAAGAATTCATTGAGACTACCATTCAAAAGATTACTGTTGATTGTAAGCAGTACGTTTGTAAT
GGTTTTAAGAAGTGCGAAGACTTGCTCAAAGAATACGGCCAGTTTTGCTCTAAAATTAACCAGGCTCTTCATGGTGCAA
ATCTACGTCAAGATGAGTCTATTGCTAATCTATTTTCAAGTATTAAAACCCAGAACACCCAGCCTCTTCAGGCAGGATT
GAATGGTGATTTTAACTTGACAATGCTTCAAATACCTCAAGTTACTACTGGTGAACGTAAGTACAGGAGTGCTATCGAA
GACCTTCTTTTCAATAAGGTCACTATTGCTGATCCTGGCTATATGCAAGGCTATGACGAATGTATGCAGCAAGGTCCTC
AGTCAGCTCGAGACTTGATCTGTGCACAATATGTTGCTGGCTACAAAGTGTTGCCACCCCTATATGACCCCTATATGGA
AGCTGCTTATACTTCTTCCCTATTGGGCAGTATTGCTGGTGCTAGTTGGACAGCAGGTTTGTCATCTTTCGCAGCAATA
CCATTTGCACAAAGTATCTTTTATCGCTTGAATGGTGTTGGCATCACTCAGCAGGTTCTGTCTGAGAATCAAAAGATTA

TTGCCAACAAGTTCAATCAAGCTCTTGGTGCTATGCAAACTGGCTTTACTACAACTAACCTAGCTTTCAACAAGGTTCA
GGATGCAGTAAATGCTAATGCCATGGCTCTTTCCAAACTAGCTGCGGAATTGTCTAACACTTTCGGTGCTATTTCATCT
TCTATCAGTGACATCCTTGCGAGGCTTGACACTGTTGAACAAGAGGCACAAATTGATCGGTTGATCAATGGCCGTCTCA
CATCCCTTAATGCATTCGTTGCGCAACAACTTGTGCGTACTGAAGCTGCTGCCAGATCTGCCCAATTGGCCCAAGATAA
GGTCAATGAGTGCGTCAAATCGCAATCCAAACGCAATGGATTCTGCGGAACTGGTACACATATTGTCTCATTTGCCATT
AACGCTCCTAATGGCCTCTATTTCTTTCATGTTGGTTATCAGCCAACATCCCATGTTAATGCAACTGCCGCCTATGGCC
TTTGCAACACAGAAAATCCACCAAAGTGCATAGCACCCATTGATGGATATTTCGTTTTAAATCAAACTATCAGCACTGT
AGCAAACAGTGACCAGCAATGGTATTATACTGGTAGTTCTTTCTTCCATCCTGAACCCATTACAGAAGCAAATTCTAAG
TATGTGTCCATGGATGTAAAATTCGAAAACCTCACTAATAAACTTCCTCCACCTCTCCTTAGCAATTCCACAGATTTGG
ATTTCAAGGAAGAGCTGGAAGAATTCTTCAAAAACGTCTCCTCACAAGGACCAAACTTTCAGGAGATTTCTAAGATTAA
CACTACACTGCTTAACCTCAACACAGAGTTGATGGTGCTTAGTGAAGTTGTTAAACAACTAAATGAATCCTACATCGAC
TTGAAAGAGTTGGGGAATTATACTTTTTACCAGAAATGGCCATGGTACATATGGCTTGGCTTTATTGCAGGGCTTGTCG
CTCTTGCTCTTTGTGTGTTCTTCATTCTATGTTGTACTGGTTGCGGTACTAACTGCTTGGGGAAACTAAAATGTAATCG
TTGTTGTGACTCGTATGATGAGTATGAGGTCGAGAAGATCCATGTTCATTGA

>gi|520688506|gb|KC522095.1|_Pipistrellus_bat_coronavirus_HKU5_isolate_21S_spike_glycoprotein_gene_complete_cds

ATGATACGCTCAGTGTTAGTACTGATGTGCTCGTTAACTTTTATAGGAAACCGCACAAGCTGTCAAAGTGTTGAT
ATTGGCACGCCTGCTTCGGGTTCTTGTTTACGGTCTCAAGTTAGACCAGAATATTTTGACATTGTCCGTAATACATGGC
CAATGCCTATTGACACGAGTAAGGCTGATGGTGTCATTTACCCTAATGGCAAGTCATACTCTAATATTTCTCTAACCTA
TACAGGACTGTATCCAAAGGCCAATGACCTTGGTAAGCAGTATTTGTTTTCTGATGGACACAGTGCTCCTAATCAGCTT
AATGATCTATTTGTCAGTAATTATTCTGCACAAGTGGAGTCTTTTGATGATGGTTTCGTTGTTCGTATTGGAGCAGCAT
CCAATAAGACTGGAACCACTGTCATCTCTCAAACAACTTTTAAACCCATTAAGAAAATATACCCTGGCTTTATGCTGGG
GCACTCAGTGGGTAATTATACACCTACCAACATTTCAGGTCGTTATCTTAACCACACCCTTGTCATCCTACCGGATGGC
TGTGGTACTCTAGTGCACGCATTCTATTGCGTTCTACAACCCCGCACACAGGCTAACTGTCCTGGTGCTTCTTCATTCA
CATCTGTCACATTGTGGGACACTCCTGCAACAGATTGCGCACCAAGTGGTGTTTACAATCCTTTGGCAAATTTGAATGC
TTTTAAGTTGTATTTTGACCTGATCAATTGTACGTTCAAATACAACTATACTATAACAGAGGATGAAAACGCTGAGTGG
TTCGGCATTACCCAAGATACACAAGGCGTTCACCTCTATTCCTCTCGAAAAGAGAATGTGTTCAGAAATAACATGTTCC
ACTTTGCTACCTTACCTGTGTATCAGAAAATCCTCTATTACACAGTCATTCCTCGCAGTATCCGAAGTCCCTTCAATGA
CAGAAAAGCTTGGGCTGCCTTCTACATTTATAAACTACATCCACTCACATATCTGTTAAATTTTGATGTGGAGGGCTAT
ATAACAAAAGCGGTAGACTGTGGCTACGATGATTTCGCACAGCTACAATGTTCGTATGAAAACTTCGATGTTGAAACAG
GTGTTTATTCCGTTTCATCCTTCGAAGCTTCTCCTAGAGGTGAGTTCATTGAACAGGCAACAACCCAAGAGTGTGATTT
CACACCTATGTTGTCTGGCACACCTCCTCCTATATATGATTTCAAAAGGTTGGTCTTCACCAATTGCAATTACAACTTA
ACAAAACTCCTCTCACTGTTTCAAGTGAGTGAGTTTTCTTGTCATCAAGTTTCACCCAGTAGTCTTGCTACTGGTTGTT
ACTCTTCTCTTACAGTAGACTATTTTGCATACTCCACTGATATGAGTTCTTACCTGCAACCTGGCTCCGCTGGAGAAAT
TGTGCAGTTTAATTACAAACAAGACTTTAGCAGTCCCACGTGTAGAGTGCTTGCTACTGTTCCAACCAATCTTACTTCA
ATTACTAAACCTAGTAATTATGCTTATCTTACGGAGTGTTATAAAACCAGTGCATATGGCAAGAATTACTTGTACAATG
CGCCTGGCGGTTACACTCCATGCTTGTCTTTAGCCTCTCGTGGGTTCTCTACTAAATACCAGTCACATAGTGATGGAGA
GCTTACCACCACGGGTTACGTCTATCCTGTGACTGGCAACCTTCAAATGGCTTTCATTATTTCTGTTCAGTATGGAACT
GACACTAATAGTGTCTGCCCCATGCAAGCACTAAGAAACGATACTAGCATTGAAGACAAGCTAGATGTTTGTGTTGAAT
ACTCCCTTCATGGTATAACCGGAAGAGGAGTTTTCCATAATTGCACATCTGTTGGATTGAGAAACCAGCGGTTTTGTATA
TGATACTTTCGACAACCTAGTTGGTTACCATTCAGATAATGGCAATTACTATTGTGTCAGACCTTGTGTCAGTGTGCCT

GTCTCTGTGATTTATGACAAGGTATCTAATTCTCATGCTACATTATTTGGTAGTGTTGCATGTTCGCACGTTGCCACAA
TGATGTCACAATTTTCACGTATGACCAAAACTAATTTGCTCGCACGTATAACTCCTGGCCCGTTGCAAACTACTGTAGG
TTGTGCAATGGGCTTCATCAATTCCTCAATGGTAGTTGACGAATGTCAACTTCCTCTCGGTCAATCACTTTGTGCTATT
CCACCAACTACTTCTACACGCTTTCGACGTGCTACTTTTGGTGTATCTGATGTGTTTCAAATCGCCACTCTAAACTTTA
CTAGTCCTTTAACACTCGCACCAATAAATTCAACTGGATTTGTTGTTGCGGTGCCGACTAATTTCACATTTGGTGTTAC
TCAAGAATTCATTGAGACTACCATTCAAAAGATTACTGTCGATTGCAAGCAGTACGTTTGTAATGGTTTTAAGAAGTGC
GAAGACTTGCTCAAAGAATACGGCCAGTTTTGCTCTAAAATTAACCAGGCTCTTCATGGTGCAAACCTACGTCAAGATG
AGTCTATTGCTAATCTATTTTCAAGTATTAAAACCCAGAATACCCAGCCTCTTCAGGCGGGATTGAATGGTGATTTTAA
CTTGACAATGCTTCAAATACCTCAAGTTACTACGGGTGAACGTAAGTACAGAAGTGCTATTGAAGACCTTCTTTTCAAT
AAGGTTACTATTGCTGATCCTGGCTATATGCAAGGCTATGATGAATGTATGCAGCAAGGTCCTCAGTCAGCTCGAGACT
TGATCTGTGCACAATATGTTGCTGGCTACAAAGTGTTGCCACCCTTATACGATCCCTATATGGAAGCTGCTTACACATC
CTCCCTATTGGGCAGTATTGCTGGTGCTAGTTGGACAGCAGGTCTGTCATCTTTCGCAGCAATACCATTTGCACAAAGT
ATTTTTTATCGCTTGAATGGTGTTGGCATCACTCAGCAGGTTCTGTCTGAGAATCAGAAGATTATCGCCAACAAGTTCA
ATCAAGCTCTTGGCGCCATGCAAACTGGTTTTACTACCACTAACCTAGCTTTTAGCAAGGTTCAGGACGCAGTAAATGC
TAATGCTATGGCTCTCTCTAAACTAGCTGCGGAATTGTCTAACACTTTCGGTGCTATTTCATCTTCCATCAGTGACATC
CTTGCAAGGCTCGACACTGTTGAACAAGAGGCACAAATTGATCGCTTGATCAATGGACGCCTCACATCACTTAACGCAT
TCGTTGCGCAACAACTTGTGCGTACTGAAGCTGCTGCCAGATCCGCCCAATTGGCCCAAGATAAGGTCAATGAGTGCGT
CAAATCGCAATCCAAACGGAATGGGTTCTGCGGAACTGGTACACATATTGTCTCATTTGCCATTAATGCTCCTAATGGC
CTCTATTTCTTTCATGTTGGTTATCAGCCGACATCACATGTTAATGCAACTGCCGCTTATGGCCTTTGCAACACTCAAA
ACCCACCAAAGTGCATTGCACCTATTGATGGATACTTCGTCCTAAATCAAACTACTAGCACTGTCGCAGACGGTGACCA
ACAATGGTATTACACTGGTAGTTCTTTCTTTCATCCGGAACCCATCACAGAAGCCAATTCTAAGTATGTGTCTATGGAT
GTAAAATTCGAAAACCTTACTAATAGACTTCCTCCACCACTCCTCAGTAATTCCACAGATAAGGACTTTTCTGATGAGT
TGCAGGAATTCTTCAAAAATGTCTCCTCACAAGGACCTAACTTTCAGGAAATTTCTAAGATTAACACTACACTGCTCAA
CCTCAACACAGAGTTGATGGTGTTGAGTGAAGTTGTTAAACAATTAAATGAATCCTATATCGACTTGAAAGAGTTGGGG
AATTATACTTTTTACCAGAAATGGCCATGGTACATATGGCTTGGCTTTATTGCAGGGCTCGTTGCTCTTGCTCTTTGTG
TGTTCTTCATTTTATGTTGTACTGGTTGCGGTACTAACTGCTTGGGGAAACTAAAATGTAATCGTTGTTGTGACTCGTA
TGATGAGTATGAGGTCGAGAAGATCCATGTTCATTGA

>gi|520688508|gb|KC522096.1|_Pipistrellus_bat_coronavirus_HKU5_isolate_22S_spike_glycoprotein_gene_complete_cds

ATGATACGCTCAGTGTTAGTACTGATGTGCTCGTTAATTTTTATAGGAAACCGCACAAGCTGTCAAAGCGTTGAT
ATTGGCATGCCTGCTTCGGATTCTTGTTTACGGTCTCAAGTTAGACCAGAATATTTTGACATTGTCCGTAATACATGGC
CAATGCCTATTGATACGAGTAAGGCTGAAGGTGTCATTTACCCTAATGGCAAGTCATACTCTAATATTTCCTTAACTTA
TACAGGACTGTATCCAAAGGCTAATGACCTTGGTAAACAGTATTTGTTTTCTGATGGACACAGCGCTCCTAATCAGCTT
AATGATCTATTTGTCAGTAATTATTCTGCACAAGTGGAGTCTTTTGATGATGGTTTCGTTGTTCGTATTGGAGCCGCTT
CCAATAAGACTGGAACCACTGTCATCTCCCAAGCAACTTTTAAACCCATTAAGAAAATATACCCTGGTTTTATGCTAGG
GCATTCAGTGGGTAACTACACACCTGCCAACATTACAGGTCGTTATCTTAATCACACTCTTGTCATCCTACCGGATGGC
TGTGGTACTCTAGTACATGCATTCTATTGCGTCTTACAACCTCGCACACAGGCTAATTGTCCTGGTGCTTCTTCATTCA
CATCTGTCACATTGTGGGACACCCCCTCAACTGATTGTACACCATCTGGTGTTTACAATCCTTTGGCAAATCTGAATGC
TTTCAAATTGTATTTTGACCTGCTCAATTGCACGTTCAAATACAACTATACTATAACAGAGGATGAAAACGCTGAGTGG
TTCGGAATAACCCAAGACACACAAGGCGTCCACCTCTATTCCTCTCGAAAAGAGAATGTGTTCAGAAATAACATGTTCC
ACTTTGCTACTTTACCTGTGTACCAGAAGATCCTTTACTACACAGTCATTCCTCGCAGTATCCGAAGTCCTTTCAATGA

CAGAAAAGCTTGGGCTGCCTTCTACATTTACAAACTACATCCACTCACATATCTGTTAAATTTTGATGTGGAGGGCTAT
ATAACTAAAGCGGTAGACTGTGGCTATGATGATTTCGCACAGCTACAATGTTCATACGAAAACTTCGATGTTGAGACAG
GTGTCTATTCTGTTTCATCGTTCGAAGCTTCTCCTAGAGGTGAGTTCATTGAACAGGCAGTAACTCAAGAGTGTGACTT
CACACCTATGTTAACTGGCACACCTCCTCCTATATATGATTTCAAAAGGTTGGTTTTCACCAATTGCAATTACAACTTA
ACAAAACTCCTTTCGCTTTTTCAGGTGAGTGAGTTTTCTTGTCATCAAGTTTCACCTAGTAGTCTTGCTACTGGCTGTT
ACTCATCTCTTACAGTGGATTATTTTGCATATTCCACTGATATGAGTTCTTATCTGCAACCTGGCTCCGCGGGAGAAAT
AGTGCAGTTTAATTACAAACAAGACTTTAGCAATCCCACGTGTAGAGTGCTTGCTACTGTTCCACAAAATCTTACTACA
ATTACTAAACCTAGTAATTATGCTTATCTTACAGAGTGTTATAAAACCAGTGCATATGGCAAGAACTACTTGTACAATG
CACCTGGTGGTTACACTCCGTGTTTGTCGTTAGCCTCTCGTGGGTTCTCCACTAAATACCAGTCACACAGTGACGGGGA
GCTTACTACCACAGGTTACATCTACCCTGTAAATGGCAACCTCCAAATGGCTTTCATTATTTCAGTACAGTATGGAACT
GACACTAATAGTGTCTGTCCCATGCAAGCTCTAAGAAACGACACTAGCATTGAAGACAAGCTAGATGTCTGTGTTGAAT
ACTCCCTCCATGGTATAACAGGAAGGGGAGTTTTCCATAATTGTACATCTGTTGGATTGAGAAACCAGCGGTTTGTGTA
TGATACTTTCGACAACCTAGTTGGTTACCATTCAGATAATGGTAATTACTATTGTGTCAGACCCTGTGTCAGTGTACCT
GTCTCTGTGATTTATGACAAGGTATCTAATTCTCATGCTACATTATTTGGTAGTGTTGCTTGTTCGCACGTTACCACTA
TGATGTCACAATTTTCACGCATGACCAAAACCTATTTGCTCGCGCGTACTACTCCTGGCCCTTTGCAAACTACTGTTGG
TTGTGCAATGGGCTTCATCAATTCCTCAATGGTAGTTGACGAATGTCAGCTTCCACTTGGTCAATCACTTTGTGCTATT
CCACCAACCACTTCTACACGCTTTCGACGTGCTACTTCTGGTGTATCTGATGTGTTTCAAATCGCCACTCTAAATTTCA
CTAGTCCACTAACACTTGCACCAATAAACTCCACTGGATTTGTTGTTGCAGTGCCGACTAATTTCACATTTGGTGTCAC
TCAAGAATTTATTGAGACTACCATTCAAAAGATCACTGTAGATTGTAAGCAGTACGTTTGTAATGGTTTTAAGAAGTGC
GAAGACTTGCTCAAAGAATATGGCCAGTTTTGCTCTAAAATTAACCAGGCTCTTCATGGTGCAAACCTACGTCAAGATG
AGTCTATTGCTAATCTATTTTCAAGTATTAAAACCCAGAATACCCAGCCTCTTCAGACGGGATTGAATGGTGATTTTAA
CTTGACAATGCTTCAAATACCTCAAGTTACTACTGGTGAACGTAAGTACAGAAGTGCTATCGAAGACCTTCTTTTCAAC
AAGGTTACTATTGCTGATCCTGGCTATATGCAAGGCTATGATGAATGTATGCAGCAAGGTCCTCAGTCAGCTCGAGACT
TGATCTGTGCACAATATGTTGCTGGCTACAAAGTGTTGCCTCCCCTCTATGACCCTTATATGGAAGCTGCTTACACTTC
TTCCCTATTGGGCAGTATTGCTGGTGCTAGTTGGACAGCTGGTCTGTCATCTTTCGCAGCAATACCATTTGCACAAAGT
ATCTTTTATCGCTTGAATGGTGTTGGCATCACGCAGCAGGTTCTGTCTGAGAATCAGAAGATCATTGCCAACAAGTTCA
ATCAAGCTCTTGGCGCCATGCAAACTGGCTTTACTACTACTAACCTAGCTTTCAGCAAGGTTCAGGATGCAGTAAATGC
TAATGCTATGGCTCTTTCAAAACTAGCTGCGGAATTGTCTAACACTTTCGGTGCTATTTCATCCTCAATCAGTGACATC
CTTGCTAGGCTTGACACTGTTGAACAAGAGGCTCAAATTGATCGATTGATCAATGGTCGCCTCACATCTCTTAACGCAT
TCGTTGCGCAACAACTTGTGCGTACTGAAGCTGCTGCCAGATCCGCCCAATTGGCCCAAGATAAGGTCAATGAGTGCGT
TAAATCGCAATCCAAACGGAATGGATTCTGCGGAACTGGCACACATATTGTTTCATTCGCCATTAATGCTCCTAATGGC
CTTTACTTCTTTCATGTTGGTTACCAACCAACATCCCATGTTAATGCAATTGCCGCCTATGGCCTTTGCAACTCTGAAA
CACCACCAAAGTGCATTGCACCTATTGATGGTTACTTCGTCCTAAATCAAACTACTAGCACTATCGCAAACAGTGACCA
ACAATGGTATTATACTGGTAGTTCATTCTTCCATCCGGAACCCATCACAGAAGCAAATTCTAAGTATGTGTCCATGGAT
GTAAAATTCGAAAATCTCACTAATAAACTTCCTCCACCTCTCCTTAGCAATTCAACAGACTTGGATTTCAAGGATGAGC
TGGAAGAATTCTTCAAAAATGTCTCCTCGCAAGGACCAAACTTCCAGGAGATTTCTAAGATTAACACTACACTGCTTAA
CCTCAACACAGAGTTGATGGTGCTGAGTGAAGTTGTTAAACAATTAAATGAATCCTATATCGACTTGAAAGAGTTGGGG
AATTATACTTTTTACCAGAAATGGCCATGGTACATATGGCTTGGCTTTATTGCAGGGCTTGTTGCTCTTGCTCTTTGTG
TGTTCTTCATTTTATGCTGTACTGGTTGCGGTACTAGCTGTTTGGGGAAACTAAAATGTAATCGTTGTTGTGACTCGTA
TGATGAGTATGAGGTCGAGAAGATCCATGTTCATTGA

>gi|520688510|gb|KC522097.1|_Pipistrellus_bat_coronavirus_HKU5_isolate_24S_spike_glycoprotein_gene_complete_cds

ATGATACGCTCAGTGTTAGTACTGATGTGCTCGTTAACTTTTATAGGAAACCTCACAAGAGGTCAAAGTGTTGAT
ATGGGACCCAATGGCACTGGTTCATGTTTAGATTCTCAGGTACAACCTGATTATTTGAATCTGTGCGCACTACTTGGC
CCATGCCAATTGACACGAGTAAGGCTGAAGGTGTCATTTACCCTAATGGCAAGTCATACTCTAATATTTCTTTAACTTA
TACGGGACTGTATCCTAAGGCCAATGACCTTGGTAAACAGTATTTATTTTCTGATGGACATAGTGCTCCAGGACGACTT
AATAACTTATTTGTCAGTAATTACTCCTCACAAGTGGAGTCTTTTGATGATGGCTTTGTTGTCCGTATTGGTGCTGCTG
CCAATAAAACTGGAACCACTGTCATCTCCCAATCCACCTTTAAACCCATTAAGAAGATTTATCCAGCCTTTTTGCTTGG
ACATTCTGTGGGTAATTATACACCGTCCAATAGGACAGGTCGTTATCTCAACCACACACTTGTCATCCTACCGGATGGT
TGTGGTACAATTCTACATGCATTTTATTGCATTTTGATACCTCGCACGCAACAAAATTGTGCTGGCGATAGCAACTTTA
AATCCCTCTCCCTTTGGGATACTCCTGCATCTGATTGTAATCCCGGTTCTTATAATGAACAGGCCACTCTAGGTGCTTT
CAAAGTGTATTTTGACCTCATTAATTGCACGTTCAGATATAACTATACTATAACAGAGGATGAAAATGCTGAGTGGTTT
GGCATCACCCAAGACACACAAGGCGTTCACCTCTATTCCTCTCGAAAAGAGAACGTGTACAGAAACAACATGTTCCACT
[truncated: 479,374 more chars]
